# Transcriptional Profiling Uncovers Human Hyalocytes as a Unique Innate Immune Cell Population

**DOI:** 10.3389/fimmu.2020.567274

**Published:** 2020-09-11

**Authors:** Stefaniya Konstantinova Boneva, Julian Wolf, Dennis-Dominik Rosmus, Anja Schlecht, Gabriele Prinz, Yannik Laich, Myriam Boeck, Peipei Zhang, Ingo Hilgendorf, Andreas Stahl, Thomas Reinhard, James Bainbridge, Günther Schlunck, Hansjürgen Agostini, Peter Wieghofer, Clemens A. K. Lange

**Affiliations:** ^1^Eye Center, Medical Center, Faculty of Medicine, University of Freiburg, Freiburg, Germany; ^2^Institute of Anatomy, Leipzig University, Leipzig, Germany; ^3^Heart Center Freiburg, University of Freiburg, Freiburg, Germany; ^4^Department of Ophthalmology, University Medical Center Greifswald, Greifswald, Germany; ^5^National Institute for Health Research Moorfields Biomedical Research Centre, Moorfields Eye Hospital and University College London Institute of Ophthalmology, London, United Kingdom

**Keywords:** hyalocytes, vitreous macrophages, viterous body, innate immunity, myeloid cells, immune privilege

## Abstract

**Purpose:**

To decipher the transcriptional signature of macrophages of the human vitreous, also known as hyalocytes, and compare it to the profiles of other myeloid cell populations including human blood-derived monocytes, macrophages, and brain microglia.

**Methods:**

This study involves a total of 13 patients of advanced age with disorders of the vitreoretinal interface undergoing vitrectomy at the University Eye Hospital Freiburg between 2018 and 2019. Vitreal hyalocytes were analyzed by fluorescence-activated cell sorting (FACS) and isolated as CD45^+^CD11b^+^CX3CR1^+^Mat-Mac^+^ cells using a FACS-based sorting protocol. RNA extraction, library preparation and RNA sequencing were performed and the sequencing data was analyzed using the Galaxy web platform. The transcriptome of human hyalocytes was compared to the transcriptional profile of human blood-derived monocytes, macrophages and brain microglia obtained from public databases. Protein validation for selected factors was performed by immunohistochemistry on paraffin sections from three human donor eyes.

**Results:**

On average, 383 ± 233 hyalocytes were isolated per patient, resulting in 128 pg/μl ± 76 pg/μl total RNA per sample. RNA sequencing revealed that *SPP1*, *FTL*, *CD74*, and *HLA-DRA* are among the most abundantly expressed genes in hyalocytes, which was confirmed by immunofluorescence for CD74, FTL, and HLA-DRA. Gene ontology (GO) enrichment analysis showed that biological processes such as “humoral immune response,” “leukocyte migration,” and “antigen processing and presentation of peptide antigen” (adjusted *p* < 0.001) are dominating in vitreal hyalocytes. While the comparison of the gene expression profiles of hyalocytes and other myeloid cell populations showed an overall strong similarity (*R*^2^ > 0.637, *p* < 0.001), hyalocytes demonstrated significant differences with respect to common leukocyte-associated factors. In particular, transcripts involved in the immune privilege of the eye, such as *POMC*, *CD46*, and *CD86*, were significantly increased in hyalocytes compared to other myeloid cell subsets.

**Conclusion:**

Human hyalocytes represent a unique and distinct innate immune cell population specialized and adapted for the tissue-specific needs in the human vitreous. Vitreal hyalocytes are characterized by a strong expression of genes related to antigen processing and presentation as well as immune modulation. Thus, hyalocytes may represent an underestimated mediator in vitreoretinal disease and for the immune privilege of the eye.

## Introduction

In the course of evolution, the eye has developed a unique relationship to the immune system, the so-called immune privilege, which protects against immune-mediated inflammatory damage, thus obtaining a free optical axis, and preserving optimal vision (1). While the concept of the immune privilege is simple, the study of its nature has revealed its highly complex character. Several mechanisms, including local and systemic responses, maintain in concert the privilege of the ocular immune system. These include physical barriers such as an efficient blood retina barrier and the lack of efferent lymphatics, an inhibitory ocular microenvironment consisting of soluble and cell-bound immunosuppressive factors, and finally an active regulation of the systemic immune response [for a detailed review see (1)]. The cellular mechanisms involved in these processes, however, are not yet fully understood.

Vitreal macrophages, also known as hyalocytes, belong to the family of resident innate immune cells in the posterior eye segment and reside mainly in the posterior vitreous cortex abutting the inner retinal surface (2). Although already first described by Hannover in 1840 (3), little is known about the biology and function of hyalocytes in the human eye. Hyalocytes are relatively scarce and have long been regarded as resting cells. In general, they have been studied less extensively compared to other, more abundant intraocular cells, such as endothelial cells, glia cells, or retinal pigmented epithelium (RPE). Recent evidence indicates that hyalocytes actively contribute to the maintenance of vitreous transparency, avascularity, and the synthesis of vitreal extracellular matrix (ECM) proteins (4). Also, hyalocytes have been postulated to inhibit intraocular inflammation, in order to guarantee vitreous transparency, e.g., by contributing to the vitreous cavity-associated immune deviation (VCAID), which belongs to the unique mechanisms maintaining the immune privilege in the human eye (2, 5). Furthermore, macrophage-like (6) hyalocytes are regarded as important modulators of immunological and inflammatory processes within the vitreous cavity and were suggested to play pivotal roles in pathological conditions such as uveitis, proliferative diabetic retinopathy, or proliferative vitreoretinopathy (2, 7). Animal studies show that vitreal hyalocytes can migrate to sites of damage and take part in immune response modulation during inflammation (2, 8). However, these assumptions are merely based on pre-clinical examinations and the exact molecular mediators, through which hyalocytes modulate the human ocular immune privilege, are still unknown.

In this study, we establish a fluorescence-activated cell sorting (FACS)-based isolation protocol for human hyalocytes in patients undergoing vitrectomy for vitreoretinal interface diseases and provide an elaborated transcriptional profile of this cell population, prior to comparing them to other myeloid cell subtypes. We show for the first time that vitreal hyalocytes share common myeloid cell markers but represent a distinct myeloid cell subset with a unique transcriptional profile well-suited to suppress ocular inflammation and immune reaction in the eye.

## Materials and Methods

### Patients’ Characteristics

A total of 13 consecutive patients undergoing 23 gauge vitrectomy for idiopathic macular pucker (MP, *n* = 7) or idiopathic macular hole (MH, *n* = 6) in the University Eye Hospital Freiburg between 2018 and 2019 were included in this study (see Table 1). Patients with MP and MH were treated with a meticulous removal of the vitreous body. The epiretinal membrane (ERM) and the internal limiting membrane (ILM) were removed in MP patients and the ILM only in MH patients. Diagnosis was made based on a thorough funduscopic exam and spectral domain optical coherence tomography (HRA2, Heidelberg Engineering, Figure 1A). Only patients with no history of previous vitreoretinal surgery, intraocular inflammation, or concomitant vitreoretinal disease were included in this study. Ethics approval was granted from the local Ethics Committees and a written informed consent was obtained from each patient.

**TABLE 1:** Patients’ characteristics.

#	Diagnosis	PVD	Lens status	No of isolated cells	RNA conc. (pg/μl)
1	MP	Yes	Pseudophakic	291	142
2	MP	No	Pseudophakic	464	161
3	MP	No	Phakic	180	93
4	MH	No	Pseudophakic	315	301
5	MH	Yes	Phakic	550	110
6	MH	No	Phakic	164	174
7	MP	No	Pseudophakic	944	109
8	MH	No	Phakic	210	78
9	MP	Yes	Pseudophakic	186	26
10	MP	No	Phakic	306	240
11	MP	No	Pseudophakic	701	73
12	MH	No	Pseudophakic	240	78
13	MH	No	Phakic	424	73

**FIGURE 1:**
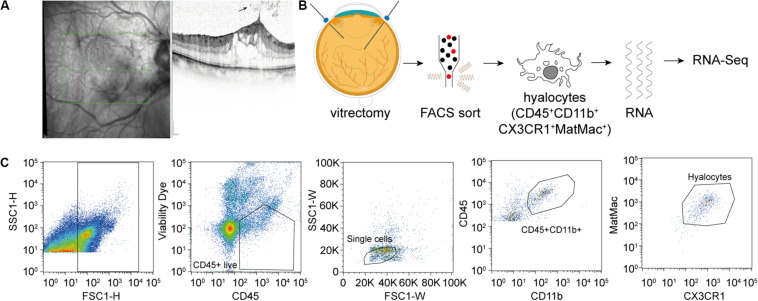
Experimental Setup. **(A)** Study subjects were diagnosed by funduscopic examination and optical coherence tomography (SD-OCT). The arrow points at a cell in the vitreous cavity of a patient with macular pucker, most likely one of a group of hyalocytes. **(B)** After performing a standard vitrectomy the content of a single vitrectomy collection bag was treated according to a FACS protocol developed for the project, hyalocytes were sorted and then subjected to RNA sequencing analysis. **(C)** Flow cytometric gating strategy for human hyalocytes characterized as CD45^+^CD11b^+^CX3CR1^+^MatMac^+^ cells. Cell populations were defined by fluorescence intensity after exclusion of dead cells and doublets by viability dye and physical parameters (FSc, SSc): FSc, forward scatter; SSc, sideward scatter; H, Height; and W, Width.

### FACS Sort

Vitreous tissue samples and the corresponding ERM and ILM which are rich in hyalocytes (9, 10) were collected in vitrectomy collection bags and immediately placed on ice in the operating theater and processed for cell isolation within 2 h of surgical resection. The content of each vitrectomy bag was centrifuged for 10 min at 250 × *g* at 15#x00B0;C. After removing the supernatant, the pellet was transferred to FACS tubes by washing with phosphate-buffered saline (PBS) buffer (PAN) and Hank’s balanced salt solution (HBSS, Gibco) and again centrifuged for 10 min at 250 × *g* at 15#x00B0;C. The vitreous pellet was digested with Collagenase D (5 mg/mL, Roche) and DNase I (1 mg/mL, Roche) in HBSS for 20 min at 37#x00B0;C. Cells were then filtered through a 70 μm cell strainer. Following another centrifugation step (7 min at 250 × *g* at RT), 0.5 μL of Fixable Viability Dye (eFluor^TM^ 780, eBioscience^TM^) per 1 mL of cells was added. The pellet was stained for CD45 (BV421, anti-human, 1:100, and BioLegend®), CD11b (FITC, anti-human, 1:100, and BioLegend®), and CX_3_CR1 (PE-Cy7, anti-human, 1:200, and BioLegend®). To exclude any potential contamination with blood-derived monocytes due to possible surgically induced micro bleedings, we further used the Anti-Human Mature Macrophages (MatMac) Antibody, an ED2-like (ectodermal dysplasia 2) marker for resident macrophages, which is absent in monocytes (11–14) (eFluor660, anti-human, 1:100, and eBioscience^TM^). After an incubation step of 20 min at 4#x00B0;C cells were re-suspended in FACS buffer and processed for sorting on the MoFlo Asrios EQ Cytometer (Beckman Coulter). Sorted cells were stored in RNAprotect Cell Reagent (QIAGEN) at 2–8#x00B0;C until sequencing was performed.

### Total RNA Extraction

RNA extraction, library preparation and RNA sequencing were performed at the Genomics Core Facility “KFB – Center of Excellence for Fluorescent Bioanalytics” (University of Regensburg, Regensburg, Germany; www.kfb-regensburg.de). In brief, total RNA was extracted from isolated hyalocytes and stabilized in RNAprotect Cell Reagent according to the RNeasy Plus Micro Kit protocol (QIAGEN). After pelleting, the RNAprotect buffer was removed and replaced by RLT Plus buffer and the samples homogenized by vortexing for 30 s. Genomic DNA contamination was eliminated using gDNA Eliminator spin columns. Next, ethanol was added and the samples were applied to RNeasy MinElute spin columns followed by several wash steps. Finally, total RNA was eluted in 12 μl of nuclease-free water.

### RNA-Seq Libraries

In total 13 samples were analyzed using RNA sequencing. The SMARTer Ultra Low Input RNA Kit for Sequencing v4 (Clontech Laboratories, Inc.) was used to generate first strand cDNA from 750 pg total-RNA. Double-stranded cDNA was amplified by LD PCR (12 cycles) and purified via magnetic bead clean-up. Library preparation was carried out as described in the Illumina Nextera XT Sample Preparation Guide (Illumina, Inc.). 150 pg of input cDNA were tagmented (tagged and fragmented) by the Nextera XT transposome. The products were purified and amplified via a limited-cycle PCR program to generate multiplexed sequencing libraries. For the PCR step 1:5 dilutions of index 1 (i7) and index 2 (i5) primers were used. The libraries were quantified using the KAPA SYBR FAST ABI Prism Library Quantification Kit (Kapa Biosystems, Inc.). Equimolar amounts of each library were pooled, and the pools then used for cluster generation on the cBot with the Illumina TruSeq SR Cluster Kit v3. The sequencing run was performed on a HiSeq 1000 instrument using the indexed, 50 cycles single-read (SR) protocol and TruSeq SBS v3 Reagents according to the Illumina HiSeq 1000 System User Guide. Image analysis and base calling resulted in bcl files, which were converted into fastq files with the bcl2fastq v2.18 software. The sequence data are available at the Gene Expression Omnibus database under accession number GSE147657. The password is available from the corresponding author upon request.

### Bioinformatics

Sequencing data was analyzed on the Galaxy web platform (usegalaxy.eu) (15). Quality control was performed with *FastQC Galaxy Version 0.72* (16). Sequencing adapter were trimmed using *Trim Galore! Galaxy Version 0.4.3.1*^1^, followed by quality control with *FastQC.* Reads were mapped to the human reference genome (Gencode, release 31, https://www.gencodegenes.org/human/releases.html) with *RNA STAR Galaxy Version 2.6.0b-2* (default parameters) (17) using the Gencode annotation file (Gencode, Release 31). Samples were then checked for contamination with rRNA using *SortMeRNA Galaxy Version 2.1b.6* (18) as well as with non-human reads (bacteria, virus, funghi, arachea, and protozoa) using *RNA STAR Galaxy Version 2.6.0b-2* (17). Reference genomes were downloaded from NCBI FTP server (ftp://ftp.ncbi.nlm.nih.gov/genomes/refseq/, Download between 6th and 13th July 2019).

Three BAM files for each sample (one for each lane) were combined in one BAM file per sample using *Convert, Merge, Randomize BAM datasets Galaxy Version 2.4.0* (19). Reads mapped to the human reference genome were counted using *featureCounts Galaxy Version 1.6.4* (default parameters) (20). Samples with less than 10 million assigned reads were excluded from further analysis. Next, we compared the human hyalocytes’ transcriptome to the transcriptional profile of human brain microglia [raw data from (21), GEO accession: GSE99074, samples with origin Netherlands], human macrophages and monocytes [raw data from (22), GEO accession: GSE58310]. For analysis of the similarity between the four cell populations the expression of each gene in each group was calculated as a percentile. The similarity was defined as 1 min Δ-percentile. The Pearson coefficient *R*^2^ was used to quantify the deviation from the diagonal with the incline 1, i.e., the bigger R^2^ the stronger the similarity. Differential gene expression was analyzed using *DESeq2 Galaxy Version 2.11.40.6* (default parameters) (23). Transcripts per million (TPM) were calculated based on the output of *featureCounts* (assigned reads and feature length), as described previously (24). The output of *featureCounts* or DESeq2 was imported to RStudio (Version 1.2.1335, R Version 3.5.3). Genesymbols and genetypes were determined based on ENSEMBL (download: 22.09.2019) (25). Genes without a hit in the ENTREZ Gene (NCBI) search were removed from analysis. Data visualization with volcano plots and MA plots was performed using the *ggplot2* package (26). Heatmaps were created with the R package *ComplexHeatmap 1.20.0* (27). Gene enrichment analysis was performed using the R package *clusterProfiler 3.10.1* (28). Gene ontology (GO) analysis for clusters related to biological processes (BP) was performed based on the 100 highest expressed genes using the R function enrichGO of the clusterProfiler package with default parameters. Genes associated with the 5 most disease relevant BP were illustrated using the R function cnetplot of the clusterProfiler package with default parameters.

### Immunohistochemistry

For immunohistochemical analysis, three randomly chosen and macroscopically normal eyes from three body donors were analyzed at the Institute of Anatomy at Leipzig University. Eyes were enucleated in accordance with the consent of the body donors, secured by contract during lifetime, and no data other than age, sex, body weight, and cause of death were disclosed. Following formalin fixation and paraffin embedding (FFPE), human ocular donor samples were cut in 7 micrometer sections and deparaffinized according to a standard protocol. Sections were blocked with 2% bovine serum albumin (BSA) and 2% normal goat serum (NGS) or normal donkey serum (NDS) in PBS Triton-X 0.1% for 60 min at room temperature. Primary antibodies against FTL (1:800, Sigma-Aldrich), CD74 (1:1000, abcam), IBA1 (1:500, abcam), and HLA-DP, DQ, DR (1:200, DAKO, clone CR3/43) were incubated in PBS containing 2% BSA and 2% NGS (CD74 and FTL), or NDS (IBA1, HLA-DP, DQ, and DR) in PBS Triton-X 0.1% overnight at 4#x00B0;C. The primary antibody was omitted for negative control. Following extensive washing with 2% BSA and 0.2% NGS/NDS in PBS Triton-X 0.1%, sections were incubated with an Alexa Fluor^®^ 568-labeled donkey anti-mouse, Alexa Fluor^®^ 647-labeled donkey anti-goat or Alexa Fluor^®^ 647-labeled goat anti-rabbit secondary antibody 1:500 in PBS Triton-X 0.1% (Thermo Fisher Scientific) at room temperature for 90 min in the dark. After washing at least three times with 2% BSA and 0.2% NGS/NDS in PBS Triton-X 0.1%, slides were counterstained with 4′,6-Diamidin-2-phenylindol (DAPI) 1:10000 for 10 min, washed three times with PBS followed by autofluorescence quenching with TrueBlack^®^ Lipofuscin Autofluorescence Quencher (Biotium) according to the manufacturers’ instructions. Slides were imaged using a confocal Fluoview FV1000 (Olympus) equipped with a 0.75 NA U Plan S Apo 20x and 40x 0.95 NA U Plan S Apo 40X2 (Olympus).

## Results

### Patients

A total of 13 patients undergoing vitrectomy for macular pucker (MP, *n* = 7, mean age 73 ± 3.7 years) or macular hole (MH, *n* = 6, mean age 73 ± 9.7 years, *p* = 0.833) were included in the study (Table 1). All patients revealed idiopathic ERMs or full-thickness macular holes on SD-OCT (Figure 1A). Posterior vitreous detachment (PVD) was present in 3 out of 13 patients prior to surgery and had to be induced in the remaining 10 patients intraoperatively.

### FACS-Based Characterization and Isolation of Human Hyalocytes

Fluorescence-activated cell sorting characterization revealed that hyalocytes express the pan-leukocyte marker CD45 and characteristic myeloid cell markers including CD11b and CX_3_CR1 beside the macrophage-specific marker MatMac (Figure 1C). Based on the observed staining pattern, hyalocytes were defined as CD45^+^CD11b^+^CX3CR1^+^MatMac^+^ cells and isolated by flow cytometry (Figures 1B,C). The cell number of isolated hyalocytes ranged from 180 to 944 per vitrectomy collection bag in patients with macular pucker (average 438.9) and 164 to 550 cells in patients with macular hole (average 317.2, *p* = 0.351; Table 1). The mean concentration of extracted RNA of the isolated hyalocytes was similar in patients with macular pucker (120.6 pg/μL, 26–240 pg/μL) and macular hole (135.7 pg/μL, 73–301 pg/μL, and *p* = 0.744; Table 1).

### Transcriptional Profiling of Human Hyalocytes

Following RNA-Seq a mean total number of 36.8 million raw reads (25.2–47.7) per sample was obtained from isolated CD45^+^CD11b^+^CX_3_CR1^+^MatMac^+^ cells. Four samples [from three patients with MP (patients #3, 9, and 10) and one patient with MH (patient #8)] were excluded due to a low number of assigned reads. Hierarchical clustering of expressed genes from all hyalocytes revealed no strong patterns that were related to diagnosis, sex or age (Supplementary Figures S1A,B). The expression profile of vitreal hyalocytes in patients with macular pucker or macular hole (Supplementary Figure S1A) was very similar (Supplementary Figure S1B) and only a negligible number of transcripts were differentially expressed between these two groups (Supplementary Figures S1C,D). The comparable transcriptional profile of hyalocytes derived from eyes with macular pucker and macular hole (Supplementary Figures S1B–D) implies no or at least a similar affection of hyalocytes due to the underlying vitreoretinal interface pathology. This prompted us to process the samples together for further analysis.

**FIGURE 2:**
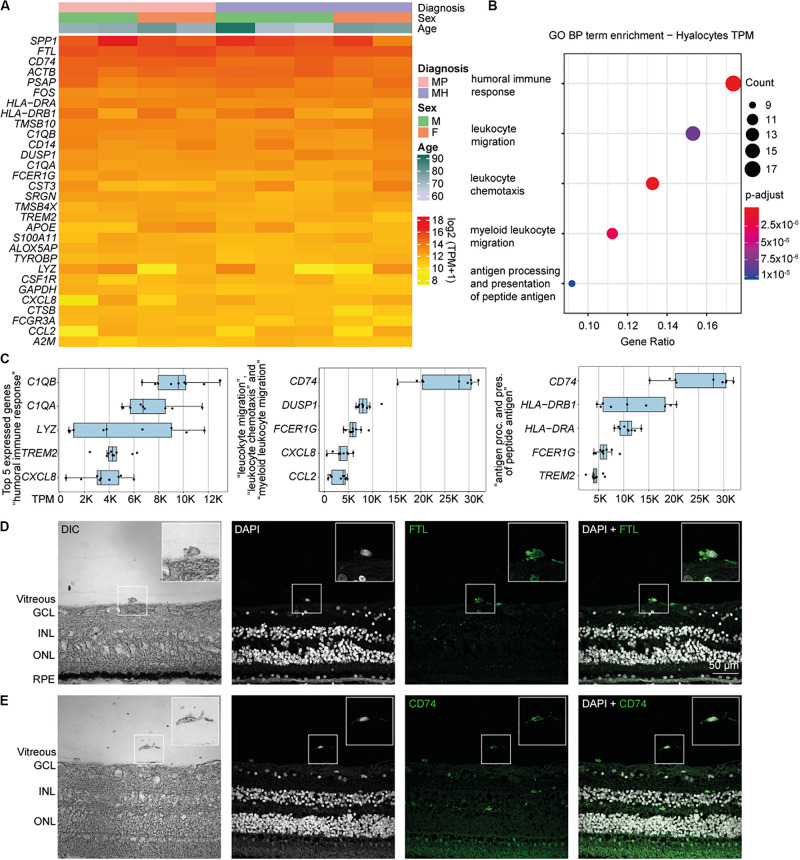
High throughput sequencing characterization of human hyalocytes. **(A)** Heatmap of the most prominent transcripts in human hyalocytes sorted by transcripts per million (TPM). The color bars on the top of the heatmap reveal diagnosis of study subjects (pink for macular pucker, lilac for macular hole), sex (grass-green for male, orange for female), and age (age scale bar legend to the right). **(B)** Most significant Gene ontology biological process (BP) clusters of the 100 most highly expressed mRNA transcripts in hyalocytes. Color coding of the dots according to the *p*-adjusted value, size of the dots according to the count of transcripts associated with the respective GO term. **(C)** Top five most highly expressed transcripts in the five enriched GO terms in **B**. **(D)** Immunohistochemical staining for FTL. Higher magnification of a vitreal cell is presented in the upper right corner. Nuclei are counterstained with DAPI (4’,6-Diamidin-2-phenylindol). DIC, differential interference contrast; GCL, ganglion cell layer; INL, inner nuclear layer; ONL, outer nuclear layer; and RPE, retinal pigment epithelium. **(E)** Immunohistochemical staining for CD74. Higher magnification of a vitreal cell is presented in the upper right corner. Nuclei are counterstained with DAPI (4’,6-Diamidin-2-phenylindol). DIC, differential interference contrast; GCL, ganglion cell layer; INL, inner nuclear layer; and ONL, outer nuclear layer.

Relative expression values for the 30 most strongly represented transcripts common to all samples demonstrated that human hyalocytes express high levels of numerous genes associated with antigen presentation, including *CD74*, *HLA-DRA*, and *HLA-DRB1*, regulation of myeloid cell migration and chemotaxis, such as *TREM2*, *CXCL8*, *CCL2*, and humoral immune response, e.g., *C1QA*, *C1QB*, and *LYZ* (Figures 2A–C and Supplementary Figure S3A). Other genes are associated with a myeloid gene expression signature including *TYROBP* encoding the adaptor molecule DAP12 associated with both TREM2 and the colony-stimulating factor 1 receptor (CSF1R) essential for mediating macrophage survival pathways (Figure 2A). According to mean TPM in all samples, *SPP1* (secreted phosphoprotein 1) is the most prominent transcript in human hyalocytes (44917.0 TPM), followed by *FTL* (ferritin light chain, 37850.2 TPM), *CD74* (Cluster of differentiation 74, 25094.1 TPM), *ACTB* (beta actin, 23141.3 TPM), *PSAP* (prostatic specific acid phosphatase, 17229.7 TPM), and *FOS* (Fos proto-oncogene, 11907.0 TPM; Figure 2A). Furthermore, human hyalocytes express numerous common leukocyte-associated genes, such as *PTPRC* (CD45), *ITGAL* (CD11a), and *ITGB2* (CD18), or preferentially in myeloid cells, e.g., *ITGAM* (CD11b), CD14, and *FCGR1A* (CD64) supporting the notion of a myeloid-cell derived origin (Supplementary Figure S2). In addition, a plethora of factors involved in vitreous metabolism were found to be strongly expressed in hyalocytes in our analysis (Supplementary Figure S3). For a full list of genes expressed in hyalocytes refer to Supplementary Table S1.

### Biological Pathways and Molecular Functions of Human Hyalocytes

To gain more insight into the biological pathways and molecular functions (MF) hyalocytes are involved in, we performed a GO cluster analysis of the top 100 most highly expressed genes in hyalocytes. GO enrichment analysis of BP showed that hyalocytes express genes that are involved in “humoral immune response” (GO:0006959), “leukocyte chemotaxis and migration” (GO:0030595 and GO:0050900), “myeloid leukocyte migration” (GO:0097529), and “antigen processing and presentation of peptide antigen” (GO:0048002; adjusted *p* < 0.00, Figure 2B, and Supplementary Figure S4A). *CD74* was the most highly expressed factor in two of the most enriched clusters (Figure 2C). When sorted according to the lowest adjusted *p* value, the GO enrichment analysis revealed MF such as “amide and peptide binding” (GO:0033218 and GO:0042277), “amyloid-beta binding” (GO:0001540), “MHC class II protein complex binding” (GO:0023026), and “MHC protein complex binding” (GO:0023023) to be the most enriched in hyalocytes (Supplementary Figure S4B and Supplementary Table S3). All top expressed genes enriched in the 5 most relevant GO BP and MF terms are illustrated in the cnetplots in Supplementary Figure S4 and summarized in Supplementary Tables S2, S3.

### CD74 and FTL Expression in Human Hyalocytes

Our RNA-Seq analysis revealed that both *FTL* and *CD74* are strongly expressed in human hyalocytes. To validate these findings on a protein level we assessed FTL and CD74 localization by immunofluorescence microscopy in human body donor eyes. In healthy eyes, hyalocytes could be clearly identified in the vitreous by differential interference contrast (DIC) and we observed a strong FTL and CD74 immunofluorescent signal in these cells (Figures 2D,E, negative controls omitting the primary antibody are provided in Supplementary Figure S5A). More hyalocytes positive for CD74 are shown in Supplementary Figure S6A.

### Transcriptome Comparison of Hyalocytes and Other Myeloid Cell Types

**FIGURE 3:**
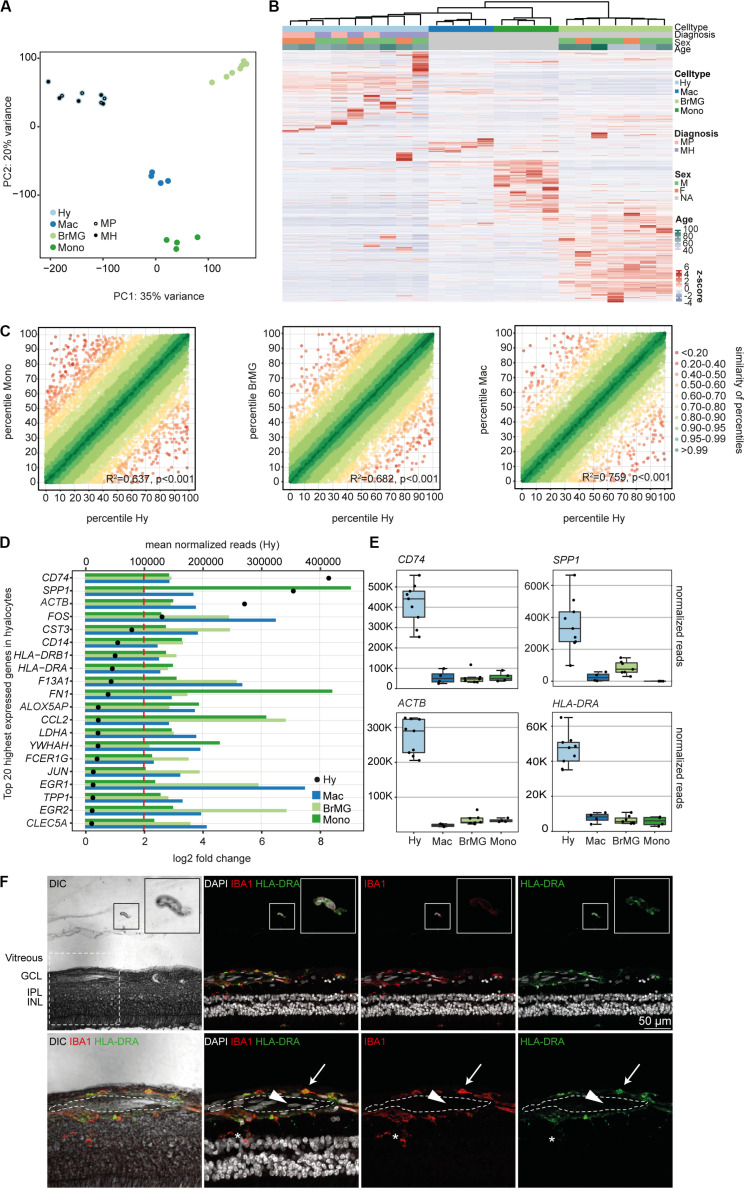
The transcriptional profile of human hyalocytes differs significantly from other myeloid cell populations. **(A)** Principal component analysis (PCA) plot illustrating the distribution of the four analyzed populations. Hy, hyalocytes; Mac, macrophages; BrMG, brain microglia; Mono, monocytes; MP, macular pucker; and MH, macular hole. **(B)** Unsupervised heatmap showing the transcriptional profile of hyalocytes, macrophages, brain microglia, and monocytes (all genes of all data sets). Rows and columns are clustered according to similarity of the expression. The color bars on the top of the heatmap reveal cell type (hyalocytes in light blue, macrophages in dark blue, brain microglia in light green, and monocytes in dark green), diagnosis of study subjects (pink for macular pucker, lilac for macular hole), sex (grass-green for male, orange for female), and age (age bar on the right side). Color coding of the transcripts according to the *z*-score (deviation from a gene’s mean expression in standard deviation units). Hy, hyalocytes; Mac, macrophages; BrMG, brain microglia; Mono, monocytes; MP, macular pucker; MH, macular hole; and NA, not applicable. **(C)** Graphical presentation of the percentiles of normalized counts to illustrate the similarity between the transcriptomes of monocytes (Mono) and hyalocytes (Hy), brain microglia (BrMG), and hyalocytes or macrophages (Mac) and hyalocytes. The expression of each gene in each cell population was calculated as a percentile (the gene with the highest mean of normalized counts getting the percentile 100, the one with the lowest – the percentile 0). Colors code for the similarity defined as 1 min Δ percentile. The Pearson coefficient *R*^2^ quantifies the deviation from the diagonal with the incline 1. The higher *R*^2^, the higher the similarity between the two compared cell populations. **(D)** Top 20 most highly expressed genes in human hyalocytes (Hy). Transcripts are sorted according to their expression level in hyalocytes (mean normalized reads counts, black dot, and upper *x*-axis). The color bars code for the log2 fold change (lower *x*-axis) of the upregulated transcripts in hyalocytes in comparison to monocytes (dark green), brain microglia (light green), and macrophages (blue). The dashed red line distinguishes the log2FC of 2, according to which factors are defined as differentially upregulated. **(E)** Box plots, illustrating the normalized reads count of *CD74*, *SPP1, ACTB*, and *HLA-DRA* in hyalocytes (Hy), macrophages (Mac), brain microglia (BrMG), and monocytes (Mono). **(F)** Immunohistochemical staining for HLA-DRA and IBA1. A vitreal cell is presented in higher magnification in the upper right corner. Higher magnification of the section within the dashed white square in the lower panel. The lumen of an intraretinal vessel is traced with the white dashed line. The arrow points at a perivascular macrophage, the arrow head to an intraluminal leukocyte, the asterisk is positioned between two microglial cells (positive for IBA1). Nuclei are counterstained with DAPI. DIC, differential interference contrast; GCL, ganglion cell layer; IPL, internal plexiform layer; and INL, inner nuclear layer.

Immunohistochemical studies (29) and our sequencing data (Supplementary Figure S2) indicate that vitreal hyalocytes belong to the myeloid cell lineage. To determine potential similarities and differences between hyalocytes and other myeloid cell populations, we conducted a detailed comparison of the hyalocyte transcriptome with published RNA sequencing profiles of human brain microglia, human monocytes and monocyte-derived macrophages obtained from public databases. A first assessment of the data in a principal component analysis (PCA) showed that the transcriptional profile of hyalocytes is distinct from the one of microglia, macrophages, and monocytes (Figure 3A). Hierarchical clustering analysis of all expressed genes in the four cell types is illustrated in Figure 3B, showing considerable cell-dependent gene expression patterns. However, no obvious difference was found in the expression profiles of hyalocytes in patients with macular pucker and macular hole (Figures 3A,B and Supplementary Figure S1). To analyze the level of similarity between the four groups, we correlated the transcriptional profile of hyalocytes with the profiles of the other groups. We found a robust correlation between the transcriptional profiles of hyalocytes and blood-derived monocytes (*R*^2^ = 0.63, *p* < 0.001), brain microglia (*R*^2^ = 0.68, *p* < 0.001) and, most pronounced, macrophages (*R*^2^ = 0.75, *p* < 0.001, Figure 3C), indicating a general similarity in the patterns of gene expression and thus close relationships. However, when focusing on distinct myeloid cell markers, we found considerable differences, indicating tissue- and cell-specific immunogenic properties of the myeloid cell subsets. While *CX3CR1* or *FCGR1A* (CD64) were highly and similarly expressed in hyalocytes and brain microglia, they were barely detectable in macrophages and monocytes (Supplementary Figure S2). Expression of *HLA-DRA* (MHCII) molecules and *CD14*, in contrast, was remarkably increased in hyalocytes compared to a low expression in microglia, macrophages or monocytes (Supplementary Figure S2). On the other hand, *CD11b* expression was significantly reduced in hyalocytes compared to microglia, macrophages or monocytes (Supplementary Figure S2). Interestingly, *SALL1* expression was, as expected, high in microglia but almost not detectable in hyalocytes, macrophages, or monocytes (Supplementary Figure S2). Furthermore, we found 91 collectively upregulated genes in hyalocytes compared with other myeloid cell subsets. Among these factors, *CD74*, *SPP1*, *ACTB*, *FOS*, and MHCII-related genes such as *HLA-DRA* emerged as the transcripts with the highest log2 fold change in comparison to the other cell types (Figures 3D,E). Immunohistochemical staining for HLA-DRA and IBA1 (ionized calcium-binding adapter molecule 1) revealed that hyalocytes express HLA-DRA on the protein level, while microglia cells were at most slightly positive for HLA-DRA, but positive for IBA1 supporting our sequencing data (Figure 3F and Supplementary Figure S6B). Negative controls omitting the primary antibodies are provided in Supplementary Figure S5B. These data indicate that hyalocytes express numerous myeloid cell markers underscoring their common myeloid cell identity, but at the same time are characterized by a distinct immune cell profile specialized and adapted for the tissue-specific environment.

### Immune Privilege Factors Expression in Hyalocytes

**FIGURE 4:**
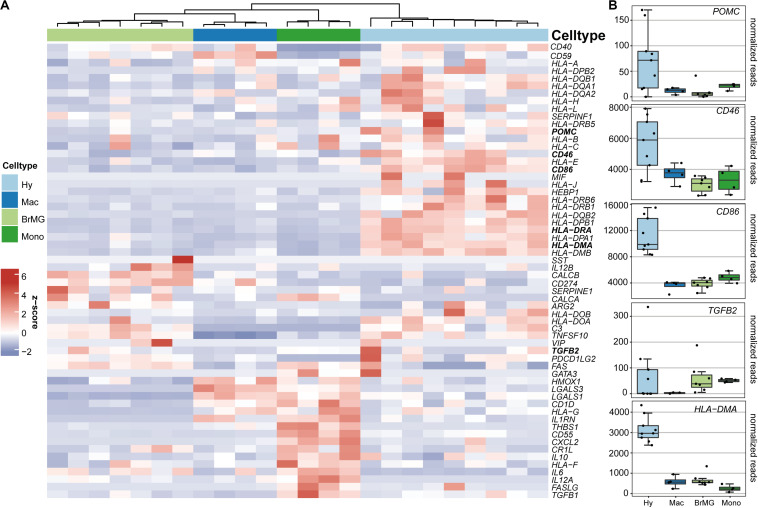
Immunomodulatory transcripts in human hyalocytes and other myeloid cells. **(A)** Heatmap representing the expression pattern of 40 established immune privilege factors in hyalocytes (Hy, light blue), macrophages (Mac, dark blue), brain microglia (BrMG, light green), and blood-derived monocytes (Mono, dark green). **(B)** Boxplots illustrating the expression of *POMC*, *CD46*, *CD86*, *TGFB2*, and HLA*-DMA* in hyalocytes (Hy, light blue box), macrophages (Mac, dark blue box), brain microglia (BrMG, light green box), and blood-derived monocytes (Mono, dark green box).

To determine if hyalocytes contribute to the expression of factors that have been associated with the immune privilege of the eye, we next explored the expression pattern of 60 established immune privilege factors (30–36) in hyalocytes, macrophages, brain microglia, and blood-derived monocytes (Figure 4A). In contrast to the other myeloid cell subsets, hyalocytes expressed immunosuppressive factors, such as *POMC*, *CD46*, and *CD86* indicating that hyalocytes contribute to the immunosuppressive environment in the eye. *TGFB2*, a well-known immune privilege factor in the eye (36), was expressed in hyalocytes to a similar extent as in microglia and more abundantly compared to macrophages. Interestingly, hyalocytes abundantly expressed MHCII-related genes such as *HLA-DRA*, *HLA-DMA*, and *HLA-DMB*, supporting the notion that hyalocytes are antigen-presenting cells (APCs; Figure 4B).

## Discussion

In 1874, the so-called “subhyaloidal cells” were already assumed to have macrophage characteristics (37). More than 100 years later, vitreal hyalocytes were characterized as macrophage-like cells with phagocytic properties and receptors for IgG and complement factors on their surface (38). Due to their scarcity vitreal hyalocytes have eluded comprehensive investigation in the past (39). In particular, the transcriptional profile and the exact function of hyalocytes remain unknown to date. In this study, we devised a protocol to isolate hyalocytes from patients with vitreoretinal interface disease, deciphered the hyalocyte transcriptional profile using RNA sequencing and compared it to reported transcriptomes of other myeloid cell subsets including monocytes, macrophages, and microglia. We show that vitreal hyalocytes share common myeloid cell markers but at the same time represent a unique myeloid cell subset with a specialized transcriptional signature, which may contribute to reducing ocular inflammation and immune responses in the eye.

Morphological studies using light and electron microscopy have demonstrated that hyalocytes are morphologically very similar to cells of the monocyte/macrophage lineage (29, 40). The hypothesis that hyalocytes originate from the myeloid cell lineage has been supported by immunohistochemical studies demonstrating CD45, CD64, CD11a, and major histocompatibility complex (MHC) class II expression in this cell type (29). In line with these observations, our transcriptional analysis strongly supports a myeloid cell identity. The GO enrichment analysis revealed BP contributing to “innate immune responses” and “antigen processing and presenting processes” as highly enriched in hyalocytes. In more detail, hyalocytes expressed common leukocyte-associated genes such as *PTPRC* (CD45), *ITGAL* (CD11a), *Cx3CR1*, *FCGR1A* (CD64), *CD163*, and genes encoding for the major histocompatibility complex class II, which supports previously published immunohistochemical studies (29). Interestingly, hyalocytes express very little *CD68*, which is also consistent with earlier reports (29), but remarkable since CD68 is regarded as a selective marker for monocytes and tissue macrophages (41).

Beyond the already known factors involved in hyalocyte biology, our sequencing analysis revealed a plethora of strongly expressed factors which so far were unknown in hyalocytes. These most abundantly expressed genes in hyalocytes were strikingly similar to the top expressed transcripts in brain microglia indicating a close relationship between both populations (42). Among them *SPP1*, *CD74*, and *FTL* emerged as the most prominent factors expressed in human hyalocytes and brain microglia (42). The *SPP1* gene encodes for the secreted phosphorylated glycoprotein Osteopontin (OPN). It is implicated in various immune processes in neurodegenerative disease (43) and has an essential role in the interaction between the innate and adaptive immune system (44, 45). In the human eye *SPP1* is distributed in the aqueous humor (AqH) (46) and the vitreous fluid (47) and reported to mediate photoreceptor survival in experimental models for retinal degeneration (48). Our results demonstrate that human hyalocytes represent a source for *SPP1* expression in the posterior segment of the eye where it may act as a neuroprotective molecule for retinal homeostasis. Ferritin light chain (*FTL*), on the other hand, encodes the light subunit of the ferritin protein (49), the main iron storage in the human body, which has been linked to anti-inflammatory responses in murine macrophages (50) and neurodegeneration in mice (51). The vitreous humor of patients with vitreoretinal diseases has been shown to contain more iron than a healthy vitreous, leading to various complications due to an increased susceptibility to oxidative damage (52). *FTL* in hyalocytes may therefore act in neuroprotective iron reduction of vitreous iron levels. Finally, *CD74* (Cluster of differentiation 74) is a transmembrane glycoprotein, which acts as MHC class II chaperone regulating T- and B-cell development, innate immune cell motility and inflammatory processes (53). CD74 is a high affinity receptor for MIF (macrophage migration inhibitory factor) on APCs and implicated in a number of inflammatory processes in neurodegenerative affections such as Alzheimer’s disease (54). In the eye, increased levels of soluble CD74 and MIF have been detected in the vitreous and in endothelial and stromal cells in neovascular membranes of patients with proliferative diabetic retinopathy suggesting that MIF-CD74 signaling is implicated in disease progression (55). Hyalocytes expressing CD74, as shown in this study, may thus be an additional cellular target for MIF signaling which may induce a pro-inflammatory cell phenotype. These hypotheses need to be investigated in further *in vivo* studies to determine the exact function of these factors in hyalocytes and to define hyalocyte-specific markers that will enable future tracing of hyalocytes in neurodegenerative eye disease.

Since hyalocytes belong to the innate immune system and have been reported to be potential candidates for inhibiting intraocular inflammation to maintain vitreous transparency, it is tempting to speculate about their role in the ocular immune privilege. Our study demonstrates that hyalocytes express a variety of factors which are known to play a key role in the immune privilege of the eye, including α-melanocyte-stimulating hormone (*α-MSH*), *CD86*, *CD46*, and *TGFβ2*, just to name a few. In the ocular microenvironment, and in the vitreous body in particular, hyalocytes or any other potential APCs are exposed to a number of immune-modulating molecules that are either stimulatory or suppressive. Animal studies suggest that TGFβ2 (56, 57) and α-MSH (58, 59) are the dominant immunosuppressive factors which suppress the generation of Th1-responses and the inflammatory activity of innate immune cells, e.g., by inhibiting their production of IL-12 (57). The human AqH is rich in TGF-β2, α-MSH and vasoactive intestinal peptide (60) and it has been postulated these factors mediate the ocular immune privilege by inducing regulatory T-cells (61, 62). Our study demonstrates that vitreal hyalocytes strongly transcribe *TGFβ2* and *α-MSH* and much less *IL-12*, which suggests that hyalocytes are a source of immune-suppressive factors and contribute to the ocular immune privilege.

Furthermore, the ocular immune privilege is achieved in part by molecules that typically have a role in immune co-stimulation. For example iris and ciliary body pigmented epithelial cells express cell surface CD86, thus preventing T-cells from proliferating and secreting cytokines through direct cell-to-cell contact (63, 64). Conversely, myeloid dendritic cells isolated from uveitic AqH were characterized by elevated major histocompatibility complex classes I and II (MHC I/II), but reduced CD86 compared to matched peripheral blood dendritic cells (65). *CD86* expression in hyalocytes may thus serve as an additional protective immunosuppressive mechanism to reduce T-cell activation in the steady state to prevent leakage of cells and proteins into the optically clear vitreous. Future studies are needed to investigate how the above-mentioned immunosuppressive properties can be brought together with the high expression of antigen-presenting molecules in hyalocytes, such as MHCII, which are classically considered pro-inflammatory. One explanation may be that hyalocytes capture antigens in the vitreous cavity and present them to natural killer T cells in the spleen, thus inhibiting intraocular inflammation, as described in preclinical models for VCAID (2, 5).

Last but not least, human tissue and organs are protected from the autologous complement system through the activity of membrane regulatory proteins, such as the membrane cofactor protein (MCP, CD46) (66). *CD46* is expressed ubiquitously in humans and functions as a key regulator of the alternative complement pathway and a co-factor in the factor I-mediated proteolytic cleavage of C3b and C4b. Also, it is responsible for the suppression of adaptive T helper type 1 (Th1) immune responses by regulating the production of interferon (IFN)-γ *versus* interleukin (IL)-10 within these cells. Sohn and colleagues (66) have shown that the soluble form of CD46 is present in normal human vitreous. They hypothesize that the expression of CD46 in retinal cells is polarized toward the outer limiting membrane end of Müller cells. In this study we provide an alternative explanation, and reveal vitreal hyalocytes as a possible source of CD46 production which may mediate a suppression of the alternative complement factor, thereby contributing to an immune privileged microenvironment.

Besides their function in modulation of ocular inflammation, hyalocytes are involved in ECM synthesis (2). In line with this assumption, we found a strong expression of genes involved in the formation of proteoglycans, such as versican (*VCAN*), and several collagen types, such as *COL5A1* and *COL9A2*, in our analysis. Furthermore, Fibronectin, which is suggested to play a role in attaching the vitreous to the ILM (67), was among the most strongly represented ECM factors in our analysis. Although hyalocytes are located in the region with the highest hyaluronan concentration in the vitreous (68), our analysis showed that hyalocytes are not actively engaged in hyaluronan synthesis. Interestingly, enzymes, important for hyaluronan degradation, such as hyaluronidases (e.g., *HYAL2*), were found to be strongly expressed in our samples, which is in line with previous reports (69).

We acknowledge that our study has some limitations, which include the fact that hyalocytes were obtained from patients of advanced age with vitreoretinal disease, namely macular hole or macular pucker. However, since both conditions are disease of the posterior pole and represent a frequent consequence of PVD, we assume that hyalocytes in these conditions represent the most physiological state clinicians can access. Furthermore, hyalocytes of macular pucker and macular hole vitreous bodies are similar on a transcriptional level indicating that the disease itself does not affect hyalocyte RNA expression patterns or yet affects them in a similar way. Since mRNA degradation occurs within a few hours (70, 71), we did not attempt to analyze hyalocytes from post mortem eyes, in which the typical death to preservation time is in a range of more than 24 h (72).

In summary, this study reports on the first transcriptional profile of human hyalocytes obtained from patients with vitreoretinal interface disease. Vitreal hyalocytes share common myeloid cell markers, but represent a distinct myeloid cell subset with a unique transcriptional profile. Our data suggest that hyalocytes contribute to the ocular immune privilege and modulate vitreoretinal disease, thus deserving further scientific scrutiny.

## Data Availability Statement

The sequence data have been submitted to the Gene Expression Omnibus database under accession number GSE147657. The password is available from the corresponding author upon request.

## Ethics Statement

The studies involving human participants were reviewed and approved by Ethikkommission, Albert-Ludwigs-Universität Freiburg. The patients/participants provided their written informed consent to participate in this study.

## Author Contributions

SB, JW, and ASc conducted experiments and analyzed data. GP, YL, D-DR, MB, and PZ performed experiments. ASt, HA, and CL performed surgical procedures. IH, ASt, TR, JB, GS, HA, PW, and CL contributed to design of the study and interpretation of the data. All authors approved the final version of the manuscript.

## Conflict of Interest

The authors declare that the research was conducted in the absence of any commercial or financial relationships that could be construed as a potential conflict of interest.

## Footnotes

1 https://www.bioinformatics.babraham.ac.uk/projects/trim_galore/

## Supplementary Material

FIGURE S1 Transcriptional analysis does not reveal significant differences of hyalocytes from macular pucker and macular hole eyes. **(A)** 13 patients undergoing vitrectomy for macular pucker (right-hand side panel) or macular hole (left-hand side panel) were included in this study. **(B)** Heatmap depicting the transcriptional profile of the 13 samples. The color bars on the top of the heatmap reveal diagnosis of study subjects (pink for macular pucker, lilac for macular hole), sex (grass-green for male, orange for female), and age (age bar on the right side). Color coding according to the *z*-score (deviation from a gene’s mean expression in standard deviation units). The transcripts are sorted according to the normalized counts (log2-scaled). **(C)** Volcano plot illustrating the genes differentially expressed between hyalocytes in macula pucker and macular hole patients [log2FC < -2 and > 2 (positive log2FC referring to macular hole samples), *p*-value < 0.05]. The darker colored genes are filtered for StdErr ≤ 1. **(D)** Graphical presentation of the percentiles of normalized counts to illustrate the similarity between the transcriptomes of maclar pucker (MP) and macular hole (MH) eyes. The expression of each gene in each cell population was calculated as a percentile (the gene with the highest mean of normalized counts getting the percentile 100, the one with the lowest – the percentile 0). Colors code for the similarity defined as 1 min Δ percentile. The Pearson coefficient *R*^2^ quantifies the deviation from the diagonal with the incline 1.

FIGURE S2 Hyalocytes express numerous leucocyte-associated transcripts. Box plots, illustrating the normalized reads count *PTPRC* (CD45), *ITGAL* (CD11a), *CX3CR1*, *HLA-DRA*, *SALL1*, *ITGB2* (CD18), *HEXB*, *CD14*, *CD68*, *FCGR1A* (CD64), and *ITGAM* (CD11b) in hyalocytes (Hy), macrophages (Mac), brain microglia (BrMG), and monocytes (Mono).

FIGURE S3 Hyalocytes express numerous factors involved in vitreous homeostasis. Heatmap depicting the expression of genes coding for extracellular matrix proteins in hyalocytes (Hy) and microglia (BrMG). The transcripts are sorted according to the normalized counts (log2-scaled). Significance level for the comparison is shown in the bar on the right side, red coding for a significantly higher expression in human hyalocytes.

FIGURE S4 Biological processes and molecular functions of human hyalocytes. All top expressed genes enriched in the 5 most disease-relevant GO biological processes (BP, **A**) and molecular functions (MF, **B**) terms are illustrated in cnetplots.

FIGURE S5 Negative controls for immunohistochemistry. Negative controls were performed by omitting primary antibodies. **(A)** Negative control for FTL and CD74 immunohistochemical stainings shown in Figures 2D,E. Nuclei are counterstained with DAPI. GAR647, goat anti-rabbit Alexa Fluor 647. **(B)** Negative control for HLA-DRA and IBA1 immunohistochemical staining shown in Figure 3F. Nuclei are counterstained with DAPI. DAM568, donkey anti-mouse Alexa Fluor 568. DAG647, donkey anti-goat Alexa Fluor 647.

FIGURE S6 Immunohistochemical stainings for CD74 **(A)** and HLA-DRA **(B)**. The arrows point at vitreal cells positive for CD74 (first and second row in **A**) or HLA-DRA **(B)**. Vitreal cells are presented in higher magnification in the upper right corner (third and fourth row in **A**). Nuclei are counterstained with DAPI. DIC, differential interference contrast; GCL, ganglion cell layer; INL, inner nuclear layer; and ONL, outer nuclear layer.
