# Supplementary material for: Transcriptional Profiling Uncovers Human Hyalocytes as a Unique Innate Immune Cell Population
Source: Front Immunol. 2020 Sep 11;11:567274. doi: 10.3389/fimmu.2020.567274 (PMC7517040; doi:10.3389/fimmu.2020.567274)
Supplement: Supplementary file 1 [file Data_Sheet_1.PDF]

ENSEMBL	Symbol	S1	S2	S3	S4	S5	S6	S7	S8	S9	sum	mean
ENSG00000118785	SPP1	33961,20555	76834,29365	63701,51706	56019,73734	46814,16672	45173,56854	32687,58928	12496,14982	36564,52256	404252,7505	44916,97228
ENSG00000087086	FTL	39593,74537	33996,46506	32093,45669	34040,18074	39157,83048	43869,15656	49183,79393	34414,51361	34302,87256	340652,015	37850,22389
ENSG00000019582	CD74	27932,88383	30430,75121	20535,69258	19188,98335	20339,12538	29753,45873	31949,97008	15199,50621	30516,15448	225846,5258	25094,05843
ENSG00000075624	ACTB	26790,35898	17495,38165	24343,82414	20462,7149	23010,45916	26206,5308	20966,30202	17437,51524	31558,51463	208271,6015	23141,28906
ENSG00000197746	PSAP	25202,91185	11322,08364	21058,49615	11455,44632	21175,88559	14190,63494	16493,58466	20962,95962	13205,53473	155067,5375	17229,72639
ENSG00000170345	FOS	9505,1205	8294,307047	14519,79227	13284,26016	11208,5026	9769,195944	11426,26729	15150,62198	14005,16292	107163,2307	11907,02564
ENSG00000196126	HLA-DRB1	18256,34552	5395,566853	14477,35736	4641,415362	20531,64696	19370,63356	7437,460664	10693,28203	5842,748517	106646,4568	11849,60631
ENSG00000204287	HLA-DRA	10824,32713	13509,91651	8906,982914	10966,82011	10121,11016	11588,00022	12234,26511	8121,146678	9230,327475	95502,89631	10611,43292
ENSG00000034510	TMSB10	10946,50219	11634,80353	7290,193919	11829,24086	7335,813224	10689,89557	9962,507663	13766,4345	10014,74904	93470,14049	10385,57117
ENSG00000170458	CD14	12200,5281	7196,391093	7816,166157	6288,881223	13858,92996	8361,772257	13138,75372	10114,94698	7020,442596	85996,81209	9555,201344
ENSG00000173369	C1QB	11673,39286	9597,578573	7714,693002	6654,891189	10186,95157	8998,313584	10071,76929	12965,5703	7955,277474	85818,43785	9535,381983
ENSG00000120129	DUSP1	8031,905921	7235,438232	7853,593735	9426,972691	7011,622835	6570,981937	7977,744031	8859,192056	11885,3144	74852,76584	8316,973982
ENSG00000173372	C1QA	8541,091493	5697,138348	6874,766841	5034,547842	9102,157905	6541,364467	6726,855308	11524,04097	5131,649013	65173,61218	7241,512465
ENSG00000101439	CST3	8746,68948	3362,493264	5470,248487	4223,826254	10154,83082	2984,829949	3447,015367	13513,08279	3504,556453	55407,57286	6156,396985
ENSG00000158869	FCER1G	5688,435165	7613,11882	4011,679603	6583,465199	4431,227351	5922,353584	5917,800399	9225,088709	5316,675717	54709,84455	6078,871616
ENSG00000122862	SRGN	6373,588088	6655,738323	5021,914919	5673,030808	3379,895095	5524,399589	5907,724704	6520,448758	3867,65396	48924,39424	5436,043805
ENSG00000090382	LYZ	6687,07107	10254,15138	11695,49263	1128,351466	3538,855011	789,4860436	3789,218252	9030,941573	737,5915547	47651,15899	5294,573221
ENSG00000130203	APOE	2529,944526	2439,793435	4223,774679	3173,49746	7884,580693	7648,446776	9892,234755	3166,434498	2996,667322	43955,37415	4883,930461
ENSG00000205542	TMSB4X	3768,53614	5023,214462	4167,905959	5612,476876	3652,11786	5796,445973	4553,548954	4639,860137	4161,30334	41375,4097	4597,267745
ENSG00000095970	TREM2	4270,564128	4154,609291	3941,478554	4375,367186	3817,140708	6252,631752	5875,563666	2438,275165	4590,154516	39715,78497	4412,864996
ENSG00000198502	HLA-DRB5	4696,274759	353,9474401	3799,654377	1005,539018	14733,29994	4458,614474	2347,198595	2095,748201	2577,022216	36067,29902	4007,477669
ENSG00000163191	S100A11	2977,506899	5601,647515	3147,728424	5163,617687	3109,216441	4225,479079	3744,003774	3025,334669	4110,100361	35104,63485	3900,514983
ENSG00000132965	ALOX5AP	4100,60341	4956,952753	2834,424484	3362,33353	3068,09926	4174,561636	4398,485032	3559,830361	3569,987073	34025,27754	3780,586393
ENSG00000011600	TYROBP	3210,974673	3785,029798	2546,567412	3750,616608	3069,326558	3813,565274	4388,003232	4171,053322	3006,943456	31742,08033	3526,897815
ENSG00000169429	CXCL8	501,2497581	5994,931773	4940,57171	3325,726382	3223,557109	1730,90896	4007,82165	4757,136154	3026,086048	31507,98955	3500,887727
ENSG00000108691	CCL2	768,6399722	4732,719803	1535,469127	1224,397295	4353,350872	3420,297498	3897,91971	4786,30977	4102,47308	28821,57713	3202,397459
ENSG00000182578	CSF1R	3838,490122	1936,910802	3001,008605	2041,533763	3491,966675	3140,061389	4042,185928	2353,007171	4337,753802	28182,91826	3131,435362
ENSG00000164733	CTSB	3779,684901	3549,024463	2861,025734	1418,14967	3482,316231	3184,884461	3805,9677	2376,672191	2909,563573	27367,28892	3040,809881
ENSG00000111640	GAPDH	4065,732074	3929,802309	2224,884958	2642,347783	2649,615991	2800,745032	3184,621357	2458,675906	2957,333203	26913,75861	2990,417623
ENSG00000203747	FCGR3A	3334,547461	4250,839354	2962,583712	2607,144413	2115,248459	3286,97009	2940,705604	1533,538115	3094,363444	26125,94065	2902,882295
ENSG00000101474	APMAP	3838,928069	2610,34912	1946,485087	1570,767592	3442,107764	2181,060739	2800,25431	3818,404483	3139,520496	25347,87766	2816,430851
ENSG00000175899	A2M	2677,59694	1803,322154	2938,516131	2597,90523	3851,625646	2721,385658	2524,127392	2470,132968	3235,604255	24820,21637	2757,801819
ENSG00000124491	F13A1	2745,540524	1732,631836	2885,480844	2312,417985	2986,877845	2661,007548	3494,656403	3019,002392	2812,107527	24649,7229	2738,8581
ENSG00000163220	S100A9	1775,087698	1206,872981	8708,102577	1878,913971	3116,112427	1380,271761	1520,752977	4345,820071	613,001618	24544,93608	2727,21512
ENSG00000125730	C3	1410,855238	1795,501757	1915,485826	2499,154224	2636,504041	4989,111137	4712,323971	270,2040592	4185,028026	24414,16828	2712,685364
ENSG00000159189	C1QC	3712,078816	2215,996349	1892,017701	1680,797359	3042,523623	2626,17012	3509,307221	3226,450439	2202,811482	24108,15311	2678,683679
ENSG00000128245	YWHAH	1698,919974	3411,524034	2111,579598	2779,851989	2823,096265	3227,418497	2868,874945	871,9494106	4130,578809	23923,79352	2658,19928
ENSG00000171860	C3AR1	2963,810172	2388,151408	2415,64058	2034,684562	2897,886033	2539,708059	2152,708232	2597,724062	2081,654333	22071,96744	2452,440827
ENSG00000067560	RHOA	2460,174459	2467,915422	2530,086281	2617,184063	2112,870504	2341,411353	1990,045858	2758,824396	2365,11647	21643,62881	2404,847645
ENSG00000162511	LAPTM5	2142,74602	2753,771823	2546,084141	1545,401129	2411,360821	3084,250894	3236,96354	1511,216084	2385,606102	21617,40055	2401,933395
ENSG00000169508	GPR183	1040,196741	4143,072387	1878,386592	2533,004973	1777,898242	2416,288162	2447,155484	1748,655809	2443,578458	20428,23685	2269,804094
ENSG00000196154	S100A4	2990,423883	2798,200661	2377,523236	3045,436633	734,1789925	2133,511803	2033,653912	2136,297902	1616,825453	19866,05247	2207,339164
ENSG00000143119	CD53	2486,934517	2589,838045	2278,739278	1422,047111	1500,049995	2001,720359	2077,140357	2357,87789	2171,692609	18886,04016	2098,448907
ENSG00000184009	ACTG1	2554,339159	1884,429946	1539,989315	2338,301689	1553,216367	2137,713157	1783,036963	2308,736277	2757,334002	18857,09688	2095,232986
ENSG00000111348	ARHGDIB	2346,505918	2773,20808	2451,452313	1987,680475	1308,615883	2019,770356	1909,029258	1554,434111	2481,533436	18832,22981	2092,469979
ENSG00000168329	CX3CR1	881,4025324	1851,390291	1900,227845	2305,223072	1466,535824	3002,640993	2357,057164	567,4795569	4000,962914	18332,92019	2036,991133
ENSG00000165168	CYBB	1437,241575	922,4618298	2317,359758	1691,446226	3795,619912	1698,379648	1882,367025	3015,448892	1439,375643	18199,70051	2022,188945
ENSG00000159388	BTG2	1140,920435	2601,376161	2554,150115	2345,859369	1319,239198	2429,202847	1960,19519	781,2014911	2801,921126	17934,06593	1992,673993
ENSG00000135929	CYP27A1	3178,995596	1760,676284	1616,254387	1192,008347	2240,421543	1081,672442	2118,61051	2597,616697	1915,533749	17701,78956	1966,865506
ENSG00000074800	ENO1	2355,84702	2360,625402	1812,771661	1806,514749	2357,06622	1509,861719	1833,945336	1164,725484	2323,398552	17524,75614	1947,195127
ENSG00000129538	RNASE1	1293,020922	248,582242	1230,504704	275,1502687	702,981708	1684,842588	2688,86513	8922,343544	193,7402804	17240,03139	1915,559043
ENSG00000128016	ZFP36	1142,501165	2485,049342	2152,202139	1939,746463	1491,499126	1727,45102	2017,388154	1660,721394	2284,52531	16901,08411	1877,898235
ENSG00000139318	DUSP6	1089,139521	2086,907704	1869,360339	1156,322623	2349,013994	881,3166395	1378,580481	3732,908777	1916,208156	16459,75824	1828,862026
ENSG00000169385	RNASE2	2875,71811	1052,45735	1211,143848	1589,891297	2291,36943	1748,743565	2067,12372	2599,877535	936,2018559	16372,52671	1819,169634
ENSG00000169714	CNBP	2020,349114	1984,556931	2629,198383	1911,717681	792,9658239	1544,681711	1442,615815	2165,400996	1474,813191	15966,29964	1774,033294
ENSG00000019169	MARCO	2169,770445	422,2688352	668,5428066	577,9728514	2477,03009	232,3865317	38,86623079	8974,342194	370,2627683	15931,44275	1770,160306
ENSG00000090104	RGS1	357,3390373	2063,785114	1327,05816	2678,202249	879,0181965	2332,033202	2596,595278	570,9202064	3094,796211	15899,74765	1766,638628
ENSG00000169413	RNASE6	1823,532183	1719,730624	2044,010064	946,3530299	1388,814761	1767,026272	2115,15626	1718,338053	2128,427114	15651,38836	1739,043151
ENSG00000142669	SH3BGRL3	2411,731608	1533,245752	1764,665902	1615,354301	1122,037344	2064,412427	1637,846172	1261,762133	2086,530304	15497,58594	1721,953994
ENSG00000139644	TMBIM6	2120,897685	1728,922052	1492,76122	1649,50892	1741,540115	1676,646264	1709,061595	1512,274982	1783,718028	15415,33096	1712,814551
ENSG00000068697	LAPTM4A	1248,07948	1696,960756	1839,05592	1550,588365	2403,485906	1610,062343	1573,492107	1937,29555	1139,602219	14998,62265	1666,513627
ENSG00000118640	VAMP8	1689,230458	1574,697601	996,3800643	1816,049114	1393,536564	1638,158398	1619,7589	2153,547299	1251,920316	14133,27871	1570,364301
ENSG00000160255	ITGB2	2161,50373	939,0239936	1463,270406	1172,820848	1618,460003	1224,052439	1981,002831	1589,491772	1740,285341	13889,91136	1543,323485
ENSG00000155659	VSIG4	2921,686936	1627,471376	1263,600064	1361,156726	2291,240728	770,6699184	510,0559917	2366,580021	516,5147237	13628,97648	1514,330721
ENSG00000110955	ATP5F1B	1601,447951	1797,078579	1507,650778	1738,283975	1052,320305	1439,893267	1229,755019	1666,199327	1428,975404	13461,60461	1495,733845
ENSG00000112149	CD83	432,2877902	3574,926733	1318,773037	1163,152674	841,9076947	1419,426226	1499,749405	1264,632967	1773,259581	13288,11611	1476,457345
ENSG00000171223	JUNB	1180,396507	1348,036285	1644,487733	1396,50117	1158,791851	1804,073533	1460,803767	745,373713	2519,286709	13257,75127	1473,083474
ENSG00000166710	B2M	1489,802571	1965,654211	1154,996108	1185,131543	1139,145183	1294,724765	1208,490444	2557,266765	837,5780837	12832,78967	1425,865519
ENSG00000135404	CD63	1544,220328	1490,005926	1293,721281	1294,070983	1101,90301	1578,324392	1379,649469	2088,401686	976,7353495	12747,03242	1416,336936
ENSG00000265972	TXNIP	1825,123894	1130,634613	1843,103911	1408,689553	1422,600802	978,9868236	949,317185	1573,142004	1550,49831	12682,0965	1409,121833
ENSG00000133872	SARAF	1587,368533	1287,440319	1394,270082	1565,173501	1094,527814	1277,455952	1228,81195	2018,413693	1204,210877	12657,67272	1406,40808
ENSG00000204472	AIF1	1445,336745	1784,233695	1048,854919	1468,415243	810,1090819	1526,030268	1110,558803	2141,045862	1300,178083	12634,7627	1403,862522
ENSG00000150991	UBC	1833,717859	1707,565128	1329,355317	1319,93736	1122,77206	1178,824867	1141,36801	1308,005484	1606,966915	12548,513	1394,279222
ENSG00000125356	NDUFA1	1011,112773	1912,661345	550,0176305	2158,756571	880,3071347	1276,841493	1100,324147	2465,308733	973,3766784	12328,70651	1369,856278
ENSG00000197747	S100A10	2279,201335	1789,026467	1158,891342	1827,576186	1080,54636	984,479262	938,2998741	1125,828447	1113,242706	12297,09198	1366,343553
ENSG00000028137	TNFRSF1B	1899,521469	831,112446	1352,816861	1065,081589	1525,156423	1094,31595	1436,798034	1367,918644	1578,711604	12151,43302	1350,159225
ENSG00000164111	ANXA5	1511,415677	1122,896191	981,9112419	1479,523608	887,91045	1459,445859	1372,452401	1557,617715	1515,363951	11888,53709	1320,948566
ENSG00000130066	SAT1	1083,301881	1666,602359	959,2427755	1293,915745	1015,966242	1425,515896	1246,06405	1528,755923	1613,918578	11833,28345	1314,809272
ENSG00000109971	HSPA8	1268,531015	1499,803532	1641,682016	1089,264953	1368,038615	1267,407627	1287,306458	1194,171038	1201,429353	11817,63461	1313,070512
ENSG00000117115	PADI2	301,7174394	778,7290586	2082,163601	1311,58965	1493,569859	1264,681023	1813,799119	124,573832	2395,623621	11566,4472	1285,1608
ENSG00000146278	PNRC1	738,4822073	1377,902156	1435,796942	1225,051333	1232,186059	1392,440004	1268,290348	1458,020441	1400,184594	11528,35408	1280,928232
ENSG00000102265	TIMP1	2038,425167	943,6892382	903,1469606	1366,931143	1451,619665	603,0608514	712,3868781	2693,064577	654,9611878	11367,28567	1263,031741
ENSG00000163131	CTSS	1478,589652	1097,531355	1301,883706	1332,748938	1207,555892	929,9713706	1158,358378	2083,093279	775,2963615	11365,02893	1262,780993
ENSG00000129562	DAD1	1001,976268	1387,220834	1064,433651	1261,471448	1679,971683	1200,835028	1207,054271	1480,225283	1047,431976	11330,62044	1258,957827
ENSG00000120738	EGR1	893,5610729	1331,991325	1276,209559	1493,243438	1576,018873	1271,751905	1049,239152	843,5192913	1574,393156	11309,92777	1256,658641
ENSG00000067082	KLF6	567,372317	1522,1066	1538,422196	713,7776557	1117,774556	871,4696948	1512,204658	1911,288656	1495,127552	11249,54389	1249,949321
ENSG00000131171	SH3BGRL	1011,348882	1813,990006	1279,615616	1414,766319	1085,388824	1247,384555	877,8828637	1430,496687	956,6113997	11117,48515	1235,276128
ENSG00000143546	S100A8	893,6044858	1050,599015	3282,411472	1372,031825	1201,895074	501,4558732	466,9580482	2021,918364	267,8192307	11058,69339	1228,74371
ENSG00000119655	NPC2	1406,004429	1242,875724	840,108644	1262,53041	839,595651	1576,542774	1427,230732	1462,374299	997,3109846	11054,57365	1228,285961
ENSG00000171659	GPR34	1124,541957	940,167667	823,8371781	1119,066921	2014,861974	1066,789503	1074,706161	1844,668988	1043,19146	11051,83181	1227,981312
ENSG00000044574	HSPA5	1152,926251	1504,358	637,560579	1002,812837	1070,941449	1211,515336	1660,201929	1231,313304	1444,209011	10915,8387	1212,870966
ENSG00000136156	ITM2B	1020,15867	1589,366247	1007,10014	1170,064013	1264,827683	1207,090103	1100,85977	1245,225353	1297,801018	10902,493	1211,388111
ENSG00000130208	APOC1	402,1431596	850,6048013	971,0109063	1475,098691	1764,081055	1753,854049	2130,753282	811,1886783	631,8177869	10790,55241	1198,950268
ENSG00000030582	GRN	1356,135899	928,9857205	1426,34397	717,1269095	1100,587247	1248,707783	1477,160367	1124,734006	1333,359144	10713,14105	1190,349005
ENSG00000102144	PGK1	1589,565361	1681,201516	924,8449532	914,3042489	1463,947252	896,5335191	1028,962877	852,6147779	1281,476204	10633,45071	1181,494523
ENSG00000161921	CXCL16	937,4482126	1186,429945	1345,511263	1204,056553	684,8048803	1365,896098	1410,496772	914,2851191	1571,344656	10620,2735	1180,030389
ENSG00000143933	CALM2	1661,772178	1126,901491	1157,884932	1217,938715	1401,808244	862,3500555	763,5738194	1357,771738	995,9821279	10545,9833	1171,775922
ENSG00000027697	IFNGR1	809,5044455	1580,049442	1037,696232	1249,971691	1049,846218	1076,722684	1086,771408	1286,446205	1231,67982	10408,68814	1156,520905
ENSG00000136167	LCP1	1174,319773	987,7266178	1879,362834	1064,196107	1121,138623	1045,216571	910,0265575	936,6974258	1252,535147	10371,21966	1152,35774
ENSG00000132475	H3F3B	809,5772226	1481,03211	1184,338426	1259,668482	708,6921829	775,1058684	997,0329952	1526,226307	1613,65924	10355,33283	1150,592537
ENSG00000165457	FOLR2	2197,399548	248,4479549	144,4868311	220,5785061	1279,229438	445,5917537	284,9665167	5314,489294	77,04230831	10212,23215	1134,692461
ENSG00000136235	GPNMB	1566,271072	1129,03764	1253,303652	687,935188	2124,976981	597,1682016	894,994004	997,5403815	823,3862477	10074,61337	1119,401485
ENSG00000152518	ZFP36L2	1036,332906	601,0504946	1162,954923	830,1448732	1259,293846	895,3690208	1035,223671	1275,658172	1873,109506	9969,137412	1107,681935
ENSG00000177606	JUN	461,1006735	326,2394334	1924,344719	683,2958233	2307,076596	784,7945217	811,8355804	1180,696849	1483,46474	9962,848936	1106,983215
ENSG00000010610	CD4	1309,492305	555,2310539	1095,328211	727,8923408	1699,996406	978,7886645	1186,667948	967,4474435	1080,699362	9601,543736	1066,838193
ENSG00000112799	LY86	775,2738505	1075,019839	1217,058339	1431,780355	1087,50225	1169,235137	985,197636	986,9088953	818,9296879	9546,905989	1060,767332
ENSG00000134333	LDHA	1468,660604	1623,329359	944,0182063	1081,250922	564,2510971	675,4852115	893,5033679	1146,396708	1115,356149	9512,251625	1056,916847
ENSG00000137154	RPS6	753,2700481	1129,772944	957,0545486	1564,359975	758,8574449	935,3713096	869,3697838	1444,3424	1054,887869	9467,286323	1051,920703
ENSG00000111669	TPI1	1276,268686	1429,395484	776,5940321	870,3631042	940,2229285	828,6311581	976,9027109	982,6144973	1314,210241	9395,202842	1043,911427
ENSG00000106565	TMEM176B	1533,17289	1529,994677	646,9814734	725,4493331	1022,187527	1441,803991	1098,610365	465,0127693	748,9267383	9212,139764	1023,571085
ENSG00000130303	BST2	1022,501137	953,2314154	794,0485116	1015,063121	1037,885577	971,5036222	1181,654743	1302,530448	884,8387555	9163,25733	1018,139703
ENSG00000108518	PFN1	1139,679422	1098,073203	787,4936976	865,4058795	660,2196186	1215,898248	1100,754317	726,8895292	1446,460225	9040,87414	1004,541571
ENSG00000131238	PPT1	816,0802399	1230,090885	829,7880785	867,8344419	976,7037247	1155,504559	993,5533955	767,9616275	1373,284532	9010,801484	1001,200165
ENSG00000116741	RGS2	469,5428077	1292,038632	2328,349593	1015,496937	1153,544711	782,8309395	765,0805877	178,6688825	938,3113208	8923,864411	991,5404901
ENSG00000137575	SDCBP	1305,776271	1163,726177	1271,94662	993,4865644	745,6744615	831,2547086	769,7369792	1159,460796	656,4735643	8897,536141	988,6151268
ENSG00000118705	RPN2	1295,521292	799,9569669	954,604251	904,1880083	1282,448646	947,5951864	1022,164971	834,2860914	847,3913095	8888,156723	987,5729692

ENSG00000100979	PLTP	656,2991963	69,22516807	1,422172879	186,6901943	1336,9744	100,8783318	767,3885988	5511,274068	85,35336147	8715,505491	968,389499
ENSG00000173812	EIF1	726,4401902	1450,468374	707,6484827	1032,288433	675,3154336	960,0649298	750,051463	1150,911898	1205,15545	8658,344654	962,0382948
ENSG00000110876	SELPLG	1069,043668	1152,236494	1037,242502	756,6486513	721,9788923	924,7485673	848,6603972	559,2479188	1517,547885	8587,354976	954,1505529
ENSG00000118515	SGK1	328,14223	1578,393517	1227,040657	980,5169627	717,8397391	832,7243127	1249,178205	464,9299024	1202,031945	8580,797471	953,4219412
ENSG00000163823	CCR1	1617,043011	475,8861801	1147,74474	608,059355	1299,256324	498,2960518	900,29468	1360,451315	632,5524488	8539,584105	948,8426784
ENSG00000114013	CD86	944,1223144	1055,014334	1185,1965	781,4383746	585,2247177	817,0987291	871,6482738	1288,762112	932,2606606	8460,766016	940,0851129
ENSG00000122877	EGR2	469,6347771	1397,987283	949,6083511	1049,257092	505,9186413	735,3315069	1119,879683	1075,950129	1154,928578	8458,496042	939,8328935
ENSG00000104904	OAZ1	1002,888665	954,1048001	904,4061486	810,4476277	852,6428727	1060,070082	950,7043346	973,3202147	948,055251	8456,639996	939,6266663
ENSG00000133800	LYVE1	2219,799236	328,6360559	512,701275	425,8102459	822,6499149	127,1115741	188,6873897	3438,305635	329,1307542	8392,832081	932,5368979
ENSG00000144746	ARL6IP5	1189,130776	1271,339409	617,9753277	972,7615002	830,5383788	981,5672319	767,7016439	830,030941	919,4129321	8380,458141	931,1620156
ENSG00000275395	FCGBP	917,5528054	561,2755545	718,8319572	519,5547326	1416,077413	1641,901553	878,2051986	291,4279408	1410,560909	8355,388064	928,3764516
ENSG00000107798	LIPA	976,2095075	649,8235794	725,8969176	944,1354008	2055,509221	679,7550698	589,8465229	1017,92223	504,0008733	8143,099323	904,7888136
ENSG00000108654	DDX5	687,1767518	1263,149165	684,4526233	797,144339	684,549587	1016,797387	848,3682952	852,5193952	1124,638105	7958,795648	884,3106275
ENSG00000108298	RPL19	758,3845676	952,8862218	660,9328077	1433,734704	816,5284722	869,1226974	758,0944534	798,2218343	828,8691573	7876,774916	875,1972129
ENSG00000142583	SLC2A5	794,7472281	855,2660119	671,3387599	497,1526664	1579,516243	761,1113622	964,7886802	862,0525898	826,7435162	7812,717057	868,079673
ENSG00000051523	CYBA	807,3475924	828,2480224	809,4151545	706,6821784	698,9158194	1067,631756	1192,218863	899,3752416	795,7750411	7805,609669	867,2899632
ENSG00000106066	CPVL	787,5324885	1396,251404	704,6162221	737,9860568	624,58033	872,5722627	1251,431162	844,2127755	575,7565371	7794,939239	866,1043598
ENSG00000183283	DAZAP2	736,6176922	913,1199078	1059,041751	800,1874575	975,1542398	848,3387575	666,8230779	889,7226664	830,5400064	7719,545556	857,7272841
ENSG00000110077	MS4A6A	1043,891967	1031,219217	726,5596232	627,3523832	485,996232	834,3373881	709,359121	1610,747264	553,7526752	7623,21587	847,0239856
ENSG00000126264	HCST	883,9845129	523,1612269	617,6327811	856,0199575	1068,565085	818,7267447	932,7378514	1181,586577	716,4034782	7598,818215	844,3131351
ENSG00000111897	SERINC1	758,6475978	639,5079445	963,1899238	555,0191284	1606,663155	740,4124489	723,4318214	919,0839235	685,2235615	7591,179505	843,4643894
ENSG00000166598	HSP90B1	739,2992137	742,0023142	776,5088388	774,690652	878,9392288	591,9774187	963,462494	1221,165108	824,6518847	7512,697153	834,7441281
ENSG00000165312	OTUD1	159,4569438	1497,773743	1043,207279	902,5521027	1004,50062	554,69455	634,9662932	834,4796596	874,6128697	7506,244061	834,0271178
ENSG00000147065	MSN	799,8234297	620,3498987	1206,917437	930,8148075	974,721908	722,0587918	717,8235864	463,3921211	1014,131599	7450,033579	827,7815087
ENSG00000130522	JUND	425,2671616	968,0746605	679,8053306	450,0182895	581,3733268	829,7309947	935,6059324	1321,915637	1253,940303	7445,731636	827,3035152
ENSG00000148175	STOM	1034,667969	834,1916069	613,7640647	626,3908068	1066,572363	711,4751873	726,103638	1041,829814	736,8773929	7391,872843	821,3192047
ENSG00000127022	CANX	810,7057116	650,7390878	1002,98387	845,3452299	652,7766001	750,0704752	819,6095375	950,849118	844,3437165	7327,423347	814,1581497
ENSG00000112306	RPS12	397,7589669	668,8863038	536,3841347	2169,966283	831,75894	570,1715594	534,0834163	1159,882214	382,7449504	7251,636769	805,7374188
ENSG00000113732	ATP6V0E1	669,4320371	951,4124789	812,1718119	789,1296373	609,9864781	785,6262796	877,2785799	927,6401128	722,3642948	7145,04171	793,8935234
ENSG00000231389	HLA-DPA1	611,8032708	1086,656185	680,6836975	831,1060395	615,0231536	1051,777228	1101,096026	303,8756305	855,159128	7137,180358	793,0200398
ENSG00000258227	CLEC5A	378,050907	840,5982197	1307,361478	1078,876698	790,7717837	964,202034	763,3360959	155,653968	854,8759984	7133,727183	792,6363536
ENSG00000123095	BHLHE41	759,9442347	671,2594751	747,3870746	723,1568406	1031,321473	823,3968865	739,0834139	616,9712371	1005,326491	7117,847127	790,871903
ENSG00000096384	HSP90AB1	458,4532448	850,136113	672,8424342	997,1568761	988,4168062	668,6530289	886,7131517	742,1471679	848,8446358	7113,363459	790,3737176
ENSG00000142089	IFITM3	996,7258492	825,15983	399,4605419	869,1787077	435,8707269	262,9199756	560,0925854	2459,455204	239,2915256	7048,154947	783,1283274
ENSG00000042493	CAPG	911,3485388	709,6507171	523,0944235	659,2949834	433,0491406	1179,275095	1059,054667	152,3086163	1376,063274	7003,139456	778,1266062
ENSG00000167552	TUBA1A	844,707139	1424,662578	556,3100785	771,2278843	482,1757562	684,7335192	838,1007576	545,4561804	769,7016759	6917,075569	768,5639521
ENSG00000100906	NFKBIA	535,2867497	1250,764348	972,0517747	640,5167413	554,7934725	610,4362601	584,2592367	935,743908	800,166	6884,018491	764,8909435
ENSG00000143761	ARF1	826,9400476	582,5407426	721,934905	641,4021854	907,0426923	815,3435351	775,6521621	798,8439468	789,3237695	6859,023987	762,1137763
ENSG00000122884	P4HA1	559,6416382	883,6635651	962,4600323	1045,595886	508,3144521	471,5014449	714,284386	987,7782125	725,1198936	6858,359511	762,0399456
ENSG00000171051	FPR1	704,6389602	1371,514286	675,5537949	456,6865014	460,318361	1062,556828	1163,381814	183,8153743	754,4910278	6832,956947	759,2174385
ENSG00000177575	CD163	793,6359405	716,7366932	743,0814998	608,2348023	1417,874886	496,3332064	697,0693914	913,7683937	420,1928165	6806,92763	756,3252922
ENSG00000092010	PSME1	897,0488438	1199,693881	685,0894669	802,0518166	423,4189942	600,0245688	543,9167151	1046,190075	513,1708025	6710,605164	745,622796
ENSG00000161016	RPL8	766,2614764	865,6552759	632,1826244	1003,977227	589,8243655	567,5079245	644,2050163	947,8375529	678,3096783	6695,761142	743,9734602
ENSG00000131724	IL13RA1	903,7387033	743,4741521	745,8833753	882,91417	631,5834971	641,7706544	597,3736947	679,9845971	821,8812654	6648,604109	738,7337899
ENSG00000085514	PILRA	994,6720428	733,9545098	311,5053371	696,9192915	829,0381857	601,6134279	785,4821086	883,0407186	808,8526278	6645,07825	738,3420277
ENSG00000067225	PKM	1096,613872	845,5029395	581,3549497	453,2493177	969,9651272	594,9048832	744,2728526	398,5956124	876,3907055	6560,85026	728,9833622
ENSG00000142534	RPS11	693,9932596	767,6968264	596,0098503	944,1068137	615,7757149	751,4842445	651,483995	727,9410203	713,3129826	6461,804707	717,9783008
ENSG00000132963	POMP	577,3622761	939,0472233	488,8418841	1019,288399	528,8509362	809,9205102	688,3704056	791,5060571	560,0819269	6403,269619	711,4744021
ENSG00000118508	RAB32	726,3239542	783,7803251	861,6077998	611,0533584	224,5749138	1042,043049	932,0148324	593,2308855	574,6409369	6349,270055	705,4744506
ENSG00000135047	CTSL	936,934494	753,461646	796,5660828	545,2582893	560,3940051	438,7850997	648,8957019	1240,02173	419,8606097	6340,177659	704,4641843
ENSG00000173391	OLR1	238,5540118	980,2927684	1710,07319	894,4512917	337,7373027	828,0332577	521,294958	62,69285219	761,6850106	6334,814643	703,8682937
ENSG00000112335	SNX3	795,6450999	753,8193318	561,4346419	921,4087861	309,4147315	735,3432672	668,3285682	874,7116369	697,4327915	6317,538855	701,9487617
ENSG00000197956	S100A6	827,4509827	780,9532196	782,4094856	1297,956671	390,1843534	509,1797308	442,6996472	599,9548327	556,3911846	6187,180107	687,4644564
ENSG00000075785	RAB7A	927,519686	580,3986391	764,6754729	638,1718462	666,4227635	661,6271986	595,5338246	601,0333576	729,3638082	6164,746597	684,9718441
ENSG00000118680	MYL12B	690,0090344	852,4472901	650,4906975	668,2744588	408,003626	756,6449748	605,4008462	744,3329388	714,5279335	6090,1318	676,6813111
ENSG00000148908	RGS10	405,4128183	585,7450441	370,2764741	614,446565	776,0208745	1052,112676	898,9742469	530,925871	831,2036765	6065,118246	673,9020274
ENSG00000163902	RPN1	748,4260748	565,6342216	898,8607438	676,1934083	587,539848	691,7613251	701,684525	611,0331019	575,4914503	6056,624699	672,9582999
ENSG00000137161	CNPY3	575,6677231	721,4147502	601,5866044	587,2561081	1100,636533	584,4665234	720,868099	353,2098473	809,6047682	6054,710957	672,7456619
ENSG00000133574	GIMAP4	914,8704774	708,6153761	809,3074725	525,8479955	476,8273418	527,1356055	468,28708	794,36533	792,0833674	6017,340046	668,5933385
ENSG00000103187	COTL1	680,0757941	520,4596165	771,2806799	457,2247662	508,3736131	883,907554	821,6534461	331,0699246	1013,593353	5987,638748	665,2931942
ENSG00000163563	MNDA	643,8429648	763,1122699	868,5333759	1007,788857	440,1628346	637,8339949	558,7612118	433,3996105	594,341767	5947,776887	660,8640986
ENSG00000183291	SELENOF	464,1106123	890,9234196	952,1544599	669,3497804	724,4352058	579,0663563	510,0902002	617,7820288	495,6470176	5903,559081	655,951009
ENSG00000084070	SMAP2	722,9052488	723,0377175	819,6591755	575,6366116	698,588832	574,4440629	487,3865604	720,1473163	558,365322	5880,170847	653,3523163
ENSG00000168653	NDUFS5	419,6903076	736,4118798	517,9154764	1088,974976	306,6916238	712,3861076	693,9791538	894,2234738	491,4080489	5861,681048	651,2978942
ENSG00000115414	FN1	932,41612	390,7652556	799,2089312	1561,699461	780,0418504	227,597008	154,1486403	791,7109361	209,1636975	5846,7519	649,6391
ENSG00000092841	MYL6	719,5538499	770,566455	437,1446642	882,5025785	353,1179653	637,0764562	579,5754759	708,6885875	681,3700441	5769,596077	641,0662307
ENSG00000115738	ID2	901,473906	834,7176858	393,9202849	927,8343042	163,8991043	848,745431	636,0262921	164,8439957	897,4043999	5768,865404	640,9850449
ENSG00000104870	FCGRT	770,5444124	533,1354361	635,9635766	306,8940463	994,8235777	632,6707422	794,1042268	532,9078935	517,4576425	5718,501554	635,3890616
ENSG00000104765	BNIP3L	694,2199536	831,3112912	522,326549	606,7728694	655,3094512	463,3419745	493,7464716	715,2646061	674,628699	5656,921866	628,5468739
ENSG00000143162	CREG1	497,8923155	539,2216484	613,5695855	580,0760771	425,891985	713,1226371	1116,174299	759,4162672	394,6552138	5640,020028	626,668892
ENSG00000084234	APLP2	871,1687395	429,4655403	720,1111153	321,4923076	712,845675	443,9992011	626,4606071	1032,657834	479,3460682	5637,547089	626,3941209
ENSG00000171867	PRNP	772,5137031	767,6517028	739,4602142	332,2321844	573,1269091	502,3290418	573,3083181	1029,898831	333,2330487	5623,753953	624,8615503
ENSG00000092964	DPYSL2	730,5748725	544,7369415	820,6432774	736,8838016	417,1492834	559,015859	522,5350929	566,8927469	707,4954984	5605,927374	622,8808193
ENSG00000102760	RGCC	365,4528461	539,4079846	707,6347762	634,0973218	572,8447787	479,8520466	555,2366731	380,8017783	1338,455827	5573,784033	619,309337
ENSG00000069849	ATP1B3	638,4522659	731,1659582	580,4972262	655,8770588	369,1349595	669,3480584	684,2136446	696,8545579	518,2682153	5543,811945	615,979105
ENSG00000105372	RPS19	564,029453	566,0697133	637,3273657	674,2823495	534,3752216	689,5399041	627,9461496	532,1396905	663,4999688	5489,209816	609,9122018
ENSG00000087074	PPP1R15A	283,5356907	720,8776772	630,0579927	305,0342623	1147,535794	453,723476	649,8667277	728,6809924	558,0246475	5477,337261	608,593029
ENSG00000107738	VSIR	795,3465743	520,4650271	384,0365466	353,2977523	761,6525559	728,4868443	830,6413557	347,6841112	748,9762717	5470,587039	607,8430043
ENSG00000087253	LPCAT2	442,3284154	426,0182979	686,9631817	1007,901662	506,3552469	603,5755106	556,5966881	547,5670228	663,0991796	5440,405205	604,4894672
ENSG00000026297	RNASET2	564,5441888	788,1122452	577,1642275	465,1286267	441,9076915	719,0833133	665,864469	393,2097856	823,8960767	5438,910624	604,3234027
ENSG00000254999	BRK1	369,3667949	648,4373063	758,0396438	810,2598211	529,9325821	673,8854815	555,2334953	562,0260992	514,8756695	5422,056894	602,450766
ENSG00000135821	GLUL	586,0219175	530,8807845	561,0999983	691,3101204	667,3813052	672,386044	609,8480486	596,3334201	484,8018059	5400,063444	600,0070494
ENSG00000185201	IFITM2	998,4266156	634,8170618	544,7905504	712,1174193	517,38062	229,8877596	371,2880985	1019,785749	317,2519376	5345,745812	593,9717568
ENSG00000172757	CFL1	588,2104567	642,9464531	577,0516121	537,7529477	441,8400999	688,1116417	635,6093835	557,4850888	667,7482973	5336,755981	592,9728867
ENSG00000134996	OSTF1	533,4122949	321,2011828	281,5628599	693,5845496	864,1691277	702,6356948	536,3167034	853,2677308	521,0465803	5307,196724	589,6885249
ENSG00000179344	HLA-DQB1	441,5916779	988,8009644	301,6174769	22,50723782	391,045611	609,8837615	1442,832062	221,8843864	875,4568742	5295,620052	588,402228
ENSG00000115956	PLEK	586,4787484	633,9194804	653,3616492	465,7750358	502,4284118	638,6392464	616,2335401	466,4230838	724,3665386	5287,625734	587,5139705
ENSG00000182117	NOP10	504,8838742	673,743076	696,4555549	469,5887539	398,6593656	581,5854512	564,616494	764,6518331	630,3350458	5284,519449	587,1688276
ENSG00000051108	HERPUD1	444,0477556	489,2452613	456,509001	702,0295178	814,6477161	587,6026905	620,9566885	430,0479911	719,9992391	5265,085861	585,0095401
ENSG00000120708	TGFBI	842,4947911	484,9627958	621,5558647	468,7159023	545,7265024	499,1721874	699,1999686	494,945337	583,4766803	5240,25003	582,2500033
ENSG00000130755	GMFG	483,4885046	720,196957	629,8612473	756,6738276	314,6159449	614,6341393	528,8194829	678,4859636	511,8813074	5238,657375	582,0730416
ENSG00000100097	LGALS1	741,9571506	602,377108	601,8125854	788,1850264	201,6039436	702,0489726	764,933936	97,55097354	719,3497066	5219,819403	579,9799336
ENSG00000183255	PTTG1IP	678,5663539	563,7274279	469,5875294	614,3812464	381,1499163	642,3933695	667,7561576	496,1656738	686,1136239	5199,841299	577,7601443
ENSG00000140968	IRF8	382,4041879	481,8176564	401,8549806	353,2407219	900,1131567	719,3606088	592,5336429	389,0867033	965,5888373	5186,000496	576,2222773
ENSG00000166340	TPP1	602,9548824	447,2882395	581,4409945	584,1941595	748,888766	527,9181669	514,1543411	608,8798144	558,0540604	5173,773425	574,8637139
ENSG00000100300	TSPO	545,2519958	463,4972125	651,1615399	453,9754762	391,1906281	717,3123949	725,8220471	436,8614535	773,4456532	5158,518401	573,1687113
ENSG00000143226	FCGR2A	478,3272317	594,4638508	601,7322316	588,4049305	716,7483354	590,5842057	585,4888639	445,2845198	539,7732828	5140,807452	571,200828
ENSG00000165502	RPL36AL	447,0523183	718,8793568	553,3518056	661,4316849	300,3878638	584,912623	542,9198816	833,1734873	479,8128712	5121,921893	569,1024325
ENSG00000101336	HCK	679,4111104	495,0914225	438,6367733	401,0229623	884,7857172	593,5649416	389,5991777	538,5057522	697,8861482	5118,504005	568,7226673
ENSG00000266412	NCOA4	546,7616848	450,5627038	850,2279272	624,7342393	312,9842763	566,8374104	611,448703	544,4596049	603,7847789	5111,801328	567,9779254
ENSG00000140319	SRP14	585,1483644	770,9918357	365,3254667	766,1467309	304,6678003	602,2429812	486,5495418	711,4643531	515,1795366	5107,716611	567,5240678
ENSG00000127528	KLF2	346,1016143	415,5005125	554,7204453	385,9717043	746,3523155	305,1011646	615,4761231	1126,064678	590,4172717	5085,70583	565,0784255
ENSG00000100644	HIF1A	406,0418188	308,4856324	659,7372763	724,2959167	803,4305102	606,1485426	488,8982787	614,4248277	443,9606788	5055,423482	561,7137202
ENSG00000131669	NINJ1	747,0804481	570,3681023	467,3567102	453,129015	370,7019792	640,7053607	442,1817346	586,1279829	743,3147195	5020,966052	557,8851169
ENSG00000177885	GRB2	525,6827867	559,311462	570,8140964	631,3674714	280,8366192	664,6625372	747,3147064	418,8587457	607,3508484	5006,199274	556,2443637
ENSG00000189067	LITAF	274,6569009	568,1324867	553,5979165	534,4525843	626,3265437	675,7085306	778,238413	171,8129145	776,6988946	4959,625185	551,069465
ENSG00000160888	IER2	348,1579696	493,6111908	607,4212751	573,9044001	467,0044299	705,6722678	536,6413778	267,6530729	926,5767988	4926,642783	547,4047536
ENSG00000196735	HLA-DQA1	370,523032	824,5550374	387,534837	39,74107091	516,9799527	669,3666648	921,8727511	269,8048093	925,9862667	4926,364422	547,3738247
ENSG00000204592	HLA-E	725,609999	512,4246846	695,996716	189,9413727	519,9953189	506,873278	498,3602578	805,9572768	460,8001553	4915,959059	546,2176732
ENSG00000114353	GNAI2	501,3075326	459,9513417	711,2611662	349,4872489	543,792708	600,1235377	625,1997718	425,79428	687,0423426	4903,959929	544,8844366
ENSG00000084207	GSTP1	571,7056127	772,9954362	623,3096411	621,4102927	444,2458766	459,0973991	636,9375671	340,8092792	418,7624934	4889,273598	543,252622
ENSG00000142227	EMP3	730,9342315	469,9412682	467,41213	580,5604906	584,1936434	419,9706027	394,1065079	803,6891821	416,657735	4867,465791	540,8295324
ENSG00000101182	PSMA7	547,603318	515,9284716	310,6649736	711,9116316	346,2040691	673,3014842	596,7718474	602,024623	558,6003346	4863,010753	540,3345281
ENSG00000110079	MS4A4A	771,7615907	552,1213077	400,2686965	443,6603149	626,3224483	436,4585775	433,2261429	927,8189893	265,6482367	4857,286305	539,6984783
ENSG00000135046	ANXA1	804,9276299	741,8615408	331,4162015	520,4636075	235,6482054	355,107502	393,8804786	1010,96801	450,4054948	4844,67867	538,2976301
ENSG00000108798	ABI3	731,2767273	489,4299623	373,6581084	361,8864899	611,0688463	559,8751724	551,9148319	435,7314984	694,7158951	4809,557532	534,3952813
ENSG00000180353	HCLS1	508,4887021	581,1937049	431,8980018	464,2316522	527,2886279	532,2950762	548,2459785	503,0157475	701,458413	4798,115904	533,1239894

ENSG00000010278	CD9	354,901225	701,868928	328,7707005	814,9440935	434,1312907	487,5739397	767,0108339	205,2423112	658,3830579	4752,82638	528,09182
ENSG00000127884	ECHS1	622,1921539	331,3454003	401,188193	449,5355866	813,2873395	603,3584324	522,3181558	391,7603252	606,1478193	4741,133406	526,7926007
ENSG00000165678	GHITM	674,6687317	578,1600808	516,2820982	532,7148537	541,5408111	437,2043199	398,1655078	642,2804566	410,5476071	4731,564467	525,7293852
ENSG00000163466	ARPC2	508,6963984	582,0512917	521,7833661	534,2458367	204,8871688	567,1773391	504,4619918	656,2537021	647,7100744	4727,267169	525,2519077
ENSG00000026025	VIM	843,6033713	470,0773781	535,2835484	585,4400812	485,391485	366,9841949	299,3010412	659,3369809	479,5529659	4724,971047	524,996783
ENSG00000221869	CEBPD	144,3112888	413,6625897	812,1154131	527,6205823	683,2796582	314,1484014	683,116252	483,8181064	645,2577601	4707,330052	523,0366724
ENSG00000126709	IFI6	339,3706522	169,419886	346,6497671	648,6186483	438,4475458	237,8314324	499,1990644	1909,613546	95,35118482	4684,501727	520,5001919
ENSG00000185650	ZFP36L1	373,2958225	461,6615366	456,5661509	409,6577964	458,6404272	737,2410103	654,5855305	451,7219844	673,9817774	4677,352036	519,7057818
ENSG00000143753	DEGS1	537,4742293	646,4568171	184,903361	505,8682132	630,8782009	516,6561172	463,4092311	658,8427579	522,9687706	4667,457698	518,6064109
ENSG00000173334	TRIB1	373,6290885	847,0775211	703,3934098	534,7289062	212,5443176	531,4886028	391,1343992	309,5556817	730,4643377	4634,016265	514,8906961
ENSG00000127951	FGL2	263,7053337	202,2417083	1100,513918	641,6870294	463,3598685	591,8173633	438,710312	526,141363	400,9459166	4629,122812	514,3469792
ENSG00000177156	TALDO1	347,0812798	546,3381722	397,8930829	362,4545674	1041,033206	522,5013174	578,5360613	353,8502954	475,2607659	4624,948748	513,8831942
ENSG00000135077	HAVCR2	691,5237479	523,0104703	430,9903154	501,8042771	326,8059079	590,1395825	574,0306752	448,5350916	531,5025385	4618,342606	513,1491785
ENSG00000179163	FUCA1	647,9225592	403,0644525	296,312934	407,2727132	214,7336941	607,3121403	612,8248067	1044,089293	381,6799119	4615,212505	512,8013894
ENSG00000182853	VMO1	547,987524	403,7156639	282,1342225	313,5768997	857,767679	579,7572581	766,1034095	617,6616262	246,0209878	4614,725271	512,7472523
ENSG00000100219	XBP1	557,5321319	612,3520246	364,6423227	392,0056801	433,5976667	620,1921459	515,1825092	666,2163259	452,4638581	4614,184665	512,687185
ENSG00000157514	TSC22D3	454,4414203	818,997712	601,6291903	370,2144982	323,4016978	271,8761234	415,8738637	810,140143	542,7217577	4609,296406	512,1440452
ENSG00000155368	DBI	684,3640552	533,8259428	474,6380008	976,8681612	147,2825632	271,7785852	214,734797	619,1203276	682,1285865	4604,741019	511,6378911
ENSG00000126945	HNRNPH2	632,4793485	503,7005428	647,4902306	580,0575394	613,7531517	348,2243332	343,0761081	660,4373826	268,0042836	4597,22292	510,8025467
ENSG00000121316	PLBD1	333,524889	759,0394386	731,409532	339,8197374	565,5156246	406,5682343	380,7430243	588,103965	456,0478029	4560,772248	506,752472
ENSG00000170315	UBB	495,3741965	602,2856399	347,6037388	621,6723454	534,5778098	544,8130782	539,6540128	424,4076308	441,7437695	4552,132222	505,7924691
ENSG00000102409	BEX4	368,2168403	455,555273	505,1101799	669,2425225	826,6272697	507,8482287	440,3893894	338,4939472	434,3029248	4545,786575	505,0873973
ENSG00000137462	TLR2	428,1031693	530,5274535	502,7299662	575,2373285	807,3777371	368,9978889	488,4372192	383,1951569	455,6214337	4540,227053	504,4696726
ENSG00000167996	FTH1	448,5058255	490,1218295	508,5204805	403,1210985	474,1641802	485,6444326	497,4360218	668,1241854	523,4120617	4499,050116	499,8944573
ENSG00000244038	DDOST	648,0927452	605,5614078	289,1156487	471,9077582	414,1531966	559,3155899	579,6258998	413,3771657	496,2737342	4477,423146	497,4914607
ENSG00000080824	HSP90AA1	568,9969487	650,1217243	423,7501509	524,9526414	587,2611945	412,9428264	446,1751783	406,4949634	454,9131908	4475,608819	497,2898687
ENSG00000089220	PEBP1	591,4671346	507,592452	391,9996772	663,712409	541,048292	482,9378544	451,2456788	482,3384374	358,435829	4470,777764	496,7530849
ENSG00000167851	CD300A	520,4174259	659,0956155	582,5961009	329,5715883	573,4189097	529,3425133	473,8907212	321,2660168	478,6080805	4468,206972	496,4674413
ENSG00000169403	PTAFR	547,5603568	431,397657	380,9014379	333,6098297	620,1775619	532,9404148	639,2486806	370,7965846	602,4112557	4459,043779	495,4493088
ENSG00000198189	HSD17B11	730,7997495	644,8286177	603,1643657	478,9029551	284,1217263	333,5286379	457,8698651	512,7970411	411,8588452	4457,871804	495,3190893
ENSG00000184076	UQCR10	357,3958728	515,6576925	180,4732335	654,8798798	697,2405207	520,1934697	546,92196	480,909386	503,4058106	4457,077826	495,2308695
ENSG00000117450	PRDX1	397,2680827	549,8337289	285,1883595	689,8121403	523,155941	523,434632	585,8062974	523,1148306	376,8829075	4454,49692	494,9441022
ENSG00000084733	RAB10	324,7595269	344,5338507	715,4145733	390,0456572	747,9158449	439,6584877	485,6395472	533,1806815	468,227445	4449,375614	494,3750683
ENSG00000176340	COX8A	331,2808665	468,4343038	325,8106828	718,8917013	503,7823013	620,3174801	566,6484022	474,2471829	437,9055707	4447,318492	494,1464991
ENSG00000204103	MAFB	754,705671	764,311514	309,4774014	357,7942836	498,3563888	403,5411283	396,6995006	500,8682128	446,492294	4432,246394	492,4718216
ENSG00000112308	C6orf62	502,904844	472,5343004	518,6243915	362,6502677	339,2974696	605,9482004	430,8896234	604,7551984	577,5516007	4415,155896	490,5728773
ENSG00000177105	RHOG	435,6247851	405,1110081	501,5659124	473,9279715	708,6341038	575,2132907	489,6055048	283,0685203	537,136621	4409,887718	489,9875242
ENSG00000110321	EIF4G2	557,7823304	418,6301797	625,5125551	596,7830427	439,0248989	392,4953455	434,5103401	457,3899818	470,7600829	4392,888757	488,0987508
ENSG00000197061	HIST1H4C	97,26657422	229,3076415	565,5457186	1350,863363	321,1418942	695,6802113	511,9766421	369,9347917	227,715394	4369,43223	485,49247
ENSG00000170540	ARL6IP1	481,6171645	582,7076854	500,0985423	437,3117632	581,3707262	504,8661647	506,8888567	375,2911994	399,2200589	4369,372161	485,4857957
ENSG00000168003	SLC3A2	685,6299241	421,9430314	437,6924965	341,866139	266,1825275	557,6812645	740,4155011	295,5915251	603,3013119	4350,303721	483,3670801
ENSG00000067167	TRAM1	494,0814519	471,5041125	479,1136056	504,0573947	465,261943	523,43562	442,1378898	523,6879965	435,5763807	4338,856395	482,095155
ENSG00000228474	OST4	264,9472006	571,5893635	270,5672963	844,1246428	353,7307792	510,7280586	437,8914587	616,8111927	440,1465136	4310,536506	478,9485007
ENSG00000110108	TMEM109	733,2787168	478,8160376	264,7119801	403,7959574	217,1173911	521,2557847	570,5171811	353,4737582	693,5545228	4236,52133	470,7245922
ENSG00000128524	ATP6V1F	449,5638631	539,0991393	386,6914763	508,5150096	475,5244098	559,7848986	441,7807097	434,9420882	392,911742	4188,813336	465,423704
ENSG00000072694	FCGR2B	829,5518202	761,0056223	364,914185	420,2579233	379,7434484	410,5981195	301,5362783	440,9768789	256,5134678	4165,097744	462,7886382
ENSG00000124762	CDKN1A	246,6322691	649,3432173	596,6090911	456,4223244	319,4379975	768,3563593	349,465728	156,9835044	615,9136548	4159,164146	462,1293495
ENSG00000177600	RPLP2	259,5741751	427,3564314	376,470299	787,7462703	340,2512573	493,3736636	444,5497407	633,8207343	387,7005947	4150,843167	461,2047963
ENSG00000198951	NAGA	464,2185232	375,2975053	316,1592329	407,6668923	536,7382804	540,3957289	505,4362079	503,8547601	498,7667325	4148,533863	460,9482071
ENSG00000223865	HLA-DPB1	523,6493464	597,7995337	381,783468	417,5359341	390,1328644	605,8817882	511,7805576	225,0777014	471,9476698	4125,588863	458,3987626
ENSG00000111229	ARPC3	514,0355289	622,1213302	383,3131593	463,5108551	258,6310678	451,3074624	379,2296226	595,1533737	455,069185	4122,371585	458,0412872
ENSG00000157557	ETS2	366,6035859	421,2637827	492,6620834	409,8251558	469,8793905	404,2802089	519,3472893	463,7252736	557,5808936	4105,167664	456,1297404
ENSG00000234745	HLA-B	148,9313294	685,5154378	353,511237	399,570043	761,6681798	567,4457122	511,5455456	465,8141838	185,9074356	4079,909104	453,3232338
ENSG00000139679	LPAR6	418,8209492	455,1876961	580,7661498	517,3089883	348,4629511	444,9211878	446,8221299	382,0364699	445,510189	4039,836711	448,8707456
ENSG00000175567	UCP2	560,7687073	297,5911215	397,9895709	261,4063167	703,5745781	556,8313792	438,5568866	305,4848476	515,5092478	4037,712656	448,6347395
ENSG00000111843	TMEM14C	386,9849058	575,8659003	515,9782004	430,611843	325,4077958	528,3768328	561,6245822	338,9393179	366,7690262	4030,558404	447,8398227
ENSG00000086598	TMED2	458,0231177	347,1924723	286,6828414	666,1228089	487,7552636	418,9534234	444,6946152	550,7817511	358,9412482	4019,147542	446,5719491
ENSG00000167004	PDIA3	420,5033791	465,1987365	421,2837463	449,7280124	221,3749929	435,4317386	519,0030791	565,7547073	500,8742093	3999,152601	444,350289
ENSG00000049245	VAMP3	559,1588734	463,1900411	294,864364	518,7192525	278,4376408	495,4248871	348,1308187	525,4550422	503,2748303	3986,65575	442,96175
ENSG00000107223	EDF1	343,3993901	405,5044322	391,94739	417,2397221	551,9007729	413,5545144	457,5252041	510,2537061	479,8246954	3971,149827	441,2388697
ENSG00000086730	LAT2	296,4028025	420,7676006	540,1804696	360,6012267	491,7026328	511,1286898	516,0985383	180,5939419	650,5650538	3968,040956	440,8934395
ENSG00000106799	TGFBR1	252,3604121	248,1355096	797,2674607	780,5099812	512,0925611	402,6164757	420,8828903	103,3152935	442,3594003	3959,539985	439,9488872
ENSG00000140379	BCL2A1	158,8830043	1214,016113	201,5023206	447,2725103	13,74612602	421,5240011	423,987665	525,2961008	535,0868171	3941,314658	437,9238509
ENSG00000142507	PSMB6	389,3159692	385,6278522	342,7204538	481,2960934	607,5464711	417,0122788	392,192337	475,8988519	449,2682342	3940,878542	437,8753935
ENSG00000169976	SF3B5	321,8458908	463,1543638	636,5864604	399,1213176	240,9663163	568,836263	407,0091001	618,4323724	269,249507	3925,201591	436,1335101
ENSG00000196419	XRCC6	482,3313379	436,056957	385,9425644	460,1280361	513,6647815	427,9463507	395,1475936	337,1678753	476,9382466	3915,323743	435,0359715
ENSG00000135677	GNS	622,8368847	444,0513658	189,237569	646,0097217	542,9160548	363,1080554	414,4444406	339,2384241	348,8032049	3910,645721	434,5161912
ENSG00000198668	CALM1	510,6733047	492,1766349	308,1133448	536,3360807	216,6135554	517,4695916	425,7414918	569,4026398	328,0314757	3904,558119	433,8397911
ENSG00000135535	CD164	284,7336332	376,6728752	427,5650757	564,5414317	555,9753002	422,1397664	438,2226448	457,9656468	372,0993553	3899,915729	433,3239699
ENSG00000002549	LAP3	369,8116819	320,2118469	508,8100729	379,9240806	494,1305576	390,6898204	432,8975074	723,0275002	276,0937899	3895,596858	432,8440953
ENSG00000181631	P2RY13	128,6489271	550,7336052	818,5583964	423,1582011	637,8395119	330,1410463	348,4581119	219,0548146	436,6889169	3893,281531	432,5868368
ENSG00000057608	GDI2	377,0050608	384,145239	450,1745095	434,9582234	619,793982	445,9005545	365,9070989	488,4831585	309,6019017	3875,969728	430,6633031
ENSG00000005022	SLC25A5	278,3155462	484,379986	402,9370695	482,2682424	134,6402104	511,3886779	488,2450025	582,0323347	485,9498132	3850,156883	427,7952092
ENSG00000101608	MYL12A	431,212291	601,7288273	325,6950189	478,8498532	275,2312258	400,435019	367,9818643	508,1258835	460,2922957	3849,552279	427,728031
ENSG00000131236	CAP1	528,0945979	457,6130778	430,8910187	338,9281238	399,0404154	454,1008155	374,8977198	340,8310405	523,5765291	3847,973338	427,5525932
ENSG00000143384	MCL1	156,2962457	446,4739137	652,1180359	437,3988925	675,6766571	343,3601182	414,9046394	228,1518016	490,701147	3845,081451	427,2312723
ENSG00000100316	RPL3	450,8292439	457,6062413	497,53126	400,1002944	516,2600593	376,5219011	339,0096486	361,6539693	439,955143	3839,467761	426,607529
ENSG00000159840	ZYX	570,2608588	340,6150292	402,9732932	325,8692061	376,2654874	477,1135195	464,2056636	257,1058067	604,1291909	3818,538055	424,2820062
ENSG00000183726	TMEM50A	381,2404231	408,0925257	409,9513819	451,1314312	503,9576408	409,7605406	390,1251951	491,590031	369,2233233	3815,072493	423,8969436
ENSG00000167468	GPX4	409,7723499	416,8884291	251,2697774	411,3699971	563,8447792	503,5661288	513,9995621	349,7467413	390,2869017	3810,744667	423,4160741
ENSG00000165025	SYK	433,0869561	326,9170796	401,8706537	310,2171272	476,2270826	549,3837369	450,5853957	211,0722151	645,5402137	3804,900461	422,7667178
ENSG00000170348	TMED10	378,294764	385,4947035	525,7839887	357,6536082	373,4648534	387,8611666	437,5512002	532,6557223	415,6422221	3794,402229	421,6002477
ENSG00000169223	LMAN2	649,0505692	330,3539125	400,6862589	265,1474857	465,130101	388,7572554	403,2065512	437,7343533	445,3287284	3785,395216	420,5994684
ENSG00000103257	SLC7A5	256,7039984	178,7613334	301,9620093	267,8910497	621,6713627	479,030223	736,3612537	168,4772386	773,5802974	3784,438766	420,4931962
ENSG00000126432	PRDX5	490,0550949	432,4569234	349,4145198	351,9588029	320,7003169	544,611114	458,1126075	444,1655349	381,2014713	3772,676386	419,1862651
ENSG00000085662	AKR1B1	418,2628111	630,0676554	350,8604862	361,9812223	309,7806496	506,9489844	438,3563925	226,439014	522,7505626	3765,447778	418,3830864
ENSG00000165119	HNRNPK	358,1572623	391,3978517	363,2018683	506,9666955	402,3949066	445,7279635	361,0935188	487,4477349	430,2009585	3746,58876	416,28764
ENSG00000009307	CSDE1	370,6890323	302,6691112	632,4071307	564,2301095	227,6648594	333,4578621	332,6766158	513,6661969	461,2390324	3738,69995	415,4111056
ENSG00000008018	PSMB1	457,145228	474,051855	299,2764563	546,2113371	391,9705667	333,0174442	294,3661777	522,0836384	364,9928545	3683,115558	409,235062
ENSG00000179639	FCER1A	1234,826294	465,8535693	40,32501007	417,7199379	152,5864236	1089,455718	152,2875961	3,403617863	124,908006	3681,366173	409,0406859
ENSG00000159346	ADIPOR1	716,6708851	516,2142649	174,1282342	464,3686988	91,01951202	424,8227456	497,689579	370,4518601	415,035343	3670,401123	407,822347
ENSG00000118181	RPS25	279,4062075	521,7603983	428,5448107	597,6594577	267,2106832	338,2239312	316,8614342	611,8744361	302,2365244	3663,777883	407,0864315
ENSG00000137642	SORL1	260,5115717	239,4322373	428,4428432	822,3861566	548,2796732	350,3886396	308,1871495	79,96966012	620,0884891	3657,68642	406,4096023
ENSG00000166033	HTRA1	235,2623685	402,0938259	701,3746764	609,4281296	168,0670098	421,1916507	527,2542174	24,96616732	565,38642	3655,024466	406,1138295
ENSG00000277632	CCL3	53,80911887	707,6866843	400,4104237	297,6125815	346,1373902	301,726263	440,4898871	641,1846126	460,8844478	3649,941409	405,5490455
ENSG00000118855	MFSD1	430,7533007	290,7650385	373,9196625	361,7246654	590,7198449	359,1819564	409,8249432	427,219592	401,9148819	3646,023886	405,1137651
ENSG00000100060	MFNG	732,5037722	379,6474669	426,7478837	266,853618	498,8924175	283,7714433	379,504446	340,971107	336,7475446	3645,639699	405,0710777
ENSG00000154589	LY96	343,7952193	524,7985109	217,9876888	408,8124561	327,1696885	432,13282	492,1661623	664,1353746	232,3846959	3643,382616	404,8202907
ENSG00000168374	ARF4	204,3923326	412,1843693	615,4125891	560,6833982	262,9376996	393,7099543	378,5384313	429,8832755	383,5098754	3641,251925	404,5835473
ENSG00000086061	DNAJA1	339,0925737	702,4154568	364,4581658	449,0076718	135,9477953	399,2300984	400,0660099	373,6585795	459,5312996	3623,407651	402,6008501
ENSG00000131469	RPL27	283,0526237	380,3751768	266,5919705	741,977487	386,2374752	391,0281361	335,3513563	580,951594	253,0851703	3618,65099	402,0723322
ENSG00000147416	ATP6V1B2	264,5258662	415,0234266	456,1340755	332,7866343	800,2651402	258,6860178	341,2657857	296,9545998	439,0525767	3604,694123	400,5215692
ENSG00000104763	ASAH1	464,8580938	396,197329	426,2072294	321,3073663	652,80087	268,9438715	291,0914966	543,117709	239,0214929	3603,545459	400,3939398
ENSG00000164032	H2AFZ	313,1383808	421,1128768	331,2494131	850,0502807	346,6196191	340,2994031	255,4730725	433,9319144	310,0443964	3601,919357	400,2132619
ENSG00000077549	CAPZB	274,2757162	318,2271838	391,6095065	433,736926	297,5830453	473,9131385	432,3477541	477,033664	490,7518694	3589,478804	398,8309782
ENSG00000005893	LAMP2	326,6952566	328,4569668	731,8734094	316,7012079	336,4371185	343,7105107	375,2303125	491,6454966	303,791465	3554,541744	394,9490826
ENSG00000133112	TPT1	288,189	468,888838	333,5455253	505,4291433	327,746014	449,2070519	370,5595113	460,7627977	330,7858447	3535,113726	392,790414
ENSG00000185825	BCAP31	519,8408932	304,7240675	471,8501708	274,2291426	427,7290173	367,463986	456,2460671	379,3497328	333,3980351	3534,831113	392,7590125
ENSG00000148180	GSN	365,2684542	288,9985706	381,2197582	379,5258219	485,2552161	543,8999846	496,2329994	147,6428913	546,5250652	3534,568761	392,7298624
ENSG00000114978	MOB1A	418,8625461	313,8593784	596,750756	605,3367268	171,4172865	397,1806461	344,7706514	353,2673909	329,9352933	3531,380676	392,3756306
ENSG00000103811	CTSH	700,5406038	319,9773256	379,8872636	290,6068064	193,9429769	317,7418445	314,2507959	562,9501339	437,269236	3517,166986	390,7963318
ENSG00000091317	CMTM6	446,6322286	323,6891761	260,6108473	536,8887629	502,8762889	361,7599041	291,4737295	459,9697156	326,4962497	3510,396903	390,0441003
ENSG00000117410	ATP6V0B	376,8200306	382,5514611	498,9738486	394,2623543	168,9704028	443,8453681	478,2515341	448,4687739	314,8890751	3507,032849	389,6703165
ENSG00000082996	RNF13	340,2170317	578,9949267	257,7146997	273,7843172	506,0247736	382,8951384	340,1678465	469,4261641	355,894812	3505,11971	389,4577455
ENSG00000164096	C4orf3	385,5361587	561,648784	208,4850742	581,5500093	329,7572568	269,3244328	237,9697445	502,0149494	423,617056	3499,903466	388,8781628
ENSG00000182220	ATP6AP2	394,6894299	372,3976292	389,3175257	439,4134054	433,6832664	371,37243	414,5377025	359,4340029	322,5292522	3497,374644	388,5971827
ENSG00000106991	ENG	376,7971841	228,5510212	307,7914336	164,9825202	636,6464995	425,2117322	735,0297569	180,2065011	439,2778917	3494,49454	388,2771711

ENSG00000125304	TM9SF2	403,43568	374,593015	369,1640127	303,0227846	418,2120112	382,038837	329,5299741	494,9938534	410,8540424	3485,84421	387,3160234
ENSG00000165672	PRDX3	350,0720767	495,5746643	474,8449634	309,1990643	297,581864	313,5557688	327,5076438	531,3943297	376,5880846	3476,31846	386,2576066
ENSG00000100612	DHRS7	368,9162962	528,6086521	318,8771403	311,0499097	406,0935213	440,1302741	431,8666429	261,1745058	403,4396804	3470,156623	385,5729581
ENSG00000130592	LSP1	616,4494015	379,8388621	331,681385	277,8022289	518,6218703	284,6200706	304,4845879	194,4416783	551,9287161	3459,868801	384,4298668
ENSG00000168899	VAMP5	389,8270165	445,3193723	287,1288111	391,7327593	485,0359718	282,8880339	375,1404104	497,7432093	297,8249332	3452,640518	383,6267242
ENSG00000049860	HEXB	604,5628095	400,0475463	488,0033482	246,8346001	359,8336292	311,9714334	400,479283	336,2987104	301,5049462	3449,536306	383,2818118
ENSG00000089327	FXYD5	283,8435109	550,9767125	295,9981876	419,6057757	329,2410746	333,3732737	382,9507126	346,332522	505,3187432	3447,640495	383,0711661
ENSG00000168461	RAB31	525,4928044	432,4254936	261,685064	345,1336781	444,5030501	359,2709311	364,7942213	311,7887893	392,4293895	3437,523421	381,9470468
ENSG00000135940	COX5B	370,6980342	518,2341917	428,2552531	453,2409725	140,5567619	347,1342046	365,6141041	485,2182824	306,9498425	3415,901647	379,5446274
ENSG00000059377	TBXAS1	367,5714631	319,752173	360,7402401	246,9956959	584,4607937	387,644996	421,3890388	325,2707041	395,6980969	3409,523201	378,8359113
ENSG00000204257	HLA-DMA	340,3585711	360,7148335	319,897733	407,9697619	255,0874606	504,5129957	540,1696952	351,9390394	323,9039388	3404,554029	378,283781
ENSG00000179144	GIMAP7	474,7411174	370,6279046	337,2855731	255,3465814	544,5925828	203,3566212	270,5777011	706,8686753	235,5995039	3398,996261	377,6662512
ENSG00000164587	RPS14	286,1645282	385,5398651	379,2825672	580,6599031	308,3803532	382,3256127	313,3054925	467,3679007	283,5969658	3386,623189	376,2914654
ENSG00000141682	PMAIP1	49,28841432	381,2775285	770,8817242	461,3937448	190,243924	212,0092202	427,2166562	545,993225	340,0118253	3378,316262	375,3684736
ENSG00000165280	VCP	479,1234248	289,9675117	311,3423285	551,7235977	302,5953046	387,8021931	412,7025601	244,463946	380,7615522	3360,482419	373,3869354
ENSG00000169397	RNASE3	817,8859637	226,0586029	370,2540485	228,3181028	635,9373146	330,4385653	261,0479963	312,0770915	161,5832263	3343,600912	371,5112124
ENSG00000184840	TMED9	504,3005643	399,6854321	250,1820891	261,8974397	112,5921803	470,6692672	563,7937477	411,2826974	357,2975557	3331,700963	370,1889959
ENSG00000157303	SUSD3	410,4489825	263,0264134	197,5470656	226,5031735	255,4930051	441,812744	449,4191701	377,7145533	708,5213997	3330,486507	370,0540564
ENSG00000115053	NCL	186,3231673	276,2031689	673,1203101	340,6045181	483,4578441	298,8774783	363,1997189	334,5534909	362,4811184	3318,820815	368,7578683
ENSG00000113140	SPARC	350,1818978	1005,604721	322,9025455	403,0126908	192,1329148	170,7366762	344,9961014	294,1942756	218,9232789	3302,685102	366,9650114
ENSG00000116489	CAPZA1	287,7802456	291,5420888	255,63034	308,6423991	206,942126	470,4834694	440,5642101	630,9929338	408,5964741	3301,174287	366,797143
ENSG00000138085	ATRAID	438,4377033	528,7138694	382,48079	351,0150525	329,5912858	378,249819	286,9224977	319,4691807	249,8204366	3264,700635	362,744515
ENSG00000255302	EID1	395,6624472	573,0397574	207,9407268	343,2578017	378,1829645	380,8434692	325,5239858	269,1790733	387,6250571	3261,255283	362,3616981
ENSG00000142676	RPL11	287,5590401	349,2199121	308,6112269	846,8856077	226,3561493	327,2038602	288,1712364	357,0283988	263,5679712	3254,603403	361,6226003
ENSG00000197629	MPEG1	282,898583	287,8814044	548,9512445	413,4897087	36,70404418	425,9442181	413,4702206	315,854384	528,1271163	3253,320924	361,4801026
ENSG00000143110	C1orf162	302,3303253	395,7225688	316,2425331	371,0404841	297,232041	385,3464948	422,1478331	282,3257775	477,2388296	3249,626887	361,0696541
ENSG00000198034	RPS4X	300,7255077	341,4463489	153,4108601	865,3601188	268,6640453	367,0983852	296,6324197	470,3899594	184,3242299	3248,051875	360,8946528
ENSG00000153234	NR4A2	141,8067657	422,8158866	463,9314597	417,7724991	203,2552732	411,8249667	467,5297483	243,4693255	474,816058	3247,221983	360,8024425
ENSG00000179218	CALR	341,1401866	282,467936	193,6847337	296,1237	116,4419445	425,1687771	653,3333573	515,0103982	405,1098105	3228,480844	358,7200938
ENSG00000166794	PPIB	352,4331834	344,3330551	262,4155408	314,733185	320,21784	372,6259525	462,0932478	407,2074341	388,2257022	3224,285141	358,2539045
ENSG00000145337	PYURF	328,0401862	645,0952466	344,3475826	296,1641817	101,352777	342,5781321	292,5726964	500,3147342	366,0226701	3216,488207	357,3875785
ENSG00000165629	ATP5F1C	264,0506636	547,9019378	476,9502565	347,7625571	218,457985	331,1023034	276,8175609	417,4814553	328,9354608	3209,46018	356,6066867
ENSG00000136888	ATP6V1G1	308,7286995	381,8174118	471,2291275	294,208562	397,6804999	317,9024809	289,5361241	484,1938294	264,1141013	3209,410836	356,601204
ENSG00000152291	TGOLN2	286,1362992	319,8264477	307,6094507	422,7807183	374,511246	422,2939664	363,1636969	200,4376541	498,9412387	3195,700718	355,0778576
ENSG00000273749	CYFIP1	414,3714775	305,0907009	244,2669374	289,0229433	351,7034583	391,7872962	452,6522348	211,4793924	524,0072435	3184,381684	353,8201871
ENSG00000104774	MAN2B1	512,6061971	164,6074541	407,7627689	213,3510008	313,6765433	329,7835468	517,2207415	268,1475901	453,5844783	3180,740321	353,4155912
ENSG00000175489	LRRC25	484,5251178	308,4682589	306,2706159	397,2619411	242,2230807	290,1213381	449,9251337	253,9186108	446,8127142	3179,526811	353,2807568
ENSG00000179010	MRFAP1	270,2915631	362,8754073	414,6143849	429,2896305	415,5213145	379,4384263	313,5576847	328,3187906	260,8851181	3174,79232	352,7547022
ENSG00000100823	APEX1	480,172667	331,5520383	383,2175278	297,7370442	401,5794787	349,1511553	323,9210215	180,8104314	413,5725631	3161,713927	351,3015475
ENSG00000211445	GPX3	461,4559362	491,424064	260,2217245	337,7630775	397,505186	409,9491891	275,145883	299,0840393	218,6429674	3151,192067	350,1324519
ENSG00000166913	YWHAB	336,3151413	522,9905053	312,0816161	397,9860622	255,9285887	335,7868925	274,1241919	383,9677256	330,3729249	3149,553649	349,9504054
ENSG00000161203	AP2M1	295,3264832	304,7324454	338,7663275	295,6641249	495,9386077	327,9838608	364,6976177	337,2433538	386,5029335	3146,855755	349,6506394
ENSG00000146066	HIGD2A	187,6174058	385,8128778	469,2508093	246,5715815	516,1628285	348,0570508	408,4500563	241,0050286	319,9742876	3122,901926	346,9891029
ENSG00000158710	TAGLN2	633,9109705	242,8048992	521,3104872	375,4022272	194,3595545	274,3161606	268,8001322	335,483427	273,0531534	3119,441012	346,6045569
ENSG00000116288	PARK7	368,1625919	434,4862446	335,1355313	467,8758952	201,3575206	383,317803	332,9648652	337,1893835	257,409451	3117,917066	346,4352295
ENSG00000139687	RB1	198,4438725	353,0896517	530,4973588	421,8152178	264,1061281	375,2273923	309,1058474	442,6962805	219,7493984	3114,731147	346,0812386
ENSG00000138069	RAB1A	551,0769438	435,5992286	262,6253274	277,7110536	260,151555	353,6053674	306,8399124	305,7990896	346,9546454	3100,363123	344,4847915
ENSG00000102879	CORO1A	385,8577865	293,0239923	256,1229079	237,3110116	468,9687306	369,3903109	404,9258907	202,968636	480,3165405	3098,885726	344,3206362
ENSG00000074842	MYDGF	391,194469	371,8512719	190,6764565	291,8801832	281,4300168	378,5885567	436,1552125	441,8922345	303,3573448	3087,025746	343,0028606
ENSG00000143621	ILF2	369,6561549	310,1244273	445,4320648	428,5057765	383,8952233	276,0528923	246,7231739	250,639223	371,3642581	3082,393194	342,4881327
ENSG00000136250	AOAH	414,4993089	314,2074206	730,5887217	208,8223201	434,3173696	151,2575058	274,3546433	402,8289215	148,9419565	3079,818168	342,2020187
ENSG00000121691	CAT	538,4079344	244,2422242	322,1741969	286,5983194	429,8573768	316,1989505	437,8665679	206,8101233	296,8663224	3079,022016	342,1135573
ENSG00000171858	RPS21	169,0513044	357,9540407	304,0071932	813,0290521	309,2015905	282,336242	268,6227764	347,9769432	221,0066751	3073,185818	341,4650909
ENSG00000025708	TYMP	548,9930983	266,6523223	254,1973572	356,3936861	319,3153481	239,7868696	389,4459694	322,1737033	371,6930445	3068,651399	340,9612665
ENSG00000166927	MS4A7	241,1856864	453,2186073	288,4723283	394,495247	217,0408898	447,0230429	357,55435	338,2676539	320,2377243	3057,49553	339,7217256
ENSG00000143870	PDIA6	402,6298965	277,8842966	324,4117907	217,986643	418,5764088	295,1274462	419,4276473	370,3187692	322,0252103	3048,388109	338,7097898
ENSG00000142168	SOD1	419,8763256	615,9695871	246,5885871	365,3606603	186,2288593	236,0055356	330,2995615	358,7481783	278,8158078	3037,893103	337,5436781
ENSG00000137076	TLN1	521,0223929	227,6329008	416,0694434	303,9485569	280,2105894	256,4255815	318,1859523	224,0705041	481,6089555	3029,174877	336,5749863
ENSG00000081237	PTPRC	416,8891431	401,8620289	243,4448314	379,4486345	128,9501472	287,42994	359,0438656	320,919436	471,7112318	3009,699259	334,4110287
ENSG00000148834	GSTO1	301,8636947	388,8138286	398,9696631	315,2514211	187,3102734	384,1158532	288,3369072	486,3699814	258,6025233	3009,634146	334,403794
ENSG00000021355	SERPINB1	391,6873276	288,7344609	393,6606915	389,1330898	374,7928404	322,4556729	327,1205505	179,8032533	339,3666065	3006,754493	334,0838326
ENSG00000182054	IDH2	456,4999862	217,4484876	317,8891111	243,4094912	417,1838116	325,5715013	376,6339355	205,3767088	434,1041901	2994,117223	332,6796915
ENSG00000172216	CEBPB	537,1532709	252,3770052	312,656553	233,4540928	386,2900353	295,0086839	255,8157894	360,5047889	357,6310377	2990,891257	332,3212508
ENSG00000073849	ST6GAL1	388,3975112	219,7918934	362,46365	392,6022943	329,9053315	409,0729251	280,9826627	207,7968202	388,9266776	2979,939766	331,1044184
ENSG00000172270	BSG	536,5877862	197,3107054	294,6720828	232,0032853	292,7228848	303,8125467	415,6018872	326,2573594	380,79693	2979,765468	331,085052
ENSG00000138279	ANXA7	379,7827864	364,9242105	408,3257638	270,0521994	267,7696839	354,8932741	289,5199415	297,7605694	341,0433036	2974,071733	330,4524147
ENSG00000126267	COX6B1	252,436651	312,3126784	288,5749477	501,8109944	273,5139421	336,6441353	288,0630232	459,3638174	260,7613995	2973,481589	330,3868432
ENSG00000171766	GATM	304,9136398	309,7170023	443,9947845	282,305704	475,6427093	283,2771397	297,810638	293,1974117	282,3716854	2973,230715	330,3589683
ENSG00000133639	BTG1	188,1409978	593,507725	378,2219336	295,3786086	275,2262489	239,003806	365,9877892	341,786161	295,6209515	2972,874221	330,3193579
ENSG00000111679	PTPN6	236,4251432	237,2540117	204,003762	277,761087	365,7134325	403,2663748	426,0330219	363,8804301	454,5429715	2968,880235	329,8755816
ENSG00000163513	TGFBR2	175,0428358	139,8283006	378,6769716	319,012976	525,3162859	340,6034097	430,7974771	313,575661	339,9284604	2962,782378	329,198042
ENSG00000155090	KLF10	252,5100742	807,5137386	522,4949152	279,9195134	103,5717173	366,1669441	179,4544267	83,36532926	364,105487	2959,102146	328,7891273
ENSG00000111885	MAN1A1	415,7196947	364,914651	348,4999607	413,1383765	364,1987192	257,7267727	295,2478438	228,3672938	263,3628178	2951,17613	327,9084589
ENSG00000143256	PFDN2	282,0243984	275,914232	345,5726834	544,1397618	352,7563621	286,6699659	215,1785887	361,2964112	276,5252602	2940,077664	326,675296
ENSG00000172531	PPP1CA	381,9090243	351,3863856	289,9339772	277,8647429	144,5410855	418,8131193	380,4813274	330,683858	361,3176069	2936,931127	326,3256808
ENSG00000185624	P4HB	384,1750104	261,5294369	312,4154085	173,9899047	306,6675706	357,638628	389,0995461	381,0325581	368,4955322	2935,043595	326,115955
ENSG00000106153	CHCHD2	230,340724	413,168218	288,7964452	454,3517554	449,9449363	305,0301459	255,3345667	308,8984624	227,0654008	2932,930655	325,8811839
ENSG00000122406	RPL5	337,7120017	307,2415331	319,6359814	535,9802343	298,7332367	270,6703624	248,2739553	322,9671197	287,1446267	2928,359051	325,3732279
ENSG00000136942	RPL35	192,063012	337,3173956	288,8457706	565,7274238	289,1656563	317,2296695	318,7028172	346,3643121	259,7008406	2915,116898	323,9018775
ENSG00000138071	ACTR2	123,7528483	170,6806796	497,576064	433,6310689	420,8292525	380,3310092	309,5627584	312,8752427	250,9590719	2900,197996	322,2442217
ENSG00000084623	EIF3I	337,4364743	452,9656744	193,0551644	416,4370928	358,284806	283,0578709	359,5361456	173,5499815	324,07324	2898,39645	322,04405
ENSG00000140931	CMTM3	356,7525576	254,5760584	406,6894244	185,8211282	415,2332518	288,3114633	304,9375024	357,4152959	327,3588598	2897,095542	321,8995046
ENSG00000110324	IL10RA	422,2591113	233,887869	287,2438149	227,1506265	300,5648056	308,6608396	311,5696877	377,0510462	414,6795149	2883,067316	320,3408129
ENSG00000126821	SGPP1	314,9495797	164,1324833	310,6444272	421,2817066	567,6339331	306,9544603	299,9478111	177,4996238	318,7691248	2881,81315	320,2014611
ENSG00000110848	CD69	44,87429023	589,1170638	305,2676934	393,9823275	334,2633622	338,0310832	276,7258805	69,77396588	529,0588498	2881,094516	320,1216129
ENSG00000115234	SNX17	284,8516491	164,0741466	322,32999	247,7739326	752,5547861	290,4646279	258,8346031	219,9973176	340,0690412	2880,950094	320,105566
ENSG00000138413	IDH1	533,8002514	159,8287626	538,4723587	106,7285397	227,9761225	249,2348676	300,320854	540,9019991	223,1260513	2880,389807	320,0433119
ENSG00000129625	REEP5	396,1043821	208,494912	304,2112356	332,0453363	471,443437	225,945657	247,4992281	368,3505622	321,2329546	2875,327705	319,4808561
ENSG00000108700	CCL8	25,06822692	439,8524224	78,04081665	428,3329339	1314,403534	99,50785169	112,5157386	365,3180311	8,408369337	2871,447924	319,0497694
ENSG00000170525	PFKFB3	109,6132452	141,175842	456,9803688	271,9032913	493,1551988	384,2211082	405,5930065	112,5769061	483,5535617	2858,772529	317,6413921
ENSG00000196664	TLR7	401,9349422	205,9656394	235,1687052	624,5682559	1,173812386	396,8966344	403,4060549	297,2068654	292,1160007	2858,43691	317,6041012
ENSG00000142657	PGD	452,5156333	214,4814198	265,0527316	178,419084	667,4867011	246,8000718	343,6976091	225,7424273	257,8320789	2852,027757	316,891973
ENSG00000137818	RPLP1	205,8021886	299,2273427	280,4590113	512,161114	156,2580942	352,7895566	433,0115424	357,4627332	242,0805253	2839,252108	315,4724565
ENSG00000125740	FOSB	165,5331535	372,3470129	332,4447753	280,9526766	216,3888523	302,0630165	348,0316088	472,9504669	328,2184124	2818,929975	313,2144417
ENSG00000105697	HAMP	162,1983484	438,002743	234,56392	464,157916	225,483656	361,3859312	341,6896463	230,3343683	355,848687	2813,665216	312,6294685
ENSG00000110700	RPS13	209,5542807	426,2380162	227,7728187	558,2651348	275,5781424	316,7597677	262,0342584	353,8696432	178,9047373	2808,976799	312,1085333
ENSG00000110958	PTGES3	244,5154865	301,7484997	349,8672737	368,6407026	155,3112054	386,8318095	313,3620619	383,5140832	293,6692177	2797,46034	310,8289267
ENSG00000099795	NDUFB7	281,3198661	158,3792001	266,5491416	513,5785575	257,7591332	304,5311894	394,4693429	358,2281293	258,7956245	2793,610184	310,4011316
ENSG00000168385	SEPT2	224,1466638	317,5327534	422,7723896	418,4579316	69,99336214	364,0228267	305,768036	282,4682593	386,3028281	2791,465051	310,1627834
ENSG00000140105	WARS	601,3753752	243,7577842	186,5300696	408,6448611	373,2991822	247,064759	182,0662165	267,1134059	279,6762092	2789,527863	309,9475403
ENSG00000149131	SERPING1	746,9131704	317,0371691	189,177686	176,5638268	233,2320425	192,0197349	168,930497	629,045434	133,0275273	2785,947088	309,5496764
ENSG00000070756	PABPC1	393,3903354	377,0282138	207,999095	393,0069572	327,8560442	284,2123793	286,1280274	224,5393324	290,5336397	2784,694024	309,4104472
ENSG00000165704	HPRT1	305,5958191	414,3559071	304,3640662	171,5825515	514,6163293	264,4071163	202,4493384	280,0084487	318,7752072	2776,154784	308,4616427
ENSG00000136238	RAC1	237,0973599	398,4936111	161,2284574	435,9297527	261,4607397	336,7449512	316,5899796	254,7543924	362,5805699	2764,879814	307,2088682
ENSG00000059804	SLC2A3	74,92057613	277,0197043	292,3118023	383,6014904	595,0194957	173,4258804	246,2285514	252,8741559	460,4377436	2755,8394	306,2043778
ENSG00000003056	M6PR	411,1809252	248,2463873	321,6349932	244,3399469	405,697642	292,1136848	338,4724075	167,751998	320,4750908	2749,913076	305,5458973
ENSG00000244405	ETV5	162,9418504	214,1698878	380,254433	369,8198445	434,2006661	294,2592058	291,10508	186,2522229	414,5088898	2747,51208	305,27912
ENSG00000178741	COX5A	254,2441049	324,9654556	134,3780116	357,1621259	324,1807679	367,4806665	327,9639296	386,0053884	265,8754546	2742,255905	304,6951006
ENSG00000186480	INSIG1	570,369954	348,3642094	218,2714969	347,579684	67,00247845	239,5021448	322,0290603	158,5180624	460,7975415	2732,434632	303,603848
ENSG00000077984	CST7	301,0339651	148,6668328	585,9109157	29,28563127	105,5990978	192,1657858	620,4514958	127,2043285	621,7828091	2732,100862	303,5667624
ENSG00000140749	IGSF6	98,98243611	260,639902	379,0856383	220,5190296	342,2337352	384,7957737	319,6290661	248,278641	471,8797045	2726,043927	302,8937696
ENSG00000079246	XRCC5	429,4605016	254,1041798	316,550283	436,5156018	62,12082693	273,9361892	303,6046712	289,1226858	341,2799399	2706,694879	300,7438755
ENSG00000084090	STARD7	194,7251758	124,7157966	343,0352513	289,310323	402,8885523	351,4041644	357,4511611	334,9262019	300,158554	2698,61518	299,8461311
ENSG00000163932	PRKCD	411,6821226	193,7901602	438,1459178	140,514568	266,4035397	306,5898773	278,9890918	230,7549985	430,432901	2697,303103	299,7003448
ENSG00000118418	HMGN3	219,9597656	473,0828779	209,9928558	501,5406958	90,31062456	244,120888	232,998654	361,7183136	357,7637194	2691,488395	299,0542661
ENSG00000112695	COX7A2	203,0793986	341,8043185	252,2358442	544,4064767	328,4748384	270,4463251	222,2822479	314,1019536	213,6944923	2690,525895	298,9473217
ENSG00000123416	TUBA1B	314,2288084	334,1646526	257,9910432	328,176472	197,5447925	367,8906884	349,9758645	185,6498633	342,1052798	2677,727465	297,5252739

ENSG00000167658	EEF2	371,0307546	205,4577225	235,5330011	312,9143117	403,3262425	269,8058346	337,531107	206,3469781	332,1758159	2674,121768	297,1246409
ENSG00000117748	RPA2	345,7007099	286,4151803	384,6256695	308,617186	151,0070231	292,1232163	171,6429075	418,005566	305,2723793	2663,409838	295,9344264
ENSG00000254087	LYN	309,0608313	316,8634537	180,7051033	345,2604888	76,78730059	406,2440516	368,329925	300,8434122	358,7284104	2662,822977	295,8692197
ENSG00000095585	BLNK	235,2032932	293,5256203	341,2576482	224,739617	305,7657433	298,7298236	256,6735082	196,1813985	490,1670245	2642,243677	293,5826308
ENSG00000140497	SCAMP2	204,6339348	256,2364953	289,7108592	299,6055378	264,5978052	333,5798055	352,3708804	294,8137211	345,74887	2641,297909	293,4775455
ENSG00000136810	TXN	193,4514533	359,4695724	284,8607945	677,6558796	125,8689208	255,436581	262,990785	242,7549001	222,4638074	2624,952694	291,6614104
ENSG00000100220	RTCB	318,7143386	409,4613133	179,9639947	333,4218411	116,9787846	351,3964578	284,1747302	302,2335895	322,7195475	2619,064597	291,0071775
ENSG00000178952	TUFM	358,9474071	306,4653253	457,5167708	138,3155994	158,3625526	291,7504849	339,1274102	244,9490078	317,897579	2613,332137	290,3702375
ENSG00000116701	NCF2	173,8700521	327,3017446	514,1872323	177,6104523	260,7526964	307,0592265	295,3352214	294,3215	256,1736899	2606,611815	289,6235351
ENSG00000187840	EIF4EBP1	217,5648784	405,2600683	114,9826773	220,9051774	477,8711842	277,7116812	310,6816516	140,0591974	430,1924612	2595,228977	288,3587752
ENSG00000111269	CREBL2	242,6168443	256,3142168	113,1907746	235,4535633	623,7401067	295,229453	268,9838224	308,1079075	237,307339	2580,944028	286,7715586
ENSG00000145741	BTF3	209,0556861	465,3084323	200,0059488	300,0699323	215,1502747	324,4234382	253,9413732	358,1605324	253,8722254	2579,987843	286,6653159
ENSG00000121966	CXCR4	172,6492626	469,8513374	126,9909085	759,691529	350,0750143	139,8005489	253,3665742	5,497337679	301,6663424	2579,588855	286,6209839
ENSG00000134153	EMC7	412,8482327	434,1610484	236,6276876	279,2651174	160,267333	267,8336032	232,2756559	335,6687239	212,5971581	2571,54456	285,7271733
ENSG00000011422	PLAUR	327,8187363	293,4701989	148,5013722	242,434761	384,0954284	306,3222203	320,5869735	223,7495007	319,195676	2566,174867	285,1305408
ENSG00000166428	PLD4	265,0553481	215,220754	276,1473507	248,5558035	251,7991998	458,8995713	352,7088464	20,79128209	476,8293816	2566,007538	285,1119486
ENSG00000198898	CAPZA2	317,2816356	379,8144272	264,5926731	301,0044586	141,6643173	315,543359	247,447047	375,6481884	221,1813474	2564,177454	284,908606
ENSG00000169313	P2RY12	90,14611235	251,8428898	1044,331905	227,3881973	460,5358908	131,907387	172,6482567	29,68400902	153,0559578	2561,540606	284,6156229
ENSG00000148248	SURF4	548,1651093	257,7120936	185,1048903	249,1190512	180,8618102	330,328199	311,3995177	236,6275989	250,636558	2549,954828	283,3283142
ENSG00000125538	IL1B	0,048808324	589,8950784	72,54777777	285,2922768	30,39043802	325,1225675	369,3998954	273,0140903	604,0612253	2549,772158	283,3080175
ENSG00000277443	MARCKS	184,2803258	348,7843334	275,1977097	290,2111985	195,6087172	284,7050213	304,4174279	395,6600129	270,7666161	2549,631363	283,2923737
ENSG00000071553	ATP6AP1	384,7409767	307,611886	289,4508442	134,5655217	333,313572	255,6610119	347,9219409	160,2312561	327,015098	2540,512107	282,279123
ENSG00000174125	TLR1	132,6922005	283,0354945	173,1478421	426,2263824	240,7836817	392,5246044	343,3633771	217,3279148	331,3450559	2540,446553	282,2718393
ENSG00000163154	TNFAIP8L2	298,175867	312,137665	321,4294927	329,2107063	408,1163089	276,3743545	303,2741444	80,75387099	202,2164232	2531,688833	281,2987592
ENSG00000241839	PLEKHO2	212,1883181	140,4484703	374,8734579	191,0203899	553,7802897	248,5314578	324,5840946	188,3503676	290,8815226	2524,658369	280,5175965
ENSG00000153310	FAM49B	163,7527603	322,6982677	271,4354514	293,9090176	213,6143769	318,7764979	260,1462184	342,0497116	336,0582153	2522,440517	280,2711686
ENSG00000092820	EZR	641,5097331	177,2436508	164,9505924	308,8811828	127,1829259	201,42691	221,2280111	339,9528464	336,1353867	2518,511239	279,8345821
ENSG00000185043	CIB1	283,1270067	260,126886	197,3432089	269,6209894	286,4157573	395,5028625	288,9568108	176,890244	357,0475306	2515,031296	279,4479218
ENSG00000100504	PYGL	319,1072324	183,7356644	87,56434266	305,5338473	612,0639503	251,6395571	248,1254476	321,4213941	181,5539827	2510,745418	278,9717132
ENSG00000137491	SLCO2B1	183,5633577	149,1347208	358,0360042	121,3797607	232,7967065	453,5252408	477,1048466	149,0483041	379,7366386	2504,32558	278,2583978
ENSG00000176788	BASP1	424,9544706	246,5257065	336,1408932	287,8066565	101,0750212	255,6692811	229,5417164	212,0262812	401,7437171	2495,483744	277,2759715
ENSG00000182899	RPL35A	186,2028293	335,9321616	215,4815177	435,3392452	239,1036708	258,284719	201,3821025	417,799384	195,7694952	2485,295125	276,1439028
ENSG00000178913	TAF7	402,1661056	300,2442006	183,2443179	405,536839	216,0244342	254,8064751	247,7152056	135,7900408	332,312419	2477,840038	275,3155598
ENSG00000115866	DARS	326,8020486	439,285663	222,2904832	151,4433207	437,7878626	195,5873423	171,1487147	249,2166435	283,8539686	2477,416047	275,2684497
ENSG00000118816	CCNI	287,8315886	305,0402957	279,396843	252,255807	294,0949187	349,5337096	304,9588729	125,6856805	265,3597205	2464,157437	273,7952707
ENSG00000174748	RPL15	288,9764735	317,5229001	308,2723086	279,6506097	181,3488891	257,7334201	238,9009326	370,2404487	216,1861858	2458,832168	273,2035742
ENSG00000134294	SLC38A2	314,8272313	246,3907363	220,240186	333,0138015	429,5727738	259,125353	206,6434105	210,8811174	223,0500894	2443,744699	271,5271888
ENSG00000125835	SNRPB	233,0508097	262,686842	256,881453	249,7053787	335,2325728	286,773446	258,3039464	285,1872676	274,9888682	2442,810584	271,4233983
ENSG00000168894	RNF181	213,9081603	280,2303689	147,4205935	397,3911334	161,6028941	249,9538033	337,6386065	346,1437519	304,9710964	2439,260408	271,0289343
ENSG00000130255	RPL36	196,8230154	276,6431996	188,1353642	500,4987888	204,780809	254,0163255	267,0733531	277,6174174	263,3439492	2428,932222	269,881358
ENSG00000155660	PDIA4	317,707913	187,4880086	290,707508	117,9666607	98,76276862	336,197812	390,297191	467,3975917	221,9113416	2428,436795	269,8263106
ENSG00000175348	TMEM9B	210,53848	258,0807427	267,9432954	156,8380838	279,2853949	352,4351755	291,7573844	292,5369392	315,9719811	2425,387477	269,4874975
ENSG00000124562	SNRPC	350,8054425	332,7467205	143,3579882	215,097888	422,4912263	235,4299159	255,9958748	281,1755869	182,4021468	2419,50279	268,8336433
ENSG00000149806	FAU	188,7905967	288,0621099	286,9587843	402,8647218	164,2475581	280,4831907	278,82296	294,1465336	231,7078734	2416,084329	268,4538143
ENSG00000204628	RACK1	302,7256841	309,1914515	162,6578042	447,2486451	274,6439836	232,4963908	211,7076203	246,4743986	227,5456418	2414,69162	268,2990689
ENSG00000058262	SEC61A1	211,5547989	212,2598203	326,7464353	245,9762024	406,948284	295,0991357	359,5644406	181,2405117	173,1897333	2412,579362	268,0643736
ENSG00000129515	SNX6	214,1684044	235,1549818	174,7110001	150,3315993	298,4918525	284,0081906	304,0630604	530,1442294	217,5377685	2408,611087	267,6234541
ENSG00000231500	RPS18	203,018782	282,0315802	263,8840525	525,5691364	234,581688	238,0102112	212,6975257	246,1142962	200,2359477	2406,14322	267,3492467
ENSG00000101367	MAPRE1	325,8137363	251,5008689	174,469945	328,1547573	247,8219418	331,1088129	323,3726004	202,7490789	218,9971314	2403,988873	267,1098748
ENSG00000149781	FERMT3	365,2085221	144,7407078	336,3686129	137,9568147	232,1496963	268,7253503	342,8131021	211,2367098	356,6604491	2395,859965	266,2066628
ENSG00000184574	LPAR5	210,8785914	254,1107514	220,3977905	329,393916	167,4413081	333,6772443	280,3272557	61,05467289	530,3768669	2387,658397	265,2953775
ENSG00000084754	HADHA	298,0691769	277,3669269	174,8367097	171,586887	484,8211199	217,894103	250,1294958	232,3952188	279,9498294	2387,049468	265,2277186
ENSG00000169813	HNRNPF	141,2161497	161,8084064	509,131703	265,1060839	308,7770569	274,7495471	282,7228815	154,6023399	288,2820631	2386,396232	265,1551368
ENSG00000184557	SOCS3	138,8957074	308,2554156	165,323936	236,2158238	125,0475129	295,2285866	459,1094372	325,8418413	331,7911029	2385,709364	265,0788182
ENSG00000176978	DPP7	295,5300959	178,6663486	319,7312722	136,7990657	365,8597421	266,4090132	279,5637224	238,3155436	302,7000742	2383,574878	264,8416531
ENSG00000162704	ARPC5	299,4401393	240,7990694	314,0261569	308,3442869	292,4869073	221,1050531	189,6192482	298,6321796	212,8073558	2377,260396	264,140044
ENSG00000162736	NCSTN	235,5364233	185,9663109	373,8701113	210,1502446	395,1944681	231,2145219	219,8995894	327,1550184	194,3942926	2373,38098	263,7089978
ENSG00000108946	PRKAR1A	294,1793385	230,0100927	209,310334	397,0067683	159,7364937	251,8454935	253,1296348	314,7872482	254,1800625	2364,185466	262,687274
ENSG00000116815	CD58	331,3590541	460,3805753	178,8795704	284,1909505	48,71437514	209,8062001	255,2308491	248,9372527	335,0237756	2352,522603	261,3914003
ENSG00000139832	RAB20	384,645089	86,65239153	239,4281145	193,7533089	364,5984338	180,7731698	292,8196136	253,2527707	356,5244166	2352,447309	261,3830343
ENSG00000124783	SSR1	264,8063291	253,493305	207,2175203	295,4687954	154,9246178	323,4883462	249,9456574	285,3925131	316,8020812	2351,539166	261,2821295
ENSG00000182718	ANXA2	398,2236186	328,309856	204,641741	292,6647148	98,40732292	254,3185019	234,3895005	237,2283528	296,5877977	2344,771406	260,5301562
ENSG00000138135	CH25H	91,95696649	1119,325858	87,05426587	139,9081952	519,2933382	129,3039726	226,8289676	13,13114829	16,86507586	2343,667748	260,4075276
ENSG00000135926	TMBIM1	360,1671348	241,3962439	178,4246895	227,4279113	388,4505283	247,4162242	230,9363621	241,9380527	216,863515	2333,020662	259,224518
ENSG00000023318	ERP44	194,8282919	198,3180017	322,0938293	295,0714345	208,3897552	254,7665948	207,2817902	396,4115194	253,0264839	2330,187701	258,9097446
ENSG00000198961	PJA2	235,7010634	236,3346687	250,4657969	268,2208539	507,7298278	237,7316775	158,184915	164,8673145	268,9460071	2328,182125	258,6869028
ENSG00000106803	SEC61B	223,9786126	251,507881	184,1704378	290,5032885	139,6073159	294,631934	246,6856734	428,28037	252,4069393	2311,772452	256,8636058
ENSG00000187514	PTMA	201,0781572	261,6329284	224,3261545	337,0216975	153,6189605	261,5866582	287,266888	328,0868974	256,3779733	2310,996315	256,7773684
ENSG00000143815	LBR	181,1738996	189,8126032	227,3010652	482,752552	448,8025888	174,4530828	213,4094333	191,2006674	200,7207536	2309,626646	256,6251829
ENSG00000123562	MORF4L2	222,4429775	231,1813392	293,7267058	162,7887379	473,3082412	209,5184926	264,9660485	213,010501	237,3613815	2308,304425	256,4782695
ENSG00000117592	PRDX6	172,1653538	282,0654272	193,6598699	306,0989685	492,6017349	217,744171	209,0810249	203,6083185	229,2723648	2306,297233	256,2552482
ENSG00000165527	ARF6	411,839674	251,6728914	122,16777	394,9377099	188,5946319	241,3089059	213,9957224	258,2079666	223,4474703	2306,172742	256,2414158
ENSG00000142634	EFHD2	299,8954459	170,1548288	300,3589231	154,769245	299,4535781	313,0956503	290,1563321	165,4780845	312,8000858	2306,162174	256,2402415
ENSG00000143612	C1orf43	286,7139142	186,7955506	261,5303403	315,1742671	150,6410665	346,92088	273,1681797	278,9511293	206,1713105	2306,066638	256,2296265
ENSG00000066336	SPI1	318,4350353	220,0604718	209,4591466	180,4726673	216,2689501	320,5947248	342,8754264	174,5601654	316,1816135	2298,908201	255,4342446
ENSG00000164691	TAGAP	19,47548808	279,0276866	438,96246	111,801926	465,7374241	260,4905606	215,8372011	204,3700223	302,2448944	2297,947663	255,3275181
ENSG00000169919	GUSB	365,4135403	278,335942	211,5733453	188,278023	61,71323808	279,8276	278,9482978	338,4132085	292,8288126	2295,332008	255,0368897
ENSG00000118271	TTR	0	0	0	0	2290,105313	0	0	0,413905513	0,387988564	2290,907207	254,5452452
ENSG00000090487	SPG21	226,6468866	135,5857364	238,0592814	124,0145385	440,6245115	282,5853257	227,1570585	385,3548999	230,4534204	2290,481659	254,4979621
ENSG00000115484	CCT4	244,2609224	242,4297535	149,0245033	192,5373582	416,9539477	296,3171499	272,4949898	192,0704447	282,5885476	2288,677617	254,297513
ENSG00000124767	GLO1	134,7552245	375,1256504	209,6649558	192,4893828	140,0802298	264,710466	264,8403909	420,4765846	285,8691837	2288,012069	254,2235632
ENSG00000188186	LAMTOR4	134,0950209	294,7345951	341,875318	279,6694588	252,4581483	246,2949367	276,2810048	296,2987651	163,3836678	2285,090916	253,8989906
ENSG00000119013	NDUFB3	297,5494709	269,0985114	165,9286679	356,8076347	223,7545686	290,3928287	234,5295623	254,9964736	190,9291263	2283,986844	253,776316
ENSG00000122122	SASH3	252,0530934	199,1744298	251,0035553	253,1288207	214,6058133	364,9911629	245,72227	133,8133418	363,5567502	2278,049238	253,116582
ENSG00000109475	RPL34	143,8957424	287,3458038	267,7391581	459,8853958	198,2396572	201,5082483	171,2135683	373,771845	171,76973	2275,369149	252,8187943
ENSG00000079819	EPB41L2	153,5119402	182,0351601	450,3169859	347,9132995	140,1323976	290,4560455	287,9130748	106,9749893	314,0301992	2273,284092	252,5871213
ENSG00000112977	DAP	298,4445011	208,9449266	87,00834993	193,7797372	479,7494095	233,1436042	253,4045265	247,6538755	269,5078695	2271,6368	252,4040889
ENSG00000152558	TMEM123	182,2715978	203,2494629	330,5472496	441,2162526	247,5501834	223,2061248	196,0014934	203,3042938	242,202953	2269,549611	252,1721791
ENSG00000103363	ELOB	268,8392918	248,8871224	214,8924169	319,4602836	183,0608039	312,7002536	311,3399462	239,9165876	227,6949413	2264,791647	251,6435164
ENSG00000256618	MTRNR2L1	1,848744908	364,313986	1010,429915	820,9454513	33,0674878	2,563975429	2,908520252	26,38370018	1,436032368	2263,897814	251,5442015
ENSG00000115128	SF3B6	339,0249603	386,1244801	114,8984409	263,0224544	179,3991634	281,9742424	203,5407608	259,9418598	234,7448072	2262,671169	251,4079077
ENSG00000164919	COX6C	196,2988718	210,7959971	210,740075	463,5636828	284,5662236	228,6776312	213,4198531	291,3233964	158,6411132	2258,026844	250,8918716
ENSG00000146731	CCT6A	429,2467458	229,1686829	253,6225084	331,1553851	238,0110805	186,4013526	179,2992576	192,5387067	218,4638916	2257,907611	250,8786235
ENSG00000111639	MRPL51	223,7263852	318,7567004	82,51282477	315,4530889	322,1191212	244,2041125	250,4886815	317,579044	175,8043831	2250,644342	250,0715935
ENSG00000168002	POLR2G	349,0161603	286,3282872	206,5120294	408,5511853	64,76199633	190,6837648	270,4010993	266,5019067	206,3658544	2249,122284	249,902476
ENSG00000139921	TMX1	258,901466	339,0180756	157,2686888	315,1831746	189,9239259	246,7775809	205,2507297	303,6154425	231,3402022	2247,279286	249,6976985
ENSG00000116752	BCAS2	287,3328229	276,6709543	109,5530569	126,3176293	576,4268387	191,6531022	210,6422021	206,8740853	260,9636969	2246,434389	249,6038209
ENSG00000144655	CSRNP1	115,6019257	253,1991023	331,42457	221,4311967	262,7228336	315,3328267	299,6526752	187,2121544	259,609755	2246,18704	249,5763377
ENSG00000125148	MT2A	265,3528073	210,0543458	215,9364007	332,0211985	275,8211321	174,0790405	201,5212772	361,525785	203,3331754	2239,645163	248,8494625
ENSG00000162496	DHRS3	229,9116165	223,972948	204,0366018	179,8706809	514,9224505	182,7612384	256,7947563	204,0891738	240,7809566	2237,140423	248,5711581
ENSG00000125995	ROMO1	233,5540871	239,1123818	125,5266259	263,9085925	162,6771773	302,9876914	311,8885033	343,1435893	241,4416358	2224,240285	247,1378094
ENSG00000245848	CEBPA	229,4254685	162,4728151	275,628864	126,504807	259,707842	386,0402961	311,6256612	91,34273512	378,8382396	2221,586729	246,8429698
ENSG00000112697	TMEM30A	352,2359863	163,5230069	313,9328455	270,9571684	399,0898653	178,102712	174,4733443	179,1715556	190,0711346	2221,557619	246,8397354
ENSG00000163344	PMVK	321,6754962	225,0573075	311,2711288	239,8414287	264,8581475	247,2100709	225,0201463	140,1543824	244,2713272	2219,359436	246,5954928
ENSG00000162783	IER5	125,3842486	264,9406868	214,8571109	211,028357	258,2765133	219,1937013	385,4288016	174,6853229	364,9922745	2218,787017	246,5318908
ENSG00000117448	AKR1A1	233,9042508	174,9237783	294,9029502	128,8371662	157,8690382	346,8121123	309,2335129	286,7083655	284,337438	2217,528612	246,3920681
ENSG00000156136	DCK	186,3193823	221,9970346	271,7091976	253,8713143	276,2531658	230,5531443	174,7203689	364,5109651	234,4193645	2214,353937	246,0393264
ENSG00000108588	CCDC47	257,5543282	275,1011287	280,5977343	236,3636977	67,63352888	250,8092365	279,5850163	220,3909013	346,1252754	2214,160847	246,0178719
ENSG00000171700	RGS19	274,8006827	291,809366	198,0676741	161,7333644	209,3081534	312,4321955	265,912962	138,0069422	357,3971582	2209,468498	245,4964998
ENSG00000142541	RPL13A	196,1439446	209,6238853	246,4154529	424,170784	197,3706375	256,1844194	202,7439556	234,470455	240,5257228	2207,649257	245,2943619
ENSG00000117676	RPS6KA1	306,5613328	178,8220952	210,6110267	178,8052035	146,7230747	311,8577461	335,5968873	167,9690332	366,8289445	2203,775344	244,8639271
ENSG00000171729	TMEM51	369,5389425	183,6736757	55,27307598	144,9250105	218,2304994	469,1903664	368,9545375	114,8695682	273,7042616	2198,359938	244,2622153
ENSG00000093072	ADA2	330,9710385	123,873964	292,0324018	145,7266672	598,7080259	175,7081452	168,5575129	241,9036149	118,8657617	2196,347132	244,0385702
ENSG00000116209	TMEM59	281,9717633	315,4456604	261,6944576	240,1434568	175,5746821	212,8965101	233,6746078	295,1482442	177,1727366	2193,722119	243,7469021
ENSG00000120265	PCMT1	206,1229288	329,8752572	284,0658215	225,1854089	141,6434657	251,0334821	212,0856212	298,2311986	245,1047884	2193,347972	243,7053303
ENSG00000110880	CORO1C	298,8204595	273,0680626	312,7488897	127,0320299	265,7645744	272,5637475	202,1124974	163,193802	276,6359057	2191,939969	243,5488854
ENSG00000118564	FBXL5	279,6850798	280,3969045	131,4220367	205,4156809	236,1396129	290,0260551	229,7042684	358,3983063	177,4746912	2188,662636	243,1847373

ENSG00000115875	SRSF7	157,7601829	242,0238424	159,0224843	372,5983716	91,1776801	184,2038072	202,7377231	350,2034588	426,1835205	2185,911071	242,8790079
ENSG00000100650	SRSF5	127,8389222	239,8111485	147,9412474	381,1004744	244,6182401	239,3113732	245,623926	258,4507818	300,6432634	2185,339377	242,8154863
ENSG00000197405	C5AR1	239,7172413	122,9436498	298,9880417	103,1767174	530,0739702	189,3641974	208,6939058	194,7601854	295,8872262	2183,605135	242,6227928
ENSG00000155876	RRAGA	66,56853333	152,8865023	278,5049198	160,4364192	421,0421532	300,8108065	258,1261896	274,495676	269,8224839	2182,693684	242,5215204
ENSG00000127184	COX7C	171,0642265	303,8216365	158,8256025	426,7194907	200,2595538	244,6106674	227,809262	297,8777843	146,5818431	2177,570037	241,9522263
ENSG00000172243	CLEC7A	172,821445	130,9992062	392,7546983	291,2009406	221,0415613	301,569201	227,673254	225,0740467	214,2779353	2177,412289	241,9346987
ENSG00000163106	HPGDS	344,8127044	249,5968878	77,56052975	184,4220664	130,3098235	439,6295256	321,2539765	132,1112151	298,7102085	2177,406937	241,9341042
ENSG00000123349	PFDN5	197,6955454	277,3445066	148,2012244	299,5348229	106,035298	259,0757467	251,9800632	416,0738223	220,321602	2176,262631	241,8069591
ENSG00000095139	ARCN1	307,5391336	110,415188	323,633522	212,7710135	360,6650922	191,4902778	173,5617856	240,0931081	250,8505369	2171,019658	241,2244064
ENSG00000144802	NFKBIZ	59,55323405	328,9305457	145,866548	288,1691231	124,8575393	206,5027273	157,7414204	445,7675409	411,7467173	2169,135396	241,015044
ENSG00000109861	CTSC	283,2046816	184,6520645	177,424244	196,5885702	356,9429824	276,5079212	267,6773823	228,9118452	192,3703142	2164,280005	240,4755562
ENSG00000010256	UQCRC1	254,4920847	260,6099214	239,5452726	162,4735563	315,8530694	223,38896	240,1715188	227,6120757	233,7582662	2157,904725	239,7671917
ENSG00000166797	CIAO2A	168,9630142	353,0931824	230,379082	259,4340212	260,7574721	225,374102	227,1790259	286,5715718	145,1411999	2156,892672	239,6547413
ENSG00000121064	SCPEP1	180,32868	121,5790486	236,7845017	279,2877849	301,7034738	286,2205044	296,367893	166,0320069	286,7043157	2155,008209	239,4453565
ENSG00000132589	FLOT2	225,6851974	268,5374898	245,1800873	164,9509294	213,5637066	292,129862	255,8262345	145,3327257	340,9927341	2152,198967	239,1332185
ENSG00000179091	CYC1	354,4901569	175,2553164	228,416658	184,635089	219,3489217	232,1733847	204,0251145	364,6058447	185,8465223	2148,797008	238,7552231
ENSG00000163565	IFI16	131,3012136	301,7230628	238,5462013	308,5972565	220,8023423	227,3043847	209,6781313	239,2436149	270,6253948	2147,821602	238,6468447
ENSG00000144118	RALB	198,4216289	169,8700629	436,4239041	210,1141314	229,5093264	221,2141023	160,5494719	354,7702372	164,180645	2145,05351	238,3392789
ENSG00000100292	HMOX1	228,5391275	218,3392015	169,8834446	197,8293435	191,9771464	255,3247588	448,0147696	140,5676412	292,7143354	2143,189768	238,1321965
ENSG00000008282	SYPL1	158,0086281	313,3742276	330,9192476	156,895994	266,0082509	209,1632984	181,3144741	276,2263289	250,9988516	2142,909301	238,1010335
ENSG00000063177	RPL18	197,9285981	229,7664792	219,2417581	315,2597826	343,196075	266,4376749	209,9521708	164,1463913	196,6397867	2142,568717	238,0631908
ENSG00000134440	NARS	385,4816245	217,9104589	148,065702	190,2930042	387,3094551	204,7001368	251,7691548	162,0252648	194,4366403	2141,991441	237,9990491
ENSG00000140575	IQGAP1	236,1537666	121,9653453	403,8405592	197,804448	272,2015473	208,4011016	233,1238114	266,4554056	199,8007584	2139,746743	237,7496382
ENSG00000137752	CASP1	235,2879032	357,6918568	294,2920734	176,4733292	205,3903737	215,3068842	204,7968967	238,6670443	211,7283392	2139,634701	237,737189
ENSG00000088986	DYNLL1	162,4528858	293,5747794	209,5994605	198,438778	174,3515056	296,3295357	221,9596286	337,4243127	241,3912352	2135,522121	237,2802357
ENSG00000213719	CLIC1	303,4661306	229,3428744	235,4671796	202,3522512	207,8517598	251,6851488	228,8444469	270,2008719	201,2539837	2130,464647	236,7182941
ENSG00000136240	KDELR2	151,6104275	211,4432718	355,2911036	323,214111	125,9195861	259,6732716	303,4594162	220,962352	172,0380784	2123,611618	235,9568465
ENSG00000169567	HINT1	124,0261864	198,4749412	281,1389637	430,4887959	173,9939332	229,2411119	250,2277644	245,4364195	188,342395	2121,370511	235,7078346
ENSG00000256235	SMIM3	317,8936954	134,0162548	268,7859838	202,4403641	321,7247399	258,6551988	170,914262	328,4106727	105,1789628	2108,020134	234,2244594
ENSG00000120690	ELF1	188,1991818	213,4388584	397,0339491	213,1076678	284,5608267	195,6855665	167,3691166	270,910214	172,4551944	2102,760575	233,6400639
ENSG00000134308	YWHAQ	209,7421434	344,6830893	319,4558413	212,3417527	148,1143807	248,712301	163,9336548	215,5593143	236,6945711	2099,237049	233,248561
ENSG00000198258	UBL5	138,8245222	287,3049188	188,5830811	512,275758	72,1582633	242,0352639	222,6107496	230,8421707	197,9732244	2092,607952	232,5119947
ENSG00000260314	MRC1	430,0010321	97,63304514	0,535813606	77,75741533	620,1492024	120,3395336	187,9152814	501,8299841	53,33952808	2089,500836	232,1667595
ENSG00000161547	SRSF2	163,3817307	281,2069235	183,6308708	257,3024692	253,4177155	201,6553148	189,7650397	293,8534785	254,0374736	2078,251016	230,9167796
ENSG00000089248	ERP29	294,8365972	234,1955219	124,5394605	310,9995729	233,4440698	241,2750072	233,5336616	177,8429404	225,3442417	2076,011073	230,667897
ENSG00000108679	LGALS3BP	333,4853973	223,712861	102,5163311	167,0685358	277,8910731	105,9849564	344,4017617	431,6313608	86,84061634	2073,532893	230,3925437
ENSG00000110031	LPXN	125,9969371	283,5761971	142,2014126	251,2041203	387,7945411	209,8933563	297,9348216	184,5718825	185,6205233	2068,793792	229,8659769
ENSG00000119801	YPEL5	251,6889891	423,1015833	47,04778641	210,5175475	26,66350651	256,698241	297,7398875	384,0420463	168,190831	2065,690419	229,5211576
ENSG00000102316	MAGED2	210,8770349	264,1330813	238,9703195	290,1050754	199,997843	166,1924499	239,7706922	157,8630656	295,9146461	2063,824208	229,3138009
ENSG00000255112	CHMP1B	151,5064071	142,2364244	328,9522193	354,6630909	300,910868	185,8480953	193,7929129	174,5222519	226,4966457	2058,928916	228,7698795
ENSG00000173915	ATP5MD	175,4779679	238,8321061	212,6968939	477,5335953	211,0398544	176,1453201	141,0862283	289,6666663	136,1667701	2058,645402	228,7383781
ENSG00000130724	CHMP2A	288,6644861	280,610792	115,0307502	294,2718995	87,71306812	266,9843287	248,1118355	239,9491975	232,4609154	2053,797273	228,199697
ENSG00000110057	UNC93B1	393,2891917	197,4665634	122,7955075	117,5535675	264,2601372	234,2266965	245,9729427	291,7468099	178,6460207	2045,957442	227,3286046
ENSG00000131143	COX4I1	221,6834958	263,6095498	144,9649974	316,4217732	137,3669564	259,7026786	211,6133604	243,6196425	233,1613714	2032,143826	225,7937584
ENSG00000171522	PTGER4	82,03632561	235,3808584	278,5291567	413,0488735	21,27953834	235,9450639	311,6932288	121,7684947	328,4826793	2028,164219	225,3515799
ENSG00000135838	NPL	223,4310267	90,49455641	251,9421641	133,9029126	450,3586627	245,6143512	284,3573856	189,8919454	157,7921025	2027,785107	225,3094564
ENSG00000164924	YWHAZ	225,9329953	224,8356726	304,5209125	244,6208211	181,9719135	195,8663838	165,2214212	284,6363442	196,3366322	2023,943096	224,8825663
ENSG00000225921	NOL7	263,4846752	244,5491338	260,224179	214,1344546	302,4547491	183,73684	184,437142	129,4341834	232,3867681	2014,842125	223,8713472
ENSG00000167850	CD300C	331,0088506	176,7580752	160,1546516	139,6698743	678,9381091	160,4421991	113,9737096	89,0888188	162,9372938	2012,971582	223,6635091
ENSG00000105193	RPS16	158,7960746	184,711131	261,8668173	433,2652533	221,5945942	180,8636242	200,2176165	224,16167	147,1342886	2012,61107	223,6234522
ENSG00000122218	COPA	270,5237278	163,0100596	166,2553512	349,996815	176,466538	214,8480743	223,7118311	191,0385011	249,7846564	2005,635554	222,8483949
ENSG00000023892	DEF6	280,8348062	138,0738954	384,1493109	87,61911129	78,95294553	267,361131	258,91939	113,262566	395,401267	2004,574423	222,7304915
ENSG00000282608	ADORA3	235,9731874	151,6843469	299,8088302	216,3614155	163,1866465	283,5977964	210,128952	67,51342671	373,2146759	2001,469278	222,3854753
ENSG00000168615	ADAM9	214,2631818	125,1456476	276,1216261	296,5305138	347,1639855	183,6816527	230,3085582	135,1350472	191,5831788	1999,933392	222,2148213
ENSG00000108639	SYNGR2	318,5784711	257,1504159	165,6008101	146,0954043	54,56400558	307,3458818	275,3017422	223,0473885	242,1843856	1989,868505	221,0965006
ENSG00000198755	RPL10A	278,4723315	292,6801937	244,4307767	379,2900957	16,19362923	164,3702928	185,0437788	191,5118117	233,4173164	1985,410227	220,6011363
ENSG00000242574	HLA-DMB	277,8495253	218,6755266	158,0623888	187,343732	236,0382372	238,7774374	219,3350872	252,3565969	194,8709361	1983,309468	220,3677186
ENSG00000155629	PIK3AP1	235,0343211	131,4310647	379,5347132	130,2839887	109,6040882	242,4196208	269,0950387	167,0181754	317,3244417	1981,745453	220,1939392
ENSG00000124172	ATP5F1E	171,4529502	239,6443264	126,3794585	314,9604801	183,6227859	249,1760698	201,6760877	306,0470527	186,651211	1979,610422	219,9567136
ENSG00000188313	PLSCR1	138,3784935	283,0594105	115,8892102	145,5862958	273,4001973	292,4116466	242,5122553	250,9533073	235,5686181	1977,759435	219,7510483
ENSG00000136930	PSMB7	324,617283	262,5932003	77,57945715	236,4175944	102,432856	255,4540367	223,3624534	239,3248126	252,3687674	1974,150461	219,3500512
ENSG00000262655	SPON1	374,7780883	76,10048488	84,27758502	94,17607979	575,722763	92,48804765	55,26641293	394,5700713	222,7921258	1970,171659	218,9079621
ENSG00000129450	SIGLEC9	314,2433293	182,0704246	200,9033012	176,6629466	101,4128704	253,3419873	273,2278321	219,6823749	248,1369478	1969,682014	218,8535571
ENSG00000100387	RBX1	163,1329511	267,3615091	125,7743074	355,0302193	199,3032674	194,2956657	216,8137076	262,2530494	181,0981797	1965,062857	218,3403174
ENSG00000132824	SERINC3	184,9146131	164,1497381	68,04503176	400,3547373	110,771038	237,677875	223,7630722	307,4768636	261,0006332	1958,153602	217,5726225
ENSG00000171298	GAA	382,4228747	174,2718156	180,1349266	80,13434667	372,4232725	105,6111444	224,2698314	142,2458967	292,7544148	1954,268523	217,140947
ENSG00000163162	RNF149	175,0457851	320,4840282	113,7557105	272,1040275	318,7205318	181,2459836	266,2482521	152,1666216	154,0650761	1953,836016	217,0928907
ENSG00000125743	SNRPD2	231,1766193	292,4882265	67,76629279	464,5878523	107,1992664	224,3094489	188,5176133	185,7666972	191,625332	1953,437079	217,0485643
ENSG00000128340	RAC2	254,0669999	170,1527317	121,7471302	122,1419758	320,5440766	241,5337092	274,6621655	167,9021465	280,0588497	1952,809785	216,978865
ENSG00000162368	CMPK1	272,6201372	242,4717982	161,769376	180,2243386	321,8418141	195,1305196	145,2178738	253,5853211	179,0779184	1951,939097	216,8821219
ENSG00000147123	NDUFB11	239,2406426	190,4224218	260,3367923	223,7637018	201,3388196	219,7320614	175,552284	247,2433541	193,8413304	1951,471408	216,8301564
ENSG00000073756	PTGS2	0	239,9781775	363,8292236	504,276043	120,9335997	133,2011411	239,6685905	109,2662074	239,8885024	1951,041485	216,7823872
ENSG00000102524	TNFSF13B	302,7151282	310,4802106	148,9406284	144,2435574	238,3159892	231,8083703	172,6396297	213,9885186	182,6265596	1945,758592	216,1953991
ENSG00000197448	GSTK1	247,5358968	197,6385981	199,4807355	155,7312437	227,3672527	232,2118133	185,8533015	292,0903569	202,7817535	1940,690952	215,632328
ENSG00000122566	HNRNPA2B1	192,3329212	159,085434	250,9230094	341,2681442	193,5289745	174,754844	207,668496	200,0008082	220,9822843	1940,544916	215,6161017
ENSG00000259330	INAFM2	151,4076426	206,377968	158,572527	390,1200161	177,6761104	279,0363664	246,7062459	144,1049834	183,5934681	1937,595328	215,2883697
ENSG00000005020	SKAP2	173,1246269	226,477705	230,7945198	274,6607091	92,97801693	177,5778566	178,2235736	304,2389527	279,2031133	1937,279074	215,2532304
ENSG00000079215	SLC1A3	80,51190501	148,6444	278,1575803	104,8321358	577,6260933	162,7062383	197,6082627	238,7145814	146,7787059	1935,579903	215,0644336
ENSG00000100997	ABHD12	263,5247924	169,5430519	237,1749336	139,2978388	158,5672828	338,4809099	278,6595712	161,9739325	188,2968866	1935,5192	215,0576889
ENSG00000122705	CLTA	208,4304452	142,227208	163,2511357	146,1858367	374,8244726	210,4392603	266,9549688	237,9391657	177,7180461	1927,970539	214,2189488
ENSG00000130309	COLGALT1	359,1098268	109,1938423	137,9859934	184,4586766	348,3699585	199,5036161	224,1115074	163,6145568	196,1265776	1922,474555	213,6082839
ENSG00000158457	TSPAN33	320,4571479	286,2206958	108,9870983	78,28049238	86,87999467	329,5651456	318,2610064	120,7537088	271,557043	1920,962333	213,4402592
ENSG00000185862	EVI2B	208,1527948	290,3895896	319,3871297	117,4903719	339,7764229	184,9085855	150,8566146	158,7240281	149,7773282	1919,462865	213,2736517
ENSG00000090861	AARS	180,8086019	96,63365543	232,741897	261,6891219	389,3766683	197,6689152	233,4227964	77,87030715	245,8396637	1916,051627	212,8946252
ENSG00000142937	RPS8	176,7031916	256,0895909	150,7349836	424,5705718	141,7875086	190,6092386	163,5039494	257,2423009	154,6382066	1915,879542	212,8755046
ENSG00000088930	XRN2	199,6184827	218,0227354	388,0954551	287,9625703	4,531119226	223,5021883	189,9726858	198,3770907	204,6056783	1914,688006	212,7431118
ENSG00000132002	DNAJB1	192,1381256	346,4541568	199,5363998	123,7475181	91,50118202	267,0478461	267,6127492	235,3780481	189,9198707	1913,335896	212,5928774
ENSG00000150459	SAP18	160,2768774	329,5714689	220,1903611	241,8028166	133,9817215	200,1577246	156,5053965	337,2456452	132,927327	1912,659339	212,5177043
ENSG00000143333	RGS16	8,471730498	134,5164186	63,9538932	391,2941002	121,3960463	165,9794764	300,7536363	13,17347113	712,5917526	1912,130525	212,4589473
ENSG00000124126	PREX1	96,99732995	94,20143777	309,6318482	160,578042	268,7283887	215,1159109	261,2001904	136,8119684	365,6293346	1908,894451	212,0993834
ENSG00000111144	LTA4H	292,8545307	180,6925834	364,0957434	209,1003564	270,5626967	88,34374125	97,41106532	286,5699922	117,9328573	1907,563567	211,9515074
ENSG00000167601	AXL	131,8906247	166,6094625	249,5540078	218,8519674	325,332234	238,4277824	158,8052624	192,6998627	223,6746516	1905,845856	211,7606506
ENSG00000115091	ACTR3	180,9840356	186,580009	308,7104079	193,3461244	190,0649488	221,3747482	181,2441803	216,377188	220,4276413	1899,109284	211,0121426
ENSG00000122034	GTF3A	261,2157438	267,8670766	140,0350875	156,3291218	134,5156618	234,0665963	238,548003	222,5859647	242,2483376	1897,411593	210,8235103
ENSG00000138029	HADHB	114,6843377	204,8211163	289,8090394	251,9056605	145,1751767	207,4948971	212,5397604	200,4085266	267,8414691	1894,679984	210,5199982
ENSG00000156261	CCT8	159,7041081	352,653681	211,5765128	203,3746405	257,2188802	183,3408708	189,8391285	187,8165949	148,1877089	1893,387176	210,3763529
ENSG00000100348	TXN2	209,2652448	156,6599322	271,7466059	173,4822225	232,882285	227,7499735	175,202209	248,5268474	188,6623787	1884,177699	209,3530777
ENSG00000101161	PRPF6	68,1410975	295,8626204	335,6379338	119,8913569	291,5841155	163,732558	199,3854111	129,9181529	275,2626698	1879,415916	208,8239906
ENSG00000143553	SNAPIN	260,7244561	195,8964871	234,4106793	172,2103233	304,4591081	197,6098125	164,3953897	157,0848268	191,8648682	1878,655951	208,7395501
ENSG00000172936	MYD88	371,4312222	274,6623746	194,9832833	183,8123035	0,09002696	185,209275	188,9446689	272,0348758	202,2927049	1873,460735	208,1623039
ENSG00000155115	GTF3C6	194,2615805	161,5839414	73,34328344	382,4779758	189,1118437	210,4055956	193,5866903	336,175016	131,5656162	1872,511543	208,0568381
ENSG00000120727	PAIP2	182,4523768	201,7433161	350,3359069	268,1234935	176,260985	181,9342391	162,3009203	187,6089866	160,7178126	1871,478037	207,9420041
ENSG00000250722	SELENOP	143,8324352	104,1474835	131,5631003	62,31707681	538,0888803	127,7951698	141,7071325	596,6680168	24,55660154	1870,675897	207,8528774
ENSG00000154723	ATP5PF	169,3059698	146,4740441	135,6564653	530,8104525	68,90556858	202,4547566	179,3310668	252,7994268	183,7542707	1869,492021	207,7213357
ENSG00000105223	PLD3	157,0547582	92,81043003	275,4072018	130,3668568	520,1179832	127,3602017	235,5048615	271,8789625	58,41300257	1868,914258	207,6571398
ENSG00000012660	ELOVL5	287,082343	201,1944039	107,6051996	202,9722357	357,5164413	175,1350829	176,6719676	147,2353344	213,189795	1868,602804	207,6225337
ENSG00000112081	SRSF3	228,2368285	247,5359768	154,8065626	276,6247989	158,318467	168,3579791	180,8902145	260,6152939	187,2825378	1862,668659	206,9631843
ENSG00000134686	PHC2	292,2888485	200,9313319	263,3966204	163,4318353	208,9017422	216,0672616	212,6816703	88,66937641	215,8808145	1862,249501	206,9166113
ENSG00000135363	LMO2	168,1209457	194,0696244	268,2853511	196,9626569	152,2777424	184,3005267	224,4688502	249,993832	221,3344804	1859,81401	206,6460011
ENSG00000146376	ARHGAP18	172,0725553	263,0033903	227,2511523	221,8401875	93,1422023	219,0583246	216,512829	188,6038766	253,4749061	1854,959424	206,1066027
ENSG00000099194	SCD	304,0650072	133,4830815	91,79438159	301,3811394	170,6236255	246,604167	299,3107329	80,19889503	224,1851185	1851,646149	205,7384609
ENSG00000115762	PLEKHB2	309,1388072	234,7039594	101,3916711	184,6091675	198,187193	218,361795	243,2572846	143,7547531	217,5851748	1850,989806	205,665534
ENSG00000139641	ESYT1	271,4928494	169,3399961	108,5503936	175,2392356	229,605099	238,4001788	192,4655047	115,7393242	348,3480417	1849,180623	205,4645137
ENSG00000158716	DUSP23	153,4917069	175,1861067	238,5574668	195,7803022	160,6234826	263,4458854	217,4819088	221,0907652	221,6642157	1847,32184	205,2579822
ENSG00000106211	HSPB1	123,9006274	208,8122419	172,3291909	143,5575807	267,2502641	214,0852757	227,2638705	198,4802984	288,3399532	1844,019303	204,8910336
ENSG00000197249	SERPINA1	158,6321903	287,9982926	274,6910648	154,4211263	192,3059055	194,1038673	215,2857941	112,9759966	250,828354	1841,242591	204,5825102
ENSG00000145569	OTULINL	219,131792	175,8539225	287,4961791	231,0238206	180,2729254	192,0697727	235,31034	102,2751311	217,736096	1841,169979	204,5744421

ENSG00000143158	MPC2	267,4508167	271,2930244	232,7740859	118,7956423	126,3309626	178,8035518	178,6929634	352,8560185	113,3536976	1840,350763	204,4834181
ENSG00000123144	TRIR	146,6152905	226,8264516	221,7903765	256,357168	211,745126	193,4990631	217,0608032	184,4989347	180,3179072	1838,711121	204,3012357
ENSG00000034713	GABARAPL2	177,4168627	258,2537071	111,1920396	311,9083185	91,27073751	228,3613021	187,9137065	299,3671314	172,4482846	1838,13209	204,2368989
ENSG00000179632	MAF1	232,078305	208,5178783	229,5206283	118,960417	161,193961	252,2665166	222,4175947	164,1910894	248,1031834	1837,249574	204,1388415
ENSG00000221983	UBA52	144,6183608	223,1818226	184,8896896	240,7558416	251,2822004	185,8691644	195,6940896	201,514439	203,4557608	1831,261369	203,4734854
ENSG00000156515	HK1	235,9062578	154,8971497	291,8437824	202,9540296	127,8814374	231,2101969	239,4020169	119,7667857	225,2237873	1829,085444	203,231716
ENSG00000147533	GOLGA7	82,09078697	254,5940009	239,4404167	325,3776866	113,6576539	192,6681839	194,3420825	247,4920184	179,0857353	1828,748565	203,194285
ENSG00000141367	CLTC	218,9035016	161,0075359	304,801092	196,2964696	153,0406289	192,0811619	180,3609521	215,6088345	205,8035119	1827,903688	203,1004098
ENSG00000119396	RAB14	214,388999	148,5113306	104,3393268	357,483338	195,313928	183,0870199	175,7982646	232,7155241	216,087565	1827,725296	203,0805885
ENSG00000107719	PALD1	222,1095937	94,57676619	66,32930778	151,7498193	338,2404271	305,4770011	216,1845655	10,32160037	419,3036178	1824,292699	202,6991888
ENSG00000149273	RPS3	137,3122851	198,3948746	164,2544978	373,565893	169,2308473	198,7019144	197,0820047	226,8882514	158,3137042	1823,744272	202,6382525
ENSG00000156587	UBE2L6	190,9035095	247,0755578	171,61429	199,9404752	33,96116429	211,0491963	290,1881733	301,2691498	174,5803655	1820,581882	202,2868757
ENSG00000168918	INPP5D	150,0313338	143,1985154	262,9939584	219,9957469	187,1084718	252,1435593	249,7554817	68,89604506	282,6340038	1816,757116	201,8619018
ENSG00000004142	POLDIP2	182,9173649	179,263472	388,8598014	361,8884685	125,3548874	153,8556509	117,8575253	116,3327691	187,5866582	1813,916598	201,5462886
ENSG00000145685	LHFPL2	167,8564331	129,6170932	271,6726654	126,3622674	155,9912604	324,7912205	291,4271419	135,8687955	209,5403813	1813,127259	201,4585843
ENSG00000099860	GADD45B	139,307266	203,9283873	278,3701552	297,4268162	50,89458154	239,1657612	195,2477991	176,0360021	232,1777647	1812,554534	201,3949482
ENSG00000129675	ARHGEF6	105,0444645	178,5242494	276,7160743	340,9754679	137,3736866	165,2818894	289,6657816	99,606176	217,9594492	1811,147239	201,2385821
ENSG00000123131	PRDX4	213,5098683	199,6248529	98,45440702	219,8605743	257,2869077	198,3771747	199,2644734	293,3831443	129,0406041	1808,802007	200,9780008
ENSG00000006747	SCIN	167,5005956	130,2885049	250,9854721	183,9068624	181,7519026	153,8376068	127,5748146	349,9605049	262,0810023	1807,887266	200,8763629
ENSG00000184060	ADAP2	297,4258992	145,2257072	148,6266929	146,0540031	256,4578076	226,4789979	185,4618801	136,2271577	261,6223443	1803,58049	200,3978322
ENSG00000198856	OSTC	241,6272986	208,0755372	120,2401542	362,0149833	30,00053726	204,7188675	177,1026159	301,3988568	157,9005415	1803,079392	200,3421547
ENSG00000122861	PLAU	118,9822805	178,9431652	147,1437553	120,4247204	0,852432281	403,6651027	432,1897702	22,52198985	376,8606996	1801,583937	200,175993
ENSG00000141030	COPS3	274,7649811	285,2843218	103,6345726	216,2459093	208,5588419	205,0435896	149,3654221	228,8137079	127,8789159	1799,590262	199,9544736
ENSG00000102317	RBM3	181,5816399	256,6377097	325,971858	208,5867705	96,38919448	184,8734125	111,1081025	286,3161372	146,4892872	1797,954112	199,7726791
ENSG00000100353	EIF3D	291,4416828	210,3364076	178,3213538	134,197085	180,2651393	204,2880036	188,221951	196,0249126	213,4334962	1796,530032	199,614448
ENSG00000101856	PGRMC1	171,7416126	224,685949	119,3619602	241,218679	568,899077	100,6357016	114,5513401	138,2693891	113,7934673	1793,157176	199,2396862
ENSG00000100991	TRPC4AP	281,6238434	185,9694517	204,1333934	76,82810289	24,41817571	235,2760993	255,0674554	259,1643667	269,4245598	1791,905448	199,1006054
ENSG00000144713	RPL32	132,8716873	185,9694517	142,4274378	272,99971	245,8796721	208,7262094	184,1580679	255,9091368	158,8188426	1787,760216	198,640024
ENSG00000155508	CNOT8	240,6233973	118,7193588	284,8411854	182,5216909	362,3157902	149,7416833	124,2890592	186,1532144	138,1317281	1787,337108	198,5930119
ENSG00000140990	NDUFB10	249,7683375	283,8695597	252,053109	150,7903074	67,67984452	206,5905739	153,097934	230,0332951	186,5613166	1780,444278	197,827142
ENSG00000182934	SRPRA	147,5768857	169,3664514	147,4339966	145,7536972	386,8218851	208,9481323	217,1532096	155,7376035	198,61563	1777,407491	197,4897213
ENSG00000182022	CHST15	322,6602144	143,8041079	250,1948346	69,5906359	159,1687105	61,5159803	130,8396875	436,5392636	198,6850716	1772,998506	196,999834
ENSG00000163468	CCT3	150,484854	192,9872935	160,7881784	159,7078714	243,4080858	237,8309225	218,9561203	195,4900159	207,1534605	1766,806802	196,3118669
ENSG00000155926	SLA	165,4530638	120,7730755	205,1360282	118,7461392	259,6902586	228,0135345	219,8033863	185,9273398	262,4328544	1765,97568	196,21952
ENSG00000119900	OGFRL1	131,8594528	154,9355803	182,5834848	258,8306603	101,4812323	237,149425	219,8875773	127,3339833	350,5280908	1764,589487	196,0654985
ENSG00000089597	GANAB	91,08692848	63,49048153	294,062049	125,7986707	292,5058789	207,4706814	273,8396232	183,6769485	229,1491314	1761,080393	195,6755992
ENSG00000113384	GOLPH3	98,26044766	205,2626033	328,7707735	160,5387681	0,392907596	226,720252	211,6797067	341,6418669	185,5076336	1758,774959	195,4194399
ENSG00000198843	SELENOT	173,9872858	192,9844238	239,3922932	219,349449	160,4087305	246,8396295	188,7971961	182,837797	152,4525764	1757,049381	195,227709
ENSG00000265354	TIMM23	148,1899462	315,9118354	131,9821478	119,2845598	252,5893974	171,6268235	211,6125683	250,4638487	152,2692094	1753,930337	194,8811485
ENSG00000082074	FYB1	143,2247598	206,7779775	290,3201169	201,9866013	113,6617365	240,8167752	194,5335792	101,0376556	260,7477299	1753,106933	194,7896592
ENSG00000275302	CCL4	12,45925597	349,2568224	224,242442	137,6631872	169,6555298	183,1024248	294,9077051	210,4104238	169,9020296	1751,59982	194,6222023
ENSG00000247596	TWF2	280,2573956	139,6832745	267,3134208	125,4611767	92,70243535	244,621641	265,0925503	88,94837486	244,8419881	1748,922257	194,3246952
ENSG00000162976	PQLC3	408,2181202	242,5203295	116,2840573	131,6739238	170,3080551	91,97772614	140,000802	336,400141	111,2951432	1748,678298	194,2975887
ENSG00000165916	PSMC3	107,8223053	215,008414	152,1806693	220,8087702	320,4603377	206,2218899	182,031211	149,682794	193,6853914	1747,901783	194,2113092
ENSG00000159399	HK2	135,1123869	127,9246053	172,820741	261,1070953	412,3574287	135,7599409	139,0022576	62,28342854	300,8053457	1747,17323	194,1303589
ENSG00000162434	JAK1	199,8497865	159,0644307	234,7232147	238,4179515	232,6192065	136,3377075	133,1026832	220,3762763	188,7677188	1743,258976	193,6954417
ENSG00000061794	MRPS35	211,3982077	198,2583597	146,1916065	334,4834856	182,3498402	155,8527853	119,3593417	245,0009669	143,5736605	1736,468254	192,9409171
ENSG00000168906	MAT2A	149,9313354	203,2139706	130,8396854	199,3810832	277,6855378	215,8502152	191,3807227	162,8965059	201,9242147	1733,103271	192,5670301
ENSG00000136161	RCBTB2	262,6212203	152,9430534	185,8428306	172,5335911	268,0465492	180,9337911	162,987554	112,5062124	227,3146744	1725,729476	191,7477196
ENSG00000136754	ABI1	192,5244682	178,5070498	139,2536627	220,848236	237,3683518	190,2582882	155,662932	214,4142748	195,0110734	1723,848337	191,5387041
ENSG00000105058	FAM32A	221,3561405	286,7067507	62,07884563	155,3928219	31,78031348	193,1161111	207,1795383	352,2800595	213,2252655	1723,115847	191,4573163
ENSG00000138449	SLC40A1	80,85985964	167,7590481	64,26283582	97,50410829	368,9870545	173,9953646	301,9981063	350,1232785	117,3880458	1722,877701	191,4308557
ENSG00000213585	VDAC1	278,2833238	187,760961	194,3763762	191,5291811	98,81915974	195,2401277	164,4406188	213,606603	198,5857558	1722,642107	191,4046786
ENSG00000132646	PCNA	201,947484	374,2138533	65,58190503	280,4632514	146,4114285	232,605919	173,225968	45,69088104	198,1656431	1718,306333	190,9229259
ENSG00000178449	COX14	160,4576163	186,5936599	169,6538336	260,3969643	131,0593917	229,7091799	182,3847094	194,8541986	199,6413157	1714,750869	190,5278744
ENSG00000158864	NDUFS2	290,8609973	256,1203249	94,91447338	146,5173422	102,6891606	224,1470941	190,1263123	184,1870102	224,7127305	1714,275445	190,4750495
ENSG00000165264	NDUFB6	47,86258335	189,7888182	309,0673395	198,2428054	202,6189885	203,8569352	160,2326751	263,4417007	131,8088971	1706,920743	189,6578603
ENSG00000180448	AC004151.1	190,6686922	124,3563332	251,1981112	120,8466081	195,8508944	152,3994236	211,8996389	101,0691175	355,0389557	1703,327775	189,2586416
ENSG00000135624	CCT7	176,5691881	211,3176999	265,7492843	242,3330939	0,600029311	264,5051661	189,4458758	86,12830833	262,5968404	1699,245486	188,805054
ENSG00000135506	OS9	241,4144114	146,7544435	225,384909	91,77399571	154,999272	222,722672	230,5750984	175,0938828	207,9582675	1696,676952	188,5196614
ENSG00000151726	ACSL1	117,9018345	215,0597836	273,6891904	197,6943689	209,3899865	106,4795283	143,6799028	300,5145693	131,9321031	1696,341267	188,482363
ENSG00000277791	PSMB3	217,7389473	188,2503768	188,0173615	102,3186801	176,7915983	202,2745387	177,5705983	268,4780861	174,549345	1695,989532	188,4432813
ENSG00000099783	HNRNPM	201,7678728	202,8498529	159,8855967	170,3932342	255,6169753	167,41778	164,6714297	162,0977554	210,4428825	1695,143379	188,3492644
ENSG00000113312	TTC1	285,3011239	213,2130145	11,04461694	212,3294401	328,1719131	150,0728798	133,9515567	208,1840077	149,9814039	1692,249957	188,027773
ENSG00000184900	SUMO3	218,1479102	174,7045242	274,1136115	238,5854929	111,336954	160,019901	155,7816128	222,1845902	134,2829557	1689,157553	187,6841725
ENSG00000140988	RPS2	178,0042024	191,3375076	181,8278537	225,2039291	124,0669901	205,0954868	160,5158561	244,3666023	177,6748787	1688,093307	187,565923
ENSG00000110852	CLEC2B	190,4634752	259,0955111	263,9651812	144,1176111	137,3576953	170,7533039	121,1421287	237,7425357	160,202802	1684,840244	187,2044716
ENSG00000204397	CARD16	125,2391519	292,9228958	139,6487701	180,0884155	209,5561488	139,8641888	133,4980547	338,2647705	123,9710806	1683,053477	187,0059419
ENSG00000113269	RNF130	167,4630995	158,4332813	209,0327061	147,4363023	155,9528939	195,7866484	202,2777148	261,6767465	184,7590221	1682,818415	186,9798239
ENSG00000101265	RASSF2	273,8053821	110,1715907	267,9724268	223,4500831	266,1687342	110,0948025	112,8619012	93,37866055	223,6524209	1681,556002	186,8395558
ENSG00000175166	PSMD2	180,2968462	232,8402408	94,09850528	182,1284036	296,5615834	186,6159491	165,1857395	123,5798635	218,2197874	1679,526919	186,6141021
ENSG00000165030	NFIL3	96,9901911	234,3469743	0,811620727	258,1415102	254,6152113	134,4238979	218,7030313	237,0124343	237,847374	1672,892245	185,8769161
ENSG00000065427	KARS	210,6626332	192,7456148	103,1911364	243,8602629	131,2788851	199,9497947	169,1766026	195,4156704	222,6500771	1668,930677	185,4367419
ENSG00000156508	EEF1A1	190,4479041	200,0043453	201,4009997	194,4380273	170,7022004	184,3989676	158,5684612	207,0497929	161,9112285	1668,921927	185,4357697
ENSG00000117335	CD46	202,597142	212,0378747	189,4593348	290,9898413	135,1433379	116,7928152	148,4665022	247,4085186	122,3966244	1665,291991	185,0324435
ENSG00000170266	GLB1	216,1212314	129,4009375	378,9763287	167,9373885	152,9252556	197,0642582	182,1128787	127,5241134	112,1081097	1664,170502	184,9078335
ENSG00000071127	WDR1	215,7481317	177,2196967	79,0930983	200,9602692	142,7385555	198,0455498	204,7088463	168,5735567	264,1551757	1651,24288	183,4714311
ENSG00000137409	MTCH1	218,83479	190,2555842	162,2884455	137,8130656	238,6075289	185,3888818	202,9761577	178,4304382	135,5502466	1650,145138	183,3494598
ENSG00000082397	EPB41L3	215,4777547	150,6719409	159,5259062	159,1739696	258,1479211	144,2523329	179,697205	193,5816376	188,4660094	1648,994677	183,2216308
ENSG00000198931	APRT	217,8238343	119,4220888	150,4761933	193,9283782	138,428559	220,3859297	196,799833	186,0183505	224,9124302	1648,195597	183,1328441
ENSG00000154639	CXADR	175,6305885	162,0163754	186,0125924	174,6500494	280,7451041	170,0989852	143,6834312	174,1902204	177,6051967	1644,632543	182,7369493
ENSG00000184007	PTP4A2	190,645313	144,5440478	185,2786259	237,1676416	23,93046235	202,5157725	196,7909009	241,3728952	219,7576945	1642,003354	182,4448171
ENSG00000135604	STX11	103,4079606	172,2178889	227,8974955	109,4680475	133,6901754	150,1464876	271,266076	247,2254475	226,4702948	1641,789874	182,4210971
ENSG00000150782	IL18	163,5822684	194,4415502	124,5308159	155,659482	167,3584247	173,7443583	125,5184972	424,4276633	110,8658332	1640,128893	182,2365437
ENSG00000015475	BID	210,7254922	146,9775201	278,4802881	176,8324513	141,7569246	183,150947	169,3539052	133,1102812	195,7115199	1636,09933	181,7888144
ENSG00000067182	TNFRSF1A	177,8992112	151,712802	139,7188363	120,5317611	198,8820746	181,0724421	189,8815686	277,7135466	198,2362904	1635,648533	181,7387259
ENSG00000150093	ITGB1	174,8629807	170,8669332	200,0698417	195,9616451	75,02522176	197,3430695	175,412199	201,6024607	243,9931138	1635,137466	181,6819406
ENSG00000074071	MRPS34	155,2430384	80,17372473	135,3157455	166,7414924	312,6319535	227,4653668	234,9280419	160,5484271	155,1826932	1628,230483	180,9144982
ENSG00000103769	RAB11A	205,1964492	133,5989191	145,5480307	211,0449495	317,7052201	123,1477097	113,9836115	237,2962326	137,8797966	1625,400912	180,6001013
ENSG00000152601	MBNL1	148,3724574	130,4605186	260,0028096	202,5779085	274,1646189	157,6391314	164,7983538	115,8125359	170,013567	1623,841901	180,4268779
ENSG00000242372	EIF6	333,8144611	152,7307897	161,4023015	139,9865744	97,3206338	202,4292138	207,6299363	133,3083663	194,8482852	1623,470562	180,385618
ENSG00000148229	POLE3	234,5778594	186,3725754	304,2106722	221,9999132	13,08636997	179,9114693	181,9247622	144,1135801	154,4800206	1620,677222	180,0752469
ENSG00000215301	DDX3X	120,6640537	151,6018041	167,4667422	267,9295819	142,4086806	217,9742594	173,0151558	231,0671626	148,3193709	1620,446811	180,0496457
ENSG00000072506	HSD17B10	294,3606811	159,4422835	114,4330632	221,853234	93,72484738	157,5778482	166,881168	184,6284624	226,0160396	1618,917627	179,8797364
ENSG00000136051	WASHC4	51,25353339	148,1085716	85,25734135	232,1907737	470,7528085	130,8878842	155,2650536	226,427948	118,5864013	1618,730316	179,858924
ENSG00000038274	MAT2B	151,1399982	124,5933422	191,0402667	213,4229588	295,5463942	183,9071086	126,2766652	189,7666095	142,7417106	1618,435054	179,8261171
ENSG00000057252	SOAT1	145,092034	193,2689448	218,5348187	144,1195456	130,2666203	181,8976772	179,2049496	237,5543872	187,6240693	1617,563047	179,7292274
ENSG00000148290	SURF1	217,3826127	186,9977771	232,59882	252,1777712	84,77461893	149,2062695	130,3235644	214,087015	148,8463614	1616,39481	179,5994234
ENSG00000052802	MSMO1	195,7761426	257,8939363	53,21778894	304,3936397	0,251835584	223,9094705	232,2872907	159,3083907	188,1272475	1615,165742	179,4628603
ENSG00000158270	COLEC12	335,0032657	102,6483947	130,831979	33,15434173	183,9847738	84,61597573	186,0610711	411,7044611	140,2390821	1608,243345	178,693705
ENSG00000152234	ATP5F1A	181,7122621	135,275525	216,7164248	173,9088069	191,5754473	175,097411	184,4211435	181,8783593	167,4629141	1608,048294	178,6720327
ENSG00000082781	ITGB5	212,4825691	138,2073425	136,3233179	133,6115234	157,9786456	173,9832786	207,184932	276,2666292	171,811758	1607,849996	178,6499996
ENSG00000143742	SRP9	171,3267146	276,4761433	147,6299715	241,3060824	145,5881928	152,9815092	120,7228059	231,8995749	118,8748025	1606,805797	178,5339775
ENSG00000071082	RPL31	128,717456	216,3123923	106,8850057	391,8722141	124,2654221	161,3202898	130,0592602	208,5801676	137,7032219	1605,71343	178,4126033
ENSG00000084073	ZMPSTE24	135,2920753	194,0696244	78,24505258	158,2757653	466,0487326	152,6435198	111,8980832	192,3611527	115,7014842	1604,53549	178,2817211
ENSG00000087460	GNAS	245,2597168	184,86096	185,987886	149,4890984	103,3945777	195,2189541	168,4429787	160,1565742	210,5842013	1603,394947	178,1549941
ENSG00000108561	C1QBP	162,2416277	172,9429774	90,23936404	165,1656927	120,4235699	249,1145533	208,2753777	245,5263275	188,473047	1602,402537	178,0447263
ENSG00000121552	CSTA	127,8798094	236,3418507	256,0021702	264,3733564	58,88896741	181,9429311	204,3715144	118,288802	146,2554989	1594,344901	177,1494334
ENSG00000164104	HMGB2	100,5770177	202,0486206	288,1977459	314,8145207	136,6587837	88,6052161	130,8508616	76,08925717	255,698026	1593,540049	177,0600055
ENSG00000099985	OSM	0	305,6187879	99,81120731	103,5144393	91,42710137	323,8574457	323,5658444	5,13145446	339,0715138	1591,997794	176,8886438
ENSG00000137364	TPMT	181,9591482	139,6292199	290,3774526	194,226096	74,10182956	202,9069679	102,732775	225,0981712	174,6396968	1585,671357	176,1857064
ENSG00000161057	PSMC2	387,770738	140,5130775	30,09224927	164,4672112	46,38686175	164,1286089	195,3336881	237,8792661	214,1069601	1580,678661	175,6309623
ENSG00000179820	MYADM	293,5721882	131,762658	158,5223878	76,43488437	301,1306707	141,1282151	112,2460276	174,1354806	191,727194	1580,659706	175,6288563
ENSG00000128604	IRF5	175,591614	112,0258675	179,7111052	97,47009727	190,7441404	197,0256573	195,0134304	170,1996232	262,1814575	1579,962993	175,5514436
ENSG00000149932	TMEM219	223,2647556	172,3689424	79,17228734	160,4909267	119,2022469	223,5639786	210,8767254	214,2194651	176,7770243	1579,936353	175,5484836
ENSG00000062716	VMP1	80,07493327	221,5868527	245,5747738	149,0458748	112,2307814	159,2054075	247,0231485	232,9047849	130,1251036	1577,77166	175,3079623
ENSG00000101347	SAMHD1	144,2534974	128,5348119	183,3617064	181,8736005	74,75104514	228,6445467	230,8738788	179,0526357	225,5045627	1576,850285	175,2055872

ENSG00000120742	SERP1	139,9542269	227,8950204	150,969864	235,036849	189,9077096	169,8972839	141,6156525	146,3739404	172,9346709	1574,585218	174,9539131
ENSG00000099341	PSMD8	140,7445315	233,5987473	189,7068695	114,7945814	131,7342611	175,207981	178,2446488	198,8992614	207,633361	1570,564243	174,5071381
ENSG00000133048	CHI3L1	64,47923548	98,07156114	378,1620437	87,67663434	248,06127	125,3086128	172,4051157	8,05698321	387,6085362	1569,829993	174,4255547
ENSG00000026508	CD44	392,3980812	172,8020266	72,32928805	150,0037024	196,7536235	122,7579057	169,3673894	162,0794105	121,8479948	1560,339422	173,3710469
ENSG00000108528	SLC25A11	120,4114584	177,6400057	129,4840526	176,7726214	144,0248824	275,8238159	178,2785656	147,8998914	209,8388495	1560,174143	173,3526825
ENSG00000116586	LAMTOR2	119,7955845	157,3795884	135,5104655	196,5177869	212,2739298	183,4477169	196,7563273	168,8938853	188,8190327	1559,394317	173,2660353
ENSG00000168288	MMADHC	265,4626165	181,3388224	205,317394	65,2944262	83,68273894	189,2923929	176,5921975	241,6014605	150,6653434	1559,247392	173,2497103
ENSG00000099785	MARCH2	204,1629281	171,9448475	36,0019352	157,432338	207,2704588	177,5153327	147,1558632	215,3192759	242,0365809	1558,83956	173,2043956
ENSG00000229117	RPL41	120,9702854	171,5818394	177,8771572	276,4780327	104,3644843	182,5315821	176,7682204	202,7937779	143,7459112	1557,111291	173,0123656
ENSG00000136717	BIN1	69,87377451	146,6535888	203,4415705	58,37003585	108,2795388	277,1059632	203,1441837	73,67602569	415,4057066	1555,950388	172,8833764
ENSG00000131100	ATP6V1E1	140,8217822	205,094931	182,725703	151,2642455	221,7776487	158,7842872	168,8639848	197,7560997	128,6083951	1555,697077	172,8552308
ENSG00000075618	FSCN1	166,5525268	72,70787404	188,5515189	79,2187014	363,680539	153,0378177	173,772509	144,4478766	213,0778862	1555,04725	172,7830277
ENSG00000131981	LGALS3	320,5000917	153,032383	197,834859	113,3210099	255,5793332	136,1085404	63,10604238	244,0317133	71,41442079	1554,928394	172,7698215
ENSG00000129667	RHBDF2	174,2878419	84,81094457	244,3767855	138,2684029	289,0989574	128,6022624	167,5112289	98,35725645	227,288901	1552,602581	172,5113979
ENSG00000113583	C5orf15	170,6150641	174,1000209	167,0779995	295,9757861	215,0647429	120,1968411	88,71804875	102,3901985	215,4882753	1549,626977	172,1807752
ENSG00000155096	AZIN1	77,12218806	123,9665089	231,0173508	241,7531083	338,349153	123,6986293	82,99108286	171,9307158	155,7964776	1546,625215	171,8472461
ENSG00000150337	FCGR1A	197,48206	215,2827866	147,4277376	156,6654284	125,0562756	230,769548	186,6569982	147,6925685	139,2919065	1546,325309	171,8139233
ENSG00000128590	DNAJB9	162,5421032	269,6211128	201,9682043	75,87470893	116,875922	181,1419259	248,1227724	195,9432719	83,18425898	1535,27428	170,5860312
ENSG00000153071	DAB2	125,9483207	106,3603294	110,4788083	81,45553373	204,6648894	197,106151	247,4937662	310,5237118	149,5779124	1533,609423	170,401047
ENSG00000148672	GLUD1	190,8870304	166,489226	96,48409785	214,9607172	213,8554465	162,1358916	176,953	150,531303	159,6411793	1531,937892	170,2153213
ENSG00000066322	ELOVL1	178,2453499	213,5720001	204,1035044	116,9313553	140,866937	190,2079357	212,2381234	117,4849161	154,4024179	1528,05254	169,7836155
ENSG00000143368	SF3B4	245,9980769	112,1979144	263,7874531	173,9566497	138,884073	175,6471129	107,503923	118,4832868	190,8635059	1527,321996	169,702444
ENSG00000162745	OLFML2B	108,7642116	116,6005476	190,5567823	32,93205682	254,1689897	198,6068631	239,9183801	291,4403858	94,25656708	1527,244784	169,6938649
ENSG00000116560	SFPQ	123,0230283	113,6684985	171,2318442	253,8655677	198,7998041	159,4029641	168,2746736	164,9723195	171,467778	1524,706478	169,4118309
ENSG00000132432	SEC61G	64,89058708	229,7112248	83,15257033	287,4251159	269,6305978	148,5268952	113,2093875	216,1637643	111,185053	1523,895196	169,3216884
ENSG00000164258	NDUFS4	154,1997135	168,1026508	96,60637171	368,7317912	79,83788633	157,0073686	104,7367337	235,3009356	159,25179	1523,775241	169,3083602
ENSG00000078369	GNB1	189,117811	118,9235051	100,6307885	223,321627	63,96623826	213,3542277	220,7511373	182,5963809	210,4059505	1523,067666	169,2297407
ENSG00000165140	FBP1	284,0600664	158,2022381	144,0253417	96,33137073	167,8910649	178,5995397	185,5719919	132,0598185	173,7702388	1520,511671	168,9457412
ENSG00000132341	RAN	210,1366196	190,4629806	96,73016515	277,5963465	144,5088375	154,925711	114,4207699	183,873703	147,7772539	1520,432387	168,9369319
ENSG00000196262	PPIA	168,124148	152,8869211	168,1553803	169,3251324	161,6515111	197,6926344	174,2203121	167,0066406	159,6384725	1518,701152	168,7445725
ENSG00000023330	ALAS1	134,7839554	231,6636917	291,4625722	161,9525434	40,92467261	220,0801183	143,0341995	127,9521866	166,7928567	1518,646796	168,7385329
ENSG00000174574	AKIRIN1	78,42233857	97,95961462	319,5530156	64,16592939	284,5685882	173,0704081	127,5241618	169,8886058	203,2696337	1518,422296	168,7135884
ENSG00000204272	NBDY	161,910506	264,3719895	51,94824345	163,7149138	257,0835599	151,1370514	199,1942588	126,5988325	141,7528834	1517,712239	168,6346932
ENSG00000100345	MYH9	147,8744033	99,34821774	273,3532664	221,0361292	140,8257207	149,9123198	193,802377	74,94543453	216,5734772	1517,671346	168,6301495
ENSG00000198356	ASNA1	214,042818	122,0597967	162,547685	94,13876808	284,9897688	154,9636924	121,7487544	223,2233519	138,2599128	1515,974548	168,4416164
ENSG00000106537	TSPAN13	195,2975938	60,88054393	135,7996591	59,24421829	340,3015035	77,90511611	193,9030031	297,9517283	154,5011508	1515,784517	168,4205019
ENSG00000095303	PTGS1	163,8337463	160,8666563	6,247823801	305,551968	153,6059474	200,389227	257,339998	61,29949705	206,0570103	1515,191874	168,3546527
ENSG00000111237	VPS29	168,6640564	200,5845793	54,79956483	161,4692602	91,70862184	256,7971243	156,8086086	255,1066001	169,2355127	1515,173928	168,3526587
ENSG00000121933	TMIGD3	265,5637582	273,111683	173,3368492	85,43793443	45,25945962	205,3382549	234,9726522	85,6026948	145,5848787	1514,208165	168,2453517
ENSG00000119421	NDUFA8	132,503672	217,1149777	228,538365	176,5530176	0	228,2302212	223,318464	112,1997972	195,3237703	1513,782285	168,1980317
ENSG00000126524	SBDS	82,91256385	275,021445	101,1426133	213,4645886	108,0493597	166,7812445	129,2746803	214,8306372	221,8238082	1513,30094	168,1445489
ENSG00000092330	AL096870.1	294,7547636	82,65734221	152,9300545	236,0152997	222,4621944	139,3599903	109,9716	145,357094	128,3519676	1511,860306	167,9844785
ENSG00000130775	THEMIS2	251,6329455	85,35747287	162,9621485	125,4136721	148,9005001	152,3390125	182,9243944	208,8724024	190,471571	1508,874119	167,6526799
ENSG00000143947	RPS27A	133,0545472	179,6465732	193,5203309	292,6973469	111,1772839	148,6304336	120,2991905	223,2808659	105,1546936	1507,461266	167,4956962
ENSG00000132305	IMMT	200,1665362	153,5123041	83,8961513	304,5332418	245,0576305	155,9626018	146,0567839	52,55783136	164,5389807	1506,282062	167,3646735
ENSG00000110092	CCND1	89,07076494	118,1379247	111,4798037	236,0840246	142,5317451	242,6828622	244,2644993	56,91957768	263,5326772	1504,703879	167,1893199
ENSG00000008988	RPS20	85,77362172	175,5159994	143,2495713	344,3785374	135,7756198	162,5945758	135,7938403	173,4726444	147,7114597	1504,26587	167,1406522
ENSG00000077420	APBB1IP	120,7547652	164,4576358	220,1851213	171,7838891	39,43471321	193,9724749	171,2443251	223,3851175	197,743021	1502,96105	166,9956722
ENSG00000177663	IL17RA	85,62263411	93,17904828	160,9860401	150,2484144	251,533303	136,1289305	232,1117828	154,2832493	238,3960867	1502,489489	166,9432766
ENSG00000152804	HHEX	211,2671975	196,0562443	27,00441088	232,7928996	122,6011911	177,8177196	117,5102792	203,2133284	214,1435344	1502,406805	166,9340895
ENSG00000151465	CDC123	215,0925486	191,7133621	216,2873877	220,3980791	46,72591173	151,2544204	159,1038065	139,3346806	162,474179	1502,384376	166,9315973
ENSG00000117118	SDHB	167,6824042	251,1516865	180,2015589	199,8876369	22,46307942	170,4256086	146,3650664	227,8951648	135,0444469	1501,116653	166,7907392
ENSG00000140941	MAP1LC3B	113,3704281	153,2620247	134,5474452	117,2316553	222,7997459	166,4375906	146,6685159	251,2536021	192,6723326	1498,24334	166,4714823
ENSG00000109332	UBE2D3	196,6814506	205,1911004	130,6041504	176,5285894	82,23519986	155,3883707	135,0642403	289,8375687	125,6739924	1497,204663	166,3560736
ENSG00000115307	AUP1	135,9687375	184,6957728	115,4947865	121,3799352	137,6495346	239,168037	236,0682132	129,7510888	194,2493589	1494,425465	166,0472739
ENSG00000042980	ADAM28	140,0572469	272,3859329	167,6620424	185,2858765	37,13684717	186,5274188	173,4659878	167,6378557	157,7042193	1487,863428	165,3181587
ENSG00000077147	TM9SF3	138,7902399	148,0255477	160,9866066	191,4010616	205,2442533	142,1552993	148,4414149	157,8978098	192,6167519	1485,558985	165,0621094
ENSG00000126756	UXT	66,82817168	231,2300475	200,3479986	277,8627182	155,4092529	134,1460517	129,3416476	176,7197529	113,5075278	1485,393169	165,0436854
ENSG00000181789	COPG1	172,2489759	64,65706533	159,8778196	208,9248847	282,1000175	162,7985817	164,7134822	93,19374921	175,8927085	1484,407285	164,9341427
ENSG00000198833	UBE2J1	119,8287955	177,8597993	143,52959	176,4632297	96,85134488	219,7797169	160,5743034	261,0782246	125,2812651	1481,246269	164,5829188
ENSG00000112303	VNN2	109,7662527	95,16716072	320,0788192	73,10201408	402,5714569	156,9597171	78,37665055	175,722429	68,82408782	1480,568588	164,5076209
ENSG00000105677	TMEM147	99,35500417	159,6050203	120,1647236	255,5185766	176,6701765	174,5203627	204,0602078	156,7292137	133,0919568	1479,715242	164,4128047
ENSG00000181817	LSM10	135,2570048	169,5027783	189,3808642	158,016399	135,2056086	187,3930219	173,2923576	135,1578311	196,4592719	1479,665137	164,4072375
ENSG00000110934	BIN2	200,8150202	138,0162315	24,26260216	171,2670457	193,3503177	169,5101329	208,6687392	184,1698899	187,3759326	1477,435912	164,1595457
ENSG00000138757	G3BP2	83,77033167	107,659965	292,9568912	183,1957506	264,6760781	136,0683847	166,7639231	99,68699816	140,982556	1475,760879	163,973431
ENSG00000205302	SNX2	210,8629843	132,8246975	140,8957927	223,7625044	124,393376	174,3738283	133,0953591	211,0235165	123,4153933	1474,647452	163,8497169
ENSG00000013275	PSMC4	89,19765147	205,1224517	164,0862838	164,956379	94,33041435	187,0403695	179,8215357	227,1651132	162,7227614	1474,44296	163,8269956
ENSG00000261371	PECAM1	158,9969769	199,1407258	303,5121814	106,180748	15,26284349	193,0443824	183,9021298	152,3046812	161,0221709	1473,36684	163,7074267
ENSG00000081189	MEF2C	111,5942887	162,960991	191,1689596	171,6332815	179,3296447	151,0484893	155,6344458	139,2294694	210,2097286	1472,809299	163,6454776
ENSG00000206503	HLA-A	107,4502102	158,2136746	46,46678407	119,0094571	146,9599094	131,3815858	202,4655948	410,7874753	149,7744667	1472,509158	163,6121287
ENSG00000180964	TCEAL8	187,9211556	346,5253758	0	222,5811963	10,675666	261,1276294	204,6275026	73,1267251	164,2079099	1470,793161	163,4214623
ENSG00000167797	CDK2AP2	155,8257593	215,4167034	74,21063564	114,6356228	82,53122321	202,4782868	258,8330524	100,1614439	264,2720978	1468,364825	163,1516473
ENSG00000115107	STEAP3	142,1671568	136,3045371	18,2801468	164,2140097	163,0277138	148,5266978	213,5085899	300,0131474	182,1393689	1468,181368	163,1312631
ENSG00000198818	SFT2D1	97,69110056	175,9546242	117,9773097	221,4864045	45,68895893	178,7472966	166,698039	316,7422319	146,3789539	1467,364919	163,0405466
ENSG00000125734	GPR108	247,712224	119,3411329	78,47119508	64,48253029	319,8862142	192,1423143	120,3156218	179,7695686	144,0845587	1466,20536	162,9117067
ENSG00000156471	PTDSS1	240,6966945	172,6843	95,78059328	116,477725	260,7666856	134,0726138	120,1286245	172,7498271	150,5371592	1463,894223	162,6549137
ENSG00000001036	FUCA2	246,7111358	162,9205063	246,1313229	101,0854431	0,128577709	223,055573	217,3381548	117,3616509	148,184821	1462,917185	162,5463539
ENSG00000128272	ATF4	131,7559152	142,6037606	107,5873653	168,1442557	164,1312873	203,7255172	164,1495314	215,426514	165,2215502	1462,745697	162,5272997
ENSG00000112110	MRPL18	181,9045734	150,4639607	41,47446892	284,6736004	197,0703253	130,1685602	116,7951635	181,8111594	178,136732	1462,498544	162,4998382
ENSG00000103490	PYCARD	184,1546292	165,5696085	128,7751181	147,7583591	177,6117484	189,1980251	171,5013152	142,5247894	155,394562	1462,488155	162,4986839
ENSG00000023734	STRAP	230,9979198	83,5295621	183,855912	150,7886083	59,66377227	165,9854799	190,3863593	228,9919012	167,5555327	1461,755048	162,4172275
ENSG00000117984	CTSD	155,8220402	91,96425856	245,5686487	76,18724981	293,3356996	180,4055248	226,7349553	80,2744581	110,4576602	1460,750495	162,3056106
ENSG00000079459	FDFT1	255,3498233	223,0990972	38,88619049	261,3868535	33,65215377	149,6930333	197,246991	108,9156079	191,1032933	1459,333044	162,148116
ENSG00000116030	SUMO1	234,277248	252,0336544	166,8651334	187,0194758	44,30886498	155,5603328	110,8012035	184,4570044	123,8404275	1459,163345	162,1292605
ENSG00000101150	TPD52L2	197,0167645	169,373888	105,7082396	182,3876548	101,5230036	172,9089351	194,4068438	166,8363709	168,3071083	1458,468808	162,0520898
ENSG00000143549	TPM3	175,6775043	146,243189	161,0178998	156,7744708	82,74204658	181,7944674	148,8159599	207,8188373	194,4668776	1455,351253	161,7056947
ENSG00000117691	NENF	223,4380194	181,1655602	151,3056235	158,9556412	54,5495001	145,0404962	121,1026939	283,379012	130,4160326	1449,352579	161,0391755
ENSG00000078668	VDAC3	163,3191267	163,8692387	190,5050663	232,7169201	154,4397353	147,7600326	151,5031579	109,9024748	134,6087354	1448,624488	160,9582764
ENSG00000129103	SUMF2	150,7052509	90,09989664	128,0430156	118,2192594	206,3108827	214,2961046	261,8019095	169,6085746	106,7223994	1445,807293	160,6452548
ENSG00000168090	COPS6	301,4055031	167,2148565	144,641678	216,1469275	0	145,9702627	149,4898414	143,0416642	171,4683279	1439,379061	159,9310068
ENSG00000189060	H1F0	71,42939944	121,2587543	73,05279052	191,8050176	211,7322771	221,3320705	187,3490452	151,9660682	209,1566236	1439,082041	159,8980045
ENSG00000156642	NPTN	126,9748545	124,7605139	58,60195539	133,8327201	334,6241584	123,4042211	123,3542649	268,3961331	144,4090164	1438,357838	159,8175375
ENSG00000089063	TMEM230	253,8320795	203,7211263	161,9956132	174,1276986	89,92651667	133,6637816	114,6135088	177,5097529	128,64892	1438,038998	159,7821108
ENSG00000131446	MGAT1	206,2366885	127,4693699	217,5861849	117,3936893	181,0788783	128,9762681	198,4373565	138,8989811	118,4819743	1434,559391	159,3954879
ENSG00000146701	MDH2	196,4727936	188,899816	170,5596103	167,9128386	43,98967973	164,3612882	159,5861322	150,5605459	190,4403665	1432,783071	159,198119
ENSG00000106682	EIF4H	199,9811783	189,3211928	241,6536492	142,283565	37,58861145	153,432188	142,653201	107,4870201	217,5295395	1431,930145	159,1033495
ENSG00000155465	SLC7A7	221,5267419	166,8113943	118,2133261	159,5817483	326,4323168	104,9915499	115,9335108	95,18267215	121,4829927	1430,156253	158,9062503
ENSG00000155959	VBP1	141,9214143	195,6711893	123,8196179	355,4628734	102,6583411	98,04590733	112,4928912	151,6495442	148,1749299	1429,896709	158,8774121
ENSG00000178982	EIF3K	164,3421968	222,9086105	103,3235536	218,7825055	69,94962851	195,736531	176,0082391	155,7695477	121,6836415	1428,504454	158,7227171
ENSG00000158050	DUSP2	99,0953198	347,9312733	71,76469118	44,63895366	98,48272675	186,0365876	109,6645826	97,46877601	372,5601356	1427,643046	158,6270052
ENSG00000147010	SH3KBP1	103,4781751	93,52711272	247,8098511	149,9122695	164,5463276	161,3729497	147,9711566	138,591476	219,1747198	1426,384038	158,4871154
ENSG00000105281	SLC1A5	133,695667	89,08559247	197,5560848	169,9366431	272,6792864	133,8936197	155,2563562	82,21196173	192,0376879	1426,352899	158,4836555
ENSG00000150540	HNMT	170,205914	154,7623613	135,5416	46,57985564	296,2191166	143,0263592	124,8635579	278,3959866	76,27678963	1425,871541	158,4301712
ENSG00000197019	SERTAD1	179,9694992	411,6056902	140,0571516	64,3572826	35,84282572	231,112478	141,9067047	114,4787705	106,0323968	1425,362799	158,3736444
ENSG00000169019	COMMD8	88,44867873	171,8976303	188,0081557	159,8560681	220,6182854	166,7599483	128,4679167	162,2099157	138,5920311	1424,85863	158,3176256
ENSG00000113575	PPP2CA	164,5630312	182,2445271	88,55211591	191,0600029	69,71784966	199,5731624	178,1122464	199,0640852	149,8333599	1422,720381	158,0800423
ENSG00000092199	HNRNPC	151,7928321	104,6667295	151,4457182	246,547672	213,1415924	122,2140573	142,8943828	163,0698433	126,1691673	1421,941995	157,993555
ENSG00000160791	CCR5	314,7725368	98,94673478	177,295586	195,3459273	131,6373126	128,9511944	100,5266113	181,5950319	92,41714316	1421,488078	157,9431198
ENSG00000111775	COX6A1	166,4586197	142,982366	133,9037818	218,4526561	100,0379711	173,9644882	152,9257519	182,8616848	148,6494588	1420,236942	157,8041046
ENSG00000120686	UFM1	188,319221	133,3366997	193,0382111	345,3726273	90,31207934	88,97532069	85,35533083	174,5677827	120,0665232	1419,343796	157,7048662
ENSG00000135390	ATP5MC2	108,5527452	149,2887808	151,0746266	197,2540262	195,1706212	155,8503513	137,9199064	189,9315705	131,08443	1416,127058	157,3474509
ENSG00000143149	ALDH9A1	150,1969638	180,76078	55,1254368	257,3853755	157,521026	147,9051665	143,2592031	177,2040274	145,3962514	1414,754231	157,1949145
ENSG00000120594	PLXDC2	74,2145653	151,3006225	171,0748855	194,4709932	177,6012991	183,4429429	213,4203722	60,29175327	187,2218486	1413,039283	157,0043647
ENSG00000109046	WSB1	138,6731975	193,5472772	121,5718591	248,25527	76,64959402	130,5181205	133,2447667	162,6051528	207,2787183	1412,343956	156,9271062
ENSG00000126698	DNAJC8	175,0416361	223,1735111	99,96518082	218,3480769	49,54629993	146,2330775	156,2305206	153,7914688	189,188161	1411,517933	156,8353259
ENSG00000136826	KLF4	71,22251364	220,3274927	217,4044248	196,8056799	18,2402684	78,96836024	125,3675436	275,3922174	206,9585248	1410,687025	156,7430028
ENSG00000175334	BANF1	131,9187201	185,1542019	114,8340248	186,4568769	28,08606448	217,0367638	215,3795539	126,3426288	204,853553	1410,062388	156,6735986

ENSG00000101310	SEC23B	180,0920963	35,46690081	270,8800372	244,3922328	85,17268963	118,0859544	140,6068897	133,0027393	197,332134	1405,031674	156,1146305
ENSG00000186468	RPS23	139,2033107	136,9432426	139,0792262	378,1982912	51,02458063	134,2891075	141,7836379	182,9961444	100,2225767	1403,753618	155,9726242
ENSG00000110651	CD81	195,2543132	192,094695	124,1398819	116,9160902	120,2562936	179,800034	210,8254537	80,44119848	183,4587187	1403,186679	155,909631
ENSG00000130830	MPP1	169,5093188	207,8150193	192,5151279	123,0688492	65,48770602	151,0905437	168,2548781	206,9196449	117,0667771	1401,727865	155,7475406
ENSG00000197329	PELI1	205,8516769	125,5830047	140,7907028	161,6891128	138,8716578	126,6938748	98,32752002	231,8938263	170,9460922	1400,647468	155,6274965
ENSG00000167526	RPL13	146,9729027	126,0771361	123,7218619	186,3579663	156,2847892	171,6093846	161,4322596	166,9304896	159,8605293	1399,247319	155,4719244
ENSG00000163950	SLBP	199,3151635	173,7799268	140,3179799	104,9833921	127,7793242	162,698525	148,2044637	134,0580806	1398,52533	155,3917034	
ENSG00000018280	SLC11A1	150,7099779	173,5072831	74,26900293	158,8587524	90,77279511	236,091745	185,03837	124,046264	205,0181034	1398,312294	155,3680326
ENSG00000031698	SARS	222,2491402	158,2797153	92,29892608	186,3627553	90,40917083	149,5307328	117,3859874	184,06124	197,1933481	1397,771016	155,3078907
ENSG00000169896	ITGAM	235,1405874	135,6266762	79,56251778	106,933795	77,34159448	105,2108738	153,3967456	296,7985593	207,5090125	1397,520362	155,2800402
ENSG00000118971	CCND2	269,8071863	107,9673576	84,13065757	111,1794069	266,2077737	106,1411179	148,1844869	77,4700848	225,8151886	1396,90326	155,2114734
ENSG00000156171	DRAM2	152,4589123	186,2809306	7,618320112	77,33254505	230,5996966	209,265157	169,4929055	214,4981562	148,3574054	1395,904029	155,1004476
ENSG00000102390	PBDC1	175,8972338	108,2838219	179,8422456	282,2269209	181,5023715	97,94387917	106,874396	167,2807802	91,76268752	1391,614337	154,6238152
ENSG00000170891	CYTL1	54,69927634	114,4144834	202,6528325	123,7396391	239,6537694	185,7057719	264,3884448	68,15839411	137,740648	1391,153259	154,5725844
ENSG00000174600	CMKLR1	88,70345182	48,83913591	168,4266996	79,99365529	140,7695132	195,9829668	179,6577486	328,9182776	159,8358812	1391,12733	154,5697033
ENSG00000185475	TMEM179B	163,6696274	184,8824828	113,2990696	114,3325454	43,45704302	162,1599387	184,5348234	289,1302466	135,4307104	1390,896487	154,5440541
ENSG00000136404	TM6SF1	162,467998	139,9658918	41,05455802	166,3080484	95,68370445	199,0802223	231,6556712	122,5625397	227,3319917	1386,110626	154,0122917
ENSG00000083845	RPS5	120,0278323	134,9976727	109,7471886	234,1705633	210,6849391	142,5170478	139,3112672	189,7463635	102,5058922	1383,708767	153,7454185
ENSG00000168214	RBPJ	198,3560998	119,5090898	179,5019012	175,7708523	213,7317957	99,83681953	78,41726251	228,1580516	89,90046836	1383,182341	153,6869268
ENSG00000022277	RTF2	117,6006952	128,8903092	195,9475765	156,5738292	104,827321	184,7761161	156,4132984	168,5923846	167,18411	1380,80564	153,4228489
ENSG00000116459	ATP5PB	116,6797045	201,6619249	84,15586171	222,7676217	118,1036659	165,4876052	136,6659002	197,4393926	137,6969931	1380,65867	153,4065189
ENSG00000136158	SPRY2	106,1489443	266,639804	153,0491115	126,2038439	102,2697016	162,7607027	180,3367548	78,77774003	204,1246116	1380,311214	153,3679127
ENSG00000188641	DPYD	154,6706009	83,81552632	247,3792888	184,860958	56,55434021	135,4552486	177,3757421	171,4033747	167,3687233	1378,883803	153,2093114
ENSG00000124702	KLHDC3	182,2457848	137,0592096	37,17415885	113,4724654	375,8372298	166,9788469	194,7617734	83,63153244	87,63045528	1378,791456	153,1990507
ENSG00000178988	MRFAP1L1	168,3504372	80,99993906	57,26518436	194,5261196	438,4053877	112,2674341	130,8600507	55,40311686	138,8054626	1376,883132	152,9870147
ENSG00000066294	CD84	159,2253144	88,07689684	230,523745	204,2933361	139,2623295	148,9161708	158,9155302	106,6816302	137,9541446	1373,849098	152,6498997
ENSG00000114738	MAPKAPK3	191,0207444	175,6792414	185,0360506	138,232219	0,183457237	189,218576	178,8312778	107,3595836	207,9677175	1373,528868	152,6143186
ENSG00000144381	HSPD1	240,6847241	127,7950595	176,6451272	160,1810383	115,2346613	119,5538015	132,3783517	158,8458193	140,5005117	1371,819095	152,4243438
ENSG00000150593	PDCD4	163,5397225	166,1218417	77,94691581	144,6131814	246,0985841	140,0771286	122,7109229	182,7673053	124,1701638	1368,045766	152,0050851
ENSG00000173692	PSMD1	186,1861687	199,3876411	128,9951992	183,4644018	100,0433485	107,2805233	128,6149519	128,0457838	205,2301676	1367,248186	151,9164651
ENSG00000125971	DYNLRB1	67,201205	158,8141846	94,3550336	148,2874368	301,0164115	146,6217861	149,2892934	182,4106271	118,3071764	1366,303154	151,8114616
ENSG00000135334	AKIRIN2	99,92904852	258,4241021	298,6264956	155,7437388	0,803394949	147,94621	142,4804039	77,32316725	181,4394235	1362,715985	151,4128872
ENSG00000126860	EVI2A	129,2940276	154,2043081	139,1913871	291,6776756	110,8044952	164,9492407	159,3370223	88,07333717	122,8507431	1360,382237	151,1535819
ENSG00000170043	TRAPPC1	137,0447703	167,3883878	136,2600998	117,654711	211,2997324	163,6436517	136,5065781	147,8631199	142,678073	1360,339124	151,1487916
ENSG00000106049	HIBADH	208,793665	44,62969518	218,1345417	46,97065576	254,1921998	100,3267486	149,361336	247,3402122	89,68712766	1359,436182	151,0484647
ENSG00000105851	PIK3CG	35,49590224	48,55217671	140,7263064	116,8410698	468,4946174	100,700135	135,6824698	168,8982355	143,3878319	1358,778745	150,9754161
ENSG00000138629	UBL7	184,7797694	92,60500905	249,3710066	121,262089	117,5466969	146,7230405	175,5253398	107,1063771	163,6632504	1358,582579	150,9536199
ENSG00000197451	HNRNPAB	61,85687666	70,39044006	174,8203455	81,80397693	266,7100539	214,712413	228,8441419	140,4112893	118,8961462	1358,445683	150,9384093
ENSG00000128595	CALU	109,1751237	36,91074836	106,1163386	101,9135812	312,6848106	125,0738674	141,436046	319,6493333	105,4068024	1358,366652	150,929628
ENSG00000241553	ARPC4	130,7139942	74,02146122	149,1600531	194,9780187	176,2741595	202,8817806	190,2572579	133,8956036	105,9551819	1358,137511	150,9041679
ENSG00000139684	ESD	176,3410443	209,0380277	166,2037168	174,348444	56,18625813	173,5334951	117,7085037	139,5168174	145,049583	1357,92589	150,8806545
ENSG00000115758	ODC1	109,4753041	108,46733	140,1354351	260,187042	135,1033391	176,49176	127,9428753	130,2321835	168,800048	1356,835317	150,7594797
ENSG00000213639	PPP1CB	155,7426716	141,9383608	101,3469111	213,1874983	46,26123223	191,8053004	122,7692346	240,5403024	142,7727189	1356,375721	150,7084135
ENSG00000103066	PLA2G15	196,1049289	126,5558095	124,0314791	46,21942375	349,7772655	124,5207602	158,8071803	72,8289641	136,5513573	1355,397169	150,5996854
ENSG00000188243	COMMD6	96,70659234	178,7304806	126,2760822	246,883937	82,43464469	173,0693002	141,1487097	178,0491405	131,4461595	1354,745047	150,5272274
ENSG00000188404	SELL	120,0142174	19,63690956	754,5464935	103,0936791	161,3464755	47,9324222	20,12303384	107,4426569	19,39445582	1353,530344	150,3922604
ENSG00000183617	MRPL54	176,7303324	94,73443252	88,05054912	194,9727323	212,9184211	179,7224217	104,5798402	179,8100794	120,7530703	1352,271879	150,252431
ENSG00000213445	SIPA1	102,1263186	131,1313521	103,4358451	134,5060338	170,295206	170,6539558	222,3511215	86,3022355	229,5915657	1350,393614	150,0437349
ENSG00000273703	HIST1H2BM	0	2,111354331	63,47577063	1027,987918	256,377878	0,435460831	0	0	0	1350,388382	150,0431536
ENSG00000179918	SEPHS2	219,2909967	103,0102548	176,5298943	190,1521434	126,1345817	141,7166392	129,3559283	128,1065415	135,9426149	1350,239595	150,0266216
ENSG00000173113	TRMT112	158,3638781	220,7839976	108,9681988	94,09716623	156,9067416	136,3019199	142,0710221	201,3109057	131,4077871	1350,211617	150,023513
ENSG00000144021	CIAO1	168,0547478	71,57548799	136,3350672	93,01998474	430,923233	135,8647516	97,39436091	72,21851997	143,3844416	1348,770595	149,8633994
ENSG00000142166	IFNAR1	91,00902664	126,5673898	212,6776073	40,35976348	393,8860092	111,354722	104,6406883	119,9920846	147,9964019	1348,483693	149,8315215
ENSG00000111481	COPZ1	129,9830024	131,1130457	124,0142104	38,42398035	307,3412317	140,7984063	179,1927497	153,0103628	143,842729	1347,719718	149,7466354
ENSG00000064115	TM7SF3	88,40407659	82,82002183	223,8891313	118,5680181	325,5155194	126,3319816	143,8440761	125,3538112	110,5489669	1345,275603	149,475067
ENSG00000184752	NDUFA12	216,5103492	218,6411624	86,04515055	216,4028484	85,72354051	115,3878913	110,1451771	170,8381729	123,6558288	1343,350121	149,2611246
ENSG00000122042	UBL3	54,56387622	128,0769499	260,98679	176,2977634	187,258828	176,4692106	152,5449064	88,56309691	114,7127485	1339,47417	148,8304633
ENSG00000182952	HMGN4	116,0638419	121,148494	84,98487966	288,8361541	0,180279891	179,2367274	106,2592621	277,3527093	165,0414146	1339,103763	148,789307
ENSG00000243147	MRPL33	141,7401371	141,0720967	49,89851646	317,3119175	81,80941402	128,7648014	90,08966397	273,0614638	114,3950852	1338,143096	148,6825662
ENSG00000182154	MRPL41	256,2648338	92,6057423	212,067292	106,7053676	0,617040451	183,869969	192,9483628	159,5397139	133,4773992	1338,095721	148,6773023
ENSG00000135932	CAB39	100,3646938	142,7957397	117,8162903	190,1424353	93,01030511	190,0717105	191,375595	126,4930388	185,8465223	1337,916331	148,6573701
ENSG00000102393	GLA	173,4284396	106,5496123	50,58626856	159,5430988	109,2348744	185,8927736	186,6888237	254,6161744	107,0966113	1333,636677	148,181853
ENSG00000100603	SNW1	212,0643	116,9813578	176,9379906	189,5855675	36,32521193	152,198395	128,049903	167,5270649	153,6748998	1333,34469	148,1494101
ENSG00000159658	EFCAB14	173,9143127	104,7171757	137,9588684	213,1552476	44,67002737	155,4347708	134,9573547	209,9967749	156,5393911	1331,343923	147,9271026
ENSG00000100664	EIF5	160,1030848	157,958139	60,82276751	218,522846	122,2674778	137,0687044	125,0328904	155,8818518	189,287297	1326,945059	147,4383398
ENSG00000105612	DNASE2	132,830556	180,4823508	143,9352794	138,0585215	0	254,6647488	242,9051007	55,8792655	178,1450548	1326,900878	147,4334308
ENSG00000105355	PLIN3	222,0591628	111,4076656	253,0136498	80,21440256	0	159,3686482	154,5131179	160,2106994	185,1001507	1325,887497	147,320833
ENSG00000090273	NUDC	129,8341174	182,4646054	157,7302809	121,3970651	130,8862391	124,9572286	170,3189268	148,9740115	158,8961663	1325,458641	147,2731823
ENSG00000136048	DRAM1	80,27269655	121,3444635	74,85707742	109,2085719	180,1967973	121,6958291	137,7944484	384,4446846	112,2154509	1322,03002	146,8922244
ENSG00000175606	TMEM70	138,8980799	250,4277231	105,0582064	145,605566	127,5336946	136,8678802	123,9555996	187,1576072	106,5096266	1322,013984	146,8904426
ENSG00000114956	DGUOK	173,9589044	194,0574062	241,298189	101,5046018	56,52090529	129,7637363	123,5731861	172,143919	127,601839	1320,422687	146,7136319
ENSG00000105669	COPE	148,9030423	164,8956717	142,8092543	67,70695341	168,3792859	168,5651459	158,8999926	125,6226025	173,1766376	1318,958586	146,550954
ENSG00000125457	MIF4GD	146,958581	158,1126544	76,78119871	151,0328613	137,2332763	190,9231257	158,0853605	102,6186889	197,2015701	1318,947317	146,5497019
ENSG00000197756	RPL37A	94,38612315	138,2454077	103,4146107	359,5722856	124,8853433	116,2174366	106,6158515	177,220352	98,2886592	1318,84607	146,5384522
ENSG00000158985	CDC42SE2	130,1898841	138,7002776	197,2255882	154,2479158	281,8571946	147,6229567	115,7226369	19,74708571	133,0604334	1318,373973	146,485997
ENSG00000111231	GPN3	180,6566792	224,7098211	163,3789706	78,4918165	75,06429154	154,2654842	163,5033752	125,7766749	150,5120312	1316,359144	146,2621272
ENSG00000197858	GPAA1	205,9505758	131,281273	8,676245439	136,3760516	189,229011	178,3995614	182,2928814	100,3032714	183,6855162	1316,194387	146,2438208
ENSG00000166595	CIAO2B	126,3056279	184,6638302	75,5266684	150,406858	133,4833129	151,0412326	200,2790583	116,5872908	174,8009271	1313,094806	145,8994229
ENSG00000188725	SMIM15	132,0124549	144,8412466	224,5594082	281,8061585	86,17328838	110,2432122	111,0030119	103,0891777	118,9855331	1312,713491	145,8570546
ENSG00000137478	FCHSD2	78,45073097	9,202204007	429,7164237	120,7912523	219,3732327	85,91289348	105,4716289	148,0498062	115,192954	1312,161126	145,7956807
ENSG00000157240	FZD1	94,01278071	88,30239928	189,1526223	90,38850477	244,8786573	120,0188963	205,8376565	105,1145436	174,4496438	1312,155705	145,7950783
ENSG00000174444	RPL4	121,2063572	127,1630808	148,084246	210,7855892	136,4377755	132,6633868	127,0809335	154,9372281	152,779161	1311,137758	145,6819731
ENSG00000160213	CSTB	110,5200057	153,1169185	105,5406017	232,3548033	93,08764757	127,4159499	150,0542268	199,688285	137,342233	1309,120671	145,4578524
ENSG00000166226	CCT2	126,6756699	174,7043386	169,7452945	165,2588172	0	205,1721576	129,0890981	221,6176993	113,7486161	1306,011691	145,1124101
ENSG00000115271	GCA	106,6262077	179,0299959	109,8760339	134,2665594	204,5546475	125,2043559	106,3539617	224,8420071	113,5399005	1304,293669	144,9215188
ENSG00000106609	TMEM248	127,2525706	157,2684991	143,7451161	80,70206745	84,44840359	154,4279729	137,6078474	172,2989526	245,5541938	1303,305624	144,8117359
ENSG00000089127	AC004551.1	177,2896131	204,1048497	119,5522587	99,12466046	214,7001053	72,52921386	75,08920699	223,36601	116,9016376	1302,657556	144,7397284
ENSG00000115649	CNPPD1	152,5555412	158,1830703	117,1626309	157,9621067	172,8888793	171,7683977	142,9595765	104,3925316	124,1255184	1301,998253	144,6664725
ENSG00000172809	RPL38	95,78097545	142,1593785	52,05320736	309,3875123	74,65593202	150,5284261	151,1935523	207,9778814	118,0894721	1301,826337	144,6473708
ENSG00000213625	LEPROT	169,2311432	119,2481799	101,5420908	205,5790921	144,8950481	131,4739151	120,5109356	139,6948888	168,8481484	1301,023442	144,5581602
ENSG00000115310	RTN4	167,850092	134,999246	96,02112089	192,0880435	177,5588692	121,1366959	130,9091423	136,0729947	142,825458	1299,461662	144,3846292
ENSG00000180992	MRPL14	55,89670012	163,0706834	0	241,0308438	95,31437383	235,7767861	216,8525905	73,89027947	214,9760712	1296,808329	144,0898143
ENSG00000133318	RTN3	186,2009915	142,3115518	139,7579503	156,1014653	143,9074268	125,9797507	134,8936045	93,56227395	173,8781603	1296,593175	144,0659084
ENSG00000136003	ISCU	134,5323215	124,6778752	117,5419342	112,6270357	229,1103582	121,9194191	116,0014063	211,6299787	127,274933	1295,315262	143,923918
ENSG00000101365	IDH3B	189,0206632	172,9813604	178,6782872	143,2245342	0,419693271	131,7848328	147,3188861	160,905029	170,7152706	1295,048557	143,8942841
ENSG00000173660	UQCRH	155,6179702	134,3709352	124,1685901	345,5783109	25,70863838	111,2256588	137,6337497	169,2830335	91,32949901	1294,916386	143,8795984
ENSG00000081154	PCNP	173,9573978	174,0934843	195,984035	156,1235696	108,360419	134,0334962	119,0795156	70,83784249	160,3240792	1292,793839	143,6437599
ENSG00000173418	NAA20	111,1069726	203,476442	116,0590312	94,11669749	189,5342585	130,8570794	134,6588159	224,1837217	87,52620304	1291,519222	143,5021358
ENSG00000124207	CSE1L	126,7340497	82,70063792	90,48983748	161,2990146	147,0491831	174,686373	152,5785274	148,5216425	206,2407327	1290,299998	143,3666665
ENSG00000134107	BHLHE40	73,99967742	72,74845507	98,02112951	183,4773421	221,0674055	155,7060169	138,4849768	114,7091071	228,8571326	1287,071243	143,0079159
ENSG00000114686	MRPL3	140,8139019	126,5156177	159,4162115	258,1478239	109,917745	153,4443676	117,5677807	92,01050941	129,0315633	1286,865521	142,98505790
ENSG00000103381	CPPED1	177,4134179	88,91646116	253,9619884	54,82344839	147,2302798	113,7926178	110,6603306	180,3505005	156,3637879	1283,512832	142,6125369
ENSG00000054118	THRAP3	100,3348577	93,16798308	123,5597982	227,4888603	249,6323425	98,31607691	119,675304	127,9733876	142,4954585	1282,644069	142,5160076
ENSG00000002933	TMEM176A	173,2317327	233,36143	57,32290221	93,50732768	148,3154417	229,3259222	150,2973635	77,26295755	117,9112534	1280,536331	142,2818146
ENSG00000100804	PSMB5	157,4460847	169,6966652	115,9169138	198,9906379	160,9416295	177,6606175	83,74167201	102,7810638	113,192641	1280,367925	142,2631028
ENSG00000143222	UFC1	161,0406939	102,0216467	72,67194016	239,5631576	146,3831506	147,8184233	117,2848865	173,5568228	120,0037544	1280,344476	142,2604973
ENSG00000189043	NDUFA4	88,71906374	232,6583144	65,05746321	173,3283752	63,29433714	195,2960675	158,7139883	215,8660957	86,39903332	1279,332739	142,1480821
ENSG00000065518	NDUFB4	156,0214629	210,5707555	101,8914801	150,9725	75,76480915	135,5923413	113,0369374	225,688394	109,5078602	1279,046541	142,1162823
ENSG00000100591	AHSA1	95,16270313	154,7463513	113,8736411	182,0517792	141,5001186	119,8681374	162,9324318	134,6880031	171,9585349	1276,7817	141,8646334
ENSG00000093010	COMT	207,744311	116,6508027	129,3851548	131,8989881	112,5396264	142,9481696	134,2375564	153,4819271	147,8901873	1276,776723	141,8640804
ENSG00000089157	RPLP0	150,6937662	106,4365061	89,22129556	228,8742581	79,69383776	180,5546396	146,5813131	171,5972892	121,2103321	1274,863238	141,6514709
ENSG00000137547	MRPL15	152,1255871	201,2753552	125,3361152	182,5011199	4,824417026	215,6419586	119,6761521	97,93736552	175,3916801	1274,709751	141,6344168
ENSG00000105887	MTPN	56,5366096	106,1365876	187,2800878	204,4289608	209,5643617	126,1218689	132,1386389	150,4516989	101,9158348	1274,574649	141,6194054
ENSG00000141699	RETREG3	92,25234256	78,38397826	181,8621989	156,9159983	308,7224276	139,5003216	138,3465876	98,77713602	79,79299664	1274,553988	141,6171097
ENSG00000184897	H1FX	82,53268635	146,6291016	131,8388464	119,9681249	128,8653978	127,9350027	222,8836439	74,76191072	235,6180843	1271,032799	141,2258665
ENSG00000116171	SCP2	155,0105527	99,03026975	151,5030274	116,2556527	251,7695505	140,9023667	87,99494506	145,4184356	122,2087818	1270,093582	141,1215091
ENSG00000108960	MMD	65,02534422	36,27852929	194,9256003	241,1585061	173,3166717	135,9812024	175,6113103	73,68287256	171,4700453	1267,450082	140,8277869

ENSG00000069329	VPS35	154,8522379	150,3942442	147,6867453	132,4647806	56,82122832	160,6773961	151,2012251	126,8708496	185,9992781	1266,967985	140,7742206
ENSG00000160593	JAML	223,5070872	151,0738321	161,990499	53,45110287	239,9693092	38,27516623	70,04830227	259,6299088	68,47774502	1266,422953	140,7136614
ENSG00000090339	ICAM1	105,049895	126,4497947	97,55102081	103,5264052	85,92574607	177,3626409	237,2218951	180,4548407	150,9733494	1264,515588	140,501732
ENSG00000153551	CMTM7	153,4471244	216,9584032	140,0646193	53,28610785	92,6754948	168,122128	149,9556114	153,5450416	134,5665568	1262,621087	140,2912319
ENSG00000170677	SOCS6	62,3437857	111,7296603	176,2905132	178,3509991	107,9382235	215,294475	164,9658741	73,34187352	172,0647884	1262,320193	140,2577992
ENSG00000150753	CCT5	108,2271168	143,1073569	109,1113798	126,1170283	257,4588695	125,9778607	105,5303857	153,4955893	133,0065125	1262,032099	140,2257888
ENSG00000149923	PPP4C	154,2068721	133,7276056	172,5042985	120,490281	131,7946926	135,9216177	145,4409117	134,930471	132,329796	1261,346546	140,1496163
ENSG00000179115	FARSA	104,920651	114,9022622	82,57173852	28,08749521	284,461225	175,8624848	145,6260016	166,1675886	158,6643686	1261,263815	140,1404239
ENSG00000053371	AKR7A2	102,3241708	110,809945	283,130769	71,93659322	157,0761176	106,3839857	131,8107755	159,5585222	137,3239884	1260,354867	140,0394297
ENSG00000104805	NUCB1	160,4855938	143,9575962	148,4829753	72,84878475	188,9886604	120,9454245	179,7895968	108,2566583	135,8828858	1259,638176	139,9597973
ENSG00000101337	TM9SF4	246,3813903	67,04787962	113,1941123	154,0070506	326,0243469	74,91253186	103,7823854	83,05105197	90,81253886	1259,213288	139,9125875
ENSG00000184216	IRAK1	53,97081653	123,6999219	76,00630227	200,7807029	0,091681783	227,2852067	213,0465096	133,5404443	230,2561074	1258,677693	139,853077
ENSG00000123975	CKS2	59,61017641	126,2356819	58,21336488	291,010272	50,13654	190,9587644	166,0037672	196,3296294	120,0761621	1258,574358	139,8415954
ENSG00000137841	PLCB2	157,1352539	77,93595213	84,88057452	135,4206005	207,9255875	144,2150992	177,8677261	62,94848204	210,2248115	1258,554087	139,839343
ENSG00000100911	PSME2	126,7270112	197,2513038	149,6218958	130,9774105	49,42628375	125,3824487	109,9529235	219,9207172	146,9339339	1256,193928	139,5771032
ENSG00000136783	NIPSNAP3A	93,48068919	142,4633888	102,2554305	255,7929021	116,4217999	165,7182538	119,8464686	120,4017763	136,4406462	1252,821355	139,2023728
ENSG00000147471	PLPBP	139,9547567	203,7472332	66,51405928	211,7479324	0,314371937	157,1962774	170,906189	161,5050775	139,9678652	1251,853763	139,0948625
ENSG00000134970	TMED7	83,88406771	83,71585009	221,1501355	136,0336711	186,2451767	87,84463827	118,6940058	235,6638149	98,30845629	1251,539816	139,0599796
ENSG00000168610	STAT3	149,6529676	89,34009445	247,6962934	82,20103149	51,91962653	147,7982328	161,1094878	151,7554125	168,932211	1250,405357	138,9339286
ENSG00000169299	PGM2	71,21964073	119,814334	171,2333218	175,8490256	0	138,3415361	195,6955001	159,3311091	215,2625342	1246,747002	138,5274446
ENSG00000129083	COPB1	143,865745	127,4079509	14,41097375	303,2460054	148,938244	142,5000802	77,27270495	122,5525204	166,1788821	1246,373107	138,4859007
ENSG00000147443	DOK2	492,7196706	61,61616759	124,4849635	43,59570129	0	119,413345	62,2126291	266,8085092	73,93795987	1244,788946	138,3098829
ENSG00000058799	YIPF1	73,55136323	111,1551317	194,4222303	46,77861775	321,5477798	113,7579396	93,3632382	168,7877948	121,2201285	1244,584224	138,287136
ENSG00000107874	CUEDC2	157,6756741	178,1587671	85,58039002	155,3882433	0,269001904	150,2516009	176,8802733	107,6696905	232,3429556	1244,216597	138,2462885
ENSG00000100567	PSMA3	171,780668	220,416694	152,9024748	210,5452145	41,95676468	117,1573239	87,06599157	138,6825478	101,0880665	1241,595746	137,9550829
ENSG00000089693	MLF2	102,9917854	169,0691691	150,0548485	89,99185583	54,42326151	197,1970727	158,8870536	113,1744948	205,6059251	1241,395466	137,9328296
ENSG00000197157	SND1	145,2860806	114,2683454	59,61614519	80,46098508	217,762765	144,4747051	161,0647524	148,4051642	168,5684691	1239,907412	137,7674902
ENSG00000100600	LGMN	204,0538511	59,5640511	91,22566716	49,54802407	171,9613412	80,34420858	181,0838589	252,1737489	149,5498718	1239,504623	137,7227359
ENSG00000107099	DOCK8	70,69878629	118,7030241	263,4599545	159,9797933	83,57871996	108,905898	134,1724241	47,15356894	250,5233771	1237,175546	137,4639496
ENSG00000132661	NXT1	167,4737681	157,8048494	215,9449921	89,92674266	128,4978023	145,757597	140,5929332	28,31407311	162,7469545	1237,059713	137,4510792
ENSG00000102172	SMS	103,4503265	111,703775	92,20267844	116,6866175	180,9768069	141,9984896	112,4650162	202,7295989	174,6553694	1236,868678	137,4298532
ENSG00000159352	PSMD4	100,6405386	153,6036433	93,94570243	141,2859013	87,67355923	150,5089011	170,4854561	160,577882	174,7951929	1233,516777	137,0574197
ENSG00000140740	UQCRC2	132,2833	181,9479932	195,6570679	108,1352147	88,17894801	127,71648	101,1554948	158,155092	139,8925231	1233,122114	137,0135682
ENSG00000087191	PSMC5	76,77363417	114,0496472	122,3268349	90,43387879	238,9361166	125,7027552	122,1569065	225,1032401	117,2235951	1232,706609	136,967401
ENSG00000144224	UBXN4	113,4992161	155,155395	115,6859956	232,6383047	111,3709865	127,3043341	133,5036466	105,8474875	136,0197236	1231,02509	136,7805655
ENSG00000113532	ST8SIA4	49,69317154	137,3661398	245,1007324	143,8282131	178,1337016	123,8807468	147,6990749	89,54556828	113,2398164	1228,487165	136,4985739
ENSG00000196743	GM2A	63,62905476	67,88859748	241,9215061	65,30656542	264,9083188	112,0146425	182,7009389	83,88124654	146,1793476	1228,430218	136,4922465
ENSG00000168159	RNF187	179,8956616	92,73310163	76,84269718	129,4656696	270,4517817	100,0559355	146,7402583	93,29457961	138,5341471	1228,013832	136,4459814
ENSG00000164105	SAP30	85,77169901	234,3425883	108,3039347	126,5803735	173,6603547	120,4087455	116,7477956	89,10878712	173,0686865	1227,992965	136,4436628
ENSG00000177674	AGTRAP	236,9984679	117,7181922	140,1887316	164,2765158	105,1543358	78,15171724	145,9268913	129,5954875	109,6761612	1227,686501	136,4096112
ENSG00000148484	RSU1	138,768287	96,63770095	191,7429379	84,99675372	170,0716621	167,2777506	129,1803127	111,5597074	135,488691	1225,723803	136,1915337
ENSG00000146476	ARMT1	116,1605557	59,06923805	168,8194145	94,69190458	246,1209975	116,7082915	74,59676354	259,283882	90,05546273	1225,50651	136,16739
ENSG00000172086	KRCC1	76,68422429	74,34374214	186,6020255	273,0647687	0	134,6933127	100,4040399	185,4237141	193,8686743	1225,084502	136,1205002
ENSG00000177731	FLII	99,72626913	63,97490649	174,5528174	76,48522004	243,7194354	107,5735984	165,8284968	89,73278601	203,0228892	1224,616419	136,068491
ENSG00000073921	PICALM	37,26536306	191,1494769	107,7381084	205,3494888	123,7828678	173,36635	167,0563676	82,06156478	136,7035677	1224,473155	136,0525728
ENSG00000177868	SVBP	87,4497981	183,5177325	142,7909599	174,3423629	3,715924311	193,0105305	145,9647469	48,35627817	244,9108272	1224,059161	136,0065734
ENSG00000175857	GAPT	156,1891416	116,0883636	71,76693665	230,2414319	127,4960538	151,3116625	144,5611433	36,04190058	187,1525015	1220,849136	135,6499039
ENSG00000167815	PRDX2	160,1742332	192,3398123	160,9933907	67,86361925	0,176557297	136,8721473	158,5342936	165,5421948	177,6232248	1220,119473	135,5688304
ENSG00000159322	ADPGK	82,06493814	61,44073143	142,3880527	69,55626283	271,6844398	116,9290525	137,5498396	200,567264	133,8934732	1216,074054	135,1193394
ENSG00000163527	STT3B	197,5411929	196,6125001	1,442035629	160,0907915	2,077894798	126,6177087	114,1800048	268,65939	146,1335084	1213,355027	134,8172252
ENSG00000119414	PPP6C	38,50037497	102,8390655	159,3552357	138,5427875	378,2737111	80,58407034	86,07552823	148,967013	79,68436303	1212,822149	134,7580166
ENSG00000130985	UBA1	105,9675495	77,02261273	208,4958704	185,6582544	122,6255023	116,3461773	140,1868305	89,52584834	166,1849592	1212,013605	134,6681783
ENSG00000176101	SSNA1	114,4307398	101,0689077	171,539856	192,2444001	119,3989094	134,7137161	136,8377296	104,7788244	135,0167042	1210,029787	134,4477541
ENSG00000162711	NLRP3	120,0752485	119,8944785	170,3409688	140,2587198	23,62913407	100,8647021	225,3032533	110,1680101	198,0748093	1208,609324	134,2899249
ENSG00000121807	CCR2	148,2365148	168,5853936	67,31690583	168,7374842	0	184,8882934	207,6831453	0,531102975	262,5648429	1208,543683	134,2826314
ENSG00000071073	MGAT4A	62,79023713	112,922399	54,17669426	199,2486035	242,1322823	136,6919955	138,0059526	119,5064828	142,0078812	1207,486128	134,1651254
ENSG00000143183	TMCO1	156,7934539	136,2799062	171,248485	131,6357134	177,3721672	91,74997508	88,12583132	139,5352275	113,7910565	1206,531816	134,0590907
ENSG00000130770	ATP5IF1	103,5701664	99,06189846	57,47004294	304,5609569	50,3571565	136,5943621	93,77849983	221,7253076	140,9092508	1206,025905	134,0028783
ENSG00000182287	AP1S2	160,704446	136,8417841	251,0153566	161,5843838	16,58185636	77,62675093	75,36162559	246,9492374	78,96141572	1205,626856	133,9585396
ENSG00000103978	TMEM87A	93,04444101	107,5311867	198,4801448	96,86083037	339,7931157	82,04787341	65,94497513	146,153751	73,02050196	1202,87682	133,65298
ENSG00000113013	HSPA9	129,306271	162,4640347	120,7027714	214,5584774	95,45721528	148,4632323	131,0491558	58,44856658	141,1298766	1201,579605	133,508845
ENSG00000173545	ZNF622	164,2121316	145,3024898	18,72742319	4,127266734	446,8457293	123,7282742	130,1010472	69,10670217	98,53023003	1200,681294	133,4090327
ENSG00000197958	RPL12	93,45076873	124,2875883	106,0698625	198,4935437	168,7984208	97,59843546	113,296292	194,5159052	103,7952448	1200,306461	133,3673846
ENSG00000183087	GAS6	118,8786734	102,3085437	136,1016445	88,93045685	42,32989818	184,4462222	200,9819865	160,6302037	162,642742	1197,250371	133,027819
ENSG00000214253	FIS1	116,5950643	134,2950741	120,4838535	116,4395068	181,6594588	152,323471	112,3990495	134,9351187	127,4524061	1196,583003	132,953667
ENSG00000213593	TMX2	123,3432488	150,9116982	102,6539913	166,4285657	111,4172934	133,1408445	151,9232427	115,424769	139,4055138	1194,649167	132,7387964
ENSG00000159720	ATP6V0D1	183,4411182	123,4189637	88,10428591	92,26783555	79,43208024	188,9582328	193,8975067	110,0368177	134,3490799	1193,905921	132,6562134
ENSG00000109390	NDUFC1	89,06259291	107,0633952	161,4031796	232,7039882	74,74677337	109,4103752	119,3939738	218,7575576	81,28990078	1193,831737	132,6479707
ENSG00000165169	DYNLT3	112,8485992	121,9530443	26,28337474	268,378312	146,5245614	89,60234671	95,75155785	200,5907278	131,7008528	1193,633377	132,6259308
ENSG00000101457	DNTTIP1	223,3456345	75,44188105	40,81808527	128,2301027	177,4417867	123,05479	107,9878634	124,6655603	191,5235061	1192,50921	132,5010233
ENSG00000145781	COMMD10	109,7859497	116,2058606	230,9092581	96,82089053	165,6446229	81,90091609	73,10100114	219,7477936	98,21901075	1192,335303	132,4817004
ENSG00000102007	PLP2	165,3750374	71,82007523	274,1400055	75,69717605	1,92925771	128,2209906	186,7129499	78,86811243	208,9525366	1191,716141	132,4129046
ENSG00000135211	TMEM60	202,1125421	219,2221369	0	189,5274559	40,53892439	108,9674939	147,0735477	181,6071397	99,81038995	1188,859631	132,0955145
ENSG00000198492	YTHDF2	201,2781455	173,1525707	168,1000599	128,4732136	0,963039803	146,1561396	119,4138397	112,1829344	136,8556048	1186,575548	131,8417275
ENSG00000038945	MSR1	86,34889752	100,7763585	160,8740435	188,7870554	47,16520718	188,2103854	189,6349475	111,2023141	112,4785404	1185,477749	131,7197499
ENSG00000100100	PIK3IP1	54,68574397	121,3509919	217,4162351	71,38851736	256,714774	93,44188328	147,3328366	111,0711726	110,9218008	1184,323956	131,5915506
ENSG00000114573	ATP6V1A	13,73463944	90,56868181	163,8213445	211,0023436	161,729245	104,7156474	116,4451009	156,433253	164,6681559	1183,118412	131,4576013
ENSG00000172340	SUCLG2	151,4039892	164,5298486	173,7150716	124,3874491	44,16332236	160,7560733	127,4193857	75,06960078	160,8264079	1182,271149	131,363461
ENSG00000064726	BTBD1	84,7074607	86,76506551	155,7595374	129,6225312	279,6103484	126,6714717	86,79693871	97,56895002	133,0213534	1180,523657	131,1692952
ENSG00000136522	MRPL47	86,58525399	209,816222	114,8443329	128,372389	85,97850649	167,8482185	114,2054774	147,3689933	122,7672282	1177,786622	130,8651802
ENSG00000104388	RAB2A	128,1413316	113,0205104	156,3389505	120,2484674	178,7881327	105,4149283	118,9185908	139,2174251	116,6858261	1176,774163	130,7526848
ENSG00000178695	KCTD12	60,44422827	138,9600148	219,6600975	147,6326073	106,4372475	150,4255895	118,2343723	123,8399753	110,283864	1175,917996	130,6575552
ENSG00000134955	SLC37A2	122,1028438	135,1863293	137,3974779	78,92351217	245,1987615	138,5409305	148,9455534	78,62797118	90,75004407	1175,673424	130,6303804
ENSG00000136536	MARCH7	81,03448541	124,9453022	55,21583073	219,5621741	175,8465192	124,5120883	102,3038155	146,1941741	143,9955879	1173,609978	130,4011086
ENSG00000114850	SSR3	116,4273145	150,0366627	124,2409433	130,8904422	111,2977211	162,6834421	133,2529093	119,1906836	125,1562056	1173,176325	130,3529249
ENSG00000143314	MRPL24	109,6554584	112,7936555	59,69602126	132,5457305	239,1998261	117,7524624	123,2496182	152,643225	125,1037651	1172,639762	130,2933069
ENSG00000067829	IDH3G	144,6590336	150,0095105	235,0093071	63,96124147	69,65585014	113,1513797	140,7096436	120,4012594	134,3402907	1171,897516	130,2108351
ENSG00000004779	NDUFAB1	78,38616812	138,2282404	76,49839933	193,3413539	113,9061455	162,9096102	156,1084363	108,6506427	141,4258419	1169,454838	129,9394265
ENSG00000134590	RTL8C	109,2704719	165,9710432	65,67015039	202,6057339	62,33623724	125,7324015	130,1449252	142,6940131	163,5008778	1167,925854	129,7695394
ENSG00000120438	TCP1	206,2567998	110,2660529	88,23861426	120,6617187	194,451522	134,7874786	105,2994922	77,14425701	130,4312988	1167,537234	129,7263594
ENSG00000244509	APOBEC3C	142,8234932	133,0568472	122,1682583	128,4936978	49,71142984	151,0388646	124,0941445	132,4737676	183,5972451	1167,457748	129,7175276
ENSG00000158615	PPP1R15B	60,97292849	82,95627194	253,9825776	170,4834708	187,9238404	87,57706045	112,3005995	126,3555648	84,77767768	1167,329992	129,7033324
ENSG00000136758	YME1L1	105,9113079	48,87605373	202,7909352	116,7893008	206,4130278	117,1439505	71,19393617	190,5123089	106,3898496	1166,020671	129,5578523
ENSG00000164405	UQCRQ	85,57027807	156,7989964	56,64878482	236,3473237	31,24873114	167,2570106	141,3096886	164,1744335	125,2662537	1164,621501	129,402389
ENSG00000115524	SF3B1	74,67900914	101,0576387	152,8584135	152,9144479	147,1166851	125,788619	98,0864591	143,2843845	168,3894541	1164,175111	129,3527901
ENSG00000160789	LMNA	473,6023784	28,90636021	201,7295808	98,69590571	35,52615916	130,8282686	76,09052632	82,54950899	35,78576556	1163,714354	129,3015949
ENSG00000134248	LAMTOR5	148,3895679	178,8736207	90,21548438	124,4576784	143,1657874	102,2698362	127,7821807	137,1275781	110,9787022	1163,260436	129,2511595
ENSG00000166197	NOLC1	171,8169058	102,4613134	259,3547321	66,18678071	116,600129	105,3135147	112,1015787	86,56220409	141,0935562	1161,490715	129,0545239
ENSG00000157020	SEC13	112,6588288	93,65033795	132,3763966	138,9066482	174,4189848	114,4721302	126,9395943	126,2775968	141,7338812	1161,434399	129,0482665
ENSG00000142347	MYO1F	186,1263667	71,8579158	121,8703253	116,8392891	0,462076613	165,2507382	167,0344892	125,3865642	206,0420328	1160,869798	128,9855331
ENSG00000054148	PHPT1	92,70989726	115,9398102	90,87810928	211,0789125	31,63442371	153,86868	135,0440608	169,7269367	158,9840907	1159,864921	128,8738801
ENSG00000141429	GALNT1	193,7080686	120,2088014	167,1351278	46,42463848	2,311915509	101,4365485	138,2474513	233,7408782	151,8749672	1155,088397	128,3431552
ENSG00000172081	MOB3A	155,5582159	96,97101523	182,816345	117,2790971	45,31205661	141,2941703	157,9200777	57,79615475	199,656107	1154,60324	128,2892488
ENSG00000109919	MTCH2	114,6573091	107,3126436	181,2032717	230,5936985	79,4022473	160,3245559	131,5239162	51,84186898	97,47105004	1154,330561	128,2589513
ENSG00000170542	SERPINB9	15,86606281	52,42457641	68,70664286	265,5326703	207,4265698	133,192995	109,449621	24,08670997	277,1324679	1153,818316	128,2020351
ENSG00000125166	GOT2	119,5268097	107,3711768	168,8186258	151,7888869	177,7875969	148,8374009	123,9985392	60,25519706	95,0289602	1153,413193	128,1570215
ENSG00000091542	ALKBH5	113,5112729	127,0342272	112,6278713	130,2863328	155,7738409	124,0758631	131,5330473	98,27698467	157,9256757	1151,045116	127,8939018
ENSG00000166295	ANAPC16	125,1449939	178,2859043	104,2103933	145,3728848	94,92861954	122,8597749	115,6044872	142,06241	121,9778978	1150,447366	127,8274851
ENSG00000124226	RNF114	115,1534323	62,32122114	109,8588632	190,421765	105,5740988	149,2697879	152,8672433	134,6271341	128,8970115	1148,990557	127,6656175
ENSG00000148110	MFSD14B	117,5647293	76,96958229	224,7843741	152,8463912	3,980882908	128,7888111	110,1708574	184,303061	147,3543896	1146,763079	127,4181199
ENSG00000114391	RPL24	88,59997172	157,5257434	80,37850838	209,5054685	92,17990392	123,7675158	113,0574917	183,64488	96,330684	1144,990167	127,2211297
ENSG00000099942	CRKL	109,2454182	42,86657957	94,81088363	197,3864156	273,4725126	77,89287278	101,8888619	127,6088568	118,389314	1143,561715	127,0624128
ENSG00000145850	TIMD4	400,0044328	15,91872032	169,0617752	176,5958337	9,475396896	48,37234492	36,58171949	254,3648683	33,09931465	1143,474406	127,0527118
ENSG00000143653	SCCPDH	208,7033897	29,30650538	0	52,04074458	364,4850161	121,2751296	130,1000173	135,044487	100,5185767	1141,473866	126,8304296
ENSG00000125037	EMC3	101,3891768	127,0377673	165,0954405	203,431506	63,83377392	138,1292975	131,2296259	84,50457792	125,0873314	1139,738497	126,6376108
ENSG00000014641	MDH1	126,3393388	161,8524457	68,35302233	91,03963743	180,99812	126,0787388	137,6150555	83,30394508	163,5747346	1139,155038	126,5727821
ENSG00000122986	HVCN1	136,073266	138,2152772	121,6539804	78,11068654	205,460416	111,2068725	104,9895457	93,80829506	149,1609832	1138,679323	126,5199247

ENSG00000146386	ABRACL	220,0871636	129,9439244	69,67838893	332,9961725	5,685238138	75,14859015	79,56386049	141,8224001	82,70742328	1137,633162	126,4036846
ENSG00000188229	TUBB4B	173,3654412	189,028921	116,9508811	94,536088	81,62563677	158,0915215	115,183859	69,10413636	139,2252297	1137,111715	126,3457461
ENSG00000182481	KPNA2	105,0073118	138,4600945	100,1966742	228,6153203	33,57258158	157,4554646	146,0080002	115,8695632	111,5831803	1136,768191	126,3075768
ENSG00000068724	TTC7A	129,5173331	43,99321394	156,4509164	110,9518771	161,052714	99,84674122	134,6621697	90,15617239	208,4172977	1135,048436	126,1164928
ENSG00000111275	ALDH2	178,6502158	180,0299266	123,6801304	84,26780009	229,2546113	97,46798459	63,96754456	81,18977495	96,10576098	1134,613745	126,0681939
ENSG00000163382	NAXE	60,6631158	59,92619971	111,1632781	181,2910869	167,0184535	139,3030864	128,521085	128,0369845	157,4840163	1133,407306	125,9341451
ENSG00000145494	NDUFS6	93,51984518	126,2170821	96,74703652	297,0810324	76,22894765	113,0844661	137,4034166	103,161171	89,91334158	1133,356337	125,9284819
ENSG00000107551	RASSF4	66,54220362	138,6102671	65,79341231	93,60192476	198,2513676	182,5523823	127,5877514	71,27293435	189,1004536	1133,312697	125,923633
ENSG00000187713	TMEM203	199,7128219	96,16834974	129,3219478	90,08667598	105,4205932	130,6532557	136,1997669	106,7006325	137,6948388	1131,958882	125,7732092
ENSG00000104951	IL4I1	248,681364	159,1656522	28,85645116	102,2823267	236,341084	79,66367571	58,83779096	145,0203813	70,68871729	1129,537443	125,5041604
ENSG00000130340	SNX9	198,9171713	49,44175331	194,4721676	137,4733972	1,161691424	153,459233	150,7159261	114,2304533	127,9323052	1127,804098	125,3115665
ENSG00000139572	GPR84	191,4215158	112,2180177	55,65044566	96,42971406	0,175779512	131,4778383	269,4879182	69,54814686	200,6742184	1127,083594	125,2315105
ENSG00000266094	RASSF5	85,82283271	92,90413048	137,3961269	160,0171762	188,2587231	108,2842396	118,7435682	65,22482791	169,6755304	1126,327156	125,1474617
ENSG00000179262	RAD23A	106,6139362	106,7514676	99,20844522	75,29925651	181,7442136	122,0064822	133,5493012	122,9886023	177,5283034	1125,690008	125,0766676
ENSG00000143878	RHOB	45,23124164	87,94876731	180,1245414	112,3164456	217,8626237	103,379356	117,6945568	89,57395738	170,3331954	1124,464685	124,9405206
ENSG00000169764	UGP2	132,8406263	141,9696607	194,5988972	85,69391952	148,5413123	88,71022375	92,48636071	135,3556766	104,0580435	1124,254721	124,9171912
ENSG00000175550	DRAP1	112,6747809	128,9323903	99,11717815	109,2532617	29,82218848	153,9735491	147,4750011	163,1064938	170,8449737	1120,199817	124,4666464
ENSG00000173846	PLK3	149,1575335	80,27334899	219,0753891	66,97880021	88,51108648	117,2394848	122,3778862	97,23337589	177,0206279	1117,867533	124,2075037
ENSG00000126254	RBM42	87,47425147	90,79876431	132,6895033	52,99132757	279,0242374	131,4649538	121,5458293	99,01053904	121,9881071	1116,987513	124,1097237
ENSG00000214194	SMIM30	156,1940516	143,0187662	104,8270042	166,0752714	148,6742728	128,2813737	107,8377677	83,5499232	77,94864987	1116,407081	124,0452312
ENSG00000131174	COX7B	98,79346526	140,5818811	109,6492363	122,4937558	53,03123272	139,4372339	130,029875	225,7478314	96,51494318	1116,279455	124,0310505
ENSG00000065548	ZC3H15	75,00597394	81,70090023	147,8089819	115,2908331	116,1850872	155,4383625	102,0016579	184,3270159	136,4061884	1114,165001	123,7961112
ENSG00000086589	RBM22	28,3095354	96,73146031	257,5985082	108,9714502	204,4376977	128,2141396	100,530174	93,40971605	95,74696771	1113,949649	123,7721832
ENSG00000134108	ARL8B	154,472366	120,2146055	82,66600522	129,8428894	266,8857207	116,1046981	67,80556663	86,26100932	88,02980863	1112,282669	123,5869633
ENSG00000184602	SNN	186,29563	51,75534928	185,4052315	134,0649364	0,330071271	156,1003006	169,1773686	130,6957802	98,44627359	1112,270941	123,5856602
ENSG00000181481	RNF135	106,3021824	92,64389674	176,1200716	118,7335459	0,159892049	129,5668529	145,3020185	173,3089584	169,7111385	1111,848557	123,5387286
ENSG00000171115	GIMAP8	197,4005949	44,85051375	72,66672122	88,91117653	276,1386502	70,76472626	107,8715065	154,9134101	98,18558202	1111,702882	123,5225424
ENSG00000171490	RSL1D1	127,8627906	138,7271497	161,7716058	104,5094965	25,52636864	103,3912065	109,9952505	183,7713831	155,9277053	1111,482957	123,4981063
ENSG00000102580	DNAJC3	80,6914228	108,9836537	19,4934166	72,81906268	402,224496	83,43016657	75,14752621	156,646083	111,6076521	1111,04348	123,4492755
ENSG00000134243	SORT1	137,9482058	61,78400638	134,1017175	210,4461869	96,20273815	151,0409753	152,4285133	37,65223515	129,2801107	1110,884689	123,4316321
ENSG00000060762	MPC1	85,81829304	183,1552083	76,95697436	110,2138023	9,059239425	139,4035182	115,8936831	270,5887209	119,7303598	1110,819799	123,4244222
ENSG00000067955	CBFB	153,8716194	85,53798971	82,76062706	174,7793166	0,077196376	144,2979779	133,9393097	146,4651931	187,7241634	1109,453393	123,2725992
ENSG00000124299	PEPD	144,7442008	115,7792788	84,2253607	84,58599373	98,53978405	156,5303455	181,7553205	113,4629065	129,5367705	1109,159961	123,2399957
ENSG00000168310	IRF2	127,2872755	33,10287232	162,8039798	78,70546791	193,595939	83,54975622	91,58925538	218,1729213	119,9829991	1108,790466	123,1989407
ENSG00000139278	GLIPR1	96,28736363	109,2862161	155,283891	139,2073383	194,767775	78,9477865	86,79879972	128,2894915	119,9173937	1108,786056	123,1984506
ENSG00000064601	CTSA	170,9283504	92,49433746	108,5622415	41,79593685	178,8841993	114,8568098	163,0942908	143,3801633	94,19270459	1108,189034	123,1321149
ENSG00000132386	SERPINF1	65,70024811	106,9877612	111,1920396	102,454067	313,597292	174,9552001	121,4838164	54,48302401	57,12138929	1107,974838	123,1083153
ENSG00000133561	GIMAP6	210,1182251	83,05343831	125,314821	89,69014588	201,8143306	114,9035683	103,1851593	81,57418661	97,03154741	1106,685423	122,965047
ENSG00000110711	AIP	113,4014071	164,7736641	120,9339403	66,21914763	0,216989452	167,3140499	193,2470551	113,8052589	166,6442532	1106,555766	122,9506406
ENSG00000140678	ITGAX	83,11722229	53,02949648	124,8827756	140,9801347	218,9530619	126,2974916	135,2255882	28,91419244	195,1467299	1106,546693	122,9496326
ENSG00000157191	NECAP2	81,01281774	76,40343741	97,52006013	107,3101003	188,7485304	129,8077126	149,061487	177,3312189	97,71277224	1104,908137	122,7675707
ENSG00000132109	TRIM21	72,86106585	198,0232135	0	96,82238334	156,2671683	193,0050171	133,8350208	61,30543139	191,993384	1104,112684	122,6791871
ENSG00000196072	BLOC1S2	98,2140844	119,0104652	222,6896568	131,3190442	145,4481561	122,9933825	85,77326028	76,16681374	100,999381	1102,614687	122,512743
ENSG00000105568	PPP2R1A	154,3313275	99,00238152	86,96018475	98,77020622	184,2820897	117,7018377	104,4316671	112,1953064	144,3550891	1102,03009	122,4477878
ENSG00000132274	TRIM22	140,9670448	107,4101787	84,55338899	109,0448312	82,10645021	116,3662844	85,68036299	226,5900567	147,0763864	1099,795434	122,1994927
ENSG00000103507	BCKDK	216,7361787	150,5832301	73,35286888	96,61263971	114,327893	106,6231411	136,0638767	88,98612288	115,8841599	1099,170111	122,1300123
ENSG00000140157	NIPA2	82,9922535	114,8305684	132,3676991	154,9231618	151,0240392	139,4974236	112,8696025	57,03649454	153,025125	1098,566368	122,0629297
ENSG00000105329	TGFB1	248,5064203	73,86740707	96,32293732	81,79400841	103,032797	101,9648078	134,1889314	108,478056	149,3430052	1097,49837	121,9442634
ENSG00000138801	PAPSS1	135,1651608	113,5079632	193,7333696	37,45937672	68,51874581	134,204353	135,7465831	176,4682767	102,3866117	1097,190441	121,910049
ENSG00000074319	TSG101	241,4031713	114,0997718	103,5979796	76,41771881	136,0568991	117,3218131	96,37729549	110,6092102	101,1239087	1097,007768	121,889752
ENSG00000005486	RHBDD2	156,9906054	171,5435641	63,03983835	25,11089714	169,3412009	168,6873228	148,0969902	71,20242773	122,8608808	1096,873727	121,8748586
ENSG00000024862	CCDC28A	158,9318212	166,8992675	93,70530211	45,36493918	20,01716892	137,3964165	85,33980776	182,4297015	205,7013929	1095,785818	121,7539797
ENSG00000198771	RCSD1	115,521394	142,2318435	130,0871964	82,86642855	0,175930211	109,1982167	172,7387633	133,1046286	209,4728162	1095,397217	121,7108019
ENSG00000164961	WASHC5	26,9035826	84,16382325	179,4643721	103,3221419	125,68737	100,946641	73,9703322	281,4556037	119,3975138	1095,311381	121,7012645
ENSG00000116649	SRM	146,4033248	93,29755969	104,2058449	123,8545629	109,972637	89,26234301	145,3441966	115,1461534	166,516129	1094,002751	121,5558613
ENSG00000140264	SERF2	83,93660526	123,9796344	95,08152834	156,4118182	84,80589846	137,1410007	144,5461944	136,9502189	129,8674351	1092,720334	121,4133704
ENSG00000162493	PDPN	44,5953505	128,5374519	84,88628866	69,54511867	153,3539438	187,975296	226,4355331	2,050174009	195,3218373	1092,700994	121,4112215
ENSG00000114784	EIF1B	147,9907653	153,0212155	20,74917626	46,68926491	140,8714129	153,7970581	130,6512139	150,9608427	146,5914866	1091,315436	121,2572707
ENSG00000070961	ATP2B1	134,5716596	60,70127022	152,8260968	192,3546774	143,7907699	75,53623855	76,91055113	165,8264279	84,57357803	1087,091269	120,7879188
ENSG00000129055	ANAPC13	251,0395619	152,4283265	141,2878926	52,87279265	0	89,95321221	100,8443194	187,2789389	111,0824911	1086,787535	120,7541706
ENSG00000175756	AURKAIP1	120,5081903	98,16342843	103,9302467	57,53389494	125,8740337	154,4787515	161,02903	157,9908837	106,1184756	1085,626935	120,625215
ENSG00000147526	TACC1	114,8738305	54,64323338	167,0930705	64,29494206	258,0666572	115,3736109	116,2822764	63,9745704	130,3452471	1084,947438	120,5497154
ENSG00000113811	SELENOK	68,82193439	190,7933462	96,35426508	141,8497353	28,46663445	138,751669	127,000458	177,7713175	114,8901109	1084,699471	120,5221634
ENSG00000072135	PTPN18	146,2862102	138,7489833	37,53604267	115,0353595	180,059982	121,3937472	141,6247467	83,9921781	119,2828603	1083,96011	120,4400122
ENSG00000139726	DENR	108,1259132	181,8285656	33,82117483	167,8390591	86,67300866	125,9621707	91,62963837	145,6311168	141,3087142	1082,819361	120,3132624
ENSG00000107581	EIF3A	51,39163895	85,56678949	117,7530119	154,775118	245,4804324	98,87812429	89,1597374	130,2938726	109,4246317	1082,723357	120,3025952
ENSG00000145817	YIPF5	76,90375716	123,5570493	0,902044669	50,45119168	238,3111003	132,0375412	108,3360655	179,3353643	172,3276703	1082,161784	120,2401983
ENSG00000171202	TMEM126A	131,8132372	216,778033	121,18488911	244,005105	0,369842989	134,3004256	80,63180965	160,9634917	92,06197447	1082,108809	120,2343121
ENSG00000169020	ATP5ME	80,09508788	125,1521138	80,53071537	243,0166931	73,21065778	100,0447898	105,1419842	165,2172838	108,6763462	1081,085672	120,1206302
ENSG00000127837	AAMP	209,3892016	90,26308729	22,84040304	54,18140237	213,1831525	128,3957648	134,3811231	134,6994415	93,7518762	1081,085452	120,1206058
ENSG00000055211	GINM1	114,4410722	115,5989956	156,6572291	148,4430078	97,04154033	115,2525473	45,78273003	202,2259553	84,99380952	1080,436887	120,048543
ENSG00000149269	PAK1	216,6741823	120,895901	109,5768512	92,004537	89,05248471	105,225076	125,5898465	30,84545574	189,2099932	1079,074328	119,8971475
ENSG00000004059	ARF5	191,2687223	145,6047792	97,17879625	121,1024238	52,34261165	129,4557048	124,2328164	50,51575032	166,4981172	1078,199722	119,7999691
ENSG00000169692	AGPAT2	154,8223478	78,15929479	104,2425371	78,19503333	158,1920615	120,1764931	167,6590463	111,6479243	104,6908357	1077,785574	119,7539526
ENSG00000143575	HAX1	86,03836204	103,3995369	66,94741131	149,1748904	157,1451705	161,445234	107,8870187	97,52036424	147,814821	1077,372809	119,7080899
ENSG00000113845	TIMMDC1	85,51861966	111,7749436	153,3472634	148,6277969	69,71879027	125,1925963	117,3814022	151,5399555	113,2098046	1076,311172	119,5901303
ENSG00000150316	CWC15	67,48573767	125,7344711	189,2176168	239,8425718	0,394057289	95,63828416	92,19820665	153,0103008	112,5003637	1076,02161	119,5579567
ENSG00000132823	OSER1	108,1088965	113,0273971	105,5558046	112,5419855	104,589591	129,6891035	99,24931998	189,8272393	113,1275629	1075,7169	119,5241001
ENSG00000083937	CHMP2B	66,57793929	101,0053199	175,8576734	132,0595761	131,2382027	101,283239	85,58092009	155,2427014	126,2849201	1075,130492	119,4589436
ENSG00000169252	ADRB2	209,9221201	79,048542	66,77012543	133,4610776	180,5496561	110,5428007	91,46980346	74,5709146	126,6865489	1073,021589	119,224621
ENSG00000163875	MEAF6	145,7803154	146,4899656	66,32619511	175,7775324	61,07160958	126,5621692	90,02775184	135,6042059	123,7555563	1071,395258	119,0439176
ENSG00000133606	MKRN1	179,2223609	171,6329078	100,0499997	84,00114448	100,9806815	110,853139	72,55934483	144,8302118	106,6160436	1070,745834	118,9717593
ENSG00000211460	TSN	146,2970371	134,9556551	88,04284364	158,5795943	41,50892185	119,7047642	110,5680537	136,7600633	132,5240047	1068,940938	118,7712153
ENSG00000120705	ETF1	128,4289972	114,538064	174,7408513	91,73270542	121,7374433	108,7908234	95,40944355	125,2472982	97,73634607	1068,361972	118,7068858
ENSG00000135698	MPHOSPH6	197,5901932	114,7079123	1,39752335	110,7900326	138,0337537	119,8074548	119,6211666	192,8541786	73,21741417	1068,019629	118,6688477
ENSG00000120697	ALG5	116,9735345	120,9102878	230,2587283	30,99406915	81,93224073	166,0593087	101,1748665	127,3750462	92,21519505	1067,893277	118,6548086
ENSG00000159069	FBXW5	164,3682983	66,83199213	171,9020831	36,43900677	11,88336737	144,7562227	181,4634674	103,3234986	186,6363982	1067,604335	118,6227038
ENSG00000068305	MEF2A	44,84219823	101,5261118	195,890669	152,1524083	36,97484588	100,8400891	134,0041264	113,9939613	186,275915	1066,500325	118,5000361
ENSG00000115415	STAT1	137,2053026	97,36925759	208,6802527	56,98926754	198,9296414	74,58164203	123,8949569	87,28975276	80,4424068	1065,38248	118,3758312
ENSG00000170759	KIF5B	125,1698368	56,49906446	73,07284566	109,6051445	110,4411944	138,3625198	138,8814977	128,4898139	184,7693348	1065,291252	118,3656947
ENSG00000083444	PLOD1	104,8968679	43,09425001	129,0621888	57,28797939	250,5722401	92,81012712	144,951252	121,8632747	120,4509039	1064,989084	118,3321204
ENSG00000134001	EIF2S1	168,124712	74,29347897	54,34197424	161,3676817	271,1607458	105,4405376	82,99437938	47,26704998	99,6654376	1064,655997	118,2951108
ENSG00000113068	PFDN1	145,5034994	144,6424611	62,27476243	48,77402355	117,5960041	150,750844	143,2200775	120,3112873	131,5407203	1064,61368	118,2904089
ENSG00000054983	GALC	221,5735016	131,2844888	125,2478623	103,3125857	100,7618995	80,14741933	106,2474427	94,92145014	99,93113178	1063,427782	118,1586424
ENSG00000115806	GORASP2	135,7265189	81,49693095	144,8686935	178,0791081	138,9910828	115,1676819	104,829729	54,56910924	108,5849287	1062,313783	118,0348648
ENSG00000185787	MORF4L1	163,9861828	152,5200168	60,07984217	138,7108765	90,65208922	113,7737324	116,2277574	118,2919617	107,9400214	1062,18248	118,0202756
ENSG00000114902	SPCS1	114,3435777	147,2922176	58,7878241	143,690027	139,972391	100,5636458	98,12706106	151,5701619	107,6755773	1062,022484	118,0024982
ENSG00000091527	CDV3	97,78760538	109,970763	128,2005769	151,5299051	68,25453633	124,3854427	140,9278632	98,74447232	141,8000028	1061,601168	117,9556853
ENSG00000107968	MAP3K8	5,800377321	168,7228451	60,86001598	50,15412079	305,306071	84,67713539	87,72577378	218,5359124	79,36920002	1061,151452	117,9057169
ENSG00000135956	TMEM127	123,9120919	51,42160862	204,8940796	81,96481062	177,7820785	100,817971	138,8509438	71,77493433	109,3420877	1060,760606	117,8622896
ENSG00000115165	CYTIP	177,5459896	291,5172631	39,42980127	128,154234	0,530611026	80,70000273	135,5924953	111,4142558	94,02164484	1058,906298	117,6562553
ENSG00000079785	DDX1	118,6255432	124,3121143	77,88954508	136,968737	0,374763937	148,5100936	156,0441097	196,6400335	99,0082438	1058,373184	117,5970205
ENSG00000160410	SHKBP1	141,2388328	85,13201782	139,5553369	31,10790622	138,5715377	116,862788	149,837219	116,4164491	138,841619	1057,563707	117,5070785
ENSG00000130520	LSM4	126,7153679	89,94132456	74,97845522	144,7245815	94,01789735	160,8165907	132,4293996	75,27231812	158,4818643	1057,377799	117,4864221
ENSG00000196683	TOMM7	53,50574175	131,0848384	74,29736512	159,3029628	142,3344228	98,63672778	85,84435051	220,2268077	92,12723092	1057,360448	117,4844942
ENSG00000133313	CNDP2	129,1380558	102,7476959	110,655925	103,8045327	117,7414646	137,0846315	119,5268888	71,16278201	164,6868692	1056,548845	117,3943162
ENSG00000159461	AMFR	73,26175976	143,257131	153,8172621	87,12919794	80,98053861	181,048298	133,6971812	74,16866095	127,8843285	1055,244358	117,2493731
ENSG00000100138	SNU13	68,76640934	161,8745863	110,6412078	181,5044784	59,01453293	126,119246	103,9942007	115,4884054	125,4638633	1052,86693	116,9852145
ENSG00000122958	VPS26A	163,2502197	133,8089379	148,8247637	94,76489109	71,10751963	133,9997074	61,70511245	121,6846609	123,6555047	1052,801317	116,9779242
ENSG00000166523	CLEC4E	64,69928232	145,1420983	161,9446407	36,41454229	212,9202274	109,5338613	77,34401556	217,4701489	26,77970119	1052,248518	116,916502
ENSG00000100055	CYTH4	197,3183448	115,4078948	62,48741035	68,11091647	75,13264208	113,9530096	153,962939	114,424956	150,986968	1051,785081	116,865009
ENSG00000174021	GNG5	115,6103036	104,8321117	86,7455628	182,0530364	76,08962112	133,2579632	134,146472	91,39679527	127,1928326	1051,324699	116,8138554
ENSG00000011260	UTP18	103,3679042	165,6667185	128,5516551	152,3327377	0,531435505	128,9597194	113,7263461	116,623965	141,1553497	1050,915831	116,7684257
ENSG00000120280	CXorf21	44,55928957	248,4830718	9,141124549	149,1056998	0	142,7457037	212,9951889	73,03405535	170,2173808	1050,281514	116,6979461
ENSG00000067064	IDI1	158,7617388	155,8864654	40,94528501	122,1499973	0,082650758	118,1802612	125,8168554	189,9713578	137,5563672	1049,350979	116,5945532
ENSG00000170581	STAT2	140,5294174	56,371253	125,0417096	138,0454633	183,6146324	81,42948924	105,064702	117,5020631	101,4389779	1049,037708	116,5597453
ENSG00000124795	DEK	127,075464	146,1852924	163,9350149	120,2986184	49,88706927	109,4016494	84,65655867	112,4088348	134,8278191	1048,676321	116,5195912

ENSG00000125691	RPL23	55,17263173	116,4294815	96,2691452	317,5850402	77,84427675	93,75844106	85,14134129	140,9593015	62,12718628	1045,286846	116,1429828
ENSG00000186517	ARHGAP30	145,7393418	64,017314	100,0157465	91,92495946	29,64279872	106,4789444	194,3253977	173,8305071	138,7976989	1044,772709	116,0858565
ENSG00000136868	SLC31A1	143,4742736	102,0450994	78,88259369	72,82849826	149,6497591	164,2659821	104,0099059	81,82189291	146,2931808	1043,271186	115,9190206
ENSG00000164332	UBLCP1	92,58053233	113,5493732	80,35803105	203,938216	70,27797621	124,0119252	75,34332969	110,9498122	172,1891043	1043,1983	115,9109222
ENSG00000131467	PSME3	188,5362477	115,8548316	170,8239506	120,7432025	0,139408984	134,5769216	101,7011076	58,42893847	151,0724447	1041,877054	115,7641171
ENSG00000100365	NCF4	83,14793348	96,1670505	68,40868001	99,99205369	191,1871865	102,7030189	164,1369372	120,5739355	115,3759115	1041,692707	115,7436341
ENSG00000042753	AP2S1	106,1498551	111,0963921	97,06464724	79,78117475	79,24189902	172,5314213	133,422677	152,4187618	108,7376659	1040,444494	115,6049438
ENSG00000013364	MVP	132,3601628	61,08624949	115,6552928	71,08682829	153,9158722	161,2636757	133,3364903	63,0629072	148,5622175	1040,329696	115,5921885
ENSG00000122033	MTIF3	85,31195661	87,81943234	67,45742889	135,7578096	121,1993702	114,8850531	81,55962941	253,1339045	92,74935609	1039,873941	115,541549
ENSG00000134809	TIMM10	119,5642917	91,56911085	150,0949246	90,11671803	77,7470968	103,4803081	123,5300691	177,1401805	105,616387	1038,859087	115,4287874
ENSG00000118292	C1orf54	33,07774696	105,1454457	141,1788692	187,5855369	83,69983664	149,319519	171,3114612	65,04617471	99,80104582	1036,165636	115,1295151
ENSG00000100216	TOMM22	110,7359315	89,09392762	141,4105911	185,9550346	136,2737512	102,4205072	84,81610435	77,53551722	107,2322509	1035,473616	115,052624
ENSG00000108604	SMARCD2	43,58491821	64,05534657	51,29306058	175,0514492	184,6560475	116,3240198	125,8735575	115,8379349	158,6215497	1035,297884	115,0330982
ENSG00000187688	TRPV2	213,190601	36,87844722	141,5799262	99,01791553	59,48116644	153,8389997	109,1417469	76,13384445	145,9947835	1035,257431	115,0286034
ENSG00000000419	DPM1	192,5501721	133,7377664	0	138,7905996	130,0201872	154,2267333	81,81039874	105,673358	97,61946572	1034,428681	114,9365201
ENSG00000156411	ATP5MPL	80,17931304	164,1829933	38,42067547	154,4979557	146,1708298	114,5584597	83,91632161	163,0401892	87,94377159	1032,910509	114,7678344
ENSG00000185651	UBE2L3	120,7152188	97,88091249	140,438617	56,42939784	145,4996817	114,9443	135,6047257	90,48638105	130,450894	1032,450128	114,7166809
ENSG00000147140	NONO	90,06098637	117,1981191	215,9966302	98,07249896	59,98864464	114,3378445	115,014471	81,85935715	139,6658388	1032,194391	114,6882656
ENSG00000138463	SLC49A4	122,5897014	48,914525	27,1118459	162,8290796	76,86109952	96,73955059	123,8462428	257,0267677	115,9722579	1031,89107	114,6545634
ENSG00000157107	FCHO2	120,2757451	90,29406728	132,506749	148,9828172	218,7734361	61,44734891	106,4853699	58,6035264	93,73501865	1031,104079	114,5671198
ENSG00000108055	SMC3	42,98256232	116,9789076	210,6146294	209,7856158	131,1304055	59,8054266	85,31982576	86,10622414	88,24992754	1030,973525	114,5526139
ENSG00000128228	SDF2L1	65,49551646	146,8577526	103,6847395	159,419196	0	124,5504329	201,0605792	131,986612	97,22282657	1030,277655	114,475295
ENSG00000117280	RAB29	69,47834896	189,9110221	149,8842491	74,72471865	35,78297233	126,0053432	125,7917212	96,40496219	161,2827914	1029,266129	114,3629032
ENSG00000204525	HLA-C	100,0572586	113,3747587	66,08541643	51,72903166	145,2513713	149,2996654	95,24134096	149,8620276	155,9717123	1026,872583	114,0969537
ENSG00000075975	MKRN2	162,8182365	131,6069925	67,61678083	85,28577368	165,3881871	84,00157127	104,5812173	117,289387	108,0923557	1026,680502	114,0756113
ENSG00000106105	GARS	84,23610535	124,2380081	162,3348112	215,7367262	88,72315752	110,5101488	91,36250208	99,99884659	48,52013085	1025,660437	113,9622707
ENSG00000184584	TMEM173	121,8836825	130,1589659	81,16926192	95,77042315	61,97359839	135,6884333	139,5393271	103,3727148	153,2710898	1022,827497	113,6474996
ENSG00000116133	DHCR24	95,66607419	102,5274207	55,33655977	75,33269141	88,01673914	154,0293075	192,6476404	99,49196586	159,5576434	1022,606042	113,6228936
ENSG00000139508	SLC46A3	97,08534016	83,86538721	0,809793721	64,54270823	246,1324681	105,1001323	172,2558795	187,0945724	65,3612769	1022,247559	113,5830621
ENSG00000137312	FLOT1	117,2352339	102,7105934	94,95835092	67,17949111	77,91215754	160,388906	159,2311089	90,57123311	150,894539	1021,081614	113,4535127
ENSG00000168439	STIP1	113,0814556	77,96097662	96,69911109	103,8809197	47,34768542	134,6665192	155,6726601	108,9149798	181,5764819	1019,80079	113,3111988
ENSG00000004700	RECQL	18,67389164	62,86684628	352,4219778	176,1431914	94,74469165	94,96284134	59,89639559	41,70872948	118,0781953	1019,49676	113,2774178
ENSG00000162517	PEF1	72,25845102	93,72304726	121,0457651	112,0975712	294,4990012	98,14323363	114,2948368	34,63394252	78,58557397	1019,281423	113,2534914
ENSG00000120063	GNA13	85,95280166	92,73018321	184,0521321	142,6601416	8,87881966	160,131698	162,3251691	73,62972092	108,7452128	1019,105879	113,2339865
ENSG00000077097	TOP2B	32,37726489	58,60615191	362,1927994	84,01380883	26,20298415	69,3947614	128,1690652	126,0178157	132,1281446	1019,102796	113,233644
ENSG00000109107	ALDOC	122,4157609	230,1586387	64,38666152	96,1023911	68,74378682	99,21604931	89,20306529	91,08456685	157,1015012	1018,412422	113,1569357
ENSG00000074695	LMAN1	94,89843416	96,3455852	178,1911969	227,4905907	47,33015385	85,74324203	91,67395757	103,9752923	92,7089406	1018,357393	113,1508215
ENSG00000102699	PARP4	27,43145723	68,1559014	156,8151841	254,0262213	13,84649799	124,2613388	146,0671497	74,52959052	150,5716652	1015,705006	112,8561118
ENSG00000162191	UBXN1	84,74605464	77,20202135	118,6794972	132,6316399	159,2768563	101,4334805	128,2949011	81,69511819	131,2375916	1015,197161	112,7996845
ENSG00000136827	TOR1A	130,1334904	124,4130591	87,53800516	87,97907598	160,2465472	109,3482665	110,155294	79,50926812	122,627095	1011,950101	112,4389002
ENSG00000109787	KLF3	111,742819	68,31264956	171,1973139	56,53398431	237,9796799	80,34570684	58,14885568	120,8323718	106,6606979	1011,754079	112,4171199
ENSG00000129128	SPCS3	48,30493129	116,7354175	224,1669944	160,3284747	90,76539436	84,47372605	91,03902402	115,2093579	80,33664281	1011,359963	112,3733292
ENSG00000081087	OSTM1	45,48924784	127,4996327	57,57972754	93,25056463	262,071506	92,84760912	90,76276411	164,3175089	77,13066934	1010,94923	112,3276922
ENSG00000196576	PLXNB2	147,2268878	55,72164708	185,5620314	41,77972037	113,322413	94,21969529	153,354544	59,58358337	159,8443563	1010,614879	112,2905421
ENSG00000183684	ALYREF	72,57915592	41,39676367	85,65460483	184,0138639	152,4082073	134,7101125	121,8831878	132,4677332	83,47744044	1008,59107	112,0656744
ENSG00000138768	USO1	93,2748771	109,9335445	98,88045369	40,38008543	284,4448886	90,47695356	98,25956907	82,10449149	110,4628305	1008,217694	112,0241882
ENSG00000136628	EPRS	144,6948297	76,77031902	168,4696223	208,5322904	1,214374556	137,2354442	103,2425426	39,23250924	127,9593282	1007,35126	111,9279178
ENSG00000110422	HIPK3	98,87710295	34,28959433	121,7610037	119,472677	376,5367666	66,413004	70,57321957	42,09357548	77,21599749	1007,232941	111,9147712
ENSG00000100129	EIF3L	115,6192844	118,4668334	128,7663866	85,70280474	125,1394229	117,9061896	94,24878058	118,0672861	103,2624881	1007,179476	111,9088307
ENSG00000099622	CIRBP	66,22952304	111,4170434	125,4140742	116,4127297	68,57520909	133,8122993	106,5355033	165,0616142	113,3231856	1006,781182	111,8645758
ENSG00000159348	CYB5R1	105,6597383	134,3394403	52,99865291	94,3313312	95,71141618	120,7696122	167,3402982	104,0188906	130,6093802	1005,77876	111,7531956
ENSG00000128789	PSMG2	82,86708714	117,9863676	141,6600287	189,2464241	123,1334524	82,68468393	100,5490772	113,8975258	53,48392123	1005,508568	111,7231742
ENSG00000131507	NDFIP1	105,2278587	108,8991523	82,1799527	77,05005253	122,0387263	113,5709429	123,8765728	172,6631297	99,50404542	1005,010433	111,6678259
ENSG00000129518	EAPP	169,4292692	206,3141069	41,53883716	109,4868176	0	107,8706075	107,2178443	172,8200583	88,83114325	1003,508684	111,5009649
ENSG00000100075	SLC25A1	140,6031323	156,5391266	18,63398362	68,79076119	0	161,0967631	216,2935288	77,85626878	162,0462673	1001,859832	111,3177591
ENSG00000121774	KHDRBS1	127,2756923	169,4046101	114,2040136	53,82151987	0,709317945	135,3407103	126,6740541	185,2633989	88,78051981	1001,473837	111,2748708
ENSG00000055130	CUL1	53,68566129	86,10673977	87,50516473	113,9749022	30,56109077	154,9899962	150,7648192	159,3475427	163,5306083	1000,466525	111,1629472
ENSG00000166974	MAPRE2	144,5331041	92,37448514	46,3749971	219,8947165	35,44687716	105,9357825	110,4463764	101,8572575	143,1138511	999,9774473	111,1086053
ENSG00000121895	TMEM156	111,0031592	206,2872296	104,4866648	46,95199972	0,841024689	128,286047	87,46682785	106,7914996	207,4633309	999,5777834	111,0641982
ENSG00000145416	MARCH1	82,18930184	107,0406602	146,6324349	93,22146727	154,4539703	81,22788239	94,76796691	135,9978755	103,1509725	998,6825319	110,9647258
ENSG00000038002	AGA	94,84291095	58,5692123	43,43105577	2,604341397	554,3230855	67,6380163	53,8005224	75,72723102	47,15721736	998,093593	110,8992881
ENSG00000174238	PITPNA	72,99676605	86,93316098	98,78055357	77,72415109	297,6381805	103,6941104	124,2376594	51,25051194	84,4933499	997,7484439	110,8609382
ENSG00000134516	DOCK2	137,7725748	46,64574687	136,9699065	131,9489501	110,9431257	95,01237795	126,2016882	76,1175162	135,8325642	997,4444504	110,8271612
ENSG00000173020	GRK2	95,2852629	36,79743291	138,1900462	75,05536455	139,9046227	109,9452852	153,0084282	71,87277173	177,0918618	997,1510762	110,794564
ENSG00000167863	ATP5PD	115,0984569	164,9019357	111,587802	117,1824971	20,11769581	132,0198368	94,32604037	135,6101011	104,5135039	995,3578697	110,5953189
ENSG00000141580	WDR45B	99,19030965	110,6028973	173,7571821	121,1115467	3,649980668	119,6317371	75,91273898	105,0449811	186,3187466	995,2201202	110,5800134
ENSG00000132792	CTNNBL1	161,6387662	104,5934803	79,61435815	107,0034696	77,48558351	103,3541297	95,88740416	134,3884078	129,6176444	993,5832437	110,3981382
ENSG00000133246	PRAM1	146,0597767	83,67725132	78,38515097	103,7079753	61,87166409	109,397129	148,4980076	38,35490878	223,5692439	993,5211077	110,3912342
ENSG00000071051	NCK2	39,11097123	128,5278965	180,5235466	70,43051583	75,32415006	164,683675	173,7184193	51,86989682	108,8217283	993,0107997	110,3345333
ENSG00000086300	SNX10	131,8673826	184,1475475	190,9038997	82,25607926	0,306240087	160,2513188	104,5348332	26,7691449	110,6732417	991,7096877	110,1899653
ENSG00000115902	SLC1A4	145,9726806	68,84152776	44,85487838	196,5000882	92,61207331	121,8095806	134,4182955	30,32514328	155,8047707	991,1390383	110,1265598
ENSG00000119917	IFIT3	111,8587512	115,2048798	264,8316513	202,4123098	0	55,65230618	81,8005239	151,6065576	6,959172099	990,3261518	110,0362391
ENSG00000132471	WBP2	108,3719447	114,1801182	93,80991672	110,2896114	132,6985547	107,7696597	115,1519717	100,9848187	106,5028968	989,7594927	109,973277
ENSG00000116863	ADPRHL2	295,6312194	140,6486808	82,333294	72,22730672	0,217515168	94,36101243	105,635228	94,06677573	102,0973929	987,2184253	109,6909361
ENSG00000266472	MRPS21	120,9989579	94,11931265	134,1622416	113,0559214	90,81887878	87,75769688	102,9147269	161,4920921	81,54143573	986,8612638	109,6512515
ENSG00000196562	SULF2	138,7847422	118,6640127	27,68526448	65,89029477	155,7416771	58,14471616	93,08967549	181,1259932	147,5996039	986,7259801	109,63622
ENSG00000102158	MAGT1	145,8891441	155,7451683	15,72285628	111,4152627	44,3172615	117,5569594	132,5154576	119,1385843	142,8441904	985,1448846	109,4605427
ENSG00000117394	SLC2A1	85,407541	106,7118883	0	141,9565802	295,0328497	92,85934903	79,65400628	79,46097983	102,758492	983,8416862	109,3157429
ENSG00000007520	TSR3	164,0985867	105,4839604	53,98787046	36,66028569	65,88768132	135,4707849	152,8456772	139,5471603	126,7135379	980,6955448	108,9661716
ENSG00000101843	PSMD10	95,78766299	195,1041582	45,12814996	182,5453044	0	89,27238562	104,5236113	134,109427	134,2138483	980,6845478	108,9649498
ENSG00000215021	PHB2	49,34222716	107,2997199	27,69789702	130,3108637	152,7578822	151,7029483	122,1565897	110,0126291	129,3265715	980,6073286	108,9563698
ENSG00000170860	LSM3	94,56219694	123,0946604	107,8321134	188,3796706	89,69920156	87,86987674	64,54680967	148,6414503	75,90992217	980,5359017	108,9484335
ENSG00000185432	METTL7A	67,10258413	127,2293003	220,4881403	89,36830828	0,26431615	106,563761	54,26992034	156,299766	158,0789727	979,6650693	108,8516744
ENSG00000149357	LAMTOR1	104,0571675	96,06426566	33,71336022	132,7001898	176,7531654	136,6540241	132,9854821	90,84492016	75,569289	979,341864	108,8157627
ENSG00000134352	IL6ST	73,44179855	46,43518673	206,4586798	100,613555	210,3164415	91,03188804	82,75808297	49,77585064	117,9703831	978,8018664	108,7557629
ENSG00000161970	RPL26	86,70968679	109,7963064	69,46737054	276,7177506	109,4644001	71,89678394	76,29075499	114,5278585	63,74838926	978,6193011	108,7354779
ENSG00000143799	PARP1	119,6809183	81,23026482	95,04651582	150,4653578	106,7903481	93,52247321	112,8412007	69,09546435	148,4081375	977,0806806	108,5645201
ENSG00000141480	ARRB2	176,5850139	134,2910462	59,24593434	83,02198371	74,34954042	117,518371	126,7353318	80,5749004	124,7546526	977,0767744	108,564086
ENSG00000119705	SLIRP	60,13408114	119,1402501	84,20757844	163,5430533	159,4258037	97,17529407	74,72900427	149,3297989	67,52918103	975,2140449	108,3571161
ENSG00000118217	ATF6	113,3137125	112,4532447	56,87563317	90,08627366	88,63773521	129,1587679	90,68837323	169,0409798	124,6854338	974,940154	108,3266838
ENSG00000185721	DRG1	82,28530855	105,8919702	8,782170744	126,9753782	167,8192991	99,51722613	151,8416743	128,4620565	103,1574597	974,7325434	108,3036159
ENSG00000108861	DUSP3	87,51530748	58,43638945	75,37783352	133,0000761	106,3626101	118,2656542	161,6023728	116,3827974	117,2279844	974,1710256	108,2412251
ENSG00000178562	CD28	98,24507112	36,28287862	114,8339488	252,0679929	136,0015258	30,56694351	16,79787045	225,5207468	63,11553336	973,4325112	108,1591679
ENSG00000135387	CAPRIN1	84,38599103	56,07543531	152,4229526	113,0383507	98,83135882	93,43529124	111,4126884	151,7928118	111,0379332	972,4328131	108,0480903
ENSG00000168175	MAPK1IP1L	98,3930458	74,43689592	215,1216797	97,46710766	101,5972055	80,24324279	72,00061208	163,6537132	69,49924435	972,412747	108,0458608
ENSG00000113282	CLINT1	137,1100624	28,12796106	81,89237841	127,3533136	288,2301757	82,01735167	86,10322721	52,04551998	89,3627593	972,2427493	108,0269721
ENSG00000048740	CELF2	24,87309175	102,6843659	69,27979148	194,4771326	175,1752996	84,46658892	108,2007317	79,34282383	132,8926216	971,3924474	107,9324942
ENSG00000135766	EGLN1	72,46651455	106,4448077	27,09014592	205,3358912	97,87374176	124,1189242	111,5502924	107,931346	118,2868987	971,0985625	107,8998403
ENSG00000134061	CD180	179,7703913	123,0482255	177,6855855	30,89050246	51,51068489	69,5489644	101,3635586	148,9520677	88,13610654	970,906087	107,8784541
ENSG00000181804	SLC9A9	213,7461079	125,8761134	21,60003739	45,2003173	88,42414121	130,6017185	80,41775795	161,0878738	102,0983122	969,0523796	107,6724866
ENSG00000138363	ATIC	111,6678801	126,8879932	122,5929653	38,63407779	195,0551339	89,67665904	91,03139157	74,82524648	118,1589528	968,5303001	107,6144778
ENSG00000154518	ATP5MC3	68,31178453	108,6805629	86,26403072	172,7250982	105,6695423	99,96234599	90,40155927	138,802013	97,63795167	968,4548886	107,6060987
ENSG00000175895	PLEKHF2	112,8662419	112,2849934	131,9899171	77,94161579	130,2469389	143,8382162	66,29221216	106,0279433	86,88928314	968,3773618	107,5974846
ENSG00000116679	IVNS1ABP	168,1736086	104,4231389	138,7605473	24,72469085	139,7607202	87,01191048	91,65246784	131,9555799	81,50997605	967,97264	107,5525156
ENSG00000139970	RTN1	29,47396743	124,6042821	61,42441131	161,1389434	161,5104149	123,6542361	134,4548333	9,991541998	161,5157265	967,7683571	107,5298175
ENSG00000140391	TSPAN3	100,0889907	83,82195841	71,89703947	66,90561716	167,8218	101,5046604	108,8756784	183,5531779	82,39210387	966,8610264	107,4290029
ENSG00000196352	CD55	154,5635035	124,7214313	143,5069908	125,785851	79,44594243	49,31262232	31,367583	187,1418522	69,5823472	965,4281238	107,2697915
ENSG00000081041	CXCL2	16,10549001	66,55599001	35,59258778	6,906004335	0	5,577823394	18,93722464	809,0683295	6,513933899	965,2573835	107,2508204
ENSG00000129187	DCTD	131,4914833	98,94795675	115,2502603	141,8353988	0,414446096	118,5690994	81,74033747	157,7810759	118,8752856	964,9053436	107,2117048
ENSG00000172053	QARS	122,5785301	88,2023547	63,83553198	123,7263435	164,4014605	92,74482376	96,41713371	106,3039551	106,1020439	964,3121771	107,1457975
ENSG00000055044	NOP58	96,00182864	86,40495116	45,84888621	120,1862986	136,1224405	94,61058238	83,5063694	195,3944174	104,9005416	962,9763159	106,9973684
ENSG00000141424	SLC39A6	122,4311547	75,06228675	119,4038872	128,9442448	124,5161829	95,45601739	96,88776112	90,79548801	108,9949737	962,4919966	106,9435552
ENSG00000131508	UBE2D2	113,3754225	108,583937	70,90212311	133,7695328	73,21715923	111,9756423	102,4298014	135,2031203	112,8353885	962,2921273	106,9213475
ENSG00000085871	MGST2	46,4955602	94,42453156	47,93666902	135,5677296	129,2913724	131,1802416	107,4329829	176,2595486	93,62633751	962,2149734	106,9127748
ENSG00000072042	RDH11	129,1503404	109,441733	46,31316309	81,63116703	120,9686592	89,38288981	155,7310923	90,63210764	136,7651926	960,0163452	106,6684828
ENSG00000049541	RFC2	70,1902108	104,0551045	50,7932881	166,2855627	98,42841731	120,5871989	102,8845373	105,2129966	141,0449861	959,4823024	106,6091447
ENSG00000176087	SLC35A4	90,34324414	78,20808336	61,95115693	140,8390803	186,9419443	96,05349178	138,8153125	59,14851222	106,8821282	959,1829538	106,5758838

ENSG00000112531	QKI	50,06560199	96,15988451	116,3213199	164,1738423	80,8035152	111,4001045	106,3650822	94,63551626	138,5714653	958,4963321	106,4995925
ENSG00000080815	PSEN1	72,88916438	87,28700073	120,4757421	117,3643386	141,307536	72,6304308	84,57611099	184,6673128	76,73385613	957,9401049	106,4377894
ENSG00000145293	ENOPH1	168,9565622	122,8072338	133,318348	121,5107659	0,34480801	106,1732111	96,07545825	119,1341144	89,57748841	957,8979901	106,43311
ENSG00000136938	ANP32B	167,5953602	90,19321821	24,52765579	155,8632977	158,2421684	82,95528835	84,20602495	79,86974573	114,290642	957,7434013	106,4159335
ENSG00000183431	SF3A3	74,54632929	120,7121022	88,87653396	149,0291175	76,9142348	105,5368512	103,5919665	78,57791719	159,1197497	956,9048023	106,3227558
ENSG00000147955	SIGMAR1	176,9149714	113,1910902	27,59547431	126,5463333	82,42041956	106,7539366	123,7562564	77,04121064	121,6973129	955,9170053	106,2130006
ENSG00000177700	POLR2L	71,3950476	129,5464015	43,69669407	179,1320918	57,08884554	116,2552798	130,2618278	114,7423765	113,719586	955,8381505	106,2042389
ENSG00000138459	SLC35A5	123,6615296	53,98384923	182,2876954	52,15456721	246,05651	56,83846186	65,43792377	96,0915768	78,87314087	955,3852547	106,1539172
ENSG00000142230	SAE1	132,1105077	78,64235338	101,6640998	119,0957628	225,7703042	99,95672554	83,27742295	26,27129954	88,21690044	955,0053764	106,1117085
ENSG00000100632	ERH	61,23168676	157,5605284	34,46653518	342,2866693	0,322657271	108,3710595	85,33786283	73,92471457	91,33697007	954,8386839	106,0931871
ENSG00000127483	HP1BP3	89,4781993	41,94926183	86,86408659	161,4539562	89,36699717	129,2856264	82,48292833	148,3428895	125,6133681	954,8373134	106,0930348
ENSG00000158435	CNOT11	180,9188365	70,26074614	64,1967364	49,44437713	280,4359787	122,437339	76,20163391	26,65978376	83,91293752	954,468369	106,052041
ENSG00000137767	SQOR	126,6019878	125,4550806	76,84386959	76,74558076	167,0427739	91,89674582	90,31645455	95,2766111	103,8238137	954,0029178	106,0003242
ENSG00000153879	CEBPG	45,63867087	102,4043289	133,986956	46,70581646	232,2399688	123,5739281	86,4000585	24,19602233	158,5377138	953,6834638	105,9648293
ENSG00000174744	BRMS1	124,4828925	90,76724028	157,4557124	56,15187799	75,70894887	85,7270641	108,7509361	155,8852017	98,39368383	953,3235578	105,9248398
ENSG00000166441	RPL27A	93,62381748	93,19665467	99,96027327	175,8707854	100,9491214	110,7044165	94,63658697	101,8064249	82,12583995	952,8739204	105,87488
ENSG00000189159	JPT1	113,488209	65,5003359	117,1988908	114,8389686	162,3725823	91,57718268	141,2370091	72,95560622	72,89810739	952,0668919	105,7852102
ENSG00000271605	MILR1	32,11931733	83,86820267	0	217,9726633	0	162,8043214	161,3288685	178,5001711	115,4377077	952,031252	105,7812502
ENSG00000171155	C1GALT1C1	120,6042884	152,1646527	103,1662867	59,74949087	0	96,62822858	100,417904	232,4738771	85,21391978	950,4186482	105,602072
ENSG00000161929	SCIMP	64,72610935	83,11202968	128,8168545	79,52713394	134,8064203	98,16408327	108,1956452	102,1088697	150,3742866	949,8314325	105,5368258
ENSG00000198682	PAPSS2	112,5094624	56,34725351	237,1987913	51,44566607	2,726584328	32,26377612	35,8925662	418,4047793	2,002754587	948,7916338	105,4212926
ENSG00000138668	HNRNPD	84,18120746	95,91041029	99,54881069	228,0424409	60,80460322	98,98546798	88,87706514	66,21944706	126,080666	948,6501188	105,4055688
ENSG00000105366	SIGLEC8	57,28000818	57,24759825	246,0706836	51,10079446	293,2057134	85,44054612	50,05360138	0,312615485	107,4873926	948,1989535	105,3554393
ENSG00000099250	NRP1	119,0444931	79,1486671	127,50606	114,4109956	36,87172822	121,3154495	138,4943311	129,0249192	82,21313349	948,0297773	105,3366419
ENSG00000123136	DDX39A	166,6938483	52,37148439	124,9462632	21,74458122	168,2428278	110,8230805	98,02369688	65,22640682	139,9172646	947,9894537	105,3321615
ENSG00000108100	CCNY	84,88218205	70,37190998	106,4043011	121,0690436	124,3506225	115,2247325	105,9760057	106,8989367	110,3273559	945,50509	105,0561211
ENSG00000109790	KLHL5	64,71551073	84,96723564	78,73580953	42,60991032	243,0186946	98,0497965	94,24909853	143,8906359	94,91034487	945,1470366	105,0163374
ENSG00000109220	CHIC2	109,0741345	139,3990447	101,2271407	105,3897951	0,291255103	79,2549308	102,1672452	157,3115094	150,4256523	944,5407077	104,9489675
ENSG00000170606	HSPA4	5,331223583	37,68283302	107,8248953	135,5307101	282,4654444	105,1330644	85,21260298	101,2935444	83,52529901	943,9996172	104,8888464
ENSG00000162244	RPL29	101,3277879	83,64779595	58,75672646	170,7868857	90,91362467	112,2193988	112,0575657	107,0941966	106,1980127	943,0019945	104,7779994
ENSG00000064666	CNN2	154,3486543	114,4023423	53,61044766	66,6674523	116,2199079	60,94023106	111,4410943	122,7328394	142,6014661	942,9644354	104,7738262
ENSG00000124214	STAU1	59,94224884	109,4443777	145,7799884	106,6939784	157,5415501	74,64760193	80,69452471	90,96232477	117,118717	942,8253118	104,758368
ENSG00000164609	SLU7	152,2829529	89,45563475	71,11254367	78,7342538	75,90490306	86,97346231	67,69451966	223,9388807	94,54050389	940,6380264	104,5153363
ENSG00000164758	MED30	127,0797924	91,61518336	5,405168591	167,6144802	175,5685763	74,42932816	88,09062893	100,0482441	110,7335529	940,5849548	104,5094394
ENSG00000183978	COA3	117,3043736	135,3901865	94,43549712	136,0753596	75,5058116	96,14393337	69,85013304	164,2761185	50,81665653	939,7980698	104,4220078
ENSG00000103194	USP10	57,07894859	105,716421	73,55562328	138,9634028	0,070650707	118,9412043	134,6035256	140,5468296	169,7225175	939,1991234	104,3554582
ENSG00000170017	ALCAM	57,44264484	106,4873718	56,80412386	170,3219517	17,40812157	119,1579515	144,7479161	90,72106597	175,672726	938,7638733	104,307097
ENSG00000186283	TOR3A	54,27606725	94,39862596	121,5731822	204,8469047	93,3438277	89,90253645	104,5326649	77,4840363	98,34186321	938,6997087	104,2999676
ENSG00000075415	SLC25A3	107,3197601	133,2571698	103,2426087	141,1149014	26,44132085	117,1967248	101,648458	108,9810051	95,97335019	935,1752989	103,9083665
ENSG00000168476	REEP4	177,9183595	97,03285087	69,80171064	119,0674156	54,3589219	101,6989369	131,6164957	46,08001912	137,2935032	934,8682134	103,8742459
ENSG00000075884	ARHGAP15	79,43971733	84,98967915	165,8715916	127,8057547	106,9088293	89,16982685	74,86853824	75,34145435	130,2472608	934,6426523	103,8491836
ENSG00000276085	CCL3L1	5,966244451	305,0138068	57,71540517	69,0971444	98,27450785	89,22352321	179,1945245	4,948645667	124,4880721	933,9218741	103,7690971
ENSG00000133142	TCEAL4	106,52155	106,8095417	44,60046068	141,2447857	74,59033942	107,795752	123,8757924	82,41179841	145,7692143	933,6192346	103,7354705
ENSG00000184014	DENND5A	103,063448	116,3776153	49,60239684	129,0124596	158,282058	70,33081952	93,19903611	65,02743464	148,7117618	933,6070298	103,7341144
ENSG00000033867	SLC4A7	65,41943695	20,46757779	122,7437084	117,1439565	108,6016861	150,8654412	141,4672774	103,0889874	103,7076163	933,505688	103,7228542
ENSG00000140299	BNIP2	130,1951535	62,78742278	58,7075551	200,7192112	21,04503605	91,37677515	110,7489416	144,3811224	113,4808856	933,4421033	103,7157893
ENSG00000099284	H2AFY2	24,87223679	64,12091306	176,6871437	178,5486731	0,929393226	98,24104542	137,1601145	57,97997846	194,1191936	932,6586918	103,6287435
ENSG00000171657	GPR82	69,08822655	61,25727428	94,83829778	135,8938023	284,8696117	49,30313405	64,13432281	126,9753085	45,36940417	931,7293911	103,5254879
ENSG00000137876	RSL24D1	119,614337	135,7440614	48,37697507	134,5597221	43,11832886	113,0877826	103,5500514	159,3664187	73,21036695	930,6280443	103,403116
ENSG00000170275	CRTAP	23,67662265	140,098688	55,05625261	112,2858345	132,7581654	102,9165982	105,3800862	163,6625879	94,60268079	930,4375162	103,3819462
ENSG00000175203	DCTN2	81,77242643	142,7504838	76,20227518	87,12270435	61,8257851	118,0298151	111,307686	80,47339361	170,8624536	930,3470232	103,3718915
ENSG00000166181	API5	49,54711251	135,2528773	177,0322626	109,2325082	44,16582116	108,3359324	99,11373893	78,2215564	128,9058011	929,8076105	103,3119567
ENSG00000023902	PLEKHO1	64,32856093	110,8730159	23,10560183	77,54441092	85,43194513	161,0801737	142,4672181	88,32856444	175,5295031	928,6889941	103,187666
ENSG00000151491	EPS8	115,8632338	57,14501641	86,38866308	68,28918832	155,0429015	52,88156789	61,8683335	221,8632096	109,0923438	928,4344579	103,1593842
ENSG00000132718	SYT11	49,60229269	69,28640257	282,7543616	39,17549406	221,9709549	72,90204229	88,0132491	0,255063348	103,6106914	927,5705519	103,0633947
ENSG00000110719	TCIRG1	106,830856	44,92527681	135,1459818	33,99609955	59,59299103	132,0837803	186,0975964	68,27722264	159,6793319	926,6291365	102,9587929
ENSG00000116044	NFE2L2	39,9159998	166,0579623	91,712355	12,6993569	83,33604051	129,2528527	107,6597841	200,7895468	95,16810098	926,5919991	102,9546666
ENSG00000088298	EDEM2	233,0824407	110,7295521	77,31505938	37,81064122	0,190614407	155,9594646	143,052162	55,77398751	112,5535467	926,4674686	102,9408298
ENSG00000127125	PPCS	103,8553014	136,6644016	68,84325429	167,5581856	50,20488502	111,1095158	78,14315812	98,97541852	110,8433977	926,1975179	102,9108353
ENSG00000108788	MLX	119,249267	65,22543134	105,6050247	96,31128905	204,2222793	76,43027741	76,44881699	99,69674085	81,99239618	925,1815228	102,797947
ENSG00000138674	SEC31A	72,80028461	78,73805101	61,71355537	85,29433019	132,8004852	90,38798225	114,8356518	158,1621841	130,3744238	925,1069483	102,7896609
ENSG00000137947	GTF2B	58,37274421	81,37748497	51,54306114	54,3786833	264,0737554	71,38624655	111,1535905	125,8112597	106,766029	924,8628547	102,7625394
ENSG00000181744	DIPK2A	83,99621661	53,41337818	238,4134036	105,191938	187,9689414	67,06572975	74,3172187	42,09472621	71,26318259	923,7247351	102,6360817
ENSG00000075142	SRI	130,211162	128,0904905	43,56355575	114,6093905	24,57967602	107,4874989	102,1648915	176,2525661	96,54193899	923,5011704	102,6112412
ENSG00000123983	ACSL3	42,45805857	109,0809962	70,53283762	194,1497647	129,4551207	87,3829351	108,9190742	29,28768775	152,0796071	923,3460819	102,5940091
ENSG00000135829	DHX9	90,5433633	62,51191084	102,6047263	129,7596271	1,156872381	164,7572478	184,7166654	69,02261566	117,8162338	922,8892626	102,5432514
ENSG00000101558	VAPA	100,9947753	114,7954724	75,25117831	87,65921579	184,9697173	89,46322848	68,02646535	111,5633881	89,8258191	922,5492602	102,5054734
ENSG00000130638	ATXN10	105,5740324	74,16137237	219,4779483	87,08011343	0,490310668	113,292945	95,5160339	110,9827044	115,9592402	922,5347006	102,5038556
ENSG00000145220	LYAR	167,7038755	93,8031476	62,73856401	119,7084497	91,33805548	77,98661223	42,9670538	172,244755	92,19729816	920,6878115	102,2986457
ENSG00000116918	TSNAX	72,68517752	61,9747979	119,8981321	128,1462994	80,69927783	101,0248887	73,40386469	163,8667338	117,6918752	919,3910472	102,1545608
ENSG00000169756	LIMS1	85,55996795	85,4355414	94,15051094	146,1316445	100,4660323	119,4957532	92,0643203	57,02132995	137,8483277	918,1734282	102,0192698
ENSG00000156482	RPL30	58,74627151	106,3959479	109,9509948	175,5273644	112,2298615	99,24673324	72,3806781	122,9080671	60,41060769	917,7965262	101,9773918
ENSG00000144566	RAB5A	81,43203038	133,8218636	77,1142032	64,76618032	130,4148654	132,1735176	92,49564958	137,8930555	67,44151737	917,5528829	101,9503203
ENSG00000173141	MRPL57	100,0185506	97,38610035	61,99466605	127,1371484	154,8724556	111,077614	92,43112232	104,620349	67,74700812	917,2850144	101,9205572
ENSG00000140280	LYSMD2	98,29444271	126,0582188	32,9304066	103,7334878	84,89278082	117,1289717	128,3493204	112,6721924	113,1541526	917,2139739	101,9126638
ENSG00000276070	CCL4L2	6,922746258	308,2577324	66,06255752	82,18424397	53,46918311	89,66534389	228,6667611	20,10518127	61,5940872	916,9278367	101,8808707
ENSG00000111652	COPS7A	163,8272701	127,839531	2,055486647	109,8867006	0	142,309213	113,8952521	94,59271246	161,8958789	916,3020448	101,8113383
ENSG00000205155	PSENEN	82,72719983	113,6495894	58,70514232	176,4527165	114,6850724	86,31256639	87,15525637	108,929129	87,52154103	916,1382132	101,7931348
ENSG00000148303	RPL7A	89,39695959	101,721429	77,63072469	178,8535527	119,5453838	87,77396015	75,9101115	92,42718466	91,42851699	914,6878232	101,6319804
ENSG00000115268	RPS15	82,59108401	102,7647628	77,39736027	128,8190913	97,97170201	99,29790716	121,9475337	92,15676286	111,4905201	914,4367243	101,6040805
ENSG00000205220	PSMB10	62,35866723	109,1697442	89,24084358	69,48637393	121,6935829	124,9941307	118,3711496	73,36216413	145,4997681	914,1764243	101,5751583
ENSG00000178057	NDUFAF3	77,85236467	85,48816437	96,04817717	138,1861883	133,0830005	101,2231744	90,15924843	116,4802214	75,1326943	913,6532335	101,5170259
ENSG00000102054	RBBP7	73,55192549	87,49438639	61,78589051	102,2983707	264,6742926	60,6902326	88,45047349	93,42644022	81,13186247	913,5038745	101,5004305
ENSG00000067334	DNTTIP2	67,95754714	72,18933336	168,7181686	145,5654806	80,7751748	68,72114071	63,8867293	145,7023931	97,17513688	910,6911045	101,1879005
ENSG00000104979	C19orf53	103,5176842	134,4070176	35,29906019	100,275763	90,30441124	140,6844071	129,1390552	57,97097676	118,3083124	909,9066877	101,1007431
ENSG00000084463	WBP11	56,75986631	90,77458352	245,730601	113,4952999	64,95260228	90,4020164	50,1594975	113,7479207	83,69809143	909,7204791	101,0800532
ENSG00000085832	EPS15	72,27828732	53,0977856	31,3094961	245,3029254	4,137298875	117,6080667	87,97208287	211,5264926	86,32366926	909,5561048	101,0617894
ENSG00000123338	NCKAP1L	103,3733677	65,53735817	71,89049719	111,712786	88,62313593	108,9102384	130,7220258	109,4877416	119,0227301	909,2798809	101,0310979
ENSG00000090013	BLVRB	137,1536441	79,855196	131,1723735	111,6968541	79,7330245	59,42918822	98,50423933	135,5779487	76,14315773	909,2656262	101,029514
ENSG00000115446	UNC50	85,85519867	129,020792	111,1920396	91,97080271	77,94300022	86,22886027	73,25666918	158,1047908	95,25676007	908,8289135	100,9809904
ENSG00000103035	PSMD7	64,78565007	130,7452848	99,41828049	125,2024165	32,84044167	108,3547787	104,6128055	137,8747714	103,2098421	907,0442712	100,7826968
ENSG00000170876	TMEM43	114,3482286	55,17479565	137,1223221	156,6194248	13,06978738	110,4057785	95,19165726	134,3199866	90,42588346	906,6778643	100,7419849
ENSG00000196305	IARS	160,563914	37,64349927	80,03993932	104,500914	310,4491334	61,26653238	62,28590728	7,991864287	81,78653196	906,5282359	100,7253595
ENSG00000179271	GADD45GIP1	110,8770609	98,22273378	76,0556065	144,701645	20,76660787	115,3589651	145,8973844	83,12231913	111,360833	906,3031557	100,7003506
ENSG00000164125	GASK1B	78,89199083	75,1342051	145,1281887	49,43856816	195,0992638	97,41982721	98,3700141	111,5426332	54,85774249	905,8824336	100,6536037
ENSG00000119523	ALG2	130,9624946	96,96448084	34,91130536	79,68620268	106,6470277	134,7953608	112,1628725	84,44438948	125,0865987	905,6607327	100,6289703
ENSG00000163541	SUCLG1	105,1819614	97,92788086	72,26681093	137,1131162	200,267135	70,9963328	90,59066498	63,91153937	65,7294764	903,9849179	100,4427687
ENSG00000101160	CTSZ	138,0315628	120,8933614	5,845058316	55,37690904	229,8703486	79,02048983	75,711682	100,6653964	97,78526062	903,200069	100,3555632
ENSG00000145088	EAF2	53,10213274	231,9352554	97,84271281	90,66384711	0	92,79744121	74,14800411	166,3374009	95,86320612	902,6900005	100,2988889
ENSG00000128512	DOCK4	12,01104979	63,07714673	152,2979932	229,347751	67,61366025	108,6015228	91,20849684	10,0575788	168,4562398	902,6714392	100,2968266
ENSG00000204370	SDHD	98,35470943	158,9417238	91,1639076	125,7393868	54,3766067	88,81900985	75,45327464	125,0837706	84,35444947	902,2868388	100,2540932
ENSG00000008324	SS18L2	5,588064246	68,58485673	116,074895	216,3066507	112,881323	106,2185587	110,0040353	87,82736964	78,74852639	902,2342798	100,2482533
ENSG00000089006	SNX5	121,5648725	84,28957629	53,68759362	161,3249428	57,03073758	121,1420494	91,62978603	98,71904092	112,8445688	902,233168	100,2481298
ENSG00000086065	CHMP5	113,8859257	145,6781506	36,62222132	55,22086874	0,750977713	94,57804937	109,9374093	219,7369144	125,0635945	901,4741116	100,1637902
ENSG00000185728	YTHDF3	109,5018349	73,39859714	259,3067644	139,7608877	36,65178227	89,53733216	77,77002568	46,91388067	68,30153707	901,1426421	100,1269602
ENSG00000135678	CPM	194,0472305	68,53901688	139,0084954	54,33403784	97,79816814	87,6122342	73,31730216	104,2831323	81,68861072	900,6282281	100,0698031
ENSG00000136146	MED4	118,9402965	169,8914962	100,2048703	167,0957661	6,853388213	95,90166075	81,83773139	65,44229984	94,0266756	900,1941829	100,0215759
ENSG00000153786	ZDHHC7	57,81022592	72,14765749	58,87797384	95,79295115	101,6301735	153,8886803	124,6438236	77,77393292	156,3709907	898,9364095	99,88182328
ENSG00000111786	SRSF9	98,64105452	113,7154301	156,6318176	67,65242373	76,0026842	100,8933872	114,5362462	54,99790501	114,3748286	897,4457772	99,71619747
ENSG00000175040	CHST2	125,1715036	80,27428298	96,77626816	63,94229822	199,1533063	97,85599683	114,9589353	7,498997911	111,2745957	896,906185	99,65624278
ENSG00000167600	CYP2S1	92,7940327	70,37121418	124,6965649	75,30854593	192,9942413	90,5077971	114,5558468	13,64123537	121,7284446	896,5979228	99,62199143
ENSG00000125753	VASP	140,4755983	60,85868459	93,18383194	92,85390984	56,53715972	122,7213056	111,1075608	92,36850969	125,7011667	895,8077272	99,53419191
ENSG00000111361	EIF2B1	158,9848188	148,0066954	74,03605614	66,18698005	37,12960529	111,8421175	74,47963187	121,8972846	102,9918692	895,5550588	99,50611765
ENSG00000078808	SDF4	86,44495098	66,79349461	137,0124888	39,17626397	165,1833928	110,7362375	115,9048512	67,99556082	104,3092713	893,561312	99,28459022
ENSG00000159063	ALG8	81,19832757	102,2383912	20,1961711	105,7844492	252,8746891	77,33814735	114,9324285	64,91536403	72,37266488	891,8506329	99,09451476
ENSG00000104549	SQLE	122,9945272	106,3225074	27,37829546	225,9618084	19,35314049	89,91158547	126,0240919	59,17887376	114,7024877	891,8273178	99,0919242
ENSG00000166848	TERF2IP	77,38179872	94,70345404	196,3562256	79,46516178	0	101,6397932	127,3568258	76,5688591	138,0699399	891,5420581	99,06022867

ENSG00000143774	GUK1	107,6482922	102,8870786	57,71396341	92,88732852	89,68438095	111,5551116	130,5422544	96,3680072	102,1860878	891,4725047	99,05250052
ENSG00000162734	PEA15	111,9596851	176,6908618	43,78731424	127,1197827	32,50743256	125,3494022	87,86500441	56,28928128	129,7481601	891,3169244	99,03521382
ENSG00000065154	OAT	94,10959189	58,09215704	47,09580189	86,33651314	191,5364224	118,3116745	118,5450215	102,7975307	74,38249239	891,2072075	99,02302305
ENSG00000172354	GNB2	118,3287827	105,5834027	68,15262456	82,48724494	132,8681546	113,0312027	105,1489855	81,3811732	84,22464977	891,2062207	99,02291341
ENSG00000043462	LCP2	46,95276541	92,60877383	60,31912076	43,80075316	120,7735557	127,2894862	113,8020865	137,2663557	145,8999822	888,7128793	98,74587548
ENSG00000122068	FYTTD1	74,45501209	67,60424493	93,4227153	57,29533316	294,5427785	78,36475258	61,93280412	72,22110466	88,3694683	888,2082136	98,68980151
ENSG00000102401	ARMCX3	99,31447272	129,0421515	85,31543024	120,7108953	115,6394068	56,86908892	78,4374569	114,6660176	88,03487066	888,0297907	98,66997674
ENSG00000137207	YIPF3	149,1242339	66,4407059	83,03776207	50,84930659	112,9396937	97,8670671	120,8522981	117,384084	89,16724368	887,662395	98,629155
ENSG00000112033	PPARD	54,07703841	55,95065292	69,41706308	34,24881313	243,4941021	99,79947412	155,4796149	11,0466122	163,663361	887,1767318	98,57519243
ENSG00000108179	PPIF	63,53319762	80,31209288	1,561101267	167,2484749	218,3477682	80,1290318	79,99849322	88,70145448	106,6651886	886,4968029	98,49964477
ENSG00000139370	SLC15A4	105,9819017	118,4277488	131,0085434	150,3668809	85,64178406	82,98563045	67,00317656	28,40238602	115,0154776	884,8335294	98,3148366
ENSG00000137193	PIM1	176,3954087	118,1417723	93,48571646	32,89675257	24,77081398	100,5319534	124,7249637	141,4434436	71,70118302	884,0920078	98,23244531
ENSG00000187474	FPR3	169,2177387	70,53321413	84,02421128	102,9092235	0,129225456	210,1494171	159,1518245	38,28712873	48,61835973	883,0203431	98,11337146
ENSG00000090581	GNPTG	124,3506381	79,84003373	114,7499676	55,00412386	89,73552658	89,88511459	111,1273629	119,9379012	97,41735144	882,04802	98,00533556
ENSG00000148572	NRBF2	54,29169898	65,73075857	75,16137342	80,71002897	235,1927092	67,9002781	76,8926548	126,7158336	99,13118021	881,7265159	97,96961288
ENSG00000143337	TOR1AIP1	87,33452326	87,87472099	141,0861033	60,24707682	100,6948408	88,43387474	104,4215909	116,8463369	93,86070849	880,7997762	97,8666418
ENSG00000155849	ELMO1	112,6144817	99,29939899	154,2397414	60,20317909	52,34785492	93,65192637	104,7421612	49,13358303	154,1341533	880,36648	97,81849778
ENSG00000198736	MSRB1	113,7707774	61,41036071	99,23258054	77,90548152	262,3872261	68,61021531	63,05509499	89,15758845	44,8030559	880,3323809	97,81470899
ENSG00000146729	NIPSNAP2	100,0896949	145,8624152	81,31323724	87,3169416	62,85033612	112,0358805	85,85980779	94,02562105	110,7975462	880,1514806	97,79460896
ENSG00000263465	SRSF8	76,04298082	71,69536596	95,51252656	48,4556507	345,2093158	61,66041046	52,61156672	60,8695256	67,77769146	879,8350341	97,75944823
ENSG00000050130	JKAMP	73,60995385	134,9956276	92,91564688	190,6924791	0	72,27598686	99,19367167	118,3487485	97,45193501	879,4840496	97,72044995
ENSG00000166986	MARS	91,46618776	76,49753987	87,76861691	114,5024846	173,5802919	83,61628667	92,31745685	64,08399481	95,35608414	879,1889435	97,68766039
ENSG00000132294	EFR3A	87,78177046	104,1475307	159,7308127	119,7216187	0,869955287	138,2856083	90,49588222	97,01712593	81,03772774	879,088032	97,676448
ENSG00000131473	ACLY	119,0489	70,53658604	61,3819343	124,5052634	0,137909963	82,31815096	127,3735878	129,0522101	164,1501541	878,5046967	97,61163297
ENSG00000177383	MAGEF1	68,53547887	145,8718705	64,73741914	168,7464567	0,2195095	118,8552542	68,41047442	109,0676445	134,0457801	878,4898879	97,60998755
ENSG00000175582	RAB6A	68,64172981	94,41928271	32,64676408	128,6021433	72,76237786	124,026289	104,9686438	150,3693253	101,8056317	878,2421874	97,58246527
ENSG00000183605	SFXN4	77,90122032	23,11073984	177,8298813	94,28860164	236,0794122	76,38281369	55,74966572	67,21902337	69,44372192	878,00508	97,55612
ENSG00000101782	RIOK3	96,65350751	106,240776	183,1256871	39,44115967	114,7016697	77,55704471	76,84282689	64,4401844	118,7948116	877,7976676	97,53307418
ENSG00000118503	TNFAIP3	52,05420852	79,40204898	101,4005018	48,65593457	219,8299622	60,9164207	85,29485654	139,2099462	90,98158105	877,7454605	97,52727339
ENSG00000100280	AP1B1	142,0722654	47,51631039	132,5172202	53,73218343	69,24807839	108,8379308	136,8020522	60,57065722	126,1743314	877,4710294	97,49678105
ENSG00000102241	HTATSF1	97,34389626	106,7301709	48,86262296	219,6807708	86,63490302	82,05920831	46,3275072	72,14397923	117,5912692	877,3743279	97,48603643
ENSG00000110917	MLEC	64,61749006	53,22856106	86,01061777	110,2022672	167,7889977	108,0042291	134,3183486	71,18868362	81,66508411	877,0242792	97,44714213
ENSG00000077348	EXOSC5	141,3900076	96,82905559	104,4457389	35,85948481	102,6425139	88,89799176	67,32106995	93,96129149	145,6341169	876,981271	97,44236344
ENSG00000013306	SLC25A39	125,0112123	72,62496032	23,25698503	69,11890706	48,88724343	170,8982458	142,8710672	76,47039339	147,0011204	876,1401349	97,34890388
ENSG00000109436	TBC1D9	150,38743	53,06929347	3,373198945	112,1159676	255,3902057	46,53208962	45,63337318	126,6166981	82,67962698	875,7978836	97,31087595
ENSG00000100227	POLDIP3	158,3141979	72,28814468	107,384093	76,06433232	132,5291314	85,04621617	89,54800407	73,14825495	80,24496414	874,5673386	97,17414873
ENSG00000114023	FAM162A	128,5615111	124,7747509	145,6958705	104,8417878	23,29104018	72,61048176	75,55345064	74,04940216	124,0610056	873,4393007	97,04881119
ENSG00000149485	FADS1	115,3787554	83,53553805	58,50353403	86,2386265	102,3994009	100,6491729	129,2356685	81,30046984	116,0314504	873,2726165	97,03029072
ENSG00000078618	NRDC	168,2229002	91,1754062	61,85087984	162,0008221	11,87691267	76,77007934	117,0969982	62,00831763	121,912904	872,9152201	96,99058001
ENSG00000130706	ADRM1	168,6766454	78,1141961	40,61987441	51,8946308	125,5737811	135,4704009	113,2278953	55,68948594	103,5083572	872,7752672	96,97502968
ENSG00000141959	PFKL	113,4091097	61,46297694	94,01993265	49,75479798	114,1805495	109,1076726	122,9963483	83,81177021	123,7172345	872,4603924	96,9400436
ENSG00000134644	PUM1	95,92474377	30,2120423	110,8393689	205,366584	109,2376877	79,20908216	107,6380435	67,37451535	66,58026746	872,3823351	96,93137056
ENSG00000160307	S100B	102,4138087	111,2061783	57,25835926	43,05571563	83,11801368	123,9276678	170,9759065	11,70975212	168,1468535	871,8122554	96,86802838
ENSG00000100266	PACSIN2	118,2892751	32,88533963	82,14988884	117,186023	69,64894854	84,01088439	150,7681019	81,26747481	135,5725508	871,7784871	96,86427634
ENSG00000067704	IARS2	138,1008093	56,80589893	3,361307122	152,9009537	145,2102856	78,64201459	83,53562854	86,70411264	126,4295688	871,6905792	96,8545088
ENSG00000187608	ISG15	49,13903786	79,78720513	73,26149261	128,6650672	56,48182388	108,9614627	118,5367961	211,7449992	44,23976726	870,8176519	96,75751688
ENSG00000151500	THYN1	116,8807104	86,30148375	29,77889687	112,4273864	119,1409308	97,08783158	86,33116756	91,38020602	129,1892059	868,5178192	96,50197992
ENSG00000119929	CUTC	32,71813695	133,1732032	142,8681201	167,1614681	60,72132552	86,84381749	70,78711253	111,3741511	62,74394307	868,391278	96,48791978
ENSG00000188021	UBQLN2	149,4672879	68,27161563	125,9782151	57,73880289	110,7066845	80,13127854	89,99455964	107,9575821	77,15047118	867,3964974	96,3773886
ENSG00000011304	PTBP1	87,28979064	47,13814036	126,6923682	61,60385497	131,4277334	100,3707165	122,3896279	83,628798	106,1401539	866,6811839	96,29790932
ENSG00000123689	G0S2	17,94006775	33,05860275	241,4872778	45,87213024	75,84103349	46,4019307	2,005233616	285,5467667	117,9573817	866,1104247	96,23449163
ENSG00000121858	TNFSF10	82,25228098	132,2397484	113,8256203	116,0216539	50,72104783	120,6359381	93,45552482	55,31249953	100,4575796	864,9218934	96,1024326
ENSG00000175445	LPL	67,41631124	19,66764126	252,2075553	84,51488647	97,71196263	152,6570085	66,9418087	57,91203749	65,77284327	864,8020549	96,08911721
ENSG00000119335	SET	85,87799366	88,72390864	80,17060949	174,7755042	95,65473	89,67933354	97,32233789	96,51269113	55,81796811	864,5350767	96,05945296
ENSG00000152795	HNRNPDL	54,88658237	122,8086443	102,1358581	166,3960795	9,574718989	107,7388624	104,2989319	66,9799799	129,6106407	864,4302983	96,04781092
ENSG00000041357	PSMA4	110,4269555	67,93646328	118,3621296	158,7472572	34,19678056	104,0202858	55,5772217	138,8543314	75,51841812	863,6398432	95,95998258
ENSG00000133789	SWAP70	29,24720723	52,04758511	86,19238331	173,7873371	85,63495344	99,33411336	104,23877	100,6649246	132,2270098	863,3742839	95,93047599
ENSG00000163328	GPR155	85,44584077	100,9889937	129,2398143	162,8282606	118,5194206	49,92906788	93,57383236	50,65614235	71,86033632	863,041709	95,89352322
ENSG00000180879	SSR4	110,3535148	122,328834	44,33146677	79,36061537	52,57668594	88,95554015	111,7478687	181,3430666	71,86875317	862,8663454	95,87403838
ENSG00000164054	SHISA5	113,980915	78,47637867	140,8972886	66,85619525	96,24719724	78,54428674	108,2262319	99,8246796	79,25573173	862,3089047	95,81210053
ENSG00000144455	SUMF1	175,7661404	59,04221171	90,11134833	70,94545392	140,9784592	62,69431931	78,61771523	84,7024093	98,383038	861,2410954	95,69345505
ENSG00000135842	FAM129A	66,52130404	50,89225716	111,8260878	129,9976164	169,2551586	104,5854068	89,51622846	18,56463116	120,0528375	861,2115279	95,69016977
ENSG00000144034	TPRKB	93,82585995	145,2708735	151,5514365	104,654428	51,05188316	90,29036759	82,19624583	82,16472636	60,01199376	861,0178147	95,66864607
ENSG00000132676	DAP3	56,02421114	98,01092723	101,5463483	62,44694902	78,89940774	107,3283766	102,8812966	145,9155261	106,5336429	859,5866856	95,50963173
ENSG00000107521	HPS1	146,2360361	58,27194569	78,97340673	58,57623463	231,0790298	81,40603848	72,71172607	43,57173414	88,69867975	859,5248314	95,50275905
ENSG00000104915	STX10	52,75408007	40,31087975	117,6307769	89,64191499	82,38108433	119,7216634	134,3426773	71,01767431	151,2848639	859,0856149	95,45395721
ENSG00000004961	HCCS	165,8153125	81,71292704	162,8965753	140,3235459	17,47306864	74,56416443	62,22643013	80,95153297	71,94314797	857,9067049	95,32296721
ENSG00000198053	SIRPA	98,75260656	70,15712157	59,40261389	86,90967583	52,64884254	123,5162995	138,2698903	74,84981916	152,512961	857,0198303	95,22442559
ENSG00000161011	SQSTM1	142,627522	62,95198383	63,78601077	56,58496994	189,0669264	76,86712983	115,7957118	68,27957819	80,85001991	856,8098528	95,20109475
ENSG00000103365	GGA2	77,71243417	78,05469958	42,51937123	107,3729509	73,16730878	123,2184856	115,7296514	38,0174384	200,0937772	855,8861173	95,09845748
ENSG00000101972	STAG2	40,88309686	38,84407135	227,0832908	235,7880716	59,26075482	51,6558031	85,07216477	50,12821948	66,98828475	855,7037575	95,07819528
ENSG00000205352	PRR13	92,2502209	111,7574837	73,0386804	117,2544202	50,35784234	98,64159288	88,9044385	131,5185743	91,21220959	854,9354629	94,99282921
ENSG00000168710	AHCYL1	92,04689577	67,36834759	70,44738953	157,4938208	70,39702369	102,8085401	84,70181896	131,9113169	76,73781923	853,9129726	94,87921918
ENSG00000232810	TNF	48,53909272	86,77062339	149,4267278	60,95739574	181,270893	97,78638877	110,5155844	41,55047303	75,64551532	852,4626942	94,71807713
ENSG00000136710	CCDC115	66,0598238	50,36721353	182,0018351	93,1490844	80,80144703	70,28779162	83,75992434	130,2388498	94,63941145	851,3053811	94,58948678
ENSG00000125868	DSTN	108,3395105	133,0762441	109,8475906	111,4297748	0,493428885	74,90823785	94,36996391	152,096153	66,69842212	851,2593258	94,58436953
ENSG00000126067	PSMB2	49,02416703	148,6387854	86,68460684	103,8438216	73,83799292	89,88044272	84,93077906	123,402132	89,80392905	850,0466566	94,44962851
ENSG00000143344	RGL1	190,048959	3,133799059	106,0044751	9,394642818	284,9924323	24,1750944	49,7151061	152,3549391	29,18193271	849,0013806	94,33348673
ENSG00000108599	AKAP10	78,0691107	21,66636124	96,10677942	67,94339078	369,0621075	37,23473575	43,58661461	90,47857036	42,86107023	847,0087406	94,11208229
ENSG00000078061	ARAF	138,0299951	67,05446339	113,7870448	50,34162735	166,5045691	50,96998198	82,1324889	81,32335891	96,62967693	846,7732064	94,08591183
ENSG00000134910	STT3A	42,84157394	72,43062007	99,94198608	119,4731353	105,5056543	109,0211797	102,3042285	117,7729048	77,07364227	846,3649249	94,04054721
ENSG00000102226	USP11	61,74626279	32,82436706	143,3953139	97,36157906	90,44391321	121,6068793	102,109847	48,85339307	147,929458	846,2710134	94,03011261
ENSG00000105254	TBCB	90,4065021	74,09082244	149,4153334	118,2833016	71,22728605	96,06384711	90,61539542	59,85636548	95,81592121	845,7747747	93,97497497
ENSG00000089820	ARHGAP4	93,99727433	59,25073228	63,21805477	104,56104	153,2363563	102,0141915	105,4130675	48,6177025	115,1715545	845,4799737	93,94222193
ENSG00000111726	CMAS	89,41091516	54,87809931	0	130,2031155	275,0687558	63,87641251	45,25576311	111,8187898	74,37626778	844,888119	93,87645767
ENSG00000012779	ALOX5	135,3513181	68,65297857	122,6689659	96,11921048	50,7385911	100,0801066	78,78056771	82,5643477	108,5184251	843,4745113	93,71939014
ENSG00000154719	MRPL39	0,126030501	182,2649959	9,737417979	121,8712142	0	101,1514859	85,91421399	205,8670742	135,9361135	842,868546	93,65206067
ENSG00000131779	PEX11B	6,508837153	56,50023518	161,9662858	134,568328	122,0748805	79,47869885	98,15606881	142,9062187	40,56603367	842,7255867	93,6361763
ENSG00000164144	ARFIP1	65,98034756	98,80238139	170,8643194	38,81323703	110,8730196	115,6982401	76,65182147	76,86144894	88,06922337	842,6140389	93,6237821
ENSG00000071537	SEL1L	64,23741521	55,12727815	162,0507873	148,7818284	0,075184244	98,62934838	100,0426587	134,2076962	78,12829828	841,2804949	93,47561055
ENSG00000106052	TAX1BP1	81,6969861	64,07321115	147,3158037	66,47934159	75,50676532	87,83394634	102,44348	96,91305155	118,9093297	841,1719154	93,46354616
ENSG00000033627	ATP6V0A1	78,33472668	74,31119236	85,18294772	41,53999048	182,3615598	95,48755692	103,1135919	84,93872589	95,83267954	841,1029713	93,4558857
ENSG00000186395	KRT10	27,94462703	148,2652945	71,32637769	105,5562195	101,1107644	108,0758218	74,42368745	107,08952	97,09041617	840,8827286	93,43141429
ENSG00000164951	PDP1	93,2441744	82,34696327	146,0337993	196,399665	90,93286806	69,8695505	55,57055033	41,74655136	64,23710094	840,3812232	93,37569147
ENSG00000165283	STOML2	33,97059341	127,389721	229,8030386	88,71211207	0,397693845	87,64047075	148,9525979	44,09453531	79,33979439	840,3005573	93,36672859
ENSG00000110048	OSBP	113,8492175	41,35731747	78,96545178	136,4788748	183,2747473	64,4595954	99,79815161	21,86983959	99,96235237	840,0155479	93,33506088
ENSG00000156599	ZDHHC5	89,23435801	45,38244028	113,9800406	120,6535613	96,79753815	87,78822919	98,47477937	91,38124102	95,11614046	838,8083283	93,20092537
ENSG00000068079	IFI35	150,9573922	79,50485051	74,25013288	78,29371767	0,546047961	100,9927028	90,68458866	163,1238058	99,46980614	837,8230446	93,0914494
ENSG00000130811	EIF3G	106,197512	87,99730298	145,9614621	73,29213961	54,86089139	93,10774124	94,39943293	80,50139937	100,6735245	836,9914062	92,99904513
ENSG00000119977	TCTN3	68,12400223	50,66660815	1,897475078	62,74421512	264,296249	89,10679748	124,4581605	79,04593595	96,41184773	836,7512912	92,97236569
ENSG00000062650	WAPL	50,83858755	104,1306476	193,0620979	133,2742133	2,741355285	110,2539683	83,51918283	59,52867018	98,8688022	836,2175251	92,91305834
ENSG00000143727	ACP1	83,29243715	100,8110789	87,87111302	135,0463943	81,06042603	84,07199416	65,69095748	105,6437439	92,69988791	836,1880329	92,90978143
ENSG00000196976	LAGE3	43,31093279	82,22267864	66,49260534	103,9340573	199,4256531	98,9519351	108,4148285	24,37346652	107,5128986	834,6390559	92,73767288
ENSG00000157637	SLC38A10	144,0012837	52,46182802	163,3708764	58,44389023	27,47769231	99,81492518	97,85955956	75,30581645	115,0758281	833,8117	92,64574444
ENSG00000185627	PSMD13	112,4899486	110,044934	60,23861358	88,1188274	0,082612731	123,5837238	114,5310047	132,5691019	90,72149074	832,3802574	92,48669527
ENSG00000143079	CTTNBP2NL	82,10525512	47,54600381	47,10312626	105,4926366	140,8017497	95,09865508	77,17846768	99,87901457	136,5294964	831,7344053	92,41493392
ENSG00000170248	PDCD6IP	72,27399785	66,0093301	101,9060733	110,8655573	126,6419726	75,38311383	81,28041804	110,7568411	86,14815547	831,2654596	92,36282884
ENSG00000232388	SMIM26	130,4670501	85,22910567	68,35576204	149,9544344	34,66890664	101,8431045	95,64541702	77,34215287	86,82993254	830,3358657	92,25954064
ENSG00000171148	TADA3	90,5806869	65,86388287	108,5921313	67,23666918	95,08048299	86,32522449	97,7625473	99,03671851	118,6889457	829,1672892	92,1296988
ENSG00000091640	SPAG7	133,1471383	92,24625723	82,212414	127,2715978	0	104,9491977	61,37921861	175,6921548	51,81743813	828,7154165	92,07949072
ENSG00000127540	UQCR11	92,70283488	86,70384748	60,74717184	131,5053662	59,65686829	113,8160531	88,77451661	115,1959536	79,57396719	828,6765792	92,07517546
ENSG00000139192	TAPBPL	221,1200791	122,3797397	30,60571353	106,4038479	87,07586582	64,2721337	69,30779632	55,14152758	71,87853261	828,1852362	92,0205818
ENSG00000104442	ARMC1	0,044197301	17,09709155	166,5116241	58,23146698	286,43274	81,57229583	85,39615752	48,41992346	83,76410963	827,4696063	91,94106737
ENSG00000164442	CITED2	76,29226627	56,59961843	80,04265043	63,83387791	127,3655271	54,89355015	100,7483196	144,7826841	122,1815802	826,7400742	91,86000825
ENSG00000068912	ERLEC1	100,7986793	117,7259244	157,9724868	39,96573492	122,6153646	79,75485284	72,70695936	109,379961	25,59691684	826,5168801	91,83520889
ENSG00000114450	GNB4	29,62395903	54,62008381	132,3236675	108,2642657	177,1203828	96,32976131	92,42606514	28,30795992	105,1125592	824,1287043	91,56985603
ENSG00000010671	BTK	75,83603721	130,4664682	41,43878197	157,3594743	0,078495638	121,3413748	93,46511289	89,84931191	113,9452448	823,7803017	91,53114464

ENSG00000107372	ZFAND5	48,16716279	79,09524815	75,5444855	54,6615375	89,25837288	136,3214293	102,3298023	109,9577733	127,6067204	822,942532	91,43805911
ENSG00000109445	ZNF330	72,75473453	150,2483368	59,08173259	126,3517937	0,283215727	92,17981174	85,0981747	136,2912293	100,3981607	822,6871896	91,40968774
ENSG00000241399	CD302	47,10854435	64,19097181	147,1378596	137,7956832	107,1979517	74,06358832	68,25930743	101,9589615	74,21097333	821,9238412	91,32487124
ENSG00000068028	RASSF1	92,35674574	99,74329083	51,39952414	130,9988509	121,1034449	91,71706059	50,21538674	70,96761958	113,0822852	821,5842087	91,2871343
ENSG00000170881	RNF139	28,99084282	24,43071556	1,441378291	126,2152136	68,49834604	115,5229201	107,1144871	240,2381689	108,5481354	821,0002079	91,22224532
ENSG00000130748	TMEM160	92,97337401	101,9348977	67,05474143	63,73984112	83,33720067	112,4645741	117,9995794	94,33780783	87,10669059	820,9487069	91,21652299
ENSG00000184432	COPB2	94,66496058	121,6127102	105,7521829	64,03745715	52,42011141	103,1810639	74,24755154	98,25598955	106,5901285	820,7621557	91,19579507
ENSG00000146070	PLA2G7	195,8619736	0,132350444	0,137206367	69,8059014	337,8470068	23,25696139	13,62461651	150,2123577	29,72076664	820,5991408	91,17768232
ENSG00000138678	GPAT3	50,88459735	65,86982613	58,69577028	154,126648	0	73,86523881	86,37124152	108,6247778	222,1130117	820,5511116	91,17234573
ENSG00000122565	CBX3	179,7716252	142,9905996	81,0966735	95,85586464	46,00266038	54,40084406	65,66453532	53,04703698	101,5244225	820,3542622	91,15047358
ENSG00000131495	NDUFA2	76,16460215	89,02026078	51,00038106	77,50891047	31,6158589	125,770921	129,686405	136,1266061	101,7909379	818,6848834	90,96498705
ENSG00000069345	DNAJA2	83,75023347	24,80489518	136,9407113	128,8600309	94,07704553	84,91565368	83,62999305	80,15591105	101,3128183	818,4472924	90,93858805
ENSG00000100982	PCIF1	120,0192364	86,27324782	82,32217217	143,4152721	107,9395647	83,11259926	54,01093305	26,73554023	114,480048	818,3086138	90,92317931
ENSG00000112759	SLC29A1	47,56042451	79,22653653	150,0210058	114,6693834	41,20486994	110,8327798	113,2647147	39,71209088	120,7370264	817,2288319	90,80320355
ENSG00000172301	COPRS	117,579146	68,4452386	116,3121952	61,11819927	0,520837625	109,6044491	94,80789409	129,8734848	118,5967655	816,8582102	90,76202336
ENSG00000069509	FUNDC1	138,2475388	67,83544632	127,1587565	50,85844771	138,275967	55,17153625	94,3273379	91,74356727	53,07788504	816,6964828	90,74405364
ENSG00000111328	CDK2AP1	55,20913831	98,94604417	77,94696653	117,7173048	105,8821757	107,0284258	88,31012914	93,47783933	71,90875891	816,4267826	90,71408696
ENSG00000133226	SRRM1	114,525103	75,92053853	42,73917705	139,7889463	103,5375494	72,83981138	74,62966478	91,05512003	100,8760472	815,9119577	90,65688419
ENSG00000133398	MED10	111,3872983	100,697103	92,75945363	105,9698368	73,01799815	111,8828299	64,88916312	63,28601964	91,82652737	815,7162299	90,63513666
ENSG00000115520	COQ10B	40,80318495	132,5933623	161,252967	36,81081199	103,8300903	92,8904184	96,89149077	83,51909715	66,29572855	814,8871515	90,54301683
ENSG00000091483	FH	18,90645795	126,0592876	58,91828428	193,6912276	102,7726673	129,8161188	85,63848057	39,02891951	58,73860671	813,5700505	90,39667228
ENSG00000171222	SCAND1	51,32914174	45,27247196	87,59994101	86,93474855	133,8594446	134,5737594	125,2188884	66,88154573	80,72591924	812,3958607	90,26620674
ENSG00000125877	ITPA	72,75405951	91,81999488	50,95824678	78,19987395	147,0125881	93,80525912	98,91610623	52,91717327	125,4607573	811,8440592	90,20489547
ENSG00000148450	MSRB2	97,96607487	91,32274283	143,6956247	87,15412744	0	75,78734782	96,49613204	161,6274186	56,81513187	810,8646001	90,09606668
ENSG00000114520	SNX4	157,7509539	86,93821125	80,88933456	81,90351862	0,795681408	84,48695911	49,62286764	193,5263014	74,25625447	810,1700824	90,01889804
ENSG00000106263	EIF3B	116,8820282	51,83623704	106,7071997	75,73668677	146,834051	60,65824338	81,13849953	73,38521379	96,75864638	809,9368058	89,99297842
ENSG00000270170	NCBP2AS2	121,5832178	192,322538	52,08132889	91,47720374	0	73,42019774	96,48235875	76,72158924	105,7402627	809,8286968	89,98096631
ENSG00000189266	PNRC2	85,53634908	168,3772603	142,7628521	104,166224	0	85,02691879	74,47388508	44,48847597	104,9296326	809,7615979	89,97351088
ENSG00000198301	SDAD1	130,3334083	70,20389232	76,60179181	47,53785272	182,7991615	69,2442596	63,36413501	70,70599311	98,75259204	809,5430864	89,94923182
ENSG00000105339	DENND3	54,15052965	47,81591494	93,72117039	77,15991273	125,1648258	96,02908703	117,9609284	51,28831017	144,9526863	808,2433653	89,80481837
ENSG00000139083	ETV6	34,64329973	73,03316384	176,4535158	102,4561639	134,1043883	84,42092949	75,56594836	55,99873448	71,21270981	807,8888537	89,76542819
ENSG00000115216	NRBP1	31,27696743	99,27644327	202,3949679	29,86870604	0	90,84763798	111,7672483	116,5729005	125,6530343	807,6579057	89,7397673
ENSG00000119408	NEK6	61,68894987	56,06770945	158,2706334	97,31179863	42,42121682	124,6863601	101,1939949	63,85859259	102,1125495	807,6118052	89,73464502
ENSG00000114480	GBE1	23,1003259	94,3195683	188,1490758	29,00524229	134,8910657	54,1858022	76,42602678	92,00446462	114,7605272	806,8420988	89,64912208
ENSG00000136560	TANK	99,18747262	100,9317441	92,82612017	93,96278257	116,5731976	56,05689856	72,37202516	115,6136002	59,26168903	806,78553	89,64283667
ENSG00000181274	FRAT2	126,5286432	60,21987078	198,9699398	83,95196652	114,0802676	44,00791167	81,87038383	42,25620543	54,67062178	806,5558107	89,6173123
ENSG00000113141	IK	173,195098	147,9862215	12,60103	127,2033763	10,4079149	73,44121887	61,07238107	107,1436336	93,31456543	806,3654396	89,59615996
ENSG00000105518	TMEM205	116,1086203	73,79748695	95,71792948	89,52376775	137,5201049	59,81458525	79,4541268	90,11851841	64,2246699	806,2798098	89,58664553
ENSG00000135269	TES	104,9664241	97,59187395	35,17910791	130,6203669	0,084161599	88,22383373	124,0000914	63,81019068	161,1076367	805,583687	89,50929856
ENSG00000057757	PITHD1	66,54984635	76,50623104	50,65813125	73,48927955	234,0130879	99,10351061	105,3766019	44,40262021	55,24935729	805,3486661	89,48318512
ENSG00000132507	EIF5A	106,2557756	64,41400002	89,5541101	99,61204803	67,81102196	180,7405724	159,4431184	16,36789478	20,9726243	805,1711655	89,46346283
ENSG00000179933	C14orf119	115,627952	125,3769762	50,66258459	126,014672	0,122565714	80,63367277	88,98544365	109,7313427	107,8924691	805,0476787	89,44974208
ENSG00000006712	PAF1	153,8146546	98,78915793	39,69750887	28,8401814	180,3148081	75,38743748	45,38944291	106,6665972	75,90363225	804,8034208	89,42260231
ENSG00000115685	PPP1R7	59,50902928	85,31468556	71,60873219	127,0513854	125,847856	85,74167333	77,68475197	66,68118884	105,2851761	804,7244787	89,41383096
ENSG00000132383	RPA1	67,43845752	124,6967713	99,20629898	47,60685443	64,60453967	116,8805574	98,58056857	111,6690745	73,70242635	804,3855488	89,37617209
ENSG00000090432	MUL1	27,01070332	96,74315983	127,4266269	84,68565074	0,147906731	90,88182332	116,7370181	127,5194357	132,7256465	803,8779711	89,31977457
ENSG00000167283	ATP5MG	66,98236847	116,8424642	83,22302348	137,6909094	71,92169509	89,92965641	87,43472555	86,95646514	60,40329552	801,3846032	89,04273368
ENSG00000155097	ATP6V1C1	47,36571297	55,91065461	97,82276941	134,1021139	40,99991767	69,24519369	97,57135714	162,5347266	94,1779078	799,7303538	88,8589282
ENSG00000138107	ACTR1A	46,4855719	56,85416844	50,22153883	42,71373805	187,6205936	118,7926857	114,1497005	76,16676497	106,6771015	799,6818635	88,85354039
ENSG00000196531	NACA	91,09213357	102,3751581	71,91151841	129,5549449	62,11355574	88,97302809	67,73743044	110,4089922	75,41194314	799,5787046	88,84207829
ENSG00000184500	PROS1	58,37353602	108,4586121	46,10874897	109,7134748	140,6389787	87,10000518	74,16670249	138,5105957	36,15199616	799,2226502	88,80251669
ENSG00000166068	SPRED1	53,87335571	101,3749755	95,08712805	62,75907002	114,9176136	94,79381079	93,62515225	101,3901163	79,71017291	797,5313951	88,61459946
ENSG00000169241	SLC50A1	97,44694493	64,85827865	7,008899932	46,83448262	115,4087145	120,7460308	90,0276442	153,9203755	99,75351794	796,004889	88,44498767
ENSG00000072401	UBE2D1	75,8158208	71,50453336	28,19740659	91,02731585	214,4385785	56,92449097	53,36094579	132,4586844	72,25584616	795,9836224	88,44262471
ENSG00000163634	THOC7	76,11687407	187,8067182	33,53562086	146,2071327	9,650385644	94,95510994	61,91017525	110,9005667	73,95607747	795,0386608	88,33762898
ENSG00000112118	MCM3	65,99184922	64,94178851	65,49668086	141,0478644	10,03561351	71,50064032	110,8951482	51,8737006	212,6797051	794,4629907	88,27366563
ENSG00000162819	BROX	70,00242701	105,4261117	87,022284	188,8372788	48,09867485	103,077776	73,34219608	55,47054186	63,14303528	794,4203255	88,26892506
ENSG00000124193	SRSF6	37,90027081	107,4656966	43,4325106	159,915137	50,98054702	79,27516844	91,69299095	116,1276212	106,8185162	793,6084588	88,17871765
ENSG00000138777	PPA2	141,8289947	86,56825779	24,62280618	71,30723013	137,7649193	83,06255812	75,71485552	100,1985855	70,73382723	791,8020343	87,97800382
ENSG00000119535	CSF3R	103,8717103	83,34702103	140,2267625	50,21195027	92,75108432	82,66988043	93,28776494	42,6518934	102,2387381	791,2568053	87,91742281
ENSG00000139433	GLTP	47,96811708	67,45035386	149,4636353	70,38690068	45,4943228	123,5722752	95,87267087	79,99235071	110,8178389	791,0184654	87,8909406
ENSG00000131871	SELENOS	94,05205556	120,014594	101,3392059	64,01863932	64,40798924	90,06321886	84,73754726	104,1950406	66,8117753	789,640066	87,73778511
ENSG00000126088	UROD	103,0657355	67,41817972	14,00594961	136,6524687	173,1857495	84,95247449	69,0413815	62,90476875	78,18561808	789,4123231	87,71248035
ENSG00000100442	FKBP3	85,13741829	97,39846402	45,08117381	173,7644749	51,4158567	76,61358933	75,07151106	103,817489	80,89210094	789,192078	87,68800867
ENSG00000108651	UTP6	131,5239156	87,47775958	32,24534335	72,87225966	110,6360869	93,11042853	76,9163776	82,55691449	101,4766233	788,8157091	87,6461899
ENSG00000173726	TOMM20	59,82009502	50,53985342	182,4901348	172,2110115	0,313366093	86,62288691	79,59592615	103,9936597	53,08356163	788,6704952	87,63005502
ENSG00000100412	ACO2	139,1680212	76,88635764	0,549232105	123,6686135	0,088692898	109,8558301	100,0292002	93,19740087	144,9042901	788,3476388	87,59418209
ENSG00000170385	SLC30A1	127,9554976	71,7108081	3,915212662	31,70362155	335,1073079	36,79706834	63,23551435	70,93156761	46,6114305	787,9680286	87,55200318
ENSG00000153187	HNRNPU	79,18000584	74,45609642	80,43542843	113,9272328	125,8315198	68,11973438	85,59760834	68,09194913	92,15550498	787,7950802	87,53278668
ENSG00000147434	CHRNA6	105,8821757	74,2658313	173,3627813	82,01962222	24,90029184	108,2636814	95,7943752	0,254572086	122,9908803	787,7342113	87,52602348
ENSG00000134575	ACP2	43,67970855	29,65408895	114,1035924	62,29459897	288,6178727	76,83921677	68,42501429	57,5215236	46,38007124	787,5156875	87,50174305
ENSG00000157916	RER1	74,87795723	125,610552	139,2587945	66,73587415	19,12684397	108,9836219	102,290631	69,52543106	81,08122135	787,4909272	87,49899191
ENSG00000132842	AP3B1	35,94819184	26,33734009	99,51763598	56,89881662	198,3252419	66,99972947	68,24487552	176,9700864	58,06750436	787,3094222	87,47882469
ENSG00000143543	JTB	118,1762155	130,4523496	55,52099143	133,988877	19,14324281	78,25336917	77,72048905	94,98321576	78,5019723	786,7407226	87,41563584
ENSG00000169490	TM2D2	120,9566011	69,58995312	134,5712001	158,5613882	43,48277796	59,92981824	76,18750937	59,97741304	63,23391254	786,4905737	87,38784152
ENSG00000111667	USP5	99,64889157	79,05095883	95,93213689	31,62213577	124,7976692	81,62374279	100,0271775	28,02240714	145,7239585	786,4490781	87,3832309
ENSG00000106635	BCL7B	102,4557739	88,91024213	34,42478006	40,31162611	0,222363803	137,3513567	124,3049729	131,2531115	127,0430716	786,2772987	87,3641443
ENSG00000162772	ATF3	46,60489382	173,5622105	45,27497652	90,55218673	72,00128943	89,27960241	102,2707434	99,21216099	67,20835867	785,9664224	87,32960249
ENSG00000106952	TNFSF8	133,5867364	50,74951825	176,4877091	75,4251131	0,123577957	57,2636987	66,27562272	149,4119139	75,84789243	785,1717826	87,24130917
ENSG00000182180	MRPS16	35,91174621	94,14720905	71,23613131	126,825231	51,54120776	96,37539942	97,53813926	116,4917856	94,91874992	784,9855996	87,22062218
ENSG00000117614	SYF2	125,1177903	126,0047088	99,83117787	61,68937115	111,5520169	61,0346887	40,60410161	88,27936498	70,7329992	784,8462194	87,20513549
ENSG00000113369	ARRDC3	89,05975057	89,075858	98,58953159	55,48466862	1,440792547	96,38455971	88,62207402	200,8458856	64,83191619	784,3350368	87,14833743
ENSG00000114209	PDCD10	38,25058415	89,57170934	119,170705	99,59350176	56,36226466	61,6528769	89,11266938	142,9697045	87,44717277	784,1311885	87,12568761
ENSG00000160948	VPS28	72,68863481	115,8225461	38,42600997	76,63969671	72,79538402	115,0237031	108,325938	78,90451334	104,535587	783,1620129	87,01800144
ENSG00000167461	RAB8A	91,96569105	81,67699942	78,52895516	67,6811666	85,79202289	92,09029577	72,34073289	102,3938388	110,3546172	782,8243197	86,98047997
ENSG00000170802	FOXN2	28,36424247	85,20674282	248,8231203	108,5721775	34,65399123	64,94784706	61,36190443	95,05569372	55,63191227	782,6176318	86,95751465
ENSG00000164088	PPM1M	70,75845685	97,16151764	44,15930045	90,35425098	77,65779957	116,1420034	64,63096115	99,30442783	122,4206645	782,5893823	86,95437582
ENSG00000171135	JAGN1	98,08187518	68,05175971	73,20194633	200,2796801	0,201638148	111,5075577	74,60588523	57,88634759	98,50596127	782,3226513	86,92473903
ENSG00000133321	PLAAT4	105,5449217	24,95783232	73,77164165	54,99706388	171,5476875	87,93260565	110,8964704	91,7591376	59,61384599	781,0212066	86,78013407
ENSG00000183160	TMEM119	48,97804929	34,10725818	119,3463553	83,01731447	0,451152691	323,7965841	84,3299342	10,89686859	76,05465406	780,9781708	86,77535231
ENSG00000109180	OCIAD1	82,63959507	115,5274506	10,20164555	81,978086	40,90923213	97,17418377	91,9867947	161,2918837	98,71864484	780,4274564	86,71416182
ENSG00000103855	CD276	111,6928187	46,02729334	99,74222085	27,84512224	113,0021453	105,3208331	115,3768595	63,89285691	97,49992668	780,4000766	86,71111962
ENSG00000148730	EIF4EBP2	43,20902982	112,7191508	159,4183027	93,49212012	75,93674904	108,0600325	85,30508622	31,40694327	70,80576352	780,353178	86,70590866
ENSG00000134802	SLC43A3	78,31521938	72,5414045	97,48073429	104,2700087	115,9161782	73,01975348	86,77647048	50,9880216	100,6788665	779,9866571	86,66518412
ENSG00000007255	TRAPPC6A	93,45208925	121,281805	126,0295626	96,76688233	0	108,2953282	56,48182148	58,08281787	118,7186788	779,1089854	86,56766505
ENSG00000163219	ARHGAP25	78,10520692	71,70782027	176,6183167	85,9094423	5,73059908	63,63151429	69,61624254	100,6698123	126,1588369	778,1477914	86,46086571
ENSG00000175315	CST6	48,2430707	34,42363515	0	91,67985594	0	239,3372171	289,7262565	41,30432094	32,96264331	777,6769996	86,40855551
ENSG00000014257	ACPP	40,91989519	58,64040002	93,7858091	153,6346112	0,083905968	107,6092899	69,0086826	205,5951526	48,28535812	777,5631048	86,39590053
ENSG00000137845	ADAM10	48,4756734	75,91679131	145,4528824	130,8349093	49,15139027	73,78187359	84,78807466	42,16509689	126,4021531	776,9688449	86,32987166
ENSG00000102218	RP2	34,46954639	52,10651299	82,64273213	75,67114263	140,2499591	109,4561399	87,6596779	100,0234367	94,37989604	776,6590438	86,29544931
ENSG00000115677	HDLBP	135,1669142	42,11769903	85,70291045	86,4561688	97,31022221	64,87703883	104,1837124	65,59778339	94,66557228	776,0780216	86,23089129
ENSG00000177119	ANO6	69,10533201	55,71232084	108,018982	101,2370666	155,4649713	83,91227103	41,36635718	77,20171316	84,0427989	776,0618131	86,22909034
ENSG00000167978	SRRM2	12,23425855	57,02333536	89,46633766	101,3640529	179,4064737	69,2477409	99,77450335	49,02121273	118,4809196	776,0188347	86,22431497
ENSG00000242247	ARFGAP3	82,50369379	89,62644305	119,1585942	88,33362415	0	95,55192089	75,71155078	128,5191581	95,50682874	774,9118137	86,10131264
ENSG00000176390	CRLF3	94,08251574	79,19599638	143,171154	105,1946149	1,145771557	58,83246521	72,7716488	105,8023171	114,3964122	774,5928958	86,06587731
ENSG00000125257	ABCC4	117,7328041	40,42794555	145,7068012	70,27614799	53,35365504	96,90454358	106,34952	17,23207271	126,59704	774,5805302	86,06450335
ENSG00000125144	MT1G	93,48707305	12,15577067	28,53929016	136,3820134	104,383499	31,41240252	52,53024566	222,4926088	92,67546577	774,058369	86,00648545
ENSG00000150779	TIMM8B	41,98955823	113,1439657	92,73950129	135,8383616	25,87124662	74,844737	82,85776642	121,858374	84,79446814	773,9379789	85,99310877
ENSG00000165071	TMEM71	232,6522291	84,90090339	42,38111953	40,17396218	56,04475451	68,81405072	58,85658131	121,780807	67,87971717	773,4841249	85,94268055
ENSG00000100647	SUSD6	78,27589746	69,10449694	51,62078999	93,64487343	98,40017707	91,11995493	94,98482781	64,19450703	132,0874202	773,4329449	85,93699388
ENSG00000109606	DHX15	100,9254289	53,12928682	157,6446043	112,7650656	133,6466093	54,42988427	35,29333243	59,92818938	64,20193967	771,9643405	85,77381562
ENSG00000101294	HM13	127,3216692	79,83459642	61,33313799	67,47360657	84,88534967	99,20853643	85,9886912	76,51525108	88,82104961	771,3818881	85,70909868
ENSG00000128951	DUT	68,52210417	89,33201092	79,36884154	114,6295724	83,96351902	98,08322607	63,34988183	89,03925472	85,03211866	771,3205293	85,70228104
ENSG00000168701	TMEM208	136,5660725	90,63109572	112,3120489	111,1200423	3,416339239	67,58975928	96,33200644	101,1458917	51,89558736	771,0088435	85,66764928
ENSG00000125870	SNRPB2	51,50969989	134,2766304	44,87332119	111,1574988	165,9266252	71,05909286	43,68368921	101,4869972	46,96045176	770,9340064	85,65933405
ENSG00000140030	GPR65	115,5354374	113,6527387	81,65010666	74,32973672	119,4139467	72,93298018	101,2162576	22,19500642	68,67793231	769,6041427	85,51157141
ENSG00000198171	DDRGK1	113,9927995	39,95784172	106,0633675	52,13002222	88,21118671	123,4367446	103,4171405	61,32519266	80,34412972	768,878425	85,43093611

ENSG00000120694	HSPH1	47,42242343	46,21374638	100,6559643	83,22550561	164,3208874	87,02889727	89,41443564	69,74965662	80,58517855	768,6166952	85,40185502
ENSG00000137168	PPIL1	129,1429547	79,3119824	0,349880553	20,36245239	264,8746127	112,3472502	74,77684355	34,93581331	52,51421554	768,6160053	85,40177837
ENSG00000179051	RCC2	60,42200067	41,08574407	175,483882	150,586156	50,88745758	82,44685927	114,6120267	29,32515652	63,46756693	768,3168498	85,36853887
ENSG00000176393	RNPEP	94,1652847	54,48210827	108,3760328	75,44834721	61,22148551	108,4301251	89,30001343	34,52586943	142,1685552	768,1178217	85,34642463
ENSG00000144848	ATG3	115,8736924	114,084307	82,24317116	74,29665453	38,25650795	88,42591201	74,18169032	88,51601817	92,23899559	768,1169491	85,34632768
ENSG00000107175	CREB3	54,43677439	89,70866914	0,170018409	51,70821515	143,8666607	91,02249988	85,41191408	166,7330387	84,7960279	767,8538183	85,31709092
ENSG00000143641	GALNT2	70,83308	76,04078763	72,27050032	73,32029175	116,4151784	97,45525317	115,3344377	24,50837301	121,6485016	767,8264036	85,31404484
ENSG00000120860	WASHC3	91,0859321	93,06895491	67,2169387	131,3228468	141,2649029	79,02027082	51,24299329	62,31488759	51,0307458	767,5684729	85,28538588
ENSG00000175416	CLTB	49,43146181	86,26834333	95,43210853	54,77202998	28,88439281	114,8110955	107,9327917	117,1673332	112,7474978	767,4470547	85,27189496
ENSG00000160014	CALM3	84,18840902	135,4968983	59,05276714	61,48017945	147,4859609	63,79435864	77,70469702	84,25315534	53,97506033	767,4314862	85,27016513
ENSG00000124380	SNRNP27	23,10071982	122,833631	86,45664649	154,3326523	41,83709903	101,8027998	76,19495122	54,5208327	106,2959814	767,3753139	85,26392376
ENSG00000134882	UBAC2	81,08692365	136,6050381	7,911230277	103,795772	65,92533637	104,2753201	104,345086	80,32941148	82,89005219	767,1641701	85,24046335
ENSG00000079739	PGM1	7,264574896	60,50788327	79,90990336	48,51329672	213,5150657	41,30469349	104,2354708	101,2295253	109,8577676	766,3381812	85,1486868
ENSG00000166902	MRPL16	143,0402087	71,85020444	56,18474376	102,2355725	0,661358273	87,15652303	82,66484581	116,3823408	106,1002245	766,2760217	85,14178019
ENSG00000141543	EIF4A3	86,29318376	90,79550175	52,89848408	83,55127112	166,9538196	64,45734381	64,87239292	70,21450291	86,01637043	766,0528704	85,1169856
ENSG00000101463	SYNDIG1	49,64079152	35,72268268	133,0670147	178,34695	0,148579869	109,6231655	64,31846973	0,273425377	193,1512056	764,2922849	84,92136499
ENSG00000120333	MRPS14	66,1181564	63,77934235	43,50806484	118,8994372	214,4460575	51,80708062	49,04661397	106,6205601	49,79311217	764,0184251	84,89093613
ENSG00000044115	CTNNA1	66,05739556	76,10009651	99,26061745	92,52405498	0,12201049	101,8198658	113,2785376	92,31960114	122,3893009	763,8714804	84,87460894
ENSG00000140374	ETFA	57,52589974	113,053313	80,58274675	89,48415779	52,51549993	80,56535853	80,99604782	142,533965	66,39222075	763,6492093	84,84991215
ENSG00000090238	YPEL3	81,08951403	73,92660612	70,9587678	71,77557459	136,715292	67,49081618	60,74326038	97,85287604	103,0013891	763,5538963	84,83932181
ENSG00000068308	OTUD5	51,02926565	47,17865926	139,9961022	51,8673641	236,1457781	65,77301259	64,65634313	50,31951794	56,08508634	763,0511293	84,78345881
ENSG00000152315	KCNK13	52,48270148	84,69491746	56,95616707	0	149,0439778	105,0984431	120,9207306	50,81387845	142,8964086	762,9072245	84,76746939
ENSG00000125991	ERGIC3	94,73315716	88,97925652	48,73436865	94,05290917	93,42312645	73,73803409	75,19454528	115,5311811	77,62152652	762,008105	84,66756722
ENSG00000108828	VAT1	152,8544895	65,44954763	108,9143031	70,69184684	80,29893114	52,51122407	78,08410448	101,3421654	51,85042904	761,9970412	84,66633791
ENSG00000125901	MRPS26	114,2253133	65,97982286	0,27360246	74,73629686	86,24476411	127,8621866	114,4793285	48,88525799	93,28910075	761,9756734	84,66396371
ENSG00000114942	EEF1B2	43,55772063	87,5137076	62,27334854	111,5956889	123,7689702	90,53605883	85,96191937	73,16149171	83,46110139	761,8300072	84,64777857
ENSG00000120656	TAF12	97,95999767	115,8850749	38,42001368	71,01182877	135,6871159	59,06615311	65,98332821	84,27000524	93,51968772	761,8032052	84,64480058
ENSG00000172725	CORO1B	77,38495152	62,61848097	110,8371936	68,72381629	28,38154408	112,9325608	133,4079145	53,03577158	114,3268356	761,6490689	84,62767432
ENSG00000109854	HTATIP2	61,53555237	98,45614376	29,71614215	90,77215956	142,6455451	87,13716023	114,8387737	100,2999456	35,88027244	761,2816948	84,58685498
ENSG00000116688	MFN2	84,62556592	37,20450031	173,7864725	73,03870594	0,072213461	69,52697403	102,0313739	70,29957218	149,4470627	760,0324409	84,44804899
ENSG00000105701	FKBP8	95,0404547	79,99553816	68,82941082	73,03217274	60,89329026	99,75822189	107,8448437	54,50587935	119,920122	759,8199337	84,42443707
ENSG00000113712	CSNK1A1	55,30012198	46,71802542	110,7019528	69,17449093	138,4050367	73,42736698	77,8781371	103,8337298	84,15373377	759,5925956	84,39917728
ENSG00000071859	FAM50A	94,68095709	105,4916019	28,64763559	68,40340831	0,28144008	156,5488514	104,1325091	89,34163463	111,857468	759,385506	84,37616734
ENSG00000159228	CBR1	128,7215351	91,16828003	32,56338302	61,45284912	80,91138097	72,32346772	112,5135998	104,1206066	73,12491733	756,9000196	84,10000218
ENSG00000095015	MAP3K1	142,2676449	64,8792102	30,30812602	214,1480291	0,231958108	71,52093973	89,89257993	65,35270932	77,69013643	756,2913338	84,03237042
ENSG00000169032	MAP2K1	34,89331613	128,0433334	68,70005639	90,58796006	37,16326788	74,7678949	100,736755	130,2750809	90,22875849	755,3964231	83,9329359
ENSG00000186469	GNG2	53,63134221	49,0970146	150,2548019	60,25352212	188,9884919	45,09937921	49,76327637	72,36511849	84,78027588	754,2332226	83,8036914
ENSG00000079999	KEAP1	187,2705309	54,85916654	38,04429566	40,45843155	59,90317807	78,05644541	102,5394261	104,4110346	88,2679956	753,8105045	83,75672272
ENSG00000101911	PRPS2	172,9169547	108,4040356	150,7825337	99,15150594	0,260418812	80,66935582	71,65183934	53,67465056	15,97013262	753,4814271	83,72015857
ENSG00000155307	SAMSN1	100,2546505	61,59250185	125,6673774	87,76674941	0,069260857	116,3133937	105,3612007	77,36693635	78,63978204	753,0318529	83,67020587
ENSG00000119392	GLE1	90,82914936	78,45833402	98,91268053	140,0481732	0	69,71348871	71,43306682	132,567313	70,92413354	752,8863392	83,65403769
ENSG00000103342	GSPT1	83,94197829	58,2627887	98,40095135	127,2600671	0,089177438	104,64685	83,45475149	126,0771574	69,87127756	752,0049993	83,55611103
ENSG00000122694	GLIPR2	77,06391787	97,16307499	211,9200165	102,7511537	0,11755075	59,1412825	71,05193923	69,87257958	62,17189714	751,2534123	83,47260136
ENSG00000143418	CERS2	84,69871133	60,62642121	101,4591447	110,2399699	74,46817254	83,41972364	70,21907685	85,02784414	81,03772774	751,196792	83,46631022
ENSG00000174123	TLR10	5,194649774	133,8802874	16,01450072	205,7538467	0,085139294	142,0742065	89,03098712	0,313356631	158,749412	751,0963862	83,45515402
ENSG00000125652	ALKBH7	122,4921918	65,256882	99,18777548	49,41769418	82,14033448	111,1130303	88,85090299	69,85566797	62,4022632	750,6967424	83,41074916
ENSG00000048544	MRPS10	66,84843817	126,1544267	0,264742951	76,41667113	179,0457461	77,02991061	61,48046268	107,1552098	54,95747159	749,3530797	83,2614533
ENSG00000156976	EIF4A2	84,92266165	98,94571031	47,524167	91,43365831	79,43937805	39,89432138	93,13050744	135,0307654	78,58306456	748,9042341	83,21158156
ENSG00000117758	STX12	105,113483	121,6197864	55,06945923	82,10424352	15,30572004	119,249591	89,48962643	39,72864966	121,2085053	748,8890646	83,20989607
ENSG00000063322	MED29	7,880603424	52,02024721	179,1829999	49,53341891	166,9639056	81,15990662	49,04303199	93,1420515	69,15332133	748,0794865	83,11994294
ENSG00000169251	NMD3	165,3867798	73,52014174	53,20958149	169,3478167	45,28613083	63,82224485	63,00574281	39,36168601	74,7737394	747,7138637	83,07931819
ENSG00000117362	APH1A	126,5708753	93,65282065	40,74053279	82,49542243	135,6643293	101,5145337	58,92372919	44,71760632	62,55530796	746,8351576	82,98168418
ENSG00000179958	DCTPP1	152,4486782	113,8034675	72,26427621	123,1230177	0,681437462	102,8414707	96,90023743	52,54021129	70,17733953	744,780136	82,75334844
ENSG00000105220	GPI	105,7773995	101,1056025	77,0692016	58,44161603	74,71527894	68,91192118	92,23479181	47,61599245	118,8338858	744,7056898	82,74507664
ENSG00000165389	SPTSSA	23,55780872	36,16190009	113,7191314	267,8945476	59,89789211	54,84647164	58,1631817	85,15331079	43,84358226	743,2378263	82,5819807
ENSG00000138138	ATAD1	45,09160901	63,23854156	156,024354	76,21546525	177,7514893	59,03626797	58,11700596	34,04121655	73,35564329	742,8715928	82,54128809
ENSG00000110330	BIRC2	50,41464758	5,384139896	118,4420942	159,65858	170,3668835	79,98998458	46,64993588	44,76761352	67,00644691	742,6803261	82,52003624
ENSG00000174080	CTSF	79,69066928	77,62733978	155,9750641	19,49152822	80,22970223	51,74353958	113,8519088	69,15807816	94,68256556	742,4503958	82,49448842
ENSG00000144744	UBA3	98,37276069	63,86821142	119,3567741	14,23512769	133,641404	62,38819543	65,1797901	103,814075	80,88352053	741,739859	82,41553989
ENSG00000060982	BCAT1	15,50157497	73,14885574	51,18827578	194,8497349	85,28845005	71,10245652	73,11683298	59,40006417	118,0202751	741,6165202	82,40183557
ENSG00000198399	ITSN2	39,07858627	81,52212696	34,89202873	66,30813071	238,4956295	55,36792828	63,99800997	52,08563512	109,2942033	741,0422789	82,33803099
ENSG00000128463	EMC4	81,92946576	84,0922268	127,2642298	81,89072881	27,7114324	71,9630128	82,93179649	95,15326129	88,09417827	741,0303324	82,3367036
ENSG00000168216	LMBRD1	92,54071062	120,3842789	133,6806601	36,43351127	58,73601955	81,09767267	57,04699398	97,24424539	63,71006466	740,8741571	82,31935079
ENSG00000196504	PRPF40A	50,48639662	99,25977981	62,6737849	76,83571819	79,79971395	63,43435011	78,91894414	128,2790236	101,0501487	740,73786	82,30420667
ENSG00000106638	TBL2	71,34868114	88,28863566	84,56338613	84,82606196	113,1892285	84,39218787	89,78521902	55,49828956	68,69365732	740,5853471	82,28726079
ENSG00000114316	USP4	110,9899086	51,01905903	0,125235065	114,6170297	103,4909992	99,08790562	87,39020871	57,56181083	116,2971104	740,5792672	82,28658524
ENSG00000156052	GNAQ	47,59675238	97,28186909	70,74255951	128,3222231	143,2352967	51,707837	60,51118591	72,07850123	69,05113658	740,5273616	82,28081795
ENSG00000143891	GALM	161,450323	95,22321028	0,531307529	63,03933637	0,274554696	94,75168257	143,9839207	103,8292104	77,00979746	740,0933431	82,23259368
ENSG00000147649	MTDH	55,79349156	66,25245457	95,1541832	114,2667252	50,74883502	86,5574606	82,25029509	82,10518647	106,6564602	739,7850919	82,19834354
ENSG00000106268	NUDT1	89,67000897	61,11024082	90,31364898	117,1900985	58,16571756	75,14726551	74,21513188	85,88456987	86,75205555	738,4487376	82,04985973
ENSG00000099246	RAB18	62,18771838	69,63160407	157,550347	107,1330323	59,49705707	65,26580295	67,14064046	89,52477597	60,41579858	738,3467768	82,03853075
ENSG00000137965	IFI44	129,0149131	74,73241219	0	98,07467642	0	53,81103592	73,4475496	224,2350377	84,71610363	738,0317286	82,0035254
ENSG00000141380	AC091021.1	48,5747684	73,10186363	115,6893088	99,37953134	180,2017225	49,5247392	60,4071032	60,88084805	49,8494578	737,6093429	81,95659366
ENSG00000111845	PAK1IP1	85,32835901	64,96677481	5,293120729	70,07380474	138,1765462	85,40636384	41,43898687	170,9668018	75,90054948	737,5513075	81,95014528
ENSG00000131013	PPIL4	67,04603551	56,9523069	146,5541338	78,99051952	160,1506803	47,9673436	33,52514162	124,1315042	21,7031653	737,0208307	81,89120341
ENSG00000178035	IMPDH2	90,73173464	82,86970576	0,100245257	111,4125532	154,8880565	55,58273558	81,26387712	63,03638161	96,77691243	736,6621967	81,85135518
ENSG00000114125	RNF7	53,00074174	98,28415671	107,0877557	110,5314496	54,29597916	93,5988102	76,28531469	82,23581113	61,11007502	736,430094	81,825566
ENSG00000040531	CTNS	22,32416318	34,24768335	44,42118709	55,4429228	196,679737	112,1532062	100,47227	52,21994195	118,4101591	736,3712707	81,81903008
ENSG00000103502	CDIPT	89,36808974	67,05782287	104,8789384	74,03150129	55,84678398	89,06022299	80,68038997	63,71016137	111,5821777	736,2160884	81,8017876
ENSG00000198561	CTNND1	18,92746634	104,7820182	38,54691075	89,24225262	49,14497852	97,68319929	104,3494452	80,59929324	152,0063167	735,2818809	81,69798677
ENSG00000123146	ADGRE5	156,5855913	32,18501153	222,4563891	53,6220888	1,534690352	69,63805143	38,27987461	107,0136476	53,93761943	735,2529642	81,6947738
ENSG00000183576	SETD3	37,8504881	34,79744814	48,24182204	49,66116852	140,7801704	108,1649942	111,3410081	116,5377601	87,69117624	735,0660358	81,67400398
ENSG00000154640	BTG3	104,5847662	91,27990896	112,9520177	50,91966113	9,655371911	104,4675444	65,67332642	77,66501977	117,7457802	734,9433966	81,6603774
ENSG00000147669	POLR2K	75,78109312	115,3988285	26,91737958	89,9937121	55,38094795	105,2188415	62,84766182	128,3816311	74,53856333	734,458659	81,60651767
ENSG00000170855	TRIAP1	63,82841964	101,8597534	162,1386403	174,388151	0	88,30534593	70,69377317	28,10216024	44,91400715	734,2302508	81,58113898
ENSG00000112078	KCTD20	123,1118818	79,46699121	86,74903874	109,1585645	0,287800563	79,40519026	77,08411325	69,48712175	109,2321201	733,9828221	81,5536469
ENSG00000164674	SYTL3	31,568334	103,5161451	84,75705366	88,38992061	20,46077158	65,94028791	115,5921429	85,57525057	137,7163801	733,5162864	81,5018096
ENSG00000080608	PUM3	124,3887247	83,26679288	134,0743501	128,084288	0,558286113	32,88550102	38,47496772	127,1396121	64,50095002	733,3734726	81,4859414
ENSG00000168036	CTNNB1	120,1791017	81,81762874	2,316697823	110,995556	44,58811224	62,10888329	174,0195293	49,09169057	87,93690364	733,0541033	81,45045592
ENSG00000130741	EIF2S3	100,4194726	100,3972007	53,06211642	49,68058693	68,11001661	103,831319	68,5613429	80,48320512	108,0651304	732,6103908	81,40115453
ENSG00000023697	DERA	85,84240737	136,4063996	0,259463202	109,4572592	0	108,1692135	66,45957863	98,20238125	127,5093452	732,4418639	81,38242933
ENSG00000172428	COPS9	81,23027731	95,30427495	80,65107437	91,84603672	34,37796797	92,85128004	93,74857403	87,11014372	75,08673924	732,2063683	81,35626315
ENSG00000163755	HPS3	72,0528824	77,83661639	159,0298552	96,39078106	0	111,5440994	61,95298203	39,2972056	113,6586947	731,7631168	81,30701298
ENSG00000147677	EIF3H	71,0928967	106,5233062	22,12089069	108,4052203	135,5760818	66,91606257	65,58233238	86,12663502	68,6598047	731,0032303	81,22258115
ENSG00000188820	CALHM6	65,36232947	47,25267961	22,50091864	71,5041035	139,5895337	99,4624115	86,64435237	95,00690348	103,2654791	730,5887114	81,17652349
ENSG00000182149	IST1	164,6032773	42,01097439	36,07402514	74,91100155	89,75638293	85,39952565	75,047525	69,38025316	92,99468563	730,1776507	81,13085008
ENSG00000183578	TNFAIP8L3	48,24576212	117,1636913	54,40015433	81,87921599	167,5175039	67,31588983	67,25971825	69,40380203	56,90618944	730,0919273	81,12132525
ENSG00000182511	FES	28,30176561	56,53827037	119,6730126	47,69111387	118,2814561	76,51944256	96,38701373	67,47093263	119,1505309	730,0135385	81,11261539
ENSG00000159377	PSMB4	97,63520636	127,0677619	7,456407361	145,3868282	34,30628758	59,02512752	61,70188091	128,908356	68,12914864	729,6170045	81,06855605
ENSG00000132406	TMEM128	58,17929866	61,51312473	0,159027517	104,9412398	159,0142893	58,84700089	42,20626729	163,7049974	81,01547481	729,5807204	81,06452449
ENSG00000105401	CDC37	88,52039347	67,34401317	74,47527307	51,6380193	89,95646524	77,44675178	110,044328	66,36933621	103,6146291	729,4092094	81,04546771
ENSG00000106605	BLVRA	135,0950077	38,87396377	86,55393527	126,2403694	0	41,54597637	66,14580929	227,9109373	6,939903083	729,3059022	81,03398913
ENSG00000105383	CD33	116,8925619	109,6352981	54,71206634	48,56229151	84,68562631	59,92217984	57,30686795	110,218593	87,30898544	729,2444704	81,02716338
ENSG00000140307	GTF2A2	97,62761588	91,55660053	112,094347	84,1279116	31,83189202	76,27536813	65,16311874	98,38782067	70,64952064	727,7141952	80,8571328
ENSG00000197930	ERO1A	105,751377	62,5510746	97,2506877	76,0441044	38,15237369	61,40603409	61,24760543	141,3396766	83,90050972	727,6434432	80,84927147
ENSG00000112667	DNPH1	111,0964916	48,05104642	97,40422668	34,65216463	104,8623224	84,01711604	77,18934825	109,9686241	60,21427322	727,4556132	80,82840147
ENSG00000143507	DUSP10	85,09657888	98,17013445	13,66792846	105,0385572	94,76536491	89,4989846	66,21922014	42,66311183	132,0055949	727,1254753	80,79171948
ENSG00000138326	RPS24	57,02467125	78,3915202	51,83159539	211,8166308	60,37855078	61,97626863	56,67483878	88,78037063	60,15116535	727,0256118	80,78062353
ENSG00000060138	YBX3	57,26461366	80,71319919	80,90152747	57,78040442	110,4720708	72,84207549	74,98238609	90,76314301	100,0911035	725,8105236	80,64561373
ENSG00000147403	RPL10	46,97737948	85,13855837	78,18010245	101,3455217	78,07550299	85,00123225	80,50904619	92,93258199	77,49703685	725,6569623	80,62855137
ENSG00000092201	SUPT16H	95,24345968	55,61031915	102,9734975	137,8795532	29,71827778	64,80130496	56,33436066	72,64771579	110,4305422	725,639031	80,626559
ENSG00000085365	SCAMP1	18,54722095	46,10802255	98,48390699	59,87656992	230,2376772	40,75212357	37,77501398	140,2363129	53,33514188	725,35199	80,59466555
ENSG00000132912	DCTN4	115,5994688	48,22126665	79,53427797	124,4696832	46,3377474	101,8681116	68,90407511	75,74711685	64,4105759	725,0923235	80,56581373
ENSG00000188612	SUMO2	70,09400127	91,9435532	85,27047839	121,7384798	47,94669696	80,923528	59,54968863	102,8413439	64,5022876	724,8100577	80,53445086
ENSG00000037474	NSUN2	55,82253918	81,2802394	280,3529767	44,31060684	66,2012905	59,72298638	43,88954948	12,78288465	80,26571558	724,6287887	80,51430986
ENSG00000174718	RESF1	58,38879709	41,96022872	58,6828286	66,74532904	108,7727374	82,30622501	84,82368367	139,7112752	82,96819615	724,3593008	80,48436676
ENSG00000071967	CYBRD1	122,0980423	85,49229663	63,9590715	159,2248221	4,443836564	106,5651461	96,98963406	34,50012886	50,59394883	723,866927	80,42965855

ENSG00000127337	YEATS4	4,921062941	102,8304245	64,14925361	87,46669278	235,5133574	50,49047309	28,81379326	96,66277916	52,6965153	723,544352	80,39381689
ENSG00000146232	NFKBIE	66,45118082	78,22972726	189,9871734	38,51849102	44,24617531	96,29370163	71,26675967	42,76061432	95,76748339	723,5213068	80,39125632
ENSG00000155463	OXA1L	32,22482483	111,0848446	69,23231284	109,5497514	149,3328451	64,0473118	71,23098901	56,35830849	59,71719473	722,7783828	80,3087092
ENSG00000022840	RNF10	90,51267063	119,8003919	47,02104047	44,66852955	8,947504869	103,1457619	113,6676143	70,11933534	124,8636634	722,7465125	80,30516805
ENSG00000086232	EIF2AK1	20,05819483	102,9212193	15,7920231	123,1506654	14,45101545	77,54778776	123,5969585	106,6752528	137,7267563	721,9198734	80,21331926
ENSG00000185608	MRPL40	84,43735704	180,9446779	15,70269261	66,2628765	0	87,38801368	70,23784489	142,4315922	74,43318729	721,8382421	80,20424913
ENSG00000138185	ENTPD1	81,62330602	54,75375894	123,8352505	90,1375348	74,00665471	89,58292084	71,63110351	61,16172454	74,9656984	721,6979522	80,18866136
ENSG00000181061	HIGD1A	49,07710474	77,64111644	62,56626705	154,3800357	65,82034955	108,1637909	62,77261983	89,75983774	51,14940106	721,330523	80,14783589
ENSG00000081320	STK17B	39,67630579	62,84960983	260,3258649	140,5217896	2,679981659	24,81330891	60,86247448	81,1743767	47,26830524	720,172017	80,019113
ENSG00000143401	ANP32E	61,1987332	56,15733982	178,2002798	86,78530778	26,59760993	75,47665625	83,1828276	64,43703217	88,10310862	720,1388952	80,0154328
ENSG00000159199	ATP5MC1	64,95154165	112,8964979	108,6407882	89,00726476	38,31502651	71,94745468	67,1254281	94,64454725	71,56690169	719,0954508	79,89949453
ENSG00000112685	EXOC2	138,7249548	39,06597877	120,216644	7,268529353	126,2308956	76,03957694	72,84319675	51,71731407	86,62906028	718,7361506	79,85957229
ENSG00000186635	ARAP1	98,08170653	56,0800517	100,6511423	53,39139884	81,57075802	76,79959558	90,30649558	51,26552201	110,1206315	718,267302	79,807478
ENSG00000096060	FKBP5	60,8821475	61,10650397	139,2777594	135,8197478	56,90195157	47,67509421	44,84962281	116,3112267	55,41471858	718,2387725	79,80430806
ENSG00000116711	PLA2G4A	38,63055922	55,32349707	2,220751051	178,8206686	79,58214381	83,61800861	102,9824225	90,90106894	85,79021469	717,8693345	79,76325939
ENSG00000136936	XPA	126,7086455	156,2333094	0	146,482318	12,34282879	66,34612805	53,18627821	67,42091012	88,41143886	717,1318568	79,68131743
ENSG00000138385	SSB	113,1385832	108,975795	64,8473559	43,47195263	90,80969908	57,94067429	63,0579033	113,6318319	60,89470536	716,7685007	79,64094452
ENSG00000167635	ZNF146	103,8384023	43,52618574	4,089914626	101,3907969	3,37809555	80,31416484	67,51671749	236,628131	75,8895712	716,5719796	79,61910885
ENSG00000143207	COP1	101,3674453	57,71119418	53,24014345	113,8193736	15,3895236	89,6878339	64,82283372	152,204	68,11851223	716,36086	79,59565111
ENSG00000213523	SRA1	86,36796751	54,87593936	49,4405123	61,16143692	172,8966966	101,5689308	72,5259899	44,5639081	72,8391405	716,240522	79,58228022
ENSG00000126261	UBA2	147,3131918	54,35235557	118,7549457	84,64806445	103,1484031	42,14094191	54,27927343	50,98917625	59,63299624	715,2593485	79,47326095
ENSG00000107854	TNKS2	57,54402859	70,10697599	38,29908445	39,00229595	123,2920367	91,0355215	71,70020414	146,5833575	77,14327767	714,7067825	79,41186472
ENSG00000099817	POLR2E	84,15028083	62,21775361	97,492815	83,42417976	7,15285454	99,85295694	110,6045452	56,99487407	112,6527585	714,5430185	79,39366872
ENSG00000165475	CRYL1	199,9674434	78,01959282	35,65494683	50,17284467	48,468834	52,95181568	75,363391	124,9343546	48,53398895	714,067212	79,34080133
ENSG00000163399	ATP1A1	98,90609428	93,74099879	109,4806876	77,30875433	53,64309036	67,87354498	86,21020476	64,38865147	62,49367047	714,045697	79,33841078
ENSG00000102178	UBL4A	77,87329214	41,13246215	139,1519796	74,27780054	189,2514582	67,0085436	41,7097492	33,62091522	49,94399744	713,9701982	79,33002202
ENSG00000115541	HSPE1	41,86003192	75,45630611	51,23494968	241,1823545	43,53536855	66,98309624	59,53474338	70,14710786	63,81784034	713,7517986	79,30575539
ENSG00000064225	ST3GAL6	33,23567418	95,73454043	111,3600301	72,78625087	37,69363517	86,72838194	72,4726854	91,17429622	112,3118296	713,4973239	79,27748044
ENSG00000142875	PRKACB	59,38892413	54,50494147	72,28308666	38,38419389	87,83171987	138,0040726	72,70940487	111,7487482	77,40383387	712,2589256	79,13988062
ENSG00000081721	DUSP12	133,958406	44,89324065	108,3838411	102,1822545	0	32,48532858	58,3202054	125,0850491	106,5295048	711,8378302	79,09309224
ENSG00000100242	SUN2	94,38297779	48,55399139	71,11218436	89,59209792	68,62471144	81,81751355	86,77929573	36,918641	133,6067231	711,3881363	79,04312626
ENSG00000060558	GNA15	75,00589749	80,9659541	85,85148866	66,50696253	0	122,2900003	117,0192113	90,5438769	72,90190789	711,0852991	79,00947768
ENSG00000196209	SIRPB2	134,7207471	92,20377476	111,7111125	85,11228214	15,6231112	55,79789558	79,08569	44,04481422	92,73866983	711,0380974	79,00423304
ENSG00000172775	FAM192A	64,76167314	67,79113368	106,6645514	82,05615603	29,65797049	91,18020882	83,09847845	68,71701151	116,5869488	710,5141323	78,9460147
ENSG00000111670	GNPTAB	65,58328635	44,36112279	143,7965398	40,9262652	83,55159518	76,18238881	82,15777827	72,11141114	101,7839609	710,4543485	78,93937205
ENSG00000104660	LEPROTL1	47,38756136	102,7852935	82,45563534	142,1400074	46,17320181	63,56691898	79,38816432	86,98213021	59,41192088	710,2908338	78,92120376
ENSG00000180957	PITPNB	90,86079156	95,51480487	54,46870725	121,0811541	0	106,2293496	76,37324557	72,22015016	93,33995737	710,0881605	78,8986845
ENSG00000083223	TUT7	49,40889838	95,39090685	120,1941683	37,2708671	124,9709772	62,16390029	47,06774345	81,73415052	91,0528809	709,254493	78,80605478
ENSG00000147274	RBMX	59,13915907	95,91478548	64,41463361	129,3140855	116,6124628	57,1745561	54,73595238	61,06466761	70,14268607	708,5129886	78,7236654
ENSG00000182827	ACBD3	192,1617845	78,5224971	63,89901969	99,67730571	10,80654627	85,61337087	59,43147517	71,721518	46,55224952	708,3857668	78,70952965
ENSG00000116685	KIAA2013	100,8631971	57,69833955	81,58940424	69,50843273	33,27392041	100,1228107	72,39114248	85,60481024	107,1123967	708,1644542	78,68493935
ENSG00000123358	NR4A1	0,559285183	105,6762509	77,92588107	58,26772771	47,16032976	136,2081486	111,3323453	24,41456229	145,7984437	707,3429745	78,59366383
ENSG00000006007	GDE1	85,98501265	116,8463886	38,43597268	88,56335129	46,87342865	105,8955435	81,7986538	93,03351377	49,3191796	706,7510446	78,52789384
ENSG00000131408	NR1H2	40,29615218	48,25551724	90,73395366	48,96195591	142,8400112	79,41337716	71,59327745	65,28248478	119,3037225	706,6804522	78,52005024
ENSG00000104324	CPQ	93,335582	118,7241163	64,77049372	97,03566749	0	91,82441854	69,07794281	61,53647815	110,1472706	706,4519696	78,49466328
ENSG00000159592	GPBP1L1	81,50171906	40,36740187	147,2363312	68,34930654	44,35220017	69,01594284	92,25014992	84,82028183	78,13404845	706,0273819	78,44748688
ENSG00000140367	UBE2Q2	100,269631	70,0287778	60,69087831	90,36533129	125,5792652	56,6387353	86,64358336	43,57755979	71,98416699	705,777929	78,41976989
ENSG00000147684	NDUFB9	127,1890239	86,20249394	56,59799385	96,7226087	35,77181559	71,07567083	69,51899788	106,55026	55,58196039	705,2108251	78,35675834
ENSG00000120509	PDZD11	70,51103441	135,8996784	0,199554989	70,80647552	46,14647529	99,3319605	94,84584321	73,0609094	114,3363026	705,1382344	78,34869271
ENSG00000168883	USP39	106,9968346	131,5951126	12,0952636	83,17178548	139,4498931	66,64963213	56,40292941	40,54155458	67,98230994	704,8853155	78,32059061
ENSG00000213024	NUP62	116,3450988	59,81971862	72,77352757	99,60607502	37,93999285	70,96673339	76,09504687	65,31846102	105,7658115	704,6304657	78,29227397
ENSG00000149658	YTHDF1	0,188534711	40,81512665	65,81001096	65,68138631	191,3202627	98,22154541	78,66355812	48,75095148	114,7193598	704,1707362	78,24119291
ENSG00000143353	LYPLAL1	52,51105176	66,73918533	121,078717	98,48276838	128,3347627	72,4906804	71,0756794	33,81870276	59,14734609	703,6788938	78,18654376
ENSG00000163221	S100A12	23,36761402	0	679,7616441	0	0	0	0	0	0	703,1292581	78,12547313
ENSG00000128699	ORMDL1	34,84556209	100,3775893	79,84903767	52,29118657	181,0375432	65,83424431	72,1133453	60,61128204	55,74931864	702,7091091	78,07878989
ENSG00000198730	CTR9	69,61038073	21,79525096	87,48905715	34,14313792	173,3971357	98,74123404	64,90969515	46,50136375	104,6473745	701,2346299	77,91495888
ENSG00000173511	VEGFB	70,51826631	88,51289323	19,61154096	46,10695668	57,87719615	116,1910964	112,9639041	121,0524047	68,08358033	700,9178388	77,87975987
ENSG00000167792	NDUFV1	66,09780152	58,64446354	103,0125975	72,03188146	61,92416392	90,25509126	81,72815377	59,65725927	107,3110233	700,6624356	77,85138173
ENSG00000139163	ETNK1	87,88801839	37,22102968	100,9972866	129,2410197	66,20284324	63,03427111	59,18903221	82,69940952	73,78499456	700,257905	77,80643389
ENSG00000141447	OSBPL1A	105,9411387	56,90795468	93,61790854	85,79752559	47,9479394	44,34476244	40,59931779	118,1563885	106,7303916	700,0433272	77,78259191
ENSG00000177189	RPS6KA3	115,5596203	56,38411629	158,8337674	65,38491505	23,37430049	59,72780761	69,9094142	56,33201897	94,52425637	700,0302166	77,78113518
ENSG00000116096	SPR	58,97250666	75,03562142	95,90122756	21,1181941	203,9432957	95,70013375	84,24928328	12,69158833	52,26136772	699,8732185	77,76369094
ENSG00000172586	CHCHD1	93,13220606	39,59750372	27,74177657	162,5785007	0,968624524	89,72372388	75,3837261	146,6122356	63,53620148	699,2744987	77,69716652
ENSG00000103024	NME3	53,96059132	76,82407477	67,90794114	58,50721922	137,4339107	83,47196489	111,8379153	42,30477162	67,00763883	699,2560278	77,6951142
ENSG00000125944	HNRNPR	80,41465016	44,7521457	58,85770422	95,64006676	103,5215506	68,30712079	62,34469816	95,44364064	89,56020166	698,8417787	77,64908653
ENSG00000104812	GYS1	108,3891431	70,43390429	92,02642277	38,3926961	146,6615382	49,37023056	48,19409829	33,42523685	111,6130847	698,5063548	77,6118172
ENSG00000129071	MBD4	64,20848444	97,39722828	207,2426125	29,23466938	0,321022833	96,74821174	42,43409619	48,54119243	112,3053478	698,4328656	77,60365173
ENSG00000104823	ECH1	41,27162351	65,43456537	85,57876256	69,79415734	109,7780069	83,63967849	99,53725426	63,27470442	80,05199744	698,3607503	77,59563892
ENSG00000099800	TIMM13	32,05650928	72,03093392	52,9565453	137,2103983	102,9441513	72,38646556	63,64000013	90,35320206	74,38328355	697,9614894	77,5512766
ENSG00000097033	SH3GLB1	76,40076665	59,93349748	68,87895201	106,8236918	7,306562408	92,80479723	85,26366407	102,3814123	98,06840901	697,861753	77,54019477
ENSG00000159128	IFNGR2	90,61529153	72,83316489	88,27746387	51,9225338	100,5771766	69,5591303	91,85615184	75,01757749	57,07246241	697,7309527	77,52566141
ENSG00000109519	GRPEL1	55,1525634	106,8600425	64,98770255	108,1705776	58,89350571	70,57848207	81,65627772	77,25079408	74,15528312	697,7052287	77,52280319
ENSG00000176597	B3GNT5	76,15972761	87,22453	56,45134318	32,88583557	100,1551201	97,0551349	97,39586264	30,82700334	119,5364831	697,6910405	77,52122672
ENSG00000168286	THAP11	102,4320945	54,66849671	78,23943183	88,13035566	0,169875848	84,52492484	108,0801677	89,8769519	91,42875268	697,5510517	77,50567241
ENSG00000197965	MPZL1	72,10297848	26,36280804	64,8843066	99,29481987	72,34168628	99,04408455	90,53956197	69,83579628	102,1643211	696,5703631	77,39670701
ENSG00000122359	ANXA11	89,69361137	43,56165701	133,6185322	47,96936864	81,20536462	89,14307269	70,56877125	50,11053996	90,37996032	696,2508781	77,36120868
ENSG00000157593	SLC35B2	154,9474874	87,71636313	10,35220005	11,70508138	91,7930578	84,18399554	81,02739172	96,76712684	77,23287553	695,7255794	77,30284215
ENSG00000153147	SMARCA5	30,44100742	33,64118882	134,5879586	165,0623526	37,25194246	80,6945378	79,97077876	64,25341946	68,89964366	694,8028295	77,20031439
ENSG00000169740	ZNF32	9,004534005	124,3370781	84,15561901	91,32724111	101,5859897	77,1597819	74,42281336	78,76111609	53,58999033	694,3441637	77,14935152
ENSG00000149792	MRPL49	24,67812242	100,2050137	140,3438487	80,65262851	145,978949	81,55474452	39,96311548	32,7216049	48,15114441	694,2491716	77,13879685
ENSG00000213465	ARL2	77,44694932	63,84333335	21,69800478	89,15598543	59,41218055	106,6160751	93,93336097	92,94992989	88,77205016	693,8278695	77,0919855
ENSG00000116473	RAP1A	72,93297608	68,44116127	40,85282366	133,2504163	61,4758338	95,58152711	79,65506254	60,9858448	80,32520513	693,5008507	77,05565008
ENSG00000086062	B4GALT1	46,31056461	21,06400123	44,5811789	85,93124852	83,97239411	152,9451103	63,46479352	117,3140476	77,85001229	693,4333511	77,04815012
ENSG00000144909	OSBPL11	114,2366994	93,65725362	66,92561322	56,57941924	6,716857034	101,5742221	89,25912483	71,36179329	92,96368013	693,2746629	77,0305181
ENSG00000203950	RTL8A	119,4549966	60,62340872	68,56109951	57,3438126	61,6035626	65,83961226	66,05789811	88,29002674	105,4844292	693,2588463	77,02876071
ENSG00000119471	HSDL2	125,5218439	81,89398854	82,29286873	48,36804942	202,0918495	48,46374874	63,77853989	0,399678945	40,29390882	693,1044754	77,01160838
ENSG00000159596	TMEM69	85,61213218	16,83895154	0	198,5955609	69,61732562	115,4012817	96,35093754	51,42599413	59,24254328	693,0847268	77,00941409
ENSG00000100596	SPTLC2	67,13454425	56,45557806	100,0278383	94,92176808	77,57378827	72,71176647	60,66983898	83,84646141	79,37555173	692,7171355	76,96857062
ENSG00000002919	SNX11	117,9215601	72,35223685	43,40066437	22,35965257	180,3554823	66,9439515	70,98155221	81,46320869	36,23131036	692,009619	76,88995767
ENSG00000116005	PCYOX1	47,77609107	17,69627456	57,83351309	104,5697801	166,8818729	72,00586932	74,28939166	80,74910219	69,97768096	691,7795759	76,86439732
ENSG00000186417	GLDN	112,3233989	86,96701156	208,7783022	63,30700861	0	67,76431994	39,38815486	37,45854591	75,54099147	691,5277334	76,83641482
ENSG00000137509	PRCP	130,2136402	91,35155205	38,39946355	61,82796587	62,12513065	65,23527942	58,26189244	106,2137937	77,43957157	691,0682894	76,78536549
ENSG00000171492	LRRC8D	81,25463291	56,92214759	0	112,7204672	104,9523238	91,26449489	84,79332618	87,64824575	71,24372041	690,7993587	76,7554843
ENSG00000174780	SRP72	79,12964057	81,41068194	81,49930073	125,4729336	0,223540331	82,25818509	64,43387731	98,57501804	77,39266941	690,395847	76,71064967
ENSG00000114395	CYB561D2	64,97899419	57,20191277	47,1739957	62,91960318	174,0497772	73,80873211	75,72730009	62,57030401	71,63020322	690,0608225	76,67342472
ENSG00000169504	CLIC4	110,1446201	83,31790824	75,29794488	52,22204327	0,160786901	87,50510829	84,4895363	135,8872399	60,24306341	689,2682513	76,58536125
ENSG00000105438	KDELR1	42,69345	92,48748131	61,19526243	60,93133988	81,45758871	104,47043	136,9326395	58,01747754	50,68541517	688,8710845	76,54123162
ENSG00000175215	CTDSP2	99,89654994	51,78220878	70,467341	119,7072822	0,066111477	75,07514682	99,55396372	83,70360179	88,6123882	688,8645939	76,54051043
ENSG00000103994	ZNF106	41,49175411	53,88719894	72,62133455	63,921172	104,3632118	55,4211349	69,36479421	90,35193516	137,2339607	688,6564964	76,51738848
ENSG00000124098	FAM210B	123,147813	30,94532463	121,6064162	170,0750133	28,37824809	76,4471418	58,0630189	21,88495004	57,15916347	687,7070894	76,41189883
ENSG00000100225	FBXO7	74,53558602	112,8770513	54,74129868	91,46963609	58,32867957	84,03591703	82,32656099	55,60886866	73,63195155	687,5555499	76,3950611
ENSG00000168291	PDHB	37,28910415	98,96867122	71,83755367	100,2362039	69,58520961	58,14080629	62,50906937	121,2597491	67,72740551	687,5537728	76,39486365
ENSG00000168066	SF1	60,32277302	56,97793003	57,06691248	115,9176972	74,74599885	67,42548052	91,24542061	54,9433231	108,7631606	687,4086964	76,37874405
ENSG00000149600	COMMD7	66,95882485	94,53458285	79,60730389	100,5771032	45,30662493	87,06452864	64,04619501	71,61120658	77,38810293	687,0944729	76,34383032
ENSG00000163660	CCNL1	47,38110115	67,30428711	77,25738574	72,34077655	87,87662624	63,12166631	58,41578981	94,18274762	118,9724329	686,8528134	76,31697927
ENSG00000111911	HINT3	46,4920643	22,49973474	26,75297193	132,7825495	221,194805	49,49873446	44,15046269	107,3291227	35,93964325	686,6400886	76,29334318
ENSG00000090054	SPTLC1	71,48344361	106,054628	28,76686423	74,0778087	46,75751204	66,15534588	97,74857432	114,3050945	81,01002253	686,3592938	76,26214376
ENSG00000170571	EMB	51,44680497	40,11895574	71,93530806	112,2418765	70,38429695	92,61229711	73,03139553	102,3464319	72,13966151	686,2570282	76,25078091
ENSG00000133961	NUMB	112,1536134	55,53286551	73,52815315	81,81210758	128,809812	49,22048536	55,53238558	69,16072343	60,44976447	686,1999104	76,24443449
ENSG00000165637	VDAC2	89,80040987	64,40340221	83,48252707	95,43925141	83,58660873	72,12737841	57,56456336	60,31278275	79,47946425	686,1963881	76,24404312
ENSG00000173456	RNF26	72,38342728	35,11726948	82,18715521	14,51199177	217,1198632	57,02990042	51,41267282	101,9640216	54,14688772	685,8731895	76,20813217
ENSG00000145675	PIK3R1	16,27842882	28,58582127	150,530459	141,1786844	90,89211118	82,22089711	69,92009928	38,89095693	66,85002106	685,347479	76,14971989
ENSG00000163938	GNL3	104,5827019	62,68792281	12,44815144	70,67324667	9,648927173	47,39570212	91,44841966	147,7669097	138,380471	685,0324528	76,11471698
ENSG00000172322	CLEC12A	60,19361491	15,4824005	165,1147148	28,44960565	56,69115994	20,17831338	12,87072816	290,6525188	34,93184018	684,5648964	76,06276626
ENSG00000089639	GMIP	49,18183185	66,99955605	115,3654674	15,04048781	111,490111	89,71625555	96,00330497	25,86779992	114,8461753	684,5109898	76,05677664
ENSG00000185989	RASA3	84,02064191	31,67164148	169,2331199	66,50426473	35,27248741	88,67095115	54,20843873	46,77984663	108,0439677	684,4053597	76,04503996
ENSG00000112079	STK38	98,46423796	0,299182148	87,93010662	6,623454028	1,502583859	72,98214211	60,75773957	241,8578257	113,8925549	684,3098269	76,03442521

ENSG00000140612	SEC11A	92,75195443	90,61754409	90,50044009	62,62059296	37,8603527	86,27833341	61,29586685	87,54229711	74,5145743	683,9819559	75,9979951
ENSG00000155099	PIP4P2	82,77360757	45,25129988	27,81739484	107,995054	94,41235249	67,85673168	63,65388182	135,3767841	58,70883863	683,845945	75,98288278
ENSG00000162385	MAGOH	24,27968817	52,92017463	4,078944959	201,9709451	0,790427459	82,52925239	62,32092059	158,7928406	95,71845828	683,4016522	75,93351691
ENSG00000113580	NR3C1	30,40025999	98,11286806	97,40378973	155,0912495	32,49737393	69,14600828	62,08459938	69,83094888	68,54878275	683,1158805	75,9017645
ENSG00000086666	ZFAND6	96,91838464	95,01791037	90,62809354	65,57554669	20,41945273	75,37292064	86,97458294	93,30143523	57,3444538	681,5527806	75,72808674
ENSG00000010270	STARD3NL	55,84974403	63,27361824	176,5186415	51,25079768	28,32964973	87,26713177	91,3613319	72,19706512	55,26668659	681,3146666	75,70162962
ENSG00000136603	SKIL	60,66170943	68,36208278	10,50194668	240,3136678	28,1497945	68,82027006	72,151084	45,56107568	86,69016652	681,2117975	75,69019972
ENSG00000198937	CCDC167	44,64630497	75,00475527	128,7844864	46,98654263	0	134,0046966	105,7284965	90,11851841	55,88391928	681,1577201	75,68419112
ENSG00000163320	CGGBP1	70,46376997	29,79816044	95,03740015	115,5050645	94,96910587	94,21259023	60,98982908	53,52324989	66,4968623	680,9960324	75,66622582
ENSG00000186141	POLR3C	68,20918509	72,32401495	108,1692283	95,08270183	132,1613813	52,92158268	43,01839954	47,20493822	61,22236181	680,3137937	75,59042152
ENSG00000138073	PREB	93,90308189	93,08167189	86,71732851	17,95349607	0,447219853	80,57923402	105,0794499	78,287891	123,8783948	679,927768	75,54752977
ENSG00000154122	ANKH	116,2462255	24,35350343	136,6799352	37,28523387	122,4592234	66,56026523	54,0031425	71,03236981	50,28616022	678,9060592	75,43400658
ENSG00000104783	KCNN4	126,73933	35,52645183	27,35703089	86,92722127	107,5031656	76,5703631	51,27180723	86,20032196	80,69364275	678,7893346	75,42103718
ENSG00000170791	CHCHD7	62,10835081	107,1013819	89,94310056	102,7103403	0	86,32101604	68,03974192	85,73498325	75,71994344	677,6788582	75,29765092
ENSG00000077585	GPR137B	112,9235474	72,7598145	96,51584711	35,96762979	0,311328602	113,6275298	84,02759555	93,95972098	67,10453102	677,1975447	75,24417163
ENSG00000168995	SIGLEC7	170,9332006	94,49938329	13,74253059	37,16694939	82,26481146	73,33198765	75,17165265	39,30278997	90,51262343	676,925929	75,21399211
ENSG00000164062	APEH	118,2248126	51,14392047	69,79342146	48,70786193	0,081156507	97,14673542	116,1415774	50,55462197	125,0316603	676,825768	75,20286311
ENSG00000107290	SETX	34,88698868	21,511433	90,64518671	81,56232836	150,7932815	61,9995294	57,82114386	96,67272744	80,25642997	676,1490489	75,1276721
ENSG00000134851	TMEM165	64,44925871	74,41625315	139,3450184	97,92466059	14,31782483	77,23504571	75,30356191	78,29536432	54,58318688	675,8701745	75,09668606
ENSG00000162236	STX5	98,35242725	98,01380154	99,14664585	70,63857753	72,07808892	56,38166253	74,65957283	39,52916847	66,94427231	675,7442172	75,0826908
ENSG00000166401	SERPINB8	93,40116561	63,79110977	162,676152	33,88419404	29,19207016	57,50888088	73,70419809	72,75416858	88,80600689	675,717946	75,07977178
ENSG00000111335	OAS2	115,6970461	35,95773948	13,75699724	92,06039174	183,5718982	50,61543935	73,29117893	69,31529721	40,8541924	675,1201807	75,01335341
ENSG00000152332	UHMK1	33,38223355	32,29997709	74,60495304	195,7576615	119,446968	66,43240328	42,04504313	60,52726615	49,79754349	674,2940493	74,92156103
ENSG00000124570	SERPINB6	102,7931918	79,4615184	62,85664688	48,46697657	67,41104992	75,33277757	66,69383529	99,64851248	71,51733453	674,1818434	74,90909371
ENSG00000197872	FAM49A	96,607584	16,73426579	133,938649	79,79399187	50,55858205	73,36244113	73,4904239	35,66364275	113,868519	674,0180995	74,89089994
ENSG00000171310	CHST11	40,78030188	50,0597819	52,71002801	75,40265132	116,6080991	71,91502816	86,37198301	67,7971109	112,1892361	673,8342204	74,87046893
ENSG00000182782	HCAR2	69,9190305	94,65373382	77,79647821	136,5105937	44,82403259	24,34865119	29,24174485	145,1592127	50,74307775	673,1965554	74,79961727
ENSG00000167565	SERTAD3	72,12095418	110,710144	86,34230347	28,09851729	0	89,86822905	109,1075885	89,76090525	86,78871684	672,7973585	74,75526206
ENSG00000184743	ATL3	79,75059196	84,53529371	12,72856243	145,6085825	111,6572043	67,04097119	64,98042457	58,91300513	47,51313063	672,7277665	74,74752961
ENSG00000062485	CS	72,45092038	65,89398002	129,8051111	80,79567562	59,07682739	64,04702938	72,38899173	46,97632502	81,1790152	672,6138758	74,73487509
ENSG00000064419	TNPO3	60,49593419	39,45288624	172,1621828	39,95497045	0,076800159	68,37247248	60,91053329	142,886804	88,03465501	672,3472386	74,70524873
ENSG00000151414	NEK7	47,01771881	33,1858911	91,08002143	195,4859381	0	68,84041714	63,93143613	97,90653852	74,65891645	672,1068776	74,67854196
ENSG00000158019	BABAM2	100,9037272	87,82599118	70,56039659	18,8470132	61,82919951	70,48264771	82,08097486	89,03808806	90,40293684	671,9709751	74,66344168
ENSG00000130560	UBAC1	48,18900374	75,87905541	11,01096062	51,15341563	170,3367195	92,22823599	113,2560331	46,03234156	63,69563586	671,7814015	74,64237794
ENSG00000000938	FGR	120,1020739	36,62968777	137,6749455	22,99190492	100,2386044	47,87957506	47,78408859	52,66239855	105,4522402	671,4155189	74,60172432
ENSG00000134046	MBD2	75,55528533	79,98044885	51,96849195	81,59380669	64,8003163	60,51680198	61,41117375	134,5913574	60,56517864	670,9828609	74,55365121
ENSG00000069974	RAB27A	77,61845839	69,7753054	111,0828916	120,6178091	135,814755	27,95280821	38,20317444	61,03868952	28,62494177	670,7288334	74,52542593
ENSG00000172893	DHCR7	163,7152264	79,47574185	0,200466418	60,52938714	70,01065549	73,70258311	121,7001898	18,58694442	82,55874355	670,4799382	74,49777091
ENSG00000128805	ARHGAP22	102,166162	38,8199092	66,39064726	57,47263041	141,6245652	78,29570338	50,99833486	35,26973712	99,3482957	670,3859852	74,48733168
ENSG00000155304	HSPA13	147,6175259	46,74567976	232,9999337	0,314152389	0,086471838	63,91931681	79,27622229	56,49134191	42,6019478	670,0525924	74,45028804
ENSG00000121579	NAA50	70,36804766	61,47523954	101,6842394	44,8671974	0,111561834	101,845076	80,06090771	126,9796707	81,9248882	669,3168284	74,36853649
ENSG00000196843	ARID5A	60,80916814	61,29390286	73,30056527	3,297409115	48,40674539	118,9002504	125,1179862	63,09899071	114,8167225	669,0417407	74,33797119
ENSG00000173744	AGFG1	68,2032154	46,14309282	83,74295869	39,22002998	201,2306538	37,66406827	45,33677483	105,9262405	41,47202729	668,9390615	74,32656239
ENSG00000197992	CLEC9A	86,05421718	87,84771161	0,0972979	82,01596332	51,78733897	109,058475	55,37909998	0	196,5146461	668,75475	74,30608333
ENSG00000093144	ECHDC1	68,10092314	94,43549298	104,2670244	52,68348853	31,06531242	76,67079681	80,86160579	95,41618326	65,12486483	668,6256922	74,29174358
ENSG00000141552	ANAPC11	63,11596222	62,99039784	59,84821103	101,2119271	65,9021266	69,75083346	89,95104258	76,72121198	78,50134949	667,9930623	74,22145136
ENSG00000130312	MRPL34	80,21587233	51,8471483	70,23696451	73,51370874	0	123,6948962	107,3865297	83,29459739	76,49910229	666,6888194	74,07653549
ENSG00000100380	ST13	49,86167077	65,25453562	97,7804599	132,0619359	55,05069658	72,64097513	68,8353442	66,54341184	57,86719556	665,8962255	73,9884695
ENSG00000177889	UBE2N	69,71796063	100,6996158	31,00080378	121,7901066	40,04938665	55,28494864	66,76016321	90,12860894	90,33252191	665,7641161	73,97379068
ENSG00000120805	ARL1	78,35703147	50,58565392	98,0675486	109,079808	133,9686718	58,93439533	70,07015907	31,46995881	34,98509001	665,5183171	73,94647967
ENSG00000059573	ALDH18A1	42,73225236	45,79600002	140,7278873	150,7022692	0,095535393	86,99381396	66,45688508	45,00731539	86,76792939	665,2798881	73,91998756
ENSG00000127870	RNF6	81,78068705	119,5229987	12,1405086	120,0700333	0	75,93340892	85,13452964	80,04223202	90,62214481	665,2465431	73,91628256
ENSG00000133935	ERG28	83,50394239	72,40750553	47,19221582	59,65926878	94,9281464	84,56209416	71,67010229	71,6589966	78,77125669	664,3535287	73,81705874
ENSG00000205581	HMGN1	87,63334165	70,35935464	86,00732932	68,72702227	59,54428112	67,98319149	63,00890135	61,14546836	99,92660093	664,3354911	73,81505457
ENSG00000148943	LIN7C	63,91459081	105,1081198	128,713627	41,49523553	97,30735555	96,59271287	28,2450768	25,86425053	76,57245187	663,8134208	73,75704675
ENSG00000088448	ANKRD10	36,49437845	70,57605569	79,44029127	130,0386001	32,74006084	112,38869	53,20734	47,36011553	101,3338039	663,5793359	73,73103732
ENSG00000114354	TFG	54,33563188	79,17494153	48,72447648	19,34013233	172,1490943	81,5594061	65,952354	44,34919075	97,87470093	663,4599283	73,71776981
ENSG00000143390	RFX5	50,61182655	30,87199652	147,2942958	69,24506413	160,7650053	64,685589	40,68346788	22,48050263	76,63230999	663,2700578	73,69667309
ENSG00000161638	ITGA5	64,5950103	46,85130436	169,9206538	98,25263841	49,66260473	54,62936638	78,56553499	15,84367288	84,83039145	663,1511773	73,68346414
ENSG00000011007	ELOA	46,92248104	26,80922613	98,12578897	52,27411845	213,8984286	60,93543719	44,1042996	45,54125091	74,359293	662,9703239	73,66336932
ENSG00000113407	TARS	130,2066775	57,39870726	115,0228951	99,61893137	0,879266456	113,8567861	64,88558442	19,82145097	61,07128635	662,7615855	73,64017617
ENSG00000100994	PYGB	86,27138505	47,29793808	95,82078816	76,71113481	0,241775724	95,98686417	99,11480445	63,32834844	97,76127653	662,5343154	73,61492393
ENSG00000135521	LTV1	42,2424585	69,60404859	82,50893386	115,6112327	100,7777237	30,17369905	52,81535832	111,9875383	56,26546087	661,9864538	73,55405043
ENSG00000174917	MICOS13	62,75897487	77,81035386	8,84618438	124,556339	59,85292373	74,18547362	87,16601274	99,98586428	65,95847446	661,1206009	73,45784455
ENSG00000165512	ZNF22	149,0986372	73,44260202	0	183,262462	0	49,22881591	44,92589634	110,9304825	49,57674703	660,465643	73,38507144
ENSG00000104687	GSR	2,81077408	62,45728431	141,9226546	73,81808415	106,1799508	62,8267703	94,95617019	62,1972895	53,0916356	660,2606135	73,36229039
ENSG00000197771	MCMBP	150,1031669	44,82440435	30,53148087	85,19526921	16,75881864	60,44675345	112,7978285	89,38255051	70,15706216	660,1973345	73,35525939
ENSG00000129824	RPS4Y1	125,7738284	100,1030348	178,0669184	23,08410382	67,86473241	0	0,058115022	0,765249114	164,4126251	660,128607	73,347623
ENSG00000167460	TPM4	77,02577745	77,69035475	57,48314141	82,37347148	37,19312823	90,95097879	62,3717788	79,73762791	94,49494743	659,3212063	73,25791181
ENSG00000099624	ATP5F1D	47,10027125	68,07873813	94,30805492	64,97269265	33,05350434	102,7627538	95,12090532	70,23020154	83,62680326	659,2539252	73,25043614
ENSG00000108883	EFTUD2	132,6849933	76,27244127	59,74121698	27,83340663	53,02050586	88,92793838	82,63765489	41,72334439	96,31876295	659,1602647	73,24002941
ENSG00000174695	TMEM167A	73,67610968	141,3188259	6,570958147	65,32760515	10,62958399	73,52583831	112,9104036	87,95740286	87,05945	658,9761777	73,2195753
ENSG00000101079	NDRG3	43,24728909	53,79290431	65,65787614	92,12765514	131,7498714	76,59368648	54,88364829	37,60467011	103,2987418	658,9563428	73,21737142
ENSG00000136044	APPL2	46,09347596	67,61520953	132,543184	43,6430156	0	85,14003848	73,26500715	81,89425897	128,6461186	658,8403083	73,2044787
ENSG00000125970	RALY	76,83247437	63,74306032	99,60367715	61,96033471	70,45644316	77,26888166	72,51326788	54,14148178	81,58150279	658,1011238	73,12234709
ENSG00000117153	KLHL12	4,609130861	64,28375772	42,31510059	42,91889405	216,8218229	60,2064322	77,4711343	52,9373509	96,52575929	658,0893828	73,12104253
ENSG00000139168	ZCRB1	77,10481949	48,72309518	70,00811353	67,85593679	0,294600117	89,76048018	100,3042154	124,2856843	79,50694123	657,8438862	73,09376513
ENSG00000130726	TRIM28	87,41672818	42,03340689	112,0054509	51,87188534	68,22065714	74,47866808	97,96359874	27,93269451	95,55601494	657,4791048	73,05323386
ENSG00000169738	DCXR	93,35169323	93,18709887	20,16890308	64,20482025	49,27953657	89,41027399	88,06671568	56,46226837	103,1829272	657,3142372	73,03491525
ENSG00000153066	TXNDC11	63,54952937	48,91779731	54,66337454	82,43891555	140,2332248	86,39565213	85,36192205	32,48913968	63,21899406	657,2685495	73,02983884
ENSG00000196290	NIF3L1	123,6634926	119,2041205	108,3745795	61,2732404	0,180189434	43,12176555	51,18282986	96,16259366	53,99153858	657,15435	73,01715
ENSG00000198911	SREBF2	98,59384772	39,4973592	2,023412579	56,32410582	130,0351503	68,88055573	88,0417814	74,56206162	99,19521232	657,1534867	73,01705408
ENSG00000054611	TBC1D22A	51,48847478	72,49671762	100,2088184	77,17587584	52,83723765	70,89048348	77,28878024	43,20828305	111,3680477	656,9627187	72,99585763
ENSG00000099282	TSPAN15	59,22439944	99,02339422	36,59564487	85,43627233	0	144,068709	130,4399227	27,67124632	74,24046288	656,7000517	72,96667241
ENSG00000164022	AIMP1	141,5299425	75,2537945	96,55799111	66,17461495	55,4646685	60,47161344	33,89357827	68,70814926	58,63547127	656,6898238	72,96553598
ENSG00000080546	SESN1	52,81935589	32,77814725	70,40425471	49,88387818	134,1518958	61,75074384	52,87345762	126,6633579	75,30395113	656,6290424	72,95878249
ENSG00000143393	PI4KB	64,39370909	31,69933835	21,60255901	34,38640886	178,5056406	95,97026761	76,37224571	69,0424133	83,99881304	655,9713955	72,88571061
ENSG00000133706	LARS	129,3157768	82,5052308	55,99352586	58,15710479	17,35380629	67,55456544	70,1285115	53,97260872	120,8048215	655,7859517	72,86510574
ENSG00000122203	KIAA1191	62,37127464	38,7683658	18,89185139	12,11899515	308,6092761	50,19545918	62,09316351	31,9943205	70,61290062	655,6556069	72,85062299
ENSG00000155957	TMBIM4	107,7900155	86,01025722	63,49799215	131,1297588	14,23622841	71,74619814	62,78065923	56,15071331	61,18514946	654,5269722	72,72521914
ENSG00000096401	CDC5L	85,78509083	22,85716136	120,9732333	5,142605652	137,6972086	74,08953329	70,54564893	52,31042344	84,95755937	654,3584647	72,70649608
ENSG00000116906	GNPAT	82,80729524	55,91430851	115,5906207	63,53338541	0	70,21054764	77,87548358	87,041795	100,8164667	653,7899028	72,64332254
ENSG00000148362	PAXX	60,08945657	49,57577698	73,21706202	50,73203835	167,5706214	48,81154898	64,43817491	40,00124992	99,26358636	653,6995155	72,6332795
ENSG00000172115	CYCS	63,26504933	112,8037357	64,24040878	112,9918109	41,09265125	63,69149579	62,2425183	73,91592188	58,79371694	653,0373089	72,55970098
ENSG00000116161	CACYBP	62,47422174	94,5124762	101,739114	92,43495664	18,62857568	62,70838689	60,05108552	82,91995798	77,41201848	652,8807932	72,54231035
ENSG00000135018	UBQLN1	32,72191912	73,80055048	104,237477	77,44072786	142,6115535	47,42718248	43,94293701	40,42013058	90,17629419	652,7787723	72,5309747
ENSG00000250317	SMIM20	58,68189655	149,5407308	13,45064995	76,88840753	0,222777632	84,08743249	91,76769319	107,2067486	70,78769521	652,634032	72,51489245
ENSG00000108669	CYTH1	61,58233213	64,00965207	48,68415971	29,51250615	67,06244536	82,7627174	78,59290721	117,2331049	102,9418124	652,3816373	72,48684859
ENSG00000188554	NBR1	122,776215	46,92883785	25,40020417	124,7396464	0,299086591	101,638734	103,0931723	59,20695523	67,97051868	652,0533702	72,45037446
ENSG00000172500	FIBP	96,30876514	74,05293531	56,1266111	17,97673103	57,72870723	117,8148175	79,33107779	47,50058501	104,3689238	651,2091539	72,35657266
ENSG00000242485	MRPL20	77,27555385	78,49476692	68,91736462	94,58539195	51,91435379	68,77464094	87,78842167	72,24897483	50,84416963	650,8436382	72,3159598
ENSG00000056097	ZFR	106,922278	57,50143024	33,82891767	99,92680389	105,9160733	47,53603881	49,58774309	77,6430951	71,77501931	650,6373993	72,29304437
ENSG00000156299	TIAM1	25,62780186	42,5663787	85,57178177	110,2801352	95,2347302	75,93433296	84,42290893	50,66233115	80,0979152	650,398316	72,26647955
ENSG00000102531	FNDC3A	36,09929065	44,72516603	46,87167686	202,0907143	29,34332738	76,83439932	96,49056449	61,29651369	56,61341839	650,3650711	72,26278568
ENSG00000142185	TRPM2	18,14228328	45,35019062	69,11153244	44,44261611	138,5052893	76,48465214	121,8400196	13,99603537	122,2346254	650,1072442	72,23413825
ENSG00000164535	DAGLB	44,60604247	42,13324866	159,2001512	41,00080616	48,88774242	104,249323	89,24530176	31,97380855	88,41936904	649,7157932	72,19064369
ENSG00000165795	NDRG2	46,69416707	54,51628744	78,0185205	59,58303261	36,57766756	131,0583681	107,7216811	1,794412883	133,7356497	649,699787	72,18886522
ENSG00000103653	CSK	81,40792669	62,89895494	88,56433788	37,8555564	17,75942332	93,77396491	77,20406424	54,51808838	135,623228	649,6055437	72,17839375
ENSG00000174684	B4GAT1	10,84201404	44,75713504	99,03127592	91,78878529	223,3077692	42,65563407	73,9568524	30,95251554	32,13190993	649,4238915	72,15821016
ENSG00000100364	KIAA0930	94,77780478	53,87462283	66,47772404	58,78133922	100,7206601	81,85407532	83,69280654	26,87934649	82,22952722	649,2879066	72,14310073
ENSG00000163719	MTMR14	68,18679347	48,38049711	62,62518927	88,2210788	92,1808611	68,9111882	70,9171074	68,84282234	80,92768481	649,1932225	72,13258028
ENSG00000145592	RPL37	31,57534312	81,64323585	47,81672598	138,9705922	47,61434081	80,39646851	67,21401989	105,9527755	47,82542967	649,0089315	72,1121035
ENSG00000107341	UBE2R2	43,84467976	90,55856693	125,7336834	158,2395479	30,72191618	35,68726499	61,68420834	38,30590586	63,4709253	648,2466986	72,02741096
ENSG00000060688	SNRNP40	46,68993964	118,5840232	35,61463578	71,69502261	67,81380913	72,81796858	75,34030226	55,17160646	103,9918153	647,7191229	71,96879144
ENSG00000173163	COMMD1	63,17331549	67,03550002	57,98999557	52,03427378	99,23774895	83,09400957	53,60814352	93,93624829	77,30655922	647,4157944	71,93508827
ENSG00000136807	CDK9	84,72265995	58,27619469	82,5600894	42,44542254	7,421762542	103,4175928	78,46078094	70,05212831	119,9329557	647,2895869	71,92106521
ENSG00000178927	CYBC1	79,59561909	46,68494129	58,42306626	67,09962522	70,51347626	92,87049139	95,14732376	51,09248045	85,69432704	647,1213508	71,90237231

ENSG00000138663	COPS4	147,4239134	88,65379083	39,44417557	50,63572698	0,302117955	88,72447273	66,60553519	82,46964809	82,70847752	646,9678583	71,88531759
ENSG00000174173	TRMT10C	31,07321512	171,8274453	0,295409424	63,50064738	16,79189359	96,88401902	59,73507836	112,5443984	94,30575386	646,9578603	71,8842067
ENSG00000115241	PPM1G	108,7088311	37,51974419	108,9654155	136,6397915	0,269675251	64,57845977	72,70683819	24,06920139	92,71392212	646,1718789	71,79687543
ENSG00000062194	GPBP1	60,37885286	52,49318821	137,1860995	101,398557	0,776830283	59,60168276	48,99003847	83,98715444	100,733869	645,5462726	71,72736362
ENSG00000106245	BUD31	109,1000498	73,64304299	81,38002055	59,35336406	27,0775232	79,81039536	64,42642574	97,84983191	52,68521882	645,3258724	71,70287471
ENSG00000241837	ATP5PO	63,20902735	78,75085239	55,68371068	52,92781977	62,19417374	98,44725387	64,71419332	94,02270659	75,15938219	645,1091199	71,6787911
ENSG00000101017	CD40	89,21922099	61,65037675	34,21124289	20,85827656	80,00917149	103,5381794	100,3961264	85,77961072	68,39622703	644,0584322	71,56204803
ENSG00000146409	SLC18B1	83,10018724	88,34354926	159,9069768	0,733994303	1,136447919	78,93003174	60,35281792	111,0743483	59,72704689	643,3054003	71,47837782
ENSG00000159479	MED8	94,89117936	81,51358139	95,19584751	66,00574062	0	75,71510786	73,08275625	56,94619396	99,59009274	642,9404997	71,4378333
ENSG00000107021	TBC1D13	115,7835255	77,48901351	104,1629829	99,43540879	2,832013181	58,37047255	48,57945696	27,40312109	108,7847489	642,8407434	71,42674926
ENSG00000130826	DKC1	160,3504985	58,88533573	128,5732364	50,10100512	0,170886292	37,18481162	49,8239315	62,18742396	95,37733727	642,6544663	71,40605182
ENSG00000159256	MORC3	59,11894517	58,66652374	75,56824788	69,63152412	128,112157	89,47986159	44,36887833	30,8880003	86,65877162	642,4929097	71,38810108
ENSG00000101557	USP14	75,66886435	79,22886223	90,7718546	56,39717006	0	66,75773831	71,29615324	109,500949	92,75253191	642,3741238	71,37490264
ENSG00000167862	MRPL58	15,41109611	31,46070398	0,250884566	49,6906856	151,0368003	75,46834897	101,1236521	119,8869099	97,95520669	642,2842881	71,3649209
ENSG00000134825	TMEM258	37,33435872	96,83876666	48,2025465	110,3105157	72,57264546	54,18591847	57,00966193	108,672907	57,12296953	642,2502899	71,36114332
ENSG00000243317	STMP1	65,54045829	94,12686343	81,29819271	49,3946628	82,75570443	79,22280477	31,91432239	102,9343191	54,95542683	642,1427547	71,34919497
ENSG00000112773	TENT5A	79,28748925	54,44350208	2,066595813	50,82477037	169,3245442	31,54850054	29,9113975	179,9293465	44,76527829	642,1014245	71,34460273
ENSG00000116514	RNF19B	56,68093819	89,58613949	3,408185122	66,98330766	0,137592928	137,2417376	79,4645436	36,96815851	171,4629983	641,9336014	71,32595571
ENSG00000180530	NRIP1	24,06173969	40,70885961	82,99817714	72,01277103	95,77031317	79,45041033	83,87330655	94,30432226	68,63438706	641,8142868	71,31269854
ENSG00000008952	SEC62	132,1455079	102,819334	65,35758129	90,05021323	28,3108557	58,26123178	54,56883259	57,93441585	52,15798588	641,6059582	71,28955091
ENSG00000139793	MBNL2	99,60120132	36,76845274	148,2787081	75,97312117	45,14578721	54,42461868	57,00872868	37,09646428	86,8646948	641,161777	71,24019744
ENSG00000179021	C3orf38	28,60853701	71,91043513	17,40041123	153,8867816	40,2202832	68,42093129	61,09893123	116,0931512	82,85823776	640,4976996	71,16641107
ENSG00000122223	CD244	30,18026974	47,14992594	184,9351853	56,26946666	142,5779928	65,87034665	27,49142269	40,60376306	45,09463901	640,1730118	71,13033464
ENSG00000196776	CD47	106,6846506	58,62754718	121,4220316	20,20121279	52,89869486	55,4715601	58,58738643	98,47175007	67,7838373	640,1486709	71,1276301
ENSG00000125657	TNFSF9	10,26516117	103,7122057	0	69,62524414	193,1850182	75,44159026	91,76073852	52,78055209	42,42396133	639,1944714	71,02160793
ENSG00000136819	C9orf78	66,08679596	106,5942016	32,05575568	64,63245203	53,17390557	73,79587918	67,20130941	53,78715259	121,6986738	639,0261259	71,00290288
ENSG00000144827	ABHD10	76,75378292	11,25381164	103,0712727	105,0293454	129,832978	73,03135762	34,20787307	34,61591471	71,12038096	638,9167169	70,99074632
ENSG00000132463	GRSF1	21,07656486	42,63374614	154,72884	61,35479819	90,93992573	75,74924469	66,96832999	46,81441132	78,62922278	638,8950837	70,98834263
ENSG00000016864	GLT8D1	75,65514693	53,44410998	46,95460831	30,34929654	74,04485409	69,95172346	80,03169736	147,6168171	60,67154507	638,7197989	70,96886654
ENSG00000182247	UBE2E2	102,5742843	101,3495681	33,62178303	41,06042488	3,656550107	88,8678683	89,54423879	71,47011164	106,1406704	638,2854995	70,92061106
ENSG00000126561	STAT5A	85,14085334	37,46718249	163,4352928	34,23807887	4,743297396	74,24743396	84,38828378	73,56390007	81,0050535	638,2293762	70,91437514
ENSG00000077721	UBE2A	101,4562234	94,49598044	32,24505085	79,77885146	0	80,83639967	68,00024523	104,581557	76,41586406	637,8101721	70,8677969
ENSG00000171566	PLRG1	85,9055308	79,02569813	122,7214236	68,57672912	102,4720245	50,94761478	56,74311326	25,23558113	45,76679063	637,3945059	70,82161177
ENSG00000125844	RRBP1	47,08674659	20,01461061	86,34109665	44,33651574	98,52580138	57,23677202	122,4144643	90,23041342	70,58440266	636,7708234	70,75231371
ENSG00000158195	WASF2	53,65076941	47,01098963	97,7162925	92,64316912	91,28071311	62,87721733	69,72537196	68,75086407	53,09433298	636,7497201	70,7499689
ENSG00000128989	ARPP19	34,3423185	109,0090074	87,6901223	46,73968968	0,103836213	98,84595729	113,7341306	78,34504709	67,65383014	636,4639393	70,71821548
ENSG00000091140	DLD	83,90753561	19,1304759	76,16938032	87,81367138	1,126201622	56,81022687	67,01424044	153,1708648	91,13837686	636,2809738	70,69788598
ENSG00000107263	RAPGEF1	30,49403547	34,96338847	48,98992319	69,05348585	71,75170548	121,0327372	100,5626254	31,71265772	127,6666341	636,2271929	70,69191032
ENSG00000160710	ADAR	60,62510564	35,22286308	21,71405788	88,63873041	90,71507003	101,2480808	100,9355803	80,36676046	56,59200157	636,0582501	70,6731389
ENSG00000174851	YIF1A	53,87140174	75,58288361	92,58100525	63,98192341	7,503948646	100,7491093	88,87258911	80,3188501	72,27805045	635,7397616	70,63775129
ENSG00000182512	GLRX5	24,74214118	96,90391377	50,75348541	40,75743112	106,6224194	72,39481613	77,49947224	95,06220598	70,89873609	635,6346213	70,62606904
ENSG00000176485	PLAAT3	49,85290971	61,95835689	67,17555024	111,8771265	71,10896137	68,8986798	79,28468668	53,65850166	71,57118958	635,3859624	70,59844027
ENSG00000166888	STAT6	72,76000895	33,4718227	64,29575323	53,95325797	131,778128	58,4028873	60,22507166	67,46334689	92,64314169	634,9934184	70,55482427
ENSG00000135972	MRPS9	24,53791115	82,24585131	179,9499383	70,47669626	76,77143743	57,14798432	42,25791389	42,99804651	57,91827102	634,3040502	70,4782278
ENSG00000109113	RAB34	90,1264984	127,7794454	97,87794584	45,18764925	0	68,86596835	75,17583622	76,33108538	52,89001845	634,234177	70,47046411
ENSG00000132205	EMILIN2	88,34443936	43,60571713	157,3172271	44,89420725	0,832704148	53,01239172	88,61863455	115,2868788	42,00148641	633,9136865	70,43485406
ENSG00000162961	DPY30	90,3890753	56,28521984	66,00114644	99,96921596	67,21098958	65,08565376	62,48192313	51,89944767	72,63359494	631,9562666	70,21736296
ENSG00000072849	DERL2	50,44910326	56,87987907	136,9649712	19,86752274	156,8009698	55,70207831	40,13949835	50,99139104	63,21052794	631,0059418	70,11177131
ENSG00000185963	BICD2	61,96283326	69,44744729	66,17547332	51,67710338	73,34953525	81,35999434	80,98751889	46,89014102	98,94713259	630,7971793	70,08857548
ENSG00000175602	CCDC85B	29,0520979	38,53221655	9,484885267	95,88161796	70,6924296	107,3410949	124,2353878	49,65191336	105,4837544	630,3553978	70,03948864
ENSG00000125445	MRPS7	73,35244112	65,36313858	21,05530055	50,82232147	31,30270132	104,2239571	76,7831668	102,7952442	104,5700901	630,2683612	70,02981791
ENSG00000101109	STK4	88,79318027	54,87925954	138,7769679	52,71846563	32,08934675	64,22403511	68,40963459	29,85200594	100,392126	630,1350217	70,01500242
ENSG00000101997	CCDC22	57,20140868	85,56585563	130,1749646	64,7393515	54,5766987	46,47086114	38,93734276	31,89675522	120,018157	630,0313952	70,00348835
ENSG00000113328	CCNG1	40,90137539	22,08533915	6,464653465	128,5290169	2,899850956	69,66529384	60,46567679	203,2129898	95,62451873	629,848715	69,98319056
ENSG00000114503	NCBP2	108,0332665	55,61152809	61,45911013	93,8875794	0,140527311	81,54321438	54,10616547	117,7953007	56,76118593	629,337878	69,92643088
ENSG00000163694	RBM47	110,0963603	28,21391129	143,3822912	11,75795473	44,7215666	52,27432952	58,91701451	116,1780713	63,68897673	629,2304061	69,91448956
ENSG00000110756	HPS5	71,78515528	70,69311863	1,72846323	88,91177745	125,2696666	68,57705945	41,60239673	52,23876188	108,1913922	628,9977914	69,88864349
ENSG00000147400	CETN2	96,56028167	124,7527874	54,35606707	128,4373915	4,656607136	70,82178663	86,94417197	0,342774448	60,92064423	627,7925121	69,75472356
ENSG00000131051	RBM39	79,55586679	70,38278203	58,61896022	99,83998232	52,95298882	55,03015861	55,13504452	64,22655803	91,47522774	627,2175691	69,69084101
ENSG00000074201	CLNS1A	81,61023851	40,99209148	44,07001569	136,1498692	29,95253021	66,94954238	58,49999582	82,96843562	85,8406955	627,0334144	69,67037937
ENSG00000103051	COG4	20,58442802	61,04592929	0	51,54271103	375,4239886	28,95363485	39,02871986	34,08751659	23,87733096	626,4433592	69,60481769
ENSG00000198286	CARD11	34,99267517	49,84581476	132,1099387	45,56799429	67,59680105	61,57760304	67,09344076	1,053010094	166,3813989	626,2186768	69,57985298
ENSG00000156110	ADK	100,3290661	78,15193972	51,16091018	88,83590216	88,087563	81,70470612	56,19368512	27,50162073	54,22469196	626,1900851	69,57667612
ENSG00000134375	TIMM17A	95,17723443	93,35994295	114,1590646	49,29764646	42,12251995	51,37356613	42,57540661	66,01147182	71,70790471	625,7847577	69,53163974
ENSG00000160075	SSU72	51,67330686	63,59604309	66,17043258	87,45602054	69,96537752	88,69825455	65,99024977	54,82568052	77,22340678	625,5987722	69,51097469
ENSG00000121067	SPOP	133,5540564	42,58650881	56,88259753	130,0745752	0,056921468	46,59907147	63,30196018	80,71004415	71,72868628	625,4944215	69,49938017
ENSG00000225973	PIGBOS1	51,85770093	103,7616701	102,1902401	65,89803839	50,83232015	66,22906932	75,00110477	45,42634075	63,96754025	625,1640247	69,46266941
ENSG00000123143	PKN1	106,0332967	46,689076	41,64269828	38,86265579	79,91651778	82,37695982	82,59358372	55,59852146	91,10756563	624,8228751	69,42476939
ENSG00000104164	BLOC1S6	59,96063895	62,82363948	41,0114503	51,3289586	156,3099696	59,47645747	62,4957188	67,76528051	63,56758616	624,7396999	69,41552221
ENSG00000125827	TMX4	226,1242823	30,30245876	114,8464371	10,14769232	0,711991467	61,40993894	53,13880006	84,51102177	43,42181975	624,6144425	69,40160472
ENSG00000126581	BECN1	82,55245395	31,93958342	89,53958287	62,04019898	94,67847897	64,58975835	61,83531641	63,83043797	73,30709407	624,312905	69,36810055
ENSG00000124784	RIOK1	0	108,6248715	175,9595116	51,83198135	0	70,20161817	28,55090208	90,58852429	98,16994867	623,9273577	69,32526196
ENSG00000132334	PTPRE	67,11542035	38,13893335	28,3794547	73,71674316	186,6708008	53,29517324	67,01802068	44,73439939	64,66168719	623,7306329	69,30340365
ENSG00000100883	SRP54	4,452399597	100,3729086	20,65442703	142,4011007	179,0527126	61,50976287	47,73851986	32,17144137	35,09323262	623,4465052	69,27183391
ENSG00000129255	MPDU1	83,04191847	87,69628939	44,35852643	79,47016552	0,218308536	74,4832589	86,49444163	67,69389012	99,758346	623,215145	69,24612722
ENSG00000072778	ACADVL	56,18052752	39,77625105	116,7285727	71,02628354	0,074505714	90,04607234	89,33904137	55,6665703	103,6036526	622,4414771	69,16016412
ENSG00000168297	PXK	39,42873874	52,68684798	77,3609166	143,6263299	79,68465452	64,61030116	75,88213205	17,46600348	71,67969154	622,425616	69,15840177
ENSG00000184293	CLECL1	65,76514667	70,73589078	55,07914803	117,5041286	54,67100296	93,80069212	55,69978713	77,37660123	31,31638086	621,9487784	69,10541982
ENSG00000103018	CYB5B	49,42889592	63,51038246	110,9423689	37,95142902	46,66499138	82,31837248	77,97591254	42,79422039	110,2748595	621,8614326	69,09571474
ENSG00000163479	SSR2	78,5693077	69,10418548	52,13957599	121,6884768	64,87145128	67,7828248	70,18632451	56,3574236	40,92931839	621,6288886	69,06987651
ENSG00000106785	TRIM14	60,95702823	11,54561431	128,5169806	53,3200459	179,3949701	72,073177	65,31050122	17,3627359	32,65283725	621,1338905	69,01487672
ENSG00000186074	CD300LF	25,59299809	57,8223841	48,10946646	45,28024779	114,8311993	127,3695728	85,23389513	29,66418607	86,00024168	619,9041914	68,87824349
ENSG00000275052	PPP4R3B	22,19672128	36,32007376	34,81094339	81,13531217	207,2016878	49,1250375	54,68045015	80,3868444	53,80660539	619,6636758	68,85151953
ENSG00000147894	C9orf72	107,6531561	80,12076187	114,8006884	83,5717196	42,19574408	27,17139419	25,20503195	91,58443872	47,0486379	619,3515728	68,81684142
ENSG00000122545	SEPT7	74,98720048	92,61790889	45,49738462	84,48206449	35,49685592	73,23432205	61,60770474	78,74559334	72,6106759	619,2797104	68,80885671
ENSG00000134697	GNL2	70,12561196	101,6205457	41,88807477	108,923439	0,187463481	95,72985425	63,99052429	40,1903189	96,14089276	618,7967251	68,75519168
ENSG00000035681	NSMAF	69,16308777	50,15168041	4,009761557	134,2736701	4,714509065	68,0582933	77,90548491	84,13496038	125,6045377	618,0159852	68,6684428
ENSG00000197045	GMFB	129,9248532	48,53108801	72,56150608	19,71404567	41,68822163	69,54794313	62,20405762	95,15464393	78,64387852	617,9702377	68,66335975
ENSG00000124731	TREM1	54,50856606	126,3103124	82,24737948	107,3239592	1,706890373	66,21214833	85,87614643	55,02568507	38,51953891	617,7306262	68,63673624
ENSG00000105939	ZC3HAV1	37,03960111	73,92532753	86,014956	69,78530324	140,4920467	50,47176505	34,28700251	61,10693859	62,76069306	615,8836338	68,43151486
ENSG00000065911	MTHFD2	26,19834863	68,00864084	99,6739094	86,36503929	67,77207871	59,38649013	72,57087432	49,81873301	85,67748217	615,4715965	68,38573294
ENSG00000067900	ROCK1	46,78628865	81,56843325	84,00897438	106,3059689	33,07448246	63,56285119	47,88831543	67,99642252	83,51766513	614,7094019	68,30104466
ENSG00000010327	STAB1	80,06519658	18,94316359	37,9374049	19,71552509	30,01690829	97,08934013	212,7236428	55,89424919	62,13606808	614,5214986	68,28016651
ENSG00000180304	OAZ2	72,3345086	81,44581674	109,1482281	69,69831351	0,187259832	77,66717259	76,43369332	65,04447881	62,14265801	614,1021296	68,23356995
ENSG00000135912	TTLL4	83,40046701	31,36163744	38,90637643	36,21553644	91,05111438	51,22852895	81,217378	121,0949292	79,09647309	613,5724409	68,17471566
ENSG00000105968	H2AFV	76,84397308	80,23368754	39,10101203	123,0224064	29,94536677	66,83237171	69,85545542	64,91797252	62,69238338	613,4446289	68,16051432
ENSG00000087245	MMP2	57,83045851	44,27248918	51,39028604	56,66713643	61,73525112	107,8101453	125,2037142	12,70902183	95,33223646	612,950739	68,10563767
ENSG00000117395	EBNA1BP2	60,89403788	78,46221963	95,93865253	67,853352	51,83555652	48,45464751	68,88359985	77,11067835	63,35795508	612,7906993	68,08785548
ENSG00000124541	RRP36	44,60329903	99,05424511	78,44608339	29,75779004	60,06706657	86,73947413	68,90767786	56,45170161	88,1856709	612,2130086	68,02366763
ENSG00000101220	C20orf27	51,18362186	96,37567539	60,20024504	64,93661273	24,23857532	102,5004474	88,01107346	38,58490602	86,11273641	612,1438936	68,01598818
ENSG00000111731	C2CD5	22,9158655	57,8812218	90,68591778	71,76773454	179,5322053	50,2469227	34,10105043	18,57747236	86,25544317	611,9638336	67,99598151
ENSG00000164329	TENT2	134,8692021	37,48535586	90,23754847	72,95567554	0,065199263	61,0266573	50,13801756	80,86888108	83,17205472	610,8185919	67,86873244
ENSG00000169967	MAP3K2	72,35134205	44,5971314	32,60976135	79,26908856	112,060401	59,02310575	38,97779257	93,26133779	78,58265358	610,7326141	67,85917934
ENSG00000101096	NFATC2	120,0532086	41,13063989	109,8366937	52,7475359	0,780125001	60,46945953	81,19381127	42,63028635	101,7827079	610,6244682	67,84716314
ENSG00000120889	TNFRSF10B	36,49576657	63,52985112	132,3318435	67,49934672	81,85872922	54,80678731	69,2466004	18,53335598	86,060665	610,3629458	67,81810509
ENSG00000113161	HMGCR	52,07917882	67,34020684	9,431467644	92,89454534	117,820869	51,6046534	84,99800251	47,41950612	86,73843369	610,3268634	67,81409593
ENSG00000180370	PAK2	63,50500059	54,26988806	47,34972021	62,17493653	147,3126505	62,47446118	52,84962469	48,90642403	71,13742479	609,9801306	67,77557007
ENSG00000047315	POLR2B	96,88416659	47,51998779	63,79716412	43,67372007	31,5774719	58,21374334	69,30579873	90,08282387	108,8705395	609,925416	67,76949066
ENSG00000130202	NECTIN2	57,06019143	62,12696795	62,6235231	63,24639817	61,99624061	80,16203172	108,4704768	55,63168828	58,55506407	609,8725821	67,76362023
ENSG00000122557	HERPUD2	108,1888374	41,55127112	48,47095996	36,7366862	0	78,15073068	76,26400648	145,9775631	74,3157073	609,6557622	67,73952914
ENSG00000155962	CLIC2	20,5382677	58,16037748	36,41147775	48,41997592	244,048189	35,92781855	46,21196031	87,14629967	32,58858031	609,4529467	67,71699408
ENSG00000127952	STYXL1	52,45652946	121,6423275	6,442339572	59,4437664	100,9354039	81,97356896	64,096284	41,87459756	80,18525986	609,0500772	67,6722308
ENSG00000090266	NDUFB2	52,87340858	70,84930292	38,06833377	116,8251708	18,49289795	86,2943926	76,63085714	69,10369863	79,90522838	609,0432908	67,67147675
ENSG00000104408	EIF3E	69,22650789	110,516478	69,25199468	99,50725926	64,34812249	52,88140613	46,69878302	49,74402149	46,73243108	608,907004	67,65633378
ENSG00000096746	HNRNPH3	79,7598322	41,07218799	64,15323334	86,20129907	95,10967109	37,23955993	69,58897997	68,44847825	66,7912962	608,364538	67,59605978
ENSG00000014216	CAPN1	105,6553586	37,56047082	78,79721858	51,37168108	52,72889639	68,24892914	66,94630451	63,9169738	83,09330143	608,3191344	67,59101493
ENSG00000113643	RARS	89,41924901	36,09754757	80,53857126	64,29828757	73,98851326	70,40396143	76,67019409	45,35376417	70,9773812	607,7474696	67,52749662

ENSG00000243449	C4orf48	38,61714584	67,53205928	57,65513164	67,64980823	87,78428813	76,50321037	123,1468073	47,72943753	40,26674649	606,8846348	67,43162609
ENSG00000173801	JUP	86,16543669	25,01284137	133,9839328	34,68925097	185,8080849	21,25078815	33,76680323	51,0829643	34,67229736	606,4323998	67,38137776
ENSG00000146425	DYNLT1	61,45743155	103,6065093	37,37152025	69,09961061	1,824166317	53,03744816	53,83369815	164,6018452	61,36144911	606,1936787	67,35485319
ENSG00000185236	RAB11B	69,17447951	54,92904549	57,78711456	48,93237072	88,49626747	67,47078022	65,85969787	67,54660713	85,80008347	605,9964465	67,3329385
ENSG00000108671	PSMD11	74,26456254	50,46354456	118,4839643	97,66181246	6,439069304	69,49833137	47,43887464	75,20267159	66,38312881	605,8359595	67,31510661
ENSG00000115539	PDCL3	26,23721397	103,0911476	82,1964653	71,535698	1,406465571	73,4251769	67,32154889	105,6872964	74,84810982	605,7491224	67,30545804
ENSG00000015171	ZMYND11	89,69695383	36,22446643	125,4581881	55,04250974	0,067758027	61,76255997	88,56311547	42,33303233	106,5636946	605,7122784	67,30136427
ENSG00000120053	GOT1	0	30,1991609	91,85728518	16,03931981	150,0767617	75,90545774	84,37155163	83,97182079	73,06589758	605,4872554	67,27636171
ENSG00000119321	FKBP15	67,75766716	54,39751023	134,7384704	54,80652911	63,00936819	56,69903978	61,65892507	44,32988026	67,61122459	605,0086148	67,22317942
ENSG00000135241	PNPLA8	5,593728158	47,92807828	120,0513384	62,6924679	95,08100628	87,47374139	66,21860991	62,73189304	57,23142507	605,0022884	67,22247649
ENSG00000159140	SON	73,81192912	84,22825752	49,05147464	72,12703952	91,28916798	60,71924821	51,37561111	67,37918792	54,85997458	604,8418906	67,20465451
ENSG00000132591	ERAL1	56,32916362	47,16426252	24,69555344	71,52414036	0,160320331	95,62078327	106,6497822	102,5232252	99,72657133	604,3938022	67,15486692
ENSG00000171720	HDAC3	62,77152773	66,48292974	55,5960198	130,6372931	0,545297787	75,82941109	57,15568269	60,78275776	94,37455719	604,1754769	67,13060855
ENSG00000136986	DERL1	58,04088787	46,30982774	110,9282103	42,10081126	69,97472964	62,03156448	80,43525166	68,804897	65,18258094	603,8087609	67,08986233
ENSG00000007080	CCDC124	56,40490898	53,75754728	116,4805231	64,88027966	39,99804054	75,61367042	66,09665292	52,71833859	77,76239192	603,7123534	67,07915038
ENSG00000163599	CTLA4	0	31,57560001	271,2254424	183,1422111	0,167811936	30,31356799	44,55955716	0	42,64048712	603,6246777	67,06940864
ENSG00000151366	NDUFC2	56,97826904	105,6493458	51,44201658	69,14436773	36,66056579	67,74802003	58,26576234	79,66602301	78,06845658	603,6228269	67,06920299
ENSG00000198728	LDB1	95,9200074	16,81894266	37,26328209	67,80568336	170,9348015	42,33990345	64,71390959	47,72943753	59,28437449	602,8103421	66,9789269
ENSG00000158042	MRPL17	70,90777217	64,67924343	54,96091588	77,84483645	97,35304202	57,58759033	70,28764205	48,34565509	60,83289064	602,7995881	66,97773201
ENSG00000112062	MAPK14	110,1168721	50,26685308	106,2888921	48,11701791	78,94303992	60,97092768	44,55477096	57,41661873	45,91980397	602,5947964	66,95497738
ENSG00000182944	EWSR1	72,97970326	69,9308935	54,70226236	83,74380262	56,72290447	60,71819382	62,63795869	63,42449282	77,46304783	602,3232594	66,9248066
ENSG00000100575	TIMM9	48,02242712	93,54671472	41,02954851	82,18714595	156,2262755	40,07162234	62,38073003	32,20336887	46,33038302	601,9982161	66,88869067
ENSG00000136689	IL1RN	3,063052108	158,3651923	51,13945924	21,23702957	90,66173827	62,06360025	30,59688072	141,5713212	43,26525304	601,9635267	66,8848363
ENSG00000117133	RPF1	40,25216074	93,32278344	51,95435911	145,1664444	0	72,81228366	42,84620071	66,23120807	89,27125524	601,8566954	66,87296615
ENSG00000004455	AK2	72,03280215	67,60905328	17,57094392	53,67232409	29,53454906	103,3066381	94,82025439	37,79197516	125,3904247	601,7289648	66,85877387
ENSG00000106723	SPIN1	125,653232	46,21252092	10,37535348	78,23259487	3,296000287	63,58269909	96,57863206	88,75837761	89,03803402	601,7274444	66,85860493
ENSG00000101361	NOP56	113,3176643	71,66704366	50,93493935	94,83273117	1,305881972	75,78258867	68,03280224	29,57222493	94,91313299	600,3590093	66,70655659
ENSG00000006715	VPS41	69,53191506	55,06734574	58,73728098	46,85519668	174,462934	46,27175785	72,05803897	23,95565363	53,17435325	600,1144762	66,67938624
ENSG00000155380	SLC16A1	52,39941099	68,19650642	118,4764578	26,28016028	114,1847758	69,58749339	76,88364659	4,416576486	69,48252885	599,9075566	66,65639518
ENSG00000102738	MRPS31	1,920897085	52,39121	180,8702904	24,81584975	82,17096308	73,42558479	40,52384546	58,96172368	84,08592651	599,1662907	66,5740323
ENSG00000181163	NPM1	62,29166157	79,15372521	68,04267313	113,5626091	56,28801604	60,93789729	50,64045531	55,59035044	52,57883585	599,086224	66,565136
ENSG00000054116	TRAPPC3	87,53016611	61,42861811	67,780801	93,43943094	0,101819547	89,517388	72,57144238	66,89262297	59,80601156	599,0683006	66,56314451
ENSG00000147383	NSDHL	84,41296908	99,00834372	56,4037776	115,5643875	0	65,41664522	81,53057145	71,18580362	25,41888807	598,9413863	66,54904292
ENSG00000164163	ABCE1	55,40398761	74,30285995	40,18132547	99,75943784	70,39944147	55,41848333	51,86133806	90,71892428	60,53235783	598,5781558	66,50868398
ENSG00000117500	TMED5	77,82743972	79,21120207	34,88709092	56,09067959	71,2698803	65,69057358	55,60415755	90,86860922	66,8853785	598,3350114	66,48166794
ENSG00000105202	FBL	41,48826239	48,16020956	69,95009039	227,7383829	2,687502656	55,40699334	60,09430307	44,51129815	47,98282521	598,0198676	66,44665196
ENSG00000175573	C11orf68	27,13605318	65,61925706	25,12261458	68,14322987	136,1443265	70,10306058	63,47149424	58,17509992	83,87087772	597,7860136	66,42066818
ENSG00000145050	MANF	80,08923657	32,85667815	65,44139133	97,56318186	16,42627449	94,19933289	81,76895745	68,91564417	60,51905215	597,7797491	66,41997212
ENSG00000158604	TMED4	31,51310919	39,84067924	55,24199898	114,0249146	0,381127665	83,37347633	91,51444486	100,2087319	81,55748152	597,6559643	66,40621826
ENSG00000113070	HBEGF	0,050538652	86,80985153	140,5705383	77,32053094	0,480424806	106,038499	77,04465871	43,32118745	66,00970122	597,6459307	66,40510341
ENSG00000123609	NMI	56,31908338	48,0035822	35,14690907	37,56563858	147,1556366	45,76843495	45,28469454	111,8540002	70,01287089	597,1108504	66,34565004
ENSG00000110344	UBE4A	46,80842951	58,22791608	135,7773841	119,1983978	5,57025385	77,65704913	67,715056	28,08894818	57,2857209	596,3291555	66,25879506
ENSG00000183155	RABIF	107,4564058	36,30399919	157,0644757	5,798554991	109,5086827	40,07798705	22,20256904	79,00513734	38,85186556	596,2696773	66,25218637
ENSG00000136045	PWP1	83,47727317	93,04642157	53,23655961	67,09490609	33,29905885	70,64808437	82,51329965	66,93539028	45,99767705	596,2486706	66,24985229
ENSG00000142512	SIGLEC10	48,65364884	90,35537408	50,01822937	50,50813303	111,1227946	86,48337586	55,03864891	19,00809535	84,05653775	595,2448378	66,13831531
ENSG00000235750	KIAA0040	74,77865244	63,39333629	11,0212374	134,6001893	127,3145892	47,15939405	56,19449524	30,56883664	50,17505227	595,2057828	66,13397587
ENSG00000035403	VCL	31,3654247	50,63890654	153,2896663	34,91493805	117,6332782	44,56605163	31,53082503	68,8515582	61,82484388	594,6154925	66,06838806
ENSG00000138639	ARHGAP24	67,56773801	37,06969455	83,92385199	123,3390223	77,48177724	57,69977744	29,30298224	48,41055354	68,74196014	593,5373575	65,94859527
ENSG00000149313	AASDHPPT	52,63571602	39,08449943	87,8904261	34,55660583	196,1596571	53,47942128	48,65815974	33,192078	47,64581006	593,3023735	65,92248595
ENSG00000183520	UTP11	43,21914281	34,41988407	93,15334284	53,09768191	105,2604213	72,62574786	51,99169924	79,74995773	59,36534873	592,8832265	65,87591406
ENSG00000068394	GPKOW	138,806634	128,0871315	70,75247393	38,55794257	0	68,04535112	57,74056895	53,81021305	35,98114213	591,7814572	65,75349525
ENSG00000104872	PIH1D1	38,01580853	68,74610269	100,393293	51,62298056	51,31790758	78,99554928	80,79671634	44,04471569	77,55228639	591,4853601	65,72059557
ENSG00000186298	PPP1CC	21,50907775	52,10636608	114,9596239	55,99560272	137,2821187	59,43567431	32,9209733	74,59419212	42,14243468	590,9460636	65,66067373
ENSG00000141560	FN3KRP	125,241098	61,02416442	39,09095142	80,30132031	0,121982861	67,17574981	85,66418727	57,3546413	74,86887752	590,8429729	65,64921921
ENSG00000108349	CASC3	54,22230747	31,91765642	129,0808971	90,35201303	0	55,077726	51,70134283	76,17430043	102,0579913	590,5842345	65,6204705
ENSG00000214078	CPNE1	69,06356181	20,9578836	30,51001086	61,90696737	95,45280574	81,39182948	81,37488273	54,67556453	94,9321343	590,2656404	65,58507116
ENSG00000125629	INSIG2	73,45121409	44,80347343	23,99777563	33,16186259	147,5563978	65,56617981	63,65634059	56,13204932	81,8665693	590,1918626	65,57687362
ENSG00000276045	ORAI1	108,5564444	21,33755969	54,07216779	67,82925069	3,810265701	76,79415288	123,3070504	60,91565635	73,0404767	589,6630247	65,51811385
ENSG00000136485	DCAF7	49,11323477	30,19249545	99,73783831	55,86358936	105,9538823	61,59796214	66,5725879	59,17855842	61,22302506	589,4331737	65,49257486
ENSG00000168209	DDIT4	94,1682232	25,32597274	110,1634862	89,30734525	66,26365558	33,52136774	49,66279451	51,1578347	69,48886519	589,0595451	65,45106056
ENSG00000169564	PCBP1	88,07587547	40,91080801	55,5960198	39,96032754	77,1589691	85,60985758	75,0671072	43,61736291	82,83444993	588,8307775	65,42564195
ENSG00000179085	DPM3	42,02914586	42,67854985	44,69133424	109,9115166	97,57373739	77,35380586	64,9540947	79,68679281	29,28128486	588,1602622	65,35114025
ENSG00000197713	RPE	75,14780579	58,45389045	103,9170566	63,32654081	94,08614578	69,41589272	45,47987907	47,74090118	30,33260014	587,9007125	65,32230139
ENSG00000144136	SLC20A1	57,19258628	59,55456765	73,02481027	115,7177495	0,32688653	57,50841763	74,55582808	28,93483105	120,5820145	587,3976915	65,26641016
ENSG00000100796	PPP4R3A	16,86958333	30,70570603	58,44540985	116,873446	0,060743833	106,1917494	73,96212906	93,73118568	90,47129416	587,3112473	65,25680526
ENSG00000111716	LDHB	42,34945929	48,54062355	66,34992641	231,806129	38,76495093	36,04091643	48,03456894	39,656579	35,51207432	587,0552278	65,22835865
ENSG00000178573	MAF	67,23222132	51,83542308	14,41731332	38,9594297	101,5421655	75,68973731	64,82364917	104,4794539	67,7374594	586,7168527	65,19076141
ENSG00000170852	KBTBD2	61,34777601	35,99184372	63,33361018	102,544755	130,9853469	37,97128663	49,51550834	65,74625931	38,89365363	586,3300397	65,14778219
ENSG00000134758	RNF138	21,04983055	51,98091739	65,29882092	98,81806506	70,75832932	53,03338614	29,8390779	149,2031085	46,21554142	586,1970772	65,13300858
ENSG00000197142	ACSL5	43,19992943	53,4258376	61,09524838	64,86414737	127,5901561	48,60600229	47,21428452	82,84266069	57,03505169	585,8733181	65,09703534
ENSG00000151348	EXT2	44,25258483	26,47940296	64,8409879	75,08174354	82,34023105	32,26562105	76,66840188	111,1450361	72,53147473	585,6054841	65,06727601
ENSG00000141504	SAT2	99,2381654	58,42655875	32,31539799	106,4068369	0	66,84893596	58,42045282	88,08906913	75,33401329	585,0794302	65,00882558
ENSG00000125249	RAP2A	40,04174823	39,29379478	135,7025381	62,27542876	0,063865826	93,20852068	80,99898722	46,77679455	86,49481572	584,8564939	64,98405487
ENSG00000178789	CD300LB	70,807048	71,34686074	76,62075496	123,227285	3,167519668	65,73009985	44,82071397	72,62031289	56,27987297	584,620468	64,95782978
ENSG00000096968	JAK2	44,84334763	28,03105187	0,135160502	179,7364088	4,714509065	86,85432135	54,29591352	97,79149437	87,74245047	584,1446575	64,90496195
ENSG00000175216	CKAP5	89,73185679	33,25865286	110,9250641	14,27226905	95,89927343	42,75044141	50,50849509	69,89515448	76,70685693	583,9480641	64,88311824
ENSG00000106615	RHEB	14,67051394	74,30258083	61,44988885	71,56693756	74,40533286	80,77163655	74,79210846	74,91596054	57,02168982	583,8966494	64,87740549
ENSG00000131043	AAR2	136,4429532	72,63440183	0,089642083	72,95408674	62,76740018	47,29601735	84,60447088	48,48362729	58,61267294	583,8852725	64,87614139
ENSG00000100284	TOM1	48,688037	46,0990421	49,11660635	49,17261576	120,0525844	69,65473863	89,02001405	38,45404832	73,33796994	583,5956566	64,84396184
ENSG00000135148	TRAFD1	203,6775111	77,31955056	0,784448714	39,49503207	3,50088933	71,67609317	61,15793504	25,51585552	100,0748718	583,2021873	64,80024303
ENSG00000119541	VPS4B	46,29621655	99,24050386	98,82305257	49,76813073	11,90123026	75,24611787	69,93189999	54,82993055	76,83666182	582,8737442	64,76374935
ENSG00000171497	PPID	64,02379504	110,4114118	52,4803446	72,18233002	0	73,72338027	65,27848354	71,70797848	72,83174521	582,639469	64,73771877
ENSG00000176783	RUFY1	78,00081312	55,61867617	26,89826072	57,838154	121,5924165	60,78608148	54,21951307	53,75651327	73,22371874	581,9341471	64,65934967
ENSG00000116221	MRPL37	104,0997105	30,08576384	50,60132049	47,08908918	87,20822233	80,18902726	68,22357009	36,29724555	78,08616138	581,8801106	64,65334562
ENSG00000161091	MFSD12	76,92642094	45,21411419	41,88093363	62,98940691	55,25929669	80,32079148	88,68309477	40,78899293	89,51548937	581,5785409	64,61983788
ENSG00000141741	MIEN1	62,31363739	70,82002751	64,54815259	46,75565328	48,65804698	86,25395225	58,70065115	70,72629918	72,53214787	581,3085682	64,58984091
ENSG00000164615	CAMLG	42,38891641	84,97544729	102,0498106	81,97036051	16,54157266	58,76795646	44,67460462	98,62809754	51,19125763	581,1880238	64,57644708
ENSG00000106560	GIMAP2	69,97060759	0,127869337	132,030605	64,45604046	114,9107634	38,92608667	39,96861315	75,79352741	44,75560026	580,9397133	64,54885703
ENSG00000149182	ARFGAP2	96,21234036	56,90881246	0,187887867	68,12175325	61,28907026	71,34883706	88,94018972	30,3186837	107,2119005	580,5394752	64,50438613
ENSG00000163347	CLDN1	31,32170594	126,827914	127,88004	39,12586168	32,36809161	55,05962909	67,92274864	3,369922546	96,20170034	580,0776139	64,45306821
ENSG00000116478	HDAC1	72,27855586	97,56149928	10,65380107	62,9638857	69,72277045	52,33336648	71,81483955	50,57331883	92,07978222	579,9818194	64,44242438
ENSG00000118363	SPCS2	55,60301829	72,60681504	59,06145535	84,20924281	59,09072161	45,48084844	55,75524807	90,47085847	57,60993066	579,8881387	64,43201542
ENSG00000189403	HMGB1	56,21486016	85,6061453	67,54454068	129,1112764	34,11056698	59,32769063	44,06786739	49,84856197	53,87520676	579,7067163	64,41185736
ENSG00000155366	RHOC	80,73236049	94,92812615	42,16629605	96,00437745	15,95879843	65,36744916	62,82861461	52,99422309	68,37749404	579,3577395	64,37308216
ENSG00000187446	CHP1	82,69542251	58,76433496	91,20451689	70,07627897	31,20783304	67,80181409	55,05817457	69,48586041	52,59451872	578,8887542	64,32097268
ENSG00000143889	HNRNPLL	75,35149495	47,46276131	107,0776342	23,26948779	59,26771308	44,77795571	40,48385291	113,8584383	66,79446822	578,3438065	64,26042294
ENSG00000184203	PPP1R2	73,88873307	54,85317511	41,83538089	71,15482325	76,61828495	76,59155063	47,41568666	78,36775318	55,64875369	576,3741414	64,04157127
ENSG00000113558	SKP1	63,61055735	83,94266043	58,55191185	85,46636902	48,98463384	57,03135477	59,88111139	65,35727175	52,51899608	575,3448665	63,92720739
ENSG00000108219	TSPAN14	50,87701756	35,67432278	53,03516562	75,51346434	84,37425404	70,27248251	57,30997165	63,30142897	84,98096887	575,3390763	63,92656404
ENSG00000197646	PDCD1LG2	57,76065788	27,21102749	0	214,2645753	20,34746329	44,82742432	83,08098939	55,18886011	72,53122201	575,2122198	63,91246887
ENSG00000140259	MFAP1	88,68807044	38,78037772	108,1921659	0	138,1832206	58,33653626	55,65977134	22,79866985	64,52433436	575,1631465	63,90701628
ENSG00000125810	CD93	57,47222872	97,44780365	35,61607016	54,09294115	99,71007949	68,83761692	75,62775437	47,08482387	38,97185716	574,8611755	63,87346394
ENSG00000136960	ENPP2	37,30043957	6,136702038	0,082621518	50,37219973	147,5109061	166,2064493	77,49363944	64,37802319	24,47020638	573,9511873	63,77235414
ENSG00000198805	PNP	54,69041673	50,79084998	72,93703491	63,54734342	41,03952586	51,42867115	52,23130757	97,23829506	89,80262009	573,7060648	63,74511831
ENSG00000138867	GUCD1	77,0066675	70,09938797	123,2466414	52,025675	6,422724869	78,87071575	57,92856353	50,41516585	57,48972791	573,5052697	63,72280775
ENSG00000159335	PTMS	46,38047218	41,06200925	61,10057621	75,09415771	37,92660513	78,73907898	84,30943761	84,62614666	64,03424728	573,272731	63,69697011
ENSG00000177485	ZBTB33	75,71171449	47,1380817	61,58151261	138,9885146	0,135184469	29,04137706	44,96433505	108,590016	67,0908394	573,2415754	63,69350837
ENSG00000167085	PHB	93,03654989	55,75229705	25,20507411	54,32179383	162,6227954	53,47352355	47,33742593	37,7098334	43,77416783	573,233461	63,69260677
ENSG00000215717	TMEM167B	61,81195403	61,4956183	86,11670316	98,34978054	60,06229119	65,8709242	60,32121995	23,40638388	55,74583047	573,1807057	63,68674508
ENSG00000156273	BACH1	59,74643095	20,07742083	55,04484611	146,3972509	10,39969924	60,64198225	77,85838368	46,20739061	96,68091975	573,0543244	63,67270271
ENSG00000176209	SMIM19	109,0112253	44,55811343	39,40792823	93,60463216	0,088539828	72,52451985	46,8038985	74,62476919	92,23595891	572,8595854	63,65106504
ENSG00000169189	NSMCE1	76,82630882	65,58560002	19,00211091	86,04931739	0	67,07306092	92,10091927	82,93951761	83,17190156	572,7487365	63,6387485
ENSG00000148925	BTBD10	27,02263424	68,05206125	0,159073018	50,32050726	268,4904544	52,12297946	28,79176145	46,13793103	31,37323266	572,4706348	63,60784831
ENSG00000198431	TXNRD1	138,7920189	36,32504889	25,6437677	57,5923543	0,321118518	51,67706487	72,42125124	123,7036712	65,86334277	572,3396384	63,59329316
ENSG00000092020	PPP2R3C	163,4325561	113,9759679	24,25039952	34,17890167	46,94650761	39,29800095	49,17475606	63,29836396	37,23665758	571,7921114	63,53245682
ENSG00000155827	RNF20	92,88211547	9,200950983	84,72112991	20,29864998	53,72986247	50,45916303	58,59025947	85,70734626	115,7777887	571,3672663	63,48525181
ENSG00000164751	PEX2	28,09945005	86,12490081	80,83744074	51,72488428	102,0474884	60,43432156	48,70844645	69,35867457	43,90899922	571,2446061	63,4716229

ENSG00000152492	CCDC50	76,97868339	49,87330242	80,1277711	53,33496105	16,07288576	82,85591906	62,9606109	58,65276544	90,32266459	571,1795637	63,46439597
ENSG00000167208	SNX20	29,90675294	42,34311932	73,62846796	110,2280093	44,03093345	80,64696728	85,40710947	30,7447815	74,17700324	571,1131444	63,45701605
ENSG00000033800	PIAS1	61,53240387	35,83189311	58,71220353	120,0310569	57,72008012	52,53219077	69,44182072	58,41479687	56,70595446	570,9224003	63,43582226
ENSG00000120533	ENY2	69,27386126	63,47819165	54,0061572	121,9286364	32,97910214	64,51534513	45,94167227	72,33126957	45,99591847	570,4501541	63,38335046
ENSG00000169288	MRPL1	88,92133089	106,2356204	2,561241698	110,5928332	0	33,83434365	38,38097379	129,2916582	60,3887041	570,206706	63,35630066
ENSG00000171204	TMEM126B	16,0117161	96,66874258	90,32052057	103,2507598	0,148641367	64,89465852	61,69650431	98,33710846	38,6923844	570,0210361	63,33567068
ENSG00000169230	PRELID1	58,71959675	59,45757393	57,71108577	38,14755879	56,60004744	77,54516085	85,91990126	46,69184106	88,7821158	569,5748817	63,28609796
ENSG00000162607	USP1	53,85926	43,38508453	0	162,1991765	90,96283058	55,23205552	27,40305778	87,96784816	48,46708218	569,4763953	63,27515503
ENSG00000129559	NEDD8	66,6370882	70,16866176	49,69435622	75,7241978	77,57014778	46,35156856	46,94783746	80,7108463	55,65089946	569,4556036	63,27284484
ENSG00000123505	AMD1	41,11618203	47,69852001	62,18652563	127,478265	2,044215411	83,51013374	56,10186184	73,54485038	75,42824556	569,1087995	63,23431106
ENSG00000072274	TFRC	68,92456745	68,77288404	17,11963658	119,1850795	9,583839981	71,56676403	31,84248354	119,1328117	62,90263353	569,0307004	63,22563338
ENSG00000170310	STX8	56,68286529	66,04597455	0,241372011	48,86394459	0,311824205	111,0772486	78,87744171	131,5999898	75,30683971	569,0015005	63,22238895
ENSG00000172716	SLFN11	74,87324163	29,53954832	0,650571947	39,69422048	0	115,6825502	84,29710202	99,98103765	123,7423434	568,4606157	63,16229063
ENSG00000169139	UBE2V2	44,82190345	69,12050647	35,55412281	83,92992659	122,1032336	39,59601535	44,33627723	86,58177789	42,15453661	568,1983	63,13314444
ENSG00000138398	PPIG	41,88167772	89,46562931	34,20015884	62,57620385	108,7008422	39,9681373	61,55952058	64,1215368	65,66711719	568,1408238	63,1267582
ENSG00000149100	EIF3M	77,18231705	76,68933381	64,83708096	64,58404015	53,55346392	45,91011382	34,43257159	90,72496874	60,15624308	568,0701331	63,11890368
ENSG00000165660	ABRAXAS2	0	45,09867142	122,7627848	68,69963121	101,4765306	55,52810841	54,98615226	96,05226007	22,70409291	567,3112317	63,0345813
ENSG00000158109	TPRG1L	100,7613593	36,40745191	38,0905675	0,326032871	203,2656144	54,79678772	44,26212878	40,18612816	48,996605	567,0926756	63,01029729
ENSG00000197586	ENTPD6	65,27227766	42,93694877	69,09060467	42,41968988	58,31362383	74,00103815	65,64404267	64,51134789	84,70721909	566,8967926	62,98853251
ENSG00000163346	PBXIP1	0,156957227	37,1123568	127,7654571	43,63597082	95,02487033	83,22378716	85,9492952	26,25629126	60,46888923	565,5938751	62,8437639
ENSG00000134242	PTPN22	55,78019731	80,55589106	49,76072486	48,33418587	1,437428455	85,15045831	44,80883994	119,8440693	79,84333579	565,5151309	62,83501454
ENSG00000136940	PDCL	52,86709193	87,93217162	195,0365613	12,27148911	0	79,0803511 5	30,14158595	95,02487563	12,87402029	565,228147	62,80312745
ENSG00000084652	TXLNA	7,06207008	55,21187448	16,52443295	109,4457412	108,2127753	61,31618889	98,72753539	44,32501238	64,32310216	565,1487328	62,79430364
ENSG00000132388	UBE2G1	85,02674542	38,81235824	44,64584538	43,86283006	53,08831701	71,64237284	50,89025803	127,9806058	48,84664047	564,7959732	62,75510814
ENSG00000169599	NFU1	69,74334031	73,71769142	21,38308454	7,838738427	134,5260525	69,24897535	52,19839938	92,79841974	43,05684107	564,5115427	62,72350475
ENSG00000138433	CIR1	13,26615771	54,50589429	61,40403697	72,58228998	80,68279586	45,00154724	38,69129181	132,3609831	65,14569723	563,6406942	62,6267438
ENSG00000013561	RNF14	39,32267857	33,75965839	0,167189314	26,8363356	183,590564	58,07535423	50,3088741	110,8956427	60,35913514	563,3154321	62,59060356
ENSG00000169245	CXCL10	0	24,1898315	0	0	0	512,6989821	26,05511575	0	0,316334506	563,2602639	62,58447377
ENSG00000172466	ZNF24	38,97853352	69,55685467	93,2558813	80,73091303	28,53943099	43,29939948	61,63908933	83,43051869	63,76777056	563,1983916	62,57759906
ENSG00000164978	NUDT2	6,759839135	43,23258715	2,331613132	174,6051368	125,8417483	50,71637387	39,28987097	67,74986473	52,65362793	563,180662	62,57562911
ENSG00000123329	ARHGAP9	36,46376607	45,4762126	47,95944263	64,08660865	57,18655667	87,00742241	82,56820597	60,56030175	81,84799945	563,1565162	62,57294624
ENSG00000066455	GOLGA5	71,38265335	19,66095788	67,73867623	87,60250629	0	77,64925587	72,50266922	117,4305686	48,59263203	562,5599195	62,50665772
ENSG00000121766	ZCCHC17	69,85225755	150,3326049	14,03647559	79,3264056	29,91088328	44,21978931	42,45482343	75,34114553	56,78912471	562,2635099	62,47372333
ENSG00000108061	SHOC2	4,435397197	9,92894288	222,9991627	75,70590254	0,972998534	66,22937449	60,1430379	35,73677079	86,02063009	562,1722171	62,46357968
ENSG00000167977	KCTD5	80,21587233	54,39179361	3,627261078	51,38221327	0	97,81442067	83,34921224	82,11867366	109,2371907	562,1366376	62,4596264
ENSG00000157601	MX1	56,12277462	8,278292946	61,65505677	77,25686168	45,13977465	44,94374257	90,36019151	152,2929048	25,79763703	561,8472366	62,42747073
ENSG00000115232	ITGA4	16,76484837	35,18514324	83,59754432	66,32362598	79,71775743	54,56642207	83,90990384	49,53106855	91,99873836	561,5950522	62,39945024
ENSG00000168792	ABHD15	91,0038746	41,15810769	13,29400817	24,65880344	135,7488992	80,81726546	88,67719913	30,28037274	55,77524494	561,4137753	62,37930837
ENSG00000145740	SLC30A5	68,18033958	56,89639593	177,4082077	17,090979	109,0260154	36,74793653	26,60035136	12,47946643	56,96445127	561,3941432	62,37712702
ENSG00000117632	STMN1	55,10323035	54,85600706	11,97000906	178,2310768	51,85273302	83,20759353	78,11015226	0,211579681	47,77805583	561,3204376	62,36893751
ENSG00000155438	NIFK	48,03268242	58,14331665	78,8847016	78,012592	28,90638123	81,42223374	65,23426707	43,56036804	78,99431234	561,1908551	62,35453945
ENSG00000082153	BZW1	74,68618929	60,36096972	61,33784744	54,15570654	48,90394733	57,12987576	61,28414856	59,50683121	83,80908104	561,1745969	62,35273299
ENSG00000088205	DDX18	106,2302667	47,7353202	84,98187708	102,0948191	18,25943447	24,01113623	32,78169535	103,8532824	40,70153088	560,6493623	62,29437359
ENSG00000137656	BUD13	39,24055821	18,32405468	79,78472089	82,97613315	153,2998347	32,4262585	28,06648429	46,21567755	80,18070915	560,5144311	62,27938124
ENSG00000069275	NUCKS1	22,35377336	60,87143559	101,4245888	153,9552765	23,27665261	52,27636717	47,3359153	56,56687278	42,33346894	560,394351	62,266039
ENSG00000066654	THUMPD1	77,14751331	10,43530626	68,8001814	24,56425234	141,1391049	52,26655301	40,37593209	86,86550929	58,37541893	559,9697715	62,2188635
ENSG00000183648	NDUFB1	48,27792426	75,59637475	40,41336835	72,52677818	24,12101919	90,17676065	68,86175534	89,21587307	50,7526075	559,9424613	62,21582903
ENSG00000196950	SLC39A10	43,3648233	44,11530954	86,96673908	169,379505	22,01327714	50,89811244	58,13682435	16,48112271	68,40613643	559,76185	62,19576111
ENSG00000124357	NAGK	65,45803522	63,38121009	48,08490191	87,22272839	16,26664826	71,53343512	69,10246452	63,6579576	74,92766948	559,6350506	62,18167229
ENSG00000109270	LAMTOR3	34,91075144	83,64948562	30,57514057	19,17533675	170,9344306	42,39405706	42,00423699	90,54415023	45,34583733	559,5334266	62,17038073
ENSG00000124733	MEA1	63,25908212	78,65617172	41,00137357	57,77413425	49,39800467	77,53858573	66,37409674	54,21448821	71,2709401	559,4868771	62,16520857
ENSG00000125818	PSMF1	54,3420506	62,37064185	52,1028233	87,76743427	83,70513124	62,88300692	50,98254008	52,87718492	52,39433371	559,4251469	62,15834965
ENSG00000014123	UFL1	78,2019675	57,89362417	47,218034	42,13821026	50,793889	90,10124952	93,95762469	63,11671514	35,94916205	559,3704763	62,15227515
ENSG00000146007	ZMAT2	54,95687973	41,87868986	102,6317253	68,68983603	0	85,24434156	49,16331993	105,403521	51,38471725	559,3530306	62,15033674
ENSG00000159202	UBE2Z	111,3167983	36,22145653	132,7397533	40,22462163	27,7114324	54,72291113	53,19590804	67,24328994	35,41327432	558,7894456	62,08771618
ENSG00000138670	RASGEF1B	35,16960154	98,95685322	40,42900192	79,02616731	9,063459529	50,26358923	82,86241606	128,6627772	34,32950097	558,763367	62,08481855
ENSG00000091490	SEL1L3	45,8400368	82,23785784	88,85853224	15,93575884	54,94983108	63,12273311	102,1804361	1,766321355	103,8386088	558,7301162	62,08112402
ENSG00000131475	VPS25	37,16144523	47,32577333	0,11626102	88,06964638	107,389813	90,55345153	80,97060739	49,19895694	57,90700924	558,692964	62,076996
ENSG00000100485	SOS2	2,3053714	22,76968625	34,56747345	141,2834448	187,1566812	29,32328558	53,94068533	46,84485449	40,41653644	558,6080189	62,06755766
ENSG00000128294	TPST2	69,35828739	44,14083554	49,45978906	30,00771081	122,8573684	62,83146099	78,18981488	30,25677437	71,26849856	558,37054	62,04117111
ENSG00000111875	ASF1A	88,83363791	112,062435	74,83666628	12,79982301	0	78,37336242	53,16375211	71,47290761	64,9637322	556,5063165	61,83403517
ENSG00000173221	GLRX	40,14897968	74,04145772	34,76424741	80,09434443	39,89554331	74,5785222	62,62437987	73,63030658	75,12627116	554,9040524	61,65600582
ENSG00000105373	NOP53	82,58930193	47,35477489	77,1673173	52,36366709	22,35619833	77,75985653	66,05067882	61,0903103	67,77438531	554,5064905	61,61183228
ENSG00000157500	APPL1	55,30936401	45,41574855	48,93130268	106,3389871	36,40867047	46,28984558	64,57148007	61,94549037	88,64552719	553,856416	61,53960178
ENSG00000165102	HGSNAT	106,7618001	65,66987869	9,987867895	65,33596801	3,05495111	88,3407895	91,87675307	36,06152936	86,66181014	553,7513479	61,52792754
ENSG00000137806	NDUFAF1	110,8369807	73,8666652	79,22277177	42,51516104	0	59,35994318	59,29281087	101,9425793	26,32652303	553,3634351	61,48482613
ENSG00000116898	MRPS15	89,7268495	84,66656238	44,17776634	116,2951546	0,125434	55,40010874	44,26302316	52,16780458	66,34130135	553,1640046	61,46266718
ENSG00000048392	RRM2B	22,68837516	48,13796541	116,2926838	35,46232351	46,79920388	61,42399265	58,77746807	51,22093152	111,1796336	551,98258	61,33139778
ENSG00000168994	PXDC1	117,1503939	53,32492484	25,95529623	43,03212192	0	119,436838	103,409057	28,98314517	60,4714836	551,7632606	61,30702896
ENSG00000065413	ANKRD44	49,52977071	32,50902933	181,7728902	86,48328216	20,99938849	35,59846461	42,63213058	21,22860468	80,53445897	551,2880198	61,25422442
ENSG00000158092	NCK1	86,66232365	31,29134411	70,47880187	119,8596045	37,86702216	65,82223606	37,27675744	56,55168733	45,4666265	551,2764036	61,25293373
ENSG00000183258	DDX41	47,71722353	41,46989037	105,415484	25,98729936	58,2910447	82,57794804	83,86716949	18,19486486	87,73984724	551,2607716	61,25119685
ENSG00000151304	SRFBP1	58,62759845	63,09223531	48,42983402	98,13369344	0,115434761	59,14330748	58,19790713	110,2510707	54,66074184	550,6518232	61,18353591
ENSG00000025293	PHF20	79,65252608	35,51952677	19,68970362	79,41499227	105,9079326	54,25981355	36,1783781	74,35961839	65,55241045	550,5349019	61,17054465
ENSG00000102753	KPNA3	153,186462	24,76915681	54,5820706	103,7065105	0,170117263	68,4155173	66,74712593	14,08768881	64,70733959	550,3719888	61,1524432
ENSG00000135316	SYNCRIP	39,15430509	60,1644634	78,0919108	114,6460187	51,07741279	42,7869324	41,94577949	53,19976401	69,21961878	550,2862054	61,14291172
ENSG00000120254	MTHFD1L	35,24207892	54,73920536	58,40848525	74,73102838	26,60659744	100,1873867	96,43298371	0,263242037	103,6017558	550,2127636	61,13475151
ENSG00000100030	MAPK1	94,16417353	39,24202348	97,10666831	46,61197279	82,84159962	45,80981873	46,79680343	53,48865838	43,90410962	549,9658279	61,10731421
ENSG00000120992	LYPLA1	72,52921084	82,77327612	35,17685956	121,9067745	58,2208031	46,443651	57,97723692	30,48107536	44,34343949	549,8523269	61,09470298
ENSG00000143436	MRPL9	24,75086933	58,09743335	78,92078672	117,8706265	2,88945149	57,49659641	65,90797899	81,46921234	62,0556491	549,4586042	61,05095602
ENSG00000172336	POP7	60,04613236	46,79975633	54,61958037	73,61173268	3,547813261	84,50330142	72,16954332	104,8250165	49,16466725	549,2875435	61,03194927
ENSG00000104671	DCTN6	82,83725827	65,90741665	44,79434537	78,77282252	0	46,44819307	63,53495139	125,1977989	41,41505876	548,9078449	60,98976055
ENSG00000107020	PLGRKT	45,56836275	35,12625423	38,39802924	28,09027612	80,11441931	66,49334397	32,1405844	129,6452097	93,28483025	548,8613099	60,98458999
ENSG00000185614	INKA1	135,7089163	21,39979424	55,06144268	48,42350971	2,071831975	68,27858349	76,7299338	45,75247858	95,42504124	548,8515321	60,98350356
ENSG00000105246	EBI3	22,37783929	67,67596658	114,2020152	58,37796804	0	80,04921137	91,30563969	42,88283002	71,9119505	548,7834207	60,97593563
ENSG00000151552	QDPR	98,26669608	30,3140099	85,02489514	117,738952	0,355093153	64,0893856	49,17080578	45,41569369	58,00812676	548,3836581	60,93151756
ENSG00000243927	MRPS6	81,51412717	88,41845966	65,64051483	38,16624365	65,72195603	36,65330784	43,83973922	94,80757776	33,5332714	548,2951976	60,92168862
ENSG00000154059	IMPACT	49,13562674	70,8692296	53,99490893	98,59675307	96,30746183	58,21780449	45,48812077	32,05809223	43,31104614	547,9790438	60,88656042
ENSG00000134014	ELP3	25,56084638	60,26830461	68,5120492	56,12048475	134,8517336	49,44205909	36,61273396	67,54669032	48,82927421	547,7441761	60,86046401
ENSG00000136436	CALCOCO2	60,53127144	35,55705149	54,10821079	54,29752142	55,11956518	43,86187453	43,75576441	165,7922629	34,479626	547,5031482	60,83368313
ENSG00000144840	RABL3	72,61953343	31,88622845	122,0168838	60,88854126	97,44628344	33,68508933	57,21704294	45,64286191	26,02803557	547,4305011	60,82561123
ENSG00000145912	NHP2	32,52379832	86,83538943	68,11432889	74,65159538	40,90610416	68,34197266	64,06465296	56,45835657	55,26164801	547,1578464	60,79531627
ENSG00000119048	UBE2B	64,65016211	91,66203285	87,6764943	84,12397692	0,218242201	38,61668239	37,43903556	92,97551186	49,6381322	547,0002704	60,77780782
ENSG00000117899	MESD	53,73495947	56,75762255	59,77224664	41,93160289	61,61117863	52,49940314	45,39148027	111,6961918	63,51937475	546,9140602	60,76822891
ENSG00000144843	ADPRH	37,19644816	45,20357011	67,46212586	59,98960088	31,35956004	94,7290075	89,80032646	18,32963148	102,688761	546,7590314	60,75100349
ENSG00000167770	OTUB1	74,22428893	48,89470879	94,96958779	48,89865902	63,46473809	50,3701526	50,97820086	55,1137116	59,38311853	546,3971662	60,71079624
ENSG00000167778	SPRYD3	141,8161579	25,70694312	74,73273813	63,51954478	0,207402566	82,83551462	73,36916613	39,21703934	44,65039172	546,0548983	60,67276647
ENSG00000119487	MAPKAP1	42,25536652	31,66311112	77,52597331	17,14792965	13,90441753	83,58289845	100,2683101	81,1258284	97,73415242	545,2079875	60,57866528
ENSG00000138760	SCARB2	42,08697625	22,22125204	99,63774888	82,89875068	76,34621191	47,36965177	62,85546666	81,03738655	30,35958736	544,8132361	60,53480401
ENSG00000126062	TMEM115	91,22574555	43,39649835	35,4896282	42,47778656	0	82,36471914	114,9301095	67,12197195	67,74092506	544,7473843	60,52748715
ENSG00000180817	PPA1	78,59738741	59,48330083	7,96381236	78,29059439	69,58510645	78,04156047	57,14899171	45,03018497	70,51087566	544,6518143	60,51686825
ENSG00000131966	ACTR10	77,33239652	29,343591	0	60,40460541	108,3843239	55,61845032	47,77751191	88,60129651	77,11779434	544,5799699	60,50888554
ENSG00000110442	COMMD9	83,85352966	71,95740263	80,45904564	66,06499888	0	74,19665961	58,83325185	59,55040446	49,5346389	544,4499316	60,49443685
ENSG00000144867	SRPRB	66,15606323	40,41374642	27,99751715	26,96744235	147,4272016	46,75661707	74,80192999	61,97624424	51,79693743	544,2936995	60,47707772
ENSG00000164323	CFAP97	111,9672179	7,456674085	197,508107	28,87989986	37,24995642	31,51193657	29,6404469	37,85846547	61,68181989	543,7545241	60,41716935
ENSG00000132405	TBC1D14	32,70843822	16,83383483	114,2560375	23,42579165	132,4030811	65,75465508	61,97352501	41,54185378	54,80394251	543,7011597	60,41123996
ENSG00000123595	RAB9A	32,96179587	34,30900945	53,94231383	43,73555995	163,1119338	37,36512988	33,4706032	102,5002412	42,29368448	543,6902716	60,41003018
ENSG00000143106	PSMA5	64,80308568	42,78861704	28,65124494	82,68553346	63,13053413	52,22682088	72,60386494	80,62050226	56,16882161	543,6790249	60,40878055
ENSG00000036054	TBC1D23	41,71889596	46,52077204	61,54659842	96,03576635	105,6677003	44,23427277	56,65072757	64,66967096	25,95962821	543,0040326	60,3337814
ENSG00000100297	MCM5	42,97544711	51,17129253	123,0409987	56,97630775	0,098821558	72,47744648	57,6597288	37,41712288	100,9012626	542,7184284	60,3020476
ENSG00000168538	TRAPPC11	97,06674561	21,43850608	103,2728668	118,6961526	0	38,51647697	64,09920745	33,71665849	65,67149837	542,4781124	60,27534582
ENSG00000076685	NT5C2	39,82918261	39,94046859	117,9097123	144,7389502	0,278601662	57,08405025	52,97690317	38,83695654	50,82304042	542,4178657	60,26865175
ENSG00000163510	CWC22	59,2607289	99,52603181	29,42297869	126,2469159	1,019062265	27,02402697	71,24464652	47,63358692	80,89913881	542,2771168	60,25301298
ENSG00000114383	TUSC2	56,82360894	46,73523213	67,41364297	49,16952724	113,9794862	48,65514001	42,58893133	75,46113875	41,28204779	542,1087554	60,23430615
ENSG00000103642	LACTB	22,48052663	84,41129223	39,02589356	29,41232006	85,1525221	89,21010446	67,0831478	67,94922561	56,99482074	541,7198532	60,1910948
ENSG00000103479	RBL2	141,7545227	42,83336022	27,49968233	56,04285847	0,494107791	49,94666233	53,29209066	84,79092851	84,74069549	541,3949085	60,15498983
ENSG00000211455	STK38L	33,18135363	98,49715609	29,77056284	134,9349009	39,29728607	76,92371486	59,30726461	4,862872114	64,61165702	541,3867681	60,15408535

ENSG00000115935	WIPF1	71,78621891	52,79936763	66,98829707	67,51524395	52,79866509	61,80946603	69,51009622	44,59653027	53,40464147	541,2085266	60,13428074
ENSG00000130598	TNNI2	157,487714	58,54843672	2,04022091	61,52320043	0	37,13961534	18,53277381	102,16188	103,6648491	541,0986904	60,12207671
ENSG00000131873	CHSY1	23,43853743	79,44422413	45,86401845	68,74464745	3,928937291	52,17092777	70,65040223	137,491567	59,35807399	541,0913357	60,12125953
ENSG00000104894	CD37	45,83069939	73,14135306	47,56592898	53,55406592	38,23337449	62,12176845	69,47456018	58,36803824	92,76693746	541,0567262	60,11741402
ENSG00000101665	SMAD7	28,49273427	20,29845063	51,53098199	68,27962677	193,5082008	62,92932464	44,92301575	5,449248779	65,50600646	540,9175901	60,10195446
ENSG00000085719	CPNE3	57,68983045	30,96548554	95,9747232	97,17765345	62,6336815	51,74085226	40,22798235	52,08869092	52,08226719	540,5811669	60,0645741
ENSG00000113387	SUB1	45,14073598	60,33629076	18,63000663	113,0924733	27,82715254	70,08137246	56,10010635	91,3990715	57,44274118	540,0499508	60,00555008
ENSG00000112514	CUTA	30,85039902	39,87826121	65,64242209	80,39467768	63,17099626	77,64897993	69,15582265	60,06299975	53,19807167	540,0026303	60,00029225
ENSG00000150867	PIP4K2A	52,73776673	57,60904311	110,0269839	87,13163566	48,31840023	49,64969863	40,44734949	54,55470827	38,83631791	539,311904	59,92354489
ENSG00000131165	CHMP1A	107,88706	71,91080911	13,08672544	38,20833557	29,03255456	93,99803777	77,35979236	29,07395058	78,38137417	538,9386396	59,88207107
ENSG00000166452	AKIP1	71,02196821	72,28175655	0	82,81849194	0	78,77145642	72,2818217	83,3269779	77,97473652	538,4772092	59,83080102
ENSG00000112576	CCND3	56,64693057	64,51675838	104,2545149	46,32247912	78,91672983	39,86200343	35,27096027	75,80289717	36,70805145	538,3013251	59,81125835
ENSG00000204568	MRPS18B	73,19015119	41,66349802	83,21999548	35,79130841	58,86502686	43,81474536	43,98959847	108,3268702	49,39688441	538,2580784	59,80645315
ENSG00000105248	YJU2	96,48246868	59,80145326	77,82776153	29,40993719	82,88984041	50,72805403	56,86784832	34,86599753	49,24710003	538,120461	59,79116233
ENSG00000138744	NAAA	85,5084714	59,29713311	107,9705409	67,92105495	4,63325032	48,96953192	47,93886836	44,20254174	71,38559264	537,8269854	59,75855393
ENSG00000100528	CNIH1	57,53582398	64,91184103	126,0575225	98,72793719	31,26805459	35,99852919	47,07324869	34,95930571	41,21282711	537,74509	59,74945444
ENSG00000140632	GLYR1	112,1331949	43,07303177	91,35978142	71,15970411	4,11102061	43,28501567	60,46483872	53,62850533	58,19713844	537,412231	59,71247011
ENSG00000115159	GPD2	31,59297067	45,02091083	77,36110501	60,23537306	97,73610713	50,43311041	34,53279759	102,1972089	38,28464297	537,3942265	59,71046962
ENSG00000163453	IGFBP7	14,17150948	439,8640271	0,144031139	9,058364403	50,23924168	2,406992823	2,470400319	10,27257723	8,570124602	537,1972688	59,68858542
ENSG00000184588	PDE4B	43,81022032	31,91880695	120,2505176	101,1890053	29,55782683	29,91220924	69,98518742	39,85196296	70,32199314	536,7977298	59,6441922
ENSG00000100368	CSF2RB	41,51755358	40,78552667	126,1216321	41,93784562	12,59430259	53,99574681	81,61857669	68,20593277	69,75762249	536,5347393	59,61497103
ENSG00000181191	PJA1	145,5354293	30,96923966	69,65109314	0	113,3691568	45,82900434	58,0456268	27,77473747	44,9561366	536,1304241	59,57004712
ENSG00000121851	POLR3GL	67,67016721	72,10665601	73,8312687	46,25076027	60,0842713	33,43863087	53,00099612	65,50304237	64,13808748	536,0238803	59,55820893
ENSG00000232112	TMA7	22,68648603	84,51534507	47,8350302	88,42923634	58,64430455	49,52554528	42,19063842	80,07018493	61,67689677	535,5736676	59,50818529
ENSG00000211456	SACM1L	69,20151526	15,89774704	36,78869785	25,71295988	135,3849151	70,52952007	68,14764523	54,8097136	58,76569874	535,2384128	59,47093475
ENSG00000139505	MTMR6	81,425561	61,89676901	101,0137062	47,18412328	7,022024967	51,59046657	74,86099753	58,43642983	51,77589563	535,205974	59,46733044
ENSG00000168958	MFF	16,31502747	106,3411932	46,28481648	66,2732407	35,03585779	75,18581744	45,35293464	79,94904658	64,06416703	534,8021013	59,4224557
ENSG00000109062	SLC9A3R1	119,8418592	35,42363319	93,41272245	30,79010389	0	60,2214962	47,74202457	78,97089493	68,01394744	534,4166818	59,37963131
ENSG00000113621	TXNDC15	53,44119327	60,3001193	128,4569004	22,50037578	108,5878685	21,74326646	37,03340853	57,17490807	44,33489686	533,5729372	59,28588191
ENSG00000132286	TIMM10B	46,00556016	48,71231383	72,72681077	40,06997435	0	65,22078542	94,23917542	110,1080058	56,39437795	533,4770037	59,27522263
ENSG00000160310	PRMT2	81,62713583	52,90188085	24,20397547	77,73480435	47,04472868	78,33999347	66,57205304	36,98537642	67,75832694	533,1682751	59,24091945
ENSG00000167986	DDB1	54,8222651	52,68653923	92,14193388	74,42725906	33,92952837	41,89985389	81,24541283	46,04765586	55,71398969	532,9144379	59,21271532
ENSG00000139218	SCAF11	66,92577893	46,99888281	36,70089942	120,6045729	42,47489279	43,69116434	50,51890603	42,23723649	82,62788023	532,7802139	59,19780154
ENSG00000132485	ZRANB2	67,14341304	24,2053962	93,79388696	27,11420599	20,60333342	65,96484715	40,72437255	116,265242	76,91100811	532,7257054	59,19174505
ENSG00000130313	AC010618.1	37,0327314	41,92079156	102,3828111	30,22803051	39,57879956	67,99996876	80,23032497	52,47394012	80,75162271	532,5990207	59,17766897
ENSG00000184678	HIST2H2BE	4,890086836	50,71965818	193,344408	12,48777226	59,74380262	23,51786206	33,45494267	102,2630225	52,00992009	532,4314752	59,15905279
ENSG00000074964	ARHGEF10L	101,7536103	20,83376365	47,49496537	34,9378612	72,96127086	43,55309079	55,81016276	49,10405567	105,8909277	532,3397083	59,14885648
ENSG00000100243	CYB5R3	82,73675199	38,2068815	95,47324083	42,44638279	0,06194886	69,94746411	89,5845617	51,81387302	61,61756353	531,8886683	59,09874093
ENSG00000143851	PTPN7	55,85411036	57,0244416	44,76110297	89,4230255	0,182416632	62,58868352	71,6578784	41,90577228	108,0276178	531,4250491	59,04722768
ENSG00000182087	TMEM259	49,78289205	31,66350584	56,5476758	15,92408925	105,3031345	54,28827851	94,74862084	32,62539267	90,45667991	531,3402693	59,0378077
ENSG00000172269	DPAGT1	67,68494311	67,18785343	30,85157917	52,97770696	61,55329088	66,6470328	55,03585017	40,05267485	88,87546756	530,8663989	58,98515544
ENSG00000119314	PTBP3	32,59175678	22,8469091	152,3340389	149,2007119	20,13743228	41,05979681	44,81844727	22,70008074	45,09129725	530,7804711	58,9756079
ENSG00000135446	CDK4	72,82025238	61,70559278	87,32923601	90,82965987	0,232138036	66,1711867	49,0688758	32,78714035	69,31702867	530,2611106	58,91790118
ENSG00000181038	METTL23	66,79181964	50,2941369	30,20891358	29,03821818	136,4046255	44,46819532	59,85079055	65,08559663	47,96665726	530,1089536	58,90099485
ENSG00000126457	PRMT1	56,17809581	77,15512096	38,19618124	57,83441993	23,06612289	79,94565112	72,99045586	49,6065239	73,74185402	529,3099257	58,81221397
ENSG00000068120	COASY	110,3721802	35,84537154	54,32745982	28,21618277	43,90995744	44,39918459	67,06065612	60,071562	84,9491916	529,1517461	58,79463845
ENSG00000115514	TXNDC9	14,3812617	50,46706678	0,098714524	144,2740609	71,79800296	52,49514341	44,24433227	89,53180931	60,84889685	528,1392887	58,68214319
ENSG00000066044	ELAVL1	55,82539324	39,43944385	101,4176173	65,62728247	74,38200173	60,649586	40,73009916	43,66558491	46,36820011	528,1052087	58,67835653
ENSG00000165476	REEP3	94,75197815	45,91578257	76,11804053	50,93610818	11,98206182	67,22321162	68,37712053	60,51102818	52,02989885	527,8452304	58,64947005
ENSG00000110696	C11orf58	64,47988284	47,93991086	43,00601029	51,82367413	66,7051633	49,94001405	50,3361446	98,47218498	54,93452123	527,6375063	58,62638959
ENSG00000166478	ZNF143	30,56570994	31,8188399	0,065840857	26,76097679	320,415865	25,30706097	44,72592365	4,461101456	43,27028219	527,3916007	58,59906675
ENSG00000069248	NUP133	55,58861151	41,75801251	92,0200525	30,68056353	133,2295964	23,30005468	25,66766864	62,21798714	62,83513399	527,2976809	58,58863121
ENSG00000006015	REX1BD	37,26198345	51,40051396	26,64319643	38,01752894	50,93714696	79,16445322	79,34043843	85,69727359	78,3092171	526,7717521	58,53019467
ENSG00000143457	GOLPH3L	52,25869465	24,85179305	86,04542956	4,695890365	0,21542744	49,61393071	58,44493869	184,4447301	66,11097043	526,681805	58,52020055
ENSG00000161955	TNFSF13	42,42515582	53,60483745	36,88488132	56,97921018	71,94973608	62,03422705	85,55797683	62,13795909	54,95373879	526,5277226	58,50308029
ENSG00000068366	ACSL4	89,70055387	56,13635325	42,85616803	116,6580681	16,23446411	59,43297042	40,60427268	30,7512762	73,93302024	526,3071469	58,47857188
ENSG00000104325	DECR1	35,29305919	120,525792	0,936145341	116,6660534	0	78,58403108	61,19412785	58,78174359	54,19296371	526,1739162	58,46376847
ENSG00000104131	EIF3J	56,20134112	48,19181453	118,0546877	74,60533319	22,62429032	55,33889719	35,67097063	51,67333019	63,59314961	525,9491945	58,43879939
ENSG00000166128	RAB8B	85,99355226	22,06930042	75,39181991	60,08202923	1,548223777	50,97938159	80,67179292	103,7990617	45,19606783	525,7312296	58,41458107
ENSG00000160752	FDPS	87,50431916	42,76713449	40,22412406	93,13090675	26,27218169	64,43649079	66,17733218	49,2363503	55,68397682	525,4328162	58,38142403
ENSG00000196182	STK40	121,5372318	41,20464724	72,00629398	23,24608539	0,064242852	80,43249783	69,73816127	43,03333903	73,97290019	525,2353996	58,35948884
ENSG00000158714	SLAMF8	45,64699428	63,02771368	38,74927119	71,10437005	0,113001115	73,34938964	103,3610224	48,45264583	81,34440292	525,1488111	58,3498679
ENSG00000136718	IMP4	0,078275354	33,89103757	90,28413471	83,32888466	46,69179131	57,83880763	69,12079673	39,36801374	104,4995597	525,1013014	58,34458904
ENSG00000110315	RNF141	66,35347231	63,80580245	42,70058517	36,81434019	121,0214644	52,58252143	27,56338667	48,26667229	65,50347712	524,6117221	58,29019134
ENSG00000164342	TLR3	21,55190917	21,85333374	99,34357196	58,52021739	87,53623616	74,34392737	41,53732288	15,92218915	103,8684162	524,477124	58,275236
ENSG00000197226	TBC1D9B	30,83324727	21,45509946	20,20704284	65,43274836	111,4006217	52,75357655	84,30670177	37,585384	99,92915505	523,903577	58,21150856
ENSG00000034152	MAP2K3	61,47479996	15,66465293	57,68289128	49,24863121	114,199948	46,83914774	55,88620392	50,83608731	71,92157246	523,7539348	58,19488165
ENSG00000185009	AP3M1	21,57261868	38,5068603	91,50021526	82,35870477	92,33062659	73,49793647	53,77906314	30,51713022	39,67156818	523,7347236	58,19274707
ENSG00000122378	PRXL2A	58,28881675	78,05728776	72,82055354	48,94531809	0,220317511	89,39322944	82,92203383	23,566269	69,40740517	523,6212311	58,18013679
ENSG00000183624	HMCES	97,68160856	11,56199785	22,37425159	73,4143317	97,94114791	40,53869643	49,63810116	63,16607615	66,3785279	522,6947392	58,07719325
ENSG00000132819	RBM38	23,58826483	42,85102041	12,36481375	53,30929589	76,29749787	86,80008295	92,53262527	29,64967198	105,2923567	522,6892297	58,07658107
ENSG00000147853	AK3	11,34618619	71,0774585	26,18515608	72,9667995	57,76930659	65,68473344	53,95652502	108,5659876	54,76023808	522,312391	58,03471011
ENSG00000089041	P2RX7	53,4309104	77,40630042	134,8799939	16,93771923	0	29,40679713	60,08820804	59,52112026	90,57756035	522,2486098	58,02762331
ENSG00000186407	CD300E	199,2534163	39,85701546	106,2603735	17,16860382	0,86096374	21,70007809	15,30778158	109,5873832	12,22804768	522,2236634	58,02485148
ENSG00000089289	IGBP1	50,18103178	91,05895175	0	57,67446863	101,3389631	39,60847995	43,6184416	96,08129592	42,47350777	522,0351405	58,0039045
ENSG00000197498	RPF2	55,01935107	21,26266242	22,96418972	35,26148305	229,4616423	22,63186092	30,77574181	75,91064389	28,62093306	521,9085083	57,98983425
ENSG00000100439	ABHD4	70,91429729	23,19065947	82,43783279	54,62324284	89,82350322	30,04326659	62,51936936	38,32066729	69,72669275	521,5995316	57,95550351
ENSG00000162521	RBBP4	50,94995271	62,03792209	79,81287108	69,07750595	34,09151748	54,94145391	46,62408641	54,67697648	69,2754196	521,4877057	57,94307841
ENSG00000148803	FUOM	59,84930476	63,12949451	91,93042643	44,79041296	0,282769718	69,49954867	72,91110631	69,46931459	49,60785122	521,4702292	57,94113657
ENSG00000177963	RIC8A	58,77150093	29,48158116	98,63407551	40,3990574	68,60031437	61,77283851	57,37663499	39,24682821	67,13827244	521,4211035	57,93567817
ENSG00000111666	CHPT1	42,92942602	88,48551731	82,82331442	74,56529403	35,54610292	52,89420823	49,27920067	72,58419143	21,4354777	520,5427327	57,83808141
ENSG00000145779	TNFAIP8	52,79809734	71,90593724	18,5298279	28,0690691	78,40127864	47,04635681	55,99404894	129,8663013	37,79855203	520,4094693	57,82327437
ENSG00000145335	SNCA	44,76095379	44,62353165	50,48517493	73,7170556	56,63213859	49,35157663	95,52499616	57,37740248	47,64226991	520,1150998	57,79056664
ENSG00000155792	DEPTOR	79,15041691	73,38945492	64,33646752	87,40874673	0,410107585	41,87236862	43,60655239	83,64635988	46,26708244	520,087557	57,78750633
ENSG00000143924	EML4	17,27443081	58,00007112	74,20344993	101,4191839	67,53335636	64,94225351	30,06842287	43,9841081	62,49017011	519,9154467	57,76838297
ENSG00000168961	LGALS9	71,35042346	62,27238959	40,62187306	50,14116243	102,8833421	61,35265113	44,42185538	50,71598105	35,80976917	519,5694474	57,7299386
ENSG00000105171	POP4	71,1005401	46,9933171	56,4606747	72,38098762	41,93039086	68,12808964	58,33729689	49,47314207	54,74448393	519,5489229	57,7276581
ENSG00000163956	LRPAP1	60,32401505	67,62552135	27,12517384	32,10895845	73,67137125	60,40515411	63,3404308	77,43511488	57,09401454	519,1297543	57,68108381
ENSG00000170340	B3GNT2	0,369980411	64,71422877	1,263987858	163,2783034	0	72,82888204	90,05425718	52,12521509	73,97458634	518,6094411	57,62327124
ENSG00000174446	SNAPC5	93,19298926	73,64158322	95,09254598	39,51963468	0,145039395	52,73490463	47,12738641	51,51362805	65,60172071	518,5694323	57,61882582
ENSG00000167642	SPINT2	89,32908071	50,62267848	20,77334073	19,5508801	45,83427433	83,33050401	83,52351501	55,88470562	69,44117843	518,2901574	57,58779527
ENSG00000158526	TSR2	49,41864345	51,18117666	1,432184023	73,81207697	79,73537189	65,45245519	50,08347158	69,79984363	77,19287591	518,1080993	57,56756659
ENSG00000134884	ARGLU1	53,88033836	74,97589731	43,21357582	76,07407352	45,08375164	67,36262046	35,16987864	46,33969121	75,2320091	517,3318361	57,48131512
ENSG00000119203	CPSF3	7,215263276	114,7205464	42,76036791	7,291454798	17,63436657	87,5464112	79,8571348	49,66379555	110,5196899	517,2090304	57,46767004
ENSG00000213203	GIMAP1	55,63086675	34,3771795	104,4815757	18,71203487	148,6029438	33,34121241	28,823912	30,99761421	62,02632524	516,9936645	57,4437405
ENSG00000198176	TFDP1	5,125812627	43,47280824	47,07248471	35,33494448	90,45081662	94,21097384	61,72979475	38,12706548	101,3618051	516,8865019	57,43183354
ENSG00000145907	G3BP1	77,80866721	36,41941562	94,24345448	46,27252492	42,00222102	59,1907313	48,92999083	54,94813097	56,95538269	516,770519	57,41894656
ENSG00000013583	HEBP1	79,0817864	65,94040434	100,3907125	53,13086706	0,104760077	49,07623243	53,6945674	37,59319759	76,93005108	515,9425789	57,32695321
ENSG00000057657	PRDM1	31,31906494	28,5620886	74,74575995	13,45264758	93,15775062	32,02688614	107,6442508	79,10848313	55,39603005	515,4129618	57,26810686
ENSG00000163947	ARHGEF3	96,36335299	62,54206796	85,07185923	32,17169159	0,054461259	48,96299397	43,32665241	38,68600032	107,5790378	514,7581176	57,1953464
ENSG00000166326	TRIM44	29,81398762	46,09170109	115,0774408	79,1146402	39,56402923	37,78462436	37,3338136	54,37913803	74,89443279	514,0538078	57,11708975
ENSG00000167699	GLOD4	55,66521825	114,0429031	28,08080243	74,18693817	10,44728885	59,72543873	58,51400634	41,10528173	72,20915206	513,9770297	57,10855885
ENSG00000109320	NFKB1	32,67448257	31,62549144	54,07475742	135,9044875	9,600563328	57,71256419	40,70534595	54,14574923	96,91257554	513,3560171	57,03955746
ENSG00000132155	RAF1	79,5318793	45,82368066	48,4259966	20,01820501	57,89265848	62,6049723	63,54264042	63,41716053	72,069704	513,3268973	57,03632192
ENSG00000165233	CARD19	33,0462442	58,46574434	10,7331706	94,23122217	85,99245498	44,03466961	47,38723765	47,92480587	91,47604617	513,2915956	57,03239951
ENSG00000188811	NHLRC3	112,9851544	35,18605431	53,47939804	10,82988036	81,80864594	32,95430865	50,82243437	65,32385661	69,42059203	512,8103247	56,97892497
ENSG00000104679	R3HCC1	33,32476463	54,03622055	108,1268598	27,58171975	82,74723599	56,96473413	47,13486236	48,49724071	53,77536253	512,1890005	56,90988894
ENSG00000149573	MPZL2	71,15586365	40,16472997	45,56762382	98,64883428	79,55135432	6,37040611	17,8146695	134,8925924	17,99653032	512,1626044	56,90695605
ENSG00000115946	PNO1	42,97745508	82,37974756	90,05286857	18,95003047	0	75,76372723	64,78658207	78,90224654	58,22719937	512,0398569	56,89331743
ENSG00000169021	UQCRFS1	78,54224088	57,69484383	76,38597665	43,88374978	0	69,56846606	54,23766134	65,20889553	66,24470982	511,7665439	56,86294932
ENSG00000165006	UBAP1	101,8851575	55,04069194	0,100281421	52,90240106	1,036414264	85,52893727	56,71512482	60,913443	97,21408993	511,3365412	56,81517124
ENSG00000105856	HBP1	57,14285294	59,55423678	0,657266943	112,2464605	64,36264906	42,00069139	54,15160293	71,14968777	50,0063073	511,2717557	56,80797285
ENSG00000114988	LMAN2L	67,6622821	29,99151939	59,82369794	48,06443423	132,0343567	51,99633799	61,97095025	30,49414613	28,81341431	510,851139	56,76123767
ENSG00000075240	GRAMD4	133,3201607	17,78129974	59,38485281	19,06033889	65,65380901	47,15638305	39,96310717	55,4881961	72,61703533	510,4251828	56,7139092
ENSG00000171988	JMJD1C	22,06685452	40,4783435	127,8578954	53,53351477	117,1105406	26,16042062	40,03989631	45,61903499	36,74639622	509,6128969	56,62365521
ENSG00000152102	FAM168B	69,31345955	43,41581602	50,8905609	73,74353652	20,21894535	54,33685861	65,97869528	76,48329088	54,26650062	508,6476637	56,51640708
ENSG00000107651	SEC23IP	41,99976483	55,93837825	42,54544398	79,17499357	91,75871956	51,29336805	43,76351455	51,20535671	50,80168093	508,4812204	56,49791338

ENSG00000142208	AKT1	33,89902069	44,77841679	72,29674139	58,93362041	61,48303381	59,48522917	67,10785541	35,05057588	75,39088451	508,425378	56,49170867
ENSG00000186815	TPCN1	50,81456518	12,04304125	93,73229048	92,88782838	42,46211087	52,97809089	83,45042132	31,83953612	48,20766801	508,4155525	56,49061695
ENSG00000254415	SIGLEC14	37,16071671	33,22797119	79,30844323	69,93358109	37,42963818	42,55343322	70,93111468	8,72905918	129,0749622	508,3489196	56,48321329
ENSG00000111252	SH2B3	73,02879744	46,20046077	37,37711996	71,36447563	0,12640533	61,17259032	67,94653495	37,50973179	113,4965562	508,2226724	56,46918582
ENSG00000056050	HPF1	35,49291885	66,18694939	37,92414852	49,54461543	103,0379446	33,71881622	36,79404251	79,47160485	65,86965346	508,0406939	56,44896599
ENSG00000104205	SGK3	0,098155616	32,70130595	118,6764664	105,2553551	32,5993352	52,77993176	27,81211484	73,45971467	64,49399449	507,8763741	56,43070823
ENSG00000166887	VPS39	45,56470284	26,1915097	20,73038086	91,7051814	141,2139247	53,06596372	49,60839198	43,23258107	36,55085421	507,8634905	56,42927672
ENSG00000177954	RPS27	84,38831869	69,17286379	27,66372	76,38965923	28,45182461	46,16642134	36,36821217	87,79662422	51,35469118	507,7523352	56,41692614
ENSG00000171792	RHNO1	80,64183631	55,47415811	105,4516034	36,71248508	0,271647158	56,47485591	45,84127864	80,73401309	45,61815165	507,2200293	56,35778103
ENSG00000105819	PMPCB	33,60421586	48,72977524	134,1376948	72,37794609	54,72921576	41,97355097	32,26265671	38,5586009	50,09827342	506,4719298	56,27465886
ENSG00000172172	MRPL13	88,46197342	56,50794822	23,84196145	58,52283583	58,27447884	48,44478469	49,09708644	84,15914858	38,90016301	506,2103805	56,24559783
ENSG00000092531	SNAP23	58,22869734	43,75864834	59,56716407	73,52730231	33,01524911	53,69696382	50,449708	82,04398951	51,63814853	505,925871	56,21398567
ENSG00000035141	FAM136A	124,9135015	100,3577479	0,180155605	27,73385342	4,189316761	43,27868252	55,45739885	72,91832161	76,84386339	505,8728416	56,20809351
ENSG00000187764	SEMA4D	6,296273778	17,7057041	65,23501899	56,24106938	45,11794547	94,54325165	60,15664435	15,98500556	144,4247974	505,7057107	56,18952341
ENSG00000134755	DSC2	40,86812875	54,42178298	60,37536576	16,4844966	109,7885937	61,69405691	44,96062037	46,29519091	70,72719708	505,6154331	56,17949256
ENSG00000214941	ZSWIM7	38,14670912	82,1505776	61,37505116	34,87290399	104,2689855	48,62560865	46,72654412	37,70492134	51,56351341	505,4348149	56,15942388
ENSG00000140395	WDR61	64,93886377	44,87685394	39,8716586	78,37487621	85,13304009	53,22618112	32,3800036	60,35901324	46,2547897	505,4152803	56,15725336
ENSG00000181467	RAP2B	71,25774485	33,23278291	42,90699887	61,74200812	61,70921725	67,0118998	59,96312957	61,60778609	45,95534289	505,3869104	56,15410115
ENSG00000137393	RNF144B	2,657210035	33,05789296	92,17658934	108,1052339	122,5510299	27,95633292	48,49094957	32,11760144	38,2466756	505,3595157	56,1510573
ENSG00000166562	SEC11C	76,65613942	50,9847465	31,05168663	62,28729481	29,56310026	48,58867322	65,23360389	105,0361459	35,42727537	504,8286658	56,09207398
ENSG00000065150	IPO5	28,89315412	7,185595243	60,68680899	143,0620207	91,59076481	44,34009515	43,51542192	8,889083037	76,39609239	504,5590364	56,06211516
ENSG00000005194	CIAPIN1	52,9758016	65,23229857	32,86788046	57,38564029	97,97974921	51,20030803	41,53909898	38,19092991	67,07614653	504,4478536	56,04976151
ENSG00000197324	LRP10	39,35922984	46,48598316	39,95134044	35,99861549	53,52960276	70,1355613	83,07694222	65,13061228	70,73029346	504,3981809	56,04424233
ENSG00000136247	ZDHHC4	71,15833732	36,9383132	80,02575804	12,61760349	51,28808308	49,48864827	48,4667518	82,12093272	72,20688608	504,311314	56,03459045
ENSG00000080822	CLDND1	59,45809402	86,30981462	0,800326581	84,39067797	45,0792462	65,24966827	50,1881328	58,98352165	53,61368963	504,0731717	56,00813019
ENSG00000053372	MRTO4	70,5300903	32,83585525	27,62400045	60,84399251	146,9628127	26,61319892	44,32696001	44,47163194	49,80628629	504,0148284	56,0016476
ENSG00000076242	MLH1	32,51529833	94,06227013	52,18029604	53,41335975	10,77759329	49,36920049	54,60019827	47,80636014	109,0769651	503,8015415	55,97794906
ENSG00000115233	PSMD14	32,09885327	67,4114343	25,32276651	58,14020977	112,3869425	49,87983143	59,86055566	56,63172112	41,84152314	503,5738371	55,95264857
ENSG00000074696	HACD3	71,14594108	52,08622443	31,34819272	56,71859752	87,52667476	64,17516528	60,09982917	39,31304976	41,0780021	503,4916768	55,94351965
ENSG00000130164	LDLR	89,63257223	42,76791942	1,044496355	45,37117594	0,511828578	69,6059144	128,0714272	38,53214703	87,79430166	503,3317829	55,92575365
ENSG00000115325	DOK1	37,81481319	38,16780191	17,68715618	53,57339918	50,76407371	96,58618355	80,12159279	29,36955323	99,17097138	503,2555451	55,91728279
ENSG00000182544	MFSD5	110,2445581	87,89156609	3,769586774	40,74265586	4,452445314	55,79635048	74,13493076	47,62559439	78,1981105	502,8557983	55,87286648
ENSG00000138119	MYOF	34,42259643	19,07629777	3,503400541	119,6182475	104,8589385	28,41389618	79,74533704	67,17668593	45,9034343	502,7188341	55,85764824
ENSG00000168101	NUDT16L1	27,25543658	53,55060253	78,09789048	82,39387738	0	62,49726269	84,57719672	33,7145521	80,52151062	502,6083291	55,8453699
ENSG00000104312	RIPK2	48,21311441	92,65318539	49,43483944	11,60090113	0	81,62648511	67,1854867	75,93837887	75,88176081	502,5341518	55,83712798
ENSG00000205544	TMEM256	9,968313507	35,77906748	42,09293853	116,9708507	78,06903095	50,24578185	49,8495322	74,80602653	44,58087491	502,3624166	55,81804629
ENSG00000144028	SNRNP200	26,89141457	34,68014606	110,9137613	62,22510835	10,4961956	52,02129988	63,69963226	54,63925187	86,79371459	502,3605245	55,81783606
ENSG00000002834	LASP1	37,4512815	35,92442035	47,1239454	25,86807566	143,6775325	54,95890652	52,63321391	39,30959308	64,95811032	501,9050792	55,76723103
ENSG00000241468	ATP5MF	23,7127357	95,23460926	43,37711435	86,36660918	11,36547826	69,208412	57,13725888	64,19871597	51,22011845	501,821052	55,75789467
ENSG00000116754	SRSF11	38,20120882	36,2986987	31,69229029	83,36492107	75,78215104	33,42492086	48,482222	93,83046966	60,09907601	501,1759584	55,68621761
ENSG00000115756	HPCAL1	43,00485216	31,08331988	72,55917928	33,13459995	98,97756218	38,89069475	59,10403005	57,00618801	67,25278191	501,0132082	55,66813424
ENSG00000096092	TMEM14A	0	90,64719436	89,47132741	150,2753042	0	104,6177017	39,49521584	0	26,33452745	500,841271	55,64903011
ENSG00000163814	CDCP1	81,01179795	27,11085373	125,3644116	61,18419964	2,759046883	45,35262999	35,2620217	71,33690341	51,34481362	500,7266785	55,63629761
ENSG00000163568	AIM2	50,88088052	82,98951977	41,8305165	0	110,3797782	66,13404525	41,51099565	66,8274819	39,4700725	500,0232903	55,55814337
ENSG00000164221	CCDC112	114,0647274	98,60541	38,84129678	50,62917418	12,88672032	48,57718378	43,90863953	37,88087611	54,59130934	499,9853374	55,55392638
ENSG00000131652	THOC6	17,14211312	45,12282044	76,66870472	130,0466244	0	53,78713858	71,43714064	35,35294566	70,04215705	499,5996447	55,51107163
ENSG00000144959	NCEH1	59,40078589	20,63488769	37,46487191	68,92417174	76,85772693	68,66931107	72,65170638	63,15918626	31,57999349	499,3426414	55,48251571
ENSG00000102898	NUTF2	38,79755742	56,97293576	59,13831479	67,55848829	63,21399504	45,86728686	67,54531372	47,81666528	52,38222712	499,2927843	55,47697603
ENSG00000004487	KDM1A	24,02769184	28,29292427	86,40888739	80,05523394	66,80642516	51,32858467	46,22105613	40,02874632	76,11508302	499,2846327	55,4760703
ENSG00000135124	P2RX4	56,98826154	48,47523595	66,43720959	20,14818409	60,87260217	45,44798859	50,45571262	103,6784524	46,6267718	499,1304187	55,45893541
ENSG00000007202	KIAA0100	25,33786432	39,55907757	94,81795187	55,96309031	57,99782787	60,85065129	64,88251354	29,95815999	69,63205378	499,0000905	55,4444545
ENSG00000106554	CHCHD3	35,74990455	13,76691716	163,2362717	70,79041026	0	40,94525901	28,29304744	100,0557413	45,75684504	498,5943964	55,39937738
ENSG00000116729	WLS	38,32833717	34,51749452	144,3825766	45,87867685	79,81284693	38,80796849	54,7578529	11,41218287	50,38987339	498,2878097	55,36531219
ENSG00000120008	WDR11	41,39416305	32,20609061	32,53096703	87,12454788	43,80382089	40,76872395	44,21402472	122,4586125	53,65196604	498,1529167	55,35032407
ENSG00000168118	RAB4A	118,2862503	11,42236315	0,335927612	101,6148587	0,108494726	78,14338039	71,41574184	92,74130309	24,03090983	498,0992296	55,34435885
ENSG00000114650	SCAP	69,49932735	40,17012788	16,13593132	26,01880233	147,8047391	42,79690767	56,72903211	23,70006201	75,16025861	498,0151884	55,33502093
ENSG00000109323	MANBA	47,93382234	55,60127482	41,54433056	58,1954099	62,81422396	41,42466243	66,52288913	97,38857517	26,36637198	497,7915603	55,31017336
ENSG00000099797	TECR	58,71256619	40,77358435	43,58901366	58,21163359	88,88143954	51,43787402	52,36615653	55,51121485	47,82421102	497,5076938	55,27863264
ENSG00000089060	SLC8B1	39,02796654	11,72725059	89,2920708	12,02891727	54,97130229	73,32129653	92,47133806	71,52580399	52,72241767	497,0883637	55,23204042
ENSG00000104043	ATP8B4	1,72522887	25,50323967	56,81942913	90,76625854	126,9029673	52,80847654	43,81815388	51,56860252	47,05625046	496,9686069	55,2187341
ENSG00000128185	DGCR6L	24,35577655	62,96922186	47,93590962	33,46479744	86,20065791	69,15336863	79,47904046	35,50596739	57,81533925	496,8800791	55,20889768
ENSG00000133104	SPART	52,96039284	42,67947354	112,7831715	58,62450914	79,03443923	42,22670478	56,12944298	1,163145861	50,9698451	496,5711249	55,17456944
ENSG00000130479	MAP1S	31,33714044	28,21116928	64,63474023	38,61393299	76,07497096	73,52128941	73,02358062	35,70274164	75,3482758	496,4678414	55,16309349
ENSG00000120949	TNFRSF8	170,4949015	53,24855563	4,667032092	24,23163195	45,87886756	54,05585946	28,50401561	52,87646542	62,11135083	496,06868	55,11874223
ENSG00000105879	CBLL1	161,2828638	23,63998127	88,51345665	39,02297765	53,70623793	24,43127636	44,24694821	40,19054981	20,96948712	496,0037788	55,11153098
ENSG00000197265	GTF2E2	107,1830792	110,7904543	0,646465346	9,810345944	0	52,79557499	75,37946302	58,78661492	80,31739238	495,7093901	55,07882112
ENSG00000124831	LRRFIP1	67,67851251	66,03133684	50,36785724	46,31210852	26,40809889	39,37520647	53,88826409	61,12214538	84,05133229	495,2348622	55,0260958
ENSG00000079805	DNM2	40,8941647	35,18578354	73,3877387	43,32417342	43,62411267	83,80070042	82,36819757	16,49346089	75,88421935	494,9625513	54,99583903
ENSG00000136279	DBNL	52,17498531	42,39315009	50,08809601	27,84107976	36,86183084	66,84138671	80,66305923	33,94390211	104,1372465	494,9447365	54,99385962
ENSG00000102103	PQBP1	79,25975964	60,34377286	23,72881268	57,98554991	52,69590745	65,31083019	53,15339285	17,25019964	85,15507317	494,8832984	54,98703316
ENSG00000133597	ADCK2	23,37112041	74,32103025	0	76,18294435	27,25136334	116,2440986	71,6108808	0,212498114	105,6516564	494,8455923	54,98284359
ENSG00000141522	ARHGDIA	50,30010996	57,91867202	36,54833997	46,37835549	39,22188115	69,53817215	66,55382828	42,06575712	86,2974286	494,8225447	54,98028275
ENSG00000151651	ADAM8	87,21456199	86,6408856	24,45384453	20,98217411	22,31761338	109,6201102	99,18030653	2,775012114	41,35992308	494,5444315	54,94938128
ENSG00000038427	VCAN	90,24630484	49,65321953	76,81477596	54,54070732	65,27630488	21,13726519	35,93659053	83,76802192	16,8398879	494,2130781	54,91256423
ENSG00000066583	ISOC1	43,34118839	68,89951496	0,286282285	1,209276401	146,8276666	47,92782365	60,02159548	64,317336	61,15135311	493,9820369	54,88689299
ENSG00000163840	DTX3L	60,56997909	20,96602671	15,03637151	90,34104443	0	58,99490167	48,94387084	154,7336628	44,01288998	493,598747	54,84430522
ENSG00000101916	TLR8	70,36552418	26,48772342	22,7339571	53,64962147	204,4321778	41,35377343	60,09778135	0,075909618	14,34514851	493,5416169	54,83795743
ENSG00000067066	SP100	41,88844035	70,27012653	129,5693287	17,50151096	0,076224185	47,81051216	56,71642501	59,52217411	70,16251895	493,517261	54,83525122
ENSG00000013374	NUB1	52,92081527	33,20183701	67,14470327	33,22568795	63,58059611	84,19708808	54,79327195	44,94545217	59,17825871	493,1877105	54,7986345
ENSG00000197093	GAL3ST4	40,6942989	49,70156558	31,51122446	42,44989936	75,58632096	93,27386895	79,99502478	21,46204912	58,47560612	493,1498582	54,79442869
ENSG00000142867	BCL10	68,94542494	40,76130014	122,6772286	59,10307134	2,955059898	43,50751589	32,65815051	59,5326029	62,98176859	493,1221228	54,79134698
ENSG00000136144	RCBTB1	0,067026201	60,5980701	105,2366443	124,0150035	0	53,16939085	35,53462275	66,32142018	47,60539659	492,5475744	54,72750826
ENSG00000214160	ALG3	35,55286232	54,26314725	68,77279008	46,02519327	57,84861104	47,12933241	64,51000615	37,48867928	80,85182132	492,4424431	54,71582701
ENSG00000163931	TKT	39,37589697	74,35846877	30,512865	51,26979558	30,57525697	53,13259155	79,01646671	51,74559424	82,42025309	492,4071889	54,71190988
ENSG00000187554	TLR5	41,73958088	93,76127871	126,9297465	54,32630404	0	43,78940735	33,06718436	72,85541674	25,93151397	492,4004325	54,71115917
ENSG00000163754	GYG1	68,99180996	25,27912334	106,726623	32,62616173	0,306850649	78,58424638	66,43387844	73,8795601	39,22309114	492,0513448	54,67237164
ENSG00000142864	SERBP1	40,15966182	44,92321679	91,65810716	119,5895311	28,95958295	41,02374604	39,229911	47,91215133	38,49608786	491,951996	54,66133289
ENSG00000126903	SLC10A3	102,6233782	47,85266668	24,24748527	10,4513341	0	95,54440866	83,20017565	56,4694588	71,46034501	491,8492524	54,64991693
ENSG00000127507	ADGRE2	39,51746004	41,23590814	100,1654742	92,30250143	42,3860094	44,11739242	54,61923038	18,73558645	58,55821339	491,6377759	54,62641954
ENSG00000111653	ING4	128,9097559	79,77942121	30,47622348	80,23581906	0	53,71117599	32,74548716	34,88530318	50,35487167	491,0980577	54,56645085
ENSG00000100568	VTI1B	103,7182298	38,35248788	145,6889458	27,59541167	12,20302328	35,8686701	33,43410199	42,22092168	50,8435874	489,9253795	54,43615328
ENSG00000107566	ERLIN1	21,68616325	39,40327873	4,777658533	52,02255581	134,0390025	45,86184661	77,66868733	52,05929881	62,28600145	489,804493	54,42272144
ENSG00000181788	SIAH2	26,71408714	68,08492938	88,41444156	72,23285857	10,69536781	49,09534057	52,60268214	45,29439089	76,4958194	489,6299175	54,40332416
ENSG00000111321	AC005840.1	48,05271927	56,496835	77,6410031	53,53600032	49,10061016	40,40546966	65,90711148	42,86892942	55,5031802	489,5118586	54,39020651
ENSG00000178719	GRINA	65,30796905	46,65183078	40,55937387	44,98523089	75,40190391	45,72545352	64,00028797	65,45975418	41,29922717	489,3910314	54,37678126
ENSG00000067057	PFKP	28,59510439	24,81544617	33,88414242	34,43740302	154,7296145	31,93345454	28,60018731	15,94462803	136,3900657	489,330046	54,37000512
ENSG00000077238	IL4R	36,2624279	31,44425222	110,360556	17,07341353	63,23007714	59,94661558	77,53401209	18,30755762	75,07769943	489,2366115	54,3596235
ENSG00000095380	NANS	74,8964175	55,4664044	40,0819003	52,49391499	49,30492317	71,39773753	58,11686033	41,07815654	46,32029525	489,15661	54,35073445
ENSG00000005483	KMT2E	47,48908737	65,93893838	47,54962369	50,25916855	73,44967175	33,80758258	52,68367532	55,05452983	62,75415555	488,986433	54,33182589
ENSG00000161642	ZNF385A	40,4038959	25,72611199	71,87783623	35,1305974	92,85769733	51,1979477	46,33600811	58,00015372	67,05440738	488,5846558	54,28718397
ENSG00000135905	DOCK10	28,66607097	35,22859832	139,5946223	104,8140932	11,22142698	42,53437599	53,85255705	4,927918983	67,67354056	488,5132044	54,27924493
ENSG00000182810	DDX28	98,02331624	10,13722637	106,8858472	23,69948824	72,18775863	45,89504196	30,19617949	52,71109284	48,49660286	488,2325538	54,24806154
ENSG00000089048	ESF1	13,15639297	71,28775189	46,76219949	102,1508498	59,51792602	38,43388671	29,49489148	94,11631338	33,28594429	488,206156	54,24512845
ENSG00000146094	DOK3	42,422499	33,35791003	39,13507925	67,84417544	29,7092887	64,43095794	97,29608992	42,13503194	71,86476551	488,1957977	54,24397752
ENSG00000006125	AP2B1	45,02860109	26,88131499	45,92109658	84,21692633	77,63297155	56,76060699	54,67736244	37,74032012	58,92933462	487,7885347	54,19872608
ENSG00000198355	PIM3	61,59921352	51,23732486	65,25829439	59,1199351	0,816918886	54,1209661	80,43116369	57,72833209	57,32663426	487,6387829	54,18208699
ENSG00000100938	GMPR2	71,19632656	108,9511039	2,138308454	53,76407444	38,08224488	73,74816294	42,01877327	49,65596385	47,8912192	487,4461775	54,16068639
ENSG00000181222	POLR2A	22,26416667	21,50270976	93,12582849	72,1123706	108,6593833	53,79434498	61,49623094	24,79742356	29,42847983	487,1809381	54,13121534
ENSG00000117419	ERI3	71,18745735	89,44485389	18,56527771	42,0681521	0	45,69242813	62,03685715	97,05403096	60,65870332	486,7077606	54,07864007
ENSG00000078140	UBE2K	0,204203474	15,27063514	172,2081347	57,82883221	65,30670365	37,15371988	38,34353968	55,11495489	45,17003312	486,6007567	54,06675075
ENSG00000166130	IKBIP	34,62874956	109,8203483	52,28403015	64,30922026	0	46,38960156	76,97603361	30,16217527	72,00827298	486,5784317	54,06427019
ENSG00000112414	ADGRG6	94,46862892	31,583349	4,297815764	41,45149012	102,3143806	56,72641812	52,25832664	43,40536689	59,94553822	486,4513143	54,05014604
ENSG00000103966	EHD4	43,65758094	51,55527774	46,34179931	52,12726921	60,73580002	64,57938394	67,61740425	51,64090583	47,59599388	485,8514151	53,98349057
ENSG00000215114	UBXN2B	68,83450358	47,85537112	88,77513643	39,81783937	13,29311394	42,95814932	41,84714	61,71856228	80,27025428	485,3700703	53,93000781
ENSG00000088833	NSFL1C	60,13418667	36,58161416	30,21985458	67,56102313	108,7635123	46,20905513	55,6173038	42,60081241	37,56199807	485,2493602	53,91659558
ENSG00000076641	PAG1	15,97059011	74,41226329	152,1413351	35,08170483	27,04051167	64,55506337	46,87352147	21,4034547	47,74719687	485,2256415	53,91396016
ENSG00000149541	B3GAT3	72,69881214	30,9694541	21,59629352	36,0994377	99,51449969	66,79158655	63,69952044	37,61490299	56,20950071	485,1940079	53,91044532

ENSG00000175220	ARHGAP1	64,97606922	36,49734811	18,64666245	63,08831607	78,52407349	49,27074111	71,84616699	40,61838139	61,60591328	485,0736721	53,89707468
ENSG00000166002	SMCO4	71,04656125	40,48798736	52,52969321	27,83935534	0	64,65412166	84,17629868	54,07654315	90,23470385	485,0452645	53,89391828
ENSG00000116266	STXBP3	40,18843588	43,05199119	47,60385605	158,5007254	0,426650169	47,25016085	45,93338529	47,78178064	53,8425006	484,5794861	53,84216512
ENSG00000170515	PA2G4	49,25866926	54,30250002	59,40077534	47,74089619	72,12444496	66,08616826	31,99843942	36,55086278	66,96704294	484,4297992	53,82553324
ENSG00000120337	TNFSF18	2,558645121	47,13850898	106,7343906	2,98879702	5,18645853	57,09372933	80,9183665	171,9642047	9,62551709	484,2086179	53,80095754
ENSG00000087365	SF3B2	60,01226339	52,06800247	64,62079483	34,57821307	66,25067882	58,33547996	47,31877554	34,57840038	66,42661301	484,1892215	53,79880239
ENSG00000146535	GNA12	52,42258383	21,06171822	0,090976959	95,64626789	58,58945995	68,74252663	76,93296253	17,73564689	92,84723163	484,0693745	53,78548606
ENSG00000185811	IKZF1	32,93187368	24,55719507	57,63064083	75,67830462	5,651255012	68,67149579	64,2963705	70,10775499	84,54061379	484,0655043	53,78505603
ENSG00000156469	MTERF3	32,38682969	65,65831318	104,1846568	84,32212949	0	36,20369608	78,65408375	33,2085823	49,3251736	483,9434648	53,77149609
ENSG00000125651	GTF2F1	74,66207264	55,56767457	0,289965333	7,258274676	87,40691226	43,90053855	55,58039046	63,19158975	95,83115178	483,68857	53,74317445
ENSG00000132514	CLEC10A	102,0256465	40,94351318	0	21,13689547	0	49,59173982	20,88631286	237,4203818	11,51661317	483,5211029	53,72456699
ENSG00000140262	TCF12	44,64848642	45,78795746	33,13631076	53,17592908	121,5433816	53,30707181	50,92666239	24,80183235	56,11203488	483,4396668	53,71551853
ENSG00000117054	ACADM	75,28674261	48,43710811	39,81107418	46,20780336	0,300328281	87,48201431	56,55739824	54,92274329	74,33083778	483,3360502	53,70400557
ENSG00000124228	DDX27	31,1802277	60,88105884	105,9044577	41,50477514	32,83849251	58,2222383	40,99571229	37,84526383	73,52806333	482,9002897	53,65558774
ENSG00000100554	ATP6V1D	63,76033497	11,99599247	0	79,25738574	83,54323801	52,77729906	58,90931057	67,3006595	64,98283155	482,5270519	53,61411688
ENSG00000143321	HDGF	61,90022015	24,81444748	83,98418886	60,08464576	51,31646972	64,35018285	28,61843718	47,12784795	60,29642203	482,492862	53,610318
ENSG00000101444	AHCY	11,64180051	35,25647253	31,83850132	158,0083899	82,90874951	48,01777605	31,51257953	47,43526534	35,8425111	482,4620458	53,60689397
ENSG00000230989	HSBP1	54,4949932	70,18141798	67,24054048	85,25245574	8,030660002	51,45405182	47,73800348	55,99174719	42,04716593	482,4310358	53,60344842
ENSG00000169826	CSGALNACT2	70,71894436	23,71614503	134,375506	0,346527191	64,57438942	46,57834783	43,58969303	46,42759262	50,94119814	481,2683436	53,4742604
ENSG00000197601	FAR1	51,41747127	55,71549755	48,83083706	113,2352762	0,047863194	58,06759622	36,5725198	51,0867784	66,28352574	481,2573654	53,4730406
ENSG00000183386	FHL3	78,34525096	103,5638264	103,4796177	25,65079341	14,69443038	59,19464189	39,06860637	0	57,21921369	481,2163808	53,46848675
ENSG00000205250	E2F4	58,03217411	46,08224584	7,034535319	36,1361561	0,798926679	77,55719673	62,49092453	110,6349331	82,4319252	481,1990176	53,46655751
ENSG00000107625	DDX50	65,47749392	83,61954325	99,94440966	20,31870704	42,5482036	41,23038549	54,46085573	8,371665645	65,22631296	481,1975773	53,46639748
ENSG00000162231	NXF1	116,1994911	43,68021926	26,20929574	98,9143236	0,173934877	63,99101836	44,58511475	18,67163362	68,61979277	481,0448241	53,4494249
ENSG00000104472	CHRAC1	6,492496531	72,93786931	34,6528898	87,2414968	0	72,78981429	46,08804328	97,98059793	62,81106365	480,9942716	53,44380796
ENSG00000149177	PTPRJ	18,77101164	24,72369585	56,43400554	29,83663951	120,5022273	37,4419815	63,60902382	85,56263074	44,08542244	480,9666383	53,44073759
ENSG00000185515	BRCC3	0	41,71285042	19,65604094	67,07882125	80,01707653	41,50371435	43,76872047	148,3944051	38,7228926	480,8545217	53,42828018
ENSG00000004478	FKBP4	28,7996904	45,2875894	20,08262191	31,28389488	94,03968957	66,61488001	77,93595869	54,20842121	62,09282412	480,3455702	53,37173002
ENSG00000150681	RGS18	84,42986187	33,41005285	10,02355944	53,00678349	91,79870058	45,01679339	30,10212892	71,13603863	60,71030628	479,6342254	53,29269172
ENSG00000166938	DIS3L	24,82371957	20,668046	48,27321807	76,4180426	94,13953235	22,12751437	49,71727283	87,04604501	56,23942773	479,4528185	53,27253539
ENSG00000171608	PIK3CD	38,61290324	48,78388883	56,23697201	76,64861857	0	62,51778838	87,20703271	30,11066732	79,24599023	479,3638613	53,26265126
ENSG00000168591	TMUB2	76,22752163	43,73844973	75,47208403	33,06536481	48,99257925	72,10780524	45,46565819	14,78892915	69,44817468	479,3065667	53,25628519
ENSG00000111540	AC034102.1	73,13520033	45,20401534	72,84995697	51,86707464	1,265854939	64,71148164	64,87921842	50,03361727	55,25551773	479,2019373	53,2446597
ENSG00000135486	HNRNPA1	38,89769451	43,54082742	49,50480066	134,2844234	34,55087291	42,41420987	37,02766719	59,68973142	39,04492025	478,9551477	53,21723863
ENSG00000182957	SPATA13	27,7177877	39,90850363	47,52274419	57,0204847	139,9056073	48,36833888	44,88113143	20,27002073	53,35798684	478,9526054	53,21695616
ENSG00000148841	ITPRIP	16,3206044	34,32629223	21,75529558	81,75676425	69,74637576	49,30018233	80,08708006	29,52896629	96,0818369	478,9033978	53,21148864
ENSG00000179526	SHARPIN	73,13903654	17,53569674	44,04930782	34,41003797	46,71237885	54,84647164	66,49972681	62,9286097	78,53234286	478,6536089	53,18373432
ENSG00000119777	TMEM214	33,70667649	67,73193728	60,76036794	10,68403542	91,66312499	49,17496213	71,84703672	30,02114832	63,03411277	478,6234021	53,18037801
ENSG00000110717	NDUFS8	45,3699215	35,86688261	51,62575351	44,36413784	35,09395395	71,78576135	73,40139254	57,27629912	63,81155187	478,5956543	53,17729492
ENSG00000187239	FNBP1	74,31892612	23,40148364	29,39958138	81,08752014	64,69556937	38,66219197	49,04749342	44,95912757	72,84620502	478,4170186	53,15744651
ENSG00000162910	MRPL55	32,20914603	68,67051222	87,55046596	59,90712832	0	85,06655762	50,494158	26,10538206	67,69700997	477,7003602	53,0778178
ENSG00000137944	KYAT3	70,60084039	29,54934609	76,71035518	36,59743724	0	72,37811133	41,99485156	79,14780625	70,24253802	477,2212861	53,02458734
ENSG00000136271	DDX56	79,78994734	36,582795	0,273333431	27,19676235	65,85587183	67,53881883	79,72979553	65,38835468	54,82198297	477,177662	53,01974022
ENSG00000174547	MRPL11	74,57327493	47,84101065	40,72808067	85,09419416	7,354908311	61,29347024	51,39391307	44,2488196	63,46157338	475,989245	52,88769389
ENSG00000114767	RRP9	75,45728885	77,90377175	1,081634626	75,97912036	118,0756122	28,51251805	51,66904074	2,357185631	44,71404654	475,7502187	52,86113541
ENSG00000023041	ZDHHC6	67,85461324	78,12461689	0,074167582	57,5755667	58,35180984	49,04720357	56,97718918	30,6806909	77,05589531	475,7417532	52,8601948
ENSG00000182179	UBA7	73,52940735	46,55652309	3,787860689	120,2021334	0,078926932	62,53552506	83,83072731	23,02148525	61,96245589	475,5050449	52,83389388
ENSG00000115652	UXS1	42,15087682	42,60949339	2,691534491	55,43428479	147,574146	46,20832807	34,19481923	39,71049053	64,83325923	475,4072325	52,82302584
ENSG00000169727	GPS1	71,98313456	42,99856281	47,24080967	32,60097408	34,77401385	59,05931333	69,16666718	44,18320862	73,01005538	475,0167395	52,77963772
ENSG00000108344	PSMD3	92,13687127	43,25569515	29,07201520	38,24728277	19,50386006	68,53483544	76,37654976	47,63381409	59,99769617	474,7586199	52,75095777
ENSG00000120437	ACAT2	78,80667584	51,02120402	19,65711796	57,23695768	37,67434557	63,94831952	71,59309709	35,35703677	59,44149061	474,736245	52,74847167
ENSG00000173085	COQ2	28,76221388	38,66503246	96,30096436	9,312387342	73,14398393	69,85708493	54,00561514	90,88737383	13,80114765	474,7358035	52,74842261
ENSG00000136518	ACTL6A	37,28327875	77,70854583	74,42106249	58,33333674	0,097905546	55,09244341	56,7147793	18,37749503	96,67261846	474,7014655	52,74460728
ENSG00000060491	OGFR	57,55827989	36,95364017	44,69437576	47,28128421	83,93666079	76,49811363	49,60641157	17,35800472	60,74991594	474,6366867	52,73740963
ENSG00000087302	RTRAF	32,80414102	70,79699483	24,95682317	67,19382597	40,86495022	63,38516783	49,52497357	68,50326861	56,46935478	474,4995	52,72216667
ENSG00000164307	ERAP1	22,42408594	39,62224142	60,03950281	102,0093179	89,31608102	43,22654421	41,01276931	44,75111323	32,04653688	474,4481928	52,71646587
ENSG00000198624	CCDC69	68,71998629	55,08280309	69,76225921	46,84260726	0	51,13981144	59,57364883	62,26160339	60,85805971	474,2407792	52,69341991
ENSG00000137075	RNF38	82,00542699	33,33208636	44,42790438	77,92339662	134,0986326	34,19304	29,70159537	10,06700295	28,37051016	474,1195955	52,67995505
ENSG00000224877	NDUFAF8	31,71177771	30,43882301	86,98555749	110,2981297	32,98839261	64,43129192	37,66921095	30,35358611	48,99463955	473,871409	52,65237878
ENSG00000115419	GLS	24,74172262	34,78997152	38,21625254	127,6396075	118,475534	31,92601003	29,32790807	24,47912538	44,02673286	473,6228645	52,62476272
ENSG00000167088	SNRPD1	59,51804665	56,1984159	57,51706696	79,37532444	18,02235301	41,80182157	50,45135727	67,96268357	42,69653751	473,5436069	52,61595632
ENSG00000205423	CNEP1R1	47,38028903	60,20038202	152,2968996	67,32596999	0	35,85860478	32,88415834	34,16652302	43,32563731	473,4384641	52,60427379
ENSG00000104852	SNRNP70	18,93721285	21,75362084	28,60512547	46,34882555	129,8772666	63,86352899	66,19068966	42,53886512	55,19054494	473,30568	52,58952
ENSG00000125703	ATG4C	0,145485145	57,48358667	85,01766185	29,14164671	0	61,87037552	63,01703132	128,1017194	48,25091031	473,0284169	52,55871299
ENSG00000125454	SLC25A19	49,21574678	50,78201932	30,76558997	52,09256731	23,82565118	63,74248333	109,7297837	36,17242099	56,60584665	472,9321092	52,54801214
ENSG00000134109	EDEM1	20,20938462	56,91599527	10,19164086	67,58669002	49,68050311	66,29527571	72,07513505	52,13773865	77,62685374	472,719217	52,52435745
ENSG00000136877	FPGS	72,96053501	45,02002545	35,42814286	17,32486094	118,8531983	54,31872608	64,76196989	13,28126728	50,7283944	472,6771202	52,51968003
ENSG00000135372	NAT10	22,66514012	47,86234668	54,61747879	29,00386842	83,06353715	58,68923978	60,20271526	37,42438568	79,0745486	472,6032605	52,51147339
ENSG00000116791	CRYZ	6,06661777	24,54959805	0,136097968	39,47559714	220,4814679	37,36541752	37,68717194	73,60965886	33,16575636	472,5373835	52,50415372
ENSG00000166352	C11orf74	0,102517348	56,75732973	110,8902973	84,79501549	73,33404359	31,62881415	26,81353771	45,95596088	41,48134724	471,7588634	52,41765149
ENSG00000136021	SCYL2	59,95136663	33,87170032	46,52200026	64,87622681	51,17220962	55,39962847	55,56519315	40,37123309	63,91967676	471,6492351	52,40547057
ENSG00000166908	PIP4K2C	97,56456972	47,304833	63,95480301	4,508424842	0,217712969	41,99312869	50,50760864	96,35588177	68,78405689	471,1910195	52,35455773
ENSG00000138964	PARVG	50,04299724	37,97719315	44,30398515	43,65127646	59,02674371	70,09004992	63,76122544	25,9487533	76,35638347	471,1226078	52,34695643
ENSG00000129636	ITFG1	47,95864443	38,68560902	62,7512347	45,10111594	39,14704741	41,68049495	51,59301297	90,7525569	53,36210955	471,0318259	52,33686954
ENSG00000180573	HIST1H2AC	62,50976844	45,33335973	98,15963927	42,55054742	1,291789721	36,20590523	44,45621476	91,91944804	48,57858736	471,00526	52,33391777
ENSG00000135090	TAOK3	57,87884942	71,8983107	5,701073052	77,47281291	24,20940515	53,60745276	54,5865041	56,4110665	69,17189207	470,9373667	52,32637407
ENSG00000112367	FIG4	12,28404416	70,17079067	0,077388669	117,9001848	1,099747485	75,56506222	79,95589921	50,68748516	63,12159164	470,862194	52,31802156
ENSG00000006534	ALDH3B1	49,2375071	31,24141625	93,63893464	66,74989163	40,81127519	40,86418068	61,80729907	29,6722861	56,82539302	470,8481837	52,31646485
ENSG00000144048	DUSP11	29,33523494	27,08657902	0	82,53614744	162,2311471	54,53888879	14,2676478	35,52358076	64,60578878	470,1250146	52,23611274
ENSG00000165118	C9orf64	0	77,59955485	56,2218283	134,8258783	0	62,77371856	58,17923528	60,47168556	19,77234242	469,8442433	52,20491592
ENSG00000009844	VTA1	0	55,49039462	88,74068467	96,27774345	41,31484539	43,91212701	30,37357031	75,34709414	37,84529621	469,3017558	52,14463953
ENSG00000113552	GNPDA1	19,69513879	46,59805407	53,43340625	35,32739237	59,59591445	50,83342499	75,74490868	79,20747779	48,53884921	468,9745666	52,10828518
ENSG00000126458	RRAS	76,7894007	71,78029948	30,27006522	48,5405352	0	60,07107088	44,63191483	85,45721574	51,26800614	468,8085082	52,08983424
ENSG00000005075	POLR2J	31,3373213	93,60233656	35,08088334	41,59553839	0	65,50712807	68,80033703	56,08852438	76,39595048	468,4080195	52,04533551
ENSG00000155254	MARVELD1	57,7366998	50,27165353	36,28337097	66,80121124	48,68487596	18,35794871	49,87548053	94,39587319	45,92410355	468,3312175	52,03680194
ENSG00000183207	RUVBL2	29,01528375	32,12236365	56,36753069	39,01831396	59,0901422	71,56097658	71,70748513	55,02563741	54,00128345	467,9090168	51,98989076
ENSG00000128626	MRPS12	76,75345683	54,90407558	19,70754433	67,66783856	52,59464006	64,77012719	61,62325541	23,73457248	46,11490198	467,8704124	51,98560138
ENSG00000133731	IMPA1	35,40206786	49,06298571	1,135900518	67,11448949	233,8951712	18,00346326	36,1172261	15,67782625	11,22251417	467,6316445	51,95907162
ENSG00000136527	TRA2B	50,05451034	58,42806804	46,63453969	56,23942436	35,48906927	59,30631055	40,90923543	63,64224321	56,91585255	467,6192534	51,95769483
ENSG00000196636	SDHAF3	30,90450664	20,1426687	0,241406947	50,53278275	142,9920913	55,25549819	35,73970377	99,28819832	32,03693849	467,1337951	51,90375501
ENSG00000006625	GGCT	77,16687862	31,45706024	25,63555492	63,84230872	107,4649107	42,57053066	34,39807722	42,0816921	42,32263964	466,9396528	51,88218365
ENSG00000101052	IFT52	99,2146115	63,07982536	0	63,02677986	0	78,58235026	49,50697994	90,86300657	22,66421003	466,9377635	51,88197372
ENSG00000114098	ARMC8	67,89848448	48,81992345	149,829241	74,98436009	1,604505673	42,58955503	24,65323847	22,81635925	33,72336873	466,9190362	51,87989291
ENSG00000124659	TBCC	18,1407492	93,25235399	0	74,11457509	65,55672958	53,38706676	31,05701287	47,8475797	83,49293018	466,8489974	51,87211082
ENSG00000147872	PLIN2	50,7823191	78,39314112	67,678252	80,39984051	5,303272427	40,79062672	32,57571382	74,05268795	36,7910098	466,7668634	51,86298483
ENSG00000152240	HAUS1	70,74171123	52,51483249	71,4955894	106,1472148	0	53,08512081	25,87566545	56,44988836	30,25816432	466,5681868	51,84090965
ENSG00000167005	NUDT21	54,40490192	41,51000946	120,0517763	116,6946969	0,242237803	29,32112721	25,05174926	57,39419975	21,81267782	466,4833764	51,83148627
ENSG00000166136	NDUFB8	35,15078307	53,25281342	49,78332938	62,13359075	44,37320308	41,16640623	35,27480069	103,9736583	41,18559748	466,2941824	51,81046471
ENSG00000111711	GOLT1B	62,77330119	83,36534075	0	31,88516581	0,379115906	63,71071235	55,83082073	77,70314058	89,85770092	465,5052982	51,72281092
ENSG00000164543	STK17A	27,20149812	74,5494211	35,65854164	103,3546767	41,70641878	57,04544552	28,08345281	21,57156863	75,80366684	464,9746902	51,66385446
ENSG00000105185	PDCD5	12,04915432	76,8455732	44,721939	106,6014835	28,12975121	52,60579261	37,66464	60,75532386	45,15407999	464,5277377	51,61419308
ENSG00000108424	KPNB1	70,14363926	56,42322825	45,38970583	81,89879981	44,90732656	35,19381095	32,34923186	48,45969826	49,54609393	464,3115347	51,59017052
ENSG00000090863	GLG1	58,96214316	46,08067112	20,94060274	59,19161319	29,64863843	50,51037046	78,10441473	40,04274045	80,44437375	463,925568	51,54728534
ENSG00000053501	USE1	54,82497874	57,24627645	36,13398243	9,982292323	138,1221317	37,19509021	18,32248781	68,77823577	43,26990194	463,8753773	51,54170859
ENSG00000005700	IBTK	13,65697095	24,51074367	197,7965976	49,49953527	52,67935964	34,40375303	17,16780664	51,35208613	22,55139587	463,6182487	51,51313875
ENSG00000196704	AMZ2	34,62460976	77,00915918	66,36072347	53,22857051	52,22004698	49,49794551	59,67808689	24,4100186	46,29302952	463,3221904	51,48024338
ENSG00000187601	MAGEH1	0	62,90882765	0,950684333	201,501769	1,228172169	28,1435178	46,51499312	43,8469946	77,79621863	462,8911773	51,43235304
ENSG00000164754	RAD21	75,26243539	52,52846352	59,21443	56,29992346	48,89148711	42,12070843	46,24084264	41,25095618	40,66893373	462,4781805	51,3864645
ENSG00000164938	TP53INP1	147,2277011	27,9403679	39,35957808	101,8677563	0	45,5423504	73,26328973	7,78802011	19,29931987	462,2883836	51,36537595
ENSG00000160209	PDXK	61,51235369	27,41747449	71,8053026	52,7039581	23,66582192	53,762158	62,76676531	29,98654865	78,38254935	462,0029321	51,33365912
ENSG00000120709	FAM53C	62,62162636	46,85164432	62,40852314	49,84816491	39,77745009	58,40165244	73,02939193	35,47567262	33,44762966	461,8617555	51,31797283
ENSG00000136147	PHF11	53,25592811	46,69608357	58,29820483	62,60280673	0,074274569	63,89358714	52,29011768	82,2840164	42,43527623	461,8302953	51,31447725
ENSG00000055208	TAB2	15,6673236	38,12488417	33,46839084	32,78210421	131,5915344	46,53452683	70,34135465	36,43596295	56,86489334	461,810975	51,31233056
ENSG00000091039	OSBPL8	23,93651897	49,26722985	112,4855326	79,68757813	0,117129009	50,46942744	44,37431942	35,37631824	65,74739981	461,7582734	51,30647482
ENSG00000165092	ALDH1A1	111,0449863	24,45379508	51,09352524	46,58240568	0	36,1030762	20,80863605	171,1511439	0,392633498	461,6302019	51,29224466
ENSG00000133835	HSD17B4	9,834090026	57,90801071	89,14965665	34,54990474	41,52212394	41,94438436	59,62899874	51,31279481	75,53064774	461,3806117	51,26451241
ENSG00000113648	H2AFY	59,31068591	53,77963634	69,95754098	60,84067597	22,01696382	45,89911894	39,0352144	58,76256015	51,38294711	460,9853436	51,22059374

ENSG00000115275	MOGS	46,9269155	65,76362112	10,00542209	23,51618214	97,09400071	73,18767348	57,23883728	47,29713536	39,56498228	460,59477	51,17719666
ENSG00000057663	ATG5	63,57065587	52,63264723	54,17048083	35,80646159	0	39,95936436	46,37188221	97,58191472	70,16486738	460,2582722	51,13980802
ENSG00000087111	PIGS	36,40169939	46,37861801	56,98050479	45,75092304	16,24333116	50,61916799	68,25092937	54,35405134	85,00518552	459,9844106	51,10937896
ENSG00000116350	SRSF4	45,54252266	40,92469318	81,75885264	61,6335166	49,99751385	40,62039557	43,15803266	28,81827707	67,1546257	459,6084299	51,06760332
ENSG00000160131	VMA21	64,92223578	87,17632847	3,373412828	153,1186248	0,070291161	46,27570401	39,9324251	9,184127733	55,54661177	459,5997616	51,06664018
ENSG00000129460	NGDN	36,405534	35,84447111	97,02120798	71,94918814	33,41884716	36,90467373	35,29780026	55,17895099	57,19583492	459,2165083	51,02405648
ENSG00000100258	LMF2	68,67765903	44,18000328	39,18022929	47,7600394	72,64367904	54,13069387	65,41650588	24,04140573	43,05090728	459,0811228	51,00901364
ENSG00000148719	DNAJB12	80,90169022	75,75204133	20,36923139	21,97889782	32,50632926	63,76591849	48,92171171	56,55606014	57,6097517	458,3616321	50,92907023
ENSG00000115204	MPV17	78,12080702	42,90272001	41,51169478	55,89807011	53,11747471	54,24169788	50,83500207	38,35978179	43,21551131	458,2027597	50,91141774
ENSG00000170473	PYM1	32,72178193	51,26571905	39,85998926	38,93418781	0	89,36829069	67,16782528	49,30424345	89,37008573	457,9921232	50,88801369
ENSG00000198464	ZNF480	14,5370615	16,99413584	86,94051403	52,30550379	143,3059887	33,25746697	48,3853386	23,53425388	38,63489842	457,8951617	50,87724019
ENSG00000197766	CFD	57,74847504	49,15205239	43,90356719	35,65968965	76,70186461	32,17741954	57,0879743	82,74384424	22,50841677	457,6833037	50,85370041
ENSG00000108094	CUL2	28,77178147	53,45127105	31,05470311	51,30487552	132,4276463	50,25346592	39,32070051	51,87315534	19,17320834	457,6308075	50,8478675
ENSG00000166734	CASC4	28,54192623	14,30342266	56,84800617	36,54191411	103,2115442	54,66525828	60,14805782	63,66853243	39,54960474	457,4782666	50,83091851
ENSG00000135452	TSPAN31	55,45421247	43,7626536	89,20138389	93,91870158	5,402834859	47,39399449	47,40680607	34,35865052	40,41834478	457,3175823	50,81306469
ENSG00000135336	ORC3	33,05671741	51,89845163	29,84009737	105,0388824	0	67,55155495	64,14666472	69,41902679	35,91991607	456,8713114	50,76347904
ENSG00000186577	SMIM29	38,14205723	44,97773099	65,39831911	37,61602796	0	74,9531705	70,66951711	55,07242792	69,91653732	456,7457881	50,74953201
ENSG00000131773	KHDRBS3	45,49396431	74,15307914	43,72576158	67,93484014	86,39422717	17,3279263	21,94207055	22,24299309	77,51128375	456,726146	50,74734956
ENSG00000235568	NFAM1	69,93117693	34,01904694	32,39994965	18,92978499	129,4601145	51,76683801	42,34111427	28,06166002	49,64852942	456,5582148	50,72869053
ENSG00000196372	ASB13	48,96123385	74,11184152	0,462837328	21,46218406	102,1266671	42,04393902	50,43046644	58,75859442	58,17373658	456,5315003	50,72572225
ENSG00000138756	BMP2K	31,8095506	48,24289557	66,62795289	52,01370343	100,168427	42,50435986	44,97135466	37,73830088	32,18372558	456,2602704	50,69558561
ENSG00000131828	PDHA1	103,789722	39,03068508	20,54070618	65,16114879	52,4329196	68,24009476	55,43258603	5,662911573	45,95492683	456,2457009	50,69396676
ENSG00000183484	GPR132	66,79995184	15,4721898	31,54948191	12,62989982	146,5925686	49,92349733	32,51937312	20,09079988	80,47145552	456,0492178	50,67213531
ENSG00000176046	NUPR1	74,1437837	80,95298663	33,46147473	38,81373256	75,82354877	44,88416201	24,08128156	63,07025672	20,79882651	456,0300532	50,67000591
ENSG00000171604	CXXC5	100,5014704	28,26266141	63,79991205	64,63211492	0	41,48857373	34,88042168	72,96552505	49,41215836	455,9428376	50,66031529
ENSG00000099995	SF3A1	42,20831683	60,1734309	43,4109534	73,34007936	32,94238049	52,71432702	51,81301442	30,46857383	68,84078448	455,9118607	50,65687342
ENSG00000186222	BLOC1S4	108,964085	47,59230305	41,94628581	71,48682359	5,996396811	46,78739825	32,58969662	45,77448554	54,59313425	455,7306089	50,63673432
ENSG00000167613	LAIR1	26,34277945	49,26955596	50,49173896	49,23850407	37,95046562	74,66078734	69,98729212	34,75888339	62,86222349	455,5658304	50,6184256
ENSG00000150712	MTMR12	84,21479929	0	118,4296891	35,24488341	110,7978077	22,55999332	20,96851647	37,63232717	25,10257208	454,9505886	50,5500654
ENSG00000154511	DIPK1A	0	109,8245785	155,942249	10,88987762	0	53,99742483	40,47037237	28,11526731	55,67579412	454,9155637	50,54617375
ENSG00000156831	NSMCE2	35,71888113	94,5803444	41,35778805	26,1720135	64,83566462	51,42063009	32,63654908	57,22671547	50,70236114	454,6509475	50,51677194
ENSG00000123091	RNF11	34,81564241	42,65112148	4,287610781	69,25844763	104,5502871	51,82039864	39,6731384	67,53097202	39,53418951	454,1218079	50,45797866
ENSG00000144468	RHBDD1	0,043635741	40,68975485	0,160542939	46,37755613	232,8612305	27,23654717	21,52162206	53,10044378	31,52897252	453,5203057	50,39114508
ENSG00000188428	BLOC1S5	70,99492689	52,94523569	46,45648606	139,4502351	57,51108959	17,46162272	17,20127087	7,015595913	44,36190871	453,3983716	50,37759684
ENSG00000011295	TTC19	30,12726442	68,91789761	57,12045641	89,47819743	16,25342032	35,73627222	46,21718539	52,39794926	57,00644068	453,2550837	50,36167597
ENSG00000053254	FOXN3	46,17001121	47,02753287	30,73830515	66,15019704	51,75354775	55,06793546	54,5073236	22,55209185	79,28755373	453,2544987	50,36161096
ENSG00000064961	HMG20B	54,84957193	29,4073285	76,25173573	44,44757021	65,9204615	46,19858942	49,54402791	29,6423024	56,85737524	453,1189628	50,34655143
ENSG00000141232	TOB1	54,42617936	54,13894409	24,92007234	40,15976602	23,07697019	73,51135033	46,63455021	70,25502903	65,46279721	452,5856588	50,28729542
ENSG00000148154	UGCG	4,632248718	49,7885737	36,58113411	98,10058055	51,190173	53,07540017	51,42992369	47,24628259	60,42962242	452,4739389	50,2748821
ENSG00000184988	TMEM106A	56,14368141	40,18007046	130,4804546	0,583583952	3,31307769	54,63870823	69,48748328	58,75213445	38,81111326	452,3903074	50,26558971
ENSG00000178896	EXOSC4	46,33621395	26,11772728	0	17,69688861	181,3724961	51,59268294	41,37080932	47,92016455	39,95834317	452,365326	50,262814
ENSG00000111817	DSE	40,21637029	39,92861128	57,75984133	99,24583518	39,70444562	54,32959801	54,62564967	28,94078612	37,5508161	452,3019536	50,25577262
ENSG00000135823	STX6	23,98358293	65,28638786	0,901278691	48,42424571	12,86324993	85,8113991	67,0841068	59,48536448	88,11713292	451,9567484	50,21741649
ENSG00000079950	STX7	50,17346769	44,6057907	87,88764831	72,78536455	0,065744348	41,18656411	34,16419243	78,21783654	42,78995109	451,8765598	50,20850664
ENSG00000092094	OSGEP	39,52723335	46,36921886	39,56146907	63,69074713	78,44766032	56,32583284	37,75831498	24,03876997	65,97533071	451,6945772	50,18828636
ENSG00000160570	DEDD2	98,84503691	34,04007147	16,58794205	37,68730154	66,74590582	42,43930753	49,16758354	45,33898855	60,74800804	451,6001455	50,17779394
ENSG00000124942	AHNAK	59,01276884	38,50081631	81,75465256	81,44667141	31,43616008	22,93564995	28,38648629	69,90288447	37,86434333	451,2404332	50,13782592
ENSG00000111790	FGFR1OP2	38,62513567	45,98152245	14,40923355	66,29806034	25,02348613	46,97604024	45,12155379	79,80073427	88,7224475	450,9582139	50,10646821
ENSG00000129534	MIS18BP1	14,60332555	51,31165314	44,07652449	113,141405	4,194492895	50,78190932	45,53067408	73,22430016	53,64274019	450,5070248	50,05633609
ENSG00000206527	HACD2	16,76432658	40,17762868	61,03950823	130,4424887	0	52,99347219	28,80119903	76,98954476	43,052011	450,2601792	50,0289088
ENSG00000169446	MMGT1	57,0404	43,11218353	15,4681275	82,5805444	106,063608	43,67469435	35,81510012	23,86766539	42,40959965	450,031923	50,003547
ENSG00000130332	LSM7	43,02080304	57,57260026	34,78873125	66,70401783	0,425999457	59,33812441	65,63750464	62,71593215	59,41356792	449,6172809	49,95747566
ENSG00000135617	PRADC1	31,85806903	72,19207695	68,90105018	68,76491496	0	62,74823093	58,713681	51,77749633	34,54839196	449,5039113	49,94487904
ENSG00000158669	GPAT4	42,27441459	25,02748735	89,88221616	20,64078146	77,9844619	45,91328089	26,94633028	33,58870084	87,22309384	449,4807673	49,94230748
ENSG00000175467	SART1	27,63615923	57,73839279	35,82935306	50,39175321	26,03660823	73,18415598	63,89616951	39,78452337	74,97792418	449,4750396	49,94167106
ENSG00000136634	IL10	66,8953524	30,48482136	87,97328423	113,6974205	0,166643871	47,86695068	17,00108443	39,86681557	45,18959521	449,1619682	49,90688536
ENSG00000001561	ENPP4	73,9283412	23,3267373	68,65701411	37,08378192	106,0958803	37,22627496	47,63225567	23,69395155	31,37460669	449,0188437	49,89098263
ENSG00000119537	KDSR	38,00074697	59,08164761	47,84732081	57,04537832	33,97214161	53,37357326	63,94096378	38,24851968	57,2925336	448,8028256	49,86698063
ENSG00000168496	FEN1	18,97475774	66,97450321	65,25829439	94,85306857	0	79,63405752	61,07973396	10,37305967	51,23885009	448,3863251	49,82070279
ENSG00000198663	C6orf89	11,73474459	15,37980682	78,77365203	102,5609539	0,048926096	59,15367564	66,82064411	53,21167014	60,66597648	448,3500498	49,8166722
ENSG00000124160	NCOA5	0,609316817	11,47754219	148,7331485	3,48021213	0	79,86762253	82,66097555	35,56476269	85,89060738	448,2841878	49,8093542
ENSG00000073737	DHRS9	60,40393211	128,565151	45,88524834	17,11733433	0	54,37442634	20,81134573	75,25096551	45,69346494	448,1018683	49,78909648
ENSG00000189091	SF3B3	45,85894251	25,98057556	47,48677432	67,69261865	104,5015851	35,8118835	35,03589248	28,13058456	57,08912983	447,5879865	49,7319985
ENSG00000143977	SNRPG	40,69020469	42,71423017	32,14735839	98,66418824	48,85952957	43,73493093	33,50143218	69,49615904	37,61179617	447,4198294	49,71331438
ENSG00000179388	EGR3	22,63806284	125,3069708	37,0603311	41,26847601	36,74657944	69,88484455	44,82329762	20,74653752	48,92641407	447,401514	49,71127933
ENSG00000103544	VPS35L	38,83357555	26,71262067	96,99561558	30,91038862	50,81401081	44,87926115	54,4685377	36,33528468	67,14982872	447,0991235	49,67768038
ENSG00000085760	MTIF2	58,26759112	36,31917429	5,437666429	35,26566343	175,9399222	25,02918896	34,84460613	40,54532038	34,92196062	446,5710935	49,61901039
ENSG00000198791	CNOT7	55,85331759	54,38324634	69,46326575	53,75627756	55,07716261	45,0829124	31,9381112	49,6210579	31,18674846	446,3620998	49,59578887
ENSG00000168092	PAFAH1B2	41,48928006	49,88864429	52,34690176	56,39348477	87,8530069	45,2483391	43,2344834	25,04847018	44,75637592	446,2589864	49,58433182
ENSG00000147251	DOCK11	2,563101481	32,35577475	53,35431151	48,29684696	156,7699553	24,9569399	46,16695894	34,16932368	47,53313075	446,1663432	49,57403814
ENSG00000181924	COA4	61,24212567	57,75175083	25,10704924	54,50749122	38,25707955	44,80123493	56,25534107	53,72048782	53,79994276	445,4425031	49,49361145
ENSG00000135185	TMEM243	36,04472328	79,5405933	40,03933536	64,95445545	0,329465635	43,27881405	39,94330753	94,78520622	46,09221088	445,0081117	49,44534574
ENSG00000134717	BTF3L4	42,78248167	54,05216175	60,70883465	91,02892944	31,87862515	44,51803638	49,15028137	39,27660377	31,43268349	444,8286377	49,42540419
ENSG00000100122	CRYBB1	78,96576696	28,86250695	104,2425371	46,43374114	129,6813347	20,73882209	19,27799372	0	16,29383572	444,4965384	49,38850427
ENSG00000266028	SRGAP2	45,33799131	13,68507815	70,59896485	68,00123581	93,49656717	34,8332828	34,88355387	32,21078272	50,82769001	443,8751467	49,31946074
ENSG00000182004	SNRPE	43,80753563	63,29698175	8,15230098	116,793491	77,86635173	29,67246853	32,69467305	43,84612531	27,69827976	443,8282078	49,31424531
ENSG00000198830	HMGN2	54,20837029	43,97562435	72,436959	47,62124654	42,79230671	44,43011367	46,07267443	45,0733888	47,20466699	443,8153508	49,31281675
ENSG00000087077	TRIP6	79,66894952	19,32554956	45,2956213	54,98747213	36,1508846	49,1298065	46,39404364	60,05547956	52,48544558	443,4932524	49,27702804
ENSG00000137996	RTCA	22,68897604	14,12667765	56,10859423	45,36505321	114,8182857	37,47601436	52,63391603	69,96029862	30,16869789	443,3465137	49,26072374
ENSG00000001167	NFYA	38,30302054	0,140720021	1,385888712	15,9532535	115,0570767	27,90571495	51,42604529	114,1044059	78,85432341	443,130449	49,23671655
ENSG00000086504	MRPL28	92,6433972	45,74809272	16,81404938	64,41885796	0	54,92174581	60,714112	49,42397377	57,97776122	442,6619901	49,18466556
ENSG00000090989	EXOC1	44,82355664	82,58593742	14,96855759	82,79714212	0	51,25391022	63,35468599	38,72616787	63,78410546	442,2940633	49,14378481
ENSG00000118523	CCN2	0,129264817	441,3218205	0	0,446424251	0,153600318	0	0	0	0	442,0511099	49,11678999
ENSG00000157837	SPPL3	59,36405659	43,48901638	73,59647312	21,63029073	31,92635307	65,65165484	61,04878746	31,4108847	53,75287609	441,870393	49,09671033
ENSG00000213281	NRAS	70,59511403	49,52507121	0	19,42234439	0	38,73547907	93,79820936	117,324895	52,45460095	441,855714	49,09507933
ENSG00000215845	TSTD1	33,83260897	51,74670177	30,88667766	59,72947622	33,90165605	71,52034668	64,04542427	31,19390654	64,7435253	441,6003256	49,06670285
ENSG00000077254	USP33	30,03426347	48,29501765	76,4666274	8,712919794	63,43475524	54,03245581	68,75658118	28,08550531	63,64128524	441,4594111	49,05104568
ENSG00000173457	PPP1R14B	57,14495043	39,13773463	36,04687653	69,40429662	32,27292751	53,63574735	54,25948615	47,03411214	52,45848759	441,394619	49,04384655
ENSG00000063046	EIF4B	52,1978102	55,75748066	45,09374531	64,66058677	13,08856646	46,89011906	39,97248781	66,12992484	57,57432125	441,3650424	49,04056026
ENSG00000162627	SNX7	42,54782396	138,0276188	0	54,40879248	0,17373853	67,18294761	55,48131759	0,15986191	83,2578034	441,2399043	49,02665603
ENSG00000140526	ABHD2	56,26872561	6,098978747	92,16304831	76,01532385	0,285353629	45,64683129	69,42741553	41,9114961	53,38380437	441,2009774	49,02233083
ENSG00000214212	C19orf38	30,08580305	86,90426936	110,5114182	17,14907694	0	59,65083879	43,89844734	47,90449594	45,02467292	441,1290226	49,01433584
ENSG00000070413	DGCR2	96,70537323	22,67605853	90,85808052	23,32353653	64,32721008	30,57727489	44,99471317	11,55489299	56,03579351	441,0529335	49,0058815
ENSG00000106028	SSBP1	30,22211413	60,31173831	40,78903965	83,49919187	46,48677332	44,32063446	34,50118878	54,86210106	45,79856006	440,7913416	48,97681574
ENSG00000146109	ABT1	40,9739162	68,06050767	89,73193817	65,96521274	0	33,09191591	41,26920876	37,50089893	64,15904405	440,7526424	48,97251583
ENSG00000017260	ATP2C1	24,56232996	60,02006565	38,49403716	81,28034249	0,462943041	59,57459358	65,77156862	23,31203676	87,20607936	440,6308966	48,95898851
ENSG00000173473	SMARCC1	0	22,43895541	36,75287772	134,2665143	4,145113291	80,9749811	45,40564612	50,23264215	66,30411078	440,5208409	48,9467601
ENSG00000177971	IMP3	81,18142939	35,4386503	17,99013506	61,4443371	32,90160719	51,68130635	40,84511782	74,20285025	44,77705683	440,4624903	48,9402767
ENSG00000082213	C5orf22	88,56697702	29,03901545	1,609146738	29,13962693	129,6668984	37,57598445	36,25505493	65,83187476	22,72652841	440,4111071	48,93456746
ENSG00000115966	ATF2	62,98244442	34,87571137	1,691758558	48,32129159	63,50591907	50,6299882	59,40805321	62,28326746	56,68141194	440,3798458	48,93109398
ENSG00000057704	TMCC3	7,750450031	60,3648913	0	144,2515216	0	77,757844	42,75470926	0,152227227	107,3354991	440,3671425	48,9296825
ENSG00000134049	IER3IP1	43,94451039	102,3031727	11,1638594	38,58854101	74,23289619	37,7835568	41,853085	49,56908821	40,83091559	440,2696253	48,91884726
ENSG00000174738	NR1D2	3,051948346	59,29395859	0,815753786	91,34751053	0,185974905	97,20360734	58,71742967	64,5126007	65,09280057	440,2215844	48,91350938
ENSG00000171488	LRRC8C	15,19612443	54,387724	46,85164411	67,19231391	41,78064891	51,19963037	55,15814493	39,54279241	68,86022627	440,1692493	48,90769437
ENSG00000086827	ZW10	67,00479008	0,091955418	90,56278945	112,4812288	0	29,01728193	50,94542973	43,40069937	46,58909732	440,0932721	48,89925245
ENSG00000128928	IVD	35,06153098	41,80309145	33,24247939	27,58901219	85,41938334	59,44907204	52,2045876	33,877133	71,38978266	440,0360726	48,89289696
ENSG00000110721	CHKA	50,55207897	43,98718392	56,49943719	62,79399827	0	49,21257756	62,00700441	34,43269674	80,29221687	439,7771939	48,86413266
ENSG00000164168	TMEM184C	0	51,50010825	28,14017555	52,16484424	0,990774204	48,97742194	71,73537233	101,7530894	84,40398083	439,6657668	48,85175187
ENSG00000144560	VGLL4	64,01502039	17,17622386	62,07247921	61,51538416	117,0063382	28,94483083	25,96895637	36,38986106	26,22626629	439,3153603	48,81281781
ENSG00000132581	SDF2	40,36266743	30,09757144	30,28414644	66,79997999	84,39046689	34,10826682	50,53456949	57,02277201	45,68076283	439,2812033	48,80902259
ENSG00000101166	PRELID3B	40,44321843	56,10757179	20,77362575	88,15738454	23,71648698	43,71227568	59,3552735	52,60306984	54,32635566	439,1952622	48,79947357
ENSG00000137500	CCDC90B	52,55819574	58,76972963	60,07861307	65,66127771	34,5926363	46,50351327	45,76291792	44,01765158	30,87527957	438,8198148	48,7577572
ENSG00000171161	ZNF672	13,37011465	27,38044481	92,21419482	54,05629653	32,35033728	63,77423397	66,00524282	38,24596839	51,16864954	438,5654828	48,72949809
ENSG00000136997	MYC	48,83149796	58,03990052	23,41268126	47,42719482	85,09745821	29,43166024	25,35141336	41,31333345	79,52106266	438,4262025	48,7140225
ENSG00000188921	HACD4	86,30884264	40,21251999	54,91639153	68,97888108	35,39436157	34,04408254	28,96417291	35,16412392	54,38272497	438,3661011	48,70734457
ENSG00000205358	MT1H	112,444657	2,966993085	0	51,97045137	32,28580948	12,62113654	25,68410669	95,51981274	104,6189966	438,1119635	48,67910706
ENSG00000120725	SIL1	87,61905355	59,39982451	13,42021096	24,47290559	1,264180436	53,35670797	87,89548528	68,79552977	41,54326334	437,7671614	48,64079571

ENSG00000170502	NUDT9	33,16484347	35,34370133	17,05826531	0,653562867	155,9474112	48,25639504	45,4898699	40,65772856	61,15363022	437,7254078	48,63615643
ENSG00000171603	CLSTN1	63,12449318	11,7486113	41,33260314	28,09998319	108,7526466	43,49934177	61,09034113	34,82848952	45,19442087	437,6709307	48,63010341
ENSG00000165732	DDX21	48,95216757	61,68398313	10,50879924	93,34472548	17,98115183	52,34540415	42,38692093	58,73801683	51,53475854	437,4759277	48,60843641
ENSG00000165685	TMEM52B	29,32125432	92,76222244	63,10124345	32,08400805	8,954779646	66,38325167	43,76537519	3,693593618	97,31774038	437,3834688	48,5981632
ENSG00000012061	ERCC1	57,96727295	49,86787197	39,28413164	51,94365527	0,708445654	57,39625223	54,25196744	53,74895135	71,59223993	436,7607878	48,52897643
ENSG00000171824	EXOSC10	69,21171856	13,11393858	35,35650733	31,48989929	84,57934439	55,6081519	45,48849292	44,78599046	57,07855132	436,7125948	48,52362164
ENSG00000198876	DCAF12	111,3280427	44,30712539	12,04687799	50,49378999	0,083225386	52,25318047	50,71153164	45,5639786	69,60092236	436,3886745	48,4876305
ENSG00000122692	SMU1	62,97685498	79,36041258	19,86685124	21,28141189	34,22084204	52,87004644	43,16559208	73,30127808	49,32641721	436,3697065	48,48552295
ENSG00000166946	CCNDBP1	61,78390124	64,66835614	107,3491892	26,74044368	39,77004188	32,45913912	38,59320355	27,75551063	37,08552691	436,2053124	48,46725693
ENSG00000164308	ERAP2	90,15327498	56,68482864	95,99339716	13,67605836	1,354795363	53,85052267	56,1083309	0,311646722	67,60786986	435,7407246	48,41563607
ENSG00000164902	PHAX	18,6515505	79,08065541	69,60888879	19,31004318	0,686461714	42,2704992	48,24200641	74,262056	83,48376	435,5959212	48,3995468
ENSG00000105835	NAMPT	19,22478428	52,64416972	125,5272091	92,05296374	9,194580783	31,29501645	31,55592353	49,37614077	24,71777695	435,5885653	48,39872948
ENSG00000107262	BAG1	62,17047372	31,42792564	34,66399651	64,63425357	15,99350005	56,06196498	58,57529143	65,59170547	46,13975585	435,2588672	48,36209636
ENSG00000125352	RNF113A	56,06615408	75,50783595	0	30,66419879	0,265423165	57,26551317	44,51275636	116,7388938	53,98202753	435,0028028	48,33364476
ENSG00000129235	TXNDC17	39,57467641	71,78325742	24,09552655	47,58063755	39,95467371	63,53182496	47,01821638	47,41701114	53,97603805	434,9318622	48,32576246
ENSG00000060339	CCAR1	36,73640379	35,44838126	58,26617028	52,49706784	3,615476165	65,96444616	57,05079463	44,50150307	80,63880295	434,7190461	48,30211624
ENSG00000170291	ELP5	86,20071932	49,30790392	0,301279009	64,19679108	0	89,36873191	37,48698142	63,15024791	44,11169261	434,1243544	48,23603937
ENSG00000108784	NAGLU	20,69725704	65,4138576	25,85641125	0,088904591	124,6816953	54,09758838	54,80509902	38,50378947	49,89678349	434,0413861	48,22682068
ENSG00000198900	TOP1	65,64042464	38,84971131	38,48840685	47,44907546	62,70611613	33,9463526	55,8131284	35,04340887	55,84354526	433,7801695	48,19779661
ENSG00000092108	SCFD1	44,29142583	8,365410138	52,45705718	103,1668032	0	50,57356341	34,23654014	91,92427171	48,36365136	433,378723	48,15319144
ENSG00000119922	IFIT2	34,12413476	101,6227904	134,964319	4,931760688	0	37,91427268	41,48466794	65,85981071	12,40801523	433,3097715	48,14553016
ENSG00000143569	UBAP2L	31,86151177	12,29386219	83,69412816	47,92328335	52,02729156	45,97202305	47,96067069	45,5376973	65,93675094	433,207219	48,13413545
ENSG00000140350	ANP32A	58,12869513	46,81111004	30,28892383	63,26220421	63,69290452	42,29136254	50,14489011	30,26647483	48,1121763	432,9987415	48,11097128
ENSG00000171227	TMEM37	46,36871723	39,32593279	0,121335704	17,08435015	130,88745	22,3048702	20,64701666	111,2025541	44,61614459	432,5583714	48,06204127
ENSG00000105755	ETHE1	85,72436147	20,06848931	46,15518625	79,7574828	0,903440358	59,6860691	69,99508458	27,01666275	42,6628809	431,9696575	47,99662861
ENSG00000164081	TEX264	59,34968616	54,07153201	79,12267585	13,99176639	30,99977379	70,20864243	46,58814015	33,8683813	43,39032728	431,5909254	47,95454726
ENSG00000138246	DNAJC13	49,08347508	37,31155557	26,55676958	66,6291903	92,25927308	35,59410711	42,34460841	32,83757852	48,51694257	431,1335002	47,90372225
ENSG00000095787	WAC	30,71060744	38,32485758	73,18987017	70,43617217	34,70958852	44,92163629	59,48207895	38,63384138	40,68251735	431,0911699	47,89901887
ENSG00000157778	PSMG3	67,61427065	30,37118572	41,83884143	24,3628063	131,5542834	33,18211534	30,66341277	25,11977069	45,9875323	430,6942186	47,85491318
ENSG00000065268	WDR18	50,58389489	51,53514045	23,82349597	27,89801171	120,3660834	24,98865283	53,179831	18,50545732	59,76735846	430,647926	47,84976955
ENSG00000184787	UBE2G2	50,38419166	45,91396027	73,9477289	59,29516101	20,66360841	48,21668046	42,25359478	32,05605672	57,88492792	430,6159101	47,84621224
ENSG00000167721	TSR1	98,32406473	12,28278954	100,6142576	79,38634845	0,129528419	38,08911887	48,7173354	11,14360038	41,72748787	430,4145313	47,82383681
ENSG00000113194	FAF2	68,846629	22,8128913	79,63069345	4,184473766	0,484966296	48,19920704	60,49008579	93,26242351	52,3909001	430,3022702	47,81136336
ENSG00000104969	SGTA	12,46020874	39,45177366	24,64442268	25,94583822	17,80588192	88,34257601	73,50377304	33,03455256	114,9333788	430,1224056	47,7913784
ENSG00000143437	ARNT	53,76049149	46,78346	52,76793442	23,95680908	0,055694408	50,17456957	54,2511299	86,04212762	62,19863447	429,990851	47,77676122
ENSG00000169554	ZEB2	35,10314597	45,94946717	63,14401482	79,8591396	18,60222564	33,42974307	45,47035218	87,45478697	20,91496499	429,9278404	47,76976005
ENSG00000103111	MON1B	34,37101571	22,3644797	0,080086459	57,20873399	166,6259873	43,25815958	40,14255364	3,760347282	62,10946869	429,9208324	47,76898137
ENSG00000154822	PLCL2	27,96716957	39,75361048	111,7307607	72,6673563	0,208789269	40,54520101	37,15478048	58,5945018	41,23919923	429,8613689	47,76237432
ENSG00000125871	MGME1	48,29306898	82,81032634	0,124598879	39,88293426	0	58,60041884	54,66479727	84,71921677	60,72707967	429,822441	47,758049
ENSG00000205629	LCMT1	65,40000505	47,16168819	86,36171278	31,36430378	0,137909963	34,40541913	35,46319676	75,75631214	53,45585452	429,5064023	47,72293359
ENSG00000100599	RIN3	50,87528549	37,76734948	56,32653458	63,24876113	33,90009995	47,36750789	46,26320794	33,93467032	59,65681933	429,3402361	47,70447068
ENSG00000141452	RMC1	20,73491326	77,66572494	17,63239463	21,36457883	92,74297696	51,92088274	46,0057884	28,76598662	72,41398473	429,2472311	47,69413679
ENSG00000198909	MAP3K3	76,6811296	25,77359654	22,7136187	51,22990577	0,235216992	53,49237359	44,68609335	52,64660786	101,7636762	429,2222186	47,69135762
ENSG00000135916	ITM2C	33,58666246	37,36661582	39,71144271	107,4617373	46,68947417	24,86398288	131,9172311	6,688438112	0	428,2855846	47,58728717
ENSG00000129084	PSMA1	49,34783253	64,5782763	40,33700921	57,94163477	57,1150287	39,25360812	28,77341452	39,43993323	51,12046114	427,9071985	47,54524428
ENSG00000100941	PNN	33,65203287	27,8158249	26,58767542	97,53243995	33,83548579	29,88206199	46,48044419	63,28808633	68,66991413	427,7439656	47,52710729
ENSG00000188042	ARL4C	58,82131345	35,76553775	23,66373523	70,57706687	88,42414121	40,07144837	69,67469067	15,12754145	25,57056827	427,6960433	47,52178259
ENSG00000173757	STAT5B	30,04866629	50,40919871	133,2338306	38,16684986	51,74813042	45,55013507	39,07932785	24,6531762	14,52871835	427,4180334	47,4908926
ENSG00000162639	HENMT1	32,07829509	17,1319085	58,32311228	58,61894524	0	39,93890222	62,01109978	122,4487536	36,38792171	426,9389384	47,43765982
ENSG00000138750	NUP54	152,7765354	31,64492158	0	66,10003992	0	34,54047884	40,2410486	53,40273016	47,96339713	426,6327516	47,40363907
ENSG00000075790	BCAP29	67,56757589	41,61318945	45,65233877	68,51521274	0,213531658	46,34605122	46,9562534	51,94844788	57,41815494	426,2307559	47,35897288
ENSG00000109099	PMP22	40,29930919	76,38391667	0,087113788	0	6,189741407	110,1737737	142,1420402	44,94158643	5,882324898	426,0998063	47,34442292
ENSG00000101464	PIGU	66,92438582	35,28966803	98,14084825	57,29958407	0,398281932	33,91813269	37,43236892	59,73475176	36,61966594	425,7576874	47,30640971
ENSG00000101132	PFDN4	31,64392329	82,15021689	49,91839987	18,11424666	0	77,00611391	44,48689913	70,52304023	51,39301419	425,2358542	47,24842824
ENSG00000158850	B4GALT3	16,27643422	25,91809564	30,79933745	80,02453077	59,54519661	50,11437728	37,60884502	88,51229289	36,40489716	425,204007	47,24488967
ENSG00000111802	TDP2	22,94322403	98,1776218	0,093375915	40,93269504	84,44147788	45,42059792	47,33059551	45,84127524	39,95356878	425,1344321	47,2371591
ENSG00000187778	MCRS1	55,88449807	65,28817788	44,24154289	46,00969347	0	36,02106357	58,21423254	55,52840938	63,77254168	424,9601595	47,2177955
ENSG00000116747	RO60	20,66937601	40,03570403	37,13731052	75,73916867	59,53728537	45,93437193	40,61037114	55,39915953	50,02460773	424,6873549	47,18748388
ENSG00000153015	CWC27	33,85493064	36,54846688	58,92253238	14,35247378	66,96718324	32,54108709	18,07823136	115,7895211	47,17220555	424,226632	47,13629245
ENSG00000167315	ACAA2	39,09832448	48,06935855	51,24551825	92,01670344	3,72255989	47,4801	37,7941122	60,9154782	43,85524641	424,1974014	47,1330446
ENSG00000052841	TTC17	58,86034976	24,35496566	33,61551126	39,98569768	119,4727093	35,72012416	26,50249753	31,50478304	53,71714724	423,7337856	47,08153174
ENSG00000130021	PUDP	0,620087569	38,85011933	96,95959892	98,67427385	0	48,60006792	49,78415635	90,11851841	0	423,6068224	47,06742471
ENSG00000146833	TRIM4	92,50154336	85,25062567	61,49549522	1,547150739	0,14684831	59,63418398	33,45191432	48,10251729	41,22745383	423,3577327	47,03974808
ENSG00000105643	ARRDC2	80,16648778	36,08380592	51,97514467	35,33904767	63,66069006	38,60632139	39,83013343	8,836292079	68,85579583	423,3537188	47,03930209
ENSG00000187193	MT1X	55,12429619	33,69242271	42,01088931	67,53788862	0	44,09551517	40,83053074	93,56253823	46,05808427	422,9121652	46,99024058
ENSG00000005175	RPAP3	25,25100003	40,04588001	145,1731582	26,33736192	9,934466592	38,95022137	19,04334759	61,39288974	56,76134905	422,8896745	46,98774162
ENSG00000137124	ALDH1B1	58,27234711	7,312181587	0,178363875	60,52485936	86,98548106	50,6194926	76,28048737	34,13536437	48,35467743	422,6632548	46,96258386
ENSG00000198417	MT1F	46,34492664	45,89687583	13,35865486	23,24744644	136,317296	28,66705429	30,35135461	67,79974927	30,50612461	422,4894826	46,94327584
ENSG00000105063	PPP6R1	45,01749971	12,82665335	51,84248894	28,67677741	83,17436267	60,67816429	44,50151194	19,80806672	75,36827629	421,8938013	46,87708904
ENSG00000157181	ODR4	42,92534502	54,51057601	4,913961976	74,44302492	0	51,73249016	49,84311249	108,1436972	35,31677099	421,8289787	46,86988652
ENSG00000205060	SLC35B4	14,05579149	26,66838724	73,35364947	34,53033601	144,3048167	42,73598141	27,99202169	17,77953597	20,50481807	421,725338	46,85837089
ENSG00000005249	PRKAR2B	28,15152349	26,04664196	9,848661414	73,16219959	104,0615122	30,86979994	21,9911633	99,70409522	27,68947481	421,5250719	46,8361191
ENSG00000176022	B3GALT6	31,06996464	32,0894368	65,25030022	21,12683743	0	67,57792311	87,86487738	39,66153545	76,82008236	421,4609574	46,82899527
ENSG00000166228	PCBD1	58,27776847	92,84056347	24,31080436	19,82731707	0	37,93940786	79,41957216	54,48025128	54,35511189	421,4507965	46,82786628
ENSG00000141759	TXNL4A	47,64747724	53,35085223	13,05446123	104,6840908	55,82601626	38,83013497	38,26400196	29,51552779	39,79117604	420,9637385	46,77374873
ENSG00000084093	REST	34,03442391	36,00754404	36,28401142	68,93547055	56,23160816	47,61382077	47,7959764	42,6754402	51,30975011	420,8880456	46,7653384
ENSG00000151135	TMEM263	15,1807809	33,57398785	87,97650364	55,71136832	101,1524453	27,83734508	38,55160159	38,60152142	21,7886338	420,3741879	46,7082431
ENSG00000123472	ATPAF1	33,00636034	47,0913465	47,69290407	25,83150532	11,1967245	67,01302667	36,09178301	83,46717806	68,87811923	420,2689477	46,69654974
ENSG00000152944	MED21	21,13550933	41,56496312	66,51010628	51,2242943	75,44236917	31,61071561	37,10218518	42,85095851	52,70427256	420,1453741	46,68281934
ENSG00000128708	HAT1	36,03166389	54,346708	90,38240192	39,29003224	0	43,12600507	43,63944576	61,37892614	51,94620927	420,1413923	46,68237692
ENSG00000141002	TCF25	47,1521123	44,96519545	66,8519982	30,85235924	6,130285648	52,27005743	64,84487107	49,52163595	57,33786265	419,9663779	46,66293088
ENSG00000103202	NME4	51,70331667	48,06802021	41,18504604	37,66291994	34,78491884	42,59804984	54,53594512	54,276019	55,06939241	419,8836281	46,65373645
ENSG00000106348	IMPDH1	37,55068796	38,44198527	15,9409669	39,76016271	58,74150952	56,3606656	64,89372168	31,85237639	76,32800915	419,8700852	46,65223169
ENSG00000135899	SP110	76,75492497	66,98667591	47,09262274	36,64112389	0,261556841	58,67893376	41,08627915	49,86601776	42,01512055	419,3832556	46,59813951
ENSG00000137074	APTX	37,80755371	4,953797837	98,89606055	54,95542616	89,28179596	47,72829998	32,35273538	23,63361515	29,77272078	419,3820055	46,59800061
ENSG00000154305	MIA3	18,13922064	24,53017411	54,52844482	24,33337479	106,8688541	31,41763063	47,38342917	59,61163247	52,21271571	419,0254764	46,55838627
ENSG00000185127	C6orf120	58,81243808	56,80548558	53,88665586	69,26957706	0	60,96451637	49,58595048	23,54052037	45,85189788	418,7170417	46,52411574
ENSG00000165175	MID1IP1	60,12526136	24,19645489	44,96089728	43,99404981	5,306222262	62,03041878	77,1361144	44,81355348	55,99913679	418,562109	46,50690101
ENSG00000164654	MIOS	1,868063355	29,39417814	59,62039656	64,08724652	48,78093515	54,38921348	30,66678803	66,83499295	62,86229551	418,5041097	46,50045663
ENSG00000092203	TOX4	52,65748415	32,93635342	25,48118239	60,27520637	132,029253	35,65755449	20,01226139	42,96441563	16,45911945	418,4728303	46,49698114
ENSG00000170915	PAQR8	25,85459093	29,40293684	1,380513006	41,46761615	61,5650455	78,40708422	62,43607105	48,50837215	69,33249281	418,3456227	46,48284696
ENSG00000103671	TRIP4	31,57174602	74,24154899	10,64038656	5,826299273	10,99690082	55,09183743	70,5195295	71,7786094	87,43975131	418,1066093	46,45628992
ENSG00000196189	SEMA4A	40,23812715	40,82918359	55,09515475	79,92602826	0,053037593	56,40616564	78,29155999	7,759429366	59,47857241	418,0772587	46,45302875
ENSG00000109736	MFSD10	56,9919255	25,66770901	22,08584705	40,63058954	113,21255	23,98133097	48,60273869	34,26650748	52,48059101	417,9197893	46,43553214
ENSG00000116786	PLEKHM2	48,98117191	37,81489745	23,06414464	8,331740844	90,69151287	44,8688981	76,9090037	27,01666275	60,15061606	417,8286483	46,42540537
ENSG00000171840	NINJ2	0	52,10962697	61,07961159	21,53223179	153,6069175	35,40181739	44,66204689	22,1042655	27,31435703	417,8108747	46,42343052
ENSG00000079332	SAR1A	31,90937709	35,44717791	43,3012101	36,45692587	23,37328523	25,36411333	59,43236004	139,075006	23,22409535	417,5835509	46,39817232
ENSG00000039123	MTREX	85,04284834	60,45552263	43,79708508	62,16065568	1,188194169	45,2221991	39,61596744	21,34522966	58,09350877	416,9212109	46,32457899
ENSG00000108039	XPNPEP1	42,66146585	38,31172502	68,75759435	55,72800459	57,75078099	23,68025717	33,96006146	43,80443303	52,19034753	416,84467	46,31607444
ENSG00000183307	TMEM121B	1,669400662	3,226802526	33,39709613	18,11976939	177,043744	40,61847025	36,60892109	38,78646038	67,16725761	416,637922	46,29310245
ENSG00000135900	MRPL44	35,07125145	88,58687747	0	102,4274057	0	61,68847967	39,074834	54,58332998	34,99064185	416,4228201	46,26920223
ENSG00000064995	TAF11	54,58652901	38,4777758	37,67343643	108,1703532	4,473312685	35,05036598	45,63694188	50,21551724	42,04002048	416,3242527	46,2582503
ENSG00000109171	SLAIN2	36,71128992	31,29598986	80,58160814	49,94426171	89,29532293	32,57172438	20,65306131	49,308425	25,9348144	416,2964977	46,25516641
ENSG00000148291	SURF2	77,7485819	23,35170571	10,29555922	7,835885123	167,516291	31,55456414	32,90613312	16,86903198	48,1824317	416,2601839	46,25113155
ENSG00000120029	ARMH3	69,01363328	51,85472005	63,47807611	56,17770045	0,075651473	57,38891235	44,53707803	30,21036493	43,49599457	416,2321313	46,24801458
ENSG00000079257	LXN	24,85711923	81,2328746	0	112,4935566	114,7010108	19,55902618	28,55116534	8,832726616	25,98539448	416,2128738	46,24587487
ENSG00000011009	LYPLA2	48,52585866	51,87705197	42,36411207	53,96873794	0	82,58586846	61,52454182	21,74557025	53,42742674	416,0191679	46,22435199
ENSG00000124151	NCOA3	53,32046987	8,123584415	161,7945236	12,33837708	0,554654634	34,71171743	24,35767168	86,64240115	34,11344623	415,9568461	46,21742734
ENSG00000125450	NUP85	76,35550499	40,37878761	57,06273551	36,22840683	61,40889228	14,53674416	38,58581171	51,79544347	39,47404469	415,8263713	46,20293014
ENSG00000115816	CEBPZ	93,49751425	36,64810935	19,86702871	23,77539632	25,35909752	63,96438521	24,00799063	92,20554561	36,3543738	415,6794414	46,1866046
ENSG00000100325	ASCC2	97,41957566	61,7494135	10,72905645	21,68434692	0	40,84188342	97,54941786	20,07626458	65,27794744	415,3279058	46,14754509
ENSG00000123268	ATF1	72,8320913	24,82445372	74,94657792	10,32675518	82,35593109	27,67130467	35,29130186	43,3184539	43,67064923	415,2375189	46,1375021
ENSG00000112406	HECA	40,41391458	55,42361213	36,77845093	35,21651623	55,59134467	35,14506726	47,49027621	43,89407114	65,24049011	415,1937433	46,13263814
ENSG00000142327	RNPEPL1	63,93285374	39,26244844	67,87318352	26,25719569	49,0238251	45,06855691	53,84046869	9,86745251	60,02119803	415,1471614	46,12746237
ENSG00000105928	GSDME	84,18668765	47,83608725	17,70655604	27,03895767	45,45949042	44,32067679	56,30672988	7,778519706	83,98049633	414,6142017	46,06824464
ENSG00000204482	LST1	45,57828699	52,19423413	36,31655831	56,2823356	37,78527407	53,82456237	51,1562511	45,77699401	35,68644312	414,6009397	46,06677108
ENSG00000150977	RILPL2	18,60276688	42,48529003	25,7565016	33,2701653	133,448336	35,01385888	38,8812965	45,30628657	41,7330002	414,497502	46,055278

ENSG00000101811	CSTF2	71,3059483	44,62902974	0	138,7596596	0	47,71730807	20,13721378	36,71495195	54,47079139	413,7349028	45,97054475
ENSG00000107789	MINPP1	26,89637933	0	70,76192112	55,5254404	0	39,75914715	58,26447966	85,75746316	76,69172033	413,6565512	45,96183902
ENSG00000169180	XPO6	24,72523987	18,25566908	84,98754619	60,49702596	42,49430508	41,82402507	59,77822187	22,59021884	58,37132809	413,52358	45,94706445
ENSG00000121281	ADCY7	22,44771166	24,79459051	55,27391076	64,5836739	61,58678594	34,33560718	56,64338511	33,08174581	60,66392274	413,4113336	45,93459262
ENSG00000104814	MAP4K1	24,36894122	78,13681146	80,01060262	57,59345574	0,916349942	32,08987424	37,90683161	28,66745419	73,64624881	413,3365698	45,92628554
ENSG00000130935	NOL11	40,17609322	7,843807153	44,5731903	32,96318314	39,45234972	36,99284581	27,92156684	109,1901875	74,0969883	413,210212	45,91224578
ENSG00000178074	C2orf69	97,18052431	47,28214183	1,566452777	121,5531791	0	42,77290754	26,24890017	30,8012499	45,77436062	413,1797162	45,90885736
ENSG00000197971	MBP	37,40625891	16,42126673	141,3718827	31,70748926	8,565575547	39,46559407	42,91536953	27,83354294	67,24020024	412,92718	45,88079777
ENSG00000173039	RELA	71,27857107	63,36383589	28,0577092	52,96133941	27,4321332	41,78916339	43,92266064	20,3986901	63,69643583	412,9005387	45,87783764
ENSG00000108468	CBX1	49,93995864	51,88424378	77,8052051	29,91665773	72,79090789	24,96289229	24,85499678	42,4441248	38,03626261	412,6352496	45,84836107
ENSG00000139291	TMEM19	71,82876078	57,61250742	14,31031952	50,31283188	39,2438868	44,18826704	41,52046899	32,39155132	61,04176001	412,4503537	45,82781708
ENSG00000132394	EEFSEC	60,72195864	26,32137428	0,812807307	39,34733744	0	72,90640213	85,0095566	51,34572227	75,68937311	412,1545318	45,79494797
ENSG00000215305	VPS16	69,31141811	34,83295304	81,14639037	18,07308237	0	38,27188095	43,19103581	39,58535396	87,73016569	412,1422803	45,7935867
ENSG00000158473	CD1D	25,55398465	40,34626632	101,1734049	24,20568562	0	30,04505783	72,39743968	48,83919706	69,29031456	411,8513506	45,76126118
ENSG00000133193	FAM104A	0,477895543	40,45026377	109,8030182	65,44004897	79,04674557	41,55634938	27,97488427	22,46787856	24,62672133	411,8438056	45,76042285
ENSG00000180233	ZNRF2	39,74256854	29,2332301	66,49303572	54,21129211	5,593073683	71,96121399	50,69694578	41,85051384	51,93653914	411,7184129	45,74649032
ENSG00000144043	TEX261	139,4811243	18,45719312	0,398633029	17,27509273	63,3433906	45,08611991	60,03623438	44,38437546	22,65337628	411,1155398	45,67950442
ENSG00000116774	OLFML3	0,053566314	3,992188587	44,83549984	63,82315933	0	94,27672969	65,60394885	107,6460005	30,76580145	410,9968946	45,66632162
ENSG00000151702	FLI1	69,51161561	47,07352082	40,97314442	65,60762257	0,089488548	34,43895101	45,96097385	27,33722313	79,97930326	410,9718432	45,66353813
ENSG00000109775	UFSP2	1,795049975	44,78685675	23,41517032	56,8270913	0,258543947	41,02972145	31,20950303	132,1500376	79,14198555	410,6139599	45,62377333
ENSG00000107949	BCCIP	43,59217308	60,89256719	26,15337675	91,93082735	37,94057665	36,60660411	30,27920601	46,74296818	36,45832577	410,5966251	45,62184723
ENSG00000162980	ARL5A	20,32494508	44,48420326	64,60421286	75,35705783	61,82100826	37,29964014	29,53341247	38,29914121	38,82287119	410,5464923	45,61627692
ENSG00000160679	CHTOP	37,82465484	44,38048037	9,686062116	27,8794524	59,05488474	43,00402148	38,63370934	77,89444205	72,12497033	410,4826777	45,60918641
ENSG00000071242	RPS6KA2	19,79766415	20,27882779	61,7546384	37,85470423	67,02715007	40,9397569	80,51938043	5,126121033	76,67210784	409,9703508	45,5522612
ENSG00000172349	IL16	47,31732521	48,68859205	25,88106428	50,7241711	22,86427217	52,41808447	45,58596319	10,81958729	105,3396651	409,6387249	45,51541388
ENSG00000140943	MBTPS1	36,62306024	29,64856065	68,23689017	31,51842549	74,66177443	34,21885459	44,99052979	44,19087948	45,38533428	409,4743091	45,49714546
ENSG00000072786	STK10	53,47771738	0,060155244	62,14406475	75,48528317	97,68480541	14,7951664	23,57406686	37,69511555	44,236267	409,1526418	45,46140464
ENSG00000197442	MAP3K5	35,80370162	41,72682844	9,401568995	59,098326	0	55,45063736	55,69042287	26,93452963	124,9688091	409,074824	45,45275822
ENSG00000115350	POLE4	38,28696467	53,10483126	64,87494639	42,700656	4,507751578	69,03299217	31,32383463	64,82723733	40,26242649	408,9216405	45,43573783
ENSG00000177879	AP3S1	31,78016781	67,47034963	71,74904569	92,1156585	1,226075597	44,86212931	28,74380088	27,86525714	43,0194408	408,8319254	45,42576948
ENSG00000166477	LEO1	95,19224934	18,81343343	0	69,49079293	117,1771687	30,97915333	22,10284611	25,42763175	29,51391395	408,6971895	45,41079883
ENSG00000182446	NPLOC4	30,30131255	50,14311616	74,5942404	38,29234428	44,72029773	55,7730838	39,99531233	19,08484766	55,61759131	408,5221462	45,39134958
ENSG00000135441	BLOC1S1	39,23764818	56,51060268	66,64136431	62,55506336	7,806903095	54,3016501	45,99063498	35,11864969	39,84028708	408,0028035	45,33364483
ENSG00000197345	MRPL21	36,10606556	58,46919365	51,36481829	91,81216451	0	38,64395026	46,01319078	57,19734638	28,28814321	407,8948726	45,32165251
ENSG00000134684	YARS	64,93779882	32,06471555	0,77856092	56,65453723	52,12714448	56,4079798	49,70226376	41,16348789	53,77147308	407,6079615	45,2897735
ENSG00000116857	TMEM9	78,46644841	98,86838227	20,35730758	39,34997958	32,43878795	29,93079299	32,75227089	43,00118128	32,43659639	407,6017473	45,28908304
ENSG00000136732	GYPC	23,82430444	34,54863675	17,71555078	42,00698815	97,81450378	60,22245926	39,32029523	54,47729778	37,48857529	407,4186115	45,26873461
ENSG00000136295	TTYH3	29,40878517	17,46839088	49,17337491	38,27959752	52,68314455	51,56459342	80,93598213	24,77988142	62,97556096	407,2693109	45,25214566
ENSG00000198522	GPN1	67,97151937	41,37375228	60,47885229	65,05960125	0	46,01737565	37,2458647	41,19405847	47,92518433	407,2662084	45,25180093
ENSG00000134287	ARF3	64,23270308	71,35248848	58,3206526	25,57564352	6,836178409	48,24779023	49,59864076	50,12059357	32,85621992	407,1409106	45,23787895
ENSG00000171100	MTM1	92,90139417	39,99428736	0	45,02687684	0,178843398	46,80694308	58,14250196	67,55148903	56,31853028	406,9208661	45,21342957
ENSG00000128654	MTX2	6,072585542	69,33899129	3,076075644	3,647314729	27,19002942	57,8802769	37,41456609	131,2500728	71,00484485	406,8747573	45,20830637
ENSG00000134480	CCNH	36,43227602	33,40006522	63,53432458	28,09599125	42,2703143	43,44022122	56,78785968	38,50742243	64,1342956	406,6027703	45,17808559
ENSG00000154473	BUB3	50,46261259	62,91086926	11,18721132	66,42271341	26,35725081	45,56198712	29,71383031	55,53725942	58,26601059	406,4197448	45,15774943
ENSG00000108439	PNPO	29,60911297	27,91221738	86,22383093	32,8041099	58,40985668	55,08129556	50,87478636	28,32657601	37,16219207	406,4039779	45,15599754
ENSG00000138794	CASP6	27,37566694	67,89802576	0,286134945	69,6990488	47,6851909	64,98093097	46,68813692	20,2376292	61,40682825	406,2575927	45,13973252
ENSG00000089053	ANAPC5	57,05937121	24,95717612	32,788908	60,3647596	25,16371391	34,68707138	50,17175687	69,86592699	51,19534266	406,2540268	45,13933631
ENSG00000151176	PLBD2	51,15483224	12,9894016	49,00561015	20,76971017	72,65580977	51,34276775	66,38200506	34,65043337	47,13242919	406,0829993	45,12033326
ENSG00000169217	CD2BP2	73,70353985	30,8366911	91,18765434	14,1254861	0	55,36310383	47,44420849	28,83404252	64,21790683	405,7126331	45,07918145
ENSG00000188846	RPL14	28,18651846	51,04210111	45,18062819	57,50491383	36,8443108	43,54527541	43,09860813	58,8009577	41,38780075	405,5911144	45,06567938
ENSG00000213741	RPS29	20,2913331	34,34505456	49,21476003	86,98715964	51,06801009	47,25375897	38,5268475	51,08394228	26,61692193	405,3877881	45,04308757
ENSG00000143155	TIPRL	12,61626233	62,19615131	1,745010038	61,17966919	5,072281672	48,79416521	57,69429893	65,02902506	90,99829716	405,3251609	45,03612899
ENSG00000168827	GFM1	47,29734093	46,19452526	8,063638832	31,89462046	85,28644025	42,78185708	42,89773973	37,27998498	63,62239226	405,3185398	45,03539331
ENSG00000164808	SPIDR	11,70162103	67,85136608	71,34463153	34,02589277	87,75013373	30,48831515	26,5305062	46,27056202	29,00667266	404,9697012	44,99663346
ENSG00000162729	IGSF8	37,42784968	32,17290202	48,47492467	35,43561383	109,7303602	41,28561805	34,07836178	23,7117398	42,51812797	404,835498	44,981722
ENSG00000139343	SNRPF	31,68207167	62,38729414	44,67047926	79,83255385	51,92629597	34,23984297	25,60534873	45,49854873	28,88774401	404,7301793	44,97001993
ENSG00000136100	VPS36	20,76502293	42,35754571	58,49807262	80,07868792	65,97843873	32,23197668	29,17155136	28,61140522	46,26430072	403,9570019	44,88411132
ENSG00000103274	NUBP1	47,07229541	63,77802048	18,05804224	15,21564558	0	52,95560095	47,49260649	89,4117065	69,96574956	403,9496672	44,88329636
ENSG00000182158	CREB3L2	28,58524023	34,73941434	115,1122313	57,9822579	29,19588513	51,57978415	39,70544059	1,800937804	45,22188001	403,9230714	44,88034127
ENSG00000099804	CDC34	92,78859223	36,95093032	0	17,22115725	76,41202195	60,88059705	58,36291578	38,0331234	22,86285094	403,5121889	44,83468766
ENSG00000114541	FRMD4B	37,8147424	48,78791145	17,39702253	40,977111	53,2215981	56,71561452	38,22022791	60,82993288	49,52699482	403,4911556	44,83235062
ENSG00000140564	FURIN	37,64990761	28,95616273	29,37236887	21,56077242	86,06071281	45,72007439	61,40940171	38,19689527	54,39506199	403,3213578	44,8134842
ENSG00000173193	PARP14	117,4438297	26,72546312	13,42010211	67,2274555	42,18949519	38,82120171	34,74431522	28,75301765	33,97116691	403,2960472	44,81067191
ENSG00000087502	ERGIC2	41,91463821	43,07945624	62,7912479	34,75549953	8,752950741	42,94424127	43,00793907	75,37719537	50,5607171	403,1838854	44,79820949
ENSG00000127838	PNKD	63,51657667	32,32807679	42,30831368	31,30817018	71,90663753	37,1890606	32,33571828	61,51029276	30,72347021	403,1263167	44,79181297
ENSG00000165487	MICU2	53,42419677	43,5025504	32,63110796	90,37365815	0,795387691	53,00686753	43,97714294	27,71917884	57,57537609	403,0054664	44,77838515
ENSG00000104903	LYL1	29,35973255	46,76795385	4,723611813	25,34892507	81,05763059	69,68350609	50,72718456	42,37698846	52,85215302	402,897686	44,76640956
ENSG00000114331	ACAP2	60,83371658	32,57528984	77,76227715	43,64593663	10,68031624	33,65759807	27,95265507	73,31740314	42,22414227	402,649335	44,738815
ENSG00000173110	HSPA6	129,2941825	21,63353716	42,13965832	17,06326373	40,71523729	16,62628285	35,53660783	57,24726265	42,21991058	402,4759429	44,71954922
ENSG00000102900	NUP93	58,8248468	41,39169416	49,50809818	30,16833384	75,90446301	38,170023	33,00604077	43,52123001	31,96299533	402,4577251	44,71752501
ENSG00000121892	PDS5A	34,54592692	3,691236973	33,27834518	70,66454368	113,2026537	43,14980092	29,75345303	31,27269137	42,60886343	402,1675152	44,68527947
ENSG00000114742	WDR48	81,73276149	34,05148643	62,38092145	94,03156518	0,144166015	41,96052321	31,93423946	5,43870625	50,43446369	402,1088332	44,67875924
ENSG00000111605	CPSF6	66,78899508	58,99124002	21,20245724	22,3010264	41,62194621	51,17465366	45,68366995	43,14120875	51,18282483	402,0880221	44,67644691
ENSG00000163156	SCNM1	33,97095218	34,49299848	28,4365553	40,11925184	43,33608321	48,88391265	61,63994258	61,53268516	48,72423371	401,1366151	44,57073501
ENSG00000011275	RNF216	38,73460356	31,32253315	95,1896677	41,88521774	48,31283383	35,62537124	42,27357163	25,63155656	42,15025469	401,1256101	44,56951223
ENSG00000077312	SNRPA	68,45893257	35,21286784	45,01080226	55,00371755	56,92768521	38,63960732	43,45869978	23,03071359	35,22632764	400,9693538	44,55215042
ENSG00000184990	SIVA1	47,51563091	35,15500953	46,96682155	47,45275495	54,67638623	37,9254247	43,85757217	43,67130571	43,70085277	400,9217585	44,54686206
ENSG00000088832	FKBP1A	59,69365966	39,01949943	47,24690856	76,04274968	15,60774728	40,30268569	37,10065696	48,59709006	36,77987053	400,3908678	44,48787421
ENSG00000125656	CLPP	49,08511931	52,6511171	19,7562166	53,37576474	11,61610274	70,29468626	59,44835776	31,91440925	52,1555616	400,2973354	44,47748171
ENSG00000102871	TRADD	79,90153967	70,3363737	46,43662031	19,93336667	0,16526348	42,14571484	47,98244174	59,00074192	34,38117899	400,2832413	44,4759157
ENSG00000143153	ATP1B1	1,989639519	3,258984471	86,4347303	29,24726927	2,788556203	41,95375488	72,1814469	115,1277764	47,24592548	400,2280834	44,46978705
ENSG00000196449	YRDC	0	114,4827046	43,17115174	53,51426157	0	65,26893358	67,48783009	6,615843614	49,67975217	400,2204773	44,46894193
ENSG00000078674	PCM1	5,220088249	47,09423705	26,49903747	136,8711874	52,03413057	23,54563394	28,77696157	51,49243416	28,45915592	399,9928664	44,44365182
ENSG00000106034	CPED1	4,346666161	24,84929117	3,726662787	78,55558978	0,140225514	81,02258021	77,31049742	43,13755971	86,88802655	399,9770993	44,44189992
ENSG00000160208	RRP1B	26,30637935	32,2236734	30,31191688	81,04867211	56,58443363	41,26123908	39,23402729	45,85857128	47,02725042	399,8561634	44,4284626
ENSG00000177830	CHID1	49,49477248	27,6924726	61,78367842	22,93646334	35,46765834	59,97068473	49,94490823	52,29303734	39,97066747	399,5543429	44,39492699
ENSG00000006451	RALA	39,43483459	24,04961778	35,65042342	80,27085975	0,200680381	47,60831581	39,54471486	55,11859089	77,26728839	399,1453259	44,34948065
ENSG00000164117	FBXO8	70,76922099	59,44927618	0	9,935958108	92,27148241	34,89435973	27,41593731	62,08877283	41,99734578	398,8223533	44,31359482
ENSG00000198612	COPS8	11,05951558	62,14532363	25,59730641	46,43282016	54,79956757	36,11064932	54,04737334	51,924303	56,58517648	398,7020355	44,30022617
ENSG00000177854	TMEM187	43,83822705	35,13090375	61,8765862	21,27945648	112,1042061	42,14306093	48,30943264	29,15599125	4,472242515	398,3101069	44,25667854
ENSG00000131504	DIAPH1	79,37658993	33,55865303	48,4344986	66,72859147	0,292163424	49,58195404	45,08425489	26,21074197	48,96268377	398,2301311	44,24779235
ENSG00000123933	MXD4	45,753106	31,01598866	38,62519926	44,68506694	28,8945149	53,73446002	66,59621288	30,96171417	57,73058709	397,9968499	44,22187221
ENSG00000030066	NUP160	66,67472008	19,97933046	62,78538383	61,3087656	0,12562414	40,7811912	43,34988266	58,14199655	44,64130549	397,7882	44,19868889
ENSG00000154767	XPC	19,65256275	25,7076532	82,50180512	34,69706415	53,66554353	32,98847232	44,92664243	32,47740754	71,15466057	397,7318116	44,19242351
ENSG00000006744	ELAC2	62,00238068	13,78622108	68,800273	57,01663959	0,153863557	57,00954692	52,64664887	28,97555334	57,33054672	397,7216739	44,1912971
ENSG00000198858	R3HDM4	83,09292793	16,32247549	74,54792644	41,91549305	49,06441904	43,59571123	41,07381852	12,0686281	36,01287023	397,69427	44,18825223
ENSG00000197579	TOPORS	26,95920856	34,07759623	3,768309258	71,81870398	21,99388483	37,79275059	60,13828549	87,10802325	54,03090614	397,6876683	44,1875187
ENSG00000197798	FAM118B	74,99941736	85,05579573	0,078658772	8,638764015	0,203235734	51,26608652	47,5040476	59,74750669	69,83706326	397,3305757	44,14784174
ENSG00000087088	BAX	38,66841816	33,7898307	38,682967	63,03995328	27,62442633	57,78331257	55,14486567	16,98793872	65,51682579	397,2385382	44,13761536
ENSG00000173530	TNFRSF10D	31,16254767	117,2694575	30,19642376	34,69291034	35,04824671	42,47498498	37,87180884	1,215176627	67,29379024	397,2253466	44,13614963
ENSG00000116521	SCAMP3	28,5266065	56,21978053	104,7263272	13,46347535	19,41175905	45,38422	45,47143482	27,87449669	56,08881528	397,1669154	44,12965727
ENSG00000105135	ILVBL	30,27054701	44,78582551	38,8063386	46,04461535	44,88969279	57,93280643	52,10057233	49,65932603	32,59594452	397,0856686	44,12062984
ENSG00000183513	COA5	18,91623617	68,8840842	129,2062797	16,47412394	0	50,50761025	32,96100159	44,60147283	35,46998212	397,0207908	44,1134212
ENSG00000133943	DGLUCY	62,98369043	39,89411621	44,36426704	4,34168011	59,70354039	52,91102696	40,34466166	49,71180058	42,49319401	396,7479774	44,0831086
ENSG00000171681	ATF7IP	0,630152505	14,04853454	126,1438238	31,79335084	51,89764468	40,79520815	31,95502655	45,64802351	53,78544056	396,6972052	44,07746724
ENSG00000239697	TNFSF12	45,46759714	38,16568499	30,11087414	21,7104453	82,873279	55,91125662	42,67698367	40,80719934	38,90037096	396,6236912	44,06929902
ENSG00000205937	RNPS1	48,85914331	40,97092076	37,86655486	30,06153379	15,34224589	50,93321078	48,59467277	48,36756714	69,67888754	396,6147368	44,06830409
ENSG00000174579	MSL2	34,90818949	26,36324969	2,671403649	85,3548152	122,4490604	33,20664472	26,04013728	38,10592684	27,40815464	396,5075819	44,05639799
ENSG00000138382	METTL5	37,28976386	81,14454244	24,7416842	31,78579621	0,125346437	49,39676158	41,12273709	86,61653061	44,23990513	396,4630675	44,05145195
ENSG00000115761	NOL10	22,37507928	22,57817154	123,7721609	82,03534394	0,093617712	39,09884289	27,47525518	38,504758	40,35718276	396,2904122	44,03226802
ENSG00000138600	SPPL2A	8,771738533	26,49756247	48,58173016	94,02740726	54,99475602	37,18294217	38,45989294	44,60558612	42,69041307	395,8120287	43,97911431
ENSG00000214114	MYCBP	45,55659469	69,81555141	36,58951898	67,65677848	0,129505064	46,78762026	50,58630767	40,63403805	37,66525262	395,4211672	43,93568525
ENSG00000126247	CAPNS1	51,22465866	56,66600634	24,53705877	61,44500983	30,56319509	46,5102968	58,51434115	32,49241578	32,96932267	394,9223051	43,88025612
ENSG00000106628	POLD2	82,69163028	35,12333641	0	39,82877555	0,2915704	43,59923409	60,71473711	66,53405094	65,73786322	394,521198	43,83568867
ENSG00000164211	STARD4	50,8575865	70,05469214	100,6968726	13,40482276	0,126360852	33,94686853	26,99687122	39,35682657	58,95166778	394,3925689	43,82139655
ENSG00000105854	PON2	86,64092033	14,43948856	77,16044891	65,64103029	15,04108333	50,49827647	28,56049441	33,0090723	23,40003038	394,390845	43,821205
ENSG00000176871	WSB2	37,54848885	17,94971361	61,84695876	74,78703886	0	46,04234159	54,85249783	48,24802827	52,94371248	394,2187803	43,8020867

ENSG00000142186	SCYL1	43,19461431	20,85270575	9,413151413	26,62974869	42,10112243	60,40508853	64,61048157	42,82839444	83,89568602	393,9309932	43,77011035
ENSG00000139842	CUL4A	72,83788674	63,69788201	20,28013009	15,69684347	68,44055044	39,56116785	32,34317786	35,4414652	45,55178423	393,8508879	43,76120977
ENSG00000151694	ADAM17	23,20777229	34,94576206	54,3030891	73,20743101	91,93409084	40,65194968	26,4246355	22,53102679	26,58901872	393,794776	43,75497511
ENSG00000139597	N4BP2L1	49,7294328	67,68891065	26,91166992	35,52129838	53,09932272	32,6997056	43,57155755	38,70446198	45,80918738	393,735547	43,74839411
ENSG00000158825	CDA	33,35489426	12,75309513	0	0,318213384	114,304486	11,33047841	7,1915922	93,89177345	120,3469553	393,4914881	43,72127646
ENSG00000107745	MICU1	89,75622681	28,19250001	51,95889701	48,56967206	0	37,81658524	29,38064368	39,97469067	67,49721824	393,1464337	43,68293708
ENSG00000131844	MCCC2	66,54272645	35,49460869	67,92919914	79,15373401	0	27,51432858	37,084008	26,66031053	52,71193607	393,0908515	43,67676127
ENSG00000170889	RPS9	48,45088	34,26535782	33,97247492	56,3823249	44,05531189	43,90616795	42,24128975	51,20814511	38,47333907	392,9552914	43,66169904
ENSG00000172059	KLF11	63,28618589	48,21186157	114,48375	27,37715667	0	36,83691516	43,0706821	20,78702438	38,79556266	392,8491384	43,64990426
ENSG00000152133	GPATCH11	45,59499645	44,46909407	14,79349537	99,6590757	2,666714423	44,10593054	50,59187397	42,53118196	48,39369837	392,8060608	43,64511787
ENSG00000003147	ICA1	47,44061127	38,1647942	0,056928138	117,7760975	50,89275841	31,08912097	23,40845227	31,33139561	52,56073055	392,7208889	43,63565432
ENSG00000205707	ETFRF1	43,13943299	73,07032606	14,20336272	47,08680604	0	39,96386154	44,2148088	99,55915802	31,4266017	392,6643579	43,6293731
ENSG00000184182	UBE2F	35,12126288	45,68157717	103,8981776	23,21200694	18,6422509	35,15519063	26,17172569	68,69109344	36,04668271	392,619968	43,62444089
ENSG00000115944	COX7A2L	41,86881646	50,99721179	34,12137522	74,43979026	27,35828556	40,96703625	33,98108189	42,68235984	46,05545396	392,4714112	43,60793458
ENSG00000167969	ECI1	37,68331647	41,09167355	65,24376855	10,18004739	36,87567773	61,82809881	54,43313405	43,7279203	41,29922717	392,362864	43,59587378
ENSG00000089234	BRAP	0,292095175	21,76926032	0	44,32980346	127,0332028	53,02296781	24,84614392	68,55665641	52,44895409	392,299084	43,58878711
ENSG00000161204	ABCF3	44,40160805	14,47050164	58,67104451	46,65459216	99,10733788	31,00268699	41,75918674	6,028669851	49,93264541	392,0282732	43,55869703
ENSG00000165609	NUDT5	0,85235616	61,90385417	0,167997642	58,55792943	45,21524254	70,9565178	58,76682552	40,67194213	54,84793617	391,9406016	43,54895573
ENSG00000183011	NAA38	16,33207403	32,70697582	32,04711383	73,59360066	32,8990852	53,79498884	61,21764011	49,31859217	39,88574359	391,7958142	43,53286825
ENSG00000147457	CHMP7	27,08282258	52,2243359	52,24700456	44,10012129	0,152881031	40,18864119	56,61922176	30,38478782	88,53225135	391,5320675	43,50356305
ENSG00000177981	ASB8	75,00054755	37,27475674	67,35200636	40,42384702	0	43,12213227	43,32810535	49,33066231	35,65924461	391,4913022	43,49903358
ENSG00000138604	GLCE	38,42721299	32,51676743	70,52225136	53,87933337	38,78252795	37,13594206	28,12200805	21,87945326	69,99378071	391,2592772	43,47325302
ENSG00000187735	TCEA1	26,20595571	62,92876511	45,64712358	48,49712679	71,79042533	34,35136077	33,89606159	30,82128048	36,80976719	390,9478665	43,43865184
ENSG00000239306	RBM14	40,68509142	26,42598324	42,99971559	70,25427307	0,091216038	69,6175445	66,41609649	22,57731741	51,78400684	390,8512446	43,42791607
ENSG00000204843	DCTN1	17,32726283	22,70691906	98,36751467	32,09507686	53,21916257	22,0281693	39,49569029	45,48658622	59,80060047	390,5269877	43,39188752
ENSG00000184983	NDUFA6	35,42987956	60,02896471	33,37331697	60,44256474	35,5059717	43,11800298	47,40581047	45,97147585	29,13695476	390,4129417	43,37921575
ENSG00000071462	BUD23	21,68505168	49,01084896	55,25309369	20,29932204	39,19371969	53,71626426	44,41371772	43,46728639	63,32912082	390,3684253	43,37426947
ENSG00000168564	CDKN2AIP	32,25448544	25,97938681	101,8452617	76,03833631	36,22480813	38,88464575	29,90662708	38,57904794	10,32223523	390,3013944	43,3668216
ENSG00000005844	ITGAL	31,77711475	26,55732726	100,7205396	26,97241935	0,055487877	47,06610303	79,27196965	28,59137173	49,21831569	390,2306489	43,35896099
ENSG00000111142	METAP2	30,38362176	16,2998811	48,77495391	69,25729007	78,52447709	30,62566646	40,25890635	40,09949661	36,0023323	390,2266257	43,35851396
ENSG00000213977	TAX1BP3	23,63496254	60,19118032	73,67177134	18,51674693	34,58554173	45,29202565	36,0693134	35,41224909	62,57685218	389,9506432	43,32784924
ENSG00000183049	CAMK1D	30,4739076	49,82140791	73,44486751	41,33505418	28,73104282	44,28952738	42,47260953	13,53869091	65,2859616	389,3930694	43,26589661
ENSG00000162869	PPP1R21	47,5480485	43,12538921	49,08803149	49,73297915	0,179648595	50,79403349	29,81411091	64,63227409	54,43378629	389,3483017	43,26092241
ENSG00000204632	HLA-G	55,97005255	42,89055248	41,15303791	11,39647932	50,51681821	42,53634752	99,56494298	3,561121261	41,74431243	389,3336647	43,25929607
ENSG00000073614	KDM5A	29,89467249	48,16842479	45,01571696	78,46824644	46,30421376	40,64404335	29,7720935	29,26932609	41,23945242	388,7761898	43,19735442
ENSG00000058272	PPP1R12A	57,34151965	66,83208819	33,66970686	74,22922617	0,211886615	35,43639593	46,87158727	34,89839119	38,89555924	388,3863611	43,15404013
ENSG00000137275	RIPK1	91,9519262	26,22778035	40,41767841	40,94942043	0	39,99238367	62,35824239	31,52668188	54,80261544	388,2267288	43,1363032
ENSG00000116750	UCHL5	26,54246625	99,35061646	52,87506161	56,64494419	0	31,85702261	27,0116283	48,01991613	45,72923735	388,0308929	43,11454366
ENSG00000100889	PCK2	69,11622186	47,94422489	25,28298346	69,32934513	0,085728704	58,89449214	48,34306895	15,46077232	53,50463258	387,96147	43,10683
ENSG00000152193	RNF219	19,82060522	5,046299403	31,46794961	85,56464807	131,1746712	35,38668702	24,3284362	38,25390203	16,79878744	387,8419862	43,09355402
ENSG00000079277	MKNK1	45,65149582	36,45003396	17,32639279	55,4203899	0,133500945	77,50717152	51,3697617	33,98521574	69,94001092	387,7839733	43,08710814
ENSG00000225663	MCRIP1	47,45017919	41,59583362	49,61265857	19,60864007	20,14373761	61,67100073	51,11754267	43,63226173	52,52184325	387,3536974	43,03929972
ENSG00000141349	G6PC3	52,19640015	37,59055066	21,74487261	29,1838941	13,41463024	55,81390748	52,65839027	69,70274331	55,04748671	387,3528755	43,03920839
ENSG00000008869	HEATR5B	20,26914064	3,320039331	79,87119377	55,68386567	128,4680016	20,96539334	17,01266823	24,70838718	36,97529133	387,2739811	43,03044234
ENSG00000102096	PIM2	45,80932548	46,42226586	39,96235089	40,54368131	70,62688356	27,82201993	60,4402541	15,79766857	40,25216238	387,0805121	43,00894578
ENSG00000151876	FBXO4	20,85666801	37,72136962	166,5667083	0,952317426	0,119149815	55,43196773	19,31593698	55,14551674	30,70722233	386,8168569	42,97965077
ENSG00000106355	LSM5	50,5954882	78,01532953	4,085330528	79,08636184	14,15053149	46,82247372	23,86475111	61,23068663	28,65876084	386,5097139	42,94552377
ENSG00000123545	NDUFAF4	32,97389219	23,99386814	41,12970852	79,77564569	3,100700915	61,20719863	55,25027963	47,98304159	40,91832771	386,332663	42,92585145
ENSG00000065135	GNAI3	40,2954107	37,36995379	40,13233461	66,47969783	33,23816358	43,3261492	33,30576325	48,34975308	43,81991934	386,3171454	42,92412726
ENSG00000213672	NCKIPSD	15,59112271	34,23089363	39,86190869	7,605216398	158,1540678	38,63670964	39,12948273	11,65333217	41,38348674	386,2462205	42,91624672
ENSG00000175224	ATG13	0,021584141	67,63873335	64,79981739	63,43541305	0,102590356	47,34885477	51,3638011	34,69067378	56,7812757	386,1827436	42,90919374
ENSG00000109667	SLC2A9	91,92483719	38,99953049	48,1893684	5,039178749	19,61384284	27,91042442	42,27146218	68,20073753	43,96610545	386,1154872	42,90172081
ENSG00000111725	PRKAB1	70,85340064	44,15777724	32,15956458	28,33057652	0,41605806	76,72197142	55,96533346	13,56302098	63,91742358	386,0851265	42,89834739
ENSG00000161791	FMNL3	25,86283043	47,6519216	32,3463984	37,03467698	43,15299875	63,58153372	62,66501859	13,49818435	59,97238874	385,7659515	42,86288351
ENSG00000187325	TAF9B	48,39958314	24,11807387	73,68759479	122,3102879	0	37,00382149	32,75532616	8,698825909	38,66423078	385,6377441	42,84863823
ENSG00000110713	NUP98	13,54868606	17,30035724	0,22453966	91,58016677	116,3578547	44,6269968	47,39342811	14,5799582	39,93148217	385,5434698	42,83816331
ENSG00000108829	LRRC59	55,68457813	23,50672159	79,96520028	42,70367536	0	42,31816031	47,78374538	41,55906125	51,21013202	384,7312743	42,74791937
ENSG00000138172	CALHM2	49,4464126	23,55301352	137,6484817	12,84525454	0	57,01174257	34,79904317	29,76887996	39,4654777	384,5382708	42,72647453
ENSG00000154832	CXXC1	43,59429753	35,26387035	83,47770048	5,437403963	58,70270546	44,75244776	33,00168955	19,05029111	60,93046435	384,2108706	42,69009673
ENSG00000107819	SFXN3	63,6289382	74,75087932	0	56,90872908	0,077546435	50,69390327	72,63786657	44,6668191	20,58718763	383,9518696	42,66131884
ENSG00000141574	SECTM1	98,64800172	23,72617495	53,13635124	24,41033028	0,485130081	31,15375908	56,90869837	68,63129315	26,73779753	383,8375364	42,64861516
ENSG00000166024	R3HCC1L	165,2441273	56,16640047	0,467062614	0,073070561	0,10056498	36,51798188	37,56028701	45,24853081	42,31117967	383,6892052	42,63213392
ENSG00000105402	NAPA	41,74794676	32,09221477	44,50612833	19,67330476	48,28205974	54,03028095	47,49790776	46,45828717	49,3195516	383,6076818	42,62307576
ENSG00000160087	UBE2J2	39,54145451	32,49343847	39,52603275	28,46361989	30,71400033	51,72381943	51,79112813	60,44876851	48,70977398	383,412036	42,60133733
ENSG00000162645	GBP2	75,85863619	50,00516048	23,39912781	34,24703806	0,049718613	43,82630214	52,18231723	46,61675541	57,14594019	383,3309961	42,5923329
ENSG00000127948	POR	70,60330039	41,09557962	69,61004465	23,7959568	0,713242388	33,77524153	56,71292233	40,52499413	46,35397369	383,1852555	42,5761395
ENSG00000025772	TOMM34	74,06179515	14,67781856	46,91706688	63,084634	19,6577266	36,1589194	73,32351384	22,10712768	33,15660205	383,1452042	42,57168935
ENSG00000156735	BAG4	36,19866446	9,980748184	121,7933185	98,26434581	23,44830699	18,83700871	20,27249906	20,47608674	33,54435045	382,8153289	42,53503654
ENSG00000172465	TCEAL1	21,70857667	125,9480225	5,382500355	25,26228674	1,121541357	61,45219987	28,44830138	64,60088672	48,87032222	382,7946378	42,53273754
ENSG00000162735	PEX19	39,56448028	40,57293209	35,08244692	87,32927978	19,32037888	45,8569247	53,18637028	23,40670776	38,28580004	382,6053207	42,5117023
ENSG00000167272	POP5	20,02753997	62,19216201	29,42265199	37,94544499	39,16744479	47,33757826	39,79909177	56,87184917	50,20336336	382,5471263	42,50523626
ENSG00000125354	SEPT6	6,605794362	25,54755645	44,51242578	71,031138	0,172514432	60,65233733	67,24566873	38,62565801	68,14868471	382,5417778	42,50464198
ENSG00000175354	PTPN2	21,21579932	63,45227646	24,48826749	43,63881544	26,89880192	41,23975909	37,56338226	71,74955681	52,18539648	382,4320553	42,49245059
ENSG00000153827	TRIP12	26,90089721	15,62777736	82,63915282	93,691278	29,25636005	48,1501634	32,9964856	10,49498388	42,51419967	382,271298	42,47458866
ENSG00000153574	RPIA	39,75716614	42,00349036	48,14437456	105,6405247	0	42,2783709	24,56810141	41,9194432	37,92786169	382,239333	42,471037
ENSG00000129691	ASH2L	34,0574078	55,76083217	39,88614618	15,85036479	0,668408285	59,43023534	61,52504605	59,99260422	54,87346554	382,0445104	42,44939004
ENSG00000162384	CZIB	15,76864303	50,06825535	87,88713609	59,78794267	28,70388882	40,0410295	28,38682689	38,22300392	33,01003948	381,8767658	42,43075175
ENSG00000125089	SH3TC1	69,14794626	9,687613059	45,75750144	25,54952397	28,11692861	62,83661593	32,77307323	24,74908467	83,1672391	381,7855263	42,42061403
ENSG00000153113	CAST	50,28116836	45,02062823	35,37278895	39,84876179	64,56499516	25,21712814	26,79146389	62,24847854	32,30815185	381,6535649	42,40595165
ENSG00000163848	ZNF148	44,9158808	45,72158371	8,491353506	34,12834026	40,44911982	40,49699248	56,33943033	30,49972933	80,21595684	381,2583871	42,36204301
ENSG00000130175	PRKCSH	41,50714281	28,75545258	68,39192911	33,5891905	53,66603614	28,58312297	41,24053886	40,87256846	44,50099653	381,106978	42,34521977
ENSG00000198087	CD2AP	27,88976019	6,553611319	61,06421075	50,2466993	89,8990737	22,60964754	23,39907391	78,36257482	21,05948593	381,0841375	42,34268194
ENSG00000123728	RAP2C	48,69444567	27,02256679	21,26365497	19,95981472	106,1306579	38,59697243	35,43516176	42,60824559	41,2942127	381,0057325	42,33397028
ENSG00000117505	DR1	26,21094109	30,36079214	59,5144578	46,76527966	57,39306443	40,62341352	39,01352108	22,44232944	58,60331984	380,927119	42,32523544
ENSG00000137145	DENND4C	16,91678087	7,941541226	100,1936274	112,549654	0	18,09933564	39,8865092	44,81365318	40,1658007	380,5669023	42,28521136
ENSG00000120963	ZNF706	23,3421171	43,81381525	15,98018965	58,44830487	60,33058131	40,1030976	42,34390347	50,7295055	45,32412818	380,4156429	42,26840477
ENSG00000211450	SELENOH	35,20450171	37,22578743	40,52117712	22,86718649	32,2504691	54,63172569	55,52266318	38,41929028	63,45388852	380,0966895	42,2329655
ENSG00000106524	ANKMY2	0	45,89733288	0	10,15916597	66,00406601	56,91355455	47,75002112	79,2764799	73,98660217	379,9872226	42,22080251
ENSG00000125952	MAX	38,54442557	26,09852614	49,22517975	56,29889975	1,320074073	51,706189	62,12355578	54,39469467	40,21674783	379,9282926	42,21425473
ENSG00000157693	TMEM268	0,315339254	26,07557847	6,090966276	45,79430585	117,5825175	50,86031661	66,87212603	27,51322881	38,78266325	379,8870421	42,20967134
ENSG00000106397	PLOD3	62,56526537	21,75018869	14,70534821	28,80361384	42,80787491	38,88117729	69,71557329	47,13834687	53,51567111	379,8830596	42,20922884
ENSG00000198952	SMG5	0,455838826	25,68177527	18,0288752	72,56393053	101,1541494	32,37508425	32,43209374	26,29023143	70,81369184	379,7956705	42,19951894
ENSG00000134453	RBM17	32,56039132	25,37501978	9,423054203	39,06653575	51,84999042	60,12045223	32,47027267	64,19930863	64,31129655	379,3763216	42,15292462
ENSG00000112096	SOD2	14,42958343	21,32929183	62,79917562	72,54281322	51,31811755	41,2883177	27,27649114	80,78361985	7,521632117	379,2895825	42,14328694
ENSG00000166224	SGPL1	26,64519814	29,90897276	44,00299456	36,00665626	78,14620311	33,57661284	34,51020753	55,70510609	40,66478807	379,1667394	42,12963771
ENSG00000023287	RB1CC1	43,13191515	34,2706116	97,01735492	65,91067334	2,641610715	33,40876318	20,89798778	49,95799123	31,2714298	378,5083377	42,05648197
ENSG00000187555	USP7	16,9702143	19,88368193	0,596523818	80,01010428	59,85292373	41,6358803	41,04561171	46,24823346	72,0077727	378,2509462	42,02788291
ENSG00000068903	SIRT2	34,28151121	34,7671263	47,16371859	26,07672786	24,86819742	60,77701075	44,65243981	49,00242703	56,61391488	378,2030738	42,02256376
ENSG00000152700	SAR1B	48,36433897	31,78567884	57,45986117	24,2617813	96,78704894	19,77467237	25,36596914	51,71646778	22,59830374	378,1072222	42,01191358
ENSG00000120262	CCDC170	44,84356405	13,92047939	90,63010959	0,624476191	34,44407945	56,65760673	21,435285	71,78070502	43,69035511	378,0266605	42,00296228
ENSG00000122882	ECD	82,21529828	66,47187092	0,22618397	13,87123814	0,19480203	65,60825084	50,02478656	16,75922542	82,56485042	377,9365066	41,99294517
ENSG00000087087	SRRT	52,22615236	14,51653891	0,114442198	64,9383253	0	63,24956526	55,29682602	46,04862129	80,9109499	377,3014212	41,92238014
ENSG00000161980	POLR3K	37,51765889	27,82203918	13,53846962	138,5483216	39,35274286	18,2686077	42,70906484	22,39048419	36,99221407	377,139603	41,90440033
ENSG00000136869	TLR4	35,66924218	53,51934174	30,81921411	60,15831568	37,70504286	42,24927769	40,87212367	44,22551981	31,66263715	376,8807149	41,87563499
ENSG00000070190	DAPP1	32,6912411	5,491361222	28,16138258	58,66773285	0,078239116	48,00201435	40,92136972	102,1539545	60,69366817	376,8609636	41,8734404
ENSG00000112299	VNN1	54,94573307	116,6379424	65,22035024	0	0,186409313	38,23737143	33,73663741	43,30896875	24,50299333	376,776406	41,86404511
ENSG00000184178	SCFD2	66,84834754	23,47086777	0,17505044	2,957701223	157,5096777	35,73137105	32,31572153	43,48918426	13,92678599	376,4247075	41,8249675
ENSG00000174720	LARP7	39,5549692	48,4480791	13,49553033	84,23576507	7,818625171	30,14291258	30,39421456	78,63945789	43,57812597	376,3076799	41,81196443
ENSG00000042445	RETSAT	39,9702201	59,33693062	46,34145883	39,9554666	72,55079223	37,08300121	46,51295834	2,856312636	31,48696327	376,0941018	41,78823354
ENSG00000213614	HEXA	54,41226718	39,54834035	25,48132538	38,11293499	48,92268161	40,12586305	45,73682628	49,39094122	33,65898476	375,3901648	41,71001832
ENSG00000177946	CENPBD1	63,01212032	9,113322879	40,69773998	37,77588031	0	34,21227905	50,1881328	75,37363664	64,92481448	375,2979265	41,69976961
ENSG00000053918	KCNQ1	53,11127096	25,32485722	35,44293144	25,64168741	77,76707742	43,83740221	32,3621477	41,98235454	39,66007413	375,129803	41,68108922
ENSG00000028528	SNX1	59,53963337	33,55541425	16,61235651	44,72866354	30,15574827	44,16824629	44,59202013	45,36766358	56,06341611	374,783162	41,64257356
ENSG00000146416	AIG1	39,91822812	40,90064896	17,63401175	52,52334517	79,8038983	39,61366251	46,84094979	24,96444564	32,60847538	374,6276656	41,62529618
ENSG00000157538	VPS26C	30,3000983	22,31393485	0	62,70182435	74,3254778	38,52992642	43,00606584	49,20458833	54,20939844	374,5913143	41,62125715
ENSG00000173992	CCS	67,93626496	30,04392158	15,96244266	35,32733083	0,167655249	62,35201412	56,52608328	23,60246911	82,51169126	374,429873	41,60331923
ENSG00000137824	RMDN3	38,85615964	45,61664843	57,29194844	16,02142785	54,07127642	29,65237918	55,27662456	28,94694686	48,51488213	374,2482935	41,58314372

ENSG00000172543	CTSW	94,81272352	16,96699823	71,18000169	72,83341692	0	15,69822133	36,65425429	14,46017622	51,21494937	373,8207416	41,53563795
ENSG00000161920	MED11	36,29114112	28,7424939	26,21386279	42,3089952	56,76688424	42,43438766	43,15702736	36,98894413	60,89814807	373,8018845	41,53354272
ENSG00000184924	PTRHD1	19,86430037	26,85616276	33,49681944	45,73508162	152,1144728	19,90580732	17,80278054	42,5755546	15,12366065	373,4746401	41,49718224
ENSG00000100029	PES1	68,82924601	31,86666579	51,08889824	25,64967168	29,11335057	41,01036787	47,699236	27,73694183	50,41297313	373,4073511	41,48970568
ENSG00000113742	CPEB4	50,43927836	16,65543529	30,66577755	46,16915491	82,85704049	18,7740488	39,69273068	49,56356646	38,47297948	373,290012	41,476668
ENSG00000138095	LRPPRC	23,76359892	29,33304488	2,494835352	68,85061256	63,81606108	28,57275289	41,18950269	74,60150188	40,58674549	373,2086557	41,46762842
ENSG00000081870	HSPB11	33,57493829	64,96394563	54,97752267	67,1689845	0	37,17094194	41,48740891	41,86593717	31,70187895	372,9115581	41,43461756
ENSG00000124201	ZNFX1	61,38260226	29,68509937	53,69123111	3,883293519	18,37882069	56,23906701	34,71256294	81,08736925	33,84635272	372,9063989	41,43404432
ENSG00000147155	EBP	38,80328002	47,92583259	60,06131098	56,08330902	0	51,80013481	52,37392818	30,27737553	35,21413177	372,5393029	41,39325588
ENSG00000088356	PDRG1	86,01797431	42,4752274	0	79,86716903	11,37725479	53,4949279	33,65964451	15,70281798	49,76166462	372,3566805	41,3729645
ENSG00000138594	TMOD3	44,04549083	18,71344658	25,779569	43,85729868	99,91249909	34,31502616	18,85831363	36,6424337	50,16737386	372,2914515	41,36571684
ENSG00000114030	KPNA1	0,017781898	15,2403608	52,37069175	93,83587696	74,96758298	38,57840663	30,65149556	32,58462786	33,94137474	372,1881992	41,35424435
ENSG00000133265	HSPBP1	56,41368328	35,51303831	88,76891024	37,56885652	0	39,04679009	36,59310098	39,3410791	38,9360042	372,1814627	41,35349586
ENSG00000090659	CD209	44,50469911	47,93023065	48,30329314	21,9317797	0	36,72284401	30,95541268	131,4551655	10,2733465	372,0767713	41,34186348
ENSG00000173875	ZNF791	49,96067648	88,77510662	0	77,62835137	0,162203045	27,57682576	50,15413	17,11374454	60,63375697	372,0047948	41,33386609
ENSG00000113048	MRPS27	55,18982292	21,16802151	34,9663187	128,4436482	1,434043327	28,31003697	31,79635199	38,51940359	32,13994534	371,9675926	41,32973251
ENSG00000171311	EXOSC1	13,50734249	48,49802991	0,747928517	89,63057872	104,1387193	23,21260087	25,54418418	48,99717354	17,612361	371,8889185	41,32099095
ENSG00000136451	VEZF1	80,52258783	40,33318503	37,9655295	48,01246022	18,49312807	38,29484917	34,47688748	36,5206534	37,26009046	371,8793712	41,31993013
ENSG00000197006	METTL9	21,55928176	21,86631872	44,81744441	67,85134964	35,68966227	56,31367194	34,10598698	57,64664611	31,99457603	371,8449379	41,31610421
ENSG00000149196	HIKESHI	6,294439733	63,34493506	17,65210066	46,4408009	60,14931164	34,16800982	30,4812866	61,89030787	50,67065099	371,0918433	41,23242703
ENSG00000173226	IQCB1	39,5730403	41,21881286	77,08002745	0,598670869	0,117704865	28,54901762	29,73289087	105,2711897	48,91339147	371,054746	41,22830511
ENSG00000213064	SFT2D2	35,12037601	24,86116431	42,6251021	50,73541763	19,56145301	52,92217558	35,51469235	64,60221152	44,99947887	370,9420714	41,21578571
ENSG00000169087	HSPBAP1	12,57173343	31,81262297	61,10967465	40,2065094	64,56860863	47,99066268	30,71661204	35,01574756	46,7944136	370,786585	41,19850944
ENSG00000136193	SCRN1	29,00783276	44,82334029	51,15605988	62,515671	0	69,76034996	62,44472542	0	50,94800495	370,6559843	41,18399825
ENSG00000125149	C16orf70	33,11006133	24,09457033	28,7999499	67,10381409	77,36228699	43,7792536	50,54571799	15,2337814	30,62650987	370,6559455	41,18399395
ENSG00000141562	NARF	40,49206099	54,07535979	19,55233175	28,63398067	87,65932686	22,39656604	33,66895511	28,08213966	55,57324975	370,1339706	41,12599673
ENSG00000160712	IL6R	71,37533231	21,06740958	75,12742796	10,71560023	31,88816857	40,93129368	41,37623842	31,03104725	46,48323058	369,9957486	41,11063873
ENSG00000189077	TMEM120A	19,34215304	45,18192701	94,79121375	9,393659086	3,05249911	45,29358744	61,48046268	35,52171601	55,84687994	369,9040981	41,10045534
ENSG00000100564	PIGH	50,68207994	65,21375785	41,78785802	63,3712047	0,123535446	33,84415961	31,11940467	42,39838104	41,36315786	369,9035391	41,10039324
ENSG00000153774	CFDP1	71,40937312	54,9952408	8,085178291	68,5394416	11,62959358	27,21231948	45,48534674	42,30751434	40,17851351	369,8425215	41,0936135
ENSG00000153029	MR1	39,92758981	27,8812606	29,91527381	40,80947078	61,35116764	39,73831635	44,30654321	39,55458719	46,26282171	369,7470311	41,08300346
ENSG00000168522	FNTA	36,39455315	41,69385506	34,93981448	43,71132929	45,21456499	35,96848071	24,5305449	76,59498596	30,67773053	369,7258591	41,08065101
ENSG00000197548	ATG7	67,48909785	42,25672638	14,47649106	26,91178103	102,4946592	26,22112137	39,95789018	13,80395737	36,1046793	369,7164037	41,07960041
ENSG00000132357	CARD6	2,120850115	3,512503393	0	88,44322297	34,77769884	75,21279477	45,58021651	97,62312486	22,38852841	369,6589399	41,07321554
ENSG00000143157	POGK	57,57870821	13,71180682	17,60409008	63,90559451	0,467600967	37,01958299	53,14072537	53,85989577	72,28831347	369,5763182	41,06403535
ENSG00000167528	ZNF641	36,55613286	9,492872318	38,47895399	48,62123258	128,1109665	24,4343167	40,65866553	23,39635833	19,80403701	369,5535359	41,06150399
ENSG00000203879	GDI1	46,20142448	48,97570778	10,57762934	41,65229142	0	56,97652035	54,80971885	43,25323106	66,82837273	369,274896	41,030544
ENSG00000065882	TBC1D1	33,48349336	29,02522148	36,18202506	44,10046555	33,82501699	45,94446739	37,18976747	69,74418597	39,65822855	369,1528718	41,01698576
ENSG00000064703	DDX20	12,12131346	82,53108293	37,22992194	61,76473347	73,04521542	9,595186441	26,17241482	49,63914094	16,83766164	368,9366711	40,99296345
ENSG00000122008	POLK	25,8738473	33,92636838	68,26617258	31,12305134	104,1657803	25,34668997	33,32095143	21,41201481	25,37146921	368,8063453	40,97848281
ENSG00000122644	ARL4A	9,399625456	47,86914367	31,63622884	15,86717558	49,08440607	18,88187316	33,31876041	118,9933129	43,59746868	368,6479947	40,96088831
ENSG00000116237	ICMT	36,46965911	27,68798249	43,98992605	7,758962585	87,62175473	30,64783973	49,49817374	15,34400843	69,38017827	368,3984852	40,93316502
ENSG00000170185	USP38	26,81839074	20,14422371	59,40142349	43,12614772	134,524961	31,91653318	16,49950173	0,297038907	35,58458517	368,3128056	40,92364507
ENSG00000118557	PMFBP1	81,43667549	36,89249448	13,77444876	39,75234985	77,67944028	14,08097145	22,48809324	33,21183845	48,82104214	368,1373541	40,90415046
ENSG00000185896	LAMP1	20,08315022	19,6167288	32,19940182	54,26865697	58,17441463	42,59748999	69,06222768	35,85799261	35,99744518	367,8575079	40,87305643
ENSG00000163636	PSMD6	55,1074064	62,92798665	37,26511685	49,76727566	14,91894646	32,15278961	32,76009326	49,47644626	33,30692359	367,6829847	40,85366497
ENSG00000170619	COMMD5	54,49719587	67,47213852	17,05808629	12,34454114	32,13221754	33,38687299	58,1112165	37,26875993	55,30160484	367,5726336	40,84140374
ENSG00000149084	HSD17B12	36,70051962	54,28172682	13,90621024	48,52795342	23,73638499	45,6705631	42,65275801	48,17766828	53,88007651	367,533861	40,83709567
ENSG00000134186	PRPF38B	26,09400365	30,38043078	38,56948893	59,76555205	15,59360141	34,75653634	36,04453247	60,48069079	65,84124096	367,5260774	40,83623082
ENSG00000112237	CCNC	39,97292101	52,38567417	58,27176406	27,86097144	47,88233898	40,16159771	25,69793431	47,59200188	27,62852042	367,453724	40,82819155
ENSG00000085377	PREP	64,5655797	42,09922621	0,124696691	1,609445285	139,9499226	21,82495306	34,37524067	38,8724752	23,7596765	367,1812159	40,79791288
ENSG00000179029	TMEM107	44,28691085	24,41507936	0,703079605	66,24432014	87,99117778	38,29109105	22,61084073	53,48798564	29,02566614	367,0561513	40,78401681
ENSG00000120699	EXOSC8	18,10294827	60,84426027	95,06607693	48,81462788	0	28,29634504	24,24994614	58,98876168	32,35451406	366,7174803	40,7463867
ENSG00000115561	CHMP3	52,09966961	35,83376494	22,45543754	47,17573483	87,69446753	33,80141397	28,2974121	33,04816447	26,21285896	366,618924	40,735436
ENSG00000118707	TGIF2	107,3330054	8,571365218	47,29789754	8,603695362	0,090389515	50,36198226	38,30745349	60,88046902	44,812728	366,2589858	40,69544287
ENSG00000116001	TIA1	10,42930532	27,31338975	101,9021946	64,88817364	1,902393519	39,70101737	32,7396477	43,11103333	44,10584939	366,0930046	40,67700051
ENSG00000168275	COA6	53,31361522	34,75478472	21,98436635	41,04337474	101,7710083	32,94884915	21,72025958	36,440719	22,04681943	366,0237965	40,66931072
ENSG00000136816	TOR1B	0,05198162	44,82869351	44,5609653	15,34911615	0	56,38790842	73,02917157	108,3261585	23,39863335	365,9326285	40,65918094
ENSG00000142892	PIGK	46,50610055	58,25791007	0,100827022	19,40212905	55,88009636	47,42021561	53,9972974	27,50625072	56,65723684	365,7280636	40,63645151
ENSG00000065978	YBX1	39,96974741	50,70608661	21,77988404	72,70824329	23,05968313	36,65182107	36,80955837	50,90346946	32,8447462	365,4332396	40,60369329
ENSG00000151748	SAV1	38,3547079	20,05111581	72,53157467	17,10213244	48,09496038	40,49043204	24,55920175	64,79569494	38,85281647	364,8326364	40,5369596
ENSG00000130150	MOSPD2	53,5915396	12,09379	17,87574632	83,53045258	74,53144202	42,96838403	25,64228068	44,06958556	10,4776052	364,780826	40,53120289
ENSG00000165782	PIP4P1	52,05228703	19,31919748	17,83516807	7,753261144	96,50752782	49,86478811	29,13234372	31,71407235	60,45020985	364,6288556	40,51431728
ENSG00000139613	SMARCC2	68,2211793	24,07114153	108,024296	21,43424892	0,860724037	31,68550397	40,31071022	29,62715427	40,29138025	364,5263385	40,5029265
ENSG00000117533	VAMP4	8,819329335	20,97380265	80,06005257	50,76352294	116,2486389	8,052619246	27,6054862	40,69130053	11,14482755	364,3595799	40,48439777
ENSG00000177646	ACAD9	61,81319854	12,70374625	116,7297989	39,74026999	0	33,43517977	22,41473692	42,00115834	35,30525568	364,1433444	40,4603716
ENSG00000065809	FAM107B	52,87470602	39,65399574	0,039603946	68,77471193	78,84315896	23,94463801	24,48986392	54,04457665	21,4468846	364,1121398	40,45690442
ENSG00000152952	PLOD2	26,10862528	57,21155816	0,036275623	24,72123079	141,9974101	41,07169046	15,30924709	6,85620465	50,78462973	364,0968719	40,45520798
ENSG00000139112	GABARAPL1	29,52863853	46,37738516	63,51087384	28,52064983	62,46678198	32,3797844	57,05448356	24,94768755	19,25870697	364,0449918	40,44944354
ENSG00000177239	MAN1B1	7,912792006	36,35109228	55,90529745	49,98439954	81,82095172	31,05932673	42,04598984	21,6586692	36,94904857	363,6875673	40,4097297
ENSG00000178726	THBD	19,63812235	23,362092	99,78599319	16,06632966	105,3143438	23,41886943	42,20004839	17,6113824	16,23726004	363,6344412	40,4038268
ENSG00000136770	DNAJC1	71,97475374	37,90601768	47,11148665	46,9473368	26,68142383	30,51688519	50,69202972	24,28495014	27,46647397	363,5813577	40,39792864
ENSG00000137210	TMEM14B	49,49145793	37,97982223	26,61450169	65,19309957	37,81362284	35,21507983	24,98983352	50,57741978	35,70260293	363,5774403	40,39749337
ENSG00000213699	SLC35F6	38,02936453	35,39034599	36,83129451	45,00493027	11,87241491	48,5145997	65,88639423	45,22092749	36,57871069	363,3289823	40,36988692
ENSG00000029364	SLC39A9	51,993214	48,30913591	47,39447751	48,47293028	0,35217467	43,69737801	56,45989162	40,18173222	26,32261614	363,1835504	40,35372782
ENSG00000055950	MRPL43	41,70025824	49,98112848	48,78072266	39,50904597	36,18312769	43,64845847	36,31019404	33,3856364	33,60113445	363,0997064	40,34441182
ENSG00000066084	DIP2B	42,75943015	16,786399	107,5826757	48,77878233	62,31041504	37,44247968	24,41283438	5,01190485	18,00831435	363,0932355	40,34369283
ENSG00000105122	RASAL3	21,33211107	13,1363077	29,92437249	31,62168579	6,558872174	60,53204969	76,34980237	9,86901695	113,7281683	363,0523866	40,33915406
ENSG00000082898	XPO1	18,54679621	33,59790862	45,96596102	61,15524174	40,42679542	38,13335855	31,06751729	42,98358783	50,96969448	362,8468612	40,31631791
ENSG00000130803	ZNF317	47,44656389	15,9886573	95,81722905	42,5931892	5,691244728	37,59938585	39,6941601	16,23371092	61,66658574	362,7307268	40,30341409
ENSG00000120910	PPP3CC	49,88368959	33,57555917	19,56120738	54,90525509	91,42074724	18,37091932	38,34657266	35,33450015	21,28356171	362,6820123	40,29800137
ENSG00000135317	SNX14	63,54938566	10,30299311	0,039124574	67,13429044	0	66,27082571	34,41825152	62,1337906	58,69663561	362,5452972	40,2828108
ENSG00000104320	NBN	14,33537577	19,90592821	32,37682439	76,50871169	0,058336183	50,39843704	33,69110767	72,35636729	62,85452556	362,4856138	40,27617931
ENSG00000158122	PRXL2C	100,8704519	38,60275405	3,140511125	17,0138363	31,41409102	52,75078052	40,54093038	25,49188562	52,55053373	362,3757747	40,26397497
ENSG00000140450	ARRDC4	38,16825345	6,931292469	78,22039713	45,93020848	164,5292335	9,966071077	18,16261822	0,162695503	0	362,0707698	40,23008553
ENSG00000132376	INPP5K	54,62531744	34,93673073	57,98193056	9,785692069	44,1520891	44,90137394	49,40712667	15,58593359	50,69194721	362,0681413	40,22979348
ENSG00000142546	NOSIP	48,09357145	45,95277214	66,81207181	16,58485008	0,591766195	65,02850994	41,88251666	24,03584778	52,89276022	361,8746663	40,20829625
ENSG00000198373	WWP2	87,3809656	23,88441776	48,16471599	7,9018578	74,97051744	34,8200567	38,78933059	14,77150646	31,1702653	361,8536336	40,20595929
ENSG00000138231	DBR1	29,12225871	21,63804115	60,5222494	105,7777539	0	59,8013123	40,10285928	22,37753084	22,34863243	361,690638	40,18784867
ENSG00000167261	DPEP2	71,4513676	28,36264724	37,90637713	23,42297942	0,307991031	31,4936029	28,87467618	78,4050003	61,4175471	361,6421889	40,18246543
ENSG00000129355	CDKN2D	78,82281264	13,38033933	39,02448495	19,27064683	71,68014817	23,73304989	19,95170283	69,25275367	26,46118148	361,5771198	40,17523553
ENSG00000159131	GART	14,83616879	31,5847821	127,3163916	29,35560545	9,405349129	44,15862724	41,56983777	40,41439427	22,89813299	361,5392894	40,17103215
ENSG00000010244	ZNF207	26,24441514	34,30944121	66,55441172	53,73935532	9,094741347	39,60729948	35,31775102	52,71426788	43,93226809	361,5139512	40,1682168
ENSG00000136521	NDUFB5	37,98998506	82,94087415	57,52781813	12,75563579	14,19098447	48,88865571	30,77453212	44,35061134	31,97155297	361,3906498	40,15451664
ENSG00000184207	PGP	15,9034594	46,01776899	39,55125721	23,34731713	26,07454103	62,96936079	48,86303358	43,37938217	55,13378677	361,2399071	40,13776745
ENSG00000186132	C2orf76	8,168138955	78,68259462	27,54757738	101,5866536	0	41,31749883	33,50719934	39,76271823	30,61564331	361,1880242	40,13200269
ENSG00000160094	ZNF362	34,1159799	31,29908098	37,21187495	43,10362021	85,53449738	39,32041446	47,97831253	6,639938118	35,91714703	361,1208656	40,12454062
ENSG00000090674	MCOLN1	41,92001177	57,85580295	51,33993238	23,33779207	25,04205197	43,09147874	50,5938984	22,29055824	45,54601481	361,0175413	40,11306015
ENSG00000158717	RNF166	24,03875487	41,67978745	62,9472459	42,57860191	0,081729072	57,73265175	47,08706815	12,85942445	71,94468238	360,9499459	40,10554955
ENSG00000184575	XPOT	68,76356706	40,29456285	26,43089466	49,33524656	0	31,30088886	49,34823577	39,27296089	56,13478972	360,8811464	40,09790515
ENSG00000196872	KIAA1211L	0,032117018	47,13144189	17,72455466	44,03451006	52,51283085	50,03764258	36,45439827	14,04610276	98,90747965	360,8810777	40,09789753
ENSG00000105953	OGDH	59,54510587	14,82243686	19,20783793	30,35059397	64,31030077	44,86909838	71,83838778	10,90878035	44,90993696	360,7624789	40,08471988
ENSG00000116455	WDR77	66,91637561	26,46466668	69,77973434	44,41239605	6,954976612	44,4281954	32,5708832	33,96645834	35,23124016	360,7249264	40,08054738
ENSG00000141428	C18orf21	39,92835005	94,24793072	85,66465922	2,5835146	0	19,9174826	14,05329832	81,17730402	22,77079914	360,3433387	40,03814874
ENSG00000148057	IDNK	0	100,6641136	41,22196617	51,0650033	0	32,01141226	12,9343727	96,36156903	26,02261117	360,2810482	40,03122758
ENSG00000151893	CACUL1	24,10509587	31,25369435	21,89154898	33,23208608	101,4305875	34,47666184	24,06044816	49,54121357	40,07250097	360,0638373	40,00709303
ENSG00000178252	WDR6	38,60385054	14,00364518	54,76794937	28,38602571	40,96269101	46,60760922	57,76744924	35,32360479	43,55339879	359,9762238	39,9973582
ENSG00000178980	SELENOW	32,50835257	42,40812882	36,17766913	46,09121979	44,54754575	49,53922898	62,75907422	12,9705642	32,96934303	359,9711265	39,99679183
ENSG00000185885	IFITM1	67,64458238	30,93946155	13,66141512	24,53234804	0	4,254117351	24,397009	194,1656113	0,198553977	359,7930987	39,97701096
ENSG00000240857	RDH14	51,73583851	30,43225726	53,10716897	33,5628845	0	44,14518497	26,73957895	47,29422877	72,6512256	359,6683675	39,96315195
ENSG00000101343	CRNKL1	28,1905422	45,56124197	4,625900487	84,10253615	46,55081784	46,17305518	52,58380019	3,617632664	48,22161789	359,6271446	39,95857162
ENSG00000166508	MCM7	0	28,12250799	6,781548242	100,1262112	9,497864777	47,66240918	62,50630995	23,50560535	81,31315008	359,5156067	39,94617853
ENSG00000170776	AKAP13	53,52950393	12,03201282	72,52429505	36,54118974	32,79515229	29,0752559	40,7902317	32,35912481	49,85232156	359,4990878	39,94434309
ENSG00000110871	COQ5	23,62424355	37,41765615	0	93,16870545	0	29,58661224	35,71966269	96,71255635	43,24777128	359,4772077	39,94191197
ENSG00000149743	TRPT1	55,3844173	41,37394662	53,08036279	38,963141	43,22410238	37,16096628	36,95981273	8,821556327	44,40134107	359,3696465	39,92996072
ENSG00000167881	SRP68	50,17555524	48,91224492	30,29816402	50,83103702	0,095700877	50,18214645	38,66927088	27,64995448	62,50188171	359,3159556	39,92399507
ENSG00000162600	OMA1	16,09461699	19,76086822	10,16818006	65,19865288	191,6323841	9,683323062	14,91605345	5,776828103	26,04250621	359,2734131	39,91926812

ENSG00000147224	PRPS1	17,16772781	53,66929091	93,68448628	72,62010349	0,547643984	39,53263837	41,61616044	16,502832	23,73564047	359,0765238	39,89739153
ENSG00000187098	MITF	50,73493746	9,409801424	48,07844074	16,16990164	105,7198837	41,98015846	51,63136486	21,94921163	13,3024805	358,9761804	39,88624226
ENSG00000127419	TMEM175	51,88560583	35,45128845	60,7673857	25,42369474	7,434119701	44,14512723	35,46277118	32,92489066	65,40279213	358,8976756	39,87751951
ENSG00000105726	ATP13A1	56,69132068	26,97779903	20,38542896	29,78011314	0	37,43358627	56,31830191	63,92064789	67,27737485	358,7845727	39,86495253
ENSG00000123200	ZC3H13	21,9489392	55,84876729	37,28968303	20,79345438	48,62867841	31,96990947	42,86648362	44,55152864	54,85776546	358,7552095	39,86168994
ENSG00000075234	TTC38	51,39786599	19,18511795	0,077691475	58,26915727	60,32130882	34,03531674	52,46048538	29,82961579	53,08416595	358,6607254	39,85119171
ENSG00000122299	ZC3H7A	83,15678637	26,90146852	3,618815495	44,12300759	2,286725542	49,77082533	51,9671235	47,31849191	49,38726034	358,5305046	39,83672273
ENSG00000104613	INTS10	29,08958402	47,3239432	24,82415208	49,48222924	44,55054431	48,20883031	30,54286867	31,15611516	53,27150994	358,4497769	39,82775299
ENSG00000085449	WDFY1	0,082649355	22,92772982	25,23866715	95,52551121	83,08480606	26,16456679	30,90436072	44,09810454	30,32500227	358,3513979	39,81682199
ENSG00000069998	HDHD5	29,66493982	42,58981525	20,66829342	43,0310108	34,99504414	53,24196789	57,70211784	19,41370028	56,96632662	358,2732161	39,80813512
ENSG00000068438	FTSJ1	24,83432777	73,19753763	0,387158912	58,05621475	0	44,07721798	57,92954743	43,03012822	56,75048748	358,2626202	39,8069578
ENSG00000136159	NUDT15	74,54164373	71,94728589	73,15265763	4,981730681	0,185303169	40,23631117	27,19171282	35,80560329	30,11135603	358,1536044	39,79484493
ENSG00000124486	USP9X	24,80425775	10,59153321	25,65305323	70,80202816	101,9851189	25,00616592	51,74143991	4,459652515	43,07352975	358,1167793	39,79075326
ENSG00000138495	COX17	31,23786659	44,93724127	29,05224791	76,40637378	12,23512805	40,94294158	35,93119084	44,72733096	42,5256545	357,9959755	39,77733061
ENSG00000108826	MRPL27	46,8843062	52,38535763	26,69345321	68,94904296	0	39,44496492	26,25736749	62,80445091	34,4000682	357,8190115	39,75766795
ENSG00000047249	ATP6V1H	47,07402182	54,74215597	22,16651651	10,94045544	2,040440582	44,76074924	49,46048354	78,65948267	47,62681253	357,4711183	39,71901314
ENSG00000166275	BORCS7	102,7306354	60,20322602	10,47044686	67,06452818	0	23,03632823	27,33659375	39,53500734	26,83382209	357,2105878	39,69006532
ENSG00000146859	TMEM140	35,40939442	32,69176136	102,9956528	69,71803472	0	18,79315655	46,81404951	21,71467975	29,00587745	357,1426065	39,68251184
ENSG00000171530	TBCA	18,84063556	47,45497769	57,88297342	59,24288964	31,26246462	30,2617506	27,7667125	51,41966484	32,71278071	356,8448496	39,64942773
ENSG00000160695	VPS11	30,33978954	40,71836365	35,67105384	36,62012764	21,46072329	45,50112312	43,88381681	40,8917672	61,66568201	356,7524471	39,63916079
ENSG00000162302	RPS6KA4	35,40599659	20,91887944	32,42005883	35,84685887	6,603159434	57,52612589	72,6719417	30,63919158	64,48732753	356,5195399	39,61328221
ENSG00000128050	PAICS	21,52978864	24,96998804	101,029883	91,1554135	0	20,3277426	31,6638579	34,11164353	31,57190487	356,3602221	39,59558023
ENSG00000106591	MRPL32	77,17529145	32,57949257	25,74503001	36,7546882	25,45264297	31,07735341	27,42561754	62,97740417	36,97006459	356,1575849	39,57306499
ENSG00000141968	VAV1	19,94477261	21,39662446	83,4391668	16,70564903	6,164392979	60,07571567	58,2967003	33,03579183	57,07742878	356,1362425	39,57069361
ENSG00000090520	DNAJB11	35,75395809	37,29003212	42,97511214	32,32661524	10,08862752	43,19291231	68,1537504	51,02680025	35,1522649	355,960073	39,55111922
ENSG00000122482	ZNF644	47,29657525	60,30021307	6,214705001	68,25350664	23,18867876	33,75376935	24,18983518	50,40821914	42,18452097	355,7900234	39,53222482
ENSG00000132603	NIP7	30,8018385	41,03522177	19,3953231	76,1793718	62,07343692	20,70384937	20,65605592	53,911542	31,0266538	355,7832932	39,53147702
ENSG00000107164	FUBP3	45,9762436	26,63958081	41,80436745	27,97848887	10,55108333	52,56473216	42,27949246	50,39339838	57,41737172	355,6047588	39,51163986
ENSG00000167397	VKORC1	43,19416312	58,03621372	47,21853736	34,43388478	28,20466543	39,77308346	36,95359093	32,89257339	34,87804596	355,5847581	39,50941757
ENSG00000136490	LIMD2	58,30050011	24,49455442	37,85584594	26,96675911	56,08613269	25,38770559	27,67689075	43,26042959	55,31193064	355,3407488	39,48230543
ENSG00000121481	RNF2	28,39609802	47,23033693	27,47063958	36,20756688	69,41468113	41,54135791	17,12127162	67,00055747	20,88751236	355,2700219	39,47444688
ENSG00000166199	ALKBH3	0	23,99758236	53,14325422	82,44668105	8,902493697	32,97298436	35,4796359	73,8618445	44,27520089	355,079677	39,45329744
ENSG00000106077	ABHD11	16,76805808	31,86309481	21,98095147	34,19855499	33,41722871	43,48901093	51,41597703	55,00024911	66,82030923	354,9534344	39,43927049
ENSG00000156853	ZNF689	40,94773098	9,799851891	109,5478197	0	0,086347089	54,25303783	62,02620885	28,84052609	49,42203742	354,9235599	39,4359511
ENSG00000163159	VPS72	54,32666061	41,88796921	45,19852434	35,93955866	0,079013761	41,46851818	32,29929339	47,40227459	56,22419541	354,8260081	39,42511202
ENSG00000072518	MARK2	52,74382609	19,43310563	39,38879531	39,00880348	52,81140329	45,4272541	37,88168295	22,87376109	45,23855872	354,8071907	39,42302118
ENSG00000105849	TWISTNB	41,64343994	52,90846456	2,39395222	119,6282432	0	17,65006135	13,91996085	78,08902014	28,38537308	354,6185153	39,40205726
ENSG00000147592	LACTB2	94,79607228	51,90740581	0	93,86635991	42,0085738	21,72638504	24,48359998	0	25,58756466	354,3759615	39,37510683
ENSG00000166526	ZNF3	3,343154218	22,9878374	55,29876969	43,92212976	108,1192748	18,13882227	9,854417668	51,59994076	41,08222349	354,3465701	39,37184112
ENSG00000178209	PLEC	23,94147821	13,91920688	84,19078273	37,27203716	70,19182851	33,96133163	41,04314668	10,56005945	39,13594589	354,2158171	39,35731302
ENSG00000137309	HMGA1	35,06648395	28,57723078	34,64298612	67,84085072	14,50668199	43,77700649	56,61531955	24,85004268	48,31849849	354,1951008	39,3550112
ENSG00000103174	NAGPA	43,86725538	11,88156565	40,0445438	12,41867768	41,38626673	39,57166928	61,86575086	52,05971304	51,03662701	354,1320694	39,34800772
ENSG00000120675	DNAJC15	38,76077395	57,02733609	40,73995474	59,10107319	6,215581918	46,39959597	35,33979467	46,74058881	23,74281308	354,0675124	39,34083471
ENSG00000146085	MMUT	30,57104434	27,37004409	82,71567364	37,52095673	0	55,41961799	38,91522761	54,27762773	27,21132496	354,0015171	39,3335019
ENSG00000117305	HMGCL	84,89900932	34,73267912	56,01091547	29,44095751	3,637117966	40,97649282	34,68938106	31,29382098	38,23920981	353,919584	39,32439823
ENSG00000196365	LONP1	28,08249543	38,07247278	57,78268724	48,697599	0,494360432	31,84438994	53,12440999	12,80149652	82,92738624	353,8272976	39,31414417
ENSG00000137055	PLAA	64,14025351	14,12601317	49,00608706	91,06949725	0,0675668	30,77851903	7,648614782	62,66755297	34,23212517	353,7362298	39,30402553
ENSG00000188529	SRSF10	48,75767756	52,13074985	42,25016197	32,88029504	0,034943811	34,07971465	39,30028206	54,20966049	49,98343754	353,626923	39,29188033
ENSG00000081791	DELE1	31,4309987	35,43943434	0,092660033	37,53114718	82,65688767	46,12314032	51,75651195	17,67824936	50,82902384	353,5380534	39,28200593
ENSG00000087053	MTMR2	0	16,6520608	154,3223396	11,21521463	70,13326738	17,21739111	23,19119881	20,19652366	40,60741956	353,5354156	39,28171284
ENSG00000101307	SIRPB1	83,03514694	7,880055494	34,13710623	1,842095536	115,3232495	9,778672966	5,088026926	26,14832729	70,26835344	353,5010343	39,2778927
ENSG00000182199	SHMT2	65,84761583	67,85300612	0,060535736	44,43622608	0	41,9112084	55,27580175	8,347214685	69,57103044	353,302639	39,25584878
ENSG00000100209	HSCB	25,56616257	55,14061762	29,05981778	0	0	43,246292	49,96377045	105,9026881	44,2248258	353,1041743	39,23379714
ENSG00000124209	RAB22A	58,69274162	23,90301015	77,64002765	29,0334512	29,26114229	31,69239378	26,98708954	33,37866989	42,2196125	352,8081386	39,20090429
ENSG00000015285	WAS	54,96083755	42,45675516	36,77603157	11,72618323	32,57849187	42,78416384	53,32884334	29,42130547	48,23735857	352,2699706	39,14110784
ENSG00000168300	PCMTD1	59,57342858	58,80596096	24,67391292	4,66782489	49,43199272	38,15286237	51,45260235	30,99939204	34,28631047	352,0442873	39,11603192
ENSG00000091409	ITGA6	34,88379642	20,54023667	38,32065448	88,74122385	16,85872218	53,30897219	52,09543144	0,24622546	47,02941801	352,0246807	39,11385341
ENSG00000198369	SPRED2	15,01498313	23,23410246	125,9368514	47,21003355	37,31430231	33,24239166	25,27640294	13,3254267	31,26137686	351,815871	39,09065233
ENSG00000149547	EI24	30,3866	52,92175252	46,42291541	54,26490735	0,514347669	42,63013099	65,41277692	12,49423171	46,47493954	351,5226021	39,0580669
ENSG00000134987	WDR36	3,05931798	10,9624407	76,79309161	13,97379044	103,7575762	38,55210501	24,46802582	46,52670635	33,40188279	351,4949369	39,05499299
ENSG00000127666	TICAM1	25,52390805	16,23281166	23,99603993	35,78592514	74,07805806	55,67552238	38,54133517	25,56052148	55,55949844	350,9536203	38,9948467
ENSG00000167604	NFKBID	20,88520082	26,49558225	91,35618638	25,94662974	2,695065984	33,61790297	50,768796	39,88359695	59,27562624	350,9245873	38,99162082
ENSG00000130725	UBE2M	43,32771688	47,30194759	23,68372225	41,75529632	43,93354969	41,68152118	41,73207656	29,23560892	38,26550459	350,916944	38,99077155
ENSG00000213462	ERV3-1	52,12367804	50,40664215	44,29322842	21,07411969	19,43106654	33,59796441	20,39173687	51,07373363	58,52298483	350,9151546	38,99057273
ENSG00000127084	FGD3	31,03766448	20,04843074	24,84524776	16,01130731	1,4196809	49,90435016	61,98035995	73,8905762	71,71286396	350,8504815	38,98338683
ENSG00000198538	ZNF28	25,0881483	31,27949357	46,66343754	79,83081733	106,4430406	24,18545813	18,75230234	1,769691408	16,60954693	350,6219361	38,9579929
ENSG00000087274	ADD1	46,26882976	38,65931762	16,28748595	29,90681345	23,89229888	42,44661613	43,19890315	49,06429958	60,83884792	350,5634124	38,95149027
ENSG00000265241	RBM8A	21,18524205	44,12220198	57,73624258	50,59849194	0,385802194	40,81667545	38,64081845	41,8294072	55,23817851	350,5530604	38,95034004
ENSG00000099901	RANBP1	18,15289323	32,96637577	20,63791644	90,98836452	11,97058475	47,69031296	36,41898598	49,70823039	41,75512773	350,2887918	38,92097686
ENSG00000138698	RAP1GDS1	8,330025927	3,213353615	42,99876531	54,02470068	61,83919332	37,74587973	41,47645746	57,50926101	43,00099289	350,1386299	38,90429222
ENSG00000106868	SUSD1	145,4028322	10,51285872	0	0,873318108	126,6312156	18,68130089	5,789092536	0,236983912	41,67440542	349,8020074	38,86688971
ENSG00000120451	SNX19	14,23612835	6,808806921	2,489272231	59,28012426	55,63854956	39,93088218	56,53244964	54,39685839	60,14022642	349,4532979	38,82814421
ENSG00000183864	TOB2	70,42079808	21,61937342	52,89370232	42,67768532	25,3144363	35,00900444	21,53314843	40,72391007	39,2585259	349,4505843	38,8278427
ENSG00000100109	TFIP11	37,33287277	23,87430612	30,73326701	27,79274288	85,02286185	42,30170333	32,52165154	26,0773417	43,65820621	349,3149534	38,8127726
ENSG00000101844	ATG4A	25,14207308	64,7681565	0	29,86444021	0	40,41484154	32,57856246	80,49191891	75,81166641	349,0716591	38,7857399
ENSG00000161395	PGAP3	32,77750704	28,52574469	34,71870813	31,94092118	86,33110303	26,68139145	43,68406143	14,79148934	49,55564907	349,0065754	38,77850837
ENSG00000153130	SCOC	15,19748828	79,58202354	49,90745342	26,4914917	46,61567081	29,31245016	30,2213256	34,12495534	37,50001323	348,9528721	38,77254134
ENSG00000204356	NELFE	31,85481618	38,6967299	17,64286235	32,72777165	48,16272291	45,81814566	42,42385612	43,69176374	47,88338806	348,9020566	38,76689517
ENSG00000119707	RBM25	19,16499247	38,88560457	49,81146282	63,83635302	45,24075126	32,46567048	21,22458013	50,6718769	27,37776463	348,6790563	38,74211736
ENSG00000185909	KLHDC8B	45,93872715	18,15609121	0	0	216,5291861	32,17335458	6,704080383	10,04545438	19,08971647	348,6366102	38,73740114
ENSG00000174243	DDX23	21,38435936	15,06970887	52,09245636	49,427152	33,61557242	33,07817284	47,95015083	55,3464201	40,5800864	348,5440792	38,72711991
ENSG00000181192	DHTKD1	2,283068112	45,77910634	73,76053618	24,04839804	59,27643664	46,24816076	48,55310864	0,24962007	48,04983739	348,2482722	38,69425246
ENSG00000112343	TRIM38	54,36001417	23,00475006	60,83212827	32,05582561	1,220191267	35,42643552	37,22277634	65,43432453	38,57824921	348,134695	38,68163277
ENSG00000153339	TRAPPC8	20,74388192	1,396724471	47,96401057	63,34362867	125,3306583	29,76127871	26,71435609	10,04033412	22,82997973	348,1248526	38,68053917
ENSG00000125977	EIF2S2	53,08970753	48,67679501	7,612913509	64,519133	22,05847189	34,85679957	31,51484865	47,96213793	37,80915164	348,0999587	38,67777319
ENSG00000148308	GTF3C5	22,5487934	39,7883313	42,09943593	27,53183613	68,88418337	38,01347568	50,36977137	1,379877092	56,96464951	347,5803538	38,62003931
ENSG00000088812	ATRN	0,568538232	13,45142858	25,22770024	15,38061588	120,8659201	48,87845516	45,21852918	33,34107767	44,13616531	347,0684303	38,56315892
ENSG00000173198	CYSLTR1	24,70102736	44,69836107	102,2139865	9,936808805	29,02872176	44,76337097	36,73731654	8,784680437	46,00260602	346,8668794	38,54076438
ENSG00000136897	MRPL50	8,923495929	39,14423083	24,49824613	84,24069774	16,68561723	36,6535057	40,20617054	63,49177391	32,86853961	346,7122776	38,5235864
ENSG00000109929	SC5D	37,54467928	26,83020212	24,99443102	48,14346309	68,65167261	29,12593673	34,03198681	48,21439333	29,13209858	346,6688636	38,51876262
ENSG00000067048	DDX3Y	24,96494568	32,48643463	125,7228175	0,045618003	86,76581181	0,058010691	0,065806118	0,462146248	75,9956321	346,5672228	38,5074692
ENSG00000116977	LGALS8	23,23461195	69,3137405	0,028628229	51,19270099	68,56889017	28,22701058	36,98208962	27,08814786	41,91595096	346,5517708	38,50575232
ENSG00000143198	MGST3	38,77228502	50,51805938	18,05558765	60,59965257	9,455156356	48,09181396	41,05990124	45,71586043	34,27497337	346,54329	38,50481
ENSG00000106261	ZKSCAN1	30,56185299	40,61497685	73,86582338	23,74434937	22,48182308	32,12417213	21,04749924	40,25159912	61,6846922	346,3767884	38,48630982
ENSG00000174109	C16orf91	50,06947267	45,62416121	8,574510516	38,68588181	0,357330888	56,95437708	48,93967179	19,72743687	77,1124376	346,0452804	38,4494756
ENSG00000158796	DEDD	42,63878563	42,67839704	37,14941415	48,56109949	0	56,46017499	33,75823537	18,39001315	66,40669778	346,0428176	38,44920196
ENSG00000114867	EIF4G1	22,51011286	34,78019645	16,49633626	47,08648567	26,31296029	38,55908886	41,0585972	75,67539871	43,5525034	346,0316797	38,44796441
ENSG00000228300	C19orf24	19,17720028	33,26188932	31,43191005	33,11483933	35,466284	44,32725332	56,74898604	34,84066766	57,63370204	346,0027321	38,44474801
ENSG00000081177	EXD2	24,5618012	25,81664155	12,82711937	21,87304194	135,0919575	10,384312	34,89850205	36,03493687	44,15449867	345,6428111	38,40475679
ENSG00000203705	TATDN3	37,09628238	35,196996	0,20890288	52,2262136	1,70922678	37,82029679	31,55616566	99,66012507	50,13945503	345,6136642	38,40151825
ENSG00000107897	ACBD5	0,241998169	34,04685657	15,07235952	0	49,62411246	53,25384102	49,88959895	77,5625409	65,860504	345,5518116	38,39464573
ENSG00000166855	CLPX	32,45791968	35,84392237	141,6624456	24,22523936	10,25862234	31,17089392	20,44837859	17,0632756	32,29598052	345,426678	38,380742
ENSG00000221821	C6orf226	11,9369212	71,24778458	54,39825994	85,26062007	13,53944056	16,38255246	38,06909535	36,18762768	18,35109286	345,3733947	38,37482163
ENSG00000125107	CNOT1	83,15189544	19,42305889	19,87950943	3,967769865	34,41915437	27,03252231	46,19426563	52,19471466	59,00297385	345,2658644	38,36287383
ENSG00000124383	MPHOSPH10	79,42413232	40,88318899	0,702131134	5,572204969	85,51203018	33,18211534	34,77444104	35,35763685	29,829327	345,2372078	38,35968976
ENSG00000070761	CFAP20	22,64689265	59,17295223	1,207559075	9,181465223	128,5459839	25,08118188	34,22909144	35,31142642	29,79034175	345,1668946	38,35187717
ENSG00000169442	CD52	30,25447186	0,574179872	250,0035151	19,27678078	0	1,421075603	9,806567705	30,42545268	3,38264548	345,1446891	38,3494099
ENSG00000104142	VPS18	80,78733413	27,35327923	26,49400794	55,1143369	0	58,72139051	36,87189702	33,62575644	25,96961828	344,9376205	38,32640227
ENSG00000179593	ALOX15B	23,98899129	17,45461043	0	0,086660569	110,8004639	7,273396255	20,71031385	145,0795411	19,44259532	344,8365727	38,31517475
ENSG00000117154	IGSF21	28,39862357	50,65788528	41,33498999	32,95024572	9,590676205	48,24699263	59,19816851	6,536786697	67,64739881	344,5617674	38,28464082
ENSG00000137714	FDX1	33,98065314	20,04242049	11,405672	50,70969131	51,97152728	63,58850029	38,71063042	38,78191966	35,34867058	344,5396852	38,28218724
ENSG00000112992	NNT	24,07217953	20,06482496	0,13573247	135,6275784	0,613726269	31,71581581	44,99900335	47,43543108	39,8372184	344,5015102	38,27794558
ENSG00000198642	KLHL9	71,18869266	61,94033242	26,92171537	44,46405084	0	23,70981271	44,80455463	39,35122695	31,83150592	344,2118915	38,24576572
ENSG00000078902	TOLLIP	68,5970767	25,54586997	6,365247127	24,55396803	66,62551764	34,10830843	53,30863847	30,59681605	34,5103604	344,2118028	38,24575587
ENSG00000119986	AVPI1	74,51124866	62,40395638	34,57061595	44,97570108	0	38,24992932	28,19660552	42,77625674	18,38191057	344,0662242	38,22958047
ENSG00000148218	ALAD	30,0387646	9,977376747	40,08085148	34,72097336	106,1738821	32,91370591	49,42693766	18,10874981	22,17380144	343,6150431	38,17944923
ENSG00000177426	TGIF1	14,62953226	61,86637973	34,31644374	12,53579603	48,83159965	28,04861367	39,61578471	62,69495565	41,07068	343,6097855	38,17886505

ENSG00000168393	DTYMK	13,16469749	21,90739142	24,79689086	26,10437428	60,32695689	47,30422445	50,05214995	50,2745382	49,26985751	343,2010811	38,13345345
ENSG00000254858	MPV17L2	27,74489166	33,65904263	0	16,13274784	80,90657512	62,24560747	39,19845969	36,68055855	46,48339246	343,0512754	38,11680838
ENSG00000130193	THEM6	38,1386152	25,70982604	78,76866067	26,91026262	53,60139924	48,76185648	34,39582083	19,16193868	17,57428398	343,0226637	38,1136293
ENSG00000205138	SDHAF1	0	29,52682723	52,01239095	57,70003467	0	45,00528625	51,333878	46,74007311	60,22958018	342,5480704	38,06089671
ENSG00000139132	FGD4	12,79513723	19,65214207	4,655231689	49,24598354	70,68152212	29,64194046	27,11500166	84,96355942	43,729622	342,4801402	38,05334891
ENSG00000143033	MTF2	30,64676886	29,91460607	51,29478718	59,14879482	31,7959158	31,59800217	32,47742299	39,26952199	36,30724339	342,4530633	38,05034036
ENSG00000172292	CERS6	33,11869571	7,554338906	85,45555867	20,93677948	67,30044261	27,49519294	31,91773948	34,2710068	34,05860172	342,1083563	38,01203959
ENSG00000169282	KCNAB1	60,0296225	38,50725746	0,040569191	0,076163157	136,1103703	23,58390276	21,71742758	42,7751695	19,23017868	342,0706611	38,00785124
ENSG00000144647	POMGNT2	55,16016713	24,19401798	9,99104811	59,00588718	109,1738841	32,98334565	27,71527364	5,195901099	18,57769429	341,9972192	37,99969102
ENSG00000214046	SMIM7	29,24315861	25,78973722	94,98422983	30,25458676	2,744087533	37,15074043	23,48921332	51,43142687	46,80121724	341,8883978	37,98759976
ENSG00000166170	BAG5	60,06007314	44,15618594	0,527376397	58,16706415	5,927381177	40,855991	32,56372874	36,86122666	62,75978176	341,878809	37,98653433
ENSG00000185088	RPS27L	24,74275066	56,45414574	19,18298927	51,86175019	58,92376219	37,28052959	24,0703991	36,69415027	32,47312604	341,6836031	37,96484478
ENSG00000213619	NDUFS3	17,5902472	58,75475214	17,81630873	34,51974602	0,196722839	59,75689081	41,72034595	49,50635673	61,74522912	341,6065995	37,95628884
ENSG00000172594	SMPDL3A	28,94838518	0,114151554	0,236679522	1,999501721	188,9609546	5,226642263	15,0094352	92,42039628	8,623784984	341,5399313	37,94888126
ENSG00000181610	MRPS23	20,84610475	37,74753348	37,17150407	72,27968335	25,0520938	34,01960093	37,11592985	39,73088467	37,52474066	341,4880756	37,94311951
ENSG00000171314	PGAM1	68,53518795	34,94291418	34,25502795	35,2364946	26,86312325	32,98615797	35,96157547	36,68604254	35,9986177	341,4651416	37,94057129
ENSG00000108312	UBTF	42,3533131	22,31640842	29,9724002	28,24650018	88,73946993	31,55456414	37,12256784	21,83051197	39,25286907	341,3886048	37,9320672
ENSG00000254986	DPP3	86,06121261	22,51934323	40,0509421	3,018106319	0,451493013	27,36669306	70,9852268	34,14850572	56,63715992	341,2386828	37,9154092
ENSG00000031003	FAM13B	21,81869726	12,72177821	83,78601103	20,96525134	78,70202309	33,43556276	34,1873481	26,0639487	29,31832622	340,9989467	37,88877186
ENSG00000241685	ARPC1A	37,46664028	43,75973561	32,71145959	42,96265926	17,21677322	44,51063414	26,12699407	40,32421841	55,88895416	340,9680687	37,88534097
ENSG00000139350	NEDD1	32,97819759	57,07705581	53,12951477	49,69633171	0	49,18209567	30,69988854	22,28563854	45,68085031	340,7295729	37,85884144
ENSG00000164985	PSIP1	23,1460883	12,2063455	52,76016114	67,82606917	88,12863769	18,67232024	30,45595099	23,46947773	24,05753469	340,7225855	37,85806505
ENSG00000066136	NFYC	21,29170645	41,98951521	0,092814724	77,36569531	23,68137383	43,37495211	42,20746561	44,40731667	46,29098685	340,7018267	37,85575853
ENSG00000152422	XRCC4	48,28084377	81,76527357	54,22481751	32,47060783	28,04767054	28,76775321	20,86483473	13,21244347	32,9884522	340,6226968	37,84696631
ENSG00000100347	SAMM50	37,74228138	40,28404775	15,61198684	35,94470867	82,91637483	36,88339654	32,04289134	5,773583707	53,34751965	340,5467907	37,8385323
ENSG00000187051	RPS19BP1	46,33398528	28,72166967	18,48654787	45,87164312	1,174546336	56,75055158	42,74677433	46,11133785	54,30021038	340,4972664	37,8330296
ENSG00000121797	CCRL2	44,35810132	28,59822736	46,19060822	0,087240045	91,25019465	16,308161	57,51247916	35,24193482	20,87744282	340,4243894	37,82493215
ENSG00000137628	DDX60	0,089600101	13,47467211	69,43321476	79,52598692	61,0063302	17,26493105	41,86684218	29,1934483	28,21032298	340,0653486	37,78503873
ENSG00000138448	ITGAV	23,95341528	20,51840852	0,541080485	78,0900289	0,043688265	73,04505804	56,91957032	34,73180856	52,00085173	339,8439101	37,76043445
ENSG00000145996	CDKAL1	8,668663767	57,85854109	0,120808387	65,82909724	21,06947771	30,60801382	30,31790725	56,14946367	69,13716778	339,7591407	37,75101564
ENSG00000136908	DPM2	32,36361089	45,01841431	0	38,06169275	65,56502848	37,01611654	48,25782	45,56263427	27,87994429	339,7252615	37,74725128
ENSG00000108953	YWHAE	30,8531073	35,91706834	34,82288659	49,03143639	23,36990514	47,23592883	33,06864498	43,18586267	42,22868375	339,713524	37,74594711
ENSG00000214274	ANG	26,94629057	51,00323358	19,05038441	46,89530662	0	55,92230627	37,72884109	21,25872742	80,70677646	339,5118664	37,72354071
ENSG00000204228	HSD17B8	81,94942865	40,05921072	78,49697096	15,9986038	0	19,48517999	29,90484597	47,93869373	25,67827596	339,5112098	37,72346775
ENSG00000130816	DNMT1	17,15342533	41,41474542	25,76622396	34,20176678	58,17880152	46,4400573	42,6773322	12,21083232	61,39233831	339,4355231	37,71505813
ENSG00000157450	RNF111	16,98178952	21,5827985	112,8023964	15,77019621	49,65933986	29,13427618	36,09827404	17,85386606	39,49335649	339,3762933	37,70847703
ENSG00000115211	EIF2B4	32,81937127	40,46479163	24,25364653	40,50086013	96,55360248	21,62025115	33,40550685	23,01472741	26,54950318	339,1822606	37,68691785
ENSG00000125458	NT5C	20,40436277	24,66276713	52,5859287	40,68066466	37,949799	34,74057086	42,31452162	44,61836626	41,09717616	339,0541572	37,67268413
ENSG00000128585	MKLN1	42,43162288	17,66229735	79,71900928	40,09083267	84,35384869	23,53213555	20,57385978	6,673371722	24,00673967	339,0437176	37,67152418
ENSG00000005059	MCUB	17,2151283	38,92018904	25,10031274	46,02144278	67,47975198	34,39552603	20,11760597	68,03884766	21,64288614	338,9316906	37,65907674
ENSG00000138496	PARP9	40,30516899	26,67019893	1,959358496	61,21958399	70,47350294	43,603037	46,88642973	35,82357305	11,8727192	338,8135723	37,64595248
ENSG00000139436	GIT2	18,93907241	28,95030019	21,42574957	37,10012497	47,02603348	48,68688295	52,91599059	21,7815278	61,95341871	338,7791007	37,6421223
ENSG00000163798	SLC4A1AP	60,04374317	20,90755753	56,05513807	64,09368783	0	33,90221886	26,02419024	29,82605084	47,82683692	338,6794235	37,63104705
ENSG00000137100	DCTN3	72,64620483	33,62529359	37,75772467	29,37262682	1,344290265	40,76786631	35,42691727	65,99856201	21,71522199	338,6547078	37,62830086
ENSG00000130348	QRSL1	41,62260721	10,17887292	46,08362543	0,382813093	154,4343732	18,71171918	17,64822018	24,72351064	24,74318268	338,5289245	37,61432494
ENSG00000155252	PI4K2A	79,46922587	56,90576225	4,180650808	22,30000499	3,429148172	45,38876126	46,27635783	23,97997339	56,4327468	338,3626314	37,59584793
ENSG00000145819	ARHGAP26	17,56116551	3,234957106	67,10534852	55,00651609	54,14600082	31,40678128	34,06229202	43,54310276	32,10450472	338,1706688	37,57451876
ENSG00000136448	NMT1	59,75087342	22,75729076	13,50510602	24,02370286	40,17119307	39,25729345	39,18948891	39,58872686	59,88666612	338,1303415	37,57003794
ENSG00000198000	NOL8	27,62982505	23,19947543	8,212420998	47,51633661	104,6683204	23,62207441	27,13124607	32,81376043	43,23192437	338,0253838	37,55837598
ENSG00000109079	TNFAIP1	55,09765729	26,06265776	58,25502203	59,44674402	0,184683745	41,5949633	43,87751389	20,05208484	33,35584277	337,9271697	37,54746329
ENSG00000065559	MAP2K4	22,44557084	19,96152567	89,73853957	49,2252527	0,440326291	25,98074191	23,18640747	65,17241601	41,70071729	337,8514978	37,53905531
ENSG00000104331	IMPAD1	20,66638859	9,502341518	97,81659606	84,42114613	0	39,76279211	20,55216883	28,5807035	36,4424077	337,7445444	37,5271716
ENSG00000165506	DNAAF2	73,13282428	16,28942289	63,32654972	17,61289062	0	28,16504221	30,02310678	61,33614989	47,85720435	337,7431907	37,52702119
ENSG00000105708	ZNF14	0	79,90866894	171,8729468	0,088064453	0	10,86285922	25,83088171	28,99527086	20,0084511	337,5671431	37,50746034
ENSG00000112851	ERBIN	0,133124034	1,33861022	51,50903753	67,73683953	73,74986334	35,89099202	18,80335535	49,74631606	38,53004446	337,4381825	37,49313139
ENSG00000196083	IL1RAP	19,6765365	38,4418839	39,97256658	51,74450266	57,26368148	38,15455207	35,00967474	6,918598835	50,15186909	337,3338659	37,48154065
ENSG00000100422	CERK	39,87725201	10,11651359	45,22110332	19,47749912	0,072843315	54,35001651	84,16437889	26,40795529	57,52571866	337,2132807	37,4681423
ENSG00000262919	CCNQ	29,64542501	28,94078832	39,28520182	46,99007891	59,95999515	33,93400585	34,41642977	42,56044519	21,38841908	337,1207891	37,45786546
ENSG00000167447	SMG8	37,5762693	15,57967911	66,67966127	0,069545569	134,9562193	15,34407519	20,73337043	13,03419936	33,08781367	337,0608332	37,45120369
ENSG00000205643	CDPF1	58,70881059	6,224432501	27,67914242	29,32928242	121,095225	28,17540643	23,30053814	15,54273775	26,79277902	336,8483543	37,42759492
ENSG00000132670	PTPRA	39,83178614	34,86026002	33,65120464	9,809146505	61,71453849	32,78116787	38,66024774	17,44966613	68,03421581	336,7922334	37,42135926
ENSG00000169188	APEX2	43,80760292	36,01166102	50,20634699	33,91590747	0	37,90485544	44,19933585	56,51767533	34,19713716	336,7605222	37,4178358
ENSG00000171861	MRM3	43,92483591	33,63594863	0	31,54881305	88,46774721	26,32837867	44,8153478	15,9807146	51,86850042	336,5702863	37,39669848
ENSG00000158201	ABHD3	13,51876906	36,84865853	80,88791444	43,55935476	1,821670724	37,91039463	33,26974448	64,38026506	24,33959526	336,5363669	37,39292966
ENSG00000048405	ZNF800	21,12887316	28,69809081	47,12177752	24,86381943	60,44184426	36,22939402	31,18889661	55,55727063	31,21502955	336,444996	37,38277733
ENSG00000094914	AAAS	63,56761997	7,676068901	30,21229606	30,57535001	65,16737547	45,16052088	54,18175424	10,34111092	29,35130665	336,2334031	37,35926701
ENSG00000197894	ADH5	28,97372841	20,38893137	31,1342281	60,32713654	40,000351	47,15460904	35,73815868	22,74878342	49,76531412	336,2312407	37,35902674
ENSG00000136108	CKAP2	0,531728421	43,57970927	0,427943852	113,1655531	0	18,82776723	39,32157975	41,78576563	78,55663422	336,1966815	37,35518683
ENSG00000170638	TRABD	65,51479546	42,0965081	38,61569405	7,448191473	0	53,25134907	48,74759172	9,432004782	71,0260258	336,1321605	37,34801783
ENSG00000011201	ANOS1	18,35005518	45,96503645	77,87524942	21,22261301	87,67498316	31,64998434	28,09883459	0,093316738	25,08744356	336,0175164	37,33527961
ENSG00000143493	INTS7	28,45736057	32,67445701	46,76495845	7,54487159	93,79130706	22,05495462	17,33874659	60,24257699	26,69406488	335,5632978	37,28481086
ENSG00000106636	YKT6	56,13144208	16,70914541	14,84760173	55,32637983	3,003141034	66,46269393	42,37627614	41,62744619	39,05434067	335,538467	37,28205189
ENSG00000126858	RHOT1	23,97917895	20,28760068	60,02424789	30,71472153	0,275565947	53,21563924	61,98419256	17,74894086	67,28237355	335,5124612	37,27916236
ENSG00000153814	JAZF1	1,774431314	23,36324144	45,63365176	13,66568796	109,8943536	31,36637253	28,14095046	14,2013917	67,30280351	335,3428843	37,26032048
ENSG00000124688	MAD2L1BP	79,41342756	95,70387344	0	44,96964456	0,382039939	30,97585767	35,83913207	3,866787492	44,18797631	335,338739	37,25985989
ENSG00000148688	RPP30	28,15664405	47,88867442	58,6070228	92,75444831	0	31,48485692	20,00837875	27,41193704	28,99732972	335,309292	37,256588
ENSG00000078124	ACER3	16,84376297	33,92272638	43,56404536	24,41822198	22,46079867	59,45030734	41,88289452	60,27827931	32,18521862	335,0062552	37,22291724
ENSG00000076248	UNG	32,90643124	56,51142269	11,80359594	46,25064801	0	51,89956879	33,38380863	21,36818785	80,8689386	334,9926018	37,2214002
ENSG00000146282	RARS2	5,802157311	12,55363355	0,093627517	63,0146099	59,99403874	52,91927118	36,04789262	45,5196154	58,83992285	334,7847691	37,19830767
ENSG00000162076	FLYWCH2	15,92420812	24,44942004	0,623273765	54,02017038	71,66246921	35,95969151	53,26393765	36,30334935	42,5030163	334,7095363	37,18994848
ENSG00000162909	CAPN2	24,36074896	13,68039558	81,40267546	21,33679117	0,083661629	26,90128396	30,18010898	111,5818068	25,00323043	334,5307029	37,1700781
ENSG00000086102	NFX1	60,30889274	35,45958439	33,02618304	25,9985589	5,326960466	30,22887921	44,15867408	40,87659637	59,1400772	334,5244064	37,16937849
ENSG00000180611	MB21D2	28,36714924	13,48151094	0,272262585	0,085189349	160,6239089	20,20393246	38,38253382	29,23531433	43,86778831	334,51959	37,16884333
ENSG00000125772	GPCPD1	15,6992781	32,77409859	101,0064529	52,20779484	3,005766596	32,29161616	28,67131275	39,1587132	29,60408931	334,4191224	37,15768027
ENSG00000135341	MAP3K7	35,32995973	36,4102385	44,87382078	34,31651031	45,79317986	29,25277469	26,8099	34,66510972	46,93559813	334,3870917	37,1541213
ENSG00000159176	CSRP1	41,79778723	42,64596067	38,36437885	32,77492994	21,23102354	63,88473747	40,94384647	22,19336648	30,42205622	334,2580869	37,13978743
ENSG00000239672	NME1	41,57958111	25,26992671	10,67288338	117,5649435	0,208910729	25,57667413	33,83100575	42,57781077	36,75847445	334,0402105	37,11557894
ENSG00000147202	DIAPH2	0,696216242	10,72568	86,56678685	40,13753693	10,60430858	38,72404979	34,18573862	53,25013606	59,00383828	333,8942914	37,09936571
ENSG00000185215	TNFAIP2	36,80937008	22,21641447	54,1820936	44,43100998	0	49,84239856	64,44283891	22,58483831	39,36268483	333,8716488	37,09684986
ENSG00000133805	AMPD3	39,70740006	27,94708318	113,8437561	9,25737213	0,158597472	35,24348037	51,42196442	26,89979624	29,1094803	333,5889303	37,0654367
ENSG00000239713	APOBEC3G	47,68065634	53,87783444	54,82026138	31,36074299	36,14559822	27,16303395	33,00745106	14,26247808	35,26761911	333,5856756	37,06507506
ENSG00000168386	FILIP1L	47,76763084	22,57607556	29,24371361	21,35043075	22,99617669	35,13268184	39,98131365	64,87780175	49,61784162	333,5436663	37,06040737
ENSG00000072310	SREBF1	22,75126774	15,59431059	30,94822024	35,61453701	97,49392806	42,2524445	36,70358778	13,62065278	38,53480194	333,5137506	37,0570834
ENSG00000136875	PRPF4	27,18568252	52,9159275	1,615716047	24,26630265	0,099395943	57,12503665	71,26075872	20,85222292	78,08331603	333,404359	37,04492877
ENSG00000138303	ASCC1	9,468080869	44,83434087	110,5252058	32,74533068	1,09291053	34,86914018	24,52803483	43,89231097	31,44020114	333,3955559	37,04395066
ENSG00000198964	SGMS1	20,75035394	46,13061032	61,45597704	9,587522031	14,03370748	14,31020699	14,78764461	136,5878525	15,71145284	333,3553278	37,03948086
ENSG00000126804	ZBTB1	35,15330141	35,32591938	5,787963447	25,23902556	3,534755291	48,23748738	56,19208887	75,18121336	48,47558351	333,1273382	37,01414869
ENSG00000136261	BZW2	38,29344882	14,64456582	65,05432682	89,85226733	0,392263837	28,95053482	26,99907745	20,57320628	48,31400579	333,073697	37,00818855
ENSG00000198863	RUNDC1	31,92476845	11,7808867	34,53568184	26,43907037	114,7485105	20,68214673	33,15455273	15,69859413	43,99057895	332,9547904	36,99497671
ENSG00000170035	UBE2E3	53,40188627	52,38416343	12,33805416	41,23720177	33,44840593	39,10669612	38,13893078	27,66583971	35,18210896	332,9032871	36,98925413
ENSG00000105397	TYK2	51,69396017	10,88720656	14,89838712	32,78542203	70,57219089	47,76696066	39,2731587	14,29460037	50,6804262	332,8523127	36,9835903
ENSG00000107731	UNC5B	15,99240654	6,898551676	74,07655612	25,09797008	0,05249489	70,69570693	76,97690154	9,467208776	53,51814777	332,7759443	36,97510492
ENSG00000187676	B3GLCT	68,92938556	14,03791783	61,77335533	33,89215045	0	19,60728044	31,707104	66,05771672	36,57814255	332,5830529	36,95367254
ENSG00000120159	CAAP1	47,19814343	64,40499306	0,087968386	85,2993129	12,84186148	21,47386895	27,63523768	23,21407405	50,40204734	332,5575073	36,95083414
ENSG00000008083	JARID2	33,13106522	18,85239238	52,93375043	36,18989238	102,43247	20,96467729	23,77184541	24,92547747	19,22755499	332,4291256	36,93656951
ENSG00000175324	LSM1	20,73713082	25,27830311	51,41636676	77,0661172	0	42,56655608	46,63962818	38,83986265	29,8285886	332,3725534	36,93028371
ENSG00000013288	MAN2B2	24,21709078	21,12469567	44,45557213	23,02022421	57,83495597	35,85592706	50,53783658	45,56565682	29,63769556	332,2496548	36,91662831
ENSG00000143862	ARL8A	77,96540329	25,66017115	23,78665151	25,23472413	3,272970006	37,46638846	51,26813059	55,88108635	31,71153317	332,2470587	36,91633985
ENSG00000133027	PEMT	70,37184134	20,11065001	17,77779703	0,151706381	53,86763135	28,64851237	49,02096692	65,70268726	26,5803747	332,2321674	36,91468526
ENSG00000105993	DNAJB6	46,67169151	29,8194094	40,91490009	26,85813569	28,40626712	47,02331678	35,29350371	32,53394213	44,68454531	332,2057117	36,91174575
ENSG00000166171	DPCD	0,089361662	55,02381669	11,17838364	28,85561221	141,6507397	37,77384508	29,38276255	0	28,13525115	332,0897726	36,89886363
ENSG00000171960	PPIH	27,90215931	54,88937208	7,305147829	80,48201845	0	56,22142641	31,9315921	29,70711879	43,56982784	332,0086628	36,88985142
ENSG00000164414	SLC35A1	49,18292897	94,40104517	63,15194826	11,77740608	0	19,30353751	17,55577865	48,05718384	28,42687527	331,8567037	36,87296708
ENSG00000164548	TRA2A	15,60100602	24,25175086	30,91303149	46,90298747	50,25194279	37,98578335	40,28896611	33,83289087	51,74736783	331,7757268	36,86396964
ENSG00000173409	ARV1	57,59979399	20,56806871	44,34600167	32,04895453	0,337992981	49,89028636	27,86917492	43,22861636	55,79768528	331,6865748	36,85406387
ENSG00000128039	SRD5A3	30,8489536	35,86891355	0,323910626	43,17497832	0,083690874	62,67561054	68,24697154	53,4424586	36,81415612	331,4796438	36,83107153
ENSG00000196655	TRAPPC4	69,58729976	52,79371675	2,595923103	14,06205992	26,82901484	45,28648621	24,70349727	44,93652971	50,61967533	331,4142029	36,82380032

ENSG00000146463	ZMYM4	16,04574126	26,8597845	83,20863693	18,93800326	51,61111273	32,11032477	22,41303433	34,6552273	45,5684882	331,4103533	36,82337258
ENSG00000126391	FRMD8	17,26871845	18,18805407	81,02344492	50,21186658	0,264770859	42,20138778	54,05492553	24,52475774	43,5270916	331,2650176	36,80722418
ENSG00000101138	CSTF1	69,44789977	26,03211918	0	24,40829242	102,6477642	34,56470348	9,959207603	31,11592177	32,79416758	330,970076	36,77445289
ENSG00000125826	RBCK1	53,28675442	36,51635588	4,14483249	18,74011125	21,32929737	50,13599932	52,7262255	38,43026282	55,65235073	330,9621898	36,77357664
ENSG00000197903	HIST1H2BK	57,90588797	26,04003675	27,72504924	48,62567768	35,81749766	25,25672821	34,57843795	40,76226948	34,14503034	330,8566153	36,76184614
ENSG00000030110	BAK1	7,104656125	21,81987342	19,98139048	42,82070334	86,04534243	31,72702258	53,65955428	41,3790123	26,29745094	330,8350059	36,7594451
ENSG00000134748	PRPF38A	20,66101447	39,09707995	49,93447034	42,69169337	38,48829633	40,67275487	26,82746461	17,76593188	54,69188202	330,8305878	36,75895421
ENSG00000274523	RCC1L	39,21816288	29,39592947	60,68393009	5,783353822	37,57211656	36,45604188	37,76668954	30,39746021	53,45966453	330,733349	36,74814989
ENSG00000175197	DDIT3	29,59778584	16,20751186	0,212685615	16,17118052	0	33,51215933	43,10342698	106,6896614	84,88934491	330,3837565	36,70930628
ENSG00000168936	TMEM129	97,0672492	30,07054388	29,55860437	22,28787988	0,521669876	37,69380331	46,55014932	42,4323297	24,08121701	330,2634465	36,6959385
ENSG00000154582	ELOC	29,0338105	71,95379167	19,15549932	40,13977416	0,145568521	37,79559956	27,08267199	70,45341812	34,42718309	330,1873169	36,68747966
ENSG00000156973	PDE6D	14,98263449	33,51775001	41,53951904	44,25915469	0,254332537	70,44149485	64,37907616	27,02917319	33,69455361	330,0976886	36,67752095
ENSG00000085491	SLC25A24	54,79611684	11,72074755	46,49533324	56,95101735	18,52717621	26,73908194	54,56448426	40,82532346	19,34174332	329,9610242	36,66233602
ENSG00000116406	EDEM3	15,88290007	0,653837013	53,01314516	11,75412937	88,53501012	39,56132591	53,34743454	29,51520264	37,63678287	329,8997677	36,65552975
ENSG00000175938	ORAI3	32,61379223	35,6371988	19,15652329	49,89479825	15,50147665	41,1478333	56,39276186	29,90301463	49,54030734	329,7877063	36,64307848
ENSG00000203760	CENPW	19,00761895	35,75226668	0	144,7450614	0	23,58225807	50,34595712	17,04127958	39,03945814	329,5138999	36,61265555
ENSG00000100335	MIEF1	48,98342655	30,59409716	87,45978759	45,5902272	7,843070084	30,08570352	25,94105342	17,71356715	35,22597219	329,4369049	36,60410054
ENSG00000152219	ARL14EP	56,30321743	37,53393012	4,985959616	48,49105944	49,73724838	26,07692126	32,69003288	34,0943951	39,45114785	329,3639121	36,59599023
ENSG00000134824	FADS2	43,04353032	10,82426723	35,16568105	20,25071015	43,96663174	41,23788901	83,85693657	14,74790877	36,2605884	329,3541432	36,5949048
ENSG00000102893	PHKB	59,2580448	37,12979649	17,25640874	43,45973312	42,40636856	25,23322762	43,01567041	32,31114533	28,90980836	329,2886005	36,58762228
ENSG00000067248	DHX29	11,41971224	24,75493955	43,94538329	49,35167208	59,19278118	40,63047007	23,26941615	25,57898307	51,13056893	329,2739266	36,58599184
ENSG00000147883	CDKN2B	20,51325997	10,6586445	83,67200979	0,130467487	59,43395326	3,857420908	2,979920385	133,9086085	14,03141243	329,1856972	36,57618858
ENSG00000151490	PTPRO	96,536825	67,45346717	0,299978524	13,23447173	3,358653274	47,60479737	44,6066588	15,37273528	40,69013785	329,157725	36,57308055
ENSG00000171631	P2RY6	41,69222737	15,71355282	69,2153667	23,62590313	0,090321313	50,44916681	56,42378109	4,986437136	66,84117879	329,0379352	36,55977057
ENSG00000136026	CKAP4	30,95582067	23,84459684	37,00335196	17,72307929	100,7997684	6,969511405	35,52596962	40,56070796	35,63832656	329,0211327	36,55790364
ENSG00000178234	GALNT11	32,94604261	27,33831927	27,50285728	16,84365485	91,20926963	19,19742315	31,74605287	27,31028942	54,84804458	328,9419537	36,54910596
ENSG00000168283	BMI1	57,17790194	50,39483324	6,577913605	43,04388091	46,65668184	37,2590988	20,86889936	18,87123469	47,99449438	328,8449387	36,53832653
ENSG00000168264	IRF2BP2	31,15615915	41,15219095	3,177587922	23,25436586	65,0729501	29,50145733	41,48701494	49,87295653	43,98315239	328,6578352	36,51753724
ENSG00000168256	NKIRAS2	46,19159915	44,5855589	12,683312	41,41078046	55,1791066	34,31910396	22,69553526	32,59652091	38,91213929	328,5736565	36,50818406
ENSG00000163803	PLB1	22,62355167	38,68002117	63,91312749	5,125887563	77,6425234	41,32818387	35,99451114	12,79027211	30,4595602	328,5576386	36,50640429
ENSG00000123124	WWP1	21,94156013	10,83402021	61,59786284	33,68762772	4,080881163	25,53074689	28,67440668	119,3235938	22,79083772	328,4615372	36,49572635
ENSG00000198055	GRK6	80,63623521	16,49347166	3,062438547	19,58359805	28,84809268	53,61535277	47,42934056	30,86685081	47,85857195	328,3939522	36,48821691
ENSG00000102100	SLC35A2	34,94305853	26,42040001	115,1260704	25,50125519	0,863032812	30,56830385	40,53914599	7,764550585	46,39959902	328,1254164	36,45837959
ENSG00000145901	TNIP1	20,65934265	32,26220854	4,654595132	30,40412702	65,83511652	27,6660827	56,59485198	31,60033668	58,41990628	328,0965675	36,45517417
ENSG00000145703	IQGAP2	41,73402009	37,65847866	16,0753616	67,48890103	25,60561023	24,5449811	23,808745	61,27402567	29,88000136	328,0701247	36,45223608
ENSG00000105323	HNRNPUL1	38,54555134	23,44142199	30,03697539	68,19483518	13,88609785	40,31756334	30,89297033	27,11957248	55,21712354	327,6521114	36,40579016
ENSG00000009954	BAZ1B	13,29173716	22,40846743	67,80194482	34,22461565	69,58538214	26,08509067	30,74556294	15,07081172	48,40959695	327,6232095	36,40257883
ENSG00000152229	PSTPIP2	55,27272573	58,37945973	0,134941796	13,49008485	1,30746678	54,96794592	56,06574787	58,94888522	28,86936268	327,4366206	36,38184673
ENSG00000018510	AGPS	26,60120466	18,59611337	74,91216153	22,1406529	0	37,09008933	37,90086961	67,58973844	42,30379034	327,1346202	36,34829113
ENSG00000107669	ATE1	0,528358639	32,8614584	98,89676598	4,333710242	95,11592249	26,9169607	29,85404228	19,35237415	19,20197459	327,0615675	36,34017416
ENSG00000070501	POLB	26,79213235	55,90101122	66,21152869	29,29640119	0	35,71966629	39,51123704	31,54660265	42,04856775	327,0271472	36,33634969
ENSG00000141506	PIK3R5	50,14873855	49,33441275	27,22027348	32,43436335	48,65741281	34,65864432	41,77329599	2,817454346	39,68984482	326,7344404	36,30382671
ENSG00000084676	NCOA1	4,656018973	42,55236188	87,05381693	70,63651519	2,828679819	26,46343845	39,59008275	8,458941205	44,47572281	326,697578	36,29973089
ENSG00000163939	PBRM1	38,67766937	27,10861737	50,18845168	32,32631341	0,446386007	50,62901999	32,97267788	34,22773643	60,06226947	326,6391416	36,29323796
ENSG00000226479	TMEM185B	18,24452179	50,12380853	168,0929981	11,63235956	1,394748982	33,25495686	16,73785246	16,68354199	10,32480679	326,489595	36,27662167
ENSG00000106400	ZNHIT1	28,09396606	29,25866229	23,17887121	25,34915871	80,59190807	41,27079377	36,84267479	31,57354036	30,28857145	326,4481467	36,2720163
ENSG00000113851	CRBN	45,15711278	57,86257092	25,94434984	32,43899102	29,64455667	34,23910637	35,62872219	34,48511476	31,02816875	326,4286933	36,26985481
ENSG00000165322	ARHGAP12	26,89859771	38,65435367	0	18,76376459	58,96591509	75,99810856	58,19220181	28,10196305	20,62527083	326,2001753	36,24446392
ENSG00000121749	TBC1D15	40,95983622	17,04610016	66,05701878	6,660549978	48,93287529	27,2047366	15,23579211	78,68254744	25,47471007	325,8541666	36,20601852
ENSG00000117868	ESYT2	0,489651046	31,50611835	44,84181647	77,49379071	1,214260498	32,46686264	31,00520743	54,56034279	52,10405457	325,6821045	36,1869005
ENSG00000135845	PIGC	11,8285595	36,02596824	32,24037129	32,71050878	53,26623834	44,0099635	22,86081456	58,11853924	34,60835716	325,6693206	36,18548007
ENSG00000166454	ATMIN	15,98261668	46,01126507	33,13199454	52,32040152	0,172128555	37,16354506	42,87922035	46,88063524	51,07142008	325,6132271	36,17924746
ENSG00000066027	PPP2R5A	51,25186022	39,38463167	64,17095131	31,30954921	35,0736311	19,02373939	25,20865118	33,78110875	26,16560626	325,3697291	36,15219212
ENSG00000121073	SLC35B1	63,09019271	34,06054758	4,9706293	44,67763145	0,060579882	44,8921331	45,65443761	52,00665511	35,92781959	325,3406263	36,14895848
ENSG00000085063	CD59	33,71357041	47,16396804	15,46525671	38,88471804	46,34651602	42,10163371	33,72705206	44,92709561	22,96485553	325,2946661	36,14385179
ENSG00000163714	U2SURP	35,75930126	34,87437778	53,08074745	27,41248879	52,19769279	29,99462666	26,39639383	22,40959593	43,11010802	325,2351325	36,13723694
ENSG00000165632	TAF3	33,75577671	22,22140883	119,0039931	52,88282152	17,31130717	16,84630471	21,00180326	7,93042962	34,15763773	325,1114827	36,12349808
ENSG00000197170	PSMD12	19,43335119	55,26475891	0,312220253	82,45193507	0,134450596	40,80990224	42,65333637	34,63934066	49,33177997	325,0310753	36,11456392
ENSG00000110218	PANX1	82,92007121	14,10284012	52,24310416	28,63697302	0	39,26705444	47,99724131	40,89880867	18,8974374	324,9635303	36,10705892
ENSG00000275379	HIST1H3I	0	4,566895379	4,734454241	199,3517811	106,5993678	1,211026107	3,663367358	4,8238623	0	324,9507543	36,10563936
ENSG00000155313	USP25	34,92367529	5,244244665	0,248735868	63,34085604	91,85660261	36,30818194	25,1485978	20,52808383	47,29904459	324,8980226	36,09978029
ENSG00000138641	HERC3	8,998548834	40,66955002	32,9874504	22,91465123	19,94238612	33,5677878	37,43045203	80,27345714	48,05501982	324,8393034	36,09325593
ENSG00000169895	SYAP1	34,62266413	43,49711116	5,868564648	55,81626746	71,91270252	34,15048505	21,67781427	30,46850871	26,68550654	324,6996245	36,07773605
ENSG00000139826	ABHD13	55,60345674	30,78611332	0	11,78701184	61,77930241	24,91937516	29,00010685	65,17888702	45,54341653	324,5976699	36,06640776
ENSG00000130640	TUBGCP2	49,57180602	23,34093024	77,86476464	11,83140264	4,852309264	35,88945176	51,65850099	31,29619329	38,27033101	324,5756898	36,06396554
ENSG00000025800	KPNA6	23,87129573	31,46362479	48,64744927	55,74217709	65,49548848	28,50093184	23,85904932	1,329359934	45,65788237	324,5672588	36,06302876
ENSG00000160799	CCDC12	39,73248072	20,98208331	47,12914594	34,03022386	61,64070563	26,03476866	29,63886892	26,20400336	38,85810752	324,2503879	36,02782088
ENSG00000173890	GPR160	16,52179031	43,82325315	57,10616048	0,952969549	44,92043214	45,78541137	29,72796271	56,56834641	28,46450957	323,8708357	35,98564841
ENSG00000176171	BNIP3	60,8131367	50,5063711	6,36874593	30,72417447	78,02798056	12,74317857	24,76419135	24,69726908	35,1799614	323,8250092	35,98055657
ENSG00000100077	GRK3	0,717524077	21,22048117	19,82848443	21,58626359	124,2151845	24,63186466	18,52200005	34,7623999	57,63458151	323,1187839	35,9020871
ENSG00000177565	TBL1XR1	43,28979892	35,91249402	21,51392992	70,86151038	57,44395058	29,28422486	26,90426493	24,34900933	13,51844047	323,0776234	35,89751371
ENSG00000083642	PDS5B	80,0758829	25,5392699	19,16159172	26,85135061	108,930778	9,599588233	9,105566674	26,9369197	16,72858284	322,9295306	35,88105895
ENSG00000164967	RPP25L	70,9887571	19,88965385	14,76451075	107,050246	0	32,36167842	37,45726029	10,28825577	30,12347476	322,9238369	35,88042633
ENSG00000133704	IPO8	35,37527532	15,94911294	0,163301571	89,90357621	110,2826819	21,51549996	14,29945332	15,43225033	19,70644575	322,6275973	35,84751081
ENSG00000148444	COMMD3	32,63738471	62,53259871	20,10879118	54,91948656	0	39,83454081	37,47253801	38,35909378	36,7467805	322,6112143	35,84569047
ENSG00000116670	MAD2L2	28,00364366	38,07551866	50,59505412	32,02811963	1,296453221	44,57224579	42,2386587	47,21913946	38,57824921	322,6070824	35,84523138
ENSG00000072210	ALDH3A2	54,38763609	14,74220381	44,68690456	30,22756094	71,30071075	23,84466706	34,31633983	19,0369613	30,04859366	322,591578	35,84350867
ENSG00000132952	USPL1	15,80395432	35,21162265	10,46832373	51,27054038	88,71079193	46,00174234	14,90954774	30,81906796	29,26402799	322,459619	35,82884656
ENSG00000094880	CDC23	35,6823221	46,29748138	59,49834008	55,73697681	0	35,72684429	68,87821085	0,143169223	20,45278018	322,4163249	35,8240361
ENSG00000109743	BST1	13,08042636	36,03304364	40,55695685	43,53470082	19,05265844	21,71604407	37,83817342	70,93098046	39,24507713	321,9880612	35,77645124
ENSG00000196998	WDR45	43,90749511	33,61618687	32,55346974	13,74602381	56,2274713	29,0830765	26,85359896	50,70545126	35,13618171	321,8289552	35,7587728
ENSG00000134900	TPP2	12,27032269	31,44028228	33,98482957	48,45977636	60,97605945	24,60450267	38,54149514	34,23471978	37,28343743	321,7954254	35,75504726
ENSG00000132128	LRRC41	60,31429354	16,79392727	32,8821451	19,82936014	0,041728741	57,44986272	49,54080736	44,2703993	40,57699458	321,6995188	35,74439097
ENSG00000130764	LRRC47	22,52080361	36,33505479	63,99275732	46,23797842	0,079644609	41,7260133	47,22181269	28,65378485	34,86940737	321,637257	35,737473
ENSG00000215712	TMEM242	27,69058907	26,38692809	73,02664099	52,64773282	23,43079572	20,22079811	30,36792704	49,02442227	18,80875656	321,6045907	35,73384341
ENSG00000107672	NSMCE4A	26,05942949	31,71083772	7,845896104	29,75380461	50,57850794	27,39421038	36,44057907	68,82887689	42,96028838	321,5724306	35,73027006
ENSG00000114779	ABHD14B	36,7139887	66,16601078	38,25414206	10,97891541	0,11360884	44,29880631	38,62152263	45,78625137	40,56757367	321,5008198	35,72231331
ENSG00000143164	DCAF6	67,38390814	21,17581923	11,54944274	14,56535221	34,11218013	35,36057962	27,53741508	61,04603095	48,59830562	321,3290337	35,70322597
ENSG00000169249	ZRSR2	41,10614946	28,40213771	2,944420885	55,78003309	0	32,91052364	30,14429257	81,13703873	48,6762196	321,1008157	35,67786841
ENSG00000077232	DNAJC10	57,56198394	20,27645353	1,19462942	48,45781863	92,9275952	23,69609297	27,68343722	18,56645561	30,63030736	320,9947739	35,66608599
ENSG00000119285	HEATR1	42,19288641	28,05414591	50,95739371	72,05157339	82,83364568	12,04824627	20,20320212	0	12,63452795	320,9756214	35,66395794
ENSG00000179222	MAGED1	16,65330923	42,16461637	74,94742695	60,89639367	0,179081221	32,7905046	37,29071702	0,054925959	55,85281121	320,8297863	35,64775403
ENSG00000125779	PANK2	71,55871439	54,67434399	1,437544838	20,43373767	8,118348697	25,56807499	41,823444	45,45428226	51,62403396	320,6925248	35,63250275
ENSG00000099203	TMED1	18,84871768	27,89587687	39,54281663	28,25410553	57,94677966	35,4013014	41,55705264	47,17988252	23,97065017	320,5971831	35,62190923
ENSG00000180398	MCFD2	80,0427197	27,10945146	29,12437473	12,14562194	15,27777332	40,99325394	38,41142129	47,57508037	29,70319792	320,3828947	35,59809941
ENSG00000167645	YIF1B	32,28256739	24,12880585	16,18558696	33,31437427	39,84281012	41,73232576	51,19232051	41,20812201	40,41168291	320,2985938	35,58873264
ENSG00000137770	CTDSPL2	36,09583104	26,24479208	86,41744157	22,83919364	50,47852487	32,1198012	11,84779679	33,37471055	20,7141645	320,1322562	35,57025069
ENSG00000177697	CD151	57,23885252	15,08040524	17,84669357	17,0204118	28,49700066	71,7923271	63,54070229	10,52231288	38,46746198	320,0061681	35,55624089
ENSG00000198887	SMC5	8,212033737	43,79733849	70,9888627	80,50037515	0	26,49646661	14,18806207	28,21148896	47,34781971	319,7424474	35,5269386
ENSG00000122140	MRPS2	48,38727283	20,45888637	101,5349388	15,0376487	4,518118431	36,69694168	41,39909899	27,62561725	24,06048726	319,7190103	35,52433447
ENSG00000071994	PDCD2	19,11049373	35,11186165	46,87372886	54,23385976	33,7157915	33,35632657	27,22140464	36,63558052	33,34676635	319,6058136	35,51175707
ENSG00000158417	EIF5B	52,49002912	31,12697344	3,0749229	117,0475004	10,63882865	25,6090833	23,43495457	26,25830956	29,67873261	319,3593345	35,4843705
ENSG00000013297	CLDN11	12,83549059	38,72683227	37,83866299	60,82889279	86,02654953	13,20349445	13,31357221	43,20794988	13,34589174	319,3273365	35,48081516
ENSG00000157869	RAB28	9,048944574	49,7408843	78,85072596	36,30297485	12,12688684	69,47178583	21,74969735	34,96268499	7,028864417	319,2834491	35,47593879
ENSG00000186575	NF2	19,76871901	13,72498104	75,13604349	4,112364314	90,15503155	28,11455379	22,80165169	21,98288545	43,44194858	319,2381789	35,47090877
ENSG00000165630	PRPF18	61,70621459	35,48082865	2,110477975	49,80971316	53,94553104	31,22069963	36,47076895	25,62480648	22,77728527	319,1463257	35,46070286
ENSG00000189241	TSPYL1	61,68324439	35,86426642	30,93295299	48,40553465	2,543463934	28,69877859	53,84868103	20,07270732	37,05792817	319,1075575	35,45639528
ENSG00000064545	TMEM161A	19,98841441	31,21921488	72,77808748	28,63047573	57,90099967	20,78306828	35,87051857	20,44444292	30,98386092	318,5990828	35,39989809
ENSG00000011454	RABGAP1	16,13321249	12,98075686	44,62384061	17,27970655	106,5854273	12,20170671	21,5538588	52,24989828	34,80941309	318,4178207	35,37975785
ENSG00000187164	SHTN1	15,96805836	43,99712411	56,02046832	38,35604995	22,76035805	23,40280069	24,60142961	46,61001413	46,57423753	318,2905408	35,36561564
ENSG00000136273	HUS1	67,73671919	31,28859833	40,24537808	29,06934651	0,086222699	35,5323492	35,7582915	49,66435517	28,64669083	318,0279515	35,33643906
ENSG00000135966	TGFBRAP1	14,69792617	22,36866317	0,048717157	63,29022167	59,41236593	42,59708988	44,04435244	40,07373304	31,33269443	317,8657639	35,31841821
ENSG00000114062	UBE3A	13,8456107	50,87436215	19,13513208	52,45988407	6,922815341	28,99194422	32,66752732	66,32933018	46,58854519	317,8151513	35,31279458
ENSG00000165879	FRAT1	29,14967973	10,22360967	139,4622694	37,13570609	0,406701633	30,81464845	41,25506198	6,985404221	22,38015878	317,8132399	35,31258221
ENSG00000105447	GRWD1	26,79767538	23,12451398	89,04238465	39,75375806	0,249257361	39,46961663	31,78668831	10,20603037	57,27284809	317,7027728	35,30030809
ENSG00000151332	MBIP	52,180162	18,8004908	55,70971309	7,165611339	7,029177721	34,94639959	35,84866629	70,27647244	35,72509835	317,6817916	35,29797685
ENSG00000184967	NOC4L	57,97559023	23,23012377	29,31176291	17,69707501	0	41,64191207	52,3621237	28,46317562	66,51833446	317,2000978	35,24445531

ENSG00000253729	PRKDC	18,86799303	15,80989026	21,1681025	54,60052073	98,205848	26,2043364	21,7214968	31,18520437	29,3048515	317,0682436	35,22980484
ENSG00000127995	CASD1	21,80473824	21,35960039	11,15091272	65,53138811	77,14882564	17,31652217	45,47179774	7,16101569	49,92417034	316,815065	35,20167389
ENSG00000165898	ISCA2	20,43769234	33,72579877	21,25035713	30,34722344	0	62,16904001	37,54682264	60,45193003	50,87859708	316,8074614	35,20082905
ENSG00000154813	DPH3	3,34225793	29,23442718	21,61239375	49,96006998	0	42,33017842	61,50176685	67,47479775	41,18850538	316,6443972	35,1827108
ENSG00000186088	GSAP	10,3498078	23,51860001	63,64036009	36,44343221	56,65630948	45,6949242	33,61763756	16,06022758	30,56604255	316,5473415	35,17192683
ENSG00000070785	EIF2B3	22,19478039	30,89410907	0,081495192	92,33319095	0	53,89296951	28,76511276	45,43354212	42,87927676	316,4744767	35,16383075
ENSG00000163512	AZI2	57,10013592	36,63866296	79,289923	29,20541329	4,924124465	26,34676888	22,54545028	29,66113863	30,7274942	316,4391116	35,15990129
ENSG00000170949	ZNF160	0,532642124	14,90202867	12,63991285	129,8389869	63,62933308	19,04531	14,66842678	39,44560223	21,63951399	316,3417566	35,14908407
ENSG00000151502	VPS26B	67,41784177	28,3391979	90,29076204	22,28036123	0,111302508	35,94343503	40,55960213	5,888730089	25,48801677	316,3192495	35,14658327
ENSG00000188986	NELFB	28,97277477	16,99640237	34,36446893	56,09074651	58,67471657	22,60041714	49,20017743	21,98560705	27,36480289	316,2501136	35,13890152
ENSG00000106305	AIMP2	57,03845392	20,53603939	48,96585989	41,96656594	0,196453798	45,74339752	38,64321494	18,07628925	44,83496517	316,0012398	35,11124887
ENSG00000186625	KATNA1	73,84649617	28,06275478	0	54,9641299	0,477761697	35,75722407	23,31355615	62,86316163	36,6748126	315,959897	35,10665522
ENSG00000072501	SMC1A	28,81247909	21,04930046	8,61727701	39,82220139	54,42939477	42,03152344	28,62695862	54,46308019	38,07691052	315,9291255	35,10323616
ENSG00000150347	ARID5B	30,46361504	45,24131006	53,43818341	55,61251114	22,95728581	30,56845557	30,16444283	23,19831724	24,24915239	315,8932735	35,09925261
ENSG00000184162	NR2C2AP	91,39072975	12,85407581	65,85370238	26,32621539	0	32,3682842	21,40129409	33,70653615	31,69957403	315,6004118	35,06671242
ENSG00000130304	SLC27A1	38,55124627	20,36231912	21,24494418	19,81502793	84,92053049	34,60177922	49,44485414	16,63450468	29,9701303	315,5453363	35,06059293
ENSG00000175193	PARL	18,32248983	50,48352471	13,7274123	88,01378112	27,39300477	26,4813178	35,40609016	36,8606462	18,76492663	315,4531935	35,05035483
ENSG00000139624	CERS5	37,79443522	43,06799942	51,36185856	52,04007643	9,79072867	29,72083082	30,71225196	34,27236587	26,54556934	315,3061163	35,03401292
ENSG00000162747	FCGR3B	5,855333168	46,89863635	59,09424074	41,64941472	62,17214473	53,61276723	4,727996869	35,35755593	5,879964622	315,2480543	35,02756159
ENSG00000197062	ZSCAN26	0	26,87819571	0	24,73232968	96,11567363	24,49344688	41,32435756	77,17275102	24,45849219	315,1752467	35,01947185
ENSG00000198060	MARCH5	5,550734013	22,5382969	34,11683151	54,57758328	76,19996253	36,92478263	24,88802178	14,7081938	45,6183339	315,1227403	35,01363781
ENSG00000075239	ACAT1	58,10017548	39,32813361	23,37943202	16,7289973	41,93134628	36,04038853	45,41109258	25,74123153	28,22311083	314,9023382	34,98914869
ENSG00000136824	SMC2	22,21115189	35,97330078	68,74314658	5,404217403	44,12649864	22,92489267	35,54669529	17,00691051	62,96376767	314,9005814	34,98895349
ENSG00000117000	RLF	60,20660253	21,98661137	53,34791373	52,79829958	0,115249532	22,57576525	27,34093563	34,46445265	41,92878856	314,7646188	34,97384654
ENSG00000145332	KLHL8	42,8016479	19,06965815	68,64452811	55,17458329	0	14,30341387	41,71538778	33,74440579	38,99390588	314,4475308	34,93861453
ENSG00000185104	FAF1	36,69170434	23,15917129	18,30124642	27,89436011	53,13740824	31,51619645	33,12523138	52,57013056	37,74045816	314,1359069	34,90398966
ENSG00000198252	STYX	33,14546533	12,80710336	5,188069277	2,094601442	146,659482	31,46407686	20,69769474	40,84842336	21,14689507	314,0518114	34,89464571
ENSG00000131069	ACSS2	116,3850851	8,788190552	31,78126132	20,08721984	0,06842941	40,90859113	30,93723506	11,77425002	53,29630641	314,0265688	34,89184098
ENSG00000108950	FAM20A	30,16832943	29,28261609	25,31997989	15,15542079	109,6932406	30,81464845	17,03116139	43,73005602	12,65837015	313,8538228	34,87264697
ENSG00000163297	ANTXR2	87,11879154	12,26863497	39,60036175	14,36315395	37,31091349	13,16013965	22,96320162	60,83825254	26,08535103	313,7088005	34,85653339
ENSG00000131373	HACL1	78,30050192	49,76645304	9,190176758	25,45290423	0	42,52307089	34,70455992	39,58725097	34,12762651	313,6525442	34,85028269
ENSG00000146842	TMEM209	0,194187925	50,71094155	54,53625416	35,8233709	0	43,42315801	56,43343268	25,83178778	46,6905056	313,6436386	34,84929318
ENSG00000244462	RBM12	67,86672899	31,72733603	41,93343481	14,32767393	0,375085466	30,79569642	32,74360052	56,99527903	36,21539213	312,9802273	34,77558081
ENSG00000176142	TMEM39A	79,63761454	15,40205455	74,48179915	46,07612642	0,183629219	38,91136075	17,79296339	6,533221379	33,95729877	312,9760682	34,77511868
ENSG00000078747	ITCH	0,816323611	1,408308824	97,40149026	90,09774558	1,724453985	14,63916853	29,25883792	25,33794478	52,15080982	312,8350833	34,7594537
ENSG00000185130	HIST1H2BL	0	0,549471312	5,126682153	135,2798127	159,6895629	0,33998069	8,741785426	2,031360046	0,761666075	312,5203212	34,72448014
ENSG00000005812	FBXL3	44,80355812	20,69043698	27,85300879	75,1534028	0,15225447	40,09427447	29,80718317	47,16491142	26,73710566	312,4561359	34,71734843
ENSG00000134152	KATNBL1	32,45022513	29,78133006	42,09054433	39,08135647	81,49035737	18,83542315	19,20411729	19,66065338	29,84322489	312,4372321	34,71524801
ENSG00000101745	ANKRD12	32,83027666	44,13571785	32,84105186	43,60988676	43,01318049	15,52363822	21,59466524	46,56515868	32,24394675	312,3575225	34,70639139
ENSG00000136270	TBRG4	24,65995354	31,74453801	27,8794447	31,02621618	50,55988835	38,25406714	39,35137071	37,98033027	30,74490115	312,2007101	34,68896778
ENSG00000136444	RSAD1	73,0897394	57,4914078	9,608690876	35,00577977	0,086806271	57,46914586	27,05330823	20,7669779	31,62580413	312,1976602	34,68862891
ENSG00000172590	MRPL52	32,54544504	45,06588237	7,527005481	27,61216662	7,041520438	43,68513784	48,93069592	59,85462754	39,91101785	312,1734991	34,68594435
ENSG00000117697	NSL1	40,51554783	38,63472389	17,54038989	44,93493325	29,36751223	35,3907468	28,23014609	46,53773061	30,93198049	312,0837111	34,6759679
ENSG00000075089	ACTR6	40,10592644	53,09394442	0,583226014	0,068432986	84,19907759	41,24920711	31,1619209	33,53741873	27,87941535	311,8785696	34,65317439
ENSG00000089876	DHX32	0	29,74827661	0,070410359	15,79621553	83,04820572	37,06512884	48,75164369	51,30607647	45,9908947	311,7768519	34,64187243
ENSG00000101084	RAB5IF	31,54835052	29,0606024	9,611014061	12,66506193	60,64884026	44,23546626	53,9756016	30,75853687	39,17110875	311,6745827	34,63050919
ENSG00000169689	CENPX	27,93226094	33,97749707	50,65737924	30,84861555	0	51,90801191	59,25215548	27,62614281	29,13335424	311,3354172	34,59282414
ENSG00000113916	BCL6	37,57398961	28,36812265	28,00405984	45,76032826	70,23845463	21,85681789	25,34348519	40,07966452	14,057461	311,2823836	34,58693151
ENSG00000108774	RAB5C	47,1775849	61,86398191	13,08852331	32,79407323	13,91726803	49,57487352	42,44450927	20,70452017	29,68428697	311,2496213	34,58329126
ENSG00000136950	ARPC5L	18,79463884	9,656083106	20,40574134	33,53845657	69,8339376	47,88887006	39,44842848	26,3615389	45,14886428	311,0765592	34,56406213
ENSG00000174132	FAM174A	36,0107596	57,92822719	0	14,72238906	0	52,96328729	39,30844848	82,66996975	27,45616773	311,0592491	34,56213879
ENSG00000109572	CLCN3	30,04352722	7,625360337	58,51852385	7,067055818	95,49352351	30,18784099	32,54536372	6,630657947	42,94397665	311,0558301	34,56175889
ENSG00000013441	CLK1	42,64416298	26,19182012	0,268839554	36,89428751	2,153316018	32,25147574	39,14577283	73,6926608	57,51536473	310,7577003	34,52863336
ENSG00000065613	SLK	13,55951729	62,07145242	7,196000073	30,20155898	78,57232601	22,32101225	27,59254221	24,5512678	44,61704312	310,6827201	34,52030224
ENSG00000147654	EBAG9	27,17609285	54,92503887	31,86840581	66,49198755	0	25,51083575	31,35906152	39,76705064	33,52406704	310,62254	34,51361556
ENSG00000132768	DPH2	11,39775977	11,89708533	45,72914939	36,8693104	91,55658844	39,07109144	28,26294168	13,19482744	32,60242746	310,5811813	34,50902015
ENSG00000071246	VASH1	24,83292917	14,84934238	21,05151795	24,89051606	16,85460984	57,65409768	67,71194159	17,84154525	64,89061736	310,5771173	34,50856859
ENSG00000105341	DMAC2	28,28364938	17,99446428	30,32971635	41,69241776	57,62602882	36,05269779	34,59670419	44,27415913	19,68335684	310,5331946	34,50368828
ENSG00000139697	SBNO1	22,18859386	8,19269231	67,17186252	7,597315939	114,2089308	18,91988873	19,23547685	24,52722437	28,45816624	310,5001517	34,50001685
ENSG00000258289	CHURC1	31,40214116	38,45485753	23,86801738	28,82645815	53,36258078	38,86802186	38,37943353	21,15661739	36,11007557	310,4282033	34,49202259
ENSG00000119950	MXI1	16,50013849	23,46866667	13,88253415	80,66146328	18,96811803	13,93120807	28,03806302	79,76971276	35,2060705	310,425975	34,491775
ENSG00000171806	METTL18	0,095943219	41,71097779	92,48353769	10,93441798	0	14,74760682	32,90110928	49,30293547	68,20272373	310,379252	34,48658355
ENSG00000156256	USP16	20,56738536	38,6154619	17,47749832	49,17162412	15,6412277	22,43609836	30,23275299	74,57120113	41,64737661	310,3606265	34,48451406
ENSG00000129116	PALLD	40,59753779	29,59940325	11,93931112	79,4553181	50,24383467	26,79993123	29,16243696	19,51025586	22,95556866	310,2635976	34,47373307
ENSG00000076513	ANKRD13A	27,46717312	20,21097524	43,79604825	46,86179136	0,04885953	55,98071159	33,9879498	33,7627701	48,1430991	310,2593781	34,47326423
ENSG00000153140	CETN3	47,43490225	102,9057515	0	4,536538515	46,36385527	19,0161511	23,67212317	44,89484465	21,18147743	310,0056439	34,44507154
ENSG00000124641	MED20	77,90630397	17,42350397	40,36562755	100,0595677	0	16,60118034	22,54485221	17,64064958	17,34869915	309,8903845	34,43226495
ENSG00000159593	NAE1	110,7302929	24,4630593	27,91407675	48,42822389	0,224917041	18,35753771	21,74120575	25,80011028	32,12545312	309,7848767	34,42054186
ENSG00000120802	TMPO	13,96273631	27,74097964	62,56412329	55,38757054	0,125375611	21,87555957	34,19661556	43,95283494	49,91664999	309,7224455	34,41360505
ENSG00000114446	IFT57	76,11711202	35,89011623	38,73806943	19,83422005	66,7596643	19,41944233	15,9645534	24,29799767	12,64227238	309,6634478	34,40704976
ENSG00000148153	INIP	21,4677379	47,61746773	25,45159117	75,16323415	0,165644623	39,18183361	37,73369763	19,58525919	43,16131179	309,5277778	34,39197531
ENSG00000134070	IRAK2	0	23,52397028	12,15302094	3,878660363	133,2429704	26,01570687	38,10553365	26,09961172	46,42100542	309,4404796	34,38227551
ENSG00000118260	CREB1	48,25274899	45,33129263	34,00284099	34,6278692	28,5352218	17,40884967	27,25142803	33,6916881	40,28819268	309,3901321	34,37668134
ENSG00000157017	GHRL	31,26173143	32,86818121	40,40408415	13,52715227	77,60001157	26,04410216	16,53484995	4,482639482	66,2708529	308,9936051	34,33262279
ENSG00000138617	PARP16	0,122027379	40,99721563	55,89532542	0,632143949	129,2436485	19,91820113	50,96493432	2,846273529	8,154235028	308,7740049	34,30822276
ENSG00000135930	EIF4E2	27,66236236	32,68428041	28,28569428	58,47650308	0,164355855	52,03846616	35,83225957	30,95146063	42,61286392	308,7091463	34,30101625
ENSG00000172845	SP3	14,95927092	27,7903658	14,67130639	72,90362293	93,43353117	19,8826819	17,0688607	25,78552277	21,98045438	308,475617	34,27506855
ENSG00000163161	ERCC3	11,94961293	31,77460414	54,69164379	35,13420342	48,31158579	31,85435794	19,06934516	40,84331332	34,60072987	308,2293963	34,24771071
ENSG00000176018	LYSMD3	47,10556027	13,47024176	83,72443887	2,750379862	0	15,92503405	43,75563271	60,24743292	41,22050023	308,1992207	34,24435785
ENSG00000112679	DUSP22	31,14695547	36,78346114	12,66212551	32,30710906	0,063158203	38,13667319	41,3526912	59,97335098	55,72780874	308,1533335	34,23925928
ENSG00000001497	LAS1L	57,3998464	12,61788263	17,95505739	34,40849459	45,17080556	36,30499827	27,45575524	21,28403011	55,53116697	308,1280372	34,23644857
ENSG00000128791	TWSG1	107,1573269	46,25222775	56,66155794	14,06165929	0,080973516	33,70584659	36,38215264	0,149012206	13,66087176	308,1116286	34,2346254
ENSG00000170006	TMEM154	31,60137131	5,67322644	55,72725712	33,78603754	78,96315956	24,23820368	13,49261308	27,8491802	36,76959957	308,1006485	34,23340539
ENSG00000143499	SMYD2	31,15554413	47,82569525	34,17182235	20,12118745	66,99022267	38,51677328	20,04343	2,297969993	46,75623285	307,878878	34,20876422
ENSG00000109814	UGDH	17,3073225	41,43709914	27,76347821	0,12965713	1,962878492	56,6775262	45,29401053	96,79064723	20,40848766	307,7711071	34,19678968
ENSG00000237441	AC005747.1	80,83667458	11,20355684	17,66962609	44,69480258	19,8759115	44,28662523	27,7401239	37,75460306	23,68163111	307,7435549	34,19372832
ENSG00000103266	STUB1	44,3292442	48,4691536	19,30750472	26,34114929	0,154925601	50,388717	40,70478415	21,38273733	56,52364029	307,6018562	34,17798402
ENSG00000173786	CNP	27,66181632	27,16506705	15,93707569	37,66362105	36,04255383	57,60877168	52,96056507	15,46752933	36,82834766	307,3353477	34,14837196
ENSG00000183621	ZNF438	0	12,06544739	42,67095496	0,794974143	88,36967881	18,50795278	44,78352839	84,87374877	15,06981409	307,1360993	34,12623326
ENSG00000031691	CENPQ	31,07270781	22,79438025	67,85843887	4,496294795	0	43,45503904	37,54741124	57,69793712	42,16506393	307,0872731	34,12080812
ENSG00000051009	FAM160A2	5,870849874	34,70672425	54,8799393	5,785087064	69,6093547	22,45538	19,79018961	32,79228183	61,17709633	307,066903	34,11854477
ENSG00000041802	LSG1	66,16005406	25,91499438	0,057900458	26,63156375	38,89629703	37,25298931	16,83042987	61,9435765	33,25185641	306,9396618	34,10440686
ENSG00000168890	TMEM150A	10,00893925	42,4522068	34,13002831	18,46357332	9,746647353	44,70740581	63,89054709	41,10413845	42,27332849	306,7768149	34,08631276
ENSG00000178974	FBXO34	19,33553232	34,97794574	0,069200921	73,27225575	48,09689763	35,47851267	44,82198816	12,2565971	38,16862855	306,4775588	34,05306209
ENSG00000087470	DNM1L	63,31205041	29,42123841	2,595804275	88,90135567	0,147947353	36,6133338	35,3908352	17,28859794	32,59077299	306,2619361	34,02910401
ENSG00000187109	NAP1L1	33,50629017	46,60910591	11,08253027	37,76986015	20,65518095	39,94641301	25,06212163	38,90952117	50,65022103	306,1912443	34,02124937
ENSG00000215251	FASTKD5	0	93,02885717	0,293434306	1,469021673	0,126360852	17,57180985	48,07782954	94,52614475	50,94104182	306,0345	34,00383333
ENSG00000010165	EEF1AKNMT	0,039444158	42,55555627	93,31307943	36,37151565	0,281219689	37,76403625	28,03513552	25,01331887	42,49583751	305,8691433	33,98546037
ENSG00000108406	DHX40	20,80137046	18,79627295	22,87475925	61,14697	54,9682117	21,2936365	24,00634527	46,8292809	35,05469452	305,7715415	33,97461573
ENSG00000148334	PTGES2	20,60339884	52,92143429	19,89311443	28,99277496	0	45,18016835	54,11055749	33,35460041	50,67905165	305,7351014	33,97056682
ENSG00000117616	RSRP1	8,464886351	14,62230521	1,404357112	16,47806303	69,52899817	29,82939046	39,18850488	64,27025986	61,91202124	305,6987863	33,96653181
ENSG00000177917	ARL6IP6	25,53854374	72,9403888	30,21342372	65,52592226	0,107231276	27,6930464	22,55393845	30,58665749	30,52122641	305,6803785	33,9644865
ENSG00000165525	NEMF	37,8277678	35,2225449	42,72361097	19,76342963	51,14367476	34,873508	26,58128661	16,66056643	40,76951572	305,5659048	33,9517672
ENSG00000176974	SHMT1	71,39408915	39,77384427	24,77806404	26,62748058	32,30627527	36,57278885	21,85089699	11,11886678	41,09502827	305,5173342	33,94637047
ENSG00000136450	SRSF1	37,61427282	36,45729321	37,07363022	73,61787642	15,84068038	28,41229388	28,95302247	26,29301177	21,18541051	305,4474917	33,93861019
ENSG00000132424	PNISR	19,26586799	32,83019061	97,1434455	37,03639645	5,252478082	18,78722472	28,04644529	23,55687516	43,35450101	305,2734248	33,91926942
ENSG00000163933	RFT1	25,48387795	25,64453514	104,7181829	16,52572298	0,065544359	39,12328255	32,0603148	28,88814708	32,49515757	305,0047654	33,88941837
ENSG00000165806	CASP7	5,610965067	0,684035715	15,12816865	36,75869682	124,1846377	18,15234768	12,30519696	66,40535952	25,7593394	304,9887475	33,88763861
ENSG00000117528	ABCD3	0,059220341	12,18986493	59,77207615	52,86873228	68,16430948	14,65128019	16,12838755	45,92846242	34,98430289	304,7466362	33,86073736
ENSG00000204237	OXLD1	48,44982672	15,25057626	5,038389294	21,85330412	114,9176136	29,13804503	31,83809675	16,52172838	21,48850414	304,4960842	33,83289825
ENSG00000110911	SLC11A2	49,19844405	19,34745629	0,301407697	39,89257226	57,06234384	52,5288095	24,89618318	28,56492023	32,69946203	304,4915991	33,8323999
ENSG00000185803	SLC52A2	48,56950094	44,00770557	0	14,50109869	43,99977688	34,13110011	52,80600583	13,20879162	53,09037799	304,3143576	33,8127064
ENSG00000119686	FLVCR2	124,3969301	23,75596131	23,8349535	15,78364307	0	40,95389388	21,18429842	14,46913076	39,74314631	304,1219573	33,79132859
ENSG00000035862	TIMP2	12,55454413	49,41086385	44,66039226	49,84307344	50,30068186	23,09140638	31,3397403	24,98937081	17,91490531	304,1049783	33,78944204
ENSG00000167325	RRM1	3,391331021	75,06455708	10,11314322	59,11567225	0,084837596	48,75803388	46,80313117	18,73477349	41,97242506	304,0379048	33,78198942
ENSG00000171812	COL8A2	53,79005535	50,94066269	0	31,63186884	0	59,41368264	47,37721039	11,25275139	49,51661463	303,9228459	33,7692051
ENSG00000099940	SNAP29	49,06107665	66,20521261	11,17564662	18,17273021	1,16667628	53,63038337	40,07407761	17,37716812	47,05596209	303,9189336	33,7687704

ENSG00000147419	CCDC25	12,47261853	11,00537461	58,11738124	37,45393173	71,25348063	18,09591551	17,01389082	49,228007	29,07802729	303,7186274	33,74651415
ENSG00000102181	CD99L2	75,75194146	23,31062676	56,04943472	18,83549357	0,06231434	37,31044723	50,20555016	29,98738137	12,15758093	303,6707676	33,74119639
ENSG00000144567	RETREG2	26,73392299	32,03529094	49,03226063	12,78236528	31,95671522	35,95947301	32,78810614	44,07735112	38,18450132	303,5499866	33,72777629
ENSG00000163683	SMIM14	15,44212512	40,50681969	32,53819452	59,48693768	27,620194	26,18169052	23,4523985	33,00801869	45,01660054	303,2529793	33,69477547
ENSG00000164253	WDR41	11,17299836	4,956552865	49,59342474	15,34696672	65,77870682	34,47854584	24,05389784	56,91563059	40,91173967	303,2084635	33,68982927
ENSG00000141378	PTRH2	35,35477276	32,41463419	1,17291181	29,2313231	108,1140787	24,08153518	31,81758208	14,50008229	26,42627764	303,1131977	33,67924419
ENSG00000108592	FTSJ3	0	49,15593161	41,02396541	15,09251102	47,75352408	43,07171198	49,18514168	26,73192793	30,92680405	302,9415178	33,66016864
ENSG00000164024	METAP1	24,07235159	51,93029899	0,068232719	15,62791699	86,47381175	34,20836633	38,00451391	0,729973271	51,82171068	302,9371762	33,65968625
ENSG00000109133	TMEM33	18,30398664	45,25015081	43,72388463	56,76923756	5,899000046	30,99794632	36,66841539	32,41085926	32,87783548	302,9013161	33,65570179
ENSG00000117640	MTFR1L	18,97679756	43,61875213	21,91424965	25,38680795	40,63845227	47,42346483	36,14549844	32,60454936	36,16264915	302,8712213	33,65235792
ENSG00000156671	SAMD8	63,37235157	3,968037416	55,72796623	21,71118873	0,651755975	28,74424483	43,30066462	50,60543356	34,74081995	302,8224629	33,64694032
ENSG00000185905	C16orf54	91,17220106	10,85510627	45,01351544	5,586385209	0,559155379	29,96594301	38,87675526	33,05631913	47,67335857	302,7587393	33,63985993
ENSG00000051382	PIK3CB	0	28,38547797	30,52469367	5,185459368	165,4685542	20,79694363	21,81745263	14,26972209	16,24085532	302,6891589	33,63212877
ENSG00000119760	SUPT7L	30,26762936	13,78064119	56,04606288	13,11551924	64,0218962	25,73612862	44,33505519	18,28835897	37,05035148	302,6416431	33,62684924
ENSG00000100983	GSS	4,894269495	42,38163435	60,50287498	24,55112878	0	42,47848126	30,51926132	52,49494911	44,78374496	302,6063443	33,62292714
ENSG00000140474	ULK3	73,01814143	30,06486854	41,13535898	21,68270136	0	54,43793254	41,99007127	9,779509113	30,46317497	302,5717582	33,61908424
ENSG00000047410	TPR	11,79518948	45,29102933	26,8976695	42,63698613	10,2937336	27,86222964	23,41774213	61,29761896	52,81953791	302,3117367	33,59019296
ENSG00000128739	SNRPN	33,66368392	50,62985657	15,38004646	37,50558329	42,99738724	37,15503255	27,21707143	27,95538349	29,75938164	302,2634266	33,58482518
ENSG00000009790	TRAF3IP3	36,71499414	37,85127072	31,02616589	49,19045134	0	34,33813742	38,93627722	24,72929667	49,42318483	302,2097782	33,57886425
ENSG00000143420	ENSA	13,91988872	28,06875115	22,21614117	78,38024906	49,49062071	28,54278081	21,37998011	41,20656813	18,74366891	301,9486488	33,54984986
ENSG00000119004	CYP20A1	12,85838568	26,99708348	22,66093045	24,32229221	86,77604791	29,09766529	43,68437671	18,35151382	37,12101695	301,8693125	33,54103472
ENSG00000102910	LONP2	47,92680347	38,02515859	20,7151987	35,14249281	29,79121121	29,57630548	27,83609153	37,73266077	35,07136842	301,817291	33,53525455
ENSG00000105483	CARD8	37,32324784	13,14441456	28,83052543	19,10772792	69,32959892	33,39799112	39,98838859	20,37250719	40,32134245	301,815744	33,53508267
ENSG00000122729	ACO1	34,58241915	1,384187691	37,01495428	46,83359703	48,57950077	26,83556912	28,5982676	46,84254425	31,06871709	301,739757	33,52663966
ENSG00000085224	ATRX	20,18886167	22,81141587	37,1101845	33,04688768	50,0855827	24,00906716	19,66782947	68,15877563	26,51780804	301,5964127	33,51071252
ENSG00000112592	TBP	42,89506277	21,28478344	5,516429919	52,68229858	3,563288815	53,60958669	22,08451055	46,04416105	53,87785288	301,5579747	33,50644163
ENSG00000117475	BLZF1	61,94742242	13,19651728	0	10,31444533	56,87594584	44,95221123	18,16363707	57,00428796	39,06669041	301,5211575	33,50235084
ENSG00000137815	RTF1	51,90661197	27,13950226	13,00773427	69,55251143	16,43102793	12,39297375	24,44170765	54,51884026	32,08026624	301,4711758	33,49679731
ENSG00000028116	VRK2	22,03963293	23,54121213	45,03104407	61,7789971	20,84936171	45,38929966	27,60986915	9,919587697	45,26423369	301,4232381	33,4914709
ENSG00000132669	RIN2	19,06919325	18,89602152	51,71175781	11,63846315	76,96306087	24,18568301	30,28698815	43,41621841	25,15993049	301,3273167	33,48081296
ENSG00000123353	ORMDL2	39,68901149	30,12607293	5,584421631	28,63683687	41,28248534	54,13079456	44,90418876	18,56235852	38,37232882	301,2884989	33,47649988
ENSG00000140688	C16orf58	19,60081682	8,249379714	54,54820124	20,2499426	68,47917659	30,30351165	34,94946886	23,72021979	41,15614504	301,2568623	33,4729847
ENSG00000123213	NLN	64,92546687	16,30079807	40,40482287	17,19847488	43,2687559	33,92832618	27,01185043	23,77292299	34,28451247	301,0959307	33,45510341
ENSG00000151929	BAG3	1,045176863	13,95861158	46,65046437	0	111,644114	47,04926802	29,87171355	11,66805717	39,17187346	301,059279	33,451031
ENSG00000083799	CYLD	22,26880341	26,43332825	20,7508689	65,44524722	21,95623696	42,55989242	49,24279511	9,744172054	42,62390132	301,0252456	33,44724952
ENSG00000106399	RPA3	24,45260781	38,82749259	12,12722585	38,18757518	0	43,47965918	34,35682049	73,20270741	36,28377338	300,9178619	33,43531799
ENSG00000164897	TMUB1	26,85375318	8,538676942	20,84493808	28,81408297	79,12759408	36,89742153	29,83706406	24,94806442	45,05370237	300,9152976	33,43503307
ENSG00000141458	NPC1	56,1000913	28,01607296	41,47503177	83,16042004	0,037003353	20,54863608	33,26482888	9,77173837	28,53285092	300,9066737	33,43407485
ENSG00000164830	OXR1	51,93558672	15,83321882	77,47630107	26,83439208	0,202919928	22,8510824	32,98492553	46,81817647	25,85941862	300,7960216	33,42178018
ENSG00000166619	BLCAP	26,27008015	26,20424426	23,91568522	27,55584749	65,94546305	36,51761582	26,89902784	44,30149558	23,1215841	300,7310435	33,41456039
ENSG00000132300	PTCD3	32,97473581	27,94525438	51,21336408	21,85842719	0,21285244	36,74518551	40,39393579	54,02244485	35,23061111	300,5968112	33,39964569
ENSG00000167740	CYB5D2	37,11973688	12,53459395	89,17785027	12,43686405	0	27,22089205	41,34425697	55,27434333	25,29624982	300,4047873	33,3783097
ENSG00000133106	EPSTI1	22,27990948	34,47073234	65,65614442	27,45010496	0,072932076	22,03605141	37,30474534	74,82423696	16,26723215	300,3620891	33,37356546
ENSG00000160883	HK3	83,65049447	11,6054195	0	16,5058746	42,93324129	33,59387888	45,34248681	38,50402799	28,06822476	300,2036483	33,35596092
ENSG00000164091	WDR82	0,132112756	27,756612	38,5448887	74,27909401	0,062793765	41,92005304	46,48918229	26,63583417	43,8700481	299,6906188	33,29895765
ENSG00000184117	NIPSNAP1	39,36659471	28,06442192	40,51421723	13,01697134	0	59,77541156	33,1369315	15,51437161	69,98784911	299,376769	33,26408544
ENSG00000143363	PRUNE1	62,86697696	81,22425605	0	40,34735982	0	45,21010645	20,47440462	0	49,22930691	299,3524108	33,26137898
ENSG00000102974	CTCF	76,73653757	46,19386722	40,32444341	16,36975796	0,248745577	20,02878523	25,96066339	31,81405444	41,65271848	299,3295733	33,25884148
ENSG00000196227	FAM217B	31,44269831	4,391679882	35,33601482	66,7270901	0	22,17081595	26,85480771	52,02822336	60,35774825	299,3090784	33,25656426
ENSG00000198836	OPA1	50,76527236	17,83268715	1,596880816	15,88324912	45,70298374	27,45625761	24,32616357	44,93647289	70,46235798	298,9623252	33,21803614
ENSG00000198019	FCGR1B	26,66657129	31,94242567	18,40601306	43,65628011	22,50455778	32,95652011	43,20808063	50,69333152	28,89603036	298,9298105	33,21442339
ENSG00000147905	ZCCHC7	0,037853349	48,76530362	67,47563024	39,61087039	50,01737313	25,76767474	22,6299196	37,82780495	6,796992047	298,9294221	33,21438023
ENSG00000154174	TOMM70	14,97921751	12,04573307	0	133,7854835	16,5992701	33,60097256	32,81746757	25,39445163	29,53120903	298,753805	33,19486722
ENSG00000125912	NCLN	63,77408994	24,59841582	3,66201328	19,62481007	22,62225476	36,83810889	40,45103398	15,67090883	71,04456277	298,2861983	33,14291093
ENSG00000166685	COG1	64,34348918	6,707332332	64,20153499	15,21343655	74,76831284	16,88125296	7,810923433	18,45760138	29,78018375	298,1640674	33,12934082
ENSG00000090530	P3H2	13,90987109	47,05596197	0,199519181	54,78089364	14,0476627	31,05055293	46,37876797	11,85840903	78,86722146	298,1488638	33,12765153
ENSG00000134283	PPHLN1	29,42646818	44,3410465	22,38934734	60,83831493	0,088634523	43,5967406	27,42695781	32,70364333	36,84822038	297,6593736	33,07326373
ENSG00000130731	METTL26	40,29615218	49,96525794	24,60807434	70,29559698	0,588717283	41,52297493	25,36831849	25,0985819	19,90487342	297,6485475	33,07206083
ENSG00000148700	ADD3	46,38893531	54,31709985	32,12856671	3,071700511	54,68293414	10,90682586	16,45827012	50,36588653	29,17969399	297,499913	33,05554589
ENSG00000111644	ACRBP	86,28757018	41,51630399	69,10016665	0	0	15,789785	27,98130984	52,45179392	4,355777866	297,4827074	33,05363416
ENSG00000118985	ELL2	0,340147599	14,82552139	52,83499068	56,31320358	29,70752883	46,61274925	36,53961554	28,58993515	31,42761955	297,1913116	33,02125684
ENSG00000213316	LTC4S	15,5927854	43,86551176	58,76760482	35,02482881	30,1273106	29,22921234	31,47284335	15,52377163	37,57467439	297,1785431	33,01983812
ENSG00000151117	TMEM86A	49,04059764	20,91798304	1,244784794	0	37,15592767	84,77354002	67,27433545	31,30678674	5,124686096	296,8386414	32,98207127
ENSG00000176476	SGF29	50,01446854	33,45462807	0	23,21879011	50,04651249	32,49472689	29,65447583	47,7605519	30,18693511	296,8310889	32,98123211
ENSG00000077380	DYNC1I2	44,48025743	45,00445071	10,0653071	41,8496349	21,87595679	21,06127875	35,70359181	45,11364383	31,62755512	296,7816764	32,97574183
ENSG00000136040	PLXNC1	10,63331634	22,9309116	1,080556549	74,1218656	54,37053193	44,08627504	60,60293321	6,982171665	21,8392355	296,6477974	32,96086638
ENSG00000095485	CWF19L1	38,7540786	19,10974354	14,92791498	6,875325554	62,27107196	42,00847475	15,27054229	61,11173808	36,28321062	296,6121004	32,95690004
ENSG00000037241	RPL26L1	16,87661013	34,784744	31,28470186	46,85172654	0,617040451	41,47764418	40,85331257	38,03971825	45,66332076	296,4488187	32,93875764
ENSG00000085265	FCN1	34,57142207	0	213,5039558	0,068775692	0	1,464945788	0	38,66972799	7,959952522	296,2387799	32,91541999
ENSG00000113812	ACTR8	65,32070485	24,50401246	70,18720627	43,33755679	0	29,24917537	23,83811338	14,56347571	25,22070584	296,2209507	32,91343896
ENSG00000205531	NAP1L4	23,79991488	30,16597501	18,54982584	31,66345557	54,69636414	35,12837403	31,39171191	27,38794204	43,17357671	295,9571401	32,88412668
ENSG00000065615	CYB5R4	21,12500962	23,3276015	21,61200645	41,27876846	47,51654001	29,66076208	32,17288318	50,83080344	28,27973273	295,8041075	32,86712305
ENSG00000159618	ADGRG5	10,70925714	21,14654494	0,049374796	24,14691328	35,01874436	48,00503187	49,16297556	0,234767011	107,0845681	295,558177	32,83979745
ENSG00000165934	CPSF2	21,30775756	12,99783583	8,935439739	72,80076195	114,7749703	14,559126	16,04026753	7,486123339	26,38350776	295,28579	32,80953222
ENSG00000136381	IREB2	22,08678988	28,84208128	59,46607505	49,32233807	0,03324239	13,74525281	20,82461402	82,89157437	18,06339428	295,2753622	32,80837357
ENSG00000122971	ACADS	17,89695912	20,24682791	62,69440521	19,40260064	71,02516361	31,85940932	27,84489162	20,64424473	23,57807546	295,1475776	32,79417529
ENSG00000071054	MAP4K4	23,37217254	15,96163398	70,41883621	42,78015928	47,76824763	25,61966685	22,36458054	12,01730933	34,82000304	295,1226094	32,79140104
ENSG00000095794	CREM	22,27283372	43,14550168	23,60806596	43,22207686	21,55083339	34,06130457	30,33832558	58,79288497	18,0335369	295,0253636	32,78059596
ENSG00000100350	FOXRED2	15,76947322	22,93414632	25,76935829	58,48461866	125,6299669	15,4100195	16,94085709	13,86438745	0,033323744	294,8361511	32,75957235
ENSG00000197982	C1orf122	26,75422475	49,03717438	17,56844193	33,80107918	25,91569893	42,46299386	42,96698057	29,32588273	26,8232094	294,6556857	32,73952064
ENSG00000185591	SP1	25,59326627	5,838549055	23,11055629	56,26743297	121,7347601	25,59575518	15,58483401	0,327041512	20,57808208	294,6302775	32,7366975
ENSG00000058668	ATP2B4	30,45217652	12,991964	43,55145466	100,5247896	0,147769794	28,33160758	23,5813493	31,23849064	23,8019669	294,621569	32,73572989
ENSG00000113593	PPWD1	70,1741967	60,84132753	0,07359812	46,90888212	0,095080101	16,86779493	24,21706964	40,15606738	35,27984021	294,6138567	32,73487297
ENSG00000180104	EXOC3	22,57867682	33,92415016	36,22422845	35,53701063	61,53159115	20,21812907	22,81977257	11,49337626	50,25708771	294,5840228	32,73155809
ENSG00000130227	XPO7	10,06966942	11,00785314	78,11409546	82,07495009	0,051910602	27,96353461	23,83174563	30,37819962	31,02814877	294,5201073	32,72445637
ENSG00000214517	PPME1	51,28672098	28,51322855	38,20477551	26,70298553	41,15322979	21,88161174	21,06554419	30,27597663	35,3273877	294,4114606	32,71238451
ENSG00000155189	AGPAT5	0,460407448	48,93290508	79,48019828	7,908388422	2,130748728	38,09877258	56,49786598	9,69684507	51,17037825	294,3765098	32,70850109
ENSG00000171067	C11orf24	24,33399727	36,40364503	29,40723939	44,47319579	0	54,55596101	44,6870533	22,72162468	37,79188757	294,3746041	32,70828934
ENSG00000120899	PTK2B	31,3948552	19,10426464	52,02401089	30,70799637	11,1024889	50,0264716	36,81101598	14,5878874	48,56599959	294,3249906	32,70277673
ENSG00000138942	RNF185	0,043825572	2,255254641	60,94923284	13,47054103	59,3668791	30,84358459	39,1912841	23,86258913	64,30246551	294,2856565	32,69840628
ENSG00000152404	CWF19L2	3,671214646	20,51686904	128,0059154	32,35273072	15,84489575	18,99562593	12,613605	39,58571055	22,67939967	294,2659668	32,69621853
ENSG00000163348	PYGO2	1,567194783	25,32126966	74,42644026	15,097766	0	55,06202546	37,80547121	41,30432094	43,46901463	294,0535029	32,67261144
ENSG00000108559	NUP88	29,8545955	25,05874763	0,046306863	22,21185608	0	41,23581217	42,61769231	89,11770195	43,83786033	293,9805728	32,66450809
ENSG00000166145	SPINT1	31,05829911	16,95759684	4,316461164	34,91902258	0	62,48580162	62,34550658	0	81,85222325	293,9349111	32,65943457
ENSG00000104267	CA2	92,75945344	21,37654211	0,415515843	31,2029865	0	41,91164045	21,8182092	42,31274731	42,13261965	293,9297145	32,65885717
ENSG00000182973	CNOT10	0	14,88448164	19,33125153	107,3139506	21,63894395	31,73733071	27,21199318	39,48031667	32,13771887	293,7359971	32,63733301
ENSG00000143294	PRCC	42,09270133	19,95751069	52,23649612	32,2403076	1,146776726	43,64780831	32,23826266	19,56146666	50,49035953	293,6116896	32,62352107
ENSG00000132153	DHX30	49,1928911	27,70061984	14,14309904	42,48954067	49,2951895	28,45160716	25,53476798	19,75609566	36,71812746	293,2819384	32,58688205
ENSG00000160294	MCM3AP	39,66455105	14,40177514	90,16198822	26,42068865	14,52230814	32,32262545	34,87472133	9,564302529	31,24629617	293,1892567	32,57658407
ENSG00000112651	MRPL2	11,76309832	26,80184893	24,71218752	20,31230341	86,34700926	26,97670823	30,5729183	22,37396657	43,31567953	293,1757201	32,57508001
ENSG00000123384	LRP1	23,35655013	17,22933625	22,87709918	24,06627099	53,62895493	24,73651144	44,26593795	44,54103845	38,37504608	293,0767454	32,56408282
ENSG00000063978	RNF4	12,82355494	24,89541326	55,82179834	42,02145649	6,537639057	31,48142157	31,50375371	38,54530104	49,13572806	292,7660665	32,52956294
ENSG00000116120	FARSB	23,91972921	38,23492122	31,42452732	14,37637742	0	45,18375398	31,12581174	76,07635704	32,0973868	292,4388647	32,49320719
ENSG00000212747	RTL8B	0	20,77901975	16,60197037	39,15312551	156,161182	17,52461175	14,73935786	9,785821348	17,61230616	292,3573948	32,48415498
ENSG00000152778	IFIT5	25,27885943	17,37033061	0	39,18234292	118,7253826	24,54274106	28,58817691	0,4100702	38,05494685	292,1528506	32,46142784
ENSG00000139620	KANSL2	117,2825102	25,10366628	0,574678291	1,541257972	0,106059522	29,20339744	26,0130458	31,42348811	60,54008094	291,7881846	32,4209094
ENSG00000082014	SMARCD3	83,20093367	48,54205888	37,2743611	17,318693	0,483613466	23,71519017	12,45530806	27,77989727	40,93771989	291,7077755	32,41197506
ENSG00000167130	DOLPP1	72,65190976	26,71834943	0	3,031416148	129,6545908	12,6768135	17,82822412	20,81826364	8,138051467	291,5176189	32,39084654
ENSG00000105700	KXD1	40,27655288	44,6685968	5,54337746	19,03708472	31,35291773	35,30441503	35,70045925	39,77750752	39,81779818	291,4787096	32,38652329
ENSG00000156709	AIFM1	50,91868594	8,516306718	0,420417573	21,38940657	0	48,87988557	58,10498436	66,56667331	36,6521272	291,4484872	32,38316525
ENSG00000152382	TADA1	51,22127275	47,80324622	0	40,48990985	0,123833635	19,62321649	28,16592625	89,67310505	14,29095671	291,391467	32,37682966
ENSG00000183735	TBK1	86,87318768	35,41894486	0,087113788	58,50798318	0	50,48548103	20,99691548	3,572545465	35,26482897	291,2070004	32,35633338
ENSG00000128815	WDFY4	25,34344561	11,37025589	1,951022774	42,67521116	58,89023596	28,41997962	56,43321264	22,20129899	43,90043233	291,185095	32,35389944
ENSG00000062598	ELMO2	25,76919691	19,46945855	75,93150498	21,20071948	59,00915809	32,945711	37,39194675	0,100177222	19,35373061	291,1716036	32,3524004
ENSG00000101577	LPIN2	35,22209625	25,98611442	51,05840347	13,5839678	33,11274986	28,8147634	38,10238759	10,44756353	54,66074184	290,9887882	32,33208757
ENSG00000196924	FLNA	66,15459466	15,90866094	52,07695194	39,56548269	2,680234846	16,67726534	22,4862749	26,19127087	49,23133193	290,9720681	32,33022979
ENSG00000129351	ILF3	18,09707433	9,341909638	58,00709741	38,56496767	31,83250217	39,91969238	35,86980731	25,24277861	34,07692084	290,9527504	32,32808337

ENSG00000169255	B3GALNT1	5,645379559	22,26511746	24,22011865	39,99489503	96,39567603	22,84029945	14,54402774	45,53198941	19,30749438	290,7449977	32,30499975
ENSG00000154781	CCDC174	49,25116744	24,43138583	3,908606566	36,1757431	67,35978649	27,2474063	18,20660611	12,63754251	51,47885952	290,6971039	32,29967821
ENSG00000108107	RPL28	33,58739209	19,95691217	22,77407994	48,51365675	45,94508295	26,44326947	27,45398726	30,97973011	35,00873476	290,6628455	32,29587172
ENSG00000100744	GSKIP	14,39305852	44,79797374	9,677870755	47,59335096	0,235899415	52,27618127	25,5775956	44,27982381	51,80263255	290,6343866	32,29270962
ENSG00000090060	PAPOLA	13,4349442	59,0433309	22,71136168	54,8809656	7,679703044	30,72321818	33,05316673	35,69397373	33,07561481	290,2962789	32,2551421
ENSG00000138785	INTS12	32,53317136	42,72932565	52,14355975	18,34646729	51,42098759	17,88104753	18,99602152	28,3205403	27,85074904	290,22187	32,24687445
ENSG00000197520	FAM177B	39,70788718	16,1729904	82,97754011	0,10024394	93,26294992	35,94835392	16,43695091	2,15804366	3,427058421	290,1920185	32,24355761
ENSG00000117228	GBP1	12,77384709	47,87068204	26,47157257	39,71449634	0	41,56295402	38,42561175	47,23406508	35,9308373	289,9840662	32,2204518
ENSG00000169762	TAPT1	51,9479108	17,38533463	2,149884756	27,14288792	4,628996421	57,82704298	60,66501873	20,78526282	47,35996063	289,8922997	32,21025552
ENSG00000014824	SLC30A9	33,73172483	9,42352077	47,8037731	26,71330411	0	49,36525285	45,12330734	33,41524728	43,83489302	289,4110233	32,15678037
ENSG00000004897	CDC27	15,19077542	30,05988131	18,24957274	57,28027432	19,31361929	31,53370721	36,11133236	47,45396779	33,98834405	289,1814745	32,13127494
ENSG00000153250	RBMS1	19,43729959	22,47038157	40,09918392	21,31256967	60,40175339	17,10098829	19,65467503	69,18421154	19,45565987	289,1167229	32,12408032
ENSG00000163655	GMPS	12,65432503	30,9900566	44,93985171	45,96504287	57,89310751	28,8095719	23,76277822	12,8554692	31,1899868	289,0601898	32,11779887
ENSG00000198689	SLC9A6	21,84170204	43,13979131	55,40983085	36,03803869	0	28,04811089	51,48233448	17,1746299	35,80004006	288,9344782	32,10383091
ENSG00000006695	COX10	57,45250574	39,73100254	0	37,59372112	0	46,23737384	24,16846559	36,66865314	47,04752755	288,8992495	32,09991661
ENSG00000136813	ECPAS	43,31752896	43,03474401	0	74,67638402	0,34003318	30,08344224	33,65081931	28,27603176	35,47999058	288,8589741	32,09544156
ENSG00000123106	CCDC91	44,44074646	46,47557427	25,93497141	53,08736776	0,047659926	21,90856321	29,77318562	36,92447324	30,11527587	288,7078178	32,07864642
ENSG00000111729	CLEC4A	68,18259941	22,60681968	0	34,83206921	40,08884353	27,97555392	40,55013072	22,69964242	31,62727701	288,5629359	32,06254843
ENSG00000101928	MOSPD1	74,78808236	8,773356152	87,92074586	72,02943639	0	26,95500908	11,51363708	0,497080959	6,010831484	288,4881794	32,05424215
ENSG00000158517	NCF1	34,65678599	23,08096939	33,16169292	21,40541097	49,27233756	23,19489287	30,33468165	23,57519878	49,76306359	288,4450337	32,04944819
ENSG00000144895	EIF2A	26,07648967	18,2999536	40,34359777	38,57683875	0,060815841	32,0582503	37,69421998	52,76880562	42,4881973	288,3671688	32,04079654
ENSG00000110060	PUS3	65,93232362	28,28164379	0	126,9623906	0	12,4639289	30,08791046	0,986371843	23,57754387	288,2921131	32,03245701
ENSG00000174231	PRPF8	31,0616173	17,54509383	73,76032785	33,54878245	16,0557843	25,51570069	31,09097292	28,43951833	31,05636329	288,074161	32,00824011
ENSG00000182985	CADM1	19,01055196	50,08951949	30,50539527	66,36469083	17,34142984	30,99246318	36,47830384	6,148596382	31,02221607	287,9531669	31,99479632
ENSG00000172239	PAIP1	29,54476802	31,78799293	27,29795723	39,55192478	23,19278731	26,12687937	38,88803271	40,24461114	31,29412097	287,9290745	31,99211939
ENSG00000138375	SMARCAL1	21,54482999	17,64092106	0,109183071	39,40671553	61,4985265	43,98650404	46,25055247	37,70276585	19,59928956	287,7392881	31,97103201
ENSG00000040633	PHF23	2,819845995	46,89786902	63,77369999	24,91914686	0,657002456	49,83995005	42,83345033	21,88388464	34,06846237	287,6933117	31,96592352
ENSG00000146457	WTAP	27,14476492	25,33822736	9,969658937	46,34413474	23,29532163	39,71194998	30,26705186	49,51623568	35,81885257	287,4061977	31,93402196
ENSG00000169718	DUS1L	33,77884235	17,25334423	26,96856436	19,39939968	51,36236895	32,02607898	31,41464222	32,66339366	42,31811126	287,1847457	31,90941619
ENSG00000197943	PLCG2	24,7764846	33,92121601	0,062350676	45,88553722	5,155182664	47,55896457	39,73774403	29,81940359	60,20732568	287,124209	31,90268989
ENSG00000025796	SEC63	27,25117426	28,16643831	0,872119762	57,15343099	75,2786482	20,55085693	27,73002225	30,60148156	19,50027999	287,1044523	31,90049469
ENSG00000124422	USP22	40,06871706	12,38430663	42,20769099	64,1460333	4,186260269	23,30631091	43,80445706	32,57050186	24,32431242	286,9985905	31,88873228
ENSG00000114439	BBX	49,72213782	20,65405921	38,87326813	28,74409828	10,29760191	39,60815063	37,31824456	17,53314935	44,24247431	286,9931842	31,88813158
ENSG00000103510	KAT8	34,25454431	20,46420783	49,80921788	42,28914712	0,250868	32,04629721	33,21917976	41,20333238	33,39784691	286,9346414	31,88162682
ENSG00000196235	SUPT5H	7,654293612	16,2424951	30,14046591	32,92141778	35,83630937	41,2646404	39,36324141	27,69047846	55,81035269	286,9236947	31,88041053
ENSG00000055917	PUM2	0,779590218	7,877487821	41,86831241	39,33843412	80,54155256	26,48672721	34,0017767	32,48468233	23,35732302	286,7358864	31,85954293
ENSG00000133275	CSNK1G2	36,5287124	32,76424333	28,37881994	22,84302475	0,189959028	44,31887926	54,28972842	25,1693085	42,12390098	286,6065766	31,84517518
ENSG00000135828	RNASEL	0	10,24411886	145,2304191	31,2269937	0,117262871	6,880210038	28,33068884	34,52704052	30,00852417	286,5652581	31,84058423
ENSG00000115289	PCGF1	53,02733328	37,05402639	0	32,61687183	48,18434915	23,49765622	30,86397502	39,9780354	21,31267457	286,5349219	31,83721354
ENSG00000100401	RANGAP1	6,41400515	21,25882723	68,71534982	43,57048099	0,201449631	31,3331493	39,95437087	34,84762455	40,10226004	286,3975176	31,8219464
ENSG00000047932	GOPC	4,897706482	27,17342222	50,2742746	76,28457663	0,310386813	32,73041412	19,303128	32,20094856	43,20891486	286,3837723	31,82041914
ENSG00000119446	RBM18	37,05059027	20,69379177	33,86478111	31,68783083	29,0970911	38,47618263	17,57936907	60,85948443	17,06096827	286,3700895	31,81889883
ENSG00000110583	NAA40	14,53642726	8,732787277	44,89964701	19,40310899	114,1847758	25,64242912	20,80936794	14,36942491	23,79059397	286,3685623	31,81872914
ENSG00000108389	MTMR4	16,96077174	19,83712723	60,95108792	50,19725665	0,192169922	30,25232322	46,07572727	14,36672033	47,41261779	286,2458021	31,80508912
ENSG00000214063	TSPAN4	39,27269757	23,33666884	7,143569444	31,47134181	68,59963333	17,96393429	30,43789449	43,13708077	24,61184885	285,9746694	31,77496326
ENSG00000155229	MMS19	18,9838718	22,84534479	114,1247465	3,078878756	0	27,52221262	27,32348892	26,31779741	45,76023167	285,9565725	31,7729525
ENSG00000148356	LRSAM1	17,27472503	29,48640551	33,4977164	56,49107698	33,07336816	23,71780066	43,92180236	11,27943885	37,12672793	285,8690619	31,7632291
ENSG00000133773	CCDC59	4,896704109	44,96980672	12,56362585	55,48020495	59,71681702	24,47629031	29,70914911	27,70841314	26,30800685	285,829018	31,75877978
ENSG00000179041	RRS1	0,351419932	9,977376747	131,7173143	67,66104574	0	17,55565405	10,50449291	12,48735284	35,5485499	285,8032065	31,75591183
ENSG00000189050	RNFT1	22,89554101	42,04756871	48,2519148	37,69316638	0	43,93042631	22,29599309	38,30280071	30,23628326	285,6536943	31,73929936
ENSG00000161813	LARP4	17,21229873	16,32916026	57,70691404	24,09273866	82,873279	26,91547324	26,3871285	11,89434094	22,19986194	285,6111953	31,73457726
ENSG00000080503	SMARCA2	32,67628957	17,82325959	6,607539863	75,17228604	6,025526927	35,45730744	28,27215333	45,34923454	37,81894274	285,20254	31,68917112
ENSG00000164347	GFM2	31,28000229	40,3799839	61,04221766	51,05678047	0	17,33892957	16,5566492	35,94755305	31,59063272	285,1927489	31,68808321
ENSG00000145495	MARCH6	17,79651996	22,90458375	54,00812219	56,3549619	27,47831689	24,04416327	17,11059623	40,08092074	25,40972814	285,1879131	31,6875459
ENSG00000138613	APH1B	51,13828241	35,37129452	49,53898512	27,56524167	0,329889346	27,76860837	27,08573215	28,65425608	37,62683585	285,0791255	31,67545839
ENSG00000135392	DNAJC14	45,45573225	0,319825859	62,731196	12,94715523	91,66406735	23,27180799	19,90404837	7,015428557	21,72347231	285,0327339	31,67030377
ENSG00000151116	UEVLD	6,959580226	15,8989001	31,30111359	71,15978274	75,66538245	20,54703969	34,60387579	0,059915606	28,1718391	284,3674293	31,59638103
ENSG00000205730	ITPRIPL2	3,432484737	9,767000272	60,00484395	64,70766507	35,6704118	35,07759908	37,28694947	22,6063434	15,78696264	284,3402604	31,59336227
ENSG00000162616	DNAJB4	22,04206055	0,144863317	80,87103366	69,28662346	6,596432437	14,2068214	17,21743076	55,2509447	18,5746119	284,1908222	31,57675802
ENSG00000047579	DTNBP1	19,30967318	19,15300001	30,96763881	49,92989029	56,24449058	30,55103018	26,02317174	31,50604821	20,39927423	284,0842172	31,56491303
ENSG00000117408	IPO13	30,2101969	8,511605287	67,79601389	13,63323507	73,56528519	16,71535976	19,44394341	36,15756802	18,02769893	284,0609064	31,56232294
ENSG00000127463	EMC1	12,36960097	30,04798918	77,49976307	52,52940074	0,860206569	28,80367958	23,5730555	28,2875666	30,05828464	284,0295469	31,55883854
ENSG00000134452	FBH1	33,3412029	15,98569743	74,07528402	19,42550665	0,053792322	28,03282363	44,11007627	22,61962213	46,37811655	284,0221219	31,55801354
ENSG00000178053	MLF1	64,65185861	84,85778959	5,581929698	0,069862108	0,096149275	37,35764257	38,29616559	35,65331478	17,41533676	283,980049	31,55333878
ENSG00000160633	SAFB	19,6942003	23,51387352	38,91563353	40,20089717	0,612480175	50,6031975	38,1796448	16,24934059	55,8384804	283,807748	31,53419422
ENSG00000083290	ULK2	32,55584447	2,853152615	89,58012918	1,163471715	0,327529173	14,2237888	32,80436325	65,36362746	44,91730397	283,7892106	31,53213452
ENSG00000091164	TXNL1	42,3088196	44,36198975	7,111290485	47,97397028	33,37819062	29,72497187	30,15419593	28,34301991	20,39919338	283,7556418	31,52840465
ENSG00000049656	CLPTM1L	42,57123269	28,70171661	31,15550354	39,96301413	0	30,42580587	39,88841521	24,87467427	46,11400194	283,6943642	31,52159603
ENSG00000167081	PBX3	9,40896024	38,42441114	52,35527146	73,80386453	0,158585799	31,13799739	41,00156577	17,07257425	20,3122165	283,6754471	31,51949412
ENSG00000116871	MAP7D1	21,15719999	21,11154051	14,02821217	10,59383126	80,39436539	24,33103316	37,61015454	43,19283191	31,20359936	283,6227683	31,51364092
ENSG00000170873	MTSS1	34,16463918	2,741454382	13,67056265	30,39232265	73,29213981	33,90355903	22,27841277	44,73075678	28,42896199	283,6028092	31,51142325
ENSG00000109466	KLHL2	0,038460313	28,32245611	115,4653911	0,531300534	0	30,57247583	55,59782337	13,96083945	39,07081379	283,5595605	31,50661783
ENSG00000121931	LRIF1	66,58012865	24,27213422	12,06538408	19,59620169	46,45352561	19,56626733	8,670422714	76,23961466	9,81770606	283,261385	31,47348722
ENSG00000172046	USP19	39,44536969	27,37097715	26,43502948	28,36546662	0,062662283	41,13748552	43,30488862	47,56735617	29,50974267	283,1989782	31,46655313
ENSG00000099330	OCEL1	39,93287955	42,71799356	24,23723145	29,49211231	1,352972442	44,20114934	48,68897168	5,335326709	47,21181836	283,1704554	31,46338393
ENSG00000152359	POC5	28,00454021	0	0	80,36918301	24,16336132	35,46290459	21,90636307	39,00431235	54,22320226	283,1338668	31,45931854
ENSG00000197622	CDC42SE1	10,25702726	56,58924141	3,321941909	26,25564634	26,52182614	61,14581962	43,93260908	12,63612113	42,41955755	283,0797904	31,45331005
ENSG00000129003	VPS13C	16,93407806	12,96956433	30,29098475	67,81952012	63,22787161	17,85161765	24,17324165	21,66784049	28,03308451	282,9678032	31,44086702
ENSG00000185477	GPRIN3	13,15844815	8,331388988	27,69050993	28,06294933	71,76125313	30,02275671	38,52452853	7,755231882	57,53336464	282,8404313	31,42671459
ENSG00000152642	GPD1L	0	17,64532945	0,347330403	32,65762593	89,96634807	46,53926423	57,3526403	0,275247453	38,00526549	282,7890513	31,4210057
ENSG00000156304	SCAF4	60,4083776	48,12106051	3,014866351	15,74188289	0	43,91667867	33,74367343	56,3323462	21,44746342	282,7263491	31,41403879
ENSG00000171159	C9orf16	14,0780568	19,46584393	50,28194168	55,56510046	11,94885955	40,14774996	43,72105943	13,99299439	33,3916619	282,5932681	31,39925201
ENSG00000008513	ST3GAL1	35,16581287	35,65331384	13,91156618	55,24086561	0,689674449	32,10628309	30,26199264	42,06956141	36,78299899	281,8820691	31,3202299
ENSG00000144485	HES6	60,86183168	49,95071035	9,247035022	16,15820467	0	40,50120015	25,36375698	34,32866797	45,46296707	281,8743739	31,31937488
ENSG00000135052	GOLM1	64,22110008	0,063345618	0,065669761	66,82105185	0,084837596	13,1693725	23,44602708	91,56620546	22,39114726	281,8287572	31,31430635
ENSG00000145191	EIF2B5	12,41947214	16,90080078	13,0827785	8,947791021	47,11454637	38,30477427	40,90022762	37,15840618	66,90268392	281,7314808	31,30349787
ENSG00000043093	DCUN1D1	18,05350969	20,78323634	15,59755192	57,38584201	57,37683986	28,71680928	16,37736858	30,75728321	36,14418812	281,192629	31,24362545
ENSG00000137776	SLTM	13,83979181	36,8211624	9,619511863	55,26282416	29,02625281	26,41422975	43,69521956	25,65736737	40,70079743	281,0371572	31,22635079
ENSG00000164305	CASP3	29,86766367	56,42173855	0,095888271	6,840654733	0	40,57626729	24,62663639	45,82086793	76,73621496	280,9859318	31,22065909
ENSG00000175274	TP53I11	26,88924654	22,74369858	32,6612424	61,99203958	0,29335674	32,4818801	23,21500084	35,58526112	45,11427491	280,9760008	31,21955564
ENSG00000140368	PSTPIP1	58,17148199	33,24768757	39,69366677	13,84100022	45,25138235	27,81267277	15,1081107	18,78477622	28,86693967	280,7777183	31,19752425
ENSG00000101158	NELFCD	26,72867191	22,39483837	1,147583768	71,34487556	0,114041773	40,04193023	25,58016931	30,95528657	62,47016317	280,7775607	31,19750674
ENSG00000130349	C6orf203	15,54581285	19,63725197	42,04842822	18,42696856	65,12332067	9,402081505	14,65826023	79,22363214	16,52689213	280,5926483	31,17696092
ENSG00000031081	ARHGAP31	16,56998905	15,14442243	39,78338373	41,7651675	0,191345664	35,05974783	36,14091085	44,01568728	51,7494632	280,4201175	31,15779084
ENSG00000115318	LOXL3	0,03343894	9,434516046	70,06402583	43,59502231	0	39,10041252	50,69901001	31,29586079	36,06713875	280,2894252	31,14326947
ENSG00000156983	BRPF1	38,21218557	5,581191895	12,89153645	6,050527985	120,2522499	21,68924099	29,80407192	12,79023705	33,01600624	280,287248	31,14302756
ENSG00000123178	SPRYD7	37,19019128	43,94193064	27,26599057	1,716677464	80,22153234	11,36169918	14,82080776	39,22922348	24,45348977	280,2015425	31,13350472
ENSG00000132356	PRKAA1	47,10836928	28,1502115	23,0838266	67,54967573	0	42,04011718	27,1090859	31,41721318	13,73389659	280,192396	31,13248844
ENSG00000157954	WIPI2	36,63796314	32,08868419	26,17957962	27,21764123	9,474658526	41,79624124	50,47865745	31,11963087	25,17813844	280,1711947	31,13013275
ENSG00000117091	CD48	54,47224935	17,46960787	10,76382682	62,86816843	55,9534708	3,008212669	4,742732279	65,49793978	5,368645696	280,1448537	31,12720597
ENSG00000198246	SLC29A3	41,48732659	17,11020118	12,98399138	38,72278862	0,198115599	54,03229705	43,12321635	20,29517204	52,18603154	280,1391403	31,12657115
ENSG00000166886	NAB2	41,14475168	35,7085292	29,34288357	44,96362897	0	39,6074533	16,57734551	20,21183129	52,55149826	280,1079218	31,12310242
ENSG00000177479	ARIH2	24,01333005	39,46707747	16,29670033	18,45455041	42,77875177	37,16794259	25,21301046	37,13627857	39,45500573	279,9826474	31,10918304
ENSG00000185100	ADSSL1	55,8912864	45,08500123	13,57409315	44,86726057	0	15,85846551	23,5080345	32,95762959	48,17528032	279,9170513	31,10189458
ENSG00000160446	ZDHHC12	22,87613801	23,03376965	21,73727276	35,17998502	44,82052532	31,12421065	46,30222575	20,747625	34,07663505	279,8983872	31,0998208
ENSG00000184887	BTBD6	29,81780491	49,21167978	29,05310399	23,8899496	0	50,70260517	46,15965391	20,5830897	30,37875845	279,7966455	31,08851617
ENSG00000198265	HELZ	26,96475617	5,420983037	39,76462768	85,15333323	3,458338578	21,47949431	28,6949369	34,982231	33,7557969	279,6744978	31,0749442
ENSG00000197008	ZNF138	32,18390049	67,74113686	105,1447532	43,85538696	0	14,49534512	9,157133002	5,333329862	1,760651264	279,6716368	31,07462631
ENSG00000013523	ANGEL1	10,05990235	18,64535891	0,043339585	42,47210999	68,53131772	47,95731357	30,51663828	18,03119717	43,37577858	279,6329561	31,07032846
ENSG00000141551	CSNK1D	35,79006713	15,56702468	25,24111748	24,80586478	11,69477483	40,87564644	31,58383267	31,47281349	62,56136104	279,5925025	31,06583361
ENSG00000122952	ZWINT	0	12,18314514	74,72834802	103,5177455	11,78425255	30,43206203	22,27533871	0,13901328	24,47200735	279,5319126	31,0591014
ENSG00000142961	MOB3C	86,43647247	20,16957235	0,046987846	33,12409836	0	41,44959979	16,45790697	49,31942581	32,51371713	279,5177807	31,05753119
ENSG00000126246	IGFLR1	11,5922317	33,47210623	29,40052794	86,9388431	0	18,37593314	70,11074839	4,949768828	24,63331117	279,4734705	31,05260783
ENSG00000108262	GIT1	27,16504136	11,32525218	17,95647844	43,29622028	67,29869117	32,03384338	30,34890196	11,43473659	38,55449592	279,4136613	31,04596236
ENSG00000240344	PPIL3	9,800925825	67,17352782	53,99338916	78,17303803	4,190115456	14,91714863	28,80564588	0	22,25821423	279,312005	31,03466723
ENSG00000103152	MPG	15,01098384	19,04922096	56,39933375	15,5524157	79,13125858	21,67525295	26,06096601	25,46395222	20,9231184	279,2665024	31,02961138
ENSG00000082641	NFE2L1	25,99420321	19,00649567	32,41447355	14,72555016	69,58932488	22,33574312	31,84212233	33,08277414	30,26363562	279,2543227	31,02825808

ENSG00000017797	RALBP1	44,6510031	27,87796444	36,33342669	43,12679782	7,503015435	18,54913673	26,26123228	38,3404367	36,58687287	279,2298861	31,0255429
ENSG00000176986	SEC24C	12,25144548	30,50597386	6,127713746	13,89248417	63,33027461	34,12433121	44,20643492	18,70384344	56,0351741	279,1776755	31,01974173
ENSG00000149187	CELF1	61,89287725	22,31951206	7,925571134	41,01761991	13,53734325	26,25488843	30,6353126	35,91405173	39,54483979	279,0420162	31,00466846
ENSG00000126934	MAP2K2	45,54096266	30,77794921	32,27881974	13,55506362	26,81721907	32,57373431	32,85328976	24,73841682	39,89524275	279,0306979	31,00341088
ENSG00000188938	FAM120AOS	29,40578337	37,3770493	52,39530257	19,44665229	16,60352202	33,61063043	34,29901722	11,72677882	44,15320571	279,0179417	31,00199352
ENSG00000198894	CIPC	26,23148779	10,93730412	0	61,78084771	85,2997843	46,17545362	31,27831839	0,125378322	17,17079422	278,9993685	30,99992983
ENSG00000129521	EGLN3	24,00438335	35,16198262	13,82534532	55,13709546	37,8686252	14,42896713	22,4547394	6,645511074	69,37361009	278,9002597	30,98891774
ENSG00000141298	SSH2	2,392345991	22,91541195	33,87170866	53,68321941	29,19102896	23,848992	48,61283578	19,62410711	44,73432357	278,8739734	30,98599705
ENSG00000131374	TBC1D5	25,65218934	9,779604405	72,94233319	51,98322992	20,55469117	18,50648637	17,44715682	31,37500996	30,59235947	278,8330606	30,98145118
ENSG00000164885	CDK5	0	37,62374719	21,0115279	47,26754318	0	53,54976477	49,65398456	40,47895223	29,22521332	278,8107331	30,97897035
ENSG00000187837	HIST1H1C	26,12659399	41,34900001	3,463926467	20,72847929	0	44,96641798	32,24704794	70,51329556	39,36935674	278,764118	30,97379089
ENSG00000180917	CMTR2	1,773785385	4,031678714	0	23,09250435	86,10863783	29,21269057	42,44676887	43,31996309	48,60639062	278,5924194	30,95471327
ENSG00000111145	ELK3	38,52603697	47,34634799	5,506927025	64,33474288	0	34,18958267	33,36689492	15,72481469	39,41835152	278,4136987	30,93485541
ENSG00000116604	MEF2D	19,41524573	12,15948607	38,47339112	32,53983623	52,98270149	34,01284378	29,59448234	30,43681656	28,67730937	278,2921127	30,92134586
ENSG00000100897	DCAF11	43,6197915	34,45730269	16,45992397	19,8064028	0	40,6023679	43,29220432	31,5343855	48,37863013	278,1510088	30,90566765
ENSG00000065243	PKN2	0,701107476	12,86863883	2,982532597	34,88212942	116,11311	24,48846848	31,56314717	29,12921807	25,30228859	278,0306406	30,8922934
ENSG00000162851	TFB2M	40,23403647	41,93476347	0	4,680447975	0	59,14749259	21,23993288	59,64121678	50,94111513	277,8190053	30,86877837
ENSG00000143819	EPHX1	36,63286562	25,33877752	6,317729522	15,29403559	66,58279792	22,42571192	28,86618085	66,13961439	10,00370036	277,6014137	30,84460152
ENSG00000162144	CYB561A3	34,80804973	23,30459395	35,15082879	42,50011948	42,29297847	23,17143093	23,17122202	13,37703402	39,60002334	277,3762807	30,81958675
ENSG00000160179	ABCG1	15,84920542	4,993985952	25,6135886	23,19043068	107,9523408	27,02382698	33,94524911	19,92000579	18,61197813	277,1006114	30,78895683
ENSG00000178202	POGLUT3	30,23435476	0	55,90000775	0,158526716	96,72465195	14,24586564	41,74721658	20,0750041	17,99000357	277,0756311	30,78618123
ENSG00000006327	TNFRSF12A	52,0873558	67,03550002	13,8388361	51,36928288	0	36,00115869	26,3433381	4,827777772	25,34270758	276,8729569	30,76366188
ENSG00000135596	MICAL1	55,24448724	15,09058179	1,828549109	24,75468477	43,62325357	31,50385019	48,58513514	9,998531717	46,15600663	276,7850802	30,7538978
ENSG00000204899	MZT1	24,99318717	0,120189153	36,88126818	61,16942704	0	33,91090228	23,62059141	65,31673881	30,65509646	276,6674005	30,74082228
ENSG00000180185	FAHD1	0,049723781	48,26379534	34,94188806	29,62243048	0,118169642	42,52857494	34,96984903	49,90768887	36,26422761	276,6663478	30,74070531
ENSG00000072958	AP1M1	30,567005	26,56977302	40,25305031	22,31501522	45,87988364	31,93296605	27,13532752	23,82090404	28,16600971	276,6399345	30,7377705
ENSG00000162542	TMCO4	18,48812113	5,754404047	85,36882665	0,965472279	76,35898409	25,20989523	10,52291882	40,83564833	13,11102747	276,615298	30,73503312
ENSG00000163686	ABHD6	45,21949091	24,20674506	3,020680931	21,15688983	72,74406612	27,08932793	28,4571887	22,83303782	31,84608838	276,5735157	30,73039063
ENSG00000163376	KBTBD8	0	83,56790374	33,04535383	62,91970882	0,151654368	16,88532474	45,59426549	0,27908325	34,10063933	276,5439336	30,72710373
ENSG00000148832	PAOX	69,10790098	41,2342809	31,87505135	18,09149157	0	41,29329909	31,95311122	10,42704635	32,54379101	276,5259725	30,72510805
ENSG00000117143	UAP1	32,88644366	14,19896538	0	39,93360391	0,313457267	59,95372018	47,5670185	41,14809278	40,22979708	276,2310988	30,69234431
ENSG00000131558	EXOC4	5,410486212	20,13613879	82,5895668	46,24512963	0,075859219	20,50225758	36,20457827	27,78051497	36,88272236	275,8272538	30,64747265
ENSG00000168397	ATG4B	35,85404471	20,52065924	32,83163567	47,09251274	29,53874332	36,81625181	31,81177595	15,97913471	25,33761715	275,7823753	30,64248614
ENSG00000156603	MED19	35,42282956	45,42842623	8,293492564	47,54382595	18,74987701	28,72719096	28,94440797	23,23780656	39,40698767	275,7548445	30,63942716
ENSG00000132744	ACY3	44,30813232	14,66859957	39,94316521	19,41310533	0	62,20133948	29,10252777	11,32780793	54,7644019	275,7290795	30,63656439
ENSG00000159210	SNF8	30,76691505	26,40715333	44,25527572	15,89376221	52,47068456	33,02470493	21,33233728	23,70099934	27,78881585	275,6406483	30,6267387
ENSG00000140382	HMG20A	30,27875405	9,254940187	15,36857021	0,774287758	109,3112744	15,62456313	23,16194478	46,4971959	25,30972473	275,5812551	30,62013946
ENSG00000103249	CLCN7	16,57201064	31,76337742	13,63827325	22,89984232	31,94040322	38,95840644	59,68193937	16,99204388	43,10332564	275,5496222	30,61662469
ENSG00000176095	IP6K1	49,19811464	17,79019039	0	17,76365019	81,76830195	32,54399774	34,14240765	16,77590881	25,44667767	275,429249	30,60324989
ENSG00000146576	C7orf26	20,85814044	43,50909106	61,43319368	28,83312311	0	31,30618077	33,71669751	21,34966369	34,38570016	275,3917901	30,59908778
ENSG00000118308	LRMP	33,69697143	40,56611196	56,10067353	10,02685076	0	22,64014224	19,57033469	50,39002179	42,3707578	275,3618642	30,59576269
ENSG00000205846	CLEC6A	31,35409582	0,159418549	50,73715243	6,825885186	142,4086806	8,384303817	28,34644837	0	7,071449123	275,2874339	30,58749265
ENSG00000165219	GAPVD1	11,11286545	13,52026085	30,31768272	78,71139417	1,792293055	41,9251461	38,25348691	15,83660716	43,78475864	275,2544951	30,58383278
ENSG00000070061	ELP1	52,29982763	60,34966085	50,21635209	33,39243813	0,118598924	16,5198279	20,20039196	15,87788295	26,14617519	275,1211556	30,56901729
ENSG00000079385	CEACAM1	63,73258785	11,85562337	18,96698274	21,03242244	0,13068324	12,43725575	6,460717605	123,19149	17,17795366	274,9857166	30,55396851
ENSG00000029363	BCLAF1	35,44353677	14,76707753	28,22665854	32,1891881	16,87660327	30,30004343	16,54423658	62,8425334	37,76195465	274,9518323	30,55020358
ENSG00000176853	FAM91A1	36,59692879	20,19464051	33,10653802	18,25071831	0,939451095	35,33851882	48,00799293	37,71585522	44,67685913	274,8275028	30,5363892
ENSG00000011405	PIK3C2A	36,11943236	13,89653781	3,110815971	116,8877554	18,49431933	26,71441489	24,3879209	3,015700095	32,10516845	274,7320652	30,52578502
ENSG00000176024	ZNF613	54,55354368	9,673433172	46,60657772	0,135445095	130,5797241	8,353659974	10,25776138	10,2912401	4,19637878	274,647764	30,51641822
ENSG00000159692	CTBP1	38,82137373	27,54861596	40,1294079	24,56115596	16,06386327	32,84080905	29,92319603	36,99159852	27,76250524	274,6425257	30,51583619
ENSG00000134072	CAMK1	43,35732396	23,11058122	17,50813883	27,10263825	15,07896863	31,65439364	37,80284773	21,78555712	57,22566947	274,6261188	30,51401321
ENSG00000104853	CLPTM1	31,14153076	17,31476311	18,22283671	40,25414917	40,95138216	34,51721517	34,89528174	17,77018086	39,54026107	274,6076008	30,51195564
ENSG00000076321	KLHL20	0,035605695	15,79535816	3,340477156	11,00550435	130,0574134	22,79120316	27,9677036	41,96612439	21,36972264	274,3291126	30,48101251
ENSG00000130717	UCK1	37,15833705	34,76654845	41,73844258	0,583312164	0	39,74683339	31,74156313	28,56215338	59,82540806	274,1225982	30,45806647
ENSG00000099331	MYO9B	16,89133038	9,191437679	52,56137577	29,06272134	55,05169402	16,72781548	30,61082587	30,32637315	33,66459923	274,0881729	30,45424144
ENSG00000152213	ARL11	23,65792782	26,21147622	0	29,46260037	79,21986559	28,96713676	31,79717615	16,58218426	32,49642535	273,9173335	30,43525928
ENSG00000143374	TARS2	45,77423467	23,08507286	0	20,03253546	0	43,78704453	63,08908035	24,96292539	53,01435011	273,7452434	30,41613815
ENSG00000198604	BAZ1A	51,99946761	27,86614706	13,25262358	37,48948843	1,212919733	20,88430096	21,26881368	68,12154568	31,26412657	273,7195012	30,41327791
ENSG00000115380	EFEMP1	0,038139972	19,35603534	0,07016156	0,065859408	251,7085853	0	2,470137783	0	0	273,7089194	30,41210216
ENSG00000196954	CASP4	27,45609919	42,08200031	37,36986777	14,25061227	28,39534056	32,63447353	38,04584261	25,58203103	27,77241353	273,5886808	30,39874231
ENSG00000100393	EP300	7,987199168	14,33362556	44,05555186	45,17040614	87,81363784	25,92530137	23,13667771	11,53073072	13,49501346	273,4481106	30,3831234
ENSG00000204634	TBC1D8	21,87720202	15,91954163	42,22715931	32,41132603	1,607988996	31,83391994	56,09751494	26,85123351	44,59700128	273,4228877	30,38032085
ENSG00000152904	GGPS1	48,99489493	14,85375742	62,68966574	18,08528047	23,381767	33,75707455	17,91962736	15,3549274	38,35005684	273,3870434	30,37633815
ENSG00000181852	RNF41	56,75026021	35,72765521	23,85602502	26,30505557	17,19501159	14,08602877	20,17275798	25,88075548	53,33456873	273,3081185	30,36756873
ENSG00000272886	DCP1A	37,93860935	55,63221437	3,097778881	47,29499867	1,177048648	23,92679572	16,75816463	43,59223646	43,76621641	273,1840631	30,35378479
ENSG00000109917	ZPR1	47,05880339	26,2139388	9,294885713	23,18025348	51,74589694	33,70866633	30,34540333	28,23066651	23,30322644	273,0817409	30,34241566
ENSG00000100410	PHF5A	50,41209554	34,35429951	0	78,15024198	3,58520009	23,87897651	18,26731123	41,51049891	22,72831845	272,8869422	30,32077136
ENSG00000141526	SLC16A3	39,32738698	24,47410584	22,06534638	17,63740573	35,69217152	29,80672506	53,54981707	13,15125905	37,06599899	272,7702166	30,30780185
ENSG00000104886	PLEKHJ1	22,62600811	34,67701054	16,95947328	20,68421174	43,43355024	39,3837137	23,90809549	35,27948014	35,77196218	272,7235054	30,30261171
ENSG00000184260	HIST2H2AC	1,162389005	11,68824103	52,03217237	121,7696548	0	21,69599849	26,0592228	26,26531178	11,91323861	272,5862289	30,28735877
ENSG00000133119	RFC3	27,96973124	83,12589646	46,29759521	3,012895088	9,424008284	22,81415558	23,70678071	33,06658023	23,08450504	272,5021478	30,27801643
ENSG00000198721	ECI2	42,40600203	24,12633851	36,91022061	30,71499264	0	32,46470337	34,88179354	25,69004281	45,13241598	272,3265095	30,25850106
ENSG00000150768	DLAT	21,30124169	34,69028153	1,49606346	64,221007	0	34,78936332	33,9712677	36,93534806	44,74011704	272,1446898	30,23829887
ENSG00000105404	RABAC1	22,39255089	36,6784912	30,53460155	39,07264646	18,97926908	32,83825922	39,78644725	22,77087952	28,98435399	272,0374992	30,22638879
ENSG00000115207	GTF3C2	0,025879529	0,183690358	54,65339161	34,76749619	46,74247854	26,99350023	43,1485574	28,06911209	37,27115561	271,8552616	30,20614017
ENSG00000161618	ALDH16A1	18,83531192	23,78160689	0	19,44645049	86,16003795	47,28939515	34,58704946	10,1528163	31,54814575	271,8008139	30,20009044
ENSG00000135049	AGTPBP1	37,23457471	31,81437782	55,26958032	5,547311669	0	16,59441686	30,76423812	58,67404651	35,85983716	271,7583832	30,19537591
ENSG00000175105	ZNF654	19,63282547	17,06429836	50,00700193	96,45083203	0	18,89780966	23,40121271	1,894025299	44,33111488	271,6791203	30,18656893
ENSG00000010295	IFFO1	38,59810656	19,22294177	71,32489566	14,84465741	0,069021246	24,3620374	27,10525719	35,88228534	40,18399722	271,5931998	30,1770222
ENSG00000175283	DOLK	0	27,45568805	71,02458031	8,48975037	0,171826575	31,43568822	22,27248477	57,23316433	53,44199038	271,525173	30,16946367
ENSG00000033100	CHPF2	31,76735897	17,21720334	20,62539617	26,18657771	0	43,20382437	46,72688226	45,49860038	40,17467033	271,4005135	30,15561261
ENSG00000197471	SPN	47,44978364	12,45904986	25,45577715	37,96317566	19,60503516	30,92562362	43,00188837	7,475287561	46,87702851	271,2126495	30,13473884
ENSG00000135549	PKIB	10,39105323	58,59746505	45,27706158	0,152060009	0	32,34096032	60,39539291	33,02420066	30,92026929	271,098463	30,12205145
ENSG00000113460	BRIX1	29,61296755	49,39035444	36,89019474	40,72064461	0	37,3803714	13,24562076	25,12384159	38,71957079	271,0835659	30,12039621
ENSG00000134698	AGO4	40,61236541	14,12414415	1,647733309	6,81953187	56,02291716	29,23495006	32,90997392	47,18586038	42,51211031	271,0695866	30,11884295
ENSG00000175110	MRPS22	43,01400859	46,18499333	24,17218252	30,8351252	0	26,04876204	21,8209273	56,60733394	22,37616991	271,0595028	30,11772254
ENSG00000197568	HHLA3	16,44656784	9,874296239	0	60,00223229	85,51817089	19,9581463	14,78537464	10,00497463	54,44603517	271,035798	30,11508867
ENSG00000064763	FAR2	6,814078588	31,66563261	48,59899716	90,12889808	0	43,35567464	28,89226274	3,175493903	18,30644915	270,9374869	30,10416521
ENSG00000067208	EVI5	2,264102315	11,95558217	0,134354809	24,46659501	106,2251986	26,56253192	34,15716022	24,39530217	40,46585707	270,6266843	30,06963159
ENSG00000198408	OGA	16,84798414	22,57025253	56,53962549	45,29545992	22,20017372	26,00723267	30,85250885	8,891229566	41,28782957	270,4922964	30,05469961
ENSG00000136478	TEX2	38,05181513	4,540781616	20,05222619	23,04055252	102,0408763	31,70800333	29,33251879	0,508696277	21,19230741	270,4677776	30,05197529
ENSG00000168411	RFWD3	45,87619555	0	124,0059921	38,31573047	0,111250788	27,26628282	8,90108613	16,17368701	9,787456254	270,4376811	30,04863124
ENSG00000105671	DDX49	49,54744664	11,43618331	0,168167029	25,17793615	58,54941178	22,88421748	43,75901174	24,48774018	34,40353044	270,4136447	30,04596053
ENSG00000156675	RAB11FIP1	7,79001588	25,24477901	76,11015207	5,38066288	52,95880441	27,81879072	20,34548083	13,45552134	41,24546334	270,3496705	30,03885228
ENSG00000136631	VPS45	0,728572889	48,84491157	0,041883396	63,06157892	0	49,99565367	59,32287205	0,647227569	47,68669993	270,3294	30,0366
ENSG00000197147	LRRC8B	25,20656055	13,7776603	4,603104038	18,14122373	118,2337556	15,87796339	27,45284142	30,1748357	16,65445518	270,1223999	30,01359999
ENSG00000145016	RUBCN	38,14731347	22,21878679	28,44935029	6,933903625	38,65534907	31,27858974	37,13759625	38,62337794	28,46343182	269,907699	29,98974433
ENSG00000173442	EHBP1L1	43,04620148	20,43024596	40,0889167	17,98215182	33,86894986	31,59028061	33,02145081	19,82705559	29,89332892	269,7485817	29,97206464
ENSG00000105258	POLR2I	20,06833436	23,81912226	12,46876433	7,114322087	66,9594714	30,78903402	32,33316383	34,29312134	41,84407186	269,6894055	29,9654895
ENSG00000101146	RAE1	26,94385574	50,80535237	5,863103825	34,45525631	0,127301504	48,31461849	23,10593317	29,51772812	50,5296996	269,6628491	29,96253879
ENSG00000102978	POLR2C	38,00434974	70,80236118	2,123143146	33,02613972	30,36727718	25,16265157	33,95160102	14,51171996	21,64660802	269,5958515	29,95509461
ENSG00000027075	PRKCH	21,54566347	9,231615858	83,26810439	52,00968916	1,789487187	37,52247131	25,44930517	3,891868066	34,85411562	269,5623202	29,95136891
ENSG00000255398	HCAR3	6,788308275	47,05346281	72,32425598	33,21212592	7,057995685	9,782280143	14,03247981	62,93255451	16,34962386	269,533087	29,94812078
ENSG00000176946	THAP4	49,10035349	13,3319902	46,23268453	23,9373121	0,125741436	34,39042766	25,78819499	36,44498906	40,14961633	269,5013098	29,94458998
ENSG00000176533	GNG7	34,63643085	26,54287286	42,31461252	8,093231724	0	43,50316925	39,90983284	20,93382214	53,55454117	269,4885134	29,94316815
ENSG00000145833	DDX46	29,91020747	59,86222816	27,81310931	34,35667455	13,53761947	21,34037184	12,24034309	37,58446412	32,72756248	269,3725805	29,93028672
ENSG00000109084	TMEM97	10,52460103	29,38667874	16,03913852	58,17362186	0,12260756	50,13003086	53,41806214	19,40414668	32,16940485	269,3682922	29,92981025
ENSG00000166801	FAM111A	0	27,36749122	50,62232795	44,37448398	0,049732384	33,20049624	32,70117225	38,66738811	42,15712553	269,1402177	29,90446863
ENSG00000145388	METTL14	14,31992539	30,82182101	48,53878885	32,16849716	5,882007128	13,66008404	23,90872253	84,95232081	14,83945615	269,0916228	29,8990692
ENSG00000151690	MFSD6	35,27834248	0	0,507010627	71,47480982	31,2612684	26,9594371	28,89708028	24,76478603	49,88982251	269,0325572	29,89250636
ENSG00000106829	TLE4	24,89900572	23,45055235	44,71720903	34,26225696	21,71285983	24,78424763	22,08412708	39,55366754	33,52819267	268,9921188	29,8880132
ENSG00000124222	STX16	1,18541338	32,02558895	0,054516591	108,1813172	0	28,99123825	25,56651793	36,48453844	36,41119352	268,9003242	29,87781381
ENSG00000076928	ARHGEF1	44,00684173	21,76557921	51,14756569	25,22935431	0	34,56150156	31,86156069	15,49629456	44,76155238	268,8302501	29,87002779
ENSG00000163605	PPP4R2	38,56309332	36,77735682	51,6435194	12,81924003	30,64903237	18,41596274	15,21043763	44,82252936	19,91212739	268,8132991	29,86814434
ENSG00000100201	DDX17	22,80942794	24,247311	42,12662298	73,97116315	3,592253099	24,94384283	19,47883918	30,83879679	26,60261812	268,6108751	29,84565279
ENSG00000064652	SNX24	5,132713684	45,69374325	0	0,150221632	139,760195	26,98315367	20,04491487	11,03350888	19,63318183	268,4316328	29,82573697
ENSG00000134371	CDC73	17,85916422	23,2164088	70,61916119	26,22768562	30,5896289	29,44350393	18,09025264	29,10419473	23,21561045	268,3656105	29,81840116

ENSG00000196975	ANXA4	16,57297669	32,24492406	38,40448384	15,02853335	27,30721999	23,68651	22,25793774	65,51925918	27,28885643	268,3107013	29,81230014
ENSG00000092036	HAUS4	28,74612274	49,0027129	17,73642223	56,72946277	35,50157666	39,46829001	21,54590971	7,288040875	12,22385554	268,2423934	29,80471038
ENSG00000128487	SPECC1	28,41886427	43,50739238	27,80614035	29,68812668	10,05718183	26,04635918	36,25758643	15,41512533	51,01402783	268,2108043	29,80120048
ENSG00000100749	VRK1	41,15520801	9,972223144	14,51511411	34,6998426	0	38,89113443	18,99494508	79,81571259	30,09010184	268,1342818	29,79269798
ENSG00000204020	LIPN	25,24821565	36,73791814	0	130,7944735	0	3,742343835	38,88846547	19,87576346	12,73134071	268,0185208	29,77983564
ENSG00000060237	WNK1	4,076119398	24,457891	65,91978015	51,37429422	20,47249616	21,82305417	31,47225792	21,06566313	27,0423228	267,7038789	29,74487544
ENSG00000100104	SRRD	22,35694713	32,28382497	0	12,68727591	120,39875	18,40036264	16,26843851	22,87565077	22,07438433	267,3456343	29,70507048
ENSG00000065600	TMEM206	17,83872214	13,34268703	0,095394681	8,685893115	0	64,90667723	61,1850864	30,61679246	70,53749267	267,2087457	29,68986064
ENSG00000029725	RABEP1	29,2202761	20,9681802	32,17307563	8,945497075	0	32,99878874	20,93838378	67,45377699	54,41914741	267,1171259	29,67968066
ENSG00000005007	UPF1	0,05271299	21,3266428	29,64043614	40,62696872	52,36434862	36,67395422	30,6526939	4,764405392	50,91330308	267,0154659	29,66838509
ENSG00000180628	PCGF5	30,56025858	35,06621199	52,23831985	30,41939249	4,709313296	26,4275269	31,47324858	21,2683462	34,81268469	266,9753026	29,66392251
ENSG00000188976	NOC2L	54,14340844	27,83062275	34,17047787	20,5356767	21,78041412	25,90481026	36,7578247	18,13214955	27,507969	266,7633534	29,6403726
ENSG00000140455	USP3	54,80774948	32,42984777	2,900101496	26,38252899	54,11740399	20,25801478	19,86128774	24,47190576	31,5010334	266,7298734	29,6366526
ENSG00000108840	HDAC5	40,80263112	27,87719402	0	28,87479438	0,047140659	34,47577355	45,77081289	30,53635279	58,305748	266,6904474	29,63227193
ENSG00000145632	PLK2	57,84930175	53,95153219	0	61,42949477	5,950516626	21,38593403	23,22024651	16,78126919	26,11529568	266,6835907	29,63151008
ENSG00000120688	WBP4	1,83509487	62,6562965	34,22368053	17,37381112	28,72338802	27,58228599	36,83195257	20,75594017	36,65565829	266,6381081	29,62645645
ENSG00000119328	ABITRAM	37,22406929	43,64608397	0	0	0	17,34669	64,25074843	69,48940806	34,3724063	266,3294058	29,59215621
ENSG00000167515	TRAPPC2L	29,52462864	33,94582793	17,12850711	43,20657545	16,25403172	25,16725531	22,8562357	46,71839841	31,4394184	266,2408787	29,58231985
ENSG00000078304	PPP2R5C	22,4725287	25,25852979	14,47387456	19,39344552	35,26082385	22,94347397	33,74501266	46,0652011	46,61445418	266,2273443	29,58081604
ENSG00000107201	DDX58	14,72025387	47,8494776	11,80217231	27,49939711	43,88354451	24,63629038	33,88239784	28,41452423	33,52446964	266,2125275	29,57916972
ENSG00000070610	GBA2	17,80732521	19,8747766	59,55476199	28,79357413	0,074769424	38,10105478	19,64474072	23,04717471	59,24027183	266,1384494	29,57093882
ENSG00000036549	AC118549.1	12,0277841	27,7727005	62,61341261	24,30333522	69,3278648	9,945793896	18,31632844	2,934587633	38,84869021	266,0904974	29,56561082
ENSG00000174891	RSRC1	30,68279909	19,44121204	84,74671769	21,36637763	1,052719002	25,84145586	32,34226459	17,4354765	33,12331998	266,0323424	29,55914915
ENSG00000183527	PSMG1	0	30,91895826	2,210577328	83,20868773	0	27,50883121	44,2513656	46,11631539	31,47937117	265,6941067	29,52156741
ENSG00000149474	KAT14	0,177735322	22,64478429	103,5804462	26,88532554	1,774045728	18,65567059	26,23926727	28,83820855	36,89825095	265,6937344	29,52152605
ENSG00000247077	PGAM5	0,076472961	16,62317561	29,47208033	20,46807746	65,78975219	17,46427123	37,11407341	57,60865815	21,06762196	265,6841833	29,52046481
ENSG00000148396	SEC16A	20,64799076	13,03748071	21,23517147	57,49725054	50,6544744	19,87600393	34,62855378	17,85506084	30,20122432	265,6332107	29,51480119
ENSG00000131462	TUBG1	26,7957698	40,19902908	28,62917963	10,18599984	0,149135192	62,56096497	38,71447536	20,7207723	37,50889112	265,4642173	29,49602414
ENSG00000120733	KDM3B	22,36328948	18,18951068	71,96690063	47,13535745	0	28,19973979	30,2199885	6,548246003	40,79557806	265,4186106	29,49095673
ENSG00000178802	MPI	21,94344921	35,57527526	14,49533189	21,24223116	49,46261419	35,95641761	32,75479624	13,08651753	40,78809812	265,3047312	29,47830347
ENSG00000176014	TUBB6	42,28306471	50,02017393	16,7449756	14,38723324	51,02836101	28,65251883	36,54091039	4,414355408	21,21638705	265,2879802	29,47644224
ENSG00000130254	SAFB2	24,28114354	29,05094451	34,59739019	21,41938808	25,95814738	29,14049179	22,39201339	38,38048566	40,05366224	265,2736668	29,47485186
ENSG00000072803	FBXW11	16,98876238	28,99222271	1,644852657	46,50696876	6,310475014	36,47236275	40,02361855	30,09875566	58,21658139	265,2545999	29,47273332
ENSG00000137267	TUBB2A	26,02075616	33,90124829	44,84575968	44,63361408	0	37,11157637	25,84057181	22,87333105	29,98210485	265,2089623	29,46766248
ENSG00000163545	NUAK2	50,58976385	3,740428946	52,18690612	40,49383215	0	33,36533539	30,26456715	19,49381494	35,05213396	265,1867825	29,46519806
ENSG00000143771	CNIH4	9,648513844	43,72453047	49,37013939	47,16309953	14,95735147	18,09044599	20,04074262	29,14835379	32,89739029	265,0405674	29,44895193
ENSG00000026950	BTN3A1	40,13064623	10,21447229	0	0,612412831	91,09228146	23,75285924	43,67337066	25,23448674	30,19712404	264,9076535	29,43418372
ENSG00000075188	NUP37	34,91832568	38,66841343	0,079696129	39,12528432	70,73215355	22,07067305	18,8493274	10,23134555	29,94430777	264,6195269	29,40216965
ENSG00000173145	NOC3L	87,2677086	21,8045578	13,3261069	18,88037563	0,080447478	32,22322357	24,00350885	20,20802799	46,71142428	264,5053811	29,38948679
ENSG00000107937	GTPBP4	20,31759511	32,59232937	32,01698575	29,41433303	41,6555228	37,48641196	36,44604645	7,989595816	26,50612873	264,424949	29,38054989
ENSG00000005302	MSL3	42,41293624	24,12729653	39,67895891	23,69225536	0,036719585	31,41783109	21,89957635	52,50463373	28,59908139	264,3692892	29,37436547
ENSG00000111647	UHRF1BP1L	0	34,89961331	67,8010443	0,981548957	0	27,88475988	39,40062938	59,21570417	33,97585383	264,1591538	29,35101709
ENSG00000189306	RRP7A	53,23333788	27,8961765	25,74973548	19,77612439	32,95060959	30,82443872	23,35509162	18,14491573	32,05037393	263,9808038	29,33120043
ENSG00000164011	ZNF691	11,50399728	4,477813361	0,136532465	55,36537771	130,3476738	25,58738757	12,26355406	19,15092287	5,111692165	263,9449513	29,32721681
ENSG00000118762	PKD2	0,08196198	11,10188357	0,050258561	10,2845461	59,86374508	41,60512925	51,19988055	24,01639778	65,72334073	263,9271436	29,32523818
ENSG00000033178	UBA6	5,059978786	16,57083714	63,3057764	65,64545732	54,74276001	13,27808174	20,55610541	11,12262219	13,55402406	263,8356431	29,31507145
ENSG00000198382	UVRAG	2,427313822	28,76339451	17,7095952	49,87104606	10,70606341	16,35165499	34,91170142	47,77042073	55,22773729	263,7389274	29,30432527
ENSG00000108064	TFAM	18,08062867	36,65250361	22,9457257	21,09413748	28,62334065	17,43286067	33,06601707	59,49323622	26,00162438	263,3900745	29,26556383
ENSG00000183943	PRKX	13,23271426	16,04197335	20,37888822	8,776021309	37,6817384	42,40336965	44,24132134	38,35949955	42,02574023	263,1412663	29,23791848
ENSG00000141524	TMC6	49,64389263	15,4964981	6,413198731	10,59512182	32,85041078	41,56855146	46,86502884	8,271346309	51,40846238	263,1125111	29,23472345
ENSG00000155975	VPS37A	53,98486296	17,69803986	58,02791294	6,783334489	0	27,81533174	21,9729333	31,7883815	44,78368874	262,8544855	29,20605395
ENSG00000101210	EEF1A2	0,823490848	9,38581304	6,525627979	0,328151299	236,3506776	0,799820428	4,339259019	3,185912829	1,051740956	262,790494	29,19894378
ENSG00000116251	RPL22	28,37574703	41,04038329	6,018433011	63,6535908	8,006050248	30,72813253	21,89342985	40,09131087	22,94696609	262,7540437	29,19489375
ENSG00000276180	HIST1H4I	33,98236172	22,7941422	54,35005026	9,477545445	46,34669982	15,25761872	28,70109604	33,19667216	18,52720353	262,6333899	29,18148777
ENSG00000124532	MRS2	51,07672708	6,16418391	28,37747358	39,7528054	0	21,42971213	24,51719131	74,03073303	17,27036648	262,6191929	29,17991032
ENSG00000151287	TEX30	42,131449	0	13,55399419	44,99277709	74,02023812	11,17837219	29,42101313	31,63735221	15,56958897	262,5047849	29,16719832
ENSG00000149636	DSN1	33,54334683	25,45342096	3,044688926	48,58592079	0,262225296	38,46411691	49,29028011	0	63,78084372	262,4248435	29,15831595
ENSG00000122643	NT5C3A	44,92242716	87,84623465	10,73742872	33,82075656	6,473363424	22,88094363	20,06807313	12,85811193	22,81206239	262,4194014	29,15771127
ENSG00000183696	UPP1	35,33611816	30,67825503	2,54916248	10,88178606	22,26842402	32,74741514	42,81880544	37,37229388	47,76012155	262,4123818	29,15693131
ENSG00000196418	ZNF124	0,033453746	25,76347754	31,50892421	41,88130542	62,01277021	16,52861623	9,527634243	48,50080103	26,6200354	262,377018	29,153002
ENSG00000116663	FBXO6	70,11182097	5,563753316	15,38103141	10,37724106	41,18985068	24,6714287	38,11116136	40,56631866	16,38877113	262,3613773	29,15126414
ENSG00000204308	RNF5	31,91154908	32,1199379	17,7591545	30,09897666	21,3494901	49,31680851	39,85659171	12,90072846	26,87931067	262,1925476	29,13250529
ENSG00000015676	NUDCD3	43,91417069	7,26392965	46,05371295	48,50494474	0	33,89318463	27,92739018	9,218633441	45,34858029	262,1245466	29,12494962
ENSG00000104419	NDRG1	24,49028082	40,37280038	3,707155957	38,11887291	28,40622319	27,17602748	39,16237788	6,929606119	53,67470057	262,0380453	29,11533837
ENSG00000106299	WASL	30,75548631	0,061458171	10,1303772	62,07896026	110,542026	15,0966076	10,6691481	4,392666724	18,27370114	262,0004315	29,11115905
ENSG00000163528	CHCHD4	18,93145976	15,58613206	10,03991328	43,7345866	0	43,25659505	32,99614826	61,90986254	35,44928021	261,9039778	29,10044197
ENSG00000181826	RELL1	24,1690851	50,39593357	62,9314225	13,49877182	11,58993492	21,92980333	18,99622017	23,75929615	34,44379067	261,7142582	29,07936203
ENSG00000066557	LRRC40	69,2567969	46,59238412	0,195554062	4,589077992	0	23,45974387	22,50786821	55,44048143	39,61413735	261,6560439	29,07289377
ENSG00000204394	VARS	29,99082244	9,758909527	35,83484949	22,27710157	31,94191746	34,42362306	43,16776899	14,66724866	39,53979581	261,602037	29,066893
ENSG00000155256	ZFYVE27	29,26095944	30,25967685	73,00631479	22,41939382	0,092105038	27,87161522	29,09083871	14,83099495	34,7479135	261,5798123	29,06442359
ENSG00000170242	USP47	29,02222199	16,96614555	1,021436105	35,3822026	10,71752479	29,82762295	36,1297882	60,79782821	41,52315648	261,3879269	29,04310298
ENSG00000106459	NRF1	0	16,33980141	38,46774858	40,60677803	96,77603108	19,45491832	19,67803702	15,48285301	14,57354375	261,3797112	29,04219013
ENSG00000101407	TTI1	29,29625777	15,94941235	43,69857102	24,3313695	0,080303565	29,19759031	42,28170624	37,38816886	39,02278274	261,2461624	29,02735137
ENSG00000140983	RHOT2	26,38205889	12,69188128	23,13355237	28,85216984	38,87247112	31,70163299	29,84027682	26,02452656	43,73071416	261,229284	29,025476
ENSG00000173465	ZNRD2	36,94984572	34,45250798	34,88594868	4,418221681	30,58222099	39,57727402	28,74320355	18,10149523	33,31909762	261,0298155	29,00331283
ENSG00000121769	FABP3	11,04609435	15,15812476	25,73889805	86,2153571	0,350017098	25,38787577	24,58044516	49,27533024	22,82325712	260,5753997	28,95282218
ENSG00000182541	LIMK2	0,073473535	22,55521848	59,56072785	41,02219212	65,36288494	27,07811195	17,93595346	8,943690887	18,01235337	260,5446066	28,94940073
ENSG00000134077	THUMPD3	16,96963628	33,4921444	36,57912303	75,49971095	0,137172476	24,65210632	23,29204788	23,09760346	26,62049116	260,3400359	28,92667066
ENSG00000087263	OGFOD1	56,58187748	30,15789288	7,852004174	13,5725736	7,979015323	32,97637022	32,84789952	38,58674419	39,75566322	260,3100406	28,92333785
ENSG00000083520	DIS3	27,26545137	11,38397047	0,025655754	24,73283054	133,1071574	20,31964331	19,32717897	5,214980253	18,81620996	260,193078	28,910342
ENSG00000018699	TTC27	0	16,04334941	1,049590847	77,45441302	72,80396299	31,0811275	43,9856784	0,095972863	17,54287532	260,0569703	28,89521893
ENSG00000105656	ELL	30,41876067	37,40248314	37,30517798	19,89642446	0,146042108	26,54974052	27,65551848	27,14427923	53,3203422	259,8387688	28,87097431
ENSG00000170027	YWHAG	5,709981078	26,27140244	23,33382207	66,20212581	61,35530481	23,37525534	18,6596904	24,79374083	10,08236194	259,7836847	28,86485386
ENSG00000169410	PTPN9	13,14689974	5,839978686	52,26618404	39,78109668	35,11297564	23,37201822	31,04780227	29,13122172	30,07733916	259,7755162	28,86394624
ENSG00000176624	MEX3C	20,94582891	9,266320074	14,39172662	5,357119473	176,9027118	11,67847977	7,055906199	7,963801742	6,161705661	259,7236003	28,85817781
ENSG00000120948	TARDBP	26,43377138	23,27636865	17,49108842	24,49891872	36,49736841	24,81416631	36,06419525	32,00387509	38,54072995	259,6204822	28,84672024
ENSG00000185950	IRS2	5,891755943	24,92050467	61,48554421	37,30384752	63,09654711	17,55527559	18,96823435	11,18194466	19,18621527	259,5898693	28,84331881
ENSG00000182670	TTC3	17,4771843	18,9926179	22,01718595	43,99571	28,37578012	15,56423593	27,56982048	47,60021976	37,99305281	259,5858073	28,84286747
ENSG00000165282	PIGO	27,58517284	9,410448886	27,41626444	26,13331276	59,67857064	33,50891936	25,32380816	21,58894178	28,84758752	259,4930264	28,83255849
ENSG00000189369	GSPT2	47,18834737	19,90490222	0,892869697	44,60665697	14,73894267	33,6913341	14,10533711	55,77999659	28,52034425	259,428731	28,82541455
ENSG00000082516	GEMIN5	65,32774494	45,75137245	34,96914782	6,426286582	0,063628197	27,66156142	29,38904658	41,86050953	7,771045226	259,2203427	28,8022603
ENSG00000117222	RBBP5	37,26296089	30,68655042	4,860233338	17,24161617	0,489260957	52,47807297	22,13679563	46,36888345	47,64321609	259,1675899	28,79639888
ENSG00000161999	JMJD8	15,12255711	14,69271233	25,80941558	13,10632293	87,31967639	27,08357588	24,01505974	26,40458873	25,59986342	259,1537721	28,79486357
ENSG00000204161	TMEM273	36,57733435	25,62404755	29,53075027	0,379725891	0	50,62224242	44,4912989	34,46200922	37,45778802	259,1451966	28,79391074
ENSG00000185222	TCEAL9	16,15214306	45,36951775	9,998271037	28,55215528	72,58758835	13,19552206	10,86901052	40,1744155	22,12458598	259,0232095	28,78035662
ENSG00000166681	BEX3	23,64600596	31,03495371	1,029555922	135,7828294	3,325162429	5,683973461	15,91614397	42,52815267	0	258,9467775	28,77186417
ENSG00000184436	THAP7	46,13622494	21,2352817	29,64219881	7,975782482	65,86108453	19,19007365	21,35479774	13,54334108	33,82548721	258,7642721	28,75158579
ENSG00000188486	H2AFX	28,36834133	28,40909666	38,75187253	15,84363538	0	55,92029351	45,86585866	8,803399259	36,73174759	258,6942449	28,74380499
ENSG00000161179	YDJC	27,31017725	18,95644698	0	0,148765664	66,13167969	38,30888459	35,98151482	21,8531413	49,90519498	258,5958053	28,73286725
ENSG00000112146	FBXO9	33,55750424	33,46848335	56,11357216	19,46464962	0,04398794	32,37329112	22,13097478	31,20591028	30,07144242	258,4298159	28,71442399
ENSG00000155903	RASA2	18,83524049	13,02007709	4,823949657	1,044959017	73,22570278	40,88935788	20,60116915	50,67067705	35,16601973	258,2771529	28,69746143
ENSG00000176108	CHMP6	30,96411127	39,87947593	22,18065946	7,145528105	0	39,55847084	31,93036305	45,55566275	40,97749098	258,1917624	28,6879736
ENSG00000102038	SMARCA1	0,069269113	0,430207197	0,127426128	0,17941896	143,1366963	12,51079068	61,51295901	0	40,16806567	258,1348331	28,68164812
ENSG00000146802	TMEM168	49,42515851	43,96925105	0,834461836	62,10872504	0,089835533	15,16955992	20,3075184	36,32920386	29,8935119	258,127226	28,68080289
ENSG00000168298	HIST1H1E	21,64448492	8,179397881	65,6239491	106,9348901	11,4307971	19,36356863	22,96406607	0	1,97184639	258,1130002	28,67922225
ENSG00000164896	FASTK	27,04501935	25,65142918	19,50609367	22,83609633	53,23056344	26,03117327	21,73060415	18,49738841	43,47001441	257,9983822	28,66648691
ENSG00000108848	LUC7L3	14,22697851	24,52035989	65,12168985	30,72464426	0,30897527	26,26509621	19,98642457	42,4821236	34,21805437	257,8543465	28,65048295
ENSG00000185000	DGAT1	14,63186354	16,74670444	20,59111844	31,203553	49,02984601	33,25440753	42,8864945	19,91726151	29,19738392	257,4586329	28,60651477
ENSG00000027847	B4GALT7	8,209163843	8,575266915	23,42144745	30,54404889	85,17814606	23,43427375	23,71538461	32,17638778	22,17357031	257,4276896	28,60307662
ENSG00000072756	TRNT1	33,23286594	19,22562427	49,58797009	44,25370821	55,02507672	13,86877451	12,73733636	6,036894891	23,44702763	257,4152786	28,60169762
ENSG00000175115	PACS1	25,85025762	10,75506543	62,54171432	37,88918809	17,55248481	26,67296614	27,76160003	5,721497169	42,6479581	257,3927317	28,59919241
ENSG00000123179	EBPL	73,23050732	26,12665642	0,178192371	41,98376835	0,230203553	20,41976329	31,5284424	48,92973404	14,65328349	257,2805512	28,58672791
ENSG00000161542	PRPSAP1	16,61584744	68,39765675	0,120181625	30,79777262	35,81342258	33,425996	24,01276854	27,33382933	20,64961359	257,1670885	28,57412094
ENSG00000105640	RPL18A	25,00484107	31,24357115	17,3488868	45,64307744	23,23520314	30,44622951	31,21492084	25,44715106	27,56040611	257,1442871	28,57158746
ENSG00000119878	CRIPT	36,93682828	87,321526	0,976892187	28,22295508	0	21,37855928	16,51054349	36,25619425	29,46258807	257,0660866	28,56289852
ENSG00000204264	PSMB8	46,66142622	25,86529567	20,86437387	33,21261378	11,78610656	26,42069115	34,48285665	32,06271488	25,61754288	256,9736217	28,55262463
ENSG00000184281	TSSC4	42,16774856	18,75683039	37,84460167	36,40724918	0	30,57396863	31,70968902	17,52210381	41,79624683	256,7784381	28,53093757

ENSG00000103549	RNF40	6,648587119	15,74404201	52,62998961	18,36105371	39,58385911	33,21772393	30,75686967	16,21018881	43,61965746	256,7719714	28,53021905
ENSG00000127922	SEM1	25,22454957	34,6787652	19,3787693	47,8267238	22,12839164	19,03134379	27,78037638	36,7245381	23,98697933	256,7604371	28,52893746
ENSG00000138660	AP1AR	26,45751281	4,048601627	30,91358244	0,151530183	70,90590267	30,78305996	24,04483714	37,89827357	31,35215721	256,5554576	28,50616196
ENSG00000131748	STARD3	24,81497922	13,92945455	51,44436897	28,53697603	0	40,23019623	45,38972878	23,32014292	28,86782104	256,5336678	28,50374086
ENSG00000100811	YY1	23,70534942	28,59638431	22,63195059	61,53810463	23,8265114	25,85267017	17,54327618	20,72694344	32,10688079	256,5280709	28,50311899
ENSG00000107771	CCSER2	22,96077047	24,92345514	32,23961974	39,3075122	6,991367159	35,29341803	20,06274183	36,08734593	38,65184081	256,5180713	28,50200792
ENSG00000163832	ELP6	28,61100878	63,33868936	36,19532539	15,0293429	0	15,85909924	27,17755047	46,87230538	23,28775356	256,3710751	28,48567501
ENSG00000065000	AP3D1	9,368750114	19,11519657	11,29606811	28,32627438	32,73043896	38,66045831	47,16973587	29,84768298	39,85353845	256,3681437	28,4853493
ENSG00000177666	PNPLA2	21,81261957	21,97770505	43,39130754	14,50773939	54,2753999	26,67734671	34,35614155	11,38537273	27,84544604	256,2290785	28,46989761
ENSG00000169981	ZNF35	38,48828305	15,5356915	2,61456075	88,74537887	0,13510818	28,83773004	28,74771715	11,68579735	41,39245342	256,1827203	28,4647467
ENSG00000070831	CDC42	25,43408576	24,16542414	16,71650893	39,5485234	33,30324459	34,85220138	30,6012698	34,47707678	16,96139925	256,059734	28,45108156
ENSG00000123130	ACOT9	28,70229609	30,0178184	22,57141419	22,81718388	0,05751402	39,56451773	47,88529807	24,34335379	39,79449076	255,7538869	28,41709855
ENSG00000104976	SNAPC2	27,19684689	30,90817797	25,71807791	16,75366116	0,544667658	53,84964117	50,8097755	23,38768425	26,49581359	255,6643461	28,40714957
ENSG00000126653	NSRP1	30,40447812	32,91797338	39,15782917	49,4614569	21,81904336	15,6037413	25,40212922	23,9265979	16,78264475	255,4758941	28,38621045
ENSG00000176623	RMDN1	35,00147584	39,84458755	22,03012312	31,72390271	3,428175386	45,57161914	19,81943105	22,02103566	35,98433203	255,4246825	28,38052028
ENSG00000134291	TMEM106C	0,043485056	43,828678	0	4,505352079	71,2034494	43,16062197	31,44882278	24,6280728	36,52753229	255,3460144	28,37177938
ENSG00000196591	HDAC2	44,23036856	21,47514355	37,60064732	14,05675921	34,5462972	24,12969321	16,81611767	36,31267503	26,12008449	255,2877863	28,36530958
ENSG00000134759	ELP2	20,79478197	37,14227343	25,51083187	47,89312825	39,67826734	18,69119155	27,87347249	15,7748148	21,86133142	255,2200931	28,35778812
ENSG00000065989	PDE4A	32,17971336	7,336035199	1,521038696	14,85654617	87,52228775	24,3149041	26,05151525	25,70372412	35,70166111	255,1874258	28,35415842
ENSG00000112297	CRYBG1	12,18521328	4,102070963	0,025617925	51,14816063	127,2515851	12,61415775	17,09014383	8,831077742	21,92273049	255,1707577	28,35230642
ENSG00000106392	C1GALT1	0,232328872	45,58929329	19,62421967	37,97849214	51,53256213	17,60076329	17,45813972	40,76982556	24,16838182	254,9540065	28,32822295
ENSG00000123737	EXOSC9	3,528372484	49,12047253	0,118013203	46,13857649	27,06150022	23,03237469	25,24854059	36,05250853	44,61736716	254,9177259	28,32419177
ENSG00000198677	TTC37	35,08182083	11,87581271	3,53657482	39,46223406	65,44087605	28,24955295	30,74947299	9,229662686	31,28849045	254,9144975	28,32383306
ENSG00000163743	RCHY1	29,76176205	21,64741204	46,15358841	51,15358908	0	27,1421111	18,51953568	47,56445793	12,93706941	254,8795257	28,3199473
ENSG00000052723	SIKE1	11,20382073	22,51956058	46,55011159	63,83919806	32,41441	15,42568647	20,43629432	26,23742409	16,17564743	254,8021533	28,31135036
ENSG00000147162	OGT	18,49892332	3,616875538	19,03195037	22,77114943	84,32987046	19,86993622	28,52764037	22,184295	35,93108728	254,761728	28,30685867
ENSG00000144659	SLC25A38	82,58627939	9,604790993	42,39504464	44,9987567	0,132613568	13,17236853	20,29394587	11,47003299	29,99074233	254,644575	28,29384167
ENSG00000171425	ZNF581	18,02236164	34,76825934	0,170018409	24,09858175	69,18778339	35,82044867	13,35280597	31,52770185	27,62122625	254,5691873	28,28546525
ENSG00000085415	SEH1L	20,68921562	36,04733118	68,90490384	30,97062383	0	33,44805348	20,86583233	9,142832228	34,36414084	254,4329334	28,27032593
ENSG00000014919	COX15	36,75564754	6,355292796	0,133100359	21,23961967	67,92024383	20,97217728	34,30383508	5,300243242	60,82168924	253,801849	28,20020545
ENSG00000180776	ZDHHC20	1,979022544	30,45100216	117,3461021	41,9443375	0,386563555	25,18126083	21,2493709	4,920356854	10,33592069	253,7939371	28,19932634
ENSG00000147535	PLPP5	44,7953325	17,19194199	46,28483852	26,01716348	2,62640816	31,9525489	35,51220269	20,62195253	28,76951283	253,7719016	28,19687796
ENSG00000172409	CLP1	59,52215101	35,53247815	0,253169489	0	0	32,94035129	35,53850023	58,68373467	31,22830907	253,6986939	28,18874377
ENSG00000036257	CUL3	0,262686781	16,22136741	47,30856443	56,58694147	30,7146164	24,02494748	21,58242391	18,92710821	38,04159582	253,6702519	28,18558355
ENSG00000172037	LAMB2	0,066035209	2,109201457	0,080984734	31,50984617	115,9743032	17,23149908	39,58758035	39,22857575	7,823691906	253,6117178	28,17907976
ENSG00000100461	RBM23	38,22487478	25,96896563	17,90158532	10,25354236	53,39894992	25,62846307	28,53550768	25,96911235	27,6741933	253,5551944	28,17279938
ENSG00000204070	SYS1	27,07397724	28,02083901	0,648620231	34,35643832	32,55999051	36,03024691	31,86946433	22,35940574	40,51454186	253,4335241	28,15928046
ENSG00000046651	OFD1	8,962701931	40,40779754	53,22223302	22,53437667	0	32,02176703	33,86438443	38,04849348	24,30797828	253,3697324	28,15219249
ENSG00000119820	YIPF4	22,1869185	14,96372139	40,72377788	52,12729904	14,05484948	26,89489208	21,46702142	36,50196383	24,31083276	253,2312764	28,13680849
ENSG00000103254	FAM173A	12,01297424	21,03624478	0	16,10372751	65,36239788	30,54420659	44,09835936	28,86117823	35,08922309	253,1083117	28,12314574
ENSG00000163781	TOPBP1	3,020556314	2,49639321	58,45802739	123,5011237	0,049167243	13,76530798	15,09972917	4,704982889	31,83105139	252,9263392	28,10292658
ENSG00000148926	ADM	58,03945789	69,88991486	32,40112121	0	0,463377474	19,40975349	21,10060207	36,9518226	14,62791982	252,8839694	28,09821883
ENSG00000186591	UBE2H	21,74988377	27,79143596	65,82951752	35,10965751	0,165670095	26,45690728	29,44298426	17,37792128	28,89294695	252,8169246	28,0907694
ENSG00000100949	RABGGTA	58,56866688	35,70184035	0,235244089	12,66031471	1,215630609	40,38951416	31,21716018	17,71017428	55,09864454	252,7971898	28,08857664
ENSG00000101346	POFUT1	85,90484055	18,88735921	6,151343939	27,40691489	0	35,24016145	43,5440906	12,92489893	22,64796329	252,7075729	28,07861921
ENSG00000103591	AAGAB	66,46815083	0	0	26,54960608	0	36,74878153	42,32536897	36,72453059	43,87975545	252,6961935	28,07735483
ENSG00000140750	ARHGAP17	14,93862957	27,02269172	48,81423731	17,75928233	31,54925395	32,1500956	23,33801389	24,39136311	32,65403905	252,6176065	28,06862295
ENSG00000160214	RRP1	32,88103455	17,22012845	43,55086942	30,93653898	0	40,35557194	27,98068095	14,45796979	45,20472925	252,5875233	28,06528037
ENSG00000088827	SIGLEC1	24,66133711	1,156077606	26,54311622	9,926514377	33,9718091	5,343810104	32,25061666	118,7067119	0	252,559993	28,06222145
ENSG00000095261	PSMD5	48,70988616	68,33079839	1,390216453	9,846602815	0	21,42241591	38,19158321	29,06982897	35,48785604	252,4491879	28,04990977
ENSG00000213341	CHUK	3,918411147	8,040764018	58,18937318	0,907205474	74,91369854	17,23278046	27,17342407	33,50777883	28,53792751	252,4213632	28,04681814
ENSG00000152270	PDE3B	0,259952814	34,54986377	82,17939104	16,69840195	8,7107472	28,38432282	25,02715465	33,82222048	22,66732914	252,2993839	28,03326487
ENSG00000141127	PRPSAP2	34,53773499	20,27774314	28,53788088	22,04773761	54,16348231	24,63775569	24,59578963	18,98567857	24,45762217	252,241425	28,026825
ENSG00000196363	WDR5	59,08677315	32,58490549	4,136380641	16,46572311	0,296873141	43,47780613	30,04511395	26,31457427	39,7913331	252,199483	28,02216478
ENSG00000129219	PLD2	20,01464512	23,75098842	37,96913604	50,22134238	0	43,67513526	23,78535267	25,71262363	27,03851514	252,1677387	28,01863763
ENSG00000143622	RIT1	21,10608268	30,9445696	61,81924935	0	0,088934508	42,56645801	30,91703327	19,63949881	45,05788641	252,1397126	28,01552363
ENSG00000122873	CISD1	30,2440699	18,5059862	0	8,056473971	66,91874291	34,17046749	24,61096232	42,24953424	27,34029841	252,0965354	28,01072616
ENSG00000125676	THOC2	42,99380253	60,03646416	9,385919465	18,28093624	2,900336286	22,8758672	32,68308638	25,9473286	36,76422884	251,8679697	27,98532997
ENSG00000123992	DNPEP	30,64813594	41,86016496	46,08632821	5,819294616	31,96017682	18,18628455	31,12340026	17,72792231	28,38724493	251,7989526	27,9776614
ENSG00000174837	ADGRE1	70,48022031	32,62261722	0	20,06109192	0,096875517	23,81020416	56,69350043	0	47,92804837	251,6925579	27,96583977
ENSG00000112282	MED23	6,043608872	15,23780286	131,3162116	5,938326247	26,16248188	17,51601025	17,50434473	22,78245335	9,184712318	251,6859521	27,96510579
ENSG00000219545	UMAD1	11,31718293	27,37163807	0	53,13281466	84,41942227	30,15751954	6,587861248	19,46323012	19,02053959	251,4702084	27,94113427
ENSG00000087076	HSD17B14	2,717816019	9,645395687	0	20,41487661	96,56128162	42,07444481	38,81456314	13,37190246	27,74327581	251,3435562	27,9270618
ENSG00000115282	TTC31	26,22334991	17,94982765	0	4,354038642	74,37554126	26,08841224	25,34530264	38,3366371	38,56297095	251,2360804	27,91512004
ENSG00000181929	PRKAG1	20,51405453	36,91438625	27,10704598	40,72431606	0	47,46533468	35,56592107	28,27332103	14,52481185	251,0891914	27,89879905
ENSG00000164631	ZNF12	0	5,291281716	3,134524702	148,2930583	0,067490611	16,52557896	20,39688727	50,36317126	6,985398319	251,0573911	27,89526568
ENSG00000117360	PRPF3	18,13438616	30,04697708	0,051401646	44,34157575	50,6005117	21,32171797	23,34008025	15,03086235	48,11115591	250,9786688	27,88651876
ENSG00000091106	NLRC4	56,20770699	9,627062305	89,16204336	46,22158605	0	12,83310427	22,52371324	0	14,32607827	250,9012945	27,87792161
ENSG00000124786	SLC35B3	0,106867447	20,48043565	7,07728682	55,63783228	0	29,15755185	16,90388843	76,76644656	44,68728258	250,8175916	27,86862129
ENSG00000090776	EFNB1	20,99901663	19,72706813	39,21850624	21,40883382	20,0053369	46,06115617	53,21947779	1,900774079	28,13298854	250,6731583	27,85257314
ENSG00000008838	MED24	59,493547	15,25197243	52,34586466	8,882733263	0,051582525	31,48059061	39,54063608	8,211028466	35,34340642	250,6013615	27,84459572
ENSG00000168818	STX18	42,85562455	18,89877249	3,481896921	5,609931217	80,02768938	25,74420988	16,27900737	14,57890408	43,08276082	250,5587967	27,8398663
ENSG00000100462	PRMT5	0,046126548	16,69748718	32,66860138	18,39926103	0	39,80638379	46,72582571	65,86500995	30,18020447	250,3889001	27,8209889
ENSG00000138814	PPP3CA	44,84328656	19,39181547	60,84314079	12,51366654	4,822219061	23,32868842	17,57017885	50,32899417	16,64882523	250,2908151	27,81009056
ENSG00000235162	C12orf75	8,921864365	17,02646937	30,86373917	40,88917909	22,00311715	60,00627616	19,89447893	0	50,65404655	250,2591708	27,80657453
ENSG00000099968	BCL2L13	24,25815739	16,44828244	71,76254709	22,06811062	0,234349741	27,56514802	21,79600782	8,625282368	57,48580759	250,2436931	27,80485479
ENSG00000213995	NAXD	43,41708704	14,12760801	0,195279311	69,47267676	0	43,12392932	32,08373786	30,87305759	16,8417291	250,135105	27,79278944
ENSG00000118246	FASTKD2	15,62358498	20,22261626	94,71091599	37,99452802	0,073597201	17,93581908	15,41540404	24,9205699	23,15702132	250,0540568	27,78378409
ENSG00000165156	ZHX1	25,24868439	8,442908662	1,114727859	78,94319668	0	30,77711426	31,73562682	34,74642415	39,02970184	250,0383847	27,78204274
ENSG00000064490	RFXANK	18,41339355	20,35436677	57,19526607	14,49059587	17,6474062	29,23154787	36,14227302	32,4757905	24,05684108	250,0074809	27,77860899
ENSG00000114054	PCCB	16,28398788	29,25315498	80,96939666	27,5272826	0,047895111	29,87186964	20,61609083	27,23507105	18,09388654	249,8986353	27,76651503
ENSG00000108010	GLRX3	16,59915692	8,268013107	22,17840836	37,01062658	0,138414932	40,7022864	25,50996343	62,66093938	36,7466818	249,8144909	27,75716566
ENSG00000100372	SLC25A17	46,1021721	24,80431136	51,49844401	13,08272623	0	27,57551074	17,58283168	61,13329427	7,966847658	249,746138	27,74957089
ENSG00000163125	RPRD2	15,71978814	7,763781875	86,49895733	35,89423661	0,24659021	26,98088001	10,72629183	34,90399471	30,92479325	249,659314	27,73992377
ENSG00000137563	GGH	0	32,68132153	32,37236596	22,55459636	91,56523232	21,6613809	21,59995589	0,956049382	26,14621959	249,5371219	27,72634688
ENSG00000105639	JAK3	1,108987015	15,38874534	39,65898374	46,99357314	3,373480509	57,32474645	44,73322126	0,727508955	40,20809832	249,5173447	27,72414941
ENSG00000166037	CEP57	15,06636868	28,62295959	30,41691831	28,14761168	17,90277566	13,97190887	22,12207515	70,12602355	23,13594222	249,5125837	27,72362041
ENSG00000163378	EOGT	57,17697268	14,09432321	0	109,0430966	0	31,63588443	6,337830377	0	31,17649022	249,4645975	27,71828861
ENSG00000067113	PLPP1	80,27993108	21,47907494	0	20,49721764	0,185590461	31,81024494	32,48611697	14,00291191	48,69467018	249,4357581	27,71508424
ENSG00000187866	FAM122A	23,35195048	27,95710034	0,182282032	16,54014047	63,58146652	29,37433161	28,76904443	10,83392025	48,78725098	249,3774871	27,70860968
ENSG00000107036	RIC1	1,143311267	16,19901037	59,69890381	2,186860455	105,0474199	22,17033431	12,07821727	25,42396102	5,386542201	249,3345605	27,70384006
ENSG00000032219	ARID4A	51,99417627	33,09257771	0,661157741	15,34303167	0,047452107	38,27693802	26,36070664	21,39441571	62,10436771	249,2748236	27,69720262
ENSG00000088451	TGDS	0,058775018	38,48479114	30,92271813	14,00584461	0	21,94072193	24,6450866	58,0927361	61,08141242	249,232086	27,69245399
ENSG00000197535	MYO5A	33,39138727	4,459398831	12,3708421	37,7700764	89,2263866	15,18845671	14,59698735	26,58639691	15,61122234	249,2011545	27,68901717
ENSG00000145715	RASA1	19,49534312	1,332509939	18,22384823	54,46119883	77,01259222	13,19166665	21,89495664	20,21006529	23,12425191	248,9464328	27,66071476
ENSG00000144635	DYNC1LI1	25,48584956	47,11017636	1,182313524	30,47727333	0,058746531	49,34163724	28,71470546	29,89208504	36,5430263	248,8058133	27,64509037
ENSG00000101421	CHMP4B	14,24325604	15,56629589	21,34304131	26,71244434	62,09460626	24,95908176	17,07399025	57,75385699	9,048707077	248,7952799	27,64391999
ENSG00000112561	TFEB	44,6000923	22,07613846	1,870262706	10,81069123	0	37,42388008	49,31730472	18,78000995	63,86828961	248,7466691	27,63851878
ENSG00000196396	PTPN1	15,48126114	26,21036625	30,40514817	37,90369465	26,21620267	27,75440481	39,5568431	21,34209902	23,8229395	248,6929593	27,63255104
ENSG00000110066	KMT5B	15,54520236	39,73049329	4,637295442	15,59805919	39,06526555	19,39339341	20,53864448	69,66993893	24,47955234	248,657845	27,62864944
ENSG00000058804	NDC1	33,45562423	16,92930424	8,186278339	37,42647835	15,74943459	23,26966139	29,00172293	49,98522907	34,53122882	248,534962	27,61499577
ENSG00000135775	COG2	32,01401547	30,98845515	38,01719424	56,56849029	0	14,1871277	15,4631352	21,61439342	39,5259185	248,37873	27,59763666
ENSG00000070882	OSBPL3	2,110952464	14,02201336	34,09027793	66,41922521	8,080033713	50,49719865	34,37288363	14,58340223	24,03207594	248,2080631	27,57867368
ENSG00000165733	BMS1	31,14135874	41,85074908	37,86892185	16,61320756	33,37076273	14,60457841	14,81258205	16,65161953	41,29390442	248,2076844	27,5786316
ENSG00000185947	ZNF267	9,594703643	47,4070277	1,371321303	38,49247636	7,42922987	18,16462075	34,86174406	33,70600856	57,16762055	248,1947528	27,57719476
ENSG00000166928	MS4A14	93,54823146	20,88375038	2,636855657	74,84130949	0	10,51954231	22,51951291	21,19862303	1,948465785	248,096291	27,56625456
ENSG00000205981	DNAJC19	34,72256281	48,00534413	5,426270755	27,06854728	22,23203971	34,42204938	23,89744472	30,40808904	21,71491713	247,8972649	27,54414055
ENSG00000134313	KIDINS220	0,009836647	18,03090002	14,1324344	8,764643073	68,61150806	24,00854134	44,1294235	31,554974	38,65249568	247,8947567	27,54386186
ENSG00000138061	CYP1B1	19,31488497	7,930014038	116,626237	0,305187105	55,80272588	1,081121552	0	46,81846504	0	247,8786356	27,54207062
ENSG00000063601	MTMR1	76,70150964	14,74611505	9,75214633	27,33879396	0,05675056	35,29008158	26,23218224	12,74115589	44,81697108	247,6757063	27,51952292
ENSG00000114120	SLC25A36	26,17490234	18,37052422	10,33152624	14,02794526	80,11753808	20,43090945	18,39744509	21,80451295	37,83658454	247,4918882	27,49909868
ENSG00000113649	TCERG1	35,80942488	16,67810007	10,52713251	19,16437729	15,33070068	23,34247958	18,31515519	49,63723306	58,60755603	247,4121593	27,49023992
ENSG00000125351	UPF3B	25,65103163	46,82258879	2,796943547	13,4659724	64,92322983	10,554519	26,26691468	20,37733458	36,49371827	247,3522527	27,48358363
ENSG00000082146	STRADB	12,65200245	23,29467801	27,64926386	45,09074632	54,93582801	19,531179	15,0074681	41,91537007	7,195191136	247,271727	27,47463633
ENSG00000155329	ZCCHC10	47,35860792	35,9408777	3,367480683	37,21815777	0	28,52702374	21,72073352	34,47676027	38,63632272	247,2459643	27,47177381
ENSG00000127914	AKAP9	26,18088141	20,58553081	55,50442833	7,881067327	72,59262347	11,27025719	11,10009362	22,08340849	19,99304432	247,191335	27,46570389
ENSG00000138381	ASNSD1	33,68823185	48,12528452	0,335589657	63,84244047	0	31,24593557	26,35634338	13,69608067	29,7653585	247,0552646	27,45058496

ENSG00000156467	UQCRB	13,81226832	35,24625158	21,14537243	38,46205699	17,15188262	28,22956081	20,78041834	52,61783592	19,53157995	246,977227	27,44191411
ENSG00000155100	OTUD6B	36,13431046	3,2363128	66,54187936	42,97520955	10,38433929	13,01586621	20,91698017	16,70036411	37,01042133	246,9156833	27,43507592
ENSG00000157326	DHRS4	45,4662774	12,92849699	15,59369123	14,75849184	66,26276396	26,99796589	13,6696931	34,92771506	16,28418763	246,8892831	27,43214257
ENSG00000160445	ZER1	0	19,69647186	82,69430862	31,58686535	0	30,7902695	44,44578351	3,705101558	33,85213541	246,7709358	27,41899287
ENSG00000163428	LRRC58	48,85615823	13,10570271	51,04610557	56,97860206	12,336445	9,156721649	18,08053155	25,83215424	11,14891362	246,5413346	27,39348163
ENSG00000162636	FAM102B	32,38776295	3,928925563	34,16033516	55,42426305	7,758475247	30,94631506	24,46335992	32,44266663	25,02468145	246,536785	27,39297611
ENSG00000113658	SMAD5	63,28590935	24,76754094	13,74525489	58,10421503	0,119176176	16,53599885	12,28332343	28,91302466	28,74042457	246,4948679	27,38831866
ENSG00000135776	ABCB10	9,594883939	15,14110169	0,130262464	55,0849138	89,94766935	14,14982425	13,55244214	37,23969704	11,62629368	246,4670884	27,38523204
ENSG00000143376	SNX27	28,09529909	18,96618061	26,08298697	22,85972131	19,1260016	28,41458144	32,09025187	26,10098912	44,70725986	246,4432719	27,38258576
ENSG00000165240	ATP7A	19,22093248	18,7110077	27,13615252	0,071685433	79,74908793	27,39347709	12,88024196	36,88642288	24,38810132	246,4371093	27,38190103
ENSG00000162613	FUBP1	21,36243683	29,90923827	26,59110724	44,67118344	23,89468999	24,68454221	27,30408037	30,15084211	17,81056505	246,3786855	27,3754095
ENSG00000142687	KIAA0319L	34,07410622	15,08107101	0,086697193	5,913694195	36,40083208	25,63084697	44,76704225	44,10832568	40,24517163	246,3077872	27,36753191
ENSG00000089009	AC004086.1	19,43675431	22,55118238	13,5645787	59,73280957	30,6869049	24,47432838	20,39915674	30,68798659	24,74053096	246,2742325	27,36380362
ENSG00000119844	AFTPH	58,69389254	37,05725231	6,459048598	6,198732707	0,062271119	35,5008933	35,04988813	17,70492134	49,49891767	246,2258177	27,35842419
ENSG00000137200	CMTR1	25,06098357	0,457406748	29,87390374	61,27718604	3,743643827	28,14442118	37,12266646	0,814186766	59,7061894	246,2005877	27,35562086
ENSG00000131375	CAPN7	86,16758953	26,64169979	1,589593132	48,35410448	0,064173971	20,4275174	19,75328136	17,8916501	25,14042483	246,0300346	27,33667051
ENSG00000259956	RBM15B	60,72710433	22,95237229	9,190465229	31,98659411	0	28,30298183	33,83001403	20,70204492	38,29717737	245,9887541	27,33208379
ENSG00000140332	TLE3	15,45313102	15,87543265	40,11063166	27,51032026	49,57266059	25,38429197	32,39545852	7,454001246	32,09728602	245,8532139	27,31702377
ENSG00000254685	FPGT	0	29,31600336	54,93099564	72,22530278	0	28,81604753	21,55208095	6,482209219	32,50091095	245,8235504	27,31372783
ENSG00000166847	DCTN5	24,50795899	20,78243208	30,42153886	43,43087057	45,19053833	20,31531385	19,4195586	22,9790498	18,77074785	245,8180089	27,3131121
ENSG00000163374	YY1AP1	42,99746892	42,51881745	17,7871689	25,04474861	0	33,10912928	21,04971177	33,49936527	29,76665954	245,7730697	27,30811886
ENSG00000101363	MANBAL	56,23234439	1,966811737	33,43917083	0,127596565	0	56,95316618	34,78810672	33,1242476	29,0811184	245,7125624	27,30139582
ENSG00000150433	TMEM218	16,01928063	46,81609035	13,94000496	29,48988559	29,66162592	27,89646865	37,03502978	16,93599	27,82215636	245,6165322	27,2907258
ENSG00000137814	HAUS2	18,81423133	13,08009757	108,673753	16,30464766	0,166837418	15,80100731	23,19962469	19,64953526	29,70090762	245,3906418	27,26562687
ENSG00000243943	ZNF512	46,56953041	6,703550002	0	29,77030847	72,55806708	22,39792785	5,418037064	46,63633328	15,29010024	245,3438544	27,26042827
ENSG00000143756	FBXO28	10,66810507	2,316176745	61,69126449	72,1255478	27,44087533	29,68200849	14,38115101	15,97283375	10,89764491	245,1756076	27,24173418
ENSG00000141497	ZMYND15	31,95652527	33,31577792	14,51718164	14,89987373	38,95163013	32,51561661	35,06688762	3,413384342	40,47561274	245,11249	27,23472111
ENSG00000163993	S100P	23,09970507	0	221,3271349	0,554002069	0	0	0	0	0	244,975442	27,21949356
ENSG00000165775	FUNDC2	30,19516753	37,5090652	40,4274604	27,14302787	10,35300226	23,42208954	21,51665789	29,52763819	24,85563751	244,9497464	27,21663849
ENSG00000035928	RFC1	8,276334277	48,1689826	34,1734725	47,88403389	0,106896128	28,96459391	25,69536789	25,86822184	25,80663868	244,9445417	27,21606019
ENSG00000074706	IPCEF1	0,481471018	26,01047015	39,23344328	94,96382602	1,822286325	31,34562583	18,28603493	19,88690458	12,85178924	244,8818514	27,2090946
ENSG00000163110	PDLIM5	36,12210305	9,69566655	62,31366331	37,50499094	0,032382105	22,3956838	27,20975189	36,02302905	13,49021194	244,7874826	27,19860918
ENSG00000137474	MYO7A	49,29489939	17,35734737	35,11327566	22,38419064	0,035328829	36,28324762	36,5363682	25,64809383	22,12240944	244,775161	27,19724011
ENSG00000099337	KCNK6	8,501194556	27,38804476	11,31209547	28,17488539	52,05108347	23,6707697	32,60845366	22,39326349	38,65776396	244,7575545	27,19528383
ENSG00000103043	VAC14	31,82909059	18,2109733	48,23920509	28,09583835	22,08952617	30,65586366	21,27309677	4,033098649	40,15632409	244,5830167	27,17589074
ENSG00000114796	KLHL24	19,35612859	29,79011122	0,096409283	15,29410776	57,83245509	31,01472862	20,9600728	31,09522982	38,84514593	244,2843891	27,1427099
ENSG00000163904	SENP2	18,44731974	6,078690793	70,96281603	33,36087819	69,12330053	18,22166289	13,19717516	7,537369746	7,327094655	244,2563077	27,13958975
ENSG00000090621	PABPC4	20,3872268	54,40333119	13,60799069	48,13176839	9,690137165	30,57921275	23,07011265	17,6861911	26,55341788	244,1093886	27,1232654
ENSG00000186501	TMEM222	0,641443441	27,37732217	55,33518056	22,85221404	0,481390807	52,22698896	28,45227591	37,20710948	19,51471525	244,0886406	27,12096007
ENSG00000111247	RAD51AP1	0	81,13444412	0	74,97545205	0	26,78696958	14,96030151	19,63489221	26,59101921	244,0830787	27,12034208
ENSG00000159200	RCAN1	10,22014342	6,776707642	2,474837182	75,7625673	51,77398227	30,327996	32,32248357	17,8408095	16,54578458	244,0453115	27,11614572
ENSG00000198680	TUSC1	37,26215826	37,76463841	0	19,07419405	0	49,56295493	34,79172461	33,01125065	32,42351607	243,890437	27,09893744
ENSG00000103005	USB1	46,54900338	34,77897448	32,62440363	4,577806572	0,043450398	33,86462709	24,03140176	17,19139311	50,14370776	243,8047682	27,08941869
ENSG00000164603	BMT2	0,663898041	0,589035392	71,58140211	0,827960622	0	3,928075651	52,46050249	96,78337345	16,51162658	243,3458743	27,03843048
ENSG00000071794	HLTF	12,31661524	26,61834596	23,58031716	29,19491582	34,934077	39,24677487	11,2056837	20,95385206	45,25844093	243,3090227	27,03433586
ENSG00000064313	TAF2	16,02337035	18,1199237	59,0377593	60,93547369	0,275143689	28,14327435	20,91813074	23,13953377	16,68802667	243,2806362	27,03118181
ENSG00000096717	SIRT1	0,794621756	7,285190186	1,827213622	40,99263295	0,078684825	37,95149914	35,76619236	41,63012189	76,92026305	243,2464198	27,02737998
ENSG00000145824	CXCL14	16,60303485	3,258434431	2,955737761	5,152640005	209,2882487	0,504032132	1,016468512	3,178864194	1,223293564	243,1807542	27,0200838
ENSG00000114744	COMMD2	36,14922947	4,217964046	15,04897369	38,807081	0,073364156	27,42015456	18,45528887	73,30989588	29,61395043	243,0959021	27,01065579
ENSG00000128335	APOL2	17,95460687	1,961823237	3,660844148	33,60005526	107,724753	26,51073306	16,59718761	0,676921853	34,37372024	243,0606453	27,00673836
ENSG00000167842	MIS12	30,92094667	39,49509979	8,364938043	49,09577919	0	38,15365179	37,71661231	23,02679913	16,24758953	243,0214165	27,00237961
ENSG00000185187	SIGIRR	42,83483282	21,32250458	8,985054687	32,61687183	11,60763223	40,86069981	33,12617747	17,95840041	33,5438088	242,8559826	26,98399807
ENSG00000130449	ZSWIM6	18,51227723	25,26890903	14,20630444	23,9277088	54,66812896	33,04380641	24,94399315	23,11485014	25,12531816	242,8112963	26,97903293
ENSG00000162642	C1orf52	0	43,20065557	64,6181809	55,4677563	18,27089251	22,8490882	10,16236315	20,86955163	7,336046932	242,7745352	26,97494835
ENSG00000079150	FKBP7	0	42,74601365	28,42978285	48,20519498	0,349789814	9,426737313	7,903001431	97,62839495	8,085530514	242,7744455	26,97493839
ENSG00000271601	LIX1L	39,86956425	25,39656127	20,4776119	35,63256356	3,4193101	22,94729099	24,8677105	46,28236613	23,84199935	242,7349781	26,97055312
ENSG00000147586	MRPS28	12,22523604	40,28770832	31,82160737	22,11556747	11,46330075	32,14117942	31,90274815	37,82630162	22,80890174	242,5925509	26,95472787
ENSG00000164190	NIPBL	1,858856768	29,06531625	26,9564534	42,3740557	35,22616359	16,57913024	24,51477211	45,02926529	20,66445874	242,2684721	26,91871912
ENSG00000068796	KIF2A	54,15797788	17,97348974	1,048102594	42,41423131	4,212522491	27,732853	33,77238723	31,28538427	29,39131217	241,9882607	26,88758452
ENSG00000073050	XRCC1	32,45884764	26,9769922	62,20196491	9,127820736	0	37,60447879	25,1375947	7,164669721	41,26340824	241,9357769	26,88175299
ENSG00000177951	BET1L	20,78356349	23,9858149	24,29091868	40,81325243	36,48738215	39,29009521	28,99960821	1,537699642	25,70524545	241,893578	26,87706422
ENSG00000120526	NUDCD1	18,29118844	43,89764767	0,133259875	7,818042144	0	31,7345734	18,10477945	95,99393476	25,7922187	241,7656444	26,86284938
ENSG00000169857	AVEN	71,93389911	11,24560676	49,20671687	50,16887039	0	12,10894187	8,405692177	29,87589227	8,705229257	241,6508487	26,8500943
ENSG00000075945	KIFAP3	37,24641828	17,39852672	8,09704989	67,04215197	0,072140929	17,73090133	26,54083158	48,92130901	18,5174518	241,5667815	26,8407535
ENSG00000110906	KCTD10	22,55498137	34,67759365	16,79768184	12,93758783	38,76436647	22,43331731	41,42166936	16,45175721	35,45692869	241,4958837	26,83287597
ENSG00000198715	GLMP	16,80457513	25,95670182	25,70775332	25,10869534	10,76007618	28,8706391	44,71529838	38,55545948	24,89733012	241,3765289	26,81961432
ENSG00000223496	EXOSC6	29,03383422	39,05535233	19,16732815	41,94771042	4,034247038	32,52014403	22,33337887	23,93618599	29,33660966	241,3647907	26,81831008
ENSG00000183828	NUDT14	47,47837345	10,63611274	0	6,945555169	39,3604822	49,53071497	22,06863355	15,52145672	49,75953336	241,3008622	26,81120691
ENSG00000111832	RWDD1	15,17029591	36,88266475	21,51064683	37,13664629	27,48317309	30,88023688	23,88790879	26,35816901	21,98354976	241,2932913	26,8103657
ENSG00000132329	RAMP1	37,41534618	9,818625984	25,44717558	10,00971132	0	14,46474078	16,51789026	33,12988253	94,30050215	241,1038748	26,78931942
ENSG00000144228	SPOPL	1,588670193	10,4056342	5,887952004	90,64948793	70,12452937	4,79673436	6,459743438	22,83615518	28,15856398	240,9074707	26,76749674
ENSG00000183023	SLC8A1	23,09878306	11,00847551	23,57289499	60,46663949	33,22189471	25,94386721	31,15867564	9,586551635	22,72851593	240,7862982	26,75403313
ENSG00000105810	CDK6	7,050183232	9,382782874	33,01297097	131,4039587	12,74193564	8,904499141	7,788166611	19,64818921	10,79304436	240,7257307	26,74730341
ENSG00000084112	SSH1	39,26920732	17,65111391	39,08318354	3,769180401	32,13729004	32,03404793	30,76776266	5,159488707	40,83049884	240,7017734	26,74464148
ENSG00000164306	PRIMPOL	0	20,67068125	155,5851325	0	0	27,0073151	18,17497817	0,118477794	19,1244001	240,6809849	26,74233166
ENSG00000101888	NXT2	52,8689725	47,08237469	26,49157601	9,282608431	0	24,74580077	13,02205143	57,37310376	9,583469491	240,4499571	26,7166619
ENSG00000064012	CASP8	27,72690162	20,62061218	28,1319084	15,27494536	39,66506059	30,07601646	29,29315635	8,303063826	41,23365474	240,3253195	26,70281328
ENSG00000142444	TIMM29	55,11091401	0	34,88377713	29,96732201	0	38,10831174	10,68430838	43,87282493	27,69477587	240,3222341	26,70247045
ENSG00000135605	TEC	40,34395605	0,066968531	0,416553595	5,930340332	79,19600147	4,102184589	10,43497017	92,676828	7,008697518	240,1765003	26,68627781
ENSG00000231925	TAPBP	29,88737553	15,2739114	22,48691861	19,09532053	47,51772861	24,84064608	31,19387012	21,82854936	27,93579053	240,0601108	26,67334564
ENSG00000028203	VEZT	25,46090724	20,8310178	25,87631312	61,85522213	0	28,18170759	21,88502597	29,51520264	26,33143745	239,9368339	26,65964822
ENSG00000086189	DIMT1	16,01610171	36,55888331	13,76139104	58,31966564	2,914441993	19,71214773	23,99026961	46,98274325	21,59813503	239,8537793	26,65041992
ENSG00000158828	PINK1	46,93088487	24,41185186	32,43963911	16,14309071	18,42771581	23,00022479	30,32264593	24,88685756	23,26446223	239,8273729	26,64748587
ENSG00000188342	GTF2F2	22,65820918	20,21469624	10,40114007	47,44272265	38,91767158	22,67015363	23,82127977	18,59152362	35,02650641	239,7439032	26,63821146
ENSG00000166501	PRKCB	8,001254283	28,61374581	20,23542665	33,56424984	44,57385335	14,78618526	5,217555751	74,3671519	10,22608502	239,5855079	26,62061199
ENSG00000214022	REPIN1	9,361119456	28,08886944	22,30298493	14,31241526	0	41,46872233	65,25860675	13,93099059	44,85261303	239,5763218	26,61959131
ENSG00000135837	CEP350	30,74979848	16,33934493	0,097461643	36,33804499	20,85052416	36,08825531	31,89327211	34,38502897	32,76194916	239,5036797	26,61151997
ENSG00000167207	NOD2	13,67536374	30,97697484	62,00254499	33,6751524	0	21,14617564	25,79507917	12,11545337	40,10044995	239,4871941	26,60968823
ENSG00000161911	TREML1	0	10,66782064	101,7823289	42,75603427	0	38,93248867	25,38172596	0	19,92555431	239,4459527	26,60510586
ENSG00000182636	NDN	103,7662438	0	71,40244904	0,135129453	0	36,00027532	5,717964379	16,59873281	5,77462006	239,3954148	26,59949054
ENSG00000164934	DCAF13	54,55356073	13,26281227	58,35194816	30,77665479	0	11,65107431	13,63956213	25,75514566	31,35490842	239,3456665	26,59396294
ENSG00000214087	ARL16	15,12851304	39,1320054	15,62768365	20,99938764	29,73056281	18,1434747	23,57702041	44,27848652	32,70383546	239,3209696	26,59121885
ENSG00000107959	PITRM1	15,93600516	6,377929791	75,47962281	17,75958059	0	21,84723026	30,02370747	29,40247405	42,47387921	239,3004293	26,58893659
ENSG00000164167	LSM6	28,16929129	18,21341887	38,1129947	34,46310985	41,82928858	13,9824008	15,90869115	33,50066181	15,10149099	239,2813481	26,58681645
ENSG00000112578	BYSL	34,55961941	11,35139081	62,427126	39,20086609	0	13,1906189	35,10837375	15,52377163	27,82420829	239,1859749	26,57621943
ENSG00000119787	ATL2	61,56260254	10,93797832	3,731918713	30,1804308	6,181024825	33,89601627	19,7815532	27,18549454	45,70992777	239,166947	26,57410522
ENSG00000167508	MVD	20,53463553	17,45168413	2,35726181	12,83377446	0,076132614	47,51859002	56,49755887	21,57596922	60,28093684	239,1265435	26,56961594
ENSG00000112365	ZBTB24	34,14293923	23,02940896	116,4504419	19,81006602	0,065069314	12,47560959	10,52595827	11,25597005	11,31444673	239,0699101	26,56332334
ENSG00000122490	PQLC1	37,03917591	15,87885742	48,3188865	16,00214486	0,137645666	35,00719528	41,09405853	17,73125314	27,81643041	239,0256477	26,5584053
ENSG00000155506	LARP1	10,88830407	28,98154265	39,11686268	41,05246705	50,15631601	20,39677344	14,10422144	7,923626897	26,17372942	238,7938437	26,5326493
ENSG00000141179	PCTP	21,30453454	34,56660477	0,709132906	34,79132995	42,56886514	27,65176278	18,79921131	29,50308639	28,73035523	238,624883	26,51387589
ENSG00000115267	IFIH1	15,42728689	38,02130844	0	34,3456362	83,79175201	28,23043257	16,83407936	3,489823322	18,28296513	238,4232839	26,49147599
ENSG00000104299	INTS9	0	41,98431316	35,36382911	37,55761306	45,90554517	24,11707262	17,77816121	22,36580559	13,29910893	238,3714489	26,48571654
ENSG00000139636	LMBR1L	23,06516566	34,27712354	41,41889684	15,79331912	0,653264919	23,76030419	34,09091376	26,44788005	38,81662934	238,3234974	26,4803886
ENSG00000160746	ANO10	57,39172043	57,56405714	26,55191034	1,628684351	2,241512937	23,41011731	33,3195283	20,24982029	15,95882752	238,3161786	26,4795754
ENSG00000119185	ITGB1BP1	34,98307012	39,43028513	0	67,16789777	2,419929531	28,32256026	24,89490714	18,06058957	23,01513536	238,2943749	26,47715277
ENSG00000047621	C12orf4	0	26,31401623	60,53337922	29,2979989	0	32,83226335	35,59063148	0,133996175	52,82985082	238,2321362	26,47023735
ENSG00000151729	SLC25A4	41,86192402	23,70377506	15,17094128	46,51237667	30,55368175	19,27991761	19,99610188	27,0162315	14,11492574	238,2098755	26,46776395
ENSG00000141027	NCOR1	17,72635687	38,94012297	9,095168131	30,0336088	10,77072868	30,71779828	27,70219418	24,64744989	48,57297447	238,2064023	26,46737803
ENSG00000145246	ATP10D	7,057518178	18,58309906	18,4971064	35,87241647	0,045086948	44,15950064	45,74586869	35,55334895	32,68938829	238,2033336	26,46703707
ENSG00000144736	SHQ1	60,80173313	12,54976273	0,047656454	78,86651127	0,492532203	18,28913095	8,668702862	34,15944526	24,05522447	237,9306993	26,43674437
ENSG00000181904	C5orf24	41,30023237	31,19554438	36,44295446	2,12952662	3,367311562	20,77123212	26,6235016	38,50001646	37,59527669	237,9255963	26,43617736
ENSG00000159111	MRPL10	36,9732829	29,70754025	44,74165433	42,84569233	0,388795607	23,11132537	22,77574053	11,68624598	25,68719525	237,9174725	26,43527473
ENSG00000164761	TNFRSF11B	25,20514694	3,842799364	62,41272923	77,90654098	0	32,36313001	17,78162348	0,151342423	18,1259546	237,769467	26,41882967
ENSG00000060642	PIGV	38,02338724	38,69109877	58,27554411	43,82349662	0	22,41902234	21,81447523	7,327792006	7,253619438	237,6284357	26,40315953
ENSG00000121057	AKAP1	50,11550948	35,40076967	15,82508429	2,4920756	0,403502857	21,68643868	41,89393295	26,36051044	43,32938581	237,5072098	26,38968998
ENSG00000112996	MRPS30	5,599404641	42,95478642	0,134941796	15,49565302	76,70471778	32,02772136	20,93202464	24,54198487	19,03573602	237,4269706	26,38077451

ENSG00000162882	HAAO	26,10897974	15,14815408	32,83550692	14,16668241	88,65961335	13,26797715	15,18899837	13,93982952	17,58928934	236,9050309	26,32278121
ENSG00000120837	NFYB	31,06110322	31,03419352	33,81835864	41,06570023	0	18,01682523	34,19124173	17,80663928	29,43802843	236,4320903	26,27023225
ENSG00000196116	TDRD7	0,1810141	11,11369574	0	45,57297472	0	43,00796454	38,5669468	38,39500844	59,57418467	236,411789	26,26797656
ENSG00000164574	GALNT10	5,226099195	26,26238339	22,24688292	32,39312804	45,81348983	32,78377821	30,99700561	11,93165978	28,71980365	236,3742306	26,2638034
ENSG00000198700	IPO9	9,20322276	6,802661629	0,420332811	4,822386963	100,5191239	30,57861268	19,20421244	33,72631044	31,05248091	236,3293446	26,25881606
ENSG00000139410	SDSL	44,10125193	35,82987652	19,71204754	3,902066673	0	47,78032527	46,90762533	18,80910708	19,27496492	236,3172653	26,25747392
ENSG00000127445	PIN1	17,72325293	36,73017332	18,49145516	27,92061326	0	37,82906366	35,36231677	25,16219464	37,00663791	236,2257076	26,24730085
ENSG00000071189	SNX13	30,20738878	4,148377879	26,51460805	37,33318646	39,67835436	24,52582347	19,44186409	34,13430543	20,09243373	236,0763413	26,23070459
ENSG00000175701	MTLN	24,97634493	23,14048465	17,75494538	44,01925949	31,16671016	24,75311383	31,3217016	7,088796183	31,80503623	236,0263925	26,22515472
ENSG00000187912	CLEC17A	22,88940279	18,21161037	0,12420916	41,50708264	0,160463602	50,26245353	54,8862623	0,14764726	47,8318127	236,0209444	26,22454937
ENSG00000189180	ZNF33A	16,9128141	5,916731625	36,91374703	3,676606049	103,396065	14,75397791	17,77899427	22,57654761	14,03174593	235,9572295	26,21746994
ENSG00000068745	IP6K2	26,11654955	31,8966924	24,46659833	39,15880487	0,191563412	23,65936745	26,01323028	23,14922112	41,20743479	235,8594622	26,20660691
ENSG00000136738	STAM	65,31050088	26,87912543	12,38458552	35,63373503	0,3478136	19,04155287	28,2839901	27,04282901	20,92460844	235,8487409	26,20541565
ENSG00000090316	MAEA	16,09425282	16,65478261	41,15498054	28,11009094	52,94798914	17,75501641	23,1240506	21,66784049	18,16644551	235,6754491	26,18616101
ENSG00000165886	UBTD1	39,2685636	4,884189437	90,63467708	0	0	30,22050577	36,79548898	5,416960123	28,43553346	235,6559185	26,18399094
ENSG00000198918	RPL39	18,03669405	21,79913817	16,98186695	62,53610763	15,18824192	19,95916712	21,63891534	44,25462958	15,19609722	235,590858	26,176762
ENSG00000119865	CNRIP1	40,58860593	53,43190363	20,29223383	27,50554693	0	23,28733574	40,95113993	18,53339316	10,99993508	235,5900942	26,17667714
ENSG00000110514	MADD	18,9655868	32,24370692	42,43133952	21,73938289	9,271980534	10,76852058	23,69628551	22,2764877	54,18116995	235,5744604	26,17494004
ENSG00000149308	NPAT	26,26875657	13,74539336	88,52540204	0,346527191	0,047691573	21,30617897	16,29614671	31,02486577	37,93440966	235,4953718	26,16615243
ENSG00000138080	EMILIN1	9,620986308	1,693671047	0,070232466	49,97188245	108,6970227	24,22847735	22,85601551	1,5862195	16,71585698	235,4403643	26,16004048
ENSG00000108622	ICAM2	38,53482638	27,12012829	13,88614741	28,08088379	42,63344243	15,70599662	15,01155992	44,22096895	10,20064168	235,3945955	26,15495505
ENSG00000122783	CYREN	25,67260964	20,61080211	29,04659557	36,28864522	20,71961765	33,42532197	26,52774356	5,837408259	37,19724605	235,32599	26,14733223
ENSG00000154845	PPP4R1	25,3864272	16,48886976	43,14486347	16,39869291	0,044166467	24,05713565	11,77393473	53,48073925	44,43311269	235,2079421	26,13421579
ENSG00000169814	BTD	15,96493529	35,21659487	69,92341873	19,97224891	0,06149273	18,21039035	20,1311132	34,34482577	21,32143321	235,1464531	26,12738367
ENSG00000075914	EXOSC7	20,36870867	25,42778055	3,765817552	54,79104231	49,78509167	11,16309593	20,76535411	29,46977967	19,55395786	235,0906283	26,12118092
ENSG00000180596	HIST1H2BC	19,93065081	17,04019905	46,99663544	108,2535986	0	23,67309188	14,44262814	0	4,67958869	235,0163926	26,11293251
ENSG00000024048	UBR2	6,102542277	25,17622852	50,12446581	21,2821399	25,59321852	14,09489175	27,46437697	58,27460303	6,790546006	234,9030128	26,10033475
ENSG00000198960	ARMCX6	28,95931788	23,80851025	7,036057146	47,70004558	12,98536714	35,26183008	24,34799934	28,27744591	26,50683624	234,8834096	26,09815662
ENSG00000105520	PLPPR2	13,38895982	14,58273022	110,2408092	0	53,32451133	12,67266855	7,360312785	3,836392779	19,36256159	234,7689463	26,08543847
ENSG00000123064	DDX54	8,474605464	16,83830095	67,57375629	29,9889722	0,070915786	15,13639148	37,04149809	24,14312598	35,48846505	234,7560313	26,08400348
ENSG00000124459	ZNF45	3,570081633	0,060913676	36,62618296	93,65680597	0	4,522778102	9,94755699	55,62290198	30,56630671	234,573528	26,06372534
ENSG00000166822	TMEM170A	10,1427411	27,478299	0,34922123	20,69871837	0,58005346	32,24715624	42,01860006	73,00160671	27,91700272	234,4333989	26,04815543
ENSG00000023191	RNH1	37,70591945	19,96337918	27,63214165	18,78986595	22,40223345	24,45684796	31,81848669	15,81819939	35,81338597	234,4004597	26,04449552
ENSG00000110075	PPP6R3	3,602636122	5,35961132	54,39104887	62,71236716	4,626800365	20,63643898	21,99696609	10,74260484	50,30499483	234,3734686	26,04149651
ENSG00000078070	MCCC1	0,105156973	11,50690559	48,94152056	1,331609706	19,07629719	15,77901565	63,09897466	29,81652674	44,70490457	234,3609116	26,04010129
ENSG00000164611	PTTG1	45,06538031	17,37831697	0	69,75881853	0	44,43883295	29,54111614	21,58281479	6,586965346	234,352245	26,03913834
ENSG00000132612	VPS4A	25,34483144	32,26585378	13,02725622	30,57113427	32,3939714	23,88106786	26,72544598	15,31083355	34,67432472	234,1947192	26,02163547
ENSG00000162604	TM2D1	27,36755984	30,91702539	74,43375747	13,67117615	1,106779953	28,03275154	14,85354003	17,79170895	26,01042698	234,1847263	26,02052514
ENSG00000153561	RMND5A	10,71289418	35,73355101	36,71199931	29,89473385	48,55552771	21,78723996	26,89186147	12,84968405	11,00736207	234,1448536	26,01609485
ENSG00000100601	ALKBH1	43,52324129	40,21659081	0	18,97208983	0,126138933	29,19606566	39,97245912	29,36422395	32,76956592	234,1403755	26,01559728
ENSG00000174485	DENND4A	55,40578676	14,28677135	41,26655522	43,25581194	2,264979322	17,3672749	23,25624435	9,269462593	27,72914787	234,1020343	26,01133714
ENSG00000169045	HNRNPH1	9,340801098	11,87970886	25,77473703	60,25175732	23,03201116	23,03146824	35,09992832	17,98567899	27,70054177	234,0966328	26,01073698
ENSG00000137727	ARHGAP20	15,52462284	13,32242358	43,73553557	1,318633574	102,0145833	29,19969795	8,06532099	2,086955163	26,42281082	234,0581035	26,00645595
ENSG00000108239	TBC1D12	41,9642417	14,95489265	24,33982661	38,11663912	0	53,84123035	37,90759489	7,782232987	15,09113503	233,9977933	25,99975481
ENSG00000102081	FMR1	28,10768435	0,120256531	103,3815437	30,19227422	0,040264328	25,6148519	23,69010417	5,853645214	16,9614384	233,9620628	25,99578476
ENSG00000238227	TMEM250	21,32751892	13,53298827	23,24657518	27,86981536	57,64005155	32,81212697	30,33672346	9,501932986	17,55944254	233,8271752	25,98079725
ENSG00000162972	MAIP1	40,75519086	35,82931898	0	43,70339476	0	19,61504355	9,734767138	67,95883347	16,13568943	233,7322382	25,97024869
ENSG00000059378	PARP12	31,7849124	12,67114941	0,044229133	31,46996193	0	37,03620352	29,48602711	52,36480974	38,82521619	233,6825094	25,96472327
ENSG00000160908	ZNF394	3,246481892	20,33407572	19,62744167	10,90890615	87,49606362	19,67189627	11,30704653	38,02817982	23,05006222	233,6701539	25,96335043
ENSG00000143751	SDE2	12,84837809	50,84408128	20,77704276	3,337768674	0,18014424	17,35257349	56,48839878	28,26139642	43,53665709	233,6264408	25,95849342
ENSG00000166471	TMEM41B	36,62965446	19,86933474	0	31,16824965	5,87616362	32,65467639	54,81628109	15,37084594	37,23012379	233,6153297	25,95725885
ENSG00000275714	HIST1H3A	0,367665622	17,61516789	23,6722712	0	74,27005134	51,67044725	32,05446438	11,2556787	22,60906597	233,5148123	25,94609026
ENSG00000163521	GLB1L	76,9475003	0,078427025	24,63526469	12,74528832	0	30,03762708	37,8355988	51,22267353	0	233,5023797	25,94470886
ENSG00000174282	ZBTB4	33,24333983	20,81467581	12,37582632	28,85093163	22,25451176	35,68754903	41,65975146	3,664310273	34,91441514	233,4653113	25,94059014
ENSG00000161981	SNRNP25	5,037019022	28,82964641	0	46,21789419	60,79179312	32,96523877	21,03473213	6,047168556	32,49277432	233,4162665	25,93514072
ENSG00000120885	CLU	58,21959562	50,37794832	0	7,217518191	111,1114887	0,025077173	2,427479216	0,049944765	3,960755689	233,3898077	25,93220085
ENSG00000075856	SART3	20,08343973	7,829732358	17,39878058	40,28313933	24,78132052	30,47957825	17,05358185	20,63863935	54,77812251	233,3263345	25,92514828
ENSG00000198160	MIER1	13,49821246	25,11906806	90,08396535	17,00035674	3,168811869	26,53205524	21,42990198	8,467560536	27,85563733	233,1555696	25,9061744
ENSG00000128284	APOL3	18,18141081	25,85541401	0,188429147	41,34455198	58,54449682	21,56471993	20,43331453	18,98276875	28,02972459	233,1248306	25,90275895
ENSG00000169122	FAM110B	17,27239984	18,2269294	37,00402693	1,425297252	41,12088387	23,93166927	33,53152105	32,82285508	27,67206967	233,0076524	25,88973915
ENSG00000130414	NDUFA10	22,3311393	22,13730466	15,0450117	1,958391458	16,7031415	38,02263534	35,7046653	45,89757101	35,18510797	232,9849682	25,88721869
ENSG00000110011	DNAJC4	11,843365	10,69893267	38,12694624	6,228230601	49,38345969	25,77022139	27,31114102	18,36401586	45,15401788	232,8803304	25,87559226
ENSG00000116337	AMPD2	12,97827853	5,030364358	16,50035763	11,54951815	91,47681205	28,40151745	30,21365316	9,928050175	26,6662385	232,74479	25,86053222
ENSG00000116212	LRRC42	52,02281661	31,22540851	0,128456608	85,12943256	0	27,98399284	6,493843431	15,42231577	14,25624894	232,6625153	25,85139058
ENSG00000183161	FANCF	0	14,62427575	22,22334125	29,69991581	81,50703028	9,61067365	10,370854	43,43110169	21,1532627	232,6204551	25,84671724
ENSG00000116691	MIIP	18,74820976	31,46224815	19,11667466	31,449093	0	33,64085426	30,51139587	30,45479885	37,22200818	232,6052827	25,84503141
ENSG00000100079	LGALS2	0	0	232,595593	0	0	0	0	0	0	232,595593	25,84395478
ENSG00000131148	EMC8	23,82593927	13,73719274	84,96332794	23,49128595	0	29,9967643	20,3760574	5,177164326	30,82806027	232,3957922	25,82175469
ENSG00000130204	TOMM40	43,28208589	11,4387446	19,01969098	20,02188032	22,68110794	26,28832406	34,58254303	18,85582297	36,19116486	232,3613647	25,81792941
ENSG00000125347	IRF1	22,96383319	38,92161457	49,3202027	26,67052003	13,6204262	15,76342455	21,00313349	18,73510469	25,35139626	232,3496557	25,81662841
ENSG00000139131	YARS2	0,070218667	32,89474351	0	27,28177011	57,73915876	27,75455744	35,85699544	20,42183898	30,22596784	232,2452507	25,80502786
ENSG00000196850	PPTC7	19,2709171	3,207327354	71,34089084	4,039098991	0	23,37631872	23,10772869	60,27452006	27,5582442	232,175046	25,79722733
ENSG00000171307	ZDHHC16	0	21,17607108	35,03335897	50,74451135	0,118169642	43,8934201	34,22668813	23,05104584	23,91115176	232,1544169	25,79493521
ENSG00000141646	SMAD4	46,78520526	18,3283833	8,727257546	36,04931105	54,70364758	16,34705294	14,65716825	26,48915378	10,02662027	232,1138	25,79042222
ENSG00000112763	BTN2A1	0,159609792	30,44654345	0,807441534	6,545767781	105,0705669	34,12842336	14,74367514	9,161771743	30,96624123	232,0300409	25,78111566
ENSG00000163166	IWS1	12,24629806	25,02917367	50,28583556	4,531432265	0	34,01446942	25,39064364	34,4302299	45,99096282	231,9190453	25,76878281
ENSG00000100373	UPK3A	42,51727311	29,93097971	0	0,722144764	0	9,948512441	18,86684882	100,2887202	29,48856257	231,7630416	25,75144907
ENSG00000188994	ZNF292	14,21523301	40,25468018	32,60341246	18,96304236	9,956020958	27,41917612	27,06802264	30,4175014	30,81393115	231,7110203	25,74566892
ENSG00000107165	TYRP1	0	0,143353114	0	0,06975006	231,4441044	0	0	0	0	231,6572076	25,73968973
ENSG00000101236	RNF24	17,11953875	22,97658426	33,78381894	34,71623092	33,87280848	22,00854589	23,52158369	7,824261737	35,81265402	231,6360267	25,7373363
ENSG00000146433	TMEM181	40,81947883	27,31168053	64,87924172	9,536955472	18,78871001	18,5302722	31,84478927	4,413968249	15,4125038	231,5375726	25,72639695
ENSG00000075413	MARK3	3,773090393	17,9143345	50,61857837	20,16222332	5,897105071	24,59454956	35,60472924	36,19877053	36,70526297	231,4686439	25,71873822
ENSG00000167333	TRIM68	0	33,78563988	53,45268476	44,59325965	0	30,26483487	32,20818113	26,56526142	10,4849942	231,3548559	25,7060951
ENSG00000135632	SMYD5	0	28,29679252	41,81270217	0,155133574	80,06485041	15,33834404	3,767093797	13,94818924	47,95326437	231,3363703	25,70404114
ENSG00000114405	C3orf14	24,69617453	50,60196661	16,06386125	9,659895222	0	40,11066087	16,73881856	35,803294	37,54687075	231,2215418	25,69128242
ENSG00000011243	AKAP8L	14,42973553	32,83802403	19,26540556	24,45462648	0	38,57853733	34,8075798	29,86207255	36,9570757	231,193057	25,68811744
ENSG00000048828	FAM120A	4,379412067	25,86685869	22,71104314	70,76141666	0,099121596	20,85903055	24,72697131	34,74898433	26,95620962	231,109048	25,67878311
ENSG00000196968	FUT11	10,03312825	6,914524672	3,950986178	27,23260446	119,6572359	20,91989201	21,37453981	2,415359275	18,60351989	231,1017905	25,67797672
ENSG00000119401	TRIM32	0	10,7114926	0	25,05804121	125,4061259	18,30283346	11,02014556	24,91371739	15,68387318	231,0962293	25,67735881
ENSG00000213563	C8orf82	31,67229222	17,69147485	38,07058337	12,86850258	35,06212593	30,79379914	16,55656497	21,03065725	27,31033533	231,0563356	25,67292618
ENSG00000161526	SAP30BP	31,17712253	31,5189662	27,54850114	37,4735092	0,044155606	29,84472811	16,33752386	31,24359799	25,79868728	230,9867919	25,6651991
ENSG00000162998	FRZB	0	8,439812669	0	0,395805801	222,1163108	0	0	0	0	230,9519293	25,66132547
ENSG00000116584	ARHGEF2	20,54971715	20,70741606	50,8289116	42,95237649	15,45519516	23,79471187	17,28215059	16,71121843	22,63450271	230,9162001	25,65735556
ENSG00000048140	TSPAN17	11,90919358	27,04557853	0	29,97124636	60,2546212	22,04771731	41,57482909	15,01687296	23,09272223	230,9122413	25,6569157
ENSG00000108479	GALK1	19,33964412	31,90620824	0	17,14896063	74,53148579	17,61831444	30,68402792	12,34414849	27,32091732	230,8937069	25,65485633
ENSG00000164597	COG5	18,59657667	28,47049227	1,411733112	40,86450647	48,60620096	22,10312159	28,22971329	12,9566879	29,65379138	230,8928236	25,65475818
ENSG00000144231	POLR2D	19,44586432	14,01814746	45,1943899	16,78938174	45,73212671	25,79842748	20,51927682	23,8555787	19,53844135	230,8916345	25,65462605
ENSG00000153317	ASAP1	0,455481585	19,91505571	4,357054843	37,59552624	10,17514136	31,88587645	36,18580108	35,05937056	55,25651362	230,8858214	25,65398016
ENSG00000118257	NRP2	18,64958468	8,278131695	22,74491743	41,4423927	61,22261499	12,40508702	11,16014179	33,81390004	21,07758485	230,7943552	25,64381724
ENSG00000103932	RPAP1	18,33657932	0,608894692	42,49000378	28,66359216	71,20170404	11,25535845	18,96478904	12,28695099	26,98282604	230,7906985	25,64341095
ENSG00000107872	FBXL15	48,77730337	12,82283379	26,90821629	12,98133888	26,86803055	22,07448861	40,35567177	11,2141311	28,52561352	230,5276279	25,61418088
ENSG00000170445	HARS	28,32319543	30,24077551	14,99201915	6,169822726	37,20930965	45,46672685	28,83417566	17,73320474	21,52738675	230,4966165	25,61073516
ENSG00000136286	MYO1G	40,96297791	23,37821728	15,27503355	28,58700249	0,205986889	39,87411474	34,17076611	15,20067243	32,64093445	230,2957059	25,58841176
ENSG00000126768	TIMM17B	17,16361283	39,14328963	7,9205883	28,31378725	0,420512345	34,38661836	38,98297591	42,04592858	21,83442748	230,2117407	25,5790823
ENSG00000198298	ZNF485	24,0028945	36,41157084	0,116504652	36,08907863	32,81124234	14,60235587	2,524130736	60,79663669	22,74400021	230,0984145	25,5664905
ENSG00000106009	BRAT1	36,41327703	23,1454806	7,081764847	12,82581622	9,417888493	32,34671966	44,8256064	14,55833401	49,35112205	229,9660093	25,55177881
ENSG00000167202	TBC1D2B	21,01896497	26,91582097	0	19,08302488	33,21552194	39,01779003	20,87102521	38,68551784	31,10449835	229,9121642	25,54579602
ENSG00000112855	HARS2	4,225732549	11,53609035	63,83302085	46,44768841	0	29,48827405	26,88890955	29,85372807	17,49026157	229,7637054	25,5293006
ENSG00000129925	TMEM8A	32,76109933	16,28956595	0,0627778	33,23562007	42,41609635	39,71662405	29,582436	14,62628167	21,02735042	229,7178516	25,52420574
ENSG00000133895	MEN1	42,88465324	22,67145031	0,654686997	2,519626462	70,87623657	21,49100734	24,64490271	15,40886584	28,49413236	229,6455618	25,51617354
ENSG00000167083	GNGT2	44,46533666	50,167479	0	1,081435656	0,425242797	29,46901895	23,69747537	32,67174232	47,64449032	229,6222211	25,51358012
ENSG00000163935	SFMBT1	0,053198581	19,91831087	0	70,68807004	61,00130758	28,79560441	18,46386157	13,90140145	16,71670003	229,5384545	25,50427273
ENSG00000184164	CRELD2	6,699771671	17,23584589	23,68078288	24,65368201	22,66647024	30,38749382	37,67753823	33,20339604	33,28325907	229,4882398	25,49869332
ENSG00000198081	ZBTB14	43,4297031	49,69427029	59,83548463	5,100321656	0	36,75335652	28,85454305	0,478428428	5,247114649	229,3932223	25,48813581
ENSG00000187796	CARD9	6,541583146	16,29105748	13,77659246	3,973035887	36,34597607	50,77126172	36,69150327	13,02205192	51,93382441	229,3468864	25,48298737
ENSG00000171421	MRPL36	31,95528197	40,17324589	1,394882217	31,61147721	15,4459158	31,04133371	26,89292421	29,60883221	21,04928353	229,1731767	25,4636863
ENSG00000246705	H2AFJ	50,16006428	18,05723838	19,13391027	40,11991862	2,889205496	24,17469369	26,35774257	25,304435	22,87086225	229,0680705	25,45200784

ENSG00000119899	SLC17A5	23,84569598	55,09340135	0,565492791	25,78258976	0,52182148	52,69987223	28,76957046	6,145833281	35,59222847	229,0165058	25,44627842
ENSG00000170430	MGMT	54,29052983	19,6782623	48,72211326	15,59133447	27,84027523	22,21630826	11,30328715	13,06145525	16,28684093	228,9904067	25,44337852
ENSG00000006576	PHTF2	26,05897072	10,8236824	0,032151295	58,45836755	0	16,23413693	29,83657213	51,55635341	35,96128056	228,961515	25,44016833
ENSG00000105127	AKAP8	15,79344651	5,679916669	16,5649221	38,56929909	26,16475576	32,17027709	26,16885882	38,61223298	29,15748511	228,8811941	25,43124379
ENSG00000149136	SSRP1	14,09284318	24,4996399	28,24983174	42,86465339	41,48241139	19,81920571	25,00694797	18,14600434	14,69293162	228,8544693	25,42827436
ENSG00000103707	MTFMT	0,660448298	18,67305886	104,3235337	17,33484097	0	10,97368908	23,43428986	17,81188665	35,41480931	228,6265567	25,40295074
ENSG00000087589	CASS4	3,21955842	38,56293511	73,21038314	16,21513454	12,96478798	21,04566992	18,14356941	26,20530601	18,95495206	228,5222966	25,39136629
ENSG00000084072	PPIE	32,63359245	23,78759411	26,03803182	9,343353693	20,73457747	31,20459118	15,37481828	32,7118349	36,6811436	228,5095375	25,38994861
ENSG00000132254	ARFIP2	39,85427513	16,80935354	36,94539719	17,97857694	40,76579029	16,58481104	35,0523292	10,26387493	14,23937021	228,4937785	25,38819761
ENSG00000168569	TMEM223	26,1792436	36,6931158	12,3140307	50,12698196	0	29,03435092	39,07190163	15,07245396	19,97034998	228,4624286	25,38471428
ENSG00000177058	SLC38A9	24,645598	9,634026298	53,71824422	14,28443758	50,43031112	16,8110683	16,78057257	29,43687749	12,70151342	228,442649	25,38251655
ENSG00000164244	PRRC1	40,82852908	16,56658429	0,889389108	29,21988076	32,88477054	17,18777929	14,17493601	64,16205195	12,50732332	228,4212443	25,38013826
ENSG00000104412	EMC2	53,02212795	5,708915334	0,045878874	11,5416031	78,35507361	18,51057103	21,18437855	17,06981674	22,97393055	228,4122957	25,37914397
ENSG00000070540	WIPI1	17,49561118	3,07303497	0,245060328	18,47937963	67,01135451	36,12686963	35,69064983	16,11875939	34,07823388	228,3189533	25,36877259
ENSG00000142039	CCDC97	45,75160695	58,06941433	1,161881291	25,76630627	0,28144008	16,9464565	19,92841219	29,95318572	30,3918294	228,2505327	25,3611703
ENSG00000034693	PEX3	20,1836734	50,97540496	48,13082986	20,1924945	0,126896658	33,82699744	15,51753223	13,54431443	25,74269849	228,240842	25,36009355
ENSG00000082515	MRPL22	13,26236391	37,79661171	31,31500735	14,22650792	15,62270464	18,59560035	21,59500775	51,97565006	23,72508795	228,1145416	25,34606018
ENSG00000109501	WFS1	38,60635674	25,37040417	6,097945889	19,42702226	39,15041676	28,27060348	39,03397876	7,8343671	24,29635456	228,0874497	25,34304997
ENSG00000076944	STXBP2	27,86382789	18,52472068	43,42863375	10,28868853	37,12547158	24,40831546	21,701628	16,58742113	27,95703151	227,8857385	25,32063762
ENSG00000111011	RSRC2	32,27839291	29,28251688	1,907239101	16,02310822	0,246392825	23,52534192	30,96226146	51,31276646	42,26972359	227,8077434	25,31197148
ENSG00000067992	PDK3	23,22119651	36,2402172	3,855422988	55,88301227	0,026493364	21,73789629	15,88396088	30,39851757	40,48725786	227,7339749	25,30377499
ENSG00000179295	PTPN11	5,787945822	4,085318811	4,63102258	54,72835221	28,22623998	36,85326779	27,90611684	30,5356734	34,56009655	227,314034	25,25711489
ENSG00000070495	JMJD6	50,91026262	3,773484337	0	9,267584958	3,248843573	41,27612773	39,64433677	17,16111843	61,99035447	227,2721129	25,25245699
ENSG00000100441	KHNYN	57,72107416	4,544779663	29,58561871	5,333167477	42,33863348	22,00010638	18,37333501	17,62537324	29,60018139	227,1222695	25,23580772
ENSG00000105698	USF2	11,13254204	19,91634421	38,10475994	23,42532179	2,891445591	28,78722164	23,5635708	37,38007789	41,78554054	226,9868244	25,22075827
ENSG00000114251	WNT5A	8,31445211	22,14881196	45,77257815	13,29897059	15,2929601	28,68725532	35,50215018	4,288457277	53,36460907	226,6702447	25,18558275
ENSG00000166200	COPS2	51,96626335	39,59119666	25,2406374	30,01568607	1,577803075	24,98267371	22,68522312	14,3858479	16,20682627	226,6521575	25,18357306
ENSG00000167925	GHDC	39,17820717	21,95041504	25,38409961	27,20372092	0,08935495	23,28279802	33,71683468	23,10328775	32,69308068	226,6017988	25,17797765
ENSG00000121680	PEX16	37,74010278	15,01026764	36,55452113	9,075247543	20,22192189	31,37957579	26,72322739	7,333261353	42,47568744	226,5138129	25,16820144
ENSG00000101391	CDK5RAP1	21,93700643	10,6857552	35,66812744	37,19480369	1,33676335	17,25586248	35,19323304	22,57402782	44,64060323	226,4861827	25,16513141
ENSG00000159363	ATP13A2	56,35362801	35,62975748	41,64674241	6,656251018	0	13,29684995	27,82656326	16,16632093	28,9047418	226,4808549	25,16453943
ENSG00000171206	TRIM8	15,09205343	19,7832706	20,91696825	17,36467291	0,075270916	33,7141698	43,49778435	32,3785706	43,4914362	226,3141971	25,14602189
ENSG00000008405	CRY1	50,55978943	1,233610205	49,70158608	34,86772245	11,94054564	13,18402743	19,52110561	21,62819315	23,55144134	226,1880213	25,13200237
ENSG00000006652	IFRD1	18,79263061	16,88913697	82,70112835	16,47016524	6,545160247	14,71894958	18,83232	19,57277926	31,60536542	226,1276357	25,12529285
ENSG00000118961	LDAH	0,034087654	15,4848527	62,07992285	34,43423418	0	19,83591375	21,96367643	33,09563455	39,198398	226,1267201	25,12519113
ENSG00000101193	GID8	60,06266914	25,79859905	0,363056289	32,43227481	2,032445821	31,05857553	20,01953708	16,68716144	37,62239132	226,0767105	25,1196345
ENSG00000182473	EXOC7	24,88633953	20,3306558	0,027479251	24,14344961	35,67745453	29,11143472	31,83890295	28,74480224	31,26836506	226,0288837	25,11432041
ENSG00000141385	AFG3L2	36,47732526	11,82026933	5,287664926	26,43227173	42,28739176	11,77203669	20,60865357	28,28448064	43,01067612	225,98077	25,10897445
ENSG00000138035	PNPT1	22,04826319	4,459742205	0,052538291	42,16582939	0,067873283	54,93769238	27,95870751	41,78051893	32,45552572	225,9266909	25,10296566
ENSG00000006194	ZNF263	7,95318793	4,774041545	26,65622064	13,48890779	78,23972694	26,09276132	27,21822445	26,16434886	15,15100494	225,7384244	25,08204716
ENSG00000025770	NCAPH2	38,12500381	21,70873784	0,062341354	20,36451473	38,2554009	32,44539648	28,30750258	27,19656566	19,25568363	225,721147	25,08012744
ENSG00000145022	TCTA	42,46718622	62,0816571	0,202388132	31,15641488	0	42,09671349	20,05150191	0,240578498	27,40001511	225,6964553	25,07738393
ENSG00000103343	ZNF174	0	31,36055226	0,096186885	2,437800801	113,8241068	20,26520193	12,02604912	33,27213119	12,41120374	225,6932328	25,07702586
ENSG00000173852	DPY19L1	70,45247536	7,132799301	0,309949139	40,19180001	16,53153389	17,41558937	18,72661693	44,73867193	10,18337108	225,682807	25,07586745
ENSG00000090376	IRAK3	11,7365967	15,43880368	46,12624593	19,48404898	1,435895811	42,92048906	34,91697364	16,18481988	37,39594655	225,6398202	25,07109114
ENSG00000197323	TRIM33	19,02256143	25,85375803	22,43175518	31,73386613	45,20752902	15,58218869	20,60253161	17,20296582	27,88471872	225,5218746	25,05798607
ENSG00000120685	PROSER1	13,73170147	10,24420249	49,11778253	55,07796503	0,146998585	23,4298604	24,70373708	8,521235265	40,4962336	225,4697165	25,05219072
ENSG00000173535	TNFRSF10C	39,23374024	7,894973236	68,75096666	17,87991261	45,94705173	11,72387373	14,57551501	6,01433367	13,43109235	225,4514592	25,05016214
ENSG00000155287	SLC25A28	47,31494918	31,50302187	3,038033869	18,67895173	0,196239094	28,46771644	21,8030413	36,11306749	38,2864147	225,4014357	25,04460396
ENSG00000156384	SFR1	43,03148701	44,0984084	0,153410651	0	0	19,22804697	30,8136301	30,81867655	57,23088269	225,3745424	25,04161582
ENSG00000185298	CCDC137	18,65953049	15,71363751	31,30039624	37,68211228	0,300642564	22,98719167	36,76401524	25,58827752	36,25135177	225,2471553	25,0274617
ENSG00000055147	FAM114A2	35,07303449	34,89997824	54,12152778	18,88108355	0,330324276	21,51775991	17,75396542	13,22143525	29,43116013	225,230269	25,02558545
ENSG00000168028	RPSA	21,22274323	23,04827629	27,16510561	52,46735483	29,94942922	17,9959285	19,51546735	17,75166518	15,87944191	224,9954121	24,99949024
ENSG00000089280	FUS	11,94718409	12,72520627	44,28001577	58,76936703	13,62013435	25,23428396	27,405073	13,49630637	17,44061986	224,9181907	24,99091008
ENSG00000170222	ADPRM	59,79128136	21,1865284	73,09847047	16,26816817	0	9,422082134	9,7588036	22,02897117	13,30752876	224,8618341	24,98464823
ENSG00000161202	DVL3	20,19395346	8,884730216	5,543887024	48,26969222	40,7165304	24,85532195	22,89518307	13,18002201	40,30371345	224,8430338	24,98255931
ENSG00000115548	KDM3A	17,89601841	45,50691738	33,56914347	50,64088799	0,027003349	28,08216469	7,009884704	7,180659449	34,78246439	224,6951438	24,96612709
ENSG00000129245	FXR2	3,489045732	11,45376819	33,37558335	27,11169212	43,94650137	16,47230385	30,85336033	35,40097883	22,57106228	224,674296	24,96381067
ENSG00000121039	RDH10	0,170073799	0,100597261	0	0	216,8447512	2,769844556	2,212382519	0	2,510026187	224,6076756	24,9564084
ENSG00000151718	WWC2	13,17420692	1,447677265	158,0000863	17,00955843	0	2,637450679	5,25615568	24,94282128	2,136807645	224,6047642	24,95608491
ENSG00000067596	DHX8	16,9364276	30,25876771	5,432315374	7,473853569	65,77571999	17,16480725	39,05097315	17,31465738	25,19138126	224,5989033	24,9554337
ENSG00000167930	FAM234A	45,89838875	11,47971609	30,75444538	14,99288385	55,18660746	12,41151678	19,87508864	12,73193129	21,18935112	224,5199294	24,94665882
ENSG00000109534	GAR1	21,08161729	9,010881877	34,81828124	68,15664051	22,30770884	21,03448758	23,38194578	6,393336846	18,262922	224,447822	24,93864688
ENSG00000145780	FEM1C	53,76533732	11,15493313	12,14973576	85,42105101	1,197688837	19,77414105	11,45244385	10,4402707	18,920657	224,2762587	24,9195843
ENSG00000075303	SLC25A40	30,79279135	49,36681984	0,820161582	7,082814023	0	19,93929236	17,34965448	80,39881196	18,20447838	223,954824	24,88386933
ENSG00000107864	CPEB3	36,65657359	0,033860588	50,58332146	0,29655446	72,78490409	5,719609476	6,115866669	18,35979413	33,34864353	223,899128	24,87768089
ENSG00000166479	TMX3	18,89135777	28,98540487	0,597244741	42,88766066	0,626900504	24,79635717	25,84550682	27,51033058	53,60525444	223,7460176	24,86066862
ENSG00000130772	MED18	3,820842392	0,132941002	39,14047998	4,786610838	90,98119194	16,20445395	15,116158	28,83316008	24,6935389	223,7093771	24,85659745
ENSG00000112039	FANCE	26,15807055	14,14419168	22,92406463	12,01929304	0,133401762	39,13566724	32,06671189	27,37255147	49,70635068	223,6603029	24,85114477
ENSG00000126749	EMG1	28,3680264	20,78819927	31,79205361	36,23748566	0,115046466	20,19734716	13,92774949	50,33529933	21,55260005	223,3138074	24,81264527
ENSG00000150961	SEC24D	43,33765862	11,65012117	30,74033957	28,38193927	0,036201365	25,35488249	35,47825416	19,95265181	28,32660702	223,2586555	24,80651728
ENSG00000162852	CNST	49,74194244	22,91387056	1,499302742	0	4,842306319	15,49207138	33,07527377	44,72256156	50,90183696	223,1891657	24,79879619
ENSG00000115368	WDR75	24,25564517	48,78694724	0	57,98554991	0,071253481	22,38476035	18,57160073	32,65008227	18,4740293	223,1798684	24,79776316
ENSG00000107929	LARP4B	43,62852718	10,94627673	72,28868576	19,96246066	0,951230636	15,30368673	21,18216029	16,91301892	21,86259338	223,0386403	24,78207114
ENSG00000104343	UBE2W	39,38184179	17,40415622	24,77050387	30,25582654	2,765481624	22,88799375	27,05410899	33,98859413	24,49330404	223,001811	24,77797899
ENSG00000096080	MRPS18A	49,31100398	32,99566668	28,57739335	11,58356196	0	34,11245502	24,13642679	24,19069264	18,09253527	222,9997357	24,77774841
ENSG00000100934	SEC23A	27,76176881	10,46558846	25,0374685	27,41926407	5,598252681	33,16295708	21,57336309	23,92407319	48,0282352	222,9709711	24,77455234
ENSG00000117862	TXNDC12	16,23534487	29,01848908	16,8494701	49,34956466	24,19652536	24,55856351	24,28458039	17,96587529	20,450853	222,9092663	24,76769625
ENSG00000087586	AURKA	0,051608801	27,65672269	4,936805037	2,317045539	151,226752	15,01581381	16,4979774	0	5,204718178	222,9074434	24,76749371
ENSG00000115008	IL1A	0	8,837071566	0	64,74234068	0	49,05453963	26,70084278	0	73,49862463	222,8334193	24,75926881
ENSG00000178381	ZFAND2A	18,15181778	20,45449716	24,739132	47,01634132	0	26,40312992	25,52311883	35,20192894	25,33900413	222,8289701	24,75877445
ENSG00000178685	PARP10	61,56634886	19,37840412	37,27596554	9,774789368	0	25,24546816	27,95162046	6,298686435	35,27171689	222,7629998	24,75144443
ENSG00000138802	SEC24B	37,78488115	16,54372946	0,372841673	14,79914782	0,068809646	14,46432504	43,63453776	54,32321497	40,70177715	222,6932647	24,74369607
ENSG00000196628	TCF4	22,5731868	16,60229503	17,79080625	30,04296603	19,83483048	27,7233068	28,64427983	38,32983987	21,1371368	222,6786479	24,74207199
ENSG00000126882	FAM78A	7,635759338	14,97314354	42,08889452	32,77213339	33,07142508	31,40819046	24,16601781	15,18474486	21,26585068	222,5661597	24,7295733
ENSG00000126226	PCID2	22,70410304	30,83869231	21,95165606	0,543353303	44,40793572	26,46915496	23,13252477	30,53992399	21,96936304	222,5567072	24,72852302
ENSG00000108587	GOSR1	12,09804569	11,64533496	10,26735221	45,22343345	46,42473353	23,33354229	21,4273535	22,90074172	29,23004663	222,550584	24,72784266
ENSG00000148300	REXO4	41,45007364	51,32484403	0,942660471	7,27550419	8,254020378	32,19440354	24,53621036	11,7034097	44,81604154	222,4971679	24,72190754
ENSG00000137497	NUMA1	19,9707349	10,30366987	33,65195234	7,095840744	61,23680636	20,71702616	23,36497578	8,898986645	37,17174595	222,4117387	24,71241542
ENSG00000100418	DESI1	58,36757151	9,921090469	0	40,20913006	39,78838394	25,43130206	17,03886598	14,17395685	17,41720813	222,347509	24,70527878
ENSG00000123104	ITPR2	13,97139937	8,752352293	50,5036818	17,33438181	9,991982809	38,44487661	29,28152259	26,86904133	27,17749848	222,3267371	24,70297079
ENSG00000105472	CLEC11A	2,423670241	15,70706277	81,99831372	7,096557887	0	40,49421328	47,42323567	13,36485698	13,73760904	222,2455196	24,69394662
ENSG00000179950	PUF60	44,57538958	17,59929057	11,99250427	20,97486153	26,48359457	22,60470431	26,50977147	23,02807274	28,47316741	222,2413564	24,69348405
ENSG00000164182	NDUFAF2	12,18633634	17,58780861	1,195613329	47,69779106	24,32731739	8,741525009	19,56257865	58,98079377	31,77376026	222,0535244	24,67261382
ENSG00000136243	NUPL2	16,38883027	19,59309651	0	50,54415983	72,94575079	13,95295751	18,01906193	7,516778995	22,97459306	221,9352289	24,65946988
ENSG00000005243	COPZ2	36,19851291	21,69852198	10,72592022	15,66169194	0	46,67902078	15,33078012	42,85378636	32,6675559	221,8157902	24,64619891
ENSG00000148655	LRMDA	16,50628769	27,94719438	8,047937138	26,33850683	17,36577787	30,19624424	30,39562972	30,87158635	34,11548836	221,7846526	24,64273918
ENSG00000135108	FBXO21	9,872528289	16,39953231	0,800057847	18,47460174	55,29655638	30,44121245	28,47412773	31,14615653	30,62228637	221,5270597	24,61411774
ENSG00000186566	GPATCH8	1,458805961	11,12553847	78,73771316	22,99293501	24,74765029	18,18081394	18,41399861	18,8684009	26,98849569	221,514352	24,61270578
ENSG00000092929	UNC13D	28,03108883	14,52781734	0,071718292	17,97462286	51,69958427	24,52702798	43,18302964	12,70246402	28,79278551	221,5101388	24,61223764
ENSG00000179387	ELMOD2	52,06771968	0,648371165	11,04862718	48,62215863	0	27,55564815	27,15331954	23,47049218	30,69823921	221,2645757	24,58495286
ENSG00000205336	ADGRG1	6,631483642	23,3490089	47,99402333	2,50116675	0,33178662	53,24696762	19,88050531	0,038160823	67,28592763	221,2590306	24,58433674
ENSG00000263528	IKBKE	26,30782818	6,719464523	45,53564797	6,745351493	25,95573901	21,92592955	26,90777629	12,72578392	48,43480981	221,2583308	24,58425897
ENSG00000132541	RIDA	57,03237971	24,00202272	15,9694646	10,45831562	0	23,3848575	7,124233948	54,96206233	28,30528195	221,2386184	24,58206871
ENSG00000174442	ZWILCH	0,404955147	14,81386436	1,891021082	31,89743144	87,05879815	27,94247396	10,21661124	12,87407406	34,09676455	221,195994	24,57733267
ENSG00000164815	ORC5	23,00743651	14,32874813	27,61886053	51,92073474	0,289299846	26,24095856	24,15741892	33,62908193	19,96203247	221,1545716	24,57273018
ENSG00000133678	TMEM254	19,67060166	76,79100003	6,680429615	4,790194794	0	28,46396409	35,59739059	24,37451249	24,75058825	221,1186815	24,56874239
ENSG00000176903	PNMA1	0,175031549	50,62603785	12,12808926	51,38101816	2,773108435	17,03946463	39,23975634	23,85763094	23,75104204	220,9711792	24,55235325
ENSG00000165494	PCF11	8,85312659	7,518000002	13,63222674	61,56922122	43,19804022	14,0051135	25,81024717	23,47676005	22,803598	220,8663335	24,54070372
ENSG00000104064	GABPB1	39,36807011	35,28308509	22,5733355	39,34478391	0,885616627	24,34446193	17,21708449	24,33004214	17,4814238	220,8279036	24,53643373
ENSG00000142552	RCN3	33,80688633	34,48214799	98,26425108	11,79799944	0	8,280915455	6,851873679	0	27,28222582	220,7662998	24,52958887
ENSG00000176915	ANKLE2	1,774106121	11,90138309	21,96967737	28,87156354	79,84046157	15,68743841	13,71040567	9,159288167	37,83771758	220,7520415	24,52800461
ENSG00000072422	RHOBTB1	0,06323941	12,17552082	34,66753275	26,97256276	0	19,85788865	19,1920512	90,85394888	16,79968563	220,5824301	24,5091589
ENSG00000074054	CLASP1	14,49305148	22,5877852	33,65339961	44,81130852	38,63470484	8,751888004	14,29308252	27,9846865	15,30223808	220,5121447	24,50134942
ENSG00000153936	HS2ST1	5,819771507	13,72124428	36,01088813	56,85332466	65,76890084	5,205651006	5,389851321	17,08684001	14,64048149	220,4969532	24,49966147
ENSG00000136937	NCBP1	0,02744471	49,28436326	0,403894078	11,37384561	42,78625278	26,18530533	36,39232114	9,722193959	44,28453273	220,4601536	24,49557262
ENSG00000155729	KCTD18	0,061564706	31,7903125	1,868678604	72,13052771	65,03473114	23,28628791	18,6582486	0,538495934	7,041648634	220,4104957	24,49005508

ENSG00000166548	TK2	40,28069473	12,86662687	40,94671286	10,95570196	31,05773138	38,87986733	17,65930604	5,116227786	22,34617132	220,1090403	24,45656003
ENSG00000117308	GALE	18,87449353	31,59397523	33,03429748	21,64011926	0	29,28080469	19,98642457	30,07748276	35,61862551	220,106223	24,456247
ENSG00000100726	TELO2	25,59504358	12,20839963	10,09882879	22,16008277	0,169435028	28,76722668	50,61435898	17,22718552	53,09274321	219,9333042	24,4370338
ENSG00000151576	QTRT2	14,89716825	6,026037002	24,43629378	1,619909204	98,94236733	26,16171249	14,73738938	5,989998368	27,06704378	219,8779196	24,43087995
ENSG00000183337	BCOR	27,89107794	10,16830618	70,99164045	17,80490288	18,74607021	19,12709715	25,02357745	14,58070259	15,53071359	219,8636564	24,42929516
ENSG00000198839	ZNF277	4,818672906	34,26926907	83,86494765	29,1227874	0,178932507	16,28519363	18,28603493	18,19283493	14,81586625	219,8345393	24,42605992
ENSG00000004864	SLC25A13	46,39216711	23,27270189	6,144034533	32,42345641	0	29,96228744	37,30290464	27,43230372	16,78128975	219,7111455	24,4123495
ENSG00000197879	MYO1C	38,08523265	14,45571848	24,82822498	8,466061969	0,265816094	34,11953261	52,05486387	13,1668351	34,23005894	219,6723447	24,4080383
ENSG00000121940	CLCC1	35,95116518	40,83709752	8,399882121	6,728379684	0	41,07657107	25,0485695	39,14091976	22,46331857	219,6459034	24,40510038
ENSG00000137710	RDX	11,05687102	25,4684592	16,46106355	12,51435326	40,34176895	30,52458898	23,6286765	27,08416542	32,47083937	219,5507863	24,39453181
ENSG00000099917	MED15	28,41880888	11,32120648	34,16470274	10,36294675	47,21769113	24,6610863	18,83143502	10,38381232	34,12739781	219,4890874	24,38767638
ENSG00000167900	TK1	3,849429171	51,15771492	35,55734599	9,899971936	0	52,96549034	44,8156904	0	21,1532627	219,3989055	24,37765616
ENSG00000075568	TMEM131	25,14261144	14,84423586	36,76703502	50,97855507	14,48365491	10,39326339	14,45958511	29,66867647	22,5432601	219,2808774	24,36454193
ENSG00000131269	ABCB7	19,46894932	5,024101605	2,094113166	39,91896849	61,59868217	13,81252821	4,944166067	65,55076315	6,784816054	219,1970882	24,35523203
ENSG00000176102	CSTF3	25,81569909	18,597041	20,69605878	4,336388898	87,53141959	8,345158633	17,23722709	18,08494088	18,4075771	219,051511	24,33905678
ENSG00000068323	TFE3	0,071837685	34,1630882	0	33,30688884	34,48621039	29,53815592	30,36101687	24,97698894	32,11562199	219,0198088	24,33553431
ENSG00000052749	RRP12	9,779931411	8,394174466	61,69730653	28,88059749	39,07387493	18,46044522	29,3926279	3,525565206	19,80706868	219,0115918	24,33462131
ENSG00000183751	TBL3	54,35662494	17,69083654	0	16,63053837	29,88035021	18,70822444	27,29449861	22,35605773	32,07212711	218,9892579	24,33213977
ENSG00000107862	GBF1	19,45483179	0,928665262	38,09093361	8,958466226	0	43,34510625	29,28459789	26,37107676	52,47134908	218,9050269	24,32278076
ENSG00000159873	CCDC117	14,77572154	20,14474111	0,706837977	53,80351302	0	30,5665117	37,73391168	22,30395139	38,75024574	218,7854342	24,30949269
ENSG00000120158	RCL1	0	17,61705894	0,067144951	54,8971891	47,27513541	23,04313565	22,85135998	25,62065125	27,33829277	218,7099681	24,30110756
ENSG00000117859	OSBPL9	24,19710783	9,161670596	27,70871424	8,610625561	39,32709385	24,40288926	37,52018494	9,778264239	37,93635328	218,6429038	24,29365598
ENSG00000113300	CNOT6	53,85554256	8,628833059	5,837624076	73,75877587	0	25,16607025	10,48271545	1,248053209	39,64576061	218,6233751	24,29148612
ENSG00000138434	ITPRID2	19,72160674	23,86560703	18,94340375	39,95524701	2,447264615	31,98296817	34,13673979	24,87215278	22,68152703	218,6065169	24,28961299
ENSG00000198162	MAN1A2	0,031263178	17,80771326	38,30241976	11,39076852	75,114893	12,52867704	23,29773931	17,70612454	22,3584882	218,5380868	24,28200965
ENSG00000171443	ZNF524	31,24979185	7,815380988	26,04254918	15,75388119	0	39,83696012	37,26669125	11,46546036	49,00879838	218,4395133	24,27105704
ENSG00000124155	PIGT	34,25418981	18,86940346	13,45133436	18,28257569	8,241083377	27,2592099	37,30337098	27,00238719	33,76878497	218,4323397	24,27025997
ENSG00000162174	ASRGL1	32,4891022	9,004332319	18,79063033	16,50046721	0	39,14418753	48,64506112	19,52633402	34,28394196	218,3840567	24,26489519
ENSG00000120306	CYSTM1	20,0178785	15,73908347	2,694475919	24,3089664	45,83244886	22,78255092	24,45894463	48,57761816	13,81088857	218,2228554	24,24698394
ENSG00000121644	DESI2	1,102103028	37,59203081	34,80382925	49,21605781	4,001512324	18,923755	33,55365442	16,73594852	22,13892931	218,0678205	24,22975783
ENSG00000205765	C5orf51	8,020213182	14,71247123	60,46079614	15,25278855	32,32508629	18,5336721	10,34899636	41,94865946	16,42860597	218,0312893	24,22569881
ENSG00000153201	RANBP2	3,612204876	9,244020493	34,73903668	55,14025522	34,51312587	21,90737268	18,31199658	9,080972747	31,46048129	218,0094664	24,22327405
ENSG00000137038	DMAC1	17,11626332	31,257022	20,88926697	30,99081077	15,27039403	23,04989317	22,62881354	39,36628829	17,37201729	217,9407694	24,21564104
ENSG00000164494	PDSS2	8,517071185	19,28488754	0,14179041	34,07261081	0,091588254	24,66867284	40,03146782	62,78341056	28,34384604	217,9353455	24,21503838
ENSG00000185339	TCN2	16,79544139	8,846452918	16,65031342	0	101,5697597	30,02545671	20,73756929	4,021945409	19,28708303	217,9340219	24,21489132
ENSG00000196199	MPHOSPH8	36,72334708	24,14315829	16,76148081	34,81613534	12,87526736	19,83897098	19,16943349	28,74222048	24,83506185	217,9050757	24,21167507
ENSG00000180543	TSPYL5	32,70529385	52,72086085	0	36,83651167	0	23,64345782	40,65004076	0,073577058	31,24340332	217,8731453	24,20812726
ENSG00000101189	MRGBP	34,09871526	11,98454434	0	35,36654997	46,7165989	19,95508753	21,38284364	22,21307958	26,13466719	217,8520864	24,20578738
ENSG00000125733	TRIP10	62,51413222	33,23184848	2,650086242	17,93682465	19,64064833	22,8928292	14,60567839	18,98382237	25,36390068	217,8197706	24,20219673
ENSG00000108578	BLMH	29,22435338	35,04336747	0,108607189	83,5970616	0,210461541	20,0622479	14,70642688	8,197927344	26,64804598	217,7984993	24,19983325
ENSG00000145945	FAM50B	23,42679278	27,94292917	26,82764816	30,00484308	0	21,80344334	31,367583	13,73983571	42,55007645	217,6631517	24,18479463
ENSG00000102445	RUBCNL	2,450441686	14,75769239	10,65476546	54,49501927	0,235294049	50,76510357	27,21092868	0,216500945	56,8650784	217,6508244	24,18342494
ENSG00000187049	TMEM216	30,8101925	6,011643826	33,39524032	13,57656594	0	33,89797316	39,22274761	25,71910339	34,90518439	217,5386511	24,17096124
ENSG00000137513	NARS2	30,0203772	32,81458043	0,088359853	29,52728892	0	34,17357079	20,65913286	39,91263053	30,06862042	217,264561	24,14050678
ENSG00000110851	PRDM4	0,094998263	44,95255659	0,757280236	64,19481563	5,343115152	32,02088037	24,6365677	1,938844906	43,07339766	217,0124565	24,11249517
ENSG00000227057	WDR46	34,00654112	14,94406914	20,68917139	22,0897333	0	27,73954616	36,15846957	26,80774269	34,48157697	216,9168503	24,10187226
ENSG00000149823	VPS51	35,33138548	24,39652624	14,35471003	18,01948493	0	28,38838761	44,71166134	13,1361283	38,57838669	216,9166706	24,10185229
ENSG00000170899	GSTA4	0	15,05985531	59,40650378	29,55855495	98,28300183	8,212613031	6,389969312	0	0	216,9104982	24,10116647
ENSG00000175221	MED16	19,14830775	15,94117528	0	20,39520171	65,32502902	29,6296956	18,98994366	6,652519716	40,62916629	216,711039	24,07900434
ENSG00000118518	RNF146	17,83345959	19,95013171	1,030142898	20,82719296	0	31,82948065	23,92806503	53,31412919	47,99799007	216,7105921	24,07895468
ENSG00000152520	PAN3	6,090098566	24,73728149	24,94468772	33,06874285	50,71291491	20,63434439	7,920907294	26,01027767	22,5871403	216,7063952	24,07848836
ENSG00000104856	RELB	11,47358113	17,24786075	23,87163317	37,3753014	0,952566425	31,63082944	33,36445831	32,42991777	28,34529186	216,6914403	24,07682669
ENSG00000197056	ZMYM1	4,732185018	0,051516234	10,78808453	50,83344174	112,4614014	7,458804025	8,605746114	0,063484067	21,60175715	216,5964202	24,06626891
ENSG00000173230	GOLGB1	26,97447861	8,678173454	125,8583305	8,972732376	0,030266965	10,64120934	13,9904972	5,737001341	15,71041398	216,5931038	24,06590042
ENSG00000124356	STAMBP	21,40765818	37,29034834	8,526553595	27,84346428	54,00298889	21,32643103	19,43860615	8,159938631	18,4059072	216,4018963	24,04465514
ENSG00000110931	CAMKK2	0,18959991	30,42905173	20,15191015	40,88817949	0	16,28343036	13,2414668	29,57465389	65,55021628	216,3085079	24,03427866
ENSG00000143319	ISG20L2	2,010212837	31,13545459	39,19825797	31,24078943	0,204742042	35,91260868	22,98620297	25,30694236	28,29020607	216,285417	24,031713
ENSG00000041880	PARP3	44,02230764	7,0515485	92,75497155	4,634091079	0	14,84582346	6,55633907	38,0315742	8,251382478	216,148038	24,01644866
ENSG00000108175	ZMIZ1	16,18896192	19,65779707	35,15696853	55,13504141	1,066014113	19,37140672	28,55446073	8,64949709	32,26444077	216,0445884	24,00495426
ENSG00000127526	SLC35E1	36,75158324	21,44400357	15,32894373	45,56518829	0,197046662	27,56072817	24,68099535	22,11962537	22,33217469	215,9802891	23,9978099
ENSG00000157379	DHRS1	1,740412715	32,40026592	0,056168943	41,81075669	66,83112882	15,58868825	13,23409038	22,76786978	21,51748985	215,9468713	23,99409682
ENSG00000169018	FEM1B	4,023536968	3,446736098	0	11,39708113	66,22760553	17,14815509	41,45589799	39,22393541	32,88391377	215,806862	23,97854022
ENSG00000060971	ACAA1	26,11367588	18,11260344	25,66210877	38,99703673	22,69121538	24,81887743	36,23207448	7,858632411	15,29977702	215,7860016	23,97622239
ENSG00000198783	ZNF830	0	11,0228168	40,61639516	17,72212517	0,481390807	34,32376989	51,18606484	42,22711631	18,10301245	215,6826914	23,96474349
ENSG00000163389	POGLUT1	20,42727737	8,682765557	28,18321988	13,20909515	0,863263323	23,22560392	30,1611375	53,87316973	37,00111756	215,62665	23,95851667
ENSG00000130511	SSBP4	18,64747123	24,25359718	27,94756207	28,86604124	0,241504736	29,73447794	43,41256612	10,44413226	32,05758774	215,6049405	23,9561045
ENSG00000160013	PTGIR	35,06005848	7,395443278	31,22039781	23,59321067	40,23096721	1,698260097	5,886438664	70,46514803	0	215,5499242	23,94999158
ENSG00000140521	POLG	40,30457529	11,83770703	0,418364697	23,16997851	0,900796511	27,41131267	34,80857538	23,26303561	53,42310565	215,5374513	23,94860571
ENSG00000111737	RAB35	26,02501615	13,6139791	29,333888	22,27493686	34,58897185	25,43576786	31,53933184	19,07934785	13,64477951	215,536019	23,94844656
ENSG00000138614	INTS14	0,071786494	19,74442281	0,066028527	6,631839022	69,77628258	31,329907	44,28715282	22,2905979	21,14500812	215,3430253	23,92700281
ENSG00000138764	CCNG2	30,25879146	18,65712946	34,7849962	28,57055008	0,096849391	24,51968609	22,4344202	38,36356023	17,6173281	215,3033112	23,92259013
ENSG00000178567	EPM2AIP1	12,73821572	16,76299976	49,84211226	85,9935838	0,044193643	10,2902942	9,743019874	14,11036118	15,68923385	215,2140143	23,91266825
ENSG00000119953	SMNDC1	19,44324677	29,50205502	4,464513312	61,22362059	0,066294544	20,76562138	24,99215216	31,53676784	23,19221877	215,1864904	23,90961004
ENSG00000112167	SAYSD1	0,361879574	43,11554569	0,760807661	33,38682193	57,98955867	44,27309265	26,356067	0,452185518	8,456239296	215,152198	23,90579978
ENSG00000214706	IFRD2	31,874886	33,37327695	23,739141	0	0	31,37640756	29,64371799	34,36092216	30,49044506	214,8587967	23,87319963
ENSG00000074603	DPP8	16,72264664	20,14150564	2,58624125	80,49605034	6,189862644	23,3811889	19,41466307	0,873737472	45,02833181	214,8342278	23,87046975
ENSG00000074582	BCS1L	32,19876025	14,99817639	80,62163273	15,90784397	0	23,96104215	18,12060433	16,72219918	12,26510046	214,7953595	23,86615105
ENSG00000088826	SMOX	17,4910742	40,13938291	0	2,214315806	0	36,88778946	64,43876861	16,37591543	37,21977194	214,7670183	23,86300204
ENSG00000009780	FAM76A	15,05861413	1,794703521	0,068909296	21,47506484	132,376744	20,27613146	14,46299067	6,634903314	2,579921975	214,7279833	23,85866481
ENSG00000127220	ABHD8	67,72717066	14,13884737	26,48654042	9,172552299	0	36,83491065	19,44151768	12,37983347	28,53887391	214,7202465	23,85780516
ENSG00000070476	ZXDC	16,37082728	8,012429802	18,12996143	32,57681836	51,30250605	17,67992365	19,08847321	14,96851909	36,56579139	214,6952503	23,85502781
ENSG00000113318	MSH3	3,344739904	29,03988459	0,384557449	41,82179138	1,206521387	29,67372963	22,1184854	31,08435853	55,93759949	214,6116678	23,84574086
ENSG00000106780	MEGF9	30,46197218	17,83357112	95,83694841	5,425790742	1,995097458	8,190188786	14,20005503	30,21116046	10,44280458	214,5975888	23,84417653
ENSG00000198146	ZNF770	23,75428165	14,14906307	7,564084326	31,75932215	16,04441309	18,18030956	24,63730733	50,66784874	27,77959599	214,5362259	23,83735844
ENSG00000136856	SLC2A8	30,39138259	21,91282638	0,141537729	37,33336449	0,091425036	44,68772662	36,22285043	18,67527342	25,07603279	214,5324195	23,8369355
ENSG00000165983	PTER	0	49,9040195	28,19383467	2,151100215	0,538272609	31,95529719	28,24101152	16,67443983	56,87284281	214,5308184	23,83675759
ENSG00000152683	SLC30A6	29,60761643	5,055542331	59,39833538	8,247667404	0	44,80505593	28,15544973	8,856379351	30,36752785	214,4935744	23,83261938
ENSG00000143952	VPS54	59,67300119	3,495149992	0	0,622756503	0,197788255	34,93655801	44,20370243	44,28441973	27,02229588	214,435672	23,82618578
ENSG00000163558	PRKCI	59,34058235	5,476074076	33,48462251	29,94113675	0,186457706	23,74663325	2,432100586	35,91431436	23,89822881	214,4201504	23,82446116
ENSG00000116353	MECR	26,23821994	15,38295513	25,30313497	19,22748793	48,16313801	19,67068286	17,78722708	18,6195919	24,02452493	214,4169627	23,82410697
ENSG00000253719	ATXN7L3B	30,56392132	29,93852684	0,255899618	23,09432376	0,56672942	16,64778669	19,66867908	58,83855923	34,55904557	214,1334715	23,79260795
ENSG00000114745	GORASP1	33,74384067	5,443419759	22,350382	59,85321109	15,0971729	24,23949282	19,79527137	16,26819802	17,31913723	214,1101258	23,79001398
ENSG00000111674	ENO2	19,94334792	57,18608878	0	0,243807498	1,006636419	21,43156947	22,33319154	23,15589121	68,80792754	214,1084604	23,78982893
ENSG00000196378	ZNF34	0,068006557	0,120675968	148,9983339	0,117432482	0,646476224	25,76019305	13,04394541	0	25,34270758	214,0977712	23,78864124
ENSG00000100304	TTLL12	44,91151289	17,28451569	0,060129807	22,68999779	6,913575875	28,20802797	41,74202263	17,86905513	34,41155593	214,0903937	23,78782152
ENSG00000198815	FOXJ3	32,47474851	23,52463893	32,81261609	19,14633079	6,717831056	30,16993376	27,36475918	18,78340349	22,90741646	213,9016783	23,76685314
ENSG00000117523	PRRC2C	10,08612149	37,08165668	52,15191443	33,3786149	0,633453648	19,83013596	21,64502658	15,20096931	23,83891748	213,8468105	23,76075672
ENSG00000169136	ATF5	18,18342966	33,74954635	32,3925634	15,51895423	13,16267617	13,65377498	20,60802479	54,84391162	11,6957355	213,8086167	23,75651297
ENSG00000132780	NASP	9,189648144	15,40266547	5,925153844	47,47101309	3,632691902	35,68356093	36,02134772	24,07429111	36,4352286	213,7816008	23,7535112
ENSG00000164151	ICE1	2,611056975	0,957539685	17,64393901	10,5504177	118,1476444	2,541885347	15,73979859	19,14624899	26,43940272	213,7779334	23,75310371
ENSG00000113441	LNPEP	0,474555447	13,11505707	45,89662065	38,93276749	54,97382664	17,94796363	10,69759078	21,92446382	9,785317356	213,7481629	23,74979588
ENSG00000124535	WRNIP1	2,377545734	27,73405779	20,50614091	4,643929133	0,092627687	31,15370127	33,23653107	60,00153096	33,93844152	213,6845061	23,7427229
ENSG00000137942	FNBP1L	13,57858008	3,329807521	0,095888271	32,26808844	57,66444152	24,98101646	18,13455299	31,3451211	32,18177102	213,5792674	23,73102971
ENSG00000168172	HOOK3	3,432527406	7,569659929	36,15274265	51,99170483	26,54716076	18,85446483	26,86178766	16,33516474	25,82964073	213,5748537	23,7305393
ENSG00000179562	GCC1	0	39,03879703	0	32,20965353	0	38,72137704	41,53709742	13,77365193	48,23926041	213,5198374	23,72442637
ENSG00000147687	TATDN1	12,61890282	33,11087904	4,305258963	44,18091135	27,28331266	26,66792024	17,43664723	36,30769107	11,59109745	213,5026208	23,72251343
ENSG00000170854	RIOX2	55,21685678	16,75887501	22,50507487	17,9772061	30,37883861	15,96407003	23,14271095	8,74114699	22,74458892	213,4293682	23,71437425
ENSG00000198832	SELENOM	5,547517028	43,07772326	25,73082184	18,91307786	0,112682003	36,02451179	42,55833978	12,23447724	29,15696929	213,3561201	23,70623557
ENSG00000116698	SMG7	40,22589918	13,86329927	7,620471633	36,33073231	53,41209569	17,19549322	15,29721253	4,171651989	25,02682517	213,143681	23,68263122
ENSG00000166900	STX3	0	22,8221571	0	33,59876999	66,83085079	14,07560996	15,64086925	26,40582751	33,74635127	213,1204359	23,68004843
ENSG00000138078	PREPL	5,208152871	17,52970514	17,86600906	35,81334927	62,37096038	14,22439508	12,04995339	28,73520279	19,21606995	213,0137979	23,66819977
ENSG00000185753	CXorf38	21,82985765	28,67568166	28,71695237	18,50266184	0,059358271	24,40667988	37,37149393	15,40207406	37,84510999	212,8098696	23,64554107
ENSG00000138430	OLA1	18,50796025	35,2994801	25,75519664	25,69781203	3,710262894	23,74890608	21,03368672	28,30251087	30,72150476	212,7773203	23,64192448
ENSG00000166889	PATL1	76,96704499	17,25758201	15,9028984	28,77206871	17,6452218	13,20395593	11,63531106	4,573488824	26,7944813	212,752053	23,639117
ENSG00000198121	LPAR1	24,63403869	18,63261313	41,64020237	14,11646292	0	32,71139045	30,5002418	8,26285045	42,2276902	212,72549	23,63616556
ENSG00000153012	LGI2	57,11723649	31,33483881	17,84722114	5,343241204	18,35673966	7,969838327	13,96690532	51,18047343	9,471162259	212,5876566	23,62085074
ENSG00000126216	TUBGCP3	13,34309675	15,64901573	36,08985557	35,38894592	0	23,0278753	21,08483228	27,44503444	40,5385419	212,5671979	23,61857754

ENSG00000130714	POMT1	17,03636564	12,55189188	0,045818378	10,92425969	93,04974066	28,74106084	18,4472616	7,243744434	24,47525487	212,515398	23,612822
ENSG00000142197	DOP1B	0,050202847	7,542421708	23,61137718	47,10121911	62,59701126	9,388738061	22,28731036	14,60059717	25,33522358	212,5141013	23,61267792
ENSG00000100697	DICER1	43,42554467	10,49099577	19,44549253	27,931546	0	28,08361071	25,62681012	28,11408473	29,22086608	212,3389506	23,59321673
ENSG00000141295	SCRN2	35,67398601	10,37161454	22,70927784	27,32030078	0	36,89975147	20,79318273	29,08793792	29,3735417	212,229593	23,58106589
ENSG00000278637	HIST1H4A	9,20224629	12,89144231	89,09618557	13,38128075	13,81221317	4,254117351	44,6384835	14,82719213	10,12625282	212,2294139	23,58104599
ENSG00000163702	IL17RC	23,13256533	23,15234891	3,000225738	20,69087904	40,49959949	25,50783756	33,49560405	22,92662748	19,81271295	212,2184006	23,57982228
ENSG00000029639	TFB1M	17,31283843	12,06671613	0,40571165	13,32920084	119,2399526	8,232974373	10,07180967	18,08508336	13,47172553	212,2160126	23,57955696
ENSG00000111358	GTF2H3	3,026751541	13,36010466	16,00786749	44,09892535	0,089914257	37,21979888	26,29410813	36,23694055	35,73613648	212,0705474	23,56339415
ENSG00000169220	RGS14	34,21910089	20,19206964	5,026900575	29,29796206	0	18,09119379	41,78957886	22,92083235	40,32944775	211,8670859	23,54078732
ENSG00000144747	TMF1	17,55411635	39,96200456	16,37450716	39,8774345	0	24,43253974	31,75219374	29,02945575	12,8502146	211,8324664	23,53694071
ENSG00000136636	KCTD3	49,8481153	18,43910505	0,609483972	56,22298337	0	17,95683539	10,40375884	50,4510422	7,89023505	211,8215592	23,5357288
ENSG00000099256	PRTFDC1	32,64092361	19,15300001	0,239225559	0	44,65790436	27,05684534	18,78815643	55,0250334	14,23437219	211,7954609	23,53282899
ENSG00000111846	GCNT2	11,79302073	9,487828707	57,48353899	19,99005813	69,82841101	4,79150916	10,73247108	23,3110806	4,261031375	211,6789498	23,51988331
ENSG00000160818	GPATCH4	32,14973602	43,14928737	0	14,58460079	5,260906075	22,99639501	33,19859585	33,14027103	27,18377123	211,6635634	23,51817371
ENSG00000111801	BTN3A3	31,51475316	9,051806648	32,22957669	27,11731443	16,12157224	21,58186364	30,64687148	11,38825392	31,9926675	211,6446797	23,51607552
ENSG00000162896	PIGR	51,94857907	10,24238655	1,144474332	30,199702	5,50340241	28,46132949	23,64765635	0,453478363	60,02179539	211,622804	23,51364489
ENSG00000160691	SHC1	50,95022465	9,880865964	21,38308454	19,0502964	24,06798821	22,04753633	24,59126037	19,40599141	20,15976785	211,5370157	23,50411286
ENSG00000128609	NDUFA5	29,36316984	34,29395054	15,44984129	42,16159853	0,037801847	21,13176819	18,16017963	34,57389054	16,31536838	211,4875688	23,49861875
ENSG00000079462	PAFAH1B3	20,12677791	26,26620015	20,17755475	0	0	32,50397486	47,21716511	32,36815002	32,74251626	211,4023391	23,48914878
ENSG00000107537	PHYH	23,35792653	34,54005627	31,60922654	9,967607854	6,486872989	23,58219627	30,83813312	26,02771542	24,98499293	211,3947279	23,4883031
ENSG00000100234	TIMP3	13,88654603	24,64133522	3,865028532	21,31469703	145,6598852	0	1,717278061	0,143573568	0	211,2283437	23,46981596
ENSG00000157349	DDX19B	40,50110828	26,15007122	36,20749188	16,04155748	0	23,70151096	23,30718241	16,95285707	28,19343527	211,0552146	23,4505794
ENSG00000131725	WDR44	80,19432951	32,9912932	0	28,44079447	0	28,03343871	6,001793606	6,3696335	28,96941268	211,0006957	23,44452174
ENSG00000172164	SNTB1	29,58155122	31,73184693	20,1304399	0,26884675	54,81379032	24,98182513	21,73661772	7,198162495	20,51588044	210,9589609	23,43988455
ENSG00000141971	MVB12A	26,72077164	18,63913903	2,203500785	21,08163667	26,60535451	31,3611456	29,3784192	19,94696475	34,95954398	210,8964762	23,43294179
ENSG00000143537	ADAM15	34,17430737	19,6708059	32,17205884	6,261805284	0,109087953	35,75745874	31,29135558	26,24806786	25,00911585	210,6940634	23,41045149
ENSG00000172939	OXSR1	26,75734862	0,044167682	45,33030769	76,46241473	0	18,61062473	12,52429932	10,28695985	20,57138247	210,5875051	23,39861168
ENSG00000117226	GBP3	17,67189447	0,18777451	40,55498643	37,39824333	0	3,950251826	32,50981992	35,48083591	42,81758111	210,5713875	23,39682083
ENSG00000160058	BSDC1	37,39811923	31,98376911	2,127737961	12,73259484	0,057266392	48,73342087	40,61629309	1,159234649	35,59267633	210,4011125	23,37790138
ENSG00000136143	SUCLA2	4,163664917	33,81808827	0,560443748	70,72834213	8,084971552	17,87701331	25,88668812	20,35607215	28,91362225	210,3889064	23,37654516
ENSG00000156502	SUPV3L1	14,8957395	54,30486197	48,24555327	15,23796862	0	16,58718216	12,42686773	11,34638669	37,33426983	210,3788298	23,37542553
ENSG00000117385	P3H1	37,90047863	13,51410317	54,14983306	9,448576526	0,318460723	31,31359835	19,267466	8,986101156	35,40587751	210,3044951	23,36716612
ENSG00000047849	MAP4	11,70012248	3,678585961	46,023072	19,52253909	38,47602123	19,71874351	26,67598506	19,01718049	25,33422779	210,1464776	23,34960862
ENSG00000163811	WDR43	0,883902924	15,88069852	36,72074425	47,12474194	9,890392953	24,01922558	29,35693851	19,32836856	26,76950881	209,9745221	23,33050245
ENSG00000109065	NAT9	51,83812859	11,52302001	0	0,257777204	73,17164051	21,55321397	8,95552629	21,46314924	21,20550588	209,9679617	23,32977352
ENSG00000116260	QSOX1	26,79785347	19,45445947	24,15394133	15,49969502	19,93806968	25,39812623	35,31727435	19,12929741	24,24259292	209,9313099	23,3257011
ENSG00000134265	NAPG	9,922630065	24,99588156	12,78240928	45,42668282	6,489688992	24,99493748	31,74361235	20,63368475	32,91967383	209,9092011	23,32324457
ENSG00000100142	POLR2F	12,63567272	27,8549338	21,85602662	39,76577524	11,27366714	20,17063518	21,93224268	31,49690227	22,91258494	209,8984406	23,32204895
ENSG00000124198	ARFGEF2	3,196442365	5,642619905	0,030466911	19,161161	107,9241891	31,40372271	10,1074851	2,535118339	29,88128542	209,8824908	23,32027676
ENSG00000198546	ZNF511	34,13025995	26,47448319	8,865245621	30,66470431	0,156888398	23,26662085	23,57022401	32,04738925	30,69025138	209,866067	23,31845188
ENSG00000105197	TIMM50	19,91835664	23,11568966	30,84373178	27,86402398	0	26,11384494	22,76679411	27,33712009	31,88745947	209,8470207	23,31633563
ENSG00000164134	NAA15	37,02709347	16,78662149	9,808693476	60,02260436	0	23,67453283	22,13647724	22,94304582	17,36541904	209,7644877	23,3071653
ENSG00000177613	CSTF2T	2,760850865	10,97389915	0,067717442	4,449561077	95,18145824	27,28127632	23,32128217	11,43037968	34,22654588	209,6929708	23,29921898
ENSG00000164463	CREBRF	23,14504727	33,72157968	34,68834609	1,269447699	0,029118426	23,03080413	33,09454228	24,4885425	36,18077515	209,6482032	23,2942448
ENSG00000165424	ZCCHC24	25,07041343	19,05820195	78,31433	16,63454006	0,071098306	11,20085263	22,23241876	3,794338728	33,22498705	209,6011809	23,2890201
ENSG00000113615	SEC24A	18,69451855	24,93488905	8,425950058	35,14444453	72,45397131	11,28674339	15,79780068	3,897596051	18,96445105	209,6003647	23,28892941
ENSG00000205744	DENND1C	41,49281002	9,059241234	3,170420754	9,994926938	0	27,84812779	45,873487	9,74166898	62,38876151	209,5694442	23,2854938
ENSG00000137106	GRHPR	17,64978157	37,55323887	21,58153006	18,05528471	0,347885801	31,59326786	39,18994455	23,64166502	19,85517786	209,4675963	23,27417737
ENSG00000138182	KIF20B	35,73188213	12,44623501	51,57096859	11,73083815	14,88678763	11,11559695	23,51538553	27,1543806	21,26750452	209,4195791	23,26884212
ENSG00000184939	ZFP90	1,077053248	18,84447698	45,41200191	73,35192729	5,580015982	22,23178077	10,4633135	17,99372841	14,43853951	209,3928376	23,26587084
ENSG00000106330	MOSPD3	27,62044629	46,16595756	5,900520969	40,92494295	0	23,57573643	26,26295275	21,82115069	17,09437351	209,3660811	23,2628979
ENSG00000134779	TPGS2	23,03331719	12,05104078	16,41210916	32,61378574	55,52067974	19,46533401	17,05940648	22,6667671	10,37462845	209,1970687	23,24411874
ENSG00000166747	AP1G1	32,53418844	3,12588124	18,68390829	30,98899881	4,284553556	37,941845	28,01942205	35,02967546	18,53387449	209,1423473	23,23803859
ENSG00000125534	PPDPF	19,66015972	24,44599431	10,82353662	35,18781234	44,67654136	21,90082636	24,665108	14,43493837	13,23693179	209,0318489	23,22576098
ENSG00000117543	DPH5	39,17681462	18,77896836	72,25610654	35,23061442	0	10,16691076	20,9539679	0	12,45234274	209,0157253	23,22396948
ENSG00000162599	NFIA	15,95957695	24,35357905	27,02802545	36,67938403	12,31569488	15,06856943	7,790177953	51,77244072	18,03485189	209,0023004	23,22247782
ENSG00000094975	SUCO	64,12723736	6,419918675	35,39016968	12,5938973	0,090984936	31,33679628	20,59913229	16,74358082	21,56516524	208,8668826	23,2074314
ENSG00000070081	NUCB2	4,9728354	6,714691637	19,58951168	51,34853351	41,3034913	11,48965213	26,77627251	35,36839803	11,11990272	208,6832889	23,1870321
ENSG00000106683	LIMK1	16,81153408	9,715289858	10,22973084	16,31307031	40,87143385	39,48568002	28,25667465	6,432159101	40,53344866	208,6490214	23,1832246
ENSG00000198363	ASPH	13,59821766	12,18523281	20,70256337	25,52469124	49,60325609	24,69582286	25,13537454	22,00479611	15,10171022	208,5516649	23,17240721
ENSG00000115392	FANCL	22,49116703	44,02331345	3,918459107	52,7928141	0	20,15414551	22,6803751	30,68712401	11,78877194	208,5361702	23,17068558
ENSG00000162512	SDC3	41,66502359	8,919089883	23,88307432	0,221600828	0,457474576	40,46198495	22,35911507	44,80627257	25,75283019	208,526466	23,16960733
ENSG00000133884	DPF2	54,9461148	26,16171078	8,181569541	9,856871578	2,579986979	26,68411129	29,92096677	11,79302976	38,39679597	208,5211575	23,1690175
ENSG00000197694	SPTAN1	18,28851142	8,967794164	31,98243563	38,34631987	39,90194915	7,991012009	30,31658736	5,678199331	26,91088777	208,3836967	23,15374408
ENSG00000130844	ZNF331	7,228793824	24,33285011	49,54809965	28,50230789	0,080465504	31,85869689	36,65994016	8,144253289	21,92430965	208,279717	23,14219077
ENSG00000167118	URM1	15,07679163	30,64480001	10,52668402	27,86856531	31,84012677	28,36657026	24,80955318	17,30847735	21,7031653	208,1447338	23,12719265
ENSG00000007944	MYLIP	22,18452714	12,13272722	47,14441132	10,10783267	0,350700725	21,38691028	25,81290684	67,01195819	1,996398188	208,1283726	23,12537473
ENSG00000082212	ME2	20,10435031	22,66986527	47,16061691	21,69944244	15,81318989	23,46146923	21,31819631	10,10198149	25,69608454	208,0251964	23,11391071
ENSG00000115760	BIRC6	0,186159287	16,12635839	100,8218782	34,98030738	1,347344346	12,69088631	9,063541734	13,87758571	18,90934769	208,0034091	23,1114899
ENSG00000204178	MACO1	0,113931066	9,906226191	19,70102737	27,60834991	45,66812677	19,38889625	15,26203586	40,85795607	29,47201698	207,9785665	23,10872961
ENSG00000050393	MCUR1	35,00216785	30,1919835	0,27536414	16,7580824	22,58936497	26,2958814	26,34939117	24,60392768	25,89639505	207,9625582	23,10695091
ENSG00000163444	TMEM183A	43,22256293	17,99566172	31,11614778	27,26957882	17,43116352	25,22761122	12,73968205	15,87526154	17,07505195	207,9527215	23,10585795
ENSG00000198746	GPATCH3	0	25,65779246	0,624393753	15,23879007	0,322657271	38,53358857	52,98387784	37,25924369	37,31958466	207,9399283	23,10443648
ENSG00000151239	TWF1	15,14180184	21,8905346	37,18655444	11,2151604	0,09133203	17,84846743	14,07233378	63,2800685	27,13018916	207,8564422	23,09516024
ENSG00000123575	FAM199X	23,64571233	15,264117	29,75232959	51,95426173	2,119660205	15,88598124	8,508415746	32,37599973	28,27675313	207,7832307	23,08702563
ENSG00000009335	UBE3C	20,10434722	12,45412654	12,39824424	18,91530798	36,86654314	22,30745573	27,68108355	26,67860209	30,27190775	207,6776182	23,07529092
ENSG00000099219	ERMP1	0	6,736410541	38,32446218	23,94302233	81,52760382	18,68018901	7,073082441	21,5632852	9,82185205	207,6699076	23,07443418
ENSG00000133317	LGALS12	19,70428587	16,06489516	22,53230758	15,05836197	0	26,82342804	60,35841522	9,025085104	37,9880116	207,5547905	23,06164339
ENSG00000175826	CTDNEP1	30,78390609	31,83931943	14,86919346	27,91489485	22,88761271	21,4626353	21,51463963	22,68917175	13,39561428	207,3569875	23,03966528
ENSG00000185085	INTS5	0	14,20295526	43,32596977	26,60980715	0,546602043	38,33367663	12,56613066	30,17667283	41,46895002	207,2307644	23,02564048
ENSG00000163584	RPL22L1	8,178095243	15,42698977	19,24581865	40,45700728	0,875469387	19,8995621	19,14783711	55,26038842	28,72420651	207,2153745	23,0239305
ENSG00000144741	SLC25A26	34,69085409	18,97620308	31,88455494	40,58988494	14,85580261	13,84006178	14,00549176	19,09177501	19,15648768	207,0911159	23,01012399
ENSG00000008130	NADK	21,86626929	28,45097344	0	13,67917641	1,09604995	30,91834446	52,26053326	35,29776798	23,45603324	207,025148	23,00279423
ENSG00000168556	ING2	10,76557683	48,45460261	35,17299212	19,17073283	2,265305462	23,94776042	27,32878845	27,46469133	12,27483524	206,8452853	22,98280948
ENSG00000177200	CHD9	3,579994736	22,69718699	35,15174907	40,05969621	0,500466764	19,35154267	23,22539168	15,79695066	37,4080312	206,77101	22,97455666
ENSG00000134255	CEPT1	25,40050576	21,4054911	30,94171451	0,061994529	0,042660673	23,88733891	26,873724	52,08917452	26,02911021	206,7317142	22,97019047
ENSG00000177556	ATOX1	38,8520593	22,11058294	20,85248691	18,87657098	9,185293124	22,07285206	24,7396169	32,10368293	17,87872633	206,6718715	22,96354128
ENSG00000112701	SENP6	34,38602633	19,30566034	10,00699139	28,43155894	0,062909266	22,64068306	24,46325296	29,43434835	37,86274948	206,5941801	22,9549089
ENSG00000136205	TNS3	28,86309523	11,19157787	22,77905605	56,56104941	0,122107291	26,96540485	37,19101051	1,825760531	20,8848268	206,3838885	22,93154317
ENSG00000166272	WBP1L	27,87626383	7,39418767	16,07621407	31,71327663	0	19,35941109	36,56948397	27,37802047	40,0022005	206,3690582	22,92989536
ENSG00000134594	RAB33A	80,11258826	17,30863704	0	78,97079655	0	2,633501218	15,53442206	0	11,79977919	206,3597243	22,92885826
ENSG00000141644	MBD1	19,83690889	18,15752184	0	31,73533475	41,11666545	20,74498152	26,30889646	17,27249299	31,15840862	206,3312105	22,92569006
ENSG00000126456	IRF3	25,37096297	25,8671513	27,32909249	16,98759587	48,65219208	8,658485553	21,2644764	8,796492177	23,36552217	206,291971	22,92133011
ENSG00000163918	RFC4	15,52954814	77,74954891	0	9,925454489	21,44879549	21,31317051	29,18012059	4,63071466	26,47863364	206,2559864	22,91733182
ENSG00000178950	GAK	9,253092021	8,351238802	17,60876905	19,92093472	52,58210666	23,48255936	29,52340508	16,70160863	28,75685218	206,1805665	22,90895183
ENSG00000219607	PPP1R3G	72,39880816	28,74316121	0	2,073866723	0	19,00178197	37,53626399	13,40054594	32,950168	206,104596	22,90051066
ENSG00000240065	PSMB9	18,87750388	20,40385501	20,06986335	31,033908	25,0672221	24,80209041	17,6665836	28,41064137	19,76498364	206,0966514	22,89962793
ENSG00000160404	TOR2A	47,28684348	22,07395885	0,146691345	14,73353155	0	35,28335747	30,52338947	30,95099511	25,05740709	206,0561744	22,89513049
ENSG00000162695	SLC30A7	14,9353471	27,06363443	21,72123564	54,55361446	0,167031415	29,40089751	21,15488156	17,86652022	19,16812387	206,0312862	22,89236514
ENSG00000213722	DDAH2	24,87601015	12,48227677	11,99914096	16,8950703	30,09110173	29,13791339	29,04454998	18,45846928	32,95374221	205,9382748	22,88203053
ENSG00000170633	RNF34	10,5031293	9,318772624	1,590477362	65,41345093	0,076100348	20,3213209	21,43019445	42,78353904	34,4991637	205,9361487	22,8817943
ENSG00000059758	CDK17	0,02336641	10,69748508	1,332516324	108,7800667	0,111061556	12,31437712	38,08539689	2,55477476	31,9850285	205,8840734	22,87600815
ENSG00000141101	NOB1	57,17891655	9,827193721	1,323084717	35,89253101	0	14,68794254	17,02001172	32,08407985	37,85926299	205,8730231	22,87478034
ENSG00000175390	EIF3F	27,28674603	27,29470046	7,074034965	25,75871714	40,02041533	25,70197779	18,3728691	18,9550526	15,37061684	205,8351303	22,87057003
ENSG00000168884	TNIP2	45,00687138	21,56316903	10,14223742	27,88098947	0	20,01304053	37,74386756	27,06463323	16,39822255	205,8130312	22,86811457
ENSG00000183486	MX2	21,14198995	10,75126844	19,29644831	21,60735976	58,2039052	16,3944267	8,613170397	39,31444062	10,38360591	205,7066153	22,85629059
ENSG00000143013	LMO4	30,46741445	22,40699613	35,74103099	39,56623301	0,331228134	18,45491196	12,03552345	26,88095264	19,71255309	205,5968439	22,84409376
ENSG00000198860	TSEN15	13,54467604	14,25252173	0,027260969	37,74434515	78,92344929	10,44567915	12,2061949	20,93367957	17,51480905	205,5926158	22,84362398
ENSG00000182307	C8orf33	10,96966238	84,82231429	2,618236632	6,29408967	0	32,93056243	36,23161207	3,94730014	27,53756188	205,3513395	22,8168155
ENSG00000175087	PDIK1L	23,89270207	39,04523861	77,72469873	18,08233596	0,393769235	15,9725314	17,94005624	0	11,90069836	204,9520306	22,77244784
ENSG00000099385	BCL7C	31,50713809	10,5476503	41,39994653	11,92540471	17,63742364	23,91707722	15,54827898	29,00130583	23,41040776	204,8946331	22,76607034
ENSG00000055070	SZRD1	13,3520306	16,85076255	1,348279687	17,44335448	30,89834612	27,32521308	35,66723061	23,41333081	38,56452508	204,863073	22,76256367
ENSG00000171735	CAMTA1	10,10150437	24,12546926	7,045643224	28,95764284	54,83042755	24,27591898	13,8609051	29,16286095	12,49861495	204,8589872	22,76210969
ENSG00000107960	STN1	45,8691548	39,55390441	20,26982302	15,17387126	0,054668525	17,25025482	18,77566393	30,28187085	17,62557214	204,8547838	22,76164264
ENSG00000111707	SUDS3	0,29906734	30,00797482	25,30729221	56,19632325	1,873769113	25,76121405	20,02378274	12,60383741	32,73547055	204,8087315	22,75652572
ENSG00000131931	THAP1	0	10,34666532	0,790357714	78,85281117	0	24,19248458	31,08728778	14,22667106	45,29159763	204,7878753	22,75420836
ENSG00000125630	POLR1B	0,0238759	2,033625534	36,32320143	47,78379453	0,056741593	10,90516668	49,12553053	29,81168242	28,6301721	204,6937907	22,74375452

ENSG00000136811	ODF2	33,58800577	27,38881997	35,35082441	14,77529498	0,280867779	17,12826226	28,63463388	17,52187054	29,88428358	204,5528632	22,72809591
ENSG00000136280	CCM2	27,74785369	15,37661543	14,24495383	15,66373932	8,149812455	35,82988296	32,60758994	19,95669972	34,87456702	204,4517144	22,71685715
ENSG00000167747	C19orf48	21,99828332	31,45892016	0	3,526148305	48,30880944	16,71335048	25,74145632	17,75984316	38,9273121	204,4341233	22,71490259
ENSG00000169641	LUZP1	37,39431185	1,971224064	5,123261298	6,727356458	73,58600293	10,61635291	22,09826411	31,33962144	15,49041732	204,3468124	22,70520137
ENSG00000173559	NABP1	6,807965736	14,52775721	38,11504617	21,98083264	19,66484587	25,41654858	20,03926424	34,27367092	23,38225476	204,2081861	22,68979846
ENSG00000276231	PIK3R6	3,900721802	10,98449271	86,49829679	0,146428156	0	32,49314886	39,39430458	8,715165361	22,05754178	204,1901	22,68778889
ENSG00000068784	SRBD1	3,354684621	30,02977801	5,069210923	37,80841753	0,071182863	35,84589269	21,73658477	32,4212311	37,83238422	204,1693667	22,68548519
ENSG00000173674	EIF1AX	16,29556675	20,1076126	21,66405846	46,70109423	24,24490884	20,93615569	21,83081445	22,6827535	9,683892189	204,1468567	22,68298408
ENSG00000187824	TMEM220	10,59669338	18,95644698	34,0739574	24,62071739	17,4030736	17,26264552	21,13824578	47,19148185	12,8736331	204,116895	22,679655
ENSG00000176261	ZBTB8OS	22,03963293	20,04800001	0,236176805	39,01831396	19,73061547	18,74775421	24,305213	36,12227218	23,84267193	204,0906505	22,67673894
ENSG00000093000	NUP50	13,17355388	13,08803837	28,95907296	68,97224863	0,087206785	33,13779731	13,57383023	3,530626753	29,3798169	203,9021918	22,65579909
ENSG00000166189	HPS6	93,60983256	22,57292035	0,10493775	30,63449494	0	17,5367918	17,76193828	0	21,60842917	203,8293449	22,64770498
ENSG00000122741	DCAF10	13,32310061	45,50425692	55,82400975	25,68121328	1,126167664	15,42842295	17,93298075	13,98896794	15,01835363	203,8274735	22,64749705
ENSG00000128513	POT1	23,49811655	32,37529321	4,10111389	77,34684341	0	22,14204237	8,021420242	12,94393507	23,3529376	203,7817023	22,64241137
ENSG00000006282	SPATA20	12,82613613	21,15191719	28,49074651	11,83179012	0	31,83294558	47,98293941	14,48785625	35,17050543	203,7748366	22,64164851
ENSG00000197070	ARRDC1	10,80500387	16,626219	35,93937955	10,60263485	11,93900009	30,20535565	34,66159304	19,18089838	33,78581896	203,7459034	22,63843371
ENSG00000149532	CPSF7	21,30468781	23,01152353	35,18502247	5,922480371	44,85994482	15,77744715	18,147051	17,46332456	22,04541252	203,7168942	22,63521047
ENSG00000053770	AP5M1	35,02970292	25,66287212	0	4,168866635	23,88784021	27,02944413	13,02987087	46,34561182	28,42157046	203,5757792	22,61953102
ENSG00000115365	LANCL1	2,243362107	0,051035782	0,052908279	42,61176403	0,068351264	46,38801621	41,5289127	58,30088391	12,30959074	203,554825	22,61720278
ENSG00000237190	CDKN2AIPNL	36,4483499	13,2397014 8	51,11552331	29,17377412	1,834312078	27,30650808	16,59170679	0	27,73986116	203,4497369	22,60552632
ENSG00000175906	ARL4D	23,36563957	32,79556782	0	21,1658281	0	15,03498889	17,65171971	73,49970418	19,903715	203,4171633	22,60190703
ENSG00000083635	NUFIP1	16,32688924	24,27147415	83,47390903	19,5701231	33,64147092	6,674563431	3,929961548	10,06496096	5,447225653	203,400578	22,60006423
ENSG00000114127	XRN1	10,49273867	5,370896346	85,49129233	9,756206695	36,08558847	14,59437054	16,22045938	10,83798907	14,31616926	203,1657108	22,57396786
ENSG00000110367	DDX6	1,529299092	10,83142087	56,65342098	28,29717095	6,704864253	32,61180678	27,21333496	16,63416031	22,4241616	202,8996398	22,54440442
ENSG00000185404	SP140L	57,47550732	14,7596747	36,90001314	4,756867679	15,76303549	22,1997161	15,41175405	9,181987097	26,38033821	202,8288938	22,53654375
ENSG00000012822	CALCOCO1	7,268523741	26,15725512	52,3594792	14,75053857	1,574014852	20,88332725	35,3793058	25,14095327	19,21541925	202,7288171	22,52542412
ENSG00000205758	CRYZL1	40,65821085	20,31576106	47,16256172	24,58542257	0,058137857	9,051621232	19,37812801	22,57461348	18,92463372	202,7090905	22,52323228
ENSG00000169964	TMEM42	17,32039104	6,723195704	54,85377401	25,57519614	19,17788243	13,18208424	13,40093419	22,27286079	30,1977905	202,704109	22,52267878
ENSG00000142252	GEMIN7	38,50755012	39,6262068	11,75790497	31,33944865	0	20,27314619	24,17678982	16,04721398	20,96232314	202,6905837	22,52117596
ENSG00000155906	RMND1	37,77764267	8,911577903	0,058844221	24,96668258	13,15142566	23,63628665	10,06631156	41,75898324	42,33083361	202,6585881	22,5176209
ENSG00000168724	DNAJC21	12,55565018	23,40209586	36,53652201	31,34714848	1,803855382	19,09811996	17,97497745	25,79575004	34,13150341	202,6456228	22,51618031
ENSG00000169902	TPST1	0	33,36240928	0	61,5825145	0,256055289	13,24918687	40,30259149	39,11025897	14,77496416	202,6379806	22,51533117
ENSG00000149925	ALDOA	28,78664089	22,39081564	19,73403154	15,05906646	31,36317658	20,54816897	24,15848578	16,2854626	24,25116774	202,5770162	22,50855736
ENSG00000183530	PRR14L	22,44457117	10,62667932	53,97648971	31,33252553	35,45863785	13,57181955	8,021856267	10,52107692	16,54032474	202,4939811	22,49933123
ENSG00000171863	RPS7	17,31926311	23,28955409	24,29338974	32,05612331	20,00099493	21,154787	16,08824458	25,44535531	22,69830376	202,3460158	22,48289065
ENSG00000083099	LYRM2	19,5964717	33,93889523	21,11687438	31,25203903	20,82260793	15,05148402	21,26125511	18,66431563	20,56630102	202,270244	22,47447156
ENSG00000139428	MMAB	22,49835017	45,80424993	12,14834929	23,80413776	28,82249468	16,4587262	17,38821151	13,17784915	22,04958739	202,1519561	22,46132845
ENSG00000144579	CTDSP1	24,00762398	24,95927227	27,46281071	24,89563646	28,26141861	18,2161179	14,81117945	17,8254315	21,66309155	202,1025824	22,45584249
ENSG00000101654	RNMT	8,916865528	22,2363532	46,90068127	30,53335352	5,554954823	15,20807349	28,71692789	24,65717698	19,32463122	202,0490179	22,44989088
ENSG00000135002	RFK	22,58063853	22,25273579	26,87811712	8,904709578	0,464216058	34,82921103	32,60022845	13,32673023	40,16744897	202,0040358	22,44489286
ENSG00000124596	OARD1	21,15409122	40,57655017	0,085152427	15,46666184	0,715044884	26,45319503	18,44869333	64,7305578	14,31778231	201,947729	22,43863656
ENSG00000134250	NOTCH2	10,9181747	19,30676217	55,05205473	17,96300979	37,31723182	14,70968334	8,240876296	21,88527994	16,54248522	201,935558	22,43728422
ENSG00000004534	RBM6	19,21230437	35,37755118	26,20226169	8,901604148	8,56096073	26,29929268	25,5272562	10,86509059	40,89183652	201,8381581	22,42646201
ENSG00000115828	QPCT	0,225875292	0	201,5251839	0	0	0	0	0	0,069449373	201,8205085	22,42450095
ENSG00000105186	ANKRD27	3,561658246	37,44217507	31,90980624	25,63140087	61,44666319	8,391705816	4,475945083	13,5979571	15,31949292	201,7768045	22,41964495
ENSG00000035687	ADSS	53,00507376	45,50855218	15,18752067	3,564063879	0,208728592	20,10598968	24,32104372	6,241861926	33,59387877	201,7367132	22,41519035
ENSG00000132466	ANKRD17	1,455640774	12,33604618	26,40834025	48,27795411	12,04812217	19,67449789	21,71401527	32,9556482	26,7920248	201,6622896	22,40692107
ENSG00000082068	WDR70	24,92390575	36,45573151	3,90965049	9,934731239	0,051018261	23,64090189	35,52526945	32,9542643	34,13830847	201,5337814	22,39264237
ENSG00000171428	NAT1	2,876470254	12,76056473	56,79542631	52,31944922	0	14,58033939	5,493307683	40,15115462	16,45024737	201,4269596	22,38077329
ENSG00000178307	TMEM11	25,93859297	29,98263525	16,04539103	31,14817618	18,88378736	23,01388779	15,47050331	22,76793031	18,14095699	201,3918612	22,37687347
ENSG00000071243	ING3	6,509378429	17,17140116	26,24773627	39,74240382	0,069061066	18,69684576	20,79912042	60,17721835	11,9728048	201,3859701	22,3762189
ENSG00000054282	SDCCAG8	1,192753988	36,73661305	16,40401261	7,94427098	38,13219534	25,31843418	15,09133856	36,39065149	24,16947818	201,3797484	22,3755276
ENSG00000146757	ZNF92	0	37,10621072	0,150333018	8,841475506	96,42592325	16,29816314	25,04404975	9,879165601	7,620130754	201,3734217	22,37482464
ENSG00000163050	COQ8A	29,82674981	1,478787483	46,8469732	17,83741714	41,4525828	8,958362549	15,93120799	17,50281857	21,49975158	201,3346511	22,37051679
ENSG00000184209	SNRNP35	17,58056834	19,98023075	12,16838955	25,43354519	51,91134902	21,43280602	16,91652531	7,585851939	28,16623363	201,1754997	22,3528333
ENSG00000143811	PYCR2	13,92801484	21,73721933	2,160866955	35,42398739	30,00954477	23,30670511	31,21083217	18,25546662	25,12694513	201,1596623	22,35107359
ENSG00000115084	SLC35F5	5,210709333	3,182159876	97,92999457	22,22173504	0,080411451	9,757988837	23,06563873	26,11809278	13,57991829	201,1466489	22,34962766
ENSG00000130305	NSUN5	37,22436729	16,25817048	2,035589477	7,184501351	34,71259197	24,29856132	33,00303402	15,67967192	30,59336424	200,9898521	22,33220579
ENSG00000148090	AUH	20,27745922	21,13785475	45,05789027	18,37407483	0	26,45164199	24,72732407	19,31681263	25,59710736	200,9401651	22,32668501
ENSG00000137672	TRPC6	0,026907153	16,90211325	30,39169886	39,40043776	0	46,82483335	33,512375	0,058838064	33,72108372	200,8382872	22,31536524
ENSG00000081019	RSBN1	17,46225688	19,89732395	45,57585433	41,32714072	0,15799276	15,88790242	12,47527278	17,15410425	30,82452217	200,7623703	22,30693003
ENSG00000130713	EXOSC2	22,6665856	0,063241038	0	19,81628817	60,47403897	15,22151282	16,89706594	46,13614716	19,41745504	200,6923347	22,2991483
ENSG00000172315	TP53RK	37,86067045	28,11912968	21,73219612	18,84302799	0	16,92965068	19,57888697	25,21054942	32,32620827	200,6003196	22,2889244
ENSG00000106853	PTGR1	39,76172045	8,179397881	73,88224381	1,661124507	1,14307971	17,119155	15,67547119	18,23087269	24,79596836	200,4490336	22,27211484
ENSG00000198740	ZNF652	35,26485054	5,326316687	12,36869453	55,35612571	0,244574944	19,39703678	24,22531609	12,26471096	35,92470232	200,3723286	22,26359206
ENSG00000118197	DDX59	17,02992146	14,22794286	37,25311531	39,11066173	0	30,54861412	20,28579291	13,11248284	28,74089075	200,309422	22,25660244
ENSG00000002330	BAD	13,18236243	34,22421313	24,08726853	21,84642937	0	33,998069	24,5357675	24,56978342	23,8292672	200,2731606	22,2525734
ENSG00000065457	ADAT1	21,45729682	18,70897147	0	23,79010357	43,86901766	17,75235774	17,28503459	12,44838206	44,86664815	200,1778121	22,24197912
ENSG00000181396	OGFOD3	48,01842775	12,26820831	3,776431701	11,44952028	45,70281028	23,2647873	17,30017226	22,59999072	15,78708746	200,1674361	22,24082623
ENSG00000134463	ECHDC3	17,15122267	15,46265081	0	0	99,76400376	13,36396407	17,34189486	0	36,99919322	200,0829294	22,2314366
ENSG00000102854	MSLN	43,09103599	55,43120945	0	29,94501061	0	2,948951518	18,32603767	0,638397542	49,69311956	200,0737623	22,23041804
ENSG00000170142	UBE2E1	13,9607684	33,67040079	5,606625289	60,437769	28,03780422	10,20074788	9,795992089	22,78859086	15,47713393	199,9758325	22,21953694
ENSG00000182872	RBM10	13,50040722	17,81548488	62,71645999	3,46732932	13,19911804	21,99945165	16,35335681	11,77120597	39,14494178	199,9677557	22,21863952
ENSG00000115993	TRAK2	0,301577775	18,33754498	43,97529772	48,46530396	0,38224326	29,04980295	18,24628431	15,65123816	25,51804297	199,9273361	22,21414845
ENSG00000169715	MT1E	21,25890158	29,5669886	0,264239638	27,28407529	0	22,71019301	32,20246164	35,17934559	31,44551422	199,9117196	22,21241329
ENSG00000170871	KIAA0232	1,689081564	6,373065539	44,77641552	52,80717217	0	17,2176878	13,07997578	21,42245519	42,42172646	199,78758	22,19862
ENSG00000070214	SLC44A1	7,026666365	24,91526471	22,791993	29,56205471	47,76629158	20,1867864	14,83337231	17,37414749	15,3294371	199,7860137	22,19844596
ENSG00000049239	H6PD	7,716836352	22,46742861	22,27777427	8,034367311	43,45934854	23,10395114	21,10690705	23,68096675	27,89392604	199,7415061	22,19350067
ENSG00000054967	RELT	26,28009925	24,31350607	26,64331642	22,98522081	0,116087778	25,66290963	33,76565424	15,6485095	24,30092719	199,7162309	22,19069232
ENSG00000136867	SLC31A2	23,76951556	11,49334897	25,0665435	5,59221741	19,7492866	29,98868896	26,68717939	33,93868991	23,37127197	199,6571923	22,18413247
ENSG00000128040	SPINK2	0	0	29,88646142	163,2533585	6,512494291	0	0	0	0	199,6523142	22,18359046
ENSG00000172331	BPGM	40,35170024	13,51798976	33,17367861	3,699747572	0	42,99691196	38,24893146	0	27,52197415	199,5109338	22,16788153
ENSG00000186470	BTN3A2	17,38136357	36,3259682	57,39606133	17,45248126	3,416767302	24,54967636	25,28293114	1,07923815	16,52086381	199,4053511	22,15615013
ENSG00000066697	MSANTD3	4,227193953	20,4424273	0,170906916	31,60417461	51,0028603	11,42452173	16,27681528	44,18660832	20,05291402	199,3884224	22,15426916
ENSG00000119318	RAD23B	22,89752632	15,57816085	36,09583336	36,71077358	9,264033718	22,47867124	11,75009396	31,23118826	13,3754705	199,3817518	22,15352798
ENSG00000126107	HECTD3	37,95640123	26,59215813	5,535486987	1,183261912	0,283215727	20,34628918	34,21243477	20,39156539	52,83728019	199,3380935	22,14867706
ENSG00000184277	TM2D3	30,36440759	15,3752181	29,12083431	22,75008985	10,83934738	18,6638937	16,20333193	39,78327734	16,18774147	199,2881417	22,14312685
ENSG00000102309	PIN4	6,622790041	32,67173062	11,29015088	15,20823395	54,7163947	16,14182756	17,18811111	29,41914002	15,99184164	199,2502205	22,13891339
ENSG00000158941	CCAR2	33,34885872	12,84488002	1,957007125	24,44019046	44,62862632	24,47777716	24,19538083	10,5188843	22,78283813	199,1944431	22,1327159
ENSG00000112640	PPP2R5D	7,555528533	6,744676076	54,65805014	10,96567225	27,31937132	16,64195049	21,55318587	32,23257438	21,43505901	199,1060681	22,12289645
ENSG00000047188	YTHDC2	32,27794513	25,6354175	0,385904811	21,1307823	28,71610889	20,45283555	14,89851328	40,52066654	15,08444239	199,1026164	22,12251293
ENSG00000177989	ODF3B	24,29852723	18,41218427	36,8465581	28,69037313	0,936421232	22,4242457	19,08501356	28,29013898	20,11116212	199,0946243	22,12162493
ENSG00000125686	MED1	11,902064	6,541568311	37,395557	46,31838304	12,47399456	19,92931416	30,60963025	0,038386915	33,78831405	198,9972123	22,11080136
ENSG00000144476	ACKR3	0,073000276	0,129537198	52,10448232	110,2725448	0	4,969302297	11,39537315	18,99599688	1,077371144	198,9176081	22,10195645
ENSG00000084092	NOA1	14,57964929	13,05650857	0	73,05708712	0	21,39333856	33,71520193	17,43049793	25,55599157	198,788275	22,08758611
ENSG00000179409	GEMIN4	14,8034194	23,56215592	40,09839952	38,45692074	7,810978388	18,50507737	9,409729092	36,97049363	9,151655786	198,7688298	22,08542554
ENSG00000146223	RPL7L1	21,67292088	23,48689052	11,7827395	21,08464195	32,51326706	23,85933892	21,24110371	20,4379429	22,68046302	198,7593085	22,08436761
ENSG00000094841	UPRT	47,43454617	32,85448198	0,150707562	2,121997625	0,194696418	17,89981283	21,08745076	72,46450667	4,534070752	198,7422708	22,08247453
ENSG00000110104	CCDC86	7,324928919	20,53990186	0,249533302	16,78666054	73,49982016	17,47472262	18,84683024	17,40170074	26,58127997	198,7053784	22,07837537
ENSG00000068001	HYAL2	27,54922415	14,61710518	16,59958067	12,68966739	60,27261172	23,83017783	18,39058474	16,96644353	7,734847342	198,6502426	22,07224917
ENSG00000276966	HIST1H4E	0	3,42615871	37,76192333	49,13368721	41,78031932	39,72035327	15,69433877	0,88886232	10,12344876	198,5290917	22,05878797
ENSG00000141858	SAMD1	54,75504549	8,88672117	37,46528068	21,90792982	0	18,69518209	17,57582644	10,6591796	28,5791382	198,5243035	22,05825594
ENSG00000175198	PCCA	2,239289466	25,46024479	34,90008103	0,859280926	52,82299745	13,40852493	29,90422155	30,3774918	8,4661149	198,4382469	22,04869409
ENSG00000257923	CUX1	16,13604011	12,21091349	21,10864923	29,74350259	25,3090438	21,16258749	35,26447327	8,48174291	28,98986199	198,4159149	22,04621276
ENSG00000109083	IFT20	29,12636348	23,34921042	26,3594392	19,2446619	24,14890198	31,05360655	21,77359341	9,11331779	14,22500496	198,3940997	22,04378885
ENSG00000112893	MAN2A1	4,987991753	17,45249243	6,917151849	50,97231666	18,87532745	24,27925409	30,00031214	27,54786703	17,34987	198,3825834	22,04250927
ENSG00000100413	POLR3H	27,06663048	11,74314866	33,78391262	17,00288515	53,00507444	17,54895932	17,15021056	4,957269614	16,05281371	198,3109046	22,03454495
ENSG00000088992	TESC	10,12111299	5,568888891	56,43313536	33,73458394	0,186457706	17,74536802	24,62501843	43,0628642	6,7545465	198,231976	22,02577511
ENSG00000063854	HAGH	19,64256262	32,65096113	10,37843151	33,87373022	0,430522829	30,26113447	27,99928799	19,75024218	23,23479384	198,2216668	22,02462964
ENSG00000198925	ATG9A	18,26505839	12,60108468	7,943969806	0,110472047	54,96231649	26,72691551	16,1485948	30,42739985	31,00064766	198,1864592	22,02071769
ENSG00000237765	FAM200B	6,305423439	22,65596721	67,86476986	13,37989178	0	20,52786043	15,42006156	38,41464767	13,54206546	198,1106874	22,0122986
ENSG00000177409	SAMD9L	7,798573507	18,60117931	0,272148349	62,5878995	2,461085255	23,32029784	19,3382246	33,55181762	30,15132389	198,0825499	22,00917221
ENSG00000164610	RP9	25,11885341	12,83583483	16,67003224	48,59073172	0	10,47305061	23,0347953	45,19357578	16,03305084	197,9499247	21,99443608
ENSG00000138175	ARL3	14,14937268	31,19231404	27,47899257	33,96102043	0	35,13807728	21,0426248	19,90881197	15,07518738	197,9464012	21,99404457
ENSG00000204392	LSM2	25,5418245	34,68881399	7,349804317	9,609503314	19,32927282	27,41676197	22,03728211	24,02593173	27,90330221	197,902497	21,98916633
ENSG00000129472	RAB2B	1,218294843	1,341828191	0,386405475	33,36949911	95,5450342	19,60300114	9,662296904	3,21523766	33,53194133	197,8735389	21,98594876
ENSG00000004399	PLXND1	19,72289334	16,43877326	12,16055886	21,75797899	12,83352893	28,79327117	44,28440451	26,63700303	15,21048247	197,8388945	21,98209939

ENSG00000104907	TRMT1	18,86636138	9,644822239	20,16264689	40,72261048	0	23,37741182	36,51470126	11,49252212	37,0146165	197,7956927	21,97729919
ENSG00000165669	FAM204A	48,8848681	31,61016323	2,720034251	35,12018363	5,784892848	24,29629693	12,90365859	15,83657649	20,63449372	197,7911678	21,97679642
ENSG00000105699	LSR	0	32,4602627	0	18,61264565	62,43916117	19,51554452	22,50376668	1,699766294	40,34322654	197,5743736	21,95270817
ENSG00000106460	TMEM106B	11,55333023	6,258842754	15,226833	12,52065495	80,11243339	15,23864308	16,46582764	24,57126535	15,53869246	197,4865229	21,94294698
ENSG00000155542	SETD9	8,806886818	27,49667135	34,55525391	36,76767256	1,722068628	16,4625397	11,43163851	46,31690729	13,91620952	197,4758483	21,94176092
ENSG00000196428	TSC22D2	13,88674566	11,29889209	22,66504404	42,97515897	10,24204707	23,24680571	24,41491901	24,70618169	23,98687128	197,4226655	21,93585172
ENSG00000174606	ANGEL2	10,94555179	14,58528304	6,952351799	12,5171759	81,81617372	11,90420292	12,36355192	20,99932676	25,27228231	197,3559002	21,92843335
ENSG00000025156	HSF2	0,111850904	18,16061659	0	64,70260288	0	23,39449657	18,11007753	71,9080406	0,962935349	197,3506204	21,92784671
ENSG00000155868	MED7	12,08449088	35,25642292	0	54,51811429	0	17,87005131	22,96070917	28,8058953	25,84177147	197,3374553	21,92638393
ENSG00000088682	COQ9	49,96200391	9,498894655	0	18,12467988	28,18727642	23,04847095	23,8216421	7,421197742	37,26397043	197,3281361	21,92534845
ENSG00000111490	TBC1D30	49,21424162	28,4963777	0,119240793	18,94401907	40,01320391	20,17614949	17,27655832	1,417413694	21,64389525	197,3010998	21,92234443
ENSG00000185900	POMK	38,4015675	0	59,97972301	53,79959253	0	9,192701107	23,66355755	0,176043989	11,88150382	197,0946895	21,89940995
ENSG00000134326	CMPK2	21,88328348	0	0,06461648	24,74697109	0	19,12869504	17,09103081	85,94985613	28,16641853	197,0308716	21,89231906
ENSG00000241973	PI4KA	30,1903209	13,39887263	30,14476922	30,74398228	1,164841682	21,38060382	30,77636442	12,94856243	26,19805451	196,9463719	21,88293021
ENSG00000241878	PISD	4,92423491	17,36865414	29,06509232	38,86376571	1,220511439	18,37554168	42,01575492	11,89088808	33,17891731	196,9033605	21,87815117
ENSG00000170906	NDUFA3	10,07403804	41,81931799	6,065020341	22,24536551	6,239213867	40,15371129	29,35245343	18,69124826	21,92613515	196,5665039	21,84072265
ENSG00000164172	MOCS2	8,448111771	22,3079867	0	32,99676883	0	21,06318969	12,65141784	84,61544083	14,47190889	196,5548245	21,83942495
ENSG00000125863	MKKS	23,43931011	41,09733212	0,315886476	16,67908218	42,54318613	13,39615957	21,85037316	16,52172838	21,69969337	196,5427515	21,8380835
ENSG00000137185	ZSCAN9	38,33844438	16,21155412	0	0	74,73134282	22,65877242	15,40863726	0,089186118	29,0933827	196,5313198	21,83681331
ENSG00000124787	RPP40	23,10559184	21,5934944	1,416820076	49,07492642	0	16,15133545	12,02264139	42,94638874	30,21663639	196,5278347	21,83642608
ENSG00000136504	KAT7	8,440550853	17,4238067	14,18729656	43,72496734	0,61815568	22,48981481	17,35354364	44,82011175	27,3675359	196,4257832	21,82508703
ENSG00000121879	PIK3CA	11,87297341	0,471885507	66,09940863	2,971309235	55,94947218	11,61030537	9,24791681	20,3528538	17,81510141	196,3912263	21,82124737
ENSG00000126883	NUP214	27,87076824	17,98030819	41,7611819	17,77351677	10,7085682	14,71635479	31,14680156	9,553167698	24,86767966	196,378347	21,81981634
ENSG00000085433	WDR47	19,16640971	0	54,92982286	61,05984597	0	6,977021765	17,72981178	5,453701883	31,05145376	196,3680677	21,81867419
ENSG00000074590	NUAK1	27,68635456	9,489043382	68,503372	5,473379176	0,307463649	27,2256226	20,7863949	21,7837857	15,06290306	196,318319	21,81314656
ENSG00000115170	ACVR1	28,89658281	32,19048071	0,418015186	28,77478417	2,160105518	23,90941895	11,35207766	26,83228067	41,73395588	196,2677015	21,80752239
ENSG00000121022	COPS5	5,691656666	44,36286851	11,93538412	27,17024417	0,461650009	35,74574194	23,80694306	23,10790651	23,91468939	196,1970844	21,79967604
ENSG00000198912	C1orf174	10,57222999	35,87040885	23,18612494	3,151675855	0	33,95344072	25,76316525	26,50705738	37,1862458	196,1903488	21,79892764
ENSG00000150403	TMCO3	43,46557788	12,94086717	35,96255399	9,677291708	0,034593733	24,90017132	19,90652508	28,36115978	20,76697484	196,0157155	21,77952394
ENSG00000115839	RAB3GAP1	0,227953795	0	9,183533214	13,97373902	2,004427375	42,84792688	39,31239698	65,54856785	22,76472712	195,8632722	21,7625858
ENSG00000082269	FAM135A	7,24942581	18,33363	35,43990307	39,94481007	7,155897765	29,39354442	17,92771861	9,299225717	31,08265095	195,8268064	21,75853405
ENSG00000136933	RABEPK	8,752949844	20,02589474	30,97749412	46,26397815	0	23,70847994	13,79767838	17,48845109	34,80865472	195,823581	21,75817566
ENSG00000149262	INTS4	23,58689804	21,21446515	53,33259193	33,41791252	0	18,48616282	25,64417532	6,535709264	13,60076741	195,8186825	21,75763138
ENSG00000003400	CASP10	36,37552431	31,05619375	11,08554488	21,18937606	0	30,67969996	23,00344465	16,96097412	25,43832366	195,7890814	21,75434238
ENSG00000146072	TNFRSF21	27,9943366	12,38152212	0	3,919468325	88,10616755	9,737727868	20,59179301	0	32,72346275	195,4544782	21,71716425
ENSG00000144401	METTL21A	12,15908828	25,40209592	8,914336802	14,35564585	63,23384793	17,93825741	18,10626592	15,4572216	19,82034843	195,3871081	21,70967868
ENSG00000185669	SNAI3	13,72153458	11,40374023	12,94045288	23,54413277	0	38,90316971	21,34437832	10,44477081	63,01692422	195,3191035	21,70212261
ENSG00000111596	AC092881.1	27,66706797	17,82120061	24,41488899	20,60552754	0,205312681	20,3697828	27,52761518	31,87927293	24,82292218	195,3135909	21,7015101
ENSG00000111530	CAND1	3,683181067	34,59387218	31,13458988	45,32736414	1,137117402	28,73502772	15,13405035	2,96855797	32,59841061	195,3121713	21,70135237
ENSG00000103496	STX4	11,88761333	26,58275809	31,80832699	17,82465794	12,22150946	22,58511426	34,65518418	13,03810871	24,61148766	195,2147606	21,69052896
ENSG00000108405	P2RX1	21,33010336	20,86152915	21,06336787	10,05122051	0,091007994	44,86228721	40,44558092	6,95034696	29,5300987	195,1855427	21,68728252
ENSG00000037637	FBXO42	6,340303664	19,8822129	0,123423287	64,53137123	16,30359361	21,54682814	15,97452223	25,85818294	24,58974964	195,1501877	21,68335418
ENSG00000089123	TASP1	18,98662331	35,91270143	63,23399559	1,170919803	0,247923743	0	8,835328867	52,01179247	14,690664	195,0899492	21,67666102
ENSG00000135093	USP30	26,27243294	0,052147413	57,1421557	79,72167349	0,279360204	1,226099167	6,978707692	9,896328938	13,33671076	194,9056163	21,65617959
ENSG00000166579	NDEL1	48,49595059	27,37037135	11,35682365	24,89624711	0,225718128	20,81442559	10,61490049	21,30897965	29,71675378	194,8001703	21,64446337
ENSG00000129347	KRI1	29,00388033	14,01351442	7,947872528	59,62306075	27,94165082	11,15920682	12,46764021	12,00313053	20,55766546	194,7176219	21,63529132
ENSG00000164010	ERMAP	0	9,938130073	33,58116231	56,56632317	30,13573782	14,79272624	9,498016391	10,51382715	29,66396413	194,6898873	21,6322097
ENSG00000173653	RCE1	20,68625508	24,62071787	13,61473436	13,23705923	4,196699159	14,23673396	31,16858999	37,29111313	22,52497081	194,5059037	21,61176708
ENSG00000156928	MALSU1	4,394802367	15,94508388	23,82067844	38,01208017	15,20024913	17,70689354	25,67401236	31,49038854	22,24753227	194,4917207	21,61019119
ENSG00000143514	TP53BP2	0,050547105	8,072513801	0	6,589928276	96,70166302	14,70690846	47,63633612	0,221063434	20,39058682	194,369547	21,59661634
ENSG00000197343	ZNF655	10,34264708	44,46158864	2,250047224	30,00395018	39,36998649	19,74890807	8,73707228	21,82968515	17,47634241	194,2202275	21,58002528
ENSG00000140553	UNC45A	19,93176239	17,59480715	0,185369498	19,66234471	46,93720879	29,71697846	28,24672185	13,13276889	18,78789862	194,1958604	21,57731782
ENSG00000062822	POLD1	26,90086801	16,63654745	0,05636255	24,21956173	0,072813776	16,18065438	34,59846949	25,25827899	50,45589889	194,1794553	21,57549503
ENSG00000165832	TRUB1	28,21514406	19,59470576	85,81158376	6,887697301	28,53782082	9,680806087	3,730293283	6,243276074	5,473668064	194,1749952	21,57499947
ENSG00000116157	GPX7	16,07559262	0	0	40,40482467	0	46,08627131	57,54431302	9,569322582	24,49395654	194,1742807	21,57492008
ENSG00000185414	MRPL30	11,01583362	20,16698236	41,05462386	13,26602182	2,640570164	18,57780197	16,86996061	50,39821345	20,14638771	194,1363956	21,57071062
ENSG00000105516	DBP	9,352044792	25,24075062	32,58110268	18,02301475	12,73155101	24,23754757	22,03016679	15,5522382	34,22143327	193,9698497	21,55220552
ENSG00000051128	HOMER3	30,0278165	16,14180363	37,61098359	12,73411477	19,30965161	23,02966801	16,02295005	17,86393775	21,18063813	193,921564	21,54684045
ENSG00000133316	WDR74	9,286522002	7,702689901	13,28701674	34,46774682	62,65742851	16,17305834	13,9148873	18,59521112	17,7663499	193,8509106	21,53899007
ENSG00000179331	RAB39A	39,60139093	0,284500796	77,71645208	0	0,38102657	18,48340642	42,99938168	0,175296853	14,09870169	193,740157	21,52668411
ENSG00000273802	HIST1H2BG	49,35227895	60,83012779	1,268488066	9,185455429	2,106947771	30,39170277	7,279569458	3,446514394	29,80328845	193,6643731	21,51826367
ENSG00000046647	GEMIN8	49,06106399	14,0486939	0,688069552	7,535250917	90,6682904	8,692509588	12,2157584	0	10,65704067	193,5666774	21,5074086
ENSG00000104695	PPP2CB	7,002684982	23,12799069	56,27402004	16,89423782	14,89023956	16,88535581	12,15754658	29,23356817	16,89513839	193,360782	21,48453134
ENSG00000176731	RBIS	5,885108771	20,67499293	15,85645726	47,0134612	11,86698409	20,49406809	23,14932561	32,23751878	16,08427809	193,2621948	21,4735772
ENSG00000159216	RUNX1	20,92882374	15,68494684	23,34647293	27,92979342	31,06018554	26,15323397	22,24371303	5,452785659	20,38178118	193,1817363	21,46463737
ENSG00000113273	ARSB	36,15174794	10,45569997	3,154960108	43,01512616	0,04035482	24,55379588	27,07079868	17,34048129	31,34685132	193,1298162	21,45886846
ENSG00000172831	CES2	17,53482197	26,59840166	0,05202697	16,70218254	0,134425432	27,20150949	30,10536508	21,70738035	53,07913026	193,1152438	21,45724931
ENSG00000198001	IRAK4	21,36862929	44,63475823	17,2348649	27,30337538	0	19,06984245	20,43991729	26,18117199	16,87112682	193,1036864	21,45596515
ENSG00000160688	FLAD1	17,63395313	51,70690472	0	14,34850196	0	31,00244914	42,48945555	11,92162535	23,8146203	192,9175102	21,43527891
ENSG00000173436	MICOS10	13,01536302	28,08640658	18,8701819	28,1885958	13,43414164	19,89110381	20,65223348	29,96824454	20,78903616	192,8953069	21,43281188
ENSG00000159579	RSPRY1	21,20237456	28,7263384	12,9399782	13,95558464	51,39566016	23,82670877	12,94469421	3,272709483	24,60364986	192,8676983	21,42974425
ENSG00000132694	ARHGEF11	32,7209258	22,47000001	16,15079905	16,61820509	18,13643901	27,34280454	22,0238985	12,07286073	25,29173901	192,8276717	21,42529686
ENSG00000168769	TET2	17,72159904	17,62764272	15,25397654	30,14199688	26,42044806	14,82459445	18,22571959	41,6402915	10,95404717	192,8103159	21,42336844
ENSG00000134574	DDB2	13,7317141	18,01484951	27,24738565	13,00145677	46,05387311	19,58266558	19,49824845	0,809723261	34,8391071	192,7790235	21,4198915
ENSG00000185722	ANKFY1	57,60134379	11,48048918	8,168987723	6,69581452	0,075741889	23,89980116	36,66902208	19,69969863	28,37870124	192,6696002	21,40773336
ENSG00000175073	VCPIP1	22,31142415	4,336165329	18,12063924	49,77054206	0,108211393	10,64853942	23,16336583	41,6196211	22,45614366	192,5346522	21,39273913
ENSG00000171953	ATPAF2	27,64781916	10,28529552	35,99274107	11,65349788	26,19171703	19,65949207	26,2328571	15,59046582	19,27743505	192,5313207	21,39236897
ENSG00000095321	CRAT	40,7538225	30,70113236	0	8,702400676	0,943060773	37,25145564	21,05450161	0,173547567	52,65976826	192,2396894	21,35996549
ENSG00000141698	NT5C3B	0,166055572	30,64480001	17,7174129	24,56420823	0	12,94467137	47,56842257	45,02624876	13,47897854	192,110798	21,34564422
ENSG00000054267	ARID4B	19,20099096	24,96653139	18,07510803	33,65078513	8,626864108	16,61652936	21,03069956	22,38110048	27,49179116	192,0404002	21,33782224
ENSG00000158411	MITD1	9,442216843	30,9562309	20,14629289	39,09478714	10,51075734	10,94508799	16,26305349	32,54454277	22,10300102	192,0059704	21,33399671
ENSG00000075426	FOSL2	21,68970492	21,86469255	31,51631393	23,46301643	5,164633742	14,50153882	23,54539601	22,00744048	28,20653026	191,9592671	21,32880746
ENSG00000100083	GGA1	12,36193505	15,23166532	24,41737683	13,9924556	67,81449807	18,81253825	15,82310361	5,58031839	17,79186467	191,8257558	21,31397287
ENSG00000070010	UFD1	26,9233922	27,69960354	34,00650833	11,63766397	4,323244892	15,02744271	20,74389117	27,69406889	23,75818302	191,8139988	21,31266653
ENSG00000123684	LPGAT1	12,72148149	19,70645416	17,55164447	30,63536903	0,040855238	33,35198965	17,98550834	28,90832565	30,91099153	191,8126196	21,31251328
ENSG00000145390	USP53	2,174980599	20,44162026	17,09752073	1,812351706	47,27128821	46,62156624	26,13352773	1,189011414	28,98894707	191,730814	21,30342377
ENSG00000139514	SLC7A1	4,736946869	6,661696412	23,71942572	22,50258691	81,23119227	15,58109214	8,568148322	0,859611258	27,79962287	191,6603228	21,29559142
ENSG00000144591	GMPPA	35,6804592	14,03068605	12,77290402	22,90276971	24,98736088	24,64388161	19,1546981	8,180300621	29,20843692	191,5614971	21,28461079
ENSG00000165802	NSMF	27,8102516	7,860840393	32,87997506	30,43887224	13,0136243	12,63244212	28,88985047	15,40503175	22,3984408	191,3293287	21,2588143
ENSG00000163608	NEPRO	18,0447908	27,30999415	0,042832065	42,05515923	21,24824904	19,27528118	15,29230086	27,44287086	20,617796	191,3292742	21,25880824
ENSG00000023228	NDUFS1	11,91606274	19,20649122	33,58869311	36,50174649	0,058997461	26,91583613	20,13874065	24,45552368	18,51748246	191,2995719	21,25550799
ENSG00000205339	IPO7	0,062823131	19,2113933	2,889205574	41,62086416	44,83992595	15,10577174	20,32633289	27,65832577	19,57363594	191,2882785	21,25425316
ENSG00000141994	DUS3L	22,06905551	22,9793778	37,8402229	13,08629523	0,098957714	20,34450445	24,13289935	14,38651465	36,30895131	191,2467789	21,2496421
ENSG00000117751	PPP1R8	14,81581439	35,05373216	0,296248773	58,67560885	0,510291357	17,62247333	25,71807516	5,399641245	33,14207964	191,2339649	21,24821832
ENSG00000132313	MRPL35	9,520953603	11,98752471	0,363372678	18,2142845	0	32,444735	41,26579808	57,36171316	19,96939102	191,1277728	21,23641919
ENSG00000155016	CYP2U1	9,623088706	28,80714731	0	28,8262624	81,83269673	18,30060922	9,325497649	0,16744995	14,22102611	191,1037781	21,23375312
ENSG00000114021	NIT2	21,21051257	18,10670562	20,78975377	17,81801799	15,49195554	20,78827516	16,40117282	32,12657783	28,3221559	191,0551272	21,22834747
ENSG00000128563	PRKRIP1	17,13077086	27,56871213	10,07828281	58,52681113	0,097163837	23,65662173	15,7856343	14,12572015	24,03534568	191,0050626	21,22278474
ENSG00000115540	MOB4	38,91624841	10,44709091	1,702892055	63,93897932	0	13,78183913	13,89673856	36,43605866	11,84025871	190,9601058	21,21778953
ENSG00000135069	PSAT1	108,0241931	0	0,252021849	2,957105333	0	25,721404	26,95962709	0	27,04294363	190,957295	21,21747722
ENSG00000125482	TTF1	17,20263417	61,13470795	7,954640469	1,055102541	6,925439386	26,62826052	11,66893601	43,68108591	14,68274235	190,9335493	21,21483881
ENSG00000010322	NISCH	38,40296229	11,2145021	26,16608082	17,62218103	0,951094793	22,90378851	26,86078615	21,76886303	25,04067576	190,9309345	21,21454828
ENSG00000092931	MFSD11	25,3036222	47,22099895	0,042201321	38,86097011	0,163557405	16,22080027	27,35318001	15,90219491	19,86275265	190,9302778	21,21447531
ENSG00000138386	NAB1	4,174325156	4,608947822	0,455052341	74,05705131	45,26629959	10,62615316	6,598296567	38,74340233	6,388830723	190,918359	21,213151
ENSG00000172057	ORMDL3	53,88751161	8,790369406	2,667186451	14,46547834	49,00537891	23,61307248	16,6870191	0,088068915	21,69530297	190,8993882	21,21104313
ENSG00000115963	RND3	3,994297221	31,26085087	7,657214747	0	0	19,06540573	57,63815196	48,26882247	23,01104686	190,8957898	21,21064332
ENSG00000130723	PRRC2B	12,59864564	8,656723167	34,41658368	21,52344988	51,59235072	13,89953015	21,53958	8,240862095	18,20965924	190,6773846	21,18637606
ENSG00000165097	KDM1B	29,00807258	6,291280486	21,34507692	23,56113884	45,6397161	6,399323701	12,55595395	37,94182912	7,895008678	190,6374004	21,18193337
ENSG00000181915	ADO	24,66431151	21,36560188	13,79553841	15,61149421	30,79281932	26,11936567	17,3313031	24,68921086	16,19175656	190,5614015	21,17348906
ENSG00000138190	EXOC6	11,88290779	6,841019863	46,90605578	48,07473051	0	24,35212566	21,39458592	6,456092793	24,6374931	190,5450114	21,17166793
ENSG00000162694	EXTL2	11,40386125	30,05327523	53,51895804	5,199202483	0	11,65299692	25,90657664	24,03160491	28,55972708	190,3262026	21,14735584
ENSG00000186522	SEPT10	0	17,91397978	0,653916958	20,68574134	0,084478368	38,32608476	31,1093225	31,79198732	49,57655993	190,142071	21,12689677
ENSG00000166260	COX11	21,59055163	32,27788256	15,42429649	27,10699335	0	26,49250269	17,77238519	37,12805174	12,34584635	190,13851	21,12650111
ENSG00000141279	NPEPPS	32,84382325	22,58283976	6,276969977	31,73533475	31,55976309	16,96714093	15,74122125	12,12841787	20,30215278	190,1376637	21,12640707
ENSG00000055332	EIF2AK2	22,02085597	25,11103823	40,53499444	32,17704606	5,276720142	19,90371468	13,52367849	9,218857087	22,22622781	189,9931329	21,1103481
ENSG00000172296	SPTLC3	20,62773593	17,86145843	5,900597856	61,29386116	0,042115344	9,533972392	17,33365206	15,61687941	41,47600937	189,6862819	21,07625355
ENSG00000114302	PRKAR2A	23,66983282	7,804883423	10,96905717	29,28422521	12,39265337	35,34778979	27,89902956	13,93129985	28,38586042	189,6846316	21,07607018

ENSG00000104361	NIPAL2	22,72319141	15,35383197	0	10,14695406	0	25,03752192	27,05915651	42,96946124	46,37875217	189,6688693	21,07431881
ENSG00000010292	NCAPD2	1,75166813	11,68717837	54,86562386	52,2273249	0	16,10383959	28,58474024	0	24,27207285	189,4924479	21,05471644
ENSG00000103353	UBFD1	20,47127465	24,67166103	37,9951007	13,52060159	15,73856542	18,61974632	19,33701364	3,760393383	35,36933814	189,4836948	21,05374387
ENSG00000134531	EMP1	76,43305004	10,44086685	1,455021583	48,26947251	5,318222254	17,11422775	5,558305273	23,07515747	1,75573122	189,420055	21,04667277
ENSG00000118263	KLF7	13,83521528	13,11469726	54,2334311	40,00514618	0,310185742	31,27616788	15,48397641	6,136336171	15,0089828	189,4041388	21,04490431
ENSG00000073060	SCARB1	30,23773078	21,40702184	0	31,65197535	0,231958108	24,94766972	31,46078212	26,1666955	23,28783447	189,3916679	21,04351865
ENSG00000113391	FAM172A	0,027905923	1,634106372	6,365564593	20,19053635	0	47,12289325	28,50018622	63,03580946	22,44572956	189,3227317	21,03585908
ENSG00000134184	GSTM1	0,120310964	0,106744427	0,110660867	0	77,62771716	39,49622805	0,199793522	36,96342097	34,62427246	189,2491484	21,02768316
ENSG00000089159	PXN	32,40977387	10,81913432	17,55075424	12,7669257	19,83330127	26,06530776	34,19318833	17,23043522	18,36044061	189,2292613	21,02547348
ENSG00000104133	SPG11	1,287620656	12,91732743	73,78093237	9,069873411	25,60147838	10,19007001	11,12539601	20,76889841	24,39696184	189,1385585	21,01539539
ENSG00000144674	GOLGA4	22,46181157	18,33065363	10,35596904	12,88027722	13,11111825	15,8694386	8,180053311	61,08900219	26,77699264	189,0553165	21,00614627
ENSG00000100151	PICK1	10,75163292	27,9736335	18,63376092	1,193937198	48,14524819	16,47339059	20,98348494	21,84753832	23,04479869	189,0474253	21,00526948
ENSG00000087008	ACOX3	28,17115943	13,17702497	52,61817786	0	0	28,21256297	27,92579412	2,491576302	36,43480962	189,0311053	21,00345614
ENSG00000089057	SLC23A2	6,706209222	7,19700824	1,551310677	48,7822893	0,238584602	20,41365852	40,278544	11,81064293	52,00541801	188,9836655	20,99818505
ENSG00000100028	SNRPD3	6,061410218	25,07408585	58,23803811	31,33353042	6,422643834	16,1436581	12,85477852	16,04051299	16,096569	188,865227	20,98502523
ENSG00000117682	DHDDS	15,35895601	25,72403639	36,8789575	36,15308047	0,192109955	13,6385121	10,537897	24,15801938	26,21337359	188,8549424	20,98388249
ENSG00000147454	SLC25A37	23,37716057	20,3889865	21,03276137	33,90588277	0,853330829	12,59476239	23,89836364	13,43061962	39,37268743	188,8545551	20,98383946
ENSG00000156873	PHKG2	41,62302922	34,56545954	10,13739875	12,47858949	0,14391566	18,61673583	22,77774261	13,41866264	35,05413965	188,8156734	20,97951926
ENSG00000034677	RNF19A	1,42021213	20,06941149	75,62274736	10,34465381	0,122733268	21,60353032	18,0100955	31,39467183	10,19424704	188,7823028	20,97581142
ENSG00000065357	DGKA	23,65872571	20,5793276	14,78266124	21,08565451	32,18472726	15,15466987	12,61164458	15,54331745	33,09091375	188,685472	20,96505244
ENSG00000105393	BABAM1	19,08641846	30,8555498	20,67887602	19,81528672	0	25,32691485	21,92449428	14,72296601	36,21883018	188,6293363	20,95881515
ENSG00000124766	SOX4	49,44920417	6,048315791	14,63053153	23,27547108	23,4420542	4,791334153	15,41578413	33,70974655	17,81611398	188,5785556	20,95317284
ENSG00000171236	LRG1	11,07323945	8,038305702	38,3740107	16,32050729	41,99894278	5,342050397	6,222436894	49,77308252	11,41766199	188,5602377	20,95113753
ENSG00000104231	ZFAND1	13,41133209	33,53365782	54,75165689	29,53630348	0	19,56752649	11,76098271	0,078413519	25,79976638	188,4396394	20,93773771
ENSG00000213859	KCTD11	8,702703023	35,27260603	33,01923296	38,93532344	0	18,83866823	17,57241561	17,9489981	18,12246847	188,4124159	20,93471287
ENSG00000172262	ZNF131	18,3653641	4,376671913	48,73465098	54,93851685	0,168678977	17,08590603	18,07798231	3,0265261	23,54725194	188,3215492	20,92461658
ENSG00000169925	BRD3	20,52607228	6,941430485	13,05811111	13,81331163	40,68536438	23,88764848	24,36051385	13,64796643	31,32578815	188,2462068	20,9162452
ENSG00000110697	PITPNM1	2,814850742	2,3725697	59,07400737	10,28833958	43,92806945	11,95009432	26,52756786	0	31,18598115	188,1414802	20,90460891
ENSG00000105552	BCAT2	39,76069924	21,90837339	11,84452871	17,30778878	17,35248393	18,62075658	26,45541812	4,935108849	29,7158507	187,9010083	20,87788981
ENSG00000171823	FBXL14	2,962952366	5,952097671	37,43424196	5,019836729	47,56347768	24,85896543	27,85135006	4,523151683	31,69635471	187,8624283	20,87360314
ENSG00000153214	TMEM87B	19,25445723	20,40922947	3,030040545	49,08774846	0,674906112	26,19148364	32,13983509	6,458409138	30,59604464	187,8421543	20,87135048
ENSG00000262814	MRPL12	17,52223664	21,79152032	34,4375742	19,65417053	13,88065823	28,44651117	20,64229446	10,47958985	20,81333698	187,6678924	20,85198804
ENSG00000172469	MANEA	11,67540247	22,42266436	0	58,37100299	0	31,87145375	19,19526852	23,3023221	20,80478409	187,6428983	20,84921092
ENSG00000099899	TRMT2A	39,66939108	23,19285888	0,14060703	28,11287283	0,27247158	25,4698331	29,57469636	8,774817802	32,43148719	187,6390359	20,84878176
ENSG00000196459	TRAPPC2	15,11848022	14,53332275	25,58126996	11,36543364	89,50006541	9,073871397	10,23157448	5,248428533	6,908000746	187,5604471	20,84004968
ENSG00000181026	AEN	44,0486302	16,7364401	8,372894548	21,17695159	0	21,26631555	13,79417807	18,63394398	43,44504545	187,4743995	20,83048883
ENSG00000103145	HCFC1R1	13,91946638	27,69366086	7,759388667	9,65077017	22,30388086	31,02863542	21,10143057	24,44247324	29,56943969	187,4691459	20,8299051
ENSG00000237541	HLA-DQA2	19,5333218	66,50766144	6,931262311	7,704772872	18,61567313	0,762056455	66,11068018	0	1,219465369	187,3848936	20,82054373
ENSG00000138592	USP8	19,83230153	14,57561766	21,95182217	13,00819189	69,15809461	9,114994137	13,93236766	4,562465566	21,23085604	187,3667113	20,81852347
ENSG00000244754	N4BP2L2	31,03290788	20,1897013	16,81648476	15,95457362	13,23398962	23,67356106	17,62946941	25,95158278	22,77058215	187,2528526	20,80587251
ENSG00000126777	KTN1	31,37749362	21,73753272	7,79634707	27,68481782	5,761243995	16,61182245	18,18741643	25,69712446	32,35239266	187,2061912	20,80068791
ENSG00000186153	WWOX	30,9110964	16,08081003	30,71540219	35,85243336	0	28,96364555	14,55960928	3,528978745	26,56586147	187,177837	20,79753745
ENSG00000186350	RXRA	22,12459372	15,90956728	42,78333965	13,15384196	8,939489143	15,246957	27,91961171	17,65973465	23,39663124	187,1337663	20,7926407
ENSG00000134318	ROCK2	12,80246451	10,33337969	40,28776096	1,637560147	33,13685108	26,29059541	12,52488703	22,61652561	27,48152143	187,1115459	20,79017176
ENSG00000083750	RRAGB	43,91910898	7,67525725	48,76127975	0	0	11,9333846	20,58173886	16,37015289	37,78310948	187,0240318	20,78044798
ENSG00000096872	IFT74	17,47436258	31,60033392	2,047484157	33,89015506	0	25,25523142	22,61115239	32,45122195	21,44558445	186,7755259	20,75283621
ENSG00000064102	INTS13	16,79555215	16,56712586	0,908728666	8,871277332	0	37,09801715	45,69269364	28,6254202	32,13887227	186,6976873	20,74418748
ENSG00000037897	METTL1	51,22392226	56,3277237	1,998829679	0,134018991	0,184446606	17,29832926	18,81732765	13,74689264	26,82222535	186,5537161	20,72819068
ENSG00000151503	NCAPD3	15,95000757	33,51039479	66,61274261	7,615613698	0,078805693	15,67357983	16,4375221	17,04018507	13,5398388	186,4586902	20,71763224
ENSG00000168438	CDC40	17,88966454	17,02060051	11,24990829	54,15879574	0,542746915	17,91444178	17,1507725	26,96745589	23,56240543	186,4567916	20,71742129
ENSG00000143315	PIGM	22,58393527	0,91425032	6,555575569	40,85102174	42,03904744	16,00415991	10,42763805	34,2686794	12,80516598	186,4494737	20,71660818
ENSG00000080189	SLC35C2	13,62504497	25,60359523	14,53372858	18,72042566	42,16416396	31,05980309	20,85203623	8,402332023	11,33210502	186,2932348	20,69924831
ENSG00000133983	COX16	35,88051214	11,85562337	46,12770201	21,3647632	2,156273453	17,65969566	19,24517647	9,379147113	22,62214763	186,2910411	20,69900456
ENSG00000091947	TMEM101	27,68780677	27,79682668	5,689919491	0,430727921	0,11855977	25,68902624	32,14426465	44,94521024	21,7199303	186,2222721	20,69136356
ENSG00000068831	RASGRP2	32,81016829	15,91046384	12,38208528	28,73544263	34,17637361	11,31704295	13,858656	13,36076654	23,58210309	186,1331022	20,6814558
ENSG00000173011	TADA2B	14,57649853	24,66258603	43,07612233	13,55330929	0,123940481	14,1431967	11,17216416	26,17247049	38,58039108	186,0606791	20,67340879
ENSG00000196151	WDSUB1	0,070777785	12,43375082	64,57991995	0,122217787	0,336409876	36,29051022	14,63330804	42,87137012	14,71103619	186,0493008	20,67214453
ENSG00000075292	ZNF638	15,95419445	20,95867004	6,195163341	31,712155	28,29986725	20,11037293	28,10358406	11,86745921	22,83972412	186,0411904	20,67124338
ENSG00000089335	ZNF302	24,08399795	13,69485175	43,03388257	9,956183451	19,62585933	8,605457178	6,906852537	49,24998522	10,82134957	185,9784196	20,66426884
ENSG00000113734	BNIP1	23,84326627	19,01170738	82,02691445	0,32082169	4,856917581	19,31090319	26,43104535	0,135424003	9,825492366	185,7624923	20,64027692
ENSG00000186432	KPNA4	22,83405682	12,74417434	13,09326553	35,73118525	6,467483446	23,40852553	13,95897556	28,41652771	29,0929818	185,747176	20,63857511
ENSG00000065526	SPEN	0,067424959	3,848546592	53,95464106	4,831769813	40,27286784	19,3214816	22,27245352	14,62099856	26,52186185	185,7120458	20,63467176
ENSG00000102119	EMD	5,909769843	24,82304654	14,72468841	17,56789928	40,35627827	21,76549645	20,68397057	21,10200951	18,76865868	185,7018176	20,63353529
ENSG00000145365	TIFA	30,85410853	6,615345397	62,83290771	15,572717	0	28,10656151	13,00103769	11,33539635	17,37944452	185,6975187	20,63305763
ENSG00000204252	HLA-DOA	16,60055931	14,05610184	20,84676438	31,61063274	10,62851016	23,92740948	33,01188319	5,221313708	29,78578574	185,6889605	20,63210673
ENSG00000178951	ZBTB7A	18,40679206	8,672842139	22,76632265	8,749451671	52,90856373	21,31729368	18,44823427	9,952257746	24,31997517	185,5417331	20,61574813
ENSG00000109103	UNC119	11,4924186	17,56936662	0,054208288	36,53496719	30,32330262	18,02109813	22,0209241	9,987784315	39,46701692	185,4710868	20,60789853
ENSG00000135945	REV1	23,08768791	7,040927792	18,10502382	29,18683137	0,323570961	24,51606926	24,56434225	31,98439886	26,64860675	185,457459	20,60638433
ENSG00000095370	SH2D3C	28,01488108	21,6692109	10,63147742	28,69833666	2,405497799	14,33972141	25,05068858	18,20674475	36,38222757	185,3987862	20,59986513
ENSG00000103569	AQP9	0	21,63371314	129,4949024	1,855179108	18,42766987	0,7692917	1,822907451	5,617898366	5,629971927	185,2515339	20,58350377
ENSG00000128829	EIF2AK4	45,6054425	13,12974258	10,66657036	16,5641905	15,60729392	17,31349111	20,43027094	22,29909619	23,51761547	185,1337136	20,57041262
ENSG00000152256	PDK1	13,05705798	41,24014232	22,07703265	17,26943156	12,90921757	18,70576817	15,09892929	10,1463816	34,50204536	185,0060065	20,55622295
ENSG00000135272	MDFIC	28,82954909	13,68279818	0,846854833	7,700860827	0	16,04771001	36,07391495	74,24082058	7,572573451	184,9950819	20,5550091
ENSG00000133794	ARNTL	34,50874763	17,82032026	44,55286154	37,01040229	0,106784877	14,18355361	12,81565835	0,638663303	23,29298517	184,929977	20,54777523
ENSG00000256683	ZNF350	22,95603121	0	57,53000727	1,493683254	0,158131899	15,8166622	17,26529227	51,36213225	18,33096477	184,9129051	20,54587835
ENSG00000124588	NQO2	17,62390113	39,78746584	13,73305386	6,524503172	31,8166087	22,23529013	17,20630526	20,61285025	15,3076561	184,8476344	20,53862605
ENSG00000158006	PAFAH2	3,185466575	37,73065298	46,43989005	0	2,554986467	18,58023586	17,25836978	31,43264265	27,61987841	184,8021228	20,5335692
ENSG00000049246	PER3	6,007853285	4,800665516	63,32550718	47,71511265	1,285888224	18,96940289	13,29220211	2,815160533	26,57245003	184,7842424	20,53158249
ENSG00000152457	DCLRE1C	11,57905228	37,09595852	68,48071037	3,699661858	0,336953491	11,4504589	5,755519817	21,87509908	24,47082544	184,7442398	20,52713775
ENSG00000168404	MLKL	42,58281014	32,08093852	15,82318202	33,14405949	0,063091627	14,74890229	11,39637415	12,13296286	22,75738335	184,7297045	20,52552272
ENSG00000119711	ALDH6A1	0,904853717	16,77894551	0,083227575	15,70297302	0,053760111	41,17960122	52,98679396	26,11818138	30,90950393	184,7178439	20,52420487
ENSG00000108590	MED31	21,80237944	27,6159535	0	32,07506332	0	15,74851226	17,20974179	56,14407934	14,11269272	184,7084224	20,52315804
ENSG00000121486	TRMT1L	0	24,28229346	0,056569007	57,24214543	0	23,76903663	12,868752	30,59579329	35,73971582	184,5543056	20,50603396
ENSG00000171574	ZNF584	22,01638216	31,10797713	0	0,071060723	84,30264747	23,90160541	8,610709059	0	14,42436243	184,4347444	20,49274938
ENSG00000123810	B9D2	11,17204676	14,30635671	22,24688292	67,62034403	0,547435278	17,32450382	14,34493125	13,85205885	22,94751266	184,3620723	20,4846747
ENSG00000180871	CXCR2	0	0	89,08509706	2,922731514	66,03561529	11,56315158	10,89179298	0	3,816387825	184,3147762	20,47941958
ENSG00000163812	ZDHHC3	1,454178561	31,9650068	13,97676887	28,6142792	20,84248026	21,75835836	20,9382419	20,80468295	23,92921279	184,2832097	20,47591219
ENSG00000076003	MCM6	34,05926284	0	0,201678427	22,65433032	0,086848257	15,4074151	30,79864256	0,079911625	80,90054174	184,1886309	20,46540343
ENSG00000171469	ZNF561	48,82808256	0,127605647	11,37672359	53,47842436	29,45174007	11,71164445	14,03181854	4,350582028	10,7899472	184,1465685	20,46072983
ENSG00000165458	INPPL1	20,82539961	14,27588987	0,084569546	26,71269211	21,57764363	30,58771204	33,09485179	9,952242831	27,02605344	184,1370549	20,45967276
ENSG00000151445	VIPAS39	0	40,92884656	0	8,544825961	26,43571624	18,54426744	23,15852948	25,66568901	40,84096782	184,1188425	20,45764917
ENSG00000139977	NAA30	17,04492435	3,614057316	37,10281353	47,22233314	11,98311611	10,74662856	18,24097603	27,93257401	10,21401981	184,1014429	20,45571587
ENSG00000143179	UCK2	10,19087611	9,828801836	48,91717378	54,39881487	1,028400751	14,41977664	10,24027624	14,66705555	20,38351032	184,0746861	20,4527429
ENSG00000111196	MAGOHB	3,778544958	46,71289648	10,51257659	47,99176016	18,92435902	19,30309045	10,31973386	14,27171412	12,25150663	184,0661823	20,45179803
ENSG00000021776	AQR	34,03391231	12,41786345	1,593661501	55,23327621	0	20,15978417	17,83358378	24,94681345	17,82222624	184,0411211	20,44901346
ENSG00000128944	KNSTRN	0	15,33707663	0,076075561	36,56231719	59,75464306	13,7577736	8,138059301	26,67712026	23,70128062	184,0043462	20,44492736
ENSG00000150527	MIA2	33,86689135	19,66730867	16,3796984	10,77015978	0,306675954	20,44955301	26,75128912	15,20644893	40,59386035	183,9918856	20,44354284
ENSG00000150776	NKAPD1	35,60485383	19,33604345	16,37450105	33,50668049	34,57013353	11,4162621	10,55215303	8,038373493	14,56416708	183,9631681	20,44035201
ENSG00000050748	MAPK9	34,82883505	16,94789191	0,062083774	29,05105189	0,240614769	26,86435915	27,91031841	26,75210066	21,20995486	183,8672105	20,42969005
ENSG00000106366	SERPINE1	2,368504242	27,57071349	78,68841046	0,899775774	4,84076938	23,61235167	9,479106587	10,5656194	25,75052127	183,7757723	20,41953025
ENSG00000171862	PTEN	16,62775997	19,16165165	32,17025086	28,90110027	18,20082584	20,77442039	12,27611975	21,82199556	13,81802221	183,7521465	20,41690517
ENSG00000089177	KIF16B	22,0765087	28,2693418	0,036003121	0,03379549	0,046511792	37,045698	13,91045256	61,19951192	21,13369338	183,7515168	20,4168352
ENSG00000166881	NEMP1	19,56789589	18,76526039	0	63,25282369	0,125346437	23,71392013	11,12372228	12,34083725	34,8340602	183,7238663	20,41376292
ENSG00000164040	PGRMC2	8,917679592	5,796411589	33,47058261	42,36103889	37,96119287	14,45351544	18,28080497	8,500154244	13,98067223	183,7220524	20,41356138
ENSG00000138399	FASTKD1	7,751982894	3,297600142	22,85570737	29,52250942	0	22,06505737	16,13567868	52,30526095	29,71193522	183,645732	20,40508134
ENSG00000154928	EPHB1	52,12207094	0,038537223	66,87822445	12,22546734	42,16715035	5,031206048	3,678635776	1,282226692	0,213678094	183,6371969	20,40413299
ENSG00000169371	SNUPN	22,77832834	17,38804779	0	32,80240579	10,52022379	15,90212297	20,62623774	44,73460015	18,81722467	183,5691912	20,39657681
ENSG00000171243	SOSTDC1	0	0	0	0,105513536	183,2617624	0,06708879	0,050736082	0	0	183,4851008	20,38723342
ENSG00000198837	DENND4B	4,410537298	19,84800763	25,43704298	9,194132684	34,41975919	13,98220874	30,41904973	22,85150967	22,82154244	183,3837903	20,37597671
ENSG00000180773	SLC36A4	21,27594501	11,24047729	15,31767978	19,6460466	43,53500333	19,60031223	14,35755885	24,84470432	13,55230588	183,3700333	20,37444814
ENSG00000141627	DYM	20,20083428	7,095267919	1,149311324	42,14660836	1,23731237	18,52088852	17,87986815	41,03108398	33,9113555	183,1725304	20,35250338
ENSG00000104626	ERI1	35,76007397	13,14462741	1,784993588	20,88297134	36,05236408	16,94184746	19,45428797	0,258758471	38,85749526	183,1374195	20,34860217
ENSG00000196230	TUBB	22,2803304	18,97388841	13,95099624	17,08115482	26,08303846	24,35906535	20,69008904	18,23158306	21,37698464	183,0271304	20,33634782
ENSG00000090372	STRN4	26,87025729	13,62303209	13,10368412	14,04272287	11,61477451	23,50598979	19,25510398	27,12877751	33,7283331	182,8726753	20,31918614
ENSG00000159459	UBR1	3,652954972	5,374723813	1,903973281	31,59184523	78,09576692	7,520120822	20,09446292	17,34049251	17,25931643	182,8336569	20,31485077
ENSG00000114268	PFKFB4	5,171460499	40,48254856	0,045518274	19,61178211	8,350203212	15,8656913	38,11158766	10,71328711	44,21729112	182,5693699	20,28548554
ENSG00000174840	PDE12	20,25384617	0,029949961	12,10904039	53,59761178	0	13,69461814	8,801001731	43,66179977	30,22367211	182,3715401	20,26350445

ENSG00000070770	CSNK2A2	15,19416681	19,05846397	6,70351106	23,03557948	65,93866029	16,98647281	11,28896796	9,226631557	14,78175695	182,2142109	20,24602343
ENSG00000119638	NEK9	29,64632107	16,44737175	43,29875891	21,82541609	26,56668808	7,617084098	14,35445899	11,08614116	11,12315617	181,9653963	20,21837737
ENSG00000124370	MCEE	16,19041829	106,9926207	0	0	0	22,57773242	12,8251694	17,29491271	5,950750713	181,8316043	20,20351158
ENSG00000145982	FARS2	13,72027725	13,47642317	0,057493299	27,36174398	55,18600496	24,12308902	9,835213201	16,33378731	21,71732887	181,8113611	20,20126234
ENSG00000198586	TLK1	5,639533259	20,84123361	48,05907931	32,96020732	8,247675614	13,35399326	13,64765015	25,6828994	13,23946517	181,6717371	20,18574856
ENSG00000109111	SUPT6H	52,47454057	11,94237305	20,74004545	3,973124851	0,854390803	15,88371147	39,82142722	15,72299998	20,17994689	181,5925603	20,17695114
ENSG00000018610	CXorf56	4,802379068	49,47115971	51,28625082	15,17063806	0	24,3721351	19,35550083	8,324901895	8,788801363	181,5714168	20,17460187
ENSG00000145431	PDGFC	54,82104064	27,40430911	6,163946843	25,4931469	0,239491525	30,23345454	20,500321	4,572535696	12,05306722	181,4813135	20,16459039
ENSG00000205413	SAMD9	0,110219235	32,15357025	0,162205747	22,00151938	8,958293179	33,78718674	22,98916659	27,04212872	33,99730692	181,2015968	20,13351075
ENSG00000214367	HAUS3	0,042765125	52,4749379	41,41970344	2,658457361	0,050816123	19,57965487	6,338320153	35,44211153	23,06316684	181,0699333	20,11888148
ENSG00000138821	SLC39A8	32,76394318	28,9659568	0,042898163	0,281874201	0,831290607	17,15433432	23,29333478	73,68486884	4,015202642	181,0337035	20,11485595
ENSG00000135655	USP15	16,03866959	16,54311032	30,72125309	18,22164103	1,997357842	21,1980709	23,77203375	25,54239808	26,98019087	181,0147255	20,11274727
ENSG00000121671	CRY2	2,437267269	0	27,0358064	14,43559618	94,99238217	12,73765073	7,872251476	3,784008757	17,71537011	181,0103331	20,11225923
ENSG00000076053	RBM7	30,81620769	27,10325606	33,949884	24,12448909	0,592077115	19,5684003	13,38244124	10,22524217	21,23623546	180,9982331	20,11091479
ENSG00000136715	SAP130	1,174882095	16,06521931	20,2779903	48,51135018	0	16,99701312	28,26166865	0,151124888	49,55340612	180,9926547	20,11029496
ENSG00000197712	FAM114A1	16,03862965	23,61587313	9,416283378	15,42789421	26,90985485	28,54302264	18,93259988	5,63048021	36,47490768	180,9895456	20,10994951
ENSG00000126003	PLAGL2	0,026716862	5,83123515	13,26991279	16,60830814	101,3986413	8,770732396	14,70771395	4,956866025	15,34482424	180,9239508	20,10266121
ENSG00000235173	HGH1	17,54105666	10,28020448	0	30,01169037	0,254964531	25,38425934	38,92831742	25,45413601	33,05257638	180,9072052	20,10080058
ENSG00000076043	REXO2	22,50930642	29,06865154	11,4979603	24,47808494	0,097085034	18,43450852	21,81060381	33,85636688	19,14234252	180,89491	20,09943444
ENSG00000158290	CUL4B	0,021492045	0,076274214	0,079072706	85,95155756	0,051076311	21,6620551	31,63972389	4,60568733	36,74109884	180,828038	20,09200422
ENSG00000172175	MALT1	18,36608914	14,56206229	43,43212963	23,08958741	0,311544671	9,787385457	15,13002182	34,6573603	21,1046324	180,4408131	20,04897923
ENSG00000100815	TRIP11	8,697744956	6,876761668	0,857632392	21,2582003	36,00870074	12,76481298	22,34320316	42,72201649	28,8660574	180,3951301	20,04390334
ENSG00000136731	UGGT1	22,42225756	6,946316682	0,293925561	62,65290385	25,94734203	22,03077268	21,83486701	2,824227073	15,37667499	180,3292874	20,03658749
ENSG00000023572	GLRX2	6,180690008	12,30603382	58,68468756	34,74931143	23,82551167	8,335603854	11,19332752	6,54483926	18,46494432	180,2849495	20,03166105
ENSG00000160194	NDUFV3	16,75035181	22,53542826	10,69491393	29,91165666	0,396376978	31,31424792	16,9746996	15,31815876	36,36637246	180,2622064	20,02913404
ENSG00000123685	BATF3	21,94014903	5,636305179	31,163203	41,33925055	0	23,37860209	15,92188721	15,04898575	25,82608348	180,2544663	20,02827403
ENSG00000177733	HNRNPA0	22,30465448	30,57544007	14,23986984	39,38246179	11,64683297	17,49229092	19,25335486	10,03497139	15,2493823	180,1792586	20,01991763
ENSG00000101493	ZNF516	21,45195067	10,6621349	8,582956229	44,44545825	63,09208731	9,073382342	12,70857071	4,630676974	5,505308852	180,1525262	20,01694736
ENSG00000227500	SCAMP4	15,27784578	19,13890656	2,168203863	14,55398902	32,2386609	28,83596631	34,06810339	10,60113844	23,24790934	180,1307236	20,01452484
ENSG00000213853	EMP2	25,27242207	8,925669906	21,39791331	6,061942891	43,99729483	26,02180451	28,32218659	0,267341883	19,71880576	179,9853817	19,99837575
ENSG00000112312	GMNN	26,05986013	22,20077141	16,95274432	63,1260298	0,145039395	21,30838586	9,982897982	16,94878436	3,227544611	179,9520579	19,9946731
ENSG00000135114	OASL	23,02964625	0	112,3934321	0	0,204218108	8,255431027	14,34150511	11,9320984	9,722991214	179,8793222	19,98659136
ENSG00000013810	TACC3	11,58846	12,7172931	18,33543447	17,75197706	34,93974626	24,03093942	24,2465833	13,17387266	23,06653983	179,8508461	19,98342734
ENSG00000153922	CHD1	10,68502454	16,08286788	8,549546016	16,42562789	19,44058747	17,89933021	33,28804727	17,25465548	40,18928818	179,8149749	19,97944166
ENSG00000161677	JOSD2	25,25163433	14,69999076	8,996713904	9,479143727	33,91929231	23,23186411	30,08304526	17,67846761	16,44876749	179,7889195	19,97654661
ENSG00000054219	LY75	36,10864744	23,57091517	0,03157071	23,26336798	36,46236035	14,92347266	11,21466795	13,58515769	20,45261557	179,6127755	19,95697506
ENSG00000198276	UCKL1	31,78778096	23,32035605	0	14,25516031	46,45413735	12,50189589	21,39101736	8,19602224	21,64805644	179,5544266	19,95049184
ENSG00000145476	CYP4V2	22,23260879	10,02066449	52,59621408	43,38675118	2,79440291	13,26673087	12,07557204	11,26596816	11,81639094	179,4553034	19,93947816
ENSG00000162430	SELENON	10,11733294	8,491264423	27,22582738	23,19553156	15,1087679	26,79487712	30,02660286	10,83759608	27,61844948	179,4162497	19,93513886
ENSG00000117151	CTBS	30,79308283	23,39571821	16,15603979	18,51456699	10,40995472	21,96508645	16,64734719	23,97008761	17,55544737	179,4073312	19,93414791
ENSG00000149929	HIRIP3	26,90852187	25,03386264	0	32,52560101	0	15,70051756	28,72635431	23,53642302	26,94798212	179,3792625	19,93102917
ENSG00000039650	PNKP	19,18389423	20,23601893	33,08850023	11,48228711	0,079013761	22,66896989	27,57862743	14,0316549	30,99486554	179,343832	19,92709245
ENSG00000211584	SLC48A1	17,47369791	10,26276385	55,57338298	27,6765313	0,073110249	26,68350073	24,36862718	6,65981722	10,51818672	179,2896182	19,92106868
ENSG00000182362	YBEY	0,167714285	33,03414208	0	28,38138458	67,35945023	9,759445688	10,72276977	8,80180868	21,03922894	179,2659443	19,91843825
ENSG00000116991	SIPA1L2	23,15802621	0,105277582	30,77753746	44,32582084	0,046998762	22,4731641	8,982105955	9,254416627	40,0831133	179,2064608	19,91182898
ENSG00000111880	RNGTT	14,01005869	8,217921745	35,68518618	14,13313953	0,069220806	26,03145903	25,82928192	27,89713581	27,33247812	179,2058818	19,91176465
ENSG00000072736	NFATC3	28,65712616	6,48583307	6,408126288	37,39495094	23,53064069	16,74931895	26,68702383	14,18399574	19,09960285	179,1966185	19,91073539
ENSG00000137496	IL18BP	25,58707789	29,52477262	6,494561646	34,10322939	0	20,72443012	25,62810621	9,019495482	28,07258363	179,154257	19,90602856
ENSG00000168876	ANKRD49	30,78443035	4,650838854	5,347456966	22,79375212	2,151760739	21,45169866	20,06100862	37,09703754	34,81325583	179,1512397	19,9056933
ENSG00000213903	LTB4R	29,78215055	8,467642108	34,61521501	17,7657855	54,93332171	5,276439393	11,099839	3,330247601	13,81882316	179,089464	19,89882934
ENSG00000038219	BOD1L1	24,55951653	16,80324978	43,25147423	11,71262778	6,067551466	10,06215955	13,87435143	20,14438449	32,58348118	179,0587964	19,89542183
ENSG00000110395	CBL	12,81994279	12,42497854	3,575382874	41,0516949	14,34634816	22,30041653	24,37800914	17,59127808	30,53645157	179,0245026	19,8916114
ENSG00000141510	TP53	10,99855419	20,22379009	18,13126173	37,02083764	0,063135995	26,92254963	23,18377618	13,01289436	29,43876874	178,9955686	19,88839651
ENSG00000047365	ARAP2	0	11,39285234	22,81642399	32,48301325	20,8644778	17,85003951	25,60495389	38,10354351	9,825473979	178,9407783	19,8823087
ENSG00000160551	TAOK1	12,904024	11,41735371	19,9235442	36,02070556	41,74900051	10,45490678	12,25146116	23,76093092	10,31021343	178,7921403	19,86579336
ENSG00000153446	C16orf89	0	26,07476634	0	49,23301408	0	15,33087574	24,46276913	43,48243946	20,05020535	178,6340701	19,84823001
ENSG00000177150	FAM210A	19,33672972	41,70206709	4,513242368	14,99498145	32,29833056	17,82803249	13,29486962	11,6474479	22,86853169	178,4842329	19,83158143
ENSG00000157657	ZNF618	10,1429187	2,788842066	19,7027497	18,19307797	69,27122856	29,35070584	8,554795364	1,27286043	19,203902	178,4810806	19,83123118
ENSG00000172795	DCP2	8,46413898	6,166114354	26,36532793	28,90457217	0,739057219	19,01677244	18,05263904	27,17156116	43,53491368	178,415097	19,82389966
ENSG00000186665	C17orf58	18,03740535	20,03231136	32,25311666	0	0	17,17271412	22,46526395	48,26882247	20,16698489	178,3966188	19,82184653
ENSG00000145354	CISD2	14,60672078	23,55529535	34,13483583	12,69346775	24,70629188	17,47389418	20,18744951	10,92431662	20,10021423	178,3824861	19,82027624
ENSG00000151881	TMEM267	18,66153343	7,132339458	53,25457881	26,52315606	0	16,2338088	27,93085605	0,20926825	28,24772979	178,1932707	19,79925229
ENSG00000102471	NDFIP2	14,43326836	0,053692831	48,76062609	0	101,2489987	3,853749879	0,025124215	7,675292317	2,121195789	178,1719482	19,79688313
ENSG00000121989	ACVR2A	46,51899547	4,948650436	55,35459787	0,121402749	0,055694408	8,979961425	24,30403919	16,24499967	21,58794125	178,1162825	19,79069805
ENSG00000132604	TERF2	36,47681507	11,37485588	2,466987034	16,95104734	3,569503438	18,99402236	27,8600258	28,32799008	32,02999168	178,0512387	19,78347096
ENSG00000061987	MON2	30,0730229	7,216760462	12,13363864	25,76109622	53,50658524	3,84368827	10,553799	10,17261407	24,77721525	178,0384201	19,78204667
ENSG00000115694	STK25	11,94864818	24,59499632	30,2231921	9,078391315	0,18930814	23,89829472	30,34239051	15,85110471	31,88879441	178,0151204	19,77945782
ENSG00000144320	LNPK	6,755962085	10,51189869	1,010168843	87,1219957	0,039546035	24,42709405	26,81840264	5,858381827	15,30813772	177,8515876	19,76128751
ENSG00000131378	RFTN1	1,109010269	13,32557224	60,85368491	21,55763892	0,22590745	18,96021478	27,07254595	11,64038736	22,91418643	177,6591483	19,73990537
ENSG00000151883	PARP8	24,97571784	27,65280721	5,309794052	12,75750964	48,15956749	13,62883795	23,28353845	9,026122391	12,76499272	177,5588877	19,72876531
ENSG00000143632	ACTA1	2,223700705	86,45113314	10,97045207	7,854230005	47,80226818	1,109769744	9,651564	8,177979263	2,983489321	177,2245864	19,69162071
ENSG00000167680	SEMA6B	16,63343968	14,7578153	31,24683016	10,40575575	17,58737964	14,35466977	21,00925802	34,75419542	16,46960785	177,2189516	19,69099462
ENSG00000047056	WDR37	11,13181204	14,479668	24,87921886	7,262690126	0	25,10780061	27,31070893	44,33330448	22,67327572	177,1784788	19,68649764
ENSG00000132434	LANCL2	12,46986944	0,051221012	70,62340624	26,41748549	0,137198679	9,25422892	10,30606356	33,57997897	14,30680964	177,146262	19,682918
ENSG00000167112	TRUB2	18,47425187	1,931063787	5,815084375	21,1181941	69,95156518	12,5741555	18,76031027	14,95794339	13,51147556	177,094044	19,677116
ENSG00000182580	EPHB3	33,42445735	19,05998323	37,24810598	20,44854657	0	24,79502438	25,14946169	5,470770985	11,48720226	177,0835524	19,67595027
ENSG00000100519	PSMC6	17,47702148	23,66275171	19,69908443	36,94747133	0,048016786	18,9669131	16,22273179	27,70189515	16,32586778	177,0517536	19,67241706
ENSG00000058729	RIOK2	16,02229042	10,25866643	24,46062813	31,13725785	0,130849897	27,74747849	30,31037716	17,45782922	19,46834584	176,9937234	19,66596927
ENSG00000110002	VWA5A	44,36793266	16,66726785	0	34,84294913	0	42,20964071	20,18342058	0,361243475	18,22764579	176,8601002	19,65112224
ENSG00000149809	TM7SF2	10,46422966	0	0,08156693	20,82579609	46,78644038	27,70044546	17,41416286	33,64459945	19,90433704	176,8215779	19,64684199
ENSG00000010704	HFE	0	0,377997533	0	0	49,16904501	19,49997439	29,40538177	76,45120478	1,866652294	176,7702558	19,64113953
ENSG00000095383	TBC1D2	0,126030501	15,87830026	62,36584372	26,26034496	12,28008277	17,43513984	17,26655945	18,64837843	6,458386234	176,7190662	19,63545179
ENSG00000103275	UBE2I	27,55229371	19,02870798	2,793185001	16,68738429	25,3344383	22,34947794	22,32568185	24,07185043	16,57329255	176,716312	19,63514578
ENSG00000149418	ST14	26,32319708	13,87615307	6,010745581	0,380371683	84,80618347	17,3730463	18,29013586	6,342160067	3,205807357	176,6078005	19,62308894
ENSG00000156531	PHF6	3,95873501	25,32229217	0,069356312	29,361695	12,45442575	12,93589202	25,6700779	39,53178022	27,2880934	176,5923478	19,62137198
ENSG00000162441	LZIC	1,799264901	18,46883899	35,08486686	8,651626745	5,723250812	14,43625645	21,07644155	39,94995687	30,94605033	176,1365535	19,57072817
ENSG00000032389	EIPR1	25,11967928	13,55800243	2,31048394	17,21493079	45,70586903	20,85732964	15,88204549	15,90663373	19,3409108	175,8958851	19,54398724
ENSG00000141503	MINK1	19,38595878	17,08062836	29,1234077	6,10451816	41,04852773	14,61869417	19,14359802	6,806153838	22,44808597	175,7595727	19,52884141
ENSG00000179583	CIITA	24,4898569	7,621707378	26,74405057	20,64148135	2,387542556	26,84846689	16,32679899	12,60799622	38,08792724	175,7558281	19,52842534
ENSG00000101901	ALG13	6,385473553	13,16476356	36,49592164	30,2745757	6,315696886	18,94557758	11,50808736	36,4817842	16,16056715	175,7324476	19,52582752
ENSG00000107758	PPP3CB	6,76348844	8,685400474	55,0066627	21,36040134	0,140996287	15,30780743	6,55184694	32,23910091	29,47860031	175,5343048	19,50381165
ENSG00000137414	FAM8A1	0	12,87944012	45,82024907	21,75378332	38,75369162	14,70938037	16,16830982	4,754454209	20,60591995	175,4452285	19,49391428
ENSG00000162378	ZYG11B	7,034394101	14,50829247	9,925441019	46,74316508	0,087525601	33,70374499	19,29607104	11,75809087	32,31818027	175,3749055	19,48610061
ENSG00000164713	BRI3	11,73246112	13,82713174	6,815624143	13,40471557	82,59913117	10,81534213	10,23001929	18,06812872	7,847576442	175,3401303	19,4822367
ENSG00000100532	CGRRF1	21,66793316	63,62991381	0,061134836	14,97779379	0,078979006	20,46972367	28,64266651	7,049076985	18,71953094	175,2967527	19,47741697
ENSG00000162924	REL	0,413058508	19,01629674	3,778165354	10,02529211	4,608266109	30,47362833	32,22024775	45,23716972	29,43628287	175,2084075	19,46760083
ENSG00000109016	DHRS7B	33,19853446	18,18567677	0,05014071	8,283649987	37,76434473	14,81344435	15,38957896	23,48326472	24,03534568	175,2039804	19,46710893
ENSG00000141582	CBX4	16,77844801	7,327297823	34,51914754	15,97561069	8,198463437	22,38157209	26,1703944	13,60142288	30,14943582	175,1017927	19,45575474
ENSG00000172977	KAT5	18,66612355	8,837477495	26,81480054	6,082889279	23,19060228	13,91479381	26,76162494	22,48938375	28,18572658	174,9434222	19,43815803
ENSG00000163703	CRELD1	4,879695506	31,37076867	8,240807593	15,54006598	3,802197378	25,77805943	37,22928591	14,25644111	33,69636521	174,7936868	19,42152075
ENSG00000176148	TCP11L1	16,77130356	15,82886667	73,29978029	9,475292374	0	21,62707704	18,41166138	4,553474487	14,77774023	174,745196	19,41613289
ENSG00000112242	E2F3	0,087347151	0	46,70494149	49,82399997	0,138388263	14,76891839	14,5777669	30,81509262	17,7968635	174,7133183	19,41259092
ENSG00000189319	FAM53B	12,97612584	10,30210348	0	20,09035546	93,28370877	7,415565345	8,469679537	4,046344008	18,06406577	174,6479482	19,40532758
ENSG00000083312	TNPO1	8,781243816	23,17165986	33,56092704	14,66220572	2,428698416	23,86774014	25,62414896	18,98129602	23,55473164	174,6326516	19,40362796
ENSG00000160783	PMF1	26,92406596	4,362627779	36,29184626	15,32475248	26,64880175	18,96120877	20,2146646	8,12973936	17,77341741	174,6311244	19,40345826
ENSG00000083720	OXCT1	62,06590268	5,42824049	6,644402366	36,14904038	7,532709425	24,72472253	12,57763572	2,740189096	16,45421648	174,3170592	19,36856213
ENSG00000105711	SCN1B	17,96908776	6,108629868	10,1950393	12,37881529	93,1395103	5,129542176	14,93582247	7,486375857	6,955577689	174,2984007	19,36648897
ENSG00000149428	HYOU1	10,82016646	16,07532643	33,11429381	15,91355342	0	25,00581526	44,14125237	5,305436537	23,91964821	174,2954925	19,36616583
ENSG00000142686	C1orf216	0	4,309267958	0,364683633	42,61909392	0,117782074	28,40450018	33,5794657	53,53711917	11,33730834	174,269221	19,36324677
ENSG00000173482	PTPRM	0,045791082	0,081255152	0,112315192	18,92437493	124,9293753	8,3960807	11,08954955	7,943780512	2,703222142	174,2257446	19,35841607
ENSG00000171865	RNASEH1	0,076254283	29,63318655	14,26138439	12,64075234	30,44495229	14,1770518	15,61783779	35,07219716	22,28907828	174,2126949	19,3569661
ENSG00000139531	SUOX	31,50148478	33,14295089	32,47171683	0,052103629	0	18,45291358	24,95376411	5,278507468	28,3148755	174,1683168	19,3520352
ENSG00000135720	DYNC1LI2	14,36568536	3,944727162	16,52966097	15,54835675	41,99841699	18,89030055	10,85786035	25,4903165	26,53637449	174,1616991	19,3512999
ENSG00000196511	TPK1	28,76506599	8,385393038	36,84668689	15,08366117	0	13,462301	16,31916898	23,01534301	32,22924764	174,1068677	19,34520752
ENSG00000081307	UBA5	17,30898876	32,44255047	8,713599119	11,73385634	0	27,55860466	13,85742351	35,81684195	26,65061452	174,0824793	19,3424977
ENSG00000134321	RSAD2	42,65785487	5,735354089	0	54,20948964	8,517823961	1,897325528	3,241715482	57,73157946	0	173,991143	19,33234923
ENSG00000101442	ACTR5	0,059328846	17,68663369	0	2,151407329	14,52261754	31,59270895	20,936353	45,66665401	41,372194	173,9878974	19,3319886

ENSG00000112339	HBS1L	0,326754996	32,27657556	0,125227543	53,97843965	0,064711693	24,47027787	13,48644978	21,822555	27,42739017	173,9783823	19,33093136
ENSG00000101152	DNAJC5	41,99872683	12,59660154	1,456755151	31,74391419	1,881956052	36,0202637	15,02919942	14,90449762	18,29595185	173,9278663	19,32531848
ENSG00000119508	NR4A3	0,143595728	5,435884702	39,93157266	57,93963167	3,469459944	19,70746477	19,93138153	6,018367954	21,2808102	173,8581692	19,31757435
ENSG00000188786	MTF1	40,59122351	9,196508702	4,210238074	15,97161129	43,96256808	17,09155146	12,21879062	14,72458456	15,6791283	173,6462046	19,29402273
ENSG00000107443	CCNJ	30,45526448	19,8456109	69,6960711	4,088903893	0	8,119538831	11,40364396	26,0492053	3,942742035	173,6009805	19,28899783
ENSG00000112715	VEGFA	19,11002643	26,25491277	2,638990615	18,56976086	46,51033793	12,2556077	14,58937129	9,639869235	23,97936557	173,5482424	19,28313804
ENSG00000172663	TMEM134	17,13201989	19,06848236	7,029707187	17,05023171	0	35,73328612	22,31719415	15,5907688	39,61697759	173,5386678	19,2820742
ENSG00000162928	PEX13	20,27591921	4,310235196	21,63635288	19,55908524	0,303821948	23,74963585	13,18393844	46,46211939	23,89904517	173,3801533	19,26446148
ENSG00000105607	GCDH	7,438476367	14,27142579	27,57573696	8,802154316	51,77681708	21,30885468	14,6726925	7,678764313	19,82920165	173,3541236	19,26156929
ENSG00000178096	BOLA1	14,58356637	27,16291308	28,15951721	17,50282513	0,245802562	17,14749971	24,47573212	30,30679812	13,73814128	173,3227956	19,2580884
ENSG00000206053	JPT2	18,69866541	16,19649665	0,408108367	68,35314247	0,075318277	19,31215815	22,78886483	8,524213885	18,9432487	173,3002167	19,25557964
ENSG00000163960	UBXN7	18,36651108	15,61598738	5,783528333	3,990469959	72,16196726	10,5473515	16,96811372	11,92819724	17,91212147	173,2742479	19,25269422
ENSG00000105176	URI1	5,020376889	16,53739208	5,867788433	51,53561539	0,329586584	26,73815829	18,60866892	19,59074253	28,89353971	173,1218688	19,2357632
ENSG00000138621	PPCDC	27,44167963	16,44604267	38,4724457	12,24654814	33,80493132	8,450378707	10,43913162	13,12926682	12,63756351	173,0679881	19,22977646
ENSG00000138796	HADH	20,27977376	18,90261328	0	50,97364513	28,37640683	14,63351603	13,46475431	16,29480409	9,964301376	172,8898148	19,20997942
ENSG00000116138	DNAJC16	14,55870849	16,4711774	70,15239915	3,961409167	7,968274325	17,24400482	15,34128665	18,79267014	8,399229723	172,8891599	19,20990665
ENSG00000164941	INTS8	21,88087211	31,77070227	21,91045784	25,11159112	0,091308808	12,14905825	24,7718568	7,435407888	27,47768947	172,5989446	19,17766051
ENSG00000112249	ASCC3	6,943022555	14,58312131	22,89052729	48,22325675	7,683786815	27,32411851	17,25203695	0,507017493	27,18546008	172,5923478	19,17692753
ENSG00000169439	SDC2	0	34,86371255	54,31638565	0,063892011	39,83355698	3,04683968	15,05398205	12,62188848	12,78718726	172,5874446	19,17638274
ENSG00000100395	L3MBTL2	40,01798608	6,520553815	49,8044043	23,53900383	2,197299051	14,18595298	14,3501525	6,843012694	15,10331009	172,5616753	19,17351948
ENSG00000138468	SENP7	3,780126846	28,04674766	19,86193015	27,53769666	34,30594409	11,98415986	13,496496	28,36281701	5,143032277	172,5189505	19,16877228
ENSG00000162702	ZNF281	19,38660641	15,11893606	17,26372831	38,59385528	1,173826492	21,55651211	10,73995283	24,7741545	23,84302676	172,4505988	19,16117764
ENSG00000180354	MTURN	3,30465219	17,80300882	2,405428799	22,86670561	72,77271783	11,43310771	12,27856301	18,40368737	11,1402651	172,4081364	19,1564596
ENSG00000132825	PPP1R3D	22,68939944	0	14,26908551	4,22595759	62,99097161	22,22463943	10,02247231	23,03880872	12,75367295	172,2150075	19,13500084
ENSG00000100196	KDELR3	0	35,6027538	0	77,20292505	0	5,203677262	38,30354098	0	15,83532835	172,1482254	19,1275806
ENSG00000186818	LILRB4	14,4949615	15,63596682	15,24321971	16,98881314	29,96429557	26,08739314	24,32871324	15,19539493	14,09708134	172,0358394	19,11509327
ENSG00000173542	MOB1B	20,01987563	31,41170345	37,2071988	1,571361624	0,530454272	33,70618238	21,38455835	1,57689488	24,45293407	171,8611635	19,09568483
ENSG00000132773	TOE1	25,50551617	41,16514166	19,92308597	10,29132851	0	22,37470882	12,87693823	0	39,72292079	171,8596402	19,09551557
ENSG00000132664	POLR3F	0	15,97398951	2,603785116	44,87416761	0,134551346	20,88645701	7,85071017	53,60740642	25,9029248	171,833992	19,09266578
ENSG00000149480	MTA2	20,88808405	5,852430417	3,653340893	22,47433365	7,248089332	39,28743767	25,05873094	37,99881204	9,333761034	171,79502	19,08833556
ENSG00000107951	MTPAP	0,216335821	25,81610523	24,97244268	23,65910054	13,23876409	17,71533836	17,99276295	29,13280188	19,00148766	171,7451392	19,08279325
ENSG00000140598	EFL1	1,765310405	12,81477273	65,42097487	14,46559651	0	22,06032732	24,75827069	0,140371524	30,23756076	171,6631848	19,0736872
ENSG00000172071	EIF2AK3	54,25615371	43,87332543	0,050819031	2,098928498	0	16,40907166	18,35032278	4,228595928	32,31079391	171,5780109	19,06422344
ENSG00000162227	TAF6L	12,49896804	11,609379	26,76512746	7,250535643	53,25846589	12,22216849	13,82403335	16,0145994	18,13429717	171,5775744	19,06417494
ENSG00000134086	VHL	29,37563669	24,56748446	1,451593206	4,521303856	0	41,86160528	29,30519978	16,70604388	23,77670912	171,5655763	19,06284181
ENSG00000175395	ZNF25	35,17749014	53,66619311	0	6,742505804	0,168718601	12,5105227	14,7664168	20,4920662	27,94028054	171,4641939	19,0515771
ENSG00000126602	TRAP1	9,854403412	39,37026087	8,688554126	13,24057463	0,069287583	23,49572898	34,44805758	19,50858145	22,62572941	171,301178	19,03346423
ENSG00000127452	FBXL12	21,00919366	12,05856574	12,31013827	21,85655125	31,80649706	15,83355315	26,05889147	15,65395479	14,67377278	171,2611182	19,02901313
ENSG00000173281	PPP1R3B	4,661305508	26,68335344	0,096890937	66,48430339	0	9,483908881	16,42178796	26,72039723	20,50206494	171,0540123	19,00600137
ENSG00000188917	TRMT2B	24,59506206	0	92,75143623	10,81062287	0,088561663	11,865368	17,11099719	0	13,79526589	171,0173139	19,00192377
ENSG00000100852	ARHGAP5	13,60106165	1,994810985	0,102123475	22,40762319	47,39639129	15,29888125	24,92582	22,09371465	23,17960849	171,000035	19,00000389
ENSG00000119729	RHOQ	15,52505863	14,19182441	15,01542565	21,44671024	28,28665573	12,83585875	11,81655524	37,39507014	14,46501574	170,9781745	18,99757495
ENSG00000048162	NOP16	59,02921663	21,64248848	18,0657926	12,67294251	0	21,21707585	13,15202642	7,389177219	17,79243435	170,9611495	18,99568328
ENSG00000240682	ISY1	27,84972975	21,89848124	9,013006622	17,49320283	31,16671016	16,54252215	11,50093731	13,21093834	22,24540284	170,9209312	18,99121458
ENSG00000066926	FECH	11,35224191	22,58971022	13,94337022	29,86533453	0,081878145	20,0484737	18,65176846	30,43674539	23,91937114	170,8888937	18,98765486
ENSG00000188010	MORN2	0,110784876	39,7101789	35,66460214	27,35608605	0	20,31309847	21,06503374	7,994384698	18,6664029	170,8805718	18,9867302
ENSG00000187037	GPR141	8,627420124	4,755113323	21,88251644	2,370084112	59,49049253	17,07902995	21,59913155	14,4350741	20,57816164	170,8170238	18,97966931
ENSG00000189079	ARID2	22,39277115	7,720785705	26,56520059	6,628085157	63,26007345	18,99886134	10,1530684	0,328083304	14,74655096	170,79348	18,97705334
ENSG00000179151	EDC3	49,82580813	14,33181844	0,109247435	10,66505693	14,81915581	23,70151096	26,134516	0	31,11441088	170,7015246	18,96683606
ENSG00000054277	OPN3	29,41867984	13,53670677	13,21226571	32,51176912	0,192866564	17,10785753	24,86495838	17,12509977	22,65690685	170,6271105	18,95856784
ENSG00000163734	CXCL3	0	33,71411358	21,04581191	6,232336031	6,797177458	5,532844829	3,731880087	93,36749609	0,167504752	170,5891647	18,95435164
ENSG00000127054	INTS11	21,41652248	22,08420122	13,08738422	19,64948288	4,325138284	16,97454927	23,23185466	25,32527707	24,48561845	170,5800285	18,9533365
ENSG00000155636	RBM45	33,57661719	8,015114133	0,232424832	19,69011828	65,60801255	6,069053287	12,69390483	14,29764956	10,37228709	170,5551817	18,95057575
ENSG00000171103	TRMT61B	26,42970737	13,90077229	0	25,0315528	0	13,98664745	26,56518173	37,30196426	27,28270167	170,4985276	18,94428084
ENSG00000117593	DARS2	0,060948066	15,53767169	27,09539819	57,18257712	3,331421138	12,55816815	22,38493285	11,55054955	20,78842519	170,4900919	18,94334355
ENSG00000178028	DMAP1	24,02007642	29,30932382	0	0,046603853	33,22430558	22,52045692	13,96106748	25,2001358	22,20598739	170,4879573	18,94310636
ENSG00000213015	ZNF580	21,122983	24,30165592	10,35486544	14,52978929	18,34202501	13,82588139	26,83831602	10,40538961	30,68894185	170,4098475	18,9344275
ENSG00000089195	TRMT6	6,231363739	27,00557812	28,21685382	27,10743987	0	17,4550382	6,898712412	30,0474468	27,41273049	170,3751634	18,93057372
ENSG00000095319	NUP188	2,499403793	8,370058702	10,0945391	30,24392443	0,089321612	30,80518481	21,8609545	22,10841902	44,28329022	170,3550962	18,92834402
ENSG00000166411	IDH3A	22,21367468	17,29187748	27,29688793	6,501384388	0	28,54741566	23,59349787	16,36917541	28,51843893	170,3323523	18,92581693
ENSG00000147168	IL2RG	20,2575765	14,28147609	17,92770204	10,30504066	33,82991341	21,03938473	15,6837915	8,380586857	28,617671	170,3231428	18,92479364
ENSG00000179715	PCED1B	2,135634498	23,7620107	0	7,37554932	75,03334441	13,94206527	19,84137412	13,37893971	14,83648707	170,3054051	18,92282279
ENSG00000135597	REPS1	23,74774538	27,28335077	25,96835542	21,14220061	0,037400286	19,07574221	19,03877044	13,73082612	20,24530955	170,2697008	18,91885564
ENSG00000153044	CENPH	23,36831638	41,22471688	0,12532917	32,82274928	19,10544184	11,29508435	13,74629877	18,175391	10,3899769	170,2533046	18,91703384
ENSG00000145723	GIN1	20,91407994	23,41177453	0,066677884	21,4055556	67,87829776	5,611271604	19,77308902	9,035629815	2,095175473	170,1915516	18,9101724
ENSG00000070423	AC004156.1	12,73846352	16,9336489	0,080527259	11,03606787	16,85314075	26,24193942	18,35530629	28,33390266	39,57045014	170,1434469	18,90482743
ENSG00000257218	GATC	27,10135235	12,69059738	13,84864624	9,98596257	47,41067192	24,34557376	15,48490284	5,686826796	13,34088486	169,8954187	18,87726874
ENSG00000130733	YIPF2	32,52658034	12,56582844	6,955022334	15,1296689	10,69651139	19,37160824	25,31331559	37,26902986	10,0377788	169,8653439	18,8739271
ENSG00000162222	TTC9C	34,46481213	36,53270758	0,627730747	14,53457894	3,108657687	12,61344994	30,1279909	22,8829358	14,96844402	169,8613077	18,87347864
ENSG00000089818	NECAP1	8,583286825	31,86570838	21,70681251	9,118615504	37,81260406	9,650575378	9,82624797	24,14786481	17,11438094	169,8260964	18,86956626
ENSG00000163795	ZNF513	34,62153581	9,051206754	0	10,56951796	55,30713205	12,60080329	22,97642367	5,99522633	18,66306848	169,7849143	18,86499048
ENSG00000132185	FCRLA	25,05436332	0	115,1813723	0	0	7,750936866	18,61941415	0,100896051	3,064339604	169,7713223	18,86348026
ENSG00000167674	HDGFL2	15,54824117	11,63658703	16,34414021	14,1852191	24,38245563	22,80012358	26,78613014	11,48734264	26,53711424	169,7073538	18,85637264
ENSG00000167186	COQ7	27,64531999	0,653208283	41,10448723	3,114692754	0	21,29960388	10,14766144	45,88250998	19,78439226	169,6318758	18,8479862
ENSG00000165675	ENOX2	46,72102944	25,1911858	0,0649638	3,170978892	3,273097488	21,59667942	21,49328192	6,332235227	41,7383753	169,5818273	18,84242525
ENSG00000101596	SMCHD1	27,94968336	11,21301488	61,56471191	23,48100013	0,144397886	11,88792311	13,268475	11,24035561	8,817808455	169,5673703	18,84081893
ENSG00000124019	FAM124B	0,04241105	0	0,078018551	5,272893116	118,2275827	2,514502145	5,141360784	34,87044552	3,390408068	169,5376219	18,83751355
ENSG00000086475	SEPHS1	18,83046246	23,95604378	1,001987998	60,39663804	9,523456968	24,77552381	8,689886537	3,31795781	19,04400511	169,5359625	18,83732917
ENSG00000131979	GCH1	29,29220293	0	7,41280264	42,73088986	8,594265971	11,5711992	16,7293776	38,18355002	14,93125905	169,4455473	18,82728303
ENSG00000033011	ALG1	22,93375891	13,4866062	13,7982654	10,88900619	0	20,11478988	49,02519671	8,781590747	30,40976479	169,4389788	18,8265532
ENSG00000160991	ORAI2	18,48917489	8,159898805	20,0720302	8,081536982	23,37642776	33,50476488	26,43383244	3,599764353	27,55822654	169,2756568	18,80840632
ENSG00000186047	DLEU7	11,71836804	21,00832726	20,33458685	15,57613173	40,67287288	20,07285858	15,02969067	11,05117608	13,74348033	169,2074924	18,80083249
ENSG00000139974	SLC38A6	33,35177934	22,03692754	0,054008179	13,53597012	45,49147807	16,600728	15,06521569	23,04760244	0	169,1837094	18,79818993
ENSG00000164209	SLC25A46	8,752353488	2,496606289	24,35586356	55,59461007	4,21214046	18,39461613	12,27394179	22,93463625	20,13524594	169,132012	18,79244577
ENSG00000109066	TMEM104	16,43626918	2,798190649	52,9406209	10,36827714	0	15,51561885	27,21923116	21,08733543	22,71333174	169,0788751	18,78654167
ENSG00000126522	ASL	14,51985765	15,44229359	14,70379866	11,92378914	6,294391979	20,14813889	23,16979527	25,44191036	37,40197616	169,0459517	18,78288352
ENSG00000168685	IL7R	29,26826003	0,04454186	34,72442432	1,560408486	0,059654077	47,45814171	47,35358709	8,507866772	0,030871515	169,0077559	18,77863954
ENSG00000139637	C12orf10	18,85859922	22,41667121	0	2,922471716	56,16598362	21,56837497	20,57713445	6,873039004	19,55105414	168,9333283	18,77036981
ENSG00000163481	RNF25	56,16569991	10,23659809	0	0,27670729	0,380824541	12,22776785	22,28661783	25,7549742	41,58389841	168,9130882	18,76812091
ENSG00000099308	MAST3	5,890902341	11,08679268	55,94903646	18,30243841	0,053029761	30,55086963	24,80873812	2,04935792	20,17087919	168,8620445	18,76244939
ENSG00000110074	FOXRED1	91,67606125	19,71841471	0	1,568285158	0,063481977	11,1154514	14,39459881	16,58890175	13,63378235	168,7589774	18,75099749
ENSG00000037749	MFAP3	2,461968246	3,439923029	0,071322668	52,22046958	52,10544457	11,45091601	12,42631126	21,32246151	13,20833526	168,7071521	18,74523913
ENSG00000100714	MTHFD1	33,7052821	25,94001492	16,23524005	15,43209794	0,117666298	15,66963425	20,00043136	22,33031768	19,25752381	168,6882084	18,74313427
ENSG00000163900	TMEM41A	10,86085435	5,506377021	27,35544813	40,0674263	39,2758918	7,215999933	9,53910346	14,21920785	14,62244211	168,662751	18,74030566
ENSG00000083535	PIBF1	60,42253256	15,35523284	0	6,243957277	22,08499842	21,3988516	9,517610731	16,13033065	17,43283961	168,5663537	18,72959485
ENSG00000182557	SPNS3	10,06575347	6,284578127	37,49073703	15,7719742	0	22,64897675	26,72435525	0	49,51831679	168,5046916	18,72274352
ENSG00000132323	ILKAP	21,79632363	11,40549253	0,526810042	22,47260573	22,08092701	19,80865382	27,714967	7,09714169	35,5726445	168,4755659	18,71950733
ENSG00000101000	PROCR	29,29603874	0	0	0,922575043	86,97543306	20,43330025	20,33255233	0	10,51341552	168,473315	18,71925722
ENSG00000204160	ZDHHC18	38,49721681	16,25549488	18,22653396	3,966593936	61,36568993	14,16013347	6,204576857	0,060519152	9,734817833	168,4715768	18,71906409
ENSG00000166266	CUL5	3,177881553	7,150998174	2,37227759	12,16795218	0,109453686	34,30976114	24,53191648	42,70169358	41,43454162	167,956476	18,66183067
ENSG00000158773	USF1	44,76391905	10,6751738	0,35699499	8,377621188	0,307463649	62,1454429	8,164165438	27,58336501	5,569031061	167,9431771	18,66035301
ENSG00000023608	SNAPC1	45,63038057	9,100075591	27,87305273	10,86809767	12,88003526	18,87528736	14,90353346	13,89023057	13,90444478	167,925138	18,65834867
ENSG00000196357	ZNF565	43,31097307	24,14838149	0	3,366477031	77,31065736	1,55291964	0,571329617	11,53553512	6,064811883	167,8610852	18,65123169
ENSG00000220205	VAMP2	4,382719421	24,40663068	14,06625617	29,06433997	12,41010945	22,93364343	17,18877736	15,90490359	27,40963827	167,7670183	18,64077982
ENSG00000180423	HARBI1	0,397360629	1,712401954	2,610632034	2,54857601	103,2024492	5,173019484	13,76308676	16,63344555	21,71234727	167,7533189	18,63925766
ENSG00000162402	USP24	14,36574751	13,89440789	28,4846945	59,27084884	0,477907401	14,43420637	8,359123009	2,116232363	26,29334895	167,6965168	18,63294631
ENSG00000094916	CBX5	5,506622737	12,44601118	21,81250943	55,61222194	15,64184002	11,41890191	12,75729071	14,8759278	17,19061793	167,2619437	18,58466041
ENSG00000168904	LRRC28	9,152294505	12,94618127	0,02995798	25,98382547	84,68037317	5,095863171	6,909725148	16,13178781	6,309047798	167,2390563	18,58211737
ENSG00000114904	NEK4	0,047273759	3,98459096	69,04933477	23,38741443	1,34816534	11,39288959	10,55889858	29,87504771	17,55847012	167,2020853	18,57800947
ENSG00000115808	STRN	2,653464135	3,045581912	27,9122934	22,90976712	89,21009257	2,647447709	5,280752598	3,983358664	9,440604469	167,0833626	18,56481806
ENSG00000148335	NTMT1	32,97592843	9,118067352	17,80394163	22,65628865	0	30,94733438	20,25585749	14,39992173	18,89756648	167,0549061	18,56165624
ENSG00000163312	HELQ	20,883546	0	0	1,546489612	0,773960221	26,10231056	27,00721855	27,68457718	62,98354661	166,9816487	18,55351653
ENSG00000006757	PNPLA4	17,58748067	18,15913281	8,231014364	27,00382469	0	41,58708387	35,01839814	19,37464483	0	166,9615794	18,5512866
ENSG00000145041	DCAF1	11,36252648	31,28127582	6,088044218	49,26104864	1,205972182	16,5208079	14,82048454	24,07458741	12,29208273	166,9068299	18,54520332
ENSG00000186130	ZBTB6	14,04565197	15,503697	129,2583724	0	0	4,938055711	0,550979745	0	2,493671316	166,7904282	18,5322698
ENSG00000254004	ZNF260	40,82236741	23,68082284	3,742327665	5,152189411	0,064461953	20,34049971	18,06268287	42,70559839	12,17626649	166,7472167	18,52746853
ENSG00000111331	OAS3	8,350798599	0,065713025	8,140821426	46,42538698	0	26,40826359	26,81292231	38,34352842	12,13775228	166,6851866	18,52057629

ENSG00000091592	NLRP1	12,15921387	13,74910615	42,87965574	5,6734706	14,17164758	20,2381931	16,57256654	12,44574642	28,77900692	166,6686069	18,5187341
ENSG00000115145	STAM2	16,70333787	11,91126653	4,020373332	12,97743306	12,77262806	30,65526004	30,07140188	11,50864196	36,01187777	166,6322205	18,51469117
ENSG00000157426	AASDH	34,31969772	1,843692308	79,73859737	7,34475326	0,077163202	23,81355076	3,828273989	3,976006829	11,66033187	166,6020673	18,51134081
ENSG00000100319	ZMAT5	32,48877269	22,37309813	4,864651732	0	0	29,55282148	27,1329593	27,88041663	22,30158267	166,5943026	18,51047807
ENSG00000011028	AC080038.1	0,04585361	1,423906843	34,16232441	16,15255191	0	35,14051966	37,65442523	0,100268417	41,81617244	166,4960225	18,49955806
ENSG00000128694	OSGEPL1	37,36644309	31,73100978	1,551661172	19,88145355	0	27,64404529	25,10807369	18,81346686	4,356993545	166,453147	18,49479411
ENSG00000135968	GCC2	35,46240391	12,41585078	12,03422353	22,73014347	3,163435661	21,8599239	13,19696234	19,60415895	25,94183499	166,4089375	18,48988195
ENSG00000178764	ZHX2	21,00548916	0,180648102	0,062425353	18,6926245	108,2272045	5,476949197	8,650211526	3,561836793	0,542556656	166,3999458	18,48888287
ENSG00000171984	SHLD1	54,01251977	14,11273685	44,1460386	0,955368328	0	26,19640685	25,26633688	0	1,701112332	166,3905196	18,48783551
ENSG00000104998	IL27RA	78,88484028	5,635526782	2,167302467	12,91406993	0	13,83525487	15,01390752	12,20927724	25,64052019	166,3006993	18,47785548
ENSG00000151092	NGLY1	22,68576439	12,64056328	0,604815881	27,62952227	15,62701744	28,2567391	18,13882716	12,14216228	28,52956404	166,2549758	18,47277509
ENSG00000180667	YOD1	0	12,50040914	44,58935494	48,33759382	47,80156828	0	7,638663058	0	5,386442679	166,2540319	18,47267021
ENSG00000186352	ANKRD37	35,61460853	28,68274391	5,552199048	13,78218463	6,057020244	15,464368	18,71195364	18,03970342	24,33409857	166,23888	18,47098667
ENSG00000103319	EEF2K	27,19237542	10,38507622	4,60415357	22,6165308	42,84366196	13,9050832	19,18774193	8,394601715	17,10343462	166,2326594	18,47029549
ENSG00000130881	LRP3	22,47154951	5,467167604	8,475637137	6,833313963	23,10819951	27,4345211	27,76494627	27,13445102	17,4861206	166,1759067	18,46398963
ENSG00000011523	CEP68	0	4,882093704	28,8521615	7,323004042	49,8091825	20,56806291	22,23340685	9,081873188	23,4059287	166,1557134	18,46174593
ENSG00000138642	HERC6	0	19,07034421	21,88139567	65,26752601	0	11,85689259	10,89445486	27,721595	9,431879432	166,1240878	18,45823198
ENSG00000165355	FBXO33	21,72729202	30,96890476	0,14429281	18,82686814	0,093204657	28,72108867	36,14639723	5,917463057	23,49007432	166,0355857	18,44839841
ENSG00000131023	LATS1	30,16161296	8,620203253	1,833123592	20,37210176	36,57991923	7,971209997	24,68922807	9,533262958	26,26187314	166,022535	18,44694833
ENSG00000174013	FBXO45	0,05057248	8,839353082	0,186064323	28,29415387	52,04079513	5,580325622	10,91776651	27,37033315	32,65375642	165,9331206	18,4370134
ENSG00000130695	CEP85	12,39429754	9,381061228	0,054029174	23,98877415	14,2390629	23,25005361	26,60605096	25,56131348	30,45065467	165,9252977	18,43614419
ENSG00000196453	ZNF777	0	12,341971	37,33276235	0,246785915	67,47605777	14,74993147	16,0991252	0,83337848	16,81524335	165,8952555	18,43280617
ENSG00000170088	TMEM192	3,977375068	13,27279199	32,51564505	23,78193444	23,77187424	15,6101289	20,71455786	17,45961474	14,78449058	165,8884129	18,43204587
ENSG00000134215	VAV3	38,32574424	0	4,223349437	5,071553024	36,0788771	1,135440574	16,8301294	44,63987382	19,45970292	165,7646705	18,41829672
ENSG00000133243	BTBD2	27,30362599	6,631371806	32,92361872	10,53573249	39,39176272	8,010823606	14,90276824	13,23072461	12,81915783	165,749586	18,41662067
ENSG00000145730	PAM	15,56203141	22,4007391	29,79031583	13,03789948	0,040596602	10,67184243	22,31119514	44,22728102	7,647313374	165,6892144	18,40991271
ENSG00000167657	DAPK3	18,01064283	10,72870217	2,976400045	16,10165101	0	32,583734	46,17195086	9,310638702	29,79618799	165,6799076	18,40887862
ENSG00000137700	SLC37A4	14,26585407	9,49290301	0	0,042181535	0	27,67858312	32,79753101	52,29476909	29,08170604	165,6535279	18,40594754
ENSG00000152689	RASGRP3	9,939080236	0	2,379213797	27,30968894	46,70632842	28,63188388	28,248042	1,752234659	20,64385037	165,6103223	18,40114692
ENSG00000072858	SIDT1	18,89819544	19,42865112	45,87016522	8,482005378	0	14,18267833	12,51589912	29,18975336	16,80919538	165,3765433	18,37517148
ENSG00000123080	CDKN2C	0	30,29702519	0	39,68494147	0	45,79307833	23,946624	15,65677374	9,97585706	165,3542998	18,37269998
ENSG00000152818	UTRN	12,80983884	6,609909616	60,85335363	17,06333201	1,77050555	13,88268452	19,06454853	5,113545074	28,14930891	165,3170267	18,36855852
ENSG00000171475	WIPF2	33,3920318	18,78281399	0,718648539	20,65507678	49,5593703	7,945243671	16,12594197	5,613685869	12,44620314	165,2390161	18,35989067
ENSG00000181444	ZNF467	16,47705836	9,148707003	54,55958327	8,994592863	0	14,18088995	11,51873255	24,98889624	25,36350708	165,2319673	18,35910748
ENSG00000156697	UTP14A	26,17721655	41,33336434	0	12,76902249	0,527209507	22,16471125	26,55537978	11,31902115	24,14595139	164,9918765	18,33243072
ENSG00000140199	SLC12A6	39,62595215	0,906605808	1,271587565	29,58093387	42,49700432	14,88179596	7,673454075	0,098578332	28,42402061	164,9599327	18,32888141
ENSG00000165195	PIGA	0	28,42194907	58,13790395	51,64209037	0	6,010682181	2,64001806	0,888686545	17,11163992	164,8529701	18,31699668
ENSG00000106771	TMEM245	12,00552256	15,01121162	11,23270104	0,466788863	32,23479571	22,10278755	15,01207697	24,13149425	32,62043556	164,8178141	18,31309046
ENSG00000110446	SLC15A3	13,72044051	16,27225227	13,96520086	16,31595248	0,16551738	25,38745343	21,74382155	21,3724002	35,71885072	164,6618894	18,29576549
ENSG00000172922	RNASEH2C	15,700569	19,13422624	34,33198635	14,3230333	0	20,751019	27,91782529	9,911093674	22,58867748	164,6584303	18,29538115
ENSG00000040487	PQLC2	30,88596549	23,75941773	51,9530937	0,097146305	0	19,58066002	12,42558016	10,45679011	15,49874199	164,6573955	18,29526617
ENSG00000221923	ZNF880	18,93453238	18,45774456	0,074745926	55,70916102	0	16,32784917	19,66905178	17,14812356	18,23984421	164,5610526	18,2845614
ENSG00000070367	EXOC5	10,12896385	16,70512621	13,3284236	3,661290936	0,057587803	26,55207754	31,20662042	26,97100623	35,92655269	164,5376493	18,28196103
ENSG00000038532	CLEC16A	6,045388158	3,083322528	40,62154285	9,918154429	58,44298901	7,313154862	13,54594657	8,971313021	16,59158444	164,5333959	18,28148843
ENSG00000178996	SNX18	3,043104369	19,70565645	13,03423368	19,60737711	2,806449084	19,82249902	24,5887155	21,40326099	40,35891564	164,3702118	18,26335687
ENSG00000170144	HNRNPA3	9,367703204	37,9123365	4,515506332	36,24457297	10,23839173	9,515176453	12,24963129	17,91017795	26,39987893	164,3533753	18,26148615
ENSG00000196505	GDAP2	0	30,93847813	30,64222317	39,38781966	0,034244068	11,34336641	17,50387105	22,08778791	12,35196205	164,2897524	18,25441694
ENSG00000168944	CEP120	13,32227914	19,8328572	0,183576093	24,34014539	0	18,27015926	20,98422426	0,218216653	67,13425635	164,2857143	18,25396826
ENSG00000114626	ABTB1	33,5610317	10,32488286	35,85965859	2,458851101	26,663967	16,60453538	20,14048546	10,57605358	8,061904378	164,25137	18,25015223
ENSG00000145782	ATG12	9,952472456	28,72841999	11,28598575	32,87068531	6,763576133	20,15176397	13,24464089	22,21033069	18,98916761	164,1970428	18,24411587
ENSG00000096063	SRPK1	13,50330563	3,245592051	33,81288114	40,82470389	40,24778194	10,16487395	8,737174942	9,628622335	3,999088345	164,1640242	18,24044713
ENSG00000132950	ZMYM5	16,99284195	15,7603591	3,879486083	19,32851664	0,578289118	33,44037657	19,96884243	35,02996623	19,15326478	164,1319429	18,23688254
ENSG00000172985	SH3RF3	23,01942865	2,448996382	29,98716904	23,02235171	0	28,76202657	21,05946982	7,288579122	28,53511138	164,1231327	18,23590363
ENSG00000124575	HIST1H1D	0	0	80,97318199	12,92921045	0	23,91503808	32,5921433	0	13,67339278	164,0829666	18,23144073
ENSG00000160993	ALKBH4	19,58719011	17,02859157	0	0,340498009	60,92033124	21,50559019	19,15619249	11,06718647	14,46999891	164,075579	18,23061989
ENSG00000113838	TBCCD1	24,54768541	26,19868865	0	7,67907518	6,658153375	31,10212989	20,23476808	23,14403386	24,50242554	164,06696	18,22966222
ENSG00000146540	C7orf50	28,6016581	20,26874623	24,59852116	22,98499978	0,072388136	16,62115634	22,58516559	11,85594699	16,40813883	163,9967212	18,22185791
ENSG00000118939	UCHL3	13,96087162	22,66186982	5,253167225	32,46277637	25,82630095	21,86013373	11,85546444	16,82527719	13,26778322	163,9736446	18,21929384
ENSG00000088854	C20orf194	1,415389961	1,663918277	2,571177593	52,822453	0	23,52390919	25,32617403	18,91845258	37,68717674	163,9286514	18,2142946
ENSG00000124275	MTRR	21,20850115	47,12498678	0,042779332	5,82264871	0	27,3198395	30,81727453	0,101703468	31,46063012	163,8983636	18,21092929
ENSG00000204120	GIGYF2	1,295297825	4,017333186	1,139602374	41,34971348	44,91646065	16,57127108	15,30969987	25,02396546	14,1850655	163,8084094	18,20093438
ENSG00000196510	ANAPC7	17,87978295	19,32193144	0,046260626	19,62765993	54,80292667	20,84581218	15,72294225	7,698592021	7,855719197	163,8016273	18,20018081
ENSG00000140396	NCOA2	9,390625932	15,61862185	25,74558415	10,68871412	3,085633794	8,569863938	16,59733436	69,98913809	4,103514979	163,7890312	18,19878124
ENSG00000129484	PARP2	7,120200229	22,84706007	0	26,24638905	0,087675181	24,66785674	10,29248817	39,6908709	32,80445205	163,7569924	18,19522138
ENSG00000111300	NAA25	33,2681321	4,413971522	35,34406631	7,246729753	0,181335863	15,81696469	16,32051986	23,72641663	27,42090174	163,7390385	18,1932265
ENSG00000131042	LILRB2	17,60973579	10,20487927	12,84107544	21,50487717	33,55533992	10,93006686	16,33419545	22,54934057	18,19442331	163,7239338	18,1915482
ENSG00000185745	IFIT1	21,71354754	0	38,61838826	18,83210805	0,07783215	11,29083682	12,37299546	60,65844792	0	163,5641562	18,17379513
ENSG00000154727	GABPA	7,654339092	29,9307519	0,051202818	1,05738984	51,00011515	15,1272307	14,46756821	12,29467354	31,97285905	163,5561303	18,17290337
ENSG00000136840	ST6GALNAC4	31,73723721	21,69169997	18,58127513	10,00927931	0	28,79648065	21,63445907	12,04774724	19,0575621	163,5557407	18,17286008
ENSG00000127191	TRAF2	24,53227546	15,73002183	31,02151719	10,32348467	0	28,24780351	14,27588771	23,261151	15,97316631	163,3653077	18,15170085
ENSG00000114544	SLC41A3	0	0,081353762	0,168677245	23,7105658	84,93086294	14,72355694	13,32360928	13,13317001	13,25058639	163,3223824	18,14693138
ENSG00000148840	PPRC1	0	20,84620437	0,957451546	60,08737155	0,176702087	31,26640194	18,25359759	0,216785021	31,48752788	163,292042	18,14356022
ENSG00000137959	IFI44L	16,36539286	9,269752737	0,488636893	15,08530322	0,105210217	5,832793712	10,51304149	104,7451764	0,834857424	163,2401649	18,1377961
ENSG00000164039	BDH2	19,82728378	26,79876886	34,50088702	13,31454119	16,32665596	11,80756262	13,28594346	14,51554114	12,79856158	163,1757456	18,1306384
ENSG00000146192	FGD2	4,340378382	6,251195556	6,070389728	7,289546802	77,07992105	10,39593128	12,61368083	20,90000265	18,20805983	163,1491061	18,12767846
ENSG00000174227	PIGG	21,86451692	9,521144512	40,42558555	4,093942624	5,436681759	18,51062127	22,93209481	27,46799873	12,85698484	163,109571	18,12328567
ENSG00000204574	ABCF1	25,71165181	8,170697067	40,99217207	13,1163933	7,987489821	11,99309107	22,21405344	19,18225581	13,72354213	163,0913465	18,12126072
ENSG00000112200	ZNF451	11,42489449	2,630124407	31,04286106	38,59815513	17,48110859	7,621333912	17,37504963	15,23936686	21,59189216	163,0047862	18,11164292
ENSG00000134330	IAH1	18,46679621	27,14098105	10,9159416	28,61981441	0,243139839	16,37201527	14,20779487	30,59372181	16,38897057	162,9491756	18,10546396
ENSG00000100290	BIK	0	12,68810726	0	32,3767897	0	46,92949015	19,26253257	0	51,5914636	162,8483833	18,09426481
ENSG00000137054	POLR1E	28,42015535	46,03157985	0,222473068	9,501833809	1,005931491	10,68891671	20,08328643	30,80882522	15,98919659	162,7521985	18,08357761
ENSG00000007168	PAFAH1B1	6,949725722	12,42999173	56,54979018	11,14346953	29,62424793	14,69557122	10,85404624	7,236673823	13,20530101	162,6888174	18,07653527
ENSG00000177311	ZBTB38	2,800898031	8,773023383	10,12930361	32,02375452	2,445720818	25,54531312	24,04928782	33,10609618	23,81309437	162,6864918	18,07627687
ENSG00000184517	ZFP1	0,040199673	40,80266668	81,27164905	0,416496368	0,191070786	23,96633231	9,312642359	1,23066878	5,389005301	162,6207313	18,06897015
ENSG00000198890	PRMT6	0,244007842	5,195831144	36,16626893	36,83787877	11,34927412	14,6583047	27,43849475	15,93098607	14,79055368	162,6116	18,06795556
ENSG00000106244	PDAP1	19,45963736	26,05912982	4,009328351	24,55226066	6,166166593	23,1317631	21,84528102	21,78689456	15,57230475	162,5827662	18,0647518
ENSG00000168765	GSTM4	10,50148544	18,86873163	15,72648019	2,59704794	40,44540157	20,80038311	23,74888072	13,61665059	16,25791997	162,5629812	18,06255346
ENSG00000100023	PPIL2	24,43305615	10,90889347	19,39227448	0,136098565	33,05997978	14,88414093	27,30603522	10,34089141	22,1009295	162,5622995	18,06247772
ENSG00000120162	MOB3B	31,26206048	24,0649913	0,039042149	12,27713715	0,050437857	22,78940225	12,93472243	46,96626156	12,1113464	162,4954016	18,05504462
ENSG00000162419	GMEB1	29,38456057	19,76089055	0	14,01833661	31,46934959	23,40060929	17,3276879	7,92961108	19,1342216	162,4252672	18,04725191
ENSG00000110107	PRPF19	25,88396209	7,532594727	18,09394272	40,67331738	13,90211795	18,07453354	15,30230839	7,471285185	15,40762535	162,3416873	18,03796526
ENSG00000075336	TIMM21	7,327278008	10,42229825	23,80241371	35,34697366	10,84884629	18,93110871	14,36498894	26,57718861	14,55446114	162,1755573	18,01950637
ENSG00000107371	EXOSC3	17,99530448	35,03674827	0,735640355	32,28239514	0	19,37361349	17,6397258	17,27048021	21,82451717	162,1584249	18,01760277
ENSG00000185359	HGS	15,32285128	10,81718489	14,46648413	16,95043772	21,88406718	18,88610343	23,35078574	12,44415373	28,01854333	162,1406114	18,01562349
ENSG00000167994	RAB3IL1	38,13719358	0	16,05661222	0	46,43659721	14,21157221	16,26543523	21,47228237	9,514951583	162,0946444	18,01051604
ENSG00000164880	INTS1	19,51262653	9,18011471	6,51802742	44,09238067	1,027857343	23,83457756	26,14655862	8,911984703	22,68866063	161,9127882	17,9903098
ENSG00000167543	TP53I13	17,29339791	15,13799475	20,13378835	13,07529741	6,286521529	21,27431027	36,40561493	7,158210544	25,13198047	161,8971162	17,98856846
ENSG00000050344	NFE2L3	14,01187955	17,26129073	31,65471729	18,43463451	0,083457481	13,64916202	24,1434894	24,18937689	18,39893527	161,8269431	17,98077146
ENSG00000130165	ELOF1	13,50688782	21,71866667	0	10,12975459	6,19613301	32,28358214	36,62182228	19,79598416	21,55516817	161,8079988	17,97866654
ENSG00000145860	RNF145	28,59114178	15,10986563	33,25639676	18,7190023	10,97433322	14,4191897	15,96252382	14,39474384	10,35165935	161,7788564	17,97542849
ENSG00000127824	TUBA4A	0,876334626	76,7375618	20,1861494	4,868680817	44,821781	0,627498399	3,06873984	8,248366663	2,342997003	161,7781095	17,97534551
ENSG00000064999	ANKS1A	11,09590446	8,74033114	35,23000313	17,50211056	13,01579231	18,37159951	23,03291574	6,260292465	28,51775301	161,7667023	17,97407804
ENSG00000121390	PSPC1	37,80117037	19,59435002	0,346248826	19,88024407	11,18281739	16,94581767	13,70093308	21,60823931	20,67962133	161,7394421	17,97104912
ENSG00000132423	COQ3	60,31899851	22,40689365	0	1,441629694	0,248009352	10,65585886	11,26460163	33,31729755	22,07569187	161,7289811	17,96988679
ENSG00000154328	NEIL2	0	1,593238266	43,27438261	27,44235918	0	32,63006589	17,63145188	25,91643667	13,19587606	161,6838106	17,96486784
ENSG00000164031	DNAJB14	9,134208743	20,98412722	40,42177846	20,62130678	22,34447623	12,03715132	11,35714493	14,31798203	10,41714894	161,6353247	17,95948052
ENSG00000175213	ZNF408	0,049093753	0	29,53199882	11,61406482	80,38726013	7,546290038	12,96282182	7,085341604	12,37769235	161,5545633	17,95050704
ENSG00000170439	METTL7B	0	25,56521253	62,31286062	11,20463137	0	20,64825955	9,314391459	32,46627847	0	161,511634	17,94573711
ENSG00000156521	TYSND1	0,032700838	3,539636876	5,293713203	0,90347535	100,4068091	17,70047919	11,62114306	9,438944582	12,54795822	161,4848604	17,94276227
ENSG00000114166	KAT2B	5,045718537	38,54123164	0	68,90165516	0,344576418	10,283845	10,20906175	10,27257723	17,86780036	161,4664661	17,94071845
ENSG00000115947	ORC4	0	44,00356098	17,27436622	1,404921905	0,040282394	22,07178283	21,20962314	31,09754371	24,26532271	161,3674039	17,92971154
ENSG00000170836	PPM1D	0,02995848	32,85324268	0,110222085	21,98599608	3,132666904	21,182873	32,88496806	29,61071065	19,56473103	161,355369	17,92837433
ENSG00000183597	TANGO2	25,67186242	15,59117038	22,0776788	22,63103402	0	25,70711226	15,4672701	12,98768318	21,21976696	161,3535781	17,92817535
ENSG00000144668	ITGA9	9,592871777	3,696274245	26,65516548	15,64978808	0,217120642	27,56482858	23,74378109	16,50175047	37,7086414	161,3302218	17,9255802
ENSG00000163882	POLR2H	39,91509352	5,540123969	0	19,9889793	0	24,52270126	19,3430105	25,31301042	26,70140752	161,3243265	17,92492517
ENSG00000131196	NFATC1	35,14470595	8,811900753	0	9,754847418	40,03835855	11,30709488	22,55366577	18,72996997	14,87919885	161,2197421	17,91330468
ENSG00000141873	SLC39A3	26,3112579	12,41398149	34,96723467	7,896406187	0,081101523	27,65176278	21,761792	13,9546667	16,07480082	161,1130041	17,9014449

ENSG00000005889	ZFX	3,661969124	11,77961426	18,48689161	20,99144921	17,36575921	22,74684127	20,45123371	38,31973296	7,300599248	161,1040906	17,90045451
ENSG00000151779	NBAS	12,09289914	11,02897687	9,134475143	3,149768331	0	16,8006016	32,12837047	62,34196378	14,39495249	161,0720078	17,89688976
ENSG00000158793	NIT1	37,04080936	19,57705167	15,89923075	13,3424842	0	19,54718708	12,83144144	24,125033	18,66302714	161,0262647	17,89180718
ENSG00000139405	RITA1	28,53365475	18,58064964	31,06057407	14,80401978	0,155529468	14,44262708	12,33510843	8,300218673	32,75856846	160,9709503	17,88566115
ENSG00000074416	MGLL	26,99814989	12,35593991	42,15800809	14,51229203	33,85385221	4,186620173	1,23892555	18,57282656	7,073743062	160,9503575	17,88337305
ENSG00000162654	GBP4	48,30321612	0,96061293	0,678993891	42,788067	0	12,15758989	22,51560102	20,60844152	12,83161137	160,8441337	17,87157042
ENSG00000095906	NUBP2	15,76168475	21,13915101	5,113440268	14,39968629	29,03719768	19,8207299	24,55130006	7,013468686	23,89294283	160,7296015	17,85884461
ENSG00000144357	UBR3	2,567813533	9,939080397	27,37536302	39,67311052	0,035686927	9,447158944	17,29378421	37,7620742	16,58453513	160,6786069	17,85317854
ENSG00000171049	FPR2	38,22576367	2,293076415	88,26347419	0	0,198133816	9,382529165	14,12190219	3,737328902	4,306512516	160,5287209	17,83652454
ENSG00000197063	MAFG	17,18410611	8,64644114	38,24149043	19,66596355	3,631602699	15,28998446	20,03790648	14,12275198	23,54552391	160,3657708	17,81841897
ENSG00000165171	METTL27	5,419137707	13,31463725	0	9,357668055	67,86083214	12,35747744	21,11362828	17,54721496	13,3296816	160,3002774	17,81114194
ENSG00000154144	TBRG1	14,25571421	10,98051796	43,55215807	5,944430588	0,050191131	26,7822105	21,62193143	17,73401453	19,37687011	160,2980385	17,81089317
ENSG00000115421	PAPOLG	51,78998919	4,870134216	5,376664257	19,11081722	0,63530642	16,45604376	19,87341147	11,14568774	31,03652265	160,2945769	17,81050855
ENSG00000229809	ZNF688	6,998173964	12,77289051	19,21860426	2,675813501	43,71659133	10,2630757	18,88488292	19,56592378	26,12794309	160,2238991	17,80265545
ENSG00000115825	PRKD3	10,98959148	9,397010741	9,76892187	2,801917923	71,5851251	19,38646625	8,536979833	10,61235579	16,98090051	160,0592695	17,78436328
ENSG00000126767	ELK1	27,74955741	19,25455792	0	25,41773884	28,42925806	11,66940195	14,25295708	6,807068783	26,47137045	160,0518105	17,7835345
ENSG00000138032	PPM1B	5,848755374	16,83199606	0,312996593	25,30389831	35,27994997	19,3114774	16,05243234	22,32351758	18,72851653	159,9935402	17,77706002
ENSG00000196387	ZNF140	0,084356478	11,47611835	25,55306455	12,7699848	57,80362377	6,79202212	8,498548725	18,99968019	17,94833365	159,9257326	17,76952585
ENSG00000179833	SERTAD2	24,66324902	3,205395283	4,65220121	25,95209183	43,78784187	10,41899382	13,77893828	7,215418513	26,21520118	159,889331	17,76548122
ENSG00000104833	TUBB4A	0	109,5884696	0,863292233	0,648285651	46,50683701	0,206100095	0,155863766	1,539291464	0,288581556	159,7967214	17,75519126
ENSG00000118482	PHF3	14,86643791	8,371363271	24,83017458	14,28127439	1,487960524	16,88201667	25,99356464	27,00024217	26,07366896	159,7867031	17,75407812
ENSG00000179094	AC129492.1	15,2362272	19,2241194	23,7803598	18,48382568	12,25779672	19,4059929	20,21287268	10,28773718	20,88849086	159,7774224	17,75304694
ENSG00000163104	SMARCAD1	0,263300535	0	17,43705357	49,97154828	0	24,04691552	12,20319719	12,24801026	43,59871685	159,7687422	17,75208247
ENSG00000174106	LEMD3	0	24,80481751	0,797361345	3,087434264	33,220626	20,93959233	18,33243467	31,87052659	26,68753394	159,7403266	17,74892518
ENSG00000105011	ASF1B	0	20,40508581	0	47,57598873	8,803698689	43,04523645	14,54635616	0	25,24779107	159,6241569	17,73601743
ENSG00000106266	SNX8	14,61173518	5,689273741	17,79072633	12,66101877	20,1730376	27,29589662	26,27239647	13,79205151	21,33194864	159,6180849	17,73534276
ENSG00000187808	SOWAHD	27,60224046	0	0	16,96481259	0	32,05207426	40,63218801	5,933499974	36,36377773	159,548593	17,72762145
ENSG00000153208	MERTK	0,225058297	0,114102979	0,650591721	2,220723188	31,09805101	38,65363436	55,90103728	29,8094163	0,790836265	159,4634514	17,71816127
ENSG00000147679	UTP23	20,2575765	0,255665523	41,71821852	5,523223147	0,273926424	12,21233459	18,85404684	39,25643317	21,05126511	159,4026898	17,71140998
ENSG00000125875	TBC1D20	15,31613755	23,4929428	0,429792285	7,620502608	0,246773779	22,26012032	38,62615271	43,59624598	7,694384447	159,2830525	17,69811694
ENSG00000154930	ACSS1	22,11628649	16,50626741	19,54272701	39,39094312	0,785091854	14,08836792	23,11333581	11,40609484	12,29567947	159,2447939	17,69386599
ENSG00000177030	DEAF1	46,56866643	17,82794969	5,61954378	18,75543393	3,065244072	19,04319512	20,48897829	0	27,84358274	159,2125941	17,69028823
ENSG00000145919	BOD1	15,72508416	27,90377603	0	8,663351486	0,53387147	16,27863934	36,87012234	25,54400026	27,62832343	159,1471685	17,68301872
ENSG00000165501	LRR1	8,098736467	39,14055339	0	22,51203702	0	40,55591875	6,615211404	0	42,10901594	159,031473	17,67016366
ENSG00000095574	IKZF5	3,73641865	16,41242076	0,394377927	0,26442539	8,006268678	26,49727086	18,20769309	57,32711589	28,17454371	159,020535	17,66894833
ENSG00000135070	ISCA1	9,900508013	8,412944943	0,085506029	19,82495808	0	21,99552709	19,2971904	58,34187689	21,15140364	159,0099151	17,66776834
ENSG00000102189	EEA1	32,15752046	21,37210621	13,03630809	19,1009543	0,495334541	15,83914767	16,094815	23,76524476	17,11985205	158,9812831	17,66458701
ENSG00000173041	ZNF680	0,271732729	6,50947132	35,69102512	22,89808804	0	5,101727858	8,821956449	60,60839037	19,04918498	158,9515769	17,66128632
ENSG00000169955	ZNF747	18,92721325	2,111890753	31,00438604	19,49056975	0	16,14424565	22,34621513	22,33119791	26,58322968	158,9389482	17,65988313
ENSG00000105053	VRK3	2,730635121	13,769824	43,81840458	12,20094113	0,073326706	17,49725633	23,63376851	25,90849902	19,18232098	158,8149764	17,64610848
ENSG00000136111	TBC1D4	0,018943283	4,168184532	0,0348477	37,4867094	4,862065259	39,35098547	24,64736245	12,21990691	36,01839331	158,8073983	17,64526648
ENSG00000175387	SMAD2	20,70064645	17,12233823	18,48316586	7,392656174	59,18252366	11,7967494	6,978611229	9,325392176	7,806104955	158,7881881	17,64313201
ENSG00000129158	SERGEF	21,95051691	12,98355123	40,46182132	22,2646022	0,106028209	16,06692328	20,67093158	12,97543474	11,30333144	158,7831409	17,64257121
ENSG00000104824	HNRNPL	6,203043511	9,709877441	23,80008471	27,19905687	0,263244057	18,65612261	25,86296537	29,30844647	17,62830962	158,6311507	17,62568341
ENSG00000104964	TLE5	20,02257845	9,919938093	6,296264983	20,73492343	37,95881912	24,92729691	15,98574445	10,22862761	12,31259519	158,3867882	17,59853203
ENSG00000104381	GDAP1	63,69616355	19,52169053	0	31,23234518	0	14,04425662	21,98517663	0	7,888845467	158,368478	17,59649755
ENSG00000196821	ILRUN	4,648534365	14,25848473	2,260000805	48,04735415	0,157819179	8,931683431	22,27642897	27,8084903	29,8922554	158,2810513	17,58678348
ENSG00000088247	KHSRP	11,82258903	8,81466141	17,42325407	28,70685015	27,62442633	16,61650965	20,48551333	12,09348516	14,66244752	158,2497367	17,58330407
ENSG00000130311	DDA1	4,079623506	23,35223166	14,94907129	4,828972968	23,69096785	21,27614406	22,94689285	23,45344416	19,66501411	158,2423625	17,58248472
ENSG00000188706	ZDHHC9	16,91815849	0,153428571	0,053019283	5,773117882	71,713917	19,96749455	17,80467805	11,34431092	14,42676608	158,1548908	17,57276565
ENSG00000117318	ID3	0,303029219	24,55578477	0	22,67483068	0,240051833	27,61479519	18,11603724	60,299891	4,348013556	158,1524335	17,57249261
ENSG00000168301	KCTD6	0	9,384101105	82,42118943	0	0,23273982	15,9136522	20,49159019	13,2773449	16,38051006	158,1011277	17,56679197
ENSG00000113597	TRAPPC13	12,94084017	13,04817601	0	41,68167025	46,84834675	12,4414528	5,678734151	7,57541145	17,86721483	158,0818466	17,56464962
ENSG00000171552	BCL2L1	18,42144555	15,85873024	20,55073152	16,69227961	1,842184134	20,32579787	25,2530813	10,96795487	28,1517665	158,0639716	17,56266351
ENSG00000145391	SETD7	23,79155363	18,38225554	37,35432488	17,06315226	1,367722747	20,446726	9,620353855	12,67979729	17,30792562	158,0138118	17,5570902
ENSG00000100311	PDGFB	14,26329502	7,557312419	30,67315636	27,3353842	17,76013371	18,35118317	19,88309436	4,041475699	18,13498369	158,0000186	17,55555763
ENSG00000122707	RECK	37,3367113	3,322283715	0,099831244	33,12641893	27,79307968	15,52153178	10,97217664	4,450097426	25,1955691	157,8176998	17,53529998
ENSG00000105676	ARMC6	41,91714744	13,56592054	0,068603183	10,81862679	0,177254463	18,17973743	30,81021232	13,0477618	29,21624745	157,8015114	17,53350127
ENSG00000145882	PCYOX1L	12,81149399	22,86688691	28,21689033	22,07944561	0,118932784	10,11013284	18,17958942	23,4187771	19,9110283	157,7131773	17,52368636
ENSG00000118496	FBXO30	20,6355834	4,591979893	24,96937581	61,95439846	0,039677112	10,13684111	10,89599834	0,438096874	24,02391239	157,6858634	17,52065149
ENSG00000086619	ERO1B	14,17806697	30,0102966	0,518522848	7,836323617	73,88670942	12,90518756	15,1191633	2,958563559	0,173331955	157,5861658	17,50957398
ENSG00000117643	MAN1C1	37,14982897	19,3991498	17,18512023	0	0,119361027	18,19759261	22,75591995	25,51660686	17,25452977	157,5781092	17,5086788
ENSG00000179152	TCAIM	10,21461138	17,97223059	30,33203871	0,925195495	0,041074865	27,23111947	13,59034707	48,03641031	9,161597975	157,5046259	17,50051398
ENSG00000275700	AATF	14,76873539	20,3637506	13,97397261	23,41940657	8,52274642	18,93564079	18,00167654	14,97114545	24,53895828	157,4960327	17,49955918
ENSG00000221978	CCNL2	24,41745841	0,703887415	3,121549894	6,583309116	18,69694657	17,59034407	19,92667224	41,73200167	24,58258651	157,3547559	17,48386177
ENSG00000196588	MRTFA	12,93132232	10,47627931	23,525151	12,13486971	13,39954599	21,59955189	21,81355238	11,52522893	29,94850001	157,3540015	17,48377795
ENSG00000119912	IDE	0,019864673	15,26297963	0,036542671	23,29102771	69,44418362	16,96837501	22,18451381	1,6506525	8,159949841	157,0180895	17,44645438
ENSG00000125812	GZF1	4,288198179	22,582446	49,9773814	0,716591838	0,06574836	17,40511909	28,14008728	12,40188326	21,43597749	157,0134329	17,44593699
ENSG00000181218	HIST3H2A	15,70199226	9,587200003	45,65706654	35,86033729	0	6,302748165	21,30892789	2,953605073	19,51907608	156,8909533	17,43232814
ENSG00000115970	THADA	28,00115005	16,9332993	20,05679195	24,22181199	3,611378942	15,15596618	11,11762435	17,07942385	20,70189109	156,8793377	17,43103752
ENSG00000141655	TNFRSF11A	11,27239499	2,325189106	59,24267368	8,441587283	15,73010691	5,828542772	16,87816584	25,56781089	11,42558386	156,7120553	17,41245059
ENSG00000120925	RNF170	4,866484109	23,47072833	20,65913205	15,03985238	36,71242919	14,71411721	13,84214398	12,55160207	14,85544455	156,7119339	17,4124371
ENSG00000110768	GTF2H1	14,69735377	12,86617954	2,082848004	9,587668375	0	29,16583625	21,08046212	39,51883156	27,68952508	156,6887047	17,40985608
ENSG00000137288	UQCC2	15,88771835	25,16154405	7,825415454	32,05343258	0,995634048	20,06212348	21,67433758	14,5168524	18,43007738	156,6071353	17,40079281
ENSG00000137574	TGS1	9,647017564	12,47494443	35,49293587	30,88865815	22,09097314	18,01045289	9,469459019	9,650841595	8,838397544	156,5636802	17,39596447
ENSG00000130177	CDC16	0	25,9404865	0,842576708	13,57731871	13,69708232	28,03591205	17,55760645	25,20617312	31,6394433	156,4965991	17,38851102
ENSG00000161904	LEMD2	0,022281122	11,82165409	32,05255639	35,12734176	0,211806277	23,28912843	17,37196133	16,41941157	20,16854031	156,4846813	17,38718681
ENSG00000133195	SLC39A11	29,82694768	5,283113023	1,991618119	16,35809441	1,479438201	25,25953613	19,08012034	23,67433763	33,35450749	156,307713	17,36752367
ENSG00000163406	SLC15A2	0	0,044809826	0,510992829	1,395374196	75,61632743	2,495312818	3,879012604	62,78477662	9,503515343	156,2301217	17,35890241
ENSG00000163866	SMIM12	27,99426747	14,65611203	15,03249253	27,48942511	1,56334424	24,66910674	18,87456383	16,43062472	9,474810606	156,1847473	17,35386081
ENSG00000005448	WDR54	14,37135497	23,34523008	19,63355944	22,71776527	0	23,49432992	28,12114784	0,115536562	24,36798806	156,1669121	17,35187913
ENSG00000114554	PLXNA1	17,90848046	7,913913197	19,78677788	13,37577858	26,71067978	9,111134558	17,51815308	19,74636396	24,0629132	156,1341947	17,34824385
ENSG00000180767	CHST13	33,78660048	7,943837901	26,41510164	5,688353275	0	0	49,65511183	10,52809969	22,02317648	156,0402813	17,33780903
ENSG00000163659	TIPARP	0	13,68641459	1,505725536	40,11875234	0,074816155	14,8973872	28,59677984	43,71373966	13,43515484	156,0287702	17,33653002
ENSG00000183665	TRMT12	0	13,918997	3,816114615	27,42559793	0	18,36333281	13,88732117	48,90601707	29,65890446	155,9762851	17,33069834
ENSG00000115286	NDUFS7	12,52499097	16,5430955	13,75733054	16,51841577	28,60995442	17,86831989	19,62156748	12,09879787	18,42014216	155,9626146	17,3291794
ENSG00000085117	CD82	23,27080341	17,3273241	19,68366138	14,9880946	33,34218331	16,67731967	17,20984499	6,872122721	6,533846537	155,9052007	17,32280008
ENSG00000102921	AC026470.1	22,69560812	6,248635597	14,9489938	23,22020581	0,137945279	22,85784642	24,22653786	15,61208136	25,93760682	155,8854611	17,32060678
ENSG00000197594	ENPP1	11,04613871	5,573390302	63,58456326	14,54151129	2,632350697	10,69530979	7,811186448	10,4183607	29,54062102	155,8434322	17,31593691
ENSG00000165997	ARL5B	5,058094416	7,853531383	0,89170743	41,08725053	73,37617846	8,79302917	7,157233722	3,318171389	8,294405457	155,829602	17,31440022
ENSG00000085998	POMGNT1	0,048831983	25,56209081	6,153380766	19,60490599	0	25,78865322	21,77332955	25,04020232	31,71011917	155,6815138	17,29794598
ENSG00000261236	BOP1	18,56917192	24,15442125	0	1,902155503	0,093495845	25,78719456	32,40473037	13,33438114	39,43371925	155,6792698	17,29769665
ENSG00000111912	NCOA7	18,99009525	18,75977116	25,77323005	17,40248948	14,79299409	14,49303848	16,47341615	17,58687969	11,37389465	155,645809	17,29397878
ENSG00000175792	RUVBL1	11,01388999	19,62205307	21,63868409	36,55371875	0,837591936	14,46275873	22,31396575	8,140443427	20,99578058	155,5788863	17,28654292
ENSG00000166788	SAAL1	37,80228562	19,59028311	0	1,361695888	0	20,1300504	13,50463456	38,2597102	24,91288997	155,5615497	17,28461664
ENSG00000129932	DOHH	18,89812159	17,95534811	24,22573979	23,89655602	0	20,5039659	35,08968418	0,325391007	14,64078954	155,5355962	17,28173291
ENSG00000130347	RTN4IP1	15,00388645	19,69634752	4,506263003	5,683993874	0	18,07030091	22,50075863	48,376692	21,65385011	155,4920925	17,27689917
ENSG00000111199	TRPV4	5,748553234	5,920666669	0	0	70,40958465	24,23115366	23,79897492	16,70193345	8,652072731	155,4629393	17,27365992
ENSG00000105829	BET1	2,161192372	11,24928299	51,41889703	12,78800314	0	20,97610838	16,0307251	21,92815208	18,85399501	155,4063561	17,2673729
ENSG00000147316	MCPH1	9,584506784	9,326678264	38,87455706	26,32039744	32,66040871	11,94895611	11,23135964	7,572106713	7,871146826	155,3901176	17,26556862
ENSG00000267534	S1PR2	7,134509513	15,23617733	18,92370699	12,82112557	15,67380984	26,73332652	23,73018357	14,8754513	20,25283265	155,3811233	17,26456925
ENSG00000178691	SUZ12	5,651364649	24,72260236	14,34243222	25,73103245	1,315649674	24,97142858	26,02872557	11,04643112	21,44710286	155,2567695	17,25075216
ENSG00000140694	PARN	14,07913008	27,29364391	22,84562856	17,73480924	0	16,4409447	15,44728452	22,60081327	18,77682442	155,2190787	17,2465643
ENSG00000108582	CPD	6,534837628	11,98172486	33,19762333	21,10840882	0,086119315	17,89075683	14,14174006	35,36785659	14,90040462	155,2094721	17,2454969
ENSG00000161533	ACOX1	5,471056143	11,84407097	40,52618387	18,92613079	1,516909725	28,65291278	13,52220691	0,677937201	33,9799036	155,117312	17,23525689
ENSG00000188906	LRRK2	13,82989007	10,8408391	3,313942683	41,03856987	27,12327976	9,938663943	17,19700737	3,841408893	27,96298654	155,0865882	17,23184314
ENSG00000146963	LUC7L2	23,80541764	17,32139583	32,08478343	18,63231057	3,5252197	18,62422402	13,07148171	7,062803454	20,9503996	155,0780359	17,23089288
ENSG00000134057	CCNB1	0,24843497	0	70,83750981	38,71661563	0,295205542	10,70610791	5,982144888	22,40924934	5,881702513	155,0769706	17,23077451
ENSG00000175581	MRPL48	11,33501545	14,61147698	4,69004271	5,949269827	35,94450549	29,54312823	15,79106778	17,93055793	19,23719132	155,0322557	17,22580619
ENSG00000102030	NAA10	17,0883271	19,51813519	18,90939148	22,95051986	3,111937109	22,14057956	22,56073994	11,38195326	17,35265405	155,0142375	17,22380417
ENSG00000198157	HMGN5	2,251647576	19,31155133	19,55985796	70,63388799	0	14,48970682	9,971152212	14,22400456	4,538470536	154,980279	17,220031
ENSG00000163788	SNRK	8,74497501	11,10056411	0,039819524	14,09146045	62,39930939	21,50824694	17,93717108	11,02868561	8,0397722	154,8900043	17,21000048
ENSG00000001461	NIPAL3	21,96860787	0,085613665	53,10496782	24,71606294	3,439823203	14,7440753	19,26923043	2,743071122	14,77515455	154,8466069	17,20517854
ENSG00000168259	DNAJC7	1,138651237	4,890216666	8,317849418	57,50428948	0,392178161	21,36164354	25,21190465	16,0580302	19,97083957	154,8456029	17,20506699
ENSG00000107362	ABHD17B	0,051068121	15,58649004	58,80890232	3,968257471	0	17,38164203	26,62905323	18,76073246	13,62916368	154,8153093	17,20170104
ENSG00000163807	KIAA1143	13,35543962	17,47858129	10,71203986	27,41418061	37,04088148	10,210371	11,4020202	16,5977486	10,5465051	154,7577678	17,19530753
ENSG00000130222	GADD45G	36,20714354	14,59276191	1,050567268	0	56,73134267	15,29938803	10,62182705	0	20,08771934	154,5907498	17,17674998

ENSG00000119812	FAM98A	0,092159323	0	49,16501039	9,070925118	0,146012418	18,98915525	19,3090143	19,48079378	38,27252024	154,5255908	17,16951009
ENSG00000169026	SLC49A3	19,31746327	20,1816407	0	31,18587442	36,12907196	15,31096094	17,1786158	10,12299545	5,060873526	154,4874961	17,16527734
ENSG00000111203	ITFG2	0,072475094	18,39055444	44,30793904	31,620897	14,23839337	12,36040428	11,39362887	10,19566291	11,82524634	154,4052013	17,15613348
ENSG00000049449	RCN1	13,70500149	11,06981947	5,113644602	21,60039255	44,7072534	11,81780151	7,434284912	29,47405661	9,30226443	154,224519	17,13605766
ENSG00000110435	PDHX	0	32,80460639	0	2,845301585	2,292239632	29,38735215	41,37851374	18,63088519	26,78957848	154,1284772	17,12538635
ENSG00000169131	ZNF354A	27,04190507	35,94396784	0	6,121128761	0	14,90081587	19,76241824	4,872359575	45,40285346	154,0454488	17,11616098
ENSG00000100106	TRIOBP	8,030150057	15,12889093	17,16573844	11,89764195	28,06618155	12,17729754	18,58286203	19,29386683	23,50133979	153,8439691	17,09377435
ENSG00000107077	KDM4C	5,811382016	14,80586458	21,9195777	8,771135928	34,96203914	9,00028315	15,27813691	30,88921415	12,35016122	153,7877948	17,08753275
ENSG00000174903	RAB1B	21,8529133	15,12595898	20,81286895	13,91653198	25,59863507	14,03858726	16,34331504	10,33667109	15,72547496	153,7509566	17,08343962
ENSG00000065970	FOXJ2	0,143983393	34,32149469	0	16,94813476	0,057029942	35,7272895	28,09483375	13,64347905	24,7618282	153,6980733	17,0775637
ENSG00000059588	TARBP1	0	0,034293644	0,035551874	33,50539896	54,19602251	8,190495282	20,02647069	19,14399016	18,49195477	153,6241779	17,0693531
ENSG00000102034	ELF4	47,12577111	6,026360984	0,257627525	13,36112822	40,77099068	13,37740517	20,49503801	0	12,0998315	153,5141532	17,05712813
ENSG00000013375	PGM3	18,63117761	22,19576064	23,86510077	34,19581106	0	15,97427323	22,71821737	0	15,93041198	153,5107527	17,0567503
ENSG00000119402	FBXW2	18,9776744	23,2753063	6,562767935	23,66872673	21,48266828	12,91413648	5,233962549	28,33050432	12,99787517	153,4436222	17,04929135
ENSG00000111581	NUP107	9,771928795	7,478722945	3,361970838	46,24245632	0,088638169	18,10008019	10,63780305	27,11822626	30,61891981	153,4187464	17,04652738
ENSG00000130005	GAMT	6,288413261	28,00817396	0	29,42712364	0,149445502	23,68178436	33,26034186	6,737949983	25,83135183	153,3845844	17,0427316
ENSG00000205133	TRIQK	8,809539515	23,04968412	20,67073907	32,59746857	14,04638811	8,512663439	6,754946488	21,52444089	17,41274673	153,3786169	17,04206855
ENSG00000043591	ADRB1	26,80054285	14,19188293	0	2,743795737	9,062903277	21,5747718	8,267936353	55,0145179	15,63377685	153,2901277	17,03223641
ENSG00000163923	RPL39L	14,77460565	32,44699904	0	50,01464179	0	26,98255265	22,22756124	0	6,656651814	153,1030122	17,0114458
ENSG00000228594	FNDC10	18,9243935	9,847022602	0	0	58,33123922	24,76160679	13,941148	10,73445629	16,53719054	153,0770569	17,00856188
ENSG00000179862	CITED4	6,889539696	16,09667022	69,07256259	15,46575077	0	14,24612095	10,10627294	2,510900969	18,64114053	153,0289587	17,00321763
ENSG00000047230	CTPS2	27,42994268	0,048479841	7,538784098	36,75074041	20,64714852	7,559115048	16,24240783	28,49706901	8,299419816	153,0131073	17,00145636
ENSG00000136802	LRRC8A	3,236019869	10,51034608	38,95973471	23,34943941	0,068664922	19,63645258	20,03207021	11,43568962	25,76266322	152,9910806	16,99900896
ENSG00000106603	COA1	5,331058204	32,14528038	22,01263728	42,73858806	0,151910974	19,3843068	7,802089173	11,12630777	12,29542982	152,9876085	16,99862316
ENSG00000071894	CPSF1	17,98460242	15,35907462	8,901970816	18,43515502	1,778396545	23,6076126	14,22880787	20,07261817	32,61057332	152,9788114	16,99764571
ENSG00000225830	ERCC6	16,7265652	2,209795214	18,87616048	15,47994233	48,67019752	10,36706979	10,08521973	5,315751397	25,20824074	152,9389424	16,99321582
ENSG00000175931	UBE2O	0,094252656	7,609830659	16,99176654	14,52577357	54,15042312	12,93548859	15,86721336	14,94246446	15,79391153	152,9111245	16,99012494
ENSG00000039523	RIPOR1	33,95440375	5,10063546	29,95030046	13,99874065	15,58364131	13,33892436	17,11721872	10,8033296	13,03604674	152,883241	16,98702678
ENSG00000139173	TMEM117	0,043927491	0	65,29299999	9,254089215	0,417578538	16,20521912	20,82661615	30,7380993	10,10270339	152,8812332	16,98680369
ENSG00000087842	PIR	24,6637476	24,55121816	0,110660867	50,06793701	0	8,321947717	22,12713259	16,04817565	6,880464399	152,771284	16,97458711
ENSG00000151327	FAM177A1	19,9498392	17,01270651	35,40640975	17,70685755	0,085657136	12,72277887	13,2129163	18,2852283	18,24110767	152,6235013	16,95816681
ENSG00000151806	GUF1	0	15,26060229	0	75,83075556	0,160931007	12,67656434	7,646858683	23,32217987	17,65604424	152,553936	16,95043733
ENSG00000077235	GTF3C1	32,39950741	3,617899327	2,764894602	61,2730026	0	12,64203581	12,76190196	8,688125629	18,35640274	152,5037701	16,94486334
ENSG00000019991	HGF	0,061962305	9,318312458	40,692525	49,3248686	0,073627379	7,942618075	7,202807373	18,79971143	19,07046323	152,4868958	16,94298843
ENSG00000179119	SPTY2D1	6,480924628	29,68244212	34,72984115	5,042649111	7,974978479	30,2908444	18,86627979	6,945903776	12,41287164	152,4267351	16,9363039
ENSG00000010017	RANBP9	9,397423549	40,31764733	5,6087748	5,336978474	11,21621954	31,36617868	18,51883839	14,24759329	16,38613615	152,3957902	16,93286558
ENSG00000164823	OSGIN2	3,603281062	10,82936542	45,08592368	12,16388603	0,694319631	19,03249151	24,90538921	19,73379372	16,28901849	152,3374688	16,92638542
ENSG00000154001	PPP2R5E	13,29457111	17,31361129	28,30045651	8,239453684	16,38395582	21,13625596	11,89946202	21,55164481	14,20560307	152,3250143	16,92500159
ENSG00000115073	ACTR1B	7,419675323	11,12622407	41,04339636	23,18820487	0	23,11962739	13,49283307	18,28130387	14,58753823	152,2588032	16,9176448
ENSG00000171940	ZNF217	3,031501499	13,83874746	20,64275448	32,08389916	11,09714457	10,65628522	17,5589447	18,87128334	24,41471867	152,1952791	16,91058657
ENSG00000168763	CNNM3	13,83533168	21,45318193	7,130116349	25,17599211	0	19,41776581	26,04463151	11,95559077	27,18088257	152,1934927	16,91038808
ENSG00000111906	HDDC2	14,57724255	20,31102786	13,78569784	27,83070042	21,52997494	16,90664844	15,5559345	15,43300035	6,218030721	152,1482576	16,90536196
ENSG00000120942	UBIAD1	0	45,48177963	0,054698957	0,821519007	5,582504102	36,45942695	29,20728114	11,83374484	23,18510333	152,110658	16,90118422
ENSG00000119688	ABCD4	24,37212634	12,53252791	19,65987923	12,10794435	4,387336036	17,53571776	24,16028137	17,70317454	19,60116313	152,0601507	16,8955723
ENSG00000241127	YAE1	10,86107227	15,0829875	48,64651732	31,08795596	0	11,98963152	12,74308059	17,94156441	3,702411186	152,0552208	16,89502453
ENSG00000165689	ENTR1	14,44651727	17,68814341	37,82584521	2,74405805	0,114441537	18,61074665	36,70546997	11,68841204	12,20024971	152,0238839	16,89154265
ENSG00000089154	GCN1	17,19156492	0,527401449	63,56708475	2,836249724	0,074351458	24,16523617	13,13152232	8,756858104	21,75904935	152,0093182	16,88992425
ENSG00000155330	C16orf87	4,06256246	12,29975084	18,99171131	34,82266914	24,08715223	11,26610069	12,08451784	16,48509483	17,89957497	151,9991343	16,8887927
ENSG00000114491	UMPS	35,74593771	24,70104687	22,72095058	6,217699741	0,04780585	19,72286275	22,68220326	2,771216421	17,36744657	151,9771697	16,88635219
ENSG00000171055	FEZ2	11,37809511	15,36011457	17,01220789	25,16513181	23,26348814	20,6228072	12,10062947	12,93937107	13,99359023	151,8354355	16,87060394
ENSG00000115904	SOS1	1,484514944	0,057895282	63,86069855	14,11296797	17,95006176	12,84226314	20,72434502	6,421053887	14,22489305	151,6786936	16,85318818
ENSG00000168071	CCDC88B	28,35811548	15,38203809	7,856683037	10,34364989	0,086016178	21,73726598	17,97155893	11,87190063	38,0595872	151,6668154	16,85186838
ENSG00000005238	FAM214B	0,02357787	8,242155409	73,73477427	1,099273571	0,056033319	12,86590055	21,76985632	9,899116393	23,80714539	151,4978331	16,83309257
ENSG00000090263	MRPS33	4,350371052	31,13412356	0,052999066	31,1430494	7,326134801	21,92107334	16,55395038	24,69596768	14,31496568	151,492635	16,83251499
ENSG00000116717	GADD45A	13,8943226	26,60525819	30,6139122	29,41448805	0,186554567	18,96120877	6,517939325	0	25,29443576	151,4881195	16,83201327
ENSG00000087157	PGS1	5,975111533	25,8705607	31,93088919	11,29789629	1,703997828	24,66681567	30,61099045	3,527771183	15,79952641	151,3835593	16,82039547
ENSG00000077713	SLC25A43	35,44897501	2,55013526	0	9,834463643	0	40,20657864	23,15831418	16,64393912	23,50084589	151,3432518	16,81591686
ENSG00000133657	ATP13A3	20,61324217	18,57208059	42,78552904	37,26798754	0,135475156	12,78064726	6,229023575	3,490330425	9,464795725	151,3391115	16,81545683
ENSG00000100342	APOL1	0	8,915207164	31,59762421	35,88875468	0,102051021	11,88106432	9,412949033	11,36191608	42,14422415	151,3037906	16,81153229
ENSG00000154856	APCDD1	13,64245678	10,79433014	36,28677391	10,32840139	0,120976096	20,45587707	6,382354769	38,57017943	14,71245839	151,293808	16,81042311
ENSG00000091157	WDR7	1,357626511	21,75001696	38,52470468	11,85463469	26,90978908	14,67370774	16,93297841	2,526580101	16,66153032	151,1912315	16,79902572
ENSG00000174652	ZNF266	0	6,111073932	0,824102572	1,837229764	103,6696555	14,78654575	14,9253202	0,244902403	8,712093781	151,110924	16,79010266
ENSG00000152137	HSPB8	15,58657984	0	0,097195839	26,64091349	89,90495116	9,455742658	6,054162873	3,3505603	0	151,0901062	16,78778957
ENSG00000103485	QPRT	13,36013283	10,74923998	39,613309	0,143292133	24,55251731	19,08746064	14,05598447	13,24641594	16,27815503	151,0865073	16,7873897
ENSG00000204977	TRIM13	9,500405196	16,03022827	20,18926608	23,23790967	11,39985281	25,53416237	20,56560407	9,5914184	14,9668889	151,0157358	16,7795262
ENSG00000161944	ASGR2	0	10,76875502	3,684073601	14,88062907	50,47836941	27,9182858	8,213519725	18,97676432	15,97235171	150,8927487	16,76586096
ENSG00000136141	LRCH1	0,017550589	18,68585366	1,323714757	40,09488634	0	10,1357681	22,17954069	35,19262467	23,18224912	150,8121879	16,75690977
ENSG00000080371	RAB21	17,59880711	20,60188722	10,30646553	19,28395313	13,1580954	18,75385008	17,03638169	17,97540986	16,09712525	150,8119753	16,75688614
ENSG00000123836	PFKFB2	17,77509366	3,689060536	8,932733134	57,92571812	0,141162556	13,17371718	25,0916004	13,54080333	10,51961447	150,7895034	16,75438926
ENSG00000123989	CHPF	32,32646875	7,865498669	0	5,798554991	50,11684813	14,10854386	25,61453593	5,580672696	9,361158159	150,7722812	16,75247569
ENSG00000176396	EID2	0	9,876316762	0	24,18734264	0	25,97126891	21,79748555	23,53002445	45,40633264	150,768771	16,75208566
ENSG00000159788	RGS12	34,52857314	7,778164888	15,68248323	6,392954303	2,898004159	13,34039243	25,39508574	12,08350327	32,65656447	150,7557256	16,75063618
ENSG00000163870	TPRA1	6,235797295	12,25764746	10,92735715	0	38,32631188	20,92326554	34,19077706	3,644066735	24,2307899	150,736013	16,74844589
ENSG00000284024	HSPA14	18,01419679	1,470315598	0,903265959	11,55236078	77,23506851	12,23101936	9,453593252	9,260757579	10,56803945	150,6886173	16,7431797
ENSG00000174886	NDUFA11	21,39093633	17,45499691	10,79980546	13,0494449	13,95207645	19,85166851	19,75788241	15,36428553	19,04936455	150,670461	16,74116234
ENSG00000078142	PIK3C3	39,63539622	17,71115914	0	11,95450279	25,23528676	11,35235192	14,46589391	8,168290232	22,13742029	150,6603013	16,74003347
ENSG00000038358	EDC4	0,024610842	0,218356678	64,51492525	9,264466525	0,116976398	18,0231848	36,76239858	5,919837529	15,76971305	150,6144696	16,73494107
ENSG00000055609	KMT2C	14,61972609	6,785536345	38,46169909	34,58549479	0,535623261	11,17673419	15,16448131	10,87539444	18,36844418	150,5731337	16,73034819
ENSG00000102572	STK24	17,67982416	12,87756562	30,29078498	8,426317802	19,26870642	20,31810408	17,45385094	4,897556312	19,25097287	150,4636832	16,71818702
ENSG00000102710	SUPT20H	29,48128119	3,776993324	19,75583435	9,570559299	0	12,398147	11,74024967	27,53369533	36,17318205	150,4299422	16,71443802
ENSG00000159792	PSKH1	0	20,54882345	32,58068778	2,54857601	0	24,05765688	36,13595838	0,165506921	34,34879375	150,3860032	16,70955591
ENSG00000134602	STK26	33,347283	22,4731613	3,603600921	42,40095005	0	13,95509524	8,435298172	0	26,16530778	150,3806965	16,70896627
ENSG00000197961	ZNF121	19,90216453	9,392920704	14,49130843	53,58325433	1,7334318	5,674503244	5,052728857	31,94519816	8,586206725	150,3617168	16,70685742
ENSG00000105875	WDR91	29,93652718	24,27147415	0,23042117	18,73090915	0	12,45979629	34,36289597	7,285775444	23,04595468	150,323754	16,70263934
ENSG00000170909	OSCAR	15,15456299	21,48738676	17,07363372	17,15359478	29,46693846	17,11649424	11,92088567	7,927892695	13,01995789	150,3213472	16,70237191
ENSG00000135457	TFCP2	1,066105411	5,885531227	44,5262087	9,750117943	0,469189368	19,03181565	27,83493908	7,425495899	34,18760169	150,177001	16,68633344
ENSG00000123636	BAZ2B	27,96519525	12,26352013	17,21672564	24,16422568	2,23484024	17,07698137	14,8107914	17,02194172	17,37578242	150,1300039	16,68111154
ENSG00000078043	PIAS2	5,403269336	12,47812216	22,74887038	12,73797196	25,74341322	13,7099758	13,84574237	28,58678294	14,84864429	150,1027924	16,67808805
ENSG00000152082	MZT2B	8,159138251	13,73950744	8,576796442	22,71500054	26,51336125	19,92755136	16,17634032	16,74930039	17,50950706	150,0665031	16,6740559
ENSG00000118454	ANKRD13C	8,088227505	18,9052251	2,472332704	32,61687183	0,116144095	13,36084528	26,13288933	25,00701449	23,26086809	149,9604184	16,66226871
ENSG00000068650	ATP11A	10,40927691	5,467105514	27,34661864	1,140034841	28,84341726	11,40461548	18,08095733	25,40878929	21,81494167	149,9157569	16,65730633
ENSG00000149231	CCDC82	17,1508272	15,20911708	21,83636196	21,68806264	35,36622248	6,324783725	10,54463889	6,202924649	15,56503537	149,887974	16,65421933
ENSG00000196544	BORCS6	23,29743555	18,22324272	9,068088666	18,94945252	0,139463123	24,22616576	24,11953955	16,93878171	14,86772178	149,8298914	16,64776571
ENSG00000118900	UBN1	14,24337738	21,88786295	12,90587058	27,03641533	11,12924204	13,48472118	10,40328212	17,234688	21,45135276	149,7768123	16,64186804
ENSG00000092847	AGO1	19,96313155	3,609975091	34,68247023	31,22202504	1,473700498	14,40519478	9,249936643	25,8114835	9,339155691	149,757073	16,63967478
ENSG00000130635	COL5A1	32,61524963	7,668731793	10,18606136	14,03904323	16,88227967	1,008304515	5,875990166	41,61071639	19,86526416	149,7516409	16,63907121
ENSG00000178537	SLC25A20	35,50859564	0	0	12,92301771	0,378416799	27,53521162	21,6168591	47,18006733	4,602100393	149,7442686	16,63825207
ENSG00000137486	ARRB1	13,66230281	15,37586105	13,21305082	18,02372448	0,544777825	18,33942762	23,81753774	17,31038693	29,35803679	149,6451061	16,62723401
ENSG00000134222	PSRC1	0	30,61796568	23,46522026	26,77896587	0	19,52572812	11,82189818	0	37,36458977	149,5743679	16,61937421
ENSG00000163964	PIGX	24,50533546	17,12827891	0,062523639	18,07646698	0,161546353	18,28524125	10,49819242	48,75507789	12,03864112	149,511304	16,61236711
ENSG00000175376	EIF1AD	28,20548975	11,122211	44,61718451	5,646924608	0	20,14664051	24,70185376	4,370039366	10,66927735	149,4796209	16,60884676
ENSG00000136319	TTC5	35,26225786	6,598200581	15,17509225	0,215827109	30,52053555	12,0590504	13,46548848	19,302681	16,87068306	149,4698163	16,60775737
ENSG00000174405	LIG4	0,241115568	20,78146342	35,04057322	30,2748808	0	18,64461234	30,05911077	0	14,36106019	149,4028163	16,60031292
ENSG00000097007	ABL1	0	5,637676743	27,62450064	11,82950936	41,58637769	21,71989646	20,71249732	10,80099851	9,402222217	149,3136789	16,59040877
ENSG00000197217	ENTPD4	0,021421969	3,725250356	32,62936234	0,776812371	0,916375923	30,3644109	23,58571877	29,51145131	27,76895867	149,2997626	16,58886251
ENSG00000217555	CKLF	11,06322007	14,31938329	22,26713971	15,28301315	7,423618447	22,57870036	17,18327544	18,49978199	20,64961359	149,2677461	16,58530512
ENSG00000146858	ZC3HAV1L	0	6,984446208	46,27754762	29,25545015	0	7,985503409	14,42269388	16,46559566	27,78226607	149,173503	16,57483367
ENSG00000105821	DNAJC2	9,937333979	16,8338476	30,71626206	39,49433332	0	15,21547937	7,016309946	8,52448258	21,42251144	149,1605603	16,57339559
ENSG00000196507	TCEAL3	7,342465906	10,46975271	24,60214325	8,829903696	0,467398103	21,80950314	37,45058066	10,75166706	27,41336988	149,1367844	16,57075382
ENSG00000173905	GOLIM4	6,1458786	1,32714999	0,598192595	44,07875082	8,114340854	17,35152578	30,37532247	12,08820238	28,95478419	149,0341477	16,55934974
ENSG00000134905	CARS2	8,094174876	8,838716753	36,17257325	15,45207113	21,98889513	12,30498145	15,54552887	17,60460134	13,0178045	149,0193473	16,55770525
ENSG00000198218	QRICH1	1,673334022	23,45235962	0,834775072	32,17610329	2,35906794	39,2978881	13,06982625	20,03197547	16,04530785	148,9406376	16,54895973
ENSG00000099875	MKNK2	13,60313507	19,20802682	17,14414707	19,09158404	14,51327359	12,71101909	18,40840343	21,45514621	12,63656683	148,7713022	16,53014469
ENSG00000109536	FRG1	6,703611268	37,17310537	9,591426705	15,91655113	18,36522244	16,66261491	13,6834558	19,54511305	11,10727829	148,748379	16,52759766
ENSG00000117906	RCN2	16,92572698	24,34700112	16,87365311	18,89610916	5,549437833	25,85597615	18,4384742	5,673555851	16,18015374	148,7400882	16,52667646
ENSG00000021300	PLEKHB1	0,030632591	12,44770282	24,73814382	24,70233796	45,06259046	13,01589158	8,368080119	8,172109718	12,16874654	148,7062356	16,52291507
ENSG00000154978	VOPP1	33,61156299	31,17696724	2,133904523	3,761841049	21,71783677	13,73010202	12,77960318	8,352118819	21,39966508	148,6636017	16,51817797

ENSG00000142002	DPP9	22,63753984	8,407623982	2,204070943	3,166077899	22,90862746	17,08137485	26,66470655	18,26043982	27,28309109	148,6135524	16,51261694
ENSG00000170234	PWWP2A	14,49385464	7,83465229	49,71068814	3,512662959	30,76423799	9,796769559	3,723610893	14,55792496	14,21497034	148,6093668	16,51215186
ENSG00000168137	SETD5	19,18242995	3,919914509	24,51101354	12,26452323	18,80648862	8,811313011	21,54634865	30,05340302	9,491791293	148,5872258	16,50969176
ENSG00000129696	TTI2	17,3945375	1,079681194	54,45038888	1,60689468	12,16338431	13,67524412	19,79233565	18,47052533	9,904184631	148,5371763	16,5041307
ENSG00000163606	CD200R1	17,90796223	5,652350645	0	0	0	27,4669438	25,96494017	38,81969084	32,67841163	148,4902993	16,49892215
ENSG00000170471	RALGAPB	11,87972422	19,55179971	1,848652388	21,85648034	28,06183313	2,62703787	14,40360869	23,85846929	24,32145525	148,4090609	16,48989565
ENSG00000106546	AHR	8,596278893	16,45288139	37,91108542	25,150359	2,720925361	6,305879577	20,71172168	24,9949884	5,56316133	148,407281	16,48969789
ENSG00000130475	FCHO1	9,421189425	13,93923991	1,464594831	32,90328622	0	18,76473681	18,17931575	9,749386607	43,83419027	148,2559398	16,4728822
ENSG00000165915	SLC39A13	10,49424797	13,41002796	0,0607076	16,12679577	0	28,04428617	33,75834222	15,65938003	30,64296229	148,19675	16,46630556
ENSG00000263001	GTF2I	13,16853155	8,279355749	22,40465984	22,13895786	18,61832078	12,75557738	15,60747472	11,98603669	23,18354387	148,1424584	16,46027316
ENSG00000217128	FNIP1	4,452263099	4,659228911	15,01554551	14,19339071	13,65569098	22,77040149	23,74488907	28,95775107	20,61581291	148,0649738	16,45166375
ENSG00000166387	PPFIBP2	25,27247197	5,827984288	0,085095949	0,026626018	39,57621895	16,86193208	17,87312076	22,2874744	20,12073063	147,931655	16,43685056
ENSG00000056586	RC3H2	11,44796851	12,73112151	1,202024124	26,24469216	31,18170133	13,37271102	13,44441247	17,06040273	21,21572338	147,9007572	16,43341747
ENSG00000204577	LILRB3	11,48699199	19,2350257	17,85739394	9,428860164	28,54869113	12,87849766	15,54945496	16,09718718	16,71424826	147,796351	16,42181677
ENSG00000076108	BAZ2A	11,01703739	8,79234622	23,60208388	31,66686585	6,973156162	22,04948221	16,67498184	4,116056163	22,87760816	147,7696179	16,41884643
ENSG00000183172	SMDT1	13,00720683	14,87096014	7,065929726	26,53065022	6,846261639	18,97763962	23,050009	15,65316842	21,68746245	147,6892881	16,40992089
ENSG00000129657	SEC14L1	16,95543483	14,31851352	1,288436148	31,49562749	7,524962021	12,51218235	20,57248201	23,67540065	19,30966184	147,6527009	16,40585565
ENSG00000133059	DSTYK	9,735265083	4,9989895	20,13920717	0,400254238	64,40810199	16,26804593	16,35925863	6,862121992	8,398727791	147,5699723	16,39666359
ENSG00000145241	CENPC	4,659954039	7,637754711	41,1931432	14,8649397	26,75628582	8,963389207	16,15637279	9,295416859	17,98091352	147,5081698	16,38979665
ENSG00000163930	BAP1	20,17796938	13,81062018	0,53027149	19,74433784	49,70406002	13,04638066	11,40881145	8,824662041	10,24164811	147,4887612	16,38764013
ENSG00000198825	INPP5F	13,30408441	0,176177398	0,219169592	46,52940349	40,72515625	6,191669354	6,578536461	23,88160475	9,841806633	147,4476083	16,38306759
ENSG00000163607	GTPBP8	31,91253034	16,46977784	17,00139654	7,502051021	31,0684544	10,40735452	5,443825173	12,52300373	15,10670895	147,4351025	16,38167806
ENSG00000158062	UBXN11	32,44508426	7,410386907	22,51496975	3,882960932	30,30817694	9,276037771	12,48301772	15,73497941	13,27475159	147,3303653	16,37004059
ENSG00000172123	SLFN12	21,26584155	2,786869102	31,62414047	0	12,30683713	9,274214821	17,62223764	3,341473155	49,07183453	147,2934484	16,36593871
ENSG00000151532	VTI1A	0,071888949	4,145868222	5,389005249	68,61646394	2,56268465	22,19902459	16,40008264	6,942905041	17,94807042	147,2759937	16,3639993
ENSG00000108771	DHX58	0	0	0,072693541	13,30604604	85,3655455	12,75567718	8,957479246	8,641071326	18,17641196	147,2749248	16,36388053
ENSG00000180340	FZD2	22,03695822	11,17258334	0,131619365	18,28521602	18,19392852	28,28021194	20,61468049	20,18279319	8,359573682	147,2575648	16,36195164
ENSG00000174839	DENND6A	0,029542634	17,35190656	9,999675115	55,45187046	0,210626125	14,30431365	12,78006705	25,58203103	11,51776101	147,2277936	16,35864374
ENSG00000167981	ZNF597	15,13034895	10,66112639	0	0,951796369	108,2657847	1,755027074	7,757512776	0	2,643813375	147,1654096	16,35171218
ENSG00000213079	SCAF8	2,619139612	12,37728991	30,32879022	28,42148875	0,65520442	23,00529799	29,87388857	4,581833084	15,22442775	147,0873603	16,34304003
ENSG00000163961	RNF168	11,67171604	20,30998879	16,1682833	29,47535528	1,410411144	17,56225667	15,51073302	8,280948578	26,65873996	147,0484328	16,33871475
ENSG00000100865	CINP	15,4956199	12,9454783	15,81323916	10,35146232	0	14,80900919	10,91760935	43,0974726	23,57858198	147,0084728	16,33427475
ENSG00000185345	PRKN	5,020196494	0	7,329410239	0,963198184	118,359183	9,682248456	5,116737041	0,406581468	0,065335392	146,9428902	16,3269878
ENSG00000186010	NDUFA13	19,92114983	15,94197268	12,93408141	19,07870198	5,437156291	18,50258278	21,08161044	15,37472508	18,66700667	146,9389872	16,32655413
ENSG00000161800	RACGAP1	5,085289987	13,80450234	0,867332602	9,18826075	54,42387537	20,2627582	24,80325039	2,20927948	16,27762052	146,9221696	16,32468552
ENSG00000198498	TMA16	30,30197292	44,95275457	2,448444794	9,88273979	0,210873483	20,99455624	11,12508522	16,00754658	10,96745478	146,8914284	16,32126982
ENSG00000166527	CLEC4D	0	9,94087415	39,45961405	5,218699492	16,81721174	16,99571761	8,99714088	46,42202705	2,991675724	146,8429607	16,31588452
ENSG00000131389	SLC6A6	38,57402137	5,425213716	1,549198516	38,94740595	0,870166083	13,48752083	32,01363208	2,361962622	13,57710842	146,8062296	16,31180329
ENSG00000104960	PTOV1	26,76497193	12,90787555	29,42057069	14,83679242	12,16735048	11,52079483	16,39406049	9,754525978	12,99865813	146,7656005	16,30728894
ENSG00000113368	LMNB1	0	9,373529868	8,87577525	28,29848059	46,75546313	22,28581377	19,99651038	0,090953638	11,04950421	146,7260308	16,30289232
ENSG00000004799	PDK4	3,658046385	29,26902114	57,50468597	0,287120351	0	9,16451469	29,71084446	0	17,09591688	146,6901499	16,29890554
ENSG00000177628	GBA	23,4867581	6,321330046	10,59189522	6,222955809	27,07163491	21,73937929	21,15248196	19,65578431	10,44367792	146,6858976	16,29843306
ENSG00000205659	LIN52	55,62276965	16,48652059	0	6,855550524	0	23,27781114	25,59023835	0,089500154	18,67525996	146,5976504	16,28862782
ENSG00000136122	BORA	20,94414306	13,53742929	0	27,00173773	65,42718473	6,035003731	7,750911071	0	5,886014032	146,5824237	16,28693596
ENSG00000119906	SLF2	8,282615255	13,02369224	6,656128108	25,67301668	18,50197052	12,36986483	26,31288851	17,96166113	17,80009814	146,5819354	16,28688171
ENSG00000011021	CLCN6	51,38092205	8,923849752	7,108508588	2,809528663	1,537882538	12,07840366	19,26651984	19,1638303	24,30807932	146,5775247	16,28639163
ENSG00000124608	AARS2	17,3826162	6,189466236	1,272705528	17,96976838	0	25,32019484	26,71211189	18,28043201	33,39706676	146,5243618	16,28048465
ENSG00000143498	TAF1A	0,052269308	16,41685715	63,8459999	4,242111313	0	9,296960715	15,58071573	31,66045493	5,399898952	146,495268	16,277252
ENSG00000173928	SWSAP1	40,37990309	23,40669478	32,35397827	12,24101128	0	7,38912901	12,21920098	13,73540364	4,745487208	146,4708082	16,27453425
ENSG00000168887	C2orf68	0,259952814	9,917517636	63,50551719	15,48642117	0,123556698	20,72098378	15,04435256	6,991820369	14,41885112	146,4689733	16,27433037
ENSG00000011638	TMEM159	0,118147436	12,36933386	5,433543764	28,35806213	0,421169909	42,3532473	19,17861603	15,37205377	22,66775408	146,2719283	16,25243648
ENSG00000175104	TRAF6	3,996650596	31,00670194	3,761571028	25,42262269	18,28941598	16,28695291	10,897042	21,95038338	14,63178426	146,2431248	16,24923609
ENSG00000144029	MRPS5	25,56422557	18,03631375	0,332114814	32,85847828	8,795590942	13,79614831	14,58573871	11,05396403	21,20471073	146,2272851	16,24747613
ENSG00000115355	CCDC88A	8,503364268	13,45371004	20,63024776	24,22385555	12,65407403	18,89235693	16,13939867	16,43313741	15,18332468	146,1134693	16,23482992
ENSG00000230124	ACBD6	20,79983095	18,954127	6,697869797	7,885015873	26,72346994	11,19763876	12,34326083	21,37795525	20,06410138	146,0432698	16,22702998
ENSG00000023516	AKAP11	4,282609214	3,921390823	36,55935946	15,47450984	31,5472899	12,04796926	9,61749393	18,89625817	13,62687057	145,9737512	16,21930569
ENSG00000171777	RASGRP4	6,285737341	27,37189434	3,522100226	26,82334188	0	37,83855849	12,38805812	11,29623312	20,39291268	145,9188362	16,21320402
ENSG00000163257	DCAF16	0	17,87242152	44,96397365	17,77131887	0	24,14935928	7,633473272	16,1923916	17,24945919	145,8323974	16,20359971
ENSG00000107816	LZTS2	19,20329843	10,48485597	3,09951844	7,293584902	47,11806459	19,7916848	20,46774318	8,882921015	9,424323414	145,7659947	16,19622164
ENSG00000135048	CEMIP2	5,813201119	19,64558851	5,737432343	7,049248287	0	9,26904778	38,5874827	45,7409424	13,89731937	145,7402625	16,1933625
ENSG00000170364	SETMAR	0	5,637973088	27,97725134	49,89000636	0	11,81420984	15,12538345	30,84797056	4,428638742	145,7214334	16,19127037
ENSG00000100034	PPM1F	6,874789566	12,62438795	27,58034286	22,29417664	13,34810966	12,99131287	12,15031365	3,799227855	34,02304754	145,6857086	16,18730095
ENSG00000084774	CAD	0,876988981	11,78262253	36,64477676	20,953916	0,127603059	16,78166913	20,04731468	4,109395901	34,33857334	145,6628604	16,18476227
ENSG00000162545	CAMK2N1	2,088284278	42,71743955	0,106710211	0,100166977	46,45781642	9,426013571	7,850926724	3,805388493	33,10279706	145,6555433	16,18394925
ENSG00000277157	HIST1H4D	9,20224629	20,6263077	44,54809279	0	51,79579938	1,595294007	15,6837915	2,118170305	0	145,569702	16,17441133
ENSG00000132635	PCED1A	20,58230187	31,55291639	0	3,149215211	37,61446672	19,80915075	14,54806867	0,142428693	18,10401467	145,502563	16,16695144
ENSG00000072110	ACTN1	10,15367471	19,05206193	22,62872065	29,90954254	0,033796117	26,52758045	23,34414139	5,504133112	8,307650864	145,4613018	16,16236686
ENSG00000165526	RPUSD4	19,51366595	15,37496398	0	29,89550334	0	19,02625579	16,3870833	33,35923593	11,88579817	145,4065065	16,1562785
ENSG00000132382	MYBBP1A	11,69322488	8,299736259	0,089627631	21,41155926	0	13,90835078	60,56072449	7,351276853	22,08107156	145,3955717	16,15506352
ENSG00000182923	CEP63	24,72189307	1,98814964	28,74825482	1,331685475	35,06453423	8,131775016	9,677586513	14,7637592	20,92003703	145,347675	16,14974167
ENSG00000198814	GK	9,333382887	11,65834783	14,0053418	48,05799961	0,938168612	26,09864082	16,92760777	6,720912084	11,58289577	145,3232972	16,14703302
ENSG00000151148	UBE3B	12,42569858	21,33853064	13,50501565	39,14129387	0,201864835	12,8966063	12,62461463	8,756396634	24,35569938	145,2457205	16,13841339
ENSG00000007376	RPUSD1	16,31710387	26,63020947	18,07021228	0,565406229	0	28,64039569	20,5718782	9,78535014	24,63187926	145,2124351	16,13471502
ENSG00000065491	TBC1D22B	40,46201589	11,77284345	24,8110487	8,290828194	0	17,34824632	11,12634082	10,02184564	21,36537142	145,1985404	16,13317116
ENSG00000138758	SEPT11	6,500209487	3,863085932	0,880492804	33,99326582	19,48415398	17,4776545	19,7814164	19,68359588	23,47055293	145,1344277	16,12604753
ENSG00000139496	NUP58	13,11223999	19,50123637	5,883176698	7,756508624	14,57889398	18,62603588	23,07833331	17,13364424	25,42316062	145,0932297	16,12146997
ENSG00000150938	CRIM1	1,018489403	0,737667125	45,96039601	32,15924859	9,731245646	11,38780987	8,56028675	18,86249594	16,61626403	145,0339034	16,11487815
ENSG00000148296	SURF6	9,209049277	4,556105575	23,86825046	24,23727675	1,301740072	21,04891775	4,889192819	10,25589845	45,64060493	145,0070361	16,1118929
ENSG00000125447	GGA3	5,803192096	13,85131115	0	24,40370771	0,054083967	35,73074167	19,76535652	23,09060833	22,30718046	145,0061819	16,11179799
ENSG00000167995	BEST1	13,25348237	8,703504147	0,031995868	1,862105021	89,53137624	10,35031452	11,75562273	1,787571901	7,700822746	144,9767955	16,10853284
ENSG00000122257	RBBP6	12,88226631	13,25990989	10,98651656	25,48319609	1,57703169	15,53778289	30,07694546	7,539270801	27,49996272	144,8428824	16,0936536
ENSG00000104957	CCDC130	7,222541242	10,73566667	8,023634142	16,4400372	0	30,53537306	20,44442095	18,15236451	33,28163977	144,8356775	16,09285306
ENSG00000101974	ATP11C	8,078815443	17,34035616	3,039879576	63,55159905	0,048483535	12,49873117	5,06488852	20,11961522	15,07948696	144,8218556	16,09131729
ENSG00000170322	NFRKB	15,36151658	5,587173483	0	13,22180376	48,86492842	10,50199546	17,15190332	8,241304131	25,87476443	144,8053896	16,08948773
ENSG00000161013	MGAT4B	6,379918543	26,0578395	20,58924482	20,65636287	0,326767554	20,01796403	20,02501932	12,26727057	18,3986366	144,7190238	16,07989153
ENSG00000181704	YIPF6	10,44502667	8,348287177	16,95394423	7,51764332	15,68636105	14,39767667	16,50004831	32,9743603	21,8058768	144,6292245	16,06991384
ENSG00000160584	SIK3	52,45972284	4,272592309	3,487479264	12,61663613	27,62442633	9,997175776	15,53442206	7,111000308	11,47074689	144,5742019	16,06380021
ENSG00000092068	SLC7A8	16,84891691	5,147108999	0,742393855	0,217772471	52,03038337	27,85946255	13,29889181	24,76466714	3,629451361	144,5390485	16,05989427
ENSG00000124102	PI3	0	0	68,61214784	0	0	0	0	70,16487187	5,73392174	144,5109415	16,05677127
ENSG00000214026	MRPL23	15,08566033	16,22371765	17,3795961	16,31391774	24,6252029	11,87863961	15,47818886	15,21665067	12,30647559	144,5080495	16,05644994
ENSG00000148459	PDSS1	0	0,140168322	34,58403741	37,64665603	0,3754496	3,642574083	11,80588592	29,70974679	26,52174625	144,4262644	16,04736271
ENSG00000160932	LY6E	31,90621223	9,148134954	14,33104377	9,990307974	6,942757642	4,088016484	13,17486049	52,23321253	2,606641897	144,421188	16,04679866
ENSG00000239789	MRPS17	32,30887916	11,61948667	47,25661683	15,78035323	0	11,29772022	3,226379966	15,57762961	7,345362547	144,4124282	16,04582536
ENSG00000160049	DFFA	44,56906492	19,02290416	15,02141516	5,592870399	0,108412843	11,56991493	13,10573128	16,65888989	18,7108876	144,3600912	16,04001013
ENSG00000073169	SELENOO	22,82965456	23,26242489	27,4098024	0,993827665	0	22,25715312	31,96493435	5,173964874	10,46025707	144,3520189	16,03911321
ENSG00000157895	C12orf43	9,032179061	5,939301631	0,996019899	6,417130483	82,05894331	15,23999434	6,35525623	0,753433867	17,52526651	144,3175253	16,03528059
ENSG00000115425	PECR	15,74462454	38,25805758	0,086977503	20,98256836	0	14,63916853	35,96083358	0	18,60791212	144,2801422	16,03112691
ENSG00000160785	SLC25A44	18,39931259	11,83898588	28,96817157	20,25081525	0,147724205	18,01743819	24,13751211	7,249352914	15,18778655	144,1970993	16,02189992
ENSG00000181004	BBS12	57,47574897	16,49022501	0	0	0	0	8,630427966	44,30192395	17,22506385	144,1233897	16,01370997
ENSG00000146247	PHIP	7,587356415	13,82795068	5,288383457	10,51327897	0,439197973	16,94276374	27,87661269	34,91138419	26,56754198	143,9544701	15,99494112
ENSG00000062725	APPBP2	5,699114546	8,459080286	32,8854111	40,78922859	0,072622354	13,83986105	11,53210635	13,83214469	16,7994733	143,9090423	15,98989358
ENSG00000102606	ARHGEF7	10,54229933	14,17269919	49,53469303	14,2352357	0	10,34826337	12,67710951	9,350332341	23,04130489	143,9019374	15,98910415
ENSG00000123892	RAB38	0	3,745000001	0	0	115,6097675	0	18,57521675	0	5,969930185	143,8999144	15,98887938
ENSG00000188542	DUSP28	8,189060314	0,072294958	54,48679751	12,73368304	4,260224282	17,93748753	6,156807879	13,45258013	26,60676822	143,8957039	15,98841154
ENSG00000174885	NLRP6	0,706784708	30,18374245	22,96998012	27,33836965	0	10,24330969	17,29276169	21,22529495	13,90806528	143,8683085	15,98536762
ENSG00000180098	TRNAU1AP	29,93468507	20,02823568	0,128963164	20,57942272	0	21,59031165	18,21953789	18,31915139	15,04533432	143,8456419	15,9828491
ENSG00000121058	COIL	2,757492165	30,0703371	0	9,003728387	0	21,68837665	15,48605292	22,69407945	42,11452379	143,8145905	15,97939894
ENSG00000058091	CDK14	0,021534925	4,012385636	39,25869718	27,14586454	18,42415779	8,488228165	8,117932269	28,30143581	9,958428442	143,7286648	15,96985164
ENSG00000114982	KANSL3	14,24780755	13,68223184	0,030835286	16,52732895	55,80961474	4,214478321	5,093970218	19,64647013	14,32760211	143,5803391	15,95337101
ENSG00000166821	PEX11A	33,78662712	16,40477705	0,175326458	61,46907159	0	3,819448786	10,28769673	0	17,58245244	143,5254002	15,94726669
ENSG00000106484	MEST	11,12683176	16,55611849	0,521865017	66,2951187	0	10,59003681	10,59980902	0,068926693	27,75680224	143,5155087	15,94616764
ENSG00000105705	AC004475.1	27,65795946	10,52060825	0,221453973	10,0819329	25,74832011	17,81035667	21,16577797	9,147660802	21,09789239	143,4519625	15,93910695
ENSG00000033327	GAB2	17,01806132	0,435359705	8,206054582	20,99034113	54,38444258	16,40739283	13,01928316	1,902132566	11,05478206	143,4178499	15,93531666
ENSG00000125821	DTD1	15,6837112	13,18935128	27,26053871	19,01011184	0	16,78352934	15,42906635	18,24673482	17,76537521	143,3684187	15,92982431
ENSG00000185527	AC139530.1	29,39411101	33,05860275	0	24,06797483	0	10,60615559	5,270899792	11,7689024	29,19233044	143,3589768	15,9287752
ENSG00000065833	ME1	0	0	45,05309573	0,078171053	44,00211948	23,80801865	26,53746387	0	3,785968698	143,2648375	15,91831528
ENSG00000141642	ELAC1	27,70994847	5,943701765	0,210060528	34,1778808	30,21291162	16,92538629	11,28284849	5,077206201	11,7031815	143,2431257	15,91590285

ENSG00000132549	VPS13B	1,64525747	18,49531743	65,08818542	8,975688586	0,773404614	7,543194442	13,51080388	10,4768079	16,63235208	143,1410118	15,90455687
ENSG00000053900	ANAPC4	0,060492622	9,839745133	36,68565758	4,247940606	1,916827021	20,89933072	13,79604398	37,61151402	18,05394559	143,1114973	15,90127747
ENSG00000101216	GMEB2	7,290839365	8,606193835	0	15,05288819	77,82010095	11,86815747	7,027601981	11,85322237	3,586717023	143,1057212	15,90063569
ENSG00000001629	ANKIB1	0,061352241	1,378995264	70,87758918	34,18392955	0,243008217	5,590977615	8,86392114	11,09050924	10,76496299	143,0552454	15,89502727
ENSG00000095564	BTAF1	20,19192825	0,141481071	5,206855831	2,891246256	0,37896588	18,62417897	15,70654862	47,59722302	32,18790183	142,9263297	15,8807033
ENSG00000148341	SH3GLB2	15,94489291	12,71057617	17,29726474	9,611781424	29,25010801	13,63515159	17,75071911	14,20858942	12,4915919	142,9006753	15,87785281
ENSG00000101019	UQCC1	12,97757149	18,88889989	0,083327368	13,84457149	0	13,82588139	32,00697018	23,42559209	27,71542111	142,768235	15,86313722
ENSG00000272047	GTF2H5	10,58672868	16,37562512	19,21792356	2,897361251	8,924533769	19,51889138	12,93679563	32,62850257	19,62873381	142,7150958	15,85723286
ENSG00000170584	NUDCD2	25,98847935	22,616586	10,95006179	8,293993971	3,505972147	19,56874664	14,14372116	17,96777816	19,64162927	142,6769685	15,8529965
ENSG00000088543	C3orf18	0	15,29169853	23,39351634	2,573957826	87,45464194	8,183012414	0	0	5,671641709	142,5684688	15,84094097
ENSG00000176973	FAM89B	13,36986531	11,58638272	3,050536066	18,07574241	9,852333124	29,81383477	25,47271486	14,50467237	16,82561107	142,5516927	15,83907697
ENSG00000073008	PVR	14,397383	6,991567401	27,94388335	43,7744308	0,058891037	12,95071081	9,711708217	8,07391777	18,52979428	142,4322867	15,82580963
ENSG00000109381	ELF2	12,14533559	14,7854	32,53925925	13,1381979	2,265461132	13,80003862	16,70983393	17,79559996	19,2359835	142,4151099	15,8239011
ENSG00000162623	TYW3	9,755405125	13,07017981	39,62541272	27,58584653	0	17,10862966	9,894107636	11,09561481	14,21226654	142,3474628	15,81638476
ENSG00000185658	BRWD1	1,839832653	20,26575892	34,16821162	17,37457293	0,743852644	15,94230155	9,419466169	22,119117	20,46809228	142,3412058	15,81568953
ENSG00000106733	NMRK1	21,58163907	4,995142046	0	1,957856213	0,092915276	23,18026169	30,51542357	40,01122318	20,00314444	142,3376055	15,8152895
ENSG00000138101	DTNB	7,422307627	1,726075008	18,67204695	20,22921578	34,72575172	28,65010518	14,23963606	5,918783185	10,6628987	142,2468202	15,80520225
ENSG00000181381	DDX60L	26,81354894	5,271959363	8,36958837	30,24493083	13,53042402	7,274624661	6,337511011	16,44561643	27,84028604	142,1284897	15,79205441
ENSG00000186660	ZFP91	0,156970814	36,58169946	4,764548095	32,02958743	1,989570872	19,27389144	14,81488338	19,39358005	12,93460906	141,9393406	15,77103785
ENSG00000140848	CPNE2	8,906090245	13,8628409	7,149804795	13,01808197	0,092831212	14,2172305	17,72509666	53,34273941	13,5715762	141,8862919	15,76514354
ENSG00000111678	C12orf57	16,81795388	8,611426359	36,78695662	19,21614154	8,351570752	19,29192752	7,016890106	19,39427694	6,380113168	141,8672569	15,76302854
ENSG00000143643	TTC13	23,89355177	24,82796297	0,065997174	14,24858598	0	24,61873467	19,571227	5,334651138	29,29774235	141,8584531	15,76205034
ENSG00000164073	MFSD8	0,033200169	0,353477315	40,12587609	21,69919541	29,35114265	7,162787723	7,498172098	24,1391835	11,49418785	141,8572228	15,76191364
ENSG00000167470	MIDN	13,05229328	7,819813449	11,6340736	17,71708977	17,82796348	26,92538077	19,32891134	14,63857501	12,90830698	141,8524077	15,76137863
ENSG00000100503	NIN	2,760918122	8,759736464	33,84600197	19,54676233	22,04624246	12,07680585	8,408703826	12,36443021	22,03048009	141,8400813	15,76000904
ENSG00000120696	KBTBD7	16,9177445	0,056641741	8,45566841	75,34814328	0,075859219	9,707906579	12,74846424	10,74924449	7,694532819	141,7542053	15,75046725
ENSG00000108091	CCDC6	33,23558482	16,3595794	3,139287717	33,34907815	0	9,414014268	14,86082985	22,43551051	8,881523651	141,6754084	15,74171204
ENSG00000158863	FAM160B2	6,645223891	27,23418145	48,49580522	11,4119754	0,086534348	7,176132173	19,00195269	4,737555848	16,83814274	141,6275038	15,73638931
ENSG00000125124	BBS2	36,84491865	8,464868446	19,2581717	2,46800062	0	12,02398234	30,43900082	11,48667026	20,63693111	141,622544	15,73583822
ENSG00000166313	APBB1	13,601104	30,78212892	20,67357716	19,9532656	0	16,83150778	12,70494781	15,18587543	11,73058712	141,4629938	15,71811042
ENSG00000166557	TMED3	18,44805298	13,3014344	12,9589445	21,03555531	10,59982238	19,32333044	14,96233548	16,34045149	14,48440675	141,4543337	15,71714819
ENSG00000189114	BLOC1S3	48,1218584	13,1757697	1,042686043	11,25563469	0,538811016	15,93139821	18,07224587	20,8225834	12,47806732	141,4390547	15,71545052
ENSG00000104643	MTMR9	27,924169	5,902697527	38,23714831	15,0587176	0	11,98330796	13,2427635	14,27269729	14,72796095	141,3494621	15,70549579
ENSG00000104738	MCM4	0,021670812	5,191333717	3,986520852	47,48694719	0	19,1060079	15,02477086	24,59422638	25,82608348	141,2375612	15,69306235
ENSG00000137936	BCAR3	12,38324809	28,36825323	26,23515026	24,73960736	0,051903099	13,4761879	9,937525934	9,599269847	16,43851303	141,2296588	15,69218431
ENSG00000144597	EAF1	37,86229414	13,16402975	41,05430266	27,26820981	0,146309856	7,536781138	5,060730441	2,625167678	6,473773744	141,1915992	15,68795547
ENSG00000078967	UBE2D4	19,29343871	20,9281561	8,371829068	18,53938844	17,67025871	16,92534197	13,22561082	14,99745439	11,23356497	141,1850432	15,68722702
ENSG00000277203	F8A1	17,64279868	20,66243083	4,219195898	13,86169451	26,62459304	13,4626796	9,595220953	13,88866834	21,15590884	141,1131907	15,67924341
ENSG00000089351	GRAMD1A	16,40294818	21,29973478	13,98789121	5,752689612	0	21,41592264	24,43102897	13,56883784	24,20821411	141,0672673	15,67414082
ENSG00000169379	ARL13B	7,239047559	8,326990373	3,078258198	17,0857759	40,45907795	17,33394085	21,23390549	9,227349502	17,0456247	141,0299705	15,66999672
ENSG00000113719	ERGIC1	28,42726928	23,1767395	19,9833162	8,605086727	4,91976578	11,15365222	15,01816346	14,04714424	15,66985012	141,0009875	15,66677639
ENSG00000216490	IFI30	9,992379886	19,10397356	19,21193517	26,49425083	0	36,31063389	14,23852744	8,881133854	6,739315014	140,9721496	15,66357218
ENSG00000140400	MAN2C1	0	10,28602587	42,56227513	3,117263506	3,935966049	22,16626403	18,30348662	20,6068905	19,9615949	140,9397666	15,65997407
ENSG00000197696	NMB	10,49789679	29,91016439	28,28760303	22,21266418	0	10,0650252	5,401853824	17,45936071	17,09351575	140,9280839	15,65867599
ENSG00000069399	BCL3	19,87782878	9,18650679	9,25402398	16,7828248	0,464276073	16,89134831	30,25238128	26,48602868	11,71301611	140,9082348	15,65647053
ENSG00000172113	NME6	7,474331065	9,97497107	18,23071468	33,61452208	0,049478857	14,2868711	20,65817673	14,29546076	22,27700115	140,8615275	15,65128083
ENSG00000151422	FER	0,044460631	16,03526635	38,01140155	3,416434386	49,52889974	6,419195538	7,623280194	11,39932418	8,338828877	140,8170914	15,64634349
ENSG00000170296	GABARAP	9,29434571	15,667959	28,78112762	17,11926025	17,30243413	17,68805738	6,624010352	18,9690782	9,33519179	140,7814644	15,64238494
ENSG00000172943	PHF8	0,38552891	8,625754856	14,12256077	32,41787815	34,53742753	7,269515008	21,07177844	11,91250521	10,43131894	140,7742678	15,64158531
ENSG00000176749	CDK5R1	40,0741559	28,38990781	0	0	0	12,31301899	28,69800147	0,585876893	30,64490527	140,7058663	15,63398515
ENSG00000145439	CBR4	16,70792024	4,4078137	0,108798473	13,94036166	66,90409008	16,13650815	9,035828215	0,064664299	13,27475159	140,5807364	15,62008182
ENSG00000113595	TRIM23	44,54742221	0,115230769	0	15,5305088	0	10,73035745	22,99660361	27,1930467	19,40726468	140,5204342	15,61338158
ENSG00000129292	PHF20L1	13,46486199	13,79097934	21,90490207	11,96158092	19,79676695	13,28349229	11,1191685	18,18345877	16,95755784	140,4627687	15,6069743
ENSG00000185022	MAFF	31,88645797	23,51200571	9,165267922	9,812382755	0,128004827	11,47269716	17,68794023	16,5482291	20,23743096	140,4504166	15,60560185
ENSG00000137713	PPP2R1B	36,60833468	17,23770001	0,082732172	17,97810911	0,05344011	7,604234766	24,87001224	20,75050409	15,12768567	140,3127528	15,59030587
ENSG00000198040	ZNF84	5,746011195	21,29362942	5,547922144	0,41113704	55,79147533	10,05571458	8,448159959	19,02081334	13,97753256	140,2923956	15,58804395
ENSG00000109920	FNBP4	11,10053955	9,389912062	16,87056683	39,77918649	0,047277191	13,08325901	11,74426093	16,8349365	21,38360459	140,2335432	15,5815048
ENSG00000118689	FOXO3	13,20001074	13,45953977	18,19374311	6,906101675	7,912759408	17,67261293	20,5627389	23,91019361	18,36657938	140,1842795	15,57603106
ENSG00000101773	RBBP8	19,78899754	0,580851123	17,62693814	39,72090102	0,848643267	21,56380083	6,251278849	14,64115354	19,14094745	140,1635118	15,57372353
ENSG00000119397	CNTRL	18,42146492	6,065014975	0,059037931	14,87969431	0,038135026	21,65276614	15,14917357	38,10682174	25,73472072	140,1068293	15,56742548
ENSG00000177042	TMEM80	3,266453014	2,371189389	0,955962021	47,81564506	33,25652505	15,11982902	8,66056578	8,279126968	20,36048856	140,0857849	15,56508721
ENSG00000162777	DENND2D	14,84626844	8,473560284	37,63576598	13,83420413	7,099523078	17,49304268	7,83602343	14,64858948	18,09199333	139,9589708	15,55099676
ENSG00000151461	UPF2	2,00418713	11,85460296	4,821283812	22,40642105	6,045338666	7,729892538	16,91854672	32,81309087	35,36171713	139,9550809	15,55056454
ENSG00000165752	STK32C	16,20408878	17,65978835	0,391190682	16,6710496	0,202148912	13,82199007	26,41480674	18,41431026	30,12879168	139,9081651	15,54535167
ENSG00000160953	PWWP3A	7,451483183	7,928054276	13,22910556	10,5156055	33,27182576	16,76577112	18,52326287	9,569085289	22,63804843	139,892242	15,54358244
ENSG00000126814	TRMT5	18,18530234	12,62509769	0,390695852	13,01924329	50,78875994	14,34082989	6,612983064	12,77153985	11,13381309	139,868265	15,54091833
ENSG00000133703	KRAS	20,21843708	20,34383293	7,575738112	20,97628459	0,04918071	14,4507158	17,01117895	25,11519926	14,10010591	139,8406733	15,53785259
ENSG00000168303	MPLKIP	26,54938365	22,12057748	0,870842237	9,332487017	15,28159755	18,32141338	17,80266948	22,64432162	6,889493842	139,8127862	15,53475403
ENSG00000149761	NUDT22	14,78211441	7,494428382	12,84938915	16,26899062	13,41503316	23,76520757	20,06311409	8,880262497	22,27561111	139,794151	15,53268344
ENSG00000136098	NEK3	1,379520659	16,57057304	0,130140496	36,64817059	0,084063095	25,70992551	26,81517161	16,24327228	16,13976118	139,7205985	15,52451094
ENSG00000196535	MYO18A	0,130553427	1,496904898	14,72387445	23,84432711	38,2339538	15,3814883	22,22226589	0,32940244	23,23235917	139,5951295	15,51056994
ENSG00000136643	RPS6KC1	28,04491127	21,52585593	0,314304748	1,137981637	0	23,74362316	27,60306771	18,09357428	19,06195148	139,5252702	15,5028078
ENSG00000163041	H3F3A	8,923088897	12,13351156	10,17000362	53,34261195	8,413215851	12,83197978	9,019690105	8,377513105	16,28244563	139,4940605	15,49934005
ENSG00000125355	TMEM255A	6,345233916	31,0677745	0	6,966382163	8,191379919	20,59905812	16,06592639	20,89840189	29,24023821	139,3743951	15,4860439
ENSG00000154721	JAM2	8,99497932	10,64091341	36,13227115	29,35353926	0,060386336	17,32478025	12,08920468	0,833448548	23,81285522	139,2423782	15,47137535
ENSG00000198874	TYW1	61,10545856	18,49731172	0	0	0	24,72644116	16,60019322	2,891834053	15,40349554	139,2247343	15,46941492
ENSG00000106993	CDC37L1	27,35292608	16,56370836	13,01932109	4,822342569	0,090915834	22,84945664	15,15173376	26,0164938	13,22098045	139,0878786	15,45420873
ENSG00000167113	COQ4	10,94183907	15,17574366	0,05784022	2,877560061	58,88152796	13,77409906	16,70849199	7,838023449	12,76099716	139,0161226	15,44623585
ENSG00000086544	ITPKC	0	1,667944906	39,93493721	13,60324512	0	3,734953741	10,07181871	55,98595161	13,98252863	138,9813799	15,44237555
ENSG00000136160	EDNRB	12,20632819	18,31283631	32,71520423	0,465290611	7,876508151	31,41887169	19,50965219	0,058921998	16,37093117	138,9345446	15,43717162
ENSG00000107882	SUFU	5,216781536	3,632513621	31,46256911	10,37658384	48,85881785	7,709464526	11,02091918	5,487187283	15,05180865	138,8166456	15,42407173
ENSG00000087884	AAMDC	4,27744796	18,76855996	16,5958268	10,74359134	17,55850053	8,282720504	14,33577648	36,90391207	11,19096403	138,6572997	15,40636663
ENSG00000140548	ZNF710	4,831672923	6,537434854	13,55458516	18,40728338	40,33254572	13,29539194	18,05328518	2,047056673	21,57810341	138,6373592	15,40415103
ENSG00000094631	HDAC6	10,6755157	15,5543605	23,28299641	11,46912829	0,101617867	19,84291749	24,17806907	5,142586648	28,38007164	138,6272636	15,40302929
ENSG00000127314	RAP1B	15,59672498	15,43012573	13,81196167	11,20759344	15,82782031	11,67431743	13,69899406	28,15116663	13,20887193	138,6075762	15,4008418
ENSG00000118579	MED28	12,53286724	11,55953026	42,58882122	18,93039006	1,145562703	13,98717494	13,48245729	11,29356205	13,05939379	138,5797596	15,39775106
ENSG00000244045	TMEM199	15,37953604	4,175933759	13,10939269	2,804521552	31,82353304	12,08210385	18,3017703	21,23652737	19,44478858	138,3581072	15,37312302
ENSG00000119041	GTF3C3	11,27660057	27,28975881	0,113770845	21,78611248	0,14697853	5,070118579	14,34435719	26,55197275	31,76885299	138,3485227	15,37205808
ENSG00000111452	ADGRD1	10,92415051	10,20244055	29,48273777	15,58376428	0	7,902114028	21,25272032	16,86073924	26,13821858	138,3468853	15,37187614
ENSG00000241258	CRCP	5,122392226	9,316798308	20,8092447	7,076202701	18,05732275	24,60538214	17,11925716	8,960937424	27,14199209	138,2095295	15,35661439
ENSG00000164118	CEP44	0	16,96957424	0,157073089	24,18044121	78,88512196	3,585862161	6,894753376	6,534939886	0,84010294	138,0478689	15,3386521
ENSG00000196214	ZNF766	20,35729235	8,902651458	23,24413452	14,68851066	5,151980045	18,06677547	17,60596133	15,80162572	14,19431698	138,0132485	15,33480539
ENSG00000090686	USP48	6,969193424	5,788049912	35,38780032	37,22932406	0,226882727	18,25592847	16,75227766	9,15070983	8,218968027	137,9791344	15,33101494
ENSG00000196152	ZNF79	43,45577662	0	0	43,86700051	0	22,70279395	7,140144871	0,143542384	20,66755417	137,9768125	15,33075694
ENSG00000137338	PGBD1	52,7424637	12,54212581	0	0,33669029	0	21,24725773	4,492647372	14,70966784	31,89366124	137,964514	15,32939044
ENSG00000137831	UACA	12,34060499	33,1153907	0,025280111	18,93653235	29,5889863	11,82920081	6,949020752	13,0719386	12,03371443	137,890669	15,32118545
ENSG00000197119	SLC25A29	18,93448327	12,0638692	38,78878899	3,539408063	0,144688776	21,19002934	14,99712537	3,772084104	24,41013951	137,8406166	15,31562407
ENSG00000100221	JOSD1	0,803408027	23,30591587	37,78370277	19,48266223	0,083013764	23,01117569	7,134928727	20,08883293	6,100371281	137,7940113	15,3104457
ENSG00000198435	NRARP	8,353708185	13,70661492	28,20850683	25,78721256	0	16,0822066	20,3812088	3,878633697	21,39240544	137,790497	15,31005523
ENSG00000140464	PML	17,63374519	10,82567449	34,98635519	16,71946468	0,028373038	14,3141621	22,9984333	5,404120682	14,78608264	137,6964113	15,29960126
ENSG00000198056	PRIM1	6,837861127	22,40893193	0,226645005	28,29543548	0	27,93357855	16,93056661	8,217084639	26,7443222	137,5944255	15,2882695
ENSG00000135870	RC3H1	1,378746083	12,60556326	50,36396475	17,52717548	1,872354235	16,23351663	19,22177575	2,182223421	16,16548217	137,5508018	15,28342242
ENSG00000143801	PSEN2	0	31,89577725	0	17,35333975	0	34,31240637	21,7088436	13,9356022	18,33846462	137,5444338	15,28271487
ENSG00000180182	MED14	13,55118585	25,15564688	12,96742084	33,84489637	0,111434901	15,35891251	10,88845765	17,46504592	8,188934473	137,5319354	15,28132616
ENSG00000115919	KYNU	11,14139398	13,66690965	4,621845471	18,7450707	24,58355381	16,27525494	16,23589837	12,80666022	19,43493043	137,5115176	15,27905751
ENSG00000141446	ESCO1	5,832839435	22,83139074	0	60,03726189	0	17,2032159	12,1790502	0,187569291	19,23520297	137,5065304	15,27850338
ENSG00000102225	CDK16	19,99074155	20,61051662	13,40740027	16,26099137	0	14,73405826	21,51688437	9,764794293	21,14285194	137,4282387	15,2698043
ENSG00000204859	ZBTB48	15,11105707	23,96036054	9,676912537	0,925711662	0	24,13244752	28,90547116	11,94253537	22,74662534	137,4011212	15,26679124
ENSG00000118007	STAG1	21,57896712	34,0519485	1,063292486	3,293709357	5,403017978	31,60402927	16,02974279	9,563769836	14,71512053	137,3035979	15,25595532
ENSG00000184857	TMEM186	17,01143341	9,92614909	0	25,5377536	0	13,71370386	10,752782	32,84238095	27,51885624	137,3030591	15,25589546
ENSG00000168061	SAC3D1	23,53640943	17,09934876	1,411862805	3,092344507	0	38,48151638	33,06695544	11,37500486	9,12451887	137,1879611	15,24310678
ENSG00000116984	MTR	0,09782994	1,931264709	32,55136386	33,21311009	0,145309356	15,7223667	7,69656969	16,63270219	31,0872187	137,0957352	15,23285947
ENSG00000125384	PTGER2	0	0	0	33,87391151	0	3,836471753	8,09321817	84,98803887	6,257712515	137,0493528	15,22770587
ENSG00000168924	LETM1	0,429450528	4,53916397	0,034348214	2,61160669	53,11549465	22,91956317	23,37937238	9,349995794	20,62154665	137,000542	15,22228245
ENSG00000232838	PET117	11,42191108	8,619682018	11,59256712	25,1640158	29,95246226	11,53157788	12,64514206	17,79925559	8,234728615	136,9613424	15,21792693
ENSG00000132964	CDK8	21,95331637	12,42942002	22,86382386	1,327538028	39,5861621	4,454919386	3,475435991	14,47635895	16,38895278	136,9559275	15,21732528
ENSG00000240230	COX19	32,4724577	25,23689413	0,041396887	8,004855513	0	19,29652426	13,8082763	15,3530283	22,72226281	136,9356959	15,21507732

ENSG00000108523	RNF167	4,680903246	36,44933164	19,75441666	7,3221549	1,046989919	23,82243703	15,36371412	14,45049129	14,01976316	136,910202	15,21224466
ENSG00000085274	MYNN	25,0796078	9,873989881	12,66192074	10,70152165	10,21573463	9,873561371	11,40838446	34,88881559	12,19516446	136,8987006	15,21096673
ENSG00000151233	GXYLT1	0,080678361	9,520257878	0,074207181	73,00049476	0,047933468	5,381242677	18,68992132	20,42060928	9,649532456	136,8648774	15,2072086
ENSG00000140932	CMTM2	0	0	55,78922925	0	67,70504491	1,297302511	0	12,05756024	0	136,8491369	15,20545966
ENSG00000136861	CDK5RAP2	0,044973384	3,298572223	13,1544154	8,956696549	63,84311864	9,908548331	4,169896946	13,04692042	20,39149342	136,8146353	15,20162615
ENSG00000175309	PHYKPL	0,035812435	5,909990759	36,69508594	7,111629833	0,042554514	16,75030351	14,18399061	43,9717999	12,11228667	136,8134542	15,20149491
ENSG00000204217	BMPR2	6,044422827	8,449852944	35,50670172	28,41779219	0,030177945	17,28816093	11,35559223	7,025205511	22,64516448	136,7630708	15,19589675
ENSG00000162413	KLHL21	35,34161151	20,24373019	0	10,66275364	0	15,74720008	22,76320181	5,554326885	26,4186985	136,7315226	15,1923914
ENSG00000226742	HSBP1L1	20,94013805	17,26122949	3,005190259	38,33884885	0	21,93117697	12,14626801	20,29696361	2,739752171	136,6595674	15,18439638
ENSG00000171703	TCEA2	11,2985765	13,49670468	43,36907559	10,73604637	0,675032974	15,21193466	18,21258996	4,140784054	19,48516169	136,6259065	15,18065628
ENSG00000244187	TMEM141	14,07304522	19,12769882	10,46556515	32,40143674	15,65505799	13,91720161	11,68514981	3,928548359	15,34399953	136,5977032	15,17752258
ENSG00000018189	RUFY3	11,93781542	9,151896016	44,9260766	16,28589368	0,11455105	16,49403394	7,564240111	13,45629339	16,65801364	136,5888139	15,17653487
ENSG00000133858	ZFC3H1	14,29846661	0,027729266	18,65657541	13,16817659	14,66922743	14,2576721	8,122484781	36,768107	16,58588922	136,5543284	15,17270316
ENSG00000157259	GATAD1	28,5672185	22,00313083	0,921190165	13,91763002	3,626877499	13,77133712	21,56181025	6,257242876	25,89592539	136,5223626	15,1691514
ENSG00000130544	ZNF557	0,47230253	0	80,41584388	17,44399466	0,187072865	15,23991927	6,143884984	13,13934988	3,420686119	136,4630542	15,16256158
ENSG00000092148	HECTD1	0,288815298	10,52751502	51,91234627	3,532606967	0	15,94760421	28,11766459	0,710498791	25,26399725	136,3010484	15,14456093
ENSG00000155011	DKK2	58,38759074	0,371130796	0	43,45906613	0,082841417	14,54348769	10,21707663	1,600721088	7,459583593	136,1214981	15,1246109
ENSG00000088035	ALG6	14,87460312	14,14868632	29,94254341	17,99387834	0,06534162	10,26375475	9,223073895	20,6220991	18,93632687	136,0703074	15,11892305
ENSG00000175727	MLXIP	12,9339665	14,19211336	15,97563736	23,06794627	4,11737015	16,2704344	13,75215638	18,34688614	17,36783191	136,0243425	15,11381583
ENSG00000168116	KIAA1586	14,67847936	12,5058186	5,007039416	24,67509892	2,674259746	26,41548262	10,89642639	5,739294515	33,53486619	135,9937044	15,1104116
ENSG00000104447	TRPS1	20,93602018	1,81635206	37,63476954	11,10010595	0,616260233	11,22353883	19,5027494	19,13010818	14,03248669	135,9923911	15,11026567
ENSG00000184719	RNLS	33,40837042	16,73788268	0	39,16748366	0,074972347	8,486031585	20,77201947	11,17544884	6,169018171	135,9912272	15,11013635
ENSG00000164828	SUN1	8,556556364	24,70979444	26,03864218	29,5285908	0	14,57492914	1,397644205	12,77122632	18,34940346	135,9267869	15,10297632
ENSG00000140006	WDR89	50,66842444	0,083559364	0	37,48551676	0,111909487	18,40578539	16,96918794	0	12,16197248	135,8863559	15,09848398
ENSG00000156030	ELMSAN1	4,202496588	17,85757313	11,21486012	26,51148882	9,774261202	12,32886368	17,02838235	18,45277583	18,51022541	135,8809271	15,09788079
ENSG00000186111	PIP5K1C	13,84583428	12,86218986	19,3623938	6,782885308	0,154725352	24,04190319	29,62418868	9,775889833	19,43074368	135,880754	15,09786155
ENSG00000180011	ZADH2	17,54046911	20,82885737	26,07266445	27,3319777	28,25739191	2,610955821	7,392687319	2,78724865	3,041677942	135,8639303	15,09599225
ENSG00000117009	KMO	0	3,69788833	0	27,64699529	49,22314111	7,952323303	8,039723594	21,28429858	17,97204008	135,8164103	15,09071225
ENSG00000175470	PPP2R2D	5,376363648	12,87749053	16,54643446	3,459365194	49,35922931	21,32238202	6,195437119	6,749948552	13,92843254	135,8150834	15,09056482
ENSG00000055483	AC022966.1	19,5862862	3,368107419	9,752631518	15,45548794	33,87020892	14,37232933	15,23034566	9,338757133	14,79125001	135,7654041	15,0850449
ENSG00000105732	ZNF574	12,36329792	17,22908546	49,89233732	20,62219011	0,230745904	7,42671033	14,56520025	0	13,41406682	135,7436341	15,08262601
ENSG00000149089	APIP	12,51289372	15,47071919	0,053283515	23,45763908	29,04880254	8,840931632	11,03908039	18,9380747	16,35107413	135,7124989	15,07916654
ENSG00000028839	TBPL1	19,25242833	15,05937409	13,57076887	19,62578991	0	6,222881325	15,5375049	35,08285248	11,32265972	135,6742596	15,07491774
ENSG00000166669	ATF7IP2	19,8611922	8,097544362	0	34,50008661	17,58782518	17,57494296	9,462791429	1,602796369	26,98569436	135,6728735	15,07476372
ENSG00000167965	MLST8	13,75937994	8,917566517	29,58320319	1,077254482	0	21,38572113	27,97182631	9,70083134	23,27344063	135,6692235	15,07435817
ENSG00000166166	TRMT61A	20,94974029	7,084883434	0	22,5489347	0,111631191	20,73237545	17,82404157	20,64574077	25,76547993	135,6628273	15,07364748
ENSG00000130779	CLIP1	11,06521179	3,843190475	27,06557675	5,750439389	1,528653798	21,13328322	14,81927732	29,51993712	20,89884649	135,6244163	15,06937959
ENSG00000006756	ARSD	3,290623016	11,67825961	32,12636498	10,54732889	0,102225318	19,12718006	23,98329859	19,51755558	15,23592326	135,6087593	15,06763992
ENSG00000175643	RMI2	14,68839942	25,76717641	0,615851784	34,75760808	0	20,4459171	10,18078296	3,202772047	25,93925429	135,5977621	15,06641801
ENSG00000082701	GSK3B	2,908822903	5,424648114	27,53897989	25,91433661	15,80716009	14,95148037	8,245720691	17,38063137	17,40886991	135,58065	15,06451666
ENSG00000164169	PRMT9	6,385492232	27,54697673	2,250851004	8,715439435	0	6,926934503	21,36678478	50,08357944	12,22674489	135,502803	15,055867
ENSG00000197555	SIPA1L1	19,35754168	11,06096552	23,65025657	2,838077793	0,810126586	14,18233338	23,38163696	12,29944974	27,8949461	135,4753343	15,05281493
ENSG00000144711	IQSEC1	15,64389957	7,611535019	8,846434738	25,39893525	25,361508	8,473971111	5,681349873	29,43759481	9,017599647	135,472828	15,05253645
ENSG00000158552	ZFAND2B	22,85780851	13,09934432	9,816836927	4,991398867	0,105684974	18,84681654	19,05317578	21,87986395	24,66650428	135,3174342	15,03527046
ENSG00000128923	MINDY2	7,064826765	0,255265285	0,058806875	7,341728712	58,95392699	13,03909017	10,52443128	7,969016437	30,05704808	135,2641406	15,02934895
ENSG00000168228	ZCCHC4	40,62959347	13,98155974	0	0,769021689	0,569898616	15,27084429	3,413384684	38,6543271	21,90890764	135,1975372	15,02194858
ENSG00000101452	DHX35	0	0	0,166854801	10,2327441	93,19236743	2,323677545	12,70267867	0	16,50977509	135,1280976	15,01423307
ENSG00000147121	KRBOX4	30,30412801	26,41398807	0,075435576	0,070810034	0,097453879	9,815062611	10,96375764	34,79202502	22,49322902	135,0258899	15,00287665
ENSG00000186687	LYRM7	1,214910521	27,46526034	1,162161533	13,92995844	0,230980892	20,24861356	11,23765476	26,61966849	32,90191687	135,0111254	15,00123616
ENSG00000092208	GEMIN2	18,909375	9,725861447	0,201654044	41,92752766	0	15,70644197	7,236047439	25,52867714	15,7736983	135,009283	15,00103144
ENSG00000127415	IDUA	20,40625745	19,95536817	1,644041985	13,37468574	0,088496191	14,5141091	30,88833457	9,689924479	24,41010261	134,9713203	14,99681336
ENSG00000080802	CNOT4	4,058978038	4,022704673	21,24865737	15,77018063	4,156096718	25,74351855	21,70946879	9,395124866	28,70411353	134,8088432	14,97876035
ENSG00000106080	FKBP14	10,61121919	9,616057457	40,60618044	10,82738066	6,135879901	10,1240964	12,15596908	27,11226174	7,606936135	134,795981	14,97733122
ENSG00000184831	APOO	0,064220387	22,22171271	16,30312523	21,06997245	0	22,98633574	17,75674482	13,90268686	20,45654453	134,7613427	14,97348253
ENSG00000131116	ZNF428	17,12521599	20,37395751	9,486764486	25,03497181	22,661635	9,18757862	11,31091209	11,91521943	7,658839295	134,7550942	14,97278825
ENSG00000112305	SMAP1	10,78583021	17,97924009	10,28813632	10,59793576	9,407308848	14,99215978	20,08249005	22,39426773	18,22287457	134,7502434	14,97224926
ENSG00000154655	L3MBTL4	0	10,44402815	0,034926511	8,130653813	74,90075642	8,254879806	8,118761274	5,065085719	19,7545116	134,7036033	14,96706703
ENSG00000028310	BRD9	0,854536686	26,56428234	12,54680309	8,486915985	42,45928425	11,46727203	5,663180762	13,42639148	12,80626366	134,6689303	14,96321447
ENSG00000087301	TXNDC16	0,09578105	0,623190788	0	49,45249783	18,36180969	2,208401929	21,44633395	25,83156349	16,64883276	134,6684115	14,96315683
ENSG00000066117	SMARCD1	43,0147302	13,313125	7,847958402	25,35174305	5,38396627	12,46913602	10,13827644	4,117564704	13,02662539	134,6631255	14,9625695
ENSG00000141540	TTYH2	12,36049234	17,24418278	16,41326647	6,623960431	2,633618682	22,18172622	27,32129456	20,75313004	9,12108731	134,6527588	14,96141765
ENSG00000159842	ABR	15,74828497	4,056872366	15,50299441	19,52917483	4,479576619	14,56691125	27,14181977	6,089611779	27,51952366	134,6347696	14,95941885
ENSG00000167670	CHAF1A	1,991054676	13,88437541	6,040251804	57,18131205	0	10,58514082	16,61916325	10,23538495	18,04335168	134,5800246	14,95333607
ENSG00000172493	AFF1	10,05245015	9,993179916	2,797153703	40,81895298	6,123032726	15,24157539	21,80212142	15,42417948	12,25932524	134,511971	14,94577456
ENSG00000121542	SEC22A	38,99834881	31,06662203	0	0,094765943	0	19,99814987	13,06982625	13,69786154	17,58114422	134,4119527	14,93466141
ENSG00000186908	ZDHHC17	1,030299345	11,63980046	0,094765943	61,70519115	0,081617623	15,74265131	10,45110834	15,1699506	18,47905761	134,3944424	14,93271582
ENSG00000028277	POU2F2	13,27547672	21,28111112	13,20650603	11,33249339	2,216774953	14,99632638	16,47213023	12,7482472	28,78244751	134,3115135	14,9235015
ENSG00000173540	GMPPB	3,698196998	8,908900626	13,64749522	15,51986426	37,17947858	20,37584656	16,76783879	4,362010755	13,7000477	134,1596795	14,90663105
ENSG00000148680	HTR7	27,33221151	25,40861694	0,720570533	26,52939114	0	12,80646157	18,93619066	15,51291316	6,851484692	134,0978402	14,89976002
ENSG00000171241	SHCBP1	25,89068563	10,46513717	41,28060741	1,943807098	0,058156687	11,74136389	8,330823663	13,11035936	21,15791177	133,9788527	14,88653919
ENSG00000135249	RINT1	59,03087261	17,64586111	0	2,922822454	0,201129959	19,00239553	15,5300719	0	19,62031333	133,9534309	14,88371454
ENSG00000171763	SPATA5L1	24,59939522	0	0	0,153626715	70,30119684	5,030553253	10,78520369	15,36904965	7,713971045	133,9529964	14,88366627
ENSG00000104714	ERICH1	5,461827855	14,99342046	22,41359464	20,82428229	18,41628422	13,56533806	12,40361639	7,213688427	18,65928938	133,9513417	14,88348241
ENSG00000146676	PURB	0,180891966	4,260390904	11,31402296	7,269491076	71,3623498	7,348525924	11,40142858	8,090954151	12,70123147	133,9292868	14,88103187
ENSG00000154451	GBP5	16,68284453	0,131716566	85,4456819	13,84304421	0	4,889910157	5,932221272	0,121736915	6,846857016	133,8940126	14,87711251
ENSG00000141076	UTP4	7,312672259	4,788250002	0	10,62378111	32,96186014	22,8127043	24,8252014	19,11799998	11,44947325	133,8919424	14,87688249
ENSG00000165209	STRBP	0	0,894137829	57,95114327	20,23800964	4,479544495	5,757795733	8,708697861	0	35,85183003	133,8811589	14,87568432
ENSG00000164051	CCDC51	22,25902597	0,111262241	0	1,299261284	7,599582846	13,28661465	3,540241733	40,44738482	45,26635211	133,8097256	14,86774729
ENSG00000165688	PMPCA	11,75227172	20,03147017	15,86264716	15,41033367	0	18,29877353	18,59247442	6,842869553	26,99749296	133,7883332	14,86537035
ENSG00000103356	EARS2	39,53136918	5,381558466	0,097026213	6,557528158	0	14,73795524	16,66368633	23,4129903	27,2769503	133,6590642	14,85100713
ENSG00000101290	CDS2	43,89890138	9,953067461	0,259133991	0,840299071	0,3043369	21,10438777	23,09504755	17,75385727	16,44269231	133,6517237	14,85019152
ENSG00000103047	TANGO6	13,15715411	0	36,38588221	0,490621156	8,466323316	14,27781214	19,61721563	4,779209828	36,42204768	133,5962661	14,84402956
ENSG00000170153	RNF150	4,912603006	9,788692702	0,252433799	15,37838708	78,56103882	14,34315919	2,632005693	4,921110522	2,784660023	133,5740908	14,84156565
ENSG00000107833	NPM3	12,86393867	3,75629957	0,29954752	66,92082323	0,38698011	16,98438016	14,06133031	4,272860787	14,01859543	133,5647558	14,84052842
ENSG00000169116	PARM1	100,7703984	0,05326619	2,20882081	0	0	19,37930455	8,499281528	0,065640558	2,54735996	133,524072	14,836008
ENSG00000087338	GMCL1	8,927014498	5,864921098	6,080104373	31,84345459	0,073409146	18,51741105	19,51817716	20,26377152	22,37604285	133,4643063	14,82936736
ENSG00000180257	ZNF816	21,1372188	0,243029003	75,91964033	8,592722729	0,325484177	11,62877758	11,22030764	0,099829174	4,267170904	133,4341803	14,82602004
ENSG00000154429	CCSAP	0,386410281	2,399864473	24,52373576	3,83666576	0	25,93264248	32,68605421	12,35762966	31,27639251	133,3993951	14,82215502
ENSG00000134508	CABLES1	0,045419468	13,4997851	0	35,80307061	0,701612271	23,63739506	22,85392882	22,49577083	14,3281133	133,3650955	14,81834394
ENSG00000086200	IPO11	0,02004119	17,35454855	2,470114938	10,58966873	54,24865792	7,085305796	14,41078349	0,043824213	27,08823978	133,3111846	14,81235385
ENSG00000197362	ZNF786	48,32212073	0	0	0	0	7,456085226	3,950915376	47,78219212	25,79465641	133,3059699	14,81177443
ENSG00000147548	NSD3	7,582786918	4,995233062	29,39350079	21,24108255	12,72046395	11,80800103	17,53045636	16,4512848	11,49578685	133,2185963	14,80206626
ENSG00000170312	CDK1	0	44,95181329	14,35511577	11,61628607	0	36,33910113	23,68328322	0,084058654	2,174749434	133,2044076	14,80048973
ENSG00000166167	BTRC	55,75529902	22,82836021	0,210176999	17,87442061	0,054304785	14,07467617	9,183069815	9,793614885	3,316178683	133,0901012	14,78778902
ENSG00000156990	RPUSD3	20,34398109	18,40091885	25,67729411	3,756917174	0	11,66461796	16,32897365	17,30023914	19,59937146	133,0723134	14,7858126
ENSG00000120868	APAF1	7,758079374	1,118953718	20,28256413	7,358181504	0,18164772	19,88912831	17,54807628	30,1686593	28,76531908	133,0706094	14,78562327
ENSG00000236287	ZBED5	20,01641849	26,22321255	4,802845879	26,55878189	0,159095157	17,38303307	12,04815349	6,807047243	19,01895964	133,0175474	14,77972749
ENSG00000165271	NOL6	0,025210305	14,53889724	0,510140322	23,85591693	0,119825673	11,98519848	33,17825763	29,76887996	19,00632602	132,9886526	14,77651695
ENSG00000114648	KLHL18	1,476023411	5,31425255	0	23,8254613	0,457539338	21,72526663	31,01234423	25,11938884	24,02008421	132,9503605	14,77226228
ENSG00000082258	CCNT2	0,016640301	24,03563353	0	75,80073373	0	7,545542141	5,043130842	4,184558448	16,29047811	132,9167171	14,76852412
ENSG00000198855	FICD	20,84782963	0	0	36,18714977	0,172032356	15,49823351	19,23377544	13,37567471	27,59876498	132,9134604	14,76816227
ENSG00000063244	U2AF2	8,703087545	8,210423332	16,34779683	13,18942277	51,22770288	10,7650362	8,964344498	3,774100771	11,65210273	132,8340176	14,75933529
ENSG00000166135	HIF1AN	27,29865642	8,684604868	0,18006484	43,13859484	0,232622562	13,17110739	10,67409907	6,706675402	22,73924628	132,8256717	14,75840796
ENSG00000095002	MSH2	11,49480134	7,279168762	59,88567422	14,92463962	19,10671108	5,094009868	3,570034851	0,095938032	11,35826172	132,8092395	14,75658217
ENSG00000180329	CCDC43	18,31322503	8,358892472	0,486830296	3,56443573	0,251570958	21,96644413	20,83115144	23,14778056	35,86740237	132,787733	14,75419255
ENSG00000118507	AKAP7	40,13823127	43,96057611	15,43325174	0,056811447	0,078188013	5,924087651	11,06368048	8,561226548	7,485653521	132,7017068	14,74463409
ENSG00000117625	RCOR3	11,28509431	0,884762429	23,05290306	12,39814222	1,856414924	20,14576294	15,02828768	19,2256804	28,82133704	132,698385	14,744265
ENSG00000077514	POLD3	0,32921693	9,444362385	21,49955014	14,35426603	0,325996317	23,28410962	23,73639905	23,69674186	16,02706937	132,6977117	14,74419019
ENSG00000232119	MCTS1	21,56315836	18,40922873	11,69871654	22,35061748	0,066725667	14,56572789	8,91720587	26,98364779	8,09754914	132,6525775	14,73917527
ENSG00000115486	GGCX	9,995447043	19,53391342	1,771546615	19,55363726	5,326982553	25,26668051	16,8332354	13,39746791	20,89012112	132,5690318	14,72989242
ENSG00000149218	ENDOD1	0,03248991	18,39116277	0,179303439	29,73458107	0,154425948	33,24632498	3,72283505	13,00140311	33,92468231	132,3872086	14,70968984
ENSG00000158623	COPG2	85,21190431	18,78743175	0	12,04121289	0,433821626	3,126066664	4,425868198	0,23950319	8,037334498	132,3036831	14,70040924
ENSG00000003402	CFLAR	14,14371817	13,75362454	11,56240903	7,661243082	31,8075994	11,84601075	16,07520602	7,246952346	18,11422796	132,2109913	14,69011014
ENSG00000166997	CNPY4	0,06165262	13,89393473	0,113414973	0,212921236	83,51570752	6,159878613	3,173872127	0,80889735	24,18810306	132,1283822	14,68093136
ENSG00000023909	GCLM	13,25629039	15,27015813	8,555598328	40,01067064	0,198553746	8,989625793	19,47033165	3,653902332	22,70848586	132,1136169	14,67929076
ENSG00000167720	SRR	10,59446805	19,7890775	15,89923075	19,97933846	3,09203296	22,14181743	11,6519189	15,34305649	13,60131375	132,0922543	14,67691714
ENSG00000198792	TMEM184B	30,92455556	6,916361113	1,772832264	8,986281013	18,01695153	14,39020308	18,51114167	6,181599052	26,31245405	132,0123793	14,66804215

ENSG00000104093	DMXL2	16,37295674	3,651096981	27,66311697	28,31032027	9,337524278	14,88833233	7,533490637	11,6790084	12,51808979	131,9539364	14,66154849
ENSG00000165792	METTL17	0	14,76619241	0	34,25174762	0	31,39166102	17,45698708	13,36889687	32,66621273	131,9016977	14,65574419
ENSG00000176407	KCMF1	21,2973357	22,58073319	9,907925215	24,36441028	1,892938853	8,620353759	10,99125147	15,96602767	16,18693354	131,8079097	14,6453233
ENSG00000165416	SUGT1	11,15668695	21,11116661	17,52331176	25,22613316	0,118897346	13,51278038	10,92527391	17,43850817	14,75499803	131,7677563	14,64086181
ENSG00000165801	ARHGEF40	26,9800787	7,232045646	6,615342849	9,808148911	0,043826891	23,16349765	23,07588561	10,8074767	24,01897328	131,7452742	14,63836381
ENSG00000198885	ITPRIPL1	26,27868694	17,52260706	0	3,085000775	0	23,60980262	25,59573196	7,316156589	28,28446642	131,6924524	14,63249471
ENSG00000136463	TACO1	12,12818029	13,61067853	0	2,603689551	0	31,02275859	23,62434885	26,80748986	21,76944079	131,5665865	14,61850961
ENSG00000136152	COG3	0	5,242138658	56,76511632	12,34586376	0,412982224	12,1291616	18,3866496	0,977135242	25,28025636	131,5393038	14,6154782
ENSG00000155561	NUP205	0,059484544	2,814769716	33,77635109	6,471159979	1,743517789	14,47717275	17,99489212	27,14237492	27,01980668	131,4995296	14,61105884
ENSG00000029559	IBSP	0	0	0	0,165883646	0	0,105473984	130,9741164	0	0,236295642	131,4817697	14,60908552
ENSG00000256053	APOPT1	9,761466162	18,34440438	0	8,295236985	45,57468302	15,9496943	12,70020146	11,76521858	9,074906479	131,4658114	14,60731237
ENSG00000182606	TRAK1	0,395207919	21,03860266	0,051929778	44,89465937	0,134174311	22,64107534	16,44263738	9,475379435	16,352272	131,4259382	14,60288202
ENSG00000173171	MTX1	16,41103208	11,77927029	19,41959813	3,422880997	30,1273106	15,53829989	10,4112051	16,63261246	7,597142765	131,3393523	14,59326137
ENSG00000123374	CDK2	8,513819134	8,898975562	44,48253706	10,33804633	0,369557543	25,48202066	10,87818417	0	22,32835758	131,291498	14,58794423
ENSG00000079616	KIF22	9,637153741	20,21014611	3,223331389	1,210273537	0	25,77924081	11,46458971	15,40291004	44,34973827	131,2773836	14,58637596
ENSG00000160741	CRTC2	3,90152223	19,35505831	5,788036486	14,4883384	0	34,40732948	24,66213078	9,815796425	18,83231	131,2505221	14,58339135
ENSG00000141867	BRD4	11,7709549	12,06228794	18,06767203	24,87869228	18,56019714	9,013747531	9,877429593	10,11004631	16,90491809	131,2459458	14,58288287
ENSG00000115526	CHST10	0	24,13814404	0	22,85138475	0	25,51903069	21,08370104	12,85308942	24,74373568	131,1890856	14,57656507
ENSG00000113749	HRH2	26,95457089	4,438342667	19,09002265	24,71967183	8,536867092	5,784520899	10,09683778	16,35008161	15,21722713	131,1881425	14,57646028
ENSG00000133641	C12orf29	10,11342052	23,27111963	43,64222351	12,6489198	0	19,74322891	10,94799769	0,310024614	10,46204035	131,138975	14,57099723
ENSG00000173950	XXYLT1	31,61672244	7,652681248	27,45800459	11,94420658	0	16,17289906	17,57886786	6,30740557	12,33006949	131,0608568	14,56231743
ENSG00000131788	PIAS3	14,11748777	4,017108616	26,7221818	18,94846153	1,86806878	14,4645301	10,80830575	27,36432748	12,72232747	131,0327993	14,55919992
ENSG00000113456	RAD1	1,58476374	24,39814949	0	24,43615567	22,86257914	7,033432129	12,60263305	20,89010264	17,21207821	131,0198941	14,55776601
ENSG00000104427	ZC2HC1A	0,073017913	34,72435662	0,738772913	35,17799368	0	10,50219162	11,9437813	23,3914782	14,45822183	131,0098141	14,55664601
ENSG00000009950	MLXIPL	15,28366519	6,738355558	5,195589701	16,74535974	30,20614842	7,167061257	12,24736366	12,42132019	24,93392594	130,9390897	14,54878774
ENSG00000048028	USP28	6,367834854	25,17787271	0,048606417	3,011314622	0,313968825	21,00354215	14,98447924	27,67584505	32,30135393	130,8848178	14,54275753
ENSG00000083814	ZNF671	0	14,1966461	60,52284715	0	0,101675408	6,670244024	2,095908717	21,98531239	25,30922345	130,8818572	14,54242858
ENSG00000165516	KLHDC2	20,27880423	15,48849977	0	22,71154234	0	11,39502932	23,39041796	18,98207755	18,5875933	130,8339645	14,53710716
ENSG00000215271	HOMEZ	20,91628065	12,64637912	0	19,99212671	44,12720072	8,634305401	9,069051825	0,298549483	15,01145563	130,6953495	14,5217055
ENSG00000196814	MVB12B	9,903248432	4,342380568	22,43817095	16,83664436	0	18,57677889	30,20876035	4,347823257	24,00674332	130,6605501	14,5178389
ENSG00000221914	PPP2R2A	19,60124375	13,9742709	25,78612539	29,49150388	15,10050828	8,181370181	11,03141298	1,136816173	6,322760123	130,6260117	14,5140013
ENSG00000154945	ANKRD40	0,218955495	0,943575658	0,632949925	33,3798001	0,074336068	29,91267073	23,34875356	18,19407886	23,8511372	130,5562576	14,50625084
ENSG00000126249	PDCD2L	13,87314561	13,25560735	15,70509034	17,32195453	52,75172939	3,163549132	11,43055991	3,03365069	0	130,5352869	14,50392077
ENSG00000184992	BRI3BP	10,5053746	21,56176907	1,658839916	24,79719213	0,107151288	19,33106973	27,51624467	3,598645894	21,37664757	130,4529349	14,49477054
ENSG00000102977	ACD	7,829806175	18,5527974	18,11226211	5,829139985	0,111423376	18,73765123	21,95633486	18,76187585	20,52788145	130,4191724	14,49101916
ENSG00000182628	SKA2	10,56325053	13,18731148	0,515892544	48,5873352	0	26,22341624	14,63109037	2,452963075	14,25608955	130,417349	14,49081655
ENSG00000140577	CRTC3	21,08227174	18,41988776	5,268668666	9,505212647	0,066404871	22,50284911	8,607524625	16,40563635	28,52304243	130,3814982	14,48683313
ENSG00000071889	FAM3A	15,35411448	17,46236642	8,560783747	8,854796117	20,21709193	18,84311964	13,97943283	7,648348169	19,43040337	130,3504567	14,48338408
ENSG00000213339	QTRT1	17,0495017	17,82401633	17,18127725	19,3228815	0,104698992	18,52587489	18,29013586	9,344651035	12,67874234	130,3217799	14,48019777
ENSG00000168062	BATF2	17,28511679	13,52616839	36,73484789	0	0	7,661944928	18,05168187	25,94175468	11,09137327	130,2928878	14,47698753
ENSG00000243364	EFNA4	1,08193726	0	0	0	88,27949132	14,12285736	8,584286835	0	18,18545922	130,254032	14,47267022
ENSG00000163728	TTC14	12,28012486	9,605593168	25,58120658	20,80873848	31,72962589	10,92069619	6,321163562	0,836543209	12,11531126	130,1990032	14,46655591
ENSG00000198168	SVIP	9,442594783	22,11746165	0,106894866	33,5638335	40,32390785	7,145394007	7,816263712	4,892032628	4,752468268	130,1610003	14,46233336
ENSG00000163281	GNPDA2	10,84191927	12,15077035	0,971958388	42,99040561	0	15,82531655	5,106554771	34,47611012	7,797756179	130,1607912	14,46231014
ENSG00000160714	UBE2Q1	26,97630235	13,31110645	6,767057302	17,00125252	0,171416488	11,04750618	15,72122298	19,32135299	19,82658603	130,1438033	14,46042259
ENSG00000075131	TIPIN	0	28,5861497	2,131282248	35,82020827	0	11,93296225	21,94242024	17,37224451	12,34905697	130,1343242	14,45936935
ENSG00000103335	PIEZO1	2,288337547	6,150263729	1,378576474	24,7255068	0,031802829	10,10861466	26,02298243	15,94817918	43,43331317	130,0875768	14,4541752
ENSG00000003756	RBM5	4,416136469	7,96563812	14,63899827	28,59009753	4,468187014	12,27367017	20,93592538	11,37781063	25,36389169	130,0303553	14,44781725
ENSG00000165819	METTL3	31,22247075	15,32632145	17,78579374	17,71918101	0,061272401	8,747034329	18,17510866	10,59916374	10,36884709	130,0051932	14,44502146
ENSG00000143942	CHAC2	3,232311672	19,86995723	7,007901655	46,24668763	0	21,03984395	0	32,56383438	0	129,9605365	14,44005961
ENSG00000153914	SREK1	31,34053119	7,967816011	7,620068503	19,78470778	17,26678096	10,96064939	10,11879706	8,550719072	16,1291143	129,7391843	14,41546492
ENSG00000110660	SLC35F2	13,70971586	0,384929658	69,91403006	0,224750193	0	2,286450669	16,57087359	12,42805867	14,19327445	129,7120832	14,41245368
ENSG00000117481	NSUN4	0,463967269	9,024618567	9,815310533	20,09088373	32,78198633	19,10283565	14,90589915	5,541061477	17,97452784	129,7010905	14,41123228
ENSG00000154814	OXNAD1	45,1472271	7,74290271	25,81406662	2,739924262	0,17676007	2,01433678	12,24853938	23,69153503	10,06223993	129,6375319	14,40417021
ENSG00000089050	RBBP9	20,25031067	21,90907833	0,07233414	8,555245069	23,82903287	21,32704265	6,823653237	1,461719398	25,29214966	129,520566	14,391174
ENSG00000075420	FNDC3B	9,288468731	4,447566948	5,132325454	17,48836178	19,02859388	10,99512453	21,13144889	25,09755196	16,90528257	129,5147247	14,39052497
ENSG00000124067	SLC12A4	24,18351866	14,83214968	15,04134303	10,5342512	2,553378525	10,4168969	12,05107913	12,18522266	27,70452495	129,5023647	14,38915164
ENSG00000010219	DYRK4	23,19999263	17,94202212	16,66934815	18,27358626	0	13,50052042	13,34033867	16,72315574	9,748116422	129,3970804	14,37745338
ENSG00000247315	ZCCHC3	6,442698749	30,29988612	0,248640518	29,48549653	13,91928459	21,27058676	7,818515023	4,630418805	15,23786333	129,3533904	14,37259894
ENSG00000198954	KIF1BP	18,2585013	35,39828384	0,050968115	1,77018593	0,131689601	13,6585715	21,55586745	13,4500319	25,04532341	129,319423	14,36882478
ENSG00000144182	LIPT1	52,91190671	38,12110927	0	0	0,157576807	12,01195487	6,166159361	17,2539331	2,691064166	129,3137043	14,36818937
ENSG00000131263	RLIM	1,587030698	9,632502927	1,957372698	21,45652196	1,114340148	20,90960365	22,82095548	32,3769877	17,41132832	129,2666436	14,3629604
ENSG00000167182	SP2	15,14424994	6,552618893	25,26397934	12,75299986	28,98971923	8,290973355	10,71424307	13,15568706	8,266649261	129,13112	14,34790222
ENSG00000137842	TMEM62	0	0	45,9785878	22,87167076	2,875361696	21,74386403	11,87024422	13,0892749	10,69028668	129,1192901	14,34658779
ENSG00000072364	AFF4	0,187531955	5,25302562	31,78745924	1,850438611	27,34526805	11,19208837	19,30826136	18,86356365	13,31123039	129,0988672	14,34431858
ENSG00000108433	GOSR2	15,12578314	27,49360755	0,309597771	21,90502432	0,224979666	18,22406307	13,51998287	17,0208534	15,23925959	129,0631514	14,34035015
ENSG00000148634	HERC4	18,16530808	3,401056479	0,087707484	31,86149908	31,31825857	10,5951602	9,049825802	17,89063286	6,684704846	129,0541534	14,33935038
ENSG00000140044	JDP2	13,5441812	15,16572424	7,239296395	15,17500092	15,14731286	16,85878364	17,11961392	15,09894333	13,68832829	129,0371848	14,33746498
ENSG00000148158	SNX30	1,037135008	6,510311087	20,10442892	45,17559873	0,154048362	8,924668119	9,042130995	26,56290235	11,44799271	128,9592163	14,32880181
ENSG00000196405	EVL	24,96885216	11,84408949	7,929407517	7,692130047	13,70416113	18,04408104	12,26932745	9,362627986	23,01362201	128,8282988	14,31425543
ENSG00000119616	FCF1	11,93732126	17,28490668	22,36958033	7,970417383	3,02605892	14,88896264	9,700360616	23,87593357	17,69594019	128,7494816	14,30549795
ENSG00000198554	WDHD1	0	13,98234983	62,16028521	9,683931879	0,337410782	10,13234026	4,44038444	17,48933351	10,50588702	128,7319229	14,30354699
ENSG00000126773	PCNX4	5,450181639	3,004766991	16,57135696	30,36467697	36,21805175	6,126994349	6,870641989	11,60313743	12,51230556	128,7221136	14,30245707
ENSG00000272602	ZNF595	12,19191929	17,47120184	43,93627932	4,250404269	0	2,955902359	19,16065136	25,06222986	3,689495734	128,718084	14,30200934
ENSG00000204842	ATXN2	19,85020099	10,78806884	10,67858596	8,490189128	36,14240414	9,731061455	9,176120378	13,35432965	10,50622911	128,7171897	14,30190996
ENSG00000171612	SLC25A33	5,08263239	10,40654593	17,33329983	15,79787427	24,06505653	16,99885857	14,7058371	15,81640233	8,462868802	128,6693757	14,29659731
ENSG00000118200	CAMSAP2	26,11451806	4,344334835	0	2,348649636	52,68781224	9,642738931	15,1816359	7,180589162	11,0642844	128,5645632	14,28495146
ENSG00000049167	ERCC8	24,38072533	0	77,96504979	2,538464327	0	5,298906084	8,083716324	0	10,26795213	128,534814	14,281646
ENSG00000048471	SNX29	1,217015836	10,01468484	20,09998592	27,73545486	12,20472044	16,54847508	15,51305901	8,422440521	16,77701148	128,532848	14,28142756
ENSG00000138439	FAM117B	2,532715447	31,5029038	14,96299002	2,481090008	0	1,497339612	11,24278881	32,64405961	31,65830363	128,5221909	14,28024344
ENSG00000136891	TEX10	16,76694933	9,449938329	36,39304435	1,472788265	0	9,410126322	15,6837915	19,92433103	19,39312553	128,4940947	14,27712163
ENSG00000129315	CCNT1	0,040945826	14,82210216	34,12138866	31,46350459	0,145962963	9,575654474	12,05235949	8,505970441	17,70086779	128,4287564	14,26986182
ENSG00000129422	MTUS1	7,750550502	11,43119934	46,73120898	3,106886042	0,777440142	21,56423066	8,31593541	2,503709046	26,19801517	128,3791753	14,26435281
ENSG00000111181	SLC6A12	21,66490568	0	0	20,50909365	0	11,02928266	27,56545173	29,41368309	18,16227377	128,3446906	14,26052117
ENSG00000120314	WDR55	10,90752523	14,39407219	0,434626865	30,39424981	0,046790559	21,8548004	16,88740755	13,51875625	19,83170055	128,2699294	14,25221438
ENSG00000182518	FAM104B	29,81092891	22,53294118	0	13,46963254	0	18,52302957	13,60642656	12,6937649	17,62530043	128,2620241	14,25133601
ENSG00000179941	BBS10	0,126100615	0,074587483	0	60,3888453	0	13,98356182	31,13199893	3,033196308	19,48932932	128,2276198	14,24751331
ENSG00000143748	NVL	50,09732895	14,7579779	3,132170129	2,94011239	0,077815285	18,29869633	22,75594104	1,93320332	14,17507602	128,1683214	14,2409246
ENSG00000204138	PHACTR4	27,09540368	18,54309854	14,81648744	13,37305118	1,423316759	13,78620072	14,83283274	16,39302282	7,873776398	128,1371903	14,23746559
ENSG00000163874	ZC3H12A	15,44837927	5,812201231	29,13799808	12,83148371	0,23236334	21,20177218	9,376786377	17,21124729	16,83501334	128,0872448	14,23191609
ENSG00000168874	ATOH8	24,98461135	8,422704822	11,78185876	4,126230773	17,46139513	16,83021448	16,8310568	18,80377433	8,796522139	128,0383686	14,2264854
ENSG00000080839	RBL1	0,126898363	6,395056099	69,51836872	2,629509317	1,869775582	10,81177423	2,212694804	18,59180049	15,85657261	128,0124502	14,22360558
ENSG00000197785	ATAD3A	11,86629115	11,18350473	11,05036562	14,96401288	23,87096078	14,43405799	14,71792751	11,41285896	14,47289633	127,9728759	14,21920844
ENSG00000115255	REEP6	29,55216579	14,26568276	26,97639384	0	0	5,557595673	3,275826402	26,53243079	21,78890261	127,9489979	14,21655532
ENSG00000186184	POLR1D	20,01776962	18,72927619	4,810717146	17,364629	0	15,28863744	16,80026479	19,74943445	15,16945298	127,9301816	14,21446462
ENSG00000139990	DCAF5	10,72985246	5,366072576	2,412847152	15,16222517	0,043293254	34,44219567	25,23020058	3,585185181	30,94081341	127,9126854	14,2125206
ENSG00000132275	RRP8	24,12616393	6,237662479	0,04016473	20,99996834	0	15,31814167	17,71196566	23,87188033	19,602364	127,9083111	14,21203457
ENSG00000093167	LRRFIP2	10,43362288	7,43582837	25,60000349	32,87966468	0	16,35168884	10,15611456	14,42594233	10,60338505	127,8862502	14,20958336
ENSG00000144815	NXPE3	17,6407316	6,396463164	0,733534143	18,78379277	17,28494821	13,76458869	23,00421857	7,463900934	22,79434863	127,8665267	14,20739186
ENSG00000119522	DENND1A	22,05660338	20,07927614	26,39943608	9,439047931	1,540675884	18,50640598	14,53380991	2,158650158	13,11943078	127,8333362	14,20370403
ENSG00000198130	HIBCH	8,194491407	25,06928914	0,381631108	26,86727498	1,331160577	20,2946628	9,284528961	23,90705891	12,45100259	127,7811005	14,19790005
ENSG00000165410	CFL2	12,62938326	16,69584539	0,116163852	28,96854364	47,92235765	4,067455286	6,117093774	0,276167629	10,97630944	127,7693199	14,1965911
ENSG00000164219	PGGT1B	4,367184036	23,72773086	30,23023224	16,66314318	0	10,7597186	9,37071106	12,14229715	20,50605683	127,767074	14,19634155
ENSG00000187147	RNF220	16,64446979	13,81262084	12,56893603	23,24629742	9,972397945	11,37241901	15,99626923	8,732450633	15,36730302	127,7131639	14,19035155
ENSG00000156017	CARNMT1	8,429721917	0	48,53164951	1,56345336	0,074197839	2,22813791	16,30595844	28,74234565	21,7716897	127,6471543	14,18301715
ENSG00000134419	RPS15A	4,52494597	11,48205001	9,447744044	50,12420608	13,01189478	10,75599337	8,6226804	12,46736203	7,123328298	127,560205	14,17335611
ENSG00000155093	PTPRN2	0,031041613	12,83424117	33,00585399	0,964837622	0,073771065	14,04173328	19,12468906	26,60853543	20,80656422	127,4912674	14,16569638
ENSG00000116922	C1orf109	16,66510353	9,433953995	0	42,95952446	0	20,9539745	17,74242178	0	19,69956371	127,454542	14,16161577
ENSG00000161217	PCYT1A	16,99448617	1,581859421	0,209348637	6,681402638	0	23,76101017	29,81239343	14,06016297	34,33740188	127,4380653	14,15978504
ENSG00000049089	COL9A2	0	13,29577177	0	0,990500497	0	35,34702204	17,92008063	5,487644063	54,36506808	127,4060871	14,1562319
ENSG00000113716	HMGXB3	1,013355256	6,165168132	19,39602479	7,124362232	10,43579638	18,22552719	15,48595987	23,7419057	25,78646461	127,3745641	14,15272935
ENSG00000179921	GPBAR1	8,918552612	9,853770605	5,984723735	11,52608091	55,72053924	0	0	21,46475476	13,86605456	127,3344764	14,14827516
ENSG00000159885	ZNF222	0,075517527	0	0	0,391206858	115,0396525	3,399467089	0	0	8,358914045	127,264758	14,14052867
ENSG00000170846	AC093323.1	0,503841858	17,4340728	31,43592673	16,6029756	23,94782166	10,69498577	5,299108215	17,81169939	3,511409701	127,2418417	14,13798241
ENSG00000141985	SH3GL1	12,71215113	8,731896882	5,205054771	6,585333145	15,98243608	20,03533423	24,75357703	11,11910078	22,08976303	127,2146471	14,13496079
ENSG00000132467	UTP3	0,074807213	10,22125446	0	45,46985696	0,355561923	18,39800455	22,60950537	0,817907345	29,25702677	127,2039246	14,1337694
ENSG00000107140	TESK1	23,02163438	9,7112188	0	6,169967972	37,83579016	11,81883186	13,51970054	8,703454637	16,1871709	126,9677693	14,10752992

ENSG00000168434	COG7	50,13870957	17,8679182	0	12,65102933	0,082517818	16,04971342	11,50336913	1,214832969	17,42311146	126,9312019	14,10346688
ENSG00000188770	OPTC	126,6482266	0	0	0	0,264251319	0	0	0	0	126,9124779	14,10138644
ENSG00000189042	ZNF567	18,20108271	14,53098053	0	9,997720238	0,076019802	10,04454381	14,44874272	47,91441125	11,68425426	126,8977553	14,09975059
ENSG00000148843	PDCD11	6,381624726	16,60159672	2,282683295	27,48115999	5,476636139	14,66206609	19,39622181	8,527899422	26,0650232	126,8749114	14,09721238
ENSG00000148339	SLC25A25	18,42961174	5,975611833	22,52675032	13,74454891	0	12,47018314	22,49620013	10,77795084	20,4446235	126,8654804	14,09616449
ENSG00000133065	SLC41A1	0,071077409	4,07804547	0,087168422	73,72292635	0	9,988971692	21,85599543	3,678402994	13,31637828	126,798966	14,088774
ENSG00000148429	USP6NL	20,58857585	2,209027884	11,37209108	13,06328453	25,96913928	9,777999768	4,982767726	24,12763319	14,66933893	126,7598582	14,08442869
ENSG00000087269	NOP14	0	1,2054712	0,234318712	21,701813	23,71245363	11,18812543	21,32889881	16,99059451	30,33909228	126,7007676	14,07786306
ENSG00000167264	DUS2	0,723882973	8,606234254	0	71,18681103	0	10,21293849	9,67699327	2,690964143	23,59249265	126,6903168	14,07670187
ENSG00000093183	SEC22C	0,037041444	21,82207232	25,17799892	2,878312013	0	31,84409402	19,62252036	13,3648005	11,86739038	126,61423	14,06824777
ENSG00000115942	ORC2	15,47127606	14,76137576	48,57858358	3,177222294	4,619073174	9,218834994	20,52798778	2,096737952	8,159271086	126,6103627	14,06781807
ENSG00000119431	HDHD3	0	40,25314586	0	0	0,511806474	16,39401423	39,16104899	0,156976042	30,10625373	126,5832453	14,06480504
ENSG00000170653	ATF7	17,6374517	1,276067126	22,31499605	7,123835249	47,80738228	7,147556467	8,548010095	7,903945197	6,796140827	126,555385	14,06170944
ENSG00000020426	MNAT1	39,63875429	6,155439259	2,509492769	33,04593049	0,092627687	8,430328504	3,592263826	27,35865261	5,704342572	126,527832	14,058648
ENSG00000135482	ZC3H10	20,05652678	1,987899558	29,51648068	8,642710506	32,67827929	6,050786308	5,311072286	20,11134699	2,111134714	126,4662371	14,05180412
ENSG00000177000	MTHFR	0,012615676	6,827793457	16,70943991	3,311246964	43,83284746	15,73504885	10,19223852	11,282997	18,51017708	126,4144049	14,04604499
ENSG00000138111	MFSD13A	0,085712178	3,193977312	0	19,09280302	69,46062504	14,02193757	13,73554967	0,281141152	6,430310754	126,3020567	14,03356186
ENSG00000167985	SDHAF2	23,95211694	10,65251124	15,17066351	20,41826647	0,288216326	14,44726932	15,25582287	11,27084199	14,84087397	126,2965826	14,03295363
ENSG00000197763	TXNRD3	26,7278575	22,25628375	0,073715221	56,3248659	0,095231382	5,323569287	6,421578912	0,262875551	8,772389542	126,2583671	14,02870745
ENSG00000174276	ZNHIT2	27,19064633	2,053154671	0	0	52,24527798	15,3715006	9,126861822	20,24101486	0	126,2284563	14,02538403
ENSG00000183688	RFLNB	4,423562687	2,44371334	34,7763007	30,12173968	15,76903873	6,643754107	17,81048071	7,026639519	7,082800352	126,0980298	14,0108922
ENSG00000143258	USP21	0,040146273	0,071238576	30,05789062	0,277295403	0,190816973	12,73861761	11,30033011	34,6763162	36,73480674	126,0874585	14,00971761
ENSG00000144524	COPS7B	10,86122796	24,04702408	18,78863181	14,06622761	2,782480134	19,96615908	13,9204638	0,108946445	21,53809844	126,0792594	14,0088066
ENSG00000168389	MFSD2A	0	29,77868481	1,109977935	3,190869417	7,617916421	18,17687626	33,16023998	10,47296982	22,49452541	126,00206	14,00022889
ENSG00000188785	ZNF548	0	0,783404642	0	8,936419354	72,74447214	8,886623975	3,502823747	12,97925099	18,09899584	125,9319907	13,99244341
ENSG00000172992	DCAKD	0	1,135393084	0,294262631	13,90302793	0,760305312	20,89981506	44,76023488	28,09976386	16,06647775	125,9192805	13,99103117
ENSG00000011198	ABHD5	7,246261304	29,09104492	39,25378004	8,226589476	0,142714615	10,26364278	8,626189205	11,90597461	11,12764976	125,8838467	13,98709408
ENSG00000183354	KIAA2026	9,443299167	3,597527128	11,18855994	47,66047788	0,042264039	18,0808749	13,54081375	1,24442817	21,08462369	125,8828687	13,98698541
ENSG00000184922	FMNL1	5,313822715	5,628866695	12,22829958	16,25828295	23,76830906	16,41580783	15,13226895	5,301798115	25,79338015	125,840836	13,98231512
ENSG00000102981	PARD6A	43,36304682	7,208118282	0	17,36059307	0	19,17783548	13,91304085	10,6591796	14,11334477	125,7951589	13,97723987
ENSG00000110200	ANAPC15	14,57118926	25,01914604	3,95323762	12,12809108	31,51465079	8,689731905	9,356961977	3,323830197	17,1471297	125,7039686	13,96710762
ENSG00000198042	MAK16	2,703291459	39,65052673	0	7,740598205	0,406609536	22,05378363	15,62696617	8,305760189	29,12268873	125,6102247	13,95669163
ENSG00000129197	RPAIN	1,179597994	13,93039193	6,77178976	20,93098854	22,3783879	9,892111101	7,261405486	29,26324972	13,95724891	125,5651713	13,95168571
ENSG00000185163	DDX51	33,46376789	10,51620066	13,83551918	23,67279026	0,226251405	19,023925	4,327926084	3,695204645	16,80200091	125,563586	13,95150956
ENSG00000144580	CNOT9	13,45681202	11,50845794	4,099536343	23,08897109	1,357978984	26,91459157	11,00741805	13,18239625	20,94243284	125,5585951	13,95095501
ENSG00000175634	RPS6KB2	16,11153118	11,75859158	9,799814324	7,807856506	0	19,97203847	18,05996008	14,83119899	27,19783155	125,5388227	13,94875808
ENSG00000189144	ZNF573	0,193359655	14,63944381	51,99158601	33,27769563	0,076587234	6,192013419	3,532114273	2,959746606	12,64342943	125,5059761	13,94510845
ENSG00000141376	BCAS3	25,53694702	4,944111767	15,33710496	6,032536821	34,61036381	6,753611164	11,24703919	11,22460519	9,818853918	125,5051738	13,94501932
ENSG00000188690	UROS	14,18841384	2,447372431	0,087488491	15,08342121	22,00216583	14,75998416	17,03300562	29,70860516	10,13850101	125,4489578	13,93877308
ENSG00000131355	ADGRE3	0,106528425	17,8635312	63,19956827	0	0	7,60252907	28,61448883	0	8,057498146	125,4441399	13,93823777
ENSG00000067533	RRP15	0,709799558	20,28851492	35,00777684	26,53407575	0,091181298	10,86833281	9,605003199	4,278827712	18,04907827	125,4325904	13,93695448
ENSG00000034533	ASTE1	17,461274	9,154541585	0,162229413	0	0	16,26669208	27,20293454	24,6837611	30,47731121	125,4087439	13,93430488
ENSG00000197355	UAP1L1	0	0	0,105555382	1,634868836	70,77349705	5,512502549	19,00993449	8,093043328	20,25363656	125,3830382	13,93144869
ENSG00000248333	CDK11B	14,85978376	10,37820241	13,8671265	16,15881723	19,35610607	14,03200118	8,525363728	15,44175301	12,73432306	125,3534769	13,92816411
ENSG00000177853	ZNF518A	0,166497736	5,761199892	48,98024148	27,86405063	3,066562431	7,053217979	6,532152993	16,11061935	9,811931899	125,3464744	13,92738604
ENSG00000140522	RLBP1	0	0	0	0	125,34135	0	0	0	0	125,34135	13,92681667
ENSG00000134744	TUT4	14,86012341	2,648479537	28,45181827	3,350483037	7,493161881	13,58092627	9,914317239	24,82491936	20,1919797	125,3162087	13,92402319
ENSG00000103876	FAH	17,01383768	22,89425657	9,648066742	5,904817557	0,049856663	10,50329349	16,23467297	26,10263347	16,89982953	125,2512647	13,91680719
ENSG00000101251	SEL1L2	15,20047161	18,76373142	0,143030666	17,92375565	0	27,36009253	23,33806278	2,295274845	20,22461511	125,2490346	13,9165594
ENSG00000198585	NUDT16	3,848170742	11,44438754	7,197663681	18,67268349	9,962714577	27,40392368	14,40525792	12,97481016	19,32761528	125,2372271	13,91524745
ENSG00000176619	LMNB2	13,97667743	19,9801824	0	16,54119765	2,729160192	33,18211534	19,46592547	7,411044054	11,91897993	125,2052825	13,91169805
ENSG00000104341	LAPTM4B	0	0	5,130067513	98,75862348	0	5,345257867	10,91046365	1,033577002	3,778549874	124,9565394	13,88405993
ENSG00000104365	IKBKB	16,53465011	3,601943067	24,90222931	10,77073465	0,031947117	15,79257335	24,9690537	8,671665992	19,67417147	124,9489688	13,88321875
ENSG00000159685	CHCHD6	20,72779815	22,02976258	3,52128247	17,8489722	0	14,6515312	14,71313339	16,62338215	14,79776869	124,9136308	13,87929232
ENSG00000122025	FLT3	49,5587445	0,06447271	21,05403491	8,532617587	3,022148108	0	9,593547866	32,97195132	0	124,797517	13,86639078
ENSG00000101199	ARFGAP1	1,897454972	10,22266022	15,05550309	8,487294938	47,80989974	12,44956824	15,66006413	2,499505049	10,59971844	124,6816688	13,85351876
ENSG00000186654	PRR5	34,91602768	5,372049229	12,72948364	6,024257189	0,068520806	15,89145383	10,84488846	23,51690397	15,28331446	124,6468993	13,84965547
ENSG00000139946	PELI2	17,84751452	0,816458013	36,26214753	7,234254905	0,63305977	12,89529322	11,38080255	22,66442226	14,86176516	124,5957179	13,84396866
ENSG00000108854	SMURF2	22,26958777	9,033482049	2,04255445	1,157620849	0,099575085	32,3632582	26,19691113	13,97234758	17,41747526	124,5528124	13,83920138
ENSG00000111445	RFC5	20,13877411	24,9242487	0,171117328	8,914675956	0	7,608702964	12,47365874	16,17085141	34,09188282	124,493912	13,83265689
ENSG00000131368	MRPS25	13,83584184	12,78055335	6,314200722	7,724569161	0,066862324	14,08587283	20,74430363	11,81222062	17,09331261	124,4677371	13,82974856
ENSG00000102125	TAZ	14,96694961	10,76695332	21,48683382	18,90326987	0,060083243	10,27052257	13,225081	17,30400195	17,47461026	124,4583056	13,82870063
ENSG00000187742	SECISBP2	2,571636126	19,81075615	33,62502573	19,43783414	5,708991808	11,63216924	11,96782133	0,505097168	19,05244078	124,3117725	13,81241916
ENSG00000131876	SNRPA1	0	17,39378469	20,98677771	19,84215261	15,85637554	13,43021022	8,993921318	7,835324986	19,95735344	124,2959005	13,81065561
ENSG00000047648	ARHGAP6	22,63675133	4,698440772	15,64152581	7,759788855	44,3579294	3,214210333	7,385161447	13,00698754	5,572643745	124,2734392	13,80815992
ENSG00000165275	TRMT10B	16,71390471	20,58312632	5,334579708	7,465690132	26,1882646	12,61985545	9,324906042	4,381198034	21,65831767	124,2698427	13,8077603
ENSG00000100629	CEP128	0,937069721	8,11335251	82,08927974	1,785506081	0	4,008958659	12,26129407	5,12274268	9,935128957	124,2533324	13,80592582
ENSG00000153975	ZUP1	0,05689404	0	18,83901273	29,9642949	0	23,61227334	18,04580829	0	33,72666556	124,2449489	13,80499432
ENSG00000114737	CISH	12,20826794	13,74280204	0	0,153130854	0,07024991	29,27451882	16,54284111	14,99624798	37,22747238	124,215531	13,80172567
ENSG00000150907	FOXO1	26,00373109	20,59344233	0,088585118	25,11228882	0	16,81311772	15,99366883	7,423721163	12,17063745	124,1991925	13,79991028
ENSG00000069943	PIGB	16,66807484	17,00239895	0	21,60095783	0	30,46442558	13,50836467	15,56869164	9,247317048	124,0602306	13,78447006
ENSG00000172009	AC006538.1	7,14725917	11,59865005	40,26797014	2,180025391	0	18,17616209	22,65600928	6,144720431	15,82738203	123,9981786	13,7775754
ENSG00000151292	CSNK1G3	0,030222114	40,91846921	0,500364178	7,097431309	10,62987925	24,75358804	18,4441388	0,132173827	21,48385797	123,9903947	13,77671052
ENSG00000137040	RANBP6	43,58895361	19,83419988	0	17,82078269	0,078358617	14,62532686	22,20301969	0,144200117	5,677179384	123,9720208	13,77466898
ENSG00000126001	CEP250	6,104065605	0,975663152	0,565731931	7,949545325	87,28228396	2,660299102	8,42659214	0,896646375	9,06596356	123,9267912	13,76964346
ENSG00000153823	PID1	0	1,913022593	59,0831661	0	2,348568945	0	2,983836671	47,34526205	10,22045381	123,8943102	13,76603446
ENSG00000095209	TMEM38B	25,71314279	0	0	23,61141902	11,41313752	16,95356368	10,93815518	16,33576321	18,9086839	123,8738653	13,76376281
ENSG00000113569	NUP155	22,55603299	45,09048702	3,035379985	4,558811524	0,039213534	11,21409117	14,55006471	7,504956327	15,30118779	123,8502251	13,76113612
ENSG00000198242	RPL23A	6,567081252	22,9458432	14,19790828	14,73020018	5,1486386	11,22423704	14,44707676	19,3937851	15,15415191	123,8089223	13,75654692
ENSG00000141905	NFIC	4,024064784	7,742494238	26,00834106	12,22009523	13,99682697	15,38748232	15,23412867	14,09260782	15,07478678	123,7808279	13,75342532
ENSG00000108384	RAD51C	17,58142104	18,38283982	4,489461058	14,57761312	0,118364384	16,10437206	12,79929722	22,81675738	16,78376635	123,6538924	13,73932138
ENSG00000213516	RBMXL1	0,623810101	33,78789504	1,584710609	42,01017185	2,117854584	5,93586101	20,94049771	1,55896002	15,08838484	123,6481458	13,73868286
ENSG00000115364	MRPL19	0,152005715	20,49951135	9,320893003	23,652427	1,725947728	21,08419869	6,212513365	21,75320893	19,23481386	123,6355196	13,73727996
ENSG00000141401	IMPA2	11,19777206	3,08935533	23,65633207	12,09360841	0,188068888	31,49650906	14,0287593	0,086523846	27,78695057	123,6238795	13,73598661
ENSG00000197780	TAF13	2,26665856	0	15,28890544	57,03293016	0,25651253	19,79076165	17,92433314	0,708074073	10,35430624	123,6224818	13,73583131
ENSG00000118046	STK11	16,79392674	9,347333873	11,35330593	10,29690477	0,12394761	20,48113769	24,49645115	14,06589853	16,65605624	123,6149625	13,73499584
ENSG00000180694	TMEM64	15,67739992	23,75363544	0	35,33957492	6,056667239	10,40120822	1,474864209	14,57965128	16,30510374	123,588105	13,73201166
ENSG00000244617	ASPRV1	0	10,59265595	6,001407741	0,239719775	99,14085574	0	1,729035351	0,151784367	5,719667888	123,5751268	13,73056965
ENSG00000164327	RICTOR	15,26730138	3,307216692	28,39338825	25,26482657	14,40183349	13,56447625	7,209447437	7,223680353	8,853102427	123,4852728	13,72058587
ENSG00000173914	RBM4B	23,3480094	9,998303429	15,09423516	13,49978195	26,36262546	10,9807979	6,725279972	9,702809487	7,7093174	123,4211602	13,71346224
ENSG00000172667	ZMAT3	0,053499937	4,746716235	0,738130905	28,22291901	42,94280401	10,96965842	18,9016202	2,544504105	14,29462098	123,4144738	13,71271931
ENSG00000127663	KDM4B	17,51441223	1,621349141	8,045351054	0,496377423	43,08727358	12,46668237	8,584049482	4,804198481	26,79301041	123,4127042	13,71252269
ENSG00000161277	THAP8	30,96731695	35,85021809	0,304635725	1,000846478	0	24,00021493	27,98160281	0	3,258678747	123,3635137	13,70705708
ENSG00000198862	LTN1	1,225772993	1,87300631	29,28249127	0,029397811	0,080918779	20,59863176	16,83586812	18,01828872	35,40631694	123,3506927	13,70563252
ENSG00000136169	SETDB2	0	19,9560608	42,33389648	26,60927189	0	7,726593195	11,74288073	0,098962135	14,86104266	123,3287079	13,70318977
ENSG00000196465	MYL6B	5,471324743	22,95539062	0	23,29919534	29,20102753	6,719912895	11,74239069	13,58644739	10,3216387	123,2973279	13,6997031
ENSG00000119596	YLPM1	11,51414994	3,536924943	13,45695424	40,39381729	6,203704448	16,66326515	8,157461826	3,385101361	19,98456744	123,2959467	13,69954963
ENSG00000107796	ACTA2	8,835093849	65,50377489	0,840665622	0	25,46162683	2,22998087	2,951251089	6,773020369	10,55378436	123,1491979	13,68324421
ENSG00000122390	NAA60	10,91845326	10,61767887	0,983464767	6,000545749	23,40689928	21,24395873	18,3877463	10,74620515	20,81258509	123,1175372	13,67972635
ENSG00000158769	F11R	6,031285373	8,636069159	10,05144401	20,9841009	0,638620407	17,04672987	22,08932342	8,814200082	28,78950276	123,081276	13,67569733
ENSG00000145725	PPIP5K2	7,787400573	6,707431618	34,83201704	4,910472886	0,603034669	20,21284617	16,24502967	13,25947628	18,47703988	123,0347488	13,67052764
ENSG00000167114	SLC27A4	29,90388809	9,881975228	0	4,848590423	0	21,37466705	36,2927501	11,25667464	9,439092491	122,997638	13,66640423
ENSG00000119682	AREL1	0	20,5415072	0,266665848	6,758491188	0	29,69428713	41,57698544	6,656691986	17,49713044	122,9917592	13,66575103
ENSG00000099377	HSD3B7	8,55686364	7,430440966	21,54624864	6,706762399	0	19,12302631	22,77614059	22,02897117	14,77815719	122,9466109	13,66073454
ENSG00000145868	FBXO38	33,95743161	5,626001607	0,063742284	26,44651657	0,123521283	17,42422475	16,03983263	0,037885183	23,20418055	122,9233365	13,6581485
ENSG00000120253	NUP43	0,410254943	0,485324887	45,53339178	30,28498687	10,23728741	18,31772883	10,24769001	1,420420087	5,970634878	122,9077197	13,6564133
ENSG00000004975	DVL2	0,085196713	6,097572263	26,59121789	9,268316143	24,97152616	12,28505304	13,41714319	0	30,17692074	122,8929461	13,65477179
ENSG00000108106	UBE2S	6,316519031	17,62705538	1,291082989	12,11916638	5,388687667	23,53215397	8,113658482	26,20810075	22,2431529	122,8395775	13,64884195
ENSG00000103245	CIAO3	13,42333831	7,255139265	22,35792494	9,961565005	25,51180959	14,55355936	13,26940996	6,123694728	10,37838251	122,8348238	13,64831375
ENSG00000277775	HIST1H3F	1,470662488	8,481377132	31,78847847	15,23707881	30,58178585	6,862481275	19,53795924	5,627839349	3,165269236	122,7529318	13,63921465
ENSG00000164331	ANKRA2	0,379811129	12,90162586	0	38,13304656	37,00780419	10,60397941	12,07398527	0	11,54450569	122,6447581	13,62719534
ENSG00000167785	ZNF558	0	8,533282745	0,068048984	0,031938185	65,6697195	3,249166741	14,12885012	12,65924353	18,28893561	122,6291854	13,62546504
ENSG00000147394	ZNF185	49,33389578	18,890303	0	0	0	15,3084572	18,74754129	10,506336	9,809853836	122,5963871	13,62182079
ENSG00000140718	FTO	7,273154835	14,53653483	0,091017386	12,64458738	30,65576102	12,14509811	15,75788584	14,45139252	14,9866413	122,5420732	13,61578591
ENSG00000148399	DPH7	0,030682349	2,395583351	0,05644266	0,317890324	57,96922765	9,061917794	20,83953941	20,12799396	11,73568902	122,5349665	13,61499628
ENSG00000184634	MED12	10,21649919	4,622515288	2,021673447	10,82396932	24,32809748	10,77022195	29,48764032	10,41369546	19,79859921	122,4829117	13,60921241
ENSG00000128872	TMOD2	39,09717223	15,00855179	30,95060869	2,497954479	0,061943517	9,729986387	8,353867728	6,013082686	10,70681129	122,4199788	13,60221987

ENSG00000188419	CHM	0,08740778	26,55362703	6,045842042	25,32675193	0	20,21099459	15,53138075	17,54621315	11,11553124	122,4177485	13,60197206
ENSG00000163516	ANKZF1	46,00735302	5,609115217	3,861465667	17,44115127	0	13,77391289	8,755651539	11,93430943	15,00378853	122,3867476	13,59852751
ENSG00000008294	SPAG9	27,22667477	4,907496074	14,31019062	11,25208481	3,93150577	11,75766079	21,69464457	5,881878897	21,37095225	122,3330886	13,5925654
ENSG00000198692	EIF1AY	24,11458771	31,45986181	40,28396662	0,064860794	0,178532211	0	0	0,246409073	25,96215399	122,3103722	13,59004136
ENSG00000132024	CC2D1A	12,2351881	6,864520344	18,9218444	11,91010997	0,498873347	16,49557431	26,16973982	5,967363692	23,23542549	122,2986395	13,58873772
ENSG00000005801	ZNF195	4,258089687	15,89023551	4,177653268	23,08330917	36,18467253	10,48363306	3,782002152	12,86637886	11,55292189	122,2788961	13,58654401
ENSG00000175538	KCNE3	8,30142379	11,15739081	27,2158922	0,567712754	0	16,37898813	25,08041719	20,12981864	13,44443158	122,2760751	13,58623057
ENSG00000165724	ZMYND19	24,13220652	25,89498412	0	16,25389265	0	19,69841295	6,976402239	13,12344645	16,16056715	122,2399121	13,58221245
ENSG00000115523	GNLY	6,636780226	6,679381189	47,83335037	20,35480956	22,12851457	9,842613695	2,302962501	0,108303693	6,274071384	122,1607872	13,5734208
ENSG00000127989	MTERF1	0	25,16915706	55,36863321	1,387384323	0	5,86963799	2,386245578	16,96095633	14,93613154	122,078146	13,56423845
ENSG00000221963	APOL6	1,020915927	10,94946468	0,552369794	29,21745816	10,84666993	21,13237549	16,09361139	16,28371043	15,95344215	122,050018	13,56111311
ENSG00000068400	GRIPAP1	38,39061129	7,682478003	13,28746686	5,95027862	0,05249489	10,06474044	21,12875639	8,549469149	16,92477465	122,0310603	13,5590067
ENSG00000114999	TTL	15,92761133	12,81899454	17,44223507	24,15265881	3,173384513	16,78091084	14,73259397	1,113352325	15,72547496	121,8672164	13,54080182
ENSG00000134256	CD101	16,69795491	9,176228321	0	0	0,09381336	40,22074587	29,76151031	0,08632042	25,77965082	121,816224	13,535136
ENSG00000115138	POMC	3,144043083	14,9936359	0	33,25309169	27,08558837	14,2393993	3,018467025	7,94933615	18,12547356	121,8090351	13,53433723
ENSG00000164430	CGAS	12,99681238	4,111425013	0	12,62790246	0	18,12535289	23,41671984	19,23708671	31,25641079	121,7717101	13,53019001
ENSG00000007923	DNAJC11	8,948314336	10,18127209	0	23,4086855	0	20,2043374	22,11713899	1,170140117	35,6483099	121,6781983	13,51979981
ENSG00000155363	MOV10	17,71352979	7,621181444	0,042707036	38,28436023	2,648278082	15,67598781	18,31261567	8,528653763	12,81995522	121,647269	13,51636323
ENSG00000197223	C1D	8,692761116	33,92022509	11,02294724	2,25886183	3,409661111	20,61720375	10,47629971	17,25530973	13,90864786	121,5619174	13,50687971
ENSG00000169193	CCDC126	17,16875105	17,00928955	0,783704818	3,163294025	0	23,85519992	20,37522166	21,14706705	18,02402133	121,5265494	13,50294993
ENSG00000175130	MARCKSL1	18,37567095	7,458024582	6,65282255	30,38052744	0,23228819	10,1950225	16,96203065	25,00701449	6,250982638	121,514384	13,50159822
ENSG00000164944	VIRMA	23,54660308	9,580943855	5,237119266	5,44875817	0,23330142	10,3472768	11,93569284	32,56812164	22,56030173	121,4581188	13,49534653
ENSG00000140995	DEF8	5,478189767	20,50377518	0,889197575	5,763224877	10,88566503	21,2031948	21,92147309	17,41513486	17,32491571	121,3847709	13,48719676
ENSG00000167074	TEF	7,391627234	0,171081667	30,62392413	20,92141332	0,152750975	20,50064761	19,21281137	5,973402433	16,40287255	121,3505313	13,48339237
ENSG00000180228	PRKRA	14,00252392	25,46831352	6,940558456	11,75382768	0	15,75830188	17,05233856	12,3328217	17,98669044	121,2953762	13,47726402
ENSG00000167566	NCKAP5L	10,79628057	4,905440793	17,4897244	5,063880224	20,19758736	11,34098256	22,53502996	6,045032879	22,78859705	121,1625558	13,4625062
ENSG00000165861	ZFYVE1	22,0954021	3,951566317	0,159605798	2,696744235	0,20619189	16,44816818	26,5588875	21,56520335	27,42347726	121,1052466	13,45613851
ENSG00000100239	PPP6R2	0,282502469	5,263583849	9,042538273	2,195190476	0,067137323	23,82114842	23,80863469	17,17345481	39,43462381	121,0888141	13,45431268
ENSG00000100425	BRD1	16,58341647	21,08467009	25,56466551	6,869057451	4,849621512	19,39877512	9,02957432	16,77590881	0,921290452	121,0769797	13,45299775
ENSG00000157045	NTAN1	7,173375494	16,21429444	11,7035984	28,60773386	0	11,5795175	10,17518657	19,04737264	16,48934953	120,9904284	13,44338094
ENSG00000187626	ZKSCAN4	0,0282661	25,83111299	0	48,07724467	0	10,70691152	17,62592954	9,395071879	9,316660675	120,9811974	13,44235526
ENSG00000125814	NAPB	0	0	0	0,64809986	99,28220936	8,400134281	3,26021497	0	9,373993481	120,964652	13,44051688
ENSG00000111364	DDX55	15,96759704	0	3,258339159	1,460797618	38,51278052	11,37805215	13,7192018	12,19763711	24,41755392	120,9119593	13,43466215
ENSG00000065485	PDIA5	18,83773281	9,625948368	10,25252394	0,064159079	0	11,42241492	11,44565851	39,56765045	19,60367791	120,819766	13,42441844
ENSG00000118873	RAB3GAP2	25,81575736	25,43239893	0,161256946	2,901239687	0	5,80678998	16,44955721	20,95765989	23,28696634	120,8116263	13,42351404
ENSG00000135473	PAN2	14,02784204	10,31032134	0	35,33121189	1,282210396	7,951495643	20,33163853	12,88703889	18,68163338	120,8033921	13,42259912
ENSG00000089682	RBM41	0	10,81182298	36,54155702	29,6390653	0,117724157	4,106293572	10,38559542	3,953732737	25,22224561	120,7780368	13,41978187
ENSG00000165671	NSD1	9,616925236	1,990339509	32,90618354	17,46527907	0,695380254	16,673515	17,94631483	7,571436873	15,88206258	120,7474369	13,41638188
ENSG00000101846	STS	43,40286748	0	57,37788886	4,321572958	0	6,589608858	8,966289635	0	0,028499697	120,6867275	13,40963639
ENSG00000151657	KIN	17,50026154	10,67718291	21,77294533	7,726050152	4,295163138	15,87475763	14,75045035	17,39890299	10,68021146	120,6759255	13,40843617
ENSG00000107863	ARHGAP21	3,697847418	8,100022771	20,05862134	23,92761554	4,152578251	10,42518055	16,10554997	17,830908	16,37568406	120,6740079	13,4082231
ENSG00000148481	MINDY3	13,33001849	0,854164666	42,64041705	6,393897932	0,175994875	14,06641988	8,30114914	19,67547168	15,2101785	120,6477122	13,40530136
ENSG00000032742	IFT88	0	1,491871707	0,04548848	81,17123003	0	3,502285124	4,024248907	23,14285631	7,268420688	120,6464012	13,40515569
ENSG00000116539	ASH1L	9,768376326	1,282496675	19,79785026	14,43030276	1,449244996	19,72675641	11,78657853	19,63197677	22,70824781	120,5818305	13,39798117
ENSG00000138074	SLC5A6	36,42554379	17,65859986	3,13399181	1,120694236	0,064265845	17,69554469	32,3779919	0,118265772	11,97293272	120,5678306	13,39642562
ENSG00000145604	SKP2	0	12,71875007	29,05134733	16,66121157	1,310306383	12,02062557	15,10745202	19,20430246	14,48217622	120,5561716	13,39513018
ENSG00000085788	DDHD2	26,20324606	16,86238294	22,73578575	9,547592651	0,033606358	9,082695805	8,359992175	9,771413376	17,94812006	120,5448352	13,39387058
ENSG00000204569	PPP1R10	22,62882248	6,082421378	6,359021872	1,153691737	28,85642689	10,78004132	12,9281234	14,16511122	17,54145376	120,4951141	13,38834601
ENSG00000164187	LMBRD2	19,08784148	4,237876438	0,033283058	39,02152578	0,12899337	22,78489362	14,12142147	18,87180181	2,047159968	120,334797	13,370533
ENSG00000121741	ZMYM2	6,790987608	11,59756484	1,939206154	53,22935522	0,026370799	21,53986632	12,21718756	1,455872672	11,40899491	120,2054061	13,35615623
ENSG00000109689	STIM2	16,77604833	35,1724541	0	11,20159681	4,025405316	21,05042376	16,41318319	3,506877714	11,92289876	120,068888	13,34098755
ENSG00000114416	FXR1	6,35036001	24,63726816	4,869450951	3,076545094	19,93081189	12,63122464	13,56778729	24,6838017	10,26741777	120,0146675	13,33496306
ENSG00000054965	FAM168A	0,881334633	22,98262864	4,652386594	14,62321019	0,136598533	23,70752123	18,4538589	16,75844136	17,79055716	119,9865372	13,33183747
ENSG00000198515	CNGA1	0,042197869	0,074879084	66,91394731	2,33172834	12,83637124	0,926615899	2,417607626	34,41834507	0	119,9616924	13,32907694
ENSG00000125386	FAM193A	18,84810682	8,014590605	0,679071948	8,811570489	19,76462404	9,178843516	18,01190712	22,88683166	13,62002103	119,8155672	13,3128408
ENSG00000141096	DPEP3	0	14,37885799	0,163806776	74,26729094	0	4,692815369	5,323431882	20,8370756	0	119,6609105	13,29565672
ENSG00000165028	NIPSNAP3B	20,23754357	11,47819219	24,98364655	8,064420174	0,701650616	14,91132359	5,905797155	22,18548251	11,19040338	119,6584597	13,29538441
ENSG00000134028	ADAMDEC1	22,74716138	0,109984413	7,525302105	0,214056583	0,147300058	16,53661614	0,102928902	72,24020693	0	119,6235565	13,29150628
ENSG00000040933	INPP4A	3,873160907	6,608489424	43,88826618	25,03092006	0,408490417	13,80176709	13,40621049	1,378167985	11,21815665	119,6136292	13,29040325
ENSG00000124164	VAPB	11,42073279	12,26081512	4,96346138	30,17330268	0	8,511048007	16,86766957	24,19336701	11,20170819	119,5921048	13,28801164
ENSG00000164136	IL15	10,9361383	11,81377273	24,45914279	0	32,51025999	9,142355293	9,144231916	15,31343539	6,205895805	119,5252322	13,28058136
ENSG00000100614	PPM1A	15,06410073	9,188035276	0,280151271	13,32396612	30,85388812	16,73457652	10,7588149	14,26416122	9,021524303	119,4892185	13,27657983
ENSG00000163029	SMC6	0,015381776	20,00693058	26,3718905	15,16631418	0	6,704651766	12,31203176	16,07774056	22,79571044	119,4506516	13,27229462
ENSG00000144233	AMMECR1L	22,50312846	2,252820837	0,058386914	0,822101369	0,678861139	12,85885377	9,750897467	39,213512	31,30621946	119,4447814	13,27164238
ENSG00000149179	C11orf49	12,75623374	4,687376963	2,3673787	19,454139	24,52065096	8,626457596	13,81617133	15,99593603	16,96581878	119,1901631	13,24335146
ENSG00000256269	HMBS	20,33414012	15,56497098	0,056419748	10,27428152	0	14,95114594	20,85654595	15,49226407	21,53812953	119,0678979	13,22976643
ENSG00000080200	CRYBG3	0,013763601	0	1,139366468	5,299981723	84,19423937	5,576191165	3,737025099	13,21256719	5,789189418	118,962324	13,218036
ENSG00000256060	TRAPPC2B	20,90786518	19,35045361	13,42388975	15,00765453	0,584564638	12,87314838	9,12263944	16,49483462	11,19314377	118,9581939	13,2175771
ENSG00000105618	PRPF31	2,261288001	9,529925181	15,18334996	14,25234055	22,70514952	16,57243695	15,20852509	7,169923276	15,99128325	118,8742218	13,20824686
ENSG00000141569	TRIM65	13,83242916	5,362840002	1,069154227	36,33017723	0,092081421	15,86785772	25,06189452	6,862871787	14,39119224	118,8704983	13,20783315
ENSG00000086712	TXLNG	0	24,85805747	0	21,61279588	0	15,23174543	14,05380374	26,25675351	16,76998922	118,7831452	13,19812725
ENSG00000170468	RIOX1	24,69777544	6,513123921	0	19,33647403	0,591383355	27,2533224	10,69261372	29,52009105	0	118,6047839	13,17830932
ENSG00000185798	WDR53	0	3,581195327	0,154691207	2,323294153	0	29,45212797	20,73716672	14,15885459	48,19392286	118,6012528	13,17791698
ENSG00000066422	ZBTB11	4,645794244	17,57241263	0,187419161	25,61497593	12,29987268	6,286525358	12,80758378	16,79798166	22,35370791	118,5662733	13,17403037
ENSG00000104691	UBXN8	6,057571043	10,41570852	37,14463722	4,135389591	0,223193003	14,07507378	14,89424078	14,78638214	16,80588502	118,5380811	13,1708979
ENSG00000068097	HEATR6	0	10,63093797	1,027863965	0,375214631	0,368855323	22,63036626	24,30536629	51,52009793	7,635436412	118,4941388	13,16601542
ENSG00000088970	KIZ	0,087061977	1,132921836	97,26901101	0,100224688	0	5,28925595	10,40967609	0	4,068850305	118,3570019	13,15077798
ENSG00000153064	BANK1	36,79384989	19,81387826	1,402568859	17,83735178	2,162654471	11,01749923	10,00658572	8,174813519	11,13143232	118,3406341	13,14895934
ENSG00000110536	PTPMT1	12,02266584	14,38572044	6,594418407	18,18926283	5,373656656	20,70854644	16,57673729	9,767821157	14,65067475	118,2700038	13,14111153
ENSG00000197111	PCBP2	4,575641807	19,65562731	6,593522408	29,49756967	0,045308799	16,15984926	16,98582718	9,672069097	15,07687532	118,2622909	13,14025454
ENSG00000108961	RANGRF	4,895131162	16,05075353	0,783042532	19,47824458	35,65885456	12,38487404	8,129063763	6,98101199	13,87305025	118,2340264	13,13711404
ENSG00000063241	ISOC2	19,81440786	11,74033712	8,261205301	14,44376901	0	19,34619701	28,3788387	1,949024219	14,25048197	118,1842612	13,13158458
ENSG00000123427	EEF1AKMT3	0	0,169817606	0	21,40030349	4,321237052	25,53278666	41,59830196	19,35731317	5,708395396	118,0881553	13,12090615
ENSG00000141084	RANBP10	59,66246044	11,67098854	17,17999682	1,131688242	0	6,424666074	5,966448563	5,169437937	10,85256179	118,0582484	13,11758316
ENSG00000160199	PKNOX1	2,716460856	11,11834038	12,00045303	35,16339311	0,047122102	14,06353924	16,67779115	15,52231665	10,72988713	118,0393036	13,11547818
ENSG00000187630	DHRS4L2	22,76268135	4,795862482	13,58964713	10,05989369	17,55622325	13,51815112	10,07353222	14,97199551	10,71055077	118,0385375	13,11539306
ENSG00000122965	RBM19	14,60615301	0,457112172	30,89720841	6,49446067	0,061220174	14,53768095	23,50697151	13,4066531	14,06680121	118,0342612	13,11491791
ENSG00000181555	SETD2	0,149629241	10,06295851	2,669974215	22,99555673	12,76593699	20,12481003	11,18163173	4,351697938	33,713321	118,0155164	13,11283515
ENSG00000007392	LUC7L	0,04893477	18,14821179	0,225048655	10,05546048	41,40085657	16,60180317	9,48747491	9,042008081	12,96953548	117,9793339	13,10881488
ENSG00000083457	ITGAE	7,553239673	20,18578825	9,389512583	20,19657256	8,21368508	17,81741879	10,547718	9,459577895	14,55356741	117,9170802	13,1018978
ENSG00000141425	RPRD1A	27,24357836	8,671258709	21,99605616	9,550924799	0,042539391	12,1258974	7,030024525	18,59232641	12,52625813	117,7788639	13,08654043
ENSG00000198026	ZNF335	28,19929536	4,477918093	20,96364166	0,622508886	29,27200643	8,465947254	6,2361	8,846624604	10,69017769	117,77422	13,08602444
ENSG00000119669	IRF2BPL	17,04800005	6,178980319	8,140559788	13,46639931	13,7061184	17,20436129	20,35956419	7,455796771	14,09685575	117,6566359	13,07295954
ENSG00000104081	BMF	7,351624973	8,31944153	31,74290831	4,359295518	0,131858837	14,83356909	26,30569472	12,19338132	12,38521894	117,6229933	13,06922147
ENSG00000066427	ATXN3	5,659806165	18,78242463	28,7039978	11,50111735	0,037156497	5,493159291	5,335572318	31,93232137	10,13358688	117,5791423	13,06434915
ENSG00000100354	TNRC6B	6,883774871	10,48540074	22,7410462	19,46769587	7,847503051	8,561841942	8,049726771	17,92786807	15,57549612	117,5403536	13,06003929
ENSG00000101849	TBL1X	29,70024085	14,89350088	0,030574142	0,573988066	4,186808127	21,27713731	18,24370643	5,524203065	23,09795096	117,5281098	13,05867887
ENSG00000221909	FAM200A	32,73596836	37,66874678	0	0	0	13,92299479	18,08999241	0	15,0463543	117,4640566	13,05156185
ENSG00000151650	VENTX	10,50829914	13,5215974	0	25,67965183	0	14,70862372	5,765818429	25,93488066	21,31302126	117,4318924	13,04798805
ENSG00000177842	ZNF620	0,039944639	0,496165477	100,4490604	3,172881002	0,1898586	4,122546712	6,699711093	0	2,210704071	117,3808719	13,04231911
ENSG00000090470	PDCD7	4,530981562	36,22196415	0,515573599	9,679195348	0	13,23181725	24,62864322	7,150052466	21,25601646	117,2142441	13,02380489
ENSG00000123415	SMUG1	32,36830044	21,39975146	9,178370615	7,922863838	0,059584792	15,74595153	8,618668898	4,057102704	17,85384709	117,2044414	13,02271571
ENSG00000185344	ATP6V0A2	22,97445656	3,759549001	0,101497042	21,66518666	0,052448889	15,36253916	13,04730194	12,71644641	27,50919385	117,1886195	13,02095772
ENSG00000064933	PMS1	0	19,81320609	3,210991242	20,04615958	4,872515243	20,59432718	9,01803633	15,69170388	23,9208395	117,1677791	13,01864212
ENSG00000101473	ACOT8	11,17537982	19,83043731	40,34989034	4,854325435	0	7,582959293	15,44604914	6,830296527	11,05517898	117,1245168	13,0138352
ENSG00000108799	EZH1	0,064872884	11,74176474	9,507333093	16,3924519	17,93532088	9,069524943	16,93167188	12,38893198	23,08454226	117,1164146	13,01293495
ENSG00000173275	ZNF449	0	0,050308068	0	60,70532242	0	6,661325218	4,6139184	27,02991959	18,02676398	117,0875577	13,00972863
ENSG00000244165	P2RY11	0,776736578	12,1541	13,50931322	16,33891897	14,76745034	19,53713333	14,71638006	7,566025144	17,71621053	117,0822682	13,00914091
ENSG00000146834	MEPCE	1,082049541	8,932501248	0	4,468064634	45,28101017	11,20877806	13,98455133	17,18012851	14,87003618	117,0071197	13,00079107
ENSG00000177302	TOP3A	26,31205462	12,16159606	0,709822724	36,10700402	3,886364454	11,216717	10,93898687	5,38402627	10,25952348	116,9760955	12,99734395
ENSG00000108423	TUBD1	0	11,50444225	0	36,09091608	7,973206761	10,10496462	6,023178254	17,94439146	27,21065193	116,8517513	12,98352793
ENSG00000003509	NDUFAF7	36,25660364	12,41194139	0,076138071	0,071469453	0	17,67713348	20,58524483	12,58022593	17,15428047	116,8130373	12,97922636
ENSG00000138036	DYNC2LI1	9,456681447	0	2,046626974	0,169511677	57,62366801	5,640528485	6,57510185	28,69299731	6,55976248	116,7648782	12,97387536
ENSG00000169905	TOR1AIP2	6,436368871	3,068376352	31,17630163	27,74403973	11,39602712	11,37361127	7,757145446	8,875308632	8,920160478	116,7473395	12,97192661
ENSG00000175063	UBE2C	11,39792018	9,382884918	0,864634834	26,17465919	0	40,38103461	28,29424283	0	0,144515181	116,6398917	12,95998797
ENSG00000076662	ICAM3	13,76688843	11,63743975	9,671474032	3,743686867	0	20,94710294	18,40651571	2,725966662	35,59614164	116,495216	12,94391289
ENSG00000127720	METTL25	19,61451699	8,315051046	1,144264952	32,50946171	0	15,70777963	10,15745004	19,4960022	9,537129436	116,481656	12,94240622

ENSG00000168758	SEMA4C	13,89522489	5,308047256	12,82507162	8,664507458	0,057330387	18,77883124	23,33539859	3,006827961	30,5293856	116,400625	12,93340278
ENSG00000167772	ANGPTL4	18,92698057	0	0	0,105428273	96,05487395	1,273656952	0	0	0	116,3609397	12,92899331
ENSG00000112739	PRPF4B	10,81025912	14,66525828	14,34587057	3,683860716	6,262934954	10,84550098	15,71914469	27,7550924	12,21485032	116,302772	12,92253023
ENSG00000112304	ACOT13	10,24828176	16,41561175	8,287526847	12,53010461	0,245187216	14,72579083	14,96277969	24,44042659	14,42277289	116,2784822	12,91983135
ENSG00000257727	CNPY2	10,96164789	12,7009957	9,115611063	15,29827443	26,76430839	6,264924062	8,228925385	18,05952423	8,802932324	116,1971435	12,91079372
ENSG00000131323	TRAF3	27,37656109	11,55252959	18,77198296	13,76832251	0	14,93857789	12,43618564	5,358614553	11,85297316	116,0557474	12,89508304
ENSG00000175471	MCTP1	10,66236387	11,00160742	24,58108411	13,44714113	0	8,989281805	8,635231717	28,20816227	10,53063676	116,0555091	12,89505656
ENSG00000070614	NDST1	4,766894974	3,806432178	8,067561232	13,30740933	41,76487927	12,59594494	15,48918715	10,87550154	5,198230802	115,8720414	12,87467127
ENSG00000111859	NEDD9	4,215750065	17,00639966	6,467149711	16,5996861	0,138668807	14,943003	10,05313018	35,2476298	11,17698459	115,8484019	12,87204466
ENSG00000105443	CYTH2	32,26865609	17,18085461	5,77822128	9,813369344	0,038478254	11,76822048	14,33101617	12,49795375	12,12691868	115,8036887	12,86707652
ENSG00000122550	KLHL7	10,56448088	0,075896405	9,795788939	23,85564585	18,9062731	21,10863409	13,06908638	12,2054093	6,1808566	115,7620715	12,86245239
ENSG00000015532	XYLT2	27,53239016	4,410230265	24,38421921	19,59873439	1,312564117	11,40028817	16,18824093	0,301932171	10,63047542	115,7590748	12,86211942
ENSG00000188636	RTL6	22,25248854	0,544300056	0,820756889	34,81380082	0,066270076	12,58336354	12,15573432	17,13454761	15,26143244	115,6326943	12,84807714
ENSG00000100908	EMC9	12,06225171	10,11220616	27,78053786	19,84483465	0	10,21950755	11,98710903	8,099904546	15,30225922	115,4086107	12,82317897
ENSG00000168575	SLC20A2	1,38784615	13,60745517	3,591585961	6,092815409	19,50996611	19,60244827	17,53928365	15,22550311	18,8334505	115,3903543	12,82115048
ENSG00000182093	WRB	0,05198162	19,24743631	21,45173794	6,432865444	27,26015515	11,56675045	5,078663823	13,10977644	11,12394045	115,3233076	12,81370085
ENSG00000198113	TOR4A	11,7381503	7,393030451	25,72532203	2,815169949	0	21,55922622	25,539274	7,44685911	13,09970692	115,316739	12,812971
ENSG00000198743	SLC5A3	0,013065067	3,361628049	10,71927409	3,699925284	70,54427254	6,971514809	5,033566838	7,942314523	6,973663381	115,2592246	12,80658051
ENSG00000119718	EIF2B2	23,62840049	9,520126199	8,590996437	15,40841186	0,06606283	9,705585613	15,85690178	16,04759489	16,41028894	115,234369	12,80381878
ENSG00000108506	INTS2	53,26616963	9,138025736	0,096666315	25,52789134	0,083254328	9,846553295	10,71886342	0,15320949	6,398100975	115,2287345	12,80319272
ENSG00000043514	TRIT1	13,02238654	6,48498944	0,085100291	17,57406839	35,40053532	9,472622855	8,892203232	7,637474314	16,64169838	115,2110788	12,80123098
ENSG00000116809	ZBTB17	0	0	2,819137796	1,53853169	60,21994637	16,90409649	10,06130021	1,168990215	22,48567593	115,1976787	12,79974208
ENSG00000127081	ZNF484	26,44434987	0	63,68478497	6,149082393	0,07358966	3,841781797	12,80414454	0,203136005	1,942248491	115,1431177	12,79367975
ENSG00000096996	IL12RB1	21,62238273	4,117583063	0	12,93139823	16,71091402	11,50333919	16,31873327	8,456910755	23,39296616	115,0542274	12,78380305
ENSG00000185305	ARL15	14,83269549	17,14697527	0	35,36355577	6,237304683	17,46427123	11,16025585	3,826084466	8,949977257	114,98112	12,77568
ENSG00000155324	GRAMD2B	0,329086572	16,66929084	8,310234596	48,61211861	0	9,624193026	4,123554764	11,64469472	15,60065837	114,9138315	12,7682035
ENSG00000158186	MRAS	25,76461791	32,5643848	12,61526815	13,3010957	0	11,85075548	5,373290025	9,926765531	3,504296841	114,9004744	12,76671938
ENSG00000150456	EEF1AKMT1	41,64286824	0,200255415	0	0	0,268198314	28,7462687	27,54912443	0,246777123	16,23901651	114,8925087	12,7658343
ENSG00000162298	SYVN1	5,966338034	5,317954514	3,23703172	25,35285234	37,83279786	8,150628768	10,43303161	12,86626591	5,680898061	114,8377988	12,75975542
ENSG00000155846	PPARGC1B	2,309627001	4,452043449	28,83539393	17,49149027	34,63287339	11,05727251	5,831075248	4,845382362	5,363868903	114,8190271	12,75766967
ENSG00000077454	LRCH4	10,84972623	8,891599272	5,194794599	11,84234885	8,628509167	16,8544818	18,72270515	7,057194641	26,68752926	114,728889	12,74765433
ENSG00000176771	NCKAP5	1,769777676	14,77683092	1,860370839	23,79329686	0,075105624	24,27155671	19,62811008	0,691068843	27,79049742	114,656615	12,73962389
ENSG00000160216	AGPAT3	18,2952384	4,986156559	13,04689381	12,24688716	16,650923	7,450146075	15,73091292	4,910233525	21,30859029	114,6259817	12,73622019
ENSG00000010404	IDS	11,6088818	8,886134713	13,19014672	12,71826377	20,01537411	13,85265844	14,75565665	2,204319258	17,35687339	114,5883089	12,73203432
ENSG00000103528	SYT17	36,55093326	10,83940491	13,70557943	3,907972449	0,142790275	13,28164193	18,7748184	3,810179904	13,47356497	114,4868855	12,72076506
ENSG00000187210	GCNT1	9,915048794	8,521646095	4,597444076	5,274547644	46,38908908	17,80879934	11,75902906	10,07270004	0,140625409	114,4789295	12,71988106
ENSG00000118620	ZNF430	24,57675948	5,809547067	0,08138782	4,545639288	0	10,2252017	12,89418432	24,62175302	31,6681821	114,4226548	12,71362831
ENSG00000173868	PHOSPHO1	3,690279199	0,451607579	63,20389619	13,29395028	7,409161926	6,007709304	9,984796947	2,226085507	8,138121396	114,4056083	12,71173426
ENSG00000164889	SLC4A2	31,91886427	8,855416119	11,78753128	10,61664097	0	17,9279857	17,25424249	4,801559105	11,19498205	114,357222	12,706358
ENSG00000164303	ENPP6	0	8,541034456	13,01055285	12,38239641	68,01055948	9,311124456	2,990625465	0	0,040270102	114,2865632	12,69850702
ENSG00000112234	FBXL4	0,074457044	16,05284701	0	16,90709493	0	4,94584375	10,94271927	43,91954895	21,4279804	114,2704914	12,69672126
ENSG00000148400	NOTCH1	7,144338476	11,97175017	28,58447488	12,63102847	0,067509642	11,69592373	13,16147105	9,597169035	19,40741482	114,2610803	12,69567559
ENSG00000162775	RBM15	0,453008135	20,09629551	0	4,805226513	68,13236457	5,755356194	9,564761926	0,707568667	4,576520356	114,0911019	12,6767891
ENSG00000101040	ZMYND8	4,875173821	9,987190561	4,885158461	36,03469223	8,100749808	9,16043971	11,56795323	21,66784049	7,799460607	114,0786589	12,67540655
ENSG00000134597	RBMX2	23,26864135	18,47927639	13,8690717	10,11521079	0	10,12376096	8,646914481	19,09546496	10,47304522	114,0713859	12,67459843
ENSG00000137955	RABGGTB	21,56899193	11,77650676	18,97026351	16,52882018	0,121323494	10,98597062	9,812966844	13,11691273	11,17590573	114,0576618	12,67307353
ENSG00000172732	MUS81	14,95018311	17,27566851	25,71622061	3,879539821	0	13,88678836	11,92981497	4,791543839	21,59340099	114,0231602	12,66924002
ENSG00000142856	ITGB3BP	5,674604687	12,27981316	0,152764297	32,07315771	30,19508187	12,4303766	8,389205199	0,484241901	12,32394964	114,0031951	12,66702167
ENSG00000052795	FNIP2	8,105284229	9,376789731	0,911327162	10,4079336	0,065407074	22,49711763	21,30973219	12,06668442	29,26223814	114,0025142	12,66694602
ENSG00000126746	ZNF384	4,608274496	10,40272959	50,11260615	3,726923011	0	13,48163536	9,033088947	15,88075802	6,743707043	113,9897226	12,66552474
ENSG00000162377	COA7	18,2148711	0	9,985302495	29,44010803	0,259728213	15,51911855	19,60095833	4,62034834	16,30861478	113,9490498	12,66100554
ENSG00000136842	TMOD1	0	22,1749641	31,98737265	0,922784187	35,6577538	7,176219177	4,368934303	0,089889708	11,52693337	113,9048513	12,65609459
ENSG00000116793	PHTF1	53,10999136	2,430854952	0	4,439897755	13,37299635	9,626052986	12,49453515	9,585828458	8,786885781	113,8470428	12,64967142
ENSG00000100722	ZC3H14	1,892660257	15,94958368	18,62644058	7,219888572	0,017102464	16,19757511	11,14999712	24,75348008	17,98457631	113,7913042	12,64347824
ENSG00000156253	RWDD2B	1,789879427	13,02199823	1,81094527	22,4078006	25,94749196	9,776785539	19,95190059	6,458004577	12,54752949	113,7123357	12,63470396
ENSG00000154447	SH3RF1	26,15162837	10,09452705	28,143453	1,525935524	0	12,97692376	26,04793442	3,562799613	5,196366577	113,6995683	12,63328537
ENSG00000185049	NELFA	22,04915825	2,484887954	0	5,935336406	28,19692919	18,98117634	13,61464091	10,57836021	11,83656031	113,6770496	12,63078328
ENSG00000185482	STAC3	31,50846906	8,207120041	1,196471014	15,22432659	0	18,40806016	13,261092	3,002514004	22,84196854	113,6500214	12,62778016
ENSG00000139190	VAMP1	0,886402789	0	0	3,207022034	99,60997753	2,780624191	0,245333051	6,830211731	0	113,5595713	12,61773015
ENSG00000065183	WDR3	0,441155812	8,715397823	10,84760799	3,148689845	15,48161456	11,81846459	14,83519398	20,61196553	27,59846175	113,4985519	12,61095021
ENSG00000119383	PTPA	9,511582862	9,86938016	2,718510925	12,48070913	33,71516045	16,25475245	11,02104268	7,168032937	10,57448206	113,3136537	12,59040596
ENSG00000107560	RAB11FIP2	20,20381074	4,532906103	0,299041139	18,60670481	22,7932296	6,323316893	10,85597002	15,58988969	14,0520192	113,2568882	12,58409869
ENSG00000196498	NCOR2	7,493354238	7,943526872	17,24570632	12,39791221	14,13342743	15,42167006	17,70500857	4,066886985	16,83589962	113,2433923	12,58259914
ENSG00000145349	CAMK2D	1,292946618	1,092524786	4,379422991	31,00884188	0,146319792	11,64956786	9,696131863	29,84367614	24,13001158	113,2394435	12,58216039
ENSG00000185808	PIGP	9,593413738	25,72105516	17,92854103	6,619705906	14,09637204	11,01463629	10,81613489	10,46814041	6,966605719	113,2246052	12,58051169
ENSG00000181830	SLC35C1	42,43171407	20,83234185	0,123409589	6,023804075	0,159430651	10,97477289	18,85536399	0	13,81988567	113,2207228	12,58008031
ENSG00000176845	METRNL	10,70203968	8,110528959	14,25276052	9,336293816	4,768805431	19,21649616	18,7441846	8,897721663	19,126219	113,1550498	12,57278331
ENSG00000100226	GTPBP1	21,32616457	4,914209452	10,22367899	29,47351789	0	11,66607222	24,30880139	2,801340306	8,433877834	113,1476626	12,57196252
ENSG00000100813	ACIN1	14,09922997	7,270150826	18,12515684	11,6575779	13,24516951	7,794701079	12,46439632	11,06284776	17,38103589	113,1002661	12,56669623
ENSG00000153560	UBP1	3,715833705	5,65955451	0,142407838	25,85288119	25,64599662	12,81727201	12,09708836	12,05273627	15,06194663	113,0457171	12,56063524
ENSG00000160124	CCDC58	0,259640156	0,153575029	0,318419357	61,87117954	4,319283155	11,59283526	8,120359402	9,651868811	16,60484391	112,8920046	12,54355607
ENSG00000164080	RAD54L2	19,82027277	7,848168235	29,70032255	2,275850164	9,396567697	10,04591046	11,26960127	10,66901279	11,8608859	112,8865918	12,54295465
ENSG00000130147	SH3BP4	0	18,54475371	16,43947233	55,09650275	0	0,88985207	7,951471993	0	13,8499596	112,7720125	12,53022361
ENSG00000185813	PCYT2	15,00808191	14,61815166	0	6,620794629	30,92878355	15,10683517	8,313751156	12,21702403	9,943071875	112,756494	12,52849933
ENSG00000166133	RPUSD2	21,61324353	20,57658578	0	20,75738247	0	10,26525866	23,44062048	9,824089191	6,272040125	112,7492203	12,52769114
ENSG00000132640	BTBD3	0,048526195	0,086108542	2,945839199	42,31604437	0,807264867	13,50620783	12,30930842	30,93181645	9,727997152	112,679113	12,51990145
ENSG00000140961	OSGIN1	0,087778432	0	19,05411076	0	66,441439	1,927511783	9,730054791	14,01203801	1,403430026	112,6563628	12,51737364
ENSG00000166347	CYB5A	15,79399665	5,58207378	0,149919156	8,246569686	32,07305847	8,05304277	9,081069224	24,80665074	8,860334683	112,6467152	12,51630168
ENSG00000167384	ZNF180	0	29,32014762	0	35,89697121	0,300328281	4,613444847	5,03665142	0,069085212	37,37912491	112,6157535	12,5128615
ENSG00000158882	TOMM40L	0	12,71046855	0	33,38001346	4,473965128	19,86288885	17,3851326	15,86407222	8,865969005	112,5425098	12,50472331
ENSG00000133103	COG6	7,135829111	15,72797615	23,83574851	2,172566584	4,239613089	16,90650838	13,09743742	12,60636414	16,79016052	112,5122039	12,50135599
ENSG00000181982	CCDC149	7,238546434	10,38203737	19,14703249	14,29668771	11,54333156	9,696297532	9,218442259	17,00106886	13,98406831	112,5075125	12,50083473
ENSG00000256043	CTSO	2,498555767	9,051986224	0	31,72926147	0	15,08797528	13,57136901	23,44799205	17,09301462	112,4801544	12,49779494
ENSG00000265808	SEC22B	1,806502284	9,112197701	26,80104784	3,841526635	18,36532041	15,48180387	9,833185196	19,31253616	7,879036753	112,4331568	12,49257298
ENSG00000100105	PATZ1	4,579896752	2,308782505	0	51,71967552	0	14,74252757	14,64936888	8,649225807	15,71390433	112,3633814	12,48482015
ENSG00000101413	RPRD1B	0,054839619	22,23569117	10,18907276	21,49600408	1,694258048	11,25940041	8,21897838	23,98363764	13,18562697	112,3175091	12,47972323
ENSG00000110925	CSRNP2	20,71630577	0,205079924	0	0,049891965	52,52866537	7,232812904	7,67696104	9,540271452	14,35602199	112,3060104	12,4784456
ENSG00000255529	POLR2M	0,208972824	19,65330886	10,33669083	15,11562468	27,64564977	8,642238092	9,581861556	10,96709689	10,13760224	112,2890458	12,47656064
ENSG00000130119	GNL3L	23,02604585	9,407957083	4,766094578	34,69454471	0	14,2984135	10,7134881	2,626367631	12,74569087	112,2786023	12,47540026
ENSG00000177427	MIEF2	15,82289386	5,08000717	0	0,801643547	62,42724303	10,49152904	4,175919908	6,683136414	6,708745428	112,1911184	12,46567982
ENSG00000196177	ACADSB	0	0,042868425	34,53086122	0	0	6,074104247	14,04144403	35,34144295	22,1351973	112,1659182	12,4628798
ENSG00000140463	BBS4	25,53724929	15,38519673	10,38739521	2,704967191	0	10,11942524	6,896633485	19,19834397	21,86429674	112,0935078	12,45483421
ENSG00000186188	FFAR4	0,483851581	4,755227588	0	0	51,30250605	14,87474136	21,16925552	8,789885047	10,61980127	111,9952684	12,44391871
ENSG00000172171	TEFM	11,61069108	22,32825133	27,98739831	0	9,378921432	12,8106577	14,24719894	0	13,5762221	111,9393409	12,43770454
ENSG00000104972	LILRB1	14,87570115	10,4943754	17,20723456	6,044020977	10,68460739	11,42954252	11,47472285	14,51589554	15,17796078	111,9040612	12,43378458
ENSG00000115317	HTRA2	7,200537023	5,718456516	8,521882408	0	29,79682374	14,98226134	18,68884985	8,698544097	18,20689022	111,8142452	12,42380502
ENSG00000172889	EGFL7	7,956289797	8,78468331	26,99572724	15,16353792	0	2,782431669	2,740381666	47,29699153	0	111,7200431	12,41333813
ENSG00000091732	ZC3HC1	14,70454905	9,350684161	0	14,12567282	0,11926853	17,35696834	20,46028994	25,67973723	9,875603973	111,672774	12,40808601
ENSG00000169375	SIN3A	41,35427807	7,945426647	2,120622292	23,69850287	5,825242396	5,875416713	7,244292035	4,590474599	12,99866064	111,6529163	12,40587958
ENSG00000119559	C19orf25	16,82291425	10,85998709	0,217554372	12,20181157	20,37646004	11,26413033	15,17132952	15,96895679	8,690533912	111,5736779	12,39707532
ENSG00000167332	OR51E2	0	0	0	0	111,3054371	0	0,086322898	0	0,063930692	111,4556907	12,38396563
ENSG00000100558	PLEK2	0	45,45938306	0,177170235	0,166306549	0	10,04557348	37,74505845	17,69056958	0	111,2840614	12,36489571
ENSG00000131153	GINS2	0,093886655	0	27,8066455	38,09861387	0,111561834	10,56591122	5,184080198	15,70565046	13,62528091	111,1916307	12,35462563
ENSG00000239998	LILRA2	16,10264073	9,624839038	17,357516	8,927107936	24,43798568	6,482578151	7,365429846	9,328027612	11,53506177	111,1611868	12,35124297
ENSG00000180855	ZNF443	33,70622568	0,302075103	56,47286277	1,175824144	0	7,35165154	3,298131145	0,248167155	8,583973804	111,1389113	12,34876793
ENSG00000101751	POLI	13,76923477	19,64845825	40,56261185	14,65710131	0	4,537096333	6,120504	7,326708814	4,493596621	111,1153119	12,34614577
ENSG00000112699	GMDS	29,81361034	18,56710079	1,318378463	3,403230639	0,170318967	8,498146684	10,29469644	24,68268645	14,36707761	111,1152464	12,34613849
ENSG00000104219	ZDHHC2	0,336763449	12,33572716	0,309751782	20,31155724	31,09836725	11,83189086	20,81185704	0,105200435	13,96363555	111,1047508	12,34497231
ENSG00000137504	CREBZF	0,054093635	7,679026314	23,33500383	6,818776856	0,064277348	8,879051805	11,81267131	23,83481846	28,60712532	111,0848449	12,34276054
ENSG00000256812	CAPNS2	0	11,08461024	0	52,64928129	36,05313909	10,28776214	0	0	0,914599027	110,9893918	12,33215464
ENSG00000128915	ICE2	23,47357834	10,86971631	0,885508123	24,49626901	0,179446617	13,0572971	12,50784821	7,368216153	18,01585275	110,8537326	12,3170814
ENSG00000073803	MAP3K13	4,759671259	12,27530419	11,64499304	23,8700433	9,459459224	8,503509406	6,759310967	19,40031577	14,16998635	110,8425935	12,31584372
ENSG00000166261	ZNF202	53,10176236	0	0	1,337864196	0,070817894	11,45113426	4,874315797	19,74397041	20,19353851	110,7734034	12,30815594
ENSG00000188493	C19orf54	21,39654494	26,4122741	2,678609091	0,419060452	0,096123539	6,750109116	18,16901756	11,23265259	23,57902879	110,7334202	12,30371335
ENSG00000178921	PFAS	17,93430973	7,458748264	0,042957827	8,427663374	0,721453879	15,05014813	22,47258767	7,200011439	31,41957895	110,7274593	12,30305103
ENSG00000188868	ZNF563	0	0	0	3,299493883	99,2713403	5,128251054	1,895055945	0	0,979170296	110,5733115	12,2859235
ENSG00000143556	S100A7	0	18,20717285	0	0	92,36562303	0	0	0	0	110,5727959	12,28586621

ENSG00000124813	RUNX2	17,63448554	10,86708301	0,305398058	27,80715733	0,043837591	16,20220476	16,98566872	0,121008753	20,59919949	110,5660433	12,28511592
ENSG00000182150	ERCC6L2	16,17373573	7,200876741	0,053705583	22,13107686	0,069381287	18,99188885	7,526765473	32,55827462	5,798727463	110,5044326	12,27827029
ENSG00000118412	CASP8AP2	2,829508911	16,44096708	19,56168852	7,728094952	0	10,51788994	9,643295618	19,77511975	23,97285944	110,4694242	12,27438047
ENSG00000257365	FNTB	12,45570832	8,386100188	8,91481367	12,17189386	21,22534109	9,718059224	8,945539175	15,93932979	12,70829651	110,4650818	12,27389798
ENSG00000101191	DIDO1	17,51066699	2,119939011	22,66790507	5,402990589	0,621918792	14,65349796	13,68921851	17,09273006	16,69414209	110,4530091	12,27255656
ENSG00000124571	XPO5	0	5,121920058	27,13919608	17,81409625	0,211718867	14,75021517	15,01619938	7,324808241	23,03085661	110,4090107	12,26766785
ENSG00000138376	BARD1	15,40641317	15,61682806	17,03417405	12,23348072	0,047427039	6,398030692	5,484770192	25,65973662	12,51739651	110,3982571	12,26647301
ENSG00000197562	RAB40C	32,88154455	2,949433765	23,13177294	8,860058918	0	20,15365207	10,92089929	3,31856811	8,176891463	110,3928211	12,26586901
ENSG00000108443	RPS6KB1	10,40517116	15,85093024	36,9099209	0,881422546	0,13997046	8,98852936	13,56259792	19,96259243	3,645943207	110,3470782	12,26078647
ENSG00000148606	POLR3A	0,017003552	25,49566671	1,908043889	16,20750602	0,363683794	6,870157784	25,87905014	21,5282564	12,06632647	110,3356948	12,25952164
ENSG00000174130	TLR6	0,12519517	15,15102271	34,91448468	0,172947788	0,357034839	23,91751479	14,76121553	0,711789458	20,17058361	110,2817886	12,25353206
ENSG00000188677	PARVB	17,88097527	12,45992814	13,10482974	13,3234392	1,309762751	14,70178824	12,54194863	7,097000707	17,57295226	109,9926249	12,22140277
ENSG00000181284	TMEM102	14,97620029	5,91952748	14,75422789	19,97764249	0	18,93671683	21,56981753	8,225942733	5,586743741	109,946819	12,21631322
ENSG00000171606	ZNF274	18,82308561	13,19311413	2,03489572	15,46884405	0	8,859979195	13,62662312	18,6372551	19,29164068	109,9354376	12,21504862
ENSG00000224051	CPTP	34,42127285	12,0690137	0	9,191447246	3,935534711	23,24696143	13,84838889	1,293285979	11,92909184	109,9349967	12,21499963
ENSG00000255561	FDXACB1	7,744895483	14,64926725	0	8,964817488	45,10459698	9,718220207	11,23614913	0,093053947	12,35138813	109,8623886	12,20693207
ENSG00000109762	SNX25	15,9785492	10,1564932	12,55818557	40,46062143	0,070845836	6,447895761	13,73762759	3,846052374	6,59940304	109,855674	12,206186
ENSG00000196865	NHLRC2	23,72421149	9,246275865	0	24,71477127	12,92733557	8,219395941	11,93997279	5,152459175	13,90992415	109,8343462	12,20381625
ENSG00000076924	XAB2	4,124444976	1,671205244	10,15616093	21,6464432	23,23111546	14,94015294	20,65555003	7,385599618	5,991183826	109,8018562	12,20020625
ENSG00000173065	FAM222B	0	0,048629307	11,54469399	20,4432189	43,96161427	14,41261629	8,100732353	2,996323608	8,223893985	109,7317227	12,19241363
ENSG00000138593	SECISBP2L	0	6,623104278	33,09980906	28,27327582	0,125521686	8,891863601	9,7505198	13,97503763	8,964285241	109,7034171	12,18926857
ENSG00000173153	ESRRA	10,80062558	7,579062381	9,753687684	5,679871117	0	22,53106597	17,97674347	21,90014678	13,46451412	109,6857171	12,1873019
ENSG00000113360	DROSHA	0,097114763	11,99401234	31,51393924	20,69368629	1,846362686	2,047225625	20,30426067	1,104280046	20,04180362	109,6426853	12,18252059
ENSG00000100271	TTLL1	33,57113856	2,009629551	0	33,80420977	0	10,74688425	14,10533711	0,707568667	14,62496722	109,5697351	12,17441501
ENSG00000134056	MRPS36	7,172663393	20,12354972	11,76824024	7,364425197	8,062292484	17,45306901	12,71604391	13,77693835	11,08641858	109,5236409	12,16929343
ENSG00000161021	MAML1	19,75301577	0,134812469	18,2618064	14,29960394	15,70800713	9,231156606	15,37100933	2,381211061	14,23359488	109,3742176	12,15269084
ENSG00000152127	MGAT5	0,051720674	11,74746469	26,76727935	4,733446773	32,36769634	10,63796282	6,742330447	2,111162097	14,184977	109,3440402	12,1493378
ENSG00000077152	UBE2T	0	0	39,71144271	33,95945493	0	21,79333847	5,544391184	0,200020925	8,099848428	109,3084966	12,14538852
ENSG00000166920	C15orf48	0	22,52011104	1,582804834	13,00031902	0	4,13300369	3,393503641	62,55914232	2,116401677	109,3052862	12,1450318
ENSG00000004866	ST7	0	16,70418867	20,86293831	23,72487977	2,396957788	13,87919983	15,50234152	9,116112364	7,056765011	109,2433833	12,1381537
ENSG00000106100	NOD1	0,199412777	13,72163607	7,825832699	17,25537735	0,052656531	14,37729484	5,648004681	22,33582634	27,79522767	109,211269	12,13458544
ENSG00000154127	UBASH3B	34,81072383	2,452883089	4,124425833	27,42087752	0,040062198	7,125788937	8,51025902	18,54178798	6,157565497	109,1843739	12,1315971
ENSG00000135250	SRPK2	15,83266582	17,26959159	0,043666368	11,88676447	14,21577453	11,6497059	10,34745905	18,06334111	9,779859403	109,0888282	12,12098092
ENSG00000116990	MYCL	19,81777976	1,80067707	0,109808453	33,86021693	0	12,190151	11,1765988	7,962278735	22,09749287	109,0150036	12,11277818
ENSG00000120278	PLEKHG1	1,526665956	21,72434701	0,135020448	20,43069648	45,8752252	1,627838376	8,629589572	0	8,954718773	108,9041018	12,10045576
ENSG00000105364	MRPL4	7,165685538	9,091792632	18,30434067	8,719203082	1,058823221	23,3579264	12,70117366	13,55837169	14,93164933	108,8889662	12,09877402
ENSG00000103522	IL21R	5,401752755	4,276508526	14,21749727	7,175113875	0,13166546	28,58954026	29,60225798	3,997925107	15,46733659	108,8595978	12,09551087
ENSG00000223501	VPS52	17,95972507	4,296956155	11,13652745	0	31,73150775	16,09993578	12,37110106	3,971392532	11,29225475	108,8594005	12,09548895
ENSG00000051620	HEBP2	13,42945566	18,64975271	5,477969493	16,18240472	7,666632231	11,23486421	13,368796	11,04427747	11,77698209	108,8311346	12,09234829
ENSG00000168495	POLR3D	0,027066196	4,082405518	6,223806622	13,03973812	32,67628721	6,389176785	10,18055891	18,82109842	17,37630031	108,8164381	12,09071534
ENSG00000166816	LDHD	0	9,548083089	30,52007021	0,110612537	0,152232956	27,64004097	19,25403145	5,042664023	16,5442008	108,811936	12,09021511
ENSG00000102543	CDADC1	19,05018067	12,70255916	0,071959643	0,472830656	35,60497508	6,313449334	8,08752593	0,08553833	26,41204782	108,8010666	12,0890074
ENSG00000033170	FUT8	21,83209439	10,40551045	45,80448198	5,023971899	0,053599633	7,428813793	6,460785752	3,748213005	8,016364916	108,7738538	12,08598376
ENSG00000172869	DMXL1	12,74719288	7,056368423	0,081280731	39,80784298	10,29050268	8,234889004	10,26325303	7,777772715	12,44410924	108,7032117	12,07813463
ENSG00000158467	AHCYL2	59,7347269	14,70128201	0	1,674123488	0	4,282031507	11,58103239	0,433641165	16,23243903	108,6392765	12,07103072
ENSG00000124782	RREB1	9,371062398	5,905526836	18,62437663	10,3896098	20,98009057	10,3369578	11,80965684	12,77384123	8,433860377	108,6249825	12,0694425
ENSG00000179761	PIPOX	5,150596273	7,733513374	0,072884137	0,889395561	42,93592011	15,44264676	9,93498696	11,5227576	14,86187449	108,5445753	12,06050836
ENSG00000158792	SPATA2L	11,93944946	14,55876338	16,44452774	6,872274453	0	11,42820018	27,91053982	1,071100705	18,2985028	108,5233585	12,05815095
ENSG00000136371	MTHFS	13,97286967	10,18052909	4,936572487	24,12809624	17,3731083	11,37904751	8,835938873	5,261052514	12,40494239	108,4721571	12,0524619
ENSG00000084444	FAM234B	21,0789112	5,035155292	26,92802747	27,37678422	4,281580237	7,09632422	0,691863232	14,77352761	1,163271823	108,4254453	12,0472717
ENSG00000105983	LMBR1	31,40092745	0,226873678	0,540954588	3,64280149	0	14,41663104	16,19999379	24,12767846	17,83145412	108,3873146	12,04303496
ENSG00000127947	PTPN12	0	0,38825991	0,503131401	45,10278746	0,454990551	11,20084074	18,80351763	18,18137711	13,6904135	108,3253183	12,03614648
ENSG00000165496	RPL10L	0	0,330224138	0,684680047	0,964045473	103,0472751	0,408646741	0,309040227	2,043965613	0,457750055	108,2363574	12,02626193
ENSG00000128309	MPST	16,83228694	11,741129	20,06720241	8,91656496	0,106247794	12,98323892	10,4496741	11,68252391	15,42306198	108,20193	12,02243667
ENSG00000111224	PARP11	0,024372673	11,11491839	0,538025998	27,52443119	35,15876584	9,392679423	6,556836705	8,314160086	9,562103323	108,1862936	12,02069929
ENSG00000178718	RPP25	20,7659328	23,21728043	0	0,770224589	0	19,48080426	26,99525674	7,586231462	9,264897142	108,0803041	12,00892268
ENSG00000129993	CBFA2T3	12,55037559	9,428971872	16,33808016	11,88451324	0,300667735	10,52915415	14,91693498	17,59514274	14,47063511	108,0144756	12,0016084
ENSG00000100263	RHBDD3	8,168138955	13,50999066	0	14,45285478	0	13,67364446	18,08581362	16,64852175	23,37809106	107,9170553	11,99078392
ENSG00000158156	XKR8	0,661074316	0	37,49640416	12,84222442	0	20,92783797	9,834532038	0	26,15266409	107,914737	11,99052633
ENSG00000110429	FBXO3	15,95366301	15,81361305	15,43341221	11,9292946	24,35282064	5,425486479	6,542684032	4,752353173	7,690097941	107,8934251	11,98815835
ENSG00000122591	FAM126A	7,952090863	8,081802472	23,46697297	7,306683198	2,427306652	14,7498954	12,80751027	16,84193016	14,24067082	107,8748628	11,98609587
ENSG00000079263	SP140	1,164054916	18,44617127	25,24828201	0	28,56470598	2,199459455	14,79030427	11,12893205	6,259252273	107,8011622	11,97790691
ENSG00000080819	CPOX	0	11,61506189	3,784382536	11,70655053	0	26,79821582	9,472388926	23,31035934	19,09066999	107,777629	11,97529211
ENSG00000160113	NR2F6	7,736228767	9,920051573	59,21100763	1,755167991	0,134199381	6,94394043	7,126864897	7,161885245	7,778329781	107,7676757	11,97418619
ENSG00000001630	CYP51A1	10,87619296	8,661997169	4,804983292	17,59776819	9,5656132	15,32639501	18,7725518	7,022553858	15,11418302	107,7422385	11,97135983
ENSG00000121864	ZNF639	14,38198568	1,690879308	18,42601624	17,67883242	7,899637975	13,64948431	22,08012882	2,955933219	8,966638191	107,7295362	11,96994846
ENSG00000182919	C11orf54	15,88480875	17,36571256	8,380641044	11,32147282	11,97249423	15,58802515	12,06868882	4,63841792	10,46479859	107,6850599	11,96500665
ENSG00000171130	ATP6V0E2	4,65575451	22,9972179	2,964681844	8,580590188	0,053194718	26,46803305	23,4362448	6,85244252	11,64465693	107,6528165	11,96142405
ENSG00000153046	CDYL	9,307775177	17,45685533	10,72435279	29,68550018	0,944631852	13,09246112	9,323639314	3,838893485	13,23983335	107,6139426	11,95710473
ENSG00000198742	SMURF1	22,84957482	5,297898026	0,044292559	12,01564813	0	8,406558858	13,57462642	12,68877163	32,662319	107,5396894	11,94885438
ENSG00000101945	SUV39H1	41,63616674	4,61764727	4,126782942	1,007171814	5,864449177	23,44816939	13,2623035	0,098110026	13,35358028	107,4143811	11,93493124
ENSG00000185420	SMYD3	30,19120164	31,0836277	3,222408239	4,937569414	2,265146449	4,072813339	4,342052062	9,745172209	17,52013754	107,3801286	11,9311254
ENSG00000136709	WDR33	4,302633744	8,021263262	7,915042269	14,30442845	1,18268556	14,12658838	18,48266229	15,25780199	23,76795317	107,3610591	11,92900657
ENSG00000227345	PARG	0	23,10757731	6,124342208	24,77584961	0,070017068	13,52127531	21,20935423	3,801060535	14,4937822	107,1032585	11,90036205
ENSG00000175029	CTBP2	10,27814301	16,20105427	14,20574855	12,43783752	6,301447469	12,74041965	12,90337984	5,977470469	16,03632256	107,0818233	11,89798037
ENSG00000130159	ECSIT	13,5389938	12,52467941	6,846218998	16,39846125	0	21,27600601	14,65152497	9,541210442	12,15302099	106,9301159	11,88112399
ENSG00000156711	MAPK13	6,896004149	7,586047486	27,07085127	4,748319128	0	22,36042461	20,67388609	6,013025966	11,51963089	106,8681896	11,87424329
ENSG00000119632	IFI27L2	14,90353943	12,3860849	12,49348759	17,9168834	0	15,53469819	13,23625849	8,869342324	11,48489744	106,8251918	11,86946575
ENSG00000183549	ACSM5	12,12545079	17,59195559	0	17,77755315	0	10,5499534	15,48195618	0	33,24884792	106,775717	11,86396856
ENSG00000132530	XAF1	16,17328557	11,26885503	0,042022691	8,914785224	24,80978335	5,818783642	7,511149127	20,78016328	11,43454793	106,7533758	11,8614862
ENSG00000150787	PTS	17,43955115	10,02960806	11,37514472	23,85786532	0	11,40370013	9,065311711	14,89490857	8,615008226	106,6810979	11,85345532
ENSG00000065923	SLC9A7	0,287685455	7,442418239	0,752050368	4,261763613	7,988386213	26,36614976	9,756009783	7,151690038	42,53241051	106,538564	11,83761822
ENSG00000125962	ARMCX5	15,93876514	20,96650948	35,49043414	0,371929306	0,292500544	8,817483307	8,661055294	11,70751675	4,276248629	106,5224426	11,83582695
ENSG00000116731	PRDM2	14,55354027	2,651225488	7,329329718	32,41680496	0,427403465	10,38934674	17,00046611	3,932664449	17,74585029	106,4466315	11,8274035
ENSG00000142082	SIRT3	10,31350734	10,47844661	22,2764531	18,70198449	0	10,57668722	8,766880752	17,13761128	8,099200142	106,3507709	11,81675233
ENSG00000124587	PEX6	25,77383759	7,506428285	0	7,455284989	0	23,31845624	28,78756535	0,095363512	13,35520463	106,2921406	11,81023784
ENSG00000189308	LIN54	0,022347023	8,16877433	21,54119667	10,68899556	0	18,27911559	5,863446453	26,58331924	15,11617676	106,2633716	11,80704129
ENSG00000163913	IFT122	3,026602339	8,264650688	0	3,141184833	52,06384461	7,420865355	9,075635264	14,88258637	8,293275693	106,1686452	11,79651613
ENSG00000180901	KCTD2	13,31055415	5,841320328	6,424241727	0,098857729	16,19056167	15,43134934	15,4966199	23,78578058	9,575725338	106,1550108	11,7950012
ENSG00000125846	ZNF133	42,74875156	18,03756543	0	11,01255237	0	12,0778986	4,254799316	5,907142103	12,09064179	106,1293512	11,79215013
ENSG00000163002	NUP35	0,037542999	35,70785392	0,069063379	23,7920834	1,784434993	5,68836263	2,867893303	31,27840254	4,894343195	106,1199804	11,79110893
ENSG00000163319	MRPS18C	7,856755935	17,85409125	2,056572865	16,67710175	1,918836026	11,96765566	10,39139823	24,85389472	12,5272625	106,1035689	11,78928544
ENSG00000101544	ADNP2	24,5484558	5,925004421	0,196556549	35,47092018	0	8,739853607	15,48140211	0,116823252	15,50637414	105,9853901	11,77615445
ENSG00000153006	SREK1IP1	8,252137547	13,05845201	2,16863907	14,95903814	8,702020825	10,49198091	14,13395111	25,54423252	8,655263567	105,9657157	11,77396841
ENSG00000091127	PUS7	0	19,17460276	0	27,7828351	0,081010048	20,02304501	14,03863855	0	24,86058825	105,9607197	11,7734133
ENSG00000076258	FMO4	3,328950416	20,78047758	50,52195347	0	0,14127362	6,13516688	4,787813613	0	20,25156832	105,9472039	11,77191155
ENSG00000005810	MYCBP2	4,338509535	5,400745858	9,476396694	17,79065005	30,14545707	4,908719759	10,4617472	11,07855934	12,28746945	105,888275	11,76536388
ENSG00000198380	GFPT1	0,017043827	10,91803091	25,61580655	3,296268572	23,41190384	12,42551578	10,06196775	0,931746468	19,20092651	105,8792102	11,76435669
ENSG00000129353	SLC44A2	0,091545176	0	17,00887924	30,50915798	0	19,16414577	18,11617273	10,74312709	10,24557766	105,8786057	11,76428952
ENSG00000143252	SDHC	12,99807027	10,24469082	4,966734819	12,62291367	16,6878067	14,5991934	11,26220282	10,47776277	12,00915027	105,8685256	11,76316951
ENSG00000106144	CASP2	4,785796128	8,492277808	1,59439957	38,457	8,642105446	8,709270922	17,91315837	2,554481694	14,71478075	105,8632707	11,76258563
ENSG00000050405	LIMA1	11,60180889	0,042447681	5,500635171	20,11640536	0,284246907	22,35078372	12,61257889	0,104617561	33,21524832	105,8287725	11,7587525
ENSG00000156966	B3GNT7	10,46437587	6,558020887	5,118072721	1,218987241	32,17156329	26,12441892	14,44684504	1,543662448	8,171322771	105,8172692	11,75747435
ENSG00000092439	TRPM7	3,283420316	9,29183506	28,53899078	3,056185082	24,04753171	5,561622725	8,999810567	3,523211867	19,30522033	105,6078284	11,73420316
ENSG00000175482	POLD4	10,73706412	6,936220696	4,369038884	5,21186426	16,10972603	15,75444245	15,28322315	11,79350618	19,41225953	105,6073453	11,73414948
ENSG00000129493	HEATR5A	23,34565112	9,608377916	0	18,59806751	13,21991344	9,664851492	13,24231622	9,414182411	8,46080212	105,5541622	11,72824025
ENSG00000139117	CPNE8	1,357634033	8,431808987	18,51390894	14,11698984	0	10,85547719	16,00236851	35,1732108	1,088944466	105,5403428	11,72670475
ENSG00000145216	FIP1L1	6,243795525	9,843127153	8,774686579	19,7124134	1,21000768	15,88787221	14,70755266	14,94251015	14,20461981	105,5265852	11,72517613
ENSG00000086758	HUWE1	10,05143102	0,493730595	24,08872945	18,64786458	3,678170352	6,825827858	10,49015435	15,41989955	15,80534898	105,5011567	11,72235075
ENSG00000116062	MSH6	0,123714649	14,17179133	0	24,18745921	0	18,48817033	21,18374749	9,678880264	17,66666204	105,5004253	11,72226948
ENSG00000196470	SIAH1	13,37581606	9,221390553	22,07048932	12,36788082	0	10,41902545	9,005047751	18,28062051	10,67061372	105,4108842	11,71232047
ENSG00000126803	HSPA2	0,104629095	26,17830847	61,59157464	4,607126088	4,164942935	2,125217704	2,845181494	1,086767662	2,702286285	105,4060344	11,7117816
ENSG00000179476	C14orf28	13,65597588	11,32216478	49,81311763	6,113564029	0	6,407463055	9,239061522	2,892578603	5,933308126	105,3772336	11,70858151
ENSG00000100417	PMM1	19,87117451	9,809426541	0	14,39605654	0	17,38829457	13,14994581	12,61100881	17,97092137	105,1968282	11,68853646
ENSG00000204084	INPP5B	0,016921676	13,99262845	0,249030324	25,10001603	0	20,91996745	11,78831003	1,924143058	31,17559803	105,166615	11,68517945
ENSG00000173926	MARCH3	2,728513043	6,908913372	40,88771998	1,376406845	0	16,49344341	18,25161859	0,335194327	18,17367087	105,1554804	11,68394227
ENSG00000185361	TNFAIP8L1	0	5,669219075	0	6,886273134	53,14875008	12,7125963	9,463424351	9,612281616	7,635619599	105,1281642	11,68090713

ENSG00000105486	LIG1	12,29381924	3,262068128	24,51770946	23,88729542	0,382272323	9,965735675	14,67254947	0,050248566	16,08069817	105,1123965	11,67915516
ENSG00000171813	PWWP2B	9,24817244	3,444211561	0	2,267285386	70,14120491	1,441610602	9,954200877	4,119509153	4,493455771	105,1096507	11,67885008
ENSG00000151413	NUBPL	20,7851694	6,32377049	5,67680044	24,30580066	18,12147809	6,120548284	6,761181263	8,271748067	8,71691198	105,0834087	11,6759343
ENSG00000197860	SGTB	20,65995754	11,14332042	0	1,412429581	0,250824196	14,22421742	19,5424369	30,63746383	7,171657311	105,0423072	11,67136747
ENSG00000117174	ZNHIT6	0	11,7579597	0	4,416922833	9,263067351	14,41654052	10,98345805	40,43202269	13,75198351	105,0219547	11,66910607
ENSG00000130529	TRPM4	0,640686853	11,16581618	48,99588662	0,434628213	0,108757584	9,220045677	14,57234171	3,452450811	16,37835796	104,9689716	11,66321907
ENSG00000140829	DHX38	0,838172737	18,31524596	5,154306114	0,289468276	0,056912447	19,5096114	17,5777771	14,76744026	28,45130595	104,9602402	11,66224892
ENSG00000108733	PEX12	0	10,87188815	0	48,0645689	0	18,49894232	3,4645775	0	24,03467782	104,9346547	11,65940608
ENSG00000172687	ZNF738	0	16,61725071	8,328725117	31,0716423	38,16486046	7,605119905	1,670795714	0	1,475350113	104,9337443	11,65930492
ENSG00000116213	WRAP73	10,45145157	15,73586796	40,78303784	3,5206055	0	11,75761357	7,971327092	7,661372603	6,962549826	104,843826	11,649314
ENSG00000111450	STX2	0,974121326	5,905894173	0	2,873578823	0,192918368	56,86325433	13,85127759	2,840157443	21,26527491	104,766477	11,64071966
ENSG00000159055	MIS18A	21,49802097	17,7283967	0	22,79715244	0,211993827	17,04158226	15,70230837	0,195061728	9,544656103	104,7191724	11,6354636
ENSG00000197857	ZNF44	18,17515361	0,486646098	33,06775307	22,25761126	0,118501086	3,449056701	2,484151104	15,21056663	9,444106395	104,693546	11,63261622
ENSG00000133997	MED6	0,286533436	23,51565301	1,515416515	2,04097041	0	19,22973975	13,26375162	14,88091202	29,95393106	104,6869078	11,63187865
ENSG00000175854	SWI5	2,640767254	11,14221127	0	17,63211091	14,22523857	17,33201753	17,54148331	6,416205195	17,75465805	104,6846921	11,63163245
ENSG00000169660	HEXD	16,9379609	12,16411628	0	11,37747742	29,65626038	8,987888542	7,349726561	6,403488646	11,74586036	104,6227791	11,62475323
ENSG00000104897	SF3A2	4,657888558	12,49407169	2,889398878	11,22300966	29,47595599	12,90415597	10,29846094	9,001084993	11,65703993	104,6010666	11,62234073
ENSG00000059691	GATB	15,38004701	14,93611005	0,023676016	4,733766254	31,62656833	11,06447335	11,58417851	4,728132812	10,51035608	104,5873084	11,62081205
ENSG00000182831	C16orf72	3,836308427	6,773651382	22,97529859	17,57209826	0,067869008	13,45136149	12,30674732	18,10999583	9,412914445	104,5062447	11,61180497
ENSG00000172366	MCRIP2	8,339834245	10,25767364	9,858628735	6,498679383	12,09222249	11,47060052	19,34928013	12,44314271	14,18195219	104,4920192	11,61022436
ENSG00000172671	ZFAND4	0,048495048	7,142421697	0	0,460572003	72,37670623	7,401017382	4,046780605	0	12,91263545	104,3886284	11,59873649
ENSG00000080845	DLGAP4	7,857268266	10,12892753	9,267057572	12,05365795	22,59551723	14,55422171	10,34998188	5,338685697	12,222015	104,3673328	11,59637031
ENSG00000144120	TMEM177	0,058388938	9,53209583	4,189035495	2,318973886	72,01777607	2,115552176	8,775166187	0	5,242193248	104,2491818	11,58324243
ENSG00000109586	GALNT7	26,41715574	0,078784193	20,01032004	10,84833228	0	7,897021721	23,15127619	0,097086695	15,69880275	104,1987796	11,57764218
ENSG00000025039	RRAGD	8,491031672	5,498179813	0,054806802	31,74228693	0,070803932	17,63126988	8,435603946	28,66545933	3,554241455	104,1436838	11,57152042
ENSG00000140830	TXNL4B	0	14,76402483	29,63823461	1,910090521	0	6,60051626	24,31936098	9,990860571	16,91664716	104,1397349	11,57108166
ENSG00000178966	RMI1	0	36,70035929	0	2,232121254	5,017610372	26,54001526	28,80057521	0,188442867	4,610392768	104,089517	11,56550189
ENSG00000128311	TST	11,43191318	13,99995525	0	18,48926565	0	7,513265328	28,40963831	9,154287433	14,85187978	103,8502049	11,53891166
ENSG00000174842	GLMN	29,75476185	32,15861101	13,46913882	11,83622565	0,12340809	2,679655363	2,328315439	0	11,43179639	103,7819126	11,53132362
ENSG00000113163	COL4A3BP	2,549334801	3,503674827	11,9694816	17,16261025	19,62098823	9,083096409	9,56695717	17,68948975	12,6028902	103,7485232	11,52761369
ENSG00000188483	IER5L	17,16785532	5,242171156	2,973597518	2,021259486	20,92975717	17,25812712	17,63341811	7,435082486	13,02502369	103,6862921	11,52069912
ENSG00000183734	ASCL2	2,039829518	18,09813715	7,654959529	23,95191452	0	9,675130823	16,39515137	8,564178856	17,26004419	103,639346	11,51548288
ENSG00000167136	ENDOG	9,502149422	13,11436856	13,59553323	7,520396648	0	9,998104622	24,21741779	6,637550265	18,99043066	103,5759512	11,50843902
ENSG00000213463	SYNJ2BP	6,059840088	14,21072808	18,11971738	8,282476167	22,84877094	9,756679798	4,889378107	7,023548977	12,3511434	103,5422829	11,5046981
ENSG00000197933	ZNF823	0,061452042	20,06430582	0	57,19558817	0,438126323	9,985671148	5,102494185	1,88128668	8,767058391	103,4959828	11,49955364
ENSG00000168246	UBTD2	31,91123497	0,358958501	4,744640244	49,86407982	0,120186594	4,164422106	12,26149161	0	0,062197631	103,4872115	11,49857905
ENSG00000083123	BCKDHB	0,111356353	11,85594695	0	39,86773623	0	16,50547373	18,70805491	0,243503734	16,16056715	103,4526391	11,49473767
ENSG00000001460	STPG1	0,053264212	0,031505346	0,195967641	0,061317113	83,79831267	1,130632528	0	13,23912437	4,913108625	103,4232325	11,49147028
ENSG00000101126	ADNP	5,049553374	12,70965755	22,76149406	12,21345123	1,829844095	1,887358142	12,83101037	30,11069824	4,030088112	103,4231552	11,49146169
ENSG00000069424	KCNAB2	22,47480482	6,834330399	10,40894644	16,99607921	17,47750573	8,039719576	6,869678341	7,971465338	6,335233872	103,4077637	11,48975153
ENSG00000133111	RFXAP	19,46220273	10,69777277	0,120546444	28,96762944	0	20,72083525	6,910118024	0	16,52148182	103,4005865	11,48895405
ENSG00000158711	ELK4	8,101876256	6,890158461	3,731737451	7,56457841	39,63247461	12,77601624	6,89249806	8,871822518	8,938755477	103,3999175	11,48887972
ENSG00000142599	RERE	8,251951163	10,79563009	25,09072546	16,18328836	11,3352329	4,17868942	8,42287794	3,764080726	14,68995554	103,3623915	11,48471017
ENSG00000133812	SBF2	15,61845404	2,015023082	14,77188932	6,473963705	5,50445568	8,984720203	8,291353722	27,98455811	13,71098873	103,3554066	11,48393407
ENSG00000159086	PAXBP1	0	6,566589703	26,06306554	13,40094258	0,100508688	7,40630758	9,112664751	25,61723347	15,03208646	103,2993988	11,47771098
ENSG00000119673	ACOT2	30,31051982	10,53294944	5,227373362	5,888210877	1,200560108	16,57025818	15,83453417	2,899760099	14,83355025	103,2977163	11,47752403
ENSG00000148331	ASB6	20,72784548	6,207527912	0	8,637613828	0,155394869	31,4088608	21,55418512	3,07414245	11,3791791	103,1447496	11,46052773
ENSG00000147408	CSGALNACT1	0,068895397	16,70793618	0,380215941	0,237934627	57,1422594	11,64904049	9,305398483	7,633138946	0	103,1248195	11,45831327
ENSG00000165644	COMTD1	23,24042321	1,52739114	22,69584352	0	0	15,75100412	14,92938128	13,80296295	11,17431621	103,1213224	11,45792471
ENSG00000135953	MFSD9	0	27,45366363	0,04984402	49,92247049	0	8,954470789	7,041817091	0,236998076	9,430619653	103,0898837	11,45443153
ENSG00000163069	SGCB	0	0,059049108	0,183646839	32,40857205	0	8,184093632	27,71344263	0,072766916	34,45998057	103,0815517	11,45350575
ENSG00000106608	URGCP	26,65181329	3,253319286	15,47884076	0,866450746	0,137592928	24,55815561	9,246025743	0,126603283	22,73831013	103,0571118	11,4507902
ENSG00000176994	SMCR8	10,9640308	2,003712668	35,24587973	0,974928795	0,649242273	22,97592535	4,279631669	19,91289427	5,980597981	102,9868435	11,44298262
ENSG00000140386	SCAPER	10,9721112	4,619155902	15,32362303	23,9358956	0	4,401417539	8,926657557	14,54367476	20,23341982	102,9559554	11,4395506
ENSG00000087266	SH3BP2	8,24061813	5,991677111	30,25864707	13,442332	7,895528665	10,37090112	9,772225887	9,021780116	7,931659307	102,9253694	11,43615216
ENSG00000181472	ZBTB2	0	19,27027587	0,895843052	11,35231117	0,347197108	29,30035322	34,4507047	0	7,246996196	102,8637413	11,42930459
ENSG00000154889	MPPE1	3,6836915	11,28094129	1,603030715	1,98351619	13,36689148	12,76402471	19,58521172	16,28353832	22,31132561	102,8621715	11,42913017
ENSG00000109670	FBXW7	6,343818456	7,303759981	17,267552	23,05816973	13,17151772	4,20689742	9,530962832	12,5056592	8,871314574	102,8403569	11,42670632
ENSG00000113810	SMC4	4,460760354	12,08675356	5,686266111	21,8298123	18,82683833	11,21786359	7,376991592	9,754376626	11,58695245	102,8266149	11,42517944
ENSG00000139719	VPS33A	31,4603617	10,05577232	8,681081431	22,46137176	0	13,373814	10,51586394	0,044099101	6,225474055	102,8178383	11,42420426
ENSG00000154620	TMSB4Y	0	37,90489006	55,09702785	0,195164528	0	0	0	0	9,591181778	102,7882642	11,42091825
ENSG00000135297	MTO1	29,44346468	7,308318949	0,207258793	12,99162619	27,07289898	10,8719465	5,373884653	0,465362244	9,006728153	102,7414891	11,41572102
ENSG00000135218	CD36	0,041666518	0	102,1219169	0,143897964	0,231049889	0,060996536	0,0115322	0,060741648	0,068325927	102,7401276	11,41556973
ENSG00000160959	LRRC14	16,51616351	3,543148778	15,64488322	6,427598885	3,397849504	14,588222	15,31024606	7,492725104	19,79735384	102,7181909	11,41313232
ENSG00000100522	GNPNAT1	7,875837723	5,683807982	27,65936646	0	0,089555497	7,199112306	9,105203644	33,53787256	11,49375001	102,6445062	11,40494513
ENSG00000248487	ABHD14A	14,04349711	15,10976788	17,18379922	4,608607625	0	13,95379114	12,45205986	16,39666904	8,831226668	102,5794185	11,39771317
ENSG00000173575	CHD2	8,36184976	4,777233439	16,33344219	25,53779263	7,097184632	9,640842313	10,5033568	8,265250229	11,99434224	102,5112942	11,3901438
ENSG00000222009	BTBD19	21,37871142	4,113542047	16,74198323	17,19798696	0	6,551582432	8,733020267	11,921929	15,83919224	102,4779476	11,38643862
ENSG00000039319	ZFYVE16	11,25768874	11,23461317	0,107674667	0,13476306	11,54554784	22,01073177	20,79292074	12,99126221	12,39376743	102,4689696	11,38544107
ENSG00000132361	CLUH	12,22024615	3,147753914	30,17494118	9,189449106	4,007543589	10,19508471	17,0385074	7,327027367	9,130720442	102,4312739	11,38125265
ENSG00000135164	DMTF1	11,79423059	24,06074016	11,27624486	6,92723199	0,076270053	8,791481127	6,44872472	30,21176399	2,822135785	102,4088213	11,37875792
ENSG00000101246	ARFRP1	14,02730878	21,34026407	8,56500895	6,91082534	0,047085032	17,75049569	17,33915431	4,895647847	11,47682077	102,3526108	11,37251231
ENSG00000160007	ARHGAP35	0,322512556	4,268334194	16,83454401	11,95033454	27,30506881	17,97057203	15,02966093	1,821871337	6,842192994	102,3450914	11,37167682
ENSG00000176953	NFATC2IP	23,56987814	8,972336009	25,33698009	4,229165674	0,062585839	10,95854105	12,59513964	2,073134268	14,47776149	102,2755222	11,36394691
ENSG00000253958	CLDN23	16,2327169	20,39807746	0	0	0	10,78533486	12,610665	32,60165857	9,59650092	102,2249537	11,35832819
ENSG00000182858	ALG12	8,476934452	11,52054747	19,8184686	17,57516996	0,06737665	9,431689445	16,4547396	9,237289036	9,62357226	102,2057875	11,35619861
ENSG00000085382	HACE1	0,016359269	0,058058244	37,01586248	20,19795462	0	4,849610881	5,840883703	29,90617067	4,245276194	102,1301761	11,34779734
ENSG00000198646	NCOA6	2,03598182	11,75364197	18,4021677	2,273483566	13,96729505	15,76694423	7,528445323	13,08916233	17,26087344	102,0779954	11,34199949
ENSG00000162813	BPNT1	25,04756006	7,752258274	1,250151425	1,844063425	1,384327179	15,02948366	18,33890172	18,36337301	13,01462957	102,0247483	11,33608315
ENSG00000156639	ZFAND3	8,968221893	5,914897061	11,97183199	7,948649436	9,053383421	17,64542636	17,39714687	8,850925916	14,25083627	102,0013192	11,33347991
ENSG00000153487	ING1	2,199950387	16,70640514	3,394246669	16,54331007	25,57897335	11,37583478	11,54924158	9,155748035	5,469496898	101,9732069	11,33035632
ENSG00000129480	DTD2	13,24191047	0	62,86641268	8,178952692	0	9,562085816	8,119414811	0	0	101,9687765	11,32986405
ENSG00000171316	CHD7	13,14004962	14,7147255	27,52035645	8,20212758	0,021099738	8,412504565	7,217124267	3,844068412	18,87947338	101,9515295	11,32794772
ENSG00000152464	RPP38	13,78003131	9,169622801	1,008217976	0,135199469	0	12,20689217	21,12842378	22,08604104	22,43639362	101,9508222	11,32786913
ENSG00000137073	UBAP2	2,646569317	6,84241827	2,696040508	27,71512725	4,632683927	9,748418066	10,18412676	19,88207991	17,58717089	101,9346349	11,32607054
ENSG00000151062	CACNA2D4	21,36160419	16,41979017	7,859279229	4,859970718	0	12,29378922	19,61997554	8,855274488	10,6582221	101,9279056	11,32532285
ENSG00000168394	TAP1	13,93173419	9,263594592	26,32277732	6,625196025	0	11,42901163	12,63842678	8,73365038	12,91836388	101,8627548	11,31808387
ENSG00000077063	CTTNBP2	1,322678262	3,23201722	26,48569174	40,06319742	0,15459214	13,42711512	8,173845706	3,413874137	5,573528936	101,8465407	11,3162823
ENSG00000197102	DYNC1H1	1,614244863	7,062698037	34,84101869	8,092821387	5,163858216	10,62054984	9,883743025	12,13452664	12,37663376	101,7900945	11,3100105
ENSG00000129028	THAP10	81,71929737	0	0	0,127471898	0	7,213503335	7,171484535	0	5,53817189	101,769929	11,30776989
ENSG00000087152	ATXN7L3	4,760510338	1,922164875	7,393924534	3,396437134	55,34786495	11,11078188	5,491250146	8,664479322	3,61105297	101,6984661	11,29982957
ENSG00000163682	RPL9	1,263768213	3,523968938	14,00417464	29,30452522	4,076005558	3,105454841	27,55750223	1,842327338	16,91195951	101,5896865	11,28774294
ENSG00000119326	CTNNAL1	0,096741723	2,74665301	0	83,44239425	0	2,76163572	5,582706834	0,105772909	6,781851325	101,5177558	11,27975064
ENSG00000166716	ZNF592	17,8417487	4,649104275	5,939745705	17,20450382	0,087696592	11,58740536	20,97300771	6,21329956	17,0189123	101,5154181	11,2794909
ENSG00000005469	CROT	22,33729263	0	0	12,99696198	0	6,564447401	27,92149585	1,260947335	30,42075028	101,5018955	11,27798839
ENSG00000120616	EPC1	2,664451848	7,008850791	24,82756865	12,6698889	4,104745965	7,21797742	11,95114362	15,86882293	15,16610376	101,4795539	11,27550599
ENSG00000139323	POC1B	0	23,14784971	30,17870387	4,136157084	0,040953078	17,70923706	7,7265343	2,072516959	16,44621978	101,4581718	11,2731302
ENSG00000101695	RNF125	6,457290513	0,044240554	28,38965208	11,1072799	2,014518469	11,90745766	12,68987189	2,235244558	26,55388357	101,3994392	11,26660435
ENSG00000122176	FMOD	0	47,90143898	0	31,04730581	0,118364384	0	0	19,05934387	3,246494951	101,3729474	11,26366082
ENSG00000173821	AC124319.1	3,268685078	13,51503256	10,35249651	19,28927779	14,65631972	7,456394137	11,44032954	8,51242008	12,87330303	101,3642584	11,26269538
ENSG00000197885	NKIRAS1	0	20,76884481	0,080040339	0,976721759	0	27,18200926	27,45679595	12,84441884	12,04015765	101,3489886	11,26099873
ENSG00000099821	POLRMT	22,69644935	3,7624668	0	7,163976575	0,070971846	10,09890467	14,28278878	14,56263015	28,6850067	101,3271949	11,25857721
ENSG00000130429	ARPC1B	17,01320116	8,673600304	8,155384047	7,982464118	3,331832765	10,10939572	17,55577865	13,83715466	14,58624912	101,2450605	11,24945117
ENSG00000177051	FBXO46	0,171375754	7,070373124	0	0,517874914	64,65541236	7,479382392	12,0952404	4,496983057	4,689634387	101,1762764	11,24180849
ENSG00000139372	TDG	11,22889194	7,574942889	3,300480193	4,27324421	47,41674817	6,826617383	6,678052471	3,990919034	9,853479891	101,1433762	11,23815291
ENSG00000074356	NCBP3	5,676912064	3,643233697	32,38694767	28,16750507	0,024396572	3,832173647	2,770234912	13,1545283	11,45127688	101,1072088	11,23413431
ENSG00000070814	TCOF1	4,081927296	11,79084064	19,1978563	1,756446634	8,00550514	14,38790909	21,09270465	1,819859931	18,95995205	101,0930017	11,23255575
ENSG00000111860	CEP85L	3,365848519	26,67768308	14,09360281	14,97304001	0	16,17223377	14,08014545	1,927651805	9,777245974	101,0674514	11,22971682
ENSG00000130813	C19orf66	7,797458611	2,841403364	0,896503429	19,05468485	15,38720638	15,6317498	10,28966556	17,05075861	12,1157719	101,0652025	11,22946694
ENSG00000134460	IL2RA	48,36148501	0	0,07016156	0,065859408	0	3,601289651	1,551753223	39,03164503	8,3494904	101,0316843	11,2257427
ENSG00000103415	HMOX2	8,591421252	10,79873024	8,396200798	9,117656797	27,96799338	8,008120818	10,92338691	6,653701685	10,5113076	100,9685195	11,21872439
ENSG00000185875	THNSL1	0	52,31356705	0	26,88297208	0,187333095	9,693262697	10,43949815	0,172370666	1,260305055	100,9493088	11,21658987
ENSG00000130958	SLC35D2	0,592280784	10,15954795	11,83975859	6,340985795	36,31525709	11,44509858	9,475026378	7,684524826	7,090042397	100,9425224	11,21583582
ENSG00000132182	NUP210	0,018439362	0,032720195	0,169603477	26,77807366	0,175286171	16,47970829	15,75460301	5,362757472	36,14879274	100,9199844	11,2133316
ENSG00000095932	SMIM24	0	27,38700001	0	26,65090195	0	10,07867989	8,962166571	15,66145146	12,1581837	100,8983836	11,21093151
ENSG00000149476	TKFC	7,503523912	12,29313742	0,034166679	14,43224417	23,30556322	13,05097948	15,99222398	6,010854965	8,131927474	100,7546213	11,19495792
ENSG00000027001	MIPEP	0	31,26212811	0	0,462104441	0,105996913	19,49008546	12,96181116	22,04197528	14,42669284	100,7507942	11,19453269

ENSG00000182551	ADI1	5,648993296	10,57532	11,27508065	13,46131832	12,48520801	9,954634602	10,27215055	18,28198729	8,753892264	100,708585	11,18984278
ENSG00000007341	ST7L	9,952274881	16,53564381	19,95367755	7,723778232	2,923255772	6,159235766	8,913531232	14,3454573	14,18833218	100,6951867	11,18835408
ENSG00000254470	AP5B1	18,02898931	3,009700271	0,115560216	9,274554283	0	23,58820089	18,28200015	2,541276034	25,84313519	100,6834163	11,18704626
ENSG00000074266	EED	10,18947436	17,4506987	0,285861715	19,58833143	0,105514189	11,9674002	11,83369372	14,4659176	14,77052572	100,6574176	11,18415751
ENSG00000152620	NADK2	15,48770355	10,96093919	0,055429731	0,676401729	0	15,97902464	12,20927657	33,53762609	11,74742723	100,6538287	11,18375875
ENSG00000145687	SSBP2	17,47600981	14,1865273	0,02463489	8,949116906	0	12,23303791	9,740518507	24,1295714	13,85119862	100,5906153	11,17673504
ENSG00000085982	USP40	0,016158102	1,949706587	58,34847458	0,334818188	0	16,39236237	5,125071303	3,851301097	14,5267117	100,5446039	11,17162266
ENSG00000170190	SLC16A5	6,701893228	7,031240533	27,80700893	5,659644965	0,116256893	10,52718454	12,42923975	0	30,26247999	100,5349488	11,17054987
ENSG00000032444	PNPLA6	4,466737449	6,804495819	1,70539938	5,384603491	19,17763786	15,52230856	21,20329648	4,284776182	21,87039386	100,4196491	11,15773879
ENSG00000118096	IFT46	0,950879312	8,858388047	35,27592237	0,136830086	0	3,828036379	12,73125812	27,63729078	10,91495044	100,3335555	11,14817284
ENSG00000125637	PSD4	17,58197683	1,077195953	19,17032821	11,783033	4,728746094	17,18103874	12,0317853	7,571332153	9,20798868	100,333425	11,14815833
ENSG00000146828	SLC12A9	12,56283814	4,33798989	11,39902664	15,85954626	0	20,44757922	19,86155463	4,083563917	11,73415858	100,2862573	11,14291748
ENSG00000173327	MAP3K11	8,240053839	6,219330685	23,47422591	6,323899792	3,671749936	15,3769246	15,66745886	7,883130835	13,42430148	100,2810759	11,14234177
ENSG00000080345	RIF1	14,98960962	10,06676451	0,122417747	8,575260368	3,835120732	14,92336686	14,24198088	14,71548856	18,77289268	100,2429019	11,13810022
ENSG00000108591	DRG2	6,735074057	17,52846138	30,71146961	0,775333528	0,097006359	14,29645434	9,761063105	5,712537094	14,55847959	100,1758791	11,13065323
ENSG00000112357	PEX7	22,51811451	42,29969325	0	0,142431755	0	17,02575787	11,30054846	2,344786778	4,362118154	99,99345078	11,11038342
ENSG00000079134	THOC1	15,96527382	25,68232437	2,779114789	1,256043447	0,044324555	9,440233999	22,45519395	13,25490428	9,106525468	99,98393868	11,10932652
ENSG00000141219	C17orf80	0	13,3409702	36,48377023	0,391710234	0,308057081	9,535499583	11,16667119	23,52654437	5,221079652	99,97430254	11,10825584
ENSG00000135686	KLHL36	1,932476076	3,643452094	15,66398	5,00544661	0,035879463	10,19432557	10,15395833	24,79332203	28,42751779	99,85035797	11,09448422
ENSG00000196867	ZFP28	0	14,4183037	0	0,187076982	69,92853779	8,04097719	3,184434867	0	4,077217939	99,83654847	11,09294983
ENSG00000213676	ATF6B	10,78468939	10,12274842	28,03546879	12,51107346	0	14,90406393	9,646244869	6,829041765	6,913552082	99,7468827	11,08298697
ENSG00000188647	PTAR1	0,037000629	0,547139243	1,815083898	42,19002487	45,98885275	2,870800054	1,904789565	0,080909542	4,307901757	99,74250231	11,08250026
ENSG00000196678	ERI2	11,57747014	0,039660109	1,521263668	4,322543582	35,37528224	12,61322828	29,85974918	0	4,425571674	99,73476887	11,08164099
ENSG00000176463	SLCO3A1	8,740740927	6,374846956	17,96751011	12,87781397	0,876640148	14,59773595	11,95843326	10,13538421	16,19401346	99,72311898	11,08034655
ENSG00000171723	GPHN	0	0,044638255	0,092552056	4,34384842	74,78875403	4,640082718	2,966004186	0,440066013	12,40626859	99,72221427	11,08024603
ENSG00000265491	RNF115	5,042029169	7,322441815	0,427313652	17,57813148	20,91253253	11,37175306	12,89987951	16,79213554	7,343786078	99,69000284	11,07666698
ENSG00000139233	LLPH	10,51615884	15,43287499	2,753658661	10,27380339	15,4454316	12,52390811	7,504620485	11,43572923	13,7491471	99,63533241	11,07059249
ENSG00000133055	MYBPH	0,166238252	20,64899891	0	24,11280293	6,716169659	21,44608665	11,24954022	0	15,23164566	99,57148228	11,06349803
ENSG00000137764	MAP2K5	15,85895077	15,29651243	0,110506897	0,155596288	0	12,53150848	24,78980521	12,87322108	17,95297353	99,56907467	11,06323052
ENSG00000108469	RECQL5	5,292145242	11,86848004	0	13,42478594	29,90597832	12,20765708	9,96205237	0,339255203	16,5154507	99,51580489	11,05731165
ENSG00000116198	CEP104	15,41631155	15,29611968	0,070633998	10,64161185	0	9,40110751	11,23829044	18,13590817	19,31424917	99,51423237	11,05713693
ENSG00000117602	RCAN3	0,867964892	24,00430626	0,311549565	40,02855102	0	9,320557755	8,26156571	0,277753909	16,4288534	99,50110251	11,05567806
ENSG00000167705	RILP	14,9353471	10,06241121	0	2,89621272	24,10567013	8,155223908	14,05904354	13,44791845	11,78730585	99,4491329	11,04990366
ENSG00000122779	TRIM24	0,016246702	0,951369315	0,089661359	56,13706958	0,386106378	3,692451282	18,46778674	13,99755076	5,694684319	99,43292644	11,04810294
ENSG00000173638	SLC19A1	6,106292753	3,150400001	22,44129504	10,31198297	0	11,35605104	12,95465822	17,29377176	15,81390968	99,42836148	11,04759572
ENSG00000175806	MSRA	4,783829086	14,32077864	8,195643324	12,29635574	6,161755802	12,42926022	9,369340886	13,25571247	18,59363466	99,40631083	11,04514565
ENSG00000118655	DCLRE1B	0	4,015324367	0	39,20647334	0,091146584	17,43324334	11,17768694	22,05692671	5,42445433	99,40525561	11,0450284
ENSG00000011485	PPP5C	9,483210484	5,204059217	11,69572027	16,74508337	0,050873713	14,29007375	13,11759527	19,19226132	9,609573683	99,38845109	11,04316123
ENSG00000198093	ZNF649	0	13,6719164	48,96893338	0,299649718	0,082479913	11,20296499	5,849902939	0	19,2932081	99,36905544	11,04100616
ENSG00000005339	CREBBP	15,85041811	2,596262588	3,086275305	7,579443492	33,40358756	5,504649911	6,9570756	14,12004155	10,22083895	99,31859306	11,03539923
ENSG00000133256	PDE6B	23,11522296	3,954119152	0	11,80004801	0	10,21038351	7,548942507	32,48471958	10,19488394	99,30831964	11,03425774
ENSG00000114388	NPRL2	12,57542416	19,61658907	0,302398802	19,94091049	0,195331815	21,29719668	14,33166697	0	11,01836874	99,27788673	11,0308763
ENSG00000168067	MAP4K2	14,72562134	6,208127406	18,50776591	9,795727627	0	11,28495747	14,73804554	6,353679716	17,64873449	99,2626595	11,02918439
ENSG00000257103	LSM14A	6,015142767	5,478048443	4,391008303	30,83077522	0,151271079	9,225019429	8,905539571	16,98105191	17,26165045	99,23950717	11,02661191
ENSG00000175294	CATSPER1	0,056237652	8,482348347	0,103453703	23,98620719	0	11,29945498	25,44895085	23,48827778	6,363185727	99,22811624	11,02534625
ENSG00000196961	AP2A1	22,47825891	6,775598219	7,405598338	4,594555921	12,11292877	9,143482503	10,87428672	13,48789625	12,218357	99,09096263	11,01010696
ENSG00000139197	PEX5	0	19,49501786	0	18,92349123	0	8,162510151	10,51682812	22,15741998	19,8105349	99,06580225	11,00731136
ENSG00000204856	FAM216A	10,73768674	7,740590676	0	30,80616465	0,132908047	9,149028841	19,6424687	0,978340688	19,87773684	99,06492519	11,00721391
ENSG00000119965	C10orf88	0	15,65538761	0	16,08097476	0	13,23840476	13,14528616	23,79386117	17,11982236	99,03373682	11,00374854
ENSG00000038210	PI4K2B	12,73970587	0,149216472	12,76202458	45,52204482	0	12,09476102	7,121855239	0,367762457	8,273634826	99,03100528	11,00344503
ENSG00000213390	ARHGAP19	21,70973984	0	36,826616	5,923608958	0	9,738878234	3,377343135	0,321486526	21,12508709	99,02275439	11,00252827
ENSG00000078177	N4BP2	0	5,274834983	37,20190296	6,542656585	0,073207123	5,614342214	13,83741711	21,95936582	8,505258231	99,00898502	11,00099834
ENSG00000242259	C22orf39	13,90748303	7,492373328	16,25520959	12,43979211	5,275815832	12,21090374	14,927048	4,073692834	12,01859261	99,00091107	11,00010123
ENSG00000176386	CDC26	10,42585709	11,42001704	0	11,55759683	0	16,11056707	9,832428061	28,70896583	10,92283649	98,97826841	10,99758538
ENSG00000101574	METTL4	16,02447875	10,72755381	31,46944517	23,06657882	0,125477828	4,782537588	5,480011006	3,290490976	3,993557344	98,96013129	10,99557014
ENSG00000147813	NAPRT	17,73698274	11,81450476	6,466932158	4,598783479	23,67112458	11,75467956	8,491471182	0,69883941	13,69119604	98,9245139	10,99161266
ENSG00000122642	FKBP9	11,57666589	22,20251036	0	11,00480257	0	6,291056884	24,22507087	11,50671738	12,08056326	98,88738721	10,98748747
ENSG00000149483	TMEM138	10,11597825	7,233805831	3,05523506	11,42812628	1,674486459	17,0745606	11,32471606	9,574623605	27,38953742	98,87106957	10,9856744
ENSG00000185220	PGBD2	0	9,474203783	64,8696369	1,64379743	0	1,545045086	7,079399724	13,93780427	0,305417456	98,85530465	10,98392274
ENSG00000254521	SIGLEC12	33,8471201	0	0	13,159374	0	32,11172452	19,61008369	0	0	98,72830232	10,96981137
ENSG00000166377	ATP9B	0,252963993	7,235919783	10,53547181	11,3920988	32,36722995	8,876560251	6,914562959	3,827854572	17,32277749	98,7254396	10,96949329
ENSG00000105514	RAB3D	15,54536208	7,217325732	16,16900667	21,24858256	1,228732467	8,590716449	12,02042414	13,94397696	2,713087916	98,67721498	10,964135
ENSG00000125245	GPR18	0	0,421606918	59,73366907	0	0	17,99973238	4,471688981	1,731837356	14,31836414	98,67689884	10,96409987
ENSG00000184903	IMMP2L	0	13,36386397	4,72672112	9,868253217	0	11,04124917	3,935891388	36,23058583	19,34198634	98,50855103	10,94539456
ENSG00000196757	ZNF700	0,728463131	0	2,680133524	44,10500776	0,541002625	7,048325193	4,801064225	15,1328877	23,46179356	98,49867771	10,94429752
ENSG00000107902	LHPP	11,56969277	10,79148172	9,072724703	8,836570919	22,7367671	9,201420941	10,31473895	5,027470721	10,85434903	98,40521686	10,93391298
ENSG00000143793	C1orf35	24,34325349	8,34053575	8,130337157	12,6591016	0,083360618	13,97993021	11,35873479	2,914696742	16,56570672	98,37565708	10,93062856
ENSG00000119231	SENP5	23,73520691	14,33139901	0,82979134	9,940379985	0,765709046	13,1124777	9,791501389	20,61986853	5,177813556	98,30414747	10,92268305
ENSG00000138646	HERC5	6,153537093	0,112570109	39,15294845	5,751085712	0	19,67663221	8,796618574	2,011461473	16,61851773	98,27337135	10,91926348
ENSG00000100299	ARSA	7,856003896	7,208438572	5,496607156	4,058086697	9,494553997	19,94170673	17,47831552	12,03982872	14,61667827	98,19021956	10,9100244
ENSG00000123360	PDE1B	27,79351476	8,553464099	11,01807912	6,092142749	35,00018941	1,103518156	4,632541103	0,044853342	3,910168447	98,1484712	10,90538569
ENSG00000099290	WASHC2A	31,67408707	5,142591633	12,70889255	2,780206045	0,139138916	9,5458042	12,90676991	10,05002462	13,17702967	98,12454462	10,90272718
ENSG00000198015	MRPL42	1,935660598	10,71324936	18,55009322	11,39620848	8,543115867	10,28212431	7,607509455	19,7930185	9,261718232	98,08269803	10,89807756
ENSG00000105723	GSK3A	10,52128427	11,38400049	8,092579301	3,481665115	29,18592351	7,124253294	12,2061143	7,134564898	8,927125524	98,0575107	10,89527897
ENSG00000137101	CD72	13,87932294	7,048032489	2,052422467	18,18684407	0,424238089	16,07166839	22,82626241	12,78408988	4,77514691	98,04802764	10,89422529
ENSG00000164346	NSA2	18,27213672	14,53651713	1,400544634	14,51391636	1,592218043	12,33796913	10,77193902	12,0533367	12,54707476	98,02565251	10,89173917
ENSG00000075151	EIF4G3	0,047241737	11,26106982	0,086904991	31,92347438	3,817214394	9,526545203	18,99837353	5,165192281	17,17860205	98,00461839	10,88940204
ENSG00000021574	SPAST	24,41016911	10,65221644	9,226947437	3,464468577	2,346184154	9,615470726	6,055364811	27,85539031	4,308349305	97,93456087	10,88161787
ENSG00000198231	DDX42	2,908359769	12,55588053	6,720326299	14,20887551	4,298789968	9,521214882	12,33941301	20,00988579	15,35453018	97,91727594	10,87969733
ENSG00000110063	DCPS	18,64189297	12,89831426	9,243658578	23,18244475	0,091158153	12,12901284	7,691606848	0,125815929	13,89308308	97,89698741	10,87744305
ENSG00000154359	LONRF1	0,105875334	23,15536977	6,427285526	25,18395008	0,125807512	10,92702344	9,824003924	15,39597039	6,640863644	97,78614963	10,86512774
ENSG00000156232	WHAMM	15,07271162	7,798781576	15,89852996	11,5620221	16,89387619	13,47234041	3,894160216	2,38650771	10,77423719	97,75316697	10,861463
ENSG00000187266	EPOR	0	8,104389343	44,17688281	3,222466375	0	5,661556891	12,88548096	9,128026727	14,55606496	97,73486806	10,85942978
ENSG00000196937	FAM3C	3,427250056	3,79110928	7,532892226	31,74260516	2,432902349	11,63084455	8,019753179	17,71401805	11,2766962	97,56807105	10,84089678
ENSG00000139746	RBM26	1,597394888	11,04675104	4,613777576	26,37191059	0,106436735	8,572538196	4,524468601	24,45124393	16,26753791	97,55205947	10,83911772
ENSG00000070269	TMEM260	24,82316857	0,465062934	44,52778345	0,258607507	0,088978578	6,392243478	4,911871273	4,625756458	11,44273062	97,53620287	10,83735587
ENSG00000151743	AMN1	7,514583686	0,085477208	27,29291377	17,5509339	0	15,76071146	6,039534629	18,43355095	4,857958185	97,53566378	10,83729598
ENSG00000198841	KTI12	0	40,67498251	0	7,764109513	0	6,195027368	10,02884217	27,95391616	4,879284424	97,49616214	10,8329069
ENSG00000151065	DCP1B	0	16,11178612	0	5,037573262	0	23,53698854	18,53477139	7,382811378	26,84897697	97,45290766	10,82810085
ENSG00000130766	SESN2	7,463867009	10,45614385	0	6,70803372	0,103731237	24,77636284	20,11439464	5,726768934	22,06323531	97,41253755	10,82361528
ENSG00000172661	WASHC2C	15,8742953	14,62507226	15,3566698	6,818539322	9,573108811	7,419711866	14,21498325	2,665729587	10,8535412	97,40165139	10,82240571
ENSG00000197563	PIGN	0,238557971	4,24726317	0	21,07747124	0,094489697	21,01493228	9,831359488	24,32657791	16,56706882	97,39772057	10,82196895
ENSG00000163349	HIPK1	3,221093275	6,741655009	1,093931303	37,87949784	0,314051196	12,76793244	21,38262435	4,984692864	8,999782397	97,38526068	10,82058452
ENSG00000177683	THAP5	0,597747511	10,23564201	47,94277024	7,741346161	0	3,08457164	7,147044228	10,2607253	10,32889097	97,33873806	10,81541534
ENSG00000184730	APOBR	1,750622725	5,930470776	21,66458907	12,02307018	21,08562716	8,51831555	7,66432781	0,087002256	18,54550604	97,26953157	10,80772573
ENSG00000159921	GNE	36,87802307	0,090573214	0,187792669	1,983123435	0,060651502	7,033196209	8,031287929	38,22794777	4,770929131	97,26352493	10,80705833
ENSG00000155393	HEATR3	2,878867788	21,01926772	0	0,621397042	16,9616938	14,25665404	23,45565643	0	17,99823435	97,19177117	10,79908569
ENSG00000026103	FAS	20,69710936	10,09498387	0	46,77930703	0	0	8,660107175	0	10,86159309	97,09310052	10,78812228
ENSG00000139722	VPS37B	7,697680827	7,948121053	13,46974921	14,86690217	1,721010586	8,657740424	12,9389888	10,38106469	19,36313606	97,04439382	10,78271042
ENSG00000108395	TRIM37	0,027564861	15,06525648	1,673357035	34,93729868	0	12,5900766	19,59186505	1,808288403	11,32300957	97,01671667	10,77963519
ENSG00000164252	AGGF1	23,39330973	2,504505963	0	8,037543931	3,925171866	14,01271763	9,475104563	4,727998581	30,83899962	96,91535188	10,76837243
ENSG00000176658	MYO1D	13,21387013	0,589467025	41,62232552	17,8141977	7,719185082	3,566226368	9,638597805	0	2,678296242	96,84216587	10,76024065
ENSG00000120457	KCNJ5	0,024228086	0	40,7811112	16,19077043	22,62837809	9,09754966	7,906018996	0,105959457	0	96,73401592	10,74822399
ENSG00000242802	AP5Z1	22,79793916	5,204470828	0,03310075	12,89450041	0,513147238	12,72284013	13,59585641	3,81664123	25,11738542	96,69588157	10,74398684
ENSG00000140836	ZFHX3	1,163065206	6,743446729	9,006022807	13,54475189	17,08246957	15,9176747	12,47011265	6,698297717	14,04639442	96,67223569	10,74135952
ENSG00000111615	KRR1	5,497741987	10,47917	9,311699603	15,36911128	19,3476372	6,746340562	8,884611766	8,670563696	12,34711239	96,6539885	10,73933206
ENSG00000156170	NDUFAF6	15,95357579	4,313388413	0,651210602	11,98108426	18,7326658	9,924059172	8,308514879	13,00476511	13,75780908	96,62707312	10,73634146
ENSG00000187091	PLCD1	15,58743717	13,92299676	21,77961833	0	0	12,26830644	19,10848589	2,52766359	11,41616328	96,61067145	10,73451905
ENSG00000010810	FYN	0,081560151	10,92684982	23,55572826	13,52031173	14,68258202	1,477546628	2,979729919	23,0960877	6,269279526	96,58967576	10,7321862
ENSG00000147439	BIN3	18,94831368	11,71801654	28,94714023	4,109212198	0	10,48371951	5,927732221	0,195138524	16,24327872	96,57255163	10,73028351
ENSG00000102312	PORCN	0	4,987031553	35,54378549	13,17240656	0	4,683626377	9,83427378	12,83986861	15,49235373	96,5533461	10,72814957
ENSG00000188352	FOCAD	0,075284262	17,47358191	0,027698296	9,359963218	0	16,53154411	14,03977509	12,24807285	26,77700989	96,53292963	10,72588107
ENSG00000163815	CLEC3B	2,607999697	3,26670457	6,773119163	7,152532299	0,364586337	0,168437134	3,311907241	72,46077826	0,377353345	96,48341804	10,72037978
ENSG00000171448	ZBTB26	0,064896101	0,115156539	0	58,72017466	0	4,417631847	0,026942309	33,13569745	0	96,4804989	10,72005543
ENSG00000124279	FASTKD3	0	14,64128527	0	4,840585808	0,251394849	21,54456561	6,587521806	9,368288404	39,22486977	96,45851152	10,71761239
ENSG00000156374	PCGF6	12,45858428	28,70403059	0	22,20723188	0	11,58285276	12,68051228	3,625113274	5,189862989	96,44818806	10,71646534
ENSG00000157827	FMNL2	0,942429784	7,505039248	1,733675701	20,83828136	0	14,96577	18,20789424	8,44432725	23,80328137	96,44069896	10,71563322

ENSG00000275126	HIST1H4L	2,905972513	4,297147437	66,82213918	4,181650234	13,81221317	0,531764669	2,8150395	1,059085152	0	96,42501185	10,71389021
ENSG00000185262	UBALD2	14,28681759	13,16333455	6,065020341	11,22811103	16,32352465	9,150219685	11,25430857	5,206847731	9,686546009	96,36473015	10,70719224
ENSG00000133028	SCO1	0,528204733	9,789435094	9,932680496	32,07531584	10,35614235	8,731287753	12,47515487	6,224323342	6,171425441	96,28396992	10,69821888
ENSG00000136010	ALDH1L2	28,46157172	1,04511261	37,04253926	8,713408506	11,19759744	0,052431447	3,304285579	0,034808234	6,382172786	96,23392759	10,69265862
ENSG00000166432	ZMAT1	0	6,843613597	0	3,036535013	62,92392801	6,911112359	4,430121482	8,914130094	3,17035194	96,2297925	10,69219917
ENSG00000166329	CCDC182	0	12,2468702	5,011660438	0	0	31,50705663	17,49345975	0	29,932012	96,19105902	10,68789545
ENSG00000174437	ATP2A2	5,024192855	5,872007423	5,905433896	23,36929456	5,643462288	8,956394901	20,04604213	14,83289401	6,503614202	96,15333627	10,68370403
ENSG00000143398	PIP5K1A	21,0240794	9,128238301	7,251654756	8,979445934	0,066441728	17,89562742	10,49261611	12,10472606	9,180577511	96,12340722	10,68037858
ENSG00000182325	FBXL6	17,01393092	5,879261632	21,49712765	9,818886452	0	9,438468364	9,740215403	7,342990389	15,30824687	96,03912768	10,67101419
ENSG00000059769	DNAJC25	0,058030173	36,14356453	23,6987642	0,200410887	0	9,174778435	13,92508677	3,17237488	9,634900348	96,00791022	10,66754558
ENSG00000154237	LRRK1	17,83158298	11,08379544	0,905886778	8,54815279	0,184784054	12,60620642	12,50327532	10,91939741	21,34615878	95,92923997	10,65880444
ENSG00000184515	BEX5	1,076067136	7,637818924	0,282787486	40,87892786	7,306562408	11,81458837	19,14603235	0	7,751482618	95,89426716	10,65491857
ENSG00000185946	RNPC3	15,6677918	26,08400409	3,724212772	5,661760771	0	9,156852857	7,582668965	16,21617144	11,79977919	95,89324188	10,65480465
ENSG00000100147	CCDC134	28,55632831	0	0	1,02730305	0	9,014039207	26,08202177	22,1156994	9,072822347	95,86821409	10,65202379
ENSG00000173848	NET1	0	0,056308694	0,408622573	58,79530192	0,301652703	8,91917422	12,35732585	5,065460611	9,95188223	95,8557288	10,65063653
ENSG00000134058	CDK7	13,69752275	7,88000083	0	2,700072171	24,6744669	8,103248365	8,465092763	16,00200513	14,30772067	95,83012959	10,64779218
ENSG00000158545	ZC3H18	11,93438018	4,712949123	0,964315792	10,22860273	20,97067055	8,863400374	13,11577014	7,145150801	17,87977643	95,8150161	10,6461129
ENSG00000167895	TMC8	24,02710667	6,680218357	0,712900052	5,927081306	0,131568984	8,297048441	27,25917087	4,842419015	17,90717544	95,78468913	10,64274324
ENSG00000180787	ZFP3	0	11,39885783	67,22358119	6,331087212	0	4,058770876	5,73636168	0,397555124	0,558992948	95,70520686	10,63391187
ENSG00000147124	ZNF41	27,89101879	0,056049749	0,058106208	45,48908211	0,225199128	7,594986684	2,858751293	0,414424625	11,03269488	95,62031347	10,62447927
ENSG00000175054	ATR	22,99005457	0,908003225	0,08964931	20,00718865	0,057908174	8,52092537	16,93439779	1,598490208	24,43890009	95,54551739	10,6161686
ENSG00000100711	ZFYVE21	14,31858208	20,48617665	0,033132312	9,858925739	0,428030444	10,99478911	12,35262148	17,1715699	9,83502454	95,47885225	10,60876136
ENSG00000168010	ATG16L2	4,46999223	10,04076746	22,54774603	11,79245982	0	4,323529526	15,28796861	12,37335685	14,55096025	95,38678078	10,5985312
ENSG00000134202	GSTM3	22,39617721	4,516075791	0	5,987770996	22,90791902	9,081421041	8,162175282	14,60868614	7,707739976	95,36796546	10,59644061
ENSG00000187715	KBTBD12	1,116836462	8,89505088	19,20728769	60,68150922	0	1,625455977	3,60150317	0	0,063886738	95,19153014	10,57683668
ENSG00000157978	LDLRAP1	0,027998994	16,49493126	2,00875002	3,190978011	8,650227998	20,19700739	14,34411615	6,000108878	24,27678307	95,19090177	10,57676686
ENSG00000143442	POGZ	9,667203773	1,483748569	28,98268385	6,079448005	1,045871883	8,536317411	10,41424462	22,6470056	6,314560023	95,17108373	10,57456486
ENSG00000110047	EHD1	5,56745609	0,302814229	22,44559805	2,320567956	31,1769182	6,136161928	6,287686033	19,40440148	1,521611842	95,16321582	10,57369065
ENSG00000224531	SMIM13	14,19270553	10,69934316	1,877090908	22,05159495	0,146968505	16,77098934	13,78710217	6,558656241	9,01281477	95,09726557	10,56636284
ENSG00000141933	TPGS1	20,82853006	4,417495883	7,022012385	5,588393196	25,34135321	10,0220557	12,02337888	2,631137413	7,092989181	94,9673459	10,55192732
ENSG00000168872	DDX19A	3,928451181	17,15398038	0,460525205	4,522385185	38,0306713	8,9012811	8,07474419	6,35855992	7,507754245	94,93835271	10,54870586
ENSG00000100483	VCPKMT	20,07454296	26,4879932	0	8,380393345	0	11,86805585	3,297030622	5,788634759	18,81074289	94,70739363	10,52304374
ENSG00000185246	PRPF39	0,061277604	0,108735604	2,36722712	45,34089076	0,364068879	2,186574916	13,15250642	19,76443581	11,34221476	94,68793187	10,52088132
ENSG00000076351	SLC46A1	9,53702621	3,202601208	9,191446572	3,871049409	17,15673449	7,509153585	19,86008901	17,65585758	6,682437185	94,66639524	10,51848836
ENSG00000160813	PPP1R35	5,941791876	5,990661307	0	8,161505014	0	18,97815355	24,89223745	7,087068472	23,58374099	94,63515865	10,51501763
ENSG00000213190	MLLT11	1,965364477	16,77943804	25,99028656	3,393755498	3,877588187	15,71570125	9,175499125	0,648705899	17,01122768	94,55756672	10,5063963
ENSG00000186446	ZNF501	0,038647205	31,61469617	22,03934289	1,40144104	0	17,86402885	7,637324557	0,253530359	13,64141992	94,49043099	10,49893678
ENSG00000182552	RWDD4	11,57746202	6,523425565	0	27,76105576	0	8,373845375	10,06860689	15,5978554	14,57619783	94,47844884	10,49760543
ENSG00000146373	RNF217	0,437521803	3,630062291	26,27748334	18,49965467	34,64996711	2,479765252	0,922936944	3,283918153	4,246590852	94,42790041	10,49198893
ENSG00000139154	AEBP2	12,41728853	3,374603199	15,51650834	1,475824109	0,05317109	10,90676578	6,78067385	21,08636343	9,493196651	94,36681195	10,48742355
ENSG00000134815	DHX34	12,86388115	1,373387143	25,33340358	0,138255903	0,126851834	11,89680222	9,462351071	13,4811701	19,69408572	94,37018872	10,48557652
ENSG00000132740	IGHMBP2	21,8076044	0	22,0148199	10,89751634	0,055547957	6,467048001	8,869287196	13,28893852	10,9236935	94,32445582	10,48049509
ENSG00000275074	NUDT18	0	9,019599775	0	0,297531328	0	31,49841622	7,797187108	0	45,66696186	94,27969629	10,47552181
ENSG00000122126	OCRL	25,82300706	0,83794375	1,691655208	0,472086303	23,09456563	14,7900547	15,20915044	1,413042558	10,94293667	94,27444233	10,47493804
ENSG00000136932	TRMO	11,57687455	20,54287985	0,264553984	15,95533868	0,170886292	16,93448427	10,89618634	1,650993556	16,27207238	94,26426991	10,47380777
ENSG00000242498	ARPIN	22,71056423	3,652225147	20,25792107	0,364519173	17,14065103	8,092727781	7,872934685	1,654096205	12,48352076	94,22916008	10,46990668
ENSG00000104129	DNAJC17	9,665921364	13,05453234	10,6687458	10,24638049	17,8665444	5,807459774	9,140518145	3,640181803	14,13331761	94,22360173	10,46928908
ENSG00000146281	PM20D2	0,064261353	15,16601574	27,89398478	10,2088104	3,283447655	3,421927693	18,67515041	0,07026038	15,49050324	94,21976166	10,46886241
ENSG00000149499	EML3	13,43205073	7,765861205	13,7724202	2,328927824	0,065413031	11,90687928	20,63746991	13,18126963	11,10339878	94,19369059	10,46596562
ENSG00000137757	CASP5	0	33,68618092	0,34922123	40,32035412	0	11,77631606	5,595724606	1,24535641	1,167377652	94,14053099	10,460059
ENSG00000104825	NFKBIB	2,405805127	17,76844579	3,22954504	3,592908428	0	18,84698462	14,9549406	5,829525503	27,50912378	94,13727889	10,45969765
ENSG00000087206	UIMC1	3,645419682	29,41855801	0,291567127	8,484355163	5,424053503	11,4505097	14,5816161	5,545367191	15,2825334	94,12397988	10,45821999
ENSG00000174197	MGA	3,004918152	0	1,464628021	32,52263011	2,929744836	12,80211917	12,38993761	11,47563254	17,41488572	94,00449615	10,44494402
ENSG00000151151	IPMK	13,54117374	4,928919089	3,467337522	1,498887922	26,52271362	9,584855244	3,233028414	14,1545088	17,01991784	93,95134219	10,43903802
ENSG00000082805	ERC1	0	7,795288048	0,041231103	21,65427444	0,18643005	10,76622327	11,94778302	18,7223383	22,82422433	93,93779257	10,43753251
ENSG00000140365	COMMD4	5,951692339	8,543837147	1,599236445	6,890007443	10,85987552	17,54769875	19,2490257	8,627661668	14,63962869	93,90896371	10,4343293
ENSG00000130818	ZNF426	0,040050509	10,19835065	0	17,25504271	0,142771353	15,32463185	8,995421364	30,87150412	11,03355976	93,86133233	10,42903693
ENSG00000164284	GRPEL2	24,24471478	8,859183529	0,817773543	17,00594539	0,162533398	6,720523474	5,423134965	13,08577717	17,53745186	93,85703811	10,42855979
ENSG00000174206	C12orf66	12,0908608	20,33749126	32,48275536	9,48221778	0,538766108	4,922834248	7,111170675	0,385571257	6,443753398	93,79542087	10,42171343
ENSG00000125834	STK35	22,31852794	11,63025543	5,24044694	11,9094347	0,152707672	6,937437297	8,003068661	20,98294631	6,480261443	93,6550864	10,40612071
ENSG00000167491	GATAD2A	0,227825696	3,087161185	17,52615756	29,00469633	0,246105772	13,73492165	16,06212858	2,490940408	11,23332186	93,61325903	10,40147323
ENSG00000152503	TRIM36	0,049439087	20,74777786	0,045473597	9,476127002	0	27,46630192	23,09081032	1,351360083	11,37029271	93,59758258	10,3997314
ENSG00000167840	ZNF232	22,71526116	13,33992055	49,74590171	1,307476609	0	6,116245312	0,359256498	0	0	93,58406188	10,3982291
ENSG00000187566	NHLRC1	28,90460204	24,45245108	0,123656628	0,116074277	0	9,889687401	24,27917901	0	5,787035836	93,55268627	10,39474292
ENSG00000196715	VKORC1L1	1,736332887	3,653280697	4,791186867	8,566479796	2,888502885	15,90475653	14,9320405	17,19431351	23,79518834	93,46208201	10,38467578
ENSG00000146090	RASGEF1C	0,150358777	5,336159206	0	7,789103719	52,88497141	14,8026352	7,365919988	0,109596871	4,992891643	93,43163682	10,38129298
ENSG00000101868	POLA1	1,550218923	0,507844697	41,0652419	6,753998711	0,056678905	14,53288669	6,970574	12,62076473	9,327524319	93,38573287	10,37619254
ENSG00000139629	GALNT6	15,17893033	12,18993168	3,51873543	0,781348774	0,04887948	7,971475919	30,56919753	10,07449886	13,00192084	93,33491885	10,37054654
ENSG00000179364	PACS2	14,44735861	14,64012376	0,899393671	6,943913827	0	12,10477555	12,25982052	13,3103951	18,70040231	93,30618335	10,36735371
ENSG00000159147	DONSON	21,69174811	0,152743948	31,90714323	4,384837226	0	13,75106175	7,218743111	0	14,18594929	93,29222668	10,36580296
ENSG00000140743	AC092338.1	0,038255841	0	57,84801047	0	2,272895838	20,37129866	5,399988972	0	7,339761386	93,27021117	10,3633568
ENSG00000166225	FRS2	0	6,923854462	2,494988461	2,702309182	45,86905919	5,956469199	6,462363137	21,03429103	1,822024728	93,26535939	10,36281771
ENSG00000196670	ZFP62	0,108013274	8,81667763	0	36,43266503	0	5,020405824	1,016438239	7,715651088	34,14049766	93,25034874	10,36114986
ENSG00000142733	MAP3K6	0	7,638307971	0	0,571769865	64,38362313	8,824327486	0	11,78242781	0	93,20045626	10,35560625
ENSG00000063176	SPHK2	4,09902197	3,926859046	6,522748204	0,60793637	47,9301496	6,598872996	7,391662095	2,474547435	13,57740444	93,12920216	10,34768913
ENSG00000171174	RBKS	7,764006485	3,826955282	0	11,25504489	32,80236353	7,366787421	2,507019003	11,94720606	15,6198314	93,08921407	10,34324601
ENSG00000132781	MUTYH	11,01727108	5,693473013	0,142225684	12,94995781	0	12,01143341	12,71073202	8,453173894	30,09487045	93,07313736	10,34145971
ENSG00000243335	KCTD7	0	4,678750035	23,40691646	3,414747189	45,61955826	3,289700794	0,149270719	6,639266922	5,773157004	92,97136738	10,33015193
ENSG00000064201	TSPAN32	22,86777421	5,796900608	17,14931272	4,058835619	7,574322011	8,04839075	10,6185016	6,533770779	10,2894199	92,9372282	10,32635869
ENSG00000188763	FZD9	0	0	0	10,82578808	77,87536098	2,341766053	0	1,837317955	0	92,88023307	10,3200259
ENSG00000068971	PPP2R5B	15,20100383	15,00318334	1,240982585	1,475525154	0	24,73845206	15,90784566	0	19,30370127	92,87069391	10,31896599
ENSG00000135913	USP37	0,113984267	9,59301121	0,179728512	26,59962133	0,116094033	12,01421418	15,88659915	6,053219448	22,28956673	92,84603886	10,31622654
ENSG00000105963	ADAP1	22,08607985	11,81032313	4,502146576	4,741461123	3,546489654	10,74830519	14,373453	8,288601634	12,70055238	92,79741254	10,31082362
ENSG00000142751	GPN2	0	6,209490538	15,78541843	1,891594661	36,3022566	12,72894276	8,539865529	5,522768648	5,800686861	92,78102402	10,30900267
ENSG00000183808	RBM12B	4,839981523	3,238259912	44,15278548	9,111145083	1,461360438	3,201477392	8,284533604	0,260253007	18,22359571	92,77339215	10,30815468
ENSG00000129465	RIPK3	34,34969305	11,45976959	0	0,161619681	0	17,21277275	19,89495509	0	9,669314259	92,74812442	10,30534716
ENSG00000085231	AK6	14,68738257	16,41430001	14,80828565	12,9248165	0	9,148340211	5,042265679	13,43355485	6,252780189	92,71172665	10,30130285
ENSG00000138686	BBS7	31,60523429	0,055527438	0	0	0	11,20042411	13,56295575	31,47647568	4,810688607	92,71130586	10,30125621
ENSG00000010318	PHF7	1,61711669	3,746335911	0	2,482140068	50,70773859	14,94378857	2,685453004	8,349268204	8,176363049	92,70820408	10,30091156
ENSG00000175866	BAIAP2	20,71013848	5,056332428	16,61720071	9,333599403	0,558503176	8,78900314	14,65934478	4,978359056	11,9225024	92,62498356	10,29166484
ENSG00000073331	ALPK1	0,110335481	4,503114881	0	5,059505024	21,12284749	15,03505713	15,96117804	6,728790885	24,06385279	92,58468173	10,28718686
ENSG00000183784	C9orf66	0,393773474	13,276086	19,73930345	5,354691661	0	10,53829396	14,10008617	9,041206408	20,09806039	92,5415015	10,28238906
ENSG00000143486	EIF2D	15,28715914	21,10961692	0,103389573	3,137948492	0,044522383	15,13887732	10,32882475	6,55461577	20,82881926	92,53377361	10,2815304
ENSG00000149257	SERPINH1	10,22480378	13,4903474	22,79250931	0,33234832	0	11,54321319	17,43919755	8,312157565	8,373597485	92,5081746	10,27868607
ENSG00000137976	DNASE2B	0	0	0,1883334	75,66407123	0	0	0	16,56650271	0	92,41890733	10,26876748
ENSG00000134470	IL15RA	1,289410319	18,37802313	0	0	51,30250605	3,607744442	9,5665516	8,185827436	0,051155112	92,38121809	10,26457979
ENSG00000239305	RNF103	4,815480392	11,51096814	8,382597138	15,94335373	0,094579284	8,476863808	10,52470391	24,23650963	8,394173971	92,37923	10,26435889
ENSG00000137801	THBS1	6,484653229	6,060864164	3,794224981	7,237113294	9,489675175	13,31560099	22,40052335	23,48907004	0,08117341	92,35289864	10,26143318
ENSG00000115998	C2orf42	22,22948891	0,590946557	0	29,54387729	0,098930452	21,16159697	9,74728199	0,182057613	8,754753529	92,30893331	10,25654815
ENSG00000109184	DCUN1D4	3,151026982	0,070332328	49,79945117	4,482948416	0,376779061	18,49494953	4,887172276	0,736706577	10,18804542	92,18741177	10,24304575
ENSG00000158158	CNNM4	1,128577375	13,43872052	0,054634453	2,102659976	0,423487668	27,97784489	23,37772695	10,13124853	13,55130892	92,18620929	10,24291214
ENSG00000169067	ACTBL2	1,838854475	63,14868864	8,257819691	1,587650168	9,125750003	0,653191247	1,392118931	3,78450471	2,394586543	92,18316441	10,24257382
ENSG00000205269	TMEM170B	17,02989448	5,3984274	20,57259084	3,815267877	0	22,09404148	8,862374142	1,56093261	12,83430038	92,16782921	10,24086991
ENSG00000117713	ARID1A	12,45744964	6,056284587	14,94629179	23,16475243	7,998414426	6,27668472	6,761968454	6,488865025	7,98700469	92,13771577	10,23752397
ENSG00000179630	LACC1	13,39381481	0	36,55509674	2,912870893	0	9,667930458	14,62279824	14,75483668	0,124478582	92,03182641	10,22575849
ENSG00000172728	FUT10	0	0,382650018	46,51183247	5,725116282	0	9,943980338	8,415420056	13,32112242	7,724266802	92,02438838	10,22493204
ENSG00000147246	HTR2C	0	1,495215614	0	89,7267106	0	0,274119086	0,025912914	0	0,422203995	91,94416221	10,21601802
ENSG00000173614	NMNAT1	24,45303378	0,058165293	1,628083009	0,339611681	0	14,14378669	9,335428173	33,68855678	8,264324743	91,91099014	10,21233224
ENSG00000147050	KDM6A	0	11,64365038	0,116814722	17,39810168	0,251518099	20,33502656	14,76328322	5,878300893	21,47687083	91,86356638	10,20706293
ENSG00000096654	ZNF184	1,355251755	0,858878924	0	11,95185822	0	26,35863102	14,7895843	26,24848582	10,29834989	91,86103993	10,20678221
ENSG00000167562	ZNF701	16,09523784	20,40575143	0	29,02498468	0,09974102	9,008614844	6,603701684	1,560168768	9,0587598	91,85696006	10,2063289
ENSG00000164162	ANAPC10	11,79611487	15,59516719	17,45828132	15,00289549	0,079415644	10,86013505	3,523824008	7,234193318	10,23347723	91,78350412	10,19816712
ENSG00000133422	MORC2	23,13558039	4,181008317	0,041280086	18,75445912	0,106658017	11,38263832	8,235504417	17,71411923	8,224274374	91,77552227	10,19728025
ENSG00000180891	CUEDC1	0,133348545	7,098711615	15,25799696	3,546063015	32,13423826	6,851936983	8,503460552	15,10458048	3,116028877	91,74636529	10,19404059
ENSG00000110172	CHORDC1	0,789657906	6,112255701	1,653003562	33,66599546	22,00197575	7,055571872	6,907142342	3,602359012	9,895963119	91,68392472	10,18710275
ENSG00000169221	TBC1D10B	28,65626928	11,64825257	4,172309178	10,2430817	0,051828192	12,18768705	11,824524	4,864241	8,01964355	91,66783651	10,18531517
ENSG00000213983	AP1G2	12,77330183	14,30712716	18,70128548	5,826299273	0	8,756191086	11,51554591	5,749187778	14,011917	91,64085552	10,18231728

ENSG00000144036	EXOC6B	15,90091618	11,59660901	0,163565813	5,808790746	30,252228	3,758492029	8,674765525	8,84658595	6,561218792	91,56317204	10,17368578
ENSG00000100095	SEZ6L	0,168815049	5,729049855	3,377219468	42,37779297	0	11,74649663	3,994866038	1,938032654	22,21542006	91,54769273	10,17196586
ENSG00000123159	GIPC1	8,270240692	5,254133786	13,83639682	5,700606427	4,529410444	21,63563602	23,51155771	2,455932596	6,32045605	91,51437054	10,16826339
ENSG00000065308	TRAM2	0	2,43523315	43,19401283	11,66376004	0,050960344	10,9947835	8,706540705	10,50338344	3,955864672	91,50453868	10,16717096
ENSG00000183401	CCDC159	0	3,562910434	34,74751237	8,090011516	0,530193222	15,2683454	9,570817648	13,17185038	6,539383043	91,48102401	10,16455822
ENSG00000133612	AGAP3	6,299059986	4,580175769	19,33731815	4,942037752	23,23346521	6,578275809	8,417250346	7,457369211	10,6254406	91,47039284	10,16337698
ENSG00000167553	TUBA1C	7,668102808	23,97123473	3,541530089	17,41069038	1,395835837	12,53912802	11,29801953	3,567637309	10,07287348	91,46505218	10,16278358
ENSG00000172890	NADSYN1	4,097630306	10,45330062	2,204970348	12,57904569	0,066245627	11,91560927	7,460481525	19,48513498	23,16367833	91,42609671	10,15845519
ENSG00000138722	MMRN1	0,054849572	0	0	49,62973746	3,715009059	3,613297496	14,61922925	0	19,76516553	91,39728836	10,15525426
ENSG00000166529	ZSCAN21	0	0	2,987636578	17,19244459	0,167811936	16,82364259	6,038992615	39,37421248	8,771261098	91,35600189	10,15066688
ENSG00000143970	ASXL2	1,171939464	2,058987945	14,30136423	16,10932347	23,96323	6,994157	7,563096876	15,24926477	3,895884249	91,307248	10,14524978
ENSG00000123395	ATG101	7,22723267	3,604284465	8,428905783	12,80612411	0	18,04841535	20,55218942	9,399670411	11,21237224	91,27919445	10,14213272
ENSG00000100296	THOC5	0,076725347	0,034036811	12,42053755	24,80836456	0,045584862	13,79429141	12,10427168	6,962698427	21,01919921	91,26570986	10,14063443
ENSG00000204256	BRD2	12,4921882	7,859896531	17,19774218	6,555978019	9,65343657	11,51938895	9,897970267	6,115025767	9,966374354	91,25800084	10,13977787
ENSG00000083812	ZNF324	11,33862863	8,142730229	17,42283459	0,46069028	0	7,17656979	11,36410316	17,91020696	17,42311146	91,23887511	10,13765279
ENSG00000164466	SFXN1	1,36687029	8,217663564	18,61457123	7,151316301	5,13925469	11,58036562	8,334197832	12,75878038	18,04018489	91,20320481	10,13368942
ENSG00000142556	ZNF614	38,24121324	8,917402457	0,29661757	2,877106247	0	13,92669255	4,663578052	19,09856561	3,172908792	91,19408452	10,13267606
ENSG00000108509	CAMTA2	9,911769414	6,011154104	12,4634053	6,412882139	0	12,56313899	12,79289004	9,986564478	21,04738097	91,18918544	10,13213172
ENSG00000151353	TMEM18	4,924609458	27,48225954	0,361055743	19,96526904	0,127211315	8,737290968	15,1559983	4,135796925	10,26994597	91,15943727	10,12882636
ENSG00000152242	C18orf25	45,25270092	10,02347667	0	1,19882415	0,262485413	3,378152079	9,367368422	15,00877486	6,636682742	91,12846525	10,12538503
ENSG00000172932	ANKRD13D	8,325525947	2,785157008	19,45818847	7,109623725	0,108119085	18,53163449	8,083893135	4,824951535	21,87746205	91,10455545	10,12272838
ENSG00000127616	SMARCA4	15,94619725	4,605557233	6,430646224	11,2788473	10,81130895	9,93696888	11,3557887	7,518444019	13,20407856	91,08783711	10,12087079
ENSG00000241058	NSUN6	0	1,703472135	1,76597233	54,3269179	0,103701283	5,317953801	6,123155099	1,71753457	19,96387707	91,02258418	10,11362046
ENSG00000171208	NETO2	33,14696517	0,035843069	1,374851445	0,104639075	25,8741285	10,95573117	7,966636506	0,132509518	11,32816658	90,91947104	10,10216345
ENSG00000221944	TIGD1	2,59244212	34,75725506	0,211955851	0	0	17,141352	12,05433613	0,125975817	24,01905111	90,90236808	10,10026312
ENSG00000070759	TESK2	35,51257762	20,03591926	0	2,358556565	0	6,498455852	13,60931591	10,553198	2,295784096	90,8638073	10,09597859
ENSG00000070950	RAD18	0,02163668	13,0922712	0	25,03242168	0	5,13125495	10,06062227	23,6565412	13,86398026	90,85872823	10,09541425
ENSG00000063761	ADCK1	7,422775056	0,151979218	36,97298124	0,443683123	0	17,86681064	13,60664473	8,427860687	5,898774937	90,79150963	10,08794551
ENSG00000183475	ASB7	24,72311171	21,363917	0	1,112007489	0	20,53516124	4,765873274	1,224512016	17,04540088	90,76998361	10,08555373
ENSG00000104517	UBR5	11,30415272	4,429357737	1,092339204	21,81736907	22,76170713	7,485413881	5,249995699	6,468265075	10,06184053	90,67044105	10,07449345
ENSG00000124574	ABCC10	7,773215951	2,155215779	33,99558772	9,260336059	0,088813538	13,15057248	8,036803756	2,369878189	13,76555791	90,59598139	10,06622015
ENSG00000187257	RSBN1L	3,76200169	8,913204175	10,40008993	26,3112457	0,649308491	8,560893457	6,264791264	9,329376524	16,33588346	90,5267947	10,05853274
ENSG00000128908	INO80	15,59795454	8,678105791	2,496827236	23,139657	7,577615664	9,248793198	10,80446495	3,674635909	9,114799496	90,36885379	10,04098375
ENSG00000138767	CNOT6L	0,177454497	3,921795026	1,661885934	33,31677481	6,479221166	12,57569227	10,72934087	9,771578435	11,66624908	90,29999208	10,03333245
ENSG00000123444	KBTBD4	35,33477334	3,615767087	0	8,556556017	0	9,50820651	7,805846582	7,392498752	17,99800829	90,21165658	10,0235174
ENSG00000146918	NCAPG2	29,71178078	19,49286435	1,233168728	7,841491795	0,051390604	6,078006244	4,057855614	0	21,72819243	90,19475054	10,02163895
ENSG00000137492	THAP12	13,00761631	8,464421656	15,92887991	9,626277587	14,29550055	9,338779926	7,364701282	5,236349004	6,87971406	90,14224028	10,01580448
ENSG00000002016	RAD52	13,8848615	1,40790549	44,25124726	10,71141185	0	6,454277805	1,347533399	0,236587996	11,70966155	90,00348685	10,00038743
ENSG00000130939	UBE4B	0,148991579	1,527539878	23,84513776	25,12728338	0	12,2506276	4,384863706	3,185609328	19,50492642	89,97497965	9,997219961
ENSG00000112419	PHACTR2	26,03776227	2,436785817	7,504273877	7,369599692	0,095986513	18,5806838	10,23973843	10,45120024	7,235827711	89,95185834	9,994650926
ENSG00000203880	PCMTD2	1,739801331	14,18338295	18,18257948	13,10552767	21,1527603	5,869020901	5,066547696	3,47361551	7,070416666	89,8436525	9,982628056
ENSG00000090857	PDPR	25,37668129	2,556192394	0,294442088	25,95530368	0,034580409	10,67195621	13,96665419	1,272737862	9,627869907	89,75641804	9,972935338
ENSG00000115129	TP53I3	24,70579399	15,30914533	0	0	0,155327657	7,391344896	15,08683058	16,00721088	11,09291526	89,7485686	9,972063177
ENSG00000104375	STK3	11,06593595	17,67899096	9,924709976	21,986111	0,028492347	9,701372186	7,635333596	0,498116216	11,22097774	89,74003997	9,971115552
ENSG00000142188	TMEM50B	8,677502488	7,638859132	8,605036711	7,784735671	16,2722619	10,2345017	12,74967707	11,4147428	6,336624357	89,71394184	9,96821576
ENSG00000075539	FRYL	5,183739085	5,87635681	3,597011357	20,40238224	6,209069341	10,19526478	9,174858262	19,2682235	9,783011689	89,68991707	9,965546341
ENSG00000164898	FMC1	0	8,682308738	16,7457891	27,11523079	0	7,495959791	6,424685675	13,68516658	9,516237586	89,66537826	9,962819806
ENSG00000127603	MACF1	1,606099244	3,126425539	27,50528435	13,56587732	11,98850874	4,907392055	8,635921621	4,631388548	13,62634238	89,59323979	9,954804421
ENSG00000174177	CTU2	0	3,502138559	0	15,33604195	31,91476171	10,45773652	7,766193179	1,688762696	18,89070272	89,55633734	9,950704148
ENSG00000162341	TPCN2	7,977246168	8,399061478	12,12501017	9,064485847	0,035042695	12,06121972	9,060113747	13,73586317	16,99240743	89,45045043	9,938938936
ENSG00000178163	ZNF518B	15,92094881	18,44934879	0,025467714	1,458271503	0,065802573	5,320082624	18,72571423	16,62011705	12,78705801	89,37281131	9,930312368
ENSG00000103197	TSC2	6,34367358	4,093342954	28,28049313	3,478587334	0,018774443	12,3253309	15,67067248	0,069099659	19,08210841	89,36208289	9,929120322
ENSG00000154124	OTULIN	0,736782443	5,322995358	3,452968492	16,66057999	0	10,36218833	19,6202156	20,67613558	12,51346447	89,34533026	9,927258918
ENSG00000159884	CCDC107	14,0857009	9,74059356	15,24222613	4,113436885	7,384185244	10,8029505	9,97570837	6,34144475	11,59152401	89,27777035	9,919752261
ENSG00000131381	RBSN	0	7,244336324	0	1,680050966	29,78667155	10,24242798	15,34916699	5,54826379	19,38003123	89,23094883	9,914549869
ENSG00000138166	DUSP5	4,768775406	5,394572783	22,36999613	10,29329288	2,833274496	18,58722043	7,2262992	9,456821695	8,064346134	89,18359916	9,909288795
ENSG00000144589	STK11IP	17,53275184	5,695134634	0,04879412	0,137406516	0	24,11338556	27,28764988	3,364087224	10,99355415	89,17276391	9,908084879
ENSG00000151466	SCLT1	0,774063057	31,95010469	2,074016329	2,365354094	3,839118493	9,251862803	4,736574894	19,20788911	14,42483586	89,17077272	9,907863636
ENSG00000196923	PDLIM7	9,32654305	10,10019787	19,49077617	6,21696672	0	9,223528771	8,911326053	2,999179192	22,89872058	89,16723841	9,907470934
ENSG00000123096	SSPN	23,48295043	20,28460226	0,081507139	4,590558122	11,42479207	5,837639409	5,591992513	10,56072947	7,274742918	89,12951434	9,903279371
ENSG00000132716	DCAF8	3,58506692	10,10789667	13,00684528	29,74940728	0,236666365	8,506552569	10,02175052	2,830927493	11,07191618	89,11702928	9,901892142
ENSG00000168612	ZSWIM1	22,04719052	0,581022752	0	1,884687429	15,56305709	4,613620226	14,95312732	13,7233569	15,63822307	89,00428532	9,889365035
ENSG00000107938	EDRF1	0,039041615	1,42020695	49,26865984	7,516922792	0	4,222243071	7,277635844	14,04378927	5,209752659	88,99825204	9,888694671
ENSG00000134982	APC	12,86383395	0,538870579	4,379750756	25,79984074	0,02886797	10,32273202	7,382980951	5,47182644	22,14023682	88,92894022	9,880993358
ENSG00000008311	AASS	0	0,040237395	34,41380277	9,828133047	0,053889187	6,149446646	6,420375632	21,42072827	10,56960263	88,89621558	9,877357286
ENSG00000155744	FAM126B	1,816629811	5,777298363	46,43851771	22,03994087	0	5,025238389	2,125453711	0,644876205	5,005234206	88,87318926	9,874798807
ENSG00000121964	GTDC1	7,735853956	18,42269214	0	18,2223337	0,050715653	9,407300741	8,735612721	12,20288651	14,09399555	88,87139097	9,874598997
ENSG00000085978	ATG16L1	33,36077589	10,90837308	2,988044722	2,847975579	0,05938772	11,02961003	8,112932001	7,540924478	11,98613258	88,83415608	9,870461787
ENSG00000147854	UHRF2	0,091406737	6,460927313	8,323428712	2,946337046	33,34481868	8,329818248	6,640984404	12,05941259	10,60481214	88,80194587	9,866882874
ENSG00000154079	SDHAF4	22,35177191	20,48306945	0,579125206	7,429398582	0,249387182	15,78454792	9,148878375	8,031395738	4,646163057	88,70373743	9,855970825
ENSG00000170627	GTSF1	0	18,27275119	0	0	0	10,97744307	4,126005371	28,93266221	26,36016441	88,66902626	9,852114029
ENSG00000173599	PC	0,020545285	16,84318206	7,407763345	0,567635567	0,244131572	16,71512404	26,40762392	1,976766957	18,47094599	88,65371874	9,850413193
ENSG00000187189	TSPYL4	0	15,90890335	9,908859988	24,96985403	0	2,778406787	8,9450141	11,69959713	14,40532852	88,61596391	9,846218212
ENSG00000136682	CBWD2	12,25658631	5,077963079	16,15701385	22,62513834	1,368718599	8,47732699	4,034896336	13,45981683	5,157484479	88,61494481	9,846104979
ENSG00000163644	PPM1K	14,07317688	2,664677338	0,024446407	16,20086994	2,526550294	15,75793007	10,8576912	14,79124042	11,58782731	88,48440987	9,831601096
ENSG00000213020	ZNF611	4,777698513	0,562693061	0	40,62963441	0,150720849	11,86070295	10,19841395	0,277365334	20,01984082	88,47706988	9,830785542
ENSG00000169957	ZNF768	14,79850249	7,583585482	0	0,330436861	0	13,16639444	21,97981755	11,99551406	18,59249716	88,44674804	9,827416449
ENSG00000088325	TPX2	0,041916941	0,148761165	0,077109597	48,42316172	0,996165166	18,4549629	20,25623668	0	0	88,39831417	9,822034908
ENSG00000184232	OAF	24,22541589	8,468611057	0,286282285	0,08957603	0	6,720716804	13,99857806	21,09880864	13,46159155	88,34958033	9,816620036
ENSG00000151498	ACAD8	20,22109672	3,625560916	0	7,674558076	0	19,42638478	12,57501655	1,888460729	22,8487082	88,25978597	9,806642885
ENSG00000136878	USP20	30,07978938	0,048567651	0,050349592	5,860521074	0	14,66480011	22,38512534	2,453870181	12,69047979	88,23350312	9,803722569
ENSG00000137171	KLC4	18,08522959	7,81401808	18,62730911	7,603995676	19,0835409	4,860711925	4,985964572	0	7,153512701	88,21428255	9,80158695
ENSG00000072415	MPP5	0	12,20639552	0	21,57794654	33,14741584	8,748115734	6,960209427	0,151176744	5,420434997	88,21169481	9,801299423
ENSG00000213965	NUDT19	10,6444588	8,947104149	11,89871	18,20476567	9,319868811	10,4567839	5,751252158	7,350415051	5,637407147	88,21076569	9,801196188
ENSG00000127481	UBR4	9,516025962	6,099820042	7,958069417	15,97712224	15,17377556	7,393687352	5,209911195	6,109977811	14,75558536	88,19397494	9,799330549
ENSG00000174796	THAP6	0	0	0,936091255	19,03833125	34,01211465	5,028298663	5,915256286	9,411844704	13,79441705	88,13635385	9,792928205
ENSG00000196502	SULT1A1	19,94679887	3,220473006	27,17708679	16,25534975	3,103833288	4,786061086	2,718124826	3,746087251	7,176024656	88,12983952	9,792204391
ENSG00000131626	PPFIA1	12,30420146	3,596915073	0,023750863	11,45937944	0,398883121	11,75152666	15,08345498	20,32748536	13,17947825	88,12507521	9,791675023
ENSG00000100526	CDKN3	0	7,30234205	0	49,60038461	0	14,36807282	16,46968955	0,179975255	0,202447192	88,12291147	9,791434608
ENSG00000169155	ZBTB43	26,6993436	7,604707887	0,039418619	21,31289639	0	6,728647892	9,803481698	0,046856859	15,75953209	87,99488504	9,777209449
ENSG00000184863	RBM33	4,303636417	12,25138593	15,30456987	6,517413316	0,027346752	9,993547531	10,78708535	13,18517464	15,60986248	87,98002229	9,775558032
ENSG00000163681	SLMAP	0,010681457	4,188830283	3,635139486	15,34586123	4,315401301	16,3248408	16,08844152	13,22018557	14,81832736	87,94770901	9,771967667
ENSG00000138180	CEP55	0	0	0	52,56990164	0	26,07093746	8,707357189	0	0,568164238	87,91636052	9,768484503
ENSG00000127993	RBM48	1,504688652	10,64086719	31,83195745	0,038209837	0	6,025160788	10,4543299	9,096748967	18,26080194	87,85276472	9,761418302
ENSG00000125633	CCDC93	5,902024769	16,09184477	7,513907201	8,314976872	0,133583711	5,492637815	9,536678675	15,69205696	19,17226367	87,84997444	9,761108271
ENSG00000150281	CTF1	0	10,95772597	0	13,485822	0	15,75941081	10,17938391	25,81520059	13,62576666	87,82330993	9,758145548
ENSG00000177614	PGBD5	3,906296177	1,953241895	5,729002662	14,44101352	7,241688027	15,5785271	21,11049203	0,645781335	17,20281391	87,80885666	9,756539629
ENSG00000139546	TARBP2	19,84018984	3,933623473	0	0,382789694	0	16,5099278	15,36934874	8,725411563	23,03769848	87,79898959	9,755443288
ENSG00000147133	TAF1	3,436861045	4,691250462	21,65480329	26,30974191	3,604402589	9,028835252	9,450711932	1,886458857	7,683709807	87,74677515	9,749641683
ENSG00000196981	WDR5B	0,609316817	28,68831076	4,558275817	0,07014381	0	3,567969391	14,77311974	19,18652328	16,28655007	87,74020968	9,748912187
ENSG00000203710	CR1	27,57290553	3,345648416	8,659953811	2,903194504	1,284295431	1,806385521	2,652396751	38,13009553	1,373577571	87,72845306	9,747605895
ENSG00000147130	ZMYM3	3,277412608	11,67014778	0,241162608	18,82685835	26,84554213	3,862290753	7,129820774	7,59674613	8,276565769	87,7265469	9,7473941
ENSG00000004660	CAMKK1	27,539413	1,98650458	22,72816811	0,210884947	0	7,352448779	5,999726274	5,741656682	16,02125192	87,5800543	9,731117144
ENSG00000161835	GRASP	0	18,9969536	0	1,109181614	35,36474558	6,817437796	5,466826368	6,906000526	12,90326545	87,56441094	9,729378993
ENSG00000141034	GID4	10,93448851	1,286669866	27,95807521	3,555927677	0	21,11299661	10,45184723	9,196355526	3,067716107	87,56407675	9,729341861
ENSG00000102547	CAB39L	67,18062992	0	0	0,158334329	0	5,067252646	8,374840121	0	6,691076247	87,47213326	9,719125918
ENSG00000221886	ZBED8	21,0254472	6,32673699	0	45,90355035	0,249214117	9,095722109	0	0	4,836394577	87,43706535	9,715229483
ENSG00000130338	TULP4	16,21194401	9,843878066	18,78091023	0,849605127	1,14005569	9,831737879	9,079619467	5,998120355	15,67252723	87,40839805	9,712044228
ENSG00000100836	PABPN1	4,327469903	6,779648093	10,04056085	30,76555299	5,744398252	5,821423744	11,62122012	4,859331875	7,288098913	87,24770474	9,694189416
ENSG00000184220	CMSS1	1,866080309	24,03492883	0,077141695	19,18905738	2,092817647	10,10611117	12,13442783	10,22435251	7,452427492	87,17734486	9,686371652
ENSG00000221883	ARIH2OS	11,68186457	6,519662097	0	0	0	17,06686107	15,3317862	20,18864565	16,33688257	87,12570215	9,680633572
ENSG00000178105	DDX10	11,07956892	9,721160124	3,585381244	0,485121961	0	11,12368147	10,83249555	11,78752176	28,50539268	87,1203237	9,680035967
ENSG00000020129	NCDN	8,08258696	4,635360994	0	8,278595899	25,78167428	7,929425569	5,818026377	8,06429695	18,4070076	86,99697463	9,666330514
ENSG00000101181	MTG2	17,9278811	10,86157843	7,311052709	17,08174819	0,068941744	12,13513721	6,190415689	2,53741269	12,84406758	86,95823534	9,662026149
ENSG00000198625	MDM4	1,578644225	7,356366645	0,757576578	37,57103332	0,521973172	10,38448286	10,88518959	4,322545215	13,54005368	86,91786528	9,657540587
ENSG00000173120	KDM2A	6,754195625	1,523974377	23,61799897	8,797537916	11,1045883	8,310711866	10,58780569	8,339645187	7,877128388	86,91358631	9,657065146
ENSG00000145916	RMND5B	7,375074581	11,10297223	5,664963076	5,554690529	0	7,27904659	17,86616747	13,12473749	18,9127302	86,88038217	9,653375796

ENSG00000006459	KDM7A	0,741212012	16,06907699	19,29676311	0	1,991268096	7,111969699	11,10475321	14,19973669	16,3492622	86,864042	9,651560223
ENSG00000197863	ZNF790	10,6647834	0,765324858	0	12,93165027	0	4,520137663	15,91982157	28,97947167	13,06808706	86,8492765	9,649919611
ENSG00000001084	GCLC	8,931008745	20,93405622	13,47139272	2,686006563	6,147281549	2,283525171	23,23363423	3,974692924	5,158820882	86,820419	9,646713222
ENSG00000127124	HIVEP3	8,689503585	10,24898311	25,01345711	9,491788478	4,49357335	8,009084933	4,521221536	7,727085271	8,615654161	86,81035154	9,645594616
ENSG00000170779	CDCA4	0	14,29806129	0	33,53839904	0	9,771096439	5,242492981	8,152384873	15,75221219	86,75464682	9,639405202
ENSG00000085511	MAP3K4	22,49984567	7,62860782	1,626046843	28,72296716	0,047742295	8,205096322	3,30272876	5,798639047	8,869835349	86,70150927	9,63350103
ENSG00000135749	PCNX2	6,36047965	1,105380343	0,683541142	16,30489752	42,72426102	5,351567022	6,197746204	1,338275532	6,59945342	86,66560186	9,629511318
ENSG00000087995	METTL2A	7,9428403	7,047187616	23,21925885	0,203696311	1,308260628	11,6564808	12,73313283	10,31801928	12,23501695	86,66389357	9,629321508
ENSG00000168803	ADAL	38,62739261	0,935107237	0,096941621	26,75322843	0,062618578	2,372217487	5,62264609	2,765624977	9,397644544	86,63342157	9,62593573
ENSG00000171453	POLR1C	14,71303649	6,673106996	15,75473041	8,152736718	0	5,515283476	11,39603379	13,44547559	10,97186553	86,62226899	9,624696555
ENSG00000221838	AP4M1	0,160006958	3,691069463	23,60652683	2,376155	0,152103999	12,29748027	9,858003637	18,75401611	15,70367691	86,59903919	9,622115465
ENSG00000134247	PTGFRN	0	2,479734896	6,555326009	2,493521644	20,64593762	27,13180054	19,35816929	0,250450561	7,676767566	86,59590812	9,621767569
ENSG00000148337	CIZ1	2,41707357	9,010064905	5,981850231	7,206489517	10,70327313	14,81548993	12,60478709	6,198024684	17,64365718	86,58071024	9,620078915
ENSG00000156127	BATF	0	0	3,120184784	3,993902672	0	11,34286596	40,20171862	16,52172838	11,37835851	86,55875892	9,61763988
ENSG00000124802	EEF1E1	6,223902815	26,96830461	7,854801647	0,749813145	0	11,44210874	11,53750179	8,070959264	13,70707109	86,5544631	9,617162566
ENSG00000143740	SNAP47	6,361341653	2,934076597	23,19317239	0,356901941	0,109154268	12,88454479	14,68269843	14,16148147	11,83429982	86,51767135	9,613074594
ENSG00000126947	ARMCX1	0,070579435	38,19865018	0	2,193755041	0	21,62029468	8,321712818	10,18621273	5,902645266	86,49385014	9,610427794
ENSG00000147364	FBXO25	14,71736105	8,955258265	8,384606937	24,77490666	0	7,789297053	10,05912698	7,626746444	4,175778652	86,48308205	9,609231339
ENSG00000131732	ZCCHC9	14,34519452	19,13059884	5,581276841	2,034580699	0	8,635111887	5,331877656	17,06923205	14,34604733	86,47391982	9,608213314
ENSG00000107242	PIP5K1B	33,20752721	0	10,48157482	0,973637965	19,81775911	7,493996984	7,121155921	0	7,372544678	86,4681967	9,607577411
ENSG00000125122	LRRC29	0	0	19,80620584	11,06208122	43,49838418	2,00960442	0,178796341	5,061876168	4,76699309	86,38394126	9,598215696
ENSG00000149346	SLX4IP	3,983951215	1,943057972	41,0584235	7,885176537	0,055368107	2,813777897	2,611547151	13,65347889	12,37830676	86,38308803	9,598120892
ENSG00000151247	EIF4E	5,360359335	8,253748919	12,39024114	21,10926289	3,165605691	8,727790343	7,554799972	9,137630395	10,67491221	86,37435089	9,597150099
ENSG00000148297	MED22	12,69713682	14,73593218	0,07696016	4,551191417	4,722614414	15,89287363	15,11062968	4,436898263	14,07226574	86,2965023	9,588500255
ENSG00000164164	OTUD4	0,049898923	12,63233638	21,60197577	10,62696099	0,039528623	10,08064264	13,87976705	4,691916259	12,6011511	86,20417773	9,57824197
ENSG00000170037	CNTROB	8,241513318	8,172438245	0,044590969	4,77166941	0,057606279	17,37882685	11,93517916	24,06437178	11,50734001	86,17353601	9,574837335
ENSG00000172534	HCFC1	9,877275122	3,511347909	9,038749196	27,48055609	0,398532396	14,01153578	7,894981494	6,527283655	7,424786152	86,16504779	9,573894199
ENSG00000178761	FAM219B	2,922776741	12,03029911	12,69340831	1,925143382	0,071608682	14,3248813	12,65961874	14,8250803	14,71207764	86,16489419	9,573877133
ENSG00000173273	TNKS	0,06390354	0	49,96118215	19,23724157	0,126556788	3,11053097	4,227151022	5,286766763	4,139237457	86,15257027	9,572507807
ENSG00000105204	DYRK1B	6,248933373	6,888359651	0	24,85094996	26,88881348	1,81919492	8,687012335	5,072460466	5,647591686	86,10331587	9,567035097
ENSG00000205364	MT1M	9,462892879	0	0	0	59,73579472	0,324678232	0,736616431	7,113072882	8,728603786	86,10165893	9,566850992
ENSG00000164649	CDCA7L	0	0	25,19953132	15,5462029	0,092223303	0,639100835	9,634213988	34,45208896	0,524990176	86,08835148	9,565372387
ENSG00000169604	ANTXR1	5,390871916	2,293211974	16,94710683	0,031879655	0	24,66868318	29,27896568	0,242224484	7,220426889	86,07337061	9,563707846
ENSG00000197024	ZNF398	32,76855613	4,216767093	9,663271112	1,77095414	0	15,1876426	14,80886388	1,312767691	6,337424861	86,0662475	9,562916389
ENSG00000161654	LSM12	5,913256056	5,055683388	8,405659343	19,58636771	7,920771123	10,91905254	8,748553921	9,991795887	9,520419497	86,06155947	9,562395497
ENSG00000106588	PSMA2	7,507004172	12,76384285	5,723428559	10,54780592	1,831540167	11,00011568	5,972520526	23,78080284	6,915709272	86,04276999	9,560307776
ENSG00000114126	TFDP2	9,653192713	2,028476087	14,74367004	15,17752395	13,97591175	7,460885815	5,114996303	11,77643579	6,092304252	86,0233967	9,558155189
ENSG00000105379	ETFB	9,285218945	7,560240272	18,65695424	6,237489433	12,87672463	9,50820651	9,690016446	3,696249376	8,45792478	85,96902463	9,552113847
ENSG00000102882	MAPK3	12,02624329	7,892977926	14,84984993	5,902862603	2,251213811	10,49093862	11,86650455	9,186243087	11,49827216	85,96510598	9,551678443
ENSG00000167625	ZNF526	1,839791542	5,775945682	7,224488641	2,504878005	0	2,991129573	12,27970002	41,23662479	12,09677668	85,94933493	9,549926103
ENSG00000165650	PDZD8	16,59241298	3,537493405	13,45610993	3,362973592	1,931523213	7,812310492	9,205911309	13,01081918	16,99286752	85,90242161	9,544713513
ENSG00000077458	FAM76B	12,72826561	16,53935069	3,502982511	6,01392165	4,644531306	10,53618817	12,96103745	3,561307725	15,40760423	85,89518935	9,543909928
ENSG00000178467	P4HTM	10,66499903	7,583956006	0	1,059183477	26,8033258	14,4082029	10,64616671	6,663208511	11,77814807	85,88747833	9,543053148
ENSG00000184357	HIST1H1B	0	0	4,490133757	54,4093486	15,82015605	2,923534391	7,738258361	0,485219629	0	85,86665079	9,540738976
ENSG00000278705	HIST1H4B	1,035003909	14,08051599	1,26931552	7,148903414	36,89563791	12,12132067	8,593858356	3,017667283	1,697228514	85,85945157	9,539939063
ENSG00000152348	ATG10	25,55974942	11,27723012	0	10,49490531	0	9,20201913	6,636447312	5,886529486	16,79274361	85,84962439	9,538847155
ENSG00000151725	CENPU	0	13,01062246	0	18,05493221	0	33,05826593	10,51890237	0	11,04514215	85,68786511	9,520873901
ENSG00000104946	TBC1D17	8,055409688	9,378755165	11,48000409	6,542617357	0,378337065	10,80201606	14,4082029	7,449746887	17,19060752	85,68569672	9,520632969
ENSG00000005189	REXO5	0	0	61,89347853	13,06577656	0	0,483939456	6,770640181	0	3,388062511	85,60189724	9,511321915
ENSG00000110455	ACCS	8,844611156	14,82006935	24,81113139	16,8850816	0,123280996	12,95731418	1,550611724	1,020910095	4,52972986	85,54274035	9,504748928
ENSG00000128203	ASPHD2	20,71604856	9,707217807	0	11,30460127	0	12,20944303	12,4725701	3,137657614	15,99272744	85,54026582	9,50447398
ENSG00000048707	VPS13D	0,101865754	3,537697805	1,164496755	24,52547527	4,444010419	12,15090462	15,15817907	3,898135066	20,52830904	85,5090738	9,5010082
ENSG00000111877	MCM9	0	0,793954386	41,8215898	8,561406817	0	9,068255753	3,725151502	5,473748037	16,03253449	85,47664078	9,497404531
ENSG00000099810	MTAP	16,75446204	9,757287555	21,33335643	9,227317501	0,098253774	7,660030046	8,187325358	1,649912683	10,75418316	85,42212855	9,491347617
ENSG00000136925	TSTD2	0	2,128862033	1,667488244	38,39445463	0,063358776	8,898520698	6,176115495	18,71374315	9,344788082	85,3873311	9,487481234
ENSG00000048649	RSF1	11,80851346	5,894354004	3,620452369	13,87564001	8,076722363	8,51688348	10,67374679	15,36709679	7,544849284	85,37825855	9,486473173
ENSG00000090020	SLC9A1	0	9,608962715	0,044817687	1,053006354	21,91225682	11,22139332	11,44321833	13,17471157	16,91968338	85,37810419	9,486456021
ENSG00000066777	ARFGEF1	0,327936856	5,116010672	18,24880657	3,256083416	0	15,96246077	15,19167868	17,21044497	9,998976467	85,3123984	9,479155377
ENSG00000119772	DNMT3A	8,59897091	4,684677206	6,290398247	21,99060976	17,47785044	5,176078724	10,30276353	5,745492896	5,024968364	85,29181008	9,476867786
ENSG00000164124	TMEM144	10,87185216	3,713315591	46,73602443	0,621072102	0,271970442	7,215844621	5,280532203	0	10,55603421	85,26664576	9,474071751
ENSG00000182134	TDRKH	0	0,052659466	0	0	0	22,156165	38,21769146	10,96689747	13,86914345	85,26255685	9,473617427
ENSG00000166140	ZFYVE19	9,106475068	0,056108391	0,116334003	0,436802636	0,075144914	4,547873101	22,31633861	22,33320052	26,13284431	85,12112156	9,457902395
ENSG00000006831	ADIPOR2	22,2610153	0,873726291	0,095345601	12,48514113	8,930208136	6,231249579	6,369270841	10,08700961	17,75278904	85,08575552	9,453972836
ENSG00000164053	ATRIP	47,10554155	8,788303446	0	0,1921819	0,06612365	12,12787681	2,610596118	0	14,16690485	85,05752831	9,450836479
ENSG00000175575	PAAF1	15,99255126	23,61487439	0,245163247	15,5502385	0,09049201	7,734271561	6,354929251	0,208160872	15,26671696	85,05739806	9,450822006
ENSG00000142192	APP	14,92922674	3,353897721	0,61616868	37,18199448	7,505306458	2,626829903	8,085247803	4,237682072	6,502862796	85,03921666	9,448801851
ENSG00000044090	CUL7	59,25746792	2,811012439	0	2,874550182	0,191427261	3,655456914	7,200944783	0	8,948899704	84,9397592	9,437751023
ENSG00000151553	FAM160B1	0,194479499	10,52552253	0,089440186	6,842406831	0	26,93110873	9,264942469	2,445300854	28,64236942	84,93557052	9,437285614
ENSG00000101266	CSNK2A1	6,955907785	11,95254114	8,483557538	8,626241751	16,50653744	8,828557938	7,900597744	3,35773364	12,24372475	84,85539973	9,428377748
ENSG00000103248	MTHFSD	14,81821566	0	13,89292932	26,00605022	0,052326613	8,509474428	8,811991844	6,692467563	6,038725698	84,82218134	9,424686816
ENSG00000132436	FIGNL1	14,76885196	27,32555543	1,270071966	4,716941337	0,142676815	8,766828249	10,46832329	0	17,31466407	84,77391312	9,41932368
ENSG00000120896	SORBS3	9,765059019	13,0178829	2,91794757	10,91330466	1,060212525	9,551355162	9,857354277	8,725597076	18,92909469	84,73780788	9,415311987
ENSG00000072682	P4HA2	13,24128148	6,150045874	0	0,153455054	42,74605421	7,591062613	6,153978881	0	8,612506443	84,64838455	9,405376061
ENSG00000136699	SMPD4	9,117858162	6,180900341	0,376922168	5,484057093	0	16,3098533	10,49697557	14,47191394	22,1503855	84,58886608	9,398762898
ENSG00000110497	AMBRA1	23,86889243	4,543002738	0	18,66979031	0	12,97358869	10,07294728	3,488231457	10,97204152	84,58849444	9,398721604
ENSG00000148690	FRA10AC1	4,813617786	18,49595026	17,88036657	9,105396084	4,927856499	7,338117887	7,194329255	7,084789183	7,810016802	84,56044033	9,395604481
ENSG00000147119	CHST7	13,66530718	17,22012845	0	2,0519133	0	5,436126366	18,69173579	23,81900552	3,653601355	84,53781795	9,393090883
ENSG00000116874	WARS2	0	0,139403171	11,34465182	0,135656342	0	7,54727084	12,68727818	39,76896407	12,85031841	84,47354282	9,385949203
ENSG00000204611	ZNF616	0	15,24589729	0,849745564	0	56,64499242	2,873934995	3,809879655	0,134678854	4,847840809	84,40696959	9,378552176
ENSG00000156858	PRR14	3,366712196	13,05976581	18,21993913	4,326382999	0	8,811313011	11,83191732	6,334755958	18,44021192	84,39099834	9,376777594
ENSG00000155545	MIER3	18,03992237	0	4,341178016	3,124156385	0,06231434	6,189618947	10,92939557	24,13898194	17,51078633	84,33635389	9,370705988
ENSG00000111962	UST	0	25,8709315	2,001502461	14,38604674	0,221631892	6,143572065	11,87334966	2,855019921	20,95122284	84,30327708	9,367030787
ENSG00000154229	PRKCA	0,270443974	4,295070963	30,79546868	5,860821353	0,064271596	11,0755517	9,307821497	15,99687705	6,63559395	84,30192076	9,366880085
ENSG00000242616	GNG10	9,436546877	9,153890095	20,36823373	12,16682646	0	10,08448967	11,59634209	7,153454418	4,33280818	84,29259153	9,365843503
ENSG00000165714	BORCS5	3,614420273	1,315630789	39,38264512	10,40219157	0,110124975	6,664914305	9,984529639	10,08225681	2,650065405	84,20677888	9,356308765
ENSG00000077150	NFKB2	22,21460023	8,987364449	0,056969049	8,524392467	1,447440243	7,25022885	7,532478565	14,22957514	13,95623014	84,20127913	9,355697681
ENSG00000123600	METTL8	8,595032459	3,300536829	9,740616929	19,03784061	0,142591837	4,677248738	12,19331497	7,904976253	18,44813602	84,04029465	9,337810517
ENSG00000109618	SEPSECS	1,792303611	0,042978362	1,470322691	37,30629866	0	15,15112969	9,532447086	13,71735102	5,034150066	84,02698118	9,336331243
ENSG00000067365	METTL22	24,25751225	1,299452387	19,36498145	19,44208982	0,043508304	4,000024808	8,543049017	0,960798355	6,034270411	83,94568681	9,327298534
ENSG00000132196	HSD17B7	6,293638251	10,10162411	3,37439216	18,18571506	0	8,958754456	12,26342508	15,07633648	9,646282445	83,90016805	9,322298894
ENSG00000164023	SGMS2	18,34702017	21,73934487	33,75082538	5,94665168	0	0,412875044	2,300700259	1,255088731	0,146048343	83,89855448	9,322061608
ENSG00000181523	SGSH	18,21675248	8,051103441	6,259873119	0,156694175	1,563490276	14,24723763	14,46647875	15,03102747	5,887046419	83,87970376	9,319967084
ENSG00000048991	R3HDM1	20,83558008	6,959877186	3,519626475	15,52791062	8,593721892	6,974210112	10,24669083	6,150149585	4,988536683	83,79630346	9,310700384
ENSG00000180008	SOCS4	0	7,042343841	24,82247051	0,148174318	0,101964095	8,620566592	6,448077823	34,38508489	2,163460367	83,73214244	9,303571382
ENSG00000162894	FCMR	9,198419285	5,728523853	0,244056277	0,534546334	0	7,768712986	15,75264045	30,26807715	14,19547624	83,69045258	9,298939176
ENSG00000143093	STRIP1	0	9,273974404	2,157525278	0,781667952	0	24,28565767	10,3704373	9,133744173	27,60873581	83,61174259	9,290193621
ENSG00000197728	RPS26	15,89862799	2,220367268	12,05076903	14,87062447	0	3,959872508	6,111560253	15,45139721	13,03550863	83,59872735	9,288747483
ENSG00000205208	C4orf46	0	3,693415979	0,07657854	41,90773834	0	14,30578802	7,846216354	0,455144032	15,30801933	83,5929006	9,288100066
ENSG00000183891	TTC32	0	4,166617545	17,69597623	3,400656311	1,980096725	13,7219274	16,98094719	1,325050897	24,31375555	83,58502783	9,287225315
ENSG00000166164	BRD7	10,30299345	5,999408857	0,720155697	15,85521042	9,176696502	9,612341467	11,71667137	9,64999679	10,54851905	83,5819936	9,286888178
ENSG00000149311	ATM	2,540362088	13,14311906	4,499260738	8,120202363	15,02565055	9,260318356	10,29535054	9,221622129	11,41190058	83,5177864	9,279754045
ENSG00000167550	RHEBL1	27,4534986	0	0	19,68562549	0	3,193044201	3,863597598	19,84133662	9,442548476	83,47965098	9,275516775
ENSG00000104689	TNFRSF10A	13,56973949	5,815346664	0,188397221	14,85499008	0	18,15964631	6,8879003	13,54882503	10,45426049	83,47910559	9,275456176
ENSG00000180979	LRRC57	6,127110888	9,12092581	21,27503592	9,229521532	10,35660647	6,154681241	8,644061571	4,316744412	8,222689364	83,44737721	9,271930801
ENSG00000109189	USP46	3,261745876	13,83666982	0,187507655	24,52407406	0,121118901	9,437970974	13,96465752	0	18,09365804	83,42740285	9,269711428
ENSG00000112394	SLC16A10	0,02689518	0,668147726	0,024737928	4,435221158	0,223709424	28,3333983	45,60121348	0,323465359	3,787385744	83,4241743	9,2693527
ENSG00000171791	BCL2	8,862413409	5,766249556	11,57758168	10,24666029	0,061048456	13,3828421	12,32840661	6,656437952	14,51703816	83,39867821	9,266519802
ENSG00000149577	SIDT2	18,69495086	19,79770423	0	22,23174047	0	2,709939891	4,638843529	7,330024067	7,978622356	83,3818254	9,264647267
ENSG00000103657	HERC1	5,013692655	0,974547395	22,37333231	0,428288838	3,368240036	13,94664207	10,95907115	11,00221785	15,2956514	83,3616837	9,2624093
ENSG00000124177	CHD6	22,7947184	10,77612142	0,752689451	11,71362648	2,430965265	4,657885651	7,617266842	9,677113243	12,93801142	83,35839818	9,262044242
ENSG00000187672	ERC2	13,39192511	0	0	19,38986555	0,178798876	30,50160839	18,39731779	0,699201848	0,763372571	83,32209013	9,258010014
ENSG00000100426	ZBED4	0,043844645	0	0	27,70990881	0,104197749	14,08061619	17,85672359	2,492760411	21,03007216	83,31812354	9,257569283
ENSG00000117480	FAAH	0,055473778	38,68568503	0	13,69812826	0	1,705395062	4,375800859	0	24,76589118	83,28637417	9,254041575
ENSG00000205808	PLPP6	29,20581342	2,459182745	0	0,088632804	0	30,15018972	6,520706792	0	14,58238677	83,00691225	9,22299025
ENSG00000185238	PRMT3	9,811300959	13,59538137	0	4,521032753	4,25727526	20,69721584	14,69121888	0	15,42224469	82,99566975	9,221741084
ENSG00000213930	GALT	2,777389658	5,293477197	10,6600038	10,71686644	0	13,17646967	15,57346758	9,897291335	14,84410616	82,93907183	9,215452426
ENSG00000126106	TMEM53	14,04594184	6,701667511	24,82383361	13,84911828	0,201694772	5,544329859	8,209656657	2,041437935	7,515276385	82,93295685	9,214772983

ENSG00000153094	BCL2L11	0	19,13573468	26,43650881	0	1,834434561	16,3268371	9,745817555	4,964461651	4,467464478	82,91125884	9,212362093
ENSG00000119650	IFT43	10,82983237	8,419607385	3,997796246	15,63608429	11,19014394	10,02144423	9,082464109	5,861015819	7,840121745	82,87851013	9,208723347
ENSG00000103160	HSDL1	2,813228709	20,91887944	0,246436258	0,173493999	0,397958269	19,1208998	6,812994535	0,512642281	31,88058693	82,87712022	9,208568913
ENSG00000112983	BRD8	0,042892583	12,59650895	0,039452185	22,73830179	0	8,147184153	7,140732775	18,7587038	13,39909641	82,86287265	9,20698585
ENSG00000064393	HIPK2	7,680309378	4,737369865	9,840160974	16,30217227	17,7466869	5,681869065	5,164346657	5,689853966	9,969234776	82,81200385	9,201333761
ENSG00000158481	CD1C	68,00594478	0	0,113832964	0	0	0	12,69089763	0	1,978709901	82,78938528	9,198820586
ENSG00000124120	TTPAL	0	12,84467064	0,15758509	14,79223212	0,050895343	17,75261981	17,19526801	0,889775621	19,09562481	82,77867144	9,19763016
ENSG00000115840	SLC25A12	0,567342985	9,84850637	1,860460995	26,53648207	0,175865594	9,289475646	0,389150556	0	34,06882868	82,7361129	9,192901434
ENSG00000072952	MRVI1	25,21780303	5,95484182	22,70772497	10,16629771	0	3,94306955	5,11658237	2,016938269	7,602812275	82,72606999	9,191785555
ENSG00000169733	RFNG	9,891584574	10,4759229	11,57950736	0,742635115	0	17,59982832	17,14057834	1,282410999	13,99258421	82,70505182	9,189450202
ENSG00000105967	TFEC	0,017481556	16,34785216	4,920286342	6,822224	0	4,491332132	17,49094749	21,67473389	10,92203069	82,68688826	9,187432029
ENSG00000125744	RTN2	15,78068246	11,5499653	3,187252452	2,749237414	0	16,50365514	12,90863038	15,05233388	4,952835735	82,68459275	9,187176972
ENSG00000126012	KDM5C	0,986971758	1,136845881	2,420819013	15,51796171	20,73968619	17,88952133	8,726995706	3,672757311	11,58479525	82,67635414	9,186261571
ENSG00000160336	ZNF761	0,064660064	0,344213094	0	34,89181838	0	26,0544209	11,03301379	1,13114101	8,986160469	82,50542771	9,167269746
ENSG00000127334	DYRK2	10,47300985	11,77746093	9,398737284	13,57719375	0	5,766851729	16,17607819	4,94224864	10,35525703	82,4668374	9,162981934
ENSG00000008283	CYB561	0	4,658055893	1,417194613	0,344891394	0	14,69260961	6,704702409	47,41885788	7,228924394	82,46523618	9,162804021
ENSG00000160961	ZNF333	14,06527763	8,20394812	1,102049862	15,67453911	3,187891654	6,162842244	7,407323746	9,768082104	16,80194793	82,3739024	9,152655822
ENSG00000083093	PALB2	9,422525578	0	0	21,82245311	0	15,27341943	9,145299109	0,069609136	26,6222109	82,35551726	9,150613029
ENSG00000153767	GTF2E1	0	17,98222315	0	0,851716534	0	22,59731594	25,05683485	3,922072018	11,91182457	82,32198706	9,146887451
ENSG00000118894	EEF2KMT	5,26568165	0,085723146	24,97199738	5,839337667	0	17,60943461	13,15673558	2,640941237	12,65515001	82,22500129	9,136111254
ENSG00000153989	NUS1	15,02913724	6,373683905	11,36032098	21,70831002	0	6,399803313	8,188535012	9,43901913	3,720030471	82,21884007	9,135426674
ENSG00000198331	HYLS1	0	0,08941047	38,83749966	0,261021982	0	3,485283872	14,51757426	25,01121935	0	82,20200959	9,133556621
ENSG00000176410	DNAJC30	8,695698835	0	0	3,990152529	22,68871394	10,01472294	10,24968909	22,07329505	4,487239974	82,19951237	9,133279152
ENSG00000048052	HDAC9	2,173219118	3,617295657	20,07165985	9,800374633	0,021341745	2,928355695	6,323102249	24,25189819	12,95524874	82,14249588	9,126943987
ENSG00000137760	ALKBH8	0,100606239	6,129300046	19,43269667	0,231633355	0	1,435977029	3,926168844	42,82596259	8,001381563	82,08372633	9,120414037
ENSG00000275835	TUBGCP5	12,41874828	0	0	0,163698227	51,14158225	5,022073605	5,096740274	3,938680541	4,255522223	82,0371054	9,115233934
ENSG00000151689	INPP1	18,59302324	7,573361859	0	24,00660141	5,724189014	10,71737786	11,26285698	0	4,157640319	82,03505068	9,115005631
ENSG00000068354	TBC1D25	9,677782387	5,070763996	0,070090797	14,34287052	0,090549053	9,328809532	17,68479969	12,33089158	13,40194286	81,99850041	9,11094449
ENSG00000121274	TENT4B	2,504902633	31,01544254	8,942876465	10,66936966	0,044095965	10,36941104	4,621942485	1,217219674	12,59666998	81,98193045	9,109103384
ENSG00000106290	TAF6	1,674870857	3,764555174	0,136936009	21,53034889	0,088452597	13,0766957	13,01059354	6,348250312	22,29242767	81,92313075	9,102570083
ENSG00000106346	USP42	14,38029045	3,260776585	1,572285628	27,41440698	0,710922737	10,11134878	5,322553676	7,709516917	11,43145322	81,91355497	9,101506108
ENSG00000125843	AP5S1	7,705510797	1,416156985	26,07170843	4,324363994	8,436744302	12,29750587	9,939827795	7,522185565	4,196861913	81,91086564	9,101207294
ENSG00000189046	ALKBH2	16,76073579	12,88018341	0	15,0407933	0,156819887	13,25835613	3,670966045	7,791906832	12,33566436	81,89542575	9,09949175
ENSG00000185122	HSF1	12,04896727	8,69376513	12,73311292	3,147943143	0	9,38231348	13,55221494	11,27401635	11,03518464	81,86751787	9,096390875
ENSG00000184384	MAML2	1,421169039	2,822943572	0,858445	4,541821568	28,43097893	15,06795945	7,46763346	4,081729638	17,13941116	81,83209182	9,092454647
ENSG00000103540	CCP110	0,014899484	10,39043641	33,68542118	2,958738127	0,035408947	7,377802218	7,249616895	17,91944509	2,125635632	81,75740399	9,084155999
ENSG00000179454	KLHL28	23,35141062	0,066404656	9,534483336	3,651021809	0,088934508	16,00350907	7,395230068	6,178259001	15,41817359	81,68742666	9,07638074
ENSG00000188060	RAB42	27,47464921	14,38216182	0	0	0	10,33170409	17,73694239	2,553358022	9,207850422	81,68666596	9,076296218
ENSG00000103042	SLC38A7	12,53012393	0	0,584605886	0,078394164	0	12,66074402	14,6071064	18,26647452	22,94815102	81,67559993	9,075066659
ENSG00000131115	ZNF227	18,51246092	12,53692129	0	15,08264896	1,204379211	13,48493936	9,851487894	0,130374657	10,81568832	81,6189006	9,068766733
ENSG00000241852	C8orf58	24,44904382	12,76481953	0,167508345	0	0,21640105	15,4963178	7,825356651	12,34525049	8,343215372	81,60791306	9,067545895
ENSG00000167904	TMEM68	21,21963227	21,23494496	0	6,330123013	0,083768963	4,450598629	12,29240481	3,699757229	12,24670925	81,55793912	9,061993236
ENSG00000163946	TASOR	0,938288356	10,78136096	5,064987553	13,04143654	0	14,52393078	11,63512545	14,12688501	11,44514922	81,55716387	9,061907096
ENSG00000155008	APOOL	2,697987717	14,36253902	10,61090727	10,08075706	7,738410948	4,468885782	3,051302517	18,56373975	9,954531284	81,52906135	9,058784595
ENSG00000066629	EML1	0	0,210072076	27,59578599	11,38943929	0,080384453	13,77791247	13,42469361	0,036982044	14,97588092	81,49115085	9,054572317
ENSG00000175048	ZDHHC14	3,665096486	1,108024794	1,69804588	15,37669272	31,93733084	3,889926385	3,640578264	7,658293067	12,50440578	81,47839422	9,053154913
ENSG00000141505	ASGR1	7,176542577	14,09390982	13,42433242	14,82901537	0	3,983978629	5,52364055	16,83911483	5,603163558	81,47369776	9,052633084
ENSG00000144749	LRIG1	0	0,038131684	36,76357964	27,86720221	0	0	7,547490108	0,845822272	8,404322253	81,46654817	9,051838685
ENSG00000147804	SLC39A4	13,00037296	6,286659251	0	4,510499263	15,26945243	12,98803243	16,10447734	1,378725595	11,92696735	81,46518662	9,051687402
ENSG00000184708	EIF4ENIF1	0,396225685	8,744716162	20,09000715	2,865067732	3,119178916	7,749019069	18,30032702	0,108303693	20,04048044	81,41332587	9,045925097
ENSG00000178694	NSUN3	14,26150791	4,96447144	33,51577622	5,096156086	0,040541606	5,113297295	6,954835517	5,297099637	6,147327955	81,39101366	9,043445962
ENSG00000092871	RFFL	7,136565955	6,300328949	0,261259492	0,858338732	33,75164872	13,1176948	2,609051781	0,194099253	17,09560937	81,32459705	9,036066338
ENSG00000147421	HMBOX1	0	14,87656506	0,796254417	4,445296567	13,58940195	11,93115409	16,89808307	1,245418994	15,66232564	81,23550979	9,026167754
ENSG00000013016	EHD3	20,54477396	0,055573472	2,246885774	1,08159575	0	13,47915981	8,76342008	18,14821977	16,90914472	81,22877333	9,025419259
ENSG00000198799	LRIG2	0,012120845	10,6250219	1,293241818	4,248800822	50,35192621	4,684408679	2,405342853	0,901161089	6,57402072	81,09604493	9,010671659
ENSG00000159958	TNFRSF13C	50,53621772	1,223771806	3,946979093	9,130077712	0,091054144	6,520319318	1,145266722	5,194458211	3,298493042	81,08663777	9,009626419
ENSG00000174010	KLHL15	21,53903861	0	0	6,198869773	0	15,55411657	2,800677054	10,64218473	24,32716754	81,06205427	9,006894919
ENSG00000178814	OPLAH	17,04715062	4,166133305	0	0	0	11,39444402	20,53972785	6,696489772	21,21688511	81,06083068	9,006758964
ENSG00000120370	GORAB	23,46959831	0	0	14,17749001	0	11,52095709	2,194816303	29,6891912	0	81,0520529	9,005783656
ENSG00000131747	TOP2A	0,023707338	0,042068089	6,192841866	45,76801092	0,169023004	16,2682947	11,41713093	0,673933068	0,437354539	80,99236446	8,999151607
ENSG00000089685	BIRC5	0,118828758	13,846389	2,113085942	40,69628044	0	13,65554943	10,55726778	0	0	80,98740135	8,99860015
ENSG00000125485	DDX31	4,141508608	2,755877029	12,88151559	4,328528248	31,72874067	2,303482583	7,330037427	0	15,48162519	80,95131534	8,994590593
ENSG00000124789	NUP153	0,025059796	0	23,69515272	17,56875617	1,667544144	4,98006872	5,618074567	17,42590257	9,954962967	80,93552164	8,992835738
ENSG00000160999	SH2B2	11,28258455	4,161599834	11,31043188	10,17909087	0,301273106	9,395103966	13,52595441	0	20,73623109	80,89226972	8,988029969
ENSG00000157911	PEX10	6,460784494	3,396889946	18,48798758	0,344332244	0	15,23802605	13,51066124	7,063921871	16,38235377	80,8849572	8,987217466
ENSG00000169499	PLEKHA2	6,781827128	5,593353984	12,30000438	8,288231652	0,624256158	17,54016685	7,058697565	11,12240248	11,51261642	80,82155662	8,980172957
ENSG00000070366	SMG6	19,17479895	3,298491674	38,06180954	1,950592638	0	4,788297303	7,954686075	0,750419154	4,78333214	80,76242747	8,973603052
ENSG00000163539	CLASP2	0,02979701	5,058285397	0,036542671	9,896114128	0,236044132	13,84950916	19,4547465	12,20614085	19,98454781	80,75172766	8,972414185
ENSG00000152061	RABGAP1L	9,205229835	13,58145654	8,250669356	10,21268739	6,435557974	8,589235257	9,376508539	5,016864817	10,00293957	80,67114927	8,96346103
ENSG00000125484	GTF3C4	0,016713922	0,415218228	1,106877952	1,875984222	48,41989649	13,28605879	5,842606987	4,203072145	5,447331977	80,61376071	8,957084523
ENSG00000188283	ZNF383	0	3,822863254	0,033026031	19,22058741	28,11672335	3,626891543	2,265829923	12,79810728	10,66459193	80,54862072	8,949846747
ENSG00000171621	SPSB1	0,044588543	0,079121275	0,082024225	0,076994681	66,22852286	5,042428268	1,25877583	0	7,732180478	80,54463616	8,949404018
ENSG00000184867	ARMCX2	0,127771057	2,191690531	0	26,32883904	0	14,07527722	13,4381979	0,372530516	23,93795397	80,47226024	8,941362248
ENSG00000183765	CHEK2	0,033632444	8,474552418	0	0,522683012	0	15,13984786	16,75544162	14,3411397	25,19041444	80,45771149	8,939745721
ENSG00000156381	ANKRD9	17,0439642	0	15,77328808	1,520431317	0	10,47299007	19,98334366	5,776186946	9,84924688	80,41945115	8,935494573
ENSG00000171943	SRGAP2C	6,304024628	2,530679525	7,87058959	7,602126876	25,49336677	5,832173193	7,757159084	9,536546812	7,473516283	80,40018276	8,93335364
ENSG00000163877	SNIP1	16,27913808	24,52575482	0,097229835	10,35890858	1,507313924	8,066306457	5,683248686	0,231153947	13,61834432	80,36739864	8,92971096
ENSG00000073969	NSF	7,215869408	3,321639162	5,942831287	17,25663868	0	13,35903345	14,11384554	7,196277295	11,95655947	80,36269429	8,929188255
ENSG00000164366	CCDC127	6,978407664	4,505426865	10,63092637	16,64970099	0,037018611	9,252409236	9,221765459	17,4737587	5,613135865	80,36255006	8,929172228
ENSG00000184640	SEPT9	14,74910852	3,514695796	0,759093662	5,659664737	18,38036263	11,98667348	10,24946849	7,631164234	7,366000883	80,29623243	8,921803603
ENSG00000012983	MAP4K5	4,600741784	0	1,641614758	7,396581957	0,282769718	6,9673733	12,02012367	18,1695648	29,16961163	80,24838162	8,916486847
ENSG00000174165	ZDHHC24	4,624654037	10,58617231	15,61112415	7,02747598	0,329717713	13,98878772	6,623911942	8,191338935	13,2239683	80,20715109	8,911905677
ENSG00000134146	DPH6	8,245040231	9,66768525	18,29012099	0,055832882	0	6,425551382	8,859571961	22,16566533	6,442096204	80,15156423	8,905729359
ENSG00000260027	HOXB7	20,07555356	12,63496335	0,181924149	0,170768962	0,470049139	9,772219832	11,7423151	21,19279294	3,892073765	80,13266081	8,903628979
ENSG00000115750	TAF1B	1,216487613	7,690110191	0	0	14,09366959	12,43807594	18,27605591	25,18784251	1,168846052	80,07108779	8,896787533
ENSG00000189283	FHIT	18,03943108	2,619695665	2,046159722	24,06105427	0,144185309	6,372635909	15,61662323	0	11,16770456	80,06748975	8,89638775
ENSG00000182173	TSEN54	10,20601234	4,059209317	0	2,886616895	0	26,70991235	22,2084314	5,386997316	8,602502778	80,0596824	8,895520266
ENSG00000163017	ACTG2	0,47581392	54,24756811	4,44943218	1,232440184	7,538547202	1,001297104	1,316928176	3,641629975	6,144492733	80,04814959	8,894238843
ENSG00000204673	AKT1S1	15,06627966	7,057598399	9,78769043	6,231147206	18,15305687	7,200929684	5,533204462	3,340631849	7,612677834	79,9832164	8,887024044
ENSG00000146592	CREB5	14,79928883	11,776119	0,690131228	16,77614639	0	7,314780156	7,744551783	7,750969224	13,10996784	79,96195445	8,884661605
ENSG00000058063	ATP11B	0,082241521	13,74712986	0,423611776	13,49124991	0	6,627754615	11,82728938	20,86122229	12,86583087	79,92633022	8,880703357
ENSG00000138640	FAM13A	31,92589582	2,658085661	7,151966224	3,949072639	1,576149637	3,854297923	2,981285225	15,55280371	10,26620971	79,91576654	8,879529616
ENSG00000073111	MCM2	0	3,014109439	0	2,988438733	0	16,57346376	20,86293537	0	36,45981614	79,89876345	8,877640383
ENSG00000146242	TPBG	0	5,709280709	0,120790889	0,075589506	35,00667816	14,68298991	11,55839095	0	12,70561392	79,85933405	8,873259339
ENSG00000166169	POLL	22,94538739	11,42819225	0	12,27646098	0,066257849	8,448582873	6,69020774	2,255734132	15,70437402	79,81519724	8,868355249
ENSG00000131653	TRAF7	8,550252829	8,541836938	6,254397415	12,78808073	10,23992992	9,646393522	9,419358851	4,931859217	9,397897206	79,77000663	8,86333407
ENSG00000007129	CEACAM21	16,8155415	4,882707134	36,41722086	0	0	5,790505712	0,253860055	6,852714855	8,742400896	79,75495101	8,861661223
ENSG00000144791	LIMD1	18,62059888	1,734588514	10,57008581	16,59147914	1,274872941	6,688253763	11,60075963	4,222972542	8,444903505	79,74851472	8,86094608
ENSG00000123643	SLC36A1	0,021677029	14,5014394	1,993832298	27,66187724	0,051515929	12,47125838	8,639472007	2,986282851	11,38379931	79,71115445	8,856794938
ENSG00000197635	DPP4	0,032302388	0,057319795	0	5,41057985	57,42195846	3,865808649	6,437126909	0	6,475627005	79,70072306	8,855635895
ENSG00000106948	AKNA	1,339910729	3,439987927	21,31853792	6,8673576	15,48124864	7,058359597	8,841273277	4,239137929	11,1146774	79,70049102	8,855610113
ENSG00000196812	ZSCAN16	0	20,54567319	0	1,428103848	0	17,90121938	11,18343851	0	28,62530484	79,68373976	8,853748862
ENSG00000163171	CDC42EP3	0,30495118	5,205139729	9,777120529	15,19571349	0,069021246	10,71419446	5,269126954	27,65793853	5,429304514	79,62251063	8,846945626
ENSG00000179195	ZNF664	17,8281872	1,94550978	36,39171643	3,827595053	0,113286291	8,557217442	4,630923926	2,084760678	4,221119749	79,60031655	8,844479616
ENSG00000108963	DPH1	6,772021405	14,48535036	3,91113219	7,025346719	0	12,99733717	11,37667419	6,600655769	16,36689018	79,53540799	8,837267554
ENSG00000117139	KDM5B	0,309437456	2,434640678	31,53348159	8,942482129	9,351874799	4,230777398	2,627498646	9,29434994	10,79951972	79,52406236	8,836006929
ENSG00000116199	FAM20B	2,57432828	6,253394312	2,482786197	1,942122702	0,23759017	14,95555149	12,92888138	17,37978704	20,74866069	79,50310227	8,83367803
ENSG00000167700	MFSD3	0,082709672	14,38309579	0	11,42572412	0,786245303	7,900503653	33,23899326	3,436374813	8,239500987	79,4931476	8,832571956
ENSG00000170632	ARMC10	12,45217162	2,930513662	9,357144315	12,09141303	12,87328458	7,833155097	7,130554387	7,511518038	7,311994319	79,49174904	8,83241656
ENSG00000104883	PEX11G	30,89507619	5,42797571	0	9,859891078	0	12,76235205	18,03318537	0	2,508050233	79,48653063	8,831836737
ENSG00000171503	ETFDH	0,975340875	18,56046339	3,340958234	5,865664909	0	26,69782928	6,171587664	1,323797511	16,5454282	79,48107007	8,831230007
ENSG00000126453	BCL2L12	10,02403154	10,76924098	10,41170948	18,25131817	0,162056653	12,80266634	6,171600265	0,149113072	10,73481717	79,47655367	8,830728186
ENSG00000132906	CASP9	20,56296815	0,050890492	0	9,310262901	0	19,39664362	13,33524121	1,94410165	14,8493805	79,44948852	8,827720947
ENSG00000171291	ZNF439	23,73889894	21,47401844	0	10,77415393	0	4,556407234	11,15666674	0	7,745092248	79,44523755	8,827248616
ENSG00000166311	SMPD1	10,8054257	9,97828676	6,896259296	7,901350927	0,39305094	13,0137811	14,87700033	7,233153612	8,339701656	79,43801032	8,826445591
ENSG00000150995	ITPR1	0,316617946	3,197478093	25,84168234	11,19188651	12,24943364	3,77278627	9,301830862	2,545407096	11,01198195	79,42910471	8,825456078
ENSG00000171302	CANT1	7,586283738	4,002119404	9,555173009	6,313894682	9,014483763	8,516893015	12,51298426	13,45052514	8,447569194	79,3999262	8,822214023

ENSG00000130962	PRRG1	0,293418584	0	0,044980599	3,588911463	69,84778089	0	0,22332907	5,400306039	0	79,39872665	8,822080738
ENSG00000137331	IER3	1,455138829	10,12980889	10,29555922	19,13523147	8,512415819	8,725667367	14,68467589	1,468598078	4,955907261	79,36300283	8,818111426
ENSG00000218739	CEBPZOS	1,929071115	9,698753195	5,323023172	27,64800369	0,076407988	10,48413644	11,37241733	3,796482265	8,896907981	79,22520318	8,802800353
ENSG00000156931	VPS8	0,174930238	14,56337508	0,1072661	1,611020488	0	18,74216528	22,45296796	13,48387247	8,049883128	79,18548075	8,79838675
ENSG00000078596	ITM2A	0	0,130737201	0	76,84286917	0	0	0	0	2,174703332	79,1483097	8,794256633
ENSG00000089916	GPATCH2L	0,075599958	4,585500161	13,41414859	18,98830943	13,3115385	9,37949001	3,703563263	6,056539351	9,559849761	79,07453902	8,786059891
ENSG00000123453	SARDH	15,4122153	0	41,6046807	10,14577178	0,070167554	8,071851035	3,75087159	0	0	79,05555795	8,783950883
ENSG00000116830	TTF2	3,049583283	4,091554441	3,940651137	22,01430136	0,141412696	7,137519395	13,29061599	18,67192772	6,677887441	79,01545346	8,779494829
ENSG00000215012	RTL10	24,32251578	5,321059828	16,0585299	21,70942167	0	2,95093044	1,198819871	2,185735049	5,245117195	78,99212974	8,776903304
ENSG00000128283	CDC42EP1	4,104972195	16,67969922	0	0	38,03252712	0	8,397620646	6,374525121	5,341258317	78,93060262	8,770066958
ENSG00000133121	STARD13	1,279376403	13,78120702	10,73998951	12,04171657	2,355290345	8,566572825	6,493456247	13,98810774	9,68458505	78,93030171	8,770033523
ENSG00000198865	CCDC152	8,65219414	1,765607215	28,56995578	13,22229903	6,682691169	10,21207386	2,909561091	6,621935221	0,266027086	78,90234459	8,766927176
ENSG00000014164	ZC3H3	14,37601237	0,50443268	3,809993294	2,384248626	22,87311409	4,770876567	14,16219818	6,571394248	9,439664797	78,89193485	8,765770539
ENSG00000159251	ACTC1	0,755224495	58,67427077	2,114146776	1,757710607	9,832422933	0,288414736	0,708871932	1,723257197	2,948021757	78,8023412	8,755815689
ENSG00000164086	DUSP7	17,77928217	2,270385607	16,08351957	9,804048261	2,913989583	7,755565854	4,847063452	3,963582747	13,34265912	78,76009637	8,751121819
ENSG00000119280	C1orf198	0	31,27717188	0	2,831303735	0,208748814	8,486803381	16,5073349	0	19,44528619	78,7566489	8,750738766
ENSG00000256222	MTRNR2L3	1,767375095	1,742313191	14,08865999	13,73341972	5,133584101	9,055548046	9,131044304	19,32365892	4,709560993	78,68516435	8,742796039
ENSG00000120539	MASTL	0,041616792	0	0,076557449	33,56007522	0	8,910097069	9,01893601	0,091003737	26,82004342	78,5183297	8,724258856
ENSG00000100580	TMED8	0	0,138199717	13,46740333	17,71842953	0,277632425	15,3704039	11,47840945	15,54034495	4,453994736	78,44481803	8,716090892
ENSG00000186017	ZNF566	18,13979694	9,971026388	0	21,53991844	0,221643291	12,77416722	3,658986692	3,314032848	8,803407242	78,42297907	8,713664341
ENSG00000182359	KBTBD3	6,438876523	2,573339732	0,160065316	31,05176281	0,068928511	10,66795455	5,442666993	15,72893911	6,278116683	78,41065023	8,71229447
ENSG00000103227	LMF1	7,433472746	0	18,84038567	1,74315846	23,37993474	11,18430342	6,934167504	4,33462083	4,559819932	78,4098633	8,712207034
ENSG00000135365	PHF21A	0,013504072	1,174169616	16,49497638	15,20371791	1,92556323	8,495689048	9,183217329	2,126120542	23,74982367	78,3667818	8,7074202
ENSG00000173917	HOXB2	15,78380961	4,247112159	0	15,63822622	0	11,71885495	5,102603399	19,66198839	6,205491754	78,35808648	8,706454053
ENSG00000140525	FANCI	0,019457967	25,17068221	29,60205278	5,745542191	0	6,815023689	8,595186277	0,170195502	2,177701974	78,29584259	8,699538065
ENSG00000148985	PGAP2	20,18271309	6,524481356	0	4,278753881	0	20,66751696	17,35545719	0,087393432	9,19156299	78,2878789	8,698653211
ENSG00000229833	PET100	5,44542597	13,28631532	5,008650432	11,98890424	4,852939762	9,640749727	7,347361784	13,09830718	7,61803312	78,28668753	8,698520837
ENSG00000187118	CMC1	7,4888178	11,12485085	6,545055022	8,967859186	0	9,876192119	5,634431504	20,26590056	8,381002381	78,28410943	8,698234381
ENSG00000164916	FOXK1	8,958714589	5,360384864	6,299411674	4,798209132	35,48429859	4,911743	2,996506479	1,638810231	7,784964194	78,23304275	8,692560306
ENSG00000186834	HEXIM1	2,140733084	19,44029501	0,849383636	2,754313621	0	22,12141023	13,38350208	10,4637613	7,072492653	78,22589161	8,691765735
ENSG00000100731	PCNX1	8,714962017	2,021291775	23,91477011	4,402253236	0,06445424	10,9184346	9,855970441	11,68332811	6,637772886	78,21323742	8,690359713
ENSG00000204291	COL15A1	0,249844623	8,101088534	27,61834442	1,764929183	2,21310976	20,64842289	8,015164753	1,19200806	8,408207306	78,21111953	8,690124393
ENSG00000213799	ZNF845	20,88805657	1,745229196	0,043077654	0,242617364	0,055651254	13,47236048	9,332986109	27,44660285	4,92480324	78,15138472	8,683487191
ENSG00000135778	NTPCR	14,56395559	5,243094489	11,58980985	9,463706976	9,10591977	8,016115917	7,613375765	4,731212743	7,795378915	78,12257002	8,680285558
ENSG00000243056	EIF4EBP3	3,052738801	15,09024243	0	6,024472718	17,61904248	7,182276048	7,061100935	10,01316871	12,06795599	78,11099812	8,678999791
ENSG00000174456	C12orf76	29,94742193	14,78198341	1,318222165	3,093479248	0	9,867434022	3,693949707	9,467103792	5,912130031	78,0817243	8,675747144
ENSG00000075340	ADD2	2,921335896	8,928897975	0,02193483	2,264881812	57,12782809	1,754282118	4,970102317	0,026073903	0,014664761	78,0300017	8,670000189
ENSG00000020922	MRE11	3,517855657	21,93540357	0,180764793	2,273721329	0,046705364	13,7881205	10,32939526	0,386774757	25,52398589	77,98272712	8,664747458
ENSG00000183878	UTY	24,38221916	0,116697639	36,35426819	0	0	0	0	0,035951971	17,08631393	77,97545089	8,663938988
ENSG00000233927	RPS28	2,56723952	2,27775177	17,70991664	11,20579646	7,965325385	7,594773922	11,90162187	3,430656199	13,24342844	77,8965102	8,6551678
ENSG00000164330	EBF1	1,789561472	3,175532924	14,39720077	15,86293466	22,51218448	2,095822854	1,862341893	13,82680568	2,288967115	77,81135185	8,645761761
ENSG00000205309	NT5M	0	4,922545642	23,95646952	5,189425808	0,366259605	9,814190157	10,94106414	1,011018565	21,60785675	77,80883018	8,645425575
ENSG00000168488	ATXN2L	3,041715543	6,551102952	4,314073448	13,19108891	0	15,2705033	17,25506256	4,417302915	13,76429375	77,80514338	8,645015931
ENSG00000172578	KLHL6	1,031960646	9,715474157	5,325996395	24,87334245	0,272497424	13,04561018	10,05619184	0,062683215	13,34400241	77,72775871	8,636417634
ENSG00000125246	CLYBL	19,59189217	6,826916842	0,153856427	0,361055728	28,67176172	5,877003962	4,513945803	0,137166695	11,57201259	77,70561194	8,633956882
ENSG00000170545	SMAGP	7,744232944	9,741130071	0,090165455	25,39101277	0	6,673017032	10,41855498	6,537952844	11,09171589	77,68778199	8,631975777
ENSG00000135636	DYSF	3,926821362	0,255708189	17,03203849	0	50,6420375	5,181615341	0,01495653	0,63022447	0	77,68340188	8,631489098
ENSG00000196247	ZNF107	6,30858149	0,299516336	0,038813194	32,2069977	0	11,69852572	0,525566875	23,0224587	3,529059904	77,62951992	8,625502214
ENSG00000132846	ZBED3	5,016796277	2,318707845	27,85810442	4,150139343	19,68602958	11,29811061	2,479957149	0,816391767	3,960287226	77,58452422	8,620502691
ENSG00000197050	ZNF420	0,074128315	0	8,181901368	33,92090668	0,088083773	10,98745541	7,139837386	0	17,18521925	77,57753219	8,619725799
ENSG00000135040	NAA35	0	8,469192532	0	3,153292744	2,268526085	28,16059775	17,6781874	0,18150759	17,55869367	77,46999777	8,60777753
ENSG00000117036	ETV3	0,540726658	15,43205786	22,09085922	15,17289997	1,552767068	6,110021238	7,96933971	2,906760024	5,680414055	77,45584579	8,606205088
ENSG00000163785	RYK	0	12,22916789	3,247188928	16,92656353	0,044627506	9,154255754	10,35321243	9,608759637	15,88945039	77,45322608	8,605914009
ENSG00000116574	RHOU	32,58500326	4,801533923	0,070108474	0,131619155	0,271715669	3,556721064	8,797163253	21,75117834	5,437123981	77,40216712	8,600240791
ENSG00000107679	PLEKHA1	26,11328756	6,539776122	6,26979211	8,594122819	4,716168842	7,131401842	2,369634803	11,47552717	4,176239286	77,38595055	8,598438951
ENSG00000144354	CDCA7	0	11,17826335	0,35330465	54,85338688	7,941847022	2,952145493	0	0	0	77,2789474	8,586549711
ENSG00000150760	DOCK1	0,575341156	11,52661509	4,028697087	6,79418013	0,264640783	19,9695855	16,33487496	5,275914244	12,48563592	77,25548487	8,583942763
ENSG00000168273	SMIM4	2,375377775	3,458502218	2,577007127	16,51220919	13,46147982	13,10700243	8,749039676	7,991162455	8,988949082	77,22072478	8,580080531
ENSG00000186106	ANKRD46	14,6696688	7,003021308	2,101567759	12,19489916	0,12340809	10,71862145	0,129350858	18,45210214	11,81498509	77,20762465	8,578624962
ENSG00000134444	RELCH	0,84814165	0	28,16206241	17,59907546	0,033593783	6,332227811	6,255910847	8,593153393	9,353173899	77,17733926	8,575259918
ENSG00000165338	HECTD2	4,906187359	3,737736971	0	1,287731043	0	9,925905498	13,41609178	36,10462547	7,771763659	77,15004178	8,572226864
ENSG00000197808	ZNF461	1,075294978	0	0	58,48919167	0	4,479377228	5,330782209	0,069157507	7,701467436	77,14527103	8,571696781
ENSG00000071575	TRIB2	16,13866164	3,097542721	0	0	0	11,42714521	11,78677269	24,12719706	10,53184302	77,10916235	8,567684706
ENSG00000170832	USP32	12,01330624	13,06636085	0,992577543	3,041775066	0,037714508	17,31937757	4,38790386	9,612515774	16,62898939	77,1005208	8,566724534
ENSG00000213047	DENND1B	0	8,91572658	0,021054313	33,85454908	1,22398617	7,803564957	13,35195156	7,608279067	4,307281361	77,08639309	8,565154788
ENSG00000164949	GEM	0,114131851	29,06221829	1,784615439	0	6,37406514	15,16252249	7,012692272	1,497437617	16,07207462	77,07975772	8,564417525
ENSG00000100814	CCNB1IP1	14,08449069	7,650790763	0	15,88299845	1,561380619	14,96801942	4,500566257	12,66067229	5,706700276	77,01561877	8,557290974
ENSG00000146733	PSPH	23,6896731	0	0	8,408114678	0,130020834	11,71345491	15,8087167	8,613790319	8,545441104	76,90921164	8,545467961
ENSG00000112137	PHACTR1	5,262825733	15,46880979	14,4724413	13,4685136	0,609326068	12,10474559	4,056104678	3,363952554	8,099044728	76,90576405	8,545084895
ENSG00000164077	MON1A	0	1,663928018	0,431244336	0	27,63299736	13,69289898	9,810277277	7,638031424	16,03019956	76,89957696	8,54439744
ENSG00000172339	ALG14	16,17530432	4,505653357	14,84905622	11,12377083	0,148995972	6,74587139	3,227532656	12,06440906	8,057654425	76,89824822	8,544249803
ENSG00000145012	LPP	1,603718999	2,677744665	18,69167143	9,3807835	11,32130885	4,099846246	11,52253489	6,780799427	10,79343617	76,87184418	8,541316021
ENSG00000184156	KCNQ3	0	0,674518041	21,4587263	5,456230111	2,117271887	18,67651488	10,93833804	0,363657255	17,16607685	76,85133337	8,539037041
ENSG00000100124	ANKRD54	8,7086502	1,89348565	28,747828	0	0,736231864	13,68100883	10,37488167	2,258094994	10,4141787	76,81435991	8,534928878
ENSG00000125898	FAM110A	8,049607916	7,388188834	0,61274085	2,684121708	19,52586196	10,05703349	8,57365237	8,983158705	10,92411593	76,79848177	8,533164641
ENSG00000175352	NRIP3	0	0	61,6235291	0	0	4,104506142	7,881837494	0,160288415	2,974986774	76,74514792	8,527238658
ENSG00000065029	ZNF76	0,022281122	17,19872752	5,861273099	4,655430836	1,641498645	19,3749892	12,32132721	1,559113265	14,08509473	76,71973562	8,524415069
ENSG00000171150	SOCS5	1,694697989	7,231600002	0,927837447	46,33424783	0,143838802	3,566302116	5,008762252	2,249954999	9,503233382	76,66047482	8,517830535
ENSG00000004468	CD38	0,749272189	12,23982004	0,040539609	8,714322471	0	1,52433518	0,439155311	41,97294856	10,94968572	76,63007909	8,514453232
ENSG00000100241	SBF1	19,32846804	7,489366063	6,999498895	1,297497229	11,74008893	9,127539133	9,57089604	2,272349438	8,788975397	76,61467917	8,51274213
ENSG00000167522	ANKRD11	11,64651041	8,623607111	0,612134186	2,391937341	17,41610655	8,148115074	5,622294285	5,347330534	16,79823382	76,60626932	8,511807702
ENSG00000162241	SLC25A45	4,732425256	4,096376831	0,070777874	2,092794831	39,82067211	8,42753916	6,005963696	4,417012042	6,932276388	76,59583818	8,510648687
ENSG00000154309	DISP1	0,084231087	0,099643999	0,98134929	0,048482901	0	7,182676398	22,3571253	18,72585342	27,10693422	76,58629662	8,509588513
ENSG00000040341	STAU2	13,70514212	7,238996433	3,814318627	0,902385566	0,040062198	20,32237988	17,09050372	1,879982479	11,56875942	76,56253044	8,506947827
ENSG00000152556	PFKM	15,16110749	2,331347779	0	17,89741683	0	14,95164319	10,66653664	0,364818733	15,18370704	76,55657771	8,506286412
ENSG00000134864	GGACT	0,608440607	3,855939029	12,31203199	11,33194742	0	3,1970114	8,949278785	28,70038521	7,589935623	76,54497007	8,504996674
ENSG00000172954	LCLAT1	0,02010251	12,16395131	4,91836546	20,61931288	0,143322153	10,72669154	5,374676986	14,94582319	7,590113388	76,50235942	8,500262158
ENSG00000284976	BX255925.3	8,860933987	7,440589531	20,22996532	18,71627828	0	6,862269926	2,496268385	0	11,87082498	76,47713041	8,497458935
ENSG00000100918	REC8	0,039310762	11,09120136	17,93419993	1,900670991	0	11,35132198	7,801095044	0,171922252	26,15581908	76,4455414	8,493949044
ENSG00000126787	DLGAP5	0	0,074546011	0	50,63458779	0	20,66386944	0	0	5,063374808	76,43637804	8,492930894
ENSG00000135045	C9orf40	7,263077195	0	0	20,53295206	14,51134263	16,01943892	9,126085399	0,421651936	8,537398726	76,41194687	8,490216319
ENSG00000204852	TCTN1	6,953760774	7,900487873	29,75154001	9,155136537	0	4,007418407	12,35761937	0,103207049	6,152961383	76,3821314	8,486903489
ENSG00000118162	KPTN	22,72017091	11,1567357	18,40057393	4,934940418	0	4,07912529	2,610257498	10,46766715	1,933155314	76,30262621	8,478069579
ENSG00000112159	MDN1	0,914399405	1,20616515	29,98028914	4,317709497	31,50018927	1,723607791	2,916039632	0,69010111	3,045359099	76,29386009	8,477095565
ENSG00000172766	NAA16	9,267073756	0,046191559	0	6,472805571	0	10,74631815	9,726382326	38,82108097	1,184551477	76,26440381	8,473822645
ENSG00000011566	MAP4K3	2,295603121	0,044277081	0	19,86311481	0	5,369628969	14,46140881	30,93731833	3,222239901	76,19359101	8,465954557
ENSG00000196267	ZNF836	31,26180043	0,442270028	0	0,860765703	0,169235411	0,430022701	6,356296272	20,78841412	15,76454949	76,07335416	8,452594906
ENSG00000189376	C8orf76	1,116580571	13,64926437	13,23714757	10,81873253	0,147420994	4,018359616	8,550112936	15,59933303	8,92612607	76,06307769	8,451453077
ENSG00000160563	MED27	2,820835128	20,16105264	0	4,735661971	0	16,38888507	10,05194	8,053111057	13,82887008	76,04035595	8,448928439
ENSG00000118495	PLAGL1	6,696414846	4,421441431	1,655211857	12,2205891	7,97764837	10,5819525	5,057317859	10,59448974	16,76938733	75,97445304	8,441605893
ENSG00000116497	S100PBP	0,016442935	7,498639175	32,97043611	5,820638715	0	9,026690789	1,119539415	9,672132607	9,808004712	75,93252446	8,436947163
ENSG00000167323	STIM1	5,10766445	7,145438993	9,317986488	3,513289981	0,453304051	14,75277779	14,34198045	9,593261638	11,57305132	75,79875515	8,422083906
ENSG00000137198	GMPR	15,90993912	0	0	4,091722272	26,06498291	9,440252348	3,148001161	14,80441611	2,331407985	75,7907219	8,421191323
ENSG00000187531	SIRT7	19,26450379	2,01084643	0	0,467930342	24,88179907	7,924975379	10,82063998	0	10,3618374	75,73253239	8,414725822
ENSG00000064687	ABCA7	0,884954938	1,057569739	5,083178576	11,78838537	30,64538368	5,155581444	15,6106867	0,118477794	5,375266928	75,71948517	8,41327613
ENSG00000140992	PDPK1	2,465189403	1,548249107	9,299123465	17,33826932	9,2157375	7,192347427	8,797067545	9,327636953	10,45823157	75,64185229	8,404650255
ENSG00000135315	CEP162	0,027564861	18,29352573	0,101415578	0,999568491	0	8,141179338	1,716576961	33,51361174	12,81466352	75,60810621	8,400900069
ENSG00000145214	DGKQ	30,0522929	6,887853906	10,20878148	0,052365036	0,072068542	7,52474219	12,51429962	0,066312376	8,16784836	75,54656441	8,394062712
ENSG00000112972	HMGCS1	9,873378165	9,144423121	4,865805264	9,725507579	0,054190062	8,862583998	11,09485658	14,95855896	6,954872118	75,53417585	8,392686205
ENSG00000146587	RBAK	30,11788497	5,587094412	0	24,89737364	0,464442863	2,193385983	3,822346657	0	8,439071855	75,52160038	8,391288931
ENSG00000172007	RAB33B	2,193928333	15,19897972	3,427757784	0,311378251	0	16,69664913	11,30430795	15,31250084	11,01477002	75,46027203	8,38447467
ENSG00000083844	ZNF264	0,819942452	1,390778009	1,885437952	1,311754181	59,00279442	3,309738603	2,242687071	1,081057873	4,359948735	75,4041393	8,3782377
ENSG00000149212	SESN3	9,375629845	7,91263634	9,570108392	21,37233406	2,343621529	5,460785084	7,630513901	0,031252678	11,68901327	75,3858951	8,376210567
ENSG00000088256	GNA11	8,703584181	5,759613367	4,245996739	4,068675817	30,74069018	3,088550115	5,350206679	9,253219393	4,169349187	75,37988566	8,375542851
ENSG00000104218	CSPP1	13,05394304	4,647895436	0,078348393	1,066391525	47,97680808	4,185173792	2,387048312	0	1,964274122	75,3598827	8,3733203
ENSG00000140153	WDR20	11,89830776	4,134831606	12,44234067	6,551860716	1,813229949	13,65230319	5,753006489	7,485280869	11,61540764	75,34656889	8,371840988
ENSG00000020633	RUNX3	8,586207531	7,46979704	13,21373453	6,230594132	2,778933005	6,969491394	15,8953129	0,876678048	13,31290586	75,33365444	8,370406049

ENSG00000186314	PRELID2	15,09700027	14,01821433	6,050915643	14,02980608	0,066812566	4,630062606	5,719112807	13,4018113	2,31659851	75,3303341	8,370037123
ENSG00000169570	DTWD2	0,309072243	13,61962094	27,90698454	0	0	11,22660626	12,91700708	5,857370892	3,484424314	75,32108627	8,369009585
ENSG00000148187	MRRF	6,093720339	7,386965838	30,20372802	2,672011974	5,532395306	4,134608844	6,765278436	4,880909334	7,612381338	75,28199943	8,364666603
ENSG00000105325	FZR1	19,86647957	3,871194595	5,251351893	6,251859321	0,055155513	8,434403454	13,6627961	3,603264367	14,27173416	75,26823897	8,363137664
ENSG00000130703	OSBPL2	10,35432285	3,000592628	20,77340358	3,659929513	0,08257474	10,36384763	10,94391338	3,469727581	12,5350609	75,18337279	8,353708088
ENSG00000178425	NT5DC1	0,041360496	14,71533352	13,619389	0,357102743	0	8,423816061	9,100763098	17,90777183	10,98750481	75,15304155	8,35033795
ENSG00000204104	TRAF3IP1	24,95584197	3,545006937	12,16991331	5,259417563	0	13,5561958	3,290400991	0,071615641	12,28504319	75,1334354	8,348159489
ENSG00000256045	MTRNR2L10	0,395060315	0,350512418	3,815413123	1,193820145	34,7381675	0,325314856	2,378195835	30,45181308	1,336151467	74,98444874	8,331605416
ENSG00000134278	SPIRE1	0,555213364	0	0	21,01221626	19,29734497	10,49005943	9,911618388	3,743441212	9,958095958	74,96798959	8,329776621
ENSG00000196605	ZNF846	1,236078288	22,6193405	0	0,066701169	0,091798963	18,44864542	15,97245024	6,588419291	9,928916962	74,95235083	8,328038981
ENSG00000177728	TMEM94	6,298362094	5,098996574	18,99300179	1,38472955	0	7,997458424	13,6914558	7,708313294	13,70476842	74,87708594	8,319676216
ENSG00000047634	SCML1	1,484440752	22,53622049	20,73346199	0,047468615	0,130659466	8,782968496	3,583562329	8,475763875	9,094545114	74,86909113	8,318787904
ENSG00000132481	AC087289.1	22,34226244	0	0	8,213851347	33,1141772	4,589577162	1,316540845	0,630399811	4,609230123	74,81603892	8,312893213
ENSG00000274997	HIST1H2AH	3,123732727	8,314480623	0,718294829	0,67425058	40,82990146	13,28999452	7,781105861	0	0	74,73176059	8,303528955
ENSG00000226124	FTCDNL1	0,931090867	13,02320696	19,04249319	4,255916585	0	11,66605722	10,95989489	4,670876453	10,17137545	74,72091162	8,302323514
ENSG00000167799	NUDT8	6,117836869	6,513570852	18,00680803	17,0787399	0	6,60507694	8,635609895	9,364542399	2,382647721	74,70483261	8,300536957
ENSG00000131944	FAAP24	15,366096	13,91623038	0	6,495849579	0,151526389	11,20071404	1,905878461	18,12510286	7,527960396	74,68935811	8,298817568
ENSG00000143674	MAP3K21	0,01898374	0,336861809	22,42000296	26,81467453	0	8,879133879	5,028270843	0	11,18347791	74,68140567	8,297933963
ENSG00000184445	KNTC1	0,04002929	3,456841679	5,301872087	20,96696043	23,27525617	0,161149346	12,39746592	0,350129343	8,664632562	74,61433682	8,290481869
ENSG00000171928	TVP23B	14,14853585	10,8731441	6,38589855	9,309375182	0	8,691104001	5,699226706	9,43112932	10,02652661	74,56494033	8,28499337
ENSG00000148600	CDHR1	0,039102231	2,91420986	22,40672812	3,308529889	0,092927298	14,08168435	6,217510306	4,360761533	21,13586403	74,55731762	8,284146402
ENSG00000067646	ZFY	0	0,096506028	55,17582314	0	0	0	0	0	19,2635589	74,53588807	8,281765341
ENSG00000204946	ZNF783	0,022476658	6,221947347	33,82235921	3,609542264	0	7,329382907	3,396638469	9,092725716	10,9744265	74,46949907	8,274388785
ENSG00000163421	PROK2	0	0,166238066	65,31584471	8,573808837	0,222639518	0,102858386	0,077786939	0	0	74,45917645	8,273241828
ENSG00000197724	PHF2	1,625447533	7,061603118	7,681571726	17,46986755	0	9,138620416	5,67781176	17,77188883	8,030830803	74,45764174	8,273071304
ENSG00000147459	DOCK5	9,904551758	5,450381514	1,303928165	9,351162702	0,336904318	11,77737848	9,518763695	17,21508504	9,577680552	74,43583623	8,27064847
ENSG00000186814	ZSCAN30	13,51974638	0,129678153	11,02375438	24,57603013	0	6,118090424	8,980587177	2,437009868	7,639682937	74,42457945	8,269397716
ENSG00000106415	GLCCI1	4,456697057	8,33682813	1,49429953	24,71736212	2,139157233	5,953786495	11,19261715	6,384977115	9,747289632	74,42301446	8,269223829
ENSG00000160218	TRAPPC10	4,109357833	1,974436952	5,326536915	9,694178623	5,138406806	8,732135616	19,47514567	9,151856903	10,76109057	74,36314589	8,262571765
ENSG00000196693	ZNF33B	0,072745491	0,161356361	29,10611218	32,40882101	0	4,273060948	4,318752552	2,18725493	1,834085308	74,36218878	8,26246542
ENSG00000100294	MCAT	0,063279133	22,45745394	0	10,38057897	0	12,2279152	6,357583824	8,302376068	14,55330807	74,34249521	8,260277246
ENSG00000148358	GPR107	0,049604083	1,93646701	1,733763611	35,4327939	0,117885176	10,81986035	5,354352717	6,833584994	12,05897666	74,3372885	8,259698723
ENSG00000078699	CBFA2T2	8,56458347	0	6,466704217	8,829353058	0,075947455	7,842024721	3,860829715	27,98753184	10,6708958	74,29787027	8,255318919
ENSG00000197461	PDGFA	0,400437552	0,452178752	31,13966418	9,994859302	2,681918529	7,354263096	7,284594077	8,039964182	6,939583462	74,28746314	8,254162571
ENSG00000118960	HS1BP3	11,41724312	3,226871249	2,090790488	6,173230852	10,90244026	14,9971817	10,85264752	6,619988259	7,99298889	74,27338234	8,252598037
ENSG00000159079	CFAP298	7,411493842	8,982732189	9,157959952	9,079358731	13,55912986	10,19476429	6,084254485	3,730614218	5,916269078	74,11657665	8,235175183
ENSG00000063180	CA11	0,155303773	3,582575541	0,142846916	15,28601598	0,184541389	12,78858505	21,34156212	0	20,62839096	74,10982174	8,234424637
ENSG00000090615	GOLGA3	8,071567929	10,12952822	1,021311716	7,669497134	20,4678392	6,330078527	6,54833386	5,696611009	8,018625266	73,95339286	8,217043651
ENSG00000137720	C11orf1	5,20329512	28,38611953	0	11,50965312	0	10,48154469	11,89004569	0,188067483	6,293607328	73,95233296	8,216925884
ENSG00000136068	FLNB	12,76944974	2,998147363	1,075897745	9,46338139	0,154437016	8,300285446	17,00576246	9,331366084	12,81424559	73,91297283	8,212552537
ENSG00000120798	NR2C1	15,00803777	13,50265727	0	0,080859924	0	2,930555585	18,66308006	15,30832595	8,408302526	73,90181909	8,211313233
ENSG00000258102	MAP1LC3B2	7,098095699	7,977084554	7,689428625	6,264618388	11,99557553	6,148043291	7,923752699	10,34763781	8,438751272	73,88298787	8,209220874
ENSG00000138709	LARP1B	5,738376101	9,560337134	4,193159721	23,3960684	15,26628371	4,4627845	4,301461804	5,019259253	1,94080229	73,87853291	8,208725879
ENSG00000174370	C11orf45	0,080592304	21,09383734	0,074128026	0	0	3,229725893	10,9075542	22,11708705	16,35449396	73,85741877	8,206379864
ENSG00000100281	HMGXB4	7,573140488	12,50079254	19,80943762	2,215731553	0	9,060717476	3,029170519	2,255713831	17,35929054	73,80399458	8,200443842
ENSG00000129250	KIF1C	8,89611077	8,65881721	0	19,31612367	0,0837299	7,407758321	11,65771212	11,78747699	5,979673601	73,78740259	8,198600288
ENSG00000129317	PUS7L	4,241473594	0,114615089	0,099016919	15,00139093	14,81292705	6,784403436	7,329605488	21,16268976	4,210243933	73,7563662	8,1951518
ENSG00000187954	CYHR1	11,1630676	7,753569636	7,327972365	5,598890842	6,861651213	8,204834896	10,60222382	1,553079606	14,62162278	73,68691276	8,187434751
ENSG00000163590	PPM1L	6,339794075	0,344821733	0,245762827	17,74240383	40,49525092	3,120324301	4,659001236	0,106231978	0,627355243	73,68094615	8,186771794
ENSG00000164506	STXBP5	4,488432792	7,428534085	28,87245697	5,713541904	0,034188646	8,229189876	3,069866272	12,30006815	3,485506939	73,62178564	8,180198404
ENSG00000152484	USP12	0,859930642	1,420689561	0	23,09295007	0,070470475	12,01354058	19,10615731	8,753662993	8,278485195	73,59588682	8,177320758
ENSG00000183647	ZNF530	25,53133372	12,28783822	0	0,415915481	0	3,074254559	4,599828868	27,32224457	0,333257362	73,56467278	8,173852531
ENSG00000127774	EMC6	6,850867841	5,557347152	0	4,393982733	19,53748287	11,60514397	9,751580207	2,140120256	13,72182872	73,55835375	8,173150416
ENSG00000143772	ITPKB	13,18673826	0,609760091	0	14,7971937	0,510400145	10,72900972	16,24551911	8,359487353	9,086299554	73,52440793	8,169378659
ENSG00000105287	PRKD2	0,05549415	4,234339333	1,990676434	9,007670809	0,395649147	18,6748409	6,727384676	10,19335427	22,18146153	73,46087124	8,162319027
ENSG00000005436	GCFC2	3,401024738	5,593447028	0	1,193664111	0,065712268	11,35417304	12,60443042	31,92487679	7,311436832	73,44876523	8,160973915
ENSG00000117519	CNN3	1,303145417	56,46108949	11,3868959	0	0	0	3,967441328	0,237466452	0,066779203	73,42281779	8,158090865
ENSG00000107890	ANKRD26	8,467976822	3,309144441	0	17,51395812	13,97397313	4,241260178	12,6560554	6,449736553	6,810393903	73,42249855	8,158055394
ENSG00000167065	DUSP18	3,633445182	0,060256629	15,86672925	3,752772668	27,51889482	8,910702884	4,708661898	0,074254959	8,895575111	73,42129339	8,157921488
ENSG00000153037	SRP19	1,034511846	10,07094134	3,806136185	7,989071201	15,80977675	8,566078078	6,692864529	15,04974402	4,382422509	73,40154646	8,155727384
ENSG00000089737	DDX24	10,34089998	7,788486119	13,95229496	11,91442373	2,71205301	4,491363352	6,341302942	7,102404437	8,658586666	73,3018152	8,144646133
ENSG00000125755	SYMPK	0,739344155	2,169760257	2,667855673	23,15220936	22,74050677	3,215805307	5,324343219	7,555099728	5,735572008	73,30049648	8,144499609
ENSG00000198892	SHISA4	6,837190769	18,42900344	0	8,070153854	0	11,30776554	20,48055247	4,542055911	3,619004443	73,28572643	8,142858492
ENSG00000204386	NEU1	12,2530554	5,894452926	0,980021087	2,002197026	12,06491951	8,085646106	11,44897264	13,91087043	6,590575551	73,23071067	8,13674563
ENSG00000165029	ABCA1	0,040246068	1,642560192	38,00509166	11,11938812	9,245746385	2,710186977	3,196909205	5,016362841	2,227386409	73,20387786	8,133764206
ENSG00000088888	MAVS	0,01283753	4,282617961	10,72151601	10,24143728	0,762716724	12,14976783	15,05089702	3,059839254	16,84633755	73,12796715	8,125329684
ENSG00000184954	OR6C70	0	0	73,12149569	0	0	0	0	0	0	73,12149569	8,124610632
ENSG00000256294	ZNF225	0,06304154	0	44,91904812	3,483487354	0	1,615038294	5,914955159	13,09607172	4,00586997	73,09751215	8,121945795
ENSG00000102796	DHRS12	17,67950869	4,329771673	3,149916136	12,49235431	6,30744692	6,580022872	9,596880918	6,271704256	6,686262983	73,09386876	8,121540973
ENSG00000103489	XYLT1	0,096126317	0,657927481	23,97338367	11,7615183	0,261081456	10,34302577	5,689721526	9,879405008	10,35295512	73,01514466	8,112793851
ENSG00000111271	ACAD10	0,016919782	8,496717728	0	24,74660435	0,040210228	17,90816213	9,553222009	0,813969375	11,38260527	72,95841087	8,106490097
ENSG00000049759	NEDD4L	0,017175559	11,0328943	1,45340811	0,860094824	35,55255506	5,09159533	5,447791183	5,445898192	8,04813878	72,94955133	8,105505704
ENSG00000147852	VLDLR	9,366956239	8,710545322	0,207248982	5,641687064	10,05942205	4,36467275	8,405670341	1,055813934	25,1186747	72,93069138	8,103410154
ENSG00000100206	DMC1	0	6,152546618	30,84182335	21,52353552	0,312911016	0,674629124	0,255095069	0	13,11667293	72,87721363	8,097468182
ENSG00000125505	MBOAT7	10,76505766	7,397719579	7,353970873	5,275896123	7,600371267	10,50265367	10,52698931	6,493801025	6,95344131	72,86990081	8,096655645
ENSG00000179241	LDLRAD3	21,10593544	6,974622516	4,739332835	13,0039725	0,156991275	17,87845122	8,803492147	0,14445227	0,040622191	72,8478724	8,094208044
ENSG00000196476	C20orf96	22,71025917	2,169935261	0,160682138	2,262441976	0	4,027886833	17,62386744	20,4372825	3,437623533	72,82997886	8,092219873
ENSG00000079387	SENP1	0	3,7278492	0,045466159	36,23385565	0	11,69569162	13,64700209	0	7,477632398	72,82749711	8,091944124
ENSG00000123094	RASSF8	0	22,62965036	8,756427846	3,630280523	0	16,26659677	13,86612673	0,390328272	7,268967533	72,80837803	8,089819781
ENSG00000081181	ARG2	1,786333366	0	26,41054355	0,22848947	0	16,92520332	10,71222187	10,56117488	6,184034892	72,80800134	8,089777927
ENSG00000165655	ZNF503	0,23574192	5,85645554	6,504994516	6,513197494	23,53032259	11,80268689	5,598208261	3,608489817	9,103870202	72,75396723	8,083774137
ENSG00000147044	CASK	13,58850761	5,594416572	0,08364916	9,343877419	1,044626985	5,907844398	6,173147886	24,57668206	6,4406413	72,75339339	8,083710377
ENSG00000173166	RAPH1	5,317864688	9,851715199	8,873740898	9,474665228	0,494396892	6,195593726	7,589540819	9,069749358	15,86299691	72,73026372	8,081140413
ENSG00000171163	ZNF692	32,30666311	0	0	18,13063071	0,091401767	7,72757331	3,959867948	0,084101442	10,40627002	72,70650831	8,078500923
ENSG00000231767	AL136454.1	2,026386963	13,35573181	4,792760327	1,499626291	31,64637346	0,953509062	1,201823103	7,596196954	9,612751152	72,68515912	8,076128791
ENSG00000175155	YPEL2	15,85610286	8,813769914	1,565455103	5,21809971	0,123819403	11,05943391	15,24217227	0,265836338	14,48154719	72,62623671	8,069581856
ENSG00000180425	C11orf71	21,74400858	5,842403996	2,018920374	4,382475742	0	10,468257	8,429237011	19,64908232	0,084360655	72,61874567	8,068749519
ENSG00000116580	GON4L	15,0895907	3,405831371	5,714306372	4,709781442	0,270080879	5,157410757	8,429693902	17,11953137	12,64135281	72,53757961	8,059731067
ENSG00000151665	PIGF	6,951450519	9,588042591	0,111683447	1,572528975	14,21176293	7,732272759	6,830546399	12,14735353	13,36541884	72,51105998	8,056784442
ENSG00000117595	IRF6	0	6,203244822	0	0,314948672	58,80540726	0	6,183912963	0	0,972039746	72,47955346	8,053283718
ENSG00000169951	ZNF764	38,05675792	6,977032447	0	0	0	2,877986233	3,909612015	0,530733324	20,11894571	72,47106765	8,05234085
ENSG00000187815	ZFP69	0	0	0	0	0,226358363	12,65375341	0,158172495	44,46755762	14,87709318	72,38293507	8,042548341
ENSG00000152749	GPR180	0,085046472	2,414606034	0,031289971	18,09274475	0	5,84534112	8,092582197	20,71725057	17,09101854	72,36987966	8,04109774
ENSG00000165443	PHYHIPL	0	0	0	0	72,35685136	0	0	0	0	72,35685136	8,039650151
ENSG00000181513	ACBD4	8,243849989	9,941705512	0	7,532286078	0,095105281	11,51180379	15,05245244	11,11366263	8,859209218	72,35007493	8,038897214
ENSG00000171109	MFN1	0,254180943	0,405934062	0,093517275	0,526698014	0	10,93977226	21,82276254	12,72826173	25,57148111	72,34260793	8,038067548
ENSG00000165572	KBTBD6	40,91483586	0	0,268424198	9,87702878	0,138708977	5,190713292	4,022416978	11,4229022	0,502481906	72,33751219	8,037501354
ENSG00000180155	LYNX1	16,88033257	7,902233376	11,12584626	8,625090757	2,216774953	3,73315316	2,398477075	10,52758479	8,918560706	72,32805364	8,036450404
ENSG00000139180	NDUFA9	7,634784871	10,73496988	1,57836838	11,80246733	6,324560702	6,514437041	8,005661641	9,858022898	9,872705254	72,325978	8,036219778
ENSG00000146414	SHPRH	0,239099004	4,773097312	29,2651969	16,1609817	0,020293713	3,937751029	8,224773119	4,369444439	5,335105861	72,32574308	8,036193676
ENSG00000167987	VPS37C	15,695336	5,624408897	6,884521026	0,263770508	2,994915061	8,511459962	14,11025973	10,18777287	7,984308513	72,25675256	8,028528062
ENSG00000122483	CCDC18	1,824164531	5,569432631	14,40976192	0,046322559	0	12,07586303	8,174615985	10,2655866	19,79547548	72,16122273	8,017913637
ENSG00000065802	ASB1	10,77175392	1,810058054	0,187646887	0,140912636	39,17469613	5,82378155	5,437555903	5,799445629	3,010879259	72,15672997	8,017414441
ENSG00000143633	C1orf131	18,81554122	14,71239129	0	0,24102621	0,066343533	8,398207651	1,413946102	12,14788083	16,30834991	72,10368675	8,011520749
ENSG00000180818	HOXC10	0	7,803373263	0	28,5036518	0,302924962	21,34235592	0,10583748	0	14,03058941	72,08873284	8,009859204
ENSG00000114473	IQCG	5,847744366	5,744874132	0,446673967	9,573672073	0	11,13031587	16,61625045	13,4067587	9,282371827	72,04866138	8,00540682
ENSG00000178971	CTC1	12,33987395	5,974699255	0,064857699	8,249344157	0	15,09685602	10,5388053	8,287847879	11,44738261	71,99966687	7,999962986
ENSG00000121578	B4GALT4	9,990424105	14,46616114	0,079559273	0,672127868	0,102781208	9,401915909	8,815960649	4,681313993	23,74941963	71,95966378	7,995518197
ENSG00000164338	UTP15	0,095579109	8,098532893	0,747258331	2,970796675	0,056786455	22,56215939	7,678212917	13,5852289	16,16312259	71,95767725	7,995297473
ENSG00000140471	LINS1	25,02206368	2,881380892	0	5,438960501	0,325456084	4,338935331	5,669231728	19,9783717	8,301016337	71,95541626	7,995046251
ENSG00000170745	KCNS3	32,17379797	0	0,088868318	0,250257329	0	11,29761919	12,03359962	0	16,1011533	71,94529573	7,993921748
ENSG00000163872	YEATS2	9,909616196	3,406808054	2,852610476	23,46170608	11,33649005	6,258940833	3,874963342	5,296253819	5,521632405	71,91902126	7,991002362
ENSG00000137872	SEMA6D	0,017659293	12,50305692	0,129942783	6,159736458	50,57106913	1,066387954	1,12904237	0,038615703	0,21718654	71,83269715	7,981410794
ENSG00000157184	CPT2	11,29322731	15,57450808	0,02755279	12,31093745	0,035594959	6,824550434	6,690756132	6,779656604	12,15766723	71,69445099	7,96605011
ENSG00000185504	FAAP100	3,698016954	7,613970486	0,051929778	2,144804574	0,067087155	6,601709852	21,75162864	11,79021154	17,94930918	71,66866816	7,963185351
ENSG00000179335	CLK3	3,25239207	8,145388742	0,054391254	0,331864665	28,01900308	6,654927012	7,223919227	8,712235577	9,245507663	71,63962929	7,95995881
ENSG00000147650	LRP12	0	11,35427923	0,028432044	26,92885234	0	7,924745763	1,398820312	9,192820125	14,78864624	71,61659606	7,957399563

ENSG00000180447	GAS1	9,993958251	6,479743086	0,265163846	20,57611246	0	8,598862958	8,018930598	8,720530717	8,863904083	71,517206	7,946356223
ENSG00000219200	RNASEK	6,672853596	10,94151258	6,526083934	1,166841697	0	11,68514962	10,52015081	14,40687326	9,557227529	71,47669303	7,941854781
ENSG00000152766	ANKRD22	0,151299695	12,48420826	0	30,30633998	0,08989175	12,33427817	1,538935236	0	14,46765167	71,37260476	7,930289418
ENSG00000180346	TIGD2	39,69177252	0	0,086409729	0	0	4,074272788	17,04399599	0	10,45639432	71,35284535	7,928093928
ENSG00000135631	RAB11FIP5	10,49081604	4,027068859	0,078770218	0,998192734	33,37788436	5,900191946	13,56387976	0,093634051	2,817452236	71,3478902	7,927543355
ENSG00000153207	AHCTF1	0,031487929	6,369699523	8,138404648	14,10973659	13,73162513	2,523749135	12,05289082	7,711746523	6,660888066	71,33022837	7,92558093
ENSG00000010626	LRRC23	10,74410021	0	21,67734717	0	0	2,609340656	4,43997243	26,74224711	5,115041897	71,32804947	7,92533883
ENSG00000124788	ATXN1	3,317102691	2,845207614	6,39859873	16,97864561	13,42759281	11,60591616	8,312092508	2,467314029	5,957689435	71,31015959	7,923351065
ENSG00000161960	EIF4A1	4,888286281	2,521553508	7,789925663	21,98586959	0	7,956967662	4,76678711	16,46890359	4,92841069	71,3067041	7,922967122
ENSG00000127946	HIP1	0,016874436	0,029943272	35,63608639	7,896524636	0,441127076	0,500232895	4,763809367	10,40564467	11,53887955	71,2291223	7,914346922
ENSG00000240849	TMEM189	6,039850069	7,505676966	5,432721642	14,77238729	0,815044227	11,79845735	13,00423817	3,499748288	8,295255187	71,16337919	7,907042132
ENSG00000101639	CEP192	0,030524305	4,874816687	1,094962515	2,951693481	13,60156332	7,121704383	11,21515441	20,12443634	10,13605919	71,15091463	7,905657181
ENSG00000104518	GSDMD	12,86742912	6,907886335	4,154668146	5,285408532	11,86465037	10,89756787	5,058292637	9,292456864	4,787786513	71,11614639	7,901794043
ENSG00000156162	DPY19L4	4,912787611	0,075151906	20,29535196	14,33387192	0	10,11366324	11,58701637	0,046305293	9,74027457	71,10442286	7,900491429
ENSG00000132478	UNK	12,38342944	10,5514361	8,22901376	13,47065031	0,064822661	4,791641204	5,02787251	9,304655692	7,212455287	71,03597697	7,89288633
ENSG00000124074	ENKD1	4,933065534	6,942513323	1,043077294	0	36,51814408	7,159368225	2,0715552	7,191446498	5,160466284	71,01963644	7,891070716
ENSG00000116031	CD207	70,77740555	0	0,148971114	0	0	0,088912421	0	0	0	71,01528909	7,890587676
ENSG00000116824	CD2	0	0,164001223	38,59417888	32,23783784	0	0	0	0	0	70,99601794	7,888446438
ENSG00000143387	CTSK	70,83308	0	0,15240137	0	0	0	0	0	0	70,98548137	7,887275708
ENSG00000167766	ZNF83	0,075291764	16,29961336	0,03462632	23,85728343	0	7,233271281	7,126864897	0,32928208	16,01965538	70,97588851	7,886209835
ENSG00000189164	ZNF527	18,59533436	0,083325668	0,043191439	4,378647803	0,111596502	4,0730067	10,02047089	15,96723905	17,61441557	70,88722798	7,876358664
ENSG00000185112	FAM43A	19,75222375	2,150296713	3,901083987	2,929502522	6,911644761	2,527907744	12,97969952	3,709770606	16,02125192	70,88337152	7,875930169
ENSG00000164576	SAP30L	2,925882363	14,61721359	5,340993859	2,487315814	0	8,896009475	13,34313629	8,957267097	14,31079118	70,87860967	7,875401074
ENSG00000213762	ZNF134	0	16,85344737	0	6,09305253	0	4,745836695	33,10717459	0,385796343	9,691937725	70,87724525	7,875249472
ENSG00000181991	MRPS11	7,410539713	12,76866667	5,186195877	0	0	9,050203811	11,68202807	9,628867994	15,12397072	70,85047285	7,872274761
ENSG00000154153	RETREG1	25,3394696	0,058700088	26,16712841	3,313097313	0	4,939544315	0	0,072336814	10,94411438	70,83439092	7,87048788
ENSG00000140406	TLNRD1	9,481447571	5,755782871	6,02433651	24,45087275	1,778910427	6,917221155	8,623657494	1,705028728	6,022271207	70,75952871	7,862169857
ENSG00000156113	KCNMA1	0,21197196	4,41587235	0,912458523	19,69237688	28,37153516	9,276730428	0,788501694	7,064053991	0,005213964	70,73871495	7,859857217
ENSG00000139055	ERP27	0	18,85708258	0	0	0	8,043613468	10,22746979	22,88571858	10,69335344	70,70723786	7,856359763
ENSG00000188315	C3orf62	1,386335511	0,064737325	4,563652035	9,701590074	48,98633786	2,443395746	1,454026059	0,159553147	1,929357909	70,68898567	7,854331741
ENSG00000173706	HEG1	0	1,668486954	0	22,07629301	0	14,3032101	5,666564572	11,07638956	15,89133249	70,68227669	7,853586299
ENSG00000174943	KCTD13	6,551712571	0	0	0	44,20849275	5,035378296	2,642298723	6,702838084	5,496544713	70,63726514	7,848585015
ENSG00000163291	PAQR3	30,92144065	4,95077949	0,896493103	9,340895648	0	12,15937388	0,880103111	8,205583068	3,266511477	70,62118042	7,846797825
ENSG00000102078	SLC25A14	0,050219532	44,55666336	0	1,387490726	0,238695608	11,13789847	0	1,317784915	11,92036517	70,60911778	7,845457531
ENSG00000198793	MTOR	14,21454333	8,075899829	0,022937544	7,98802505	0,148163026	9,555704476	16,42016227	1,881342121	12,2681094	70,57488704	7,841654116
ENSG00000073417	PDE8A	30,37692506	0,737021989	0,764063241	0,489008573	0	10,59224197	17,68247557	0,330269433	9,589531946	70,56153778	7,840170865
ENSG00000141441	GAREM1	0,018969441	4,308583481	21,1817625	10,80950811	0	10,05960439	10,6947471	5,102115466	8,375458385	70,55074888	7,838972098
ENSG00000124508	BTN2A2	14,28913649	20,7875011	2,563430109	0,489406017	0,056129657	15,14407265	10,31531645	0	6,884280366	70,52927283	7,83658587
ENSG00000158301	GPRASP2	0	0	56,2953408	0	0	8,389440482	5,744805138	0	0,046753842	70,47634026	7,830704474
ENSG00000178585	CTNNBIP1	28,23989101	12,12621803	0	3,047758014	0	9,490542123	8,266988032	4,552258193	4,675384208	70,39903962	7,822115513
ENSG00000115295	CLIP4	14,93433091	0,10338337	3,000941822	3,118744716	0,046153135	0,042645052	2,112403739	45,56693111	1,40919481	70,33472866	7,814969852
ENSG00000132840	BHMT2	0	0,1435834	0	0	70,09282152	0	0	0	0,049758105	70,28616303	7,809573669
ENSG00000170412	GPRC5C	4,889149007	4,3880827	2,465532421	3,129263905	42,70829486	3,5441235	0,92476572	6,604563498	1,601925051	70,25570066	7,806188962
ENSG00000189362	NEMP2	0	0,082189119	0,042602314	0,71982062	13,81432998	13,04400396	5,980271763	15,5468831	20,96291807	70,19301893	7,799224325
ENSG00000159882	ZNF230	0,024035402	5,715130907	31,30426437	14,5678664	0	1,425031198	6,625759539	7,831199389	2,690000561	70,18328777	7,798143085
ENSG00000162073	PAQR4	7,270797983	2,70668237	11,59809296	2,546135351	0	14,45855968	19,92664761	3,780207031	7,879092129	70,16621511	7,796246124
ENSG00000161640	SIGLEC11	9,667647603	7,69017397	0,613255812	1,72695697	0	10,14391802	6,129184198	16,55817719	17,62994113	70,1592549	7,795472766
ENSG00000134690	CDCA8	25,6875409	13,15077141	0	11,6043401	0	4,82699101	14,86244581	0	0	70,13208923	7,792454359
ENSG00000188566	NDOR1	10,92301375	1,796437941	24,11251916	4,876429356	0	6,669183972	6,945995107	0,699085827	14,08921096	70,11187608	7,790208453
ENSG00000166750	SLFN5	5,138948581	8,917969836	1,588606524	0,293350168	0,067288278	12,23267977	29,563227	0,061913916	12,24003234	70,10401641	7,789335156
ENSG00000061273	HDAC7	6,818849281	3,889247382	12,03983104	5,992463156	15,04763272	6,600994943	7,974595033	4,127103784	7,600089448	70,09080679	7,787867421
ENSG00000197037	ZSCAN25	0	0	3,486994899	18,60931602	0,101231161	8,628753038	13,91756889	12,90068713	12,38976815	70,03431929	7,781591032
ENSG00000197296	FITM2	0,130605506	18,1928669	0,060064844	2,424417439	0	5,771737867	9,59734173	4,855131934	28,95318551	69,98535174	7,776150193
ENSG00000196141	SPATS2L	2,977975467	4,591736154	15,98259251	11,38298559	0	11,1263096	12,73548477	8,31381338	2,862459589	69,97335706	7,774817451
ENSG00000113555	PCDH12	37,62254384	0	0	0	0,052656531	15,93422694	8,941140961	7,412974902	0	69,96354317	7,773727019
ENSG00000197302	ZNF720	12,70030774	4,452643237	0	1,594957057	12,80472085	10,14123329	6,378330855	17,03340375	4,790054007	69,89565078	7,766183421
ENSG00000165060	FXN	17,03071481	14,2233843	1,328093086	8,662670356	0,087986657	5,223449493	4,611184565	12,91297648	5,80556942	69,88602917	7,765114353
ENSG00000144306	SCRN3	0	0	7,17757495	1,574173351	4,159662653	18,9772233	19,43821263	4,465331993	14,082	69,87417888	7,763797653
ENSG00000185670	ZBTB3	11,1967471	17,04155482	27,12817833	0,157189744	0,649007607	10,49434371	0	0	3,078782748	69,74580407	7,749533785
ENSG00000132122	SPATA6	0,025448058	0,361255642	0	0,747035124	0	23,30236123	8,029425086	31,10692544	6,165672767	69,73812334	7,748680371
ENSG00000105722	ERF	0,878281078	4,101284798	16,92200664	9,817987726	17,79658668	4,618495711	3,377604532	8,18758029	4,036434103	69,73626156	7,748473506
ENSG00000111602	TIMELESS	0,051652904	22,77671749	0,760157509	19,44413757	0	10,74689943	2,444644992	0,056474888	13,37231001	69,6529948	7,739221644
ENSG00000163517	HDAC11	11,75721703	0,820162875	0	0,299294561	30,27544182	4,123183898	5,348859498	6,064178643	10,90706984	69,59540817	7,73282313
ENSG00000076650	GPATCH1	22,22214274	12,90522995	0	0,058140591	0	10,42486244	6,877384508	0,515383684	16,52245151	69,52559543	7,725066159
ENSG00000178385	PLEKHM3	6,401229708	4,446761129	5,326478955	8,654485324	29,68474615	6,629009982	0	0,028392728	8,351755555	69,52285953	7,72476217
ENSG00000169398	PTK2	21,02484435	1,795158202	0,121371023	0,778513605	0,078398532	7,29224191	8,947818757	18,51510821	10,96794714	69,52140173	7,724600192
ENSG00000136935	GOLGA1	0,025105594	0,044549261	1,016042894	0,130055644	0	20,70092093	26,84927523	9,442556174	11,30085183	69,50935756	7,723261951
ENSG00000118513	MYB	0,054949298	0,048753091	3,386303024	65,7556136	0,065294099	0	0,045625575	0,060079012	0,067580554	69,48419825	7,720466472
ENSG00000133250	ZNF414	2,955440353	6,79823977	14,59873754	8,316652577	13,00679255	8,472796565	6,54390721	2,154227532	6,596508215	69,44330232	7,71592248
ENSG00000041988	THAP3	13,69346772	7,243521613	14,81377247	8,458599373	0,088191931	11,73434334	5,885273431	4,21969487	3,240447712	69,37731246	7,708590274
ENSG00000160803	UBQLN4	4,707615615	7,041834194	8,445327415	3,695011226	5,825027077	4,100776355	10,75736884	14,80319638	10,00049515	69,37665225	7,708516917
ENSG00000139579	NABP2	0,68655416	3,532993186	7,830425323	14,10779735	0,326322165	7,010306058	11,17318722	19,96719558	4,728489435	69,36327049	7,707030054
ENSG00000124374	PAIP2B	0,095943219	8,725255558	0,08824765	20,62628847	35,79774867	1,896120877	0,378402589	0,209799725	1,474972399	69,29277915	7,699197684
ENSG00000005882	PDK2	16,2110957	3,179936144	0,050717041	7,521935046	0	12,28967235	11,05677255	10,97228449	7,934334284	69,21674761	7,690749734
ENSG00000134369	NAV1	0,080761005	3,785730817	28,17809225	9,204137527	0,01599419	7,182340024	5,54342713	2,35467558	12,87094563	69,21610415	7,690678239
ENSG00000112658	SRF	0	4,246162611	18,59332735	11,96440205	0,169755397	4,783996776	12,87027277	2,96774133	13,61673881	69,21239709	7,690266343
ENSG00000205593	DENND6B	2,464177813	5,192489895	6,563484586	4,476716908	20,86261245	6,284705046	6,521814437	3,199383446	13,60622577	69,17161035	7,685734483
ENSG00000085872	CHERP	6,95887416	5,081993019	11,78469248	2,255796954	0	9,571233602	11,8482375	7,251431906	14,36718751	69,11944713	7,67993857
ENSG00000110492	MDK	13,76234705	19,29254828	5,696313504	0,831759937	0	10,35052323	5,027955016	14,14428477	0	69,10573178	7,678414642
ENSG00000103061	SLC7A6OS	11,0371392	2,563499045	18,17766613	3,692005377	0	5,805284041	17,65701039	3,664475127	6,467316836	69,06439614	7,673821794
ENSG00000168826	ZBTB49	0,261527467	15,22162963	0	0,090320171	0,49722055	9,820252244	2,605822056	26,07790979	14,47402821	69,04871009	7,672078899
ENSG00000188321	ZNF559	0,066833512	21,74231167	0,040981881	0,192344814	0	13,59962858	13,31838995	6,186818528	13,89122612	69,03853506	7,67094834
ENSG00000159082	SYNJ1	0,131074844	8,472888479	2,583458169	23,73311913	0,044500315	5,28364538	4,05796241	14,53584529	10,1789545	69,02144852	7,669049835
ENSG00000142694	EVA1B	5,24082904	0,664265896	0	1,077353322	25,79962616	10,41222447	12,8474989	5,457218291	7,519801479	69,01871756	7,668746396
ENSG00000278828	HIST1H3H	2,321019275	3,03475182	6,516914204	0,632825322	29,03132921	1,341233441	17,34472657	4,274012191	4,507191324	69,00400336	7,667111485
ENSG00000143224	PPOX	0	7,263952595	0	30,04203729	0	4,986095638	9,37376237	3,916261541	13,41241568	68,99452511	7,666058346
ENSG00000136514	RTP4	0	10,1826076	0,370393203	0	0	16,02733752	25,16093933	17,17115008	0	68,91242773	7,656936414
ENSG00000183741	CBX6	8,054212235	5,743514821	2,388621025	14,29780683	1,833601399	6,590968116	9,625121359	12,09810247	8,262416993	68,89436525	7,654929472
ENSG00000121060	TRIM25	2,36980055	8,918310918	17,14517819	16,80793648	0,340180811	7,962869485	6,787890817	1,63460948	6,904938676	68,8717154	7,652412823
ENSG00000186416	NKRF	15,83474126	7,068769774	0,916015265	3,152773199	4,654643014	5,868102559	7,910805203	16,33299158	7,103975148	68,842817	7,649201889
ENSG00000100099	HPS4	18,61202354	7,659778893	1,675821409	3,751152018	0,133228545	13,74124884	8,227371983	2,666277812	12,3070318	68,77393485	7,641548316
ENSG00000105619	TFPT	1,340496998	4,108627421	6,725324975	6,102511503	8,688327638	14,85167259	12,04110444	4,263671839	10,64121216	68,76294957	7,64032773
ENSG00000144362	PHOSPHO2	14,3189452	0	2,80221874	28,75896225	0	7,470435913	3,541501306	1,998596174	9,866851653	68,75751125	7,639723472
ENSG00000111727	HCFC2	14,18846827	8,645868341	5,352953093	0,116853997	1,179367955	5,200958517	8,25980242	8,385412222	17,42224451	68,75192933	7,639103259
ENSG00000170734	POLH	0	0,075746328	0,157050903	1,84276112	5,883846739	21,32466452	11,94449206	0	27,50948522	68,73804689	7,637560765
ENSG00000204560	DHX16	0,03799612	6,674895149	13,42021096	8,004542847	0	10,34594494	7,477110557	10,46888497	12,19661612	68,62620166	7,625133518
ENSG00000127980	PEX1	0,790448252	7,697365402	0,070931385	47,27324112	1,374524913	1,206545403	2,673328074	1,053950522	6,4730927	68,61342778	7,623714197
ENSG00000112624	BICRAL	12,58702693	3,369897999	3,168558778	13,04102898	0,997111399	17,8202943	2,732000507	5,746268235	9,098141891	68,56032902	7,617814335
ENSG00000185340	GAS2L1	16,38914022	7,120932384	0,062035282	3,493884953	0	10,70032508	19,7403669	2,802167723	8,211919529	68,52077207	7,613419119
ENSG00000066135	KDM4A	0	2,637971066	15,81869776	0,755020181	28,81807439	4,54461842	2,880202451	5,545487534	7,49265493	68,49272673	7,61030297
ENSG00000136104	RNASEH2B	10,75378942	4,365173483	19,19036475	7,791029442	0,177571338	8,525548253	7,277861485	1,784706119	8,609830898	68,47587519	7,608430577
ENSG00000135709	KIAA0513	0,136798072	0,182058617	28,56240012	8,326769587	13,53243653	5,951527986	6,162059985	2,953981083	2,649842911	68,45787489	7,606430544
ENSG00000173581	CCDC106	2,621704253	4,949095609	10,4665818	0	20,1498215	8,451701582	8,800060199	4,147203874	8,849834394	68,43600321	7,604000357
ENSG00000071564	TCF3	0	3,167031497	4,788043043	19,90399659	0,117820716	9,525705683	13,17273827	5,745745433	11,98124725	68,40232848	7,60025872
ENSG00000166012	TAF1D	0,376879095	10,41359074	0,46219969	20,82521412	5,544570131	4,610816502	5,766866803	10,83134687	9,535118482	68,36660244	7,59628916
ENSG00000196345	ZKSCAN7	0	5,225200572	0	35,90336271	0	2,24736285	3,548234199	21,43741372	0	68,36157405	7,59573045
ENSG00000196787	HIST1H2AG	0	13,46668712	0,617733553	29,45665935	4,628626101	4,055591875	8,866570128	0	7,268663982	68,36053211	7,595614679
ENSG00000165533	TTC8	8,211218479	5,699678122	24,83472516	0,917463551	0,172183575	6,125194697	9,124021266	9,241817055	4,009793912	68,33609582	7,592899535
ENSG00000186532	SMYD4	16,70087316	7,478063747	2,625162511	5,351919945	0,052990636	5,973466241	9,034900696	10,82432846	10,2836721	68,32537751	7,591708612
ENSG00000101940	WDR13	5,125320423	10,55945376	0	0,994416824	0	14,83753125	12,51196781	8,898898414	15,36916743	68,29675592	7,588528435
ENSG00000204130	RUFY2	0,074283186	6,063429767	14,92900372	3,014364952	25,90659916	6,52468779	2,667613056	4,304542725	4,79633399	68,28085834	7,586762038
ENSG00000136874	STX17	9,342675543	4,573337524	0,093574988	11,4481121	0,040295954	10,94652369	11,85435587	7,48965245	12,4078412	68,19636932	7,577374369
ENSG00000125945	ZNF436	29,11072841	0	17,77306943	12,92135165	0	4,159897452	3,277014522	0,552335257	0,34948155	68,14387826	7,571542029
ENSG00000010539	ZNF200	9,492275111	0	9,305561792	13,50263672	0,287370666	7,036484848	8,534258378	12,251375	7,733277534	68,14322705	7,571469673
ENSG00000162373	BEND5	0	11,14128759	2,855071045	0,243636764	39,06367291	6,583753044	3,221693866	0	5,032258773	68,14137398	7,571263776
ENSG00000033030	ZCCHC8	0,06624751	0	0,060933823	0,085796243	17,90864553	13,36522072	6,298269625	19,95500293	10,38817694	68,12829331	7,569810368
ENSG00000140876	AC092724.1	7,19574146	4,911025643	0	40,30314321	0,219241479	4,254117351	3,293787714	5,44672364	2,496107137	68,11988763	7,568876404

ENSG00000160753	RUSC1	7,264663648	14,17512507	9,421318514	1,201579364	0	8,220656683	6,656005824	11,32083801	9,790404378	68,05059149	7,561176833
ENSG00000164296	TIGD6	0	4,807973832	40,95272599	0	0,261049825	4,381936724	0,668850813	0	16,97701451	68,0495517	7,5610613
ENSG00000161847	RAVER1	6,737607288	1,884910625	8,207084666	3,406461809	12,0451154	10,12990868	9,601164188	4,711961095	11,30980041	68,03401416	7,559334907
ENSG00000277972	CISD3	5,333981841	7,652569285	5,741233738	5,781135374	13,35059583	9,906038939	7,962630908	3,84659091	8,444396994	68,01917382	7,55768598
ENSG00000127804	METTL16	3,53504693	5,252389749	1,120134717	11,59516285	13,94826362	6,462601376	11,89538518	6,028747986	8,175249972	68,01298238	7,556998043
ENSG00000101190	TCFL5	0,351829035	3,121559954	0,323608963	19,44102255	28,532913	0,772575444	5,294877224	0	10,16855244	68,00693861	7,556326513
ENSG00000196653	ZNF502	0	0	0	0,079723488	0,109721217	12,52059653	20,81588499	28,87390355	5,564607144	67,96443652	7,551604058
ENSG00000037757	MRI1	3,40064181	2,166178881	21,65453743	9,485806924	0,414446096	9,956549323	10,17229178	9,628935753	1,072397705	67,9517857	7,550198411
ENSG00000104522	TSTA3	5,10961464	10,33030433	0	7,593728474	11,04823925	10,80625985	9,493736318	9,066802157	4,481332438	67,93001745	7,547779716
ENSG00000120662	MTRF1	0	0	0	4,211915758	0	15,24770465	18,29299295	12,30204196	17,85403976	67,90869508	7,545410564
ENSG00000111412	C12orf49	15,6369316	7,446672277	1,057080349	3,788638719	0	8,029781311	18,96947172	7,996227147	4,968471211	67,89327434	7,543697148
ENSG00000104154	SLC30A4	7,38043156	12,79571219	0	6,567264499	0	9,550297338	12,99100995	5,105144078	13,47653575	67,86639537	7,540710597
ENSG00000054523	KIF1B	3,462239937	5,43244797	21,00538107	12,64916158	0,486389448	5,711368611	4,722839246	9,100003891	5,243976362	67,81380811	7,534867568
ENSG00000160703	NLRX1	0	1,512078948	22,41606437	0,588575131	0	6,206053527	8,042365265	8,136640666	20,85533342	67,75711133	7,528567926
ENSG00000179832	MROH1	14,48321988	3,393891224	14,20112952	0	17,45687402	7,136756899	4,787274889	2,818528366	3,477271443	67,75494624	7,52832736
ENSG00000169598	DFFB	0,039046659	3,395079588	0,071829483	1,753051509	0,092795231	11,18931796	5,219825181	24,33432862	21,61006073	67,70533496	7,522814995
ENSG00000100116	GCAT	16,80602354	0	0	1,612240813	0	7,648919173	14,61037611	21,98709099	5,03481548	67,69946612	7,522162902
ENSG00000113790	EHHADH	0,03248991	7,667788865	0	2,86125214	13,89833533	26,82536093	4,505169951	7,530867374	4,355465691	67,67673019	7,519636688
ENSG00000063587	ZNF275	0,018548002	5,990161288	0	6,341613474	44,0797278	7,351628598	2,00210423	0,040559048	1,847743747	67,67208618	7,519120687
ENSG00000188895	MSL1	4,739441989	13,44878762	0,601281815	20,7774368	0,04854908	10,65398458	6,259098622	0,84875717	10,27595344	67,65329112	7,517032347
ENSG00000137522	RNF121	2,32477801	9,075575388	0,171064676	13,9700571	0	17,4078482	21,31065331	0	3,373829174	67,63380586	7,514867318
ENSG00000169612	RAMAC	9,655627518	19,36105176	1,953853731	6,669264527	2,753616938	5,406670551	7,776755402	1,68912239	12,35018423	67,61614705	7,512905228
ENSG00000166233	ARIH1	3,428026321	2,496470234	3,973227221	19,00155904	7,770049107	6,613796921	6,301486645	9,937012958	8,042139044	67,56376749	7,507085277
ENSG00000203666	EFCAB2	0,035113412	2,398853725	0	1,061080993	44,31077379	1,484270292	4,898109859	6,219402804	7,125520199	67,53312507	7,503680563
ENSG00000151229	SLC2A13	7,523114313	3,772126311	23,99010133	1,770286169	0,035829347	2,449841899	9,463790381	13,945308	4,561333381	67,51173113	7,501303459
ENSG00000127920	GNG11	7,815211183	0	0	58,76375205	0,352652251	0,543078811	0	0	0	67,4746943	7,497188255
ENSG00000125611	CHCHD5	8,924327398	12,198	6,507647968	4,720292172	0	9,621842437	7,309923572	8,702586375	9,443119381	67,42773931	7,491971034
ENSG00000166949	SMAD3	7,191113974	5,259376851	0	15,38201887	3,117740721	5,814872302	5,688526306	16,53943655	8,366081388	67,35916696	7,484351885
ENSG00000133315	MACROD1	16,14922129	0	0	8,216463895	0,171334705	13,06070857	12,51111612	3,941251998	13,30008509	67,35018166	7,483353518
ENSG00000205726	ITSN1	8,81038273	4,7221991	11,2480452	2,447615175	0,247690047	7,651660311	11,07125102	14,66200835	6,468908284	67,32976022	7,481084469
ENSG00000133302	SLF1	7,442135055	4,883833602	6,156633403	0	23,28534261	5,826089026	4,168328092	12,95160989	2,599630794	67,31360247	7,479289164
ENSG00000104983	CCDC61	0	9,221266618	7,303959324	14,92209283	0	12,37277485	4,945121277	2,936628691	15,58295801	67,2848016	7,476089066
ENSG00000241360	PDXP	3,632136166	0,972850794	19,91875539	5,44354142	0	8,276718112	10,92530782	4,195994508	13,90688262	67,27218683	7,474687426
ENSG00000102575	ACP5	22,57081494	4,96048921	0,285693832	2,860541003	0,246055185	17,33563751	11,90657142	2,943233558	4,138411767	67,24744842	7,471938714
ENSG00000106665	CLIP2	16,79465399	6,561506222	5,16818776	5,885751307	0,049093307	10,79609494	11,97242539	2,484470432	7,494835826	67,20701918	7,467446576
ENSG00000183340	JRKL	5,703780387	4,990322937	6,266392795	0	0	2,174450547	13,87902493	24,33869292	9,791651632	67,14431614	7,460479571
ENSG00000143303	RRNAD1	0,667575888	6,599897762	2,894712296	1,235097702	0	16,80571004	7,047560459	20,74991446	11,14258101	67,14304963	7,460338847
ENSG00000137601	NEK1	0,054565685	0,322751565	6,323813037	11,6522479	0,043225511	9,445799564	10,91899976	27,08545263	1,207957656	67,05481332	7,450534813
ENSG00000182327	GLTPD2	4,345825779	6,609909616	9,821811221	9,005151137	0	7,770667931	13,91823732	0	15,57629028	67,04789329	7,449765921
ENSG00000197150	ABCB8	0,194229525	5,945307843	0,029775075	7,54631996	19,30987642	9,169864779	8,816252977	11,11358764	4,877077759	67,00229198	7,444699109
ENSG00000121417	ZNF211	0	16,16375494	0,060933823	6,920896959	0,157438642	15,20180207	20,90255421	0,217295858	7,373568727	66,99824523	7,44424947
ENSG00000164620	RELL2	0	5,813376696	3,766668008	0,202040244	43,37772869	5,973551542	5,877626857	0	1,942646574	66,95363861	7,439293179
ENSG00000122376	SHLD2	9,796182681	14,60179208	1,834852138	7,196933529	0	9,504071226	11,18052463	0	12,83664098	66,95099727	7,438999697
ENSG00000162408	NOL9	0,041565279	5,494864257	2,523268675	6,710884369	0	6,776982709	3,192409768	25,35861486	16,84401845	66,94260837	7,438067597
ENSG00000012211	PRICKLE3	24,26200902	7,554328777	0	7,422657921	0,392907596	4,810317596	8,030650352	6,236319792	8,08249372	66,79168477	7,421298308
ENSG00000143156	NME7	10,22731564	4,923908711	22,41147703	0,410705626	1,444509177	8,385476857	11,58592782	0,346730921	7,052937274	66,78898906	7,420998784
ENSG00000102967	DHODH	0,021833633	2,944486636	0	4,524230162	35,38768442	8,438162549	3,571399423	3,62852581	8,270586456	66,78690909	7,420767677
ENSG00000214655	ZSWIM8	2,185051632	3,573878035	11,28977392	8,792980409	0,090309957	10,57672103	12,06900591	11,79975068	6,402860191	66,78033177	7,420036863
ENSG00000092140	G2E3	15,12889392	0,028773688	0,477270263	14,5881663	0	9,257806619	3,514084843	17,587246	6,182253665	66,7644953	7,418277256
ENSG00000197044	ZNF441	0,031826152	0	0	38,469773	9,908255697	0,349432554	3,14468608	14,82362318	0	66,72759667	7,414177408
ENSG00000156795	WDYHV1	5,517221647	0	24,58404121	11,75001306	0	2,187461965	3,766654997	15,549862	3,317341574	66,67259645	7,408066272
ENSG00000104980	TIMM44	0,456428908	13,94222697	1,979146336	12,38526309	0,232438539	9,020380869	9,826479076	1,497114545	17,32161761	66,66109594	7,406788438
ENSG00000158161	EYA3	1,623074311	20,74510804	6,836994963	1,259181851	0,33541489	9,142643859	8,066507957	2,314687973	16,2586777	66,58229154	7,398032393
ENSG00000101412	E2F1	17,63892907	8,67225056	0,31001498	0,873016644	0	7,216184935	15,90534692	0	15,95931102	66,57505412	7,397228236
ENSG00000076067	RBMS2	0,288184844	13,32588131	0,997909263	3,863968661	0	7,612455225	13,28741232	16,71819497	10,46611557	66,56012216	7,395569129
ENSG00000280789	PAGR1	4,758773779	6,713932372	2,511436103	7,476453841	4,449572027	8,222723471	7,805459477	8,785431196	15,83101506	66,55479733	7,394977481
ENSG00000198551	ZNF627	0	20,68584309	0	9,791052576	0,18846368	6,486663849	8,790495577	0,260116427	20,335345	66,5379802	7,393108911
ENSG00000198142	SOWAHC	16,55782566	12,05393041	0	0	0,077613474	4,984130141	11,41625454	6,855785278	14,58013564	66,52567514	7,391741683
ENSG00000111276	CDKN1B	0	0	0	28,99277496	0	2,328569498	1,760987116	28,21254202	5,216744485	66,51161808	7,390179786
ENSG00000163507	CIP2A	0	5,498548867	42,63089214	0,9107678	0,078341523	4,705142882	7,089183243	0	5,594856037	66,5077325	7,389748055
ENSG00000185436	IFNLR1	0	5,482255467	0	0,21128338	0	13,46763993	12,24224697	0,334447943	34,76157623	66,49944992	7,388827769
ENSG00000114857	NKTR	0,017460347	9,976512107	17,61766529	7,884279376	0,10373723	7,025966418	4,972710599	8,380656025	10,47930012	66,45828751	7,384254168
ENSG00000103148	NPRL3	3,269603814	5,436681497	17,53937075	12,35779196	0,706389477	5,171369163	9,131673429	3,8498202	8,970349615	66,4330499	7,381449989
ENSG00000149679	CABLES2	15,92948948	0	0	17,23480678	0	6,881891821	9,11607432	0	17,18527947	66,34754187	7,371949097
ENSG00000137494	ANKRD42	11,93333031	3,549172815	0	3,531392428	4,753340462	12,48523971	10,9907831	12,08906954	6,937459413	66,26978778	7,363309754
ENSG00000138081	FBXO11	1,217600018	3,25985468	19,962382	3,873080624	0,050765839	5,441228979	10,25188746	14,01334044	8,170521406	66,24066145	7,360073494
ENSG00000100902	PSMA6	6,480148973	10,0151408	6,024468791	10,54809381	5,188602672	5,049230585	5,32275002	11,17362509	6,427216033	66,22927677	7,35880853
ENSG00000157036	EXOG	6,640733599	5,819763589	0	2,761464305	0	8,42200775	2,160565358	32,30257207	8,100574872	66,20768154	7,356409061
ENSG00000138018	SELENOI	7,986667205	4,358248131	35,49512022	0,579719892	0,293945603	3,10403324	6,103327656	0,850042152	7,432122383	66,20322648	7,355914053
ENSG00000105655	ISYNA1	7,776396602	14,45223326	9,734382273	0,158912896	0	8,740112597	8,176203121	3,722922959	13,41218812	66,17335182	7,352594647
ENSG00000149150	SLC43A1	0	0	0	41,58974921	0,101848424	6,634540929	12,16983935	5,622823043	0,052707465	66,17150842	7,352389825
ENSG00000101057	MYBL2	0	37,18828171	2,903970775	8,177717143	0,129365109	10,99695465	6,734538713	0	0	66,1308281	7,347869789
ENSG00000133114	GPALPP1	14,77376913	12,56374618	0,358948661	4,308002255	0,066245627	9,625323072	4,386022489	6,552613172	13,4045361	66,03920669	7,337689632
ENSG00000165813	CCDC186	3,021204345	15,60662091	0	11,53527934	0	9,011470844	5,769712035	17,17687189	3,839548278	65,96070764	7,328967515
ENSG00000171643	S100Z	0,077333967	37,60025999	0	10,28249388	0	7,387011348	2,56848172	6,426056072	1,616883766	65,95852075	7,328724527
ENSG00000135205	CCDC146	3,321111443	8,899981165	1,184485732	4,213347874	0	7,292772602	6,331200875	1,407996587	33,30149614	65,95239242	7,328043602
ENSG00000156958	GALK2	13,06757625	8,873553864	0	6,076519223	0	8,653649854	10,64524266	10,70998598	7,921825068	65,94835289	7,327594766
ENSG00000126775	ATG14	0,927242219	6,687627081	7,758351931	10,58821651	6,326496688	17,99663421	8,146133986	4,186027775	3,310805029	65,92753543	7,325281714
ENSG00000089022	MAPKAPK5	0,032993574	4,371456188	10,29780716	4,386898045	0,052273296	14,29680661	11,98086431	8,20074145	12,29508652	65,91492715	7,323880794
ENSG00000133116	KL	0	0,052950632	0	3,864558273	0	3,341800716	12,1654686	46,3939529	0,03669955	65,85543068	7,317270075
ENSG00000140853	NLRC5	11,74870657	4,935925724	0,426418689	15,86339705	0	7,594969885	6,584507977	5,255579671	13,41407968	65,82358524	7,313731694
ENSG00000156313	RPGR	19,91239795	3,200549058	27,60556724	0,062290517	0,085728704	5,723102968	7,218512774	1,498748337	0,44365367	65,75055122	7,305616802
ENSG00000106443	PHF14	9,033725668	5,954046262	8,526754157	20,40904826	8,229723766	2,987358615	4,054055279	1,034739234	5,396440188	65,62589143	7,291765714
ENSG00000154957	ZNF18	0,050759345	0	0	0,964153416	0,120630683	10,31019707	21,53689609	0,110995824	32,46227463	65,55590706	7,283989674
ENSG00000119138	KLF9	0	35,49319228	7,949962251	10,08444346	0,138789388	3,847201779	2,036619882	0,127704181	5,853716547	65,53162977	7,281292197
ENSG00000113761	ZNF346	0,022601043	1,16304487	0	35,55365867	0	6,526246137	10,49026557	0,098843723	11,61888219	65,4735422	7,274838022
ENSG00000197818	SLC9A8	0	8,415628566	17,60887468	13,9746344	0	8,551106602	3,793373124	6,422209418	6,688976471	65,45480327	7,272755918
ENSG00000008256	CYTH3	0,02601766	0,738683196	16,12935492	2,830389704	0,123663066	21,08161254	13,32906247	6,59958847	4,511769911	65,37014193	7,263349103
ENSG00000241794	SPRR2A	0	0	0	0	63,65451268	1,715471251	0	0	0	65,36998393	7,263331548
ENSG00000204922	UQCC3	2,962952366	3,915298291	10,20536033	13,4985135	0,749097919	13,22024203	4,920405176	9,649737057	6,202637372	65,32424404	7,258249338
ENSG00000057019	DCBLD2	12,61934021	4,469033335	26,19617422	8,859760219	0,063673323	1,588505522	4,749630476	6,181001219	0,510748421	65,23786695	7,248651883
ENSG00000161513	FDXR	6,130075942	6,854421786	0	17,5455026	0,09978259	10,41839131	8,611050849	0,183625767	15,38823663	65,23108748	7,247898609
ENSG00000120647	CCDC77	1,830353391	8,384158313	0,548129773	2,352089912	0,404639484	17,99311888	13,32459582	1,396202398	18,95111016	65,18439813	7,242710904
ENSG00000012174	MBTPS2	0,050679901	0,029976747	0,06215318	1,31269691	0,080294587	8,40217901	8,90706102	24,30698104	22,0023999	65,1544223	7,239380255
ENSG00000134262	AP4B1	0,02584412	24,03051275	0,047542346	1,561950421	0,061419111	8,285597468	13,60495818	0	17,51349988	65,13132427	7,236813808
ENSG00000127527	EPS15L1	21,04184675	2,957799313	6,890978613	6,994580033	0	3,542154407	2,1132472	13,40394047	8,133954065	65,07850085	7,230944539
ENSG00000172375	C2CD2L	17,52790799	2,240335721	5,21815941	0,028313257	0	9,469288557	14,4993385	3,728862307	12,36153626	65,073742	7,230415777
ENSG00000153898	MCOLN2	41,39955495	0	4,280344348	2,501700802	0	8,387112245	8,056055599	0,096000746	0,323962561	65,04473125	7,227192361
ENSG00000103121	CMC2	5,840593217	7,489765592	4,458643321	11,69828186	5,45096653	8,94393141	7,244902982	8,298998214	5,612765636	65,03884876	7,226540751
ENSG00000166123	GPT2	0,332528763	6,406410565	37,88254414	0,369131526	0,846709075	4,459400914	3,727427342	0	11,01291086	65,03706319	7,226340354
ENSG00000278311	GGNBP2	14,43374539	11,02407605	3,379871374	6,587230168	9,065821523	7,162960499	4,770587585	4,460270824	4,098006777	64,98257018	7,220285576
ENSG00000115459	ELMOD3	7,015327499	5,577826424	3,676276278	10,35256546	0,098944081	14,39918216	10,00789671	2,367075012	11,46979501	64,96488864	7,21832096
ENSG00000182185	RAD51B	11,70162103	5,809683565	14,12764981	1,57042582	5,043111134	6,102100922	6,443842114	1,060643255	13,07413009	64,93320774	7,21480086
ENSG00000257704	INAFM1	6,020341461	7,905381676	12,84688904	17,04913778	0	3,833790219	3,299219885	10,26848457	3,702121958	64,92536659	7,213929621
ENSG00000196455	PIK3R4	0,028103138	9,62458732	0,206791965	6,745389896	8,281676632	6,325398591	15,96089029	0,921799984	16,7977329	64,89237072	7,210263413
ENSG00000109272	PF4V1	0	0	0	0	20,3000495	0	3,3773979	40,91518198	0,250129909	64,84275929	7,204751032
ENSG00000139668	WDFY2	2,192171375	6,339797891	2,042078633	14,12679627	0	8,439429298	6,32037724	16,23716993	9,132281729	64,83010237	7,203344707
ENSG00000108021	TASOR2	0,013787461	0,562706752	0,507262954	31,71217027	0,982985974	6,251922279	6,307860669	17,305606	1,170019164	64,81432152	7,20159128
ENSG00000182013	PNMA8A	32,01287252	0	0,300844263	2,259177269	0	17,64146554	12,18718863	0,089403292	0,3016989	64,79265042	7,19918338
ENSG00000213928	IRF9	7,806450145	3,251062732	10,62390995	4,883074116	0	3,891945526	4,100769944	22,29605938	7,886476037	64,73974783	7,193305314
ENSG00000138780	GSTCD	0,480097127	8,732185865	11,48130671	8,165876092	0,057048061	2,451101451	10,14525798	8,661112572	14,55477927	64,72876512	7,192085014
ENSG00000151093	OXSM	7,323614087	7,797344106	0,89815864	0,737699847	0,145039395	3,21636013	4,053968724	34,69829869	5,77955663	64,65004025	7,183337805
ENSG00000011478	QPCTL	2,116777086	8,239338457	0	1,061190588	0	11,02072064	10,77241892	20,75481468	10,66539012	64,63065049	7,181183388
ENSG00000165804	ZNF219	0,430902123	1,006085848	14,14306672	3,093781029	23,66090366	4,257948164	1,770105239	6,992536998	9,232357628	64,58768741	7,176409712
ENSG00000181433	SAGE1	0	52,0302628	0	0,26508802	0,364833263	9,944521529	0	0	1,950979408	64,55568502	7,172853891
ENSG00000113108	APBB3	2,766231758	1,685045356	3,113984715	28,8026584	0,68683683	5,031714211	11,38146181	3,159893405	7,921327179	64,54915366	7,172128185
ENSG00000122678	POLM	10,43706644	7,707213216	1,669550144	2,910471617	0	12,88280325	7,428102023	3,969183994	17,54021231	64,544603	7,171622556

ENSG00000083168	KAT6A	14,69608665	8,196528852	4,19105977	5,717375702	3,778149184	5,621291958	9,733915911	3,72274375	8,883191265	64,54034304	7,171149227
ENSG00000139675	HNRNPA1L2	0	4,885969389	0,202609402	13,31300891	36,3828997	3,144077984	2,011914945	0,240841522	4,334612764	64,51593461	7,168437179
ENSG00000170175	CHRNB1	9,139189638	7,922939984	12,3204476	10,1193619	0,082898786	4,251171287	10,02140694	4,881766464	5,74871514	64,48789774	7,165321971
ENSG00000220008	LINGO3	5,461827855	7,777434184	23,6921905	0	0	6,66307537	8,566247566	5,013286611	7,297855404	64,47191749	7,163546388
ENSG00000197951	ZNF71	4,310447397	3,129047651	28,0683309	0,211454599	0,291019078	6,69558081	2,704627902	0,053555035	19,00634612	64,47040949	7,163378833
ENSG00000061656	SPAG4	0	15,20269375	0	0	0	13,99870491	7,225746798	15,48912035	12,52804681	64,44431263	7,160479181
ENSG00000145247	OCIAD2	0,547722568	0	0	29,42480583	0	18,97648159	8,287206785	0,133078763	7,035671806	64,40496734	7,156107483
ENSG00000180090	OR3A1	0	0	52,85301521	0	0	0	11,55051793	0	0	64,40353314	7,155948126
ENSG00000100605	ITPK1	9,8811625	5,331247424	9,334236019	7,070991948	0	7,770186066	9,054560041	6,618424327	9,278640803	64,33944913	7,148827681
ENSG00000198879	SFMBT2	0,698391063	16,21837477	2,290200755	0,367034024	14,82942931	12,0853178	8,496634559	0,033199494	9,317533871	64,33611565	7,148457294
ENSG00000155850	SLC26A2	0,842819822	4,206271208	0	8,489634312	0	9,793466531	18,12219645	0	22,86909913	64,32348745	7,147054161
ENSG00000101194	SLC17A9	0	2,111354331	12,98020971	6,546070595	14,46728792	9,904202162	11,87844015	4,659121351	1,769642768	64,31632899	7,146258776
ENSG00000129933	MAU2	7,04981109	4,974624229	1,76960776	9,017396358	0	13,23240958	5,226028743	4,177010266	18,82803072	64,27491875	7,141657639
ENSG00000203791	EEF1AKMT2	6,881646542	0	14,57018907	3,407983858	9,750914537	5,360231727	5,110236928	5,167476481	14,02072921	64,26940835	7,141045372
ENSG00000051180	RAD51	0,342987677	35,82182362	0	5,584211519	0	13,07272054	0,528895693	1,714316822	7,171120671	64,23607654	7,137341837
ENSG00000198718	TOGARAM1	0,044046999	7,086535564	21,76952582	0,076059553	0	9,672206182	9,033571319	9,182305316	7,367409744	64,2316605	7,136851167
ENSG00000160917	CPSF4	26,90279915	0	5,687739561	13,13266335	0	5,150563529	9,767328291	2,409094918	1,13640865	64,18659745	7,131844161
ENSG00000225697	SLC26A6	34,99752115	9,466805275	0	0,040943822	0	7,757940037	5,276329668	6,584840746	0,058323089	64,18270379	7,131411532
ENSG00000181220	ZNF746	14,54819132	3,733501608	0	11,55156617	0,08547365	11,25419835	14,27462066	2,398727671	6,32537253	64,17165196	7,130183551
ENSG00000196233	LCOR	6,318535776	8,336928442	14,48658779	11,24166436	0,055206386	6,765223746	2,93182021	6,705205674	7,313867748	64,15504013	7,128337792
ENSG00000178999	AURKB	15,10431407	0	0,744257293	38,19128589	0,480746375	4,886255271	0	4,718387399	0	64,12524629	7,125027366
ENSG00000134852	CLOCK	11,87082086	4,533745774	0,192824139	29,2994704	0,031138259	3,697131554	2,295520684	1,461212429	10,69991249	64,0817766	7,1201974
ENSG00000126262	FFAR2	9,070853241	0	44,88557206	1,821323694	5,848819908	2,316108563	0	0	0,086480466	64,02915793	7,114350881
ENSG00000011132	AC005954.1	5,832716809	1,389585061	11,02780235	4,849399099	0	13,01550092	6,771272385	11,63252498	9,498231839	64,01703345	7,113003717
ENSG00000143367	TUFT1	0,116898327	0	0	46,1581724	0	5,518943887	4,820804401	0	7,332263514	63,94708253	7,105231392
ENSG00000175595	ERCC4	3,947451078	2,123583216	18,5320066	9,68482057	15,1542509	3,353511657	2,981032711	3,163735221	4,986662004	63,92705396	7,103005995
ENSG00000141026	MED9	0,201985725	4,166617545	23,5017427	2,703085785	0	5,488770959	5,261997215	13,25050897	9,346667202	63,92137611	7,102375123
ENSG00000203797	DDO	0	20,39530845	0	0	0	24,54206851	0	18,9656798	0	63,90305676	7,10033964
ENSG00000160685	ZBTB7B	21,66749294	3,053441321	2,067246843	1,576646372	0,250372444	12,7237951	5,452696278	10,6740425	6,392118358	63,85785215	7,095316906
ENSG00000078579	FGF20	0,410626551	9,472407612	30,36630429	12,76312376	0	6,221646626	1,363807957	0	3,232113431	63,83003022	7,092225581
ENSG00000187116	LILRA5	0	0,152353409	8,213048379	3,706462707	21,01653799	2,922288567	0,926769498	20,46441356	6,335676896	63,737551	7,081950111
ENSG00000113638	TTC33	5,183335397	11,02066935	0	4,004873426	0,036991918	10,04897189	6,255422403	21,10315532	6,068536007	63,72195571	7,080217301
ENSG00000100162	CENPM	0,086348898	14,096608	0	2,982113995	0	18,3923725	8,603679909	11,89564443	7,646256916	63,70302466	7,078113851
ENSG00000115020	PIKFYVE	3,01133714	4,428214597	4,257283553	4,525858033	2,91561433	17,66406546	11,58739758	12,651568	2,64049861	63,6818373	7,0757597
ENSG00000156876	SASS6	0	3,847959216	0,349924596	32,78110582	0	5,597184612	7,296990607	9,81653549	3,930288991	63,61998933	7,068887704
ENSG00000166321	NUDT13	25,41486789	9,253611268	0	10,97148247	0,142450433	5,725593087	3,782524884	7,733296106	0,58975493	63,61358107	7,068175675
ENSG00000120519	SLC10A7	17,62617693	7,418314643	0,083140451	3,550932061	0	14,0677878	2,02643874	9,784065256	9,032468931	63,58932481	7,065480534
ENSG00000112130	RNF8	0,046042221	5,514803475	6,775870786	7,274695361	0,054710168	9,958678736	13,24663926	15,30349003	5,407782717	63,58271275	7,064745861
ENSG00000135974	C2orf49	12,00334239	3,09282394	0	18,90997748	0,078153981	7,040818815	10,8131124	0,575294133	11,04158881	63,55511195	7,061679106
ENSG00000004766	VPS50	0,015808199	12,39865719	3,693218179	3,930812464	0	14,85714548	14,75349442	2,661728392	11,23750286	63,54836719	7,060929687
ENSG00000143324	XPR1	0,118810962	0,150590812	2,872533877	24,97097589	3,630302012	9,895374169	3,903771983	12,99023909	5,009902881	63,54250168	7,060277964
ENSG00000100027	YPEL1	15,62433587	12,60227302	0,059384768	0,222973702	0	4,430425569	5,628876249	8,753233577	16,19854328	63,52004603	7,057782893
ENSG00000102901	CENPT	4,031395083	17,33921767	9,527939788	2,858298308	0	6,800574875	5,78582273	5,89644723	11,26243059	63,50212628	7,055791809
ENSG00000121210	TMEM131L	3,900085118	0,083886125	0	5,55093955	0,112347112	19,20441526	16,19161957	1,395547211	17,03520445	63,4740444	7,0526716
ENSG00000081014	AP4E1	11,87678948	0,036462061	0	34,84337028	0	6,068798631	3,326994118	2,02196839	5,25646949	63,43085245	7,047872495
ENSG00000164087	POC1A	0,06724992	0	0	0,232251869	0	18,75446661	18,81775785	0,147055882	25,39158092	63,41036306	7,045595895
ENSG00000234284	ZNF879	0	0	54,40764235	0	0	3,204556594	0	0	5,791251402	63,40345034	7,044827816
ENSG00000128534	LSM8	3,169052929	7,404926688	11,93247522	6,301393459	7,261606091	9,268907948	8,096964634	5,211660808	4,735011631	63,38199941	7,042444378
ENSG00000143379	SETDB1	15,78639967	5,93518878	1,175722962	0,220726046	0,050629852	13,63680618	3,608620856	3,400778715	19,52004217	63,33491523	7,037212803
ENSG00000182400	TRAPPC6B	5,600391765	3,549199207	5,703099317	9,095020072	0	5,965899846	7,224301048	16,10986972	10,08564846	63,33342943	7,037047715
ENSG00000167380	ZNF226	14,95387762	6,89530136	0	0,037696471	0	9,803153116	8,990650776	7,542424396	15,08895486	63,3120586	7,034673178
ENSG00000121236	TRIM6	13,53285888	6,493852676	0	3,291308963	0	4,520267983	11,52149365	10,25314122	13,69000618	63,30292956	7,03365884
ENSG00000140691	ARMC5	4,850799203	8,414194412	13,98673537	4,188135751	0	6,612486466	10,18244353	7,389339292	7,641660429	63,26579445	7,029532717
ENSG00000141556	TBCD	2,679067516	8,953252865	7,146123546	6,399528076	1,140729432	11,91291132	10,40874513	2,39215392	12,19115844	63,22367025	7,02485225
ENSG00000176894	PXMP2	5,529732354	5,472285716	14,28046955	21,85169738	0	4,436736041	7,240371023	0	4,315929089	63,12722115	7,014135683
ENSG00000172803	SNX32	0	0	24,31312819	13,27842966	11,99279362	3,693741967	3,292224455	2,452204583	4,088327948	63,11085043	7,012316714
ENSG00000140326	CDAN1	3,494848	2,85466887	23,42012948	11,15966393	0	1,674914578	3,155182725	11,88788092	5,458047644	63,10536315	7,011707016
ENSG00000150756	ATPSCKMT	0,230000869	19,75353547	0,169242069	10,08789704	0	3,181846676	11,84042707	0,100588909	17,70775083	63,07128893	7,007920992
ENSG00000128881	TTBK2	1,298140496	8,716015791	10,15452416	4,160096337	0,972766348	7,20345434	4,350337337	19,05222141	7,134113075	63,0416693	7,004629922
ENSG00000184005	ST6GALNAC3	37,77764267	1,003859401	0,077088214	0,108542003	0,099588891	2,898604085	0,034794879	15,85279539	5,153813707	63,00672924	7,000747693
ENSG00000183018	AC118754.1	0	6,130089973	0	11,09679218	4,767047022	16,23215573	1,480475894	0	23,29934276	63,00590357	7,000655952
ENSG00000115841	RMDN2	0,025055641	8,002911626	0,230459376	8,782921546	0,059545273	1,84314519	5,367492233	30,68203578	8,011954202	63,00552087	7,00061343
ENSG00000167257	RNF214	6,78917472	0	0	5,44724703	14,17890909	9,374116995	10,96121575	3,299097112	12,94642213	62,99618283	6,999575871
ENSG00000181007	ZFP82	8,173580028	21,36276526	0,037034386	0,347635191	0	3,359766541	6,218353784	18,0493169	5,422380546	62,97083264	6,996759182
ENSG00000197021	CXorf40B	8,191982563	10,28734946	0,46368657	4,026102611	0	9,617001735	10,35993567	0,413387699	19,60766061	62,96710692	6,996345213
ENSG00000135679	MDM2	3,196722906	3,512512837	0,460405193	21,92301676	3,703915027	15,30079473	4,741858516	0,248765014	9,877862284	62,96585327	6,996205919
ENSG00000078487	ZCWPW1	2,459304043	15,75879747	0	2,831120882	0	12,55080011	7,298304423	12,14979422	9,858043376	62,90616453	6,989573836
ENSG00000162769	FLVCR1	46,37779095	0	0	0,934522643	8,443903596	3,978546997	1,328555368	0,05145353	1,76528151	62,88017314	6,986685904
ENSG00000157014	TATDN2	7,783333414	14,87004272	0,399109977	10,72399662	0	8,366990677	7,385906119	5,93026862	7,337802387	62,79745054	6,977494504
ENSG00000083817	ZNF416	0	20,65062162	0	0	0	13,82044883	6,852800058	0	21,46910709	62,79297759	6,97699751
ENSG00000196652	ZKSCAN5	0,049904416	0	3,85573453	7,238618846	11,68199403	16,54722396	9,13679267	4,910685449	9,359839712	62,78079362	6,975643735
ENSG00000167703	SLC43A2	11,17015721	6,54806506	2,778228408	4,256246521	5,315996054	9,683070618	8,789784714	3,52056441	10,70641383	62,76852683	6,974280759
ENSG00000137817	PARP6	0,079053398	6,312524198	0	1,501587612	0,046968028	4,708683644	15,09713647	11,32276441	23,67440658	62,74312434	6,97145826
ENSG00000167378	IRGQ	22,2631239	9,428034657	0	0,132199298	0,218330657	10,87690173	13,76880834	1,707585667	4,331208848	62,72619309	6,969577011
ENSG00000186642	PDE2A	0	0,037335283	0	0,290654386	52,60256956	1,224347057	0,192171213	8,281568108	0,077630126	62,70627573	6,96736397
ENSG00000132600	PRMT7	12,2703445	4,762936775	1,85163314	4,359032141	6,606730003	5,964220351	9,976072073	2,969648788	13,89231246	62,65293023	6,961436692
ENSG00000177932	ZNF354C	1,452180683	0	0,051373147	53,57581903	0	1,747718143	5,657874886	0	0,068692117	62,55365801	6,950406445
ENSG00000197046	SIGLEC15	24,44312177	0	0,463557697	31,47464674	0	1,152797226	3,870819025	1,102060814	0	62,50700327	6,945222586
ENSG00000158079	PTPDC1	0,071177848	0,126303344	1,614894593	25,97468581	0	10,52408117	4,038533217	1,037634063	19,05444796	62,441758	6,937973111
ENSG00000112029	FBXO5	33,1534084	0	6,416034442	2,097765615	0,279396428	11,05783667	3,286437638	6,084246445	0,048196712	62,42332235	6,935924706
ENSG00000155755	TMEM237	0	4,137669153	18,684683	5,800829826	0	6,400368789	4,971558859	16,72265956	5,63834094	62,35611012	6,92845668
ENSG00000047346	FAM214A	0,660878208	0,027272376	0	1,645440239	31,59445424	5,467354236	0	16,19909088	6,729186527	62,32367671	6,924852968
ENSG00000153391	INO80C	4,457538958	8,489845037	4,646668321	13,2904933	0	7,634822999	5,872554379	9,422421296	8,479133366	62,29347765	6,921497517
ENSG00000166803	PCLAF	0	0,071676557	7,356329805	46,31403987	0	6,164546956	1,978815715	0	0,397426404	62,2828353	6,920315033
ENSG00000130024	PHF10	10,25591636	1,299919425	6,505719468	4,972682565	0,480264182	10,12326321	12,31211717	8,672388348	7,642635319	62,26490604	6,918322894
ENSG00000108256	NUFIP2	3,22309942	0,345130827	5,111337666	12,69050304	0,132064923	8,541870272	14,1423763	10,14665308	7,859648823	62,19268435	6,910298261
ENSG00000079335	CDC14A	0,023212069	0	23,65606372	4,2487108	0	6,473315896	10,44622426	3,654575862	13,67442153	62,17652414	6,908502683
ENSG00000170634	ACYP2	4,931489212	3,835230058	0,055999214	12,9311047	0,072344388	12,39984367	6,369565605	16,109018	5,466074184	62,17066903	6,907852114
ENSG00000160293	VAV2	16,6748201	0	0,048922932	20,11431166	0	6,628247257	7,971627922	3,37296813	7,261162961	62,07206097	6,896895663
ENSG00000180198	RCC1	0,893915413	7,2414892	0,357484695	18,12048435	0	6,742250802	9,681352778	1,529789664	17,49481151	62,06157841	6,895730934
ENSG00000142405	NLRP12	0,118797618	1,159419026	40,15632326	9,590180867	0,070581278	0	0,049320099	9,871472929	1,022740296	62,03883537	6,89320393
ENSG00000178229	ZNF543	24,5708245	14,82411057	0	0,636426767	0	7,193954545	9,8948148	0	4,885396385	62,00552757	6,889503063
ENSG00000083828	ZNF586	20,23999249	19,03416752	0,101452591	0	0	3,875283543	7,532799129	0,060298279	11,15757406	62,00156762	6,889063069
ENSG00000181885	CLDN7	4,244678951	9,253696632	0	17,90524906	0	14,44726932	4,632131037	1,591177693	9,918775065	61,99297776	6,88810864
ENSG00000106617	PRKAG2	6,393245669	12,83007318	1,218394541	6,179077441	0,04372284	11,41285394	14,76436061	1,005766627	8,134391521	61,98188637	6,886876263
ENSG00000130559	CAMSAP1	0,033730038	0,418971875	0,062049129	29,03484037	9,69938005	4,25886525	4,873178073	0,55318287	13,0465918	61,98078945	6,886754383
ENSG00000085644	ZNF213	1,243032787	2,788377154	0,12943354	0,040498987	42,91797418	6,051371337	2,161602802	3,94900849	2,653714116	61,93501339	6,881668154
ENSG00000137221	TJAP1	7,845824022	1,113777778	9,938169983	6,580469616	11,98656683	5,340838918	4,597414628	9,803725486	4,714397761	61,92118503	6,88013167
ENSG00000175768	TOMM5	5,280069857	6,097516364	21,35343522	6,295436159	6,074922375	4,094853391	4,488539331	3,482166072	4,689970473	61,85686008	6,872984453
ENSG00000066933	MYO9A	2,98949062	1,860631005	0,109440984	8,655028193	31,51115049	1,616648336	8,527289788	0,260184699	6,274149325	61,80401344	6,867112605
ENSG00000142784	WDTC1	1,099264852	5,352860093	4,937897207	0,662159469	26,06351306	4,462828911	10,48593199	5,646065187	3,081197629	61,7917184	6,865746488
ENSG00000130812	ANGPTL6	0	3,811280148	24,72633618	8,853364567	0	7,835300046	4,027016616	0	12,52610673	61,77940428	6,864378254
ENSG00000258890	CEP95	1,127835941	10,00658603	9,336353858	0,067156087	9,51978043	1,793397046	11,60895235	8,504299769	9,805311303	61,76967281	6,863296979
ENSG00000174944	P2RY14	0,052287395	0	0	0	0,124262125	4,190820796	0	57,39728474	0	61,76465505	6,86273945
ENSG00000162877	PM20D1	0,173623789	38,92218232	0,212929988	0,199873592	0	6,481378332	4,32490612	0,126554794	11,31734854	61,75879747	6,862088608
ENSG00000068489	PRR11	0,037036905	1,511584804	0,340661886	13,62234059	0,264057016	8,742836763	6,2735166	27,53621396	3,325195129	61,65344365	6,850382628
ENSG00000134769	DTNA	11,87690333	4,202320407	12,981499	1,404430764	0	5,344752629	12,15574532	0,104617561	13,53322784	61,60349685	6,844832983
ENSG00000116285	ERRFI1	0	0	0	0,124879146	0,687470768	31,08590131	22,57805448	6,562830606	0,533658355	61,57279466	6,841421629
ENSG00000127399	LRRC61	28,94750261	0	0	0,26517782	0	9,357761186	17,78771475	0	5,193881466	61,55203783	6,839115315
ENSG00000171467	ZNF318	4,877541035	1,681400288	8,372550267	0,295053939	20,70980071	4,331957903	8,009567863	8,933418098	4,336673577	61,54796368	6,838662631
ENSG00000269028	MTRNR2L12	0,144052022	3,578634892	8,744845821	12,93481285	14,72073815	3,637696153	3,827502978	11,65498475	2,303150419	61,54641803	6,838490892
ENSG00000197372	ZNF675	21,29133464	2,924466913	6,923896349	12,53718522	0,052928157	4,914963289	7,175016775	0,438306722	5,259032023	61,51713009	6,835236677
ENSG00000173681	BCLAF3	0,017509915	6,089899423	41,29437855	1,028017281	0	4,979239787	2,297139334	1,225249845	4,522337158	61,4537713	6,828196811
ENSG00000154217	PITPNC1	11,71160069	12,17424351	0,177759368	6,07368786	0,045928833	3,45868065	6,611302824	15,63637166	5,561847578	61,45142298	6,827935886
ENSG00000120137	PANK3	0,029205754	6,76314863	0,053726343	3,303293552	21,62062513	2,437032051	11,3248251	7,057019658	8,81819119	61,40706741	6,82300749
ENSG00000143409	MINDY1	15,02338743	5,657004221	1,099604822	4,472777782	0,094703993	9,581860838	7,941160253	0,435699588	17,00652511	61,31272404	6,812524894
ENSG00000100379	KCTD17	0	8,854653269	7,483312069	2,154165261	0	17,44859978	13,73598394	0	11,60706923	61,28378355	6,809309283

ENSG00000128394	APOBEC3F	0,02897614	5,193162418	0,15991185	27,46947288	0,48203697	3,149596758	8,108054053	0,063362333	16,60680333	61,26137673	6,806819637
ENSG00000168040	FADD	3,365308744	4,515155895	8,002686174	5,102476418	0	15,14012867	12,26760443	5,384593713	7,470202416	61,24815646	6,805350718
ENSG00000116903	EXOC8	0,118518095	0	22,72896103	1,483747895	6,337368394	5,627947014	13,1128798	0	11,80672024	61,21614247	6,801793607
ENSG00000166825	ANPEP	0,031060755	0	53,08191407	7,777095851	0,221449666	0	0,025790407	0,067920774	0	61,20523152	6,800581281
ENSG00000164180	TMEM161B	0,535294791	10,89553866	22,27200418	2,718639035	0,112247617	6,758807615	3,987127658	10,50036915	3,40789622	61,18792492	6,798658325
ENSG00000152454	ZNF256	0	0	0	2,601073603	0	16,76395949	16,2025576	0	25,59915848	61,16674918	6,796305464
ENSG00000152147	GEMIN6	6,372758424	9,280183822	18,98649312	4,066829767	0,082309774	8,784172179	4,256156116	0,378678166	8,902572348	61,11015372	6,79001708
ENSG00000142632	ARHGEF19	11,72822275	1,274170011	0,073384398	9,781617316	0	9,329185015	13,51422795	0,087231934	15,30731651	61,09535588	6,788372876
ENSG00000139437	TCHP	16,30605562	11,75749658	3,960617712	1,383353079	0	9,858613902	12,943372	0	4,823716891	61,03322578	6,781469531
ENSG00000115525	ST3GAL5	15,88978639	5,165434848	0,750375727	4,658409669	0,132190506	10,00552742	14,50991263	1,317683858	8,585425231	61,01474627	6,779416253
ENSG00000213551	DNAJC9	5,856513309	8,725035029	2,246885774	10,49147877	0,074428506	12,03496411	7,593230455	5,821127096	8,165691756	61,00935481	6,778817201
ENSG00000150045	KLRF1	0	51,92591113	0	0	0	3,291876522	0	0	5,752392356	60,97018001	6,774464445
ENSG00000130023	ERMARD	13,74054404	8,60919944	20,39085329	0,489214857	0	3,421638329	1,058573225	3,87197759	9,364190834	60,9461916	6,771799067
ENSG00000114346	ECT2	0,082438937	27,30669577	2,274796227	5,646710671	0,065305972	4,223946306	11,75072213	9,554300097	0	60,90491611	6,767212901
ENSG00000153179	RASSF3	0	3,983245934	11,27389225	3,630078637	30,90731029	1,721307563	3,106433591	4,441115385	1,840498452	60,90388211	6,767098012
ENSG00000010072	SPRTN	0,025585942	9,4435381	0	1,502165448	0	17,50123422	3,44161764	6,490079552	22,40479578	60,80901668	6,756557408
ENSG00000135723	FHOD1	0,664539795	3,383818739	8,238415935	4,440384845	8,995104789	6,979029995	7,74893255	8,971646001	11,37110653	60,79297918	6,754775464
ENSG00000122085	MTERF4	2,904218052	13,59866855	16,25140245	6,222739885	0,505901146	1,502510757	5,529885834	7,148664924	7,124927046	60,78891864	6,754324293
ENSG00000162946	DISC1	10,02252042	9,287112509	1,098480168	10,02818436	0,055651254	6,55620596	8,059422379	6,912857061	8,740805751	60,76123986	6,751248874
ENSG00000256525	POLG2	19,29920676	0,157452731	6,202726812	3,141025825	0	6,478598562	13,33536705	5,238833425	6,875120906	60,72833208	6,747592453
ENSG00000147874	HAUS6	11,66756058	7,190749322	0,108037349	9,668014346	0,093047686	6,297680914	4,941440721	12,88519301	7,848939793	60,70066372	6,744518191
ENSG00000059728	MXD1	1,332418825	4,644248151	4,420719571	7,107817762	0	9,95306719	24,41841133	4,422443432	4,389384088	60,68851035	6,743167817
ENSG00000204604	ZNF468	0,079861131	3,802595086	29,57808153	0,183870325	0	10,7119222	7,780420482	0	8,545044008	60,68179476	6,74242164
ENSG00000100154	TTC28	0,011950223	0,021205378	0,087933602	0,392073113	42,7703455	2,269871868	3,512573945	6,428383045	5,158729088	60,65306576	6,739229529
ENSG00000244005	NFS1	0,125444605	6,989588911	0	18,4123135	0,178873091	9,503428352	14,43648349	5,157039573	5,800954041	60,60412556	6,733791729
ENSG00000152942	RAD17	9,737348627	6,83410724	1,938307976	11,10495083	0	10,53147205	5,279468165	5,164281531	10,00952657	60,59946298	6,733273665
ENSG00000153790	C7orf31	10,61943105	22,96166529	0	0	0,18694302	5,3979235	5,976332836	8,17055802	7,255850687	60,5687044	6,729856044
ENSG00000145425	RPS3A	3,446381436	5,99791316	7,437186859	17,73900047	1,417569246	3,85669323	2,256264742	6,231880001	12,14523325	60,52812239	6,725346933
ENSG00000253710	ALG11	2,864472735	10,09749286	0,118148631	4,613607167	20,84981991	5,204097484	4,660875135	3,595343815	8,49927142	60,50312916	6,722569907
ENSG00000154274	C4orf19	0	0,119974049	19,83795337	0,116749429	0	20,71098474	10,66638169	0,073922722	8,938926686	60,46489269	6,71832141
ENSG00000119514	GALNT12	17,99389929	0	1,141421758	3,296714777	0	6,812499991	6,142735774	13,568065	11,50534377	60,46068035	6,717853372
ENSG00000109881	CCDC34	0,877133271	8,906361177	0	8,751124592	13,54942033	6,259767002	2,872748653	9,590168035	9,648916506	60,45563957	6,717293285
ENSG00000159714	ZDHHC1	10,28747105	0	0,157050903	2,800996902	30,12935313	4,827341639	4,111457207	2,520263651	5,617394882	60,45132937	6,716814374
ENSG00000101417	PXMP4	0,660563782	6,845380663	0	4,790727808	16,82872903	3,075104237	9,762947673	9,013427615	9,423936258	60,40081707	6,711201897
ENSG00000168734	PKIG	14,15512645	3,370803732	3,381753029	0,317439142	0	7,266156644	8,293456657	6,431816399	17,18289014	60,39944219	6,711049132
ENSG00000221823	PPP3R1	0,44919908	1,275348395	12,56033741	14,03960476	12,81037606	5,671734935	4,736840715	4,714880868	4,088181524	60,34650375	6,705167083
ENSG00000139289	PHLDA1	11,01165145	13,59153278	1,378015115	18,98238073	0,08901166	4,89363208	4,882598122	0,081902235	5,389526114	60,30025029	6,70002781
ENSG00000138160	KIF11	0	0,16037201	2,105909841	42,91693661	0	11,47746613	3,6020191	0	0	60,26270369	6,695855966
ENSG00000156804	FBXO32	6,089377966	4,387448548	33,4553466	6,351358408	0	1,705098557	0,967114121	3,842781989	3,392735701	60,19126189	6,687917987
ENSG00000182208	MOB2	6,150815308	6,017708253	4,892939036	4,822560587	0	13,9810233	11,70504307	3,271629381	9,322993857	60,16471279	6,684968088
ENSG00000186918	ZNF395	0,078052981	7,479167358	2,680248888	13,028778	15,45788319	3,97065602	1,641829413	10,63899176	5,183735642	60,15934325	6,684371473
ENSG00000174953	DHX36	1,618223511	2,236210766	5,584892057	9,471009787	0,068066251	10,58167344	9,060689252	7,045847014	14,47743	60,14404207	6,682671341
ENSG00000160193	WDR4	0	7,44294814	3,858014522	16,77302978	0	6,786699997	19,4300295	0	5,837399896	60,12812183	6,680902426
ENSG00000173889	PHC3	3,885164504	3,185563096	11,00813679	10,30229701	2,058892465	7,472300931	4,850026428	4,980247929	12,34656251	60,08919165	6,67657685
ENSG00000100307	CBX7	6,981806198	8,654375522	4,169515346	11,32220862	4,328455713	6,021396315	6,03238907	5,664339714	6,894266328	60,06875283	6,67430587
ENSG00000076554	TPD52	10,21291401	0	5,629461563	13,36985352	0,043810851	9,695152646	8,020794677	1,854335745	11,2228899	60,04921292	6,672134769
ENSG00000249115	HAUS5	0,029687735	7,902023578	2,839875274	5,690330487	0	13,46189945	8,652276332	10,32202284	11,1361865	60,03430219	6,670478021
ENSG00000197580	BCO2	23,3265397	4,423612315	0,245673972	3,920366388	0	4,814314864	5,470499083	9,734410591	8,048148114	59,98356503	6,664840559
ENSG00000171634	BPTF	0,899527175	2,376547921	10,07560647	6,799945763	14,60792966	4,224854291	8,132874222	4,764517211	8,088300262	59,97010298	6,663344775
ENSG00000139971	ARMH4	0,01997496	8,967604233	16,68248049	7,208910733	0,427238319	11,95257955	6,750353619	0,087358775	7,861320175	59,95782086	6,661980095
ENSG00000278588	HIST1H2BI	0	0	1,459213118	42,46185938	16,02361738	0	0	0	0	59,94468988	6,660521097
ENSG00000122515	ZMIZ2	0,082192315	6,490246943	9,941360624	4,399773844	9,47359304	5,707829196	12,83025424	3,729408363	7,278188525	59,93284709	6,659205232
ENSG00000105136	ZNF419	0	0	6,283016723	9,742701974	37,40060319	2,278991438	1,410131104	0,041263058	2,761705313	59,9184128	6,657601422
ENSG00000162433	AK4	4,047960655	7,572233837	0,917062876	4,476319174	25,73249215	1,379304082	0,728516048	5,581370367	9,466449934	59,90170912	6,655745458
ENSG00000166925	TSC22D4	12,44149191	6,725480918	3,865044997	5,193089734	0,587433275	8,006044732	7,422863153	3,333173118	12,31207877	59,88670061	6,654077845
ENSG00000075651	PLD1	13,74800682	0,041701711	26,28489894	0,608713005	0,055850318	3,896189282	6,322299777	0,025694756	8,87323209	59,8565867	6,650731855
ENSG00000144535	DIS3L2	0,1999346	0,473038723	0,245197229	0,391275873	30,09276398	3,453726392	11,65389959	7,432373178	5,868664988	59,81087455	6,645652728
ENSG00000116745	RPE65	0	0,102933589	0	0,701168838	58,72709138	0	0	0,126846283	0,071342235	59,72938232	6,636598036
ENSG00000169105	CHST14	27,86286124	12,97945328	0,155556854	0,511064584	0,401922263	7,473867613	2,387236311	1,294371557	6,655947076	59,72228078	6,635808975
ENSG00000152465	NMT2	9,809341527	1,657755797	33,81303522	0,080659961	0,05550503	0,23078751	2,016833776	1,68536951	10,36948911	59,71877744	6,635419716
ENSG00000067836	ROGDI	4,964534089	6,916505568	0,226429622	7,014000132	0,097506799	9,369915817	11,24225076	8,07469755	11,80778882	59,71362916	6,634847684
ENSG00000170260	ZNF212	0	0	0,087360182	14,02258977	0	21,0125589	14,39241709	9,034508917	1,109705821	59,65914068	6,628793409
ENSG00000117266	CDK18	0	3,611340069	42,48444201	0,114595948	0	4,177517083	7,255274651	0,048372796	1,958856625	59,65039918	6,627822131
ENSG00000117479	SLC19A2	0	29,12449772	0	0,119585231	0	9,884681472	8,280351883	11,20630522	1,0220707	59,63749223	6,626388025
ENSG00000109846	CRYAB	4,638288922	40,71946366	0	0	0,331518617	0,727510029	0,781836825	0,076259997	12,35259599	59,62747404	6,625274894
ENSG00000150401	DCUN1D2	0,069556074	0,246851093	0	15,9743851	0	13,09719858	7,017097969	23,1950617	0	59,60015051	6,622238945
ENSG00000120913	PDLIM2	10,85634549	8,571539557	3,882027537	6,213740516	0,086967438	7,459815242	7,687438004	4,26780197	10,53150296	59,55717871	6,617464301
ENSG00000084693	AGBL5	16,64704753	11,87681772	0,105235699	2,864703488	0	12,46763183	5,604958992	5,816849286	4,151029648	59,5342742	6,614919355
ENSG00000167671	UBXN6	17,48124955	9,096488663	1,48340819	3,083279669	0,547539611	10,62434796	10,33031893	3,967481943	2,904405341	59,51851986	6,613168874
ENSG00000061936	SFSWAP	2,960618644	7,787918679	11,00206752	8,991556671	0,106069964	5,014723545	1,494723853	7,937977205	14,21706683	59,51272291	6,612524768
ENSG00000130956	HABP4	0,036964425	6,100099317	0	16,97864561	0	3,246782323	10,49678414	9,861305586	12,77467533	59,49525673	6,610584081
ENSG00000105321	CCDC9	0	3,049485851	0	5,797021388	0	6,494251614	12,84237463	20,18788286	11,10851998	59,47953632	6,608837369
ENSG00000105865	DUS4L	14,31949933	13,36320626	0	20,17049185	0,195953552	6,819896387	2,145182104	1,141916476	1,318300176	59,47444614	6,608271793
ENSG00000155621	C9orf85	7,444618905	16,4993871	21,07349634	0,419762678	0	1,467939951	2,472570387	2,059817332	8,034788315	59,47238101	6,608042334
ENSG00000204209	DAXX	5,830790502	5,477608951	7,481306166	2,876715025	0	7,370216929	12,97828661	7,714425701	9,702104438	59,43145432	6,603494925
ENSG00000120334	CENPL	0,030751032	10,04031909	24,21153487	0	0,146160986	7,866740816	2,476724095	0,201730505	14,40934574	59,38330714	6,598145238
ENSG00000179403	VWA1	0	1,270564265	17,3977673	0,618207245	0,070901785	0	39,93251238	0,065238809	0	59,35519178	6,595021309
ENSG00000125910	S1PR4	0,090811641	4,35086178	24,3900808	13,17219824	0,431631661	2,891470387	6,258436031	7,744560176	0	59,33005071	6,592227857
ENSG00000137692	DCUN1D5	10,89189361	6,216774507	8,976019724	5,490635704	0	6,131671019	3,619428814	14,43707537	3,555102194	59,31860094	6,59095566
ENSG00000103423	DNAJA3	1,346339408	2,957866781	9,592298706	0,77494477	19,19762848	5,772026852	2,963307233	8,832150086	7,858592867	59,29515519	6,588350576
ENSG00000058600	POLR3E	7,837486903	10,93937951	12,30778427	2,860767168	0,037857637	2,221235847	7,777414634	5,085752357	10,16807348	59,23575181	6,581750201
ENSG00000196646	ZNF136	0,027090457	0	1,943568279	28,16114283	0,515048465	12,76006407	8,502650681	0,059238897	7,263273907	59,23207759	6,581341955
ENSG00000142396	ERVK3-1	2,767703066	7,526980293	2,047633618	8,2597374	21,09091678	2,675444299	4,32139507	2,894509451	7,621816362	59,20613634	6,578459593
ENSG00000124215	CDH26	9,569284572	1,679386222	0	0,090791571	0	15,81750105	9,560891687	7,185859593	15,29321591	59,1969306	6,577436734
ENSG00000186431	FCAR	2,77058756	6,608850017	0,25065834	1,49016066	17,37839625	3,3910187	2,111914215	19,76449622	5,362568722	59,12865069	6,569850076
ENSG00000112276	BVES	0,078282112	0	26,8331679	0	0	0,372446468	4,831615271	9,072542952	17,93959695	59,12765166	6,569739073
ENSG00000177465	ACOT4	0	21,90999408	0	0	0	16,50676779	3,984028323	13,01033649	3,698046802	59,10917348	6,567685943
ENSG00000160505	NLRP4	0,038716518	13,94640687	0	30,0178846	0,092010644	7,098915273	0	0	7,904310197	59,0982441	6,566471567
ENSG00000164068	RNF123	0	8,529655212	0	6,154956268	0	9,862052073	12,98843712	0,089077926	21,46791611	59,0920947	6,5657883
ENSG00000137449	CPEB2	23,39192476	0	0,735909416	1,34521305	1,401043777	7,628603917	1,206277401	21,73124089	1,605473649	59,04568686	6,560631873
ENSG00000141040	ZNF287	6,462400296	0,033530324	0,104281643	31,29131432	0,044906533	3,257216524	7,421216336	0	10,41130949	59,02617547	6,558463941
ENSG00000145348	TBCK	0,149587498	12,42738751	1,375891419	0,587056728	0	8,749423861	9,021849826	18,9720351	7,710155397	58,99338735	6,554820816
ENSG00000172159	FRMD3	0,069893881	5,457098984	28,92947376	0,040230493	0,110736214	0,102319196	0,077379175	21,90670121	2,292279029	58,98611194	6,554012438
ENSG00000145979	TBC1D7	5,734919248	11,82409302	0,100474735	5,501641031	0	7,815787409	11,86677236	3,941327974	12,15839296	58,94340874	6,549267638
ENSG00000076770	MBNL3	26,71903442	0,461593737	6,106948646	10,9302215	0,382697602	3,168879775	5,34835418	5,823709486	0	58,94143934	6,549048816
ENSG00000172765	TMCC1	2,488180937	4,283418532	0,068316564	15,13410027	0,308899336	13,45551495	20,1665267	0,527850747	2,489219824	58,92202785	6,546891984
ENSG00000135637	CCDC142	0,678871259	2,135498878	0	15,93211301	0	6,064527921	5,354972307	12,68566443	16,01536296	58,86701076	6,540778973
ENSG00000183137	CEP57L1	0,023493559	4,127185636	20,09650902	16,75468665	0,111665902	5,777980283	5,559552957	2,106314874	4,247425183	58,80481406	6,533868229
ENSG00000142731	PLK4	0	0	0	49,05274584	0	8,075129654	1,652768383	0	0	58,78064388	6,531182653
ENSG00000186001	LRCH3	0,035867688	8,422531374	0,153956855	22,4413575	0,028413446	12,94309903	6,184667841	1,777794967	6,76393704	58,75162574	6,527958415
ENSG00000138134	STAMBPL1	11,68639397	13,82481291	0,043963324	0,330140724	0	10,73184025	5,218835571	0,156777432	16,72412955	58,71689373	6,524099303
ENSG00000133739	LRRCC1	0	2,091089383	0,055584903	8,19179858	0	9,255958988	13,54808624	14,73443482	10,7769429	58,6538171	6,517090789
ENSG00000198342	ZNF442	0,045818851	0	0	1,424144798	53,41029549	0,025153211	3,709326067	0	0	58,61473842	6,512748713
ENSG00000106477	CEP41	1,277432051	17,55678938	3,258878264	0,645106006	0,343680557	11,96133355	6,614234744	6,377316001	10,56771436	58,60248491	6,511387213
ENSG00000130734	ATG4D	14,52731123	13,77199913	0	3,89455186	0	4,80689116	11,45646578	3,771421754	6,363489349	58,59213026	6,510236696
ENSG00000128607	KLHDC10	2,731605333	5,254491571	0,084453926	35,51537859	0,109104524	11,87055774	1,886915216	0,050195134	1,044557394	58,54725943	6,505251048
ENSG00000185129	PURA	11,04099504	3,617650521	6,551871644	13,12733658	0,029187056	5,25886906	4,364540157	5,10261442	9,39503713	58,48810161	6,498677957
ENSG00000119333	WDR34	0	11,2240699	0	30,56266024	0	10,51276696	2,601919327	0	3,56848161	58,46989803	6,496655336
ENSG00000179912	R3HDM2	4,966195275	1,181042182	0	0,574649275	25,49047099	5,08721233	7,120542304	5,261875207	8,752385769	58,43437333	6,492708148
ENSG00000110801	PSMD9	3,943961783	7,945853661	1,900935256	3,776925208	12,72914927	7,904105432	6,149104486	4,368635723	9,701129154	58,41979997	6,491088886
ENSG00000185973	TMLHE	0	9,67906466	8,545600028	0	0	8,292238264	9,829587691	13,6315728	8,367148067	58,34521151	6,482801279
ENSG00000074370	ATP2A3	0	5,057081098	0,040327883	7,722433602	0,312593247	6,089565633	16,74636667	0,0479377	22,27030718	58,28661301	6,476290335
ENSG00000121350	PYROXD1	6,284171906	16,4923963	0	9,073223824	0	10,72635215	4,560165832	0	11,13845136	58,27476138	6,474973487
ENSG00000186364	NUDT17	0	0	36,40415297	3,149486718	0	5,306735404	2,725969796	0	10,65505107	58,24139596	6,471266218
ENSG00000176401	EID2B	17,25820459	8,914104024	0	0	0	5,604485969	1,681907936	18,24920132	6,477224637	58,18512847	6,465014274
ENSG00000044459	CNTLN	3,004729352	7,270661608	0,577868969	16,46173285	0,032458202	4,30371796	5,171228434	17,91944509	3,376279011	58,11812148	6,457569053
ENSG00000086288	NME8	40,89092933	0	0	0	0,147239665	12,92456317	1,028867011	0	3,124111281	58,11571046	6,457301162

ENSG00000119689	DLST	5,183212186	6,249571682	11,796429	2,180195478	0,236884922	8,828135348	8,772991551	4,57725984	10,25670121	58,08138122	6,453486802
ENSG00000122435	TRMT13	0	4,086343978	0	15,21173896	0	12,08008795	10,80489555	9,911438406	5,979097112	58,07360196	6,45262244
ENSG00000196935	SRGAP1	4,314975884	6,879891958	17,73010696	5,371898067	0,027382199	5,939383588	3,77415524	6,563340056	7,453699635	58,05483359	6,450537065
ENSG00000197619	ZNF615	27,45782113	0	0	0,15982134	0	11,65235573	8,863359508	0	9,903214029	58,03657174	6,448507971
ENSG00000054598	FOXC1	1,896944146	0	0	17,88482251	0	0	0,157507321	38,07920324	0	58,01847722	6,446497468
ENSG00000169696	ASPSCR1	2,823095724	5,962651734	5,859711105	6,751479462	13,03237175	5,472292313	8,090175991	2,977380165	7,020902759	57,99006101	6,443340112
ENSG00000151240	DIP2C	0,705876775	0,68890856	0,194777601	0,822754212	39,88330991	6,9363525	4,556962893	0,23153187	3,950025348	57,97049967	6,44116663
ENSG00000150990	DHX37	3,55715241	0,040462049	0,167786388	1,417482886	19,9419429	5,332571727	8,008744596	10,42112941	9,058160056	57,94543242	6,43838138
ENSG00000106772	PRUNE2	0,020583065	0,018262072	15,18354828	0,053313691	28,29796339	4,135617454	3,477928565	0,562614192	6,164084747	57,91391546	6,434879495
ENSG00000163535	SGO2	0,047699044	0,084640783	0,087746243	17,46155764	0	10,65745024	5,742802443	0,260759602	23,5534655	57,89612149	6,432902388
ENSG00000204642	HLA-F	7,319144674	6,711317731	5,089322786	5,32150122	3,661917002	6,613353231	9,043168772	8,95963949	5,168385324	57,88775023	6,431972248
ENSG00000143178	TBX19	27,56478914	0	0	0	0,188513146	1,611205076	7,343801584	8,759551527	12,38976815	57,85762862	6,428625402
ENSG00000172830	SSH3	0	2,747508724	11,08533211	3,251751276	0	13,23241376	8,304461188	7,961176232	11,27122359	57,85386688	6,428207431
ENSG00000124107	SLPI	0	0	56,90196657	0	0	0	0	0	0,935469072	57,83743564	6,426381738
ENSG00000247626	MARS2	0	0	0	1,123602077	0	0,054955474	10,68097891	18,93513752	26,96282768	57,75750166	6,417500184
ENSG00000157350	ST3GAL2	4,620928687	1,981141509	15,46077103	8,032887879	0,036851467	7,542163723	10,82817334	3,967249297	5,282656405	57,75282333	6,416980371
ENSG00000156026	MCU	0	7,985698216	0,981178377	10,07359818	12,43800003	2,964649617	10,71189466	0	12,58656129	57,74158037	6,415731153
ENSG00000151914	DST	1,148721837	0,397595848	16,91694356	13,20396474	12,25483082	5,672067624	4,244970821	0,558970824	3,341767552	57,73983362	6,415537069
ENSG00000114993	RTKN	0	14,41456995	11,19418896	4,072395557	0	5,626254624	6,527358312	11,14182068	4,762540937	57,73912901	6,415458779
ENSG00000151746	BICD1	1,585322275	2,667250234	0,151217026	30,86284048	0,558155956	6,859218745	9,09728161	5,23847154	0,707684146	57,72744202	6,414160224
ENSG00000163249	CCNYL1	2,215132648	1,538099427	9,744362934	10,69905462	0,076294358	8,212696913	8,023490532	3,720635666	13,46370599	57,69347309	6,410385899
ENSG00000102897	LYRM1	0	12,08989814	0,07736713	9,513632528	4,19786718	7,665225642	5,098432639	10,02431613	9,000082069	57,66682146	6,407424607
ENSG00000103313	MEFV	15,33369352	0,166927993	41,24419398	0,649765448	0	0,103285273	0	0,137138231	0	57,63500444	6,403889382
ENSG00000138443	ABI2	0,012961964	1,690550438	4,530469961	23,40088488	8,733043683	5,294110013	3,761526937	1,955737276	8,249749123	57,62903428	6,403226031
ENSG00000167536	DHRS13	2,866596191	1,362510163	21,18750754	3,35891905	0	4,159004972	4,420364	4,813240652	15,42425405	57,59239662	6,39915518
ENSG00000188167	TMPPE	0	0,124398979	0,064481582	14,58717905	0	7,119799743	11,14709746	9,351198617	15,17466385	57,56881928	6,396535475
ENSG00000110619	CARS	3,297122146	3,152411678	15,592587	7,825189534	6,869639148	4,479602101	6,38789874	5,925896399	4,018002923	57,54834967	6,394261074
ENSG00000148773	MKI67	0,011764155	0,041750409	0,930567867	53,83243929	0,251619921	2,079533114	0,361417072	0,025724762	0	57,53481659	6,392757399
ENSG00000162885	B3GALNT2	0,017976513	2,16912396	14,35205365	14,58951202	0	9,098831294	4,851041863	6,800521078	5,637742467	57,51680285	6,390755872
ENSG00000080007	DDX43	0	15,94677093	0	14,65603932	0	2,862627425	9,857067198	0,121304907	14,05447268	57,49828247	6,388698052
ENSG00000119681	LTBP2	0	14,14849284	25,8443714	0,057487326	0	7,273893978	4,312265211	5,569121924	0,286610631	57,49224331	6,388027035
ENSG00000164291	ARSK	5,464359996	8,639716739	0,064436741	0,241942489	5,410904092	1,076841944	10,03413643	24,43408137	1,981673626	57,34809343	6,372010381
ENSG00000196187	TMEM63A	0	0,336796715	1,960632582	9,277687986	0,381670818	9,04092418	12,87434711	8,141141519	15,31662642	57,32982733	6,369980815
ENSG00000160219	GAB3	14,10212458	0,356030728	0,421821091	1,088878877	0	8,087825913	7,187412797	10,96852225	15,08769187	57,3003081	6,3667009
ENSG00000127511	SIN3B	5,956962225	5,865701722	12,16182728	12,42689067	0,040915751	2,816529102	2,501687148	7,153078253	8,257963277	57,18155542	6,353506158
ENSG00000092470	WDR76	0,298195502	1,587421806	12,75389699	15,38319165	0	9,371860401	8,263586247	0	9,4436072	57,10175979	6,344639977
ENSG00000213185	FAM24B	21,6972697	10,024	0	18,84407208	0	0,70480279	0	0,280743048	5,526447136	57,07733476	6,341926085
ENSG00000170191	NANP	40,09218138	0	0,073094951	11,73281111	1,983031393	0,82889849	0,626856773	0,173775739	1,514920376	57,02557022	6,336174469
ENSG00000198324	PHETA1	2,472800175	0	24,15060664	9,704514081	0,213696842	5,479343652	4,143768775	4,424146248	6,414220941	57,00309735	6,333677483
ENSG00000147164	SNX12	14,15023841	5,99315453	0,428485702	6,43539051	8,580072303	2,109845484	8,751495219	4,456728271	6,08745834	56,99286877	6,332540974
ENSG00000011143	MKS1	0	0	2,862963256	0,255944065	0,176124346	18,87751535	10,15331279	8,426972786	16,22397301	56,9768056	6,330756178
ENSG00000159445	THEM4	4,683403877	14,45655298	12,34220419	3,422961322	0	4,448517697	6,312403902	4,382294971	6,831607781	56,87994672	6,31999408
ENSG00000164116	GUCY1A1	0,05241435	0	0	24,43719845	0,062281919	3,844482805	8,160141259	0	20,24134859	56,83786738	6,315318598
ENSG00000198924	DCLRE1A	0,032979173	0,1755622	6,127453077	6,150366053	0	5,214125501	5,011145953	0	34,11107054	56,8227025	6,313633611
ENSG00000163661	PTX3	0	18,64469321	0	0,138500517	0	0	4,528653172	33,49947049	0	56,81131739	6,312368599
ENSG00000168675	LDLRAD4	5,004258412	2,511981265	3,615947346	18,8862178	0	7,464441484	7,000105043	5,146095956	7,174813528	56,80386084	6,311540093
ENSG00000148483	TMEM236	4,767586454	6,417904626	0	0	36,92106011	3,760439182	1,45604737	0,479324849	2,999155119	56,80151771	6,311279746
ENSG00000102786	INTS6	13,71955316	5,045598026	0,948068078	7,23199761	0,39418602	11,96085893	4,948181191	6,787710744	5,755566764	56,79172053	6,31019117
ENSG00000182768	NGRN	5,558135105	3,798736419	10,94722771	3,018353618	0	9,035161378	9,311614189	8,890038403	6,2077666	56,76703342	6,307448158
ENSG00000177463	NR2C2	0,076699384	1,292961171	20,50576485	11,98610026	0,121518498	2,947400483	9,807510936	0,391344552	9,637417998	56,76671814	6,307413126
ENSG00000172456	FGGY	0	3,964642682	0,022833916	6,130064296	24,15946009	3,938570451	3,5041821	9,852780352	5,190390798	56,76292469	6,306991632
ENSG00000168268	NT5DC2	0	12,46002626	16,91397977	2,718639035	0,149663489	5,358645424	8,078835713	0,06885488	10,99821053	56,7468551	6,305206122
ENSG00000131067	GGT7	0,816262367	11,22539096	7,132507604	3,788049935	0	11,48266094	3,55824034	3,235179763	15,49766745	56,73596831	6,303996479
ENSG00000232629	HLA-DQB2	4,251383281	0,794102666	5,899873769	7,598797385	5,672142821	9,089868714	10,71390298	1,794067247	10,91596059	56,73009945	6,303344383
ENSG00000140403	DNAJA4	0	11,26920559	7,88685761	0,950150112	0,108972096	7,72789365	15,49580492	6,918520758	6,372525267	56,72993	6,303325556
ENSG00000100298	APOBEC3H	0,120791823	0,214342126	0	30,03568053	0,574128765	5,437516902	3,410064978	0	16,93565431	56,72817943	6,303131048
ENSG00000213654	GPSM3	7,501943224	5,558863068	5,914470191	3,985913415	6,46529127	8,236695297	8,761703489	2,343507571	7,90836265	56,67675018	6,297416686
ENSG00000140859	KIFC3	11,71106923	6,452166877	3,677445059	0,027180727	0,112224232	11,64830507	13,68410808	6,471010281	2,82641586	56,60992542	6,289991713
ENSG00000130821	SLC6A8	18,32209002	8,755077659	5,396719182	0,230263832	0	5,504975012	1,749409853	10,26411646	6,363258969	56,58591099	6,287323443
ENSG00000197912	SPG7	5,30028468	4,765909799	4,215094813	14,05837177	5,395539614	5,436971798	4,439269134	3,753269777	9,1612302	56,52594159	6,280660176
ENSG00000162066	AMDHD2	3,253411279	5,588850666	0	4,840983267	0	12,46419558	8,161606571	11,50390615	10,68425953	56,49721104	6,277467893
ENSG00000119457	SLC46A2	28,44434271	13,16234564	0,112770831	0,105855973	0	5,586441325	4,377463916	4,423667638	0,22618238	56,43907042	6,271007824
ENSG00000129680	MAP7D3	1,668032443	5,452415398	9,932186543	1,553861141	0	7,66297217	9,512783341	0,671907617	19,97478961	56,42894827	6,269883141
ENSG00000076382	SPAG5	0	17,52326984	0,639279645	21,95294248	0	5,850430455	0,3847308	7,59910849	2,457533545	56,40729525	6,26747725
ENSG00000127580	WDR24	0	4,33316297	18,80610366	0,714694535	0	7,861552936	5,979687145	10,0460797	8,602591691	56,34387264	6,260430293
ENSG00000101752	MIB1	10,67850041	0,240534038	21,4725981	3,771112461	4,044680782	4,431778586	3,864281131	0,362282492	7,464980412	56,33074841	6,258972045
ENSG00000157483	MYO1E	13,01017872	5,270966178	0	17,82358381	3,115559614	1,366493878	9,162944298	1,306352342	5,183946481	56,24002532	6,248891702
ENSG00000145287	PLAC8	5,294556719	3,270873087	0,937903267	23,4997239	1,304865194	5,296392664	4,200797516	6,003223391	6,415153766	56,2234895	6,247054389
ENSG00000170921	TANC2	4,69205393	4,397500657	11,46397417	7,314760724	0,32981144	5,862667103	5,788996775	2,774575219	13,59753484	56,22187486	6,246874985
ENSG00000105778	AVL9	0,030709369	6,266697604	13,65232162	4,931639203	0,145962963	10,44105987	4,691750661	8,528354574	7,503355052	56,19185091	6,24353899
ENSG00000124225	PMEPA1	4,218020116	3,8827261	11,25111357	1,820900032	10,02421612	4,833751973	9,062232632	0	11,08853988	56,18150042	6,242388936
ENSG00000114115	RBP1	0	0	0	0	49,84422739	1,699468135	1,895710522	2,707786469	0	56,14719252	6,238576946
ENSG00000169372	CRADD	0	12,22817821	16,96744758	0	0	3,220434487	2,288747871	11,82331357	9,605257582	56,1333793	6,237042144
ENSG00000166341	DCHS1	0,014105346	5,181125177	0,077843769	0,097427415	34,4267447	2,710197977	2,728888953	3,238647399	7,6503609	56,12534164	6,236149071
ENSG00000051825	MPHOSPH9	3,578317085	17,85184511	18,91149548	0,734386089	0,592488181	6,456729548	4,103600726	0,865851448	3,030106341	56,12482001	6,236091112
ENSG00000171649	ZIK1	0	20,37280001	0	1,895058475	0	5,12870684	9,766591951	0	18,89612685	56,05928412	6,228809346
ENSG00000072609	CHFR	8,020056562	0,078280493	7,774418603	1,157882762	0	11,65355426	8,102422327	2,045078774	17,20981984	56,04151361	6,226834846
ENSG00000138002	IFT172	0,018000068	0,063881358	0,463576103	26,23336731	0,256665307	7,5297117	13,6156608	0,865939307	6,973395416	56,02019737	6,224466375
ENSG00000186529	CYP4F3	0,054779978	0,048602864	55,62625145	0	0,065092902	0	0,090969971	0,059893886	0,033686156	55,97927721	6,21991969
ENSG00000072062	PRKACA	14,58472793	5,84814909	5,655519592	2,165964145	0,350700725	6,156837807	4,227271928	7,744560176	9,225779507	55,9595109	6,217723433
ENSG00000227877	MRLN	0	0	0	0	54,82617359	0	0	1,123544942	0	55,94971853	6,216635392
ENSG00000146909	NOM1	1,221604767	0,688161171	24,2916011	5,625186158	0,092164133	3,768275642	5,602935395	9,285919452	5,36577281	55,94162063	6,215735626
ENSG00000013563	DNASE1L1	4,242213504	11,29155567	5,381996108	10,48286684	0	6,384264689	5,071041976	4,238391113	8,725611162	55,81794106	6,201993451
ENSG00000100577	GSTZ1	10,18844732	5,673775734	7,410587217	11,82239753	0	3,272593563	4,784821985	9,361983563	3,243704991	55,75831191	6,19536799
ENSG00000198873	GRK5	0,043174449	4,098742001	1,389900495	29,18744073	0	8,366633368	5,628240607	0,472049382	6,557727286	55,74390832	6,193767591
ENSG00000090661	CERS4	14,66803765	14,00495295	0	8,421404699	0,796269495	6,662582903	3,833042909	1,790973266	5,54014023	55,71740411	6,190822678
ENSG00000121775	TMEM39B	5,69307855	5,580278437	0,398966773	9,643452596	0,257709036	8,691404449	7,338236138	6,639517682	11,46953779	55,71218146	6,190242384
ENSG00000162086	ZNF75A	3,429645843	1,430001985	0,034476014	4,821941116	10,9120424	2,037101215	5,461997586	12,78625636	14,7515533	55,66501582	6,185001758
ENSG00000144199	FAHD2B	0,056765804	6,648148763	7,518620258	2,744620319	21,85463631	3,614881085	4,383448864	0	8,796642301	55,61776371	6,179751523
ENSG00000108688	CCL7	54,28073801	0,310709154	0	0	0,832253864	0,19224864	0	0	0	55,61594967	6,179549964
ENSG00000152475	ZNF837	12,48176268	0	32,47177109	0,127534201	0	6,730487716	0,245298792	1,292021769	2,180017857	55,52889411	6,169877123
ENSG00000143919	CAMKMT	10,5179616	9,734749628	4,732760824	6,271847161	0	10,21882871	7,822261559	2,068318525	4,141297066	55,50802508	6,167558342
ENSG00000145936	KCNMB1	0,350488401	12,70520411	3,500080769	5,44695275	0	6,899197275	5,861934	3,613101633	17,08827367	55,46523261	6,162803623
ENSG00000105427	CNFN	0	16,18475368	0	11,49305761	0	10,28483183	9,006027093	0	8,488911295	55,45758151	6,161953501
ENSG00000100321	SYNGR1	2,286255032	5,911487898	3,885312623	12,44516954	12,62603463	5,570196595	3,959366384	4,475626707	4,284646047	55,44409545	6,16045505
ENSG00000184497	TMEM255B	4,873196666	3,82692027	6,027289096	5,084776505	10,98987401	7,809431564	8,402569235	1,541755907	6,886038289	55,44185155	6,160205728
ENSG00000132326	PER2	8,817715197	5,111554425	0,809022407	7,632120183	0,052258082	7,822326375	6,810307601	9,953427747	8,410690982	55,419423	6,157713667
ENSG00000171169	NAIF1	0,208716258	0,074072376	0	2,739096418	0	13,10785219	10,01683037	12,87051769	16,37708304	55,39416834	6,154907594
ENSG00000167107	ACSF2	0,65167574	4,625530448	0,095904812	0	0	6,124696121	16,38451636	11,79919881	15,7089522	55,39047449	6,154497165
ENSG00000178150	ZNF114	0,100272442	15,56896152	0	0,60602018	34,67259616	0	3,080558914	0,54816617	0,801594157	55,37816954	6,153129949
ENSG00000186564	FOXD2	1,616155836	3,154611766	0	6,641981172	36,18061229	0,035488893	1,74450729	2,827247637	3,140508077	55,34111296	6,149012551
ENSG00000144426	NBEAL1	0,009227563	0,08187042	0,050924542	10,38895966	38,59592541	0,749718043	2,34452379	1,372102503	1,725004359	55,3182563	6,146472922
ENSG00000171105	INSR	7,701323516	13,02213019	7,725234031	1,734587589	7,526514829	1,562448419	0,729815429	6,010120007	9,282888796	55,2950628	6,143895867
ENSG00000139160	ETFBKMT	0,022002122	0,585633372	40,43420485	0	0	6,860600434	5,535455824	0,769795149	1,082390928	55,29008267	6,143342519
ENSG00000006062	MAP3K14	13,32406484	0,741748271	0	7,398571202	0,062088095	5,822933449	11,32356731	3,5991317	13,0131123	55,28521716	6,142801907
ENSG00000106344	RBM28	10,94327781	4,512411069	4,853175811	8,255978649	0	5,238951149	7,655066266	7,435090168	6,383862009	55,27781293	6,141979214
ENSG00000112473	SLC39A7	7,914050977	3,581510019	5,667081104	4,494134888	12,62276072	6,58976983	5,600960339	4,297391563	4,507349749	55,27500918	6,141667687
ENSG00000278053	DDX52	6,105958197	5,375222714	0	2,793384489	25,44126166	5,049755168	4,964558686	0,277442962	5,266431676	55,27401555	6,141557284
ENSG00000113504	SLC12A7	7,500158237	3,840808129	0,370393203	2,911832661	16,20933611	3,233100845	12,2879006	1,816179335	7,088416656	55,25812577	6,139791753
ENSG00000081665	ZNF506	0,03168933	12,25856265	0,029147541	17,42849731	0,188275948	1,043790983	3,565320328	14,03229525	6,586570675	55,16415002	6,129350002
ENSG00000100376	FAM118A	12,86497777	11,30995829	0	0,649799159	0,055893781	7,927555961	2,070016372	4,885802944	15,3883813	55,15238557	6,128042842
ENSG00000100523	DDHD1	9,490727437	4,916335155	17,55190884	13,16017343	0,420277998	1,655037518	1,321549975	1,381107477	5,219942445	55,11706027	6,124117808
ENSG00000221994	ZNF630	0	16,04767139	0	0	0	3,746076677	0	29,03437576	6,26903394	55,09715777	6,121906419
ENSG00000203778	FAM229B	7,345241381	8,829450415	0	0,409149313	0,844651217	9,820657421	5,262769708	22,53846129	0	55,05038074	6,116708971
ENSG00000123607	TTC21B	21,42300621	13,28965653	0,032040122	2,01505801	0	7,515313122	10,67278758	0,03808605	0,021420761	55,00736838	6,11192982
ENSG00000110665	C11orf21	0,035987276	2,107331746	3,376276506	18,14551383	0	2,2916917	12,81895033	4,249456215	11,9501217	54,9753293	6,108369923
ENSG00000101224	CDC25B	0	12,92849699	0,708804147	6,83487532	0	10,57612624	11,51744355	5,591498245	6,763538247	54,92078274	6,102309193

ENSG00000110628	SLC22A18	6,509465892	3,934363432	6,327237016	8,295336852	0	6,085884586	4,980105352	7,210385597	11,39690863	54,73968735	6,082187484
ENSG00000135245	HILPDA	16,13306093	5,440562321	1,745768738	4,285888472	0,173486735	5,530352557	8,425302487	9,098922874	3,860579931	54,69392504	6,077102782
ENSG00000109794	FAM149A	0	2,518006517	0,053273304	0,100013405	0	17,96463699	14,66786173	0	19,37533693	54,67912889	6,075458765
ENSG00000140022	STON2	0,483584776	0	0,960760711	2,939359673	4,459088788	3,950251826	1,622184272	37,73011191	2,47414725	54,6194892	6,068832134
ENSG00000278845	MRPL45	4,87739518	17,93907747	0	0	0	10,51545909	10,38222818	3,102671995	7,743605095	54,560437	6,062270778
ENSG00000140854	KATNB1	0,099067245	9,316996944	0,060747399	15,68118838	0	7,17882303	8,719310659	5,632407401	7,838369493	54,52691055	6,058545616
ENSG00000174799	CEP135	8,167666262	6,486305326	12,35467107	10,99309384	0,124693591	4,570221905	7,246492554	0,879629057	3,699722434	54,52249603	6,058055115
ENSG00000147889	CDKN2A	8,656831029	2,911220606	5,522358169	0	21,48566493	5,442204179	1,912922595	3,969183994	4,593573085	54,49395858	6,054884287
ENSG00000111850	SMIM8	11,51894293	2,022556801	5,750216905	11,00400064	9,521745123	3,678474501	2,452048774	4,191798514	4,332878919	54,47266311	6,052518123
ENSG00000071655	MBD3	11,73117845	3,492050324	8,04873469	8,281031562	2,134090845	5,642931487	6,742305108	1,504064285	6,861447023	54,43783378	6,048648198
ENSG00000196792	STRN3	3,53047859	3,99647694	1,175746642	12,5606161	0	3,675763029	4,624586255	23,89244909	0,973214417	54,42933106	6,047703451
ENSG00000168056	LTBP3	9,171111543	5,155575453	1,727590438	2,407149212	9,241226328	6,959930971	5,823928792	6,096578736	7,83233547	54,41542694	6,046158549
ENSG00000120217	CD274	0	0,130546251	0,067667989	2,858343198	0	12,88351362	11,48413944	26,94634375	0	54,37055425	6,041172695
ENSG00000141012	GALNS	9,478132676	3,542461558	17,2041956	1,30436431	0,213709559	6,358391538	7,242693528	1,612451472	7,387852706	54,34425295	6,038250328
ENSG00000162722	TRIM58	0,146141751	0	0	6,712640546	35,14767436	0	3,567531684	6,135728913	2,624138516	54,33385577	6,037095085
ENSG00000101003	GINS1	0	11,10224746	0,60576777	16,11099722	0,0869534	8,034410494	0	14,16148147	4,229920846	54,33177866	6,036864295
ENSG00000174151	CYB561D1	10,14485729	0	0	0,963028905	0,063113804	18,22392099	10,14347148	0,871092884	13,9139927	54,32347806	6,035942007
ENSG00000188033	ZNF490	0,022248317	1,144893699	0,122782729	14,29149154	0	4,983179866	4,174955099	14,25461253	15,29567225	54,28983603	6,032204004
ENSG00000133134	BEX2	0	7,06836905	34,03837947	0,221883482	0	9,311307876	0	0	3,634753412	54,27469329	6,030521476
ENSG00000256087	ZNF432	0,025243998	37,22452424	0,046438373	0,479499619	0	3,714002218	6,875086685	0,055201231	5,805763392	54,22575975	6,025084417
ENSG00000162714	ZNF496	19,54349988	1,756888995	0,052792726	3,196335744	0	3,812587557	4,777666236	2,039525865	19,0417242	54,2210212	6,024557911
ENSG00000156239	N6AMT1	0,031060755	2,976293526	12,51338986	8,04527157	18,52795542	7,161607627	0	3,871484142	1,069620272	54,19668318	6,021853686
ENSG00000170903	MSANTD4	5,117879547	0	0	15,32507682	0	5,976418581	9,997342428	1,293792355	16,48169041	54,19220013	6,02135557
ENSG00000047644	WWC3	0	7,312278327	0,117225232	1,063693318	0	9,818435872	24,1981017	0,975418319	10,68473821	54,16989098	6,018876775
ENSG00000197782	ZNF780A	1,345816673	0,538167587	0,662521561	1,832960183	13,55925492	3,309054402	11,48938063	0,373044544	21,05110507	54,16130557	6,017922841
ENSG00000141499	WRAP53	0	3,742496954	20,92910908	5,437213939	0,070595153	6,97952888	14,33030527	0,844436677	1,826680974	54,16036693	6,017818548
ENSG00000196437	ZNF569	17,98504998	5,024989635	1,27462964	3,693457575	0	7,045245781	9,980594591	3,425557717	5,668763538	54,09828846	6,010920939
ENSG00000128059	PPAT	0,027782786	2,514293437	0	8,635465238	4,357741827	4,36205414	7,86640618	11,9683048	14,28274431	54,01479272	6,001643635
ENSG00000175970	UNC119B	27,93149759	0,155534803	0,053747119	1,412641007	0	10,00852667	7,374899638	0,063889128	6,971041245	53,9717772	5,996864134
ENSG00000154146	NRGN	6,10421926	1,326341303	7,791692799	13,55226991	20,1318985	0,820662374	0,310314094	2,996521222	0,919273812	53,95319327	5,994799253
ENSG00000102763	VWA8	19,35640316	7,691302949	0,800990462	1,640455636	0,047035696	8,626915383	6,179023554	1,601320105	8,008317725	53,95176467	5,994640519
ENSG00000043143	JADE2	3,870707136	10,5334713	3,715032203	9,748405956	0,109076909	5,795195805	8,574716422	0,802918864	10,80043574	53,94996033	5,994440037
ENSG00000145423	SFRP2	0,075706699	53,87021144	0	0	0	0	0	0	0	53,94591814	5,993990904
ENSG00000185917	SETD4	11,69701627	6,527526807	0,038016972	3,318784551	0,147340348	13,90907871	3,757932537	4,609453759	9,937908945	53,9430589	5,993673211
ENSG00000119969	HELLS	0,580621897	9,061350348	0,191710413	23,41987802	12,84331703	2,729760228	3,362357665	0,097665389	1,647900197	53,93456119	5,992729021
ENSG00000111913	RIPOR2	0,079231633	3,121199875	24,51565261	7,798497039	0,414250521	1,270078869	0,684192247	9,529100888	6,509305625	53,9215093	5,991278811
ENSG00000135426	TESPA1	13,6011497	0	0	6,983786989	0,14239395	2,499842155	10,29831829	11,59534466	8,76912615	53,88996189	5,987773543
ENSG00000183955	KMT5A	2,940280015	2,579415829	8,933772311	5,932933397	2,355383968	11,28075849	6,748076448	5,996079822	7,11043756	53,87713784	5,986348649
ENSG00000167632	TRAPPC9	0,078109465	6,486635792	8,046565462	5,368144314	0	10,61704197	8,029167322	3,347729517	11,87358118	53,84697502	5,982997225
ENSG00000224470	ATXN1L	3,516253924	7,054718197	8,938789432	2,99013418	0	4,384446952	6,968943838	16,38263057	3,542210375	53,77812747	5,975347496
ENSG00000175463	TBC1D10C	0,030264484	4,296286803	6,736549565	3,971772095	0	9,569847004	8,041359151	3,838414764	17,2707363	53,75523016	5,972803351
ENSG00000267508	ZNF285	0,132051183	0	0,040486469	0,152015715	47,43950057	0,894070978	1,699496195	0	3,38345693	53,74107804	5,971230894
ENSG00000029993	HMGB3	3,513301464	1,918236076	1,562483949	26,93350274	2,11029726	8,096300498	6,090792816	0	3,513705327	53,73862013	5,970957793
ENSG00000151474	FRMD4A	0,02057886	2,592684462	8,328424592	7,355786428	0,293436641	10,89049407	9,508952711	4,589993992	10,13639278	53,71674454	5,968527171
ENSG00000124444	ZNF576	2,995297149	10,09095145	0,159712783	10,26948909	0,206330162	7,768866418	5,803138021	7,87879032	8,542213032	53,71478837	5,968309819
ENSG00000197429	IPP	21,57557136	0,217119028	4,276616907	0	19,96712921	0,089560365	7,585790653	0	0	53,71178752	5,967976391
ENSG00000181873	IBA57	8,67076295	0,65528348	30,08054444	4,973833827	2,667930911	2,432706403	4,022900185	0,161502721	0,018166815	53,68363173	5,96484797
ENSG00000162927	PUS10	22,57474608	4,661205857	0	6,718623709	0,046937334	3,664734997	3,247042966	0	12,7525061	53,66579705	5,962866339
ENSG00000179889	PDXDC1	5,427776077	4,787232308	0	2,689765413	17,47883469	5,730174009	6,333518353	4,810790197	6,359466543	53,61755759	5,957506398
ENSG00000171853	TRAPPC12	1,841007714	3,150150768	4,838117681	3,462859448	8,32068275	7,471660911	8,92609563	6,973165028	8,632270751	53,61601068	5,95733452
ENSG00000173825	TIGD3	0	0	45,78173228	7,719969663	0	0,081809949	0	0	0	53,58351189	5,953723543
ENSG00000159231	CBR3	7,873496668	14,24000602	0,278537173	4,706242027	0	7,314694764	3,143044389	10,59509635	5,400349846	53,55146724	5,950163026
ENSG00000134490	TMEM241	0,029098897	0	29,65549294	0,653216767	0,207462474	7,603835404	4,131605387	9,035568763	2,218849293	53,53512992	5,948347769
ENSG00000151611	MMAA	1,75235181	0	28,63676994	9,371566865	1,374689984	6,070810339	3,206683783	0	3,096744573	53,50961729	5,945513032
ENSG00000071626	DAZAP1	2,676994133	5,333114446	7,432138284	12,98861193	3,395633756	6,257034031	5,809190461	4,453199258	5,15062093	53,49653723	5,944059692
ENSG00000011376	LARS2	7,808955826	6,003179106	0,022306219	11,95585544	0,086451021	6,057559974	6,051008629	0,132576861	15,31571003	53,43360311	5,937067012
ENSG00000177548	RABEP2	11,38872465	5,544065364	0	11,77630054	0	7,561509165	7,364199121	0	9,792268959	53,4270678	5,936340867
ENSG00000012963	UBR7	0	6,877395272	4,753150738	6,46946218	10,02950104	7,659593453	3,432645162	7,156747544	7,044054563	53,42254995	5,935838883
ENSG00000176532	PRR15	0	12,474909	3,552915376	12,4064175	0	5,428566927	3,014880166	0	16,43734578	53,31503476	5,923892751
ENSG00000104635	SLC39A14	0,087133095	0	0	21,81667656	0,051768422	6,314025119	16,33266683	0,190534564	8,519416763	53,31222135	5,92358015
ENSG00000198829	SUCNR1	0,036142208	0	0	0	0	19,04737546	8,312672079	20,78552769	5,067329237	53,24904668	5,916560742
ENSG00000125430	HS3ST3B1	4,92537714	7,24168188	0,103550046	0,048600293	0,735759539	6,458429974	9,885258044	1,969436797	21,84189897	53,20999268	5,912221409
ENSG00000148343	MIGA2	12,34876926	2,212533165	0,044109822	2,028850167	0,056984694	5,765537337	11,80639589	10,43422389	8,493144782	53,190549	5,910061
ENSG00000163602	RYBP	0,481710759	9,588445741	1,50259513	4,66536545	22,29863603	5,035955135	2,608530811	3,480669041	3,503136109	53,16504421	5,907227134
ENSG00000104047	DTWD1	8,978362127	8,511309152	3,095670614	13,18943123	4,669773323	2,323367639	1,823988555	6,896907151	3,668349599	53,15715939	5,906351044
ENSG00000137274	BPHL	4,156847878	9,463835297	0,048093443	5,372190654	0,062131063	9,013135136	11,93921844	7,374750728	5,723301205	53,15350385	5,905944872
ENSG00000174950	CD164L2	0	0	0	0	53,12922517	0	0	0	0	53,12922517	5,903247241
ENSG00000051341	POLQ	0,016106435	9,74594138	0	40,57814197	0,114831872	0,070735697	2,126389127	0,422640675	0	53,07478715	5,897198573
ENSG00000122026	RPL21	2,150083709	7,988223213	0	9,165790571	0,479036295	4,795103373	9,428406451	7,052405176	11,98210126	53,04115005	5,893461116
ENSG00000144712	CAND2	0	2,927628782	33,83326782	0,88737507	0	7,928785391	5,210151606	0	2,195430548	52,98263922	5,886959913
ENSG00000237441	RGL2	6,903344176	8,445924985	3,876615951	0	0	7,260333003	7,391255432	3,257469889	15,81862681	52,95357024	5,883730027
ENSG00000167394	ZNF668	3,488674653	3,547403892	13,41226937	0	18,1654788	7,703759593	3,710406705	1,457169299	1,398067223	52,88322953	5,875914392
ENSG00000176834	VSIG10	5,385833041	5,003008254	6,283726672	17,0710957	0	7,282523819	5,402382433	1,976046929	4,445557284	52,85017413	5,87224157
ENSG00000091009	RBM27	0	7,162256305	12,2221142	23,45438922	0	2,09130979	5,083581879	0,297510115	2,537820157	52,84898167	5,872109074
ENSG00000159761	C16orf86	0,506515879	2,546600001	23,13912556	13,99427797	0	7,229622895	4,696375555	0	0,726774109	52,83929197	5,871032441
ENSG00000112874	NUDT12	2,670170512	5,860345968	0	1,152700423	0,08349629	8,987948006	5,71778309	12,90700008	15,38278585	52,76223021	5,862470024
ENSG00000243279	PRAF2	0	10,64823827	0	0	0	4,791641204	12,38094732	15,30894206	9,616007049	52,7463759	5,860708434
ENSG00000111671	SPSB2	12,32549516	2,62455465	9,296234958	7,342786806	0	5,07475255	4,349501721	2,021418643	9,701612908	52,7363574	5,859595266
ENSG00000137413	TAF8	13,13509592	2,281690867	0,179651076	5,649281548	0,116044014	5,450530579	13,50116993	2,740571058	9,668663319	52,72269832	5,858077591
ENSG00000172167	MTBP	16,42308952	3,896383092	0,033382983	4,543721787	0	0,338715	6,539464884	14,24594809	6,69556343	52,71626879	5,857363199
ENSG00000168566	SNRNP48	0,025888397	0,045938324	0,095247593	0,491739716	0,061524335	4,462559627	8,57677959	26,83330221	12,09896839	52,69194818	5,854660909
ENSG00000160299	PCNT	0,025961785	1,197782322	22,08844528	0,022415168	0,092548117	10,88872782	7,49092696	0,312239519	10,53678419	52,65583116	5,850647907
ENSG00000128159	TUBGCP6	0,039147816	2,882873835	0	11,99895285	4,140085657	10,2512105	10,58046452	0	12,75889337	52,65162855	5,85018095
ENSG00000107643	MAPK8	3,545803136	3,381571636	11,35023146	11,4094991	0	4,613391062	2,282693656	9,598109724	6,435661046	52,61696081	5,846328979
ENSG00000126790	L3HYPDH	0	16,73488578	0	0,747022544	0	9,2621135	10,09366255	5,77054355	10,00261843	52,61084636	5,845649595
ENSG00000168060	NAALADL1	5,935420142	3,245790021	0	5,994800553	0	11,96785781	5,641205638	7,918021998	11,84496115	52,54805731	5,838673035
ENSG00000090971	NAT14	6,337633838	2,303392623	0,140464931	17,93186283	0	7,880542804	3,740651636	6,511848476	7,700563328	52,54696047	5,838551163
ENSG00000125648	SLC25A23	0,030958937	6,647240732	2,619767374	8,660410958	0	12,84863716	11,15634585	2,843321417	7,729326782	52,53600921	5,837334357
ENSG00000104826	LHB	15,35091512	0	32,21039242	0	0	2,896851339	0	0	2,064961359	52,52312023	5,835902248
ENSG00000214013	GANC	0	6,756179663	0,609048966	0,309672692	4,229154917	13,08624596	6,002049842	11,0707948	10,45110989	52,51425673	5,834917415
ENSG00000151948	GLT1D1	0	0,231289822	30,45152896	0	0	0,143108606	0	21,47159639	0,213739531	52,51126331	5,834584812
ENSG00000163006	CCDC138	2,867867973	7,587178746	17,74545145	1,845813312	0	2,089625966	4,178014851	13,85362317	2,340717068	52,50829254	5,834254727
ENSG00000274750	HIST1H3E	0	2,602348638	4,669318572	9,058213004	11,66227181	1,981761275	13,02006431	0,493370015	9,018308285	52,50565591	5,833961768
ENSG00000206560	ANKRD28	5,896474911	2,425548422	5,670044587	7,867851309	0,031847955	1,662637032	4,918223372	22,12469894	1,895384007	52,49270993	5,832523326
ENSG00000196547	MAN2A2	10,26183636	3,872582404	0,569456313	0,507811586	0,220701142	9,312608423	16,66854662	5,990670742	5,044487187	52,44870077	5,827633419
ENSG00000179134	SAMD4B	8,627387676	3,960394441	0,069003376	3,303384313	1,426307727	10,95499898	7,116785618	5,659671133	11,32563515	52,44356842	5,827063158
ENSG00000260916	CCPG1	5,019827831	1,494762003	1,423106309	16,44573105	1,307367617	6,889347042	3,982505418	9,961906757	5,898882789	52,42343682	5,824826313
ENSG00000167196	FBXO22	3,042408918	16,58165993	0	13,11186659	9,721479871	4,449171205	3,884793293	0	1,603616045	52,39499585	5,821666206
ENSG00000259431	THTPA	16,55219451	9,728178041	1,056593327	0	0	7,968752647	10,53534578	3,767913175	2,761368506	52,37088598	5,818997331
ENSG00000154370	TRIM11	2,332173039	5,623147504	13,19815974	0,153708161	0,042308853	6,978095651	10,14050987	9,888122072	4,006822994	52,36304788	5,818116432
ENSG00000140090	SLC24A4	0,062910313	7,460792536	0	0,561267292	36,55463743	1,381436942	2,176490633	0,022927739	4,062007668	52,28247055	5,809163395
ENSG00000061938	TNK2	4,985121918	0,520360955	9,278590535	6,329685974	21,53450817	1,867419649	3,579301146	0,384748187	3,786897892	52,26672442	5,807413825
ENSG00000118849	RARRES1	0	0	0	9,64520606	0	0,572387401	5,874658226	36,15400394	0	52,24625562	5,805139514
ENSG00000119640	ACYP1	0	5,056476984	0,154176428	6,801965505	0,398355566	7,269515008	0,4871283	30,60597492	1,443067838	52,21666055	5,801851172
ENSG00000042088	TDP1	0,024266994	6,889789628	0,624975331	6,662704506	0	12,44262716	17,00609607	1,48581466	7,073329979	52,20960432	5,801067147
ENSG00000196422	PPP1R26	0,170842929	2,071570003	36,56116621	0,049168075	0	8,097011375	3,380866304	0,560375185	1,295707806	52,18670789	5,798523099
ENSG00000139626	ITGB7	14,59841505	0	15,17617705	0	4,921628866	6,970406166	2,170571908	2,746816221	5,553266111	52,13728137	5,793031264
ENSG00000152684	PELO	11,65048326	1,594987875	0	11,87967512	0	11,72875045	5,884561441	0	9,311459249	52,04991739	5,783324154
ENSG00000158234	FAIM	0	12,51153733	7,441019349	10,76549011	0	8,597036268	4,868446085	0	7,850098681	52,03362783	5,781514203
ENSG00000197081	IGF2R	0,025406698	1,743231072	9,472168367	4,913644088	0,181138703	12,5527814	9,950149626	2,09264732	11,08225633	52,01342361	5,77926929
ENSG00000108510	MED13	0,04769528	4,654875722	8,773931949	11,15969038	2,69203555	6,702928689	6,276981811	10,24704372	1,437146625	51,99232973	5,776925525
ENSG00000235718	MFRP	0,036892229	0,065464355	0	0,063704828	51,81603211	0	0	0	0	51,98209352	5,775788169
ENSG00000186104	CYP2R1	12,31370461	4,21569575	0	0,231120438	0	12,01345407	13,14160032	1,243885662	8,806721826	51,96618267	5,774020297
ENSG00000085721	RRN3	4,50859258	2,838846969	5,484689152	9,229413202	13,73909751	3,712628401	3,471869148	3,736868304	5,231964174	51,95396944	5,772663271
ENSG00000145386	CCNA2	0	0,095833452	0,993495708	25,64586062	0,256695884	15,83206718	4,215229167	0	4,848747722	51,88792974	5,765325526
ENSG00000153832	FBXO36	22,61882625	0,677982301	0,070285739	0,065975973	0,090800896	13,38191504	0,063448967	6,600336494	8,317277988	51,88684965	5,765205516
ENSG00000166444	ST5	4,344084558	1,109267845	9,238715953	7,757427376	8,410128954	6,78206887	6,404599754	0,625559762	7,166988308	51,83884138	5,759871264

ENSG00000102908	NFAT5	0,59887733	0,539521127	7,745680461	2,290996667	17,42927649	5,664893784	5,102659072	2,820610905	9,643033379	51,83554922	5,759505468
ENSG00000050426	LETMD1	0,400823795	5,423296421	0,138252702	21,80225915	0	12,26726081	10,1923844	0	1,602125192	51,82640247	5,758489163
ENSG00000166451	CENPN	0	0	16,2830271	7,020671962	0	8,323428596	6,364946775	11,48371255	2,34391634	51,81970332	5,757744813
ENSG00000112511	PHF1	8,656500989	7,978641271	5,411181015	8,344694683	0	3,460315143	7,990185214	1,929679065	8,01062596	51,78182334	5,753535926
ENSG00000122507	BBS9	3,317358775	13,02836837	0,044871687	0	7,420023474	4,60639535	7,392521579	13,17471156	2,789947792	51,77419859	5,752688732
ENSG00000265681	RPL17	11,94913272	6,197929442	7,552581765	1,693008757	0	9,688208859	5,749451547	5,627839349	3,315996342	51,77414878	5,752683198
ENSG00000169683	LRRC45	12,5820625	0	0,308609602	0	0,099671813	5,571795665	13,30271075	4,95239152	14,90692338	51,72416523	5,74712947
ENSG00000115568	ZNF142	0	0,191694309	12,67881778	3,768148761	0	6,570950779	8,090809227	16,53590201	3,826408237	51,6627311	5,740303456
ENSG00000102755	FLT1	14,23914967	7,682540392	0	1,017573717	0,028580783	2,759674535	0,039942804	21,93254511	3,919564537	51,61957155	5,73550795
ENSG00000075711	DLG1	0,027472152	0,341240615	2,653205199	4,269456906	0,032644082	9,742589996	11,10881769	11,47404825	11,96067065	51,61014553	5,734460615
ENSG00000162664	ZNF326	10,67180586	2,318300874	3,516493789	9,64139058	1,111211908	10,67517089	5,275509045	3,27788204	5,090990463	51,57875545	5,730972828
ENSG00000107185	RGP1	0	1,356536257	15,00061242	3,336858163	0,050466209	5,875416713	8,234210939	5,34007522	12,37932147	51,57349739	5,730388599
ENSG00000276600	RAB7B	10,12596608	7,201860556	0	4,459821866	0	10,48756494	12,62872848	6,454500505	0,201678266	51,56012069	5,728902299
ENSG00000167535	CACNB3	5,703880061	0,899679575	0	6,529758207	25,9560701	0,139167267	4,56064397	4,527133736	3,169757545	51,48609046	5,720676718
ENSG00000167232	ZNF91	0,131935888	13,13656481	0,053934827	11,39122415	0	7,194608827	7,899713375	3,109444417	8,545911094	51,46333739	5,718148599
ENSG00000010818	HIVEP2	0,397099576	3,850373159	6,339668142	0,416320466	17,32392461	6,384170478	0,329720253	12,93207083	3,436111205	51,40945872	5,71216208
ENSG00000105771	SMG9	4,930011031	11,1217218	0	4,718475135	0,04541193	10,04946462	5,632519962	7,270563322	7,614349167	51,38251697	5,709168553
ENSG00000164047	CAMP	3,245327692	0	48,1334668	0	0	0	0	0	0	51,3787945	5,708754944
ENSG00000070404	FSTL3	0,099644293	11,66987933	35,74422638	0	0	0	2,275261543	0,108946445	1,470595626	51,36855362	5,707617068
ENSG00000133393	FOPNL	2,653279251	7,846964886	0	6,327018615	8,707699606	6,519897245	3,357065739	7,874067871	8,080283577	51,36627679	5,707364088
ENSG00000251369	ZNF550	1,604083117	17,55283783	0,655742321	1,025889422	24,49651803	1,663345668	4,365686802	0	0	51,36410318	5,707122576
ENSG00000114026	OGG1	6,488774075	10,07106104	0,063661994	2,748885568	0,082243798	6,174388804	11,70941493	4,237795896	9,76795531	51,34418141	5,704909046
ENSG00000110811	P3H3	0	0	0	0,226899978	45,28003795	2,416523617	0	3,376179277	0	51,29964082	5,699960091
ENSG00000119685	TTLL5	12,03513716	2,77606864	0,045321611	21,05858196	0,234200729	5,653429613	3,313963281	0	6,150948728	51,26765172	5,696405746
ENSG00000157353	FCSK	0,041795207	0	0	14,57858246	0	5,804919915	15,07865005	7,814176607	7,916018374	51,23414261	5,692682512
ENSG00000134461	ANKRD16	3,259247603	19,27818301	0	0,170545735	0,117358674	7,048488553	6,560540235	6,479109167	8,320579592	51,23405257	5,692672508
ENSG00000139178	C1RL	3,06530782	6,462550715	9,267864316	22,69227636	0	2,099290286	3,729586089	0,663656492	3,172716287	51,15324837	5,683694263
ENSG00000163170	BOLA3	1,524878806	2,444002605	7,781994958	15,37409844	0	9,019227314	9,067191961	1,398323365	4,53726861	51,14698606	5,682998451
ENSG00000214113	LYRM4	10,41755602	5,537190513	0	12,21362478	0,076060053	7,941499594	6,683424441	4,059134791	4,192026818	51,12051701	5,680057445
ENSG00000135541	AHI1	7,995268289	4,842223834	6,586599999	5,912605245	9,62438318	4,083835221	3,983185143	4,332820416	3,719495615	51,08041694	5,675601882
ENSG00000176371	ZSCAN2	16,91397531	9,56472079	0,11397298	14,97781732	0	4,489585101	2,366394125	1,219315747	1,409659968	51,05544135	5,672826816
ENSG00000044446	PHKA2	0,033472272	4,751657992	28,01660096	5,635432501	0	5,953596949	2,056662879	1,354090043	3,252159765	51,05367336	5,672630374
ENSG00000163463	KRTCAP2	6,753765704	3,994797179	5,66713218	3,341822728	9,386239999	3,989485901	8,264120142	4,404642693	5,197485071	50,9994916	5,666610177
ENSG00000142627	EPHA2	19,26741881	0	23,69644408	0,063012551	0	7,892872159	0,030299525	0	0,044879624	50,99492675	5,666102973
ENSG00000172530	BANP	1,334309675	7,234638217	0,136365023	0,672017963	0,044041886	8,952741446	6,401233519	16,65545833	9,481500278	50,91230634	5,656922927
ENSG00000127554	GFER	2,797307861	5,857229917	0	5,603194568	0	13,82076259	10,54489647	6,850920319	5,435718349	50,91003007	5,656670008
ENSG00000177054	ZDHHC13	0,077902086	11,88823921	0	0,605339547	6,248339763	6,13691655	7,066701261	8,389690656	10,49114277	50,90427184	5,656030205
ENSG00000256771	ZNF253	22,75410137	11,90591892	0	0	0	0,045755813	3,667913732	0,273387673	12,24967425	50,89675176	5,65519464
ENSG00000163867	ZMYM6	0,063173315	5,072502301	4,474178015	0,245444688	0	8,635395819	10,2023326	15,74813574	6,429264096	50,87042657	5,652269619
ENSG00000132256	TRIM5	0,094365469	8,70735887	0,752235905	1,629485687	0	3,660807896	11,70081364	23,38629329	0,928458896	50,85981965	5,651091072
ENSG00000176225	RTTN	0,015640488	5,411963983	20,55755118	12,53157669	4,088695302	2,842022506	3,467430383	0,256508747	1,673513164	50,84490244	5,649433605
ENSG00000157873	TNFRSF14	2,429821043	5,217105967	0,04469852	0,881111829	20,73053509	4,401872513	6,980661661	4,835109446	5,289408979	50,81032505	5,645591672
ENSG00000143578	CREB3L4	0,052071182	5,081946935	0	10,96969914	0	8,804351762	15,04606324	0	10,82290223	50,77703449	5,641892721
ENSG00000185338	SOCS1	3,113079685	10,74956793	6,81020287	0,290573468	0,199954088	13,02527356	6,986098664	7,727311713	1,862604343	50,76466632	5,640518481
ENSG00000149091	DGKZ	6,55601623	2,361499027	10,97798481	8,442227323	1,897626765	4,245046739	6,606762638	3,09389926	6,581382359	50,76244515	5,640271684
ENSG00000168781	PPIP5K1	6,961378611	13,54822737	15,69769971	0,064627857	0	5,074911758	3,589306092	0	5,822807447	50,75895885	5,639884316
ENSG00000078804	TP53INP2	2,495005833	10,52292128	0	1,685868465	2,835816917	6,709472951	5,704561637	17,86987611	2,935120955	50,75864414	5,639849349
ENSG00000117155	SSX2IP	0,017491674	0	35,36290413	1,177967821	0,041569342	7,105789256	0	0,114747506	6,884001288	50,70447102	5,633830113
ENSG00000142453	CARM1	1,356772801	5,537388105	0	2,928563127	1,612199966	11,35863656	10,44882002	6,527102568	10,92969446	50,69917761	5,633241957
ENSG00000156535	CD109	1,220377743	0,959412186	0,284175117	21,44671024	3,010390357	3,35823903	9,940657054	1,114735302	9,347422711	50,68211974	5,631346638
ENSG00000105866	SP4	0,02427479	6,590478074	10,94058221	12,11408958	0,057689565	5,836854024	7,860791884	0,053081858	7,195021987	50,67286398	5,63031822
ENSG00000164074	ABHD18	0	17,6309262	0,124338795	7,275211011	2,248835065	9,078452609	8,755049258	0,344869833	5,181645991	50,63932877	5,626592085
ENSG00000182903	ZNF721	1,655358364	2,14010047	0	0,612523415	0	13,52729428	14,54984601	0,258556	17,88663712	50,63031566	5,625590629
ENSG00000130402	ACTN4	1,435847493	1,317866973	7,013259378	6,796962805	4,647818782	6,713616063	11,28492993	9,419334002	1,979034067	50,60866949	5,623185499
ENSG00000269343	ZNF587B	0,05689404	0,050478539	0,104661182	42,29386542	0	0,843295476	3,590265524	0,186615908	3,428644424	50,55472052	5,617191169
ENSG00000197841	ZNF181	8,585279789	2,56759017	0	2,706794342	0	6,195830665	3,243886656	18,19341299	9,046152137	50,53894675	5,615438528
ENSG00000273559	CWC25	13,30098428	5,341186543	5,163022027	0,280952866	0	7,011564076	4,154199418	7,560414062	7,704001192	50,51632447	5,612924941
ENSG00000013392	RWDD2A	18,32250751	10,81389865	0,073754338	0,900014505	0	6,867086751	7,856460162	0	5,6705625	50,50428441	5,611587157
ENSG00000162923	WDR26	7,695955631	0,195438776	0,075978526	7,41724782	12,23669468	10,00663281	9,327969289	2,137468503	1,388430651	50,48181669	5,609090744
ENSG00000196782	MAML3	15,40845094	1,108972904	4,400426553	10,0474106	0,153644128	2,48439968	2,916666303	8,152478634	5,777886745	50,45033649	5,605592944
ENSG00000136720	HS6ST1	7,473502289	4,474424207	12,23917203	6,452553654	0	4,769846591	5,398200854	2,790159195	6,837537912	50,43539583	5,60393287
ENSG00000151849	CENPJ	0,181841842	3,457220218	3,584065227	1,345719312	11,17419205	9,8114558	3,753109467	6,532572677	10,57507287	50,41524946	5,601694385
ENSG00000164292	RHOBTB3	0,051357393	0,637927042	12,59680974	4,25678445	2,847879004	10,05575604	4,065312672	11,9041795	3,979267329	50,39527317	5,599474797
ENSG00000168807	SNTB2	0	2,878283748	26,20754148	2,193174976	0,145465334	2,049730669	2,12187802	13,08353579	1,71261099	50,39222101	5,599135668
ENSG00000276368	HIST1H2AJ	0	0	0	44,5005383	0	3,000966504	0	0	2,881341431	50,38284624	5,598094026
ENSG00000132570	PCBD2	14,35034146	7,36633301	1,821930645	1,782988949	0,100158288	6,547579585	10,77809573	3,45594653	4,172540801	50,375915	5,597323889
ENSG00000173598	NUDT4	6,897287409	7,370047036	4,109846671	6,472885855	0	6,79907404	6,971957325	3,278771259	8,445892757	50,34576235	5,593973595
ENSG00000117877	CD3EAP	0,784239509	6,372126197	6,150338164	11,19005491	1,569483933	4,758429542	4,93518924	0	14,56923024	50,32909173	5,592121303
ENSG00000120832	MTERF2	0,124747307	12,54379197	0	16,51487181	0,098821558	8,263570276	4,868276503	1,363929145	6,494911483	50,27292005	5,585880006
ENSG00000115590	IL1R2	19,16349814	0,181360839	5,076403566	0	10,08006629	2,132093985	6,152586453	4,469862259	3,016784941	50,27265647	5,585850719
ENSG00000109458	GAB1	0,209066903	7,393182926	30,52039628	2,604450285	1,490556061	1,590406754	2,281504209	0,097964592	4,058907147	50,24643516	5,58293724
ENSG00000277224	HIST1H2BF	0,631733155	1,34519398	2,789097983	28,14432418	1,501327518	8,184551861	4,720873696	2,762830832	0,155390069	50,23532328	5,581702586
ENSG00000141252	VPS53	0,853200983	1,937386574	16,57319505	6,018022765	14,41693947	2,842049211	2,323413488	0,031622046	5,23774351	50,2335731	5,581508122
ENSG00000177034	MTX3	15,36103162	0,067721177	0,140411718	30,24855495	0	0,02095095	2,360787911	0,584175268	1,40810599	50,19173959	5,576859954
ENSG00000159450	TCHH	1,360967255	7,091675485	0	10,55676881	0	3,605204812	12,07169885	4,818345373	10,65396075	50,15862133	5,573180148
ENSG00000162444	RBP7	0,175302286	0	49,01737244	0	0	0,962358334	0	0	0	50,15503306	5,572781451
ENSG00000176715	ACSF3	2,780732214	4,347356275	6,750782264	0,481956787	0,024566804	8,773353112	7,441700496	9,629574891	9,916560909	50,14658375	5,571842639
ENSG00000114861	FOXP1	4,914365674	7,172071801	7,245926751	7,263592745	1,790012264	3,954125255	4,511108521	7,686199288	5,608703674	50,14610597	5,571789552
ENSG00000170946	DNAJC24	2,096311251	0,031259268	0,61571717	3,194004702	24,90964534	4,293792029	3,30570005	10,24663033	1,451587432	50,14464757	5,571627508
ENSG00000177688	SUMO4	0,141754757	20,87784804	0,521538647	2,447795259	0	10,89469078	4,590378	7,129451269	3,486801544	50,09025829	5,565584254
ENSG00000147324	MFHAS1	4,31241582	0,07808445	0	9,080293962	21,33371539	4,807251713	5,553724655	0,14433659	4,735460308	50,04528288	5,560586987
ENSG00000119938	PPP1R3C	38,35746481	0,211052342	0,109397914	0,308069628	0,141329218	6,13758135	0,098756656	0	4,680904142	50,04455606	5,560506229
ENSG00000258484	SPESP1	0	49,75123482	0	0	0	0	0,080275324	0,211410472	0	50,04292062	5,560324513
ENSG00000166860	ZBTB39	13,69349141	0	0,04434909	1,207261369	0	4,261583097	3,803344461	11,22887091	15,8034774	50,04237774	5,560264193
ENSG00000100478	AP4S1	14,70728661	3,600965528	0,038485408	1,589524973	4,971861309	4,639891526	2,605641672	10,97941246	6,895592963	50,02866245	5,558740272
ENSG00000025434	NR1H3	9,631882427	0	15,63408144	1,266115889	0,079205457	5,122955611	7,167361268	3,425325248	7,665041832	49,99196917	5,554663241
ENSG00000196220	SRGAP3	8,775656159	0,694301311	0,381921506	7,307944226	0,018976831	7,59239904	7,929746199	6,495543179	10,78310513	49,97959358	5,553288175
ENSG00000019995	ZRANB1	12,65768504	0	11,92072175	1,523898567	0	8,609742926	9,48407482	0,551367541	5,209780701	49,95727135	5,550807928
ENSG00000112425	EPM2A	14,39109641	4,335324812	0,01493152	14,54855797	0,057869293	5,810479455	1,604014557	5,857195428	3,324213993	49,94368343	5,549298159
ENSG00000169239	CA5B	0	3,870155599	25,27981421	14,05411328	0	1,713228203	1,958173452	0	3,053099404	49,92858415	5,547620461
ENSG00000198033	TUBA3C	0,099349488	37,37416438	0,913807031	1,029329288	8,26371728	0,109079932	0,247476	1,520737655	0,366561188	49,92422224	5,547135804
ENSG00000170396	ZNF804A	7,337083546	0,828512029	2,944834419	0,575888269	0,079257899	0,915419205	5,621381019	21,73239928	9,885016965	49,91979263	5,546643625
ENSG00000143322	ABL2	0	4,774521806	2,390956035	22,01035926	8,670409956	4,293596485	5,727293712	1,421063102	0,630986382	49,91918673	5,546576304
ENSG00000103037	SETD6	23,21115501	0	0	7,245338995	0	5,292932931	4,052212377	0	10,06455172	49,86619103	5,540687892
ENSG00000163625	WDFY3	1,497120628	4,756434387	7,470632703	7,709160972	8,692451191	5,866320818	5,776280116	7,664921446	0,429996106	49,86331837	5,540368708
ENSG00000068654	POLR1A	0,022355288	2,875996007	1,78890958	17,29401119	0,318766958	5,301676835	11,53632833	7,577092679	3,148077047	49,86321391	5,540357101
ENSG00000196584	XRCC2	0	0	0,57528994	44,44318793	0	0,892730752	1,687825244	0	2,192312038	49,79134591	5,532371768
ENSG00000276234	TADA2A	0	7,943891079	0	13,42400969	14,58895107	2,472323655	4,562962225	0	6,79162384	49,78376156	5,531529063
ENSG00000152086	TUBA3E	0	37,0947431	0	2,062727072	0,236572821	0,43718202	0,991860332	4,788906776	4,162570328	49,77456244	5,530506938
ENSG00000137507	LRRC32	0,101666677	0	0	0,05851872	49,61121464	0	0	0	0	49,77140004	5,53015556
ENSG00000180488	MIGA1	15,3229222	0,317703792	0,054893384	16,35996336	0	1,277747332	7,408299619	3,066829517	5,945327134	49,75368634	5,528187371
ENSG00000148120	AOPEP	0,014067266	6,71478291	5,615498183	18,80130984	11,03228346	2,39398058	1,039551252	1,199678657	2,923856104	49,73500826	5,526112029
ENSG00000166676	TVP23A	0	12,86821449	0,0481601	0	0,186651529	8,910651209	7,97775673	4,465331993	15,22962665	49,6863927	5,5207103
ENSG00000198520	ARMH1	11,62035695	0	1,520863763	0,872426967	0,109154268	3,782156004	1,105969492	17,67674282	12,93582176	49,62349203	5,513721337
ENSG00000183044	ABAT	0	1,242292804	22,46621823	0,564154686	3,068748689	2,86965684	8,926181346	1,054614598	9,375558109	49,56742531	5,507491701
ENSG00000152953	STK32B	0	0	0,578905924	0,889214226	0,067988933	17,74696627	5,938580651	11,07287362	13,26469877	49,55922839	5,506580932
ENSG00000158813	EDA	0,021581058	0	26,24176599	0	0	12,96102334	3,40465054	6,087697688	0,769715674	49,48643429	5,498492699
ENSG00000203814	HIST2H2BF	0	0	5,131672666	31,43303042	10,11282191	2,336037532	0,314068416	0	0,058149725	49,38578066	5,487308963
ENSG00000213096	ZNF254	0	3,774163905	2,523102714	22,20796219	0	4,779586464	3,350496895	9,693095048	2,982540873	49,31094809	5,478994232
ENSG00000116954	RRAGC	1,624527245	3,878519939	0,434683501	0,153011127	27,16545913	2,756528346	4,193789439	3,939896133	5,122040586	49,26845544	5,474272827
ENSG00000148288	GBGT1	0,037589694	0,20010597	10,8564367	0,519273581	0,178665444	8,501885274	8,302265749	15,9463448	4,715508774	49,25807599	5,473119554
ENSG00000167077	MEI1	5,174431306	2,70504149	11,53313284	4,856845932	0,204102042	9,547283826	3,226787453	2,910904119	9,083717485	49,24224649	5,471360721
ENSG00000182095	TNRC18	0,540882571	0,597198218	2,940769421	9,858742677	23,16610936	2,784531633	4,301440749	1,629569137	3,370426907	49,18967067	5,465518963
ENSG00000121289	CEP89	9,459754126	3,51404549	0,030107235	0	12,13524025	8,265879485	5,992896828	3,721996645	6,058681166	49,17860122	5,464289025
ENSG00000163666	HESX1	0	10,59636839	0	9,962018454	0	0	6,302930275	22,13225502	0,124478582	49,11805072	5,457561192
ENSG00000147206	NXF3	0,042638423	24,06014561	0	0	0	10,01830232	0	11,84119359	3,146385817	49,10866575	5,456518417
ENSG00000187994	RINL	7,014177881	8,935939065	0	6,583934137	0	6,160925962	9,646969111	2,123716573	8,626534597	49,09219733	5,454688592

ENSG00000108839	ALOX12	4,618851405	0	0	0,098466028	38,21552715	2,754741651	0,852251312	1,371615186	1,12209221	49,03354494	5,44817166
ENSG00000150637	CD226	14,62538798	0,863770994	0,020351424	2,368836434	17,69427528	1,858431674	7,954997255	0,09676684	3,537601273	49,02041915	5,446713239
ENSG00000087510	TFAP2C	0	31,18345819	0	10,60781969	0	0,165855292	0,752571135	0,220216306	6,068963406	48,99888402	5,444320447
ENSG00000137992	DBT	9,465299835	6,784801559	2,188819677	10,59622696	0,031771878	8,997892906	7,237605633	0	3,683059452	48,9854779	5,442830878
ENSG00000166349	RAG1	0,019335966	0	46,0276709	0,100166977	0,275714044	2,207895071	0,337156362	0	0	48,96793932	5,440882147
ENSG00000185090	MANEAL	8,511861171	12,17211863	0	0	0	1,759157871	8,10693	10,94879283	7,451103113	48,94996361	5,438884845
ENSG00000197136	PCNX3	12,79473598	1,244512544	8,333823841	6,088046322	0,225236793	10,07284485	4,831835414	0,082898788	5,268604369	48,94253889	5,438059877
ENSG00000003393	ALS2	5,399908302	4,726779844	0,479367866	15,84908736	0,068809646	3,512764653	0,120205338	11,45979244	7,246554375	48,86326982	5,429252203
ENSG00000006530	AGK	5,183655771	6,140466718	0,025772307	3,628800871	16,94704516	7,229554149	3,501443532	0,30635506	5,841087618	48,80418118	5,422686798
ENSG00000116095	PLEKHA3	9,765540575	4,926790709	1,976486448	5,382185678	4,778121472	4,508376108	5,0435452	6,676150713	5,717348133	48,77454504	5,419393893
ENSG00000139187	KLRG1	0	0	0,107204049	22,03808694	0	12,92477304	6,677557199	0	7,023894787	48,77151602	5,419057335
ENSG00000106367	AP1S1	7,826798245	5,839466747	0	11,97935728	0	11,5229241	8,418845114	0,19448768	2,953417364	48,73529653	5,415032948
ENSG00000112294	ALDH5A1	0,054700659	0	31,29477313	2,644770783	0	2,492412284	11,96793936	0,119614323	0,134549518	48,70876006	5,412084451
ENSG00000173272	MZT2A	3,900346588	5,866430189	11,07000394	2,950592142	3,178031348	5,669019214	5,706000841	4,792438418	5,482198297	48,61506098	5,401673442
ENSG00000121690	DEPDC7	23,11800221	0	0	0	0,108363773	2,453113536	12,72119238	0,498543403	9,701704391	48,6009197	5,400102188
ENSG00000116704	SLC35D1	0	13,29236138	0,089772356	0,294936997	0,347925925	7,072564549	18,43662233	0	9,032747167	48,56693071	5,396325634
ENSG00000108187	PBLD	0	15,08687588	1,021195259	8,021782862	0	4,073983612	6,695632566	5,941688859	7,725638494	48,56679753	5,396310836
ENSG00000140263	SORD	4,422506014	2,909794805	5,164707528	9,05249583	4,900814866	7,09252712	6,910976853	4,45505336	3,636260465	48,54513684	5,393904094
ENSG00000102931	ARL2BP	1,894565831	3,630809429	4,600473052	3,66408189	11,16614224	6,489982439	5,285610776	5,302861665	6,430998012	48,46552534	5,385058371
ENSG00000177721	ANXA2R	0	3,371441745	0,116504652	11,48288866	0	9,665368886	11,83186282	0	11,9951234	48,46319016	5,384798907
ENSG00000139517	LNX2	32,57586134	0	0	0,108384205	0,59666466	6,029979423	4,898945465	0,068626078	4,168520126	48,4469813	5,382997922
ENSG00000167173	C15orf39	4,163542501	2,955241606	1,866923344	12,80634885	8,225010011	3,599919522	3,997246671	6,202405349	4,6085585	48,42519635	5,380577372
ENSG00000197620	CXorf40A	7,222413151	2,197027703	0	7,533822911	17,72468557	1,456491602	4,503812151	3,867747571	3,915611472	48,42161213	5,380179125
ENSG00000147601	TERF1	6,364711536	4,159358471	9,158991033	5,524778108	3,537502965	4,37694343	6,946896779	2,993066735	5,344770908	48,40701996	5,378557774
ENSG00000010361	FUZ	2,817315724	4,908362036	8,480748782	3,980364019	0	7,367554423	7,400622972	10,86513663	2,582951665	48,40305625	5,378117361
ENSG00000132017	DCAF15	12,75840684	3,078969268	0	10,83924413	0,12128252	7,284152304	2,796704462	5,244993135	6,276478294	48,40023096	5,37780344
ENSG00000173207	CKS1B	0,129154334	5,385758121	11,64190158	11,59710998	0	6,66478385	5,4155998	2,824227073	4,685873852	48,34440859	5,371600955
ENSG00000097021	ACOT7	9,013870122	0	0	1,265446046	0	10,01739818	11,37873525	0	16,62884741	48,30429301	5,367143667
ENSG00000165699	TSC1	4,954022686	6,535961252	0,694950247	6,686458724	0,612132174	8,653744853	2,238504787	14,98220369	2,903851911	48,26183032	5,362425591
ENSG00000100211	CBY1	0	5,481608179	0	19,89240224	0	8,055283537	7,267466828	0	7,519343959	48,21608474	5,357342749
ENSG00000158480	SPATA2	0,0352157	5,311598697	9,976447272	8,026897378	0	2,513210787	1,637459471	14,47720781	6,236751155	48,21478827	5,357198696
ENSG00000197948	FCHSD1	3,907173846	5,299249014	3,479329302	4,984923757	0	7,404770469	9,38134564	2,829808549	10,92674711	48,21334769	5,357038632
ENSG00000126217	MCF2L	0,026100798	1,296826324	4,981507085	6,411261791	10,01770154	6,025166034	6,306566476	0,014268701	13,12915236	48,20855112	5,35650568
ENSG00000196371	FUT4	0,249398532	8,054438689	0	14,59926661	6,69752142	2,245365125	6,336676282	0,327216934	9,692606212	48,2024898	5,3558322
ENSG00000118600	RXYLT1	0,83261847	5,978563303	4,025083442	11,83636353	0	8,822769007	5,94874716	5,038661396	5,715423545	48,19822986	5,355358873
ENSG00000172264	MACROD2	4,046187839	13,30386017	8,173023561	5,114581231	3,865195809	1,553415445	4,325805986	1,966183986	5,821933407	48,17018744	5,352243049
ENSG00000148737	TCF7L2	5,538553339	4,018747887	0,082498916	10,72547677	0,213157764	5,514760229	5,12009813	10,54213266	6,398040238	48,15346594	5,350385104
ENSG00000117834	SLC5A9	0,033320964	0	0,061296604	0,115076064	47,90873498	0	0,027667107	0	0	48,14609572	5,349566191
ENSG00000132953	XPO4	0,014391483	0	0	6,113333691	2,565125303	5,719964644	9,511846121	7,363970362	16,85008469	48,13871629	5,348746254
ENSG00000066651	TRMT11	0,026408698	4,076958058	0	0,228010289	0,062760843	3,189472813	3,15758962	26,91060966	10,42585349	48,07766347	5,341962607
ENSG00000102858	MGRN1	0	2,343116152	6,468461803	4,970189992	19,81581333	3,274229349	5,358821249	3,179439138	2,627579696	48,03765071	5,337516746
ENSG00000000460	C1orf112	0	2,739809381	0	0,874154019	0	9,726750728	2,206764633	32,48996501	0	48,03744377	5,337493753
ENSG00000163001	CFAP36	0	15,53005022	0	3,573431892	0	20,25955116	4,653678505	0,565651185	3,446511825	48,02887479	5,336541643
ENSG00000139737	SLAIN1	0	0	6,593946534	37,68386494	0	2,315031303	0,379328911	0,230535745	0,821182308	48,02388974	5,335987749
ENSG00000276903	HIST1H2AL	25,37734775	13,64590331	1,414656992	0	0,913785095	6,33246476	0,319262931	0	0	48,00342084	5,333713426
ENSG00000119403	PHF19	6,920744971	7,766927816	0	4,629874436	0	6,64246167	9,190502026	5,512253932	7,327888352	47,9906532	5,3322948
ENSG00000154114	TBCEL	0	4,430836469	34,41092678	0,037170224	0	1,867084838	3,932118667	0	3,309232946	47,98736993	5,331929992
ENSG00000091651	ORC6	0,070137188	10,88996287	0,193534532	23,79843236	0,250023817	3,080261345	3,814484449	0	5,865659557	47,96249611	5,329166235
ENSG00000162367	TAL1	0,026510626	0,376339649	2,584727236	9,292947341	0,063003078	21,71391056	3,301850842	0,115941954	10,46609362	47,94132491	5,326813879
ENSG00000117597	UTP25	0,014514511	10,43103545	0,026700615	4,461283784	0	6,27881733	12,88327585	0,095216953	13,74525234	47,93609684	5,326232982
ENSG00000167920	TMEM99	0	6,924920409	10,59195974	0	0,152039603	12,01130763	6,055723052	0	12,19568626	47,93163669	5,325737411
ENSG00000167874	TMEM88	0	0	0	0	44,32536523	0,189612088	0	3,398755552	0	47,91373287	5,323748096
ENSG00000197714	ZNF460	0,03773048	0	0	0	0	6,296731001	9,116570939	20,6263775	11,83292464	47,91033456	5,323370506
ENSG00000145908	ZNF300	0	0	0	0	0	2,670517104	0	22,3985345	22,83582296	47,90487456	5,322763841
ENSG00000177602	HASPIN	0,054025946	0	0	10,26201187	0	12,39732232	0,089717792	16,30317137	8,770733264	47,87698256	5,319664729
ENSG00000176542	USF3	0,27498648	0,683139467	0,080937574	24,74874595	8,103540408	2,42742946	4,40214733	3,559784248	3,503730475	47,7844414	5,309382377
ENSG00000168016	TRANK1	0,049650261	6,211273536	0	8,294877211	19,85264547	0,449732958	6,864074348	0,92284995	5,129327377	47,77443112	5,308270124
ENSG00000234545	FAM133B	8,33106239	5,769079339	4,547859082	12,0467514	0,804835371	2,714359502	2,755735665	6,590918088	4,206745574	47,76734641	5,307482935
ENSG00000068878	PSME4	0,174492576	0,619265589	0,080248296	27,64524702	0,034557115	9,818610919	5,143414303	0,508752221	3,737675438	47,76226348	5,306918164
ENSG00000130299	GTPBP3	0	5,86264532	0	0	0,079310411	9,123616078	7,897315508	11,96803646	12,8056791	47,73660287	5,304066985
ENSG00000121406	ZNF549	0,048855665	0,202284082	0,059915961	22,04688222	0	0,447005541	3,840238634	11,32430138	9,733959459	47,70344294	5,300382548
ENSG00000103429	BFAR	10,15632307	4,693267213	0,324364176	0,365369859	15,3368752	4,336519158	3,015972975	5,78356886	3,686570453	47,69883097	5,299870107
ENSG00000148384	INPP5E	17,7749842	0,658825553	4,140674079	7,693414485	0,110294085	3,286619225	0,655096942	3,09528695	10,27407064	47,68926616	5,298807352
ENSG00000147475	ERLIN2	1,669198019	15,86352566	2,125817008	6,346692425	0	8,123714908	4,184565507	3,860626917	5,487555304	47,66169574	5,295743972
ENSG00000141956	PRDM15	8,320145232	0,020619963	1,068825358	2,167100681	24,52294506	2,436859439	4,660271482	0	4,373195618	47,56996284	5,285551426
ENSG00000185418	TARSL2	3,290174041	5,055876096	4,243018346	4,509987215	4,836599897	11,47036086	7,209967451	0,741716201	6,17402588	47,53172599	5,281302887
ENSG00000152443	ZNF776	5,652100553	0,098328566	0,050968115	18,75440228	0,131689601	2,889898201	11,20352983	0,060585729	8,655118566	47,49662144	5,277402382
ENSG00000101882	NKAP	3,800316651	3,678310175	0,3883883	4,77259448	0	4,720433022	5,099772163	13,30468156	11,73196006	47,49645641	5,277384046
ENSG00000197386	HTT	10,7859788	3,264408063	0,056168943	4,587056535	4,952469643	2,40535136	7,99242719	7,678313854	5,76428393	47,48645832	5,276273147
ENSG00000177706	FAM20C	1,375363326	7,58692244	2,200079929	6,711821666	2,131683077	4,33324023	12,33849525	1,111473615	9,671079414	47,46015895	5,273350994
ENSG00000162757	C1orf74	10,16221814	0	27,4181908	0,111415446	0	1,806455895	6,48245524	0,634908435	0,833214553	47,44885851	5,27209539
ENSG00000213889	PPM1N	5,583626687	10,22089304	2,378670625	3,349223711	4,050722959	3,032982149	9,174804518	2,827522554	6,794855346	47,41330159	5,268144621
ENSG00000163820	FYCO1	0,03397652	9,164156046	0,062502552	6,277693599	0	4,439203739	11,07298602	0,037148349	16,27593489	47,36360171	5,262622412
ENSG00000142544	CTU1	10,16406943	4,162128882	0	4,418466174	0	7,725845293	7,672221618	5,59531723	7,605196349	47,34324497	5,260360553
ENSG00000089902	RCOR1	3,924633308	3,287432168	0	18,39467396	0	4,362546218	2,489571033	9,061764233	5,786155638	47,30677656	5,256308507
ENSG00000007038	PRSS21	0	9,678740217	3,533046505	0	0	0	3,125595459	29,7340714	1,22826883	47,29972241	5,255524713
ENSG00000151923	TIAL1	3,143428594	3,90091013	3,59049754	9,614327412	0,048826314	6,744699175	9,024310758	4,85206435	6,317012994	47,23607727	5,24845303
ENSG00000175782	SLC35E3	4,78247226	2,74599928	10,17105035	11,66902496	0,277210948	3,440817333	3,209075494	7,754125298	3,175228767	47,22500468	5,247222743
ENSG00000151470	C4orf33	12,06299638	7,797714614	0,079253057	0,892721225	0	9,294776428	3,291025102	7,01849616	6,782139658	47,21912262	5,24656918
ENSG00000103852	TTC23	6,759174843	11,51423032	0	0,042442254	0	2,158888441	13,95928873	4,890947567	7,889710851	47,214683	5,246075889
ENSG00000156875	MFSD14A	0	3,824096436	6,79611787	4,961751639	9,877256683	4,844926858	4,004825538	6,058902087	6,752318466	47,12019558	5,235577286
ENSG00000087448	KLHL42	11,30244985	12,28778194	0,073210455	2,370886654	0	3,58300429	9,797722791	0,174050338	7,513152797	47,10225911	5,233584345
ENSG00000171408	PDE7B	14,21828127	0	5,427653843	1,185868425	7,011889398	0,949496652	0,887014635	9,344051059	8,07076296	47,09501824	5,232779804
ENSG00000198315	ZKSCAN8	19,31743311	0	2,310388685	4,509562226	0,094753969	7,22301983	4,998949903	0,130778853	8,507749766	47,09263634	5,232515149
ENSG00000160588	MPZL3	0,682899893	0	0	0,196536511	0,991788342	8,247625957	2,961137637	27,04536003	6,952330359	47,07767873	5,230853192
ENSG00000116273	PHF13	0,166421333	0	0,153072742	3,520323171	0	13,61270701	13,09285678	6,641443675	9,824485759	47,01131047	5,223478941
ENSG00000164983	TMEM65	0,016736136	9,32512881	12,13026459	5,664332154	0	3,270803262	3,182268914	9,917794639	3,499159241	47,00648775	5,222943083
ENSG00000137960	GIPC2	0,077413202	14,35493802	0,996854863	0,401027113	0	0	3,053197116	28,10048064	0	46,98391095	5,22043455
ENSG00000173588	CEP83	0,021821021	12,23579379	0	1,0173638	0,051858129	12,38639251	11,75888021	0,047716183	9,392965025	46,91279068	5,212532298
ENSG00000198959	TGM2	18,36598028	20,27596151	0,279798791	0,22981188	0,09036677	0,584486179	1,910154778	1,621407666	3,53080333	46,88877118	5,209863464
ENSG00000007541	PIGQ	4,282485808	8,956159922	0	7,923126759	0	7,304767294	7,397415829	5,309361092	5,690138966	46,86345567	5,20705063
ENSG00000114933	INO80D	6,949425117	5,466193977	0,638234416	22,92916391	1,099364911	2,05469162	1,09992777	0,873618143	5,728101953	46,83872182	5,204302424
ENSG00000165912	PACSIN3	10,80536567	3,795839566	24,51875283	0,094713239	0	1,023767624	0	6,596697355	0	46,83513629	5,203904032
ENSG00000138207	RBP4	11,60794507	0,183910837	0	1,07380648	0,492616656	0	0	31,0490643	2,42186826	46,8292116	5,203245734
ENSG00000147789	ZNF7	0,029353258	5,417010103	13,17543593	8,084523786	0,139517305	6,848484372	2,120419364	7,445689556	3,519820498	46,78025417	5,197806019
ENSG00000169871	TRIM56	11,86511893	0,746779445	22,57604972	0,281306234	1,968032529	3,085391194	0,450886109	0,623405437	5,175853194	46,77282279	5,19698031
ENSG00000205978	NYNRIN	12,58440547	0,485450408	0,035947252	19,26727926	0	3,604419615	1,833460173	5,68314981	3,268476274	46,76258826	5,19584314
ENSG00000006634	DBF4	12,82548913	10,91241654	0,725954557	6,927979739	0,468923886	3,466249263	3,631676639	1,653970632	6,147697799	46,76035819	5,195595354
ENSG00000072571	HMMR	0	21,27678129	0	0	0,09133203	17,64739264	5,105608627	0,084037276	2,552317447	46,74756931	5,194174368
ENSG00000275342	PRAG1	0,493019806	1,038886216	5,158276714	4,043853603	18,30737879	2,706534693	4,861207806	4,784024122	5,343466485	46,73664824	5,192960915
ENSG00000164251	F2RL1	0	0	0	9,261855754	0,245131428	0	0	27,96852312	9,26061169	46,73612199	5,192902444
ENSG00000144554	FANCD2	10,69569926	0,192032943	0	5,637291764	3,600605581	3,940821767	13,70319334	0	8,96180413	46,73144878	5,192383198
ENSG00000100427	MLC1	0	0	0,064691668	23,25764377	0,16714803	1,351377748	10,74542289	0	10,98557521	46,57185931	5,174651035
ENSG00000186448	ZNF197	0,907230443	3,219716324	9,38769565	7,049650346	0,044917766	10,39664777	7,4858472	1,405225178	6,648168276	46,54509895	5,171677662
ENSG00000141753	IGFBP4	0,068530871	0	0	5,561879277	0,162865099	6,771860274	5,80406978	28,17310598	0	46,54231128	5,17136792
ENSG00000188375	H3F3C	0,542587327	9,868780973	10,97946531	2,108092254	5,802617381	0,89359377	2,590500571	10,08507657	3,670218573	46,54093273	5,171214747
ENSG00000196323	ZBTB44	4,314705381	1,555573605	4,863139875	14,97206662	0,130209406	2,255854469	6,323559154	2,186523153	9,939217562	46,54084922	5,171205469
ENSG00000197183	NOL4L	7,773045866	2,043419137	3,589497468	10,10819228	5,701485114	6,550025943	4,780833988	1,049220684	4,878274505	46,47399499	5,163777221
ENSG00000167193	CRK	6,246696819	1,38557628	0	0,706270847	13,34319609	8,36901285	9,107713568	1,870077523	5,396134259	46,42467823	5,158297581
ENSG00000162585	FAAP20	3,280799308	6,902519423	2,979449577	6,071590651	0	8,815329286	7,172360495	4,177351623	7,014361229	46,41376159	5,157084621
ENSG00000198482	ZNF808	0,120695344	1,338568291	0	3,595150409	0	5,963239578	7,76673381	17,22113062	10,39078	46,39629805	5,155144227
ENSG00000148019	CEP78	0,157900283	1,569064995	19,190406	2,562997661	0,12508448	2,681383058	5,322983782	2,90036611	11,84601657	46,35620294	5,150689215
ENSG00000091831	ESR1	3,588521564	0,464516241	24,21841919	1,7139514	0	2,766373843	2,834720219	10,58993417	0,160975766	46,33741239	5,148601377
ENSG00000189143	CLDN4	0,03213751	0	0,118239089	0,055494465	44,52690923	0	0	1,5460571	0	46,27883739	5,142093044
ENSG00000130052	STARD8	11,41893481	0,289465995	0,050014411	9,107841863	0,064612728	10,23881393	3,137887036	6,836987273	5,082524539	46,22708258	5,136342509
ENSG00000188372	ZP3	0,1130644	12,43906024	0	9,469020777	0	4,158626502	7,932841791	9,889549346	2,224874191	46,22703724	5,136337471
ENSG00000219626	FAM228B	1,257465678	5,496720521	0,282098741	7,20258911	8,673632543	4,175545263	2,750313752	11,80362977	4,56412202	46,2061174	5,134013044

ENSG00000170325	PRDM10	0	14,00198189	0,251717566	2,441588956	0	1,377162952	10,20653621	0,94751838	16,94103523	46,16754119	5,129726799
ENSG00000176473	WDR25	1,110580296	12,96846089	0,065903295	4,639668823	0,085139294	8,535465895	7,91254346	6,580489254	4,229792826	46,12804403	5,125338225
ENSG00000141456	PELP1	2,090780284	2,853875628	0,972106169	2,935865611	12,44926253	2,850523821	9,653031472	4,471442376	7,827198825	46,10408672	5,122676302
ENSG00000214530	STARD10	7,861008123	9,901739626	0,149854501	2,03965344	0,096797181	7,334052447	5,88459239	12,46922896	0,350653816	46,08758049	5,120842276
ENSG00000174004	NRROS	1,562135465	1,385985528	0,766313161	6,923498637	0,123748292	2,000989037	2,377969766	26,18881824	4,674981436	46,00443956	5,111604395
ENSG00000166971	AKTIP	0,029087694	18,1170052	0	10,34698841	0,069127535	5,493093204	6,569380232	0,127212538	5,223020645	45,97491546	5,10832394
ENSG00000077684	JADE1	0,033387223	5,332032702	2,610285949	1,643094736	0,277709103	10,41065042	4,366234487	11,27974827	9,998592173	45,95173506	5,10574834
ENSG00000121594	CD80	0,04703099	0,500732026	0	0,081212255	0,223540331	9,862720246	2,421151754	27,66476771	5,147942883	45,9490982	5,105455355
ENSG00000159917	ZNF235	0	0,402951745	0	0,043569039	0	5,56821271	5,426083818	20,19353009	14,30543442	45,93978183	5,104420203
ENSG00000117399	CDC20	0	0	4,973798988	30,47699753	0	1,236907878	4,614714	0	4,618452343	45,92087074	5,102318971
ENSG00000175592	FOSL1	0,160159587	0,284199258	0	3,180447491	11,22837043	7,737218203	2,992138283	5,953775991	14,37922218	45,91553142	5,101725714
ENSG00000163735	CXCL5	0	0,110074713	45,53122311	0,107116164	0	0	0	0	0,076291676	45,82470566	5,091633962
ENSG00000138030	KHK	0	11,16525705	0,182282032	0,769972056	0	8,377124201	11,97110381	5,308620921	8,043193751	45,81755382	5,090839313
ENSG00000119979	FAM45A	7,998699735	2,898371643	2,739590866	3,940362834	4,395493683	3,771294304	4,228216561	8,45651969	7,326329284	45,7548786	5,0838754
ENSG00000071539	TRIP13	0	11,79533956	0	2,594412221	0	8,098075754	4,234009396	19,01566809	0	45,73750503	5,081945003
ENSG00000157193	LRP8	9,997474177	0,078381175	11,07803376	0,940718509	0,034991478	8,713417212	9,156901361	0,257573472	5,468737185	45,72622833	5,080692037
ENSG00000203485	INF2	0,031852987	1,582625633	5,742421944	1,760101009	22,02850017	3,32240826	3,768870639	1,149277058	6,307185937	45,69324364	5,077027071
ENSG00000168148	HIST3H3	0,942477572	27,87338878	12,13634528	0	0	1,724642169	0,260853081	2,747896611	0	45,68560349	5,076178166
ENSG00000180884	ZNF792	0,273875071	12,08886679	0	0	0,081358754	8,833027985	6,850555055	0	17,55731758	45,68500123	5,076111248
ENSG00000105327	BBC3	8,173276149	4,890640253	1,573472258	3,036035868	13,89039551	5,478179419	3,511591053	2,078204827	3,038999735	45,67079507	5,074532786
ENSG00000113240	CLK4	0,228897608	4,874078265	0	5,467710904	0,045331677	10,01076189	0,886939989	7,090870548	17,05508984	45,65968071	5,073297857
ENSG00000146143	PRIM2	0,064743175	9,995010286	0	15,03674125	0,076931779	10,34275446	4,918824069	0	5,215487236	45,65049226	5,072276918
ENSG00000109685	NSD2	7,819878575	0	7,76104381	8,2336729	0,072523359	1,499368568	6,657712876	6,906644669	6,671221151	45,62206591	5,069118434
ENSG00000157881	PANK4	5,412651092	4,776066159	7,998252995	0	0	11,4958591	8,202601466	2,263693455	5,456445164	45,60556943	5,067285493
ENSG00000131634	TMEM204	0	6,58690686	0	24,43761533	0	6,877562289	4,173782794	0	3,519099961	45,59496723	5,06610747
ENSG00000139645	ANKRD52	2,640534197	9,857938049	1,040887696	13,76770262	0	4,216948733	3,744320585	1,237301796	9,088829127	45,5944628	5,066051423
ENSG00000149016	TUT1	2,998519408	4,007021559	0	16,81183202	0	6,42182046	3,596283401	4,776001833	6,920301662	45,53178035	5,059086705
ENSG00000182141	ZNF708	0,139755441	0,123996301	25,00214069	4,162893236	0	6,137732317	6,962515533	0	2,964950298	45,49398382	5,054887091
ENSG00000160305	DIP2A	0,043933877	8,965352525	0,626354344	13,92108383	0,052204905	4,498084395	8,827975096	0,744546561	7,794261037	45,47379657	5,052644064
ENSG00000169495	HTRA4	9,890603296	0	0	0	4,41827685	11,1042512	3,457843795	16,58677455	0	45,45774969	5,050861077
ENSG00000177225	GATD1	0	4,866460982	16,59808576	7,434989294	0	5,23928137	2,778108984	0	8,533424707	45,4503511	5,050039011
ENSG00000167619	TMEM145	0	0	0	0	45,26124029	0	0,04720479	0	0,13983937	45,44828445	5,049809383
ENSG00000138688	KIAA1109	0,103348705	4,139368443	2,675235931	14,77399294	0,824549316	1,864163783	5,099722336	9,249585109	6,70935906	45,43932563	5,048813959
ENSG00000101460	MAP1LC3A	6,296273778	9,77601042	5,308647724	0,226506054	0	6,480881902	10,56477622	0	6,775654458	45,42875055	5,047638951
ENSG00000196466	ZNF799	0	11,93510386	0,109982235	0,516191839	0	4,430846262	3,524586972	14,6424022	10,2206396	45,37975297	5,042194775
ENSG00000159259	CHAF1B	0,236850424	8,405705332	0	5,214609602	0,14072004	8,028979711	10,49683224	0	12,81700154	45,34069889	5,037855432
ENSG00000113119	TMCO6	21,70434524	3,904980584	0	0	0,102546414	5,921993743	7,237294993	1,415339381	5,041524733	45,32802509	5,036447232
ENSG00000196284	SUPT3H	0	6,445721156	18,01524872	0,150539408	0	6,955481869	7,43170428	0,571905982	5,754094247	45,32469567	5,036077296
ENSG00000169972	PUSL1	15,48591452	2,398993087	0,226091988	0,424457055	1,898547398	9,715788144	5,663768545	0,13437762	9,371683108	45,31962147	5,035513496
ENSG00000197892	KIF13B	0,217562236	1,302948619	3,952205133	11,64615742	5,17041364	3,821929959	6,966183285	5,708938807	6,50538083	45,29171993	5,032413325
ENSG00000119782	FKBP1B	7,11850631	4,477028028	0	14,47045476	0	9,398631358	5,835829395	0	3,989549673	45,28999953	5,03222217
ENSG00000086289	EPDR1	0	8,391494247	0	36,88757015	0	0	0	0	0	45,2790644	5,031007155
ENSG00000144115	THNSL2	19,502686	3,293922875	1,858675829	3,043091758	0,111683266	4,901727503	5,228743427	1,130393482	6,068693777	45,13961791	5,015513102
ENSG00000103168	TAF1C	0	0,048079971	0	5,053071052	21,76469954	3,24264889	1,282375636	1,599737013	12,12984295	45,12045505	5,013383894
ENSG00000140650	PMM2	2,375018792	1,510164506	5,898202493	12,33759343	0	5,236993973	5,242309877	8,525947584	3,991988167	45,11821882	5,013135425
ENSG00000142207	URB1	0,013200889	1,030684721	4,783967808	15,66020159	11,01164125	5,855496898	3,441747554	0,11546591	3,182136662	45,09454327	5,010504808
ENSG00000180263	FGD6	0	9,63752111	0	0,129895945	0	5,500619479	6,096129233	17,50210971	6,180081485	45,04635696	5,005150774
ENSG00000167775	CD320	5,03922921	0	14,63695124	8,129172092	0	3,931185231	4,019014584	3,334793792	5,952960125	45,04330628	5,004811808
ENSG00000145743	FBXL17	0,018598224	0,660041846	17,10646763	4,945721365	0	5,125360585	3,227483002	10,73658164	3,202278538	45,02253283	5,002503648
ENSG00000160055	TMEM234	1,819589622	17,04515094	12,14362796	0	0	5,900488166	4,075765475	0	4,0073319	44,99195407	4,999106007
ENSG00000156802	ATAD2	0,085132716	1,397359719	5,520449853	20,13976987	0,354059549	9,066662502	5,937750923	0	2,486678819	44,98786395	4,99865155
ENSG00000137177	KIF13A	1,383846131	7,174206862	3,893418517	3,959239604	0	2,785534014	7,874282822	10,76923577	7,141525546	44,98128927	4,997921029
ENSG00000183077	AFMID	0,037329686	0,662406127	0	3,86761326	6,29875136	17,70587182	5,734192544	4,244712824	6,381587598	44,93246522	4,992496136
ENSG00000179981	TSHZ1	4,835684244	0,55046661	3,22256847	2,331745939	0,260198678	4,868526765	5,77275649	16,4798305	6,553216339	44,87499404	4,986110449
ENSG00000178149	DALRD3	0	2,327874119	0,075415111	16,77742512	0,09742744	4,366067808	4,186883027	11,74360509	5,24363492	44,81833263	4,979814736
ENSG00000103495	MAZ	7,05641617	3,359410024	5,223999582	1,411664156	2,965378339	6,613747363	6,966604425	3,857578949	7,35554288	44,81034189	4,978926877
ENSG00000161671	EMC10	4,260002132	6,28691701	1,163854155	1,165321807	15,40316217	5,557109008	2,393135287	3,320332461	5,239253466	44,7890875	4,976565277
ENSG00000126870	WDR60	0,022597663	8,581185587	0,041570226	4,838632698	5,316679631	3,597582417	6,886139053	5,583835222	9,894027505	44,76225	4,973583333
ENSG00000120800	UTP20	0	2,994207778	0	14,51662172	0	10,35493729	4,866197323	0,889504818	11,13596808	44,75743701	4,973048557
ENSG00000171827	ZNF570	28,45117373	6,310738715	0	0,083552666	0,172486812	5,604728095	4,057798121	0,052903389	0	44,73338153	4,970375725
ENSG00000177888	ZBTB41	0,38827757	8,393138989	0,551934324	15,8779633	0,041943184	2,906632388	4,235099971	1,389353472	10,91810333	44,70244653	4,966938503
ENSG00000166484	MAPK7	8,491972023	9,805038494	0,053498864	9,039317827	0	3,608139948	4,467284723	3,179701381	6,044661714	44,68961497	4,965512775
ENSG00000185507	IRF7	3,392512812	3,673178083	0,105776293	1,390064553	9,292234734	6,376319729	8,020934466	5,406653883	7,00106762	44,65874217	4,962082464
ENSG00000167106	FAM102A	0,045979179	0	0	25,367024	0	3,155153216	11,03329559	2,614117984	2,40330966	44,61887963	4,957653292
ENSG00000123066	MED13L	1,540871626	4,104946581	4,798660846	11,9349607	1,864597024	3,694401023	4,499792444	8,817130495	3,357413621	44,61277436	4,956974929
ENSG00000104221	BRF2	8,322031428	0	1,510761408	19,19195647	0	6,492153001	6,455357661	0	2,626092163	44,59835213	4,955372458
ENSG00000178700	DHFR2	0	15,25169136	0	0,224026593	0	16,45217567	5,817040505	4,397283363	2,433277068	44,57549455	4,952832728
ENSG00000181090	EHMT1	7,253143941	2,586190194	1,390652199	2,375557277	11,66953975	4,800549407	4,263774578	1,325431608	8,90368006	44,56851902	4,952057668
ENSG00000241343	RPL36A	0,266845105	2,976349143	0,210378481	0,592435613	31,07399521	0,209271666	2,184019402	3,751149228	3,281850797	44,54629465	4,949588294
ENSG00000166165	CKB	0	5,063529167	0	3,284955618	0	2,576034972	14,79528464	2,911928627	15,90964773	44,54138075	4,949042306
ENSG00000172183	ISG20	2,594082371	4,565711655	3,685709617	4,588668081	10,87627449	3,102863542	8,7207572	2,674836694	3,683210909	44,49211456	4,943568284
ENSG00000166435	XRRA1	0,066524574	9,355163331	20,92652297	0,028718355	0	3,359844388	2,817075471	4,109520815	3,804474262	44,46784417	4,940871574
ENSG00000129473	BCL2L2	0	0,768131774	28,26916261	3,301397387	0	0	5,960514698	0,709933423	5,456940138	44,46608003	4,940675559
ENSG00000142920	AZIN2	3,26478323	5,401461853	1,827862043	10,10404713	0	9,091227718	2,370329829	0,68977052	11,71603167	44,465514	4,940612667
ENSG00000157600	TMEM164	1,744554667	7,942836096	23,60487245	2,972827449	0,054552262	2,117042145	4,517160669	0,150585402	1,270407641	44,37483878	4,930537642
ENSG00000101350	KIF3B	0,098829673	0	0,090902583	3,711795747	0,058717715	3,960586036	8,123978331	13,66905585	14,64650486	44,3603708	4,928930089
ENSG00000068383	INPP5A	0,484976424	0	23,49337023	0	0	0	5,30203596	13,78651846	1,292326713	44,35922779	4,928803087
ENSG00000135898	GPR55	0,036447316	0	0	0,188809678	39,67096826	1,920817096	2,542090663	0	0	44,35913301	4,928792557
ENSG00000064651	SLC12A2	0,028369581	0,503411246	30,06036553	2,228957354	0,101131383	4,469758332	3,816050649	0,093053947	3,052956106	44,35405413	4,928228237
ENSG00000152926	ZNF117	9,738582283	1,780757834	2,340118689	8,615662336	0,100771923	9,062929267	0,98582994	0,185446395	11,54261442	44,35271309	4,928079232
ENSG00000135587	SMPD2	0,073604759	3,526465661	0	12,70993544	0	9,697646958	10,81746165	0	7,513522332	44,3386368	4,9265152
ENSG00000081377	CDC14B	0	0,05126018	26,72987761	0,049882427	0	7,342439019	4,293479149	0,410595429	5,435770963	44,31330478	4,923700531
ENSG00000116668	SWT1	0,18573079	3,361662243	0,136666715	0	4,678768374	7,137254406	7,865028186	0,649822158	20,2841337	44,29906657	4,922118508
ENSG00000198440	ZNF583	12,89876831	0	0	0,194244398	13,85676869	7,307475773	2,008147994	3,443734947	4,588518354	44,29765847	4,921962053
ENSG00000284753	EEF1AKMT4	3,315646092	10,44327044	0	16,46051677	0	0,819086775	6,676150414	3,625173533	2,956417524	44,29626155	4,921806839
ENSG00000121297	TSHZ3	0,018008649	0,031955905	0,033128364	0,031097006	42,49835771	0,731580424	0,882224954	0	0,022148316	44,24850133	4,916500148
ENSG00000137070	IL11RA	8,243681331	7,219932103	10,41367732	4,276620984	0	3,457279636	0,52879091	4,796755604	5,308657391	44,24539528	4,916155031
ENSG00000131759	RARA	9,143734721	7,405988196	0	5,061357298	0,378576368	5,107093443	7,48642293	3,971066909	5,681582486	44,23582235	4,915091372
ENSG00000196730	DAPK1	0,033926935	1,23415155	1,029787075	10,69165534	0	8,083205017	11,94418967	4,859331875	6,342315084	44,21856254	4,913173615
ENSG00000064607	SUGP2	4,550448219	3,73650874	2,265071662	8,905315028	0,042408779	6,681094315	14,52065722	0,03902156	3,467613429	44,20813895	4,912015439
ENSG00000206562	METTL6	5,262688758	9,19773049	0,04864895	19,81900227	0	5,720053134	1,668838858	0	2,47188234	44,1888448	4,909871645
ENSG00000138031	ADCY3	0	0,708199406	0,137659343	10,68205244	0	10,87264757	20,54581864	0	1,196436839	44,14281424	4,904757138
ENSG00000197888	UGT2B17	16,80623102	0	0	27,26186302	0	0	0	0	0	44,06809404	4,896454893
ENSG00000178295	GEN1	0	0	0,023493923	4,675305495	19,63734364	4,7815675	0,668072255	10,33306203	3,942484541	44,06132938	4,895703265
ENSG00000099992	TBC1D10A	7,698126134	4,223607494	3,550192835	0,296222477	10,03918772	4,214274618	6,338504072	3,42297764	4,272333845	44,05542683	4,895047426
ENSG00000204619	PPP1R11	4,916694844	4,52793328	1,373871988	6,448145995	6,507896155	4,919918255	4,754230043	6,532479094	4,056781582	44,03795124	4,893105693
ENSG00000171793	CTPS1	0,039361962	3,492341757	0,048273005	7,499303342	6,4233927	7,807895509	8,48670562	1,06156803	9,149516205	44,00835813	4,88981757
ENSG00000107736	CDH23	8,731519497	0,634758057	9,646374554	1,431934546	14,00765149	1,767380114	0,006750435	3,839988518	3,909506118	43,97586333	4,886207036
ENSG00000165113	GKAP1	1,510217343	0,15763786	0	21,16932775	0,105560712	4,730548483	5,790370995	3,982309603	6,500804277	43,94677702	4,882975224
ENSG00000158987	RAPGEF6	4,074891793	11,20773304	12,61842247	2,228208772	1,581731198	2,356303022	2,593993381	3,029602327	4,243147565	43,93403357	4,881559285
ENSG00000185924	RTN4RL1	0	10,16476315	0	0,433206931	0,29810532	5,554836685	19,06010301	8,411726677	0	43,92274177	4,880304641
ENSG00000197122	SRC	0	3,440906928	0,042977752	0	0,055522193	22,13680082	7,643059803	4,138095233	6,378776738	43,83613947	4,870682163
ENSG00000204767	INSYN2B	0,052882089	0	42,94950611	0,091315827	0,12567543	0	0,285409329	0,231275288	0	43,73606407	4,859562674
ENSG00000164048	ZNF589	0,05474029	0	0	21,78790496	0,065045742	7,212196778	3,158916165	11,43144401	0	43,71024795	4,856694217
ENSG00000063245	EPN1	6,416366193	3,455281223	2,430116669	3,377223786	6,80888165	6,489122806	7,172378765	2,1196132	5,401533799	43,67051809	4,852279788
ENSG00000072864	NDE1	6,381079795	1,805834365	6,527017249	7,931587324	0	5,496127586	5,321184448	2,044917236	8,118522997	43,626271	4,847363445
ENSG00000185899	TAS2R60	0	0	0	43,6254921	0	0	0	0	0	43,6254921	4,8472769
ENSG00000213626	LBH	0	5,443306268	0,245348719	13,53038814	0	3,148347219	9,579155974	4,37468536	7,299355906	43,62058758	4,846731954
ENSG00000178199	ZC3H12D	0	6,332181496	0	0	0	9,68407198	9,168484085	6,478146875	11,92420877	43,5870932	4,843010356
ENSG00000100399	CHADL	17,81195479	7,850073963	0	0	0	2,045122671	7,636483997	7,509876534	0,715895694	43,56940765	4,841045294
ENSG00000145649	GZMA	0	0	13,34056279	0	23,64724888	0	6,581591076	0	0	43,56940274	4,841044749
ENSG00000049618	ARID1B	9,406148511	3,517049311	11,57913388	4,485486055	0,040190427	3,015408113	3,367256711	1,989544926	6,156475434	43,55669336	4,839632596
ENSG00000123815	COQ8B	7,325571678	3,811750685	0,202646327	8,227036742	0,065448796	6,017168719	8,02625962	0	9,856267174	43,53214974	4,836905527
ENSG00000133169	BEX1	0	42,83526291	0	0	0	0,626077648	0	0	0	43,46134056	4,829037839
ENSG00000100056	ESS2	7,098647497	2,144063969	0	0,130402286	0	5,168295493	8,715830159	7,706286765	12,47645319	43,43997936	4,826664373
ENSG00000174775	HRAS	6,751748902	2,139430852	0	7,633735959	0	6,089271166	4,738507219	7,382048849	8,699198915	43,43394186	4,82599354
ENSG00000168421	RHOH	0	0,12173517	0,084134413	0,197438691	32,11841216	6,427528395	4,063355183	0,050005231	0,365618158	43,4282274	4,8253586

ENSG00000035720	STAP1	3,717738284	9,758114816	0,14248083	5,349768828	0	1,275581061	10,86852184	0	12,28816062	43,40036628	4,82226292
ENSG00000166503	HDGFL3	1,420955049	5,710833481	2,87362663	6,353566762	0,044727555	6,236170491	5,258533655	6,872907308	8,633796588	43,39911752	4,822124169
ENSG00000177873	ZNF619	17,54141317	12,59328844	0	0,809279939	0	0,036754669	6,393038627	6,002577434	0	43,37635227	4,819594697
ENSG00000130768	SMPDL3B	35,91926069	0,100691701	0,104386068	0,097985345	2,02281755	0	0	1,489002933	3,628997055	43,36314135	4,818126816
ENSG00000124496	TRERF1	1,680743543	7,45609545	0	19,30351856	0,464456211	6,673330232	4,981814786	0	2,788178555	43,34813733	4,816459704
ENSG00000126070	AGO3	0,31432256	2,028209711	17,39921765	6,19247186	7,164355074	3,521668271	1,933689228	1,234072275	3,531901005	43,31990764	4,813323071
ENSG00000159314	ARHGAP27	5,029289087	3,446290813	7,065483045	1,977159313	0	6,683526013	4,982228798	2,376999095	11,74686919	43,30784535	4,811982817
ENSG00000172432	GTPBP2	0,095922918	6,808514602	3,41152047	0,220850592	4,86320836	4,318028128	3,982342319	11,04711419	8,533367612	43,28086919	4,808985466
ENSG00000065057	NTHL1	0	8,055294653	0	1,503329072	1,034494978	11,94829256	11,61762333	0	9,101125988	43,26016058	4,806684509
ENSG00000156345	CDK20	0,079156925	4,401143357	0	0,820120402	31,85467287	2,665230148	0	0,173093016	3,245093806	43,23851052	4,804278947
ENSG00000156970	BUB1B	0,033760181	10,18412422	4,098902264	15,56515599	0,080231801	7,487474641	2,775148094	0	2,989488294	43,21428549	4,801587276
ENSG00000174373	RALGAPA1	2,554190457	0,094032122	4,874107501	3,257569468	13,44990655	4,317072798	2,639998569	8,296786027	3,727879462	43,21154296	4,801282551
ENSG00000152760	TCTEX1D1	0	0	0,082732172	4,581893899	0	3,555226644	4,854506893	21,04553495	9,071080254	43,19097482	4,798997202
ENSG00000162300	ZFPL1	6,78060253	6,789492951	3,207462681	4,683448262	2,186933752	4,62635262	5,26814535	4,024523579	5,599222145	43,16618387	4,796242652
ENSG00000188783	PRELP	0,026193547	43,04030024	0	0	0	0,086276951	0	0	0	43,15277074	4,794752304
ENSG00000031823	RANBP3	3,308397988	5,114326933	0,784094302	4,240850494	0	7,019722184	6,960274965	5,814228119	9,910150348	43,15204533	4,794671704
ENSG00000020256	ZFP64	0	0	0,040939632	4,227223447	0,158667544	7,916793351	6,984946318	2,919893086	20,85641384	43,10487722	4,789430802
ENSG00000167972	ABCA3	0,251721863	4,123147105	0,071240415	4,881664056	1,242462025	9,71567575	13,29625379	7,113404324	2,309983683	43,00555301	4,778394779
ENSG00000235109	ZSCAN31	19,93671842	12,06287392	0	0	0	4,384564363	3,239903671	1,533184396	1,837094935	42,99433971	4,777148856
ENSG00000104863	LIN7B	0,431005621	6,730318314	0	0,446551582	0	6,413297962	3,865030193	19,79214466	5,286504387	42,97485272	4,774983635
ENSG00000178338	ZNF354B	0,121146903	5,302647784	0	0	0,191938825	7,138322514	2,078877762	15,36494248	12,76391134	42,9617876	4,773531956
ENSG00000114770	ABCC5	10,27791211	3,279519403	0	0,43623093	0	4,058349347	11,45958686	1,192064872	12,24804621	42,95170973	4,772412192
ENSG00000197128	ZNF772	0,024788479	0	0,18240164	1,369737399	0	4,681203936	2,655130057	33,98662628	0,030486634	42,93037443	4,770041603
ENSG00000000457	SCYL3	11,76743656	4,557985473	7,06763291	0,227460388	0,7826185	5,616324912	10,90095286	0,048007347	1,863055359	42,83147431	4,759052702
ENSG00000204954	C12orf73	0	7,013409698	0,062678715	5,059843927	0	4,650929091	6,4253505	19,59510513	4,148546946	42,75100052	4,750111169
ENSG00000151164	RAD9B	0,19019581	0	0,087470138	16,99607921	0,339003344	6,525746409	3,118991891	0	15,43847762	42,69596443	4,743996047
ENSG00000189190	ZNF600	13,93064135	7,354267952	0	1,948394411	0,051567711	0,357360518	3,711500344	9,869383981	5,470783611	42,69389988	4,743766653
ENSG00000272391	POM121C	4,432301868	1,707134152	3,318316325	8,513908335	7,267271776	4,338271105	5,00683112	3,193148959	4,880689705	42,65787334	4,739763705
ENSG00000198648	STK39	0	0	12,39306034	21,71520459	0	1,658657481	1,254364303	0,089282509	5,473458775	42,584028	4,731558667
ENSG00000273820	USP27X	0,057719851	0,10242246	0,106180328	34,08699821	1,920414665	1,394206527	3,258969662	0,631082062	0,99383167	42,55182544	4,727980604
ENSG00000169609	C15orf40	0,010872046	7,466073389	13,28000473	7,434365778	0,051675306	4,476326805	3,303988885	4,350638597	2,166136888	42,54008242	4,726675825
ENSG00000078403	MLLT10	0,777896695	1,233511939	5,297758732	8,688305836	9,912116175	3,416340462	2,006425901	4,017331544	7,185522712	42,53520999	4,726134444
ENSG00000173276	ZBTB21	1,874355875	0	13,30939199	2,912943155	0	4,650929091	2,381166063	3,278933937	14,10792255	42,51564266	4,723960296
ENSG00000006468	ETV1	13,39014105	6,525764745	0,028911087	0	0,074699437	10,88815121	4,045325317	1,374662787	6,146562047	42,47421768	4,71935752
ENSG00000142303	ADAMTS10	4,37041966	2,67906994	9,940042102	8,141354159	0	1,046937907	2,881089131	3,011848819	10,39176873	42,46253045	4,718058939
ENSG00000012048	BRCA1	0,032475945	0	0,716905478	17,52464637	0	6,846084403	1,523549056	5,042092047	10,74418966	42,42994296	4,714438107
ENSG00000136059	VILL	20,74386926	0	0	10,11344636	0	0,461147372	0	2,296101843	8,81020419	42,42476902	4,713863225
ENSG00000128581	IFT22	17,51741611	3,504275897	0	0,378898801	0,086911312	10,15860985	2,064855415	6,717450066	1,979004593	42,40742204	4,711935782
ENSG00000035115	SH3YL1	4,533993835	9,414389972	0	6,941127403	0,032164581	7,23676228	3,011737481	3,25551298	7,973173683	42,39886222	4,710984691
ENSG00000105617	LENG1	5,977785221	0,869461738	10,63607383	0	0	4,949342755	6,997696856	8,357294509	4,579875387	42,36753029	4,707503366
ENSG00000172731	LRRC20	0,041685675	6,879229244	0,076684165	0,431892372	0	4,256464451	12,97968952	0,091154363	17,58492611	42,34172589	4,70463621
ENSG00000189134	NKAPL	0	9,488856623	0,169603477	0	0	6,377282692	4,286966804	10,88680088	11,11223867	42,32174915	4,702416572
ENSG00000139631	CSAD	6,367985324	0,488641458	19,50293177	0	0,245410621	3,968248418	1,000332943	10,31196714	0,423340597	42,30885828	4,700984253
ENSG00000143476	DTL	0,306948143	0	2,484485955	26,50162245	0	0	5,989341463	0,06712057	6,946122303	42,29564088	4,699515653
ENSG00000084764	MAPRE3	8,575420815	4,284362536	0,07657854	0	0,494652262	12,02051837	12,37420905	2,366748968	2,099092951	42,29158349	4,699064832
ENSG00000177125	ZBTB34	0,022874746	3,612570089	16,91613681	3,989469033	0,054362329	2,712434497	7,046547559	2,951201859	4,979538971	42,2851359	4,698348433
ENSG00000106123	EPHB6	12,20235594	7,251163946	3,340981471	0,490018732	0	5,795186341	2,992437965	3,226778875	6,945250316	42,24417359	4,693797065
ENSG00000171262	FAM98B	0	1,056755802	0,162300452	15,04441906	20,65279692	1,138198381	0,329654937	0	3,797768664	42,18189421	4,686877135
ENSG00000186073	C15orf41	9,772186074	0,144504204	0,089883628	0,984341896	0,038706353	4,738769436	5,260612109	16,41844531	4,687226365	42,13467538	4,681630598
ENSG00000185379	RAD51D	10,14622462	0,521861642	0	17,08173047	0	3,727997743	1,842540261	4,501674044	4,307483089	42,12951187	4,681056874
ENSG00000160226	CFAP410	1,121552974	4,511047998	0	9,468199639	5,271558844	6,48537138	7,656939278	0,926502993	6,682259913	42,12343302	4,680381447
ENSG00000078401	EDN1	0	0	0	1,410459322	0	12,1477523	0	13,47718383	15,06863694	42,1040324	4,678225822
ENSG00000148950	IMMP1L	0	3,917993849	0,071258677	5,418029465	8,745492572	2,551816099	2,637417899	12,36694357	6,383859007	42,09281114	4,676979015
ENSG00000198205	ZXDA	0	0,78232531	22,03294731	0	0	3,348061723	1,433772333	5,54339537	8,946659716	42,08716176	4,676351306
ENSG00000117010	ZNF684	0	14,18100226	0	6,310907157	0	3,798662027	7,485330996	0,42622969	9,828709982	42,03084211	4,670093568
ENSG00000178177	LCORL	0,162725073	4,980965946	2,937331777	10,25615999	0,16918985	9,691767488	7,954842694	0,200155546	6,291613993	42,0201129	4,668901434
ENSG00000135469	COQ10A	0	4,216849215	0	0,16092197	0	15,09208145	4,874888015	15,58941993	2,063051126	41,9972117	4,666356856
ENSG00000132680	KHDC4	4,094919821	6,696426145	0,393878993	2,44944273	1,780958411	1,55743191	1,244480799	13,57789934	10,17119649	41,96663464	4,662959404
ENSG00000257093	KIAA1147	0,430631291	2,074106935	6,563785754	3,930490186	16,52067402	3,107023963	3,439409291	0,044841168	5,851050776	41,96201339	4,662445932
ENSG00000171456	ASXL1	2,784429936	3,666463441	0,040652252	8,604977592	6,748552821	9,729473714	4,220265627	3,938347068	2,215047026	41,94820948	4,660912164
ENSG00000099910	KLHL22	0	15,71485566	0,060338637	0,623026855	0	7,346593802	13,45394921	0,143448911	4,598763521	41,9409766	4,660108511
ENSG00000140470	ADAMTS17	0,774095132	1,661113177	29,3412399	0,777147292	0	2,549733666	6,816115248	0	0	41,91944441	4,657716046
ENSG00000137871	ZNF280D	6,96109126	0,110683563	0,550773745	2,175714723	12,45185898	6,040333883	4,226194622	5,319470046	4,081166922	41,91728774	4,657476416
ENSG00000139610	CELA1	0	0	0	0,27409136	25,27402872	6,971032635	7,248811198	0	2,147386287	41,91535019	4,657261133
ENSG00000083544	TDRD3	1,910817539	10,34509136	7,317153573	1,043739898	0,880417201	2,93287084	6,443508663	8,356796804	2,661801803	41,89219768	4,654688631
ENSG00000139146	SINHCAF	8,212825634	3,222513723	6,731356657	8,471610835	3,736110755	3,392610896	2,723212587	3,437704918	1,933470546	41,86141655	4,651268505
ENSG00000102554	KLF5	21,35838455	0	0	20,28469767	0	0	0	0,179632817	0	41,82271504	4,646968337
ENSG00000170892	TSEN34	8,276524762	2,649624507	7,769630096	3,609772376	0	5,574070759	6,199127075	3,171873319	4,564835527	41,81545842	4,646162047
ENSG00000008853	RHOBTB2	5,902039006	0,203755319	5,998962622	0,039655771	0,109154268	10,74132305	12,05125377	2,611337008	4,151890391	41,80937121	4,64548569
ENSG00000059122	FLYWCH1	2,602569515	8,185353845	0	6,446665247	3,327137226	8,690647859	3,44264719	1,883936244	7,218412256	41,79736938	4,644152154
ENSG00000137822	TUBGCP4	0,486973462	6,384904845	0	11,11852546	0	3,995161143	8,536160802	7,039963035	4,22567511	41,78736386	4,643040429
ENSG00000154642	C21orf91	0	13,17460694	6,873624798	11,47979818	0,115323552	4,049198404	3,424848497	2,387533002	0,268564339	41,77349771	4,641499745
ENSG00000134198	TSPAN2	0	0	38,36174234	3,287490158	0,105220493	0	0	0	0	41,75445299	4,639383666
ENSG00000185019	UBOX5	11,48159676	0	0	3,855096712	0	13,55158151	10,96342707	1,883016742	0	41,73471879	4,637190976
ENSG00000173214	MFSD4B	0,177538435	6,795811013	0,046656613	13,00733257	0,120549695	4,622550475	3,285225208	8,92916505	4,741301005	41,72613007	4,636236674
ENSG00000181690	PLAG1	5,096217508	0	0,142043995	15,63343167	3,715958218	7,439270877	3,750646102	0	5,935313052	41,71288142	4,634764602
ENSG00000018869	ZNF582	4,555870936	18,24966448	0	0,077891037	0	7,478357338	3,071214097	0	8,266009498	41,69900739	4,633223043
ENSG00000105576	TNPO2	4,020156027	0,610367336	2,016927735	13,28990196	0,613090128	5,924534749	5,854925152	7,52162908	1,824357666	41,67588984	4,630654426
ENSG00000186812	ZNF397	0,052017408	0,092303614	5,773310145	14,04228377	0,041206832	6,110991982	4,160748818	5,308185824	6,056272212	41,63732061	4,626368957
ENSG00000198919	DZIP3	0,021764449	0,888271065	1,161086399	3,156926093	0,206894739	17,39635602	9,523673479	0,523517247	8,752961657	41,63145115	4,625716794
ENSG00000076716	GPC4	17,46044706	15,57267514	0	5,577557432	0	0	2,783169292	0,133266613	0,074953225	41,60206877	4,622452085
ENSG00000100592	DAAM1	12,93580968	4,756066096	0,181652417	4,579515984	0,771070153	4,259280115	3,631049563	5,860956668	4,597584802	41,57298548	4,619220609
ENSG00000108819	PPP1R9B	2,713936107	3,087059637	4,032407607	1,502043372	15,13205394	2,330312038	3,784622034	4,793317466	4,150843348	41,52659555	4,614066172
ENSG00000104756	KCTD9	13,10107617	0	0,063422336	20,71762974	0,081934187	0,794917295	3,091671425	3,618722163	0,042401671	41,5117749	4,612419433
ENSG00000185010	F8	0,041377484	4,062757577	0	22,00656412	0,032778162	5,724187477	5,485605059	0	4,155932636	41,50920252	4,612133613
ENSG00000170469	SPATA24	0	0	0	34,02557393	0	0	2,60528098	0,54889463	4,322012146	41,50176169	4,611306854
ENSG00000185324	CDK10	0	5,893665439	0,041006063	3,849166169	0	6,240920056	3,146475356	12,23470666	10,08873288	41,49467262	4,61051918
ENSG00000158805	ZNF276	0	2,805133585	0,074565477	1,644842249	11,12609339	5,896768083	3,988260285	4,564747915	11,39107573	41,49148671	4,61016519
ENSG00000077044	DGKD	9,851339015	2,661935729	0	10,54849196	0,110260222	3,837456794	6,035314302	0,642539841	7,798288213	41,48562608	4,609514009
ENSG00000105793	GTPBP10	3,257941587	7,168610389	0	11,27651146	0,172057082	5,818634637	1,791402785	6,585894025	5,413697084	41,48474905	4,609416561
ENSG00000198133	TMEM229B	18,55411272	0,042048299	0,043591046	0,163672557	0	8,481550573	14,10729623	0	0,058286505	41,45055793	4,605617548
ENSG00000166912	MTMR10	2,2569128	4,581290112	2,799301744	0,088572632	10,80847095	5,969626996	5,167594574	3,065810651	6,707970197	41,44555066	4,605061184
ENSG00000067369	TP53BP1	16,78489365	1,32099823	0,663334852	2,24961259	0,027643564	5,964147434	7,330612115	0	7,081366217	41,42260866	4,602512073
ENSG00000204519	ZNF551	0,032130676	13,79765342	1,89142317	1,331583966	0	5,079975132	11,12505389	0,14052076	8,021867749	41,42020876	4,602245418
ENSG00000125817	CENPB	0,575161342	3,247394465	1,923737709	6,952246728	0,248524251	14,1799005	7,467438444	3,087104956	3,729791796	41,41130019	4,601255577
ENSG00000073578	SDHA	5,999598242	4,25233748	1,268591439	2,292298123	9,013788855	3,445034222	5,554191536	4,41082081	5,173594003	41,41025471	4,601139412
ENSG00000176890	TYMS	0,124833185	3,876484925	0,229640726	6,682349618	3,708359586	8,497691661	7,929351836	0,27297362	10,05613152	41,37781668	4,597535187
ENSG00000157617	C2CD2	1,609768966	0	0	0,149447294	4,360419186	10,79464004	5,547714565	9,954672538	8,9410698	41,35773239	4,595303599
ENSG00000060749	QSER1	1,816266149	8,266420041	0,229545912	19,20083032	0,658991728	3,8969729	2,62475784	0,121271518	4,501649865	41,31670627	4,590745141
ENSG00000083097	DOP1A	0	2,188914286	0,027673479	1,272853535	0	19,27502668	7,319623151	0,164477137	11,06383478	41,31240304	4,590267005
ENSG00000106086	PLEKHA8	0,027762368	0,098527283	0,025535559	0,071909326	36,02391661	1,600276553	1,740402364	0,212478594	1,468197769	41,26900643	4,585445159
ENSG00000139266	MARCH9	6,868662303	0,803622378	0	3,453934479	0	9,696072664	2,130864779	14,02943968	4,270199812	41,2527961	4,583644011
ENSG00000181938	GINS3	13,46822912	0	1,851385576	0,715590528	0,070346237	6,207428041	5,038475624	0,064727633	13,83382536	41,25000812	4,583334235
ENSG00000012817	KDM5D	0	4,675412661	22,47223668	2,068067536	0	0	0	0	12,01924037	41,23495725	4,581661917
ENSG00000182183	SHISAL2A	0,112601021	2,897212371	7,353422887	0	0	10,07579136	10,00396835	0,24622546	10,52484031	41,21406175	4,579340195
ENSG00000091073	DTX2	5,724339349	2,871810394	0,220531614	2,691118342	9,686629465	7,403784165	5,076546555	1,048582523	6,487304228	41,21064663	4,578960737
ENSG00000196313	POM121	4,9231707	2,975715538	1,216976609	2,470673248	2,120628941	5,523595865	3,091409117	10,12632914	8,760633253	41,20913241	4,57879249
ENSG00000157456	CCNB2	0,054889419	0	0	19,24075548	0	12,6557287	2,96243065	0	6,278142598	41,19194685	4,576882983
ENSG00000094804	CDC6	0,028452376	0	1,046808883	22,35462501	0	4,560900809	6,4731446	0	6,683616222	41,1475479	4,571949767
ENSG00000108306	FBXL20	10,9508339	0,873347689	2,716172142	3,02177479	0,162452521	4,653241543	10,67059731	0,597909287	7,499099695	41,14542888	4,57171432
ENSG00000162688	AGL	0,444606313	8,776990808	0	0,095967258	0	10,88171614	3,860862245	1,012733136	16,06249825	41,13537415	4,570597127
ENSG00000169228	RAB24	9,293483038	5,064183053	5,249987342	1,389968388	0	2,731699568	4,070950239	5,76060263	7,559858533	41,12073279	4,56897031
ENSG00000132879	FBXO44	0	4,105377943	0	4,660874227	0,101819547	9,502108447	9,569469608	6,089664556	7,060797841	41,09011217	4,565568019
ENSG00000157224	CLDN12	0,045053837	0,039973465	0	26,45136892	0	0	6,078988954	8,423421444	0,027705206	41,06651183	4,562945759
ENSG00000185619	PCGF3	2,523597409	4,044700877	0,074876794	5,728269476	0	4,379592328	8,584367496	5,295853675	10,38738138	41,01863943	4,557626604
ENSG00000142621	FHAD1	9,605935535	2,345326358	0	0,183398264	0,028045103	5,739819249	6,06529758	5,960982827	11,0883829	41,01718781	4,557465313
ENSG00000114423	CBLB	0,061232908	0,086925034	33,31976387	1,586038017	0	3,025356979	0,345732336	0,053559376	2,515306688	40,99391521	4,554879467

ENSG00000078246	TULP3	0,047533995	4,63913495	0,087442623	0,16416167	0,112965569	10,69885757	7,459544746	0	17,77204869	40,98168981	4,55352109
ENSG00000224578	HNRNPA1P48	1,256636762	2,926705822	3,467527222	17,49512044	3,546379057	3,535516447	2,021611378	4,121844917	2,608033317	40,97937536	4,553263929
ENSG00000153982	GDPD1	0,617881199	9,251001792	0,213120444	13,60356116	0	4,15518439	8,144509156	0	4,986937091	40,97219523	4,552466137
ENSG00000109321	AREG	0	18,28983192	0	0,635651582	0,656122185	0	4,66119991	0,201239079	16,52472123	40,9687659	4,5520851
ENSG00000155111	CDK19	0	3,402194115	5,454154983	11,43403747	0	11,93601271	3,577832881	1,901781684	3,233170368	40,93918422	4,548798246
ENSG00000107562	CXCL12	4,401278757	10,63390193	8,508194571	0,042938123	0,059094544	1,556179508	1,507213137	13,64802969	0,581057088	40,93788735	4,54865415
ENSG00000090612	ZNF268	0,289463314	2,225797376	1,321366913	14,42130864	4,178451717	3,5901189	5,358860579	0,117216945	9,427475234	40,93005961	4,547784401
ENSG00000198945	L3MBTL3	20,30372815	0	2,881467875	1,377908139	0	0,648975461	0,122697371	1,292527156	14,28470238	40,91200653	4,545778504
ENSG00000170631	ZNF16	18,29369903	0	0,955440478	0,548078045	0,068573142	7,318186599	0,023958437	7,192961752	6,458672342	40,85956982	4,539952202
ENSG00000247746	USP51	0,720063985	1,521114137	0	33,39399267	0,081487983	1,694117529	1,224239681	1,124692197	1,096439659	40,85614784	4,539571982
ENSG00000176444	CLK2	16,89563242	2,712207218	2,431755923	0	0	1,587444009	6,002544587	5,058593707	6,147465608	40,83564348	4,537293719
ENSG00000187741	FANCA	0,015520806	5,040056082	0,114207107	4,824188109	14,56978525	3,663801766	3,092941627	1,357578338	8,15082837	40,82890746	4,536545273
ENSG00000186866	POFUT2	7,836508612	2,698802116	10,71707649	4,451296053	0	3,735959762	2,825329892	2,818445646	5,738352189	40,82177076	4,535752306
ENSG00000148660	CAMK2G	0,401178506	4,310837465	1,558000555	4,541346166	23,83523511	1,810823404	2,331749533	0,487366619	1,535015523	40,81155288	4,534616987
ENSG00000178502	KLHL11	12,84092799	0,036225615	0	10,22306709	0,048516285	2,017286126	1,000101268	0,044641255	14,58752086	40,79828649	4,533142944
ENSG00000143669	LYST	0,03081688	6,548384727	9,325527946	2,647397774	0,036618491	4,686165978	3,691056468	12,29821731	1,497081193	40,76126677	4,529029641
ENSG00000104731	KLHDC4	9,315127779	2,423042141	0	3,081633412	0,032130048	6,457108425	5,680235125	2,217284832	11,53954428	40,74610604	4,527345116
ENSG00000157933	SKI	0,046140632	7,041286109	2,291744327	10,03902498	0,109654211	4,686023922	7,547375696	1,816128921	7,150125742	40,72750454	4,525278282
ENSG00000179988	PSTK	0	10,56263845	0,590673633	6,866709858	0,352191117	3,389804199	3,240325671	11,77422944	3,827502747	40,60407511	4,511563901
ENSG00000197380	DACT3	0,027645549	4,856578487	0	10,88422507	0	5,129690352	8,011186584	0	11,66215828	40,57148431	4,507942702
ENSG00000197114	ZGPAT	0	6,914606299	9,25211124	2,034275664	0	9,352680186	6,508655903	0,693565809	5,795513738	40,55140884	4,505712093
ENSG00000277075	HIST1H2AE	6,828179028	0	0	10,76549011	0	2,881445116	0	20,95280593	1,947551478	0	40,49402655	4,499336283
ENSG00000166446	CDYL2	9,845731233	24,06441519	0,062998323	0	0,529011677	1,880006535	4,08045159	0	0,021059096	40,48367365	4,498185961
ENSG00000170989	S1PR1	0,607566912	0,051338694	0,372556135	0	0	8,449590925	20,03489506	10,5020368	0,462570322	40,48055485	4,497839427
ENSG00000173264	GPR137	0,374871175	6,186357233	0,344802901	9,645078446	0	8,27289157	5,229227431	7,377601359	3,042885257	40,47371537	4,497079486
ENSG00000237651	C2orf74	4,388689841	2,12389703	1,834852138	6,544903653	8,296444873	2,956822159	3,892478947	3,053520756	7,360258308	40,45186771	4,494651967
ENSG00000101255	TRIB3	32,71568791	0	0	0,430585767	0	0	6,66690543	0,436217251	0,184006458	40,43340281	4,492600312
ENSG00000135503	ACVR1B	0,053414836	0	7,615220103	4,150609352	0	4,369154459	2,927197565	13,02349038	8,277363664	40,41645036	4,490716706
ENSG00000162601	MYSM1	1,049731695	7,090365493	2,95889181	4,063861229	2,2935238	3,030075518	6,340293527	6,442085686	7,142336934	40,41116569	4,490129521
ENSG00000157625	TAB3	5,967862991	0	0,415515843	0,34898077	13,50469556	4,359541548	2,704655099	8,786632352	4,313171589	40,40105575	4,489006195
ENSG00000144648	ACKR2	0,055351857	4,567253848	7,229519058	7,550865565	10,45781854	4,071798036	1,700513657	0,847268122	3,914349828	40,39473851	4,488304279
ENSG00000075399	VPS9D1	3,619414866	3,77325569	0,083227575	5,156200097	12,25730534	3,725536902	6,048120794	2,275447621	3,449845623	40,38835451	4,487594945
ENSG00000092200	RPGRIP1	21,52013429	1,421593785	0	0,286217522	0	2,881445116	9,381602521	0	4,892486144	40,38347937	4,487053264
ENSG00000146670	CDCA5	0	0	0	9,498273877	6,351989342	9,484249835	6,17541752	5,082305576	3,76361837	40,35585452	4,483983835
ENSG00000164109	MAD2L1	6,436647473	5,394917892	0	11,72954028	0,065092902	6,074666756	2,410704222	6,109176343	2,122227824	40,34297369	4,482552632
ENSG00000108176	DNAJC12	0	13,41336793	0	0	0	2,404501112	6,041815893	14,21223946	4,257353713	40,32927811	4,481030902
ENSG00000164465	DCBLD1	17,57305801	0	5,299517945	0,038265871	0	3,065659578	8,924052063	2,277522316	3,106981015	40,28505679	4,476117422
ENSG00000181036	FCRL6	24,38541895	12,01981323	0	0,097472908	0	0	0	0	3,748865223	40,25157031	4,472396701
ENSG00000143443	C1orf56	4,732334474	10,81310511	0	3,470177697	2,464985276	4,911123892	2,099246224	0,425269714	11,32140977	40,23765215	4,470850239
ENSG00000164818	DNAAF5	4,677887115	4,432099175	0,077876481	8,187341968	0,251518099	3,578964675	5,747135252	5,369156721	7,887868924	40,20984841	4,467760935
ENSG00000067221	STOML1	8,582872344	4,179302545	9,356377399	0	0	3,553640197	4,571064878	6,793214	3,16319382	40,19966518	4,466629465
ENSG00000170522	ELOVL6	9,422407791	0	1,066663392	19,38149325	0,049214409	0,04547364	0,687791322	9,464276362	0,025468894	40,14278906	4,460309895
ENSG00000196912	ANKRD36B	0,044634639	11,76165618	3,243306427	3,969325415	0,053037593	4,680094543	3,706109349	2,635277898	10,04575796	40,13920001	4,459911112
ENSG00000269335	IKBKG	3,468392242	4,103051291	0,157540436	6,802498629	0	5,45356387	6,186409115	7,958894372	6,003543083	40,13389304	4,459321449
ENSG00000244482	LILRA6	5,9487973	3,650203828	5,318235889	2,496066718	3,303141486	2,868946691	6,785923033	6,200207117	3,555562604	40,12708467	4,458564963
ENSG00000243477	NAA80	6,768206547	12,01001646	0	0	0	6,066200245	5,849165386	0,151021283	9,258350515	40,10296043	4,455884493
ENSG00000196642	RABL6	0,846266705	2,739906466	2,621938446	4,97360828	6,774471225	4,613155159	7,482854345	4,545179746	5,496148871	40,09352924	4,454836583
ENSG00000117620	SLC35A3	2,278881652	2,073376584	13,75037287	0,858574087	10,63468456	2,201829425	2,848627225	0,97852847	4,453793816	40,07866868	4,453185409
ENSG00000164904	ALDH7A1	5,061200313	7,858353804	0,031034956	2,767536294	0,280654549	6,483052567	7,081082151	2,527047882	7,988267397	40,07822991	4,453136657
ENSG00000253251	SHLD3	0,615521673	31,26502954	0	0	0	0	0,511080782	3,196668423	4,447447325	40,03574774	4,448416416
ENSG00000108666	C17orf75	9,447700256	11,06751202	0	0	0,772534756	0,12508448	10,86422446	4,282860514	0	3,463179708	40,02309619	4,447010688
ENSG00000187747	OR52B6	0,14991128	0	0	39,86506556	0	0	0	0	0	40,01497684	4,446108538
ENSG00000225614	ZNF469	0,011372813	1,331931362	1,945672402	4,340078978	16,89233566	2,909397484	5,297573286	1,790568891	5,440979692	39,95991057	4,439990064
ENSG00000162746	FCRLB	0,058298831	9,931185188	0	0,40267743	0	4,416601	15,10291033	0	10,0380066	39,94967939	4,438853265
ENSG00000144451	SPAG16	0,750337095	19,97182149	0,033665992	0,094805005	0	5,766784003	4,118016225	1,240580307	7,945237055	39,92124717	4,43569413
ENSG00000139625	MAP3K12	0,189575424	4,406799901	0,069747861	0,065471076	29,10424443	1,914913192	0,031481704	0	4,080183339	39,86241693	4,429157437
ENSG00000240038	AMY2B	0,121033697	0,035795221	16,25354203	0,069666259	22,2440982	0,022147988	0,55273274	0,044110875	0,496186149	39,83931317	4,426590352
ENSG00000139567	ACVRL1	0	2,872950001	3,19897733	0,258864062	0,498774364	6,549861915	9,335590179	12,85023318	3,871802547	39,83305113	4,425894569
ENSG00000197417	SHPK	0,039372217	2,445276186	10,93668446	5,778914237	0	6,527487515	4,31529021	3,443820401	6,343380515	39,83022574	4,425580638
ENSG00000005381	MPO	0	0	0	30,63728857	0	0	0	9,183944304	0	39,82123287	4,42458143
ENSG00000197016	ZNF470	0,015626739	6,57183599	0,028746649	8,931693546	0,408510131	7,291829896	4,281821051	10,55887088	1,72969876	39,81863364	4,424292626
ENSG00000163214	DHX57	0,018924304	11,45102342	4,699726157	2,908354758	0,134922057	7,230667588	6,002462971	6,414196364	0,930978195	39,79125582	4,421250646
ENSG00000167992	VWCE	0,031705953	0	23,03863598	0,656991124	0	0,034811283	0	13,51966862	2,495631017	39,77744398	4,419715998
ENSG00000137216	TMEM63B	2,766091802	2,423882487	0,251281445	4,481595044	0	5,136666443	5,614265703	0	19,06764319	39,74142611	4,415714013
ENSG00000167914	GSDMA	0,19944202	0	0	0,114797613	0	39,41562315	0	0	0	39,72986278	4,414429198
ENSG00000122481	RWDD3	0	7,931548524	5,048937968	4,468528366	0,186360946	4,046599432	4,818269106	2,229190129	10,99455295	39,72398742	4,41377638
ENSG00000175893	ZDHHC21	1,731375094	0,142235307	10,05635622	7,723409496	0,114295844	2,44659136	2,955061925	14,19753871	0,335178324	39,70204228	4,411338031
ENSG00000008382	MPND	4,793649143	6,150646171	0	2,928991906	0	9,149778022	8,389182764	4,837987811	3,446641213	39,69687703	4,410764115
ENSG00000109906	ZBTB16	4,126371515	5,091836474	0,043625251	2,702716309	5,354075098	3,541092033	5,926956989	8,608307942	4,28741977	39,68240138	4,409155709
ENSG00000170684	ZNF296	11,53052547	4,922859439	0	0,748522589	0	1,522988656	11,58962906	0,189578065	9,169707926	39,67381121	4,408201245
ENSG00000163738	MTHFD2L	0,030779218	4,778984623	21,57254664	7,945774255	0,146294956	0,270350262	4,561860528	0,302873114	0,056781706	39,6662453	4,407360589
ENSG00000154330	PGM5	0,097945664	3,024157896	10,95488075	7,915320723	1,629389938	3,03258897	6,115743432	0,685371154	6,11939547	39,57479399	4,397199333
ENSG00000162889	MAPKAPK2	8,176900963	4,346521098	1,855409481	4,851711089	0,085606089	4,429555326	6,221174984	3,623358786	5,980758166	39,57099598	4,396777331
ENSG00000160392	C19orf47	3,626653696	1,76011159	1,82468988	1,926904428	17,45863744	2,994898615	4,11800064	4,066886985	1,791751087	39,56853436	4,396503818
ENSG00000172613	RAD9A	2,066035627	7,267940995	12,66879799	3,630181945	0	5,133716573	5,14642043	0	3,655412527	39,56850608	4,396500676
ENSG00000088876	ZNF343	0	7,796504418	0,179612384	19,10788097	0	12,4709867	0	0	0	39,55498448	4,394998276
ENSG00000100246	DNAL4	0	4,303513582	0,114395102	8,375690543	0	9,558634049	8,467956563	0	8,718725736	39,53891557	4,393212842
ENSG00000188177	ZC3H6	0,051223922	5,817317968	3,203838429	0,508602069	2,799899483	3,388512626	5,220841781	10,3610839	8,174097039	39,52541721	4,391713024
ENSG00000189057	FAM111B	0,078194344	9,157760417	1,006913683	2,768003612	0,650406933	4,421420285	14,67337388	0	6,731827456	39,48790061	4,387544513
ENSG00000177192	PUS1	7,513348332	2,292211794	3,976686692	5,917925104	0,313256754	5,267921312	7,09218206	3,631782581	3,469221543	39,47453617	4,386059575
ENSG00000013503	POLR3B	0	9,785383392	3,789577692	10,06963828	13,63260805	1,252680529	0,36841121	0,415815311	0,155911512	39,47002597	4,385558442
ENSG00000123739	PLA2G12A	0,52097113	13,19909864	1,384310012	2,798766247	0,343916436	4,989074994	5,695550409	9,113690428	1,388242553	39,43362085	4,381513428
ENSG00000176454	LPCAT4	8,034377428	7,812729479	0,118239089	0,22197786	0	6,386604505	7,951969148	2,740737587	6,086849092	39,35348419	4,372609354
ENSG00000111554	MDM1	0,069221517	0,573215453	0	2,470295988	0	14,08555209	11,66764883	0,050455729	10,41466998	39,33105958	4,370117732
ENSG00000106692	FKTN	0,932887941	0,03065531	0,0635601	19,71853415	0	7,074956569	5,967263989	2,795490796	2,740848448	39,3241973	4,369355256
ENSG00000267680	ZNF224	1,871865177	4,000992681	13,81291877	0,440768538	0	2,942670702	1,907488155	7,07010899	7,27271019	39,3195232	4,368835912
ENSG00000153406	NMRAL1	2,731171003	11,28301554	2,51210482	5,158273997	1,825505722	4,497999255	5,952842637	0,653217163	4,671093048	39,28522318	4,365024798
ENSG00000215421	ZNF407	0,012396273	10,40452552	10,76346241	7,384952111	0,294600117	0,544415346	0,072050232	5,665367072	4,116370879	39,25813995	4,36201555
ENSG00000174628	IQCK	5,054168897	8,377759266	0,111347927	7,107381643	0	2,492147657	1,080557636	15,02276123	0	39,24612426	4,360680473
ENSG00000167487	KLHL26	0,031852987	4,973966275	0	5,280303027	0	4,161753505	11,79590389	0,069653155	12,92777242	39,24120526	4,360133918
ENSG00000133030	MPRIP	2,114249474	2,79160725	0,367496	4,872587661	8,980905818	3,902380536	5,273430832	3,986183226	6,930599073	39,21943987	4,357715541
ENSG00000161558	TMEM143	11,58099747	0	0	0	0	7,064032502	10,89807596	7,034513927	2,637617404	39,21523726	4,357248584
ENSG00000119574	ZBTB45	5,429122299	7,492990022	0	0,694437723	0	6,402404158	6,636654287	4,397000233	8,099099939	39,15170866	4,350189851
ENSG00000124217	MOCS3	10,35814723	9,97786526	0,326651115	1,022071973	0	3,866697734	3,292797589	0,258859826	10,04575796	39,14884868	4,349872076
ENSG00000124613	ZNF391	0,031259944	14,81049111	0	1,673352133	0	1,819044387	12,53665088	0	8,265825877	39,13662433	4,348515815
ENSG00000141568	FOXK2	1,967191487	3,329622272	4,398243104	11,18367406	0,035962101	5,250124398	4,975590975	5,029646932	2,959102448	39,12915777	4,347684197
ENSG00000128011	LRFN1	6,123084348	3,806807455	0	0	0	5,348788187	7,199421594	14,36672033	2,253684535	39,09850645	4,344278494
ENSG00000184979	USP18	17,53138138	0	0	0	0	5,610879062	3,241366022	12,5717238	0,087292871	39,04264313	4,338071459
ENSG00000146872	TLK2	6,423478655	3,914253184	9,998583497	0,457085673	1,174272006	4,591942856	7,707274861	0,810361546	3,928321912	39,00557419	4,333952688
ENSG00000166025	AMOTL1	10,77208953	2,406059821	0,027714865	1,482880713	0,035804341	0,115790034	6,517451941	14,75919105	2,87200508	38,98898738	4,332109709
ENSG00000116017	ARID3A	1,193474093	5,498824444	2,965840047	8,06269909	3,383676442	3,103495615	5,26777201	3,983345902	5,459257686	38,91838533	4,324265037
ENSG00000122512	PMS2	0,048502831	0,086067084	0,044612438	17,54642182	0,288170071	3,035436646	6,242305075	7,000058243	4,563395588	38,8549698	4,317218866
ENSG00000196705	ZNF431	5,453325151	0,929730965	14,83137522	2,769618327	0,025411657	2,336272627	1,820083361	3,577447554	7,088258952	38,83152382	4,314613757
ENSG00000196268	ZNF493	0	0	0	5,840438615	0,096072109	0,843312188	2,37200913	18,77000352	10,90485212	38,82668768	4,314076409
ENSG00000156398	SFXN2	0,075235534	9,779139411	0,069200921	2,435912758	0,044699719	7,909354105	7,871178408	4,729894855	5,875655546	38,79027126	4,31003014
ENSG00000128891	CCDC32	0,384416345	3,503705598	10,31817897	4,646621888	0	4,546809283	6,659445235	5,50214821	3,223517384	38,78484292	4,309426991
ENSG00000090061	CCNK	0	5,969584673	2,603949832	2,698232706	13,06279138	2,422052215	3,113862254	5,225850824	3,594841489	38,69116538	4,299018375
ENSG00000164975	SNAPC3	0,090431221	10,10948295	0	11,36027493	0,483551448	8,76218336	5,199772885	0	2,669249871	38,67494666	4,297216296
ENSG00000196712	NF1	2,679107427	2,213925839	1,782843244	9,252467584	2,554724868	2,733580088	3,500959872	9,646302155	4,288238958	38,65215004	4,294683337
ENSG00000124171	PARD6B	26,74238244	0,057173134	0,11854162	7,510921444	0,459425427	1,308889411	0,508301985	0,070455132	1,862427835	38,63851843	4,293168714
ENSG00000204713	TRIM27	3,614542141	2,552134253	7,449936278	3,333170621	0	7,189112027	5,798190671	3,145025314	5,539320263	38,62143157	4,291270174
ENSG00000186260	MRTFB	1,649837935	0	13,71623362	2,07765814	11,74096441	1,681323253	3,61016699	0,099660564	4,035755098	38,61160001	4,290177779
ENSG00000072818	ACAP1	0,162922448	6,613205663	0	2,531983581	0,77437745	11,62715632	7,62629646	0,445329606	8,816438792	38,59771032	4,28863448
ENSG00000169071	ROR2	0	0	34,7947879	0,133130089	0,061074412	0	0,042676984	0	3,508327206	38,53999659	4,282221843
ENSG00000182318	ZSCAN22	0,519503466	0,460923077	0	18,72631747	3,318017688	3,600551836	4,070911073	0,923001585	6,908347304	38,5275735	4,2808415
ENSG00000188295	ZNF669	0	0,135425253	0	0	0,181372496	12,90415597	4,689295236	0,33377229	20,27416607	38,51818731	4,27979859
ENSG00000161798	AQP5	0	0	0	0	38,50411017	0	0	0	0	38,50411017	4,278234463

ENSG00000197928	ZNF677	8,227993204	0,315528669	0,029736853	15,85484227	8,413215851	2,289523363	1,838835632	1,166489166	0,357855948	38,49402096	4,27711344
ENSG00000277149	TYW1B	0,046324516	0,164403434	0	6,2394016	0	4,374098589	12,03930531	0	15,61065209	38,47418553	4,274909504
ENSG00000102870	ZNF629	0,024841455	0,088161105	0	7,506759914	0,059036256	1,881938154	8,43619362	1,520987653	18,94210814	38,46002629	4,273336255
ENSG00000170425	ADORA2B	24,10897776	0	0	0,716011298	0	0	2,508422945	0,259062773	10,85501051	38,44748529	4,27194281
ENSG00000075391	RASAL2	0,028225529	0,869071554	8,437815271	9,18899655	6,088255517	6,266629861	1,95509742	0,88560748	4,714506019	38,4342352	4,270470578
ENSG00000125967	NECAB3	0,069604132	8,275225245	15,10900584	0,420669006	0,082707863	3,247903966	5,374823066	0	5,821079463	38,40101858	4,266779842
ENSG00000102024	PLS3	0,110595685	36,82945744	0,067816565	1,145847656	0	0,040475866	0,030609986	0,161226918	0	38,38603012	4,265114457
ENSG00000197353	LYPD2	10,4301909	0	14,03939894	0	0	0	0	13,90717877	0	38,37676861	4,264085401
ENSG00000011258	MBTD1	0	0	3,79204478	19,14152723	0	5,034591554	6,462141264	0,091991806	3,828686706	38,35098334	4,261220371
ENSG00000181896	ZNF101	0,046070296	6,580924088	0,042375015	3,341240528	0,054743528	2,756745863	4,70513745	17,42840859	3,371301242	38,3269466	4,258549622
ENSG00000004948	CALCR	0	0,063435533	0,131525952	0,8642275	0,169916036	4,003519949	8,311259276	20,24664135	4,528552589	38,31907819	4,257675354
ENSG00000146243	IRAK1BP1	13,76861692	9,341674933	3,948263499	3,286598903	0,144359189	3,267969285	0,672492735	0	3,859870153	38,28984561	4,25442729
ENSG00000168806	LCMT2	13,81433419	0,192713813	0,479482706	7,801419393	1,342109545	6,367417562	4,851447364	0,332476926	3,098777718	38,28017921	4,253353246
ENSG00000087095	NLK	3,041856738	7,421837704	0	9,082660051	0	4,313883731	2,867742962	0,27715208	11,26224469	38,26737796	4,251930884
ENSG00000263155	MYZAP	8,840957175	0	0	10,44543393	0	0,766330608	4,105072448	13,22755775	0,858413498	38,24376541	4,249307268
ENSG00000144161	ZC3H8	1,88543317	1,836030128	0	13,34055865	0	1,666174386	4,849279417	8,296059592	6,362670041	38,23620539	4,248467265
ENSG00000141577	CEP131	0,076203011	3,921390823	0	5,000266785	0	1,924328424	12,02186741	3,332673399	11,94928471	38,22601457	4,247334952
ENSG00000102962	CCL22	0,207213672	0	0	0	0,123111945	18,88320585	8,430642808	1,01951015	9,556728948	38,22041337	4,246712597
ENSG00000171617	ENC1	0,072982647	0,874164936	19,26594745	3,623221695	0	2,283724432	5,54481303	0	6,552425079	38,21727927	4,246364364
ENSG00000166575	TMEM135	11,88409203	0,026559231	0,798476909	2,73961047	0,071140559	2,432128105	3,902306284	16,33189869	0	38,18621228	4,242912475
ENSG00000051596	THOC3	6,695603044	4,426323199	4,26670873	6,272112045	0	4,516534668	2,97960244	4,880441049	4,144275185	38,18160036	4,24240004
ENSG00000182118	FAM89A	8,367071565	1,227960866	0	4,128030406	0	14,64323158	2,298374108	0	7,50504274	38,16971127	4,24107903
ENSG00000165682	CLEC1B	10,66420649	0	1,59062182	12,37130397	0	1,039766557	2,01709798	10,44425336	0	38,12725018	4,236361131
ENSG00000142655	PEX14	0,773075907	3,566690997	0	1,334933363	17,88238785	5,658614485	3,765821697	3,493680625	1,648025095	38,12323002	4,235914447
ENSG00000102984	ZNF821	6,795978046	4,650316874	0	1,994212034	0	4,389168696	5,974777714	3,982309603	10,32480679	38,11156976	4,234618862
ENSG00000125841	NRSN2	0,051520822	18,19306445	0	3,202747728	0	8,824428901	5,432912432	0,11266095	2,217738929	38,03507421	4,226119356
ENSG00000020577	SAMD4A	7,387903825	7,557329994	2,444032095	2,422814472	0,059016852	0,17722576	4,14454178	11,32220334	2,519694016	38,03476213	4,226084682
ENSG00000070718	AP3M2	9,029073295	0	0,088585118	2,037255219	0	8,115765941	3,018804992	11,74106255	3,997654638	38,02820175	4,22535575
ENSG00000189227	C15orf61	4,866326523	5,926101875	8,542432004	5,766822213	0	3,946094542	3,882159294	1,391010598	3,67703075	37,9979778	4,221997533
ENSG00000165259	HDX	0	0,775831083	0,114899462	0	0	3,200259126	7,519922075	13,24834102	13,08453746	37,94379023	4,215976692
ENSG00000154134	ROBO3	1,796616153	8,219794874	0,039819524	1,270847892	0	4,135287258	6,23667171	8,804015121	7,400849906	37,90390244	4,211544715
ENSG00000104228	TRIM35	0	9,206864791	0,131650532	4,81954251	0	15,55779976	4,872634252	0,234739214	3,080572238	37,90380329	4,211533699
ENSG00000131791	PRKAB2	0	6,678994874	0,050912106	12,13873325	0	1,306621758	4,917701657	0	12,76418422	37,85714787	4,206349763
ENSG00000151458	ANKRD50	0,017175559	0,030477609	2,338091308	16,66804452	0,040818088	3,281250324	13,07755108	0	2,344733345	37,79814184	4,199793538
ENSG00000173933	RBM4	0,733880446	5,6566608	4,176662589	11,04884776	0	2,165474214	4,436877729	2,858517279	6,712926438	37,78984726	4,198871918
ENSG00000234127	TRIM26	0,20714266	4,533358009	7,494094512	5,723042623	0	5,761573603	4,930522528	3,095228985	6,029290876	37,7742538	4,197139311
ENSG00000168395	ING5	0,029459123	1,385274004	0	19,10148805	0,035005122	3,428505923	4,194982345	4,1227824	5,452753992	37,75025096	4,194472329
ENSG00000196911	KPNA5	0,092649032	4,532837524	0	2,856868864	20,50842057	3,560312805	3,593658454	0,144711644	2,457985886	37,74744478	4,194160531
ENSG00000130856	ZNF236	0,474019267	2,253041677	1,65056523	8,389910118	0,201163759	6,579917561	4,793343403	4,109145977	9,286079872	37,73718686	4,193020763
ENSG00000118965	WDR35	11,51263536	0,068438489	0,035474745	5,694216521	7,882620889	6,500069812	4,067057724	0,210843905	1,755059041	37,72641649	4,191824054
ENSG00000117586	TNFSF4	0,076260697	0	0	0	0	9,000952307	9,973039258	0	18,5705836	37,62083586	4,180092874
ENSG00000163251	FZD5	5,431307626	2,133250747	16,15199041	0,111209678	0	5,727556491	4,991006245	0,985811325	2,085789922	37,61792244	4,17976916
ENSG00000165304	MELK	0,048950622	0,086861678	1,710923836	19,27216528	5,35128181	6,180666123	4,877369563	0	0,060202955	37,58842186	4,176491318
ENSG00000104974	LILRA1	5,139528749	4,036706999	0,697468885	3,637231316	6,00698426	2,497678043	3,917668577	4,329641671	7,305369289	37,56827779	4,174253088
ENSG00000184470	TXNRD2	2,045271716	1,078976467	6,169049874	2,959023611	0	4,167489184	7,680295899	5,318541996	8,112797826	37,53144657	4,17016073
ENSG00000122547	EEPD1	0	1,698176061	1,291020092	6,830470097	0	12,46868594	9,085143314	5,510730596	0,627727329	37,51195343	4,167994826
ENSG00000089094	KDM2B	4,555930498	3,894668135	0,061175198	4,632208323	0,052687433	3,419958337	12,2874775	3,732902245	4,867019399	37,50402706	4,167114118
ENSG00000204371	EHMT2	6,053544606	4,304103994	0,038136932	6,909102771	0	3,823978171	5,198523839	5,349331728	5,736790714	37,41351275	4,157056973
ENSG00000111186	WNT5B	16,53662849	0,126482076	0	0,923119014	0	4,891231624	10,26844462	0	4,646163057	37,39206888	4,15467432
ENSG00000186166	CCDC84	0	1,206665232	0	17,05190403	0	3,7655306	3,314125381	5,560041637	6,472447851	37,37071473	4,152301637
ENSG00000183598	HIST2H3D	19,85394359	0	0	1,269756567	4,368826549	7,266156644	4,579209197	0	0	37,33789255	4,148654728
ENSG00000181450	ZNF678	10,69554348	6,286222997	0	8,01976254	0,040282394	3,554561991	2,885184303	2,742810768	3,106128078	37,33049655	4,14783295
ENSG00000269556	TMEM185A	3,720636385	7,953804966	4,257547076	0,657649219	0	7,494603047	8,416582953	0	4,756056793	37,2568808	4,139653422
ENSG00000189339	SLC35E2B	0,792002991	5,36603407	0	1,896013514	0	3,675922247	7,428082788	4,526500925	13,57044886	37,25500539	4,139445044
ENSG00000169016	E2F6	0,037155292	3,626213426	9,090571223	0	0	3,549107493	2,15955821	14,94958456	3,838482388	37,2506726	4,138963622
ENSG00000184508	HDDC3	5,053382355	2,818232372	6,321351381	2,044397852	0	4,502064187	5,778396715	3,157219258	7,564553649	37,23959777	4,137733086
ENSG00000164659	KIAA1324L	0,056174933	0,033227014	33,5849562	0,129335799	0	1,130741229	0	0	2,279901574	37,21433675	4,134926305
ENSG00000256229	ZNF486	19,6977739	11,16996864	0,06505502	1,893045685	0	2,64028065	0,557906929	1,159962208	0	37,18399303	4,131554781
ENSG00000110888	CAPRIN2	0	0	0,33124416	23,16450859	0	0,869883863	8,253053297	0	4,539863807	37,15855371	4,128728191
ENSG00000160211	G6PD	0	3,423089363	5,427396175	9,666677407	2,247293757	3,197775821	3,831634669	2,894921117	6,466249461	37,15503777	4,12833753
ENSG00000006025	AC003665.1	0,09197235	8,52733993	13,32375703	0,317632349	0	0,984557942	4,028338413	4,675960861	5,203250167	37,15280869	4,128089854
ENSG00000197980	LEKR1	0,053948794	3,494174582	0	0	23,91125371	4,116667291	1,276652789	3,89301704	0,398100369	37,14381458	4,127090508
ENSG00000182534	MXRA7	1,74665994	13,99077304	7,987761297	2,649658379	0,077588321	3,710002093	3,90358168	1,427825721	1,646258488	37,14010896	4,126678773
ENSG00000142102	PGGHG	11,69239587	0	0,105695855	0,992148192	0	4,699748275	1,598196244	6,470486778	11,55357657	37,11224779	4,123583087
ENSG00000166192	SENP8	0	4,305772783	30,68828873	0	0	0	2,039963246	0,066325686	0	37,10035044	4,12226116
ENSG00000144559	TAMM41	0	2,353313756	6,709055553	13,52017506	0	4,956315962	2,7105827	3,569251025	3,262116172	37,08081022	4,120090025
ENSG00000213923	CSNK1E	0,290318099	4,207159143	9,390618137	2,214145638	0,172486812	3,931278475	3,414978617	7,141957511	6,307951124	37,07089356	4,118988173
ENSG00000125900	SIRPD	15,23958368	10,10012394	0	0	0	2,822294136	1,219638707	3,613500738	4,064687	37,0598282	4,117758689
ENSG00000122912	SLC25A16	0,019213041	0	13,50138561	1,327069165	0	3,881442608	4,211591564	13,82237415	0,28355477	37,04663091	4,116292323
ENSG00000145331	TRMT10A	14,60804422	0,550222982	1,330958066	2,201229836	0,081878145	5,447132477	3,032342725	0,150676957	9,618595658	37,02108107	4,113453452
ENSG00000173402	DAG1	11,50300025	2,014066735	0,120459381	0,075382052	0	4,16993216	7,466961835	5,107106561	6,550130209	37,00703919	4,111893243
ENSG00000184635	ZNF93	18,06609619	0,961254705	0	0,046770922	0	2,944102365	3,710809245	0,059228279	11,1594524	36,94771411	4,105301568
ENSG00000136895	GARNL3	20,66762421	0,027657762	0,028672522	1,103489836	0,222249124	3,200131804	4,257837981	0,64757677	6,785937997	36,941178	4,104575334
ENSG00000117411	B4GALT2	11,37736004	4,336336693	0,073695678	6,779328608	3,617833141	5,762005713	1,164226304	1,401631251	2,414231069	36,9266485	4,102960944
ENSG00000145428	RNF175	10,80836428	7,085301141	0	0,059438491	0	0,90702821	3,744103301	10,23669731	4,064069735	36,90500247	4,10055583
ENSG00000115464	USP34	0,116719889	6,09514505	9,81557315	6,996656422	0,257573961	2,846796654	1,696012764	6,034418861	3,035065964	36,89396272	4,099329191
ENSG00000105131	EPHX3	0	0	0	28,00277776	0	5,665239204	3,18266208	0	0	36,85067905	4,094519894
ENSG00000110171	TRIM3	0,581969314	2,303693676	0	0,115953921	24,20359677	3,440598539	1,096541192	1,272596468	3,82649483	36,84144471	4,093493856
ENSG00000120875	DUSP4	0,747969276	1,327253128	0,03822083	4,699914983	0,098753621	3,649895816	9,453834997	5,76999726	11,03886947	36,82470938	4,091634375
ENSG00000057935	MTA3	9,626514615	3,522848583	0,36783756	8,903357829	0,610975025	5,708076552	1,043609567	2,061312047	4,935999316	36,78053109	4,086725677
ENSG00000131849	ZNF132	0	0	0	1,509167002	0	4,318089041	0	14,42903982	16,4993876	36,75568347	4,08396483
ENSG00000105556	MIER2	10,04992837	1,317236176	0	1,544771861	10,31349032	3,719874374	2,038754607	1,91459757	5,852327821	36,7509811	4,083442344
ENSG00000172785	CBWD1	5,630654997	7,26651574	4,531644521	2,209752784	6,209160048	2,341715973	1,815202404	2,891593957	3,842839169	36,73907959	4,082119955
ENSG00000141384	TAF4B	29,9420204	0,049701946	0	1,64444655	0,133129765	0,2767739	0,32559493	1,102469363	3,169208536	36,64334539	4,071482821
ENSG00000105954	NPVF	0	0	0	36,61860568	0	0	0	0	0	36,61860568	4,068733964
ENSG00000166704	ZNF606	0	6,356738967	0	2,153098022	0	1,499388316	3,697553988	13,71941819	9,176704505	36,60290199	4,06698911
ENSG00000169118	CSNK1G1	0,125689807	0,920013101	11,5319255	9,468320455	0,709423301	5,865002711	4,096244983	0,549693604	3,323518593	36,58983206	4,065536895
ENSG00000075407	ZNF37A	2,589157617	2,297198333	5,100165576	3,165246091	0	3,371041286	2,216420573	12,57605405	5,255553662	36,57083719	4,063426354
ENSG00000275066	SYNRG	2,694803409	2,14619027	4,996342438	4,873180035	0,37820425	5,183613469	5,664356068	5,034353798	5,597708211	36,56875195	4,063194661
ENSG00000185347	TEDC1	8,234601119	0	14,19366883	2,804914627	0	1,981613338	2,1355058	4,933331853	2,275218696	36,55885427	4,062094919
ENSG00000188343	FAM92A	3,867844325	0,072246262	0	20,38829116	3,289774064	0,447017585	1,724097247	4,763101	2,002926281	36,55529792	4,061699769
ENSG00000138442	WDR12	0,033218415	0,029472631	2,535980239	1,434023822	0,473665697	8,443239944	9,115837487	8,135562005	6,311999932	36,51300017	4,057000019
ENSG00000125449	ARMC7	3,791113257	1,42124735	7,563416121	0	0,126896658	3,927918247	12,28101836	1,984942632	5,384952235	36,48150486	4,05350054
ENSG00000113231	PDE8B	0	1,042602823	0	0	35,01189173	0,406175087	0	0	0	36,46066964	4,051185516
ENSG00000081692	JMJD4	5,813291942	2,547815065	0	4,656313568	0	4,267918518	6,920495716	1,148671729	11,06895857	36,42346641	4,047051823
ENSG00000239642	MEIKIN	3,701035482	5,285953407	1,328459254	4,832129159	0	5,153733565	8,394669763	3,750452081	3,885679976	36,33211269	4,03690141
ENSG00000145375	SPATA5	0,146958979	6,780152689	20,68123275	0,602694447	0,0436564	5,001923538	2,913303357	0,040169532	0,112962875	36,32305456	4,035894951
ENSG00000155275	TRMT44	1,325531322	0	0,035859146	12,58896807	0,046325792	8,710733323	1,391956728	8,695646514	3,524179408	36,31920031	4,035466701
ENSG00000115596	WNT6	0	1,871846423	0	0	25,27819815	0	2,99260245	0	6,162450128	36,30509715	4,033899684
ENSG00000163684	RPP14	13,73240319	0,576374015	0,033195617	5,889265608	9,520431622	3,170013407	2,592114573	0,157838341	0,621411825	36,29304819	4,03256091
ENSG00000178607	ERN1	6,300910209	3,455438145	1,509648918	2,353794874	3,999744351	6,0017357	5,820788598	4,866488476	1,967263445	36,27581271	4,030645857
ENSG00000105298	CACTIN	0	2,479456961	0	0	0	5,634488314	5,864282498	8,388638476	13,90411944	36,27098536	4,030109484
ENSG00000170113	NIPA1	0,017397026	0,030870596	0,096009705	3,574863226	0	3,533669893	7,049219666	20,39062033	1,56191528	36,25456572	4,02828508
ENSG00000161653	NAGS	0	5,701105601	0	10,17110107	0,254512787	11,46440909	3,556919405	0	5,070936292	36,21898424	4,024331582
ENSG00000128973	CLN6	2,386822739	4,892569174	17,2223509	1,350153736	0,146698342	1,875078686	1,77681298	2,384672124	4,150167436	36,18532612	4,020591791
ENSG00000142528	ZNF473	0,021906432	5,558757033	0	0,945690688	0,052061111	5,026864385	8,912795402	0,383223621	15,24922175	36,15052042	4,016724491
ENSG00000188659	SAXO2	0	0	35,88348874	0,250899014	0	0	0	0	0	36,13438775	4,014931973
ENSG00000125968	ID1	0	20,65976481	0	0,423252189	0	6,862481275	3,052806131	0	5,12472162	36,12302602	4,013669558
ENSG00000084731	KIF3C	0,094429352	1,885078895	2,562228689	16,0612998	0	4,276713819	4,469182268	3,200584781	3,571179853	36,12069745	4,013410828
ENSG00000165480	SKA3	0	0,089440294	0	30,46272218	0	1,051467964	4,268837179	0,220436669	0	36,09290429	4,010322699
ENSG00000146826	MAP11	5,418307315	0,082176525	0	7,916813511	0	8,186209883	5,99858161	0,202534212	8,258579752	36,06320281	4,007022534
ENSG00000168795	ZBTB5	32,44527605	0	0,47416648	0,166909365	0	0,884384737	0	2,043198818	0,039626124	36,05356157	4,005951286
ENSG00000198455	ZXDB	18,4081082	5,868733969	0,188652934	0,088542577	0,121858684	0	0,276741486	0	11,09907971	36,05171757	4,005746396
ENSG00000113272	THG1L	0	0,357761174	4,357926835	3,133308568	0	5,368020661	7,909904185	12,45467182	2,417616534	35,99920978	3,999912198
ENSG00000068024	HDAC4	10,58045288	0,621813435	2,453744141	1,405394836	0	4,070816065	3,322598558	5,067256608	8,438722249	35,96079878	3,995644308
ENSG00000165359	INTS6L	0,16806403	0,074556374	0,154583678	7,364076163	0,149777927	5,627996763	0,139547151	0,091876704	22,14242591	35,9129047	3,990322745
ENSG00000160256	FAM207A	1,295233463	2,106830001	1,191343281	10,62378111	0	5,214332411	6,632003263	1,652172838	7,168365859	35,88406222	3,987118025

ENSG00000089692	LAG3	10,38390702	0	5,912534828	0	0	5,809041789	10,18214736	3,568174322	0	35,85580532	3,983978368
ENSG00000186716	BCR	12,18495925	1,212039067	6,304980855	1,95874991	0,115947225	6,33430485	3,473752835	4,267457487	0	35,85219147	3,983576831
ENSG00000111684	LPCAT3	0,418588838	4,285243982	12,02428299	0,05560089	0	3,853452559	6,282874072	4,083785407	4,831296765	35,83512551	3,981680612
ENSG00000109944	JHY	6,78060253	0,057295299	0	0	0	9,394509151	7,6944413	3,036044103	8,855507365	35,81839975	3,979822194
ENSG00000108641	B9D1	0	0	0	5,906082054	27,02929962	0,588174938	0,102648213	2,162647838	0	35,78885266	3,976539184
ENSG00000111424	VDR	0	15,50063025	0,930846295	3,173160601	0,063291777	6,900757156	4,887018571	0	4,32353559	35,77924024	3,975471138
ENSG00000127080	IPPK	0,030023956	6,180105704	15,29912327	0	0	3,889818619	7,329282259	0	3,027898833	35,75625084	3,972917676
ENSG00000198131	ZNF544	0	11,74236031	0,038522741	0,216963671	0,049766843	3,701718798	5,477155568	1,007422462	13,52126167	35,75517207	3,972796896
ENSG00000086015	MAST2	0,172675868	0,987318182	8,047163861	4,008777543	0	7,014756481	13,33401065	0	2,170883704	35,73558629	3,970620699
ENSG00000136982	DSCC1	0	0	0	25,39053633	0,144397886	1,801200471	8,374778895	0	0	35,71091358	3,967879287
ENSG00000012232	EXTL3	12,2191528	0,20283056	0	0,256592638	0,027164716	1,204796926	12,13892244	7,323549794	2,333625015	35,70663489	3,967403876
ENSG00000184208	C22orf46	0,028292561	0,853475754	14,41686714	0,097710157	5,311792894	6,896114591	3,288868467	1,917893951	2,853288677	35,6643042	3,962700466
ENSG00000197312	DDI2	0,012408488	3,302783709	0,365222662	7,949324649	0,088467123	4,741080694	5,780001335	2,496303187	10,91150135	35,6470932	3,960788133
ENSG00000110080	ST3GAL4	0	0,120108399	25,33883545	1,285681846	0	1,412004908	7,474828289	0	0	35,63145889	3,959050988
ENSG00000157216	SSBP3	3,266786777	4,257850776	4,414070828	12,67983232	2,061129954	0,761785698	3,024538326	3,729795195	1,422204112	35,61799399	3,957554888
ENSG00000121104	FAM117A	8,112251688	5,786222107	0	1,854011662	0	1,964730514	10,16970059	4,173910327	3,521302527	35,58212942	3,953569935
ENSG00000171970	ZNF57	19,50652805	0	0	1,122783884	0	4,854526341	6,154740898	0	3,918450771	35,55702995	3,950781105
ENSG00000196417	ZNF765	13,68391528	0,065156812	1,148305933	0,042270367	0,145438823	3,144587312	3,851713578	0,13382252	13,33711475	35,55232537	3,950258375
ENSG00000150457	LATS2	0	0,238858008	10,05344024	0	0,127959217	2,7784775	5,118957069	16,89554977	0,331100164	35,54434198	3,949371331
ENSG00000065060	UHRF1BP1	0	0,027063181	1,655311449	0,869080961	0,07249042	4,052317275	17,93156945	0,100050838	10,82291515	35,53079873	3,947866526
ENSG00000168528	SERINC2	0,218873944	3,728509851	0,322109037	7,181003067	0,208063466	8,170567219	5,524765488	0	10,17525861	35,52915068	3,947683409
ENSG00000138378	STAT4	0,065614664	0,058215805	21,60592172	2,662601782	0,077967334	2,017149869	5,175719297	0,143480055	3,712088591	35,51875912	3,946528791
ENSG00000163472	TMEM79	0	14,8574723	0,091682091	0	0,355327384	3,775669457	7,117710127	0,108982377	9,19425407	35,50109781	3,944566423
ENSG00000158636	EMSY	0,153893478	2,025344819	13,47081698	1,439427425	0,213343189	3,773575028	3,631111195	3,000636402	7,775807136	35,48395566	3,94266174
ENSG00000170448	NFXL1	0,03362496	7,458333336	0	3,94828177	0,319641782	0,443018896	18,34312597	0,147055882	4,755752128	35,44883473	3,938759414
ENSG00000204463	BAG6	2,703484786	5,150750284	0,157050903	7,174483294	0,541043378	3,405697337	4,371376915	5,911729551	6,01988735	35,4355038	3,9372782
ENSG00000170604	IRF2BP1	2,92202761	1,693082873	10,75061156	0	1,84235519	4,779586464	4,010693328	4,955214509	4,47381131	35,42738285	3,936375872
ENSG00000186453	FAM228A	0	0	24,12979377	10,98191914	0,314877284	0	0	0	0	35,4265902	3,9362878
ENSG00000178104	PDE4DIP	4,928886224	2,203534519	0,181140419	9,219582783	3,055157747	2,955073806	3,565659437	5,538544139	3,760931324	35,4085104	3,934278933
ENSG00000186806	VSIG10L	27,00835975	1,137701406	0	1,245513005	0	2,947761506	0,299451866	0	2,75985289	35,39864043	3,93318227
ENSG00000148835	TAF5	13,67831186	0	0	0,48039578	0	0,559992741	2,733474554	8,821051664	9,12410051	35,39732711	3,933036345
ENSG00000198039	AC092161.1	1,589350662	0,061983819	0,064257998	12,03340902	0,041506882	2,761341082	1,11664535	9,242390816	8,441710963	35,35259659	3,928066288
ENSG00000106804	C5	20,6322714	0	0,042550145	2,516287066	0	1,90468288	1,67088916	1,669116903	6,912705482	35,34850303	3,927611448
ENSG00000145990	GFOD1	2,361544572	0,050185664	10,74356924	7,691794591	2,453264139	3,928071861	3,663367358	2,504697732	1,947857991	35,34435315	3,92715035
ENSG00000146555	SDK1	9,245629629	0,853119432	5,737393134	0,383164427	0	4,209348128	4,288796631	1,994791809	8,596425039	35,30866823	3,923185359
ENSG00000163380	LMOD3	0,954159356	3,13543031	2,145309464	7,078684999	15,2011869	1,823619529	1,085688093	2,704679575	1,173493008	35,30225123	3,922472359
ENSG00000140939	AC074143.1	0,05050487	1,971632354	16,63049389	7,325714528	0,480103666	1,996250254	3,899980239	2,871423381	0,062114479	35,28821766	3,920913073
ENSG00000119778	ATAD2B	0,129511012	0,408558423	0,767681447	4,447896434	0	3,491690073	5,74718881	13,02732225	7,256173139	35,27602159	3,919557954
ENSG00000106351	AGFG2	0,055079486	0	11,70282538	0,427996131	1,70166869	3,870339679	3,109889767	5,480143183	8,87402749	35,22196981	3,913552201
ENSG00000166004	CEP295	2,206227121	1,560284596	5,764293208	6,018178636	6,990862018	5,090358363	2,654894879	2,13251255	2,792023504	35,20963487	3,912181653
ENSG00000105088	OLFM2	0,060444228	0	0,444768158	0	0	13,20648191	8,78292324	0	12,71190212	35,20651966	3,911835517
ENSG00000122484	RPAP2	3,288480861	3,318744839	0,048917192	8,20396213	0,06319525	5,128746711	4,018465584	4,787502239	6,344596197	35,202611	3,911401223
ENSG00000183779	ZNF703	9,706438948	0,323452352	1,425109072	2,045931646	12,23772083	2,451633974	3,216217799	1,49472814	2,297861102	35,19909387	3,91101043
ENSG00000221968	FADS3	5,954150226	0,639041945	0,088331776	0,041457733	0,285285623	6,089187038	5,561840265	2,834996289	13,70079224	35,19508313	3,910564793
ENSG00000010803	SCMH1	0,101941019	5,366457839	0,125019159	2,5230951	0,403775064	7,573610765	3,188249947	0,148610105	15,75537191	35,18613091	3,909570101
ENSG00000105229	PIAS4	0	2,940814962	18,60496725	4,91637888	0	1,679634635	3,387275555	0,092923107	3,553870505	35,1758649	3,908429433
ENSG00000135914	HTR2B	0	12,60390968	0	0	0	12,84468981	9,713832155	0	0	35,16243165	3,90693685
ENSG00000125459	MSTO1	0,32319276	2,48515637	3,963594567	2,852426053	1,792174047	4,888999884	3,637685481	9,423038998	5,785621297	35,15188946	3,905765495
ENSG00000160539	PLPP7	10,63229124	4,624200967	0	0,059998844	0	1,373368766	6,924092821	0	11,49522062	35,10917327	3,901019252
ENSG00000106003	LFNG	13,13439556	6,153574299	3,349157819	3,592908428	0	0,809087723	5,290917614	0,189578065	2,558988258	35,07860776	3,897623085
ENSG00000203326	ZNF525	0,021008004	13,53198193	0	4,208043522	0	2,952391744	3,017707137	5,145095449	6,175075604	35,05130339	3,894589266
ENSG00000133247	KMT5C	0	0	11,93172535	0,218538505	19,47475785	0,660029514	0,709317204	1,383729345	0,661514003	35,03961177	3,893290197
ENSG00000006607	FARP2	0,010514966	3,153294692	0	6,863365138	2,249013904	5,345250645	3,518512546	10,16297257	3,724431036	35,0273555	3,891928389
ENSG00000167967	E4F1	0	6,086504385	0,087031966	2,532556109	0	3,947778281	10,05648246	4,086463812	8,204245348	35,00106237	3,88900693
ENSG00000168014	C2CD3	1,73096191	0	2,874664933	0,705734559	0,057134284	5,345142277	7,116406548	13,56329941	3,60723502	35,00057894	3,888953215
ENSG00000137265	IRF4	6,372287285	7,295140285	0	0,310584054	0,122128054	2,59543837	9,344670195	2,921713571	6,035824818	34,99778663	3,888642959
ENSG00000130382	MLLT1	1,744193365	0,056273242	0,641716913	4,545142265	3,617553417	4,456779395	6,688240153	6,17181039	7,059437677	34,98114682	3,886794091
ENSG00000112195	TREML2	0	0	6,095732686	1,654541451	1,612945369	2,805357175	11,90062066	0,349204299	10,55667167	34,97507331	3,886119257
ENSG00000130517	PGPEP1	0,281734983	2,249693169	0	4,239454908	0,526074439	9,633374809	10,32636372	0,132015409	7,573449969	34,96216141	3,884684601
ENSG00000187790	FANCM	0,035647693	0,031627978	0,032788405	0,954114675	0,084717514	8,219207622	6,215798471	15,59021314	3,792338801	34,9564543	3,884050478
ENSG00000255823	MTRNR2L8	0,579856372	0,205788181	6,186817245	12,01542477	3,858515421	2,291934291	2,985096157	5,832689987	0,998408024	34,95453045	3,883836716
ENSG00000173818	ENDOV	2,500105953	7,504870539	7,090351346	0,071952289	0,099025932	9,676020122	2,248882278	1,50342489	4,253484447	34,9481178	3,8831242
ENSG00000159110	IFNAR2	1,768524234	4,939763764	0	7,578085522	0	2,876646323	4,677264218	10,38426794	2,658402789	34,88295479	3,875883866
ENSG00000249141	AL159163.1	0	2,234516667	0	0	13,16764322	4,147764418	7,73733714	3,855069954	3,716930445	34,85926184	3,873251316
ENSG00000171466	ZNF562	0,185668033	0,061774382	0,064040877	14,02660745	0,082733269	5,376613682	7,737112317	4,060016188	3,239683655	34,83424985	3,870472206
ENSG00000005187	ACSM3	0	1,249663116	23,83744124	0,799135075	1,434557426	1,966183932	3,241177684	0	2,276681764	34,80484024	3,867204471
ENSG00000180479	ZNF571	0	0	0,447921526	19,97165855	0	5,640852672	3,275248757	0	5,450220279	34,78590178	3,865100198
ENSG00000165417	GTF2A1	0,044274999	0,314259596	0,040723718	19,99252735	0,526102465	5,201415677	2,646898302	2,565636109	3,43051008	34,7623483	3,862483144
ENSG00000174989	FBXW8	0,029009516	2,36792705	12,75431823	7,513965481	0	6,465703028	4,480223028	0	1,141694896	34,75284123	3,861426803
ENSG00000177791	MYOZ1	0	0	0	0	0	9,905109057	1,46558467	23,37272411	0	34,74341784	3,86037976
ENSG00000146350	TBC1D32	0,240482679	0	0	17,66747224	1,194980248	2,256307033	2,650270323	0,430253343	10,29791928	34,73768514	3,859742793
ENSG00000243646	IL10RB	2,003233372	3,080730005	4,115039026	2,075488088	7,061745751	5,132010769	3,93654157	4,964549401	2,29947089	34,66880887	3,852089875
ENSG00000188191	PRKAR1B	3,768511226	0,776184814	5,570743467	9,412484054	0,559746782	4,063719314	4,88918016	0	5,627950797	34,66852061	3,852057846
ENSG00000100448	CTSG	0	0,392020468	1,828816441	22,50754898	0	0	0	0	9,91724863	34,64563452	3,849514946
ENSG00000141337	ARSG	19,47667747	0	0	0	0,054519135	5,062703191	7,466884795	0,100329305	2,482844081	34,64395798	3,849328664
ENSG00000066855	MTFR1	0	7,704231154	0	0,112739242	0,07757994	7,705935189	10,27290037	1,784589369	6,985805763	34,64378103	3,849309003
ENSG00000186767	SPIN4	0	2,409826088	0,067520063	16,79566876	0,087227968	7,898584657	1,645712394	4,65513843	1,083389977	34,64306833	3,849229815
ENSG00000073350	LLGL2	3,763912305	0	15,00790268	5,740635426	0	2,559247738	2,109944893	0	5,404564436	34,58620747	3,842911941
ENSG00000175787	ZNF169	22,98390065	4,869775257	0	0	0,135874969	1,192698054	2,634734554	0,062511269	2,672027184	34,55152193	3,839057992
ENSG00000167110	GOLGA2	2,366190519	0,038520615	0,479207181	15,29399075	0	4,83836332	9,192625962	0,332285875	2,002368793	34,54355302	3,838172558
ENSG00000100479	POLE2	0,051137249	13,88349442	0	8,388772449	0	9,825499466	1,486112223	0,894577509	0	34,52959332	3,83662148
ENSG00000131584	ACAP3	16,99061085	0,554330539	0,031926048	1,018926052	0	5,049535182	3,458467863	3,073986444	4,290243594	34,46802657	3,82978073
ENSG00000149554	CHEK1	0,046084346	1,635510827	14,58145075	11,8172747	0,054760223	1,69503029	1,511460236	0,856570242	2,238773294	34,43691491	3,826323879
ENSG00000222011	FAM185A	19,43562122	0,098256504	0,101861524	1,24300281	0	6,93067268	2,620670181	0,121082656	3,881733884	34,43290146	3,82587794
ENSG00000049247	UTS2	0	34,28504635	0	0,145692336	0	0	0	0	0	34,43073869	3,825637632
ENSG00000172380	GNG12	14,13356557	0	0	0,055341458	0	15,51782913	4,417407235	0,28032625	0	34,40446964	3,822718849
ENSG00000131943	C19orf12	0,590056152	8,326462256	0	2,086315342	0	5,892324312	11,01189972	1,474605731	5,010737399	34,39240091	3,821377879
ENSG00000165392	WRN	0,056666464	1,37422775	0,938182834	3,653089644	0,134669078	10,49384398	10,72771339	1,032608023	5,970319528	34,38132069	3,820146743
ENSG00000088727	KIF9	6,147746294	4,033589061	9,978772784	3,033090303	0	4,622807522	2,74533376	1,299144453	2,509722267	34,37020644	3,818911827
ENSG00000100888	CHD8	8,034036926	3,314502841	3,290176325	3,18805677	1,37113556	5,170601183	2,538988248	5,882312494	1,578804036	34,36861438	3,818734932
ENSG00000158488	CD1E	28,74050345	0	0	0	0,326025912	0	4,841115851	0,449978984	0	34,3576242	3,8175138
ENSG00000135763	URB2	0	5,551779598	0	0,087138078	0,119925711	6,426991784	10,37031463	0,717256533	11,01611545	34,28952178	3,809946865
ENSG00000154262	ABCA6	0,032019474	0	0	0,110581286	0,279014901	0,738265739	0	33,09480271	0	34,25468411	3,806076012
ENSG00000165434	PGM2L1	0	7,297419497	0	19,49138503	0	6,423319982	0,457190916	0,451516148	0,126973256	34,24780483	3,805311648
ENSG00000250571	GLI4	3,015291073	3,420849854	13,4579832	0	0,117473844	4,396060423	5,622975297	1,405184618	2,796512929	34,23233124	3,80359236
ENSG00000074755	ZZEF1	0,03804395	0,894481748	1,609653141	8,425204052	0,067809204	7,831881453	9,41343377	4,346728639	1,602528547	34,2297645	3,803307167
ENSG00000164002	EXO5	0	0	0	15,10587337	0	8,531624093	8,196962181	0,106867583	2,284012887	34,22534011	3,802815568
ENSG00000158373	HIST1H2BD	0	12,31421355	0,319150515	0,599161825	1,236914612	0,190482866	15,26963857	0	4,267428755	34,19699069	3,799665632
ENSG00000196110	ZNF699	0	1,480220812	0	3,601089906	1,189459152	5,541037754	8,934956019	2,827345182	10,61833566	34,19244449	3,799160499
ENSG00000183020	AP2A2	2,492901093	1,724922599	4,787787553	2,463693991	3,42797405	3,150200823	6,001434224	6,479781413	3,644427548	34,17312329	3,797013699
ENSG00000184154	LRTOMT	0	0	2,846971729	0,083512554	26,60768477	1,141647431	1,164551009	0,42302393	1,903372928	34,17076435	3,796751594
ENSG00000118922	KLF12	0,027668328	0,883741829	0,458083107	27,83019733	0,065754379	1,260695584	2,57304352	0,6050253	0,459384427	34,1635938	3,795954867
ENSG00000196890	HIST3H2BB	4,218823039	0	0	0,551892924	3,4939524	14,66806706	2,229168335	8,526441886	0,471691681	34,16003733	3,795559703
ENSG00000074657	ZNF532	5,904831102	1,211586311	10,39721398	0,720513276	7,001454888	3,359581588	1,973761394	1,023017237	2,503664136	34,09562391	3,788402657
ENSG00000100784	RPS6KA5	5,454280479	3,248521837	0,029802741	7,618607471	0,064169384	1,458580583	2,022268592	8,762148849	5,419582667	34,0779626	3,786440289
ENSG00000169062	UPF3A	0,787718005	2,003493658	3,864189039	14,96238777	0	2,046855073	2,943265825	1,837342513	5,61899129	34,06424317	3,784915908
ENSG00000126351	THRA	3,061731637	0,038531686	0	3,224659838	23,22214313	1,740404095	2,127532573	0	0,640942166	34,05594513	3,783993903
ENSG00000236383	CCDC200	10,58971092	0,571905546	10,33320295	0,159009735	0	3,235306797	2,714318578	4,732000205	1,642153914	33,97760865	3,77528985
ENSG00000123405	NFE2	20,01591562	0	5,764293208	3,864885362	0	0	4,300929705	0	0	33,9460239	3,771780433
ENSG00000262165	AC233723.1	0	0	0	17,63615804	0	7,390793739	2,31281718	6,598539751	0	33,93830871	3,77092319
ENSG00000144214	LYG1	0	0	0	33,90006074	0	0	0	0	0	33,90006074	3,766673416
ENSG00000103199	ZNF500	0,023373638	0	3,654804086	14,57038296	0	2,745929112	7,530160683	2,913344215	2,414711194	33,85270589	3,761411765
ENSG00000232859	LYRM9	2,471697067	6,578948701	0	5,480604485	0	4,903305148	4,780932725	6,387582718	3,212588582	33,81565943	3,757295492
ENSG00000181751	C5orf30	0,344706178	0	0	2,827353588	0	7,001643956	0,035777112	17,24252234	6,359162439	33,81116562	3,756796179
ENSG00000115042	FAHD2A	6,600109462	5,336394155	0	7,215003701	0,06324719	3,009649419	4,176451699	1,74586774	5,662460083	33,80918345	3,756575939
ENSG00000148411	NACC2	2,994569236	6,168455102	0,115568223	1,699548754	6,419922805	6,207851401	3,060252	3,251227036	3,863217619	33,78061218	3,753401353
ENSG00000135378	PRRG4	14,14872563	2,273619701	0,050149756	0,32952279	0	7,333229531	0,475352151	7,451618427	1,676407381	33,73862537	3,748736152

ENSG00000159403	C1R	0,025720948	25,51342605	0	0,266486783	1,161401414	1,242564319	0,961049351	4,555778718	0	33,72642758	3,747380843
ENSG00000180376	CCDC66	0	6,278926731	0,059993547	4,477032585	0	2,058888132	4,887751144	6,453939432	9,505908229	33,7224398	3,746937756
ENSG00000135094	SDS	0,402291037	0	23,58899342	0	0	4,251285992	3,799600736	0	1,669835641	33,71200683	3,745778537
ENSG00000124523	SIRT5	6,107588764	7,293858001	0,042720163	2,005032846	0,220757672	1,733812696	1,118377018	6,957051752	8,225572217	33,70477113	3,74497457
ENSG00000116885	OSCP1	18,97012186	0,096177188	0,099705918	7,019412875	0	0	0,405033353	7,111217378	0	33,70166857	3,744629841
ENSG00000137218	FRS3	0	2,157895732	8,851010849	0	0	7,778872386	3,643819999	9,249393073	2,015830018	33,69682206	3,74409134
ENSG00000198824	CHAMP1	4,38664702	4,584440251	0,198026785	4,507692348	3,197840983	2,570653637	5,184170441	5,178671762	3,872492094	33,68063532	3,742292813
ENSG00000137094	DNAJB5	10,69650449	0	0	2,258985521	0	4,731450838	5,260765915	7,572341982	3,154753391	33,67480214	3,741644682
ENSG00000133131	MORC4	0,050211188	0,222746303	0,046183768	0	0	7,525101751	1,334125477	16,74406763	7,750037729	33,67247385	3,741385983
ENSG00000101546	RBFA	0	0,168748899	4,828351625	8,670463599	0,045200446	5,074420408	6,459076877	3,535171585	4,888851247	33,67028468	3,741142743
ENSG00000065883	CDK13	0,153455369	1,949687953	2,156722679	9,984594446	0,452215607	5,998066994	6,223059362	1,409360208	5,314645856	33,64180847	3,737978719
ENSG00000183691	NOG	0,239604499	0,141724102	0,734619712	29,92753144	1,51846741	0,08769058	0,066316243	0,873241457	0	33,58919544	3,732132827
ENSG00000254901	BORCS8	3,178610291	1,796930753	0,051746109	4,614449476	7,353486534	5,126797419	2,756049735	2,767973853	5,915814465	33,56185864	3,729095404
ENSG00000100360	IFT27	3,377299557	4,218490699	3,054344951	3,681564756	0,044839249	4,205249978	7,974079035	1,361510891	5,615539816	33,53291893	3,725879881
ENSG00000138050	THUMPD2	0,039526699	0,070139158	0,727125553	1,365079647	8,36030899	4,339800594	12,27462835	5,704598864	0,631965679	33,51317353	3,723685948
ENSG00000198848	CES1	5,453769319	14,242476	9,938001496	0	0	3,558858132	0	0,225015027	0	33,41811997	3,713124442
ENSG00000214413	BBIP1	2,23695184	6,001757226	6,419483574	0,370821849	0	3,752832798	4,160551037	7,356904156	3,103311184	33,40261367	3,711401518
ENSG00000121413	ZSCAN18	9,222710886	3,26018322	0,066926712	8,700973632	0	1,418039117	3,473958873	1,909336613	5,279857862	33,33198691	3,703554102
ENSG00000130921	C12orf65	5,136480611	0	0,313658786	4,011553206	0,253256377	4,188715547	5,39752486	8,901692863	5,111434675	33,31431692	3,701590769
ENSG00000162543	UBXN10	10,89452334	12,72734413	0,099956886	0	0	1,96873392	0,879781184	2,614009524	4,109874549	33,29422353	3,69935817
ENSG00000132199	ENOSF1	5,333314259	7,274440564	0,048811255	7,537103305	0,031529196	2,17038419	4,406332994	6,237351362	0,24474959	33,28401672	3,69822408
ENSG00000184454	NCMAP	4,665256403	0	0	28,44732478	0,086617835	0,040017023	0,030262984	0	0	33,26947902	3,69660878
ENSG00000117245	KIF17	0,033805497	2,159532887	11,69133302	3,210609307	0	3,15489911	1,487679552	2,069836217	9,437843525	33,24553912	3,693948791
ENSG00000213366	GSTM2	4,077797322	2,853200308	4,848490099	1,688806878	11,9364431	2,001992479	1,624122332	1,848635689	2,36487457	33,24436278	3,693818087
ENSG00000276043	UHRF1	0,029370373	2,345265307	1,242670996	11,66473161	0,767792608	5,25625345	4,633501085	0	7,296598153	33,23618359	3,692909287
ENSG00000184205	TSPYL2	0	0	0,224721179	3,480539273	0	2,615405211	6,161942336	17,96420749	2,77943465	33,22625014	3,691805571
ENSG00000183814	LIN9	0,136074354	3,380448448	0	0,117485356	0,161691825	2,128974082	8,247937178	19,04350502	0	33,21611626	3,690679584
ENSG00000177469	CAVIN1	0,042327891	0	2,647429514	20,10003306	0	0	10,33285087	0,092558702	0	33,21520004	3,690577782
ENSG00000121897	LIAS	5,073503608	2,650572817	0,26056933	5,381019502	0,030602262	2,163128951	1,582412368	11,20689884	4,846104458	33,19481213	3,688312459
ENSG00000005206	SPPL2B	5,316061321	0,578281709	1,835965069	0,386882965	15,15080075	2,795366234	3,027252922	2,27148712	1,803605554	33,16570364	3,685078183
ENSG00000235194	PPP1R3E	8,797533224	3,818171955	0	4,185871078	0	4,6053044	0,655847085	9,708153299	1,373415179	33,14429622	3,68269958
ENSG00000133216	EPHB2	5,602053754	2,420904922	1,732378373	1,480215979	0,028692677	5,593980774	11,08742307	2,983309854	2,212458598	33,14141801	3,682379778
ENSG00000169469	SPRR1B	0	0	0	27,82182282	2,900787903	2,412270097	0	0	0	33,13488082	3,681653424
ENSG00000100916	BRMS1L	0,89645167	13,3483121	0	0	0	3,937006205	6,990351228	0,34091779	7,621768646	33,13480764	3,681645293
ENSG00000173486	FKBP2	2,389729636	3,957815499	0,146536689	5,502055342	2,839622106	3,148540201	5,952730564	4,180511134	4,996400968	33,11394214	3,679326904
ENSG00000160613	PCSK7	6,138464647	0,613157064	7,789874092	4,399029299	0,01610176	4,202998513	3,887369386	0,96302053	5,099675901	33,10969119	3,678854577
ENSG00000079974	RABL2B	9,267428525	2,23832334	6,393068716	0,444522955	0	3,815660623	2,821479357	3,377525392	4,717398947	33,07540785	3,675045317
ENSG00000143412	ANXA9	0,18959921	25,23293602	0	0,327396455	0	0,104084427	1,416854439	0	5,712973395	32,98384394	3,664871549
ENSG00000138835	RGS3	4,940974942	5,054514611	0,060461125	4,38895919	0,026036217	3,716839571	4,011630277	1,820708122	8,960192657	32,98031671	3,664479635
ENSG00000186272	ZNF17	0,040167616	3,991481128	0	21,43245804	0,095459208	2,954813567	4,068947502	0	0,395207916	32,97853498	3,664281664
ENSG00000156869	FRRS1	0	0	0	18,78890039	0	1,131780084	10,39774353	2,170616159	0,469546544	32,95858671	3,66206519
ENSG00000251247	ZNF345	0,031886594	0,735565731	0,058657966	2,202447559	0	3,115855946	0,05295224	18,68674068	8,039362116	32,92346883	3,658163204
ENSG00000108773	KAT2A	0,04002929	0,071030993	0	0,967705866	0	2,94463805	7,910447421	16,01841745	4,923086683	32,87535575	3,652817305
ENSG00000148516	ZEB1	0,01746943	8,245753991	0	14,53996043	0,041516479	1,764598041	6,440326868	0,343804752	1,460990002	32,85442	3,650491111
ENSG00000156504	FAM122B	0,651930255	3,044300637	0	3,851220107	0,815434928	10,77439258	1,737895153	1,575641671	10,38107277	32,8318881	3,647987567
ENSG00000107829	FBXW4	0,212874622	5,774037836	8,615201297	0,420100583	0,794987516	3,005021514	3,282580632	5,053939853	5,647579968	32,80632382	3,645147091
ENSG00000141570	CBX8	6,777080139	8,060511033	0	0,063256964	3,30823433	4,786268758	3,467543721	0	6,307518332	32,77041328	3,641157031
ENSG00000163449	TMEM169	0	0	4,367383033	0	0,09898499	3,338332993	24,55455781	0	0,4098049	32,76906372	3,64100708
ENSG00000188747	NOXA1	0	4,634158524	0	0	0	13,44686438	5,234161645	2,363060078	7,088305975	32,7665506	3,640727845
ENSG00000173137	ADCK5	0	4,204998543	0,101378592	0	0	9,86266375	5,353766902	3,13322347	10,09888104	32,7549123	3,6394347
ENSG00000185189	NRBP2	12,55681678	0	0	8,461612472	0	1,34503913	2,517544156	0	7,872298977	32,75331151	3,639256835
ENSG00000181827	RFX7	0,014271871	0	5,434631705	8,453031383	0	9,464486997	3,70912485	0,749001664	4,914717249	32,73926572	3,637696191
ENSG00000108984	MAP2K6	0,018140525	0,064379832	2,436080099	13,26602182	0,129334049	1,951889138	2,018370287	7,576590924	5,276434876	32,73724155	3,637471283
ENSG00000140265	ZSCAN29	4,168410376	0,08083871	1,298972425	0,629327645	0,054132882	1,225447431	9,078347808	12,95040512	3,249652786	32,73553518	3,637281687
ENSG00000087299	L2HGDH	11,3332928	0	0	1,75017361	0	6,955092773	0,707683275	7,454926218	4,532842007	32,73401068	3,637112298
ENSG00000131370	SH3BP5	0	0,174269064	9,816021693	0	9,335811326	3,055112439	0,842630046	8,446984178	1,046795836	32,71762458	3,63529162
ENSG00000173517	PEAK1	1,349647836	1,840538042	0,057471903	11,46931351	0,980059452	3,889815456	2,879425829	9,632664989	0,568666997	32,66760402	3,62973378
ENSG00000176896	TCEANC	0,144437556	0	0	0,299294561	0,137303591	10,49826054	11,8250571	4,421796927	5,258131389	32,58428166	3,62047574
ENSG00000100324	TAB1	2,94082577	5,036385157	4,969546915	9,565844283	0	1,65197225	4,429366778	0,07477587	3,911230272	32,57994729	3,619994144
ENSG00000158169	FANCC	0,165161058	7,431525492	0,043403872	12,85424069	0,11214538	2,629389263	8,071477365	0,180579434	1,059161225	32,54708378	3,616342642
ENSG00000174791	RIN1	0	2,094469779	10,95997415	5,968942138	0,13357543	1,542780144	3,500195239	3,257026609	5,080795755	32,53775924	3,615306582
ENSG00000092969	TGFB2	0	0	0	17,76945612	0,118716543	6,828385719	4,666252017	0	3,13328021	32,51609061	3,612898957
ENSG00000141258	SGSM2	0	1,920984547	0,96728312	1,682417705	0	4,227994432	4,776887064	3,550878067	15,38735406	32,513799	3,612644333
ENSG00000152433	ZNF547	21,93813778	10,2206592	0	0,219395999	0	0,092999202	0	0	0	32,47119218	3,607910242
ENSG00000146410	MTFR2	0,431744488	1,641685715	0	0	0	19,02892737	6,145485649	0,134871252	5,082333466	32,46504794	3,607227549
ENSG00000148604	RGR	0	0	0	0	32,40923187	0	0	0	0,037605529	32,4468374	3,605204155
ENSG00000180424	DEFB123	0	0	0	32,43259571	0	0	0	0	0	32,43259571	3,603621746
ENSG00000162739	SLAMF6	0	18,16256811	1,113540091	0	0	0,060419001	13,02223038	0	0	32,35875758	3,595417508
ENSG00000152223	EPG5	0	3,161499564	0,445091862	3,34239851	16,02176517	4,081351887	0,483983086	0,384785523	4,436510866	32,35738647	3,595265163
ENSG00000162078	ZG16B	0,056701903	0	0	0,09791181	0	0,062255376	0,0941616	30,25367147	1,743400772	32,30810293	3,589789215
ENSG00000096070	BRPF3	0,045701065	0	0,0420354	4,143077625	0,054304785	4,817001471	11,36499756	11,64240953	0,112412837	32,22194027	3,580215586
ENSG00000185164	NOMO2	2,488514029	2,878159977	2,942885918	3,107739001	4,910738338	2,293132511	4,446162331	2,234963991	6,858914438	32,16121053	3,573467837
ENSG00000119723	COQ6	2,147099045	7,499634163	10,64356945	0,50735188	0,053711867	1,985169928	3,058878869	2,915889842	3,335564265	32,14686931	3,571874367
ENSG00000128918	ALDH1A2	0,037187294	0	0	0,03210717	29,20840353	0,551197929	2,254050507	0,040658862	0	32,12360529	3,569289477
ENSG00000130810	PPAN	1,622031749	0,557081718	15,11179901	2,891246256	0	1,953240861	2,38949732	3,203659242	4,375887644	32,1044438	3,567160422
ENSG00000103091	WDR59	7,971797814	8,242186559	0,206962422	4,384995319	0	3,864541193	4,737499241	0	2,668504627	32,07648718	3,564054131
ENSG00000166046	TCP11L2	1,142008545	5,674105201	4,369699258	9,189517697	3,256809634	2,307099918	2,655055393	0,899005776	2,556232395	32,04953382	3,561059313
ENSG00000130590	SAMD10	11,59542338	14,44683429	0	0,745528499	0	2,776462712	2,45819426	0	0	32,02244314	3,558049238
ENSG00000169621	APLF	0	7,828963506	2,380233863	0,073255187	0	0	0,422696234	19,01714945	2,295689775	32,01798801	3,557554224
ENSG00000115009	CCL20	14,12514117	1,345756588	1,569523771	0,163698227	0	0,104084427	0	0	14,69050302	31,9987072	3,555411911
ENSG00000278463	HIST1H2AB	0	0	0	29,87805053	0	2,110821587	0	0	0	31,98887211	3,554319124
ENSG00000123838	C4BPA	0,061178369	0	0,2250851	0,21128338	0	0	0	31,43810663	0	31,93565348	3,548405942
ENSG00000187068	C3orf70	20,96002188	0,861470317	0,155317837	1,129904206	0	4,31056953	2,48872568	0,092313051	1,895068603	31,89339111	3,543710123
ENSG00000116205	TCEANC2	7,898284265	2,225702006	3,426776368	1,694104454	0	7,567420288	5,0010775	1,357801477	2,718662144	31,8898285	3,543314278
ENSG00000167513	CDT1	0,054750207	8,646607974	0	7,279707625	0	3,787089251	4,909708644	0	7,204919523	31,88278322	3,542531469
ENSG00000154222	CC2D1B	0	0,035776117	0,037088739	0,382959936	14,70968452	6,264535451	6,3948855	0,176349336	3,868186454	31,86946606	3,541051784
ENSG00000198752	CDC42BPB	2,762738043	0,231870785	0,171698641	0,032234092	0,842894787	7,460339706	8,168358859	6,449494956	5,716588517	31,83621839	3,537357598
ENSG00000152439	ZNF773	6,92566718	3,188607953	0	0,581794815	1,779351133	3,740335063	2,129250029	8,30895791	5,17966896	31,83363304	3,537070338
ENSG00000109118	PHF12	0,704449377	0,964933999	1,023072254	2,966382716	17,798743	2,103225598	1,087744761	2,378199226	2,76634228	31,79309321	3,532565912
ENSG00000187187	ZNF546	0,050213969	0	3,417788102	0,462445679	0,039778195	2,333921493	0,542018492	22,6560697	2,264412655	31,76664829	3,529627588
ENSG00000162129	CLPB	2,334505115	3,085315901	3,355086486	3,002389875	0,606812712	6,100831474	8,117005707	0,026587912	5,129172605	31,75770779	3,528634199
ENSG00000061918	GUCY1B1	0,07735376	0	0,071149245	9,483687329	0,091916443	1,825993038	13,77700856	0	6,421622858	31,74873123	3,527636803
ENSG00000100926	TM9SF1	3,510217222	1,671139944	1,023722744	2,513254713	2,95025743	2,961010292	7,393152707	4,961198889	4,738296602	31,72225054	3,524694505
ENSG00000243678	NME2	4,018898156	2,037553192	3,274081713	5,452668542	1,637314023	4,286443471	5,86354515	1,380995533	3,742350183	31,69384996	3,521538885
ENSG00000115977	AAK1	1,855199616	4,554841365	1,409873706	7,257821819	1,185349534	5,823532701	4,414163755	1,795623178	3,388820778	31,68522645	3,520580717
ENSG00000179627	ZBTB42	0,162615626	4,616919022	0	10,45986312	0	2,142509465	8,20266093	0	6,099885853	31,68445402	3,520494891
ENSG00000153395	LPCAT1	3,804018999	2,443301622	4,550716678	0	0	5,022157997	6,08458444	3,16400667	6,601498088	31,6702845	3,518920499
ENSG00000100139	MICALL1	0,101280543	0,04492996	0	2,011228021	17,45041677	5,448805803	1,030168611	1,993238008	3,581157852	31,66122557	3,517913952
ENSG00000150455	TIRAP	1,767375095	3,210834309	0	0	0,40001954	2,956913648	6,498881023	7,913498415	8,901587811	31,64910984	3,51656776
ENSG00000172399	MYOZ2	0	0	0	0	0	25,80973161	2,174110489	0	3,649662162	31,63350426	3,514833807
ENSG00000100578	KIAA0586	11,11374146	0	0	4,801876785	1,430747181	3,913529698	1,824965305	1,567229024	6,981144263	31,63323371	3,514803746
ENSG00000253846	PCDHGA10	0,045494677	0,080729189	0,334764533	7,188183103	22,21847206	0,149851492	1,227694051	0,049741768	0,33571553	31,6306464	3,514516267
ENSG00000160325	CACFD1	11,03278882	1,599710796	0,157943238	0,074129254	0	6,740117179	4,063527798	5,256913574	2,692662681	31,61779334	3,513088149
ENSG00000125503	PPP1R12C	6,545607188	2,061837755	0,14249909	0,802568165	3,727863761	4,911616458	6,930374611	1,439802037	5,025453825	31,58762289	3,509735877
ENSG00000226372	DCAF8L1	0	0	0	2,188925156	29,39839875	0	0	0	0	31,58732391	3,509702656
ENSG00000064886	CHI3L2	0	5,696491656	0,084364218	8,948604592	0	1,611271155	0,951975205	10,83063226	3,440557772	31,56389686	3,507099651
ENSG00000196586	MYO6	0,017200976	6,623427891	0	19,03919394	0,040878491	3,456076897	1,985244866	0,376134966	0,021154983	31,559313	3,506590334
ENSG00000163808	KIF15	0,028614007	0,253874266	0	13,39014924	0	0,439831107	0,095035283	17,2694453	0,07038308	31,54733228	3,505259142
ENSG00000214753	HNRNPUL2	4,157214739	4,10926625	1,437120157	9,009387048	2,055510305	1,133436362	3,359157707	5,490973242	0,789230061	31,54129587	3,50458843
ENSG00000126970	ZC4H2	0,642650793	8,915604874	0,214946916	4,186662071	0,069421524	7,63323357	3,832266085	0	6,035611007	31,53039684	3,503377427
ENSG00000188735	TMEM120B	7,404400077	0	12,53526071	5,435501897	0	2,002944588	0,386108253	1,642591056	2,111642341	31,51844892	3,50204988
ENSG00000151131	C12orf45	1,500479741	2,880128121	0,03222086	4,768651688	0,069375926	4,532060031	4,784762294	8,451730303	4,416027015	31,43543598	3,49282622
ENSG00000112290	WASF1	0,046352936	11,18629203	0,085269969	13,68707996	0,220317511	6,208923423	0	0	0	31,43423582	3,492692869
ENSG00000158163	DZIP1L	0	0,05315005	12,12202612	0	0	4,669831891	5,595802716	0,065497437	8,914739027	31,42104724	3,491227471
ENSG00000134986	NREP	0,01618406	0,057436436	17,11883441	9,27818481	0,076923539	0	0,41657709	1,804879827	2,647272942	31,41629312	3,490699235
ENSG00000106462	EZH2	0,033416756	0	0,245891286	23,36989489	0	2,531585536	1,415078932	3,43441501	0,369884719	31,40016713	3,488907459
ENSG00000121577	POPDC2	8,961567585	1,754712475	0	6,189868518	0	6,107137793	2,822441006	1,756911811	3,800542378	31,39318157	3,488131285

ENSG00000198420	TCAF1	4,190863687	5,269351507	0	3,432266702	0	3,681058754	3,519532292	4,60827923	6,68576237	31,38711454	3,487457171
ENSG00000103995	CEP152	0,129549785	7,454774528	2,689580872	2,268999779	0,043982553	1,259823118	0,384171255	9,186607082	7,943715404	31,36120438	3,484578264
ENSG00000103264	FBXO31	0,021868389	0,620878727	7,804361679	0,037761936	0	4,033715902	8,025743378	0,047819764	10,70432936	31,29647913	3,47738657
ENSG00000214787	MS4A4E	0,26225368	8,190383897	0,385949461	7,789103719	0,498601239	2,188338047	1,742038625	4,817164816	5,418642093	31,29247558	3,476941731
ENSG00000101247	NDUFAF5	0,253328702	6,742883489	0,737862794	4,374433774	0,652211239	1,761554042	3,943954275	3,646874942	9,165105108	31,27820836	3,475356485
ENSG00000144893	MED12L	1,84412615	2,63488454	7,908301413	9,635921877	4,06956245	1,235881941	1,740019661	0,340411235	1,855666995	31,26477626	3,473864029
ENSG00000239388	ASB14	0	0,165929456	14,10542581	0,322939325	0	2,053348722	0,543497725	10,73503391	3,335117046	31,261292	3,473476888
ENSG00000112144	ICK	0	4,112738778	0,133238584	11,67307763	0,057376185	3,976128216	5,83349842	3,959513111	1,484634305	31,23020523	3,470022803
ENSG00000189298	ZKSCAN3	0	8,372460452	0	13,25315025	0,14950772	2,486586495	6,9473581	0	0	31,20906302	3,467673669
ENSG00000171224	FAM241B	0,111933756	0	0	0	0	1,966347576	20,16819411	0	8,948165887	31,19464133	3,466071258
ENSG00000101966	XIAP	0,033487107	4,248676566	1,016436927	8,095489517	0,039791417	3,180335709	5,783452422	2,63615389	6,15713132	31,19095488	3,465661653
ENSG00000111642	CHD4	2,365374307	1,645172344	1,508741196	11,53507872	3,502759007	3,11906142	3,483916243	1,013682697	3,011435821	31,18522175	3,465024639
ENSG00000154760	SLFN13	1,03666808	0,201199914	0,029797417	10,01336916	0,038494752	5,851064394	4,613176533	1,452226098	7,928707886	31,16470424	3,462744915
ENSG00000129968	ABHD17A	4,578362297	2,227600913	3,079108443	1,912701394	2,632397688	3,972773216	4,496411963	4,359863165	3,905229088	31,16444817	3,462716463
ENSG00000169519	METTL15	13,5995628	0	10,68604392	0,100643472	0,046170936	3,11428956	0,887229141	0,169932922	2,556644109	31,16051686	3,462279651
ENSG00000130363	RSPH3	0,046063274	8,132945895	0	8,312057566	0	3,818396141	1,109172269	7,050882404	2,662638789	31,13215634	3,459128482
ENSG00000111266	DUSP16	0,369592591	0	6,993210037	0,319102869	0,062738914	5,130358504	8,504977082	4,618232949	5,129935451	31,1281484	3,458683155
ENSG00000170271	FAXDC2	0	5,883023097	2,246952301	0	0,051835673	7,854888735	5,433184122	0	9,65715185	31,12703578	3,458559531
ENSG00000183508	TENT5C	6,280683429	0,094850371	0,540817313	4,753502367	0,762187922	10,32906314	1,952845634	0	6,409634214	31,12358439	3,458176043
ENSG00000102595	UGGT2	0	5,32644812	8,985908197	2,092630888	0,26584889	2,620179373	8,405970423	0,326153799	3,095531216	31,11867091	3,457630101
ENSG00000150636	CCDC102B	1,067275348	1,662896125	0	0,494450426	0	5,030191473	5,122561358	10,98602438	6,755144909	31,11854402	3,457616002
ENSG00000155085	AK9	0,014964406	2,150871658	21,49955014	2,273943134	0,106689704	1,708724498	1,006446513	0,261782189	2,061280501	31,08425274	3,45380586
ENSG00000119509	INVS	0,084762626	0	0,038981924	0	0	6,514508691	3,923697102	0,973092963	19,54633175	31,08137505	3,453486117
ENSG00000189195	BTBD8	4,037221577	15,88528184	0,080726034	0,416768509	0	0,048180798	0,382587044	0,047979464	10,17339029	31,07213555	3,452459506
ENSG00000158089	GALNT14	0,034786043	0	24,82879337	5,466179256	0,165339568	0	0	0,532468226	0,042782349	31,07034881	3,452260979
ENSG00000235942	LCE6A	0	0	0	0	0	0	0	31,04401192	0	31,04401192	3,449334657
ENSG00000101670	LIPG	0	1,946175415	0,021237688	0,15948352	0,054873182	12,48543953	1,514578077	0	14,85181926	31,03360668	3,44817852
ENSG00000163710	PCOLCE2	0	7,969122561	0	0,147712977	22,87049166	0	0,035513822	0	0	31,02284102	3,446982335
ENSG00000167637	ZNF283	0,230863372	13,48158389	0,386083471	0,579855499	1,047426165	3,548642891	1,097865405	9,086950607	1,548721019	31,00799232	3,44533248
ENSG00000170837	GPR27	0	0	24,65127972	0	0	0,067801625	6,153019959	0,135036603	0	31,00713791	3,445237545
ENSG00000160111	CPAMD8	17,20162492	0,078873027	0	0,307012422	0,035211054	0,894703571	0,012302219	12,31151443	0,163998304	31,00523994	3,44502666
ENSG00000276410	HIST1H2BB	0,396615671	0	0	7,533555697	0	21,33758073	0	1,734564659	0	31,00231676	3,444701862
ENSG00000235453	SMIM27	4,30390703	1,471382807	6,246743797	4,363689149	0	3,641622274	3,016271373	5,612293412	2,282410908	30,93832075	3,437591195
ENSG00000163626	COX18	0,021661492	2,421580563	0,039848065	0,261832687	0	5,826847949	7,769951752	0	14,57254124	30,91426375	3,434918194
ENSG00000196369	SRGAP2B	1,003223706	0,556311204	3,806366501	4,40305213	9,437390463	3,235600459	2,082495137	3,427744476	2,956065015	30,90824909	3,434249899
ENSG00000134897	BIVM	0,418062444	0,269760563	16,22016674	6,56275087	0,090321313	1,230976361	2,556110888	0	3,529027272	30,87717645	3,430797384
ENSG00000179604	CDC42EP4	7,716417398	7,927290885	0	0	0	4,904943657	4,798844619	0,136627896	5,379059979	30,86318443	3,429242715
ENSG00000198794	SCAMP5	0,03107992	5,184152202	0,343044137	0,053668238	0	2,79816275	22,3998865	0	0,038224295	30,84821805	3,427579783
ENSG00000157823	AP3S2	0,376263194	2,960970446	6,500346582	0,903964403	0,038878158	5,101072186	4,659123512	9,229416306	1,066348996	30,83638378	3,426264865
ENSG00000175137	SH3BP5L	8,711825353	0,140535639	6,264750658	2,324892331	0,062738914	4,173850986	5,238890522	1,905021092	2,013012645	30,83551814	3,426168682
ENSG00000205189	ZBTB10	0,044374678	7,585457912	0,163261609	4,904024581	0,421829533	2,53348179	3,635397247	0,485172133	11,04236874	30,81536822	3,423929802
ENSG00000099949	LZTR1	4,853803445	3,451184109	5,040042086	1,606202977	0	3,100958736	4,198727394	3,62424036	4,929560803	30,80471991	3,422746657
ENSG00000097046	CDC7	0	0,074504585	0,386190746	10,29529491	0	9,173716244	3,62570562	0	7,177734536	30,73314665	3,414794072
ENSG00000114631	PODXL2	0	0,862985747	0,127806942	1,439641239	0	4,65312422	4,788063244	18,83856845	0	30,71018984	3,412243316
ENSG00000114670	NEK11	0,084655782	0	28,75835198	0	0	1,611083098	0,210874508	0	0,034705233	30,6996706	3,411074511
ENSG00000104450	SPAG1	0,029893288	10,76811593	5,93904069	0	0	4,332383012	6,676858419	0,065367867	2,867661471	30,67932068	3,408813408
ENSG00000112031	MTRF1L	1,265995007	0,686423552	0	6,193941682	7,354513318	2,857198668	2,686355724	4,690832818	4,930533754	30,66579452	3,407310502
ENSG00000115137	DNAJC27	0	0,143034495	16,95362268	0	0,319272353	0,32450504	5,688999762	3,760279573	3,469751927	30,65946583	3,406607314
ENSG00000205268	PDE7A	5,718797354	2,63627825	0	0,210856545	0,822221982	3,843315716	8,466078967	1,913627798	7,033383537	30,64456015	3,404951128
ENSG00000188559	RALGAPA2	0,051887912	0,230184565	0,23863001	2,419175659	0,030828186	5,981838975	1,76642926	9,55931404	10,35405554	30,63234414	3,403593794
ENSG00000129566	TEP1	0,026510626	2,634377545	0,024384219	11,94807515	1,449070785	4,657138995	3,037702775	0,144927442	6,700256198	30,62244374	3,402493749
ENSG00000104973	MED25	5,720460006	0,244177703	2,350553718	6,313764183	0	6,841895774	5,647552345	2,23528002	1,257189952	30,6108737	3,401208189
ENSG00000185198	PRSS57	0,138633551	0	0,765082841	29,68434573	0	0	0	0	0	30,58806213	3,39867357
ENSG00000219481	NBPF1	4,368001265	2,612913365	0	3,995646925	5,6167254	4,170860387	3,667942067	1,867014834	4,276357088	30,57546133	3,397273481
ENSG00000103064	SLC7A6	0	0,671415129	6,191175109	5,158176883	0	5,990972327	5,919659839	1,611238666	4,996400968	30,53903892	3,393226547
ENSG00000214300	SPDYE3	0,32921693	0,843826192	6,930997384	8,148296229	0	3,293310891	6,104947163	3,039582078	1,844518861	30,53469573	3,39274397
ENSG00000166407	LMO1	9,837696976	0,31453607	0	0	0	0	0	20,34934287	0	30,50157591	3,38906399
ENSG00000099139	PCSK5	13,17831659	0,232157576	0,371952907	2,444019046	1,441558021	5,850290308	5,431616104	1,300411521	0,234045207	30,48436728	3,38715192
ENSG00000023171	GRAMD1B	8,736585456	2,314662608	0,09300726	4,766812638	0	6,505861747	2,392869688	0,287449771	5,322692153	30,41994132	3,37999348
ENSG00000017427	IGF1	0	0,495214233	0,034225572	8,385130309	8,931512381	4,044606524	2,332679159	3,336079111	2,860233352	30,41968064	3,379964516
ENSG00000187231	SESTD1	4,23984065	4,655161422	0,048747058	2,287899821	0,062975457	6,357117231	3,366411363	0,144863905	9,25566196	30,41867887	3,379853207
ENSG00000090006	LTBP4	4,15609981	1,058281204	6,582656513	5,406644762	0	3,192162058	6,391125693	0	3,598656142	30,38562618	3,376180687
ENSG00000278570	NR2E3	2,049588505	0,16531566	0,085690536	0,080436182	26,1256905	0,716013586	1,160329827	0	0	30,3830648	3,375896089
ENSG00000213380	COG8	1,338697673	4,858948995	4,253655206	1,330943495	0	5,723358149	7,612428197	1,596730796	3,667038762	30,38180127	3,375755697
ENSG00000169359	SLC33A1	1,941560121	0,854635857	6,617255344	3,690514581	0,178843398	4,990537267	3,33671102	3,225357332	5,534672327	30,37008725	3,374454139
ENSG00000152767	FARP1	0,900957361	1,694946396	3,775152056	7,577111441	5,907973259	3,448456008	2,479760343	0,136814577	4,437375559	30,358547	3,373171889
ENSG00000116641	DOCK7	8,712309357	3,363633967	0,178822836	0,419644539	0,086631765	4,055710463	2,138928143	1,859956556	9,512006387	30,32764401	3,369738224
ENSG00000159374	M1AP	0,05037019	0	0	0,086978325	0	6,249298389	5,729811828	13,98839669	4,212521172	30,3173766	3,368597399
ENSG00000198270	TMEM116	6,903912666	0,570586306	0,069590712	1,796397998	6,922531171	4,008103806	4,8529644	0	5,141079162	30,26516622	3,362796247
ENSG00000173548	SNX33	0	7,667896856	0	0,032584288	4,843243578	1,118777615	4,606428273	7,79871794	4,177369382	30,24501793	3,360557548
ENSG00000178904	DPY19L3	0	7,411772194	0,128921296	0,411454834	8,527417757	2,215980479	2,502191019	9,16426451	0	30,16200209	3,351333566
ENSG00000167258	CDK12	0,485049197	0,251914507	2,132783974	6,414591875	3,205151478	3,052453263	8,064757111	1,112400094	5,412581718	30,13168322	3,347964802
ENSG00000088766	CRLS1	2,026063517	2,097200001	0	1,846467232	9,696842391	3,27495738	4,602915345	1,969070049	4,602902693	30,11641861	3,346268734
ENSG00000160606	TLCD1	0	0,20070509	0	27,34348536	0	0	2,535702817	0	0	30,07989327	3,342210363
ENSG00000023445	BIRC3	3,818968931	0,035666667	0	0,069416061	19,82359405	2,383392163	1,635553676	1,758098258	0,543844572	30,06853438	3,340948264
ENSG00000167702	KIFC2	0	0	0	0	29,23331467	0,634243266	0,197502209	0	0	30,06506014	3,340562238
ENSG00000008438	PGLYRP1	0,213433009	0	29,83967164	0	0	0	0	0	0	30,05310465	3,33923385
ENSG00000089775	ZBTB25	3,929443247	3,924884947	0,068193662	5,889120369	0,205562417	1,872242995	3,160099947	2,377810282	8,601613509	30,02897138	3,336552375
ENSG00000142409	ZNF787	2,295765588	3,928287576	2,036207996	5,026148453	3,020250628	4,59112285	5,344232806	1,434333446	2,319300061	29,9956494	3,332849934
ENSG00000166035	LIPC	0	0,107428686	0	27,91251531	0	0,465294085	1,482922594	0	0	29,96816068	3,329795631
ENSG00000181585	TMIE	0	2,538765555	20,05266719	0	0	0,074801883	0	4,767315672	2,513704088	29,94725439	3,32747271
ENSG00000167962	ZNF598	7,042607996	1,426590764	2,129662388	1,888016415	4,509030645	4,272223831	1,44188081	2,320566233	4,904228296	29,93480738	3,326089709
ENSG00000166188	ZNF319	0,989961623	5,646415538	0,065039799	0,244206808	0	4,231224347	10,36289827	0	8,348744098	29,88849048	3,320943387
ENSG00000157741	UBN2	0,154435476	9,655949143	2,23934912	2,290279325	8,894819493	3,042126121	2,293073279	0,059595028	1,251341259	29,88096824	3,320107583
ENSG00000131089	ARHGEF9	3,324849532	0,934631908	2,745283092	4,395970816	0,078233435	3,650490505	5,940502249	2,951379842	5,857046741	29,87838812	3,319820902
ENSG00000161036	LRWD1	0,051232606	6,181948129	0,424109322	8,625584005	1,156676268	4,050029335	3,190464452	6,10567348	0,063009501	29,8487271	3,316525233
ENSG00000133818	RRAS2	6,208825531	0	1,045642733	1,359036326	16,8336348	1,392189446	2,977015979	0	0	29,81634481	3,312927201
ENSG00000268799	AC106774.4	0	0	10,61946446	0	7,263051418	0	8,176718265	3,71274795	0	29,77198209	3,30799801
ENSG00000198453	ZNF568	0,064050258	2,50042344	0	1,161308566	6,126727172	6,786211996	4,33435713	6,127588144	2,599527492	29,7001942	3,300021577
ENSG00000179456	ZBTB18	6,770186858	6,31568459	0	3,874610478	0	6,923559652	2,891021475	0,25378999	2,616886514	29,64573956	3,293971062
ENSG00000171045	TSNARE1	0	6,215654538	0	0	0	5,045137178	7,943535476	0	10,42259908	29,62692627	3,291880697
ENSG00000170004	CHD3	0,015485814	0,851855094	6,295716527	12,75527597	1,324885379	1,564231713	1,002939731	1,456106416	4,34238646	29,6088831	3,2898759
ENSG00000143554	SLC27A3	6,160449224	3,284371848	4,421255688	1,765008055	0	4,428326179	2,339666154	4,349412955	2,853950251	29,60244035	3,289160039
ENSG00000118058	KMT2A	0,390990456	3,040741416	13,71031058	4,934467496	0,057357857	0,773772375	2,020030261	3,187709292	1,478223416	29,59360315	3,288178128
ENSG00000204220	PFDN6	4,86132889	2,270081274	0	2,827605549	12,40433096	2,472084448	2,93174836	0	1,825109768	29,59228925	3,288032139
ENSG00000164741	DLC1	0,01181105	0	20,40200977	0,142765736	3,031475268	0	0	5,888626027	0,101682481	29,57837033	3,286485592
ENSG00000132004	FBXW9	0,071854765	3,315117452	0	13,40036959	0	1,814523663	2,923464702	0	8,041860926	29,5671911	3,285243456
ENSG00000134324	LPIN1	0,063700603	4,65704848	3,398289719	3,167914707	5,843494282	3,427037458	5,31035382	0,027858913	3,650808506	29,54650649	3,282945165
ENSG00000197375	SLC22A5	4,064907437	0,041936503	0,195638168	0,14283272	20,7248003	0,025947854	1,471735205	2,86817618	0	29,53597437	3,28177493
ENSG00000110090	CPT1A	0,045993173	0	0,465344862	20,29185391	0	2,701633192	0,992916948	0	5,034344995	29,53208708	3,281343009
ENSG00000152455	SUV39H2	0,084774514	0	0	2,854548109	0,3022027	8,004661765	11,86072984	4,078294802	2,34588878	29,53110051	3,28123339
ENSG00000129009	ISLR	0	27,50696809	0	0	0	2,017149869	0	0	0	29,52411796	3,280457551
ENSG00000182197	EXT1	0,01815142	6,216385107	0	14,63743341	0,086274485	3,288317736	3,074588316	0,515993919	1,651969087	29,48911348	3,276568164
ENSG00000164403	SHROOM1	12,03414499	5,277908147	0	0,059035062	0,081248313	0,713190262	7,465768624	3,737947596	0,084093449	29,45333645	3,272592938
ENSG00000161551	ZNF577	0,865213654	1,999460986	0,740293206	17,12928661	0,047818581	1,192965532	0,434384638	1,099981916	5,93916982	29,44857495	3,272063883
ENSG00000111785	RIC8B	0,038900906	0,207086112	0,429368154	4,769309614	0,184897692	1,430815889	10,9497857	0,297727117	11,12352077	29,43141195	3,270156884
ENSG00000135439	AGAP2	0,022459954	0,079709275	14,87408377	4,731579504	0,160129701	2,293472705	0,484873459	4,862220895	0,911553989	29,40635293	3,267372547
ENSG00000081760	AACS	1,045780494	6,653813582	0,069326043	7,06065085	0,06717081	5,854815538	4,302555184	0,020601943	4,322012146	29,39672659	3,266302954
ENSG00000160117	ANKLE1	0	0	0,155774782	6,872481819	0	1,534056887	5,097561821	12,03600274	3,69714292	29,39302097	3,265891219
ENSG00000169710	FASN	2,075115658	1,29453814	11,60114349	8,286316112	0,462333495	2,224956774	2,301837439	0,141802196	0,976985258	29,36502857	3,262780952
ENSG00000176714	CCDC121	3,984983404	0	0	0,642245015	0	6,592086908	3,088228987	11,38628257	3,659425192	29,35325207	3,261472452
ENSG00000180953	ST20	0,561749333	0	7,061452329	0	17,13262129	1,541919858	1,088342409	0	1,957491251	29,34357647	3,260397385
ENSG00000169291	SHE	0	0,030679863	0	0,119421041	0,082177927	4,859623299	6,330940093	17,08883576	0,808028358	29,31970634	3,257745149
ENSG00000049883	PTCD2	9,554329107	8,167098476	1,789719168	2,692271715	0,029642389	2,91695855	4,153000671	0	0,0153402	29,31836028	3,257595586
ENSG00000187605	TET3	0,09851557	0	0	5,422412071	12,26226471	0,892355803	3,077709228	6,758950081	0,80269462	29,31490208	3,257211343
ENSG00000174915	PTDSS2	9,243597224	3,376992805	0	2,432661687	0	3,663383686	5,910280605	3,242733734	1,428653344	29,29830308	3,255367009
ENSG00000183856	IQGAP3	0	0	0,082462207	15,67467587	0,106531457	6,988816936	6,401927352	0	0,027565488	29,28197931	3,253553257

ENSG00000064547	LPAR2	8,584096283	11,71712942	0	0,789383116	0	2,955717837	1,686996061	0,111070443	3,435818059	29,28021121	3,253356802
ENSG00000176170	SPHK1	0,034218879	1,821616848	0	8,872395028	1,057184794	6,123963769	6,676976454	0	5,176431667	29,16278744	3,240309715
ENSG00000180357	ZNF609	1,858939484	2,98870294	5,32458578	8,014185151	0	3,246433841	3,718943955	1,282234534	2,715887916	29,1499136	3,238879289
ENSG00000167395	ZNF646	0,05249933	0,031052924	3,315801991	2,568555048	8,400896764	4,419158384	4,446314023	1,301074151	4,605808427	29,14116104	3,237906782
ENSG00000176531	PHLDB3	0,663784628	0	0	9,857416128	12,14673875	2,623668246	0,578712271	0,725751301	2,530745526	29,12681685	3,236312983
ENSG00000177542	SLC25A22	0	0	6,836072075	4,656510972	0	6,715857947	3,768205836	0,071911767	7,077941545	29,12650014	3,236277794
ENSG00000273611	ZNHIT3	1,785215356	4,651483776	6,277104075	0	1,557411205	3,920124289	2,47675846	5,089690512	3,362857663	29,12064534	3,23562726
ENSG00000169224	GCSAML	0	0	14,43358206	14,66365349	0	0	0	0	0	29,09723555	3,233026172
ENSG00000143545	RAB13	4,229557958	5,068478373	1,111516208	7,208672508	1,044325823	2,894841033	1,550705666	4,083887785	1,891567657	29,08355301	3,23150589
ENSG00000197776	KLHDC1	0,100606239	12,85367777	0,092536651	0	0,836825165	0	3,717330076	7,479877028	3,959446546	29,04029947	3,226699941
ENSG00000001631	KRIT1	3,45868811	0	9,678223949	5,131216457	2,720587442	2,19288641	3,114511787	0,37283075	2,366517611	29,03546252	3,226162502
ENSG00000198108	CHSY3	0	0,135940177	23,39401593	0	0	0,378503217	2,289952827	0	2,826563076	29,02497523	3,224997248
ENSG00000138778	CENPE	5,888256036	8,773388186	0,500387726	2,707711511	0	2,002732501	6,164573703	0,524832541	2,459849141	29,02173134	3,224636816
ENSG00000109089	CDR2L	0	0	0	7,231795111	0	7,507265914	7,309640382	0	6,937709542	28,98641095	3,220712328
ENSG00000166783	MARF1	0	0,319626444	1,121504159	1,291994189	17,15571608	2,205853074	2,116866045	3,27222935	1,499586829	28,98337617	3,22037513
ENSG00000204351	SKIV2L	5,033371651	2,378522448	0	4,298530537	0,19503125	3,243725975	5,905555091	0,71781586	7,199702347	28,97225516	3,219139462
ENSG00000157833	GAREM2	0,025430927	0	0,701733673	12,64713441	0,181311449	5,612256129	1,604803977	0	8,194511753	28,96718232	3,218575813
ENSG00000152207	CYSLTR2	0,562585892	6,089598662	0,879683858	0	4,345242043	5,713599533	4,204143663	0,922657951	6,227173122	28,94468472	3,21607608
ENSG00000160190	SLC37A1	1,278729661	3,32536767	7,827569172	2,093875635	0,052395323	2,783734508	5,711517885	0	5,856849841	28,9300397	3,214448855
ENSG00000151835	SACS	0,018986125	0,016845207	1,449450196	19,29391036	0	4,148285559	1,899632492	0,249102576	1,797987463	28,87419998	3,208244442
ENSG00000198707	CEP290	0	1,019253217	0,812807307	1,449638748	0	1,358332207	6,163454905	9,629623361	8,422868699	28,85597844	3,206219827
ENSG00000239900	ADSL	2,35877842	1,960819013	0,156366249	1,798033154	15,90803345	2,35648548	1,782098654	0,139404261	2,378291876	28,83831055	3,204256728
ENSG00000120937	NPPB	0	0,378731639	0	0	28,40477736	0	0	0	0	28,783509	3,198167667
ENSG00000196526	AFAP1	0	0,026718015	0,083094888	0,025999898	1,89649559	4,843742425	1,750283627	11,85297373	8,296058388	28,77536656	3,197262951
ENSG00000117016	RIMS3	12,67755029	0,923481196	0	6,218728558	0	7,72527551	1,227220495	0	0	28,77225605	3,196917338
ENSG00000130749	AC008755.1	6,992858158	0,123264787	0,809319933	7,317054911	0	3,177876507	3,826018398	0,202534212	6,322085189	28,7710121	3,196779122
ENSG00000126016	AMOT	0	0,09461609	0	5,923365102	0,042239184	5,717689835	1,121588477	5,285709384	10,53611194	28,72132001	3,191257779
ENSG00000116127	ALMS1	0,02044383	6,783813031	0,282060575	10,85537505	0	2,222166961	1,400433253	0	7,153261934	28,71755464	3,190839404
ENSG00000183826	BTBD9	0,015844665	0,112463878	11,28009839	0,109441113	0,188275948	4,0359918	3,986317563	4,712079394	4,248143216	28,68865596	3,18762844
ENSG00000185621	LMLN	0,069540069	8,699498852	0	3,542375403	5,040536144	3,683932502	2,049791434	2,471036227	3,121674212	28,67838484	3,186487205
ENSG00000198816	ZNF358	2,248885113	3,733133943	5,604975592	0	1,206828035	5,416187814	4,939302556	2,220875634	3,301162422	28,67135111	3,185705679
ENSG00000184056	VPS33B	0	1,269864906	7,621587958	5,593321988	0	1,364668253	3,47138755	4,200439418	5,141815546	28,66308562	3,184787291
ENSG00000187650	VMAC	0	4,877472051	0	10,53464183	0	5,962181328	2,50495339	0,146599187	4,617304901	28,64315269	3,182572521
ENSG00000152128	TMEM163	0,044418157	11,23169753	0	0,460202777	0	10,04631946	3,761897079	1,311248284	1,77542974	28,63121303	3,181245892
ENSG00000235034	C19orf81	0	3,32157883	0	5,289222458	0	6,539268226	4,097567149	3,721109995	5,650738853	28,61948551	3,179942834
ENSG00000137166	FOXP4	2,204206055	0,999556888	7,70414861	5,666983241	0,640241972	3,764583588	5,714289026	0,053555035	1,86750152	28,61506594	3,179451771
ENSG00000130558	OLFM1	0,024679172	0	27,69359144	0,34092435	0,058650587	0	0,040983287	0,053966122	0,364226403	28,57702136	3,175224596
ENSG00000179943	FIZ1	4,298355216	6,046532942	0	3,884219961	0,052928157	3,374452408	4,826493243	2,435037344	3,642975308	28,56099458	3,173443842
ENSG00000131351	HAUS8	10,07767948	3,246975241	0	0,188639056	0,129809341	5,577330068	1,201866545	1,911062021	6,213917698	28,54727945	3,171919939
ENSG00000264364	DYNLL2	0,932370202	3,860425446	0,490048654	17,13499833	0,158271284	1,169929144	2,06444501	1,941733906	0,764463438	28,51668542	3,168520602
ENSG00000253873	PCDHGA11	0	0	0	0,044911355	0	3,455280513	5,830807167	16,83453218	2,335076786	28,500608	3,166734223
ENSG00000005471	ABCB4	0,058668527	0	0	7,429234426	0	10,24192379	3,572340241	4,190835721	3,006446911	28,49944961	3,166605512
ENSG00000114374	USP9Y	0,124377665	0,029427349	17,61781246	0,100227438	3,349977074	0,036415842	0,027539581	0,417032213	6,791801132	28,49461076	3,166067862
ENSG00000176438	SYNE3	0,05823464	0,575728804	7,835598474	7,599350449	0,079083361	1,543651589	1,616387232	3,110785677	6,057098722	28,47591895	3,163990994
ENSG00000157870	PRXL2B	3,855090204	1,113612578	0	0,541840647	9,055173865	1,378076579	3,3126252	3,626840403	5,568228641	28,45148812	3,161276457
ENSG00000173083	HPSE	0,032240361	8,695878817	0	0,556720663	0	4,707938276	5,48782122	0	8,961236192	28,44183553	3,160203948
ENSG00000130487	KLHDC7B	2,382162076	3,114696537	0	3,030980829	0	7,846424287	3,279249498	2,810167448	5,975152656	28,43883333	3,15987037
ENSG00000131477	RAMP2	11,79069027	9,378957851	0	1,170111994	0	0,446396171	0	5,630723572	0	28,41687985	3,157431095
ENSG00000174501	ANKRD36C	0,934086803	0,406560065	5,997937755	3,043328372	0,251306888	5,727726931	3,775524336	3,622678953	4,616901008	28,37605111	3,152894568
ENSG00000132141	CCT6B	0,070530021	3,629459978	0	20,58250208	0	0,309751368	0,146406455	0	3,599827619	28,33847752	3,148719725
ENSG00000103404	USP31	0,0377242	1,963592911	0,069396713	0,19542438	13,02947894	6,281876708	2,526738815	0,082491779	4,144700671	28,33142512	3,147936124
ENSG00000040275	SPDL1	0,158230964	0,449243142	2,445060556	12,51394957	0	8,443197935	1,813079143	0	2,452006472	28,27476778	3,141640864
ENSG00000022567	SLC45A4	1,913458667	0	0	0	19,35763704	3,392223687	2,375890348	0	1,230628691	28,26983843	3,141093159
ENSG00000198932	GPRASP1	0,15580704	0	0	25,90516613	0,158690916	0	0,055444247	0	1,943600395	28,21870873	3,135412081
ENSG00000198589	LRBA	2,29051053	0,01600179	0,033177788	3,005338074	0,085723588	1,247522389	8,917775581	0,867644624	11,74502996	28,20872433	3,134302703
ENSG00000159023	EPB41	0,211628869	0,053647177	0,75080905	14,22593519	0,053886492	2,082906572	3,608535032	2,264269282	4,945248329	28,19686618	3,132985131
ENSG00000064655	EYA2	0,083532654	5,410272528	4,225789233	1,081819961	0	5,548695351	2,878396229	1,004637989	7,961915686	28,19505963	3,132784403
ENSG00000088882	CPXM1	0,12719745	0	6,08373954	21,96422345	0	0	0	0	0	28,17516044	3,130573382
ENSG00000158406	HIST1H4H	0,139723135	4,462834953	2,955867904	1,447628153	0,332055055	3,374972434	5,278687107	10,08260816	0,085920722	28,16029763	3,128921959
ENSG00000099991	CABIN1	2,832920796	0,656930763	2,487252696	3,168575533	0,803309373	3,711250651	3,461527055	1,337506771	9,680331209	28,13960485	3,126622761
ENSG00000186187	ZNRF1	1,164496691	0,212714642	12,53808697	9,610592333	0	1,598186652	1,834278426	0	1,179443024	28,13779874	3,126422082
ENSG00000131437	KIF3A	0,02202778	6,293128573	0,04052188	0,646631861	0	5,56259951	7,919628827	0,770692869	6,881197763	28,13642907	3,126269896
ENSG00000213638	ADAT3	10,63774775	2,420054152	0,125442283	0	0	4,791641204	4,246513944	3,876939872	2,01277822	28,11111743	3,123457492
ENSG00000196236	XPNPEP3	6,992060038	8,738281181	0,058069793	6,132271703	0,037509666	5,094809855	0,537318113	0,345137422	0,155292686	28,09075046	3,121194495
ENSG00000275111	ZNF2	2,004219281	0,671026026	0	0	0	2,947860597	0,941969459	16,20750131	5,301927813	28,07450449	3,119389387
ENSG00000069493	CLEC2D	0,021726897	0,539753846	1,878513969	13,6939275	0,258172209	3,864487912	2,561723529	4,560994749	0,694753354	28,07405396	3,119339329
ENSG00000204086	RPA4	0,193731501	0	0,534577113	0,669064037	0,920814211	5,423999623	0,0804297	20,12261789	0,119132386	28,06436647	3,118262941
ENSG00000142945	KIF2C	0	14,26756665	0	1,35978182	0	8,281877389	4,129577575	0	0	28,03880344	3,115422604
ENSG00000005156	LIG3	0,07018062	9,713635424	4,023707602	1,292657201	0,027797627	4,803046342	3,632317065	0,537125623	3,912861217	28,01332872	3,11259208
ENSG00000146067	FAM193B	0,181592102	0,32223073	0,858994297	0,806322668	0	7,177590614	6,677391059	1,361447145	10,62435913	28,00992774	3,112214194
ENSG00000149782	PLCB3	0	1,080346495	5,5439222	4,888588364	0	6,082942176	6,571774037	0,732228091	3,107425735	28,0072271	3,111914122
ENSG00000117650	NEK2	0	0	0,089815864	16,27155092	0	4,181268169	7,418762764	0	0	27,96139772	3,106821969
ENSG00000179859	RNF227	0,051171883	0	0	1,855616142	0	5,112720108	0,212445533	12,42066949	8,307396187	27,96001934	3,106668816
ENSG00000114812	VIPR1	5,013825814	0,036313922	11,33152895	0	0	2,157017249	0	9,218515833	0,176181698	27,93338347	3,103709274
ENSG00000100150	DEPDC5	0,034876365	2,413602909	0,62553943	0,75279838	16,01741736	2,508139813	0,463337444	3,069641992	2,037438072	27,92279176	3,102532418
ENSG00000144369	FAM171B	0	0	0,242523206	1,730156872	25,81686049	0	0	0,057657401	0,032428289	27,87962626	3,097736251
ENSG00000173960	UBXN2A	0	1,99988915	0	0,839510009	0,052517921	4,755553237	3,798239065	9,326392444	7,093586182	27,86568801	3,096187556
ENSG00000198768	APCDD1L	0	0	27,57235381	0	0	0	0	0,167648182	0,094290473	27,83429246	3,092699162
ENSG00000266173	STRADA	7,1241816	0,888868509	0,895884297	12,32593388	0	1,588830569	1,698355323	0,395547825	2,892086765	27,80968877	3,089965419
ENSG00000253953	PCDHGB4	23,2158001	0	0	0	0,052000803	0,288289447	1,507969527	0,047847461	2,637266027	27,74917336	3,083241485
ENSG00000125741	OPA3	1,617044811	0,750872688	2,807879788	1,174324818	0	3,517655992	6,261595726	4,593499838	7,025701112	27,74857477	3,083174975
ENSG00000172888	ZNF621	16,57603375	0,029983451	0,03108354	1,45887831	0,200781361	3,561984874	3,240931085	0,036948962	2,576872276	27,71349761	3,079277512
ENSG00000154065	ANKRD29	8,218418133	4,930770767	0	0,447293863	0,111926926	3,981647002	0	7,415081459	2,606543089	27,71168124	3,079075693
ENSG00000180902	D2HGDH	11,12629202	0,306098174	1,798196987	5,113451747	0	2,178049808	2,196208247	2,514722736	2,475124916	27,70814463	3,078682737
ENSG00000149256	TENM4	0,027760668	1,149414575	23,69554794	0,111852102	0,043982553	1,280142845	0,284286729	0	1,081164704	27,67415211	3,07490579
ENSG00000188171	ZNF626	1,326500986	0,078461448	6,507215192	4,50480278	0	2,864293789	4,51583053	2,997358184	4,867085221	27,66154813	3,073505348
ENSG00000158470	B4GALT5	0,064002783	6,359996614	0,353214865	8,233653371	0,912623996	3,197344871	6,138002264	1,67946413	0,708436553	27,64673945	3,071859938
ENSG00000182195	LDOC1	0	0	0	7,746802287	0,520083334	7,929107938	11,44769141	0	0	27,64368497	3,071520552
ENSG00000115112	TFCP2L1	6,759403129	0,028626241	0,534177622	0,167141011	0	6,075299222	0,05357973	13,54615927	0,476173432	27,64055966	3,071173296
ENSG00000060069	CTDP1	8,029341179	2,820644101	0	0	0	7,76412274	1,472140106	1,158637728	6,366151943	27,6110378	3,067893088
ENSG00000167733	HSD11B1L	6,416262947	11,92339887	2,230532389	0,087240045	0	0,221879741	4,362726355	0,441905139	1,926192641	27,61013812	3,067793125
ENSG00000069956	MAPK6	0,284532692	0	6,106576786	1,59680904	0,845247628	3,202102767	2,835042807	10,78462106	1,953823473	27,60875626	3,067639584
ENSG00000080618	CPB2	0,087651143	5,910322508	8,062067836	10,89751634	0	0,962358334	1,673908142	0	0	27,5938243	3,065980478
ENSG00000163818	LZTFL1	0	12,24938385	0	0,036341918	0,050016371	3,142595882	4,875518472	1,380645825	5,849765186	27,5842675	3,064918611
ENSG00000188807	TMEM201	3,376772529	1,690051283	4,021771952	5,49454824	0,102884269	2,257771779	4,978553497	4,686008048	0,958383441	27,56674504	3,062971671
ENSG00000038382	TRIO	0,769936278	0,270243897	5,276329986	4,003145747	3,961150943	2,331666	4,123800945	1,443107294	5,358956269	27,53833736	3,059815262
ENSG00000235106	BRD3OS	0,076139018	6,665269737	0	5,434319256	0,120630683	3,148789917	7,101696655	0	4,962982372	27,50982764	3,056647515
ENSG00000174307	PHLDA3	0,048155058	3,417998726	0	0,498919646	0	8,300815979	8,396676138	0,315903028	6,514696447	27,49316502	3,054796113
ENSG00000181016	LSMEM1	0	0	0,643472451	26,67737973	0,138548435	0	0	0	0	27,45940062	3,051044513
ENSG00000146574	CCZ1B	3,635246096	3,848402134	0	4,636624754	3,583321568	4,331454367	3,344274841	2,303466777	1,752789781	27,43558032	3,048397813
ENSG00000102683	SGCG	0	0	0	0	0	6,946994591	15,45201133	0	5,035250603	27,43425652	3,048250725
ENSG00000161888	SPC24	0	0,094449454	0,097914794	25,09166892	0	1,92851322	0,088390512	0	0,130923933	27,43186083	3,047984537
ENSG00000171848	RRM2	0,033011594	3,602563191	0,425092451	4,446297765	0,078452767	12,34135475	6,372878687	0,108280033	0	27,40793124	3,045325693
ENSG00000197937	ZNF347	15,6720743	1,99689585	0,091330664	2,629067754	0,039329487	1,617132989	4,603281264	0	0,732720929	27,38183324	3,042425915
ENSG00000213023	SYT3	0	3,278531219	0,144630645	0,067881107	0	8,718505852	1,827871642	0	13,34381898	27,38123944	3,042359938
ENSG00000158747	NBL1	6,345299269	1,938735262	0,077302586	3,990940935	0	2,445289367	6,45495312	1,929679065	4,186198082	27,36839769	3,040933076
ENSG00000261652	C15orf65	0	18,7667592	0	0	0	0	5,507001523	0	3,086419112	27,36017984	3,040019982
ENSG00000185252	ZNF74	0	0,042895857	0	0,208714585	0,229798459	4,405879976	5,780747979	9,25068778	7,432671663	27,3513963	3,039044033
ENSG00000126467	TSKS	10,8528927	0	0,117439839	0	0,151718438	2,032702461	2,226343027	11,16804622	0,78515641	27,33429909	3,037144343
ENSG00000159164	SV2A	0	0,112052654	0,290409631	16,95586651	0	0,103997436	4,535388098	0	5,319885824	27,31760015	3,035288905
ENSG00000176125	UFSP1	0	0	0	1,048984823	0	7,503493419	6,809445154	11,95542154	0	27,31734493	3,035260548
ENSG00000161265	U2AF1L4	1,208884565	2,051869218	0	0,363039965	0,12491045	5,136014374	8,248310521	6,551224469	3,619967043	27,3042206	3,033802289
ENSG00000101935	AMMECR1	0,095008218	0	0	1,394496878	1,072498162	8,449391206	7,553463872	0,207755151	8,529893823	27,30250731	3,033611923
ENSG00000133069	TMCC2	14,69058451	0,046056682	0	0	0,061682848	0,883412552	2,370617746	7,43506155	1,787599665	27,27501555	3,030557283
ENSG00000130584	ZBTB46	2,458646806	2,162435485	0,039329386	2,510410056	0	2,746397492	9,124469531	0	8,230045483	27,27173424	3,030192693
ENSG00000165959	CLMN	0,019246077	0,051227536	19,631913	0,083084434	2,8358005	1,341822788	1,981573097	1,157352176	0,153856256	27,25587586	3,028430651
ENSG00000141068	KSR1	1,613103481	1,695428971	0,388049079	4,435337473	7,962041093	3,583058111	2,730303661	0,786878179	4,028862036	27,22306208	3,024784676

ENSG00000158477	CD1A	0	0	0	0,576970646	0	0	0,138717891	26,48591061	0	27,20159914	3,022399905
ENSG00000167280	ENGASE	0	3,170964848	0	0,084540734	0,290877646	6,85358773	7,07333153	0,053529008	9,664139584	27,19097108	3,021219009
ENSG00000138755	CXCL9	0	0	0	0	0,262129593	21,4957134	0	5,426845087	0	27,18468808	3,020520898
ENSG00000163629	PTPN13	23,36039572	0	0,556194385	0,824352279	0,11345331	0	0	0,069594475	2,231097677	27,15508784	3,017231983
ENSG00000011426	ANLN	0	0,04471269	0	11,44337141	0	13,39014826	2,154984858	0	0,092969746	27,12618696	3,014020773
ENSG00000185730	ZNF696	6,930295364	1,336699901	0,138574327	5,853476499	0	2,812043673	1,845151941	1,153061801	7,041044712	27,11034822	3,012260913
ENSG00000182584	ACTL10	11,6459189	6,267374607	0	0	0	6,602884607	0	0	2,582832274	27,09901039	3,011001154
ENSG00000153048	CARHSP1	0	3,461757936	3,987521592	4,585194103	0	5,087091378	3,509659636	1,421988456	4,998561653	27,05177475	3,00575275
ENSG00000151623	NR3C2	3,522177673	6,20943333	8,204346799	0	0,054354101	0,678005989	3,513245258	4,851241569	0	27,03280472	3,003644969
ENSG00000137434	C6orf52	1,538456036	0	1,886742753	0	23,5620107	0	0	0	0	26,98720949	2,998578832
ENSG00000276547	PCDHGB5	0,031150396	12,8792179	0	0	0	1,778468355	6,673128356	0	5,593401825	26,95536683	2,995040759
ENSG00000188643	S100A16	0	25,06441976	0	0	0,422242848	1,463056232	0	0	0	26,94971884	2,994413205
ENSG00000172717	FAM71D	0,30208688	0,119121279	0,740950952	0,5795979	24,88775505	0,147410552	0,083609728	0	0,08256176	26,9430941	2,993677122
ENSG00000176208	ATAD5	0	0,113635542	0,196341361	7,593248307	0,304379892	3,632736176	4,431075434	0	10,65880605	26,93022276	2,992246974
ENSG00000178226	PRSS36	3,034513495	0	0	0,070810034	0	2,791439825	14,67509175	4,842189049	1,512997468	26,92704162	2,991893513
ENSG00000175548	ALG10B	4,536806971	0,571629775	19,90157753	1,019820473	0	0,221056906	0,590684633	0,02935109	0,016507952	26,88743532	2,987492814
ENSG00000132510	KDM6B	0,083279455	3,805266741	1,263894085	0,467367687	7,718701654	1,485834594	3,5611585	4,82585618	3,661622029	26,87298093	2,985886769
ENSG00000119862	LGALSL	0	0	3,604623709	0	0	2,193579961	0,925156275	13,52656124	6,615436847	26,86535804	2,985039782
ENSG00000037042	TUBG2	3,462612432	0	0,298582276	0	0	4,455171233	4,088005805	0,118308116	14,43920348	26,86188335	2,984653705
ENSG00000168152	THAP9	0,034882403	0,123795937	0	1,445623128	0,082898786	1,225562893	4,692103828	7,551482499	11,66903372	26,82538319	2,980598132
ENSG00000198393	ZNF26	0,021730022	2,956219922	0,213195359	11,74470047	0,705772181	2,576695756	2,050876388	5,084339765	1,469881899	26,82341176	2,980379084
ENSG00000174306	ZHX3	0,013003233	9,921784703	2,368129231	0,493982397	0,030902465	2,698313312	2,710012334	5,061298771	3,518306075	26,81573252	2,979525836
ENSG00000047457	CP	20,10676569	5,270324945	1,375749924	0	0	0	0,035483691	0	0	26,78832426	2,976480473
ENSG00000181019	NQO1	0	0	0	13,17409835	0,120874299	0	13,47190707	0	0	26,76687972	2,974097746
ENSG00000174371	EXO1	0,109579819	0	0	1,478260135	24,85094996	0,173612542	0	0,079872992	0,044923017	26,73719847	2,97079983
ENSG00000115109	EPB41L5	0,01640901	9,05550679	0,301856987	0,510026012	0,077992734	1,837645654	1,75759288	12,95329339	0,161447734	26,67177119	2,963530133
ENSG00000103222	ABCC1	4,667970104	0	0,844031984	7,027159712	5,641582514	2,255945795	1,441045397	2,48643833	2,30621427	26,67038811	2,963376456
ENSG00000186310	NAP1L3	0	17,57808489	0	2,175282092	0	0	0	6,886662074	0	26,64002905	2,960003228
ENSG00000074935	TUBE1	0,213222197	13,31818598	0	1,804122161	0	4,096846469	0,017704294	5,734930408	1,416073402	26,60108491	2,955676101
ENSG00000221882	AC087498.2	0	0	7,692792567	0	0	0	13,98285675	0,74143882	4,170079034	26,58716717	2,954129685
ENSG00000183060	LYSMD4	0	7,060084257	0	2,427515123	0,063036255	5,329407677	0,198215374	0,696018046	10,79782322	26,57209995	2,95245555
ENSG00000213123	TCTEX1D2	6,306380156	0	0	18,84763059	0	0	1,409779011	0	0	26,56378976	2,951532195
ENSG00000157540	DYRK1A	0,558978683	3,606887121	1,485302625	13,04528625	0,187858072	0,998079544	1,570547443	4,29664946	0,777745787	26,52733499	2,947481666
ENSG00000140416	TPM1	0,010977884	11,84383117	1,009735194	4,62536388	0,208713428	5,098450704	1,175857089	0,120027086	2,416747366	26,5097038	2,945522644
ENSG00000120458	MSANTD2	0,033572666	0,297869362	2,099827453	0	5,026528587	6,929835241	4,292919602	2,863130001	4,954806193	26,4984891	2,944276567
ENSG00000166866	MYO1A	0,033957432	0	0	0,117274146	0	11,70694856	3,270687306	10,98973393	0,375869371	26,49447074	2,943830083
ENSG00000197530	MIB2	7,928694252	2,293903274	0	2,410828441	0	7,039892156	2,461605692	0,829195903	3,497738813	26,46185853	2,940206504
ENSG00000173705	SUSD5	0,032835848	0,058266406	0	1,701010265	0	0,108155526	0	24,55641506	0	26,4566831	2,939631456
ENSG00000103550	KNOP1	2,765880515	3,007006188	4,944734939	6,62595901	0,092579928	2,908460741	2,296569624	1,107411544	2,683012438	26,43161493	2,936846103
ENSG00000135298	ADGRB3	0	0,163252359	1,480868107	0,11914839	0,054660204	10,95969411	4,163246937	0	9,476192536	26,41706264	2,935229182
ENSG00000163737	PF4	0,250183064	0	0	0	26,16088057	0	0	0	0	26,41106363	2,934562626
ENSG00000187079	TEAD1	0,02823705	4,033529105	13,97302562	2,779275633	0	2,743732792	2,813498989	0,030873079	0	26,40217227	2,933574697
ENSG00000118420	UBE3D	0,118018253	3,266959701	10,33415551	1,059717173	0	8,759415015	0,5291626	1,909728053	0,406412264	26,38356857	2,931507618
ENSG00000204444	APOM	0	2,167679871	0	0	0	6,572043863	4,868695179	12,02065929	0,751198554	26,38027675	2,931141862
ENSG00000120756	PLS1	0	0	0,052568097	0,24672369	0	0,345124119	0	21,05833004	4,674279021	26,37702497	2,930780552
ENSG00000175691	ZNF77	0	0,110029545	0	0,214144419	0	9,326938452	3,501018702	5,288037806	7,931078505	26,37124743	2,930138603
ENSG00000165055	METTL2B	3,761531467	0	3,542197142	0,734592461	0,372473373	4,105358766	6,655560654	2,643868224	4,543588113	26,3591702	2,928796689
ENSG00000133874	RNF122	5,731223567	8,451056627	0,445480928	0,975718388	1,726526646	0,797647003	1,13942075	1,412113536	5,658788338	26,33797578	2,926441754
ENSG00000091436	MAP3K20	11,70220226	0,019424949	0,181238836	10,92584869	1,040618784	0,781236416	0,018178837	1,62775649	0,026926474	26,32343174	2,924825749
ENSG00000171611	PTCRA	0	0	0	10,94675835	0	5,255728885	3,437543342	0	6,682837273	26,32286785	2,924763095
ENSG00000072121	ZFYVE26	0	0,068299032	2,708288025	2,276368538	10,15334875	2,525001772	8,03764353	0,547077099	0	26,31602675	2,924002972
ENSG00000113966	ARL6	0	10,52344179	0	0,28605018	0	9,675995046	0,385131473	0,072447833	5,337841354	26,28090768	2,920100853
ENSG00000248098	BCKDHA	2,953738387	6,313427812	2,346344683	0,81143706	1,276295131	3,390442705	4,682144775	1,614740223	2,889661608	26,27823238	2,919803598
ENSG00000159267	HLCS	0,038309183	0	0	0,396909582	0	12,28188323	10,46513861	0	3,086055827	26,26829643	2,918699603
ENSG00000108309	RUNDC3A	0	0	0	0,1064172	19,91842812	1,590089132	1,125753387	3,503792315	0	26,24448016	2,916053351
ENSG00000136830	FAM129B	4,516625244	1,393850553	0	1,582451654	5,75583672	4,312165736	4,348116335	1,860796785	2,455412142	26,22525517	2,913917241
ENSG00000171695	LKAAEAR1	2,785448307	0	0	16,23328183	0	4,969671652	1,15640859	0	1,070544483	26,21535486	2,912817207
ENSG00000134830	C5AR2	2,243776483	0	0,081734813	2,608582516	0	4,244110607	2,951375054	11,07601902	3,005456844	26,21105534	2,912339482
ENSG00000164037	SLC9B1	0	7,746124289	0	0	0	1,67192385	2,233768087	13,65248633	0,873984317	26,17828687	2,908698541
ENSG00000135525	MAP7	0,083225943	4,775064074	7,450907738	5,173669406	0	5,147583525	3,455213843	0,060663589	0	26,14632812	2,905147569
ENSG00000186409	CCDC30	0,03357018	4,884695795	0	0,057968374	13,90835861	0	0	7,04718272	0,192672639	26,12444832	2,90271648
ENSG00000248771	SMIM31	0	0	0	0	26,11763944	0	0	0	0	26,11763944	2,901959938
ENSG00000073711	PPP2R3A	0	0	13,53300675	9,586050448	0,138388263	0,809839681	0,225637077	1,358240997	0,453575327	26,10473854	2,900526504
ENSG00000170265	ZNF282	0,361604663	0	4,582495649	8,672393466	0,09548459	3,440846845	1,401157656	4,041475699	3,50840284	26,10386141	2,900429045
ENSG00000064042	LIMCH1	0,389531944	6,418423865	0,614207547	1,129068662	0,958792923	1,924574909	4,632075413	9,795611374	0,239537038	26,10182368	2,900202631
ENSG00000148842	CNNM2	2,675260654	0,098557461	0,476809775	0,543481324	11,37366879	2,947443472	3,76626211	2,064709991	2,117584731	26,06377831	2,895975368
ENSG00000261609	GAN	0,637349107	1,357152424	16,88335427	1,523856188	0,396145495	2,551476652	0,716461567	0,278739172	1,640070536	25,98460541	2,887178379
ENSG00000167685	ZNF444	2,115505679	0,525547809	8,834030865	0,073060332	4,072311484	4,436360094	4,163022355	0,046259914	1,717187522	25,98328605	2,887031784
ENSG00000141564	RPTOR	3,02162469	0,364394098	1,969767737	5,572286392	0	3,156520388	3,958246739	2,822582211	5,08723736	25,95265961	2,883628846
ENSG00000215440	NPEPL1	1,337979295	3,777154549	4,25137259	1,540265479	8,093875384	1,313217605	2,541725266	0,044329832	3,041759554	25,94167955	2,882408839
ENSG00000112941	TENT4A	10,81738667	0,970306168	0	3,448479841	0,056500557	4,933464285	0,730396835	1,091744166	3,888858946	25,93713746	2,881904163
ENSG00000163138	PACRGL	4,570764346	7,035795921	0	1,553184373	0	3,345621445	3,307466235	4,415427894	1,693208111	25,92146832	2,880163147
ENSG00000197299	BLM	0,02270291	0	0,083527674	9,447915997	0,161861873	7,577646532	3,317725125	0,049644617	5,193427455	25,85445218	2,872716909
ENSG00000213533	STIMATE	1,914811877	0,896638156	2,348300731	3,122769847	9,606805075	2,54034146	2,07571475	1,512020196	1,831644711	25,8490468	2,872116311
ENSG00000233822	HIST1H2BN	0,662160616	1,762481651	8,83121001	5,335898696	6,556829329	1,90841087	0,206177285	0,482650455	0,101796525	25,84761544	2,871957271
ENSG00000249992	TMEM158	0,083348357	0	23,30555711	0,287848841	0	0	1,591736148	0,546775346	0	25,8152658	2,868362867
ENSG00000131061	ZNF341	9,802664837	2,456862746	0	0	5,133070974	4,074036518	4,32259894	0	0	25,78923401	2,865470446
ENSG00000186787	SPIN2B	5,933928977	7,987981045	0	0,353331042	0	2,69590646	2,718382278	3,579521381	2,516540586	25,78559177	2,865065752
ENSG00000085840	ORC1	0	0	0	1,003274494	0	4,040116575	6,030294713	0,105874581	14,58904132	25,76860168	2,863177965
ENSG00000185838	GNB1L	2,464816863	0,920790958	6,88089242	2,613456606	0,102766502	1,756672296	4,470183527	5,011598821	1,542294791	25,76347278	2,862608087
ENSG00000115661	STK16	0	0	4,836650074	1,135019349	0,082215555	4,330083733	4,078934786	2,647712881	8,63709799	25,74771437	2,860857152
ENSG00000197153	HIST1H3J	0,974906908	12,45562839	1,076051996	0	4,170397266	2,783016125	1,295177621	2,984570287	0	25,73974859	2,859972066
ENSG00000136630	HLX	1,798615782	3,906136793	1,432123445	1,019820473	4,657235849	2,210569062	3,477237838	2,876406788	4,358099296	25,73624533	2,859582814
ENSG00000206418	RAB12	0,915451761	0,324889338	1,459507789	2,740028005	1,305354556	4,087457665	7,651865966	4,537469829	2,702130372	25,72415528	2,858239476
ENSG00000164300	SERINC5	2,962331137	2,586126849	0	11,07858944	0,112941884	3,026359193	2,735908277	2,736589877	0,467587434	25,7064341	2,856270455
ENSG00000167220	HDHD2	2,648229706	3,381145118	2,792277122	2,788362627	0	3,439479791	2,413406685	5,296557505	2,939226896	25,69868545	2,855409494
ENSG00000100416	TRMU	0,016391211	2,239606683	12,81500619	2,745492194	0,038954067	1,457723909	3,810792164	0	2,540043585	25,66401	2,851556667
ENSG00000105352	CEACAM4	0	0	10,18988633	8,034654643	0	1,337988522	1,839740938	2,180286736	2,043766741	25,62632391	2,847369323
ENSG00000181027	FKRP	0	3,799689826	1,42245278	0,051355043	0	5,387767203	4,716558436	0,065033373	10,13176278	25,57461944	2,841624382
ENSG00000137726	FXYD6	0	0,430231849	0,223008503	4,919351707	0	0,698781009	2,113820274	17,09829892	0	25,48349226	2,83149914
ENSG00000112659	CUL9	3,209340661	3,416937756	0,045414164	3,474301655	0,029334875	2,01933311	8,394069752	1,025691355	3,855988944	25,47041227	2,830045808
ENSG00000198920	KIAA0753	0,024837372	0	0	5,103757721	0,118053104	0	0	15,20737655	5,009669568	25,46369431	2,829299368
ENSG00000124839	RAB17	19,42442118	3,04130813	0	0	0	2,369653269	0,263537769	0,34702223	0	25,44594258	2,827326953
ENSG00000146083	RNF44	2,739164423	2,049068065	0,098802239	7,094908675	0,319102135	2,004961652	3,411580024	5,461243074	2,245879423	25,42470971	2,824967746
ENSG00000203783	PRR9	0	0	0,227479623	25,19666694	0	0	0	0	0	25,42414656	2,824905174
ENSG00000124610	HIST1H1A	11,89296158	0	1,286944903	4,429451729	0	0,256034841	7,551455167	0	0	25,41684822	2,824094247
ENSG00000162396	PARS2	0	0	0	14,17643326	4,267950418	4,859010948	0	2,103785447	0	25,40718007	2,823020008
ENSG00000063015	SEZ6	0,206798435	1,153298925	10,1627133	5,713532191	1,404174164	0,194616512	0,637776859	1,227420681	4,687038392	25,38736945	2,820818828
ENSG00000113758	DBN1	0,029519549	3,509574918	16,0195603	0,764607271	0,561230775	0,03241074	0,049021423	0,129101218	4,28401829	25,37904449	2,819893832
ENSG00000172840	PDP2	0	0,448826877	20,00765418	0,873526936	0	0,019836272	1,860153176	0	2,133102121	25,34309956	2,815899952
ENSG00000111110	PPM1H	0,105465222	0	0,34922123	15,77119542	0	1,5284894	7,566050171	0	0	25,32042145	2,813380161
ENSG00000188732	FAM221A	4,883031313	7,694071872	0,037272741	7,277349788	0,04815199	0	1,985183585	0	3,388995311	25,3140566	2,812672955
ENSG00000118690	ARMC2	0	3,908040434	0	6,440567074	0	3,003082841	3,185424508	3,573105338	5,198809626	25,30902982	2,812114425
ENSG00000234444	ZNF736	9,769555807	2,709473845	0	4,94663459	5,427064711	0,04450789	0,79660141	0	1,612010432	25,30584868	2,811760965
ENSG00000168517	HEXIM2	5,412694631	0	9,820690445	0,768208954	0	1,302536422	2,893574879	0	5,106675784	25,30438111	2,811597902
ENSG00000143702	CEP170	0,93934718	1,323673828	3,710128389	4,579898997	1,805600588	3,276047588	2,374290038	2,960287743	4,332284777	25,30155913	2,811284348
ENSG00000188517	COL25A1	0	0	0	23,54502458	0	1,517937573	0	0,037789864	0,148779238	25,24953126	2,805503473
ENSG00000198521	ZNF43	0	1,519130305	5,110282777	5,913198638	0	3,126768875	3,104480408	4,966642823	1,504134184	25,24463801	2,804959779
ENSG00000179965	ZNF771	0	1,046500621	0	2,360774118	17,07353226	2,825512749	0,600975513	0	1,318761911	25,22605717	2,802895241
ENSG00000182979	MTA1	3,102040139	1,655197531	0,758200098	0,074916731	6,495666141	2,286450669	3,170080165	6,214029335	1,467349803	25,22393061	2,802658957
ENSG00000214954	LRRC69	3,856879749	0	1,637317337	0,426922406	18,09689186	0,542901102	0,164228183	0,108126495	0,364881915	25,19814905	2,799794338
ENSG00000164932	CTHRC1	0	25,04707508	0	0,149532937	0	0	0	0	0	25,19660802	2,799623113
ENSG00000131080	EDA2R	0	0	0,153750055	0	0	6,331764266	0	0	18,70800169	25,19351601	2,799279557
ENSG00000150764	DIXDC1	0	20,54631379	0,03266895	2,575924065	0,042204436	0	1,445069049	0	0,54602927	25,18820956	2,798689951
ENSG00000136052	SLC41A2	6,160594028	3,175434286	1,780885899	0,202628596	0	4,734780582	2,167901291	2,052787063	4,906838872	25,18185062	2,797983402
ENSG00000112742	TTK	0	0	0	16,35473634	0	7,717613636	0	0	1,095841035	25,16819101	2,796465668
ENSG00000130707	ASS1	11,64294561	0	2,096243369	11,29292349	0	0	0	0,108339202	0	25,14045167	2,793383519

ENSG00000183208	GDPGP1	3,478315316	1,113777778	8,804146437	5,103089673	0,559373119	1,292138448	2,062942461	2,173159149	0,546796621	25,133739	2,792637667
ENSG00000165115	KIF27	0,039051705	0	20,94099983	1,989296227	0,092807221	1,221979955	0,016212732	0,042697321	0,768456999	25,11150199	2,790166888
ENSG00000198556	ZNF789	0,045480984	3,510662755	11,42040136	0,235607201	0	3,195869649	2,586822496	3,878690183	0,223742991	25,09727762	2,788586402
ENSG00000179598	PLD6	0	0	0	21,15415426	0	0,772275764	3,163526602	0	0	25,08995663	2,787772959
ENSG00000109911	ELP4	0,016637858	2,676794657	5,070507182	9,327656816	0,013180076	4,262392325	2,772163536	0,048509497	0,893525688	25,08136764	2,786818626
ENSG00000170464	DNAJC18	0,023254935	0,206326562	3,892919207	2,449528078	0,055265858	9,957698509	1,641270886	0	6,835536907	25,06180094	2,784644549
ENSG00000173451	THAP2	0,028837895	11,61607519	0,053049637	7,469512632	0	3,799478856	0,670452156	0	1,418675743	25,05608211	2,784009124
ENSG00000153815	CMIP	2,008469244	1,244229493	1,421054379	6,628548706	0,08473084	2,100794561	3,068916496	4,885701824	3,610231302	25,05267685	2,783630761
ENSG00000087237	CETP	15,01747635	7,986535538	0	0	0	0	1,99805306	0	0	25,00206495	2,778007217
ENSG00000102032	RENBP	0,073820503	7,466582318	6,382542576	0,254943795	0	2,917821574	2,145315887	2,582781182	3,177639609	25,00144744	2,777938605
ENSG00000146776	ATXN7L1	0,856202148	2,650710549	0,301605894	6,95197461	0,649398811	3,720237163	0,363024469	4,063209872	5,444328501	25,00069202	2,777854668
ENSG00000157445	CACNA2D3	2,209499762	18,23979276	0	0	0	2,88295556	1,302828198	0	0,354443463	24,98951974	2,776613305
ENSG00000173611	SCAI	0,336730179	0,041208237	0,021360081	1,924831532	3,918448672	0,841409103	0,173541261	15,0059036	2,713296391	24,97672905	2,775192117
ENSG00000142046	TMEM91	5,52917649	0,323452352	3,353197816	2,308230575	0	2,668445142	5,751354181	3,321618089	1,718725377	24,97420002	2,774911114
ENSG00000182621	PLCB1	0,034714121	4,096357226	15,19854435	1,034029017	4,042444194	0,009528519	0,518829767	0,037954809	0	24,972402	2,774711334
ENSG00000131149	GSE1	0,033891426	4,951485797	2,743224661	3,374831834	0,080543707	4,440489418	1,99799497	1,679840804	5,668763538	24,97106615	2,774562906
ENSG00000123700	KCNJ2	0,135476574	10,96223346	0,697816279	0,233938475	1,931778065	2,677416515	7,109310545	0,177748557	1,033036075	24,95875455	2,77319495
ENSG00000015153	YAF2	3,220457775	2,844942339	0,230816578	10,66465483	0,066263962	1,76028382	2,199406133	2,499827893	1,457418064	24,9440714	2,771563489
ENSG00000127903	ZNF835	0	0,154370754	0	0	0	4,823537204	5,092491886	0	14,87200149	24,94240134	2,771377927
ENSG00000123352	SPATS2	13,34368197	0,039202047	0	0,762967762	3,622676962	1,988986446	4,952776263	0,096618295	0,108682177	24,91559192	2,768399102
ENSG00000176055	MBLAC2	0,026636801	0,094532699	7,595084672	2,25380112	0	2,924565075	3,627205085	2,621109737	5,765730287	24,90866548	2,767629497
ENSG00000267796	LIN37	1,875485806	3,232917732	0	9,808194081	4,966519203	0,82366953	2,0911722	0	2,108896707	24,90685526	2,767428362
ENSG00000249437	NAIP	3,145158519	1,268410596	0,087663229	4,608123172	0	6,261105544	4,378865786	2,987214633	2,148966409	24,88550789	2,765056432
ENSG00000089169	RPH3A	0,014705194	3,496596731	21,10008536	0,02539266	0,209683267	0,016145443	0	0	0	24,86260866	2,762512073
ENSG00000171587	DSCAM	0	0,031193811	9,410447744	8,04417965	0	0,038601809	3,751265161	0,038440503	3,459218656	24,77334733	2,752594148
ENSG00000109756	RAPGEF2	0,011383094	0,100995104	4,250844451	2,280109759	0,081156507	0,349943213	2,561390582	14,01391047	1,091979566	24,74171275	2,749079194
ENSG00000186376	ZNF75D	0,02209865	8,156410649	0	0,152638183	0	4,173240596	10,44057018	0,048323277	1,739423432	24,73270497	2,74807833
ENSG00000109680	TBC1D19	6,025433581	0,064799903	0	0,126116469	0,086785293	8,459915825	4,487580748	0	5,479283644	24,72991546	2,747768385
ENSG00000172828	CES3	5,427469766	3,972071024	0	0,211797869	0	8,21472823	6,161489518	0,73757716	0	24,72513357	2,747237063
ENSG00000169248	CXCL11	0	0	0	0	0,376433483	24,34746409	0	0	0	24,72389757	2,74709973
ENSG00000107331	ABCA2	0	1,957410968	7,682077994	0,04535239	0,093625847	5,551018687	5,669989801	0,775330957	2,939434001	24,71424065	2,746026738
ENSG00000133816	MICAL2	0,022452167	0,03984082	0,192745351	5,324412339	0,124502144	5,538295711	3,983283781	5,040555039	4,427327879	24,69341523	2,743712804
ENSG00000137404	NRM	3,768527679	3,409140343	4,893537195	1,148369081	0	1,78485706	3,74263582	1,615816956	4,271289265	24,6341734	2,737130378
ENSG00000100003	SEC14L2	0,025668519	0,865414982	12,1825829	0,398915368	8,662254292	0,169095203	0	0	2,24139682	24,54532809	2,727258676
ENSG00000164056	SPRY1	0,038130348	1,488550089	2,314747228	5,99169384	0,181235197	5,986679906	4,052536587	0	4,455064248	24,50863744	2,723181938
ENSG00000117569	PTBP2	14,97439527	0,145087269	1,181796819	1,532894649	0,055517901	2,077569253	1,862124429	0,587461935	2,082998047	24,49984557	2,722205063
ENSG00000072134	EPN2	12,33084967	0,440764119	0,032638265	1,654395753	0,126494379	2,201232261	3,594547494	0,659550035	3,447663557	24,48813553	2,720903948
ENSG00000123908	AGO2	0,01907119	3,840994133	0	8,578729208	0,589200234	2,533625264	2,335694323	2,502186414	4,069460669	24,46896144	2,718773493
ENSG00000138769	CDKL2	0	0	0	0	24,43900099	0	0	0	0	24,43900099	2,715444555
ENSG00000093217	XYLB	0	13,7508718	0	0	0,056840383	1,890718823	6,732263778	0,575305515	1,41193939	24,41793969	2,71310441
ENSG00000156650	KAT6B	1,939163193	2,607566457	1,864891878	5,604942676	0,066308825	2,889841096	3,200951076	3,853968336	2,384920751	24,41255429	2,712506032
ENSG00000103326	CAPN15	2,458004778	0,805231231	0,034782295	2,775209314	0	3,923560936	6,358293851	3,886492661	4,162478414	24,40405348	2,711561498
ENSG00000158691	ZSCAN12	0,082498947	10,78423695	0,151763475	0,047485892	0,065353511	4,468565123	1,14167727	0	7,643551235	24,3851324	2,709459155
ENSG00000145147	SLIT2	2,962952366	2,931705251	0,100471708	0,070733254	0,032449403	3,328105903	3,061081561	10,95775606	0,923607005	24,36886251	2,70765139
ENSG00000169057	MECP2	2,534142206	0,145999129	1,997896824	2,656802801	0,175980501	4,019939379	5,192064267	3,724270689	3,895835298	24,34293109	2,704770122
ENSG00000187097	ENTPD5	6,100330389	0,232515464	0,589224605	1,533580639	5,155459467	3,660614902	3,602481832	0,063673681	3,402142714	24,34002369	2,704447077
ENSG00000139354	GAS2L3	0,040703184	6,572638925	0,037438397	0	0	5,809663292	9,05752161	2,091639687	0,725865205	24,3354703	2,703941144
ENSG00000123552	USP45	9,68489063	3,830124209	1,208459007	0,097230769	0,044605334	0,144252147	1,698704035	0	7,6176068	24,32587293	2,70287477
ENSG00000135299	ANKRD6	0	13,07461292	0	0,031260929	0,086047093	2,504460604	2,209672793	0,870918951	5,499471786	24,27644508	2,697382786
ENSG00000184677	ZBTB40	0,016480594	0	0,060634769	3,415006757	0,117499469	6,315060668	5,596833437	0,252267638	8,4927138	24,26649713	2,696277459
ENSG00000137875	BCL2L10	23,94397973	0	0	0	0,272265006	0	0	0	0	24,21624474	2,69069386
ENSG00000163485	ADORA1	0,10816791	0,063980434	0	0	0,085687793	8,986328063	8,053333168	0	6,917694458	24,21519183	2,69057687
ENSG00000122417	ODF2L	0,055463597	1,525491283	0,025507442	0,023943382	0,164763049	3,364492334	3,384866729	7,398241372	8,270835319	24,21360451	2,690400501
ENSG00000101986	ABCD1	0,499185462	2,467564418	0	3,386367061	0	3,797386961	7,460718184	4,522228626	2,061063366	24,19451408	2,688279342
ENSG00000197321	SVIL	8,713530944	0,323810406	5,958514751	2,783446443	4,011477025	0,467494832	0,732341678	0,764817858	0,411454512	24,16688845	2,685209828
ENSG00000087903	RFX2	17,91260118	2,388333188	0	0,946872021	0,11846851	0,985173867	0,331129093	1,199069796	0,265670199	24,14731786	2,683035318
ENSG00000101624	CEP76	0	8,316843458	0,045141296	0,042373331	0	4,687957185	7,987068877	0,053659397	2,927429792	24,06047334	2,673385926
ENSG00000114735	HEMK1	4,846429113	3,092087929	4,178645503	2,888083374	0,036974779	2,417127063	1,82795902	1,547981758	3,224209936	24,05949848	2,673277608
ENSG00000164197	RNF180	0	0,098672309	0	14,93114574	0	6,196843987	0,023085618	0	2,80394017	24,05368783	2,672631981
ENSG00000126368	NR1D1	15,34088303	0,203910266	0	0	1,092372752	4,100451515	3,148685138	0	0,141328154	24,02763085	2,669736762
ENSG00000179361	ARID3B	1,167628363	5,697801676	0	2,436289203	0	6,303106262	3,696244487	0,053192944	4,667105196	24,02136813	2,669040903
ENSG00000180822	PSMG4	2,929961151	3,629105588	2,001200559	0,676256893	3,964150256	3,551359052	2,396678448	2,696000983	2,158516913	24,00322984	2,667025538
ENSG00000145414	NAF1	3,321459058	0,168395646	0	7,15563778	0,075176375	1,041933703	2,757878346	6,640510463	2,840024309	24,00101568	2,66677952
ENSG00000132359	RAP1GAP2	0,461436952	2,883630112	0	10,21983769	0,095357818	2,114632948	2,048971168	4,738042126	1,431107049	23,99301587	2,665890652
ENSG00000188508	KRTDAP	0	0	23,97105241	0	0	0	0	0	0	23,97105241	2,663450268
ENSG00000179542	SLITRK4	0,032714997	14,42591189	0,030090939	0,169474978	0	3,48415803	2,227444734	0,214614354	3,379759227	23,96416915	2,662685461
ENSG00000135999	EPC2	1,53293048	0,276625172	1,194893823	4,531367337	2,778591714	2,225073071	2,804529429	2,670293101	5,943509825	23,95781395	2,661979328
ENSG00000058056	USP13	0,018529806	0,29592618	0,306783678	2,23978519	0,044036486	7,60889708	8,108260572	0	5,332689909	23,9549089	2,661656545
ENSG00000119684	MLH3	0,018068943	5,643069714	0	1,778463895	0,042941234	1,74580064	8,956808347	0,118534462	5,644507552	23,94819479	2,660910532
ENSG00000124006	OBSL1	4,572510063	3,780923632	0	3,034752175	0,073922919	5,122804962	3,590032142	0,136037286	3,634297986	23,94528117	2,660586796
ENSG00000050730	TNIP3	0,040306901	0	0,074147799	0,208803661	0	0	0	23,62135612	0	23,94461448	2,66051272
ENSG00000171320	ESCO2	0,151622296	8,172395086	0,244056277	9,360015394	0	5,743298529	0,204579746	0	0,04661897	23,9225863	2,658065144
ENSG00000149716	LTO1	0	1,158133639	0	11,36949902	0,091239213	3,182481845	1,944535125	3,69388236	2,478897972	23,91866917	2,657629908
ENSG00000165895	ARHGAP42	12,38026043	0,028792226	0,029848609	1,989303468	0,038560887	3,242319871	0,094308206	0,035481002	6,046547434	23,88542214	2,653935793
ENSG00000144504	ANKMY1	0,026332765	4,368960008	0	9,276071241	0,093870578	1,792533895	2,558164824	0,028791023	5,732305383	23,87702972	2,653003302
ENSG00000162390	ACOT11	0,01546996	4,227463965	0,085374723	6,411178737	1,323529026	3,05732021	6,846405299	0	1,902605674	23,86934759	2,652149733
ENSG00000147604	RPL7	0,503701902	0,893806667	0,205911184	11,88703773	0	0,553035256	1,905290227	5,874392311	1,996129313	23,81930459	2,646589399
ENSG00000070087	PFN2	8,800184218	3,759337828	0	5,909554561	0	0	0	4,81085647	0,501069081	23,78100216	2,642333573
ENSG00000074660	SCARF1	5,425107216	0,757157725	3,644353859	0	2,028094229	2,576666883	4,023756109	2,132695877	3,186154577	23,77398648	2,641554053
ENSG00000186185	KIF18B	0	1,670487219	11,82385813	9,417202091	0,077146626	0	0,781662648	0	0	23,77035671	2,641150746
ENSG00000157106	SMG1	0,029073144	6,376472066	0,181840145	3,012178405	0,082911546	3,843241772	3,350639157	2,364969584	4,512434799	23,75376062	2,639306735
ENSG00000095066	HOOK2	3,453955901	0	0	3,376805554	0	2,788413054	4,533801913	3,443183729	6,153237796	23,74939795	2,638821994
ENSG00000026652	AGPAT4	0	0	10,35183833	1,069770993	0,36807401	0,982501992	0,014288843	4,064108107	6,857336661	23,70791893	2,634213215
ENSG00000214756	CSKMT	0	5,303907694	0,087277896	8,60225191	0	2,135740548	3,427290073	0,933724053	3,209280604	23,69947278	2,633274753
ENSG00000215883	CYB5RL	0,01692926	0,450608335	2,179991814	3,800307719	13,96076487	3,085498066	0,014056726	0	0,187387262	23,69554405	2,632838227
ENSG00000179088	C12orf42	0	0,109490404	16,45860121	0	0,366596103	2,371118899	1,767548572	0	2,618091065	23,69144625	2,632382917
ENSG00000212719	C17orf51	0,010214315	0,036250101	0,018790057	10,86494149	0,07282362	0,515877148	3,392465378	7,996185965	0,766299707	23,67384778	2,630427531
ENSG00000134909	ARHGAP32	0,035303459	6,494210889	0,021647854	2,885504742	0,111865911	2,24814581	5,452258022	0,694784933	5,673377208	23,61709883	2,624122092
ENSG00000117525	F3	0,680678246	0	0	4,611117015	0	0	0	18,31931074	0	23,611106	2,623456223
ENSG00000274180	NATD1	0,030645015	0,217515311	0	0	0	9,589234305	3,104315657	0	10,628416	23,57012629	2,618902921
ENSG00000131018	SYNE1	3,437909822	1,140930705	0	2,090244131	0,873158318	3,374830896	5,967903296	0,286935192	6,386658665	23,55857102	2,617619003
ENSG00000103150	MLYCD	3,213329808	0,911652964	6,15175097	3,677639501	0	3,681887682	4,296876054	0,817047827	0,79269395	23,54287876	2,615875417
ENSG00000127418	FGFRL1	8,422099977	1,049842009	0	10,45662957	0,082707863	0,534948888	2,918586719	0,076101927	0	23,54091695	2,615657439
ENSG00000176422	SPRYD4	6,384558423	3,834512769	0	1,017668208	0,033347344	3,974828562	2,446723904	2,147870715	3,675857484	23,51536741	2,612818601
ENSG00000067606	PRKCZ	1,512087582	0	13,98028568	0,101729035	0	3,363489274	1,271824028	0,042941464	3,236313708	23,50867077	2,61207453
ENSG00000171132	PRKCE	0,045433124	0,967439567	2,563055633	2,641254002	10,58133468	1,912178425	1,156872173	2,848002887	0,782276402	23,49784689	2,610871877
ENSG00000104529	EEF1D	1,738012953	1,884704154	3,937311306	2,417608391	2,294680782	3,427758454	3,126736708	1,829882589	2,830250552	23,48694589	2,609660654
ENSG00000188001	TPRG1	0	0,056226043	0	5,526196778	11,52127993	2,852729564	1,341787992	0	2,18230347	23,48052378	2,608947086
ENSG00000112984	KIF20A	0,07845824	0	0,072165135	5,4869504	0	12,36145782	5,472226318	0	0	23,47125791	2,607917546
ENSG00000116678	LEPR	0,026580575	0,047166579	0,171139903	0,137696557	0,063169313	3,793909406	2,262217947	12,72914165	4,184407186	23,41542912	2,601714346
ENSG00000062038	CDH3	0,099480297	0,044131336	0	0,30061633	20,56828584	0,054611776	0,103250767	0	2,171675952	23,34205229	2,593561366
ENSG00000148814	LRRC27	5,400560395	0,652867478	0,024172183	4,084199602	0,062455225	1,832229847	4,69149937	2,758410303	3,830054416	23,33644882	2,592938758
ENSG00000165138	ANKS6	0,03410304	1,180042654	0,031367648	3,032758111	3,201341215	1,198180649	10,09482585	0	4,508801883	23,28142105	2,586824562
ENSG00000183763	TRAIP	0	0	13,38327983	0,240050575	0,110124975	3,815790633	5,694452357	0	0	23,24369837	2,582633153
ENSG00000141542	RAB40B	0,612875449	1,740051915	8,02732927	0	0,233041883	4,602645785	3,501118933	0,69689315	3,829089606	23,24304599	2,582560666
ENSG00000197603	CPLANE1	0,029930788	0,442595405	11,47085447	4,444818661	0,071131165	1,621204632	4,398834431	0,458149077	0,294488085	23,23200672	2,58133408
ENSG00000256223	ZNF10	0	6,823321063	1,103692867	0,565100107	5,508931799	1,676771755	0,317015818	1,431227147	5,802463157	23,22852371	2,580947079
ENSG00000101335	MYL9	0,053814306	0,381968661	6,533720275	0	11,89385023	1,65437897	0,893663333	0,823732896	0,992769884	23,22789855	2,580877617
ENSG00000233493	TMEM238	6,951086251	4,558414001	0	0	0,359117542	3,650032687	2,007525312	2,313041973	3,345237401	23,18445517	2,576050574
ENSG00000153107	ANAPC1	0,216119237	0,038349828	1,948087078	6,325583266	0	3,618615982	2,386663924	3,000943226	5,59506478	23,12942732	2,569936369
ENSG00000115504	EHBP1	0,073542071	0,0652493	6,49375581	2,85729988	0	2,281043335	1,144941587	1,447334765	8,750773101	23,11393985	2,568215539
ENSG00000242114	MTFP1	0,094208585	1,003024938	0,173304301	3,904263959	0	1,965274911	5,006297536	7,210230588	3,707661292	23,06426611	2,562696235
ENSG00000205356	TECPR1	0,400027983	0	3,924713509	1,093704945	0	7,320122511	3,252319437	1,858831121	5,206818515	23,05653802	2,561837558
ENSG00000138587	MNS1	0,782332704	15,48410594	0,553524689	0	0,143017739	1,91613171	3,247937706	0,26318962	0,666116567	23,05635667	2,561817408
ENSG00000198208	RPS6KL1	0	0	0,036494696	2,26095685	18,85873926	0,522758808	1,284847827	0	0,07319674	23,03699418	2,55966602
ENSG00000189068	VSTM1	0	0	20,57646284	0	0	0,590429099	0	0	1,851851467	23,01874341	2,557638157
ENSG00000105221	AKT2	5,648429712	3,031868308	0,240355257	2,707406454	0,023885437	3,332556978	4,155917881	1,538438292	2,336215012	23,01507333	2,55723037

ENSG00000172006	ZNF554	0	8,127249522	0,059334066	2,951879115	0	0,637436581	6,829228316	0	4,403194018	23,00832162	2,55648018
ENSG00000105501	SIGLEC5	10,05269474	0,908955933	0	0	0	1,687226204	4,040570014	1,120117178	5,197402742	23,00696681	2,556329646
ENSG00000185467	KPNA7	0	0	0	0	22,90024908	0	0,072736424	0	0	22,9729855	2,552553945
ENSG00000036565	SLC18A1	0,038647205	10,42393453	0	0	0	0	9,72314849	1,352161913	1,425932396	22,96382453	2,551536059
ENSG00000105991	HOXA1	0,119031564	12,25067822	0	3,699747572	0	6,730519654	0	0,130143587	0	22,9301206	2,547791178
ENSG00000008086	CDKL5	0,008849814	0,706669986	1,937313721	0	12,68215977	2,244529617	1,344718639	0,019351951	3,983591634	22,92718513	2,547465014
ENSG00000189171	S100A13	1,568385887	8,470189533	0	1,530755717	0	1,347649089	4,812715803	1,938469936	3,186898848	22,85506481	2,539451646
ENSG00000125375	DMAC2L	0	2,705867753	13,18910623	1,616840597	0	1,688918856	1,288356069	0,906742639	1,431233729	22,82706587	2,536340652
ENSG00000169679	BUB1	0,050102974	5,601109418	0	5,839890844	0,119070803	9,956835008	0	1,259946129	0	22,82695518	2,536328353
ENSG00000143971	ETAA1	0,404038959	0	0,07432623	4,604734846	0	6,454542489	7,66576761	0,574284676	3,031186593	22,8088814	2,534320156
ENSG00000069869	NEDD4	0	0,029395089	0	9,496865991	0,826734092	5,547327987	0,316357996	0,144895667	6,43800713	22,79958395	2,533287106
ENSG00000214226	C17orf67	1,365830959	2,533798686	0,114207107	5,25300483	0	4,771462765	8,144746284	0	0,610834913	22,79388554	2,532653949
ENSG00000013573	DDX11	10,85424321	0	0	0,093558614	0	2,934715591	5,323529086	0,315940784	3,242929634	22,76491692	2,529435214
ENSG00000118777	ABCG2	0,026974397	0	0	11,73788176	0	1,184652458	7,95108316	0	1,857801722	22,75839349	2,528710388
ENSG00000143190	POU2F1	2,073752451	5,737178344	2,236880592	1,39981335	0,067204331	4,284634692	2,809796593	2,411630241	1,727349	22,7482396	2,527582177
ENSG00000170100	ZNF778	0	7,706733614	0	0,162028282	0,063712861	5,430764021	2,904972647	6,419335606	0,032971972	22,720519	2,524502111
ENSG00000145757	SPATA9	0	0	20,71075242	0,08917805	0	0	0	1,919818061	0	22,71974853	2,524416503
ENSG00000181035	SLC25A42	0,367828342	3,678867892	0	0,057741751	0	4,80953431	5,164302224	0	8,554121848	22,63239637	2,514710708
ENSG00000150477	KIAA1328	0,040705011	3,05773853	4,39296915	0,093718227	0,032245447	2,502736544	1,892701426	5,370266384	5,239813953	22,62289467	2,513654964
ENSG00000115827	DCAF17	0,019734958	0,700383963	0,181520242	1,431274511	0,046900554	2,578471807	4,276839056	5,005930499	8,373651585	22,61470717	2,512745242
ENSG00000165185	KIAA1958	0,011518452	4,966728106	0,211891226	5,78794707	0	3,224879721	5,183696924	0,050374963	3,130732634	22,56776909	2,507529899
ENSG00000113522	RAD50	1,687747027	0,837547184	3,709909508	2,988464924	4,894739811	1,381933814	1,769529528	1,814027914	3,482973158	22,56687287	2,507430319
ENSG00000198075	SULT1C4	0,039454457	0,490076763	0	10,96880703	0	0,519824261	0,524158045	0	9,995922608	22,53824316	2,50424924
ENSG00000147536	GINS4	0	2,018326052	0	11,54494747	0	2,284430559	2,971483905	0	3,718977589	22,53816558	2,50424062
ENSG00000213171	LINGO4	0	0	0	0	22,53119828	0	0	0	0	22,53119828	2,503466475
ENSG00000155367	PPM1J	0	3,731969382	17,41002708	0	0	1,385474544	0	0	0	22,527471	2,503052334
ENSG00000169635	HIC2	0,083348357	0,406723938	12,87938683	1,655130837	0,099039587	2,310668539	2,474111621	0	2,613947224	22,52235693	2,502484103
ENSG00000188396	TCTEX1D4	0	0,351431193	0	0	0	4,131456038	11,18215279	0	6,820055863	22,48509588	2,498343987
ENSG00000143382	ADAMTSL4	6,742651396	3,376788247	1,988041753	2,961482143	0	1,031782193	2,165299573	2,774170809	1,444702443	22,48491856	2,498324284
ENSG00000203668	CHML	0	2,870074926	0,145140373	5,040905396	0	7,947941737	5,290029661	0	1,188680276	22,48277237	2,498085819
ENSG00000143570	SLC39A1	3,434331151	2,849206613	0,574339047	3,23473109	1,5899537	3,476874541	2,592362231	0,780246913	3,94951287	22,48155816	2,497950906
ENSG00000127955	GNAI1	0,012492607	5,408949076	0,298755067	6,730465615	0,415645304	3,579915718	3,205219295	0,027317673	2,79630184	22,47506219	2,497229132
ENSG00000165507	DEPP1	18,05819508	0,360042968	0	0	0	1,392334481	0	2,662111319	0	22,47268385	2,496964872
ENSG00000177025	C19orf18	0	9,377643719	0	0	1,569912753	3,263814624	1,645512551	0,72226135	5,890217646	22,46936264	2,496595849
ENSG00000181315	ZNF322	0	2,173121566	9,505941317	1,392615994	0,070985875	1,016649116	2,13292124	0,849110373	5,326694155	22,46803964	2,496448849
ENSG00000174928	C3orf33	7,18526731	6,895451725	0,134876322	0,126606004	0	0	4,809391668	0,160327301	3,156054478	22,46797481	2,496441645
ENSG00000170365	SMAD1	0,14910826	0	13,40135239	7,540410177	0,050622715	0	1,220390881	0,093158886	0	22,45504331	2,495004812
ENSG00000117724	CENPF	1,708780547	2,802477856	1,547905974	6,259041625	0,092294408	4,107612153	3,138639334	0,028307596	2,738422156	22,42348165	2,491497961
ENSG00000133056	PIK3C2B	0,032103372	0,085449968	0	2,965800115	1,564034336	7,155268126	8,476644482	0	2,132082473	22,41138287	2,490153652
ENSG00000122696	SLC25A51	0,088967071	3,236332648	2,454931695	2,150774003	0	8,986619403	1,551296422	0	3,939049044	22,40797029	2,489774476
ENSG00000250312	ZNF718	0	1,691414154	0	0,051436029	0,070789975	3,82644145	8,186611451	0	8,572459336	22,39915239	2,488794711
ENSG00000152990	ADGRA3	0,016316874	0	2,911572141	1,775067854	0,038777404	1,612347684	2,56061902	9,526620184	3,953327382	22,39464855	2,488294283
ENSG00000109805	NCAPG	0	0	0,057350959	5,760272804	0,074090683	10,9876821	2,743935464	0	2,76066631	22,38399832	2,487110924
ENSG00000162989	KCNJ3	0,369538791	0,491803139	3,682230779	4,573142004	6,147518557	1,386250998	1,022786485	3,299631915	1,401328984	22,37423165	2,486025739
ENSG00000126005	MMP24OS	1,916257237	4,177574639	4,330849368	0,898145746	0,650575258	2,584838695	2,70488578	2,095146714	2,996438493	22,35471193	2,483856881
ENSG00000105948	TTC26	0,755703994	3,862017205	0,055607141	0,260987172	0	6,770505631	8,257599366	0,066100134	2,304957868	22,33347851	2,481497612
ENSG00000253276	CCDC71L	0	3,62833711	1,880730189	1,07459616	0,052819171	3,831145836	3,72775512	5,346051247	2,78810785	22,32954268	2,481060298
ENSG00000243667	WDR92	2,750167789	0,527578948	0,615302728	5,51903783	0	1,468957393	0,33944261	6,013811608	5,073527785	22,30782669	2,47864741
ENSG00000167302	TEPSIN	1,332768578	0,112617388	5,020220183	0,164385746	6,259293577	4,633789732	1,844376993	0,208169614	2,73188924	22,30751105	2,478612339
ENSG00000178372	CALML5	0	0	5,177743404	16,70712876	0,418064659	0	0	0	0	22,30293682	2,478104091
ENSG00000125510	OPRL1	1,660555722	0,220996154	0	4,802923983	7,991351904	2,324571267	2,137132029	0,544672364	2,603893581	22,286097	2,476233
ENSG00000184227	ACOT1	6,273867109	5,08929063	2,30825705	1,083359918	0	3,444169742	2,902338641	1,175923728	0	22,27720682	2,475245202
ENSG00000174327	SLC16A13	0	0	0	7,501724234	0	1,430948913	7,995953117	0	5,342976203	22,27160247	2,474622496
ENSG00000130684	ZNF337	0,031124731	0,110460144	10,36341873	1,612368535	0,147937196	0,751808998	0,697775276	4,696186438	3,827940727	22,23902077	2,471002308
ENSG00000161956	SENP3	0,416971773	3,403568434	1,610811832	6,264167437	0	2,380615339	2,492788053	4,011898723	1,641028894	22,22185048	2,469094498
ENSG00000110013	SIAE	6,1825556	2,701516649	0	0,480897484	0,088246109	0,285384946	5,84264109	3,532012803	3,105433964	22,21878143	2,468753492
ENSG00000196663	TECPR2	0,016072173	0,998188684	2,601813307	0,582815834	0,038195867	7,252632102	6,125386342	0,070290272	4,526574516	22,2119691	2,467996566
ENSG00000104881	PPP1R13L	0	0,266648767	0	1,470803405	7,213777203	1,451882533	2,919655697	0,065718888	3,178759132	22,20624562	2,467360625
ENSG00000178301	AQP11	0	0,335737062	7,193141126	10,89044134	0	1,10791704	0,576032409	0	2,094263815	22,1975328	2,466392533
ENSG00000156011	PSD3	7,243939886	6,63839861	0	0,03427042	0	1,743215936	3,773667721	1,323647795	1,403490285	22,16063065	2,462292295
ENSG00000164989	CCDC171	9,442861641	3,056563721	0	0	0,647907654	3,10214954	1,502269023	2,167850205	2,240400096	22,16000188	2,462222431
ENSG00000139718	SETD1B	0,381684145	0,387022372	5,215888314	8,599492788	0,302360211	1,935689913	1,705334077	0,87437581	2,749473447	22,15132108	2,461257898
ENSG00000138658	ZGRF1	0,049800254	0,029456443	0,091611589	0,114658892	0,039450461	2,369369985	1,626441742	10,81725817	7,002675727	22,14072326	2,460080362
ENSG00000132801	ZSWIM3	0	0,09659294	0,100136923	0	0	6,394968194	9,356037004	6,18969651	0	22,13743157	2,459714619
ENSG00000267710	EDDM13	0	1,824871119	0	3,773623622	13,74758775	0,493991509	0	2,248810328	0	22,08888433	2,454320481
ENSG00000141664	ZCCHC2	2,391784313	0,838353102	0,081479267	3,033831165	4,070115771	1,491331027	1,422037959	3,841887009	4,902643968	22,07346358	2,452607065
ENSG00000198353	HOXC4	3,27670193	1,647421034	0	1,697444721	7,268009531	3,837469067	1,859155624	0	2,485117934	22,07131984	2,452368871
ENSG00000104859	CLASRP	0	0	2,14438566	7,257022233	0	0,505208821	3,922539815	1,542832938	6,677798303	22,04978777	2,449976419
ENSG00000014138	POLA2	2,469659111	3,708141883	0,049924587	1,405899648	0	3,665050455	2,704101983	1,602322795	6,441878376	22,04697884	2,449664315
ENSG00000213337	ANKRD39	0	1,942317195	0	0,198959188	9,44685872	4,364403276	3,80285604	0	2,26728506	22,02267948	2,446964387
ENSG00000196604	POTEF	0,1436071	3,440168212	3,170122298	0,619945295	12,71288045	0,078836102	0,50677017	0,157013337	1,192172987	22,02151595	2,446835105
ENSG00000235098	ANKRD65	5,886682594	0	0,105136195	0	0	2,070744717	5,314930856	3,124381312	5,482612987	21,98448866	2,442720962
ENSG00000188501	LCTL	0	0	0,079016515	0	18,37440524	0	0,49931343	2,160316956	0,845237168	21,95828931	2,439809923
ENSG00000130513	GDF15	0	18,37293053	0,133196023	0	0	0,079497162	0	0,316659863	3,027686515	21,92997009	2,436663343
ENSG00000092853	CLSPN	0,047266366	8,135681579	0,028983432	4,461822108	0,149772721	0,916824165	0,758761261	0,034452567	7,382705139	21,91626934	2,435141037
ENSG00000132881	CPLANE2	0	0	0,353214865	19,72761244	0	0	1,833429248	0	0	21,91425655	2,434917395
ENSG00000163026	WDCP	0	0	0	0,471975406	0	0,38583855	6,419412335	0	14,59879658	21,87602287	2,430669208
ENSG00000104356	POP1	0,06222383	0,05520733	0	3,384579658	0,517566975	7,754107661	6,174059985	0,204097942	3,711573535	21,86341692	2,429268546
ENSG00000259207	ITGB3	1,719510363	2,379958581	0	0	0,081729072	9,326334193	4,054799077	0	4,27184769	21,83417898	2,426019886
ENSG00000105499	PLA2G4C	14,73859339	0,486572509	0	0,118373646	0,10860957	3,763282399	2,618313294	0	0	21,83374481	2,425971645
ENSG00000175182	FAM131A	2,810571959	0,841229848	2,273674759	3,56684223	0,04023726	3,1044332	2,347736184	3,184019362	3,664872596	21,83361735	2,425957484
ENSG00000183780	SLC35F3	0,219191428	9,801535251	0,080644067	0,529894059	0	0	0	0	11,16049612	21,79176092	2,421306769
ENSG00000196456	ZNF775	0	3,030487234	0	1,747576222	0	7,083666314	3,939001633	0	5,990030228	21,79076163	2,421195737
ENSG00000100445	SDR39U1	4,534779952	1,297879962	2,242498378	0,505198402	0	2,783914162	2,469729026	4,478300044	3,478250498	21,79055042	2,421172269
ENSG00000184110	EIF3C	2,618788177	1,728213979	3,344360973	2,466586698	1,080130104	2,138635334	2,19240626	1,798412141	4,41857959	21,78611326	2,420679251
ENSG00000155970	MICU3	2,03493818	1,921956126	0	0,736784246	0,078001204	1,873881405	2,779751056	0	12,35209292	21,77740514	2,419711682
ENSG00000182504	CEP97	0	0,090568115	0	14,30706289	0	3,007386045	0,01412636	0	4,331257612	21,75040103	2,416711225
ENSG00000182704	TSKU	0	0	0	1,365470836	20,31390035	0	0,062531937	0	0	21,74190313	2,415767014
ENSG00000144040	SFXN5	7,046465684	0	0,050145233	0,353028287	0,194345202	3,576497504	1,561730479	3,963903444	4,995243406	21,74135924	2,415706582
ENSG00000120784	ZFP30	0,019612014	10,89272499	0	0,20319401	0	0,279926995	1,872691522	0,514628788	7,93556208	21,7183404	2,413148933
ENSG00000168802	CHTF8	6,856440484	1,510883156	2,80288356	2,011954134	0	3,296562467	1,748845078	0,783949152	2,700616723	21,71213475	2,412459417
ENSG00000174749	FAM241A	0,47841429	1,728187925	1,603005998	6,107365416	0,690298306	4,577361004	3,67444776	2,316479329	0,525346343	21,70090637	2,411211819
ENSG00000198429	ZNF69	0	3,384938247	0	6,498892237	0	7,471919524	4,323610895	0	0	21,6793609	2,408817878
ENSG00000198947	DMD	5,338014871	0,79407467	0,117601311	0,041396347	0,03798176	2,675977044	1,685323338	6,832359381	4,137566678	21,6602954	2,406699489
ENSG00000174502	SLC26A9	0	0	0,049392342	0,092727425	21,50366236	0	0	0	0	21,64578213	2,405086903
ENSG00000121310	ECHDC2	0,03917488	0,023171621	0	8,816589619	0	3,914067356	3,773217727	1,370623854	3,677744003	21,61458906	2,401621007
ENSG00000197238	HIST1H4J	0,405122173	1,437758714	6,707294614	0,699557573	0	1,334401421	0,672763174	8,85883559	1,494744147	21,61047741	2,401164156
ENSG00000189221	MAOA	0,08200646	14,50333901	0,050285836	0	0	3,151340549	3,268402281	0,537972342	0	21,59334647	2,399260719
ENSG00000242110	AMACR	0	2,212849186	4,071918099	3,445396818	0	2,7041336	5,591415662	0,068173008	3,489175475	21,58306185	2,398117983
ENSG00000183718	TRIM52	8,635547699	1,920553795	0	0,079529099	3,776152152	2,680055034	2,791628844	0	1,670976046	21,55444267	2,394938074
ENSG00000163412	EIF4E3	5,690414717	0,71947798	0,077159539	0,144856574	0,066454023	0	4,852572056	7,643286628	2,321362001	21,51558352	2,390620391
ENSG00000174007	CEP19	0	0	0	0	0	2,690857361	3,488517108	1,378086704	13,95140714	21,50886831	2,389874257
ENSG00000205903	ZNF316	0,118332475	0,454952493	0,689326792	2,758513827	1,124878754	3,139785124	1,768571634	0,345011295	11,10907168	21,50844407	2,389827119
ENSG00000154743	TSEN2	0,053330006	0,047316393	0,049052426	4,097972956	0	3,45464056	3,963153243	0	9,838354805	21,50382039	2,389313376
ENSG00000178935	ZNF552	0,556111167	0,44854801	0	0,174596838	0	5,051141679	5,708921095	0,110550207	9,450877011	21,50074601	2,388971778
ENSG00000220201	ZGLP1	4,10254943	0,551505553	0	0,214672953	0	3,548889341	6,709643422	5,301089318	1,07028026	21,49863028	2,388736698
ENSG00000165730	STOX1	0	0,086508431	18,74980739	2,066679666	0,091752055	0	0	0,506542515	0	21,48329006	2,387032229
ENSG00000002587	HS3ST1	0,040648439	5,085140822	0	0	0,048300947	8,836662861	4,877057962	0	2,574605487	21,46241652	2,384712946
ENSG00000009413	REV3L	0,048647266	0,046742535	0,246099082	0,693026492	1,271724094	1,281884536	3,130446915	9,494176306	5,20519837	21,4359456	2,381771733
ENSG00000160352	ZNF714	0,018811225	0,033380057	0,103814303	9,127689265	0	4,91556296	5,513634657	0,863827451	0,856009128	21,43272905	2,381414338
ENSG00000105649	RAB3A	0	0,147492849	9,938782417	0	0	1,73393892	7,039589584	0	2,555645246	21,41544902	2,379494335
ENSG00000164402	SEPT8	2,156202202	4,243523675	0,108177493	0,033848096	0	1,678690489	3,922473734	7,072474204	2,193803804	21,4091937	2,3787993
ENSG00000123154	WDR83	3,076042151	2,842896523	0,117888083	0,442637786	0	3,025510941	5,693522275	0,140133404	6,068779565	21,40741073	2,378601192
ENSG00000204520	MICA	7,459888932	1,131400844	0	1,43128889	0	3,150200823	3,441169443	2,509629627	2,27407137	21,39764993	2,377516659
ENSG00000105429	MEGF8	0,664397514	0,023579142	10,73102915	1,422614177	0,221053711	1,050436293	2,747283914	0,52302341	3,987561681	21,37097899	2,374553221
ENSG00000145545	SRD5A1	0,039604395	0	0,036427742	4,650393991	2,776560739	1,369724326	2,186810923	7,491178113	2,800727305	21,35142753	2,372380837
ENSG00000180061	TMEM150B	0	0	0,162276765	0,152326313	0	1,549660962	4,614495573	8,873315882	5,967051211	21,31912671	2,368791856

ENSG00000204406	MBD5	0,39870863	0,972810686	2,635860107	2,409689739	10,37852066	0,574558396	2,291530222	0,422306711	1,225900543	21,3098857	2,367765077
ENSG00000214943	GPR33	0,225034357	0	0	14,57194571	0	0	0	0	6,503936371	21,30091644	2,366768494
ENSG00000127423	AUNIP	0	0	11,65946428	9,102382835	0,447405576	0,068899741	0	0	0	21,27815244	2,36423916
ENSG00000172197	MBOAT1	0	0,448397993	1,549498879	2,181730557	0,400354005	2,866905172	6,854009217	0,552566166	6,42278951	21,2762515	2,364027944
ENSG00000188707	ZBED6CL	0,029194469	6,734632923	0	0,100824952	0	1,185991371	0,290889487	12,89563034	0,035905433	21,27306897	2,363674331
ENSG00000115183	TANC1	0,020262899	0,017978009	0,074550479	11,58155905	0	1,924406957	5,535332112	0,221545134	1,844135789	21,21977043	2,35775227
ENSG00000203667	COX20	1,554091208	1,232162364	3,406320688	4,168107909	1,493048863	1,270649931	1,866954612	3,832173321	2,358664834	21,18217373	2,353574859
ENSG00000109576	AADAT	0	0	0	17,57137876	0	2,681383058	0	0,890059441	0	21,14282126	2,349202362
ENSG00000274736	CCL23	0	5,420660379	0	12,65991791	0	1,1179958	1,183682377	0	0,751401033	21,1336575	2,348184166
ENSG00000135924	DNAJB2	0	3,416692152	6,753509031	2,881544911	0,061012155	2,565046293	2,899076648	1,291198616	1,231398975	21,09947878	2,344386531
ENSG00000007264	MATK	2,373655653	0,064799903	0,134354809	8,702036369	0	1,283020409	1,364467119	4,631513996	2,515081017	21,06892928	2,340992142
ENSG00000187624	C17orf97	0	1,581958703	0	0,096214961	13,24179728	3,792940913	2,035654354	0	0,274109915	21,02267613	2,335852903
ENSG00000106070	GRB10	0,015427317	0	0	16,46327864	1,283217354	0	0	0,809640594	2,447595852	21,01915976	2,335462196
ENSG00000107249	GLIS3	0,012615676	0	1,833396879	15,57593144	0,029981428	0,983440553	2,534965799	0	0,031031311	21,00136309	2,333484788
ENSG00000177576	C18orf32	1,184664753	3,690456099	1,646572015	1,591066918	1,376408699	5,318387825	3,388136143	0,921071965	1,877891688	20,9946561	2,332739567
ENSG00000177432	NAP1L5	0	0	0	20,98380475	0	0	0	0	0	20,98380475	2,331533861
ENSG00000076201	PTPN23	1,466847381	1,929724854	1,163096648	0,131013376	0	6,525353226	5,291803124	0,553028565	3,919106578	20,97997375	2,331108195
ENSG00000186666	BCDIN3D	6,790567063	6,31520141	0	0,706375134	0	0	1,154843337	5,993263677	0	20,96025062	2,328916736
ENSG00000270647	TAF15	2,157621611	2,005481558	2,83508515	6,830526277	0	1,635698359	2,388692365	1,685030941	1,421569523	20,95970578	2,328856198
ENSG00000138623	SEMA7A	0	0,352818421	6,656891844	1,854011662	0	4,366067808	3,400906367	0	4,25490722	20,88560332	2,320622591
ENSG00000092978	GPATCH2	0,017092022	0	0,125768623	0,419939963	0	3,847038596	7,110127399	4,559780255	4,309301783	20,88404864	2,320449849
ENSG00000156860	FBRS	0,334963858	2,674733168	1,810067145	5,097233502	0,049753054	3,74666445	2,503148768	1,052922562	3,604670701	20,87415721	2,319350801
ENSG00000126214	KLC1	3,210442232	3,84432975	1,328459254	2,805752415	0,055963463	2,20628059	1,694576194	2,214225713	3,504352376	20,86438199	2,318264665
ENSG00000104147	OIP5	0	0	0	0	12,55864844	0,138143694	0,417886202	0	7,737157463	20,8518358	2,316870645
ENSG00000185261	KIAA0825	0,014525672	0	0,053442295	1,530042627	0,06904115	3,428893011	0,844268311	14,89703087	0	20,83724394	2,315249327
ENSG00000236609	ZNF853	14,31326834	0	0	0,135059511	0,092939323	1,331063115	2,954917239	0,085516192	1,923877042	20,83664076	2,315182307
ENSG00000166398	KIAA0355	5,024188433	2,30072235	3,354096918	0,314843112	1,107347295	2,75813266	1,446634744	0,177200465	4,335339171	20,81850515	2,313167239
ENSG00000070047	PHRF1	2,559867921	0,602566292	4,853239412	0,360843526	0	1,978881554	4,099203587	2,741721563	3,598065773	20,79438963	2,310487736
ENSG00000186063	AIDA	3,177892436	1,710735823	0,45979695	4,377689791	0	2,195414058	3,468815889	3,982080091	1,405266709	20,77769175	2,308632416
ENSG00000175455	CCDC14	0,013228624	0,563372844	1,606118054	1,599006235	0,157190555	4,793004492	4,514427598	2,950566916	4,555460583	20,7523759	2,305819545
ENSG00000244687	UBE2V1	7,935029966	2,226168535	0,17308848	3,195343192	0,223609927	2,548232187	2,552115512	1,234500252	0,65574738	20,74383543	2,304870604
ENSG00000154133	ROBO4	0	0	14,30808984	2,374497138	0	3,066738941	0,927691919	0,046983445	0	20,72400128	2,302666809
ENSG00000107404	DVL1	0,038925958	0,483512107	0	2,755882009	0	4,188365924	8,04794247	0,851196722	4,356525896	20,72235109	2,302483454
ENSG00000167548	KMT2D	1,349926016	1,073353381	3,446763248	3,923259564	2,577021173	1,060985382	3,47286689	1,758231284	2,032200195	20,69460713	2,299400793
ENSG00000165948	IFI27L1	0	5,235370121	0	0,22150677	8,231047236	0,774624934	3,248595183	2,103785447	0,867704476	20,68263417	2,298070463
ENSG00000143373	ZNF687	0,860211977	0,424006958	11,51656957	0,825221299	0	3,095738148	2,718127053	0,156752641	1,057949842	20,65457748	2,294953054
ENSG00000174516	PELI3	5,696475919	7,854388693	0	0,066463315	0	4,141425501	1,949488093	0,925822782	0	20,6340643	2,292673811
ENSG00000123485	HJURP	0	0	0	7,51186789	0	5,818376676	7,289823006	0	0	20,62006757	2,291118619
ENSG00000149639	SOGA1	0,030272568	1,145982505	2,450308719	1,150030606	0,023981138	6,204335423	2,195207144	3,287796365	4,120270143	20,60818461	2,28979829
ENSG00000166896	ATP23	0	2,014893298	5,096719579	2,745032796	0	2,942207402	3,394147845	0	4,412947177	20,6059481	2,289549788
ENSG00000227124	ZNF717	4,229356804	8,521907667	0	0	0	0,542475074	2,888147846	0	4,423759751	20,60564714	2,289516349
ENSG00000137804	NUSAP1	0	1,025934609	6,768211106	4,084199602	0	4,501226081	3,054929823	0	1,163560835	20,59806206	2,288673562
ENSG00000130270	ATP8B3	0,021544136	0	1,307861886	1,004454564	0	7,096260766	4,185922111	6,925275367	0,05299302	20,59431185	2,288256872
ENSG00000163464	CXCR1	0	0,215548232	11,06110522	6,712153687	1,732078179	0,133368631	0,353011384	0	0,373485776	20,58075111	2,286750124
ENSG00000140945	CDH13	4,756042451	1,386312281	2,849138429	2,840108567	0	3,130104304	2,480955318	0	3,1353699	20,57803135	2,286447928
ENSG00000204851	PNMA8B	6,092725515	0,876599346	0	2,085205028	0	2,53114574	2,597823801	1,860419832	4,522962947	20,56688221	2,285209134
ENSG00000119636	BBOF1	0	9,800172581	0,060116803	0,112861148	0,077663828	3,982714968	2,225036164	0,285843051	3,978980473	20,52338902	2,280376558
ENSG00000183048	SLC25A10	2,363805188	2,250714995	0	7,267551753	0	2,025615587	4,739245657	0	1,843574811	20,49050799	2,27672311
ENSG00000112320	SOBP	8,172794639	0,237744649	0	1,817786397	0,727787293	4,476118156	1,684592171	2,678633606	0,68265347	20,47811038	2,275345598
ENSG00000163482	STK36	0	8,694237883	0	0,976218143	0,055980911	2,586291141	6,102376241	0	2,056913964	20,47201828	2,274668698
ENSG00000138834	MAPK8IP3	0	0,104284686	0,05405544	7,737984176	0	2,710060951	1,573716366	7,196630347	1,084180004	20,46091197	2,273434663
ENSG00000181894	ZNF329	0,105059493	13,54691912	0,193265422	2,963108634	0,499352318	0,115349184	2,907771309	0	0,129209633	20,46003512	2,273337235
ENSG00000154310	TNIK	0,131997354	0,140535639	0	6,404850442	0	2,97097032	0,909681827	7,418036675	2,467563888	20,44363614	2,271515127
ENSG00000149548	CCDC15	0	0	0	1,082044265	0,14891874	5,159980615	0	0	14,02615262	20,41709624	2,268566249
ENSG00000172878	METAP1D	0,033654916	0,059719822	0,061910935	7,90359834	0	2,327921232	3,6607157	6,32903626	0	20,3765572	2,264061912
ENSG00000108557	RAI1	5,774827124	0	0,143170632	0,05375669	0,147967673	2,358432178	2,326396761	4,52696719	5,034779085	20,36629733	2,262921926
ENSG00000105609	LILRB5	3,682472222	1,031756306	1,465763669	0	1,842415779	0,756610867	0,482784518	10,40701716	0,688614733	20,35743526	2,261937251
ENSG00000273899	NOL12	5,518674312	0,971799833	2,557385912	0,581957011	0,901047639	1,572612101	2,098751023	2,303000889	3,834023599	20,33925232	2,259916924
ENSG00000164929	BAALC	0	9,399068606	0,347997119	10,58374208	0	0	0	0	0	20,33080781	2,258978646
ENSG00000113083	LOX	0	18,03082135	0	2,163873956	0,064740859	0,05981993	0	0	0	20,3192561	2,257695122
ENSG00000182568	SATB1	1,283747303	0,679396975	1,677948284	3,208455981	0,40142806	1,644392779	4,87145413	0,886477713	5,664447992	20,31774922	2,257527691
ENSG00000198846	TOX	0	0	0	0,064017413	10,48454061	0,040704263	0	9,728201203	0	20,31746349	2,257495944
ENSG00000223547	ZNF844	0,160560716	1,343152095	10,92848294	4,475660615	0	0,201470038	2,761566202	0,050157038	0,394937965	20,31598761	2,257331957
ENSG00000143951	WDPCP	0,033333214	0,650638677	4,455857469	0,191863952	0,15843421	1,378521703	1,328509398	6,025571525	6,08100753	20,30373768	2,255970853
ENSG00000162836	ACP6	0,05039959	3,44316418	1,692032621	3,502920946	0,029943929	2,282601947	1,192663875	2,70012404	5,408191134	20,30204226	2,255782474
ENSG00000256806	C17orf100	0	0	0	0	0	5,83436547	9,635959208	0,133562881	4,657431035	20,26131859	2,251257622
ENSG00000277462	ZNF670	0,036004425	0	0	0,3730307	0,427826474	0,079061509	3,168895686	0	16,16249241	20,2473112	2,249701244
ENSG00000177084	POLE	5,367887091	0	0,368207729	5,090187957	5,275734863	1,848001487	0,007554358	0,159159284	2,126006336	20,2427391	2,249193234
ENSG00000104368	PLAT	0,022833268	0,688790269	1,890163864	1,222270204	3,418616677	3,685230398	4,701819634	0	4,577362214	20,20708653	2,245231837
ENSG00000158483	FAM86C1	0,050827639	6,223275481	0,093501547	1,404291824	0	2,120619547	2,447789861	6,668709738	1,187717431	20,19673307	2,244081452
ENSG00000182749	PAQR7	0,73332397	0,325316348	0,421565209	4,115443943	0,762457627	0,251608397	3,044472721	9,922057077	0,620052091	20,19629738	2,244033043
ENSG00000065328	MCM10	0	0	0,968394184	2,966257504	3,818998434	3,467877841	5,084148033	0	3,884580774	20,19025677	2,243361863
ENSG00000052126	PLEKHA5	0,481320499	0,256227425	0,088542793	1,038919313	0,085790144	7,147445612	1,568628932	6,972858767	2,545437317	20,1851708	2,242796756
ENSG00000203965	EFCAB7	0	0	7,066464648	0,045745963	0	3,635838374	1,209829639	0	8,178028943	20,13590757	2,237323063
ENSG00000126953	TIMM8A	0,048887276	0,086749272	0	3,376706239	0	2,361716394	5,601716537	0	8,658006861	20,13378258	2,237086953
ENSG00000137411	VARS2	7,763019374	1,33309233	0	0,463307838	0,042509179	1,512205777	3,950652026	2,69886188	2,353879958	20,11752836	2,235280929
ENSG00000250479	CHCHD10	0,88888571	1,182979412	1,021985658	3,261687183	2,640570164	3,659792133	2,583212718	2,915599125	1,913125965	20,06783807	2,229759785
ENSG00000129810	SGO1	0,040382301	7,309055053	0,371432521	2,998451071	0	4,123378844	4,291876669	0,883042671	0,049665025	20,06728416	2,229698239
ENSG00000112584	FAM120B	3,634273138	1,022933488	3,365823059	0,822319542	0	4,568113407	3,600326329	0	3,051717649	20,06550661	2,229500735
ENSG00000125618	PAX8	0	7,239215577	0,113138013	5,734834607	0	2,61099266	4,221495365	0,134487003	0	20,05416323	2,228240358
ENSG00000205213	LGR4	0	0	0	8,211958288	0	4,594848387	6,100760439	1,143911957	0	20,05147907	2,227942119
ENSG00000174032	SLC25A30	0,111208839	0	0	3,994294577	0,052858043	2,466436304	6,168250057	0	7,248944422	20,04199224	2,226888027
ENSG00000162139	NEU3	4,881969352	0,037179978	0,077088214	3,256260082	0,049794446	0,184038355	8,872694027	2,107597075	0,566919508	20,03354104	2,225949004
ENSG00000147099	HDAC8	3,479345978	3,948088694	6,50984503	0,741033439	0,094141264	1,014832259	0,75102392	2,454293799	0,966259007	19,95886339	2,217651488
ENSG00000135976	ANKRD36	0,352935217	1,637949695	5,119108901	2,930010045	0,09677979	1,788471901	2,907954155	1,365432097	3,706245773	19,90488758	2,211654175
ENSG00000176472	ZNF575	7,128742005	8,405444447	0	0	0	0,92687473	0,038941754	0	3,403148608	19,90315154	2,211461283
ENSG00000160326	SLC2A6	0	3,113515153	0,078725602	1,404068116	0	0	3,83766521	7,205738232	4,26325922	19,90297153	2,211441281
ENSG00000111341	MGP	0,057260542	19,71184086	0	0,098876459	0	0	0	0	0	19,86797786	2,207553095
ENSG00000239779	WBP1	2,936665083	4,644622731	3,053435815	2,204773761	1,517184378	1,471956954	1,802277688	0,418801733	1,805859743	19,85557789	2,206175321
ENSG00000102934	PLLP	0,052077164	0,184819299	0	0,059950597	18,44061361	0,438362236	0,115308749	0,075918338	0,469686788	19,83673678	2,204081864
ENSG00000046653	GPM6B	2,594060429	0	0,048201855	6,198732707	0,062271119	0,949375591	1,588232051	5,385963126	2,997004781	19,82384166	2,202649073
ENSG00000256892	MTRNR2L7	0,196886737	0,87342671	0,543283581	1,019941269	8,89020626	0	0,73565667	7,319071854	0,242145306	19,82061839	2,202290932
ENSG00000105464	GRIN2D	4,836250347	3,316894955	0,054580817	0,051234042	0,211535957	3,094738815	3,646104287	2,919606428	1,678566665	19,80951231	2,201056924
ENSG00000160285	LSS	9,520091216	0	0,153622602	0,468659114	0	2,246371445	5,009799109	1,689151561	0,718942059	19,80663711	2,200737456
ENSG00000164692	COL1A2	0,067726143	15,98372446	0,523268383	0,842027526	0,032190529	0	2,316859842	0,029619449	0	19,79541633	2,199490703
ENSG00000135973	GPR45	0	0	19,35439098	0,399288408	0	0	0	0	0	19,75367939	2,194853265
ENSG00000168000	BSCL2	4,95444494	3,31373468	0	0,131619155	2,626584799	1,924813752	2,37333541	0,41668924	3,984099469	19,72532144	2,191702383
ENSG00000230626	AC011005.1	10,64833	0,212305622	1,320570541	0	0,56867386	0,262724587	1,788175753	3,139520832	1,765762682	19,70606388	2,189562653
ENSG00000204524	ZNF805	0,014859924	6,671248503	0,164016186	0,256598461	0,070629864	1,696794176	5,552330554	0,389931636	4,8613605	19,6777698	2,186418867
ENSG00000137269	LRRC1	0	0,054711692	0,170157171	12,77788082	0	2,268110312	2,124880138	2,224921593	0,037920123	19,65858185	2,184286872
ENSG00000119720	NRDE2	0,573408708	1,032916346	0,831079466	3,270501033	0	2,48012131	4,335518285	2,982707244	4,13514656	19,64139895	2,182377661
ENSG00000198722	UNC13B	0,016868784	5,492750281	0,217220439	0,218465317	0,120267094	5,797054087	4,594135845	0,055330638	3,111964539	19,62405702	2,18045078
ENSG00000166987	MBD6	0,054297726	0,433574919	6,64235594	2,625289001	1,354917066	3,189441557	1,780840142	0,890499194	2,637778523	19,60899407	2,178777119
ENSG00000141338	ABCA8	0,376231191	1,311382714	2,867303173	2,366651913	2,714297626	1,932623648	2,67765247	3,378976993	1,966542428	19,59166216	2,176851351
ENSG00000163617	CCDC191	0,057195523	0,456714232	0	0,790113473	0	0,973358796	3,134383767	9,317704497	4,85367526	19,58314555	2,175905061
ENSG00000179922	ZNF784	0	6,64491837	0,675364672	0,126790561	0,34899664	6,368773353	1,950947825	0	3,431568438	19,54735986	2,171928873
ENSG00000168096	ANKS3	10,81281791	2,249284698	0	0,033164079	0	0,885643648	2,136886691	0	3,424980393	19,54277742	2,171419713
ENSG00000167123	CERCAM	0	0,575317049	5,230191663	0,12919705	0	2,217982623	7,537745643	0	3,834100559	19,52453459	2,169392732
ENSG00000197013	ZNF429	0,01995913	10,76676371	0	3,791156678	0,094866607	0,043827916	3,115628374	0	1,669206646	19,50140906	2,166823229
ENSG00000181722	ZBTB20	3,3994189	1,615313254	0,741350038	3,610451926	2,839408279	2,139471857	0,862136129	0,860506686	3,422813397	19,49087047	2,165652274
ENSG00000146112	PPP1R18	2,102865084	2,507089803	2,417743849	2,439705133	0	2,561350499	2,973747812	1,293285979	3,192406014	19,48819417	2,165354908
ENSG00000165568	AKR1E2	13,31673626	1,775040133	0	0	0	4,393163802	0	0	0	19,4849402	2,164993355
ENSG00000187240	DYNC2H1	0,030197956	1,125296163	1,592478585	8,934224417	0,334908446	2,04459477	0,409542118	0,022011362	4,989085297	19,48233911	2,164704346
ENSG00000140563	MCTP2	0,057078859	0,101285034	0,021000234	2,77946902	16,46780601	0,01253385	0	0,02496295	0	19,46413596	2,162681774
ENSG00000093009	CDC45	0	0	0,195347926	7,243100138	4,164047399	2,739914654	5,114040236	0	0	19,45645035	2,161827817
ENSG00000181104	F2R	0	0,067508056	0,069984919	10,31389502	0	0	2,590273722	0	6,410114187	19,4517759	2,161308434
ENSG00000081386	ZNF510	0	0	0	2,115331664	0,060651502	8,182044992	9,090824595	0	0	19,44885275	2,160983639

ENSG00000136870	ZNF189	5,986320306	0	0,161945878	0,30403143	0	1,93312644	8,844689876	0,096252423	2,111277125	19,43764348	2,159738164
ENSG00000178623	GPR35	11,22196379	0	0,054612986	0,256321193	0,141107089	2,412059465	2,908742471	2,421980157	0,036512087	19,43329924	2,159255471
ENSG00000117262	GPR89A	5,570520947	1,542985794	0	0,89152392	4,391295249	2,804458588	1,263502714	0,415939934	2,506471708	19,38669885	2,154077651
ENSG00000138286	FAM149B1	0,768393107	0,143525759	0,706760589	3,002864688	0,576664058	2,242334838	1,242446751	7,782213821	2,884811533	19,35001514	2,150001683
ENSG00000099849	RASSF7	2,043659269	2,34650915	4,533486101	1,349305756	0	2,24381846	2,49543222	0,788626653	3,548382287	19,34921989	2,149913322
ENSG00000083454	P2RX5	0	0	10,01170252	0	0	0,535727148	1,807570605	0	6,970398625	19,3253989	2,147266544
ENSG00000213921	LEUTX	0	0	19,32481972	0	0	0	0	0	0	19,32481972	2,147202191
ENSG00000164334	FAM170A	0	0	0,605181638	0	0	0	0,728419925	17,98446485	0	19,31806641	2,146451824
ENSG00000173064	HECTD4	0,041693342	0,295935253	0,664718346	2,375844982	7,74515439	2,044699465	0,611600073	4,588946905	0,88026266	19,24885541	2,138761713
ENSG00000163322	ABRAXAS1	0,039254597	3,064878037	4,946522089	1,118438	0,0466447	0,323244402	1,694884339	3,86272387	4,127777024	19,22436706	2,136040784
ENSG00000171843	MLLT3	0,074622504	0,893806667	0,343185307	15,81716945	0,93104548	0,040965574	0,6970574	0,367149519	0,022944015	19,18794592	2,131993991
ENSG00000179299	NSUN7	3,202037859	0,909110019	0	0,132701292	5,296359753	3,48752526	1,084757914	4,537243595	0,535580756	19,18531645	2,131701828
ENSG00000272325	NUDT3	0,259482798	1,354252526	3,088667766	2,161279588	0	4,122626451	2,458711556	1,368466391	4,355191229	19,16867831	2,129853145
ENSG00000172273	HINFP	0	6,842582556	0	0,238757093	0,073021054	3,879568182	3,814114403	0	4,307951106	19,15599439	2,128443822
ENSG00000182405	PGBD4	0	8,079990614	2,988580713	0,079023312	0	1,984696481	6,022727929	0	0	19,15501905	2,12833545
ENSG00000103351	CLUAP1	0,799836483	5,919478701	0,179434611	1,751693607	0	0,942430335	7,143353526	0,81051598	1,607502839	19,15424608	2,128249565
ENSG00000167716	WDR81	0,993017778	0,338862631	2,107772771	1,450921128	7,805916503	2,075716807	1,617335254	0,62637666	2,137246749	19,15316628	2,128129587
ENSG00000133985	TTC9	0	0,052638791	19,04496556	0,051223984	0	0	0	0	0	19,14882833	2,127647592
ENSG00000172638	EFEMP2	0,055997988	5,465188069	0	0,483481517	0	3,28931475	4,091676567	2,449024032	3,305774715	19,14045764	2,126717515
ENSG00000262180	OCLM	0	0	0	6,198681523	0	4,316677901	0	0	8,619578522	19,13493795	2,126104216
ENSG00000173068	BNC2	7,830176408	0,017499315	1,015916305	0,034057949	0,07030951	3,324058412	5,437075015	0	1,394788883	19,1238818	2,124875755
ENSG00000175305	CCNE2	0	0	14,54069201	2,729817792	0,163346619	1,169712958	0,51363793	0	0	19,11720731	2,124134145
ENSG00000178665	ZNF713	0,116597663	0	8,751225338	6,966319538	0	0	0,52279305	2,753621396	0	19,11055698	2,123395221
ENSG00000070018	LRP6	0,014573302	0,051719934	0,026808766	0,629122805	0	4,272169349	4,102077592	0,73295352	9,266337353	19,09576262	2,121751402
ENSG00000143228	NUF2	0	0	0	7,606076	0	5,527062639	5,821942829	0	0,110557122	19,06563859	2,118404288
ENSG00000120075	HOXB5	0	0	1,361979907	0,255693263	0	0	0	14,89464979	2,549584823	19,06190778	2,117989753
ENSG00000196218	RYR1	2,631302139	0,80618954	0,87059223	7,273164027	0,022494052	0,374117179	2,184826326	1,655794889	3,224521558	19,04300194	2,115889104
ENSG00000205784	ARRDC5	0	0	0	11,46966921	0	1,721904642	0,842596857	0	4,992214273	19,02638499	2,114042776
ENSG00000122711	SPINK4	0	0	0	7,214791924	11,032797	0,76456487	0	0	0	19,01215379	2,112461533
ENSG00000170917	NUDT6	1,857071192	0	0	2,335785693	0,381402336	2,013783362	2,493796615	3,659797213	6,259737209	19,00137362	2,111263735
ENSG00000103260	METRN	2,949298208	0,363434535	0	2,970796675	0	3,687901136	5,64599488	1,164448191	2,216656812	18,99853044	2,110947826
ENSG00000163013	FBXO41	0	5,18811759	0,173499001	2,117185539	0	1,11835865	2,615596735	1,979885063	5,799729193	18,99237177	2,11026353
ENSG00000183439	TRIM61	15,92118075	0	0,103492219	0	0	2,964894893	0	0	0	18,98956786	2,109951985
ENSG00000180549	FUT7	3,801155996	0	5,163717009	4,54414623	0	0,706275675	4,709993113	0	0	18,92528802	2,10280978
ENSG00000187609	EXD3	5,046993317	0,212389703	0,513758599	2,755748907	0,047408256	0,941802614	0,662549608	4,144063883	4,587894345	18,91260923	2,101401026
ENSG00000042317	AL049834.1	0,043978629	7,101548896	0	0,835356438	0	1,64172282	4,308934568	0	4,976100131	18,90764148	2,100849054
ENSG00000108846	ABCC3	0,07858064	0,055775767	0,809510429	0,027138323	0,074699437	15,87495898	1,004806611	0	0,947111762	18,87258195	2,09695355
ENSG00000135540	NHSL1	0,414901464	0,061352706	0,063603729	2,806073403	0,041084263	0,986998054	4,80866734	5,027776854	4,656262256	18,86672007	2,09630223
ENSG00000134207	SYT6	13,14519855	0	0	0,102247247	0	0	0	0	5,607438172	18,85488396	2,094987107
ENSG00000156076	WIF1	0	18,7075814	0	0,123841943	0	0	0	0	0	18,83142334	2,092380372
ENSG00000164237	CMBL	6,809570984	6,536029908	0	4,650008765	0	0,815619386	0	0	0	18,81122904	2,09013656
ENSG00000196943	NOP9	0,824921229	0	0	0,776977592	4,871404214	2,278011227	4,213478477	4,099684461	1,721655128	18,78613233	2,087348036
ENSG00000187180	LCE2C	0	18,77867427	0	0	0	0	0	0	0	18,77867427	2,086519364
ENSG00000111676	ATN1	0,118147436	1,362723222	0,217341751	2,040148355	0,070194985	2,367371403	5,714344675	3,939896133	2,942448848	18,77261681	2,085846312
ENSG00000175662	TOM1L2	4,919789659	0	0	0,033185168	0	3,122824031	3,686079956	2,395366953	4,608937027	18,76618279	2,085131421
ENSG00000107815	TWNK	0	7,392548491	0	1,830015541	0,434241285	3,250001623	0	0	5,842816903	18,74962384	2,083291538
ENSG00000111837	MAK	5,864205771	0	7,104082037	1,852354292	0	3,572612986	0	0	0,35181547	18,74507056	2,082785617
ENSG00000147606	SLC26A7	0,289807711	0	17,94853124	0	0	0	0	0	0,504937011	18,74327597	2,082586218
ENSG00000105137	SYDE1	0	0	15,1472506	0	0,090594738	1,088212663	1,80418994	0	0,609486577	18,73973452	2,082192724
ENSG00000198756	COLGALT2	16,22222114	0,034350756	0,035611081	0	0	0	0	0	2,404624488	18,69680746	2,077423052
ENSG00000166863	TAC3	0	0	18,57919185	0	0	0,10560826	0	0	0	18,68480011	2,076088901
ENSG00000177096	PHETA2	0,308263098	0	0	3,193818539	0	2,843020898	6,296552769	2,831140725	3,184640529	18,65743656	2,073048506
ENSG00000160360	GPSM1	8,029035771	0	0	0,10157064	0	4,682178595	0	0	5,823528627	18,63631363	2,070701515
ENSG00000182700	IGIP	1,048772035	1,008052632	0	3,772917504	0	2,302980822	7,583371714	2,197800767	0,698671136	18,61256661	2,068062957
ENSG00000116128	BCL9	13,91525236	0,042353815	0,043907771	0,783093432	0,113447336	1,467539456	0	0,83508973	1,409040131	18,60972403	2,067747115
ENSG00000111261	MANSC1	0,793092568	13,88558993	0	0,182599702	0	0	0,021950723	0,231234827	3,47893245	18,5934002	2,065933355
ENSG00000102048	ASB9	0,063948612	0	0	0	0	2,597838061	7,805390945	8,110539533	0	18,57771715	2,064190795
ENSG00000158113	LRRC43	18,55456628	0	0	0	0	0	0	0	0	18,55456628	2,061618475
ENSG00000173597	SULT1B1	12,88572024	0	0	0	0	2,788413054	0	0	2,841846532	18,51597983	2,057331092
ENSG00000123342	MMP19	0	0,573934076	0	0,111701616	0	10,54696945	6,284258923	0,990172077	0	18,50703614	2,056337349
ENSG00000166965	RCCD1	0	0,05396297	0	2,153015488	0	7,412386441	1,262531012	3,657456473	3,964526335	18,50387868	2,05598652
ENSG00000171365	CLCN5	1,477209035	0	0,024262905	0,182201257	2,726998881	3,229297251	1,850788698	7,412209466	1,5896796	18,49264709	2,054738566
ENSG00000066739	ATG2B	0	0,150472503	3,938831369	2,78213497	0	6,761647381	2,033084083	1,738397346	1,082018894	18,48658655	2,054065172
ENSG00000090097	PCBP4	0,040425514	0	0	0	0	1,686624375	1,544043679	15,20458684	0	18,47568041	2,052853379
ENSG00000112208	BAG2	0	0	3,260028224	0,392324424	0	1,396931634	1,66010964	8,098155842	3,660486305	18,46803607	2,052004008
ENSG00000123843	C4BPB	0	0	0	16,30334112	0,673135037	0	1,489494725	0	0	18,46597088	2,051774542
ENSG00000104497	SNX16	0,032793093	8,612199656	0	0,453012109	0,155866989	2,268308666	6,725514758	0,215126672	0	18,46282194	2,05142466
ENSG00000180035	ZNF48	0	0	0	1,533271752	0	6,026666248	3,049626125	0	7,842882083	18,45244621	2,050271801
ENSG00000143458	GABPB2	0,065778897	0,145903798	2,359609067	0,908686385	0,820706104	4,008286868	4,574225837	0	5,52139521	18,40459217	2,044954685
ENSG00000116194	ANGPTL1	0	18,18417121	0	0,072522228	0	0	0	0,091838401	0	18,34853184	2,03872576
ENSG00000215193	PEX26	1,764024621	0,013377002	0,027735605	1,106484053	9,656490662	1,55605829	1,539820488	0,346177397	2,327137794	18,33730591	2,037478435
ENSG00000184305	CCSER1	2,596897338	0,435212335	0,053080027	0,124563192	4,251534777	0,744490844	1,449485409	5,647105937	3,034156512	18,33652637	2,037391819
ENSG00000196458	ZNF605	0,809570765	1,188880104	0,025677083	0,385641935	0	0,030650393	0,092717777	15,56637996	0,188833525	18,28835154	2,03203906
ENSG00000122432	SPATA1	0,211837249	1,44095	3,11753382	0,54869504	9,816998237	0,620226455	0,556994464	1,312473749	0,65133127	18,27704029	2,030782254
ENSG00000204469	PRRC2A	2,005965886	0,294176632	1,280873742	2,404666798	2,088121749	2,384452611	1,624300513	2,030097178	4,118595447	18,23125056	2,025694506
ENSG00000100285	NEFH	0,039944639	4,749012426	4,4823648	0,068975674	0	2,806840314	3,051353567	0,087347229	2,898478671	18,18431732	2,020479702
ENSG00000185267	CDNF	0	0,157452731	2,121985488	0,153220772	0	0,487112674	6,18878913	4,268679087	4,801671744	18,17891163	2,01987907
ENSG00000101134	DOK5	0,058030173	6,899198927	0	0	0	5,925377739	4,722001742	0	0,570957058	18,17556564	2,019507293
ENSG00000131711	MAP1B	5,217773293	0,684253908	0	10,57057154	0,028637763	0	0,010005609	0,13175222	1,526490574	18,1694849	2,018831656
ENSG00000166333	ILK	7,590226712	3,848191735	0,797876289	0,37447614	0,618457306	0,571448599	0,756279269	1,043277911	2,560457253	18,16069121	2,017854579
ENSG00000106012	IQCE	0,490671839	0,029022838	2,647716063	0,11297109	0	2,747517939	3,544513113	3,075806127	5,511629733	18,15984874	2,017760971
ENSG00000186951	PPARA	0,605859766	3,793218399	0,210288296	0,23687266	0,271667707	2,208961457	4,328199667	4,349467792	2,136974914	18,14151066	2,015723407
ENSG00000102384	CENPI	0,034971204	0	3,666944143	10,44706101	0,083109822	3,686048453	0	0	0,215050361	18,13318499	2,014798332
ENSG00000162618	ADGRL4	1,515611579	11,48453131	4,973349562	0,141466508	0	0	0	0	0	18,11495896	2,012773218
ENSG00000163739	CXCL1	0	0	0	0	0	0	0	18,10251103	0	18,10251103	2,011390114
ENSG00000169891	REPS2	0,039990447	0,023654023	0,098087544	0,966766843	0	5,429853913	5,168899528	0,029149133	6,311830546	18,06823198	2,007581331
ENSG00000163393	SLC22A15	0,08590709	0	0,105355353	4,944759799	0	0	6,181975804	0,187853648	6,550588051	18,05643975	2,006271083
ENSG00000162194	LBHD1	1,393345536	1,834403927	0	4,501555183	0	3,059623961	1,903327463	0	5,362020422	18,05427649	2,006030721
ENSG00000088305	DNMT3B	0	0,173742117	1,500972457	15,94915719	0,38781592	0,035833818	0	0	0	18,04752151	2,005280167
ENSG00000095059	DHPS	0,050640272	0,269579759	0	13,37903858	0,240695404	2,724402902	0,336381587	0,110735445	0,934215092	18,04568904	2,00507656
ENSG00000165244	ZNF367	0	0	0	8,987996142	1,16881218	0	0	0,268864579	7,611289629	18,03696253	2,004106948
ENSG00000104953	TLE6	0	5,657004221	0,078194121	4,697563537	0	1,213410688	0	0	6,377855335	18,0240279	2,002669767
ENSG00000164045	CDC25A	0	0,065130435	2,835842642	9,253462786	2,093471221	0,161195605	3,596186343	0	0	18,00528903	2,00058767
ENSG00000007545	CRAMP1	1,624262673	0	7,10471052	1,121898852	0	2,159829555	3,401620132	0,749821663	1,842262194	18,00440559	2,00048951
ENSG00000124615	MOCS1	1,898374003	0	1,289432233	0,151295888	0	2,404965839	9,748564643	0,127728863	2,370674617	17,99103609	1,99900401
ENSG00000139324	TMTC3	1,381794491	1,646839089	4,249183989	2,635347089	0,049012903	3,758858432	3,664617312	0,045098208	0,532656881	17,96340839	1,995934266
ENSG00000278540	ACACA	5,778917539	0,803066964	0	4,207982235	0	1,809192282	3,921550079	0	1,44144515	17,96215425	1,995794917
ENSG00000198346	ZNF813	0,024193175	0	1,691201371	0,29243433	0	2,151578084	3,816740807	0,105806778	9,84873501	17,93068955	1,992298839
ENSG00000177370	TIMM22	0,854805239	3,792077311	0	5,576234529	0	2,502736544	2,64189574	1,246139161	1,28492253	17,89881105	1,988756784
ENSG00000181544	FANCB	0	4,029966046	0	4,502635242	0,099949219	0,461760581	1,990483976	5,793870791	0,982767582	17,86143344	1,984603715
ENSG00000206190	ATP10A	0,0179701	0,031887501	0	0,58957837	0,042706332	0	1,656226288	15,40377577	0,110504532	17,8526489	1,983627655
ENSG00000174059	CD34	0,016391211	0,029085801	0	17,71833107	0,038954067	0	0	0	0,040318152	17,8430803	1,982564477
ENSG00000106483	SFRP4	0	17,35126397	0,079241761	0,074382832	0,102371021	0,047294919	0	0,188389149	0	17,84294366	1,982549295
ENSG00000166851	PLK1	0	0,056332353	0,05839918	12,88229391	0	3,067254359	0,711701463	1,041285402	0	17,81726667	1,979696297
ENSG00000075886	TUBA3D	0,384505269	9,040410944	0,707328496	0	2,741355285	0	0,319262931	4,624402344	0	17,81726527	1,979696141
ENSG00000091513	TF	0,046427704	16,27100239	0,20284284	0,220468909	0,317217278	0,031859317	0,024093696	0,101523794	0,578138425	17,79357435	1,977063817
ENSG00000229676	ZNF492	0	0,063151672	0,0654687	17,63738524	0	0	0	0	0	17,76600561	1,974000623
ENSG00000165914	TTC7B	0,014210135	1,172522381	0,02614069	4,478148661	0,118197423	2,652322554	4,076546897	2,610165852	2,604018414	17,75227301	1,972474779
ENSG00000196659	TTC30B	0,78997311	0	0,069200921	0,389746041	0	0,784740094	6,278201825	0	9,438060877	17,74992287	1,972213652
ENSG00000135437	RDH5	0	0	0	0	14,32617752	0,945517869	1,261853825	0	1,183735811	17,71728502	1,968587225
ENSG00000087116	ADAMTS2	0,034519833	0	0	0	0	4,927098794	4,815309144	7,925900534	0	17,70282831	1,966980923
ENSG00000183010	PYCR1	0	0,557467776	0,481601003	3,526148305	0	0	1,043412324	3,892853533	8,178277314	17,67976026	1,964417806
ENSG00000166483	WEE1	0	0	0,060097308	5,048898546	0,077638643	2,277661144	3,173717186	0	7,031270435	17,66928326	1,963253696
ENSG00000131127	ZNF141	0,105067609	1,077208282	2,877729702	5,342071096	0,110975755	2,307161913	1,909584008	1,506152618	2,412099486	17,64805047	1,960894497
ENSG00000073536	NLE1	0	0,048975708	0,304635725	1,000846478	0	5,909143828	3,483377254	6,81992806	0,067889141	17,63479619	1,959421799
ENSG00000144677	CTDSPL	0	0	9,272310994	1,55424168	0	0	1,452005204	4,217595739	1,138610416	17,63476403	1,959418226
ENSG00000119927	GPAM	0,015405298	5,467264759	0	1,010860336	0	4,651382261	0,306992351	0,875858778	5,28608214	17,61384592	1,957093991

ENSG00000100890	KIAA0391	1,428429915	5,415070469	1,393483739	6,839172207	0,102869534	1,710908267	0,610998466	0,047326635	0,053235899	17,60149513	1,955721681
ENSG00000166592	RRAD	0	8,709171771	0	0	0	2,072586842	6,818187935	0	0	17,59994655	1,955549616
ENSG00000100802	C14orf93	0,496957479	1,133792812	0	2,084047248	0	3,741464732	3,389496871	5,743988254	1,004103832	17,59385123	1,954872359
ENSG00000106479	ZNF862	0,287077788	4,775742106	0,033006424	3,377097154	0,596965756	2,639755079	2,800810071	2,00096924	1,081272808	17,59269643	1,954744047
ENSG00000196312	MFSD14C	3,372392708	1,523659557	1,602454659	1,396753138	0,029574038	1,311654069	0,371978255	4,353910138	3,627244155	17,58962072	1,954402302
ENSG00000135537	AFG1L	1,68099333	0	0,986911594	0,092639636	0,127497352	2,257954594	0,50485104	8,016459922	3,914873487	17,58218096	1,953575662
ENSG00000135622	SEMA4F	0	6,286933036	0,044641095	0,041903802	0	0,63944979	8,241105795	0	2,327931385	17,5819649	1,953551656
ENSG00000162878	PKDCC	0	0	0,071040148	0,60015711	0	6,52957547	3,911929595	0	6,459270899	17,57197322	1,952441469
ENSG00000099904	ZDHHC8	2,235847491	0,048383616	0	1,224162638	0	0,957982399	7,357967864	3,398551127	2,347393822	17,57028896	1,952254328
ENSG00000135119	RNFT2	0,023294369	4,174863883	0	1,649195924	4,539484889	2,404130447	3,442842469	0,050937963	1,260559115	17,54530906	1,949478784
ENSG00000011465	DCN	0,012042602	15,15083504	0,022153339	0,145564618	0	0,11899866	0	0	2,088329586	17,53792384	1,948658205
ENSG00000170893	TRH	0	0	0	17,537715	0	0	0	0	0	17,537715	1,948635
ENSG00000153944	MSI2	0,049212524	0,57385931	3,375491106	11,39936453	0,083539021	0,856800689	0,30354784	0,04612002	0,830057976	17,51799301	1,946443668
ENSG00000143147	GPR161	0	3,631288668	6,56314382	1,185645376	0	1,138196223	1,743885584	1,265920029	1,986954978	17,51503468	1,946114964
ENSG00000115306	SPTBN1	0,073620697	1,64023418	0,346102002	13,23532026	0	0,107774965	0,006792093	2,092829773	0	17,50267397	1,944741552
ENSG00000142330	CAPN10	0	1,97797881	0	0	0,096329813	2,091683773	6,428335143	2,215897046	4,686044285	17,49626887	1,944029874
ENSG00000168237	GLYCTK	0	4,30682812	1,507610049	0,489865409	0	4,222171539	3,166856523	3,722041436	0,077532967	17,49290604	1,943656227
ENSG00000196839	ADA	0,053795148	10,21402421	0,098960519	2,415204464	0	1,358470368	3,350044464	0	0	17,49049918	1,943388797
ENSG00000204590	GNL1	3,390637371	2,760194079	0	0,181079094	0	2,091632299	3,497380293	3,095674297	2,471935006	17,48853244	1,943170271
ENSG00000141013	GAS8	7,696816244	4,488931079	0,049506696	0,046471055	0	1,506934891	1,899372791	0	1,787304043	17,4753368	1,941704089
ENSG00000149930	TAOK2	1,180685016	0	0	1,300094993	0	7,402193095	4,233966588	0,112252712	3,240897342	17,47008975	1,941121083
ENSG00000204947	ZNF425	0	0	0	0,071196446	0,195971374	0,497958075	0	16,6795075	0	17,4446334	1,938292599
ENSG00000197566	ZNF624	0	0	0,054580817	0	0,070511986	0,032576198	1,15788447	0	16,12883622	17,44438969	1,938265521
ENSG00000149503	INCENP	1,323150484	0	0,187234014	10,60377872	0	1,37824233	3,098728451	0	0,834515143	17,42564914	1,936183238
ENSG00000138092	CENPO	0,027385025	0,291564335	7,556544916	4,634220281	0	2,405372623	0,864058104	0,059883031	1,582962404	17,42199072	1,935776747
ENSG00000198853	RUSC2	0	3,717658478	0	0,091588268	0	5,590528383	2,157966398	0	5,805665315	17,36340684	1,929267427
ENSG00000204227	RING1	0	2,366393823	3,219846706	1,151395365	1,386554218	1,738720881	3,114266376	1,093550692	3,280247497	17,35097556	1,927886173
ENSG00000131015	ULBP2	0	0	0	0	0	0	0	17,3261571	0	17,3261571	1,925128566
ENSG00000204022	LIPJ	0	0	16,8212339	0,115253964	0,317241645	0	0,027709879	0	0,041043843	17,32248324	1,92472036
ENSG00000100330	MTMR3	1,022357693	0	1,571936337	0,685076173	0	3,065902811	2,787384938	6,139549674	2,045569113	17,31777674	1,924197415
ENSG00000278677	HIST1H2AM	0	3,411475828	0	4,647696749	2,741355285	1,688657269	3,511892244	0,840800426	0,472891914	17,31476972	1,923863302
ENSG00000206535	LNP1	0	0	13,24407801	1,09292136	0,188019656	2,779653641	0	0	0	17,30467267	1,922741408
ENSG00000172244	C5orf34	0	0	0	0	0	0	6,219254647	0	11,06670908	17,28596372	1,920662636
ENSG00000105738	SIPA1L3	0,86123683	2,201706917	0,107411167	4,0582043	0,034690644	2,452117295	2,33923629	0,255359017	4,972902499	17,28286496	1,920318329
ENSG00000022976	ZNF839	0	4,413070484	0	0,51346775	0	4,74878216	3,793289107	0,177335188	3,590594706	17,23653939	1,915171044
ENSG00000181074	OR52N4	0	0	0	0	0,56288016	0	0,098330981	16,57352063	0	17,23473177	1,914970197
ENSG00000274349	ZNF658	2,496148184	0,664403056	0	2,640060656	0	0,102773432	1,891252165	9,347416013	0,076748512	17,21880202	1,913200224
ENSG00000155592	ZKSCAN2	0	0,714229072	0	3,276580731	0	3,36703352	0,38194926	0	9,476192536	17,21598512	1,912887235
ENSG00000168306	ACOX2	0	0	0	2,86666726	0	0	0	14,34793048	0	17,21459774	1,912733082
ENSG00000166845	C18orf54	0	0,173695223	0,04501702	0	0	2,60620663	1,889674636	10,54179916	1,956279182	17,21267185	1,912519094
ENSG00000080298	RFX3	0,054117851	2,472793089	1,119984283	1,985806504	0,064306123	1,827110837	2,448968786	3,43185691	3,793804913	17,1987493	1,910972144
ENSG00000089505	CMTM1	2,166090091	0	0	1,424900885	0	2,491489889	3,340165835	1,804420847	5,962311636	17,18937918	1,90993102
ENSG00000126243	LRFN3	1,307831348	0,530449061	0	0	9,76828132	0,533342606	3,78520784	0,65367867	0,597429473	17,17622032	1,908468924
ENSG00000103126	AXIN1	3,769243371	1,245186414	0	2,527299077	0,238236395	1,320768821	2,447145921	0,876833137	4,734315016	17,15902815	1,906558684
ENSG00000164106	SCRG1	0,118448419	13,39921811	0,054473858	0,255668209	0	0,682759575	0,319638314	0,064753002	2,257982438	17,15294193	1,905882436
ENSG00000157985	AGAP1	0,010827642	0,057640155	0,039836644	4,30030411	10,42151079	0,558741552	1,762122748	0	0	17,15098365	1,90566485
ENSG00000124614	RPS10	1,409852653	0,796011027	0,943104662	3,762421177	1,066082611	1,61829655	3,458681756	2,662534683	1,418675743	17,13566086	1,903962318
ENSG00000175513	TSGA10IP	0	0	0,404432734	1,518535255	0	5,63226982	1,277825884	0	8,291891391	17,12495508	1,902772787
ENSG00000110046	ATG2A	2,89372981	1,917011618	0	0,033312265	2,980038332	2,266364319	2,242543914	2,910760265	1,874361708	17,11812223	1,902013581
ENSG00000166323	C11orf65	0,160372057	6,260678166	0,073754338	6,092405881	0	2,11294977	0	2,3671354	0,049309239	17,11660485	1,901844983
ENSG00000042062	RIPOR3	0	2,990265314	0	8,208507024	0,239092904	0,138074714	0,167071015	5,279913196	0,092799528	17,1157237	1,901747077
ENSG00000104848	KCNA7	0	0	0,063581907	0	17,00304924	0	0	0	0,042508354	17,1091395	1,9010155
ENSG00000149657	LSM14B	0,089493971	1,826255849	0	4,945169788	0	3,979495621	1,89487324	2,935456626	1,430858626	17,10160372	1,900178191
ENSG00000106624	AEBP1	0,06079685	12,56831342	0	4,199315624	0,216727545	0,033375694	0	0	0	17,07852914	1,897614348
ENSG00000177685	CRACR2B	5,294593858	0,634805871	0,065809683	4,756627141	0,085018358	2,553074689	3,68331467	0	0	17,07324427	1,897027141
ENSG00000156510	HKDC1	0	0	0	0	0,08503849	0	0	16,97946984	0	17,06450833	1,896056482
ENSG00000167216	KATNAL2	1,579663085	0	0,058118356	0,163664002	5,706237345	0,346875552	5,58753517	0	3,613574445	17,05566796	1,895074217
ENSG00000204316	MRPL38	0,41253227	1,756868141	0,910663715	1,282235747	2,549018865	2,400562535	1,54140457	1,26292218	4,921406678	17,0376147	1,8930683
ENSG00000176732	PFN4	0	0	0,26323873	0	0	0,157112289	5,346747102	11,2648148	0	17,03191292	1,892434769
ENSG00000078237	TIGAR	0,336014611	1,050535155	2,08985462	0,38681593	0	6,552800202	4,304573017	0,944698573	1,338157933	17,00345004	1,889272227
ENSG00000088179	PTPN4	0,022764473	0,080789997	0	11,16383441	0,081150394	1,674602085	2,315473888	0,273785797	1,371870984	16,98427202	1,887141336
ENSG00000170085	SIMC1	0	4,359244944	0,050213168	0,188536831	0	5,96390982	1,654504017	0	4,767017009	16,98342579	1,88704731
ENSG00000123119	NECAB1	15,4716563	0,043234763	0,179284166	1,135963288	0	0,026751141	0,040461249	0,053278711	0	16,95062962	1,883403291
ENSG00000085185	BCORL1	2,607961325	0,603621311	0,06952979	4,046515364	0	3,029382716	5,037015579	0,371924851	1,162121825	16,92807276	1,880896973
ENSG00000131781	FMO5	0,05050487	0	0,046453894	0	0	6,071927857	3,396756983	0	7,360565805	16,92620941	1,880689934
ENSG00000163577	EIF5A2	2,427150476	1,016239307	0,100335715	4,238250806	0,129621925	3,413428216	1,494503142	0	4,091910434	16,91144002	1,879048891
ENSG00000214309	MBLAC1	0	3,630900475	0	6,183293237	0	2,358918152	0,339797785	3,132081209	1,258270293	16,90326115	1,878140128
ENSG00000131845	AC005261.1	0	0	0	6,170950799	0	0,035997088	0,735018217	3,7997466	6,12902395	16,87073665	1,874526295
ENSG00000151687	ANKAR	5,852319774	0,884803404	0	0,906338918	0,062368451	3,976321567	1,917573674	0	3,259899053	16,85962484	1,873291649
ENSG00000171444	MCC	6,557177198	0	0	0,048595768	0	0,370784416	5,199208282	0,061539169	4,603331309	16,84063614	1,871181793
ENSG00000188305	PEAK3	0	0,209159126	0	0,101768711	0	10,54735726	1,663803154	0,257749273	4,059050408	16,83888793	1,870987548
ENSG00000101353	MROH8	0	5,87727437	0	6,190308835	0,185207603	2,86642822	0	0,08520747	1,629391892	16,83381839	1,870424266
ENSG00000124635	HIST1H2BJ	0,159063759	0	0	1,098673577	7,560369313	2,444997972	5,547109415	0	0	16,81021404	1,86780156
ENSG00000159423	ALDH4A1	0	0	0,076536371	0,50290331	0,098875975	1,59880787	5,803693771	7,55123048	1,176891523	16,8089393	1,867659922
ENSG00000168785	TSPAN5	0	0	0,037504061	0,422452738	14,58369944	0,604369343	1,083391965	0,044581026	0,025073735	16,80107231	1,866785812
ENSG00000141255	SPATA22	0,369851047	5,437844757	1,263545904	3,010788168	2,511311485	0,52209622	0,833544164	2,079658117	0,714794316	16,74343418	1,860381576
ENSG00000124159	MATN4	0	0	0	0	16,74037996	0	0	0	0	16,74037996	1,860042218
ENSG00000125319	C17orf53	0	0	0,27751757	0	13,02622699	3,257478877	0,167015417	0	0	16,72823886	1,858693206
ENSG00000059145	UNKL	0,020164207	2,755128637	3,560994062	1,392767412	0	2,169633909	3,063927243	1,455076158	2,30634195	16,72403358	1,858225953
ENSG00000156787	TBC1D31	0	0,280320363	7,652605815	0,151547784	0	0,154174075	4,488890958	0	3,9936818	16,72122079	1,857913422
ENSG00000183570	PCBP3	1,372122932	0,903231693	0,081423579	0,267508029	0,052594836	2,284064764	1,010671977	6,291226388	4,436582181	16,69942638	1,855549182
ENSG00000183742	MACC1	0	7,964613864	0,056168943	2,478065024	1,95921876	1,558868825	2,002860422	0,30045576	0,319194876	16,63944647	1,848827386
ENSG00000258429	PDF	0,898523855	1,875774747	7,972846491	0	0	2,089115341	0,877721805	1,27134671	1,625100754	16,6104297	1,8456033
ENSG00000167363	FN3K	1,617210493	0,979897677	0	1,294117598	3,187156471	3,334668339	2,259841532	3,450112947	0,4851123	16,60811736	1,845346373
ENSG00000142459	EVI5L	6,235848885	0	0	0	0	5,970870083	2,629016873	0	1,753829945	16,58956579	1,843285087
ENSG00000105374	NKG7	0	0	2,716417905	2,549853172	0	3,602835542	7,356566697	0,358778032	0	16,58445135	1,842716816
ENSG00000166532	RIMKLB	1,476478764	0,155950913	0,194007281	8,346762593	0,041772426	3,068486879	0	0,422796353	2,853523431	16,55977864	1,839975404
ENSG00000163520	FBLN2	0,031363755	16,02841345	0	0,487425233	0	0	0	0	0	16,54720244	1,838578049
ENSG00000188306	LRRIQ4	0,082709672	1,027363985	0	15,42472756	0	0	0	0	0	16,53480122	1,837200136
ENSG00000197859	ADAMTSL2	0	0	0	0	0	7,946112718	5,445900662	0	3,139165119	16,5311785	1,836797611
ENSG00000164294	GPX8	0,149429489	7,954768853	0	0,064508028	8,345376757	0	0	0	0	16,51408313	1,834898125
ENSG00000066827	ZFAT	0	0	0,187402314	4,896195591	0	2,162429988	5,399453613	3,824130388	0,041763263	16,51137516	1,83459724
ENSG00000130396	AFDN	0,770972299	0,87947449	0	1,616579653	0,13087374	3,760800973	3,502561017	3,80529604	2,031849733	16,49840795	1,833156438
ENSG00000130244	FAM98C	3,417898945	2,565952154	0,483653935	0	0	0,978269636	4,093203523	0,071864847	4,16315611	16,49164015	1,832404461
ENSG00000145734	BDP1	0	4,541114518	0,381101749	0,357733433	7,414039584	0,30773736	1,305296196	0,639550776	1,543725145	16,49029876	1,832255418
ENSG00000166439	RNF169	0,019269392	0,068386126	2,764912997	4,791460896	0,824294282	0,550073322	0,479993619	0,421365172	6,564586415	16,48434222	1,83159358
ENSG00000243725	TTC4	3,379192807	2,69064507	0	1,57099612	2,985782319	1,33185096	1,906515939	0,378938724	2,23783083	16,48175277	1,831305863
ENSG00000149591	TAGLN	0,038578139	6,914052083	0	2,664641048	0	4,066228073	0,640645045	0	2,135076207	16,45922059	1,828802288
ENSG00000204815	TTC25	0,044839932	0	0	0	0,106563069	2,461581257	0	0	13,84198133	16,45496559	1,82832951
ENSG00000058453	CROCC	0	2,809826029	0,908329338	0,176406743	0,040463948	1,396472913	6,531526015	0,223392384	4,397495175	16,45211254	1,828012505
ENSG00000160050	CCDC28B	0	0	7,00375693	0,648170625	0	2,413887028	2,626951593	2,227912272	1,516845285	16,43752373	1,826391526
ENSG00000147118	ZNF182	0	0	0	0,145489253	0,300349213	2,775197826	13,11719389	0,092120036	0	16,43035022	1,825594469
ENSG00000198483	ANKRD35	0,044933265	0	0	0	0	8,436131019	4,290540642	0	3,647300169	16,4189051	1,824322788
ENSG00000091138	SLC26A3	0,134042493	0,079285038	15,5346655	0,077154043	0,106184962	0,147170825	0	0	0,329709974	16,40821283	1,823134759
ENSG00000100014	SPECC1L	0,020253394	2,012592415	0	4,371648817	0	1,623304128	5,011413877	0,221441206	3,138541993	16,39919583	1,82213287
ENSG00000182389	CACNB4	0,033331989	0,310520679	12,24804782	0,050362436	0,980275634	1,463863035	0,114164579	0	1,193951684	16,39451785	1,821613095
ENSG00000196367	TRRAP	0,022698363	2,107865713	0,027836982	3,605950981	0,485488366	1,968796649	5,396505868	0,066179565	2,689247193	16,37056968	1,818952187
ENSG00000050165	DKK3	0	13,99278277	2,307800782	0,05157837	0	0	0	0	0	16,35216192	1,81690688
ENSG00000152580	IGSF10	4,613079585	0,06859606	0	9,612339816	0,030623138	0	1,957966296	0,056354493	0	16,33895939	1,815439932
ENSG00000128191	DGCR8	2,582203873	1,356288996	0	0,28536976	0	1,927874097	5,025674269	1,806887587	3,353621455	16,33792004	1,815324449
ENSG00000102001	CACNA1F	1,352317463	0,038704099	8,18532624	0	0	2,083461341	0,597650253	0	4,077464115	16,33492351	1,814991501
ENSG00000103253	HAGHL	1,343332102	1,724385853	8,465064486	0,987081425	0	1,098329901	1,091665457	0,499995563	1,124851279	16,33470606	1,814967134
ENSG00000240445	FOXO3B	0	0	12,25534669	1,488736888	0	0,774478833	0,065077973	0	1,735081639	16,31872202	1,813191336
ENSG00000137747	TMPRSS13	0	1,598164277	0	0	0	1,019752101	0	13,10906368	0,588450527	16,31543058	1,81282562
ENSG00000165996	HACD1	0,039208762	7,027073692	5,048937968	4,197708465	0	0	0	0	0	16,31292889	1,812547654
ENSG00000056558	TRAF1	0,236321268	1,018412422	0	0,058296464	0,080231801	5,448805803	4,036579046	0	5,397687197	16,276334	1,808481556
ENSG00000112877	CEP72	0,042092081	4,406790531	0,077431782	0,218051511	0	0	0	2,20903332	9,31821003	16,27160926	1,807956584

ENSG00000196781	TLE1	0	0,052086636	12,52746367	0,405493356	0,279034609	2,900534558	0	0,064186979	0,036100723	16,26490053	1,80721117
ENSG00000239264	TXNDC5	3,272282702	0,611219512	0,063364509	0,773228783	2,865081829	2,080027745	3,174653944	0,828534361	2,584143574	16,25253696	1,80583744
ENSG00000111057	KRT18	0,098475445	4,193814273	0	3,485934819	0,117014514	2,000225265	5,069508364	0	1,271677083	16,23664976	1,804072196
ENSG00000102805	CLN5	1,424071741	1,892296924	0,012184628	5,478559342	0	1,658087643	2,276878997	2,027739084	1,466309021	16,23612738	1,804014153
ENSG00000116191	RALGPS2	0,049393301	0	0,151438276	9,325198482	0	1,446158873	5,222234346	0,036002895	0	16,23042617	1,803380686
ENSG00000039560	RAI14	0,099664009	0	0,036667999	1,273525137	0	1,597608772	0	13,20692177	0	16,21438769	1,801598632
ENSG00000125247	TMTC4	0,024092884	0,128256696	0,044320807	0,790459904	0,229029045	5,290515839	2,480599676	0,052684083	7,17073635	16,21069528	1,801188365
ENSG00000103512	NOMO1	1,517384539	0,324964335	2,021323434	1,80702891	0	3,446895638	2,346051914	0,915331212	3,828901688	16,20788167	1,800875741
ENSG00000227507	LTB	0,984433685	0,87342671	3,802985068	0,339980423	6,316725501	1,080850663	0,8173963	0,645800458	1,331799182	16,19339799	1,799266443
ENSG00000275713	HIST1H2BH	0	0	0	14,06008844	0,348657808	0	0,791803066	0	0,992384342	16,19293365	1,79921485
ENSG00000174996	KLC2	0,496439181	1,076678956	6,291208665	2,095480723	0	2,452775454	2,244222036	0	1,526390491	16,18319551	1,798132834
ENSG00000107438	PDLIM1	0	11,19766772	0,124822676	1,288857082	0	0	3,549452589	0	0	16,16080006	1,795644452
ENSG00000111371	SLC38A1	0,015088424	0	5,384736815	9,014827879	0,035857967	0,049698625	1,64119955	0	0,018556817	16,15996608	1,795551786
ENSG00000183230	CTNNA3	0	0,048067043	0	0,023387557	16,02944663	0	0	0,029616794	0	16,13051803	1,792279781
ENSG00000167291	TBC1D16	0,117453591	0	0,048014526	5,566192135	0,124058223	1,10330032	6,80502362	0,941734237	1,412427149	16,1182038	1,790911534
ENSG00000205403	CFI	8,090227476	2,310146462	1,197452734	0,053525123	0	2,314239839	1,956050304	0,06778145	0,114367091	16,10379048	1,789310053
ENSG00000117472	TSPAN1	15,74865441	0,339420253	0	0	0	0	0	0	0	16,08807466	1,787563852
ENSG00000135931	ARMC9	0,046923891	0,083265293	0,086320287	2,32278313	0	3,125527892	3,909178132	6,498557895	0	16,07255652	1,785839613
ENSG00000143199	ADCY10	0,073844553	0	0,13584302	0,042504475	0,058497726	0	0,020438236	15,71703758	0	16,04816559	1,78312951
ENSG00000092621	PHGDH	0,032789535	0,029092112	6,303335216	4,98259255	0	0	1,728841506	0	2,964027208	16,04067813	1,78229757
ENSG00000102221	JADE3	0	15,20634376	0,053987201	0	0	0	0	0,770094156	0	16,03042512	1,781158346
ENSG00000070731	ST6GALNAC2	3,463153814	0	5,652920024	0	0,115919155	0,535540919	2,956531387	0	3,299405657	16,02347096	1,780385662
ENSG00000102043	MTMR8	0	0	0,495861753	0,186182643	0	3,314660113	3,670555556	0	8,354142635	16,0214027	1,780155856
ENSG00000128641	MYO1B	0	0,112511067	0,087479314	0,76640924	0	2,40172659	0	12,65169591	0	16,01982212	1,779980236
ENSG00000173621	LRFN4	6,118827772	0,521170068	0	2,789392343	0	0	5,446396012	0	1,143856146	16,01964234	1,77996026
ENSG00000255423	EBLN2	0	1,277627159	0,331125788	0	0	0,790520913	0,224187609	1,771239492	11,62232569	16,01702666	1,779669628
ENSG00000019102	VSIG2	0,104430249	0	0,768431511	11,90166436	0,248180748	0,343974934	0,346842659	2,283583742	0	15,9971082	1,777456467
ENSG00000077942	FBLN1	0,124456201	13,17705368	0	2,543086232	0	0,045548545	0,034446213	0,045358211	0	15,96994908	1,774438787
ENSG00000141098	GFOD2	0,035174714	0	0,129413454	2,186605932	0	2,375116496	3,022853669	3,499714344	4,715379639	15,96425825	1,773806472
ENSG00000167779	IGFBP6	7,409582217	3,386637445	0	5,040348692	0	0,123261944	0	0	0	15,9598303	1,773314478
ENSG00000170956	CEACAM3	0,154667933	0	15,36430434	0,089025921	0,122523897	0	0	0,225475652	0	15,95599775	1,772888639
ENSG00000204514	ZNF814	0,698074315	0,019979286	0,082849296	2,119209614	10,9172161	0,284326299	0,476789131	0,541655651	0,80315165	15,94325134	1,771472371
ENSG00000162650	ATXN7L2	0	6,918801608	0	0,149618678	0	3,488181085	1,58276795	0	3,800760299	15,94012962	1,771125513
ENSG00000154102	C16orf74	0	0	13,89263218	0	0	0	0	0	2,045078649	15,93771083	1,770856759
ENSG00000110944	IL23A	0	0	0	0	0	6,196999469	4,686497969	0	5,048462405	15,93195984	1,77021776
ENSG00000105662	CRTC1	0,303783456	2,040713097	1,59666915	0,149876493	7,992995271	0,619424899	0,576543743	2,419897034	0,213493995	15,91339714	1,768155237
ENSG00000244734	HBB	0,811176376	0	0	12,80662452	0	2,290176672	0	0	0	15,90797757	1,767553063
ENSG00000237489	C10orf143	4,503809403	3,818789313	0	1,800608399	0	2,971823573	1,952702275	0	0,845873174	15,89360614	1,765956237
ENSG00000171747	LGALS4	0	0	0	0,234442924	15,487549	0	0	0	0,166978007	15,88896993	1,765441103
ENSG00000173480	ZNF417	0,658745854	0,152468916	0	0,148370911	0,204198754	1,037727261	4,613579304	3,256747064	5,812106932	15,883945	1,764882777
ENSG00000196172	ZNF681	12,86195869	0	0	0	0,055274364	0,127682451	1,313218805	0	1,516063673	15,87419798	1,763799775
ENSG00000135625	EGR4	0,127143938	0	0	0	0	3,001327218	0	0	12,7442083	15,87267946	1,763631051
ENSG00000125388	GRK4	0,063813586	1,641916808	0,176085367	6,336047736	0,151654368	0,035031794	2,728767778	2,930374121	1,805097134	15,86878869	1,763198743
ENSG00000131848	ZSCAN5A	4,360946933	0,63374773	0,03457894	0,194751816	0,357375338	5,076999859	2,871817526	0	2,334929562	15,8651477	1,762794189
ENSG00000139618	BRCA2	0,01231244	0,415114316	0,022649727	3,635613188	4,359856091	3,10921801	1,502822358	0,0269237	2,771116563	15,85562639	1,761736266
ENSG00000221995	TIAF1	0	0,100484167	0	1,955667788	10,22781833	0,310868609	0,141057147	0	3,099183152	15,83507919	1,759453244
ENSG00000187792	ZNF70	1,11046673	5,447841789	0	1,54154668	0	2,65361297	2,621498291	0,04761305	2,410113401	15,83269291	1,759188101
ENSG00000140025	EFCAB11	0	1,67952108	0,10045053	6,69466991	0	4,816242952	2,070517404	0,238810817	0,223857531	15,82407022	1,758230025
ENSG00000129911	KLF16	1,069899523	2,802563049	0,187444436	3,694965925	0	3,635936442	2,11514383	0,557037369	1,754451323	15,8174419	1,757493544
ENSG00000156500	FAM122C	0,018986125	6,805463503	0	1,245825988	0	1,042282804	1,087756365	3,487436069	2,124894274	15,81264513	1,75696057
ENSG00000216937	CCDC7	0,01578508	0,028010237	0	2,153333646	0	1,039865727	1,992216699	8,560302177	2,019015807	15,80852937	1,756503264
ENSG00000110274	CEP164	4,593311391	3,638713556	0	2,043612937	0	1,63388983	1,313468891	1,229905338	1,340239343	15,79314129	1,754793476
ENSG00000168661	ZNF30	0	0	0	7,034312612	0	2,054994393	0	0	6,702661459	15,79196846	1,754663163
ENSG00000133627	ACTR3B	0,100339024	2,759761621	0,138436304	1,602688257	0	5,838818436	1,062248827	0,109706032	4,164887202	15,7768857	1,7529873
ENSG00000178852	EFCAB13	0,520446709	0,963672956	2,039686261	5,118670511	0,107552424	2,01239244	0,67638993	0,940140279	3,395219484	15,77417099	1,752685666
ENSG00000131778	CHD1L	3,548516075	1,433914439	2,262102642	0,36401066	0	1,504420482	1,604479949	1,382892866	3,672856171	15,77319328	1,752577031
ENSG00000134901	POGLUT2	0	0	0	0,781911311	9,531373128	0	5,424873087	0	0	15,73815753	1,74868417
ENSG00000125378	BMP4	0	0,086609173	0	0	15,54320112	0,107177375	0	0	0	15,73698767	1,748554186
ENSG00000149260	CAPN5	0,049245746	0	0	8,631220441	0	1,567999584	4,824995658	0,64611615	0	15,71957758	1,746619731
ENSG00000215790	SLC35E2A	0,215508398	2,537840789	0,072080928	4,228817817	1,39680102	0,086042047	1,54540147	4,798220091	0,819237879	15,69995044	1,744438938
ENSG00000079313	REXO1	1,706392893	2,018634881	0	0,093541844	0	2,825148734	4,565419859	1,954533201	2,531696665	15,69536808	1,743929786
ENSG00000104884	ERCC2	0	0	0	7,484181442	0,055677138	2,752314993	1,906370936	0,614761986	2,881341431	15,69464793	1,74384977
ENSG00000154269	ENPP3	0,0777918	0	9,015570778	0,402988378	0	1,964449557	0	0	4,209650955	15,67045147	1,741161274
ENSG00000121570	DPPA4	0,030246311	0,053671337	0,166921597	12,69159304	0,503167093	1,494390703	0,075342473	0,462978778	0,185995319	15,66430665	1,740478517
ENSG00000177707	NECTIN3	0	1,59835843	0	5,038138035	0	0,021499362	0	8,991999375	0	15,6499952	1,738888356
ENSG00000008197	TFAP2D	0	0,14447306	14,97737602	0,140589965	0,38698011	0	0	0	0	15,64941916	1,738824351
ENSG00000099204	ABLIM1	0,014769873	0,078626332	0,543407485	11,50246052	3,334587677	0,113515203	0,024527482	0	0	15,61189457	1,734654952
ENSG00000167555	ZNF528	1,11605697	0	2,998144092	0,214131867	0	7,410542758	0,647209215	0,658544859	2,549125804	15,59375556	1,732639507
ENSG00000245680	ZNF585B	0	0,889300876	0,221263013	5,157775433	0	0,022009893	2,596626664	0	6,706056521	15,5930324	1,732559156
ENSG00000165698	SPACA9	0	3,609421184	0	0,092431801	0	5,759559517	0,666686142	0,234101713	5,200805972	15,56300633	1,729222926
ENSG00000178919	FOXE1	0,043648345	0	15,33627929	0	0,103731237	0,047923332	0	0	0	15,53158221	1,725731356
ENSG00000164920	OSR2	0	0	0,094873754	0	0	2,88786328	2,269599862	10,26264356	0	15,51498046	1,723886718
ENSG00000124145	SDC4	0	1,026184463	0	0,099860304	1,099479655	7,111360349	5,281950448	0,8852055	0	15,50404072	1,722671191
ENSG00000101187	SLCO4A1	0,075725668	0,94061338	0	11,96469565	0	0	1,729107557	0	0,791628885	15,50177114	1,722419016
ENSG00000175279	CENPS	0,17289539	0	0	1,940591916	0	0,664401623	1,650925421	7,561431751	3,508544185	15,49879029	1,72208781
ENSG00000175764	TTLL11	0,042746979	2,12389703	0,07863652	8,488690829	0	2,769087419	0	0,093475125	1,892637851	15,48917175	1,721019084
ENSG00000197381	ADARB1	0,045112122	2,561611305	0,027662464	1,739739606	0,714732894	2,063769737	2,334854422	3,715703665	2,237777808	15,44096402	1,715662669
ENSG00000140948	ZCCHC14	0,04089596	1,777937484	0,188078551	1,377058729	0,048595067	3,659461976	1,562012252	6,081069172	0,704154618	15,43926381	1,715473757
ENSG00000108511	HOXB6	8,356344921	0	0,640507141	1,102259877	0	2,421122087	0	2,91858489	0	15,43881892	1,715424324
ENSG00000099956	SMARCB1	3,703466331	0,827548684	1,29527792	1,026370551	0,108658863	2,670633186	2,004488209	1,919619878	1,878146398	15,43421002	1,714912224
ENSG00000156265	MAP3K7CL	0	7,03596976	0	0	0	2,066042316	4,18513449	0,07347889	2,066338918	15,42696437	1,714107153
ENSG00000175591	P2RY2	0	0	0	2,371307004	13,0542553	0	0	0	0	15,4255623	1,713951367
ENSG00000188997	KCTD21	0	0	0	2,104314311	0	5,728263359	3,573121854	0,083274841	3,934250976	15,42322534	1,713691704
ENSG00000283268	TEX54	6,231363739	0	0	5,918112826	0	1,710418317	1,552210293	0	0	15,41210517	1,71245613
ENSG00000249709	ZNF564	0	0	8,218809227	3,100360295	0	0,275065891	0,38136879	2,921775673	0,513530042	15,41090992	1,712323324
ENSG00000225528	Z82206.1	0,457218066	1,216983359	0,841089558	3,158063233	1,901530103	2,760992956	2,27782751	1,249752525	1,546378022	15,40983533	1,712203926
ENSG00000197837	HIST4H4	0,058615427	0,728081459	4,63659591	0,303648147	0,975105817	5,470286664	2,579490922	0,64087387	0	15,39269822	1,710299802
ENSG00000185133	INPP5J	0,03133124	0	12,1612691	0	0,07445937	0,687997415	2,393379752	0	0	15,34843688	1,705381876
ENSG00000232434	AJM1	1,067797513	2,93385366	0	3,449789954	0	0,983285843	2,087835477	0,753213056	4,066848903	15,34262441	1,704736045
ENSG00000170456	DENND5B	0	5,766494626	0,024500273	0,528953324	0,063302934	3,626460693	1,028445344	4,281145904	0	15,3193031	1,702144789
ENSG00000188672	RHCE	0	0	0	0	0	0	0,206280858	14,80368593	0,305542988	15,31550977	1,701723308
ENSG00000145708	CRHBP	0	0	0,788436261	9,515456934	0,291019078	0	0,05083887	0	4,668753801	15,31450495	1,701611661
ENSG00000114107	CEP70	0,068546414	0	0,063048333	0,710188182	0	6,81102617	3,984088564	0	3,667191526	15,30408919	1,700454354
ENSG00000163864	NMNAT3	5,866111655	2,251211431	0,171288669	2,230900576	0	1,456813198	1,343324051	0	1,946786338	15,26643592	1,696270658
ENSG00000197165	SULT1A2	1,500195196	1,331029784	8,906402088	1,295254838	0	0,673824544	1,132403718	0	0,419328796	15,25843896	1,695382107
ENSG00000074621	SLC24A1	0,016634805	0	0	7,870561761	0	0	5,566330229	0	1,800362611	15,25388941	1,694876601
ENSG00000146830	GIGYF1	1,24523674	0,814078647	3,335600436	1,207159056	0,155754319	0,575661969	1,614407915	3,630624133	2,659940105	15,23846332	1,693162591
ENSG00000184083	FAM120C	0	1,249439937	0	5,397223023	0,081626899	1,885561731	4,662893347	0	1,943161729	15,21990667	1,691100741
ENSG00000186792	HYAL3	0	0	0	0	0	13,24248121	1,977415735	0	0	15,21989875	1,691099861
ENSG00000132522	GPS2	0,342432501	0,173610877	0,17998064	0,253416939	4,185248147	2,202115133	1,381026639	0,641828231	5,835905685	15,19556479	1,688396088
ENSG00000047578	KIAA0556	0,123557294	0,027406173	0,085235108	1,546834477	0	4,612395428	4,078042271	0,067545905	4,653761034	15,19477769	1,688308632
ENSG00000204516	MICB	6,642816366	4,887506077	0	0	0	1,897476217	1,210757314	0	0,531369613	15,16992559	1,685547287
ENSG00000168350	DEGS2	0	1,697960993	10,77278499	0	0	1,680958224	0,921641243	0,083696699	0	15,15704215	1,684115794
ENSG00000122386	ZNF205	2,659027209	0	0	0	0	2,705837732	7,808239545	0,141817411	1,834536486	15,14945838	1,683273154
ENSG00000198553	KCNRG	0	12,54079508	0	1,445178321	0	0	1,15818768	0	0	15,14416108	1,682684565
ENSG00000156298	TSPAN7	0	0	0,089382668	6,963859451	5,427178293	1,066949046	1,533078012	0	0,059757724	15,14020519	1,682245022
ENSG00000213145	CRIP1	2,128317897	2,913414085	0	1,575060209	0	1,268531572	1,918661012	1,595659883	3,739366645	15,1390113	1,682112367
ENSG00000205129	C4orf47	1,548717415	0	0	0	0	5,101203676	0,91852366	0,483798781	7,074684596	15,12692813	1,680769792
ENSG00000138606	SHF	2,840656416	4,985885167	2,385607715	0,266586611	0	1,18652844	0	0,877737919	2,582256542	15,12525881	1,680584312
ENSG00000163945	UVSSA	0,095803316	1,445005389	0,070495175	0,430121685	2,937054648	2,208915305	3,253494312	0,02094938	4,654116294	15,1159555	1,679550612
ENSG00000126822	PLEKHG3	0,050691235	0	0	9,365998753	0	2,462778604	2,346518285	0,886775089	0	15,11276197	1,679195774
ENSG00000121621	KIF18A	0	0	0	0,404371225	0	4,481091512	3,527724632	0,219460638	6,459575824	15,09222383	1,676913759
ENSG00000162063	CCNF	0	5,780204789	1,365887315	2,853782414	0,170764404	0	4,9122162	0	0	15,08285512	1,675872791
ENSG00000135951	TSGA10	0,020489569	4,944720273	0,037692217	0	0	1,439763361	0	6,317460884	2,318356617	15,07848592	1,675387324
ENSG00000104177	MYEF2	0,0217394	2,025241693	0,559879353	4,073003128	0,051664155	3,174522616	2,454893562	0,190150809	2,513246021	15,06434074	1,673815637
ENSG00000113739	STC2	0	5,032038187	0	0,127742318	0	1,868118439	1,699418661	0,05392209	6,277779065	15,05901876	1,673224307
ENSG00000111752	PHC1	1,675174243	0	0,181271665	6,125633574	2,575998021	1,217146389	0,327278336	0	2,938883707	15,04138594	1,671265104

ENSG00000131591	C1orf159	0	2,968987663	0	0,20637059	0,05680442	1,889522544	3,096072729	0	6,820055863	15,03781381	1,670868201
ENSG00000167034	NKX3-1	0,322688833	0,245401464	1,611233033	1,512435789	1,205092424	0,151840064	6,353897228	1,814466814	1,814243048	15,0312987	1,6701443
ENSG00000160957	RECQL4	0	0	0	1,724782646	0,254332537	7,167524914	1,658720159	0	4,211819202	15,01717946	1,668575495
ENSG00000151090	THRB	1,33640822	0,366714989	0,329480424	2,141151323	0,622103693	1,951355251	0,835096119	7,351024296	0,067777725	15,00111204	1,666790227
ENSG00000197054	ZNF763	2,70883624	0	0	0	0	1,807812962	0,970245098	0,232291436	9,275995136	14,99518087	1,666131208
ENSG00000196597	ZNF782	0,065387525	9,978456083	0,060142817	0,056454992	0,155394869	3,733167455	0,298609617	0,071491685	0,562927588	14,98203263	1,664670292
ENSG00000285938	AC072022.2	0,660448298	0,703169581	1,214947985	0,228090013	0	2,03037419	2,412891	5,776828103	1,949439045	14,97618822	1,664020913
ENSG00000138380	CARF	0,014943688	6,5497502	0,109960482	0,154826923	0	0,032814592	1,451743359	0,065354938	6,561235013	14,94062919	1,660069911
ENSG00000079691	CARMIL1	0,330754204	1,604234934	4,624212941	4,797578695	0,628835621	0,435778547	0,952058553	0,723262588	0,840689069	14,93740515	1,659711684
ENSG00000177694	NAALADL2	3,090036031	2,236826075	0	0,809040967	0,106043863	2,572067113	0,018525075	3,488272786	2,606735511	14,92754742	1,65861638
ENSG00000141371	C17orf64	0	0	0	1,079135544	0	0	9,236442968	0	4,611576235	14,92715475	1,65857275
ENSG00000178538	CA8	0,039218939	0,034796522	0	2,742763164	0	0,43060103	0,048846483	11,62052528	0	14,91675142	1,657416824
ENSG00000154803	FLCN	0,55589419	0,438409156	0	3,146364909	0,073394143	3,780713121	2,987388857	0	3,931149613	14,91331399	1,657034888
ENSG00000068885	IFT80	0	0,151492655	0,078525452	1,253077562	3,043369003	0,421806551	1,772179831	3,220336887	4,934907654	14,87569559	1,652855066
ENSG00000196878	LAMB3	4,812645676	0	0	0,729966574	0	3,28465097	6,048064207	0	0	14,87532743	1,652814159
ENSG00000124785	NRN1	0	0,117297463	0	0	0	4,209454702	9,00137115	0	1,544656134	14,87277945	1,65253105
ENSG00000124467	PSG8	0	0	0	0	14,75103841	0	0,030677343	0	0,090878495	14,87259425	1,652510472
ENSG00000188037	CLCN1	0,045707977	0	0,084083515	3,630674178	0,108625996	4,315883121	3,263898534	0,09994996	3,316680222	14,8655035	1,651722611
ENSG00000173218	VANGL1	0	0,059290658	5,470476243	7,587163993	0	1,155596057	0	0,584516648	0	14,8570436	1,650782622
ENSG00000176244	ACBD7	0	0,075618161	0	14,49638748	0,202547965	0,046788093	0,035383624	0	0	14,85672532	1,650747258
ENSG00000243244	STON1	0	0,034782981	0,108177493	0,440025252	0,046584193	2,690209118	0	10,54441609	0,988417099	14,85261222	1,650290247
ENSG00000164953	TMEM67	0,054743595	0,032380389	0,335684216	0,03151008	0,086732893	0,701228135	2,621225388	10,65403086	0,314195304	14,83173086	1,647970095
ENSG00000198881	ASB12	0	0	0	14,82819114	0	0	0	0	0	14,82819114	1,647576793
ENSG00000138346	DNA2	0,030682349	8,384541729	0	2,119268829	3,500028839	0	0,789762496	0	0	14,82428424	1,647142694
ENSG00000139651	ZNF740	0,233504193	0,088788742	0,03068213	0,403210778	9,988699854	0,036624851	1,523370477	2,407139234	0,102564306	14,81458456	1,646064952
ENSG00000133138	TBC1D8B	0	0,028180977	0,029214934	0,082270617	0,113226761	2,702694628	0,118679242	0	11,73870309	14,81297025	1,645885583
ENSG00000153443	UBALD1	1,220330423	2,837481482	0	2,543225873	0,20000977	2,725904769	3,074739409	0,552104541	1,65610936	14,80990563	1,64554507
ENSG00000186230	ZNF749	0,065176006	0,404786284	2,098189231	3,376342134	0	5,903654336	0,784696922	0,42756252	1,723398848	14,78380628	1,642645142
ENSG00000122335	SERAC1	0,590329294	0	0,277803315	3,105522093	0,032626287	2,260978151	2,268428824	1,981346548	4,254867231	14,77190174	1,641322416
ENSG00000197457	STMN3	0	1,327873886	9,269062396	3,618114537	0	0	0	0	0,552201618	14,76725244	1,640805826
ENSG00000119661	DNAL1	0,017009294	4,014282532	0,125159882	0,440570083	0,040422956	1,456666477	0,296586782	6,025484009	2,342954806	14,75913682	1,639904091
ENSG00000073792	IGF2BP2	0	0,113259557	0,234830073	14,16267972	0,151686396	0	0,026498486	0,069785548	0	14,75873978	1,639859975
ENSG00000213424	KRT222	0	0	0	0	0	0,157062711	0	14,59792977	0	14,75499248	1,639443609
ENSG00000066382	MPPED2	0	0	0,033912419	14,51584097	0	0	0	0,201558233	0	14,75131162	1,639034625
ENSG00000128731	HERC2	0,044922134	0,425137195	4,434900256	5,003311459	0,320275269	1,413893831	0,870328815	0,65487701	1,574580355	14,74222632	1,638025147
ENSG00000196123	KIAA0895L	0	6,166761449	0	0,109109335	0	2,393441311	3,279069935	0,069085212	2,719894744	14,73736199	1,637484665
ENSG00000122674	CCZ1	3,454090171	2,001370861	0	1,217236703	0	1,393122554	2,419269347	2,055260877	2,196289176	14,73663969	1,63740441
ENSG00000166337	TAF10	1,246272748	0,691087629	0,907495168	0,851849573	0,802152586	2,651148391	3,01818668	1,589719569	2,969712469	14,72762481	1,636402757
ENSG00000204348	DXO	1,705399427	0,864625554	4,929917112	2,944852595	0	0,802469537	0,55629732	0	2,921408452	14,72497	1,636107778
ENSG00000182492	BGN	0	14,61173129	0	0,108542003	0	0	0	0	0	14,72027329	1,635585921
ENSG00000162881	OXER1	0	0	0	0,439531119	0	2,515208069	5,072354803	5,751528126	0,939145817	14,71776794	1,635307548
ENSG00000143624	INTS3	0	0	0	0,274222291	0	5,357578573	4,315402648	1,325905401	3,44456151	14,71767042	1,635296714
ENSG00000167695	FAM57A	0	9,310486114	0	0,381483881	0	0,667038137	0	4,347823257	0	14,70683139	1,634092377
ENSG00000253506	NACA2	2,139274488	0,690198198	1,431042981	0,671647296	0,462184739	2,135271257	2,099246224	3,402157709	1,674292993	14,70531589	1,633923987
ENSG00000248905	FMN1	0,082763208	0	7,16957236	2,23465523	0,160927001	1,007821667	1,049542709	1,497189088	1,489807314	14,69227858	1,632475397
ENSG00000180934	OR56A1	0	0	0	0,030633362	14,62946551	0	0	0	0,021818094	14,68191696	1,631324107
ENSG00000106258	CYP3A5	0,169320187	0,245826424	0,028316196	0,02657991	0,18290595	3,53216976	1,610396438	2,322500271	6,550157554	14,66817269	1,629796965
ENSG00000129173	E2F8	0	14,07518481	0	0,568073239	0	0	0	0	0	14,64325805	1,627028673
ENSG00000196550	FAM72A	2,117065886	4,040204601	4,4823648	0	0	0,877137598	0,829172165	1,310208436	0,982535143	14,63868863	1,626520959
ENSG00000213793	ZNF888	0	0,060337984	0	0,058716241	0	8,773399083	0	0	5,729291978	14,62174529	1,624638365
ENSG00000163697	APBB2	0,034990098	0,124178141	0,064367112	0,060420263	0,027718242	0	0	14,3079494	0	14,61962325	1,624402584
ENSG00000258818	RNASE4	2,665831794	11,20370474	0	0	0	0	0,058249922	0	0,69023778	14,61802424	1,624224915
ENSG00000189292	ALKAL2	0	9,316111786	0,139969838	4,073003128	0	1,086020895	0	0	0	14,61510565	1,623900627
ENSG00000250305	TRMT9B	0	0	0	0,053364573	14,4685028	0	0	0,067578138	0,012669338	14,60211485	1,622457205
ENSG00000186300	ZNF555	0,017690303	9,793994852	1,041367732	0	0,168165555	0,252497951	2,526445458	0	0,783244533	14,58340638	1,620378487
ENSG00000066379	ZNRD1	3,045256598	1,606515251	0,227107924	2,273943134	0,293396685	2,213948327	1,435118176	1,259826782	2,226919112	14,58203199	1,620225777
ENSG00000099381	SETD1A	1,897182306	0,462894225	1,526883783	2,006562109	0,281793426	1,327909512	1,693416282	3,163293882	2,216625187	14,57656071	1,619617857
ENSG00000185278	ZBTB37	0,015527186	0,041328915	7,640739465	1,836626157	0,036900693	0,485866362	1,095867059	0,305580673	3,112719187	14,5711557	1,6190173
ENSG00000274070	CASTOR2	0	0	8,973678911	0	0	1,947592977	1,564927105	1,939454542	0,13635108	14,56200461	1,618000513
ENSG00000137834	SMAD6	0,052984071	0,783491118	4,061186579	1,219892354	0	1,047121452	2,639628303	0	4,756941138	14,56124502	1,617916113
ENSG00000217442	SYCE3	0	0	0	6,936921615	0	0	0	0	7,601084756	14,53800637	1,615334041
ENSG00000091622	PITPNM3	0,169930358	0,301537251	12,19142408	0,19562176	0,033653598	0,046643401	0,011758067	0	1,584859294	14,5354278	1,615047534
ENSG00000198513	ATL1	0,034658388	10,33207707	0,063756903	0,059847471	0	0,114158654	1,611545549	0	2,301768854	14,51781289	1,613090321
ENSG00000244057	LCE3C	0	14,51121412	0	0	0	0	0	0	0	14,51121412	1,612357125
ENSG00000127586	CHTF18	0,025542693	0	0,093975693	3,131572548	0	4,85167846	4,02964217	0,111708779	2,2618238	14,50594414	1,611771572
ENSG00000114739	ACVR2B	0,028865439	1,365893665	0,070800407	9,171353453	0,091465786	1,014162073	0,455384204	0,378721567	1,91704149	14,49368809	1,610409787
ENSG00000130300	PLVAP	0,065843386	0	0	0	0	4,626700178	1,421450384	0,575920815	7,773972173	14,46388694	1,607098549
ENSG00000188739	RBM34	0	2,189659068	0,094583225	2,752291328	0	1,806443843	0,896521596	2,473480941	4,236718949	14,44969895	1,605522106
ENSG00000186318	BACE1	0,021016769	1,827393325	4,136838747	2,649270257	0,049946807	2,122916976	0,872533602	0,505532718	2,248768767	14,43421797	1,603801996
ENSG00000152413	HOMER1	0,024554854	1,960739357	0	0,76321572	0	3,046456803	3,384477594	0	5,25467905	14,43412338	1,603791487
ENSG00000157404	KIT	0,027163504	0	0	13,64948366	0,451882581	0	0	0,29699314	0	14,42552289	1,602835876
ENSG00000165406	MARCH8	1,447573322	0,214056945	0	1,145669793	6,54591582	1,523127966	2,337126993	0	1,211612506	14,42508334	1,602787038
ENSG00000186844	LCE1A	0	0	0	11,74825715	0	0	2,675907555	0	0	14,4241647	1,602684967
ENSG00000175697	GPR156	0	0	0	0	0	0	0,041423021	14,29083141	0,061355735	14,39361017	1,599290019
ENSG00000160191	PDE9A	0,457824111	5,636008712	0	2,025815936	0	2,733236162	2,542193813	0,062570454	0,914980038	14,37262923	1,596958803
ENSG00000214827	MTCP1	0	0	12,07775602	0	0	2,288421748	0	0	0	14,36617777	1,596241975
ENSG00000164970	FAM219A	0	1,91428715	3,454538512	2,207805179	0	1,228317331	2,952633408	0	2,604406579	14,36198816	1,595776462
ENSG00000198939	ZFP2	0,630343677	0	0	0,197902901	0,136184127	5,222062141	0,04758071	0	8,104797141	14,3388707	1,593207855
ENSG00000110921	MVK	1,770099163	0,537275894	0,985441165	3,458755829	0,055351039	1,380883345	2,668758603	1,018602243	2,463440338	14,33860762	1,593178624
ENSG00000089163	SIRT4	6,43023705	0	0	0	0	0	0	0	7,90836265	14,3385997	1,593177744
ENSG00000121152	NCAPH	0,023087941	0	3,015521318	6,937003144	0,05486899	1,292809689	2,760539008	0,252432825	0	14,33626292	1,592918102
ENSG00000106246	PTCD1	2,212644015	0,046191559	2,44220242	0	0	0,800257734	1,534606989	1,309215341	5,986787195	14,33190525	1,592433917
ENSG00000228716	DHFR	0,171288337	1,823681705	0,063019746	7,690216889	0	1,542129595	1,56446798	0	1,432505499	14,28730975	1,587478861
ENSG00000137266	SLC22A23	0	4,791478761	3,748887725	0,32257639	0,08071871	3,188436572	1,86132657	0,037135825	0,250635903	14,28119746	1,586799717
ENSG00000121988	ZRANB3	0,0498331	0,117903485	0	1,663650492	0	5,106624324	5,213563317	1,01705704	1,103189206	14,27182096	1,585757885
ENSG00000183150	GPR19	0,05758787	14,20416845	0	0	0	0	0	0	0	14,26175632	1,584639591
ENSG00000108821	COL1A1	0,015389609	13,65424178	0,141552143	0,026574496	0,182868695	0,016896891	0	0,067305136	0,132490646	14,2373194	1,581924377
ENSG00000144550	CPNE9	0,06524636	0	0	7,886635674	0	6,23239213	0	0	0	14,18427416	1,576030463
ENSG00000183111	ARHGEF37	4,448622197	1,467468957	0,052458973	0,147726915	0	5,573142603	0,828734029	1,30951612	0,350719989	14,17838978	1,575376643
ENSG00000100084	HIRA	0	0	0	8,740320154	0,083535134	0,849042263	2,130573211	1,537262468	0,821373325	14,16210656	1,573567395
ENSG00000197852	INKA2	0	0,041943063	0,652229233	0,081631464	0,112347112	5,47585354	2,237387431	0	5,55243012	14,15382196	1,572646885
ENSG00000130518	IQCN	0,0165076	3,954464716	0,030367063	8,095426348	0,039230669	0	2,001165444	0	0	14,13716184	1,57079576
ENSG00000090924	PLEKHG2	0	1,970767223	1,15875864	2,690641467	0,039394202	1,292195145	3,633629624	0	3,343443358	14,12882966	1,569869962
ENSG00000108244	KRT23	0	0	14,08100432	0	0	0	0	0	0	14,08100432	1,564556036
ENSG00000130508	PXDN	0,026825949	0	0,666204746	10,97844647	2,390716818	0	0	0	0,01649623	14,07869022	1,564298913
ENSG00000177494	ZBED2	0,136504581	0	13,93667163	0	0	0	0	0	0	14,07317621	1,563686246
ENSG00000176155	CCDC57	1,036089256	1,7736262	2,959859084	0,252578825	0,057936201	1,967319091	2,024204759	1,679227051	2,308652451	14,05949292	1,56216588
ENSG00000110203	FOLR3	0,108245394	0	11,94756872	0	1,286237616	0	0	0,710102939	0	14,05215467	1,561350519
ENSG00000101447	FAM83D	0	0	0	3,900846085	0,145097997	9,183736973	0,811121338	0	0	14,04080239	1,560089155
ENSG00000255150	EID3	0	0	0	0	0	0,678570866	0,940813669	0	12,4151051	14,03448964	1,559387737
ENSG00000108474	PIGL	1,814700135	1,174916261	0,541343912	0,677533202	0,05827938	5,196485119	1,099545266	0,107249129	3,317610751	13,98766316	1,554184795
ENSG00000212916	MAP10	0	2,74548123	0	9,350912059	0	1,88749234	0	0	0	13,98388563	1,55376507
ENSG00000102743	SLC25A15	0,108297112	0	0	0,249340635	0	0	3,836646559	0	9,767375752	13,96166006	1,551295562
ENSG00000065054	SLC9A3R2	0	0	0,079061462	0,296854351	0	0,141561926	4,817546877	7,612400446	1,004290081	13,95171514	1,550190571
ENSG00000152154	TMEM178A	10,39190818	0	0	0	0	0	3,552962476	0	0	13,94487065	1,549430072
ENSG00000137880	GCHFR	2,448787101	1,26738285	2,815463528	0,880941845	0	2,576599098	1,440240138	2,008042612	0,501948743	13,93940591	1,548822879
ENSG00000185480	PARPBP	0	0,113020864	0	1,814721636	0,15136672	4,5804606	2,670706751	0	4,585216988	13,91279356	1,545865951
ENSG00000188869	TMC3	0,030361778	0	0,055852943	0	13,78168587	0	0	0	0,037341073	13,90524167	1,545026852
ENSG00000170929	OR1M1	0,058388938	0,103609737	0	13,61136846	0	0	0	0,127679508	0	13,90104664	1,544560738
ENSG00000187980	PLA2G2C	3,986240039	3,089568224	1,770037016	2,057098041	0,108889491	0,352144948	0,494576809	0,801539278	1,239728166	13,89982201	1,544424668
ENSG00000162929	KIAA1841	0	0,033551301	0,173911473	0,261196171	0	3,446090557	2,810208887	0,041345667	7,092491153	13,85879521	1,539866134
ENSG00000148344	PTGES	0	0	0	13,66079455	0,189908801	0	0	0	0	13,85070335	1,538967038
ENSG00000179057	IGSF22	1,435878176	2,658710185	0,114844081	0,646812579	0	2,741757103	3,213865145	2,457270074	0,575851649	13,84498899	1,53833211
ENSG00000134899	ERCC5	0,450230127	1,331537189	1,87119687	0,374327375	4,517700268	2,160389324	1,093815518	0,875130172	1,168975035	13,84330188	1,538144653
ENSG00000164935	DCSTAMP	0	0	0	0	0,129271973	2,866705429	10,8397695	0	0	13,83574691	1,537305212
ENSG00000275464	FP565260.1	1,419433932	0	2,248501779	2,995730954	0	2,229457195	1,342278829	2,62968149	0,969844865	13,83492904	1,537214338
ENSG00000138741	TRPC3	1,4602433	0,145571118	3,742620225	1,501580201	1,208756114	0,288226843	0,381451607	4,843503433	0,262324081	13,83427692	1,53714188

ENSG00000106004	HOXA5	0	0,155896512	0,161616337	2,57900847	0,417578538	0,675217463	1,386009481	8,452977309	0	13,82830411	1,536478234
ENSG00000155714	PDZD9	0	13,09483847	0	0	0	0	0	0	0,698146214	13,79298469	1,532553854
ENSG00000184148	SPRR4	0	0	0	0	0	0	12,02911305	1,759971065	0	13,78908412	1,532120458
ENSG00000178498	DTX3	0	0,13121703	0,068015684	8,810625519	0	1,014867731	1,627092634	0	2,13721227	13,78903087	1,532114541
ENSG00000056972	TRAF3IP2	0	0,170270511	0	6,892872075	0	3,097390751	2,183062673	0	1,439755888	13,7833519	1,531483544
ENSG00000253293	HOXA10	0	0	4,782453316	8,066538193	0	0,713593878	0	0	0,19983497	13,76242036	1,529157818
ENSG00000065361	ERBB3	0,01671762	0	0,030753413	0,086603045	13,54785728	0	0,013880997	0	0,061681554	13,75749391	1,528610434
ENSG00000155974	GRIP1	7,153027013	0,244878539	6,008093464	0,15886452	0	0,126263757	0	0,050294455	0	13,74142175	1,526824639
ENSG00000284773	AC114490.3	0	0	0	0	0	0	0,039668142	13,68540258	0	13,72507072	1,525007857
ENSG00000109771	LRP2BP	0,061593983	0,145729348	7,327192826	3,970749613	0	1,84846023	0,136380796	0	0,227257976	13,71736477	1,524151641
ENSG00000255393	OOSP4B	0	0	0	13,65268239	0	0	0	0	0	13,65268239	1,51696471
ENSG00000121900	TMEM54	0	0	0	6,445112287	0	0	1,328196881	5,829826526	0	13,60313569	1,511459522
ENSG00000197361	FBXL22	6,606383554	4,229135032	0,07691757	0	2,682947881	0	0	0	0	13,59538404	1,510598226
ENSG00000169592	INO80E	3,553970981	0,71125199	0,049156516	0,553708169	6,223433979	0,762807249	0,865312634	0,116864569	0,75587445	13,59238054	1,510264504
ENSG00000141696	P3H4	0	2,766602227	0	0,070848486	0	1,486573183	3,236405523	5,92144487	0,100921272	13,58279556	1,509199507
ENSG00000137802	MAPKBP1	0,029180375	0,02588993	0,026839828	0,125970346	0	1,938319956	5,05176484	0,031904467	6,352193578	13,58206332	1,509118147
ENSG00000166839	ANKDD1A	0	0	1,159861331	4,66603372	0	2,571234847	2,792107527	0,984803996	1,40317263	13,57721405	1,508579339
ENSG00000109072	VTN	0	0,140830882	0	0	0	6,796756819	5,930845525	0	0,683259273	13,5516925	1,505743611
ENSG00000062582	MRPS24	0,34778037	3,188493288	0,533141732	4,704236213	0,413253789	3,245661454	0,433154196	0,253497942	0,427725022	13,54694401	1,505216001
ENSG00000125848	FLRT3	0	0	0	0	5,326921742	0	0,12085372	8,020565499	0,071603361	13,53994432	1,504438258
ENSG00000240563	L1TD1	0,078888317	0	13,27861084	0	0,093739896	0	0	0,086252824	0	13,53749187	1,504165764
ENSG00000016391	CHDH	13,3315734	0	0,035697971	0	0	0,042612194	0,064451179	0	0,047732509	13,52206725	1,502451917
ENSG00000169583	CLIC3	0	0	0	8,646103661	0	2,678250601	2,025434416	0	0,15789849	13,50768717	1,50085413
ENSG00000221829	FANCG	0	0	0	0,722011551	0	5,876190874	1,041535683	0	5,862341876	13,50207998	1,500231109
ENSG00000166278	C2	1,999469215	1,603894508	0	1,182413334	0,520743219	1,142759093	0,977927184	5,150874172	0,90952621	13,48760694	1,498622993
ENSG00000115896	PLCL1	0,037210187	0,066028564	0,102676717	0,16063468	0	3,390932742	0,015448206	9,642082308	0,0686456	13,48365901	1,498184334
ENSG00000134323	MYCN	0,05776398	0	1,806445597	11,47076532	0,137277348	0	0	0	0	13,47225225	1,496916916
ENSG00000174938	SEZ6L2	3,308990598	0	0,067635061	0,126975657	0,786388779	2,825727584	6,258252569	0,080397705	0	13,45436795	1,494929773
ENSG00000182667	NTM	0,021723774	0,038548304	13,14770738	0,037512216	0,154881056	0	0	0,047503532	0	13,44787627	1,494208474
ENSG00000126461	SCAF1	0,067701869	5,10575045	0,747258331	0,233812701	0	1,858317391	2,164250798	0,814242886	2,456304842	13,44763927	1,494182141
ENSG00000177352	CCDC71	0	0	0	7,430309159	0,401024615	4,075971734	0	0,184497246	1,348969732	13,44077249	1,493419165
ENSG00000263002	ZNF234	0,057930064	0	0	2,300749249	0	3,180191234	2,116424998	1,076746722	4,702269684	13,43431195	1,492701328
ENSG00000082512	TRAF5	0,033498242	6,063064511	0	1,041194756	0,039804649	3,861806818	0,681450484	1,062137271	0,638577055	13,42153379	1,491281532
ENSG00000107186	MPDZ	0,013404646	0,023786215	0,049317857	0,208322077	13,12484941	0	0	0	0	13,41968021	1,491075579
ENSG00000093134	VNN3	12,92651528	0,069719709	0	0,135691614	0,280122888	0	0	0	0	13,41204949	1,490227721
ENSG00000215375	MYL5	0	1,308009757	0,581143064	0,121224146	0	3,969521347	3,526576114	0,230267991	3,669443529	13,40618595	1,489576217
ENSG00000108556	CHRNE	0,901255889	2,817737575	0	2,593787024	0	1,319368403	1,567933714	1,876935913	2,32242175	13,39944027	1,488826696
ENSG00000049130	KITLG	0	12,3591639	0	0,042052373	0,05787551	0,695193392	0,242650118	0	0	13,3969353	1,488548366
ENSG00000140950	MEAK7	1,148566309	0	1,397227419	4,5744393	0	2,074644946	0,230728819	0	3,96436127	13,38996806	1,487774229
ENSG00000132763	MMACHC	0	0,159323867	0,220225866	5,994940989	0,213379407	1,774444671	0,298206374	0	4,711498287	13,37201942	1,485779936
ENSG00000140320	BAHD1	0	0,78735034	0,816238132	2,202791123	0	2,040008926	5,226971071	0,303206614	1,978179169	13,35474538	1,483860597
ENSG00000119630	PGF	0	9,147504483	0	0,494535617	0	0,314441134	0	0	3,394164604	13,35064584	1,483405093
ENSG00000119242	CCDC92	0,588050378	1,891309255	2,805830988	0,539449662	0	1,311466312	2,502387748	1,285893976	2,418287472	13,34267579	1,482519532
ENSG00000179750	APOBEC3B	0	0	0	0,825743591	0	2,975189667	9,529392304	0	0	13,33032556	1,481147285
ENSG00000102804	TSC22D1	6,494269808	1,155515258	0,550391531	0,425470492	0,083651885	1,835721499	2,718085459	0,038485275	0	13,30159121	1,477954578
ENSG00000151773	CCDC122	1,367137733	0,049509232	0	2,312569937	0,066306784	0	0,370665678	7,382308469	1,750031875	13,29852971	1,477614412
ENSG00000169231	THBS3	0	0,05800173	0	4,741193568	0	3,552919553	1,899831979	0	3,015031185	13,26697802	1,474108669
ENSG00000223638	RFPL4A	0	0	11,81751145	0	0	0	0	0	1,436494858	13,25400631	1,472667368
ENSG00000160072	ATAD3B	0,216378063	0,314147097	0,217115412	6,8953137	0,51422715	0,2807651	1,747634148	0,086028265	2,975673293	13,24728223	1,471920248
ENSG00000204438	GPANK1	0,69893881	0,826833179	0	2,494290537	1,88251564	1,483628345	1,586273084	2,547290838	1,719209272	13,23897971	1,470997745
ENSG00000065665	SEC61A2	0,03858799	0	1,348728774	2,86522061	0,641936363	5,507756632	2,691396294	0,084380635	0,047458254	13,22546555	1,469496173
ENSG00000160181	TFF2	0,216181074	0	11,13511126	1,866487658	0	0	0	0	0	13,21777999	1,468642221
ENSG00000136213	CHST12	1,837780581	3,011522835	0	0,939080946	0,044566585	0,494149149	1,884079197	2,132365042	2,871418717	13,21496305	1,468329228
ENSG00000176222	ZNF404	0,060956261	0	0,336401895	0,315774475	0	4,684848878	0,101226569	0	7,646770985	13,14597906	1,46066434
ENSG00000196139	AKR1C3	0	0	0	13,14349016	0	0	0	0	0	13,14349016	1,460387795
ENSG00000024422	EHD2	0	0	0,216945967	12,82952919	0,093422878	0	0	0	0	13,13989803	1,45998867
ENSG00000198018	ENTPD7	0,017459338	0,557661005	7,001694001	1,266235579	0	2,300319954	0,115974888	0,801747651	1,073636755	13,13472917	1,459414352
ENSG00000213139	CRYGS	13,00422699	0	0	0,125449507	0	0	0	0	0	13,1296765	1,458852945
ENSG00000165246	NLGN4Y	10,90796994	0,312192339	0	0	0	0	0	0	1,904120848	13,12428313	1,458253681
ENSG00000197162	ZNF785	0,030515059	0,054148223	0,056134915	1,211935464	0,072519698	4,388991427	2,508393148	3,336374874	1,463653952	13,12266676	1,458074084
ENSG00000160447	PKN3	0	0	0	2,076405633	0	3,422853041	2,847396276	4,771969882	0	13,11862483	1,457624981
ENSG00000196700	ZNF512B	0	0,044824808	0,046469425	4,274762205	0	0,526964386	2,957425077	2,099049409	3,168897571	13,11839288	1,457599209
ENSG00000135835	KIAA1614	2,718024042	1,257075967	0,239362887	4,418818456	0	0,634942888	1,416523075	2,181399269	0,248933344	13,11507993	1,457231103
ENSG00000075643	MOCOS	0	0	0,044102824	4,057056562	0	7,791453388	0	0,052424967	1,149930885	13,09496863	1,454996514
ENSG00000157227	MMP14	0,101689482	1,263118439	1,496528124	0,117063694	0	2,791227737	5,319401693	0,667095358	1,334026181	13,09015071	1,45446119
ENSG00000160796	NBEAL2	5,559503596	0,089076323	0	2,405429963	0	2,177051002	0,791939642	0,137212261	1,929309466	13,08952225	1,454391362
ENSG00000177732	SOX12	0,979118168	0,173742117	1,440933558	1,803438269	4,964043782	0,752510175	1,327871764	0,142736314	1,485166593	13,06956074	1,452173416
ENSG00000143994	ABHD1	0,032637272	0	1,74112805	7,664612645	0	1,146682172	0,704585018	0	1,766144056	13,05578921	1,450643246
ENSG00000204152	TIMM23B	0	5,556890133	0	0,171667746	0	2,51048899	1,774744828	0,652173489	2,384215253	13,05018044	1,450020049
ENSG00000168234	TTC39C	0,021022617	0,447649416	0,038672802	0,471919125	5,595598879	0,83093778	3,264183651	0,459703071	1,913278053	13,04296539	1,449218377
ENSG00000089091	DZANK1	0,022829819	4,37518296	0	2,523015316	0,054255559	0,726908404	5,25083577	0	0,084233202	13,03726103	1,448584559
ENSG00000177455	CD19	4,287566365	0	0	1,083467023	0	1,377804097	1,389290873	0,114337221	4,758699878	13,01116546	1,445685051
ENSG00000169607	CKAP2L	0	4,122241583	0	0,620818937	0,065724294	2,854244914	1,882973504	0,725698172	2,721032537	12,99273394	1,443637104
ENSG00000196632	WNK3	0	0	0,02423118	1,910611739	0,657380438	1,446221903	0,721848144	2,275482116	5,945403912	12,98117943	1,44235327
ENSG00000170955	CAVIN3	1,552838234	1,549953758	0	2,681412713	0	1,704925644	5,479757595	0	0	12,96888794	1,440987549
ENSG00000152104	PTPN14	0,016116742	4,118221844	0,207536337	1,419334866	0,095754464	3,574438619	0,903290039	0,017621297	2,616439947	12,96875416	1,440972684
ENSG00000171033	PKIA	0,037636506	0	0,761589312	9,293574438	0	0	0	0	2,869859124	12,96265938	1,440295487
ENSG00000143850	PLEKHA6	0,091449147	0,064909707	0	4,737381529	0	1,686817773	0,865626837	0	5,511062449	12,95724744	1,43969416
ENSG00000177082	WDR73	0,015698169	0	5,140292709	0,379502352	0,037307037	1,913159569	1,89000604	3,329747875	0,250987408	12,95670116	1,439633462
ENSG00000142623	PADI1	0	7,000239855	0,113391841	0	0	4,906594661	0,127952613	0	0,79599775	12,94417672	1,438241858
ENSG00000168026	TTC21A	0,019683544	0,034927967	6,155609851	0,033989185	0,046778369	0,280947961	0,294186007	0	6,076263787	12,94238667	1,438042964
ENSG00000140104	CLBA1	0,028920683	4,618713877	0	0,049939708	0	1,587661021	0	6,640311883	0	12,92554717	1,436171908
ENSG00000107281	NPDC1	0,055555357	0,098581618	1,635177053	8,250149932	0	0,182989607	0	1,336316266	1,366518546	12,92528838	1,436143153
ENSG00000143590	EFNA3	2,876075676	2,068996914	0,142993878	2,01338715	0	1,36551915	1,355389389	1,189836406	1,912001258	12,92419982	1,436022202
ENSG00000174137	FAM53A	2,673576976	0	0,196730431	0	0	1,722119692	2,545517469	5,300672468	0,482262738	12,92087977	1,435653308
ENSG00000058866	DGKG	7,200986648	1,832405468	0,455912691	0,095101585	0,032721416	1,723353599	0	0,782806265	0,778946699	12,90223437	1,433580381
ENSG00000254995	STX16-NPEPL1	0,156631843	1,250727132	1,152547702	1,758048546	5,397465006	0,30095207	0,650273812	1,883793855	0,337114707	12,88755467	1,431950519
ENSG00000183323	CCDC125	0,83823312	0	6,308171686	2,467236901	0	1,024914052	1,803280112	0,208292087	0,2342997	12,88442766	1,431603073
ENSG00000173930	SLCO4C1	1,907886471	0	3,070997345	0,102953235	7,793041953	0	0	0	0	12,874879	1,430542112
ENSG00000143515	ATP8B2	0,05947674	0,105540016	0,145883022	6,812655464	0,04711592	2,742683372	2,946626795	0	0	12,85998133	1,428886814
ENSG00000083896	YTHDC1	0,021488989	1,449003982	1,857944348	1,595592137	0,970311903	1,321244638	1,962704283	0,704851893	2,960012869	12,84315504	1,427017227
ENSG00000075218	GTSE1	0	0	0	5,709736862	0	3,953133119	3,050579431	0,080339063	0,04518515	12,83897363	1,426552625
ENSG00000138772	ANXA3	0	0,054778754	0	0	0	3,728327566	0	9,045604095	0	12,82871042	1,425412268
ENSG00000131187	F12	11,95132357	0	0,100390068	0	0	0	0,77031262	0	0	12,82202626	1,424669585
ENSG00000197969	VPS13A	0,007482203	3,226307488	0,72949818	3,720997855	0,035563235	1,248683287	2,789472127	0,638094085	0,423298674	12,81939713	1,424377459
ENSG00000112787	FBRSL1	2,400882387	0,972150565	0,355700702	3,72843747	0	0,884573346	1,993503889	0,140940315	2,338439926	12,8146286	1,423847622
ENSG00000204116	CHIC1	7,469509402	1,932137196	0	1,616977073	0	0	1,699698978	0,095239823	0	12,81356247	1,423729164
ENSG00000197121	PGAP1	12,1731578	0,021012617	0,021783567	0,122687082	0,056283605	0,039004132	0,009832328	0,258940967	0,087381799	12,79008389	1,421120432
ENSG00000164849	GPR146	4,737008485	0,50434232	0	0,408988988	0	0,832153363	0,47198871	3,211119622	2,621659405	12,78726089	1,420806766
ENSG00000196368	NUDT11	0	0	2,218236825	10,41109726	0,150826351	0	0	0	0	12,78016044	1,420017827
ENSG00000169908	TM4SF1	0	0,076721602	0,159073018	12,54279706	0	0	0	0	0	12,77859168	1,41984352
ENSG00000163909	HEYL	0	0	0	0	0,528502638	2,848599551	0,830929351	8,104845904	0,45584136	12,7687188	1,418746534
ENSG00000011451	WIZ	0,768932275	1,118850194	1,98031823	1,4871116	0,036547684	1,013091248	2,885843174	1,244257989	2,231822677	12,76677507	1,418530563
ENSG00000079432	CIC	0,159164283	0,790812724	1,903171101	1,923893851	0,416083102	0,943666647	2,563855531	2,262296913	1,800914267	12,76385842	1,418206491
ENSG00000135736	CCDC102A	0,059235818	0	0	0	0,140775203	1,690974126	2,85271629	0	8,013766151	12,75746759	1,417496399
ENSG00000135643	KCNMB4	1,741191761	0,055173251	0	0,107380648	0	3,208970002	3,949991933	1,903738249	1,759041157	12,725487	1,413943
ENSG00000136383	ALPK3	0,027625333	3,627515175	0,101638062	0,04770292	0	3,306079134	2,007066554	0,030204257	3,567437813	12,71526925	1,412807694
ENSG00000140043	PTGR2	0	2,02634564	0,034437574	2,327467563	4,004036027	0,041107675	0,8704582	0,777782059	2,624690726	12,70632547	1,411813941
ENSG00000113448	PDE4D	0,465356525	0,016515275	0,034242436	1,639281067	6,148969991	1,961987603	1,530126	0,162815751	0,732580526	12,69187518	1,410208353
ENSG00000268655	AC008687.4	3,614261941	0	3,957575441	0	0	3,212391576	0	0	1,905032688	12,68926165	1,409917961
ENSG00000184270	HIST2H2AB	0	0	1,048981506	8,861942534	0	1,252155296	0,473472951	0	1,051961447	12,68851373	1,409834859
ENSG00000180626	ZNF594	0	1,517784906	5,140009377	2,65858276	0,067758027	2,786045533	0,497146598	0	0	12,6673272	1,4074808
ENSG00000105173	CCNE1	0	0	0,225908248	5,089345299	0	0	7,341620402	0	0	12,65687395	1,406319328
ENSG00000168487	BMP1	0	0,037942833	0	0,036923019	0,457345108	1,76076033	4,349827556	0,280544418	5,732919464	12,65626273	1,406251414
ENSG00000049769	PPP1R3F	0	2,351579968	0	0	0,165759309	0,536060022	1,766372087	2,211539916	5,618715536	12,65002684	1,405558538
ENSG00000140905	GCSH	1,684942452	0,072924123	0,226799102	0,49674866	9,08292941	0,045121179	0,443599216	0,089865262	0,505429759	12,64835916	1,40537324
ENSG00000167964	RAB26	0,06073576	0	0,223456671	0,943896612	2,020757875	1,200317677	0	7,437433996	0,746971553	12,63357014	1,403730016

ENSG00000186854	TRABD2A	0,017228431	0	6,402004332	0	0,08188748	0,737716622	0,972749125	0,037673534	4,364882407	12,61414193	1,401571326
ENSG00000278619	MRM1	0	1,895511692	0	0,1418896	4,296131556	0,631524762	2,524416685	1,976498229	1,111643146	12,57761567	1,397512852
ENSG00000276644	DACH1	0,027978258	0,347527125	8,492263716	3,285239451	0,332454677	0,030718492	0	0,061180257	0	12,57736198	1,397484664
ENSG00000154710	RABGEF1	0,245974884	0,77595804	1,709409182	2,029336934	0,649516264	1,560381622	2,065075097	2,629611317	0,873939153	12,53920249	1,393244721
ENSG00000005100	DHX33	0	0	0,096037346	7,031586809	0,062034469	2,894622257	1,127043922	0	1,316238973	12,52756378	1,391951531
ENSG00000182993	C12orf60	0,065529302	6,395407634	0,120546444	0,113154803	0,389329513	2,158420339	0,027205189	2,006107522	1,208888913	12,48458966	1,387176629
ENSG00000198182	ZNF607	0,064261353	7,92509845	0	0,277413326	0	0	4,215248236	0	0	12,48202137	1,386891263
ENSG00000169914	OTUD3	0,899859607	0	6,500333469	3,297217544	0,104318821	1,518135996	0	0	0,161957754	12,48182319	1,386869244
ENSG00000255833	TIFAB	0,02575602	0	0	6,182028544	0	0,028278605	1,069288665	2,309155832	2,850892621	12,46540029	1,385044476
ENSG00000279170	TSTD3	0,0499407	0,443092736	1,690407106	0,551914812	4,367613708	2,719665743	0,43954839	2,074908052	0,122841247	12,45993249	1,384436944
ENSG00000119599	DCAF4	0,189531266	1,53745637	0	4,675416137	0	1,426932034	3,979259768	0	0,632697352	12,44129293	1,382365881
ENSG00000204564	C6orf136	0	0,126601511	0	2,83357149	0	1,645005718	3,317440317	2,496200699	2,018163367	12,4369831	1,381887011
ENSG00000162971	TYW5	0,013074111	0,278396262	0,024050882	0,361218169	0	1,736907751	4,885070895	2,458675619	2,669192135	12,42658582	1,380731758
ENSG00000157212	PAXIP1	0,015670494	0,139034533	0,317098526	1,136499941	0,037241267	0,498953305	2,862539981	0,068533562	7,342893808	12,41846542	1,379829491
ENSG00000182218	HHIPL1	0	0	0	0,066599024	0	4,192227436	2,081564977	0,042168781	6,024121574	12,40668179	1,378520199
ENSG00000115604	IL18R1	0	0	0	5,874483692	0	0,038114077	0	0	6,489471947	12,40206972	1,378007746
ENSG00000121211	MND1	0	0	0,183970946	5,526088145	0	2,745045941	0	0	3,935862815	12,39096785	1,376774205
ENSG00000218891	ZNF579	0	0,565359639	2,093223637	0,157189744	5,949236394	1,849003415	1,43610621	0	0,335867209	12,38598625	1,376220694
ENSG00000128271	ADORA2A	0	0	0	0	12,17057065	0,197983982	0	0	0	12,36855463	1,374283848
ENSG00000185453	ZSWIM9	0,040156941	0	7,756553386	0,970791827	0	1,146339355	1,267040291	0	1,185308672	12,36619047	1,374021164
ENSG00000196155	PLEKHG4	0,020620984	0,439097162	0	0,035607939	0,441055934	0,022640635	3,612751099	1,037117229	6,720705295	12,32959628	1,369955142
ENSG00000130427	EPO	0	0	0	0	12,3163056	0	0	0	0	12,3163056	1,3684784
ENSG00000185033	SEMA4B	0,021568737	0,459278333	0,039677433	0,297956009	0,10251714	3,575863129	4,853334281	0,047164511	2,917944255	12,31530383	1,368367092
ENSG00000077463	AC016586.1	0,164907862	1,755749728	0	0	0,261271402	1,629532765	2,190824276	4,808069371	1,487313019	12,29766842	1,366407603
ENSG00000182890	GLUD2	2,303329998	0,860463699	1,003538263	1,884007037	2,016705011	0,86515744	0,754935812	1,192904576	1,416399488	12,29744132	1,366382369
ENSG00000182156	ENPP7	9,095809478	3,121471324	0	0,074087159	0	0	0	0	0	12,29136796	1,365707551
ENSG00000189007	ADAT2	0	0	7,300019273	0,761377583	0,049898227	0,092210964	0,976287146	1,193733327	1,910885459	12,28441198	1,364934664
ENSG00000184785	SMIM10	0,203790385	0	0	11,96465157	0	0,111874967	0	0	0	12,28031692	1,364479658
ENSG00000088038	CNOT3	2,348295043	1,022092362	1,059592812	0	0	1,313468867	1,765892372	1,307980256	3,460270976	12,27759269	1,364176965
ENSG00000167984	NLRC3	0	0	0,083115592	10,49357276	1,664321096	0	0	0	0,027783902	12,26879335	1,363199261
ENSG00000035499	DEPDC1B	0	0,070452444	1,241634704	10,69518025	0,188711268	0	0	0	0,04882988	12,24480855	1,360534283
ENSG00000130822	PNCK	6,97524732	4,424263997	0	0	0	0,619190917	0	0	0,21902949	12,23773172	1,359747969
ENSG00000102886	GDPD3	0	0	0,176832124	0	9,823189772	0	2,234840519	0	0	12,23486241	1,359429157
ENSG00000108001	EBF3	0,032686691	0,116003461	0,120259615	0,056442781	10,3315235	0	1,574146497	0	0	12,23106254	1,359006949
ENSG00000008128	CDK11A	0	0	6,341335591	0,667090397	0,706229189	0,913568564	0,863610938	2,664270849	0,073095977	12,22920151	1,358800167
ENSG00000283208	AC001226.2	0,049181634	0	10,81159376	0,339703791	0	0	1,020914011	0	0	12,2213932	1,357932577
ENSG00000171044	XKR6	0,029009516	0,51476675	0,960576268	6,71247583	0	0,286656784	1,589756558	0	2,104999964	12,19824167	1,355360186
ENSG00000244274	DBNDD2	0,601380708	1,649209118	1,307431725	0,566429033	0,389780256	2,401021371	2,315118282	1,554142322	1,412003244	12,19651606	1,355168451
ENSG00000079482	OPHN1	7,435036114	0,165744839	0	0	0	2,994553616	1,411521846	0	0,183801728	12,19065814	1,354517571
ENSG00000109193	SULT1E1	0	1,544003839	0	0	0	1,273785618	0	8,56212411	0,802600144	12,18251371	1,353612635
ENSG00000206432	TMEM200C	1,747189339	1,587981388	0,026130861	0,098114298	0,067515988	2,666921284	1,521495848	0	4,454865875	12,17021488	1,352246098
ENSG00000140092	FBLN5	0	0	0	1,836461715	0	0,100086815	0	10,23264094	0	12,16918947	1,352132163
ENSG00000174827	PDZK1	10,90677103	0	0	1,157372871	0	0	0	0	0,074938114	12,13908201	1,34878689
ENSG00000124882	EREG	0,052900602	0,281612463	0,048657465	0,045673897	0	0,029040885	2,21818721	9,427767286	0,032530461	12,13637027	1,348485586
ENSG00000162062	TEDC2	0	2,259623596	1,268869834	3,298335353	0	5,184705522	0	0,116023373	0	12,12755768	1,347506409
ENSG00000081026	MAGI3	0	1,848376041	0,066075612	0,124048003	6,231419204	3,154943222	0	0,07854399	0,618457645	12,12186372	1,346873746
ENSG00000107614	TRDMT1	0,009778721	2,377237689	0,053966239	0,135085731	1,85914731	2,276130348	0,479049349	2,480451034	2,44139287	12,11223929	1,345804366
ENSG00000183918	SH2D1A	0	0	0	4,346075671	7,740613258	0	0	0	0	12,08668893	1,342965437
ENSG00000145362	ANK2	0,240463485	1,265375988	0	0,229091286	1,89175176	2,758500041	3,587030453	0,435164049	1,672455534	12,07983259	1,342203622
ENSG00000178917	ZNF852	1,821422057	0,2394125	5,70851989	2,155043317	0	1,629478878	0,280067705	0,147515432	0,082967197	12,06442698	1,340491886
ENSG00000008516	MMP25	0	0	11,39155375	0	0	0	0,502354132	0,15564511	0	12,04955299	1,338839221
ENSG00000162614	NEXN	0,034064601	0	6,015800158	0,117644263	0,080955262	0,710617889	0,028284565	3,798954676	1,256852044	12,04317346	1,338130384
ENSG00000149050	ZNF214	0	0	0,053872112	0	0	2,475797366	1,50758926	8,00471336	0	12,0419721	1,3379969
ENSG00000196150	ZNF250	2,362065465	2,296173169	0,075568873	1,170430089	0	2,097279276	0,886836654	0,853371861	2,29876764	12,04049303	1,337832559
ENSG00000162366	PDZK1IP1	0	0	0,194391678	0	0	3,944726999	0	0,231073124	7,667793576	12,03798538	1,337553931
ENSG00000177181	RIMKLA	0,014204791	1,260302689	0	0,441514339	0,067515988	1,606391183	1,167659604	0,031061719	7,442246521	12,03089683	1,336766315
ENSG00000165684	SNAPC4	0	0	0	0	0,213548588	2,992638747	1,268381949	0	7,551741738	12,02631102	1,33625678
ENSG00000273983	HIST1H3G	0,12535095	4,559859811	1,729448977	0	2,08529473	0,275256038	2,393876098	0,548211643	0,30833102	12,02562927	1,33618103
ENSG00000203910	C1orf146	0	0	0	0	11,90444892	0	0,115534376	0	0	12,01998329	1,335553699
ENSG00000078053	AMPH	0,110118835	9,330501369	0,253215612	0	0	0	2,26299196	0,060199411	0	12,01702719	1,335225243
ENSG00000171914	TLN2	0,018645268	0,446655192	3,532845974	0,080490769	0,044310882	1,74007021	2,879570825	0,876592412	2,39631829	12,01549982	1,335055536
ENSG00000285976	AL135905.2	0,534589283	2,002632862	1,748302509	0,717981455	1,341044282	1,56519412	1,923483863	0,974158513	1,205372491	12,01275938	1,334751042
ENSG00000149948	HMGA2	0	0,017408427	0,523367063	11,36709524	0,069944337	0,010771316	0	0,021452611	0	12,01003899	1,334448777
ENSG00000128322	IGLL1	0,168274578	0	0	11,0417918	0,799816353	0	0	0	0	12,00988273	1,334431414
ENSG00000165495	PKNOX2	0,02311266	0,123038544	0,042517605	0,119731558	11,69960791	0	0	0	0	12,00800828	1,334223142
ENSG00000066185	ZMYND12	0	9,466728109	0	0,478560247	0,164657287	0	1,725864264	0	0,170423221	12,00623313	1,334025903
ENSG00000152377	SPOCK1	0	11,46090807	0,089671	0,168345145	0	0	0,283320105	0	0	12,00224432	1,333582702
ENSG00000100036	SLC35E4	9,343008257	0,049637542	0	0,144910204	0,199435881	1,474214676	0,650346038	0	0,137613123	11,99916572	1,333240636
ENSG00000178078	STAP2	7,320613329	0	0	0,377346312	0,129832806	3,119070856	1,043318017	0	0	11,99018132	1,332242369
ENSG00000112186	CAP2	0	0,080138075	1,827723305	0	0	0	0	10,07302029	0	11,98088167	1,331209074
ENSG00000232593	KANTR	8,326531898	1,863785434	0,168014566	0,183997665	0,108527514	0,050139189	0,164310901	1,06516633	0	11,9304735	1,325608166
ENSG00000100068	LRP5L	0,096721082	0,057209729	0	0,167016199	0,15323983	0,035398032	2,917914698	6,274520271	2,220483304	11,92250315	1,324722572
ENSG00000080986	NDC80	0	0	0	5,433086134	0,404184066	4,668277341	1,412159055	0	0	11,9177066	1,324189622
ENSG00000269313	MAGIX	0,029588911	4,672926965	4,082339421	0,051093592	0	0	2,456830468	0,582320562	0,036390547	11,91149047	1,323498941
ENSG00000115163	CENPA	0	0	0	3,882562475	0	2,553780042	5,472025767	0	0	11,90836828	1,323152032
ENSG00000130827	PLXNA3	0,012904404	1,076231768	0,023738693	1,158720639	0	2,082737385	1,264346642	1,834179922	4,427940198	11,88079965	1,32008885
ENSG00000144134	RABL2A	2,163034134	1,160401465	3,099977802	0	0	2,153965543	1,085961595	1,484975586	0,711463051	11,85977918	1,317753242
ENSG00000101384	JAG1	3,331648418	0	0,149483813	0,028063559	0,038623096	1,356120007	2,509946413	4,442280161	0	11,85616547	1,317351719
ENSG00000164241	C5orf63	0,021016769	1,939274549	6,069940965	0,907284334	0	2,284443268	0,017450672	0	0,594502088	11,83391265	1,314879183
ENSG00000230510	PPP5D1	0,185476766	0	0	6,725835747	0	2,589170845	2,266078239	0	0,065175003	11,8317366	1,3146374
ENSG00000204344	STK19	2,293028386	1,08504603	0,210910546	5,015442102	0	0,587442608	0,888510191	1,50425448	0,235010777	11,81964512	1,313293902
ENSG00000096093	EFHC1	0,01328795	1,827383486	0,073332773	5,770765574	0,047368652	0,007294696	1,335025516	0,116227424	2,622965778	11,81365185	1,312627984
ENSG00000164743	C8orf48	0	0	0	0	0,252899678	0	0	0	11,51724927	11,77014895	1,307794327
ENSG00000125735	TNFSF14	0,063252646	0	11,69401421	0	0	0	0	0	0	11,75726686	1,306362984
ENSG00000205710	C17orf107	0	0,235902053	0	6,045121112	0	2,578668787	1,692561663	0	1,199009821	11,75126344	1,305695937
ENSG00000284308	C2orf81	2,724531157	0,805768949	4,269477313	0	0	2,21583408	0,544612459	0,441314948	0,744627134	11,74616604	1,30512956
ENSG00000009830	POMT2	0,015098978	0	0,027775789	0,130363197	0,215298287	3,133203337	4,764061367	0,132068172	3,305423757	11,72329288	1,302588098
ENSG00000167523	SPATA33	1,130830933	0,746653672	0	4,813628143	0	2,425424372	1,506692118	0	1,099683564	11,7229128	1,302545867
ENSG00000165288	BRWD3	0	0,063370569	0,766448989	1,685573335	0,96187147	1,646819967	0,336063596	3,227815218	3,030583749	11,71854689	1,302060766
ENSG00000006704	GTF2IRD1	1,558080676	3,012367806	0,128337996	0,562186772	0,828987863	0,995769851	1,390252986	0,406813872	2,774255565	11,65705339	1,295228154
ENSG00000283930	AL117339.5	0	0	0	1,649399334	0	1,342386461	4,663499066	0	3,994173045	11,64945791	1,294384212
ENSG00000182168	UNC5C	0	0,05019036	0,052031839	0,244206808	0,033609503	0	0,011742661	11,25673211	0	11,64851328	1,294279253
ENSG00000273542	HIST1H4K	0	0	8,909618557	1,672660094	0	0	0	1,059085152	0	11,6413638	1,293484867
ENSG00000135801	TAF5L	0	1,756388647	0	0,406947871	0,112014205	6,003002775	1,937236879	1,391411934	0	11,60700231	1,289666923
ENSG00000118298	CA14	0,091693307	0,325415049	0	4,987531371	0,21791113	1,510108404	0	0	4,454452445	11,58711171	1,287456856
ENSG00000026751	SLAMF7	0	0	0,172551272	0,16197081	0,965968974	10,12696464	0	0	0,153814626	11,58127033	1,286807814
ENSG00000188878	FBF1	5,792946097	0	0,289132347	0,852982086	0,053360705	1,207967052	1,025389043	1,571159905	0,773209899	11,56614713	1,285127459
ENSG00000106178	CCL24	0	0	0	0	0	11,56264959	0	0	0	11,56264959	1,284738844
ENSG00000136943	CTSV	0,066656626	0,236561094	0	0,805710111	0,079205457	1,536886683	3,791229705	0	5,041712007	11,55796168	1,284217965
ENSG00000073584	SMARCE1	1,554467159	0,900097672	0,707366792	1,285602744	0,991607724	1,518077285	1,854542536	1,288104432	1,438876702	11,53874305	1,282082561
ENSG00000184986	TMEM121	0	1,672403327	0	5,424843547	0,186651529	0	0	0	4,250128368	11,53402677	1,28155853
ENSG00000181374	CCL13	0	0	0	0	0	11,28917654	0	0	0,225815944	11,51499249	1,27944361
ENSG00000184619	KRBA2	0,04082966	1,376573359	6,083865987	0	0	1,120714514	0,406820855	0,624977566	1,857963071	11,51174501	1,279082779
ENSG00000183397	C19orf71	0	0,397836795	0	2,710007155	3,729707413	1,476948754	0,74463105	0,490259002	1,93015676	11,47954693	1,275505214
ENSG00000170270	GON7	0	1,430090667	4,447681584	0	0	1,327284614	1,895996128	0,881158847	1,486772178	11,46898402	1,274331557
ENSG00000141576	RNF157	0	0	8,395961539	0,197852881	2,8137606	0	0,031712456	0	0,023486228	11,4627737	1,273641523
ENSG00000225940	C5orf67	0	0	0	0	0	2,812043673	0,654343322	0	7,996004218	11,46239121	1,273599024
ENSG00000112232	KHDRBS2	0,129764337	7,253304425	0	1,008336098	0,154193878	0,07123683	1,346826234	0,141878303	1,356543958	11,46208406	1,273564896
ENSG00000196860	TOMM20L	0	4,843287358	0	0	0	0,817293481	0	0	5,798167362	11,4587482	1,273194245
ENSG00000083838	ZNF446	0	0	0	2,703165489	0	1,685055624	0,89202958	6,175092466	0	11,45534316	1,272815907
ENSG00000111696	NT5DC3	0,017252035	2,847038019	0,095209533	0,208533488	0,122999501	1,723697052	4,956357446	0,188625738	1,294284491	11,4539973	1,272666367
ENSG00000121957	GPSM2	0	0	0,030446889	2,829415387	0,118001383	3,507200581	4,177763519	0	0,77351236	11,43634012	1,270704458
ENSG00000121270	ABCC11	0	0	0	0,072031739	11,20228635	0,091600042	0,069272785	0	0	11,43519091	1,270576768
ENSG00000173626	TRAPPC3L	0	0	0,100827022	0	11,33232723	0	0	0	0	11,43315426	1,270350473
ENSG00000197496	SLC2A10	0	0	0,057457648	0,431475774	0,222685537	3,635080845	0	0	7,068160417	11,41486022	1,268317802
ENSG00000157764	BRAF	0,315995987	0,16020775	0,011863268	3,195985734	0,214563229	1,904657952	2,040124466	1,508898034	2,062141336	11,41443776	1,268270862
ENSG00000185869	ZNF829	0,078073144	0,069269439	0	2,123340661	5,009647969	0,557178377	1,6044385	0	1,968408012	11,4103561	1,267817345

ENSG00000144792	ZNF660	3,387401093	0,041170275	0	1,882994596	0,055138576	3,260640844	2,273222659	0,050734618	0,456555252	11,40785791	1,267539768
ENSG00000124406	ATP8A1	0,0674661	0,21549049	0,049643736	6,034660097	2,308818917	1,348143806	0,851481849	0	0,531037478	11,40674247	1,26741583
ENSG00000057294	PKP2	0,021455427	10,01296976	0	0,037048839	0	1,224953853	0	0	0,105549636	11,40197752	1,266886391
ENSG00000079156	OSBPL6	0	3,64120404	0,193579456	0,249850689	0,125040927	0,014442077	3,669745086	0,661559458	2,831053395	11,38647513	1,265163903
ENSG00000164850	GPER1	0	5,237483831	0	0,083552666	0	0	0,642819504	0	5,415316531	11,37917253	1,264352504
ENSG00000125885	MCM8	0,058943143	3,416709922	3,939647743	2,34098469	1,307410114	0,280436549	0,032627833	0	0	11,37675999	1,264084444
ENSG00000056736	IL17RB	0,049642106	0	0	3,514564375	0	5,014380111	2,184601707	0	0,61053391	11,37372221	1,263746912
ENSG00000196517	SLC6A9	10,04670008	0	0	0,354050169	0	0	0	0	0,972641229	11,37339148	1,263710165
ENSG00000163624	CDS1	0,035068594	4,355985149	0,129023021	0	0	0	6,842777447	0	0	11,36285421	1,262539357
ENSG00000171160	MORN4	0	0,106744427	0,221321735	6,128647891	0	0,198141612	4,695147774	0	0	11,35000344	1,261111493
ENSG00000091972	CD200	0	0	0	11,34120585	0	0	0	0	0	11,34120585	1,260133983
ENSG00000055163	CYFIP2	0	0	4,104297518	4,548590241	0,136832746	0	0	0,062951908	2,478425711	11,33109813	1,259010903
ENSG00000204131	NHSL2	0,051206564	0,060576528	9,215764041	1,621080267	0,060846754	0,121813933	0,063776855	0,111973761	0,010496245	11,31753495	1,257503883
ENSG00000083807	SLC27A5	1,995977631	3,374746668	0	0,227606831	0,447498495	0,909665467	2,892462482	0,617634706	0,833704025	11,29929631	1,255477367
ENSG00000163155	LYSMD1	6,32639949	0,855317385	0	0	0	1,190745766	0,800448689	0,790513319	1,333826715	11,29725136	1,255250151
ENSG00000125746	EML2	0,01837434	0,032604815	0	1,586423727	0,043667016	4,075138192	2,05964188	0,040179301	3,43490654	11,29093581	1,254548423
ENSG00000186603	HPDL	0	0	0	11,2126754	0	0	0,077022917	0	0	11,28969831	1,254410924
ENSG00000152409	JMY	0,066451438	2,358328937	0	2,467063304	0	1,805754958	2,910536505	0	1,675397408	11,28353255	1,253725839
ENSG00000078687	TNRC6C	0,108800699	2,968361625	0,075055377	4,555970216	0,032320902	3,150673358	0,124216871	0,059478817	0,200716251	11,27559412	1,252843791
ENSG00000277117	FP565260.3	3,691113135	0,159750968	2,152958765	0	1,604636025	1,334401421	0,710138906	0	1,605465936	11,25846516	1,250940573
ENSG00000135919	SERPINE2	0,016870668	3,59239031	3,413845137	2,388820801	0	0,018523007	0	0,18445605	1,639150972	11,25405695	1,250450772
ENSG00000259494	MRPL46	1,605048236	0,963334271	0	2,77156799	0,056094586	2,099149752	1,352304422	1,445199608	0,957971764	11,25067063	1,250074514
ENSG00000120324	PCDHB10	0	11,16579149	0,084492431	0	0	0	0	0	0	11,25028392	1,250031547
ENSG00000173715	C11orf80	0,120406829	8,11904064	0	0,900970696	0,095383145	1,498262844	0,399905441	0	0,098723252	11,23269285	1,248076983
ENSG00000182698	RESP18	0	0	11,21674084	0	0	0	0	0	0	11,21674084	1,246304537
ENSG00000158428	CATIP	0,316461928	7,159812568	0,291078638	0,478153095	0	1,433258909	0,624067096	0,086501196	0,827065675	11,2163991	1,246266567
ENSG00000116016	EPAS1	0	0,367771225	2,058829426	0,21473184	0	6,758392714	0,326969731	1,450268298	0,025489854	11,20245309	1,24471701
ENSG00000125864	BFSP1	0	0	4,205572545	1,238492908	0	5,659957378	0	0,098022714	0	11,20204554	1,244671727
ENSG00000108515	ENO3	0,035207495	1,811770271	0	0	0,502028251	0,154623091	0,17540121	7,467882817	1,039216341	11,18612948	1,242903275
ENSG00000168589	DYNLRB2	0	9,971473972	0,187951385	0,352853245	0	0	0	0,670252672	0	11,18253127	1,242503475
ENSG00000234511	C5orf58	0	3,452833544	0	2,458558178	0	0	1,182195339	0	4,085821784	11,17940884	1,242156538
ENSG00000274290	HIST1H2BE	0,116913401	0,518649903	0,107535822	6,258401712	1,111388913	0	1,019294767	2,045242971	0	11,17742749	1,241936388
ENSG00000090905	TNRC6A	0,337924626	1,612822024	0,450152844	3,199310685	0,055385185	1,612024419	0,754679665	0,356730718	2,794576792	11,17360696	1,241511884
ENSG00000170782	OR10A4	0	0	10,89570237	0,262246206	0	0	0	0	0	11,15794858	1,239772064
ENSG00000166780	BMERB1	1,33535567	0,61149829	0,158483523	0,297531328	0	1,466142497	2,467917021	2,166475215	2,648895699	11,15229924	1,23914436
ENSG00000170419	VSTM2A	0,025324379	0,04493749	0	0,30610773	10,77292443	0	0	0	0	11,14929403	1,238810448
ENSG00000186973	FAM183A	0	10,81474259	0,329751007	0	0	0	0	0	0	11,1444936	1,238277066
ENSG00000120833	SOCS2	0,023330334	0,082798209	0,042918033	4,068925804	1,275235985	3,842301452	0,019371674	0,153049823	1,63551826	11,14344957	1,238161064
ENSG00000267855	NDUFA7	0,106943079	0,37953574	0,393460862	1,662006208	0,762457627	0,352251755	1,953536662	4,209357548	1,315262012	11,13481149	1,237201277
ENSG00000185158	LRRC37B	1,44350171	0	0,0442573	1,620198059	0,114350435	0,554708185	6,512231847	0	0,828483143	11,11773068	1,235303409
ENSG00000000971	CFH	0,018552556	0,131684223	0	0,288325939	0	0	0	10,66964902	0	11,10821174	1,234245749
ENSG00000188322	SBK1	10,36498499	0,053778981	0,055752126	0,261667644	0,360125895	0	0	0	0	11,09630964	1,232923293
ENSG00000249222	ATP5MGL	0	0,335596996	1,739550056	3,918923273	0,898917503	1,245886684	0,785171039	1,240680479	0,930395606	11,09512164	1,232791293
ENSG00000130055	GDPD2	0,114564496	0	0	1,285881224	0	0,188677684	0	3,507266069	5,988231385	11,08462086	1,23162454
ENSG00000123219	CENPK	0	0	0	3,209457799	0,250023817	1,655640473	0,058236404	0	5,908789406	11,0821479	1,231349766
ENSG00000176058	TPRN	0,028614007	2,132543837	0,736928874	0,09882029	0,340009035	1,507992365	3,064887867	2,940811337	0,211149239	11,06175685	1,229084095
ENSG00000139133	ALG10	0	1,403884817	0	0,745173654	0	3,198181036	3,851897389	0,707737056	1,149930885	11,05680484	1,228533871
ENSG00000150773	PIH1D2	0	10,0797114	0	0,105470887	0,145156646	0,067061672	0,659302472	0	0	11,05670308	1,228522564
ENSG00000048342	CC2D2A	0,079065807	0	0,11635835	0,109223514	0,03758032	2,708460649	2,744171137	0,242051274	5,017622724	11,05453378	1,228281531
ENSG00000260007	AC107871.1	0	0	0	0,143449684	10,90777582	0	0	0	0	11,0512255	1,227913945
ENSG00000143248	RGS5	0	0,040480374	0,041965595	0	0	1,077016123	0,151534217	5,487289014	4,23653757	11,03482289	1,226091433
ENSG00000149289	ZC3H12C	0	2,989196533	0	3,555265963	0,148273139	0	0,388533233	3,785945189	0,153465336	11,02067939	1,224519933
ENSG00000198003	CCDC151	0,05636351	0,100015666	0	0,291982441	9,510386239	0,99014145	0	0	0,069319852	11,01820916	1,224245462
ENSG00000170954	ZNF415	0,039976341	0	0	0,414182499	0	0,57059193	5,476879571	0,087416552	4,424917197	11,01396409	1,223773788
ENSG00000163467	TSACC	0	0	0	10,99905274	0	0	0	0	0	10,99905274	1,222116971
ENSG00000068137	PLEKHH3	3,806866344	0,055828024	0,057876348	0	0	2,763449123	0,28735658	1,92633102	2,089467458	10,9871749	1,220797211
ENSG00000143494	VASH2	0,016631144	0	0	0,832832298	0,158097091	0	0,013809193	9,964678792	0	10,98604852	1,220672058
ENSG00000165972	CCDC38	0	0	0	10,94905683	0	0,033469957	0	0	0	10,98252679	1,220280754
ENSG00000144649	GASK1A	0	0	0	9,457571119	0	1,269914365	0,084739081	0	0,167353915	10,97957848	1,219953164
ENSG00000254469	AP002495.1	0	0,07344344	5,025112718	3,787881034	0	0,681637538	1,409006741	0	0	10,97708147	1,219675719
ENSG00000111206	FOXM1	0	0	0,624164337	1,384835546	0,073304254	4,538072521	3,867323971	0,067449391	0,41729164	10,97244166	1,219160184
ENSG00000124140	SLC12A5	0,542770037	1,715579078	0,156010831	0	1,612380929	3,277613817	2,394203215	0,333809755	0,938724156	10,97109182	1,219010202
ENSG00000155511	GRIA1	0,060139521	0,035572035	0,073754338	0,103847828	10,5762927	0,044010797	0,016645043	0,043835841	0	10,9541071	1,217123011
ENSG00000185024	BRF1	0,401676158	1,323705476	0	0,396346889	0,02727406	3,528135602	2,42993352	0,702678506	2,13129983	10,94105004	1,215672227
ENSG00000176248	ANAPC2	4,263654797	0,098256504	0,76396143	0	0,197389635	1,884656606	0,827580057	0,484330623	2,417571103	10,93740076	1,215266751
ENSG00000017483	SLC38A5	0	0	0	1,688479179	1,340657824	1,982007885	4,121972082	0	1,803886105	10,93700307	1,215222564
ENSG00000131969	ABHD12B	0,033203817	0	0,061081103	0	0	1,822792537	5,045280324	0	3,961132204	10,92348998	1,213721109
ENSG00000136425	CIB2	0	4,87303728	0	1,945461134	0	1,159673183	0	0	2,944446299	10,9226179	1,213624211
ENSG00000152782	PANK1	0,118704297	4,072332025	0	0,717417448	0,564206665	3,084485715	2,053389827	0,043261923	0,267650137	10,92144804	1,213494226
ENSG00000113657	DPYSL3	0	0,597614153	0,154885137	3,307575246	0	4,483445031	0,297115983	0	2,07100178	10,91163733	1,212404148
ENSG00000237440	AC008554.1	0,033662413	0,029866563	0	1,395063353	0	2,346919497	0,139753099	3,09161324	3,870940038	10,9078182	1,2119798
ENSG00000168778	TCTN2	0,118471635	0	0,163453606	8,131842603	0,211162804	0	2,213314363	0	0,03642621	10,87467122	1,208296802
ENSG00000160271	RALGDS	0	0,806389383	0,363467703	0,818833602	0,046955746	1,279906384	2,526468505	0,475258923	4,54410299	10,86138324	1,20682036
ENSG00000129596	CDO1	0,079783828	0	0	0	0	7,445828416	3,312310771	0	0	10,83792302	1,204213668
ENSG00000180481	GLIPR1L2	0,04007175	0	0	0,069195167	0	0	1,463986902	9,200644283	0,049283087	10,82318119	1,202575688
ENSG00000185245	GP1BA	0	4,373039779	0	0,415171002	0,142847073	0	0	1,971566632	3,918005442	10,82062993	1,202292214
ENSG00000116771	AGMAT	0	0	9,251033989	0	0,116031516	0	1,216190617	0,106763996	0,120094683	10,8101148	1,201123867
ENSG00000114853	ZBTB47	2,062849072	0,439257008	0,202388132	2,279737674	0	1,811910193	1,004858866	0,060144625	2,942964586	10,80411016	1,200456684
ENSG00000114315	HES1	1,247634629	2,344126276	0	3,041495576	0,174413571	2,256190455	0,182812528	1,283864274	0,270781723	10,80131903	1,200146559
ENSG00000197124	ZNF682	0	0	0	3,257090482	0	1,803738905	1,212517805	0	4,527366458	10,80071365	1,200079294
ENSG00000155158	TTC39B	0,074267974	4,744321102	1,958247748	0,641222906	0	0,883370535	0,010277714	0	2,481404254	10,79311223	1,199234693
ENSG00000092096	SLC22A17	7,700351661	0	0	0,182983853	0	2,13302518	0,762559288	0	0	10,77891998	1,197657776
ENSG00000130589	HELZ2	1,928644777	0	6,404646736	0,605517925	0,059525533	0,233754335	0,644717436	0,246470339	0,646904851	10,77018193	1,196686881
ENSG00000154040	CABYR	0	0,152440023	0	0,667542573	0,612480175	3,348394243	1,497940291	0	4,490322454	10,76911976	1,196568862
ENSG00000214688	C10orf105	0,030895639	0	10,68498438	0	0	0	0,051306617	0	0	10,76718664	1,196354071
ENSG00000113356	POLR3G	0,075123326	0	0	0,064860794	0	1,443417893	1,65297728	1,14990901	6,37504849	10,76133679	1,195704088
ENSG00000152672	CLEC4F	0,183238364	0	0,786524128	0	0	0	9,788105932	0	0	10,75786842	1,195318714
ENSG00000144218	AFF3	2,194822609	0,02105221	0,06547384	0,225350139	0,253753465	0,625242026	6,215889102	0	1,152694925	10,75427832	1,194919813
ENSG00000099725	PRKY	3,229363251	0,31447459	0	0,408029671	0,421169909	0	0,016350056	0,043058974	6,320816043	10,75326249	1,194806944
ENSG00000282988	AL031777.3	0	4,193690049	0	0,247331729	0,170197887	1,258089681	1,962332207	2,818873088	0,088078921	10,73859356	1,193177062
ENSG00000197647	ZNF433	0,049077808	4,528543035	0,090282591	0	0,349903419	1,400998699	0,448253866	2,361013474	1,508984429	10,73705732	1,193006369
ENSG00000182108	DEXI	3,861313148	0,240414226	0	0	0,107327419	4,537004713	1,98742606	0	0	10,73348556	1,192609507
ENSG00000116761	CTH	0	0	0	0	0,297529033	0,137456982	4,521921659	0	5,774022026	10,7309297	1,192325522
ENSG00000205084	TMEM231	0	0	5,374281914	0,347913299	0	0,663642307	0	0	4,336418853	10,72225637	1,191361819
ENSG00000149506	ZP1	10,60751543	0	0,114113341	0	0	0	0	0	0	10,72162877	1,191292085
ENSG00000140987	ZSCAN32	0	0	8,317514773	1,408872754	0	0,037325214	0,536318629	0	0,418102412	10,71813378	1,190903754
ENSG00000100985	MMP9	0,129375489	0	0	0	0,307463649	0,071023363	10,2052068	0	0	10,7130693	1,190341033
ENSG00000183971	NPW	0	2,600515087	0	0	0	1,251480643	3,920947875	2,136430393	0,801062596	10,71043659	1,190048511
ENSG00000124313	IQSEC2	0,11521965	2,146771636	0	0,696357157	0,027382199	1,265044428	0,908858676	0,050390327	5,455654676	10,66567875	1,185075417
ENSG00000233608	TWIST2	0	0	0	4,886422745	0	0	2,886690356	0	2,883653904	10,65676701	1,184085223
ENSG00000241106	HLA-DOB	0	0	3,813751358	0	0	2,782037109	1,08383925	1,175326206	1,794249961	10,64920388	1,183244876
ENSG00000204498	NFKBIL1	1,552505863	1,377441781	1,903973281	0,595741951	0	1,231071631	0,859385836	1,320229436	1,803305296	10,64365507	1,182628342
ENSG00000129467	ADCY4	0,28097912	3,390415769	0,034458919	0,129383897	0	0	0,062214123	5,079197517	1,658726863	10,63537621	1,181708468
ENSG00000132005	RFX1	0,579813993	2,106725014	3,91092045	1,04888899	0,262464858	0,848802512	0,687759905	0,362252404	0,814967392	10,62259552	1,180288391
ENSG00000171119	NRTN	0	9,67148783	0	0,470577051	0	0,448811299	0	0	0	10,59087618	1,17676402
ENSG00000166086	JAM3	0,055443247	3,73854192	0	3,733797105	0	2,343627666	0,644499963	0,060619073	0	10,57652897	1,175169886
ENSG00000080823	MOK	4,662887626	0	0	0,807554081	0,032688653	1,902855214	3,106492837	0,030077787	0,033833337	10,57638637	1,175154042
ENSG00000284695	AC108941.2	0	1,1235	0	0	0	2,548905508	0	5,076508719	1,816935274	10,5658495	1,173983278
ENSG00000126545	CSN1S1	0	0,537358718	0	0	0,359837217	0,166243063	0	0	9,49716697	10,56060597	1,173400663
ENSG00000128000	ZNF780B	0,686940575	0,899707977	2,707891429	3,502103783	0	0,808093511	0,52963989	0,071530375	1,347734278	10,55364182	1,172626869
ENSG00000131697	NPHP4	0	0,101823498	0	0,396346889	0,109096238	0	1,600897378	3,362818565	4,982442649	10,55342522	1,172602802
ENSG00000056277	ZNF280C	0,097742931	0	0,119870677	0,225040944	0,077429397	1,788600439	0,02705268	0,071245056	8,13429151	10,54127363	1,171252626
ENSG00000177303	CASKIN2	0	0,696231608	0	1,897051919	0	1,895464439	1,04250925	4,461467244	0,546891272	10,53961573	1,171068415
ENSG00000106089	STX1A	0	4,066057949	0	0	0	1,123142274	3,431492968	0	1,9123119	10,53300509	1,170333899
ENSG00000160233	LRRC3	9,617302359	0	0,047558614	0,535709101	0,245760508	0	0,085865069	0	0	10,53219565	1,170243961
ENSG00000182585	EPGN	0,083302409	0	0,15324151	0	10,29443892	0	0	0	0	10,53098284	1,170109204
ENSG00000185920	PTCH1	0,18215969	2,068722561	0,016754873	0,031455003	0,086581289	0,880003059	1,845263155	0,019916495	5,399193764	10,53004989	1,170005543

ENSG00000135362	PRR5L	0,022840171	4,336637547	0	0,236639941	0	2,332177091	1,327527697	0,699226715	1,573066089	10,52811525	1,169790583
ENSG00000168913	ENHO	0	0	0	9,64545626	0	0	0,854369965	0	0	10,49982622	1,166647358
ENSG00000152583	SPARCL1	0,043460043	0,077118781	0	0	0	3,435594341	5,990243058	0,950343881	0	10,4967601	1,166306678
ENSG00000125434	SLC25A35	0	0	0	0,330130282	0	0,965572688	4,762284868	4,0969873	0,329181593	10,48415673	1,164906303
ENSG00000180044	C3orf80	0	0	0	10,4642797	0	0	0	0	0	10,4642797	1,162697745
ENSG00000170390	DCLK2	0	0	0	10,16322818	0	0	0	0,299306673	0	10,46253485	1,162503872
ENSG00000185442	FAM174B	0	0,060000448	0,062201857	4,787797699	0,080357472	3,823850839	0,617665541	0,073939263	0,956471249	10,46228437	1,162476041
ENSG00000143167	GPA33	0,051293473	0,091019009	0	9,034408489	0	1,013710245	0,255540391	0	0	10,44597161	1,160663512
ENSG00000159433	STARD9	0,036366618	0	0,026759732	0,766125984	4,407728788	0,44719832	2,470031083	0,047713886	2,227367349	10,42929176	1,158810196
ENSG00000188056	TREML4	0,055719237	0	0	10,19878588	0	0,122352933	0,046264872	0	0	10,42312292	1,158124769
ENSG00000163687	DNASE1L3	0,096004175	0,085178526	0	0,331556512	8,099537963	1,791918554	0	0	0	10,40419573	1,156021748
ENSG00000160298	C21orf58	6,112657139	0	0,15690316	2,749267842	0,1351336	0,655526267	0,590170894	0	0	10,3996589	1,155517656
ENSG00000115687	PASK	0,042422956	0,075278495	0,026013485	0,048836791	0,100819074	1,816539161	3,346345182	4,01988525	0,921754228	10,39789462	1,155321625
ENSG00000139998	RAB15	0	0,213871984	0,055429731	0,312185413	0	6,815070549	2,977262115	0	0	10,37381979	1,152646644
ENSG00000134245	WNT2B	2,930866087	0,368386456	2,965360681	0,168698868	0,058043889	0,227935979	0,18251705	1,335197056	2,132714253	10,36972032	1,152191147
ENSG00000151882	CCL28	0	0	0	7,901876994	0	0	1,19257831	0	1,273484127	10,36793943	1,15199327
ENSG00000154783	FGD5	0,056015286	0,066265167	0	1,160714084	0	3,013574049	6,046389408	0	0,02296386	10,36592186	1,151769095
ENSG00000105926	MPP6	0,032257567	1,602727293	1,928562967	3,175001292	0	0,017708462	0,174096949	1,763446299	1,66625124	10,36005207	1,151116896
ENSG00000184349	EFNA5	0,131514857	1,540241254	1,596752527	1,635101669	2,000306589	0,779736392	1,113835845	1,15033792	0,388191169	10,33601822	1,148446469
ENSG00000256061	DNAAF4	0	2,650346967	1,51591056	0,444674463	4,406350213	0	0,55593535	0	0,760108476	10,33332603	1,148147336
ENSG00000263956	NBPF11	0,940153206	0	4,814519959	0,394890663	0	0,697454921	1,054904422	1,444644149	0,968764451	10,31533177	1,146147975
ENSG00000040199	PHLPP2	0	0,042926759	0	0,751913799	0	2,05844388	4,469246597	0,052899154	2,930582317	10,30601251	1,145112501
ENSG00000196388	INCA1	0	4,834498392	0	0,33603989	0	5,127950858	0	0	0	10,29848914	1,144276571
ENSG00000165949	IFI27	1,311786965	3,297622861	0	1,824720102	0	0,640117971	0,665625103	2,549772404	0	10,28964541	1,143293934
ENSG00000121005	CRISPLD1	0	0	0	10,25859004	0	0	0,027557727	0	0	10,28614777	1,142905308
ENSG00000196914	ARHGEF12	0,042470649	0,742865093	0,156256379	1,770577801	0,04325675	1,745305191	3,8941848	0,106138141	1,775936413	10,27699122	1,141887913
ENSG00000204179	PTPN20	0	9,878289395	0,061691101	0	0	0	0,111380676	0,219996383	0	10,27135756	1,141261951
ENSG00000135476	ESPL1	0,050993443	2,352651969	0,093806558	3,316721274	3,797193361	0,615866033	0	0	0,041810241	10,26904288	1,141004764
ENSG00000136848	DAB2IP	0	1,116401447	2,893405216	0,34307217	0,078693446	1,108857804	1,594670594	1,267142529	1,852967407	10,25521061	1,139467846
ENSG00000134627	PIWIL4	0,033595058	0,059613606	0	0,116022665	0,079839382	0	9,95840563	0	0	10,24747634	1,138608482
ENSG00000115641	FHL2	0	3,561197707	0,041952928	0,196902335	0,054198241	0,125196632	0,700634211	5,535502112	0,028048072	10,24363224	1,13818136
ENSG00000268043	NBPF12	5,402743176	0,811210671	0,382260862	0,825289386	0	0,205334872	1,57010454	0,136317891	0,894475836	10,22773723	1,136415248
ENSG00000105146	AURKC	0,077572162	0	0	0,133950192	0	0,851697006	2,962851782	0	6,201244326	10,22731547	1,136368385
ENSG00000110987	BCL7A	0,690528122	0	2,709935466	4,690072658	0	0,935063418	1,032048428	0,05033276	0,113234743	10,22121559	1,135690622
ENSG00000181045	SLC26A11	0,277380203	1,968817459	0,056695921	0	0	3,180826884	1,381888217	0	3,335609619	10,2012183	1,1334687
ENSG00000163209	SPRR3	0,104865073	0	0	0	0	0	0	10,08960512	0	10,19447019	1,13271891
ENSG00000113971	NPHP3	0,062487572	0,52669265	0,114950935	4,423998329	1,670659506	0,102911554	1,452773409	1,639704253	0,192129145	10,18630735	1,131811928
ENSG00000113389	NPR3	0	0	0,083569526	10,06713751	0	0,016625972	0	0	0,018623762	10,18595677	1,131772975
ENSG00000077782	FGFR1	0,34933812	5,462797574	0,04016473	0,245062467	0,233496451	0,119860263	0,045322328	3,246575725	0,443067132	10,18568479	1,131742755
ENSG00000134940	ACRV1	0	0	6,283456125	3,892791212	0	0	0	0	0	10,17624734	1,130694149
ENSG00000136854	STXBP1	0,048365701	0	0,088972612	0,75165308	0,038314045	2,602032943	0,870113259	0	5,769906965	10,1693586	1,129928734
ENSG00000095951	HIVEP1	1,002369074	0	0,151556619	0,379369341	0,163161082	0,226138905	2,405655616	5,825016456	0	10,15326709	1,128140788
ENSG00000105085	MED26	0	1,509242027	0	0,048955905	0	0,622553759	1,76553	4,215675533	1,98747688	10,1494341	1,1277149
ENSG00000100505	TRIM9	9,223741932	0,029976747	0,03107659	0	0	0,092739283	0	0,147762803	0,623297448	10,1485948	1,127621645
ENSG00000185716	MOSMO	0,266765572	0,731570214	0,044612438	4,103936368	0	0,878679029	1,832418586	1,750014561	0,536870069	10,14486684	1,127207426
ENSG00000142549	IGLON5	0,173956912	3,601293171	0	0,10012854	0	1,846280401	0	2,916344993	1,497612037	10,13561605	1,126179561
ENSG00000187151	ANGPTL5	0	7,133845442	0	0	0	0	0	0	2,982334395	10,11617984	1,124019982
ENSG00000160469	BRSK1	0,432756125	0,628294194	1,592179687	1,494550753	2,991867054	0,259167784	0,163330294	2,06467837	0,483849316	10,11067358	1,123408175
ENSG00000165490	DDIAS	0	3,666298184	0,104131897	0,09774676	0	5,00310973	1,057532298	0	0,174046191	10,10286506	1,122540562
ENSG00000221890	NPTXR	7,283373989	0	0	0,492330141	0,184794619	0	1,850848808	0,283391567	0	10,09473912	1,12163768
ENSG00000269743	SLC25A53	4,050568981	1,060470778	2,198758656	0,496873395	1,104653345	0,097208482	1,029198563	0	0,027222282	10,06495448	1,118328276
ENSG00000139714	MORN3	0,039188426	4,102795126	0	0,609028727	4,749739279	0	0,553162771	0	0	10,05391433	1,117101592
ENSG00000104332	SFRP1	0	0	0	0,11026198	9,93965731	0	0	0	0	10,04991929	1,116657699
ENSG00000188312	CENPP	2,431776161	0,086302543	0	3,05137992	0	1,957961961	1,211493389	0	1,296000853	10,03491483	1,114990536
ENSG00000183495	EP400	0,008078619	1,290178028	0,148612723	3,417752942	0,038398026	1,12647117	1,254367928	1,02460331	1,708933538	10,01739628	1,113044032
ENSG00000163596	ICA1L	0,014258404	1,846986791	0,052458973	2,511357559	5,455550511	0,015654895	0,082873403	0,031178955	0	10,01031949	1,112257721
ENSG00000215045	GRID2IP	0,036553113	0	9,615663801	0,063119249	0	0	0	0,15986191	0,134866852	10,01006493	1,112229436
ENSG00000169509	CRCT1	0	0	5,807383683	0	0	0	0	3,681722201	0,517678335	10,00678422	1,111864913
ENSG00000005513	SOX8	10,00642705	0	0	0	0	0	0	0	0	10,00642705	1,111825228
ENSG00000143369	ECM1	2,428562743	0	0,082732172	4,038279369	0	3,456470348	0	0	0	10,00604463	1,111782737
ENSG00000100092	SH3BP1	1,729802117	0,32887408	0,113646811	1,226799758	0,440454876	1,967050991	1,846660651	0,945642671	1,405625782	10,00455774	1,111617526
ENSG00000135338	LCA5	0,025337118	8,812178407	0,046609675	0,087503345	0	0	1,030859535	0	0	10,00248808	1,111387564
ENSG00000178934	LGALS7B	0	0	0	0	9,479810901	0	0	0,513097155	0	9,992908055	1,110323117
ENSG00000180113	TDRD6	0,066855689	0,05931689	0	0,086583887	0,873861955	0,550527298	0,111023411	8,223402023	0,02055597	9,992127122	1,110236347
ENSG00000099326	MZF1	2,009989656	2,164051413	0	1,286930827	0	2,033278626	0,356289988	0	2,138721001	9,989261511	1,109917946
ENSG00000130158	DOCK6	0,024220319	0,709142972	6,928338739	0,690082879	0	0	0,030165972	1,588882357	0	9,970833237	1,107870036
ENSG00000262874	C19orf84	0	5,311556013	0	0	0	2,03988414	0,68563023	1,805653374	0,126944346	9,969668102	1,1077409
ENSG00000122786	CALD1	0,015350525	2,015695653	0	0,265070068	0	0,067415919	2,498190276	1,443385453	3,662558444	9,967666337	1,107518482
ENSG00000110484	SCGB2A2	0,100139543	0,177695162	0	9,683471556	0	0	0	0	0	9,961306261	1,106811807
ENSG00000139926	FRMD6	3,257800111	5,894795456	0,619964112	0,027711871	0	0	0,026650453	0,070185762	0,059211934	9,956319699	1,106257744
ENSG00000066279	ASPM	0	0	3,191358594	5,727724002	0	0,944751631	0,080666084	0	0	9,944500311	1,104944479
ENSG00000100276	RASL10A	0	2,950668502	0	3,349921728	0	0	0	3,636143797	0	9,936734026	1,104081558
ENSG00000129910	CDH15	0	0	0	9,92319696	0	0	0	0	0	9,92319696	1,10257744
ENSG00000039068	CDH1	0,247600476	1,581699493	0,865414039	1,154390351	1,059170205	1,549549873	1,562468496	0,974573524	0,913550003	9,90841646	1,100935162
ENSG00000104918	RETN	0,292849943	0	0	0	0	0	0	9,605656032	0	9,898505976	1,099833997
ENSG00000171295	ZNF440	0,047023672	8,135629379	0	0	0	1,006770265	0,390447587	0,0514135	0,260248748	9,89153315	1,099059239
ENSG00000115361	ACADL	0	0	7,769630096	1,215535596	0	0	0	0,89792002	0	9,883085712	1,098120635
ENSG00000156050	FAM161B	0	0	0	0,051608974	0,14205599	0,459404287	0,893380528	0	8,307221921	9,853671701	1,094852411
ENSG00000274618	HIST1H4F	0,484328752	2,578288462	4,454809279	0	2,302035528	0	0	0	0	9,819462021	1,091051336
ENSG00000154479	CCDC173	0	0,242882246	2,014348543	5,436145304	0,162643814	0,225421979	0	1,646186704	0,084169621	9,811798212	1,090199801
ENSG00000024526	DEPDC1	0	0	0	7,424978952	0	2,33241495	0,02125175	0	0,031478069	9,810123722	1,090013747
ENSG00000174667	OR7D4	0,030306974	0	0	0	9,723399161	0,033275286	0	0	0	9,786981421	1,08744238
ENSG00000187801	ZFP69B	0	0	0	0,20337878	0,139952277	0	0	0	9,415451245	9,758782302	1,084309145
ENSG00000077157	PPP1R12B	0,035860879	3,932596612	0,857596774	0,928858659	1,176093332	1,315063188	0,434730373	0,345035858	0,732130682	9,757966359	1,084218484
ENSG00000042286	AIFM2	0,02942757	0	0,487209521	0,10162998	0,20980577	2,455541155	6,401796881	0,064349478	0	9,749760354	1,083306706
ENSG00000183722	LHFPL6	0,03310924	8,577723929	0,121814241	0,857586463	0,15736965	0	0	0	0	9,747603524	1,083067058
ENSG00000150048	CLEC1A	0,095367984	0,169228148	0	0	0	5,916028769	3,444594157	0	0,117290326	9,742509384	1,082501043
ENSG00000204536	CCHCR1	0	1,759326129	0	0	0,069300954	2,881503648	0,411616296	2,35933597	2,259423177	9,740506174	1,082278464
ENSG00000255154	HTD2	0	0	9,576473443	0	0	0	0	0,155938918	0	9,732412361	1,081379151
ENSG00000188827	SLX4	0	0,062365857	1,163772946	2,548963585	1,127593167	0,405177592	0,01459127	0	4,408965059	9,731429476	1,081269942
ENSG00000102003	SYP	0,02897614	1,028348994	0	0,200141879	0	1,590705433	0,745844735	5,132348987	0,997833677	9,724199845	1,080466649
ENSG00000154277	UCHL1	0	0,117503068	0	9,60496839	0	0	0	0	0	9,722471458	1,080274606
ENSG00000159214	CCDC24	0	2,257721022	0	0	0	1,117556509	1,936813994	0,27822164	4,120649806	9,710962971	1,078995886
ENSG00000160318	CLDND2	0	0	0	9,706058744	0	0	0	0	0	9,706058744	1,078450972
ENSG00000137812	KNL1	0,058969979	0,05232039	1,952640695	7,077065653	0	0,372287148	0,085687056	0,096712556	0	9,695683477	1,077298164
ENSG00000198729	PPP1R14C	0,204295499	0,120839117	2,505453799	0	0,161837559	0	0	0	6,700190077	9,69261605	1,076957339
ENSG00000149582	TMEM25	0	2,909842649	0,402213925	0	0	0	1,906223167	0	4,4705349	9,688814641	1,07653496
ENSG00000137878	GCOM1	9,651597311	0	0	0	0	0	0,012179221	0	0,018039849	9,68181638	1,075757376
ENSG00000138193	PLCE1	0,093494553	2,778888477	0,021498848	1,231015735	0,055547957	0,166808778	0,698674703	4,548905879	0,071866405	9,666701333	1,074077926
ENSG00000109452	INPP4B	0,053384643	0,037891896	0,39282145	1,124640249	0	2,778266564	3,076252752	2,194647732	0	9,657905286	1,073100587
ENSG00000160949	TONSL	0,021262216	0,037729281	0	0,073430414	0	1,867573679	4,360654567	0	3,294872915	9,655523071	1,072835897
ENSG00000196754	S100A2	0	0	4,789701769	1,332150477	0	2,011678977	1,521337784	0	0	9,654869007	1,072763223
ENSG00000256713	PGA5	2,645602164	0	0	0	0	0,854328407	0,301507835	5,444853842	0,382794073	9,629086321	1,06989848
ENSG00000257017	HP	4,557081039	0	0	5,06904206	0	0	0	0	0	9,626123099	1,069569233
ENSG00000147488	ST18	0,010496705	0,149009169	0,308952597	0,670644211	0,074836943	1,0833283	2,919738901	1,285380368	3,111212272	9,613599467	1,068177719
ENSG00000079101	CLUL1	0	0,069719709	0	0	0	0,043138475	0,26098873	0,343665697	8,891253276	9,608765887	1,067640654
ENSG00000203805	PLPP4	0	1,802635295	0	0	0	0	7,80235678	0	0	9,604992075	1,067221342
ENSG00000078399	HOXA9	0	0	2,107992838	7,484781665	0	0	0	0	0	9,592774503	1,065863834
ENSG00000123473	STIL	0	0	0,076252942	0,715772801	0	5,802660411	2,994354378	0	0	9,589040532	1,065448948
ENSG00000151014	NOCT	0,060155482	0	0	5,505395563	0	0	3,796076924	0	0,221950464	9,583578433	1,064842048
ENSG00000105784	RUNDC3B	0	0,056930361	0	0,332401242	0	0	0	9,190430646	0	9,579762249	1,064418028
ENSG00000146263	MMS22L	0,048537328	3,728124904	0,714306683	0,802213706	0,807450079	0,753689125	0,184235792	0	2,53275901	9,571316628	1,063479625
ENSG00000177675	CD163L1	0,026557218	0	0	0	0	7,406201491	0,22051025	1,509894333	0,391943456	9,555106748	1,061678528
ENSG00000053702	NRIP2	0,053699563	0,095288557	0	0	0	0,94334372	0	8,4546158	0	9,54694764	1,06077196
ENSG00000152582	SPEF2	0	2,541866544	1,153944649	0,0646679	0,044500315	0,483134889	0,031095497	3,255210423	1,969005905	9,543426122	1,06038068
ENSG00000099840	IZUMO4	0	0	0	3,014411259	5,704394175	0,419268605	0	0	0,402555644	9,540629683	1,060069965
ENSG00000149115	TNKS1BP1	0	0,937690586	0	0,291996055	0,100466511	3,573958429	0,9301899	3,697687145	0	9,531988627	1,059109847

ENSG00000250486	FAM218A	0,069476124	8,260006439	0	1,199701033	0	0	0	0	0	9,529183597	1,058798177
ENSG00000176273	SLC35G1	0	0	0	4,546970563	0	4,300215563	0	0,677417476	0	9,524603602	1,058289289
ENSG00000172748	ZNF596	0	2,588587884	0,462683254	0	0,059773226	4,114626486	2,234574821	0	0,061866352	9,522112023	1,058012447
ENSG00000131188	PRR7	9,223520286	0	0	0,297701055	0	0	0	0	0	9,521221341	1,057913482
ENSG00000136866	ZFP37	0,047563919	0	0,874976704	6,077805515	0,226073366	0	2,290613552	0	0	9,517033057	1,057448117
ENSG00000197208	SLC22A4	0	1,379635429	0,102161007	0	0	7,133971876	0,507230302	0,364315951	0	9,487314564	1,054146063
ENSG00000197465	GYPE	0	0	0	0,260024887	0	7,439936175	0	0	1,759384117	9,45934518	1,051038353
ENSG00000213186	TRIM59	0,347735908	0,034280491	0,213229429	2,201701397	0	0,212107615	0,978482518	4,520135352	0,950378534	9,458051243	1,050894583
ENSG00000187607	ZNF286A	0,067120479	1,111633995	0	2,588487311	0	0,245647878	2,30357139	0	3,136882372	9,453343424	1,050371492
ENSG00000188070	C11orf95	0,025405274	6,626912242	0,046735054	0	0	0,362615585	1,793036015	0	0,593659032	9,448363202	1,049818134
ENSG00000122970	IFT81	0,021584141	0	0	0,484524307	0,102590356	3,080756316	3,028779618	0,047198196	2,681116805	9,446549739	1,049616638
ENSG00000215041	NEURL4	0	0,999556888	0	2,791200701	0	1,478943552	1,037112955	2,624196727	0,512056868	9,443067693	1,049229744
ENSG00000117425	PTCH2	0	0	0,104229508	0,978383857	0	0,777608627	1,105569105	3,159385629	3,309977431	9,435154157	1,048350462
ENSG00000168038	ULK4	0,0962641	0	0	0,041556773	0	1,162615922	0	8,104303774	0,029598108	9,434338677	1,048259853
ENSG00000128242	GAL3ST1	6,73772496	0	0,179631728	0,168617108	0	0	0	0	2,341846323	9,427820119	1,047535569
ENSG00000164877	MICALL2	0	3,499753718	0,914927122	1,332660749	0	1,242775855	0	0	2,425644088	9,415761532	1,046195726
ENSG00000184481	FOXO4	0,043838286	0	1,935457608	0,151398303	1,562739523	4,187473215	1,383194841	0,095861493	0,05391544	9,413878709	1,045986523
ENSG00000178568	ERBB4	0,27865184	0,041205071	0,085433761	0	8,967591338	0,012747643	0,00964044	0	0,01427941	9,409549503	1,0455055
ENSG00000121742	GJB6	0	0	0	0	0	0	0	9,397680361	0	9,397680361	1,044186707
ENSG00000179104	TMTC2	0	2,738444072	4,442070547	0,125404289	0	0	0,135676197	1,548353735	0,401926877	9,391875717	1,043541746
ENSG00000188266	HYKK	6,195877613	0	0	0,777272572	0,125851601	1,279142347	0,989340273	0	0	9,367484406	1,040831601
ENSG00000163827	LRRC2	0,053642375	0	0	0,046314337	0	0	0,022270204	9,208063028	0,032986603	9,363276548	1,040364061
ENSG00000079308	TNS1	0,018562812	2,223399669	0,068295584	0,240404436	0,110287311	0,988472817	1,240754465	3,7141162	0,75338557	9,357678865	1,039742096
ENSG00000183844	FAM3B	0,083973643	0	0	0	0	5,531889193	1,150464283	0	2,581918898	9,348246017	1,038694002
ENSG00000006837	CDKL3	3,215118525	0	0	0	0,114041773	0,737614504	1,952380523	2,203596671	1,121335003	9,344086999	1,038231889
ENSG00000109943	CRTAM	0	7,306594254	0	0	0	0	1,967060076	0	0,069371602	9,343025932	1,038113992
ENSG00000158555	GDPD5	0,061023956	0	0,028064624	0,07903129	0,072512376	0,820759037	5,991667545	1,901541681	0,375257996	9,329858504	1,036650945
ENSG00000168546	GFRA2	0,026157274	0	0	0,225839514	0,062163327	1,09132801	0,086875771	7,836166825	0	9,328530722	1,036503414
ENSG00000165046	LETM2	0,024715501	2,499851816	0,045466159	0	0	2,008076983	0,759306883	1,567321305	2,401353494	9,306092141	1,034010238
ENSG00000104880	ARHGEF18	2,399349879	0,645089014	0	0,878850756	0	0,851505767	0,945806833	2,437849255	1,132665252	9,291116756	1,032346306
ENSG00000071205	ARHGAP10	0,603936661	0,024922576	0	0,072758149	0,033378338	3,300015189	1,492722604	3,25551298	0,500934022	9,28418052	1,031575613
ENSG00000122574	WIPF3	4,335241434	0,13263413	1,23750415	0,043023079	0,177634399	1,313059799	0,124125638	0,326893224	1,59340794	9,283523793	1,031502644
ENSG00000118242	MREG	0,041536715	0	0	2,72554399	0	0	0	0,181657266	6,334515877	9,283253849	1,03147265
ENSG00000078081	LAMP3	0,039923533	0,141686658	0,073442562	0,413635363	0	4,471037998	3,878475256	0,261903224	0	9,280104594	1,031122733
ENSG00000158806	NPM2	0	2,079627727	0	4,173947727	0	1,849705896	0	0	1,171112356	9,274393707	1,03048819
ENSG00000143036	SLC44A3	0	0	0	0	9,262704125	0	0	0	0	9,262704125	1,029189347
ENSG00000166436	TRIM66	0,012858285	3,080256128	0,141923127	0,155424168	0,886181818	2,131764558	0,074735562	2,333736309	0,442792939	9,259672895	1,028852544
ENSG00000122859	NEUROG3	0	0,389741279	0	0	0	8,319643745	0,547109006	0	0	9,25649403	1,028499337
ENSG00000280670	CCDC163	0	0,680810301	0	4,542937534	0	0,661957325	3,367720192	0	0	9,253425351	1,028158372
ENSG00000069020	MAST4	0,010297143	0,073088109	4,962915566	3,431717213	0,562841804	0,113056611	0,059849562	0,022516836	0,012664158	9,248947002	1,027660778
ENSG00000164236	ANKRD33B	0,030092715	0,053398785	0,359826873	3,845302822	0,321821954	1,602442093	0,349812735	0	2,683236655	9,245934633	1,02732607
ENSG00000186827	TNFRSF4	4,475400176	0	0	0	0	0,385390422	4,371788571	0	0	9,23257917	1,02584213
ENSG00000149243	KLHL35	0	0	0	0	0	0	5,426013167	3,801865685	0	9,227878852	1,025319872
ENSG00000198198	SZT2	0,040137133	0,142444714	2,183421131	2,128747801	0,027253361	1,32204679	1,947232518	0,06269154	1,368074119	9,222049107	1,024672123
ENSG00000185818	NAT8L	5,713940843	0	0	1,600010064	0,061168037	0,536927433	1,303643375	0	0	9,21568975	1,023965528
ENSG00000107779	BMPR1A	3,455998856	0,164475464	0,024358579	0,137189787	0,062936828	1,439287337	0,40680006	2,866545933	0,651407369	9,209000213	1,023222246
ENSG00000168282	MGAT2	1,293465994	0,099792333	1,034537026	0,582660903	0	2,469826226	2,568242747	0,122975276	1,037475934	9,208976439	1,023219604
ENSG00000130669	PAK4	0,021238309	0,942171469	0,703252534	0,036673925	1,867512167	1,422423778	2,310135417	0,046441963	1,854547165	9,204396727	1,022710747
ENSG00000105610	KLF1	0	5,492666668	0	0	0	3,295554596	0	0,410222927	0	9,198444191	1,022049355
ENSG00000019485	PRDM11	0	0,038470875	0	6,345549239	0,02576166	1,416309801	1,233101541	0,071112174	0,066659441	9,196964732	1,02188497
ENSG00000137869	CYP19A1	0	0	0,065895484	0	0,510775217	4,22789783	3,866574176	0,078329873	0,440551197	9,190023776	1,021113753
ENSG00000159640	ACE	0,018064623	0,833435984	0	0,686260543	0	1,804884935	3,269878347	1,224563251	1,333029448	9,170117131	1,018901903
ENSG00000119866	BCL11A	0,077273126	0,390262529	3,356930627	2,43260125	0,127136255	0,437259407	0,602131245	1,481769361	0,263176572	9,168540371	1,018726708
ENSG00000177595	PIDD1	0,283896888	1,694492571	0,332341707	1,604382423	0	0,935106581	3,45016626	0	0,857020683	9,157407113	1,017489679
ENSG00000103546	SLC6A2	0,02766073	0	0	9,122932482	0	0	0	0	0	9,150593212	1,016732579
ENSG00000165548	TMEM63C	0	1,517069307	0	0	0	0,234668425	7,394526874	0	0	9,146264607	1,016251623
ENSG00000198089	SFI1	0	3,61223	0,182671332	0,085735165	0	1,526366403	0,556546569	0,325711747	2,854714939	9,143976154	1,01599735
ENSG00000160284	SPATC1L	2,396524267	1,149344192	0	1,342143033	0	0,711146921	1,452078424	0,566540193	1,513537901	9,131314931	1,014590548
ENSG00000054654	SYNE2	0,009632853	2,897307899	1,577116645	0,307725953	0,045785369	2,633501218	0,163966457	0,337027671	1,155098867	9,127162932	1,014129215
ENSG00000197256	KANK2	1,088122727	0,035106311	0	1,059044804	0	0,02172173	0,95277288	5,970145368	0	9,126913819	1,014101535
ENSG00000171502	COL24A1	0,031586658	1,008895485	0	1,990829133	2,965121848	2,670383446	0,458973832	0	0	9,125790402	1,013976711
ENSG00000204334	ERICH2	0	0	0,462529283	0	0	0	0	0	8,658407028	9,120936311	1,013437368
ENSG00000160051	IQCC	0	0,10820904	1,68268825	0,105300635	0	4,954553138	0	2,266903813	0	9,117654876	1,013072764
ENSG00000066056	TIE1	3,673530651	0	0,248751766	4,942392509	0,214238653	0	0	0	0,027717602	9,106631182	1,011847909
ENSG00000159713	TPPP3	0	3,298940409	0	2,708667556	0	1,403318988	1,688374325	0	0	9,099301278	1,011033475
ENSG00000178460	MCMDC2	0	4,17784986	0	4,024492841	0,113036683	0,783336056	0	0	0	9,09871544	1,010968382
ENSG00000125650	PSPN	0,038637323	1,09697571	0,071076476	1,734673828	0	0,593901323	3,43270916	0,084488511	2,043313848	9,095776178	1,010641798
ENSG00000140443	IGF1R	0,010839292	1,365618105	0,737770867	3,013451755	0,051519624	0,630748193	0,864009172	0,284428291	2,132949112	9,091334413	1,010148268
ENSG00000161996	WDR90	0,014345032	0,025454908	0,580554602	4,48350393	0,10227384	1,685250779	0,011910987	0	2,18767503	9,090969108	1,010107679
ENSG00000214193	SH3D21	0	0	1,242646844	0	0	4,932075704	1,542438145	0	1,370794643	9,087955337	1,009772815
ENSG00000118307	CASC1	0,049014133	0	7,123070976	0,084636709	0	1,829698219	0	0	0	9,086420037	1,009602226
ENSG00000046604	DSG2	0	0	0	8,531229355	0,059299462	0,054792132	0	0,436505373	0	9,081826322	1,009091814
ENSG00000141293	SKAP1	0	0	8,509594867	0,295844642	0,271441831	0	0	0	0	9,07688134	1,008542371
ENSG00000014914	MTMR11	0,066102612	0	0	0	0,235641432	0,036288403	0,933068961	3,613676373	4,186831101	9,071608881	1,007956542
ENSG00000107104	KANK1	0,025379673	0,022517803	0,023343979	3,111586026	2,985609396	1,853048934	0	1,02671137	0	9,048197181	1,005355242
ENSG00000131095	GFAP	0,013457171	1,767077033	0	4,972845718	0	1,034263102	0	0	1,257844482	9,045487506	1,005054167
ENSG00000283599	BX276092.9	0	0	0	9,032924861	0	0	0	0	0	9,032924861	1,003658318
ENSG00000124743	KLHL31	0	0	0	4,562747998	4,352186282	0	0,021722703	0,057208201	0,032175644	9,026040828	1,002893425
ENSG00000156172	C8orf37	0	0	0	0,078124244	0,215040445	0,149021476	0	0	8,568971386	9,011157551	1,001239728
ENSG00000145103	ILDR1	0	5,37179299	0	2,439458861	0	0,221583408	0,293252863	0	0,682574873	9,008662995	1,000962555
ENSG00000100764	PSMC1	0,394480198	0,656245717	0,317484205	0,936624154	0	1,949022927	0,20471583	3,881757034	0,667094712	9,007424777	1,000824975
ENSG00000080603	SRCAP	0,022150479	0,334096599	0,998316099	1,568210783	0,315846563	1,118716876	1,784025536	0,944513935	1,920577517	9,006454387	1,000717154
ENSG00000158402	CDC25C	0	0,481980827	0	7,817105291	0,107584644	0,596443056	0	0	0	9,003113818	1,00034598
ENSG00000115274	INO80B	0,314377054	0,743805825	0,192773994	0,723814077	1,992330332	0,460223514	0,870113259	3,437252783	0,257762167	8,992453005	0,999161445
ENSG00000185015	CA13	0,026998494	6,467602289	0	0,139861519	0,128325011	0,029642769	0	0,0590378	2,1250987	8,976566582	0,997396287
ENSG00000188488	SERPINA5	0,040285409	8,935683821	0	0	0	0	0	0	0	8,97596923	0,997329914
ENSG00000170537	TMC7	0,080406476	3,709662293	0	0,27768887	0,06369591	4,826061472	0	0	0	8,957515021	0,995279447
ENSG00000067715	SYT1	0,070955034	1,217110346	0,565619234	0,081682571	0,168626174	0	6,834195576	0	0	8,938188936	0,993132104
ENSG00000136929	HEMGN	2,895366774	0	0,253631477	4,999666484	0,327661991	0	0	0,452237091	0	8,928563817	0,992062646
ENSG00000186051	TAL2	0	0	0	4,375434025	0	4,552424361	0	0	0	8,927858386	0,991984265
ENSG00000142156	COL6A1	0	0	0,05202697	0,048836791	0	1,242078059	0	6,122594457	1,460893493	8,92642977	0,99182553
ENSG00000165072	MAMDC2	0	0	0	0	0	4,02179442	4,719558323	0,184137402	0	8,925490144	0,991721127
ENSG00000153904	DDAH1	0,164716122	0	0	8,760406821	0	0	0	0	0	8,925122944	0,991680327
ENSG00000198901	PRC1	5,034282999	0,29130038	0	2,220522145	0,260088751	0,931238073	0,181742288	0	0	8,919174636	0,991019404
ENSG00000164746	C7orf57	0	2,003698462	0	0,344090076	0	0,072927726	2,647286126	0	3,839466614	8,907469004	0,989718778
ENSG00000170959	DCDC1	0,022965132	0,061126596	0,021123108	0,218106742	0	0	0,095342198	8,486845275	0	8,90550905	0,989501006
ENSG00000113889	KNG1	0,1939387	0,401496043	1,129758691	3,125638198	2,304497598	0,10646668	0,241547163	0,918855482	0,477039202	8,899237758	0,988804195
ENSG00000130653	PNPLA7	0	0,571963637	3,247101207	0,159025886	0	1,685226782	2,82929545	0	0,396422242	8,889035203	0,987670578
ENSG00000172062	SMN1	0,585510768	2,251111399	1,077094345	0,168508217	0	1,446427372	0,931810667	0,746865345	1,680239788	8,887567901	0,987507545
ENSG00000021762	OSBPL5	0,080883485	0	0	0,384087344	0	0	4,818678614	1,1054281	2,486906494	8,875984036	0,986220448
ENSG00000103021	CCDC113	0	0,090481525	0	0	7,634353018	0,923747095	0,211692816	0	0	8,860274454	0,984474939
ENSG00000109471	IL2	0	0	0	0	0	0	0	8,85883559	0	8,85883559	0,984315066
ENSG00000160654	CD3G	0,039392745	0,06990146	2,246450226	0	3,838326183	0,043250932	2,61669097	0	0	8,854012516	0,983779168
ENSG00000144445	KANSL1L	0,053214193	0,503612161	0	6,187212686	0	1,558028658	0	0,15515181	0,392679587	8,849899095	0,983322122
ENSG00000186020	ZNF529	0,018479952	0	0	3,574014572	1,097950172	1,562322906	1,779938671	0,161640977	0,636382857	8,830730108	0,981192234
ENSG00000184428	TOP1MT	0,023460731	0	0	4,456271884	0,055754936	0,025758512	0,058439838	0	4,183782911	8,803468813	0,978163201
ENSG00000132793	LPIN3	0,231561733	0,821801226	1,277929527	1,142447349	1,729550335	1,125925542	0,027467236	0,434020886	1,993537564	8,784241398	0,976026822
ENSG00000125520	SLC2A4RG	0,841255787	0,746393876	0	3,177704979	0	0,923649696	1,440682309	1,264711288	0,387988564	8,7823865	0,975820722
ENSG00000164619	BMPER	0,037824924	0,0335597	0	1,012388512	7,685744649	0	0	0	0	8,769517784	0,974390865
ENSG00000136153	LMO7	0,010517893	0,27995615	0,483712847	3,523448533	0,174972005	2,436634766	0,008733231	0,022999552	1,82392703	8,764902007	0,973878001
ENSG00000103196	CRISPLD2	0,056936914	0,033677719	1,396534032	0	0	2,208806974	4,774869454	0,29051017	0	8,761335264	0,973481696
ENSG00000146700	SSC4D	0	0	0	0	2,927934588	2,646570702	3,113407742	0	0,065879661	8,753792693	0,972643633
ENSG00000198488	B3GNT6	0	0	0	0	8,752293492	0	0	0	0	8,752293492	0,972477055
ENSG00000133424	LARGE1	0,014139662	4,566468047	0,052022101	0,219744996	0,033603213	2,049237029	1,162305873	0,061838602	0,591258703	8,750618226	0,972290914
ENSG00000058404	CAMK2B	4,94503277	0,295781101	0,272562911	0,287831201	2,464834216	0,101673353	0,046134452	0,242996373	0,091112402	8,747958779	0,97199542
ENSG00000137502	RAB30	0,012771346	6,16418391	0	0,110266639	0,121405525	0,701109604	1,124058079	0,47476231	0,031414219	8,739971633	0,971107959
ENSG00000266967	AARSD1	0,04079659	0,579140389	0	3,099659525	0,09695398	0,179169089	0,372617077	4,014458839	0,351221829	8,734017319	0,970446369

ENSG00000129195	PIMREG	0	0	0,099385091	0,466455085	0	3,440405237	4,710184076	0	0	8,716429489	0,968492165
ENSG00000143776	CDC42BPA	0,043005499	0	0,158224176	2,432051346	0,357712244	1,664417739	1,472970813	1,410606478	1,176829632	8,715817927	0,968424214
ENSG00000070444	MNT	0,85098471	0,554712792	1,277922533	1,079606837	1,774744779	0,40042778	0,461447032	1,519064786	0,790290923	8,709202171	0,96768913
ENSG00000178188	SH2B1	0	1,163387264	1,478410603	0,401719345	0	0,626953894	2,230193445	0,277481792	2,523038861	8,701185204	0,966798356
ENSG00000174669	SLC29A2	0,111110714	0	0,408794263	0,287795928	0	4,147764418	3,736432681	0	0	8,691898003	0,965766445
ENSG00000155666	KDM8	0,074825734	0	3,785319496	0	0	1,273391403	0,931941065	0	2,622741216	8,688218914	0,965357657
ENSG00000174099	MSRB3	0	0,651796894	0	8,03418963	0	0	0	0	0	8,685986524	0,965109614
ENSG00000167525	PROCA1	0	0	0	0,054034992	0	0,240499904	2,156561929	0	6,234652435	8,68574926	0,965083251
ENSG00000141294	LRRC46	0,053604317	0	0	0	2,293052771	2,236467511	0	0,937735559	3,164467211	8,68532737	0,965036374
ENSG00000170417	TMEM182	0	0	0	0,128666161	0	5,126756808	0	0	3,421237754	8,676660723	0,964073414
ENSG00000188549	CCDC9B	0	0	0,03701466	0,034745003	0	2,93822992	1,921316668	2,551958045	1,187833964	8,671098261	0,963455362
ENSG00000106333	PCOLCE	0	1,522623431	0	0	0	1,04127977	1,349949776	2,468877522	2,277258641	8,659989141	0,962221016
ENSG00000268163	AC004076.1	0	0	8,653077011	0	0	0	0	0	0	8,653077011	0,961453001
ENSG00000172915	NBEA	0,075877766	0	0	0,174699121	0,661192228	1,805035984	1,501569795	0,248883681	4,183840865	8,651099439	0,961233271
ENSG00000176723	ZNF843	0,198481485	0	0	2,056405229	0	2,796653767	0,082401707	0,217010443	3,295439646	8,646392276	0,960710253
ENSG00000162959	MEMO1	0,492122548	1,797886974	0,21301157	0,249937715	0,068796464	1,017076332	2,427682669	1,329334467	1,032480679	8,628329419	0,958703269
ENSG00000174038	C9orf131	0	0	5,959311926	0	0,093886939	0,997632226	1,574529656	0	0	8,625360747	0,958373416
ENSG00000122687	MRM2	0,425214409	0,188633134	0	0,367126239	5,936871084	0,175072715	0,794395349	0	0,7190685	8,60638143	0,956264603
ENSG00000042429	MED17	0,018195132	0,113003853	1,037613855	0,754056519	0	1,738015674	1,004669124	2,407139234	1,532870172	8,605563563	0,956173729
ENSG00000177191	B3GNT8	0,08063531	0	0	6,822739464	0	0,088532858	1,606877251	0	0	8,598784883	0,955420543
ENSG00000185522	LMNTD2	2,242151325	0,925265701	3,357247572	0	0	0,229000106	0,432954907	0	1,410843164	8,597462775	0,955273642
ENSG00000069535	MAOB	0	0	0	0,901329791	7,690945639	0	0	0	0	8,592275431	0,95469727
ENSG00000076984	MAP2K7	0,969222319	0,104234014	0,86446678	0,45644602	0,139598656	1,386619008	0,755992282	1,734058955	2,167306382	8,577944416	0,953104935
ENSG00000250510	GPR162	0	0	0	0,243409491	0	2,424688155	1,170432201	0	4,738624013	8,57715386	0,953017096
ENSG00000126215	XRCC3	2,596678249	2,000260025	0	0	0	0,265209394	0,51812712	0	3,193579509	8,573854297	0,952650477
ENSG00000144395	CCDC150	0,031227644	0	0,517011963	6,389914133	0,074213173	0,600007252	0	0,955999989	0	8,568374154	0,952041573
ENSG00000171757	LRRC34	0	0,055275613	0	0,053789935	0	2,052078871	0	6,402978633	0	8,564123052	0,951569228
ENSG00000142224	IL19	0	0	0	0,100863925	0,277632425	2,437032051	0	0	5,747089981	8,562618383	0,951402043
ENSG00000144283	PKP4	0,047593881	3,800437797	0,350211148	1,972421855	0,678647324	1,010269128	0,395182148	0,242839052	0,058534338	8,556136671	0,950681852
ENSG00000123411	IKZF4	0,066539221	0,07871481	0,979234166	1,914978531	0	0,316576765	1,970545358	3,201039404	0,027278221	8,554906475	0,950545164
ENSG00000188124	OR2AG2	0	0	8,489449046	0,061774378	0	0	0	0	0	8,551223424	0,950135936
ENSG00000106327	TFR2	0,029834269	0	0	1,957656275	0	0,949932226	1,907446311	0	3,705922754	8,550791835	0,950087982
ENSG00000196793	ZNF239	0	0	0	0,311998774	0	3,835318234	4,400713119	0	0	8,548030127	0,949781125
ENSG00000165621	OXGR1	0,05682985	3,932883792	0	0	0	0	0	0	4,543070308	8,53278395	0,948087106
ENSG00000172005	MAL	0	8,304466624	0	0	0	0	0	0,227415394	0	8,531882018	0,947986891
ENSG00000007312	CD79B	0	0	0	8,279667463	0	0,0797647	0	0,158862773	0	8,518294936	0,946477215
ENSG00000175414	ARL10	0	0	0,02429896	1,573821088	0,470870903	0,26104811	3,323208968	0,086652422	2,761705313	8,501605765	0,944622863
ENSG00000168813	ZNF507	0	0	0	8,26522386	0,191716812	0	0,011163834	0	0,033071719	8,501176225	0,944575136
ENSG00000124256	ZBP1	0,083872657	2,232449584	2,262927725	0,193106364	0,066441728	0	0	0	3,644723656	8,483521716	0,942613524
ENSG00000187860	CCDC157	0,022944211	0,040713939	0	6,299523377	0	0,050382805	1,657442892	0,200689078	0,197528949	8,46922525	0,941025028
ENSG00000115468	EFHD1	0	7,918960003	0,311753382	0,097545785	0,134249549	0	0	0	0	8,462508718	0,940278746
ENSG00000153060	TEKT5	0	8,308625355	0	0,147005619	0	0	0	0	0	8,455630974	0,939514553
ENSG00000205078	SYCE1L	0,12147152	0,862192926	0	2,30730283	0	1,467054939	2,622370283	1,062490571	0	8,442883069	0,938098119
ENSG00000134020	PEBP4	0,113191439	0	0	0	0	8,326598232	0	0	0	8,43978967	0,937754408
ENSG00000186496	ZNF396	0,024853712	0	0	0	0	0,627622247	4,540044908	0	3,240087395	8,432608262	0,936956474
ENSG00000196074	SYCP2	0	0,041475947	5,460707281	0,121083515	0,111095914	1,616762	0,058222892	1,02222604	0	8,43157359	0,93684151
ENSG00000091137	SLC26A4	0	0,046231379	0,047927603	0,089977577	0,061916818	0	0	8,146921234	0,032042504	8,425017115	0,936113013
ENSG00000214021	TTLL3	0,043572829	0,025772972	0,427497269	0,927969441	0	0,845180754	1,748673408	2,985471871	1,411176015	8,415314559	0,935034951
ENSG00000257335	MGAM	0,042103809	2,241365284	0	0,945153154	2,067919349	1,217324748	0,407863065	0	1,484424716	8,406154124	0,934017125
ENSG00000050767	COL23A1	0	5,059283021	0	0,072401491	0	0	1,322939128	1,925395649	0	8,380019288	0,931113254
ENSG00000213988	ZNF90	0	1,409419186	0,091320663	1,585840023	0	0,84481404	2,98837026	0,054276374	1,404227994	8,37826854	0,930918727
ENSG00000075073	TACR2	6,894979017	0	0	0	0	1,386922271	0,087405317	0	0	8,369306605	0,929922956
ENSG00000104835	SARS2	1,21599345	1,82578892	0	0,484558913	0	0,359447241	1,359164847	0,818160489	2,3007926	8,363906461	0,92932294
ENSG00000142794	NBPF3	0,199646967	0	0,12242224	0,076610386	0,105436742	1,826672527	4,752105939	0,048507717	1,227700162	8,359102678	0,928789186
ENSG00000176182	MYPOP	2,789488383	1,697101266	0	0	0,378815973	1,225078098	0,992645032	0	1,27426413	8,357392881	0,928599209
ENSG00000159208	CIART	0	0,282353808	0	0	0	2,504090838	3,611290707	0	1,956965836	8,35470119	0,928300132
ENSG00000013725	CD6	0	0,045005371	0,046656613	5,12410071	0	1,086020895	0,021059136	0	2,027530035	8,350372759	0,927819195
ENSG00000115594	IL1R1	0	0,037644532	0,234154232	0,879185651	0	0	0	7,190419481	0	8,341403896	0,926822655
ENSG00000145736	GTF2H2	0	0,494498848	4,773978187	1,172944215	0	0,382458683	0,433853154	0,266602348	0,813988917	8,338324351	0,926480483
ENSG00000161010	MRNIP	1,498794183	0,031661589	0	2,372416229	0	0,705252186	1,05188258	0,74132209	1,931100952	8,332429809	0,925825534
ENSG00000253626	EIF5AL1	0,550923956	0,488800521	0,651515857	2,174458122	0,654641353	1,209764622	1,176284363	1,032608023	0,387180255	8,326177071	0,925130786
ENSG00000115461	IGFBP5	0,116850117	6,096021344	1,934597039	0,04035493	0,055539366	0	0,019404629	0	0,028742116	8,291509541	0,921278838
ENSG00000067141	NEO1	0	0	0,13664992	0,609286533	0,0441339	0,040779299	5,258127469	0,121826681	2,078411396	8,289215196	0,921023911
ENSG00000183066	WBP2NL	0,513482522	1,865712946	1,079534365	1,013339707	0	1,073854865	1,400882348	0,106936753	1,232962365	8,28670587	0,920745097
ENSG00000119725	ZNF410	0,302873484	0,78549152	3,300102933	0,643687881	0,055368107	1,406888948	0,851170923	0,305674901	0,630376733	8,28163543	0,920181714
ENSG00000105672	ETV2	0	0	7,49202869	0	0	0	0	0	0,690879265	8,260003948	0,917778216
ENSG00000100565	LRRC74A	0,065557731	3,140925814	0,120598741	0	0,311598735	0,719785582	2,122925357	1,290200047	0,483765351	8,255357359	0,917261929
ENSG00000127311	HELB	0	0	0,261941236	3,480141731	0,728855551	1,274755083	2,501039526	0	0	8,246733128	0,916303681
ENSG00000140993	TIGD7	0	1,489677778	0,201434854	0,063027772	0	0,561050259	3,636821217	0	2,28941368	8,241425561	0,915713951
ENSG00000111665	CDCA3	0	0,047433575	0	1,338601497	0	4,108876833	2,685637745	0,058452957	0	8,239002608	0,915444734
ENSG00000230797	YY2	0	0	0	0,285590997	0	0	2,426095438	5,183760089	0,339012262	8,234458787	0,914939865
ENSG00000183496	MEX3B	0	0,061755412	0	6,4903218	0	0,458527619	1,069185234	0,152203854	0	8,231993919	0,914665991
ENSG00000165204	OR1K1	0	0	0	8,231387211	0	0	0	0	0	8,231387211	0,914598579
ENSG00000188603	CLN3	0,015328725	0,897614729	0	0,238224262	0	0,58905155	2,481914662	0,033519433	3,940142337	8,195795698	0,910643966
ENSG00000153956	CACNA2D1	0	0	0,278320268	0,029028254	0	0,018457067	0	7,866614468	0	8,192420057	0,910268895
ENSG00000081913	PHLPP1	1,702936494	0,029625677	0,061425279	0,836052841	0,039677112	2,218006826	2,578442355	0,438096874	0,287465618	8,191729076	0,91019212
ENSG00000119227	PIGZ	0	0	0,086923108	0	0	7,107488746	0	0	0,987927104	8,182338957	0,909148773
ENSG00000116525	TRIM62	0	0,067440141	1,048717677	4,397043083	0,090321313	0,542464159	1,988086246	0	0,046742083	8,180814703	0,908979411
ENSG00000239857	GET4	0,168212138	1,144205195	2,062932089	1,258684479	0	1,231247322	0,768185706	0,735661375	0,793037108	8,162165412	0,906907268
ENSG00000003096	KLHL13	0	0,115197766	0	8,033942366	0	0	0	0	0	8,149140132	0,905460015
ENSG00000258659	TRIM34	0,561749333	0,598086246	1,240059921	0	1,869013232	0,308383972	0,513075707	1,596895679	1,450846456	8,138110546	0,904234505
ENSG00000166263	STXBP4	0,016738917	0,059405594	6,389462259	0,260140102	0,019890199	0	0,069493399	0,164713991	1,152855746	8,132700206	0,903633356
ENSG00000196381	ZNF781	0	0	0,231008393	0,216843469	0,099478544	3,033268161	0,034756325	1,830662424	2,677013617	8,123030932	0,902558992
ENSG00000078589	P2RY10	0,042256871	0,599870246	0	0	0	0	0	0,092403403	7,379811567	8,114342087	0,901593565
ENSG00000182871	COL18A1	1,583707111	0,15787918	0	0,061454304	0	2,031877058	0,87173217	1,828830037	1,575712389	8,111192249	0,901243583
ENSG00000266524	GDF10	0,395134103	7,712713488	0	0	0	0	0	0	0	8,107847591	0,900871955
ENSG00000235169	SMIM1	3,080745579	0,820006116	0	0,797966283	0	0,845619657	2,558008807	0	0	8,102346443	0,900260716
ENSG00000104611	SH2D4A	4,638708579	0,395101179	0	0,192240895	0	0,814884954	1,294143896	0,081147978	0,684601629	8,10082911	0,900092123
ENSG00000137843	PAK6	0,023402597	0	2,71221097	0	0	1,798627903	0	2,354032849	1,208851469	8,097125789	0,899680643
ENSG00000196684	HSH2D	0	0	0	2,918048568	4,438768917	0	0,738495185	0	0	8,09531267	0,899479186
ENSG00000198795	ZNF521	0,02427479	0	0,089310875	7,628942229	0,173068695	0,106609206	0	0,053081858	0	8,075287654	0,897254184
ENSG00000106976	DNM1	0,014024183	0,024885568	0	0,24216703	0,033328774	0,523522933	1,245969886	5,980022336	0	8,063920711	0,89599119
ENSG00000139211	AMIGO2	0,038197819	0,271124368	0	0,197877888	0,090777943	0,461328702	6,755606854	0	0,234891964	8,049805538	0,894422838
ENSG00000161692	DBF4B	0	1,801121747	0,272300685	1,862256326	0,050254344	0,81260428	1,264184705	1,803379252	0,18204949	8,048150829	0,894238981
ENSG00000116852	KIF21B	1,517324249	0,154485103	1,052434943	0,107380648	0,059113999	2,539865619	1,528362892	0	1,086016715	8,044984166	0,89388713
ENSG00000120149	MSX2	0	6,390074642	0	0,234653754	0,161473715	0	0	0	1,253461256	8,039663368	0,89329593
ENSG00000084110	HAL	0	0	3,349157819	0	0,069785764	0	0,438877959	4,173775144	0	8,031596686	0,892399632
ENSG00000186026	ZNF284	0,034850224	0,618408672	0,064109801	0,902680955	0	2,48712811	0,954917195	0,609657874	2,357370555	8,029123385	0,892124821
ENSG00000164114	MAP9	0,017061146	3,814597721	0,502165698	0,029460876	0,446008012	0,074928566	2,74824934	0,373077303	0,02098301	8,026531672	0,891836852
ENSG00000198598	MMP17	0,037749331	0	0	0,195554565	0	0	0,06268815	2,806588882	4,921241909	8,023822837	0,891535871
ENSG00000181634	TNFSF15	0	0,068886834	0	0	0,046129421	3,239358723	3,013866677	0,509340374	1,145874511	8,02345654	0,891495171
ENSG00000243660	ZNF487	0,087718985	0,155655186	0,053788719	0	0,138977377	2,439861422	3,010511062	0	2,121699848	8,008212598	0,8898014
ENSG00000147180	ZNF711	0	0	0	4,693075083	0,071766096	3,216092314	0,025074007	0	0	8,0060075	0,889556389
ENSG00000006453	BAIAP2L1	0,062688476	0	0,057660257	4,762969874	0,223470779	1,996020213	0,468464214	0,274162678	0,154197488	7,99963398	0,88884822
ENSG00000184402	SS18L1	0,088025575	3,123984467	0,323860309	0,65867081	0	0,257725168	1,437427104	0,192486156	1,912595278	7,994774867	0,888308319
ENSG00000162426	SLC45A1	0,052323605	0,649928671	0	0	0	1,83834434	4,865885452	0	0,5791616	7,985643667	0,887293741
ENSG00000196436	NPIPB15	5,060694787	0	0	0	0	0	0,958351537	0	1,965474384	7,984520708	0,887168968
ENSG00000111641	NOP2	0,741528296	0,858145936	0,118617495	1,781506121	1,149298727	0,601766547	0,508627333	0,352501139	1,863619916	7,975611509	0,886179057
ENSG00000172201	ID4	0,078032828	6,854133232	0	0	0,092723352	0	0,939488672	0	0	7,964378084	0,884930898
ENSG00000233436	BTBD18	0	0	0	0,65479291	0	0	2,518852336	0	4,780243045	7,953888291	0,883765366
ENSG00000150625	GPM6A	0,128787418	0,304706818	1,184574285	1,927360608	2,703583771	0,471336866	0,231692374	0,891797838	0,105594615	7,949434594	0,88327051
ENSG00000188092	GPR89B	2,438688969	0,990608206	0	0,507359468	0	0,612930383	1,073438578	0,835241956	1,48156862	7,939836179	0,88220402
ENSG00000153933	DGKE	0,442612198	0	0	0,716526246	0,065742342	5,558194149	0,390479752	0	0,765500549	7,939055235	0,882117248
ENSG00000166793	YPEL4	4,133517852	0	0	0,175516799	0	1,004391384	1,125294457	0,814973149	0,666713981	7,920407622	0,880045291
ENSG00000131037	EPS8L1	0	1,857564876	0	0,048855079	0,067237885	0,962970956	0,916184787	2,041629045	2,018179796	7,912622424	0,879180269
ENSG00000120215	MLANA	0	0	7,393567342	0	0	0,376702574	0,081394961	0	0,060281065	7,911945942	0,879105105
ENSG00000181350	LRRC75A	0	3,26559946	0	0,070618396	0,097190133	0,359210992	3,259851657	0	0,855044893	7,907515531	0,878612837

ENSG00000105750	ZNF85	0	0,057442588	2,679756738	2,180047988	0	0,710842231	0,376303481	1,698892378	0,199064398	7,902349802	0,878038867
ENSG00000128596	CCDC136	0	0,25791151	0	5,647037764	0	0,984080048	0	0	1,012949945	7,901979267	0,877997696
ENSG00000184271	AC139768.1	0,849497256	0	0,091924636	1,553184373	0,059377901	1,124724478	2,987388857	0	1,229143666	7,895241166	0,877249018
ENSG00000196263	ZNF471	0,022300852	2,136904959	0,041024218	0,038508703	0,31799074	0,12242516	0	0,048765432	5,156308469	7,884228533	0,876025393
ENSG00000132692	BCAN	0	0	6,767196533	0	0	0,139274356	0,974271203	0	0	7,880742093	0,87563801
ENSG00000148824	MTG1	2,078197373	0,656715524	0,157110079	0,835699806	0	0,843930967	0,68550106	1,805313199	0,805288246	7,867756254	0,874195139
ENSG00000107130	NCS1	0,055331589	0	0	0,143318368	0,39449016	2,156655245	2,297149982	0,060496991	2,756054248	7,863496583	0,873721843
ENSG00000133026	MYH10	0,234773942	0,119028743	2,066881215	4,893797659	0,119559719	0	0,01392413	0,366701329	0,041248812	7,855915549	0,872879505
ENSG00000118972	FGF23	0,159770111	0,623717911	1,234422093	0,993197197	2,657879886	0,38592014	0,344917385	1,257733604	0,19649664	7,854054968	0,872672774
ENSG00000129295	LRRC6	0	5,937876459	0	0,060824003	0	1,237561411	0,614190436	0	0	7,85045231	0,872272479
ENSG00000205086	C2orf91	1,595749828	1,538923325	0	0	0,329768175	1,180722653	0	0	3,199836816	7,845000797	0,871666755
ENSG00000204183	GDF5OS	0	0,463913495	0,801557379	1,805778371	1,863931881	0,09568084	0,0723589	2,096182378	0,643067551	7,842470794	0,871385644
ENSG00000124104	SNX21	0	3,813692007	0	0	0	2,766532422	1,261472195	0	0	7,841696624	0,871299625
ENSG00000100403	ZC3H7B	0,05744557	0,025483938	0,079256824	1,661532342	3,310625509	0,126143757	1,681364457	0,125616638	0,759494436	7,826963471	0,869662608
ENSG00000126705	AHDC1	0,018212676	0,872584549	1,842702838	2,924786385	0,043282818	0,099982269	0,710751549	0,876167348	0,425585624	7,814056056	0,868228451
ENSG00000143320	CRABP2	0	0	0	0	7,633737037	0	0	0	0,171762035	7,805499072	0,867277675
ENSG00000225190	PLEKHM1	1,685734732	0,290416983	0,963431514	0,310872384	0	0,862526555	1,481237538	0,679980156	1,529766673	7,803966534	0,867107393
ENSG00000243989	ACY1	1,342636761	0,680707426	1,352558129	0,717612581	0,075971555	0,631772875	1,167906623	0,768940183	1,061530802	7,799636935	0,866626326
ENSG00000188916	INSYN2A	0,062442385	0	5,398787046	0	0	0	0	2,321234565	0	7,782463996	0,864718222
ENSG00000181638	ZFP41	0,043736779	1,746221419	0	0	0	1,272541326	2,43314392	0	2,286100491	7,781743935	0,864638215
ENSG00000124780	KCNK17	0	0	0	7,777869435	0	0	0	0	0	7,777869435	0,864207715
ENSG00000143126	CELSR2	0,058889544	0	0,021666415	0,020337878	0,083971366	2,935440445	0,54764915	0	4,099342619	7,767297417	0,863033046
ENSG00000174990	CA5A	0,965325513	0,527060442	0,034149889	2,147744877	0,220588171	1,89553853	0,724460148	0,852472471	0,365300289	7,732640331	0,859182259
ENSG00000167595	PROSER3	0,907570995	0	0,439355301	2,928146546	0	1,468467251	0,555266208	1,253426524	0,176241368	7,728474193	0,858719355
ENSG00000172361	CFAP53	5,716353824	0	0	0	0	1,091516952	0	0	0,917005866	7,724876643	0,858319627
ENSG00000145014	TMEM44	0	3,821361545	0	0,28605018	0	0,836646188	2,778448483	0	0	7,722506397	0,858056266
ENSG00000128849	CGNL1	0,087216074	0,030952557	0	0,090361875	0	0	0	0,724720857	6,779118208	7,712369572	0,856929952
ENSG00000178033	CALHM5	0,030879855	0,027397773	0	0,026661385	7,595517653	0	0,012820101	0	0,01898912	7,712265887	0,856918432
ENSG00000179913	B3GNT3	0	0	0	7,711246533	0	0	0	0	0	7,711246533	0,85680517
ENSG00000029153	ARNTL2	0,064919334	0	0,03980812	7,548169106	0	0,047518424	0	0	0	7,700414984	0,855601665
ENSG00000214654	B3GNT10	0,120119691	0	0,331454808	0,311130733	0	2,571745823	3,391090054	0	0,960256276	7,685797384	0,853977487
ENSG00000089486	CDIP1	1,237452029	1,317496959	0	0,061051702	0,420118791	1,35865002	1,086196136	1,159690808	1,043593012	7,684249457	0,853805495
ENSG00000105877	DNAH11	2,756128252	2,254687277	0,739034516	1,145442265	0,022203385	0	0	0,020429984	0,735388737	7,673314415	0,852590491
ENSG00000179256	SMCO3	0	0	0,132119819	0	0	0	0	7,538431198	0	7,670551017	0,852283446
ENSG00000147144	CCDC120	0,05682985	0,756323806	0	3,238380655	0,135057368	0,592760616	0,141561112	2,050458956	0,698933893	7,670306257	0,852256251
ENSG00000161381	PLXDC1	0,016327452	2,462676392	0,030035667	0,028193946	0,038802544	3,26263911	0,786307861	0	1,044194399	7,669177371	0,852130819
ENSG00000105976	MET	0,020891825	0	7,6480077	0	0	0	0	0	0	7,668899526	0,852099947
ENSG00000234616	JRK	0,013457171	0	0,59413326	0,116187984	0,223868804	0,059100749	3,173352417	1,559625263	1,919867894	7,659593541	0,851065949
ENSG00000160877	NACC1	0	0,056617821	1,936939034	0,495864605	0	1,12101741	0,158957346	0	3,884882961	7,654279179	0,850475464
ENSG00000159733	ZFYVE28	0	1,560261015	0	0	0,074629581	1,517054317	2,737818965	0,137337725	1,622101815	7,649203419	0,849911491
ENSG00000187902	SHISA7	0	0,132241657	6,397700782	1,115290044	0	0	0	0	0	7,645232484	0,849470276
ENSG00000137337	MDC1	0	0,316620047	0	0,308110046	4,381780561	0,827158477	1,218158563	0,520232854	0,073148723	7,645209272	0,849467697
ENSG00000274641	HIST1H2BO	0,396615671	0	0,729606559	0,6848687	0,942565728	3,048225819	0	0,867282329	0,975572295	7,644737101	0,849415233
ENSG00000188215	DCUN1D3	0	0,129287367	0,044676969	0,964561944	0,115434761	4,959718301	1,310763674	0	0,119477031	7,643920048	0,84932445
ENSG00000182132	KCNIP1	0,045094172	0	0	0,700810138	0	3,416242851	1,872132677	0	1,608340539	7,642620376	0,849180042
ENSG00000186891	TNFRSF18	0	0	2,405807308	0	0	0	5,232043742	0	0	7,63785105	0,848650117
ENSG00000169989	TIGD4	0,183386615	0	0	0,950005975	0,145274087	2,349057518	4,009771937	0	0	7,637496132	0,848610681
ENSG00000272333	KMT2B	0,058803608	0,365209456	2,271653693	0,380778735	0,104811035	1,049147532	1,989655036	0,321465675	1,084812855	7,626337626	0,847370847
ENSG00000101445	PPP1R16B	0	0,041844881	0	0,407201895	0	2,071293092	5,051708124	0,051565944	0	7,623613937	0,847068215
ENSG00000075035	WSCD2	0,022419966	0	0	0,348429491	7,193007154	0	0	0,0490259	0	7,612882511	0,845875835
ENSG00000117707	PROX1	0,067701869	0,180202957	0	0,058453175	0	4,348462696	2,234518681	0,185055201	0,520403568	7,594798148	0,843866461
ENSG00000101871	MID1	0	5,856140508	0	0,084217851	0	1,213762153	0	0,426596537	0	7,580717049	0,842301894
ENSG00000164128	NPY1R	0	0	0	0,065561551	0,360922153	0	0	0	7,144351233	7,570834938	0,841203882
ENSG00000154016	GRAP	5,301272032	0,258907628	0	0	0	1,228176139	0,363448017	0	0,418707968	7,570511783	0,841167976
ENSG00000266338	NBPF15	0	0	0	0	0	3,689681125	0,425643795	3,300609137	0,140102919	7,556036976	0,839559664
ENSG00000174233	ADCY6	0,017967963	0	0	0	0,042701254	0,769383174	4,535431739	0	2,187729573	7,553213702	0,839245967
ENSG00000157999	ANKRD61	0	0	0	0	0	0	7,286662081	0,262875551	0	7,549537632	0,838837515
ENSG00000154920	EME1	0	0	0	1,425874178	0	0	0	0,100314076	5,980489181	7,506677435	0,834075271
ENSG00000175322	ZNF519	0,995470916	0,016984988	3,187077844	0,016528471	0,068243024	0,630558979	0,810665349	1,151194097	0,623922574	7,500646242	0,833405138
ENSG00000280071	GATD3B	1,654140181	0,690642627	1,521462229	0,084009973	0,462482347	0,587577702	0,565545605	0,319157663	1,615536414	7,500554741	0,833394971
ENSG00000110318	CEP126	0	1,080293737	0	0	0	0,081020914	0,689313263	0	5,649589067	7,500216982	0,833357442
ENSG00000187800	PEAR1	0	0,048488608	0,050267649	7,360914293	0	0	0	0	0,033606966	7,493277515	0,832586391
ENSG00000185219	ZNF445	0,048007171	0	0	2,79089616	0,17113512	2,301629307	0,983247333	0	1,190693063	7,485608154	0,831734239
ENSG00000106701	FSD1L	0,030598475	0	0,056288367	2,245567768	2,581486839	2,116506294	0,215955821	0,066909905	0,169344811	7,48265828	0,831406476
ENSG00000105497	ZNF175	0,020384537	0,072343721	3,674902158	2,006379813	0	0,760955026	0,016925716	0,579475162	0,350984933	7,482351066	0,831372341
ENSG00000082196	C1QTNF3	0	7,273426736	0	0,072968393	0,100424369	0	0,035086782	0	0	7,48190628	0,83132292
ENSG00000158292	GPR153	0	4,40728871	0	0,497255788	0	0	0,537986178	0	2,03643164	7,478962316	0,830995813
ENSG00000177990	DPY19L2	0	0	0	0,323511304	7,123833642	0,029385508	0	0	0	7,476730454	0,830747828
ENSG00000101425	BPI	0	7,475085626	0	0	0	0	0	0	0	7,475085626	0,83056507
ENSG00000159784	FAM131B	0	0,041701711	0	0	0,279251588	2,631862959	1,678141299	0	2,83249754	7,463455096	0,829272788
ENSG00000165474	GJB2	0,061079455	0	6,966356563	0	0,435469938	0	0	0	0	7,462905956	0,829211773
ENSG00000180881	CAPS2	0,045181812	2,405220512	0,955829313	0	0	0,322445432	0	0,691595746	3,028445347	7,448718163	0,827635351
ENSG00000154917	RAB6B	4,64187715	1,815660116	0	0,30165893	0,059309256	0,054801181	0,455878993	0,054572183	0,061386135	7,445143944	0,827238216
ENSG00000120915	EPHX2	0	6,714040691	0	0,116671127	0	0,074183133	0,532961392	0	0	7,437856343	0,826428483
ENSG00000108924	HLF	0,13380511	0,079144628	0,123072653	7,047092732	0,052998457	0	0	0	0	7,43611358	0,826234842
ENSG00000116661	FBXO2	7,118392358	0	0,175769901	0	0,113537004	0	0	0	0	7,407699263	0,823077696
ENSG00000148488	ST8SIA6	0	0	0	7,351841986	0,050844902	0	0	0	0	7,402686888	0,822520765
ENSG00000168938	PPIC	0	0	0,529990656	0	0,342342748	0,948964214	4,784378723	0,787498969	0	7,393175309	0,821463923
ENSG00000169676	DRD5	7,263684083	0	0	0,111989259	0	0	0	0	0	7,375673343	0,81951926
ENSG00000165891	E2F7	0,048040239	0,042623112	0	1,493190126	0	2,030696933	3,749550535	0	0	7,364100946	0,818233438
ENSG00000170561	IRX2	0	0	7,033329954	0	0,316961644	0	0	0	0	7,350291597	0,816699066
ENSG00000164176	EDIL3	0,051883458	7,089076054	0	0	0	0,085447507	0	0,113453929	0	7,339860948	0,815540105
ENSG00000122824	NUDT10	0	0	0	7,339587064	0	0	0	0	0	7,339587064	0,815509674
ENSG00000175265	GOLGA8A	0,022386751	0,556146371	0,864826975	0,309256266	0	0,467007549	1,95176072	1,174878462	1,982362904	7,328625998	0,814291778
ENSG00000160207	HSF2BP	0,158313851	5,197094816	0	0	0	0	0,065725685	1,904023176	0	7,325157527	0,813906392
ENSG00000278129	ZNF8	0,016587329	0	0,061027464	1,804489945	0,039420147	0,10927151	2,065922042	3,228175248	0	7,324893686	0,813877076
ENSG00000204271	SPIN3	0	0	0,017548141	0	0,226701308	1,267292455	5,36225079	0,438048477	0,011731994	7,323573165	0,813730352
ENSG00000198088	NUP62CL	0,080937638	0	0	0,838569817	0	3,554591895	0,537633988	2,300829876	0	7,312563214	0,812507024
ENSG00000175793	SFN	0	0,820006116	0,212523011	0,398983142	3,020101656	0	1,342954624	1,515754897	0	7,310323446	0,812258161
ENSG00000067191	CACNB1	0	0	0	4,044363422	0	1,379276318	0	0	1,885437453	7,309077193	0,812119688
ENSG00000183117	CSMD1	0,024235857	0,315376851	0,564728349	6,263555391	0,115194079	0,017739703	0	0	0	7,300830231	0,811203359
ENSG00000147145	LPAR4	1,941671322	0,258408609	0,178593061	0,335284259	4,037620939	0,213184165	0,120915836	0,21229333	0	7,297971521	0,810885725
ENSG00000203943	SAMD13	0	0	0	0,151047742	0,103941402	0,048020427	1,961041368	0,669476693	4,357038583	7,290566215	0,810062913
ENSG00000243910	TUBA4B	0,360215901	0,479395709	0,165661561	0,466510682	2,354167441	0,197748006	0,299094951	1,969216731	0,996793028	7,28880401	0,809867112
ENSG00000004809	SLC22A16	0,07735376	0,068631175	0,426895468	0,06678653	0	0	6,647647485	0	0	7,287314419	0,809701602
ENSG00000145555	MYO10	0,172920121	1,898588042	0,178931547	3,620468107	0,231158481	0,094928094	0,080763338	0,472657084	0,531673644	7,282088459	0,80912094
ENSG00000128965	CHAC1	0,147353067	0	0	0	0	0,242677587	2,630533533	0	4,258794026	7,279358213	0,808817579
ENSG00000146648	EGFR	0	0,021408543	0,110970099	0	0	0,900752033	1,512656298	0,026382001	4,688822438	7,260991413	0,806776824
ENSG00000247595	SPTY2D1OS	2,581389512	0	0	0	0	1,308099159	0,329751201	0	3,01196729	7,231207163	0,803467463
ENSG00000171476	HOPX	0,019490593	0,172927899	0	5,822488147	0,185279269	1,027177568	0	0	0	7,227363476	0,803040386
ENSG00000124091	GCNT7	0	1,169223838	0	0,379265951	0	3,376087317	1,033428122	0	1,260586876	7,218592104	0,802065789
ENSG00000107831	FGF8	0,323115974	0,573361369	0,990663218	0,743934354	0,767892108	0,473016612	0,35772012	2,590720059	0,397390996	7,217814809	0,801979423
ENSG00000198523	PLN	0	0	0	6,809276687	0	0	0	0,33165062	0,062176822	7,20310413	0,800344903
ENSG00000154265	ABCA5	0,057421557	0,020378629	0	2,39953883	0,191049004	1,45004684	0,762853516	0	2,316372523	7,197660899	0,7997401
ENSG00000204923	FBXO48	0,291668061	3,105346904	0,048776996	0,045786098	0	0,37845894	0,814599453	0	2,510998809	7,195635261	0,799515029
ENSG00000131242	RAB11FIP4	0,029579258	0,017495889	0	0,32348718	0,210887243	0,930987185	2,734378891	0	2,946672642	7,193488288	0,799276476
ENSG00000175066	GK5	0,236440817	0,485804497	0,068676628	2,21331651	0	2,240742368	0,826618345	0,027211938	1,071337936	7,17014904	0,796683227
ENSG00000206384	COL6A6	0,015546355	0,055173251	6,06294043	0,13422581	0,886709981	0	0,01290847	0	0	7,167504298	0,796389366
ENSG00000162999	DUSP19	0	0	0	7,134940712	0	0,030861342	0	0	0	7,165802054	0,796200228
ENSG00000185760	KCNQ5	0,019961766	0	0,073442562	1,861359132	0	0	0	0	5,20468464	7,1594481	0,795494233
ENSG00000197020	ZNF100	1,036897786	0	0,381491444	0,835564877	1,09520446	0,379484393	0,363515753	2,267391817	0,793489269	7,153039799	0,7947822
ENSG00000092377	TBL1Y	0	0	0	0	0	0	0	0	7,15066619	7,15066619	0,794518466
ENSG00000257108	NHLRC4	0	1,407233779	0	0	0,171334705	0	3,17267538	0	2,394015316	7,14525918	0,793917687
ENSG00000197779	ZNF81	0,12528414	0,03175909	0	0,834447982	0,212671765	0,628821326	4,220487302	1,056701803	0,022011906	7,132185314	0,792465035
ENSG00000172752	COL6A5	0,047291019	0,027972251	6,930653417	0,027220423	0,074925421	0,017307592	0	0	0	7,125370124	0,791707792
ENSG00000253368	TRNP1	0	6,097215768	0	0	0	0	1,014414003	0	0	7,111629771	0,790181086
ENSG00000158321	AUTS2	0,093675189	0,19946919	0,528457435	0,970539642	1,395092325	0,322262971	0,922995375	0,860328874	1,812612277	7,105433278	0,789492586

ENSG00000188610	FAM72B	0	2,121796242	0,091651863	1,978735383	0	1,367545143	0,992841401	0	0,55147336	7,10404339	0,789338154
ENSG00000107554	DNMBP	0,01780075	0,06317399	0	0	0	3,381143806	2,941288263	0	0,700564108	7,103970918	0,789330102
ENSG00000253159	PCDHGA12	0	0	0	0	0	1,254586417	1,128284204	0	4,709783111	7,092653731	0,788072637
ENSG00000186281	GPAT2	0	0,707623153	0,146717153	2,892136418	0,063180426	1,10918401	1,258235208	0	0,91550011	7,092576478	0,788064053
ENSG00000108370	RGS9	1,585900189	0	0	2,553469472	0	1,364744497	0	1,030997091	0,553506874	7,088618122	0,787624236
ENSG00000162576	MXRA8	0	6,736864166	0	0,288166731	0	0	0	0	0,05131047	7,076341366	0,786260152
ENSG00000062282	DGAT2	0,057354721	0,033924848	6,893231199	0	0,090869823	0	0	0	0	7,075380592	0,786153399
ENSG00000203724	C1orf53	0	4,696754779	0	0	0	2,377699348	0	0	0	7,074454126	0,786050458
ENSG00000124243	BCAS4	0,418478436	0,424331267	0,733166554	3,303409938	0,236791206	0,503223429	0,41365664	0,566484159	0,465657909	7,065199539	0,785022171
ENSG00000148426	PROSER2	0	0	0	7,052296611	0	0	0	0	0	7,052296611	0,783588512
ENSG00000008441	NFIX	0,431607469	0	0,132329466	1,076532742	1,424617353	1,105719489	0	0	2,860538347	7,031344867	0,781260541
ENSG00000107518	ATRNL1	0,03035263	0	0	6,132247068	0,324601575	0,533206634	0	0	0	7,020407907	0,780045323
ENSG00000142765	SYTL1	0,027061349	0	0	0,140187128	0,128623762	0	0	0	6,722958005	7,018830243	0,779870027
ENSG00000050438	SLC4A8	0,278272814	2,528794532	0	1,106643865	0,921841906	0,092584027	1,029248817	0	1,057831767	7,015217729	0,779468637
ENSG00000278535	DHRS11	1,320356353	2,442639525	0	1,018708769	0	0,277597172	1,189623895	0	0,760108476	7,009034189	0,778781577
ENSG00000004139	SARM1	1,360743545	1,134864836	0	0,540432635	0,06467673	1,583657914	0,982973335	1,071197157	0,267766264	7,006312415	0,778479157
ENSG00000285713	AC098588.1	0	1,844362964	0	0	0	0,790050365	0	4,370827613	0	7,005240941	0,778360105
ENSG00000254726	MEX3A	0,022926805	0	3,289705313	3,642242097	0	0	0	0,050134208	0	7,005008424	0,778334269
ENSG00000123191	ATP7B	0,046875371	0,19408479	0,287436769	0,647548277	0,037133445	0	1,141700881	2,323394746	2,325243428	7,003417706	0,778157523
ENSG00000185897	FFAR3	0	2,457366039	0	0	0	2,325431264	0,270555972	1,246922896	0,701307631	7,001583802	0,777953756
ENSG00000130038	CRACR2A	0,751552654	0	0	4,856161889	0,086423281	0,905015179	0,010065003	0,026506864	0,357798535	6,993523405	0,777058156
ENSG00000068985	PAGE1	0,458605677	0,406892261	2,109105455	1,583823822	2,179772639	0,251761118	0	0	0	6,989960972	0,77666233
ENSG00000077616	NAALAD2	0,021081274	0,037408203	0	0,400430346	0,100200207	0,092584027	0	2,397125769	3,940941879	6,989771705	0,776641301
ENSG00000171532	NEUROD2	0,072626035	0,343661647	1,558683669	0,794258974	2,876622416	0,132898572	0,040201965	0,899933939	0,267961983	6,9868492	0,776316578
ENSG00000285043	AC093512.2	2,3772218	0,716318789	0	2,555908033	0	0,443216144	0,558639056	0	0,33098223	6,982286053	0,775809561
ENSG00000171517	LPAR3	0	0	0,078614281	0,295175311	0,101560391	0	0	0,09344869	6,412125486	6,980924159	0,77565824
ENSG00000141736	ERBB2	0,014641078	0,025980234	0,053866893	2,654604431	0,03479484	0,643001944	1,969401587	0	1,584584242	6,98087525	0,775652806
ENSG00000250565	ATP6V1E2	0,398934186	0,758463134	1,991937349	1,230129052	0,13543939	0,657009638	1,348634532	0	0,455592078	6,97613936	0,775126596
ENSG00000134042	MRO	0	0	0	2,786454932	0	4,144010785	0,022709562	0	0	6,953175279	0,772575031
ENSG00000163958	ZDHHC19	2,737284936	0	0	0	0,147845839	1,024560992	0,258275694	1,632447431	1,147673048	6,94808794	0,772009771
ENSG00000198300	PEG3	0	0,027448255	0	0	0,110282795	0,815201933	3,544867605	0	2,435085971	6,932886559	0,770320729
ENSG00000170801	HTRA3	0,091057891	0	0	6,289484172	0,541002625	0	0	0	0	6,921544689	0,769060521
ENSG00000182324	KCNJ14	0	0,061783871	0,128101428	0,180369798	0	4,013965565	1,069677946	0	1,455940497	6,909839105	0,767759901
ENSG00000198948	MFAP3L	0,034575122	3,589133281	2,957573413	0,089555534	0,041084263	0	0,114833847	0,037802833	0,042522943	6,907081237	0,767453471
ENSG00000198870	STKLD1	2,982445474	0,060830762	0	0,828740845	0	0	1,053176562	0	1,981575895	6,906769538	0,767418838
ENSG00000142530	AC020909.1	0	6,506638572	0	0	0	0	0,16024308	0	0,23735188	6,904233533	0,767137059
ENSG00000146707	POMZP3	0	0,432254702	5,078626204	0	0	0,267453912	0,809051039	0	0,299591385	6,886977242	0,765219694
ENSG00000117560	FASLG	0	0,148555125	6,006218205	0,144562313	0,397914174	0	0	0,183066242	0	6,880316059	0,764479562
ENSG00000069966	GNB5	0	0	0,020897617	4,707893092	0	0,249452077	0,688568203	0,372614533	0,838279307	6,877704828	0,764189425
ENSG00000162994	CLHC1	0,06825229	0	0	0,117856809	0	6,169815183	0,207794889	0	0,307785568	6,871504739	0,763500527
ENSG00000173988	LRRC63	0	3,004953307	0,103840157	0,097472908	0	0,557786773	0,046869754	0	3,054630923	6,865553821	0,762839313
ENSG00000111254	AKAP3	0,082304232	0,146046841	0	0	0	0	0	0	6,630145539	6,858496612	0,762055179
ENSG00000175262	C1orf127	0,042292351	0,150093479	0,155600391	0	0	0	4,740692645	0	1,76848076	6,857159627	0,761906625
ENSG00000197363	ZNF517	1,622702059	1,010331575	0,366590183	0,786540994	0	0	2,812918144	0	0,245087727	6,844170682	0,760463409
ENSG00000168263	KCNV2	0	0	0	0	0,164884087	0	0	6,675445808	0	6,840329896	0,760036655
ENSG00000174473	GALNTL6	0,032700838	0,029013417	0,030077916	2,089286748	0	0	0	3,32508275	1,327187889	6,833349557	0,759261062
ENSG00000132470	ITGB4	0,022350328	1,348443722	0,452267577	2,470024904	0,053116039	1,69321547	0,70520228	0	0,082464068	6,827084389	0,758564932
ENSG00000182601	HS3ST4	0,043889216	0	0	0	0	6,216225499	0,364421528	0,191945726	0	6,816481969	0,757386885
ENSG00000066923	STAG3	0,052817396	0	0,097161866	2,158497401	0,041840562	3,11215226	0,336224821	0	1,017684556	6,816378863	0,757375429
ENSG00000023839	ABCC2	0,019281686	0	0	0,199771577	0,045823343	0	0,016009995	0,0843268	6,450204678	6,815418079	0,757268675
ENSG00000204231	RXRB	0	0,136807143	0	1,065040713	1,557397505	0,761834281	1,632394626	1,264417988	0,379278617	6,797170872	0,755241208
ENSG00000205464	ATP6AP1L	0,08361354	0	0	0	3,328382257	1,652450065	1,162887293	0	0,565586318	6,792919474	0,75476883
ENSG00000152661	GJA1	0	0,078564899	5,619873082	0,764532595	0	0	0,036762476	0,290449371	0	6,790182423	0,754464714
ENSG00000178127	NDUFV2	0,27869895	0,791271118	0	0,866254065	0	0,979184518	2,128969115	1,462639178	0,274211025	6,781227969	0,753469774
ENSG00000108352	RAPGEFL1	0,026459564	5,117751359	0	0,045689892	0	0,029051055	1,559865798	0	0	6,778817668	0,753201963
ENSG00000147127	RAB41	0	0	0	0,388296093	0,534401105	3,456470348	0,186711804	2,212731479	0	6,778610828	0,753178981
ENSG00000196811	CHRNG	0	0	0,254910682	0,299100154	0,082328643	1,939807293	1,150576176	0,227258987	2,811983143	6,765965079	0,751773898
ENSG00000154548	SRSF12	0,033298936	3,48620053	0,306280409	0	0	2,815142002	0	0	0,122860195	6,763782072	0,751531341
ENSG00000067445	TRO	0,04500017	0	0	1,670667646	0	1,902190948	0,018682301	0	3,126959056	6,76350012	0,751500013
ENSG00000159339	PADI4	0	0,090253113	0	5,620948628	0	0,055843345	0,929097039	0	0,062553525	6,75869565	0,750966183
ENSG00000012124	CD22	0	4,784366587	0	0	0	1,722348242	0	0,16079541	0,090436264	6,757946503	0,750882945
ENSG00000056998	GYG2	0	0	0	6,557603615	0,198352688	0	0	0	0	6,755956303	0,750661811
ENSG00000183775	KCTD16	0,042408074	6,584557639	0	0,018307372	0,100783707	0	0,008803082	0	0	6,754859874	0,750539986
ENSG00000172824	CES4A	0	0	0	1,798311334	0	2,896669844	1,758256433	0	0,298857261	6,752094871	0,750232763
ENSG00000177169	ULK1	1,130354662	0,31670315	0,072960656	0	0,047128286	0,827375579	1,613660438	2,124841707	0,609732685	6,742757163	0,74919524
ENSG00000146063	TRIM41	0	0,690710015	3,8840401	1,303554631	0	0,259012687	0,597431134	0	0	6,734748566	0,748305396
ENSG00000150627	WDR17	0,058059902	0	0,035601959	1,002567782	1,333812593	0,382478276	0,739195091	0,846400019	2,332602354	6,730717977	0,747857553
ENSG00000221916	C19orf73	0	0	0	0	0	1,1179958	0,845487412	0	4,758873212	6,722356424	0,746928492
ENSG00000270885	RASL10B	0	0	2,540080859	0,074510273	4,101856566	0	0	0	0	6,716447697	0,746271966
ENSG00000142515	KLK3	6,54736467	0	0	0	0,077412706	0,035764298	0,054093698	0	0	6,714635371	0,746070597
ENSG00000197241	SLC2A7	0	0,174231319	0	0	6,533652492	0	0	0	0	6,707883812	0,745320424
ENSG00000088320	REM1	0	0	0	0	6,706411936	0	0	0	0	6,706411936	0,745156882
ENSG00000185518	SV2B	0,012133497	0,043061185	0	0,272374713	0,028835518	0	0,020149403	6,314712307	0,014922637	6,70618926	0,74513214
ENSG00000131503	ANKHD1	0,040752581	0,45799155	1,449374842	0,187655501	0,225981913	0,611500687	1,048969874	1,336709416	1,336544569	6,695480932	0,743942326
ENSG00000166926	MS4A6E	0,285923502	0	0	0	0,339751696	0,313927297	0,237408386	0,937846455	4,571437634	6,686294969	0,742921663
ENSG00000162522	KIAA1522	0,251014237	0	4,802314002	0,04334468	0	0,551197929	0,333475965	0,603784093	0,092614546	6,677745454	0,741971717
ENSG00000091262	ABCC6	1,61711669	0	0	0	0	1,008308433	2,619145522	0	1,424111282	6,668681926	0,740964658
ENSG00000148948	LRRC4C	0	0,300383122	0	0,09743651	0,067049578	0,061953165	2,248908117	0,246777123	3,643369089	6,665876704	0,740652967
ENSG00000172345	STARD5	0	0	0	1,852354292	0	2,277050035	0,356281113	0	2,169528735	6,655214175	0,739468242
ENSG00000164406	LEAP2	0,125663676	0	0	0,216993742	0	0	3,912796216	0	2,395526898	6,650980533	0,738997837
ENSG00000100065	CARD10	0	0,046600973	0	6,122040593	0,124823616	0	0	0	0,355285322	6,648750504	0,738750056
ENSG00000179348	GATA2	0	0,060135008	0,43638948	6,14446565	0	0	0	0	0	6,640990138	0,737887793
ENSG00000152284	TCF7L1	4,126467852	0,121030016	1,944794193	0	0	0	0	0,447440173	0	6,639732233	0,737748026
ENSG00000103494	RPGRIP1L	0,048796503	1,839999193	0	1,895870486	0,086974459	0,508969235	2,228422785	0,026675916	0	6,635708578	0,737300953
ENSG00000176302	FOXR1	0,244317818	0	0	0	6,386892427	0	0	0	0	6,631210245	0,736801138
ENSG00000103226	NOMO3	0,623665866	0,74863632	0,067487278	0,665165631	0	1,490335358	0,670149867	0,240666109	2,120608533	6,626714961	0,736301662
ENSG00000176105	YES1	0	0	1,744188856	1,688402777	0	3,188085592	0	0	0	6,620677225	0,735630803
ENSG00000164715	LMTK2	0	0,760520563	0,030323999	5,7498489	0,078350069	0	0	0	0	6,619043532	0,735449281
ENSG00000005102	MEOX1	0	6,116926704	0	0,487911321	0	0	0	0	0	6,604838025	0,733870892
ENSG00000170293	CMTM8	0	0	0	4,014384225	0	0	2,5737504	0	0	6,588134625	0,732014958
ENSG00000152380	FAM151B	0	0	0	0,503573447	0	5,443190358	0,524642752	0,106283232	0	6,577689789	0,730854421
ENSG00000197043	ANXA6	0	0	1,522344463	2,42929604	0	0	2,565293389	0,060320293	0	6,577254185	0,730806021
ENSG00000204920	ZNF155	0	0	0	0,075523871	0	0,288122564	2,614721825	0,095639528	3,496388986	6,570396774	0,730044086
ENSG00000257594	GALNT4	0,869903007	0	0,051621188	0,920661935	0	1,263200305	1,421298097	0,797706477	1,242428004	6,566819013	0,729646557
ENSG00000118193	KIF14	0,020717106	0	0,457329475	5,723827247	0	0	0	0,362417952	0	6,564291779	0,729365753
ENSG00000172602	RND1	1,033430954	0	0	0,066092952	0	1,008574934	4,449302553	0	0	6,557401394	0,728600155
ENSG00000173262	SLC2A14	0,027105035	0,336680538	0,049861901	4,540034536	0,579741324	0,029759745	0,337588337	0,355624647	0,300021292	6,556417354	0,728490817
ENSG00000164512	ANKRD55	0	0,181914518	0	0	0,121817348	6,246972189	0	0	0	6,550704056	0,727856006
ENSG00000139734	DIAPH3	0	0,036030906	0	3,856872777	0,048255515	0,022293816	0,758689188	0	1,823004048	6,54514625	0,727238472
ENSG00000077327	SPAG6	1,208572676	0	0	0,26928274	0,185303169	4,879722844	0	0	0	6,542881429	0,726986825
ENSG00000204479	PRAMEF17	0	0	0	5,952889154	0	0	0	0	0,588867308	6,541756462	0,726861829
ENSG00000283288	SMIM33	6,000149876	0	0	0,402366962	0,138441612	0	0	0	0	6,54095845	0,726773161
ENSG00000012171	SEMA3B	0	4,555810681	0	0,04523838	0	0	0	0	1,933216251	6,534265312	0,726029479
ENSG00000230601	TEX48	0	0	0	0	0	0	0	6,534016872	0	6,534016872	0,726001875
ENSG00000267221	C17orf113	0	2,648492793	0	0	0,287600808	1,062961517	0,100483448	2,381669333	0,049611992	6,530819891	0,725646655
ENSG00000166507	NDST2	0,941600032	0	0,477834291	0,056066819	0	1,105119871	1,267104771	0,994001707	1,677171022	6,518898513	0,724322057
ENSG00000269713	NBPF9	0	0	2,919313443	0	0	1,008742175	1,900225015	0,620980284	0,061633823	6,51089474	0,723432749
ENSG00000064932	SBNO2	0,594383485	0,283962784	0,336435823	0,671088437	0,325976589	1,154597054	2,239863718	0,199960404	0,702899101	6,509167395	0,723240822
ENSG00000134030	CTIF	0	0,066085521	0,753612098	0,128618595	1,593127729	0,102224631	0,726692003	0	3,137519846	6,507880424	0,723097825
ENSG00000118596	SLC16A7	0	0	0	6,417214219	0	0	0,073251307	0	0,01356247	6,504027995	0,722669777
ENSG00000213920	MDP1	0,992283297	1,006161351	1,825385265	0,244779526	0,673766496	0	1,412424	0	0,348680154	6,50348009	0,722608899
ENSG00000249715	FER1L5	0,02885166	0,895939857	0	5,554988003	0	0	0	0	0,017741911	6,497521432	0,721946826
ENSG00000198105	ZNF248	4,415770552	1,030041981	0	0	0	0,318664848	0	0,090666639	0,63742119	6,492565211	0,721396135
ENSG00000144026	ZNF514	2,656995328	0,914380222	0	2,057671368	0	0,141441242	0,414490653	0	0,297069249	6,482048063	0,720227563
ENSG00000166979	EVA1C	0,069428243	0	0	4,675609469	0	0,190570384	1,527665425	0	0	6,463273522	0,718141502
ENSG00000160221	GATD3A	0	1,518357871	0	0	0	1,899819112	2,241951321	0,374218083	0,420943425	6,455289812	0,717254424
ENSG00000161267	BDH1	1,112800038	0	0	0,507999358	0	1,2920072	1,200114061	1,314578015	1,022517686	6,450016358	0,716668484

ENSG00000254505	CHMP4A	1,87870581	0,222247824	0	0,648822979	0	1,993952227	0,935957719	0	0,770188654	6,449875214	0,716652802
ENSG00000105707	HPN	0,178829078	6,267224262	0	0	0	0	0	0	0	6,44605334	0,716228149
ENSG00000176153	GPX2	0	0	0	6,440689833	0	0	0	0	0	6,440689833	0,715632204
ENSG00000187535	IFT140	0	0	0	0,128381291	0	0,04081441	6,265800097	0	0	6,434995799	0,714999533
ENSG00000171357	LURAP1	2,778669228	0	0	0,423366643	0	3,230276237	0	0	0	6,432312108	0,714701345
ENSG00000184381	PLA2G6	0,178882001	2,737762357	0,699269263	0,038611272	0	1,080210917	1,3181997	0	0,357502928	6,410438438	0,712270938
ENSG00000147996	CBWD5	0,701567805	0,949511332	0	0,677593182	1,017330148	0,261112019	0,681260065	1,404112893	0,716594239	6,409081684	0,712120187
ENSG00000155858	LSM11	0,023010594	0	0	3,059539066	0	1,237950093	1,471176422	0,050317431	0,566001286	6,407994891	0,711999432
ENSG00000167693	NXN	0	1,593644083	0,047203277	0	3,353959047	1,40864813	0	0	0	6,403454537	0,711494949
ENSG00000105559	PLEKHA4	0	0,768916059	0,072466136	0	0	1,384029837	2,191478687	0	1,986367939	6,403258658	0,711473184
ENSG00000183625	CCR3	0	0	0	0,146263999	0	2,278480454	0	0	3,958614264	6,383358716	0,70926208
ENSG00000213398	LCAT	0	0	0	4,274363393	0	0	0,191192887	0	1,911564229	6,377120509	0,708568945
ENSG00000085999	RAD54L	0,378249238	0	0	0,979730818	0	1,765006135	2,198478909	0	1,046695057	6,368160157	0,707573351
ENSG00000163704	PRRT3	0	0	0	5,346757638	0,181693672	0,839416022	0	0	0	6,367867332	0,707540815
ENSG00000141391	PRELID3A	0	3,043609536	3,26045519	0	0	0,062773582	0	0	0	6,366838308	0,707426479
ENSG00000233954	UQCRHL	0,831801306	0,738005505	0,765082841	1,196949425	0	0,761057691	0,690662378	1,212603917	0,170501397	6,366664461	0,707407162
ENSG00000171962	DRC3	0,577072556	0	1,895663523	0,533827689	0,146938438	0,090513135	0,359366745	2,028035398	0,735072169	6,366489653	0,707387739
ENSG00000184486	POU3F2	0	0,164875179	0	5,775974023	0,368023716	0	0,051432807	0	0	6,360305725	0,706700636
ENSG00000286132	AC022415.2	0	0	4,553578764	0	0	0,726846336	1,075459989	0	0	6,355885089	0,706209454
ENSG00000100453	GZMB	0	0	1,773083551	0	4,581230165	0	0	0	0	6,354313716	0,706034857
ENSG00000164684	ZNF704	0,010068668	0,053599813	0,777929381	0,886706004	0,047856815	0,386918323	0,02508069	3,060394782	1,102101578	6,350656054	0,70562845
ENSG00000175175	PPM1E	0,092140592	0	0,042375015	0,039776673	5,638583363	0	0,5355441	0	0	6,348419743	0,705379971
ENSG00000161647	MPP3	0,067294843	0,119413048	0,06189715	2,440273644	0,159927652	0,960515474	0	0	2,52430146	6,333623271	0,703735919
ENSG00000161973	CCDC42	0	0	0	0,297192454	0	0	0	0	6,032603513	6,329795967	0,703310663
ENSG00000135318	NT5E	0	0	1,839709457	0,057563418	0,07922293	1,57382634	1,300927775	1,385011424	0,081997142	6,318258486	0,702028721
ENSG00000163121	NEURL3	0,060589643	0	0	0,10462509	0	1,530049424	0	0	4,620081949	6,315346107	0,701705123
ENSG00000174776	WDR49	0,038835922	0	0,071441814	4,358975417	0,184588816	0,298477008	1,35434437	0	0	6,306663347	0,700740372
ENSG00000119411	BSPRY	0,064604776	0	0	2,119608601	0	4,11407159	0	0	0	6,298284967	0,699809441
ENSG00000166582	CENPV	0,37114877	0	0	0,091556131	0	1,455355936	0,220123389	0,115941954	4,042976976	6,297103156	0,699678128
ENSG00000015133	CCDC88C	0,328342621	0	0	4,199822543	0,057800989	0	0	1,701900235	0	6,287866388	0,698651821
ENSG00000175536	LIPT2	0,573595587	0	0	0	0,151462481	1,119598999	4,127661702	0	0,313532724	6,285851493	0,698427944
ENSG00000072954	TMEM38A	0	0	0	0	0	0	0	0	6,28406386	6,28406386	0,698229318
ENSG00000140545	MFGE8	0	0,039392096	0,735072981	4,906666189	0	0,268108762	0,331785805	0	0	6,281025834	0,697891759
ENSG00000188897	AC099489.1	1,846948856	0	0	4,165587023	0	0,179332959	0,052161941	0	0,030904884	6,274935662	0,697215074
ENSG00000118473	SGIP1	0,011344637	0	5,050389186	0,097948564	0,080882329	0	0	0	1,004575796	6,245140513	0,693904501
ENSG00000139263	LRIG3	0,051284769	0	0,047171237	0,354230408	0,243758726	0	0	0,112144771	5,424334266	6,232924177	0,692547131
ENSG00000197816	CCDC180	5,540543001	0	0,039276595	0,01843412	0	0	0,629346067	0	0	6,227599782	0,691955531
ENSG00000155760	FZD7	0	0	1,656787322	0,608555266	1,302836381	0,945849362	0,292623215	1,370031894	0,048159244	6,224842684	0,691649187
ENSG00000153976	HS3ST3A1	0	0,562929321	0,06484257	0,060866567	0,167537925	0	1,434113895	3,930992056	0	6,221282335	0,691253593
ENSG00000156413	FUT6	0,0694921	0	0,063918165	0	0	0	0	0	6,068109028	6,201519292	0,689057699
ENSG00000104804	TULP2	0	0	0	0,116488828	0	3,703361087	0,168040623	2,212731479	0	6,200622017	0,688958002
ENSG00000163623	NKX6-1	0	6,082287753	0	0	0,11636991	0	0	0	0	6,198657663	0,68873974
ENSG00000188536	HBA2	1,461770937	0	0	4,732798814	0	0	0	0	0	6,194569751	0,688285528
ENSG00000131899	LLGL1	0,068790852	0	0	0,118786787	0	1,309157813	0,228474049	0,651843608	3,807174584	6,184227692	0,68713641
ENSG00000151846	PABPC3	0,75845282	0,870847948	0,410363299	0,77040146	0,848225668	0,538829744	0,222268081	1,365835236	0,384093787	6,169318045	0,685479783
ENSG00000131398	KCNC3	0,072221079	0	1,107137562	2,99304097	0	0,660787704	0,93947835	0,210568467	0,177645234	6,160879366	0,684542152
ENSG00000134285	FKBP11	0,060968558	0,10818721	0,056078293	0,368477874	0	0,401639484	0,78466417	0,133320382	4,23656587	6,149901841	0,683322427
ENSG00000118276	B4GALT6	0	0	0,104779532	5,016088845	0,270725626	0	0	0,685032085	0,07005146	6,146677547	0,682964172
ENSG00000267281	ATF7-NPFF	0	0	0	5,723109108	0	0	0	0	0,414527559	6,137636667	0,68195963
ENSG00000079393	DUSP13	0,128550039	0,22810889	0	2,108789671	3,666023401	0	0	0	0	6,131472001	0,681274667
ENSG00000174428	GTF2IRD2B	0,089719799	0,199006976	0,330093631	0,735900923	0	0,591042577	0,856707032	2,010956994	1,29653949	6,109967422	0,678885269
ENSG00000171680	PLEKHG5	0,085700026	0	0	0	0,152750975	1,599591552	2,063598258	2,155109897	0,052699992	6,1094507	0,678827856
ENSG00000164604	GPR85	0,14301974	0,326295024	0,714118697	0,917294394	0,825445946	0,358919582	0,186610824	0,670162049	1,959982252	6,101848508	0,677983168
ENSG00000150967	ABCB9	1,227894186	0,34296998	0,020914912	1,177947369	0,027019603	1,522917038	0	0	1,775826373	6,095489462	0,677276607
ENSG00000120054	CPN1	0	0	0	0,138280326	0	0	0	5,953775991	0	6,092056317	0,676895146
ENSG00000003987	MTMR7	0	0	0	0,112991473	0	0,335269641	0,86930946	4,769552072	0	6,087122645	0,676346961
ENSG00000187260	WDR86	0,03736661	3,713143424	0	0	0	0	0	2,287875344	0,045956113	6,084341491	0,676037943
ENSG00000170476	MZB1	0	0	0	6,084273495	0	0	0	0	0	6,084273495	0,676030388
ENSG00000166405	RIC3	0	0,034850793	5,455549122	0,576539455	0	0	0,016307555	0	0	6,083246926	0,675916325
ENSG00000185674	LYG2	5,638454129	0,444679934	0	0	0	0	0	0	0	6,083134063	0,675903785
ENSG00000158887	MPZ	0	0	0	5,70415991	0	0	0,116716588	0	0,259320729	6,080197227	0,67557747
ENSG00000255274	SMIM35	0	0	0	4,846231951	0,095281916	0	0,099870256	0	1,035494022	6,076878145	0,675208683
ENSG00000108387	SEPT4	0	5,656015728	0	0,293112178	0	0,082831042	0	0,041242457	0	6,073201405	0,674800156
ENSG00000129226	CD68	0,139723135	3,223158577	0	0	0	0,460223514	0,870113259	0	1,374731556	6,067950042	0,674216671
ENSG00000213085	CFAP45	0,057522105	0	0	0,595968728	0	0	0	5,408711992	0	6,062202825	0,673578092
ENSG00000203872	C6orf163	0,051750195	5,877084933	0	0	0,12298546	0	0	0	0	6,051820589	0,67242451
ENSG00000071282	LMCD1	0,016743554	0	0	2,949071181	0	0,716954293	0	0	2,368127431	6,050896458	0,672321829
ENSG00000185513	L3MBTL1	0,05830633	0,017243859	0,464789876	0,083901921	0,046188751	0,096025414	1,137705261	4,143713226	0	6,047874638	0,671986071
ENSG00000141965	FEM1A	0,582830305	0,279518399	1,448869483	0,598412326	0,074870748	1,020402796	0,784761797	0,068890768	1,181761478	6,0403181	0,671146456
ENSG00000162723	SLAMF9	0	0	0	0	0	0	0	0	6,037156562	6,037156562	0,670795174
ENSG00000100767	PAPLN	0,019627298	0	0	3,660342545	0,093289399	2,090313799	0,146672683	0	0,024139047	6,034384772	0,670487197
ENSG00000137825	ITPKA	0	0	0	5,859504924	0,171580288	0	0	0	0	6,031085212	0,670120579
ENSG00000123570	RAB9B	0	0	0	5,18826169	0,285618406	0,043984776	0,49895413	0	0	6,016819003	0,668535445
ENSG00000198225	FKBP1C	0,478500857	0,509452818	0,704192778	2,974559558	0	0,210146392	0,15892379	0,627804751	0,353096622	6,016677568	0,66851973
ENSG00000115602	IL1RL1	0,052596788	0	0	4,450335453	0	0	0	0,057506886	1,45546354	6,015902667	0,66843363
ENSG00000197134	ZNF257	0,087718985	3,839494583	0,053788719	0	0	0	2,015100146	0	0	5,996102433	0,666233604
ENSG00000263961	RHEX	0	0	0	5,458256296	0	0	0,145810961	0,384002984	0	5,988070241	0,665341138
ENSG00000109654	TRIM2	0,032065904	0,085350239	0	0	0,038102657	0	0,38606256	0	5,442296037	5,983877396	0,664875266
ENSG00000148200	NR6A1	0,021208501	0,301071719	0	2,636816585	0	1,280713224	0,15848884	0,046376781	1,538939623	5,983615275	0,664846142
ENSG00000213066	FGFR1OP	0,03804874	0,050637417	0,769931031	0,082127337	0,022605914	4,156641667	0,221148766	0,020800363	0,62003435	5,981975584	0,664663954
ENSG00000106069	CHN2	0,017169705	1,310090445	0	0,385428323	0,040804175	1,602363256	0,869638706	0	1,752671441	5,97816605	0,664240672
ENSG00000109674	NEIL3	0,05185675	0	0	1,790905797	0	0	4,133545598	0	0	5,976308145	0,664034238
ENSG00000239282	CASTOR1	0	1,269426803	0	1,900473231	0	0,362514006	0,548304437	0	1,89501188	5,975730357	0,66397004
ENSG00000094661	OR1I1	0,024948088	0,088539541	0,13768207	0,38771913	5,276780794	0	0	0,054554163	0	5,970223787	0,663358199
ENSG00000239474	KLHL41	0,115527959	0	0,106261506	0	0,137277348	2,346594548	3,261461229	0	0	5,96712259	0,663013621
ENSG00000182809	CRIP2	3,498942302	0	0,402286684	0,053945622	0,074243858	0,960408548	0	0	0,960546425	5,95037344	0,661152604
ENSG00000165716	DIPK1B	0,044522855	0	0	5,61233151	0	0,293300961	0	0	0	5,950155326	0,66112837
ENSG00000181404	WASHC1	0,652463604	0,347334197	0,240051899	1,013996015	0,775296939	0,071636691	0,975158021	1,712096205	0,160489225	5,948522796	0,660946977
ENSG00000063169	BICRA	0,060387866	0	1,259001381	1,633667751	1,817832238	0,132604697	0,43455823	0,30811802	0,297077164	5,943247346	0,660360816
ENSG00000110042	DTX4	0,023489907	0	0,043211581	1,419667979	0	0	4,31042179	0	0,144447787	5,941239043	0,660137671
ENSG00000103449	SALL1	0,080093942	0	0	2,120672938	0	0,351753873	0	0	3,382012684	5,934533438	0,659392604
ENSG00000167554	ZNF610	0,85180705	0	2,193755009	0,220632729	0	0,046761718	2,616912223	0	0	5,92986873	0,658874303
ENSG00000259120	SMIM6	0	0	0	0	0	5,920220753	0	0	0	5,920220753	0,657802306
ENSG00000174136	RGMB	0,022651862	0,281366212	1,125088094	0,156459286	0,592159042	2,71087586	0,526633083	0,198131955	0,306447571	5,919812964	0,657756996
ENSG00000119943	PYROXD2	0	0	0	0,877659682	0,371661105	1,030233853	0,973896497	2,650316065	0	5,903767201	0,655974133
ENSG00000186231	KLHL32	0	0	0	0	0	0	0	0,080124774	5,813336899	5,893461673	0,654829075
ENSG00000102109	PCSK1N	3,049316448	0	0	0	0	0,867992554	0	1,975692481	0	5,893001484	0,654777943
ENSG00000164663	USP49	1,079262385	0	0	0	0	2,035712598	1,194846125	0,54462249	1,03805859	5,892502188	0,654722465
ENSG00000241945	PWP2	0	2,862167862	0,040646308	0,038153966	0	1,358530823	1,486050138	0,048316211	0,054349034	5,888214341	0,654246038
ENSG00000054392	HHAT	0	0,685991573	0,083665944	0,078535734	0,054043272	0,998709265	2,832287404	1,093989539	0,055935748	5,883158478	0,653684275
ENSG00000119919	NKX2-3	0	0	5,724685946	0	0	0	0	0,158254103	0	5,88294005	0,653660006
ENSG00000254122	PCDHGB7	0	0,05399557	0	0	0	0	3,08243667	2,728114633	0	5,864546873	0,651616319
ENSG00000151693	ASAP2	1,332654888	0	4,405086314	0,107868944	0	0	0,017289559	0	0	5,862899705	0,651433301
ENSG00000237172	B3GNT9	0	0	0	0,096571049	0	4,789424494	0,975158021	0	0	5,861153564	0,651239285
ENSG00000254598	CSNK2A3	0,533582524	0,189365819	0,588940888	1,105656672	0,760842251	0,585842432	0,708871932	0,466715491	0,918732808	5,858550816	0,650950091
ENSG00000142959	BEST4	0	0	0	4,674392459	0	0	0	0	1,183735811	5,85812827	0,650903141
ENSG00000183166	CALN1	0,060462367	0	0	5,794494785	0	0	0	0	0	5,854957152	0,650550795
ENSG00000124216	SNAI1	0	0	0	3,519943939	0	1,459623842	0	0	0,872007143	5,851574923	0,650174991
ENSG00000197497	ZNF665	0,039760708	0	0	0,240303226	1,133906199	0,414721873	0,643776207	0	3,374137623	5,846605836	0,649622871
ENSG00000198046	ZNF667	0,051456494	0	0	2,487915762	1,263637209	0,018832075	1,267520948	0	0,738323301	5,827685788	0,647520643
ENSG00000139182	CLSTN3	0,06568121	0,077699797	0,080550594	1,625645307	0,104061878	1,778815224	2,090566239	0	0	5,823020249	0,64700225
ENSG00000184661	CDCA2	0,040873836	0	0	0,070580193	0	5,519881237	0,101815255	0,089379109	0	5,82252963	0,646947737
ENSG00000079337	RAPGEF3	0,074893724	0,066448538	0	0,237096045	0	2,042018497	0,435301003	0	2,96285964	5,818617447	0,64651305
ENSG00000154175	ABI3BP	0,055059417	4,811803062	0,025321561	0,142613395	0,45797464	0,10579104	0,068575514	0,150498528	0	5,817637157	0,646404129
ENSG00000176809	LRRC37A3	0,553735896	2,842494701	0	1,570868843	0,046998762	0,130279212	0,049262007	0,3459595	0,267545052	5,807143973	0,645238219
ENSG00000197557	TTC30A	0	0	0	0	0	2,166311499	3,626057645	0	0	5,792369144	0,643596572
ENSG00000197275	RAD54B	0	1,512363227	2,351777487	0,392457191	0	0	0,235890829	0	1,292784607	5,78527334	0,642808149

ENSG00000266714	MYO15B	0,034581716	0,143183614	0,042410573	2,249267841	0,082184196	0,455624438	1,148557467	0,604960685	1,020745259	5,78151579	0,642390643
ENSG00000128656	CHN1	0	0,060270173	0	0,087975379	4,278091649	1,267916298	0,028201918	0,037135825	0,020886325	5,780477566	0,642275285
ENSG00000170145	SIK2	0	0,027529979	2,026343637	1,125181615	0	0,647289724	0,038645893	0,101776561	1,812671418	5,779438829	0,64215987
ENSG00000198478	SH3BGRL2	0,16414357	0,174761243	0,845474991	1,360512577	1,326308542	0,180220049	0,218067055	1,220375331	0,282625604	5,772488962	0,641387662
ENSG00000153822	KCNJ16	0,119172374	0,338349527	0,569990739	1,15239421	1,812580655	0,261688607	0,158322185	1,094499356	0,263819984	5,770817638	0,64120196
ENSG00000184371	CSF1	0	2,969117221	0	0,049815765	0,2742402	0,380092959	1,988170591	0	0,106441307	5,767878044	0,640875338
ENSG00000163611	SPICE1	1,408157943	0,5065017	0,49007951	1,642960424	0,045223214	0,146250351	0,221204464	0,873835275	0,421261479	5,75547436	0,639497151
ENSG00000155719	OTOA	0	0,07114407	1,770104106	0,346159425	0,047640958	1,188534245	0,699091794	0,175343363	1,454622554	5,752640516	0,63918228
ENSG00000110844	PRPF40B	0	0,028392842	0,294345721	0,055259419	2,09142999	0,404060066	0,943286062	0	1,928521726	5,745295827	0,638366203
ENSG00000112852	PCDHB2	0	5,653987884	0	0	0,087037698	0	0	0	0	5,741025582	0,637891731
ENSG00000105245	NUMBL	3,217879231	0	0	0,089622179	0	0,883260841	0,560231604	0,056746448	0,925562278	5,733302582	0,63703362
ENSG00000197245	FAM110D	0	0	0	5,686685763	0	0	0,044826842	0	0	5,731512605	0,636834734
ENSG00000173200	PARP15	0,027569891	0	0,608604486	0,095214368	0	1,119994771	3,479564033	0,060287277	0,339074115	5,73030894	0,636700993
ENSG00000156453	PCDH1	0	0,045424699	0	1,635540244	0	4,047284948	0	0	0	5,728249891	0,63647221
ENSG00000135862	LAMC1	0	0	4,212817034	1,51383066	0	0	0	0	0	5,726647694	0,636294188
ENSG00000115239	ASB3	2,135075534	0,468258771	0	1,698417838	0	0,579462246	0,517896274	0	0,324545443	5,723656106	0,63596179
ENSG00000183801	OLFML1	0,087957259	0	0	0,379707472	0,20903233	0	2,117950889	0	2,920754425	5,715402374	0,635044708
ENSG00000239382	ALKBH6	0,422391532	1,311665968	0,518015558	0,425470492	0	0,96616921	0,81834831	0,076970549	1,168846052	5,707877672	0,63420863
ENSG00000260286	ARMH2	0	0	1,024496679	1,282235747	2,20588171	0	0,154140457	0,811878544	0,228312681	5,706945819	0,634105091
ENSG00000186638	KIF24	0	0	0,045340091	2,511036291	0,117148114	0	0	0	3,031259538	5,704784034	0,633864893
ENSG00000132465	JCHAIN	0,091526693	0	0	0	0,217515168	0,100490961	0	0	5,290603602	5,700136424	0,633348492
ENSG00000183251	OR51B4	0	0	0	0	0	5,690394914	0	0	0	5,690394914	0,632266102
ENSG00000173762	CD7	0,275648615	1,076089749	0	1,332757988	0	1,089525859	0,823956941	0	1,08483924	5,682818392	0,631424266
ENSG00000076356	PLXNA2	0	0,0774697	0	0,414631234	0,129692142	3,115691581	0,842812631	0	1,10071613	5,68101342	0,631223713
ENSG00000162374	ELAVL4	0,03605597	0	0,132655738	0,280173559	0,085687793	1,484525561	0,98795537	0	2,660651715	5,667705706	0,629745078
ENSG00000163959	SLC51A	0,02871187	0	0	1,983165302	0	0,788098882	1,025123366	0	1,836218727	5,661318147	0,62903535
ENSG00000027869	SH2D2A	0	0	1,770573879	0,103875388	0	0,858613653	0,84912247	0	2,071537669	5,653723059	0,628191451
ENSG00000119715	ESRRB	0,127411948	0	0	4,048232321	0	0,027978175	0,507805728	0	0,94020163	5,651629803	0,627958867
ENSG00000180209	MYLPF	0	0	0	0,93274343	0	1,186134597	1,906162327	0,29529452	1,328661464	5,648996338	0,62766626
ENSG00000163806	SPDYA	0,13015553	0	0	4,495003869	0,309317435	0,714515834	0	0	0	5,648992669	0,627665852
ENSG00000117298	ECE1	4,259548106	0	0,342340023	0,071410776	0	0	0,017168901	0,271292748	0,686625082	5,648385637	0,627598404
ENSG00000171877	FRMD5	0,067742336	0,120207113	0	4,835017461	0,053663709	0,520639885	0	0,04937755	0	5,646648055	0,627405339
ENSG00000239620	PRR20G	0	0	5,641662525	0	0	0	0	0	0	5,641662525	0,626851392
ENSG00000100557	CCDC198	0	0	5,347072759	0,127068407	0	0	0	0,160912865	0	5,635054031	0,626117115
ENSG00000181754	AMIGO1	0	0	0,054071212	0	0	3,324020502	2,147712355	0	0,108449634	5,634253702	0,626028189
ENSG00000143416	SELENBP1	0	0	0,07997126	0	0	0	1,804809148	0	3,742593947	5,627374355	0,625263817
ENSG00000182310	SPACA6	0	0,261410675	0	0,381576858	0	0,404364067	1,559587359	2,013371725	0,996496098	5,616806782	0,624089642
ENSG00000115718	PROC	0,060565359	0	0	0	0	1,928419529	1,910970988	0	1,713214434	5,61317031	0,62368559
ENSG00000228672	PROB1	0	0	0	5,145340186	0	0,032391756	0	0	0,435407705	5,613139648	0,623682183
ENSG00000131470	PSMC3IP	0	0	0	0	0	2,192366735	2,048097141	0	1,372360639	5,612824514	0,623647168
ENSG00000175311	ANKS4B	0,034955024	0,1860805	4,115365796	0,724316376	0,249214117	0	0	0	0,300931218	5,610863031	0,623429226
ENSG00000269964	MEI4	0,028565325	0	0	0	0,067886114	3,857656131	0	0	1,651188383	5,605295954	0,622810662
ENSG00000175832	ETV4	0	0	5,456792839	0,148469402	0	0	0	0	0	5,605262242	0,622806916
ENSG00000116985	BMP8B	0,814105851	0	0	0	0	3,128442288	0,701968289	0,342348288	0,616150923	5,603015639	0,622557293
ENSG00000085276	MECOM	0,065274545	0,115828078	0,930602923	3,832306322	0	0,053750727	0,054198848	0,428208943	0,120418913	5,600589298	0,6222877
ENSG00000129535	NRL	0	2,584086091	0	0,083821065	0,115360598	2,504913943	0,201526393	0,106146665	0	5,595854755	0,621761639
ENSG00000152217	SETBP1	0,053871861	0,152950873	0,47568787	0,706989593	0,076816587	1,419555736	0,921457697	0,447647542	1,325108893	5,580086653	0,620009628
ENSG00000286261	AC022137.3	0	0	0	0,655286225	0	0,499981648	2,898862517	0,497892367	1,026776366	5,578799122	0,619866569
ENSG00000187961	KLHL17	0,044418157	0	0	0	0	2,243352889	1,143321661	1,651201543	0,491657466	5,573951716	0,619327968
ENSG00000119915	ELOVL3	0,215872244	0,19153	0	0,186382125	0	4,977317301	0	0	0	5,57110167	0,619011297
ENSG00000223609	HBD	0,13117237	0	0	5,436145304	0	0	0	0	0	5,567317675	0,618590853
ENSG00000121207	LRAT	0,721961585	0,071172396	0,036891851	0,034629725	0,095319852	0,286242534	0,116561689	0,175413175	4,020303003	5,558495809	0,617610645
ENSG00000249915	PDCD6	1,918554695	1,125192383	0	0,028075637	0,038639718	0	0,931509889	0,284428291	1,219780273	5,546180887	0,616242321
ENSG00000107282	APBA1	0	0	0	0	3,921524661	0,318863692	0,789190522	0	0,503297125	5,532876	0,614764
ENSG00000128266	GNAZ	0	0	0	0,212892283	0	0,045121179	0,068246033	0	5,205926515	5,53218601	0,614687334
ENSG00000113946	CLDN16	0	0,224511862	0,077583059	0,364129186	0	0	0,245127637	0	4,616338399	5,527690142	0,614187794
ENSG00000140538	NTRK3	1,095904699	2,937032001	0,041567118	0,634047602	0,013424955	0,396944931	0,079738155	0	0,319586543	5,518246004	0,613138445
ENSG00000196420	S100A5	0	0	0	2,662601782	0	0	0	0	2,844589627	5,507191408	0,611910156
ENSG00000162738	VANGL2	0,254014781	0	0	4,581226674	0,67074625	0	0	0	0	5,505987705	0,611776412
ENSG00000064989	CALCRL	1,674641705	0,036686551	0,038032576	3,534349772	0	0	0	0,090418544	0,127135396	5,501264544	0,611251616
ENSG00000091986	CCDC80	0	5,149081327	0	0	0,082423122	0,253860572	0,009599138	0	0	5,494964158	0,610551573
ENSG00000188163	FAM166A	0	4,019896725	0	0,434650152	0,199398969	0,829092277	0	0	0	5,483038123	0,609226458
ENSG00000196208	GREB1	0,048619875	0	0	0,020988978	5,372897593	0,013345445	0,01009253	0	0,014949045	5,480893466	0,608988163
ENSG00000166105	GLB1L3	0,101359801	0,029976747	0,06215318	0	5,259295478	0,018547857	0	0	0	5,471333063	0,607925896
ENSG00000187855	ASCL4	0	2,135644248	0,123000044	0	0	1,76188223	1,443463996	0	0	5,463990519	0,607110058
ENSG00000161243	AC010605.1	0	0	0	0	0	1,3677216	4,09240151	0	0	5,46012311	0,606680346
ENSG00000105141	CASP14	0	0	0	2,608582516	2,850977255	0	0	0	0	5,459559771	0,606617752
ENSG00000118492	ADGB	0	0,034505469	0,572343532	0,067156087	1,340163264	3,415994373	0,01614597	0	0	5,446308695	0,605145411
ENSG00000153896	ZNF599	0,088901645	0	0	0,153513737	0,105638342	1,000490314	3,026494256	0	1,066041237	5,44107953	0,604564392
ENSG00000122966	CIT	0,034193069	0	0	5,077781222	0,162521138	0	0,094637451	0	0,070088446	5,439221326	0,604357925
ENSG00000160972	PPP1R16A	1,451376002	0,594330255	0	0,144589014	0,132662557	0,98063141	0,115875815	0,061033352	1,956640519	5,437138924	0,604126547
ENSG00000085563	ABCB1	0,078079868	0	0	0	0	0	0	5,292871991	0,064018781	5,43497064	0,603885627
ENSG00000154258	ABCA9	0	0	5,08274782	0,144578333	0,119387481	0	0	0	0,082378778	5,429092411	0,60323249
ENSG00000139438	FAM222A	0	0,648382053	0	0	0	0,133726956	0	0	4,643666462	5,425775471	0,602863941
ENSG00000094755	GABRP	0,032700838	0	0	5,307917683	0,077714249	0	0	0	0	5,418332769	0,602036974
ENSG00000116151	MORN1	2,7990844	0,070955808	0,024519723	0,299210961	0,063353187	1,156120275	0,298817938	0	0,704895515	5,416957808	0,601884201
ENSG00000172476	RAB40A	0,188042024	0,200205575	1,037755472	0,389649041	1,251280635	0,371626478	0,281043551	1,644771366	0,04625349	5,410627633	0,601180848
ENSG00000189167	ZAR1L	5,410608668	0	0	0	0	0	0	0	0	5,410608668	0,601178741
ENSG00000165943	MOAP1	0	0,318080664	0,439668009	0,20635427	0,993998733	1,377667897	0,644964142	0,914607344	0,514403185	5,409744246	0,601082694
ENSG00000108551	RASD1	0	0,153399314	0	0,597105205	0	1,423717763	3,230071476	0	0	5,404293758	0,600477084
ENSG00000176887	SOX11	0,083931666	0,029786936	4,014376013	0,49276767	0,438823924	0,036860826	0,041814152	0,256947564	0	5,395308751	0,59947875
ENSG00000230054	TEX53	0,26557218	0	0	3,668681541	0	1,457913679	0	0	0	5,392167401	0,599129711
ENSG00000188958	UTS2B	0	0	0,072202623	0	0	2,930368627	0,032589697	0,08582716	2,268775726	5,389763834	0,598862648
ENSG00000116117	PARD3B	0,268327182	0	2,07835588	0,121932231	0,167811936	0,992362328	0,093809594	1,544086764	0,121581837	5,388267752	0,598696417
ENSG00000159723	AGRP	0	0	0	0	0,470049139	2,388764848	0,821140916	0	1,702782272	5,382737175	0,598081908
ENSG00000143882	ATP6V1C2	1,294493752	0,45940948	0	0	0	0,04737595	1,468955914	0,094355959	2,016609893	5,381200949	0,597911217
ENSG00000115598	IL1RL2	0	0	0	0	0,962781615	0,571886323	0	0	3,843627806	5,378295745	0,597588416
ENSG00000104879	CKM	0	0	0	0	0	0,853010677	4,515641769	0	0	5,368652446	0,596516938
ENSG00000188573	FBLL1	0	0	0	0,17178076	4,728341571	0	0,247801841	0,217534277	0	5,36545845	0,59616205
ENSG00000143847	PPFIA4	0,031134351	0,469600701	0	0,403216712	1,516824893	1,230613117	0,4911788	0,953143905	0,268038664	5,363751142	0,595972349
ENSG00000156096	UGT2B4	0	0	0	5,206204601	0,14330309	0	0	0	0	5,349507691	0,594389743
ENSG00000198400	NTRK1	0,034690214	0,554012397	0	0,059902428	4,699196491	0	0	0	0	5,347801529	0,59420017
ENSG00000160349	LCN1	0	0	0	5,345658891	0	0	0	0	0	5,345658891	0,593962099
ENSG00000105072	C19orf44	0	0	0	0	0,082784127	4,704241801	0,549547328	0	0	5,336573256	0,592952584
ENSG00000172738	TMEM217	0	0	0	0	5,252764052	0	0,074907661	0	0	5,327671713	0,591963524
ENSG00000174721	FGFBP3	4,928163994	0,105360314	0	0	0	0	0	0	0,292096695	5,325621004	0,591735667
ENSG00000174514	MFSD4A	0	0	0	0,180265958	0	0,200582735	0	2,054515446	2,888806046	5,324170185	0,591574465
ENSG00000075213	SEMA3A	0,016320399	0	4,263222168	0,281817663	0,232714683	0	0,284574681	0	0,240863837	5,319513432	0,591057048
ENSG00000183963	SMTN	0,01475401	0,052361258	0,027141193	0,076430865	0,070126448	0,048597123	2,486865592	0	2,540374255	5,316650744	0,590738972
ENSG00000163491	NEK10	0	0,027983928	0	1,797297884	0	0,796481583	1,348720956	1,344912141	0	5,315396491	0,59059961
ENSG00000162897	FCAMR	0	0,075724936	0	0	0	0	4,074862519	0	1,154652214	5,30523967	0,589471074
ENSG00000172575	RASGRP1	0,099007745	0	2,640924773	0,341929533	0	0	2,219622581	0	0	5,301484632	0,589053848
ENSG00000124003	MOGAT1	0	0	0	0,248983754	0	1,266492952	0	3,783601918	0	5,299078624	0,588786514
ENSG00000111981	ULBP1	0,094120567	0	0	0	0	2,428463751	2,774336211	0	0	5,296920529	0,588546725
ENSG00000157343	ARMC12	0	5,294764459	0	0	0	0	0	0	0	5,294764459	0,588307162
ENSG00000142606	MMEL1	0	0,346436693	0	0,67425058	2,691061687	0	0,162106372	0,939219701	0,480223572	5,293298605	0,588144289
ENSG00000143995	MEIS1	0,276202834	0,098023031	0,25404871	3,815536094	0,295380907	0,060650915	0,034400566	0,422782302	0,033969388	5,290994746	0,587888305
ENSG00000035664	DAPK2	3,252673906	0,039397884	0,020421694	0,651762352	0,026382423	0,170639735	0,082958639	0,04855048	0,996679116	5,289466229	0,58771847
ENSG00000158104	HPD	0	2,050568602	0	0,234759311	0	0,67170274	2,314117684	0	0	5,271148337	0,585683149
ENSG00000114841	DNAH1	0,010893207	0,386594579	0,721401641	0,790028037	0,051775886	2,356140687	0,009044862	0,404944323	0,522492385	5,253315608	0,583701734
ENSG00000092067	CEBPE	0	0	0	0	0	0	2,018505985	1,913713711	1,315515923	5,247735619	0,583081735
ENSG00000229859	PGA3	2,309091872	0	1,379143178	0	0	0,230477086	0	1,245933079	0,073763256	5,23840847	0,582045386
ENSG00000070019	GUCY2C	0	1,855432594	0	0,062260791	0	1,38555719	1,856158574	0,078843848	0	5,238252998	0,582028111
ENSG00000087085	ACHE	0,037091451	1,711264605	0,068232719	0	1,05778363	0,040724246	1,909465043	0	0,410559328	5,235121021	0,581680113
ENSG00000234906	APOC2	0	2,019141567	0	0,982435898	0	0,874528642	0,377922687	0	0,979612693	5,233641487	0,581515721
ENSG00000180574	EIF2S3B	0,49892058	0	0,114725588	0,215381737	0,444635835	2,465035395	0,155349152	0,954619056	0,383504998	5,232172341	0,581352482
ENSG00000142065	ZFP14	0	1,406039392	0	1,668595566	0	0,381940194	0,657920912	0	1,117123231	5,231619296	0,581291033

ENSG00000107485	GATA3	0	0	0	5,22484637	0	0	0	0	0	5,22484637	0,580538486
ENSG00000095464	PDE6C	0,04565274	0	0	0,078832319	5,099252112	0	0	0	0	5,223737171	0,580415241
ENSG00000059915	PSD	1,415318942	0,538167587	0,557912893	0,087283818	0,660694592	0,582726561	0,797436464	0,055265858	0,528414598	5,223221314	0,580357924
ENSG00000237412	PRSS56	3,16130901	0	0,116309665	0	0	0	0	0	1,944001279	5,221619954	0,580179995
ENSG00000117215	PLA2G2D	0,05636351	0,100015666	0	0,09732748	0,267898204	0	0	0,123250491	4,575110209	5,21996556	0,579996173
ENSG00000149599	DUSP15	0,079720691	1,980473754	0,073326325	0,137660235	0	1,487987235	1,456263415	0	0	5,215431656	0,579492406
ENSG00000162065	TBC1D24	1,088817671	0,746485548	0	1,495574242	0,029404531	0,271695041	0,123282075	0,514063439	0,943460606	5,212783155	0,579198128
ENSG00000175556	LONRF3	0	0	0,481529496	0,064571882	0,088868484	0,492681742	3,756968615	0,327081977	0	5,211702196	0,579078022
ENSG00000108932	SLC16A6	0	0	0,273804579	2,248885524	0	0	0	2,685136845	0	5,207826948	0,578647439
ENSG00000104435	STMN2	0	0	0,117688442	0,662832281	4,409148488	0	0	0	0	5,18966921	0,576629912
ENSG00000142611	PRDM16	0	0	0,085383464	5,0226042	0,073536919	0	0	0	0	5,181524583	0,575724954
ENSG00000049249	TNFRSF9	0,023868357	0	0,043907771	0,288508106	0	0	3,46822115	0	1,350330125	5,17483551	0,574981723
ENSG00000197279	ZNF165	0	0,213319014	0	1,245513005	0	2,441802442	0,848446955	0,262875551	0,147849262	5,159806228	0,573311803
ENSG00000107742	SPOCK2	0,04714103	0	3,989107644	1,098930637	0	0	0,019571102	0	0	5,154750412	0,572750046
ENSG00000155657	TTN	1,385671334	0,211852374	0,07476603	0,48688445	2,414722582	0,140843265	0,09069433	0,108315527	0,238993729	5,152743619	0,572527069
ENSG00000090447	TFAP4	0,288122354	0,511266048	0,035334956	2,38811722	0,091297202	0,168715471	0,095693656	1,554096733	0	5,132643641	0,570293738
ENSG00000275385	CCL18	0	4,222708663	0	0	0,25706338	0	0,179628249	0,473063089	0	5,132463381	0,570273709
ENSG00000172794	RAB37	0	0	0	5,093170837	0	0	0	0	0,036641664	5,129812502	0,569979167
ENSG00000164379	FOXQ1	0	0	5,118761687	0	0	0	0	0	0	5,118761687	0,568751299
ENSG00000169435	RASSF6	0	0	0,083855233	0,039356708	0,054165542	0	0	4,884251526	0,056062299	5,117691309	0,568632368
ENSG00000085465	OVGP1	0	0,31337203	0	0,304949328	0	0	4,496794135	0	0	5,115115492	0,568346166
ENSG00000159588	CCDC17	0,045474141	4,841564853	0	0	0,108070281	0,099855899	0	0	0	5,094965174	0,566107242
ENSG00000173013	CCDC96	0	0	0	0	0,171744401	4,919395388	0	0	0	5,091139789	0,565682199
ENSG00000257950	P2RX5-TAX1BP3	0,23031247	0	0	0,044188819	0,060815841	4,607846669	0,021248151	0	0,125890955	5,090302905	0,565589212
ENSG00000173662	TAS1R1	5,084400532	0	0	0	0	0	0	0	0	5,084400532	0,564933392
ENSG00000164509	IL31RA	0,071166674	0	0,087277896	0,040963104	2,931571774	0	0,019697069	1,815574547	0,116701113	5,082952178	0,564772464
ENSG00000095981	KCNK16	0	0	0,448354998	4,629491485	0	0	0	0	0	5,077846483	0,564205165
ENSG00000152784	PRDM8	0	0	1,938832425	1,421833994	0	0,18080926	0,382865006	1,15234374	0	5,076684426	0,564076047
ENSG00000155034	FBXL18	0,27888755	0,054986568	0,057004019	0,294297623	1,178279643	2,619730218	0,128647936	0,406563602	0,057165956	5,075563114	0,563951457
ENSG00000157570	TSPAN18	0	0	0,142043995	4,933364364	0	0	0	0	0	5,075408359	0,563934262
ENSG00000058085	LAMC2	0	0	0,084970227	4,985002571	0	0	0	0	0	5,069972799	0,563330311
ENSG00000164050	PLXNB1	0,01546996	0	0	3,659714529	0	1,392779207	0	0	0	5,067963696	0,563107077
ENSG00000172927	MYEOV	0	0	0	0,139276741	0	2,61241634	2,310502511	0	0	5,062195592	0,562466177
ENSG00000011114	BTBD7	0,040001033	0,165621989	0	1,082144517	0	0,585583964	0,60891805	0,379039034	2,197426452	5,05873504	0,562081671
ENSG00000165383	LRRC18	0	0	0	0	0	0	0	0	5,057048225	5,057048225	0,561894247
ENSG00000169900	PYDC1	0	0	0	4,848965846	0	0,192695211	0	0	0	5,041661057	0,560184562
ENSG00000133433	GSTT2B	1,787477178	0	0	0	0	2,566405645	0,342503181	0	0,338210232	5,034596236	0,559399582
ENSG00000102890	ELMO3	0	4,165312623	0,107953436	0	0	0	0,243631713	0,513296416	0	5,030194187	0,558910465
ENSG00000168005	SPINDOC	0,772944095	0	0	0,667352876	0	0,666794426	1,558637665	0	1,358030853	5,023759915	0,558195546
ENSG00000146006	LRRTM2	0,024784414	0	0	0,213986366	3,416240357	1,306168227	0	0	0,060963268	5,022142632	0,558015848
ENSG00000251192	ZNF674	0,024360885	0	0	0,084131863	1,447354274	0,641923882	1,597961668	1,225212809	0	5,020945381	0,55788282
ENSG00000260861	AL049634.2	4,004489012	0	0	0	0	0	0	0	1,013971673	5,018460685	0,557606743
ENSG00000162391	FAM151A	0	0	0	3,121854133	0	0,496243001	0,750570281	0	0,648517276	5,017184691	0,557464966
ENSG00000151640	DPYSL4	0	0	0	0	4,732825213	0	0,28231833	0	0	5,015143542	0,557238171
ENSG00000179023	KLHDC7A	0	0,052117007	4,808596464	0,152148673	0	0	0	0	0	5,012862144	0,556984683
ENSG00000178201	VN1R1	0	0	1,299524475	0	0	0	3,572678387	0,14043118	0	5,012634042	0,556959338
ENSG00000286190	AC055839.2	0	0	4,783923031	0	0	0	0	0	0,228453008	5,012376039	0,556930671
ENSG00000141469	SLC14A1	0,042080359	0	0	0,072663596	4,850236927	0	0	0,046008712	0	5,010989594	0,556776622
ENSG00000144821	MYH15	0,412441121	0,592463478	1,445178575	0,576539455	0,046675012	0,043127262	0	1,889669999	0	5,006094902	0,556232767
ENSG00000121903	ZSCAN20	0	0	0,523700262	2,064670108	0,135311809	2,28173928	0	0	0	5,00542146	0,55615794
ENSG00000170379	TCAF2	4,385483873	0	0	0,244283016	0	0	0,313235343	0	0,057995482	5,000997714	0,555666413
ENSG00000214491	SEC14L6	0,133647321	0	0,16390336	0	0	4,597757727	0	0,097415851	0	4,992724259	0,55474714
ENSG00000151150	ANK3	0,017598964	0,03122893	0,053957859	0,384934549	0,01394144	1,10139014	0,949831699	2,424478173	0,014429638	4,991791391	0,554643488
ENSG00000213347	MXD3	0,791615329	0	0	0,202510652	0,348387216	0,836956732	0,559918051	1,346357376	0,901467415	4,987212771	0,554134752
ENSG00000078098	FAP	0	0,038481917	0,239362887	4,493713684	0,154614327	0	0	0	0,053342859	4,979515674	0,553279519
ENSG00000180592	SKIDA1	0,045107633	0,040021194	1,286176577	0	2,894380192	0,569543771	0,03745383	0,098637184	0	4,971320382	0,552368931
ENSG00000205076	LGALS7	0	0	0	0	4,96892197	0	0	0	0	4,96892197	0,552102441
ENSG00000175711	B3GNTL1	0,021928685	0	0	0	0	1,011209436	1,329173449	0,959032267	1,645136825	4,966480661	0,551831185
ENSG00000269526	ERVV-1	0,137248475	0	0	4,739963208	0	0	0,056980169	0	0	4,934191852	0,548243539
ENSG00000170485	NPAS2	0	2,744895714	0	0,062604361	0,028720213	0,053074401	0,010034416	2,034825792	0	4,934154896	0,548239433
ENSG00000131044	TTLL9	0	0	0,04501702	4,732747717	0	0,080604329	0,020319082	0,053511671	0	4,932199819	0,548022202
ENSG00000161594	KLHL10	0	0,113140084	0	0,110099145	0	4,690299004	0	0	0	4,913538234	0,545948693
ENSG00000270149	AL591806.3	0,142961751	0	0	2,221773672	2,208386022	0	0	0,156307742	0,175824524	4,905253712	0,54502819
ENSG00000277957	SENP3-EIF4A1	0	1,828760444	0	2,743561824	0	0,047147081	0,28524088	0	0	4,904710229	0,544967803
ENSG00000119703	ZC2HC1C	0,033461154	0	0	0,05778011	0,079521156	0	0	0	4,732584159	4,903346579	0,544816287
ENSG00000273173	SNURF	0	0	0	0	4,890111215	0	0	0	0	4,890111215	0,543345691
ENSG00000188026	RILPL1	1,533586693	0,185543997	0,032058598	0,601856705	0	0,306143378	0,969497433	1,257564379	0	4,886251183	0,542916798
ENSG00000158286	RNF207	0	0	0,042150129	0,079131152	0,108906002	0,025157025	0,019025069	4,609549691	0	4,883919068	0,542657674
ENSG00000008710	PKD1	0,008154035	0,926024606	0,195000069	1,239060963	0,01937824	1,163844969	0,609342212	0,481423123	0,240681876	4,882910095	0,542545566
ENSG00000184226	PCDH9	0,053716917	0,025996187	0,161699902	0,801086562	0,417794452	0,00536164	0,012164264	1,751269037	1,651622079	4,880711041	0,542301227
ENSG00000155961	RAB39B	0,044274999	0	0	0,152906519	1,999189366	0	2,683660778	0	0	4,880031661	0,54222574
ENSG00000198690	FAN1	0	0,519487246	0	0	0	1,874998445	0,44564858	1,707120788	0,330047101	4,87730216	0,541922462
ENSG00000162975	KCNF1	0	0	0	0	0	2,605925289	0	0	2,270376363	4,876301651	0,541811295
ENSG00000132016	C19orf57	0	0	0,072390651	0,54361453	0	0	0	0	4,258982802	4,874987983	0,541665331
ENSG00000163810	TGM4	0,027762368	0	0,051071119	4,793955073	0	0	0	0	0	4,87278856	0,541420951
ENSG00000163581	SLC2A2	0	0	0	4,819379478	0	0,048639864	0	0	0	4,868019342	0,540891038
ENSG00000137310	TCF19	0	0,834860459	0	0	0	1,643608883	0,63924879	0	1,735900151	4,853618284	0,53929092
ENSG00000196391	ZNF774	0	0,031505346	0	0,061317113	0,042194518	0,799240236	0,840301836	0	3,078881405	4,853440454	0,539271162
ENSG00000081985	IL12RB2	0	0	0	4,778596641	0	0	0	0,074708245	0	4,853304886	0,539256098
ENSG00000154654	NCAM2	0	0	0,067096331	0	3,163840284	0,440505997	0,015142449	1,116602449	0,044857959	4,848045468	0,538671719
ENSG00000070778	AL162171.1	0,019701509	4,439900132	0	0,034020205	0	0,216311052	0,016358583	0	0,121151579	4,84744306	0,538604784
ENSG00000254788	CKLF-CMTM1	0,855544632	0	0	0	0	0,587086259	2,219928025	0	1,183735811	4,846294727	0,538477192
ENSG00000242612	DECR2	0,752917642	0	0,069252641	1,430136881	0,178932507	0,950658511	0,50013087	0,08232052	0,879692059	4,844041631	0,538226848
ENSG00000125170	DOK4	0	0,056026327	0	1,417532248	0,075035007	1,97595129	1,310805809	0	0	4,83535068	0,537261187
ENSG00000165272	AQP3	0	0	0	2,420214467	2,343948232	0,056994358	0	0	0	4,821157058	0,535684118
ENSG00000189157	FAM47E	0	0,028192829	0	0	3,775812663	0,174440728	0	0	0,840227154	4,818673374	0,535408153
ENSG00000176406	RIMS2	0,033286711	0,039377634	1,979886159	1,68604727	0,052737726	0	0,027638666	0,994773278	0	4,813747443	0,534860827
ENSG00000080572	PIH1D3	0	0	0	0	0	0	4,808288228	0	0	4,808288228	0,534254248
ENSG00000182612	TSPAN10	0	0	0,234335173	0	0	0	0,528852822	1,81060037	2,232508276	4,806296641	0,53403296
ENSG00000197272	IL27	0	0	0,218881968	0	0	4,572338728	0	0	0	4,791220696	0,532357855
ENSG00000138829	FBN2	0	0	2,439528785	2,092533463	0,054337652	0	0,018984768	0,174991827	0	4,780376495	0,531152944
ENSG00000095397	WHRN	0	0,43365283	2,547526514	0,187554339	0	0,327945435	0,112731655	0,296886404	0,868285639	4,774582815	0,530509202
ENSG00000167615	LENG8	0,116795927	0,165801206	0,300797757	1,613448599	0	0,538587434	0,8340121	0,970514265	0,229830295	4,769787584	0,529976398
ENSG00000080644	CHRNA3	0	0,056320521	0	4,713381184	0	0	0	0	0	4,769701705	0,529966856
ENSG00000121454	LHX4	0,02483329	0,220330321	0	0,428816721	0	0	0,061858833	0	4,031510426	4,76734959	0,52970551
ENSG00000088899	LZTS3	3,921312306	0	0	0,048366075	0	0	0	0	0,792302134	4,761980515	0,529108946
ENSG00000149534	MS4A2	1,42493129	0	0,062411338	3,163558291	0,080628097	0	0,028170259	0	0	4,759699275	0,528855475
ENSG00000234465	PINLYP	0	0	0,425883277	0,057109865	0,078598718	0,79886905	1,949746897	1,446419643	0	4,75662745	0,528514161
ENSG00000091129	NRCAM	0,055331589	0,032728183	0,033928976	0,095545578	0	0	1,975548984	0,362981949	2,200306684	4,756371943	0,528485771
ENSG00000179855	GIPC3	0	0	0,062848768	0	0,081193204	0	0	3,511287514	1,092473339	4,747802825	0,527533647
ENSG00000187658	C5orf52	0,209584703	0	0,385547987	1,085721115	0	0,230111757	0	0	2,835383835	4,746349397	0,527372155
ENSG00000091136	LAMB1	0,037440677	0,033218781	0	0,096977815	0,133467868	0,020553838	0	4,421076969	0	4,742735947	0,526970661
ENSG00000122035	RASL11A	0,186096762	0	0,342340023	0,642696982	0,221131492	0,408646741	2,08602153	0,610408684	0,228875027	4,72621724	0,525135249
ENSG00000187122	SLIT1	0,033058537	0,029330781	0,030406924	1,341494619	0,078564328	0	0	0	3,211961334	4,724816523	0,524979614
ENSG00000007968	E2F2	0	0,098184548	0,203573855	0,286636735	0,06574836	0,911262779	2,710636978	0	0,442329694	4,71837295	0,524263661
ENSG00000029534	ANK1	0	0,372522923	0	0,193338872	1,962390942	0,414891689	0	0	1,772917643	4,716062069	0,524006897
ENSG00000165215	CLDN3	0	0,631417583	0	0	0	0	0	0	4,084538951	4,715956534	0,52399517
ENSG00000013619	MAMLD1	0	0,045005371	0	0	0,241099391	0,083540069	0,252709631	0,942831092	3,150469746	4,715655301	0,5239617
ENSG00000116544	DLGAP3	0	0,796067294	0	0,77467088	0	0,820933086	0,807082789	0,817502641	0,689682789	4,705939478	0,522882164
ENSG00000095739	BAMBI	0	0	0	0,121989235	0	0	2,756945116	0	1,824580163	4,703514514	0,522612724
ENSG00000198835	GJC2	0	0	0	4,70044527	0	0	0	0	0	4,70044527	0,522271697
ENSG00000168917	SLC35G2	0,070562956	0,062606117	0	1,035698008	0	0,154948005	0	0,154300522	3,210983574	4,689099183	0,52101102
ENSG00000196689	TRPV1	0,082140191	2,234921544	0	0,189117575	0	1,172406684	0	0	1,010218458	4,688804453	0,520978273
ENSG00000086848	ALG9	1,909947733	0,398724164	0	0,517343196	0,489503465	0,390619697	0,155477487	0,040946043	0,7829965	4,685558286	0,520617587
ENSG00000184459	BPIFC	0	0	0	0	4,674330668	0	0	0	0	4,674330668	0,519370074
ENSG00000081138	CDH7	0	0,295310573	0,91843645	0,107764995	3,337064167	0	0	0	0,012792299	4,671368484	0,519040943
ENSG00000225526	MKRN2OS	0,082619229	0,586423182	0	3,994630557	0	0	0	0	0	4,663672969	0,518185885

ENSG00000006432	MAP3K9	0,012200111	1,039142258	0,044886178	0,231736212	0	0,361665233	1,266251534	1,707396441	0	4,663277966	0,518141996
ENSG00000100336	APOL4	0	0	0	0,119230055	0	0	0	0	4,543198054	4,662428109	0,518047568
ENSG00000113327	GABRG2	0,14508303	0,023404207	0	0,785742919	3,667343323	0,014481154	0	0,014420641	0,00811061	4,658585884	0,517620654
ENSG00000133401	PDZD2	0,009490678	0,050522924	0,523766045	0,016388329	4,014754587	0,01042021	0	0,020753333	0,01167231	4,657768416	0,517529824
ENSG00000158055	GRHL3	0	0,612507107	0,211659974	2,251723131	0,911465844	0	0	0,191071572	0	0,471691681	4,650119308	0,516679923
ENSG00000215545	DEFB116	0	0	0	0	4,648770775	0	0	0	0	4,648770775	0,516530086
ENSG00000122641	INHBA	0,072965027	0,032368662	0,100668795	0	0	1,201670039	0,969350706	2,153967485	0,112171971	4,643162686	0,515906965
ENSG00000090889	KIF4A	0	0,117271813	0,060787251	0,228239645	0	0,798170279	1,920604251	1,517412184	0	4,642485424	0,515831714
ENSG00000189184	PCDH18	0	0,120314987	0	0,039027068	0,214847468	0	3,077644997	1,186124681	0	4,6379592	0,5153288
ENSG00000158560	DYNC1I1	0,066160495	0,23480035	0	2,798996006	1,100622941	0	0	0,434020886	0	4,634600678	0,514955631
ENSG00000166448	TMEM130	0	0	0	0,167875815	0	0	0	4,464374384	0	4,632250199	0,514694467
ENSG00000283761	AC118553.2	0	0,465120555	0	0	3,612977876	0,546799819	0	0	0	4,624898251	0,513877583
ENSG00000139988	RDH12	0	0	0	0	0	0,070420449	0,213022635	0	4,338522379	4,621965463	0,513551718
ENSG00000104885	DOT1L	0,026307551	0,070023155	0,048394864	0,068141097	0,187561391	2,455153033	0,065531162	1,150538188	0,550033231	4,621683671	0,513520408
ENSG00000127129	EDN2	0	0	0,674299816	1,424144798	0	2,515321054	0	0	0	4,613765667	0,51264063
ENSG00000125726	CD70	0	0	0	0,68218294	0	3,686901705	0	0	0,24293663	4,612021275	0,512446808
ENSG00000181588	MEX3D	0	0,188169825	0	1,92267876	0,504024621	0,873213562	0,528296135	0,463767814	0,130418612	4,610569328	0,512285481
ENSG00000075673	ATP12A	0,034018589	0,120730302	0	0,176228033	4,203987439	0	0	0,074388691	0	4,609353054	0,512150339
ENSG00000135314	KHDC1	0	0	2,821290557	0	0	1,78291963	0	0	0	4,604210187	0,51157891
ENSG00000268696	ZNF723	0	0	0	3,981569095	0	0	0,615385825	0	0	4,596954919	0,510772769
ENSG00000152056	AP1S3	0,45737183	0	0,747886596	1,40405569	0	0,362676559	1,371375749	0	0,250003729	4,593370153	0,510374461
ENSG00000285162	AC004593.3	0	0,371755595	1,926976325	0	0	0,361460951	1,341928684	0,589009924	0	4,59113148	0,51012572
ENSG00000183347	GBP6	0,050589411	0	0,093063307	0,087356871	0	0	0	0	4,355291784	4,586301374	0,509589042
ENSG00000077522	ACTN2	0	0	0,045863735	0	4,503041283	0	0	0	0,030662683	4,579567702	0,508840856
ENSG00000285749	AC097636.2	0	0	0	0	4,438314602	0,089151304	0	0	0,049931897	4,577397803	0,508599756
ENSG00000113249	HAVCR1	0	0	0	0,121308682	0	2,23682321	2,216584201	0	0	4,574716093	0,508301788
ENSG00000100490	CDKL1	0	0	2,350783078	0,424353512	0	0	1,040654892	0,752331102	0	4,568122584	0,507569176
ENSG00000176490	DIRAS1	1,318207944	2,183185811	0,080831666	0	0	0,048243843	0,839144413	0,096084492	0	4,565698169	0,507299797
ENSG00000147231	RADX	0	4,312386806	0	0,107602051	0	0	0,103480686	0	0,038318871	4,561788414	0,506865379
ENSG00000185437	SH3BGR	0	1,614232776	0,185939866	0	0	0,443907898	0,755339791	1,547185266	0	4,546605597	0,5051784
ENSG00000110841	PPFIBP1	0,012926482	0,068813174	0,451806833	1,294630327	0,061440127	0,411583124	1,846098982	0,11306572	0,28616231	4,54652708	0,505169676
ENSG00000196866	HIST1H2AD	0	0	0	0	0	4,221643173	0,319262931	0	0	4,540906104	0,504545123
ENSG00000166840	GLYATL1	0	0,049290809	1,890673467	0	0	2,439861422	0,092257597	0	0,068325927	4,540409223	0,504489914
ENSG00000145949	MYLK4	0,024939853	0	0	0,818247981	0	0	3,665332359	0	0,030672804	4,539192998	0,504354778
ENSG00000183850	ZNF730	0,046595921	0	0	1,931063642	0	2,557979906	0	0	0	4,535639468	0,503959941
ENSG00000095637	SORBS1	0,06899396	0	0	0,500377542	0,065586256	0	0,011457431	0	3,886298384	4,532713573	0,503634841
ENSG00000124713	GNMT	0,140437333	0	0	0	0	0,308383972	4,081284033	0	0	4,530105338	0,503345038
ENSG00000152253	SPC25	0	0	0	0	0	2,539910414	1,989414777	0	0	4,529325192	0,503258355
ENSG00000129167	TPH1	0,026859326	0	1,334067308	2,968332452	0	0	0	0	0,198201055	4,527460141	0,503051127
ENSG00000235272	RAMACL	0,455380926	0,538708187	0,139618332	0	0,180370438	0,249990824	2,961881268	0	0	4,525949975	0,502883331
ENSG00000197168	NEK5	0,23560826	0	0,078803713	4,031454604	0	0	0	0,046836934	0,131712631	4,524416143	0,502712905
ENSG00000165695	AK8	0,055331589	0	0	0,382182314	0	0	0	0	4,083043331	4,520557234	0,502284137
ENSG00000198517	MAFK	0,236638478	1,439688591	0,559691474	0,291873573	0	0,816562122	0,926291042	0	0,249458419	4,5202037	0,502244856
ENSG00000105852	PON3	0,110058682	0	0	0,063349108	0,087185614	2,900112047	0	0	1,353579915	4,514285365	0,501587263
ENSG00000215784	FAM72D	1,24730145	0	0	2,046126503	0,148211945	0,479312438	0,051783051	0	0,536906998	4,509642385	0,501071376
ENSG00000275663	HIST1H4G	0	0	1,871919858	2,635706814	0	0	0	0	0	4,507626673	0,500847408
ENSG00000198467	TPM2	0	0	0	0	0	0	0	0,101734781	4,405844278	4,507579059	0,500842118
ENSG00000116819	TFAP2E	0	0	0	1,263329543	0	1,752576514	1,491064685	0	0	4,506970742	0,500774527
ENSG00000105767	CADM4	0	2,138482943	0	0	0	1,543696106	0	0	0,823422784	4,505601832	0,500622426
ENSG00000046774	MAGEC2	0	0	0	0	4,50247671	0	0	0	0	4,50247671	0,50027519
ENSG00000169184	MN1	0	0,202345869	0,069923305	2,264433813	0,496829703	0	0	0,041558869	1,425812836	4,500904394	0,500100488
ENSG00000158786	PLA2G2F	0,023464374	0,083273913	0,043164612	3,565571082	0	0	0,681903978	0,102619431	0	4,49999739	0,49999971
ENSG00000180138	CSNK1A1L	0,062805723	0,111447215	3,235013621	0,32535533	0,298518323	0	0,104297865	0,274675451	0,077242944	4,489356472	0,498817386
ENSG00000140057	AK7	0	0	0	0	2,017514283	0,066577278	2,366414769	0	0,037288628	4,487794958	0,498643884
ENSG00000141639	MAPK4	0,079594717	0,035309718	0	0,068721352	4,303357434	0	0	0	0	4,486983222	0,498553691
ENSG00000127329	PTPRB	0,020782639	0,055317425	0,057347015	1,919958897	0,024695196	0,901315882	1,018119872	0	0,485639379	4,483176306	0,498130701
ENSG00000166289	PLEKHF1	0	0	0,120966101	0	0	1,443956281	0	0	2,911433769	4,476356151	0,497372906
ENSG00000157992	KRTCAP3	0,21541065	0	0	0	0,255964036	0	0	4,003840091	0	4,475214777	0,497246086
ENSG00000134827	TCN1	0	0	0	0	0	0	4,349638176	0	0,123897682	4,473535858	0,49705954
ENSG00000181963	OR52K2	0	0	0	0	0	4,04280693	0,117591689	0,30968563	0	4,470084249	0,496676028
ENSG00000184792	OSBP2	0	0	0,041914973	0,039344839	0	0,200133386	4,010812784	0,149472814	0,028022696	4,469701492	0,496633499
ENSG00000133466	C1QTNF6	0,021152096	0	0	0,949651363	0	0	3,495044242	0	0	4,465847701	0,4962053
ENSG00000146904	EPHA1	0,321717204	0	0	1,777713261	0,07645679	0,176613345	0,213702929	0	1,899219304	4,465422833	0,496158093
ENSG00000104524	PYCR3	1,378257042	0	1,619848563	0,132219394	0	0,210173013	0,699353257	0	0,423769623	4,463620891	0,495957877
ENSG00000171817	ZNF540	0	2,38429959	0	0,35695619	0,081878145	0	0,743782178	0	0,88982603	4,456742132	0,49519357
ENSG00000109472	CPE	0,872071264	0	0	0,167319637	0	0	3,298674968	0,105942471	0	4,44400834	0,493778704
ENSG00000167664	TMIGD2	0	0	0	0,206763054	0	0,65733192	3,579185382	0	0	4,443280357	0,493697817
ENSG00000171097	KYAT1	0,12645236	0,299182148	1,783414861	1,382918973	0	0,231395504	0,244990885	0	0,362880239	4,431234971	0,492359441
ENSG00000196739	COL27A1	0,064710829	0,076551852	1,448329686	1,508509952	0,051262229	0,047365806	0,21492313	0,471678777	0,543837514	4,427169775	0,491907753
ENSG00000151575	TEX9	0	1,843678408	0	0,125903486	0	0,780520204	1,634595399	0,039859417	0	4,424556914	0,491617435
ENSG00000244020	MT1HL1	2,390990042	0	0	0	0	0	0,397058013	1,045679011	0,588121906	4,421848972	0,491316552
ENSG00000264006	AKR1C8P	0	0	0	0,223498908	0,153797663	0	0	3,962384536	0,079591658	4,419272764	0,491030307
ENSG00000131059	BPIFA3	0	0	0	4,417278893	0	0	0	0	0	4,417278893	0,490808766
ENSG00000160408	ST6GALNAC6	0,028473821	0	0,052379894	2,851748356	0	0	0,047284843	0	1,435784325	4,415671241	0,490630138
ENSG00000002726	AOC1	0	0	0	0	0	2,08255954	0	0	2,332801535	4,415361075	0,490595675
ENSG00000157734	SNX22	0,078607892	0,046495925	0,192807421	0,497708466	0	0,287689573	0,543915086	2,349196683	0,418936152	4,415357197	0,490595244
ENSG00000179532	DNHD1	0,023094998	0,368833563	0	0,372213525	0	0,65928091	0,786227054	1,043707942	1,155090719	4,40844871	0,489827634
ENSG00000173210	ABLIM3	0	0,111339239	0	0,433386809	0,596458202	1,814108728	1,406657009	0,045734888	0	4,407684875	0,489742764
ENSG00000063127	SLC6A16	0,041986822	0	0	0,145004154	0	0	0,139450216	0	4,079431859	4,40587305	0,48954145
ENSG00000188157	AGRN	2,092321813	0,598030666	0,749133247	0,048496418	0,06674427	0,616710628	0,233194558	0	0	4,4046316	0,489403511
ENSG00000179071	CCDC89	0	0	0	0	0,305892285	4,09830215	0	0	0	4,404194435	0,489354937
ENSG00000174705	SH3PXD2B	0	0	0	3,386710612	0,261366479	0,462875214	0,136976345	0,080163651	0,067629739	4,395722039	0,48841356
ENSG00000176083	ZNF683	0	0	0,116847456	4,277622534	0	0	0	0	0	4,394469991	0,488274443
ENSG00000148848	ADAM12	0,015325616	0,027194929	0	0,026463993	0,036421657	1,985542399	0,050900743	0	2,242975269	4,384824606	0,487202734
ENSG00000139344	AMDHD1	0	0	0	0	0	4,37923845	0	0	0	4,37923845	0,48658205
ENSG00000123388	HOXC11	0	0,082126187	0	0	0	2,744003413	1,037580081	0	0,512287504	4,375997184	0,486221909
ENSG00000279765	AC013394.1	0,031920273	0,679700887	1,350558149	0,82679016	0	0,385512536	0,609593924	0,209400867	0,274804744	4,368281539	0,485364615
ENSG00000257987	TEX49	0	0	0	4,362134834	0	0	0	0	0	4,362134834	0,484681648
ENSG00000178386	AC092072.1	1,506540433	0	0	0,945987823	0	0,801984661	0,037906445	0	1,066792726	4,359212087	0,484356899
ENSG00000134569	LRP4	0,017273728	0	0	0,626387113	0	1,327588062	0	0,113317753	2,273157051	4,357723707	0,484191523
ENSG00000144642	RBMS3	0	1,140469819	0,045473597	0,042685257	0,137075238	0	0,006841722	2,900919645	0,081071606	4,354536883	0,483837431
ENSG00000069188	SDK2	0	0,067242832	0,023236655	0,523484025	0	1,386864304	0,346110587	0	2,004029204	4,350967608	0,483440845
ENSG00000172426	RSPH9	0	0	0	2,074047868	0	1,318744175	0,332434642	0,625349295	0	4,35057598	0,483397331
ENSG00000164825	DEFB1	0	0	0	0	0	0	4,349638176	0	0	4,349638176	0,483293131
ENSG00000130032	PRRG3	0,093711982	0,083144806	0,818856106	0,970920836	1,057865629	0,15433542	0,155622117	0,870912814	0,144067072	4,34943678	0,483270753
ENSG00000196967	ZNF585A	0,154509786	0,099699572	0,723502767	0,412334502	0,033381441	2,143667054	0,069977876	0,122860966	0,587356549	4,347290513	0,483032279
ENSG00000214029	ZNF891	0,009689681	0,171941007	0,623873258	1,20470139	0,759915287	0	0,587325055	0,40258139	0,583935851	4,343962919	0,482662547
ENSG00000178026	LRRC75B	0	0	0	0	0	0	3,66657896	0,67593278	0	4,34251174	0,482501304
ENSG00000176909	MAMSTR	0	2,815139109	0	0	0	1,524112909	0	0	0	4,339252018	0,482139113
ENSG00000181666	ZNF875	0	0,475105084	0	0,924670756	0,048946101	1,130643156	1,62459882	0,135110223	0	4,339074139	0,482119349
ENSG00000127564	PKMYT1	0	0	0	1,206575074	0	0,366911207	0	0	2,764906041	4,338392322	0,482043591
ENSG00000177599	ZNF491	0	0	0	0	0,104122222	2,838133959	0	1,341282675	0,053884176	4,337423031	0,481935892
ENSG00000007171	NOS2	1,348892711	0	0	0,776414436	0,082196737	0	0	0,075631625	2,04180203	4,324937538	0,480548615
ENSG00000161914	ZNF653	1,174312797	0	0	0	0	0,928315319	1,872109399	0	0,346620806	4,32135832	0,480150924
ENSG00000092051	JPH4	0	0	0,05247878	0	0	1,534758969	0,450054051	0	2,280540674	4,317832474	0,479759164
ENSG00000196440	ARMCX4	0,033954888	1,164874242	1,540748058	0,273619178	0,161389054	0,782890145	0,187956456	0	0,167040541	4,312472562	0,479163618
ENSG00000120279	MYCT1	0,049871475	0	0	4,219740513	0	0	0,04140935	0	0	4,311021339	0,479002371
ENSG00000152595	MEPE	0	0	3,927857676	0	0	0,200941352	0,101308302	0	0,075028875	4,305136205	0,478348467
ENSG00000206140	TMEM191C	1,827808717	0,06237311	0,452631006	0,303483339	0,918886477	0,694670942	0	0	0,043230175	4,303083766	0,478120418
ENSG00000159496	RGL4	0	0	0	0	0	0	0,026610887	4,274975317	0	4,301586204	0,477954023
ENSG00000166016	ABTB2	0,06073576	0	0,055864168	0,471948306	0,072169924	0,733527469	2,092853207	0,066405661	0,746971553	4,300476047	0,477830672
ENSG00000186340	THBS2	0,023566839	3,889146289	0	0,325558296	0,056007103	0	0	0	0	4,294278527	0,477142059
ENSG00000126878	AIF1L	0	0	0	4,284080542	0	0	0	0	0	4,284080542	0,476008949
ENSG00000179673	RPRML	0	0	0	4,277622534	0	0	0	0	0	4,277622534	0,475291393
ENSG00000166833	NAV2	0,029875558	0,141369184	0,073278002	1,633637972	0,141999819	0,754437182	0,727651853	0,500856403	0,269449288	4,272555259	0,474728362
ENSG00000154736	ADAMTS5	0	1,427893907	0,028466984	0,133607258	0	0,271845287	1,413388277	0	0,9896589	4,264860613	0,473873401
ENSG00000155893	PXYLP1	0,011325082	3,4766219	0,020833403	0,019555945	0,026914303	0,435199744	0	0,27241102	0	4,262861398	0,473651266

ENSG00000153132	CLGN	0,214189328	0,095018427	0	3,883511316	0	0	0	0	0,065856315	4,258575386	0,473175043
ENSG00000104490	NCALD	0,05636351	0,166692776	0,622111374	1,395027217	1,071592816	0,123767681	0,17159936	0,534085463	0,115533086	4,256773284	0,472974809
ENSG00000108465	CDK5RAP3	0	0,176496298	0	2,061029913	0	0,573329293	0,681342925	0	0,7645488	4,256747228	0,472971914
ENSG00000072071	ADGRL1	0,089024726	1,800882998	0,163768174	1,352791196	0,126941514	0,097743948	0,029567652	0	0,591240262	4,25196047	0,472440052
ENSG00000134954	ETS1	0,126823811	0,075015247	0,388837738	0,875988165	1,858630447	0,046415045	0	0,462210893	0,415938503	4,249859848	0,47220665
ENSG00000148798	INA	0	0	0	0	3,976693794	0	0	0,1016409	0,171497867	4,249832562	0,472203618
ENSG00000158859	ADAMTS4	0,409276067	0	0	3,634613691	0	0,192583374	0,012136809	0	0	4,248609941	0,472067771
ENSG00000198466	ZNF587	0,041101746	0,656406365	0,075609982	0,248408109	0,09767919	0,767164369	1,791702007	0,494326158	0,075824774	4,248222702	0,472024745
ENSG00000135472	FAIM2	0,074844265	3,098883937	0	0,12923971	0	0	0,02071493	0	0,920487976	4,244170818	0,471574535
ENSG00000171462	DLK2	0	0	4,226888804	0	0	0	0	0	0	4,226888804	0,469654312
ENSG00000157150	TIMP4	0,126558267	0	0	0	0	0	4,098277176	0	0	4,224835443	0,46942616
ENSG00000138735	PDE5A	0,031527346	0	1,884905741	0,435526768	0,074925421	0,103845552	0,628267884	0,827292888	0,23264743	4,21893903	0,468771003
ENSG00000258472	AC005726.1	3,380969884	0	0,345531509	0	0	0,103114094	0,038990159	0	0,346513093	4,215118739	0,468346527
ENSG00000049540	ELN	0,023098528	2,049388567	0	1,236469614	0,658729824	0,025360834	0,019179201	0,202038867	0	4,214265435	0,468251715
ENSG00000169682	SPNS1	0,478198008	0,070712553	0,366534941	0,206435898	0	0,700044627	1,422791212	0,174279835	0,784162541	4,203159615	0,467017735
ENSG00000197893	NRAP	0,052798942	0,046845213	0,33994771	0,364688993	3,387901343	0	0	0	0	4,192182201	0,465798022
ENSG00000134538	SLCO1B1	0,067792988	0	0,124710677	0,585318472	2,577784064	0,07443274	0	0,592973652	0,166753273	4,189765864	0,46552954
ENSG00000104889	RNASEH2A	0,207284733	0,441386008	0	0,572696789	0,197046662	0,18206922	0,137690351	0	2,44736161	4,185535374	0,465059486
ENSG00000105369	CD79A	0	0	0	0	0	2,097339293	2,086998204	0	0	4,184337497	0,464926389
ENSG00000138083	SIX3	0	0	0	4,039538862	0	0	0,138743456	0	0	4,178282318	0,464253591
ENSG00000069667	RORA	0,115399014	0,567062915	0,097978065	0,459851333	0,822744766	0,22416397	0,162153986	1,6305295	0,0982564	4,178139949	0,464237772
ENSG00000261884	AC040162.1	0,079741726	0	0	0	0	0,758781881	2,096689805	0	1,242245883	4,177459295	0,464162144
ENSG00000099338	CATSPERG	0,016442935	0,087532753	0,030248107	0,02839336	0	0,758242026	0,46419927	1,006764732	1,779596731	4,171419913	0,463491101
ENSG00000064199	SPA17	0	0	0,640876309	0,501315993	0	0,988132157	1,952564244	0	0,071410767	4,154299471	0,46158883
ENSG00000153993	SEMA3D	0	0	0,413763481	0	3,652646796	0	0	0,040986674	0,046104322	4,153501274	0,461500142
ENSG00000134871	COL4A2	0	2,821376977	0	0,933214806	0,018613878	0,257985658	0,097551183	0	0,019265695	4,148008196	0,4608898
ENSG00000170298	LGALS9B	0,056595719	0	0,104112397	3,518224377	0	0	0,328948436	0	0,139210878	4,147091807	0,460787979
ENSG00000212864	RNF208	0	0	0	0,226899978	0	1,009890467	0	0	2,908902088	4,145692533	0,460632504
ENSG00000283324	CTXND2	0	4,145401141	0	0	0	0	0	0	0	4,145401141	0,460600127
ENSG00000180745	CLRN3	0	0	0	0	0	3,329793424	0	0	0,810848701	4,140642125	0,460071347
ENSG00000170426	SDR9C7	0,084466501	0	0	3,646380305	0,401472937	0	0	0	0	4,132319743	0,459146638
ENSG00000143797	MBOAT2	0	0	0	1,050462861	0,041306366	0	0	1,330251882	1,710112926	4,132134035	0,459126004
ENSG00000126759	CFP	0,83397324	0,286425494	0	0,464545086	0,191802141	0	0,044675212	2,176620794	0,132345752	4,130387719	0,458931969
ENSG00000196497	IPO4	0,061527105	0	0,509328357	1,274926586	1,754645972	0,135106333	0,229892709	0	0,151340816	4,116767879	0,457418653
ENSG00000213906	LTB4R2	0	1,40996412	0	0	0	0,05131784	0	0	2,644274675	4,105556636	0,45617296
ENSG00000112619	PRPH2	0,050353406	0	0	0,086949342	0	0	0	3,963893512	0	4,10119626	0,455688473
ENSG00000134812	CBLIF	0,188730938	0	0	3,910765648	0	0	0	0	0	4,099496585	0,455499621
ENSG00000159905	ZNF221	0,055616699	0,098690467	0	0	0	2,320427646	1,477751426	0	0,13680274	4,089288978	0,454365442
ENSG00000153707	PTPRD	0	0,023945526	0,496481691	3,518590924	0	0	0	0,029508356	0,016596403	4,0851229	0,453902544
ENSG00000187391	MAGI2	0,019113404	0,101748798	0,087901625	0,074260523	2,645914096	0,00524635	0,182508072	0,062693126	0,905020378	4,084406372	0,45382293
ENSG00000205517	RGL3	0	0	4,082314441	0	0	0	0	0	0	4,082314441	0,453590493
ENSG00000151376	ME3	0	0	1,520995852	1,835655111	0,093568927	0,086456788	0,457682295	0,08609551	0	4,080454483	0,453383831
ENSG00000069702	TGFBR3	0	0,031262912	0,032409945	0,212958473	2,344710548	0,019343661	0,014628697	1,309872367	0,10834005	4,073526654	0,452614073
ENSG00000123560	PLP1	0,099284212	0,176177398	0,365282653	1,285816235	1,297730935	0	0,164875601	0,651316493	0,030526696	4,071010222	0,452334469
ENSG00000180801	ARSJ	0	3,699588419	0,270093373	0	0	0	0,024382109	0	0,072229507	4,066293408	0,451810379
ENSG00000106006	HOXA6	0	0	0	4,061244741	0	0	0	0	0	4,061244741	0,451249416
ENSG00000106992	AK1	2,738988361	0,10340995	0	0,201261068	0	0,895776349	0,048388096	0	0,071672396	4,05949622	0,451055136
ENSG00000162595	DIRAS3	0	0	0	0,127972033	0,176124346	0	3,753648971	0	0	4,05774535	0,450860594
ENSG00000198851	CD3E	0,106266224	0	1,563882413	0,091749288	0	0	2,294112962	0	0	4,056010887	0,450667876
ENSG00000167608	TMC4	3,40516962	0	0	0	0	0,650204742	0	0	0	4,055374362	0,450597151
ENSG00000177238	TRIM72	1,12929608	0,031808066	0,032975101	0,061906281	2,428196906	0	0,014883788	0	0,352733613	4,051799834	0,450199982
ENSG00000171916	LGALS9C	0	0	0,116945772	2,085723398	0	0	0,158355488	1,181612883	0,508204625	4,050842167	0,450093574
ENSG00000185668	POU3F1	0	0	0,127280265	0,119475721	0,164431109	0,455798288	0,459598286	2,72336182	0	4,049945489	0,449993943
ENSG00000178093	TSSK6	0	0,48284874	0	0	0	3,485516317	0,075312324	0	0	4,043677381	0,449297487
ENSG00000176293	ZNF135	0	0	0	1,748239936	0	0,206806577	0	0	2,084909567	4,039956081	0,448884009
ENSG00000162779	AXDND1	0,024816977	0	0,410875495	2,099821605	0	0,136237951	0,432727373	0,651209527	0,274695139	4,030384067	0,447820452
ENSG00000162676	GFI1	0,029810726	0	2,083891056	1,801681616	0	0,032730435	0	0	0,0733267	4,021440533	0,446826726
ENSG00000158571	PFKFB1	0	0	0	0	0	0	0	4,014025359	0	4,014025359	0,446002818
ENSG00000183579	ZNRF3	0	0	0	0,936190351	0	0,690500391	1,746644978	0	0,640114313	4,013450033	0,445938893
ENSG00000143512	HHIPL2	0	0,081502128	0	3,886265579	0	0	0,038136879	0	0	4,005904586	0,44510051
ENSG00000078269	SYNJ2	0,036217281	0	1,643407152	0,333543149	0,229523076	0,159057835	0,030071982	1,557532914	0,014847529	4,004200918	0,444911213
ENSG00000213512	GBP7	0,062134281	0,110255757	0,45720411	2,038554489	0	0,272879238	0,515914194	0,543477907	0	4,000419976	0,444491108
ENSG00000107954	NEURL1	0	0,27024995	0	0	0,144776272	1,070175056	2,023307107	0	0,486999554	3,99550794	0,443945327
ENSG00000143845	ETNK2	0	0	0	3,989477786	0	0	0	0	0	3,989477786	0,44327531
ENSG00000137868	STRA6	0,059112233	3,776155432	0	0,102073924	0	0	0	0	0,048466951	3,98580854	0,442867616
ENSG00000140398	NEIL1	0,065340402	0,772966273	0	0,037609538	0	1,745671966	1,012732573	0,047626775	0,29465433	3,976601858	0,441844651
ENSG00000110237	ARHGEF17	0	0,027063181	0	3,792353284	0,108735631	0,016745113	0,012663538	0	0,018757219	3,976317965	0,441813107
ENSG00000185842	DNAH14	0,061386511	0,013616107	0,635205629	1,272013282	0	0,909883831	0,235738703	0,167792905	0,670040272	3,965677241	0,440630805
ENSG00000148935	GAS2	0	0	0,523010534	0,061367586	0	0,117058262	0	3,263935051	0	3,965371433	0,440596826
ENSG00000186280	KDM4D	0,051206564	0	0	0,08842256	0	3,823083435	0	0	0	3,962712559	0,440301395
ENSG00000132170	PPARG	0	0,471977118	0,30580869	0	2,29139906	0,255527841	0,634943374	0	0	3,959656083	0,439961787
ENSG00000186193	SAPCD2	0,039619971	0	0	1,847206165	0	0	2,072530392	0	0	3,959356529	0,439928503
ENSG00000144771	LRTM1	2,110750828	0,170248889	0,352990602	0,662691999	0,228011138	0,105340049	0	0,209799725	0,117997792	3,957831022	0,439759002
ENSG00000182885	ADGRG3	0	0	1,458537556	0	0,110838748	2,099485693	0	0	0,286800189	3,955662186	0,439518021
ENSG00000145217	SLC26A1	3,238335081	0,062460284	0	0	0,083651885	0,541054757	0,029226725	0	0	3,954728733	0,439414304
ENSG00000139351	SYCP3	0	0	0	3,543645516	0	0,043330002	0,229379035	0,086297876	0,048536569	3,951188997	0,439021
ENSG00000149292	TTC12	0	0,859753678	0	0	0	1,877524444	1,088577003	0,124645254	0	3,950500379	0,438944487
ENSG00000197935	ZNF311	3,140207073	0	0	0	0	0,104477693	0	0	0,70219089	3,946875656	0,43854174
ENSG00000189129	PLAC9	2,924720723	0	0	0,420862862	0	0	0	0	0,599504911	3,945088495	0,438343166
ENSG00000204822	MRPL53	0	2,348689052	0	0	0	0,484410443	1,099010207	0	0	3,932109702	0,436901078
ENSG00000165182	CXorf58	0	0	0,135600048	0	0	0	3,79471248	0	0	3,930312528	0,436701392
ENSG00000204580	DDR1	0,927668489	1,087618286	0	0,14302509	0	0,491074937	0,742753555	0	0,529709447	3,921849804	0,435761089
ENSG00000119042	SATB2	2,537355813	0,024469976	0,659562199	0,119061405	0,229405256	0,257390017	0,091600899	0	0	3,918845565	0,435427285
ENSG00000018408	WWTR1	0,197577116	0,095617021	0,429542528	0,496250992	0,25611616	0,256369606	1,864232913	0,078553326	0,242994383	3,917254046	0,43525045
ENSG00000249471	ZNF324B	0	1,129718464	0	0	0,26700189	1,151300161	1,368201886	0	0	3,9162224	0,435135822
ENSG00000130943	PKDREJ	0	2,474727935	0	0,237429458	0,093362158	0,021566434	0	0	1,087104316	3,914190302	0,434910034
ENSG00000162407	PLPP3	0	3,356861154	0	0,049494494	0	0	0	0,50141816	0	3,907773809	0,43419709
ENSG00000275993	SIK1B	0	1,115863504	0,289201102	1,683101168	0	0,414258618	0,130535094	0	0,270687819	3,903647305	0,433738589
ENSG00000125878	TCF15	0	0	0	2,852665317	0	0	1,048948113	0	0	3,90161343	0,433512603
ENSG00000100167	SEPT3	0	0	0	0	0	3,82709984	0	0,046195242	0,025981619	3,899276701	0,433252967
ENSG00000226784	PGAM4	0,395060315	0	0,363372678	2,728731761	0	0	0,164013506	0	0,24293663	3,894114891	0,432679432
ENSG00000131097	HIGD1B	0	0	0	3,890085631	0	0	0	0	0	3,890085631	0,432231737
ENSG00000003436	TFPI	0,038699992	0	0,071191762	3,742280667	0	0	0	0,028208517	0	3,880380938	0,431153438
ENSG00000090565	RAB11FIP3	1,754584326	0,341201845	0	0	0	0,501399866	0,957941138	0	0,325164903	3,880292079	0,431143564
ENSG00000152495	CAMK4	0,077697517	0	0	0,210833313	0	0,999314477	2,313284364	0,072815021	0,204766992	3,878711685	0,430967965
ENSG00000146674	IGFBP3	0,033058537	0,997246555	1,155463111	0,114169755	0,942771934	0	0,631331795	0	0	3,874041686	0,430449076
ENSG00000160223	ICOSLG	0,08088565	0,287059201	0,267832233	0,223474981	1,268694883	0,017761543	0,899958489	0,707492918	0,119374707	3,872534605	0,430281623
ENSG00000076604	TRAF4	0	0	0	0,114119823	0,078529968	1,269816354	1,75598103	0	0,650239308	3,868686482	0,429854054
ENSG00000178462	TUBAL3	0	0,448648076	0	0,145529824	0	0,092532391	0,069977876	0	3,109534673	3,86622284	0,429580316
ENSG00000163202	LCE3D	0	0	0	0	0	0	0	0	3,865607663	3,865607663	0,429511963
ENSG00000244607	CCDC13	0	0	0	0	0,078788403	0,545997949	0,302802469	1,957378965	0,978568788	3,863536574	0,429281842
ENSG00000153930	ANKFN1	0,02015345	0,143047213	0,0370739	2,958051859	0	0	0	0,705115108	0	3,86344153	0,429271281
ENSG00000186191	BPIFB4	0,061728174	0	0	0,426364338	3,374061877	0	0	0	0	3,862154389	0,429128265
ENSG00000157703	SVOPL	0	0,085097429	0,088219644	0,952317426	0,113969388	0	0	2,621664293	0	3,861268181	0,429029798
ENSG00000111077	TNS2	0	0,099397751	0	0,386904695	0	1,312032239	0,589135378	1,469868334	0	3,857338398	0,428593155
ENSG00000073861	TBX21	0	0,102149333	0	0	0	0	3,250279077	0,503519341	0	3,855947751	0,428438639
ENSG00000134873	CLDN10	0	0	0	3,854283229	0	0	0	0	0	3,854283229	0,428253692
ENSG00000180089	TMEM86B	0	0,510552171	0	0,099365946	0	0	0,621140257	0	2,618553436	3,849611811	0,427734646
ENSG00000183833	MAATS1	0,021265208	0,03773459	0	3,782198478	0	0	0	0	0	3,841198277	0,426799809
ENSG00000132622	HSPA12B	0	0,085586339	0	0,16657196	0	0	0	3,585948068	0	3,838106366	0,426456263
ENSG00000166743	ACSM1	0,052633428	0,280190178	0	0,727091535	0	0,057788428	0	0	2,718757902	3,83646147	0,426273497
ENSG00000204428	LY6G5C	0,749499206	0	0	0	0	0,633004871	0,622325187	1,260719449	0,567253788	3,832802501	0,425866945
ENSG00000105997	HOXA3	0,035093026	0	0	3,211693835	0,583795355	0	0	0	0	3,830582216	0,425620246
ENSG00000180532	ZSCAN4	0,067190116	0	0	3,596702629	0,159678765	0	0	0	0	3,82357151	0,424841279
ENSG00000104866	PPP1R37	0,588666033	0,139276458	0	1,050381017	0	0,301616423	0,89610028	0,042908008	0,796381669	3,815329888	0,423925543
ENSG00000168078	PBK	0,211048283	0	0	0,121478107	0	3,475780238	0	0	0	3,808306629	0,423145181
ENSG00000128408	RIBC2	0	0	0	0,143765826	0	0	3,663871954	0	0	3,80763778	0,423070864

ENSG00000214511	HIGD1C	0	0	0	0	0	3,13038824	0,67638993	0	0	3,80677817	0,422975352
ENSG00000147262	GPR119	0	0	0	3,172461697	0	0,630359334	0	0	0	3,802821031	0,42253567
ENSG00000239521	CASTOR3	0,0310034	0,220059089	1,596931221	0,10707221	0,221040752	0,442518978	1,18416809	0	0	3,80279374	0,422532638
ENSG00000196639	HRH1	0	0	0	0,04704922	0	0,209407508	3,099429406	0,238322804	0,201060067	3,795269005	0,421696556
ENSG00000229894	GK3P	0,566260168	0,861269808	0	1,396868172	0	0,444086126	0,201504816	0	0,298468719	3,768457809	0,418717534
ENSG00000154188	ANGPT1	0	0	0,03899833	2,891956088	0,604574987	0,232758946	0	0	0	3,768288351	0,418698706
ENSG00000138615	CILP	0	3,400180346	0	0,137866313	0	0,204539281	0,022097628	0	0	3,764683568	0,418298174
ENSG00000198092	TMPRSS11F	0,144741926	0	0	1,874532864	0	1,03296815	0	0	0,712055641	3,76429858	0,418255398
ENSG00000169855	ROBO1	0,081984214	0	0	3,468436682	0,032472877	0,045006938	0,136146486	0	0	3,764047197	0,418227466
ENSG00000110693	SOX6	0,115433824	0,040966898	0,198193185	1,541477748	0,054866196	0,371769473	0,415337726	0,403871951	0,615197797	3,757114797	0,4174572
ENSG00000167371	PRRT2	0	0,142818642	0,296117283	0,764390072	1,912743235	0,088367817	0,100242609	0,351994213	0,098986164	3,755660034	0,417295559
ENSG00000198673	TAFA2	0,016327452	0,028972663	0	0,028193946	3,026598412	0,286825416	0,203355481	0,142813427	0,020080662	3,753167461	0,417018607
ENSG00000274386	TMEM269	1,209437451	0,919764464	0	0	0	0,227638569	0,258228445	0,075562444	1,062465826	3,753097198	0,4170108
ENSG00000280433	FP565260.6	0,502677929	0	0	0,916238129	0	0,429264105	0,278256142	0,732806285	0,89299752	3,75224011	0,416915568
ENSG00000261221	ZNF865	0,188652398	0,468662672	0,069408264	0	1,793346029	0,124277586	0,720553717	0,24751653	0,139210878	3,751628074	0,416847564
ENSG00000169760	NLGN1	0,014890675	0,026423138	1,123096576	0,128564729	2,406384793	0,016349091	0	0,032561546	0	3,748270549	0,416474505
ENSG00000185633	NDUFA4L2	0	0	0,099066322	0	0	0,591270765	0,804870269	0	2,251882308	3,747089665	0,416343296
ENSG00000007952	NOX1	0	2,108863548	0	0	0	1,239599275	0,394715948	0	0	3,743178771	0,415908752
ENSG00000242028	HYPK	0	0,754975129	0	1,387734888	0,056173556	0,8304612	0,628038577	0,051686934	0,029070315	3,738140599	0,415348955
ENSG00000185988	PLK5	0	0	0	3,736133707	0	0	0	0	0	3,736133707	0,415125967
ENSG00000149927	DOC2A	0,688160576	0,084215452	0	0,327807757	0,225576346	0,807667694	1,280711617	0	0,321028854	3,735168297	0,4150187
ENSG00000141837	CACNA1A	0,035012528	0,11183197	0,141698422	0,296249073	2,903960293	0,049974223	0,014535825	0,130155649	0,047366986	3,730784969	0,414531663
ENSG00000119714	GPR68	0,043497574	0	0,0800173	3,605319165	0	0	0	0	0	3,728834039	0,414314893
ENSG00000243811	APOBEC3D	0	0	0,989253021	0	1,277998371	0,065603233	0,54573889	0,261316384	0,587889355	3,727799254	0,414199917
ENSG00000148219	ASTN2	0,054684163	0	0	1,227558988	0,173277463	0,680453511	0,908108555	0,039859417	0,627708399	3,711650496	0,412405611
ENSG00000132010	ZNF20	0,036892229	0	0	2,484488284	0	0,202527559	0,214426837	0	0,771335664	3,709670573	0,412185619
ENSG00000188452	CERKL	0	0,056226043	0,05828897	0,383003737	0	0	1,131311444	2,078640601	0	3,707470795	0,411941199
ENSG00000172819	RARG	0	0	0	0,05089428	0	1,747449023	0	1,288997728	0,616226034	3,703567066	0,411507452
ENSG00000222047	C10orf55	0,120215251	0,426638027	0,110572832	0	0	0,593951945	0,898355599	0	1,55241725	3,702150905	0,411350101
ENSG00000131142	CCL25	0	0,271124368	0	0	0	0	3,425378123	0	0	3,696502492	0,410722499
ENSG00000166689	PLEKHA7	0	0,048850793	0	0,071306697	0,032712474	0,937006354	0,34287757	1,113689105	1,134242758	3,680685751	0,408965083
ENSG00000176928	GCNT4	0,099153918	0,92371752	2,462422137	0,171217175	0	0	0,020582404	0	0	3,677093153	0,408565906
ENSG00000139287	TPH2	0	0,038698514	2,808285024	0,828484549	0	0	0	0	0	3,675468086	0,408385343
ENSG00000147257	GPC3	0	0,395635559	3,076135363	0	0,132466818	0	0	0	0,068552756	3,672790496	0,408087833
ENSG00000169258	GPRIN1	3,604668785	0,063330657	0	0	0	0	0	0	0	3,667999442	0,407555494
ENSG00000185697	MYBL1	0,020874509	0,037041304	0	0,252320047	0	2,475251041	0	0	0,872880475	3,658367376	0,406485264
ENSG00000196118	CCDC189	0	0	0	0	0	0,448060971	1,791050264	0,127482472	1,290600849	3,657194556	0,406354951
ENSG00000010030	ETV7	0	0	0,117093555	0,109913637	0	2,795460433	0,634222403	0	0	3,656690028	0,406298892
ENSG00000144857	BOC	0,043884967	0,051915198	0,107639922	0,10103968	0,1390581	0,016061043	0,024292417	3,134810031	0,035981902	3,65468326	0,406075918
ENSG00000075702	WDR62	0	0,027322397	0,028324852	2,8183317	0	0,642408999	0	0,134678854	0	3,651066802	0,405674089
ENSG00000148082	SHC3	0,045424019	0,026867936	0	0,653644726	0,035983722	0,731469476	0,176010486	0,099329028	1,880811498	3,649540891	0,405504543
ENSG00000179588	ZFPM1	0,441413546	0,391638754	0,541343912	0,465804077	0	0,350022314	0,610858481	0,214498259	0,633362053	3,648941395	0,405437933
ENSG00000100288	CHKB	0,950380948	0,177518702	0,276047765	0,172747418	0	0,494271827	0,456859865	0,875033611	0,246072853	3,648932989	0,405436999
ENSG00000163435	ELF3	0	2,947845534	0,038683565	0,036311575	0	0,623376784	0	0	0	3,646217458	0,405135273
ENSG00000204583	LRCOL1	0	0	0	0,140589965	0	0	0	0	3,504648858	3,645238822	0,405026536
ENSG00000133619	KRBA1	1,783767276	0,200968334	0	1,368967452	0	0,031086861	0,258604769	0	0	3,643394692	0,404821632
ENSG00000166343	MSS51	0	0	0,114725588	0	0	0	0	0	3,528245986	3,642971574	0,404774619
ENSG00000002822	MAD1L1	1,274930126	0,259611895	0,038448146	1,082717737	0,049670476	0,137685126	0,694165046	0	0,102819653	3,640048205	0,404449801
ENSG00000149294	NCAM1	0,106775154	0,252626521	0,283719972	0,757995922	0,98681903	0,143284631	0,216718796	0,59668643	0,2918215	3,636447956	0,404049773
ENSG00000180332	KCTD4	0	0	0	0	0	0	3,632921141	0	0	3,632921141	0,403657905
ENSG00000123977	DAW1	0,051520822	0	3,411961665	0	0	0,056566852	0	0,11266095	0	3,632710289	0,403634477
ENSG00000261341	AC010325.1	0	0,322286058	0	2,50899014	0	0,797647003	0	0	0	3,628923202	0,403213689
ENSG00000114859	CLCN2	0,01773182	2,485709693	0,032619115	0,061237966	0	1,01236212	0,014723109	0	0	3,624383824	0,402709314
ENSG00000163430	FSTL1	0,07138801	0,095007204	3,118945247	0,246543025	0,084827576	0	0	0	0	3,616711063	0,401856785
ENSG00000205456	TP53TG3D	0	0	3,615073156	0	0	0	0	0	0	3,615073156	0,401674795
ENSG00000075826	SEC31B	0,057137851	0	0	1,84173917	0	0,083645363	0,015814259	0	1,616260403	3,614597046	0,401621894
ENSG00000117594	HSD11B1	0	0	0	0,172576041	0	0	0	0	3,441602264	3,614178306	0,401575367
ENSG00000137807	AC027237.1	0,045958203	0	0,042271913	0,198399464	0,054610332	0,126148553	3,090966208	0,050248566	0	3,608603238	0,400955915
ENSG00000156097	GPR61	0,029881465	3,287483489	0	0,154796307	0	0,098424309	0	0	0,03675035	3,607335921	0,400815102
ENSG00000175449	RFESD	0	0	0,054915073	0,103095604	0	1,212700778	0,793174758	1,436104402	0	3,599990615	0,399998957
ENSG00000137343	ATAT1	0,074899911	0	0	2,198708584	0	0,123353589	0,279859477	0	0,921172353	3,597993914	0,399777102
ENSG00000115457	IGFBP2	0,22306495	1,074377478	0,058620856	0,11005271	0	0,489824562	0,767321727	0,278730129	0,587873858	3,58986627	0,39887403
ENSG00000120318	ARAP3	0	0,043283616	0,94230542	0	0	1,472975257	0,891153286	0	0,239995509	3,589713089	0,39885701
ENSG00000090554	FLT3LG	0	0	0	3,025598792	0	0	0,264519323	0,232209816	0,065300957	3,587628887	0,398625432
ENSG00000263353	PPIAL4A	0	0	0	0,790712044	0	0,502759323	0	2,002633743	0,28158564	3,57769075	0,397521194
ENSG00000154162	CDH12	0,050981974	0,045233131	0	3,301302816	0,121159765	0	0,021165711	0	0,031350628	3,571194024	0,396799336
ENSG00000181458	TMEM45A	1,811817	0	0	0	0,059803088	1,65772433	0,04178862	0	0	3,571133039	0,39679256
ENSG00000157322	CLEC18A	0	0	0	0	3,567780144	0	0	0	0	3,567780144	0,396420016
ENSG00000159217	IGF2BP1	0	0	0	3,440841218	0,037287669	0,034453448	0	0,034309476	0,0192967	3,566188512	0,396243168
ENSG00000123610	TNFAIP6	0	0	0	0	0	0,841651626	0	2,723937462	0	3,565589088	0,396176565
ENSG00000100652	SLC10A1	0	0	0	0,912360051	0	0,662979327	0,501379948	0	1,485286891	3,562006218	0,395778469
ENSG00000166823	MESP1	0	0	0	0,771019342	0,303180703	0,840408156	1,641865889	0	0	3,55647409	0,395163788
ENSG00000163093	BBS5	0	0	0	0	0	1,170032276	1,681196268	0	0,698999614	3,550228157	0,394469795
ENSG00000182223	ZAR1	0,103500391	0	0	0	3,443592872	0	0	0	0	3,547093263	0,394121474
ENSG00000163508	EOMES	0	0,073342998	0	0,285486843	0,098226899	0	3,088711674	0	0	3,545768414	0,393974268
ENSG00000278318	ZNF229	0,027474649	0,048753091	0,101083672	3,368433308	0	0	0	0	0	3,545744721	0,393971636
ENSG00000180875	GREM2	0	0,064210249	3,128607436	0,187453286	0,08599558	0	0	0,079127052	0	3,545393603	0,393932623
ENSG00000169684	CHRNA5	0	0	0	0	0	1,602779515	0,13852645	0	1,795370764	3,53667673	0,392964081
ENSG00000127561	SYNGR3	0	0	0	0	0	0,488690948	2,882676258	0	0,164223731	3,535590937	0,392843437
ENSG00000213160	KLHL23	0	0	0,705917175	2,366542487	0	0,060188854	0,091035975	0	0,303395375	3,527079865	0,391897763
ENSG00000113494	PRLR	0,046474111	0	0,14961254	0,220689276	0,027611682	1,696609773	0	0	1,386061253	3,527058635	0,391895404
ENSG00000138039	LHCGR	1,420439364	0,0536284	0,444768158	0	0,071823508	1,460013075	0,075282199	0	0	3,525954705	0,391772745
ENSG00000109339	MAPK10	0,124017961	0,012575247	0,436727164	1,694859728	0,042104481	0,12060282	0,035305632	0,991784098	0,065368331	3,523345463	0,391482829
ENSG00000167377	ZNF23	0	0,036807413	0	0,107454348	0	0	0,172231067	2,766850874	0,433684403	3,517028104	0,3907809
ENSG00000178878	APOLD1	1,001423083	0,148083391	0	1,477058396	0	0,229063339	0,051968935	0,09124246	0,513175541	3,512015145	0,390223905
ENSG00000131435	PDLIM4	0	2,540025261	0	0,082391845	0	0	0	0	0,880232976	3,502650083	0,389183343
ENSG00000242852	ZNF709	0,026533902	0	0	3,115641488	0	0,203928716	0,154221655	0	0	3,50032576	0,388925084
ENSG00000163235	TGFA	0	1,036139516	0	0	0	2,463183262	0	0	0	3,499322778	0,388813642
ENSG00000180096	SEPT1	0	0	0	1,546923018	0,340637934	0,550805804	0,981864111	0,078357735	0	3,498588601	0,388732067
ENSG00000269404	SPIB	0	0	0,129836571	0	0,083866778	0	3,281802238	0	0	3,495505587	0,38838951
ENSG00000105675	ATP4A	0	2,482157882	0	1,006434718	0	0	0	0	0	3,4885926	0,3876214
ENSG00000100450	GZMH	0	0	0,584605886	0	0	0	2,902573401	0	0	3,487179287	0,387464365
ENSG00000130226	DPP6	0,111159755	0,473400618	0,368077224	0,806184268	0,686851265	0,244093831	0,184596634	0,461840281	0,150383386	3,486587261	0,387398585
ENSG00000144061	NPHP1	0,077077567	0,045590751	0,165422145	0	0	2,567008838	0,405327945	0	0,221189434	3,48161668	0,386846298
ENSG00000123297	TSFM	0,268084986	0,396425192	0	0,385770217	0,212369925	0,5886833	0,964585639	0,390815574	0,274758312	3,481493145	0,386832572
ENSG00000274211	SOCS7	0	0,119108051	0,246956223	1,303950456	0,877355462	0,257939819	0,153267479	0,440334793	0,082552592	3,481464876	0,386829431
ENSG00000156475	PPP2R2B	0,102983124	0,020304559	0	2,529128938	0,054387027	0,615600353	0,00950101	0,050043097	0,098510197	3,480458303	0,386717589
ENSG00000182040	USH1G	0	0	0	0	0	0	3,384420307	0,092844779	0	3,477265086	0,386362787
ENSG00000147138	GPR174	0	0	0	3,358662653	0,065104703	0	0,045493231	0	0	3,469260588	0,385473399
ENSG00000143479	DYRK3	0	0	0	3,430187627	0	0,038263509	0	0	0	3,468451137	0,38538346
ENSG00000101400	SNTA1	0,12904404	0,114492741	0	0	0	2,904497713	0	0	0,317415068	3,465449562	0,385049951
ENSG00000160345	C9orf116	0	0	0	0	0	0	2,759808805	0	0,697921489	3,457730294	0,384192255
ENSG00000184108	TRIML1	0	0	0	3,457728012	0	0	0	0	0	3,457728012	0,384192001
ENSG00000130224	LRCH2	0	0	0	2,95020363	0,072505056	0,033496987	0	0,400284152	0	3,456489825	0,384054425
ENSG00000103942	HOMER2	0,010290131	0,018259585	0,018929527	2,114488388	0	1,129796232	0	0	0,164521947	3,456285809	0,384031757
ENSG00000197826	CFAP299	0	0,059879857	3,352149474	0	0	0	0	0	0,041502126	3,453531458	0,383725718
ENSG00000067798	NAV3	0,079663654	0	0,041870779	0,058955033	0,027046057	0,999611849	0,774858203	1,468266266	0	3,450271841	0,383363538
ENSG00000148541	FAM13C	0,022376806	0	0	0,07727972	3,350274718	0	0	0	0	3,449931244	0,383325694
ENSG00000182687	GALR2	0	0	0	0,380094646	0	0,362514006	0	0	2,707159829	3,449768481	0,383307609
ENSG00000196932	TMEM26	0,043755775	0	0,080492283	0,226670023	0	3,098662863	0	0	0	3,449580944	0,383286772
ENSG00000172780	RAB43	0	0,10745021	0,278481365	0,052281101	0	0,531871214	1,407801762	0,662060845	0,409599628	3,449546125	0,383282903
ENSG00000184465	WDR27	0,910737777	0	0,044088834	0,455239448	0,05695758	1,434119973	0,258701715	0,288245856	0	3,448091184	0,383121243
ENSG00000079102	RUNX1T1	0,022328862	0,752818323	1,643029769	0,886812621	0,053065023	0,049031571	0,037080261	0	0	3,444166429	0,382685159
ENSG00000125398	SOX9	0,03840167	0	0	0	0,0912624	0	0	0	3,306037245	3,435701314	0,38174459
ENSG00000239961	LILRA4	0	0	0	2,151548119	0	0,684010623	0	0	0,595934794	3,431493536	0,38127706
ENSG00000174225	ARL13A	0	0	0,181094527	0	0	3,026381855	0	0,215266819	0	3,422743201	0,3803048
ENSG00000146021	KLHL3	0,025497439	0	1,149162647	1,188769816	0,212083253	0,727862144	0	0,055755432	0,062717126	3,421847855	0,380205317

ENSG00000151704	KCNJ1	0	0	0	0,056272369	0,077446095	2,86237786	0,351760689	0,07126042	0	3,419117433	0,379901937
ENSG00000187775	DNAH17	0,084429795	0,099879066	0,034514539	1,360723731	0,066883078	0,823990945	0,334940668	0,594897098	0,011537529	3,41179645	0,379088494
ENSG00000124440	HIF3A	0,090258375	0,864870864	0,398490167	0,187027816	1,201205493	0,257655895	0,074943455	0,157894907	0,177609865	3,409956837	0,378884093
ENSG00000251287	ALG1L2	0	2,146567045	0	1,044436189	0	0	0,08370269	0	0,123980335	3,398686259	0,377631807
ENSG00000182986	ZNF320	0,181841842	0	0	1,758406568	0	0,439233777	0,815330677	0	0,201277822	3,396090686	0,37734341
ENSG00000107957	SH3PXD2A	0,154246925	0,091235795	0	0,954423606	0,061095193	0,691529284	0,736428454	0,702693449	0	3,391652705	0,376850301
ENSG00000160229	ZNF66	0,050219532	1,158473247	0	0,520309022	0	0,606519224	0	0	1,049980352	3,385501376	0,37616682
ENSG00000127241	MASP1	0,011481694	3,015349594	0	0,317222065	0	0	0	0,025107102	0,014121003	3,383281458	0,375920162
ENSG00000140505	CYP1A2	0,048247309	0,085613665	0	3,249190297	0	0	0	0	0	3,383051271	0,375894586
ENSG00000134253	TRIM45	0,034594911	0	0	0,119475721	0	1,633277197	1,551144214	0	0,042547281	3,381039324	0,375671036
ENSG00000099998	GGT5	0	0	0	3,31879634	0,059319052	0	0	0	0	3,378115393	0,375346155
ENSG00000101200	AVP	0	0	0	3,37234216	0	0	0	0	0	3,37234216	0,374704684
ENSG00000139725	RHOF	0	0,173526614	0,404759892	0,759881822	0	0,671050707	0,669878815	0	0,691549913	3,370647762	0,374516418
ENSG00000182010	RTKN2	0,030860935	0,082142959	0	1,9717337	0,036670841	1,033446868	0,179371454	0,033741914	0	3,367968671	0,374218741
ENSG00000172215	CXCR6	0	0	3,166395829	0,069121848	0,095130475	0	0,033237174	0	0	3,363885326	0,373765036
ENSG00000187871	GFRAL	0	0	2,618336882	0	0,187921268	0,260456164	0,196970694	0	0,097250927	3,360935935	0,373437326
ENSG00000006042	TMEM98	0	2,636445345	0	0	0,623107361	0	0,096757534	0	0	3,35631024	0,37292336
ENSG00000272031	ANKRD34A	0	0,073043312	0	0	0	0	3,281163681	0	0	3,354206993	0,372689666
ENSG00000137135	ARHGEF39	0,542629684	0	0	0	0	1,96865009	0	0,464310868	0,377206056	3,352796698	0,372532966
ENSG00000062524	LTK	0	0	0	0	0	0,04752523	0	0	3,300625718	3,348150947	0,372016772
ENSG00000108375	RNF43	0	0	2,814988344	0	0	0,132640035	0	0	0,396208442	3,343836821	0,371537425
ENSG00000112210	RAB23	0	0	0	0	0	0	3,341454257	0	0	3,341454257	0,371272695
ENSG00000136828	RALGPS1	0,11505516	0,306243838	0,026456657	0	0	0,900057378	0	0,754775828	1,238151381	3,340740241	0,37119336
ENSG00000157110	RBPMS	0	0	0,03125479	2,64044836	0,161510026	0,093271069	0	0,037152526	0,376122937	3,339759707	0,371084412
ENSG00000175183	CSRP2	0,051732479	0	0	1,831279349	0,245886712	0,709990486	0,386591232	0,113123782	0	3,338604039	0,370956004
ENSG00000186583	SPATC1	0	1,736844046	0	0	0	0,413329788	1,187810816	0	0	3,33798465	0,370887183
ENSG00000183655	KLHL25	0,021250256	2,865812404	0,039091562	0,110083662	0,050501693	0,093326158	0	0	0,156810453	3,336876189	0,370764021
ENSG00000181323	SPEM1	0	0	0	3,332175511	0	0	0	0	0	3,332175511	0,370241723
ENSG00000180264	ADGRD2	0,054405246	0	0	3,10021752	0	0,089600671	0,045173837	0	0,03345572	3,322852994	0,369205888
ENSG00000148482	SLC39A12	0	0,095492165	0,098995762	0,185851122	0	0	0	2,941903201	0	3,322242249	0,369138028
ENSG00000163794	UCN	0,19007619	0	0	0	0	3,13038824	0	0	0	3,320464429	0,368940492
ENSG00000146938	NLGN4X	0,020313291	0,072090872	0,037367939	0,03507662	0,241374877	0,066908419	0	2,842829993	0	3,315962012	0,368440224
ENSG00000118804	STBD1	0	0	0	2,975574272	0	0	0,330185084	0	0	3,305759356	0,367306595
ENSG00000125869	LAMP5	0	0	0	0,127784023	0,351731187	0	0	0	2,821372277	3,300887488	0,366765276
ENSG00000128567	PODXL	0,087689291	0,272304367	0,443606715	0,757101334	0,520988746	0,336972011	0,564279746	0,287626201	0,026961631	3,297530042	0,366392227
ENSG00000180210	F2	0	0	0	2,96749579	0	0,065062971	0	0,259164367	0	3,291723128	0,365747014
ENSG00000166963	MAP1A	0	0	0,026111225	3,161808353	0,033732627	0,031168622	0,035357035	0	0	3,288177862	0,365353096
ENSG00000132749	TESMIN	0,130515262	0,046319226	0,14405602	2,298787995	0,124068938	0	0,130043529	0,285398659	0,128413558	3,287603189	0,365289243
ENSG00000151962	RBM46	0,150097413	0,133172088	0	0	0,089177438	0	2,86646897	0	0,046150117	3,285066027	0,365007336
ENSG00000122367	LDB3	0,203714071	0	2,914710445	0,039085526	0	0,12425897	0	0	0	3,281769012	0,364641001
ENSG00000022267	FHL1	0	1,93763311	0,730445325	0,171414009	0,058978082	0,326971082	0	0,054267461	0	3,279709068	0,364412119
ENSG00000123500	COL10A1	0	0	0	0	0	0	0	3,278463072	0	3,278463072	0,364273675
ENSG00000030419	IKZF2	0,098765079	0	0,204397132	1,343047663	0	0,189766999	0,881572594	0,026996288	0,531423879	3,275969635	0,363996626
ENSG00000102349	KLF8	0,047485449	0	0,02183833	0	0	0,560464671	1,182845458	0	1,460024529	3,272658437	0,363628715
ENSG00000141198	TOM1L1	0	0,036858007	0	0	2,665614747	0	0,017246781	0,49962615	0,051091827	3,270437513	0,363381946
ENSG00000150687	PRSS23	0	3,116295527	0	0,091204116	0	0	0	0,057748089	0	3,265247732	0,362805304
ENSG00000283755	CPHXL	0	0	0	3,264087247	0	0	0	0	0	3,264087247	0,362676361
ENSG00000145284	SCD5	0,035715096	0	0	0	0,084877699	0	3,143430676	0	0	3,264023471	0,362669275
ENSG00000269466	H3.Y	0	0	0	0	2,203175106	0	0,384878319	0,675735312	0	3,263788737	0,362643193
ENSG00000089199	CHGB	2,888328875	0,060297279	0	0,11735326	0,080755013	0,037308427	0	0,074305052	0	3,258347907	0,362038656
ENSG00000101213	PTK6	0,102343766	2,451687776	0	0,176725347	0	0,056183737	0,467380173	0	0	3,2543208	0,3615912
ENSG00000126251	GPR42	0	0,863581321	0	0	0	0,356222387	0,673485411	0,354733835	0,997565874	3,245588827	0,360620981
ENSG00000161714	PLCD3	0	0	0	0	0,319310204	2,876635666	0	0,048967778	0	3,244913648	0,360545961
ENSG00000082458	DLG3	0	0,03407141	0	0,066311302	0,0456312	0	0,031885726	0,041986603	3,022662624	3,242548866	0,360283207
ENSG00000180900	SCRIB	0	0,366425448	0	0	0	0,40306244	0,533429896	0,551894965	1,382702641	3,23751539	0,359723932
ENSG00000126838	PZP	0,241699569	0,128666987	0,622476229	1,502504652	0,114880852	0,106148801	0,100344155	0,211410472	0,208081519	3,236213237	0,359579249
ENSG00000241563	CORT	0	0	0	3,232822694	0	0	0	0	0	3,232822694	0,359202522
ENSG00000162069	BICDL2	0	0	0	3,226914398	0	0	0	0	0	3,226914398	0,358546044
ENSG00000233670	PIRT	0	0	0	0	0,415525071	0,095985292	0,036294571	2,676357504	0	3,224162437	0,358240271
ENSG00000144407	PTH2R	0,034421542	0,061080182	0	0	0,08180354	0,982613894	2,057827769	0	0	3,217746927	0,357527436
ENSG00000172818	OVOL1	0	0	0	0,06861293	0	3,097462778	0	0	0,048868399	3,214944108	0,357216012
ENSG00000136492	BRIP1	0,018910095	0,067111	0,173933237	2,122484464	0	0,830487182	0	0	0	3,212925977	0,356991775
ENSG00000102053	ZC3H12B	0,019301389	0	0,035506463	2,733001394	0,045870168	0	0,352579806	0	0,02373822	3,20999744	0,356666382
ENSG00000156509	FBXO43	0	0	0	2,690727857	0,180642627	0,333824098	0	0	0	3,205194582	0,356132731
ENSG00000259224	SLC35G6	0	0	0	2,767725128	0,211619059	0	0,221809662	0	0	3,201153849	0,355683761
ENSG00000164532	TBX20	0	1,430090667	0	0	0	1,769712818	0	0	0	3,199803485	0,355533721
ENSG00000132932	ATP8A2	0,057500217	0,076524543	0	0,124112906	2,733010216	0,031565939	0,011935914	0,157170171	0	3,191819907	0,354646656
ENSG00000105426	PTPRS	0	0	0,064910706	0,121861051	0	0,639235147	1,127987807	0	1,236806978	3,190801688	0,354533521
ENSG00000182791	CCDC87	0,10464721	0	0	0,090351445	2,984356308	0	0	0	0	3,179354963	0,353261663
ENSG00000050327	ARHGEF5	0,025972941	0	0,955586452	0	0	0,598852202	1,595875656	0	0	3,176287251	0,352920806
ENSG00000276293	PIP4K2B	0	0,262241565	0,04531053	0	0	0,459735909	1,165739026	0	1,242006098	3,175033128	0,352781459
ENSG00000100433	KCNK10	0	0,064941148	0	0,094793524	0	1,084908106	1,929611548	0	0	3,174254327	0,352694925
ENSG00000173269	MMRN2	0	0,158164766	0,054655938	0	0	0	2,960369611	0	0	3,173190314	0,352576702
ENSG00000128573	FOXP2	0	0,164161871	1,63377553	0,031949917	0,219858909	0,010157376	0,606841939	0,505746552	0	3,172492094	0,352499122
ENSG00000022556	NLRP2	0,31117336	0	0	0,330663678	0	2,260147251	0,258373882	0	0	3,16035817	0,351150908
ENSG00000119946	CNNM1	0,024546876	0	0	0,084774196	0,058336183	2,99156498	0	0	0	3,159222235	0,351024693
ENSG00000213397	HAUS7	0,190411505	0,135152218	0,070055468	0,263039289	0,814530716	0,167248565	0,158102737	0	1,358253313	3,156793812	0,350754868
ENSG00000253731	PCDHGA6	0	0	3,09257253	0	0	0,062924365	0	0	0	3,155496895	0,350610766
ENSG00000171794	UTF1	0,497893149	0,220874794	0	0	0	0,273328792	1,550292405	0	0,61234439	3,15473353	0,350525948
ENSG00000285053	TBCE	0	1,965547069	0,053622704	0	0,138548435	0,768104522	0,12101691	0	0,107550071	3,15438971	0,350487746
ENSG00000179240	GVQW3	0	1,124182647	0,050670816	0,214037074	0,261842904	0,120970162	1,34938928	0,030116165	0	3,151209048	0,350134339
ENSG00000177692	DNAJC28	0	0	0	0,210092572	2,602301032	0,333958488	0	0	0	3,146352091	0,349594677
ENSG00000137285	TUBB2B	0	2,814467558	0,265248186	0	0	0	0,0598618	0	0	3,139577544	0,348841949
ENSG00000181322	NME9	0	0	0	0	0	0,22192426	0	2,917159103	0	3,139083363	0,34878704
ENSG00000008226	DLEC1	0,036246239	0,032159031	1,600269219	0	0,086139972	0	0,707256609	0,673709241	0	3,135780311	0,348420035
ENSG00000115884	SDC1	0,041824127	0	0	0	0	0	2,535104964	0,548742708	0	3,1256718	0,347296867
ENSG00000124181	PLCG1	0,031324745	0	0	0,297500489	0	2,562259114	0,078028813	0,136996089	0,019262699	3,12537195	0,34726355
ENSG00000089723	OTUB2	0	0	0	0	0	0,899774408	0,355020982	1,86994332	0	3,12473871	0,34719319
ENSG00000203867	RBM20	0,018378809	0,032612746	0	0,158680962	2,88272413	0,020178859	0	0	0	3,112575507	0,345841723
ENSG00000166147	FBN1	0,047054422	0,033398767	0,069248328	1,446298358	1,476101252	0	0,015628116	0	0,023148349	3,110877592	0,345653066
ENSG00000131400	NAPSA	0,084395739	0	0	0,072866511	0	2,177547362	0,315340125	0,092274384	0,363285578	3,105709699	0,345078855
ENSG00000169860	P2RY1	0	0,127504517	0	2,398831594	0	0	0,576737934	0	0	3,103074046	0,344786005
ENSG00000130997	POLN	0,782854919	0,069457842	0	0	0	1,095899419	1,153787537	0	0	3,101999716	0,344666635
ENSG00000176697	BDNF	0,01952333	0,034643669	0	3,000415083	0,046397615	0	0	0	0	3,100979697	0,3445533
ENSG00000126464	PRR12	0,20004047	0,177483453	0,47838778	0,069085246	1,283581367	0,197669472	0,415244678	0,131228978	0,147614394	3,100335837	0,34448176
ENSG00000007047	MARK4	0	0	0,375874533	0	0	2,019043977	0,20735782	0,297867699	0,195451571	3,0955956	0,343955067
ENSG00000186998	EMID1	0	0	2,691607881	0,376296832	0	0	0,025848853	0	0	3,093753565	0,343750396
ENSG00000175544	CABP4	0,039912987	0	0,587385312	0,068921018	0	0,043822128	2,352982983	0	0	3,093024428	0,343669381
ENSG00000152463	OLAH	0	0	0	0,331135755	0,303821948	1,754553476	0	0,698888679	0	3,088399858	0,34315554
ENSG00000134201	GSTM5	0,049813935	0,088393605	1,053822693	0,086017793	0,059191947	1,750169261	0	0	0	3,087409233	0,34304547
ENSG00000164066	INTU	0,009420271	0,735505767	0,069317399	0,325335047	0,022387478	0,910175846	0,007821852	0,020599375	0,984786135	3,085349171	0,342816575
ENSG00000180730	SHISA2	0	2,343898602	0	0	0	0,058010691	0,614190436	0	0,064981301	3,081081031	0,342342337
ENSG00000204682	MIR1915HG	0,039776407	0,141164517	0,073171913	2,678721771	0	0	0,099081599	0	0,048919853	3,080836061	0,342315118
ENSG00000149564	ESAM	0,310501173	0	0	1,072335512	0	0	0	1,697437847	0	3,080274531	0,342252726
ENSG00000248099	INSL3	0	0	0	0	0	2,876022998	0	0	0,201350512	3,07737351	0,34193039
ENSG00000153531	ADPRHL1	0,027665795	0,024546137	0,101786927	0,453841497	0,06574836	1,078327622	0,03445725	0,030248496	1,25893836	3,075560445	0,341728938
ENSG00000111245	MYL2	0	0,196873715	0	2,8737332	0	0	0	0	0	3,070606916	0,341178546
ENSG00000187699	C2orf88	0,075593082	0,04471269	0	2,784698745	0	0	0	0,165299934	0	3,07030445	0,341144939
ENSG00000248712	CCDC153	0	0	0	0,102087236	0	0,649102413	0	1,809891997	0,508969349	3,070050995	0,341116777
ENSG00000196091	MYBPC1	0,020461824	0,217854029	0,037641178	0	0,048627968	1,34795323	1,32521136	0,044744017	0,025165406	3,067659012	0,340851001
ENSG00000111344	RASAL1	0,031396337	0,05571203	0	0	0,14922815	2,792178831	0	0	0,038613447	3,067128795	0,340792088
ENSG00000164008	C1orf50	0	0	0,157763961	0	0	0,784669773	1,139344672	0	0,984431068	3,066209473	0,340689941
ENSG00000054938	CHRDL2	0	0,085531738	0	0	2,978327305	0	0	0	0	3,063859043	0,340428783
ENSG00000152049	KCNE4	0,036641748	0	2,898434592	0,126544605	0	0	0	0	0	3,061620945	0,340180105
ENSG00000110195	FOLR1	0,572388525	0	0	0,164731676	1,587009341	0,73319068	0	0	0	3,057320222	0,339702247
ENSG00000251322	SHANK3	0,020269694	0	0	3,010115066	0	0,02225494	0	0	0	3,0526397	0,339182189
ENSG00000132330	SCLY	0,103032862	0,091414642	0,047384318	1,82363146	0,091822435	0,466636754	0,064162788	0,281628371	0,079198211	3,04891184	0,338767982

ENSG00000188227	ZNF793	0	0	0,023151503	0,130391426	0,05981803	0,746162334	1,003177469	0	1,083472688	3,046173449	0,338463717
ENSG00000181649	PHLDA2	0,657002481	0	2,115065971	0	0	0	0,272761591	0	0	3,044830043	0,338314449
ENSG00000187105	HEATR4	0,466723074	1,847497744	0,066044215	0	0	0	0	0	0,662318326	3,042583358	0,338064818
ENSG00000148513	ANKRD30A	0	0	2,882345991	0,156092707	0	0	0	0	0	3,038438698	0,3376043
ENSG00000157399	ARSE	0	0,093169562	0	0	0	0	2,877359942	0	0,064574886	3,03510439	0,337233821
ENSG00000183607	GKN2	0	0	0	0,827839386	0	0,947458877	0	1,257999623	0	3,033297886	0,337033098
ENSG00000054690	PLEKHH1	0,02793946	0	0,051396894	0,04824535	0,033199366	2,822182316	0,04639746	0	0	3,029360846	0,33659565
ENSG00000204304	PBX2	0,242164376	0,184163462	0,50912106	0,836330047	0	0,303865525	0,258524036	0,226946818	0,468020088	3,029135412	0,336570601
ENSG00000159674	SPON2	0	0,985651462	0	0,043598158	0,120005862	0,637584505	0	0	1,24208202	3,028922008	0,33654689
ENSG00000268870	AC008758.5	0	0	1,351605019	0	0	0,268898828	0,203355481	0	1,204839691	3,028699019	0,336522113
ENSG00000165152	TMEM246	1,143745534	0	0,200382122	0	1,617938108	0	0	0,059548489	0	3,021614253	0,335734917
ENSG00000134716	CYP2J2	0	0	2,898547954	0	0,120792984	0	0	0	0	3,019340939	0,335482327
ENSG00000116748	AMPD1	0	0	2,97642546	0	0	0	0,040710685	0	0	3,017136145	0,335237349
ENSG00000183723	CMTM4	0,018616554	0,891934705	0	1,125135409	0	0	0,973836506	0	0	3,009523174	0,334391464
ENSG00000221955	SLC12A8	0	0	0	0	0	1,72266621	1,28030951	0	0	3,00297572	0,333663969
ENSG00000230358	SPDYE21P	1,518112379	0,621658424	0	0	0	0	0	0	0,861730397	3,0015012	0,333500133
ENSG00000168497	CAVIN2	0	0	0,091021643	2,306890738	0	0,5975823	0	0	0	2,995494681	0,332832742
ENSG00000187682	ERAS	0	0	0	0	0	0	0	0	2,995224073	2,995224073	0,332802675
ENSG00000111961	SASH1	0,041605333	0,024609214	0,025512124	0,167634437	0,296627926	2,025156636	0,046061062	0,333588495	0,034112798	2,994908024	0,332767558
ENSG00000177535	OR2B11	0,047118981	0	0,130018755	0,366138879	0,111979277	0,439738042	0,332553109	0	1,564657344	2,992204388	0,332467154
ENSG00000139329	LUM	0,113149061	0	2,289615192	0,586151197	0	0	0	0	0	2,98891545	0,332101717
ENSG00000143869	GDF7	0,962072264	0	0	0,056314875	0	0	0	0,320914115	1,644482014	2,983783269	0,331531474
ENSG00000167711	SERPINF2	0	0,799401193	1,183901614	0,444522955	0	0,141320764	0,053437109	0,281460449	0,079150989	2,983195073	0,331466119
ENSG00000206557	TRIM71	0	0	0	2,877959279	0	0,038122835	0	0,03796353	0,021351852	2,975397496	0,330599722
ENSG00000132623	ANKEF1	0	0	0,047364133	0	0,183566643	1,950558833	0	0	0,791644753	2,973134363	0,330348263
ENSG00000124920	MYRF	0,01414893	0,075320787	1,171264462	1,710247961	0	0	0	0	0	2,970982139	0,330109127
ENSG00000149124	GLYAT	0	0	0	2,8480864	0,11528653	0	0	0	0	2,963372931	0,329263659
ENSG00000173093	CCDC63	0,069189822	2,33273718	0	0,358427163	0	0	0,114899571	0	0,085094561	2,960348298	0,328927589
ENSG00000181619	GPR135	0,030361778	0	0	1,258275947	0	1,666772922	0	0	0	2,955410648	0,328378961
ENSG00000182950	ODF3L1	0	0	0	0	0	0	0	0	2,955156715	2,955156715	0,328350746
ENSG00000107338	SHB	0	0,666467274	0	0,259421681	0	0,384879548	0,686084665	0	0,954638308	2,951491478	0,327943498
ENSG00000261456	TUBB8	0	0,179900705	0	0	2,770782782	0	0	0	0	2,950683487	0,327853721
ENSG00000161249	DMKN	0	0	0	0,070106119	0	1,025239996	0,30339414	0	1,547888821	2,946629075	0,327403231
ENSG00000188580	NKAIN2	0	0	0	2,859902033	0,085565295	0	0	0	0	2,945467328	0,327274148
ENSG00000119608	PROX2	0	0	1,704652381	0,400031718	0,157300719	0	0	0,072368499	0,61053391	2,944887227	0,327209692
ENSG00000106789	CORO2A	0,026445672	0	0,0972979	0,410993135	0,062848712	1,016253095	1,207712331	0,057828941	0,065049535	2,944429321	0,327158813
ENSG00000198720	ANKRD13B	0	0	0	1,029926763	0	0,192605731	1,718771671	0	0	2,941304165	0,326811574
ENSG00000158966	CACHD1	0,021519591	0	0,079174053	2,786972813	0,051141775	0	0	0	0	2,938808232	0,326534248
ENSG00000106013	ANKRD7	0	0	0	2,581810435	0,355327384	0	0	0	0	2,937137818	0,326348646
ENSG00000065675	PRKCQ	0	0	0	2,93007407	0	0	0	0	0	2,93007407	0,325563786
ENSG00000088538	DOCK3	0,083311595	0	1,471280709	0,172633129	0,079196723	0	1,120641404	0	0	2,92706356	0,325229284
ENSG00000136379	ABHD17C	0,041743252	0,370361879	0,153580165	1,22538524	0,198407482	0	0,935828443	0	0	2,92530646	0,325034051
ENSG00000099364	FBXL19	0,557089661	0,061783871	0,512405712	0,30061633	0	0,229369461	0,404743006	0	0,856435587	2,922443629	0,324715959
ENSG00000135253	KCP	0,073185892	0,844132704	0,06731568	0,252752125	0,347855714	0,843715247	0,379798801	0	0,112511516	2,921267678	0,324585298
ENSG00000156959	LHFPL4	0,028250247	0	0	0,292692063	0,134274647	2,326284026	0	0	0,138976648	2,92047763	0,324497514
ENSG00000134200	TSHB	0	0	0	2,915474577	0	0	0	0	0	2,915474577	0,32394162
ENSG00000240184	PCDHGC3	0	0	0	0	0	0,580534246	2,333794389	0	0	2,914328635	0,323814293
ENSG00000165985	C1QL3	0	0	0	0,109821117	0	1,396553676	0	0	1,407928199	2,914302992	0,323811444
ENSG00000197191	CYSRT1	0	0	0	0	0	0	1,633227905	0	1,28071876	2,913946665	0,323771852
ENSG00000122870	BICC1	0,092014353	0,040819303	0,338535666	0,834165697	0,218674101	0,050513191	0,706713698	0,150906333	0,480954617	2,91329696	0,323699662
ENSG00000173421	CCDC36	0	0	0	2,588879536	0	0	0,318452619	0	0	2,907332155	0,323036906
ENSG00000140986	RPL3L	0	0,09722335	0	0,094610216	1,822931687	0	0,045493231	0,838666415	0	2,898924899	0,322102767
ENSG00000161610	HCRT	0	0	0	0	0	0	0	0	2,898819238	2,898819238	0,322091026
ENSG00000141519	CCDC40	0,060680884	0,484545327	0	0,104782642	0,036052358	1,082641049	0,314903955	0,796147939	0,018657416	2,89841157	0,32204573
ENSG00000112218	GPR63	0,050648758	0	0,046586241	1,355619945	1,444414449	0	0	0	0	2,897269393	0,321918821
ENSG00000124721	DNAH8	0,040148939	0	1,144786857	1,525226022	0,143122235	0	0	0,043896987	0	2,897181041	0,321909005
ENSG00000166800	LDHAL6A	0	0	0	0,504514645	0	0,192471667	1,55261045	0	0,646797641	2,896394404	0,3218216
ENSG00000123240	OPTN	0,029410387	0	0	0,15235596	0	0,032290887	0	0	2,676652909	2,890710144	0,321190016
ENSG00000009724	MASP2	0	0	0,065391696	2,823573002	0	0	0	0	0	2,888964698	0,320996078
ENSG00000204576	PRR3	1,023640866	0	0	0,487614996	0	0,155020394	0	1,003421952	0,217059708	2,886757917	0,32075088
ENSG00000181856	SLC2A4	0,074696278	0,265093426	0,412229509	0,709412931	0,621310627	0,041006074	0,124043828	0,408347216	0,229666983	2,885806873	0,320645208
ENSG00000100429	HDAC10	0,42729711	0,270795799	0	0	1,450686901	0,167552592	0,126712111	0,066740975	0,375371687	2,885157174	0,320573019
ENSG00000175287	PHYHD1	0	0	0,097742651	0,183498576	0	0	2,602935861	0	0	2,884177087	0,320464121
ENSG00000166578	IQCD	0,061427061	0,109000813	0	0	0	0,067443324	1,8871554	0	0,755473668	2,880500266	0,320055585
ENSG00000172421	EFCAB3	0	0,174344603	0	0,084829316	2,45171274	0	0	0,107423461	0,060418245	2,878728366	0,319858707
ENSG00000075275	CELSR1	0,047199928	0,020938779	0,08682808	0	0,028042913	0,025911382	2,616006454	0,051606211	0	2,876533746	0,319614861
ENSG00000125879	OTOR	0	0	0	0	0	0	0	0	2,874786969	2,874786969	0,319420774
ENSG00000271503	CCL5	0	1,073283523	0	0	0	0	0,837026898	0,220436669	0,74388201	2,874629101	0,319403233
ENSG00000266202	AC005697.1	0	0	0	0	0	0	0	0	2,873915293	2,873915293	0,319323921
ENSG00000184378	ACTRT3	0,090431221	0	0	0	0	2,780069508	0	0	0	2,870500729	0,318944525
ENSG00000165181	SHOC1	0,048155058	0,128174952	1,550239558	0,166306549	0,114441537	0	0,039984172	0,052650505	0,769918671	2,869871001	0,318874556
ENSG00000116667	C1orf21	0,054745248	0	0,025177076	2,221527725	0	0	0,568201848	0	0	2,869651897	0,318850211
ENSG00000183840	GPR39	0	0	0	0	0	0	0	0	2,864573637	2,864573637	0,31828596
ENSG00000144290	SLC4A10	0,020014645	0	0,073637112	0,103682771	0	0	2,658973923	0	0	2,856308452	0,317367606
ENSG00000204681	GABBR1	0	0	2,396380164	0	0	0	0,459326912	0	0	2,855707076	0,317300786
ENSG00000132938	MTUS2	0	0	0	1,575806241	0,212621399	0,216106139	0,014857351	0	0,836254333	2,855645463	0,31729394
ENSG00000205085	FAM71F2	0,051441896	0	0,993630992	1,598920695	0,183379171	0,028240098	0	0	0	2,855612853	0,317290317
ENSG00000160602	AC010761.1	0,102749708	0	0	0	0	2,707516211	0	0	0,042122965	2,852388884	0,316932098
ENSG00000085733	CTTN	0,021745657	0	0,080005785	0,037550004	0,051679025	0	0	2,472671969	0,187210484	2,850862923	0,316762547
ENSG00000137809	ITGA11	0,027813468	0	0,076747681	0,096055577	0,033049654	0	0,762105827	1,855007235	0	2,850779441	0,316753271
ENSG00000111319	SCNN1A	0	0,045633424	0	0,08881381	0,061115987	0,818823472	1,58012331	0	0,253024537	2,847534541	0,316392727
ENSG00000189045	ANKDD1B	0	0,326306054	0,338278186	0,317535716	0,109253892	0	0	0	1,752735683	2,844109531	0,31601217
ENSG00000140481	CCDC33	0,107475513	0	0,118625931	0,148469402	1,941175905	0,094401466	0,017847842	0,282020968	0,132181026	2,842198053	0,315799784
ENSG00000161298	ZNF382	0,053400679	0,047379097	0	0,069158488	0,158634836	1,392482091	1,119578013	0	0	2,840633204	0,315625912
ENSG00000106304	SPAM1	0	0	0	2,839991478	0	0	0	0	0	2,839991478	0,315554609
ENSG00000241962	AC079447.1	0	0	0	0	1,261326139	0,832466516	0	0	0,745997079	2,839789635	0,315532182
ENSG00000136244	IL6	0	0,259199613	0	0	0,173570586	0	0	0,159707379	2,245608051	2,838085629	0,315342848
ENSG00000170579	DLGAP1	0,025022449	0	0	1,38266587	1,427193412	0	0	0	0	2,834881731	0,314986859
ENSG00000099889	ARVCF	0	0	0	0,092595804	0	1,383569394	1,3580004	0	0	2,834165597	0,314907289
ENSG00000021645	NRXN3	0,058362628	0	0,089468973	0,907015683	1,571933883	0,181556473	0	0	0,02392617	2,832263809	0,314695979
ENSG00000147437	GNRH1	0	0	0	1,331300891	0	0	0	1,498569467	0	2,829870358	0,31443004
ENSG00000100314	CABP7	0,090648213	0	0	0,078264839	1,507992079	0,199052881	0	0,396442193	0,557428081	2,829828287	0,314425365
ENSG00000163817	SLC6A20	0	0,120911769	0,083565339	0,117661946	2,429022908	0	0	0,049667002	0,027934244	2,828763207	0,314307023
ENSG00000118369	USP35	0	0	0	0,453484488	0	0,288339549	2,04974126	0	0,032298666	2,823863963	0,313762663
ENSG00000092421	SEMA6A	0,027584989	0,07342333	0,101489631	0,166716395	1,50779545	0,469443946	0,080165418	0,361921761	0,033925981	2,822466901	0,313607433
ENSG00000166394	CYB5R2	0,017581218	0,717541129	0	0,242871413	0	0	0,948873948	0,845789469	0,043245264	2,815902441	0,312878049
ENSG00000129038	LOXL1	0	0	0	0,215233688	0,197480089	0	0	0	2,401646012	2,81435979	0,312706643
ENSG00000163638	ADAMTS9	0	0,025106929	2,342528924	0,39091382	0	0,031069396	0,023496317	0	0	2,813115386	0,312568376
ENSG00000269058	CALR3	0	0	0	0,178722585	0	0,227274763	2,40628034	0	0	2,812277688	0,312475299
ENSG00000164070	HSPA4L	0,013210121	0	1,725376958	0,889628815	0	0,014503941	0,010968645	0,086659997	0,064986982	2,805335458	0,31170394
ENSG00000204764	RANBP17	0,041343521	0,04890871	0,076054747	1,998954662	0	0,090785541	0,469154912	0,060270783	0,016949067	2,802421943	0,311380216
ENSG00000010932	FMO1	0,041951852	0,446655192	0,15434764	0,941741996	0,199398969	0,368485456	0,034833518	0,458682076	0,15478611	2,800882808	0,311209201
ENSG00000235376	RPEL1	0,496607509	0,125888263	0,652535444	0	0,33719957	0,233676869	0,294531296	0,3102672	0,349007554	2,799713704	0,3110793
ENSG00000277481	PKD1L3	0	0	0,053467994	0	0,069074349	0,223384119	1,737615677	0	0,714931803	2,798473943	0,310941549
ENSG00000186226	LCE1E	0	0	0	0	0	0	0	2,798137215	0	2,798137215	0,310904135
ENSG00000179044	EXOC3L1	0,049625803	0	0	0	0	0	0	0	2,746500329	2,796126132	0,310680681
ENSG00000134317	GRHL1	0,019002838	0	1,153589445	1,082853894	0,13548197	0,396416116	0	0	0	2,787344263	0,309704918
ENSG00000134343	ANO3	0,021574896	0	2,460703332	0,149020545	0,102546414	0	0	0	0,053068681	2,786913869	0,309657097
ENSG00000255508	AP002990.1	2,209814002	0	0	0	0	0,574637364	0	0	0	2,784451366	0,309383485
ENSG00000078795	PKD2L2	0	0,230511068	0,119484246	0,504709815	0	0,285253517	0	1,562338381	0,079882451	2,782179478	0,309131053
ENSG00000157782	CABP1	0	0,045773643	0	1,692647497	0	0,028322051	0	1,015333256	0	2,782076447	0,309119605
ENSG00000184361	SPATA32	0	0	0	0,270679434	0	0	0	0	2,506229035	2,776908469	0,308545385
ENSG00000159915	ZNF233	0	0	0	0	0	0	2,694623274	0,081568642	0	2,776191916	0,308465768
ENSG00000041353	RAB27B	0,063367586	0	0	2,662601782	0,050198147	0	0	0	0	2,776167515	0,308463057
ENSG00000136449	MYCBPAP	0,023194255	0	0	1,802313716	0	0,942239653	0	0	0	2,767747623	0,307527514
ENSG00000146205	ANO7	0,026533902	0	0	0	0	1,048776253	0	0,058021873	1,631663936	2,764995964	0,307221774
ENSG00000198933	TBKBP1	0,317755873	0	0,844332076	0,670627271	0	0	0,850149446	0,077204338	0	2,760069004	0,306674334

ENSG00000188211	NCR3LG1	0,140829982	0,041649891	0	0,162121761	0	1,030820607	1,325253584	0	0,057734241	2,758410067	0,306490007
ENSG00000240303	ACAD11	0	0	0	0	0	0	0	2,757451189	0	2,757451189	0,306383465
ENSG00000010319	SEMA3G	0	0	0,055473977	0,104144871	0,071665844	0	0	0,065941841	2,447788958	2,745015492	0,305001721
ENSG00000243489	KRTAP10-11	0	0	0	2,742243064	0	0	0	0	0	2,742243064	0,304693674
ENSG00000008056	SYN1	1,497747419	0	0	0	0	0,711108821	0	0,531102975	0	2,739959214	0,304439913
ENSG00000197774	EME2	0,021871555	0	0	0,188837006	0	1,152657068	0,50849172	0,382613481	0,484185468	2,738656298	0,304295144
ENSG00000184898	RBM43	0	0	0	0,05550627	0,076391734	1,764630682	0,693943551	0,140580544	0	2,73105278	0,303450309
ENSG00000188848	BEND4	0,017074641	0	0,031410181	2,653576013	0	0	0,028354877	0	0	2,730415712	0,303379524
ENSG00000206561	COLQ	0	0,064225629	0	0	0	0,03973906	0	0	2,626334087	2,730298776	0,303366531
ENSG00000147576	ADHFE1	0	0	0	0,463472424	0	0	2,265746078	0	0	2,729218502	0,3032465
ENSG00000177710	SLC35G5	0	0	0	1,975283684	0	0,7535681	0	0	0	2,728851784	0,303205754
ENSG00000078018	MAP2	0,052291918	2,134186695	0,14429281	0,045148365	0,093204657	0,258360618	0	0	0	2,727485063	0,303053896
ENSG00000185800	DMWD	2,093972125	0	0,195039536	0,137310108	0,062992026	0,029102013	0	0,173882425	0,032598934	2,724897166	0,302766352
ENSG00000236782	AL391650.1	0	1,034797878	0	0	0	0,160068091	0,363155809	0	1,165462995	2,723484774	0,302609419
ENSG00000132003	ZSWIM4	0	0	0	0,609014369	0,152394459	0,492838547	0,425954872	0,490779116	0,552058415	2,723039778	0,302559975
ENSG00000185942	NKAIN3	0	0	0	0	0	0	0,858210205	0,339023154	1,525416051	2,722649411	0,302516601
ENSG00000137103	TMEM8B	0,018631474	0,016530547	0	0,112603713	0,022139051	0,450037937	1,268549069	0,020370789	0,813458053	2,722320632	0,30248007
ENSG00000249860	MTRNR2L5	0	0,362844384	0,376157103	1,059275946	0	0,22450687	0	0,447137439	0,251483792	2,721405534	0,302378393
ENSG00000105971	CAV2	0	2,527334145	0	0,119003485	0,054593728	0	0,019074237	0	0	2,720005595	0,302222844
ENSG00000127954	STEAP4	0,537520927	0	0,049440658	0,232045331	0,063871506	0	0	0	1,83450102	2,717379442	0,301931049
ENSG00000198796	ALPK2	0,047394847	0	0,261559947	0,845685751	0	0,017345591	1,272412149	0	0,27201791	2,716416195	0,301824022
ENSG00000162461	SLC25A34	0,045269793	0	0	0,390855265	0,107584644	0	0	0	2,1713644	2,715074103	0,3016749
ENSG00000105278	ZFR2	0,076190204	0,270395294	0	0,043854618	0	0,223073044	0,379574114	1,721591864	0	2,714679138	0,301631015
ENSG00000105855	ITGB8	0,697924251	0,03538427	0	0,15494951	0,047389488	0,328405734	0,720237456	0,43604456	0,29429378	2,714629049	0,30162545
ENSG00000124772	CPNE5	1,43989629	0	0,866881806	0,406863266	0	0	0	0	0	2,713641362	0,301515707
ENSG00000127530	AC005255.1	0,025330747	1,887849133	0	0,196833021	0,030099534	0,02781168	0,536332825	0	0	2,70425694	0,300472993
ENSG00000100359	SGSM3	0,285114284	0,056214256	0	0,273516745	0	0,626077648	0,341952687	0,069273494	1,051961447	2,704110561	0,300456729
ENSG00000167549	CORO6	0	0,062055543	0	0	0	1,76623155	0,871120102	0	0	2,699407195	0,299934133
ENSG00000171246	NPTX1	0,021525722	0,038196866	0	0,111510673	0,20462538	0	2,323524667	0	0	2,699383308	0,299931479
ENSG00000036672	USP2	0,133637471	0	0,184377691	0	0,714582773	0,256770736	0,221924089	1,022791056	0,164356863	2,698440678	0,299826742
ENSG00000184860	SDR42E1	0,0126941	0,045050739	0,023351823	1,293276504	0	1,29617638	0,010540182	0	0,015612107	2,696701836	0,299633537
ENSG00000261594	TPBGL	0	0	0	0	0	1,069241096	0,494154548	0	1,13118184	2,694577483	0,299397498
ENSG00000125775	SDCBP2	0	0	0	0	0	0	0	0	2,693427859	2,693427859	0,299269762
ENSG00000132631	SCP2D1	0	0	0	0	2,69203555	0	0	0	0	2,69203555	0,299115061
ENSG00000132561	MATN2	0,02177072	0,038631609	0	0,225559696	0	0	2,404200282	0	0	2,690162307	0,298906923
ENSG00000122733	PHF24	0,025995281	0,046127989	0,095640839	0,044888177	0,061778349	2,368927897	0,043168874	0	0	2,686527407	0,298503045
ENSG00000189056	RELN	0	0,125848874	0,021744376	0,387810116	2,078746725	0,012977986	0,058887828	0	0	2,686015904	0,298446212
ENSG00000179058	C9orf50	0	0	0	0	0	0,094643797	0	0,094248308	2,491382358	2,680274463	0,297808274
ENSG00000110375	UPK2	0	0	0	0,464297108	0	2,21410912	0	0	0	2,678406228	0,297600692
ENSG00000185736	ADARB2	0,042900701	0	0,05261287	0	0,101954446	2,480729736	0	0	0	2,678197753	0,297577528
ENSG00000137501	SYTL2	0,080535035	0	0,024691784	0,046355476	0,031898876	0	0,278624827	0	2,212065552	2,674171549	0,297130172
ENSG00000184058	TBX1	1,899434547	0	0	0	0	0	0,771046733	0	0	2,67048128	0,296720142
ENSG00000149489	ROM1	0	0	0	0,104583156	0	0	2,564724221	0	0	2,669307377	0,296589709
ENSG00000145975	FAM217A	0	0,085449968	0,088585118	0,415766371	0	0	2,079176948	0	0	2,668978405	0,296553156
ENSG00000165821	SALL2	0	0,049812744	0	2,617590308	0	0	0	0	0	2,667403052	0,296378117
ENSG00000236699	ARHGEF38	0,199354315	0,459874143	0	0,172120696	0,284261907	0,437758778	0,082764071	1,00263787	0,024518011	2,663289789	0,295921088
ENSG00000179431	FJX1	0	0,875565715	0	0	0,146578589	0	1,638796173	0	0	2,660940476	0,295660053
ENSG00000137674	MMP20	0	0	0	0	2,530977197	0	0	0,129379236	0	2,660356433	0,295595159
ENSG00000273045	C2orf15	0	0,127808389	0	0,870612403	0	1,660687374	0	0	0	2,659108166	0,295456463
ENSG00000040608	RTN4R	0	0	0	0,250417442	0,114880852	0	2,287846745	0	0	2,653145038	0,294793893
ENSG00000196542	SPTSSB	0,040349952	0,0716	0,148453991	0	0	2,392302041	0	0	0	2,652705984	0,294745109
ENSG00000183921	SDR42E2	0,057456491	0	0	1,488222289	0,273093188	0,126167739	0	0	0,706640769	2,651580475	0,294620053
ENSG00000137857	DUOX1	0,051608801	0,061052368	0,094938558	0,683231377	0,040883145	0	0,128555668	1,504711145	0,084629564	2,649610627	0,294401181
ENSG00000162591	MEGF6	0,703541067	0	0	0	0,039809061	0,03678319	0,013908694	0	1,854138899	2,648180911	0,294242323
ENSG00000184675	AMER1	0,053923125	0,031895087	0,099195943	0,869059033	0	0,019734813	1,567073256	0	0	2,640881258	0,293431251
ENSG00000182742	HOXB4	0	0,133937063	0	1,303371502	0	1,077341407	0,125344987	0	0	2,639994959	0,293332773
ENSG00000185567	AHNAK2	0,02412879	0,014271982	1,834656817	0,124995464	0	0	0	0,017587533	0,623181334	2,63882192	0,293202436
ENSG00000286070	GGT1	0	0	0	2,638205114	0	0	0	0	0	2,638205114	0,293133902
ENSG00000174898	CATSPERD	0	0	0,099956886	0	1,807855301	0	0,135350951	0,594093074	0	2,637256212	0,293028468
ENSG00000196275	GTF2IRD2	0,017177512	0	0,410792462	0,800868968	0	0,132019329	0,299519947	0,300497504	0,676036002	2,636911724	0,292990192
ENSG00000133742	CA1	0,037146158	0	0	0	0	0	0	2,599288633	0	2,636434791	0,292937199
ENSG00000137766	UNC13C	0	0,018569391	0,019250699	0	0,074608908	1,29833069	1,164336876	0,045766561	0,012870258	2,633733382	0,292637042
ENSG00000177076	ACER2	0	0	0	0	0	2,632551524	0	0	0	2,632551524	0,292505725
ENSG00000204128	C2orf72	0	0	0	0	0	0	2,629027386	0	0	2,629027386	0,292114154
ENSG00000077092	RARB	0	0,072529619	1,052670107	0	0	0,044877083	0	0	1,457816918	2,627893727	0,291988192
ENSG00000096433	ITPR3	0	0,054334752	0,056328288	0,370120531	0,036384756	0	2,11024064	0	0	2,627408966	0,29193433
ENSG00000128268	MGAT3	0,026103052	0	0	0,315519921	0	0,401234768	0	1,88363115	0	2,626488892	0,291832099
ENSG00000215788	TNFRSF25	0	1,001697876	0	0,060923412	0,167694393	0	0	1,388704697	0	2,619020377	0,291002264
ENSG00000081479	LRP2	0,037522024	0,049936433	0,017256198	2,381118708	0,044585951	0,030897742	0,007788834	0,02051242	0,023073626	2,612691937	0,290299104
ENSG00000140465	CYP1A1	0	0	0	2,561991717	0	0,049363456	0	0	0	2,611355174	0,290150575
ENSG00000264522	OTUD7B	0,383121591	1,776355771	0	0,064022649	0,029370863	0,027138395	0	0,081074974	0,243194926	2,604279167	0,289364352
ENSG00000266074	BAHCC1	0	0,358239145	0,144426682	1,278238575	0	0,221657417	0,307319896	0,294308232	0	2,604189948	0,289354439
ENSG00000158106	RHPN1	0	0	0	2,432233272	0,171662305	0	0	0	0	2,603895577	0,289321731
ENSG00000165661	QSOX2	0,066715484	0	0	0	0	1,757992866	0,581650546	0,072943613	0,123077167	2,602379675	0,289153297
ENSG00000011677	GABRA3	0	0	0,066983156	0,628758975	0	0	1,904730341	0	0	2,600472473	0,288941386
ENSG00000147041	SYTL5	0	0,111262241	0	0,378951208	1,490114284	0	0	0,616994006	0	2,597321738	0,288591304
ENSG00000137225	CAPN11	0,042590352	0	0	0,073544243	0	2,478371073	0	0	0	2,594505668	0,288278408
ENSG00000099617	EFNA2	0	0,331858911	1,032104328	0,64587865	0,444452404	0	0	0,136317891	0	2,590612184	0,287845798
ENSG00000154358	OBSCN	0,018719179	0,016608362	0,017217721	0,298996409	0,077851434	0,472708983	0,0116572	0,010233341	1,663352274	2,587344902	0,287482767
ENSG00000187736	NHEJ1	0	0,689976198	0	0	0,11550902	0,266823057	0,484285617	0,425132927	0,597769451	2,57949627	0,286610697
ENSG00000221926	TRIM16	0,053585309	0,047542908	0,049287252	2,081927989	0,063673323	0,235334151	0,044493026	0	0	2,575843957	0,286204884
ENSG00000163554	SPTA1	0	0	0,137532208	0,258198075	0,142140329	1,526784448	0,049661719	0	0,45974303	2,574059809	0,286006645
ENSG00000250067	YJEFN3	0	2,446716459	0	0	0	0	0,127208853	0	0	2,573925312	0,285991701
ENSG00000102678	FGF9	0,0332916	0	0,061242586	0,229949306	0,079118207	0	0	0,655190815	1,514941909	2,573734422	0,285970491
ENSG00000115593	SMYD1	0,13278609	0	0,061067684	0,114646298	0	0	0	2,177732211	0,081654887	2,567887171	0,285320797
ENSG00000173401	GLIPR1L1	0	0,968721099	0	0	0	0,959020675	0,63460428	0	0	2,562346054	0,284705117
ENSG00000138336	TET1	0,031442066	0,027896588	0	0	0	0,811256461	0	1,6501102	0,038669688	2,559375003	0,284375
ENSG00000151917	BEND6	1,454281022	0,047788629	0	1,023092041	0	0	0	0	0,033121818	2,558283511	0,284253723
ENSG00000149054	ZNF215	0	0	0	0	0	2,390802546	0	0,166103167	0	2,556905714	0,284100635
ENSG00000153162	BMP6	0	0,141724102	0	1,93080848	0	0,482298188	0	0	0	2,55483077	0,283870086
ENSG00000278803	AC236972.4	0	0	0	0	2,554343981	0	0	0	0	2,554343981	0,283815998
ENSG00000161281	COX7A1	0	0	0	0	0	0	1,284679167	0	1,268576944	2,553256111	0,283695123
ENSG00000164283	ESM1	2,357594342	0	0	0,193859565	0	0	0	0	0	2,551453907	0,283494879
ENSG00000042832	TG	0,122169883	0,048174991	0	1,125123857	0,06451986	0,014903933	0,157795962	0	1,018382848	2,551071334	0,28345237
ENSG00000278615	C11orf98	0	0	0	0	0	0,935234367	0,565818859	0	1,047612865	2,548666091	0,283185121
ENSG00000276240	OR4E1	0	0,214972207	0	2,231405448	0,095969413	0	0	0	0	2,542347068	0,282483008
ENSG00000133980	VRTN	0	0	0	0	2,538623192	0	0	0	0	2,538623192	0,282069244
ENSG00000161860	SYCE2	0,207284733	0	0	0,178967747	0	0	2,151411728	0	0	2,537664208	0,28196269
ENSG00000144810	COL8A1	0,016061923	2,337122025	0,147736022	0	0	0,035270106	0	0	0	2,536190075	0,281798897
ENSG00000185880	TRIM69	0	0,318355316	0	0	0,503888619	0,662567872	0,297932791	0,249653748	0,501474696	2,533873041	0,281541449
ENSG00000008394	MGST1	0	0	0,074715791	1,157217331	0	0	1,298375966	0	0	2,530309088	0,281145454
ENSG00000069122	ADGRF5	0	2,39666384	0	0	0,054403506	0,025134158	0	0,050058259	0	2,526259762	0,280695529
ENSG00000164796	CSMD3	0,01480968	0,052558828	0,013621801	0,306877022	0,035195525	0,016260163	0,03689038	1,311569558	0,737666894	2,525449852	0,280605539
ENSG00000061455	PRDM6	0,016371676	0,087153413	0	2,402976472	0	0,017975144	0	0	0	2,524476705	0,280497412
ENSG00000175318	GRAMD2A	0	0	2,228297387	0,26145789	0	0,033248613	0	0	0	2,52300389	0,280333766
ENSG00000256591	AP003108.2	0	0	0	0,728481051	0	0,132340263	0,050041345	0	1,605959046	2,516821706	0,279646856
ENSG00000226174	TEX22	0	0,559017373	0	0,090665384	0	0,115295745	0	1,492581438	0,258299545	2,515859484	0,279539943
ENSG00000105261	OVOL3	0	2,512709506	0	0	0	0	0	0	0	2,512709506	0,279189945
ENSG00000184194	GPR173	0	0	0	2,038554489	0,077933494	0,396053894	0	0	0	2,512541878	0,27917132
ENSG00000087250	MT3	0	0	0	0	0	0,541896277	0,341508797	0	1,618695894	2,502100967	0,278011219
ENSG00000130699	TAF4	0,138661821	0	0	0,5586903	0	0,236821786	0,025585304	1,179160875	0,360020792	2,498940878	0,277660098
ENSG00000204805	AL391987.2	0	0,191393291	0	0	2,30696494	0	0	0	0	2,498358231	0,277595359
ENSG00000151276	MAGI1	0,051887912	0,046036913	0	0,268797295	1,17147108	0,128182264	0,786276439	0,028365917	0,015953861	2,49697168	0,277441298
ENSG00000072133	RPS6KA6	0,773251726	0,030491471	0,0632204	0,741798313	0,735059786	0	0,071338601	0,075150004	0	2,490310303	0,276701145
ENSG00000124593	AL365205.1	0,742226514	0	0	0,582574179	0	0,629711946	0,280130234	0	0,248957163	2,483600035	0,275955559
ENSG00000175336	APOF	0	0	0	2,385691196	0	0,094806044	0	0	0	2,48049724	0,275610804
ENSG00000046889	PREX2	0,024911073	0	0	1,032383678	1,036029837	0	0,031026294	0,354075946	0	2,478426828	0,275380759

ENSG00000101082	SLA2	0	0	1,523456882	0,953361252	0	0	0	0	0	2,476818133	0,275202015
ENSG00000133710	SPINK5	0,137969021	0	0	0,119121194	2,049289787	0	0	0	0,169684111	2,476064113	0,275118235
ENSG00000128383	APOBEC3A	0	0	0	0	0,152232956	0	0	2,24118401	0,078781909	2,472198874	0,274688764
ENSG00000198203	SULT1C2	2,374877405	0	0	0	0	0,019753611	0,074693614	0	0	2,46932463	0,274369403
ENSG00000161040	FBXL13	0	0	0,970746168	1,497007836	0	0	0	0	0	2,467754004	0,274194889
ENSG00000163884	KLF15	0	0	0	0	0,127618174	0	2,273982563	0	0,06604354	2,467644277	0,274182697
ENSG00000181029	TRAPPC5	0,6386078	0,141649234	0	0,321631418	0,063236053	0,233718016	0,353499791	0,058185344	0,654504393	2,465032048	0,27389245
ENSG00000182508	LHFPL1	0,172697795	0	0	0	0,205210024	2,085732964	0	0	0	2,463640784	0,273737865
ENSG00000163357	DCST1	0	0	0	2,288186436	0	0	0	0	0,174613081	2,462799516	0,273644391
ENSG00000186976	EFCAB6	0,035318586	0,376031787	0	0	0,167870769	0	0,058651552	0	1,824366936	2,46223963	0,273582181
ENSG00000196411	EPHB4	0,02347531	0,124969085	0,215923644	0,851271472	0,055789582	1,108304303	0	0,051333629	0,028871605	2,45993863	0,273326514
ENSG00000091428	RAPGEF4	0,021208501	0	0	0,183112263	0,050402462	0,023285695	0	2,179708726	0	2,457717647	0,273079739
ENSG00000165566	AMER2	0	1,188370957	0,026212173	0,024604901	1,18520641	0,031289123	0	0	0	2,455683566	0,27285373
ENSG00000139445	FOXN4	0	0	0	0,147587655	0	0	2,306439926	0	0	2,454027582	0,272669731
ENSG00000174939	ASPHD1	0	0	0,120233607	0	2,329914851	0	0	0	0	2,450148458	0,272238718
ENSG00000279761	OR5D13	0	0	0	0	0	0	0	2,447663463	0	2,447663463	0,271962607
ENSG00000074855	ANO8	0	0,053574825	0,111080959	1,355506362	0	0,099446899	0,827276914	0	0	2,446885959	0,271876218
ENSG00000203795	FAM24A	0,253541226	0	0	2,1890518	0	0	0	0	0	2,442593026	0,271399225
ENSG00000135111	TBX3	0	0	0	0,350248288	0,060254621	0	0	0	2,026849656	2,437352565	0,270816952
ENSG00000285130	AL358113.1	0	0,707742254	0,031900402	1,077996223	0	0,2665536	0,331170259	0	0,021327349	2,436690087	0,270743343
ENSG00000118245	TNP1	0	0	0	0	0	0	0	2,435635633	0	2,435635633	0,270626181
ENSG00000185585	OLFML2A	0,044634639	1,306850687	0,164218047	0,34683426	0	0	0,277958201	0,292808655	0	2,433304489	0,270367165
ENSG00000242220	TCP10L	0,036447316	0	0	0	0	0,640272365	1,755253077	0	0	2,431972758	0,270219195
ENSG00000105889	STEAP1B	0	0	0	0,120840525	0,055436484	1,050066941	0	1,173200842	0,028688873	2,428233666	0,269803741
ENSG00000183773	AIFM3	0,055956516	0	2,161667128	0	0	0	0,209078502	0	0	2,426702145	0,269633572
ENSG00000153233	PTPRR	0,09000034	0,053234465	0,055187631	0,051803648	0,142591837	0	0	2,033645343	0	2,426463265	0,269607029
ENSG00000146926	ASB10	0	0	0,234088504	0	0	0	0	0	2,191032684	2,425121188	0,26945791
ENSG00000205571	SMN2	0,451675568	0	0	0	0	0,220405947	0,25002391	1,316909602	0,185167574	2,424182601	0,269353622
ENSG00000186115	CYP4F2	0	0	1,076399222	1,347195739	0	0	0	0	0	2,42359496	0,269288329
ENSG00000285458	AC093827.5	1,876022303	0	0	0	0	0	0,100496862	0	0,446567534	2,423086699	0,269231855
ENSG00000136206	SPDYE1	1,254463402	0	0,865382529	0	0	0	0,043400322	0	0,257138045	2,420384299	0,268931589
ENSG00000258311	AC009779.3	0	0	0	0	0	0,094535941	2,323524667	0	0	2,418060608	0,268673401
ENSG00000102780	DGKH	0,015390393	0,013654937	0,127403417	0,624532454	0,329180413	0,067591007	1,233170753	0	0	2,410923374	0,267880375
ENSG00000273136	NBPF26	0	0	1,516553179	0,192373175	0	0,146780221	0,037000983	0,04872229	0,465849437	2,407279284	0,267475476
ENSG00000117461	PIK3R3	0,020428629	0	0	0	0,097098159	0,515877148	1,340023824	0	0,427117869	2,40054563	0,266727292
ENSG00000103269	RHBDL1	0	0	0	0	0	0	2,397522268	0	0	2,397522268	0,266391363
ENSG00000146426	TIAM2	1,637948433	0,023251994	0,626732793	0,045254071	0,031140959	0	0,010880188	0	0,016115723	2,391324163	0,265702685
ENSG00000117020	AKT3	0	1,980996307	0	0	0	0,297675969	0,013242251	0	0,098072043	2,38998657	0,265554063
ENSG00000151789	ZNF385D	0,045107633	1,020540448	0	0,175254834	0	0	0,514990169	0,049318592	0,582504025	2,387715701	0,265301745
ENSG00000166268	MYRFL	0	0,228270715	0,473291882	0,592360896	0,917156039	0,047080186	0,035604521	0,093766903	0	2,387531143	0,265281238
ENSG00000248483	POU5F2	0,017950887	0	0	0,154986324	0,042660673	2,108865729	0,05962002	0	0	2,384083633	0,264898181
ENSG00000196296	ATP2A1	0,032943224	2,221363855	0,060601722	0	0	0	0,027353462	0	0,040515919	2,382778183	0,264753131
ENSG00000275895	U2AF1L5	0,475660579	0	0,050000917	0,516284688	0	0,164134935	0	0,891540339	0,284143437	2,381764894	0,264640544
ENSG00000235387	SPAAR	0,104214187	0,092462759	0	2,069484281	0	0	0	0,113942954	0	2,380104181	0,26445602
ENSG00000284691	AC073111.4	0	0	0	0,240881583	0	0,306320012	1,274104456	0	0,557582458	2,378888509	0,264320945
ENSG00000006210	CX3CL1	0	0	0,046640956	2,276613872	0	0	0	0,055442042	0	2,37869687	0,264299652
ENSG00000150672	DLG2	0,018087327	0,064191035	0	0,078082164	0	0,963153147	1,021243949	0	0,233573342	2,378330964	0,264258996
ENSG00000157578	LCA5L	0,025870668	0	0,047591183	0,044672997	0,061482202	2,101931634	0	0	0,095452759	2,377001443	0,264111271
ENSG00000133740	E2F5	0,025189293	0	0,046337739	1,087410296	0,478903207	0	0,271897702	0	0,464693755	2,374431993	0,263825777
ENSG00000175564	UCP3	0	0	0	0	2,366862172	0	0	0	0	2,366862172	0,262984686
ENSG00000163975	MELTF	0,018002212	0,031944484	0	0,714975508	0	0,553430563	0,448428635	0	0,597790815	2,364572218	0,262730246
ENSG00000196329	GIMAP5	0,150333846	0	0	0,475921857	0	0,495173335	0,769756638	0,164368049	0,308152085	2,363705809	0,262633979
ENSG00000284505	LYNX1-SLURP2	0	0	0	0	0	0	1,235692664	0	1,126342559	2,362035223	0,262448358
ENSG00000162148	PPP1R32	0	0	0	0	0,448896928	0,69129407	0	0,13768107	1,084104713	2,361976781	0,262441865
ENSG00000092758	COL9A3	0	1,550914274	0	0,700713627	0,074182512	0,034271964	0	0	0	2,360082378	0,262231375
ENSG00000215252	GOLGA8B	0	0,034959844	0,036242516	1,360808212	0	0,216311052	0,261737329	0,344651439	0,096921263	2,351631655	0,261292406
ENSG00000136367	ZFHX2	0	0,235625659	0,027141193	0	0	0,92334533	0,257261958	0	0,90727652	2,35065066	0,261183407
ENSG00000164411	GJB7	0	0	0	0,095615601	0	2,249428852	0	0	0	2,345044453	0,260560495
ENSG00000114805	PLCH1	0,063199737	0,112146382	0	1,891623962	0,150195543	0,092519491	0,03498406	0	0	2,344669175	0,260518797
ENSG00000283787	PRR33	0	0	0	0,471711313	0	0	0,302429243	0	1,567852553	2,341993109	0,260221457
ENSG00000135451	TROAP	0	0	0,14060703	0,263970637	0	0	1,935682917	0	0	2,340260584	0,260028954
ENSG00000177570	SAMD12	0,009826412	0,017436728	0,018076479	0,050904209	0,023352682	0,021577653	2,105042636	0,021487487	0,072511324	2,34021561	0,260023957
ENSG00000139890	REM2	0	0	0	1,747332419	0	0,592537774	0	0	0	2,339870193	0,259985577
ENSG00000185634	SHC4	0,02313389	0,041050521	2,00016299	0,079894358	0	0,050799319	0,115251377	0	0,028451703	2,338744158	0,259860462
ENSG00000087494	PTHLH	0	0,079402428	0,082315694	1,699902115	0,31902654	0	0	0,097848554	0,055033024	2,333528356	0,259280928
ENSG00000105613	MAST1	2,197046865	0	0	0,079869903	0,054961362	0	0	0	0	2,33187813	0,25909757
ENSG00000171847	FAM90A1	0	0	0	0	0	0,849817769	1,334790766	0	0,14645116	2,331059695	0,259006633
ENSG00000258366	RTEL1	0	0	0,77696301	0,040517853	0	0,257625119	0,331210504	0	0,92346098	2,329777466	0,258864163
ENSG00000049323	LTBP1	0,143954	0,072983669	1,097090755	0,852264479	0	0,022579012	0	0,089938641	0,050584247	2,329394803	0,258821645
ENSG00000260851	AC010542.3	0	0,318080664	0	0	0	0,393619399	0	1,175923728	0,440917016	2,328540807	0,258726756
ENSG00000106819	ASPN	0,059822079	0,530764054	0	0	0,142168465	0	0,049671549	0	1,545042347	2,327468493	0,25860761
ENSG00000162630	B3GALT2	0	0	0	0	0	1,41890536	0	0	0,908229797	2,327135157	0,258570573
ENSG00000152208	GRID2	0,042698664	0,075767731	0	0,073731273	0,43972196	0,171895671	0	0	1,522901708	2,326717007	0,258524112
ENSG00000168255	POLR2J3	0	0,168114107	0	0	0,225152064	0,624115022	0,039332392	0,103584504	1,165181958	2,325480047	0,258386672
ENSG00000107014	RLN2	0	0,781755102	0	0,253581122	0	1,289878147	0	0	0	2,325214372	0,258357152
ENSG00000271810	AL603832.3	0	0	2,3207986	0	0	0	0	0	0	2,3207986	0,257866511
ENSG00000069431	ABCC9	0,01300883	0	0	0,157243872	2,071356348	0	0,032404528	0,028446502	0,015999184	2,318459264	0,257606585
ENSG00000136014	USP44	0,028129295	0	0	0	0	2,254555491	0	0	0,034595406	2,317280192	0,257475577
ENSG00000129354	AP1M2	0	0	2,260000805	0	0	0	0,056671333	0	0	2,316672138	0,257408015
ENSG00000230667	SETSIP	0,166238252	0,294985699	0,30580869	0	0,395068803	0	0,414093505	0,727028751	0	2,3032237	0,255913744
ENSG00000160201	U2AF1	0,122389771	0,217177646	0,025016208	0,493127652	0,032317993	0,731607115	0,316159944	0,029736732	0,33449698	2,302030041	0,255781116
ENSG00000204311	PJVK	0,039891914	0	0	0	0	1,620562655	0	0	0,637804855	2,298259424	0,255362158
ENSG00000109265	KIAA1211	0,022403346	0,079508377	0,08242553	2,011655846	0,053242037	0	0	0,048989558	0	2,298224694	0,255358299
ENSG00000182050	MGAT4C	0,027214381	0,028974751	0	0,169175865	2,017877629	0	0,004519336	0,035705929	0,013388072	2,296855962	0,255206218
ENSG00000236444	UBE2L5	0,277776784	0,123227022	0	0,59957485	0	0,609965356	0,172982994	0	0,512444455	2,295971461	0,25510794
ENSG00000156925	ZIC3	0	2,182818338	0	0,111797333	0	0	0	0	0	2,294615671	0,254957297
ENSG00000106526	ACTR3C	0	0,91317709	0	0	0,055590951	0,333875774	0,990555253	0	0	2,293199067	0,254799896
ENSG00000158427	TMSB15B	0	0	0	0	2,080710005	0,210279565	0	0	0	2,290989569	0,254554397
ENSG00000105509	HAS1	0	0	0,124042882	0	0,320497584	0,222102512	0,167965639	0,294899212	1,161022451	2,29053028	0,254503364
ENSG00000203734	ECT2L	0,032679622	0,115978374	0	0,225722296	0,077663828	0,322922835	0,786903034	0	0,723450995	2,285320984	0,253924554
ENSG00000158715	SLC45A3	0	0	0	0	0	1,54767329	0,735700241	0	0	2,283373531	0,25370817
ENSG00000275896	PRSS2	0	0	0	2,279813122	0	0	0	0	0	2,279813122	0,253312569
ENSG00000118407	FILIP1	0,016857493	0,119652834	0,155053603	0,058218424	0,120186594	0	0,027994273	0,036862402	1,741533676	2,276359298	0,252928811
ENSG00000163462	TRIM46	0,033335665	0,11830664	0	0,863451273	0,554560511	0,439207351	0,110717257	0,072895338	0,081997142	2,274471177	0,25271902
ENSG00000127328	RAB3IP	0,011981491	0	1,124086984	0,227583628	0,455588382	0,118394798	0,278557667	0,026200013	0,02947138	2,271864345	0,252429372
ENSG00000130783	CCDC62	0	0,239465952	0	1,165148357	0,080178063	0,370418791	0	0	0,414928605	2,270139769	0,252237752
ENSG00000163132	MSX1	0,071244965	0,252844885	0,393182601	0,369073514	0,846576007	0	0,177468645	0,155791875	0	2,266182493	0,251798055
ENSG00000155761	SPAG17	0,522052851	0,028071817	0	0,081951939	0	0,277907164	0	1,314542877	0,038912588	2,263439237	0,251493249
ENSG00000135097	MSI1	0	0,087229018	0,452147201	0,84884507	0,584120921	0	0,122449901	0,107493353	0,060457554	2,262743018	0,251415891
ENSG00000186472	PCLO	0,1717616	0,035167701	0	1,24341664	0,015699814	0,007253238	0,23586711	0,057783434	0,495612392	2,26256193	0,25139577
ENSG00000130829	DUSP9	0	0	1,907411297	0,094234371	0,259384285	0	0	0	0	2,261029953	0,25122555
ENSG00000128045	RASL11B	0	0,329008589	0	1,280662452	0	0,135714173	0,513171092	0	0	2,258556306	0,250950701
ENSG00000140323	DISP2	0,011849951	0,042054893	0,065396824	0,65479291	0,056323328	0,663538222	0,009839267	0,025912372	0,728695586	2,258403354	0,250933706
ENSG00000127780	OR1E2	0	0	1,143951025	0	1,108387476	0	0	0	0	2,252338501	0,250259833
ENSG00000163141	BNIPL	0,079343959	0	0,072979811	0,411029101	0,094281319	0	0,032940491	0	1,561325469	2,251900151	0,250211128
ENSG00000139800	ZIC5	0	0	0	2,249924334	0	0	0	0	0	2,249924334	0,249991593
ENSG00000229292	RFPL4AL1	0	0	0,612290967	0	0	0	0	0	1,637414293	2,24970526	0,249967251
ENSG00000198963	RORB	0	0,055740983	0	0,027121398	2,164932695	0	0	0	0	2,247795077	0,249755009
ENSG00000163531	NFASC	0,021312196	0,037817968	0,039205505	0,539755483	1,46881793	0,015599697	0	0,124276082	0	2,246784861	0,249642762
ENSG00000172382	PRSS27	0	0	0,046956098	0	0	0,58853414	1,610767776	0	0	2,246258014	0,249584224
ENSG00000204659	CBY3	0	0	0	0	0	0	0,722755369	1,522739942	0	2,245495311	0,249499479
ENSG00000127364	TAS2R4	0	0	0	0,438915012	0	0	0	1,80641269	0	2,245327702	0,249480856
ENSG00000143786	CNIH3	0	0	0	0,655718485	0,112805887	0,677505104	0,768547659	0	0,029189025	2,24376616	0,249307351
ENSG00000198185	ZNF334	0	0	0	0	0	0	0,40208992	0	1,840868684	2,242958604	0,249217623
ENSG00000102781	KATNAL1	0	0,131249143	0,034016165	1,628448814	0	0	0,107475811	0	0,341127978	2,24231791	0,249146434
ENSG00000073910	FRY	0,008016901	0,099580561	0,014747737	0,069217193	0,114314035	0,123229247	0,153101896	1,647877837	0,009859755	2,239945163	0,248882796
ENSG00000143257	NR1I3	0	0	0	0	0	0,501998719	0,569456877	0,099980202	1,068406633	2,239842432	0,248871381

ENSG00000129673	AANAT	0	0	0	0	0,327961226	0	1,432309726	0,301766728	0,169722851	2,231760532	0,247973392
ENSG00000197608	ZNF841	0	0	0	0,24346627	0,418844813	1,431931733	0,058535261	0,077078276	0	2,229856352	0,247761817
ENSG00000188368	PRR19	0	0	0	2,229347356	0	0	0	0	0	2,229347356	0,247705262
ENSG00000167578	RAB4B	0	0	1,342216488	0,331556512	0	0,252976737	0,15942863	0	0,141687311	2,227865677	0,247540631
ENSG00000143434	SEMA6C	0,026013181	0	0	2,201035248	0	0	0	0	0	2,227048429	0,247449825
ENSG00000170264	FAM161A	0,028739173	0	0,105736059	0,049626279	0,136598533	0,12621573	0,047725497	0	1,731928412	2,226569682	0,247396631
ENSG00000136110	CNMD	0,092365874	0	0	1,913948469	0,2195095	0	0	0	0	2,225823844	0,24731376
ENSG00000137098	SPAG8	0	0	0	0,766891917	0	0	0	1,456728439	0	2,223620355	0,247068928
ENSG00000186174	BCL9L	0	0,051221012	0,29205168	0,996886245	0	0,364464495	0,287611076	0,157800653	0,071001537	2,221036698	0,246781855
ENSG00000237693	IRGM	0	0	0	0	2,055714685	0	0	0	0,163669328	2,219384014	0,246598224
ENSG00000205476	CCDC85C	1,260684103	0,015218048	0,110434773	0,088854134	0	0,62145846	0,121055371	0	0	2,217704888	0,246411654
ENSG00000160886	LY6K	0	0,794691035	0,068654013	1,35333032	0	0	0	0	0	2,216675368	0,246297263
ENSG00000006788	MYH13	0,02469127	0	0,045421585	0	2,053776795	0	0	0	0,091101236	2,214990885	0,246110098
ENSG00000184451	CCR10	0	1,535750287	0	0	0	0,253395306	0	0	0,425765229	2,214910822	0,246101202
ENSG00000163631	ALB	0,176170878	0	0	0	1,465358669	0	0,47540493	0,09630853	0	2,213243008	0,24591589
ENSG00000249242	TMEM150C	0,086422974	0,076677724	1,669311432	0	0	0,379549503	0	0	0	2,211961634	0,245773515
ENSG00000176049	JAKMIP2	0,032839416	0	0	1,984727613	0,156087164	0,036055759	0	0	0	2,209709952	0,245523328
ENSG00000177380	PPFIA3	0	0	0,044151858	0	0	1,976380758	0,15942863	0	0,02951819	2,209479436	0,245497715
ENSG00000121053	EPX	0,055575789	0,986178743	0	0,095967258	0	0,183056907	0	0	0,888563733	2,209342429	0,245482492
ENSG00000183762	KREMEN1	0,018943283	0	0	0,392530988	0,090038246	1,663889449	0	0,041423413	0	2,20682538	0,24520282
ENSG00000228049	POLR2J2	0	0,389552543	0,40384518	0	0	0	0	0,960100438	0,449991579	2,20348974	0,244832193
ENSG00000164687	FABP5	0,079364796	0	0,145997951	0	0	0	0,065898284	1,909023237	0	2,200284267	0,24447603
ENSG00000141665	FBXO15	0,024197049	0	0	0,417830224	0,057504811	1,620583696	0,020091326	0	0,059518502	2,199725607	0,244413956
ENSG00000120332	TNN	0	0,64251278	0,222028833	0	0,071708774	0	0	0	1,261737571	2,197987959	0,244220884
ENSG00000112293	GPLD1	0,062779631	0,111400914	0,076992134	0,469762453	1,293041975	0,045952244	0,034751511	0	0,102947804	2,197628665	0,244180963
ENSG00000170367	CST5	0,400292902	0	0	0	0,475652374	0	0	1,312985036	0	2,188930313	0,243214479
ENSG00000188624	IGFL3	0	0	0	0	0	0	0	0	2,186429674	2,186429674	0,24293663
ENSG00000157766	ACAN	0,013851918	0	0,025481721	0,071757716	0,032919382	0	2,024271559	0	0,017036073	2,185318369	0,242813152
ENSG00000182240	BACE2	0,031380038	0,08352466	0,028863057	0,054186476	0	1,016376703	0,573221328	0,377404241	0,0192967	2,184253202	0,2426948
ENSG00000104808	DHDH	0	0	0	0	0	2,183033904	0	0	0	2,183033904	0,242559323
ENSG00000108852	MPP2	1,903093546	0	0	0,170713101	0	0	0	0,108091124	0	2,181897771	0,242433086
ENSG00000070371	CLTCL1	0	0	0,055308416	0	0,392985771	0,01650523	1,697569156	0	0,018488512	2,180857085	0,242317454
ENSG00000121716	PILRB	0	0	0	0,045269774	0	0,863517922	0,870717085	0,401291113	0	2,180795893	0,242310655
ENSG00000188095	MESP2	0	0	1,229320504	0	0,39703432	0,550283836	0	0	0	2,17663866	0,24184874
ENSG00000197084	LCE1C	0	0	0	0	0	0	2,175786104	0	0	2,175786104	0,241754012
ENSG00000197757	HOXC6	0	0	0	0	0,207702454	0,959575342	0,036284075	0	0,967390804	2,170952675	0,241216964
ENSG00000146197	SCUBE3	0,019326074	0,068587287	0,213311241	0,667438226	0,780790155	0,403159094	0,016046852	0	0	2,168658929	0,240962103
ENSG00000186567	CEACAM19	0,087128909	0	1,068537763	0	0	0	1,012829895	0	0	2,168496567	0,240944063
ENSG00000099958	DERL3	0	0	0	0,34369728	0	0,480773536	0,396639616	0,261144284	0,685419208	2,167673924	0,240852658
ENSG00000078295	ADCY2	0,017232361	0	0,01585016	0,119026103	0,040953078	0,86086569	0,050079389	0,05652319	1,006695154	2,167225125	0,240802792
ENSG00000161055	SCGB3A1	0	0	0	0	0	0	0,46875591	1,234500252	0,462880504	2,166136666	0,240681852
ENSG00000162944	RFTN2	0	0	0	0,043438484	0,17934953	0	0	0,055008252	1,887237866	2,165034131	0,240559348
ENSG00000257949	TEN1	0	0,417992206	0	0	0	0	0	0	1,738237153	2,156229359	0,23958104
ENSG00000180616	SSTR2	0,153919603	0	0	1,727606147	0	0	0	0,084144275	0,18930127	2,154971296	0,239441255
ENSG00000085831	TTC39A	0,080704214	1,804416365	0,029692384	0,167230276	0	0	0	0,070590593	0	2,152633833	0,239181537
ENSG00000131459	GFPT2	0,03200817	0,056797712	0,176644842	1,658133709	0	0	0,186039467	0	0,039365923	2,148989822	0,238776647
ENSG00000168772	CXXC4	0	0	1,288933998	0	0,061672255	0,05698457	0	0,737703826	0	2,14529465	0,238366072
ENSG00000204186	ZDBF2	0,013862083	0,147587561	0	0,19149434	0	0	1,761027502	0,030312317	0	2,144283803	0,238253756
ENSG00000197467	COL13A1	0,025664159	0	0,047211294	0,132949206	0,243965722	0,591732695	0,660594479	0	0,441890508	2,144008063	0,238223118
ENSG00000171401	KRT13	0	0,09205012	0	0	0	2,050388178	0	0	0	2,142438298	0,2380487
ENSG00000006638	TBXA2R	0	0	1,393383955	0,747396991	0	0	0	0	0	2,140780946	0,23786455
ENSG00000164099	PRSS12	0,02639486	0,187348122	0	0,091156323	0,188183865	0,956340442	0	0	0,681707767	2,131131379	0,236792375
ENSG00000159625	DRC7	0	0	0	0	0,069718024	2,061401069	0	0	0	2,131119094	0,23679101
ENSG00000170421	KRT8	0,065881661	0,506590031	0,040398212	0,985948167	0	0,313448263	0,218811798	0	0	2,131078133	0,236786459
ENSG00000092445	TYRO3	0,098214856	0,074691365	0	1,623272358	0	0,030809764	0	0,061362037	0,241583223	2,129933602	0,236659289
ENSG00000235710	AL109810.2	0	0	0	2,129291336	0	0	0	0	0	2,129291336	0,236587926
ENSG00000127074	RGS13	0,025512506	0	0	0,044054529	0,121262044	0	0,021183578	0	1,914002677	2,126015334	0,236223926
ENSG00000286169	AHRR	0	0,045641192	0	1,510091768	0	0	0	0	0,569402111	2,12513507	0,236126119
ENSG00000180815	MAP3K15	0	0,336801364	1,0474757	0,42139149	0,257755279	0	0	0,059292045	0	2,122715878	0,23585732
ENSG00000116396	KCNC4	0,048274282	0,653169156	0,011100555	0,093779042	0,014340609	0,265011703	0,005010396	0	1,03157362	2,122259363	0,235806596
ENSG00000165409	TSHR	0	0,0541209	0	0,078999387	0	0,016743423	1,747391847	0,166734568	0,056265977	2,120256103	0,235584011
ENSG00000142609	CFAP74	0	0	0	2,053028969	0,034457642	0	0	0,031705485	0	2,119192096	0,235465788
ENSG00000234719	NPIPB2	0	0	0	0	0	0	0	0	2,118882747	2,118882747	0,235431416
ENSG00000188649	CC2D2B	0,069807778	0,289035109	0,385251138	1,245608902	0	0	0,077283851	0,050883056	0	2,117869835	0,235318871
ENSG00000232196	MTRNR2L4	0	0,304360954	0,473291882	0,148090224	0,203812453	0,094160373	0,142418084	0,750135227	0	2,116269197	0,235141022
ENSG00000189127	ANKRD34B	0,039599206	0	0	0,068379186	0	0	2,005684028	0	0	2,113662421	0,23485138
ENSG00000165805	C12orf50	0	0,047805669	0	0,046520766	0,192075705	0	0	1,826256301	0	2,112658441	0,234739827
ENSG00000105519	CAPS	0	0,060542335	0,062763626	0,176745298	0,081083211	0,824121176	0,113317076	0,373035186	0,41961283	2,111220736	0,234580082
ENSG00000129646	QRICH2	0,026166333	0	0	1,897708906	0,186554567	0	0	0	0	2,110429806	0,234492201
ENSG00000164304	CAGE1	0	0	0	0,2972771	0,10228355	1,701162279	0	0	0	2,100722929	0,233413659
ENSG00000243710	CFAP57	0,026216268	0	0,048226943	0	0	0	2,024417223	0	0	2,098860435	0,233206715
ENSG00000213967	ZNF726	0	1,236463349	0	0,165962776	0	0,501240413	0,01995076	0,052541671	0,11820418	2,094363149	0,232707017
ENSG00000122477	LRRC39	0	0	2,09427498	0	0	0	0	0	0	2,09427498	0,23269722
ENSG00000135414	GDF11	0	0,495584152	0	0,452122516	0	0,402462991	0,681195056	0,038169639	0,021467774	2,091002128	0,23233357
ENSG00000019186	CYP24A1	0,11367395	0,067237212	0,278816549	0,392580203	0,900495342	0,124807355	0	0,165714427	0,046601435	2,089926471	0,232214052
ENSG00000180938	ZNF572	0	0,081800488	0	1,671639557	0,109553857	0	0	0	0,226780381	2,089774283	0,232197143
ENSG00000147647	DPYS	0,039630362	0	0	0,205298957	0	1,087795546	0,756836516	0	0	2,089561382	0,232173487
ENSG00000182329	KIAA2012	0,03804874	0,033758278	0,06999373	0,032850935	1,898896737	0	0,01579634	0	0	2,08934476	0,232149418
ENSG00000105982	RNF32	0	0	0	0	0	0,227742727	0,373167311	0	1,488132757	2,089042795	0,232115866
ENSG00000140015	KCNH5	0,024544883	0,065331438	0,045152294	0,042383655	0	0	1,895353021	0	0,015093521	2,087858813	0,231984313
ENSG00000008118	CAMK1G	0,045405821	0,483429087	0	1,019277245	0,539539577	0	0	0	0	2,087651729	0,231961303
ENSG00000110448	CD5	0	0	0	2,086144664	0	0	0	0	0	2,086144664	0,231793852
ENSG00000101333	PLCB4	0	0	0	1,966466475	0,052046021	0	0,018184106	0,047889068	0	2,08458567	0,23162063
ENSG00000144406	UNC80	0,047079833	0,013923668	1,501190689	0,094846029	0,037295414	0,37045149	0,019545695	0	0	2,084332819	0,231592535
ENSG00000008323	PLEKHG6	0,035471965	0,503553052	0	0,980037463	0,252899678	0,311569158	0	0	0	2,083531316	0,23150348
ENSG00000184160	ADRA2C	0	1,722803121	0	0,359249621	0	0	0	0	0	2,082052742	0,231339194
ENSG00000168314	MOBP	0	0,029029122	2,016311208	0	0	0	0	0,035772931	0	2,081113262	0,231234807
ENSG00000285816	AP000944.2	0	0,063003289	0,195944619	0,429169366	0,759412096	0,077965497	0,088442433	0,465838206	0	2,079775506	0,231086167
ENSG00000124103	FAM209A	0	0	0	0	0	0	2,078410033	0	0	2,078410033	0,230934448
ENSG00000173894	CBX2	0,05329239	0	0	2,024535158	0	0	0	0	0	2,077827548	0,230869728
ENSG00000004838	ZMYND10	0	0	0,09622018	0,180640342	0,248610275	1,550566137	0	0	0	2,076036935	0,230670771
ENSG00000107317	PTGDS	1,83873482	0,098872419	0	0	0	0	0	0	0,137054957	2,074662196	0,230518022
ENSG00000174946	GPR171	0,084184162	0	0,154863565	1,017573717	0	0,092429291	0	0	0,724749669	2,073800404	0,230422267
ENSG00000000003	TSPAN6	0	1,064290188	0	0,057538032	0,9502559	0	0	0	0	2,07208412	0,230231569
ENSG00000169047	IRS1	0,045547243	0	0	1,808953406	0	0,100016423	0	0	0,112034475	2,066551548	0,229616839
ENSG00000213782	DDX47	0	0	0	0	0	0	0	0	2,064961359	2,064961359	0,229440151
ENSG00000258466	AL049779.1	0	0	0	0	0	2,063564387	0	0	0	2,063564387	0,229284932
ENSG00000206077	ZDHHC11B	0	0,058623087	1,458575071	0	0,157025598	0	0,027431205	0,361209628	0	2,062864589	0,229207177
ENSG00000122852	SFTPA1	0	0	0	1,916291123	0,146518785	0	0	0	0	2,062809908	0,229201101
ENSG00000092098	RNF31	1,105832894	0,053034415	0	0	0,071027995	0,032814592	0,099264503	0	0,698394763	2,060369162	0,228929907
ENSG00000130477	UNC13A	0	0	0	0,373164068	0,192590745	0	0	1,476736537	0,016611237	2,059102587	0,228789176
ENSG00000112936	C7	0,028931758	0,051338694	0	0,099917662	0,068756949	0,190592277	0	1,581632048	0,035582332	2,05675172	0,228527969
ENSG00000163995	ABLIM2	0	0	0	0	0,178104253	1,453671775	0,3941042	0	0,030723512	2,05660374	0,228511527
ENSG00000272822	AC073610.2	0,643708501	0	0	0	0	0	0	1,407601992	0	2,051310493	0,227923388
ENSG00000170927	PKHD1	0,008657647	0,015362782	0,031852882	0,014949867	0,102875428	0	0,007188629	1,855310394	0,01064779	2,046845418	0,227427269
ENSG00000140807	NKD1	0	0	0	1,909023663	0,020687686	0,095576114	0	0	0,021412123	2,046699587	0,227411065
ENSG00000168158	OR2C1	0	0	0	0	2,043343057	0	0	0	0	2,043343057	0,227038117
ENSG00000147912	FBXO10	0	1,245516921	0,046114814	0	0	0,082569962	0,020814587	0	0,647441435	2,042457719	0,226939747
ENSG00000196358	NTNG2	0,078234828	0	0,899495531	0,101320855	0,046481691	0,08589727	0,178640131	0,171076659	0,481093767	2,042240732	0,226915637
ENSG00000253910	PCDHGB2	0	0	0,05828897	0	0	1,982994941	0	0	0	2,041283911	0,226809323
ENSG00000164933	SLC25A32	0	0	0	0,214232327	1,572490607	0	0	0	0,254305586	2,04102852	0,226780947
ENSG00000050628	PTGER3	0,03130852	0	0	0,02703149	0,037202687	0,515623879	1,169825948	0,239618976	0,019252722	2,039864222	0,22665158
ENSG00000163354	DCST2	0	0	0	2,039218297	0	0	0	0	0	2,039218297	0,226579811
ENSG00000088367	EPB41L1	0,015617049	0	0,028728824	0	0	0	0,012967169	1,98069501	0	2,038008053	0,226445339
ENSG00000134780	DAGLA	0	0	0	0,089993093	0	0	1,947289167	0	0	2,037282261	0,226364696
ENSG00000187492	CDHR4	0	0	0	0,081516706	0	0	0,03919723	0	1,915943529	2,036657464	0,226295274
ENSG00000184163	C1QTNF12	0	0	0	2,036281853	0	0	0	0	0	2,036281853	0,226253539

ENSG00000128594	LRRC4	0	0,243599364	0	0,770418957	0	0,376812575	0,598427657	0	0,042209067	2,03146762	0,225718624
ENSG00000129757	CDKN1C	0	0,757463277	0	0	0,144922333	0,066953421	0,91140677	0	0,149997193	2,030742994	0,22563811
ENSG00000129749	CHRNA10	0	0	0,102841324	0	0	0,859322262	1,067635085	0	0	2,029798671	0,225533186
ENSG00000141854	MISP3	0	0	0	1,55186476	0	0,422881674	0,053300906	0	0	2,028047339	0,225338593
ENSG00000124678	TCP11	0,147713168	0	0,090576767	1,78547881	0	0	0	0	0	2,023768745	0,224863194
ENSG00000269858	EGLN2	0,022986092	0,081576514	0,295993412	0,754147325	0,218507784	0,176661703	0,209944273	0,150791558	0,113079721	2,023688383	0,224854265
ENSG00000169627	BOLA2B	0,332111144	0	0	0	0	0,24309242	0,827276914	0,484153212	0,136151298	2,02278499	0,224753888
ENSG00000072657	TRHDE	0,050580944	0	0	0,087342251	0,030051677	0,01388373	1,66943789	0	0,171072113	2,022368605	0,224707623
ENSG00000197483	ZNF628	0	0,526358385	0,155905832	0,219519048	0,100705985	0	0	1,019287785	0	2,021777035	0,224641893
ENSG00000062370	ZNF112	0	0,139366944	0	0	0	0,215580271	0,032606635	0,085871769	1,545501225	2,018926844	0,224325205
ENSG00000138675	FGF5	0,100773972	0	1,853818599	0	0	0	0,062756085	0	0	2,017348656	0,224149851
ENSG00000243696	AC006254.1	0,071571158	0	0	0	0	0	1,386631392	0	0,557480885	2,015683435	0,223964826
ENSG00000148225	WDR31	0	0	0	0,158687396	0	0	1,856747261	0	0	2,015434656	0,223937184
ENSG00000173567	ADGRF3	0	0	0,048252057	0,226466737	0	0	1,677003222	0,057357155	0	2,009079171	0,223231019
ENSG00000186265	BTLA	0	0	0	1,80702891	0,198957087	0	0	0	0	2,005985997	0,222887333
ENSG00000117400	MPL	0	0	0	2,005377291	0	0	0	0	0	2,005377291	0,222819699
ENSG00000173258	ZNF483	0,026335059	0	0	0,409274097	0,062585839	1,358974748	0	0,115174126	0,032388728	2,004732598	0,222748066
ENSG00000270394	MTRNR2L13	0	0,371130796	0,577121313	0,180577837	0	0,114817008	0,173661359	0,457348882	0,12861351	2,003270706	0,222585634
ENSG00000198049	AVPR1B	0	0	1,098301458	0,618573626	0,283775221	0	0	0	0	2,000650305	0,222294478
ENSG00000115474	KCNJ13	0	0	0,18675183	0	1,80946024	0	0	0	0	1,99621207	0,221801341
ENSG00000182902	SLC25A18	1,533144661	0	0	0,110308592	0	0	0,212167122	0,139689101	0	1,995309476	0,221701053
ENSG00000170011	MYRIP	0	0	0	0	0	0	0	0	1,995284748	1,995284748	0,221698305
ENSG00000161405	IKZF3	0,059674428	0	0,987983371	0	0,177271963	0,049139276	0,718459794	0	0	1,992528832	0,221392092
ENSG00000134013	LOXL2	0,079434328	0	0,146125862	0	0,125851601	0,348857004	1,231179007	0,057899872	0	1,989347674	0,22103863
ENSG00000176204	LRRTM4	0,027931714	0	1,798392507	0,096463946	0,066380322	0	0	0	0	1,989168489	0,221018721
ENSG00000167419	LPO	0,211409162	0	0,043211581	0	0,27912136	1,392689436	0	0	0,057779115	1,984210653	0,22046785
ENSG00000187944	C2orf66	0	0	0	0	0	0,198814352	0	1,781852072	0	1,980666424	0,220074047
ENSG00000148677	ANKRD1	0	0	0,561859725	0,52740773	0,181464145	0	0	0,333940947	0,375637236	1,980309783	0,22003442
ENSG00000242715	CCDC169	0,017099759	0	1,667188553	0,295275517	0	0	0	0	0	1,97956383	0,219951537
ENSG00000215915	ATAD3C	0	0	0,279658047	0,52502007	0,180642627	0	0,946707736	0	0,046742083	1,978770563	0,219863396
ENSG00000172139	SLC9C1	0	0	1,915737374	0,062009262	0	0	0	0	0	1,977746636	0,219749626
ENSG00000142871	CCN1	0	1,975667622	0	0	0	0	0	0	0	1,975667622	0,219518625
ENSG00000120210	INSL6	0	0	0	0,902889185	0	0	0	0	1,071779252	1,974668437	0,219407604
ENSG00000178409	BEND3	0,022095419	0	0	0,190769831	0	0,436670622	1,247548264	0,048316211	0,027174517	1,972574863	0,219174985
ENSG00000133063	CHIT1	0,039087059	0	0	0	0,092891242	0,085830614	0	0	1,754629608	1,972438523	0,219159836
ENSG00000120071	KANSL1	0	0,202549666	0,058328108	0,010950312	0	0,222801563	0,205352426	0,873615248	0,397757885	1,971355208	0,219039468
ENSG00000149571	KIRREL3	0	0,040658378	0	0,118696728	0,054453001	1,610049569	0,019025069	0,100207602	0,028179912	1,97127026	0,219030029
ENSG00000182472	CAPN12	0,025328624	0	0,093188099	1,793217224	0	0,055618698	0	0	0	1,967352645	0,218594738
ENSG00000241978	AKAP2	0,019220373	0,03410608	0,282859424	0,132757555	0,685164479	0,189925616	0,367058972	0,042029327	0,212747227	1,965869052	0,218429895
ENSG00000186871	ERCC6L	0,034786043	0	0	0,060067904	0,330679137	0,496509553	1,039809381	0	0	1,961852017	0,217983557
ENSG00000215126	CBWD6	0,029079298	0,309603002	0,187228009	0,753204006	0	0,03192737	0,060362904	0	0,590102495	1,961507084	0,217945232
ENSG00000170279	C7orf33	0	0	0	0	0	0	1,958773613	0	0	1,958773613	0,217641513
ENSG00000158816	VWA5B1	0,023696185	0,084096597	0	0,040918139	0	0	0	0	1,806881666	1,955592587	0,217288065
ENSG00000126091	ST3GAL3	0,294849894	0,013768524	0,014273689	0,710118288	0,018439925	0,315208798	0,077311629	0,186638267	0,324456059	1,955065073	0,217229453
ENSG00000154839	SKA1	0	0	0	0	0	0,686526524	0,562453212	0	0,704935084	1,953914821	0,217101647
ENSG00000019144	PHLDB1	0,020876432	0	0,051205176	1,045422187	0,033075528	0,122245877	0,468021961	0,015216881	0,196844118	1,952908159	0,216989795
ENSG00000176256	HMGB4	0	0	0,217087153	1,732094716	0	0	0	0	0	1,949181869	0,216575763
ENSG00000176029	C11orf16	0,173890185	0	0	1,100991454	0	0	0,673795415	0	0	1,948677054	0,216519673
ENSG00000106689	LHX2	0	0	0	1,880989004	0	0,066443963	0	0	0	1,947432967	0,216381441
ENSG00000213918	DNASE1	0,010949248	0,349725092	0	0,283604421	0,130105624	0,577038452	0,009091394	0,047885598	0,538646539	1,947046369	0,216338485
ENSG00000105507	CABP5	0	0	0,185196602	0	0	0	0	1,761143598	0	1,946340199	0,216260022
ENSG00000128833	MYO5C	0,015034382	0,160068849	0,221255675	0,493260822	1,000426941	0	0	0	0,055471054	1,945517723	0,216168636
ENSG00000151692	RNF144A	0,023427995	0,041572403	0,04309769	1,618201393	0,167031415	0	0	0,051230166	0	1,944561062	0,21606234
ENSG00000168672	LRATD2	0	0	0,048479264	0,091013245	1,628366952	0,115738107	0	0,057627235	0	1,941224804	0,215691645
ENSG00000143614	GATAD2B	0	0	0,030071408	0,564549923	0,038848717	0,574333455	0,271463289	0,178729212	0,281463794	1,939459798	0,215495533
ENSG00000126460	PRRG2	0	0	0	0	0	0	0,516642544	0	1,421179288	1,937821831	0,215313537
ENSG00000256407	AL357673.1	0	0	0	0	0	0	1,936662513	0	0	1,936662513	0,215184724
ENSG00000128602	SMO	0	0,058932308	0	0,057348346	0	0	1,820009211	0	0	1,936289865	0,215143318
ENSG00000054179	ENTPD2	0,057238853	0	0	1,877941105	0	0	0	0	0	1,935179958	0,215019995
ENSG00000164220	F2RL2	1,241518773	0,078680164	0	0,382827134	0	0,194730724	0,036816412	0	0	1,934573207	0,214952579
ENSG00000162992	NEUROD1	0,045625172	0	0,251793568	0,393923573	0,542146048	0	0	0,698382238	0	1,931870599	0,214652289
ENSG00000088808	PPP1R13B	0,047281155	0,083899249	0,028992501	0,05442949	0,037454896	0,571031397	0,06543092	0,344633466	0,697796704	1,930949778	0,214549975
ENSG00000151322	NPAS3	0,049056564	0,08704967	0,120324683	0,11294664	0,194306646	0,700196136	0,529525262	0,035757447	0,100555417	1,929718465	0,214413163
ENSG00000108797	CNTNAP1	0	0,042521725	0,132245528	0,496546098	0,056948548	0	0,139278833	0	1,06096968	1,928510412	0,214278935
ENSG00000183324	REC114	0	0	0	0	0,390345155	1,262363084	0,272761591	0	0	1,92546983	0,213941092
ENSG00000253457	SMIM18	0	0	0	0	0	0,173728353	0	0	1,751433194	1,925161547	0,213906839
ENSG00000072201	LNX1	0	0,046837031	0	0,045578162	0	0,144900067	0,986229684	0,634896112	0,064924549	1,923365604	0,213707289
ENSG00000140534	TICRR	0,035970143	0	0	0,279506638	0,042741912	0,019746558	1,269338041	0,275296593	0	1,922599884	0,213622209
ENSG00000147724	FAM135B	0,010744495	0,057197526	0	0,129873779	1,506536903	0,129765098	0,017842766	0,070485189	0	1,922445755	0,213605084
ENSG00000164161	HHIP	0	0,04491867	0,069850096	0,961649961	0	0	0	0,581215003	0,264627764	1,922261494	0,21358461
ENSG00000260456	C16orf95	0,041174542	0	0	0,284397792	0	0,813730349	0,376069115	0	0,405115035	1,920486834	0,213387426
ENSG00000101197	BIRC7	0,097490691	0	0	0	0	1,81966439	0	0	0	1,91715508	0,213017231
ENSG00000100146	SOX10	0	0	0,067915978	1,848794103	0	0	0	0	0	1,916710081	0,212967787
ENSG00000188386	PPP3R2	0	0	0	0	1,050665718	0	0,036708699	0,773398637	0,054372885	1,915145939	0,212793993
ENSG00000183474	GTF2H2C	0	0,230197739	0,119321834	0,373350944	0,051383251	0,189910518	0,269288164	0,520071576	0,159547737	1,913071764	0,212563529
ENSG00000270106	TSNAX-DISC1	0	1,912113145	0	0	0	0	0	0	0	1,912113145	0,212457016
ENSG00000203780	FANK1	0	0	0	0	0	1,91208998	0	0	0	1,91208998	0,212454442
ENSG00000172146	OR1A1	0,021935052	0,077846422	0,080702598	1,704467101	0	0,024083405	0	0	0	1,909034577	0,212114953
ENSG00000166546	BEAN1	0,061418739	0,145314727	0	0,212113514	0	0,269736746	0,084995483	0,223841327	0,906445577	1,903866113	0,211540679
ENSG00000111052	LIN7A	0	0	0	0,076802053	0	0,024416568	0,535487804	1,264355961	0	1,901062385	0,211229154
ENSG00000180305	WFDC10A	0,306512314	0	0	1,587839602	0	0	0	0	0	1,894351916	0,210483546
ENSG00000218823	PAPOLB	0,035199294	0	1,554046675	0,303907494	0	0	0	0	0	1,893153464	0,210350385
ENSG00000006118	TMEM132A	0,020782639	0	0	0,251209575	0	0,981179315	0,638481954	0	0	1,891653483	0,21018372
ENSG00000169750	RAC3	0	0	0,188716971	1,062871587	0	0	0,511080782	0	0,126168718	1,888838059	0,209870895
ENSG00000157388	CACNA1D	0,007323732	0	0	0,303515698	0,052215026	0,779979932	0,243242053	0,112104007	0,38731161	1,885692058	0,20952134
ENSG00000267041	ZNF850	0	0	0	1,429325339	0,228737288	0,021135105	0,015983482	0	0,18939773	1,884578944	0,20939766
ENSG00000221933	OR2A25	0,081549148	0,434120885	0	1,267358214	0	0	0	0	0,100294939	1,883323187	0,209258132
ENSG00000204740	MALRD1	0,019495623	0,069189008	0	0	0	0	0,016187632	1,705248703	0,071931308	1,882052273	0,209116919
ENSG00000105642	KCNN1	0	0	0	0,065545083	0,090207873	0	1,071587864	0	0,653567272	1,880908093	0,208989788
ENSG00000105808	RASA4	0,411592492	0	0	0,775342255	0	0,205411139	0,186411289	0	0,299121554	1,877878728	0,208653192
ENSG00000206559	ZCWPW2	0,100006996	0	0	0	0	0,512408576	0,44286903	0	0,81997142	1,875256021	0,20836178
ENSG00000177679	SRRM3	0,043946654	0	0,727590778	1,100351063	0	0	0	0	0	1,871888496	0,207987611
ENSG00000167748	KLK1	0	0,112664706	0	0,986728896	0	0	0,632623523	0,138838054	0	1,870855178	0,207872798
ENSG00000138792	ENPEP	0,019573908	0	0	0,033799867	0,558213796	0	0,146273703	1,112862533	0	1,870723808	0,207858201
ENSG00000147082	CCNB3	0	0,099164941	0,102803291	0	0,066404871	0	1,600860375	0	0	1,869233478	0,207692609
ENSG00000283199	C13orf46	0	0	0,072466136	0,068022673	0	1,08127331	0,065417274	0	0,581375982	1,868555376	0,207617264
ENSG00000197406	DIO3	0	0	0	1,862047868	0	0	0	0	0	1,862047868	0,206894208
ENSG00000186889	TMEM17	0,050623307	0	0	0,087415402	0	0	1,723378094	0	0	1,861416803	0,206824089
ENSG00000152822	GRM1	0	0	0,112572934	0,070446807	0,09695398	0,022396136	1,507405447	0,044605098	0	1,854380403	0,206042267
ENSG00000162571	TTLL10	0	0	0	1,85294935	0	0	0	0	0	1,85294935	0,205883261
ENSG00000114757	PEX5L	1,136724691	0	0,624613353	0,050983778	0	0,016208536	0	0	0,018156167	1,846686525	0,205187392
ENSG00000008277	ADAM22	0,012387128	0,021980654	0	1,58285008	0,058876554	0	0	0	0,167580273	1,843674688	0,204852743
ENSG00000162458	FBLIM1	0,034730078	0,061627672	0,063888784	0,059971265	0	0	0	0	1,623113732	1,843331531	0,204814615
ENSG00000072840	EVC	0	0,187669373	0	0,547875787	0,201073652	0,023223765	0	0,878815339	0	1,838657916	0,204295324
ENSG00000169992	NLGN2	0,185022563	0,046902571	0	1,232332397	0,062815732	0,116082265	0,021946883	0,173395785	0	1,838498197	0,204277577
ENSG00000161048	NAPEPLD	1,219117155	0	0	0	0	0,109714705	0,116160868	0	0,393273953	1,838266681	0,204251853
ENSG00000078328	RBFOX1	0,044791198	0	0,027465675	0,128907704	0	0	0,037191087	1,599772138	0	1,838127802	0,204236422
ENSG00000175093	SPSB4	0	0	0,073249038	0,068757569	1,608695183	0	0	0,087071032	0	1,837772822	0,20419698
ENSG00000015520	NPC1L1	0	0,051055217	0	1,738904058	0	0	0,04778002	0	0	1,837739295	0,204193255
ENSG00000165124	SVEP1	0,159702569	0,037785105	1,096800221	0,294156246	0,025302441	0,023379212	0,176805935	0,023281517	0	1,837213246	0,204134805
ENSG00000169926	KLF13	0,0320523	0,02843801	0,412739568	0,193715646	0,342778437	0,211149318	0,252830237	0,105133493	0,256231285	1,835068295	0,203896477
ENSG00000155087	ODF1	0	1,829428851	0	0	0	0	0	0	0	1,829428851	0,203269872
ENSG00000187513	GJA4	0	0,156259907	0	1,672660094	0	0	0	0	0	1,82892	0,203213333
ENSG00000197213	ZSCAN5B	0	0	0	0	0	0	1,828125284	0	0	1,828125284	0,203125032
ENSG00000130758	MAP3K10	0	0,219608518	0,113832964	0	0	0	0,436731295	0,067656545	0,989354951	1,827184273	0,203020475
ENSG00000259316	AC087632.1	0	0	0	1,825830367	0	0	0	0	0	1,825830367	0,202870041
ENSG00000229474	PATL2	0,06334545	0	0	0,054691883	0,075270916	0,973694435	0,657463488	0	0	1,824466172	0,202718464

ENSG00000169688	MT1B	0	0	0	0,663956678	0	0	0,319262931	0,840800426	0	1,824020036	0,202668893
ENSG00000165449	SLC16A9	0,13647376	0	0,75316351	0	0,486499267	0	0	0,447642223	0	1,82377876	0,202642084
ENSG00000166265	CYYR1	0,046639065	0	0,085796327	1,691245206	0	0	0	0	0	1,823680598	0,202631178
ENSG00000178342	KCNG2	0	0	0	0	0	1,765006135	0,058034381	0	0	1,823040516	0,202560057
ENSG00000197309	OR10D3	0	0	1,752615756	0	0	0	0,068788559	0	0	1,821404315	0,202378257
ENSG00000073712	FERMT2	0	0,031684036	0,03284652	0,154162221	0	0,07841691	0	1,522739942	0	1,819849629	0,202205514
ENSG00000047662	FAM184B	0	0	0	0	1,789622304	0	0	0	0,028065014	1,817687318	0,201965258
ENSG00000142949	PTPRF	0,042319988	0,050063854	0,051900691	1,461547655	0,134099157	0	0,023426126	0	0,05204813	1,8154056	0,201711733
ENSG00000180190	TDRP	0	0,148431774	0	0,072221139	0	0	0	0	1,594586823	1,815239736	0,201693304
ENSG00000073605	GSDMB	0,102361098	0	0	1,590797474	0,121631682	0	0	0	0	1,814790255	0,201643362
ENSG00000261701	HPR	0,863937774	0	0	0,949347621	0	0	0	0	0	1,813285395	0,201476155
ENSG00000181418	DDN	0	0	0	1,77832486	0	0	0,032888685	0	0	1,811213544	0,201245949
ENSG00000116675	DNAJC6	0,108587648	0,038537223	0,119853449	1,425054475	0	0,023844578	0	0,094979755	0	1,810857128	0,201206348
ENSG00000125845	BMP2	0,042626395	0	0	0,073606481	1,31693316	0,374410328	0	0	0	1,807576364	0,200841818
ENSG00000119737	GPR75	0,42868247	0	0	0,12337351	0	1,255115474	0	0	0	1,807171454	0,200796828
ENSG00000188674	C2orf80	0	0	0	0	0	0	1,802734655	0	0	1,802734655	0,200303851
ENSG00000283071	LBHD2	0	0,732628416	0	1,069405634	0	0	0	0	0	1,802034049	0,200226005
ENSG00000271698	AC233992.2	1,798935365	0	0	0	0	0	0	0	0	1,798935365	0,199881707
ENSG00000225556	C2CD4D	0	0	0	0	0	0	0	0	1,798173522	1,798173522	0,199797058
ENSG00000183682	BMP8A	0	0	0	0,138893705	0,063718514	1,177505867	0,022262302	0	0,39569877	1,798079158	0,199786573
ENSG00000204991	SPIRE2	0,170020645	0	0	0,032620949	0,044895305	0	0,015685752	0	1,533425487	1,796648138	0,199627571
ENSG00000177627	C12orf54	0,08704526	0	0,160126785	0,751540825	0	0	0,795030906	0	0	1,793743776	0,199304864
ENSG00000144724	PTPRG	0,051661734	0	0,019007186	0,089208538	0,049110091	0,907545035	0,609120928	0,06778145	0	1,793434962	0,199270551
ENSG00000080293	SCTR	0,220534983	0	0	0,990121036	0	0,581122861	0	0	0	1,79177888	0,199086542
ENSG00000170370	EMX2	0,092677443	0	0,085243821	0,320067433	0,880999797	0,050877208	0	0,303987643	0,056990654	1,790843998	0,198982666
ENSG00000105549	THEG	0	0	0	1,146966921	0	0	0,6434376	0	0	1,790404521	0,198933836
ENSG00000102383	ZDHHC15	0,04431395	0	0	1,721711709	0	0,024327064	0	0	0	1,790352724	0,19892808
ENSG00000127990	SGCE	0,072357101	0,141235491	0,026621346	1,461855585	0	0,007944387	0	0	0,080090917	1,790104827	0,198900536
ENSG00000175106	TVP23C	0,19462263	0,049336155	0,102292585	0,240050575	0	0,18315795	0,69256853	0,121595057	0,205166354	1,788789835	0,198754426
ENSG00000164309	CMYA5	0,053133112	0,77312391	0	0,146798861	0	0	0	0,813305898	0	1,78636178	0,198484642
ENSG00000167968	DNASE1L2	1,308614787	0	0	0,475724657	0	0	0	0	0	1,784339444	0,198259938
ENSG00000130940	CASZ1	0,013330149	0,023654023	0,024521886	0	0,253435104	0	0,586620289	0,029149133	0,852506983	1,783217566	0,198135285
ENSG00000114113	RBP2	0	0	0	0	0	0	0	0	1,759493702	1,759493702	0,1954993
ENSG00000163576	EFHB	0	0	0	0,062679552	0	0	0	0	1,696413127	1,759092679	0,195454742
ENSG00000185559	DLK1	0	0	0,052978864	1,292988248	0,410654708	0	0	0	0	1,756621821	0,195180202
ENSG00000122778	KIAA1549	0,048639437	0,043154744	0,35790469	0,083989692	0,115592675	1,001311117	0,010096591	0,079770154	0,014955059	1,755414158	0,195046018
ENSG00000188107	EYS	0,451472707	0	0,977082949	0,076430865	0,105189673	0,009719425	0,04410205	0,077430479	0,010887318	1,752315465	0,194701718
ENSG00000130413	STK33	0	0	0	1,75124144	0	0	0	0	0	1,75124144	0,194582382
ENSG00000179873	NLRP11	0,070088391	0	0	0,181541031	0	0	0	1,455996471	0,043099843	1,750725735	0,194525082
ENSG00000198734	F5	0	0,037460464	0,116504652	1,567505436	0	0	0	0	0,025963471	1,747434022	0,194159336
ENSG00000149516	MS4A3	0	0,257581172	0	0,375986995	0	0	0	1,110971168	0	1,744539336	0,193837704
ENSG00000092295	TGM1	0	0	0	0,349310542	0	0	0	0	1,393226941	1,742537482	0,193615276
ENSG00000183914	DNAH2	0,008769692	0,046684812	0	0,424013655	1,187957746	0,00962861	0,014563326	0,038353499	0,010785591	1,740756931	0,193417437
ENSG00000171903	CYP4F11	0,037524353	0	1,656697883	0	0	0	0	0	0,046150117	1,740372354	0,193374706
ENSG00000156150	ALX3	0	1,739444112	0	0	0	0	0	0	0	1,739444112	0,193271568
ENSG00000284292	AC004922.1	0	0,099128281	0	1,639887086	0	0	0	0	0	1,739015367	0,19322393
ENSG00000146013	GFRA3	1,596238423	0	0,139829024	0	0	0	0	0	0	1,736067446	0,192896383
ENSG00000103888	CEMIP	0,019828181	0,105553864	0,036475541	0	0,141366307	0,021770185	0,921970685	0	0,487722142	1,734686906	0,19274299
ENSG00000140511	HAPLN3	0	0	0,07888198	0	0	1,600726336	0	0	0,052737379	1,732345695	0,192482855
ENSG00000187134	AKR1C1	0,086201124	1,437840731	0	0	0,204858838	0	0	0	0	1,728900692	0,192100077
ENSG00000214534	ZNF705E	0	0,077902963	0,080761214	1,516182304	0	0,048201795	0	0	0	1,723048277	0,191449809
ENSG00000164142	FAM160A1	0,051037937	0,045282783	0,023472102	0,484722574	0,181939142	0,056036672	0,550912544	0,139506277	0,188310248	1,721220278	0,191246698
ENSG00000165059	PRKACG	0,476689497	0	0	0	0,226572582	0,104675443	0	0,208476068	0,703519958	1,719933548	0,191103728
ENSG00000111796	KLRB1	0	0	0	0	0	1,718686913	0	0	0	1,718686913	0,190965213
ENSG00000269242	AC010422.3	0	0	0	0,311378251	0	0	0	1,182939979	0,221773893	1,716092123	0,190676903
ENSG00000038295	TLL1	0	0	1,269002303	0,128777285	0,044308148	0	0	0,203846124	0,068789583	1,714723443	0,190524827
ENSG00000205359	SLCO6A1	0,051189218	0	0	0,441963033	0,121652284	0	0	0,783550804	0,314780695	1,713136033	0,190348448
ENSG00000183977	PP2D1	0	0	0	0	0	1,709740386	0	0	0	1,709740386	0,189971154
ENSG00000155966	AFF2	0,032366965	0,019144795	0,119083292	0,614797527	0,051280529	0,011845679	0,053749964	0,802140175	0	1,704408926	0,18937877
ENSG00000196159	FAT4	0,045960998	1,402774622	0	0,047618768	0,065536385	0,010092498	0	0,120603893	0,011305221	1,703892385	0,189321376
ENSG00000112182	BACH2	0,015439928	0,10959109	0,255626943	0,399920774	0,36693322	0,05856415	0,051280405	0,168813001	0,284836807	1,703298583	0,189255398
ENSG00000115257	PCSK4	0	0	0	0	0	1,006314152	0,694851522	0	0	1,701165674	0,189018408
ENSG00000157087	ATP2B2	0,029253813	0,025955087	0	0,126287375	0	0,048178466	0	1,471299013	0	1,700973754	0,188997084
ENSG00000267385	AC011498.4	0	0	0,154176428	0,434168011	0	0	0,069589757	0,733077244	0,309228822	1,700240262	0,188915585
ENSG00000156414	TDRD9	0	0	0,050670816	0	1,44013597	0	0,205839043	0	0	1,696645829	0,188516203
ENSG00000179915	NRXN1	0,012425834	0,022049338	0,022858326	0,064370111	1,520808604	0,00682142	0,025793589	0,01358583	0,007641087	1,696354139	0,188483793
ENSG00000229937	PRPS1L1	0	0	0	1,695956195	0	0	0	0	0	1,695956195	0,188439577
ENSG00000182732	RGS6	0	0,928967591	0,217463198	0,408257671	0	0,037083276	0	0	0,103848079	1,695619815	0,188402202
ENSG00000151655	ITIH2	0	0,402494746	0	1,253365234	0	0	0,037667467	0	0	1,693527447	0,188169716
ENSG00000162490	DRAXIN	0,082069556	0	0,037743394	1,523449275	0,048760019	0	0	0	0	1,692022245	0,188002472
ENSG00000215262	KCNU1	0	0	0,070090797	0,263171936	1,358235788	0	0	0	0	1,691498521	0,18794428
ENSG00000154263	ABCA10	0	0	0,066146365	0	1,580886266	0	0	0	0,044222849	1,69125548	0,187917276
ENSG00000163751	CPA3	0	0	0	1,581424088	0	0	0	0	0,102394778	1,683818867	0,187090985
ENSG00000010379	SLC6A13	0	0	0	1,598941631	0,084637648	0	0	0	0	1,683579279	0,187064364
ENSG00000152954	NRSN1	0	0,065051431	1,61851586	0	0	0	0	0	0	1,683567292	0,187063032
ENSG00000114654	EFCC1	0	0	0	1,683451449	0	0	0	0	0	1,683451449	0,187050161
ENSG00000186967	KRTAP19-4	0	0	0	1,683451449	0	0	0	0	0	1,683451449	0,187050161
ENSG00000203923	SPANXN1	0	1,681141066	0	0	0	0	0	0	0	1,681141066	0,186793452
ENSG00000007062	PROM1	0,040653907	0,07213936	0	1,474208896	0,048307444	0	0	0,044449094	0	1,679758701	0,186639856
ENSG00000171234	UGT2B7	0	0	0	0,69274772	0	0	0	0	0,986795693	1,679543414	0,186615935
ENSG00000101446	SPINT3	0	1,126647059	0	0,54818272	0	0	0	0	0	1,674829779	0,186092198
ENSG00000078814	MYH7B	0,03810631	0,067618711	0,105149451	0,03290064	0,045280235	0,020919251	1,281439527	0,08332734	0	1,674741465	0,186082385
ENSG00000105289	TJP3	0	0	0	0	0,090594738	0	0	1,583818563	0	1,674413301	0,186045922
ENSG00000196569	LAMA2	0,11671023	0,05177486	0,026837237	0,705365832	0,312034937	0,19221152	0,012113374	0,095704161	0,161480855	1,674233007	0,18602589
ENSG00000250799	PRODH2	0	0	0	0,095545578	1,57796064	0	0	0	0	1,673506219	0,185945135
ENSG00000162804	SNED1	0	0,038827396	0,060377954	0,037783808	0	0	1,535221989	0	0	1,672211146	0,185801238
ENSG00000179097	HTR1F	0	0	0,088528694	1,578905897	0	0	0	0	0	1,667434591	0,18527051
ENSG00000185271	KLHL33	0,027469655	0	0,101065297	0	0,065282229	0,331760833	0	1,141293725	0	1,666871739	0,185207971
ENSG00000133665	DYDC2	0	0,108779716	0	0,211711947	0	1,34613044	0	0	0	1,666622103	0,185180234
ENSG00000206530	CFAP44	0,037280568	0	0	1,330425035	0	0	0	0,298912849	0	1,666618452	0,185179828
ENSG00000135407	AVIL	0,025659801	0	0,047203277	0,39877989	0	0,591632214	0,426117616	0,112220943	0,063116496	1,664730238	0,184970026
ENSG00000189001	SBSN	0	0	0	0	1,265771801	0	0,063177408	0,332763915	0	1,661713124	0,184634792
ENSG00000120500	ARR3	0	0	0	1,660083702	0	0	0	0	0	1,660083702	0,184453745
ENSG00000115556	PLCD4	0,078471821	0	0	1,580876599	0	0	0	0	0	1,65934842	0,184372047
ENSG00000117122	MFAP2	0	1,655765352	0	0	0	0	0	0	0	1,655765352	0,183973928
ENSG00000198821	CD247	0,037459239	0	0	0,06468393	0,089022693	0	1,46185067	0	0	1,653016532	0,183668504
ENSG00000139292	LGR5	0	0,051338694	0	0,499588311	1,100111177	0	0	0	0	1,651038182	0,183448687
ENSG00000160767	FAM189B	0	0,180041629	0	0	0,080375457	0,33419767	0,561639803	0,369779059	0,124785042	1,650818661	0,183424296
ENSG00000006283	CACNA1G	0,015017946	0	0,027626724	1,607828307	0	0	0	0	0	1,650472977	0,183385886
ENSG00000120068	HOXB8	0	0	0	1,52667952	0	0	0,122350397	0	0	1,649029917	0,183225546
ENSG00000086548	CEACAM6	0	0,090711096	0,094039276	0,264818986	0,607438333	0,056126717	0,08489197	0,447137439	0	1,645163818	0,18279598
ENSG00000077264	PAK3	0	0	0,026611152	0,174855909	0,068756949	0,063530759	0,012011328	1,29693828	0	1,642704376	0,182522708
ENSG00000168671	UGT3A2	0	0	0	0	1,472341769	0	0,046764939	0,123158616	0	1,642265324	0,182473925
ENSG00000148498	PARD3	0	0	0,17072323	0,600955722	0	0	0,211910587	0	0,656298175	1,639887714	0,182209746
ENSG00000141404	GNAL	0	0	0	0,70548967	0,0441339	0,061168948	0,046259186	0	0,776549313	1,633601016	0,181511224
ENSG00000204970	PCDHA1	0	0	0	1,632301003	0	0	0	0	0	1,632301003	0,181366778
ENSG00000169962	TAS1R3	0,043485056	0	0	0	0	0	1,588689096	0	0	1,632174152	0,181352684
ENSG00000184185	KCNJ12	0	0,098310541	0	1,53069096	0	0	0	0	0	1,629001501	0,181000167
ENSG00000136541	ERMN	0,068796071	0,06103847	0,063277965	0	0	1,43514726	0	0	0	1,628259767	0,180917752
ENSG00000122863	CHST3	0,043585397	0,077341217	0	1,505249349	0	0	0	0	0	1,626175963	0,180686218
ENSG00000020181	ADGRA2	0	0	0,039368375	1,515130146	0	0,023496754	0	0,046797135	0	1,624792409	0,18053249
ENSG00000161634	DCD	0	0	0	0,297870976	0	1,325769449	0	0	0	1,623640424	0,180404492
ENSG00000187720	THSD4	0,028624848	0,8296368	0,105315438	0,477812355	0,090703427	0	0	0,02086472	0,070409745	1,623367332	0,180374148
ENSG00000179600	GPHB5	0	0	0	0,961086463	0,661358273	0	0	0	0	1,622444736	0,180271637
ENSG00000168970	JMJD7-PLA2G4B	0,019220373	0	0	0,26551511	0	0,696393924	0	0	0,638241682	1,619371089	0,179930121
ENSG00000076555	ACACB	0,083342611	0,018486177	0,114986597	0,179893123	0,123790949	0,011438165	0,034600574	0	1,050631839	1,617170034	0,179685559
ENSG00000145642	SHISAL2B	0	0	0	0	0	0,252527514	0,859385836	0,502944547	0	1,614857897	0,179428655

ENSG00000147117	ZNF157	0,065190065	0	0	0	1,549256007	0	0	0	0	1,614446072	0,179382897
ENSG00000205177	C11orf91	0	0	0	0	1,612199966	0	0	0	0	1,612199966	0,17913333
ENSG00000083782	EPYC	0	0	0	0,144802983	0	0	0	1,466968113	0	1,611771096	0,179085677
ENSG00000178363	CALML3	0	0	1,609729129	0	0	0	0	0	0	1,609729129	0,178858792
ENSG00000165995	CACNB2	0,055286041	0,049051861	0,101703137	1,026269451	0,098541354	0,075876053	0,149191834	0	0,050996027	1,606915758	0,178546195
ENSG00000236320	SLFN14	0	0	0,192440359	1,354802568	0	0,057428375	0	0	0	1,604671303	0,178296811
ENSG00000114923	SLC4A3	0	0	0	0,105385692	0,145039395	0	0	0	1,351065186	1,601490274	0,177943364
ENSG00000135424	ITGA7	0,047422115	0	0,043618406	0,122831465	0,112699684	0,182233491	0	1,088831934	0	1,597637095	0,177515233
ENSG00000272899	ATP6V1FNB	0	0	0,143178006	1,411185801	0	0,042727421	0	0	0	1,597091229	0,177454581
ENSG00000178222	RNF212	0	0,267874126	0	0	0	0	1,328656862	0	0	1,596530988	0,177392332
ENSG00000243444	PALM2	0,015231385	0	0	1,209858767	0	0	0	0,333065787	0,03746528	1,59562122	0,177291247
ENSG00000153157	SYCP2L	0	0	0	0,263437632	0	1,046886526	0,285015898	0	0	1,595340057	0,177260006
ENSG00000176920	FUT2	0	0	0	0	0	0	0	0	1,594541167	1,594541167	0,177171241
ENSG00000108231	LGI1	0	0,048414192	0,050190503	1,224936235	0	0	0	0,268476222	0	1,592017151	0,176890795
ENSG00000186094	AGBL4	0,020137336	0,107199627	0,037044256	1,425684163	0	0	0	0	0	1,590065382	0,176673931
ENSG00000259305	ZHX1-C8orf76	0	0	0	0	0	0,500862118	0,946945902	0	0,140261526	1,588069546	0,176452172
ENSG00000165917	RAPSN	0	0	0	0	0	0	0	1,585240463	0	1,585240463	0,176137829
ENSG00000168418	KCNG4	0,069179263	0,040918968	0	1,473309028	0	0	0	0	0	1,58340726	0,17593414
ENSG00000198670	LPA	0,047082278	0	0,043305826	0	0	0	0	1,492849736	0	1,583237841	0,175915316
ENSG00000129990	SYT5	0	0	0	1,455774195	0,069087638	0,031918156	0,024138194	0	0	1,580918183	0,175657576
ENSG00000169246	NPIPB3	0,064070626	0	0,766110088	0,16595398	0	0,070345803	0,159597624	0	0,354593746	1,580671867	0,175630207
ENSG00000175600	SUGCT	0,317348066	0	0,0972979	0,913318077	0,251394849	0	0	0	0	1,579358892	0,175484321
ENSG00000134489	HRH4	0	0	0,075867931	0	0,09801243	1,403719399	0	0	0	1,57759976	0,175288862
ENSG00000107105	ELAVL2	1,087760917	0,208670817	0	0	0,279468905	0	0	0	0	1,575900639	0,175100071
ENSG00000170465	KRT6C	0	0	1,239280277	0,089483873	0,246308328	0	0	0	0	1,575072478	0,175008053
ENSG00000160224	AIRE	0	0	0,118845703	1,450258516	0	0	0	0	0	1,569104219	0,174344913
ENSG00000018236	CNTN1	0	0,035790443	0	1,532453134	0	0	0	0	0	1,568243577	0,174249286
ENSG00000054793	ATP9A	0,036337759	0,032240231	0,902424272	0,596100098	0	0	0	0	0	1,56710236	0,174122484
ENSG00000111886	GABRR2	0	0	0	1,170111994	0,322078513	0,074399362	0	0	0	1,566589869	0,174065541
ENSG00000140832	MARVELD3	0,023442533	0	0	0	0	0,051477064	1,440397854	0,051261956	0	1,566579407	0,174064379
ENSG00000185565	LSAMP	0,029381795	0	0,027025092	0,050735947	0,034913236	0	0	0,032124691	1,39122907	1,56540983	0,173934426
ENSG00000135740	SLC9A5	0	0,494726937	0	0,144428953	0	0,520383728	0,069448523	0,060965787	0,27431221	1,564266138	0,173807349
ENSG00000166762	CATSPER2	0,037343524	0	0,034348214	0	0,044373847	0,041001007	0,325574814	0	1,079301439	1,561942846	0,173549205
ENSG00000005884	ITGA3	0	1,504571517	0,028359529	0,026620585	0	0	0	0	0	1,55955163	0,173283514
ENSG00000139910	NOVA1	0,102418086	0,025962626	0,053830383	0,176853681	0,104313771	0	0	1,023809659	0,071977739	1,559165946	0,173240661
ENSG00000113073	SLC4A9	0,124507474	0	0	1,433314884	0	0	0	0	0	1,557822358	0,173091373
ENSG00000104237	RP1	0,028869115	0,068303382	0,672689535	0,697909249	0,068608077	0,021131068	0	0	0	1,557510424	0,173056714
ENSG00000112655	PTK7	0,050454281	0	0,154691207	1,219729431	0	0	0	0,110328737	0,020684087	1,555887742	0,172876416
ENSG00000170369	CST2	0	0	0	0	0	0,667200711	0	0,885883559	0	1,55308427	0,172564919
ENSG00000163492	CCDC141	0,039132609	0,069439855	0,035993798	1,114962341	0,046499747	0	0,00812316	0,021392889	0,216576292	1,552120691	0,172457855
ENSG00000206181	ELOA2	0	0	0,182282032	1,368839211	0	0	0	0	0	1,551121243	0,172346805
ENSG00000164976	MYORG	0,023438897	0	0,043117745	1,214215796	0,111406093	0	0	0,153762014	0	1,545940545	0,171771172
ENSG00000113721	PDGFRB	0,021178777	0	0	0,511995746	0,855640956	0	0	0	0,15628299	1,545098468	0,171677608
ENSG00000137142	IGFBPL1	0,127126111	1,127910825	0	0	0,20141197	0	0,035185174	0	0,052116243	1,543750323	0,171527814
ENSG00000147174	GCNA	0	0,148103839	0	1,152985251	0,099176344	0	0	0,091255059	0,05132464	1,542845133	0,171427237
ENSG00000183145	RIPPLY3	0	0	0	1,314834961	0	0,07600118	0	0,151367186	0	1,542203328	0,171355925
ENSG00000173077	DEC1	0	0,161288421	0	0,824005183	0	0,249489589	0	0	0,3074153	1,542198493	0,171355388
ENSG00000153885	KCTD15	0	0,041975892	0,170063932	0,285933754	0	0	0,137490971	0	0,901885127	1,541349676	0,171261075
ENSG00000181378	CFAP65	0	0	0,052852952	0	1,468015431	0	0	0	0,017667699	1,538536082	0,170948454
ENSG00000120693	SMAD9	0	0	0,922876443	0,04559409	0	0	0,504249106	0	0,064947238	1,537666877	0,170851875
ENSG00000278259	MYO19	0	0	0	0,279395001	0	0,545633254	0,057577208	0	0,653838625	1,536444089	0,17071601
ENSG00000160439	RDH13	0	0,249376424	0,549367785	0,121336887	0	0,057862326	0,277137446	0	0,280865472	1,535946339	0,170660704
ENSG00000110400	NECTIN1	0	0	0	0	0	0,424486572	0,545732543	0,042271276	0,523042534	1,535532926	0,17061477
ENSG00000143450	OAZ3	0	0	0	0,112641906	0	0,680401674	0,460391894	0	0,280795523	1,534230996	0,170470111
ENSG00000179292	TMEM151A	0	0	0	0	0	0	1,532537546	0	0	1,532537546	0,17028195
ENSG00000073754	CD5L	0	0,108603483	0,225176265	0,528422387	0	0	0	0,669166803	0	1,531368938	0,170152104
ENSG00000173585	CCR9	0	0	0,099031029	0	0	0	1,430370725	0	0	1,529401754	0,169933528
ENSG00000164638	SLC29A4	0	0	0	0,179614507	1,23599223	0	0	0,113727265	0	1,529334001	0,169926
ENSG00000091181	IL5RA	0,022895541	0	0,042118197	1,462817282	0	0	0	0	0	1,52783102	0,169759002
ENSG00000228486	C2orf92	0	0	0	0,481652007	0	0,561457112	0,405303334	0,050828268	0,028587375	1,527828095	0,169758677
ENSG00000137821	LRRC49	0,151246693	0,080515064	0,055646101	0,914095097	0,071888208	0,016606003	0	0,231512559	0	1,521509724	0,169056636
ENSG00000169991	IFFO2	0	0,043395695	0	0,295605247	0	0,241656448	0,670095639	0,267385149	0	1,518138179	0,16868202
ENSG00000135373	EHF	0,034452934	0	0,126757911	0,832897776	0,409390723	0,037827309	0	0,075338479	0	1,516665132	0,168518348
ENSG00000183569	SERHL2	0	0,665364764	0	0,092497332	0	0	0,756113309	0	0	1,513975406	0,16821949
ENSG00000172867	KRT2	0	0	0,865644528	0,507853201	0,139788845	0	0	0	0	1,513286573	0,168142953
ENSG00000128165	ADM2	0,035232122	0	0	0,060838185	0	0,116048433	0	0	1,299929044	1,512047784	0,168005309
ENSG00000224916	APOC4-APOC2	0,082619229	0,293211591	0	0	0	0,997821948	0,137201019	0	0	1,510853788	0,167872643
ENSG00000178031	ADAMTSL1	0,011238329	0,099710695	0,020673814	0,213467553	1,121741542	0	0,018662849	0,024574934	0	1,510069716	0,167785524
ENSG00000162437	RAVER2	0	0,044174959	0,045795733	1,418592119	0	0	0	0	0	1,508562811	0,16761809
ENSG00000187516	HYPM	1,508089528	0	0	0	0	0	0	0	0	1,508089528	0,167565503
ENSG00000137078	SIT1	0,227747657	0	0	0	0	0	1,134622445	0	0,14005013	1,502420232	0,166935581
ENSG00000163359	COL6A3	0,038483821	0,013657719	0,807052699	0,013290632	0,182915266	0,380276878	0	0	0,066262194	1,50193921	0,166882134
ENSG00000004777	ARHGAP33	1,493868759	0	0	0	0	0	0	0	0	1,493868759	0,165985418
ENSG00000117114	ADGRL2	0	0	0	1,076853132	0,277883061	0,021396773	0	0,042614724	0,071903478	1,490651167	0,165627907
ENSG00000125462	C1orf61	0	0,021775378	0	1,250216299	0	0,18862661	0	0,026834056	0	1,487452342	0,165272482
ENSG00000154678	PDE1C	0	0	1,206772733	0,075518407	0	0,072025429	0	0,11954076	0,013446677	1,487304005	0,165256001
ENSG00000186197	EDARADD	0,227519052	0,134575659	0,139513224	0	0,270351977	0,208168854	0,283370888	0,08291959	0,139909553	1,486328797	0,165147644
ENSG00000114455	HHLA2	0	0,221788255	0,15328376	0,07194237	0	0	1,037802581	0	0	1,484816965	0,164979663
ENSG00000106927	AMBP	0	0	0	0,275102767	0	0,3498378	0,859838859	0	0	1,484779426	0,164975492
ENSG00000101680	LAMA1	0,019059163	0	0,017530434	1,316440561	0,022647256	0,083703387	0,023737844	0	0	1,483118645	0,164790961
ENSG00000136231	IGF2BP3	0	0	0	0,794881001	0,210379345	0	0,455721182	0	0,021774636	1,482756165	0,164750685
ENSG00000159167	STC1	0,511743587	0,389175617	0,067242404	0,063119249	0	0	0,091052491	0	0,359644939	1,481978287	0,164664254
ENSG00000148734	NPFFR1	0,016938748	0,060114785	0	1,403976996	0	0	0	0	0	1,481030529	0,164558948
ENSG00000198216	CACNA1E	0,016541029	0,044027475	0,015214279	0,32847159	0,098275284	0,045402708	0	0,92234486	0,010171667	1,480448893	0,164494321
ENSG00000227234	SPANXB1	0	0	0	0	0,770638503	0	0	0,709087055	0	1,479725558	0,164413951
ENSG00000187758	ADH1A	0,406393287	0	0	0,467835006	0,160967074	0	0	0,444331557	0	1,479526924	0,16439188
ENSG00000254996	ANKHD1-EIF4EBP3	0,013272777	0,023552218	0	1,237636243	0,063086086	0,043718202	0	0	0,097942831	1,479208357	0,164356484
ENSG00000174500	GCSAM	0	0,2145136	0	0,06958266	0	1,194556152	0	0	0	1,478652412	0,164294712
ENSG00000165837	ERICH6B	0	0,547228572	0	0,088753393	0,122148824	0	0,085353967	0	0,632131028	1,475615784	0,163957309
ENSG00000110799	VWF	0,218136711	0,025805216	0	0,175781428	0	0,015966757	0,941842546	0,095400222	0	1,47293288	0,163659209
ENSG00000001626	CFTR	0,048521001	1,291489886	0	0,055856786	0,076874139	0	0	0	0	1,472741812	0,163637979
ENSG00000179909	ZNF154	0,264503012	0	0,145972396	0,867804046	0,062859713	0,029040885	0,043924499	0,057839063	0	1,471943615	0,163549291
ENSG00000268279	AC090004.1	0	0	0	0	0	0,497140342	0	0	0,974476147	1,471586489	0,16350961
ENSG00000174527	MYO1H	0	0,111912354	0,058009203	0,16335662	0,149882113	0,934805002	0,052366583	0	0	1,470331875	0,163370208
ENSG00000270672	MTRNR2L6	0	0	0,384215755	1,081969487	0	0	0	0	0	1,466185242	0,162909471
ENSG00000157152	SYN2	0	0,084734397	0	0	0	0,078643029	0	0,626515217	0,675378419	1,465271062	0,162807896
ENSG00000164695	CHMP4C	0	0,144785097	0	0,140893615	0,19390796	0,985429991	0	0	0	1,465016663	0,162779629
ENSG00000184916	JAG2	0,023625793	0	0	0,040796588	0	0,907890898	0,411957	0,05166269	0,02905668	1,464989648	0,162776628
ENSG00000276023	DUSP14	0	0,109939319	0	0	0	0,20407205	0,617320207	0	0,533384853	1,464716429	0,16274627
ENSG00000132854	KANK4	0,022176485	0	0	0,497821349	0,895949254	0	0	0,048493479	0	1,464440568	0,162715619
ENSG00000205639	MFSD2B	0	0	0	0,940729967	0	0	0	0,52119017	0	1,461920137	0,162435571
ENSG00000235268	KDM4E	1,227490741	0,11463959	0,118845703	0	0	0	0	0	0	1,460976033	0,16233067
ENSG00000105717	PBX4	0	0	0	1,455235809	0	0	0	0	0	1,455235809	0,161692868
ENSG00000184221	OLIG1	0,066480673	0	0	0	0	1,386846	0	0	0	1,453326673	0,161480741
ENSG00000232263	KRTAP25-1	0	1,449416217	0	0	0	0	0	0	0	1,449416217	0,161046246
ENSG00000167618	LAIR2	0	0	0	0	0	0	0	1,266032826	0,17801391	1,444046736	0,160449637
ENSG00000177143	CETN1	0,085955956	0	0,948737539	0	0,40855238	0	0	0	0	1,443245876	0,160360653
ENSG00000185829	ARL17A	0,463974118	0	0	0,150221632	0	0,127354118	0	0,380465823	0,32097845	1,442994141	0,160332682
ENSG00000132698	RAB25	0	0	0,658330607	0,154490808	0	0	0,520007297	0	0,110033465	1,442862177	0,16031802
ENSG00000164841	TMEM74	0	0	1,273934946	0,035171179	0	0	0	0,133616889	0	1,442723014	0,160302557
ENSG00000105664	COMP	0	0	0	1,26585531	0,116144095	0	0	0	0,060105602	1,442105008	0,16023389
ENSG00000171446	KRT27	1,440762592	0	0	0	0	0	0	0	0	1,440762592	0,160084732
ENSG00000143067	ZNF697	0	0,144188206	0	0,748334754	0,321847591	0	0,22489753	0	0	1,439268081	0,159918676
ENSG00000172403	SYNPO2	0,061898851	0	0,014233492	0,146967984	0,88262376	0,033980661	0,263404179	0,016919333	0,019031902	1,439060161	0,159895573
ENSG00000273079	GRIN2B	0,043954466	0,069329886	0,026952597	0,489131849	0,046426107	0,021448638	0,02027574	0,715526842	0,006006481	1,439052605	0,159894734
ENSG00000137731	FXYD2	0	0,047814194	0	0,651406855	0	0,207092375	0	0,530297986	0	1,43661141	0,15962349
ENSG00000132429	POPDC3	0,198829698	0	0	0	0,157507694	0,800445765	0,275154237	0	0	1,431937394	0,159104155

ENSG00000143603	KCNN3	0	0,098588867	0,020441216	0,019187806	0,052815287	0	0,664303545	0	0,573979994	1,429316714	0,158812968
ENSG00000100320	RBFOX2	0	0,077252089	0	1,002343127	0	0	0,349432404	0	0	1,429027621	0,158780847
ENSG00000188130	MAPK12	0	0	0	1,344592462	0,038552608	0,017811119	0,026939416	0	0	1,427895605	0,158655067
ENSG00000165325	DEUP1	0,142995572	0,063435533	0	0,43211375	0	0,471002347	0	0,312689442	0	1,422236644	0,158026294
ENSG00000176555	OR4S1	0	0	0	0	0	0	0	1,421223946	0	1,421223946	0,157913772
ENSG00000162931	TRIM17	0,085060833	0	0	1,101611207	0	0,233479562	0	0	0	1,420151603	0,157794623
ENSG00000047936	ROS1	0	0	0	1,395018958	0	0	0	0	0,023656635	1,418675593	0,157630621
ENSG00000224109	CENPVL3	0,07769181	0	0	0	0	0	0	0	1,337712757	1,415404567	0,157267174
ENSG00000253537	PCDHGA7	0,055565571	0	0	0	0	0	0	0,060752816	1,298431301	1,414749688	0,15719441
ENSG00000204644	ZFP57	0	0,374325733	0	0	0	0	0	0	1,037765597	1,41209133	0,156899037
ENSG00000175874	CREG2	0,021406796	0	0,078759059	0,036964864	0	0,61108868	0,657657215	0	0	1,405876614	0,156208513
ENSG00000141527	CARD14	0,079456605	0,028198759	0,029233368	1,179956242	0	0,052343225	0	0,034749665	0	1,403937865	0,155993096
ENSG00000149651	SPINT4	0	0	0	0	1,4028029	0	0	0	0	1,4028029	0,155866989
ENSG00000214652	ZNF727	0	0	0	0,153889464	0	1,232881143	0,01479952	0	0	1,401570127	0,155730014
ENSG00000145721	LIX1	0,075630916	0	0	0	0	0	0	1,323061331	0	1,398692248	0,15541025
ENSG00000197641	SERPINB13	0	0,059127233	0	0,057538032	0,079187992	0,036584471	0	1,165811043	0	1,39824877	0,155360974
ENSG00000173267	SNCG	0	0	0	0,206435898	0	0	1,191174038	0	0	1,397609936	0,155289993
ENSG00000257390	AC023055.1	0,119266433	0	0	0	0	0,261895149	0,346603127	0,521601527	0,146682338	1,396048574	0,155116508
ENSG00000122543	OCM	0	0	0	0	0	1,395882256	0	0	0	1,395882256	0,155098028
ENSG00000144119	C1QL2	0	0	0	0	0	1,39347466	0	0	0	1,39347466	0,154830518
ENSG00000146047	HIST1H2BA	0	0	0	0	1,391928459	0	0	0	0	1,391928459	0,154658718
ENSG00000160062	ZBTB8A	0,021352349	0	0	0,036870846	0,101488637	0,750196193	0,035458621	0	0,446430815	1,39179746	0,154644162
ENSG00000116106	EPHA4	0	0	0	1,174449184	0,095080101	0,087853099	0,033219574	0	0	1,390601958	0,154511329
ENSG00000185630	PBX1	0,028445681	0	0,06541016	1,031509147	0,084502222	0,046847544	0,041333349	0,031101188	0,061222912	1,390372203	0,1544858
ENSG00000132849	PATJ	0,0252061	0,044727606	0,139105971	1,066373124	0,029951421	0,013837413	0,041858326	0,02755918	0	1,388619141	0,154291016
ENSG00000078900	TP73	0,022330511	0	0,246472675	0,115679758	0,053068944	0,171623177	0,778743009	0	0	1,387918075	0,154213119
ENSG00000103034	NDRG4	1,300111679	0	0	0,027048303	0	0	0,026012301	0,034252573	0	1,387424854	0,154158317
ENSG00000130595	TNNT3	0,111233398	0,197380935	0	0	0	0,061063885	1,015954105	0	0	1,385632323	0,153959147
ENSG00000065534	MYLK	0,020450747	0,072578698	0,163023471	1,047647979	0	0,007484575	0,039621614	0	0,03353571	1,384342795	0,153815866
ENSG00000143631	FLG	0,011854599	0,021035695	0,043614984	0,040940609	0,112690843	0,013015657	0	1,140591588	0	1,383743974	0,15374933
ENSG00000236396	SLC35G4	0	0	0	1,026292919	0,353114594	0	0	0	0	1,379407513	0,153267501
ENSG00000212123	PRR22	0	0,450154449	0	0	0,200961132	0	0	0	0,727994211	1,379109792	0,153234421
ENSG00000185269	NOTUM	0	0	0	0	0	0	1,378321532	0	0	1,378321532	0,153146837
ENSG00000214456	PLIN5	0	0	0	0,433536822	0	0	0,052116441	0,892139019	0	1,377792282	0,153088031
ENSG00000273777	CEACAM20	0,068905869	0	0	1,308839362	0	0	0	0	0	1,377745231	0,153082803
ENSG00000178795	GDPD4	0	0	0	0	0,337939971	0	0,669070152	0,310948464	0,058295647	1,376254233	0,152917137
ENSG00000188909	BSX	0	0	0	0,943138462	0,432671738	0	0	0	0	1,3758102	0,1528678
ENSG00000141314	RHBDL3	0	0	0	0	1,374971447	0	0	0	0	1,374971447	0,152774605
ENSG00000112902	SEMA5A	0,073664562	0,130715957	0	0,551211354	0,029177571	0,026959795	0,5300989	0	0,030199305	1,372027443	0,152447494
ENSG00000134532	SOX5	0	0,975528579	0,106454801	0,199854456	0	0,01588421	0,024024956	0,031635669	0,01779287	1,371175541	0,152352838
ENSG00000137463	MGARP	0	0,438140523	0	0,639546506	0,293396685	0	0	0	0	1,371083714	0,152342635
ENSG00000198169	ZNF251	0	1,116151041	0	0	0	0,03288614	0,074610703	0	0,147351058	1,370998942	0,152333216
ENSG00000116299	KIAA1324	0,065743124	0	0,060469893	0,17028604	0	0	0,750583887	0	0,323422271	1,370505215	0,152278357
ENSG00000127152	BCL11B	0,106316068	0	0,065192331	0,183584644	0	0,11672883	0,89747775	0	0	1,369299623	0,152144403
ENSG00000113361	CDH6	0	1,099030941	0,02234028	0	0,173166058	0	0	0	0,074679146	1,369216424	0,152135158
ENSG00000237649	KIFC1	0	0	0	0	0	1,368899148	0	0	0	1,368899148	0,152099905
ENSG00000101104	PABPC1L	0,212233948	0	0,055774498	0,104709059	0,216162245	0,033288639	0	0	0,745772561	1,367940949	0,151993439
ENSG00000137460	FHDC1	0	0	0,08432583	0,079155157	0	0	0	1,202856002	0	1,366336989	0,151815221
ENSG00000222046	DCDC2B	0	0	0,743659976	0	0	0,621387928	0	0	0	1,365047904	0,151671989
ENSG00000130762	ARHGEF16	0,028382902	0	0	1,274288005	0	0	0	0,062065095	0	1,364736003	0,151637334
ENSG00000153563	CD8A	0	0,073787012	0	0	0	0	0,93222316	0	0,357987247	1,363997419	0,151555269
ENSG00000185758	CLDN24	0,227919413	0	0	0	0	0	1,135478118	0	0	1,36339753	0,151488614
ENSG00000182963	GJC1	0,05201144	0	0	0,5688119	0	0,742371787	0	0	0	1,363195126	0,151466125
ENSG00000277865	GOLGA6L22	0	0	0	1,36243006	0	0	0	0	0	1,36243006	0,151381118
ENSG00000124701	APOBEC2	0	0	0	0	0	0,43243417	0,763068917	0	0,161465267	1,356968355	0,150774262
ENSG00000164591	MYOZ3	0	0	0	0,950897704	0	0	0,020783557	0,383144272	0	1,354825534	0,15053617
ENSG00000116254	CHD5	0	0,026564494	0	0,129252514	1,174051803	0	0,024860379	0	0	1,354729189	0,150525465
ENSG00000162849	KIF26B	0,010490875	0,726016246	0	0,108692714	0	0,034555105	0,113240372	0	0,361267886	1,354263197	0,150473689
ENSG00000166741	NNMT	0	0	0	0	0	0	1,28952037	0	0,063667873	1,353188243	0,150354249
ENSG00000030304	MUSK	0	0,126303344	0,327343499	0	0	0,898714853	0	0	0	1,352361696	0,150262411
ENSG00000151632	AKR1C2	0,023388109	0,747029253	0	0,323089274	0	0	0	0,255714725	0	1,34922136	0,149913484
ENSG00000170965	PLAC1	0	0	0,145997951	0,41113704	0	0	0,790779403	0	0	1,347914394	0,149768266
ENSG00000160683	CXCR5	0	0	0	0	0	0,374431453	0,679595569	0	0,293596402	1,347623424	0,149735936
ENSG00000006740	ARHGAP44	0,016210102	0,604052993	0	0,111965233	0	0	0,511464559	0	0,099681679	1,343374565	0,149263841
ENSG00000181773	GPR3	0,070711545	0	0	0,488413616	0	0	0	0	0,782694759	1,34181992	0,149091102
ENSG00000151612	ZNF827	0	0,124413409	0	1,05935786	0,041656135	0,115469604	0	0	0	1,340897009	0,148988557
ENSG00000101162	TUBB1	0	0	0,079513758	0,074638151	0,924501682	0	0,071779366	0,189035794	0	1,339468751	0,148829861
ENSG00000204290	BTNL2	1,338172628	0	0	0	0	0	0	0	0	1,338172628	0,148685848
ENSG00000186204	CYP4F12	0	0,100221267	1,090932924	0,146291333	0	0	0	0	0	1,337445525	0,148605058
ENSG00000110169	HPX	0,083233583	0,073847976	0,076557449	0,287452464	0,098903206	0	0	0	0,716566046	1,336560725	0,148506747
ENSG00000148346	LCN2	0	0	0	0	0	0	1,336463436	0	0	1,336463436	0,148495937
ENSG00000142173	COL6A2	0	0	0,103917794	1,170549419	0	0	0	0,061763471	0	1,336230683	0,148470076
ENSG00000164690	SHH	0,057741907	0	0,106220901	0,598246025	0,205837338	0,06339724	0,239721689	0,063132321	0	1,334297421	0,148255269
ENSG00000261949	GFY	0	0	1,333593225	0	0	0	0	0	0	1,333593225	0,148177025
ENSG00000150471	ADGRL3	0,032401666	0,019165321	0,09934247	0,018650202	0,667361597	0	0	0,49597069	0	1,332891945	0,148099105
ENSG00000081052	COL4A4	0,014997079	0	0,055176677	0,155380096	0	1,020886836	0	0,065588441	0,018444474	1,330473604	0,1478304
ENSG00000188716	DUPD1	0	0	0	0,787134162	0,541655418	0	0	0	0	1,32878958	0,147643287
ENSG00000129194	SOX15	0	0	0	0,677313367	0	0	0,651370964	0	0	1,328684331	0,147631592
ENSG00000172461	FUT9	0,01100667	0	0	0	0,052315178	0	0	1,251554921	0,013536785	1,328413554	0,147601506
ENSG00000074966	TXK	0	0	0	0,075284182	0,103611524	0,143604077	0,362003266	0	0,643438623	1,327941672	0,147549075
ENSG00000102230	PCYT1B	0	0	0,100082844	1,127351825	0	0	0	0	0,100367159	1,327801828	0,147533536
ENSG00000187144	SPATA21	0	0,059507767	0	0,752808404	0	0,515478933	0	0	0	1,327795105	0,147532789
ENSG00000178075	GRAMD1C	0	0	0,074685679	1,086644843	0,0964851	0,044575652	0	0	0,024965949	1,327357223	0,147484136
ENSG00000170950	PGK2	0	0	0	0	0,650182636	0,200254166	0,075721383	0,398834722	0	1,324992907	0,147221434
ENSG00000271303	SRXN1	0,112016731	0,099385471	0	0	0,665525468	0,307469564	0,139514824	0	0	1,323912058	0,14710134
ENSG00000110076	NRXN2	0,014282663	0	0,052548223	1,035847725	0	0,015681529	0	0,187392004	0,017565834	1,323317978	0,147035331
ENSG00000187173	LCE2A	0	0	0	0	0	0	0,410704851	0	0,912503383	1,323208234	0,147023137
ENSG00000163082	SGPP2	0	0,053811359	0	0,314190216	0	0	0,805749674	0	0,149184445	1,322935694	0,146992855
ENSG00000203730	TEDDM1	0	0	0	0	0	0	0	1,32173827	0	1,32173827	0,146859808
ENSG00000204711	C9orf135	0	0	0	0	0	0	0	1,32173827	0	1,32173827	0,146859808
ENSG00000259529	AL136295.5	0,894626629	0	0	0	0	0,089295251	0,337648902	0	0	1,321570783	0,146841198
ENSG00000113594	LIFR	0	1,058542315	0	0,042920466	0,029535122	0	0	0,190232912	0	1,321230814	0,146803424
ENSG00000215817	ZC3H11B	0,03043516	0	0,895806966	0	0	0,100248083	0,227438668	0,066552783	0	1,32048166	0,146720184
ENSG00000188522	FAM83G	0	0	0,035474745	0,499492677	0,091658382	0,677531707	0,016012038	0	0	1,320169549	0,146685505
ENSG00000167861	HID1	0,024384472	0,043269647	0	0,04210666	0	1,177999899	0	0	0,029989757	1,317750434	0,146416715
ENSG00000189431	RASSF10	0	0	0	0	0,141943693	0	0	1,175458936	0	1,317402628	0,14637807
ENSG00000188738	FSIP2	0	0,045476701	0,200367254	0,044254395	0	0	0	0,938694765	0,086678471	1,315471586	0,14616351
ENSG00000243501	AL365232.1	0	1,311207824	0	0	0	0	0	0	0	1,311207824	0,145689758
ENSG00000172154	OR8I2	0	0	0	0	0	0	0	1,308651753	0	1,308651753	0,14540575
ENSG00000140479	PCSK6	0,011414909	0,202554767	0,020998648	0,059133171	0	0,012532904	0,132692601	0,474260219	0,393088278	1,306675497	0,145186166
ENSG00000118137	APOA1	0,424467895	0	0	0	0	0	0,881111882	0	0	1,305579777	0,14506442
ENSG00000198826	ARHGAP11A	0	0	0	0	0	0,854675153	0,450486674	0	0	1,305161828	0,145017981
ENSG00000011083	SLC6A7	0	1,191184943	0	0	0,083964822	0	0,029336061	0	0	1,304485826	0,14494287
ENSG00000152527	PLEKHH2	0,107410032	0,272280666	0	0,211969923	0,036466038	0	0	0,63751592	0,037742998	1,303385577	0,14482062
ENSG00000180071	ANKRD18A	0	0	0	0	1,302392309	0	0	0	0	1,302392309	0,144710257
ENSG00000120498	TEX11	0,04482663	0	0	0,077405807	0	0	0	1,176272563	0	1,298505	0,144278333
ENSG00000138356	AOX1	0,027931714	0,148692421	0	0,868175516	0	0,184004336	0,069576894	0	0	1,298380882	0,144264542
ENSG00000141756	FKBP10	0	0	0,06505502	1,038121827	0	0,194138283	0	0	0	1,29731513	0,144146126
ENSG00000176383	B3GNT4	0	0	0	0	0	0	0,192946468	0	1,102340971	1,29528744	0,143920827
ENSG00000263639	MSMB	0	0	0,565000201	0	0,729913704	0	0	0	0	1,294913905	0,143879323
ENSG00000136883	KIF12	0	0	0,091561297	0	0	1,202250556	0	0	0	1,293811853	0,143756873
ENSG00000155495	MAGEC1	0	0,126006579	0	1,164888279	0	0	0	0	0	1,290894858	0,143432762
ENSG00000166348	USP54	0,015909725	0,028231417	0	0,247253608	0,075619613	0,40176282	0,052840738	0,347899102	0,117401467	1,286918491	0,142990943
ENSG00000196104	SPOCK3	0	0	0	0	0	0,037017085	0	0,95842244	0,290255613	1,285695138	0,142855015
ENSG00000139116	KIF21A	0	0,053008204	0	0	0,070992892	0,016399187	0	1,143146176	0	1,283546459	0,142616273
ENSG00000172425	TTC36	0	0	0	1,034430028	0	0	0,248702343	0	0	1,283132371	0,142570263
ENSG00000166342	NETO1	0	0	0	0	0	0	0	1,262192483	0,020879286	1,283071769	0,14256353

ENSG00000169758	TMEM266	0	0	0,162119031	0,355082586	0	0,032253223	0,024391589	0,706605801	0	1,28045223	0,14227247
ENSG00000244486	SCARF2	0	0	0	0,07810086	0	0	1,20175116	0	0	1,27985202	0,14220578
ENSG00000185863	TMEM210	0,132669509	0	0	1,145456429	0	0	0	0	0	1,278125938	0,142013993
ENSG00000160870	CYP3A7	0	0	0,291766045	0,068468899	0,56539104	0,043534657	0	0,260116427	0,048765815	1,278042883	0,142004765
ENSG00000091879	ANGPT2	0,022304143	0	0,041030273	0,231086324	0	0,24488646	0,240754881	0,19509052	0,301743431	1,276896032	0,141877337
ENSG00000155749	FLACC1	0,057347465	0	0	0,099026556	0	0	1,047570134	0	0,070529989	1,274474145	0,141608238
ENSG00000152932	RAB3C	0,031412654	0,027870492	0	0	0,037326426	0,017244629	0,026082597	1,133389536	0	1,273326333	0,141480704
ENSG00000089101	CFAP61	0,020649162	0,073282864	0	0,10696979	0,098146363	0,158701016	0,137163522	0,677305071	0	1,27221779	0,141357532
ENSG00000121410	A1BG	0	0	0,069390938	0	0	1,201050106	0	0	0	1,270441044	0,141160116
ENSG00000125787	GNRH2	0	0	0	1,268213728	0	0	0	0	0	1,268213728	0,140912636
ENSG00000205683	DPF3	0	0,077846422	0	0,151508187	0	0,120417025	0	0	0,917227719	1,266999352	0,140777706
ENSG00000126890	CTAG2	0	0	0	0	0	0	1,263548157	0	0	1,263548157	0,14039424
ENSG00000142178	SIK1	0	0	0	0	0	0,220458245	0,476349021	0,564523749	0	1,261331015	0,140147891
ENSG00000169064	ZBBX	0,456527404	0	0,209954758	0,131387198	0,180824543	0	0	0	0,280734928	1,25942883	0,139936537
ENSG00000136574	GATA4	0	0	0,062565856	1,115859671	0,080827716	0	0	0	0	1,259253243	0,139917027
ENSG00000163701	IL17RE	0,355706458	0,229524503	0,059486432	0,614227417	0	0	0	0	0	1,25894481	0,139882757
ENSG00000184985	SORCS2	0,047842511	0,127343043	0	0	0	0	1,052703435	0	0,029420061	1,257309049	0,139701005
ENSG00000081059	TCF7	0,059887277	0	0	0,439502087	0	0,295887286	0,459962576	0	0	1,255239227	0,139471025
ENSG00000188771	PLET1	0	0,220693004	0	0,214761296	0	0,81931149	0	0	0	1,25476579	0,139418421
ENSG00000235961	PNMA6A	0	0	0	0,118123574	0	0	1,135992141	0	0	1,254115715	0,139346191
ENSG00000147481	SNTG1	0,028511428	0	0,026224538	0	0,033879013	0	0,011836824	1,153403679	0	1,253855482	0,139317276
ENSG00000179615	OR2AP1	0	0,173218346	0	0	0	0	0	0	1,080503036	1,253721383	0,139302376
ENSG00000129595	EPB41L4A	0	0	0	0,395402106	0,04186007	0	0,511885024	0	0,303281421	1,252428622	0,139158736
ENSG00000165899	OTOGL	0,024614851	0	0	0,042504475	1,082207938	0,013512834	0	0,026912736	0,060546187	1,250299022	0,138922114
ENSG00000037280	FLT4	0	0,090875057	0,031403084	1,031713072	0,08113817	0	0,014174235	0	0	1,249303618	0,138811513
ENSG00000132535	DLG4	0,014646755	0	0,080831666	0,227625741	0	0,112568967	0,632398688	0	0,180136205	1,248208022	0,13868978
ENSG00000106125	MINDY4	0	0	0	1,102642341	0,097905546	0,045231891	0	0	0	1,245779778	0,138419975
ENSG00000111325	OGFOD2	0,065586185	0,116381076	0	0,113253027	0,233800483	0,144019598	0,245059242	0,286835562	0,040331277	1,245266451	0,138362939
ENSG00000151892	GFRA1	0,312276443	0	0	0,754926564	0,037106586	0	0	0,136571427	0	1,24088102	0,137875669
ENSG00000173918	C1QTNF1	0,0635586	0,169174763	0,058460589	0,329255488	0,151048388	0	0	0	0,469013305	1,240511134	0,13783457
ENSG00000166262	FAM227B	0	0,93537907	0	0,303412761	0	0	0	0	0	1,238791831	0,137643537
ENSG00000092054	MYH7	0,10028908	0	0	0,909181096	0,119169584	0	0	0,109651424	0	1,238291185	0,137587909
ENSG00000211452	DIO1	0	0	0	0	0	1,235968363	0	0	0	1,235968363	0,137329818
ENSG00000186862	PDZD7	0	0	0	0,118570876	0	0,050260702	0,98825466	0,050050677	0,028150034	1,235286948	0,137254105
ENSG00000240747	KRBOX1	0	0,091111791	0	0	0	0,845619657	0,298434361	0	0	1,235165809	0,137240645
ENSG00000129991	TNNI3	0	0	0	0	0	0	1,233729912	0	0	1,233729912	0,137081101
ENSG00000147036	LANCL3	0,029347557	0	0	0,050676826	0,069745104	0	0	0	1,082811355	1,232580842	0,136953427
ENSG00000183690	EFHC2	0	0,161094623	0,083502583	0,156764779	0	0	0,339210871	0,099259407	0,390785718	1,230617981	0,136735331
ENSG00000169432	SCN9A	0	0	0	0,816549298	0,052269492	0,024148254	0	0,33666283	0	1,229629873	0,136625541
ENSG00000184254	ALDH1A3	0,028324381	0,65339194	0	0,048910023	0	0,497576238	0	0	0	1,228202582	0,136466954
ENSG00000096696	DSP	0,045116611	1,067444268	0,027665217	0,051937694	0,035740201	0	0	0	0	1,227903991	0,136433777
ENSG00000241635	UGT1A1	0	0	0	1,226692292	0	0	0	0	0	1,226692292	0,136299144
ENSG00000116147	TNR	0,011799982	0,041877557	0,04341404	0,040751987	0,084128739	0,012955691	0,009797777	0,980518005	0	1,225243778	0,136138198
ENSG00000176842	IRX5	0	0	0	0	0	1,224647491	0	0	0	1,224647491	0,136071943
ENSG00000152779	SLC16A12	0	0,385182434	0,171134885	0,160641273	0,294781484	0,034046907	0	0,135618538	0,03813801	1,219543531	0,135504837
ENSG00000184545	DUSP8	0,182795045	0,108121774	0	0	0	0,234147991	0,227667941	0,466339107	0	1,219071859	0,135452429
ENSG00000172940	SLC22A13	0	0	0	1,218915464	0	0	0	0	0	1,218915464	0,135435052
ENSG00000082482	KCNK2	0	0	0	0,26694115	0,918459188	0	0,032089599	0	0	1,217489937	0,13527666
ENSG00000081923	ATP8B1	0	0,152613546	0,158212919	0,185639566	0,56207925	0	0	0	0,15866237	1,217207651	0,135245295
ENSG00000172772	OR10W1	0,102866284	0	0	0,888138103	0	0	0	0,224938439	0	1,215942826	0,135104758
ENSG00000102271	KLHL4	0,24538904	0	0	0,127119994	0,058317236	0	0	0	0,784671903	1,215498173	0,135055353
ENSG00000197428	OR51D1	0,125126059	0	0	0	1,090337556	0	0	0	0	1,215463615	0,135051513
ENSG00000134571	MYBPC3	0	0	0	0,975457849	0,083905968	0,038764153	0,117261993	0	0	1,215389963	0,135043329
ENSG00000221996	OR52B4	0	0,477120997	0	0	0	0,738036373	0	0	0	1,21515737	0,135017486
ENSG00000161609	CCDC155	0	0	0	0	0	0,029526709	1,18347172	0	0	1,212998429	0,134777603
ENSG00000187730	GABRD	0,054700659	0	0	0	0,194995951	0,960930037	0	0	0	1,210626647	0,134514072
ENSG00000145839	IL9	0	0	0,470355497	0	0	0	0,424603492	0	0,314461121	1,20942011	0,134380012
ENSG00000175868	CALCB	0,038372415	0	0	0	0	0,337045356	0,700951575	0,083909235	0,047193124	1,207471706	0,134163523
ENSG00000115657	ABCB6	0	0	0	0,701842391	0	0,095625693	0,409797434	0	0	1,207265517	0,134140613
ENSG00000145681	HAPLN1	0,977569822	0,038548304	0	0	0,154881056	0	0,036075426	0	0	1,207074608	0,134119401
ENSG00000067177	PHKA1	0,047593881	0	0,043776394	1,109487294	0	0	0	0	0	1,200857568	0,133428619
ENSG00000163288	GABRB1	0,048124386	0	0	0	0	0,264188816	0,039958704	0,315701816	0,532681115	1,200654837	0,133406093
ENSG00000126583	PRKCG	0	0,31573977	0	0,384066786	0,105716085	0,390722583	0	0	0	1,196245225	0,132916136
ENSG00000134962	KLB	0	0,044675442	0	0,043474671	0	0,856919007	0,250857045	0	0	1,195926164	0,132880685
ENSG00000241186	TDGF1	0	0	0,111683447	0,104835265	0	0	0	0,531031848	0,448002866	1,195553426	0,13283927
ENSG00000142513	ACP4	0	0	0,206369784	0	0	0,985363485	0	0	0	1,191733268	0,132414808
ENSG00000282757	DUXB	0	0,497019463	0	0	0	0	0,348851711	0	0,344479189	1,190350362	0,132261151
ENSG00000171219	CDC42BPG	0,046274865	0,041056806	0	0,119859887	0	0	0,979786699	0	0	1,186978257	0,131886473
ENSG00000197959	DNM3	0,42682834	0	0,09814815	0,644909461	0	0	0	0	0,016404495	1,186290446	0,13181005
ENSG00000135374	ELF5	0,03362496	0	0	0	0,079910446	1,070629	0	0	0	1,184164405	0,131573823
ENSG00000174564	IL20RB	0	0,109759312	0	0	0	0,814951666	0,256795604	0	0	1,181506582	0,131278509
ENSG00000176893	OR51G2	0	0,161239928	0	1,019890159	0	0	0	0	0	1,181130087	0,131236676
ENSG00000197576	HOXA4	0	0	0	0,896170491	0	0,284906543	0	0	0	1,181077034	0,131230782
ENSG00000139549	DHH	0,064535798	0	0	1,114392375	0	0	0	0	0	1,178928173	0,130992019
ENSG00000120256	LRP11	0,05347154	0	0,245913039	0	0,698919491	0	0,088797121	0,058463299	0,03288155	1,17844604	0,130938449
ENSG00000155833	CYLC2	0	0	0,244270737	0,114646298	0,473353527	0	0,055127562	0,290364295	0	1,177762419	0,130862491
ENSG00000179083	FAM133A	0,044694046	0,158616977	0	0,308707453	0,212432737	0,147214354	0	0,195465583	0,109935831	1,177066981	0,13078522
ENSG00000111339	ART4	0,086431213	0	0	0,09949856	0,410811297	0,03163214	0,071765681	0,440999423	0,035433083	1,176571398	0,130730155
ENSG00000149380	P4HA3	0,189361617	0	0,069669198	0,915561314	0	0	0	0	0	1,17459213	0,130510237
ENSG00000243716	NPIPB5	0,105724537	0	0,361193532	0,052160914	0,179469037	0,082913831	0,188111442	0	0,20432901	1,173902304	0,130433589
ENSG00000110436	SLC1A2	0,013255313	0,035281842	0,024384219	1,018561962	0	0,021830339	0,027515424	0,014492744	0,016302327	1,17162417	0,130180463
ENSG00000160145	KALRN	0,024681188	0,054745202	0,090806076	0,095892804	0	0,006774625	0	0,890513739	0,00758867	1,171002303	0,130111367
ENSG00000135722	FBXL8	0	0	0	0,08339245	0	0,318141087	0,240595076	0,528019443	0	1,170148055	0,130016451
ENSG00000076706	AP002956.1	0	0	1,086152367	0	0	0,022353891	0,016905192	0,04452096	0	1,16993241	0,12999249
ENSG00000065717	TLE2	0	0	0	0	0	1,119396662	0	0	0,044782295	1,164178957	0,129353217
ENSG00000162104	ADCY9	0	0	0	0,073759062	0	0,156327689	0,898496677	0	0,03502243	1,163605858	0,12928954
ENSG00000039139	DNAH5	0,009172114	0,244135357	0,016872844	0,126705906	0,065393179	0,211479339	0,007615802	0,481361434	0	1,162735973	0,129192886
ENSG00000186105	LRRC70	0	0	0	0,241271359	0	0,920447027	0	0	0	1,161718386	0,129079821
ENSG00000158516	CPA2	0	0,149549359	0,620145229	0,145529824	0	0	0,139955752	0	0,103651156	1,15883132	0,128759036
ENSG00000174945	AMZ1	0,344853677	0	0	0,595487034	0	0	0,035792421	0,047130875	0,13253924	1,155803247	0,128422583
ENSG00000222038	POTEJ	0	0	0	0	0,106153574	0	0,853035068	0,195350025	0	1,154538668	0,128282074
ENSG00000154025	SLC5A10	0	0	0	1,051667856	0	0	0	0,102444448	0	1,154112304	0,1282347
ENSG00000180834	MAP6D1	0	0	0,577201202	0	0	0	0,573161816	0	0	1,150363018	0,127818113
ENSG00000044524	EPHA3	0,022975608	0	1,098902626	0	0	0,025225875	0	0	0	1,147104108	0,127456012
ENSG00000112053	SLC26A8	0	0,044423791	0	0	0	0	1,101711	0	0	1,146134791	0,12734831
ENSG00000151838	CCDC175	0	0,166547826	0	0,567249945	0	0,412200191	0	0	0	1,145997961	0,127333107
ENSG00000135423	GLS2	0	0,777035499	0	0	0,08672242	0,280457386	0	0	0	1,144215306	0,127135034
ENSG00000102359	SRPX2	0,06029149	0,499266924	0	0,45114439	0	0,088262044	0	0,043946611	0	1,142911459	0,126990162
ENSG00000131386	GALNT15	0,023607338	0,293234495	0	0	0	0	0	0,825957363	0	1,142799196	0,126977688
ENSG00000164647	STEAP1	0,097869541	0	0	0,506998008	0	0,537275184	0	0	0	1,142142732	0,126904748
ENSG00000131914	LIN28A	0	0,202371321	0,139864201	0,459508131	0,090344036	0,04173851	0,031564863	0,083128193	0,093507684	1,142026939	0,126891882
ENSG00000177374	HIC1	0	0	0	0	0	0,368326391	0,680894747	0	0,091685507	1,140906645	0,126767405
ENSG00000196353	CPNE4	0	0,041721176	0,043251921	1,055595043	0	0	0	0	0	1,14056814	0,126729793
ENSG00000167281	RBFOX3	0,015305436	0,081477363	0,028155586	0,951449315	0	0	0,063542151	0	0	1,139929851	0,126658872
ENSG00000203907	OOEP	0,103006524	0	0	0,177869785	0	0	0,855285153	0	0	1,136161462	0,126240162
ENSG00000178750	STX19	0	0	0	1,13548727	0	0	0	0	0	1,13548727	0,126165252
ENSG00000140795	MYLK3	0,014196784	0,100767381	0,026116131	0,073544243	0,573562403	0,327332028	0	0	0,017460214	1,132979185	0,125886576
ENSG00000128536	CDHR3	0	0,082708822	0	0,992658242	0,036923457	0	0	0	0,019108217	1,131398738	0,125710971
ENSG00000203697	CAPN8	0,029104501	0,464807011	0,107080161	0,402056972	0	0,127820167	0	0	0	1,130868812	0,12565209
ENSG00000134504	KCTD1	0,051039373	0	0	0,23502362	0,24259235	0	0,01412636	0,520838093	0,062771849	1,126391647	0,125154627
ENSG00000180998	GPR137C	0,48782813	0	0	0,200564931	0,055206386	0,382576272	0	0	0	1,126175719	0,125130635
ENSG00000169031	COL4A3	0,016561878	0	0,030466911	0,142993739	0,03935966	0	0	0	0,896234873	1,125617061	0,125068562
ENSG00000188611	ASAH2	0,021633582	0	0	0	1,079666197	0,023752409	0	0	0	1,125052189	0,125005799
ENSG00000165966	PDZRN4	0,063989232	0	0	0,110495437	0	0	0	0,909517124	0,039349253	1,123351046	0,124816783
ENSG00000204310	AGPAT1	0,252341615	0	0,464202781	0	0	0,23088029	0,174603858	0	0	1,122028545	0,124669838
ENSG00000164199	ADGRV1	0,089356378	0,03171214	0,345194375	0,44746699	0,06370721	0,014716212	0,044516705	0,068388681	0,016484524	1,121543215	0,124615913
ENSG00000142798	HSPG2	0,016680712	0,044399271	0,015342759	0,47526516	0,05946311	0,173987248	0	0,018237916	0,317984446	1,121360622	0,124595625

ENSG00000180644	PRF1	0	0	0	0,207255738	0,142620152	0	0,69761106	0	0,073807197	1,121294147	0,124588239
ENSG00000162873	KLHDC8A	0	0	0	1,072041802	0	0,045442503	0	0	0	1,117484304	0,124164923
ENSG00000126861	OMG	0	0	0	0	0	0	0,196200676	0	0,920272068	1,116472743	0,124052527
ENSG00000185958	FAM186A	0,072859484	0	0	0,157265534	0,086576071	0	0,015124196	0	0,784068018	1,115893304	0,123988145
ENSG00000248485	PCP4L1	0	0	0	0,742347012	0,255417882	0,118001833	0	0	0	1,115766727	0,123974081
ENSG00000221947	XKR9	0	0	0	0	0,183927038	0	0,931789918	0	0	1,115716956	0,123968551
ENSG00000277535	AL772284.2	0	0	0,484708106	0	0	0,144647408	0	0	0,486085063	1,115440577	0,123937842
ENSG00000213088	ACKR1	0	0	0	1,115106729	0	0	0	0	0	1,115106729	0,123900748
ENSG00000102452	NALCN	0,049511983	0,117143731	0,091081291	0,199491571	0,509887293	0,036240842	0,013703619	0,036089402	0,060893356	1,114043087	0,123782565
ENSG00000253305	PCDHGB6	0	0	0,040987924	0	0	0	1,073028495	0	0	1,114016419	0,123779602
ENSG00000267673	FDX2	0,110219235	0	0	0,190324562	0	0	0	0	0,813332701	1,113876498	0,123764055
ENSG00000161082	CELF5	0,024911073	0	0,274955588	0,645239799	0,059201705	0,054701806	0	0,054473222	0	1,113483193	0,123720355
ENSG00000119888	EPCAM	0,059988317	0	0,110353354	0,725107115	0,142563534	0	0	0	0,073777897	1,111790217	0,123532246
ENSG00000008517	IL32	0	0	0	0,081313485	0	0,051701644	0,977487784	0	0	1,110502914	0,123389213
ENSG00000260300	AC009119.2	0	0	0	1,110361594	0	0	0	0	0	1,110361594	0,12337351
ENSG00000196482	ESRRG	0,055392438	0,122865653	0,025474716	0,119563313	0,065820664	0,547358941	0,091987047	0,03028176	0,051094169	1,1098387	0,123315411
ENSG00000164542	KIAA0895	0	0,039854637	0,165267597	0,232700631	0	0	0,671363251	0	0	1,109186117	0,123242902
ENSG00000230561	CCDC192	0,079490042	0,14105313	0	0,411785862	0,377819613	0	0	0	0,097762505	1,107911153	0,123101239
ENSG00000241489	AC244197.3	0	0,24874026	0	0,080684902	0	0,290711173	0,142256612	0	0,344798743	1,107191691	0,123021299
ENSG00000129159	KCNC1	0	0,07439606	0	0,941154101	0,024909311	0,011507982	0,008702943	0,045839574	0	1,106509972	0,122945552
ENSG00000188828	GLRA4	0,055514537	0	0,306370425	0,191722979	0,263863	0	0,046094905	0,242788073	0	1,106353919	0,122928213
ENSG00000214324	C3orf56	0	0	0	0,899775774	0,206389392	0	0	0	0	1,106165167	0,122907241
ENSG00000254126	CD8B2	0	0	0	0	0	1,032906314	0,073231711	0	0	1,106138025	0,122904225
ENSG00000276076	CRYAA2	0	0,161531325	0	0,943138462	0	0	0	0	0	1,104669788	0,122741088
ENSG00000213213	CCDC183	0,050981974	0,180932524	0	0	0	0,279876142	0,042331421	0,111482648	0,438908791	1,104513499	0,122723722
ENSG00000134363	FST	0	0,19487064	0,909091893	0	0	0	0	0	0	1,103962533	0,122662504
ENSG00000113805	CNTN3	0,059154657	0	0	0	0,070291161	0,064948357	0	0	0,909407527	1,103801702	0,122644634
ENSG00000204219	TCEA3	0,042990205	0,228855192	0,079083954	0	0	0	0	0,752055915	0	1,102985266	0,122553918
ENSG00000166317	SYNPO2L	0,029606303	0	0	0,051123624	0,07036002	0,87766175	0	0	0,072823872	1,101575569	0,122397285
ENSG00000158423	RIBC1	0	0,089589709	0,092876745	0,087181749	0	0,831493034	0	0	0	1,101141238	0,122349026
ENSG00000138696	BMPR1B	0,023940204	0,127443916	0,484439336	0,20669754	0,056894414	0,026284946	0,039756125	0,104700433	0,029443365	1,099600281	0,122177809
ENSG00000089558	KCNH4	0	0	0	0	0	0,875979814	0	0,174463869	0,049061912	1,099505594	0,122167288
ENSG00000196242	OR2C3	0	0	0,751385859	0	0,211022178	0,136487723	0	0	0	1,09889576	0,122099529
ENSG00000280969	RPS4Y2	0,190796175	0	0	0	0,906862481	0	0	0	0	1,097658656	0,121962073
ENSG00000169418	NPR1	0,030515059	0	0	0,316157077	0,725196976	0	0,025337305	0	0	1,097206416	0,121911824
ENSG00000176428	VPS37D	0	0,993118519	0	0	0	0,102413936	0	0	0	1,095532455	0,121725828
ENSG00000127529	OR7C2	0	0,279314583	0	0,815421796	0	0	0	0	0	1,094736379	0,121637375
ENSG00000133488	SEC14L4	0	0	0	1,093858506	0	0	0	0	0	1,093858506	0,121539834
ENSG00000105737	GRIK5	0,090413185	0,05347866	0	0,052041279	0,071622964	0	0,825791974	0	0	1,093348062	0,121483118
ENSG00000155886	SLC24A2	0,19681347	0,049891525	0,077583059	0,145651674	0,167046954	0,030869956	0,035018234	0,338150548	0,051868971	1,092894391	0,12143271
ENSG00000164049	FBXW12	0,043322985	0	0	0,224428017	0,823664088	0	0	0	0	1,09141509	0,121268343
ENSG00000011347	SYT7	0,050767872	0	0,0466958	0	0,965207572	0,027870078	0	0	0	1,090541323	0,121171258
ENSG00000055955	ITIH4	0	0	0	0,111107079	0	0,247258684	0,534257322	0	0,197835344	1,090458428	0,121162048
ENSG00000173947	PIFO	0,267858697	0	0	0,066076215	0	0,756239651	0	0	0	1,090174563	0,121130507
ENSG00000164362	TERT	0,032835848	0	0	1,020606159	0	0,036051842	0	0	0	1,089493848	0,121054872
ENSG00000115041	KCNIP3	0	0,034197424	0	0,96507005	0,045799967	0	0	0,042141891	0	1,087209332	0,120801037
ENSG00000102970	CCL17	0,243726727	0	0	0,841725725	0	0	0	0	0	1,085452451	0,120605828
ENSG00000184937	WT1	0,017868106	0	0,032869824	0,956483885	0	0	0	0,07814463	0	1,085366444	0,120596272
ENSG00000164627	KIF6	0	0,09035195	0,046833476	0,879235025	0,030251667	0	0,010569483	0,027835445	0	1,085077047	0,120564116
ENSG00000129946	SHC2	0	0	0	0,063165087	0	0	0,941559015	0,079989002	0	1,084713104	0,120523678
ENSG00000168925	CTRB1	0	0	0	0,119475721	0	0	0,057449786	0,907787273	0	1,08471278	0,120523642
ENSG00000198626	RYR2	0,016980624	0,0602634	0,046855843	0,718384861	0,04035482	0,037287465	0,042298123	0,111394955	0,010441989	1,08426208	0,120473564
ENSG00000165300	SLITRK5	0,042963722	0,012706345	0,158070473	0,383309682	0,255260538	0,039309713	0,029728079	0,109607258	0,05283984	1,08379565	0,120421739
ENSG00000167139	TBC1D21	0	0	0	0,20546061	0	0	0	0	0,878015066	1,083475676	0,120386186
ENSG00000181031	RPH3AL	0	0	0	0,301111906	0	0,218807223	0,082736783	0,326839335	0,153187044	1,082682292	0,120298032
ENSG00000188833	ENTPD8	0	0	1,078202076	0	0	0	0	0	0	1,078202076	0,119800231
ENSG00000178826	TMEM139	0	0	0	0	0	0	1,076578512	0	0	1,076578512	0,119619835
ENSG00000198785	GRIN3A	0	0	0	0,096380808	0,044215408	0	0	0,935729507	0	1,076325723	0,119591747
ENSG00000162409	PRKAA2	0	0,544189062	0,029692384	0,501690829	0	0	0	0	0	1,075572276	0,119508031
ENSG00000176231	OR10H4	0	0	0	0	0	0	0	1,074933531	0	1,074933531	0,119437059
ENSG00000270299	AL121758.1	0	0	0	0	0	0,611313842	0,462307782	0	0	1,073621624	0,119291292
ENSG00000117791	MARC2	0,058186589	0	0,107038929	0,703328773	0,138281687	0,063885474	0	0	0	1,070721452	0,11896905
ENSG00000184347	SLIT3	0,031790443	0	0,038987391	0,237879009	0,075550675	0,663176919	0	0,02317213	0	1,070556567	0,11895073
ENSG00000118898	PPL	0	0	0,072212001	0,067784121	0,093289399	0,021549627	0,81484824	0	0	1,069683389	0,11885371
ENSG00000052850	ALX4	0,02626183	0	0	1,043014323	0	0	0	0	0	1,069276153	0,118808461
ENSG00000033122	LRRC7	0,030653304	0,743378322	0	0	0,07284824	0	0,025452093	0,134059599	0,062832687	1,069224245	0,118802694
ENSG00000187595	ZNF385C	0	0	0	0	0,207262914	0,861790221	0	0	0	1,069053135	0,118783682
ENSG00000164742	ADCY1	0,021639778	0,019199628	0	1,027597279	0	0	0	0	0	1,068436685	0,118715187
ENSG00000154380	ENAH	0,271203497	0	0,03695561	0,034689574	0,119355737	0	0,025020672	0	0,580615963	1,067841054	0,118649006
ENSG00000178882	RFLNA	0	0	0,438039866	0	0,113179181	0,261441186	0,079086248	0	0,175713699	1,06746018	0,118606687
ENSG00000196900	TEX43	0,30838892	0	0	0	0	0	0	0	0,758557234	1,066946153	0,118549573
ENSG00000175084	DES	0	0	0,225633197	0	0,437237267	0,134667676	0	0,268209876	0	1,065748017	0,118416446
ENSG00000095627	TDRD1	0	0	0,105776293	0	0	0	0,644539377	0,314340342	0	1,064656012	0,118295112
ENSG00000162032	SPSB3	0,09752215	0	0	0,252599201	0	0,321220865	0	0,213252383	0,179909508	1,064504107	0,118278234
ENSG00000156427	FGF18	0	0	0	0	0,898692548	0	0	0,165382666	0	1,064075215	0,118230579
ENSG00000102466	FGF14	0	0	0,02020351	1,04305717	0	0	0	0	0	1,06326068	0,118140076
ENSG00000163071	SPATA18	0	0	0	0,209796965	0,072184431	0	0,580063846	0,199257026	0	1,061302267	0,117922474
ENSG00000188766	SPRED3	0	0,045726808	0,094809038	0,222488894	0	0	0	0	0,697241386	1,060266126	0,117807347
ENSG00000231068	KRTAP21-3	0	1,059849803	0	0	0	0	0	0	0	1,059849803	0,117761089
ENSG00000284638	AC005551.1	0	0	0	1,058559735	0	0	0	0	0	1,058559735	0,117617748
ENSG00000166828	SCNN1G	0	0,771187806	0,07994826	0	0,206567468	0	0	0	0	1,057703533	0,117522615
ENSG00000160951	PTGER1	0	0	0	0	0	1,056755266	0	0	0	1,056755266	0,117417252
ENSG00000186930	KRTAP6-2	0	0	0	0	0	0	0	1,055701494	0	1,055701494	0,117300166
ENSG00000248643	RBM14-RBM4	0,058638173	0	0	0	0	0,708194157	0	0	0,288469573	1,055301903	0,117255767
ENSG00000157554	ERG	0,019930173	0	0	0,894791525	0,094728975	0	0	0,043581452	0	1,053032124	0,117003569
ENSG00000164440	TXLNB	0	0	0,057960821	0,761695088	0	0	0	0	0,232501904	1,052157814	0,116906424
ENSG00000203546	AL139353.1	0	0	0	0	0	0,375193525	0,340489368	0	0,336221659	1,051904552	0,116878284
ENSG00000144935	TRPC1	0	0,223079867	0,173448481	0,054271001	0	0	0,600211655	0	0	1,051011004	0,116779
ENSG00000169884	WNT10B	0	0,412367551	0	0	0	0,637872267	0	0	0	1,050239818	0,116693313
ENSG00000185864	NPIPB4	0	0	0,607958534	0,134277615	0	0,042688943	0,14527634	0	0,1195462	1,049747631	0,116638626
ENSG00000125337	KIF25	0	0,050035828	0	0,535600806	0,402072262	0,061918484	0	0	0	1,04962738	0,116625264
ENSG00000186393	KRT26	0	0	0,160403981	0,602273456	0	0,095736051	0	0,190671995	0	1,049085483	0,116565054
ENSG00000214642	DEFB113	0	0	0	1,047931625	0	0	0	0	0	1,047931625	0,116436847
ENSG00000101489	CELF4	0,0238759	0,084734397	0	0,453513149	0,113483186	0	0,019824669	0	0,352371349	1,047802651	0,116422517
ENSG00000102098	SCML2	0,072562099	0,064379832	0,533935364	0,375896722	0	0	0	0	0	1,046774017	0,116308224
ENSG00000221836	OR2A5	0,017407046	0	0	0,931803273	0	0	0	0,076128227	0,021408423	1,046746969	0,116305219
ENSG00000132958	TPTE2	0,132147416	0	0	0,912758984	0	0	0	0	0	1,044906401	0,116100711
ENSG00000215547	DEFB115	0	0	1,041123966	0	0	0	0	0	0	1,041123966	0,115680441
ENSG00000034053	APBA2	0	0	0	0	0	0,096614108	0,511452802	0	0,432893341	1,04096025	0,11566225
ENSG00000198597	ZNF536	0	0,216767987	0	0,030134539	0,622099912	0	0,014490164	0,114482469	0,042925632	1,040900703	0,115655634
ENSG00000184731	FAM110C	0	0	0	0	0	0,040584779	0	0	1,000152517	1,040737296	0,115637477
ENSG00000111783	RFX4	0,022277837	0,118594427	0,040981881	0,076937926	0,58238139	0	0,073991055	0,097430213	0,027398868	1,039993597	0,115554844
ENSG00000143452	HORMAD1	0,06264949	0	0	0	0	0	0	0,821976536	0,154101594	1,03872762	0,11541418
ENSG00000152292	SH2D6	0	0	0	0	0	0,624751524	0,413411308	0	0	1,038162832	0,115351426
ENSG00000258315	C17orf49	0	0,204065449	0	0,099290325	0	0,378791271	0	0	0,353589274	1,035736319	0,115081813
ENSG00000152763	WDR78	0	0,321465008	0	0,039103098	0,053816506	0,1491778	0,094013436	0,099036286	0,278505203	1,035117338	0,115013038
ENSG00000187689	AMTN	0	0	0	1,034430028	0	0	0	0	0	1,034430028	0,11493667
ENSG00000150054	MPP7	0,171521647	0	0	0,16924597	0	0	0	0	0,693119833	1,03388745	0,114876383
ENSG00000115339	GALNT3	0,515955854	0,057221938	0,177964212	0	0	0	0	0,28206109	0	1,033203094	0,114800344
ENSG00000187678	SPRY4	0	0	0	0,556449298	0,054701834	0	0,019112008	0,402662078	0	1,032925218	0,114769469
ENSG00000175202	HIGD2B	0	0,240055506	0	0	0,643003657	0,148532298	0	0	0	1,031591461	0,114621273
ENSG00000140009	ESR2	0	0	0	0,894282873	0,115385309	0,017769152	0	0	0	1,027437334	0,114159704
ENSG00000172014	ANKRD20A4	0,100517009	0	0,924545784	0	0	0	0	0	0	1,025062793	0,113895866
ENSG00000205038	PKHD1L1	0,036639972	0,078020077	0,363971809	0,506153871	0	0,00804571	0	0,032048355	0	1,024879794	0,113875533
ENSG00000158445	KCNB1	0,034101116	0	0	0,618294439	0	0,36505007	0,007078721	0	0	1,024524346	0,113836038
ENSG00000099814	CEP170B	0,062866691	0,074370268	0	0,470413905	0,049801351	0	0	0,366589452	0	1,024041668	0,113782408
ENSG00000204172	AGAP9	0	0	0	0	0	0,417035383	0,052564027	0,553723615	0	1,023323026	0,113702558

ENSG00000172458	IL17D	0	0	0	0	0	0,947490519	0,075425508	0	0	1,022916027	0,113657336
ENSG00000169894	MUC3A	0,092819761	0	0,048785556	0,29766187	0,441176342	0,101910674	0,011010033	0,028995662	0	1,022359897	0,113595544
ENSG00000172379	ARNT2	0,058592699	0	0,754502014	0,033725601	0,046415606	0,021443787	0,016216923	0,042708358	0,048040978	1,021645965	0,113516218
ENSG00000142494	SLC47A1	0	0	0	0,045625979	0,188381295	0	0,548480206	0,173335146	0,064992664	1,020815291	0,113423921
ENSG00000004846	ABCB5	0	0	0,420408489	0,169127131	0,155176641	0,035845431	0	0,178478215	0,060228969	1,019264876	0,113251653
ENSG00000042781	USH2A	0,022048135	0,013041292	0	0,964498715	0	0	0,018307037	0	0	1,017895179	0,113099464
ENSG00000156222	SLC28A1	0	0	0,161288134	0,227097454	0	0	0	0,57516896	0,05391544	1,017469988	0,113052221
ENSG00000186910	SERPINA11	1,016894823	0	0	0	0	0	0	0	0	1,016894823	0,112988314
ENSG00000197077	KIAA1671	0,036768799	0	0,016909794	0	0,218454615	0,010092498	0,259504306	0	0,474819267	1,016549279	0,11294992
ENSG00000107611	CUBN	0,034162149	0,080826526	0	0,196635248	0,081187086	0	0	0,622521793	0	1,015332801	0,112814756
ENSG00000205143	ARID3C	0	0	0	0,924645551	0	0	0,088922985	0	0	1,013568537	0,112618726
ENSG00000100678	SLC8A3	0,071582459	0,095265988	0	0,741643699	0,085058632	0,019648339	0	0	0	1,013199116	0,11257768
ENSG00000156049	GNA14	0	0	0,101600913	0	0	0,90959746	0	0	0	1,011198373	0,112355375
ENSG00000213658	LAT	0,068221477	0	0	0,942428802	0	0	0	0	0	1,010650279	0,112294475
ENSG00000101349	PAK5	0,0613149	0,054400893	0	0,211754899	0	0	0	0,268155462	0,414751825	1,010377978	0,11226422
ENSG00000154553	PDLIM3	0,082872968	0,205878469	0,121961215	0,372069175	0,078779761	0,054593806	0	0,072487566	0,020384614	1,009027574	0,112114175
ENSG00000107593	PKD2L1	0	0	0	0,091620426	0	0,058255118	0,793000694	0	0,065255099	1,008131338	0,112014593
ENSG00000275793	RIMBP3	0	0,219608518	0	0	0	0,461995054	0	0,324751418	0	1,006354989	0,111817221
ENSG00000181867	FTMT	0	0,281661765	0	0	0,377224309	0	0	0,347095134	0	1,005981208	0,11177569
ENSG00000272305	AC096887.1	0	0	0,157943238	0	0	0,659871612	0	0,187746913	0	1,005561763	0,111729085
ENSG00000203896	LIME1	0	0	0	0,511637205	0	0	0,492040518	0	0	1,003677723	0,111519747
ENSG00000143921	ABCG8	0	0,026324563	0,027290408	0,102468083	0	0,781828753	0	0,064880143	0	1,002791951	0,111421328
ENSG00000188340	OR6N2	0	0	0,160914674	0,528667098	0,311824205	0	0	0	0	1,001405976	0,111267331
ENSG00000102387	TAF7L	0,222712705	0,098799558	0	0,096144058	0,132320391	0,244525537	0	0	0,205430938	0,999933188	0,111103688
ENSG00000240045	STRIT1	0	0	0	0,999750861	0	0	0	0	0	0,999750861	0,111083429
ENSG00000173114	LRRN3	0,350966487	0	0	0	0,092675495	0,214078163	0	0,341093747	0	0,998813892	0,110979321
ENSG00000233757	AC092835.1	0,065586185	0,931048611	0	0	0	0	0	0	0	0,996634797	0,1107372
ENSG00000184601	C14orf180	0,995458305	0	0	0	0	0	0	0	0	0,995458305	0,110606478
ENSG00000196553	CCDC196	0,053509409	0,189902266	0,295304638	0	0	0,058750204	0	0	0,39485805	0,992324567	0,110258285
ENSG00000135547	HEY2	0,056195824	0	0	0	0,934854145	0	0	0	0	0,991049969	0,110116663
ENSG00000154914	USP43	0,029722772	0	0	0,051324739	0	0	0	0,909929966	0	0,990977476	0,110108608
ENSG00000184908	CLCNKB	0,102831283	0	0	0,887835912	0	0	0	0	0	0,990667195	0,110074133
ENSG00000197721	CR1L	0,109183938	0,19374422	0	0	0	0	0,090657754	0,596883251	0	0,990469163	0,110052129
ENSG00000163632	C3orf49	0	0	0	0,990265558	0	0	0	0	0	0,990265558	0,110029506
ENSG00000169306	IL1RAPL1	0,082393986	0	0,151570392	0,426829293	0,097905546	0,090463782	0	0,09008576	0,050666991	0,98991575	0,109990639
ENSG00000185821	OR6C76	0	0,285561235	0	0	0	0	0	0,703800996	0	0,989362231	0,109929137
ENSG00000164270	HTR4	0	0,03806132	0	0,111114964	0,050974811	0,023550117	0	0	0,765017622	0,988718834	0,109857648
ENSG00000150551	LYPD1	0	0	0	0	0	0	0,98836497	0	0	0,98836497	0,10981833
ENSG00000184345	IQCF2	0	0	0	0,415501552	0,57184322	0	0	0	0	0,987344772	0,109704975
ENSG00000131482	G6PC	0	0	0	0,237501494	0,43582226	0	0	0,200506412	0,112770948	0,986601114	0,109622346
ENSG00000176547	OR4C3	0	0	0	0,986521643	0	0	0	0	0	0,986521643	0,109613516
ENSG00000148204	CRB2	0	0	0	0,986366261	0	0	0	0	0	0,986366261	0,109596251
ENSG00000177398	UMODL1	0,041525301	0	0,038194573	0,250967961	0	0	0,017239672	0	0,638384591	0,986312098	0,109590233
ENSG00000090975	PITPNM2	0	0,029269949	0	0,39876538	0	0,43465275	0,123265254	0	0	0,985953332	0,10955037
ENSG00000163331	DAPL1	0	0	0	0,984660282	0	0	0	0	0	0,984660282	0,109406698
ENSG00000205323	SARNP	0	0	0	0,077543826	0	0,049304778	0,857598109	0	0	0,984446712	0,109382968
ENSG00000139648	KRT71	0	0	0	0,691218476	0	0,292998811	0	0	0	0,984217287	0,109357476
ENSG00000180914	OXTR	0	0	0	0,982697069	0	0	0	0	0	0,982697069	0,109188563
ENSG00000166664	CHRFAM7A	0	0	0,121974594	0,572476908	0	0,024266576	0,018351665	0	0,244642197	0,981711939	0,109079104
ENSG00000111536	IL26	0	0	0	0,979118104	0	0	0	0	0	0,979118104	0,1087909
ENSG00000171711	DEFB4A	0	0	0	0	0	0	0	0	0,978139591	0,978139591	0,108682177
ENSG00000197702	PARVA	0	0	0,035116233	0,461481764	0,045366036	0	0,015850219	0,417426184	0	0,975240436	0,108360048
ENSG00000168952	STXBP6	0	0	0	0,129383897	0,845820417	0	0	0	0	0,975204314	0,108356035
ENSG00000172289	OR10V1	0,974906908	0	0	0	0	0	0	0	0	0,974906908	0,10832299
ENSG00000188112	C6orf132	0,066160495	0	0	0,076163157	0,104821232	0,726403576	0	0	0	0,97354846	0,108172051
ENSG00000174599	TRAM1L1	0,149392556	0	0,137409836	0	0,532551966	0	0,062021914	0	0,091866793	0,973243066	0,108138118
ENSG00000158578	ALAS2	0	0,125652296	0	0	0,673135037	0	0	0	0,174176684	0,972964018	0,108107113
ENSG00000206172	HBA1	0,180970743	0	0	0	0	0	0	0,791460042	0	0,972430785	0,108047865
ENSG00000164776	PHKG1	0,601816176	0	0,100644496	0,094473199	0,130020834	0	0,045427347	0	0	0,972382051	0,10804245
ENSG00000196092	PAX5	0,063160113	0,084057053	0,406658452	0,10906373	0,112576032	0	0,039332392	0,138112672	0,019419699	0,972380144	0,108042238
ENSG00000189013	KIR2DL4	0	0	0	0	0	0	0	0	0,970196462	0,970196462	0,107799607
ENSG00000122224	LY9	0,016572776	0	0	0	0	0,036391879	0,880686691	0,036239808	0	0,969891154	0,107765684
ENSG00000120729	MYOT	0,052233173	0	0	0	0	0,917583556	0	0	0	0,969816729	0,107757414
ENSG00000167617	CDC42EP5	0,969693502	0	0	0	0	0	0	0	0	0,969693502	0,107743722
ENSG00000248592	STIMATE-MUSTN1	0,024096726	0	0	0,124829361	0	0	0,820329072	0	0	0,96925516	0,107695018
ENSG00000283361	CFAP97D2	0	0,113957501	0	0,776262143	0	0	0	0	0,078982797	0,969202441	0,10768916
ENSG00000145020	AMT	0	0	0	0	0,124997404	0,317615096	0,524066824	0	0	0,966679324	0,107408814
ENSG00000104901	DKKL1	0	0	0	0,357200513	0,491605123	0,113559601	0	0	0	0,962365237	0,106929471
ENSG00000256029	AL590560.1	0	0	0	0	0	0,880759852	0	0,081588782	0	0,962348634	0,106927626
ENSG00000173698	ADGRG2	0	0	0	0,025690162	0,494993167	0	0	0,422925015	0,018297383	0,961905727	0,106878414
ENSG00000196131	VN1R2	0	0	0	0	0	0	0	0,452650092	0,509168554	0,961818647	0,106868739
ENSG00000196747	HIST1H2AI	0,30041863	0	0	0	0	0,659684202	0	0	0	0,960102831	0,106678092
ENSG00000105538	RASIP1	0,074807213	0,530974258	0	0,322939325	0	0	0,031057013	0	0	0,959777809	0,106641979
ENSG00000175505	CLCF1	0	0	0	0,568485783	0,39119558	0	0	0	0	0,959681363	0,106631263
ENSG00000125815	CST8	0	0	0	0	0	0	0,956745451	0	0	0,956745451	0,10630505
ENSG00000286219	AC242843.1	0	0	0,780404545	0	0	0	0	0	0,173915892	0,954320437	0,106035604
ENSG00000128422	KRT17	0	0	0	0,083365807	0,229468078	0	0,641381889	0	0	0,954215774	0,106023975
ENSG00000092529	CAPN3	0	0	0,055651671	0	0	0,199291984	0	0,066153067	0,632510686	0,953607408	0,105956379
ENSG00000120952	PRAMEF2	0	0	0	0,953477374	0	0	0	0	0	0,953477374	0,10594193
ENSG00000178809	TRIM73	0	0	0,952673499	0	0	0	0	0	0	0,952673499	0,105852611
ENSG00000147614	ATP6V0D2	0,051398153	0	0	0	0	0	0	0,89914168	0	0,950539834	0,105615537
ENSG00000179397	CATSPERE	0	0	0	0,765550591	0,081046613	0	0,028316482	0,074573362	0	0,949487048	0,105498561
ENSG00000270188	MTRNR2L11	0	0,172771907	0	0	0	0,320703434	0,242532858	0,212908871	0	0,948917071	0,10543523
ENSG00000150750	C11orf53	0,119549502	0,212137658	0	0,206435898	0	0	0	0,261419753	0,147030476	0,946573287	0,10517481
ENSG00000156466	GDF6	0	0,433419181	0	0,28117993	0,096745028	0,044695737	0	0,089017933	0	0,945057809	0,105006423
ENSG00000149926	FAM57B	0,030713531	0	0	0	0,875896445	0	0	0	0,037773683	0,944383659	0,104931518
ENSG00000153093	ACOXL	0,052587636	0	0	0,862670005	0	0,028869075	0	0	0	0,944126716	0,104902968
ENSG00000008300	CELSR3	0,010910511	0	0,020070765	0,094200352	0	0	0	0	0,818529809	0,943711437	0,104856826
ENSG00000197769	MAP1LC3C	0	0	0	0	0	0	0	0	0,943383362	0,943383362	0,104820374
ENSG00000125533	BHLHE23	0	0	0	0,941154101	0	0	0	0	0	0,941154101	0,104572678
ENSG00000163116	STPG2	0,024870074	0,088262673	0	0	0,05910427	0,737258981	0	0	0,030586985	0,940082983	0,104453665
ENSG00000187537	POTEG	0,06022741	0,106872061	0	0,103999591	0	0,264504706	0,050008104	0,131699708	0,22221585	0,939527429	0,104391937
ENSG00000270339	PPIAL4H	0	0	0	0,395356022	0,544117488	0	0	0	0	0,939473511	0,104385946
ENSG00000186766	FOXI2	0,046972512	0	0	0,40555638	0,446524765	0	0,039002279	0	0	0,938055936	0,104228437
ENSG00000112486	CCR6	0,104310104	0,123397147	0	0	0	0,305403731	0,404184226	0	0	0,937295208	0,104143912
ENSG00000052344	PRSS8	0	0	0	0	0	0	0	0	0,937041289	0,937041289	0,104115699
ENSG00000136859	ANGPTL2	0	0,221910621	0	0	0,099066908	0	0	0	0,615216074	0,936193603	0,104021511
ENSG00000096395	MLN	0	0	0	0,935250805	0	0	0	0	0	0,935250805	0,103916756
ENSG00000110887	DAO	0	0,207166109	0,501123022	0,134398648	0,092484559	0	0	0	0	0,935172337	0,103908037
ENSG00000114529	C3orf52	0,025097255	0,044534463	0,323178989	0,520049775	0	0	0,020838786	0	0	0,933699268	0,103744363
ENSG00000164756	SLC30A8	0,120330125	0,085409142	0	0,581794815	0,114386859	0,026423089	0	0	0	0,928344029	0,103149337
ENSG00000198573	SPANXC	0	0	0	0	0,927952306	0	0	0	0	0,927952306	0,103105812
ENSG00000181408	UTS2R	0	0	0	0	0	0	0,924615564	0	0	0,924615564	0,102735063
ENSG00000179002	TAS1R2	0	0	0	0,923845307	0	0	0	0	0	0,923845307	0,102649479
ENSG00000124196	GTSF1L	0,180970743	0	0	0,312496976	0,430080889	0	0	0	0	0,923548608	0,102616512
ENSG00000198915	RASGEF1A	0,02488236	0,088306274	0,04577311	0	0	0	0,764433111	0	0	0,923394855	0,102599428
ENSG00000164123	C4orf45	0	0	0	0	0	0,923007381	0	0	0	0,923007381	0,102556376
ENSG00000215187	FAM166B	0,1101389	0	0	0	0	0	0	0	0,812739893	0,922878793	0,102542088
ENSG00000183389	OR56A4	0	0	0,565287441	0	0,182571196	0	0,127575325	0	0,047241109	0,92267507	0,102519452
ENSG00000174326	SLC16A11	0	0	0	0	0	0	0,922123949	0	0	0,922123949	0,102458217
ENSG00000137962	ARHGAP29	0,090485372	0,029193468	0	0,028408816	0,762416121	0,009031605	0	0	0	0,919535381	0,102170598
ENSG00000175535	PNLIP	0,098187505	0,174231319	0	0	0	0,64682486	0	0	0	0,919243685	0,102138187
ENSG00000101850	GPR143	0	0	0	0	0	0,840408156	0	0	0,078449355	0,918857511	0,102095279
ENSG00000144852	NR1I2	0,056164494	0	0	0,484919113	0,200214203	0	0,139903734	0	0,034537544	0,915739087	0,101748787
ENSG00000127377	CRYGN	0	0	0	0	0	0,915442974	0	0	0	0,915442974	0,101715886
ENSG00000188784	PLA2G2E	0	0	0	0	0,914949152	0	0	0	0	0,914949152	0,101661017
ENSG00000166813	KIF7	0,030119707	0	0	0,884172726	0	0	0	0	0	0,914292434	0,101588048

ENSG00000186526	CYP4F8	0	0,038045119	0,709937824	0,148090224	0	0	0,01780226	0	0	0,913875427	0,101541714
ENSG00000206531	CD200R1L	0	0	0	0	0,840041035	0	0,073374463	0	0	0,913415498	0,101490611
ENSG00000135413	LACRT	0	0,912047619	0	0	0	0	0	0	0	0,912047619	0,101338624
ENSG00000206536	OR5K3	0	0	0	0,540238043	0,37175729	0	0	0	0	0,911995333	0,101332815
ENSG00000143595	AQP10	0	0	0	0,9107678	0	0	0	0	0	0,9107678	0,101196422
ENSG00000267179	AC008770.2	0,612886497	0	0	0	0	0	0	0,297822954	0	0,910709451	0,101189939
ENSG00000180116	C12orf40	0,033926935	0,060202515	0	0	0	0	0	0,816071002	0	0,910200452	0,101133384
ENSG00000285258	ATXN7	0,121244106	0,107572346	0	0,314043163	0	0	0,167785948	0	0,198819494	0,909465057	0,101051673
ENSG00000091128	LAMB4	0,021522656	0	0,039592665	0,743298603	0,102298118	0	0	0	0	0,906712042	0,100745782
ENSG00000074047	GLI2	0,102922334	0	0,643735415	0,142179526	0	0	0,017091722	0	0	0,905928997	0,100658777
ENSG00000100156	SLC16A8	0	0	0	0	0	0	0,398680129	0	0,506163928	0,904844057	0,100538229
ENSG00000138400	MDH1B	0	0	0	0,902576875	0	0	0	0	0	0,902576875	0,100286319
ENSG00000183091	NEB	0,079097364	0,035089083	0,100035368	0,076828435	0,246719285	0,037994374	0,09440958	0,19458312	0,036479836	0,901236445	0,100137383
ENSG00000179165	PXT1	0,291578525	0,129349735	0	0	0	0,480204274	0	0	0	0,901132534	0,100125837
ENSG00000129682	FGF13	0,02914801	0,012930607	0	0,025166126	0	0,744065373	0	0,079672703	0,008962074	0,899944892	0,099993877
ENSG00000165807	PPP1R36	0,098251346	0	0	0,508975893	0,116748226	0,053937119	0	0	0,12083649	0,898749074	0,099861008
ENSG00000162526	TSSK3	0	0	0	0	0	0,897298955	0	0	0	0,897298955	0,099699884
ENSG00000205329	OR6C3	0,323115974	0,573361369	0	0	0	0	0	0	0	0,896477343	0,099608594
ENSG00000172817	CYP7B1	0,04007175	0	0	0,726549253	0	0	0,016636215	0,087625184	0,024641544	0,895523946	0,099502661
ENSG00000183640	KRTAP8-1	0	0	0	0	0	0,895200953	0	0	0	0,895200953	0,099466773
ENSG00000204361	NXPE2	0	0	0	0	0,893771882	0	0	0	0	0,893771882	0,099307987
ENSG00000113763	UNC5A	0	0,496904332	0	0	0	0,07686383	0,31970666	0	0	0,893474822	0,09927498
ENSG00000065618	COL17A1	0,021385589	0,037948203	0,039340518	0,516995421	0,254116574	0,023480127	0	0	0	0,893266431	0,099251826
ENSG00000149435	GGTLC1	0	0	0	0,612044506	0,280779939	0	0	0	0	0,892824446	0,099202716
ENSG00000251503	CENPS-CORT	0	0,836450089	0	0	0	0	0,055913695	0	0	0,892363785	0,099151532
ENSG00000125831	CST11	0	0	0	0,892085383	0	0	0	0	0	0,892085383	0,099120598
ENSG00000150510	FAM124A	0	0	0	0,038933897	0,803754571	0	0	0,049303875	0	0,891992342	0,09911026
ENSG00000155875	SAXO1	0	0	0	0	0	0,018270078	0,8704582	0	0	0,888728278	0,098747586
ENSG00000112214	FHL5	0	0	0	0,73330672	0	0	0	0,154770289	0	0,888077009	0,098675223
ENSG00000185046	ANKS1B	0,054685812	0,048519316	0,12574871	0,21246854	0,259924033	0,060041826	0,079461895	0,029895464	0,016814125	0,887559722	0,098617747
ENSG00000119699	TGFB3	0,030751032	0	0	0,371702243	0	0	0,40853181	0	0,07563961	0,886624695	0,098513855
ENSG00000156968	MPV17L	0	0,044601131	0	0,130207073	0	0,165579418	0,146089874	0,274812515	0,123650381	0,884940392	0,09832671
ENSG00000176387	HSD11B2	0	0	0	0	0	0,372832757	0,093985267	0	0,417632634	0,884450658	0,098272295
ENSG00000187479	C11orf96	0,393927452	0	0	0	0,187235424	0	0,130834548	0,172280796	0	0,884278221	0,098253136
ENSG00000168143	FAM83B	0,798630498	0	0	0,083579428	0	0	0	0	0	0,882209927	0,098023325
ENSG00000167306	MYO5B	0,028603174	0,025377816	0,605105269	0,123478599	0,067976063	0,031404614	0	0	0	0,881945536	0,097993948
ENSG00000154864	PIEZO2	0,020425868	0,018122601	0,05636255	0,33507465	0,024271259	0,426101779	0	0	0	0,880358706	0,097817634
ENSG00000151577	DRD3	0	0	0,132308472	0,496782436	0,17092696	0,078967433	0	0	0	0,878985301	0,097665033
ENSG00000261857	MIA	0,130267733	0,462313793	0	0	0	0,286052718	0	0	0	0,878634245	0,097626027
ENSG00000174871	CNIH2	0,072719235	0	0	0	0	0	0	0	0,804917565	0,8776368	0,0975152
ENSG00000086570	FAT2	0,020789788	0,018445484	0	0,592340522	0,19762952	0	0	0,022730589	0,025568759	0,877504661	0,097500518
ENSG00000182793	GSTA5	0	0	0,596523818	0,279973149	0	0	0	0	0	0,876496966	0,097388552
ENSG00000138435	CHRNA1	0,031124731	0	0,057256457	0	0	0	0	0,748667403	0,038279407	0,875327999	0,097258667
ENSG00000166707	ZCCHC18	0	0	0	0,384973406	0	0	0	0,487510427	0	0,872483833	0,096942648
ENSG00000185247	MAGEA11	0,043585397	0	0	0,827887142	0	0	0	0	0	0,871472539	0,096830282
ENSG00000179363	TMEM31	0	0	0,379754234	0	0,490597735	0	0	0	0	0,870351969	0,096705774
ENSG00000130787	HIP1R	0,021367445	0	0	0,848629018	0	0	0	0	0	0,869996463	0,096666274
ENSG00000053438	NNAT	0,113702461	0	0	0,196339334	0	0	0	0	0,559357479	0,869399273	0,096599919
ENSG00000257065	AL049844.1	0	0	0	0	0	0,435460831	0	0,433641165	0	0,869101996	0,096566888
ENSG00000137948	BRDT	0,034866306	0,185608214	0,128278772	0,060206501	0,082860531	0,076562334	0,028950238	0,228727204	0,042881062	0,868941162	0,096549018
ENSG00000136274	NACAD	0	0,107839131	0	0,734584686	0	0	0,02523031	0	0	0,867654128	0,096406014
ENSG00000183559	C10orf120	0	0	0	0,245239638	0	0	0	0,621117608	0	0,866357246	0,096261916
ENSG00000213221	DNLZ	0	0,865253308	0	0	0	0	0	0	0	0,865253308	0,096139256
ENSG00000111404	RERGL	0	0	0,358915557	0,505361474	0	0	0	0	0	0,864277031	0,096030781
ENSG00000221946	FXYD7	0,505104972	0,14938273	0	0	0	0	0,209699719	0	0	0,864187421	0,096020825
ENSG00000169083	AR	0	0,020989589	0,065279084	0,674039465	0	0,051948517	0	0,051731439	0	0,863988094	0,095998677
ENSG00000102021	LUZP4	0	0	0	0	0	0	0	0,757009318	0,106441307	0,863450626	0,095938958
ENSG00000169126	ARMC4	0,031659453	0,056178923	0	0,601358626	0	0,104280689	0	0,069229953	0	0,862707645	0,095856405
ENSG00000221843	C2orf16	0,073703485	0,016348128	0,322010845	0,031817458	0	0,010115265	0,328936976	0	0,079315063	0,86224722	0,095805247
ENSG00000140937	CDH11	0,056915469	0	0,104700602	0,373466254	0,027052169	0,012497972	0,009451626	0,024891493	0,251995284	0,860970869	0,09566343
ENSG00000089356	FXYD3	0	0	0	0,860913234	0	0	0	0	0	0,860913234	0,095657026
ENSG00000170558	CDH2	0	0	0,239948294	0,619396789	0	0	0	0	0	0,859345083	0,095482787
ENSG00000187862	TTC24	0	0	0	0	0,253883027	0,117292737	0,487866261	0	0	0,859042025	0,095449114
ENSG00000235631	RNF148	0	0	0	0	0,858448308	0	0	0	0	0,858448308	0,095383145
ENSG00000068615	REEP1	0,025848541	0	0	0,089269577	0	0	0,686803049	0,05652319	0	0,858444357	0,095382706
ENSG00000108786	HSD17B1	0	0,047391658	0	0,691768225	0	0	0	0,116802604	0	0,855962487	0,095106943
ENSG00000112183	RBM24	0	0	0	0,228556182	0	0,048441044	0	0,578863476	0	0,855860702	0,095095634
ENSG00000138315	OIT3	0	0	0	0,551659566	0,303693482	0	0	0	0	0,855353048	0,095039228
ENSG00000232125	DYTN	0	0	0	0,438546176	0	0	0	0,416514161	0	0,855060337	0,095006704
ENSG00000171124	FUT3	0	0	0	0	0	0,853833782	0	0	0	0,853833782	0,09487042
ENSG00000168542	COL3A1	0	0,039368962	0,285693832	0,038310817	0	0,341028935	0,092108598	0	0,054572463	0,851083607	0,094564845
ENSG00000116983	HPCAL4	0,031759262	0	0,116847456	0,109682629	0,226429724	0	0,131851967	0	0,234358792	0,850929832	0,094547759
ENSG00000056291	NPFFR2	0,256228183	0	0	0,442450148	0,152232956	0	0	0	0	0,850911286	0,094545698
ENSG00000109819	PPARGC1A	0,013901616	0,024668077	0	0,09602023	0,033037492	0	0,011542809	0,668772814	0	0,847943039	0,094215893
ENSG00000166736	HTR3A	0,054513193	0	0	0,658926704	0	0	0	0	0,1340884	0,847528296	0,094169811
ENSG00000204624	DISP3	0,02469127	0	0,045421585	0,170545735	0	0	0,492040518	0,053992576	0,060734158	0,847425841	0,094158427
ENSG00000164821	DEFA4	0	0	0	0,845818394	0	0	0	0	0	0,845818394	0,093979822
ENSG00000132872	SYT4	0	0,062242804	0,064526485	0,605698641	0,083360618	0	0,029124961	0	0	0,84495351	0,093883723
ENSG00000198765	SYCP1	0	0	0,129957971	0,670940795	0	0	0	0	0,043442385	0,84434115	0,093815683
ENSG00000173338	KCNK7	0,193359655	0	0	0,166944961	0	0	0,481651946	0	0	0,841956563	0,093550729
ENSG00000165309	ARMC3	0	0,306491785	0,039717117	0,074563502	0,102619672	0	0,21512273	0,047211683	0,053106593	0,83883308	0,093203676
ENSG00000179314	WSCD1	0	0,112727859	0,03895461	0	0	0,581245014	0,105496355	0	0	0,838423838	0,093158204
ENSG00000156103	MMP16	0	0,066437562	0	0,280158133	0,474552418	0	0	0	0,015349069	0,836497181	0,092944131
ENSG00000167654	ATCAY	0	0	0	0,836330047	0	0	0	0	0	0,836330047	0,092925561
ENSG00000205835	GMNC	0,07944825	0	0	0,617354041	0,094405242	0,043614768	0	0	0	0,834822301	0,092758033
ENSG00000068976	PYGM	0	0	0	0,056018672	0,385484696	0,391802564	0	0	0	0,833305932	0,092589548
ENSG00000205549	C9orf92	0	0	0	0,832328468	0	0	0	0	0	0,832328468	0,092480941
ENSG00000146839	ZAN	0,017092022	0,030329375	0,094326467	0	0,690532544	0	0	0	0	0,832280408	0,092475601
ENSG00000159173	TNNI1	0,020083808	0	0	0,762967762	0,047729604	0	0	0	0	0,830781174	0,092309019
ENSG00000009694	TENM1	0,117176311	0,020792649	0,086222115	0,121402749	0,13923602	0,077191645	0,048646996	0,102492112	0,115289406	0,828450002	0,09205
ENSG00000101292	PROKR2	0,130831663	0,696472728	0	0	0	0	0	0	0	0,82730439	0,09192271
ENSG00000103534	TMC5	0,048698218	0	0	0	0	0	0	0	0,778602897	0,827301115	0,091922346
ENSG00000007402	CACNA2D2	0	0,169656438	0	0,577837653	0	0	0,01984662	0	0,058793585	0,826134296	0,0917927
ENSG00000153558	FBXL2	0,019716932	0,069974426	0,03627089	0,37451523	0,046857717	0	0,278313628	0	0	0,825648823	0,091738758
ENSG00000149968	MMP3	0,081505162	0	0	0	0,387397565	0	0	0,356455844	0	0,825358571	0,091706508
ENSG00000185055	EFCAB10	0	0,104011637	0	0	0	0	0	0	0,72089419	0,824905827	0,091656203
ENSG00000008196	TFAP2B	0,024550864	0,043564907	0	0	0,058345661	0	0	0,697912165	0	0,824373597	0,091597066
ENSG00000088726	TMEM40	0	0	0	0,079143153	0,217845036	0,050321679	0,076111818	0,400891195	0	0,824312881	0,09159032
ENSG00000162981	LRATD1	0,469680196	0,03205523	0	0,249549288	0,042930967	0	0,029998884	0	0	0,824214566	0,091579396
ENSG00000150628	SPATA4	0	0,108823864	0	0,423595738	0,291491512	0	0	0	0	0,823911113	0,091545679
ENSG00000112319	EYA4	0,033669913	0,029873217	0,092907787	0,145351479	0,120025917	0,092418993	0,013978424	0,294504962	0	0,822730692	0,091414521
ENSG00000239920	AC104389.4	0,028700963	0	0	0	0,06820846	0,630239608	0,095324089	0	0	0,82247312	0,091385902
ENSG00000204176	SYT15	0	0	0,077669768	0,07290723	0,100340191	0,023178343	0,017528686	0,323140818	0,207707764	0,822472798	0,091385866
ENSG00000165125	TRPV6	0	0	0,163476493	0	0,030170339	0	0	0,222084898	0,405948885	0,821680615	0,091297846
ENSG00000063660	GPC1	0	0	0,085243821	0,040008429	0	0,330701855	0,36552228	0	0	0,821476384	0,091275154
ENSG00000165269	AQP7	0	0	0	0	0,820371313	0	0	0	0	0,820371313	0,091152368
ENSG00000073150	PANX2	0	0	0	0	0	0,160455103	0	0	0,659030221	0,819485324	0,091053925
ENSG00000187726	DNAJB13	0	0,077163165	0	0	0	0,525184559	0,216639422	0	0	0,818987147	0,090998572
ENSG00000179954	SSC5D	0	0,476533971	0	0,03567122	0	0,13608523	0,068609888	0	0,101624893	0,818525202	0,090947245
ENSG00000053747	LAMA3	0,020590076	0,018268293	0,018938554	0,142218272	0,587193147	0	0,00854819	0,022512234	0	0,818268766	0,090918752
ENSG00000120093	HOXB3	0	0	0,037282739	0,209979862	0	0,022251955	0,016828102	0,531815291	0	0,818157949	0,090906439
ENSG00000133460	SLC2A11	0	0	0	0	0	0,111975642	0,183477426	0	0,522628015	0,818081084	0,090897898
ENSG00000196166	C8orf86	0	0,097082549	0	0	0	0,720827994	0	0	0	0,817910543	0,090878949
ENSG00000203909	DPPA5	0	0	0,421821091	0,395955955	0	0	0	0	0	0,817777046	0,090864116
ENSG00000279968	GVQW2	0	0	0	0,087181749	0,119985814	0,388030082	0	0,220804923	0	0,816002569	0,090666952
ENSG00000162753	SLC9C2	0,648923005	0	0	0,101868036	0	0	0	0,064500208	0	0,81529125	0,090587917
ENSG00000203782	LOR	0	0	0	0,209586325	0	0	0,604676299	0	0	0,814262624	0,090473625

ENSG00000126562	WNK4	0,114326136	0,50717231	0	0,098708142	0	0,031380854	0	0,062499445	0	0,814086888	0,090454099
ENSG00000169946	ZFPM2	0	0	0	0,259766027	0	0	0,015613531	0,534550694	0	0,809930252	0,08999225
ENSG00000182931	WFDC10B	0,239858049	0	0	0	0,570027845	0	0	0	0	0,809885894	0,089987322
ENSG00000161664	ASB16	0	0	0	0,585055997	0	0,223198085	0	0	0	0,808254082	0,089806009
ENSG00000175426	PCSK1	0,028208059	0	0,051891002	0,048709161	0	0,0619416	0	0,616827641	0	0,807577464	0,089730829
ENSG00000136939	OR1L4	0,807214587	0	0	0	0	0	0	0	0	0,807214587	0,08969051
ENSG00000139219	COL2A1	0,022682463	0	0	0,783353271	0	0	0	0	0	0,806035734	0,089559526
ENSG00000179636	TPPP2	0	0	0	0,527141363	0,145097997	0	0	0,133508916	0	0,805748276	0,089527586
ENSG00000204669	C9orf57	0	0,152613546	0,316425838	0,148511653	0	0	0	0,188067483	0	0,80561852	0,089513169
ENSG00000161958	FGF11	0	0,369487008	0	0	0	0	0,074096653	0,325230874	0,036583961	0,805398495	0,089488722
ENSG00000164181	ELOVL7	0,067747398	0,060108048	0	0,292462424	0,161003157	0	0	0,22221558	0	0,803536607	0,089281845
ENSG00000198838	RYR3	0,037091451	0,23694433	0,259284334	0,051239072	0,088148636	0,08959334	0,006159565	0,016221628	0,018247081	0,802929437	0,089214382
ENSG00000139220	PPFIA2	0,052879005	0,125109997	0	0	0,041889367	0	0	0,539610865	0,04335624	0,802845474	0,089205053
ENSG00000185662	SMIM23	0	0	0,168779659	0,633721857	0	0	0	0	0	0,802501516	0,089166835
ENSG00000187955	COL14A1	0,0148293	0,02631423	0	0,307283581	0	0	0	0,453982723	0	0,802409834	0,089156648
ENSG00000066230	SLC9A3	0	0,095170186	0	0,046306118	0	0,206100095	0,289461281	0	0,164903746	0,801941427	0,089104603
ENSG00000273155	AC092587.1	0	0,801620329	0	0	0	0	0	0	0	0,801620329	0,089068925
ENSG00000089250	NOS1	0,034571167	0	0	0,099494766	0,657273013	0	0,009568393	0	0	0,800907339	0,088989704
ENSG00000214929	SPATA31D1	0,058592699	0,051985653	0,377251007	0,151765204	0	0,03216568	0	0,128125075	0	0,799885318	0,088876146
ENSG00000162399	BSND	0,028780225	0,025534901	0	0,745457503	0	0	0	0	0	0,799772629	0,088863625
ENSG00000205592	MUC19	0,558567967	0,027279775	0,009426889	0,123883941	0	0,011252752	0,021274812	0,02241146	0,025209783	0,799307379	0,088811931
ENSG00000169629	RGPD8	0	0	0	0,113680645	0	0,26503287	0,291537222	0,047986432	0,080967117	0,799204286	0,088800476
ENSG00000187498	COL4A1	0,020612545	0	0	0,088983418	0,04898616	0,02263137	0,008557518	0,608493611	0	0,798264623	0,088696069
ENSG00000171551	ECEL1	0	0	0,09334456	0,087620878	0,361770526	0	0,25279449	0	0	0,795530454	0,088392273
ENSG00000072163	LIMS2	0	0,031594439	0,294782714	0	0,380824541	0	0	0	0,08759115	0,794792843	0,088310316
ENSG00000215568	GAB4	0,074128315	0	0	0,192005132	0,528502638	0	0	0	0	0,794636085	0,088292898
ENSG00000184611	KCNH7	0,030380091	0	0	0,419678286	0,144397886	0,066711129	0	0,132864724	0	0,794032115	0,088225791
ENSG00000099365	STX1B	0	0,103012678	0	0,300731819	0,068981472	0	0	0	0,321286727	0,794012695	0,088223633
ENSG00000164038	SLC9B2	0,028118826	0	0,051726851	0	0	0,030872828	0,490300888	0,122975276	0,069165062	0,793159732	0,088128859
ENSG00000278023	RDM1	0	0	0	0,792713847	0	0	0	0	0	0,792713847	0,088079316
ENSG00000168454	TXNDC2	0	0,316205189	0,08195168	0,076926584	0,317615751	0	0	0	0	0,792699204	0,088077689
ENSG00000187550	SBK2	0,553518574	0	0	0,238951442	0	0	0	0	0	0,792470016	0,088052224
ENSG00000158315	RHBDL2	0,05347154	0	0	0,738669426	0	0	0	0	0	0,792140965	0,088015663
ENSG00000005108	THSD7A	0,484284404	0,098211519	0	0,047785912	0,065766421	0	0,034466715	0,06051361	0	0,791028581	0,087892065
ENSG00000185666	SYN3	0	0	0	0,09586149	0,659657499	0	0	0	0,034137862	0,789656851	0,08773965
ENSG00000275004	ZNF280B	0	0	0,209361777	0	0	0,156195233	0,212621538	0	0,209956531	0,788135078	0,087570564
ENSG00000128052	KDR	0,023024618	0,04085662	0	0,119275472	0,547185041	0	0	0	0,056634625	0,786976377	0,08744182
ENSG00000197360	ZNF98	0	0,081477363	0,084466758	0	0	0	0,419378199	0,200811041	0	0,786133359	0,087348151
ENSG00000249884	RNF103-CHMP3	0	0,535213573	0	0	0	0	0,250439784	0	0	0,785653357	0,087294817
ENSG00000175229	GAL3ST3	0,041411502	0,146967388	0,076179802	0,143017251	0,196830662	0	0	0,181109656	0	0,785516262	0,087279585
ENSG00000185386	MAPK11	0	0	0	0,58244414	0,133600276	0	0	0	0,069139331	0,785183747	0,087242639
ENSG00000186205	MARC1	0	0,029446738	0	0,716381986	0	0,018219918	0	0	0,020409238	0,784457881	0,087161987
ENSG00000100038	TOP3B	0,227541892	0,121130252	0,041858169	0,196457593	0	0,149896621	0,018893289	0	0,02798472	0,783762535	0,087084726
ENSG00000141485	SLC13A5	0,100517009	0,029727494	0,030818193	0,202499426	0,238880849	0,018393634	0,027820473	0,073267088	0,061811482	0,783735648	0,087081739
ENSG00000173239	LIPM	0,200013992	0	0	0,345380509	0,23766879	0	0	0	0	0,783063291	0,087007032
ENSG00000005379	TSPOAP1	0,026536232	0	0,048815541	0,160377981	0,063063929	0	0,0110168	0	0,473224089	0,783034571	0,087003841
ENSG00000171561	OR2AT4	0,017181418	0	0,031606606	0,089005676	0,122496035	0	0,485047332	0,03757073	0	0,782907797	0,086989755
ENSG00000140297	GCNT3	0,116669681	0,041405497	0	0,40292615	0,221814418	0	0	0	0	0,782815746	0,086979527
ENSG00000143469	SYT14	0,021622747	0	0,258549137	0,056006648	0,128467319	0,059351283	0,017953829	0,212771776	0,026593192	0,781315931	0,086812881
ENSG00000108255	CRYBA1	0	0	0	0,328633469	0,452289096	0	0	0	0	0,780922565	0,086769174
ENSG00000172987	HPSE2	0,034180179	0	0	0,708260506	0	0,037527839	0	0	0	0,779968524	0,086663169
ENSG00000227268	KLLN	0,105992918	0,250775777	0,064994178	0,061008879	0,083964822	0	0,058672122	0,154516983	0	0,779925679	0,086658409
ENSG00000204323	SMIM5	0,029531087	0	0	0	0	0,713314967	0	0	0,03631943	0,779165484	0,086573943
ENSG00000284723	OR8S1	0	0	0	0,461423474	0,317522142	0	0	0	0	0,778945616	0,086549513
ENSG00000170962	PDGFD	0	0	0	0,612092364	0,084240568	0,038918737	0	0	0,043595243	0,778846912	0,086538546
ENSG00000270629	NBPF14	0,012608308	0,134238799	0	0,043543592	0	0,069215927	0,209378944	0,137853387	0,170572528	0,777411483	0,086379054
ENSG00000172137	CALB2	0	0	0,163421575	0,613603703	0	0	0	0	0	0,777025278	0,086336142
ENSG00000113492	AGXT2	0,076472961	0,135699393	0	0,396156338	0	0	0	0,167223971	0	0,775552663	0,086172518
ENSG00000006555	TTC22	0,049455268	0	0	0	0,705189087	0	0,020531882	0	0	0,775176237	0,086130693
ENSG00000119121	TRPM6	0,032875138	0,087504188	0	0,59606597	0,039064238	0,01804749	0	0	0	0,773557025	0,085950781
ENSG00000177098	SCN4B	0	0	0	0,230956784	0,508575029	0	0	0	0,032899013	0,772430826	0,085825647
ENSG00000147234	FRMPD3	0,03300078	0,087838611	0,455306998	0,113970288	0	0	0,082203755	0	0	0,772320432	0,085813381
ENSG00000100721	TCL1A	0,066981636	0	0	0,462650664	0,159183308	0	0	0	0,082378778	0,771194386	0,085688265
ENSG00000131910	NR0B2	0	0	0,238609527	0,223978519	0,308255401	0	0	0	0	0,770843447	0,085649272
ENSG00000074410	CA12	0,043186788	0	0	0,037287078	0,615806017	0	0	0,047218429	0,026557091	0,770055404	0,085561712
ENSG00000196778	OR52K1	0,083088657	0	0	0	0	0,06081766	0	0,181690561	0,442816127	0,768413005	0,085379223
ENSG00000172208	OR4X2	0	0	0	0,763712121	0	0	0	0	0	0,763712121	0,084856902
ENSG00000106541	AGR2	0,054670973	0	0	0,188809678	0,519707008	0	0	0	0	0,763187659	0,084798629
ENSG00000182983	ZNF662	0	0	0	0,154399393	0	0	0,0742428	0,130348942	0,403217307	0,762208442	0,084689827
ENSG00000073146	MOV10L1	0	0,039019499	0	0,075941494	0,20903233	0	0,438196736	0	0	0,762190059	0,084687784
ENSG00000132259	CNGA4	0	0	0	0	0,331442125	0	0	0	0,428810619	0,760252744	0,084472527
ENSG00000080493	SLC4A4	0,016569142	0,11760614	0,121921096	0,028611291	0,157507694	0	0,027515424	0,289854884	0	0,759585671	0,084398408
ENSG00000157103	SLC6A1	0,041418312	0,014699156	0,015238466	0,128736694	0,039372606	0	0	0	0,519579686	0,759044921	0,084338325
ENSG00000271425	NBPF10	0,010512771	0	0,038678183	0,235992394	0	0,173136124	0,026186934	0,183906814	0,090505472	0,758918692	0,084324299
ENSG00000146856	AGBL3	0,165441982	0	0	0	0	0,567642592	0	0	0,025434039	0,758518613	0,084279846
ENSG00000143171	RXRG	0	0	0,103299925	0,38786321	0,26690267	0	0	0	0	0,758065805	0,084229534
ENSG00000101440	ASIP	0	0	0,357300898	0	0	0	0,16127292	0	0,238877278	0,757451097	0,084161233
ENSG00000177182	CLVS1	0	0	0	0,423538441	0,048575347	0	0,016971504	0,268173597	0	0,75725889	0,084139877
ENSG00000104827	CGB3	0	0	0	0	0	0,756718708	0	0	0	0,756718708	0,084079856
ENSG00000139044	B4GALNT3	0,022005326	0	0	0,113995183	0,104592265	0	0	0	0,514210561	0,754803335	0,083867037
ENSG00000246922	UBAP1L	0,053108214	0,031413074	0,032565616	0,152843823	0	0	0,440968833	0	0,043544171	0,754443731	0,083827081
ENSG00000152266	PTH	0	0,576030075	0	0	0	0,178206849	0	0	0	0,754236925	0,083804103
ENSG00000130528	HRC	0	0	0	0,392826458	0	0,124885643	0,236112781	0	0	0,753824882	0,08375832
ENSG00000221837	KRTAP10-9	0	0	0	0,445661784	0,306675954	0	0	0	0	0,752337738	0,083593082
ENSG00000205279	CTXN3	0	0	0,132057054	0,619798039	0	0	0	0	0	0,751855094	0,083539455
ENSG00000163207	IVL	0	0,498405205	0	0	0,166876181	0	0	0	0,086359908	0,751641294	0,083515699
ENSG00000196338	NLGN3	0	0,26774039	0	0,06513604	0	0	0	0	0,417528383	0,750404812	0,083378312
ENSG00000166396	SERPINB7	0,057303971	0	0	0,692660153	0	0	0	0	0	0,749964123	0,083329347
ENSG00000101298	SNPH	0	0,093936591	0	0,045705899	0	0,610285884	0	0	0	0,749928374	0,083325375
ENSG00000197444	OGDHL	0,02639025	0,046828851	0,145640988	0,410131815	0,062717	0	0	0,057707748	0	0,749416652	0,083268517
ENSG00000137693	YAP1	0	0,424880368	0	0,165384234	0	0,157734663	0	0	0	0,747999265	0,083111029
ENSG00000104327	CALB1	0	0,075130849	0	0,621447824	0,050310667	0	0	0	0	0,74688934	0,082987704
ENSG00000244234	GMCL2	0	0	0	0,534337832	0	0,05662477	0,042822639	0,112776303	0	0,746561544	0,082951283
ENSG00000260097	SPDYE6	0	0	0	0	0	0,196227767	0	0	0,549516624	0,745744391	0,082860488
ENSG00000167210	LOXHD1	0,082009427	0,058209487	0	0,566449527	0,038979436	0	0	0	0	0,745647877	0,082849764
ENSG00000164035	EMCN	0	0	0,053747119	0,302708787	0,069434946	0	0	0,319445638	0	0,74533649	0,082815166
ENSG00000131126	TEX101	0,189243044	0	0,348127863	0	0	0	0	0,20690956	0	0,744280468	0,08269783
ENSG00000136696	IL36B	0	0	0,743659976	0	0	0	0	0	0	0,743659976	0,082628886
ENSG00000166596	CFAP52	0,147857701	0	0	0,595741951	0	0	0	0	0	0,743599652	0,082622184
ENSG00000171433	GLOD5	0	0,252013158	0	0,490479276	0	0	0	0	0	0,742492434	0,082499159
ENSG00000139304	PTPRQ	0	0,021490903	0,022279402	0,669224909	0,028782363	0	0	0	0	0,741777577	0,082419731
ENSG00000120329	SLC25A2	0	0,189232181	0	0,552438196	0	0	0	0	0	0,741670377	0,08240782
ENSG00000099812	MISP	0	0,089980537	0	0,087562072	0,120509242	0	0	0,443536332	0	0,741588184	0,082398687
ENSG00000129214	SHBG	0	0,272087265	0	0,1323871	0	0,168351676	0	0,167648182	0	0,740474224	0,082274914
ENSG00000204421	LY6G6C	0	0	0	0	0	0	0	0	0,740424392	0,740424392	0,082269377
ENSG00000112245	PTP4A1	0	0	0	0	0,068001807	0,282748569	0,166311745	0,187711362	0,03519154	0,739965022	0,082218336
ENSG00000161544	CYGB	0,054889419	0	0	0,189564093	0,130445893	0	0,364606849	0	0	0,739506254	0,082167362
ENSG00000249156	TAF11L12	0	0,739362133	0	0	0	0	0	0	0	0,739362133	0,082151348
ENSG00000157064	NMNAT2	0,304702289	0	0,040037462	0,375824535	0	0	0,018071487	0	0	0,738635772	0,082070641
ENSG00000176200	OR4D11	0,161442917	0	0,296987285	0,278776682	0	0	0	0	0	0,737206885	0,081911876
ENSG00000120211	INSL4	0	0	0	0	0,735896603	0	0	0	0	0,735896603	0,081766289
ENSG00000171487	NLRP5	0	0	0,068132377	0,447682554	0,088019005	0,040664357	0	0	0,091101236	0,735599531	0,081733281
ENSG00000182196	ARL6IP4	0	0	0	0	0	0	0	0	0,735099513	0,735099513	0,081677724
ENSG00000176900	OR51T1	0	0	0	0,735028097	0	0	0	0	0	0,735028097	0,081669789
ENSG00000171772	SYCE1	0	0,692157976	0	0	0	0,042826685	0	0	0	0,734984661	0,081664962
ENSG00000162643	WDR63	0	0	0	0,266056563	0	0	0	0,421150354	0,047373572	0,734580489	0,081620054
ENSG00000188523	CFAP77	0	0	0	0,243069376	0	0	0,058439838	0	0,432805129	0,734314342	0,081590482

ENSG00000204839	MROH6	0	0,446478116	0	0	0	0	0,20891825	0,07860004	0	0,733996406	0,081555156
ENSG00000121904	CSMD2	0,033289766	0,014767968	0,04592941	0,129339361	0,138449237	0	0,006910301	0,36397485	0	0,732660893	0,081406766
ENSG00000170743	SYT9	0	0,056167155	0	0,60123266	0,075223616	0	0	0	0	0,732623432	0,081402604
ENSG00000137133	HINT2	0	0	0	0	0	0	0,576272924	0	0,155195426	0,731468349	0,081274261
ENSG00000248329	APELA	0,051767924	0	0	0	0	0	0	0,679207744	0	0,730975668	0,081219519
ENSG00000169436	COL22A1	0,076852166	0	0	0,630357853	0	0	0	0	0,023629564	0,730839583	0,081204398
ENSG00000214782	MS4A18	0	0	0	0,728360013	0	0	0	0	0	0,728360013	0,08092889
ENSG00000186198	SLC51B	0,163539579	0	0	0,564794317	0	0	0	0	0	0,728333896	0,080925988
ENSG00000228278	ORM2	0	0	0,727696594	0	0	0	0	0	0	0,727696594	0,080855177
ENSG00000170381	SEMA3E	0,053648723	0,031732781	0,065794106	0,123519515	0,042499117	0	0,371214	0,039104683	0	0,727512925	0,080834769
ENSG00000101605	MYOM1	0,068958277	0	0,169139093	0	0,109253892	0,378560793	0	0	0	0,725912054	0,080656895
ENSG00000140284	SLC27A2	0	0	0,199126145	0,467290427	0	0,059423559	0	0	0	0,725840132	0,080648904
ENSG00000196834	POTEI	0,021031395	0,074639388	0,309511592	0,072633257	0,149944694	0,046182485	0	0	0,051731809	0,725674619	0,080630513
ENSG00000186523	FAM86B1	0	0,063360586	0	0	0	0	0,266831991	0	0,395231262	0,725423839	0,080602649
ENSG00000126950	TMEM35A	0	0	0	0,648285651	0	0	0	0	0,076955082	0,725240733	0,080582304
ENSG00000166143	PPP1R14D	0	0	0	0,298894587	0	0	0	0	0,425765229	0,724659816	0,080517757
ENSG00000163645	ERICH6	0,218473114	0	0	0	0,34613739	0,159913809	0	0	0	0,724524313	0,080502701
ENSG00000163064	EN1	0	0	0	0,723312473	0	0	0	0	0	0,723312473	0,080368053
ENSG00000187166	H1FNT	0	0	0,213830845	0	0	0	0	0,508360873	0	0,722191718	0,080243524
ENSG00000102302	FGD1	0	0	0	0,720980819	0	0	0	0	0	0,720980819	0,08010898
ENSG00000138472	GUCA1C	0,129820078	0	0	0,448341881	0	0,14253486	0	0	0	0,720696818	0,080077424
ENSG00000198691	ABCA4	0,123759681	0,031372645	0,357760745	0,091588268	0	0	0	0,115982649	0	0,720463989	0,080051554
ENSG00000198354	DCAF12L2	0,054142089	0,384295235	0	0,280474713	0	0	0	0	0	0,718912037	0,079879115
ENSG00000184724	KRTAP6-1	0	0	0	0	0,718235085	0	0	0	0	0,718235085	0,079803898
ENSG00000164451	CALHM4	0,312858324	0	0	0,405178532	0	0	0	0	0	0,718036856	0,079781873
ENSG00000149557	FEZ1	0,03241325	0,028758258	0,029813395	0,363808952	0	0,071175709	0,121110359	0,070878286	0	0,717958209	0,079773134
ENSG00000065320	NTN1	0,117779089	0	0	0,081351512	0	0,025862911	0	0,463587078	0,028970619	0,71755121	0,079727912
ENSG00000189420	ZFP92	0,024751936	0,043921704	0	0,042741192	0	0	0,575457706	0	0,030441691	0,717314228	0,079701581
ENSG00000148143	ZNF462	0,012396273	0	0,022803946	0,513735799	0,058920023	0,027220767	0,02058578	0	0,060983272	0,716645861	0,079627318
ENSG00000169550	MUC15	0,042127285	0	0	0,581957011	0	0	0	0,092120036	0	0,716204331	0,079578259
ENSG00000135346	CGA	0	0	0	0,571223675	0	0	0	0,144673629	0	0,715897303	0,079544145
ENSG00000283154	IQCJ-SCHIP1	0	0	0,063048333	0,236729394	0,162902038	0	0	0	0,25290976	0,715589525	0,079509947
ENSG00000115648	MLPH	0	0,072129657	0,074776086	0,070190982	0,048300947	0	0	0	0,449931056	0,715328728	0,07948097
ENSG00000154485	MMP21	0,043914726	0	0	0,379155732	0	0	0,291706671	0	0	0,714777129	0,079419681
ENSG00000231256	CFAP97D1	0,040263941	0	0,074068771	0	0,478440637	0,088414909	0,03343201	0	0	0,714620268	0,079402252
ENSG00000125813	PAX1	0	0	0,474773867	0,178264714	0,061335191	0	0	0	0	0,714373771	0,079374863
ENSG00000178586	OR6B3	0	0	0	0,713586986	0	0	0	0	0	0,713586986	0,079287443
ENSG00000145832	SLC25A48	0,03610335	0,032032254	0,066415028	0,374055632	0,085800392	0	0	0,118421181	0	0,712827837	0,079203093
ENSG00000123569	H2BFWT	0	0	0,310939708	0	0,401697475	0	0	0	0	0,712637183	0,079181909
ENSG00000129451	KLK10	0	0,28240337	0,292764717	0,137406516	0	0	0	0	0	0,712574604	0,079174956
ENSG00000170160	CCDC144A	0	0	0	0,016760982	0,530556492	0	0,048357014	0,021225242	0,09550181	0,712401541	0,079155727
ENSG00000128917	DLL4	0	0,112806899	0	0,219549831	0,07554008	0	0	0,06950664	0,234555981	0,711959432	0,079106604
ENSG00000196581	AJAP1	0,048725698	0,043231278	0	0,063103984	0,173696514	0,013374492	0,354007386	0	0,014981582	0,711120933	0,079013437
ENSG00000204315	FKBPL	0	0	0	0	0	0,370888025	0,093495031	0,24622546	0	0,710608517	0,078956502
ENSG00000254466	OR4D10	0	0	0	0,42052373	0,289377552	0	0	0	0	0,709901282	0,07887792
ENSG00000284491	THSD8	0	0	0	0	0	0	0,709272651	0	0	0,709272651	0,078808072
ENSG00000129048	ACKR4	0	0,707809943	0	0	0	0	0	0	0	0,707809943	0,078645549
ENSG00000170689	HOXB9	0	0	0	0,706320504	0	0	0	0	0	0,706320504	0,078480056
ENSG00000161896	IP6K3	0,054103319	0	0,199054851	0,093424624	0	0	0,359385126	0	0	0,70596792	0,07844088
ENSG00000187123	LYPD6	0	0,053919566	0	0,577173682	0	0	0	0	0,074742217	0,705835465	0,078426163
ENSG00000213420	GPC2	0	0	0	0,612044506	0,093593313	0	0	0	0	0,70563782	0,078404202
ENSG00000162592	CCDC27	0,071312209	0,189812648	0	0,06157031	0,338950016	0	0	0	0,043852412	0,705497595	0,078388622
ENSG00000012223	LTF	0	0,063136802	0,065453284	0,491518671	0,084557933	0	0	0	0	0,704666691	0,078296299
ENSG00000166220	TBATA	0	0	0	0,607885788	0	0,096628175	0	0	0	0,704513963	0,078279329
ENSG00000204965	PCDHA5	0	0	0,703054908	0	0	0	0	0	0	0,703054908	0,078117212
ENSG00000149798	CDC42EP2	0,279317136	0	0	0	0	0	0,11596149	0,305392391	0	0,700671017	0,077852335
ENSG00000157800	SLC37A3	0	0	0,105017038	0,098577625	0	0,01566968	0,011850239	0,46812604	0	0,699240623	0,077693403
ENSG00000125084	WNT1	0	0	0,206369784	0	0	0,492681742	0	0	0	0,699051526	0,077672392
ENSG00000168676	KCTD19	0,061427061	0	0,03766668	0,530355639	0	0	0,0680056	0	0	0,697454981	0,077494998
ENSG00000147485	PXDNL	0	0	0	0,697287582	0	0	0	0	0	0,697287582	0,077476398
ENSG00000180433	OR6K6	0	0	0,47436877	0,222640763	0	0	0	0	0	0,697009533	0,077445504
ENSG00000147799	ARHGAP39	0	0,054857201	0	0,053382769	0,073469219	0	0,513381064	0	0	0,695090253	0,07723225
ENSG00000176076	KCNE5	0	0	0	0	0,243800097	0,450537886	0	0	0	0,694337983	0,077148665
ENSG00000187475	HIST1H1T	0	0	0	0	0	0,69321968	0	0	0	0,69321968	0,077024409
ENSG00000181333	HEPHL1	0,028271388	0,050166885	0,156022506	0,390548138	0,067187566	0	0	0	0	0,692196484	0,07691072
ENSG00000124251	TP53TG5	0	0	0,09334456	0,087620878	0,120590175	0,389984566	0	0	0	0,691540179	0,076837798
ENSG00000060566	CREB3L3	0	0	0	0,689782788	0	0	0	0	0	0,689782788	0,076642532
ENSG00000261787	TCF24	0	0,310589189	0	0,100747094	0,277310844	0	0	0	0	0,688647127	0,076516347
ENSG00000065621	GSTO2	0	0,027014104	0,028005249	0,446896491	0,036179482	0,150432721	0	0	0	0,688528048	0,076503116
ENSG00000100344	PNPLA3	0	0	0	0,080734831	0	0	0,504676459	0,102238418	0	0,687649709	0,076405523
ENSG00000064835	POU1F1	0	0	0	0	0,686649221	0	0	0	0	0,686649221	0,076294358
ENSG00000177483	RBM44	0,049962166	0,132984956	0	0	0	0,41141654	0	0	0,092170535	0,686534197	0,076281577
ENSG00000187066	TMEM262	0	0,063934669	0	0,622162553	0	0	0	0	0	0,686097221	0,076233025
ENSG00000144837	PLA1A	0	0	0	0	0	0,076491737	0	0,609376796	0	0,685868532	0,076207615
ENSG00000119547	ONECUT2	0,009132755	0,048617551	0,386410146	0,047310826	0,173633527	0	0	0,019970662	0	0,685075467	0,076119496
ENSG00000132677	RHBG	0	0,158476359	0	0,077108444	0	0	0,29662017	0,097646149	0,054919185	0,684770307	0,07608559
ENSG00000203877	RIPPLY2	0	0,192815436	0,066629937	0,187633011	0	0	0	0,237608749	0	0,684687135	0,076076348
ENSG00000124116	WFDC3	0	0	0	0	0,149321223	0,482899807	0,052170616	0	0	0,684391647	0,076043516
ENSG00000170236	USP50	0	0	0	0	0,684150432	0	0	0	0	0,684150432	0,076016715
ENSG00000172464	OR5AP2	0	0	0	0,518757405	0	0,16492105	0	0	0	0,683678455	0,075964273
ENSG00000158023	WDR66	0,009821942	0,017428794	0,054204764	0,033920699	0,186736454	0,043135672	0	0	0,338232215	0,683480539	0,075942282
ENSG00000139193	CD27	0	0	0	0,185060266	0	0	0	0,234350757	0,263612088	0,683023111	0,075891457
ENSG00000085552	IGSF9	0	0	0,041883396	0,039315199	0	0,374967403	0,226856107	0	0	0,683022105	0,075891345
ENSG00000242950	ERVW-1	0,02465099	0,043742578	0	0,55336944	0	0	0	0	0,060635081	0,682398089	0,07582201
ENSG00000187957	DNER	0,076861938	0,068194812	0	0,398171375	0,09133203	0	0	0	0,047265138	0,681825293	0,075758366
ENSG00000102935	ZNF423	0	0	0,123587907	0,464039079	0,079830508	0	0,013945797	0	0	0,681403291	0,075711477
ENSG00000175065	DSG4	0,032178571	0	0,059195081	0,444522955	0	0	0,026718555	0	0,118726484	0,681341645	0,075704627
ENSG00000183560	IZUMO1R	0,365442734	0	0	0,315519921	0	0	0	0	0	0,680962655	0,075662517
ENSG00000211448	DIO2	0,020164207	0	0,111281064	0,069638371	0,479206755	0	0	0	0	0,680290398	0,075587822
ENSG00000205221	VIT	0	0,072275472	0,224781751	0,281331509	0	0	0,101458489	0	0	0,679847221	0,07553858
ENSG00000169876	MUC17	0,051824738	0,257492832	0,057201475	0,214776027	0,098530089	0	0	0	0	0,679825161	0,075536129
ENSG00000205363	INSYN1	0	0,040794462	0	0,158792013	0	0,479583289	0	0	0	0,679169764	0,075463307
ENSG00000143502	SUSD4	0,032247241	0,057221938	0	0	0	0	0,589062592	0	0	0,67853177	0,075392419
ENSG00000198844	ARHGEF15	0,02855453	0	0	0,443766964	0,203581373	0	0	0	0	0,675902866	0,075100318
ENSG00000055118	KCNH2	0,488531707	0,045625659	0	0	0	0,141152439	0	0	0	0,675309805	0,075034423
ENSG00000153820	SPHKAP	0,042892583	0	0,197260927	0,111099194	0,305805458	0	0,017807314	0	0	0,674865476	0,074985053
ENSG00000122136	OBP2A	0	0	0	0	0,351387028	0	0	0,323321495	0	0,674708522	0,074967614
ENSG00000186367	MINAR2	0	0	0,118088402	0,110847483	0,30511261	0	0	0,140371524	0	0,674420019	0,074935558
ENSG00000168703	WFDC12	0	0	0	0,67338058	0	0	0	0	0	0,67338058	0,074820064
ENSG00000133958	UNC79	0	0	0	0	0	0	0,671822284	0	0	0,671822284	0,07464692
ENSG00000163586	FABP1	0	0,108559514	0,11254255	0,31692507	0	0	0	0,133779177	0	0,671806312	0,074645146
ENSG00000082293	COL19A1	0,548595283	0,048673443	0,050459266	0,023682608	0	0	0	0	0	0,671410601	0,074601178
ENSG00000185689	C6orf201	0	0	0	0	0	0	0	0	0,671330002	0,671330002	0,074592222
ENSG00000178358	OR2D3	0	0	0	0	0,671247743	0	0	0	0	0,671247743	0,074583083
ENSG00000149646	CNBD2	0,513262678	0,053574825	0	0,10426972	0	0	0	0	0	0,671107223	0,074567469
ENSG00000155269	GPR78	0,071043992	0	0,026138232	0,53978086	0,033767517	0	0	0	0	0,6707306	0,074525622
ENSG00000084710	EFR3B	0	0,050388424	0,078355755	0,245170511	0,101226405	0,093532224	0,070734003	0,031047127	0	0,670454448	0,074494939
ENSG00000198633	ZNF534	0	0,245326624	0	0	0	0,252989595	0,114794448	0	0,056677805	0,669788473	0,074420941
ENSG00000171189	GRIK1	0,035622482	0,063211221	0	0	0	0	0,088734322	0	0,481921675	0,669489701	0,074387745
ENSG00000126549	STATH	0,069444196	0	0	0,59957485	0	0	0	0	0	0,669019046	0,07433545
ENSG00000260287	TBC1D3G	0	0	0	0	0,3478136	0,16068821	0	0,16001674	0	0,66851855	0,074279839
ENSG00000153802	TMPRSS11D	0,137623471	0,081403157	0,337559319	0	0	0,109021719	0	0	0	0,665607666	0,073956407
ENSG00000178732	GP5	0	0	0	0,664520309	0	0	0	0	0	0,664520309	0,07383559
ENSG00000198739	LRRTM3	0	0,126861063	0,043838527	0,493805345	0	0	0	0	0	0,664504935	0,073833882
ENSG00000115263	GCG	0,063545236	0,112759462	0,116896593	0,219457506	0,151016628	0	0	0	0	0,663675425	0,073741714
ENSG00000189337	KAZN	0,021805277	0,038692929	0	0,527141363	0,051820713	0,02394092	0	0	0	0,663401203	0,073711245
ENSG00000230257	NFE4	0	0	0,505112233	0,158046623	0	0	0	0	0	0,663158856	0,073684317

ENSG00000172346	CSDC2	0	0	0,185134931	0,086891433	0,239172522	0	0,041781662	0,110034821	0	0,66301537	0,073668374
ENSG00000138162	TACC2	0,02404305	0,028442535	0,117944354	0,193746467	0,190462764	0,026397864	0,026617944	0,035050073	0,019713235	0,662418287	0,073602032
ENSG00000178055	PRSS42P	0	0	0	0,562057027	0	0	0	0	0,100078903	0,662135929	0,073570659
ENSG00000143185	XCL2	0	0	0	0	0	0	0	0	0,661375524	0,661375524	0,073486169
ENSG00000198944	SOWAHA	0	0,083507319	0	0	0,111839783	0,103338883	0,156300632	0,205814119	0	0,660800735	0,073422304
ENSG00000105989	WNT2	0	0	0	0,580242327	0,079857136	0	0	0	0	0,660099464	0,073344385
ENSG00000142619	PADI3	0	0	0,087168422	0,572764134	0	0	0	0	0	0,659932556	0,07332584
ENSG00000205362	MT1A	0	0	0	0,658926704	0	0	0	0	0	0,658926704	0,073214078
ENSG00000230778	ANKRD63	0	0	0	0	0	0	0,658636913	0	0	0,658636913	0,073181879
ENSG00000101342	TLDC2	0	0	0,108883705	0,204414395	0	0	0,344023629	0	0	0,657321728	0,073035748
ENSG00000248405	PRR5-ARHGAP8	0	0,657210785	0	0	0	0	0	0	0	0,657210785	0,073023421
ENSG00000106648	GALNTL5	0,045515232	0,080765663	0	0,314379487	0,216335869	0	0	0	0	0,656996251	0,072999583
ENSG00000122728	TAF1L	0,024309937	0,043137387	0,134160279	0,335823648	0,057773092	0	0,06055518	0	0	0,655759524	0,072862169
ENSG00000072195	SPEG	0,008854481	0,047136177	0	0,581010725	0	0	0,007352064	0	0,01088987	0,655243318	0,072804813
ENSG00000144596	GRIP2	0,025420232	0,022553789	0	0	0,030205866	0,279099296	0,010553481	0,083379906	0,203213457	0,654426028	0,072714003
ENSG00000146147	MLIP	0,06215984	0,183835184	0	0	0	0	0	0,407776101	0	0,653771126	0,072641236
ENSG00000172188	OR4C11	0	0,195938619	0	0,095336125	0	0	0	0,362186227	0	0,653460971	0,072606775
ENSG00000174844	DNAH12	0,056915469	0,080796083	0	0,393122372	0	0,037493916	0,085064632	0	0	0,653392472	0,072599164
ENSG00000079112	CDH17	0,077932218	0	0,071681305	0,471001759	0	0	0,032354392	0	0	0,652969674	0,072552186
ENSG00000239887	C1orf226	0,067294843	0	0,06189715	0,522915781	0	0	0	0	0	0,652107774	0,072456419
ENSG00000117152	RGS4	0,093741049	0	0	0	0,519814474	0	0	0	0,038429802	0,651985325	0,072442814
ENSG00000069018	TRPC7	0	0,083978077	0	0	0,224940521	0,051960719	0	0	0,291021801	0,651901119	0,072433458
ENSG00000138395	CDK15	0,024511041	0,130482725	0,135270121	0,126975657	0,233004083	0	0	0	0	0,650243627	0,072249292
ENSG00000124237	C20orf85	0	0	0	0,64990031	0	0	0	0	0	0,64990031	0,072211146
ENSG00000175267	VWA3A	0,030000113	0	0	0,362625535	0	0,032938371	0,224187609	0	0	0,649751629	0,072194625
ENSG00000152939	MARVELD2	0	0,64961722	0	0	0	0	0	0	0	0,64961722	0,072179691
ENSG00000196873	CBWD3	0,012266464	0,021766539	0,090260605	0,042363012	0,116606069	0,026935722	0,061110641	0,187762154	0,090517017	0,649588224	0,072176469
ENSG00000177354	C10orf71	0,055565571	0	0	0,527722876	0,066026391	0	0	0	0	0,649314838	0,072146093
ENSG00000167774	AC010323.1	0	0	0	0	0	0	0,648984476	0	0	0,648984476	0,072109386
ENSG00000179172	HNRNPCL1	0	0	0	0	0,648226611	0	0	0	0	0,648226611	0,072025179
ENSG00000162949	CAPN13	0,58220216	0	0	0	0	0	0	0,063655282	0	0,645857442	0,071761938
ENSG00000115592	PRKAG3	0,045324106	0	0	0,078264839	0	0	0,075267146	0	0,445942465	0,644798557	0,071644284
ENSG00000270276	HIST2H4B	0	0	0	0	0,558503176	0,086008593	0	0	0	0,64451177	0,071612419
ENSG00000073464	CLCN4	0	0	0	0,538089504	0,105793944	0	0	0	0	0,643883448	0,071542605
ENSG00000128886	ELL3	0	0,6435408	0	0	0	0	0	0	0	0,6435408	0,071504533
ENSG00000183733	FIGLA	0	0	0,385013988	0	0	0	0	0	0,257405156	0,642419143	0,071379905
ENSG00000186190	BPIFB3	0,10392749	0	0	0,538380278	0	0	0	0	0	0,642307768	0,07136753
ENSG00000249459	ZNF286B	0	0	0,188973555	0,044346529	0	0,028196903	0,127943914	0	0,25268052	0,64214142	0,071349047
ENSG00000089847	ANKRD24	0	0	0	0,641547769	0	0	0	0	0	0,641547769	0,071283085
ENSG00000145864	GABRB2	0	0	0,027792451	0,130441399	0,43085488	0,03317548	0	0	0,018580936	0,640845147	0,071205016
ENSG00000188316	ENO4	0	0	0	0,164939933	0	0	0,475867252	0	0	0,640807185	0,071200798
ENSG00000136928	GABBR2	0,04115772	0	0	0,426422402	0,048906107	0	0,034174134	0,089999882	0	0,640660245	0,071184472
ENSG00000214694	ARHGEF33	0,136652714	0	0	0	0,064951626	0,030007339	0,408476393	0	0	0,640088072	0,07112089
ENSG00000026036	RTEL1-TNFRSF6B	0	0	0,03057078	0,057392494	0	0	0	0	0,551837249	0,639800522	0,071088947
ENSG00000168502	MTCL1	0,011931352	0,042343782	0,021948685	0,103014202	0	0	0,445808523	0	0,014674025	0,639720569	0,071080063
ENSG00000126785	RHOJ	0	0	0	0,255693263	0,17595176	0,081288867	0	0,080949184	0,0455283	0,639411373	0,071045708
ENSG00000111728	ST8SIA1	0	0,018514258	0,019193544	0,450415961	0	0,114555394	0	0,02281534	0,012832046	0,638326543	0,070925171
ENSG00000185372	OR2V1	0	0,19256158	0	0,187385978	0,257894106	0	0	0	0	0,637841664	0,070871296
ENSG00000147465	STAR	0	0	0,147586992	0	0	0,440431581	0	0	0,049335419	0,637353992	0,07081711
ENSG00000183072	NKX2-5	0	0	0	0,637048278	0	0	0	0	0	0,637048278	0,070783142
ENSG00000109255	NMU	0	0	0	0,267625615	0,368325684	0	0	0	0	0,635951299	0,070661255
ENSG00000080573	COL5A3	0	0	0	0,307751702	0,105887525	0	0	0,194860426	0,027398868	0,635898522	0,070655391
ENSG00000186487	MYT1L	0,025793389	0,015256579	0,023724511	0,341469911	0	0,018879756	0	0,009400431	0,200909443	0,635434021	0,07060378
ENSG00000255221	CARD17	0	0	0	0,635393607	0	0	0	0	0	0,635393607	0,07059929
ENSG00000131941	RHPN2	0,03144861	0,055804787	0,057852258	0	0,149476605	0	0,339461876	0	0	0,634044135	0,070449348
ENSG00000238083	LRRC37A2	0	0	0	0	0	0,171902611	0	0,048909794	0,412625494	0,633437898	0,070381989
ENSG00000168903	BTNL3	0,061930562	0	0	0,32082169	0,147179321	0	0,102844534	0	0	0,632776107	0,070308456
ENSG00000138115	CYP2C8	0,058775018	0	0	0,304474883	0,139680102	0	0	0,128523752	0	0,631453755	0,070161528
ENSG00000174963	ZIC4	0,035273242	0	0,097331967	0,456818933	0	0,01936398	0	0	0,021690771	0,630478893	0,07005321
ENSG00000168916	ZNF608	0,109152391	0,038737648	0,120476784	0,037696471	0	0,095874358	0,126884184	0,047736863	0,053697348	0,630256049	0,07002845
ENSG00000214891	TRIM64C	0	0	0	0,385713192	0	0	0	0,244223627	0	0,629936819	0,06999298
ENSG00000188710	QRFP	0	0	0	0,62951743	0	0	0	0	0	0,62951743	0,069946381
ENSG00000103310	ZP2	0,087524223	0	0,080503938	0,15113523	0,20800321	0,048048241	0	0	0,053821756	0,629036598	0,069892955
ENSG00000184905	TCEAL2	0,095548891	0	0	0	0,454148014	0	0,079336283	0	0	0,629033188	0,069892576
ENSG00000167780	SOAT2	0	0	0	0,125873118	0,173235669	0,240102137	0	0	0,089651	0,628861924	0,069873547
ENSG00000179059	ZFP42	0	0	0,314457126	0,19678354	0	0	0,047311588	0	0,070077874	0,628630128	0,069847792
ENSG00000105227	PRX	0	0	0	0,316723569	0	0,028768957	0,282835845	0	0	0,628328371	0,069814263
ENSG00000157131	C8A	0,064138612	0	0	0,332260154	0,152426801	0	0	0	0,078882225	0,627707792	0,06974531
ENSG00000070808	CAMK2A	0	0	0,135600048	0,424284512	0	0,02697733	0,04080336	0	0	0,627665249	0,069740583
ENSG00000167100	SAMD14	0,021537995	0	0	0,446296992	0	0	0	0	0,158933742	0,626768729	0,06964097
ENSG00000172156	CCL11	0,140046868	0	0	0	0,332824414	0,153763278	0	0	0	0,62663456	0,069626062
ENSG00000188158	NHS	0,2616885	0,051595536	0,05348857	0,100417539	0	0	0	0,158954477	0	0,626144622	0,069571625
ENSG00000168843	FSTL5	0	0	0	0,490110771	0,134905162	0	0	0	0	0,625015934	0,069446215
ENSG00000167807	AC011511.1	0	0	0	0	0	0	0,180100955	0	0,444608905	0,62470986	0,069412207
ENSG00000113600	C9	0	0	0,092413597	0,173493999	0,358162443	0	0	0	0	0,624070038	0,069341115
ENSG00000099954	CECR2	0	0,024922576	0	0	0,033378338	0	0,548109081	0	0,017273587	0,623683583	0,069298176
ENSG00000186207	LCE5A	0	0,623586047	0	0	0	0	0	0	0	0,623586047	0,069287339
ENSG00000111012	CYP27B1	0	0	0,169293605	0,397282239	0	0	0	0	0,056591511	0,623167355	0,069240817
ENSG00000162620	LRRIQ3	0	0,091839936	0	0	0	0	0	0	0,530444464	0,6222844	0,069142711
ENSG00000256574	OR13A1	0	0	0	0	0	0	0	0	0,622022574	0,622022574	0,069113619
ENSG00000125571	IL37	0	0	0	0,622014242	0	0	0	0	0	0,622014242	0,069112694
ENSG00000169208	OR10G3	0	0	0	0,098839006	0,272058744	0	0	0,250329218	0	0,621226968	0,069025219
ENSG00000096088	PGC	0	0	0,137274123	0,128856778	0,354683992	0	0	0	0	0,620814893	0,068979433
ENSG00000134853	PDGFRA	0,031656137	0,056173039	0,145585052	0,163989719	0,112847243	0,052134883	0	0	0,058399452	0,620785525	0,068976169
ENSG00000106384	MOGAT3	0,62032254	0	0	0	0	0	0	0	0	0,62032254	0,068924727
ENSG00000137203	TFAP2A	0	0	0,405909125	0,127006559	0,087397796	0	0	0	0	0,620313479	0,06892372
ENSG00000197558	SSPO	0,021440206	0,025363413	0,013146997	0,18511278	0,033968742	0	0	0,015627817	0,325213835	0,61987379	0,068874866
ENSG00000094963	FMO2	0	0	0	0	0	0	0,56654883	0,053287303	0	0,619836133	0,068870681
ENSG00000173908	KRT28	0	0	0	0,619430207	0	0	0	0	0	0,619430207	0,068825579
ENSG00000131055	COX4I2	0,218052772	0	0,401125684	0	0	0	0	0	0	0,619178455	0,068797606
ENSG00000081803	CADPS2	0	0,337020581	0,043673228	0,081990565	0	0,052132153	0	0,103828615	0	0,618645143	0,068738349
ENSG00000182447	OTOL1	0	0	0	0,363926045	0,250430643	0	0	0	0	0,614356689	0,068261854
ENSG00000259075	POC1B-GALNT4	0	0,146259273	0	0,094885445	0	0,090496678	0,045625575	0	0,236531937	0,613798909	0,068199879
ENSG00000167941	SOST	0	0	0	0	0,469230238	0	0	0,143917495	0	0,613147733	0,068127526
ENSG00000171992	SYNPO	0,059010278	0,026178073	0	0,458540422	0,035059801	0,016197459	0	0	0,018143759	0,613129793	0,068125533
ENSG00000101311	FERMT1	0	0,350250793	0	0,097381965	0,134024087	0,030959242	0	0	0	0,612616087	0,068068454
ENSG00000285868	AC008676.3	0	0	0	0,428163343	0	0	0,029411705	0,154915409	0	0,612490457	0,068054495
ENSG00000197430	OPALIN	0,039006342	0,069215798	0	0,47148808	0	0	0,032387799	0	0	0,612098019	0,068010891
ENSG00000102904	TSNAXIP1	0	0,069611111	0	0,541921027	0	0	0	0	0	0,611532138	0,067948015
ENSG00000163898	LIPH	0,073766449	0	0,203549011	0,127378557	0	0	0	0,161305622	0,045361611	0,611361251	0,067929028
ENSG00000181541	MAB21L2	0	0	0,100281421	0,376529545	0	0	0	0	0,1340884	0,610899366	0,067877707
ENSG00000165164	CFAP47	0,013205503	0,046865682	0	0,091212086	0,031383164	0	0,427627628	0	0	0,610294063	0,067810451
ENSG00000047597	XK	0	0,051824894	0	0	0	0	0,557753698	0	0	0,609578592	0,067730955
ENSG00000185306	C12orf56	0,027454682	0	0	0,426674195	0,065246647	0,030143637	0	0,06003535	0	0,609554511	0,067728279
ENSG00000187398	LUZP2	0,01705922	0	0,53349082	0,058915099	0	0	0	0	0	0,609465139	0,067718349
ENSG00000113924	HGD	0	0	0,414586277	0,194582382	0	0	0	0	0	0,60916866	0,067685407
ENSG00000149922	TBX6	0,048170408	0	0	0,083179782	0	0,317329761	0,159987672	0	0	0,608667623	0,067629736
ENSG00000074276	CDHR2	0,056511059	0	0	0,146373396	0,335749385	0	0	0	0,069501317	0,608135157	0,067570573
ENSG00000106714	CNTNAP3	0	0,056326436	0,08758957	0,082218772	0	0	0	0,381764546	0	0,607899324	0,067544369
ENSG00000153002	CPB1	0,079490042	0	0	0,091507969	0	0,378193494	0	0,057940482	0	0,607131988	0,06745911
ENSG00000185519	FAM131C	0	0	0	0,606825522	0	0	0	0	0	0,606825522	0,067425058
ENSG00000170092	SPDYE5	0	0,241932632	0	0,156953368	0,108005276	0,099795836	0	0	0	0,606687111	0,067409679
ENSG00000166206	GABRB3	0,032646674	0,019310241	0,040037462	0,450989442	0,051723685	0,011948047	0	0	0	0,60665555	0,067406172
ENSG00000165591	FAAH2	0,074844265	0,531237246	0	0	0	0	0	0	0	0,606081511	0,06734239
ENSG00000283329	FAM240B	0	0	0,606061262	0	0	0	0	0	0	0,606061262	0,06734014
ENSG00000139144	PIK3C2G	0,025503894	0	0,093832945	0,176158633	0	0	0	0,278847736	0,031366502	0,605709709	0,067301079
ENSG00000126353	CCR7	0	0	0,11083736	0,208082117	0,286377625	0	0	0	0	0,605297102	0,067255234

ENSG00000071991	CDH19	0	0,195324884	0,040498266	0,076030004	0,052318989	0	0	0,240701171	0	0,604873314	0,067208146
ENSG00000163239	TDRD10	0	0	0	0,48214149	0	0,122624225	0	0	0	0,604765716	0,067196191
ENSG00000164404	GDF9	0	0	0	0	0	0	0	0,604637818	0	0,604637818	0,06718198
ENSG00000074803	SLC12A1	0	0	0,043302453	0,040647243	0,055941669	0,051689564	0	0,411788541	0	0,60336947	0,067041052
ENSG00000197748	CFAP43	0,042068644	0,037324889	0,038694334	0	0	0,392605763	0	0,091991806	0	0,602685437	0,066965049
ENSG00000078114	NEBL	0,033201385	0,039276695	0,020358876	0,324878758	0,105205081	0	0,055135637	0,024200569	0	0,602257002	0,066917445
ENSG00000132563	REEP2	0	0	0	0	0	0,562582103	0,038677661	0	0	0,601259764	0,06680664
ENSG00000179626	OR6C4	0,176016972	0	0	0,354600043	0,069718024	0	0	0	0	0,60033504	0,066703893
ENSG00000172410	INSL5	0,211343455	0	0,388783355	0	0	0	0	0	0	0,600126811	0,066680757
ENSG00000140873	ADAMTS18	0,022902481	0	0,084261928	0	0,054428242	0	0	0,100162039	0,338005194	0,599759884	0,066639987
ENSG00000134193	REG4	0,04426203	0	0	0,458585193	0	0	0	0,096788098	0	0,599635321	0,066626147
ENSG00000174483	BBS1	0	0	0,045340091	0,29791956	0,117148114	0,10824373	0	0	0,030312595	0,59896409	0,066551566
ENSG00000106404	CLDN15	0	0	0	0,360781161	0	0	0,173481275	0	0,06424007	0,598502506	0,066500278
ENSG00000164120	HPGD	0	0,145439523	0	0,094353634	0	0	0	0,358453698	0	0,598246855	0,066471873
ENSG00000090512	FETUB	0,149318746	0	0	0,386761326	0	0	0,061991271	0	0	0,598071342	0,066452371
ENSG00000164399	IL3	0	0,255373333	0	0	0,342016707	0	0	0	0	0,59739004	0,066376671
ENSG00000228696	ARL17B	0	0,254223276	0	0	0	0	0	0,07832059	0,264299392	0,596843257	0,066315917
ENSG00000146285	SCML4	0,016667833	0,02957666	0	0,086345127	0	0,091501531	0,249113829	0	0,122995713	0,596200694	0,066244522
ENSG00000118156	ZNF541	0	0	0	0,560547744	0	0,03564137	0	0	0	0,596189113	0,066243235
ENSG00000141437	SLC25A52	0	0	0	0,197827881	0,272265006	0,125785123	0	0	0	0,595878009	0,066208668
ENSG00000254440	PBOV1	0	0,321	0	0,208248184	0	0,066205338	0	0	0	0,595453522	0,066161502
ENSG00000258817	OR4C13	0	0	0	0,253581122	0	0,161234768	0	0	0,180608865	0,595424756	0,066158306
ENSG00000114279	FGF12	0	0,218742624	0,113384133	0,070954446	0,146478943	0	0	0,044926522	0	0,594486668	0,066054074
ENSG00000187026	KRTAP21-2	0	0	0	0,593034033	0	0	0	0	0	0,593034033	0,06589267
ENSG00000231989	PPP1R2B	0,151793642	0	0	0	0,180370438	0,166660549	0	0	0,093343306	0,592167935	0,065796437
ENSG00000176246	OR4L1	0	0	0,592076888	0	0	0	0	0	0	0,592076888	0,065786321
ENSG00000150722	PPP1R1C	0,061841854	0,054868426	0	0	0	0,475291196	0	0	0	0,592001476	0,065777942
ENSG00000070159	PTPN3	0,030237233	0,026827614	0,055623832	0,208852406	0,251508034	0	0	0	0,018593949	0,591643067	0,065738119
ENSG00000132639	SNAP25	0,09002715	0	0,082806106	0,310914477	0,106975735	0	0	0	0	0,590723468	0,065635941
ENSG00000182308	DCAF4L1	0	0	0,118038259	0,055400207	0,228737288	0	0	0,070155959	0,118373581	0,590705294	0,065633922
ENSG00000187987	ZSCAN23	0	0,081675906	0,169345171	0	0	0	0	0	0,339652493	0,59067357	0,065630397
ENSG00000082438	COBLL1	0,017592476	0	0,06472556	0,45567549	0,0418089	0	0	0	0,010818239	0,590620664	0,065624518
ENSG00000234965	SHISA8	0	0	0	0	0	0,590429099	0	0	0	0,590429099	0,065603233
ENSG00000188596	CFAP54	0,035103896	0,041527335	0,172203871	0,080822355	0,02780839	0	0,204032598	0	0,028782178	0,590280623	0,065586736
ENSG00000182752	PAPPA	0	0	0,023994829	0,427946872	0,123993972	0,014321155	0	0	0	0,590256829	0,065584092
ENSG00000196611	MMP1	0	0	0	0	0	0,589230866	0	0	0	0,589230866	0,065470096
ENSG00000179813	FAM216B	0	0,081058646	0,084032678	0,315519921	0,108560321	0	0	0	0	0,589171566	0,065463507
ENSG00000187186	AL162231.1	0	0	0	0	0	0,271539405	0,136901617	0,180269813	0	0,588710836	0,065412315
ENSG00000120820	GLT8D2	0,046197056	0	0	0,079772233	0	0,405773346	0	0	0,056816424	0,588559059	0,065395451
ENSG00000088782	DEFB127	0,587978874	0	0	0	0	0	0	0	0	0,587978874	0,065330986
ENSG00000135519	KCNH3	0,033610002	0	0	0,058037138	0	0	0	0	0,496031643	0,587678784	0,065297643
ENSG00000172247	C1QTNF4	0	0	0	0	0	0,587086259	0	0	0	0,587086259	0,065231807
ENSG00000100228	RAB36	0,036793419	0	0	0,254136815	0,174880712	0,121191071	0	0	0	0,587002017	0,065222446
ENSG00000213215	OR2F1	0	0	0,326459306	0,153220772	0,070291161	0	0	0	0,036376301	0,58634754	0,065149727
ENSG00000115252	PDE1A	0,023902336	0,042414109	0,131910835	0,247644709	0,11360884	0,026243369	0	0	0	0,585724198	0,065080466
ENSG00000104369	JPH1	0	0,058418736	0	0,454788627	0	0,07229219	0	0	0	0,585499553	0,065055506
ENSG00000141161	UNC45B	0,053217317	0,047216411	0,097897552	0,321631418	0	0	0	0	0,065450439	0,585413137	0,065045904
ENSG00000185156	MFSD6L	0,069063332	0	0	0	0	0	0,516102828	0	0	0,58516616	0,065018462
ENSG00000198967	OR10Z1	0,119492781	0,127222205	0,131889973	0,206337952	0	0	0	0	0	0,584942911	0,064993657
ENSG00000168828	OR13J1	0	0	0	0,584619062	0	0	0	0	0	0,584619062	0,064957674
ENSG00000111701	APOBEC1	0	0	0	0,583747147	0	0	0	0	0	0,583747147	0,064860794
ENSG00000230453	ANKRD18B	0	0	0	0	0,551828219	0	0	0	0,031730668	0,583558887	0,064839876
ENSG00000215174	NLRP2B	0,047310761	0,083951785	0	0,245086075	0	0	0	0,20690956	0	0,583258181	0,064806465
ENSG00000130383	FUT5	0	0	0	0	0	0,583019095	0	0	0	0,583019095	0,064779899
ENSG00000182866	LCK	0,045256236	0	0	0	0,537762118	0	0	0	0	0,583018354	0,064779817
ENSG00000133101	CCNA1	0	0,119121279	0	0,46367832	0	0	0	0	0	0,5827996	0,064755511
ENSG00000123901	GPR83	0,070661946	0	0,389965068	0,122017758	0	0	0	0	0	0,582644771	0,064738308
ENSG00000214595	EML6	0,03142463	0,167286705	0,019269382	0,108526955	0	0,115008025	0	0,114527439	0,025765496	0,581808631	0,064645403
ENSG00000188993	LRRC66	0,090838936	0	0,16710556	0	0,323821048	0	0	0	0	0,581765544	0,064640616
ENSG00000122787	AKR1D1	0,1771519	0	0	0	0,210502663	0,097251217	0	0,096844832	0	0,581750612	0,064638957
ENSG00000105198	LGALS13	0,213131976	0	0	0,368032404	0	0	0	0	0	0,58116438	0,06457382
ENSG00000161682	FAM171A2	0	0	0	0,581146937	0	0	0	0	0	0,581146937	0,064571882
ENSG00000251493	FOXD1	0	0	0	0,455702016	0,125434	0	0	0	0	0,581136017	0,064570669
ENSG00000180919	OR56B4	0,13117237	0	0	0	0	0	0	0,286835562	0,161325106	0,579333039	0,064370338
ENSG00000154493	C10orf90	0	0,05102607	0,105796422	0,148963829	0,273353029	0	0	0	0	0,579139351	0,064348817
ENSG00000196335	STK31	0,049165632	0,043621604	0	0	0	0,485828922	0	0	0	0,578616158	0,064290684
ENSG00000188558	OR2G6	0,024384472	0	0,08971441	0,04210666	0,231800899	0	0,040493894	0	0,149948784	0,578449119	0,064272124
ENSG00000170819	BFSP2	0,073000276	0	0	0,504222173	0	0	0	0	0	0,577222449	0,064135828
ENSG00000117971	CHRNB4	0,121276541	0	0,223097993	0,157063588	0	0	0,075523876	0	0	0,576961998	0,064106889
ENSG00000183090	FREM3	0	0,078073082	0,080937574	0,417860638	0	0	0	0	0	0,576871294	0,06409681
ENSG00000115844	DLX2	0	0,090253113	0,09356449	0,087827322	0,241748598	0	0	0	0,062553525	0,575947048	0,063994116
ENSG00000285010	OOSP4A	0	0	0	0	0	0,575079989	0	0	0	0,575079989	0,063897777
ENSG00000184304	PRKD1	0,549913971	0	0	0	0	0	0,024031858	0	0	0,573945829	0,063771759
ENSG00000179133	C10orf67	0,106311081	0,150917124	0,039113564	0,036715207	0,101060234	0,046689342	0	0,092988481	0	0,573795033	0,063755004
ENSG00000151388	ADAMTS12	0,01573249	0,055833837	0	0,162999464	0,112165812	0,207280262	0	0	0,019348935	0,5733608	0,063706756
ENSG00000173467	AGR3	0	0	0	0	0	0	0	0,573173578	0	0,573173578	0,063685953
ENSG00000204021	LIPK	0,125925476	0,446903333	0	0	0	0	0	0	0	0,572828809	0,063647645
ENSG00000166510	CCDC68	0,088317107	0	0,054155484	0,254173948	0,139925012	0	0	0	0,036206219	0,57277777	0,063641974
ENSG00000163254	CRYGC	0	0	0	0	0,572755251	0	0	0	0	0,572755251	0,063639472
ENSG00000213231	TCL1B	0	0	0,14961254	0,42131589	0	0	0	0	0	0,57092843	0,063436492
ENSG00000121446	RGSL1	0,0333467	0	0,122687895	0,057582473	0,356621194	0	0	0	0	0,570238262	0,063359807
ENSG00000010310	GIPR	0	0	0,054839238	0,514766176	0	0	0	0	0	0,569605414	0,06328949
ENSG00000239590	OR1J4	0	0,56930361	0	0	0	0	0	0	0	0,56930361	0,063255957
ENSG00000156218	ADAMTSL3	0,030990683	0,054992207	0,085514797	0,240813656	0	0,01701298	0	0,101651323	0,038114545	0,56909019	0,063232243
ENSG00000144410	CPO	0,124988065	0,44357651	0	0	0	0	0	0	0	0,568564575	0,063173842
ENSG00000183092	BEGAIN	0,019181337	0	0	0	0,045584862	0	0	0,125831899	0,377449144	0,568047242	0,06311636
ENSG00000150656	CNDP1	0,021232341	0	0,039058606	0,036663619	0	0,233118697	0	0,185715648	0,052226085	0,568014996	0,063112777
ENSG00000105792	CFAP69	0,033069388	0,029340409	0	0,285518081	0,039295059	0	0	0,180782672	0	0,56800561	0,063111734
ENSG00000173627	APOBEC4	0,054317243	0,096384615	0,099920956	0,187588048	0,129086104	0	0	0	0	0,567296967	0,063032996
ENSG00000036448	MYOM2	0	0	0	0	0	0,211620634	0,355641531	0	0	0,567262164	0,063029129
ENSG00000197889	MEIG1	0	0	0	0	0	0,566634483	0	0	0	0,566634483	0,062959387
ENSG00000100884	CPNE6	0,403230342	0	0	0,043518175	0,119785705	0	0	0	0	0,566534221	0,062948247
ENSG00000151952	TMEM132D	0	0	0	0,421202542	0,144922333	0	0	0	0	0,566124875	0,062902764
ENSG00000171481	OR1L3	0,270323024	0	0	0	0	0	0	0,295558647	0	0,565881672	0,062875741
ENSG00000117281	CD160	0	0	0	0,065710142	0	0	0	0,49927157	0	0,564981712	0,062775746
ENSG00000243156	MICAL3	0,020277854	0,04797674	0,124342503	0,140061715	0,064254346	0,103898196	0,011224757	0,044341729	0,008313049	0,56469089	0,062743432
ENSG00000172318	B3GALT1	0	0,263193954	0,109140204	0,051223984	0,140996287	0	0	0	0	0,564554428	0,06272827
ENSG00000066405	CLDN18	0	0,072178197	0	0,491667516	0	0	0	0	0	0,563845713	0,062649524
ENSG00000159516	SPRR2G	0	0	0	0	0	0,563363588	0	0	0	0,563363588	0,062595954
ENSG00000116833	NR5A2	0,020033219	0,035548456	0,073705448	0,10377899	0,285656271	0	0	0,043806783	0	0,562529168	0,062503241
ENSG00000088881	EBF4	0	0	0	0,561603389	0	0	0	0	0	0,561603389	0,062400377
ENSG00000183378	OVCH2	0	0,128420498	0	0	0,34398232	0	0	0	0,089006955	0,561409774	0,062378864
ENSG00000172209	GPR22	0	0	0	0,421883548	0	0,089415563	0	0	0,050079904	0,561379015	0,062375446
ENSG00000196578	OR5AC2	0	0	0	0,561150483	0	0	0	0	0	0,561150483	0,062350054
ENSG00000198780	FAM169A	0	0,039002473	0	0,417495959	0,104470558	0	0	0	0	0,56096899	0,062329888
ENSG00000127743	IL17B	0	0	0	0,425321882	0	0,135216444	0	0	0	0,560538326	0,062282036
ENSG00000180336	MEIOC	0	0,105291305	0	0	0	0,192672833	0,262276852	0	0	0,56024099	0,062248999
ENSG00000181135	ZNF707	0	0	0,04393553	0	0	0,104890518	0	0	0,411229858	0,560055906	0,062228434
ENSG00000283580	AC098484.3	0,191037384	0	0	0	0	0,209747885	0,158622417	0	0	0,559407686	0,06215641
ENSG00000070915	SLC12A3	0	0,043388673	0	0	0,058109635	0	0	0,427747013	0,030072253	0,559317574	0,062146397
ENSG00000188886	ASTL	0	0	0	0,15775996	0	0,401234768	0	0	0	0,558994729	0,062110525
ENSG00000261210	CLEC19A	0	0,078795768	0	0,153355848	0,105529692	0	0,221223036	0	0	0,558904344	0,062100483
ENSG00000152977	ZIC1	0,022140743	0	0,040729685	0,152928923	0,31570773	0	0	0	0,02723026	0,55873734	0,062081927
ENSG00000163263	C1orf189	0	0	0	0	0	0	0,558473288	0	0	0,558473288	0,062052588
ENSG00000176635	HORMAD2	0	0	0,107953436	0	0	0,32215646	0	0,128324104	0	0,558434	0,062048222
ENSG00000131050	BPIFA2	0	0,228595055	0	0,222450959	0	0	0,10696533	0	0	0,558011344	0,06200126
ENSG00000151224	MAT1A	0	0,219548581	0	0,142431755	0,196024859	0	0	0	0	0,558005195	0,062000577

ENSG00000176678	FOXL1	0	0	0	0,557851362	0	0	0	0	0	0,557851362	0,061983485
ENSG00000278558	TMEM191B	0	0,129725206	0	0	0,347477061	0,080266365	0	0	0	0,557468632	0,061940959
ENSG00000186847	KRT14	0	0,237819956	0	0	0,318507798	0	0	0	0	0,556327754	0,061814195
ENSG00000130045	NXNL2	0,423448357	0	0	0	0	0,132834729	0	0	0	0,556283086	0,061809232
ENSG00000188153	COL4A5	0	0,06779823	0,210857218	0,109959956	0,151334826	0	0	0	0,015663424	0,555613653	0,06173485
ENSG00000114656	KIAA1257	0,045684945	0	0	0,078887929	0	0,417995003	0,012644395	0	0	0,555212273	0,061690253
ENSG00000251201	TMED7-TICAM2	0	0	0	0	0,192659626	0,04450391	0,168281025	0	0,149554605	0,554999165	0,061666574
ENSG00000168124	OR1F1	0	0	0	0,554002069	0	0	0	0	0	0,554002069	0,061555785
ENSG00000129244	ATP1B2	0,305362008	0	0	0	0,103671346	0	0,14488491	0	0	0,553918264	0,061546474
ENSG00000165192	ASB11	0	0,085395541	0	0,415501552	0	0,052837763	0	0	0	0,553734857	0,061526095
ENSG00000180219	FAM71C	0,081067903	0	0	0,279973149	0,192659626	0	0	0	0	0,553700677	0,061522297
ENSG00000112378	PERP	0,071991696	0,063873749	0,06621727	0,18647092	0,085544912	0	0	0,078712379	0	0,552810928	0,061423436
ENSG00000011590	ZBTB32	0	0	0	0,190533023	0	0	0	0,361921761	0	0,552454783	0,061383865
ENSG00000149133	OR5F1	0	0	0	0,552243332	0	0	0	0	0	0,552243332	0,06136037
ENSG00000196876	SCN8A	0,010117882	0,161585403	0	0,069885497	0,264499027	0	0,033604374	0	0,012443691	0,552135875	0,061348431
ENSG00000176601	MAP3K19	0,02488236	0,088306274	0,320411772	0	0,118266933	0	0	0	0	0,551867339	0,061318593
ENSG00000143375	CGN	0,045680342	0	0	0,07887998	0,162840482	0,225694557	0,037929363	0	0	0,551024724	0,061224969
ENSG00000143842	SOX13	0,224018366	0,036137736	0,037463625	0	0	0	0	0,178131842	0,075140103	0,550891673	0,061210186
ENSG00000164287	CDC20B	0	0,119280249	0	0,174111415	0	0,036901819	0	0,220485699	0	0,550779182	0,061197687
ENSG00000180245	RRH	0,021169875	0	0,194718478	0,036555754	0,251553336	0,046486572	0	0	0	0,550484016	0,061164891
ENSG00000140543	DET1	0	0	0	0,380760214	0	0,169469435	0	0	0	0,550229649	0,061136628
ENSG00000101842	VSIG1	0	0	0,073754338	0	0,476409581	0	0	0	0	0,550163919	0,061129324
ENSG00000151715	TMEM45B	0	0	0	0,431012512	0,118638105	0	0	0	0	0,549650616	0,061072291
ENSG00000174562	KLK15	0	0	0,121441721	0,113995183	0,313776795	0	0	0	0	0,5492137	0,061023744
ENSG00000105974	CAV1	0	0	0,143807604	0,404968921	0	0	0	0	0	0,548776525	0,060975169
ENSG00000175619	OR4B1	0,162484485	0	0	0	0,386147895	0	0	0	0	0,54863238	0,060959153
ENSG00000174586	ZNF497	0	0,481651014	0	0,06695791	0	0	0	0	0	0,548608924	0,060956547
ENSG00000104723	TUSC3	0,029115717	0,103330251	0	0,351935419	0	0	0	0,063667547	0	0,548048933	0,060894326
ENSG00000265763	ZNF488	0	0	0	0,445281527	0,102138095	0	0	0	0	0,547419622	0,060824402
ENSG00000160097	FNDC5	0,047236815	0	0	0,163135339	0,336777939	0	0	0	0	0,547150093	0,060794455
ENSG00000136237	RAPGEF5	0,01474969	0,052345925	0	0,356572928	0,10515887	0	0	0	0,018140217	0,54696763	0,060774181
ENSG00000254536	AL360181.3	0	0	0	0	0,223749248	0,206742152	0	0	0,115792226	0,546283625	0,060698181
ENSG00000248144	ADH1C	0,192008349	0	0	0	0	0	0	0	0,354218276	0,546226625	0,060691847
ENSG00000042304	C2orf83	0	0	0	0	0,309051241	0,047593395	0	0,189578065	0	0,546222702	0,060691411
ENSG00000149575	SCN2B	0,025937276	0,13807518	0	0,268728089	0	0	0	0,113434455	0	0,546175001	0,060686111
ENSG00000173124	ACSM6	0	0,191052369	0,099031029	0	0,255872848	0	0	0	0	0,545956246	0,060661805
ENSG00000155530	LRGUK	0,031487929	0	0,405472118	0,108745561	0	0	0	0	0	0,545705608	0,060633956
ENSG00000277556	OR13C5	0	0	0	0,545318651	0	0	0	0	0	0,545318651	0,060590961
ENSG00000276119	OR13C2	0	0	0	0,545318651	0	0	0	0	0	0,545318651	0,060590961
ENSG00000172404	DNAJB7	0,058615427	0	0	0	0	0	0,4866964	0	0	0,545311827	0,060590203
ENSG00000162641	AKNAD1	0	0	0	0,106179033	0,438393744	0	0	0	0	0,544572778	0,060508086
ENSG00000039600	SOX30	0	0,153707079	0	0,074787898	0,102928502	0	0	0	0,21306566	0,544489139	0,060498793
ENSG00000128710	HOXD10	0	0,298820951	0	0	0	0	0	0,245493735	0	0,544314686	0,06047941
ENSG00000213265	TSGA13	0	0	0	0,462036254	0	0	0	0	0,082269377	0,544305631	0,060478403
ENSG00000147697	GSDMC	0	0,100427715	0	0,19545691	0	0,124277586	0	0,123758265	0	0,543920476	0,060435608
ENSG00000198028	ZNF560	0,106565988	0	0	0,184016202	0,253256377	0	0	0	0	0,543838567	0,060426507
ENSG00000140623	SEPT12	0	0	0	0,372764249	0,171008354	0	0	0	0	0,543772603	0,060419178
ENSG00000282936	AC004706.3	0,469950884	0	0	0,073772964	0	0	0	0	0	0,543723848	0,060413761
ENSG00000144130	NT5DC4	0	0,190396686	0	0,308798787	0	0	0	0	0,043987343	0,543182816	0,060353646
ENSG00000268182	SMIM17	0,178266501	0	0,218623751	0	0	0	0	0	0,146163211	0,543053462	0,060339274
ENSG00000125508	SRMS	0	0	0,183364181	0	0,236884922	0	0	0	0,122590054	0,542839158	0,060315462
ENSG00000104499	GML	0,160755926	0	0	0	0,382039939	0	0	0	0	0,542795865	0,060310652
ENSG00000130649	CYP2E1	0	0,250808356	0	0,216948638	0,074645093	0	0	0	0	0,542402087	0,060266899
ENSG00000218819	TDRD15	0	0	0	0	0	0,081129866	0	0,430884522	0,030292832	0,54230722	0,060256358
ENSG00000215131	C16orf90	0	0	0	0,541921027	0	0	0	0	0	0,541921027	0,060213447
ENSG00000013588	GPRC5A	0,017904096	0	0,032936031	0,278248196	0,212747359	0	0	0	0	0,541835682	0,060203965
ENSG00000150244	TRIM48	0	0	0	0,541733511	0	0	0	0	0	0,541733511	0,060192612
ENSG00000133020	MYH8	0,05002833	0	0,046015577	0	0	0	0	0,382890576	0,061528397	0,54046288	0,060051431
ENSG00000151208	DLG5	0	0	0	0	0	0,379224175	0,143394665	0	0,017699669	0,540318509	0,06003539
ENSG00000140682	TGFB1I1	0	0,049628355	0,051449213	0,241472307	0,066466323	0,061414243	0,069667036	0	0	0,540097476	0,060010831
ENSG00000257591	ZNF625	0	0	0	0,118391549	0	0	0	0	0,421611893	0,540003442	0,060000382
ENSG00000121101	TEX14	0,085132716	0	0	0,049001873	0	0,311569158	0,094250015	0	0	0,539953762	0,059994862
ENSG00000158865	SLC5A11	0	0	0,081399736	0,458450907	0	0	0	0	0	0,539850643	0,059983405
ENSG00000144278	GALNT13	0,113770946	0,080753501	0,251149013	0,039291519	0,054075823	0	0	0	0	0,539040802	0,059893422
ENSG00000240204	SMKR1	0	0	0	0,538565479	0	0	0	0	0	0,538565479	0,059840609
ENSG00000221900	POM121L12	0,117779089	0	0,216664146	0,20337878	0	0	0	0	0	0,537822014	0,059758002
ENSG00000166391	MOGAT2	0,110031969	0	0,067470898	0,063333732	0,174328904	0	0,121815856	0	0	0,536981359	0,059664595
ENSG00000176681	LRRC37A	0	0	0	0	0	0,127505823	0	0,152367615	0,257088641	0,536962079	0,059662453
ENSG00000175779	C15orf53	0,107056727	0,284954304	0	0	0,084807543	0	0,05926099	0	0	0,536079565	0,059564396
ENSG00000188730	VWC2	0	0,047366543	0	0,138280326	0	0	0	0,350222117	0	0,535868986	0,059540998
ENSG00000170775	GPR37	0	0	0	0	0	0,494271827	0,041532715	0	0	0,535804542	0,059533838
ENSG00000157796	WDR19	0,051673511	0	0,031685866	0	0	0	0	0,451979347	0	0,535338723	0,05948208
ENSG00000186439	TRDN	0,022553817	0,120063582	0,041489567	0,27261863	0,053599633	0,024762773	0	0	0	0,535088002	0,059454222
ENSG00000102539	MLNR	0	0	0	0	0	0,267813683	0	0,266694566	0	0,534508249	0,059389805
ENSG00000099953	MMP11	0	0	0	0	0	0,289997124	0,243679029	0	0	0,533676153	0,05929735
ENSG00000215277	RNF212B	0	0,260458475	0	0	0	0	0,182812528	0	0,090260574	0,533531578	0,059281286
ENSG00000215455	KRTAP10-1	0	0	0	0,429522592	0	0	0,103267763	0	0	0,532790355	0,059198928
ENSG00000187527	ATP13A5	0,048526195	0	0,044633927	0,418970736	0	0	0,020146168	0	0	0,532277026	0,059141892
ENSG00000168634	WFDC13	0	0	0	0	0,532025989	0	0	0	0	0,532025989	0,059113999
ENSG00000153347	FAM81B	0	0	0	0	0	0,531977801	0	0	0	0,531977801	0,059108645
ENSG00000148053	NTRK2	0,096470924	0,065840495	0,08190741	0,140955887	0,105814725	0,024442947	0	0,016227205	0	0,531659593	0,059073288
ENSG00000189030	VHLL	0	0	0	0	0,531238968	0	0	0	0	0,531238968	0,059026552
ENSG00000154096	THY1	0,025503894	0	0	0	0,060610556	0	0,444704974	0	0	0,530819424	0,058979936
ENSG00000144339	TMEFF2	0	0,22894638	0,094938558	0,178234272	0	0,028331724	0	0	0	0,530450935	0,058938993
ENSG00000136011	STAB2	0,016283467	0,086683836	0,029954752	0,196825951	0,038698011	0,03575659	0,05408204	0,071214346	0	0,529498993	0,058833221
ENSG00000121871	SLITRK3	0	0	0,052707641	0,445281527	0	0,031458206	0	0	0	0,529447373	0,058827486
ENSG00000146955	RAB19	0,151565266	0	0	0	0	0	0,377543627	0	0	0,529108893	0,058789877
ENSG00000011332	DPF1	0	0	0	0	0,374080773	0	0	0	0,154872102	0,528952875	0,058772542
ENSG00000167355	OR51B5	0	0,067627238	0	0,460667042	0	0	0	0	0	0,528294281	0,058699365
ENSG00000177047	IFNW1	0,156429162	0	0	0	0,37175729	0	0	0	0	0,528186452	0,058687384
ENSG00000182652	AL512310.1	0,279188121	0	0,128397274	0,120524238	0	0	0	0	0	0,528109633	0,058678848
ENSG00000160973	FOXH1	0	0	0,111058769	0,416995565	0	0	0	0	0	0,528054334	0,058672704
ENSG00000214357	NEURL1B	0	0	0	0,528044002	0	0	0	0	0	0,528044002	0,058671556
ENSG00000189099	PRSS48	0	0	0	0,527674367	0	0	0	0	0	0,527674367	0,058630485
ENSG00000156042	CFAP70	0	0,036098815	0	0	0,19338586	0	0,253373045	0,044484998	0	0,527342719	0,058593635
ENSG00000148702	HABP2	0,166513026	0	0	0,359414566	0	0	0	0	0	0,525927591	0,058436399
ENSG00000258873	DUXA	0	0,266542744	0	0,259378702	0	0	0	0	0	0,525921446	0,058435716
ENSG00000187783	TMEM72	0	0	0,182341816	0,342322039	0	0	0	0	0	0,524663856	0,058295984
ENSG00000021826	CPS1	0	0	0,051597234	0,314817139	0,099986323	0	0,058222892	0	0	0,524623587	0,05829151
ENSG00000181803	OR6S1	0	0	0	0,523965812	0	0	0	0	0	0,523965812	0,058218424
ENSG00000160339	FCN2	0	0	0,523503011	0	0	0	0	0	0	0,523503011	0,058167001
ENSG00000164325	TMEM174	0	0	0	0,148174318	0	0	0	0,375280599	0	0,523454917	0,058161657
ENSG00000166682	TMPRSS5	0,056680634	0,201156789	0	0,195750168	0	0	0	0	0,069709873	0,523297465	0,058144163
ENSG00000162817	C1orf115	0,052269308	0,278251816	0,192307229	0	0	0	0	0	0	0,522828353	0,058092039
ENSG00000128683	GAD1	0,018334211	0,032533608	0,033727263	0,158295908	0,174286601	0,020129893	0	0,040091552	0,045097433	0,522496469	0,058055163
ENSG00000067842	ATP2B3	0	0	0,036896748	0,069268642	0,047666252	0	0,01665388	0,350872915	0	0,521358437	0,057928715
ENSG00000144331	ZNF385B	0,027895619	0,148500277	0,051316245	0	0	0	0	0,121999102	0,17154008	0,521251323	0,057916814
ENSG00000189433	GJB4	0,082393986	0,083546347	0	0,345528475	0	0	0,009773355	0	0	0,521242164	0,057915796
ENSG00000151360	ALLC	0	0	0,109570398	0,411407134	0	0	0	0	0	0,520977531	0,057886392
ENSG00000078549	ADCYAP1R1	0,066354173	0,117743852	0,040687954	0,190965292	0,105128086	0	0	0	0	0,520879357	0,057875484
ENSG00000184022	OR2T10	0	0,076133447	0	0,444522955	0	0	0	0	0	0,520656402	0,057850711
ENSG00000139269	INHBE	0	0	0	0,085664798	0	0,217873377	0	0,216962946	0	0,520501122	0,057833458
ENSG00000157856	DRC1	0	0	0,091320663	0,428605412	0	0	0	0	0	0,519926075	0,057769564
ENSG00000187889	FYB2	0	0,115379518	0,05980639	0	0,231788431	0,071390093	0	0	0,039984192	0,518348623	0,057594291
ENSG00000104067	TJP1	0	0	0,518205244	0	0	0	0	0	0	0,518205244	0,05757836
ENSG00000125675	GRIA3	0,046610293	0,082708822	0,042871699	0,040242902	0,110770371	0,025587689	0,038701521	0,101923062	0,028662326	0,518078686	0,057564298
ENSG00000143768	LEFTY2	0	0	0	0,228289567	0	0	0	0,28909411	0	0,517383676	0,057487075

ENSG00000119771	KLHL29	0	0,024137366	0,100091853	0,117443053	0,258613767	0	0	0	0,016729366	0,517015405	0,057446156
ENSG00000119147	C2orf40	0	0	0	0,306082082	0,210626125	0	0	0	0	0,516708206	0,057412023
ENSG00000008735	MAPK8IP2	0	0	0,047985517	0,40538836	0,061991635	0	0	0	0	0,515365512	0,057262835
ENSG00000144847	IGSF11	0	0	0	0,372840339	0,073304254	0,067732426	0	0	0	0,513877019	0,057097447
ENSG00000258839	MC1R	0	0	0,049942526	0	0	0,029807865	0	0	0,43406482	0,513815211	0,057090579
ENSG00000099937	SERPIND1	0	0	0	0,112520472	0	0	0	0	0,400704015	0,513224487	0,057024943
ENSG00000091704	CPA1	0,053264212	0	0	0,459878348	0	0	0	0	0	0,51314256	0,05701584
ENSG00000157093	LYZL4	0	0	0	0,512642386	0	0	0	0	0	0,512642386	0,056960265
ENSG00000167858	TEKT1	0	0	0,072258929	0	0	0	0,391381332	0	0,048309468	0,511949728	0,056883303
ENSG00000105550	FGF21	0,114912981	0	0	0,396859277	0	0	0	0	0	0,511772258	0,056863584
ENSG00000157429	ZNF19	0,01638055	0	0	0,056571268	0	0	0	0,358194653	0,080583858	0,511730329	0,056858925
ENSG00000102313	ITIH6	0,027489644	0	0	0,142405844	0,130659466	0,150910112	0	0,060111801	0	0,511576866	0,056841874
ENSG00000171360	KRT38	0,064660064	0	0	0,446615275	0	0	0	0	0	0,51127534	0,056808371
ENSG00000129204	USP6	0,013408214	0,07137764	0	0,162071413	0	0	0,044532505	0,087959512	0,131922997	0,511272281	0,056808031
ENSG00000185038	MROH2A	0,079115482	0,140388482	0,097026213	0,13661517	0	0,057909451	0	0	0	0,511054797	0,056783866
ENSG00000131019	ULBP3	0	0	0,089353937	0,083874952	0,230869523	0,106660609	0	0	0	0,51075902	0,056751002
ENSG00000164867	NOS3	0,025478093	0	0	0	0	0,167840745	0,317325068	0	0	0,510643907	0,056738212
ENSG00000260220	CCDC187	0	0	0	0,044880457	0	0	0,129484347	0	0,335636134	0,510000937	0,056666771
ENSG00000176787	OR52E2	0	0	0	0	0	0,508928149	0	0	0	0,508928149	0,056547572
ENSG00000183454	GRIN2A	0,01807759	0,01603912	0,016627593	0,296552489	0,128885348	0,009924068	0	0	0,022233105	0,508339313	0,056482146
ENSG00000204052	LRRC73	0	0	0	0	0,507945604	0	0	0	0	0,507945604	0,0564384
ENSG00000134438	RAX	0	0	0	0,070049658	0	0	0,437883038	0	0	0,507932695	0,056436966
ENSG00000015413	DPEP1	0	0	0,507818961	0	0	0	0	0	0	0,507818961	0,056424329
ENSG00000141934	PLPP2	0,043435059	0	0	0,412515769	0,051612179	0	0	0	0	0,507563007	0,05639589
ENSG00000171987	C11orf40	0	0	0	0	0	0,507371794	0	0	0	0,507371794	0,056374644
ENSG00000113645	WWC1	0,119976634	0,026611949	0	0,025896683	0	0	0,14942874	0	0,184444742	0,506358748	0,056262083
ENSG00000151572	ANO4	0	0,053660596	0	0,104436652	0,215599885	0	0	0,132253179	0	0,505950312	0,056216701
ENSG00000180083	WFDC11	0	0	0	0	0	0	0	0,505251632	0	0,505251632	0,05613907
ENSG00000172497	ACOT12	0,24640941	0	0	0,106373818	0	0	0	0	0,151525905	0,504309132	0,056034348
ENSG00000145248	SLC10A4	0,059235818	0	0,108969071	0	0,140775203	0,195112399	0	0	0	0,50409249	0,056010277
ENSG00000124203	ZNF831	0,403655984	0	0	0,067454108	0	0	0	0	0,032028698	0,50313879	0,05590431
ENSG00000165633	VSTM4	0,034918676	0	0	0	0	0,019169333	0,405912108	0	0,04294547	0,502945587	0,055882843
ENSG00000142149	HUNK	0,035187	0,062438468	0,032364664	0,334181479	0	0	0	0,038471832	0	0,502643443	0,055849271
ENSG00000140557	ST8SIA2	0	0	0	0,133812807	0,368325684	0	0	0	0	0,502138492	0,055793166
ENSG00000101951	PAGE4	0	0	0	0,133812807	0,368325684	0	0	0	0	0,502138492	0,055793166
ENSG00000170935	NCBP2L	0	0	0	0,502039393	0	0	0	0	0	0,502039393	0,055782155
ENSG00000178021	TSPYL6	0,148535948	0	0,182162581	0,170992775	0	0	0	0	0	0,501691304	0,055743478
ENSG00000101746	NOL4	0,345425957	0,047149991	0	0,04588271	0,063147097	0	0	0	0	0,501605756	0,055733973
ENSG00000177301	KCNA2	0,017390019	0,015429081	0,111966206	0,165158221	0,144647149	0,028639837	0,007219652	0	0,010693741	0,501143906	0,055682656
ENSG00000170484	KRT74	0,15171744	0	0	0,349310542	0	0	0	0	0	0,501027982	0,055669776
ENSG00000103599	IQCH	0,021217435	0	0,039031185	0	0	0	0,440432224	0	0	0,500680844	0,055631205
ENSG00000127472	PLA2G5	0,045215611	0	0,249533302	0,156154982	0	0,049644098	0	0	0	0,500547993	0,055616444
ENSG00000176571	CNBD1	0,058479323	0	0	0,100981027	0	0	0,33989641	0	0	0,499356761	0,055484085
ENSG00000188761	BCL2L15	0	0,029482353	0,030564057	0,229519494	0	0,127693682	0	0	0,081735689	0,498995275	0,055443919
ENSG00000127249	ATP13A4	0	0,059666667	0	0,31934632	0,119865668	0	0	0	0	0,498878655	0,055430962
ENSG00000127325	BEST3	0,022018151	0,039070669	0,121512501	0,190102706	0,052326613	0	0,073128563	0	0	0,498159203	0,055351023
ENSG00000177425	PAWR	0,039317581	0,023256028	0	0,339464408	0,031146361	0,043168407	0,021764151	0	0	0,498116936	0,055346326
ENSG00000090402	SI	0,024614851	0	0,045281007	0,127513426	0	0	0	0,269127356	0,030273094	0,496809733	0,055201081
ENSG00000112494	UNC93A	0	0	0	0,096857823	0,399908176	0	0	0	0	0,496765999	0,055196222
ENSG00000064309	CDON	0	0,303556981	0,026224538	0	0,135516054	0	0	0,031173072	0	0,496470645	0,055163405
ENSG00000181847	TIGIT	0	0	0,410740583	0,085678862	0	0	0	0	0	0,496419445	0,055157716
ENSG00000103175	WFDC1	0,017616061	0,031259268	0	0,425867294	0	0	0	0	0,021665484	0,496408106	0,055156456
ENSG00000173769	TOPAZ1	0,056659382	0	0,052114754	0,048919193	0,067326123	0	0,023522747	0,247794951	0	0,49633715	0,055148572
ENSG00000214643	DEFB133	0,496258032	0	0	0	0	0	0	0	0	0,496258032	0,055139781
ENSG00000198125	MB	0	0	0	0	0	0	0,234963169	0	0,261020396	0,495983565	0,055109285
ENSG00000145700	ANKRD31	0,048178087	0	0,132941224	0,083193041	0	0,211586898	0,020001647	0	0	0,495900898	0,0551001
ENSG00000095917	TPSD1	0	0	0	0,494507217	0	0	0	0	0	0,494507217	0,054945246
ENSG00000092850	TEKT2	0	0	0	0,399594142	0	0	0	0	0,094868056	0,494462198	0,054940244
ENSG00000133110	POSTN	0,248901013	0	0	0,047755303	0	0,151821538	0,045926183	0	0	0,494404037	0,054933782
ENSG00000164588	HCN1	0,01102514	0,019563841	0,020281636	0,019038011	0,026201484	0,01210496	0	0,385740047	0	0,493955118	0,054883902
ENSG00000127831	VIL1	0	0	0	0	0,467060387	0	0	0	0,026856434	0,493916821	0,054879647
ENSG00000175003	SLC22A1	0	0	0	0,114144783	0	0,072576805	0	0,144547055	0,162595383	0,493864026	0,054873781
ENSG00000118997	DNAH7	0,07785921	0,138159258	0,039062265	0,061111756	0	0,06217081	0,029385529	0,015477754	0,069641303	0,492867885	0,054763098
ENSG00000100170	SLC5A1	0	0,050497552	0	0,442262669	0	0	0	0	0	0,492760221	0,054751136
ENSG00000007174	DNAH9	0,133797211	0	0,065634874	0,184830866	0,042396263	0	0,007406312	0,058515064	0	0,49258059	0,054731177
ENSG00000100665	SERPINA4	0	0	0	0,262642149	0	0	0,063145612	0,166298222	0	0,492085983	0,05467622
ENSG00000163352	LENEP	0	0	0	0	0,491941839	0	0	0	0	0,491941839	0,054660204
ENSG00000185610	DBX2	0,107705325	0	0,198132644	0,185983588	0	0	0	0	0	0,491821557	0,05464684
ENSG00000282419	TEX13D	0	0	0	0,182386981	0	0,309246182	0	0	0	0,491633163	0,054625907
ENSG00000007216	SLC13A2	0	0	0,086168661	0,242654967	0,111319759	0,051429193	0	0	0	0,49157258	0,054619176
ENSG00000048540	LMO3	0	0,026808838	0,027792451	0,156529679	0,035904573	0,01658774	0,062722621	0,165184247	0	0,49153015	0,054614461
ENSG00000256797	KLRF2	0	0	0	0	0,491268868	0	0	0	0	0,491268868	0,05458543
ENSG00000183631	PRR32	0	0	0	0,206599346	0,28433693	0	0	0	0	0,490936276	0,054548475
ENSG00000198610	AKR1C4	0,157900283	0	0,145235161	0	0,18762672	0	0	0	0	0,490762164	0,054529129
ENSG00000187258	NPSR1	0	0,100277487	0	0,390329057	0	0	0	0	0	0,490606544	0,054511838
ENSG00000184389	A3GALT2	0	0	0	0	0	0	0,490597584	0	0	0,490597584	0,054510843
ENSG00000172782	AC087651.1	0	0	0	0,364816462	0,125521686	0	0	0	0	0,490338148	0,054482016
ENSG00000183287	CCBE1	0,022376806	0	0	0	0	0	0	0,440383697	0,027520587	0,49028109	0,054475677
ENSG00000074219	TEAD2	0,050708245	0	0	0,087562072	0,241018485	0	0	0,110884083	0	0,490172885	0,054463654
ENSG00000114771	AADAC	0	0	0,170330943	0,319773253	0	0	0	0	0	0,490104196	0,054456022
ENSG00000138411	HECW2	0,063827063	0,135911468	0,035224511	0,121236947	0	0,035039193	0,021198789	0,01395711	0,062799247	0,489194327	0,054354925
ENSG00000090932	DLL3	0	0	0	0,299008756	0	0,190119072	0	0	0	0,489127828	0,054347536
ENSG00000129654	FOXJ1	0	0,103649787	0	0	0	0,384794534	0	0	0	0,488444322	0,054271591
ENSG00000099999	RNF215	0,02844702	0	0	0,122804487	0,03380248	0,031233166	0	0,062205303	0,209917006	0,488409461	0,054267718
ENSG00000187372	PCDHB13	0	0	0,164777771	0,103115975	0	0	0	0	0,220327827	0,488221573	0,054246841
ENSG00000215595	C20orf202	0	0	0	0,488032995	0	0	0	0	0	0,488032995	0,054225888
ENSG00000227471	AKR1B15	0,090188344	0,080018502	0,082954372	0,077867793	0,107167276	0,049510766	0	0	0	0,487707053	0,054189673
ENSG00000147027	TMEM47	0,074807213	0	0,412841731	0	0	0	0	0	0	0,487648944	0,054183216
ENSG00000119973	PRLHR	0	0	0	0,236955117	0,130445893	0	0	0,120027086	0	0,487428095	0,054158677
ENSG00000185250	PPIL6	0	0	0	0,116462832	0	0,111076039	0,112002082	0,147482512	0	0,487023464	0,054113718
ENSG00000205754	SLCO1B7	0	0	0	0,369858221	0	0	0	0,117092334	0	0,486950555	0,054105617
ENSG00000148408	CACNA1B	0,030515059	0,054148223	0	0,368849924	0	0	0	0,033363749	0	0,486876954	0,054097439
ENSG00000157077	ZFYVE9	0	0,092319504	0,047853348	0,314433607	0	0	0	0	0,03199286	0,486599319	0,054066591
ENSG00000264424	MYH4	0	0	0	0,216867499	0,238774962	0	0	0	0,030892042	0,486534502	0,054059389
ENSG00000258643	BCL2L2-PABPN1	0	0	0	0	0	0	0,486319116	0	0	0,486319116	0,054035457
ENSG00000168754	FAM178B	0,042771178	0	0	0,443138932	0	0	0	0	0	0,48591011	0,053990012
ENSG00000143341	HMCN1	0	0,014309301	0	0,167096414	0,038328357	0	0	0,176335219	0,089258696	0,485327987	0,053925332
ENSG00000214336	FOXI3	0,053891074	0	0	0,372232489	0	0,05916925	0	0	0	0,485292812	0,053921424
ENSG00000198929	NOS1AP	0	0	0	0,332231951	0,091448317	0,021124341	0,015975341	0	0,023662659	0,48444261	0,053826957
ENSG00000133067	LGR6	0,197831862	0,058507964	0	0,227741632	0	0	0	0	0	0,484081458	0,053786829
ENSG00000185013	NT5C1B	0	0,067254076	0	0	0	0,416128861	0	0	0	0,483382936	0,053709215
ENSG00000158639	PAGE5	0	0	0	0	0,483334512	0	0	0	0	0,483334512	0,053703835
ENSG00000244242	IFITM10	0	0,447649416	0	0	0	0,034622407	0	0	0	0,482271823	0,053585758
ENSG00000163012	ZSWIM2	0	0,223451667	0	0,108722906	0,149632309	0	0	0	0	0,481806882	0,053534098
ENSG00000121380	BCL2L14	0,206919796	0	0	0,204174472	0,07024991	0	0	0	0	0,481344178	0,053482686
ENSG00000099822	HCN2	0,044184377	0	0	0	0	0	0,146748926	0,289854884	0	0,480788187	0,05342091
ENSG00000158486	DNAH3	0,040403896	0,01792393	0,018581557	0,017442177	0,192041466	0,022180558	0,016774109	0,154615105	0	0,4799628	0,0533292
ENSG00000174611	KY	0,033576396	0	0,092649739	0,144947769	0	0	0,16727519	0	0,041294639	0,479743733	0,053304859
ENSG00000237388	OR4A47	0,142021213	0	0	0	0,337516487	0	0	0	0	0,4795377	0,053281967
ENSG00000134545	KLRC1	0,112016731	0	0	0	0	0	0	0,367421684	0	0,479438415	0,053270935
ENSG00000184434	LRRC19	0	0	0,077024134	0	0,298518323	0	0	0	0,102990591	0,478533048	0,053170339
ENSG00000037965	HOXC8	0	0	0	0	0,297159737	0	0,103823196	0	0,076891404	0,477874336	0,053097148
ENSG00000176009	ASCL3	0,477442561	0	0	0	0	0	0	0	0	0,477442561	0,053049173
ENSG00000129474	AJUBA	0,025677242	0,091127273	0	0,177355973	0,183067566	0	0	0	0	0,477228054	0,053025339
ENSG00000112761	CCN6	0	0	0,086814522	0	0,112154136	0,103629342	0	0	0,174122288	0,476720288	0,052968921
ENSG00000182916	TCEAL7	0	0	0	0	0,313913936	0	0	0	0,162453254	0,476367189	0,052929688
ENSG00000178776	C5orf46	0,248129016	0	0,228226682	0	0	0	0	0	0	0,476355698	0,052928411

ENSG00000162761	LMX1A	0,072215326	0	0	0,374100322	0	0	0,029980963	0	0	0,476296612	0,052921846
ENSG00000153266	FEZF2	0	0	0	0,475941586	0	0	0	0	0	0,475941586	0,052882398
ENSG00000124818	OPN5	0	0	0	0,051324739	0,07063681	0	0,024679452	0	0,328996597	0,475637598	0,052848622
ENSG00000159899	NPR2	0	0	0	0	0	0	0	0,427470333	0,048084482	0,475554815	0,052839424
ENSG00000021461	CYP3A43	0,057303971	0	0	0	0,136184127	0	0	0	0,281905988	0,475394085	0,052821565
ENSG00000184343	SRPK3	0	0,395656408	0	0	0	0	0,079344645	0	0	0,475001053	0,052777895
ENSG00000233041	PHGR1	0	0	0	0,277295403	0	0	0	0	0,197498961	0,474794364	0,052754929
ENSG00000214376	VSTM5	0	0	0	0,474550486	0	0	0	0	0	0,474550486	0,052727832
ENSG00000184613	NELL2	0	0,08348132	0,04327212	0,121856308	0,167707445	0	0	0	0,057860063	0,474177256	0,052686362
ENSG00000204501	PRAMEF15	0	0	0	0	0,385112646	0,088960095	0	0	0	0,474072741	0,052674749
ENSG00000091656	ZFHX4	0,161819194	0	0	0,016436849	0,067864732	0,219472259	0,007903643	0	0	0,473496677	0,052610742
ENSG00000218336	TENM3	0,075492375	0,022326561	0,04629144	0,217264758	0,059803088	0,013814369	0,010447155	0,027513286	0	0,472953033	0,052550337
ENSG00000169213	RAB3B	0,011823988	0,041962754	0,108755907	0,183757024	0,028099964	0,025964097	0,01963542	0,0517112	0	0,471710355	0,052412262
ENSG00000188959	C9orf152	0	0	0,471632336	0	0	0	0	0	0	0,471632336	0,052403593
ENSG00000108379	WNT3	0	0	0	0	0,084181327	0	0	0,387288523	0	0,47146985	0,052385539
ENSG00000204518	AADACL4	0,095943219	0	0	0,165673	0	0	0	0,209799725	0	0,471415945	0,052379549
ENSG00000254535	PABPC4L	0	0	0	0,471096243	0	0	0	0	0	0,471096243	0,052344027
ENSG00000118017	A4GNT	0	0	0,470886672	0	0	0	0	0	0	0,470886672	0,052320741
ENSG00000277745	H2AFB3	0	0	0,470355497	0	0	0	0	0	0	0,470355497	0,052261722
ENSG00000165509	MAGEC3	0	0	0	0,283317019	0	0	0	0,119592677	0,067262585	0,470172281	0,052241365
ENSG00000167011	NAT16	0	0	0	0,470153107	0	0	0	0	0	0,470153107	0,052239234
ENSG00000151012	SLC7A11	0,031334488	0	0	0,243485202	0,07446709	0,120412031	0	0	0	0,469698811	0,052188757
ENSG00000089472	HEPH	0	0,037697455	0,078161141	0	0,100974987	0	0,01763958	0	0,235149543	0,469622707	0,052180301
ENSG00000146809	ASB15	0,030763553	0,109178339	0,169776119	0,159365823	0	0	0	0	0	0,469083834	0,052120426
ENSG00000284862	CCDC39	0	0,2526781	0,130974415	0	0,067681406	0	0	0	0,017512865	0,468846787	0,052094087
ENSG00000164113	ADAD1	0	0	0	0,19335678	0	0	0	0	0,275430192	0,468786972	0,052087441
ENSG00000159248	GJD2	0	0,091766598	0,190267008	0	0,122901281	0	0	0	0,063602506	0,468537393	0,05205971
ENSG00000040731	CDH10	0	0	0,075950847	0	0,392478188	0	0	0	0	0,468429035	0,052047671
ENSG00000162814	SPATA17	0,023194255	0	0	0,120154248	0	0,305591239	0,019258685	0	0	0,468198426	0,052022047
ENSG00000181240	SLC25A41	0,091637702	0	0	0,158238311	0,217778983	0	0	0	0	0,467654996	0,051961666
ENSG00000176402	GJC3	0	0	0	0,467625403	0	0	0	0	0	0,467625403	0,051958378
ENSG00000173531	MST1	0	0	0	0,102649479	0,07063681	0,293704797	0	0	0	0,466991086	0,051887898
ENSG00000235118	FAM237A	0,46687097	0	0	0	0	0	0	0	0	0,46687097	0,051874552
ENSG00000184564	SLITRK6	0	0	0	0,466580196	0	0	0	0	0	0,466580196	0,051842244
ENSG00000182393	IFNL1	0	0	0	0	0,463377474	0	0	0	0	0,463377474	0,051486386
ENSG00000196972	SMIM10L2B	0	0	0	0,46314337	0	0	0	0	0	0,46314337	0,051460374
ENSG00000131183	SLC34A1	0	0	0	0	0,317171598	0	0	0,145919438	0	0,463091037	0,05145456
ENSG00000187045	TMPRSS6	0	0,126721172	0	0,061657603	0	0,274426757	0	0	0	0,462805532	0,051422837
ENSG00000144045	DQX1	0	0	0	0	0	0	0,347924664	0,114535379	0	0,462460043	0,051384449
ENSG00000146477	SLC22A3	0	0	0	0	0	0,461717746	0	0	0	0,461717746	0,051301972
ENSG00000133454	MYO18B	0,031137558	0	0	0,430142138	0	0	0	0	0	0,461279697	0,0512533
ENSG00000119125	GDA	0,03823648	0,135699393	0,175847735	0,066026056	0,045434912	0	0	0	0	0,461244576	0,051249397
ENSG00000002746	HECW1	0,032531877	0,057727018	0,019948339	0,131076055	0,077312711	0	0	0,142275379	0	0,460871379	0,051207931
ENSG00000175984	DENND2C	0,06115361	0,108515581	0,037499002	0,035199646	0,145332878	0,02238103	0,050777148	0	0	0,460858895	0,051206544
ENSG00000186160	CYP4Z1	0,134201217	0	0	0	0,159466049	0	0,167145202	0	0	0,460812469	0,051201385
ENSG00000114378	HYAL1	0	0,173835981	0	0	0,232815262	0,053779766	0	0	0	0,460431009	0,051159001
ENSG00000279395	OR5L1	0,136135649	0	0	0	0,323529317	0	0	0	0	0,459664967	0,051073885
ENSG00000177108	ZDHHC22	0,039127543	0	0,14395655	0,135129453	0,092987453	0	0	0	0,048121834	0,459322833	0,05103587
ENSG00000141431	ASXL3	0,011081737	0,039328542	0	0,382714835	0	0,0121671	0	0	0,013629108	0,458921323	0,050991258
ENSG00000131203	IDO1	0,043322985	0	0	0,224428017	0	0,190264423	0	0	0	0,458015425	0,050890603
ENSG00000039537	C6	0	0,169745938	0	0,110122378	0,151558364	0	0,02647612	0	0	0,457902799	0,050878089
ENSG00000077279	DCX	0,029620812	0	0,136224688	0,127871692	0,0703945	0,01626096	0,012297396	0,064772041	0	0,457442089	0,050826899
ENSG00000181072	CHRM2	0,018157963	0,03222086	0,30062736	0,062709679	0,043152793	0	0	0	0	0,456868656	0,050763184
ENSG00000125731	SH2D3A	0	0	0	0,046670537	0,321156808	0,02967458	0	0,059101157	0	0,456603082	0,050733676
ENSG00000187223	LCE2D	0	0	0,456453365	0	0	0	0	0	0	0,456453365	0,050717041
ENSG00000283516	LITAFD	0,09287681	0	0	0,160377981	0	0	0	0,203094387	0	0,456349178	0,050705464
ENSG00000182938	OTOP3	0,060059845	0	0,110484936	0	0,285467045	0	0	0	0	0,456011826	0,050667981
ENSG00000188613	NANOS1	0	0,066751805	0,138401842	0,129915347	0,089399438	0	0,031234835	0	0	0,455703267	0,050633696
ENSG00000180432	CYP8B1	0	0,060378744	0	0,058755905	0	0,336229496	0	0	0	0,455364144	0,050596016
ENSG00000139223	ANP32D	0	0	0	0	0	0	0,261396525	0	0,193590127	0,454986652	0,050554072
ENSG00000178395	CCDC185	0,073568924	0	0	0,381112426	0	0	0	0	0	0,454681351	0,05052015
ENSG00000168509	HJV	0	0,110894127	0	0	0	0,34307398	0	0	0	0,453968107	0,050440901
ENSG00000159184	HOXB13	0	0	0	0	0,310744917	0,047854219	0	0,095308499	0	0,453907635	0,050434182
ENSG00000120049	KCNIP2	0	0	0	0,307253429	0	0,146520969	0	0	0	0,453774398	0,050419378
ENSG00000236334	PPIAL4G	0	0	0,360451373	0	0,093132143	0	0	0	0	0,453583516	0,050398168
ENSG00000275023	MLLT6	0,124837873	0	0	0	0,161825404	0,049841705	0,047116159	0	0,069788405	0,453409546	0,050378838
ENSG00000269720	CCDC194	0	0	0	0	0	0,453307587	0	0	0	0,453307587	0,05036751
ENSG00000163293	NIPAL1	0,025256656	0,224086579	0	0	0,120045978	0	0,083884561	0	0	0,453273773	0,050363753
ENSG00000118113	MMP8	0	0	0,161757404	0	0,104485756	0	0,03650577	0,096140404	0,054072308	0,452961642	0,050329071
ENSG00000132688	NES	0	0,385261494	0	0	0	0	0,06760255	0	0	0,452864044	0,050318227
ENSG00000109063	MYH3	0,022607805	0,080233992	0	0	0	0	0,300347892	0,04943665	0	0,452626339	0,050291815
ENSG00000111732	AICDA	0,048635523	0,172605085	0	0	0,231166748	0	0	0	0	0,452407356	0,050267484
ENSG00000088836	SLC4A11	0	0	0	0,300201305	0	0	0	0,152063768	0	0,452265073	0,050251675
ENSG00000077935	SMC1B	0,035530348	0	0,065360945	0	0	0,351092215	0	0	0	0,451983508	0,05022039
ENSG00000205420	KRT6A	0	0,091641148	0,190006903	0	0	0,170106538	0	0	0	0,451754589	0,050194954
ENSG00000259060	AL163195.3	0	0	0,450717631	0	0	0	0	0	0	0,450717631	0,050079737
ENSG00000182379	NXPH4	0	0	0	0,363926045	0	0	0	0	0,086400057	0,450326102	0,050036234
ENSG00000118526	TCF21	0	0	0	0,057335745	0,315638358	0,036455851	0	0	0,040836414	0,450266368	0,050029596
ENSG00000180777	ANKRD30B	0,025260878	0	0	0,17448009	0	0,249614709	0	0	0	0,449355677	0,049928409
ENSG00000106336	FBXO24	0	0,241641935	0	0	0,107875501	0,099675925	0	0	0	0,44919336	0,049910373
ENSG00000284373	TAF11L2	0	0,449149079	0	0	0	0	0	0	0	0,449149079	0,049905453
ENSG00000146374	RSPO3	0	0	0	0	0,144893098	0	0	0,266640765	0,037491733	0,449025596	0,049891733
ENSG00000196460	RFX8	0,092563902	0	0	0,159837657	0	0	0,038428892	0,101205074	0,056920834	0,448956359	0,04988404
ENSG00000132874	SLC14A2	0,055873755	0,148719911	0,051392143	0,19296356	0	0	0	0	0	0,448949369	0,049883263
ENSG00000188290	HES4	0	0	0	0	0	0	0,448909953	0	0	0,448909953	0,049878884
ENSG00000141433	ADCYAP1	0,03897616	0,207486717	0	0,201909962	0	0	0	0	0	0,448372838	0,049819204
ENSG00000165091	TMC1	0,052071182	0	0,031929715	0,299718556	0	0	0,043235814	0	0,021346947	0,448302214	0,049811357
ENSG00000055813	CCDC85A	0	0	0,0675693	0,380556599	0	0	0	0	0	0,448125899	0,049791767
ENSG00000086205	FOLH1	0	0	0	0,048910023	0,336567519	0,06219703	0	0	0	0,447674572	0,049741619
ENSG00000165973	NELL1	0	0,050507063	0,052360162	0,344046868	0	0	0	0	0	0,446914093	0,049657121
ENSG00000130561	SAG	0,076286363	0	0,070167463	0,131729899	0,120864129	0	0,031671098	0	0,015637065	0,446356017	0,049595113
ENSG00000226792	C13orf42	0,083555748	0,074133813	0	0,288565081	0	0	0	0	0	0,446254642	0,049583849
ENSG00000284546	SSU72P3	0	0	0	0,446042692	0	0	0	0	0	0,446042692	0,049560299
ENSG00000184672	RALYL	0	0	0	0,287499972	0,158271284	0	0	0	0	0,445771256	0,04953014
ENSG00000176399	DMRTA1	0	0,048002506	0	0,140136936	0,257155419	0	0	0	0	0,445294861	0,049477207
ENSG00000156687	UNC5D	0,027988622	0	0	0,217486087	0,199546699	0	0	0	0	0,445021408	0,049446823
ENSG00000127318	IL22	0	0	0	0,444901917	0	0	0	0	0	0,444901917	0,049433546
ENSG00000141738	GRB7	0	0	0	0,145084779	0,299514214	0	0	0	0	0,444598993	0,049399888
ENSG00000163746	PLSCR2	0	0	0	0,306141933	0	0,064884856	0	0	0,072681471	0,443708261	0,049300918
ENSG00000166569	CPLX4	0	0	0	0	0	0,234006455	0	0	0,209699884	0,443706339	0,049300704
ENSG00000080166	DCT	0,120247138	0	0,073734774	0	0	0	0,249609414	0	0	0,443591327	0,049287925
ENSG00000103723	AP3B2	0,082954859	0,088320817	0	0,114595948	0,157715214	0	0	0	0	0,443586838	0,049287426
ENSG00000130035	GALNT8	0,07828752	0	0,028803243	0,297408012	0	0	0,039002279	0	0	0,443501054	0,049277895
ENSG00000174343	CHRNA9	0	0	0	0,329879867	0,11350112	0	0	0	0	0,443380986	0,049264554
ENSG00000166111	SVOP	0,041570996	0,073766713	0	0,25124413	0	0	0	0	0,076690449	0,443272288	0,049252476
ENSG00000131480	AOC2	0	0,20033022	0,103840157	0	0	0	0	0	0,13884686	0,443017237	0,049224137
ENSG00000180758	GPR157	0,028489927	0	0	0,344371196	0	0	0	0	0,070077874	0,442938996	0,049215444
ENSG00000070729	CNGB1	0,062117253	0,036741847	0	0,214525877	0,049207665	0,045467409	0,034384854	0	0	0,442444905	0,049160545
ENSG00000088387	DOCK9	0,04079659	0,090490686	0,281432336	0	0	0,011198068	0,01693714	0	0	0,44085482	0,048983869
ENSG00000136267	DGKB	0	0	0	0,082478656	0,340539235	0,017480832	0	0	0	0,440498723	0,048944303
ENSG00000204952	FBXO47	0	0,116482189	0	0,113351423	0,156002408	0	0,054504923	0	0	0,440340942	0,048926771
ENSG00000081277	PKP1	0,072988522	0	0,134268282	0	0	0	0,020201309	0,212806033	0	0,440264146	0,048918238
ENSG00000139767	SRRM4	0,086511327	0	0,045469878	0,128045297	0,029370863	0,108553579	0	0,027024991	0,015199683	0,440175618	0,048908402
ENSG00000182111	ZNF716	0,0872295	0	0	0,150626308	0,138201864	0	0	0,063581791	0	0,439639463	0,048848829
ENSG00000203740	METTL11B	0	0,222709302	0	0,216723401	0	0	0	0	0	0,439432703	0,048825856
ENSG00000170608	FOXA3	0,071077409	0	0	0,368205514	0	0	0	0	0	0,439282923	0,048809214
ENSG00000164946	FREM1	0,070205617	0	0,025829781	0,145475734	0,166845169	0,030832666	0	0	0	0,439188967	0,048798774

ENSG00000205858	LRRC72	0	0	0,200562842	0	0	0	0	0,238408779	0	0,438971621	0,048774625
ENSG00000189377	CXCL17	0	0	0	0,438915012	0	0	0	0	0	0,438915012	0,048768335
ENSG00000182533	CAV3	0,154509786	0	0,28423323	0	0	0	0	0	0	0,438743016	0,048749224
ENSG00000147642	SYBU	0,021498161	0	0	0,296981049	0,102181688	0	0,017850382	0	0	0,43851128	0,048723476
ENSG00000198822	GRM3	0,033775273	0,059933393	0	0,116645049	0,080267667	0	0	0,147713262	0	0,438334644	0,048703849
ENSG00000197385	ZNF860	0	0	0	0,399839066	0	0	0,038452446	0	0	0,438291512	0,048699057
ENSG00000164729	SLC35G3	0	0	0	0	0	0	0,438269623	0	0	0,438269623	0,048696625
ENSG00000165553	NGB	0	0,142325902	0	0	0	0	0	0	0,295933953	0,438259855	0,048695539
ENSG00000169340	PDILT	0	0	0	0	0	0,437374104	0	0	0	0,437374104	0,048597123
ENSG00000179695	OR6C2	0,143097131	0	0	0,123548757	0,170036715	0	0	0	0	0,436682603	0,048520289
ENSG00000188152	NUTM2G	0	0	0	0	0	0,042475826	0,224857226	0,169193327	0	0,436526379	0,048502931
ENSG00000169203	NPIPB12	0,244233201	0	0	0	0	0	0,115881166	0,076295213	0	0,43640958	0,048489953
ENSG00000057468	MSH4	0,046211184	0,082000612	0	0	0	0	0,306961057	0	0	0,435172852	0,048352539
ENSG00000100867	DHRS2	0,079995008	0,354872949	0	0	0	0	0	0	0	0,434867957	0,048318662
ENSG00000164458	TBXT	0	0	0	0,297757674	0,136598533	0	0	0	0	0,434356206	0,048261801
ENSG00000234438	KBTBD13	0	0	0,266946318	0,16705184	0	0	0	0	0	0,433998158	0,048222018
ENSG00000162337	LRP5	0,048887276	0	0	0,084417656	0	0	0	0	0,300625238	0,43393017	0,048214463
ENSG00000172367	PDZD3	0	0	0,072636556	0	0	0	0,360641142	0	0	0,433277698	0,048141966
ENSG00000110975	SYT10	0,148672344	0	0	0,154034814	0	0,032646709	0,024689164	0	0,073139127	0,433182159	0,048131351
ENSG00000165066	NKX6-3	0	0	0	0,432727984	0	0	0	0	0	0,432727984	0,048080887
ENSG00000204979	MS4A13	0	0	0	0	0	0	0	0	0,432536514	0,432536514	0,048059613
ENSG00000106686	SPATA6L	0,020528538	0	0	0,07089661	0	0,315547898	0	0	0,025247456	0,432220502	0,0480245
ENSG00000198443	KRTAP4-1	0	0,432138598	0	0	0	0	0	0	0	0,432138598	0,0480154
ENSG00000258465	AL139011.2	0	0	0	0	0	0	0,431034869	0	0	0,431034869	0,047892763
ENSG00000062096	ARSF	0	0	0	0,118391549	0	0	0,227713851	0	0,084322379	0,430427779	0,047825309
ENSG00000104537	ANXA13	0	0	0,146266824	0	0,28343926	0	0	0	0	0,429706084	0,04774512
ENSG00000144820	ADGRG7	0	0	0,06814908	0,191910989	0,088040584	0	0	0,08100872	0	0,429109373	0,047678819
ENSG00000134138	MEIS2	0	0,123944717	0,102793787	0,0964907	0	0	0,02319873	0,030547709	0,051542902	0,428518544	0,047613172
ENSG00000149328	GLB1L2	0,083027786	0	0	0	0,345305329	0	0	0	0	0,428333115	0,047592568
ENSG00000189182	KRT77	0	0,271398786	0	0,066026056	0,090869823	0	0	0	0	0,428294665	0,047588296
ENSG00000004939	SLC4A1	0	0,05232039	0	0,305484848	0,070071716	0	0	0	0	0,427876954	0,047541884
ENSG00000172023	REG1B	0	0	0,349807172	0	0	0	0	0	0,077955756	0,427762928	0,047529214
ENSG00000099957	P2RX6	0	0	0	0,275829783	0,151846741	0	0	0	0	0,427676524	0,047519614
ENSG00000078898	BPIFB2	0,253541226	0	0	0	0	0	0,070173564	0	0,103941008	0,427655798	0,047517311
ENSG00000163053	SLC16A14	0	0,054367802	0,169087651	0,105813047	0,072813776	0	0,025440051	0	0	0,427522328	0,047502481
ENSG00000102891	MT4	0	0	0	0	0	0	0,426769837	0	0	0,426769837	0,047418871
ENSG00000167822	OR8J3	0	0,070452444	0,073037336	0	0,283066901	0	0	0	0	0,426556681	0,047395187
ENSG00000278870	OR51G1	0,156429162	0	0	0,270119021	0	0	0	0	0	0,426548184	0,047394243
ENSG00000230873	STMND1	0,052432537	0	0,096453886	0,090539547	0	0,057567861	0	0	0,128970522	0,425964353	0,047329373
ENSG00000151067	CACNA1C	0,031641223	0,014036643	0	0,204890573	0,056397039	0,02605516	0,065680957	0,017297522	0,009728656	0,425727774	0,047303086
ENSG00000197416	FABP12	0	0	0	0,4256688	0	0	0	0	0	0,4256688	0,047296533
ENSG00000205838	TTC23L	0	0	0	0,179049617	0,246421003	0	0	0	0	0,42547062	0,047274513
ENSG00000250374	TRIM75P	0	0	0	0,425437459	0	0	0	0	0	0,425437459	0,047270829
ENSG00000154027	AK5	0	0,077185377	0,0800173	0	0,155059388	0	0,018058482	0,095116456	0	0,425437002	0,047270778
ENSG00000166923	GREM1	0	0	0,018801495	0,035297257	0,291471796	0,022443095	0	0,044698623	0,012569937	0,425282203	0,047253578
ENSG00000185985	SLITRK2	0,063593372	0	0,116985144	0,131774249	0,06045241	0,013964361	0,010560587	0,027812016	0	0,425142139	0,047238015
ENSG00000233412	OR5H15	0	0	0	0,424629739	0	0	0	0	0	0,424629739	0,047181082
ENSG00000118520	ARG1	0	0	0,424397098	0	0	0	0	0	0	0,424397098	0,047155233
ENSG00000168032	ENTPD3	0,041812554	0	0	0	0	0	0,381896417	0	0	0,423708971	0,047078775
ENSG00000132518	GUCY2D	0	0,066371782	0	0	0,088890481	0	0,186342077	0,081790735	0	0,423395075	0,047043897
ENSG00000131016	AKAP12	0,017220578	0,061114986	0,063357288	0,178417077	0,040925076	0,018907188	0,04289584	0	0	0,422838032	0,046982004
ENSG00000214872	SMTNL1	0,091637702	0,162608854	0,168574954	0	0	0	0	0	0	0,42282151	0,046980168
ENSG00000126337	KRT36	0,081024435	0	0,149050991	0	0,192556323	0	0	0	0	0,422631749	0,046959083
ENSG00000169903	TM4SF4	0	0	0	0	0,335623871	0	0	0	0,086844169	0,422468041	0,046940893
ENSG00000274443	C8orf89	0	0	0,422462157	0	0	0	0	0	0	0,422462157	0,04694024
ENSG00000165323	FAT3	0,007837685	0,05563112	0,014418055	0,054135887	0,018626429	0,00860532	0,006507797	0,188525946	0,067475397	0,421763637	0,046862626
ENSG00000258881	AC007040.2	0	0,42170164	0	0	0	0	0	0	0	0,42170164	0,046855738
ENSG00000178690	DYNAP	0,141423089	0,125475901	0	0	0	0	0	0,154625441	0	0,421524431	0,046836048
ENSG00000179564	LSMEM2	0,421507868	0	0	0	0	0	0	0	0	0,421507868	0,046834208
ENSG00000105255	FSD1	0	0,086136203	0	0,335284259	0	0	0	0	0	0,421420462	0,046824496
ENSG00000165188	RNF183	0	0	0	0,214702393	0	0	0,206478879	0	0	0,421181271	0,046797919
ENSG00000083857	FAT1	0,028040559	0,016585761	0,051582872	0,258239599	0,066638995	0	0	0	0	0,421087786	0,046787532
ENSG00000126550	HTN1	0,079177663	0	0	0,341806359	0	0	0	0	0	0,420984023	0,046776003
ENSG00000166402	TUB	0	0	0	0	0	0	0	0,393257444	0,027647504	0,420904948	0,046767216
ENSG00000173080	RXFP4	0	0	0	0,420862862	0	0	0	0	0	0,420862862	0,04676254
ENSG00000203883	SOX18	0	0	0	0,420410808	0	0	0	0	0	0,420410808	0,046712312
ENSG00000086696	HSD17B2	0	0	0,058344023	0,191682739	0,150747211	0	0	0	0,019503256	0,420277229	0,04669747
ENSG00000115850	LCT	0,022965132	0	0	0,237934627	0,109154268	0	0	0,050218019	0	0,420272047	0,046696894
ENSG00000185873	TMPRSS11B	0,044733739	0	0	0,077245404	0	0	0,297147027	0	0	0,41912617	0,046569574
ENSG00000166091	CMTM5	0,051928031	0,092145017	0,095525807	0,179336752	0	0	0	0	0	0,418935608	0,046548401
ENSG00000129151	BBOX1	0,061327342	0	0	0,211797869	0,145745756	0	0	0	0	0,418870967	0,046541219
ENSG00000084628	NKAIN1	0	0	0,418644727	0	0	0	0	0	0	0,418644727	0,046516081
ENSG00000133937	GSC	0	0,212137658	0	0,206435898	0	0	0	0	0	0,418573556	0,046508173
ENSG00000064195	DLX3	0,111315338	0	0,30716033	0	0	0	0	0	0	0,418475668	0,046497296
ENSG00000104313	EYA1	0,016210102	0,115057713	0	0,055982616	0,23114195	0	0	0	0	0,418392381	0,046488042
ENSG00000187510	C12orf74	0,046588738	0	0	0,241345745	0,110719144	0	0,019341812	0	0	0,417995438	0,046443938
ENSG00000171714	ANO5	0,020022601	0,071059229	0	0,034574662	0,047584145	0,21983646	0	0	0,024625218	0,417702316	0,046411368
ENSG00000281593	AC006978.2	0	0	0,417387536	0	0	0	0	0	0	0,417387536	0,046376393
ENSG00000244122	UGT1A7	0,072527272	0	0	0	0,344725263	0	0	0	0	0,417252534	0,046361393
ENSG00000241484	ARHGAP8	0	0,053735872	0	0,156874734	0,143934887	0	0,025144355	0	0,037243792	0,41693364	0,04632596
ENSG00000164089	ETNPPL	0,051363212	0,18228552	0,094486777	0,088693057	0	0	0	0	0	0,416828567	0,046314285
ENSG00000122180	MYOG	0	0,177930989	0	0	0,23829963	0	0	0	0	0,416230619	0,046247847
ENSG00000139874	SSTR1	0	0	0	0,416069436	0	0	0	0	0	0,416069436	0,046229937
ENSG00000145107	TM4SF19	0	0,141201685	0	0,274813033	0	0	0	0	0	0,416014718	0,046223858
ENSG00000179709	NLRP8	0	0	0	0,331640791	0	0	0	0,083994552	0	0,415635343	0,046181705
ENSG00000172150	OR1A2	0	0	0	0,280575242	0	0	0,134914335	0	0	0,415489577	0,046165509
ENSG00000055732	MCOLN3	0	0	0,083931189	0	0,054214605	0	0,227301326	0,049884446	0	0,415331566	0,046147952
ENSG00000248746	ACTN3	0,131020726	0,154995376	0	0,075414733	0	0	0	0	0,053712868	0,415143702	0,046127078
ENSG00000283205	AL353572.3	0	0	0,41489567	0	0	0	0	0	0	0,41489567	0,046099519
ENSG00000134548	SPX	0,041731723	0	0,191922189	0,180153945	0	0	0	0	0	0,413807858	0,045978651
ENSG00000174951	FUT1	0	0,123482385	0,06400647	0	0,082688819	0,11460551	0,028890245	0	0	0,413673429	0,045963714
ENSG00000050030	NEXMIF	0,063657667	0,045183588	0,046841368	0,02198458	0,030256765	0,02795696	0,021142528	0	0,156581449	0,413604904	0,0459561
ENSG00000009765	IYD	0,072915736	0,025877437	0,053653754	0	0,242600154	0	0	0	0,017935391	0,412982472	0,045886941
ENSG00000122691	TWIST1	0	0	0	0,130663483	0	0	0,18848823	0	0,093062855	0,412214568	0,045801619
ENSG00000139899	CBLN3	0	0	0	0	0	0	0,048973588	0	0,362698131	0,411671719	0,045741302
ENSG00000260734	TLE7	0	0	0	0	0	0	0,165746806	0	0,245503992	0,411250798	0,045694533
ENSG00000151834	GABRA2	0	0,022476278	0,046601861	0,328082533	0	0,013907006	0	0	0	0,411067678	0,045674186
ENSG00000163873	GRIK3	0	0,025544632	0,026481861	0,099432209	0,171057227	0	0	0	0,088523636	0,411039566	0,045671063
ENSG00000143839	REN	0	0	0	0	0,410654708	0	0	0	0	0,410654708	0,045628301
ENSG00000178473	UCN3	0,121569244	0	0	0	0,288911941	0	0	0	0	0,410481185	0,045609021
ENSG00000212938	KRTAP6-3	0	0	0	0,410275117	0	0	0	0	0	0,410275117	0,045586124
ENSG00000077080	ACTL6B	0	0	0	0,109223514	0,300642564	0	0	0	0	0,409866078	0,045540675
ENSG00000115290	GRB14	0,051085386	0	0,093975693	0,264639934	0	0	0	0	0	0,409701012	0,045522335
ENSG00000187533	PRR27	0	0,050251499	0,052095221	0,048900857	0,134601777	0	0	0,123851037	0	0,409700392	0,045522266
ENSG00000176566	DCAF4L2	0	0	0	0,079821038	0,32956642	0	0	0	0	0,409387458	0,045487495
ENSG00000139155	SLCO1C1	0,03109271	0,275866255	0	0	0	0,102413936	0	0	0	0,409372901	0,045485878
ENSG00000140835	CHST4	0,113233848	0,100465343	0	0,195530142	0	0	0	0	0	0,409229333	0,045469926
ENSG00000188234	AGAP4	0	0	0	0,300443264	0	0	0,108350891	0	0	0,408794155	0,045421573
ENSG00000108381	ASPA	0	0,045875449	0	0,1785697	0,184320381	0	0	0	0	0,40876553	0,045418392
ENSG00000137709	POU2F3	0,090215266	0	0	0,194727593	0	0,024762773	0	0,098637184	0	0,408342816	0,045371424
ENSG00000187268	FAM9C	0	0	0	0,17814301	0,122586633	0	0,107074869	0	0	0,407804513	0,045311613
ENSG00000273111	LYPD4	0	0	0	0,241048475	0,165874154	0	0	0	0	0,406922629	0,045213625
ENSG00000003249	DBNDD1	0	0,055967856	0,058021311	0	0,149913397	0	0,026188756	0	0,116372274	0,406463595	0,045162622
ENSG00000239605	STPG4	0	0	0	0,081465805	0	0,207193977	0,117518263	0	0	0,406178045	0,045130894
ENSG00000144891	AGTR1	0	0	0,079925273	0,225073296	0	0,047702869	0	0	0,053434883	0,40613632	0,045126258
ENSG00000113100	CDH9	0	0	0,066091322	0,310193741	0	0	0,029831272	0	0	0,406116335	0,045124037
ENSG00000261732	AL031708.1	0	0	0	0	0	0,311510658	0,094232318	0	0	0,405742977	0,045082553

ENSG00000169242	EFNA1	0	0	0	0	0	0	0,405677748	0	0	0,405677748	0,045075305
ENSG00000196620	UGT2B15	0,283243806	0	0	0,122275058	0	0	0	0	0	0,405518865	0,045057652
ENSG00000185982	DEFB128	0,405122173	0	0	0	0	0	0	0	0	0,405122173	0,045013575
ENSG00000164879	CA3	0,132611295	0	0	0,114495382	0,157576807	0	0	0	0	0,404683483	0,044964831
ENSG00000167759	KLK13	0	0	0	0,403455701	0	0	0	0	0	0,403455701	0,044828411
ENSG00000012504	NR1H4	0,041651205	0,073909041	0	0,287690159	0	0	0	0	0	0,403250405	0,044805601
ENSG00000186297	GABRA5	0	0	0,231200526	0,072341274	0,099561281	0	0	0	0	0,403103081	0,044789231
ENSG00000167524	RSKR	0	0	0	0	0,154028541	0,249061556	0	0	0	0,403090097	0,044787789
ENSG00000204595	DPRX	0	0	0	0,40267743	0	0	0	0	0	0,40267743	0,044741937
ENSG00000179270	PCARE	0,040124952	0,178001859	0,184532726	0	0	0	0	0	0	0,402659538	0,044739949
ENSG00000214944	ARHGEF28	0,019352061	0,017169879	0,01779984	0,100250358	0,18396237	0,031871149	0,008034215	0	0,023800541	0,402240413	0,044693379
ENSG00000112116	IL17F	0,141621903	0	0,260524929	0	0	0	0	0	0	0,402146832	0,044682981
ENSG00000148386	LCN9	0	0	0,2515657	0	0	0,150145318	0	0	0	0,401711019	0,044634558
ENSG00000159261	CLDN14	0	0	0,096353587	0	0	0	0,304434081	0	0	0,400787668	0,044531963
ENSG00000285602	AC097625.2	0	0	0,151653082	0	0	0	0,068450809	0,180269813	0	0,400373703	0,044485967
ENSG00000170075	GPR37L1	0,04628904	0	0,085152427	0,159862138	0,055003453	0,02541133	0	0	0,028464776	0,400183164	0,044464796
ENSG00000181577	C6orf223	0	0	0	0	0,257847814	0,119124449	0,022522048	0	0	0,399494311	0,044388257
ENSG00000143061	IGSF3	0	0	0,075129756	0	0	0,022420348	0,033910901	0,26791992	0	0,399380925	0,044375658
ENSG00000002745	WNT16	0	0	0	0	0	0	0	0	0,398567909	0,398567909	0,044285323
ENSG00000227059	ANHX	0	0	0	0,302358024	0	0	0	0,095722644	0	0,398080668	0,044231185
ENSG00000188324	OR6C6	0,099349488	0,176293228	0	0	0	0	0	0	0,122187063	0,397829778	0,044203309
ENSG00000189023	MAGEB16	0	0,135016113	0	0,262774395	0	0	0	0	0	0,397790508	0,044198945
ENSG00000181961	OR4A16	0,139787762	0	0,257150878	0	0	0	0	0	0	0,39693864	0,044104293
ENSG00000182613	OR2V2	0	0	0	0,327739135	0	0	0	0,069171984	0	0,396911119	0,044101235
ENSG00000197977	ELOVL2	0	0	0,069704137	0,327150169	0	0	0	0	0	0,396854306	0,044094923
ENSG00000101276	SLC52A3	0	0	0,294470444	0,069103542	0	0	0,033228372	0	0	0,396802358	0,044089151
ENSG00000112706	IMPG1	0,158980085	0	0	0	0,188909807	0	0	0	0,048881253	0,396771144	0,044085683
ENSG00000108018	SORCS1	0,04549696	0,10764432	0,027898444	0	0,144166015	0,033302003	0,037777097	0	0	0,396284838	0,044031649
ENSG00000135773	CAPN9	0,116552696	0	0,107204049	0,100630534	0	0	0	0	0,071672396	0,396059675	0,044006631
ENSG00000240403	KIR3DL2	0	0	0	0	0	0	0	0	0,396050127	0,396050127	0,04400557
ENSG00000129221	AIPL1	0	0	0,069756612	0,065479291	0	0,083267542	0,094456963	0,08291959	0	0,395879998	0,043986666
ENSG00000170289	CNGB3	0	0	0	0,051927358	0,071466178	0,198102181	0	0	0,073968765	0,395464482	0,043940498
ENSG00000163431	LMOD1	0	0	0	0,263105596	0,090526227	0,041822681	0	0	0	0,395454504	0,043939389
ENSG00000183715	OPCML	0,019927545	0	0	0,240873641	0,047358241	0	0	0,087151409	0	0,395310836	0,043923426
ENSG00000179399	GPC5	0,085615054	0,151921813	0,157495807	0	0	0	0	0	0	0,395032674	0,043892519
ENSG00000167191	GPRC5B	0,295226543	0,080595732	0	0	0	0	0,018856377	0	0	0,394678652	0,043853184
ENSG00000113211	PCDHB6	0	0,082101041	0	0	0,109956382	0	0	0,202348174	0	0,394405596	0,043822844
ENSG00000124749	COL21A1	0,065586185	0,232762153	0	0	0	0,096013065	0	0	0	0,394361403	0,043817934
ENSG00000141449	GREB1L	0,042880412	0	0,026293993	0,098726816	0,033968742	0,062773582	0,023736347	0	0,105474757	0,39385465	0,043761628
ENSG00000184368	MAP7D2	0	0	0,191007855	0	0,164506433	0,03800059	0	0	0	0,393514878	0,043723875
ENSG00000089225	TBX5	0,034634557	0	0	0,358837921	0	0	0	0	0	0,393472477	0,043719164
ENSG00000187583	PLEKHN1	0	0	0	0,276316599	0	0	0	0,116637687	0	0,392954286	0,043661587
ENSG00000169507	SLC38A11	0,027217322	0	0	0,093996749	0	0	0,271189478	0	0	0,392403549	0,043600394
ENSG00000167880	EVPL	0	0	0	0,034745003	0,047818581	0,30928736	0	0	0	0,391850944	0,043538994
ENSG00000184276	DEFB108B	0	0	0	0,391794256	0	0	0	0	0	0,391794256	0,043532695
ENSG00000255359	CCDC179	0	0	0	0	0,391622184	0	0	0	0	0,391622184	0,043513576
ENSG00000181518	OR8D4	0,142781642	0,084454173	0	0,164368488	0	0	0	0	0	0,391604303	0,043511589
ENSG00000264324	AC006030.1	0,358435827	0	0	0	0	0,032795133	0	0	0	0,39123096	0,043470107
ENSG00000135697	BCO1	0	0,099718111	0	0,291113769	0	0	0	0	0	0,390831879	0,043425764
ENSG00000174460	ZCCHC12	0,137560829	0	0,253054255	0	0	0	0	0	0	0,390615084	0,043401676
ENSG00000171094	ALK	0,02047014	0	0,037656475	0,21208478	0,09729546	0,022475017	0	0	0	0,389981872	0,043331319
ENSG00000164675	IQUB	0,074316674	0	0	0,256657352	0,058871728	0	0	0	0	0,389845754	0,043316195
ENSG00000196767	POU3F4	0,039076951	0	0,071885208	0	0,278601662	0	0	0	0	0,389563821	0,043284869
ENSG00000167483	FAM129C	0,021852577	0,116330586	0	0,075469262	0,103866245	0,071978558	0	0	0	0,389497228	0,04327747
ENSG00000148468	FAM171A1	0,072267131	0	0,066470612	0	0,171744401	0	0	0,079013526	0	0,38949567	0,043277297
ENSG00000018625	ATP1A2	0,167953953	0	0,044137837	0,041431403	0,057020886	0	0,01992225	0	0,059017632	0,389483961	0,043275996
ENSG00000143552	NUP210L	0,051319603	0,045532688	0	0,088617753	0	0,140864813	0	0	0,063116496	0,389451353	0,043272373
ENSG00000273274	ZBTB8B	0,047107964	0,020897981	0,086658904	0,142354051	0,027988274	0,012930448	0	0,051505661	0	0,389443282	0,043271476
ENSG00000147676	MAL2	0	0	0,161710354	0,22769195	0	0	0	0	0	0,389402304	0,043266923
ENSG00000178404	CEP295NL	0	0	0	0	0	0	0	0,119809488	0,269538103	0,389347591	0,043260843
ENSG00000137440	FGFBP1	0	0,197308315	0	0	0,192005132	0	0	0	0	0,389313447	0,04325705
ENSG00000159387	IRX6	0	0	0,081782906	0,307072639	0	0	0	0	0	0,388855545	0,043206172
ENSG00000188038	NRN1L	0	0	0	0	0	0	0,388854335	0	0	0,388854335	0,043206037
ENSG00000169035	KLK7	0	0	0	0,106591084	0,146698342	0,135547857	0	0	0	0,388837284	0,043204143
ENSG00000120659	TNFSF11	0	0	0,101675237	0,286322211	0	0	0	0	0	0,387997448	0,043110828
ENSG00000127928	GNGT1	0	0	0,235476577	0	0,152103999	0	0	0	0	0,387580576	0,043064508
ENSG00000148942	SLC5A12	0	0	0	0,275720486	0	0	0	0,087289544	0,024547157	0,387557186	0,04306191
ENSG00000109832	DDX25	0,054770051	0	0,050376966	0,189151848	0,032540553	0	0	0,059883031	0	0,38672245	0,042969161
ENSG00000115221	ITGB6	0	0	0,144155626	0,180421763	0,062077363	0	0	0	0	0,386654752	0,042961639
ENSG00000178860	MSC	0	0	0,26311415	0,123490286	0	0	0	0	0	0,386604436	0,042956048
ENSG00000254827	SLC22A18AS	0	0	0	0	0	0	0,281639354	0	0,104290978	0,385930331	0,042881148
ENSG00000138347	MYPN	0,021403764	0,037980453	0	0,258717397	0	0,023500082	0,017772002	0	0,02632387	0,385697567	0,042855285
ENSG00000132746	ALDH3B2	0	0	0,085928933	0	0,222020119	0	0,07757053	0	0	0,385519582	0,042835509
ENSG00000130702	LAMA5	0,010573828	0,037525995	0,01945141	0	0,02512893	0,220579453	0	0,046243729	0,02600889	0,385512235	0,042834693
ENSG00000171195	MUC7	0	0	0,198699142	0,18651535	0	0	0	0	0	0,385214491	0,04280161
ENSG00000170790	OR10A2	0,028035356	0,099496104	0	0	0,199879894	0	0,023278355	0	0,034479874	0,385169583	0,04279662
ENSG00000099866	MADCAM1	0	0,101109351	0	0	0	0	0,283869529	0	0	0,384978881	0,042775431
ENSG00000137634	NXPE4	0,069861568	0	0,257031992	0	0	0	0,058007551	0	0	0,38490111	0,04276679
ENSG00000172986	GXYLT2	0,041208228	0	0,075805863	0	0,097932245	0,135732678	0,034216071	0	0	0,384895084	0,04276612
ENSG00000109738	GLRB	0	0	0	0,38474635	0	0	0	0	0	0,38474635	0,042749594
ENSG00000196844	PATE2	0	0	0,167761074	0	0,216727545	0	0	0	0	0,384488619	0,042720958
ENSG00000163792	TCF23	0,058993	0,104681632	0,054261194	0,101868036	0	0	0	0,064500208	0	0,384304071	0,042700452
ENSG00000181409	AATK	0	0	0	0,040969536	0,056385232	0,286546765	0	0	0	0,383901533	0,042655726
ENSG00000147081	AKAP4	0,199683608	0	0	0	0	0	0	0	0,184188823	0,383872431	0,042652492
ENSG00000100626	GALNT16	0,065918064	0,046787995	0	0,182121776	0,031331141	0	0	0,057657401	0	0,383816378	0,042646264
ENSG00000180537	RNF182	0	0,065432406	0	0,318368685	0	0	0	0	0	0,383801092	0,042644566
ENSG00000173261	PLAC8L1	0,078012685	0	0	0,13471088	0	0	0	0,170590897	0	0,383314462	0,042590496
ENSG00000255346	NOX5	0	0,0214	0,022185164	0,12494891	0,085981854	0,013241068	0,010013594	0,105485895	0	0,383256485	0,042584054
ENSG00000171557	FGG	0	0	0	0	0,199509746	0	0	0,18357476	0	0,383084505	0,042564945
ENSG00000177943	MAMDC4	0	0	0,050633898	0	0	0,332425564	0	0	0	0,383059461	0,042562162
ENSG00000173227	SYT12	0,027372624	0,04857205	0,075531229	0,141799642	0	0	0	0,089783869	0	0,383059414	0,042562157
ENSG00000116035	VAX2	0	0	0	0,087709235	0	0	0,295224311	0	0	0,382933546	0,042548172
ENSG00000185972	CCIN	0,077851917	0	0	0,134433269	0	0	0	0,170239344	0	0,38252453	0,042502726
ENSG00000181761	OR8H3	0	0	0	0	0,382446797	0	0	0	0	0,382446797	0,042494089
ENSG00000257138	TAS2R38	0	0	0	0	0	0	0,219545638	0	0,162595383	0,38214102	0,042460113
ENSG00000197584	KCNMB2	0	0,151407115	0,156962224	0,073668824	0	0	0	0	0	0,382038163	0,042448685
ENSG00000139151	PLCZ1	0,088085439	0	0	0,152104328	0,104668476	0	0,03656961	0	0	0,381427853	0,042380873
ENSG00000181652	ATG9B	0	0	0,086991112	0,040828505	0	0,233640305	0,019632347	0	0	0,38109227	0,042343586
ENSG00000205856	C22orf42	0	0	0	0,38092697	0	0	0	0	0	0,38092697	0,042325219
ENSG00000111799	COL12A1	0	0,064441721	0,022268693	0,083612905	0,143842643	0,066454609	0	0	0	0,380620571	0,042291175
ENSG00000205057	CLLU1OS	0	0	0	0,380371683	0	0	0	0	0	0,380371683	0,04226352
ENSG00000176692	FOXC2	0	0	0	0	0,123833635	0	0	0	0,256340031	0,380173666	0,042241518
ENSG00000165202	OR1Q1	0	0	0	0	0,380018563	0	0	0	0	0,380018563	0,042224285
ENSG00000173641	HSPB7	0	0,137157033	0	0,200205863	0	0,042432373	0	0	0	0,379795269	0,042199474
ENSG00000178175	ZNF366	0,353778455	0	0	0	0	0,025895205	0	0	0	0,37967366	0,042185962
ENSG00000099260	PALMD	0,056147103	0	0	0	0	0,020548746	0,279720829	0	0,0230179	0,379434579	0,042159398
ENSG00000154099	DNAAF1	0,019415466	0	0,107148952	0,16763136	0	0	0	0,084911877	0	0,379107655	0,042123073
ENSG00000206579	XKR4	0,015198448	0,04045391	0,041938159	0,078733208	0,144477764	0	0,006309798	0,033234555	0,018692132	0,379037975	0,042115331
ENSG00000269891	ARHGAP19-SLIT1	0,110866156	0	0,203947248	0,063813885	0	0	0	0	0	0,378627289	0,042069699
ENSG00000185070	FLRT2	0,043296917	0,030731727	0,039824088	0,179434383	0,05144803	0,014261246	0	0,018935536	0	0,377931927	0,041992436
ENSG00000179919	OR10A7	0	0	0	0	0,37762097	0	0	0	0	0,37762097	0,041957886
ENSG00000231824	AKAIN1	0	0,377133615	0	0	0	0	0	0	0	0,377133615	0,041903735
ENSG00000134765	DSC1	0	0	0	0,183283756	0	0,038845839	0	0,154734052	0	0,376863647	0,041873739
ENSG00000181939	OR4C15	0,135768707	0,240918239	0	0	0	0	0	0	0	0,376686946	0,041854105
ENSG00000177294	FBXO39	0,103288155	0	0,095003451	0,1783561	0	0	0	0	0	0,376647707	0,041849745
ENSG00000205922	ONECUT3	0	0,06447271	0,167095515	0,062739835	0	0,01994597	0	0,039725243	0,022342693	0,376321966	0,041813552
ENSG00000160882	CYP11B1	0	0	0,066091322	0,310193741	0	0	0	0	0	0,376285062	0,041809451

ENSG00000167910	CYP7A1	0	0,27960584	0,096621515	0	0	0	0	0	0	0,376227355	0,041803039
ENSG00000143006	DMRTB1	0	0,109894262	0,11392627	0	0	0	0	0	0,152333215	0,376153747	0,041794861
ENSG00000164488	DACT2	0	0,126303344	0,0654687	0,184362912	0	0	0	0	0	0,376134956	0,041792773
ENSG00000110723	EXPH5	0	0,147007675	0,101600913	0,09537097	0	0,015159958	0	0	0,01698159	0,376121107	0,041791234
ENSG00000176381	PRR18	0	0	0,088275674	0	0,228083545	0	0	0	0,059017632	0,375376852	0,041708539
ENSG00000157890	MEGF11	0	0	0,056569007	0,318601923	0	0	0	0	0	0,375170929	0,041685659
ENSG00000185737	NRG3	0	0	0,053540081	0,050257121	0,207502432	0	0	0,063643021	0	0,374942656	0,041660295
ENSG00000268173	AC007192.1	0	0,052147413	0	0	0	0,322657675	0	0	0	0,374805089	0,04164501
ENSG00000188800	TMCO2	0,178617696	0	0	0	0	0,196111793	0	0	0	0,374729489	0,04163661
ENSG00000065609	SNAP91	0	0,040316043	0,334361869	0	0	0	0	0	0	0,374677912	0,041630879
ENSG00000231924	PSG1	0	0	0	0	0	0	0	0	0,374615042	0,374615042	0,041623894
ENSG00000104936	DMPK	0,026022141	0	0	0,224672787	0,123684361	0	0	0	0	0,374379289	0,041597699
ENSG00000015592	STMN4	0,183497961	0,081403157	0	0	0,109021719	0	0	0	0	0,373922837	0,041546982
ENSG00000125903	DEFB129	0	0	0	0,373832342	0	0	0	0	0	0,373832342	0,041536927
ENSG00000123364	HOXC13	0	0	0	0,221412791	0,152362131	0	0	0	0	0,373774922	0,041530547
ENSG00000170577	SIX2	0	0,121551224	0,252021849	0	0	0	0	0	0	0,373573073	0,041508119
ENSG00000186943	OR13C8	0	0	0	0	0,372915413	0	0	0	0	0,372915413	0,041435046
ENSG00000163879	DNALI1	0	0	0,062383326	0,117116236	0,080591908	0	0,11263046	0	0	0,37272193	0,041413548
ENSG00000176641	RNF152	0	0,052509938	0	0,204394379	0,070325574	0,032490077	0,012285355	0	0	0,372005323	0,041333925
ENSG00000106511	MEOX2	0	0	0	0,220105419	0,151462481	0	0	0	0	0,3715679	0,041285322
ENSG00000085741	WNT11	0	0	0	0,220105419	0,151462481	0	0	0	0	0,3715679	0,041285322
ENSG00000185615	PDIA2	0	0	0,370969883	0	0	0	0	0	0	0,370969883	0,041218876
ENSG00000138075	ABCG5	0,081242242	0,144162366	0,074725833	0,07014381	0	0	0	0	0	0,370274251	0,041141583
ENSG00000254979	AP000781.2	0	0	0	0	0	0,148002299	0,055963574	0	0,165786371	0,369752244	0,041083583
ENSG00000100351	GRAP2	0,073236141	0	0	0,210771385	0,058015758	0,026803001	0	0	0	0,368826285	0,040980698
ENSG00000137561	TTPA	0	0	0	0,218447028	0,150321282	0	0	0	0	0,36876831	0,040974257
ENSG00000177669	MBOAT4	0	0	0	0	0	0	0	0	0,3687431	0,3687431	0,040971456
ENSG00000177453	NIM1K	0	0,074092843	0	0	0,099231153	0	0,10400967	0,09130549	0	0,368639156	0,040959906
ENSG00000139865	TTC6	0,018073265	0,032070566	0,099741693	0,218460091	0	0	0	0	0	0,368345615	0,040927291
ENSG00000213401	MAGEA12	0,076901054	0	0	0,132791336	0	0	0,063852586	0	0,094578383	0,368123359	0,040902595
ENSG00000183313	OR52L1	0,138126664	0	0	0	0	0	0,229379035	0	0	0,367505699	0,040833967
ENSG00000105523	FAM83E	0	0	0	0	0	0	0	0	0,367430847	0,367430847	0,04082565
ENSG00000240972	MIF	0	0,367317808	0	0	0	0	0	0	0	0,367317808	0,04081309
ENSG00000148377	IDI2	0	0	0	0	0	0,367058798	0	0	0	0,367058798	0,040784311
ENSG00000250254	PTTG2	0	0,366815322	0	0	0	0	0	0	0	0,366815322	0,040757258
ENSG00000113578	FGF1	0	0	0	0,366776069	0	0	0	0	0	0,366776069	0,040752897
ENSG00000136297	MMD2	0	0	0	0	0,291255103	0	0	0	0,075363553	0,366618657	0,040735406
ENSG00000027644	INSRR	0	0	0,053457711	0,150539408	0,138122132	0	0,02412891	0	0	0,366248161	0,04069424
ENSG00000109684	CLNK	0	0	0,136510116	0,170852823	0,058784996	0	0	0	0	0,366147935	0,040683104
ENSG00000165238	WNK2	0,057247526	0,040633732	0	0,05931239	0	0	0,180628603	0	0,028162831	0,365985083	0,040665009
ENSG00000178084	HTR3C	0	0	0	0,153762507	0,211619059	0	0	0	0	0,365381566	0,040597952
ENSG00000142279	WTIP	0,011003464	0	0,080967043	0,095002903	0,052299941	0,01208116	0,045682055	0	0,067664211	0,364700777	0,040522309
ENSG00000180347	ITPRID1	0	0,074011041	0,038363249	0,252076293	0	0	0	0	0	0,364450583	0,040494509
ENSG00000184261	KCNK12	0	0	0,124747988	0	0,214879606	0,024818336	0	0	0	0,36444593	0,040493992
ENSG00000174015	SPERT	0,071413313	0	0	0,123315205	0,169715285	0	0	0	0	0,364443803	0,040493756
ENSG00000124302	CHST8	0,049479558	0	0,27306493	0	0	0	0,041083933	0	0	0,363628422	0,040403158
ENSG00000265817	FSBP	0,019303854	0	0,035510999	0,100000629	0	0	0,208369228	0	0	0,36318471	0,040353857
ENSG00000101977	MCF2	0,059751115	0	0,054958501	0	0	0	0,248063132	0	0	0,362772748	0,040308083
ENSG00000269855	RNF225	0	0	0	0	0,362744992	0	0	0	0	0,362744992	0,040304999
ENSG00000186119	OR5D18	0	0	0	0	0,362378953	0	0	0	0	0,362378953	0,040264328
ENSG00000198128	OR2L3	0	0	0,094906145	0,267260131	0	0	0	0	0	0,362166276	0,040240697
ENSG00000137573	SULF1	0,032247241	0,085832907	0,059321404	0,16705184	0	0,017702793	0	0	0	0,362156185	0,040239576
ENSG00000120669	SOHLH2	0	0	0,16531674	0	0	0	0	0,196511786	0	0,361828526	0,04020317
ENSG00000074211	PPP2R2C	0	0,028629297	0	0,139299047	0,153370721	0	0,040189088	0	0	0,361488153	0,04016535
ENSG00000185594	SPATA8	0	0	0,186438698	0,17500669	0	0	0	0	0	0,361445388	0,040160599
ENSG00000159961	OR3A3	0,039640758	0	0,145844753	0	0,09420712	0	0,032914568	0	0,048753023	0,361360222	0,040151136
ENSG00000236279	CLEC2L	0,167962843	0	0	0,19335678	0	0	0	0	0	0,361319623	0,040146625
ENSG00000172572	PDE3A	0	0	0,03310075	0,093213256	0,08552454	0	0,149405015	0	0	0,361243561	0,040138173
ENSG00000077009	NMRK2	0,132436959	0	0	0,228689724	0	0	0	0	0	0,361126683	0,040125187
ENSG00000216588	IGSF23	0,106867447	0	0	0	0,253972802	0	0	0	0	0,36084025	0,040093361
ENSG00000175946	KLHL38	0	0,135288597	0	0,131652359	0	0	0	0	0,093767166	0,360708122	0,04007868
ENSG00000197838	CYP2A13	0	0,154193215	0	0	0,206508075	0	0	0	0	0,36070129	0,040077921
ENSG00000197410	DCHS2	0,022190597	0	0	0,242682682	0,052736435	0,024363979	0,018425327	0	0	0,36039902	0,040044336
ENSG00000237289	CKMT1B	0,130643145	0,154548703	0	0,075197399	0	0	0	0	0	0,360389247	0,04004325
ENSG00000105048	TNNT1	0	0	0	0,12314062	0	0	0,236848196	0	0	0,359988817	0,039998757
ENSG00000274808	TBC1D3B	0	0	0	0,183498576	0	0	0,176470228	0	0	0,359968804	0,039996534
ENSG00000205089	CCNI2	0	0,084801392	0,087912745	0,08252213	0	0	0	0,104501761	0	0,359738027	0,039970892
ENSG00000183873	SCN5A	0,211195766	0	0	0	0,071701616	0,033125801	0,025051479	0	0,018553112	0,359627774	0,039958642
ENSG00000157219	HTR5A	0	0	0	0,21296468	0,146548681	0	0	0	0	0,359513361	0,039945929
ENSG00000159753	CARMIL2	0	0	0,036432516	0,27358844	0	0	0,049333027	0	0	0,359353983	0,03992822
ENSG00000197403	OR6N1	0	0	0	0,095650651	0,263282656	0	0	0	0	0,358933306	0,039881478
ENSG00000179869	ABCA13	0,05545342	0,028114495	0,029146013	0,054717688	0,037653215	0,017395604	0	0	0,136401117	0,358881554	0,039875728
ENSG00000105290	APLP1	0	0	0,076033944	0,214115132	0	0	0,068638037	0	0	0,358787113	0,039865235
ENSG00000188257	PLA2G2A	0,358719456	0	0	0	0	0	0	0	0	0,358719456	0,039857717
ENSG00000082684	SEMA5B	0,035484459	0	0,032638265	0,275732626	0	0	0,014731752	0	0	0,358587102	0,039843011
ENSG00000145920	CPLX2	0,021525722	0	0,039598305	0,297361794	0	0	0	0	0	0,358485821	0,039831758
ENSG00000124191	TOX2	0	0	0,085928933	0,161319922	0,111010059	0	0	0	0	0,358258915	0,039806546
ENSG00000041982	TNC	0,015676997	0,055636892	0	0,108283006	0	0	0,143186394	0,034281001	0	0,35706429	0,03967381
ENSG00000268533	AC003002.2	0	0	0	0	0	0	0	0	0,356711175	0,356711175	0,039634575
ENSG00000145242	EPHA5	0,06147079	0	0,028270121	0	0,18260833	0	0,012760127	0,033604654	0,037800574	0,356514598	0,039612733
ENSG00000110148	CCKBR	0,292283502	0	0	0	0	0,064182041	0	0	0	0,356465544	0,039607283
ENSG00000172020	GAP43	0,07240564	0	0	0,125028737	0	0,158994324	0	0	0	0,356428701	0,039603189
ENSG00000198704	GPX6	0	0	0	0	0,355914314	0	0	0	0	0,355914314	0,039546035
ENSG00000214414	TRIM77	0,111685566	0	0	0	0	0	0	0,244223627	0	0,355909193	0,039545466
ENSG00000167771	RCOR2	0,099121398	0	0	0,25674153	0	0	0	0	0	0,355862927	0,039540325
ENSG00000176040	TMPRSS7	0,099088899	0	0	0,256657352	0	0	0	0	0	0,355746251	0,039527361
ENSG00000130287	NCAN	0	0,038748844	0	0,113122099	0,155686796	0	0	0,04775066	0	0,355308399	0,039478711
ENSG00000154556	SORBS2	0,061638249	0,01562508	0,06479345	0,015205115	0,020926376	0,01933577	0	0,038509943	0,119125444	0,355159428	0,039462159
ENSG00000198064	NPIPB13	0	0	0,267185793	0	0	0,063787227	0,024119633	0	0	0,355092653	0,039454739
ENSG00000188760	TMEM198	0	0	0	0	0	0,257425255	0,09733928	0	0	0,354764535	0,039418282
ENSG00000134775	FHOD3	0,018971823	0,033665035	0	0	0,135260845	0,166639625	0	0	0	0,354537327	0,039393036
ENSG00000173175	ADCY5	0	0	0,031328761	0,264669759	0	0,037396726	0	0	0,020945173	0,35434042	0,039371158
ENSG00000183709	IFNL2	0	0	0	0,354050169	0	0	0	0	0	0,354050169	0,039338908
ENSG00000138798	EGF	0,039682398	0,035207721	0	0,06852284	0,188612155	0,021784477	0	0	0	0,35380959	0,039312177
ENSG00000130377	ACSBG2	0	0,077789962	0	0,075699151	0,104182635	0	0	0,095861493	0	0,353533242	0,039281471
ENSG00000164418	GRIK2	0,139456031	0,035351615	0	0	0,047345754	0,131241063	0	0	0	0,353394462	0,039266051
ENSG00000158014	SLC30A2	0	0,076655803	0,079468296	0	0,102663677	0	0	0,094463856	0	0,353251632	0,039250181
ENSG00000157542	KCNJ6	0,030253881	0,013421192	0,013913614	0,104483698	0,107848504	0,041521241	0,006280111	0,016539094	0,018604187	0,352865522	0,03920728
ENSG00000179841	AKAP5	0,020115891	0,142780618	0	0,069471505	0,095611699	0	0	0	0,024739952	0,352719665	0,039191074
ENSG00000277531	PNMA8C	0	0,252072386	0	0,061324318	0	0,03899191	0	0	0	0,352388614	0,03915429
ENSG00000105583	WDR83OS	0	0	0	0	0	0	0,35195044	0	0	0,35195044	0,039105604
ENSG00000124564	SLC17A3	0	0	0	0	0,299764226	0	0	0	0,051710218	0,351474443	0,039052716
ENSG00000172551	MUCL1	0	0	0	0,262840569	0	0,041780553	0	0	0,046800937	0,351422059	0,039046895
ENSG00000185448	FAM47A	0	0	0,108628409	0,101967556	0,140335108	0	0	0	0	0,350931073	0,038992341
ENSG00000060709	RIMBP2	0,015651017	0	0,05758262	0,243233016	0	0	0	0,034224191	0	0,350690845	0,038965649
ENSG00000186118	TEX38	0	0	0	0,350248288	0	0	0	0	0	0,350248288	0,038916476
ENSG00000172350	ABCG4	0	0,086067084	0,089224875	0,041876902	0	0,053253274	0,020136468	0	0,05965223	0,350210833	0,038912315
ENSG00000182256	GABRG3	0,02381757	0,021131846	0,021907171	0,143947106	0,11320594	0	0	0,026041025	0	0,350050658	0,038894518
ENSG00000129951	PLPPR3	0	0	0,304246734	0	0	0	0,045775386	0	0	0,350022119	0,038891347
ENSG00000168229	PTGDR	0	0	0,091651863	0,258095919	0	0	0	0	0	0,349747782	0,038860865
ENSG00000162105	SHANK2	0	0	0,014805864	0,041694004	0,095637162	0	0,187119536	0	0,009898616	0,349155183	0,03879502
ENSG00000146385	TAAR8	0	0	0	0	0,34899664	0	0	0	0	0,34899664	0,03877404
ENSG00000060656	PTPRU	0	0,040129003	0,124803995	0,156201721	0	0	0	0	0,027813008	0,348947728	0,03877197
ENSG00000172519	OR10H5	0	0	0,038981924	0,109774916	0	0	0,017595054	0	0,18243243	0,348784323	0,038753814
ENSG00000219073	CELA3B	0	0,348010383	0	0	0	0	0	0	0	0,348010383	0,03866782
ENSG00000102145	GATA1	0	0	0	0,347913299	0	0	0	0	0	0,347913299	0,038657033
ENSG00000136197	C7orf25	0	0	0	0	0	0	0,347670255	0	0	0,347670255	0,038630028

ENSG00000063515	GSC2	0	0	0	0	0,347645249	0	0	0	0	0,347645249	0,03862725
ENSG00000146221	TCTE1	0	0,3475593	0	0	0	0	0	0	0	0,3475593	0,0386177
ENSG00000153165	RGPD3	0,025972941	0	0	0,089699201	0	0	0,086263549	0,113590432	0,031943369	0,347469492	0,038607721
ENSG00000145626	UGT3A1	0,035618284	0	0	0,184515009	0,126971435	0	0	0	0	0,347104728	0,038567192
ENSG00000163736	PPBP	0,346849053	0	0	0	0	0	0	0	0	0,346849053	0,038538784
ENSG00000141316	SPACA3	0	0,214170927	0	0	0	0,132516435	0	0	0	0,346687362	0,038520818
ENSG00000198650	TAT	0,028145012	0	0	0	0	0	0,186955235	0,0615449	0,069229474	0,345874621	0,038430513
ENSG00000136546	SCN7A	0,01552559	0	0,028560577	0,214474447	0	0	0	0,067899839	0,019094475	0,345554929	0,038394992
ENSG00000176435	CLEC14A	0	0	0	0,345304333	0	0	0	0	0	0,345304333	0,038367148
ENSG00000112599	GUCA1B	0	0	0	0,344847984	0	0	0	0	0	0,344847984	0,038316443
ENSG00000204010	IFIT1B	0,076628078	0	0,140963539	0	0	0	0,127251858	0	0	0,344843475	0,038315942
ENSG00000221937	TAS2R40	0	0	0	0	0,344312121	0	0	0	0	0,344312121	0,038256902
ENSG00000152402	GUCY1A2	0,009236023	0,032778192	0,03398082	0,207331744	0	0,010140613	0,007668867	0,020196477	0,022718235	0,344050972	0,038227886
ENSG00000175121	WFDC5	0	0	0	0	0,34398232	0	0	0	0	0,34398232	0,038220258
ENSG00000171509	RXFP1	0	0	0	0,229534636	0,063180426	0,029189053	0,022074302	0	0	0,343978417	0,038219824
ENSG00000101981	F9	0,275605522	0	0	0,067987226	0	0	0	0	0	0,343592749	0,038176972
ENSG00000164651	SP8	0,121928917	0,072119957	0,14953206	0	0	0	0	0	0	0,343580933	0,038175659
ENSG00000204442	FAM155A	0,053314326	0,063069975	0,032692003	0,122749606	0,042234216	0	0,029512015	0	0	0,343572141	0,038174682
ENSG00000244362	KRTAP19-7	0,343433115	0	0	0	0	0	0	0	0	0,343433115	0,038159235
ENSG00000134533	RERG	0	0,064503729	0	0,188310061	0	0	0,090548712	0	0	0,343362502	0,038151389
ENSG00000204065	TCEAL5	0	0	0	0	0,34332461	0	0	0	0	0,34332461	0,038147179
ENSG00000163032	VSNL1	0,078847154	0,06995617	0	0,068075913	0,093690984	0	0,032734237	0	0	0,343304459	0,03814494
ENSG00000101938	CHRDL1	0,038548615	0	0,212739872	0	0,091611618	0	0	0	0	0,342900105	0,038100012
ENSG00000075461	CACNG4	0,042174315	0	0	0	0,300684518	0	0	0	0	0,342858833	0,038095426
ENSG00000188000	OR7D2	0	0	0,241023207	0	0	0,047951034	0	0	0,053712868	0,342687109	0,038076345
ENSG00000173805	HAP1	0,048565184	0	0	0,041930737	0,115416212	0,026660867	0,020162354	0	0,089593374	0,342328728	0,038036525
ENSG00000117501	MROH9	0	0	0	0,068111453	0,187479792	0,086614762	0	0	0	0,342206007	0,03802289
ENSG00000242732	RTL5	0	0	0	0,273001647	0	0	0	0,069143036	0	0,342144683	0,038016076
ENSG00000215910	C1orf167	0,023251357	0	0,299409246	0	0	0	0,019306098	0	0	0,341966702	0,0379963
ENSG00000162843	WDR64	0,030595378	0	0	0	0,072710578	0,067183874	0	0,133806263	0,037628371	0,341924463	0,037991607
ENSG00000259522	AL136295.4	0	0	0	0	0	0,038131597	0	0,303778044	0	0,341909641	0,03798996
ENSG00000215018	COL28A1	0,068489456	0,040510953	0	0,157688457	0	0,075197421	0	0	0	0,341886288	0,037987365
ENSG00000183571	PGPEP1L	0	0	0,341708788	0	0	0	0	0	0	0,341708788	0,037967643
ENSG00000158856	DMTN	0	0	0	0,116671127	0	0	0,224404797	0	0	0,341075923	0,037897325
ENSG00000132437	DDC	0	0,086946174	0	0,253827796	0	0	0	0	0	0,34077397	0,037863774
ENSG00000197415	VEPH1	0	0,040981507	0,084970227	0,159520082	0,054885762	0	0	0	0	0,340357579	0,037817509
ENSG00000189051	RNF222	0	0	0	0,339980423	0	0	0	0	0	0,339980423	0,037775603
ENSG00000168646	AXIN2	0	0	0	0,149761799	0	0,126964283	0	0,063216868	0	0,33994295	0,037771439
ENSG00000162039	MEIOB	0	0	0	0,33990661	0	0	0	0	0	0,33990661	0,037767401
ENSG00000122592	HOXA7	0	0	0,230593197	0,108226866	0	0	0	0	0	0,338820063	0,037646674
ENSG00000166257	SCN3B	0,031933764	0	0	0,192999242	0,113836922	0	0	0	0	0,338769927	0,037641103
ENSG00000149970	CNKSR2	0,009484423	0,025244814	0	0,049132586	0,033809905	0,015620013	0,031500476	0,010369828	0,163304648	0,338466694	0,03760741
ENSG00000143127	ITGA10	0,096834174	0,042957706	0	0,083606208	0,115064897	0	0	0	0	0,338463524	0,037607058
ENSG00000082556	OPRK1	0,052670119	0	0	0,136424699	0,062585839	0,086743069	0	0	0	0,338423726	0,037602636
ENSG00000151320	AKAP6	0,030641908	0,013593329	0	0,06613986	0,072821158	0,050464537	0	0,06700488	0,037685597	0,33835127	0,037594586
ENSG00000178184	PARD6G	0,036047369	0,127930344	0	0	0,085667353	0	0	0	0,088667234	0,338312299	0,037590255
ENSG00000135902	CHRND	0,048247309	0,171227331	0	0	0	0	0	0	0,11867594	0,33815058	0,037572287
ENSG00000162825	NBPF20	0,015910563	0	0,029268765	0	0,037811797	0,113547644	0	0,121771096	0,019567941	0,337877807	0,037541979
ENSG00000185532	PRKG1	0,035322714	0,020893096	0	0,081326157	0,027981732	0,012927425	0	0	0,159288744	0,337739869	0,037526652
ENSG00000154319	FAM167A	0	0,053107942	0,110112933	0,103361052	0,071126469	0	0	0	0	0,337708396	0,037523155
ENSG00000136697	IL1F10	0	0	0,174063932	0,163390717	0	0	0	0	0	0,337454648	0,037494961
ENSG00000151005	TKTL2	0	0,202066315	0	0	0,135311809	0	0	0	0	0,337378125	0,037486458
ENSG00000204071	TCEAL6	0	0	0,202167345	0	0	0	0	0	0,135161107	0,337328452	0,037480939
ENSG00000187288	CIDEC	0	0	0,144330269	0	0	0	0	0	0,192987043	0,337317312	0,037479701
ENSG00000160182	TFF1	0	0	0	0	0	0,337216619	0	0	0	0,337216619	0,037468513
ENSG00000132704	FCRL2	0,059751115	0	0	0,20635427	0,07099991	0	0	0	0	0,337105295	0,037456144
ENSG00000169247	SH3TC2	0,059786576	0,017681635	0	0,137651157	0,023680682	0,010940361	0	0,087157156	0	0,336897567	0,037433063
ENSG00000178662	CSRNP3	0,036070927	0,04268078	0	0,228434916	0	0	0	0	0,029581619	0,336776242	0,037419582
ENSG00000283267	FAM237B	0,03897616	0	0	0,201909962	0	0	0	0	0,095871304	0,336757426	0,037417492
ENSG00000267795	SMIM22	0	0	0	0,148090224	0	0,188320745	0	0	0	0,336410969	0,037378997
ENSG00000171798	KNDC1	0	0,065296968	0,033846353	0,06354194	0	0	0,01527704	0	0,158398351	0,336360652	0,037373406
ENSG00000168878	SFTPB	0,036766562	0,195724088	0	0,063487828	0	0,040367537	0	0	0	0,336346015	0,037371779
ENSG00000187556	NANOS3	0	0	0	0	0	0,33585137	0	0	0	0,33585137	0,037316819
ENSG00000134376	CRB1	0,083151796	0	0,043704127	0,06153643	0,056460584	0,013042259	0	0,077926555	0	0,33582175	0,037313528
ENSG00000138759	FRAS1	0,023602422	0,069803197	0,028945707	0,176610333	0	0,017276053	0	0	0,019351957	0,335589669	0,037287741
ENSG00000104290	FZD3	0,010961161	0	0,020163942	0,151420267	0	0	0,009101286	0,143813101	0	0,335459757	0,037273306
ENSG00000178997	EXD1	0,165103055	0	0	0	0	0,13595513	0,034272148	0	0	0,335330332	0,037258926
ENSG00000178171	AMER3	0,092379991	0	0	0,159520082	0,054885762	0	0	0	0,02840387	0,335189706	0,037243301
ENSG00000205029	OR5D16	0	0	0	0	0	0	0	0,334786796	0	0,334786796	0,037198533
ENSG00000116218	NPHS2	0	0,143085379	0	0	0,191631559	0	0	0	0	0,334716938	0,037190771
ENSG00000282815	TEX13C	0	0	0	0,204855721	0	0	0	0,129709349	0	0,33456507	0,037173897
ENSG00000184828	ZBTB7C	0,039713685	0,03523548	0	0,137153732	0	0	0	0	0,122106782	0,334209678	0,037134409
ENSG00000166862	CACNG2	0	0	0	0	0,334166448	0	0	0	0	0,334166448	0,037129605
ENSG00000084734	GCKR	0	0,085151477	0	0,248588417	0	0	0	0	0	0,333739893	0,03708221
ENSG00000164078	MST1R	0	0	0	0,298097838	0	0	0	0	0,035385857	0,333483696	0,037053744
ENSG00000189233	NUGGC	0,112656986	0,066635686	0	0,064844676	0,089243922	0	0	0	0	0,33338127	0,037042363
ENSG00000166250	CLMP	0	0	0	0	0,071013949	0,131232412	0	0,130684029	0	0,33293039	0,036992266
ENSG00000114487	MORC1	0	0,142477152	0	0,069323851	0	0	0,033334307	0,087788142	0	0,332923452	0,036991495
ENSG00000095203	EPB41L4B	0,019178902	0,068064983	0	0,066235556	0,136737229	0	0	0,041938643	0	0,332155313	0,036906146
ENSG00000153789	FAM92B	0	0	0	0,139686817	0,192247078	0	0	0	0	0,331933896	0,036881544
ENSG00000197532	OR6Y1	0,02946774	0	0,162624863	0,050884355	0	0	0,024467693	0,064437318	0	0,33188197	0,036875774
ENSG00000196277	GRM7	0,036793419	0	0	0,158835509	0	0	0,01527518	0,120684648	0	0,331588757	0,036843195
ENSG00000221866	PLXNA4	0,073838539	0,147402761	0,016978995	0,063751521	0,021934861	0	0,007663714	0	0	0,331570391	0,036841155
ENSG00000178773	CPNE7	0	0,087685415	0	0,085328638	0,117435429	0	0,041030194	0	0	0,331479676	0,036831075
ENSG00000258453	OR11H2	0	0	0	0	0,33098391	0	0	0	0	0,33098391	0,03677599
ENSG00000135436	FAM186B	0	0,056379731	0	0,274321882	0	0	0	0	0	0,330701613	0,036744624
ENSG00000070886	EPHA8	0	0	0	0	0,330435722	0	0	0	0	0,330435722	0,03671508
ENSG00000116882	HAO2	0	0	0	0,139017035	0,191325276	0	0	0	0	0,330342311	0,036704701
ENSG00000169862	CTNND2	0,062356494	0,110650069	0	0,107676056	0,049397186	0	0	0	0	0,330079804	0,036675534
ENSG00000169154	GOT1L1	0	0	0	0,329463352	0	0	0	0	0	0,329463352	0,036607039
ENSG00000079557	AFM	0	0	0	0	0,32916365	0	0	0	0	0,32916365	0,036573739
ENSG00000267909	CCDC177	0	0,064118125	0,066470612	0	0	0,198362717	0	0	0	0,328951454	0,036550162
ENSG00000128606	LRRC17	0	0	0	0	0	0	0	0	0,32869919	0,32869919	0,036522132
ENSG00000081148	IMPG2	0,054278222	0	0,033283058	0,218695501	0	0	0	0	0,022251739	0,32850852	0,036500947
ENSG00000177464	GPR4	0	0	0	0,248588417	0	0	0,079689001	0	0	0,328277418	0,036475269
ENSG00000019549	SNAI2	0	0,078911713	0	0	0,211369948	0	0,036924759	0	0	0,32720642	0,036356269
ENSG00000205863	C1QTNF9B	0	0	0	0	0,327065157	0	0	0	0	0,327065157	0,036340573
ENSG00000079689	SCGN	0	0	0	0	0,242319529	0	0,084662842	0	0	0,326982371	0,036331375
ENSG00000237353	PATE4	0,052432537	0,09304025	0	0,181079094	0	0	0	0	0	0,326551881	0,036283542
ENSG00000066248	NGEF	0,032517876	0	0,05981926	0,056151275	0	0,035702728	0	0,142214146	0	0,326405284	0,036267254
ENSG00000215529	EFCAB8	0,033313618	0,177342593	0	0	0,079170534	0,036576406	0	0	0	0,32640315	0,036267017
ENSG00000151967	SCHIP1	0,039531869	0,070148332	0	0,170657276	0	0,021701841	0	0	0,024309552	0,32634887	0,036260986
ENSG00000127362	TAS2R3	0	0	0	0	0,326173971	0	0	0	0	0,326173971	0,036241552
ENSG00000120645	IQSEC3	0,019660496	0,034887067	0	0,271595082	0	0	0	0	0	0,326142645	0,036238072
ENSG00000165023	DIRAS2	0	0	0,062565856	0,234917825	0	0	0,028240003	0	0	0,325723684	0,03619152
ENSG00000145244	CORIN	0,04494663	0	0,027560985	0,103484027	0	0	0	0,131046824	0,018426187	0,325464654	0,036162739
ENSG00000111907	TPD52L1	0,039792119	0,035305069	0	0,206136912	0	0	0	0,043506855	0	0,324740955	0,036082328
ENSG00000139797	RNF113B	0	0	0	0,189632976	0	0	0	0	0,13506288	0,324695855	0,036077317
ENSG00000233087	RAB6D	0,049838579	0	0	0	0	0	0,165528142	0,108982377	0	0,324349098	0,036038789
ENSG00000162510	MATN1	0	0	0	0	0	0,324325628	0	0	0	0,324325628	0,036036181
ENSG00000103742	IGDCC4	0,079333545	0	0,072970232	0,034247929	0,094268944	0	0	0,043369808	0	0,324190459	0,036021162
ENSG00000163285	GABRG1	0,02236025	0	0,246800917	0	0	0	0	0	0,055000451	0,324161617	0,036017957
ENSG00000146399	TAAR1	0	0	0	0	0	0	0	0,323955458	0	0,323955458	0,035995051
ENSG00000204175	GPRIN2	0	0	0	0,13258891	0	0	0,19126575	0	0	0,32385466	0,035983851
ENSG00000138152	BTBD16	0,062805723	0,111447215	0	0	0,149259161	0	0	0	0	0,3235121	0,035945789
ENSG00000102878	HSF4	0	0	0	0,077268278	0	0,245647878	0	0	0	0,322916156	0,035879573
ENSG00000146411	SLC2A12	0	0	0,248863115	0	0	0	0	0,073955812	0	0,322818926	0,03586877

ENSG00000091482	SMPX	0,11361697	0	0,209007593	0	0	0	0	0	0	0,322624564	0,035847174
ENSG00000180509	KCNE1	0	0	0,098434879	0,046199535	0	0	0,177720017	0	0	0,322354431	0,035817159
ENSG00000171405	XAGE5	0	0	0	0	0,322078513	0	0	0	0	0,322078513	0,035786501
ENSG00000206199	ANKUB1	0	0,040975245	0,084957243	0	0	0	0,038346678	0,10098856	0,056799059	0,322066785	0,035785198
ENSG00000189269	DRICH1	0,116060346	0,205946237	0	0	0	0	0	0	0	0,322006583	0,035778509
ENSG00000157502	PWWP3B	0,034794053	0,061741193	0	0,060081735	0,165377639	0	0	0	0	0,321994619	0,03577718
ENSG00000133878	DUSP26	0,153333912	0	0	0	0	0,168351676	0	0	0	0,321685588	0,035742843
ENSG00000186103	ARGFX	0	0	0,218925063	0,102750531	0	0	0	0	0	0,321675595	0,035741733
ENSG00000077943	ITGA8	0,020643521	0	0	0,106940563	0,098119547	0	0	0,045141334	0,050777738	0,321622704	0,035735856
ENSG00000163705	FANCD2OS	0	0	0,146691345	0	0	0	0	0,174371803	0	0,321063149	0,035673683
ENSG00000123496	IL13RA2	0	0	0	0,320953228	0	0	0	0	0	0,320953228	0,03566147
ENSG00000175206	NPPA	0	0	0	0	0,320640663	0	0	0	0	0,320640663	0,03562674
ENSG00000185883	ATP6V0C	0	0,189365819	0	0	0	0	0	0	0,131247544	0,320613363	0,035623707
ENSG00000165757	JCAD	0,031566862	0	0	0,190781786	0,037509666	0	0,02621064	0,034513742	0	0,320582697	0,0356203
ENSG00000116962	NID1	0	0,230719325	0	0,089807253	0	0	0	0	0	0,320526579	0,035614064
ENSG00000135917	SLC19A3	0,01953595	0,034666063	0,071875914	0,101202964	0,092855215	0	0	0	0	0,320136106	0,035570678
ENSG00000148704	VAX1	0,026107562	0,092654458	0	0,045082062	0,124090374	0	0	0	0,032108936	0,320043392	0,035560377
ENSG00000171533	MAP6	0	0	0,086423162	0,121685827	0,111648544	0	0	0	0	0,319757532	0,035528615
ENSG00000145002	FAM86B2	0	0	0	0	0	0	0,183704732	0	0,136051627	0,319756359	0,035528484
ENSG00000183309	ZNF623	0,063438527	0	0	0	0	0	0,105348725	0,046240494	0,10402828	0,319056026	0,0354506
ENSG00000005961	ITGA2B	0	0	0,050077481	0,235034205	0	0	0	0	0,033479827	0,318591514	0,035399057
ENSG00000169515	CCDC8	0	0	0,083377354	0,234794518	0	0	0	0	0	0,318171872	0,03535243
ENSG00000268041	AC010616.1	0	0	0	0	0	0	0	0,318031345	0	0,318031345	0,035336816
ENSG00000180287	PLD5	0,03166277	0,028092404	0	0,027337347	0,150494517	0	0,026290274	0,034618603	0,019470563	0,317966479	0,035329609
ENSG00000159648	TEPP	0	0,086413793	0	0	0,231464739	0	0	0	0	0,317878532	0,035319837
ENSG00000258405	ZNF578	0,012655827	0,044914908	0	0,043707701	0,15038423	0,055581433	0,010508403	0	0	0,317752502	0,035305834
ENSG00000133019	CHRM3	0	0,022033032	0,045682843	0,150085852	0,059016852	0	0,010309805	0	0,030541746	0,31767013	0,035296681
ENSG00000121764	HCRTR1	0,177221154	0	0	0	0,14038997	0	0	0	0	0,317611124	0,035290125
ENSG00000153721	CNKSR3	0,007002668	0,037278187	0,03864592	0,096736633	0	0,01537704	0,005814465	0,107189489	0,008612379	0,31665678	0,035184087
ENSG00000095539	SEMA4G	0	0	0	0	0,068364276	0	0	0	0,247653847	0,316018123	0,035113125
ENSG00000133083	DCLK1	0,042048159	0	0,019337746	0,090759991	0,04996418	0,023083211	0,008728371	0,068960258	0,012928454	0,315810369	0,035090041
ENSG00000163072	NOSTRIN	0,028793935	0	0	0,049720841	0	0	0,095632875	0	0,141651313	0,315798964	0,035088774
ENSG00000172995	ARPP21	0,089839816	0	0	0,129278129	0	0	0,012432653	0,065484457	0,018415232	0,315450286	0,035050032
ENSG00000196734	LCE1B	0	0	0	0	0,31529196	0	0	0	0	0,31529196	0,03503244
ENSG00000080031	AC010327.1	0	0	0	0,120108159	0	0	0	0,152098765	0,042772502	0,314979426	0,034997714
ENSG00000072832	CRMP1	0,103802556	0	0	0,179244358	0	0	0	0	0,031915941	0,314962855	0,034995873
ENSG00000171495	MROH2B	0,04966658	0,088132128	0,091365686	0,085763344	0	0	0	0	0	0,314927738	0,034991971
ENSG00000183317	EPHA10	0,092683127	0,098678361	0	0,064017413	0,044052692	0	0,015391356	0	0	0,314822949	0,034980328
ENSG00000152611	CAPSL	0	0	0	0	0,314463697	0	0	0	0	0,314463697	0,034940411
ENSG00000135063	FAM189A2	0,063279133	0,028071817	0,029101769	0	0,037596058	0,15632278	0	0	0	0,314371557	0,034930173
ENSG00000268350	FAM156A	0	0,162313559	0	0,039487738	0	0	0	0	0,112497895	0,314299193	0,034922133
ENSG00000254834	OR5M10	0	0	0	0	0	0	0	0,3141013	0	0,3141013	0,034900144
ENSG00000185155	MIXL1	0	0	0,137003499	0	0,176992382	0	0	0	0	0,31399588	0,034888431
ENSG00000007372	PAX6	0	0,180376129	0,053426888	0,012537717	0,051765934	0	0	0,015877117	0	0,313983787	0,034887087
ENSG00000196156	KRTAP4-3	0,313507408	0	0	0	0	0	0	0	0	0,313507408	0,034834156
ENSG00000182898	TCHHL1	0,041905316	0	0	0,072361335	0,199177783	0	0	0	0	0,313444434	0,034827159
ENSG00000196071	OR2L13	0	0	0	0,12923971	0	0	0	0	0,184097595	0,313337305	0,034815256
ENSG00000104055	TGM5	0,041616792	0,073847976	0	0	0,197806413	0	0	0	0	0,31327118	0,034807909
ENSG00000168679	SLC16A4	0	0,254243363	0	0	0	0	0	0	0,058737839	0,312981202	0,034775689
ENSG00000163295	ALPI	0	0	0	0	0,214334552	0	0	0,098607749	0	0,312942301	0,034771367
ENSG00000226288	OR52I2	0	0	0	0,312871672	0	0	0	0	0	0,312871672	0,034763519
ENSG00000173557	C2orf70	0	0	0	0,312621775	0	0	0	0	0	0,312621775	0,034735753
ENSG00000145536	ADAMTS16	0,025773592	0	0	0	0,0612515	0	0	0,225437194	0	0,312462286	0,034718032
ENSG00000131746	TNS4	0,062019524	0	0,171134885	0,053547091	0	0	0,025748067	0	0	0,312449568	0,034716619
ENSG00000182771	GRID1	0,024247524	0	0,089210558	0,083740364	0,115249532	0	0	0	0	0,312447979	0,034716442
ENSG00000282034	AC106886.5	0	0	0	0,312443521	0	0	0	0	0	0,312443521	0,034715947
ENSG00000177947	ODF3	0,048588608	0	0	0,083901921	0	0	0	0	0,179273173	0,311763702	0,034640411
ENSG00000164530	PI16	0	0	0	0	0,311598735	0	0	0	0	0,311598735	0,034622082
ENSG00000167800	TBX10	0,097616648	0	0	0	0	0	0	0,213459023	0	0,311075671	0,034563963
ENSG00000177468	OLIG3	0	0	0	0,127347474	0	0	0,183704732	0	0	0,311052206	0,034561356
ENSG00000184210	DGAT2L6	0,097302364	0	0	0	0	0,213664619	0	0	0	0,310966983	0,034551887
ENSG00000073734	ABCB11	0,058759781	0	0	0,101465317	0	0,150534548	0	0	0	0,310759646	0,03452885
ENSG00000197408	CYP2B6	0	0	0	0,209698613	0	0	0,100833377	0	0	0,31053199	0,034503554
ENSG00000104371	DKK4	0	0	0,310245646	0	0	0	0	0	0	0,310245646	0,034471738
ENSG00000226430	USP17L7	0	0,132612265	0	0	0,177605115	0	0	0	0	0,31021738	0,034468598
ENSG00000101958	GLRA2	0	0,072373009	0	0,140855587	0,096927812	0	0	0	0	0,310156408	0,034461823
ENSG00000125851	PCSK2	0,021498161	0	0,039547603	0	0,102181688	0	0	0,094020363	0,052879932	0,310127747	0,034458639
ENSG00000147588	PMP2	0,085760823	0,076090238	0	0,148090224	0	0	0	0	0	0,309941286	0,034437921
ENSG00000106025	TSPAN12	0	0	0	0,30989902	0	0	0	0	0	0,30989902	0,034433224
ENSG00000173898	SPTBN2	0,015003035	0,053245036	0,027599295	0	0,213930228	0	0	0	0	0,309777594	0,034419733
ENSG00000165923	AGBL2	0	0,04890425	0	0	0,196489627	0,030259087	0	0	0,033895043	0,309548008	0,034394223
ENSG00000262179	MYMX	0	0	0	0,309531405	0	0	0	0	0	0,309531405	0,034392378
ENSG00000165105	RASEF	0	0,242151114	0	0,039273777	0	0	0	0	0,027972083	0,309396974	0,034377442
ENSG00000251692	PTX4	0	0	0	0,309164662	0	0	0	0	0	0,309164662	0,034351629
ENSG00000132464	ENAM	0,092677443	0,041113462	0	0,120025287	0,055062487	0	0	0	0	0,30887868	0,034319853
ENSG00000144285	SCN1A	0,053503094	0,031646642	0,016403877	0,107786193	0,021191877	0,078324361	0	0	0	0,308856043	0,034317338
ENSG00000196350	ZNF729	0	0,138324478	0	0	0	0	0	0,170458895	0	0,308783373	0,034309264
ENSG00000162383	SLC1A7	0	0	0,308466912	0	0	0	0	0	0	0,308466912	0,034274101
ENSG00000156574	NODAL	0	0	0	0,308251594	0	0	0	0	0	0,308251594	0,034250177
ENSG00000125823	CSTL1	0	0	0	0	0	0	0	0,307953931	0	0,307953931	0,034217103
ENSG00000187950	OVCH1	0,076357034	0,067746842	0	0,131851933	0	0	0,031700438	0	0	0,307656246	0,034184027
ENSG00000168453	HR	0,018974205	0,20201557	0	0	0,04509261	0	0	0,041491031	0	0,307573416	0,034174824
ENSG00000133107	TRPC4	0,035935926	0	0	0,186160505	0,085402507	0	0	0	0	0,307498939	0,034166549
ENSG00000115423	DNAH6	0,022363559	0,079367175	0,020569787	0,077233972	0	0,036830822	0,046422352	0,024451278	0	0,307238945	0,034137661
ENSG00000170703	TTLL6	0	0	0	0,307163007	0	0	0	0	0	0,307163007	0,034129223
ENSG00000060140	STYK1	0,043927491	0,077948256	0,080808168	0	0,104394634	0	0	0	0	0,30707855	0,034119839
ENSG00000109424	UCP1	0	0	0	0	0	0,30705227	0	0	0	0,30705227	0,034116919
ENSG00000186471	AKAP14	0	0	0	0	0,30693807	0	0	0	0	0,30693807	0,03410423
ENSG00000104938	CLEC4M	0	0,065947368	0	0,064174858	0,176644143	0	0	0	0	0,30676637	0,034085152
ENSG00000113263	ITK	0,017562828	0,062329614	0	0,121308682	0,083476881	0	0	0	0,021600014	0,306278019	0,034030891
ENSG00000144824	PHLDB2	0,012276429	0,130705338	0,112917418	0,021198714	0,0291752	0	0	0	0	0,3062731	0,034030344
ENSG00000173320	STOX2	0,039888404	0	0,024459314	0,022959523	0,189591311	0	0	0,029074753	0	0,305973305	0,033997034
ENSG00000188981	MSANTD1	0,025231353	0,089544832	0	0	0	0	0,104750653	0,055173579	0,031031311	0,305731728	0,033970192
ENSG00000179284	DAND5	0	0	0	0	0,179828514	0	0,12565882	0	0	0,305487334	0,033943037
ENSG00000091536	MYO15A	0,030216071	0	0	0,169573819	0,05385686	0,01658774	0	0,016518425	0,018580936	0,30533385	0,033925983
ENSG00000121898	CPXM2	0,031118322	0	0	0,05373455	0,147906731	0,034166099	0	0	0,038271524	0,305197226	0,033910803
ENSG00000007933	FMO3	0	0	0	0	0,208849981	0	0	0,096084492	0	0,304934473	0,033881608
ENSG00000163914	RHO	0	0,103449846	0	0,201338715	0	0	0	0	0	0,304788561	0,033865396
ENSG00000185966	LCE3E	0	0	0	0	0	0	0	0	0,304167794	0,304167794	0,033796422
ENSG00000182463	TSHZ2	0,047857663	0,042461124	0,110047545	0,020659935	0,056867386	0,026272459	0	0	0	0,304166112	0,033796235
ENSG00000104818	CGB2	0	0	0	0,303765977	0	0	0	0	0	0,303765977	0,033751775
ENSG00000166415	WDR72	0	0,125109997	0,032425067	0,030436834	0	0,038705372	0	0,077087266	0	0,303764535	0,033751615
ENSG00000196589	MBD3L2B	0	0	0	0,303412761	0	0	0	0	0	0,303412761	0,033712529
ENSG00000150394	CDH8	0,011169382	0,059459384	0	0,07714834	0,053088557	0,036789987	0	0,024424168	0,0412107	0,303290518	0,033698946
ENSG00000196090	PTPRT	0,046700323	0	0,021477254	0,120961898	0,027746082	0,025637113	0,009694069	0,051059966	0	0,303276705	0,033697412
ENSG00000172901	LVRN	0,045542667	0	0	0	0,108233135	0	0	0,149382716	0	0,303158518	0,03368428
ENSG00000280204	OR1S1	0	0	0	0,177025085	0	0	0	0	0,126083122	0,303108207	0,03367869
ENSG00000183304	FAM9A	0,095038095	0	0	0	0	0	0	0,207820483	0	0,302858577	0,033650953
ENSG00000114698	PLSCR4	0,178575479	0	0	0	0,084877699	0,039213088	0	0	0	0,302666266	0,033629585
ENSG00000139973	SYT16	0,009919297	0,052804648	0	0,068513844	0,141440544	0	0,008236204	0,021690598	0	0,302605135	0,033622793
ENSG00000113302	IL12B	0,054730377	0	0	0	0	0	0,0454438	0	0,201933925	0,302108102	0,033567567
ENSG00000133640	LRRIQ1	0	0	0	0,178051842	0,081682598	0	0	0	0,042271471	0,302005911	0,033556212
ENSG00000048545	GUCA1A	0	0	0	0	0	0	0,160790686	0,141151033	0	0,301941719	0,03354908
ENSG00000174792	ODAPH	0	0	0	0,127037475	0,174838141	0	0	0	0	0,301875617	0,033541735
ENSG00000184012	TMPRSS2	0	0,055654213	0,05769616	0,162475078	0	0	0,026041995	0	0	0,301867446	0,033540827
ENSG00000184788	SATL1	0,02147372	0,07620918	0	0,037080428	0,102065523	0,047153781	0,017830088	0	0	0,301812721	0,033534747
ENSG00000155897	ADCY8	0,204019672	0,060337984	0	0	0	0,037333613	0	0	0	0,301691269	0,033521252

ENSG00000162782	TDRD5	0,03694635	0,196681174	0,067965794	0	0	0	0	0	0	0,301593318	0,033510369
ENSG00000164707	SLC13A4	0,037706942	0	0	0	0	0	0,078272197	0	0,185498712	0,301477851	0,033497539
ENSG00000117477	CCDC181	0,038149601	0	0	0	0,090663353	0,125658099	0	0	0,046919092	0,301390145	0,033487794
ENSG00000165120	SSMEM1	0,110460943	0	0	0,19074194	0	0	0	0	0	0,301202884	0,033466987
ENSG00000162551	ALPL	0,152124736	0	0,093281912	0	0	0,05567469	0	0	0	0,301081338	0,033453482
ENSG00000257446	ZNF878	0	0	0	0,300963062	0	0	0	0	0	0,300963062	0,03344034
ENSG00000171016	PYGO1	0	0	0,032266988	0,151442237	0,083370294	0,019258337	0,014564171	0	0	0,300902027	0,033433559
ENSG00000133863	TEX15	0	0,022601315	0	0,021993845	0	0,083906226	0	0	0,172312183	0,300813569	0,03342373
ENSG00000177275	OR2AJ1	0,161356723	0	0	0,139313921	0	0	0	0	0	0,300670644	0,033407849
ENSG00000187569	DPPA3	0	0	0	0	0	0	0	0,300395061	0	0,300395061	0,033377229
ENSG00000117600	PLPPR4	0	0	0	0,23550088	0,064822661	0	0	0	0	0,300323541	0,033369282
ENSG00000156575	PRG3	0	0	0	0,300270397	0	0	0	0	0	0,300270397	0,033363377
ENSG00000128564	VGF	0	0,300158955	0	0	0	0	0	0	0	0,300158955	0,033350995
ENSG00000271321	CTAGE6	0	0	0	0,300155262	0	0	0	0	0	0,300155262	0,033350585
ENSG00000168077	SCARA3	0,037505726	0,133105982	0	0,129528406	0	0	0	0	0	0,300140114	0,033348902
ENSG00000136688	IL36G	0,110058682	0	0	0,190047323	0	0	0	0	0	0,300106005	0,033345112
ENSG00000170442	KRT86	0	0	0	0	0,300014655	0	0	0	0	0,300014655	0,033334962
ENSG00000104760	FGL1	0	0,243691609	0	0	0	0	0	0	0,056300067	0,299991676	0,033332408
ENSG00000118194	TNNT2	0	0,036939248	0	0	0,148416122	0,114279224	0	0	0	0,299634593	0,033292733
ENSG00000176971	FIBIN	0,155944861	0	0,143436584	0	0	0	0	0	0	0,299381445	0,033264605
ENSG00000163040	CCDC74A	0	0	0,090341274	0	0	0,107839179	0,040776838	0	0,06039861	0,299355901	0,033261767
ENSG00000158258	CLSTN2	0,072470128	0,036741847	0,038089901	0,089385782	0	0	0,017192427	0,045277414	0	0,2991575	0,033239722
ENSG00000249693	THEGL	0	0,299098717	0	0	0	0	0	0	0	0,299098717	0,033233191
ENSG00000273540	AGBL1	0,03335038	0,118358861	0	0,057588827	0,0528386	0,036616768	0	0	0	0,298753436	0,033194826
ENSG00000174469	CNTNAP2	0,110683443	0,032734176	0,016967594	0,079635895	0	0	0,038292844	0,020169356	0	0,298483309	0,033164812
ENSG00000170482	SLC23A1	0	0,083715891	0	0,162931611	0	0,051798494	0	0	0	0,298445996	0,033160666
ENSG00000221867	MAGEA3	0	0,151236323	0	0,147171446	0	0	0	0	0	0,298407769	0,033156419
ENSG00000105696	AC003112.1	0	0	0	0,298097838	0	0	0	0	0	0,298097838	0,033121982
ENSG00000007001	UPP2	0	0	0	0,176486287	0,121446582	0	0	0	0	0,297932868	0,033103652
ENSG00000103044	HAS3	0	0	0	0	0	0	0	0,190635327	0,107219147	0,297854475	0,033094942
ENSG00000163395	IGFN1	0	0,044262463	0,022943224	0,129218376	0,029639942	0,013693511	0	0,027272579	0,030677868	0,297707963	0,033078663
ENSG00000163564	PYHIN1	0	0	0,079902299	0,075002867	0	0,047689157	0	0,094979755	0	0,297574078	0,033063786
ENSG00000102802	MEDAG	0	0	0	0	0	0,072323704	0	0,144042968	0,081014177	0,297380849	0,033042317
ENSG00000102385	DRP2	0,03691476	0,098256504	0,033953841	0	0,087728727	0	0	0,040360885	0	0,297214718	0,033023858
ENSG00000144355	DLX1	0	0	0,077865574	0,219273088	0	0	0	0	0	0,297138662	0,033015407
ENSG00000160994	CCDC105	0	0	0	0,297023306	0	0	0	0	0	0,297023306	0,03300259
ENSG00000075290	WNT8B	0	0,126961174	0	0	0,170036715	0	0	0	0	0,296997889	0,032999765
ENSG00000123576	ESX1	0	0	0,185939866	0	0	0,110976974	0	0	0	0,29691684	0,03299076
ENSG00000170667	RASA4B	0	0	0	0	0	0,102837134	0,155541734	0	0,038398042	0,29677691	0,032975212
ENSG00000101542	CDH20	0	0	0,064994178	0	0	0	0	0,231775474	0	0,296769652	0,032974406
ENSG00000006659	LGALS14	0	0,296617257	0	0	0	0	0	0	0	0,296617257	0,032957473
ENSG00000168131	OR2B2	0	0	0	0	0,296301603	0	0	0	0	0,296301603	0,0329224
ENSG00000101470	TNNC2	0	0	0	0	0	0	0,296269969	0	0	0,296269969	0,032918885
ENSG00000279263	OR2L8	0	0	0,296038444	0	0	0	0	0	0	0,296038444	0,03289316
ENSG00000147885	IFNA16	0	0	0,296038444	0	0	0	0	0	0	0,296038444	0,03289316
ENSG00000111262	KCNA1	0,056772913	0,067161428	0	0,130712573	0	0	0	0,041381912	0	0,296028826	0,032892092
ENSG00000109511	ANXA10	0	0,295962473	0	0	0	0	0	0	0	0,295962473	0,032884719
ENSG00000171121	KCNMB3	0,084146659	0	0	0,116242332	0,039995271	0,055432869	0	0	0	0,295817132	0,03286857
ENSG00000156269	NAA11	0,111892314	0	0	0	0	0,061425612	0	0,122337863	0	0,295655789	0,032850643
ENSG00000162482	AKR7A3	0	0	0	0	0,295569994	0	0	0	0	0,295569994	0,03284111
ENSG00000130176	CNN1	0,106528425	0,189032076	0	0	0	0	0	0	0	0,295560501	0,032840056
ENSG00000122735	DNAI1	0	0,074195351	0,07691757	0,14440231	0	0	0	0	0	0,295515232	0,032835026
ENSG00000162594	IL23R	0,046253618	0,082075911	0	0	0,109922725	0	0	0	0,056885988	0,295138242	0,032793138
ENSG00000158553	POM121L2	0	0,079946929	0	0	0,21414284	0	0	0	0	0,294089769	0,032676641
ENSG00000160781	PAQR6	0,0597039	0	0	0,103095604	0	0,131102787	0	0	0	0,293902291	0,03265581
ENSG00000171936	OR10H3	0,293703733	0	0	0	0	0	0	0	0	0,293703733	0,032633748
ENSG00000257524	AL157935.2	0	0	0	0	0	0,130467033	0,065777369	0	0,097429369	0,293673772	0,032630419
ENSG00000157927	RADIL	0	0	0	0	0,055113189	0,15277217	0	0	0,085564697	0,293450057	0,032605562
ENSG00000147862	NFIB	0,011837883	0,021006032	0,043553482	0,163531516	0	0	0,009829247	0	0,043677209	0,293435367	0,03260393
ENSG00000182896	TMEM95	0	0	0	0	0,293396685	0	0	0	0	0,293396685	0,032599632
ENSG00000258947	TUBB3	0	0	0	0,1767054	0	0	0	0,074590196	0,041951811	0,293247407	0,032583045
ENSG00000240224	UGT1A5	0,064439476	0,228692537	0	0	0	0	0	0	0	0,293132013	0,032570224
ENSG00000157330	C1orf158	0,073123915	0	0	0,189403562	0	0	0,030358174	0	0	0,292885651	0,03254285
ENSG00000146215	CRIP3	0	0	0	0	0,292798648	0	0	0	0	0,292798648	0,032533183
ENSG00000143028	SYPL2	0	0,066338941	0,137545818	0	0,088846497	0	0	0	0	0,292731257	0,032525695
ENSG00000172689	MS4A10	0,065387525	0	0	0	0,155394869	0,071791682	0	0	0	0,292574076	0,032508231
ENSG00000146216	TTBK1	0	0,052417555	0	0,102017388	0,035100923	0,016216458	0	0,032297387	0,054495119	0,29254483	0,032504981
ENSG00000179213	SIGLECL1	0,064275019	0,22810889	0	0	0	0	0	0	0	0,292383909	0,032487101
ENSG00000162365	CYP4A22	0,078437877	0,069593044	0,14429281	0	0	0	0	0	0	0,292323732	0,032480415
ENSG00000116726	PRAMEF12	0	0	0	0,292200419	0	0	0	0	0	0,292200419	0,032466713
ENSG00000186377	CYP4X1	0,185260201	0	0	0,106634644	0	0	0	0	0	0,291894845	0,032432761
ENSG00000118322	ATP10B	0	0,142250398	0,049156516	0,023071174	0,031752214	0	0	0,029216142	0,016432053	0,291878497	0,032430944
ENSG00000145384	FABP2	0	0,216418079	0	0	0	0	0	0	0,074998597	0,291416676	0,032379631
ENSG00000173452	TMEM196	0,03697347	0	0	0,127690225	0	0	0	0,080850151	0,045472601	0,290986447	0,032331827
ENSG00000255804	OR6J1	0	0	0	0,125298907	0,086222699	0	0	0,079336031	0	0,290857637	0,032317515
ENSG00000188687	SLC4A5	0,018829978	0	0	0,162576308	0	0	0,109444526	0	0	0,290850812	0,032316757
ENSG00000125872	LRRN4	0	0	0	0,290789348	0	0	0	0	0	0,290789348	0,032309928
ENSG00000124939	SCGB2A1	0,290597251	0	0	0	0	0	0	0	0	0,290597251	0,032288583
ENSG00000175707	KDF1	0	0	0,149854501	0,140665755	0	0	0	0	0	0,290520255	0,032280028
ENSG00000088756	ARHGAP28	0,177627875	0,067542066	0,023340059	0,021908898	0	0	0	0	0	0,290418897	0,032268766
ENSG00000158352	SHROOM4	0,029248151	0	0	0,101010345	0	0	0	0,159892852	0	0,290151348	0,032239039
ENSG00000177283	FZD8	0,074992839	0,133072953	0	0	0	0	0	0,081993689	0	0,290059481	0,032228831
ENSG00000185101	ANO9	0,048690372	0,043199936	0	0,168155292	0	0	0	0	0,029941441	0,289987041	0,032220782
ENSG00000184388	PABPC1L2B	0	0	0,126527127	0	0,163458144	0	0	0	0	0,289985271	0,032220586
ENSG00000075388	FGF4	0,143232768	0	0,087829415	0	0	0	0	0	0,05871928	0,289781464	0,03219794
ENSG00000183837	PNMA3	0,08063531	0	0	0,208859371	0	0	0	0	0	0,289494681	0,032166076
ENSG00000139547	RDH16	0	0	0,160496593	0,075327649	0	0	0	0	0,053650844	0,289475085	0,032163898
ENSG00000196549	MME	0,031415919	0	0,05779212	0,162745306	0,037330306	0	0	0	0	0,289283652	0,032142628
ENSG00000176769	TCERG1L	0	0,050996957	0	0,049626279	0	0	0	0,188532465	0	0,289155701	0,032128411
ENSG00000013293	SLC7A14	0,071984837	0,153282393	0,026484384	0,024860421	0	0	0,011954109	0	0	0,288566145	0,032062905
ENSG00000184492	FOXD4L1	0	0	0	0	0,288332029	0	0	0	0	0,288332029	0,032036892
ENSG00000165905	LARGE2	0	0	0	0,248430633	0	0	0,03981921	0	0	0,288249843	0,03202776
ENSG00000121853	GHSR	0	0	0	0	0,288216326	0	0	0	0	0,288216326	0,032024036
ENSG00000120549	KIAA1217	0,027965313	0	0,025722226	0,217304966	0	0	0	0	0,017196865	0,288189371	0,032021041
ENSG00000167531	LALBA	0	0	0	0	0,287985198	0	0	0	0	0,287985198	0,031998355
ENSG00000261873	SMIM36	0,043100562	0	0	0	0,102429419	0,141965696	0	0	0	0,287495677	0,031943964
ENSG00000117983	MUC5B	0	0	0	0	0,038618942	0	0	0,248740937	0	0,287359879	0,031928875
ENSG00000181013	AC005666.1	0	0	0	0	0,169155696	0	0,118200972	0	0	0,287356668	0,031928519
ENSG00000157111	TMEM171	0	0	0	0	0,287294034	0	0	0	0	0,287294034	0,031921559
ENSG00000142408	CACNG8	0,017275703	0,03065531	0,127120201	0,029831368	0,082112162	0	0	0	0	0,286994744	0,031888305
ENSG00000137648	TMPRSS4	0,045570136	0	0,027943315	0,078689679	0,072198943	0,016677782	0,012612619	0,033216181	0	0,286908655	0,031878739
ENSG00000198678	OR5BS1P	0	0,286476496	0	0	0	0	0	0	0	0,286476496	0,031830722
ENSG00000161807	OR7G1	0	0,286476496	0	0	0	0	0	0	0	0,286476496	0,031830722
ENSG00000112041	TULP1	0	0	0,285987756	0	0	0	0	0	0	0,285987756	0,031776417
ENSG00000168748	CA7	0	0	0	0,285643103	0	0	0	0	0	0,285643103	0,031738123
ENSG00000162727	OR2M5	0	0,285561235	0	0	0	0	0	0	0	0,285561235	0,031729026
ENSG00000188039	NWD1	0,033900296	0	0	0,029269206	0,20141197	0	0	0	0,020846497	0,28542797	0,031714219
ENSG00000137675	MMP27	0	0	0	0	0,1877248	0	0	0	0,097149254	0,284874054	0,031652673
ENSG00000229972	IQCF3	0	0	0	0,284863509	0	0	0	0	0	0,284863509	0,031651501
ENSG00000203733	GJE1	0	0	0	0,284863509	0	0	0	0	0	0,284863509	0,031651501
ENSG00000138650	PCDH10	0,014840952	0,079004714	0,027301129	0,102508338	0	0,048883493	0,012322759	0	0	0,284861386	0,031651265
ENSG00000189108	IL1RAPL2	0	0,170411185	0	0	0,114114249	0	0	0	0	0,284525435	0,031613937
ENSG00000112280	COL9A1	0	0,037036188	0,038395041	0	0	0,183326604	0	0	0,025669409	0,284427242	0,031603027
ENSG00000130167	TSPAN16	0	0	0,178078218	0	0	0,106284803	0	0	0	0,284363021	0,031595891
ENSG00000123243	ITIH5	0,103302277	0,073322942	0	0,107028291	0	0	0	0	0	0,283653511	0,031517057
ENSG00000105388	CEACAM5	0	0	0	0,077867793	0,107167276	0	0	0,098607749	0	0,283642818	0,031515869
ENSG00000137270	GCM1	0,05435632	0	0,099992841	0	0,129178972	0	0	0	0	0,283528134	0,031503126

ENSG00000165084	C8orf34	0,073590421	0	0,054150209	0,050829838	0,104933537	0	0	0	0	0,283504006	0,031500445
ENSG00000279111	OR10X1	0	0	0,283364015	0	0	0	0	0	0	0,283364015	0,031484891
ENSG00000130876	SLC7A10	0	0	0	0,167749903	0,115434761	0	0	0	0	0,283184665	0,031464963
ENSG00000095110	NXPE1	0,068890162	0,122243902	0,063364509	0	0	0	0,028600486	0	0	0,283099059	0,031455451
ENSG00000121905	HPCA	0	0	0,283075457	0	0	0	0	0	0	0,283075457	0,031452829
ENSG00000106031	HOXA13	0,118844334	0	0,163967813	0	0	0	0	0	0	0,282812147	0,031423572
ENSG00000251258	RFPL4B	0	0,28255216	0	0	0	0	0	0	0	0,28255216	0,031394684
ENSG00000148156	ACTL7B	0	0,112712064	0,116847456	0	0	0	0,052740787	0	0	0,282300307	0,031366701
ENSG00000183421	RIPK4	0,037627134	0	0,069218152	0	0	0,082624789	0	0	0,092553049	0,282023124	0,031335903
ENSG00000203784	LELP1	0,281922706	0	0	0	0	0	0	0	0	0,281922706	0,031324745
ENSG00000165702	GFI1B	0,024755991	0	0	0,256489162	0	0	0	0	0	0,281245154	0,031249462
ENSG00000153404	PLEKHG4B	0,034074843	0,040309982	0,020894475	0,176519451	0	0	0,009431023	0	0	0,281229774	0,031247753
ENSG00000110243	APOA5	0	0	0	0	0,185303169	0	0	0	0,095896038	0,281199208	0,031244356
ENSG00000144681	STAC	0,103123683	0	0	0,178072094	0	0	0	0	0	0,281195777	0,031243975
ENSG00000154529	CNTNAP3B	0,011430452	0	0	0,019737895	0,135823579	0	0	0,099980202	0,014057982	0,28103011	0,031225568
ENSG00000167286	CD3D	0	0	0	0,28087726	0	0	0	0	0	0,28087726	0,031208584
ENSG00000142449	FBN3	0,045401273	0,026854482	0	0,156796179	0	0	0	0,033093096	0,018612571	0,280757601	0,031195289
ENSG00000153495	TEX29	0	0	0	0,280575242	0	0	0	0	0	0,280575242	0,031175027
ENSG00000131951	LRRC9	0,028129295	0	0,051746109	0	0,200549633	0	0	0	0	0,280425037	0,031158337
ENSG00000260325	HSPB9	0	0	0,144630645	0,135762214	0	0	0	0	0	0,280392858	0,031154762
ENSG00000198574	SH2D1B	0	0	0	0,280073318	0	0	0	0	0	0,280073318	0,031119258
ENSG00000228198	OR2M3	0,030425968	0,107980268	0	0,052539006	0,072307972	0,016702968	0	0	0	0,279956182	0,031106242
ENSG00000284987	U52112.2	0	0	0	0	0	0,279781748	0	0	0	0,279781748	0,031086861
ENSG00000260234	AC020636.2	0	0	0	0	0	0	0	0	0,279678739	0,279678739	0,031075415
ENSG00000182053	TRIM49B	0,279575524	0	0	0	0	0	0	0	0	0,279575524	0,031063947
ENSG00000196169	KIF19	0,053585309	0	0,098574503	0	0,127346646	0	0	0	0	0,279506458	0,031056273
ENSG00000135074	ADAM19	0	0	0,036082567	0,101610193	0,093228853	0	0	0	0,048246761	0,279168374	0,031018708
ENSG00000158022	TRIM63	0	0	0	0	0,279143057	0	0	0	0	0,279143057	0,031015895
ENSG00000133636	NTS	0	0,172438585	0	0	0	0,106694905	0	0	0	0,27913349	0,031014832
ENSG00000147571	CRH	0	0	0	0	0,278817968	0	0	0	0	0,278817968	0,030979774
ENSG00000205495	OR52J3	0	0	0	0,278776682	0	0	0	0	0	0,278776682	0,030975187
ENSG00000166106	ADAMTS15	0,026636801	0	0	0,229979706	0	0	0,022117104	0	0	0,278733611	0,030970401
ENSG00000146857	STRA8	0,152175801	0	0	0	0	0	0,126354816	0	0	0,278530617	0,030947846
ENSG00000152578	GRIA4	0,029973335	0,079780423	0,027569186	0,025878704	0	0	0,049775	0,065542907	0	0,278519556	0,030946617
ENSG00000143318	CASQ1	0	0,124312471	0	0	0	0,153834563	0	0	0	0,278147035	0,030905226
ENSG00000132855	ANGPTL3	0,04649556	0	0	0	0	0	0,231637536	0	0	0,278133096	0,030903677
ENSG00000198570	RD3	0,070844149	0	0	0	0,168362655	0,038891368	0	0	0	0,278098172	0,030899797
ENSG00000125998	FAM83C	0	0	0	0,164679694	0,113322039	0	0	0	0	0,278001733	0,030889081
ENSG00000160862	AZGP1	0	0	0	0	0,277954754	0	0	0	0	0,277954754	0,030883862
ENSG00000197472	ZNF695	0	0	0	0,277886022	0	0	0	0	0	0,277886022	0,030876225
ENSG00000183134	PTGDR2	0	0	0,096487365	0,181141947	0	0	0	0	0	0,277629312	0,030847701
ENSG00000138379	MSTN	0	0	0	0,277393665	0	0	0	0	0	0,277393665	0,030821518
ENSG00000206052	DOK6	0	0,028065941	0	0,136557973	0,112764562	0	0	0	0	0,277388476	0,030820942
ENSG00000204033	LRIT2	0	0	0	0,164265014	0,113036683	0	0	0	0	0,277301697	0,0308113
ENSG00000132000	PODNL1	0	0	0	0,187813082	0	0	0	0	0,089177794	0,276990875	0,030776764
ENSG00000171786	NHLH1	0	0	0	0,204094622	0	0	0	0	0,072681471	0,276776094	0,030752899
ENSG00000136834	OR1J1	0	0,27672033	0	0	0	0	0	0	0	0,27672033	0,030746703
ENSG00000138823	MTTP	0	0	0,051496869	0,09667839	0,066527889	0,061471129	0	0	0	0,276174276	0,030686031
ENSG00000104112	SCG3	0	0	0	0	0,113250565	0	0	0,104205162	0,058608175	0,276063902	0,030673767
ENSG00000112164	GLP1R	0	0	0,041601332	0,234302581	0	0	0	0	0	0,275903913	0,03065599
ENSG00000171773	NXNL1	0	0	0	0,275829783	0	0	0	0	0	0,275829783	0,030647754
ENSG00000146360	GPR6	0	0,275724422	0	0	0	0	0	0	0	0,275724422	0,030636047
ENSG00000225362	CT62	0,105856792	0	0	0	0,125785479	0	0,043947577	0	0	0,275589848	0,030621094
ENSG00000183615	FAM167B	0	0	0	0,275538516	0	0	0	0	0	0,275538516	0,030615391
ENSG00000154611	PSMA8	0	0	0	0,275538516	0	0	0	0	0	0,275538516	0,030615391
ENSG00000261649	GOLGA6L7	0	0	0,187507655	0,08800505	0	0	0	0	0	0,275512706	0,030612523
ENSG00000168070	MAJIN	0	0,115379518	0	0	0	0	0	0	0,159936766	0,275316284	0,030590698
ENSG00000171872	KLF17	0,096525436	0,085641009	0	0	0	0,052989644	0,040073565	0	0	0,275229655	0,030581073
ENSG00000110777	POU2AF1	0	0,038327902	0,158936592	0	0,051331839	0	0	0	0,026564683	0,275161015	0,030573446
ENSG00000187416	LHFPL3	0,081417333	0	0	0	0,193490055	0	0	0	0	0,274907388	0,030545265
ENSG00000182645	CCDC172	0,086004878	0	0	0	0	0,188856661	0	0	0	0,274861539	0,030540171
ENSG00000036530	CYP46A1	0,032316204	0	0,029724134	0,139507578	0	0,017740652	0	0,035333038	0,019872383	0,274493988	0,030499332
ENSG00000154997	SEPT14	0,038996276	0	0	0,067338058	0,092675495	0,042815633	0,032379441	0	0	0,274204902	0,030467211
ENSG00000174145	NWD2	0,05811945	0,068754359	0,071276948	0,033453202	0	0,042541174	0	0	0	0,274145133	0,03046057
ENSG00000171501	OR1N2	0,273999222	0	0	0	0	0	0	0	0	0,273999222	0,030444358
ENSG00000177551	NHLH2	0,060115596	0	0	0,034602171	0,047622005	0	0	0,131455205	0	0,273794978	0,030421664
ENSG00000197790	OR52M1	0	0	0	0,273516745	0	0	0	0	0	0,273516745	0,030390749
ENSG00000133135	RNF128	0,088420463	0	0	0,07634142	0	0	0	0	0,108745771	0,273507654	0,030389739
ENSG00000176540	OR4C5	0	0,273335372	0	0	0	0	0	0	0	0,273335372	0,030370597
ENSG00000168309	FAM107A	0	0,100786318	0,104484157	0	0,067490611	0	0	0	0	0,272761086	0,030306787
ENSG00000268223	ARL14EPL	0	0	0	0,272659326	0	0	0	0	0	0,272659326	0,030295481
ENSG00000144908	ALDH1L1	0,011020316	0,07822112	0,020272761	0,114178081	0,026190019	0	0,009150403	0	0,013553568	0,272586269	0,030287363
ENSG00000141448	GATA6	0,039776407	0	0	0	0,189058985	0,043672171	0	0	0	0,272507563	0,030278618
ENSG00000114124	GRK7	0	0	0	0,161120701	0,110872968	0	0	0	0	0,271993668	0,030221519
ENSG00000236398	TAS2R39	0,148584632	0	0	0	0	0	0,123372991	0	0	0,271957623	0,030217514
ENSG00000164778	EN2	0	0	0	0,076858608	0	0,09773819	0	0,09732977	0	0,271926567	0,030214063
ENSG00000154080	CHST9	0,039379058	0,023292391	0	0,11333173	0,031195061	0	0	0	0,064574886	0,271773125	0,030197014
ENSG00000187033	SAMD7	0	0	0,221321735	0	0	0	0,049948381	0	0	0,271270115	0,030141124
ENSG00000170624	SGCD	0	0	0	0,123268601	0,067860458	0	0	0,062440394	0,017559195	0,271128648	0,030125405
ENSG00000182263	FIGN	0	0,080911889	0	0	0,036121258	0,050063542	0	0,066472454	0,037386144	0,270955286	0,030106143
ENSG00000203727	SAMD5	0,046091374	0	0	0,119384615	0,054768574	0	0	0,050394169	0	0,270638733	0,03007097
ENSG00000186335	SLC36A2	0	0	0,065115975	0	0,08412217	0,077728075	0	0	0,043533971	0,270500191	0,030055577
ENSG00000109686	SH3D19	0,01940051	0	0	0,033500446	0,046105731	0,042601252	0,128869259	0	0	0,270477198	0,030053022
ENSG00000101955	SRPX	0	0,269896326	0	0	0	0	0	0	0	0,269896326	0,029988481
ENSG00000163885	CFAP100	0,089361662	0	0,082193997	0	0	0,098113883	0	0	0	0,269669543	0,029963283
ENSG00000152076	CCDC74B	0	0	0	0,232614196	0	0,036975836	0	0	0	0,269590032	0,029954448
ENSG00000157423	HYDIN	0,021059239	0,009342276	0	0,054547065	0	0,023121814	0	0,161176362	0	0,269246756	0,029916306
ENSG00000147432	CHRNB3	0,058411508	0	0	0	0,138816213	0	0	0	0,071838625	0,269066345	0,029896261
ENSG00000186648	CARMIL3	0	0,161118822	0	0	0,107891706	0	0	0	0	0,269010528	0,029890059
ENSG00000198798	MAGEB3	0	0	0	0,113203894	0,155799368	0	0	0	0	0,269003261	0,029889251
ENSG00000162415	ZSWIM5	0,024792546	0,087987531	0	0,042811317	0,058920023	0	0	0,054214039	0	0,268725456	0,029858384
ENSG00000189186	DCAF8L2	0,033965064	0	0	0,234601011	0	0	0	0	0	0,268566075	0,029840675
ENSG00000186777	ZNF732	0,068905869	0	0	0	0	0	0,114428027	0	0,084745336	0,268079233	0,029786581
ENSG00000184845	DRD1	0,037487118	0,06651997	0	0	0	0	0	0,163946697	0	0,267953785	0,029772643
ENSG00000188505	NCCRP1	0	0,135768101	0	0,132118974	0	0	0	0	0	0,267887076	0,029765231
ENSG00000204301	NOTCH4	0,108511899	0	0	0	0	0	0,038614177	0,101693035	0,019065093	0,267884203	0,029764911
ENSG00000166292	TMEM100	0,051999508	0,092271851	0	0	0,123577957	0	0	0	0	0,267849316	0,029761035
ENSG00000164520	RAET1E	0	0,267784953	0	0	0	0	0	0	0	0,267784953	0,029753884
ENSG00000170382	LRRN2	0,079727702	0,047158284	0,048888515	0,09178156	0	0	0	0	0	0,267556061	0,029728451
ENSG00000205277	MUC12	0,008821399	0	0	0,030465263	0,062892739	0,106539191	0,058596769	0	0	0,267315362	0,029701707
ENSG00000149090	PAMR1	0,039117414	0	0	0,135094473	0,092963381	0	0	0	0	0,267175269	0,029686141
ENSG00000144481	TRPM8	0,038120729	0	0,10518924	0,03291309	0,090594738	0	0	0	0	0,266817797	0,029646422
ENSG00000175356	SCUBE2	0	0,067102603	0,034782295	0,065299043	0,044934627	0	0,031398982	0	0,023254069	0,266771619	0,029641291
ENSG00000169129	AFAP1L2	0,026103052	0	0,048018673	0,135222823	0	0,057319253	0	0	0	0,266663801	0,029629311
ENSG00000164600	NEUROD6	0	0	0	0,266396095	0	0	0	0	0	0,266396095	0,029599566
ENSG00000174358	SLC6A19	0,028673733	0,050880835	0	0,049513278	0	0,031482083	0	0	0,105794984	0,266344912	0,029593879
ENSG00000157168	NRG1	0,028247606	0	0	0,073166177	0,050348286	0,046521332	0,005863648	0,061769243	0	0,265916292	0,029546255
ENSG00000198173	FAM47C	0,046211184	0	0	0	0,219643757	0	0	0	0	0,26585494	0,029539438
ENSG00000134874	DZIP1	0	0	0,065638748	0,123227851	0,042398765	0,019588026	0,014813498	0	0	0,265666889	0,029518543
ENSG00000273269	AC073283.3	0	0	0	0,265447584	0	0	0	0	0	0,265447584	0,029494176
ENSG00000161031	PGLYRP2	0,097271046	0	0	0,167965867	0	0	0	0	0	0,265236914	0,029470768
ENSG00000171564	FGB	0	0	0	0	0,265227136	0	0	0	0	0,265227136	0,029469682
ENSG00000204300	TMEM225	0,264873919	0	0	0	0	0	0	0	0	0,264873919	0,029430435
ENSG00000214107	MAGEB1	0	0	0	0,264774201	0	0	0	0	0	0,264774201	0,029419356
ENSG00000135960	EDAR	0,102424698	0	0	0	0,162276341	0	0	0	0	0,264701039	0,029411227
ENSG00000137860	SLC28A2	0	0	0	0,050286177	0,069207466	0	0,145080361	0	0	0,264574004	0,029397112
ENSG00000221989	OR2A2	0	0	0,264491055	0	0	0	0	0	0	0,264491055	0,029387895

ENSG00000086717	PPEF1	0	0,076328494	0	0	0	0	0	0,188121018	0	0,264449512	0,029383279
ENSG00000064787	BCAS1	0,018459635	0	0	0	0,043869722	0,040535201	0	0,161463263	0	0,26432782	0,029369758
ENSG00000136944	LMX1B	0,02591948	0,045993482	0,047680978	0,044757286	0	0,05691615	0,043042996	0	0	0,264310371	0,029367819
ENSG00000167755	KLK6	0	0	0	0,264104225	0	0	0	0	0	0,264104225	0,029344914
ENSG00000099864	PALM	0,030763553	0	0	0,159365823	0,073110249	0	0	0	0	0,263239625	0,029248847
ENSG00000196081	ZNF724	0	0	0	0	0	0,149828937	0,113308548	0	0	0,263137485	0,029237498
ENSG00000136531	SCN2A	0,044641232	0,045265583	0	0,088097902	0,045467509	0,007001923	0,010590448	0,013945329	0,00784328	0,262853207	0,029205912
ENSG00000174348	PODN	0	0,054722857	0	0,106504071	0	0,101577904	0	0	0	0,262804833	0,029200537
ENSG00000105357	MYH14	0	0	0	0,175764513	0,069114231	0	0	0	0,017883615	0,262762358	0,029195818
ENSG00000221931	OR6X1	0	0	0,262741114	0	0	0	0	0	0	0,262741114	0,029193457
ENSG00000105376	ICAM5	0,192008349	0	0,070642973	0	0	0	0	0	0	0,262651322	0,02918348
ENSG00000213949	ITGA1	0,024417306	0	0	0,028108906	0,058028257	0,026808776	0,114887194	0	0,010010046	0,262260486	0,029140054
ENSG00000016402	IL20RA	0	0	0	0,110331913	0,151846741	0	0	0	0	0,262178654	0,029130962
ENSG00000106436	MYL10	0,143232768	0	0	0	0	0	0,118929225	0	0	0,262161993	0,02912911
ENSG00000205693	MANSC4	0	0,262113392	0	0	0	0	0	0	0	0,262113392	0,02912371
ENSG00000182070	OR52A1	0,01544624	0	0,028414607	0,13336143	0	0,050877208	0	0,033776405	0	0,261875891	0,029097321
ENSG00000187054	TMPRSS11A	0,038063116	0,067542066	0	0,065726694	0,090457819	0	0	0	0	0,261789695	0,029087744
ENSG00000188996	HUS1B	0	0,261601951	0	0	0	0	0	0	0	0,261601951	0,029066883
ENSG00000163637	PRICKLE2	0,010253126	0	0,05658436	0,070819643	0	0,123830665	0	0	0	0,261487794	0,029054199
ENSG00000183024	OR1G1	0	0	0	0,26145789	0	0	0	0	0	0,26145789	0,029050877
ENSG00000054796	SPO11	0,161875277	0	0	0	0	0	0	0	0,099542861	0,261418138	0,02904646
ENSG00000186510	CLCNKA	0	0	0	0,225202797	0	0	0,036096183	0	0	0,26129898	0,02903322
ENSG00000284188	AL451007.3	0	0	0,261014177	0	0	0	0	0	0	0,261014177	0,029001575
ENSG00000168702	LRP1B	0,035471965	0,015736033	0,016313386	0,045939256	0,147524812	0	0	0	0	0,260985452	0,028998384
ENSG00000149305	HTR3B	0,09391583	0	0	0,05405738	0,074397668	0	0	0	0,038501455	0,260872333	0,028985815
ENSG00000134640	MTNR1B	0,077254893	0	0	0	0,183597926	0	0	0	0	0,260852819	0,028983647
ENSG00000161652	IZUMO2	0	0,17745996	0	0	0	0	0,083037943	0	0	0,260497903	0,028944211
ENSG00000224960	PPP4R3C	0	0	0	0	0,114697395	0	0,040073565	0,105536432	0	0,260307391	0,028923043
ENSG00000163126	ANKRD23	0	0	0,135500901	0,063596143	0	0	0,061160289	0	0	0,260257333	0,028917481
ENSG00000187908	DMBT1	0,056154058	0,033214666	0	0	0,088967557	0	0	0,081861654	0	0,260197935	0,028910882
ENSG00000215475	SIAH3	0,021358385	0	0	0	0,203034653	0	0,035468645	0	0	0,259861683	0,02887352
ENSG00000178445	GLDC	0	0	0,031097449	0	0,160696965	0,018560306	0,028072566	0	0,020790527	0,259217813	0,028801979
ENSG00000135116	HRK	0	0,078255363	0	0,076152043	0,104805937	0	0	0	0	0,259213342	0,028801482
ENSG00000136750	GAD2	0,10262178	0	0	0,044301354	0	0	0	0,11220189	0	0,259125024	0,028791669
ENSG00000270806	C17orf50	0	0	0,259068126	0	0	0	0	0	0	0,259068126	0,028785347
ENSG00000176198	OR11H4	0,091195275	0	0,167761074	0	0	0	0	0	0	0,258956349	0,028772928
ENSG00000139174	PRICKLE1	0,036753149	0	0	0,222126331	0	0	0	0	0	0,25887948	0,028764387
ENSG00000102924	CBLN1	0,058097105	0	0	0,200642041	0	0	0	0	0	0,258739146	0,028748794
ENSG00000169851	PCDH7	0,024673128	0	0,022694106	0,127815319	0,029318111	0,054179305	0	0	0	0,258679969	0,028742219
ENSG00000115353	TACR1	0	0,156869345	0	0,101768711	0	0	0	0	0	0,258638056	0,028737562
ENSG00000112212	TSPO2	0	0	0,258586139	0	0	0	0	0	0	0,258586139	0,028731793
ENSG00000125285	SOX21	0	0	0	0	0,258543947	0	0	0	0	0,258543947	0,028727105
ENSG00000007516	BAIAP3	0	0	0	0,169824259	0,0584311	0	0	0	0,030238614	0,258493974	0,028721553
ENSG00000223658	C1GALT1C1L	0	0	0	0	0	0	0	0,258353845	0	0,258353845	0,028705983
ENSG00000205328	OR6C65	0	0	0,258345817	0	0	0	0	0	0	0,258345817	0,028705091
ENSG00000283434	CSNKA2IP	0	0	0	0,158142409	0	0	0	0,100131687	0	0,258274096	0,028697122
ENSG00000205777	GAGE1	0	0	0,257627525	0	0	0	0	0	0	0,257627525	0,028625281
ENSG00000184524	CEND1	0,092819761	0,164706388	0	0	0	0	0	0	0	0,257526149	0,028614017
ENSG00000077800	FKBP6	0	0	0,175714348	0,054979978	0	0	0,02643707	0	0	0,257131396	0,028570155
ENSG00000048462	TNFRSF17	0	0	0	0,257078793	0	0	0	0	0	0,257078793	0,02856431
ENSG00000187175	KRTAP12-1	0,256990766	0	0	0	0	0	0	0	0	0,256990766	0,02855453
ENSG00000132681	ATP1A4	0,096187505	0	0	0	0,114295844	0,026402065	0,019966635	0	0	0,256852049	0,028539117
ENSG00000141750	STAC2	0	0	0	0,152148673	0,104698992	0	0	0	0	0,256847665	0,028538629
ENSG00000145861	C1QTNF2	0	0,111077879	0	0	0	0,068728491	0	0	0,07698696	0,25679333	0,028532592
ENSG00000197479	PCDHB11	0	0	0,13243454	0,124313947	0	0	0	0	0	0,256748487	0,02852761
ENSG00000255223	OR5M11	0	0	0	0,255818603	0	0	0	0	0	0,255818603	0,028424289
ENSG00000112139	MDGA1	0	0	0,059228714	0,129726195	0,025505507	0	0,01782249	0,023468364	0	0,255751271	0,028416808
ENSG00000131730	CKMT2	0	0	0	0	0,132908047	0,122805756	0	0	0	0,255713803	0,028412645
ENSG00000111432	FZD10	0,046042221	0	0	0,159009735	0	0,050551669	0	0	0	0,255603625	0,028400403
ENSG00000168356	SCN11A	0	0,208860444	0	0	0,046620478	0	0	0	0	0,255480922	0,028386769
ENSG00000180708	OR10K2	0,049986957	0	0	0,086316565	0,118795085	0	0	0	0	0,255098607	0,02834429
ENSG00000108684	ASIC2	0,026998494	0	0	0	0,128325011	0	0	0	0,099614002	0,254937506	0,02832639
ENSG00000165799	RNASE7	0	0	0	0	0,254874054	0	0	0	0	0,254874054	0,028319339
ENSG00000213694	S1PR3	0,011028359	0,117417311	0,060862669	0,038087137	0	0	0,027471245	0	0	0,254866721	0,028318525
ENSG00000188487	INSC	0	0	0	0	0,104546592	0	0	0,096196381	0,054103791	0,254846763	0,028316307
ENSG00000173253	DMRT2	0	0,075320787	0,078084297	0	0,100875714	0	0	0	0	0,254280798	0,028253422
ENSG00000169330	MINAR1	0,022222143	0,118297941	0	0,076745581	0	0	0,036903039	0	0	0,254168704	0,028240967
ENSG00000261150	EPPK1	0	0,033513561	0,052114754	0,081531988	0,022442041	0	0	0,041299159	0,023227912	0,254129415	0,028236602
ENSG00000053524	MCF2L2	0	0	0	0,215669709	0	0,032265768	0,006100269	0	0	0,254035745	0,028226194
ENSG00000141748	ARL5C	0	0,156899942	0	0	0	0,097080501	0	0	0	0,253980443	0,028220049
ENSG00000196724	ZNF418	0,033572666	0	0	0,057972667	0,079786168	0	0	0	0,082580103	0,253911605	0,028212401
ENSG00000215218	UBE2QL1	0,051267369	0	0,094310466	0,044263779	0	0	0,063852586	0	0	0,2536942	0,028188244
ENSG00000173208	ABCD2	0,096095752	0,127889666	0	0	0	0	0	0	0,029546347	0,253531766	0,028170196
ENSG00000167580	AQP2	0	0,062286179	0,064571452	0	0,083418709	0	0	0	0,043169924	0,253446263	0,028160696
ENSG00000122254	HS3ST2	0	0	0	0	0	0,25329859	0	0	0	0,25329859	0,028144288
ENSG00000172548	NIPAL4	0	0	0	0	0	0	0,208719659	0	0,044165048	0,252884707	0,028098301
ENSG00000181626	ANKRD62	0	0,055723608	0	0,162677665	0	0,034478507	0	0	0	0,25287978	0,028097753
ENSG00000176605	C14orf177	0	0	0	0	0,252721705	0	0	0	0	0,252721705	0,028080189
ENSG00000141946	ZIM3	0,057522105	0	0	0,099328121	0	0	0,095523663	0	0	0,25237389	0,028041543
ENSG00000175497	DPP10	0,030207011	0	0	0,104321829	0	0	0	0,09908083	0,018575365	0,252185034	0,028020559
ENSG00000149201	CCDC81	0	0,118020247	0,061175198	0	0	0	0	0,072718875	0	0,251914319	0,02799048
ENSG00000131620	ANO1	0	0,178137851	0,046168427	0	0	0,027555319	0	0	0	0,251861597	0,027984622
ENSG00000134588	USP26	0	0	0	0,251806972	0	0	0	0	0	0,251806972	0,027978552
ENSG00000164500	SPATA48	0,074512116	0	0	0	0,177079656	0	0	0	0	0,251591772	0,027954641
ENSG00000174332	GLIS1	0	0	0	0,251463194	0	0	0	0	0	0,251463194	0,027940355
ENSG00000120242	IFNA8	0	0	0	0,251140495	0	0	0	0	0	0,251140495	0,027904499
ENSG00000082126	MPP4	0,092065742	0	0	0,158977442	0	0	0	0	0	0,251043184	0,027893687
ENSG00000186007	LEMD1	0	0	0	0,250657997	0	0	0	0	0	0,250657997	0,027850889
ENSG00000171942	OR10H2	0	0	0	0,250657997	0	0	0	0	0	0,250657997	0,027850889
ENSG00000187753	C9orf153	0	0	0,094938558	0	0	0	0,042851889	0,112853336	0	0,250643784	0,027849309
ENSG00000156564	LRFN2	0,092422367	0	0	0	0	0	0	0,101050326	0,056833799	0,250306493	0,027811833
ENSG00000135744	AGT	0	0,126721172	0	0,123315205	0	0	0	0	0	0,250036377	0,02778182
ENSG00000004848	ARX	0,048526195	0,086108542	0	0	0,115323552	0	0	0	0	0,249958289	0,027773143
ENSG00000150893	FREM2	0,046424138	0	0,034160381	0,128262968	0	0,010194198	0,007709391	0	0,022838282	0,249589358	0,027732151
ENSG00000182489	XKRX	0,089626673	0	0,082437752	0,077382851	0	0	0	0	0	0,249447276	0,027716364
ENSG00000081051	AFP	0,139101477	0	0	0	0,110192557	0	0	0	0	0,249294033	0,027699337
ENSG00000134259	NGF	0	0	0	0,249221561	0	0	0	0	0	0,249221561	0,027691285
ENSG00000170807	LMOD2	0	0	0	0	0	0,248369127	0	0	0	0,248369127	0,02759657
ENSG00000164485	IL22RA2	0	0	0	0	0,247923743	0	0	0	0	0,247923743	0,027547083
ENSG00000256870	SLC5A8	0	0	0,066534251	0,062454518	0	0,039710526	0	0,079089174	0	0,247788468	0,027532052
ENSG00000214842	RAD51AP2	0	0	0	0,070068468	0	0	0	0,177462174	0	0,247530642	0,027503405
ENSG00000181234	TMEM132C	0,122183603	0	0	0,052746104	0,072592994	0	0	0	0	0,247522701	0,027502522
ENSG00000081800	SLC13A1	0,068671016	0	0	0	0	0	0,028509505	0,150163403	0	0,247343924	0,027482658
ENSG00000182583	VCX	0	0	0	0,247331729	0	0	0	0	0	0,247331729	0,027481303
ENSG00000186970	KRTAP15-1	0	0	0	0	0	0,247258684	0	0	0	0,247258684	0,027473187
ENSG00000183654	MARCH11	0	0	0,148098082	0	0	0	0	0	0,099012532	0,247110613	0,027456735
ENSG00000254413	CHKB-CPT1B	0	0	0	0	0	0	0,024984136	0	0,222038856	0,247022992	0,027446999
ENSG00000175894	TSPEAR	0,033858519	0	0	0,058466273	0,080465504	0	0	0,074038666	0	0,246828961	0,02742544
ENSG00000174576	NPAS4	0,044081263	0,07822112	0	0,076118721	0	0,048398651	0	0	0	0,246819756	0,027424417
ENSG00000183831	ANKRD45	0	0	0	0	0,246731393	0	0	0	0	0,246731393	0,027414599
ENSG00000151468	CCDC3	0	0	0	0,070542032	0,097085034	0,044852819	0,033920068	0	0	0,246399953	0,027377773
ENSG00000277494	GPIHBP1	0	0	0,24632707	0	0	0	0	0	0	0,24632707	0,027369674
ENSG00000115266	APC2	0,071900351	0	0	0,041385404	0,05695758	0,026314128	0,04975033	0	0	0,246307793	0,027367533
ENSG00000188676	IDO2	0	0	0,062551777	0	0,080809528	0,074667226	0,028233648	0	0	0,24626218	0,027362464
ENSG00000125999	BPIFB1	0,050119592	0	0	0,086545597	0	0	0	0,109596871	0	0,246262061	0,027362451
ENSG00000231861	OR5K2	0	0	0	0,24616507	0	0	0	0	0	0,24616507	0,027351674
ENSG00000172377	OR9I1	0,14237805	0	0	0	0	0	0	0,103779701	0	0,246157751	0,027350861

ENSG00000112541	PDE10A	0,020585402	0	0,025245672	0,047395327	0,032614435	0,015067712	0	0,105033238	0	0,245941786	0,027326865
ENSG00000173991	TCAP	0	0	0	0,245817216	0	0	0	0	0	0,245817216	0,027313024
ENSG00000214216	IQCJ	0	0	0	0	0,245718469	0	0	0	0	0,245718469	0,027302052
ENSG00000179603	GRM8	0	0	0	0	0	0,051389369	0,19431676	0	0	0,245706129	0,027300681
ENSG00000116032	GRIN3B	0	0	0	0,079529099	0,109453686	0	0	0	0,056643256	0,245626041	0,027291782
ENSG00000197253	TPSB2	0	0	0	0,150135198	0	0,095460631	0	0	0	0,245595829	0,027288425
ENSG00000135144	DTX1	0	0	0	0	0,245578442	0	0	0	0	0,245578442	0,027286494
ENSG00000177807	KCNJ10	0,045965193	0,040782053	0	0,158743711	0	0	0	0	0	0,245490957	0,027276773
ENSG00000275832	ARHGAP23	0	0	0	0	0	0	0	0,245372204	0	0,245372204	0,027263578
ENSG00000144452	ABCA12	0,062871051	0,083672353	0	0,081423437	0	0,017257185	0	0	0	0,245224026	0,027247114
ENSG00000253309	SERPINE3	0,173823509	0	0	0	0	0	0	0	0,07126017	0,245083679	0,02723152
ENSG00000261408	TEN1-CDK3	0	0	0	0	0	0,212815003	0,032188387	0	0	0,24500339	0,027222599
ENSG00000164900	GBX1	0	0	0	0	0,244797234	0	0	0	0	0,244797234	0,027199693
ENSG00000157510	AFAP1L1	0,069021272	0,040825518	0	0	0,109353697	0,025260441	0	0	0	0,244460928	0,027162325
ENSG00000255690	TRIL	0	0	0,168984863	0	0	0	0	0	0,07531774	0,244302602	0,027144734
ENSG00000083067	TRPM3	0,01517784	0,013466352	0,111683447	0,026208816	0,036070464	0,00833219	0	0,033189491	0	0,2441286	0,0271254
ENSG00000117407	ARTN	0	0	0	0,196413229	0	0	0,047222556	0	0	0,243635785	0,027070643
ENSG00000172508	CARNS1	0,026650894	0	0	0,184081111	0	0	0	0	0,032777164	0,24350917	0,027056574
ENSG00000158125	XDH	0,024919289	0	0,045841045	0,086060348	0,059221231	0,027359924	0	0	0	0,243401837	0,027044649
ENSG00000173546	CSPG4	0,036456109	0	0,100595934	0,062951743	0,043319366	0	0	0	0	0,243323152	0,027035906
ENSG00000204345	CD300LD	0,115971274	0	0	0	0	0,127329683	0	0	0	0,243300957	0,02703344
ENSG00000135454	B4GALNT1	0,01915702	0	0	0	0,182108287	0	0	0,041890792	0	0,243156099	0,027017344
ENSG00000187021	PNLIPRP1	0,030306974	0	0,055752126	0,157000586	0	0	0	0	0	0,243059686	0,027006632
ENSG00000155754	C2CD6	0,088640898	0,078645549	0	0	0	0	0	0,048457922	0,027254219	0,242998588	0,026999843
ENSG00000198398	TMEM207	0	0	0	0	0	0,114579128	0	0	0,128347046	0,242926173	0,026991797
ENSG00000058673	ZC3H11A	0	0	0	0	0	0,155565473	0	0	0,087129171	0,242694644	0,026966072
ENSG00000149633	KIAA1755	0,01872962	0	0,034454648	0,12936786	0,044511346	0	0,015551603	0	0	0,242615077	0,026957231
ENSG00000140798	ABCC12	0,017371028	0	0,063910817	0,119983895	0,041282624	0	0	0	0	0,242548363	0,026949818
ENSG00000198062	POTEH	0	0	0,242459746	0	0	0	0	0	0	0,242459746	0,026939972
ENSG00000163515	RETNLB	0	0,242443038	0	0	0	0	0	0	0	0,242443038	0,026938115
ENSG00000137975	CLCA2	0,171482718	0	0	0	0	0	0,028477152	0	0,042180327	0,242140197	0,026904466
ENSG00000196600	SLC22A25	0,040274672	0	0	0,069545569	0	0,044219237	0	0,088068915	0	0,242108393	0,026900933
ENSG00000113303	BTNL8	0	0	0	0,199491571	0	0,042280983	0	0	0	0,241772554	0,026863617
ENSG00000152785	BMP3	0,024804756	0	0,045630351	0,171329596	0	0	0	0	0	0,241764703	0,026862745
ENSG00000187323	DCC	0,023008842	0	0,042326623	0,059596873	0,082021517	0	0,009552366	0,0251568	0	0,241663022	0,026851447
ENSG00000183747	ACSM2A	0,018439362	0	0	0,222885274	0	0	0	0	0	0,241324636	0,026813848
ENSG00000182107	TMEM30B	0	0	0,124348065	0,116723317	0	0	0	0	0	0,241071382	0,026785709
ENSG00000185640	KRT79	0	0	0	0,102649479	0	0,065267733	0	0	0,073110355	0,241027566	0,026780841
ENSG00000132554	RGS22	0,047496643	0,126422442	0	0,041008168	0	0,026074269	0	0	0	0,241001523	0,026777947
ENSG00000170605	OR9K2	0,092563902	0	0	0	0,109990059	0	0,038428892	0	0	0,240982853	0,026775873
ENSG00000122012	SV2C	0	0,022691208	0	0,066243964	0,15194954	0	0	0	0	0,240884711	0,026764968
ENSG00000142748	FCN3	0	0	0	0,240826003	0	0	0	0	0	0,240826003	0,026758445
ENSG00000166073	GPR176	0,029519549	0,10476343	0	0	0,070153847	0	0	0	0,03630524	0,240742066	0,026749118
ENSG00000095777	MYO3A	0,1432158	0	0,032932129	0	0,042544431	0	0	0	0,022017121	0,240709481	0,026745498
ENSG00000106278	PTPRZ1	0,059627334	0	0	0,025740848	0,106279237	0	0,012377462	0	0,036666967	0,240691848	0,026743539
ENSG00000189132	FAM47B	0,071278571	0	0	0	0,169395067	0	0	0	0	0,240673638	0,026741515
ENSG00000137090	DMRT1	0	0	0	0,240640923	0	0	0	0	0	0,240640923	0,02673788
ENSG00000112238	PRDM13	0,046126548	0	0,08485351	0	0,109620739	0	0	0	0	0,240600797	0,026733422
ENSG00000163377	TAFA4	0	0	0	0,240493064	0	0	0	0	0	0,240493064	0,026721452
ENSG00000284609	OR8B3	0	0	0	0,240493064	0	0	0	0	0	0,240493064	0,026721452
ENSG00000187003	ACTL7A	0	0	0	0	0,240373188	0	0	0	0	0,240373188	0,026708132
ENSG00000084674	APOB	0,010190894	0	0,056240916	0,105584694	0,048437759	0,011189006	0,008461716	0	0	0,240104984	0,026678332
ENSG00000241128	OR14A2	0	0	0	0,240050575	0	0	0	0	0	0,240050575	0,026672286
ENSG00000163536	SERPINI1	0,124988065	0	0,11496282	0	0	0	0	0	0	0,239950885	0,026661209
ENSG00000076864	RAP1GAP	0,026257267	0	0	0,181362276	0	0	0	0	0,032293053	0,239912596	0,026656955
ENSG00000128578	STRIP2	0	0	0,04729966	0,088798698	0,061105588	0	0,042698769	0	0	0,239902715	0,026655857
ENSG00000188803	SHISA6	0	0	0,032599988	0,061202058	0,126346033	0,019457087	0	0	0	0,239605166	0,026622796
ENSG00000134389	CFHR5	0,053776004	0	0	0,185718843	0	0	0	0	0	0,239494847	0,026610539
ENSG00000120658	ENOX1	0,048729626	0	0	0,084145429	0	0	0	0,106557423	0	0,239432478	0,026603609
ENSG00000184838	PRR16	0	0	0,123436989	0,115868106	0	0	0	0	0	0,239305095	0,026589455
ENSG00000152591	DSPP	0,034594911	0	0	0,119475721	0	0	0	0	0,085094561	0,239165193	0,02657391
ENSG00000168594	ADAM29	0	0,090025852	0,09332889	0	0	0	0	0,055469963	0	0,238824705	0,026536078
ENSG00000165606	DRGX	0,104574789	0	0	0,090288919	0	0	0,04341534	0	0	0,238279048	0,02647545
ENSG00000189410	SH2D5	0	0,05975975	0,061952329	0,116307098	0	0	0	0	0	0,238019177	0,026446575
ENSG00000142235	LMTK3	0,079995008	0,094632786	0	0	0,063369956	0	0	0	0	0,237997751	0,026444195
ENSG00000034239	EFCAB1	0,051910193	0,138170045	0,047746496	0	0	0	0	0	0	0,237826733	0,026425193
ENSG00000131409	LRRC4B	0	0	0,237522158	0	0	0	0	0	0	0,237522158	0,026391351
ENSG00000158683	PKD1L1	0,044227484	0	0,02712001	0,050914141	0	0	0,024482016	0	0,09065684	0,237400491	0,026377832
ENSG00000049283	EPN3	0	0,059547413	0,061732201	0,115893837	0	0	0	0	0	0,237173451	0,026352606
ENSG00000132975	GPR12	0	0,101204756	0	0	0,135541628	0	0	0	0	0,236746383	0,026305154
ENSG00000198542	ITGBL1	0,024467385	0	0	0,042249834	0,116294541	0	0	0,053503006	0	0,236514766	0,026279418
ENSG00000060718	COL11A1	0,033243993	0,029495325	0,091732515	0,02870256	0,039502535	0	0,013801599	0	0	0,236478527	0,026275392
ENSG00000184292	TACSTD2	0	0	0,236478179	0	0	0	0	0	0	0,236478179	0,026275353
ENSG00000165349	SLC7A3	0	0,23645679	0	0	0	0	0	0	0	0,23645679	0,026272977
ENSG00000104415	CCN4	0	0	0,053560713	0,150829465	0	0,031967356	0	0	0	0,236357534	0,026261948
ENSG00000187244	BCAM	0	0	0	0,104186454	0	0	0	0,131936342	0	0,236122796	0,026235866
ENSG00000159871	LYPD5	0,056712543	0,050317508	0	0,097930184	0	0,031133529	0	0	0	0,236093764	0,02623264
ENSG00000153246	PLA2R1	0,059019882	0,017454889	0,036190613	0,033971485	0	0,021600127	0,024502734	0,043019733	0	0,235759462	0,026195496
ENSG00000179477	ALOX12B	0	0	0	0,190047323	0	0	0,045692036	0	0	0,23573936	0,026193262
ENSG00000184571	PIWIL3	0	0,14101604	0	0	0,094430066	0	0	0	0	0,235446106	0,026160678
ENSG00000198774	RASSF9	0,054799844	0	0,05040437	0	0,130233016	0	0	0	0	0,23543723	0,026159692
ENSG00000112309	B3GAT2	0	0	0	0,158985514	0	0	0,076448032	0	0	0,235433546	0,026159283
ENSG00000113212	PCDHB7	0	0	0,147665391	0	0	0	0	0,087764825	0	0,235430217	0,026158913
ENSG00000163217	BMP10	0	0	0,046053694	0,129689351	0,059495948	0	0	0	0	0,235238994	0,026137666
ENSG00000249662	LINC02218	0	0	0	0	0,235178482	0	0	0	0	0,235178482	0,026130942
ENSG00000241935	HOGA1	0,109289227	0	0	0,125812427	0	0	0	0	0	0,235101654	0,026122406
ENSG00000170477	KRT4	0,05252366	0	0	0	0,124823616	0,05766791	0	0	0	0,235015186	0,026112798
ENSG00000144645	OSBPL10	0,012617783	0,044779893	0,023211431	0	0,029986435	0	0	0	0,124145973	0,234741516	0,026082391
ENSG00000184144	CNTN2	0,02558161	0	0,031372959	0,147246191	0	0,009362371	0	0	0,020974722	0,234537852	0,026059761
ENSG00000163600	ICOS	0	0	0	0,098652164	0,135772228	0	0	0	0	0,234424392	0,026047155
ENSG00000125492	BARHL1	0	0	0	0,136901875	0	0	0	0	0,097506045	0,234407921	0,026045325
ENSG00000112769	LAMA4	0,013544015	0	0,024915309	0,1637129	0,032187644	0	0	0	0	0,234359867	0,026039985
ENSG00000221910	OR2F2	0	0	0	0,234232473	0	0	0	0	0	0,234232473	0,02602583
ENSG00000143858	SYT2	0	0	0	0,233842635	0	0	0	0	0	0,233842635	0,025982515
ENSG00000159307	SCUBE1	0,037397435	0,022120277	0,022931868	0,064577209	0,059250543	0	0	0,02725908	0	0,233536412	0,02594849
ENSG00000204006	C1orf185	0	0	0	0,233394432	0	0	0	0	0	0,233394432	0,025932715
ENSG00000179776	CDH5	0	0,088204605	0	0,085833873	0,059065385	0	0	0	0	0,233103864	0,025900429
ENSG00000234828	IQCM	0	0,099606984	0	0	0,133401762	0	0	0	0	0,233008745	0,025889861
ENSG00000089012	SIRPG	0	0	0	0,232769826	0	0	0	0	0	0,232769826	0,025863314
ENSG00000006071	ABCC8	0,108039016	0	0	0,1243732	0	0	0	0	0	0,232412217	0,02582358
ENSG00000261308	FIGNL2	0,0303862	0	0	0,052470335	0	0	0	0	0,149484434	0,232340968	0,025815663
ENSG00000135747	ZNF670-ZNF695	0,047266366	0	0	0,081618697	0	0	0	0,1033577	0	0,232242763	0,025804751
ENSG00000128310	GALR3	0	0,231756266	0	0	0	0	0	0	0	0,231756266	0,025750696
ENSG00000066735	KIF26A	0	0	0	0,231736212	0	0	0	0	0	0,231736212	0,025748468
ENSG00000099994	SUSD2	0	0	0	0,206804022	0	0	0,024860379	0	0	0,231664401	0,025740489
ENSG00000170786	SDR16C5	0,046084346	0	0,084775876	0	0	0	0	0,10077297	0	0,231633192	0,025737021
ENSG00000145087	STXBP5L	0,027263973	0,048379251	0,02507714	0,047078931	0	0	0	0	0,083827931	0,231627226	0,025736358
ENSG00000126778	SIX1	0,027747075	0	0	0,047913143	0	0	0	0,121349456	0,034125325	0,231134999	0,025681667
ENSG00000163630	SYNPR	0,037385099	0	0	0,19366772	0	0	0	0	0	0,231052819	0,025672535
ENSG00000196407	THEM5	0	0,230957795	0	0	0	0	0	0	0	0,230957795	0,025661977
ENSG00000178752	ERFE	0	0,149632813	0	0	0	0,046292014	0,035008463	0	0	0,230933289	0,025659254
ENSG00000124827	GCM2	0,123810382	0	0	0,106896753	0	0	0	0	0	0,230707135	0,025634126
ENSG00000181291	TMEM132E	0	0,043849877	0	0,128013888	0,058727317	0	0	0	0	0,230591082	0,025621231
ENSG00000077274	CAPN6	0	0	0	0,147754799	0	0,046973549	0,035523877	0	0	0,230252225	0,025583581
ENSG00000253485	PCDHGA5	0,184506191	0	0	0,04551456	0	0	0	0	0	0,230020752	0,025557861
ENSG00000145321	GC	0,072129151	0	0	0	0	0	0	0,157725331	0	0,229854481	0,025539387
ENSG00000082175	PGR	0,019619653	0,05222189	0,054137906	0,033878859	0,069939797	0	0	0	0	0,229798105	0,025533123

ENSG00000127252	PLAAT1	0	0,229377246	0	0	0	0	0	0	0	0,229377246	0,025486361
ENSG00000171811	CFAP46	0	0,025064685	0	0,170737037	0,033568662	0	0	0	0	0,229370384	0,025485598
ENSG00000162891	IL20	0	0	0	0	0,229175203	0	0	0	0	0,229175203	0,025463911
ENSG00000164749	HNF4G	0	0,055793175	0	0,108587172	0	0	0,026107019	0	0,038669688	0,229157054	0,025461895
ENSG00000176988	FMR1NB	0	0	0	0,229091286	0	0	0	0	0	0,229091286	0,025454587
ENSG00000182676	PPP1R27	0	0	0	0	0	0,229000106	0	0	0	0,229000106	0,025444456
ENSG00000196361	ELAVL3	0,03173925	0	0,058386914	0	0	0	0	0,138808892	0	0,228935057	0,025437229
ENSG00000185823	NPAP1	0,060235412	0,035628754	0,110807906	0	0	0,022044988	0	0	0	0,228717059	0,025413007
ENSG00000105479	CCDC114	0	0,12474622	0	0,060696668	0	0	0	0	0,043230175	0,228673063	0,025408118
ENSG00000097096	SYDE2	0,023592595	0	0	0,040739262	0,112136625	0	0	0,051590096	0	0,228058579	0,025339842
ENSG00000161180	CCDC116	0,051206564	0	0	0,17684512	0	0	0	0	0	0,228051684	0,025339076
ENSG00000169347	GP2	0,074659373	0	0	0,064460221	0,088714808	0	0	0	0	0,227834402	0,025314934
ENSG00000096006	CRISP3	0	0	0	0,116125934	0	0	0,111678088	0	0	0,227804023	0,025311558
ENSG00000205560	CPT1B	0	0	0	0	0	0	0,091762798	0	0,135918958	0,227681756	0,025297973
ENSG00000261115	TMEM178B	0	0	0,025681827	0,168749522	0,033177896	0	0	0	0	0,227609245	0,025289916
ENSG00000241149	AC115220.1	0	0	0	0	0	0	0	0,227415394	0	0,227415394	0,025268377
ENSG00000171885	AQP4	0,050853297	0	0	0	0,12085396	0	0	0,055600634	0	0,227307891	0,025256432
ENSG00000184933	OR6A2	0	0	0	0,060569864	0,166721236	0	0	0	0	0,2272911	0,025254567
ENSG00000136002	ARHGEF4	0,028481872	0	0	0,049181976	0,067687785	0	0,011824553	0	0,070058061	0,227234247	0,02524825
ENSG00000168779	SHOX2	0	0,106300099	0	0	0,071182863	0	0,049740469	0	0	0,22722343	0,025247048
ENSG00000016602	CLCA4	0	0	0	0,227097454	0	0	0	0	0	0,227097454	0,02523305
ENSG00000196570	PFN3	0	0	0	0	0	0	0,226890293	0	0	0,226890293	0,025210033
ENSG00000172016	REG3A	0	0	0	0	0,226858839	0	0	0	0	0,226858839	0,025206538
ENSG00000106631	MYL7	0	0	0	0	0	0	0	0,226635506	0	0,226635506	0,025181723
ENSG00000095587	TLL2	0,018525263	0	0	0,031989086	0,176102755	0	0	0	0	0,226617104	0,025179678
ENSG00000118514	ALDH8A1	0	0	0	0,182599702	0	0	0,043901446	0	0	0,226501148	0,025166794
ENSG00000174672	BRSK2	0	0	0	0,037800228	0	0	0	0	0,188458012	0,22625824	0,025139804
ENSG00000009709	PAX7	0	0	0	0,040311289	0,166437916	0	0,019383645	0	0	0,226132849	0,025125872
ENSG00000179520	SLC17A8	0	0	0,065070248	0,122160569	0	0,038836745	0	0	0	0,226067562	0,025118618
ENSG00000213973	ZNF99	0,038125538	0,033826416	0,10520251	0,032917242	0	0	0,015828224	0	0	0,22589993	0,025099992
ENSG00000182177	ASB18	0	0,096558156	0	0	0,129318524	0	0	0	0	0,225876681	0,025097409
ENSG00000249581	CLRN2	0	0	0	0	0	0	0	0	0,225815944	0,225815944	0,02509066
ENSG00000250673	REELD1	0,066863084	0	0	0	0,158901567	0	0	0	0	0,225764652	0,025084961
ENSG00000212722	KRTAP4-9	0	0	0	0,225527204	0	0	0	0	0	0,225527204	0,025058578
ENSG00000122420	PTGFR	0,026216268	0	0,096453886	0,045269774	0	0,057567861	0	0	0	0,225507789	0,025056421
ENSG00000177324	BEND2	0	0	0	0,225089476	0	0	0	0	0	0,225089476	0,025009942
ENSG00000179902	C1orf194	0	0	0,2250851	0	0	0	0	0	0	0,2250851	0,025009456
ENSG00000187821	HELT	0	0	0,224902993	0	0	0	0	0	0	0,224902993	0,024989221
ENSG00000185739	SRL	0,053002655	0,094051912	0,048751333	0	0	0,029096909	0	0	0	0,224902809	0,024989201
ENSG00000143340	FAM163A	0,103891764	0	0	0	0	0,057033543	0	0	0,063886738	0,224812045	0,024979116
ENSG00000188219	POTEE	0	0	0,132624093	0,062245939	0	0	0,0299309	0	0	0,224800931	0,024977881
ENSG00000157214	STEAP2	0	0,030750229	0	0,089771207	0	0,019026442	0	0	0,085250698	0,224798577	0,02497762
ENSG00000170788	DYDC1	0	0	0,224721179	0	0	0	0	0	0	0,224721179	0,02496902
ENSG00000177272	KCNA3	0	0	0,22453966	0	0	0	0	0	0	0,22453966	0,024948851
ENSG00000170178	HOXD12	0,224532794	0	0	0	0	0	0	0	0	0,224532794	0,024948088
ENSG00000154734	ADAMTS1	0	0,076014741	0	0,073971644	0,050902557	0,023516737	0	0	0	0,224405679	0,024933964
ENSG00000212122	TSSK1B	0	0	0,224358433	0	0	0	0	0	0	0,224358433	0,024928715
ENSG00000270011	ZNF559-ZNF177	0	0	0	0,12213198	0	0	0,058727045	0	0,043493218	0,224352243	0,024928027
ENSG00000174175	SELP	0	0	0	0,224299405	0	0	0	0	0	0,224299405	0,024922156
ENSG00000182870	GALNT9	0	0	0,038560147	0,036195724	0,149445502	0	0	0	0	0,224201373	0,024911264
ENSG00000080224	EPHA6	0,024020119	0	0,044186949	0,082955007	0	0,052745375	0,019944418	0	0	0,223851868	0,02487243
ENSG00000134539	KLRD1	0,034714121	0,046199518	0,015964858	0,074929639	0,041249431	0	0	0	0,010673474	0,22373104	0,024859004
ENSG00000182545	RNASE10	0	0,223451667	0	0	0	0	0	0	0	0,223451667	0,024827963
ENSG00000284732	AP002512.3	0	0	0	0,223403232	0	0	0	0	0	0,223403232	0,024822581
ENSG00000257008	GPR142	0	0	0	0	0,223331805	0	0	0	0	0,223331805	0,024814645
ENSG00000164318	EGFLAM	0,048879369	0	0	0	0,174244319	0	0	0	0	0,223123689	0,024791521
ENSG00000154143	PANX3	0	0	0	0	0	0	0	0	0,222971232	0,222971232	0,024774581
ENSG00000143107	FNDC7	0,049014133	0,173948751	0	0	0	0	0	0	0	0,222962884	0,024773654
ENSG00000125414	MYH2	0,02373713	0	0	0,122966529	0,056411804	0	0,019709446	0	0	0,222824909	0,024758323
ENSG00000151136	BTBD11	0	0	0	0,131851933	0,090732072	0	0	0	0	0,222584005	0,024731556
ENSG00000165863	C10orf82	0	0,222432186	0	0	0	0	0	0	0	0,222432186	0,024714687
ENSG00000070985	TRPM5	0	0	0	0,131718816	0,09064047	0	0	0	0	0,222359286	0,024706587
ENSG00000182346	DAOA	0,065843386	0	0	0	0,156478232	0	0	0	0	0,222321618	0,024702402
ENSG00000128591	FLNC	0,016283467	0,028894612	0	0	0,038698011	0,017878295	0	0	0,120159389	0,221913774	0,024657086
ENSG00000114270	COL7A1	0,013649225	0	0,075326556	0,117846163	0	0,014986052	0	0	0	0,221807995	0,024645333
ENSG00000127324	TSPAN8	0	0,221696569	0	0	0	0	0	0	0	0,221696569	0,024632952
ENSG00000106688	SLC1A1	0	0	0	0,131321074	0,09036677	0	0	0	0	0,221687844	0,024631983
ENSG00000126218	F10	0	0	0	0,22150677	0	0	0	0	0	0,22150677	0,024611863
ENSG00000171402	XAGE3	0	0	0	0	0	0	0,221288063	0	0	0,221288063	0,024587563
ENSG00000155052	CNTNAP5	0,1109883	0,049236504	0	0	0	0,060929334	0	0	0	0,221154138	0,024572682
ENSG00000171722	SPATA46	0,081067903	0	0	0,139986574	0	0	0	0	0	0,221054477	0,024561609
ENSG00000173612	GPRC6A	0,105597883	0	0	0	0	0	0	0,115455824	0	0,221053707	0,024561523
ENSG00000170681	CAVIN4	0,057786069	0	0	0,099783929	0	0,063445727	0	0	0	0,221015725	0,024557303
ENSG00000220948	TRIM51GP	0	0	0	0	0,220995411	0	0	0	0	0,220995411	0,024555046
ENSG00000145526	CDH18	0,023301553	0,041348034	0	0,040236696	0	0	0,11608666	0	0	0,220972943	0,024552549
ENSG00000185274	GALNT17	0,030870392	0	0,05678858	0	0,073364156	0,033893887	0,025632346	0	0	0,220549361	0,024505485
ENSG00000174498	IGDCC3	0,027404891	0,048629307	0	0	0,065128317	0	0,045509732	0	0,033704484	0,220376732	0,024486304
ENSG00000121361	KCNJ8	0	0	0	0,220291241	0	0	0	0	0	0,220291241	0,024476805
ENSG00000050820	BCAR1	0,016426848	0,058298076	0	0,02836558	0,117116276	0	0	0	0	0,22020678	0,02446742
ENSG00000173213	TUBB8P12	0	0	0	0	0,219912763	0	0	0	0	0,219912763	0,024434751
ENSG00000171435	KSR2	0,024780349	0,014657374	0	0,071317092	0,019630346	0	0,006858551	0,072249823	0,010158878	0,219652413	0,024405824
ENSG00000175820	CCDC168	0,014079062	0	0,181296999	0	0	0	0	0,015393393	0,008657716	0,219427169	0,024380797
ENSG00000102195	GPR50	0,154905762	0	0	0	0	0	0,06431078	0	0	0,219216542	0,024357394
ENSG00000197057	DTHD1	0,053795148	0,047729085	0	0	0	0	0	0,117634235	0	0,219158469	0,024350941
ENSG00000174640	SLCO2A1	0,015366135	0,027266829	0,056534492	0,026533961	0,036517952	0	0	0	0,056695095	0,218914465	0,024323829
ENSG00000056487	PHF21B	0	0,044638255	0	0,173753937	0	0	0	0	0	0,218392192	0,024265799
ENSG00000102837	OLFM4	0	0	0	0,09178156	0,126316406	0	0	0	0	0,218097966	0,024233107
ENSG00000184166	OR1D2	0	0,164605279	0	0,053393692	0	0	0	0	0	0,217998972	0,024222108
ENSG00000074181	NOTCH3	0,096371537	0	0	0,083206306	0,038171507	0	0	0	0	0,217749351	0,024194372
ENSG00000163114	PDHA2	0,103784733	0	0	0	0	0,113949572	0	0	0	0,217734305	0,024192701
ENSG00000256162	SMLR1	0	0,106744427	0,110660867	0	0	0	0	0	0	0,217405294	0,024156144
ENSG00000171053	PATE1	0,064357143	0	0	0	0,152946142	0	0	0	0	0,217303285	0,024144809
ENSG00000164930	FZD6	0	0	0	0,128381291	0,088343799	0	0	0	0	0,21672509	0,024080566
ENSG00000167768	KRT1	0	0	0,111728336	0,104877401	0	0	0	0	0	0,216605737	0,024067304
ENSG00000169181	GSG1L	0	0	0	0	0,132393564	0	0,023128172	0,060909598	0	0,216431333	0,024047926
ENSG00000138136	LBX1	0	0,092462759	0	0	0,123833635	0	0	0	0	0,216296394	0,024032933
ENSG00000135083	CCNJL	0,01640901	0	0,030185699	0,113339114	0	0,036032268	0	0	0,020180967	0,216147057	0,02401634
ENSG00000167434	CA4	0	0	0	0,215827109	0	0	0	0	0	0,215827109	0,02398079
ENSG00000143217	NECTIN4	0,08739767	0	0,080387536	0	0	0,047978767	0	0	0	0,215763973	0,023973775
ENSG00000119139	TJP2	0	0,069354543	0,017974788	0,033745228	0,046442618	0	0	0	0,048068936	0,215586112	0,023954012
ENSG00000269699	ZIM2	0	0	0	0	0	0	0	0,215547663	0	0,215547663	0,02394974
ENSG00000120328	PCDHB12	0	0	0,215210399	0	0	0	0	0	0	0,215210399	0,023912267
ENSG00000162188	GNG3	0	0	0	0	0	0	0	0	0,215100142	0,215100142	0,023900016
ENSG00000165186	PTCHD1	0,082106186	0,041627261	0,04315456	0,020254209	0,027875304	0	0	0	0	0,21501752	0,023890836
ENSG00000108576	SLC6A4	0	0	0	0,112101521	0,102854802	0	0	0	0	0,214956324	0,023884036
ENSG00000185002	RFX6	0	0	0	0	0	0	0,214688908	0	0	0,214688908	0,023854323
ENSG00000036828	CASR	0	0	0,138932161	0,065206574	0	0	0,010451506	0	0	0,214590242	0,02384336
ENSG00000145888	GLRA1	0	0	0	0,21440836	0	0	0	0	0	0,21440836	0,023823151
ENSG00000135409	AMHR2	0	0	0	0,214115132	0	0	0	0	0	0,214115132	0,02379057
ENSG00000121351	IAPP	0	0	0,128278772	0	0	0	0	0	0,085762124	0,214040896	0,023782322
ENSG00000183434	TFDP3	0	0	0	0	0,213760442	0	0	0	0	0,213760442	0,02375116
ENSG00000183813	CCR4	0,097648188	0	0	0	0,116031516	0	0	0	0	0,213679704	0,023742189
ENSG00000136573	BLK	0,061627476	0	0	0,0532086	0,073229515	0	0,025585304	0	0	0,213650895	0,023738988
ENSG00000214946	TBC1D26	0	0	0,097553992	0	0	0	0	0,115962298	0	0,213516289	0,023724032
ENSG00000204572	KRTAP5-10	0	0	0	0	0	0	0,213384918	0	0	0,213384918	0,023709435
ENSG00000163046	ANKRD30BL	0,030545901	0	0	0	0,145185988	0	0	0	0,03756752	0,213299409	0,023699934
ENSG00000214237	MINDY4B	0	0	0	0	0,212999729	0	0	0	0	0,212999729	0,023666637

ENSG00000244694	PTCHD4	0,058377659	0	0,053695209	0,100805476	0	0	0	0	0	0,212878345	0,023653149
ENSG00000091844	RGS17	0,038101505	0,033805093	0	0,098689476	0	0,041833227	0	0	0	0,212429301	0,023603256
ENSG00000140451	PIF1	0	0	0,05970363	0	0,077130056	0,035633715	0	0	0,03991549	0,212382891	0,023598099
ENSG00000177992	SPATA31E1	0,033745103	0	0,062076842	0,116540855	0	0	0	0	0	0,2123628	0,023595867
ENSG00000111863	ADTRP	0	0	0	0,125751795	0,086534348	0	0	0	0	0,212286143	0,023587349
ENSG00000168243	GNG4	0,058186589	0	0,053519465	0,100475539	0	0	0	0	0	0,212181592	0,023575732
ENSG00000164171	ITGA2	0,057412831	0,03395922	0	0,033046476	0,045480945	0,042023956	0	0	0	0,211923427	0,023547047
ENSG00000188263	IL17REL	0	0	0	0,211797869	0	0	0	0	0	0,211797869	0,023533097
ENSG00000163618	CADPS	0,010304164	0	0,037910685	0,035586086	0,048976139	0,01131337	0	0,06759657	0	0,211687015	0,023520779
ENSG00000006377	DLX6	0,087727472	0	0	0,075743099	0	0,048159819	0	0	0	0,21163039	0,023514488
ENSG00000171951	SCG2	0	0	0	0	0,139354886	0	0	0	0,072117393	0,21147228	0,02349692
ENSG00000165478	HEPACAM	0	0,070249411	0,072826853	0,068361272	0	0	0	0	0	0,211437536	0,02349306
ENSG00000188782	CATSPER4	0,074438705	0	0,136936009	0	0	0	0	0	0	0,211374714	0,023486079
ENSG00000271567	PPIAL4E	0	0	0	0	0	0,211351053	0	0	0	0,211351053	0,02348345
ENSG00000277150	F8A3	0	0	0	0	0	0	0	0	0,211309292	0,211309292	0,02347881
ENSG00000184344	GDF3	0	0	0	0,211112439	0	0	0	0	0	0,211112439	0,023456938
ENSG00000231887	PRH1	0	0	0	0	0	0,211082159	0	0	0	0,211082159	0,023453573
ENSG00000274209	ANTXRL	0	0,086469526	0	0,084145429	0	0	0,040461249	0	0	0,211076203	0,023452911
ENSG00000184949	FAM227A	0,014020279	0,124393208	0	0,072629887	0	0	0	0	0	0,211043375	0,023449264
ENSG00000187857	OR6C75	0,033073007	0,05868724	0	0	0,078598718	0	0	0	0,040675536	0,211034501	0,023448278
ENSG00000188051	TMEM221	0,062494033	0	0	0	0,148518421	0	0	0	0	0,211012454	0,023445828
ENSG00000179934	CCR8	0	0	0,210910546	0	0	0	0	0	0	0,210910546	0,023434505
ENSG00000120251	GRIA2	0	0	0,031090493	0	0,080330509	0	0	0,036957227	0,06235763	0,210735859	0,023415095
ENSG00000171864	PRND	0,037957943	0	0	0,131090166	0	0,041675603	0	0	0	0,210723712	0,023413746
ENSG00000181001	OR52N1	0	0,210720629	0	0	0	0	0	0	0	0,210720629	0,023413403
ENSG00000223614	ZNF735	0	0	0	0	0	0	0	0,210467877	0	0,210467877	0,02338532
ENSG00000212710	CTAGE1	0	0,124283662	0	0	0	0	0	0	0,086139755	0,210423417	0,02338038
ENSG00000176253	OR4K13	0,075807979	0,134519398	0	0	0	0	0	0	0	0,210327377	0,023369709
ENSG00000142677	IL22RA1	0	0	0	0,092628674	0	0	0	0,117300166	0	0,20992884	0,023325427
ENSG00000138068	SULT6B1	0	0	0,209796301	0	0	0	0	0	0	0,209796301	0,0233107
ENSG00000206026	SMIM21	0,037692834	0	0	0	0,089577835	0	0	0,08242319	0	0,209693859	0,023299318
ENSG00000125966	MMP24	0	0	0	0	0,082065252	0	0	0	0,127408493	0,209473745	0,023274861
ENSG00000179008	C14orf39	0	0	0	0	0,209336953	0	0	0	0	0,209336953	0,023259661
ENSG00000184650	ODF4	0	0	0,209322364	0	0	0	0	0	0	0,209322364	0,02325804
ENSG00000163519	TRAT1	0	0,106068829	0	0,103217949	0	0	0	0	0	0,209286778	0,023254086
ENSG00000181143	MUC16	0,031028865	0	0,006342234	0,113113496	0,008193419	0	0,008587976	0,037695023	0,004240167	0,209201178	0,023244575
ENSG00000177291	GJD4	0	0	0	0	0	0	0	0,209135802	0	0,209135802	0,023237311
ENSG00000120057	SFRP5	0	0,142401487	0	0	0	0	0,066633209	0	0	0,209034696	0,023226077
ENSG00000260691	ANKRD20A1	0,208972824	0	0	0	0	0	0	0	0	0,208972824	0,023219203
ENSG00000168079	SCARA5	0	0	0,126931552	0	0,081990306	0	0	0	0	0,208921859	0,02321354
ENSG00000146122	DAAM2	0,023328533	0,041395909	0	0	0,083161145	0,051226732	0,009685089	0	0	0,208797407	0,023199712
ENSG00000176294	OR4N2	0,040851736	0	0	0,070542032	0,097085034	0	0	0	0	0,208478802	0,023164311
ENSG00000221818	EBF2	0,10644588	0,03777712	0	0	0	0	0,017676857	0,046553194	0	0,208453051	0,02316145
ENSG00000168658	VWA3B	0,018190751	0,064558084	0	0,125645829	0	0	0	0	0	0,208394663	0,023154963
ENSG00000261740	BOLA2-SMG1P6	0	0,078472929	0,081352092	0	0	0,048554456	0	0	0	0,208379478	0,023153275
ENSG00000173406	DAB1	0	0,035524907	0	0,172850407	0	0	0	0	0	0,208375314	0,023152813
ENSG00000115616	SLC9A2	0,05050487	0	0	0,043605444	0	0,083177094	0	0	0,03105724	0,208344647	0,023149405
ENSG00000125255	SLC10A2	0	0	0	0,069048683	0,095029781	0,043903302	0	0	0	0,207981766	0,023109085
ENSG00000204347	BTBD17	0	0	0	0	0,207942989	0	0	0	0	0,207942989	0,023104777
ENSG00000143195	ILDR2	0	0,019227162	0	0,037420762	0,07725173	0	0	0,047387719	0,026652305	0,207939678	0,023104409
ENSG00000174325	DIRC1	0,20785498	0	0	0	0	0	0	0	0	0,20785498	0,023094998
ENSG00000070748	CHAT	0,040296152	0,071504533	0	0	0,095764678	0	0	0	0	0,207565363	0,023062818
ENSG00000168484	SFTPC	0,094799605	0	0	0	0,112646657	0	0	0	0	0,207446262	0,023049585
ENSG00000162438	CTRC	0	0,153839357	0	0	0	0	0	0	0,053312255	0,207151613	0,023016846
ENSG00000128709	HOXD9	0	0	0	0,139462841	0	0	0,067060573	0	0	0,206523413	0,022947046
ENSG00000099399	MAGEB2	0	0	0	0	0	0,20648485	0	0	0	0,20648485	0,022942761
ENSG00000174482	LINGO2	0	0	0	0	0,206389392	0	0	0	0	0,206389392	0,022932155
ENSG00000072080	SPP2	0	0	0	0,206272707	0	0	0	0	0	0,206272707	0,02291919
ENSG00000184363	PKP3	0	0	0	0,206272707	0	0	0	0	0	0,206272707	0,02291919
ENSG00000206538	VGLL3	0,023722225	0,063141758	0,021819474	0,040963104	0,05637638	0	0	0	0	0,206022941	0,022891438
ENSG00000172955	ADH6	0	0,060927516	0,06316294	0	0,081599078	0	0	0	0	0,205689535	0,022854393
ENSG00000160963	COL26A1	0	0	0,089642083	0	0,115807011	0	0	0	0	0,205449094	0,022827677
ENSG00000214960	CRPPA	0,046303224	0	0	0,079955561	0	0	0	0,050625796	0,028473498	0,205358079	0,022817564
ENSG00000175161	CADM2	0,015126183	0	0	0,104478468	0,035947702	0	0,012559593	0	0,037206511	0,205318457	0,022813162
ENSG00000100341	PNPLA5	0	0	0	0,205137559	0	0	0	0	0	0,205137559	0,022793062
ENSG00000183032	SLC25A21	0,040807608	0	0,07506889	0	0	0	0	0,089234288	0	0,205110785	0,022790087
ENSG00000187838	PLSCR3	0	0,094649488	0	0	0	0	0,044288857	0	0,065600608	0,204538953	0,02272655
ENSG00000125046	SSUH2	0,04813205	0	0	0,041556773	0,114386859	0	0	0	0	0,204075681	0,022675076
ENSG00000141668	CBLN2	0,027559834	0	0,050698541	0	0,065496542	0	0	0,060265287	0	0,204020204	0,022668912
ENSG00000142675	CNKSR1	0	0,067712626	0,070196995	0,06589267	0	0	0	0	0	0,203802291	0,022644699
ENSG00000163098	BIRC8	0,07473322	0	0	0,12904796	0	0	0	0	0	0,20378118	0,022642353
ENSG00000170054	SERPINA9	0	0	0	0,203775849	0	0	0	0	0	0,203775849	0,022641761
ENSG00000160401	CFAP157	0,032123846	0,057002976	0,059094409	0,05547087	0	0	0	0	0	0,203692101	0,022632456
ENSG00000162706	CADM3	0	0	0	0,203484514	0	0	0	0	0	0,203484514	0,02260939
ENSG00000111405	ENDOU	0,056405588	0,100090332	0	0	0	0	0,046834764	0	0	0,203330684	0,022592298
ENSG00000162631	NTNG1	0,016536504	0,02934362	0	0	0,039299359	0,054468344	0,027461224	0,036160491	0	0,203269542	0,022585505
ENSG00000178947	SMIM10L2A	0	0	0,053151071	0,149675894	0	0	0	0	0	0,202826964	0,022536329
ENSG00000133661	SFTPD	0	0,202677249	0	0	0	0	0	0	0	0,202677249	0,022519694
ENSG00000081842	PCDHA6	0	0	0	0,085050513	0,117052654	0	0	0	0	0,202103167	0,022455907
ENSG00000140519	RHCG	0	0	0	0,202040244	0	0	0	0	0	0,202040244	0,022448916
ENSG00000147256	ARHGAP36	0	0	0	0,084967429	0,116938308	0	0	0	0	0,201905737	0,022433971
ENSG00000128655	PDE11A	0,012318462	0,021858808	0	0,021271295	0,058550182	0,013524951	0,020456563	0,053873737	0	0,201853999	0,022428222
ENSG00000101198	NKAIN4	0,058321332	0	0	0	0	0	0	0	0,14345544	0,201776772	0,022419641
ENSG00000149635	OCSTAMP	0,073965037	0	0	0,127721476	0	0	0	0	0	0,201686513	0,022409613
ENSG00000147381	MAGEA4	0	0	0	0,08469165	0,116558761	0	0	0	0	0,201250411	0,022361157
ENSG00000101441	CST4	0,201212478	0	0	0	0	0	0	0	0	0,201212478	0,022356942
ENSG00000213999	MEF2B	0	0	0	0	0	0	0	0	0,201023821	0,201023821	0,02233598
ENSG00000126603	GLIS2	0	0	0	0,116775554	0	0	0,084227119	0	0	0,201002673	0,02233363
ENSG00000170209	ANKK1	0	0	0	0,200873729	0	0	0	0	0	0,200873729	0,022319303
ENSG00000249853	HS3ST5	0,073640629	0	0	0,127161294	0	0	0	0	0	0,200801923	0,022311325
ENSG00000121743	GJA3	0,028981697	0,102854622	0	0	0,068875631	0	0	0	0	0,200711951	0,022301328
ENSG00000167676	PLIN4	0	0	0	0,20065747	0	0	0	0	0	0,20065747	0,022295274
ENSG00000166573	GALR1	0,161395891	0	0,02699098	0	0	0	0,012182768	0	0	0,200569639	0,022285515
ENSG00000047617	ANO2	0,048316729	0	0	0,041716223	0,057412876	0,053048945	0	0	0	0,200494774	0,022277197
ENSG00000177476	OR2G3	0	0	0	0	0	0	0	0	0,19983497	0,19983497	0,022203886
ENSG00000105929	ATP6V0A4	0,073283497	0	0	0,126544605	0	0	0	0	0	0,199828101	0,022203122
ENSG00000133687	TMTC1	0,030878803	0	0,056804053	0	0,024461382	0,056505203	0,008546443	0,022507634	0	0,199703517	0,02218928
ENSG00000143125	PROK1	0	0	0,199554989	0	0	0	0	0	0	0,199554989	0,022172777
ENSG00000196376	SLC35F1	0,059062173	0	0	0	0,140362534	0	0	0	0	0,199424707	0,022158301
ENSG00000142484	TM4SF5	0	0	0	0	0	0	0	0	0,19940614	0,19940614	0,022156238
ENSG00000196344	ADH7	0,145859624	0	0	0	0	0,053381781	0	0	0	0,199241406	0,022137934
ENSG00000225180	PVALEF	0	0	0	0	0,199177783	0	0	0	0	0,199177783	0,022130865
ENSG00000156486	KCNS2	0	0	0,051978328	0	0	0	0,023461169	0,123573137	0	0,199012634	0,022112515
ENSG00000171956	FOXB1	0	0	0	0,072361335	0	0	0,034794879	0,091634655	0	0,198790869	0,022087874
ENSG00000102996	MMP15	0,035715096	0	0	0	0,084877699	0	0	0,078098456	0	0,19869125	0,022076806
ENSG00000146910	CNPY1	0	0	0	0	0,198681904	0	0	0	0	0,198681904	0,022075767
ENSG00000136872	ALDOB	0	0,067832532	0	0	0	0	0	0,083590834	0,047014046	0,198437412	0,022048601
ENSG00000151812	SLC35F4	0,071278571	0	0,065561344	0,061541268	0	0	0	0	0	0,198381183	0,022042354
ENSG00000286095	SPEGNB	0	0	0	0	0	0	0	0	0,198342073	0,198342073	0,022038008
ENSG00000151475	SLC25A31	0	0	0	0	0,198188489	0	0	0	0	0,198188489	0,022020943
ENSG00000244537	KRTAP4-2	0,19804793	0	0	0	0	0	0	0	0	0,19804793	0,022005326
ENSG00000096264	NCR2	0	0	0	0	0	0	0,197902732	0	0	0,197902732	0,021989192
ENSG00000277932	OR52E5	0	0	0,123877049	0	0	0,073935195	0	0	0	0,197812244	0,021979138
ENSG00000053108	FSTL4	0,067550546	0	0	0,077763366	0	0,024722184	0	0	0,027692821	0,197728917	0,02196988
ENSG00000155816	FMN2	0,031201852	0	0	0,053878789	0,074151877	0	0	0	0,038374256	0,197606775	0,021956308
ENSG00000149451	ADAM33	0	0,045961947	0	0	0,123111945	0,028438563	0	0	0	0,197512455	0,021945828
ENSG00000142233	NTN5	0,048510617	0	0,089239197	0	0	0	0	0	0,059661805	0,197411619	0,021934624

ENSG00000162733	DDR2	0,056300511	0,024975969	0,025892334	0,07291402	0	0	0	0	0,017310593	0,197393427	0,021932603
ENSG00000124205	EDN3	0	0,197380935	0	0	0	0	0	0	0	0,197380935	0,021931215
ENSG00000274276	CBSL	0,150959611	0	0	0	0	0	0	0	0,046415215	0,197374826	0,021930536
ENSG00000138100	TRIM54	0	0	0	0	0,129926752	0	0	0	0,06723825	0,197165002	0,021907222
ENSG00000036473	OTC	0	0	0	0	0	0,197160519	0	0	0	0,197160519	0,021906724
ENSG00000174417	TRHR	0	0	0	0	0	0	0	0,196921673	0	0,196921673	0,021880186
ENSG00000134121	CHL1	0,01292206	0	0,023771173	0,08925431	0	0,042563001	0	0,028256761	0	0,196767305	0,021863034
ENSG00000171724	VAT1L	0	0,065352669	0,067750451	0,063596143	0	0	0	0	0	0,196699262	0,021855474
ENSG00000196188	CTSE	0	0	0	0,196413229	0	0	0	0	0	0,196413229	0,021823692
ENSG00000144730	IL17RD	0,079024459	0,028045393	0	0,054583197	0	0	0	0,03456067	0	0,196213719	0,021801524
ENSG00000237452	BHMG1	0	0	0,101120443	0,094919962	0	0	0	0	0	0,196040405	0,021782267
ENSG00000138795	LEF1	0	0,050017161	0	0,146018452	0	0	0	0	0	0,196035613	0,021781735
ENSG00000074317	SNCB	0	0,133536853	0	0	0	0	0,062485225	0	0	0,196022078	0,021780231
ENSG00000064270	ATP2C2	0,020553668	0,03647198	0	0	0,048846238	0,045133454	0	0,044944854	0	0,195950195	0,021772244
ENSG00000161850	KRT82	0	0	0	0,195897128	0	0	0	0	0	0,195897128	0,021766348
ENSG00000115363	EVA1A	0	0,195367577	0	0	0	0	0	0	0	0,195367577	0,021707509
ENSG00000130957	FBP2	0	0	0	0,19531061	0	0	0	0	0	0,19531061	0,021701179
ENSG00000071203	MS4A12	0	0	0	0	0,195278707	0	0	0	0	0,195278707	0,021697634
ENSG00000170848	PSG6	0,029398944	0	0	0,101531119	0	0	0	0,064286881	0	0,195216943	0,021690771
ENSG00000285589	AC010422.8	0,030071755	0	0	0	0	0,165085151	0	0	0	0,195156906	0,021684101
ENSG00000154240	CEP112	0	0	0	0,033033925	0,090927343	0	0,047652994	0	0,023527854	0,195142116	0,021682457
ENSG00000172061	LRRC15	0	0	0,046797996	0,087856894	0,060457499	0	0	0	0	0,195112389	0,021679154
ENSG00000120156	TEK	0,031455156	0	0	0,05431619	0,07475386	0,034535924	0	0	0	0,195061129	0,021673459
ENSG00000180440	SERTM1	0,195044299	0	0	0	0	0	0	0	0	0,195044299	0,021671589
ENSG00000174992	ZG16	0	0	0	0	0,114660773	0	0,08012154	0	0	0,194782314	0,021642479
ENSG00000114200	BCHE	0,049189639	0	0	0,08493977	0	0	0	0	0,060496915	0,194626324	0,021625147
ENSG00000172179	PRL	0	0	0,194255834	0	0	0	0	0	0	0,194255834	0,021583982
ENSG00000139540	SLC39A5	0,10410649	0	0	0,089884593	0	0	0	0	0	0,193991084	0,021554565
ENSG00000286268	AF196969.1	0,105968142	0	0	0	0	0	0,08798761	0	0	0,193955752	0,021550639
ENSG00000186897	C1QL4	0,072894631	0	0	0	0	0	0,121051936	0	0	0,193946568	0,021549619
ENSG00000115386	REG1A	0	0	0	0	0	0,193481722	0	0	0	0,193481722	0,021497969
ENSG00000101890	GUCY2F	0	0	0	0	0,19338586	0	0	0	0	0,19338586	0,021487318
ENSG00000250803	AC010255.3	0	0	0	0,081313485	0,111909487	0	0	0	0	0,193222972	0,021469219
ENSG00000109061	MYH1	0	0,133559024	0	0	0,059624364	0	0	0	0	0,193183387	0,021464821
ENSG00000249961	TERB1	0,048824094	0	0	0,084308554	0	0	0	0	0,060047342	0,19317999	0,021464443
ENSG00000180269	GPR139	0	0	0	0	0	0	0	0,193010846	0	0,193010846	0,02144565
ENSG00000111816	FRK	0,022616264	0,040132006	0	0,039053352	0,053748042	0,037247005	0	0	0	0,19279667	0,021421852
ENSG00000251537	AC005324.3	0	0	0	0	0,065128317	0,060177939	0	0	0,067408967	0,192715223	0,021412803
ENSG00000151338	MIPOL1	0	0,039724741	0,027454825	0,025771356	0,070936798	0	0,012392132	0,016317756	0	0,192597607	0,021399734
ENSG00000182674	KCNB2	0,120952965	0,071542689	0	0	0	0	0	0	0	0,192495654	0,021388406
ENSG00000171790	SLFNL1	0	0	0	0	0	0,192471667	0	0	0	0,192471667	0,021385741
ENSG00000166069	TMCO5A	0,056883332	0	0	0	0,135184469	0	0	0	0	0,192067801	0,021340867
ENSG00000182836	PLCXD3	0,019541002	0,138700116	0	0,033743046	0	0	0	0	0	0,191984165	0,021331574
ENSG00000169744	LDB2	0	0	0	0,095668185	0,06583273	0,030414405	0	0	0	0,19191532	0,021323924
ENSG00000162669	HFM1	0	0	0	0	0	0	0	0	0,191792077	0,191792077	0,021310231
ENSG00000102174	PHEX	0,02324778	0	0	0	0,110497705	0	0,057909384	0	0	0,191654869	0,021294985
ENSG00000138653	NDST4	0	0,072100565	0,074745926	0	0	0,04461161	0	0	0	0,191458101	0,021273122
ENSG00000163518	FCRL4	0,038925958	0	0	0,067216634	0	0	0	0,085119672	0	0,191262265	0,021251363
ENSG00000172000	ZNF556	0	0	0,042284773	0,039691964	0,109253892	0	0	0	0	0,191230629	0,021247848
ENSG00000148357	HMCN2	0,022262521	0	0,122861115	0,025628343	0	0,008147649	0,012323364	0	0	0,191222992	0,021246999
ENSG00000165379	LRFN5	0,049221684	0,043671336	0	0	0	0,027021267	0,040869815	0	0,030268163	0,191052265	0,021228029
ENSG00000135248	FAM71F1	0,059940726	0	0	0	0	0	0	0,131072815	0	0,191013542	0,021223727
ENSG00000285932	AL133353.2	0	0	0	0	0	0	0,19097463	0	0	0,19097463	0,021219403
ENSG00000125816	NKX2-4	0	0	0	0	0	0,190921262	0	0	0	0,190921262	0,021213474
ENSG00000162267	ITIH3	0,025521123	0	0	0,04406941	0,121303004	0	0	0	0	0,190893537	0,021210393
ENSG00000168280	KIF5C	0	0	0	0,056491659	0	0	0,01358198	0	0,120705687	0,190779326	0,021197703
ENSG00000143520	FLG2	0,016561878	0	0,030466911	0,028598748	0,078719321	0,036367948	0	0	0	0,190714805	0,021190534
ENSG00000164061	BSN	0,009304265	0	0,017115947	0,03213287	0,044223575	0	0,00772553	0	0,080101327	0,190603515	0,021178168
ENSG00000162763	LRRC52	0	0	0	0,190602611	0	0	0	0	0	0,190602611	0,021178068
ENSG00000149452	SLC22A8	0,017708962	0	0	0,091738536	0,042085731	0,03888681	0	0	0	0,190420039	0,021157782
ENSG00000122584	NXPH1	0	0	0	0	0,190311363	0	0	0	0	0,190311363	0,021145707
ENSG00000186675	MAGEE2	0,134380232	0	0	0	0	0	0,055789387	0	0	0,190169619	0,021129958
ENSG00000180697	C3orf22	0,069700448	0	0	0,120357461	0	0	0	0	0	0,190057908	0,021117545
ENSG00000198074	AKR1B10	0,085228748	0	0	0	0	0	0	0	0,104820374	0,190049122	0,021116569
ENSG00000110328	GALNT18	0	0	0	0,143924421	0	0,045755813	0	0	0	0,189680233	0,021075581
ENSG00000185304	RGPD2	0	0	0	0,111542451	0,051170924	0	0	0	0,026481408	0,189194783	0,021021643
ENSG00000170500	LONRF2	0	0,019245102	0,019951202	0,149822708	0	0	0	0	0	0,189019012	0,021002112
ENSG00000196832	OR11G2	0	0	0	0,115637037	0	0	0	0,073218384	0	0,188855421	0,020983936
ENSG00000196917	HCAR1	0,09507796	0	0	0,054726295	0	0	0	0	0,038977878	0,188782133	0,020975793
ENSG00000183036	PCP4	0	0	0	0	0	0	0	0	0,188676672	0,188676672	0,020964075
ENSG00000150361	KLHL1	0	0	0	0,188673156	0	0	0	0	0	0,188673156	0,020963684
ENSG00000179528	LBX2	0,066393045	0	0,122135369	0	0	0	0	0	0	0,188528414	0,020947602
ENSG00000204961	PCDHA9	0,13498711	0	0,027591077	0,025899253	0	0	0	0	0	0,18847744	0,020941938
ENSG00000183019	MCEMP1	0	0	0	0	0,18841424	0	0	0	0	0,18841424	0,020934916
ENSG00000140287	HDC	0,038796039	0	0	0	0	0	0,064426358	0,084835576	0	0,188057972	0,02089533
ENSG00000196666	FAM180B	0	0	0	0,187993498	0	0	0	0	0	0,187993498	0,020888166
ENSG00000180251	SLC9A4	0,067490206	0	0,062076842	0,058270428	0	0	0	0	0	0,187837475	0,020870831
ENSG00000197437	OR13G1	0,05884368	0	0	0	0	0	0	0,128673897	0	0,187517577	0,020835286
ENSG00000158525	CPA5	0	0	0,187507655	0	0	0	0	0	0	0,187507655	0,020834184
ENSG00000138738	PRDM5	0,015877963	0,05635011	0,087626384	0,027417776	0	0	0	0	0	0,187272233	0,020808026
ENSG00000198797	BRINP2	0,033639931	0	0	0	0,079946025	0	0	0,073560678	0	0,187146634	0,02079407
ENSG00000263513	FAM72C	0,065786056	0	0,121018763	0	0	0	0	0	0	0,186804819	0,020756091
ENSG00000248713	C4orf54	0,02996442	0,053171128	0	0,103484027	0	0	0	0	0	0,186619576	0,020735508
ENSG00000105492	SIGLEC6	0	0	0	0,060980363	0	0,038773213	0	0	0,086864465	0,186618041	0,020735338
ENSG00000180176	TH	0,186383683	0	0	0	0	0	0	0	0	0,186383683	0,020709298
ENSG00000106633	GCK	0,036517779	0	0	0,063058235	0,086785293	0	0	0	0	0,186361307	0,020706812
ENSG00000153253	SCN3A	0,061427061	0,027250203	0,02825001	0	0	0	0,01275105	0	0,056660525	0,18633885	0,020704317
ENSG00000041515	MYO16	0,034280982	0	0	0,029597887	0,122204245	0	0	0	0	0,186083115	0,020675902
ENSG00000145623	OSMR	0,063190927	0	0	0,072744626	0,050058202	0	0	0	0	0,185993756	0,020665973
ENSG00000189139	FSCB	0	0,091266848	0,094615418	0	0	0	0	0	0	0,185882267	0,020653585
ENSG00000183960	KCNH8	0	0	0,045660332	0,085721082	0	0	0	0,054276374	0	0,185657788	0,020628643
ENSG00000130988	RGN	0	0	0	0,185652775	0	0	0	0	0	0,185652775	0,020628086
ENSG00000086967	MYBPC2	0,081264087	0	0	0,070162671	0	0	0,033737653	0	0	0,185164412	0,020573824
ENSG00000081248	CACNA1S	0	0,044299025	0	0,086216744	0	0	0	0,054590214	0	0,185105983	0,020567331
ENSG00000069011	PITX1	0	0	0,080761214	0	0,104333975	0	0	0	0	0,185095189	0,020566132
ENSG00000170516	COX7B2	0,184957859	0	0	0	0	0	0	0	0	0,184957859	0,020550873
ENSG00000168065	SLC22A11	0	0	0,053232497	0,099936796	0	0,031771462	0	0	0	0,184940755	0,020548973
ENSG00000170044	ZPLD1	0,029456252	0,104538791	0	0,050864517	0	0	0	0	0	0,184859561	0,020539951
ENSG00000120160	EQTN	0	0	0	0,184798141	0	0	0	0	0	0,184798141	0,020533127
ENSG00000197446	CYP2F1	0	0	0,184765769	0	0	0	0	0	0	0,184765769	0,02052953
ENSG00000188859	FAM78B	0	0,050185664	0	0	0,134425432	0	0	0	0	0,184611096	0,020512344
ENSG00000170777	TPD52L3	0,130831663	0	0	0	0	0	0	0	0,05363536	0,184467023	0,020496336
ENSG00000186952	TMEM232	0,029714005	0,026363386	0	0,128274002	0	0	0	0	0	0,184351393	0,020483488
ENSG00000163440	PDCL2	0	0	0	0	0	0,184345085	0	0	0	0,184345085	0,020482787
ENSG00000186994	KANK3	0	0	0	0	0	0,09232642	0	0,091940614	0	0,184267034	0,020474115
ENSG00000061337	LZTS1	0	0,04699299	0	0,137189787	0	0	0	0	0	0,184182777	0,020464753
ENSG00000114019	AMOTL2	0,05050487	0	0,046453894	0,087210887	0	0	0	0	0	0,184169651	0,020463295
ENSG00000274588	DGKK	0	0	0	0	0,095841351	0	0	0,088186434	0	0,184027785	0,020447532
ENSG00000126733	DACH2	0,021446292	0	0	0,037033065	0,101935153	0,023546775	0	0	0	0,183961284	0,020440143
ENSG00000205899	BHLHA9	0	0	0	0	0	0,183936338	0	0	0	0,183936338	0,020437371
ENSG00000206043	C18orf63	0,029473488	0,052299981	0	0,10178856	0	0	0	0	0	0,183562028	0,020395781
ENSG00000079931	MOXD1	0	0	0	0,099917662	0	0	0,048045312	0	0,035582332	0,183545306	0,020393923
ENSG00000084636	COL16A1	0	0,086936777	0	0,056400081	0	0	0	0	0,040170004	0,183506862	0,020389651
ENSG00000165762	OR4K2	0,052143054	0	0	0,090039674	0,041306366	0	0	0	0	0,183489093	0,020387677
ENSG00000165970	SLC6A5	0	0	0	0,1833954	0	0	0	0	0	0,1833954	0,020377267
ENSG00000112246	SIM1	0,048882005	0,028913306	0	0,028136184	0,077446095	0	0	0	0	0,18337759	0,020375288
ENSG00000143867	OSR1	0	0	0	0,049428864	0	0	0,071303466	0,062594159	0	0,18332649	0,02036961

ENSG00000109758	HGFAC	0	0	0	0,183305216	0	0	0	0	0	0,183305216	0,020367246
ENSG00000183760	ACP7	0,048635523	0	0	0	0	0,053398963	0,080766226	0	0	0,182800712	0,02031119
ENSG00000221977	OR4E2	0	0	0	0,182791576	0	0	0	0	0	0,182791576	0,020310175
ENSG00000151025	GPR158	0,037829658	0	0	0,097985345	0	0	0	0	0,046525603	0,182340607	0,020260067
ENSG00000134115	CNTN6	0,027439726	0	0	0,094764836	0	0	0	0,060002645	0	0,182207207	0,020245245
ENSG00000224982	TMEM233	0	0	0	0	0	0	0	0,1163502	0,065438916	0,181789116	0,020198791
ENSG00000147059	SPIN2A	0	0	0	0	0,124305138	0,057428375	0	0	0	0,181733513	0,020192613
ENSG00000122194	PLG	0	0	0	0,122696697	0	0	0,058998589	0	0	0,181695285	0,020188365
ENSG00000130643	CALY	0	0,089083721	0,092352192	0	0	0	0	0	0	0,181435913	0,020159546
ENSG00000182759	MAFA	0	0,107085463	0	0	0	0	0	0	0,074219857	0,18130532	0,020145036
ENSG00000204480	PRAMEF19	0	0	0	0,181204843	0	0	0	0	0	0,181204843	0,020133871
ENSG00000164266	SPINK1	0	0,181177027	0	0	0	0	0	0	0	0,181177027	0,020130781
ENSG00000183476	SH2D7	0	0	0	0,181141947	0	0	0	0	0	0,181141947	0,020126883
ENSG00000147573	TRIM55	0	0	0	0	0	0	0	0	0,181078131	0,181078131	0,020119792
ENSG00000158901	WFDC8	0,098829673	0	0	0	0	0	0,082060387	0	0	0,180890061	0,020098896
ENSG00000132837	DMGDH	0	0,051035782	0	0	0	0,031577955	0	0,062892	0,035372387	0,180878125	0,020097569
ENSG00000213714	FAM209B	0	0	0	0	0	0	0,180792986	0	0	0,180792986	0,02008811
ENSG00000266826	AC011195.2	0	0,180445491	0	0	0	0	0	0	0	0,180445491	0,020049499
ENSG00000103067	ESRP2	0	0	0	0	0	0	0,180435495	0	0	0,180435495	0,020048388
ENSG00000156219	ART3	0	0	0	0,079601884	0	0	0	0,100803712	0	0,180405596	0,020045066
ENSG00000114349	GNAT1	0	0	0	0	0	0,060243492	0	0,119983503	0	0,180226995	0,020025222
ENSG00000152670	DDX4	0,039464761	0	0,072598616	0,068147029	0	0	0	0	0	0,180210406	0,020023378
ENSG00000158008	EXTL1	0	0	0,122539166	0,057512668	0	0	0	0	0	0,180051834	0,020005759
ENSG00000250151	ARPC4-TTLL3	0	0	0	0	0	0,180011476	0	0	0	0,180011476	0,020001275
ENSG00000109927	TECTA	0	0	0,033159978	0,124506728	0	0	0	0	0,022169453	0,179836159	0,019981795
ENSG00000165887	ANKRD2	0	0	0	0,179831133	0	0	0	0	0	0,179831133	0,019981237
ENSG00000163888	CAMK2N2	0	0	0	0,179707283	0	0	0	0	0	0,179707283	0,019967476
ENSG00000206113	CFAP99	0,029070906	0	0	0,15059733	0	0	0	0	0	0,179668237	0,019963137
ENSG00000159409	CELF3	0	0,120811895	0	0,058782378	0	0	0	0	0	0,179594272	0,019954919
ENSG00000177459	ERICH5	0	0	0,179341999	0	0	0	0	0	0	0,179341999	0,019926889
ENSG00000172320	OR5A1	0,017546513	0	0,032278228	0	0	0	0	0	0,129479695	0,179304436	0,019922715
ENSG00000170276	HSPB2	0,179253346	0	0	0	0	0	0	0	0	0,179253346	0,019917038
ENSG00000113905	HRG	0,17914709	0	0	0	0	0	0	0	0	0,17914709	0,019905232
ENSG00000204305	AGER	0	0	0	0	0	0	0	0	0,179129178	0,179129178	0,019903242
ENSG00000087258	GNAO1	0,01115866	0	0,020527256	0,03853714	0,026518797	0	0	0	0,082342278	0,17908413	0,019898237
ENSG00000204909	SPINK9	0,179040961	0	0	0	0	0	0	0	0	0,179040961	0,01989344
ENSG00000277322	GOLGA6L6	0	0	0	0	0,178977096	0	0	0	0	0,178977096	0,019886344
ENSG00000077498	TYR	0	0	0,107204049	0	0	0	0	0	0,071672396	0,178876445	0,019875161
ENSG00000139915	MDGA2	0	0,031906473	0	0	0,08546348	0	0	0,039318725	0,022114055	0,178802734	0,01986697
ENSG00000205090	TMEM240	0	0	0	0	0,178754376	0	0	0	0	0,178754376	0,019861597
ENSG00000105270	CLIP3	0,04013561	0	0	0,138610876	0	0	0	0	0	0,178746486	0,019860721
ENSG00000149403	GRIK4	0	0,107	0,036975273	0,034708031	0	0	0	0	0	0,178683303	0,0198537
ENSG00000106823	ECM2	0	0	0	0	0	0	0,178659952	0	0	0,178659952	0,019851106
ENSG00000128276	RFPL3	0	0,178642239	0	0	0	0	0	0	0	0,178642239	0,019849138
ENSG00000141052	MYOCD	0,05107963	0	0,031321701	0,058802248	0	0	0	0,037232064	0	0,178435643	0,019826183
ENSG00000163092	XIRP2	0,069577425	0,041154478	0	0,040048342	0,027558709	0	0	0	0	0,178338954	0,019815439
ENSG00000099625	CBARP	0	0	0	0,056836196	0	0	0	0	0,121441857	0,178278053	0,019808673
ENSG00000128253	RFPL2	0	0,177990043	0	0	0	0	0	0	0	0,177990043	0,019776671
ENSG00000174450	GOLGA6L2	0	0	0,091742607	0,086117153	0	0	0	0	0	0,17785976	0,019762196
ENSG00000101204	CHRNA4	0,013676402	0	0,075476541	0,023616162	0,065004533	0	0	0	0	0,177773638	0,019752626
ENSG00000025423	HSD17B6	0	0	0,122350396	0	0	0	0,055224618	0	0	0,177575014	0,019730557
ENSG00000168269	FOXI1	0,065105804	0	0	0,112423513	0	0	0	0	0	0,177529317	0,01972548
ENSG00000164122	ASB5	0,111548157	0,065979823	0	0	0	0	0	0	0	0,17752798	0,019725331
ENSG00000135903	PAX3	0	0	0,070028996	0,065734973	0	0	0	0,041621686	0	0,177385655	0,019709517
ENSG00000171199	OPRPN	0	0	0	0	0	0	0	0	0,177334468	0,177334468	0,01970383
ENSG00000173809	AC008736.1	0,02150734	0	0	0,111415446	0	0	0,017858003	0	0,026451256	0,177232045	0,019692449
ENSG00000187714	SLC18A3	0	0	0	0,108226866	0	0,068814009	0	0	0	0,177040876	0,019671208
ENSG00000167098	SUN5	0	0	0	0,176905068	0	0	0	0	0	0,176905068	0,019656119
ENSG00000125861	GFRA4	0	0,176758075	0	0	0	0	0	0	0	0,176758075	0,019639786
ENSG00000167080	B4GALNT2	0,016863137	0	0	0,145594786	0	0	0,014001823	0	0	0,176459745	0,019606638
ENSG00000169302	STK32A	0	0,101620263	0	0,032962983	0	0	0	0,041742618	0	0,176325864	0,019591763
ENSG00000270765	GAS2L2	0	0,088965494	0	0,086574311	0	0	0	0	0	0,175539806	0,019504423
ENSG00000167791	CABP2	0	0	0	0,175359526	0	0	0	0	0	0,175359526	0,019484392
ENSG00000187135	VSTM2B	0	0	0	0,175359526	0	0	0	0	0	0,175359526	0,019484392
ENSG00000181392	SYNE4	0	0	0	0,102407761	0	0	0	0	0,072938196	0,175345956	0,019482884
ENSG00000243137	PSG4	0,024085204	0	0	0,124769672	0	0,026444147	0	0	0	0,175299023	0,019477669
ENSG00000183853	KIRREL1	0,038391913	0	0	0	0,136858819	0	0	0	0	0,175250733	0,019472304
ENSG00000173157	ADAMTS20	0,041731723	0,03702596	0	0,036030789	0	0	0,034650741	0	0,02566232	0,175101533	0,019455726
ENSG00000250821	EXOC1L	0,175099155	0	0	0	0	0	0	0	0	0,175099155	0,019455462
ENSG00000276747	PADI6	0,063067851	0,111912354	0	0	0	0	0	0	0	0,174980205	0,019442245
ENSG00000163689	C3orf67	0	0,056719619	0	0	0	0	0,013270263	0,104844389	0	0,174834271	0,01942603
ENSG00000240505	TNFRSF13B	0	0	0	0,07350281	0,101159871	0	0	0	0	0,174662681	0,019406965
ENSG00000268964	ERVV-2	0	0,088379038	0	0,086003617	0	0	0	0	0	0,174382655	0,019375851
ENSG00000105392	CRX	0,075668789	0	0	0,043554494	0	0	0	0,055155161	0	0,174378443	0,019375383
ENSG00000180739	S1PR5	0,1078206	0	0	0	0	0	0	0	0,066302719	0,174123319	0,019347035
ENSG00000185681	MORN5	0,174090519	0	0	0	0	0	0	0	0	0,174090519	0,019343391
ENSG00000266265	KLF14	0	0	0	0,088693057	0	0	0,085295943	0	0	0,173989	0,019332111
ENSG00000260802	SERTM2	0	0	0	0	0	0	0,026370393	0,069448207	0,078119597	0,173938198	0,019326466
ENSG00000166450	PRTG	0,010328815	0	0,01900069	0,05350683	0	0,056702179	0,034304944	0	0	0,173843459	0,01931594
ENSG00000186326	RGS9BP	0,052215125	0	0	0	0	0,057329156	0	0	0,064217872	0,173762153	0,019306906
ENSG00000165623	UCMA	0,173690311	0	0	0	0	0	0	0	0	0,173690311	0,019298923
ENSG00000203837	PNLIPRP3	0,063652304	0	0	0,109913637	0	0	0	0	0	0,173565942	0,019285105
ENSG00000071909	MYO3B	0,110085408	0	0	0,063364491	0	0	0	0	0	0,1734499	0,019272211
ENSG00000241322	CDRT1	0	0	0	0	0	0	0	0	0,173445191	0,173445191	0,019271688
ENSG00000102104	RS1	0,173441114	0	0	0	0	0	0	0	0	0,173441114	0,019271235
ENSG00000185742	C11orf87	0	0	0	0	0,061032893	0	0	0,112316304	0	0,173349197	0,019261022
ENSG00000100583	SAMD15	0,173291939	0	0	0	0	0	0	0	0	0,173291939	0,01925466
ENSG00000214562	NUTM2D	0	0	0	0,149425898	0	0,023752409	0	0	0	0,173178307	0,019242034
ENSG00000132517	SLC52A1	0,038411431	0,068160142	0	0,066328158	0	0	0	0	0	0,172899732	0,019211081
ENSG00000164265	SCGB3A2	0	0	0	0	0	0	0	0	0,172880486	0,172880486	0,019208943
ENSG00000213931	HBE1	0	0	0	0	0	0,17264368	0	0	0	0,17264368	0,019182631
ENSG00000116329	OPRD1	0	0	0	0,083767247	0	0,088769704	0	0	0	0,172536951	0,019170772
ENSG00000137968	SLC44A5	0	0,042568979	0	0,041424825	0	0	0	0	0,088512394	0,172506198	0,019167355
ENSG00000182685	BRICD5	0	0	0	0	0,172404005	0	0	0	0	0,172404005	0,019156001
ENSG00000152430	BOLL	0,037739903	0	0,069425599	0,065168575	0	0	0	0	0	0,172334077	0,019148231
ENSG00000146054	TRIM7	0	0,036541564	0,03788227	0	0,097878861	0	0	0	0	0,172302696	0,019144744
ENSG00000183785	TUBA8	0	0,073624931	0	0	0,098604487	0	0	0	0	0,172229419	0,019136602
ENSG00000164188	RANBP3L	0,134799795	0	0	0	0	0	0,037309049	0	0	0,172108844	0,019123205
ENSG00000111305	GSG1	0	0	0	0,172063946	0	0	0	0	0	0,172063946	0,019118216
ENSG00000159708	LRRC36	0,037608405	0	0,069183698	0,064941507	0	0	0	0	0	0,17173361	0,019081512
ENSG00000175928	LRRN1	0	0,062885084	0,065192331	0	0	0	0	0	0,043585019	0,171662435	0,019073604
ENSG00000165078	CPA6	0	0	0	0	0	0,057329156	0	0,114179187	0	0,171508343	0,019056483
ENSG00000007866	TEAD3	0	0	0	0,17116102	0	0	0	0	0	0,17116102	0,019017891
ENSG00000112530	PACRG	0	0	0	0,099898535	0	0	0	0	0,071151042	0,171049578	0,019005509
ENSG00000160856	FCRL3	0,023427995	0	0	0,121365104	0	0,02572257	0	0	0	0,17051567	0,018946186
ENSG00000268089	GABRQ	0,093451188	0	0	0	0	0,076952958	0	0	0	0,170404145	0,018933794
ENSG00000188984	AADACL3	0	0	0	0	0,088561663	0,081830124	0	0	0	0,170391787	0,018932421
ENSG00000175877	TMEM270	0	0	0	0	0	0,170339401	0	0	0	0,170339401	0,0189266
ENSG00000164393	ADGRF2	0	0	0	0,040802967	0	0,077831389	0	0,051670769	0	0,170305125	0,018922792
ENSG00000167476	JSRP1	0	0	0	0	0	0,170164694	0	0	0	0,170164694	0,018907188
ENSG00000204677	FAM153CP	0,011764613	0	0,043283911	0,050787297	0	0	0	0,064314408	0	0,17015023	0,018905581
ENSG00000127578	WFIKKN1	0	0	0	0	0	0,170025186	0	0	0	0,170025186	0,018891687
ENSG00000186723	AC114267.1	0	0	0	0,058584413	0	0,037249793	0	0,074188273	0	0,170022478	0,018891386
ENSG00000131808	FSHB	0	0	0	0	0	0	0	0,169976629	0	0,169976629	0,018886292
ENSG00000166246	C16orf71	0	0	0	0,058283443	0	0,11117528	0	0	0	0,169458723	0,018828747
ENSG00000188032	C19orf67	0	0	0	0,169438295	0	0	0	0	0	0,169438295	0,018826477
ENSG00000197887	OR1S2	0	0	0	0	0	0,169296507	0	0	0	0,169296507	0,018810723

ENSG00000204025	TRPC5OS	0,050053187	0	0	0	0,118952482	0	0	0	0	0,169005668	0,018778408
ENSG00000183035	CYLC1	0	0	0	0	0,168758244	0	0	0	0	0,168758244	0,018750916
ENSG00000107447	DNTT	0	0	0	0	0	0	0	0,168675124	0	0,168675124	0,01874168
ENSG00000181552	EDDM3B	0,16865019	0	0	0	0	0	0	0	0	0,16865019	0,01873891
ENSG00000105641	SLC5A5	0	0	0	0,070906243	0,097586289	0	0	0	0	0,168492532	0,018721392
ENSG00000271741	AC114490.2	0	0	0	0,168399467	0	0	0	0	0	0,168399467	0,018711052
ENSG00000064218	DMRT3	0	0,099422321	0	0	0	0	0	0	0,06890861	0,168330932	0,018703437
ENSG00000066032	CTNNA2	0	0,035586198	0,110675554	0	0	0,022018656	0	0	0	0,168280408	0,018697823
ENSG00000180316	PNPLA1	0,049805725	0	0	0	0,118364384	0	0	0	0	0,16817011	0,018685568
ENSG00000204478	PRAMEF20	0	0,167903569	0	0	0	0	0	0	0	0,167903569	0,018655952
ENSG00000163762	TM4SF18	0,104046755	0	0,063800803	0	0	0	0	0	0	0,167847558	0,018649729
ENSG00000134917	ADAMTS8	0	0,064752958	0	0,063012551	0	0,040065341	0	0	0	0,16783085	0,018647872
ENSG00000164764	SBSPON	0	0	0	0	0,167811936	0	0	0	0	0,167811936	0,018645771
ENSG00000143297	FCRL5	0,024345186	0,086399871	0	0,042038823	0	0	0	0	0,01497072	0,167754601	0,0186394
ENSG00000058335	RASGRF1	0	0	0,027996787	0	0	0,083548483	0	0	0,05615264	0,16769791	0,018633101
ENSG00000165828	PRAP1	0	0	0	0	0	0	0,167517132	0	0	0,167517132	0,018613015
ENSG00000263264	AC119396.1	0	0	0	0,167480728	0	0	0	0	0	0,167480728	0,01860897
ENSG00000136488	CSH1	0	0	0,167457891	0	0	0	0	0	0	0,167457891	0,018606432
ENSG00000283515	AC020915.5	0	0	0	0	0	0	0	0,167393398	0	0,167393398	0,018599266
ENSG00000080709	KCNN2	0	0,041322546	0,085677331	0,040211893	0	0	0	0	0	0,16721177	0,018579086
ENSG00000124466	LYPD3	0,061252765	0	0	0,105770156	0	0	0	0	0	0,167022921	0,018558102
ENSG00000203995	ZYG11A	0	0	0	0,166767133	0	0	0	0	0	0,166767133	0,018529681
ENSG00000205857	NANOGNB	0	0	0	0	0	0,166576884	0	0	0	0,166576884	0,018508543
ENSG00000213901	SLC23A3	0	0,074463205	0	0	0	0	0	0,09176189	0	0,166225095	0,018469455
ENSG00000165794	SLC39A2	0	0	0	0,165673	0	0	0	0	0	0,165673	0,018408111
ENSG00000135577	NMBR	0	0	0	0,165567877	0	0	0	0	0	0,165567877	0,018396431
ENSG00000152592	DMP1	0	0	0,103646569	0	0	0,061860767	0	0	0	0,165507336	0,018389704
ENSG00000184999	SLC22A10	0	0,083794375	0	0,08154218	0	0	0	0	0	0,165336555	0,018370728
ENSG00000261739	GOLGA8S	0	0	0	0	0,165149479	0	0	0	0	0,165149479	0,018349942
ENSG00000224586	GPX5	0	0	0	0	0	0	0	0,165052232	0	0,165052232	0,018339137
ENSG00000169327	OR5AU1	0,0179701	0,031887501	0	0,093091322	0	0	0	0	0,022100906	0,16504983	0,01833887
ENSG00000109132	PHOX2B	0,048871465	0	0	0	0,116144095	0	0	0	0	0,16501556	0,018335062
ENSG00000136099	PCDH8	0,058683717	0,052066408	0,053976718	0	0	0	0	0	0	0,164726843	0,018302983
ENSG00000186788	SPATA31D3	0,031580057	0	0,058094065	0	0,075050688	0	0	0	0	0,164724809	0,018302757
ENSG00000146469	VIP	0	0	0	0,164627744	0	0	0	0	0	0,164627744	0,018291972
ENSG00000197753	LHFPL5	0	0	0	0,069231885	0,095281916	0	0	0	0	0,164513801	0,018279311
ENSG00000170423	KRT78	0	0,164504295	0	0	0	0	0	0	0	0,164504295	0,018278255
ENSG00000139194	RBP5	0	0	0	0	0	0,1644307	0	0	0	0,1644307	0,018270078
ENSG00000167311	ART5	0	0	0,164387995	0	0	0	0	0	0	0,164387995	0,018265333
ENSG00000138944	SHISAL1	0,088201121	0	0	0,076152043	0	0	0	0	0	0,164353164	0,018261463
ENSG00000101203	COL20A1	0,027539743	0	0	0,071332689	0,065448796	0	0	0	0	0,164321227	0,018257914
ENSG00000134757	DSG3	0	0	0	0	0,064369518	0	0	0	0,099935395	0,164304913	0,018256101
ENSG00000236032	OR5H14	0,020517389	0,072815207	0	0,070858106	0	0	0	0	0	0,164190702	0,018243411
ENSG00000166211	SPIC	0	0	0	0	0	0	0	0	0,164175373	0,164175373	0,018241708
ENSG00000135517	MIP	0	0	0	0	0	0,093391825	0,070627825	0	0	0,16401965	0,018224406
ENSG00000007384	RHBDF1	0	0	0	0,05310032	0,073080493	0	0	0	0,037819805	0,164000618	0,018222291
ENSG00000214814	FER1L6	0,049945652	0	0	0	0,059348462	0	0	0,054608258	0	0,163902372	0,018211375
ENSG00000124249	KCNK15	0,060107626	0	0	0,10379275	0	0	0	0	0	0,163900376	0,018211153
ENSG00000244588	RAD21L1	0	0	0	0,095580577	0	0	0	0	0,068075649	0,163656226	0,018184025
ENSG00000103089	FA2H	0	0,163551083	0	0	0	0	0	0	0	0,163551083	0,018172343
ENSG00000181752	OR8K5	0,163539579	0	0	0	0	0	0	0	0	0,163539579	0,018171064
ENSG00000188883	KLRG2	0	0	0	0	0,163458144	0	0	0	0	0,163458144	0,018162016
ENSG00000173464	RNASE11	0,163274523	0	0	0	0	0	0	0	0	0,163274523	0,018141614
ENSG00000198917	SPOUT1	0	0	0	0	0	0	0	0	0,162809043	0,162809043	0,018089894
ENSG00000206262	FOXL2NB	0	0	0,162783583	0	0	0	0	0	0	0,162783583	0,018087065
ENSG00000185056	C5orf47	0	0	0	0,09498907	0	0	0	0	0,067654358	0,162643428	0,018071492
ENSG00000148735	PLEKHS1	0,081038919	0	0	0	0	0	0,022429448	0,059069461	0	0,162537828	0,018059759
ENSG00000104140	RHOV	0	0,162510303	0	0	0	0	0	0	0	0,162510303	0,0180567
ENSG00000196468	FGF16	0	0,162510303	0	0	0	0	0	0	0	0,162510303	0,0180567
ENSG00000049768	FOXP3	0	0	0	0,162475078	0	0	0	0	0	0,162475078	0,018052786
ENSG00000022355	GABRA1	0	0,162461072	0	0	0	0	0	0	0	0,162461072	0,01805123
ENSG00000186895	FGF3	0	0	0	0,162373973	0	0	0	0	0	0,162373973	0,018041553
ENSG00000183463	URAD	0,162309958	0	0	0	0	0	0	0	0	0,162309958	0,01803444
ENSG00000001617	SEMA3F	0	0	0	0,162205745	0	0	0	0	0	0,162205745	0,018022861
ENSG00000145192	AHSG	0	0	0	0	0,162056653	0	0	0	0	0,162056653	0,018006295
ENSG00000156920	ADGRG4	0,075000283	0	0	0	0,035647959	0,032938371	0	0	0,018448136	0,16203475	0,018003861
ENSG00000258984	UBE2F-SCLY	0	0	0	0	0	0,103856386	0	0	0,058167926	0,162024312	0,018002701
ENSG00000215182	MUC5AC	0	0	0,079659588	0,014955008	0,041164322	0	0,007191101	0,018938249	0	0,161908268	0,017989808
ENSG00000171388	APLN	0	0	0	0,161870332	0	0	0	0	0	0,161870332	0,017985592
ENSG00000206072	SERPINB11	0,040884895	0	0	0,07059929	0	0	0	0	0,05028315	0,161767334	0,017974148
ENSG00000124568	SLC17A1	0	0	0	0	0	0	0	0,161660747	0	0,161660747	0,017962305
ENSG00000204941	PSG5	0,064604776	0,057319795	0	0	0	0	0	0	0,039727773	0,161652344	0,017961372
ENSG00000072041	SLC6A15	0	0,030725564	0	0,089699201	0,041150171	0	0	0	0	0,161574936	0,017952771
ENSG00000167723	TRPV3	0,10815685	0	0	0,053360936	0	0	0	0	0	0,161517786	0,017946421
ENSG00000196503	ARL9	0,161500432	0	0	0	0	0	0	0	0	0,161500432	0,017944492
ENSG00000258083	OR9A4	0	0	0	0	0	0	0	0,161423824	0	0,161423824	0,01793598
ENSG00000176919	C8G	0	0	0	0	0	0	0	0	0,161325106	0,161325106	0,017925012
ENSG00000174970	OR10AG1	0,161098689	0	0	0	0	0	0	0	0	0,161098689	0,017899854
ENSG00000134668	SPOCD1	0,085312955	0,075692872	0	0	0	0	0	0	0	0,161005827	0,017889536
ENSG00000198759	EGFL6	0,057874596	0,102697051	0	0	0	0	0	0	0	0,160571647	0,017841294
ENSG00000079841	RIMS1	0,011816591	0,020968251	0	0,061214023	0	0,012973927	0,009811568	0	0,043598652	0,160383013	0,017820335
ENSG00000139364	TMEM132B	0,019246077	0	0	0,132935095	0	0	0,007990214	0	0	0,160171386	0,017796821
ENSG00000146276	GABRR1	0,039997504	0,07097459	0	0	0	0	0	0	0,049191774	0,160163868	0,017795985
ENSG00000108602	ALDH3A1	0	0	0	0,067285966	0,092603802	0	0	0	0	0,159889767	0,01776553
ENSG00000185933	CALHM1	0,09899153	0	0	0	0	0	0	0	0,060873411	0,159864941	0,017762771
ENSG00000173376	NDNF	0,084584702	0,075046739	0	0	0	0	0	0	0	0,159631442	0,017736827
ENSG00000150275	PCDH15	0	0	0,018023737	0,050755685	0,046569091	0,010757348	0	0,021424792	0,012049959	0,159580612	0,017731179
ENSG00000115665	SLC5A7	0,058479323	0	0	0,100981027	0	0	0	0	0	0,15946035	0,017717817
ENSG00000149972	CNTN5	0,066371174	0,05888701	0	0	0	0	0,013777351	0	0,020406997	0,159442532	0,017715837
ENSG00000184814	PRR23B	0,159399336	0	0	0	0	0	0	0	0	0,159399336	0,017711037
ENSG00000103647	CORO2B	0	0	0,06952979	0	0,089824298	0	0	0	0	0,159354087	0,01770601
ENSG00000137571	SLCO5A1	0	0	0,056117916	0	0,036248869	0	0	0,066707291	0	0,159074076	0,017674897
ENSG00000173702	MUC13	0	0	0,158891168	0	0	0	0	0	0	0,158891168	0,017654574
ENSG00000147689	FAM83A	0,023921255	0	0	0,082613574	0	0	0	0,052308781	0	0,15884361	0,01764929
ENSG00000178796	RIIAD1	0	0	0	0	0,158831288	0	0	0	0	0,158831288	0,017647921
ENSG00000138669	PRKG2	0,029536859	0	0	0	0	0	0	0,129176922	0	0,158713781	0,017634865
ENSG00000133105	RXFP2	0	0,080378297	0	0,078217918	0	0	0	0	0	0,158596215	0,017621802
ENSG00000125780	TGM3	0	0	0	0,096857823	0	0,061585218	0	0	0	0,158443041	0,017604782
ENSG00000181927	OR4P4	0	0	0	0	0	0	0	0,158405833	0	0,158405833	0,017600648
ENSG00000188389	PDCD1	0	0	0	0	0	0,158236125	0	0	0	0,158236125	0,017581792
ENSG00000168993	CPLX1	0	0	0	0	0	0	0	0	0,158234587	0,158234587	0,017581621
ENSG00000204950	LRRC10B	0	0	0	0	0,158201561	0	0	0	0	0,158201561	0,017577951
ENSG00000235711	ANKRD34C	0	0	0	0,158110467	0	0	0	0	0	0,158110467	0,01756783
ENSG00000131771	PPP1R1B	0	0	0	0	0,158097091	0	0	0	0	0,158097091	0,017566343
ENSG00000268606	MAGEA2	0	0	0	0	0,158062299	0	0	0	0	0,158062299	0,017562478
ENSG00000135702	CHST5	0	0	0	0	0,108102812	0,049942979	0	0	0	0,158045791	0,017560643
ENSG00000138271	GPR87	0	0,157916372	0	0	0	0	0	0	0	0,157916372	0,017546264
ENSG00000124664	SPDEF	0,157900283	0	0	0	0	0	0	0	0	0,157900283	0,017544476
ENSG00000167754	KLK5	0	0	0	0,157855399	0	0	0	0	0	0,157855399	0,017539489
ENSG00000242221	PSG2	0	0	0	0,092105533	0	0	0	0	0,065600608	0,157706141	0,017522905
ENSG00000160161	CILP2	0,034658388	0,123000917	0	0	0	0	0	0	0	0,157659305	0,017517701
ENSG00000171560	FGA	0	0	0	0,066210346	0,091123457	0	0	0	0	0,157333803	0,017481534
ENSG00000145934	TENM2	0,013685073	0,048567651	0	0,094524533	0	0	0	0	0	0,156777257	0,017419695
ENSG00000115194	SLC30A3	0,02527778	0	0	0,04364921	0,060073192	0,027753526	0	0	0	0,156753708	0,017417079
ENSG00000186075	ZPBP2	0	0	0	0	0,156478232	0	0	0	0	0,156478232	0,01738647
ENSG00000277893	SRD5A2	0,03343894	0	0,123027262	0	0	0	0	0	0	0,156466202	0,017385134
ENSG00000253767	PCDHGA8	0	0	0	0,120510322	0	0	0,014486818	0	0,02145786	0,156455	0,017383889

ENSG00000196098	OR5K4	0,156429162	0	0	0	0	0	0	0	0	0,156429162	0,017381018
ENSG00000111664	GNB3	0,043965834	0	0	0,075919399	0	0	0,03650577	0	0	0,156391003	0,017376778
ENSG00000196993	NPIPB9	0	0	0	0	0	0,156298235	0	0	0	0,156298235	0,017366471
ENSG00000117013	KCNQ4	0	0	0	0,118391549	0	0,037638516	0	0	0	0,156030065	0,017336674
ENSG00000078725	BRINP1	0,15602537	0	0	0	0	0	0	0	0	0,15602537	0,017336152
ENSG00000255298	OR8G5	0	0	0	0,155875134	0	0	0	0	0	0,155875134	0,017319459
ENSG00000083307	GRHL2	0,024935738	0,044247855	0	0	0,059260321	0,027377983	0	0	0	0,155821896	0,017313544
ENSG00000129514	FOXA1	0,034234384	0,121496149	0	0	0	0	0	0	0	0,155730533	0,017303393
ENSG00000167104	BPIFB6	0	0	0	0,155689126	0	0	0	0	0	0,155689126	0,017298792
ENSG00000135222	CSN2	0	0	0	0	0	0,15563844	0	0	0	0,15563844	0,01729316
ENSG00000149488	TMC2	0	0,155414606	0	0	0	0	0	0	0	0,155414606	0,01726829
ENSG00000183638	RP1L1	0	0,120216095	0	0	0	0	0,014063028	0	0,020830141	0,155109264	0,017234363
ENSG00000183668	PSG9	0	0	0	0,155072291	0	0	0	0	0	0,155072291	0,017230255
ENSG00000176746	MAGEB6	0,155064721	0	0	0	0	0	0	0	0	0,155064721	0,017229413
ENSG00000111087	GLI1	0,033138283	0	0,121921096	0	0	0	0	0	0	0,155059379	0,01722882
ENSG00000182253	SYNM	0	0	0	0,066743823	0,045928833	0	0	0,042260464	0	0,154933119	0,017214791
ENSG00000144227	NXPH2	0,055780942	0,098981912	0	0	0	0	0	0	0	0,154762854	0,017195873
ENSG00000178343	SHISA3	0	0	0	0	0,154658718	0	0	0	0	0,154658718	0,017184302
ENSG00000204278	TMEM235	0,058074777	0	0	0	0	0	0,096441454	0	0	0,154516232	0,01716847
ENSG00000101438	SLC32A1	0	0,105153726	0	0	0	0	0,049204052	0	0	0,154357777	0,017150864
ENSG00000176092	CRYBG2	0	0	0,040316185	0,037844086	0,052083762	0,024062448	0	0	0	0,154306482	0,017145165
ENSG00000143105	KCNA10	0,154273171	0	0	0	0	0	0	0	0	0,154273171	0,017141463
ENSG00000158296	SLC13A3	0,024956329	0	0	0,129282399	0	0	0	0	0	0,154238727	0,017137636
ENSG00000147166	ITGB1BP2	0,069158156	0	0	0	0	0	0	0	0,085055617	0,154213773	0,017134864
ENSG00000171989	LDHAL6B	0	0	0	0,154125797	0	0	0	0	0	0,154125797	0,017125089
ENSG00000164694	FNDC1	0,044119875	0,039144818	0	0	0,052425919	0	0,018316837	0	0	0,154007448	0,017111939
ENSG00000137252	HCRTR2	0,153880418	0	0	0	0	0	0	0	0	0,153880418	0,017097824
ENSG00000178217	SH2D4B	0	0	0,060641383	0,056922987	0	0,036193407	0	0	0	0,153757777	0,017084197
ENSG00000231192	OR5H1	0,050378587	0	0	0,043496412	0,059862901	0	0	0	0	0,153737899	0,017081989
ENSG00000128510	CPA4	0	0	0,070232466	0	0	0	0	0,083485237	0	0,153717703	0,017079745
ENSG00000069206	ADAM7	0	0	0	0,116410874	0	0,037008828	0	0	0	0,153419702	0,017046634
ENSG00000183770	FOXL2	0	0	0	0,08954529	0	0	0	0	0,063777118	0,153322408	0,017035823
ENSG00000283597	FAM169B	0,035698221	0	0	0	0	0,117583683	0	0	0	0,153281904	0,017031323
ENSG00000132297	HHLA1	0,024598823	0,043650008	0	0,084953597	0	0	0	0	0	0,153202429	0,017022492
ENSG00000168447	SCNN1B	0	0	0	0,067233954	0	0,08549888	0	0	0	0,152732834	0,016970315
ENSG00000146378	TAAR2	0	0	0	0	0	0	0	0	0,152708728	0,152708728	0,016967636
ENSG00000179407	DNAJB8	0	0	0,152526803	0	0	0	0	0	0	0,152526803	0,016947423
ENSG00000198681	MAGEA1	0	0	0	0,152504369	0	0	0	0	0	0,152504369	0,01694493
ENSG00000139780	METTL21C	0	0	0	0	0	0,152491339	0	0	0	0,152491339	0,016943482
ENSG00000221986	MYBPHL	0	0,152440023	0	0	0	0	0	0	0	0,152440023	0,01693778
ENSG00000197506	SLC28A3	0,029846054	0	0	0,051537621	0,070929793	0	0	0	0	0,152313468	0,016923719
ENSG00000154478	GPR26	0	0	0	0,104179521	0,047793125	0	0	0	0	0,151972647	0,01688585
ENSG00000205212	CCDC144NL	0	0	0	0,151882989	0	0	0	0	0	0,151882989	0,016875888
ENSG00000214897	PNMA6E	0	0	0	0	0,103851227	0,047978767	0	0	0	0,151829994	0,016869999
ENSG00000271383	NBPF19	0	0	0	0,15177256	0	0	0	0	0	0,15177256	0,016863618
ENSG00000181273	OR5AK2	0,15171744	0	0	0	0	0	0	0	0	0,15171744	0,016857493
ENSG00000176299	OR4M1	0	0	0	0	0	0,046801291	0	0	0,104849942	0,151651233	0,016850137
ENSG00000183379	SYNDIG1L	0,055575789	0	0	0,095967258	0	0	0	0	0	0,151543047	0,016838116
ENSG00000176230	OR4K17	0	0	0	0,050874435	0	0	0	0,064424755	0,036234456	0,151533645	0,016837072
ENSG00000204262	COL5A2	0,041292682	0,109909277	0	0	0	0	0	0	0	0,15120196	0,016800218
ENSG00000107821	KAZALD1	0	0,074215887	0,076938859	0	0	0	0	0	0	0,151154746	0,016794972
ENSG00000101638	ST8SIA5	0	0	0	0,033052755	0,06823438	0	0,007946693	0,041856301	0	0,151090129	0,016787792
ENSG00000170615	SLC26A5	0,044746986	0	0	0	0,10634218	0	0	0	0	0,151089166	0,016787685
ENSG00000217930	PAM16	0	0	0	0	0	0,071175709	0	0	0,079728238	0,150903946	0,016767105
ENSG00000106078	COBL	0,021062174	0	0,038745571	0,090924446	0	0	0	0	0	0,150732191	0,016748021
ENSG00000188626	GOLGA8M	0	0	0	0	0	0,150690805	0	0	0	0,150690805	0,016743423
ENSG00000158220	ESYT3	0,030788625	0	0,056638162	0,026582618	0,036584917	0	0	0	0	0,150594321	0,016732702
ENSG00000165194	PCDH19	0,015488988	0	0,028493245	0,053492205	0	0	0	0,033869882	0,019049459	0,150393779	0,01671042
ENSG00000112981	NME5	0	0	0	0	0,150321282	0	0	0	0	0,150321282	0,016702365
ENSG00000165462	PHOX2A	0	0,150303812	0	0	0	0	0	0	0	0,150303812	0,016700424
ENSG00000255582	OR10G2	0	0	0	0	0	0,150145318	0	0	0	0,150145318	0,016682813
ENSG00000122121	XPNPEP2	0	0	0	0	0,102663677	0,047430125	0	0	0	0,150093802	0,016677089
ENSG00000134007	ADAM20	0,054064605	0,095936315	0	0	0	0	0	0	0	0,15000092	0,016666769
ENSG00000124429	POF1B	0	0,066985261	0	0	0	0,082893119	0	0	0	0,14987838	0,016653153
ENSG00000259417	CTXND1	0,043844645	0	0	0	0,052098875	0	0	0	0,053923262	0,149866782	0,016651865
ENSG00000185052	SLC24A3	0	0	0,070678896	0	0	0	0,031901941	0	0,04725312	0,149833957	0,016648217
ENSG00000157060	SHCBP1L	0	0	0	0,087503345	0	0	0	0	0,062322777	0,149826122	0,016647347
ENSG00000127515	OR7A10	0,081814061	0	0	0	0	0	0,067931961	0	0	0,149746022	0,016638447
ENSG00000186765	FSCN2	0,14968853	0	0	0	0	0	0	0	0	0,14968853	0,016632059
ENSG00000169344	UMOD	0,044326949	0	0	0	0,105343955	0	0	0	0	0,149670904	0,0166301
ENSG00000145692	BHMT	0	0	0	0,149661586	0	0	0	0	0	0,149661586	0,016629065
ENSG00000188992	LIPI	0	0	0	0,149618678	0	0	0	0	0	0,149618678	0,016624298
ENSG00000108448	TRIM16L	0	0	0	0,120552079	0	0	0,028983676	0	0	0,149535754	0,016615084
ENSG00000189058	APOD	0,14946644	0	0	0	0	0	0	0	0	0,14946644	0,016607382
ENSG00000253148	RGS21	0	0,149465998	0	0	0	0	0	0	0	0,149465998	0,016607333
ENSG00000197651	CCER1	0	0	0	0	0,110702078	0	0,038677661	0	0	0,149379739	0,016597749
ENSG00000286106	AC253572.1	0	0	0	0	0	0,149334452	0	0	0	0,149334452	0,016592717
ENSG00000121653	MAPK8IP1	0	0	0	0,149190952	0	0	0	0	0	0,149190952	0,016576772
ENSG00000067840	PDZD4	0,034810083	0	0	0	0	0,038219437	0	0,076119458	0	0,149148978	0,016572109
ENSG00000135355	GJA10	0	0	0	0	0,149135192	0	0	0	0	0,149135192	0,016570577
ENSG00000206075	SERPINB5	0	0	0	0	0	0	0	0	0,148995074	0,148995074	0,016555008
ENSG00000240771	ARHGEF25	0	0	0	0,148978004	0	0	0	0	0	0,148978004	0,016553112
ENSG00000198753	PLXNB3	0	0	0	0,148956743	0	0	0	0	0	0,148956743	0,016550749
ENSG00000095713	CRTAC1	0	0,075469181	0	0,073440747	0	0	0	0	0	0,148909928	0,016545548
ENSG00000106809	OGN	0	0,06670199	0	0	0	0	0	0,082197654	0	0,148899644	0,016544405
ENSG00000182566	CLEC4G	0	0	0	0,148765664	0	0	0	0	0	0,148765664	0,016529518
ENSG00000101049	SGK2	0,029149416	0	0	0,050334679	0,069274217	0	0	0	0	0,148758312	0,016528701
ENSG00000188822	CNR2	0	0	0	0,148680897	0	0	0	0	0	0,148680897	0,0165201
ENSG00000160867	FGFR4	0,094247757	0	0	0,054248435	0	0	0	0	0	0,148496193	0,016499577
ENSG00000079435	LIPE	0	0,051955435	0	0	0	0,096440948	0	0	0	0,148396383	0,016488487
ENSG00000186329	TMEM212	0	0	0,102236153	0	0	0	0,046145764	0	0	0,148381917	0,01648688
ENSG00000273238	TMEM271	0	0	0	0	0,148334383	0	0	0	0	0,148334383	0,016481598
ENSG00000183034	OTOP2	0,054395454	0	0	0,093929077	0	0	0	0	0	0,14832453	0,016480503
ENSG00000169214	OR6F1	0	0	0	0,148090224	0	0	0	0	0	0,148090224	0,016454469
ENSG00000183206	POTEC	0	0,037670975	0	0,036658468	0,050452029	0,023308595	0	0	0	0,148090067	0,016454452
ENSG00000157368	IL34	0	0	0	0	0	0	0	0,147977863	0	0,147977863	0,016441985
ENSG00000109158	GABRA4	0,013366702	0	0,024589129	0	0,095298773	0,014675858	0	0	0	0,147930462	0,016436718
ENSG00000158077	NLRP14	0	0,147818082	0	0	0	0	0	0	0	0,147818082	0,016424231
ENSG00000273259	AL049839.2	0,147809492	0	0	0	0	0	0	0	0	0,147809492	0,016423277
ENSG00000285509	AP000646.1	0	0	0	0,090351445	0	0,057448261	0	0	0	0,147799706	0,01642219
ENSG00000145491	ROPN1L	0	0,147736639	0	0	0	0	0	0	0	0,147736639	0,016415182
ENSG00000171815	PCDHB1	0,018405672	0	0,033858721	0,095347737	0	0	0	0	0	0,147612131	0,016401348
ENSG00000106571	GLI3	0	0	0	0,070516613	0,032350017	0,014945552	0	0,029766198	0	0,147578381	0,016397598
ENSG00000254585	MAGEL2	0	0	0,064362144	0	0,083148308	0	0	0	0	0,147510452	0,01639005
ENSG00000183580	FBXL7	0	0	0	0	0,147481537	0	0	0	0	0,147481537	0,016386837
ENSG00000186684	CYP27C1	0	0	0	0	0	0,073885806	0	0,073577058	0	0,147462864	0,016384763
ENSG00000160678	S100A1	0	0	0	0,086117153	0	0	0	0	0,061335486	0,147452639	0,016383627
ENSG00000150051	MKX	0,147173675	0	0	0	0	0	0	0	0	0,147173675	0,016352631
ENSG00000285641	AL358472.6	0	0	0	0	0	0	0,147093004	0	0	0,147093004	0,016343667
ENSG00000281406	BLACAT1	0	0,039836874	0	0	0,106705554	0	0	0	0	0,146542428	0,016282492
ENSG00000188133	TMEM215	0	0	0	0,146537799	0	0	0	0	0	0,146537799	0,016281978
ENSG00000131864	USP29	0,077612003	0,068860298	0	0	0	0	0	0	0	0,146472301	0,0162747
ENSG00000197181	PIWIL2	0,02633965	0	0	0,090965653	0	0,028919396	0	0	0	0,146224699	0,016247189
ENSG00000170961	HAS2	0	0,063241038	0	0	0	0,039129853	0	0	0,043831727	0,146202618	0,016244735
ENSG00000186090	HTR3D	0,146141751	0	0	0	0	0	0	0	0	0,146141751	0,016237972
ENSG00000185149	NPY2R	0	0,074051919	0	0,072061578	0	0	0	0	0	0,146113498	0,016234833
ENSG00000187486	KCNJ11	0,094326199	0	0	0	0	0,051782327	0	0	0	0,146108526	0,016234281

ENSG00000178279	TNP2	0	0	0	0,14610021	0	0	0	0	0	0,14610021	0,016233357
ENSG00000173572	NLRP13	0,042209657	0	0	0	0	0	0	0	0,103824873	0,146034529	0,016226059
ENSG00000180988	OR52N2	0,146000551	0	0	0	0	0	0	0	0	0,146000551	0,016222283
ENSG00000146039	SLC17A4	0,032616139	0	0	0	0,07751296	0,035810614	0	0	0	0,145939713	0,016215524
ENSG00000150594	ADRA2A	0	0,0716	0,074226996	0	0	0	0	0	0	0,145826996	0,016203
ENSG00000146233	CYP39A1	0,145718969	0	0	0	0	0	0	0	0	0,145718969	0,016190997
ENSG00000146383	TAAR6	0,145578584	0	0	0	0	0	0	0	0	0,145578584	0,016175398
ENSG00000103460	TOX3	0,067120479	0,039701214	0	0,038634139	0	0	0	0	0	0,145455832	0,016161759
ENSG00000172969	FRG2C	0,14543847	0	0	0	0	0	0	0	0	0,14543847	0,01615983
ENSG00000102362	SYTL4	0,020343372	0,036098815	0	0	0	0	0	0,088969997	0	0,145412184	0,016156909
ENSG00000167178	ISLR2	0	0	0	0	0	0,113501335	0	0	0,031784936	0,145286271	0,016142919
ENSG00000230062	ANKRD66	0	0,145255688	0	0	0	0	0	0	0	0,145255688	0,016139521
ENSG00000147255	IGSF1	0,039907717	0,070815265	0	0,034455959	0	0	0	0	0	0,14517894	0,016130993
ENSG00000178187	ZNF454	0	0	0	0,098022154	0	0	0,047133859	0	0	0,145156013	0,016128446
ENSG00000073282	TP63	0,040414702	0	0	0,104681054	0	0	0	0	0	0,145095756	0,016121751
ENSG00000168490	PHYHIP	0,076881491	0,06821216	0	0	0	0	0	0	0	0,145093651	0,016121517
ENSG00000164185	ZNF474	0,145089362	0	0	0	0	0	0	0	0	0,145089362	0,01612104
ENSG00000151023	ENKUR	0	0	0	0,064017413	0	0	0	0,081068343	0	0,145085756	0,01612064
ENSG00000111879	FAM184A	0,01939553	0	0	0,033491846	0,092187792	0	0	0	0	0,145075167	0,016119463
ENSG00000130711	PRDM12	0	0	0	0	0,145039395	0	0	0	0	0,145039395	0,016115488
ENSG00000130294	KIF1A	0,009051247	0,016061216	0	0,015629528	0,064531454	0,00993774	0,007515444	0	0,022263734	0,144990364	0,01611004
ENSG00000167037	SGSM1	0	0	0	0,0303166	0,041723892	0	0,07288854	0	0	0,144929032	0,016103226
ENSG00000178597	PSAPL1	0,032524875	0	0	0,112326722	0	0	0	0	0	0,144851597	0,016094622
ENSG00000134762	DSC3	0,058881895	0,069656319	0	0	0	0	0,016296965	0	0	0,144835179	0,016092798
ENSG00000188176	SMTNL2	0	0	0	0	0,144805461	0	0	0	0	0,144805461	0,016089496
ENSG00000172348	RCAN2	0,040708667	0	0	0,070294982	0	0	0,033801275	0	0	0,144804924	0,016089436
ENSG00000205795	CYS1	0,053077124	0	0	0,091652608	0	0	0	0	0	0,144729731	0,016081081
ENSG00000204928	GRXCR2	0,068968768	0	0	0	0	0,075723677	0	0	0	0,144692445	0,016076938
ENSG00000212993	POU5F1B	0	0	0	0	0,144688776	0	0	0	0	0,144688776	0,016076531
ENSG00000164626	KCNK5	0	0,070880782	0,07348139	0	0	0	0	0	0	0,144362173	0,016040241
ENSG00000135824	RGS8	0,045208847	0,08022199	0	0	0	0	0,018768935	0	0	0,144199772	0,016022197
ENSG00000196136	SERPINA3	0,090539587	0,053553425	0	0	0	0	0	0	0	0,144093012	0,016010335
ENSG00000176774	MAGEB18	0	0	0	0,144083365	0	0	0	0	0	0,144083365	0,016009263
ENSG00000146352	CLVS2	0,013469166	0	0,024777618	0,046516619	0	0,059153428	0	0	0	0,143916831	0,015990759
ENSG00000115607	IL18RAP	0	0	0	0,087975379	0	0,055937484	0	0	0	0,143912863	0,015990318
ENSG00000198807	PAX9	0	0,088862303	0	0	0	0,054982793	0	0	0	0,143845096	0,015982788
ENSG00000187080	OR2AK2	0	0	0	0	0	0,143769997	0	0	0	0,143769997	0,015974444
ENSG00000189320	FAM180A	0	0	0	0	0,143647017	0	0	0	0	0,143647017	0,01596078
ENSG00000205126	ACCSL	0	0,143621853	0	0	0	0	0	0	0	0,143621853	0,015957984
ENSG00000179674	ARL14	0	0	0	0	0	0	0	0	0,143621733	0,143621733	0,01595797
ENSG00000163283	ALPP	0,052670119	0	0	0,090949799	0	0	0	0	0	0,143619918	0,015957769
ENSG00000102245	CD40LG	0	0	0	0,143607581	0	0	0	0	0	0,143607581	0,015956398
ENSG00000136487	GH2	0	0	0	0	0	0,14352126	0	0	0	0,14352126	0,015946807
ENSG00000165626	BEND7	0	0	0,055496127	0,052093227	0,035847229	0	0	0	0	0,143436583	0,015937398
ENSG00000226650	KIF4B	0	0	0	0	0	0	0,143296405	0	0	0,143296405	0,015921823
ENSG00000144152	FBLN7	0,022965132	0,040751064	0	0,079311542	0	0	0	0	0	0,143027739	0,015891971
ENSG00000121075	TBX4	0,075630916	0,067102603	0	0	0	0	0	0	0	0,142733519	0,01585928
ENSG00000100012	SEC14L3	0	0	0	0,142470639	0	0	0	0	0	0,142470639	0,015830071
ENSG00000182575	NXPH3	0	0,1424267	0	0	0	0	0	0	0	0,1424267	0,015825189
ENSG00000153294	ADGRF4	0,044562244	0	0	0	0	0,097853481	0	0	0	0,142415725	0,015823969
ENSG00000172640	OR10AD1	0	0	0	0	0	0	0	0	0,142411128	0,142411128	0,015823459
ENSG00000120327	PCDHB14	0,031298793	0,111077879	0	0	0	0	0	0	0	0,142376672	0,01581963
ENSG00000137941	TTLL7	0,042982053	0	0	0,049480416	0,03404926	0,015730594	0	0	0	0,142242323	0,015804703
ENSG00000137691	CFAP300	0	0	0	0	0	0	0,14220287	0	0	0,14220287	0,015800319
ENSG00000180525	PRR26	0,016341578	0,086993187	0	0	0,038836114	0	0	0	0	0,142170878	0,015796764
ENSG00000183318	SPDYE4	0,142154817	0	0	0	0	0	0	0	0	0,142154817	0,01579498
ENSG00000144868	TMEM108	0,035871946	0,063653887	0	0	0,042625228	0	0	0	0	0,14215106	0,015794562
ENSG00000168743	NPNT	0,024703379	0	0	0	0,117416231	0	0	0	0	0,14211961	0,015791068
ENSG00000169594	BNC1	0,031514196	0,055921168	0	0,054418139	0	0	0	0	0	0,141853503	0,0157615
ENSG00000152936	LMNTD1	0	0	0	0	0	0	0,081394961	0	0,060281065	0,141676025	0,015741781
ENSG00000166152	C16orf78	0,141489298	0	0	0	0	0	0	0	0	0,141489298	0,015721033
ENSG00000179178	TMEM125	0,141356942	0	0	0	0	0	0	0	0	0,141356942	0,015706327
ENSG00000166869	CHP2	0	0	0,14132186	0	0	0	0	0	0	0,14132186	0,015702429
ENSG00000229544	NKX1-2	0,141180851	0	0	0	0	0	0	0	0	0,141180851	0,015686761
ENSG00000143536	CRNN	0	0,14097897	0	0	0	0	0	0	0	0,14097897	0,01566433
ENSG00000147378	FATE1	0,140961353	0	0	0	0	0	0	0	0	0,140961353	0,015662373
ENSG00000126895	AVPR2	0,067100609	0	0	0	0	0,073672547	0	0	0	0,140773156	0,015641462
ENSG00000138311	ZNF365	0,140751277	0	0	0	0	0	0	0	0	0,140751277	0,015639031
ENSG00000163793	DNAJC5G	0	0	0	0	0	0	0	0,140610454	0	0,140610454	0,015623384
ENSG00000142182	DNMT3L	0	0	0	0,140363085	0	0	0	0	0	0,140363085	0,015595898
ENSG00000134709	HOOK1	0,022682463	0	0	0,117502991	0	0	0	0	0	0,140185454	0,015576162
ENSG00000176281	OR4K5	0,140176782	0	0	0	0	0	0	0	0	0,140176782	0,015575198
ENSG00000174448	STARD6	0	0	0	0,140136936	0	0	0	0	0	0,140136936	0,015570771
ENSG00000263201	DPEP2NB	0	0	0	0	0	0,140008925	0	0	0	0,140008925	0,015556547
ENSG00000129455	KLK8	0	0	0	0,139911515	0	0	0	0	0	0,139911515	0,015545724
ENSG00000171396	KRTAP4-4	0,139787762	0	0	0	0	0	0	0	0	0,139787762	0,015531974
ENSG00000118432	CNR1	0	0,042180588	0	0,041046873	0,056491669	0	0	0	0	0,139719131	0,015524348
ENSG00000122188	LAX1	0	0	0	0,081593175	0	0	0	0	0,058113359	0,139706534	0,015522948
ENSG00000134240	HMGCS2	0	0	0	0	0,139680102	0	0	0	0	0,139680102	0,015520011
ENSG00000196660	SLC30A10	0	0	0	0,045222699	0,062238742	0	0	0	0,032209103	0,139670544	0,015518949
ENSG00000173679	OR1L1	0,139529613	0	0	0	0	0	0	0	0	0,139529613	0,01550329
ENSG00000226321	CROCC2	0	0	0,139501555	0	0	0	0	0	0	0,139501555	0,015500173
ENSG00000141086	CTRL	0	0	0	0	0	0	0	0	0,139419747	0,139419747	0,015491083
ENSG00000198732	SMOC1	0	0	0,063625566	0	0	0	0	0,075631625	0	0,139257191	0,015473021
ENSG00000250506	CDK3	0	0	0	0	0	0	0,139179514	0	0	0,139179514	0,01546439
ENSG00000203757	OR6K3	0	0	0	0,13916532	0	0	0	0	0	0,13916532	0,015462813
ENSG00000170523	KRT83	0	0	0	0,13916532	0	0	0	0	0	0,13916532	0,015462813
ENSG00000205667	ARSH	0	0	0	0,13916532	0	0	0	0	0	0,13916532	0,015462813
ENSG00000049192	ADAMTS6	0	0,09392014	0	0,030465263	0	0	0,014649192	0	0	0,139034595	0,015448288
ENSG00000185008	ROBO2	0,009068629	0	0,016682476	0,062638174	0,043103588	0	0,007529876	0	0	0,139022744	0,015446972
ENSG00000007908	SELE	0	0	0,060614937	0	0,078307358	0	0	0	0	0,138922294	0,01543581
ENSG00000179148	ALOXE3	0	0	0	0,138795199	0	0	0	0	0	0,138795199	0,015421689
ENSG00000177839	PCDHB9	0	0	0	0,05910192	0	0,03757884	0	0	0,042094343	0,138775102	0,015419456
ENSG00000181499	OR6T1	0,138760855	0	0	0	0	0	0	0	0	0,138760855	0,015417873
ENSG00000168334	XIRP1	0	0,082926241	0	0	0,055530778	0	0	0	0	0,138457019	0,015384113
ENSG00000115226	FNDC4	0	0,138431595	0	0	0	0	0	0	0	0,138431595	0,015381288
ENSG00000188385	JAKMIP3	0	0,035244742	0	0,10289234	0	0	0	0	0	0,138137083	0,015348565
ENSG00000138771	SHROOM3	0	0,038779666	0,080404975	0,01886868	0	0	0	0	0	0,138053321	0,015339258
ENSG00000243709	LEFTY1	0	0	0	0,137987824	0	0	0	0	0	0,137987824	0,01533198
ENSG00000101098	RIMS4	0	0	0	0	0,137962944	0	0	0	0	0,137962944	0,015329216
ENSG00000158815	FGF17	0,137916554	0	0	0	0	0	0	0	0	0,137916554	0,015324062
ENSG00000187048	CYP4A11	0	0,137862211	0	0	0	0	0	0	0	0,137862211	0,015318023
ENSG00000136114	THSD1	0,040785579	0	0	0	0,096927812	0	0	0	0	0,137713391	0,015301488
ENSG00000073598	FNDC8	0	0	0	0	0	0	0	0	0,137460445	0,137460445	0,015273383
ENSG00000154118	JPH3	0	0,084855063	0	0	0	0,052503347	0	0	0	0,13735841	0,015262046
ENSG00000128714	HOXD13	0	0	0	0	0	0,137343193	0	0	0	0,137343193	0,015260355
ENSG00000177689	MAGEB10	0	0,137297491	0	0	0	0	0	0	0	0,137297491	0,015255277
ENSG00000242689	CNTF	0	0	0	0,137189787	0	0	0	0	0	0,137189787	0,01524331
ENSG00000165076	PRSS37	0,137186174	0	0	0	0	0	0	0	0	0,137186174	0,015242908
ENSG00000158528	PPP1R9A	0	0,023954083	0	0,06993076	0,032081253	0	0,011208713	0	0	0,137174808	0,015241645
ENSG00000144191	CNGA3	0	0,061247602	0	0	0	0,075792863	0	0	0	0,137040464	0,015226718
ENSG00000167767	KRT80	0,03462662	0	0	0,059792616	0	0	0	0	0,042586279	0,137005515	0,015222835
ENSG00000131831	RAI2	0	0	0	0	0,136963212	0	0	0	0	0,136963212	0,015218135
ENSG00000219438	TAFA5	0	0,084507406	0	0	0	0,052288237	0	0	0	0,136795643	0,015199516
ENSG00000189350	TOGARAM2	0,030502739	0,013531591	0,014028063	0,052671573	0	0	0	0,01667514	0,009378609	0,136787715	0,015198635
ENSG00000120907	ADRA1A	0	0	0,047534217	0,089239047	0	0	0	0	0	0,136773264	0,015197029
ENSG00000162621	LRRC53	0,039157961	0	0	0	0	0	0,097541072	0	0	0,136699033	0,015188781

ENSG00000160808	MYL3	0	0	0	0	0	0	0	0	0,136651855	0,136651855	0,015183539
ENSG00000260428	SCX	0,136628002	0	0	0	0	0	0	0	0	0,136628002	0,015180889
ENSG00000007350	TKTL1	0,042856089	0	0	0	0	0	0	0,093713717	0	0,136569807	0,015174423
ENSG00000204956	PCDHGA1	0	0	0	0,083472481	0	0,053074401	0	0	0	0,136546881	0,015171876
ENSG00000149654	CDH22	0,03065745	0	0	0,10587745	0	0	0	0	0	0,1365349	0,015170544
ENSG00000116981	NT5C1A	0,136504581	0	0	0	0	0	0	0	0	0,136504581	0,015167176
ENSG00000124875	CXCL6	0	0	0,136264754	0	0	0	0	0	0	0,136264754	0,015140528
ENSG00000124507	PACSIN1	0,03144861	0	0	0	0	0	0,104449808	0	0	0,135898418	0,015099824
ENSG00000215217	C5orf49	0	0	0,135865151	0	0	0	0	0	0	0,135865151	0,015096128
ENSG00000213471	TTLL13P	0	0,068719118	0	0,06687211	0	0	0	0	0	0,135591229	0,015065692
ENSG00000171459	OR1L6	0,135525175	0	0	0	0	0	0	0	0	0,135525175	0,015058353
ENSG00000197125	OR8B8	0	0	0	0	0	0,041780553	0	0	0,093601875	0,135382428	0,015042492
ENSG00000007306	CEACAM7	0	0	0	0	0,135362813	0	0	0	0	0,135362813	0,015040313
ENSG00000181562	EDDM3A	0	0	0	0	0	0	0,135350951	0	0	0,135350951	0,015038995
ENSG00000284686	AC119674.2	0	0	0	0	0,135260845	0	0	0	0	0,135260845	0,015028983
ENSG00000204963	PCDHA7	0	0	0	0,135176122	0	0	0	0	0	0,135176122	0,015019569
ENSG00000137819	PAQR5	0,048557381	0	0,044662612	0,041924	0	0	0	0	0	0,135143993	0,015015999
ENSG00000181903	OR4C6	0,135040724	0	0	0	0	0	0	0	0	0,135040724	0,015004525
ENSG00000168333	PPDPFL	0	0	0,135007333	0	0	0	0	0	0	0,135007333	0,015000815
ENSG00000161270	NPHS1	0	0	0	0	0,065784492	0	0,068952371	0	0	0,134736864	0,014970763
ENSG00000141194	OR4D1	0	0	0	0	0	0,134695008	0	0	0	0,134695008	0,014966112
ENSG00000172554	SNTG2	0,134679653	0	0	0	0	0	0	0	0	0,134679653	0,014964406
ENSG00000168875	SOX14	0,073533124	0	0	0	0	0	0,061056123	0	0	0,134589247	0,014954361
ENSG00000204390	HSPA1L	0	0	0	0	0	0	0	0	0,134573876	0,134573876	0,014952653
ENSG00000206069	TMEM211	0,134559725	0	0	0	0	0	0	0	0	0,134559725	0,014951081
ENSG00000104967	NOVA2	0	0	0	0,115932456	0	0,018428366	0	0	0	0,134360821	0,01492898
ENSG00000204149	AGAP6	0	0	0	0	0	0	0	0	0,1340884	0,1340884	0,014898711
ENSG00000124812	CRISP1	0	0,134071	0	0	0	0	0	0	0	0,134071	0,014896778
ENSG00000160868	CYP3A4	0,133963272	0	0	0	0	0	0	0	0	0,133963272	0,014884808
ENSG00000103710	RASL12	0	0	0	0	0,133949102	0	0	0	0	0,133949102	0,014883234
ENSG00000183423	LRIT3	0	0	0	0,13391582	0	0	0	0	0	0,13391582	0,014879536
ENSG00000267127	AC090360.1	0	0	0	0,133744221	0	0	0	0	0	0,133744221	0,014860469
ENSG00000112499	SLC22A2	0	0	0,046461658	0,087225464	0	0	0	0	0	0,133687122	0,014854125
ENSG00000132821	VSTM2L	0	0	0	0,133675704	0	0	0	0	0	0,133675704	0,014852856
ENSG00000112812	PRSS16	0,028565325	0	0,105096446	0	0	0	0	0	0	0,133661771	0,014851308
ENSG00000183729	NPBWR1	0	0	0	0	0,133649997	0	0	0	0	0,133649997	0,01485
ENSG00000157613	CREB3L1	0	0	0,068858087	0,064635862	0	0	0	0	0	0,133493949	0,014832661
ENSG00000215009	ACSM4	0	0	0	0	0	0	0	0,133401117	0	0,133401117	0,014822346
ENSG00000236761	CTAGE9	0	0	0	0	0	0	0,05375764	0	0,079625759	0,133383399	0,014820378
ENSG00000165555	NOXRED1	0	0	0	0	0	0,133154556	0	0	0	0,133154556	0,014794951
ENSG00000117069	ST6GALNAC5	0	0	0	0,043065683	0,059270101	0	0	0	0,030672804	0,133008589	0,014778732
ENSG00000141622	RNF165	0,039699773	0,070446274	0	0,022850948	0	0	0	0	0	0,132996995	0,014777444
ENSG00000182103	FAM181B	0	0	0	0,132960497	0	0	0	0	0	0,132960497	0,014773389
ENSG00000139880	CDH24	0	0	0	0	0	0,132799288	0	0	0	0,132799288	0,014755476
ENSG00000188089	PLA2G4E	0,025950639	0	0	0,04481109	0,061672255	0	0	0	0	0,132433984	0,014714887
ENSG00000159337	PLA2G4D	0,029410387	0,052188011	0	0,05078532	0	0	0	0	0	0,132383718	0,014709302
ENSG00000143365	RORC	0,02320494	0	0	0	0,055147043	0,025477668	0	0	0,028539085	0,132368736	0,014707637
ENSG00000165521	AL121768.1	0,013737325	0	0	0,118606807	0	0	0	0	0	0,132344131	0,014704903
ENSG00000198854	C1orf68	0	0	0	0	0	0	0,132213205	0	0	0,132213205	0,014690356
ENSG00000181616	OR52H1	0,132205224	0	0	0	0	0	0	0	0	0,132205224	0,014689469
ENSG00000072315	TRPC5	0,025879529	0	0	0,044688298	0,061503261	0	0	0	0	0,132071089	0,014674565
ENSG00000138813	C4orf17	0,046452681	0	0,085453458	0	0	0	0	0	0	0,131906139	0,014656238
ENSG00000283703	VSIG10L2	0,059131509	0	0	0	0	0	0	0	0,072724133	0,131855642	0,014650627
ENSG00000173212	MAB21L3	0,04640988	0	0,085374723	0	0	0	0	0	0	0,131784604	0,014642734
ENSG00000183246	RIMBP3C	0	0,131765111	0	0	0	0	0	0	0	0,131765111	0,014640568
ENSG00000167751	KLK2	0	0	0,131308502	0	0	0	0	0	0	0,131308502	0,014589834
ENSG00000173728	C1orf100	0,131286334	0	0	0	0	0	0	0	0	0,131286334	0,01458737
ENSG00000087303	NID2	0,023640577	0	0	0,081644235	0	0,025955973	0	0	0	0,131240785	0,014582309
ENSG00000168081	PNOC	0	0	0	0,131123103	0	0	0	0	0	0,131123103	0,014569234
ENSG00000214711	CAPN14	0	0	0	0,130991453	0	0	0	0	0	0,130991453	0,014554606
ENSG00000186795	KCNK18	0,130831663	0	0	0	0	0	0	0	0	0,130831663	0,014536851
ENSG00000112379	ARFGEF3	0,050848163	0	0,01870786	0	0	0,011165662	0	0	0,050029348	0,130751034	0,014527893
ENSG00000124157	SEMG2	0	0,130737201	0	0	0	0	0	0	0	0,130737201	0,014526356
ENSG00000226690	AC013470.2	0,080872663	0	0	0	0	0	0	0	0,049731475	0,130604138	0,014511571
ENSG00000131233	GJA9	0	0,130355858	0	0	0	0	0	0	0	0,130355858	0,014483984
ENSG00000188818	ZDHHC11	0	0,038564936	0,039979879	0	0,051649294	0	0	0	0	0,130194109	0,014466012
ENSG00000124469	CEACAM8	0	0	0	0	0	0,130176992	0	0	0	0,130176992	0,01446411
ENSG00000106038	EVX1	0	0	0	0	0	0	0,074729203	0	0,055344408	0,130073611	0,014452623
ENSG00000159495	TGM7	0,129987588	0	0	0	0	0	0	0	0	0,129987588	0,014443065
ENSG00000180638	SLC47A2	0,022584153	0	0	0	0,107343459	0	0	0	0	0,129927613	0,014436401
ENSG00000175985	PLEKHD1	0,018662538	0,033116216	0	0,03222613	0	0	0	0	0,045905032	0,129909916	0,014434435
ENSG00000197140	ADAM32	0	0	0	0,129818395	0	0	0	0	0	0,129818395	0,014424266
ENSG00000164100	NDST3	0	0,039121973	0	0,038070466	0,052395323	0	0	0	0	0,129587761	0,01439864
ENSG00000186462	NAP1L2	0	0	0	0	0	0	0	0,129582183	0	0,129582183	0,01439802
ENSG00000140527	WDR93	0	0	0	0	0,088583508	0,040925155	0	0	0	0,129508663	0,014389851
ENSG00000124493	GRM4	0,009270587	0,016450429	0,034107988	0	0,044063502	0,010178563	0,015395133	0	0	0,129466202	0,014385134
ENSG00000160180	TFF3	0,129375489	0	0	0	0	0	0	0	0	0,129375489	0,014375054
ENSG00000179698	WDR97	0,033703707	0	0	0	0,040048795	0	0,013992454	0	0,041451215	0,12919617	0,01435513
ENSG00000154415	PPP1R3A	0,129181937	0	0	0	0	0	0	0	0	0,129181937	0,014353549
ENSG00000221858	OR2A12	0	0	0,066629937	0,062544337	0	0	0	0	0	0,129174275	0,014352697
ENSG00000171496	OR1L8	0	0	0	0,128920442	0	0	0	0	0	0,128920442	0,014324494
ENSG00000126952	NXF5	0	0	0,128813762	0	0	0	0	0	0	0,128813762	0,01431264
ENSG00000137251	TINAG	0	0	0,128754099	0	0	0	0	0	0	0,128754099	0,014306011
ENSG00000285827	AP001267.5	0	0	0	0	0	0,128672698	0	0	0	0,128672698	0,014296966
ENSG00000124253	PCK1	0	0,039270943	0,04071179	0	0	0,048597123	0	0	0	0,128579855	0,014286651
ENSG00000182272	B4GALNT4	0	0	0	0,128571064	0	0	0	0	0	0,128571064	0,014285674
ENSG00000109163	GNRHR	0	0	0,128456608	0	0	0	0	0	0	0,128456608	0,014272956
ENSG00000261678	SCRT1	0,03796748	0	0	0	0,090230538	0	0	0	0	0,128198018	0,014244224
ENSG00000170454	KRT75	0	0	0,128101428	0	0	0	0	0	0	0,128101428	0,014233492
ENSG00000197614	MFAP5	0	0	0	0	0	0,127984502	0	0	0	0,127984502	0,0142205
ENSG00000173826	KCNH6	0	0	0,066012847	0,061965085	0	0	0	0	0	0,127977932	0,01421977
ENSG00000203499	IQANK1	0	0	0,055796889	0	0,072083007	0	0	0	0	0,127879896	0,014208877
ENSG00000153237	CCDC148	0	0	0	0	0,094753969	0	0,033105628	0	0	0,127859597	0,014206622
ENSG00000126752	SSX1	0	0	0	0	0	0,127820167	0	0	0	0,127820167	0,014202241
ENSG00000172260	NEGR1	0	0,020301484	0,021046343	0,059267484	0,027189396	0	0	0	0	0,127804707	0,014200523
ENSG00000069482	GAL	0	0	0	0	0	0,127623521	0	0	0	0,127623521	0,014180391
ENSG00000034971	MYOC	0	0,127443916	0	0	0	0	0	0	0	0,127443916	0,014160435
ENSG00000088002	SULT2B1	0	0	0	0	0,127256393	0	0	0	0	0,127256393	0,014139599
ENSG00000186081	KRT5	0	0	0	0	0,086890284	0,040142893	0	0	0	0,127033177	0,014114797
ENSG00000005421	PON1	0	0	0	0	0,126807042	0	0	0	0	0,126807042	0,014089671
ENSG00000170835	CEL	0,126770613	0	0	0	0	0	0	0	0	0,126770613	0,014085624
ENSG00000149295	DRD2	0	0	0	0,126759764	0	0	0	0	0	0,126759764	0,014084418
ENSG00000239389	PCDHA13	0	0	0	0	0,066002121	0	0	0,060730485	0	0,126732606	0,014081401
ENSG00000153283	CD96	0,052450736	0	0	0,045285487	0	0,028793922	0	0	0	0,126530145	0,014058905
ENSG00000175920	DOK7	0,037440677	0	0	0	0,088978578	0	0	0	0	0,126419255	0,014046584
ENSG00000168135	KCNJ4	0	0	0	0,126360763	0	0	0	0	0	0,126360763	0,014040085
ENSG00000178233	TMEM151B	0	0	0	0	0,065784492	0	0	0,060530238	0	0,12631473	0,01403497
ENSG00000173404	INSM1	0	0	0	0	0,126183255	0	0	0	0	0,126183255	0,014020362
ENSG00000113262	GRM6	0	0	0	0,126025102	0	0	0	0	0	0,126025102	0,014002789
ENSG00000163075	CFAP221	0,015651017	0,055544692	0,02879131	0	0	0	0,025990747	0	0	0,125977766	0,01399753
ENSG00000138109	CYP2C9	0,037237696	0	0	0	0,088496191	0	0	0	0	0,125733887	0,013970432
ENSG00000205649	HTN3	0	0	0	0,125630705	0	0	0	0	0	0,125630705	0,013958967
ENSG00000130540	SULT4A1	0	0	0	0	0,125609494	0	0	0	0	0,125609494	0,01395661
ENSG00000109705	NKX3-2	0	0	0	0	0	0,059232623	0	0	0,066350062	0,125582684	0,013953632
ENSG00000132915	PDE6A	0	0	0	0,035180663	0,048418167	0	0,016916588	0	0,025056832	0,125572249	0,013952472
ENSG00000174914	OR9G1	0,125541875	0	0	0	0	0	0	0	0	0,125541875	0,013949097
ENSG00000283683	MYOCOS	0	0	0	0	0	0,125452232	0	0	0	0,125452232	0,013939137

ENSG00000203989	RHOXF2B	0	0	0	0	0,125390203	0	0	0	0	0,125390203	0,013932245
ENSG00000145198	VWA5B2	0	0	0	0	0,125346437	0	0	0	0	0,125346437	0,013927382
ENSG00000182224	CYB5D1	0	0	0	0	0	0,125058224	0	0	0	0,125058224	0,013895358
ENSG00000144644	GADL1	0,08246143	0	0	0	0	0	0,017117371	0	0,025354232	0,124933034	0,013881448
ENSG00000188064	WNT7B	0,059492351	0	0	0	0	0	0	0,065046175	0	0,124538525	0,013837614
ENSG00000198883	PNMA5	0	0	0	0,076118721	0	0,048398651	0	0	0	0,124517372	0,013835264
ENSG00000254709	IGLL5	0	0	0	0	0	0,124277586	0	0	0	0,124277586	0,013808621
ENSG00000279956	AC073082.1	0	0	0	0,124077496	0	0	0	0	0	0,124077496	0,013786388
ENSG00000197587	DMBX1	0	0	0	0	0,124004676	0	0	0	0	0,124004676	0,013778297
ENSG00000111249	CUX2	0,040258578	0,035718929	0	0	0,04783769	0	0	0	0	0,123815197	0,013757244
ENSG00000100968	NFATC4	0	0	0	0,123709837	0	0	0	0	0	0,123709837	0,013745537
ENSG00000103355	PRSS33	0	0	0	0,123548757	0	0	0	0	0	0,123548757	0,01372764
ENSG00000125266	EFNB2	0	0	0	0,051989435	0,071551612	0	0	0	0	0,123541047	0,013726783
ENSG00000183196	CHST6	0,017530229	0,062213921	0	0	0	0	0,043667169	0	0	0,123411319	0,013712369
ENSG00000125551	PLGLB2	0	0	0,123327462	0	0	0	0	0	0	0,123327462	0,013703051
ENSG00000185774	KCNIP4	0	0	0	0,123111571	0	0	0	0	0	0,123111571	0,013679063
ENSG00000185069	KRT76	0	0	0	0	0	0	0,049612626	0	0,073486169	0,123098796	0,013677644
ENSG00000116690	PRG4	0	0	0	0,051731755	0,071196975	0	0	0	0	0,122928731	0,013658748
ENSG00000205301	MGAT4D	0,027008145	0,095850581	0	0	0	0	0	0	0	0,122858726	0,01365097
ENSG00000138079	SLC3A1	0	0	0,042498104	0	0,054902544	0,025364711	0	0	0	0,12276536	0,013640596
ENSG00000184394	OR4N5	0	0,122607225	0	0	0	0	0	0	0	0,122607225	0,013623025
ENSG00000120051	CFAP58	0,08657151	0	0	0	0	0	0,035941086	0	0	0,122512596	0,013612511
ENSG00000187553	CYP26C1	0	0,122495203	0	0	0	0	0	0	0	0,122495203	0,013610578
ENSG00000171540	OTP	0	0	0	0	0,08068244	0	0	0	0,041753881	0,122436321	0,013604036
ENSG00000183793	NPIPA5	0	0	0	0	0	0	0	0	0,122267449	0,122267449	0,013585272
ENSG00000184113	CLDN5	0,044068408	0,078198309	0	0	0	0	0	0	0	0,122266716	0,013585191
ENSG00000171368	TPPP	0	0	0	0	0,060063145	0	0	0	0,062166423	0,122229568	0,013581063
ENSG00000169618	PROKR1	0	0	0	0,12213198	0	0	0	0	0	0,12213198	0,01357022
ENSG00000155249	OR4K1	0	0	0	0	0	0	0	0	0,121866572	0,121866572	0,01354073
ENSG00000183783	KCTD8	0	0	0	0	0	0	0	0,121841655	0	0,121841655	0,013537962
ENSG00000101076	HNF4A	0	0,036676515	0	0,035690737	0,049120167	0	0	0	0	0,121487419	0,013498602
ENSG00000186509	OR9Q1	0,12147152	0	0	0	0	0	0	0	0	0,12147152	0,013496836
ENSG00000176927	EFCAB5	0,043623144	0	0,040124148	0,037663824	0	0	0	0	0	0,121411116	0,013490124
ENSG00000136155	SCEL	0,035935926	0	0	0	0,085402507	0	0	0	0	0,121338434	0,013482048
ENSG00000186919	ZACN	0	0	0	0,121167854	0	0	0	0	0	0,121167854	0,013463095
ENSG00000171180	OR2M4	0	0	0	0	0,121037257	0	0	0	0	0,121037257	0,013448584
ENSG00000172742	OR4D9	0	0	0	0,041589891	0	0,07933244	0	0	0	0,120922331	0,013435815
ENSG00000172650	AGAP5	0	0	0	0	0	0	0,120838201	0	0	0,120838201	0,013426467
ENSG00000166856	GPR182	0,044300959	0	0	0,076498087	0	0	0	0	0	0,120799046	0,013422116
ENSG00000213199	ASIC3	0	0	0	0	0	0,120750056	0	0	0	0,120750056	0,013416673
ENSG00000116183	PAPPA2	0	0,024541644	0	0	0,065736325	0,030369866	0	0	0	0,120647834	0,013405315
ENSG00000278522	POTEB3	0,044210231	0	0	0,07634142	0	0	0	0	0	0,120551652	0,013394628
ENSG00000121753	ADGRB2	0,077932218	0	0	0	0	0	0	0,042603735	0	0,120535953	0,013392884
ENSG00000100625	SIX4	0,02231732	0	0,041054512	0,03853714	0	0	0,018530547	0	0	0,120439518	0,013382169
ENSG00000135480	KRT7	0,037787089	0	0	0	0	0	0	0,082629299	0	0,120416389	0,013379599
ENSG00000108242	CYP2C18	0	0	0,120337705	0	0	0	0	0	0	0,120337705	0,013370856
ENSG00000205678	TECRL	0	0	0,046153096	0,043323091	0	0	0	0	0,030856139	0,120332325	0,013370258
ENSG00000165471	MBL2	0,042339751	0	0,077887391	0	0	0	0	0	0	0,120227142	0,013358571
ENSG00000138685	FGF2	0	0,07915631	0,041030273	0	0	0	0	0	0	0,120186583	0,013354065
ENSG00000070601	FRMPD1	0,051111304	0	0,047011686	0	0	0	0,021219403	0	0	0,119342393	0,013260266
ENSG00000118729	CASQ2	0	0	0	0	0	0	0	0,119333538	0	0,119333538	0,013259282
ENSG00000171804	WDR87	0	0	0	0,02836558	0,039038759	0,018035719	0,013639562	0	0,020202905	0,119282525	0,013253614
ENSG00000158458	NRG2	0	0	0,061500022	0,057728977	0	0	0	0	0	0,119228999	0,013247667
ENSG00000166884	OR4D6	0	0	0	0	0	0	0,119042061	0	0	0,119042061	0,013226896
ENSG00000269067	ZNF728	0	0	0	0,119039678	0	0	0	0	0	0,119039678	0,013226631
ENSG00000157470	FAM81A	0,027124497	0	0	0,046838086	0	0	0,045044097	0	0	0,119006679	0,013222964
ENSG00000132164	SLC6A11	0	0	0,041613787	0	0	0	0	0,049466253	0,027821336	0,118901376	0,013211264
ENSG00000286015	CR589904.2	0,118797618	0	0	0	0	0	0	0	0	0,118797618	0,013199735
ENSG00000162692	VCAM1	0	0	0	0	0,118736169	0	0	0	0	0,118736169	0,013192908
ENSG00000164736	SOX17	0	0	0,118491091	0	0	0	0	0	0	0,118491091	0,013165677
ENSG00000156395	SORCS3	0,054142089	0	0	0	0,064334923	0	0	0	0	0,118477012	0,013164112
ENSG00000116785	CFHR3	0	0	0	0,069140163	0	0	0	0	0,049243912	0,118384074	0,013153786
ENSG00000176495	OR5AN1	0	0	0	0,061382022	0,042239184	0	0,014757743	0	0	0,118378949	0,013153217
ENSG00000184599	TAFA3	0,11787096	0	0	0	0	0	0	0	0	0,11787096	0,013096773
ENSG00000197905	TEAD4	0	0,117864615	0	0	0	0	0	0	0	0,117864615	0,013096068
ENSG00000223573	TINCR	0	0	0	0,117830199	0	0	0	0	0	0,117830199	0,013092244
ENSG00000203963	C1orf141	0	0	0	0,051958378	0	0	0	0,065797405	0	0,117755783	0,013083976
ENSG00000179046	TRIML2	0	0	0	0	0,117743457	0	0	0	0	0,117743457	0,013082606
ENSG00000175097	RAG2	0,043149792	0	0	0,074510273	0	0	0	0	0	0,117660064	0,01307334
ENSG00000147655	RSPO2	0,03677551	0	0	0	0	0,080754722	0	0	0	0,117530232	0,013058915
ENSG00000285991	AL355312.6	0,029834269	0	0,054882547	0	0	0,032756284	0	0	0	0,117473099	0,013052567
ENSG00000255974	CYP2A6	0	0	0	0,117379656	0	0	0	0	0	0,117379656	0,013042184
ENSG00000185053	SGCZ	0,041146513	0,036506739	0	0	0	0,022588234	0,017082414	0	0	0,117323901	0,013035989
ENSG00000138684	IL21	0	0	0	0,079191191	0	0	0,038079008	0	0	0,117270199	0,013030022
ENSG00000186334	SLC36A3	0	0,117220546	0	0	0	0	0	0	0	0,117220546	0,013024505
ENSG00000139537	CCDC65	0	0	0,117093555	0	0	0	0	0	0	0,117093555	0,013010395
ENSG00000185176	AQP12B	0	0	0	0,078999387	0	0	0,037986779	0	0	0,116986166	0,012998463
ENSG00000164893	SLC7A13	0	0	0	0,116749429	0	0	0	0	0	0,116749429	0,012972159
ENSG00000158246	TENT5B	0	0	0,116700293	0	0	0	0	0	0	0,116700293	0,012966699
ENSG00000146530	VWDE	0	0	0	0,11662767	0	0	0	0	0	0,11662767	0,01295863
ENSG00000214866	DCDC2C	0	0	0	0,116384913	0	0	0	0	0	0,116384913	0,012931657
ENSG00000121634	GJA8	0,116060346	0	0	0	0	0	0	0	0	0,116060346	0,012895594
ENSG00000064205	CCN5	0,030337396	0	0	0,052386062	0	0,033308688	0	0	0	0,116032147	0,012892461
ENSG00000232382	OR5K1	0,04254239	0	0	0,073461423	0	0	0	0	0	0,116003813	0,012889313
ENSG00000153902	LGI4	0	0	0	0	0,079240411	0,036608689	0	0	0	0,1158491	0,012872122
ENSG00000101448	EPPIN	0,072672605	0,042985252	0	0	0	0	0	0	0	0,115657857	0,012850873
ENSG00000167346	MMP26	0,115527959	0	0	0	0	0	0	0	0	0,115527959	0,01283644
ENSG00000214050	FBXO16	0,115292907	0	0	0	0	0	0	0	0	0,115292907	0,012810323
ENSG00000117322	CR2	0,061867173	0	0	0,053415553	0	0	0	0	0	0,115282726	0,012809192
ENSG00000187754	SSX7	0,115263593	0	0	0	0	0	0	0	0	0,115263593	0,012807066
ENSG00000258417	AC100868.1	0	0	0,115201036	0	0	0	0	0	0	0,115201036	0,012800115
ENSG00000145863	GABRA6	0,042021849	0	0	0,072562562	0	0	0	0	0	0,114584412	0,012731601
ENSG00000188162	OTOG	0,014110614	0	0	0,024365951	0,033534181	0	0,011716344	0,030855782	0	0,114582873	0,01273143
ENSG00000167117	ANKRD40CL	0,041253227	0,073202839	0	0	0	0	0	0	0	0,114456066	0,012717341
ENSG00000242173	ARHGDIG	0	0	0,114442198	0	0	0	0	0	0	0,114442198	0,0127158
ENSG00000176563	CNTD1	0	0	0	0,077245404	0	0	0,037143378	0	0	0,114388782	0,012709865
ENSG00000066468	FGFR2	0,019940693	0,03538427	0	0,034433224	0	0	0	0	0,024524482	0,114282669	0,012698074
ENSG00000244414	CFHR1	0	0	0	0	0	0	0,114219692	0	0	0,114219692	0,012691077
ENSG00000284934	DIABLO	0	0,114054445	0	0	0	0	0	0	0	0,114054445	0,012672716
ENSG00000186714	CCDC73	0	0	0,03529906	0,0331346	0,045602228	0	0	0	0	0,114035888	0,012670654
ENSG00000116721	PRAMEF1	0	0	0,11397298	0	0	0	0	0	0	0,11397298	0,012663664
ENSG00000186049	KRT73	0,113959706	0	0	0	0	0	0	0	0	0,113959706	0,01266219
ENSG00000197653	DNAH10	0	0	0	0,015606159	0,021478322	0,009922881	0	0	0,066691336	0,113698698	0,012633189
ENSG00000134760	DSG1	0,040851736	0	0,037575034	0,035271016	0	0	0	0	0	0,113697785	0,012633087
ENSG00000164483	SAMD3	0,021900083	0	0	0,037816663	0	0	0	0	0,053868557	0,113585303	0,012620589
ENSG00000070669	ASNS	0	0	0,113553962	0	0	0	0	0	0	0,113553962	0,012617107
ENSG00000174529	TMEM81	0,113446374	0	0	0	0	0	0	0	0	0,113446374	0,012605153
ENSG00000155066	PROM2	0,024906967	0,088393605	0	0	0	0	0	0	0	0,113300572	0,012588952
ENSG00000105605	CACNG7	0,053968061	0	0	0	0	0,059253777	0	0	0	0,113221838	0,012580204
ENSG00000103316	CRYM	0	0	0	0,068993912	0	0,043868476	0	0	0	0,112862388	0,012540265
ENSG00000110244	APOA4	0	0	0	0	0	0,112787612	0	0	0	0,112787612	0,012531957
ENSG00000176720	BOK	0,112769083	0	0	0	0	0	0	0	0	0,112769083	0,012529898
ENSG00000120903	CHRNA2	0,0597039	0,052971553	0	0	0	0	0	0	0	0,112675453	0,012519495
ENSG00000123612	ACVR1C	0	0	0,092107389	0	0	0	0	0	0,020526455	0,112633844	0,012514872
ENSG00000106331	PAX4	0	0	0	0	0	0	0	0,11258418	0	0,11258418	0,012509353
ENSG00000271447	MMP28	0	0	0	0	0,112576032	0	0	0	0	0,112576032	0,012508448
ENSG00000137673	MMP7	0	0	0	0,112471972	0	0	0	0	0	0,112471972	0,012496886

ENSG00000274791	F8A2	0	0	0	0	0	0	0,112428613	0	0	0,112428613	0,012492068
ENSG00000185303	SFTPA2	0,112349867	0	0	0	0	0	0	0	0	0,112349867	0,012483319
ENSG00000166126	AMN	0	0	0	0,112326722	0	0	0	0	0	0,112326722	0,012480747
ENSG00000116141	MARK1	0	0	0,051316245	0	0	0	0	0,060999551	0	0,112315796	0,012479533
ENSG00000183098	GPC6	0	0	0,039031185	0,073275758	0	0	0	0	0	0,112306943	0,012478549
ENSG00000167588	GPD1	0,039537041	0	0,07273158	0	0	0	0	0	0	0,112268621	0,012474291
ENSG00000162552	WNT4	0	0	0	0,112230097	0	0	0	0	0	0,112230097	0,012470011
ENSG00000112038	OPRM1	0,02155438	0,012749239	0	0,024813139	0	0	0,005965687	0,047133116	0	0,112215562	0,012468396
ENSG00000101825	MXRA5	0,015413155	0	0	0,079845463	0	0,016922743	0	0	0	0,112181362	0,012464596
ENSG00000106328	FSCN3	0	0	0	0	0,11173539	0	0	0	0	0,11173539	0,012415043
ENSG00000134398	ERN2	0	0,056570042	0	0,055049573	0	0	0	0	0	0,111619615	0,012402179
ENSG00000101004	NINL	0,025059796	0	0	0,086545597	0	0	0	0	0	0,111605393	0,012400599
ENSG00000003989	SLC7A2	0	0	0	0,066017704	0,045429164	0	0	0	0	0,111446868	0,012382985
ENSG00000187791	FAM205C	0,111356353	0	0	0	0	0	0	0	0	0,111356353	0,012372928
ENSG00000153086	ACMSD	0,111274352	0	0	0	0	0	0	0	0	0,111274352	0,012363817
ENSG00000161649	CD300LG	0	0,056391588	0	0,054875915	0	0	0	0	0	0,111267503	0,012363056
ENSG00000183248	PRR36	0	0	0	0	0	0,037141387	0	0,073972368	0	0,111113755	0,012345973
ENSG00000267060	PTGES3L	0,052909864	0	0	0	0	0,058091939	0	0	0	0,111001802	0,012333534
ENSG00000110881	ASIC1	0	0	0,04838644	0	0,062509581	0	0	0	0	0,110896021	0,01232178
ENSG00000285382	FO393400.1	0	0	0	0,110871032	0	0	0	0	0	0,110871032	0,012319004
ENSG00000175264	CHST1	0	0	0	0	0	0	0	0,070817524	0,039829945	0,110647469	0,012294163
ENSG00000204866	IGFL2	0	0,110528442	0	0	0	0	0	0	0	0,110528442	0,012280938
ENSG00000173285	OR10K1	0	0,068125508	0	0	0	0,042152077	0	0	0	0,110277586	0,012253065
ENSG00000186038	HTR3E	0	0	0,110178398	0	0	0	0	0	0	0,110178398	0,012242044
ENSG00000088386	SLC15A1	0	0	0,066028527	0	0	0	0	0	0,044144067	0,110172594	0,012241399
ENSG00000188277	C15orf62	0	0	0	0,110099145	0	0	0	0	0	0,110099145	0,012233238
ENSG00000152969	JAKMIP1	0,039682398	0,070415441	0	0	0	0	0	0	0	0,110097839	0,012233093
ENSG00000181215	C4orf50	0,014619124	0,012970638	0	0,050488071	0	0	0	0,031967742	0	0,110045575	0,012227286
ENSG00000170923	OR7G2	0,049350284	0	0	0	0	0	0	0	0,060694488	0,110044772	0,012227197
ENSG00000274322	AL136531.2	0,109898597	0	0	0	0	0	0	0	0	0,109898597	0,012210955
ENSG00000104941	RSPH6A	0	0	0,109743426	0	0	0	0	0	0	0,109743426	0,012193714
ENSG00000196862	RGPD4	0	0,049074305	0	0	0	0	0	0,060474848	0	0,109549152	0,012172128
ENSG00000160200	CBS	0	0	0	0	0	0	0	0	0,109547022	0,109547022	0,012171891
ENSG00000160185	UBASH3A	0,038568293	0	0,070949489	0	0	0	0	0	0	0,109517782	0,012168642
ENSG00000183644	C11orf88	0	0	0	0	0,109487056	0	0	0	0	0,109487056	0,012165228
ENSG00000253313	C1orf210	0	0	0	0	0	0,109439694	0	0	0	0,109439694	0,012159966
ENSG00000146049	KAAG1	0,109341947	0	0	0	0	0	0	0	0	0,109341947	0,012149105
ENSG00000152467	ZSCAN1	0	0	0	0,109223514	0	0	0	0	0	0,109223514	0,012135946
ENSG00000273841	TAF9	0	0,109222811	0	0	0	0	0	0	0	0,109222811	0,012135868
ENSG00000152034	MCHR2	0	0	0	0,109086528	0	0	0	0	0	0,109086528	0,012120725
ENSG00000160321	ZNF208	0	0	0,023345939	0	0,030160203	0	0	0,055502573	0	0,109008716	0,01211208
ENSG00000204060	FOXO6	0	0	0	0,108813584	0	0	0	0	0	0,108813584	0,012090398
ENSG00000167646	DNAAF3	0	0	0	0	0,108757584	0	0	0	0	0,108757584	0,012084176
ENSG00000164344	KLKB1	0,051838961	0	0	0	0	0,05691615	0	0	0	0,108755111	0,012083901
ENSG00000160801	PTH1R	0,108595451	0	0	0	0	0	0	0	0	0,108595451	0,012066161
ENSG00000104413	ESRP1	0	0	0,057021559	0	0	0	0,051475008	0	0	0,108496567	0,012055174
ENSG00000188991	SLC15A5	0,051697082	0	0	0	0	0,056760375	0	0	0	0,108457457	0,012050829
ENSG00000131068	DEFB118	0	0	0	0	0	0	0,108350891	0	0	0,108350891	0,012038988
ENSG00000168907	PLA2G4F	0	0	0	0	0	0	0	0,108285947	0	0,108285947	0,012031772
ENSG00000253598	SLC10A5	0	0	0,108247702	0	0	0	0	0	0	0,108247702	0,012027522
ENSG00000169562	GJB1	0	0,108165389	0	0	0	0	0	0	0	0,108165389	0,012018377
ENSG00000160282	FTCD	0	0	0	0	0,108070281	0	0	0	0	0,108070281	0,012007809
ENSG00000137261	KIAA0319	0,020964286	0,03720061	0	0	0,049822079	0	0	0	0	0,107986975	0,011998553
ENSG00000164434	FABP7	0	0,107817451	0	0	0	0	0	0	0	0,107817451	0,011979717
ENSG00000185264	TEX33	0	0	0	0	0	0	0,107792381	0	0	0,107792381	0,011976931
ENSG00000182795	C1orf116	0	0	0	0,047434098	0	0,060320152	0	0	0	0,10775425	0,011972694
ENSG00000146166	LGSN	0	0,046048772	0	0	0,061672255	0	0	0	0	0,107721027	0,011969003
ENSG00000146755	TRIM50	0	0,10768755	0	0	0	0	0	0	0	0,10768755	0,011965283
ENSG00000119283	TRIM67	0,016203149	0	0	0,055958605	0	0	0	0,035431543	0	0,107593297	0,011954811
ENSG00000075223	SEMA3C	0	0,043957705	0	0,042776225	0	0	0,020568907	0	0	0,107302837	0,011922537
ENSG00000255872	AL138752.2	0	0	0,1072868	0	0	0	0	0	0	0,1072868	0,011920756
ENSG00000274391	TPTE	0	0	0,107080161	0	0	0	0	0	0	0,107080161	0,011897796
ENSG00000255730	AC011462.1	0	0	0	0	0	0	0,106919755	0	0	0,106919755	0,011879973
ENSG00000100078	PLA2G3	0	0	0,106915423	0	0	0	0	0	0	0,106915423	0,011879491
ENSG00000185664	PMEL	0	0	0	0	0	0,042530268	0,064327266	0	0	0,106857534	0,011873059
ENSG00000183671	GPR1	0,106641193	0	0	0	0	0	0	0	0	0,106641193	0,011849021
ENSG00000157119	KLHL40	0	0,10653238	0	0	0	0	0	0	0	0,10653238	0,011836931
ENSG00000268104	SLC6A14	0,033313618	0	0	0	0	0,073152812	0	0	0	0,106466429	0,011829603
ENSG00000104921	FCER2	0	0	0	0,106460618	0	0	0	0	0	0,106460618	0,011828958
ENSG00000154227	CERS3	0	0,052269396	0,054187154	0	0	0	0	0	0	0,10645655	0,011828506
ENSG00000171954	CYP4F22	0	0	0,106424234	0	0	0	0	0	0	0,106424234	0,011824915
ENSG00000156140	ADAMTS3	0	0	0	0,037239186	0,051251255	0	0,017906427	0	0	0,106396867	0,011821874
ENSG00000242419	PCDHGC4	0	0,053908725	0	0,052459786	0	0	0	0	0	0,106368511	0,011818723
ENSG00000169717	ACTRT2	0,106341007	0	0	0	0	0	0	0	0	0,106341007	0,011815667
ENSG00000153292	ADGRF1	0	0	0,044897052	0,021072032	0	0,040194761	0	0	0	0,106163846	0,011795983
ENSG00000184471	C1QTNF8	0	0	0	0	0	0	0	0	0,105474757	0,105474757	0,011719417
ENSG00000145451	GLRA3	0	0	0	0,027391872	0	0	0	0	0,078037591	0,105429463	0,011714385
ENSG00000179846	NKPD1	0	0,062213921	0	0	0	0	0	0	0,043119843	0,105333764	0,011703752
ENSG00000171201	SMR3B	0,105230202	0	0	0	0	0	0	0	0	0,105230202	0,011692245
ENSG00000182264	IZUMO1	0	0	0	0	0,10515887	0	0	0	0	0,10515887	0,011684319
ENSG00000110786	PTPN5	0,027489644	0	0	0,047468615	0	0,030182022	0	0	0	0,105140281	0,011682253
ENSG00000140839	CLEC18B	0	0	0	0	0	0	0	0,105133493	0	0,105133493	0,011681499
ENSG00000164855	TMEM184A	0	0	0,036518668	0,068558848	0	0	0	0	0	0,105077516	0,01167528
ENSG00000160188	RSPH1	0,104937896	0	0	0	0	0	0	0	0	0,104937896	0,011659766
ENSG00000118160	SLC8A2	0	0	0	0,049503884	0	0,03147611	0,023803895	0	0	0,10478389	0,011642654
ENSG00000100593	ISM2	0,036883224	0	0,06784967	0	0	0	0	0	0	0,104732895	0,011636988
ENSG00000132613	MTSS2	0	0	0	0,104667058	0	0	0	0	0	0,104667058	0,011629673
ENSG00000138483	CCDC54	0,10464721	0	0	0	0	0	0	0	0	0,10464721	0,011627468
ENSG00000184313	MROH7	0,016627484	0	0,030587599	0,05742407	0	0	0	0	0	0,104639153	0,011626573
ENSG00000111058	ACSS3	0,008183621	0,029043271	0	0	0,058345661	0,008985138	0	0	0	0,104557692	0,011617521
ENSG00000284512	AC092718.8	0,104502469	0	0	0	0	0	0	0	0	0,104502469	0,011611385
ENSG00000243130	PSG11	0	0	0	0,104499389	0	0	0	0	0	0,104499389	0,011611043
ENSG00000092607	TBX15	0,104358129	0	0	0	0	0	0	0	0	0,104358129	0,011595348
ENSG00000175718	RBMXL3	0	0	0	0	0,104333975	0	0	0	0	0,104333975	0,011592664
ENSG00000262209	PCDHGB3	0	0	0	0	0	0,104237012	0	0	0	0,104237012	0,01158189
ENSG00000003137	CYP26B1	0	0	0	0,104061804	0	0	0	0	0	0,104061804	0,011562423
ENSG00000171450	CDK5R2	0	0	0	0,104041058	0	0	0	0	0	0,104041058	0,011560118
ENSG00000181449	SOX2	0	0	0	0,103875388	0	0	0	0	0	0,103875388	0,01154171
ENSG00000203952	CCDC160	0	0	0	0	0	0	0	0	0,103824873	0,103824873	0,011536097
ENSG00000088280	ASAP3	0	0	0	0	0	0,066523888	0	0	0,037258725	0,103782613	0,011531401
ENSG00000184709	LRRC26	0	0	0	0	0	0	0,103780258	0	0	0,103780258	0,01153114
ENSG00000142661	MYOM3	0	0,029101585	0	0,056638805	0	0,018006357	0	0	0	0,103746747	0,011527416
ENSG00000185313	SCN10A	0	0	0	0,078713416	0	0,02502422	0	0	0	0,103737636	0,011526404
ENSG00000215397	SCRT2	0	0	0	0	0	0,10369411	0	0	0	0,10369411	0,011521568
ENSG00000166960	CCDC178	0	0	0,062118458	0	0	0	0	0	0,041529949	0,103648407	0,01151649
ENSG00000178235	SLITRK1	0	0	0	0	0	0,031973516	0	0	0,071630959	0,103604475	0,011511608
ENSG00000103154	NECAB2	0	0	0	0,103545625	0	0	0	0	0	0,103545625	0,011505069
ENSG00000115085	ZAP70	0	0,052463706	0	0,051053605	0	0	0	0	0	0,103517311	0,011501923
ENSG00000269113	TRABD2B	0,020083808	0,035638224	0	0	0,047729604	0	0	0	0	0,103451637	0,011494626
ENSG00000197454	OR2L5	0	0	0	0	0	0	0,103310277	0	0	0,103310277	0,01147892
ENSG00000095752	IL11	0,056426651	0	0	0	0	0	0,046852252	0	0	0,103278903	0,011475434
ENSG00000101180	HRH3	0	0	0,103261552	0	0	0	0	0	0	0,103261552	0,011473506
ENSG00000120055	C10orf95	0,102866284	0	0	0	0	0	0	0	0	0,102866284	0,011429587
ENSG00000066813	ACSM2B	0,018522992	0	0	0,06397033	0	0,020337163	0	0	0	0,102830485	0,011425609
ENSG00000101280	ANGPT4	0,032206004	0	0	0	0	0	0	0,0704251	0	0,102631104	0,011403456
ENSG00000241224	C3orf85	0	0	0	0,102447968	0	0	0	0	0	0,102447968	0,011383108
ENSG00000076826	CAMSAP3	0	0	0	0	0	0,034243669	0	0,068201149	0	0,102444818	0,011382758

ENSG00000124233	SEMG1	0	0	0	0	0	0,102287655	0	0	0	0,102287655	0,011365295
ENSG00000171346	KRT15	0	0	0,046570631	0	0	0,055590744	0	0	0	0,102161376	0,011351264
ENSG00000176798	OR51L1	0	0	0	0,035744517	0,049194184	0	0,017187717	0	0	0,102126418	0,01134738
ENSG00000103241	FOXF1	0	0	0	0	0,102022029	0	0	0	0	0,102022029	0,011335781
ENSG00000117090	SLAMF1	0	0,062899836	0	0	0	0,038918737	0	0	0	0,101818573	0,011313175
ENSG00000115705	TPO	0,035753122	0	0	0	0	0	0	0	0,065957597	0,101710719	0,011301191
ENSG00000141979	AC008764.1	0	0	0	0,101649776	0	0	0	0	0	0,101649776	0,01129442
ENSG00000168491	CCDC110	0	0	0	0	0,101617867	0	0	0	0	0,101617867	0,011290874
ENSG00000187905	LRRC74B	0,054532866	0	0	0,047083178	0	0	0	0	0	0,101616044	0,011290672
ENSG00000166959	MS4A8	0	0	0	0,101610193	0	0	0	0	0	0,101610193	0,011290021
ENSG00000123999	INHA	0	0	0	0	0	0,101412333	0	0	0	0,101412333	0,011268037
ENSG00000179914	ITLN1	0,101280543	0	0	0	0	0	0	0	0	0,101280543	0,011253394
ENSG00000157680	DGKI	0,009718347	0	0,017877683	0,033562927	0	0,03201053	0,008069351	0	0	0,101238837	0,01124876
ENSG00000215906	LACTBL1	0	0	0	0	0	0,101103337	0	0	0	0,101103337	0,011233704
ENSG00000145040	UCN2	0,10087488	0	0	0	0	0	0	0	0	0,10087488	0,01120832
ENSG00000286112	AL441992.2	0	0	0	0	0	0	0	0,047408116	0,053327553	0,100735669	0,011192852
ENSG00000151617	EDNRA	0	0	0	0,06157031	0	0,039148319	0	0	0	0,100718629	0,011190959
ENSG00000074527	NTN4	0,036272341	0,064364378	0	0	0	0	0	0	0	0,100636719	0,011181858
ENSG00000156886	ITGAD	0	0	0,063019746	0	0	0,037612917	0	0	0	0,100632663	0,011181407
ENSG00000178965	ERICH3	0	0	0,031492024	0	0,040683986	0	0,02842876	0	0	0,10060477	0,011178308
ENSG00000176165	FOXG1	0	0	0,051881317	0,04870007	0	0	0	0	0	0,100581387	0,01117571
ENSG00000184698	OR51M1	0,036865228	0	0	0,063658203	0	0	0	0	0	0,100523431	0,01116927
ENSG00000166509	CLEC3A	0,100505867	0	0	0	0	0	0	0	0	0,100505867	0,011167319
ENSG00000137699	TRIM29	0,075254268	0	0	0	0	0	0,02499409	0	0	0,100248358	0,011138706
ENSG00000136695	IL36RN	0	0	0	0	0	0	0	0,100162039	0	0,100162039	0,011129115
ENSG00000178772	CPN2	0,099907815	0	0	0	0	0	0	0	0	0,099907815	0,011100868
ENSG00000109991	P2RX3	0	0	0	0,099822102	0	0	0	0	0	0,099822102	0,011091345
ENSG00000184302	SIX6	0	0	0,099741693	0	0	0	0	0	0	0,099741693	0,01108241
ENSG00000167103	PIP5KL1	0	0	0	0	0	0,099735844	0	0	0	0,099735844	0,01108176
ENSG00000157551	KCNJ15	0,014611349	0,02592748	0	0	0,034724187	0	0,02426423	0	0	0,099527246	0,011058583
ENSG00000111704	NANOG	0	0,099459199	0	0	0	0	0	0	0	0,099459199	0,011051022
ENSG00000180785	OR51E1	0	0	0	0	0,099423461	0	0	0	0	0,099423461	0,011047051
ENSG00000042813	ZPBP	0	0	0	0,0994038	0	0	0	0	0	0,0994038	0,011044867
ENSG00000187833	C2orf78	0,099251606	0	0	0	0	0	0	0	0	0,099251606	0,011027956
ENSG00000133477	FAM83F	0,046101217	0	0,016961383	0,015921348	0	0	0	0,020161973	0	0,099145921	0,011016213
ENSG00000215372	ZNF705G	0	0	0	0	0,098550368	0	0	0	0	0,098550368	0,010950041
ENSG00000164438	TLX3	0,098507543	0	0	0	0	0	0	0	0	0,098507543	0,010945283
ENSG00000188282	RUFY4	0,034682252	0	0,063800803	0	0	0	0	0	0	0,098483055	0,010942562
ENSG00000111218	PRMT8	0,04548783	0	0	0	0	0,02497149	0	0	0,027972083	0,098431403	0,010936823
ENSG00000121068	TBX2	0	0,0440878	0	0	0	0	0	0,054329919	0	0,098417719	0,010935302
ENSG00000204532	ZSCAN5C	0	0	0	0	0	0	0	0	0,098227549	0,098227549	0,010914172
ENSG00000021852	C8B	0	0,098148609	0	0	0	0	0	0	0	0,098148609	0,010905401
ENSG00000171385	KCND3	0,019217928	0	0	0,033185168	0,045671823	0	0	0	0	0,098074919	0,010897213
ENSG00000106302	HYAL4	0	0,097933528	0	0	0	0	0	0	0	0,097933528	0,010881503
ENSG00000107147	KCNT1	0	0	0	0,042835915	0,029476939	0	0,010298804	0	0,015254578	0,097866235	0,010874026
ENSG00000151364	KCTD14	0	0	0	0	0	0	0	0	0,097865467	0,097865467	0,010873941
ENSG00000174607	UGT8	0,034460791	0	0,063393409	0	0	0	0	0	0	0,097854201	0,010872689
ENSG00000105398	SULT2A1	0	0	0	0	0	0	0	0	0,097608468	0,097608468	0,010845385
ENSG00000170049	KCNAB3	0	0	0	0,056972702	0	0	0	0	0,040577843	0,097550545	0,010838949
ENSG00000171060	C8orf74	0	0	0	0,097509333	0	0	0	0	0	0,097509333	0,01083437
ENSG00000205209	SCGB2B2	0	0	0	0	0	0,055488487	0,041963322	0	0	0,097451809	0,010827979
ENSG00000163749	CCDC158	0,051573574	0,04575802	0	0	0	0	0	0	0	0,097331594	0,010814622
ENSG00000175664	TEX26	0	0	0	0	0	0,097308256	0	0	0	0,097308256	0,010812028
ENSG00000050555	LAMC3	0,040285409	0	0	0,034782055	0	0,022115513	0	0	0	0,097182977	0,010798109
ENSG00000141434	MEP1B	0	0	0	0	0	0	0,097150857	0	0	0,097150857	0,01079454
ENSG00000185860	CCDC190	0,035011717	0,062127433	0	0	0	0	0	0	0	0,09713915	0,010793239
ENSG00000100346	CACNA1I	0,014002091	0	0	0	0,033276273	0,015373478	0	0	0,034441535	0,097093378	0,010788153
ENSG00000166897	ELFN2	0	0	0	0,059364117	0	0	0	0,037587825	0	0,096951941	0,010772438
ENSG00000255587	RAB44	0,035480294	0	0	0,061266723	0	0	0	0	0	0,096747017	0,010749669
ENSG00000109205	ODAM	0,096741723	0	0	0	0	0	0	0	0	0,096741723	0,01074908
ENSG00000226763	SRRM5	0	0	0	0,096714223	0	0	0	0	0	0,096714223	0,010746025
ENSG00000184307	ZDHHC23	0	0	0	0	0,050310667	0	0	0,046292318	0	0,096602985	0,010733665
ENSG00000197565	COL4A6	0	0,057776772	0	0	0,038689673	0	0	0	0	0,096466445	0,010718494
ENSG00000162755	KLHDC9	0	0	0	0	0	0,096459638	0	0	0	0,096459638	0,010717738
ENSG00000126010	GRPR	0	0	0	0	0	0	0	0	0,096343454	0,096343454	0,010704828
ENSG00000120471	TP53AIP1	0	0	0	0	0	0,096319638	0	0	0	0,096319638	0,010702182
ENSG00000188655	RNASE9	0	0	0	0,096285968	0	0	0	0	0	0,096285968	0,010698441
ENSG00000177138	FAM9B	0,021935052	0	0	0,037877047	0	0	0,036426283	0	0	0,096238381	0,010693153
ENSG00000169752	NRG4	0,030101707	0	0	0	0	0,066099831	0	0	0	0,096201539	0,01068906
ENSG00000237515	SHISA9	0	0	0	0,025632119	0,070553545	0	0	0	0	0,096185664	0,010687296
ENSG00000240583	AQP1	0	0	0	0	0	0	0	0,096168384	0	0,096168384	0,010685376
ENSG00000189325	C6orf222	0	0	0	0	0,096149275	0	0	0	0	0,096149275	0,010683253
ENSG00000196119	OR8A1	0,024821053	0,04404435	0	0	0	0,027252066	0	0	0	0,096117468	0,010679719
ENSG00000073067	CYP2W1	0	0,096073809	0	0	0	0	0	0	0	0,096073809	0,010674868
ENSG00000187823	RTL4	0	0	0	0	0	0	0,096035463	0	0	0,096035463	0,010670607
ENSG00000170214	ADRA1B	0,096034681	0	0	0	0	0	0	0	0	0,096034681	0,01067052
ENSG00000068781	STON1-GTF2A1L	0,035191097	0	0	0,060767344	0	0	0	0	0	0,095958441	0,010662049
ENSG00000132972	RNF17	0,026902363	0	0	0,046454509	0	0	0,022337606	0	0	0,095694477	0,01063272
ENSG00000205502	C2CD4B	0,095579109	0	0	0	0	0	0	0	0	0,095579109	0,010619901
ENSG00000226979	LTA	0	0	0	0	0	0,095515588	0	0	0	0,095515588	0,010612843
ENSG00000184697	CLDN6	0	0	0	0	0	0,095405737	0	0	0	0,095405737	0,010600637
ENSG00000163534	FCRL1	0	0	0	0	0	0	0,095293923	0	0	0,095293923	0,010588214
ENSG00000165810	BTNL9	0	0,048200971	0	0,046905442	0	0	0	0	0	0,095106413	0,010567379
ENSG00000123454	DBH	0	0	0	0	0,095105281	0	0	0	0	0,095105281	0,010567253
ENSG00000117598	PLPPR5	0	0,058661562	0	0	0	0,036296341	0	0	0	0,094957903	0,010550878
ENSG00000080910	CFHR2	0	0	0	0,094885445	0	0	0	0	0	0,094885445	0,010542827
ENSG00000260230	FRRS1L	0,0097121	0,017233884	0,017866193	0,016770678	0	0	0	0,02123752	0,011944632	0,094765006	0,010529445
ENSG00000167701	GPT	0	0	0	0,094678873	0	0	0	0	0	0,094678873	0,010519875
ENSG00000166819	PLIN1	0	0	0	0	0,094629128	0	0	0	0	0,094629128	0,010514348
ENSG00000170180	GYPA	0,035547064	0	0	0	0	0	0,059030972	0	0	0,094578037	0,010508671
ENSG00000197261	C6orf141	0	0	0,094551054	0	0	0	0	0	0	0,094551054	0,010505673
ENSG00000131668	BARX1	0	0	0	0	0	0,094535941	0	0	0	0,094535941	0,010503993
ENSG00000121440	PDZRN3	0,049229702	0	0,045281007	0	0	0	0	0	0	0,094510709	0,01050119
ENSG00000156463	SH3RF2	0,020961378	0,03719545	0	0,036195724	0	0	0	0	0	0,094352552	0,010483617
ENSG00000026559	KCNG1	0,027905923	0	0	0	0,066319029	0	0	0	0	0,094224952	0,010469439
ENSG00000148826	NKX6-2	0	0	0	0,094200352	0	0	0	0	0	0,094200352	0,010466706
ENSG00000081818	PCDHB4	0	0	0	0	0,093886939	0	0	0	0	0,093886939	0,010431882
ENSG00000124212	PTGIS	0,027129366	0	0	0	0	0	0	0	0,066731247	0,093860613	0,010428957
ENSG00000101327	PDYN	0	0	0	0	0	0	0	0,093766903	0	0,093766903	0,010418545
ENSG00000283900	TPTEP2-CSNK1E	0	0	0	0	0	0,093734789	0	0	0	0,093734789	0,010414977
ENSG00000165617	DACT1	0,033654916	0,059719822	0	0	0	0	0	0	0	0,093374737	0,010374971
ENSG00000107295	SH3GL2	0	0	0	0	0	0	0,093182571	0	0	0,093182571	0,010353619
ENSG00000227051	C14orf132	0,019435443	0	0	0,033560768	0	0	0,016137663	0	0,02390309	0,093036965	0,010337441
ENSG00000198643	FAM3D	0,09293393	0	0	0	0	0	0	0	0	0,09293393	0,010325992
ENSG00000240891	PLCXD2	0	0	0,092928493	0	0	0	0	0	0	0,092928493	0,010325388
ENSG00000169194	IL13	0	0,092879113	0	0	0	0	0	0	0	0,092879113	0,010319901
ENSG00000162951	LRRTM1	0,059881344	0	0	0	0	0,032873108	0	0	0	0,092754452	0,01030605
ENSG00000168348	INSM2	0	0,092750605	0	0	0	0	0	0	0	0,092750605	0,010305623
ENSG00000186732	MPPED1	0,025959555	0	0	0,044826486	0	0	0,021554773	0	0	0,092340814	0,01026009
ENSG00000278685	IQCA1L	0	0	0	0,092333678	0	0	0	0	0	0,092333678	0,010259298
ENSG00000105647	PIK3R2	0,033797936	0	0	0,058361658	0	0	0	0	0	0,092159594	0,010239955
ENSG00000114204	SERPINI2	0	0	0	0,092040555	0	0	0	0	0	0,092040555	0,010226728
ENSG00000153291	SLC25A27	0,092000347	0	0	0	0	0	0	0	0	0,092000347	0,010222261
ENSG00000138161	CUZD1	0	0	0	0,09197567	0	0	0	0	0	0,09197567	0,010219519
ENSG00000204967	PCDHA4	0,033722511	0	0	0,058231416	0	0	0	0	0	0,091953927	0,010217103
ENSG00000043355	ZIC2	0,091860529	0	0	0	0	0	0	0	0	0,091860529	0,010206725
ENSG00000164112	TMEM155	0	0	0,091833531	0	0	0	0	0	0	0,091833531	0,010203726

ENSG00000166090	IL25	0	0	0	0	0	0	0,091785173	0	0	0,091785173	0,010198353
ENSG00000112964	GHR	0	0	0,04000865	0	0,051686463	0	0	0	0	0,091695112	0,010188346
ENSG00000262246	CORO7	0	0	0	0,029675307	0,040841299	0	0	0	0,021135736	0,091652342	0,010183594
ENSG00000168398	BDKRB2	0	0,039173411	0	0	0,052464214	0	0	0	0	0,091637625	0,010181958
ENSG00000142973	CYP4B1	0,043597972	0	0	0	0	0,047868026	0	0	0	0,091465998	0,010162889
ENSG00000243284	VSIG8	0	0	0	0	0	0,091259943	0	0	0	0,091259943	0,010139994
ENSG00000284631	SCYGR4	0,091140272	0	0	0	0	0	0	0	0	0,091140272	0,010126697
ENSG00000124092	CTCFL	0,016925467	0,060067652	0	0	0	0	0,014053577	0	0	0,091046696	0,0101163
ENSG00000204510	PRAMEF7	0,091030464	0	0	0	0	0	0	0	0	0,091030464	0,010114496
ENSG00000168955	TM4SF20	0	0	0	0	0	0	0,091019465	0	0	0,091019465	0,010113274
ENSG00000179455	MKRN3	0	0	0	0,090997376	0	0	0	0	0	0,090997376	0,01011082
ENSG00000198910	L1CAM	0,041570996	0	0	0	0,049397186	0	0	0	0	0,090968182	0,010107576
ENSG00000129988	LBP	0	0	0	0	0	0,090909905	0	0	0	0,090909905	0,010101101
ENSG00000172771	EFCAB12	0	0	0	0,067000892	0	0	0	0	0,023860126	0,090861018	0,010095669
ENSG00000118434	SPACA1	0,090811641	0	0	0	0	0	0	0	0	0,090811641	0,010090182
ENSG00000197705	KLHL14	0,090811641	0	0	0	0	0	0	0	0	0,090811641	0,010090182
ENSG00000176160	HSF5	0	0	0	0	0	0	0	0	0,090789703	0,090789703	0,010087745
ENSG00000089116	LHX5	0	0	0,090754195	0	0	0	0	0	0	0,090754195	0,010083799
ENSG00000188778	ADRB3	0	0	0	0,090728433	0	0	0	0	0	0,090728433	0,010080937
ENSG00000137473	TTC29	0,090702623	0	0	0	0	0	0	0	0	0,090702623	0,010078069
ENSG00000180806	HOXC9	0,090702623	0	0	0	0	0	0	0	0	0,090702623	0,010078069
ENSG00000154438	ASZ1	0	0	0,090694975	0	0	0	0	0	0	0,090694975	0,010077219
ENSG00000176177	ENTHD1	0	0	0,09060629	0	0	0	0	0	0	0,09060629	0,010067366
ENSG00000164129	NPY5R	0,090539587	0	0	0	0	0	0	0	0	0,090539587	0,010059954
ENSG00000016490	CLCA1	0,090485372	0	0	0	0	0	0	0	0	0,090485372	0,01005393
ENSG00000176884	GRIN1	0	0	0,046648783	0,043788383	0	0	0	0	0	0,090437166	0,010048574
ENSG00000205330	OR6C1	0	0	0	0	0	0	0	0	0,090172985	0,090172985	0,010019221
ENSG00000244476	ERVFRD-1	0	0	0	0	0	0,051286113	0,038785265	0	0	0,090071378	0,010007931
ENSG00000231396	USP17L10	0,089946768	0	0	0	0	0	0	0	0	0,089946768	0,009994085
ENSG00000140600	SH3GL3	0,08986653	0	0	0	0	0	0	0	0	0,08986653	0,00998517
ENSG00000214279	SCART1	0	0	0	0	0,089611364	0	0	0	0	0,089611364	0,009956818
ENSG00000185652	NTF3	0	0	0	0,089269577	0	0	0	0	0	0,089269577	0,009918842
ENSG00000166153	DEPDC4	0	0	0,089153335	0	0	0	0	0	0	0,089153335	0,009905926
ENSG00000204435	CSNK2B	0	0	0	0,088965215	0	0	0	0	0	0,088965215	0,009885024
ENSG00000255408	PCDHA3	0,040495932	0	0	0	0,04811973	0	0	0	0	0,088615662	0,009846185
ENSG00000057149	SERPINB3	0,08852406	0	0	0	0	0	0	0	0	0,08852406	0,009836007
ENSG00000163633	C4orf36	0	0	0	0	0	0	0	0	0,088498344	0,088498344	0,009833149
ENSG00000124449	IRGC	0,088420463	0	0	0	0	0	0	0	0	0,088420463	0,009824496
ENSG00000006128	TAC1	0,08826552	0	0	0	0	0	0	0	0	0,08826552	0,00980728
ENSG00000204001	LCN8	0	0,088262673	0	0	0	0	0	0	0	0,088262673	0,009806964
ENSG00000173838	MARCH10	0	0,054500407	0	0	0	0,033721662	0	0	0	0,088222068	0,009802452
ENSG00000166603	MC4R	0,088162527	0	0	0	0	0	0	0	0	0,088162527	0,009795836
ENSG00000124602	UNC5CL	0	0	0,088079879	0	0	0	0	0	0	0,088079879	0,009786653
ENSG00000095596	CYP26A1	0	0	0	0,088034742	0	0	0	0	0	0,088034742	0,009781638
ENSG00000181786	ACTL9	0	0	0	0	0	0	0,08798761	0	0	0,08798761	0,009776401
ENSG00000224420	ADM5	0,087957259	0	0	0	0	0	0	0	0	0,087957259	0,009773029
ENSG00000006016	CRLF1	0	0	0	0,087945728	0	0	0	0	0	0,087945728	0,009771748
ENSG00000159650	UROC1	0,087752945	0	0	0	0	0	0	0	0	0,087752945	0,009750327
ENSG00000146966	DENND2A	0	0,051814879	0	0	0	0	0	0	0,035912371	0,087727251	0,009747472
ENSG00000182854	OR4F15	0	0	0	0	0	0	0	0	0,087704824	0,087704824	0,00974498
ENSG00000260643	AC092718.3	0	0	0,08749767	0	0	0	0	0	0	0,08749767	0,009721963
ENSG00000172482	AGXT	0	0	0	0	0,087291576	0	0	0	0	0,087291576	0,009699064
ENSG00000178038	ALS2CL	0	0,029599514	0	0,057607898	0	0	0	0	0	0,087207412	0,009689712
ENSG00000182326	C1S	0,046768979	0	0	0,040379909	0	0	0	0	0	0,087148888	0,00968321
ENSG00000136457	CHAD	0,086745448	0	0	0	0	0	0	0	0	0,086745448	0,009638383
ENSG00000154645	CHODL	0	0,086693178	0	0	0	0	0	0	0	0,086693178	0,009632575
ENSG00000144671	SLC22A14	0,041298325	0	0	0	0	0,045343148	0	0	0	0,086641472	0,00962683
ENSG00000261147	AC091167.2	0	0	0	0	0	0,086456788	0	0	0	0,086456788	0,00960631
ENSG00000072182	ASIC4	0,086348898	0	0	0	0	0	0	0	0	0,086348898	0,009594322
ENSG00000054803	CBLN4	0	0	0	0,086288021	0	0	0	0	0	0,086288021	0,009587558
ENSG00000139908	TSSK4	0	0	0	0	0	0,086232108	0	0	0	0,086232108	0,009581345
ENSG00000164683	HEY1	0	0	0,086168661	0	0	0	0	0	0	0,086168661	0,009574296
ENSG00000106236	NPTX2	0	0	0	0,086145584	0	0	0	0	0	0,086145584	0,009571732
ENSG00000101323	HAO1	0,086004878	0	0	0	0	0	0	0	0	0,086004878	0,009556098
ENSG00000262576	PCDHGA4	0,030920927	0,054868426	0	0	0	0	0	0	0	0,085789354	0,00953215
ENSG00000140285	FGF7	0,045965193	0	0	0,039685928	0	0	0	0	0	0,08565112	0,009516791
ENSG00000171451	DSEL	0	0	0	0	0	0,017905307	0,06770469	0	0	0,085609997	0,009512222
ENSG00000174292	TNK1	0	0	0	0	0	0	0	0,08560481	0	0,08560481	0,009511646
ENSG00000116132	PRRX1	0,036641748	0	0,033702728	0	0	0	0,015212213	0	0	0,085556689	0,009506299
ENSG00000168539	CHRM1	0	0	0,085532338	0	0	0	0	0	0	0,085532338	0,009503593
ENSG00000162728	KCNJ9	0	0	0	0	0,08546348	0	0	0	0	0,08546348	0,009495942
ENSG00000102794	ACOD1	0	0	0	0	0	0	0	0	0,085368177	0,085368177	0,009485353
ENSG00000151615	POU4F2	0	0,085286896	0	0	0	0	0	0	0	0,085286896	0,009476322
ENSG00000182255	KCNA4	0	0	0	0	0,085240338	0	0	0	0	0,085240338	0,009471149
ENSG00000143194	MAEL	0	0,085178526	0	0	0	0	0	0	0	0,085178526	0,009464281
ENSG00000161509	GRIN2C	0	0	0	0	0,041835688	0	0	0	0,043300681	0,085136368	0,009459596
ENSG00000164484	TMEM200A	0	0	0	0,085036655	0	0	0	0	0	0,085036655	0,009448517
ENSG00000197079	KRT35	0,084989072	0	0	0	0	0	0	0	0	0,084989072	0,00944323
ENSG00000173867	AC013489.1	0	0	0,084957243	0	0	0	0	0	0	0,084957243	0,009439694
ENSG00000264717	NPY4R2	0	0	0	0	0	0,084821358	0	0	0	0,084821358	0,009424595
ENSG00000147003	CLTRN	0,084750741	0	0	0	0	0	0	0	0	0,084750741	0,009416749
ENSG00000110427	KIAA1549L	0,02322634	0	0,021363365	0,040106821	0	0	0	0	0	0,084696525	0,009410725
ENSG00000142700	DMRTA2	0	0,084640783	0	0	0	0	0	0	0	0,084640783	0,009404531
ENSG00000157765	SLC34A2	0	0	0	0,051721501	0	0,03288614	0	0	0	0,084607641	0,009400849
ENSG00000183186	C2CD4C	0	0	0	0,084199734	0	0	0	0	0	0,084199734	0,009355526
ENSG00000166159	LRTM2	0	0	0	0	0,083945195	0	0	0	0	0,083945195	0,009327244
ENSG00000204387	SNHG32	0,083903704	0	0	0	0	0	0	0	0	0,083903704	0,009322634
ENSG00000165841	CYP2C19	0	0	0	0	0,057339541	0,026490592	0	0	0	0,083830132	0,009314459
ENSG00000214128	TMEM213	0	0	0,057756098	0	0	0	0,026069049	0	0	0,083825147	0,009313905
ENSG00000114248	LRRC31	0	0	0	0,083821065	0	0	0	0	0	0,083821065	0,009313452
ENSG00000146038	DCDC2	0,030197956	0,053585532	0	0	0	0	0	0	0	0,083783487	0,009309276
ENSG00000147100	SLC16A2	0	0	0	0	0,083671375	0	0	0	0	0,083671375	0,009296819
ENSG00000163083	INHBB	0	0,083637555	0	0	0	0	0	0	0	0,083637555	0,009293062
ENSG00000243708	PLA2G4B	0	0	0	0	0	0	0	0	0,083601674	0,083601674	0,009289075
ENSG00000162624	LHX8	0	0	0	0	0	0,08343504	0	0	0	0,08343504	0,00927056
ENSG00000167094	TTC16	0	0,042247046	0	0,041111545	0	0	0	0	0	0,083358591	0,009262066
ENSG00000175745	NR2F1	0	0	0	0	0,083283289	0	0	0	0	0,083283289	0,009253699
ENSG00000186212	SOWAHB	0	0,083273913	0	0	0	0	0	0	0	0,083273913	0,009252657
ENSG00000113248	PCDHB15	0	0	0	0	0	0	0	0,083211928	0	0,083211928	0,00924577
ENSG00000137877	SPTBN5	0,061367191	0,021778915	0	0	0	0	0	0	0	0,083146106	0,009238456
ENSG00000198889	DCAF12L1	0	0	0,083103169	0	0	0	0	0	0	0,083103169	0,009233685
ENSG00000277258	PCGF2	0	0	0	0	0	0,047267971	0,035746533	0	0	0,083014504	0,009223834
ENSG00000204147	ASAH2B	0	0,082926241	0	0	0	0	0	0	0	0,082926241	0,009214027
ENSG00000164398	ACSL6	0,026666571	0,015773059	0	0,030698232	0	0,009759446	0	0	0	0,082897308	0,009210812
ENSG00000260001	TGFBR3L	0	0	0	0	0	0	0,082764071	0	0	0,082764071	0,009196008
ENSG00000185974	GRK1	0	0	0,05698649	0	0	0	0,025721675	0	0	0,082708165	0,009189796
ENSG00000104044	OCA2	0	0,082353194	0	0	0	0	0	0	0	0,082353194	0,009150355
ENSG00000135638	EMX1	0	0,048611675	0	0	0	0	0	0	0,033692263	0,082303938	0,009144882
ENSG00000148584	A1CF	0,062943068	0	0	0	0	0	0	0	0,019352965	0,082296033	0,009144004
ENSG00000114646	CSPG5	0	0	0	0	0,082234381	0	0	0	0	0,082234381	0,009137153
ENSG00000167889	MGAT5B	0	0	0	0	0,05623513	0,02598036	0	0	0	0,08221549	0,009135054
ENSG00000205927	OLIG2	0	0,082201717	0	0	0	0	0	0	0	0,082201717	0,009133524
ENSG00000170374	AC073611.1	0	0	0,082096899	0	0	0	0	0	0	0,082096899	0,009121878
ENSG00000196337	CGB7	0	0	0,082096899	0	0	0	0	0	0	0,082096899	0,009121878
ENSG00000203663	OR2L2	0	0	0	0	0,08208401	0	0	0	0	0,08208401	0,009120446
ENSG00000142025	DMRTC2	0	0	0,082024225	0	0	0	0	0	0	0,082024225	0,009113803
ENSG00000186479	RGS7BP	0	0	0	0	0,08200903	0	0	0	0	0,08200903	0,009112114

ENSG00000114547	ROPN1B	0	0	0	0	0	0	0	0,081953018	0	0,081953018	0,009105891
ENSG00000165588	OTX2	0	0,081950489	0	0	0	0	0	0	0	0,081950489	0,00910561
ENSG00000143954	REG3G	0,081902748	0	0	0	0	0	0	0	0	0,081902748	0,009100305
ENSG00000237524	TEX51	0	0	0	0	0	0	0,08184627	0	0	0,08184627	0,00909403
ENSG00000274286	ADRA2B	0,081769789	0	0	0	0	0	0	0	0	0,081769789	0,009085532
ENSG00000100191	SLC5A4	0	0	0	0	0	0,081729348	0	0	0	0,081729348	0,009081039
ENSG00000249158	PCDHA11	0,024046876	0	0	0	0,057147922	0	0	0	0	0,081194798	0,009021644
ENSG00000157653	C9orf43	0	0	0	0	0	0	0	0	0,081155687	0,081155687	0,009017299
ENSG00000172116	CD8B	0,057249695	0	0	0	0	0	0,023767822	0	0	0,081017517	0,009001946
ENSG00000171596	NMUR1	0	0	0	0,081010548	0	0	0	0	0	0,081010548	0,009001172
ENSG00000275740	AC091959.3	0	0,080985201	0	0	0	0	0	0	0	0,080985201	0,008998356
ENSG00000183273	CCDC60	0	0	0,080972939	0	0	0	0	0	0	0,080972939	0,008996993
ENSG00000185231	MC2R	0,080937638	0	0	0	0	0	0	0	0	0,080937638	0,008993071
ENSG00000113296	THBS4	0	0	0	0	0	0	0	0	0,080715102	0,080715102	0,008968345
ENSG00000221813	OR6B1	0,029082096	0,051605466	0	0	0	0	0	0	0	0,080687562	0,008965285
ENSG00000100842	EFS	0	0	0	0,080635035	0	0	0	0	0	0,080635035	0,008959448
ENSG00000279486	OR2AG1	0,043211487	0	0	0,037308404	0	0	0	0	0	0,080519891	0,008946655
ENSG00000239704	CDRT4	0	0	0	0,080411394	0	0	0	0	0	0,080411394	0,008934599
ENSG00000186452	TMPRSS12	0	0,080378297	0	0	0	0	0	0	0	0,080378297	0,008930922
ENSG00000101812	H2BFM	0,080292546	0	0	0	0	0	0	0	0	0,080292546	0,008921394
ENSG00000197123	ZNF679	0	0	0	0	0	0	0,08007041	0	0	0,08007041	0,008896712
ENSG00000127533	F2RL3	0	0	0,080040339	0	0	0	0	0	0	0,080040339	0,008893371
ENSG00000180828	BHLHE22	0	0	0	0,079992328	0	0	0	0	0	0,079992328	0,008888036
ENSG00000186628	FSD2	0,023119732	0	0	0	0	0	0	0	0,056868581	0,079988313	0,00888759
ENSG00000168427	KLHL30	0,079952683	0	0	0	0	0	0	0	0	0,079952683	0,008883631
ENSG00000136378	ADAMTS7	0	0	0	0,033625641	0,046278034	0	0	0	0	0,079903675	0,008878186
ENSG00000205436	EXOC3L4	0	0	0,079879339	0	0	0	0	0	0	0,079879339	0,008875482
ENSG00000181656	GPR88	0	0	0,079604839	0	0	0	0	0	0	0,079604839	0,008844982
ENSG00000187634	SAMD11	0	0	0	0	0	0,079516212	0	0	0	0,079516212	0,008835135
ENSG00000174521	TTC9B	0	0	0	0	0	0	0	0	0,079353767	0,079353767	0,008817085
ENSG00000126259	KIRREL2	0	0,079285038	0	0	0	0	0	0	0	0,079285038	0,008809449
ENSG00000181195	PENK	0	0,079051297	0	0	0	0	0	0	0	0,079051297	0,008783477
ENSG00000187918	OR51I2	0	0	0	0,079023312	0	0	0	0	0	0,079023312	0,008780368
ENSG00000164256	PRDM9	0	0	0	0	0	0	0	0,078975757	0	0,078975757	0,008775084
ENSG00000120440	TTLL2	0,078888317	0	0	0	0	0	0	0	0	0,078888317	0,008765369
ENSG00000146950	SHROOM2	0	0	0	0,063503279	0	0	0,015267745	0	0	0,078771025	0,008752336
ENSG00000113209	PCDHB5	0	0,078680164	0	0	0	0	0	0	0	0,078680164	0,00874224
ENSG00000273154	AL121845.3	0	0	0	0	0	0	0,078664785	0	0	0,078664785	0,008740532
ENSG00000100473	COCH	0,037487118	0	0	0	0	0,041158665	0	0	0	0,078645782	0,00873842
ENSG00000260903	XKR7	0,019637501	0,034846264	0	0	0	0	0	0	0,024151595	0,07863536	0,008737262
ENSG00000165694	FRMD7	0	0	0	0,078500293	0	0	0	0	0	0,078500293	0,008722255
ENSG00000101222	SPEF1	0,078498998	0	0	0	0	0	0	0	0	0,078498998	0,008722111
ENSG00000174899	PQLC2L	0	0	0	0,078054135	0	0	0	0	0	0,078054135	0,008672682
ENSG00000149596	JPH2	0	0	0,046774373	0	0	0	0	0	0,0312715	0,078045873	0,008671764
ENSG00000124343	XG	0	0	0	0,077914295	0	0	0	0	0	0,077914295	0,008657144
ENSG00000171303	KCNK3	0,024282592	0	0	0	0	0	0	0,053098918	0	0,07738151	0,008597946
ENSG00000263761	GDF2	0,07729441	0	0	0	0	0	0	0	0	0,07729441	0,008588268
ENSG00000185792	NLRP9	0	0	0,077238149	0	0	0	0	0	0	0,077238149	0,008582017
ENSG00000240694	PNMA2	0,027158622	0	0,049960478	0	0	0	0	0	0	0,0771191	0,008568789
ENSG00000175329	ISX	0	0	0	0,077108444	0	0	0	0	0	0,077108444	0,008567605
ENSG00000250366	TUNAR	0	0,077096607	0	0	0	0	0	0	0	0,077096607	0,00856629
ENSG00000124194	GDAP1L1	0	0	0	0,077085665	0	0	0	0	0	0,077085665	0,008565074
ENSG00000170486	KRT72	0	0	0	0	0	0,077024409	0	0	0	0,077024409	0,008558268
ENSG00000184478	OR56A3	0	0	0	0	0	0	0	0	0,076923229	0,076923229	0,008547025
ENSG00000176695	AC008977.1	0	0	0	0	0	0	0,076857784	0	0	0,076857784	0,008539754
ENSG00000278195	SSTR3	0	0	0	0	0,076783738	0	0	0	0	0,076783738	0,008531526
ENSG00000204930	FAM221B	0,07668641	0	0	0	0	0	0	0	0	0,07668641	0,008520712
ENSG00000215474	SKOR2	0,07668641	0	0	0	0	0	0	0	0	0,07668641	0,008520712
ENSG00000178645	C10orf53	0	0	0	0	0	0	0	0,076560372	0	0,076560372	0,008506708
ENSG00000152092	ASTN1	0,049269831	0	0	0	0	0	0,027273195	0	0	0,076543025	0,008504781
ENSG00000145808	ADAMTS19	0,028024957	0	0	0,048392985	0	0	0	0	0	0,076417942	0,008490882
ENSG00000258539	AC068896.1	0	0	0	0,076252184	0	0	0	0	0	0,076252184	0,008472465
ENSG00000118946	PCDH17	0	0,031952097	0	0	0	0	0	0	0,044291354	0,076243451	0,008471495
ENSG00000183185	GABRR3	0	0,04701771	0	0	0	0,029091807	0	0	0	0,076109517	0,008456613
ENSG00000104722	NEFM	0,041570996	0	0	0	0	0	0,034517285	0	0	0,076088281	0,008454253
ENSG00000221888	OR1C1	0,07593496	0	0	0	0	0	0	0	0	0,07593496	0,008437218
ENSG00000164082	GRM2	0	0	0,039163158	0,03676176	0	0	0	0	0	0,075924919	0,008436102
ENSG00000110900	TSPAN11	0	0	0,039064095	0,036668771	0	0	0	0	0	0,075732866	0,008414763
ENSG00000132207	SLX1A	0	0	0	0	0	0	0	0	0,075424725	0,075424725	0,008380525
ENSG00000185551	NR2F2	0,046667872	0	0	0	0	0	0	0	0,028697734	0,075365606	0,008373956
ENSG00000178522	AMBN	0,07521681	0	0	0	0	0	0	0	0	0,07521681	0,008357423
ENSG00000134365	CFHR4	0	0	0	0	0	0	0	0	0,075059177	0,075059177	0,008339909
ENSG00000136535	TBR1	0	0	0,029581792	0,027767902	0	0,017655696	0	0	0	0,07500539	0,008333932
ENSG00000132693	CRP	0,074992839	0	0	0	0	0	0	0	0	0,074992839	0,008332538
ENSG00000159166	LAD1	0	0	0	0,074938247	0	0	0	0	0	0,074938247	0,008326472
ENSG00000109610	SOD3	0,074881353	0	0	0	0	0	0	0	0	0,074881353	0,00832015
ENSG00000135312	HTR1B	0,074770198	0	0	0	0	0	0	0	0	0,074770198	0,0083078
ENSG00000120094	HOXB1	0	0	0	0	0	0	0	0	0,07475725	0,07475725	0,008306361
ENSG00000131379	C3orf20	0	0	0	0,074723647	0	0	0	0	0	0,074723647	0,008302627
ENSG00000130307	USHBP1	0	0	0	0,050422217	0	0	0,024245475	0	0	0,074667692	0,00829641
ENSG00000135333	EPHA7	0	0	0	0	0	0	0	0	0,074657146	0,074657146	0,008295238
ENSG00000102468	HTR2A	0,026868878	0,047678165	0	0	0	0	0	0	0	0,074547043	0,008283005
ENSG00000283247	CCDC201	0	0	0,074525496	0	0	0	0	0	0	0,074525496	0,008280611
ENSG00000154975	CA10	0	0	0	0	0	0	0	0,074472519	0	0,074472519	0,008274724
ENSG00000189401	OTUD6A	0	0	0	0	0	0	0,074286757	0	0	0,074286757	0,008254084
ENSG00000172967	XKR3	0	0	0	0	0	0	0,0742428	0	0	0,0742428	0,0082492
ENSG00000197283	SYNGAP1	0	0	0	0,043026626	0	0	0,031033968	0	0	0,074060594	0,008228955
ENSG00000185352	HS6ST3	0,019358259	0	0	0,033427488	0	0,021254237	0	0	0	0,074039985	0,008226665
ENSG00000284946	AC068831.7	0	0,037512871	0	0,036504613	0	0	0	0	0	0,074017484	0,008224165
ENSG00000140675	SLC5A2	0	0	0	0	0	0	0	0,073955812	0	0,073955812	0,008217312
ENSG00000152214	RIT2	0,0738927	0	0	0	0	0	0	0	0	0,0738927	0,0082103
ENSG00000100207	TCF20	0	0	0	0	0	0	0	0	0,073865867	0,073865867	0,008207319
ENSG00000184374	COLEC10	0	0	0	0,073772964	0	0	0	0	0	0,073772964	0,008196996
ENSG00000165868	HSPA12A	0	0	0,023489953	0,022049601	0	0	0	0,027922475	0	0,073462028	0,008162448
ENSG00000204335	SP5	0,073354646	0	0	0	0	0	0	0	0	0,073354646	0,008150516
ENSG00000198445	CCT8L2	0,073354646	0	0	0	0	0	0	0	0	0,073354646	0,008150516
ENSG00000086991	NOX4	0	0	0	0,042783239	0	0	0	0	0,030471638	0,073254877	0,008139431
ENSG00000163218	PGLYRP4	0	0	0	0	0	0,072991895	0	0	0	0,072991895	0,008110211
ENSG00000273213	HIST2H3PS2	0,072929812	0	0	0	0	0	0	0	0	0,072929812	0,008103312
ENSG00000174807	CD248	0	0	0	0	0	0	0	0	0,07285242	0,07285242	0,008094713
ENSG00000224940	PRRT4	0	0,072805322	0	0	0	0	0	0	0	0,072805322	0,00808948
ENSG00000164007	CLDN19	0	0	0	0,072441692	0	0	0	0	0	0,072441692	0,008049077
ENSG00000054356	PTPRN	0,018365407	0	0,03378465	0	0	0,020164144	0	0	0	0,072314201	0,008034911
ENSG00000125531	FNDC11	0	0	0	0,072241134	0	0	0	0	0	0,072241134	0,008026793
ENSG00000160323	ADAMTS13	0,026473471	0	0	0,045713906	0	0	0	0	0	0,072187377	0,00802082
ENSG00000164363	SLC6A18	0,072129151	0	0	0	0	0	0	0	0	0,072129151	0,00801435
ENSG00000185479	KRT6B	0	0	0	0	0	0,072072362	0	0	0	0,072072362	0,00800804
ENSG00000272514	CFAP206	0	0	0	0,072001925	0	0	0	0	0	0,072001925	0,008000214
ENSG00000108813	DLX4	0	0	0	0,07192254	0	0	0	0	0	0,07192254	0,007991393
ENSG00000171004	HS6ST2	0	0	0	0	0,071794791	0	0	0	0	0,071794791	0,007977199
ENSG00000156689	GLYATL2	0	0	0	0	0	0	0,071371065	0	0	0,071371065	0,007930118
ENSG00000188269	OR7A5	0,07092728	0	0	0	0	0	0	0	0	0,07092728	0,007880809
ENSG00000177875	CCDC184	0	0	0	0	0	0,070811172	0	0	0	0,070811172	0,007867908
ENSG00000186886	OR5D3P	0	0	0	0,070810034	0	0	0	0	0	0,070810034	0,007867782
ENSG00000118733	OLFM3	0,033707466	0	0	0	0	0,037008828	0	0	0	0,070716294	0,007857366
ENSG00000165061	ZMAT4	0	0	0	0,070694927	0	0	0	0	0	0,070694927	0,007854992
ENSG00000162687	KCNT2	0	0	0	0	0	0	0,070687511	0	0	0,070687511	0,007854168

ENSG00000158748	HTR6	0	0,070563684	0	0	0	0	0	0	0	0,070563684	0,007840409
ENSG00000135127	BICDL1	0	0	0	0,047581141	0	0	0,022879346	0	0	0,070460486	0,007828943
ENSG00000171126	KCNG3	0	0	0	0,07035184	0	0	0	0	0	0,07035184	0,007816871
ENSG00000132321	IQCA1	0,025320136	0,04492996	0	0	0	0	0	0	0	0,070250096	0,007805566
ENSG00000105419	MEIS3	0	0	0	0	0,070112757	0	0	0	0	0,070112757	0,007790306
ENSG00000188338	SLC38A3	0	0	0,070055468	0	0	0	0	0	0	0,070055468	0,007783941
ENSG00000204869	IGFL4	0	0	0	0,070049658	0	0	0	0	0	0,070049658	0,007783295
ENSG00000196109	ZNF676	0	0	0	0	0	0	0	0,06982979	0	0,06982979	0,007758866
ENSG00000134817	APLNR	0	0	0	0,069806039	0	0	0	0	0	0,069806039	0,007756227
ENSG00000179242	CDH4	0	0	0	0,069434533	0	0	0	0	0	0,069434533	0,007714948
ENSG00000152078	TMEM56	0	0	0	0,035333104	0	0	0,033979778	0	0	0,069312883	0,007701431
ENSG00000140279	DUOX2	0,015366135	0,027266829	0	0,026533961	0	0	0	0	0	0,069166925	0,007685214
ENSG00000197915	HRNR	0,031386555	0	0	0	0,037295414	0	0	0	0	0,068681969	0,00763133
ENSG00000174453	VWC2L	0	0	0	0	0,068664922	0	0	0	0	0,068664922	0,007629436
ENSG00000138028	CGREF1	0	0	0,068603183	0	0	0	0	0	0	0,068603183	0,007622576
ENSG00000125850	OVOL2	0,068593087	0	0	0	0	0	0	0	0	0,068593087	0,007621454
ENSG00000169297	NR0B1	0,068593087	0	0	0	0	0	0	0	0	0,068593087	0,007621454
ENSG00000133115	STOML3	0,068530871	0	0	0	0	0	0	0	0	0,068530871	0,007614541
ENSG00000135443	KRT85	0	0	0	0	0	0	0	0	0,06850222	0,06850222	0,007611358
ENSG00000160460	SPTBN4	0	0	0	0,022175149	0,03051904	0	0	0	0,015793875	0,068488063	0,007609785
ENSG00000172365	OR5B2	0,068437758	0	0	0	0	0	0	0	0	0,068437758	0,007604195
ENSG00000173610	UGT2A1	0	0,068421026	0	0	0	0	0	0	0	0,068421026	0,007602336
ENSG00000172238	ATOH1	0,068314001	0	0	0	0	0	0	0	0	0,068314001	0,007590445
ENSG00000105143	SLC1A6	0	0	0	0,068253982	0	0	0	0	0	0,068253982	0,007583776
ENSG00000117148	ACTL8	0	0	0	0	0	0	0,068042479	0	0	0,068042479	0,007560275
ENSG00000125207	PIWIL1	0	0	0	0	0,068014686	0	0	0	0	0,068014686	0,007557187
ENSG00000142910	TINAGL1	0	0	0	0	0,068001807	0	0	0	0	0,068001807	0,007555756
ENSG00000182372	CLN8	0	0	0	0	0	0,03507994	0,013264651	0	0,019647587	0,067992178	0,007554686
ENSG00000170091	NSG2	0	0	0	0,067881107	0	0	0	0	0	0,067881107	0,007542345
ENSG00000100987	VSX1	0	0	0	0,067792927	0	0	0	0	0	0,067792927	0,007532547
ENSG00000184659	FOXD4L4	0,067762588	0	0	0	0	0	0	0	0	0,067762588	0,007529176
ENSG00000110025	SNX15	0	0	0	0	0	0	0	0,067656545	0	0,067656545	0,007517394
ENSG00000258790	AL121594.1	0	0	0	0,067652314	0	0	0	0	0	0,067652314	0,007516924
ENSG00000113319	RASGRF2	0,027394955	0	0,025197616	0	0	0,01503903	0	0	0	0,067631601	0,007514622
ENSG00000181785	OR5AS1	0	0,034114758	0	0,033197834	0	0	0	0	0	0,067312592	0,007479177
ENSG00000163362	INAVA	0	0	0	0	0,067237885	0	0	0	0	0,067237885	0,007470876
ENSG00000178125	PPP1R42	0,067160254	0	0	0	0	0	0	0	0	0,067160254	0,00746225
ENSG00000172478	MAB21L4	0	0	0	0	0	0	0	0	0,0670442	0,0670442	0,007449356
ENSG00000169836	TACR3	0	0	0	0	0,067012044	0	0	0	0	0,067012044	0,007445783
ENSG00000139352	ASCL1	0	0	0	0	0	0,066872461	0	0	0	0,066872461	0,007430273
ENSG00000138316	ADAMTS14	0	0	0	0	0	0	0	0	0,066779203	0,066779203	0,007419911
ENSG00000177511	ST8SIA3	0,014636824	0,05194537	0	0	0	0	0	0	0	0,066582194	0,007398022
ENSG00000229924	FAM90A26	0	0	0	0	0	0,066577278	0	0	0	0,066577278	0,007397475
ENSG00000133124	IRS4	0	0	0	0,066429474	0	0	0	0	0	0,066429474	0,007381053
ENSG00000165953	SERPINA12	0,066393045	0	0	0	0	0	0	0	0	0,066393045	0,007377005
ENSG00000155890	TRIM42	0	0	0	0	0	0,065994661	0	0	0	0,065994661	0,00733274
ENSG00000183862	CNGA2	0	0	0	0	0	0	0	0	0,06557746	0,06557746	0,007286384
ENSG00000196421	C20orf204	0	0	0	0	0,065532398	0	0	0	0	0,065532398	0,007281378
ENSG00000273398	AC017083.3	0,065529302	0	0	0	0	0	0	0	0	0,065529302	0,007281034
ENSG00000106536	POU6F2	0	0,065154173	0	0	0	0	0	0	0	0,065154173	0,007239353
ENSG00000143816	WNT9A	0	0	0	0,065152303	0	0	0	0	0	0,065152303	0,007239145
ENSG00000262481	TMEM256-PLSCR3	0	0	0	0	0	0,064884856	0	0	0	0,064884856	0,007209428
ENSG00000139209	SLC38A4	0	0,06469047	0	0	0	0	0	0	0	0,06469047	0,00718783
ENSG00000171345	KRT19	0,064687744	0	0	0	0	0	0	0	0	0,064687744	0,007187527
ENSG00000166173	LARP6	0	0	0	0	0,064659262	0	0	0	0	0,064659262	0,007184362
ENSG00000213759	UGT2B11	0,064577167	0	0	0	0	0	0	0	0	0,064577167	0,007175241
ENSG00000132911	NMUR2	0,064439476	0	0	0	0	0	0	0	0	0,064439476	0,007159942
ENSG00000177984	LCN15	0	0	0	0	0	0	0	0	0,064373579	0,064373579	0,00715262
ENSG00000007314	SCN4A	0	0,032529662	0	0,031655341	0	0	0	0	0	0,064185002	0,007131667
ENSG00000166984	TCP10L2	0	0	0,064168998	0	0	0	0	0	0	0,064168998	0,007129889
ENSG00000187024	PTRH1	0	0	0	0,064080298	0	0	0	0	0	0,064080298	0,007120033
ENSG00000150676	CCDC83	0,064057046	0	0	0	0	0	0	0	0	0,064057046	0,00711745
ENSG00000185272	RBM11	0,064029903	0	0	0	0	0	0	0	0	0,064029903	0,007114434
ENSG00000255819	KLRC4-KLRK1	0	0	0	0	0	0,06400871	0	0	0	0,06400871	0,007112079
ENSG00000156009	MAGEA8	0,063948612	0	0	0	0	0	0	0	0	0,063948612	0,007105401
ENSG00000259030	FPGT-TNNI3K	0,030539727	0	0	0	0	0	0	0,03339072	0	0,063930448	0,007103383
ENSG00000243135	UGT1A3	0,063921561	0	0	0	0	0	0	0	0	0,063921561	0,007102396
ENSG00000163424	TEX55	0	0	0	0	0	0	0,063885098	0	0	0,063885098	0,007098344
ENSG00000021488	SLC7A9	0	0	0	0	0	0,063860884	0	0	0	0,063860884	0,007095654
ENSG00000108830	RND2	0	0	0,063640133	0	0	0	0	0	0	0,063640133	0,007071126
ENSG00000261934	PCDHGA9	0,063638901	0	0	0	0	0	0	0	0	0,063638901	0,007070989
ENSG00000197991	AL592490.1	0	0	0,043797085	0	0	0	0,019768447	0	0	0,063565532	0,007062837
ENSG00000197177	ADGRA1	0,023237055	0	0	0,040125323	0	0	0	0	0	0,063362378	0,007040264
ENSG00000177106	EPS8L2	0	0	0	0	0,063124898	0	0	0	0	0,063124898	0,007013878
ENSG00000284461	AC027644.4	0	0	0	0,063042999	0	0	0	0	0	0,063042999	0,007004778
ENSG00000254206	NPIPB11	0,034358929	0	0	0	0	0	0,028528952	0	0	0,062887881	0,006987542
ENSG00000280571	AC006059.2	0	0	0	0	0,062837715	0	0	0	0	0,062837715	0,006981968
ENSG00000281106	TMEM272	0	0	0	0,062830478	0	0	0	0	0	0,062830478	0,006981164
ENSG00000242366	UGT1A8	0,062779631	0	0	0	0	0	0	0	0	0,062779631	0,006975515
ENSG00000112837	TBX18	0	0	0,032121574	0,03015195	0	0	0	0	0	0,062273524	0,00691928
ENSG00000184507	NUTM1	0	0	0	0,0621866	0	0	0	0	0	0,0621866	0,006909622
ENSG00000197106	SLC6A17	0,022771334	0	0	0,039321123	0	0	0	0	0	0,062092457	0,006899162
ENSG00000174279	EVX2	0	0	0	0,06208303	0	0	0	0	0	0,06208303	0,006898114
ENSG00000178297	TMPRSS9	0	0,061955176	0	0	0	0	0	0	0	0,061955176	0,006883908
ENSG00000174804	FZD4	0	0	0	0	0	0,044919609	0,016985289	0	0	0,061904899	0,006878322
ENSG00000184984	CHRM5	0	0	0	0	0,061735868	0	0	0	0	0,061735868	0,006859541
ENSG00000112077	RHAG	0	0	0	0	0	0,061653875	0	0	0	0,061653875	0,006850431
ENSG00000137273	FOXF2	0,06165262	0	0	0	0	0	0	0	0	0,06165262	0,006850291
ENSG00000176979	TRIM60	0	0	0	0	0	0	0	0	0,061640638	0,061640638	0,00684896
ENSG00000242265	PEG10	0,022590906	0	0	0,039009564	0	0	0	0	0	0,06160047	0,006844497
ENSG00000134258	VTCN1	0	0	0	0	0	0	0	0	0,061396274	0,061396274	0,006821808
ENSG00000181625	SLX1B	0,061377161	0	0	0	0	0	0	0	0	0,061377161	0,006819685
ENSG00000169918	OTUD7A	0	0	0	0	0	0	0,061286185	0	0	0,061286185	0,006809576
ENSG00000144488	ESPNL	0,029177558	0	0	0	0	0,032035253	0	0	0	0,061212811	0,006801423
ENSG00000166947	EPB42	0	0	0	0	0	0	0	0	0,061153841	0,061153841	0,006794871
ENSG00000103056	SMPD3	0	0	0	0,037361823	0	0,02375581	0	0	0	0,061117633	0,006790848
ENSG00000116996	ZP4	0,061079455	0	0	0	0	0	0	0	0	0,061079455	0,006786606
ENSG00000162670	BRINP3	0	0	0	0,06106599	0	0	0	0	0	0,06106599	0,00678511
ENSG00000135502	SLC26A10	0	0	0	0,061023147	0	0	0	0	0	0,061023147	0,00678035
ENSG00000172935	MRGPRF	0	0	0	0	0	0,061018969	0	0	0	0,061018969	0,006779885
ENSG00000262152	LINC00514	0	0	0	0,060909191	0	0	0	0	0	0,060909191	0,006767688
ENSG00000188175	HEPACAM2	0,060858063	0	0	0	0	0	0	0	0	0,060858063	0,006762007
ENSG00000154764	WNT7A	0	0	0,0607076	0	0	0	0	0	0	0,0607076	0,006745289
ENSG00000185177	ZNF479	0	0	0	0	0	0	0,06046763	0	0	0,06046763	0,006718626
ENSG00000214513	NOTO	0	0	0	0	0	0,060353065	0	0	0	0,060353065	0,006705896
ENSG00000240720	LRRD1	0	0,060256629	0	0	0	0	0	0	0	0,060256629	0,006695181
ENSG00000275591	XKR5	0,060071783	0	0	0	0	0	0	0	0	0,060071783	0,006674643
ENSG00000166796	LDHC	0	0	0	0	0	0	0,06006239	0	0	0,06006239	0,006673599
ENSG00000104888	SLC17A7	0	0	0	0	0,059962856	0	0	0	0	0,059962856	0,00666254
ENSG00000223572	CKMT1A	0	0	0	0	0	0	0	0	0,059776945	0,059776945	0,006641883
ENSG00000177103	DSCAML1	0,021479825	0,038115423	0	0	0	0	0	0	0	0,059595248	0,006621694
ENSG00000106410	NOBOX	0	0	0	0	0	0	0,059380186	0	0	0,059380186	0,006597798
ENSG00000169994	MYO7B	0	0	0	0,059303403	0	0	0	0	0	0,059303403	0,006589267
ENSG00000105251	SHD	0	0	0	0	0	0,059021906	0	0	0	0,059021906	0,00655799
ENSG00000184647	PRSS55	0	0	0	0	0	0	0	0	0,058998896	0,058998896	0,006555433
ENSG00000132031	MATN3	0	0	0	0	0	0	0	0	0,058980172	0,058980172	0,006553352
ENSG00000112337	SLC17A2	0,05884368	0	0	0	0	0	0	0	0	0,05884368	0,006538187

ENSG00000182901	RGS7	0,058683717	0	0	0	0	0	0	0	0	0,058683717	0,006520413
ENSG00000144063	MALL	0	0	0	0	0	0	0	0	0,058682198	0,058682198	0,006520244
ENSG00000179546	HTR1D	0	0	0	0	0	0,058522249	0	0	0	0,058522249	0,006502472
ENSG00000170953	OR8B12	0	0	0	0	0	0	0,058439838	0	0	0,058439838	0,006493315
ENSG00000196427	NBPF4	0,058434095	0	0	0	0	0	0	0	0	0,058434095	0,006492677
ENSG00000181264	TMEM136	0	0	0	0,058400845	0	0	0	0	0	0,058400845	0,006488983
ENSG00000187942	LDLRAD2	0	0	0	0,058114694	0	0	0	0	0	0,058114694	0,006457188
ENSG00000125798	FOXA2	0,058052467	0	0	0	0	0	0	0	0	0,058052467	0,006450274
ENSG00000152910	CNTNAP4	0	0	0,034743169	0	0	0	0	0	0,023227912	0,057971081	0,006441231
ENSG00000108947	EFNB3	0	0	0	0	0	0	0	0	0,057788098	0,057788098	0,0064209
ENSG00000068078	FGFR3	0	0	0,057339129	0	0	0	0	0	0	0,057339129	0,006371014
ENSG00000039987	BEST2	0,057109059	0	0	0	0	0	0	0	0	0,057109059	0,006345451
ENSG00000179300	RTL3	0,057065925	0	0	0	0	0	0	0	0	0,057065925	0,006340658
ENSG00000156738	MS4A1	0	0	0,057056671	0	0	0	0	0	0	0,057056671	0,00633963
ENSG00000174808	BTC	0,056958376	0	0	0	0	0	0	0	0	0,056958376	0,006328708
ENSG00000139200	PIANP	0	0	0	0	0	0	0	0	0,056903405	0,056903405	0,006322601
ENSG00000277363	SRCIN1	0	0	0	0	0	0,056891754	0	0	0	0,056891754	0,006321306
ENSG00000064692	SNCAIP	0,020788357	0	0	0,035896956	0	0	0	0	0	0,056685314	0,006298368
ENSG00000163060	TEKT4	0	0	0	0	0	0	0,056645748	0	0	0,056645748	0,006293972
ENSG00000100385	IL2RB	0	0	0	0,056467209	0	0	0	0	0	0,056467209	0,006274134
ENSG00000110675	ELMOD1	0	0	0	0	0	0	0	0,056426668	0	0,056426668	0,00626963
ENSG00000189253	TRIM64B	0,056195824	0	0	0	0	0	0	0	0	0,056195824	0,00624398
ENSG00000123572	NRK	0	0	0	0	0	0,017323857	0	0	0,03881101	0,056134867	0,006237207
ENSG00000280537	AC068946.1	0	0	0	0,056115048	0	0	0	0	0	0,056115048	0,006235005
ENSG00000264343	NOTCH2NLA	0	0	0	0	0	0,056107736	0	0	0	0,056107736	0,006234193
ENSG00000228075	BOD1L2	0	0	0	0	0	0	0	0	0,055910506	0,055910506	0,006212278
ENSG00000154165	GPR15	0,055884087	0	0	0	0	0	0	0	0	0,055884087	0,006209343
ENSG00000090534	THPO	0,055863427	0	0	0	0	0	0	0	0	0,055863427	0,006207047
ENSG00000274512	TBC1D3L	0	0	0	0	0	0	0,055814205	0	0	0,055814205	0,006201578
ENSG00000169174	PCSK9	0	0	0,055808091	0	0	0	0	0	0	0,055808091	0,006200899
ENSG00000175164	ABO	0	0	0	0	0,055445043	0	0	0	0	0,055445043	0,00616056
ENSG00000082497	SERTAD4	0,055250666	0	0	0	0	0	0	0	0	0,055250666	0,006138963
ENSG00000129007	CALML4	0	0	0,055176677	0	0	0	0	0	0	0,055176677	0,006130742
ENSG00000203859	HSD3B2	0	0	0	0	0	0	0,055103352	0	0	0,055103352	0,006122595
ENSG00000153303	FRMD1	0	0	0	0	0,040804175	0	0,014256372	0	0	0,055060547	0,006117839
ENSG00000241644	INMT	0	0	0	0	0	0	0	0	0,055049325	0,055049325	0,006116592
ENSG00000262304	AC027796.3	0	0	0	0,054899006	0	0	0	0	0	0,054899006	0,00609989
ENSG00000198547	C20orf203	0	0	0,054763613	0	0	0	0	0	0	0,054763613	0,006084846
ENSG00000188467	SLC24A5	0	0	0	0,054611757	0	0	0	0	0	0,054611757	0,006067973
ENSG00000269190	FBXO17	0	0	0	0,054452207	0	0	0	0	0	0,054452207	0,006050245
ENSG00000146151	HMGCLL1	0	0	0	0,054361453	0	0	0	0	0	0,054361453	0,006040161
ENSG00000212807	OR2A42	0	0,05421391	0	0	0	0	0	0	0	0,05421391	0,006023768
ENSG00000163145	C1QTNF7	0,054132391	0	0	0	0	0	0	0	0	0,054132391	0,00601471
ENSG00000115507	OTX1	0	0	0	0,05399027	0	0	0	0	0	0,05399027	0,005998919
ENSG00000222014	RAB6C	0	0	0	0	0	0,053954659	0	0	0	0,053954659	0,005994962
ENSG00000174740	PABPC5	0	0	0	0	0	0	0	0	0,053868557	0,053868557	0,005985395
ENSG00000132970	WASF3	0	0	0,05385124	0	0	0	0	0	0	0,05385124	0,005983471
ENSG00000115297	TLX2	0,053776004	0	0	0	0	0	0	0	0	0,053776004	0,005975112
ENSG00000218305	CDC14C	0	0	0	0	0	0,053261822	0	0	0	0,053261822	0,00591798
ENSG00000111834	RSPH4A	0,053207947	0	0	0	0	0	0	0	0	0,053207947	0,005911994
ENSG00000196616	ADH1B	0	0	0	0,024227946	0	0	0,011649984	0	0,017255945	0,053133875	0,005903764
ENSG00000099769	IGFALS	0	0	0	0	0	0	0,053097898	0	0	0,053097898	0,005899766
ENSG00000198010	DLGAP2	0	0,053065901	0	0	0	0	0	0	0	0,053065901	0,005896211
ENSG00000160183	TMPRSS3	0,052900602	0	0	0	0	0	0	0	0	0,052900602	0,005877845
ENSG00000178397	FAM220A	0	0	0	0	0	0	0,052432232	0	0	0,052432232	0,005825804
ENSG00000272674	PCDHB16	0	0	0	0,05217656	0	0	0	0	0	0,05217656	0,005797396
ENSG00000103184	SEC14L5	0,023380871	0	0	0	0	0	0	0	0,028755458	0,052136329	0,005792925
ENSG00000182521	TBPL2	0	0	0	0	0	0	0,052127267	0	0	0,052127267	0,005791919
ENSG00000070182	SPTB	0,010212934	0	0	0,017635508	0,024271259	0	0	0	0	0,0521197	0,005791078
ENSG00000164794	KCNV1	0	0	0	0	0,051955663	0	0	0	0	0,051955663	0,005772851
ENSG00000130368	MAS1	0,015355205	0	0	0	0,036491977	0	0	0	0	0,051847181	0,005760798
ENSG00000147509	RGS20	0	0	0	0	0	0,051766171	0	0	0	0,051766171	0,005751797
ENSG00000169169	CPT1C	0,051538394	0	0	0	0	0	0	0	0	0,051538394	0,005726488
ENSG00000179142	CYP11B2	0,051468178	0	0	0	0	0	0	0	0	0,051468178	0,005718686
ENSG00000069812	HES2	0	0	0,051411152	0	0	0	0	0	0	0,051411152	0,00571235
ENSG00000105825	TFPI2	0	0	0	0	0	0	0,051338106	0	0	0,051338106	0,005704234
ENSG00000180974	OR52E4	0,051276067	0	0	0	0	0	0	0	0	0,051276067	0,005697341
ENSG00000092345	DAZL	0	0	0	0	0	0	0	0	0,051183289	0,051183289	0,005687032
ENSG00000128285	MCHR1	0,051033627	0	0	0	0	0	0	0	0	0,051033627	0,005670403
ENSG00000283632	EXOC3L2	0,050981974	0	0	0	0	0	0	0	0	0,050981974	0,005664664
ENSG00000113430	IRX4	0,050810548	0	0	0	0	0	0	0	0	0,050810548	0,005645616
ENSG00000257315	ZBED6	0,024214497	0	0	0	0	0,026586103	0	0	0	0,0508006	0,005644511
ENSG00000204389	HSPA1A	0	0	0	0	0	0	0,050531749	0	0	0,050531749	0,005614639
ENSG00000187010	RHD	0	0	0	0	0	0,050078653	0	0	0	0,050078653	0,005564295
ENSG00000119614	VSX2	0,050069772	0	0	0	0	0	0	0	0	0,050069772	0,005563308
ENSG00000198914	POU3F3	0,049318071	0	0	0	0	0	0	0	0	0,049318071	0,005479786
ENSG00000165929	TC2N	0,049125673	0	0	0	0	0	0	0	0	0,049125673	0,005458408
ENSG00000205609	EIF3CL	0,048887276	0	0	0	0	0	0	0	0	0,048887276	0,00543192
ENSG00000151006	PRSS53	0	0,048744228	0	0	0	0	0	0	0	0,048744228	0,005416025
ENSG00000161905	ALOX15	0	0	0	0	0	0,048739887	0	0	0	0,048739887	0,005415543
ENSG00000120341	SEC16B	0	0	0	0	0,048680703	0	0	0	0	0,048680703	0,005408967
ENSG00000136931	NR5A1	0	0	0	0	0	0,048625609	0	0	0	0,048625609	0,005402845
ENSG00000205832	C16orf96	0	0	0	0	0	0	0	0	0,048612745	0,048612745	0,005401416
ENSG00000183628	DGCR6	0	0	0	0	0	0	0,048584833	0	0	0,048584833	0,005398315
ENSG00000170074	FAM153A	0,017719345	0	0	0,030597441	0	0	0	0	0	0,048316785	0,005368532
ENSG00000165731	RET	0	0	0	0	0	0	0	0,048105193	0	0,048105193	0,005345021
ENSG00000204381	LAYN	0,048063159	0	0	0	0	0	0	0	0	0,048063159	0,005340351
ENSG00000167741	GGT6	0,047804673	0	0	0	0	0	0	0	0	0,047804673	0,00531163
ENSG00000144460	NYAP2	0	0	0	0,047781537	0	0	0	0	0	0,047781537	0,00530906
ENSG00000162520	SYNC	0	0	0	0	0	0,047579747	0	0	0	0,047579747	0,005286639
ENSG00000118094	TREH	0,04748918	0	0	0	0	0	0	0	0	0,04748918	0,005276576
ENSG00000259803	SLC22A31	0	0	0	0	0	0	0,047418871	0	0	0,047418871	0,005268763
ENSG00000214140	PRCD	0	0	0	0	0	0	0,047400957	0	0	0,047400957	0,005266773
ENSG00000183778	B3GALT5	0,010553151	0	0	0,036445977	0	0	0	0	0	0,046999128	0,005222125
ENSG00000164175	SLC45A2	0,046885067	0	0	0	0	0	0	0	0	0,046885067	0,005209452
ENSG00000150556	LYPD6B	0	0	0	0	0	0,046880638	0	0	0	0,046880638	0,00520896
ENSG00000163673	DCLK3	0	0	0	0,046812877	0	0	0	0	0	0,046812877	0,005201431
ENSG00000283654	LMLN2	0,046797947	0	0	0	0	0	0	0	0	0,046797947	0,005199772
ENSG00000084453	SLCO1A2	0	0	0,024130217	0,022650605	0	0	0	0	0	0,046780822	0,005197869
ENSG00000137033	IL33	0,046452681	0	0	0	0	0	0	0	0	0,046452681	0,005161409
ENSG00000105409	ATP1A3	0	0	0,046415111	0	0	0	0	0	0	0,046415111	0,005157235
ENSG00000123407	HOXC12	0,04626778	0	0	0	0	0	0	0	0	0,04626778	0,005140864
ENSG00000166924	NYAP1	0	0	0	0	0	0,046227522	0	0	0	0,046227522	0,005136391
ENSG00000124134	KCNS1	0	0	0	0,046191357	0	0	0	0	0	0,046191357	0,005132373
ENSG00000146267	FAXC	0	0	0,023795591	0,022336498	0	0	0	0	0	0,04613209	0,005125788
ENSG00000172058	SERF1A	0,046000174	0	0	0	0	0	0	0	0	0,046000174	0,00511113
ENSG00000265107	GJA5	0,045958203	0	0	0	0	0	0	0	0	0,045958203	0,005106467
ENSG00000065325	GLP2R	0	0	0	0,045810213	0	0	0	0	0	0,045810213	0,005090024
ENSG00000101282	RSPO4	0	0	0	0	0	0	0,045509732	0	0	0,045509732	0,005056637
ENSG00000043039	BARX2	0	0	0	0	0	0,045330759	0	0	0	0,045330759	0,005036751
ENSG00000101115	SALL4	0	0	0	0,045199199	0	0	0	0	0	0,045199199	0,005022133
ENSG00000185483	ROR1	0,044973384	0	0	0	0	0	0	0	0	0,044973384	0,004997043
ENSG00000147113	DIPK2B	0,044966693	0	0	0	0	0	0	0	0	0,044966693	0,004996299
ENSG00000130037	KCNA5	0	0	0	0	0	0	0,044810833	0	0	0,044810833	0,004978981
ENSG00000197891	SLC22A12	0,044707269	0	0	0	0	0	0	0	0	0,044707269	0,004967474
ENSG00000140459	CYP11A1	0,044483536	0	0	0	0	0	0	0	0	0,044483536	0,004942615
ENSG00000223802	CERS1	0	0	0	0	0	0,044207454	0	0	0	0,044207454	0,004911939

ENSG00000273294	C1QTNF3-AMACR	0,04413276	0	0	0	0	0	0	0	0	0,04413276	0,00490364
ENSG00000187772	LIN28B	0	0	0	0,043906272	0	0	0	0	0	0,043906272	0,004878475
ENSG00000011105	TSPAN9	0,043768449	0	0	0	0	0	0	0	0	0,043768449	0,004863161
ENSG00000150667	FSIP1	0,043635741	0	0	0	0	0	0	0	0	0,043635741	0,004848416
ENSG00000168619	ADAM18	0	0	0	0	0	0,043363977	0	0	0	0,043363977	0,00481822
ENSG00000159263	SIM2	0	0	0	0	0	0,043363977	0	0	0	0,043363977	0,00481822
ENSG00000198842	DUSP27	0	0	0	0	0	0	0	0	0,043179954	0,043179954	0,004797773
ENSG00000272916	AC022400.7	0	0	0	0	0	0	0,043131774	0	0	0,043131774	0,004792419
ENSG00000198723	TEX45	0	0	0	0	0	0	0,042925191	0	0	0,042925191	0,004769466
ENSG00000102057	KCND1	0	0	0	0,042902824	0	0	0	0	0	0,042902824	0,00476698
ENSG00000100024	UPB1	0	0	0	0	0	0,024423756	0,018470533	0	0	0,04289429	0,004766032
ENSG00000109956	B3GAT1	0,012861569	0	0	0	0	0,014121251	0	0	0,015818072	0,042800891	0,004755655
ENSG00000087495	PHACTR3	0	0	0	0,042755198	0	0	0	0	0	0,042755198	0,004750578
ENSG00000111700	SLCO1B3	0,042662499	0	0	0	0	0	0	0	0	0,042662499	0,004740278
ENSG00000185561	TLCD2	0	0	0	0	0	0	0,042647971	0	0	0,042647971	0,004738663
ENSG00000198326	TMEM239	0	0	0	0	0	0,042628617	0	0	0	0,042628617	0,004736513
ENSG00000167131	CCDC103	0	0	0	0	0	0,042595783	0	0	0	0,042595783	0,004732865
ENSG00000165370	GPR101	0	0	0	0	0	0	0	0	0,042566771	0,042566771	0,004729641
ENSG00000196341	OR8D1	0	0	0	0	0,042214358	0	0	0	0	0,042214358	0,004690484
ENSG00000165816	VWA2	0	0,042207146	0	0	0	0	0	0	0	0,042207146	0,004689683
ENSG00000138131	LOXL4	0,042010167	0	0	0	0	0	0	0	0	0,042010167	0,004667796
ENSG00000115155	OTOF	0	0	0	0	0	0	0	0,04190673	0	0,04190673	0,004656303
ENSG00000184330	S100A7A	0	0	0	0	0	0	0	0	0,041688318	0,041688318	0,004632035
ENSG00000188779	SKOR1	0	0	0	0	0	0	0	0	0,041548518	0,041548518	0,004616502
ENSG00000104892	KLC3	0,041354836	0	0	0	0	0	0	0	0	0,041354836	0,004594982
ENSG00000284626	AC091167.7	0	0	0	0	0	0,041332979	0	0	0	0,041332979	0,004592553
ENSG00000283439	SPEM3	0,040873836	0	0	0	0	0	0	0	0	0,040873836	0,004541537
ENSG00000075043	KCNQ2	0	0	0	0,040850877	0	0	0	0	0	0,040850877	0,004538986
ENSG00000167612	ANKRD33	0,040632044	0	0	0	0	0	0	0	0	0,040632044	0,004514672
ENSG00000187017	ESPN	0	0	0,03998563	0	0	0	0	0	0	0,03998563	0,004442848
ENSG00000183798	EMILIN3	0,039860346	0	0	0	0	0	0	0	0	0,039860346	0,004428927
ENSG00000106113	CRHR2	0,039640758	0	0	0	0	0	0	0	0	0,039640758	0,004404529
ENSG00000204099	NEU4	0	0	0	0	0	0	0	0	0,039634575	0,039634575	0,004403842
ENSG00000159239	AC005041.1	0,021485934	0	0	0	0	0	0,017840229	0	0	0,039326163	0,004369574
ENSG00000189367	KIAA0408	0	0	0	0	0,039187859	0	0	0	0	0,039187859	0,004354207
ENSG00000183876	ARSI	0	0	0	0	0	0	0,038953844	0	0	0,038953844	0,004328205
ENSG00000103740	ACSBG1	0,018545725	0	0	0	0	0,020362123	0	0	0	0,038907848	0,004323094
ENSG00000145794	MEGF10	0	0	0	0	0	0,01834888	0	0	0,020553696	0,038902577	0,004322509
ENSG00000166535	A2ML1	0	0	0,038845738	0	0	0	0	0	0	0,038845738	0,004316193
ENSG00000144229	THSD7B	0	0	0	0,038588432	0	0	0	0	0	0,038588432	0,004287604
ENSG00000123307	NEUROD4	0,038479901	0	0	0	0	0	0	0	0	0,038479901	0,004275545
ENSG00000119913	TECTB	0,038430969	0	0	0	0	0	0	0	0	0,038430969	0,004270108
ENSG00000285943	AC112128.1	0	0	0	0,038165127	0	0	0	0	0	0,038165127	0,00424057
ENSG00000255639	AC005833.1	0,038149601	0	0	0	0	0	0	0	0	0,038149601	0,004238845
ENSG00000182774	RPS17	0	0	0	0	0	0	0,038102135	0	0	0,038102135	0,004233571
ENSG00000155265	GOLGA7B	0	0	0	0,038070466	0	0	0	0	0	0,038070466	0,004230052
ENSG00000112175	BMP5	0,038063116	0	0	0	0	0	0	0	0	0,038063116	0,004229235
ENSG00000135447	PPP1R1A	0,03792936	0	0	0	0	0	0	0	0	0,03792936	0,004214373
ENSG00000285772	AC000120.2	0,037768201	0	0	0	0	0	0	0	0	0,037768201	0,004196467
ENSG00000240021	TEX35	0,037711647	0	0	0	0	0	0	0	0	0,037711647	0,004190183
ENSG00000226887	ERVMER34-1	0,037571002	0	0	0	0	0	0	0	0	0,037571002	0,004174556
ENSG00000162572	SCNN1D	0,037542999	0	0	0	0	0	0	0	0	0,037542999	0,004171444
ENSG00000171944	OR52A5	0,037412867	0	0	0	0	0	0	0	0	0,037412867	0,004156985
ENSG00000144366	GULP1	0	0	0,023205618	0	0	0,013850119	0	0	0	0,037055737	0,004117304
ENSG00000104432	IL7	0	0	0	0	0	0	0,036990074	0	0	0,036990074	0,004110008
ENSG00000161681	SHANK1	0	0	0,03673584	0	0	0	0	0	0	0,03673584	0,00408176
ENSG00000165556	CDX2	0,036659527	0	0	0	0	0	0	0	0	0,036659527	0,004073281
ENSG00000143473	KCNH1	0	0	0	0	0,036655868	0	0	0	0	0,036655868	0,004072874
ENSG00000146005	PSD2	0	0	0	0	0	0,036608689	0	0	0	0,036608689	0,004067632
ENSG00000106018	VIPR2	0	0	0	0	0	0	0	0	0,036213274	0,036213274	0,004023697
ENSG00000130675	MNX1	0,035859177	0	0	0	0	0	0	0	0	0,035859177	0,003984353
ENSG00000221970	OR2A1	0	0	0	0	0	0,035818346	0	0	0	0,035818346	0,003979816
ENSG00000136542	GALNT5	0	0,024396506	0	0	0	0	0,011415734	0	0	0,03581224	0,003979138
ENSG00000205108	FAM205A	0,035765816	0	0	0	0	0	0	0	0	0,035765816	0,00397398
ENSG00000134216	CHIA	0	0	0	0	0	0,035733486	0	0	0	0,035733486	0,003970387
ENSG00000188817	SNTN	0	0	0	0	0	0,035672023	0	0	0	0,035672023	0,003963558
ENSG00000187546	AGMO	0	0	0	0	0	0	0,034969435	0	0	0,034969435	0,003885493
ENSG00000277586	NEFL	0,034906577	0	0	0	0	0	0	0	0	0,034906577	0,003878509
ENSG00000185761	ADAMTSL5	0	0	0	0	0	0	0,034650741	0	0	0,034650741	0,003850082
ENSG00000154646	TMPRSS15	0,034642497	0	0	0	0	0	0	0	0	0,034642497	0,003849166
ENSG00000161940	BCL6B	0,034555356	0	0	0	0	0	0	0	0	0,034555356	0,003839484
ENSG00000166049	PASD1	0,034531666	0	0	0	0	0	0	0	0	0,034531666	0,003836852
ENSG00000242866	STRC	0	0	0,034310059	0	0	0	0	0	0	0,034310059	0,003812229
ENSG00000251664	PCDHA12	0	0	0	0	0	0	0	0	0,034056537	0,034056537	0,00378406
ENSG00000127412	TRPV5	0	0	0	0	0	0	0,034039699	0	0	0,034039699	0,003782189
ENSG00000172324	OR5A2	0,016000696	0	0	0	0	0,017567829	0	0	0	0,033568525	0,003729836
ENSG00000168481	LGI3	0,033513101	0	0	0	0	0	0	0	0	0,033513101	0,003723678
ENSG00000175344	CHRNA7	0	0	0	0	0,017118769	0	0	0,015751481	0	0,03287025	0,00365225
ENSG00000284526	AC015802.6	0	0	0	0	0	0	0	0	0,032869919	0,032869919	0,003652213
ENSG00000181092	ADIPOQ	0,032857267	0	0	0	0	0	0	0	0	0,032857267	0,003650807
ENSG00000005981	ASB4	0,032807332	0	0	0	0	0	0	0	0	0,032807332	0,003645259
ENSG00000168621	GDNF	0,032651377	0	0	0	0	0	0	0	0	0,032651377	0,003627931
ENSG00000006047	YBX2	0,032630225	0	0	0	0	0	0	0	0	0,032630225	0,003625581
ENSG00000258461	AC012651.1	0,032357724	0	0	0	0	0	0	0	0	0,032357724	0,003595303
ENSG00000182348	ZNF804B	0,032254124	0	0	0	0	0	0	0	0	0,032254124	0,003583792
ENSG00000170324	FRMPD2	0,017536332	0	0	0	0	0	0,014560791	0	0	0,032097122	0,003566347
ENSG00000285897	AC004687.2	0	0	0	0	0	0	0	0	0,031888559	0,031888559	0,003543173
ENSG00000183148	ANKRD20A2	0	0	0	0	0	0	0,031732507	0	0	0,031732507	0,003525834
ENSG00000250120	PCDHA10	0,009923857	0	0	0	0	0	0	0,021700569	0	0,031624426	0,003513825
ENSG00000221878	PSG7	0,031514196	0	0	0	0	0	0	0	0	0,031514196	0,003501577
ENSG00000076344	RGS11	0,031494492	0	0	0	0	0	0	0	0	0,031494492	0,003499388
ENSG00000002586	CD99	0,031105511	0	0	0	0	0	0	0	0	0,031105511	0,003456168
ENSG00000285188	AC008397.2	0	0	0	0	0	0	0	0	0,031083212	0,031083212	0,00345369
ENSG00000091664	SLC17A6	0,030845187	0	0	0	0	0	0	0	0	0,030845187	0,003427243
ENSG00000224383	PRR29	0	0	0	0	0	0	0,03029221	0	0	0,03029221	0,003365801
ENSG00000196557	CACNA1H	0	0	0	0,030270879	0	0	0	0	0	0,030270879	0,003363431
ENSG00000061676	NCKAP1	0,006710061	0,011906838	0	0,011586811	0	0	0	0	0	0,03020371	0,003355968
ENSG00000277758	FO681492.1	0	0	0	0	0	0,03002363	0	0	0	0,03002363	0,003335959
ENSG00000101188	NTSR1	0	0	0	0	0	0	0,029606025	0	0	0,029606025	0,003289558
ENSG00000112782	CLIC5	0	0	0,029603844	0	0	0	0	0	0	0,029603844	0,003289316
ENSG00000086159	AQP6	0,029461995	0	0	0	0	0	0	0	0	0,029461995	0,003273555
ENSG00000126856	PRDM7	0	0	0	0	0	0	0,029404812	0	0	0,029404812	0,003267201
ENSG00000285854	AC010197.2	0,02930203	0	0	0	0	0	0	0	0	0,02930203	0,003255781
ENSG00000179796	LRRC3B	0	0	0	0,0292528	0	0	0	0	0	0,0292528	0,003250311
ENSG00000106852	LHX6	0	0	0	0	0	0,028864053	0	0	0	0,028864053	0,003207117
ENSG00000160716	CHRNB2	0	0	0	0	0	0,028158618	0	0	0	0,028158618	0,003128735
ENSG00000101144	BMP7	0,027854483	0	0	0	0	0	0	0	0	0,027854483	0,003094943
ENSG00000065371	ROPN1	0	0	0	0	0	0	0,027771211	0	0	0,027771211	0,00308569
ENSG00000019505	SYT13	0,027752171	0	0	0	0	0	0	0	0	0,027752171	0,003083575
ENSG00000166183	ASPG	0	0	0	0	0	0,027114002	0	0	0	0,027114002	0,003012667
ENSG00000163833	FBXO40	0,026655595	0	0	0	0	0	0	0	0	0,026655595	0,002961733
ENSG00000157851	DPYSL5	0	0	0	0	0	0	0	0	0,026368689	0,026368689	0,002929854
ENSG00000167614	TTYH1	0	0	0	0	0	0	0	0	0,026294075	0,026294075	0,002921564
ENSG00000189357	SPATA31D4	0	0	0	0	0	0	0,02621064	0	0	0,02621064	0,002912293
ENSG00000104321	TRPA1	0	0	0	0	0	0,026148239	0	0	0	0,026148239	0,00290536
ENSG00000143355	LHX9	0	0,02567918	0	0	0	0	0	0	0	0,02567918	0,002853242
ENSG00000145911	N4BP3	0,025248216	0	0	0	0	0	0	0	0	0,025248216	0,002805357

ENSG00000285791	AC009879.3	0	0	0	0	0	0	0,025099086	0	0	0,025099086	0,002788787
ENSG00000184408	KCND2	0,024960451	0	0	0	0	0	0	0	0	0,024960451	0,002773383
ENSG00000285253	AC090517.4	0	0	0	0	0	0	0,024850531	0	0	0,024850531	0,00276117
ENSG00000149527	PLCH2	0	0,02480729	0	0	0	0	0	0	0	0,02480729	0,002756366
ENSG00000155980	KIF5A	0,024309937	0	0	0	0	0	0	0	0	0,024309937	0,002701104
ENSG00000203690	TCP10	0	0	0	0	0	0	0	0	0,024145319	0,024145319	0,002682813
ENSG00000248383	PCDHAC1	0	0	0	0	0	0,023896093	0	0	0	0,023896093	0,002655121
ENSG00000114948	ADAM23	0,023519155	0	0	0	0	0	0	0	0	0,023519155	0,002613239
ENSG00000166006	KCNC2	0,023348358	0	0	0	0	0	0	0	0	0,023348358	0,002594262
ENSG00000137558	PI15	0	0	0	0	0	0,023175105	0	0	0	0,023175105	0,002575012
ENSG00000102290	PCDH11X	0	0,023056062	0	0	0	0	0	0	0	0,023056062	0,002561785
ENSG00000181790	ADGRB1	0	0	0	0	0	0,022554456	0	0	0	0,022554456	0,002506051
ENSG00000168631	MUCL3	0	0	0	0	0	0	0,022082072	0	0	0,022082072	0,002453564
ENSG00000169933	FRMPD4	0	0	0	0	0	0	0	0	0,021921034	0,021921034	0,00243567
ENSG00000138622	HCN4	0,021830478	0	0	0	0	0	0	0	0	0,021830478	0,002425609
ENSG00000166148	AVPR1A	0	0	0	0	0	0	0,021041478	0	0	0,021041478	0,002337942
ENSG00000144057	ST6GAL2	0,019604381	0	0	0	0	0	0	0	0	0,019604381	0,002178265
ENSG00000102287	GABRE	0	0	0	0	0	0,017260776	0	0	0	0,017260776	0,001917864
ENSG00000100033	PRODH	0,017218616	0	0	0	0	0	0	0	0	0,017218616	0,00191318
ENSG00000144619	CNTN4	0,016563693	0	0	0	0	0	0	0	0	0,016563693	0,00184041
ENSG00000186092	OR4F5	0	0	0	0	0	0	0	0	0	0	0
ENSG00000284733	OR4F29	0	0	0	0	0	0	0	0	0	0	0
ENSG00000284662	OR4F16	0	0	0	0	0	0	0	0	0	0	0
ENSG00000187642	PERM1	0	0	0	0	0	0	0	0	0	0	0
ENSG00000237330	RNF223	0	0	0	0	0	0	0	0	0	0	0
ENSG00000205116	TMEM88B	0	0	0	0	0	0	0	0	0	0	0
ENSG00000189409	MMP23B	0	0	0	0	0	0	0	0	0	0	0
ENSG00000169885	CALML6	0	0	0	0	0	0	0	0	0	0	0
ENSG00000178821	TMEM52	0	0	0	0	0	0	0	0	0	0	0
ENSG00000197921	HES5	0	0	0	0	0	0	0	0	0	0	0
ENSG00000215912	TTC34	0	0	0	0	0	0	0	0	0	0	0
ENSG00000285629	AL031847.2	0	0	0	0	0	0	0	0	0	0	0
ENSG00000173673	HES3	0	0	0	0	0	0	0	0	0	0	0
ENSG00000131686	CA6	0	0	0	0	0	0	0	0	0	0	0
ENSG00000277726	AL109811.3	0	0	0	0	0	0	0	0	0	0	0
ENSG00000171819	ANGPTL7	0	0	0	0	0	0	0	0	0	0	0
ENSG00000239810	PRAMEF11	0	0	0	0	0	0	0	0	0	0	0
ENSG00000243073	PRAMEF4	0	0	0	0	0	0	0	0	0	0	0
ENSG00000187545	PRAMEF10	0	0	0	0	0	0	0	0	0	0	0
ENSG00000232423	PRAMEF6	0	0	0	0	0	0	0	0	0	0	0
ENSG00000274764	PRAMEF27	0	0	0	0	0	0	0	0	0	0	0
ENSG00000277058	HNRNPCL3	0	0	0	0	0	0	0	0	0	0	0
ENSG00000229571	PRAMEF25	0	0	0	0	0	0	0	0	0	0	0
ENSG00000275774	HNRNPCL2	0	0	0	0	0	0	0	0	0	0	0
ENSG00000280267	PRAMEF26	0	0	0	0	0	0	0	0	0	0	0
ENSG00000179412	HNRNPCL4	0	0	0	0	0	0	0	0	0	0	0
ENSG00000204505	PRAMEF9	0	0	0	0	0	0	0	0	0	0	0
ENSG00000279169	PRAMEF13	0	0	0	0	0	0	0	0	0	0	0
ENSG00000279804	PRAMEF18	0	0	0	0	0	0	0	0	0	0	0
ENSG00000270601	PRAMEF5	0	0	0	0	0	0	0	0	0	0	0
ENSG00000182330	PRAMEF8	0	0	0	0	0	0	0	0	0	0	0
ENSG00000237700	PRAMEF33	0	0	0	0	0	0	0	0	0	0	0
ENSG00000204481	PRAMEF14	0	0	0	0	0	0	0	0	0	0	0
ENSG00000162494	LRRC38	0	0	0	0	0	0	0	0	0	0	0
ENSG00000142615	CELA2A	0	0	0	0	0	0	0	0	0	0	0
ENSG00000215704	CELA2B	0	0	0	0	0	0	0	0	0	0	0
ENSG00000215695	RSC1A1	0	0	0	0	0	0	0	0	0	0	0
ENSG00000162460	TMEM82	0	0	0	0	0	0	0	0	0	0	0
ENSG00000183888	SRARP	0	0	0	0	0	0	0	0	0	0	0
ENSG00000270136	MICOS10-NBL1	0	0	0	0	0	0	0	0	0	0	0
ENSG00000178828	RNF186	0	0	0	0	0	0	0	0	0	0	0
ENSG00000183114	FAM43B	0	0	0	0	0	0	0	0	0	0	0
ENSG00000142789	CELA3A	0	0	0	0	0	0	0	0	0	0	0
ENSG00000227868	TEX46	0	0	0	0	0	0	0	0	0	0	0
ENSG00000255054	AL020996.2	0	0	0	0	0	0	0	0	0	0	0
ENSG00000282872	C1orf232	0	0	0	0	0	0	0	0	0	0	0
ENSG00000142684	ZNF593	0	0	0	0	0	0	0	0	0	0	0
ENSG00000253304	TMEM200B	0	0	0	0	0	0	0	0	0	0	0
ENSG00000225828	FAM229A	0	0	0	0	0	0	0	0	0	0	0
ENSG00000254553	AL033529.1	0	0	0	0	0	0	0	0	0	0	0
ENSG00000142698	C1orf94	0	0	0	0	0	0	0	0	0	0	0
ENSG00000189280	GJB5	0	0	0	0	0	0	0	0	0	0	0
ENSG00000188910	GJB3	0	0	0	0	0	0	0	0	0	0	0
ENSG00000243749	TMEM35B	0	0	0	0	0	0	0	0	0	0	0
ENSG00000169218	RSPO1	0	0	0	0	0	0	0	0	0	0	0
ENSG00000274944	AL139260.3	0	0	0	0	0	0	0	0	0	0	0
ENSG00000198754	OXCT2	0	0	0	0	0	0	0	0	0	0	0
ENSG00000284895	AC119676.1	0	0	0	0	0	0	0	0	0	0	0
ENSG00000044012	GUCA2B	0	0	0	0	0	0	0	0	0	0	0
ENSG00000197273	GUCA2A	0	0	0	0	0	0	0	0	0	0	0
ENSG00000178922	HYI	0	0	0	0	0	0	0	0	0	0	0
ENSG00000284989	AL451062.3	0	0	0	0	0	0	0	0	0	0	0
ENSG00000283039	KLF18	0	0	0	0	0	0	0	0	0	0	0
ENSG00000278139	AL358075.4	0	0	0	0	0	0	0	0	0	0	0
ENSG00000250719	P3R3URF	0	0	0	0	0	0	0	0	0	0	0
ENSG00000282881	TMEM275	0	0	0	0	0	0	0	0	0	0	0
ENSG00000162456	KNCN	0	0	0	0	0	0	0	0	0	0	0
ENSG00000186790	FOXE3	0	0	0	0	0	0	0	0	0	0	0
ENSG00000285839	AL445685.3	0	0	0	0	0	0	0	0	0	0	0
ENSG00000203985	LDLRAD1	0	0	0	0	0	0	0	0	0	0	0
ENSG00000157211	CDCP2	0	0	0	0	0	0	0	0	0	0	0
ENSG00000271723	MROH7-TTC4	0	0	0	0	0	0	0	0	0	0	0
ENSG00000162398	LEXM	0	0	0	0	0	0	0	0	0	0	0
ENSG00000143001	TMEM61	0	0	0	0	0	0	0	0	0	0	0
ENSG00000162598	C1orf87	0	0	0	0	0	0	0	0	0	0	0
ENSG00000187140	FOXD3	0	0	0	0	0	0	0	0	0	0	0
ENSG00000177414	UBE2U	0	0	0	0	0	0	0	0	0	0	0
ENSG00000116783	TNNI3K	0	0	0	0	0	0	0	0	0	0	0
ENSG00000154007	ASB17	0	0	0	0	0	0	0	0	0	0	0
ENSG00000267561	AC093155.3	0	0	0	0	0	0	0	0	0	0	0
ENSG00000271949	AC093423.3	0	0	0	0	0	0	0	0	0	0	0
ENSG00000143032	BARHL2	0	0	0	0	0	0	0	0	0	0	0
ENSG00000172031	EPHX4	0	0	0	0	0	0	0	0	0	0	0
ENSG00000271092	TMEM56-RWDD3	0	0	0	0	0	0	0	0	0	0	0
ENSG00000243480	AMY2A	0	0	0	0	0	0	0	0	0	0	0
ENSG00000237763	AMY1A	0	0	0	0	0	0	0	0	0	0	0
ENSG00000174876	AMY1B	0	0	0	0	0	0	0	0	0	0	0
ENSG00000187733	AMY1C	0	0	0	0	0	0	0	0	0	0	0
ENSG00000186086	NBPF6	0	0	0	0	0	0	0	0	0	0	0
ENSG00000134183	GNAT2	0	0	0	0	0	0	0	0	0	0	0
ENSG00000198758	EPS8L3	0	0	0	0	0	0	0	0	0	0	0
ENSG00000186150	UBL4B	0	0	0	0	0	0	0	0	0	0	0
ENSG00000203857	HSD3B1	0	0	0	0	0	0	0	0	0	0	0
ENSG00000134249	ADAM30	0	0	0	0	0	0	0	0	0	0	0
ENSG00000279782	PPIAL4F	0	0	0	0	0	0	0	0	0	0	0
ENSG00000256374	PPIAL4D	0	0	0	0	0	0	0	0	0	0	0
ENSG00000280778	AC243547.3	0	0	0	0	0	0	0	0	0	0	0

ENSG00000286019	AC239811.1	0	0	0	0	0	0	0	0	0	0	0
ENSG00000177144	NUDT4B	0	0	0	0	0	0	0	0	0	0	0
ENSG00000286185	AC242842.3	0	0	0	0	0	0	0	0	0	0	0
ENSG00000263464	PPIAL4C	0	0	0	0	0	0	0	0	0	0	0
ENSG00000270882	HIST2H4A	0	0	0	0	0	0	0	0	0	0	0
ENSG00000203811	HIST2H3C	0	0	0	0	0	0	0	0	0	0	0
ENSG00000203812	HIST2H2AA3	0	0	0	0	0	0	0	0	0	0	0
ENSG00000272196	HIST2H2AA4	0	0	0	0	0	0	0	0	0	0	0
ENSG00000203852	HIST2H3A	0	0	0	0	0	0	0	0	0	0	0
ENSG00000163157	TMOD4	0	0	0	0	0	0	0	0	0	0	0
ENSG00000215853	RPTN	0	0	0	0	0	0	0	0	0	0	0
ENSG00000187238	LCE3B	0	0	0	0	0	0	0	0	0	0	0
ENSG00000185962	LCE3A	0	0	0	0	0	0	0	0	0	0	0
ENSG00000159455	LCE2B	0	0	0	0	0	0	0	0	0	0	0
ENSG00000187170	LCE4A	0	0	0	0	0	0	0	0	0	0	0
ENSG00000203786	KPRP	0	0	0	0	0	0	0	0	0	0	0
ENSG00000240386	LCE1F	0	0	0	0	0	0	0	0	0	0	0
ENSG00000172155	LCE1D	0	0	0	0	0	0	0	0	0	0	0
ENSG00000285946	AL162596.1	0	0	0	0	0	0	0	0	0	0	0
ENSG00000163206	SMCP	0	0	0	0	0	0	0	0	0	0	0
ENSG00000283227	SPRR5	0	0	0	0	0	0	0	0	0	0	0
ENSG00000169474	SPRR1A	0	0	0	0	0	0	0	0	0	0	0
ENSG00000163216	SPRR2D	0	0	0	0	0	0	0	0	0	0	0
ENSG00000196805	SPRR2B	0	0	0	0	0	0	0	0	0	0	0
ENSG00000203785	SPRR2E	0	0	0	0	0	0	0	0	0	0	0
ENSG00000244094	SPRR2F	0	0	0	0	0	0	0	0	0	0	0
ENSG00000159527	PGLYRP3	0	0	0	0	0	0	0	0	0	0	0
ENSG00000197364	S100A7L2	0	0	0	0	0	0	0	0	0	0	0
ENSG00000188015	S100A3	0	0	0	0	0	0	0	0	0	0	0
ENSG00000189334	S100A14	0	0	0	0	0	0	0	0	0	0	0
ENSG00000285779	AL358472.7	0	0	0	0	0	0	0	0	0	0	0
ENSG00000251246	AL691442.1	0	0	0	0	0	0	0	0	0	0	0
ENSG00000273088	AL713999.1	0	0	0	0	0	0	0	0	0	0	0
ENSG00000185499	MUC1	0	0	0	0	0	0	0	0	0	0	0
ENSG00000143630	HCN3	0	0	0	0	0	0	0	0	0	0	0
ENSG00000143627	PKLR	0	0	0	0	0	0	0	0	0	0	0
ENSG00000260238	PMF1-BGLAP	0	0	0	0	0	0	0	0	0	0	0
ENSG00000242252	BGLAP	0	0	0	0	0	0	0	0	0	0	0
ENSG00000132702	HAPLN2	0	0	0	0	0	0	0	0	0	0	0
ENSG00000160838	LRRC71	0	0	0	0	0	0	0	0	0	0	0
ENSG00000253831	ETV3L	0	0	0	0	0	0	0	0	0	0	0
ENSG00000158485	CD1B	0	0	0	0	0	0	0	0	0	0	0
ENSG00000186306	OR10T2	0	0	0	0	0	0	0	0	0	0	0
ENSG00000198965	OR10R2	0	0	0	0	0	0	0	0	0	0	0
ENSG00000186440	OR6P1	0	0	0	0	0	0	0	0	0	0	0
ENSG00000196171	OR6K2	0	0	0	0	0	0	0	0	0	0	0
ENSG00000196184	OR10J1	0	0	0	0	0	0	0	0	0	0	0
ENSG00000184155	OR10J5	0	0	0	0	0	0	0	0	0	0	0
ENSG00000132703	APCS	0	0	0	0	0	0	0	0	0	0	0
ENSG00000158764	ITLN2	0	0	0	0	0	0	0	0	0	0	0
ENSG00000158874	APOA2	0	0	0	0	0	0	0	0	0	0	0
ENSG00000188931	CFAP126	0	0	0	0	0	0	0	0	0	0	0
ENSG00000254706	AL512785.2	0	0	0	0	0	0	0	0	0	0	0
ENSG00000143184	XCL1	0	0	0	0	0	0	0	0	0	0	0
ENSG00000143196	DPT	0	0	0	0	0	0	0	0	0	0	0
ENSG00000180999	C1orf105	0	0	0	0	0	0	0	0	0	0	0
ENSG00000285777	AL139142.2	0	0	0	0	0	0	0	0	0	0	0
ENSG00000232113	TEX50	0	0	0	0	0	0	0	0	0	0	0
ENSG00000117601	SERPINC1	0	0	0	0	0	0	0	0	0	0	0
ENSG00000203737	GPR52	0	0	0	0	0	0	0	0	0	0	0
ENSG00000188585	CLEC20A	0	0	0	0	0	0	0	0	0	0	0
ENSG00000179930	ZNF648	0	0	0	0	0	0	0	0	0	0	0
ENSG00000116703	PDC	0	0	0	0	0	0	0	0	0	0	0
ENSG00000143278	F13B	0	0	0	0	0	0	0	0	0	0	0
ENSG00000151418	ATP6V1G3	0	0	0	0	0	0	0	0	0	0	0
ENSG00000170128	GPR25	0	0	0	0	0	0	0	0	0	0	0
ENSG00000232237	ASCL5	0	0	0	0	0	0	0	0	0	0	0
ENSG00000170498	KISS1	0	0	0	0	0	0	0	0	0	0	0
ENSG00000174567	GOLT1A	0	0	0	0	0	0	0	0	0	0	0
ENSG00000162892	IL24	0	0	0	0	0	0	0	0	0	0	0
ENSG00000284299	AL590132.1	0	0	0	0	0	0	0	0	0	0	0
ENSG00000283952	AC092017.3	0	0	0	0	0	0	0	0	0	0	0
ENSG00000162771	FAM71A	0	0	0	0	0	0	0	0	0	0	0
ENSG00000185523	SPATA45	0	0	0	0	0	0	0	0	0	0	0
ENSG00000286231	AL445423.2	0	0	0	0	0	0	0	0	0	0	0
ENSG00000255835	AL117348.2	0	0	0	0	0	0	0	0	0	0	0
ENSG00000203685	STUM	0	0	0	0	0	0	0	0	0	0	0
ENSG00000185888	PRSS38	0	0	0	0	0	0	0	0	0	0	0
ENSG00000154342	WNT3A	0	0	0	0	0	0	0	0	0	0	0
ENSG00000213029	SPHAR	0	0	0	0	0	0	0	0	0	0	0
ENSG00000135750	KCNK1	0	0	0	0	0	0	0	0	0	0	0
ENSG00000196289	BECN2	0	0	0	0	0	0	0	0	0	0	0
ENSG00000284824	GCSAML-AS1	0	0	0	0	0	0	0	0	0	0	0
ENSG00000177489	OR2G2	0	0	0	0	0	0	0	0	0	0	0
ENSG00000239395	AC118470.1	0	0	0	0	0	0	0	0	0	0	0
ENSG00000153230	OR14K1	0	0	0	0	0	0	0	0	0	0	0
ENSG00000196772	OR14A16	0	0	0	0	0	0	0	0	0	0	0
ENSG00000197591	OR11L1	0	0	0	0	0	0	0	0	0	0	0
ENSG00000238243	OR2W3	0	0	0	0	0	0	0	0	0	0	0
ENSG00000177462	OR2T8	0	0	0	0	0	0	0	0	0	0	0
ENSG00000198601	OR2M2	0	0	0	0	0	0	0	0	0	0	0
ENSG00000177212	OR2T33	0	0	0	0	0	0	0	0	0	0	0
ENSG00000177201	OR2T12	0	0	0	0	0	0	0	0	0	0	0
ENSG00000177186	OR2M7	0	0	0	0	0	0	0	0	0	0	0
ENSG00000177174	OR14C36	0	0	0	0	0	0	0	0	0	0	0
ENSG00000196944	OR2T4	0	0	0	0	0	0	0	0	0	0	0
ENSG00000198104	OR2T6	0	0	0	0	0	0	0	0	0	0	0
ENSG00000175143	OR2T1	0	0	0	0	0	0	0	0	0	0	0
ENSG00000196240	OR2T2	0	0	0	0	0	0	0	0	0	0	0
ENSG00000196539	OR2T3	0	0	0	0	0	0	0	0	0	0	0
ENSG00000203661	OR2T5	0	0	0	0	0	0	0	0	0	0	0
ENSG00000182783	OR2T29	0	0	0	0	0	0	0	0	0	0	0
ENSG00000183310	OR2T34	0	0	0	0	0	0	0	0	0	0	0
ENSG00000279301	OR2T11	0	0	0	0	0	0	0	0	0	0	0
ENSG00000177151	OR2T35	0	0	0	0	0	0	0	0	0	0	0
ENSG00000187701	OR2T27	0	0	0	0	0	0	0	0	0	0	0
ENSG00000189181	OR14I1	0	0	0	0	0	0	0	0	0	0	0
ENSG00000259823	LYPD8	0	0	0	0	0	0	0	0	0	0	0
ENSG00000118004	COLEC11	0	0	0	0	0	0	0	0	0	0	0
ENSG00000150873	C2orf50	0	0	0	0	0	0	0	0	0	0	0
ENSG00000169006	NTSR2	0	0	0	0	0	0	0	0	0	0	0
ENSG00000151379	MSGN1	0	0	0	0	0	0	0	0	0	0	0
ENSG00000250741	NT5C1B-RDH14	0	0	0	0	0	0	0	0	0	0	0
ENSG00000276087	AC008073.3	0	0	0	0	0	0	0	0	0	0	0
ENSG00000157884	CIB4	0	0	0	0	0	0	0	0	0	0	0
ENSG00000186143	PRR30	0	0	0	0	0	0	0	0	0	0	0
ENSG00000205111	CDKL4	0	0	0	0	0	0	0	0	0	0	0

ENSG00000285542	AC013717.1	0	0	0	0	0	0	0	0	0	0	0
ENSG00000284701	TMEM247	0	0	0	0	0	0	0	0	0	0	0
ENSG00000242441	GTF2A1L	0	0	0	0	0	0	0	0	0	0	0
ENSG00000170820	FSHR	0	0	0	0	0	0	0	0	0	0	0
ENSG00000177994	C2orf73	0	0	0	0	0	0	0	0	0	0	0
ENSG00000169605	GKN1	0	0	0	0	0	0	0	0	0	0	0
ENSG00000116039	ATP6V1B1	0	0	0	0	0	0	0	0	0	0	0
ENSG00000144031	ANKRD53	0	0	0	0	0	0	0	0	0	0	0
ENSG00000144035	NAT8	0	0	0	0	0	0	0	0	0	0	0
ENSG00000274049	INO80B-WBP1	0	0	0	0	0	0	0	0	0	0	0
ENSG00000187627	RGPD1	0	0	0	0	0	0	0	0	0	0	0
ENSG00000183281	PLGLB1	0	0	0	0	0	0	0	0	0	0	0
ENSG00000172073	TEX37	0	0	0	0	0	0	0	0	0	0	0
ENSG00000259916	AL845331.2	0	0	0	0	0	0	0	0	0	0	0
ENSG00000144010	TRIM43B	0	0	0	0	0	0	0	0	0	0	0
ENSG00000144015	TRIM43	0	0	0	0	0	0	0	0	0	0	0
ENSG00000204640	NMS	0	0	0	0	0	0	0	0	0	0	0
ENSG00000196228	SULT1C3	0	0	0	0	0	0	0	0	0	0	0
ENSG00000015568	RGPD5	0	0	0	0	0	0	0	0	0	0	0
ENSG00000256977	LIMS3	0	0	0	0	0	0	0	0	0	0	0
ENSG00000284337	AC013271.1	0	0	0	0	0	0	0	0	0	0	0
ENSG00000257207	AC112229.3	0	0	0	0	0	0	0	0	0	0	0
ENSG00000256671	LIMS4	0	0	0	0	0	0	0	0	0	0	0
ENSG00000183054	RGPD6	0	0	0	0	0	0	0	0	0	0	0
ENSG00000136694	IL36A	0	0	0	0	0	0	0	0	0	0	0
ENSG00000144230	GPR17	0	0	0	0	0	0	0	0	0	0	0
ENSG00000173349	SFT2D3	0	0	0	0	0	0	0	0	0	0	0
ENSG00000152093	CFC1B	0	0	0	0	0	0	0	0	0	0	0
ENSG00000136698	CFC1	0	0	0	0	0	0	0	0	0	0	0
ENSG00000173302	GPR148	0	0	0	0	0	0	0	0	0	0	0
ENSG00000284479	SMIM39	0	0	0	0	0	0	0	0	0	0	0
ENSG00000283228	AC068547.1	0	0	0	0	0	0	0	0	0	0	0
ENSG00000177519	RPRM	0	0	0	0	0	0	0	0	0	0	0
ENSG00000248672	LY75-CD302	0	0	0	0	0	0	0	0	0	0	0
ENSG00000152254	G6PC2	0	0	0	0	0	0	0	0	0	0	0
ENSG00000251569	AC093899.2	0	0	0	0	0	0	0	0	0	0	0
ENSG00000217236	SP9	0	0	0	0	0	0	0	0	0	0	0
ENSG00000128713	HOXD11	0	0	0	0	0	0	0	0	0	0	0
ENSG00000175879	HOXD8	0	0	0	0	0	0	0	0	0	0	0
ENSG00000128652	HOXD3	0	0	0	0	0	0	0	0	0	0	0
ENSG00000170166	HOXD4	0	0	0	0	0	0	0	0	0	0	0
ENSG00000128645	HOXD1	0	0	0	0	0	0	0	0	0	0	0
ENSG00000286053	ASDURF	0	0	0	0	0	0	0	0	0	0	0
ENSG00000286165	AC012488.2	0	0	0	0	0	0	0	0	0	0	0
ENSG00000270757	HSPE1-MOB4	0	0	0	0	0	0	0	0	0	0	0
ENSG00000118231	CRYGD	0	0	0	0	0	0	0	0	0	0	0
ENSG00000182187	CRYGB	0	0	0	0	0	0	0	0	0	0	0
ENSG00000168582	CRYGA	0	0	0	0	0	0	0	0	0	0	0
ENSG00000168530	MYL1	0	0	0	0	0	0	0	0	0	0	0
ENSG00000144583	MARCH4	0	0	0	0	0	0	0	0	0	0	0
ENSG00000135925	WNT10A	0	0	0	0	0	0	0	0	0	0	0
ENSG00000163497	FEV	0	0	0	0	0	0	0	0	0	0	0
ENSG00000163499	CRYBA2	0	0	0	0	0	0	0	0	0	0	0
ENSG00000163501	IHH	0	0	0	0	0	0	0	0	0	0	0
ENSG00000284820	AC068946.2	0	0	0	0	0	0	0	0	0	0	0
ENSG00000286143	AC053503.7	0	0	0	0	0	0	0	0	0	0	0
ENSG00000163081	CCDC140	0	0	0	0	0	0	0	0	0	0	0
ENSG00000286239	AC093884.1	0	0	0	0	0	0	0	0	0	0	0
ENSG00000283428	CCDC195	0	0	0	0	0	0	0	0	0	0	0
ENSG00000284629	SCYGR1	0	0	0	0	0	0	0	0	0	0	0
ENSG00000284643	SCYGR2	0	0	0	0	0	0	0	0	0	0	0
ENSG00000284704	SCYGR3	0	0	0	0	0	0	0	0	0	0	0
ENSG00000284667	SCYGR5	0	0	0	0	0	0	0	0	0	0	0
ENSG00000284725	SCYGR6	0	0	0	0	0	0	0	0	0	0	0
ENSG00000284718	SCYGR7	0	0	0	0	0	0	0	0	0	0	0
ENSG00000284635	SCYGR8	0	0	0	0	0	0	0	0	0	0	0
ENSG00000173699	SPATA3	0	0	0	0	0	0	0	0	0	0	0
ENSG00000177673	TEX44	0	0	0	0	0	0	0	0	0	0	0
ENSG00000163273	NPPC	0	0	0	0	0	0	0	0	0	0	0
ENSG00000163286	ALPG	0	0	0	0	0	0	0	0	0	0	0
ENSG00000182600	SNORC	0	0	0	0	0	0	0	0	0	0	0
ENSG00000115488	NEU2	0	0	0	0	0	0	0	0	0	0	0
ENSG00000242515	UGT1A10	0	0	0	0	0	0	0	0	0	0	0
ENSG00000241119	UGT1A9	0	0	0	0	0	0	0	0	0	0	0
ENSG00000167165	UGT1A6	0	0	0	0	0	0	0	0	0	0	0
ENSG00000244474	UGT1A4	0	0	0	0	0	0	0	0	0	0	0
ENSG00000168505	GBX2	0	0	0	0	0	0	0	0	0	0	0
ENSG00000071677	PRLH	0	0	0	0	0	0	0	0	0	0	0
ENSG00000182083	OR6B2	0	0	0	0	0	0	0	0	0	0	0
ENSG00000178602	OTOS	0	0	0	0	0	0	0	0	0	0	0
ENSG00000184945	AQP12A	0	0	0	0	0	0	0	0	0	0	0
ENSG00000154252	GAL3ST2	0	0	0	0	0	0	0	0	0	0	0
ENSG00000188011	RTP5	0	0	0	0	0	0	0	0	0	0	0
ENSG00000216921	FAM240C	0	0	0	0	0	0	0	0	0	0	0
ENSG00000272410	AC022384.1	0	0	0	0	0	0	0	0	0	0	0
ENSG00000283563	AC098650.1	0	0	0	0	0	0	0	0	0	0	0
ENSG00000187094	CCK	0	0	0	0	0	0	0	0	0	0	0
ENSG00000010282	HHATL	0	0	0	0	0	0	0	0	0	0	0
ENSG00000273291	AC092042.3	0	0	0	0	0	0	0	0	0	0	0
ENSG00000173578	XCR1	0	0	0	0	0	0	0	0	0	0	0
ENSG00000163825	RTP3	0	0	0	0	0	0	0	0	0	0	0
ENSG00000283877	AC104304.1	0	0	0	0	0	0	0	0	0	0	0
ENSG00000283473	FAM240A	0	0	0	0	0	0	0	0	0	0	0
ENSG00000206549	PRSS50	0	0	0	0	0	0	0	0	0	0	0
ENSG00000188086	PRSS45P	0	0	0	0	0	0	0	0	0	0	0
ENSG00000229453	SPINK8	0	0	0	0	0	0	0	0	0	0	0
ENSG00000213689	TREX1	0	0	0	0	0	0	0	0	0	0	0
ENSG00000183396	TMEM89	0	0	0	0	0	0	0	0	0	0	0
ENSG00000236980	C3orf84	0	0	0	0	0	0	0	0	0	0	0
ENSG00000283189	AC104452.1	0	0	0	0	0	0	0	0	0	0	0
ENSG00000145029	NICN1	0	0	0	0	0	0	0	0	0	0	0
ENSG00000176020	AMIGO3	0	0	0	0	0	0	0	0	0	0	0
ENSG00000164076	CAMKV	0	0	0	0	0	0	0	0	0	0	0
ENSG00000272104	Z84492.2	0	0	0	0	0	0	0	0	0	0	0
ENSG00000214686	IQCF6	0	0	0	0	0	0	0	0	0	0	0
ENSG00000214681	IQCF5	0	0	0	0	0	0	0	0	0	0	0
ENSG00000173389	IQCF1	0	0	0	0	0	0	0	0	0	0	0
ENSG00000180929	GPR62	0	0	0	0	0	0	0	0	0	0	0
ENSG00000114786	ABHD14A-ACY1	0	0	0	0	0	0	0	0	0	0	0
ENSG00000239732	TLR9	0	0	0	0	0	0	0	0	0	0	0
ENSG00000173366	AC097637.1	0	0	0	0	0	0	0	0	0	0	0
ENSG00000114854	TNNC1	0	0	0	0	0	0	0	0	0	0	0
ENSG00000055957	ITIH1	0	0	0	0	0	0	0	0	0	0	0
ENSG00000272573	MUSTN1	0	0	0	0	0	0	0	0	0	0	0
ENSG00000186451	SPATA12	0	0	0	0	0	0	0	0	0	0	0
ENSG00000183662	TAFA1	0	0	0	0	0	0	0	0	0	0	0
ENSG00000242120	MDFIC2	0	0	0	0	0	0	0	0	0	0	0

ENSG00000285708	AC097634.4	0	0	0	0	0	0	0	0	0	0	0
ENSG00000230301	OR5H6	0	0	0	0	0	0	0	0	0	0	0
ENSG00000197938	OR5H2	0	0	0	0	0	0	0	0	0	0	0
ENSG00000285635	AC021660.3	0	0	0	0	0	0	0	0	0	0	0
ENSG00000163530	DPPA2	0	0	0	0	0	0	0	0	0	0	0
ENSG00000144834	TAGLN3	0	0	0	0	0	0	0	0	0	0	0
ENSG00000174255	ZNF80	0	0	0	0	0	0	0	0	0	0	0
ENSG00000251012	AC083800.1	0	0	0	0	0	0	0	0	0	0	0
ENSG00000114638	UPK1B	0	0	0	0	0	0	0	0	0	0	0
ENSG00000285585	AC069444.2	0	0	0	0	0	0	0	0	0	0	0
ENSG00000189366	ALG1L	0	0	0	0	0	0	0	0	0	0	0
ENSG00000169704	GP9	0	0	0	0	0	0	0	0	0	0	0
ENSG00000261796	ISY1-RAB43	0	0	0	0	0	0	0	0	0	0	0
ENSG00000178804	H1FOO	0	0	0	0	0	0	0	0	0	0	0
ENSG00000274810	NPHP3-ACAD11	0	0	0	0	0	0	0	0	0	0	0
ENSG00000206260	PRR23A	0	0	0	0	0	0	0	0	0	0	0
ENSG00000233701	PRR23C	0	0	0	0	0	0	0	0	0	0	0
ENSG00000285558	AC112504.2	0	0	0	0	0	0	0	0	0	0	0
ENSG00000188582	PAQR9	0	0	0	0	0	0	0	0	0	0	0
ENSG00000231213	PLSCR5	0	0	0	0	0	0	0	0	0	0	0
ENSG00000163646	CLRN1	0	0	0	0	0	0	0	0	0	0	0
ENSG00000197953	AADACL2	0	0	0	0	0	0	0	0	0	0	0
ENSG00000114790	ARHGEF26	0	0	0	0	0	0	0	0	0	0	0
ENSG00000174948	GPR149	0	0	0	0	0	0	0	0	0	0	0
ENSG00000284952	AC104472.3	0	0	0	0	0	0	0	0	0	0	0
ENSG00000168811	IL12A	0	0	0	0	0	0	0	0	0	0	0
ENSG00000248710	AC079594.2	0	0	0	0	0	0	0	0	0	0	0
ENSG00000285218	AC026316.4	0	0	0	0	0	0	0	0	0	0	0
ENSG00000144962	SPATA16	0	0	0	0	0	0	0	0	0	0	0
ENSG00000275163	AC117457.1	0	0	0	0	0	0	0	0	0	0	0
ENSG00000283765	AC131160.1	0	0	0	0	0	0	0	0	0	0	0
ENSG00000284917	ECE2	0	0	0	0	0	0	0	0	0	0	0
ENSG00000090539	CHRD	0	0	0	0	0	0	0	0	0	0	0
ENSG00000283149	AC068631.2	0	0	0	0	0	0	0	0	0	0	0
ENSG00000175077	RTP1	0	0	0	0	0	0	0	0	0	0	0
ENSG00000157005	SST	0	0	0	0	0	0	0	0	0	0	0
ENSG00000198471	RTP2	0	0	0	0	0	0	0	0	0	0	0
ENSG00000188729	OSTN	0	0	0	0	0	0	0	0	0	0	0
ENSG00000253548	PYDC2	0	0	0	0	0	0	0	0	0	0	0
ENSG00000176945	MUC20	0	0	0	0	0	0	0	0	0	0	0
ENSG00000145113	MUC4	0	0	0	0	0	0	0	0	0	0	0
ENSG00000272741	AC069257.3	0	0	0	0	0	0	0	0	0	0	0
ENSG00000273331	TM4SF19-TCTEX1D2	0	0	0	0	0	0	0	0	0	0	0
ENSG00000214097	SMCO1	0	0	0	0	0	0	0	0	0	0	0
ENSG00000235608	NKX1-1	0	0	0	0	0	0	0	0	0	0	0
ENSG00000163982	OTOP1	0	0	0	0	0	0	0	0	0	0	0
ENSG00000168824	NSG1	0	0	0	0	0	0	0	0	0	0	0
ENSG00000173040	EVC2	0	0	0	0	0	0	0	0	0	0	0
ENSG00000284684	AC092442.1	0	0	0	0	0	0	0	0	0	0	0
ENSG00000109625	CPZ	0	0	0	0	0	0	0	0	0	0	0
ENSG00000215612	HMX1	0	0	0	0	0	0	0	0	0	0	0
ENSG00000233136	USP17L11	0	0	0	0	0	0	0	0	0	0	0
ENSG00000227551	USP17L12	0	0	0	0	0	0	0	0	0	0	0
ENSG00000232399	USP17L13	0	0	0	0	0	0	0	0	0	0	0
ENSG00000223569	USP17L15	0	0	0	0	0	0	0	0	0	0	0
ENSG00000249104	USP17L17	0	0	0	0	0	0	0	0	0	0	0
ENSG00000250844	USP17L18	0	0	0	0	0	0	0	0	0	0	0
ENSG00000248920	USP17L19	0	0	0	0	0	0	0	0	0	0	0
ENSG00000250745	USP17L20	0	0	0	0	0	0	0	0	0	0	0
ENSG00000249811	USP17L21	0	0	0	0	0	0	0	0	0	0	0
ENSG00000248933	USP17L22	0	0	0	0	0	0	0	0	0	0	0
ENSG00000250913	USP17L23	0	0	0	0	0	0	0	0	0	0	0
ENSG00000232264	USP17L24	0	0	0	0	0	0	0	0	0	0	0
ENSG00000230430	USP17L25	0	0	0	0	0	0	0	0	0	0	0
ENSG00000229579	USP17L26	0	0	0	0	0	0	0	0	0	0	0
ENSG00000227140	USP17L5	0	0	0	0	0	0	0	0	0	0	0
ENSG00000235780	USP17L27	0	0	0	0	0	0	0	0	0	0	0
ENSG00000231051	USP17L28	0	0	0	0	0	0	0	0	0	0	0
ENSG00000231637	USP17L29	0	0	0	0	0	0	0	0	0	0	0
ENSG00000228856	USP17L30	0	0	0	0	0	0	0	0	0	0	0
ENSG00000186146	DEFB131A	0	0	0	0	0	0	0	0	0	0	0
ENSG00000137441	FGFBP2	0	0	0	0	0	0	0	0	0	0	0
ENSG00000163394	CCKAR	0	0	0	0	0	0	0	0	0	0	0
ENSG00000215203	GRXCR1	0	0	0	0	0	0	0	0	0	0	0
ENSG00000177752	YIPF7	0	0	0	0	0	0	0	0	0	0	0
ENSG00000109182	CWH43	0	0	0	0	0	0	0	0	0	0	0
ENSG00000282278	AC058822.1	0	0	0	0	0	0	0	0	0	0	0
ENSG00000180613	GSX2	0	0	0	0	0	0	0	0	0	0	0
ENSG00000087128	TMPRSS11E	0	0	0	0	0	0	0	0	0	0	0
ENSG00000109181	UGT2B10	0	0	0	0	0	0	0	0	0	0	0
ENSG00000135220	UGT2A3	0	0	0	0	0	0	0	0	0	0	0
ENSG00000135226	UGT2B28	0	0	0	0	0	0	0	0	0	0	0
ENSG00000271271	UGT2A2	0	0	0	0	0	0	0	0	0	0	0
ENSG00000181617	FDCSP	0	0	0	0	0	0	0	0	0	0	0
ENSG00000171209	CSN3	0	0	0	0	0	0	0	0	0	0	0
ENSG00000145309	CABS1	0	0	0	0	0	0	0	0	0	0	0
ENSG00000109208	SMR3A	0	0	0	0	0	0	0	0	0	0	0
ENSG00000156194	PPEF2	0	0	0	0	0	0	0	0	0	0	0
ENSG00000272414	FAM47E-STBD1	0	0	0	0	0	0	0	0	0	0	0
ENSG00000156234	CXCL13	0	0	0	0	0	0	0	0	0	0	0
ENSG00000196475	GK2	0	0	0	0	0	0	0	0	0	0	0
ENSG00000145283	SLC10A6	0	0	0	0	0	0	0	0	0	0	0
ENSG00000170509	HSD17B13	0	0	0	0	0	0	0	0	0	0	0
ENSG00000255072	PIGY	0	0	0	0	0	0	0	0	0	0	0
ENSG00000198099	ADH4	0	0	0	0	0	0	0	0	0	0	0
ENSG00000145358	DDIT4L	0	0	0	0	0	0	0	0	0	0	0
ENSG00000250298	GIMD1	0	0	0	0	0	0	0	0	0	0	0
ENSG00000285330	AC126283.2	0	0	0	0	0	0	0	0	0	0	0
ENSG00000164093	PITX2	0	0	0	0	0	0	0	0	0	0	0
ENSG00000178403	NEUROG2	0	0	0	0	0	0	0	0	0	0	0
ENSG00000186867	QRFPR	0	0	0	0	0	0	0	0	0	0	0
ENSG00000250361	GYPB	0	0	0	0	0	0	0	0	0	0	0
ENSG00000237136	C4orf51	0	0	0	0	0	0	0	0	0	0	0
ENSG00000256394	ASIC5	0	0	0	0	0	0	0	0	0	0	0
ENSG00000151790	TDO2	0	0	0	0	0	0	0	0	0	0	0
ENSG00000164107	HAND2	0	0	0	0	0	0	0	0	0	0	0
ENSG00000150630	VEGFC	0	0	0	0	0	0	0	0	0	0	0
ENSG00000177300	CLDN22	0	0	0	0	0	0	0	0	0	0	0
ENSG00000088926	F11	0	0	0	0	0	0	0	0	0	0	0
ENSG00000272297	AC018709.1	0	0	0	0	0	0	0	0	0	0	0
ENSG00000168412	MTNR1A	0	0	0	0	0	0	0	0	0	0	0
ENSG00000205097	FRG2	0	0	0	0	0	0	0	0	0	0	0
ENSG00000260596	DUX4	0	0	0	0	0	0	0	0	0	0	0
ENSG00000185028	LRRC14B	0	0	0	0	0	0	0	0	0	0	0
ENSG00000286001	AC021087.5	0	0	0	0	0	0	0	0	0	0	0
ENSG00000286094	AC026740.3	0	0	0	0	0	0	0	0	0	0	0
ENSG00000145506	NKD2	0	0	0	0	0	0	0	0	0	0	0

ENSG00000142319	SLC6A3	0	0	0	0	0	0	0	0	0	0	0
ENSG00000186493	C5orf38	0	0	0	0	0	0	0	0	0	0	0
ENSG00000170549	IRX1	0	0	0	0	0	0	0	0	0	0	0
ENSG00000169777	TAS2R1	0	0	0	0	0	0	0	0	0	0	0
ENSG00000284439	TAF11L3	0	0	0	0	0	0	0	0	0	0	0
ENSG00000284283	TAF11L4	0	0	0	0	0	0	0	0	0	0	0
ENSG00000284234	TAF11L5	0	0	0	0	0	0	0	0	0	0	0
ENSG00000284042	TAF11L6	0	0	0	0	0	0	0	0	0	0	0
ENSG00000284465	TAF11L7	0	0	0	0	0	0	0	0	0	0	0
ENSG00000283967	TAF11L8	0	0	0	0	0	0	0	0	0	0	0
ENSG00000283988	TAF11L9	0	0	0	0	0	0	0	0	0	0	0
ENSG00000284356	TAF11L10	0	0	0	0	0	0	0	0	0	0	0
ENSG00000283740	TAF11L11	0	0	0	0	0	0	0	0	0	0	0
ENSG00000283776	TAF11L13	0	0	0	0	0	0	0	0	0	0	0
ENSG00000250782	TAF11L14	0	0	0	0	0	0	0	0	0	0	0
ENSG00000182631	RXFP3	0	0	0	0	0	0	0	0	0	0	0
ENSG00000070193	FGF10	0	0	0	0	0	0	0	0	0	0	0
ENSG00000016082	ISL1	0	0	0	0	0	0	0	0	0	0	0
ENSG00000169271	HSPB3	0	0	0	0	0	0	0	0	0	0	0
ENSG00000113088	GZMK	0	0	0	0	0	0	0	0	0	0	0
ENSG00000234602	MCIDAS	0	0	0	0	0	0	0	0	0	0	0
ENSG00000152669	CCNO	0	0	0	0	0	0	0	0	0	0	0
ENSG00000178394	HTR1A	0	0	0	0	0	0	0	0	0	0	0
ENSG00000197822	OCLN	0	0	0	0	0	0	0	0	0	0	0
ENSG00000205572	SERF1B	0	0	0	0	0	0	0	0	0	0	0
ENSG00000164326	CARTPT	0	0	0	0	0	0	0	0	0	0	0
ENSG00000285000	AC008581.2	0	0	0	0	0	0	0	0	0	0	0
ENSG00000284762	AC022414.1	0	0	0	0	0	0	0	0	0	0	0
ENSG00000164299	SPZ1	0	0	0	0	0	0	0	0	0	0	0
ENSG00000178015	GPR150	0	0	0	0	0	0	0	0	0	0	0
ENSG00000145777	TSLP	0	0	0	0	0	0	0	0	0	0	0
ENSG00000258864	AC008575.1	0	0	0	0	0	0	0	0	0	0	0
ENSG00000243414	TICAM2	0	0	0	0	0	0	0	0	0	0	0
ENSG00000113396	SLC27A6	0	0	0	0	0	0	0	0	0	0	0
ENSG00000273217	AC008695.1	0	0	0	0	0	0	0	0	0	0	0
ENSG00000281938	AC034228.4	0	0	0	0	0	0	0	0	0	0	0
ENSG00000164400	CSF2	0	0	0	0	0	0	0	0	0	0	0
ENSG00000283782	AC116366.3	0	0	0	0	0	0	0	0	0	0	0
ENSG00000113525	IL5	0	0	0	0	0	0	0	0	0	0	0
ENSG00000113520	IL4	0	0	0	0	0	0	0	0	0	0	0
ENSG00000272772	AC104109.3	0	0	0	0	0	0	0	0	0	0	0
ENSG00000152705	CATSPER3	0	0	0	0	0	0	0	0	0	0	0
ENSG00000251380	DCANP1	0	0	0	0	0	0	0	0	0	0	0
ENSG00000181965	NEUROG1	0	0	0	0	0	0	0	0	0	0	0
ENSG00000145826	LECT2	0	0	0	0	0	0	0	0	0	0	0
ENSG00000271824	SMIM32	0	0	0	0	0	0	0	0	0	0	0
ENSG00000061492	WNT8A	0	0	0	0	0	0	0	0	0	0	0
ENSG00000280987	MATR3	0	0	0	0	0	0	0	0	0	0	0
ENSG00000279686	AC142391.1	0	0	0	0	0	0	0	0	0	0	0
ENSG00000249751	ECSCR	0	0	0	0	0	0	0	0	0	0	0
ENSG00000256453	DND1	0	0	0	0	0	0	0	0	0	0	0
ENSG00000204969	PCDHA2	0	0	0	0	0	0	0	0	0	0	0
ENSG00000204962	PCDHA8	0	0	0	0	0	0	0	0	0	0	0
ENSG00000243232	PCDHAC2	0	0	0	0	0	0	0	0	0	0	0
ENSG00000113205	PCDHB3	0	0	0	0	0	0	0	0	0	0	0
ENSG00000120322	PCDHB8	0	0	0	0	0	0	0	0	0	0	0
ENSG00000279983	AC244517.10	0	0	0	0	0	0	0	0	0	0	0
ENSG00000081853	PCDHGA2	0	0	0	0	0	0	0	0	0	0	0
ENSG00000254245	PCDHGA3	0	0	0	0	0	0	0	0	0	0	0
ENSG00000254221	PCDHGB1	0	0	0	0	0	0	0	0	0	0	0
ENSG00000240764	PCDHGC5	0	0	0	0	0	0	0	0	0	0	0
ENSG00000158497	HMHB1	0	0	0	0	0	0	0	0	0	0	0
ENSG00000091010	POU4F3	0	0	0	0	0	0	0	0	0	0	0
ENSG00000173250	GPR151	0	0	0	0	0	0	0	0	0	0	0
ENSG00000196800	SPINK14	0	0	0	0	0	0	0	0	0	0	0
ENSG00000178172	SPINK6	0	0	0	0	0	0	0	0	0	0	0
ENSG00000248109	MARCOL	0	0	0	0	0	0	0	0	0	0	0
ENSG00000214510	SPINK13	0	0	0	0	0	0	0	0	0	0	0
ENSG00000145879	SPINK7	0	0	0	0	0	0	0	0	0	0	0
ENSG00000113722	CDX1	0	0	0	0	0	0	0	0	0	0	0
ENSG00000113196	HAND1	0	0	0	0	0	0	0	0	0	0	0
ENSG00000170613	FAM71B	0	0	0	0	0	0	0	0	0	0	0
ENSG00000172568	FNDC9	0	0	0	0	0	0	0	0	0	0	0
ENSG00000170231	FABP6	0	0	0	0	0	0	0	0	0	0	0
ENSG00000214360	EFCAB9	0	0	0	0	0	0	0	0	0	0	0
ENSG00000182230	FAM153B	0	0	0	0	0	0	0	0	0	0	0
ENSG00000285476	AC139491.7	0	0	0	0	0	0	0	0	0	0	0
ENSG00000175766	EIF4E1B	0	0	0	0	0	0	0	0	0	0	0
ENSG00000175325	PROP1	0	0	0	0	0	0	0	0	0	0	0
ENSG00000285891	AC113348.1	0	0	0	0	0	0	0	0	0	0	0
ENSG00000285978	AC113348.2	0	0	0	0	0	0	0	0	0	0	0
ENSG00000174339	OR2Y1	0	0	0	0	0	0	0	0	0	0	0
ENSG00000230178	OR4F3	0	0	0	0	0	0	0	0	0	0	0
ENSG00000259040	BLOC1S5-TXNDC5	0	0	0	0	0	0	0	0	0	0	0
ENSG00000265818	EEF1E1-BLOC1S5	0	0	0	0	0	0	0	0	0	0	0
ENSG00000272162	AL024498.2	0	0	0	0	0	0	0	0	0	0	0
ENSG00000112273	HDGFL1	0	0	0	0	0	0	0	0	0	0	0
ENSG00000282804	AL512428.1	0	0	0	0	0	0	0	0	0	0	0
ENSG00000164508	HIST1H2AA	0	0	0	0	0	0	0	0	0	0	0
ENSG00000197409	HIST1H3D	0	0	0	0	0	0	0	0	0	0	0
ENSG00000124557	BTN1A1	0	0	0	0	0	0	0	0	0	0	0
ENSG00000275221	HIST1H2AK	0	0	0	0	0	0	0	0	0	0	0
ENSG00000124657	OR2B6	0	0	0	0	0	0	0	0	0	0	0
ENSG00000276302	AL021997.3	0	0	0	0	0	0	0	0	0	0	0
ENSG00000232040	ZBED9	0	0	0	0	0	0	0	0	0	0	0
ENSG00000204704	OR2W1	0	0	0	0	0	0	0	0	0	0	0
ENSG00000204703	OR2B3	0	0	0	0	0	0	0	0	0	0	0
ENSG00000204702	OR2J1	0	0	0	0	0	0	0	0	0	0	0
ENSG00000204701	OR2J3	0	0	0	0	0	0	0	0	0	0	0
ENSG00000204700	OR2J2	0	0	0	0	0	0	0	0	0	0	0
ENSG00000204695	OR14J1	0	0	0	0	0	0	0	0	0	0	0
ENSG00000243729	OR5V1	0	0	0	0	0	0	0	0	0	0	0
ENSG00000112462	OR12D3	0	0	0	0	0	0	0	0	0	0	0
ENSG00000280236	OR12D2	0	0	0	0	0	0	0	0	0	0	0
ENSG00000204694	OR11A1	0	0	0	0	0	0	0	0	0	0	0
ENSG00000206474	OR10C1	0	0	0	0	0	0	0	0	0	0	0
ENSG00000204688	OR2H1	0	0	0	0	0	0	0	0	0	0	0
ENSG00000204687	MAS1L	0	0	0	0	0	0	0	0	0	0	0
ENSG00000237988	OR2I1P	0	0	0	0	0	0	0	0	0	0	0
ENSG00000213886	UBD	0	0	0	0	0	0	0	0	0	0	0
ENSG00000204657	OR2H2	0	0	0	0	0	0	0	0	0	0	0
ENSG00000204655	MOG	0	0	0	0	0	0	0	0	0	0	0
ENSG00000204618	RNF39	0	0	0	0	0	0	0	0	0	0	0
ENSG00000204616	TRIM31	0	0	0	0	0	0	0	0	0	0	0
ENSG00000204614	TRIM40	0	0	0	0	0	0	0	0	0	0	0
ENSG00000204613	TRIM10	0	0	0	0	0	0	0	0	0	0	0
ENSG00000204610	TRIM15	0	0	0	0	0	0	0	0	0	0	0

ENSG00000204599	TRIM39	0	0	0	0	0	0	0	0	0	0	0
ENSG00000248167	TRIM39-RPP21	0	0	0	0	0	0	0	0	0	0	0
ENSG00000241370	RPP21	0	0	0	0	0	0	0	0	0	0	0
ENSG00000213780	GTF2H4	0	0	0	0	0	0	0	0	0	0	0
ENSG00000196260	SFTA2	0	0	0	0	0	0	0	0	0	0	0
ENSG00000204544	MUC21	0	0	0	0	0	0	0	0	0	0	0
ENSG00000261272	MUC22	0	0	0	0	0	0	0	0	0	0	0
ENSG00000204542	C6orf15	0	0	0	0	0	0	0	0	0	0	0
ENSG00000204540	PSORS1C1	0	0	0	0	0	0	0	0	0	0	0
ENSG00000204539	CDSN	0	0	0	0	0	0	0	0	0	0	0
ENSG00000204538	PSORS1C2	0	0	0	0	0	0	0	0	0	0	0
ENSG00000204531	POU5F1	0	0	0	0	0	0	0	0	0	0	0
ENSG00000204511	MCCD1	0	0	0	0	0	0	0	0	0	0	0
ENSG00000198563	DDX39B	0	0	0	0	0	0	0	0	0	0	0
ENSG00000254870	ATP6V1G2-DDX39B	0	0	0	0	0	0	0	0	0	0	0
ENSG00000213760	ATP6V1G2	0	0	0	0	0	0	0	0	0	0	0
ENSG00000204475	NCR3	0	0	0	0	0	0	0	0	0	0	0
ENSG00000204439	C6orf47	0	0	0	0	0	0	0	0	0	0	0
ENSG00000263020	AL662899.2	0	0	0	0	0	0	0	0	0	0	0
ENSG00000240053	LY6G5B	0	0	0	0	0	0	0	0	0	0	0
ENSG00000204427	ABHD16A	0	0	0	0	0	0	0	0	0	0	0
ENSG00000204422	AL662899.1	0	0	0	0	0	0	0	0	0	0	0
ENSG00000204424	LY6G6F	0	0	0	0	0	0	0	0	0	0	0
ENSG00000250641	LY6G6F-LY6G6D	0	0	0	0	0	0	0	0	0	0	0
ENSG00000255552	LY6G6E	0	0	0	0	0	0	0	0	0	0	0
ENSG00000244355	LY6G6D	0	0	0	0	0	0	0	0	0	0	0
ENSG00000204420	MPIG6B	0	0	0	0	0	0	0	0	0	0	0
ENSG00000204410	MSH5	0	0	0	0	0	0	0	0	0	0	0
ENSG00000255152	MSH5-SAPCD1	0	0	0	0	0	0	0	0	0	0	0
ENSG00000228727	SAPCD1	0	0	0	0	0	0	0	0	0	0	0
ENSG00000204396	VWA7	0	0	0	0	0	0	0	0	0	0	0
ENSG00000204388	HSPA1B	0	0	0	0	0	0	0	0	0	0	0
ENSG00000204385	SLC44A4	0	0	0	0	0	0	0	0	0	0	0
ENSG00000204366	ZBTB12	0	0	0	0	0	0	0	0	0	0	0
ENSG00000244255	AL645922.1	0	0	0	0	0	0	0	0	0	0	0
ENSG00000243649	CFB	0	0	0	0	0	0	0	0	0	0	0
ENSG00000244731	C4A	0	0	0	0	0	0	0	0	0	0	0
ENSG00000224389	C4B	0	0	0	0	0	0	0	0	0	0	0
ENSG00000231852	CYP21A2	0	0	0	0	0	0	0	0	0	0	0
ENSG00000168477	TNXB	0	0	0	0	0	0	0	0	0	0	0
ENSG00000204314	PRRT1	0	0	0	0	0	0	0	0	0	0	0
ENSG00000285085	AL662884.4	0	0	0	0	0	0	0	0	0	0	0
ENSG00000221988	PPT2	0	0	0	0	0	0	0	0	0	0	0
ENSG00000258388	PPT2-EGFL8	0	0	0	0	0	0	0	0	0	0	0
ENSG00000241404	EGFL8	0	0	0	0	0	0	0	0	0	0	0
ENSG00000204296	TSBP1	0	0	0	0	0	0	0	0	0	0	0
ENSG00000250264	AL669918.1	0	0	0	0	0	0	0	0	0	0	0
ENSG00000204267	TAP2	0	0	0	0	0	0	0	0	0	0	0
ENSG00000248993	AL645941.2	0	0	0	0	0	0	0	0	0	0	0
ENSG00000204248	COL11A2	0	0	0	0	0	0	0	0	0	0	0
ENSG00000235863	B3GALT4	0	0	0	0	0	0	0	0	0	0	0
ENSG00000236104	ZBTB22	0	0	0	0	0	0	0	0	0	0	0
ENSG00000285064	SMIM40	0	0	0	0	0	0	0	0	0	0	0
ENSG00000213588	ZBTB9	0	0	0	0	0	0	0	0	0	0	0
ENSG00000270800	RPS10-NUDT3	0	0	0	0	0	0	0	0	0	0	0
ENSG00000196748	CLPSL2	0	0	0	0	0	0	0	0	0	0	0
ENSG00000204140	CLPSL1	0	0	0	0	0	0	0	0	0	0	0
ENSG00000137392	CLPS	0	0	0	0	0	0	0	0	0	0	0
ENSG00000286105	AL353579.1	0	0	0	0	0	0	0	0	0	0	0
ENSG00000112559	MDFI	0	0	0	0	0	0	0	0	0	0	0
ENSG00000278224	PRICKLE4	0	0	0	0	0	0	0	0	0	0	0
ENSG00000214736	TOMM6	0	0	0	0	0	0	0	0	0	0	0
ENSG00000214732	AL096814.1	0	0	0	0	0	0	0	0	0	0	0
ENSG00000137204	SLC22A7	0	0	0	0	0	0	0	0	0	0	0
ENSG00000272442	AL353588.1	0	0	0	0	0	0	0	0	0	0	0
ENSG00000249481	SPATS1	0	0	0	0	0	0	0	0	0	0	0
ENSG00000112796	ENPP5	0	0	0	0	0	0	0	0	0	0	0
ENSG00000112818	MEP1A	0	0	0	0	0	0	0	0	0	0	0
ENSG00000203972	GLYATL3	0	0	0	0	0	0	0	0	0	0	0
ENSG00000124490	CRISP2	0	0	0	0	0	0	0	0	0	0	0
ENSG00000177684	DEFB114	0	0	0	0	0	0	0	0	0	0	0
ENSG00000203970	DEFB110	0	0	0	0	0	0	0	0	0	0	0
ENSG00000180872	DEFB112	0	0	0	0	0	0	0	0	0	0	0
ENSG00000112115	IL17A	0	0	0	0	0	0	0	0	0	0	0
ENSG00000244067	GSTA2	0	0	0	0	0	0	0	0	0	0	0
ENSG00000243955	GSTA1	0	0	0	0	0	0	0	0	0	0	0
ENSG00000174156	GSTA3	0	0	0	0	0	0	0	0	0	0	0
ENSG00000256980	KHDC1L	0	0	0	0	0	0	0	0	0	0	0
ENSG00000203908	KHDC3L	0	0	0	0	0	0	0	0	0	0	0
ENSG00000118402	ELOVL4	0	0	0	0	0	0	0	0	0	0	0
ENSG00000146250	PRSS35	0	0	0	0	0	0	0	0	0	0	0
ENSG00000135324	MRAP2	0	0	0	0	0	0	0	0	0	0	0
ENSG00000271793	AL589666.1	0	0	0	0	0	0	0	0	0	0	0
ENSG00000168830	HTR1E	0	0	0	0	0	0	0	0	0	0	0
ENSG00000213204	AL049697.1	0	0	0	0	0	0	0	0	0	0	0
ENSG00000272398	CD24	0	0	0	0	0	0	0	0	0	0	0
ENSG00000112333	NR2E1	0	0	0	0	0	0	0	0	0	0	0
ENSG00000053328	METTL24	0	0	0	0	0	0	0	0	0	0	0
ENSG00000281613	AL365214.3	0	0	0	0	0	0	0	0	0	0	0
ENSG00000285446	Z84488.2	0	0	0	0	0	0	0	0	0	0	0
ENSG00000183807	FAM162B	0	0	0	0	0	0	0	0	0	0	0
ENSG00000170162	VGLL2	0	0	0	0	0	0	0	0	0	0	0
ENSG00000282218	AL132671.2	0	0	0	0	0	0	0	0	0	0	0
ENSG00000255330	AL096711.2	0	0	0	0	0	0	0	0	0	0	0
ENSG00000214338	SOGA3	0	0	0	0	0	0	0	0	0	0	0
ENSG00000184530	C6orf58	0	0	0	0	0	0	0	0	0	0	0
ENSG00000172673	THEMIS	0	0	0	0	0	0	0	0	0	0	0
ENSG00000152894	PTPRK	0	0	0	0	0	0	0	0	0	0	0
ENSG00000203756	TMEM244	0	0	0	0	0	0	0	0	0	0	0
ENSG00000180658	OR2A4	0	0	0	0	0	0	0	0	0	0	0
ENSG00000237110	TAAR9	0	0	0	0	0	0	0	0	0	0	0
ENSG00000135569	TAAR5	0	0	0	0	0	0	0	0	0	0	0
ENSG00000182747	SLC35D3	0	0	0	0	0	0	0	0	0	0	0
ENSG00000262543	SMIM28	0	0	0	0	0	0	0	0	0	0	0
ENSG00000280148	AL049844.3	0	0	0	0	0	0	0	0	0	0	0
ENSG00000118491	ZC2HC1B	0	0	0	0	0	0	0	0	0	0	0
ENSG00000203722	RAET1G	0	0	0	0	0	0	0	0	0	0	0
ENSG00000155918	RAET1L	0	0	0	0	0	0	0	0	0	0	0
ENSG00000171217	CLDN20	0	0	0	0	0	0	0	0	0	0	0
ENSG00000074771	NOX3	0	0	0	0	0	0	0	0	0	0	0
ENSG00000146453	PNLDC1	0	0	0	0	0	0	0	0	0	0	0
ENSG00000112539	C6orf118	0	0	0	0	0	0	0	0	0	0	0
ENSG00000120436	GPR31	0	0	0	0	0	0	0	0	0	0	0
ENSG00000112562	SMOC2	0	0	0	0	0	0	0	0	0	0	0
ENSG00000285733	AL031315.1	0	0	0	0	0	0	0	0	0	0	0
ENSG00000184786	TCTE3	0	0	0	0	0	0	0	0	0	0	0
ENSG00000198719	DLL1	0	0	0	0	0	0	0	0	0	0	0

ENSG00000248767	AC187653.1	0	0	0	0	0	0	0	0	0	0	0
ENSG00000164853	UNCX	0	0	0	0	0	0	0	0	0	0	0
ENSG00000225968	ELFN1	0	0	0	0	0	0	0	0	0	0	0
ENSG00000286192	AC069288.1	0	0	0	0	0	0	0	0	0	0	0
ENSG00000275572	GRIFIN	0	0	0	0	0	0	0	0	0	0	0
ENSG00000272968	RBAK-RBAKDN	0	0	0	0	0	0	0	0	0	0	0
ENSG00000155026	RSPH10B	0	0	0	0	0	0	0	0	0	0	0
ENSG00000286075	AC009412.1	0	0	0	0	0	0	0	0	0	0	0
ENSG00000169402	RSPH10B2	0	0	0	0	0	0	0	0	0	0	0
ENSG00000283321	AC019117.3	0	0	0	0	0	0	0	0	0	0	0
ENSG00000146618	FERD3L	0	0	0	0	0	0	0	0	0	0	0
ENSG00000122585	NPY	0	0	0	0	0	0	0	0	0	0	0
ENSG00000105996	HOXA2	0	0	0	0	0	0	0	0	0	0	0
ENSG00000257184	AC004080.3	0	0	0	0	0	0	0	0	0	0	0
ENSG00000005073	HOXA11	0	0	0	0	0	0	0	0	0	0	0
ENSG00000281039	AC005154.5	0	0	0	0	0	0	0	0	0	0	0
ENSG00000250424	AC004691.2	0	0	0	0	0	0	0	0	0	0	0
ENSG00000106128	GHRHR	0	0	0	0	0	0	0	0	0	0	0
ENSG00000106341	PPP1R17	0	0	0	0	0	0	0	0	0	0	0
ENSG00000256646	AC010132.3	0	0	0	0	0	0	0	0	0	0	0
ENSG00000270617	URGCP-MRPS24	0	0	0	0	0	0	0	0	0	0	0
ENSG00000164708	PGAM2	0	0	0	0	0	0	0	0	0	0	0
ENSG00000122679	RAMP3	0	0	0	0	0	0	0	0	0	0	0
ENSG00000146678	IGFBP1	0	0	0	0	0	0	0	0	0	0	0
ENSG00000164744	SUN3	0	0	0	0	0	0	0	0	0	0	0
ENSG00000249773	AC092647.5	0	0	0	0	0	0	0	0	0	0	0
ENSG00000185290	NUPR2	0	0	0	0	0	0	0	0	0	0	0
ENSG00000249319	AC068533.4	0	0	0	0	0	0	0	0	0	0	0
ENSG00000155428	TRIM74	0	0	0	0	0	0	0	0	0	0	0
ENSG00000273520	SPDYE8P	0	0	0	0	0	0	0	0	0	0	0
ENSG00000286228	SPDYE17	0	0	0	0	0	0	0	0	0	0	0
ENSG00000262461	SPDYE9P	0	0	0	0	0	0	0	0	0	0	0
ENSG00000286038	AC211486.7	0	0	0	0	0	0	0	0	0	0	0
ENSG00000286137	AC211486.8	0	0	0	0	0	0	0	0	0	0	0
ENSG00000286014	AC211486.6	0	0	0	0	0	0	0	0	0	0	0
ENSG00000006606	CCL26	0	0	0	0	0	0	0	0	0	0	0
ENSG00000243566	UPK3B	0	0	0	0	0	0	0	0	0	0	0
ENSG00000185040	SPDYE16	0	0	0	0	0	0	0	0	0	0	0
ENSG00000214415	GNAT3	0	0	0	0	0	0	0	0	0	0	0
ENSG00000164645	TEX47	0	0	0	0	0	0	0	0	0	0	0
ENSG00000285953	AC000120.3	0	0	0	0	0	0	0	0	0	0	0
ENSG00000105880	DLX5	0	0	0	0	0	0	0	0	0	0	0
ENSG00000135175	OCM2	0	0	0	0	0	0	0	0	0	0	0
ENSG00000180535	BHLHA15	0	0	0	0	0	0	0	0	0	0	0
ENSG00000248919	ATP5MF-PTCD1	0	0	0	0	0	0	0	0	0	0	0
ENSG00000272647	AC005020.2	0	0	0	0	0	0	0	0	0	0	0
ENSG00000244219	TMEM225B	0	0	0	0	0	0	0	0	0	0	0
ENSG00000282301	CYP3A7-CYP3A51P	0	0	0	0	0	0	0	0	0	0	0
ENSG00000244623	OR2AE1	0	0	0	0	0	0	0	0	0	0	0
ENSG00000213413	PVRIG	0	0	0	0	0	0	0	0	0	0	0
ENSG00000185955	C7orf61	0	0	0	0	0	0	0	0	0	0	0
ENSG00000205307	SAP25	0	0	0	0	0	0	0	0	0	0	0
ENSG00000284981	AC093668.2	0	0	0	0	0	0	0	0	0	0	0
ENSG00000270249	AC093668.1	0	0	0	0	0	0	0	0	0	0	0
ENSG00000205238	SPDYE2	0	0	0	0	0	0	0	0	0	0	0
ENSG00000205236	AC105052.1	0	0	0	0	0	0	0	0	0	0	0
ENSG00000267368	UPK3BL1	0	0	0	0	0	0	0	0	0	0	0
ENSG00000267645	AC105052.3	0	0	0	0	0	0	0	0	0	0	0
ENSG00000173678	SPDYE2B	0	0	0	0	0	0	0	0	0	0	0
ENSG00000128610	FEZF1	0	0	0	0	0	0	0	0	0	0	0
ENSG00000188050	RNF133	0	0	0	0	0	0	0	0	0	0	0
ENSG00000128519	TAS2R16	0	0	0	0	0	0	0	0	0	0	0
ENSG00000234224	TMEM229A	0	0	0	0	0	0	0	0	0	0	0
ENSG00000230268	SSU72P8	0	0	0	0	0	0	0	0	0	0	0
ENSG00000275356	C7orf77	0	0	0	0	0	0	0	0	0	0	0
ENSG00000174697	LEP	0	0	0	0	0	0	0	0	0	0	0
ENSG00000128617	OPN1SW	0	0	0	0	0	0	0	0	0	0	0
ENSG00000267697	LUZP6	0	0	0	0	0	0	0	0	0	0	0
ENSG00000105894	PTN	0	0	0	0	0	0	0	0	0	0	0
ENSG00000269955	FMC1-LUC7L2	0	0	0	0	0	0	0	0	0	0	0
ENSG00000214102	WEE2	0	0	0	0	0	0	0	0	0	0	0
ENSG00000127366	TAS2R5	0	0	0	0	0	0	0	0	0	0	0
ENSG00000257743	MGAM2	0	0	0	0	0	0	0	0	0	0	0
ENSG00000258223	PRSS58	0	0	0	0	0	0	0	0	0	0	0
ENSG00000204983	PRSS1	0	0	0	0	0	0	0	0	0	0	0
ENSG00000165131	LLCFC1	0	0	0	0	0	0	0	0	0	0	0
ENSG00000197993	KEL	0	0	0	0	0	0	0	0	0	0	0
ENSG00000179468	OR9A2	0	0	0	0	0	0	0	0	0	0	0
ENSG00000225781	OR6V1	0	0	0	0	0	0	0	0	0	0	0
ENSG00000159763	PIP	0	0	0	0	0	0	0	0	0	0	0
ENSG00000221855	TAS2R41	0	0	0	0	0	0	0	0	0	0	0
ENSG00000271079	CTAGE15	0	0	0	0	0	0	0	0	0	0	0
ENSG00000283528	TCAF2C	0	0	0	0	0	0	0	0	0	0	0
ENSG00000221938	OR2A14	0	0	0	0	0	0	0	0	0	0	0
ENSG00000225932	CTAGE4	0	0	0	0	0	0	0	0	0	0	0
ENSG00000213214	ARHGEF35	0	0	0	0	0	0	0	0	0	0	0
ENSG00000243896	OR2A7	0	0	0	0	0	0	0	0	0	0	0
ENSG00000244693	CTAGE8	0	0	0	0	0	0	0	0	0	0	0
ENSG00000106538	RARRES2	0	0	0	0	0	0	0	0	0	0	0
ENSG00000281887	GIMAP1-GIMAP5	0	0	0	0	0	0	0	0	0	0	0
ENSG00000285480	AC021097.2	0	0	0	0	0	0	0	0	0	0	0
ENSG00000285292	ABCF2	0	0	0	0	0	0	0	0	0	0	0
ENSG00000176269	OR4F21	0	0	0	0	0	0	0	0	0	0	0
ENSG00000283239	AC019257.8	0	0	0	0	0	0	0	0	0	0	0
ENSG00000104728	ARHGEF10	0	0	0	0	0	0	0	0	0	0	0
ENSG00000176595	KBTBD11	0	0	0	0	0	0	0	0	0	0	0
ENSG00000164822	DEFA6	0	0	0	0	0	0	0	0	0	0	0
ENSG00000206047	DEFA1	0	0	0	0	0	0	0	0	0	0	0
ENSG00000240247	DEFA1B	0	0	0	0	0	0	0	0	0	0	0
ENSG00000239839	DEFA3	0	0	0	0	0	0	0	0	0	0	0
ENSG00000164816	DEFA5	0	0	0	0	0	0	0	0	0	0	0
ENSG00000230549	USP17L1	0	0	0	0	0	0	0	0	0	0	0
ENSG00000236125	USP17L4	0	0	0	0	0	0	0	0	0	0	0
ENSG00000177257	DEFB4B	0	0	0	0	0	0	0	0	0	0	0
ENSG00000177243	DEFB103B	0	0	0	0	0	0	0	0	0	0	0
ENSG00000164871	SPAG11B	0	0	0	0	0	0	0	0	0	0	0
ENSG00000177023	DEFB104B	0	0	0	0	0	0	0	0	0	0	0
ENSG00000187082	DEFB106B	0	0	0	0	0	0	0	0	0	0	0
ENSG00000186599	DEFB105B	0	0	0	0	0	0	0	0	0	0	0
ENSG00000198129	DEFB107B	0	0	0	0	0	0	0	0	0	0	0
ENSG00000255251	PRR23D1	0	0	0	0	0	0	0	0	0	0	0
ENSG00000285975	AC134684.11	0	0	0	0	0	0	0	0	0	0	0
ENSG00000285687	AC134684.8	0	0	0	0	0	0	0	0	0	0	0
ENSG00000285765	AC134684.9	0	0	0	0	0	0	0	0	0	0	0
ENSG00000285814	AC084121.9	0	0	0	0	0	0	0	0	0	0	0
ENSG00000285913	AC084121.11	0	0	0	0	0	0	0	0	0	0	0
ENSG00000285620	AC084121.6	0	0	0	0	0	0	0	0	0	0	0

ENSG00000285937	AC084121.12	0	0	0	0	0	0	0	0	0	0	0
ENSG00000285720	AC084121.8	0	0	0	0	0	0	0	0	0	0	0
ENSG00000285657	AC084121.7	0	0	0	0	0	0	0	0	0	0	0
ENSG00000285607	AC084121.5	0	0	0	0	0	0	0	0	0	0	0
ENSG00000285950	AC084121.13	0	0	0	0	0	0	0	0	0	0	0
ENSG00000255378	PRR23D2	0	0	0	0	0	0	0	0	0	0	0
ENSG00000186572	DEFB107A	0	0	0	0	0	0	0	0	0	0	0
ENSG00000186562	DEFB105A	0	0	0	0	0	0	0	0	0	0	0
ENSG00000186579	DEFB106A	0	0	0	0	0	0	0	0	0	0	0
ENSG00000176782	DEFB104A	0	0	0	0	0	0	0	0	0	0	0
ENSG00000178287	SPAG11A	0	0	0	0	0	0	0	0	0	0	0
ENSG00000176797	DEFB103A	0	0	0	0	0	0	0	0	0	0	0
ENSG00000215356	ZNF705B	0	0	0	0	0	0	0	0	0	0	0
ENSG00000237038	USP17L8	0	0	0	0	0	0	0	0	0	0	0
ENSG00000225327	USP17L3	0	0	0	0	0	0	0	0	0	0	0
ENSG00000253649	PRSS51	0	0	0	0	0	0	0	0	0	0	0
ENSG00000171056	SOX7	0	0	0	0	0	0	0	0	0	0	0
ENSG00000258724	PINX1	0	0	0	0	0	0	0	0	0	0	0
ENSG00000205884	DEFB136	0	0	0	0	0	0	0	0	0	0	0
ENSG00000205883	DEFB135	0	0	0	0	0	0	0	0	0	0	0
ENSG00000205882	DEFB134	0	0	0	0	0	0	0	0	0	0	0
ENSG00000233050	DEFB130B	0	0	0	0	0	0	0	0	0	0	0
ENSG00000215343	ZNF705D	0	0	0	0	0	0	0	0	0	0	0
ENSG00000223443	USP17L2	0	0	0	0	0	0	0	0	0	0	0
ENSG00000232948	DEFB130A	0	0	0	0	0	0	0	0	0	0	0
ENSG00000104213	PDGFRL	0	0	0	0	0	0	0	0	0	0	0
ENSG00000156006	NAT2	0	0	0	0	0	0	0	0	0	0	0
ENSG00000248235	AC037459.1	0	0	0	0	0	0	0	0	0	0	0
ENSG00000284956	AC107959.5	0	0	0	0	0	0	0	0	0	0	0
ENSG00000180053	NKX2-6	0	0	0	0	0	0	0	0	0	0	0
ENSG00000172733	PURG	0	0	0	0	0	0	0	0	0	0	0
ENSG00000286131	AC083977.1	0	0	0	0	0	0	0	0	0	0	0
ENSG00000285880	AC144573.1	0	0	0	0	0	0	0	0	0	0	0
ENSG00000104755	ADAM2	0	0	0	0	0	0	0	0	0	0	0
ENSG00000176907	TCIM	0	0	0	0	0	0	0	0	0	0	0
ENSG00000254673	AC110275.1	0	0	0	0	0	0	0	0	0	0	0
ENSG00000196711	ALKAL1	0	0	0	0	0	0	0	0	0	0	0
ENSG00000172680	MOS	0	0	0	0	0	0	0	0	0	0	0
ENSG00000147570	DNAJC5B	0	0	0	0	0	0	0	0	0	0	0
ENSG00000285655	AC009879.2	0	0	0	0	0	0	0	0	0	0	0
ENSG00000169085	VXN	0	0	0	0	0	0	0	0	0	0	0
ENSG00000147596	PRDM14	0	0	0	0	0	0	0	0	0	0	0
ENSG00000258677	AC022826.2	0	0	0	0	0	0	0	0	0	0	0
ENSG00000276418	AC036214.3	0	0	0	0	0	0	0	0	0	0	0
ENSG00000205186	FABP9	0	0	0	0	0	0	0	0	0	0	0
ENSG00000170323	FABP4	0	0	0	0	0	0	0	0	0	0	0
ENSG00000147613	PSKH2	0	0	0	0	0	0	0	0	0	0	0
ENSG00000253250	C8orf88	0	0	0	0	0	0	0	0	0	0	0
ENSG00000174226	SNX31	0	0	0	0	0	0	0	0	0	0	0
ENSG00000285982	AC012213.5	0	0	0	0	0	0	0	0	0	0	0
ENSG00000174429	ABRA	0	0	0	0	0	0	0	0	0	0	0
ENSG00000205002	AARD	0	0	0	0	0	0	0	0	0	0	0
ENSG00000136999	CCN3	0	0	0	0	0	0	0	0	0	0	0
ENSG00000253117	OC90	0	0	0	0	0	0	0	0	0	0	0
ENSG00000169427	KCNK9	0	0	0	0	0	0	0	0	0	0	0
ENSG00000204882	GPR20	0	0	0	0	0	0	0	0	0	0	0
ENSG00000184489	PTP4A3	0	0	0	0	0	0	0	0	0	0	0
ENSG00000226490	AC138647.1	0	0	0	0	0	0	0	0	0	0	0
ENSG00000198576	ARC	0	0	0	0	0	0	0	0	0	0	0
ENSG00000167653	PSCA	0	0	0	0	0	0	0	0	0	0	0
ENSG00000126233	SLURP1	0	0	0	0	0	0	0	0	0	0	0
ENSG00000283992	SLURP2	0	0	0	0	0	0	0	0	0	0	0
ENSG00000167656	LY6D	0	0	0	0	0	0	0	0	0	0	0
ENSG00000261667	LY6L	0	0	0	0	0	0	0	0	0	0	0
ENSG00000176956	LY6H	0	0	0	0	0	0	0	0	0	0	0
ENSG00000264668	AC138696.1	0	0	0	0	0	0	0	0	0	0	0
ENSG00000179886	TIGD5	0	0	0	0	0	0	0	0	0	0	0
ENSG00000255181	CCDC166	0	0	0	0	0	0	0	0	0	0	0
ENSG00000181085	MAPK15	0	0	0	0	0	0	0	0	0	0	0
ENSG00000180921	FAM83H	0	0	0	0	0	0	0	0	0	0	0
ENSG00000261587	TMEM249	0	0	0	0	0	0	0	0	0	0	0
ENSG00000254402	LRRC24	0	0	0	0	0	0	0	0	0	0	0
ENSG00000170122	FOXD4	0	0	0	0	0	0	0	0	0	0	0
ENSG00000107018	RLN1	0	0	0	0	0	0	0	0	0	0	0
ENSG00000153714	LURAP1L	0	0	0	0	0	0	0	0	0	0	0
ENSG00000147869	CER1	0	0	0	0	0	0	0	0	0	0	0
ENSG00000171855	IFNB1	0	0	0	0	0	0	0	0	0	0	0
ENSG00000137080	IFNA21	0	0	0	0	0	0	0	0	0	0	0
ENSG00000236637	IFNA4	0	0	0	0	0	0	0	0	0	0	0
ENSG00000214042	IFNA7	0	0	0	0	0	0	0	0	0	0	0
ENSG00000186803	IFNA10	0	0	0	0	0	0	0	0	0	0	0
ENSG00000234829	IFNA17	0	0	0	0	0	0	0	0	0	0	0
ENSG00000228083	IFNA14	0	0	0	0	0	0	0	0	0	0	0
ENSG00000147873	IFNA5	0	0	0	0	0	0	0	0	0	0	0
ENSG00000120235	IFNA6	0	0	0	0	0	0	0	0	0	0	0
ENSG00000233816	IFNA13	0	0	0	0	0	0	0	0	0	0	0
ENSG00000188379	IFNA2	0	0	0	0	0	0	0	0	0	0	0
ENSG00000197919	IFNA1	0	0	0	0	0	0	0	0	0	0	0
ENSG00000184995	IFNE	0	0	0	0	0	0	0	0	0	0	0
ENSG00000264545	AL359922.1	0	0	0	0	0	0	0	0	0	0	0
ENSG00000205442	IZUMO3	0	0	0	0	0	0	0	0	0	0	0
ENSG00000147896	IFNK	0	0	0	0	0	0	0	0	0	0	0
ENSG00000010438	PRSS3	0	0	0	0	0	0	0	0	0	0	0
ENSG00000164972	C9orf24	0	0	0	0	0	0	0	0	0	0	0
ENSG00000122756	CNTFR	0	0	0	0	0	0	0	0	0	0	0
ENSG00000258728	AL162231.3	0	0	0	0	0	0	0	0	0	0	0
ENSG00000213927	CCL27	0	0	0	0	0	0	0	0	0	0	0
ENSG00000172724	CCL19	0	0	0	0	0	0	0	0	0	0	0
ENSG00000137077	CCL21	0	0	0	0	0	0	0	0	0	0	0
ENSG00000107159	CA9	0	0	0	0	0	0	0	0	0	0	0
ENSG00000215183	MSMP	0	0	0	0	0	0	0	0	0	0	0
ENSG00000285645	AL133410.3	0	0	0	0	0	0	0	0	0	0	0
ENSG00000196196	HRCT1	0	0	0	0	0	0	0	0	0	0	0
ENSG00000278889	OR2S2	0	0	0	0	0	0	0	0	0	0	0
ENSG00000256966	AL513165.2	0	0	0	0	0	0	0	0	0	0	0
ENSG00000283486	FAM95C	0	0	0	0	0	0	0	0	0	0	0
ENSG00000204849	SPATA31A1	0	0	0	0	0	0	0	0	0	0	0
ENSG00000273514	FOXD4L6	0	0	0	0	0	0	0	0	0	0	0
ENSG00000185775	SPATA31A6	0	0	0	0	0	0	0	0	0	0	0
ENSG00000276581	SPATA31A5	0	0	0	0	0	0	0	0	0	0	0
ENSG00000276040	SPATA31A7	0	0	0	0	0	0	0	0	0	0	0
ENSG00000283378	CNTNAP3C	0	0	0	0	0	0	0	0	0	0	0
ENSG00000170152	AL391987.1	0	0	0	0	0	0	0	0	0	0	0
ENSG00000204779	FOXD4L5	0	0	0	0	0	0	0	0	0	0	0
ENSG00000276203	ANKRD20A3	0	0	0	0	0	0	0	0	0	0	0
ENSG00000275969	SPATA31A3	0	0	0	0	0	0	0	0	0	0	0
ENSG00000286079	AL627230.3	0	0	0	0	0	0	0	0	0	0	0

ENSG00000275493	AL627230.1	0	0	0	0	0	0	0	0	0	0	0
ENSG00000187559	FOXD4L3	0	0	0	0	0	0	0	0	0	0	0
ENSG00000181778	TMEM252	0	0	0	0	0	0	0	0	0	0	0
ENSG00000204612	FOXB2	0	0	0	0	0	0	0	0	0	0	0
ENSG00000127083	OMD	0	0	0	0	0	0	0	0	0	0	0
ENSG00000204352	C9orf129	0	0	0	0	0	0	0	0	0	0	0
ENSG00000130950	NUTM2F	0	0	0	0	0	0	0	0	0	0	0
ENSG00000130948	HSD17B3	0	0	0	0	0	0	0	0	0	0	0
ENSG00000285269	AL160269.1	0	0	0	0	0	0	0	0	0	0	0
ENSG00000251349	MSANTD3-TMEFF1	0	0	0	0	0	0	0	0	0	0	0
ENSG00000241697	TMEFF1	0	0	0	0	0	0	0	0	0	0	0
ENSG00000148123	PLPPR1	0	0	0	0	0	0	0	0	0	0	0
ENSG00000136881	BAAT	0	0	0	0	0	0	0	0	0	0	0
ENSG00000186881	OR13F1	0	0	0	0	0	0	0	0	0	0	0
ENSG00000148136	OR13C4	0	0	0	0	0	0	0	0	0	0	0
ENSG00000204246	OR13C3	0	0	0	0	0	0	0	0	0	0	0
ENSG00000136839	OR13C9	0	0	0	0	0	0	0	0	0	0	0
ENSG00000179055	OR13D1	0	0	0	0	0	0	0	0	0	0	0
ENSG00000157654	PALM2-AKAP2	0	0	0	0	0	0	0	0	0	0	0
ENSG00000204193	TXNDC8	0	0	0	0	0	0	0	0	0	0	0
ENSG00000171133	OR2K2	0	0	0	0	0	0	0	0	0	0	0
ENSG00000244115	DNAJC25-GNG10	0	0	0	0	0	0	0	0	0	0	0
ENSG00000229314	ORM1	0	0	0	0	0	0	0	0	0	0	0
ENSG00000285082	AL160272.2	0	0	0	0	0	0	0	0	0	0	0
ENSG00000197233	OR1J2	0	0	0	0	0	0	0	0	0	0	0
ENSG00000171505	OR1N1	0	0	0	0	0	0	0	0	0	0	0
ENSG00000280094	OR1B1	0	0	0	0	0	0	0	0	0	0	0
ENSG00000148215	OR5C1	0	0	0	0	0	0	0	0	0	0	0
ENSG00000188394	GPR21	0	0	0	0	0	0	0	0	0	0	0
ENSG00000136918	WDR38	0	0	0	0	0	0	0	0	0	0	0
ENSG00000251184	AL672142.1	0	0	0	0	0	0	0	0	0	0	0
ENSG00000167157	PRRX2	0	0	0	0	0	0	0	0	0	0	0
ENSG00000130720	FIBCD1	0	0	0	0	0	0	0	0	0	0	0
ENSG00000283526	PRRT1B	0	0	0	0	0	0	0	0	0	0	0
ENSG00000285245	AL162417.1	0	0	0	0	0	0	0	0	0	0	0
ENSG00000171102	OBP2B	0	0	0	0	0	0	0	0	0	0	0
ENSG00000187616	MYMK	0	0	0	0	0	0	0	0	0	0	0
ENSG00000196990	FAM163B	0	0	0	0	0	0	0	0	0	0	0
ENSG00000122133	PAEP	0	0	0	0	0	0	0	0	0	0	0
ENSG00000236543	AL354761.1	0	0	0	0	0	0	0	0	0	0	0
ENSG00000204007	GLT6D1	0	0	0	0	0	0	0	0	0	0	0
ENSG00000165643	SOHLH1	0	0	0	0	0	0	0	0	0	0	0
ENSG00000107187	LHX3	0	0	0	0	0	0	0	0	0	0	0
ENSG00000187922	LCN10	0	0	0	0	0	0	0	0	0	0	0
ENSG00000204003	AL355987.1	0	0	0	0	0	0	0	0	0	0	0
ENSG00000267206	LCN6	0	0	0	0	0	0	0	0	0	0	0
ENSG00000272896	AL355987.3	0	0	0	0	0	0	0	0	0	0	0
ENSG00000184925	LCN12	0	0	0	0	0	0	0	0	0	0	0
ENSG00000284341	AL807752.7	0	0	0	0	0	0	0	0	0	0	0
ENSG00000214402	LCNL1	0	0	0	0	0	0	0	0	0	0	0
ENSG00000279073	AL807752.6	0	0	0	0	0	0	0	0	0	0	0
ENSG00000261793	AL929554.1	0	0	0	0	0	0	0	0	0	0	0
ENSG00000233198	RNF224	0	0	0	0	0	0	0	0	0	0	0
ENSG00000198569	SLC34A3	0	0	0	0	0	0	0	0	0	0	0
ENSG00000197768	STPG3	0	0	0	0	0	0	0	0	0	0	0
ENSG00000282246	AL157392.5	0	0	0	0	0	0	0	0	0	0	0
ENSG00000204683	C10orf113	0	0	0	0	0	0	0	0	0	0	0
ENSG00000223601	EBLN1	0	0	0	0	0	0	0	0	0	0	0
ENSG00000269897	COMMD3-BMI1	0	0	0	0	0	0	0	0	0	0	0
ENSG00000168267	PTF1A	0	0	0	0	0	0	0	0	0	0	0
ENSG00000120563	LYZL1	0	0	0	0	0	0	0	0	0	0	0
ENSG00000151033	LYZL2	0	0	0	0	0	0	0	0	0	0	0
ENSG00000150201	FXYD4	0	0	0	0	0	0	0	0	0	0	0
ENSG00000277288	C10orf142	0	0	0	0	0	0	0	0	0	0	0
ENSG00000264230	ANXA8L1	0	0	0	0	0	0	0	0	0	0	0
ENSG00000204174	NPY4R	0	0	0	0	0	0	0	0	0	0	0
ENSG00000265203	RBP3	0	0	0	0	0	0	0	0	0	0	0
ENSG00000265190	ANXA8	0	0	0	0	0	0	0	0	0	0	0
ENSG00000189090	FAM25G	0	0	0	0	0	0	0	0	0	0	0
ENSG00000276430	FAM25C	0	0	0	0	0	0	0	0	0	0	0
ENSG00000172538	FAM170B	0	0	0	0	0	0	0	0	0	0	0
ENSG00000107984	DKK1	0	0	0	0	0	0	0	0	0	0	0
ENSG00000183346	CABCOCO1	0	0	0	0	0	0	0	0	0	0	0
ENSG00000285551	AC067752.1	0	0	0	0	0	0	0	0	0	0	0
ENSG00000179774	ATOH7	0	0	0	0	0	0	0	0	0	0	0
ENSG00000138308	PLA2G12B	0	0	0	0	0	0	0	0	0	0	0
ENSG00000188199	NUTM2B	0	0	0	0	0	0	0	0	0	0	0
ENSG00000228570	NUTM2E	0	0	0	0	0	0	0	0	0	0	0
ENSG00000188373	C10orf99	0	0	0	0	0	0	0	0	0	0	0
ENSG00000148602	LRIT1	0	0	0	0	0	0	0	0	0	0	0
ENSG00000122375	OPN4	0	0	0	0	0	0	0	0	0	0	0
ENSG00000148671	ADIRF	0	0	0	0	0	0	0	0	0	0	0
ENSG00000188100	FAM25A	0	0	0	0	0	0	0	0	0	0	0
ENSG00000184923	NUTM2A	0	0	0	0	0	0	0	0	0	0	0
ENSG00000182333	LIPF	0	0	0	0	0	0	0	0	0	0	0
ENSG00000276490	AL583836.1	0	0	0	0	0	0	0	0	0	0	0
ENSG00000270099	AL365273.2	0	0	0	0	0	0	0	0	0	0	0
ENSG00000249967	AL355315.1	0	0	0	0	0	0	0	0	0	0	0
ENSG00000203942	C10orf62	0	0	0	0	0	0	0	0	0	0	0
ENSG00000255339	AL133352.1	0	0	0	0	0	0	0	0	0	0	0
ENSG00000075891	PAX2	0	0	0	0	0	0	0	0	0	0	0
ENSG00000107807	TLX1	0	0	0	0	0	0	0	0	0	0	0
ENSG00000107859	PITX3	0	0	0	0	0	0	0	0	0	0	0
ENSG00000148795	CYP17A1	0	0	0	0	0	0	0	0	0	0	0
ENSG00000270316	BORCS7-ASMT	0	0	0	0	0	0	0	0	0	0	0
ENSG00000214435	AS3MT	0	0	0	0	0	0	0	0	0	0	0
ENSG00000183128	CALHM3	0	0	0	0	0	0	0	0	0	0	0
ENSG00000165646	SLC18A2	0	0	0	0	0	0	0	0	0	0	0
ENSG00000254636	ARMS2	0	0	0	0	0	0	0	0	0	0	0
ENSG00000286135	AL603764.2	0	0	0	0	0	0	0	0	0	0	0
ENSG00000286088	AC073585.2	0	0	0	0	0	0	0	0	0	0	0
ENSG00000188620	HMX3	0	0	0	0	0	0	0	0	0	0	0
ENSG00000188816	HMX2	0	0	0	0	0	0	0	0	0	0	0
ENSG00000175018	TEX36	0	0	0	0	0	0	0	0	0	0	0
ENSG00000214285	NPS	0	0	0	0	0	0	0	0	0	0	0
ENSG00000283496	ZNF511-PRAP1	0	0	0	0	0	0	0	0	0	0	0
ENSG00000203772	SPRN	0	0	0	0	0	0	0	0	0	0	0
ENSG00000225899	FRG2B	0	0	0	0	0	0	0	0	0	0	0
ENSG00000188076	SCGB1C1	0	0	0	0	0	0	0	0	0	0	0
ENSG00000206013	IFITM5	0	0	0	0	0	0	0	0	0	0	0
ENSG00000161328	LRRC56	0	0	0	0	0	0	0	0	0	0	0
ENSG00000099834	CDHR5	0	0	0	0	0	0	0	0	0	0	0
ENSG00000070031	SCT	0	0	0	0	0	0	0	0	0	0	0
ENSG00000069696	DRD4	0	0	0	0	0	0	0	0	0	0	0
ENSG00000274897	PANO1	0	0	0	0	0	0	0	0	0	0	0
ENSG00000184956	MUC6	0	0	0	0	0	0	0	0	0	0	0
ENSG00000198788	MUC2	0	0	0	0	0	0	0	0	0	0	0

ENSG00000205869	KRTAP5-1	0	0	0	0	0	0	0	0	0	0	0
ENSG00000205867	KRTAP5-2	0	0	0	0	0	0	0	0	0	0	0
ENSG00000196224	KRTAP5-3	0	0	0	0	0	0	0	0	0	0	0
ENSG00000241598	KRTAP5-4	0	0	0	0	0	0	0	0	0	0	0
ENSG00000185940	KRTAP5-5	0	0	0	0	0	0	0	0	0	0	0
ENSG00000205864	KRTAP5-6	0	0	0	0	0	0	0	0	0	0	0
ENSG00000250644	AC068580.4	0	0	0	0	0	0	0	0	0	0	0
ENSG00000149043	SYT8	0	0	0	0	0	0	0	0	0	0	0
ENSG00000167244	IGF2	0	0	0	0	0	0	0	0	0	0	0
ENSG00000284779	AC132217.2	0	0	0	0	0	0	0	0	0	0	0
ENSG00000129965	INS-IGF2	0	0	0	0	0	0	0	0	0	0	0
ENSG00000254647	INS	0	0	0	0	0	0	0	0	0	0	0
ENSG00000182170	MRGPRG	0	0	0	0	0	0	0	0	0	0	0
ENSG00000184350	MRGPRE	0	0	0	0	0	0	0	0	0	0	0
ENSG00000129744	ART1	0	0	0	0	0	0	0	0	0	0	0
ENSG00000284018	SSU72P5	0	0	0	0	0	0	0	0	0	0	0
ENSG00000284306	SSU72P2	0	0	0	0	0	0	0	0	0	0	0
ENSG00000283873	SSU72P4	0	0	0	0	0	0	0	0	0	0	0
ENSG00000284438	SSU72P7	0	0	0	0	0	0	0	0	0	0	0
ENSG00000232268	OR52I1	0	0	0	0	0	0	0	0	0	0	0
ENSG00000197674	OR51C1P	0	0	0	0	0	0	0	0	0	0	0
ENSG00000280021	OR51F1	0	0	0	0	0	0	0	0	0	0	0
ENSG00000279270	OR52R1	0	0	0	0	0	0	0	0	0	0	0
ENSG00000176925	OR51F2	0	0	0	0	0	0	0	0	0	0	0
ENSG00000176922	OR51S1	0	0	0	0	0	0	0	0	0	0	0
ENSG00000176895	OR51A7	0	0	0	0	0	0	0	0	0	0	0
ENSG00000205497	OR51A4	0	0	0	0	0	0	0	0	0	0	0
ENSG00000205496	OR51A2	0	0	0	0	0	0	0	0	0	0	0
ENSG00000176742	OR51V1	0	0	0	0	0	0	0	0	0	0	0
ENSG00000213934	HBG1	0	0	0	0	0	0	0	0	0	0	0
ENSG00000284931	AC104389.5	0	0	0	0	0	0	0	0	0	0	0
ENSG00000196565	HBG2	0	0	0	0	0	0	0	0	0	0	0
ENSG00000279012	OR51B2	0	0	0	0	0	0	0	0	0	0	0
ENSG00000176239	OR51B6	0	0	0	0	0	0	0	0	0	0	0
ENSG00000167360	OR51Q1	0	0	0	0	0	0	0	0	0	0	0
ENSG00000167359	OR51I1	0	0	0	0	0	0	0	0	0	0	0
ENSG00000181609	OR52D1	0	0	0	0	0	0	0	0	0	0	0
ENSG00000175520	UBQLN3	0	0	0	0	0	0	0	0	0	0	0
ENSG00000175518	UBQLNL	0	0	0	0	0	0	0	0	0	0	0
ENSG00000258588	TRIM6-TRIM34	0	0	0	0	0	0	0	0	0	0	0
ENSG00000181023	OR56B1	0	0	0	0	0	0	0	0	0	0	0
ENSG00000181009	OR52N5	0	0	0	0	0	0	0	0	0	0	0
ENSG00000205409	OR52E6	0	0	0	0	0	0	0	0	0	0	0
ENSG00000183269	OR52E8	0	0	0	0	0	0	0	0	0	0	0
ENSG00000188691	OR56A5	0	0	0	0	0	0	0	0	0	0	0
ENSG00000255307	OR52B2	0	0	0	0	0	0	0	0	0	0	0
ENSG00000175485	OR52W1	0	0	0	0	0	0	0	0	0	0	0
ENSG00000180878	C11orf42	0	0	0	0	0	0	0	0	0	0	0
ENSG00000283977	AC084337.2	0	0	0	0	0	0	0	0	0	0	0
ENSG00000166363	OR10A5	0	0	0	0	0	0	0	0	0	0	0
ENSG00000166368	OR2D2	0	0	0	0	0	0	0	0	0	0	0
ENSG00000170748	RBMXL2	0	0	0	0	0	0	0	0	0	0	0
ENSG00000183303	OR5P2	0	0	0	0	0	0	0	0	0	0	0
ENSG00000182334	OR5P3	0	0	0	0	0	0	0	0	0	0	0
ENSG00000279000	OR10A6	0	0	0	0	0	0	0	0	0	0	0
ENSG00000170683	OR10A3	0	0	0	0	0	0	0	0	0	0	0
ENSG00000182261	NLRP10	0	0	0	0	0	0	0	0	0	0	0
ENSG00000256206	AC018523.2	0	0	0	0	0	0	0	0	0	0	0
ENSG00000110680	CALCA	0	0	0	0	0	0	0	0	0	0	0
ENSG00000006611	USH1C	0	0	0	0	0	0	0	0	0	0	0
ENSG00000129152	MYOD1	0	0	0	0	0	0	0	0	0	0	0
ENSG00000179826	MRGPRX3	0	0	0	0	0	0	0	0	0	0	0
ENSG00000179817	MRGPRX4	0	0	0	0	0	0	0	0	0	0	0
ENSG00000148965	SAA4	0	0	0	0	0	0	0	0	0	0	0
ENSG00000255071	SAA2-SAA4	0	0	0	0	0	0	0	0	0	0	0
ENSG00000134339	SAA2	0	0	0	0	0	0	0	0	0	0	0
ENSG00000173432	SAA1	0	0	0	0	0	0	0	0	0	0	0
ENSG00000170255	MRGPRX1	0	0	0	0	0	0	0	0	0	0	0
ENSG00000183695	MRGPRX2	0	0	0	0	0	0	0	0	0	0	0
ENSG00000129170	CSRP3	0	0	0	0	0	0	0	0	0	0	0
ENSG00000109851	DBX1	0	0	0	0	0	0	0	0	0	0	0
ENSG00000285283	AL035078.4	0	0	0	0	0	0	0	0	0	0	0
ENSG00000284969	AL049629.2	0	0	0	0	0	0	0	0	0	0	0
ENSG00000234776	C11orf94	0	0	0	0	0	0	0	0	0	0	0
ENSG00000180720	CHRM4	0	0	0	0	0	0	0	0	0	0	0
ENSG00000176567	OR4X1	0	0	0	0	0	0	0	0	0	0	0
ENSG00000221954	OR4C12	0	0	0	0	0	0	0	0	0	0	0
ENSG00000185926	OR4C46	0	0	0	0	0	0	0	0	0	0	0
ENSG00000221840	OR4A5	0	0	0	0	0	0	0	0	0	0	0
ENSG00000181958	OR4A15	0	0	0	0	0	0	0	0	0	0	0
ENSG00000279514	OR4C16	0	0	0	0	0	0	0	0	0	0	0
ENSG00000174982	OR4S2	0	0	0	0	0	0	0	0	0	0	0
ENSG00000186113	OR5D14	0	0	0	0	0	0	0	0	0	0	0
ENSG00000205030	OR5L2	0	0	0	0	0	0	0	0	0	0	0
ENSG00000124900	TRIM51	0	0	0	0	0	0	0	0	0	0	0
ENSG00000187612	OR5W2	0	0	0	0	0	0	0	0	0	0	0
ENSG00000167825	OR5I1	0	0	0	0	0	0	0	0	0	0	0
ENSG00000181767	OR8H2	0	0	0	0	0	0	0	0	0	0	0
ENSG00000174957	OR5J2	0	0	0	0	0	0	0	0	0	0	0
ENSG00000181718	OR5T2	0	0	0	0	0	0	0	0	0	0	0
ENSG00000172489	OR5T3	0	0	0	0	0	0	0	0	0	0	0
ENSG00000181698	OR5T1	0	0	0	0	0	0	0	0	0	0	0
ENSG00000181693	OR8H1	0	0	0	0	0	0	0	0	0	0	0
ENSG00000280314	OR8K3	0	0	0	0	0	0	0	0	0	0	0
ENSG00000150261	OR8K1	0	0	0	0	0	0	0	0	0	0	0
ENSG00000172487	OR8J1	0	0	0	0	0	0	0	0	0	0	0
ENSG00000172199	OR8U1	0	0	0	0	0	0	0	0	0	0	0
ENSG00000279961	OR5R1	0	0	0	0	0	0	0	0	0	0	0
ENSG00000150269	OR5M9	0	0	0	0	0	0	0	0	0	0	0
ENSG00000174937	OR5M3	0	0	0	0	0	0	0	0	0	0	0
ENSG00000181371	OR5M8	0	0	0	0	0	0	0	0	0	0	0
ENSG00000255012	OR5M1	0	0	0	0	0	0	0	0	0	0	0
ENSG00000172459	OR5AR1	0	0	0	0	0	0	0	0	0	0	0
ENSG00000172457	OR9G4	0	0	0	0	0	0	0	0	0	0	0
ENSG00000183908	LRRC55	0	0	0	0	0	0	0	0	0	0	0
ENSG00000186652	PRG2	0	0	0	0	0	0	0	0	0	0	0
ENSG00000186907	RTN4RL2	0	0	0	0	0	0	0	0	0	0	0
ENSG00000254462	TMX2-CTNND1	0	0	0	0	0	0	0	0	0	0	0
ENSG00000254732	AP001931.1	0	0	0	0	0	0	0	0	0	0	0
ENSG00000279051	OR6Q1	0	0	0	0	0	0	0	0	0	0	0
ENSG00000186513	OR9Q2	0	0	0	0	0	0	0	0	0	0	0
ENSG00000180475	OR10Q1	0	0	0	0	0	0	0	0	0	0	0
ENSG00000197786	OR5B17	0	0	0	0	0	0	0	0	0	0	0
ENSG00000172769	OR5B3	0	0	0	0	0	0	0	0	0	0	0
ENSG00000172362	OR5B12	0	0	0	0	0	0	0	0	0	0	0
ENSG00000198283	OR5B21	0	0	0	0	0	0	0	0	0	0	0
ENSG00000255073	ZFP91-CNTF	0	0	0	0	0	0	0	0	0	0	0

ENSG00000255151	GLYATL1B	0	0	0	0	0	0	0	0	0	0	0
ENSG00000285231	OOSP3	0	0	0	0	0	0	0	0	0	0	0
ENSG00000284873	OOSP1	0	0	0	0	0	0	0	0	0	0	0
ENSG00000149507	OOSP2	0	0	0	0	0	0	0	0	0	0	0
ENSG00000166930	MS4A5	0	0	0	0	0	0	0	0	0	0	0
ENSG00000166961	AP004243.1	0	0	0	0	0	0	0	0	0	0	0
ENSG00000229183	PGA4	0	0	0	0	0	0	0	0	0	0	0
ENSG00000168515	SCGB1D1	0	0	0	0	0	0	0	0	0	0	0
ENSG00000124935	SCGB1D2	0	0	0	0	0	0	0	0	0	0	0
ENSG00000197745	SCGB1D4	0	0	0	0	0	0	0	0	0	0	0
ENSG00000149021	SCGB1A1	0	0	0	0	0	0	0	0	0	0	0
ENSG00000254772	EEF1G	0	0	0	0	0	0	0	0	0	0	0
ENSG00000255432	AP001458.2	0	0	0	0	0	0	0	0	0	0	0
ENSG00000177363	LRRN4CL	0	0	0	0	0	0	0	0	0	0	0
ENSG00000234857	HNRNPUL2-BSCL2	0	0	0	0	0	0	0	0	0	0	0
ENSG00000197901	SLC22A6	0	0	0	0	0	0	0	0	0	0	0
ENSG00000197658	SLC22A24	0	0	0	0	0	0	0	0	0	0	0
ENSG00000149742	SLC22A9	0	0	0	0	0	0	0	0	0	0	0
ENSG00000168004	PLAAT5	0	0	0	0	0	0	0	0	0	0	0
ENSG00000133328	PLAAT2	0	0	0	0	0	0	0	0	0	0	0
ENSG00000256100	AP000721.1	0	0	0	0	0	0	0	0	0	0	0
ENSG00000126500	FLRT1	0	0	0	0	0	0	0	0	0	0	0
ENSG00000286264	AP001453.5	0	0	0	0	0	0	0	0	0	0	0
ENSG00000182450	KCNK4	0	0	0	0	0	0	0	0	0	0	0
ENSG00000219435	CATSPERZ	0	0	0	0	0	0	0	0	0	0	0
ENSG00000149735	GPHA2	0	0	0	0	0	0	0	0	0	0	0
ENSG00000273003	ARL2-SNX15	0	0	0	0	0	0	0	0	0	0	0
ENSG00000204710	SPDYC	0	0	0	0	0	0	0	0	0	0	0
ENSG00000256349	AP002748.4	0	0	0	0	0	0	0	0	0	0	0
ENSG00000173237	C11orf86	0	0	0	0	0	0	0	0	0	0	0
ENSG00000173156	RHOD	0	0	0	0	0	0	0	0	0	0	0
ENSG00000256514	AP003419.1	0	0	0	0	0	0	0	0	0	0	0
ENSG00000213402	PTPRCAP	0	0	0	0	0	0	0	0	0	0	0
ENSG00000175514	GPR152	0	0	0	0	0	0	0	0	0	0	0
ENSG00000172938	MRGPRD	0	0	0	0	0	0	0	0	0	0	0
ENSG00000284713	SMIM38	0	0	0	0	0	0	0	0	0	0	0
ENSG00000162344	FGF19	0	0	0	0	0	0	0	0	0	0	0
ENSG00000244411	KRTAP5-7	0	0	0	0	0	0	0	0	0	0	0
ENSG00000241233	KRTAP5-8	0	0	0	0	0	0	0	0	0	0	0
ENSG00000254997	KRTAP5-9	0	0	0	0	0	0	0	0	0	0	0
ENSG00000204571	KRTAP5-11	0	0	0	0	0	0	0	0	0	0	0
ENSG00000225805	DEFB131B	0	0	0	0	0	0	0	0	0	0	0
ENSG00000284922	AP000812.5	0	0	0	0	0	0	0	0	0	0	0
ENSG00000284844	AP000812.4	0	0	0	0	0	0	0	0	0	0	0
ENSG00000254550	OMP	0	0	0	0	0	0	0	0	0	0	0
ENSG00000259112	NDUFC2-KCTD14	0	0	0	0	0	0	0	0	0	0	0
ENSG00000151365	THRSP	0	0	0	0	0	0	0	0	0	0	0
ENSG00000168959	GRM5	0	0	0	0	0	0	0	0	0	0	0
ENSG00000168930	TRIM49	0	0	0	0	0	0	0	0	0	0	0
ENSG00000223417	TRIM49D1	0	0	0	0	0	0	0	0	0	0	0
ENSG00000233802	TRIM49D2	0	0	0	0	0	0	0	0	0	0	0
ENSG00000204450	TRIM64	0	0	0	0	0	0	0	0	0	0	0
ENSG00000204449	TRIM49C	0	0	0	0	0	0	0	0	0	0	0
ENSG00000255009	UBTFL1	0	0	0	0	0	0	0	0	0	0	0
ENSG00000284057	AP001273.2	0	0	0	0	0	0	0	0	0	0	0
ENSG00000257057	C11orf97	0	0	0	0	0	0	0	0	0	0	0
ENSG00000255855	KDM4F	0	0	0	0	0	0	0	0	0	0	0
ENSG00000166670	MMP10	0	0	0	0	0	0	0	0	0	0	0
ENSG00000262406	MMP12	0	0	0	0	0	0	0	0	0	0	0
ENSG00000137745	MMP13	0	0	0	0	0	0	0	0	0	0	0
ENSG00000170967	DDI1	0	0	0	0	0	0	0	0	0	0	0
ENSG00000255501	CARD18	0	0	0	0	0	0	0	0	0	0	0
ENSG00000170290	SLN	0	0	0	0	0	0	0	0	0	0	0
ENSG00000214290	COLCA2	0	0	0	0	0	0	0	0	0	0	0
ENSG00000137707	BTG4	0	0	0	0	0	0	0	0	0	0	0
ENSG00000258529	AP001781.2	0	0	0	0	0	0	0	0	0	0	0
ENSG00000254445	HSPB2-C11orf52	0	0	0	0	0	0	0	0	0	0	0
ENSG00000149300	C11orf52	0	0	0	0	0	0	0	0	0	0	0
ENSG00000255292	AP002884.3	0	0	0	0	0	0	0	0	0	0	0
ENSG00000150783	TEX12	0	0	0	0	0	0	0	0	0	0	0
ENSG00000228607	CLDN25	0	0	0	0	0	0	0	0	0	0	0
ENSG00000255663	AP002373.1	0	0	0	0	0	0	0	0	0	0	0
ENSG00000110245	APOC3	0	0	0	0	0	0	0	0	0	0	0
ENSG00000255245	FXYD6-FXYD2	0	0	0	0	0	0	0	0	0	0	0
ENSG00000223953	C1QTNF5	0	0	0	0	0	0	0	0	0	0	0
ENSG00000259571	BLID	0	0	0	0	0	0	0	0	0	0	0
ENSG00000196099	OR6M1	0	0	0	0	0	0	0	0	0	0	0
ENSG00000171014	OR4D5	0	0	0	0	0	0	0	0	0	0	0
ENSG00000196248	OR10S1	0	0	0	0	0	0	0	0	0	0	0
ENSG00000198674	OR10G6	0	0	0	0	0	0	0	0	0	0	0
ENSG00000254737	OR10G4	0	0	0	0	0	0	0	0	0	0	0
ENSG00000236981	OR10G9	0	0	0	0	0	0	0	0	0	0	0
ENSG00000234560	OR10G8	0	0	0	0	0	0	0	0	0	0	0
ENSG00000182634	OR10G7	0	0	0	0	0	0	0	0	0	0	0
ENSG00000197849	OR8G1	0	0	0	0	0	0	0	0	0	0	0
ENSG00000279116	OR8D2	0	0	0	0	0	0	0	0	0	0	0
ENSG00000284680	OR8B2	0	0	0	0	0	0	0	0	0	0	0
ENSG00000280090	OR8B4	0	0	0	0	0	0	0	0	0	0	0
ENSG00000221932	HEPN1	0	0	0	0	0	0	0	0	0	0	0
ENSG00000236027	PATE3	0	0	0	0	0	0	0	0	0	0	0
ENSG00000166118	SPATA19	0	0	0	0	0	0	0	0	0	0	0
ENSG00000080854	IGSF9B	0	0	0	0	0	0	0	0	0	0	0
ENSG00000283297	TEX52	0	0	0	0	0	0	0	0	0	0	0
ENSG00000285901	AC008012.1	0	0	0	0	0	0	0	0	0	0	0
ENSG00000111241	FGF6	0	0	0	0	0	0	0	0	0	0	0
ENSG00000272921	AC005832.4	0	0	0	0	0	0	0	0	0	0	0
ENSG00000151079	KCNA6	0	0	0	0	0	0	0	0	0	0	0
ENSG00000285238	AC006064.6	0	0	0	0	0	0	0	0	0	0	0
ENSG00000198178	CLEC4C	0	0	0	0	0	0	0	0	0	0	0
ENSG00000284393	AC092111.3	0	0	0	0	0	0	0	0	0	0	0
ENSG00000196946	ZNF705A	0	0	0	0	0	0	0	0	0	0	0
ENSG00000188393	CLEC2A	0	0	0	0	0	0	0	0	0	0	0
ENSG00000256660	CLEC12B	0	0	0	0	0	0	0	0	0	0	0
ENSG00000213809	KLRK1	0	0	0	0	0	0	0	0	0	0	0
ENSG00000183542	KLRC4	0	0	0	0	0	0	0	0	0	0	0
ENSG00000205810	KLRC3	0	0	0	0	0	0	0	0	0	0	0
ENSG00000255641	AC068775.1	0	0	0	0	0	0	0	0	0	0	0
ENSG00000205809	KLRC2	0	0	0	0	0	0	0	0	0	0	0
ENSG00000121377	TAS2R7	0	0	0	0	0	0	0	0	0	0	0
ENSG00000121314	TAS2R8	0	0	0	0	0	0	0	0	0	0	0
ENSG00000121381	TAS2R9	0	0	0	0	0	0	0	0	0	0	0
ENSG00000121318	TAS2R10	0	0	0	0	0	0	0	0	0	0	0
ENSG00000111215	PRR4	0	0	0	0	0	0	0	0	0	0	0
ENSG00000275778	AC018630.2	0	0	0	0	0	0	0	0	0	0	0
ENSG00000284826	AC006518.7	0	0	0	0	0	0	0	0	0	0	0
ENSG00000212128	TAS2R13	0	0	0	0	0	0	0	0	0	0	0
ENSG00000134551	PRH2	0	0	0	0	0	0	0	0	0	0	0

ENSG00000212127	TAS2R14	0	0	0	0	0	0	0	0	0	0	0
ENSG00000212126	TAS2R50	0	0	0	0	0	0	0	0	0	0	0
ENSG00000255837	TAS2R20	0	0	0	0	0	0	0	0	0	0	0
ENSG00000212124	TAS2R19	0	0	0	0	0	0	0	0	0	0	0
ENSG00000256436	TAS2R31	0	0	0	0	0	0	0	0	0	0	0
ENSG00000226761	TAS2R46	0	0	0	0	0	0	0	0	0	0	0
ENSG00000255374	TAS2R43	0	0	0	0	0	0	0	0	0	0	0
ENSG00000256188	TAS2R30	0	0	0	0	0	0	0	0	0	0	0
ENSG00000256537	SMIM10L1	0	0	0	0	0	0	0	0	0	0	0
ENSG00000186136	TAS2R42	0	0	0	0	0	0	0	0	0	0	0
ENSG00000197870	PRB3	0	0	0	0	0	0	0	0	0	0	0
ENSG00000230657	PRB4	0	0	0	0	0	0	0	0	0	0	0
ENSG00000251655	PRB1	0	0	0	0	0	0	0	0	0	0	0
ENSG00000121335	PRB2	0	0	0	0	0	0	0	0	0	0	0
ENSG00000111291	GPRC5D	0	0	0	0	0	0	0	0	0	0	0
ENSG00000139053	AC020613.1	0	0	0	0	0	0	0	0	0	0	0
ENSG00000177938	CAPZA3	0	0	0	0	0	0	0	0	0	0	0
ENSG00000257046	AC011604.2	0	0	0	0	0	0	0	0	0	0	0
ENSG00000257062	AC022335.1	0	0	0	0	0	0	0	0	0	0	0
ENSG00000111713	GYS2	0	0	0	0	0	0	0	0	0	0	0
ENSG00000214700	C12orf71	0	0	0	0	0	0	0	0	0	0	0
ENSG00000165935	SMCO2	0	0	0	0	0	0	0	0	0	0	0
ENSG00000174236	REP15	0	0	0	0	0	0	0	0	0	0	0
ENSG00000135406	PRPH	0	0	0	0	0	0	0	0	0	0	0
ENSG00000178401	DNAJC22	0	0	0	0	0	0	0	0	0	0	0
ENSG00000284730	C12orf81	0	0	0	0	0	0	0	0	0	0	0
ENSG00000284791	SMIM41	0	0	0	0	0	0	0	0	0	0	0
ENSG00000205426	KRT81	0	0	0	0	0	0	0	0	0	0	0
ENSG00000161849	KRT84	0	0	0	0	0	0	0	0	0	0	0
ENSG00000186442	KRT3	0	0	0	0	0	0	0	0	0	0	0
ENSG00000283536	AC021072.1	0	0	0	0	0	0	0	0	0	0	0
ENSG00000139574	NPFF	0	0	0	0	0	0	0	0	0	0	0
ENSG00000273049	AC012531.3	0	0	0	0	0	0	0	0	0	0	0
ENSG00000172789	HOXC5	0	0	0	0	0	0	0	0	0	0	0
ENSG00000197706	OR6C74	0	0	0	0	0	0	0	0	0	0	0
ENSG00000205327	OR6C68	0	0	0	0	0	0	0	0	0	0	0
ENSG00000175398	OR10P1	0	0	0	0	0	0	0	0	0	0	0
ENSG00000257411	AC034102.3	0	0	0	0	0	0	0	0	0	0	0
ENSG00000144785	AC073896.1	0	0	0	0	0	0	0	0	0	0	0
ENSG00000285528	AC097104.1	0	0	0	0	0	0	0	0	0	0	0
ENSG00000285625	AC117378.1	0	0	0	0	0	0	0	0	0	0	0
ENSG00000258830	AC137834.1	0	0	0	0	0	0	0	0	0	0	0
ENSG00000175189	INHBC	0	0	0	0	0	0	0	0	0	0	0
ENSG00000285133	AC022506.1	0	0	0	0	0	0	0	0	0	0	0
ENSG00000257921	AC025165.3	0	0	0	0	0	0	0	0	0	0	0
ENSG00000228144	AC078927.1	0	0	0	0	0	0	0	0	0	0	0
ENSG00000111537	IFNG	0	0	0	0	0	0	0	0	0	0	0
ENSG00000198812	LRRC10	0	0	0	0	0	0	0	0	0	0	0
ENSG00000258052	AC025263.2	0	0	0	0	0	0	0	0	0	0	0
ENSG00000258064	AC073612.1	0	0	0	0	0	0	0	0	0	0	0
ENSG00000111046	MYF6	0	0	0	0	0	0	0	0	0	0	0
ENSG00000111049	MYF5	0	0	0	0	0	0	0	0	0	0	0
ENSG00000231738	TSPAN19	0	0	0	0	0	0	0	0	0	0	0
ENSG00000180318	ALX1	0	0	0	0	0	0	0	0	0	0	0
ENSG00000139330	KERA	0	0	0	0	0	0	0	0	0	0	0
ENSG00000183395	PMCH	0	0	0	0	0	0	0	0	0	0	0
ENSG00000171759	PAH	0	0	0	0	0	0	0	0	0	0	0
ENSG00000257767	AC002996.1	0	0	0	0	0	0	0	0	0	0	0
ENSG00000186710	CFAP73	0	0	0	0	0	0	0	0	0	0	0
ENSG00000170890	PLA2G1B	0	0	0	0	0	0	0	0	0	0	0
ENSG00000111780	AL021546.1	0	0	0	0	0	0	0	0	0	0	0
ENSG00000135100	HNF1A	0	0	0	0	0	0	0	0	0	0	0
ENSG00000256950	AC069503.2	0	0	0	0	0	0	0	0	0	0	0
ENSG00000204671	IL31	0	0	0	0	0	0	0	0	0	0	0
ENSG00000256861	AC048338.1	0	0	0	0	0	0	0	0	0	0	0
ENSG00000187848	P2RX2	0	0	0	0	0	0	0	0	0	0	0
ENSG00000256825	AC026786.1	0	0	0	0	0	0	0	0	0	0	0
ENSG00000127863	TNFRSF19	0	0	0	0	0	0	0	0	0	0	0
ENSG00000273167	AL359736.1	0	0	0	0	0	0	0	0	0	0	0
ENSG00000240654	C1QTNF9	0	0	0	0	0	0	0	0	0	0	0
ENSG00000169840	GSX1	0	0	0	0	0	0	0	0	0	0	0
ENSG00000139515	PDX1	0	0	0	0	0	0	0	0	0	0	0
ENSG00000180660	MAB21L1	0	0	0	0	0	0	0	0	0	0	0
ENSG00000250709	CCDC169-SOHLH2	0	0	0	0	0	0	0	0	0	0	0
ENSG00000281883	AL512506.3	0	0	0	0	0	0	0	0	0	0	0
ENSG00000139656	SMIM2	0	0	0	0	0	0	0	0	0	0	0
ENSG00000151778	SERP2	0	0	0	0	0	0	0	0	0	0	0
ENSG00000285444	AL162377.3	0	0	0	0	0	0	0	0	0	0	0
ENSG00000123171	CCDC70	0	0	0	0	0	0	0	0	0	0	0
ENSG00000253797	UTP14C	0	0	0	0	0	0	0	0	0	0	0
ENSG00000204919	PRR20A	0	0	0	0	0	0	0	0	0	0	0
ENSG00000229665	PRR20C	0	0	0	0	0	0	0	0	0	0	0
ENSG00000204918	PRR20B	0	0	0	0	0	0	0	0	0	0	0
ENSG00000227151	PRR20D	0	0	0	0	0	0	0	0	0	0	0
ENSG00000234278	PRR20E	0	0	0	0	0	0	0	0	0	0	0
ENSG00000280165	PCDH20	0	0	0	0	0	0	0	0	0	0	0
ENSG00000237378	AL445989.1	0	0	0	0	0	0	0	0	0	0	0
ENSG00000285566	AL445238.1	0	0	0	0	0	0	0	0	0	0	0
ENSG00000152192	POU4F1	0	0	0	0	0	0	0	0	0	0	0
ENSG00000270181	BIVM-ERCC5	0	0	0	0	0	0	0	0	0	0	0
ENSG00000182968	SOX1	0	0	0	0	0	0	0	0	0	0	0
ENSG00000153498	SPACA7	0	0	0	0	0	0	0	0	0	0	0
ENSG00000057593	F7	0	0	0	0	0	0	0	0	0	0	0
ENSG00000126231	PROZ	0	0	0	0	0	0	0	0	0	0	0
ENSG00000139835	GRTP1	0	0	0	0	0	0	0	0	0	0	0
ENSG00000186009	ATP4B	0	0	0	0	0	0	0	0	0	0	0
ENSG00000257115	OR11H12	0	0	0	0	0	0	0	0	0	0	0
ENSG00000222036	POTEM	0	0	0	0	0	0	0	0	0	0	0
ENSG00000169488	OR4K15	0	0	0	0	0	0	0	0	0	0	0
ENSG00000169484	OR4K14	0	0	0	0	0	0	0	0	0	0	0
ENSG00000176219	OR11H6	0	0	0	0	0	0	0	0	0	0	0
ENSG00000258436	RNASE12	0	0	0	0	0	0	0	0	0	0	0
ENSG00000259171	AL163636.2	0	0	0	0	0	0	0	0	0	0	0
ENSG00000206150	RNASE13	0	0	0	0	0	0	0	0	0	0	0
ENSG00000173431	RNASE8	0	0	0	0	0	0	0	0	0	0	0
ENSG00000232070	TMEM253	0	0	0	0	0	0	0	0	0	0	0
ENSG00000259132	AL132780.3	0	0	0	0	0	0	0	0	0	0	0
ENSG00000222028	PSMB11	0	0	0	0	0	0	0	0	0	0	0
ENSG00000197616	MYH6	0	0	0	0	0	0	0	0	0	0	0
ENSG00000259371	AL136295.3	0	0	0	0	0	0	0	0	0	0	0
ENSG00000139914	FITM1	0	0	0	0	0	0	0	0	0	0	0
ENSG00000254692	AL136295.1	0	0	0	0	0	0	0	0	0	0	0
ENSG00000255526	NEDD8-MDP1	0	0	0	0	0	0	0	0	0	0	0
ENSG00000136305	CIDEB	0	0	0	0	0	0	0	0	0	0	0
ENSG00000092009	CMA1	0	0	0	0	0	0	0	0	0	0	0
ENSG00000136352	NKX2-1	0	0	0	0	0	0	0	0	0	0	0
ENSG00000136327	NKX2-8	0	0	0	0	0	0	0	0	0	0	0

ENSG00000258989	AL355916.3	0	0	0	0	0	0	0	0	0	0	0
ENSG00000125954	CHURC1-FNTB	0	0	0	0	0	0	0	0	0	0	0
ENSG00000258644	SYNJ2BP-COX16	0	0	0	0	0	0	0	0	0	0	0
ENSG00000139985	ADAM21	0	0	0	0	0	0	0	0	0	0	0
ENSG00000205669	ACOT6	0	0	0	0	0	0	0	0	0	0	0
ENSG00000258653	AC005520.1	0	0	0	0	0	0	0	0	0	0	0
ENSG00000259164	AC007375.1	0	0	0	0	0	0	0	0	0	0	0
ENSG00000197734	C14orf178	0	0	0	0	0	0	0	0	0	0	0
ENSG00000133962	CATSPERB	0	0	0	0	0	0	0	0	0	0	0
ENSG00000100604	CHGA	0	0	0	0	0	0	0	0	0	0	0
ENSG00000153485	TMEM251	0	0	0	0	0	0	0	0	0	0	0
ENSG00000259066	AL110118.2	0	0	0	0	0	0	0	0	0	0	0
ENSG00000187581	COX8C	0	0	0	0	0	0	0	0	0	0	0
ENSG00000175785	PRIMA1	0	0	0	0	0	0	0	0	0	0	0
ENSG00000140067	FAM181A	0	0	0	0	0	0	0	0	0	0	0
ENSG00000100628	ASB2	0	0	0	0	0	0	0	0	0	0	0
ENSG00000175699	CCDC197	0	0	0	0	0	0	0	0	0	0	0
ENSG00000119698	PPP4R4	0	0	0	0	0	0	0	0	0	0	0
ENSG00000140093	SERPINA10	0	0	0	0	0	0	0	0	0	0	0
ENSG00000170099	SERPINA6	0	0	0	0	0	0	0	0	0	0	0
ENSG00000258691	AL355102.2	0	0	0	0	0	0	0	0	0	0	0
ENSG00000100739	BDKRB1	0	0	0	0	0	0	0	0	0	0	0
ENSG00000140107	SLC25A47	0	0	0	0	0	0	0	0	0	0	0
ENSG00000254656	RTL1	0	0	0	0	0	0	0	0	0	0	0
ENSG00000256500	AL139300.1	0	0	0	0	0	0	0	0	0	0	0
ENSG00000227729	RD3L	0	0	0	0	0	0	0	0	0	0	0
ENSG00000258986	TMEM179	0	0	0	0	0	0	0	0	0	0	0
ENSG00000257341	AL928654.3	0	0	0	0	0	0	0	0	0	0	0
ENSG00000230031	POTEB2	0	0	0	0	0	0	0	0	0	0	0
ENSG00000280709	LINC02203	0	0	0	0	0	0	0	0	0	0	0
ENSG00000182974	AC135068.1	0	0	0	0	0	0	0	0	0	0	0
ENSG00000279408	AC135068.3	0	0	0	0	0	0	0	0	0	0	0
ENSG00000233917	POTEB	0	0	0	0	0	0	0	0	0	0	0
ENSG00000285472	AC134980.3	0	0	0	0	0	0	0	0	0	0	0
ENSG00000274102	OR4M2	0	0	0	0	0	0	0	0	0	0	0
ENSG00000183706	OR4N4	0	0	0	0	0	0	0	0	0	0	0
ENSG00000273976	GOLGA6L1	0	0	0	0	0	0	0	0	0	0	0
ENSG00000214265	AC124312.1	0	0	0	0	0	0	0	0	0	0	0
ENSG00000153684	GOLGA8F	0	0	0	0	0	0	0	0	0	0	0
ENSG00000183629	GOLGA8G	0	0	0	0	0	0	0	0	0	0	0
ENSG00000104059	FAM189A1	0	0	0	0	0	0	0	0	0	0	0
ENSG00000185115	NSMCE3	0	0	0	0	0	0	0	0	0	0	0
ENSG00000179938	GOLGA8J	0	0	0	0	0	0	0	0	0	0	0
ENSG00000261247	GOLGA8T	0	0	0	0	0	0	0	0	0	0	0
ENSG00000186399	GOLGA8R	0	0	0	0	0	0	0	0	0	0	0
ENSG00000178115	GOLGA8Q	0	0	0	0	0	0	0	0	0	0	0
ENSG00000261794	GOLGA8H	0	0	0	0	0	0	0	0	0	0	0
ENSG00000285077	AC091057.6	0	0	0	0	0	0	0	0	0	0	0
ENSG00000284906	ARHGAP11B	0	0	0	0	0	0	0	0	0	0	0
ENSG00000134160	TRPM1	0	0	0	0	0	0	0	0	0	0	0
ENSG00000249931	GOLGA8K	0	0	0	0	0	0	0	0	0	0	0
ENSG00000206127	GOLGA8O	0	0	0	0	0	0	0	0	0	0	0
ENSG00000232653	GOLGA8N	0	0	0	0	0	0	0	0	0	0	0
ENSG00000166922	SCG5	0	0	0	0	0	0	0	0	0	0	0
ENSG00000255192	NANOGP8	0	0	0	0	0	0	0	0	0	0	0
ENSG00000259288	BUB1B-PAK6	0	0	0	0	0	0	0	0	0	0	0
ENSG00000285920	AC087721.2	0	0	0	0	0	0	0	0	0	0	0
ENSG00000243789	JMJD7	0	0	0	0	0	0	0	0	0	0	0
ENSG00000285942	AC018362.3	0	0	0	0	0	0	0	0	0	0	0
ENSG00000284772	AC011330.3	0	0	0	0	0	0	0	0	0	0	0
ENSG00000262560	AC018512.1	0	0	0	0	0	0	0	0	0	0	0
ENSG00000184716	SERINC4	0	0	0	0	0	0	0	0	0	0	0
ENSG00000167014	TERB2	0	0	0	0	0	0	0	0	0	0	0
ENSG00000140274	DUOXA2	0	0	0	0	0	0	0	0	0	0	0
ENSG00000140254	DUOXA1	0	0	0	0	0	0	0	0	0	0	0
ENSG00000260170	AC090527.2	0	0	0	0	0	0	0	0	0	0	0
ENSG00000233932	CTXN2	0	0	0	0	0	0	0	0	0	0	0
ENSG00000169856	ONECUT1	0	0	0	0	0	0	0	0	0	0	0
ENSG00000198535	C2CD4A	0	0	0	0	0	0	0	0	0	0	0
ENSG00000166831	RBPMS2	0	0	0	0	0	0	0	0	0	0	0
ENSG00000249240	AC069368.1	0	0	0	0	0	0	0	0	0	0	0
ENSG00000273025	AC009690.3	0	0	0	0	0	0	0	0	0	0	0
ENSG00000140488	CELF6	0	0	0	0	0	0	0	0	0	0	0
ENSG00000260729	AC009690.1	0	0	0	0	0	0	0	0	0	0	0
ENSG00000187806	TMEM202	0	0	0	0	0	0	0	0	0	0	0
ENSG00000215186	GOLGA6B	0	0	0	0	0	0	0	0	0	0	0
ENSG00000159289	GOLGA6A	0	0	0	0	0	0	0	0	0	0	0
ENSG00000140506	LMAN1L	0	0	0	0	0	0	0	0	0	0	0
ENSG00000213578	CPLX3	0	0	0	0	0	0	0	0	0	0	0
ENSG00000167195	GOLGA6C	0	0	0	0	0	0	0	0	0	0	0
ENSG00000140478	GOLGA6D	0	0	0	0	0	0	0	0	0	0	0
ENSG00000159556	ISL2	0	0	0	0	0	0	0	0	0	0	0
ENSG00000169783	LINGO1	0	0	0	0	0	0	0	0	0	0	0
ENSG00000166426	CRABP1	0	0	0	0	0	0	0	0	0	0	0
ENSG00000259332	ST20-MTHFS	0	0	0	0	0	0	0	0	0	0	0
ENSG00000156206	CFAP161	0	0	0	0	0	0	0	0	0	0	0
ENSG00000278662	GOLGA6L10	0	0	0	0	0	0	0	0	0	0	0
ENSG00000197978	GOLGA6L9	0	0	0	0	0	0	0	0	0	0	0
ENSG00000260836	AC245033.1	0	0	0	0	0	0	0	0	0	0	0
ENSG00000214575	CPEB1	0	0	0	0	0	0	0	0	0	0	0
ENSG00000259511	UBE2Q2L	0	0	0	0	0	0	0	0	0	0	0
ENSG00000184206	GOLGA6L4	0	0	0	0	0	0	0	0	0	0	0
ENSG00000250021	ARPIN-AP3S2	0	0	0	0	0	0	0	0	0	0	0
ENSG00000275674	AC091167.6	0	0	0	0	0	0	0	0	0	0	0
ENSG00000182175	RGMA	0	0	0	0	0	0	0	0	0	0	0
ENSG00000184140	OR4F6	0	0	0	0	0	0	0	0	0	0	0
ENSG00000177693	OR4F4	0	0	0	0	0	0	0	0	0	0	0
ENSG00000130656	HBZ	0	0	0	0	0	0	0	0	0	0	0
ENSG00000206177	HBM	0	0	0	0	0	0	0	0	0	0	0
ENSG00000086506	HBQ1	0	0	0	0	0	0	0	0	0	0	0
ENSG00000161992	PRR35	0	0	0	0	0	0	0	0	0	0	0
ENSG00000127585	FBXL16	0	0	0	0	0	0	0	0	0	0	0
ENSG00000162004	CCDC78	0	0	0	0	0	0	0	0	0	0	0
ENSG00000162006	MSLNL	0	0	0	0	0	0	0	0	0	0	0
ENSG00000127588	GNG13	0	0	0	0	0	0	0	0	0	0	0
ENSG00000162009	SSTR5	0	0	0	0	0	0	0	0	0	0	0
ENSG00000116176	TPSG1	0	0	0	0	0	0	0	0	0	0	0
ENSG00000172236	TPSAB1	0	0	0	0	0	0	0	0	0	0	0
ENSG00000284395	AL032819.3	0	0	0	0	0	0	0	0	0	0	0
ENSG00000197599	CCDC154	0	0	0	0	0	0	0	0	0	0	0
ENSG00000162040	HS3ST6	0	0	0	0	0	0	0	0	0	0	0
ENSG00000179580	RNF151	0	0	0	0	0	0	0	0	0	0	0
ENSG00000196408	NOXO1	0	0	0	0	0	0	0	0	0	0	0
ENSG00000167971	CASKIN1	0	0	0	0	0	0	0	0	0	0	0
ENSG00000162068	NTN3	0	0	0	0	0	0	0	0	0	0	0
ENSG00000260272	AC093525.2	0	0	0	0	0	0	0	0	0	0	0
ENSG00000259784	AC093525.1	0	0	0	0	0	0	0	0	0	0	0

ENSG00000205923	CEMP1	0	0	0	0	0	0	0	0	0	0	0
ENSG00000215148	PRSS41	0	0	0	0	0	0	0	0	0	0	0
ENSG00000005001	PRSS22	0	0	0	0	0	0	0	0	0	0	0
ENSG00000131650	KREMEN2	0	0	0	0	0	0	0	0	0	0	0
ENSG00000213937	CLDN9	0	0	0	0	0	0	0	0	0	0	0
ENSG00000130182	ZSCAN10	0	0	0	0	0	0	0	0	0	0	0
ENSG00000285329	AC025283.3	0	0	0	0	0	0	0	0	0	0	0
ENSG00000262621	AC025283.2	0	0	0	0	0	0	0	0	0	0	0
ENSG00000103426	CORO7-PAM16	0	0	0	0	0	0	0	0	0	0	0
ENSG00000168140	VASN	0	0	0	0	0	0	0	0	0	0	0
ENSG00000232258	TMEM114	0	0	0	0	0	0	0	0	0	0	0
ENSG00000178257	PRM3	0	0	0	0	0	0	0	0	0	0	0
ENSG00000122304	PRM2	0	0	0	0	0	0	0	0	0	0	0
ENSG00000175646	PRM1	0	0	0	0	0	0	0	0	0	0	0
ENSG00000069764	PLA2G10	0	0	0	0	0	0	0	0	0	0	0
ENSG00000224712	NPIPA3	0	0	0	0	0	0	0	0	0	0	0
ENSG00000254852	NPIPA2	0	0	0	0	0	0	0	0	0	0	0
ENSG00000183426	NPIPA1	0	0	0	0	0	0	0	0	0	0	0
ENSG00000261130	AC140504.1	0	0	0	0	0	0	0	0	0	0	0
ENSG00000133392	MYH11	0	0	0	0	0	0	0	0	0	0	0
ENSG00000183889	AC138969.1	0	0	0	0	0	0	0	0	0	0	0
ENSG00000214967	NPIPA7	0	0	0	0	0	0	0	0	0	0	0
ENSG00000214940	NPIPA8	0	0	0	0	0	0	0	0	0	0	0
ENSG00000233024	NPIPA9	0	0	0	0	0	0	0	0	0	0	0
ENSG00000260342	AC138811.2	0	0	0	0	0	0	0	0	0	0	0
ENSG00000006116	CACNG3	0	0	0	0	0	0	0	0	0	0	0
ENSG00000103375	AQP8	0	0	0	0	0	0	0	0	0	0	0
ENSG00000198156	NPIPB6	0	0	0	0	0	0	0	0	0	0	0
ENSG00000261832	AC138894.1	0	0	0	0	0	0	0	0	0	0	0
ENSG00000233232	NPIPB7	0	0	0	0	0	0	0	0	0	0	0
ENSG00000255524	NPIPB8	0	0	0	0	0	0	0	0	0	0	0
ENSG00000183336	BOLA2	0	0	0	0	0	0	0	0	0	0	0
ENSG00000213648	SULT1A4	0	0	0	0	0	0	0	0	0	0	0
ENSG00000280893	AC009133.6	0	0	0	0	0	0	0	0	0	0	0
ENSG00000281348	AC120114.4	0	0	0	0	0	0	0	0	0	0	0
ENSG00000167194	C16orf92	0	0	0	0	0	0	0	0	0	0	0
ENSG00000261052	SULT1A3	0	0	0	0	0	0	0	0	0	0	0
ENSG00000261459	AC002310.4	0	0	0	0	0	0	0	0	0	0	0
ENSG00000281991	TMEM265	0	0	0	0	0	0	0	0	0	0	0
ENSG00000255439	AC135050.2	0	0	0	0	0	0	0	0	0	0	0
ENSG00000156885	COX6A2	0	0	0	0	0	0	0	0	0	0	0
ENSG00000169877	AHSP	0	0	0	0	0	0	0	0	0	0	0
ENSG00000183632	TP53TG3	0	0	0	0	0	0	0	0	0	0	0
ENSG00000205457	TP53TG3C	0	0	0	0	0	0	0	0	0	0	0
ENSG00000275034	TP53TG3E	0	0	0	0	0	0	0	0	0	0	0
ENSG00000261509	TP53TG3B	0	0	0	0	0	0	0	0	0	0	0
ENSG00000278848	TP53TG3F	0	0	0	0	0	0	0	0	0	0	0
ENSG00000259680	AC136428.1	0	0	0	0	0	0	0	0	0	0	0
ENSG00000277639	AC007906.2	0	0	0	0	0	0	0	0	0	0	0
ENSG00000177508	IRX3	0	0	0	0	0	0	0	0	0	0	0
ENSG00000159398	CES5A	0	0	0	0	0	0	0	0	0	0	0
ENSG00000103023	PRSS54	0	0	0	0	0	0	0	0	0	0	0
ENSG00000166589	CDH16	0	0	0	0	0	0	0	0	0	0	0
ENSG00000265690	AC074143.2	0	0	0	0	0	0	0	0	0	0	0
ENSG00000286140	DERPC	0	0	0	0	0	0	0	0	0	0	0
ENSG00000260914	AC026464.4	0	0	0	0	0	0	0	0	0	0	0
ENSG00000272617	AC026464.6	0	0	0	0	0	0	0	0	0	0	0
ENSG00000260371	AC026464.3	0	0	0	0	0	0	0	0	0	0	0
ENSG00000259900	AC026464.1	0	0	0	0	0	0	0	0	0	0	0
ENSG00000157315	TMED6	0	0	0	0	0	0	0	0	0	0	0
ENSG00000157335	CLEC18C	0	0	0	0	0	0	0	0	0	0	0
ENSG00000260537	AC012184.2	0	0	0	0	0	0	0	0	0	0	0
ENSG00000261611	AC010547.4	0	0	0	0	0	0	0	0	0	0	0
ENSG00000168928	CTRB2	0	0	0	0	0	0	0	0	0	0	0
ENSG00000261717	AC009163.4	0	0	0	0	0	0	0	0	0	0	0
ENSG00000260092	AC009163.2	0	0	0	0	0	0	0	0	0	0	0
ENSG00000284484	AC025287.4	0	0	0	0	0	0	0	0	0	0	0
ENSG00000286221	AC009070.1	0	0	0	0	0	0	0	0	0	0	0
ENSG00000166455	C16orf46	0	0	0	0	0	0	0	0	0	0	0
ENSG00000166558	SLC38A8	0	0	0	0	0	0	0	0	0	0	0
ENSG00000140955	ADAD2	0	0	0	0	0	0	0	0	0	0	0
ENSG00000131152	AC010531.1	0	0	0	0	0	0	0	0	0	0	0
ENSG00000124391	IL17C	0	0	0	0	0	0	0	0	0	0	0
ENSG00000205022	PABPN1L	0	0	0	0	0	0	0	0	0	0	0
ENSG00000198211	AC092143.1	0	0	0	0	0	0	0	0	0	0	0
ENSG00000268320	SCGB1C2	0	0	0	0	0	0	0	0	0	0	0
ENSG00000272636	DOC2B	0	0	0	0	0	0	0	0	0	0	0
ENSG00000184811	TRARG1	0	0	0	0	0	0	0	0	0	0	0
ENSG00000262664	OVCA2	0	0	0	0	0	0	0	0	0	0	0
ENSG00000262628	OR1D5	0	0	0	0	0	0	0	0	0	0	0
ENSG00000180042	AC087498.1	0	0	0	0	0	0	0	0	0	0	0
ENSG00000180016	OR1E1	0	0	0	0	0	0	0	0	0	0	0
ENSG00000285471	AC007846.2	0	0	0	0	0	0	0	0	0	0	0
ENSG00000161939	RNASEK-C17orf49	0	0	0	0	0	0	0	0	0	0	0
ENSG00000262526	AC120057.2	0	0	0	0	0	0	0	0	0	0	0
ENSG00000262302	AC003688.1	0	0	0	0	0	0	0	0	0	0	0
ENSG00000261915	AC026954.2	0	0	0	0	0	0	0	0	0	0	0
ENSG00000184560	SPEM2	0	0	0	0	0	0	0	0	0	0	0
ENSG00000286007	AC113189.9	0	0	0	0	0	0	0	0	0	0	0
ENSG00000248871	TNFSF12-TNFSF13	0	0	0	0	0	0	0	0	0	0	0
ENSG00000262730	AC104581.2	0	0	0	0	0	0	0	0	0	0	0
ENSG00000179111	HES7	0	0	0	0	0	0	0	0	0	0	0
ENSG00000263620	AC129492.4	0	0	0	0	0	0	0	0	0	0	0
ENSG00000263809	AC135178.2	0	0	0	0	0	0	0	0	0	0	0
ENSG00000184544	DHRS7C	0	0	0	0	0	0	0	0	0	0	0
ENSG00000214978	GSG1L2	0	0	0	0	0	0	0	0	0	0	0
ENSG00000109047	RCVRN	0	0	0	0	0	0	0	0	0	0	0
ENSG00000263429	TMEM238L	0	0	0	0	0	0	0	0	0	0	0
ENSG00000223510	CDRT15	0	0	0	0	0	0	0	0	0	0	0
ENSG00000125409	TEKT3	0	0	0	0	0	0	0	0	0	0	0
ENSG00000259024	TVP23C-CDRT4	0	0	0	0	0	0	0	0	0	0	0
ENSG00000255104	AC005324.4	0	0	0	0	0	0	0	0	0	0	0
ENSG00000266302	AC098850.3	0	0	0	0	0	0	0	0	0	0	0
ENSG00000264187	AC055811.2	0	0	0	0	0	0	0	0	0	0	0
ENSG00000214860	EVPLL	0	0	0	0	0	0	0	0	0	0	0
ENSG00000189375	TBC1D28	0	0	0	0	0	0	0	0	0	0	0
ENSG00000171931	FBXW10	0	0	0	0	0	0	0	0	0	0	0
ENSG00000189152	GRAPL	0	0	0	0	0	0	0	0	0	0	0
ENSG00000166482	MFAP4	0	0	0	0	0	0	0	0	0	0	0
ENSG00000128482	RNF112	0	0	0	0	0	0	0	0	0	0	0
ENSG00000214819	CDRT15L2	0	0	0	0	0	0	0	0	0	0	0
ENSG00000260458	KCNJ18	0	0	0	0	0	0	0	0	0	0	0
ENSG00000266728	AC015688.4	0	0	0	0	0	0	0	0	0	0	0
ENSG00000274529	SEBOX	0	0	0	0	0	0	0	0	0	0	0
ENSG00000273171	AC002094.3	0	0	0	0	0	0	0	0	0	0	0
ENSG00000109101	FOXN1	0	0	0	0	0	0	0	0	0	0	0
ENSG00000182271	TMIGD1	0	0	0	0	0	0	0	0	0	0	0

ENSG00000265118	AC134669.1	0	0	0	0	0	0	0	0	0	0	0
ENSG00000108702	CCL1	0	0	0	0	0	0	0	0	0	0	0
ENSG00000267618	AC004223.3	0	0	0	0	0	0	0	0	0	0	0
ENSG00000205045	SLFN12L	0	0	0	0	0	0	0	0	0	0	0
ENSG00000270379	HEATR9	0	0	0	0	0	0	0	0	0	0	0
ENSG00000275722	LYZL6	0	0	0	0	0	0	0	0	0	0	0
ENSG00000275152	CCL16	0	0	0	0	0	0	0	0	0	0	0
ENSG00000276409	CCL14	0	0	0	0	0	0	0	0	0	0	0
ENSG00000275688	CCL15-CCL14	0	0	0	0	0	0	0	0	0	0	0
ENSG00000275718	CCL15	0	0	0	0	0	0	0	0	0	0	0
ENSG00000274933	TBC1D3I	0	0	0	0	0	0	0	0	0	0	0
ENSG00000274226	TBC1D3H	0	0	0	0	0	0	0	0	0	0	0
ENSG00000275954	TBC1D3F	0	0	0	0	0	0	0	0	0	0	0
ENSG00000277161	PIGW	0	0	0	0	0	0	0	0	0	0	0
ENSG00000273706	LHX1	0	0	0	0	0	0	0	0	0	0	0
ENSG00000278505	C17orf78	0	0	0	0	0	0	0	0	0	0	0
ENSG00000275410	HNF1B	0	0	0	0	0	0	0	0	0	0	0
ENSG00000273513	TBC1D3K	0	0	0	0	0	0	0	0	0	0	0
ENSG00000274419	TBC1D3D	0	0	0	0	0	0	0	0	0	0	0
ENSG00000278299	TBC1D3C	0	0	0	0	0	0	0	0	0	0	0
ENSG00000278599	TBC1D3E	0	0	0	0	0	0	0	0	0	0	0
ENSG00000274611	TBC1D3	0	0	0	0	0	0	0	0	0	0	0
ENSG00000277399	GPR179	0	0	0	0	0	0	0	0	0	0	0
ENSG00000273604	EPOP	0	0	0	0	0	0	0	0	0	0	0
ENSG00000275489	C17orf98	0	0	0	0	0	0	0	0	0	0	0
ENSG00000263874	LINC00672	0	0	0	0	0	0	0	0	0	0	0
ENSG00000141744	PNMT	0	0	0	0	0	0	0	0	0	0	0
ENSG00000204913	LRRC3C	0	0	0	0	0	0	0	0	0	0	0
ENSG00000108342	CSF3	0	0	0	0	0	0	0	0	0	0	0
ENSG00000183153	GJD3	0	0	0	0	0	0	0	0	0	0	0
ENSG00000264058	AC073508.2	0	0	0	0	0	0	0	0	0	0	0
ENSG00000167916	KRT24	0	0	0	0	0	0	0	0	0	0	0
ENSG00000204897	KRT25	0	0	0	0	0	0	0	0	0	0	0
ENSG00000187242	KRT12	0	0	0	0	0	0	0	0	0	0	0
ENSG00000171431	KRT20	0	0	0	0	0	0	0	0	0	0	0
ENSG00000196859	KRT39	0	0	0	0	0	0	0	0	0	0	0
ENSG00000204889	KRT40	0	0	0	0	0	0	0	0	0	0	0
ENSG00000212899	KRTAP3-3	0	0	0	0	0	0	0	0	0	0	0
ENSG00000212900	KRTAP3-2	0	0	0	0	0	0	0	0	0	0	0
ENSG00000212901	KRTAP3-1	0	0	0	0	0	0	0	0	0	0	0
ENSG00000221852	KRTAP1-5	0	0	0	0	0	0	0	0	0	0	0
ENSG00000204887	KRTAP1-4	0	0	0	0	0	0	0	0	0	0	0
ENSG00000221880	KRTAP1-3	0	0	0	0	0	0	0	0	0	0	0
ENSG00000188581	KRTAP1-1	0	0	0	0	0	0	0	0	0	0	0
ENSG00000212725	KRTAP2-1	0	0	0	0	0	0	0	0	0	0	0
ENSG00000214518	KRTAP2-2	0	0	0	0	0	0	0	0	0	0	0
ENSG00000212724	KRTAP2-3	0	0	0	0	0	0	0	0	0	0	0
ENSG00000213417	KRTAP2-4	0	0	0	0	0	0	0	0	0	0	0
ENSG00000240871	KRTAP4-7	0	0	0	0	0	0	0	0	0	0	0
ENSG00000204880	KRTAP4-8	0	0	0	0	0	0	0	0	0	0	0
ENSG00000241241	KRTAP4-16	0	0	0	0	0	0	0	0	0	0	0
ENSG00000212721	KRTAP4-11	0	0	0	0	0	0	0	0	0	0	0
ENSG00000213416	KRTAP4-12	0	0	0	0	0	0	0	0	0	0	0
ENSG00000198090	KRTAP4-6	0	0	0	0	0	0	0	0	0	0	0
ENSG00000198271	KRTAP4-5	0	0	0	0	0	0	0	0	0	0	0
ENSG00000240542	KRTAP9-1	0	0	0	0	0	0	0	0	0	0	0
ENSG00000239886	KRTAP9-2	0	0	0	0	0	0	0	0	0	0	0
ENSG00000204873	KRTAP9-3	0	0	0	0	0	0	0	0	0	0	0
ENSG00000187272	KRTAP9-8	0	0	0	0	0	0	0	0	0	0	0
ENSG00000241595	KRTAP9-4	0	0	0	0	0	0	0	0	0	0	0
ENSG00000198083	KRTAP9-9	0	0	0	0	0	0	0	0	0	0	0
ENSG00000212659	KRTAP9-6	0	0	0	0	0	0	0	0	0	0	0
ENSG00000180386	KRTAP9-7	0	0	0	0	0	0	0	0	0	0	0
ENSG00000212658	KRTAP29-1	0	0	0	0	0	0	0	0	0	0	0
ENSG00000212657	KRTAP16-1	0	0	0	0	0	0	0	0	0	0	0
ENSG00000186860	KRTAP17-1	0	0	0	0	0	0	0	0	0	0	0
ENSG00000006059	KRT33A	0	0	0	0	0	0	0	0	0	0	0
ENSG00000131738	KRT33B	0	0	0	0	0	0	0	0	0	0	0
ENSG00000131737	KRT34	0	0	0	0	0	0	0	0	0	0	0
ENSG00000094796	KRT31	0	0	0	0	0	0	0	0	0	0	0
ENSG00000108417	KRT37	0	0	0	0	0	0	0	0	0	0	0
ENSG00000108759	KRT32	0	0	0	0	0	0	0	0	0	0	0
ENSG00000171403	KRT9	0	0	0	0	0	0	0	0	0	0	0
ENSG00000186832	KRT16	0	0	0	0	0	0	0	0	0	0	0
ENSG00000184502	GAST	0	0	0	0	0	0	0	0	0	0	0
ENSG00000267261	AC099811.2	0	0	0	0	0	0	0	0	0	0	0
ENSG00000131471	AOC3	0	0	0	0	0	0	0	0	0	0	0
ENSG00000108825	PTGES3L-AARSD1	0	0	0	0	0	0	0	0	0	0	0
ENSG00000108849	PPY	0	0	0	0	0	0	0	0	0	0	0
ENSG00000131096	PYY	0	0	0	0	0	0	0	0	0	0	0
ENSG00000073670	ADAM11	0	0	0	0	0	0	0	0	0	0	0
ENSG00000214447	FAM187A	0	0	0	0	0	0	0	0	0	0	0
ENSG00000131094	C1QL1	0	0	0	0	0	0	0	0	0	0	0
ENSG00000263715	LINC02210-CRHR1	0	0	0	0	0	0	0	0	0	0	0
ENSG00000120088	CRHR1	0	0	0	0	0	0	0	0	0	0	0
ENSG00000185294	SPPL2C	0	0	0	0	0	0	0	0	0	0	0
ENSG00000186868	MAPT	0	0	0	0	0	0	0	0	0	0	0
ENSG00000256762	STH	0	0	0	0	0	0	0	0	0	0	0
ENSG00000158955	WNT9B	0	0	0	0	0	0	0	0	0	0	0
ENSG00000262633	AC005670.2	0	0	0	0	0	0	0	0	0	0	0
ENSG00000198336	MYL4	0	0	0	0	0	0	0	0	0	0	0
ENSG00000259753	AC068234.1	0	0	0	0	0	0	0	0	0	0	0
ENSG00000189120	SP6	0	0	0	0	0	0	0	0	0	0	0
ENSG00000167183	PRR15L	0	0	0	0	0	0	0	0	0	0	0
ENSG00000159182	PRAC1	0	0	0	0	0	0	0	0	0	0	0
ENSG00000229637	PRAC2	0	0	0	0	0	0	0	0	0	0	0
ENSG00000159224	GIP	0	0	0	0	0	0	0	0	0	0	0
ENSG00000064300	NGFR	0	0	0	0	0	0	0	0	0	0	0
ENSG00000176358	TAC4	0	0	0	0	0	0	0	0	0	0	0
ENSG00000108823	SGCA	0	0	0	0	0	0	0	0	0	0	0
ENSG00000167105	TMEM92	0	0	0	0	0	0	0	0	0	0	0
ENSG00000173714	WFIKKN2	0	0	0	0	0	0	0	0	0	0	0
ENSG00000011052	NME1-NME2	0	0	0	0	0	0	0	0	0	0	0
ENSG00000141200	KIF2B	0	0	0	0	0	0	0	0	0	0	0
ENSG00000266086	AC015813.2	0	0	0	0	0	0	0	0	0	0	0
ENSG00000255713	OR4D2	0	0	0	0	0	0	0	0	0	0	0
ENSG00000213246	SUPT4H1	0	0	0	0	0	0	0	0	0	0	0
ENSG00000265303	AC099850.2	0	0	0	0	0	0	0	0	0	0	0
ENSG00000267318	AC005702.1	0	0	0	0	0	0	0	0	0	0	0
ENSG00000264813	AC113554.1	0	0	0	0	0	0	0	0	0	0	0
ENSG00000125695	AC046185.1	0	0	0	0	0	0	0	0	0	0	0
ENSG00000213218	CSH2	0	0	0	0	0	0	0	0	0	0	0
ENSG00000204414	CSHL1	0	0	0	0	0	0	0	0	0	0	0
ENSG00000259384	GH1	0	0	0	0	0	0	0	0	0	0	0
ENSG00000285947	AC127029.3	0	0	0	0	0	0	0	0	0	0	0
ENSG00000266076	AC004805.1	0	0	0	0	0	0	0	0	0	0	0
ENSG00000091583	APOH	0	0	0	0	0	0	0	0	0	0	0

ENSG00000075429	CACNG5	0	0	0	0	0	0	0	0	0	0	0
ENSG00000108878	CACNG1	0	0	0	0	0	0	0	0	0	0	0
ENSG00000187959	CPSF4L	0	0	0	0	0	0	0	0	0	0	0
ENSG00000171595	DNAI2	0	0	0	0	0	0	0	0	0	0	0
ENSG00000267426	AC087289.4	0	0	0	0	0	0	0	0	0	0	0
ENSG00000070526	ST6GALNAC1	0	0	0	0	0	0	0	0	0	0	0
ENSG00000267168	AC005837.2	0	0	0	0	0	0	0	0	0	0	0
ENSG00000187997	C17orf99	0	0	0	0	0	0	0	0	0	0	0
ENSG00000262660	AC139530.3	0	0	0	0	0	0	0	0	0	0	0
ENSG00000215644	GCGR	0	0	0	0	0	0	0	0	0	0	0
ENSG00000183979	NPB	0	0	0	0	0	0	0	0	0	0	0
ENSG00000185105	MYADML2	0	0	0	0	0	0	0	0	0	0	0
ENSG00000182459	TEX19	0	0	0	0	0	0	0	0	0	0	0
ENSG00000141579	ZNF750	0	0	0	0	0	0	0	0	0	0	0
ENSG00000206422	LRRC30	0	0	0	0	0	0	0	0	0	0	0
ENSG00000176194	CIDEA	0	0	0	0	0	0	0	0	0	0	0
ENSG00000176136	MC5R	0	0	0	0	0	0	0	0	0	0	0
ENSG00000267140	AC007998.2	0	0	0	0	0	0	0	0	0	0	0
ENSG00000274744	ELOA3D	0	0	0	0	0	0	0	0	0	0	0
ENSG00000278674	ELOA3B	0	0	0	0	0	0	0	0	0	0	0
ENSG00000183791	ELOA3	0	0	0	0	0	0	0	0	0	0	0
ENSG00000267228	AC012254.2	0	0	0	0	0	0	0	0	0	0	0
ENSG00000215472	RPL17-C18orf32	0	0	0	0	0	0	0	0	0	0	0
ENSG00000266997	AC090227.1	0	0	0	0	0	0	0	0	0	0	0
ENSG00000267699	AC091551.1	0	0	0	0	0	0	0	0	0	0	0
ENSG00000134443	GRP	0	0	0	0	0	0	0	0	0	0	0
ENSG00000166634	SERPINB12	0	0	0	0	0	0	0	0	0	0	0
ENSG00000206073	SERPINB4	0	0	0	0	0	0	0	0	0	0	0
ENSG00000197632	SERPINB2	0	0	0	0	0	0	0	0	0	0	0
ENSG00000242550	SERPINB10	0	0	0	0	0	0	0	0	0	0	0
ENSG00000221887	HMSD	0	0	0	0	0	0	0	0	0	0	0
ENSG00000187773	DIPK1C	0	0	0	0	0	0	0	0	0	0	0
ENSG00000256463	SALL3	0	0	0	0	0	0	0	0	0	0	0
ENSG00000181781	ODF3L2	0	0	0	0	0	0	0	0	0	0	0
ENSG00000197540	GZMM	0	0	0	0	0	0	0	0	0	0	0
ENSG00000070388	FGF22	0	0	0	0	0	0	0	0	0	0	0
ENSG00000172232	AZU1	0	0	0	0	0	0	0	0	0	0	0
ENSG00000196415	PRTN3	0	0	0	0	0	0	0	0	0	0	0
ENSG00000197561	ELANE	0	0	0	0	0	0	0	0	0	0	0
ENSG00000116014	KISS1R	0	0	0	0	0	0	0	0	0	0	0
ENSG00000267059	AC005943.1	0	0	0	0	0	0	0	0	0	0	0
ENSG00000104899	AMH	0	0	0	0	0	0	0	0	0	0	0
ENSG00000273734	AC005258.1	0	0	0	0	0	0	0	0	0	0	0
ENSG00000267001	AC006538.3	0	0	0	0	0	0	0	0	0	0	0
ENSG00000173976	RAX2	0	0	0	0	0	0	0	0	0	0	0
ENSG00000105428	ZNRF4	0	0	0	0	0	0	0	0	0	0	0
ENSG00000267157	AC011499.1	0	0	0	0	0	0	0	0	0	0	0
ENSG00000267740	AC024592.3	0	0	0	0	0	0	0	0	0	0	0
ENSG00000267314	AC104532.1	0	0	0	0	0	0	0	0	0	0	0
ENSG00000167769	ACER1	0	0	0	0	0	0	0	0	0	0	0
ENSG00000130545	CRB3	0	0	0	0	0	0	0	0	0	0	0
ENSG00000237247	MBD3L5	0	0	0	0	0	0	0	0	0	0	0
ENSG00000205718	MBD3L4	0	0	0	0	0	0	0	0	0	0	0
ENSG00000230522	MBD3L2	0	0	0	0	0	0	0	0	0	0	0
ENSG00000182315	MBD3L3	0	0	0	0	0	0	0	0	0	0	0
ENSG00000268861	AC008878.3	0	0	0	0	0	0	0	0	0	0	0
ENSG00000267952	AC008878.1	0	0	0	0	0	0	0	0	0	0	0
ENSG00000268614	AC008878.2	0	0	0	0	0	0	0	0	0	0	0
ENSG00000268400	AC008763.2	0	0	0	0	0	0	0	0	0	0	0
ENSG00000174788	PCP2	0	0	0	0	0	0	0	0	0	0	0
ENSG00000269711	AC008763.3	0	0	0	0	0	0	0	0	0	0	0
ENSG00000171017	LRRC8E	0	0	0	0	0	0	0	0	0	0	0
ENSG00000178531	CTXN1	0	0	0	0	0	0	0	0	0	0	0
ENSG00000181733	OR2Z1	0	0	0	0	0	0	0	0	0	0	0
ENSG00000170948	MBD3L1	0	0	0	0	0	0	0	0	0	0	0
ENSG00000170920	OR7G3	0	0	0	0	0	0	0	0	0	0	0
ENSG00000237521	OR7E24	0	0	0	0	0	0	0	0	0	0	0
ENSG00000188629	ZNF177	0	0	0	0	0	0	0	0	0	0	0
ENSG00000080511	RDH8	0	0	0	0	0	0	0	0	0	0	0
ENSG00000243207	PPAN-P2RY11	0	0	0	0	0	0	0	0	0	0	0
ENSG00000105371	ICAM4	0	0	0	0	0	0	0	0	0	0	0
ENSG00000267303	AC011511.4	0	0	0	0	0	0	0	0	0	0	0
ENSG00000130173	ANGPTL8	0	0	0	0	0	0	0	0	0	0	0
ENSG00000267477	AC008481.3	0	0	0	0	0	0	0	0	0	0	0
ENSG00000286098	AC008770.4	0	0	0	0	0	0	0	0	0	0	0
ENSG00000257355	AC008770.1	0	0	0	0	0	0	0	0	0	0	0
ENSG00000213297	ZNF625-ZNF20	0	0	0	0	0	0	0	0	0	0	0
ENSG00000269755	AC008758.6	0	0	0	0	0	0	0	0	0	0	0
ENSG00000196826	AC008758.1	0	0	0	0	0	0	0	0	0	0	0
ENSG00000269693	AC010422.6	0	0	0	0	0	0	0	0	0	0	0
ENSG00000269590	AC010422.5	0	0	0	0	0	0	0	0	0	0	0
ENSG00000283980	GNG14	0	0	0	0	0	0	0	0	0	0	0
ENSG00000132026	RTBDN	0	0	0	0	0	0	0	0	0	0	0
ENSG00000171136	RLN3	0	0	0	0	0	0	0	0	0	0	0
ENSG00000187867	PALM3	0	0	0	0	0	0	0	0	0	0	0
ENSG00000185385	OR7A17	0	0	0	0	0	0	0	0	0	0	0
ENSG00000141977	CIB3	0	0	0	0	0	0	0	0	0	0	0
ENSG00000268790	AC008764.4	0	0	0	0	0	0	0	0	0	0	0
ENSG00000269095	AC010646.1	0	0	0	0	0	0	0	0	0	0	0
ENSG00000269307	AC010463.1	0	0	0	0	0	0	0	0	0	0	0
ENSG00000269035	AC010319.2	0	0	0	0	0	0	0	0	0	0	0
ENSG00000105650	AC005759.1	0	0	0	0	0	0	0	0	0	0	0
ENSG00000284797	AC008397.1	0	0	0	0	0	0	0	0	0	0	0
ENSG00000130283	GDF1	0	0	0	0	0	0	0	0	0	0	0
ENSG00000268193	AC002985.1	0	0	0	0	0	0	0	0	0	0	0
ENSG00000064489	BORCS8-MEF2B	0	0	0	0	0	0	0	0	0	0	0
ENSG00000187664	HAPLN4	0	0	0	0	0	0	0	0	0	0	0
ENSG00000213996	TM6SF2	0	0	0	0	0	0	0	0	0	0	0
ENSG00000258674	AC011448.1	0	0	0	0	0	0	0	0	0	0	0
ENSG00000269237	AC010615.4	0	0	0	0	0	0	0	0	0	0	0
ENSG00000283201	AC092329.3	0	0	0	0	0	0	0	0	0	0	0
ENSG00000166359	WDR88	0	0	0	0	0	0	0	0	0	0	0
ENSG00000266953	AC092073.1	0	0	0	0	0	0	0	0	0	0	0
ENSG00000285526	AC020907.6	0	0	0	0	0	0	0	0	0	0	0
ENSG00000266964	FXYD1	0	0	0	0	0	0	0	0	0	0	0
ENSG00000177558	FAM187B	0	0	0	0	0	0	0	0	0	0	0
ENSG00000105695	MAG	0	0	0	0	0	0	0	0	0	0	0
ENSG00000126266	FFAR1	0	0	0	0	0	0	0	0	0	0	0
ENSG00000105679	GAPDHS	0	0	0	0	0	0	0	0	0	0	0
ENSG00000283758	PMIS2	0	0	0	0	0	0	0	0	0	0	0
ENSG00000105668	UPK1A	0	0	0	0	0	0	0	0	0	0	0
ENSG00000267120	AD000671.2	0	0	0	0	0	0	0	0	0	0	0
ENSG00000188223	AD000671.1	0	0	0	0	0	0	0	0	0	0	0
ENSG00000004776	HSPB6	0	0	0	0	0	0	0	0	0	0	0
ENSG00000267360	AC012309.1	0	0	0	0	0	0	0	0	0	0	0
ENSG00000267552	AC093227.2	0	0	0	0	0	0	0	0	0	0	0
ENSG00000167641	PPP1R14A	0	0	0	0	0	0	0	0	0	0	0

ENSG00000267748	AC011479.1	0	0	0	0	0	0	0	0	0	0	0
ENSG00000167644	C19orf33	0	0	0	0	0	0	0	0	0	0	0
ENSG00000179168	GGN	0	0	0	0	0	0	0	0	0	0	0
ENSG00000268083	AC008982.1	0	0	0	0	0	0	0	0	0	0	0
ENSG00000262484	CCER2	0	0	0	0	0	0	0	0	0	0	0
ENSG00000269547	AC011455.2	0	0	0	0	0	0	0	0	0	0	0
ENSG00000179751	SYCN	0	0	0	0	0	0	0	0	0	0	0
ENSG00000197110	IFNL3	0	0	0	0	0	0	0	0	0	0	0
ENSG00000186838	SELENOV	0	0	0	0	0	0	0	0	0	0	0
ENSG00000249861	LGALS16	0	0	0	0	0	0	0	0	0	0	0
ENSG00000105205	CLC	0	0	0	0	0	0	0	0	0	0	0
ENSG00000105219	CNTD2	0	0	0	0	0	0	0	0	0	0	0
ENSG00000160396	HIPK4	0	0	0	0	0	0	0	0	0	0	0
ENSG00000268975	MIA-RAB4B	0	0	0	0	0	0	0	0	0	0	0
ENSG00000171570	RAB4B-EGLN2	0	0	0	0	0	0	0	0	0	0	0
ENSG00000268797	AC008537.1	0	0	0	0	0	0	0	0	0	0	0
ENSG00000198077	CYP2A7	0	0	0	0	0	0	0	0	0	0	0
ENSG00000204978	ERICH4	0	0	0	0	0	0	0	0	0	0	0
ENSG00000267881	AC243967.1	0	0	0	0	0	0	0	0	0	0	0
ENSG00000285505	AC010616.2	0	0	0	0	0	0	0	0	0	0	0
ENSG00000268643	AC006486.1	0	0	0	0	0	0	0	0	0	0	0
ENSG00000221826	PSG3	0	0	0	0	0	0	0	0	0	0	0
ENSG00000204936	CD177	0	0	0	0	0	0	0	0	0	0	0
ENSG00000268361	L34079.1	0	0	0	0	0	0	0	0	0	0	0
ENSG00000267022	AC067968.1	0	0	0	0	0	0	0	0	0	0	0
ENSG00000267173	AC245748.1	0	0	0	0	0	0	0	0	0	0	0
ENSG00000213892	CEACAM16	0	0	0	0	0	0	0	0	0	0	0
ENSG00000142273	CBLC	0	0	0	0	0	0	0	0	0	0	0
ENSG00000267467	APOC4	0	0	0	0	0	0	0	0	0	0	0
ENSG00000177045	SIX5	0	0	0	0	0	0	0	0	0	0	0
ENSG00000268434	AC011530.1	0	0	0	0	0	0	0	0	0	0	0
ENSG00000188425	NANOS2	0	0	0	0	0	0	0	0	0	0	0
ENSG00000188293	IGFL1	0	0	0	0	0	0	0	0	0	0	0
ENSG00000167414	AC093503.1	0	0	0	0	0	0	0	0	0	0	0
ENSG00000130751	NPAS1	0	0	0	0	0	0	0	0	0	0	0
ENSG00000178928	TPRX1	0	0	0	0	0	0	0	0	0	0	0
ENSG00000188334	BSPH1	0	0	0	0	0	0	0	0	0	0	0
ENSG00000169393	AC010330.1	0	0	0	0	0	0	0	0	0	0	0
ENSG00000105467	SYNGR4	0	0	0	0	0	0	0	0	0	0	0
ENSG00000268465	AC008403.1	0	0	0	0	0	0	0	0	0	0	0
ENSG00000177202	SPACA4	0	0	0	0	0	0	0	0	0	0	0
ENSG00000267335	AC008687.1	0	0	0	0	0	0	0	0	0	0	0
ENSG00000267631	CGB1	0	0	0	0	0	0	0	0	0	0	0
ENSG00000189052	CGB5	0	0	0	0	0	0	0	0	0	0	0
ENSG00000213030	CGB8	0	0	0	0	0	0	0	0	0	0	0
ENSG00000283663	AC008687.8	0	0	0	0	0	0	0	0	0	0	0
ENSG00000225950	NTF4	0	0	0	0	0	0	0	0	0	0	0
ENSG00000142538	PTH2	0	0	0	0	0	0	0	0	0	0	0
ENSG00000269469	AC010619.1	0	0	0	0	0	0	0	0	0	0	0
ENSG00000269179	AC011452.1	0	0	0	0	0	0	0	0	0	0	0
ENSG00000142539	AC020909.2	0	0	0	0	0	0	0	0	0	0	0
ENSG00000204653	ASPDH	0	0	0	0	0	0	0	0	0	0	0
ENSG00000142511	GPR32	0	0	0	0	0	0	0	0	0	0	0
ENSG00000167749	KLK4	0	0	0	0	0	0	0	0	0	0	0
ENSG00000269741	AC011473.4	0	0	0	0	0	0	0	0	0	0	0
ENSG00000213022	KLK9	0	0	0	0	0	0	0	0	0	0	0
ENSG00000167757	KLK11	0	0	0	0	0	0	0	0	0	0	0
ENSG00000186474	KLK12	0	0	0	0	0	0	0	0	0	0	0
ENSG00000129437	KLK14	0	0	0	0	0	0	0	0	0	0	0
ENSG00000269403	AC008750.8	0	0	0	0	0	0	0	0	0	0	0
ENSG00000105370	LIM2	0	0	0	0	0	0	0	0	0	0	0
ENSG00000213822	CEACAM18	0	0	0	0	0	0	0	0	0	0	0
ENSG00000268500	AC018755.2	0	0	0	0	0	0	0	0	0	0	0
ENSG00000221874	ZNF816-ZNF321P	0	0	0	0	0	0	0	0	0	0	0
ENSG00000228567	VN1R4	0	0	0	0	0	0	0	0	0	0	0
ENSG00000130433	CACNG6	0	0	0	0	0	0	0	0	0	0	0
ENSG00000248385	TARM1	0	0	0	0	0	0	0	0	0	0	0
ENSG00000275183	LENG9	0	0	0	0	0	0	0	0	0	0	0
ENSG00000242019	KIR3DL3	0	0	0	0	0	0	0	0	0	0	0
ENSG00000243772	KIR2DL3	0	0	0	0	0	0	0	0	0	0	0
ENSG00000125498	KIR2DL1	0	0	0	0	0	0	0	0	0	0	0
ENSG00000167633	KIR3DL1	0	0	0	0	0	0	0	0	0	0	0
ENSG00000189430	NCR1	0	0	0	0	0	0	0	0	0	0	0
ENSG00000167634	NLRP7	0	0	0	0	0	0	0	0	0	0	0
ENSG00000088053	GP6	0	0	0	0	0	0	0	0	0	0	0
ENSG00000267110	AC010327.2	0	0	0	0	0	0	0	0	0	0	0
ENSG00000160471	COX6B2	0	0	0	0	0	0	0	0	0	0	0
ENSG00000267706	AC020922.1	0	0	0	0	0	0	0	0	0	0	0
ENSG00000180043	FAM71E2	0	0	0	0	0	0	0	0	0	0	0
ENSG00000160472	TMEM190	0	0	0	0	0	0	0	0	0	0	0
ENSG00000283567	C19orf85	0	0	0	0	0	0	0	0	0	0	0
ENSG00000231274	SBK3	0	0	0	0	0	0	0	0	0	0	0
ENSG00000197487	GALP	0	0	0	0	0	0	0	0	0	0	0
ENSG00000268133	AC003002.1	0	0	0	0	0	0	0	0	0	0	0
ENSG00000269533	AC003002.3	0	0	0	0	0	0	0	0	0	0	0
ENSG00000268107	AC003005.1	0	0	0	0	0	0	0	0	0	0	0
ENSG00000269026	AC003006.1	0	0	0	0	0	0	0	0	0	0	0
ENSG00000268750	AC010522.1	0	0	0	0	0	0	0	0	0	0	0
ENSG00000269476	AC010326.2	0	0	0	0	0	0	0	0	0	0	0
ENSG00000178591	DEFB125	0	0	0	0	0	0	0	0	0	0	0
ENSG00000125788	DEFB126	0	0	0	0	0	0	0	0	0	0	0
ENSG00000186458	DEFB132	0	0	0	0	0	0	0	0	0	0	0
ENSG00000125895	TMEM74B	0	0	0	0	0	0	0	0	0	0	0
ENSG00000286022	AL121899.2	0	0	0	0	0	0	0	0	0	0	0
ENSG00000166948	TGM6	0	0	0	0	0	0	0	0	0	0	0
ENSG00000256566	AL049650.1	0	0	0	0	0	0	0	0	0	0	0
ENSG00000241690	AL035460.1	0	0	0	0	0	0	0	0	0	0	0
ENSG00000258713	C20orf141	0	0	0	0	0	0	0	0	0	0	0
ENSG00000101405	OXT	0	0	0	0	0	0	0	0	0	0	0
ENSG00000171873	ADRA1D	0	0	0	0	0	0	0	0	0	0	0
ENSG00000286235	AL035461.3	0	0	0	0	0	0	0	0	0	0	0
ENSG00000285508	AL034430.1	0	0	0	0	0	0	0	0	0	0	0
ENSG00000285723	AL034430.2	0	0	0	0	0	0	0	0	0	0	0
ENSG00000101230	ISM1	0	0	0	0	0	0	0	0	0	0	0
ENSG00000125888	BANF2	0	0	0	0	0	0	0	0	0	0	0
ENSG00000284776	AL121900.2	0	0	0	0	0	0	0	0	0	0	0
ENSG00000125820	NKX2-2	0	0	0	0	0	0	0	0	0	0	0
ENSG00000132671	SSTR4	0	0	0	0	0	0	0	0	0	0	0
ENSG00000101435	CST9L	0	0	0	0	0	0	0	0	0	0	0
ENSG00000173335	CST9	0	0	0	0	0	0	0	0	0	0	0
ENSG00000170373	CST1	0	0	0	0	0	0	0	0	0	0	0
ENSG00000180483	DEFB119	0	0	0	0	0	0	0	0	0	0	0
ENSG00000204548	DEFB121	0	0	0	0	0	0	0	0	0	0	0
ENSG00000180383	DEFB124	0	0	0	0	0	0	0	0	0	0	0
ENSG00000101898	MCTS2P	0	0	0	0	0	0	0	0	0	0	0
ENSG00000101306	MYLK2	0	0	0	0	0	0	0	0	0	0	0
ENSG00000179772	FOXS1	0	0	0	0	0	0	0	0	0	0	0

ENSG00000101331	CCM2L	0	0	0	0	0	0	0	0	0	0	0
ENSG00000198183	BPIFA1	0	0	0	0	0	0	0	0	0	0	0
ENSG00000149609	C20orf144	0	0	0	0	0	0	0	0	0	0	0
ENSG00000261582	AL121753.1	0	0	0	0	0	0	0	0	0	0	0
ENSG00000125965	GDF5	0	0	0	0	0	0	0	0	0	0	0
ENSG00000125975	C20orf173	0	0	0	0	0	0	0	0	0	0	0
ENSG00000272897	AL109827.1	0	0	0	0	0	0	0	0	0	0	0
ENSG00000259399	TGIF2-RAB5IF	0	0	0	0	0	0	0	0	0	0	0
ENSG00000118702	GHRH	0	0	0	0	0	0	0	0	0	0	0
ENSG00000182035	ADIG	0	0	0	0	0	0	0	0	0	0	0
ENSG00000124143	ARHGAP40	0	0	0	0	0	0	0	0	0	0	0
ENSG00000277611	Z98752.3	0	0	0	0	0	0	0	0	0	0	0
ENSG00000101074	R3HDML	0	0	0	0	0	0	0	0	0	0	0
ENSG00000124232	RBPJL	0	0	0	0	0	0	0	0	0	0	0
ENSG00000254806	SYS1-DBNDD2	0	0	0	0	0	0	0	0	0	0	0
ENSG00000101443	WFDC2	0	0	0	0	0	0	0	0	0	0	0
ENSG00000243543	WFDC6	0	0	0	0	0	0	0	0	0	0	0
ENSG00000249139	EPPIN-WFDC6	0	0	0	0	0	0	0	0	0	0	0
ENSG00000180205	WFDC9	0	0	0	0	0	0	0	0	0	0	0
ENSG00000149634	SPATA25	0	0	0	0	0	0	0	0	0	0	0
ENSG00000124257	NEURL2	0	0	0	0	0	0	0	0	0	0	0
ENSG00000124208	TMEM189-UBE2V1	0	0	0	0	0	0	0	0	0	0	0
ENSG00000124089	MC3R	0	0	0	0	0	0	0	0	0	0	0
ENSG00000124227	ANKRD60	0	0	0	0	0	0	0	0	0	0	0
ENSG00000130701	RBBP8NL	0	0	0	0	0	0	0	0	0	0	0
ENSG00000130700	GATA5	0	0	0	0	0	0	0	0	0	0	0
ENSG00000243509	TNFRSF6B	0	0	0	0	0	0	0	0	0	0	0
ENSG00000273047	AL121845.2	0	0	0	0	0	0	0	0	0	0	0
ENSG00000183260	ABHD16B	0	0	0	0	0	0	0	0	0	0	0
ENSG00000196132	MYT1	0	0	0	0	0	0	0	0	0	0	0
ENSG00000125522	NPBWR2	0	0	0	0	0	0	0	0	0	0	0
ENSG00000279493	FP565260.4	0	0	0	0	0	0	0	0	0	0	0
ENSG00000276612	FP565260.2	0	0	0	0	0	0	0	0	0	0	0
ENSG00000274559	CU639417.1	0	0	0	0	0	0	0	0	0	0	0
ENSG00000273590	SMIM11B	0	0	0	0	0	0	0	0	0	0	0
ENSG00000277277	FAM243B	0	0	0	0	0	0	0	0	0	0	0
ENSG00000278961	SMIM34B	0	0	0	0	0	0	0	0	0	0	0
ENSG00000276289	KCNE1B	0	0	0	0	0	0	0	0	0	0	0
ENSG00000166351	POTED	0	0	0	0	0	0	0	0	0	0	0
ENSG00000156282	CLDN17	0	0	0	0	0	0	0	0	0	0	0
ENSG00000156284	CLDN8	0	0	0	0	0	0	0	0	0	0	0
ENSG00000188694	KRTAP24-1	0	0	0	0	0	0	0	0	0	0	0
ENSG00000197683	KRTAP26-1	0	0	0	0	0	0	0	0	0	0	0
ENSG00000206107	KRTAP27-1	0	0	0	0	0	0	0	0	0	0	0
ENSG00000186980	KRTAP23-1	0	0	0	0	0	0	0	0	0	0	0
ENSG00000182816	KRTAP13-2	0	0	0	0	0	0	0	0	0	0	0
ENSG00000198390	KRTAP13-1	0	0	0	0	0	0	0	0	0	0	0
ENSG00000240432	KRTAP13-3	0	0	0	0	0	0	0	0	0	0	0
ENSG00000186971	KRTAP13-4	0	0	0	0	0	0	0	0	0	0	0
ENSG00000184351	KRTAP19-1	0	0	0	0	0	0	0	0	0	0	0
ENSG00000186965	KRTAP19-2	0	0	0	0	0	0	0	0	0	0	0
ENSG00000244025	KRTAP19-3	0	0	0	0	0	0	0	0	0	0	0
ENSG00000186977	KRTAP19-5	0	0	0	0	0	0	0	0	0	0	0
ENSG00000186925	KRTAP19-6	0	0	0	0	0	0	0	0	0	0	0
ENSG00000206106	KRTAP22-2	0	0	0	0	0	0	0	0	0	0	0
ENSG00000186924	KRTAP22-1	0	0	0	0	0	0	0	0	0	0	0
ENSG00000244624	KRTAP20-1	0	0	0	0	0	0	0	0	0	0	0
ENSG00000206105	KRTAP20-4	0	0	0	0	0	0	0	0	0	0	0
ENSG00000184032	KRTAP20-2	0	0	0	0	0	0	0	0	0	0	0
ENSG00000206104	KRTAP20-3	0	0	0	0	0	0	0	0	0	0	0
ENSG00000187005	KRTAP21-1	0	0	0	0	0	0	0	0	0	0	0
ENSG00000274749	KRTAP7-1	0	0	0	0	0	0	0	0	0	0	0
ENSG00000182591	KRTAP11-1	0	0	0	0	0	0	0	0	0	0	0
ENSG00000206102	KRTAP19-8	0	0	0	0	0	0	0	0	0	0	0
ENSG00000170262	MRAP	0	0	0	0	0	0	0	0	0	0	0
ENSG00000265590	C21orf59-TCP10L	0	0	0	0	0	0	0	0	0	0	0
ENSG00000205929	C21orf62	0	0	0	0	0	0	0	0	0	0	0
ENSG00000249624	AP000295.1	0	0	0	0	0	0	0	0	0	0	0
ENSG00000249209	AP000311.1	0	0	0	0	0	0	0	0	0	0	0
ENSG00000159197	KCNE2	0	0	0	0	0	0	0	0	0	0	0
ENSG00000205670	SMIM11A	0	0	0	0	0	0	0	0	0	0	0
ENSG00000222018	FAM243A	0	0	0	0	0	0	0	0	0	0	0
ENSG00000243627	SMIM34A	0	0	0	0	0	0	0	0	0	0	0
ENSG00000159212	CLIC6	0	0	0	0	0	0	0	0	0	0	0
ENSG00000285815	WRB-SH3BGR	0	0	0	0	0	0	0	0	0	0	0
ENSG00000183067	IGSF5	0	0	0	0	0	0	0	0	0	0	0
ENSG00000160202	CRYAA	0	0	0	0	0	0	0	0	0	0	0
ENSG00000234289	H2BFS	0	0	0	0	0	0	0	0	0	0	0
ENSG00000205445	KRTAP10-2	0	0	0	0	0	0	0	0	0	0	0
ENSG00000212935	KRTAP10-3	0	0	0	0	0	0	0	0	0	0	0
ENSG00000215454	KRTAP10-4	0	0	0	0	0	0	0	0	0	0	0
ENSG00000241123	KRTAP10-5	0	0	0	0	0	0	0	0	0	0	0
ENSG00000188155	KRTAP10-6	0	0	0	0	0	0	0	0	0	0	0
ENSG00000272804	KRTAP10-7	0	0	0	0	0	0	0	0	0	0	0
ENSG00000187766	KRTAP10-8	0	0	0	0	0	0	0	0	0	0	0
ENSG00000221859	KRTAP10-10	0	0	0	0	0	0	0	0	0	0	0
ENSG00000212933	KRTAP12-4	0	0	0	0	0	0	0	0	0	0	0
ENSG00000205439	KRTAP12-3	0	0	0	0	0	0	0	0	0	0	0
ENSG00000221864	KRTAP12-2	0	0	0	0	0	0	0	0	0	0	0
ENSG00000189169	KRTAP10-12	0	0	0	0	0	0	0	0	0	0	0
ENSG00000286224	AP000471.1	0	0	0	0	0	0	0	0	0	0	0
ENSG00000130538	OR11H1	0	0	0	0	0	0	0	0	0	0	0
ENSG00000274252	GGTLC3	0	0	0	0	0	0	0	0	0	0	0
ENSG00000286175	AC023490.4	0	0	0	0	0	0	0	0	0	0	0
ENSG00000283809	AC007326.4	0	0	0	0	0	0	0	0	0	0	0
ENSG00000206203	TSSK2	0	0	0	0	0	0	0	0	0	0	0
ENSG00000184702	SEPT5	0	0	0	0	0	0	0	0	0	0	0
ENSG00000284874	AC000093.1	0	0	0	0	0	0	0	0	0	0	0
ENSG00000203618	GP1BB	0	0	0	0	0	0	0	0	0	0	0
ENSG00000234409	CCDC188	0	0	0	0	0	0	0	0	0	0	0
ENSG00000161133	USP41	0	0	0	0	0	0	0	0	0	0	0
ENSG00000277971	AC007731.4	0	0	0	0	0	0	0	0	0	0	0
ENSG00000099960	SLC7A4	0	0	0	0	0	0	0	0	0	0	0
ENSG00000133475	GGT2	0	0	0	0	0	0	0	0	0	0	0
ENSG00000286102	AP000552.3	0	0	0	0	0	0	0	0	0	0	0
ENSG00000274600	RIMBP3B	0	0	0	0	0	0	0	0	0	0	0
ENSG00000169575	VPREB1	0	0	0	0	0	0	0	0	0	0	0
ENSG00000169548	ZNF280A	0	0	0	0	0	0	0	0	0	0	0
ENSG00000185686	PRAME	0	0	0	0	0	0	0	0	0	0	0
ENSG00000100121	GGTLC2	0	0	0	0	0	0	0	0	0	0	0
ENSG00000100218	RSPH14	0	0	0	0	0	0	0	0	0	0	0
ENSG00000128218	VPREB3	0	0	0	0	0	0	0	0	0	0	0
ENSG00000169314	C22orf15	0	0	0	0	0	0	0	0	0	0	0
ENSG00000251357	AP000350.4	0	0	0	0	0	0	0	0	0	0	0
ENSG00000099974	DDTL	0	0	0	0	0	0	0	0	0	0	0
ENSG00000099977	DDT	0	0	0	0	0	0	0	0	0	0	0
ENSG00000285762	AC253536.7	0	0	0	0	0	0	0	0	0	0	0

ENSG00000276950	GSTT4	0	0	0	0	0	0	0	0	0	0	0
ENSG00000258555	SPECC1L-ADORA2A	0	0	0	0	0	0	0	0	0	0	0
ENSG00000100053	CRYBB3	0	0	0	0	0	0	0	0	0	0	0
ENSG00000244752	CRYBB2	0	0	0	0	0	0	0	0	0	0	0
ENSG00000196431	CRYBA4	0	0	0	0	0	0	0	0	0	0	0
ENSG00000100249	C22orf31	0	0	0	0	0	0	0	0	0	0	0
ENSG00000128250	RFPL1	0	0	0	0	0	0	0	0	0	0	0
ENSG00000128342	LIF	0	0	0	0	0	0	0	0	0	0	0
ENSG00000248751	AC004997.1	0	0	0	0	0	0	0	0	0	0	0
ENSG00000249590	AC004832.3	0	0	0	0	0	0	0	0	0	0	0
ENSG00000285404	Z82190.2	0	0	0	0	0	0	0	0	0	0	0
ENSG00000100302	RASD2	0	0	0	0	0	0	0	0	0	0	0
ENSG00000128313	APOL5	0	0	0	0	0	0	0	0	0	0	0
ENSG00000100362	PVALB	0	0	0	0	0	0	0	0	0	0	0
ENSG00000285304	Z83844.3	0	0	0	0	0	0	0	0	0	0	0
ENSG00000100101	Z83844.1	0	0	0	0	0	0	0	0	0	0	0
ENSG00000128346	C22orf23	0	0	0	0	0	0	0	0	0	0	0
ENSG00000128298	BAIAP2L2	0	0	0	0	0	0	0	0	0	0	0
ENSG00000284554	AL022318.4	0	0	0	0	0	0	0	0	0	0	0
ENSG00000285025	AL022312.1	0	0	0	0	0	0	0	0	0	0	0
ENSG00000284431	AL022238.4	0	0	0	0	0	0	0	0	0	0	0
ENSG00000205704	LINC00634	0	0	0	0	0	0	0	0	0	0	0
ENSG00000100197	CYP2D6	0	0	0	0	0	0	0	0	0	0	0
ENSG00000128274	A4GALT	0	0	0	0	0	0	0	0	0	0	0
ENSG00000138892	TTLL8	0	0	0	0	0	0	0	0	0	0	0
ENSG00000100253	MIOX	0	0	0	0	0	0	0	0	0	0	0
ENSG00000284194	SCO2	0	0	0	0	0	0	0	0	0	0	0
ENSG00000100312	ACR	0	0	0	0	0	0	0	0	0	0	0
ENSG00000182378	PLCXD1	0	0	0	0	0	0	0	0	0	0	0
ENSG00000178605	GTPBP6	0	0	0	0	0	0	0	0	0	0	0
ENSG00000167393	PPP2R3B	0	0	0	0	0	0	0	0	0	0	0
ENSG00000185960	SHOX	0	0	0	0	0	0	0	0	0	0	0
ENSG00000205755	CRLF2	0	0	0	0	0	0	0	0	0	0	0
ENSG00000198223	CSF2RA	0	0	0	0	0	0	0	0	0	0	0
ENSG00000185291	IL3RA	0	0	0	0	0	0	0	0	0	0	0
ENSG00000169100	SLC25A6	0	0	0	0	0	0	0	0	0	0	0
ENSG00000169093	ASMTL	0	0	0	0	0	0	0	0	0	0	0
ENSG00000182162	P2RY8	0	0	0	0	0	0	0	0	0	0	0
ENSG00000197976	AKAP17A	0	0	0	0	0	0	0	0	0	0	0
ENSG00000196433	ASMT	0	0	0	0	0	0	0	0	0	0	0
ENSG00000169084	DHRSX	0	0	0	0	0	0	0	0	0	0	0
ENSG00000214717	ZBED1	0	0	0	0	0	0	0	0	0	0	0
ENSG00000169059	VCX3A	0	0	0	0	0	0	0	0	0	0	0
ENSG00000177504	VCX2	0	0	0	0	0	0	0	0	0	0	0
ENSG00000205642	VCX3B	0	0	0	0	0	0	0	0	0	0	0
ENSG00000234469	CLDN34	0	0	0	0	0	0	0	0	0	0	0
ENSG00000125363	AMELX	0	0	0	0	0	0	0	0	0	0	0
ENSG00000123594	ATXN3L	0	0	0	0	0	0	0	0	0	0	0
ENSG00000165197	VEGFD	0	0	0	0	0	0	0	0	0	0	0
ENSG00000102010	BMX	0	0	0	0	0	0	0	0	0	0	0
ENSG00000130234	ACE2	0	0	0	0	0	0	0	0	0	0	0
ENSG00000182798	MAGEB17	0	0	0	0	0	0	0	0	0	0	0
ENSG00000169906	S100G	0	0	0	0	0	0	0	0	0	0	0
ENSG00000185915	KLHL34	0	0	0	0	0	0	0	0	0	0	0
ENSG00000175809	CBLL2	0	0	0	0	0	0	0	0	0	0	0
ENSG00000184735	DDX53	0	0	0	0	0	0	0	0	0	0	0
ENSG00000232030	MAGEB6B	0	0	0	0	0	0	0	0	0	0	0
ENSG00000188408	MAGEB5	0	0	0	0	0	0	0	0	0	0	0
ENSG00000120289	MAGEB4	0	0	0	0	0	0	0	0	0	0	0
ENSG00000132446	FTHL17	0	0	0	0	0	0	0	0	0	0	0
ENSG00000250349	AF241726.2	0	0	0	0	0	0	0	0	0	0	0
ENSG00000229674	AL121578.2	0	0	0	0	0	0	0	0	0	0	0
ENSG00000238205	MPC1L	0	0	0	0	0	0	0	0	0	0	0
ENSG00000188937	NYX	0	0	0	0	0	0	0	0	0	0	0
ENSG00000102055	PPP1R2C	0	0	0	0	0	0	0	0	0	0	0
ENSG00000124479	NDP	0	0	0	0	0	0	0	0	0	0	0
ENSG00000189037	DUSP21	0	0	0	0	0	0	0	0	0	0	0
ENSG00000171489	SPACA5	0	0	0	0	0	0	0	0	0	0	0
ENSG00000171478	SPACA5B	0	0	0	0	0	0	0	0	0	0	0
ENSG00000165583	SSX5	0	0	0	0	0	0	0	0	0	0	0
ENSG00000165584	SSX3	0	0	0	0	0	0	0	0	0	0	0
ENSG00000268009	SSX4	0	0	0	0	0	0	0	0	0	0	0
ENSG00000269791	SSX4B	0	0	0	0	0	0	0	0	0	0	0
ENSG00000215274	GAGE10	0	0	0	0	0	0	0	0	0	0	0
ENSG00000224659	GAGE12J	0	0	0	0	0	0	0	0	0	0	0
ENSG00000274274	GAGE13	0	0	0	0	0	0	0	0	0	0	0
ENSG00000275113	GAGE2E	0	0	0	0	0	0	0	0	0	0	0
ENSG00000236737	GAGE12B	0	0	0	0	0	0	0	0	0	0	0
ENSG00000237671	GAGE12C	0	0	0	0	0	0	0	0	0	0	0
ENSG00000227488	GAGE12D	0	0	0	0	0	0	0	0	0	0	0
ENSG00000236362	GAGE12F	0	0	0	0	0	0	0	0	0	0	0
ENSG00000216649	GAGE12E	0	0	0	0	0	0	0	0	0	0	0
ENSG00000215269	GAGE12G	0	0	0	0	0	0	0	0	0	0	0
ENSG00000224902	GAGE12H	0	0	0	0	0	0	0	0	0	0	0
ENSG00000189064	GAGE2A	0	0	0	0	0	0	0	0	0	0	0
ENSG00000130385	BMP15	0	0	0	0	0	0	0	0	0	0	0
ENSG00000187690	EZHIP	0	0	0	0	0	0	0	0	0	0	0
ENSG00000283093	CENPVL2	0	0	0	0	0	0	0	0	0	0	0
ENSG00000223591	CENPVL1	0	0	0	0	0	0	0	0	0	0	0
ENSG00000187243	MAGED4B	0	0	0	0	0	0	0	0	0	0	0
ENSG00000154545	MAGED4	0	0	0	0	0	0	0	0	0	0	0
ENSG00000155622	XAGE2	0	0	0	0	0	0	0	0	0	0	0
ENSG00000204379	XAGE1A	0	0	0	0	0	0	0	0	0	0	0
ENSG00000204382	XAGE1B	0	0	0	0	0	0	0	0	0	0	0
ENSG00000241476	SSX2	0	0	0	0	0	0	0	0	0	0	0
ENSG00000268447	SSX2B	0	0	0	0	0	0	0	0	0	0	0
ENSG00000204363	SPANXN5	0	0	0	0	0	0	0	0	0	0	0
ENSG00000179304	FAM156B	0	0	0	0	0	0	0	0	0	0	0
ENSG00000238269	PAGE2B	0	0	0	0	0	0	0	0	0	0	0
ENSG00000234068	PAGE2	0	0	0	0	0	0	0	0	0	0	0
ENSG00000204279	PAGE3	0	0	0	0	0	0	0	0	0	0	0
ENSG00000189299	FOXR2	0	0	0	0	0	0	0	0	0	0	0
ENSG00000130054	FAM155B	0	0	0	0	0	0	0	0	0	0	0
ENSG00000147160	AWAT2	0	0	0	0	0	0	0	0	0	0	0
ENSG00000204195	AWAT1	0	0	0	0	0	0	0	0	0	0	0
ENSG00000186912	P2RY4	0	0	0	0	0	0	0	0	0	0	0
ENSG00000204165	CXorf65	0	0	0	0	0	0	0	0	0	0	0
ENSG00000285171	AL590764.2	0	0	0	0	0	0	0	0	0	0	0
ENSG00000186810	CXCR3	0	0	0	0	0	0	0	0	0	0	0
ENSG00000215115	CXorf49	0	0	0	0	0	0	0	0	0	0	0
ENSG00000215113	CXorf49B	0	0	0	0	0	0	0	0	0	0	0
ENSG00000125931	CITED1	0	0	0	0	0	0	0	0	0	0	0
ENSG00000285547	AL133500.1	0	0	0	0	0	0	0	0	0	0	0
ENSG00000184911	DMRTC1B	0	0	0	0	0	0	0	0	0	0	0
ENSG00000268994	FAM236B	0	0	0	0	0	0	0	0	0	0	0
ENSG00000225396	FAM236D	0	0	0	0	0	0	0	0	0	0	0
ENSG00000269502	DMRTC1	0	0	0	0	0	0	0	0	0	0	0

ENSG00000283594	FAM236C	0	0	0	0	0	0	0	0	0	0	0
ENSG00000275520	FAM236A	0	0	0	0	0	0	0	0	0	0	0
ENSG00000186288	PABPC1L2A	0	0	0	0	0	0	0	0	0	0	0
ENSG00000131264	CDX4	0	0	0	0	0	0	0	0	0	0	0
ENSG00000187969	ZCCHC13	0	0	0	0	0	0	0	0	0	0	0
ENSG00000198934	MAGEE1	0	0	0	0	0	0	0	0	0	0	0
ENSG00000122145	TBX22	0	0	0	0	0	0	0	0	0	0	0
ENSG00000174016	TENT5D	0	0	0	0	0	0	0	0	0	0	0
ENSG00000147183	CPXCR1	0	0	0	0	0	0	0	0	0	0	0
ENSG00000153779	TGIF2LX	0	0	0	0	0	0	0	0	0	0	0
ENSG00000000005	TNMD	0	0	0	0	0	0	0	0	0	0	0
ENSG00000257529	RPL36A-HNRNPH2	0	0	0	0	0	0	0	0	0	0	0
ENSG00000268235	TCP11X1	0	0	0	0	0	0	0	0	0	0	0
ENSG00000269405	NXF2	0	0	0	0	0	0	0	0	0	0	0
ENSG00000284800	AC235565.2	0	0	0	0	0	0	0	0	0	0	0
ENSG00000269437	NXF2B	0	0	0	0	0	0	0	0	0	0	0
ENSG00000215029	TCP11X2	0	0	0	0	0	0	0	0	0	0	0
ENSG00000158164	TMSB15A	0	0	0	0	0	0	0	0	0	0	0
ENSG00000198908	BHLHB9	0	0	0	0	0	0	0	0	0	0	0
ENSG00000102128	RAB40AL	0	0	0	0	0	0	0	0	0	0	0
ENSG00000268629	TEX13A	0	0	0	0	0	0	0	0	0	0	0
ENSG00000123561	SERPINA7	0	0	0	0	0	0	0	0	0	0	0
ENSG00000147223	RIPPLY1	0	0	0	0	0	0	0	0	0	0	0
ENSG00000165376	CLDN2	0	0	0	0	0	0	0	0	0	0	0
ENSG00000080561	MID2	0	0	0	0	0	0	0	0	0	0	0
ENSG00000170925	TEX13B	0	0	0	0	0	0	0	0	0	0	0
ENSG00000260548	AL035425.2	0	0	0	0	0	0	0	0	0	0	0
ENSG00000243978	RTL9	0	0	0	0	0	0	0	0	0	0	0
ENSG00000180772	AGTR2	0	0	0	0	0	0	0	0	0	0	0
ENSG00000204019	CT83	0	0	0	0	0	0	0	0	0	0	0
ENSG00000250423	KIAA1210	0	0	0	0	0	0	0	0	0	0	0
ENSG00000101883	RHOXF1	0	0	0	0	0	0	0	0	0	0	0
ENSG00000131721	RHOXF2	0	0	0	0	0	0	0	0	0	0	0
ENSG00000101892	ATP1B4	0	0	0	0	0	0	0	0	0	0	0
ENSG00000236446	CT47B1	0	0	0	0	0	0	0	0	0	0	0
ENSG00000278646	AC008162.2	0	0	0	0	0	0	0	0	0	0	0
ENSG00000226685	CT47A12	0	0	0	0	0	0	0	0	0	0	0
ENSG00000226929	CT47A11	0	0	0	0	0	0	0	0	0	0	0
ENSG00000224089	CT47A10	0	0	0	0	0	0	0	0	0	0	0
ENSG00000226600	CT47A9	0	0	0	0	0	0	0	0	0	0	0
ENSG00000230347	CT47A8	0	0	0	0	0	0	0	0	0	0	0
ENSG00000228517	CT47A7	0	0	0	0	0	0	0	0	0	0	0
ENSG00000226023	CT47A6	0	0	0	0	0	0	0	0	0	0	0
ENSG00000237957	CT47A5	0	0	0	0	0	0	0	0	0	0	0
ENSG00000230594	CT47A4	0	0	0	0	0	0	0	0	0	0	0
ENSG00000236126	CT47A3	0	0	0	0	0	0	0	0	0	0	0
ENSG00000242362	CT47A2	0	0	0	0	0	0	0	0	0	0	0
ENSG00000236371	CT47A1	0	0	0	0	0	0	0	0	0	0	0
ENSG00000123165	ACTRT1	0	0	0	0	0	0	0	0	0	0	0
ENSG00000171054	OR13H1	0	0	0	0	0	0	0	0	0	0	0
ENSG00000224107	ETDB	0	0	0	0	0	0	0	0	0	0	0
ENSG00000169551	CT55	0	0	0	0	0	0	0	0	0	0	0
ENSG00000238210	ETDA	0	0	0	0	0	0	0	0	0	0	0
ENSG00000283644	ETDC	0	0	0	0	0	0	0	0	0	0	0
ENSG00000268940	CT45A1	0	0	0	0	0	0	0	0	0	0	0
ENSG00000269096	CT45A3	0	0	0	0	0	0	0	0	0	0	0
ENSG00000228836	CT45A5	0	0	0	0	0	0	0	0	0	0	0
ENSG00000278289	CT45A6	0	0	0	0	0	0	0	0	0	0	0
ENSG00000271449	CT45A2	0	0	0	0	0	0	0	0	0	0	0
ENSG00000273696	CT45A7	0	0	0	0	0	0	0	0	0	0	0
ENSG00000278085	CT45A8	0	0	0	0	0	0	0	0	0	0	0
ENSG00000270946	CT45A9	0	0	0	0	0	0	0	0	0	0	0
ENSG00000269586	CT45A10	0	0	0	0	0	0	0	0	0	0	0
ENSG00000102239	BRS3	0	0	0	0	0	0	0	0	0	0	0
ENSG00000102243	VGLL1	0	0	0	0	0	0	0	0	0	0	0
ENSG00000203933	CXorf66	0	0	0	0	0	0	0	0	0	0	0
ENSG00000134595	SOX3	0	0	0	0	0	0	0	0	0	0	0
ENSG00000184258	CDR1	0	0	0	0	0	0	0	0	0	0	0
ENSG00000198021	SPANXA1	0	0	0	0	0	0	0	0	0	0	0
ENSG00000203926	SPANXA2	0	0	0	0	0	0	0	0	0	0	0
ENSG00000196406	SPANXD	0	0	0	0	0	0	0	0	0	0	0
ENSG00000189326	SPANXN4	0	0	0	0	0	0	0	0	0	0	0
ENSG00000189252	SPANXN3	0	0	0	0	0	0	0	0	0	0	0
ENSG00000268988	SPANXN2	0	0	0	0	0	0	0	0	0	0	0
ENSG00000235699	CXorf51B	0	0	0	0	0	0	0	0	0	0	0
ENSG00000224440	CXorf51A	0	0	0	0	0	0	0	0	0	0	0
ENSG00000283697	HSFX3	0	0	0	0	0	0	0	0	0	0	0
ENSG00000267978	MAGEA9B	0	0	0	0	0	0	0	0	0	0	0
ENSG00000268738	HSFX2	0	0	0	0	0	0	0	0	0	0	0
ENSG00000171116	HSFX1	0	0	0	0	0	0	0	0	0	0	0
ENSG00000123584	MAGEA9	0	0	0	0	0	0	0	0	0	0	0
ENSG00000283463	HSFX4	0	0	0	0	0	0	0	0	0	0	0
ENSG00000124260	MAGEA10	0	0	0	0	0	0	0	0	0	0	0
ENSG00000268902	CSAG2	0	0	0	0	0	0	0	0	0	0	0
ENSG00000183305	MAGEA2B	0	0	0	0	0	0	0	0	0	0	0
ENSG00000198930	CSAG1	0	0	0	0	0	0	0	0	0	0	0
ENSG00000268916	CSAG3	0	0	0	0	0	0	0	0	0	0	0
ENSG00000197172	MAGEA6	0	0	0	0	0	0	0	0	0	0	0
ENSG00000225110	PNMA6F	0	0	0	0	0	0	0	0	0	0	0
ENSG00000183479	TREX2	0	0	0	0	0	0	0	0	0	0	0
ENSG00000102076	OPN1LW	0	0	0	0	0	0	0	0	0	0	0
ENSG00000268221	OPN1MW	0	0	0	0	0	0	0	0	0	0	0
ENSG00000166160	OPN1MW2	0	0	0	0	0	0	0	0	0	0	0
ENSG00000269433	OPN1MW3	0	0	0	0	0	0	0	0	0	0	0
ENSG00000278057	TEX28	0	0	0	0	0	0	0	0	0	0	0
ENSG00000268651	CTAG1A	0	0	0	0	0	0	0	0	0	0	0
ENSG00000184033	CTAG1B	0	0	0	0	0	0	0	0	0	0	0
ENSG00000203870	SMIM9	0	0	0	0	0	0	0	0	0	0	0
ENSG00000274183	H2AFB1	0	0	0	0	0	0	0	0	0	0	0
ENSG00000182712	CMC4	0	0	0	0	0	0	0	0	0	0	0
ENSG00000277858	H2AFB2	0	0	0	0	0	0	0	0	0	0	0
ENSG00000168939	SPRY3	0	0	0	0	0	0	0	0	0	0	0
ENSG00000124333	VAMP7	0	0	0	0	0	0	0	0	0	0	0
ENSG00000124334	IL9R	0	0	0	0	0	0	0	0	0	0	0
ENSG00000184895	SRY	0	0	0	0	0	0	0	0	0	0	0
ENSG00000176679	TGIF2LY	0	0	0	0	0	0	0	0	0	0	0
ENSG00000099715	PCDH11Y	0	0	0	0	0	0	0	0	0	0	0
ENSG00000168757	TSPY2	0	0	0	0	0	0	0	0	0	0	0
ENSG00000099721	AMELY	0	0	0	0	0	0	0	0	0	0	0
ENSG00000233803	TSPY4	0	0	0	0	0	0	0	0	0	0	0
ENSG00000229549	TSPY8	0	0	0	0	0	0	0	0	0	0	0
ENSG00000228927	TSPY3	0	0	0	0	0	0	0	0	0	0	0
ENSG00000258992	TSPY1	0	0	0	0	0	0	0	0	0	0	0
ENSG00000238074	TSPY9P	0	0	0	0	0	0	0	0	0	0	0
ENSG00000236424	TSPY10	0	0	0	0	0	0	0	0	0	0	0
ENSG00000129864	VCY	0	0	0	0	0	0	0	0	0	0	0
ENSG00000129862	VCY1B	0	0	0	0	0	0	0	0	0	0	0

ENSG00000129873	CDY2B	0	0	0	0	0	0	0	0	0	0	0
ENSG00000182415	CDY2A	0	0	0	0	0	0	0	0	0	0	0
ENSG00000172468	HSFY1	0	0	0	0	0	0	0	0	0	0	0
ENSG00000169953	HSFY2	0	0	0	0	0	0	0	0	0	0	0
ENSG00000286265	AC007244.1	0	0	0	0	0	0	0	0	0	0	0
ENSG00000242875	RBMY1B	0	0	0	0	0	0	0	0	0	0	0
ENSG00000234414	RBMY1A1	0	0	0	0	0	0	0	0	0	0	0
ENSG00000244395	RBMY1D	0	0	0	0	0	0	0	0	0	0	0
ENSG00000242389	RBMY1E	0	0	0	0	0	0	0	0	0	0	0
ENSG00000169807	PRY2	0	0	0	0	0	0	0	0	0	0	0
ENSG00000169800	RBMY1F	0	0	0	0	0	0	0	0	0	0	0
ENSG00000226941	RBMY1J	0	0	0	0	0	0	0	0	0	0	0
ENSG00000169789	PRY	0	0	0	0	0	0	0	0	0	0	0
ENSG00000183753	BPY2	0	0	0	0	0	0	0	0	0	0	0
ENSG00000188120	DAZ1	0	0	0	0	0	0	0	0	0	0	0
ENSG00000205944	DAZ2	0	0	0	0	0	0	0	0	0	0	0
ENSG00000169763	PRYP3	0	0	0	0	0	0	0	0	0	0	0
ENSG00000172352	CDY1B	0	0	0	0	0	0	0	0	0	0	0
ENSG00000183795	BPY2B	0	0	0	0	0	0	0	0	0	0	0
ENSG00000187191	DAZ3	0	0	0	0	0	0	0	0	0	0	0
ENSG00000205916	DAZ4	0	0	0	0	0	0	0	0	0	0	0
ENSG00000185894	BPY2C	0	0	0	0	0	0	0	0	0	0	0
ENSG00000172288	CDY1	0	0	0	0	0	0	0	0	0	0	0
