# Supplementary material for: Transcriptional Profiling Uncovers Human Hyalocytes as a Unique Innate Immune Cell Population
Source: Front Immunol. 2020 Sep 11;11:567274. doi: 10.3389/fimmu.2020.567274 (PMC7517040; doi:10.3389/fimmu.2020.567274)
Supplement: Supplementary file 2 [file Data_Sheet_2.PDF]

ID	Description	GeneRatio	BgRatio	p value	p-adjust	q value	geneID	Count
GO:0006959	humoral immune response	17/98	349/18493	3,78628E-12	1,90374E-09	1,35987E-09	C1QB/C1QA/LYZ/TREM2/CXCL8/CCL2/GAPDH/A2M/S100A9/C3/C1QC/C3AR1/GPR183/RNASE6/VSIG4/CD83/S100A8	17
GO:0030595	leukocyte chemotaxis	13/98	217/18493	1,24604E-10	4,47507E-08	3,19662E-08	CD74/DUSP1/FCER1G/CXCL8/CCL2/S100A9/C3AR1/GPR183/ITGB2/AIF1/PADI2/S100A8/CXCL16	13
GO:0097529	myeloid leukocyte migration	11/98	199/18493	8,4161E-09	2,64476E-06	1,88919E-06	CD74/DUSP1/FCER1G/CXCL8/CCL2/S100A9/C3AR1/CX3CR1/ITGB2/AIF1/S100A8	11
GO:0050900	leukocyte migration	15/98	478/18493	3,15579E-08	7,93365E-06	5,66713E-06	CD74/DUSP1/FCER1G/CXCL8/CCL2/S100A9/C3AR1/RHOA/GPR183/CX3CR1/ITGB2/AIF1/PADI2/S100A8/CXCL16	15
GO:0048002	antigen processing and presentation of peptide antigen	9/98	139/18493	5,26902E-08	1,01895E-05	7,27851E-06	CD74/HLA-DRB1/HLA-DRA/FCER1G/TREM2/HLA-DRB5/VAMP8/B2M/CTSS	9
GO:0071216	cellular response to biotic stimulus	10/98	229/18493	3,76725E-07	4,32493E-05	3,08937E-05	CD14/TREM2/CXCL8/CCL2/RHOA/CX3CR1/ZFP36/TXNIP/TNFRSF1B/HSPA5	10
GO:0019882	antigen processing and presentation	9/98	175/18493	3,78475E-07	4,32493E-05	3,08937E-05	CD74/HLA-DRB1/HLA-DRA/FCER1G/TREM2/HLA-DRB5/VAMP8/B2M/CTSS	9
GO:0002685	regulation of leukocyte migration	9/98	180/18493	4,80039E-07	4,9716E-05	3,55129E-05	CD74/DUSP1/CXCL8/CCL2/C3AR1/RHOA/CX3CR1/AIF1/PADI2	9
GO:0032103	positive regulation of response to external stimulus	11/98	297/18493	4,94391E-07	4,9716E-05	3,55129E-05	CD74/FCER1G/TMSB4X/CXCL8/S100A9/C3/C3AR1/VAMP8/ITGB2/AIF1/S100A8	11
GO:0019884	antigen processing and presentation of exogenous antigen	8/98	134/18493	5,60578E-07	5,2196E-05	3,72844E-05	CD74/HLA-DRB1/HLA-DRA/FCER1G/HLA-DRB5/VAMP8/B2M/CTSS	8
GO:0048857	neural nucleus development	6/98	62/18493	9,17845E-07	7,44343E-05	5,31696E-05	ACTB/YWHAH/RHOA/PADI2/HSPA5/CALM2	6
GO:0051235	maintenance of location	11/98	318/18493	9,68845E-07	7,61149E-05	5,43701E-05	FTL/TMSB10/SRGN/APOE/TMSB4X/S100A9/C3/ANXA5/S100A8/HSPA5/CALM2	11
GO:0002504	antigen processing and presentation of peptide or polysaccharide antigen via MHC class II	7/98	102/18493	1,15697E-06	7,90337E-05	5,6455E-05	CD74/HLA-DRB1/HLA-DRA/FCER1G/TREM2/HLA-DRB5/CTSS	7
GO:0050920	regulation of chemotaxis	9/98	210/18493	1,73772E-06	0,000112016	8,0015E-05	CD74/DUSP1/TMSB4X/CXCL8/CCL2/C3AR1/GPR183/AIF1/PADI2	9
GO:0097242	amyloid-beta clearance	4/98	20/18493	3,36593E-06	0,000201475	0,000143917	APOE/C3/MARCO/ITGB2	4
GO:0002697	regulation of immune effector process	12/98	439/18493	3,5388E-06	0,000206896	0,000147789	CD74/C1QB/C1QA/FCER1G/A2M/C3/C1QC/C3AR1/TMBIM6/VAMP8/ITGB2/B2M	12
GO:0072376	protein activation cascade	8/98	194/18493	8,91962E-06	0,00049831	0,00035595	C1QB/C1QA/A2M/F13A1/C3/C1QC/C3AR1/VSIG4	8
GO:0030901	midbrain development	6/98	92/18493	9,36891E-06	0,000512031	0,000365752	ACTB/YWHAH/RHOA/PADI2/HSPA5/CALM2	6
GO:0045807	positive regulation of endocytosis	7/98	141/18493	1,00097E-05	0,000535415	0,000382455	CD14/FCER1G/APOE/CCL2/C3/B2M/CD63	7
GO:0051651	maintenance of location in cell	6/98	95/18493	1,12742E-05	0,000583102	0,000416519	FTL/TMSB10/SRGN/APOE/TMSB4X/HSPA5	6
GO:0070997	neuron death	10/98	335/18493	1,13652E-05	0,000583102	0,000416519	FOS/APOE/CCL2/GAPDH/RHOA/CX3CR1/BTG2/ITGB2/EGR1/HSPA5	10
GO:0001819	positive regulation of cytokine production	11/98	422/18493	1,44701E-05	0,000673665	0,00048121	CD74/CD14/FCER1G/CSF1R/GAPDH/C3/C3AR1/CYBB/CD83/B2M/EGR1	11
GO:0072676	lymphocyte migration	6/98	105/18493	2,00067E-05	0,000867187	0,000619445	CCL2/RHOA/GPR183/AIF1/PADI2/CXCL16	6
GO:0002523	leukocyte migration involved in inflammatory response	3/98	11/18493	2,30886E-05	0,000964362	0,000688859	S100A9/ITGB2/S100A8	3
GO:2000257	regulation of protein activation cascade	6/98	108/18493	2,34868E-05	0,000964362	0,000688859	C1QB/C1QA/A2M/C3/C1QC/C3AR1	6
GO:0006956	complement activation	7/98	170/18493	3,35913E-05	0,001279522	0,000913983	C1QB/C1QA/A2M/C3/C1QC/C3AR1/VSIG4	7
GO:0019730	antimicrobial humoral response	6/98	118/18493	3,87602E-05	0,001426262	0,001018801	LYZ/CXCL8/GAPDH/S100A9/RNASE6/S100A8	6
GO:0043062	extracellular structure organization	10/98	402/18493	5,37909E-05	0,00175969	0,001256974	SPP1/CST3/APOE/A2M/ITGB2/TNFRSF1B/TIMP1/CTSS/APOC1/LCP1	10
GO:0046677	response to antibiotic	9/98	323/18493	5,44923E-05	0,00175969	0,001256974	PSAP/CD14/DUSP1/RHOA/CYBB/TXNIP/EGR1/S100A8/HSPA5	9
GO:0002218	activation of innate immune response	8/98	252/18493	5,81986E-05	0,001806312	0,001290277	CD14/FCER1G/S100A9/MARCO/ITGB2/UBC/CTSS/S100A8	8
GO:0051238	sequestering of metal ion	3/98	15/18493	6,26948E-05	0,001898973	0,001356466	FTL/S100A9/S100A8	3
GO:0007565	female pregnancy	7/98	191/18493	7,03355E-05	0,002056086	0,001468695	SPP1/FOS/CTSB/ARHGDIB/JUNB/TIMP1/H3F3B	7
GO:0032602	chemokine production	5/98	82/18493	7,4505E-05	0,002128474	0,001520402	CD74/CSF1R/S100A9/EGR1/S100A8	5
GO:2000146	negative regulation of cell motility	9/98	339/18493	7,8971E-05	0,002205924	0,001575726	DUSP1/APOE/CCL2/RHOA/ARHGDIB/CX3CR1/AIF1/PADI2/TIMP1	9
GO:0031349	positive regulation of defense response	10/98	427/18493	8,87947E-05	0,002453075	0,00175227	CD14/FCER1G/S100A9/C3/MARCO/VAMP8/ITGB2/UBC/CTSS/S100A8	10
GO:0002683	negative regulation of immune system process	10/98	437/18493	0,000107422	0,002842726	0,002030604	CD74/CD14/DUSP1/FCER1G/CCL2/A2M/C1QC/ZFP36/TMBIM6/PADI2	10
GO:0046697	decidualization	3/98	20/18493	0,000154087	0,003654485	0,002610455	SPP1/CTSB/JUNB	3
GO:0002696	positive regulation of leukocyte activation	9/98	372/18493	0,000159408	0,00374535	0,002675362	CD74/FCER1G/CCL2/RHOA/GPR183/VAMP8/ITGB2/CD83/AIF1	9
GO:0007566	embryo implantation	4/98	52/18493	0,000165169	0,003827072	0,002733737	SPP1/ARHGDIB/TIMP1/H3F3B	4
GO:0051271	negative regulation of cellular component movement	9/98	374/18493	0,000165931	0,003827072	0,002733737	DUSP1/APOE/CCL2/RHOA/ARHGDIB/CX3CR1/AIF1/PADI2/TIMP1	9
GO:0044706	multi-multicellular organism process	7/98	221/18493	0,000174331	0,003952636	0,002823429	SPP1/FOS/CTSB/ARHGDIB/JUNB/TIMP1/H3F3B	7
GO:0051048	negative regulation of secretion	7/98	222/18493	0,000179236	0,003952636	0,002823429	CD74/SRGN/TMSB4X/TMBIM6/VAMP8/TNFRSF1B/ANXA5	7
GO:0050867	positive regulation of cell activation	9/98	384/18493	0,000201987	0,004340141	0,003100231	CD74/FCER1G/CCL2/RHOA/GPR183/VAMP8/ITGB2/CD83/AIF1	9
GO:0040013	negative regulation of locomotion	9/98	386/18493	0,00020993	0,004421877	0,003158616	DUSP1/APOE/CCL2/RHOA/ARHGDIB/CX3CR1/AIF1/PADI2/TIMP1	9
GO:1904018	positive regulation of vasculature development	7/98	228/18493	0,000211068	0,004421877	0,003158616	CXCL8/C3/C3AR1/CX3CR1/CYBB/ITGB2/EGR1	7
GO:0035821	modification of morphology or physiology of other organism	6/98	161/18493	0,000215718	0,004481951	0,003201528	LYZ/APOE/GAPDH/S100A9/HSPA8/CALM2	6
GO:0006735	NADH regeneration	3/98	25/18493	0,000304958	0,005637245	0,004026772	GAPDH/ENO1/PGK1	3
GO:0060627	regulation of vesicle-mediated transport	10/98	498/18493	0,000308241	0,00565634	0,004040412	CD14/FCER1G/APOE/CCL2/C3/VAMP8/ITGB2/B2M/CD63/APOC1	10
GO:0050922	negative regulation of chemotaxis	4/98	62/18493	0,00032684	0,00582748	0,00416266	DUSP1/CCL2/AIF1/PADI2	4
GO:0042026	protein refolding	3/98	26/18493	0,000343414	0,00595408	0,004253092	B2M/HSPA8/HSPA5	3
GO:0032102	negative regulation of response to external stimulus	8/98	329/18493	0,000361632	0,006142857	0,004387939	SPP1/DUSP1/APOE/CCL2/AIF1/TNFRSF1B/ANXA5/PADI2	8
GO:1903409	reactive oxygen species biosynthetic process	5/98	115/18493	0,00036521	0,006161997	0,004401611	RHOA/CX3CR1/CYBB/ITGB2/AIF1	5
GO:1903706	regulation of hemopoiesis	9/98	418/18493	0,000376905	0,006316927	0,00451228	CD74/FOS/TYROBP/C1QC/RHOA/ZFP36/CD83/B2M/H3F3B	9
GO:0007596	blood coagulation	8/98	334/18493	0,000399814	0,00648473	0,004632144	ACTB/FCER1G/APOE/A2M/F13A1/ACTG1/ANXA5/H3F3B	8
GO:0050817	coagulation	8/98	340/18493	0,000449906	0,007113608	0,005081362	ACTB/FCER1G/APOE/A2M/F13A1/ACTG1/ANXA5/H3F3B	8
GO:0002687	positive regulation of leukocyte migration	5/98	121/18493	0,000461377	0,007204359	0,005146186	CD74/CXCL8/C3AR1/RHOA/AIF1	5
GO:0001893	maternal placenta development	3/98	31/18493	0,000582419	0,008512802	0,006080827	SPP1/CTSB/JUNB	3
GO:0055094	response to lipoprotein particle	3/98	31/18493	0,000582419	0,008512802	0,006080827	FCER1G/APOE/ITGB2	3
GO:1903531	negative regulation of secretion by cell	6/98	196/18493	0,000615788	0,008776038	0,006268861	SRGN/TMSB4X/TMBIM6/VAMP8/TNFRSF1B/ANXA5	6
GO:0050921	positive regulation of chemotaxis	5/98	130/18493	0,000639932	0,008991527	0,006422788	CD74/TMSB4X/CXCL8/C3AR1/AIF1	5
GO:0071402	cellular response to lipoprotein particle stimulus	3/98	33/18493	0,000701532	0,00954977	0,00682155	FCER1G/APOE/ITGB2	3
GO:0043903	regulation of symbiosis, encompassing mutualism through parasitism	6/98	203/18493	0,000739898	0,009894162	0,007067555	CD74/APOE/CXCL8/GAPDH/ZFP36/HSPA8	6
GO:0071706	tumor necrosis factor superfamily cytokine production	5/98	135/18493	0,000759147	0,010044714	0,007175096	CD14/FCER1G/CX3CR1/CYBB/ZFP36	5
GO:0051098	regulation of binding	8/98	369/18493	0,000769147	0,010123749	0,007231552	ACTB/APOE/TMSB4X/TMBIM6/B2M/S100A10/HSPA5/CALM2	8
GO:0043900	regulation of multi-organism process	8/98	371/18493	0,000796569	0,01043008	0,007450369	CD74/APOE/CXCL8/GAPDH/ARHGDIB/ZFP36/HSPA8/TIMP1	8
GO:0002699	positive regulation of immune effector process	6/98	209/18493	0,000860967	0,010995881	0,00785453	CD74/FCER1G/C3/VAMP8/ITGB2/B2M	6
GO:0051702	interaction with symbiont	4/98	80/18493	0,00086165	0,010995881	0,00785453	APOE/GAPDH/HSPA8/CALM2	4
GO:0051092	positive regulation of NF-kappaB transcription factor activity	5/98	146/18493	0,001079083	0,012880808	0,009200963	S100A9/CX3CR1/ITGB2/UBC/S100A8	5
GO:0071674	mononuclear cell migration	4/98	85/18493	0,001081086	0,012880808	0,009200963	DUSP1/CCL2/C3AR1/AIF1	4
GO:0001894	tissue homeostasis	6/98	221/18493	0,001148546	0,013367794	0,009548824	SPP1/ACTB/LYZ/CSF1R/ACTG1/B2M	6
GO:0034447	very-low-density lipoprotein particle clearance	2/98	10/18493	0,001216717	0,013675682	0,009768753	APOE/APOC1	2
GO:1900272	negative regulation of long-term synaptic potentiation	2/98	10/18493	0,001216717	0,013675682	0,009768753	APOE/CX3CR1	2
GO:0050878	regulation of body fluid levels	9/98	493/18493	0,001218517	0,013675682	0,009768753	ACTB/FCER1G/APOE/A2M/F13A1/ACTG1/VAMP8/ANXA5/H3F3B	9
GO:0006458	'de novo' protein folding	3/98	40/18493	0,001236739	0,013818497	0,009870768	CD74/HSPA8/HSPA5	3
GO:0051051	negative regulation of transport	9/98	495/18493	0,001253207	0,013883769	0,009917393	CD74/SRGN/TMSB4X/TMBIM6/VAMP8/TNFRSF1B/ANXA5/APOC1/CALM2	9
GO:0030574	collagen catabolic process	3/98	41/18493	0,001329276	0,014529566	0,010378696	CST3/CTSB/CTSS	3
GO:0051602	response to electrical stimulus	3/98	41/18493	0,001329276	0,014529566	0,010378696	CD14/BTG2/AIF1	3
GO:0051047	positive regulation of secretion	8/98	404/18493	0,001374711	0,01496114	0,010686976	SPP1/CD14/FCER1G/CSF1R/GAPDH/VAMP8/ITGB2/S100A8	8
GO:0001666	response to hypoxia	7/98	314/18493	0,001407916	0,015127045	0,010805484	RHOA/CYBB/ENO1/TMBIM6/UBC/EGR1/PGK1	7
GO:0001558	regulation of cell growth	8/98	416/18493	0,001653231	0,017103796	0,012217508	SPP1/APOE/S100A9/RHOA/ENO1/S100A8/CXCL16/H3F3B	8
GO:0002686	negative regulation of leukocyte migration	3/98	45/18493	0,001742576	0,017336116	0,012383458	DUSP1/CCL2/PADI2	3
GO:0030307	positive regulation of cell growth	5/98	163/18493	0,00175698	0,017336116	0,012383458	S100A9/RHOA/S100A8/CXCL16/H3F3B	5
GO:0061450	trophoblast cell migration	2/98	12/18493	0,001772228	0,017336116	0,012383458	ARHGDIB/TIMP1	2
GO:1901163	regulation of trophoblast cell migration	2/98	12/18493	0,001772228	0,017336116	0,012383458	ARHGDIB/TIMP1	2
GO:0006984	ER-nucleus signaling pathway	3/98	46/18493	0,001857034	0,017819024	0,012728407	CXCL8/CCL2/HSPA5	3
GO:0045185	maintenance of protein location	4/98	104/18493	0,002274245	0,020790734	0,014851146	TMSB10/SRGN/TMSB4X/HSPA5	4
GO:0042089	cytokine biosynthetic process	4/98	105/18493	0,002354827	0,021331301	0,015237282	CX3CR1/CYBB/ZFP36/EGR1	4
GO:0009620	response to fungus	3/98	51/18493	0,002499383	0,021712117	0,015509304	GAPDH/S100A9/S100A8	3
GO:0070482	response to oxygen levels	7/98	349/18493	0,002557828	0,021946689	0,015676863	RHOA/CYBB/ENO1/TMBIM6/UBC/EGR1/PGK1	7
GO:0043270	positive regulation of ion transport	6/98	260/18493	0,002609912	0,022317412	0,015941676	APOE/TMSB4X/CCL2/VAMP8/CTSS/CALM2	6
GO:0042447	hormone catabolic process	2/98	15/18493	0,002790406	0,023075924	0,016483493	SPP1/YWHAH	2
GO:0045123	cellular extravasation	3/98	54/18493	0,002943025	0,024100208	0,017215155	CCL2/CX3CR1/ITGB2	3
GO:2000242	negative regulation of reproductive process	3/98	55/18493	0,003100955	0,025066883	0,017905666	DUSP1/ARHGDIB/TIMP1	3
GO:0035966	response to topologically incorrect protein	5/98	189/18493	0,003335222	0,026703021	0,019074385	CXCL8/CCL2/TMBIM6/HSPA8/HSPA5	5
GO:0006091	generation of precursor metabolites and energy	8/98	471/18493	0,003551447	0,028024059	0,020018023	GAPDH/RHOA/CYBB/ENO1/SH3BGRL3/ATP5F1B/NDUFA1/PGK1	8
GO:0002577	regulation of antigen processing and presentation	2/98	17/18493	0,003589398	0,028024059	0,020018023	CD74/TREM2	2
GO:1903532	positive regulation of secretion by cell	7/98	373/18493	0,003692302	0,02864953	0,020464806	SPP1/CD14/FCER1G/CSF1R/GAPDH/VAMP8/ITGB2	7
GO:0060135	maternal process involved in female pregnancy	3/98	60/18493	0,003968362	0,030416043	0,021726655	SPP1/CTSB/JUNB	3
GO:0061077	chaperone-mediated protein folding	3/98	60/18493	0,003968362	0,030416043	0,021726655	CD74/HSPA8/HSPA5	3
GO:0016049	cell growth	8/98	485/18493	0,004234355	0,031797901	0,022713737	SPP1/APOE/S100A9/RHOA/ENO1/S100A8/CXCL16/H3F3B	8
GO:0032623	interleukin-2 production	3/98	62/18493	0,004352535	0,032373588	0,02312496	FCER1G/ZFP36/CD83	3
GO:0010523	negative regulation of calcium ion transport into cytosol	2/98	19/18493	0,004482144	0,032851636	0,023466436	TMBIM6/CALM2	2
GO:0043487	regulation of RNA stability	4/98	126/18493	0,004532072	0,033121016	0,023658859	ZFP36/UBC/TNFRSF1B/HSPA8	4
GO:0009612	response to mechanical stimulus	5/98	204/18493	0,004610745	0,033501197	0,023930428	FOS/RHOA/BTG2/JUNB/TXNIP	5
GO:0051701	interaction with host	5/98	209/18493	0,005103961	0,03604314	0,025746178	CD74/CXCL8/CTSB/GAPDH/VAMP8	5
GO:0050663	cytokine secretion	5/98	210/18493	0,005206922	0,036667233	0,026191978	CD14/SRGN/TMSB4X/CSF1R/GAPDH	5
GO:0019883	antigen processing and presentation of endogenous antigen	2/98	21/18493	0,005466614	0,037776747	0,026984521	CD74/B2M	2
GO:0002700	regulation of production of molecular mediator of immune response	4/98	133/18493	0,005484691	0,037776747	0,026984521	CD74/FCER1G/TMBIM6/B2M	4
GO:0051090	regulation of DNA-binding transcription factor activity	7/98	412/18493	0,006307004	0,042273698	0,030196763	FOS/TMSB4X/S100A9/CX3CR1/ITGB2/UBC/S100A8	7
GO:0034381	plasma lipoprotein particle clearance	3/98	71/18493	0,006356189	0,042273698	0,030196763	APOE/NPC2/APOC1	3
GO:0032507	maintenance of protein location in cell	3/98	74/18493	0,007127316	0,045362208	0,032402934	TMSB10/TMSB4X/HSPA5	3
GO:0030193	regulation of blood coagulation	3/98	77/18493	0,007951621	0,048996019	0,034998622	FCER1G/APOE/ANXA5	3
GO:1900046	regulation of hemostasis	3/98	77/18493	0,007951621	0,048996019	0,034998622	FCER1G/APOE/ANXA5	3
