# Supplementary material for: Transcriptional Profiling Uncovers Human Hyalocytes as a Unique Innate Immune Cell Population
Source: Front Immunol. 2020 Sep 11;11:567274. doi: 10.3389/fimmu.2020.567274 (PMC7517040; doi:10.3389/fimmu.2020.567274)
Supplement: Supplementary file 3 [file Data_Sheet_3.PDF]

ID	Description	GeneRatio	BgRatio	p value	p-adjust	q value	geneID	Count
GO:0033218	amide binding	12/99	348/17632	5,73207E-07	9,3723E-05	7,45263E-05	CD74/PSAP/HLA-DRB1/HLA-DRA/CD14/CST3/APOE/HLA-DRB5/MARCO/ITGB2/ANXA5/ITM2B	12
GO:0042277	peptide binding	11/99	285/17632	5,80328E-07	9,3723E-05	7,45263E-05	CD74/HLA-DRB1/HLA-DRA/CD14/CST3/APOE/HLA-DRB5/MARCO/ITGB2/ANXA5/ITM2B	11
GO:0001540	amyloid-beta binding	6/99	61/17632	1,16325E-06	0,000125243	9,95905E-05	CD74/CST3/APOE/MARCO/ITGB2/ITM2B	6
GO:0023026	MHC class II protein complex binding	4/99	16/17632	1,61591E-06	0,000130485	0,000103758	CD74/HLA-DRB1/HLA-DRA/HSPA8	4
GO:0023023	MHC protein complex binding	4/99	20/17632	4,22812E-06	0,000273136	0,000217192	CD74/HLA-DRB1/HLA-DRA/HSPA8	4
GO:0019955	cytokine binding	7/99	125/17632	6,59285E-06	0,000354915	0,00028222	CD74/CSF1R/A2M/CX3CR1/ZFP36/TNFRSF1B/IFNGR1	7
GO:0050786	RAGE receptor binding	3/99	11/17632	2,74207E-05	0,001193546	0,00094908	S100A9/S100A4/S100A8	3
GO:0033293	monocarboxylic acid binding	5/99	64/17632	2,95615E-05	0,001193546	0,00094908	PSAP/ALOX5AP/S100A9/S100A8/APOC1	5
GO:0005504	fatty acid binding	4/99	33/17632	3,37651E-05	0,001211792	0,000963589	ALOX5AP/S100A9/S100A8/APOC1	4
GO:0036041	long-chain fatty acid binding	3/99	13/17632	4,71425E-05	0,001522704	0,001210819	ALOX5AP/S100A9/S100A8	3
GO:0004857	enzyme inhibitor activity	9/99	371/17632	0,000239971	0,007046423	0,005603152	CST3/GAPDH/A2M/C3/TMBIM6/TXNIP/ANXA5/TIMP1/APOC1	9
GO:0044183	protein binding involved in protein folding	3/99	23/17632	0,000280254	0,007543492	0,00599841	CD74/HSPA8/HSPA5	3
GO:1901567	fatty acid derivative binding	3/99	28/17632	0,000507966	0,012621011	0,010035935	ALOX5AP/S100A9/S100A8	3
GO:0042605	peptide antigen binding	3/99	30/17632	0,00062443	0,014406481	0,011455699	HLA-DRB1/HLA-DRA/HLA-DRB5	3
GO:0019956	chemokine binding	3/99	32/17632	0,000756671	0,016293648	0,012956331	A2M/CX3CR1/ZFP36	3
GO:0001664	G protein-coupled receptor binding	7/99	274/17632	0,00089363	0,018040152	0,014345111	PSAP/CXCL8/CCL2/C3/MARCO/HSPA8/CXCL16	7
GO:0031625	ubiquitin protein ligase binding	7/99	286/17632	0,001146156	0,021776958	0,017316532	ACTG1/TMBIM6/TXNIP/UBC/TNFRSF1B/HSPA8/HSPA5	7
GO:0016892	endoribonuclease activity, producing 3'-phosphomonoesters	2/99	10/17632	0,001363787	0,024382943	0,019388751	RNASE1/RNASE2	2
GO:0005125	cytokine activity	6/99	219/17632	0,001471233	0,024382943	0,019388751	SPP1/CXCL8/CCL2/TIMP1/GRN/CXCL16	6
GO:0044389	ubiquitin-like protein ligase binding	7/99	301/17632	0,001537189	0,024382943	0,019388751	ACTG1/TMBIM6/TXNIP/UBC/TNFRSF1B/HSPA8/HSPA5	7
GO:0019864	IgG binding	2/99	11/17632	0,001660758	0,024382943	0,019388751	FCER1G/FCGR3A	2
GO:0035325	Toll-like receptor binding	2/99	11/17632	0,001660758	0,024382943	0,019388751	S100A9/S100A8	2
GO:0004859	phospholipase inhibitor activity	2/99	12/17632	0,001985627	0,027885109	0,022173591	ANXA5/APOC1	2
GO:0017017	MAP kinase tyrosine/serine/threonine phosphatase activity	2/99	13/17632	0,002338079	0,031466642	0,025021543	DUSP1/DUSP6	2
GO:0008009	chemokine activity	3/99	49/17632	0,002623348	0,032977876	0,026223242	CXCL8/CCL2/CXCL16	3
GO:0033549	MAP kinase phosphatase activity	2/99	14/17632	0,002717799	0,032977876	0,026223242	DUSP1/DUSP6	2
GO:0032934	sterol binding	3/99	50/17632	0,002779521	0,032977876	0,026223242	APOE/GPR183/NPC2	3
GO:0004252	serine-type endopeptidase activity	6/99	250/17632	0,002858763	0,032977876	0,026223242	C1QB/C1QA/CTSB/C3/C1QC/CTSS	6
GO:0004866	endopeptidase inhibitor activity	5/99	175/17632	0,003068627	0,033640209	0,026749914	CST3/GAPDH/A2M/C3/TIMP1	5
GO:0050998	nitric-oxide synthase binding	2/99	15/17632	0,003124478	0,033640209	0,026749914	CD74/ACTB	2
GO:0030414	peptidase inhibitor activity	5/99	182/17632	0,003628752	0,036627718	0,029125512	CST3/GAPDH/A2M/C3/TIMP1	5
GO:0061135	endopeptidase regulator activity	5/99	182/17632	0,003628752	0,036627718	0,029125512	CST3/GAPDH/A2M/C3/TIMP1	5
GO:0031072	heat shock protein binding	4/99	115/17632	0,004016676	0,038166027	0,030348739	ZFP36/ITGB2/HSPA8/HSPA5	4
GO:0055102	lipase inhibitor activity	2/99	17/17632	0,004017476	0,038166027	0,030348739	ANXA5/APOC1	2
GO:0008236	serine-type peptidase activity	6/99	273/17632	0,004396888	0,040576993	0,032265883	C1QB/C1QA/CTSB/C3/C1QC/CTSS	6
GO:0031406	carboxylic acid binding	5/99	192/17632	0,004551477	0,040836864	0,032472527	PSAP/ALOX5AP/S100A9/S100A8/APOC1	5
GO:0017171	serine hydrolase activity	6/99	277/17632	0,004716823	0,041176593	0,032742672	C1QB/C1QA/CTSB/C3/C1QC/CTSS	6
GO:0017127	cholesterol transporter activity	2/99	19/17632	0,005014638	0,042316496	0,033649096	APOE/NPC2	2
GO:0048306	calcium-dependent protein binding	3/99	62/17632	0,005109422	0,042316496	0,033649096	S100A11/A2M/S100A4	3
GO:0002020	protease binding	4/99	125/17632	0,005396545	0,042693899	0,033949198	CST3/A2M/UBC/TIMP1	4
GO:0008329	signaling pattern recognition receptor activity	2/99	20/17632	0,005551529	0,042693899	0,033949198	CD14/MARCO	2
GO:0038187	pattern recognition receptor activity	2/99	20/17632	0,005551529	0,042693899	0,033949198	CD14/MARCO	2
GO:0043177	organic acid binding	5/99	204/17632	0,005866174	0,04406452	0,035039084	PSAP/ALOX5AP/S100A9/S100A8/APOC1	5
GO:0042379	chemokine receptor binding	3/99	66/17632	0,006083157	0,044655906	0,03550934	CXCL8/CCL2/CXCL16	3
GO:0016894	endonuclease activity, active with either ribo- or deoxyribonucleic acids and producing 3'-phosphomonoesters	2/99	22/17632	0,006700451	0,048094352	0,038243513	RNASE1/RNASE2	2
