# Supplementary figures and images for: Transcriptional Profiling Uncovers Human Hyalocytes as a Unique Innate Immune Cell Population
Source: Front Immunol. 2020 Sep 11;11:567274. doi: 10.3389/fimmu.2020.567274 (PMC7517040; doi:10.3389/fimmu.2020.567274)

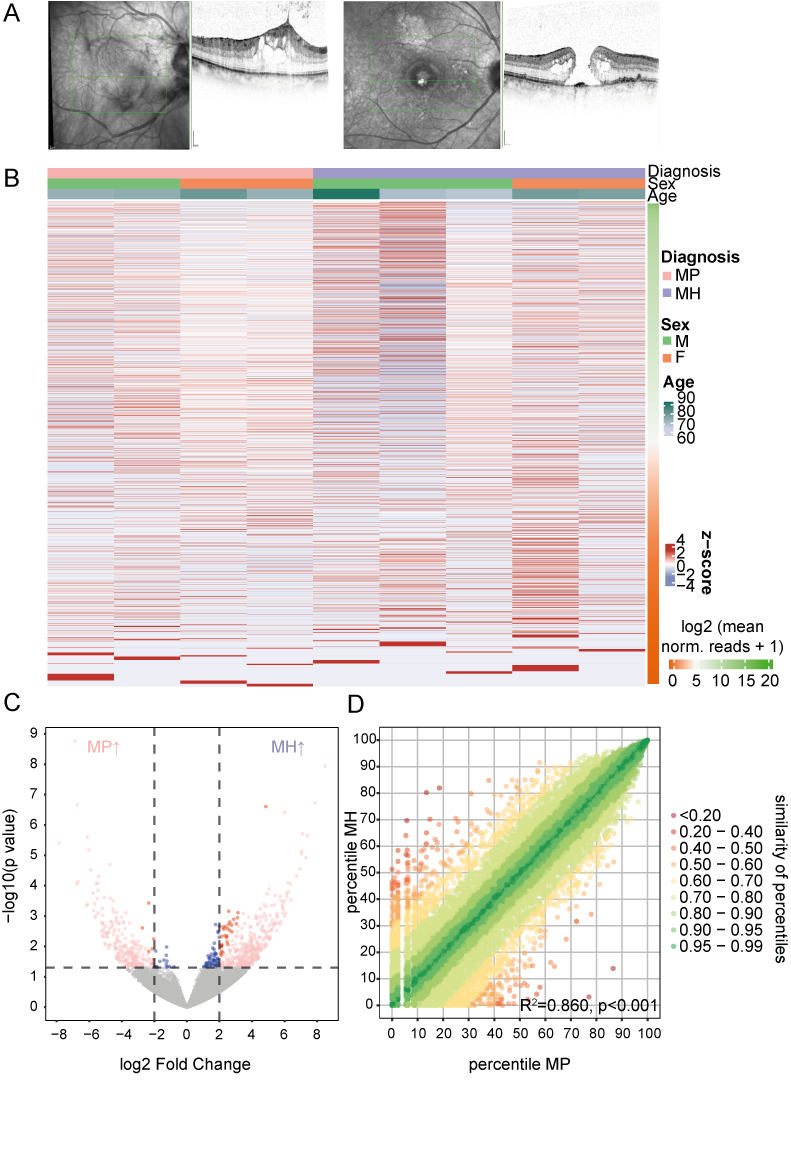

Supplement: FIGURE S1 — Transcriptional analysis does not reveal significant differences of hyalocytes from macular pucker and macular hole eyes. (A) 13 patients undergoing vitrectomy for macular pucker (right-hand side panel) or macular hole (left-hand side panel) were included in this study. (B) Heatmap depicting the transcriptional profile of the 13 samples. The color bars on the top of the heatmap reveal diagnosis of study subjects (pink for macular pucker, lilac for macular hole), sex (grass-green for male, orange for female), and age (age bar on the right side). Color coding according to the z-score (deviation from a gene’s mean expression in standard deviation units). The transcripts are sorted according to the normalized counts (log2-scaled). (C) Volcano plot illustrating the genes differentially expressed between hyalocytes in macula pucker and macular hole patients [log2FC < -2 and > 2 (positive log2FC referring to macular hole samples), p-value < 0.05]. The darker colored genes are filtered for StdErr ≤ 1. (D) Graphical presentation of the percentiles of normalized counts to illustrate the similarity between the transcriptomes of maclar pucker (MP) and macular hole (MH) eyes. The expression of each gene in each cell population was calculated as a percentile (the gene with the highest mean of normalized counts getting the percentile 100, the one with the lowest – the percentile 0). Colors code for the similarity defined as 1 min Δ percentile. The Pearson coefficient R2 quantifies the deviation from the diagonal with the incline 1. [file Image_1.TIF]

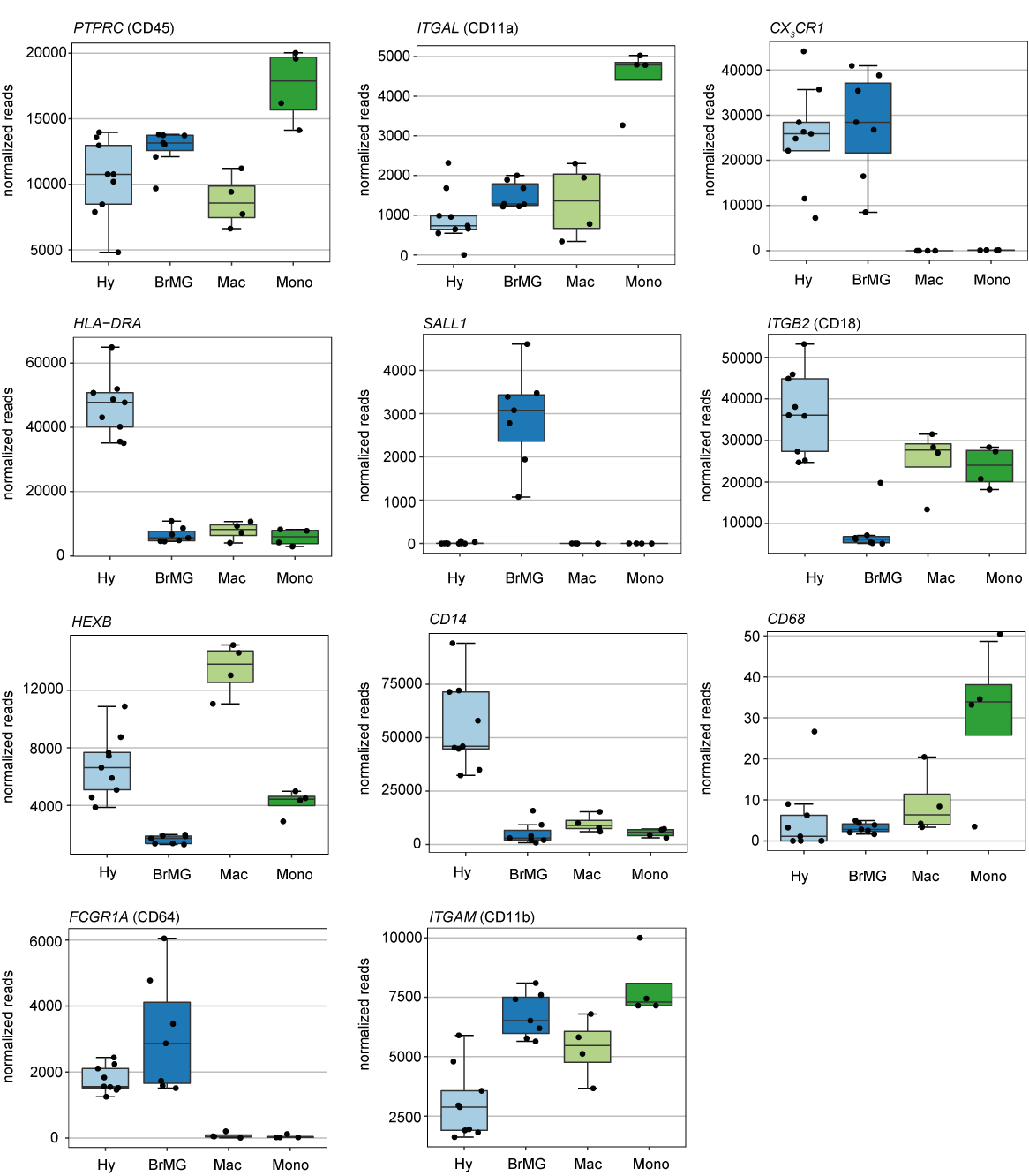

Supplement: FIGURE S2 — Hyalocytes express numerous leucocyte-associated transcripts. Box plots, illustrating the normalized reads count PTPRC (CD45), ITGAL (CD11a), CX3CR1, HLA-DRA, SALL1, ITGB2 (CD18), HEXB, CD14, CD68, FCGR1A (CD64), and ITGAM (CD11b) in hyalocytes (Hy), macrophages (Mac), brain microglia (BrMG), and monocytes (Mono). [file Image_2.TIF]

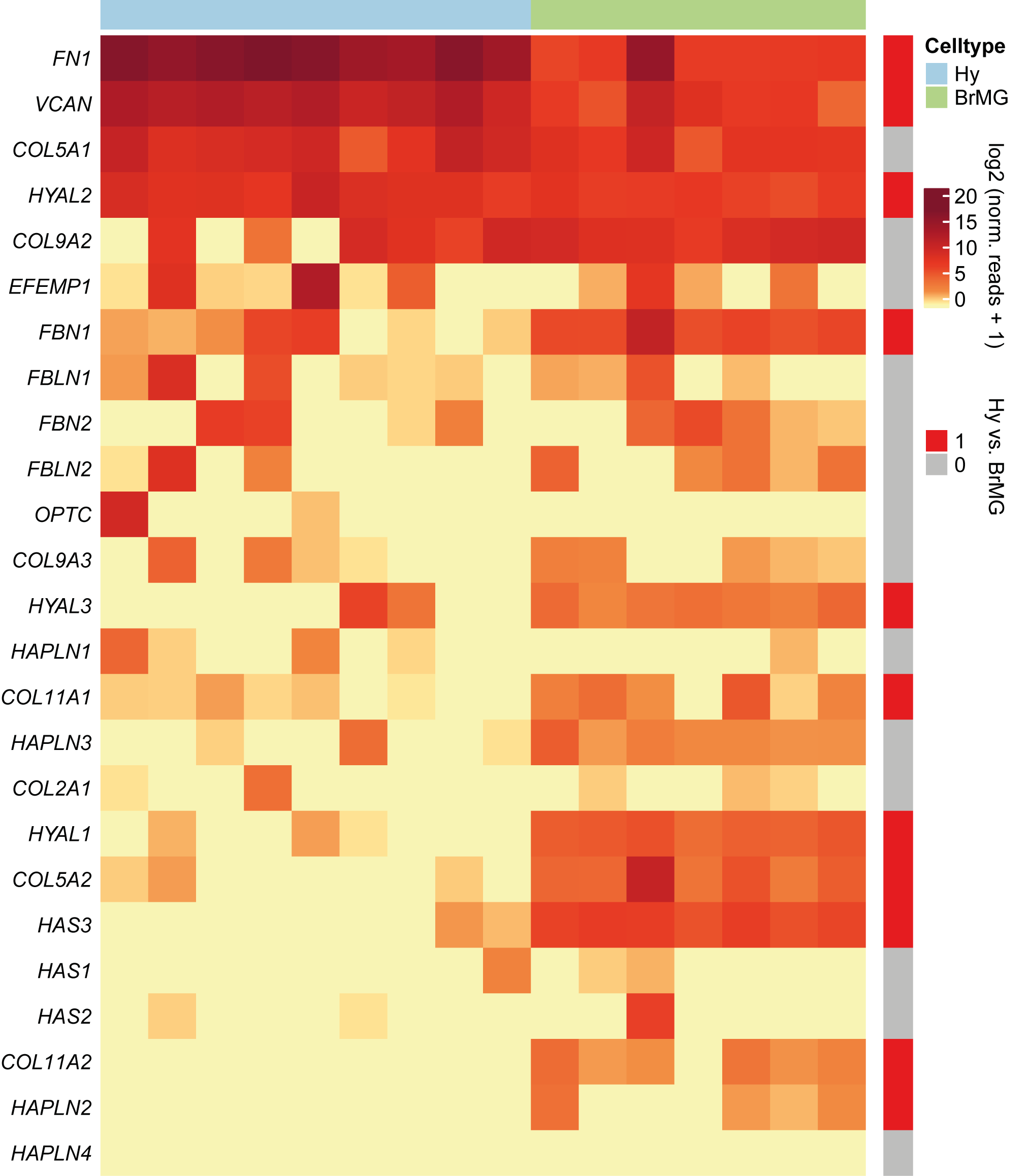

Supplement: FIGURE S3 — Hyalocytes express numerous factors involved in vitreous homeostasis. Heatmap depicting the expression of genes coding for extracellular matrix proteins in hyalocytes (Hy) and microglia (BrMG). The transcripts are sorted according to the normalized counts (log2-scaled). Significance level for the comparison is shown in the bar on the right side, red coding for a significantly higher expression in human hyalocytes. [file Image_3.TIF]

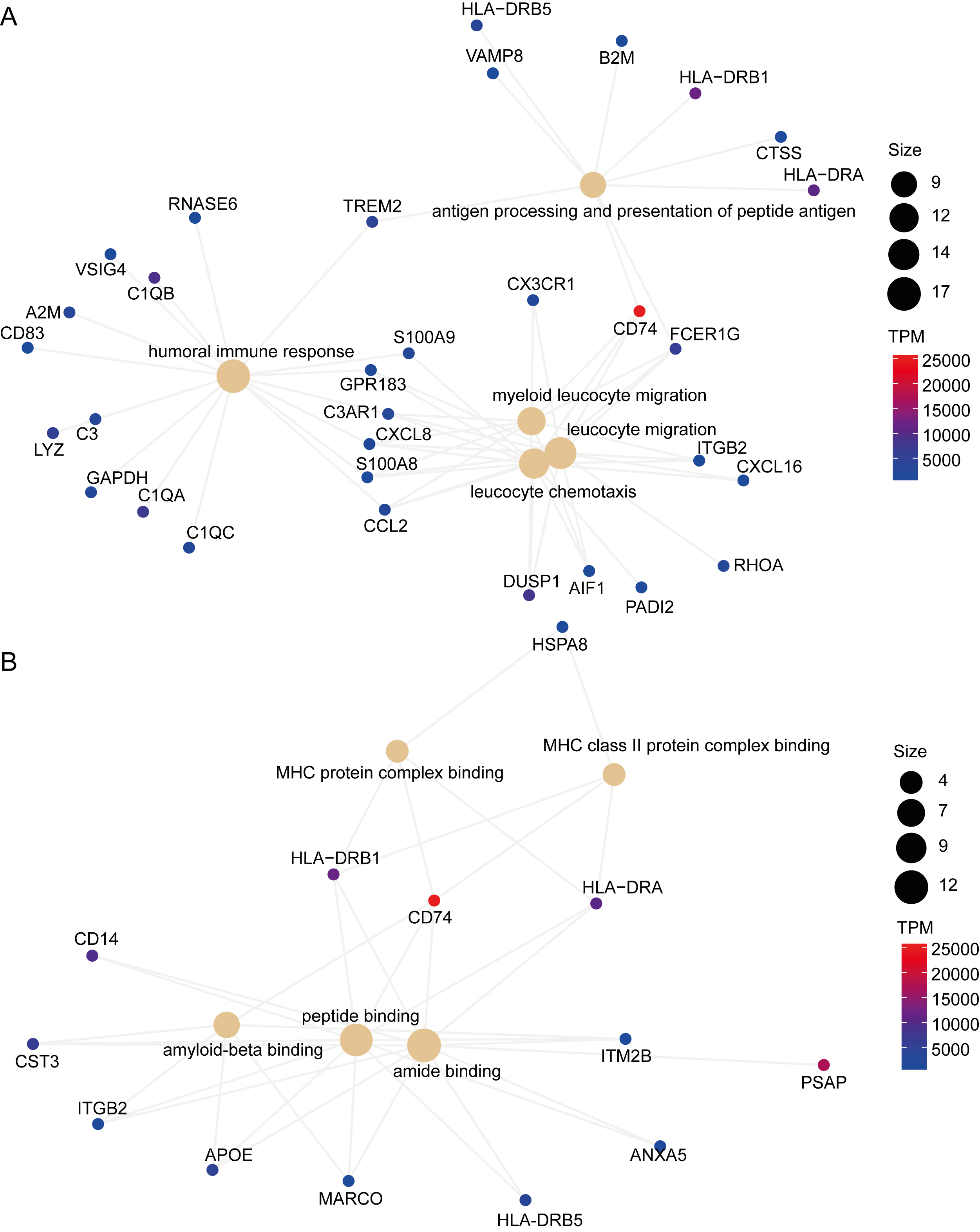

Supplement: FIGURE S4 — Biological processes and molecular functions of human hyalocytes. All top expressed genes enriched in the 5 most disease-relevant GO biological processes (BP, A) and molecular functions (MF, B) terms are illustrated in cnetplots. [file Image_4.TIF]

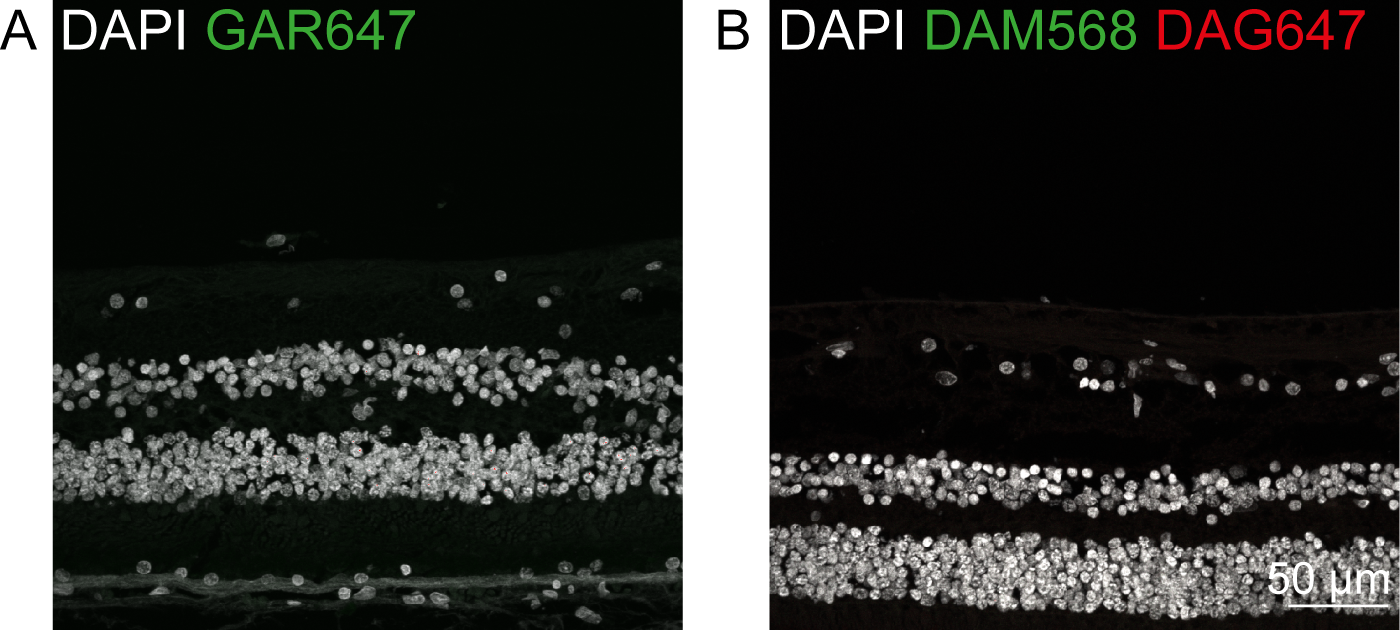

Supplement: FIGURE S5 — Negative controls for immunohistochemistry. Negative controls were performed by omitting primary antibodies. (A) Negative control for FTL and CD74 immunohistochemical stainings shown in Figures 2D,E. Nuclei are counterstained with DAPI. GAR647, goat anti-rabbit Alexa Fluor 647. (B) Negative control for HLA-DRA and IBA1 immunohistochemical staining shown in Figure 3F. Nuclei are counterstained with DAPI. DAM568, donkey anti-mouse Alexa Fluor 568. DAG647, donkey anti-goat Alexa Fluor 647. [file Image_5.TIF]

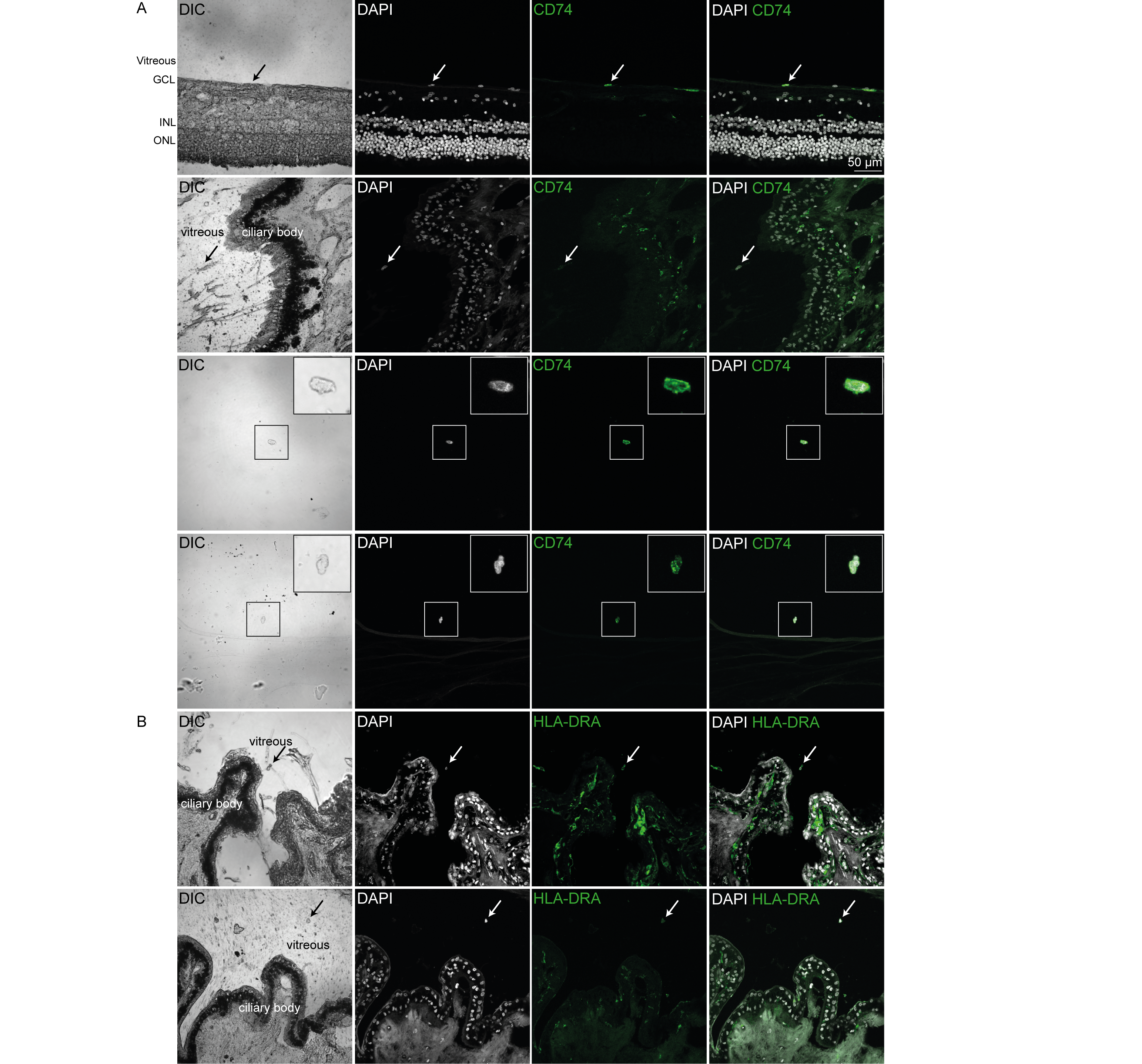

Supplement: FIGURE S6 — Immunohistochemical stainings for CD74 (A) and HLA-DRA (B). The arrows point at vitreal cells positive for CD74 (first and second row in A) or HLA-DRA (B). Vitreal cells are presented in higher magnification in the upper right corner (third and fourth row in A). Nuclei are counterstained with DAPI. DIC, differential interference contrast; GCL, ganglion cell layer; INL, inner nuclear layer; and ONL, outer nuclear layer. [file Image_6.TIF]
